# Development of a porcine (Sus scofa) embryo-specific microarray: array annotation and validation

**DOI:** 10.1186/1471-2164-13-370

**Published:** 2012-08-03

**Authors:** Stephen Tsoi, Chi Zhou, Jason R Grant, J Alexander Pasternak, John Dobrinsky, Philippe Rigault, Julie Nieminen, Marc-André Sirard, Claude Robert, George R Foxcroft, Michael K Dyck

**Affiliations:** 1 Department of Agricultural, Food and Nutritional Science, University of Alberta, Edmonton, AB, T6G 2P5, Canada; 2 International Center of Biotechnology, Minitube of America, Mt. Horeb, Wisconsin, USA; 3 Gydle Inc, 1363, avenue Maguire Suite 301, Québec, QC, G1T 1Z2, Canada; 4 Laboratory of Functional Genomics of Early Embryonic Development, Université Laval, Quebec, QC, G1T 1Z2, Canada

## Abstract

**Background:**

The domestic pig is an important livestock species and there is strong interest in the factors that affect the development of viable embryos and offspring in this species. A limited understanding of the molecular mechanisms involved in early embryonic development has inhibited our ability to fully elucidate these factors. Next generation deep sequencing and microarray technologies are powerful tools for delineation of molecular pathways involved in the developing embryo.

**Results:**

Here we present the development of a porcine-embryo-specific microarray platform created from a large expressed sequence tag (EST) analysis generated by Roche/454 next-generation sequencing of cDNAs constructed from critical stages of *in vivo* or *in vitro* porcine preimplantation embryos. Two cDNA libraries constructed from *in vitro* and *in vivo* produced preimplantation porcine embryos were normalized and sequenced using 454 Titanium pyrosequencing technology. Over one million high-quality EST sequences were obtained and used to develop the EMbryogene Porcine Version 1 (EMPV1) microarray composed of 43,795 probes. Based on an initial probe sequence annotation, the EMPV1 features 17,409 protein-coding, 473 pseudogenes, 46 retrotransposed, 2,359 non-coding RNA, 4,121 splice variants in 2,862 genes and a total of 12,324 Novel Transcript Regions (NTR). After re-annotation, the total unique genes increased from 11,961 to 16,281 and 1.9% of them belonged to a large olfactory receptor (OR) gene family. Quality control on the EMPV1 was performed and revealed an even distribution of ten clusters of spiked-in control spots and array to array (dye-swap) correlation was 0.97.

**Conclusions:**

Using next-generation deep sequencing we have produced a large EST dataset to allow for the selection of probe sequences for the development of the EMPV1 microarray platform. The quality of this embryo-specific array was confirmed with a high-level of reproducibility using current Agilent microarray technology. With more than an estimated 20,000 unique genes represented on the EMPV1, this platform will provide the foundation for future research into the *in vivo* and *in vitro* factors that affect the viability of porcine embryos, as well as the effects of these factors on the live offspring that result from these embryos.

## Background

The domestic pig is an economically-important livestock species, with pork constituting 40% of the world’s meat consumption, making it the most important meat source globally [1]. However, swine are also a well-recognized biomedical animal model for improving human health. Recent research has focused on using the pig as a medical model for renal transplantation [2], cardiovascular-related diseases [3], atherosclerosis [4] and Cystic Fibrosis [5]. As well, advances in induced pluripotent stem cell (iPSCs) technologies [6-8] make the pig an attractive model for regenerative medicine and stem cell research. As a result, there is a strong interest in the factors that affect the efficient production of viable embryos and offspring in this species using either *in vivo* or *in vitro* production methods.

During the preimplantation period of embryonic development, the mammalian embryo exhibits dramatic morphological changes and many key developmental events take place. Until recently, studies to determine the effects of various factors on embryonic development and competence have been limited to morphological and phenotypic evaluations [9-11]. Current understanding of the molecular events during the development of porcine preimplantation embryos is limited. Increased knowledge in this area will contribute to our understanding of basic reproductive biology. It will also allow us to identify the molecular markers related to embryonic quality, and facilitate improved maternal management as well as *in vitro* production and manipulation of embryos.

Powerful high-throughput genomic tools, such as microarray technologies and deep sequencing have been developed to study gene expression at the whole genome of domestic animals during development [12]. Deep sequencing allows for a detailed analysis of transcript levels, as well as data mining and identification of transcript isoforms. Alternatively, gene expression microarrays allow for efficient analysis of a large number of different predetermined transcripts in several samples, but are limited by prior knowledge and gene discovery. Although there are gene expression microarray platforms available for various species, most are based on somatic cell expression and it has been shown that the embryonic transcriptome differs significantly from that of somatic cells [13]. To date, the development of embryo-specific gene-expression microarrays has only been reported for cattle [14]. Although there have been efforts to characterize the gene expression profile of the developing porcine embryo [15-19], a full description of novel genes expressed during preimplantation development in the pig is still needed. With the on-going effort in porcine genome mapping and sequencing [20], the capacity to achieve this endeavour is now available.

To our knowledge, this is the first complete genome-wide study using 454 pyrosequencing and microarray analysis during porcine preimplantation development involving nine specific stages; from oocytes to early blastocysts. Here, we report the design of an oligo-microarray covering a total of 43,795 probes, which has been validated using gene expression profiles of porcine cumulus-oocyte-complexes (COC) and pooled embryos of 2-cells, 4-cells and 8-cells developmental stages. Further annotation of all genes incorporated into this microarray platform will also facilitate future research to define new pathways and the regulatory elements that are correlated to the factors affecting embryonic competence.

## Methods

### Embryo production and preparation

Porcine embryos of *in vitro* origin were generated by Minitube of America, Inc. (International Biotechnology Centre, Mt. Horeb, Wisconsin, USA). A total of 120 embryos were generated from eight different stages (2-cell, 4-cell early, 4-cell mid, 4-cell late, 8-cell, morula, expanded blastocyst and hatched blastocyst), as well as 30 oocytes at Germinal Vesicle (GV) and MII stages. A total of 15 embryos or oocytes were collected from each stage. Following culture and selection, five identical embryos or oocytes from each stage were placed into 50 μl of lysis buffer from Arcturus® PicoPure® RNA Isolation kit. All samples were shipped on dry ice to the University of Alberta and stored at −80°C until RNA extraction.

Porcine embryos of *in vivo* origin were generated and collected as described by Degenstein *et al.*. [21]. A total of 121 embryos and oocytes were generated at nine different stages of development: Germinal vesicle (GV) (N = 16), MII (N = 15), 2-cell (N = 13), 4-cell early (N = 19), 4-cell late (N = 15), 8-cell (N = 12), morula (N = 18), expanded blastocyst (N = 8) and hatched blastocyst (N = 8). All samples were stored at −80°C until RNA extraction.

### Normalized cDNAs preparation and sequencing

RNA was first extracted from pooled samples described above using Arcturus PicoPure RNA Isolation Kit (Applied Biosystems, Carlsbad, CA, USA). High-quality total RNA was obtained after DNase treatment using RNase-Free DNase kit according to the protocol from Qiagen (Mississauga, On, Canada). Bioanalyzer RNA 6000 Pico LabChip (Agilent Technologies, Mississauga, On, Canada) was used to evaluate the total RNA quality. RNA Integrity Number (RIN) index was used as a numerical assessment of the integrity of RNA.

A yield of 25.4 (RIN =8.4) and 50 (RIN = 7.7) ng of total RNA was obtained from *in vitro* and *in vivo* samples respectively and used for first-strand cDNA synthesis using Super SMART PCR cDNA Synthesis Kit (Clontech, Mountain View, CA, USA) with the following modifications. Reverse transcription (RT) was carried out with the SMART MMLV reverse transcriptase and the RT reaction was extended to 90 minutes at 42°C. Following second strand amplification, 3.1 μg of purified cDNA was obtained using a QIAquick Mini Elute kit (Mississauga, On, Canada). The cDNA library was normalized according to the Trimmer Direct Kit protocol (Evrogen, Russia) to minimize differences in representation of transcripts. This normalization protocol is based on denaturing-reassociation of cDNAs, followed by digestion with a duplex-specific nuclease (DSN) method [22] to remove the highly-abundant cDNA fraction. In brief, 1 μg and 880 ng of cDNAs from *in vitro* and *in vivo* generated embryos were incubated at 98°C for 2 minutes followed by incubation at 68°C for 5 hours in the provided hybridization buffer (50 mM Hepes, pH7.5 and 0.5 M NaCl). The optimal digestion was treated with 1/8 units of DSN. The normalized cDNA was then amplified from 1 μl of DSN-treated cDNA by PCR reactions (11 cycles) involving: 95°C for 1 minute, followed by 12 cycles with 95°C for 15 seconds, 64°C for 20 seconds and 72°C for 3 minutes, with a final extension of 72°C for five minutes and clean up with a QIAquick Mini Elute PCR column (Qiagen, Mississauga, ON, Canada).

In order to improve the pyrosequencing yield, an additional BAL 31 nuclease digestion was carried out on a portion of the cDNAs to remove homopolymers according to the protocol provided by USB Corporation (Cleveland, OH.) . The reaction was performed at 30°C for 2 minutes and the nuclease activity was stopped by adding 0.5 M EGTA. The reaction mix was passed through QIAquick Mini Elute PCR column. The quality of the cDNA was verified by 1% TAE agarose gel electrophoresis before 454 sequencing. Then 20 μg of normalized cDNA library was obtained and 15 μg of this sample was nebulized with the nebulization kit supplied with the GS Titanium Library Preparation kit (Roche/454 Life Sciences Corp., Bradford, CT, USA) as per the protocols of the 454 sequencing Laboratory at McGill University and Genome Quebec Innovation Centre (Montreal, QC, Canada). Sequencing runs were carried out using high-throughput pyrosequencing Genome Sequencer FLX (454 Life Sciences Corp., Bradford, CT, USA). The original 454 sequencing output data was preserved as SFF (Standard Flowgram Format) files. Sequences were deposited at the NCBI short-read archive (SRA) under accession number SRA029132.1.

### 454 sequencing analysis and microarray fabrication

Initial 454 sequence analysis and microarray probe sequences design were performed by Gydle Inc. bioinformatics service (Quebec City, Canada, http://www.gydle.com/). Raw sequences were transformed into high-quality (HQ) sequences according to Gydle Inc.'s proprietary sequence filtration process. This process utilizes the sequence and quality scores of each sequence read to identify its context (presence of 5’ or 3’-end adapters, sequencing direction, detection of artefacts), to trim the sequence to a high-quality interval, and to remove bacterial and ribosomal contaminants. EST sequences from all sources (454 and UniGene Ssc build#39, Aug. 23, 2010) were then aligned (Nuclear software, Gydle Inc.) to the available annotated porcine reference genome to characterize annotated genes and NTR’s (Novel Transcript Regions). As the porcine genome is currently in a semi-complete, semi-annotated state, a mix of porcine genome sources from Ensembl Genome Browser (Sscrofa_9 & pre-version of Sscrofa_10) and NCBI UniGene (Ssc build#39, Aug. 23, 2010) were used as the porcine reference genome for the initial probe annotation. Based on this initial analysis 60 -mer oligo-sequence probes were designed by Gydle Inc. and were used for microarrays synthesis *in situ* using the Agilent SurePrint™ technology (Agilent Technologies, Mississauga, On, Canada) with a 4 × 44 K format. This technology allows for the generation of arrays with tens of thousands of oligonucleotides that are constructed using an ink-jet oligonucleotide synthesizer [23]. A total of 43,795 probes including positive and negative controls representing 23,148 genes appear on the EMPV1 microarray (Additional file 1 and Additional file 2). The custom microarray design of the platform, including the original and updated annotation has been submitted to the NCBI GEO (Gene Expression Omnibus). The accession number of the platform is GPL14925.

### Microarray procedures

#### Samples collection for microarray platform validation

Ovaries from gilts were collected at a local slaughterhouse and the cumulus–oocyte complexes (COC) were aspirated from mature follicles and washed in saline solution. The COCs were used in this validation process as they provide tissues of both somatic (cumulus) and gametic (oocytes) origin. Individual COC samples were stored at −80°C until RNA extraction. *In vivo* collection of 2-cell, 4-cell and 8-cell stages embryos has been previously described [21]. Five morphologically identical embryos of from each stage were pooled for RNA extraction. Arcturus® PicoPure® RNA Isolation Kit (Applied Biosystems, Carlsbad, CA, USA) was used for both single COC and pooled-embryo extraction. Total RNA quality was evaluated with an Agilent 2100 Bioanalyzer using RNA 6000 Pico kit (Agilent Technologies, Mississauga, ON, Canada). The RIN value of the two COC samples were >8. The RIN value of 2-cell, 4-cell and 8-cell embryos was 5.9, 6 and 6 respectively. It should be noted that there are consistently low levels of ribosomal 28 S RNA present in 2-cell, 4-cell and 8-cell embryos which result in lower RIN values [24], so these samples were still considered suitable for RNA amplification.

### RNA amplification

RiboAmp HS^Plus^ kit (Applied Biosystems, Carlsbad, CA, USA) was used to amplify the low quantities of total RNA isolated from the samples. Five ng of total RNA from the COC samples was used for amplification of adequate antisense RNA (aRNA) for labelling. However, only 1.5 ng to 2 ng of total RNA from pooled embryos were utilized in amplification. Nanodrop ND-1000 (NanoDrop Technologies, Wilmington, DE, USA) was used to determine the aRNA quantity. The Agilent two-color RNA Spike-In® kit (Agilent Technologies, Mississauga, ON, Canada) is a mixture of 10 different viral poly-adenylated RNAs. Five ng of spiked-in RNA was also used for amplification.

### Labelling and hybridization

Two μg of aRNA from each sample were labelled with Cy3 and Cy5 using the ULS Fluorescent Labeling Kit (Kreatech Diagnostics, Amsterdam, Netherlands). The same kit was used for the spiked-in aRNA except the amount for labelling was 5 μg of each, using Cy3 for A and Cy5 for B. All the labelled probes were purified using picopure RNA extraction kit (Applied Biosystems, Carlsbad, CA, USA). Concentration and labeling efficiencies were determined using a Nanodrop ND-1000.

Samples were labelled with alternate dyes and hybridized on a single EMPV1 microarray in a dye-swap manner with the alternate dye colours used as technical replicates. A total of 110 μl of hybridization mixture was prepared according to the manufacturer's instructions (Agilent Gene Expression Hybridization Kit 60-mer oligo microarray protocol version 4.0). Briefly, a total of 825 ng of each Cy3 and Cy5 labelled aRNA plus 2.75 μl of labelled Agilent spike (0.01X) was prepared with 25X fragmentation and 10X blocking buffers. After incubating the mixture at 60°C for 15 minutes, it was immediately cooled on ice for one minute before adding an equal volume of 2X GEx hybridization buffer HI-RPM (Agilent Technologies, Mississauga, ON, Canada). Array hybridization was carried out for 17 hours at 65°C rotating at 10 rpm in an oven. Steps for washing, stabilisation and drying as indicated in established Agilent protocols were strictly followed.

### Array data acquisition and spiked-in quality control (QC) analysis

Arrays were immediately scanned at 5 μm resolution after drying using an Axon 4200AL scanner (635 nm for Cy5 and at 532 nm for Cy3) using the autoscan feature from the default setting and images were analysed with Gene Pix Pro 6.0 software (Molecular Device, Sunnyvale, CA 94089 USA). Analysed images were manually edited for any spots with hybridization artefacts and flagged for exclusion in further analysis. Data from spot intensity, background subtraction and normalization was saved as GenePix Results (GPR) format for further array QC analysis. A web-based EmbryoGENE microarray QC module (https://www.gydle.com/embryogene/qc) was created by Gydle Inc and GPR files were uploaded for analysis. Agilent spiked-in control intensities were used to identify the best normalization procedure for each dataset. Hybridization quality was evaluated graphically through the distribution of signals generated by both channels, in addition to the negative and spiked-in controls [14]. Microarray data analysis was performed using FlexArray (version 1.6 - http://genomequebec.mcgill.ca/FlexArray). All the steps of the analysis were done according to Robert *et al.*. [14]. The limma algorithm in FlexArray, based on the limma package in bioconductor [25] was used for the direct comparison of two COC samples and technical replicates of pooled embryos from 2-cell, 4-cell and 8-cell stages after dye-swaps. The threshold for positive spot selection for the COC and embryo microarray data was calculated as the mean value of all the dark corner spots plus twice the standard deviation [26].

### Bioinformatics tools and analysis

A sequence assembly program “SeqMan NGen” within LaserGene 9.0 package (DNASTAR, Madison, WI, USA) was used to compare the EMPV1 probe sequences with porcine RefSeq RNA sequences downloaded from NCBI (Index of ftp://ftp.ncbi.nih.gov/genomes/Sus_scrofa/RNA/). The porcine RefSeq sequences was newly annotated in April 2011 (26,189 RNA sequences) and was based on the mixed BAC and WGS-based assembly of the porcine genome (Sscrofa10) released by the Swine Genome Sequencing Consortium. It includes assemblies for chromosomes 1–18, X and Y located at NCBI. The default SeqMan NGen program parameters were used with some minor changes (Additional file 3). Additional porcine transcripts without GS were annotated using the public Basic Local Alignment Search Tool [BLAST®] from NCBI search with the human RefSeq RNA (46821 sequences). The resulting unique GS lists from the EMPV1 array and Affymetrix porcine genome array http://www.affymetrix.com/estore/index.jsp were initially uploaded into PANTHER (http://www.pantherdb.org/genes/batchIdSearch.jsp) to identify PANTHER-classified genes, transcripts, and proteins related to the gene ontology (GO). Then the GO terms were uploaded into the PANTHER expression analysis tool [27] to identify biological processes that differed from the reference list. The *Homo sapiens* genome (human) was used as a reference gene list, which allowed for the identification of developmental-related processes from the GO terms that were statistically over- and under-expressed using a binomial test.

## Results and discussion

### Construction of two normalized cDNA libraries and quality control of 454 sequencing

The primary goal of this research was to develop a microarray platform to study the early development of porcine embryos before implantation. Porcine microarrays have been widely used in functional genomics research; however they have not been designed specifically for the detection of the gene expression during early embryonic development. Generally, these probe sequences have not been generated from preimplantation embryonic tissues and there has been limited extensive deep sequencing projects related to porcine preimplantation embryonic development. From previous porcine EST studies using non-normalized cDNA from early embryos [18,19], only highly-expressed genes were found. Using the Sanger sequencing method, the total number of unique transcripts from these studies was low (less than 3,000 genes) and they were not able to represent the expression levels of the original samples. In order to facilitate characterization of the porcine embryonic transcriptome, cDNA normalization using duplex-specific nuclease (DSN) and 454 deep sequencing were conducted to account for low abundance of mRNA transcripts in developing embryos produced using both *in vitro* and *in vivo* procedures. Although the DSN method has been previously used to normalize cDNA libraries from a number of animal and plant models [28], this is the first time it has been successfully applied to cDNA library construction generated from pig embryo RNA and followed by 454 sequencing.

**Figure 1:**
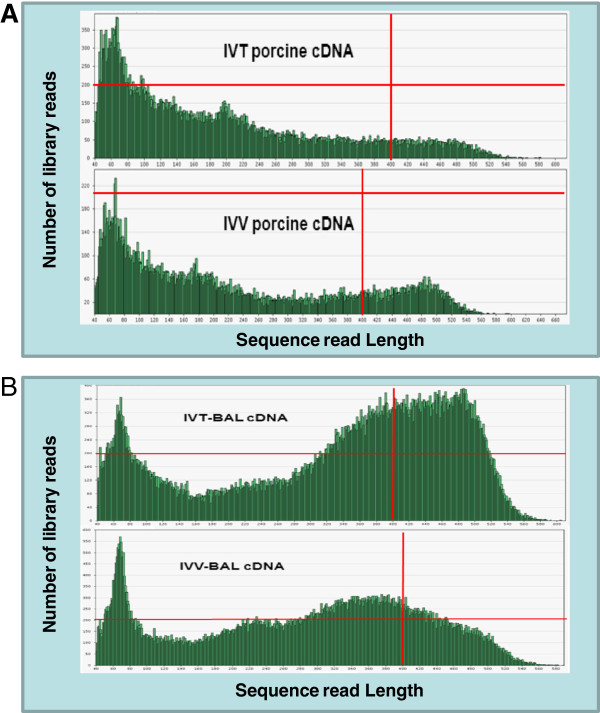
**Distribution of sequence length and number of reads from 454 sequencing of two cDNA libraries before (A) and after (B) BAL 31 nuclease digestion.** The vertical and horizontal red line indicates the read length at 400 bp and the number of reads at 200 respectively.

**Figure 2:**
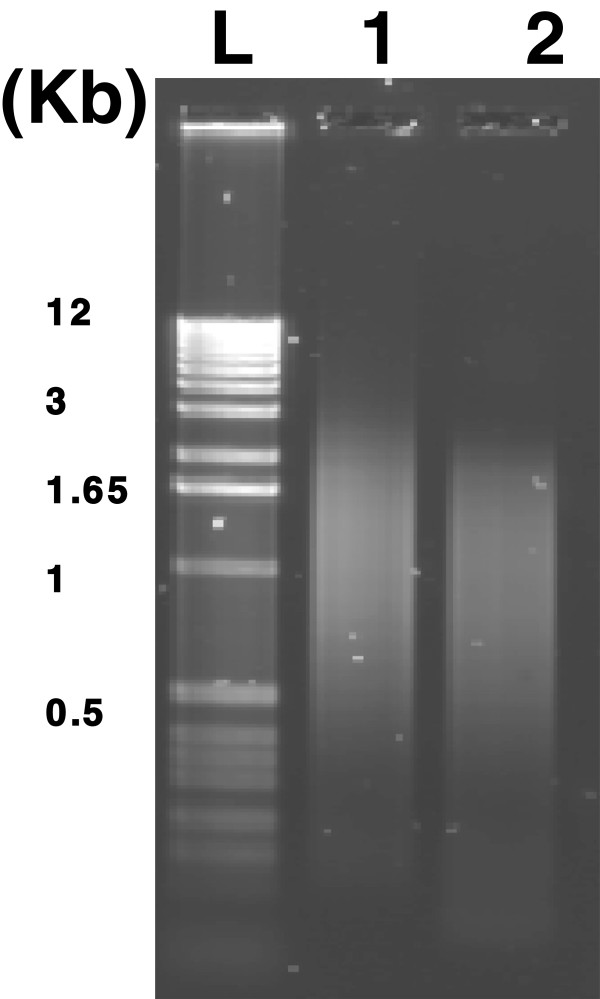
**Agarose gel electrophoresis analysis of the normalized cDNA.** Normalized cDNA as revealed by electrophoresis before (lane 1) and after (lane 2) BAL 31 nuclease digestion. The 1 kb DNA ladder (L) was loaded as size markers.

A pilot 454 sequencing test was conducted using a 1/8 plate to analyse the profile of the overall size and length distribution of the transcripts. The sequencing result did not reach the optimal yield because the number of reads for long sequences (300–400 bp) was less than for the short sequences (<100 bp) in cDNAs from both *in vivo* (IVV) and *in vitro* (IVT) sources (Figure 1A). Long homopolymeric (A:T) regions in cDNA may have resulted in sequencing reads of low quality for the 454 sequencing. To address this, methods have been developed using modified primers during the first strand of cDNA synthesis [29], however these methods are not suitable for application after library synthesis. Therefore, we adopted an old nested deletion method of cloning [30] to improve the sequencing yield. Time-series testing was first performed and demonstrated that after 2 minutes, there was visible fragment size changes detected by gel electrophoresis (Figure 2). Using this approach we were able to dramatically improve the 454 sequencing quality compared to the previous results (Figure 1A). The number of sequencing reads with longer lengths (350–450 bp) was increased in the BAL-treated IVV and IVT libraries from four to six fold respectively (Figure 1B). In general, the total initial run of 454 pyrosequencing generated 388,002 reads from two normalized cDNA pools (IVT & IVV). However, after BAL-treatment the total sequencing output almost increased 3 fold to 1,129,843 reads from two BAL-treated normalized cDNA pools (Table 1). After trimming and screening, approximately 233,570 and 886,720 high-quality (HQ) sequences remained from the normalized cDNA pools without and with BAL treatment respectively. In total, 1.5 million raw 454 sequences were produced, which yielded 1.12 million HQ EST sequences. The HQ porcine ESTs were used to augment the porcine gene catalogue for the EmbryoGENE project.

**Table 1:** Summary of 454 sequencing data before and after trimming between two normalized cDNA treated with and without BAL

**Batch ID**	**# of Raw sequences**	**# of high quality sequences**	**Library ID**	**Description**
PVT0101	49,385	14,186	PVT01	In vitro 454 library (2009)
PVT0102	45,491	20,316	PVT01	IN vitro 454 library (2009)
PVT201	113,399	86,481	PVT02	IN vitro 454 library (2010)
PVV0101	57,208	25,611	PVV01	In vitro 454 library (2009)
PVT0102	25,732	13,428	PVV01	In vitro 454 library (2009)
PVT0201	96,787	73,548	PVV02	In vitro 454 library (2010)
**Sub Total**	**388,002**	**233,570**		
PVT0301	103,807	84,146	PVT03	IN vitro BAL-treated 454 library (2010)
PVT0302	253,539	215,845	PVT03	In vitro BAL-treated 454 library (2010)
PVT0303	241,363	205,195	PVT03	In vitro BAL-treated 454 library (2010)
PVV0301	93,020	73,307	PVV03	In vitro BAL-treated 454 library (2010)
PVV0302	438,114	308,218	PVV03	In vitro BAL-treated 454 library (2010)
**Sub Total**	**1,129,843**	**886,720**		

Library ID with 01–02 and 03 represent cDNAs without and with BAL treatment respectively.

The initial sequencing annotation was performed by Gydle Inc. using the porcine databases described in the methods section. In general, the EMPV1 array features 43,795 probes including 17,409 protein-coding, 473 pseudogenes, 46 retrotransposed, 2,359 non-coding RNA (snRNA, snoRNA, etc.), 4,121 splice variants in 2,862 genes and a total of 12,324 NTR. Based on initial annotation of porcine genes at that time, 11,961 unique genes, with gene symbol,were identified from a total of 43,795 probes (Additional file 1 and Additional file 2). A large portion of the EMPV1 probe sequences, particularly for the NTR, were without gene identification and symbols.

### EMPV1 Microarray annotation and functional analysis

**Figure 3:**
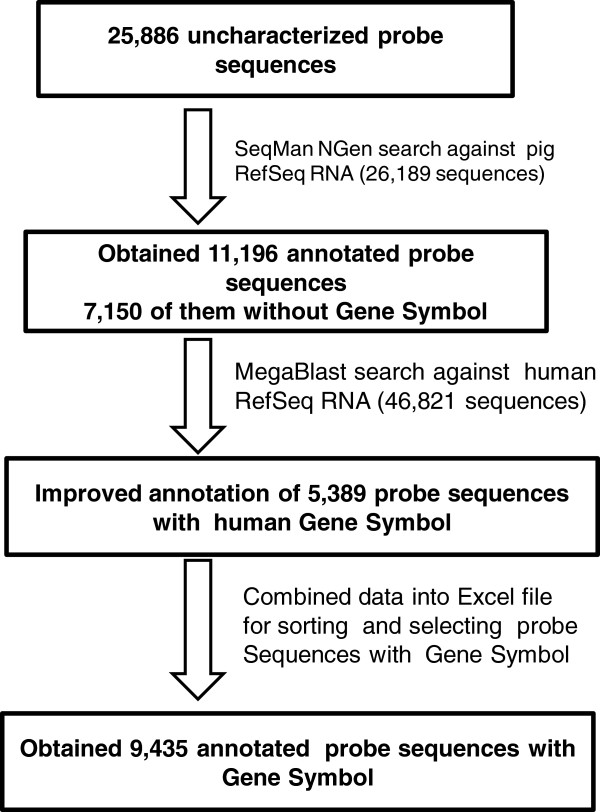
EMPV1 array probe sequences annotation work flow.

**Figure 4:**
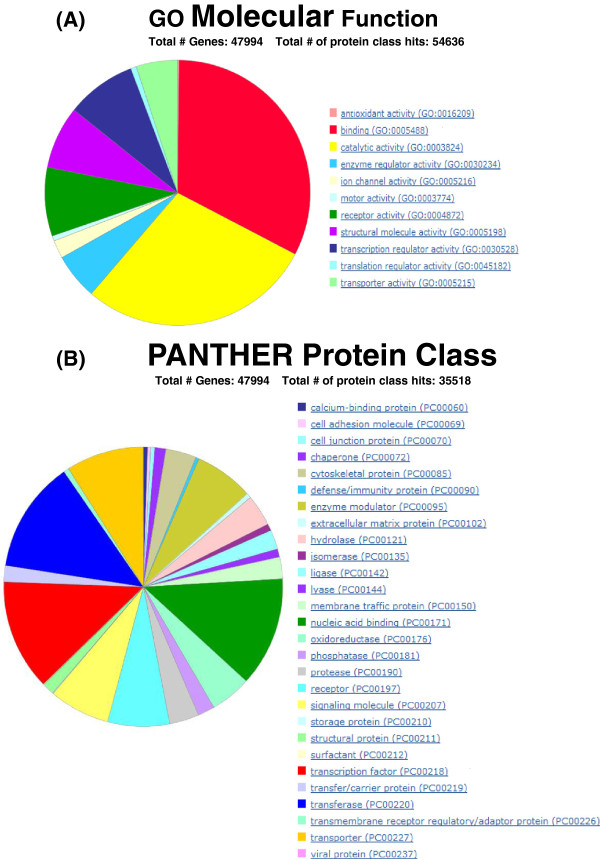
**PANTHER gene ontology of EMPV1 array probes with unique GS.** Distribution of genes associated with **(A)** GO molecular function and **(B)** PATHER protein class.

From the EMPV1 microarray, 25,886 probes were selected that included the NTR and sequences without GS (Additional file 4). They were then searched against the most recent version of the porcine genome (Sscrofa10 released in April, 2011) as described in the methods using the search parameters for LaserGene 9.0® with a minimum match setting of 98% (Additional file 3). The annotation workflow is described in Figure 3. Approximately 43% of all entries produced significant hits when queried against the nucleotide database from porcine RefSeq RNA and were thus classified as annotated. Of these annotated probe sequences, 4,044 were identified with GS (Additional file 5). However, more than 50% the annotated sequences were designated as unknown and their GS had an “LOC” prefix. Using the extended sequences and referring to each RefSeq RNA accession number corresponding to 7,148 sequences without GS (Additional file 5), the extended sequences were compared to the NCBI human RefSeq RNA database by BLAST search to yield 5,389 annotated sequences (Additional file 6). By assessing all the previously and newly annotated genes, any redundant GS were removed and a list of 16,281 unique GS (Additional file 7) was uploaded to establish GO terms in PANTHER-classified genes, transcripts, and proteins as described in the Methods. A total of 13,797 human mapped GS were found and this data, with additional GO term annotation, was exported into Excel (Additional file 8). A major portion of the GO molecular functions were related to binding (GO: 000548) and catalytic activity (GO: 0003824) and these occupied more than 50% of the total related function (Figure 4A). Most genes in these two categories are associated with transcription factors (PC00218), nucleic acid binding protein (PC00171) and transferase (PC00220) indicated by red, green and blue colour respectively in the piechart (Figure 4B), which are processes typically found in the developing embryo. However, in the context of this study one cannot associate the transcripts’ origin to a specific embryonic stage.

Normalization of the cDNA pooled from different stages of preimplantation embryos was employed as a technique to facilitate gene discovery efforts. A major goal of this study was to identify embryo-specific genes through deep sequencing after the original enrichment process of rare genes from the normalized cDNA libraries. In order to prove that the selected EMPV1 probe set was enhanced with genes related to developmental processes, we selected a commercially available Affymetrix porcine array for comparison using PANTHER as described in the Methods. The Affymetrix GeneChip® Porcine Genome Array is widely used in pig functional genomics research [31] and the probe set is frequently annotated through the human database to obtain additional GS [32,33]. To assess the level of incorporation of embryo-specific genes on the EMPV1 platform, it was compared to this popular array. After removing the common GSs from both arrays, the probe IDs with unique GS from the Affymetrix array and EMPV1 were determined to be 5,221 and 9,425, respectively (Additional file 9). Using a gene expression tool from PANTHER [27], GSs were mapped to the PANTHER ontology and compared to a reference list. In this case, each unique list was compared to the reference list (Human) using the binomial test [34] for biological processes in PANTHER.

**Figure 5:**
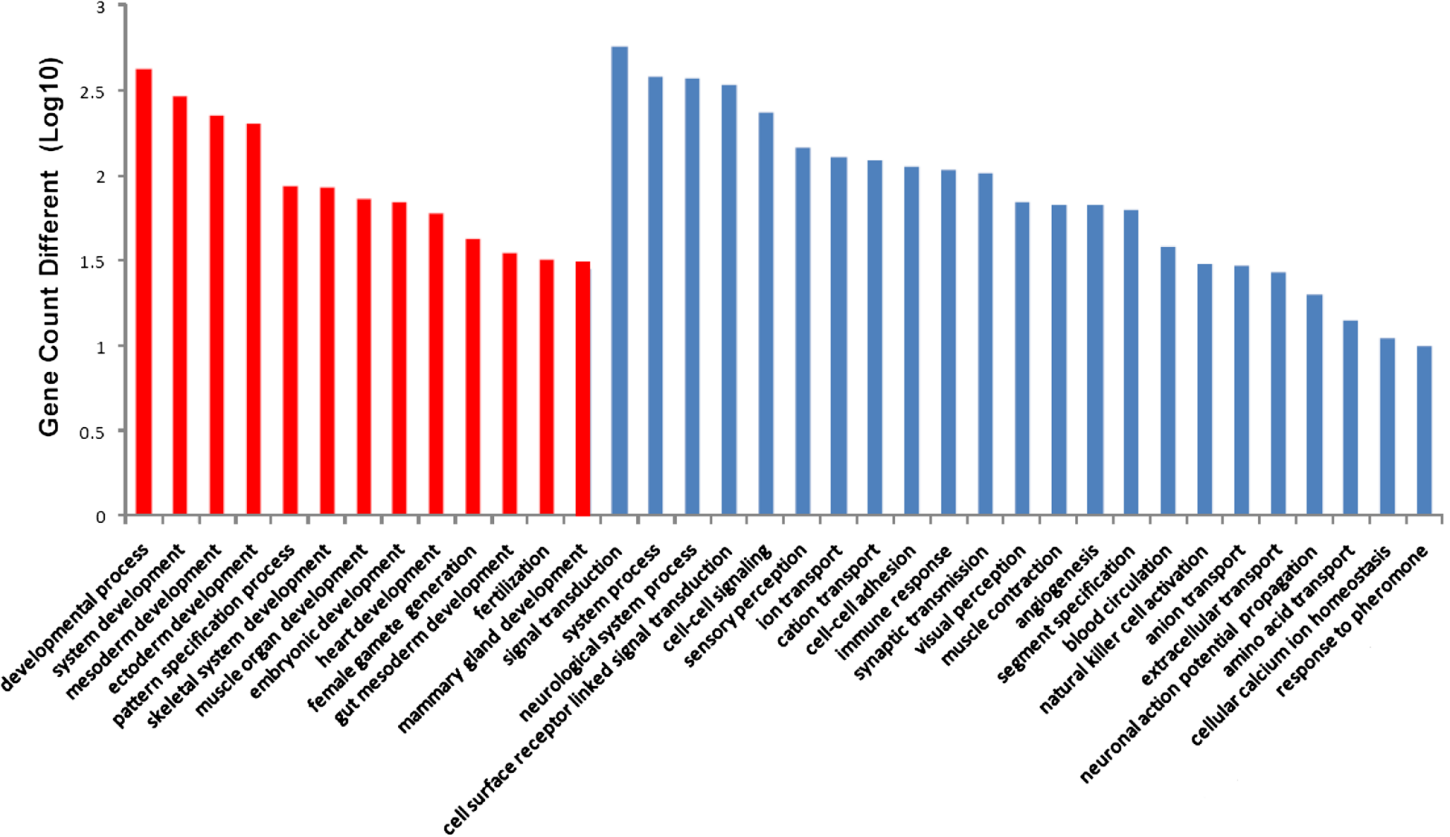
**Gene count different distributed among different categories from biological process in EMPV1 array probes with unique GS.** Red bar indicated statistical significant gene count related to development.

Within the distribution of GO in Human, 130 categories of biological processes were covered (not including the unclassified processes), the statistical significance of the gene count in the EMPV1 versus Affymetrix arrays over different categories was calculated and identified. PANTHER predicated that 37 (highlighted with yellow in Additional file 10) and 23 (highlighted with green in Additional file 10) of these were statistically significant (p value < 0.05) in EMPV1 and Affymetrix arrays respectively. None of the Affymetrix porcine array categories were related to development. However, approximately 1/3 of the 37 EMPV1 categories were significantly involved in development as indicated in Figure 5. This indicates an increased efficiency in gene discovery and an enhanced detection of genes related to early preimplantation embryonic development using the normalization method for cDNA construction and 454 deep sequencing.

Furthermore, PANTHER pathway analysis of GO terms related to several biological processes of interest such as developmental processes, system development, embryonic development, pattern specification processes, ectoderm and mesoderm development, revealed six major molecular pathways (Figure 5). These include Wnt signalling pathway, TGF-beta signalling pathway, cadherin signalling pathway, interleukin signalling pathway, PI3 kinase pathway and insulin/IGF pathway- protein kinase B signalling cascade (Table 2). The important role of these six pathways and other extrinsic regulators has been reviewed in mouse and human preimplantation embryonic development (PED) and stem cell related studies [35,36]. The manner in which these pathways influence self-renewal, pluripotency and differentiation of PED and embryonic stem cells is under active investigation [37,38], but is not well understood in pigs. However, coupling of these pathways with their distinct expression patterns, the relative concentrations of pluripotency-related molecules, and timing of embryo development, along with supportive micro-environmental conditions, will need to be the subject of on-going research to determine if and how these and other transcripts are related to porcine embryonic development [39,40].

**Table 2:** PANTHER pathway analysis of developmental processes

**Category name (Accession)**	**# genes**	**Percent of gene hit against total # genes**	**Percent of gene hit against total # Pathway hits**
Wnt signaling pathway (P00057)	109	5.20%	8.60%
TGF-beta signaling pathway (P00052)	80	3.80%	6.30%
Interleukin signaling pathway (00012)	71	3.40%	5.60%
Insulin/IGF pathway-protein kinase B signaling cascade (P00033)	56	2.70%	4.40%
Pl3 kinase pathway (P00048)	56	2.70%	4.40%

Again, the unique GS list used for this additional pathway analysis confirms the effectiveness of the gene discovery techniques in this study, As well, the representation of the newly identified embryo-specific genes on our EMPV1 microarray is expected to facilitate cost effective and fruitful functional genomics research related to early porcine embryo development in the future.

### Microarray quality assessment

**Figure 6:**
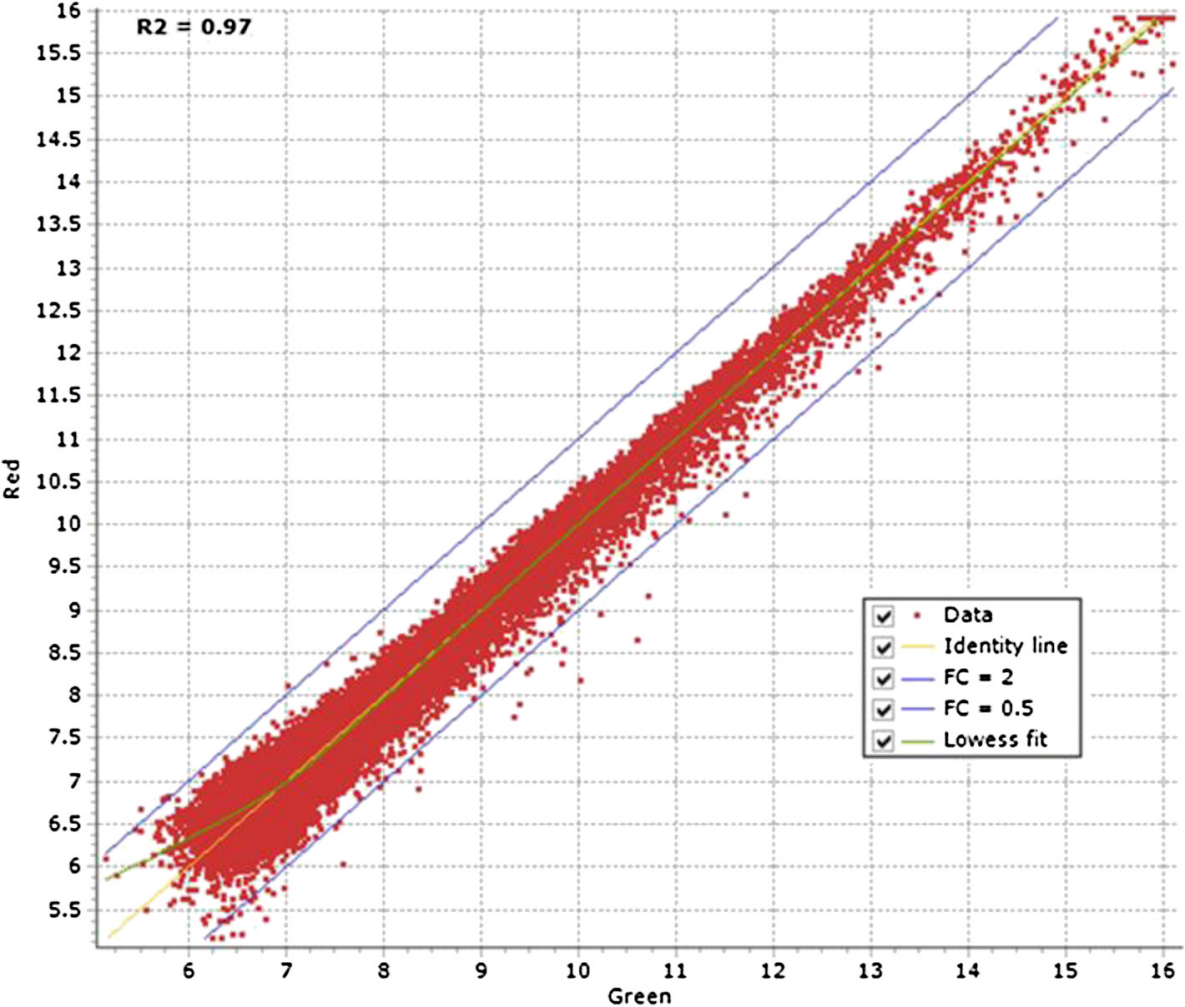
**A scatter plot of Cy3 and Cy5 normalized signal intensity.** X and Y axis show the signal intensity after the same aRNA from pooled embryos were labelled with Cy3 and Cy5 respectively. FC = fold change.

**Figure 7:**
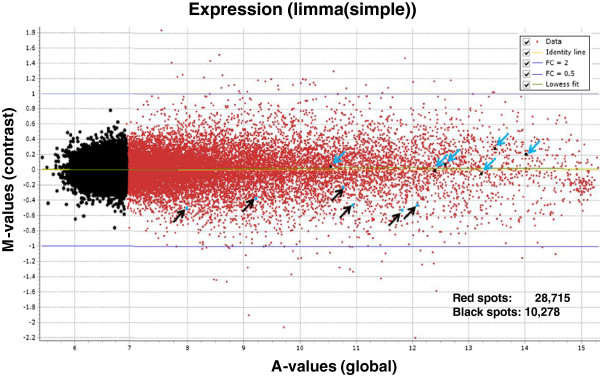
**MA plot for COC gene expression data.** Six blue arrows pointed downward represented oocyte specific markers and six black arrows pointed upward represented cumulus cells markers. 28,715 red spots represented positive signals above background signals (10,278 black spots).

With the current incompleteness of the porcine genome map and the limited ESTs resources, RNA-sequencing is not a cost effective tool to study the effect of *in vivo* and *in vitro* factors on the porcine embryonic model. However, using our normalized cDNA libraries for deep sequencing, we have enriched the porcine transcripts from different early developmental stages to construct the EMPV1 microarray platform. After 2005, high background cDNA microarrays were generally replaced by oligo-based microarrays generated by companies such as Affymetrix and Agilent. Expression analysis studies of Arabidopsis indicated that the two microarray technologies (Affymetrix and Agilent) are consistent when compared with each other [41]. Recently, the most popular commercially available porcine oligo arrays from Agilent and Affymetrix have been widely used to study gene expression related to meat quality [42], nutrition [43], disease infection [44], female reproduction [45,46] and peri-implantation embryos [47]. The Agilent two-color microarray platform was chosen as the format to construct the EMPV1 microarray as outlined in the Methods. To access the EMPV1 microarray, labelled aRNAs from technical replicates of the same sample from pooled embryos and two porcine COC samples were tested for the intra- and inter-array variability [48]. The COCs were used for this purpose as they are composed of both reproductive (oocyte) and somatic (cumulus) cells, which was optimal for validation purposes as it augmented the number of genes represented on the array from both origins would be hybridized. There are also 120 spiked-in probe sequences printed in our array corresponding to two sets of external RNA controls for the assessment of microarray performance developed by Agilent [49]. In order to perform the data analysis properly, details related to the microarray experiments were first deposited into the EmbryoGENE LIMS and Microarray Analysis (ELMA) web platform [14]. The microarray QC module within ELMA generated several QC graphics to determine the quality of the data for further analysis by FlexArray, as discussed in Methods. We first evaluated the results from pooled embryos graphically in FlexArray. The aRNA from the same sample was labelled with Cy3 and Cy5 to test the fluorescent dyes’ effect due to labelling and hybridization. The Cy3 and Cy5 signal intensity distribution curve was very narrow (r^2^ = 0.97) with very few spots over the two-fold change threshold intensity line (Figure 6). Later, inter-array variability was tested using two biological COC samples. This was based on the correlation coefficient generated from the spiked-in control (r^2^ was ≥ 0.95) within and across the arrays for the two slides in the COC test-run experiment (Additional file 11). Data was further normalized within and across arrays and a MA plot of contrast between two COC samples was generated by FlexArray after Limma algorithm. There were 72 spots that differed (fold change ≥ 2 or ≤ 0.5) and were considered to be the result of biological variation between the two COC samples (Figure 7) in this test run.

### Porcine COC transcriptome profiling

A global mRNA gene expression analysis of COC was carried out by selecting positive signals as described in the Methods. Approximately 74% of the probe sets representing 28,715 transcripts were detected in porcine COC (Additional file 12). This number is in accordance with the 16,066 transcripts (67.16% of all probe sets) detected using the Affymetrix GeneChip® Porcine Genome Array in hormonally-stimulated preovulatory ovary follicles from Large White sows [46]. The greater number of expressed genes in the present study is probably due to different physiological conditions of the female and additional cumulus cells with the oocytes. The different array platforms used for analysis should also be taken into consideration for these transcript differences. To confirm the accuracy of our microarray data as it related to biological relevance, six transcripts were identified in the COC (Figure 7) as conserved oocyte markers also present in other mammals. Zona pellucida glycoprotein 2 & 3 (ZP2, ZP3) [26,50], B-cell translocation gene 4 (BTG4) [26], myeloid leukemia factor 1 interacting protein (MLF1IP) [26] and growth differentiation factor 9 (GDF9) [26,51,52] and bone morphogenetic protein 15 (BMP15) [26,53,54] were highly expressed in COC. On the other hand, cumulus cells markers were identified in COC gene-expression profiling (Figure 7) when compared to bovine and human. This demonstrated the platforms capacity to reveal the expression of tissue specific genes even in a mix of somatic and gametic tissues. Studies from human indicated that hormone receptors and secretary proteins such as progesterone receptor membrane component 1 &2 (PGRMC1 & PGRMC2) and bone morphogenetic protein 1 (BMP1) were significantly over-expressed in cumulus oophorous cells when compared to oocytes [55]. Similarly, PGRMC1 and BMP1 transcripts were detected in our microarray. Other high-intensity spots were associated with peroxiredoxin 4 (PRDX4) and a transcriptional factor GATA6 (Figure 7) which were identified in human as cumulus cells markers [55]. Other low-intensity spots were related to factors such as secreted protein acidic, cysteine-rich (SPARC) and ADAM metallopeptidase with thrombospondin type 1 (ADAMTS1) which have been found to be exclusively expressed in bovine cumulus cells [56].

### Porcine embryo transcriptome profiling

For the microarray data from the pooled embryos, the threshold for positive spot selection was calculated similar to that for the COC experiment. Approximately 28,597 transcripts were detected from pooled porcine embryos of 2-cell, 4-cell and 8-cell stages (Additional file 13). It should be noted that in this study our intent was not to quantify gene expression between different developmental stages, but to simply identify genes, from the literature that may be present in the 2- to 8-cell stages. Very little is known regarding global gene expression during these early cleavage stages in the pig. Sequencing from the porcine EST project on early developmental stages have been generated from *in vitro*- and *in vivo*-derived four-cell embryos [19]. Most of the ESTs were poorly annotated at that time and only few highly expressed genes, such as porcine casein kinase II beta subunit (CSNK2B), cyclin-dependent kinase-2 alpha (CDK2), ribosomal protein S10 (RPS10) and eukaryotic translation initiation factor 3 (EIF3), were identified in 4-cell embryos. However, these genes were both identified and expressed in our COC and embryo expression data (Additional file 12 and Additional file 13). Later, the same group using a cDNA microarray to demonstrate the mRNA expression patterns from 4-cell embryos, detected 1409 differentially expressed transcripts (without the Benjamini and Hochberg false-discovery-rate multiple correction test) between the *in vitro*- and *in vivo*-produced embryos at the 4-cell stages [57]. However, only four genes (DSTN, PAIP1, UBE4B, NASP) were selected and confirmed by real-time PCR. This group also established that the gene expression levels from *in vitro-*produced embryos were very high for DSTN & PAIP1 and very low for UBE4B and NASP when compared to *in vivo*-produced 2-cell embryos. Our microarray profiling data for the embryos (Additional file 13) also identified three of these genes (DSTN, PAIP1, UBE4B), while the spot intensity for NASP was below the detectable threshold. Since the microarray data was obtained from a pool of three different developmental stages, further data analysis may be needed to identify if DSTN, PAIP1 and UBE4B are expressed only at the 4-cell stage.

### GO analysis for biological processes in porcine COC & embryos

**Figure 8:**
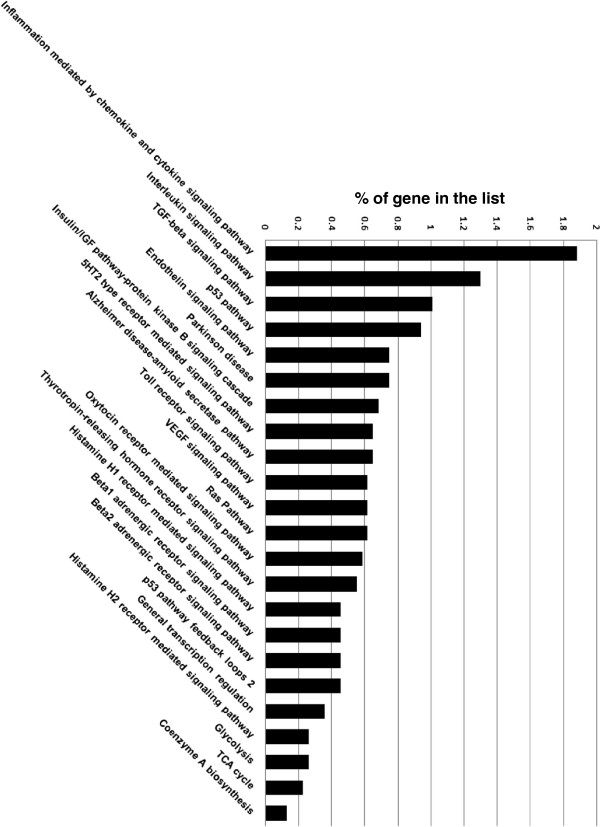
**PANTHER bar chart of gene count involved in biological pathways from early embryos.** The % of gene list (Y axis) in the category is calculated for each testing list as: # genes for the category/ # total genes in the list * 100.

To highlight differences in the biological processes between the COC and embryos, we removed the similar GS from the 28,715 COC related transcripts (Additional file 12) and 28,597 embryo related transcripts (Additional file 13). Genes expressed only in the COC or embryos were obtained after removing redundancies from both data sets. There were 793 unique GS in the COC and 4,388 in the embryos, while 7,822 appeared in both (Additional file 14). The unique GS from COC and embryos were mapped to the PANTHER ontology and compared to the human genome as a reference gene list. Using a similar binomial test in PANTHER as described previously, the analysis indicated that only one pathway related to apoptosis was found to be statistical significant (Additional file 15) in COC, but not in embryos. Studies have shown that apoptosis is important during *in vitro* culture condition in bovine [58,59] and porcine [60]. However, the gene count revealed additional unique pathways which were statistically significant in the porcine embryos. The three pathways with the highest gene counts were primarily related to biological pathways such as inflammation signalling pathway (mediated by chemokine and cytokine), interleukin signalling pathway and TGF-beta signalling (Figure 8). Of particular interest were the interleukin-signalling pathway and TGF-beta signalling, since they may play an important role during porcine preimplantation embryonic development as we have discussed in the previous section. The inflammation signalling pathway likely plays a role in the establishment of pregnancy, including cellular proliferation, attachment and development of the conceptus [61].

### Expression of porcine OR genes in COC and early developing embryos

Olfactory receptors (ORs) constitute the largest gene-family in the vertebrate genome [25,62]. Interestingly OR genes are not only expressed in olfactory sensory neurons [63], they are also expressed in various tissues including testicular cells and the placenta [64-67]. In the present study, we discovered 312 genes with OR unique GS (Additional file 7). Comparing the microarray expression data for the COCs and embryos (2-4-8 cells stages) revealed 491 array spots with an intensity higher than background that were related to OR transcripts (Additional file 16). The majority of the OR genes (428 transcripts) were only found in embryos and, in particular, one gene (OR4C16) whose spot intensity was 1.5 fold higher than the background. In addition to the embryo specific OR expression, there were 59 OR related transcripts found in both COC and embryos. The physiological significance of OR expression during early embryonic development has not been investigated and is quite novel. In a recent study, most of the known OR genes expressed in murine placenta were influenced by diet and fetal sex [67]. Based on this, one could infer that OR gene expression in the early embryo may be related to trophectoderm development that allows for proper placental OR protein expression in response to molecules from different dietary compounds. Regardless, this discovery is of significant interest and will be investigated further along with numerous other factors.

## Conclusions

Using the 454 deep sequencing of normalized cDNA libraries from *in vitro* and *in vivo* produced porcine embryos we have generated 1.12 million high quality EST sequences that provided the basis for the development of the EMPV1 microarray platform featuring 43,795 probes. The quality of this embryo-specific array was confirmed with a high level of reproducibility that is provided by the current Agilent microarray technology. Despite the current limitations for full NTR annotation, due to the incomplete porcine genome sequencing project, a significant number of NTRs were annotated using the most recent version of porcine genome and human RefSeq RNA database to enrich the orthologous genes with unique GSs for GO searchs. GO terms confirmed that many are related to relevant developmental processes. The on-going effort to complete the porcine genome sequencing project will in turn provide the necessary information needed to address the remaining unannotated NTRs on this microarray With more than 20 thousand unique genes represented on the EMPV1 microarray, this platform will provide the foundation for future research into the *in vivo* and *in vitro* factors that affect the viability of the porcine embryos, as well as the effects of these factors on the live offspring that result from these embryos.

## Abbreviations

aRNA: Antisense ribonucleic acid; BAC: Bacterial Artificial Chromosome; BLAST: Basic Local Alignment Search Tool; cDNA: Complementary Deoxyribonucleic Acid; COC: Cumulus-Oocyte Complexes; DSN: Duplex-Specific Nuclease; DSTN: Destrin; EGA: Embryonic genome activation; EGTA: Ethylene Glycol Tetraacetic Acid; EmbryoGENE: LIMS and Microarray Analysis; EMPV1: EMbryogene Porcine Version 1; EST: Expressed Sequence Tag; GEO: Gene Expression Omnibus; GO: Gene Ontology; GPR file: GenePix Results file; GS: Gene Symbols; GV: Germinal Vesicle; iPSC: Induced Pluripotent Stem Cell; IVT: In vitro; IVV: In vivo; LIMS: Laboratory Information Management System; MII Stage: Metaphase II stage; NGS: Next generation deep sequencing; NTR: Novel Transcript Regions; OR: Olfactory Receptor; PANTHER: Protein ANalysis THrough Evolutionary Relationships; PCR: Polymerase Chain Reaction; PED: Preimplantation Embryonic Development; RIN: RNA Integrity Number; RNA: Nuclear Ribonucleic Acid; RT: Reverse Transcription; SFF: Standard Flowgram Format; snoRNA: Small nucleolar ribonucleic acid; snRNA: Small nuclear ribonucleic acid; SRA: Short-Read Archive; UTR: Untranslated Region; WGS: Whole Genome Sequencing.

## Competing interests

The authors declare they have no competing interests.

## Authors’ contributions

ST drafted the manuscript. ST constructed normalized cDNA libraries. ST performed bioinformatic analysis of probe sequences re-annotation, COC microarray QC test run, the statistical analysis of the digital expression data and PANTHER GO functional analysis. CZ did the microarray experiment and analysis with pooled embryos. JRG managed microarray data in ELMA. JD generated the *in vitro* porcine embryos and JAP generated the *in vivo* embryos. PR performed 454 sequences assembly with initial annotation and oligo probes design. CR, JN, MKD and GRF designed and coordinated the study. All authors read and approved the final manuscript.

## Supplementary Material

Additional file 1 **EMPV1 initial annotation.** Excel file containing the results of the probe sequences annotation with unique gene symbol. Click here for file

Additional file 2 **EMPV1 annotation description.** A PDF file containing a detailed explanation of the terms used in additional file 1. Click here for file

Additional file 3 **Assembly parameter for gene annotation using SeqMan NGen.** Text file containing the details of the parameter set up. Click here for file

Additional file 4 **Probe sequences without GS selected from EMPV1.** PDF file containing the probe ID and sequences for re-annotation. Click here for file

Additional file 5 **Re-annotation of the probe sequences without GS.** Excel file containing the results of porcine gene annotation with probe ID, accession number, gene description, gene symbol (sheet 1) and LOC (sheet 2). Click here for file

Additional file 6 **BLAST search result from NCBI using porcine sequences.** Excel file containing porcine sequences with no GS to blast search with human RefSeq RNA database from NCBI. (XLS 36 kb) Click here for file

Additional file 7 **All GS list from EMPV1.** Excel file containing a list of unique GS from EMPV1 after re-annotation of all the unknown probe sequences. Yellow indicates olfactory receptor genes. Click here for file

Additional file 8 **EMPV1 PANTHER GO analysis.** Excel file containing genes with PANTHER Family/Subfamily, GO Molecular Function, GO Biological Process, GO Cellular Component and PANTHER Protein Class that are present on EMPV1 microarray. Click here for file

Additional file 9 **GS list from Affymetrix and EMPV1 microarray.** Excel file containing column of gene symbols only found in Affymetrix and EMPV1 microarray after removing all the common GS. Click here for file

Additional file 10 **PANTHER GO biological process difference in Affymetrix and EMPV1 porcine microarray.** Excel file containing statistical significance of the gene count differences between unique GS from EMPV1 array and Affymetrix porcine array over different categories of biological processes using PANTHER expression tool. Yellow indicates genes over-representated in EMPV1 (p-value <0.05) and Green indicates genes over-representated in Affymetrix (p-value <0.05). Click here for file

Additional file 11 **Array-array intensity correlation associated with labelled spike-in RNA mixed with labelled COC aRNA.** PDF file containing the correlation index from two biological COC samples. A: COC1 labelled with Cy3 and COC1 labelled with Cy5; B: COC2 labelled with Cy3 and COC2 labelled with Cy5; C: COC1 labelled with Cy3 and COC2 labelled with Cy5; D: COC2 labelled with Cy3 and COC1 labelled with Cy5. Click here for file

Additional file 12 **Positive spot selection in COC microarray.** Excel file containing all positive signals higher than the background intensity signal > 6.69 calculated according to Methods. Click here for file

Additional file 13 **Positive spots selection in embryos microarray.** Excel file containing all positive signals higher than the background intensity signal > 6.96 calculated according to Methods. Click here for file

Additional file 14 **Unique GS list from COC and embryos array data.** Excel file containing unique GS from microarray data with genes only found in COC, embryos and both. Click here for file

Additional file 15 **COC pathways.** Excel file containing the result of pathway analysis from PANTHER. Yellow indicates the p-value is significant. Click here for file

Additional file 16 **OR genes expression in COC and embryos.** PDF file containing the spot intensity values extracted from microarray data in COC and embryos related to olfactory receptor gene symbols. Click here for file
