# Supplementary material for: Development of a porcine (Sus scofa) embryo-specific microarray: array annotation and validation
Source: BMC Genomics. 2012 Aug 3;13:370. doi: 10.1186/1471-2164-13-370 (PMC3468353; doi:10.1186/1471-2164-13-370)
Supplement: Additional file 2 — EMPV1 annotation description. A PDF file containing a detailed explanation of the terms used in additional file 1. [file 1471-2164-13-370-S2.pdf]

EMPV1 Annotation File Description
Version 1, November 2010

The up-to-date annotation files for EMPV1 are available here:
http://www.gydle.com/embryogene/files/L14-Porcine_Oligos/EMPV1/EMPV1.annotation_probeseq.xls
http://www.gydle.com/embryogene/files/L14-Porcine_Oligos/EMPV1/EMPV1.annotation.xls
The only difference between the two files is the presence of the column Probe_Sequence in the former, which is absent in the latter.

Col. Name	Values	Description / Comment
Probe	EMPV1_00003	Name of the probe
Probe_Sequence	ATGC...	Sequence of the 60-mer oligo
Probe_Type	Gene<br>Variant<br>Spike<br>Control	**Gene** is used for both annotated genes and NTRs.<br>**Variant** is used for exon-skipping probes.<br>**Spike** is used for tomato spikes (not Agilent spikes).
TargetID	TranscriptID<br>EST_ID	ID of the sequence targeted by the probe. In the case of NTR it is the sequence ID of an EST.
Target_Location	Constitutive<br>UTR3_Alt<br>- +<br>a^b<br>NULL	Standard gene probe<br>Alternative probe in case of one or many alternative polyadenylation site (APS)<br>NTR probe: orientation of the probe relative to the target EST. For unoriented NTRs, two probes were designed, one for each strand (**+** / **-**) of the target EST;<br>Interval [**a**, **b**] on the target skipped by the probe<br>Target location is **NULL** for control probes
Alt_Probe_Count	0, 1, 2, 3, ...	Number of alternatives probes for the corresponding gene.
Paired_Probe	EMPV1_36622<br>NULL	For Variant-type (exon-skipping) probes there is one paired probe which targets the skipped segment. The paired probe is always labeled as “Control”. When the target is an unoriented EST, the paired probe is a probe on the opposite strand.
GeneID	ENSSSCG00000008829<br>genome_chr:a-b<br>NULL	GeneID has defined in EnsEMBL<br>Interval defining the position of an NTR. It is composed of a chromosome identifier (genome_chr), and an interval (a-b) on this chromosome.<br>GeneID is **NULL** for control probes.
Chromosome	1, 2, 3,..., 18, X, Y, MT<br>NULL	Chromosome carrying the gene. Some genes are assigned to more than one chromosome.<br>The value is **NULL** for control probes.
Gene_Type	protein_coding<br>pseudogene<br>NOVEL<br>snRNA,snoRNA,miRNA,etc.<br>NULL	Gene<br>Pseudogene<br>Novel Transcribed Region (NTR)<br>Other types of genes<br>**NULL** for control probes
Gene_Symbol	EFNA1<br>NULL	Primary gene symbol<br>**NULL** for control probes and NTR
Gene_Synonyms	NULL	Secondary or synonym gene symbols. A gene can have several synonym symbols, in that case they are separated by a space.<br>**NULL** for control probes or probes targeting a NTR.
Description	ENSSSCG00000008829<br>Novel Transcribed Region<br>NULL	Short description of the gene, or GeneID<br>**Novel Transcribed Region** for putative new gene<br>**NULL** for control probes

Copyright © 2010, Gydle
