# Supplementary material for: Development of a porcine (Sus scofa) embryo-specific microarray: array annotation and validation
Source: BMC Genomics. 2012 Aug 3;13:370. doi: 10.1186/1471-2164-13-370 (PMC3468353; doi:10.1186/1471-2164-13-370)
Supplement: Additional file 4 — Probe sequences without GS selected from EMPV1. PDF file containing the probe ID and sequences for re-annotation. [file 1471-2164-13-370-S4.pdf]

ProbeID	Probe_Sequence
EMPV1_00001	TCCTTCAGATCGTCAGGGATTTCTGTAAGCTTCCCTCTGTTGTGGAAGGGAACCACTCAG
EMPV1_00002	ACTGGGAATGGCAGCTCGGTGCCTGAAGGACGGAGCTCCCGGGACAGAACAGCCCCCTCT
EMPV1_00005	TACCAGCCTATACCACAGCCATAGTGACACCAGATCCCAGCCACATCCGCTACCTATGCT
EMPV1_00006	CAAATCTCAGTCACTGTGCTTGCATCCCATCAACATCTAGGCTAGCACAGAAGTCAGCAG
EMPV1_00008	CAGCACTGCCAACAGGTCTACACACAATGAGCTGGAAAAGAATCGACGAGCTCACCTTCG
EMPV1_00009	TTATGTGTGTAGGTGCATGTGCATGTGTGTGCGTGCACACACATGCACACACACACACAC
EMPV1_00012	AAGGGAGAACCGGCCAGGCCTGTGCTTGCTGCAGGGCCAGGCGAAGACGGCCCTGCTGGT
EMPV1_00013	CTTCGTGTACCGCATCCACCGCTCGCCCATGTGCGAGTACATGATCAACTTCATCCACAA
EMPV1_00014	TGAGCAGATGTGATGAAGATACACAACGGCCTGCAGCTCTGACTCGGAAAGGGTCGTCGT
EMPV1_00016	AAAACAGTCTTGGCTTCTGACGTGCTGTGTGTAGCCGCTGCTTGTGTGAATCATCAGCCC
EMPV1_00017	GCCACACAAGGTGGATGGAAGAGTTGTGGAACCAAGGAGGGCTGTCTCAAGAGAAGATTC
EMPV1_00018	GTGAGAGAACGGAAAATTACAAGCTGCGGAAAAAGCAAGACCTCGGCGATCCTGTGTCAG
EMPV1_00019	AGGATATCATCATTACGTCACCATCTGCACCCCGGCACATCCGTCAGTGCCCAAACATTG
EMPV1_00020	TTACTTTTCCGCCTGCAACTGGAGGAGAAAGACTCCTGAGTCACCGTCGGCCTTGCGCGA
EMPV1_00022	TTCCTCTGCCTATGTTCTCTCCCTTGCCAAAAAGAGCCTTAGGGAAGTTTCTACAGTTCC
EMPV1_00023	CAGAAAGAAGAGAAGAATAGAGCTTTCAGGGAAAAAATCGATTTTCAGCATGCACATGGG
EMPV1_00024	CAAGGCCTGTGGAGCTTGGGAGAGGAGGAAAGAGAGACGGAAGAGTTAGTGGTTTTAGTC
EMPV1_00025	AGCAGGTGTTCCAGGAGGTGGGCATCAACTACCACTCGCCGTTTGGCTGCTAGTTTGTCA
EMPV1_00026	TGCTTCCCGAGGCCACATGCTTCTTTATATCCCCATACGGATTACTTGACTATGGAATGT
EMPV1_00027	GGCTAACAGAAGCTGAACACTTTCTCAAAAAGACACAAATTCCTGAAAATTGGGAACATG
EMPV1_00029	ACAAGTTATTTTGGTTAACAGTGATCTGGGAGTTCCCTGGTGGCCCAGCAGTTAAGGACT
EMPV1_00030	TGACACCTGCCTCTGTGATGTCCTGGAAGCGTATGCCAGCCACTGTCGCCAAGCAGGGGT
EMPV1_00031	CTGTGTTGTAGCCCGATTTGGTGGTGTGTAGATGAGGCCCCTTCCAGGCATGCTTTCCAT
EMPV1_00032	TACTGGGTGATCTGCATTGGCTATTCTTCCTGCTCTGCCCAAGTTGTATCACCACCAAGC
EMPV1_00033	TGCAGCTGGGACAAGTGTCACTCAACAGCCTCTGTGAGCAAGAGAGTCAGGAATGATGAT
EMPV1_00034	TAGTGAGGGAGTAAGCCTTCAGCCTGGCAAGTAAAATGTTGCAAGTAAGCCCATACCTGG
EMPV1_00036	ATGGCTTGTCTTCTCTTATGAATGTTTGGTCCCTGACCGGAGAGGAAGGGGGACAGACGA
EMPV1_00037	GCTCGCCTGGTCCCGTCGTTTTCCTGTCCCTTTGATCTCTCGTTCATCTATTGTAGAGTA
EMPV1_00038	ATTTCATAGAAAGGACCCCCAGTGTAGCAGCCTCAGAAAAGAGGCTCCGAAGCACCTCCA
EMPV1_00039	TTCTTTTCATCCGTAAACTGGCCTGTCCTACAGGGGATCTTTGAGGCTTCCCCAGAACGG
EMPV1_00042	CAGAGGAGCTGAAAAAACTTGCAAAACAAGGATGGTACTGGGGACCAATCACACGCTGGG
EMPV1_00045	GGGGCAAGGAGTAGGGCGTAGCGTTTTCATAGGAACTGATGATCAGGCTTCTAGCAGAAG
EMPV1_00047	GCCTGGGAACTTCCATAAGTCACAGACTTGGAGGTTAGGTGTGAGCCCAACAAGACAAAA

EMPV1_00048	GGGCACAAACCCGTCCCCGCCTCCACCTCCTCTCCATCCTACGTTATCAATAAATAAGTT
EMPV1_00051	CAGAACAGGGAGCAGAGGAAGACCAGGCTCCAGAACAGGGAGCAGAGGAAGACCAGGCTT
EMPV1_00053	TTCTTCTTCTGCTCCTCATAGAATACATGAACCGGAGGAGTAGGTGCAGTCCAGCTCGTC
EMPV1_00054	GAACCATGAGGTTGCAAGTGTTTCAGGTTCCATTCCTGGCCTCGCTCTGTGGGTTAAGGA
EMPV1_00058	GCAAAGGCTCTGTGCACTTATATCGGGTTAGAAAAAGTGTTGCCTCGATAGCCCTCTAAC
EMPV1_00061	AACACCACCAACGACATCGCGCACATGCAGAACGAGGAGATCATGTCCCTGCAGATGAAA
EMPV1_00062	GCTGGCTGAAAGTTTAATGGGAACCTTCCAGAGGGCTGTTGGTGGTGCTTTCAGGAATTC
EMPV1_00065	AGTCTGCCCAGGGCCATTAGGCTCTCAGCGGGACTATTTTTAGAGACCCTGTGTCTGTCA
EMPV1_00068	TGGGCCTGTACCGCGGCCTCAGCTCCCTGCTCTACGGCTCCATCCCCAAGGCGGCTGTCA
EMPV1_00071	TGAGAAGAGCGGGCGCTGGGGAGCGAGACTTACTGCTTTTGTAAGCTCCGTCCAGGTGCT
EMPV1_00072	CCGGGTGTTAAGTGTCTGATAAAATCCGGACCGAAAGCGTTGTCAGAACCCTCTGCAAAG
EMPV1_00073	TCAGGTTTTAGGACACGCAGGAGTTGAAACGAGATCGGATTCCAAGCACCCTCCTCTTAC
EMPV1_00074	GCCCGTCAGTGGTCATTAACAGCGCAGACTTTTACGGTCTTTGAATCCCATCAACACTTG
EMPV1_00075	AATTATGGGAGATATCGTTCTGAGAGTGACGCATGGAGCTTTGGCATCCTCCTCTGGGAG
EMPV1_00076	CGAGCTGGAGAGCAAGTCCCAAGACTTGAAACAAGGAAGATGGGCCTCTTAAATAAAAGC
EMPV1_00077	GTGCGACCAGGAGCTTTAAAATGGAACCAATACAACTGGGCGGAGTTATCCTTACACATT
EMPV1_00078	TTACTTGCGTCTTTTTCTTGCCCGGCCCCCTCTCATCAGAACAGCAAAGGACTTGTGGTG
EMPV1_00080	TCTGTACATGCTGCTCCCTGACTTCTGCCATAAGTTTCTGCCGGGCTACGTGGGCGGCGT
EMPV1_00082	ACCAAAGCAGGAGAACGGGGGCTACCTTGTTTTTGTGAGCTTTCCAAGGGGTTCTTGAAC
EMPV1_00083	GTTTGCAGACTTTGAGTTCTGGTGAGACACAGAAGGGAATAGAAGGTGCAGCTGTGGCCA
EMPV1_00084	AACTGGCTACTTTTCAGATGAAACCTGGTGGGCCAGCTCTGTGGGGGGACCCATTTAAAA
EMPV1_00085	TGGGGGTTTCACTGGCTATTCTTCTCATTGGGGTTGTCCTACTGTGTATTTGGAAGCTGC
EMPV1_00086	GGTATTGGAATAGCAGCCTCACACTGGCTTATGGACTGTTCTGTAGATTAATGCAAGTGG
EMPV1_00087	GACCAGTCTCTGAGCTTGACTTTCATGGAGAGTGTTCCTGTTGTGGGTCTTTCCGTGTTT
EMPV1_00088	AGGATTGGTCTCCATTTCGGGATTAGTCTCCTTAAAAGGAAGTGATCATCCACCTCTACC
EMPV1_00089	GGGTACAGTGTTCTGTGAAGTGACTTATTTTTAGTTACCACATCTGATTCCCACAGTGCA
EMPV1_00092	ACCTTCCTTCATGTGCTGCTACCCACATTGTCACCATCACCCCACACTCTGACTCTCTCT
EMPV1_00093	ATCACCTTCCAGAGCCGCTGGCTGTTTCGCAGAACTTGCTCTGTCCCCAACAGCTCCTTT
EMPV1_00095	CGGAAGTGCCCTGTTAAGTTTGACGACACCAGTGATGATGAACAGGAAAGTGGTGATGGG
EMPV1_00099	AGTGGATTAAGGATCTGGCGTTGTCATGGGTTGTGGTGCAGGTCACAGATGCAGCTTGGA
EMPV1_00100	TTGCCACCCAACCCTAGGACCTGCTTTACAAAGGCCTTCGACCTCCTCATGGACCAGCTT
EMPV1_00101	CATCATGAACTTGAACTGCGGCATCGACCCCGTCATCTATTCCTTTGCATCCACGCGGTA
EMPV1_00105	TGTGGGGGACTGCCTCCAGATTCCATTTATTCAGGTCAGCAGCCTTCTATTGGCACTGAG
EMPV1_00107	GGCGCACCCACGGCATATTGAAGTTCCCAGGCTAAGGGTCAAATCAGAGATGTAACTGCT

EMPV1_00108	AACTTCCCCGCAGGTACCCTGGCATTCTGAAACCTCCTTCAACTACCCGTACTTTGGTCA
EMPV1_00109	GATGTGGCCGCTTTGGAGAGTCAGCCTTTACTCGGATTCACGGTCACTCAAGTCAAAGAT
EMPV1_00110	GTACCATTGGAGACTGGAAGAATCACCTGACTGTGGCACAAAATGAAAGATTTGACAGAA
EMPV1_00111	TTGCTGTTAGTGCAGCTCCGAATAAATAGCGAGCTTTCTCAGCCAGCGCCCTGTTGCCAC
EMPV1_00113	GGCCACATCAGCTCGGAGAACAGATTCCACTGCTGTTTCATTTGTCTTCCTTCTGGAGTC
EMPV1_00114	CGCCTAAGACTTACGGTCACTACTCTCCACAGTGTCAGTTCTGAGGGATCTTGCAGCAGA
EMPV1_00115	AGAGTGGAGGGCAGGACAGTACCTTTGAGTGCAACATCTGCCTGGACACAGCCAAGGATG
EMPV1_00117	AAAAAAGTAATGAAGAGAAATTTTAAAGTAAATAAGAAAATAACATTTACCGATGAAGGG
EMPV1_00118	TTTGATTGGGAGCCCTGGAGCAAAGGTCCTGCTGAGTTTTGCCAGGAGGAGACACTTCAT
EMPV1_00119	ATCGTTTTGGTCTCTTCATTGTCATCACCACCTTTGGCCTGGACCCTGCACTCATTCTCC
EMPV1_00124	GTGCTTGTGCACAGGGCTCGAAAACTAGCTCAGCAATACTATCTCGTTTACCGAGAACCC
EMPV1_00126	CTGTAAAACAGAAACAAATCACACACAGGGAGAGCAGACCTGTGGTTGCCTGAGGGAAGG
EMPV1_00127	CATGGCGGTTCTCCTGGAGACCACTTTGGGCGACGTCGTCATCGACTTGTACACGGAGGA
EMPV1_00129	TCAAAGGGGCATCCAGATTGACGATGTAAAGGAATCGGCGGCTAGGAAAACAGCATCGAC
EMPV1_00130	TTCCAATTCTGAGCTTTGCAACTCACCTTCTGGCCTCAATATAGCTTGCTGGGCAGGCCT
EMPV1_00131	GGCCTCAGTCTTGGGGTGTTGTTCAGGCTGTACCATCTTTCTGGAATATAATAGCATGGC
EMPV1_00132	CATGTGTGTCTGTGCCACTATTCCCCCAGAATTTTAAGTGGATTTGCTAAGTTAAAGGGT
EMPV1_00135	TTTAAATGTGCAGCCCTTATCCCAACCCTCCCAACCCTTCCATGTACCTCACCCTGTGTA
EMPV1_00139	CACTGTGTTGGTTACTGTAGCTTTGTAATATTGTCTGAAGTCTGGGAGAGTAATGCCTTC
EMPV1_00140	GGTAACCCACTTGTTTCTGGTCATCCCTGAGTGCCCAATACCCCTCCTAGGTAGAGACTT
EMPV1_00143	CCCTTAGGCTTCACCCTCTGTTCATTCTCTTGTCTGGTTGTGCATGAGCCTGTCTCTGAC
EMPV1_00144	GTGCAAGAACACCAAGGGTGGCGTCCTGTGCGAGTGCTCGGACCCTCTCGTGCTTGGGGA
EMPV1_00145	AAGGTCTAGGATCTGTCTTTGGCCACTTTTGCCTTCAAATCCTGCTACTTTAGCATCGGG
EMPV1_00146	CCACTTTTCCACATCTCTTTAATGTCCTCTCTCCTCAGAGCCAAGGGAAAGCTCTGCTTC
EMPV1_00148	CACGGTGGCCCTGCGCGAGATCCGGCGCTACCAGAAGTCCACGGAGCTGCTGATCCGCAA
EMPV1_00157	GCCAGGCACCAGCACCAAGAGAGAAGACAGAGGAAGGGGAAGATGAAAAGACACGGTTTC
EMPV1_00158	CTAAGTTTGGATCCATTCCCAAGTTTTATGTTCGAGCACCAGGAAGAGTCAATATAATAG
EMPV1_00159	TGAGGAGAGCATGATGGTGCGTTTAAGTGTCAGTAAGCGAGAGAAAGGACGGCGAAAACG
EMPV1_00160	CTGGGAGTCTCAGCGGTCTCAGATTGGTGTCGGGGGTGGGCGGATGTGCTCTCCCGCCGA
EMPV1_00163	GGCCCAGCACGTAGGAAGTGCGCCAATAGGCTTGTTCTCATTACATCTGTCTTTCCGTTA
EMPV1_00165	GCCGTTCGCTTAGGAACTCATCCTGACTGGTTGTTTTTCTGCTCTTTTATTGGGATGTTC
EMPV1_00166	CTGAGGGTGATGGTTCAGATTTTGCTGCCCTCCATAGCCCATGAAAAGTCCAACTAGGAG
EMPV1_00167	TGTCACACTCCTGGCTATCTCAGGGGGAACGGGAAAAGAAGCTTTCTGGGTACAAGAACT
EMPV1_00168	CTGCAGCTGTGCTAGGAGCTTATAACTCTCAGTTCAGCCCTGTGCCTTGTTCCAGTGTTT

EMPV1_00169	GAGGAACGAATACTCTCATACTCATTCTGAGGCCGCCATCACCCTGATACCTAAACTGGA
EMPV1_00170	CAGCCAGTCCTCTGTCCGTTTCCCTCCTGCCCTTCCAGGTTTTTATTATATGCCTTAGAG
EMPV1_00172	GAGGAGTGGTGGGAAGGAGGCATCACCCCAGAGACCTGTTAGGAGATGCTCACTGTATTT
EMPV1_00174	TCTCCACAGGTACTTTGAGCCTTGCTAAGACCATCCAGCCCATCTTCGTTGATTCATATG
EMPV1_00178	GCCGGCTCTACCCCTGGGGCATCGTGGAAGTGGAAAATCCAGGGCACTGCGACTTTGTGA
EMPV1_00179	TCGGAAGGCTCTGGTCAGGAGAATGAAAATGAGGATGAGGAATAATCAACTCTTATTGGC
EMPV1_00180	CCCCATCAGTTATGAACTTAACGACAGTGGCCCATTTATTTGTCAGGAGGGCAGGGGACT
EMPV1_00183	TGCAGCTGTGGAGATCACATCTGCGGCTTGGCTCCTGTTCAATCTCTGACCTGAGAACTT
EMPV1_00187	CTGTCCGAGTCGATGCAAGTGGGCATTTTCTATCCTATGCCTTGCACCATCCTGTCACCA
EMPV1_00189	GATGCCATTCCAAGCTTAAATCCTTTCCTCACAAAAAGTAGTGGTGATGTTCACCTTCCC
EMPV1_00191	AAGGAAATAAACCCAGAAAGGGATAGTGGCTTCGCCAAGGTCAAGGACTGGAACTCAGGC
EMPV1_00193	TTGCTATCCCAAACCCATTCACCAAAGGCGGTGGGAAGGGTCGTGTTTTCACCTCTCAGT
EMPV1_00194	TTTGTCCTGGATGACTTCGGCCGCTATTCCGTGGACCTCTGCTACGTGGTCCTGCAGAGC
EMPV1_00195	AAGAAGTCTCTGACATGTTCGCCTTCATAGCGCGGTGCCTCAACAACGACAGGATACCGT
EMPV1_00196	GCAGAGTGCATGAGCTGTACATGTAAATGTATTGATTTTTCCCTGTATAGGTTATTACAA
EMPV1_00197	GGGGCTCCTGCAAGAAACTAACTGTTGTGGTCTCAAAACTCATGGGACCATCTGTGCAAA
EMPV1_00198	ATTATGCGTCCCTCGTCTTCCACGGGGTAAAGCTGCAGAAGGACACCAACCCGGAATACA
EMPV1_00199	AGGGGTCCTCACCATTGCCCTCCCTGTGCCTGTCATCGTCTCCAACTTTAACTACTTCTA
EMPV1_00200	CTGGGCCAGCCTTTAGAACACTGTTAAGGTATGTACACTACTTTGATGCAAGAGACTCTC
EMPV1_00202	TCCCGTGAGCTGAACGACATTAGCAGAGCCAGGAAGTTGTGCGGCAGGCATTTACTGAAA
EMPV1_00203	GAGAAGAGGGGGAAAAAGGAAGTTTGTCCGGTCCTGGATCAGTTTCTTTGTCATGTAGCC
EMPV1_00204	GTTCATTTTAGGACCATAAATGTAGCTGCGCCCCTGTCGATGCTTGGCTTGACGGCGCCC
EMPV1_00205	GTGCAGCTCTTGACCTTTGACAGCGAGGAGGAGCTAATCAAGGTGGCTCAGCACTACCCG
EMPV1_00206	AACCTCATCCCTGGCACAGCAACTCACAGTCAAGAGGGAATTCAAACCTGGAGCTTCTCC
EMPV1_00207	CCCCTCCTTTTGCTGTCTTGTTCAGATACACAGGTCAATGAGTTAGCGATATTCACAGTT
EMPV1_00208	GGAAGGCTGTCCTGGGTCTGTGCATGTCCCTCTCTTGCTCGCAAAAACTAATCTGTTCTG
EMPV1_00212	TCAAACCCGGATCCTCATAGATACCAGTTAGATTCCTTTCCCCTGAGCCACGACGGGAAT
EMPV1_00213	GCATCTCACCAGCTCAGAGGTCCCAAAACTCACTTAAATCATCTCAGTCAAGTAGGAGTG
EMPV1_00216	CCATCTAATACACCCACTGATGTGAACCAGAAAGCTAGGGAGCCTCAGAACCACAGGCCT
EMPV1_00217	ATACCCATTATTTTACTAGTGTTTGCAGCCTGCGAAGCTGCACTAGGCCTGTCACTACTA
EMPV1_00222	ACGATGTTCAGAGCATCAACTGGCTGCGGGACGGGGTGCAGCTGGTGGAAAGCAACCGCA
EMPV1_00223	ATGAGTAGGTGCTGTCTCATCATTGCAGTAGAAGATATCCAATCCTGACAGCTGGACCAG
EMPV1_00224	AGTTTGGCTGCGCGGGGCCCGCTGGAAGCCTGGGGTCTTGGAGGCTGCCTAGGGCTGCAG
EMPV1_00228	AGTCTGCTACTCCAAGAGAGCCCGTCATCCGTAGGACAAGTGGATCTGACATGAGTGACA

EMPV1_00232	TCCTGACAGCAGAGTGGATGTCTGCCAGTAAAATAGTGTGTCGAGTGGGACAGGCCAAGA
EMPV1_00237	GCATGTCTTAAAAGTTGGGTCGCTTGATGTGGGGTTCAATCTGTTCGTTCATCAGGGAGA
EMPV1_00239	GCTATTTCAGTTTACATGGCAATCACTCTGCTGGTGACATGTCTCGGTGCCTCTCAACCC
EMPV1_00241	GGACAACATGAAATATGCTAGTTGGGCAGCTAGTCAAATAAACAGAGCTTATCAGTTTGC
EMPV1_00242	CGGAAACCATGTGCTGCTGAAGTAAAATAGGTGCAGAGGAAATGCGTCCCTGTCTGCTGA
EMPV1_00243	CCATCTGCAGGCAGCTTAAAAGTTCAAAGGGGTTTTCTGTAGAACCAGCCTCCCATGTGG
EMPV1_00245	CCTAAGGCCAGCATTGCGGACACGGGTGAAATCAGAATTAGACACGGGCAGATGTGAAAG
EMPV1_00247	GAGGAAGAGGACCTGTCGGGGGTCGGGGGCTCAGGCACAAAAGGACCCGGGCTTCGGAGA
EMPV1_00249	AAGAGTTGTCACTGCCCTCCCCAGCTTGGTCACAGGGTTATTGAACTGCCTGCACTTGTT
EMPV1_00250	GGGCGGCTCTCCTACAGCAGCAAACCTCCGCTCTGGGCAGCAGAACTGTGCGTGACATGA
EMPV1_00251	CTTAATGATATGGTGATGTATTTTGCAGCTGGGATGTTGGGTGGTGTTTCCCTCACTGGG
EMPV1_00253	GGACATTGAGGGCAGCCACTAGAGCTTTTCTAACTCTGCTACCTGATACCCTGAGATTGG
EMPV1_00254	CCCTCAATAACGTCTTCCAGGACAGCAATGCCTTCACCCGGGAAACGCTGCAAGAGATTT
EMPV1_00256	CTCAACGGATCCCCACTCAACACAAAAGACAAAAGAGGTGCCAAGCCATGAAACCACGTG
EMPV1_00257	CTGGATCACTTTGCTATATACACCAGAAGCTGACACAACTTTGTAAATTAACTATACTTC
EMPV1_00258	TCAGTTTGCCCGGTGAGTTCTCCATCGTGTTCTACACCTCGTGTGAGCTTATACCTCAGT
EMPV1_00259	TTAAGTAACAAAGTGGCCGGTCTGTATTCTGGGATCTGGTAGCCCCCTCACTGGTCTTCC
EMPV1_00261	GAGAAATAAAGACACAGCATGTATTATGCACTTCATTTCTCTGCTGTGTGGAGAAAGCAA
EMPV1_00262	CTTTTTAAGCTGGGAGAGGAAGGTGGTTGTGATTTGTCACTAAGAATCCCTAGCCAAGGC
EMPV1_00263	CGATGCAGAAGCGAGCTTGCCCAGGGAGGGGCCTGCTCTCCATCCGGGGTGTGCAGAGCC
EMPV1_00265	TCAGAATGTGAAAATGACAGGGTTGGACGGAACAATGGTTTTCGAATGGTGCTGGCAGAC
EMPV1_00266	TAACTCTCCGTGGCATCAATAGCTTCCGCCAGTACAAGTACGACCTGGTGGCAGTGGGCA
EMPV1_00267	GACGATGGTGTGAAATCCCTCTGTCAAGTATTGAAACACCCAGACTGCAGCTTACAGCAC
EMPV1_00268	AACGGAAGTGGCAGAGCACCCGGGACCAAAGGGCTGAACAGGATCAAAGTGACAATGGTA
EMPV1_00270	ACCGCCACGGAGTCGGACCAGGAGGAAGATGCAGAGCTCAAGGCCCAGGAGCTAGATAAA
EMPV1_00271	CAACACAGGAAGCGAATGTGGGTTCCAGCAACACGATGTTTGACGACAGAGGTCATCCGT
EMPV1_00273	CAAAAGGAGCAGGTATCAAGCACACCTATAACGGTAGCTCATAACGCCTTGCTCAACCAC
EMPV1_00274	TACTAATCCAGACACTGGCAGATACGTCCATAAAGATGAGACCATGCGCCCTTCCACTGC
EMPV1_00275	GCACGACTGAACAAGCTACTGTTTCTGAAATTTAGGCTTCTGCCTCACAGAAGTAATAAC
EMPV1_00277	TTCACCTTCCAGGTGGTGTCCAAGTGGGACCTCTGCCTGTACATCATGTCCTCTAGGAGA
EMPV1_00278	CGCTTGCAGAGAAAGAGCCTGGCGGTGGACTTTGTGGTGCCCTCGCTCTTTCGCGTCTAT
EMPV1_00279	CCACCTACTTTGATGCAGTTGTGCCGCCTTAGAATTCGGAAGTGCTTTGGGATCCGGCAA
EMPV1_00281	GCTTATAACCAGACTACCATTTGCTGGCAAGACCACATGGGCATTAGGGAGAGAGCGAGA
EMPV1_00287	TTATCTGTTCCAGCTGCTTCAGGGGCTAGCTTTTTGCCATTCTCATCGGGTCTTGCACCG

EMPV1_00289	ATATGTATGTGCTCTAGGCCTGGGGAGCTATTTAGGGGGAGTACAGACCCACCTCCATTG
EMPV1_00290	ATTCATCCCCAAACAGAACAAGCCCTCTTTATCCACCTCCAAGCATCTGTCCTCCTGAGG
EMPV1_00291	GGGGGTGGGGAGGGAACTCGACACTGAGCCACTAAAATACGGACTAATTTTTCAGACACA
EMPV1_00294	GGTAACCCCTGTCTGGTCCCTGTCTGAGCTTTTCTAATGAAACACGATACTGAATGGGGG
EMPV1_00295	TAGAGATAAAGCCTGAAGAGCATCCATCGGTTTTGTCCATAAGAGGCTCCTAGTGAGTCC
EMPV1_00298	GAACAAACAAGGAGTTCCTGCTATGGTGCAAGAGAATTGGCGGTGTTGCTATAGTGCCAG
EMPV1_00301	AGAAGGTGAAAACTACGACCGGCTGGCATTGCCCTCAACGTCCACTTCGTCAAGCTCATT
EMPV1_00302	GTGGAATGAAGCAGCATTTCCAGTTGACAAATGGATCCACAGGTAACGCCATGTGTCTAA
EMPV1_00305	CTTGCTTTTTAACCCCTGGCAGAGAAGCATGGGATAGGAGGATGGAGAGTCAGAAGATGC
EMPV1_00310	GCATGCAGGAGCCCTTGTTTGTGGAGTTTGCTGACTGCTGTTTAAGGATCGTGGAACCAT
EMPV1_00311	GCCTACAAGAAGCCGTCCAAGTACGACCTGGAGAACGTCAAGTACCTGCACCTCTTCAAA
EMPV1_00314	AAGAAATCCAGTTTCTCTCTCCGCCCGTTGTCCAAGATGCCCAAGGGGAAGAAGGCCAAG
EMPV1_00315	TGGTGACCTGTCATTCAGACTAGTGGGTAGGATGTCTGCTTTGAAAATGTTTGTCCTAGG
EMPV1_00316	CCAGCTGGGGACGCTATACCAGTTAATGGAAAGCAGAGTAAAAACTTTCCAGAAACTCTC
EMPV1_00318	GCGGCAAGACCTCATTCAATTGATGGGAGAGTGGTTAGCCAAAACATACTGTGCAAAGAG
EMPV1_00319	CAGAGCTTATTCACATGGTGTTTTGGTATGCCAAGAACCAAAAGTAAGTTAATTGAAGGC
EMPV1_00323	AGGTCCTGATGGAGCCACACCTTGGGCAGCACCACTCTCTTTGCAGAAACCAAACAAATC
EMPV1_00326	TCTGCATGTCGCCCATGTTATAGTCGTTGAGGCTGATGTAGATGTACACCAGCATGTGCC
EMPV1_00327	CCCTGGGTGAGGCTTCGTGCTGTCTTTAGAGATCACTGTGGGGGCAAGAATACATTTCTT
EMPV1_00328	CCACAGCAATCACTCCAGAAGATCTTTTCCTTCTGCTTTCAAGGCACTCAGCATATTGTC
EMPV1_00330	GAGCTCCATAGGGCCATGTTGGTTTGTTTTGCATTTAGTGGTAGCACCGGTACCCAAGGA
EMPV1_00332	CATCACACATAACAGAGAAGGGCTGCAGGGTGAGGTTACTGGAAGAGAAGGTTTCCCAGA
EMPV1_00333	TTCAGTGACCAGTGCTGAGTGGCCTGGCTCCATCAGTCGGATCTCCCTCTGGGGGTGCCG
EMPV1_00336	AATTCCTCGTCAAGTTGTAGCAGTTTGAAGGCACTGCGGGCACGCCAGCCATTCTCCTTG
EMPV1_00337	GCTTCTTTTCTGCCCCTTATCCCTAAACCCCACCTCCTGCCACTCAAACAAAGTGTGTCT
EMPV1_00342	ACTGCAACGTGATAGAGATAGAGGATACCCCCGATGACGACTCGGATGAGGACGTGATCT
EMPV1_00345	ACTAAGCCACTAAGTGCCCAGACTGCTGTGACAGAGGAAACCAGGGAATGCAGGAGCCTT
EMPV1_00346	GCCAGCAGAGTAAACCGTTTTTTCCCTGGATTCACAGAGCAGTCTAGCCTTAGCTTTTCA
EMPV1_00347	CTTCCTGTTGTGGTACAAAGTGAGGACTGTCGCCAAACACCCTGCCGAGGACATCATTTC
EMPV1_00348	GGTGATAGCACACTGAGATAAGAACCGCATGTATGTCAATCCGGACAGTGATGCCCACCC
EMPV1_00349	CCAGGCTACCAGAGCACTGTATTGAGTATGTAAGGATATTGCAGTGGCCTAAGGAGCAGC
EMPV1_00350	GCAGTTCTTCTCGAATTTGCTGAGGAGCAGCTCCACGCTGACCATGTCTTCATTTGCTTC
EMPV1_00352	TCTTCGTTGTTTAGTGTGTCCCTCTTCCTCGCTGGCGTGTGACTCTCAAATTGGCCAAAG
EMPV1_00353	TGAGAATTTCACTTGGGCCTTTTCAGAGCCTCACCTCTCATTAGCTTCTACCTCACGTAG

EMPV1_00354	ATATTACTCCTGGATTGCACTTCTGCCTCCTTGGCTGGTGCCCTTGTTGAGCTCCCCAGT
EMPV1_00355	ACATGTGTTCCGCCTGAAGAAGTGGATGCAGAAGGTCATTGATCGGTTTGGAGGTTAGGG
EMPV1_00358	ACTAGCTTCACAGCTTTAGGGGGCTGGGAAGTGAGACGGTGTGGAGGGGAGCTTTGGCAA
EMPV1_00361	GGGGCGGTGCTAATACCTACCTTAGCAGAGAGGTTGGGAGGAGTAAGAGGACAGTATGTT
EMPV1_00364	GACCAAGCTGGCCTGCACAGACACTTACGTTATCAGCCTACTAATAGTTGCAGACAGTGG
EMPV1_00367	AGACTCCAAGCAGCAGAGGCTTGCGGTGGACTTTCCAAAAGGCTCAGGAAGCAATGCACA
EMPV1_00368	GCAGCTCAATGTCCAGATGTTGTGGTAGCTCAAATTGACCCAAAGAAGTTGAAAAGGAAG
EMPV1_00369	GCAGCACAAATCCTATTTCTTCCCCTTGTATATAGGAAGAGTGAGGCCCCAGCCAAGTGC
EMPV1_00373	CACCTGTCCGCCATGTCCAGGTACCAATGGCTAAGCAGACATACTGCAAACATGTTAACT
EMPV1_00375	GCACTGCCAGGGTAATTTTGTATCCTCTGCATACTACAGCTATTTGTCAGGCCCCTGCAG
EMPV1_00377	GAAGCTGCATATTTTGTACTTTCCTGGAGTTGGGGAGGGAGTTGTGTGGCCTGTGGCCCA
EMPV1_00378	ATCCACTGATGTCCCTGAGATGGACACATCTGCTTGACAGATCCTGAGAATTCAAGGCAG
EMPV1_00383	GACTCACGGCCTGGAGGGAGGGACACGTGGCCACATGACCTAAGTTCTGTATCAGGACAA
EMPV1_00384	CCGGGTCAGAATACCAGTACTCTCATTACTGTGTAACTAGCAAGTTAGTCTCTCTGAACT
EMPV1_00385	TGCTGATAGCATCCTCAACCCTGATGGCTTTGCTGGATTTCCTTGTGACTCTTACAATGT
EMPV1_00388	CTCTACAGATGCTCTGCCCACAAATGGAGGGCCATTCGGATACATCGTGAACATAGTCAC
EMPV1_00389	AGTATGTGGCAGCCTACAAGGTGGCCTACAGCGACGATGGTGTGAGCTGGACCGAGTACA
EMPV1_00390	TTCAGTTTCCCCGGTTCCCTGAAAAGCTAGGCGCATACCCCACGAAGCTGCTCAATACAT
EMPV1_00392	CAGCGCAGGTATTTCAGGCATGGAGGAGTTCGTAAGATCAGGCAACTTTGAGATCACAGC
EMPV1_00393	CAGGTTTGTACTTTCCCTGCAGCCCAAGCATCTGTCAATACAGTTCCATTTCAGAGTGTC
EMPV1_00395	TCCTTCACTAATCATCATGGAGCCTACATATCCTCTTGCCGGACCCCAGATGAATAACAG
EMPV1_00396	ACCCTCAACCCTGGGCTCCAAGCAGCCCCCCTGTATTTATTCAGAGTTGGTACATAACAT
EMPV1_00397	GAAGATCGTAGAGCTAGCTCACAGCGGGGCCCGGCCGTGCGACATTTCCCGAATTCTGCA
EMPV1_00398	GCTCTAGTTATACTCCTAGATGCCTCCACTCGAAGGGGGCTGCTGATAAGTGGATTTCAA
EMPV1_00399	GCTCATTTTGCTGAAGTGTCATCTGAGCTTCATGGAGCCCTGATTGTTTCAGCCTTTTGC
EMPV1_00400	CTGAAAACTACTCTGAGGGATCACATTGGTGGGGAAAGACTGGTACAACTTCCCTCCCAC
EMPV1_00402	CTTCTTAGCAAATGAGCCTGAAGCTTATTAAGGAGACCATCCGTGAGACCAAGCCCTGCA
EMPV1_00404	AAGGAACGATTGTGTTTGACACTGGTAGTCAAGTCTGGACTATATTCTGCAGTTAGCCAC
EMPV1_00408	GCCCACATCATACCCTTCCTAGAGTGAAGCCCTTGTAATTCGCACTAGAGCTCAGGAAAC
EMPV1_00409	AAACCCAGAGGCACCCAAGAGAAAGAGGCTTCACCATACCGCCTGCTGACAACAGATACA
EMPV1_00410	TGTGGAAGCAGCTGGTGAATGGCCTGCCGCCCTACAAGGAGATCAGCGTGCACGTCTTCT
EMPV1_00413	GAGTGGATTCTTACCCAGTCTTCACGGGACCCTCTGCCTCTTCACGCTAGGAGTCTGTTT
EMPV1_00416	AACTCTGTACTTTCAGTCAGAGGTTCCCGCTCCCGTCCCTGTACCGGGCGTTGGTCTGGA
EMPV1_00417	ATCAACGAGCTGACCTTGAAGCTAAGTGTGCAGGACGTGCTGACGGGGGCCGAGGCCCTT

EMPV1_00418	TCAGATATTAAGCATGCCCTGGTCGGTGTGTACGGAGTGGTGCCCAAAGTCCAGTGTCTT
EMPV1_00420	TTTGAGGGCCACCCCCGCAGCATATGGAAGTTCCAGGCTAGTGGTCAATTAGGAGCTGCA
EMPV1_00422	GAAATGTGCAGACCCTGATTTGAATGCTGTTCTTTACACCAACCGGGCAGCAGCACAGTA
EMPV1_00426	CAGCCTGTGTGCTTGCCCCTTATGTCCATACTTAGCGTCATTTGTTCCCCTCACCAGAAA
EMPV1_00428	CACAGTTAGGTCACCATGCTGTGACATGGTCTGTATTCATGCTGCCCTAGATACTTTTGT
EMPV1_00431	CCAGCCCACTTCTCTCCCAACTCTGGTGTTTCCACAAAATGTGCCTGTTGCATCCAAATG
EMPV1_00432	CCAGGGTACTCACTGGGGAGGAAGCAAGGAAGGCAGGCTGAGAAAATGAAGTAAGATAAT
EMPV1_00434	GGGTTGAGCGGGGCCTTAAGAAGGACAGTTCATTGTAGGAAAGCAATTCTGTACATGAGT
EMPV1_00436	AGGAATTCAGGAACCTTCTCCCAAGATGCCAAGCCCTATTCTCTGCCTGACTGTCCAGAC
EMPV1_00437	GGTTCACGTGGAGCATGAAGACCACCAGTTCCATGGACCCCAATGACATGATGAGAGAAA
EMPV1_00438	CCAGGATTCAGAACCATTACGTGACAGTCGTGACAGTGGGCAGATCCTCTCTGATGTCAA
EMPV1_00440	GGTCGTGGCAGTGCCTCCATGGTCCAGAAGTGCAAGCTGTGTTCAAGGGAAAACTCCATT
EMPV1_00444	TAAAGGTAGAAGAATTTGCCTTCCCAACTCCCCAAGAGCGAGACGTTTGAGGGGCCGGTT
EMPV1_00445	GGTTTTGCATAAACATGGAGAAAAGCTCTACACTGGACTAAGAGAAGTTGTTACCGAACA
EMPV1_00446	GCACAGACAAGCATATTGCCCTAGTGTATGCCCTTCTTACGCCTTTCCTCAACCCTATCA
EMPV1_00447	GACTGGATGCCCAGGCGTCTGGGGTGCTGGTGCTCGGCGTGGGACACGGACGCAGGCTCC
EMPV1_00448	TGCTGATGTTGTGAGTTTCTAAGTTGTTGAGTTAATATGTTTCCTAGTGTTCCTATATAA
EMPV1_00450	CTGAACCTTGAACCTAGCAGGGCAATTACAACAACATAGCATATCTAAGCTACTCCCTCC
EMPV1_00454	CTGAGCTGCTGGTCAAGAGAGAGAACCATGGCAGCAACCCGTGCCTGATGACTGAGAGCA
EMPV1_00456	AGTGAGGCAAAGCCAGAGAGCCAGGGAAAGCCAGTGAGTGAGGGCAAGCCCAAAGAAGAA
EMPV1_00458	GGGAATAAATTCAAAGACAAGTCTACACAGGAGCCGGAGCTGGAGGAGCCGCGCTCATGG
EMPV1_00460	CATCACACGTATTCCTTCTCCTTTGTGCTGTTGCCAATCAAACCACGGTGGAGCATCCTG
EMPV1_00461	TCACCGTGTCCCTGTGGGACTGTGACCGCAAGTTCAGGGTCAAAATCAGAGGCATTGATA
EMPV1_00463	TTAATGACACTTGTCCCCAAATCCGAACCTGCTCTGTGAAGCATACGAGACAGAACCTTG
EMPV1_00464	GTGTGGTGGTTTAAGAGAATTTTCCCAACGAGTGTTTTGCCATGGGGCACCCCCTTTTGT
EMPV1_00467	GGTGAGAATGAATGTGGACTGCCTTGCTACCCCTGAGCGGGTTACTCTGGATGAATCACT
EMPV1_00470	GCAGTGCACAGTACAAAAGAACAACCTCCTCCTGCTCCCTTGCACAAAGAATTATCTGGC
EMPV1_00471	TAAGAAGGCTTTCCCTAACTCATCCAGCAAGTGCAACTAGTTCGCGCCTATCAAGGGCTG
EMPV1_00472	GGTGTCCACCACAGCGCCATGTATAACGCTCAAAACCAGAATTAACGCAAAGGCCCACCA
EMPV1_00476	TCAGTCACCTTGAGCCGGGCGAGCGCTGTGTTCCAAGCCGGGGCAGCGGGGCAGTGAGAG
EMPV1_00478	GGCCAGTAAGGTGGTTGCAGTTGATATGGTCACTTTCTATTGGAGCTGCTATTATTCCTC
EMPV1_00479	CCCTGTTAGAGACGTGGAAGGTGACTCAGATGAACCAGATTCGGTGGGGAAGCTACAGAT
EMPV1_00480	TCTCCACTTTGTAGACAAGGATATTGTGACCGAGGTACATGACCGGGCAGGGATGGGTCA
EMPV1_00481	CTGAGTCCCTTTGGAAATCCAGTTTCTCTTGGAGCTGGTGGCAGGATAGAAGCCAGTAAC

EMPV1_00482	TCCTTAGCCATCTCCGCCTCCTATGTGATGATTTTTAGGGCAGTAATGAGGCTGTCCACC
EMPV1_00485	AGGCGGACGTGGTGCTGATGTGCTACTCCGTGGCCAATTATAACTCCTTCTTGAACCTGA
EMPV1_00486	CCACAGCAGCACAATTCAGAACATGCACGACTGCAAGTTCCTACATCACAAGTAAGAAGT
EMPV1_00487	CCCCTCCTTAAATCATGGGTACATTCCTGAGTACCGAATAGGTTTTCGCTGTGAATGACT
EMPV1_00489	GGTGATGACTTCTCATTGGCTGAACTGTGGCATTTCCCATTGGCTGGGCTTTTTGCTGGA
EMPV1_00490	TTCCCAGATCCGAAGCCTCGGCCTTCAGCAACTCCCACCACGTGGCCTACTTCGAGGCCT
EMPV1_00492	CGGAAGATCTTGTCATCTGTGTTTTCAGGCTCAGGACTTAGCAAGAAGTTATGGAATTCC
EMPV1_00496	CCTGAGGAGGGCTGATTCCCTATTCCTGGTTCAGACTTAATTTCTGCGGACTGGATGTGA
EMPV1_00497	TCATCAAGCAACTTAAGGATGTATTTGAGAGGGACTGGTACTCGCCCTACGCCCGAACCT
EMPV1_00498	CCAGCTGACACTTCTGATATGGAGGCAGCTTGGAAGGAGGCAAAACCGGAAGACCTTATG
EMPV1_00499	TAGGTGAACATGAAGGACATGCCATTAATGCTGGGACTCTTGAGTACCTGCTAATATTGC
EMPV1_00502	ATAGGCATGACATCAGTGATGAGAGGGAGAGCAAGACCATCATGCTTCGCGGCCTTCCTA
EMPV1_00505	TAATGTGGATAGAGGCTGAACCATGTTCTTTGCTCCCAGGTCCATTACTGCGTGGGAAGG
EMPV1_00506	TCCCTAAGAGAATCATTGCCTATGCCTGCTCCTGCCCACTAATCTACATACGGCACCTTC
EMPV1_00507	TGCAAGTGGAGCAAGATACGGACGTTTCCGGAGAGATTACGCTCCTGCCCTCCCAGGACA
EMPV1_00509	TCTTAGCTGTGTCTCTCTCTTCGTATCTTGAGGTGGTGGTTTGCCCTGTGTCCTCAGTTC
EMPV1_00511	AAGAGAGATGGGGTGTGGGCTGTGGGTGGGGTTAAGCAGCTGTGAAACATGGAAGTTCTT
EMPV1_00512	CCAGGCTGAGGTAGTAGTTTGTACAGTTTGAGGGTCTATGATACCACCCGGTACAGGAGA
EMPV1_00513	GTAGTTAAGGGGTTTGGGTTTGCTCGGGTTTATATTTGTTTGGTCATTTGTTTTTGCTTA
EMPV1_00514	CCGCTGAGCTTCGACAGGAACTCTTTGGATGATATATTCTAGAACTTAGTGATTTAGAGG
EMPV1_00518	TTCGCCAGGTCGCCGGCCACCCCCACATCATCACTCTCATCGATTCCTACGAGTCTTCTA
EMPV1_00520	AGAGTCACAAACCCGCACGCGGCGCTCACTGACAAGGAGAAGGCTCCGCGCCTGCGCACT
EMPV1_00521	TGTGGCCATGGGCTCAGGGGGCAGCTATGGAACTGAGGATGATGCAGAGGAGAGTGACAA
EMPV1_00522	TGGACCACTTTCTGTGTGAGATGCCTGCTCTTATTGCCATGTCCTGTGAAGATACCACGC
EMPV1_00525	TCAAGGGTCTAAAGTACATCCACTCCGCTGGGGTGGTCCACAGGGACCTGAAGCCAGGCA
EMPV1_00526	GGGTATGATAAAAGTTAATGTTTTAGTGGACACTTCATTTCTTGGAATATTGAAGAAGTG
EMPV1_00528	TGAGCTCTGGGCTTCATCTTCCTATTCACCGTAGGAGGTCTAACGGGCATTGTACTAGCT
EMPV1_00533	GAGTACATTTGGAGTTCCTGCTATGGTGCTATGGGATCGGTGGGTCACAGGTTCAAGTTC
EMPV1_00534	CCGAAGACGACTGGGAGAGGGTTTATCCTTGTCTGTCCCTAAAGAGTGACCAAATGATGG
EMPV1_00536	CGGCTCTTTCCTCTAATTCAAGCCATGCACCCTACTCTTGCTGGTAAAATCACTGGCATG
EMPV1_00537	TTGCTGTGATCCGGCCAGCGTGAACCACACTTGGACCCCAGCAGGCGTTCCAGGCGAGCT
EMPV1_00539	ACACCAGGTCCTTCACCTCCTGGTGGGAGGATCGCTGTATGTCTGTGTGTATGTGTACAA
EMPV1_00540	GACTGCTCCCCGATGCCAAGAAGGCCCGGCTCCTGCACGGCACTCTCATCATGAAGGACA
EMPV1_00541	GTGACCTTTGGACCGAGTGGTTGCTGGGCAAGAGGGACATGGATTAAAGAACCTTCCTGT

EMPV1_00545	CCCAGAATGTGTATTTTGTTCCTGGTCACTGCATGGATAATTGGGTTGATCCACTCTTTG
EMPV1_00546	CCTCAGTTCTCTGTGTGTGTTCTGGGGGACCAGCAGCAATGTGATGAAGCCAATCCTATG
EMPV1_00547	ATCTTCCCCCAGCGCAACTCGGCCTCCGCGGGTGCTCCCTTCTGTCTTGTCTTGTGTTGA
EMPV1_00550	GCTGGTGCATTTGTTTTAGATCCAATGTGTGGACTTGGAACAATACTTTTGGAGGCTGCA
EMPV1_00551	TGAAGCAGTTTGAGCGTCTGGAGCAGGAGGTGTCCCGGCCCATAGACCACGACCTGGCCA
EMPV1_00552	GTCTCCGACTACTTCTGAGCAGAGGATTGCTAGGCTGGATGTTTCCAGTGTTGCTACAGA
EMPV1_00553	AACAAAAGCTACGGCTTGAAAGTAAAGCAACTTTTCCCCCAGCGCAGGACGGCACTTCGC
EMPV1_00554	CTCGCCAGTAAACTACCTGAACTTTAAAACCTTGTCTCAGAGTCAGCTTCTGGAGGATCC
EMPV1_00556	TGACCTGGATGAAGGCTAACATCTGCAGGAAATAGAGAAAAGGGCCCAACGAAATAGGAG
EMPV1_00557	CAGAGCGGGCAGCCTTGTGTGTTACCTGGTGTTTGGATCAGAGCATCATTTCCATGTGGA
EMPV1_00558	TGGAGTCACTTCCACCCCTTAGGAGCCCTGGAGAAAAGGTTTTGTGAGATCTAAACCATT
EMPV1_00559	TCTTCACTGTCTTTTCCAGAAGCTCCTCACACTTGTCTGCACCCCACACGGGACTGTTAC
EMPV1_00563	TCTCAATGGAGTAAGCCCTGCAGTGTTCATAGAACCCCCCTAGTGTGATGTGGGCTGCAG
EMPV1_00564	GCATCCTACCAACAGAGATGTGACAGATATACAACTAGACTCGTGATGGAAGCTCTGGTC
EMPV1_00566	CTGTCACTGAGGTACTGAATGTGTCTTGTGGGGGCTTTTATCTTCCAGAGTCACAGAGGC
EMPV1_00567	AAAGGTGTGTTCCAGCTTTCCAACGAGGACTACTTCATTGAGCCCCTAGATGGTGTCCCA
EMPV1_00568	GATTGTGAGGATCAAATAAGCAGTCGTGTGTTCGCCACACAGAGTAGTGCCTGGTACACA
EMPV1_00570	TCTTCAGCGCGGGCCTGTGATACTCGTGGGGCCAGGTTCCCCAGCCAGGTTAGCTGTGTA
EMPV1_00571	GGCAGGTTACTTGTGATGTGGACTTGCCTTGGAAAACACAAGACGGCATACTGAAATGTC
EMPV1_00576	GAGTAATGCTGACCTTGTGAAATGAATTTGGATATTTTCCCACATCTATTTTTGGAAGAG
EMPV1_00578	TGGTCGAAGTGACGCGGCGGCTCACTCGTGGCAACAATCAGCAGGAGCAGGAGACAGCAA
EMPV1_00579	GGGGGAGGGTAAGACATGGGGCCTTTGAAAATAAACATTCCTGGCTTTCACTGTGGCTCT
EMPV1_00582	CCATGGAAACAATTGACTCTCAGCAGGATGGAAGTATACCAGATTCTGTGGCAGAGAGCG
EMPV1_00584	GAAAAATGGGTTGGAAGGAAGGACAAGGAATTGGACCTCGAGTAAAGAGGAGACCACGCC
EMPV1_00585	CCCACTCTGTGATGGATCGCACATAAAACACAATGAAGAAACTGGCGACAACGTGGGACC
EMPV1_00586	AGAACCATTAAGACTGCAAAGGACCGAAGTGCCTGATAACGTTGTGTGTCTGCTCTTTGC
EMPV1_00587	CTGAAAGTGAGGCCGAAGATAACCTTGATGACTTAGAAAACCACCAGCGCCAAAACGCCC
EMPV1_00588	GTGGGCATATGTGGTCGCACTGGAAGTGGGAAGTCATCGTTATCACTTGCTTTCTTCAGA
EMPV1_00590	CTTTTGTTAAGCACCTGTAGAGTAAGTTCTGAAGGTTTGAGGAGTGGGGCTGCTGGGAGG
EMPV1_00591	TCTCCTACGTCAGCCGGCACTCGTAGTCGACCTCCACCTCTGCGCGCAGTGATAATCAGT
EMPV1_00592	TTCTATCTCCTGAATGCTGCCTCTCTTCTCCCCGTACTGGCTCTGGAATTAAGGCATGGG
EMPV1_00593	GTCTTCCCTGGCCTCTCAGCCCTCTTCCCTTTGTGATGGGGAGTCAGCTGAGGGTTTTAT
EMPV1_00594	CTCTGCCATGAGGATTACATTCTAGGAGTTCCTGTTGTGGCTCAGTGGGTTACGAACCTG
EMPV1_00595	GGACCTAGGCCCCAACTAATATTTTGAGACTGGCATATCCTGAAATCATTCCTTATCTCA

EMPV1_00596	CGCTGTACTCATAACATCCTGTTCTTCTGTGCTGCCTGTGATGTGGTAGATAGCGCTTCC
EMPV1_00600	ACTGCTGTTGTTTTGGGGGCCCACCAGGCCTTTGCTCCATACAACAAGCCCTCGCTCTCT
EMPV1_00602	ATGGCACAGGGTCCAGCTGTCGGCTGTAATACCCGATGGGTCGATGATGGTCCCTGTGTT
EMPV1_00605	GTCCCGAGTGGAGGTGCTCTTAGCGGTGTTGTTTCTCGAGCAGCGGCAGTTCTCACTACA
EMPV1_00607	CTACTTCTCAGGCTTCTGTGTTCAAATCATCCTTCAGCCTCTGGACTTGCCCTAACTGGC
EMPV1_00608	GAACGAGGGGGAATAATGTCCCTCACGGAGGTGTACTGTTTAGTAAACCGAGCTCGAGGA
EMPV1_00609	TGTAGAATGAATGGTAAATGCAGCGGTTTGGATTCAGGCACAGCCCCCGCCTGTTGGCAG
EMPV1_00611	AATTGTGGATGACAGAGGCGTCCCTGTGCAGGACGACTGGTCGACAGCGCCTGACGATGA
EMPV1_00613	GAGGAAGCCGTGCAGAGCGGGGCCACCTTCATCACCCACCTGTTCAACGCCATGCTGCCT
EMPV1_00616	GCGGCTTCAGGCGCGTCTATAGCAGTCAGCACTATGGCCGAAGACATCCAGACCAAAATT
EMPV1_00618	TATTCTCAGGGATACGGAAAGACCGGAGAGGAGGGAGAATGTTGAAGCACAAGCGCCAGA
EMPV1_00620	TATGGCATTGCAGCAGCATCATCTTCTCTCTGATGTCACCGTTCGGGGATTTGTTGCTGG
EMPV1_00621	ACTTGTGCCAAGTGCTTCTTGTCCCGAACGGAACTGCAGCTGCACGAGGCTTTCAAGCAT
EMPV1_00622	GGTGTCGAGCTCTCTCTGGAATATTACCCTCAAACATCTGGAGAGCCTGAGGTGCTACAT
EMPV1_00625	TGAGCCTCCTGGCTCTTGGCTGGATCTAACATGGGCAGATATTCTGAGGCTTGTCTGGTA
EMPV1_00626	ATCGGGCCAGAAGGACCGGTGATGCCCTGGAAGAAGCAGAAACGACCACCGTTTGGACCA
EMPV1_00628	TGTTGTCACTCTCTCGTTACGCTCGAGATAGAGAAATGTATATAGGCTCGGGAGCGGCTG
EMPV1_00629	AAATTGTTTTACTTGTCGAGCAACCTGGCCTGCGTCTTTGGGCTGAACTTGATCTGGCCG
EMPV1_00632	AAGGAGATATTAAACAGGGCCCCAAACGACAAAGCGTTATACTGAGAGCTGCCCAGAAAC
EMPV1_00633	GGCATCGTCAAAGCGTTGTGGTGTCTTTTTTACTGCTGCTTGCTGTGCTTATAGCTACGT
EMPV1_00635	CCTGCCTGTCCTCCGTGGTCGCGGGCTACGGCTCCCCGGGGCAGAAGGGCGTGGTCATGG
EMPV1_00638	AATATTGATGATTAAAGATGAAATATGATGAAAAAGATTAGGAACATCCCAAATTAAAAC
EMPV1_00643	TCCCGGAGCCGTGAGGGTCACAGGCCCGGAAGGGTCCATCCTGTCAGCCGTGGATGAGCT
EMPV1_00644	GCAGATTCTGATTCAGTGAGTCTGAGATTTTGCATTTCTAACAAGCTCCCAGGTAGTGCC
EMPV1_00647	AGCTGTTTCAGCTGCTATTCACTCTGGAGTTCAGGGCATGCGTCTCACATTACCTGTCTT
EMPV1_00650	GGAGCTGCTCAGGGAGTGTTAGGCAGAACTGTTTGGACTACATTGTTTTCTCTTAGATTA
EMPV1_00651	CTATCATGGGGCTTCCGTGGCCGCACTGGGCACTCAACCAGACTCTAGCTCTGCCATCTA
EMPV1_00653	AAGTTGTCTCAATGGGAACAGGAACCAAATGCATAGGCCAGTCCAAAATGAGGAAGAGCG
EMPV1_00655	CTTCTGAATAACTGAGGAGATTAAAGCATTCAACTGTAGCTCATGAATTACTCTGAAAGC
EMPV1_00658	AAAGTTCTTCCCCAAGCAAAGGCCCTGCTGTGAGACGATGAAGCACATGCTTTCGGCTTT
EMPV1_00660	TCTTAGTCGACATTTTTATAGTATCCAAAGAGGTGACATTGTGATTGCAAAAAGCCCGAG
EMPV1_00664	GGGAGTAAAGGCAGCTCTTTTATCACTCACACTATTGTAGCTCTGATCATCCCACAGTGA
EMPV1_00669	CTGTTCCATAAAGAAGTTTTGCGACAGAGTATCAGTCACAAGTATTTGATGCGAGGTCGC
EMPV1_00672	CTCTTGTCACTGCTATCTGAGTGATAACAGCAATGCTGTGGAAAATCTCAGTGTATTTCC

EMPV1_00674	CATGCGGATAGAGGGCGGTGTCGCTGGTTTTGAGGAGTATATGAACCTCGTACTAGATGA
EMPV1_00677	GGATGCACCCCTTCTCATTTCCTCAGCAGAGCACTTTAGCACATAGACAGGCAAGAGTGC
EMPV1_00679	CGTCTGGTTTAACTGACAGACGGAGACGGCGGGAGAAAACAGGGCAAAGTTAAGAGTGCT
EMPV1_00681	TACGAAATACCAGTGTCTGCTCCTCCTGGGAAGAAAACCCAGACTGCAGGGTGAGGGAAA
EMPV1_00682	TTCCCTTGGATGGCAGGAGGTCTGCGGAGCATCAACAAACCCTTCTTTTGGTTCAGAATC
EMPV1_00684	CAGATCAGTACTTTTGGCAGTGAGCACGATCGGTGAAGCCTGAAGTTAGAAGGGACCATC
EMPV1_00690	CACAGTGGAGAGTTGAGCTTAGGGAGATGTCTAGGCAAATAGTTGATAGACTGCAGTAAG
EMPV1_00692	TCCAGGAACAAGCAGGGGGGAAAGTCAAGGTGAGCTTCCTTCTTCTGCTGTCTTAAACTC
EMPV1_00693	AGTTACAAACAGGTGGACTGCAGGGTCGTCTTACAAAATGACAAGAATGAAATCTATTGG
EMPV1_00695	TTCGTCAGTTCTCCAGGGCAGCTATGACCACACTTGAACCAGCAAAGTCTTCCCAAGGGT
EMPV1_00697	GTAGAGAATCGCCACAAAGCTCTCTCCACCTGCAGTTCTCACATCACCGTGGTAGTCCTT
EMPV1_00698	GGTCATTTCATCTGAGCATTGATGTTTTCATGATACCAGTGTCCACAGAAGTGGCGGTGG
EMPV1_00701	CCCCTGGATAAAACAAGTGAAATGAATTAGTGATGGGTGCCAAGCTTGACATTTTGCATT
EMPV1_00705	CCTGCACAGATGCCAGGACAGGTACTCCAGCAACATTTAATCTGTCCAAATCCTGCATCC
EMPV1_00706	TTGGCAAGTCTGCATAGGTGTCACAAAATTACACACTGACAGCAGAGCCACCCCAAGTGC
EMPV1_00709	CCAAGAATGTGTATCTCCTTTTTAGTGGCTGCCTGGGTGACTGGCCTTATCCACTCCATG
EMPV1_00710	ACATTGGGTTACTGGATGAGGCTTCCTGTGCCTGAAGCTGACTGGTCTCTGGGAGGCCTT
EMPV1_00712	TTCAGCCACTCCATCCTCTTCGCTGTCCACCATGAGCAGCTCACTCTATGCTTCAAGTCA
EMPV1_00715	GCCTGCATGTGACCGCTGGGCTTGATGAGCATGAGAGAGATGACTCCTGGTTGAAGTAAA
EMPV1_00716	GTGGTGGTATGGAAAACATGGGTCGATTTGGATCTGGGATGAACATGGGCAGAATAAATG
EMPV1_00717	GGGTTGGGGGCTCAGTTGTTGGGCTGGTGGTAACAACGGCCATTTTCCAACTTACTTTTT
EMPV1_00719	GTGTAACCCTATAAAGACATCTGTTAAAACCATAAGGTCAGTCCTCAATCATTCACGGAC
EMPV1_00720	GGTAGCTGTTGCATAACTTCTCAAACGTTGAAATTTGCCTGAACACTGCTGGTATTCCTG
EMPV1_00721	GCCCAGAACCCAAGAATTTGGCTCAGTTATAAGACTATCTTTCACTACCCCATGGTTACC
EMPV1_00722	TGATGAAGACCCTGAAGGAAGTGTGGATCCAGAGGGACAAGGGTCCTAGATAATGTCTGC
EMPV1_00723	GGAAACTGAGTGAAGGAGTAGATCTTAAATAATAATGGCCTCATTCTTTTGGTGAAGTGC
EMPV1_00725	GAGGAGTTTCCATGTCTTGGGAAGGAGAATCCGAATGAAACCTCCAGATGCTGCAGAGTG
EMPV1_00726	CCCATTTTTTATACGACACCGATCAGCCTGCATTGGAGGGGGGCCAAGGAATTGCCATCAT
EMPV1_00727	AGGTTGAAGTTCCGACTAATACCTGCGAAGTCAACTAGTCTGGAGGGGGGAGTTTGCTGCA
EMPV1_00728	AACCCCAGGCTCCCCCTCCCGGTCTACGCCGCCGCAGCTCCGGAAACCGCTGAATTACAG
EMPV1_00732	ATGCTGGGTTCTTAACCTGCTACGCCACGAGAGGAACTCCCAACATCTCAAATGCTCAAC
EMPV1_00733	CCCCAGATCTTACACCCTGAACCATGGGGCACCACTACCCCTTTTTCAGATACTTCACTT
EMPV1_00734	AATGTGCCCCTCCAGGTGGCCAATGCCATCATCGGCCACTATGACTCCACTTACGGTGGT
EMPV1_00735	AGCTGCTACTCTGTCCTTCTCCTACTGTGGCTCCCGAGAAATACCTCAATTCTTCTGTGA

EMPV1_00737	CTCATGCTCAACAATATTTCTAGATGACAGCACAGTCAGCCAGCCTAATCTTAGAACCAC
EMPV1_00742	AGTTTGAGCGAATCCTGGGCTACATCCAGCTTGGCCAGAAGGAAGGGGCAAAACTTCTCT
EMPV1_00744	GCTGGTGGCCGGGTCTGCGCTGTGCTGGAGGGTGGCTACCACCTGGAGTCCCTCTCCCAG
EMPV1_00745	CAAAACAGAAACAGACCCACAGACCCAGAGAACAGGCTGGTGGTTGTCAAAGGGTAGGAG
EMPV1_00746	CATGTCTCTTGTAGTTTGAAGGCAGAGTAGCCATGGATGAAAAGGGGGATGGCTGAGGGC
EMPV1_00747	GGACTTGTGTATATACACATGCACATATTGCCCACCAGCTTGTTTATTGACAGTGGATGG
EMPV1_00748	ACCGGTGGACAAAGCAAACAAGACAGGAGTCTGATTAGGAAGCCTTAATTAATGAGGGGT
EMPV1_00749	GCTCATATGCTCTTGCTTTCCACCTGGATCTTGACTCTGTACCCACCTGAATTCTGAAAG
EMPV1_00753	TTCTGCTCCATCTCCTCACCACATCAGCCCCCGGAGAGTTCCAGCTCCTTCTTCAATAAT
EMPV1_00756	GTCTTTGGCAAAGTGAAAGAGGGCATGAATATTGTGGAAGCCATGGAGTGCTCTGAGTCC
EMPV1_00758	GAAAGTGCGTTTCCTCAGCTGTTCCAGCCACATTTCAAGGGCTCGGTCACCAAAACTGAT
EMPV1_00759	CTGCTTGGGCTTTGTGTGCTCAGAATCGGCTTGGGTCTAAGCTCCTCTGTCTACTTAGAA
EMPV1_00760	GTTGAGTCAGTTTGCTGGACTGCACCCGCACACACTAGGAGGGTCCTTTTTGGGGTCTCT
EMPV1_00762	GCTCTTTGCCTTGCTACAAAATTCAGTGCAAATCACTTGAACCTAGGTTTTCAGGTTCCC
EMPV1_00763	AAGGGGCAGCATGAGGAACTGCTGCGAGAGGTGAATAAGTACATAGTGGAAGACACTGAG
EMPV1_00764	TCAGAGAACAAGGCCTCCTGCTACCCAACATCAAGGGCATTTCCTTCGACCAGGCACAAA
EMPV1_00765	CTCTCTCTTCTGTGGCTTCTGAAAGTGATTATGCTATTCCCCCAGATGCGTACTCCACAG
EMPV1_00767	CCCTCAGTGACATCCCCCAAGCGCCTCATGACTCTCCTCCAGTTCCACCAATTCCAAACA
EMPV1_00772	TCAGTTACTTCCAGTTCTGGTGATTGTGATCATATCTGTGATTACTCAGCTGCTAGCTGC
EMPV1_00775	CAGTTTGGAAAGAGTTGTTGTTAGCGACTATCGGGGAACAATTCACAGATTGTGCCGCCG
EMPV1_00776	TCCAGGGGAGGCTTTCACAAAGAGCAGAGAAATCCTCGAAGGCTCAAAAGCTGGTCTCTT
EMPV1_00777	GCGACTAGTAAATAGTTCACACCTGGGTATCCGGCATATAACCGTAGAGTCTCTGCATGG
EMPV1_00778	TGCCACTCAGGCTTGGCTAGGGTTTAGGAACCTCCCTTCCTTGGGGTAACCAGGAAATGA
EMPV1_00780	GCTGTGGGAGACACACCTATAATGAAAACAAAGAAATGGGCTGTAGAGCGAACCCGAACC
EMPV1_00783	TCACCTCGATAAGCTCTTGGGCCAACTGAGGCAGCAAAGCTCTGAGGATACCCTGAAGTT
EMPV1_00786	CCGAGTCTGCCGTAACCACCGACCTTCTACTCGGCTAAGACAGTTTTGTATCATTTTGCT
EMPV1_00788	GCTCTTGGGGAGGTCAGCCAACTCTTCCTGATTATGGAGCTGATTTTCGAGTCTGTGGGT
EMPV1_00794	ACACTGCCCAGCATTATCAGTGCTCCTATCATCAAAGTCACCGGTGGTCTGAGCCCAGTG
EMPV1_00795	AGACCGACAGAAGAAACCGCCCTGTTACATCCTCTATCTGCGCACGTTGCAGTCCAAGTT
EMPV1_00801	AGGCCCCACCCCCACAATTTTTCTGATTTAGGTCCTGAGTGTCCTGTTCGTAGTAACTCC
EMPV1_00802	ATGGCCATGCTTCGGAATCTGTGCAATCTGCTGCGGGTTGGAATCAGCGACTTCCACCAC
EMPV1_00803	CTGAGAACAGCTGACACCCTCTTTGTGGGATTAGCATCCTGGTTTTACACCCCAACAGTG
EMPV1_00804	CCTGATGTGCTTTTGGTATCTAACCAGTGCCAACATCCCCAGTGAAACTATAAGTGGAGC
EMPV1_00807	CCTCTCTTTATATCAATCCACCTACCGGCCTCAGGTCAGATTTCCATACCAGCTGTGTGC

EMPV1_00808	GAAAAGGCAACGGCTTCTTCTCTTATTTTGCTGCCTCCGGTTTTCCCAGAGGACATCAGG
EMPV1_00810	GTAGGCCGCAGAGCCAAGACTCCATTGTGTAAAAACTGTCCTCGTTGTTTGTCGCCAACA
EMPV1_00812	CACTACCACCTGCCCCTACAGCACCGCTCTGGTCTTCTTCCTGCAGGGCAGTGGAGCCGA
EMPV1_00813	TGACCATCTCAGGGACCCCCGATGCCATCATCCAGTGCGTCAAGCAGATCTGTGTGGTCA
EMPV1_00815	TGGACTCTCCTTCCCAGCCCTGCCAAACCCCATACTGAACAAATCATTCTGGACACAGAG
EMPV1_00816	TGGGTCTCAGACAGCTCAGATATGGCGTTGCTATGGCTCTGGTGTATGCCGGCAGCTACA
EMPV1_00817	GTCAGAGATACAGCACGTTCAGGAATGAAATAGAAAGACAGTTAGGACCCAAATGTTTCT
EMPV1_00821	GGAAGCACATGGTATTCCTGGGGGGTGCCGTTCTAGCAGACATCATGAAGGACAAAGACA
EMPV1_00822	GCCTGGCCCCGCGCCCTACCCGGAGTCCCTCCCACCCCTGCGTGCGCCCCGCCTGGAGTT
EMPV1_00824	GCATTTTTGCCTGAGGTCTTCTTTAAATCTAAAATCTGTATAGAGACTTAAGAGGGCTGC
EMPV1_00825	ATGGTAGAAACCTGGGAAGTCACTGGGGCCAGCACATGCCAAGCCCTCCACTGTAAGTAA
EMPV1_00827	GGCATGCCCATGTGCCTGCAGCTCTGGCAGGCAAACTCCAGCCCCCGCCCCCTCCTCCTG
EMPV1_00831	GACGTAGTGAGAAGGTGGCTGTCCATAAGCCAGGTAAGAAAGTCCCCACCAGGAACCAAA
EMPV1_00833	TGAGCAAGTTCGTGGAGGAGCAGAACCTGAAGGACCTCAAGGTGTGGACCAGCCAGCTGA
EMPV1_00834	GTATCAAGGTTACATTGGTGCAGCCCTAGTTTTGGGGGGAGTAGATGTTACTGGACCTCA
EMPV1_00835	ATTCGCTGAGGAGGTTGGCATCTTCTTCACTGCCTCTGGCATGGATGAGATGGCAGTTGA
EMPV1_00837	AAGCTTCAAAGGAGGCTCAGGCTCTGGAGGAGGCTATAGTTCTGGAGGTGGCTACAGTTC
EMPV1_00839	GTCCTTGGTTGATATTGGTTACACCTCCACCACCATTCCCAAGATGATTGTGAACATCCA
EMPV1_00840	CGTTGCATAAGAATGATACAGCATGTAAATGGCATATATATGTGGGGTGCCATCTCTCTG
EMPV1_00841	GAAGAGGACATGTGACTGCATGCTGTCTAATTGTTGAAGCATTGGGATCGCCAGTCTCTA
EMPV1_00843	ACTACAAACCACAGACCCATTTAAGAGGAGGTCTGTAGGGTGCCACAAAGACAACAGAGG
EMPV1_00844	AGCCAGATCAAGTCCATCGAGAAGGGGGAGAAGGTCCTGCCTGCTTGTTACCGACAGGAG
EMPV1_00846	TTCCTGCACTTTCCCACCATGGAAAATGGGCCTGAGCTGATCCTCCGCTTGTATATCGAG
EMPV1_00848	GTCCTATGATACTACCTTCCATGACCTGGTTGTGGAAGTTGCCTTTGTTTGGGTGCACTG
EMPV1_00850	AACCTCGTTGCTGCGTGGTCCAAATGCAGATGTCAGAAATATCCAGAAGCTCATCGGCGC
EMPV1_00851	GATGATGGAGGAAAAATAAAGCATCTTACATCCTCAAGAGAATGTAGTTATTGTGGAAAG
EMPV1_00854	GGGTTAGAGCAGTTTCTGCCAAGTCAGTGAGGGCTCAGATGCCAGAATCAAGAACATAGA
EMPV1_00855	CTTCTCTGATCCTAGCTGAAAATCCTGTCGCGACCCTTCCTCATTACCTCCAGTTTACCA
EMPV1_00856	TTGTGTGGGCCCTGTTCTCATAAACTTTACAGACTAATAGGAATGCTAGACATTGAGGAC
EMPV1_00857	CACAGTCCCCACCCTTAACCCCTACTAGTTTATTCACATCCAGTACTCCTAATGGGTCCC
EMPV1_00859	AGAGACAGATTCGGCACTTGATTTGGGAAATATTCGAGCAGAACCTCTGAATTCTGTGGC
EMPV1_00861	CCTGCTTATCTTGTGAGTACATGGATAAGACATGCAGCTTTTCTGCTCTTGGCCCCTAAG
EMPV1_00863	ACATGCAGCTTATGGAAGTCCCCAGGCTAGGGTCGAATTGGAGCTGTAGCCGCTGGCCTA
EMPV1_00864	ATGTACGTCTCAGACTAACTACTTTCACAGCACGCTCTGTTTGCTCCGGTGGTAGAACAG

EMPV1_00865	ATTATTTGCCACATGTTGTAATGATCATGTTTTGAATTTATTTCAGTATACAGTATTTGA
EMPV1_00866	TTGTTTACAAAAGCTGCAGGGAGAGAACGTGCCTTGTGCGTTTAGTTAGCACAGCGGGCA
EMPV1_00867	CGCCGAGCACTTGGATTTGGCACCTTCGTTTCCAACATGGAAGAAACTTACATCGATTCC
EMPV1_00869	AATACCCAATTCAGCGGTCCAAGAAAGATTCCGAGGGTGTAGAGATGGGAGTCGCGGGTT
EMPV1_00871	TCCTAAATATGTTAAATGGTAAATGAGCTATGACAGTCATTATTCCCATCCAGCTTCTGC
EMPV1_00872	TTTCCGGGGGAGGGGGTTAACGTGACTCTTAAGGAACTGCCGAGTTTTGTGTCCTAGAAT
EMPV1_00873	ATTCCAGAAGGCGTGGTGGCGCGAAGAAGGAGAATGGAGCTCGGGGAGCAGAAGAGGAAG
EMPV1_00876	CCTCATTCACATACTCCTACCCATGCCCCTTGTTCCCCTTCTCAGAGGCATCCACACCAT
EMPV1_00877	AATCCCATAGCAGCAGAGCGCTCACTCAGAGTAACACAAATTGTCTTGGCTTCTTGTGGC
EMPV1_00878	CCCCCATGCAGCAGCTGAAATGTTTTTCCACCAGTTTTATTCTTAGTGGAGACGCAGCAA
EMPV1_00879	ACAGGTCAGAAGCAAAAATCTGGTTGCTATTCGGCTACCCTTGTGCCATGTCCAACGCTG
EMPV1_00880	TGACTCTGTTTCATACGATTGCACGTTTTCTGGAGGGACGCGGAAGACGGGGCTTGCTTT
EMPV1_00882	TTGAAAAGCTCCCACATGGTCTTGAGATAGAAGGCCAAGCACATATCCAAAGCTGGGCGC
EMPV1_00884	GTGGGCCTTCCCCTCGATGGGCAAAGAGCCAGCTTCCATTTCGAAATCTCTCGGAAAAAA
EMPV1_00885	CCCAGTCAAGTTGGGGAGTCAGTCACCTTAAACTGTCAGTGTGAAACATCTCAGAGCGCT
EMPV1_00886	ACGCAGCTCCAAAAGGAGAAGGGCTAAGGAGGGCAACCCCATCGATACCATTGACCTTAT
EMPV1_00888	TGTTGGAGAACTGCCAAGAGATTGGGAGCAAGCTGTGTGACAAGCACTGATGCAGCCTTG
EMPV1_00889	GGTTTCTTCTAACGTCATGCCCAGAGGTTCAAGGATCTTCAGAGGTGATGGGAATTGCCC
EMPV1_00890	TGATCCTCTCTCCCCCTTTTCCCTTTGTGAATGTTTCAGTTGTCCAGACAGTGCCCATCC
EMPV1_00892	TGAGCCCCAAAATCTGTGGACTTATGGTTGCCTCTTCCTGGGTCCTGGGTTCTACTGATG
EMPV1_00896	TGGCCCAAGCTGAACTTCTTAAGCGCCAGGAAGAACTAGAAAGAAAAGCAGCAGAATTAG
EMPV1_00898	TGGACCTGAACTGTAGGCAGGAGTTCCTCTCGGATGGGGACTGCGCCGACAACCTCAAGT
EMPV1_00901	GACACATTGTACCTGACCAACAAGATGAAGGACCCTCCCCCTGATAATTTGCCCATGTTC
EMPV1_00902	AGCCTTCTCTCGATTATGAGAGCTCTTATGCTGAGCAAGTGATGAATGCCAACGAAAAGC
EMPV1_00904	TGCCCACAGGGTCAGTGGGCTCATGATGGCCAACCACACCAGCATCTCCTCCCTCTTTGA
EMPV1_00906	GCCTGGGAAATGAAGCCCTATGCAGTCATGGCACTTGAAAATAACTATGAAGTTATATTC
EMPV1_00912	GGAAGTCGCAGGAAGCATCTCAGTGATCTTGCAAATCATTCTCTGTGTTCCCAGCTAGGG
EMPV1_00915	TACCAGCCTGGGAGGAGGGAGGACGGACCCGGCGGGTGGAATCTATCCCCTGATTTGTGT
EMPV1_00917	AAAAGATGTATCGTGTCCCCCATGAAAGTGAGTGGGTGTTTGTCAGGGTCCCGAGTGCCA
EMPV1_00920	GATCCTTCCAGCTGTGTTCTTTTCTCAGGATCATTTGGGCTGTGGAGGGCTCTTTGTTAC
EMPV1_00921	GTCCCCAGCCCCTACTCCCCAGGTCTGTGTTACTTGGTTTTTAAATGACTGGTTGGGCTA
EMPV1_00922	ACCAATCAGAAGGTGGCAGTGAGCCCGCAGAAGGAGTCTGTCCTGAGTGAAGAGATGGAT
EMPV1_00924	TCTCTGGAGTCCTTCCCATCACCTCGCTGAGAGCCTGTCCCAGCCACGGGGACATCTCTT
EMPV1_00925	AGATTCTGATTCTGTAGCCCTAGAGCCTGATATTATGCATTTCTAACAAGTTCCCAGGGG

EMPV1_00927	TGGGGAACAGAGTCCAGTTTAAGGAACACAAAGCAGCCTTGCTGGCTGTATACCCTAGAC
EMPV1_00929	GATGGCTTTTGTGTCTTACTGGTGGTTGGATCCTGGTTCTTCTCTTGTGTCCACGCACTG
EMPV1_00930	CGAAGTAACCTGGCTGATTGGATGCCAATCCTGTACTTGAGCTCTTGAAGGAGCGTCTTC
EMPV1_00931	TGTTGTCGGGTGGATCACGATGCAATTTTGATTAGTATCATAGGAGAAAAATTGCACGGT
EMPV1_00932	ATTGGGCGGTTTCTCTTCTAGTGCAGCTGGCATCTAACAAGCAGGACGCTGGTGTCTGCT
EMPV1_00934	AGTGAGGCAAGTACTCCTAGGGTCAGATTCGCTTTGCTTCCAGAAAGGGCCAAGGTGGAA
EMPV1_00937	CTCGTGTCCTCTCACGAGCACTCGCAAATCACACCTAGCTGCTGAACAACCATCGATAAA
EMPV1_00938	AGTCAATACTCCTAATGCTTCTACGTGTCCAGTGCCTGTTCCTAGACGTGCTTGGTTGGG
EMPV1_00940	CCAGGGCTCACGAAGGCCGTCACATCCAGAGCAGTCCCAGCTCCGAGGGGCCTGCTGTGT
EMPV1_00941	CATGAAGCACGTCACCGACCGCTACAACATGTACTACTCCTACGCGCCCACCAAACTCAA
EMPV1_00942	AGCCACAGGCAAGCCCCTCTCCCGCTGTGGGAAATGCCACCGGTTCATGAAGTACATCCA
EMPV1_00943	GCAAACCATCTGACATACGAGTAATTACAATCCCAGGAGAAGAAGAAAAAGAAAAATGAG
EMPV1_00944	GGGGGAATTTGGAAGAAAGCCTATGTATAGGGCCAGGAAAGATGTCTCCTCAGAAAGGGC
EMPV1_00945	AGCCGTTGCCGGGTGGGGACGTGGCCGGGTCTCCGGAAAGGCCCACCAGTCCCTCCGCCT
EMPV1_00949	CCACAGATTCATGTAGCTTCTTGCAGGTGGCTGAGAAAGTTTCAGATGTGCCAAACTCAA
EMPV1_00954	CAGTAGCCTTTCTGAGACCCTGAAGACAGACCTGGGCAAAGACTCGGGTGTGGAAGCCAA
EMPV1_00957	CCAAGGCTAGGCACAACTACTGGTCTCACATCCTGGTGGCATCTTGCAGTTTTTAAGAGT
EMPV1_00958	GACAAACTCAAGTGAAAGAATCTGCCTTTTCCAAGAATCTGCCCTTAGACCTTGTATATA
EMPV1_00959	GTGATGTGTGGCCTCTCTGAGCTTCATGTTTTCTTAAGTGAAAATAGTAGTTGCTACCCC
EMPV1_00960	CAAGGTTTCAGTGGGATATGGCTAAATATCCAATCAAGCAGTCTCTGAAAAATATTTCTG
EMPV1_00961	CGGTGTCTCTTGTACTTCCGGCCTCTATGTGTCTTGAACTCTGACCACATAGGTCGAATG
EMPV1_00962	TTTGGCGACAGGTCCTCTGTGAGCTCTGCTTCGCTGCCTCTGTGCGCTCTGCGTCGCTGC
EMPV1_00963	CTAAAACCGCTTACCTGTATATGACCTCGGGTTGGGCAGTAGTGCGAATCTGGCTGACAG
EMPV1_00967	CAGTAAATGTAAAGGTGATCTCTTAGAAGTTAAAGATGGTAACTTGAAAACACATCCTGC
EMPV1_00968	GAGAGGCTCTTTCACAAGCAACTTACTGCAGGAGACAATTGAACAGACCCCTAGGATAAG
EMPV1_00969	CAGGAGCAAGGCAGCAAAACGTTCCTGGACGCGGTTCTTACCACCCATGAGCGAGTGGTT
EMPV1_00970	CTATCCACCAAGAGGTTTTCCTCATTATCTGCCCCCAAGAGCTGGATTTTTCCCTCCACC
EMPV1_00971	GTTGAGCACTTCATCACCATGTCTTAAATCATAACAGAGTCAATAGTCTGTTTTGCCTGC
EMPV1_00974	TAAGTGACTCTGGCGAGAGCTCTGAGCAATTCCCTCTGTGCCCAGCCTCCCAGAGCAGAA
EMPV1_00975	AGAGCAGCCCACCCAGATCTGGCCCTGATGCTGTGTTTCTGTAATAACAGTGACATCGAT
EMPV1_00976	ACAAATACAGATGAATTAAACAGACACGGTGTGAACATGTCTTGTCGGGGGGCCAGGCGG
EMPV1_00977	GAAATTCACATAGCGAGCAGGAATCTTGGGGCAGCCACGACCTCTTTGCTTTTTGGGTCT
EMPV1_00978	TGCTGACCACCAACAGGAATTCACCCACTAAACCCCCTAGAAGTACCCCTACTAAACACC
EMPV1_00979	GACCAAGATGGGACACTACAGCACTTTATTGATGGTGGGGAACCTAGTAAGTCGAGCTGG

EMPV1_00981	CGGGGCCGGGAAGCGAGATCACAGATTCTCGCCTCCTGGAGACGCGGTTTTCCCGCTGAA
EMPV1_00983	TGAACCGGCAGACTTGTATCAGGCTGGTGTCGGTTTCATGGGTCATTGGATTTGTGCACT
EMPV1_00985	AGTCTCTTCCGCAAGTCATGTTGGGAAAGCTGCACGGCTGCGTGTCAATCAGTGAAGTTA
EMPV1_00986	AGGAGCCCTTCCCGGAATACGTGCGGCTGGTAACTGAGTTTTCAAAGAAAACGGGAGACT
EMPV1_00987	TGCTGCAGTGTGCTGTATTACTTGAAATTTGAGTGCTGTGGCAGTACAGGAAGGGGAGGG
EMPV1_00988	GTGGAGGTGTGTGAAAGGAAGAAAAGCAAGCCTTTTGGCAAACTAGAATCATCCCTGTCA
EMPV1_00989	TCTCTACTTCCCCCTAAGTCCCAGATGCTTCCGTTGACACTCATTATACTTTTAACTTCC
EMPV1_00990	TGGGAAGATGCCCACACACCTACTTGCTGCCCTGTGTGCAAGGCAATATCTCCAACAATG
EMPV1_00991	ACAACAGGAACTCCTGCAGTGAGCACTTCGGCTTTGCTCTGAGCTAGATAGGAGAGTGGG
EMPV1_00992	GATGTGTTCGGAGTGGTGTGAAAAGAACTTGTATTAACAACCTTATGTGTTTCACACCCT
EMPV1_00994	AAGCTCCGCAGCGTCAAGGAGCGCAAGCTGGAGAAGAAGAAGCAGAAGGAGCAGGAGCAA
EMPV1_00995	CCAAGCTTCAGTATGTGGGTGTTCCTGCTACCACAAGTTTCTCTTCTACCCAGAGCAAGG
EMPV1_00996	CCGACAATCGGAGCAACCTTGGCCTTTAGAGTGGTTTACAGCTTCAAAAACTGCTCTCAG
EMPV1_00998	ACGGCTCCAAGTCGGATTAATTTCTGCCGAGCCATGATGGGAACTCCAGGGTGTTTTGTT
EMPV1_00999	GACCCTTCCCTCTGGTGGTAGGTAACTCTGGGAACAGATTGGATACAGCTGCCTTAGCTG
EMPV1_01000	TGCCAGAGCTAAGGATATTGTGAAGCACTTGTTCGTAAGCGTAACCTTGGAATGTCTGGC
EMPV1_01002	CCTCAGCCACCGGAAACACTTCGCAGGTCACCGAAGACGCTCCTTGCTCCAGAAGTCTCG
EMPV1_01003	GTCCTATATGGCTGGACTTTCTGCTTCTCTGTCCCAAGTGTGTGCCATGCTTCAGCTCCA
EMPV1_01006	CTAGCTGTCAAACCATGTGTGGATTATGTAGGAGTGGAGATTGGAATGGAGAGGAGGAAC
EMPV1_01009	CAGTCATCACTCTGTCCTATGTCCAAATATTCGTCGCAGTGTTTCGTCTGCCCCAGAAGG
EMPV1_01011	GAAGGCTAGAGACCCTGCTTAGGAACATTGAAAACTCTGACAAGGCCATCAAGCTGCTGG
EMPV1_01013	TGGTCAGTTAGAGTGTGTTCGCTGGATGGTGAGTGAAACAGAAGCCATTGCTGAATTGAG
EMPV1_01014	GACGCTTCATCTGTTTCACAAATTCAGTCTCAAACTCAGTCACCGCAAAATATCCCCGAA
EMPV1_01015	TCTTGGTGTTCTTTATACTGTGACGCTTCCCGTCCTAGGGACTCTTGAGCCTACGGAGGA
EMPV1_01016	TTGTGACTGGTGGTCAGTGGGGCACTTTGAGCCATTGGGATATCTTAGGATGACACGCAC
EMPV1_01018	TGTCTAAGGTTTGTGATGCGCACTAGCTAAGACAATGTTTTCTGTACTGAGAACGTCCAG
EMPV1_01019	GTGATCTTCTGGGTGCAGGTGGATTTGATACCTCAATACAAGGCTGGGGACTGGAAGATG
EMPV1_01021	TGCCTGACGTCATCTTCGAGCTGCTGCGGGCCGGCAAAGCGGAGCCTTTGGAGAAGGTCT
EMPV1_01025	CCTGCTGAAAAGAATGGCCCTGAGATAAAGGAAGCTCTCGGGTCCTCTAGTCCCGATGTT
EMPV1_01026	GGATTGTGGTAGCCAGAAGCTTGGAGAACTAACAGGAATGAAGAGGAAGGGATGGATTCC
EMPV1_01027	GCTCCTGCTCTGGACAGTGGCAGGGACTAGGTACCCTGCGAGGCCCCTCAATTCAAACAA
EMPV1_01028	AGAACTCCCTTCGCGCCAGTCATTTCTCAGACATCATCTCATTGTGTCACATCACCACCC
EMPV1_01029	GTGGACTGCTCTCATTTACCTGTTTCCTCCTGCTGCTTGTCTCCTATACTGTCATCCTGG
EMPV1_01030	CCCTGAAGGGGTGGTGAAGGAGGACCCCAAGAGGAGATCAGCAAGGTTGTCAGCTAAACC

EMPV1_01031	GGAATGAACCATAAGGTTCTGGATGTGAGTTGGAGGCATCAGTATGAACCCATGTTTGGC
EMPV1_01032	GATATTGCTGAGAAGGAACTGGGCATTTCTCCCATCATGACAGGCAAAGAGATGGCCTCG
EMPV1_01033	CCAAGCGCCTGTCAGCAACCCTGCCGGACAAACTCTCCGTTTGCTATTTTACAAACTCAG
EMPV1_01034	GCTTTCAGCCAGAACATTAGCTTGATCCAACACTTGAGAACTCATTCTGGAGAGAAACCC
EMPV1_01035	CGCAGGTGATTTTTAAAGCCAAGTCAAAATATTCTCCAGAATTACTCAAATACCGGTTGC
EMPV1_01036	AGGGATTAAGAAAGAACTCGGTGTGCACGGCAGATCCCCGAAATTGGCGGGCTTGACTTT
EMPV1_01037	CAGAGACTTTTGGAATAACAATGAGAGCACAGTGCAGCAGAAATGGAGCTCCTACCCTCC
EMPV1_01038	AATGCAGCATTTTTGATAGGTGCTTATGCCGTAATCTATTTAAAGAAGACACCAGAAGAA
EMPV1_01040	GAGAAACTTGGCCTTGTAGGCAGCGAATGTATGGGTCCTGTTTTGTATCCATTCAGCCTG
EMPV1_01044	TGGACAAGCTGTATGCTCTCGGCCTAGTGCCCACGCGCGGGTCGCTGGAACTATGCGATT
EMPV1_01045	TGGGTAAAGGATACAGGAATGCTGAAAAGGATGCAGGCAGAGACGGAGTGTGGCGTATGC
EMPV1_01046	AACTTCACGTTGCCAGATGTTGGGGACTTCCTGGATGAGGTTCTGTTCATTGAGCTGCAG
EMPV1_01049	TCAGCAGGGCTATCCTTCTGGAGGTAATTATATTCTTGCCATGGGTTCTTGGCAGAGCTC
EMPV1_01051	GCTCTGTTGACACCAGCCAATCGTTCCTGCCTTTTGTCTAATTTGCAAGTCTCTGACACC
EMPV1_01053	CCCAGGTAGACGCCCTGAAGGACAAGGTGCGGAAACTGTACACCATCATGAACTCGTTCT
EMPV1_01056	GTGCTAGTGAATGATTTCTTTTTGGTGGCTTGTCTTGAGGATTTCATTGAAAATGCTCGT
EMPV1_01060	CACCCCAGGCGAGGCTAATAAGCATGCAACTTTATGAGGCAGAGGATGAATAGGAGTGGA
EMPV1_01062	ACTTCCTTGCAGGGGTCTCTGTCACCAGCGAGCTTCTGGATCTCATGAGCCTCCGTTACA
EMPV1_01064	ATCTGGTGTTGCTGTGATCTGTGGTGTAGTCTCAAGATGCGGCTTGGCTCCCGGGTTGCT
EMPV1_01065	TTCCAACGGGTCCGTCTACTTCGACACGGTGAAGTTTGCTAACAGCGAGGGACACTGCTA
EMPV1_01067	TATATAGAGGGGTGTATCCTCTCTGGTGGCTGTTACCTCTGCCTAAGGGCTAGTAGGCAG
EMPV1_01069	GGCTTTATTTCTCATCTTGGCTTTCACCTGGGCAGAGCCCATAATCCTTGAAACCTGACA
EMPV1_01072	ATGCTGAAGTGCATAGAGCTGGACGAGCGGCTGAAGGCCATGGACCAGGAGATCACTGTG
EMPV1_01075	GATCATCAACGCCATCAACATCAACCACGAGTTCGACCTGCTGGACGTGCGCTTCCACGA
EMPV1_01077	CGCTGGCTCTTGAACCGTAGCAAGGACCTGGGACTCCGGCTCTATGTGGAAACAGATGAT
EMPV1_01081	ATTTACCTCGAGAACTACGCGACCAGCATGGGCTCCCGGGAACAGCTTGCTATTGCAGAG
EMPV1_01082	CGCTTTAAGAAGAATCACGCCTAGTGGAGGCTTCCACCCGCCAATGTCGCCTACATACCT
EMPV1_01086	TCAAATGAGACGCCTTCCCTGACTACTGCCCCATTCCCAATCTAAAGCCCCATCATCCTG
EMPV1_01089	AGCTCGGGAGGAAGCGGCAGGATTTTCTGAAACGTCATCAGGGCAAAGTTGTGCGAAGCA
EMPV1_01090	AAATGACATGGACCTAAGACAAACAGATCACCCGGACAGGAATGGCTCAGTGTTGGGGTC
EMPV1_01091	CAAGTCACGCTGGGAGAAATATTTGCAAGACCCATGTGAGAAAGGACAGCCATCCAAACT
EMPV1_01093	GTAGTTTAGTCTTTCGTGTCCTTCTATATGAGCGGATACAAGCTAAGCGTGCTGCCAAGA
EMPV1_01094	CTGTCACCCACTGCACTACCTGGTCATCATGAACTCACACCTCCGTTGCAACTTAGTTTT
EMPV1_01097	ATGCTATGAGACTAAAAACCCACTCAGGACCCAATGCCACGCCAGAGGATAAACAGCTGG

EMPV1_01098	GAAGGCACTAGGCATTACATAACACCCTGAAAGCAGTGATTCCTTCTGATGCTGGTGAAG
EMPV1_01099	GATGCCACCAGAAACATCGGTCCATCTTCTTTCCTAGGGAGCTTGGCAATTTTATGCCCC
EMPV1_01100	AATTATCTCCCGTTGGGATGTTGCCTCTGCCCGGTTGGACCATGTACCTTTTCTTCAGTG
EMPV1_01102	GATGACTTCATGGAAGATACACTCACATAGGAAGTGGACTGCTAAGCCATTTATTGGAGC
EMPV1_01104	CGGTTTAACCATCTTCCTACGTTTCTATAAATTTGTGTATTATTACTTTAACATGCCCAC
EMPV1_01105	TTGTCTCTGGCTATGGCAAGGGCTATGTGGTGTGGCAGGAGGTATTTGACAATAAAGTCA
EMPV1_01110	GTATCTGCTCAGAACTGGGGGACAGAGTGGAATTTATGACTTGGGCCTCTGGGTTCTCTC
EMPV1_01113	CCATGGAAACTGGGCTTCCTTAATGATTTCAGCCCGATGCGCTAATGTTGGGTCCCCTCA
EMPV1_01117	CATATGTTTACTGGTTCGCCTCTGAGGTTGATAAATGAGCCATGTGTATAAAATAGGGTC
EMPV1_01118	TGAAATGATGAAAGTTGTTCAAGTTTATGCAGAAACACAAGAGATTAATTTGAAGGCTGA
EMPV1_01119	GGAGTGTTCCCTATCCAGTCATTCTTGCATGTAACGCAAGTTCCACTTGGAGCTCTAGCC
EMPV1_01121	TGCCACACCCACTTGCTTCGCCGCTTGGGATTTGATCTCCAGAATAAAAGTGCTGTGCGT
EMPV1_01122	CCTGCTTTCCAGTGTGGTTCCTGCCCAGCCCTGTTTGATCCCCAGTAAAGCCTTAATCAT
EMPV1_01123	ACAACCTATGAGGTGAGAGTTGTATTCTCCAGTTTGTGGGTGAGCGAGCCAAGGTGGGGA
EMPV1_01126	GATGTGCCATGACAGAAACTCCATGAAACTTTAATCTTAAAATCATAAGTAGACAGTGCC
EMPV1_01128	GGAGAAAAAGAAAAACCAGTGTTCTGAGGAAATAGATATGAAACAGCGAACCATTCAGCA
EMPV1_01131	TGTAAAAGGGTTGCCTCATCCAGAAATTGTTGGTGTAAAGGGGGGAGATTGGGGTCTCCC
EMPV1_01134	CCTCTTAAGGCTGTCAGCTGATTACTCTACAGAAACTTTGCAGACCAGAAGGGAGTGGCA
EMPV1_01135	TGCCAGCAACCACCTGGAGCTTCGTGTCTGAGCGGGAGACCATGGCCAACAACCTTGGAA
EMPV1_01137	AGGAGAAGAAAGCTTCAGCCTGACTGTTCCCAACCTAACGTGGCTGTAACCAGCTCAACT
EMPV1_01138	TCATTAACCTGGCCAAGAAGAGGACCAACATCATTCCCGTCATCGAAGATGCTCGGCACC
EMPV1_01140	AGTATGTTATCTCTATGCCAGACCTGGTTCGCCTGTTGGAGCAAAAGAAAGATCCCTGGG
EMPV1_01141	TAGCTGCCACCACTATAAATGGCAAGATTTTCATCCTTCATAACCTCCTTGTAAGATAGC
EMPV1_01142	GCCACCCCTTCTAGTGGTGCAGACCTTGGCCCACAATTCCACAGCCACCCTGTCTGTCAT
EMPV1_01143	TGTCCCCGAGGGCTTCACCTGTGACTGCTTCGACGGCTACCGCCTGGACATGACCCACAT
EMPV1_01144	TGTTGGCCAGAAAGAGCAGAGTAAGAGAACAGTGAACATTCGGACTCGAGATAATCATCG
EMPV1_01146	GCCAACTTCTACTGTGACATTGCCCCTTTGCTCAAGCTGTCCTGTTCCGACATCCACTTC
EMPV1_01148	TTTTACGTCTGTCCACTCCTGGCACCGGCCAAGCTGTGGCGTTTCCACGTGATTTCAATT
EMPV1_01149	GATTGATGGTATTGTTCCACTACAGCAAAGTGACCCCATCGTACCTGTATACACAGTCCC
EMPV1_01152	ACGAAGTGGGCAGGTGCAAGGAGAGCGGCAGGGTGCACGTGAGGGTGGACCTCAAGTACT
EMPV1_01154	CAGTGGAAGATCCAGAACCCATGGTGTGTCACCCTGGAGGAGTGCTTCAGGAAAGTCCAG
EMPV1_01155	AGTGGGATGGCCTCCCAGGAGTGCTCGACCACTTCCAAGGCTGAGACAGATGATGAGGAA
EMPV1_01156	GCCATTCTCATCCCAGTGGCAGTGGCAGTCATTGTTGGAGTGGTAACAAGTATCCTTTGC
EMPV1_01157	CTCCCTCTTGGCTTGACAATGAATAGAGCTAGTTATGGGGGTTGCCAAAGGTAAGGGGTG

EMPV1_01158	GGGATGGGTCAGGAAACCCAAAGCCAACAGGATCACCAGAGCTATTAACATTCCACACAG
EMPV1_01160	TGAGAGATTAGGAGTTCCTGTTGTTGCCTAGCGGTAGTGACCCTGACTGGTATCCATGAG
EMPV1_01163	GCAGAACTAGTAGACTTCCTCTTGGCCCTGGTGATGATTCTACTCCCCCTATTGGTTGTG
EMPV1_01164	CAACCACTGCAAATCAGTCTCTGTTTCCATGCCTGACACTGTCCTCCCCAAAATCCAGGT
EMPV1_01166	TCACAGGCTTGAGAAACAGCGCCTGCCCCTAGAGTTAATGCTCCCTTATCTGGTTACTGC
EMPV1_01167	GCACAGCACCAGGAATACGCTAATACAAAACAAATAATAGACTTTATATGAAGATGACAG
EMPV1_01169	TCATTGTCAGCAAGTTCATGTGTGTAAGTTGCCATCCAATGTTATTGACTGATATAAGAC
EMPV1_01170	CAGCAAAGGCTAGATGGTCTGTTGCCAAACCAAAATCACCTATTTTTACGTGGTCATCAG
EMPV1_01171	CCTAGGTTTTCCCCAGATCCTGATGAATCCAAGGAGTATGAATGGATACTGTGAGGAGAG
EMPV1_01172	TTCAGTGTGGAAAGACAGCTCTATGCACCCCTGGGGGCTGTCCACGTGCCCTGCCTGTGT
EMPV1_01173	TAAGTAACCTAGAGGTGATCTGAGGTAGGCGGGAGGATTGCGGGGGTCAAATGGGAATAC
EMPV1_01176	GCTTTTTGTACAGATGCTGCACAAGCTTGCCAAGGAACCATATGCTGCCACTGGAAGAAT
EMPV1_01177	TTAGCTCCCAAGAGCTGCAGTCGGTCAGCACCTTCGTTTCCACGGTGCCAGGGTTGGCGA
EMPV1_01178	GGTGAGAATGATTGAGGACTGCCATGCTAGCCCTGTGCGGGTTAGTCAGGATGAATCACT
EMPV1_01183	AGGCTCCTGAATGTTAGCTGAATTAGATCCCTGTCCCTCCGGCTGATGATTTAAGATTAG
EMPV1_01188	ACAGTCACAGCCCCAACCAGCCTGAGCTACAACGGCGGCCTCACCCCGGCGGCTGTCATA
EMPV1_01189	TGTGGTGGGCCATCGTTGGACTAACGGACCAGTGGGTGCAAGACAAAATCACCCAGATGA
EMPV1_01191	AGGTGGAAGCTGCCTTTGCCTTGGAGGAGAGCCCCGCTCCATCATTTGCAAGGCACCACC
EMPV1_01192	GCTACCAGGCCAGCCAGGGCAGCGACGATGACTGGGACGACGAGTGGGACGACAGCTCCA
EMPV1_01193	GACCCAACAAGAACAATATCTGCAGGGAAAGTGAATCTTGGAGCCTTTAGGACATACCCA
EMPV1_01194	TGTCAGGAGTGGGGGGGTCTGTGCAGCCAGGTAATAACCCAGCTGCTTTCTGCTCCTCTT
EMPV1_01195	CTCTAGAGGCCTATACCATTCAAGGACAGTATGCCATTCCACAGCCAGATTTGACCAAGC
EMPV1_01196	CTGCCAGAAAATTTCAAAGACAGTAGAATGAAAAGGAAGGGTTTGGTGAAAAGGATTCAG
EMPV1_01199	TCTTCCCTACTATGATGGTGTGTATGATGGCTTGGCGACCTATTCAGGCACTTATGGCCA
EMPV1_01200	TATTTGCTCTATGTTTGGGAGTGATGAACAATGGATAGGGAATCTGTCTCGGGGATGCCC
EMPV1_01201	GCCATCCCCACCACCCAACAACTGAATGTGGACCGTGTCTTTGCCTTGTATACCGATGAA
EMPV1_01202	CCTCTTTCCGCTTGGGGGTCCTTCGCAGGGAGGTCCGCAGGCCCATGAATAGATCTGAGA
EMPV1_01206	AACCTGAGGTGGCAGATTGGTCCGAAGGTGTGCAGGTGCCTTCTGTGCCCATCCAGCAAT
EMPV1_01207	CAGAGTTTGGATCTACTGCATAATGGCTGGCCATGGTTTAGGGAAGAGAATGTTGACAGG
EMPV1_01208	GGCCACGCTACACCTACTTTAAGGTGCCCACAGAGCGCGACTTGGTCTACTATGAGGCCT
EMPV1_01209	AAGCCCACCCCTTGTTTTTGCCTGGTCACTTGATAGGTAATGCTGCACACATGACGAACG
EMPV1_01217	CCCCGAAACCAGTGTACCCATTGAGAAGTCAGAGCCAGGATACCCAGAAACCAGACACAA
EMPV1_01218	TGAGCCCAAGATTGTGCATCTTCCTTTTAGTGGCTGCCTGGATAATTGGCCTTGTCCACT
EMPV1_01222	TAGGGGGACCTTTTCTTCCCCTTGCTACATTCCACGAGAACAAACACGAGAAGGTGGCAG

EMPV1_01225	GCTCCACATATGCGCCGCTCTCGTCTTTCTCGTTGATGAGGACGGAGTTGTAATAGTCAA
EMPV1_01226	TCAGGAATGGGATTGGAAGGTGAAATAGAATTGTGCCAGATGAGTTACCCCAGACGGGCC
EMPV1_01229	CCCCATTCCTATTGTTTTAGACTCAGGGAGCTAATCTCTTGATAGATCCTGACTTCAGAG
EMPV1_01230	AGCCTAAGCAGCAAGTAGAAAAATTAAATCTCAACCAAAGCCACCCATAGAGCCCTCCTC
EMPV1_01231	TGTAGGGCCCACTGATGATAGAAGAGTGAACAAGCCAAGTCACCCCCAGTGTGGTCATGA
EMPV1_01232	GAATTTCCACGGGGCTTTGAGTCGTCACAATGCTAATAAGCTCGTTTCCCTGCTGGAAAC
EMPV1_01233	CAGGCTAAGCCATGGGGGTGGGCCCCATCCAGCCTGATCGGCTATACTCGAGAAGCACTT
EMPV1_01234	CAGGAGGTGTCAGAAAAGTTGCCAAAGGGATAACTGGCTTGAGGCGGCCAAGCGTTCATA
EMPV1_01237	CTTCGGTCTTTCCCTTCAGGAATTTGGAGCCTCGGTGTCAACCTTTCTTTGAAACATACG
EMPV1_01238	TCCAAGTAAGGAAATACTCAAGGACAGGCTTCTTGTGGCTCTGCATTGTGGCAGCTATGG
EMPV1_01239	CCTCACAAAATCAAATACCTCGGAATACACCTTACCAAGGAGGTAAAGGACCTATATGCC
EMPV1_01240	CAAGTCACATTTGTTGAGTGTCTGATTGGCAGGCACATCCACCCCTCATGTAATCCATGC
EMPV1_01241	GGGATCAGAGACAAGTTCAAATTGATGCAAACAGAGAGGCACTCATGGTCTCTCCACCCC
EMPV1_01242	TGATGACAGCACACACATGCCACCCCGCTTTAGCATAGGTGCCCAAGCAGAGAAATTTGC
EMPV1_01243	ACATTCTCAGGTGTGTACCTTAGCTCTGCTGCTACCCACAGCTCGCACTCAAGTTCAGTC
EMPV1_01248	CTGGGCAAGGGTCCCAATGTGAAAAACTGGAATGCGTGGACGGCGCTGGAAACATACTTA
EMPV1_01250	GATCCGGATGCACGACTGTGACATCAAGGATGACGAGTTCCTGCACCTGCCAGCACATCT
EMPV1_01252	GTCGAGACAAAGATGTTCTGGTGAGTGAGGTACTTCTGAATCTTGTTACTTAAGGCACCG
EMPV1_01254	TCCTACAGCAGTGGTCACGGCCTTGGAGGTGGCTTCAGTTCTGGCAGTGGCAGAGCCATA
EMPV1_01255	AAAAGGAAGTCAAAGATTTCACGATGAAGGATGTGGAAGCGAAGATGCAGGCGGCCGAGG
EMPV1_01256	GAAGACGCTCCTTCAAGAGCTCAAGTACAGGATTGGCATCCAATCAGCCAGGTTACTTCG
EMPV1_01257	GCTGCCCTGAGCCCAGCAGATATCAACTATGATGAAACTTTGAGCACTCTCAGGTACTCC
EMPV1_01258	GCCTTTGGTGAATGGAGATTTAAGGGAGAGCACATTTCTGGTTGGGGCTGCAGAGTAACC
EMPV1_01259	GGCCCTGGATTAGAGCCTCGTCTTAGAGTTGTCCCACCACAAAGGACTTGACCTACGAAT
EMPV1_01260	GAAGACCCAGAAGGCGTGAAAAGATTTAGGGAGTTTTTAAAAAAAGAATTCAGTGAAGAG
EMPV1_01261	CTACATCGTGGAAGCCAGCACCATGCAGTCCATGGGCATATACACTTGCCTCAAGGCCTA
EMPV1_01264	GTGAGCAAACTGTGTGTAGGAATTCCAAAGGAGTATCTTACACCAGAATTGTCAAGTGAA
EMPV1_01267	GCTTGTGGTAGCTTCTTGGACAGTGGGCTTTCTGCATACAATGACCCAGTTAGCTTTTAC
EMPV1_01270	ACCTCTCACACAAGAGAGGCTCACTACCTGCTTATACACCCACAAATGAGGATCAGGGGT
EMPV1_01271	GCATCCTACAAGGAAGAAATAGTGCATCAGCTACCAAGTGATAAACCAGAAGATCTGGAG
EMPV1_01273	AGAAGAGTCCATTGGCCCATTTCCCAGGTGGGCCTGAAGGTGGCTGTGTTGCTGCAGATA
EMPV1_01275	GCTGCTACCACACAACTATTTTCTCACTTCTGCTTTACTGCTCAGTTCTTTCCTAATGCC
EMPV1_01277	GTCTGGAAGGGATCCTCTACCTGCTGCTGGCGCTGATGCTCTGCCATGCACTCTTCACCA
EMPV1_01278	AGGACTCTCCCGTGGATCTCTGGGAAGCGGTCAACGATGACCCTGCTGACATCGATGGTT

EMPV1_01282	AAACTTGCCAATTGAAGCTGTTTATCACAGATATATGGTACATTTGCTGGAGTTGGCAGT
EMPV1_01283	TTCAAAGGCTCTCTTTCTAGAGGTGCCACATAGTCGTTAATGCTTGGAATGGCAACGGGG
EMPV1_01284	GACTCTTCGCAGACGGTGCCGAGTTATCCAGACACAGCTCGCTGCCAGAACCAGCGCTGA
EMPV1_01285	GTGACCCTTGAACCATCTCAGCCTGCCAGCTCCTTGTCCAAAACATGAGATTCAAAGAGG
EMPV1_01286	CGGGAGAAAAAGTCAAAACTGAGACACTGCCCCTGAGACACTTCCAGCAAAGTTCAATGG
EMPV1_01287	AGGTGTTAAATTGGCCGAATATGCATGACACAAAAGAAATTCACCAGACAGATTAAGATC
EMPV1_01288	GCTCCAGAGATGCCTCCAAAAGACTTGGTTCATGGTATTTAGGCAGCGGAAGCCCTATGA
EMPV1_01290	TTCAGTATCCAAAGAACAAAGTAGGGCAGTGGTACCAGGAGGTACTCAGCAGAGATGGAC
EMPV1_01291	GATGTGATTCGCAAAATGAAGACAGACCAGAATGGTCAGTTAGAGTGTGTTCGCTGGATG
EMPV1_01292	GGCCAGACTAAATTGTCCATGAACTTTCTGCAGGTGAGGTTCATGCAAAGGAGACTTGAC
EMPV1_01299	TGGTGTCTGTCCTCGGTCCCAGAGCTGGATGTTCTGAGCTCCCTATGAGCCCGGGAGAGG
EMPV1_01302	GCTGGTAGATCGGGATAATTCCAAATTTGAGGAGTGGTGTATTGAAATGGCCGAGATGCG
EMPV1_01303	CTCAGCAACATTCTTGTGGTACAGCTTGGCTCTCCTTACTCCATTTTTCTGGGTCCTAGC
EMPV1_01304	AGGATGGCAGAATGAAGCCGAGCGGAGCAGCCACGAGAAAGATGTAAGAGCCATGGTGGA
EMPV1_01306	CACACTGCTCACCTTCTTCGACCTGGCCTGGCGGCTGCGCATGAACTTCCCCTACGTCTA
EMPV1_01307	AGAAGTCACTATGGGGTTTGTTCGTTTGGTAGCATCTGGAAGGGGAGGTTTCCTCTGCAG
EMPV1_01311	GCATGCCACAGGAAAGCTATGCACAAAAAGGCACATTTAAACACACCTATTATCCCTGTG
EMPV1_01314	ATTTACCCGACGACATTTCGGATTCTAGCCGCATCTTGGCATGGAGCTGCGGGCTGGGTT
EMPV1_01315	GACTTCAGTGCCCTTACTGTAGAATTTAAAAGCCTTACTGTTGATTGCCCATGGTGGACT
EMPV1_01317	AGAGCGTGGCCCCTGGCTCCACGGGGCATACTTTGGTTTTGGTTTTTTCTTATTACTCAA
EMPV1_01318	AGGACTCATGAAGTAGCATGACAGTCAAGGGCATGGGAGGCAGAGATAGAGCTACTTGCT
EMPV1_01319	GAAGTTGAGCTCCAGTCCTTCTATTCCGCCATCTTGATCCAGTCTCCAAAGATGATACAC
EMPV1_01320	GCATTTGGCTGCTTCTCTCGTGACCATGATTATCTTGCCTTATCCAGGAATAATTGCCCC
EMPV1_01321	GAGTAGGAGGAAGATTCCAACACACAGCAAAACCCAGAAGCCTGTGGTCCTATCAGTACC
EMPV1_01322	TGACCATCATGAGCCCAAAATTGTGCATCTTCCTTTTAGTGGCTGCCTGGATAATTGGCC
EMPV1_01323	CTGCTCGCCCTCATCATCCTGTGTGTCATCTACTGCAAGCGGCAGTTCATGGAGAAAAAA
EMPV1_01324	AATTGGAGTCCAAGAGCGCTCTGGCAGCCGCCTCCTTGGGGCAGTGGAAGCTGCAAATTC
EMPV1_01328	ACTGTGGCACTTGGGCACCTCTGGATAGAAGGAGGCTGGGAACAGGAGTGTTTAGGTTTT
EMPV1_01329	CGTCCATACAATCTGTCACATGATCTGGTCGAAACCTCTGTGAATCTCATCGAAGGTGAC
EMPV1_01330	GAGCTCACGGAACTGCAACGGATTTTAAACACAACCTTTTACCGCAGGTCTGAGGAGCCG
EMPV1_01331	TCTGTAAATTTGAGTCTAACACAGGTTCCACCGGGCTAAAGCCAAAGTCTCGAGGCAGGG
EMPV1_01332	CCCGCACCTTGTTGCTAAAGACCCAAAGCAAGATGTCTGCCTCCAGCAAGAGTTCACCTA
EMPV1_01333	GGAAGCAGAGGCTTTAGCAAAACTGCAAAAGGATAGACGGGTGACTGATAATCAGAGAGG
EMPV1_01334	CTTGTGGCCAGCACTAACAATCTTGATTTGAGGCAAACAGAGGACAAGCATCCCAGGCAT

EMPV1_01335	GACAGCATGGTAGCTTAGTCAGTAGGGAGTCATTCACTTAGCACAGGCAGCCAGAGATTG
EMPV1_01336	AGGTGAACAGCACCAAGACCTGGGAGAGACAAATGGAAACTCTGAACTACCTGATGGAGG
EMPV1_01338	GACCAGTCAGACCAGCGGGTCATTATCAAGCTGAACATCCATGTGGGAAACATCTCGCTG
EMPV1_01339	TTACCCTAATGTAAGATGGTGGCCCGTCTGGCTGTGGAACCCACCGAAAGCTGAGAGTCT
EMPV1_01340	ACCCCCTTCTCCCTTCTGGTTTGGGACAGTACAGCTGGAGGAGAAACACTTGAGGCATTA
EMPV1_01341	TGGGGCAAGTGAAAGGAGGTGCAGAGAGAGTGCATCTTATCCAAATCCTGCATTCTGTCT
EMPV1_01342	TGGTCCCACCCTTCCCTGTTGCCCAGGCCCTGACAGGTGGAAAGAGCGATCAATGAAAAA
EMPV1_01345	CATATGATCCAATTATCGAGCTGAAATGAAGAAGAAAAATTGGACAGACCATGAGGCAGT
EMPV1_01346	AGGCAGGGTAGTTCCAAGCAGAATAGGAATTGAGTATGGCCTCTGGATGTGAGCACTGTG
EMPV1_01347	GGTCTTTCATTTTTCATTCAGAGTGATGAAGAAGACTCAGCTTTAGACCAGTCTGCAGAG
EMPV1_01348	GCAAGTATGGGAAACAGGAAGCAAATCAGCACTTCCTTCAGGAACTATAGAGTTCTCGAG
EMPV1_01350	GGTGCCTGCTGTTTAAGGGTTACTCCACCAGCCCAAGCTCCTTCTGCATGTGTTTAAGTA
EMPV1_01351	CTGTGGCGTCCTTCCCAGTGTACGTTACGCTCATTCTCCAAATCAAAGCCACTCCTGCTT
EMPV1_01352	AACCCTTGGGGTGGATTCTGTGAGGCGAAGGAACTTCCAGTTGCCAAGGTTAGATTCGAA
EMPV1_01353	CAGACTTAATTCAGAGTGAGGAACCGAGCGAGGAACTGGAGGAACTGAGCGAGGAACAGA
EMPV1_01356	ACTGGGTACCTGTGTTTCCTCTATCATTCTGTCATCCTCAGTGTCAGCTTCATACCAACC
EMPV1_01358	GCAGCTAGTATTCTCACTCCAGTTGCATAGTCACAAAAGTGATCATTGGCAGGTGTGGCC
EMPV1_01359	ATCTTAAATATGGAGCCCCTGTCGGATGTGACGGCCACCCTCCTCTTTGACTTCTTAGAG
EMPV1_01360	GTTTTGATGGCAGAGCCAACAGGATTTCCTGTCAGGTTGAACATGTGGGACAAGAGAAAG
EMPV1_01361	GGTTGCAGCCCCCAGCCAACCCTAATTTATTCTCAGCATTAGCTCCTCCCCTCATTTGTA
EMPV1_01362	TCACGAGGAGCGGATGCGTCAGTTGCTGTGTTATGGATGAGTCTTCTCTGTATAGGAAGC
EMPV1_01367	GGGGGACATAATGTGTGGTTGGGGGAGTTCCTAAAAATCAAAATTTGCCATTTCACTTGG
EMPV1_01368	TGACGGGAGGCAGAGCAGCGAGCGAGGGCGTCGGCCATTTTGTGGCTGTTTCCTGTGGGA
EMPV1_01369	CTCACAGCTGGGGTCTGGCAAGTCTGTCCTGGAGCCATCTGTCTCCTTTTGTTGCTACAA
EMPV1_01372	GGTCAGCTAGGTCACAAACCAGCTCCATCTGGCTCCAAAAGCCTAGAAGAGTTTCTGAGA
EMPV1_01373	AATCTCAGCATGTTCAGCATTAATACCAAGCCCCACGTGACTACAGTGGGGCCAGTTGTG
EMPV1_01375	TTCAACTTCGCCGCCACCCTGGAGTGCCCCATCATCTTCTTTTGCCGGAACAATGGCTAT
EMPV1_01376	CCAGAAAGTGCCATCAAGTGACTCTTCCAACCTACGACCTTGAATCTGCCACCTCTTAGC
EMPV1_01378	GTCCTATATGGCTGGACTCTCTGCTTCTCTGTCCCAAGTGTGTGCCATGATTCAGCTCCA
EMPV1_01379	GTGTGTGTGTGTGTGTGCGTGCATGTGCCCACGCTCCAGAAAACTCAAAACAGGCAAACA
EMPV1_01381	ACCACGATTCTCACTCGGCAGATTATTACGAAGATTGGCATGGGTGTTGTCAGCAGGAGC
EMPV1_01383	ACATGATGTGAATGGAAGCAGCCTGTCATTATGCAAATAGTACGACACTCTGAACAGACA
EMPV1_01384	GGGGATATGTGTCTTTGAGTATGACATCTGAGAGGAGCAGCCGTAACAGTTCCTGACCTG
EMPV1_01385	GATCCAGTGTCATTCCTGTAAAATCAAAAAACTTCAAATATTCTTCTGCAACCAGTCTGC

EMPV1_01388	GTCCCTTGGCTGTAGCTCTTACATCGCCACAGTTTGGCCTTCATAGGCAGAGGGTATGAA
EMPV1_01390	CTGAAAGCCTTCCCCCTAAAATCTCGAACAAGACAGGGACGCCACTGCTATTCAACACAG
EMPV1_01391	AAGGTAGAGCTGGGCCAGAATGGTGAGGAGAAGTCACCACCCACTGCAAACCACCCTCCT
EMPV1_01393	GTTCACGCGTTCCTCACGTGTTGCAAACTGCGTTCTGCCTACTTGTTTGCGGTGATGCAG
EMPV1_01394	CTGTAAACCTCTCTTTCAGCACTTTGATGAATCCCACAGACTCGATAATTTCAGTCTGAT
EMPV1_01395	GCCGAGTTTTGAGCTTCTGCCAACAATAAGGAGGATGAAGTGAGAGAGTACAGGAGCCCT
EMPV1_01396	AATTGTAACCCACAAAAACAACAAAACTGAATGAAAAGAGATTAAGGAAATGCTGAATTC
EMPV1_01397	CTGTAAAACTGACAGAGAGGGAAAACCCCGTAAAGTGGCCGGTTGTAGTTGTGTGGTGGT
EMPV1_01398	AAGCCAGCCCAAGGAAGCAGCCTGGTGTCACCCAAGAAGAGGAAAATGGTAGAAATGTTG
EMPV1_01399	GAGATAGCACAACAGCCAATTTGAAAGTTCATGAGGGTTCAACCATCCCTGCCTTTCCTC
EMPV1_01400	ATGGCTACCATGTCGTCCGCCCAGATCATCTCCGCCTCAGCCTTCCACAATAAAATGGCC
EMPV1_01401	GAGTGAAAAGTTCCGTCAACCATCCAGCTGTTTGGGGTGATGCAAACAAACATCTGGTTG
EMPV1_01402	ACACAGTGCAGCATGGAGCTGTTTATAGTTTGGAGCCCAGACTTCGTGGTGGAAAAGGAG
EMPV1_01404	GGGACCTACTGTCCACATCCACCCTGGTCCATAAAATTCTCCCTAGACTTTGCACAGACA
EMPV1_01405	GGAGTCCTCTAAACACACCCAAAGGAGGTTACATTAAGGCGGGAGGAGACCTAATCTTTG
EMPV1_01406	CTTCCTTTGACTGCCGCTCAGGAGAGGAACACCATCCTGAAGCATTTTGGTTACCCTTAC
EMPV1_01407	TGGCTCTCATACCATCCATTCAATCCACCCTACCCATAGGGGCTCATAATTCTCTGTCCC
EMPV1_01408	CGGCCCGGAGGCCTTGGAGGACGCAGAGGACACACTGTTCTCGGAACCTGTGCCTGTTCA
EMPV1_01409	AAGCAGGGGCCTCTTTTATCTCCTACCAGCATATCCAACCCAAAGCTGTTGTTAAGGAGC
EMPV1_01410	ATTGCTGTCCTGCAAGTCACAGAAACCGTGAAAGTGTGTGCCACTCTTCCCTCCACAGTA
EMPV1_01411	TTACAGGTGAGAGACTCCACAATTGCATCCATCTTGACCTATATATCCTGTCTCCAGAAG
EMPV1_01412	CTCATGACCTGGCCCCACCAATCAGTGTCTGGCATCCTCTGTACCAGAACACCAACATTA
EMPV1_01413	AGATACGAGACCAGTTCTACGTCAGCTAGTGATCGGTATGATTCCTTGCTGGGTCGCTCT
EMPV1_01414	CCTGTAGCCCTGAGTTCAGCAATAAGTAAACATTCACATGTGTATAGGCTTCATGCTTTC
EMPV1_01415	TCTCTGAGATTGATCACACCTGGGCTCCAAATCCAACCTCGGTTAACTTCAAGGAAACTG
EMPV1_01416	GAAGACTCAGATTAATTCCCTTCCGGAGCTATGAGATGTAAAACTACTGTTGGTTTTGCC
EMPV1_01417	GGCAAGTTGGGGTAATGGCCTTTGCACTCAAGTAAGGTTTGTTTACCAGTTTTGTAGCCA
EMPV1_01423	CTCCAGATACGAGAGGAAGAAATACGCGTGGGGCTGAAGGCGCTATCAATTTGCATGGAT
EMPV1_01424	GCTAACAGCAGTCTTACTGAAGGTTTCCTGGAAACCACGCACATGCTGTTGCCACTAACC
EMPV1_01426	GTCCTTGTGGCCTATTCCTATACGTCCATCATCCTGACAATAGTGCGCATTCCTTCTGCC
EMPV1_01428	TGGTGGCATACAAAGATGGGAAACTCCTGTGTAAACCGCATATCCAAAGTCACTCGGGGG
EMPV1_01429	GCCTAGAAAAGCCAGGAGCATCAGGAGATGGCTGTGTGTAGACTCCTCTGGCAGGTGCAG
EMPV1_01430	AAGAGTCTGTGTCCTCTTACTGGGGGCTTCCTATCTGGGTGGGTGTGTGAATGCTTGGAT
EMPV1_01431	CATCATCACCGCCTTTGTCCTCACCATCATCCTTGTGGCCAGCCTCCCGTTTGTGCAGGA

EMPV1_01433	TGGTGCTTTTTAAGAGGCGCACAAAGGCTGGTGTGGCTGGAGCCGAGTGAACAAAGGGAA
EMPV1_01437	AGCAGAATCCAAGTAGTCACCCCTTTCAGGCCCTCTCCACGTGAGGACTTTGCCATTCAA
EMPV1_01438	GCACAAGAACTACATATAGAGGCCGTGAAGTCTACCGAGAAGATGAAGGATCACATGCAA
EMPV1_01439	GGCCTTTGCAGTAAAATGACTGTTGTAACTTCTAAACCCAATTTGGTGTTCACTGTATGG
EMPV1_01449	AAGTGAGGAGAACAGAATCCTTCTGAATTAATTCTATGATGCCAGCATCACCCTCAGACC
EMPV1_01450	GCTCTTCCCAAGTGCCACCGATGCTACAAGAAACAGATAAATCCAAGTCAAATACTAAGC
EMPV1_01451	TTTCCAGGGTGATGGGAATAAGGAGAAGGGGCTTGAGGCGCTTATGGACTGCCAGATTTC
EMPV1_01452	GTTACTTCGCGGTCCACCTCCTCCGACTCCAGGAAATCGACAGGCAGGAAAGTTCTAAAA
EMPV1_01454	TATAATACCTACCCCCGCCCCTCTTTGGAGCATGATATGTAAAAAGCAGCAGTGCTCGCC
EMPV1_01455	TTTCTCTGGCCATACCACCCTGAACATGCCCAATCTCGTCTGATCTCAGAAGCTAAGCAG
EMPV1_01456	GAGACCTTGCTATGCTGTACAAGTTGTGTTTGATGGATCGGTGTGAGTATGAAATAAAGC
EMPV1_01457	GAGGCAGAAACTTGGTTTTGGTGGATTTGACTCTCAGGAAGGGAGACAGTAACCTTTGGA
EMPV1_01458	TTCCCCGTATCAGGGCAATGGAAGGTCGTAGAGGAAGACCACCAAATCCAGATAGACAGC
EMPV1_01460	GAACTGACTGGTAAGTGACGTCTAATTGTATGGCTAGCAATGTAATTTATTCAGACTGTA
EMPV1_01461	TTCATGGCAAGTAGCTCCAGCTTTCCTCAACAACACCAGCCCTCTGTCCTACATCCCAGA
EMPV1_01462	CATGACAGCCAGAGAGACCGTTTTAAATGGGCCTAAATGCGAACTTGGAACTCACCTGCC
EMPV1_01463	CTTGGATATGCTGACAACTGTGCAGGAGGAAAATGAGCCAGTGATTTACAATAGAGCAAG
EMPV1_01467	TTCAGCCTGGGAAGCTCACCGAGGCCTTCAAGTACTTTTTGCAGGGAATGGGCTACATCC
EMPV1_01470	CTACAGAAGAGAAAAAGGAATTCTTCACTCCCACCAAACTTCACTATCTTCTCACTGCAA
EMPV1_01472	ATGATCCTCATCCTCATCTTCGCGGAAGTGCTCGGCCTCTACGGTCTCATCGTTGCCCTC
EMPV1_01476	GACAAGTTTGATGTTTACACCATTGCCTTTGGGATCCGGAATGTCACACACATTGATCGG
EMPV1_01479	GCTGTCTTAAGGAACCTGTGTCTCCTCTATGCTCTGTATGGAATCAGTCAGAACTCAGGG
EMPV1_01481	TCACTGGGTAAAGTTGCTGGGACCTTGGAGACCAAATATGAATGGTGTGAGCATGGTCTG
EMPV1_01483	GGCCTGATTTGAGGTGAGCAGGATGTGCAGCTTATCTTTGCTGCCTATGTTGTATTCGGC
EMPV1_01484	GGAGCCCTGCCTATCTGCTTCGTGTTTGCTCTTTAGCGGTTATTTCAAGGTCAGCCTAAC
EMPV1_01486	ACTAGAGGGAAGGCAAGCCCAGGATCCTCTCCCTGCCCTTGGAAATGTGGTCCAGCCCAT
EMPV1_01488	GTGCTGGCATTATTGTCACTCTTCCTGTCACAATCACATCTTTCCCAACAGGTACTCCTG
EMPV1_01490	AACCTGACTATTTCAAGTTATTCCCCCTATATCTTGATATGTATATTGTTGTGGATAAAA
EMPV1_01491	TTACCTGCGAGCCATTGACGTGAAGATCCTGCAGCAGCTGGTGACCTTGAACGAGGGCAT
EMPV1_01492	AGGAAAGATGCTTTATTCAGAAAATCTAGCAACCTGTGGAGAAGGTGGACTTGGGTCCCA
EMPV1_01493	GTGCTCTTCTTCCCACAAGGCAGGATTCTCACCGTAGCCGCGATGATGCTCTTCTTCATA
EMPV1_01494	GGGGAAACTAACTACACCACAGAATGCACCGTAGGACATAGATACCATGATACATAGCAT
EMPV1_01495	ACCGGATTGAAACTGTCTCAGGACCTGGATGATCTTGCCATTCTCTACCTGGCCACAGTT
EMPV1_01496	ATCTAGAGGGATAACCCGTTCCCCCTCCTCCACCTCTTTGAACCGTGTCACAGATCCAGA

EMPV1_01497	GACAAAACAATTACTTTCTATTTCTAAGCTCTAACTTGACCTTAAGTGAACCTAGTCAGT
EMPV1_01498	TTTGCCTGTGAGTACTGCCACTTCAGCACGCGGCACAAGAAGAATCTCCGCCTGCACGTA
EMPV1_01502	TTCAGACAGGTGCTTCTTGCTTCCGAGGTGCCCAGGCTCACCAGGCAGCTCCTGCAAGAC
EMPV1_01503	CTGTTTGGCCGAGGCATTTCTCACTGCAGATACGATACTGAATACGCTGCAGAACATTTC
EMPV1_01505	TGTCTCTAGTTGGTCGAGGGGACGAGACTGCTCGTTATGAGGTTGAGAACTGTGGCGACA
EMPV1_01506	TACCGGAAGGATCTAGTGTCCCCAGTGCGACACAATAGCGCATAAGTACGGAGCAGAACA
EMPV1_01508	AAAAAAGAAGATCCAGACGTCACCCACCTTCCAATGGCCCAAAACACTGCAGCTCAGGAG
EMPV1_01510	AGGCATTTCGATAAGTACTCCAAAATGAGGTTGGTGTGACTTGTCACAGGAGCTGTGGTC
EMPV1_01511	TCCACGGGACTTGGCTCCTGGCTTCACGATCCAGATGTTGCAATCTCCTTCCATGTCCAT
EMPV1_01513	TATCGAGCACTTTTTCTGTGAAATCCCACCCTTGCTAGCCCTCAGCTCCTCAGACACAAC
EMPV1_01514	AGCACACTCAGTGCTCTTCCCCTTGCCACCTACCTTCCTCTTGTTACTAAATCATCACTT
EMPV1_01519	ACCGAGACTCAGGGATCAGCTCAAGGTCACGGGGTGAGGAAGGAGCAGATTGGACTGGAT
EMPV1_01520	CTCTCTTCTAGAGGTTAGACAGAAGATCCAAGGGAGTGGGTTGGGATGGAGTTCCAGGGA
EMPV1_01521	AGAGCTGTTCTTCCGCTCGGATCCTCTGGCGTACACGGTGTGCAAGGGGAGTCGTGGTGT
EMPV1_01522	CAGCTGTCACCTCCTTTTTTCTTATCCAGTTTCCTCCATTTCCAGTCCAGCTATGAATTA
EMPV1_01525	ACAGTAGTGGACACAATCGTCTGTATTTCCATAGTGATACTTGTTTACCTCTCCGTCCAC
EMPV1_01526	CAATGGAGAAAGCCTATGAATGTCATCAGTGTGGGAAAGTCTTCAACCGGAGGCACTCCC
EMPV1_01528	AGAGTGGCTTCTTGGAGGACAGAAACATGGACTGTCCACCACCCTCCTGAATTCACTGGT
EMPV1_01530	CACTTTAAGGACTCGAAGGACACACAGAGGCCTTGACCAGAGTACAGCGCAGCAGCCCAA
EMPV1_01531	GTTGAGAGATACTTAGTGGGAAGTAGGTTTTGAAGAGGTTGATTCGGGGAGAGGGGCTGG
EMPV1_01532	GCTTCACAATAGTTACCTGAGCCACAGAAATGACAATGCTGGCTACTTACCCCACTGAGC
EMPV1_01534	TCAATGCTTGTGCCCACCTCAGGGCTCTGTTCTTGCTGTTCATTCTGCCTTGATGTTCTG
EMPV1_01535	CACTACCCTCTTCACCTCTTCATCTGCAGACCTATCACAAGTTTACACGTAACCACAGAA
EMPV1_01536	CCTTCCACCAGAGTATTCAAGATACATTATGGACTAATATTTGTGGCCTTGTCTAGCATC
EMPV1_01539	AGCTACCATCGCTGTCAACAAAACAGATGGAGGGAGAGTTGTTCCCGTTGTGGCTGGCAG
EMPV1_01541	GCACAGTCTTGGAAGATGTACAAGACCTGGTAGCCACTTTTAGACATCTTGTGGCTCTTG
EMPV1_01542	TTTTTGTGTTGGATTCCATCCATGGTTCTGTTCCAGGGGTCAGGAAACGAGGCAGTCAGC
EMPV1_01544	CAGCATTTTTGAGAAACCATGGATATTGCCAGTGGGCTGCAGGGAAAGCCCCACGTGCTT
EMPV1_01545	AACAGATTATCGAGCATAAGAGGAGCCAGACGGAGGGCCTGAAGGAGAAGCTCACACACA
EMPV1_01546	GGCACATTGTCAGAGGTGAATGAGCCTGGGAAGAGCGTTGCAAGGTATATTAAGACCTGG
EMPV1_01547	TCTCTGTCGCATGGGTGCCCCTTGTTTTCAATAAAGCCAGTCACTCCCGGTCTCGTTTGT
EMPV1_01549	CCAGAGGAGAGACACATGGAAAGTAGCAGGAGGATGCAGGCGGCATCTCGGAGCCAGTTA
EMPV1_01550	CTTCGTGCTGGGGCTGATCTTCCTCGGGCTGGGCCTTTTCATCCGTCACAGGAGTCAGAA
EMPV1_01551	GCAAATGGCAATACAGTATGTAAAGATGGTGACAGTTCAGGTTTCGGTATCCAAGTGGCC

EMPV1_01553	GAGAAACCAGGCCACGTCTTCAACATTTTGCTCTTCAGCTACATACCCAAGTTCATCATC
EMPV1_01555	GGCGGTAACATATAGGTAGCCTAGAGTATGGCACGAGGTGTTCAAGGAGTGGTTCTGGTA
EMPV1_01556	TGTCCCAGCCTACAAACTGTCCTTGGCAACATCGCCACTTACCAGCTGGGGTCATTTCTC
EMPV1_01557	GTGGAAGGCGTAGTGGCTCTTGTGGTGGGCCTCCATGGCTTCCGCCGTGGCGAAGAGCAT
EMPV1_01558	TGTATTCATTAAACGAAAGGAAGCTGCTGGAAGTAAAAACAGAAATGGTGTCATTGCACG
EMPV1_01561	AATTTTGGAGGTAGCCCTGGTTATGGAGGAGGAAGAGGAGGATATGGTGGTGGAGGACCT
EMPV1_01564	TGGCAGACGAGCAGGATGTCGACATGCACGACCCCCGACTCATACCCCTAAAAACCATGA
EMPV1_01565	GCAACGGCAGCCCCACAGTCCTAAGAGGATGCATATTCAGTCACTGTTCCCTTTTCATTG
EMPV1_01567	CCTAGTGAGACGGGAATCAGAAAGGAAGGAGTTGGCTGAAGAGATGCACTGACTACAGTC
EMPV1_01568	CTTACACCTGGTTCAGTGTGAGAGATTGCATAAAGTGCACAGAGCTGACTAAGAAGCAGA
EMPV1_01569	CAAGTCAAAATGAAGAAGCTGAGGAGCTGATGAGGAAGATTGAAAAAGAGGAGGAGCAGC
EMPV1_01571	GACAGACCGTCAGCTGTAAAGACCAAAGAGTCTGGCGTGGGGCAGATCAGTATTAACGAC
EMPV1_01572	ACCGTTCGTGAGAATGAAAGAATAACTGAAACGCCAAAACCAAGGGTCCAGGGGGTGGAG
EMPV1_01573	AGATTCTGCCTCTGGAGATCTGGCCTCTGAAGGGTTAACCTCTGGTGATCTCACTTCTGT
EMPV1_01575	CTTCTTTCCCTCTCCTTTTGTGGTCTCCTTGTTGGAATTTCATGGCAGGCACAAGGTACA
EMPV1_01577	CCTAGGCTTCCTCCTTCTAGCCGGCCTTGATCCAGTAGCCAACAAGAAAGACACAGTGAA
EMPV1_01579	CTCCCACCTCAACCTAGCCATGTGTTATCTGAAACTACAGTCCTTCTCGGCTGCCATTGA
EMPV1_01580	CCAGGACTGGCTGAAGGAGGCCCGAATGACCCTAGAAACTAAGCAGATAGTGGAAATCCT
EMPV1_01581	CGGTCCCTGAAAATGTGGTTTGTTTTCAGGATGTACGGAGTCAAGGGACTGCAGGCCTAT
EMPV1_01583	ATGACAGCTTGTTTTGGGAATGCAAAGCTAGTGGGAAGCCAAACCCTTGGTACACATGGT
EMPV1_01584	CCTAACGACAGCCGTGCTCTCCACTTAATGACCAAATGTGCCCAGACAGTAATGGAAGAA
EMPV1_01588	AACACGGCTACTTCCGAGCGCCTGAGCGTGGGGTCTATTTCTTTGCAGTGAGCATTGAAT
EMPV1_01590	TATTTGGCCTAGGGAGTTCCCCGGTGGCCTCGTGATTAAGGATTTGGCCTTGTTGCTTCT
EMPV1_01591	CCGATTCTCTGGGGCTCGTCTTTTTCTGCTGCACCGCAGGCTACTCCAAACTTCATTTCT
EMPV1_01593	CGAGCTTCTCGGGGAGGTGGACCGCACGGAGTTTGAACAATATCTGCACTTTGTGTGCAA
EMPV1_01594	CTAGGCCAACTACTTCCTACTTCACAGAGGCGAAATGGAAGCCCAGAGATGGGAATGCAT
EMPV1_01595	GGCAGAGGTGGCGGTGATGCTTCTAAAAACCAGAGCACCTAGAATTGCTCATAAACCACC
EMPV1_01597	ATGAGGCCCGGGATGGCCAGGTTGATGGCCGTCTCTATGTGCGACGACTCGAAGAGCTCG
EMPV1_01598	AGACCTTCATGCTCATCAATTCCTTGCACGATAAGAACAACGTGGGGCTCTCCTTCTACG
EMPV1_01600	AGCTGAGCTGCTGTGCTTTCTGGCTGTCAGCCCGGGATCACCAAGGCAGCAGGGATCCTG
EMPV1_01601	GAGAGGCAAAGGGGGCTTCTCTTAGCAAGTCTTTACCTTTTTATTTGGAAAGGACAGCCC
EMPV1_01609	TGTGTGTAGTCTTCCCTTCTATCCTGTCACTGCCTCAGGGGCTGGAGGTTGGCCCAAACA
EMPV1_01610	TCGGTACAAACTTCGGTTCTTCCACCTGGACACAAAGCCCCTCATTGCCACGTGCCGGAA
EMPV1_01611	ATTGGAAGAGATTAACACAGCCTTAGGTACACAAGCAGCTACTGATATGGCACATGCACG

EMPV1_01613	TGCTCTATGAAGGCCCTGTCCGTGGGCTCTGCCCCTTTGCCCCCCGAAATTCTAACACCA
EMPV1_01615	TCATGCTTCCATTCTCTCTTGGAAACTGGCACCTGTCATGCAGCATGTCCAAGCCAGCCT
EMPV1_01616	GTATGGGAGAAGCTTTCATAAAATTCTCAAGAATGGGAGTTCCTGCTGTGGTTCAGTGGG
EMPV1_01617	GACACCGGGGGTAATTGTTGGCTCAGACGTGATCGGTCCAAGAGGAGTTAGTTTCAAGTA
EMPV1_01618	GTACAGACCGTCCGCTCTCGTGCAAATATGAGGAGTTTTCTTTACTCGCAGACGTGACCT
EMPV1_01620	TCTGGCCACTGGTTAGCCCCCACCCAGTCTTCGTTCTTCTTGACTGCACTGTATTATTCA
EMPV1_01621	TCCCGGTCACCATCTGGTCCATAAGAACCCATGGAAACTATGGCTCTTTCAAGGCTTGGA
EMPV1_01625	GCAGGGCAAGGCCATGTGCTTCTAGCTTTGGTTCTCAGTTTACTTTTCATTTCTGGCCCC
EMPV1_01626	ACCAGGGAATTTGGTGTGTTGCCATGTCCATCTTCTGAGTCACTGTCATCAAACTGAGCC
EMPV1_01629	AAGTATAAGCGTATAAGGCCAGCTGTCACCTCTGAGAGACGGAAGATCCGTGGCTCCCTG
EMPV1_01631	CTCGGAGAGAACTCAGCAGTCGCCCTTTCCCTTACCATCAGTTGCTGACAAAAATTAGCG
EMPV1_01633	TTGGGTCAGTGAGCACCATGAGTCTGAGGCTAGAGTATTTTGGGGAGGAAGGGTTAGGGA
EMPV1_01634	GTTGTTTGAAGAGACAACGTTTTGGGGTGGCGCCTTACACTAGCGGCATGACTTTCAGTG
EMPV1_01635	GCCGGGCCCTGTGGATCAACGCCTGTTTCTGCAATCTGAGCTGTCGGAATCCCAATGATA
EMPV1_01638	TTCCACATAAAGCGCTCCTCTGCTGCCTGGGACTCCATCCTCCTGGACAAGCTCCACTCT
EMPV1_01639	TGCTTGTCTTGCTGTTCCCGTCCGGGGCCAGTCCCGAGCAAAGGCGCGTGGAGCCGCAGT
EMPV1_01640	GAGTCACAGTGGTGGGTCATCAACATCTAGACAGGACGGTGGAAGTGAGAGACTGTCCAG
EMPV1_01641	CAACACATCTAGATTTTAACAACAACAAAGTAAAAATACATGCCAAAAAGTCAAGGGAAG
EMPV1_01642	CCTTACGGACCCCTCCGGATAAAGTTAGCTTTCCCACTGCTGTCAGCCGACGAACCCAGA
EMPV1_01644	GTGAACCTGAAGGCGATCCTCCTGGTTCACTGGCTGCTGACCATCTGGGGCTGCATTGTG
EMPV1_01645	CCCGAGATGGTAGAAGCTGGAGCAGGTAGGAGTTCCCATTGTGGCTCAGTGGTTAATGAA
EMPV1_01647	GCTAACCAGTGTAACGTAGAGGCTTTTGGCTGCTAGATGGTGTTAGACCAAAGAACTGGT
EMPV1_01649	AGAAGAGGAAGAAAAGAAAATTATTAGTGAAGAGCACTGGTACTTGGATTTGCCTGAGCT
EMPV1_01650	GTTAATCTCTGGGAGCCTTGGGTTTCTACTCCTCAAAAAAGGAGATTGAGGGGCTGGGGG
EMPV1_01652	CCAGATTGCAAGCTCCGAGAGTCCACATGCAAGACCAATGGAGTCTTATGGAGTCTTACG
EMPV1_01654	CTGTGATTGCAGCGTTCGGCAGTGCAAGTGGTTTGTCTTTGATCATGACCTTGTTTTGCC
EMPV1_01655	ATTACGAACCTGAGCTGCTGTTAATGCCATCCAGTCAGCCGGTCAACCAGCCCATTCTGG
EMPV1_01657	AAACCGGGCAGCATCGCGACCCTGCGCGGGGCACCGAGTGCGCTGCTGTTCGCGTGGGAT
EMPV1_01658	GCTGGGAATACAGTCATAGTAAGAGATTTTAACACCCCACAAATCAATGGAAAGATCCTC
EMPV1_01663	GGAGCCTGTCATGGGGTACCTGTGGGCCTGGATCTGTCTGTAGAAACGGACAAAATTATA
EMPV1_01664	AACTCCTAGGGAACCAGTCCTGGGGTGCGGAGAGGGGCAAAATACTGTAAGATTTACCTC
EMPV1_01666	ATCTAATACTGTACAATACTGTGTACTCTATACAACATGCCCACCAAAGAGTTCACTGAG
EMPV1_01673	AATACATCAGAGAACGTGTCACCAGTTAAGCAGGCTCCACGGAAGTCCCCCTTCGACACA
EMPV1_01675	GACTGAAGAAGATTGGAGAGCTTCTAGAGCAGGACTCGGTGACCAGAGAGGAGGTGGTGT

EMPV1_01676	CACACATTGAAGACTATCTTCTCGGAAGGATATTTGCCTCCCTTGAAGGCATAGACATGC
EMPV1_01678	CCTAGACTTGGGAGGTTGGTGCGAGTAAATTCTGACAACAGCACAGAGGATGTTTCCGTA
EMPV1_01679	GAAGTACTCTCTAGAGAAGCTGCATCAAGGCATCGCCGTCTCAGACCCTCGCTTCCATTC
EMPV1_01682	CTGCATATATTGTCCTTAAGGTCTCTTCCAGTTCTAACATATAATAGTAAAGAGCCAAAG
EMPV1_01683	GAGAGAAAACTTCAGTAGGCAATTCGTAAGAGGGCTCCAGGATGTAGCTATAAACTAAGA
EMPV1_01688	GCAGCACCATGAATGGCCCTAGAGATTATCATACTAAGTGAAGTAGGCCAGAGAAAGACA
EMPV1_01689	TAGTCCTTCTCTCGTGTCCTTCAGGGGAACAGAACCCGCTCCTGACAAAGGTATCAGCCA
EMPV1_01690	GCAGTGAGACTTCCTCAGATCCCTTGGCTGGGGTGGAACCAGCCAGTCCATCTCTGTTTT
EMPV1_01694	GGAAAGCACCCATGACTTTTAGGGTAGGTTACTAAGGAATGGGGCCAAGACATAGGGCCT
EMPV1_01695	AGGGGCTGTGGGAGGGTTTGCATGAAAGGGAATGAAAACTGCAAAGCGAGACACCATGTC
EMPV1_01696	AAGAAACCAGGATGGATTCCAGGGGCTGAAATGTAGGTATGCTGGCAGGCTTTTAGACTG
EMPV1_01697	AGGGAGAATGGACTGAGCAATGATTTGGAATACAAGAAATTCAGCTTATACATTTACAGC
EMPV1_01698	AATCATTCCTACCCTGCACTATGTCATCTCAGAGGGGTTCCTGAAAGCTGCCACCATAGG
EMPV1_01699	GGAATCATGGCATGGAAGGCCATGAGCAAAGGGGATCTAGCAGTTCATCAAGTGAGGAAG
EMPV1_01701	AGGATTAGGCATTATCACTGCTGTGGCATTGGGTTCAATCCCTTGCCCTGGAACTTCTGC
EMPV1_01702	AGGTCGTAGCTTTCCATCCTTCTGCGCCAAGAGGAGTCCTGGGTACGTCTGTATTCTGGA
EMPV1_01704	TTGCCAAAGCTCCCCTCCACACCGTCTCACTTCCCAGCCCCCTAAAGCTGTGAAGCTAGT
EMPV1_01707	GCTTGAAATTGTCCCGCAAGATGCGAAAGGAAATGAATGTCTTGACAGAATGGCTGGCAG
EMPV1_01708	TGGGGTGTGGGGGGCCCCCAGGAAGGACAGTTTTCTGAAGTTGGGAGTCAGGGCTGCTGG
EMPV1_01710	AAATGTCTAGAACAGAAAAATATATAGACAGAGAGTAAATTAGTGGCCGCCAGGGACTGG
EMPV1_01713	CCTGTGAATCATGGTATTTCCATCCTATCATGTAATAGATCTGATATGTGGCAGGGACGG
EMPV1_01714	ACATGGCCAGACCTGCAGAAAAACTCAGCACAGCTCAATCTGCTGTTTTGATGGCTACAG
EMPV1_01715	CTCGGAGCAAGAGGGGACGAGACTAACGCGCGACTTTGCAAAATAGATTAAACTTCAGGG
EMPV1_01716	AACTGGCCTAGGACGGAGTCTTGCTTCTTTCGCCTCCAGTGATTCCCCTCCATCATATCA
EMPV1_01717	TGGATACCTTGGCTGGTTTGGAAAGTTCATTGTTCGACACCATGGATCCCCAGGTGGGTC
EMPV1_01719	GGGTGGGACAGATCCTGCACCCTGAGGAGTGTATGTATGCTGTGGGTCAGGGGGCCCTGG
EMPV1_01720	GTGGGATTTCTCTACAATGGAGTATCATTTGGCAATACGGAGGAAGGGTTTGGTACCTGC
EMPV1_01721	TGTATACAGGCTCTGGAGCAAGTGGGATGTCCCAGCCAGTCTTTTCCTGGCCACCAGAAC
EMPV1_01722	CATCCGGACAAGAACAAATCTCCTCAGGCGGAGGAAAAATTTAAAGAGGTCGCAGAAGCT
EMPV1_01724	ATTAACATCGACTTCAGTACCAGGGACCTCATCTCAAAGAACATCGCGGAACCGACTCGC
EMPV1_01729	CCAGCTATGTTCAATATAAGAAATATTGGGAAAACACTGGTCACAAGAACTCAAGGAACC
EMPV1_01730	TCACCTTGACACTTGCTTCCCTGGGGCGTCCTAACCCTCCCTTTGCTTCTGAGACCCATA
EMPV1_01731	CACCTGAGGTGGTAGTTTAAAAAAGATACCAGTATTTGATCCAGTTTCATCTCAGCTATG
EMPV1_01732	TGGTGGTGGGAGCCAAAGGAATAGAGCAGTTTCCCCACTTAGCCTTCTGGCAGGATCTTG

EMPV1_01733	TCGCGTTTCCTCTTATGCTGTGGGAACCAAGAAACGTCAGACGACATTGTGTGTGTACAG
EMPV1_01734	GCCACTACTCTCAGCTTTGATATTCATGGCCTCTCGATGTTTGGTGGACATGCATTTGAG
EMPV1_01735	GTCTATTTGTCCTCCTAACACGCTGACGAGTGTACTTGAAAGGGAAATGCAGTCACGGCC
EMPV1_01739	CCACTATTGCAAATGCTCTCCGATTGCAGTAATTAATATTGTAGCCATGAAAGAGGACCC
EMPV1_01740	TGGGCCTTTCCCCATGATGGCCCCTGAAGAGGAGGGGGACTCAGTGACTATCCCAGAGGA
EMPV1_01741	TCCCTCTGTTGTCCAAGGGCATCCGTGGTCTGGCATGAAGACGTGGTCCTTTCTCACCTT
EMPV1_01743	GGTGCAGCAGAAACAAATCCAACTAGGAATATGAGGTTGCAGGTTCAATCCTTGGCCTTG
EMPV1_01747	AAGAGTTCCAGAAGATCGGGTCCAGCCATCTTCCCCCTGCATGCAAATCTCACAAGAGAG
EMPV1_01748	TGTGACCTGTGCAGCAATGACGGGGGTAAGAGTATTTCTTCCAGGAGCATCCTGACTTTG
EMPV1_01749	TGGGGGCATCGTAAAACCTAATGGTGTGTGAAAGACGCAGAAGGCTACGCCAGCTGTTCT
EMPV1_01750	GAGGTCAGTTCACTTAGCAGGAGCTGTGTGGCTCTTTTCACTCATAGGAGACAAATATGG
EMPV1_01751	TCGACTGCAGTTGGTAACAGAAGTGATGGCAGAATGCCTAGACTTCAGTGGGCATATCCC
EMPV1_01753	GGTGGATACATTGAGGAAGCAGAACTGAAGACGGAGCCCAGTCTTCTGATTCCAGTTTAG
EMPV1_01754	CTCCCCAGCCCCAGCAGGTTTTAACCCAGAGTTCCTGCAGCTAATTCACTGCTACTGCCA
EMPV1_01755	GGAGCAGATCTACTGATGGAGGAAAACCAAACAAATATAGAAACGTAAGGAATGCTTCCC
EMPV1_01756	TCCAGGAAGGAGCCTCTTGAATGTGATACATACGGCAAAGCCTTTCATCCCATCTCAGCC
EMPV1_01757	TATTTTGGCCACGAGCTGGGAAATCCCTTGGCTCTGGGCTTGCCGCTGGTATAGAAACAA
EMPV1_01759	CTGAGATGGGACCCTGCTCAGCCCCCCGTCCCCATCGTGGGCATCATTGTTGGCCTGGTT
EMPV1_01760	TGGACACCAATGGGTTATGTTGTTCGGCAGACATTATCTACAGAACTGTCACCAGCCCCT
EMPV1_01761	CCAAGATGTGGAGTTTTAGCAGACTAAAGAAAGGGGTTCTCCCTCACTAGCTTGGAAGGC
EMPV1_01762	AGTCATCCCTCATGGATCTTGCTGACGTCTTCACGGCCCCAGCTCCTCCTCCGGCCACAG
EMPV1_01766	GAAGGACAGCTTCCGGATTTCGTGCCATGATGCTTCGAGATGCTCGTTGTGGCTGTCTCT
EMPV1_01769	AGGGCCTCAGCTGAAACGGGGTTGTGTTTTGCTTTTTGTTCTGTGGCCCCCACCCCTTGG
EMPV1_01770	CTCACACTGGGAATTATCTTCATCTGGATTTCCCAAAGGAACTGCCACCAATATCTTGAA
EMPV1_01774	GGTGTGATTGCAGCCTTCTGTTGTGCGATAGTCGATGGGGTCTTTGCTGCCAGACACATT
EMPV1_01776	GAGTACATGTGAAGAACCACGAAACCCTAATGGCCCATCACCAATTGCAACTGCTTCAGG
EMPV1_01779	GGGCAAGTTACTTCACCCAGCCTATGCAGAGTAGTAACAGCATTTTCCGCAAAGCCTGTG
EMPV1_01781	CAGAGCTGCCAGAACAGCTGGAAAGTGAGGGGAGATATTTCATAAAGGAGGGAACCACAG
EMPV1_01784	CGGATCTGGTGACAGTTGTGGACGACCTTCGTCTGGACATCCTGCTGTGCATGCTGAAAT
EMPV1_01786	CAGGGCCGAATAAACATCTTCGCCCAGAACAACTCCTTATCTATCAACAAGCTGACTTGG
EMPV1_01790	CCCAGTCGCTGTCTCAGCTGCTCTCCCATAGTTGGTTATTTTTTATTTGGGACAGTGGGC
EMPV1_01791	GTCAGCGATCATTCAAGTGTACCACTCTTTAACATACGTGAAGATTTTTGTTTTTCACAG
EMPV1_01792	AGAGACACATGGGGCCTGCGGGTAGTTCTTGAGCCCTTTTCCCAGAGTAGGTGACTTTAA
EMPV1_01797	AATATAAATGCGTGTTGCTGTGTGAAAAACTCTGTGCCTTAGAGTCAGGTGTGAGAGTTC

EMPV1_01799	AAGTGTCGCTGGAAGGCGGGAAAGCCAAAGCGAAGCACACGAAGCTGAAATTTGGTACCT
EMPV1_01800	ACTTGTGGCTGGCGAGTACCTCAAGTTCTTTGTCTTCACGGGCATGACTCTGGACCAAGC
EMPV1_01801	ATTGACGACAGTGAAATTACAAAGGAAGATGATGCTTTGTGGCCTCCTCCTGACAGGGTT
EMPV1_01803	GGACAGTCCTCATCCAAAGAGTTCCCTAACTACCCGACTAGTACCTACAACGCATGTATT
EMPV1_01805	TAAAAGACGAGGGCAACCAGTCCCAGAGCAAGAGATCATGGAGAAACCCACCACTTCATG
EMPV1_01807	CCGGGACAAGTGCGCTGATGTTTGGGGGCTGCGCTTTTTCACGACGATTAAGGAGATTTT
EMPV1_01808	GGACTTTCTGTCCTTGCAGATAAGGCTCAGAGCTCACAGAATCTTCCAGAGCCACAGAAC
EMPV1_01812	CGAGGTGGTAGGTGTGGATTCTGTTGAAGGAGAGGGAGAGGAAGAAGGCGTGGAAAACTA
EMPV1_01814	AATACGTGTGGAGAGGTGGCGTGAGGCTTCGGTCCCATCGTCCATTGAGCTTCAGGGTGA
EMPV1_01815	AATTGGCTGACCCAGAGTCCTTGTAACTGGGGTTTCACGTGGGTTCTCTCTGTCTGGTGT
EMPV1_01817	GTCTGAGCACGCACTGTGCTGGGATAAGAACAGAACTTGGCTACTCAACACACAATGGAC
EMPV1_01818	AAGTCCCGGTCACGAAGCAGGTGTGGGCAAGTCAGAAGTAGGTTTAAGAGAGAGATGACC
EMPV1_01829	GACTCTTTATCAGGGATGGCTCTACAGAAAGAGGCACTGCAAGAGGGGCCTATACAGGAG
EMPV1_01830	TGAAGGGCAGGTGTGTAACCCTCTGGACTCTCTCGATCTCGACAACACTCAGCTGGACTT
EMPV1_01831	GTTTCTGTGTTAATATTGCCTCAGGTTATTTCAAAGGTGATCTTTCACTGTGTGTGTGTG
EMPV1_01833	CCAACGAGGGCAGTGTTGGCAAAATAATTATTGGCAGGGAAGACAAATGAGGTCCTTTCG
EMPV1_01834	CATCATGAGCCCTTTGCTGTGTGGGTCAATGGTAGCCTTCTGTGGATTCTGGGGTATTAG
EMPV1_01835	CGGCGCTTGCGAAATCGGGAAGCTAAGGCAGCCAACCCAGGGGCAGAGGTTAAAAGGCCA
EMPV1_01836	TTCACCATCTACCATGGCAACCACATGGAGTCCCTGGATCTCATCACCTCCAACCCTGAG
EMPV1_01837	TTCACTGCTGTGCCTGGCCTTCCTGCTGGGCCGCTTCCTGCACATGTTTGTCAAGTCTCT
EMPV1_01838	ATCGTGGTGACCTCCACTCCTGCCATCACTCCGGGCACCTCGAACCTCGTCTTCACCTAC
EMPV1_01840	CCCCCCATTTGCCCGGAATCCTGTAGCTAGTTTGTACGGGAGCTAGAAATAGAATCCAGG
EMPV1_01841	CTCCTTTCTCGTCTTGGTGTCCTCCTATATCGTCATCCTGGTCAACTTGAGGAACCATTC
EMPV1_01845	CACCAGCTCGGAATCAACTCCCTCTCCCCTCGCTGGTATGAGGGCAGCAGGCATTGTGTT
EMPV1_01846	CTTACAGACGCATGAATGTAAGAGTAGGAAGGGGTGGGTGTTAGGATCACTTGGGGATCT
EMPV1_01847	TTACATGTTAATCCCTCTTTTGGGACCATTCTCTGCCTAACCCTGCAGTCCCAGATCTCT
EMPV1_01848	AGCTGCTGCTTCATTGTCTTTTGGTAATGAATATTTCTGCACAGAATTCTGAGGCCACCC
EMPV1_01850	ATGATCTCATTCAGGATTTGAGTCCTGGCCTGTGAAAGAGTGACTCAGGGGCTCTGCCTG
EMPV1_01852	AGCCAGAGGTACCATTCTGTTGGTATGTCCGCTATGGTTTGAATGTTATGGGTGGTGGGG
EMPV1_01853	ACAGATAGCTCAGGGGCCAGCTCAGAGGATGAGGATGACAGCACCCTGGAGTTGCTTGCA
EMPV1_01855	GGATCCATCATTCTATAGTAAAACCGGCTGGCACAGCTGATGACCGTGGAGAGTGGGAGA
EMPV1_01859	TATTATCACTATTGGAATTCCCTGGTGGCACAGAGGGTTGGGGATCCAGCGTTGTCACTG
EMPV1_01860	CTGGGTCTGGCGCTCAATTACTCCGTCTTCTACTACGAGATCCAAAACGCACCCGAACAA
EMPV1_01861	CTGGAGGCAACATATTCCTGATTTGGGCACACTGCTCCAGCCTCTTTACTAGGTGACTTG

EMPV1_01863	AACCGCAGCCTGGCACTGGGGAATATCTGCTGCTTCGGCTTTGACATGGACTACACTCTG
EMPV1_01864	GGTAGAGAGAGGAGCTATGGGTAAAGCCAGCAGGTGGTGAGGGCAACCATAAACTTCTCA
EMPV1_01865	TGGTGACAACTTGGGACCAAACAATGACCTTCTGCAATGAGGACTCTAGCAAGCATGCTA
EMPV1_01866	CTACAGGCCACCGGGTATTTAGAAAGACAGAGGGTGAGGTAGGGGCTCAGTCAATGAACT
EMPV1_01867	CAGGTTCTTACCCAAGTGCGGCAACAGTCGAGGGTTTAGACAAATAGGACGAGGAAGACT
EMPV1_01869	CTGGTGGACGCCGAGCTTGTGGCTCCACGGCCGCTTCATCCTCGAGGTGCAGCCGAACCT
EMPV1_01870	AAGCATGGCTCTCATCACACACCACGGCTGACTTTACAGGAGGCTACATCGACATCGAGA
EMPV1_01872	AGCTGCTAAAAACAAGTGTTGGTGACCTAGTTGAGAAGTTGGAACCCTCATACTGGTGGG
EMPV1_01874	AGCTGAAGCAGCAATCCACTAAGGTGGAAGCCTTGCACCAGAATAGAGAGATGATAGACC
EMPV1_01877	CCTGCTCCAATTTATATCCTGGATGAGGTAGATGCAGCCTTAGATCTTTCCCATACCCAG
EMPV1_01878	AACGGTTTCCTACCAGTCCCTCAAAATACTCGCAGCCTCAAAACACTTGCAGCCTCTGTG
EMPV1_01882	GCATGCTCTGCCTCTTCTTGACTCCTAACTGGGCAAGTCAACCATAATTACAGATCAAGA
EMPV1_01885	TGAGTTGTGTTGAAGCCTCCATCTCCCATCCTTGCCTGTAACCTGAAGTCACTTGTGCAG
EMPV1_01886	CCGGCATCCTAATATTACAACTTATTGGACTGTTTTCACTGTTGGCAGCTGGCTTTGGGT
EMPV1_01888	TACCTGCACCGGCGGAGCCTGTTCTACAGGTCGGGCAAGTACGACCTGGACTTCAAGTCA
EMPV1_01889	TGTTTGTCTTTTCTATGGACCGGTAGGCTTCACATACACTCGTCCTGCCTCAGCCACCTC
EMPV1_01890	TGGAGTACCTCGCCAAGAACTGCGCCAAACTCAAGTCCCTGGACATCGGCAAGTGCCCTT
EMPV1_01892	TCACTAGGCTCATCTACTTCTTCATTCCAGTCAGTGGGCTCCTATGGACCTTTTGGCAGG
EMPV1_01893	CTGAACAGAATGAAGCAGAAGGCACTGAATGGTTACACTTTGCAAGCTGTGGCGAAGATC
EMPV1_01898	TAGATCCCAAAGCACAATATCCTGGTCACTTGGCAAGCTGCCGGGCCGACCAAGGCCAAG
EMPV1_01899	TACAGTGGCTACAGCTTTGACTATGATTACTACCGGGACGACTTCTACGACAGGCTCTTC
EMPV1_01900	GGCAGAGTAGGCACTAGCTTCAAGGATCTAGACATGGTCAGGAGAGTTTTGATTCTCTTG
EMPV1_01903	TCGTCCCTTTCTCTACACACATCTGAACTTATGCTAATAGGTTCAGGACAATATCAGCCC
EMPV1_01905	CTTTGCGGACTTCGTATCTCGGGTGTGTTTGTCTAGGGGAAGTAGGGCTGTCGGATTGTA
EMPV1_01906	CCAGGTGCTTAGGGTACTTTTTCAGTAAAGGCCTGGTTGTGAGCTGTTAGTAAGCCCGGC
EMPV1_01907	GGAACCATATCTTCAGAAAAGATTAGTCAGCAAACCACTCATGGACACTCTTTGGACAAC
EMPV1_01908	GTGGCTCAGCATAAAGGAATCTGACTAGAATCCAAGAAGACACAGGTTAGATCCCTGGCC
EMPV1_01909	TCTTATAGTGGATGAAGATGACGATGGGGTAGATGTGCATGTGGTGCAGCCGCTCGATGG
EMPV1_01910	CTAGACATCTGCTGTTATCAGCATAGCTCTGTTTACTTTGCTTCCTTTTAGAAAAACCCA
EMPV1_01912	CGCTGCTCTATCTTGATGTCTCTTTGCTGGTTGTTTGGATTCCTCAGTTACTCTGTCTCC
EMPV1_01913	TCCATCACCGTTACCAAAGTTACCAACAACACCGTTACTGCCATCATCACTATGACCACC
EMPV1_01914	CATACTTGTCAGTCTGGCCCTAGCTTTCCTTGCCTGCATCGTGTTCCTGGTGGTTTACAA
EMPV1_01915	CAGGAGGGACTTTCATGTGTTTATAGTAATTTAGGGGTTGTTTGATTCAGTACAGGATGC
EMPV1_01919	TGCAGAAGCAGACCCTCAAGACCCACATGATTGTACACTCGCCCGTGAAGCCATTCAAGT

EMPV1_01921	CCGCTCCAATTCCACCCCCAGCCTGGGAATTTTGATAAGCTGCAGATGTGGCCCAAAAAA
EMPV1_01922	CAGACGAGTAAGACTTCAGACAACATTGAGGCCAGGTGTCCGTGGTCGTCAGTTTAACAG
EMPV1_01923	TGAGAGAAGTTGGGGTCCGATTGGACGCGTGCAGGGCCCTAGAGGAACCGGCCACCTGCG
EMPV1_01924	TGCACGGGGTCCAGAGATACAGTGACCTGGTCCCTACTAACCTGCCTCATGCGGTTACTT
EMPV1_01925	TCACACATCTGGAACAGGGACAGACACTATGGGCAGCCTACAGACCATGGATAGTGTGAG
EMPV1_01926	GAGTGTTGGTCTCATGAGCTTCAAGGATGCTGCCCATAGTATTCCTCAGTTTTGTGCCAC
EMPV1_01927	TTCCCCTGCTCAAACTTGCCTGCTCTGACACTTTCCTGATTGGTCTGCTGATGGTTGCCA
EMPV1_01928	AGTTCAGAGAGACTAACTTGCTAGTTACACAGTAATGAGAGTACTGGTATTCTGACCCGG
EMPV1_01930	GCAGTGGCAGAATTTCACAAAAAAATCAAAGAAGCTTTTGAAGTGTTTGACCATGAGTCG
EMPV1_01931	AAACCCCTGCTCCGAGGACCCGTCTTTAATGCTGCTTCCAGAGTGCTTTGTCCTGACCTT
EMPV1_01932	CAGAACAGAGTCTTCCTGGAAAAGGTGGACCCCAAGAGCTACGCTATCATCTGCACCCAC
EMPV1_01933	TTATTTATCGAGTGCTGAGTTCTGAAGCTCGTCTCCAGACATGCCCTATCCTGAAGGGGC
EMPV1_01934	TGCAGGACCCAGACTTCTCTTTTCTGGCTGCCCGTATGAATTATATGCAGAACCATCAGG
EMPV1_01936	TACCACGTCCACGAGTGTGTTTAATCAGCACCAAGACGATGAGGGAACACGGAGGCTATG
EMPV1_01938	CATAAAACTTCCGCCACAGAGTAGACAGATCCTCTGGGATCTGGAACACGGTTTCGCTGG
EMPV1_01939	TTTGGAACCAGAGACCAAAACTCTGACTGTTCTCCTTGGGGAGCCTGTCACCTTCCGTTG
EMPV1_01940	AAAAGATGCACGTGCAGCGGCGCCACGGAAACACTCCAGAAAAGCGAACCTTCTCCTAGT
EMPV1_01941	AGAGGTTTTGTAATTAGAAGTCGTGCGAGCCGGTTTCGCCGCGCTCCCTCTGAGTGTGAG
EMPV1_01942	AGGGCTGAGACTGCATGGAGAAGAGACCCTCTACTTCTGCCAAGCCCCATGCCCTGGGCT
EMPV1_01944	GCTGAGAGGATCTGGTGGCCTTCTTTTCCTGGTCCCCTTTCTGCCCAACAATTCCCAATT
EMPV1_01947	ATATCCAGAAAGACACTGCGGTGGAAGGCGAGGAGATTGAAGTCAACTGCACGGCCATGG
EMPV1_01949	GGAGAGGTCCTTGATGTGGGGTCTAGGAGCTATTTCTAAATGGACTGCTTTTACCTTTTA
EMPV1_01950	CCTCTTTGCACCTCCACGTGCCTTCAGTGCAATCTGACGAGCTGCTTCACAGTAAACACT
EMPV1_01951	GGCCCTGTTTCTATGACATCCATTTGGACCCTGAAACTGACCAGGTGAATGGGCTGTTCT
EMPV1_01952	CTGGACTACAGCATGAAGGCAGAAGCTGACGCCCTGCATCACAAACATGACAAGAGGAGT
EMPV1_01955	ACAAGCTCACCACCTATGAGTACATTGTGCAGCATCGTCCGCCACAGGAGGCAAAGGGGT
EMPV1_01957	ATCTATTAGGCTTTCTTGTCATTCCGTTGCCTTGGGAAGGGGGTCTGTCTTAGGGAGTGC
EMPV1_01958	TTGCCGCGCAGCCTTCCCCCCCTGGTACTTTAATGCCGAGTAGAACTCTTGTGATAAGTT
EMPV1_01959	CCAGGCATTAGGAAGTATTCCAGCATAGACTTGACTCCACCATCGGTACAGACCCTTGCT
EMPV1_01960	CCACAGGGTCATGTGCTGGACATTCGTGCTCTGTCAGTAGCATCAGCTAACCAGTCTAGA
EMPV1_01962	GGGAGAAAGTGATGAGAAGCTGAGGTAAGGACCAGAGAGTCGGAGCGATTTAAGAGTCGG
EMPV1_01963	ACTCAGGTTGTTTCTCCAAGAGCCAAGGGAGCTAATGGACCCCTGATCCACAGACCACCT
EMPV1_01964	ATTCAGAAAACCACCAGAGCACGGGACCCCAGCCTTTCTGCCCATCACCCAGGACACTCC
EMPV1_01967	CCCATGTTGTGAAACCAAAAACAAATGCCTTTTGTGAGACCAAGCTAACAAACCTCTGAC

EMPV1_01968	AAACCGAGTCCTGCAGTGAACCTCCGAACTGGAAAAGCAGAGTTTACCCAACATCCTGAG
EMPV1_01969	GAGTCACACCCTGGTCAAATATCATGGAAATCCTGGAGGAGAAGGATGGGGTTGACACGG
EMPV1_01970	TGAGCAGCTACAGAAGCAGACTCTGGATGAGCTGAAATGCACACGCTTCAGCATCAGTCT
EMPV1_01971	ATGGCTAGTACAGTGGTAGCAGTTGGACTGACCATTGCTGCTGCAGGATTTGCAGGCCGT
EMPV1_01976	CCTCTGTGATCCTTCAGAAGGTTGTAAACGTAGCCAACTTAGGGGTAGTTCCTTCTGGTC
EMPV1_01977	CTTGGCAGGATTTCTTTTACCAGATAACTCAGCACTGCAAACACTAAGCCATTCCCTGGC
EMPV1_01978	CCCTCTGAACGCCTGGGCTGTGCTTTAACAGATGATTCAGTGCTAGGGTGTTGAATGTGA
EMPV1_01979	AGAGCCGGAAGGGGCAAGACGAGTTCAGTTCACCATGGGGCTGTTTGGTAAAACCCAAGA
EMPV1_01980	CCGCAGAGAATGTTAAGGTCACTGTAGAAGATAAGCACTACCTAAAACAACTGGAGAAAA
EMPV1_01982	CTCCTCTTATGCTAATCTGCTCTGGGCTGGAGACATGAAATCCTCACAAGCCATCACGAC
EMPV1_01983	ACTACAGCATCTATGGACCTGCCCCTTATAAGAATTTACCTTAGTCCTTCACGACCTTGC
EMPV1_01984	TCTCTGTGTTGGGCGTCTGTCTGCCCGCATGCCTGCCTCTCTGTGGCTCTGAAGGAGGCA
EMPV1_01985	CACATGGCTCTTTGCATCCCCAAATCTGGCGCTTTTCCTATCCTTCCCAACTCTCAGAAC
EMPV1_01986	CCACTTAAATTGTTCATCCCATGCTATTCCTGCTGCAGTTGTTAATCCTCTCCACTTCTC
EMPV1_01987	TTTGTAGACCCAGAGCAGGACTGGAATGACAATGCAGGGAATGCACACCATTGTCCCAGG
EMPV1_01988	CTTCCTCAGAGAGTGGAGAGTTAGGATCCAGTTTAAGTTCTGAAAGTACCTGTGGTTCTG
EMPV1_01989	TGGCCCGTTTGCTGTACATGAAAGAGAAACCTCTCACTTACGAGCATGGTGTGAAGCTGG
EMPV1_01990	CTTCAGCCACCTCGGCCAGAGTCCAGAAGGGCTGTGAGTCCTTTCTTTGGGGGCTAGAAC
EMPV1_01991	AGCGCTTTGTCCATTCTCCCTGTCATCATCCGTTTGAGGTTCTTTCATTACTGCCGCCCT
EMPV1_01994	TAAAAGGCGTCTTACCACCTGGGGCGGCGGCACTGCTTCCTGGAGGTGTTTCCTGCCTCC
EMPV1_01997	TGTCCATTTGTGATTTCCACACAACTTCTGCACCTGGCTCTATAACTTCCGCCTCCCAGG
EMPV1_01998	CCAACTCTGGGTTTCACTGATTTCTCTCAGTGGTTTTATTTGCTATAGACTTGTGCTCTA
EMPV1_01999	GGGTGTCTCTTATGAAGACCTTAGAAATCCCCTTTGTAAAGACCATTGAGGGAGAGTGGG
EMPV1_02000	CCTCTCCCACCTTCAAAGGACTTCATGGAATTTGTTTCTGTGTATGAATTCACTTTGGGC
EMPV1_02001	TGGATGACCCAGGGGAGCTGGCGCTGAGAATTATGGACCTAGGACTCCTAGTGTCTTTTT
EMPV1_02002	GGACCCCTTCAGTCCAATATGCTAGAATCCTCAGAAAGATGTGCAGACGACAGGACCCAT
EMPV1_02003	AGCTGTGCAGTCTTCTGGCTGTGGGACCCTGGGCTGGTGACTTCTTGCCTCTGGCCTGTT
EMPV1_02004	GAATTTTAGACCATGGAGACACACAGGCATGGTTTGAGTGCCAGTTCTCACACTGATGAA
EMPV1_02005	GAGTTCTGAGTATAGGGCACATTCTCCATGAATAGGCTGAATCCCATACTGAACTCAAGG
EMPV1_02006	TATGGCCTGGGACAGCTAAAACATTTGTGACTACTTTCTGTGCTTAGGAATGCCTGTCCT
EMPV1_02008	GCAGAAAAAAAAAAAAAAAAACCCAACAACAACCAAAAACAGCAGAAATTTTGATGCAGGA
EMPV1_02009	AACTCTTCAGGTACTGAGCCTAACAAGTCTCCTGCATCACCCTTATCATGGGCAACAGGG
EMPV1_02010	GTTTATTTATGTACCTCTTGCACACTCATCAACCTATGCAAGTCCCCCACCCCGGCCCCT
EMPV1_02012	GGGCTGGAAAATTGTTAGACTACTTACAAGAAAACCCCAAAGGACTGGATACCCTGGTCG

EMPV1_02015	AGGAGAGAGGGAGGTCTGTTTGTCCTACTGATGTGGCCTCCAGGATACCTTCTTTTAGTC
EMPV1_02018	ACTGTAGTGAAAGAACCAGGAGTTCTTAACTGTAGTGAAAGAACCAGGAGTTCCTGCTCC
EMPV1_02019	GTATTGAATGCACACGGGGTGAGTGGTTCAACTCTAGCATCCCTTCCAACATTCAGTGCA
EMPV1_02021	CATAGATATCAAATAGACCCAGCAAAATACATGTATAAACATACTTTTCTCTTCCAGATG
EMPV1_02022	ACGCCTGAACGCATTGCCGAGAACTTCCAGGTCTTTGACTTTGAACTGAGCCCTGAGGAT
EMPV1_02023	ACTCCAACCAGTTCTGAGGAAACTCCATCCTTTTGAGGAAACTCAGGTTACACCCTCGCC
EMPV1_02024	CAGTTTATCAAATTCACAAGGTTTGGGCTGGTATGGCACCATGACTGGGTGTGGCAGCAA
EMPV1_02026	GTCAACGTCTCTGTCCCCAAGAAAACCAGGAACAATGGGACGCTGTACGCCTACGTCTTC
EMPV1_02030	TGTTTCAATGAGTTGGGAGGGCGTGACCTTGAGAAACTTGTATTGCAGACTCCCTTGGGG
EMPV1_02032	GTCCCCGCGTTGTCAGTCGTAGAAGTCAGAAACTTCCATGTCCCCACCCCCACTTTAATA
EMPV1_02034	GAAGAGTGCATTTCAAGCTGCTAAGGTGGAGAGAGTGATTGCAGAAGGGAGTGCTGCTCA
EMPV1_02038	AAAGGTCTCTTGGTCCCAGACCACTTGATCACACGCCTCATGCTGTCGGAGCTGGAGAAT
EMPV1_02039	AGAGTTTTCCACCTACCCGGGCTCTGCTTCCTGCACCAGCCTCTGTCATCTGCCGTAAGT
EMPV1_02041	AAGCTTGTACTTCGGAAAGTCAGCCTCACTCCTCAGCACTTACAGCACCTAGTTCTCCAG
EMPV1_02042	TTGTTGTGAACTTTGACCTCCCTGTGAACCAAGCAGAGGAGCCAGACTATGAGACCTACC
EMPV1_02046	GGGGAGAGCAGTGGATGACCGCAACAGCGAAGAGTGATGAGTGATACAACCTGATGATAT
EMPV1_02047	TTCTCCGATTTGTCTACTCTGCGGCTCCAGCCACCTGTCAAATACAGTTCAGCTTTCCTG
EMPV1_02049	TGCCAGCCCCTGCCACCGAGACCATCTGTGCCATCTTTAACTCAGTCCTAATTTTCTCCA
EMPV1_02052	ACAATAGAGTGGAAAGACAAAAAGATAGGGGCGTTAGAGAGGCAGAAAGAGTTCTTTGAT
EMPV1_02053	TGAGCCAGGTGCCGATGGACGGCCGGCATGTCAATGTGATAGGGAAGAATCTCGTGATGC
EMPV1_02055	CCAGTGGCCAGAAAGAAATGAGAACTTGAGGAACTCTCACTCTCTCTATCTGCTATTTAG
EMPV1_02056	ACCTCCAATGGTATCTCACCGCCCGTCTAATGAAATCCAGAAACTTCATGTCGGCCCACG
EMPV1_02057	ACAGCCAAACCCGCTGCTCCTTTATTTGGTGTCCTTTCAAATCTGCCATTGCCTGTTCCC
EMPV1_02058	GAGGACCTTTGGGGGCTGCCGTTCCCGAGTATCACGTAGGAATTTCAAAAGCCAAAATAC
EMPV1_02059	CCTTCTGCGACAGGGAGATGTCGGTCGGGATCTACACCGGCTACTTTGGCTACAAGATGT
EMPV1_02060	TAGTGCTCTTGGCTTCCCGGCCCCTCCCTCCAGGCCGCTGCCTCCTGACCCCGTACCCAA
EMPV1_02061	GATCAACAGTGCATTTGGTCTTGCCATTGTCATCGCTACTGCTGCCCTGGACTTGGTCTT
EMPV1_02062	AGAGCCACGAGCTCAGATGAGGAGGGCTGCTAGCAGAGATCAACTTAGGGACAATAGCCC
EMPV1_02064	CTCGGGCCTGGCCGCCTACGCGGTTTGGGCGCTGCTACTGCAGCCGGGTTTCCGCCGTGT
EMPV1_02065	GCGGACACTAAGAGTGAATTTTTGTATAGGTCCATTGCATAACATCGAATAAGACATGCC
EMPV1_02067	TACCTGCTGTTACTGGACCTGCTGGCAGAGCCTTCTTAAACTTGGCCCTTACTGAGTGTC
EMPV1_02068	GCTATGTCCAAACTGGGTCTTCGGCAGGTTTCAGGGGTTACTAGACTTACTATCCGGAAA
EMPV1_02072	AAGTAGCTTAGTCTGAGGAACTCTGGGCAGATGGAGCTGTGTGTGCAAAAGATATGGGAA
EMPV1_02074	TGACTCAGAGGTTTGGACGCCACATCCCAGCTCATATCCACATCTTGATGGCCAATGTCT

EMPV1_02075	ACAAAGGAGCCTGCCAAGTCCATATACCTGTTCCCAGCCCTTTCAGTCGCCCAGTCAATA
EMPV1_02076	GGAATAATTGCTTGCTGAAGTTTAGCTGAATGAGTGAATCAAGCATACCATAAAAACACC
EMPV1_02078	CCGGGAGGATATACTGAATGCCAGTGACAAGTTAACACAGGTCCTTGAAGAAGCCAACAC
EMPV1_02080	TTGGTCCTACCTGTCCTCTGAAATCAAAGCAATAGGCCGTCTTCCAGTCCCTGCTCCAGG
EMPV1_02083	CAGAAAAATAAGTTGACCTCAGAAGTGGCTGAACTTCAGAGACAGCTTCAACTAGAAGTC
EMPV1_02084	CATCGAGGGCTGCTCACAGCTGAAGCCAGACAACTATCTCCCGGCCTGGCACACGCCCTT
EMPV1_02085	TGGGTTGCCCACACTCTGCAAGAGCTGGTCACTCGGGATCAACTAATGGGGCACAGAGAT
EMPV1_02086	CTTACCTAAGGCTATGCCCATATGACATTCCAGCTGCTTTCTTAAGACCTCTGAGATTTA
EMPV1_02088	AGAGACTGAGGGCCAGTTTGTTAGTGATGGCAGCTGTGTGGGACATGTAGGAGCAGAAGA
EMPV1_02089	TGGTGCTAGGTCCCCATGGCACCCGGCTTTCTCCTGCCGGTCAGCGGGCCTTTTCAATCT
EMPV1_02092	AGCGAGCTCCTCAAGGGAATCCCTCTGGCCACCGGGGATACCAGCCCGGAGCCTGAACTA
EMPV1_02094	TGTTAGTGAGAAACAGTCTGCGGTAACTGAATCCTCAGGGGATACAGTATCCTTATTGAG
EMPV1_02095	CAGATACAACAACGCCTACGACCTCTGTAGTTAAAGCAAGTGGTGAACCGTCCCCAAAGC
EMPV1_02096	AGATCTCCATGATTTCAAAGCTCTCAGGCTGCTGCCGGGTTTGCTGAGGGCGAGGCAGAA
EMPV1_02097	GCGTGGAAATTGGAGGCTGAAAGCATGGGATTTTTTACCAAGGAAGAATGGTTAAAGGGG
EMPV1_02098	CCAGAATGTTGAACTTTTTAAAGAACTCAGTATAAATGTCATGAAACAACTCATTGGTTC
EMPV1_02099	CTCAGATAGCTAGTATGGGTACCAGAGGTTTCTCATCTTGATTTCACAGCTCTCAGCAGA
EMPV1_02103	CCATTCGGAGAGGAGGTGGCAGCGTTAAAACTGAGCTAAAGGCCAAGAATTTGAAGACTC
EMPV1_02107	CTCGGGCAAATGCGTCTGAGAATAAGTGTGCTGTCATTAAAAAGGTCCATGTACTGGCTG
EMPV1_02108	GCCCCTTCACGAGAGGTGCCACTCATTATCACGATCTCTACTCGACGCACGTTAAAACTG
EMPV1_02109	GTGAAGTCCTCCATTGTCCCTCGCCCGCCTCTGTATTTTGATCTGAAAGCTCGATTTCCA
EMPV1_02110	TTCAGGAATCCGGTAGAAGTTTAGTGAACTGAGGCCAATTCTTCACACACACCTGCTGGG
EMPV1_02112	GAGAAGCAGGACGAGACCCAGACCAAGATTAAGAGGGCCTCGCAGAAGAAGCACGTGAAC
EMPV1_02113	GAAGTAATGACAGTGGCTAGCATTCAGAGAAGCGGATGGATTCAGCATGTCTTCTGCAGG
EMPV1_02115	GCTTAACAGTTAGAGGCTACCTTGTTGGACCTGCACCTGAATACTGATCAGCCATAGAGT
EMPV1_02116	CTTACCTGTGAGATCCTGGCCCTTCTTAAACTCACCTGCTCAGATATCACCGTCAATGTG
EMPV1_02118	TGGAATTGATTACTCTCCCTCTGCAATACGACTTTCTGGAAGTATTATAGAGAAAGAAGG
EMPV1_02120	TGGCTGTTTTTGTGGTACAAGAGAGGAGTTGAGTAGTTGTGACAGAGATAGTGTGACCTG
EMPV1_02121	GGATGCAAAGCAACCTTAATGGGAACCTCAGTAAAGGAGTGAATGGAAGCTGAGAGGACA
EMPV1_02122	CCGGATTCCTGGGTGAAGGGGCTGATCGGTACTGTTAAGAGATTGGTACTTGTCACCATT
EMPV1_02123	GGAAGTTAGCCTTCTGGCCCTTGGCCTTTAGTTTTCTTACTCAACTAAAGTACATCAGGA
EMPV1_02124	CTGCCCTTATGTAAACCCATCTCCTTTGAACACCTGAAAGCAGTTAGGCTCAGAGGTTCC
EMPV1_02126	CTGCCTGGAATGAGATGAAACTGGCAGGAATGAAGGTGTGGAAACATGTGCCTAATCTCT
EMPV1_02128	CAGTCTCGCTGGATTAGGTGCCTCATCAGGAAATCAGGACGTTCTTATGTGAACTCTGTG

EMPV1_02133 TTCCTTGGCGGGGGAAGAGTCAGGTGCAAAAATCGAGCTGAGGGTGCTATTTATGGCTCT
EMPV1_02135 GACAAAATGCACACCTCGTTCACCATCAGAAGATTCATGCTGCAGAGACATCTCAGGCCC
EMPV1_02137 GTCATTTTTCCCGAGTCTGTAACGGGAGGGGAACGTGCCTGTGAACCGGACTCGTGTGAA
EMPV1_02141 AGTGCAGGAATGCCCAAGAAGGTAACTTGGTTGCAGTTGATGAAAATTCTGCGCCGGCTG
EMPV1_02142 GTACTTTCACAGTAGTTCCCAGGGCAGCAGAAAGTACCGATATCCCACCTTATGTGACGA
EMPV1_02144 GCTACATGCGTCTGGGCTCAGTGTCAGCTTCGGACAAGGATAAGGGCATTGGGATCCTCA
EMPV1_02145 TTTGTCCACTCCATCGTGCAGATTTCGCTCTTGCTGCCACTCCCCTTCTGCGGACCCAAT
EMPV1_02146 GGGGCTGGTGCTGATCCTGACTGGCGTGGGACTGGAGGTGGCCCCCTCGCCAGGTGTCTC
EMPV1_02147 TGGTATGGGATTGATATGGCTGGTTTTAGGATCTTGTGCCTGGAGATAGCCTGAGGTGGC
EMPV1_02148 AGACGGCCAATGCGATTTAGCGTCCTGCTGGGGCCCTTGCCCTCAGCTGAAAGATCATAG
EMPV1_02149 CTTCTCTGTCTTATTTCTGCCTTCATCTCCTGCTTCTCACCCAACTCAGCCACTGTTCAG
EMPV1_02150 GCTGGCCCTTCGGTCAGCCTCTCCTGCTAGGCCTCGATGGGAGGAAGGAGGTCCTAGAAA
EMPV1_02153 GGAGCTTCACCTATGAGACTGTGGAGTTGGTCCCTGCAGGAGCACAGACTCAGGCAGCTT
EMPV1_02154 AACTGTGTGCCACACTTTGCACTATTCATAACATGATGATAACCTCAAGACTATCAGGAG
EMPV1_02155 CTTGCAAAACTGAAACTCTGCACCCATTGAGCAAAAACCCCAGTTCTCTCTCCCTGCCAG
EMPV1_02156 CTACGAACACCTCTTCAAAGTGAACAACAAATCCGTGGGTGGCTCCTTCTACCTGCAGTC
EMPV1_02157 CTTCAGCAACCACCACCCTGATCAGTCAGCTGACATCAACCTCGAGCCAAAAATTACTAC
EMPV1_02160 GGACAGTGTTCCCAGAGCCAACTGGGATGGAGCAGAAACCTTAGGACCGAATTAGTCTTA
EMPV1_02161 TTCTACTGCTTCCCTCTATCCATTCCCTCCATCCACTGGTGCTTTAAAAACAATGATCAC
EMPV1_02163 GTCCTGGCTGATGCCTTCAGGAGCATCAACAATGCCGAAAAGAGAGGCAAACGCCAGGTT
EMPV1_02164 GTAGACTCACCCCTTAAGAAATGTATGGGTATCTGCAAGCTGCTTCTTATCAGCAGGAGG
EMPV1_02165 CCTTCACACACTCAGCTCCTATTTTGGGGGACTGGTTTCTTTTTATTTCCTTACCACTTC
EMPV1_02166 GAAAACTGTCTGTGAGGGGAACAAAAAGTTGGCTCTAACGTTGAAACTCTTCAAATTAGC
EMPV1_02168 GGGAAAAGAGCAGTTATTTAAAGACACCCAGCCTGTTCCTTCCTCACTTCCTTCTCAGCC
EMPV1_02170 TCCCCGTGGGTCCAGGAACTGGATTTCTTTATTTGGTCAATTTATACTTCTTATATCAGT
EMPV1_02171 TTTTTCTCCAGCCACCACAAGGCCCACCTCATAGAGCAGTCCAACCGCTGCTTCCACTGT
EMPV1_02172 GAGGATGGAATGGCTGTATGCATGTTCATGCAAAACACCTTAACAAGATTTATGAGACTT
EMPV1_02173 GGAGTCAGCAAACTCTCTCTGAAAAAGGGCTCAAGTGTAAATATTGTTGGGAGCCACGCG
EMPV1_02174 GCGCAGGACCCACTTAAGGTAAACACTGGACTACCTCTATACCAAGAGAAATGAGAGGGC
EMPV1_02175 AAATCTGACCTCCCTGAGACCACCATGCTGTGAAGAAGCCTAGAAGTTAATCACGTGGGG
EMPV1_02176 TGAGGTAGTGTCTGTGTGGGATCTGCTGTGAGTCGAATCATTGTTCAAATAAGGTGTAAC
EMPV1_02178 TCTTGCCTGGCTTCTCAAGGAAGGGGGCTGAGAGCTTTACCTGATAGCAAAGGGTTGGGA
EMPV1_02179 AACCTACCTGACACACGAGGCAAAAGGCTCCAATGATGCCCCAAATACAGACACGGCCAT
EMPV1_02181 ACAGGCAGATATGCAAGGTGCCAGCTGATGCCAGGGAGCTTTTCAGCAAAAGAAATGTCC

EMPV1_02182	ACTGAGGGTCACCATGCCCTGGCTCCTTGTTCCACTGCCAACTCTGGACATTAGCCTCTA
EMPV1_02184	GGATCACATGCATTCAAAACCACAAGACTTCGAGGAGGAGAGGCGTGAACTTACATGGGC
EMPV1_02185	AACCTGCTTCTCTGGGGGTTGGGGAGGGATCTGCCTGGGCCTGGGATTCATTTTTGTTTT
EMPV1_02186	GGTATCCAGACGGGGTTTCCAGGCTCTCTCTATACTTCATCCATTCACCAGGTTCTGTAC
EMPV1_02188	AGCTGCAATAACTCCAGATCTTTAACCACTGTACTGGGCCAGGGATTGAACCTGCATCCC
EMPV1_02191	TAGGCTTGGATTTATGAAGTGTTTACCGCTGCGCGTCTTGTTCCTGGCAGCCTGGCGTCT
EMPV1_02192	GCACCACTTCCCCCAGATGGGCCATAGCCAGAACATCTACTTCAACTCTGCCTAGTAAAT
EMPV1_02193	TGACCAATTATGCTACAGTGTGGGCATCCAAAACAAACCTTGAGCAGCGGAAAGGACGGG
EMPV1_02194	AGAACTCGCCAAGAGCTTCTTCTGCCCTTGCAGACCCATACCAGCTACTTGTAACCAACC
EMPV1_02199	CTGTGATCTTGATGCCTCCAGTGATGGTCACACCTCTAACCCATCCTAAAAAACTGCTAC
EMPV1_02202	ATGACTACTGGGACTTGAACGATGTGAATGTGGTGTGCAGGCAGCTGGGCTGTGGAGAAG
EMPV1_02204	ATATTGGGACAACGTGCGACAGAGACACAAAGTGAGCAGAGGCTGTAGGGAAAATGGCAC
EMPV1_02205	GGGAAATCCGAGACAGATTACTGTCCGAGGGGGTCTGTACCAACGATAACATACCAAGTG
EMPV1_02206	CCACAACAAGTGATTGTCCAAGGTGTCCATGAGCTGTATGATCTTGAGGAGACCCCAGTG
EMPV1_02209	AGATCTCTCTGCTGAAGGGGGCCACCTTTGAGCTGTGCCAGCTGAGATTCAACACGGTGT
EMPV1_02214	AAGACACTACCCAGGTCGTCTGGAAGGACTCTTTTACTGGCGACTCCAGGGTTTCCCCAC
EMPV1_02215	AAGCTGAACTTGAAAAAGTGCCTGCATCCGGCTTGATTCACTGCATGTCCCTGACTCCAC
EMPV1_02216	GACAGTCCTTCATCAACCTTATAATACAGCAAAGACTGTTATGTAATTTTCAGCAATTCC
EMPV1_02217	GCACACCTACAGCTCTTACCAGCTCGGATATGATTCAGTGAAGCCAACATCCTCAACATG
EMPV1_02218	GGGTAGGATGGAGGGTAGTGAGAACCCATCTACAAAGGTCTGTGTGAAAAGAACAATAGA
EMPV1_02223	AAACCCGGGCCTCACCCGGGCCTCAGAGGGCCGTTCCAACATCAGGAAAGGTCTGGGTCT
EMPV1_02224	TGTCCCTTCAAGCTGCTGCCGCCCTCCATGCCATTTACAAGGAGATGACTGAAGATTTGT
EMPV1_02228	CAGTATCTTCAGAATGCAATTGAGAGCAGATGCGCAAGATCAACCGAGATCTGTTTGACT
EMPV1_02230	TTCCTCTATTTGGCTCACAAACAGGCACCAGAGAAGCATATGGCACCTTGAACTCATGCC
EMPV1_02232	CCTCTTCTCCCTCTCTGACTGGATGTGCTTGTAAGATGGTCACACCTGGGAACCCTAACA
EMPV1_02234	CATCGTGGAGCATGTGCTCTGTTTTGGAGGGGATATCCTAAATATCACAGGGAATGGGCT
EMPV1_02235	CATGTAACCTGTATTGTCCACCTCCCACCTCTGCTCCTGAGATTGCACAGACCAACCAGA
EMPV1_02236	CCTGGACATTTGTAGGCGTGCCACCGAGATTTGTGAGTCCTCCTGCCAGAAGCCCAACTC
EMPV1_02237	AGGACACTGGGGATCCCTGGAAGACTGGGTATCGCCGCGTGAAGAAGTGGATCTGTGCTT
EMPV1_02238	CAGCTGGCGTCTGGCTGGCCTTCAGAAGCTATTTAGTTGGTGTTTTCTCAACTCAGTGGT
EMPV1_02239	AACTAATCTCAGTTATACACCAACCTACAGAAATGTAAAGGCAAAGGCTCTGGGCAGGCC
EMPV1_02240	ACAGAGATATCCCATGTAAAGACCATTATCTGAAGAATCTGCCTACACAGGATTAACATG
EMPV1_02241	TTTCTCTGAGCAGAGCTGCCCGTTTCGGGTCTCGGTGCGAGTTTCCAGTCCCGGTTTTGT
EMPV1_02245	GGAAGCAGATGAGAAGGATGATTCAGGTGCCTCCACGATGAACCTTGGTTCTGACAAATC

EMPV1_02247	ACTTGTGTGATGGATGTGGTTGCGTCCTGCTTGTGCTCCTCGTCTGTGGTGTCGTCGTAA
EMPV1_02248	TCCTGGTGAATGATGAAGGTTGCCATCAAAAGGCTGCCCATGCCCTCATAATGGTTGCTG
EMPV1_02249	AACCTTCTGACGAGCGGGTTTGGCTTGTTTAGGCTTAGTGACCCCTGTTTTGGGTGCTGT
EMPV1_02253	GTGTATGGAGTGCTTGGGCACGGTTTAGACAGTTTTGGAATAAATTCGTGAGATATCATG
EMPV1_02254	TAGTGACAACTGGGGTTTGTGCTGAAGGTTTCAATGGCGTATCCTCCTCTGTTCCTGGGG
EMPV1_02255	TGTGTGTGTGTGTGTGTGTGACCTACACCACAGCTCACAGCAACACCAGATTCCCAACCT
EMPV1_02256	GAGGAAGAAGGAGAAATTGTTATGGTACATGAGCACCGTGAACTAGATCGAAGCGGAACC
EMPV1_02257	CCCCAGGTCTCTTCACATTGTCTTCTCTCCCTGTGTGTCAGGATCCAGTATAGCCTTTTG
EMPV1_02258	AAGTTCTGTCTGGCAGCTGAGGGACTTGGGAATCGCCTGTGCTTCTTGGAACCTACTTCA
EMPV1_02261	ACCAGCTTGTCAGATTCGGGCAGGGGGCCTCCACATCTCAAAGGAAGCTTGTGTTCTTTT
EMPV1_02262	ATGTCCGGACGTGGGAAACAAGGAGGTAAAGCTCGCGCCAAGGCCAAGACCCGCTCCTCG
EMPV1_02265	AGGGGAGGTGCAGAGGAGGTACGCTGGCCACCCGGGAGCGCGGGAGTGAGGCCTGCAAGA
EMPV1_02266	CCAGCAAATTCAGGCCCCAGCTACAAGATAGGGAATTCCAGAAAAATAACCAGGACTGAG
EMPV1_02267	TGGCGCCTAAAGGAAGCTAGAGTGTTGCCTTTCCTGTGATTCTACACTGCAAAACGAGGC
EMPV1_02268	GAATTAGCAGAATCTGGATCGAACTTGAGTGCTGGACAGAGACAGCTGGTGTCCTTTGCC
EMPV1_02271	GTTGACGAGGACATTGTGAGCAAAGTGGCCGAGGAGATGACGTTTTTCCCCAAAGAGGAG
EMPV1_02273	CACACCAGAGAGTTAGCACTTGGCATTTGACTGAGGCTGGAAGTTCTTAGGAGGATGAAG
EMPV1_02276	CTTTGACATTCTAGAACTTAGAGATAACTTAAAACTTTTGTGAGGAAGATAAATATGGCC
EMPV1_02277	TAAAGTTGATGATATGTTATCAAAAGTAGAGAAAATGAAAGGAGAGCAAGAATCTCATAG
EMPV1_02278	TCCAGGGGATGAACGACCTGGTCATCCCGTTCTTCGTGGTCTTCCTCTGCGAACACACAG
EMPV1_02279	TGCCTCACCAGGAGGCTCTGGCCTAGCGGTCACCTCCCTTTGCCATGTGAGGCACAAGGA
EMPV1_02280	CTCAAAGATAGAGAGTTTGAGGAAGATGACTTTGGCTCTCCCAGCAACCCTGCTGCATCC
EMPV1_02281	AGTCCATGCTGCCATCAAGGCAATTATTGCAGCATACTATTCGCTTCCCAAGGATCAGGG
EMPV1_02284	CCCTCTTCGTTTTCTTACTTTTCTCTCTGGGACCTCAAAACCGAAGTCGTAGCCGATCTC
EMPV1_02285	GTTCCCACTGTAGTGCAACTGGATCAGCAGCGTCTCTGTAACACCAAGATACAGGTTCGA
EMPV1_02286	GAAGATGGTAGGATGTAAAAACAGGCAAATGTGGAAAGACATTCATATAATTGGAGTCCT
EMPV1_02288	CTGTGTGTCTGCTGGTGTCTCTTTTGCTCATTGGGTCTGTGGTAATGGCCGTGAGGCACT
EMPV1_02290	ACCTCTCAGTAGAAAGGCTAACAAATTTTAAGTGCCACAGGTTTTAAGGATTCTGCAGAG
EMPV1_02291	GGACTTGAGGCAGCATCTCTTTGTTTGCTATAGAAACAAAACATTGGCGATTTATGTCGG
EMPV1_02292	CTTCAGCCTTCCAGTTTATACCCTTTCAGTCCCAGCTAATGAACTGACCTCATACTTTGG
EMPV1_02294	CAGAAGGCCTTGAAGGAGAAGGAGAAGGCCTCCTGGAGCAGCCTCTCCATGGATGAGAAA
EMPV1_02295	GCGGGGAAGATCTACTACATCCTGCGGCAGAACGAGCTGGTGGACTTGTACATCCAGGTG
EMPV1_02298	GGACTTGATTGGAACAGAAACTTTTGTGCATTTTCCCCAGCCTTTAAGATAAGGTTTGGG
EMPV1_02299	AGTCTGGACTTCTCCCTGTTGCATGATGAGGGTGAGTGGTTGCTGCTCTTCAACAGCATC

EMPV1_02300	ACTGTCCGCCCCAGCTCCCACCTTCAGACCGACCGTCTCAGTAGGGCAGGGTTTATGGGT
EMPV1_02301	GACCTGGAGCAGCAGTACCGAGCCTTGCGAAAATACTATGAGAACTGTGAGGTAGTCATG
EMPV1_02302	AGTTTATTGATTTGGGAGCAAGTCCTCTGTCTGGGGGTACACTGCTGGGATGGGTCCTAG
EMPV1_02304	TGTCTTATCCAAATGCCAAGGAACACACGTGGAGACTAAAAAAGAGGGCCCCGTGATGGC
EMPV1_02305	TTTCCAAGGGAGGCAAACCCCGCCTGAGGGTGAAACCAACAGAGAAAAATGCAGACCCAA
EMPV1_02307	AGCGGCAGTGAGGTGTGCGCGTCCTGCTAATGTACAGTTGTTTTCAGGGCTCCTAAGAAT
EMPV1_02310	GTCCACTGGATGTCACTGTTTTACTGAAAGGCAACTGTTAATAGTGGATGATGACCGAGG
EMPV1_02311	TGGGATCCCCTCCAGCATCACCATGGGCTGCATTTCCTCTCAGTAAAAGGACTCTCTTTA
EMPV1_02312	TCCCTCCATCATACACCGCCTACCAACATACCACAACCTATCTATAAGCCAAAAATCCGC
EMPV1_02313	GCATGACCTTCCTGTTGATGTTGCTGCTCATCTTCTTCTACATTTTTGCTGTGGCTGGTG
EMPV1_02315	GGGGAAACCATCGATATTGCTGTGGGTAATTGTCTGGATCGTTTTGCTCGAGTCCTGAAG
EMPV1_02316	CCCTGTGGGCAGGGACTATTTCTTGTTGCCTTTGTGTCCTCAGTGTAATCCTTGTCACAC
EMPV1_02318	TAACACAAGAGGGGGCAGACAACTCAGCGGAAAAGCTTTCCATCCTTCCATGTTATTCCT
EMPV1_02319	TGTCATTCTGTCCTAGTGAAGTGGGGTCTGGGCGTGTTCACGGAAGAGCCGTTTCTTATC
EMPV1_02320	GAAGAAGGCCCAGGAGCACGGAAGATTCAGGTATGGAACACAGGCCTCCTTGTCAGGCCC
EMPV1_02322	CTTCCCAGACTTCGACACCATTGACCACTTCATCCTATCGCAGTCAGTTTTTATTGCCCC
EMPV1_02324	ATTCTTCACTATTTCAATGGCCGAGGCAGAATGGAGTGTATCCGGTGGCTCCTGGCTGCA
EMPV1_02325	GGATTTGTGACTGGACCATGAATCAAAACGGCAGGCATTTATACCCCAGTACCAGTGAGG
EMPV1_02326	CACCCGCTCGGAAGTTGGTCAACATACACCTTAGACAACATAAGAGTATTCACACAGGAG
EMPV1_02327	CTGGCTAGTTCAGGGATTAACAACATAGTGGTCCAGAGAAGGAATTGCAGCCTTGCCAGG
EMPV1_02330	CAGGTCACAGTAATAAGTGGTGAAACTGGTTGTGGAAAAACCACTCAAGTTACTCAGTTC
EMPV1_02331	CATCACGCTGTGAATGCCAGTACGTTGGAGAGGTGTCAGAGTTACACAGGCCTTCCTTGA
EMPV1_02332	AAAGAGCATTCCAGGCAGATAGAATAGCAAGTGCAGGGCCCTGAGGAGGACTCATGCTCA
EMPV1_02335	CCATCCCCAAGGGCTTCAATAGCTCTTTTCTTGTCAACATTGCCACCATCTTTGAGTCGG
EMPV1_02336	GCTGCTGTCTTTCTTGGAATGCCAAATGGCTTTGTGATAGGTGGCGAATCCAGACAGGGA
EMPV1_02337	CCAGCCTTGATAGTGGCAGAACATCCACTAGCAATAGCAATAATAATGCTTCACTCCATG
EMPV1_02338	CTGGTCAAAGTTACCAGGGCTCTCTTTCAGTGGGTGTTTGGCCCTGCCTACTTTGTGGAA
EMPV1_02340	TTAAAGTCTGCCCGCTTCTTACCCAGTCACCACGAAGAAACCACTCCCCAGGGAAAGCTT
EMPV1_02341	TTGGTGATTATTGGTACTGTTCCTGGGGGTTGAGTGGAGACTGGTGCTGTCAAACATGCC
EMPV1_02342	GGACACAGGAATGCAAACGGAAGCTGCTGAATAAACAGGTCACAGCCACACCAGATGCCT
EMPV1_02343	AAAGGTCAGTGTGAGGCACCAAGCACTATGGCAGCTGGCTCATATGGTCATTGTCAGTTC
EMPV1_02344	AGGCAAGTAGTGGAAAGTTTCATACCCCGAATTTGTGCCCTGGTTATTGACTCAAGAGTG
EMPV1_02345	TCCCAGGGTCTCCGCGTCAGGCTGGCAAAGACCGGGAGGAGCCAACTGCTGTTTCTAAAC
EMPV1_02346	ACTGCCGTTCCCTAATCTACCCAGGGAGTAAGGCTCCAGGGAAAACCATTTCTCTCTGTC

EMPV1_02347	TTATGAATCCAGATTTGTATTGGCTCAGGAACTCCGCAGCTGGTCTGTGACTAGGGCTGG
EMPV1_02349	ACAGCGTGAGTGACACCCCAGGGGGGCTGGGGAACGACCAGAATCCAGGTTCCCAGTTCA
EMPV1_02352	CTCAAAGAAGGGAATGAATCATAAAGCCAGTGAAGACTCCACCCTCTGAGTGAGTTCCCC
EMPV1_02354	CTGAAGGTCCTCCTTATTCCCCTGACTGGGCTGCTTACGGCAAAAAGCCTCAAAAGATGT
EMPV1_02356	GTGTAGGACGGAGATATGCTCATGTGGTGTTGAGGAAAGCAGACATCGACCTCACCAAGA
EMPV1_02357	AGCTAAATCTGCAGCCTTTACCACAGCTGGCCTACACCTGAGCCATGTCTGCGACCTACA
EMPV1_02358	GAGGTTTGTTCCATCCGCTTCCTGCTCACGGCTGTGTCACTGCTGAGCCTCTTTCTGGCA
EMPV1_02359	CCTTCTCTCTCCCTTCACCTGGCCCTCACCCTTTCCTTGTCATCCCCAGAAAGCATTCAG
EMPV1_02360	CTGGTGTGAGATATCAGGAATGTCTCATGATCACATTTACCATTTGTGCCATTTGCAACC
EMPV1_02366	ATACTGGTGAAGGAGACACAGTGCGGTGCTTTAGTTGTCATGCGGCAGTAGATAGATGGC
EMPV1_02367	GTGTGCATGGTTTTCTCAACTTGTTTCAGCCGGTTGGGAGTGATGGAATCTCTGGAGCTG
EMPV1_02369	ATGGGCTGCAGTACGACGCTGAGAACTGGCACTACTGTGACAGCTACGATCGGAGATTTT
EMPV1_02370	GGTTCAAGATACAAGAAGCACAGAGGGCCCCAAACAAACTGAACCCAAACAGACCCACAC
EMPV1_02371	GCCAAGAGGACAGTGACCACCACTGAGGAAGAAGTAGGACTGGTTATGGACAATTCAGAG
EMPV1_02376	CCAAAGGGGCTGGTACATGGTGGCCAAGTTAGAGGTGCAGCAAAGCAGCCATAAAAGCTA
EMPV1_02377	ATGACAGAAGAGGAAGACCTGGAGACCGCTATGATGGCATGGTTGGTTTCAGTGCTGATG
EMPV1_02378	GCACCAGCTCCTGCTGCAGAACAAGGACATGCTCCAGCACATCTCCCTCCTGGTCAAGCA
EMPV1_02381	GATTGGGACCAGACAAACCCCTTGCTCCTGTTGCTTCCCCCAGTACGATGAAAAGCCAAA
EMPV1_02384	GTATCATGACACACTGCATGTGAGTGGCGTGTACAATGGGGCTTCGGGGGAAACGCACCG
EMPV1_02387	GCATGAACTCCCACCCTGCTCCTTGGCTGTAAGTTCCCACTTTTCCTTGCATTTGGAGTT
EMPV1_02388	CTCTGGGAGTTCATGCTGCGCTGGAGGCTCCTGGCCACCGCTCTAATCGCCTTGTGCCAC
EMPV1_02389	CTCTTGGAGGCGTTAAAACAAAAGCACGGCCCCTTAAAGACCCTCAGGTTGAAGGCGGAT
EMPV1_02390	CAAACTTTTGGGGGGAACCAGACTATCCTGATGCTGAGGACTATCGCCATGCTCCAAACT
EMPV1_02391	TGTTGTCGGGTGGATCACGATGCAATTTTGATTAGTATCATAGGAGAAAAATTGCACGGT
EMPV1_02393	TGCCTGCCAGCCACGTCCTACCTTCTCACCTACCTCTCCGGTCACAGCGCAGACTTTATA
EMPV1_02394	CGAGGCCATGAGCTATGCAGAGGTCATGAGGCTGGTGAAGGGCATGAACTTCTCGGTGGT
EMPV1_02395	CTGTCGCGCCTGCACCATGAGAACATCGTGCGCTACTACAATGCCTGGATAGAGAGGCAT
EMPV1_02397	TCATGTTCCTCCCGCTCTCCGGCTCCGCGACGGACCGGTTCCTTGCTCAGGCTCCACTGA
EMPV1_02399	GCATTCTTCAGAAAGCGTTTCTAGTATCAACAGTGCCACGAGCCATTCCAGTATTGGCAG
EMPV1_02400	AAGAGCCCCCTAGGATCCTCCTGTATCACGAACCGCGTTCATTTGAAGTAGGAATGTTAG
EMPV1_02401	GTTCGTGCCGCGCTTTAACTTCACCCTGAAGGACCTGACCCGCTTCGTGGATTTCAACAT
EMPV1_02402	CTCCTCTCCAGGCAGCCCTGCTGTCCGTGGGTTTGTCTTTGAAATCATGTCTGTGCAAGA
EMPV1_02403	AGGCATTCTGCCCTTCATCAGGCAGCTGGTCACATGTGTGTCAGTATTTGCTTACTTATG
EMPV1_02404	CCAGATCCTTACTTAACCCAATGAGCAAGGCGAGGGATCAAACCTGCATCCTCATGGATA

EMPV1_02405 AAGAACCAAGAGTCCTTTGGGGAACCTGCCTCTAAAGCCCTGCTCAGATATGCACAGCTT
EMPV1_02406 GCACCTGTGATTCTAATGGACATCTTCCAGGTAAAGGCTGAAATGTTAGATATGGCAGAT
EMPV1_02407 AAAATGCCATTGTGTCTCCTTCTCCTGCACCACAAACACATCGAACAGACCATATGTCCT
EMPV1_02408 AGCTGGGCATCAAAGCTCATCCCTTACTCTTTGTTTCACTGTCAGGCTTCAGCTGCCCCA
EMPV1_02410 CTTTAAGGCCAGGAGAGCTAGCAGCGGATACTACAGGTGCCAGACTGCCTACTCCAGTCT
EMPV1_02411 CCCCCTCGAATGTCTGAATTTGCCCATCTACGTTTGTGTGTGCTGGAGTAGCAGTGATCA
EMPV1_02412 AGGACCCGACATAGTGGGTTCATTCCATGGCCTCGCTCAGTGCATTAAGGACCTGGCATT
EMPV1_02413 ATGGAGTGCATGTCCCTCACCGTAGAATCGGGATGGGGCTGTCCTGAAGTGCTGCGAACT
EMPV1_02415 CCCACATGTGACAGGACAACAGGTGGGTGTGTGAAATTGTTACAAGTCCTTGGGGCTTGT
EMPV1_02416 CTCCTCATTGCCCTCTTCTTCTTCCTCTTCCTCAAAGTCCACATCTTCCATCTCCTGAGC
EMPV1_02417 GACAGTATGCTGACTGGCCGTTTGGAGCAGGAGGTCCCTGCCACTTGGGTATTTGTGGAA
EMPV1_02418 GCCGCCAAACAGCTACTCAGCTGCTTAAGCTGGCCCACAAGTACAGACCAGAGACAAAGC
EMPV1_02420 TCCTTCGTGGGCACAAGGTCCTACATGTCGCCAGAAAGACTCCAGGGGACTCATTACTCT
EMPV1_02422 CAAATAATCCTAACAATTTGTATCAAACCACAAAAGAGCCCAAACAGAAAAAGCAATCTG
EMPV1_02423 CCAGAAAATCTTCAGTGAGGAGGCTGACCTGTCAGGAATCACAGATACAACACCCTTGTG
EMPV1_02424 CAGGGCGCTGGTCCAGGGAGAAAAACCAACCAGGGTCTTGTCTGTTAACCTCACTACTTT
EMPV1_02425 TTGCCTGCACACTGCTCTCTAGAGCAGCGTGTCAAGGTCGCCTCCCAGGCCCCAGAGGAA
EMPV1_02426 TGACTGAGCCGAAGTTAGAGCAGCCGTTCAAGCTGGTGGAGAAAGAGGTTGAGAAAGCAT
EMPV1_02427 CTCTTATTTAAGCCCCCAGAGGTGCCCTTCACTGCCCGGAAGCGCGGAATTTATGACCCA
EMPV1_02430 GCCTTTGTGGGTGCTCATCTTGATCTGTGTACTTATCCAGTCTAGAGTTCTCCAAGTTTG
EMPV1_02431 AGCTGGTGGTCTAGGCAGCTTGGCGCGGTAAGAGTGCTGAGTTTTAACTATGTCTGTAAA
EMPV1_02432 AGACCCTGACAGCTTCTGTTGGGAGAAATATCTGGAAGAAACCGGGGCCTCTGCTGTGCC
EMPV1_02434 TGGACGTTCCATTATTCTAACTGCGGTCCTTGCCCTCATCCTTGTCTACCTGTTTTCCAT
EMPV1_02438 CCTTGGGTTCTTGGACAATTGCCTTGGGAGGTGTAAGATAAAGGGACAAGGACTTGGGTA
EMPV1_02440 TGAATCCGACTAGGAACCATGAGGTTGCATGTGGCTTGGATCCCTCATTGCTGTGGCTCT
EMPV1_02444 GCAATGCTGCAAATGTAAGCAGTGGTTTCATGAAGCTTGTGTGCAATGTCTTCAAAAGCC
EMPV1_02445 ACTACAGACATGCTTGTGAGCACCTAGAGGACCCCATCTTTACAGCCCCCACAGCAACTG
EMPV1_02446 CCTTCTCAAGCTGGGTGATCTTGTTCTCCATTCTGCTTCCACCACCAATTCGCTGGACCA
EMPV1_02448 GAAGAAGGGGTCCAAGAAAGCGGTGACTAAGGCGCAGAAGAAGGATGGCAAGAAGCGAAA
EMPV1_02450 CCTGATAGTTTCAAGCCTTTCACCCCTGAGTCACTGGCAAACATCGAGAGGCGCATTGCT
EMPV1_02451 GAAGTCAGGAGGGGAAAGATTTCTTGATCTGGGTGCTAATTACATAGTGTGTTCACTTTG
EMPV1_02452 TGCAAGTCATTGGAAAGTGCCGGGAGAGGGTCTCCATGATTACCACAATTGTCTGAGGAG
EMPV1_02454 TGCAACCCATGTCAAAAACCTCGCCCATCAGCCGAAAGGGATTCGCTCCTGCCCTGTTGA
EMPV1_02455 AAGGTGAACTCATCCCCAGAAGGCCAAGGATGCATGAAATCTGAGAGGAAGGCTGGGGGC

EMPV1_02457 ATTTCTGTGGACATTGGTGTCCGGTCCAAAAGGCTTATTGGAGAGCTCAGGTTGAACAAG
EMPV1_02458 GCAGGGTGTTTGAGACCAGGTTTGGGAACCCAGGACCCCTCATAATCTCCTTAACATATA
EMPV1_02459 AATATGACTTTCACCAAAACGTCGCAGAAGTTTGGGCAGTGGGCCGACAGCAGGGCCAAC
EMPV1_02460 GGGTTCATCTCTTCTGATCGTGCTTGTGTCCTACATACTGATCCTTAAGGCGATCCTCAG
EMPV1_02461 GCTGTCATCGTTGCCATTGATCTGGGCAGGAAGAAAACCCCAAAGGAATCAGCATGGAGA
EMPV1_02462 GGTAACACATGTAAAAGTGTAGGAATTCCAATGACTACTCAGTTCTAGGACTTTCCATGG
EMPV1_02464 ACTGCATGGTTTGGGGCTGACTCTCCTGTCTCATTCATGGTTTTGCTCCCTGACCAGCTT
EMPV1_02465 GGCTCTGAGCTTCTATGGCTCTAGATTTTTCTGGTTCTAAGATTCTGGATAAAGCCTGCA
EMPV1_02466 TACAGAATCGAGTGCGGCAGACTGGCCAGACTCCATACCCCTGTGCCATCGTTCTTCCTG
EMPV1_02467 AATAATTAAAAATAAACTCCTGAGCCGACCCCAGGCCTCCAAGGATTCGAGGCAGCCAGC
EMPV1_02469 TGCAGGACCCAGACTTCTCTTTTCTGGCTGCCCGTATGAATTATATGCAGAACCATCAGG
EMPV1_02471 CCCTTCCCTGCTTCCCTTGACTGTCCCACGGCGTCTGTGCGCCCCAGCGTCATCCCGAGA
EMPV1_02473 CAAGCTGCCCCAGCTCCCAGAAACAGCAGCCGTTCTCAGAGCCCCCGGCGGAAAAAGTAG
EMPV1_02474 CATTGAGAAGCTAGTCAGCACTAACCTTTTAAGTTAGAGATCGGGAGCCTAAATCTCCCC
EMPV1_02475 TTCTGGAGTACAATGTTGTCGGAGGCAAGTACAACCGAGGTATCACGGTGCTGGTGGTGT
EMPV1_02477 TGGTGGGGAGAGCAGCAAGGTGAGAAATAAGACCTTGGGATGGGCAGTTGATGTGGGTCA
EMPV1_02481 GAGTTGCAACATCTCAAATCAGCGATGCACACCTAGCGGACACGATGATTGGCAAAGCAG
EMPV1_02482 GCTCGATTCTGGGAAAGGTTAGACCCCATTACAGAGAACTGTAAGGATTTGTCTTATTCC
EMPV1_02483 CCTCAGAAGTAGGTAAGGAGATGTGTTACATAAACTTTTAGGCACTCAGTCCTACAGTCC
EMPV1_02484 GGGTGATGTTCATCTGAGACAGTGGACACAAAATCACCTCTTGCTCTTAGATATACTGAC
EMPV1_02488 CGTCAGACCACTGGAAGCTTTCACATTTCTCTGGGAAGCAGAATCTTCTAAGCTTGTGTG
EMPV1_02492 AAGTAGGCCAGTTGTTTATGAATGATTGTGGCCAGAGACTCATGCCTGCTTTGTGACCAG
EMPV1_02494 GCTTCAATTATAAGTCCCCAGGTGTGTGTTGCTCTTGTGATGGTTTCCTGGGTTGTCGGG
EMPV1_02496 CACCCTACCAGAGTAGTGATGGGACCTACACCCTGGAAAGCTTGCAGTTGGTGATTGCAT
EMPV1_02497 TTCAGAAAACCCACCAAATCACATCTTGTTCCTCACCAACGTGCCGGAGGTGACCAACGA
EMPV1_02498 TGGGGATAGGTCCTTTGATTGCGAGGTCACCCTGTGACAAACGGGATGAAGAAGGTCAGG
EMPV1_02499 CCCAAAGAGGAAAACAGAAGAGTAAGTGTGTCCCCTCAAGTATGCAATCCAAGGACCACC
EMPV1_02500 CTCTGAGGACGGCTGTGTGTATGGCTGGGAGACTCAGAGCGGGCGGCTGCTGTGGAAACT
EMPV1_02501 TCTTTTACAGGAGCATAGCCTGATGGAGGTAAGGTGGGAGAGAATTTGAGGTAAGAAGGG
EMPV1_02505 TGAGATATTGCGAGCCGAAAGGGCAGGCCATGGTGGTGCGTGGTTCCCACAAGGTGTTGC
EMPV1_02507 GCATGGACCATCTTTCTCCATTCCTTCACTTAGAGCTGAAGTAAGTCTCTAGTAGGGAGC
EMPV1_02508 GAAACGCGGATGTAAAGAGGCAGAAAGTTGTTTTGGTTCACAGTGAGTAGCGCCGCAGCT
EMPV1_02510 ACCCTCAGACAGTTTGTTGTGTTTGGTCACGTCTCAGCTGGAAGCAACAGTTAGGGGCTC
EMPV1_02513 ACTGTACCCTCCCACGTCTGGAACCATCATCGTCAACGGCAAGAACCTGCAGACAGAACT

EMPV1_02514	GAACAGAGAGTAGAAGATGTCCGGCTCATCCGAGAGCAGCATCCTACCAAAATCCCGGTA
EMPV1_02516	CTCAGCGTTGTCCTAGCCCATGCTCAGGCCCAAATCCAAATCCATGCATAAGAGATCTAG
EMPV1_02517	GATCAGCTCTTGTGGTACACCAGGGAACTCAGATAAGTGAAAAATCCTATACATACCTGG
EMPV1_02520	TAATGCACTATTGCCTGAAACCAGTGGGGCCTGTGCTGGGGTAAAAGGGTGGCTGAGAGC
EMPV1_02521	TTCAGCTAGAAACAAGTATAAGCTGGAGGTCTGCCCCCACCCAGAGGAACTGCCAAGAGA
EMPV1_02523	CAAAGCTTGGGAGGAGGTGAGGGGGCCGTGGGGACTCACACTTGATTTTATTCATTAACT
EMPV1_02524	TGAGGAAGAAGCACAGGCACCACCTTTGCCCTTGGGACCCCCTCATCGCCACGGACTTCT
EMPV1_02525	CTGAAGGGCTCAGCAAAGCAGCCTTCAGCTCACCACTCAATGTTACTGCATTGTGTAGAG
EMPV1_02526	CCATCACCCACTGCAACCAGCCAGCTCCCGCTCGAATCTGATGCTGTGGAATGCTTAAAT
EMPV1_02529	TATGATCCCCTATTTCCTGGGTAGTCATATTGTGGGGCTCCAGGCTCATCCCACAGACCT
EMPV1_02531	TGTTTACAGCTCTTTCTGTCCTGCTGTCTGGTCGCGCCTCGTCTTCATCACCCGAACAAA
EMPV1_02533	AGAAGAAGTTCAGCTGCCCCATCTGCGACAAACGCTTCATGCGCAGCGACCACCTGACCA
EMPV1_02534	GTCCGATTTTTAAGCTATTATGGCTCCTGGTGGGTGGAGTCTCCTTTGTACACCCCCTTG
EMPV1_02535	AGACTGATTGTCATATGTCGTTTGTGGGATCCGTCTCAGTTACTTTATAGCCATACCTGG
EMPV1_02536	CTGAGATTTCATCACTGGTAGCAAATGGAGTTGCCAGAACAGCCAGGGTTTCCTTTTCAG
EMPV1_02537	GGCAGTTATGCTTGGCACTCATATTCAAAATCATAATGACAGTCTTCACTTTTGGTATCA
EMPV1_02538	CTCAGGTCTCCCCCGGTTCCTTGTCTAAAACTTCAGCCTCGCAGAGCCAGTCGTGTCACT
EMPV1_02539	TCCAGCAAGAAGAGTCTGAGTGTAAGAGAATTTCCAGAGGAACCTGAGTGCGAGTCTCTG
EMPV1_02540	CTTGACACTACAGACGGTGGAATTTGCTCTGGCCAACGGGGTGATGGCAGATGTGACACC
EMPV1_02542	CTGTTTGAAAACGGGGACGAACGGTCACCCTAGGTACTGAGGACAGCCTAACACAGTTTA
EMPV1_02546	CCTGCAAGCCATGTAGACGTGCACCTTCCCAAGGCATAGTTTTAGGTTCAAGTCAAAGTC
EMPV1_02547	CAGAGCTTCCATGTCCTCCAGGAACACTGTTTTTCACAAACCCTCCGGATGTTCAACAGC
EMPV1_02551	GAGGAACGAAGAGTAGACAGAGCGGAGGATAGAAGAGACGAAAGGGCCAGGGAGAGGGAT
EMPV1_02552	CTTCAAGGAAACTTGCTTTTTTGCTTATGGAAGCCGTGTTCACCAGCCTTGGCTCCAGCA
EMPV1_02555	CCTGAGGCAGGAGATGAGGGGTGAGAGCAATGAGTTGAAGGGAGTCAAAAGGTACAAACT
EMPV1_02559	CAAGGACGGGAAGGGTGTGTTTGCTGTGGACGGAGAAATGTTGGTTAGTGAGGCTGTGCA
EMPV1_02561	GTGTGTGTGTGTGTCTTTTCTAGGGCCACACTCACGGCACATGGAGATTCCCAGTCTAGG
EMPV1_02564	CTGCCAAGCACCCTTTGGAACTGACATTATCTTCTTGGGAGGTGAAACATCAGCCATCAC
EMPV1_02566	AATCCTCCCCAGAGCCAGATAAAAGAAGATAAAGACAGCACTGAAACCTGAAATGTGGCC
EMPV1_02567	CTCGAGACCTTTATGGCACAGTGGAAGGCACTGAGGTGTGGAGTTGGAAACCTTCAAAAC
EMPV1_02568	TGAAGGCTTGTCTGCAAATGCAATCCCATTCCGAGATTGAACCGTGAGGGGGCATAACTC
EMPV1_02569	GGCTGCCATGATCGTGTCCATGTATCTATACTGCAACCTGAAATAAGTCTGCCTCCGCCT
EMPV1_02570	CCTGCTCAGATGTCTCTGTTCTTGAACTTATCCCTTCCATCTCCTCTGGGTCCATCATCG
EMPV1_02577	CGTTGGTCTCCACCTTTTTCACATTGCTGGGGCTTATCTCTTGTACTTGAATCACCTAGG

EMPV1_02578	GGCTTCAAGTCACTCCTCACCAGACACCAGAAGACACACTCAGGAGAGAAGGCTGATGTG
EMPV1_02579	TCCAGACAGCTTTTGAAACCAACTCCTGGCACCAAAACCCTTACTCATGTCACTGCTCAC
EMPV1_02582	GGCAGGCCTCTCCCAAGCCTCTGTTTACAAAATTATAAGCTTCTTTTGTCCCCAGCCTCC
EMPV1_02583	GCAGCTTCAAACCCAGAACTTATTTTTAAGGAGCAAATTGACATTATTGAAGGAATAAAG
EMPV1_02585	AAAACTGATGCTAAACCACAAATTAGCACCCTGGGCCTTGGCCCTGAAATGGCCTCTGCT
EMPV1_02588	TTTAAGGGACTCCAGCTGCCAGCGAAATTTGAAGTTGCGTGTGTCCGTCTCCTTGAGGGT
EMPV1_02589	AGCCCTTGAGTAGCCAAAACAAGGTGGGCAGGGAATAAATAGCCAATAGCTTTTCTGGTC
EMPV1_02591	ATTAACAAAGTTTCCATCCCAATAACCTAATCACTACTACCACCAACCGCAGAAATCCCC
EMPV1_02592	TGGCTCCATCGTGTCGAAACCGAAATGGGACTCTTAGGTCAAAATGGTCATATTCCAAAG
EMPV1_02596	GAGCAGCCTAGTGTTTTAGTTAACCCCCACGACGTTTGTTCCTTGTTCTAGTTCATCCGG
EMPV1_02598	CTGCCAGCGATCCCCAGGACCTGGACATGAAGAAGCAGCGCTGTAAATGCTGTTCCATCA
EMPV1_02600	CCAGCACTGCTTAGACTGTCATGCATTGATACTCATGCTAATAAGCTGACCCTCATGATC
EMPV1_02605	GTTGTGTTGGCGGTCACCATGACCATCTGGAAATTCCCTCTAAGAGATCCTGGAGAAGTC
EMPV1_02607	CAACCACCTCAAGCCAAGAAGGCAAAAGTGAAGACTAGTACTGTGGACCTGCCAATTGAG
EMPV1_02609	GGTTTCTGGGAACCGGTTTTGCTGCTGTGTCATTTCGTAGCAGCCCTTCCTTTCCCTACT
EMPV1_02611	GGTTAAGGATCCGGCATTGCCATGAGGTCACAGATGCGGCTCAGAACTGGTGTTGCTGTG
EMPV1_02614	CTGTCACAGGAAATAGCCTGCATTGGTGTGTGTTACAGTATTTTGCTTAAAGAAGGCCTC
EMPV1_02615	GTGGCAGATACGTGGGTGGTGATGAGTGTAGTGTGTTATTTGTTGTTGCTGGAACTGAAG
EMPV1_02616	CATTTACACAGCACTTCAAATCAAGGGACAGGTAAAGTGAAACGAGACGCTGGTTCGGGA
EMPV1_02619	TCTGGACCGGATCGCGAGCTGTGAGGGGTGCGCGTGTGTGCCGAGCTCCTCGGAACCATG
EMPV1_02621	ACCACGGCATGACCATCGTGAAAAAGAAGCCCAATGTCACCGAGATCCTCCTGAGCAACT
EMPV1_02623	AGGTGACAGTACACGTGGGCAATGTGCGTCAGACGGCAGTGGTGGAAAAGATCCATGCCA
EMPV1_02626	GTTCCCAGGGCTGGCTAAAAGGTGTCCCCATGACCAAGCAAGTCCTCCTACCAAAATGTA
EMPV1_02630	GGCACGGATGGGCAGAAGCCTTTGCAGGTATCAGAAGTGCTCATATCAAATATATCTGCC
EMPV1_02631	GAAGATGGCAGAAAACATCAAGCTAACAAAAGATGCCCAGTCTCCAGATGAATCCAAGAC
EMPV1_02632	CTTAAGCAAGTCGCCAAATCCTTCCCTGGCTGTGTTAGGTAAGTTTACAGTGGAACCGGA
EMPV1_02633	TTCACCAGTAGTCCACACCTCAGTGAACTTCAGCACTTCAGACTCCTGCTTGAGAAGGCA
EMPV1_02634	TGACAAACTCTCCCTGCTTGTTTCAGGAAGGATCAGAGTGACAGTTGATGGCGAATTTCT
EMPV1_02635	CACCATGATGTCAAGGAAACACTGCCTTCGGATGACCACAGGAGCCTACCTAGCTGGAAA
EMPV1_02638	GATCAGCCTGCAGTTCGTCCGCCTGCAGCTCACCTTTGAAGAGTACACCATCATGAAAGT
EMPV1_02640	TTGCTGCCAAGAGGAATACCAATGCAAGCGAGGAGTGGAGGGCACGGCTGCTTCAAATGT
EMPV1_02641	TAAGAGAAGGAAGAATCAGCCAACTCACCGGGGACTACTCCGCAAGACATGCCATTTGTG
EMPV1_02642	GGTCGTCCCACTAGCTCACAGAGCTACGAACAGAATATCAAACAGATTGGCACCTTTGCC
EMPV1_02645	GGAATTTATTTAATGTGAGTTTGGTCTGGACTGACAGCCGTGTGCCTACGAAGGGGGACC

EMPV1_02646	GATCCGAGCCATGTCTGTGACCTCCGCCTCCGTTTGTGGCAATGCTGAATCCTTAACCCA
EMPV1_02647	TGTTCCTTTCTCTTTGTTTACTTTGGATTGCCACTGAGGTCAAGTGCACGGTTGTTGGGC
EMPV1_02649	AGGAGGTGGTTTTGGTGGAAATGACAACTTTGGTTGTGGGGGAAACTTCAGTGGTCCAGG
EMPV1_02650	TGTGCACCTTTGCTGCTTCCTCTTCTATAAATCCAGTGTTGCTATAACTTCCTTTCAGAG
EMPV1_02651	ATATCATCTGGCCTTCGAGGGGGCATCCTGATAGAGCTGGCCATGCGAGGTCGAATCTAT
EMPV1_02652	AGAGGAGGGCTGAGTTTCTCTTTGGCAGTTGGAACCCCAGTTGGAGATAGAGGTCACCAG
EMPV1_02653	GCTGCTTCACTGCCCTGGTGGAAACGGTGTCTGTATTGCAGCTGTCTCTCTGTGGAAATA
EMPV1_02654	GCAGTGAGTGAATGCCCCCAGCCACAAAAACACAAAAATGGGTATATAGTTACTGGGCAG
EMPV1_02655	AAAAGTACCAACCCTTTCGCCAGCATCAACACCACCATCATCCCAAATGGCACCACTGTG
EMPV1_02656	AGTGCTGTTTGGGGCAGAGCCTTTAGTTCAGAGGGACACTGAGGAAGAGGAAATATTCCG
EMPV1_02657	ATTATAAACAGCTGCAAGAGGACCTGGACAACAACCCTCGGATCCAGCAGGCACTGGCTA
EMPV1_02658	AGGAGCTGTGCTTATGTCTGCTGGCTGGATGCTGGCTCCTTTCCTGTGCCTATGCCTTGA
EMPV1_02661	CAAGTGTCTCAACTGACTTCATTAGTTTGGTTGATGCAGGAGGGAAAGAGAGAAGAGCCC
EMPV1_02662	GGAACCAACCCTATCAACCCCTTGACTTCAGACCTCTGTGCTTCTGGATTGTGGGAGAAT
EMPV1_02665	GTTTCTACGTCTCTAATTTCCTTTGTGGTCTGTAAACTGACAGTGACAGCAATTCCAAGC
EMPV1_02666	AGCTCAGTGAAAACTGGACCCTGGGAAGAGGTGGAGAGAAGGAGGGTTCAGCAGAAGCAT
EMPV1_02667	AGGCAGATGGACAGGGTGCTTCAAGCGCTTTGCAGACTTTTTCTGGAACGCTCCCAGCCC
EMPV1_02669	TCCATTGTGGTGAGGAGAATACTTCAATGTCCCTTGTCCTTGTTCTCATTAATTCAGCTG
EMPV1_02672	TGCTAAGGAATTGATGGAATACAAAAAGATTCAAAAGGAGCGTAGGCGCTCATTTTCCAG
EMPV1_02674	AGTCCTTAGAGATGCAGGTGTACAAAGCACCAAATGGGTACAGGGTCCTCAGATGCGGCA
EMPV1_02676	TGTGGGGCCCAAAGAAACCCATGTGTGGCAGGACTCAGGATCCCAAAGGATTTATTATGG
EMPV1_02678	CTATCTGGGTGAGGATGCCTACTTTTGCCATTTCTATTCAACAGAGGACTGGACGTCTCA
EMPV1_02679	AGGCTGGGAGCACAGAGCATCCCGCTGAGGATTCCCATAAGTCCTCAGAAGTAAACACCG
EMPV1_02680	AATCTAAATCCCCTGTCATTTCTCCTCAACCTCAGAGCAACCTGGGTTCAAGTCCCTGGG
EMPV1_02681	AACTCCTCGGAGTCAGCGGGAACAGGAAACCCATGGATGACCTTGCAGGGGTGAGTGATG
EMPV1_02684	TCCTCCTCCACTGGATGTGGGGCCAGATGTGCAGGGGGACTTTGGGGAAATAAATGTATT
EMPV1_02685	TTTGGTCAGCAACACCTTCCTTGGAAGCATCTCACCTTCATCTTCAAGTTCACTGTTGTT
EMPV1_02689	ACTGTGCAGAGCCTTTTACAGAATACTGGACCTGCATTGATTACTCCGGCCTGCAGCTCT
EMPV1_02690	TCCTTGGAGGCACTGGCTGCGAGTAGGTTCGGAGAAGCGGTTGCCACCAGTGGCTTTTTT
EMPV1_02696	TTCTGAAAGCGCGGCGACCGCATCCTGCTGGCTCATGAATTAGGAGAGAAGGACGTCTGT
EMPV1_02701	AGCCCGCTGGCCAATCAGGCGGCTCTCGTGAAGGCAGCTAGCGCGAGGCTGTGGAGCGCT
EMPV1_02705	GTGAGAACGCAGGAGCCTCAACCCACTAGGCCATCAGAGAACTCCCCAAAGATGGAATCT
EMPV1_02707	AGCCACATCATTCCCCGAAGCTTGGACCTCTTCACAGGACAGCGCCATCTGTGTTTAATA
EMPV1_02708	GGTATGGACACAGTGCAGAATGGACAAAGCCCTAACTTAAGAAGCAGTGATGCTCACGGC

EMPV1_02711	GACACCGTGCTGTGCCCAGAAGACCTGAGTGCCGATGAGCTGGTGGAGCTGGAGAACCAA
EMPV1_02713	ACCTCATCCTTCAGACTCCGGCTTCAAGAATGTGTTTTCATGCCCGGCCTTTGTTCCTCC
EMPV1_02715	ATATCCTGCATCTAATCCTCTGCCACATACCTCAGCCCAGCAGCCAAATCAGCAAGGCCA
EMPV1_02717	CTGCATAAGGAAGGCCTTCGGTGGGTGCCTGGGAACATGTCTGCAGCGGTGGGCTTATCA
EMPV1_02720	TGGAAGAGCTCTGAAGTACAGAGACAATCCCTGCCTGGTCATGGGAAGTCTGCCTCCATT
EMPV1_02721	CTCCACGTCCCGTGGCCGTGGCGCCACCCGCTCCTCATCCCGGGAGAGTGGCCGCGGGGG
EMPV1_02723	GACCATCCAGAACTGGTGCAAGAGGAGCCGGAAGCAGTGCAAGACCCACACTCACATTGT
EMPV1_02724	AGAGGTGGGAAGAGCCATTTTCGTCTTCATAGCTGTGGGCCGGGAGAGAGAATGAGCTGA
EMPV1_02725	AGCTCTGAGGAGGCCAAGGTGCAACCCCTCTTCAGCCATTCCGAATCTGGGTTCATCTGA
EMPV1_02726	TTTGCGCCCACAACAGAATCCCTCACTTCTACTGTGATGTACTCCCGATCTTGCGGTTAG
EMPV1_02728	CCAATGCTCCAGGGACACATCCAATCTCACTGTGCCGCAGCAGGAACTCCAATTTATCTT
EMPV1_02731	CAGGGAAACTGACTCCATCCAATGTCTTCAATCCTGCTGGAATCATCCCTTCCAAGCCTG
EMPV1_02733	GTGAAAGGCAGCTATTAAAGAAGTCGACGGGAACCCAGCACAGTGAGTGACACAGAGAAC
EMPV1_02737	ATCTCTTTTCACGCCCCAAACTAAAAACTTCCTCCTGCCTCTGGCTCTGTGAAAGCGGAG
EMPV1_02738	TGTTCAACATCCTGTTCGTGACGAGCGAGAACGGCAGCCGCAACACGTACCTGGTGCACT
EMPV1_02739	AGGGACAGTCAATTTTGTGGACTGAGAGGACATGTGAGGGGCTGGGCCATAGGGCTGTGA
EMPV1_02740	AATGGCATCTGCCCGTCAGCAGCCTGGTCTGTGGGCTGGAGGCCTCAATGAAAGGTGGAT
EMPV1_02741	CGAGGTCATAGCCAGAAGAAGTTTCCAGAGGTACCTCCTCCATCAGCTGCACTTGGCGGT
EMPV1_02742	GGCCCTGTGCGCCAAGCTGGCTTGTGCGAGCTCAGAACTGCACGAGCTCTTCCCCGACTT
EMPV1_02743	GTACTTCCGAGGTTGGAGCATAAGAAAAAGCTTCCACGATAGGCTTTGGGAATACTGCTT
EMPV1_02745	TGCTCAGCGGCAGCTTCTGCGGAAGGCGCCCAAGATGGATGCAGAGCCCCAGAGGAGGTT
EMPV1_02746	TAAAGCCTTATTCCTGTCCCAGCAAAGGTAACCCAACTGCATCTCTTACTGCTGGCTCCC
EMPV1_02748	CCTGGGCCTGCGTGAGTTGTAGAAGTTTATTGGTGAGATAGGGAAGTAGGAAGAAAGGCC
EMPV1_02753	AGATGACTCTTTAGGCTGACTGTCTTTTGAGCCCTCAGGAACACCTATGAAGAGAGTGCC
EMPV1_02754	GGAAAATGGAAAACCCTTTACGTTGCAGCCAGTAACAGAGAGAAGACCAGTGAGAATGGC
EMPV1_02757	CATGCAGTGTTGTGATTGATCCCCAGTTTGGCTACTCCAGAGGAAACCCTTGTATTCTTG
EMPV1_02759	TGTGGAAACTCCAGCGAGATGTTTCTCCCCCTGAGATCACGTGCTCTTTTGCGGCAATGA
EMPV1_02760	CACTCCTGTCAGCTACGTATGTGTATCTCTTTCCCCAGATGCTTGCCGACTAAGAGTTTT
EMPV1_02763	GCACTCTGAATACGTGTCCATGAGCCGCTGTGCACCACGGTCCCTTTTCTTGAGCTCTTT
EMPV1_02766	CAGAATATGGATCGCAGTTCTATTTAGGAATGTCCTAGATCTTTGAATGAGCACCGCCAC
EMPV1_02767	CGTGGAGCCGGGTTCAGGTCCTTTTAGCGACTGTTTATAGAAATGACCCACCATCAGCTT
EMPV1_02768	AAAGGCTTCAAGGAGTCTTGGCCAGCATATCCCTGGCCCTCGTGGCATTGAAGGTGTGAA
EMPV1_02770	GATAAAAGTATCACTTGCTGTGATGGGGGAGCCAGCAGCCAAGGAGAATTGGTTACAAAG
EMPV1_02771	CACTGTGTCCAGGCCAGCATTTGAGGGTGTTACAGCAGAGGAGCAAAGCAGAAATGGGAA

EMPV1_02773	TGTTTAAGCCACTGGACCCAGGGTCTCCACAGTCGCCCAAGCACTTGGAATCACAGCAGA
EMPV1_02776	TGCAATGGCAGCATACAGGAAAAGGCCGAGTGGGTTTTGTTTATGAGGGTATCTGAAAAC
EMPV1_02777	CATGCACCCCTGAGTTTGTGGCAGTATTATTCACAATAGCTAAGCCATGGACACGACCTA
EMPV1_02778	CTTCTATACCCTCATCACCACCACCCTCGCCTTCCTTACTGTCGCCACGACCGCCACCAC
EMPV1_02782	ATGCCATGACATTGTGTCAATATGCGATGATGTGTGTGATGGCACAGCGTCATCACGTGG
EMPV1_02783	CAGACCTGACGGGCTTCATCACAGAGGGACAGATCTACGTGGACCGACAGCTTCATAACA
EMPV1_02784	GGATGATGCAGTTCCCCAAAATCAGCCTTCTAGCATTTGGAGCATGGCAAAGAAAGGAAG
EMPV1_02786	TCCAGAAATAGTTTAGGAGGGAGTGGTTATCAAAGTGTGGCCCCTGGACCTGCAGCATCA
EMPV1_02789	CCATCCAGTATACGTGAGCAAATACAGAAATGACACACAAGCACAACCCTCCAGATGTGG
EMPV1_02790	TCTTCGGCCTGGTCAACACCCTTTTGGCCAACGACCCAACGTCTCTCCGCAAGAACCTCA
EMPV1_02792	AGAACATAAGGCACTACACCGGGGAGTCCGTCCTGTGCACTTCCTGGTCATTCCTAAGAA
EMPV1_02793	ACCACCTTGGTGAAGGGTTTGCGGACAGCAAGAGGCAGTATGAGACCAGGATCCAAGCTC
EMPV1_02794	GGCTGTCTTGTCCTTAGCCATCTCTCAGTGTTATATGCTAGTGCGTACCAACTGCTGGAG
EMPV1_02796	CAGCCATGGTTTTACATGGAATTATCTGTGGAGCTGTCAAAGATTCTCATATCCAAGCTG
EMPV1_02797	GAAGCTGATAGGACCTTGATATATATAACTCTCTACATTTCTGAGTGTCTAAAGAAACTC
EMPV1_02798	AACTCTCCAAGTTCTAATGAGTCAAACCCCATCTTCCTAAATCTATTCCTGGGGCACCCC
EMPV1_02799	ATCTCTGTGCACTGTGTGTTGGCAATGACCAGGGCGTGGACAAGTGTGTGCCCAACAGTA
EMPV1_02800	TGGCCAACCCAGCCACTTTTAGGCCACAGCTTTTCAAACACTAGAGACCAGTTGCCTTCC
EMPV1_02801	TGCATTGAGGACCATGGATGGGAAAGAAAAGGGAGTTCCATAAAACCACAGATATTTCAC
EMPV1_02802	CCTTTGAGCTAACCTACGAGGAGCTGCTGAAGAGGCACAAGGGCAAGTATGAGATGTATC
EMPV1_02803	TATCAAGCAGTTGTCCTCATCTGGCCGACCCACAGCGAGTGTGATTCCATCTGGTGTAGA
EMPV1_02805	TCAGCAAAAAGTGCCAATCTGCTATCTTTGAGGTTTCTGTGGAGTACTCTCTTAGGAATG
EMPV1_02806	TCTTCTGTACCGTTCCACCCGCTGTGCGTCACAAAACCCAGAAGCCCAGCTGAAGGTTCA
EMPV1_02807	TCTGGACAAGATCAACCCCATCTATGATGCCCTGTCCTACTCCAGCCCCTCTGACTCCTA
EMPV1_02808	CGCCCAAGGCTGCGTCTGGGGACAGCCCAGATGACGCCCAACCCGGGCCCAGCGCGGGCC
EMPV1_02812	AAGCGCGTGTGCGAGGAGATTGCCATTATTCCCAGCAAGAAGCTCCGCAACAAGATCGCA
EMPV1_02813	CGGCCTTGATCCACAATCAGCCACAGAGGGAAATCTTCCTGAAAAGTGAGCCAACACAAC
EMPV1_02814	TCTCCTGCTGCAGTCAAGTGACTATGTGTCAAGAAGGCGCAGGGTCCTGGACAAACAGAA
EMPV1_02815	TGCACTGATCTAGAAAGTATGCACAAGAGAGGGGAGCTCTTGACCCGCCTGCAGCAAATT
EMPV1_02816	CCAAGTACATGCTGTACCTTTCCATCTGTTTATGTCATCTTTGATTTCCCTTCATCAGTG
EMPV1_02817	GCCTCGGAGTGGTTGTTCTTCGAGCAAGTCCTGCTTGCACTTTTGCATTTGCCTAACAGA
EMPV1_02818	TGCTATGGTTTCCTTTGCTTCTGGGGAGCTCAGAACACATCTTTCAACCCCACCTGGACT
EMPV1_02819	GCTGTGCAGTCATGTTTGTCATCAACTGGTGGGCAGCTGTCATTACCTATGTCATTGAGT
EMPV1_02820	CCCTGACCCTGGAGGATGGCCTTTTGCAGCTTTTCCTGAGTTAGTGGTTAGATGCCTTAT

EMPV1_02821	CTAAGTCAATCTATGAGAGGATGTTTAAGTGGCTGGTAGCACGTATCAACCGGGCCCTGG
EMPV1_02823	GGTGGGCGGGAGGACGGCAAGGAAGGATTTAAAGATGAAAACGGGCGAATATGTCTCAAA
EMPV1_02824	CTCAGAGGGCCAGCAAGATGCAGTTTGAGATGCATGACAACGTGAAAGGCAAAGCCATGT
EMPV1_02825	TAGTGACACATGCCCCACACGCCAAGAAGGGTCTTGTGAGAACGCATGCAAGTGAACTTC
EMPV1_02826	AAGCCCAGACACTGGCTCATCCTCAAGCCCAGATGCCTTTTCCACTTCCTCCTGATATGT
EMPV1_02827	TAAAAGCAATCTAGGAGTTGTCTTGTGGTGCAGTGGGTTAAGGATCTGGCATCACTGCAG
EMPV1_02828	AATGAAGAATCCATCCATTGTTGGAGTCCTGTGCACAGATTCACAAGGACTCAATCTGGG
EMPV1_02830	CTGTGTGACTTTAACCCCAACTCCAGCAAGCAGGCCAAAGTTGTCTCCCGCATGCGTTCT
EMPV1_02831	TGGTTACTCGGTCTTTGCTGGGCTGCCCTGAGCTGCGGCCGTGTGGACTTCCTCCCCAAA
EMPV1_02833	CATCCTTGTGGTCCTCTCTGGAGTGTTTCTTGCTTTCACCGTAGTCGGAGCCCTGTTCCT
EMPV1_02834	AAGGGCTTGGGCAAGGGCGGTGCTAAGCGCCACCGCAAGGTCTTGAGAGACAACATCCAA
EMPV1_02837	GGTGCATATGGTTGCAGAGTAGTGCTGGTCTGTGGTTTTCTCGTGATGTCTTTGGCTTTG
EMPV1_02838	ATAGTACTGGTCAGTATCAGGAATGCCTGCTGTTAGCAGATATGGTGTCCTCTGAGCGCC
EMPV1_02840	GCACAGAGTCGTTCTCTGGTCTTGTGCGATGCTCCCGTGTAGTCTGTCTGCAACTATTTT
EMPV1_02842	GGATTACTTGTCTGGGTTTCAGGAGCAGTGTGATGCTATATTGAATGACGTCAACAGTGC
EMPV1_02846	GAGAATCATCTCTCCCAGATAATGGCACTCTCAAACAAGTTTCCAAGTTGTTTGAAAGGC
EMPV1_02847	GCGGGGCAGGGGCCAGCCTGGCGAATGGGCCACGCCCCGACCGCACTCATCCCTCCGCAG
EMPV1_02848	GCCCTGGGAGAGTCTTTAGGAAAAGGTGATGATCATAAAGACATGGACATCATTACCAAA
EMPV1_02849	TGAAGGTGGTGTTTTTAAGGCTCATCTCACTTTCCCAAAAGATTATCCTCTTCGGCCTCC
EMPV1_02851	CGCGGCAGGTGTTGACGCGATGTGATTTCTGCCCAGTGCTCTGAATGTCAAAGTGAAGAA
EMPV1_02852	GGGTCCCAGGTAAAGAAGAGTGAGACTGGAATACCACAAGGACAGGGGTCCCAGGTAAAA
EMPV1_02853	ACAGATGGAAGACTGCTGCGACCCCGCCCATCTCTTTGCTATGACTAAAATGAATTCCCC
EMPV1_02854	CTTGCCATCCCAAGCCCAGTGACCTTACTCGTATACCGTGCCACTCTCCACCAACTTTTT
EMPV1_02856	CAGTCCCACGATAAAGGAGATGAAGAGAAATCAAAAACAGTTACGAAAGATGGCTTGGGG
EMPV1_02857	TGGCCCTACTGTGAGAAGCGCTGCAGTTATTGCAACTTCAACAAGTACATCCCTCGTGGA
EMPV1_02861	AGAAGAGCTACCTCCCCTTCCAGTAGTGTCAGCGGGGACTTTGATGACGGGCACCATTCT
EMPV1_02862	CCCCAGCGAGGTACTCTTCAGGGATACAGGTCTAGTACAAGAAAACTCTTGACGGCTACT
EMPV1_02863	CCATGGGTGCGGCCCTTAAAAGGAAAAAGAAATGCCAAGGTACCTCCTGGTGGATGACAT
EMPV1_02867	TGTTATGGCCCACTCGTGGCATATGGAAGTTCTCAGGCCAGGGATTGAATCCGAGTCCCA
EMPV1_02870	CAGCTCACGGAAGCCAGAAGAGTATTGGGTCTTTATTGGTAGAGAGTGTATAAGTGGATG
EMPV1_02871	CGGGAGAAAAACTGGGAAGCAATGGAAGCATTGGCATCAACTGAAAAAATGCTGCAGGAC
EMPV1_02873	GCAAATGGTGCCCCATTCACCACAGACTGGCTTGGAACATCACCCCTGCACCTTGCAGCC
EMPV1_02876	TTATCTCCTGGGTGATGCTGATGGGGCCATGTCCACTGGTGACAATGAACTTTCCTGGGA
EMPV1_02877	AGAAATATGTCTGATAAAAGAGTTACTTTGATAGAGTAAAAAATAGAGGTTCAAACCCTC

EMPV1_02878	TTTGTCTCTTGGAGATTGTTGAACGCAGCTTGTCTAGGAAGGGGATGGGACTAGATTCTG
EMPV1_02879	TAGGTCTGAGATACATGGCAGAAAAGAGTTCCCATCTAAAGCCTTGGAGAAGGTGGTTGC
EMPV1_02880	GAACTACTTAAAGAGGCAAAACGTGGAAAGACGAGGGCAGAAACAATGGGACCTATGGGT
EMPV1_02883	CTGATTTCACCTACCAGAGGTCACTTGGAAACCACCATTATGGGGCCTGATGCTGTTGCA
EMPV1_02884	GAGGTAGCCAAATGCCCCTGGTGCTAAGTGCTCATTGCAGAGCTGGGGTAATCATTTTCT
EMPV1_02885	GTCCTAGTGGAGAAGAAAAACGTGGGCAGGATGTGTGGGCTGTGTGGCAACTTCAACGGC
EMPV1_02886	GAATGAGTAATAAATTGCTCAAAGGATCCGCGGAGAAGAACAAGATGAGACTTCAAAGAG
EMPV1_02887	GTGACCAATTGTCCTGGTTTGCCTGGGACTGAATTGTATTTTGAGATGCAAAACTTCTGG
EMPV1_02888	GCTGGAAATAACCTCAAGAGCATAGATGAGACTATAGCATAATAAGACAATGGTGAAGTG
EMPV1_02889	ACTTTCCTTCTGTCCAAGGAGGGGGTGGTCCATCCCCCACTGCGAATTGGATTACAGCAT
EMPV1_02894	AGTGAAACGGCACGTGAGTTGTGTGTGCGGGAGTGCCGTGTGGCTGTGTGCTGAGCAAGT
EMPV1_02897	CGGGGTATCTACTATGTGCCTAATTTAGTCCTCTTCTAGGCTTAATGGAAACTAGTAAAG
EMPV1_02899	TCCGCCTGTGCGCTGATGGTCATAATAGTAATTGTGGTGGTCCTCTTCCAGCATTTCCGG
EMPV1_02900	CTTATATGACCACCTAATGGATTTCCTTGCGGACCGAGGGGTGGACAACACTTTTGCCGA
EMPV1_02902	GTAACTCCCTCCATCGTGGTGGCTCTCCCTAACCAGGTGTAGTTCCCAAATAGGCTGAGA
EMPV1_02903	CAGCAGAACTTAATTGCTCTCTCCAGTGGCTGTCTCTAAGCTAAACACCTGGCCTCTGTC
EMPV1_02905	GGATGAAAGACAACGGACAACTAAACCCATGGCACAAAGGAGTGCACACTGTTCGCGACC
EMPV1_02906	TCAGAAGGTTATGAGACAAGAAGACATTAGTGAGATATGGTTTGAATATGAAGGCACACC
EMPV1_02907	GGCCTTTACCCAGAAGGAACTCACAGTGAGAAATGCGAACACAGCATGAGAGGGTTTGAG
EMPV1_02908	ATCGAGTACCAGATCAAGGACATGATCCTGCAGTTCATCAGCCGGGAGAGCAGCCTCATC
EMPV1_02909	ATAAGCTCAGTGAGGGCAGGATCTCGTTGGCTTGCTCTGTTCATTATTCCACTGGGACCT
EMPV1_02911	CACTTTACCTTTGTCTACTCCCAGATGATGAATCCAGCCACGATCAAGAACTTCCCTCTG
EMPV1_02912	GCTCCACCTTGAGCCATGTTTTTCACCTGTGGCCCAAACGAGGCCATGGTGGTCTCTGGT
EMPV1_02913	GGGGATTGGGAGAGAGAGGCTCTTTGTGTAAGTTATATATCTGTTGTGTCTTGAATCAGA
EMPV1_02915	ACACCCCTCCTTCGAGACTCTCAACATCTCGAGTCGGCCTTGCACAAACACAGAACAATC
EMPV1_02917	CATCATTGGAGTCCTCTACTTCCTTTGCAGGAAGGGCCACCTTCAGAGGGCCAGTTCAGT
EMPV1_02918	AAAAAGAGAAAGTTAAAGTACCAAATGTGGATGTGCCTTGAAGTAGATATCAACAGCTCG
EMPV1_02919	GGAATCCTGCCCCGTCCACCTCACAAAGAAGTTGGGAGAATTCAGTGGGTTAATGAAAGT
EMPV1_02920	GTTGCCACTGCCAACACAAGACCAAAGTGTGTGACTTGTCAATGTGCAGCGTGACAGCAT
EMPV1_02921	TCCTGCTGCTTCACATACACCGTGCGGAAGCTTCCTCGCAACTTCGTGACTGATTACTAT
EMPV1_02925	GCCTCTTCGTACTTCAGCCTGGCAGCTTTCCTCTTATTTTCTGAGTGCCTAGGAATTGCC
EMPV1_02926	TCTGTCATCTTTGGCGATGTGCAGAGCCCCGAGGAAAGCCTGGTGGTGTCTACAGAGGAA
EMPV1_02927	TTAATTGGTCCCCGAACTGATGTGGAAGCTCCGAATCTTACTGCTGAACGGAGGGCACAG
EMPV1_02932	TCTAGCAGTTTGTCTGGTGATACTCCTGGAACAAGCAGGCACAGCATAGGCCAAGGTCAG

EMPV1_02934	TCTCCAATTCGTACATCACATTTGTGTAACCTGTCCAGAAGTCAAGATTAAGCCATTAGG
EMPV1_02935	CACTGCTGCGACTGTCTTGTGTTGATACCCGTGCTAATGAGCTGACCCTCATGGTCATGA
EMPV1_02936	GGACAGGGTGGTGATGGTGGTGGGGACGGTGGTGATGGCGAGGACGGCGCTGTGGGGGAG
EMPV1_02938	GCAGGATGGAATCTGAAGGGTGTCACAAGACCAAACCCTCTGCTCAAGAGGAACAATGAG
EMPV1_02939	CAGCTAGATTTTCTTTATTGGGGGGCTTCTCGCTGCTTTAAGGGAGGCATTGGCAACACT
EMPV1_02941	TTTCATCGACTGGAATGCAGCGTGTGAAGGCCAGTTTCCCAGCGTTTACTGTCCATTGGA
EMPV1_02944	GTCCATCTGACACCTTGACTGTTGGCCTGTGTTGAGTGGCCTTTGTAACAAAAGGTGCAC
EMPV1_02946	CTCTATGTCATTTCTGTCATAGAACCTTGAAGTATTTGTCAGGTCTACCAACAAGCCCAT
EMPV1_02948	ACAATCTCCTGACCTTCAACAACGACACCCTGCGCTGGGAAAGAAGCACACCTAACTACT
EMPV1_02949	ACTTCGAAATCTGTCGTGGGGTGAGATGTGAACCCTGACTCTTCTTAGCTGTGTGGCCTT
EMPV1_02950	TTGGGTACCAATTTCTCAAACCACTATTCTGGCCCGTCAGCGTGGACAAGGTGAGTGGAA
EMPV1_02952	CAGGTAAATATTTTTTTTTGAACTTAGTAAAGATACGTAGTTATCGTATTCCAATAACTG
EMPV1_02953	CCGGGTTCCAAGTGAACGAAAATGAAACTCCTCTGTTCCAGACCTGGGCTTCAGCTATTC
EMPV1_02955	TGTGTGACATTGTGTACCGCTACGCCGCCAACCACTTCTCCGTCTACATCACCTACGTCA
EMPV1_02958	TGCTGACGCATGCCCACAATCAAAGCTCCAATGGCAGAAACTTCTATCCACCCCCTATCT
EMPV1_02960	CAGTTCATTTTGAATTTCAGATAAATAACAAACAATTTTAGGGTAAGTATATCCCGTGTG
EMPV1_02962	GAACTACACCTTCTGGCAGTGGTTTGACGGGGTCATGGAGGTGCTGAAGAAGCATCACAA
EMPV1_02965	GAGTATGCCAGAGCCTGTTCCCATGCTGGCTACCCTATTCAGGACTGGCGAGATGACTTT
EMPV1_02966	CGTGCTGCTTGGTGCTCCCTCTCCCTTGATTTAGGCTATCACTGCTTCTTTCTCCTTTTC
EMPV1_02968	TGTGTCATAAACTTGGACAAGCACCGGCTGATCGTGGGGAAGACAGACAAGGAAGGGATC
EMPV1_02969	GGTGGGAGCCTTCGTGGGCACTGCGGAGCGGGCCGGGGGAGGCCGGGATGGAGCCCCCCG
EMPV1_02970	CTCTCACACATTCTTATGCACACTCACATACATTCTCTCACAAATACTCTCTCGCAGTCA
EMPV1_02971	AGGGCTGGAGGCCCAAACGGTCTCGTTTCACCGCAGTAGCCCATTTATTTGTTTAGCAAA
EMPV1_02972	GACAGTTCTACCAAACAAACAAGCAAGCACACTTTCTCAGGCTGCCACTTCAGAGTCAAA
EMPV1_02973	GACACGGAAACGCTGAATTTATGATGCAGTTTTCTTGCGCAGTGCCGTTGGTTAGATGCC
EMPV1_02975	GACTCCTCATAAACACTCTTACCTTTTGACCCACTGATGGAGGTCTTGGGAAACGAGCCC
EMPV1_02978	GGTTCCCACTGTACCCACGGTGTCTTACTGACAGGGGCAGTTGCCAAGAGGTACCAGATT
EMPV1_02980	AAGGCCTTGGCTGGAAAAAGGTCCTGATGAGAACTCAGTGCTGAAGTGGTGCAGAGGGGC
EMPV1_02981	GGAAGAAGCATTCTTTCACTGGTTGGTGTGTATTGCTTTGTTAACCAATGGGAGGATGCC
EMPV1_02983	ATGCTAGTGTCATGTAATCAACAACCACACACATCCTGCTCCTGTGTCTGGGATGGTCCC
EMPV1_02985	TTCTGCCTGACAAATCCCGCCAAGGCAAAAGAAAAACCAGCATCAAGCGGGACACCGTTA
EMPV1_02986	CAGAGCCAGTTGGTTCCCTGTTATATTTGCGGGCGTACCTTCCTGCCAGACAGACTGATT
EMPV1_02987	TCATCAGCATCCATGCACAGCCCCTCTTCCTCCATGTGTGGAAACCATCGGTTTGAGCAA
EMPV1_02990	AGCCAGGGATCAAGCCCGAAAGTTCATGGCTCCTAGTCAGATTCATTTCCACTGTGTCAC

EMPV1_02991	AATATGAGAGAGAGTATCGTCCTGAATATGAACGTTTGAAGAAATCACTGGCCAATGCAC
EMPV1_02992	TTTTGGAGTCAGACAGAACGTGTGGTCAGCTTGCACAAAGGAGGGTAGGCAGTAATCACT
EMPV1_02993	GGAAACAATAAGCTCTGGGATTATCGAAAGGGACACTGGATTGCCTGCGACAGGTTTTGG
EMPV1_02995	GGTTCTACTCAGAAATGTAGTGGACAGTTAAGCCAGGATCTATCAGATGCTTTCTTGGAC
EMPV1_02996	AGACCTGTTCTCCACCACTTACACCAGCCCTGATGGACCAAGAGAAAGGGATGAACAAAC
EMPV1_02999	GTGTCAAGGGTGAGTTACTTGTGCATGCTTACATTAGAAAGCATTGGTCTCTATCAAGGC
EMPV1_03001	ATCTCATCTACAGCTGGATGGTACCTGTCCGTCTCCTGTCCTTCCAGTCACTTCTGTCCA
EMPV1_03002	GCACTGGCAGTACACCAAGTGCTGTGATGTTTTAGCCAAGTTTCTAATCATACAAAGTTG
EMPV1_03003	TTCTGCTTCTCCTGGGAACTAAAATCAGGCAACAGCACAAAAGCCATGGGCAGTCTCCAC
EMPV1_03004	ATGCATTTTCAGAAAGACATCCTAGGAACCACAAGCCAGGCAGCTCTGAAAACCCTGTAT
EMPV1_03005	TTGAGACAGAGGGAAAGTGGGATGCACAAGATAAGGCTTTGCCTCTTCCCTTGTTGGATC
EMPV1_03006	TGTCCTAGGTCAGCATCATAAATCAGTCAGTGTAGGTAAGTTTTCAACAGAGCAAGTCCC
EMPV1_03008	GGGTCCTCTTTACCCATCTCTCCACACCCCATTGAGCCAGAGGTTATCATGGAGATTATG
EMPV1_03010	GTCCAAGAGTGGCCCCAGTAAGATAGAAGATGCCCGTTGACCCAGCCAATACTGATGCAA
EMPV1_03011	CGCTTCCTAGAGGTTGCTGCTGTGATTGCACAGTTTAGCATTTTGCCAAAGATTGGGCCG
EMPV1_03012	AACTAACACTACTCTGATGTGTAGAGCCAGAAAGGTCTGGGTGATGGTGGGGTCTGGGGT
EMPV1_03013	TCAAAGGACTGTCATTGCTGAGCAGCACGGCTTTCAGATTAGTAGAAAGCCAATTAGACT
EMPV1_03016	AGTATCTGGAAAGTGACTCCAAAGACCTGGGGCTGGAAGAAGGAGGCACGCCCTCAGCTA
EMPV1_03017	CCAGAGATGAACCCCACCATCTGCTCAGTGTTCGAGGCAGAGATAGTCCTGCTGTTCCAT
EMPV1_03018	TGGCTGTCCCCTGACTCTGAGAAGTATGAAGGGTGATTATTGCACTGTAGGGGCTGCGTG
EMPV1_03019	GCTGAAATTCATTGGCTGCCTATTTTGGAATTGTGTCGTATTTGGTTGGACTCCAAACTC
EMPV1_03025	CCTAAAATAGGATGTTTGGGCCAGGCTTGTGATTCCCAGGTGTGAACCCCGCACACCGGT
EMPV1_03026	AGCTGCAGCAACGCTGAATCCTTTAAACCCACTGTTCTGGGCCAGGGATCCCACCTTTGT
EMPV1_03027	TTCAGGAGGACGAGGGACCAGGCACCATGTGAAGACACAGGGACAGACTTGTACAGTATA
EMPV1_03028	CTGCTCTCACTGCTCATTAGCACTATGGTTGGCCTGATCCTTAGTCTGACAATGTTGCGC
EMPV1_03030	TGCGGACAGGCTCCTTCGGTTGCACTTCAGGGAAAGATCTGCTTCTCTCAATGTGAGGGA
EMPV1_03032	TAGAATCCTTAACTCCTCACTCCCACCCTTGTGGCTGAGACCTGAACTGTGTGGCAGAGA
EMPV1_03033	TCTCACGCTCCAGCCATGGAAGATTCGTTAAGCTAAAGGCGGTACTGCGTCTTAGCAAGC
EMPV1_03034	ATTAATTATTGACTGGGTGCGTGCAAAGCTGGGGCGGCGTGCAGGTTATTTCAGGGCATC
EMPV1_03036	AGCACGTCCGGTCTATTTGGAGCAGTAAGGAGCATGGCTTTGGTCTCTGTGTCTCTGTTC
EMPV1_03038	GGGGGGAATGAAGCAATACCAAATAGATTGTACTGGTTTGCATTTGCTACCACTTGGTAT
EMPV1_03040	CCATTGTCCTCAGTACATGCTACCTATTTCGATCCTATCACTTGGGTGTCATCATTTCAG
EMPV1_03041	CCAAGGGAACGGGCTTGGCAGAATCAGTGGGGAAAGAAGACCCTGTTGAGCTTGACTCTA
EMPV1_03043	CCCTATATTAAGGGTGCCTTAGAATCATTGAGTCTTACCCATTTTAACTTTTCGCACCAC

EMPV1_03044	GGCTCGGTTAAAAGGAGCACAAACAGGTCGAAACCTCCTGAAGAAAAAATCTGATGCCTT
EMPV1_03046	AGACGAACAGCCGGTACTATTAGACGTTGTGGGTGTTGTAGTTGTTCTCCAGCTTGTGCC
EMPV1_03047	CTGCTTGACCTTCTGAATCTGTGCGTTTTATTTCAGACTTGAGCACCTAGTGAAGGTGGG
EMPV1_03048	CACGGACTGCACCCGTACCACAGCGACATCCTCTTTTGGAACTTGAAGGAACATGGGAGG
EMPV1_03050	ATATCCAGCAAGACAACAGTGAAGCTGGAACACAGCCCCAGGTACAAACTGATGGTCATC
EMPV1_03051	TTTCACAAGGCGGATGGAGCTTGAGTGGTGCCTGCCAGAAATCGAGTATTGGGTTTGACC
EMPV1_03052	TAGGCGTTGGGAAGGAGGACTATCCCCAAACTCAGGGTGATTTCTCATTTGACGAGGACA
EMPV1_03053	TAAGTCGGAGGTACCTGACCCCACCCAAAAGGCTTGGTCTAGATCAGACAGTGACGAGGT
EMPV1_03054	TGCTCGAAATCTGCGTTTTATCCACCACCTAGGCCTTGTCTCCTGAGTCAGTGGCGTTGT
EMPV1_03055	CAGCTCGGACCTCTGACCTCCCTGACGGGTCCTGTACTGAGCTGCCCCGAGGCCCTCACT
EMPV1_03056	CTGTCCCATTTCGCCCCCTCACGCAGGCCTCATCCCACAAAGTGAACAGAGTTGTAAAAA
EMPV1_03059	GCCTGGCTTTCTTCCTGTATGACCTTCTCTCACTCATGGATCGTGGCTTTGTGTTTAACC
EMPV1_03062	CTGATGCTACAGGAGTTTTAAGCCTGTGGTGAATGCTAGTACTTTACTGTAGGGAGGGTG
EMPV1_03064	GCGCCAATCGTGTTTTTTTTAATCCCTCCCAAAGGCGGGAACTAGTAATGTGCGCCGGGA
EMPV1_03066	AGATCTGACCATATGTCCTACCCTGAGCTCTCCACTTCTTCCTCGTCTTGCATAATCGCG
EMPV1_03067	GACACTGGGTGACAAGAATTACTCCATACCCTACAAATGAAGGGCTCAATCCCACCAGAC
EMPV1_03068	AGTTTTCAGAGGATTTGGGGGGATCTGGAAGGGAGGAAGGAGACTCTGCAGCACTAGCTA
EMPV1_03069	CCGCAGATGCACTTTTGGTGGCAATCGACTCTGAAGTAGTGGGAGCTGTTGATATACTAC
EMPV1_03071	GTCCCTTTAAAAAATCAAAGTCGCACCCCGCCTCACTGGCCAGCAAGAAACCTAAAAGGG
EMPV1_03072	GCTTGCTCAATAGCTTCTTCCCCAGAGAAGTCAATGTCAAGTGGAAAGTGGATGGGGTGG
EMPV1_03074	TGCGGCTGAAGCTGTGCTTCAACCCCTAGACTGGGAACCTTACATATGCTGCAGGTGCAG
EMPV1_03075	CTGGGCAGAGGATGGCTCTTGGGGATATCGCACCCCTATCTATATGCTGAACCGCATCAT
EMPV1_03077	CACAGGGTTGTGCCACCTGACGAAGATCTCCAAGCCCGCAGAGAAGAACATTTTCACTCT
EMPV1_03078	CTTTCTGGCCTTTGAGGGAATCTACTCTGTTTGGCTTTTTGTGTCTATGTACTATGGGTC
EMPV1_03080	TAGCGTCTGACCGCGACAACCAGGTTTTGTTTGCGCTCTCCTGTCACAAATGCTGCACTA
EMPV1_03081	GCTTCTAAACTCCAAAGGGGCAAAATGAGGAACTGGGTTCTGAGAGTGAAGCTTTTCAGG
EMPV1_03082	CACCTAAGCCATCATTCAGCATCTCCAGAACATCGCAGGGAGGCAAGTCAGCCTGTCATA
EMPV1_03084	GCTCAATGCCTGCTTTGTGTGGCCCGGGATAATGATTTACAAACTCAAGGAATCAGGAGG
EMPV1_03085	TGGCCAAGAAGCAAAAGCAGAATCGTCCCATCCCCCAATGGATTCGGATGAAAACTGGTA
EMPV1_03086	GCTGTGAACTGGGGATTAACCCAGAGCAAGTAGAGAAGATCAGAAAGCTACCAAACACAC
EMPV1_03087	TCACGCTCACCTGGCTCTACTTCTGGTGGGAGGTCCACAATGACTACGACGAATTCAACT
EMPV1_03089	GAATATCGGGGTCAGGGGATTGGTTCCAAAATAATCAAAAAAGTGGCTGAGGTGGCCCTG
EMPV1_03090	ACCTTGGGAGGGTAGGTTATGTCTTAGAAGATCTGCCCGGAGGCAAAGAGGCTTGGCATT
EMPV1_03091	GCTGGAAATGGTTGGTCCTAAGAAGCTGGCTGACAAAGTGAACAGCTGGTGGCAGAAAAG

EMPV1_03093   ACTGCAAAGTTCGGAGAGTCAGAGAAGTGCCCTCGATGTGGAAAGTCAGTCTATGCTGCT
EMPV1_03094   GCATTTCCTTATCTGCCAGTGATGTCTCCAAGCTCCTCTCATCCCCATCCCAGACCAAGA
EMPV1_03096   ACTACAAGGTGTTCAGTGAGAATATTTAGGCCAGTCCCTGTCAGGTGTGTGATAACCCTC
EMPV1_03097   ATACAACTATCATGAATTGGCAGCTGTGTTGGCTGTTAGTGGGAGTGTCATGGGTGGGAG
EMPV1_03098   TCGCCACTGATTGTCCAAACGCAATTCTTGTACGAGTCTGCGGCCAACCGAGAATTGTGG
EMPV1_03101   TGGCCTTGCCCTGCGCTCCAGACTCTGCCCCCCCTCCGAGCTACGGGACCTGGTCACCCA
EMPV1_03105   AGTTCTCATCCTGGGGTCTGTGATCTGAGGGATTCTGTGAGCATTTGTGATGGTGTGTGC
EMPV1_03107   AGTATGAGGGACTACAGGAGAGAACAACTCAAATAAAGTGGCCAAGGGCTCCAGGCTCTG
EMPV1_03110   CTGAAAAAGACCTTATATAATAACTCAGTAGACACTACTCTGGTGCTGGCAACATAGTGC
EMPV1_03111   ACAGCCCGCCCAAAGGCACTGCTTCTCCTGCTGGTGTATGAATGAAGGGTGTTGATTTGA
EMPV1_03112   GTGTGGGGAAACCAAAGAGCATTGAATAATTGCCTAGAACACTGTCCTGAGATCTAGCCC
EMPV1_03113   GGTGTGTTGCTTGTCATTCCCTTGATTTTCTTCACCAAGAGACTACCCTACTGCAGAGGC
EMPV1_03114   CCTCAGGGTGTTTCTGCATAGGCCGACCCTAGTCCTCTTTCCAGAAATGATCTCCAAAGA
EMPV1_03117   TGGCACACAAGCCCCGTCTTCTTCCACCCTCCCATCCACGAATACGCAGAGAAGCTTTCA
EMPV1_03119   TGTTCCAGAGAGGGGGTTATGTTGGTGATGTTGGCACCCGCCTGAGACATCTGACACATT
EMPV1_03120   CGCAGAGGCAAGAAACGCTTCGGGACCCAAGAAGAGAAACCACAATTCAACAGAGACATT
EMPV1_03121   ATAATGAGTATCAACTGTATCCCGAGACCGGAGGTGAAGTGGGACGCACATTGGAAGGCC
EMPV1_03124   TGTCTTCTTGGGCAAGTTCCGAGCGGAGGAGGTGGCCATCAAGAAAGTGAGAGAACAGAA
EMPV1_03125   CGGATCAGGAATGCTATCCTCAGGTTCAATGCCATCATCACTTTGTCCAACAGCCTCAGA
EMPV1_03128   TAGGGCACCGTCTCCTCCCTAGTCGTCTGGGTTACTTCAGTATAGTCACAGGAATTCCAT
EMPV1_03130   AACTCCAATTCAACCTCTAGCCTCGAAACTTCCACATGCCACAGGTGAGGCCCTAACAAG
EMPV1_03132   TTTTGTGACTTTAAACAAACACAGGGACTTGTGGGAGCAAGTGGGGCTCCCAGGAAACCC
EMPV1_03133   GGCAAGTGGTCGTGCAGGTTCTAGTTAAGAACACCCCCAAAATTGTTGACAATGCACGGG
EMPV1_03136   GAACAAGGCTCACACTCCTTTCCGCGAGAGCAAGCTGACGCAGGTGCTGAGGGACTCCTT
EMPV1_03137   GCCTCTGCGAAAGATGCTTCTGGTCATGGGGACTCCTTCTGTGGTTCAGTGTTGGAACTA
EMPV1_03139   TACATGGATGTGGTCAGTGACTTGTTCCTTGGCGGAGTCCCCGCGGACATACGACCTTCT
EMPV1_03141   TTCCCTGTTGGTTTTTCCATGTCTCTGTCACTGATTTGTCTGCCATCCACCATCCCTGGG
EMPV1_03142   AAGGAAGTGGCTTCTCAGTGTCCTCCACATTTGCTGCTTCGGGAGACTATGCAATGATGG
EMPV1_03143   TCTAGACCATAATCACCTTCTGGGCAGAGCTTAGGTTGTCTGGGGCTGGCTTCCTTAGGG
EMPV1_03145   GTGGCTACTGGTCTGCTGTGCATGCTTCCTCTTACTGGGATTGTCTTTTCTTACTCTCAG
EMPV1_03146   CCTTCACCAGGCCAAGAATCACAAACTCCAGGTGGATAAGGGAACATTAGAGGCCAATTG
EMPV1_03147   GACCAGTTACACTTGAGTTTCAGATAAGCAATGAATACCTTTTTTAGTATAAACATGTCC
EMPV1_03149   GATGCTGCTCTCCAGGTCCGTCCAGCTGCGAACAGCCCACTGCAGCGTCTGCGTCACCTG
EMPV1_03150   GCAGTGCACCCACCGGCATCTATTTCATACTCATTTCTCGTGTGAGTAGACAGAAGCCTA

EMPV1_03151	GCTAGTGGATTTTTTCTTGGCCCTCATGGTTATTGCTGTTCCCCTTTGTGTAGTGGTGGC
EMPV1_03152	GGCTCTGGTGCCAGTCTTCTCCTTTGGGGAGAATGACGCATTTGACCAGGTAGAGAACTT
EMPV1_03154	GCTACTGAGTTCACACTCTAAGCCACTGATATCTGGAACTGCTACAAGGAAACATGCCAG
EMPV1_03155	GAGACAGATGAGGACTTCAGCCCTTTGCCTGGAGGATATGACTATTTGGTTGACTTTCTG
EMPV1_03158	CTCATCCCAATCTGTCTTTTCTGAAGGAGCAAGGGTGTTACCTGGGCCATAACTCAAGCC
EMPV1_03159	GCTATGCAGCCATTCTAACCAACGCGGTAATCTTTGGGATCGCTTTGGCAATGGCGGGAA
EMPV1_03161	CGAGGCCTGTGCAGTACGTGGAGAGGACCCCCAACCCCCGGCTACAGAACTTTGTGCCCA
EMPV1_03162	AACATACGAAAACACTGAGTGAATATTATACTGAACCTCAGGTGCCCCTCACAGAGCTGC
EMPV1_03163	CACACTGTGATTATCAATCAAGTAAGGTGAAATATGTGAAATGATAAAGGTCAAAATGAA
EMPV1_03165	GGATAGCTTATTTAGGGGCCTGGGCCTCAGTACACATCAATTCTCATCCACAGACATGGG
EMPV1_03167	TAACTCCAGACTTAGGCTTTCATACTTTCTCTGCCCCCAAAAGCTCCTGTCAGCAGTTCT
EMPV1_03168	CCTGCAGAAAATTCCAAACCTACAGTTAACTCTTTTATGCCCACCCCCAAAGAAACCATG
EMPV1_03170	TTCGCCCAATAGTCTTGGCTACTTCCCTACAGCTGCTAATCTTAGCGGTGTCCCTCCACA
EMPV1_03171	ATCCCGGAGATAAAGCCCGCGAGGTCGGAGATCTAGAAACTGTAGAGATGCTGTAAACAG
EMPV1_03172	AGGCCATCTTCGAGATCCTCACGTCCGAGTTCTCCTACCAACACAGCCTCAGCATCCTAG
EMPV1_03173	GTTCAGAAGGTCAGGGATCCTGTGTGGCTGTGGCATAGGCTGGCAGCTGTAACTCTGATT
EMPV1_03181	GGCTGAGCAGATTGAGGACATAAACAATACGGGAACTGAATCAGCATCAGAGGAAGGAGA
EMPV1_03182	TGGTGGAAGTTAATAGTTTACTTCTCACTGATTCAAAATTAACAGAGCAGGCTGTTATTC
EMPV1_03185	TCAAAGCCGCAGCTTGAAGCCGTCCAACTGCACCCTGTGCTAGGGCCTTGGGCTTGGGGA
EMPV1_03188	AGCCGGTTAAGAACCGACAGAGTGTCCCTGTCCCAGGTTTGATCCCTGGCCTCACTCAGT
EMPV1_03189	TTCAACGTGCCAGATCGAATCACAGGAAGAACAGGACTGAGGGAACTACAAGCTGCCAAC
EMPV1_03190	GGCGAGCTTTGCCAGCGCCTCGTACGCAACGTGGAATACTACCAAAGCAACTATGTGTTT
EMPV1_03193	GACCACTGCCACCGACGATCAAGCCTGTCACCTACACTGTCACCACCCCTGTGACCACTT
EMPV1_03194	CAAGGCCCTGCGCCTGGCTAAGAACGACATCAACGAGGCCGTGGCGCTGCTCACCAACGA
EMPV1_03195	AACACGGGGATTGTCAATCACACCCACTCCCGGATGGGCTCCATAATGAGCACGGGGATT
EMPV1_03197	TTGCAGCTTCCATTTACACAGACCGGCGCAAAGACATTCACGACGGCAACCCCCAACTGT
EMPV1_03199	ACAACCTAAGGAAGTCTCAGGGGACTAGTGCTGAGGGCAGTGGTAGAAAAGAAGCTTTGC
EMPV1_03201	TGCAATAACAATGTCCAGAGAATGCGGCGGACAGAGGAGCTCATCTACCTGAGCCAGAAG
EMPV1_03202	AGACCATTGAGACAGCCACAGTAGAAGCGGTAGAGGCCCGGGAAGCCATCAGAGGGTTTT
EMPV1_03203	TTTCAGGAAACTGGTCTCAATCTCGGGGGCCAGACCACCAAGCCCATCCAGGAGAATGCA
EMPV1_03206	GCAAAAGGCTGAAACAATCAGGGCTCCATGAAGCTCAGATGACACTTCAGCAAAATGAGC
EMPV1_03207	CCGGTTAGATTTTGCTTGACTACAGCTCTCACTCTTCCAAAGCATCCATGGCACTTCCGC
EMPV1_03208	ATGAAAACGTGTACGAGTGCGTGGCCCAGAATTCAGTTGGGGAGATCACTGTCCATGCCA
EMPV1_03210	AGCTCATCAAGGTGATGTCGTCCATCGGGAACGAGCTGGCCAACAGTGTCTGGGAGGAGA

EMPV1_03211	GAGGAAGACCTGGAGTACTTTGACTGTTCCCATGTTCCCGTGTCTGCCATAAATCATACG
EMPV1_03212	CCATGTGGTTGCCAGGTATGAGTAAAACATGGTTCTGTCAAGCACCATGGAACGTCACGC
EMPV1_03214	ACTGTTTCCCATAGTAGCTGCACCAATTTACATTTCCATCAACAGTGTGGGAGGGTTCCC
EMPV1_03215	TGCCGAAGCGCCTCAAACAGACGATGGCCTCGGACCCCCGCAGCCTGCAGGAGTTCTGCT
EMPV1_03216	GGGAAAGACATTTGCAACTAACTGACTTGGGGCTGAGAGGACACAGCTTCCAGACTCATG
EMPV1_03217	TTTCTTGGTGCTTATGACCGAACTCGCAGCTTTGTCTTGGAACTTGGCCGAGAAAGGCCG
EMPV1_03218	CACACACTAGATTTGTGTTATGCTAACATCTTCAGGAGCTTCCCTGTGGAGCCCTCGGAG
EMPV1_03220	AGGGAAGAGAAGGCCCTGCACTCCAGGTGCTGAGTGTCCCACAAAACTGGGGCCATTGCC
EMPV1_03221	AATTTGGAACAAACTAGCCGCTCCCCCGTGGTCCCTAAGTTAACAGGACGAATGTGCAGA
EMPV1_03226	GGTAGGTGCCGTATCTTGTACTGCTACATCGACAAGTGTATCGACCGTTGGACATACCAG
EMPV1_03228	AGCTGCACCCCTGGAGCTTCCTACCCCAACTGCCACCATCAGAGAAACTTTTCCCAAAGT
EMPV1_03230	ACGACTATGGCTATGTCTGCGTGGAGTTTTCACTCTTGGAAGATGCCATCGGATGCATGG
EMPV1_03231	GAGCAGGTCTCAAGTCACGTCTCATGGAGATAAGCAGCGACATTGTTACGACAAAGTATG
EMPV1_03232	AGCATTCGCCAGTCGGCTTTGTAGCGAGGATGGGCTTGGAAGTGCTAGGACCAGAACTCT
EMPV1_03236	CCTCCTAGTTGACAATCAGTAAGGAAACTTGGACCTGAGTCCTACAGCCACATGGAACAG
EMPV1_03237	GGTGACTTCCTCCTGAACCTCCTCAGGGTCACCATACGACATCTCTTCTGCTTCCTGCAT
EMPV1_03239	ATTTACAGGAGTTGCAAGTGGGAGCACAGGGAGCACGGGCAGTTGCAGTGTTGGAGCTAA
EMPV1_03241	CTGGGTGAGTGTGACGTGGTGCTCCCTCTTTTGCGAGTGGAACTCTTGGAATTTCTAGGT
EMPV1_03242	CCTGCTCACCAAAAAATCCACCAGGGAGTGTGAGCCCCTGTCTGAGCCCAAGGAAGCAAG
EMPV1_03245	TAATCTCTACTATTGTGGCCTTTCTGGTTACTGTGAAGCCACTCCTGGGCCTGTCTCCAG
EMPV1_03246	TTGCATCACTTTGGTGATTCCTAGTAACAAGATAGCACCTGGCAGCAGAAATACCTACAC
EMPV1_03247	TTGTGCTTTCAGCTTCTTTACAGTGCTGCCTTGTAGCATTCAGGTCAAGCAGCATTGTAC
EMPV1_03250	CATCTACCAGCAGAAGAACAGGCTCCTCATGGAGGTGTACGGCTTCAACGACTCCTTCAG
EMPV1_03251	CTATGATAGCAAGCTGGTGTGGTTTGACCTGGATCTCTCTACCAAGCCATACCGAGTGCT
EMPV1_03253	GGGGTGCATCGAGAATATATAAAGTTTCTTTGACACACAGGAGAAATAATCACCAATATG
EMPV1_03254	GACCAAACGTATTTGGCAAAGTCTTACGTTCCTCTGGGAGCTTAGTAAAGCCGAAGGTAC
EMPV1_03256	AGAGGTCGTCCCATCAAACCTGAACCCTTTTGGGAGAGGAAGCAATCCCGGTGTTAGCAA
EMPV1_03257	GAAGGGGCTGCCCCTGCAAGAGTCCGTAACTGTAAATCTTACGGTGGTTCAGCCTCCCTT
EMPV1_03258	ATGAAATGTTCACAAGCGGGGACCCTTAACGGGGTGTCCGTGCAGAGAAAACTGGTATTG
EMPV1_03259	CGGTGAGGTCGCAGTTGGGAAGAAGGAAGGGCAGTACAGTCTATGCATGTCGTATGATAA
EMPV1_03260	CCCCTGATAATGCTGCTGCCCACACCCTTTATGCTCCAGAAACGTCAGGAGGATCAAATT
EMPV1_03261	TCCCATAGAGAAGTAAGGATAGAGGGACAACTGCCTGCGTAATAAACTCTGCTGTCCCCG
EMPV1_03262	TTTTAAGAGACTCTAGGTAACTGTGCTCCTCTCTCTGGATTGTCCCACAGAGTCCCAGCG
EMPV1_03264	GTTCACCATTCAGATCTTCAAACGCTCCAAGAGTGAGGTCGACGGCCTAAAGGATGTGGC

EMPV1_03267	ATCCTCTACCAAGAGCCATCACCCTCCGTCTTTTCCCCGGACATCCACAGAGGAGCCTTA
EMPV1_03268	CAACTTCTCGCTCAAGCAGGACTCGTCGCCCGACCACGAGAAGAGCTACCATTACTCTAT
EMPV1_03270	GGTGTACTTTCACTTGTTTATTTTTGCTTTTGGGGTCATATCTATAAAATCATTTGCAAG
EMPV1_03272	GTCCTCCTGAAAACCCAGCAGAAGAGAGCTCCTGCAGAGGACATGGAGCAGCTAGAAGCA
EMPV1_03273	GTTCACGACTAAGGAATGCTGTCTCTCGGGTGGGCTCTTTGTTCTGGATGGTGGTCACTT
EMPV1_03279	ATCCAGAGCCCAGATATGGTGTAGGCTTAAGATGAAGGCAGGCCTAAGAGAAGTCAGCTG
EMPV1_03280	CACAGGACAAAGACCTTATGGGTGCAGTGAGTGTGGGAAATCTTTTACCTCTAATAGTGC
EMPV1_03281	CTACAACCTCTGGAGACAAAAGCTCCTCAGGAAACCAAATATTCCAAGTTCCTGGCACAG
EMPV1_03282	AATCGGAGGGTGAGGAGGGCTAAATCAAACCCAACTTCGAAAAATCATAGCATACTCATC
EMPV1_03284	CCCTGGTAGTGATTCCCTGGGCCATTGGAGTCCTTCTGTCTTTGATTCATCTAGTGTTAC
EMPV1_03285	GCAATCAGTGGTTGGAAAAAGGAGATACAGATGCTGAGTGTGGAGAAAAAGGGACAGTAA
EMPV1_03287	GGTCAGGAAGCTTGTCTCAACCCTGAAGCCCCCATGGTTAAGAAAATCATCAATAAGATG
EMPV1_03288	TTACTGGGTAGGATTCGCTTTTCGAAATCCTCCAGGGACACAGCCCATTGCGAGAAGTGA
EMPV1_03291	CACATGTCAGTGTCCACATATGTGTCTGTGTACATGAGCGTGATCTATGTATGTCTGTGC
EMPV1_03292	AACAGTTTTACCATTTGGGCTGGGTCACTGGCACTGGAGGAGGAATCAGCTTGAAGCATG
EMPV1_03294	GCCTATGGATTGGCGTTTGTACTCTGCACATTTGCCGCGGATGCTGTCTTTATTCTTGCT
EMPV1_03296	GGGAGCTGTACGTTATCTTGAATCAAAAAAACGCAAACCTGATTGAAGTCAACGAAGAGG
EMPV1_03298	CCCACTAACTTCACCTACCTTGGCTTTGACCCCCAAAATCTCAATTCAGCACATGAGGCC
EMPV1_03299	ACCTACAGCATCTGCATGTTCCATCTCTCTGTCCATGGGAAGGGGCTGCTCTTGCTTCTT
EMPV1_03300	CCAACACAAAGGGAAAGAGGAGGGGCACTCTAGGCCTTTTAGAAAACATGGACTTGTTCC
EMPV1_03301	TTGGAGCTTCAGACCAAGACAGTTATGAACCAATATCTGTAAAGTGATGTACAGATGAAG
EMPV1_03302	CCTTCTGATATGGCTTTGCCACTGACTCTTCCACTTGGGACTGAGGACCACCACATGCTT
EMPV1_03303	TCTTAGTGGAAGACTACAGAGCTGGTGATATGATCTGTCCTGAATGTGGCCTGGTTGTAG
EMPV1_03306	GCTTTGGAACCAAGTACCTGAAGGAGAACTTCTCGCCCTGGGTCCAGCAGCACGGTGGAT
EMPV1_03307	AAAAGGTGGAAAGTTGCTTGCTCTGAGCAGTGCTCACTCAGATTGCGGGATCACATCTGC
EMPV1_03309	CACTAGAGGGCAGGGGAGATTCATTTTGCATGTGGCAGTTTGCATTCCTCAAAAGCAGCC
EMPV1_03311	CATCCCAATGTGCTTGTTGGTTAACCAGCATCTTAGTCTCCTAGCAAGAAAGTTTCCTGA
EMPV1_03312	AGTTCTGTCTTCTTGTCCACATCGCACCCAGGAAACCCTTCGCCCAATACCACGTTGAAT
EMPV1_03315	CCACCTGTGTGGGAATATTTGGATCCTGCTCACCTGATATTGTGACCTGTCCTGAGGGTA
EMPV1_03318	TCGGGCTTCAAGCACCCCTTTGCCATCGAAAATATTATAGCGCGCGAGTACAAGATGCCT
EMPV1_03331	GGGAAAGTCAAGAGCTGGCTTTATACCGTGTTCCAGGGCAAACTAAAATCTGGAATTTGC
EMPV1_03334	TCTCTGTGCAGCAATAACCAAAAGGAATGCCAGTCTAGAAGCAAGATGAGGGGCAGAGCG
EMPV1_03335	GGGCACCTTCGTGAGGCAATATTTATTTCCGTGGGTGACCGAGTCCTTACCTGACATTTT
EMPV1_03336	GAGCAGCATTTCTTGCTATTACAACCATCTATGCCGAGACTGGGTCAGGTTTTGTGGTAC

EMPV1_03337	GGTTAAAACCCACCTAACGCGGCCAACCAGAGACCCCAATGTCTGGCTGGTGGCATCCAT
EMPV1_03340	CCCGCTATGGACCACGACTGTGAGTACACCGATATTTGTTAGGAACAGGTGTCTTGGTGA
EMPV1_03343	CAGCCTAGAGTCATTTCATTTGAGGAGCAGGTTGCTTCCATAAGACAGCATCTTGCATCC
EMPV1_03345	AAGAAGCCTCCTAGCTTGCCCCCTGTCTCTGTATCTACTGGTGCGGGAAGTACTGGAGCA
EMPV1_03347	TGAAAATTCCCACATGACCATCTGGCTCGGCATCGTCTATGCCTACAAGGGGCTTCTCAT
EMPV1_03348	CGTTAGAAATAATGTACCCACGTAGACTAGTAAAATAGTATGTAGATGTGATCTCAGTTG
EMPV1_03349	TACGTGAAGATGTGTCTGCACGCCGCCCGCTACCCGCATGCCGCTGTCAACGGGCTATTG
EMPV1_03350	AACACACCGGGGACATCCTTCCTCGCAGATGCACTTTTCTTCTTTTCACCGCACTGTACG
EMPV1_03353	GCTGCTTTGCTGTTGGTGACAGTTATTCCAGTTTCTTGGTGCATGTAGTCACCTGCCCTC
EMPV1_03354	GGATATTGAAGAAATTATTGATGAACTCAAGGCTGGCAAAATCCCCAAACCTGGGCCAAG
EMPV1_03355	TGGGACATGCTTATACTAAAAATTGTCATTGTTTCTCTGAAATTCAAACTTAATTGGGCG
EMPV1_03356	CGTTGTTGTTGGATCTTTGTCTGGTTTTGGTATCAGGGTAATGGTGGCTTCAAAGAACTC
EMPV1_03358	GGTGTGGATTTGGAGAAACTGGATGGCTGGATAACATTGGAGTATCTCTGGTTAGTGGTA
EMPV1_03359	GATCTTTTCACTAGTTCTGTGCAGATGAACCCCACAGATTACATCAATAATACAAAATCT
EMPV1_03360	TGAGGTGGTGTCATGCCTGGAGAAAGGCCTGGTGAAAGAAGCGGAAGAAAGGGATCCTAA
EMPV1_03361	ATTCCTCGTGAAGAGATGTTACAAATGCAAGATATTGTACTGAATGAAGTTAAAAAAGTG
EMPV1_03362	CTTGTGTCCAAGGGAGTGACAACAGCCACATGTAAGAGTAGTGGAGCAAAAACCAGAAGA
EMPV1_03363	CTTCTAATAACCACCCAACTGATCACAGACTGACATTAACTTGTTTCTAATACTTCTTTC
EMPV1_03364	TCTGCTCACAACATATAAGCAAGATTACAACCCCTACCCTGTCTGCCGAGTGGACCCCAT
EMPV1_03365	TTTTAACCACAGTGGACCCGGACACCGGCATCATGAGCAGGAAGGAGCCGCTGGAGACCC
EMPV1_03368	TCTACGGTTTGGGCGCCTGGACCCTGCTTGGCTCATTATTCCTCTGGAGTCAGAAAAAGA
EMPV1_03369	TAGATGTTGCTGATGTGTGTGCAAGCATATACCTAGGATAAGAGTGGCTGAGTCATAGGC
EMPV1_03370	AAAATCAAAGCCGTGTATGACACCAACCCCGCCAAGTTCCGGACCCTGCAGAACATCCTG
EMPV1_03371	TATGGCAGAAGAAACAGCAGGTACTGGGACTGGAAGGAACCACAGCAGGCATCTCCTCCA
EMPV1_03373	AGAACCACCCCAATAGCACCAGGCCAGTTCCTCTGCCAAATGTTTCTAGGTAGATTCTGC
EMPV1_03374	GCACCTCCTGGATTTTTTCCAAGACTTGGCGTTATTGGTTTTGCTGGAGTTGTTGGACTC
EMPV1_03377	GCTCAGGCCGCCTGGACCGCAAGATTGAGTTCCCGATGCCCAACGAGGAGGCCCGGGCCA
EMPV1_03379	CAGAGCTGCTGTCTTCTACCCTTTCCAACTTTGTTTTGCCAGCCTCAGAATGTCTTGGCC
EMPV1_03381	ATGATGAGGGTGAGTGGTTGCTGCTCTTCAACAGCATCCTCCCCTTCCCAGACCTGCTGA
EMPV1_03386	GCAGCGATATTACAACCAAACCATGGAGAATGGGCTGAACAAAGCCCTGGAGCACAAGCA
EMPV1_03388	TCATGACTTTTTCCAGCATGTGGAAATGCACCTCCGATCTGAGCATCCCCCTCTCTGTGG
EMPV1_03389	AGGATCCAAGAAGAGGCCACCGAGGACATCGAAGCTGTCAACTCCAGCGCTTCCGTGAGC
EMPV1_03391	ACCATACTTTGCCTTTTAAAAGAAATGCCACCCACATTCAGAGACACAAGTGCAGCAGTT
EMPV1_03393	TCCACGTCTTCTTGGTCAGCTGCGCGCTCCCAGACAGTGTCCTTCGCAGGTTCGTGGTGC

EMPV1_03394	TTTTAAGCATTTAGCAGGATGTGAGCCCCGGTGGCTAACGGCTGGGCCGGCCAGAGAAAA
EMPV1_03395	CAGATGGCCCAGTTAAATTTGAATTTCAGATAAACAACAAATAATATTTCAGTATAAATA
EMPV1_03396	CCTTCTCCCTCCCCTACTTTTATTATCTGTTCTAATGTTTGTGTCATATGGAGGCCAGGG
EMPV1_03397	TAGTTGGTGCTTGCTGTGCTGGGGCATTTCTGCATTCCTTTGCCCAGTGTCTCCTCACTA
EMPV1_03398	GTCGTTCTCTCATATTTTTGTTGTCTCAAGACAGATGACATTAAGCACGAATGCAGTAAC
EMPV1_03399	GTGTATGTGCGATTCTGGTGGCTGTGAAGTAGTGAATCCCGTCATTGTCAGAGGAGAACC
EMPV1_03402	ACATGCAAGTTTCTCAGAGCGAGTATGTGACAACGTAGGCCAAAGCAGAAGGCGCCGAAC
EMPV1_03405	CAGACCTTGGTTAGCGTCAAGACACACCTCCCAGAATTCCCATTGTGGCTAGCAGAAACA
EMPV1_03407	TGCATTTCGGGACGTGTAGTTTTCCCGCGGCGTTGTAGCTGTCGTGTGTGCCGGCTGGTT
EMPV1_03412	TGTCTGACCAAGCACCAGAGAATTCACACCAAGGAGACCCCATACCAGTGTCCAGACTGT
EMPV1_03413	AAATGGCCTGATCTGTGACTTCAAGTTTGGACCCTGGAAAAACAGCACTTTCAAACCCAC
EMPV1_03415	AGATAAACTTCCCATGGTGAGACCAACTTTTCTGGGGCCATGTGGGATATTGAGAGCTTG
EMPV1_03416	CTTGGGTCCAAGTGGGTTGGACAGCCCCACATGCATTAAATTCTGTAAATAAAAACCACC
EMPV1_03417	ACCTGCTCTGTGCCCTAAGACCAAACCTCTCTGGGTCTCCACATCCCAATCTGAGAAATG
EMPV1_03418	ATTGGAATGTTTCAGTTTGGGGAATCTGGATGCCAAGCGAGATTGGGGCCACGCCAAGGA
EMPV1_03420	CTGCAGTGTCTTATATACTCATCATCTCTGCTGTTCTTAGGATTCCCTCTGCTCAGGGCA
EMPV1_03423	TTTTCCCAGTGGGAAACACCTGCGAAGATGGCTGGTTATTAACGTGACTCAGCGTCCCAC
EMPV1_03425	CTGGATCTTAGTTTTACCAAAGATTGCTCGGAGGGTTCCAGGGCGTAAATGCCGGGCAAT
EMPV1_03427	AGTTCCTAAGGGTACCGCCTACGGCAAGCCTGTCCACCTTGGTGTCAACCAGCTAAAGTT
EMPV1_03428	TTTGTACTAGAAGGCCCAAATCACAGAATAAAAGATGAGGAGTGGATTAGCTGACATTCC
EMPV1_03430	ACTTAACAAACAGGAGAGAAAATGCCCTGCGGTACCAAGCACCACCACAGGCTACAGTGG
EMPV1_03431	CTGCATGCAAAGAAGCCAATAGGAAAGGTTCACCATCAACACCACTACATATCACTCCCC
EMPV1_03432	TTCCTTTACAGCAATCACAACAGAATGGGCCGCCACAAACCCCATTTGGCCCTCAAACAG
EMPV1_03433	GGAACGCACTCAGAAATCACAAAAGGACAACAGAAACTCAGACACTAAGTGGCAGGTGGC
EMPV1_03434	TATTGCAACTGGGAACATGCCCAACCTAGGACCAAGTTATAGCACAAATCAGCACCCTTT
EMPV1_03435	GGCCAGAAATTGATGAGCATACACAAAAGACAGGTGTCCTTGCTGCTGTTGTTGCATCAC
EMPV1_03436	AGCCACAGCCACAGCAATGCCAAATCAAAGCCATGTCTGCGACCTACACCACAGCTTACA
EMPV1_03439	TAGGGAGCCCAGGAAATTCCTAATTTGTACAACCTTGTCTTTGCTGTAAGACACTGGGCG
EMPV1_03440	GAAGCTTGCCTGAAGCTGTTGATGCTGCCAGGCCAAAGGCTACTCTAGTGGACAGTGAGT
EMPV1_03441	GAGAATGCGGGCTCTGAAAGTGTAGAACCTCTACAAAGTGCTCCCCTGGCTCAAGGAGAA
EMPV1_03442	CACCCTGAACAACGTCATGATGGTCCTGGCGGACGTGTACTTTGGAGTGGTCAACTTTCT
EMPV1_03443	GCGTATCCAGATGCAGCTCCGTTTTATACTTTTCTATCATTTGGTGGACACACCCAGGTC
EMPV1_03444	TTGTAGAGTTCCTGCAGGGGGCTTCGAGACCCCTTCCGACAGCAGATGTCCACGCCGAAG
EMPV1_03445	GGCACATCCACAACCTCAAGCACTGGGGCCTCTACGAGGTGCTCATGGAGAAGTACGAGT

EMPV1_03446	CAGCTCTCTGCTGCTCTAGTCTCCCAGGTCCAGACCAAATACAGTGAAAACTCAGCCTCT
EMPV1_03447	AGTCTGAAGGGTGGAGACACAACTCCTGACATGGGCTGCACAGACTAGAACAGACACACT
EMPV1_03450	AGCGGGAGAACAGCGAGGACAAGTGACTCCCAATCCAGTTTCTGACTGCAGGTAGTTTGT
EMPV1_03453	CACAGTCACCCGTGGATGCCTACCTCACGGAGGAGGACCTGTTCCACCATAGGAACCCTC
EMPV1_03455	AAGGCAGACCAACCAAAAGATGATACCAAGAAGACAGAAACAGACAACCAAAGTAATGCC
EMPV1_03457	GTGCAAAGAGGACAGCTAGGATTTTGACAGGGATTACACTGAATCTGTAGATCAACTTGG
EMPV1_03458	CCCTCAGTTTAGGCCTTTTGGTTTAGAATGGGATCTGATGCTAGTTGTTCCAAGTCTATA
EMPV1_03459	TGCTGAGACTTCTGCCCATTCTGGATGCTTGTGAAGGAAGTAGCACAGCTTTGAAAGTCT
EMPV1_03463	ATCCTCGGCGATGTCTTCTTGGGTAGCTACGTGGCCGTCTTCGACCGTGGCGACAGAAAA
EMPV1_03465	TATAGAAGGGTGCCTAGTGAAGAATTACATGCAGCGGCCTTCCACAGAGCAGCTTTTGAA
EMPV1_03468	GCATGCTCCTACTCATCGTACACGATGGTGCTCAGTTTTGCCTCCCTCAGATCACTCTGT
EMPV1_03469	CACATGTGGCTCACATATCATCACCGTCCTTCTGGTCCTTGTGCCCCCCATGTTCATGTA
EMPV1_03470	TTAATGTTAAAGGATTAAATATAAAACAACAAACCATAAGAAATATCCGTAATATATACG
EMPV1_03471	GTTCAAGCACCGACGTCACGTAATGGAGCACCCCACGATTCAGATCGACCTGCAGCAGAA
EMPV1_03472	CACAGCAGTTGCACTTGCTGAGCAGGCAGCTTGAGGACCCAGATGGTAGCTTTTCAAACG
EMPV1_03473	GATGTTGTGGAAAACAGTGCAGTTAAATCAGGTGCCAAAGTTTTGCTGCCTCAGCCCCTC
EMPV1_03474	AGAGCCCTCAGAGCAGCTCCAGTGCACGGCTCTGCTGCAGGAACTGCAGAATTGGGTCGG
EMPV1_03477	ACGGTGGAGAACATAGCGCTGCCGCGGCACGAGGCCCTGCTCTTCCTCGTCTTCTGAGGC
EMPV1_03478	AGGAGGCTCTGCGGGTGTGGAGATGGCTGCAGAGATTAAGACCGAGTACCCAGAGAAAGA
EMPV1_03481	TTGCAGTTCCCTTCTGTCTCCATGGGAACCAACCACATTCTCCTGCAGCCTCCGACATGT
EMPV1_03482	AGCCCTGGTAGGAGAGTTAACCCACCCAGGATAGTGACCATGACTTACACTGCATGAGAA
EMPV1_03483	AGCAGCCTGCGCGAGAGGGAAGGCCAGTTTTCCAAGCTGCTGGCCATTCCTCAGAACTTT
EMPV1_03485	GCAGTGCTGGTGAGGCCTGATGCTGTGTCTATCTCAACCTCTCCCTAGAATGCTATTTTC
EMPV1_03487	ATGGCCGACCAGAGAATGGACATTTCTTCAACAATCAGTGATTTCATGTCCCCGGGTGCC
EMPV1_03489	TAATGTTGCAGTTACACTCAGCGGAGGCCAGTTCATTCCTCCCCAGCATAGGAACCAAGG
EMPV1_03492	CATCTGCAGGGACACTGGGTGATATTGGCAGTGACTGTTCTGCATTGAGGCTTTGTTTGG
EMPV1_03497	CATTGTGTATCAGTTCCCAGGAGATGCTGCTGTGGCTGGTCTGGGCACCAACTTTGAGAA
EMPV1_03498	TTCAAGATATTGGTGGCAGTTCCTTTGGGAAATCCAGATGAAGATAATTCCCAGTGTGAG
EMPV1_03499	TTGAACCCTGATTGTAGCAAAAGCCCAGAGAGGAGCTGTTCTGGCCCCTCCCTCAAAGAA
EMPV1_03500	CCCTTGTCCAAATTCAAAGTGGTCCCTTTTGCCCTTCTCATTACTTCTTTCAAAGGAGGG
EMPV1_03501	TCGCCTTCAATGGCTTCAATTTCTTTGACTCTTTTTACTTGTTCAACCAGGGTGGCTTGG
EMPV1_03502	CGCGCTCCCGCAGCCAGGTGCTAGGTTTGAGAGCAAAAGTGAGAGTCCGGCTGTATTCCA
EMPV1_03503	TTGACATGTTTTTTGTATGTGTGCTTTTTTTTAAGTTCTTACGAGAAGGGGAGGGGAGGG
EMPV1_03505	GAATATGCATTGACCCATGGCTTTTGGATCACCGCACGTGTCCAATGTGTAAACTTGATG

EMPV1_03507	GCTGGAGAAATTTCCCTAACTTCTCAGACCTCACTATCAAATGCCTATGAGCCTGTGACA
EMPV1_03509	TACATGGTTTGCATAAGTGGACCGAGATCCTGTGGTCTGATGTAGTGACAGAGGCCCTGG
EMPV1_03510	CTGTTTGAAGAGTGGAATTCTTTCTGGAATTAGGCTTGGAGTTGATGAGCAGCTTGTCCC
EMPV1_03511	TAAATTGTGCTGTGGCCGAAGCTGGTATATTCAGGGCTGTGATGCTCGAAGTGGTATGGG
EMPV1_03512	CTTGGGCCTCCACTGGACACTACAAGAAAATCTGGAGACCGGAAACTCCTTAGCAGTACA
EMPV1_03513	ACCACACACAGCAGAGCTTCTATAGGGACGTCCTGCAGCAGAGGAACGGGCTGTCCTTGG
EMPV1_03515	AGCAACATCTTCACAGTTGCCAAGAGGAACGTGGAGGGCCAGGACATGCTTTACCAGTCC
EMPV1_03516	CTCCAGGAAGGTGTGATGACTCTGAATTCGGTTTAGGAGCTGCTGTGCCACTGGTTTGAA
EMPV1_03517	ATCAAAGGTCCTCCATCAAAACCCCAAAGTGTCAAGACACAGAAGATGACGAGGGGGCTC
EMPV1_03519	TTGCACATCTGTTCCAAATGTGATTGCTCTCTCTTCTGCATGGCTAACTTGGTAAGAGTG
EMPV1_03521	CAACCACTTACCTCCCCTGGGAGCGTCAGCTCCTCCCGAGGCTCCAGTGTTCCCGGCTCT
EMPV1_03522	CGTGGCTGCCAGTTCTCGTACTGCTTCTGTAAATTTGCACCGATGGTTTCCAGAGCTTTC
EMPV1_03523	GATGTGGGTCTTCGTGCAGGGAGCTGGAATCTTCTCGCCCATTTAAAGTGTTCACCCCAT
EMPV1_03526	TCATCGCAGTGGGAATCTTCTTGTTCCTGATCGCATTAGTGGGGCTGATCGGAGCCGTAA
EMPV1_03527	AGGGCACCACGCTGAAGGTGTTCATGATGCGGTCAGGGTACTCCTCGCGGACCTTGCTGA
EMPV1_03528	GGCTTCCCGAGTACATGTGCAATCTGATTACTGATCTAGAACGTTGCGATCACAGAAGTG
EMPV1_03529	TTACAGGTTACACACACAGGGCCATTTATGTAAAGTCCTTCAAGACTACACACGTTTTTG
EMPV1_03534	CTCTCATTGTCAGAGCTCCCAGACTAGACCTGGCTAACAACCATAGCAGACGGAGATAGG
EMPV1_03535	GGGGCCTTGGAAGGTGTGCCAATATCCGGGTGTTTGGGTGACCAGTCTGCTGCATTGGTG
EMPV1_03536	TAGATCACCTAATGGAGGCTGCAACAATACAAACCAGTGTCCCTCTGGCCTTCCCATGAA
EMPV1_03538	TCTGGAAAATTCTATCTTCTCTAGATTCATGCTTTGCCATGGAGTCCAATTCTTCCTCAG
EMPV1_03539	CGCATGGTGTACCAGCCGTTTTAGACCAGGTAGGAGTGTTTGGGCAAAAGCTGAACGACA
EMPV1_03540	GGGAAGGCAGGGGCAACTGATTTGCACTGTTGGACTTGACCCGATAATGAGCTATTTAGA
EMPV1_03541	CCATCCAGGGGCCCAAATGCATGGGAAAGCAGATAGTAATCAGAAATGGGCATGATAAGC
EMPV1_03542	GTGGTGACTATTCCAAAGCCAGTTTAGAAAATACTAGGTACGGGCATTACAATCTTTTGG
EMPV1_03543	CCTCGCTCGGGAGGTTAAGTATCTGTGGTACAAGTTGCAGATGTGACTCGAATCCAGCAT
EMPV1_03548	AGGTGACAGTAGGGACAAGGAAACACTGAAAATGTCCTGGCGAGGGAGCGGCATCCTTAA
EMPV1_03551	CCAGTGATCCACCCTCAGGATAAGTGAAGCATTGTTGAGTCAGGATGTGCAGAAAGCTGC
EMPV1_03552	ATGTGCTTATTGATATATGGAGAGAAACAGGTGATGAGACCACTGTGTGGCAGGCCCTGA
EMPV1_03553	GAGCTCTGCATCCACATCAGCAGGAGCCGCCCATTATCATTCATCTCAAGACTCATTCTG
EMPV1_03555	GCTTGGCCTGTGTGTTCTTGACTTGTTCTCCAATCTTAACCTATGGCCCTGATCCTTGCC
EMPV1_03556	CTGAAAGGCTCTGCCGACTTTCTAGGCCTGTCCCATTACACTTCCCGCCTCATCAGCAAG
EMPV1_03559	GAGATGCACAGTTTTCAGATGATAATCTCACATTAATTAAATTTGTAAAGCTCTCCTACC
EMPV1_03560	ATGCAGATCAGTGCCACACTGGTGAAGGGAAAAGCACGAGTACAGTTCGGAGCCAACAAA

EMPV1_03561	AAAACTACCTCCTTGTCCTGACCCAGTAAACCTGGTGCTTGTGGCACCTTTGCAGATATC
EMPV1_03562	AGGCGGCAGACCTTAGGCTAGATCGGATGACTTAGGTGTCTCTCAACCTGAGGAGTGTTT
EMPV1_03565	CCAGTGGAGGATGTTCATTTGTCCGGATAGCGGTTCCCTCATGGGGTTGTGGCTGATTAA
EMPV1_03566	ATCACACCACAGCAGCGACCTGAGCCGATGCAATGACAACACCACCAGGTCTTTAACCTG
EMPV1_03567	TGAATGTCTTGCAGGCTCTTTTATACCGCTGGCGTCTTCTGTGCCTGCTTGTGTGTGTAC
EMPV1_03569	GACAACCCTGGCGCCTTCCTGTGGCAAGAGAGACAACAAAGCGCTATTAAACTAAGGTCA
EMPV1_03570	AGCAGTGGTGGCTGAACCTGCGGCTCATGCTGAAGATGCTGGCGCACTACCGCATCAGCA
EMPV1_03574	GCTGTATCATTATGGACTGTGATAAGCCCCAGTTCCTGCTATCTCTTTACAGAGTCTTTC
EMPV1_03575	TTTCTCCCAGATCTCCAAGCCCAGTTGAAAAGTCACGCTGACACAGGGTACAAACAGCTG
EMPV1_03576	ATCTTCCAAGTCCTCCGGGGAATACGCAGCCCCAAGACGATGCGTGACTCTGGCTGCTTT
EMPV1_03577	TATCAGGAAACGAGAGCGGGATGACTGGGAGAATGGGCTGACTGCAATGGAATATGCACT
EMPV1_03578	CTCAGGCATCTGCAGCTCTCACGGACCCCATGATGCCCCCAGGTTCGAGATCAACTTCTT
EMPV1_03579	TCAACAGTTTGCAGGACATGGTGACAAAGTATCAGAAAAGAAAGAATAAACCTGAGGGGG
EMPV1_03580	TCTTCAGAGATGAACAGACATGAGAAGAATCCAGTGAGTCACAAGCTAGAAGATCAGAAG
EMPV1_03584	GGTTGGGCTAGTATTTTCCCTAACAGATCCAGAAAGATGTTGGCAGCCTCCTGAAATACC
EMPV1_03586	AGGACAGGAGCAACTCATACTTCGTGGTGGCCGTGGTGAAGCGGAACAGCTCCTACGCCT
EMPV1_03587	AGGTCAAGAACATAGAAGAGTTGTCCCTCACCAACAGTGACCTAGGCCCCACCAGATTTA
EMPV1_03589	GGTACAATGTCACTGGTGGATCCTGTCAGCAGTTTGTGTATGGAGGCTGTGAAGGGAATG
EMPV1_03590	TCTAGGGGCCGTGCTGGCGATAGCGCTGCTGAAGATTGTCGCCGACAGCACAGACTTGGA
EMPV1_03591	CCCCCTTCCTATCAAACGCCAAAATCCAGGCCCCCCTGTTATGGCTGCTACTAATTATTC
EMPV1_03592	GCCCACCTCCCTCTCGGTCGCCACATCCTTTTCCACCTTGAGCACCTCCTTGACGGTGAT
EMPV1_03593	CAGTGCCTGTGGCATATAGAAGTTCCTGGATCAGGGATAAAACCTGAGCTGCAGCAGTGA
EMPV1_03594	GGGAAATTCTCTCTGCAGTCTTGTCGCAAAGTCCAAATAAGATCCATTTCATTCTCACAC
EMPV1_03596	AGTGTGTATAAAAGTAAAACCCAGATCCAGGAGGGCAGGAAAGTGGGTGAAATGCAGGAG
EMPV1_03600	TGGGCCACTTCTTCCACAGATTGGCTGAGAAGCGCAAAGGCACAGAGCCCCTCTTGAAAA
EMPV1_03604	CTGTTGATCATCCTCCTGGCGGAGCTGATCTTAATTATCCTCTTCTTTGTCTACATGGAC
EMPV1_03606	GAAGAGTAGGTTCTTGGTACTTAGGACTTGATCCTGTGGTTGGCCTTTGGCCATGCTGCT
EMPV1_03608	GCTATTTTAACACTTACAGTCCCAAAGCTGGCATTTATACATATCAGATTAGACTTCTTC
EMPV1_03609	GTACTGAATGAAGTTAAAAAAGTGGATTCTGAATACATTGCTACAGTCTGTGGCAGTTTC
EMPV1_03610	CCCAGGGGTGATTAGGGCAGAAAGAGAGGTAGTTTCTGGACAGCCAAAATGGCCAATGAC
EMPV1_03611	TCGGAGGCTCTCAGCACCAGGTCAGCTCTCTTCTCAACGCCTCCACTCACCGGCACCATC
EMPV1_03612	CCCTCAGACCACCTTGCACTTGATGAAACCGTTCTGTTTTGTGGTGTAAAACTCAGACCC
EMPV1_03613	CAACGCCATCACCCACCCGGAGATCTGCAGGGAAATGACGAAGGAGACCTTCAAGTACTT
EMPV1_03615	ACAGGCGCCGGCCAGGCTGTGGAGTGAAGAACCGGACTTCTCAGAGGCAAGGAAATCTCT

EMPV1_03616	CAAACTGTCCCACCAGCCGGTGCTGCTTTTCCTCAGGGATCCATACGTCTTGCCTGCAGC
EMPV1_03617	TAGAAGAGGGCACATCTCAGATATAGCTTGAGGAACTCACACAGAGAAGGCACCAGCTGG
EMPV1_03619	AACAACTTTATGGAGACCAAGCTCTGATGAGGCCTGCGACGTTCTGAGGTGATTTCCTAG
EMPV1_03621	TTTGAGGAGTCTCTTTCTGACAGCTCGTGGATGTGGCTGTCTCCCTGTTCGGTTTCTGGA
EMPV1_03622	TGTGAGTCGGTCCACAACCTCCGCAGCTACAAGATCCAGACGCAGCTCAATCTCATCCAT
EMPV1_03623	ACTCTTGGCAGAGAGGACTTGTATGGGGTGGGAAGCCCAACCCTTCTCCCCAGATGCCCT
EMPV1_03624	ACTGGCATGTCATACTTGAGGCCATCCACACTGCTACACTTTACCTGCTGTCTGTGCCCA
EMPV1_03625	CCAGACTAGGAATAGAACCCACTCCATAGCAAAGACCCAAGCCACAGTAGTGACAATGAA
EMPV1_03629	CAGGGTCCGTGACAGTGATAATTCGGGGTCTGTAGTTTGAGGAGATCTGTGGGTTACGAA
EMPV1_03630	CGCTACCGGCGGGAGATGCTGGCGCACGGCGGGGGCAAGGAGCCCCTGCTCATGGTGGAA
EMPV1_03637	CACGCGCATTGCCACACGCCTCTCCCCGGATCCCGTTCTCCCCGCCCTGGCCACACTCCC
EMPV1_03638	TGGAGAGAGACTCTGGGCTCTCGGCTCAGATGTGTTCATCAAATACTCCTCTCAGAGCTC
EMPV1_03640	CCTGGGCCAAGATGCGGAAACAAATCAGAAGGGAGAGGATAATAACATTAAATGGCGCCC
EMPV1_03643	ACCTGTCAAAGGTCGAGGGCTTTCCTCTCGTATTCCTCCTCGGTTCGCGAAAAAGCAAAA
EMPV1_03644	AACAGACTTCGAGAAGAGAGCTGGCTAAAATCCTTGTTTGTCCGGAAGGTCGATCCGAGG
EMPV1_03645	GGATGTTTGTAGAGTGGTGGACAGCTGGTTCCCTTTTCCCAGTGAAGTAGAAGGCAAGGT
EMPV1_03646	TGCAGAATGGTTTTCATTTAGTTAACAAGTATTTGTTGAATTCCATGCTGCATAAGACAC
EMPV1_03647	AACTGTCAGCAGAGATGATGGAAGATGAGACACCCAAGAAAACCACCCCCATCCGCCGAG
EMPV1_03648	CTTCATTCCTCATGTAGATCCATATTTCCAGCTGATGACAGTTCCCTTTGACCTGAAAGC
EMPV1_03649	GTCACGTGGCCGCACACCCATTCATAAAAGGAAAAGACACAACTCTCCTTCGGATGCAGT
EMPV1_03652	GCTTTCAGGCCAGCTCCTTCTCATCATCCAGGTCTCTGCTCAAACATTGTCTCCTTCAAG
EMPV1_03653	CCACTTACACCGTCCACACAGAGAGGTCCTACTGAATCCAGAGATGGGAACGGACAATGA
EMPV1_03654	AGCTGAAGTTGCTTCGCCAGGAGAACCGGAAGAACATGCTGCTCTCCGTGGTCATCTTCA
EMPV1_03655	TCTGGACACAGTCTTCACACGTACATTCCTCTACCGTGTACTCCAGGCCTCTCGAAAGCA
EMPV1_03656	GGTCTCCAGAGTGTTAGTCTCCAGCTTCCAGAAAGGCTTCACAAGGCTCTATTAAAAGAC
EMPV1_03657	AAGAACTGGTCTAGAGCCCTGGAGGATTAAATGGAGGAGGGAATGGGTGTGGAGGCGCCC
EMPV1_03661	CAATCGGTTTCCCAAGCAGTTTAAAGCTTCCACTGTTATTCTCCAGAGAGTGCAGCATGC
EMPV1_03662	CCCCCACAGTTCTCAGAATTCTAGCCAAAAGGAGTTTACTCCAGCCAATATGAGCCTCCC
EMPV1_03666	GCCATCTAGAAGTGTATTCTACTCTAAGGACCATCCCCACTCACCTCCAGACACACCAGA
EMPV1_03669	ATTTCCCTGGCAATGGGCATTTCTATTAGGTTTCGTAGTAGGTGCAGTCTCTCCTGCTGT
EMPV1_03671	GATCTTTGAGAGGTCTGTGTACAGTGACAGGTATATCTTTGCAAAGAATCTTTTTGAAAA
EMPV1_03672	TTAGGTTCCCCTCACAACCTCTGGCTCCGTGCCTAGCTACTAACTCTTTTCTAATCGTCC
EMPV1_03673	GTTACCATATCATCAGCTGCGGAGAGAAACTGGGGTGGGCGAACTGTAACGAGTCTGAGA
EMPV1_03674	TCACCTGTTCCTTCTCCATGGAAACAGCCTTAGTCTGTGGACATCGCTCACGCCATGTGG

EMPV1_03675	CAGGTACCTCGGGAAGCTGTTCAGCGATGATGCCAACGTGTTGGTGTGGCACGCGCTCCT
EMPV1_03678	ACTTTCGCTACGTGGTGTTTCACCCGTTCCTGGACGAGATCCTGATTGGGAAGATCAAGG
EMPV1_03679	TACGGGTTCAGGAAGGCTAACTAGCTTAAGGTGCCCATTCAGGTTCCTTTTCTCAGCTCC
EMPV1_03680	GTGGGTAAATTTGGTGGATGAGGGTAAGAGGATGAAGTAGTTCTAGTCTGTCCATGACAT
EMPV1_03681	TGACGGAGCCAGAGCTTCTGTGAAAGGCATTGCGGAAGTTTCCTGCGGCTGCAACAAATT
EMPV1_03682	GATACTAGGCAGGTTCATTAAGCACTGAGCCACAGCGGGAACTCTCTCCTCCTTTTTTGG
EMPV1_03685	CAGGAAGAACTAAAGGAACTTCTGAGAAAGGCAGCCAGGGGTGAAGTTAAGGTGACTCCG
EMPV1_03686	CCAGCTAGGTGAGGAACCAGAGCTCAAGTATCAGTAACATCTGTACCAGAATGGCTCTTG
EMPV1_03688	CCTTCTTTGCTTTCTCCCTCATCGAGGGCTACATCTCCATTGTCATGGATGCTGAGACAC
EMPV1_03689	TGAACCTCAAATTAGCCTACCTATATAAAAACGGGCCCATACCTCCAATTCCATACTCTC
EMPV1_03690	ACATCAACATCATTTAGAAACCTTTTAGAGAACAGTCCTCTTCACCCACAACTAACACAG
EMPV1_03691	TGGAACAGAAACCTGCTAAAGATCTCCTTGCCGATGGGTCTAGTGCTCCAAGCAGCCGCT
EMPV1_03692	ATCTTCCTGCAGCTCTGTCTCTGACATCCCAGGGCATCGGGAAATCCTCCCTTGAACCAT
EMPV1_03693	CATGGAAGAAATGTAAAGAGTGTCAGGGAAGTCATTCTCCAAATCGCTGTGGACTGAGAG
EMPV1_03695	AAATTAGAAAGCGGGATGGGAGCATGAGCGCAGTGTTAGGCTGGGCGGGCGTTTGATGAA
EMPV1_03696	ACATAGAAGAGGTGCCCAACTATGAAAACCAGGAGGGAGGCTCTGCGCACCAGGGCAGCA
EMPV1_03697	AGCCAAAGCAATGCCAAATCTGAGGCGTGTCTGTGACCTACACCACCGCTCACAGCAACA
EMPV1_03699	ATCCAACCCCAGAAGGATTGAATCAGAAACTCTAGGAATGGGACTCAGGAATCTGAGCTC
EMPV1_03700	AATTTCAGAAGTGATTCCTTTTTTGTACGTATCTTAAATAAAAGTTTTCTTTTTAAACAG
EMPV1_03702	ACTGGAGGCCAAGTGCAAACGCCAAAAAGCCGAAGCGGCTGAGAAGCGCCGCCTGGAAGA
EMPV1_03703	GGAGATGACCACCAGAGGGCAGGATACCCCAACTATGAAGTCTATGTGCAGTCACCAAAA
EMPV1_03704	CTCAAGCCAGCATGTTCAGCTTGTTCCTAACATCAGTCTGATTTGTGTAGCTGTTATTGG
EMPV1_03707	GTCCTGTCTACTGTCTGTCCGGCATGATGAGGGTGTTGCTCCAGGACACTTTGTGTAAAA
EMPV1_03709	TTCACCATTTTAGCGATTTTCAAGGGTGCTGCTCAGTGGCTTGCAGTACACCCCCAATGC
EMPV1_03711	TCAGCAAGGACCGCGTCCTCATAAAGCTGGCGTCCACCTGGGAAGGCATTCAGGCGGGAA
EMPV1_03713	AAGTACCACACACACTTGCTCCAGTTCGACGGGGAGGGTGGCTGGAAGTTCGAGAAGCTG
EMPV1_03714	TGAAGGCAAAGGGGTGCTCAGGGCTGATTATGTAAAGGAGATGCTGACCACGCAGGCGGA
EMPV1_03715	ATCTACCTCCTCTCCAAGCAGGGCAAGAGTTGGCACTACCTGCTGAGGGAGTACGTCCAA
EMPV1_03716	TCTGAAGAAACCCAGAGACCCCGAGATCCTTTTGCCGTGGAACAGTCTAGAACACATCTC
EMPV1_03717	GCTATTATCTCTGAGAGACTGAAGATTCGTGGTTCTCTGGCCAGGGCAGCCCTTCAGAAA
EMPV1_03718	TGGCAGACCCAGGCAGAGAGAGGGATGTGACAGACGCTGGGGTGCGGAGCTTCACGTGCA
EMPV1_03719	GTGAGCATTTTGCCCTCTTTGCTGTATGCCTTCCTCCCTACATTTCTACGTGGTATTTTC
EMPV1_03721	CTGTTCCTTTGTGCCCGACAAGGGTGCTCACTACTGCGTGCCCTGCTATGAGAACAAGTT
EMPV1_03722	AGTTGCAAGTCTGATTCAATTTGACCCCTAGCCTGGGAACCTCCACATGGCGCAGATACA

EMPV1_03723	GAAGAGATGAATGTGTGCGACAACTTGGGGGATCACCTCGTGGGCAATGTTTATGTCAAG
EMPV1_03726	GCATACATGAAGATGTTCAGGCGATGAGCACCTGTTGTCAAGACATGACAAGTCGCCTGC
EMPV1_03727	GCTAGATGAGATAGCAAGAAGGGAACAGGAAGTACAGAAAGTCTCCACCCACTCCCAGGA
EMPV1_03728	GGCTGCCTCTGTCTTGTTTTTCTTCCAGTGACCATTCCAGAAGTGGGAAGAGCGAGGGTG
EMPV1_03729	AAAAAGGACAACATGAAATATGCTAGTTGGGCAGCTAGTCAAATAAACAGAGCTTATCAG
EMPV1_03731	CACACACCTTGGTATAAATTCCATTCATTTCTATGCAATCAAAACACATACGAGCTGTCC
EMPV1_03732	AGAGAAATGCAAATCAAAACTACCATGAGATACCACCTCACACCAGTCAGAATGGCCATC
EMPV1_03733	CCATCCCAAGCCCTCTAGTGTCGTCTTCACCCAAATTACTGTTCCCTCCTACATGTTTCC
EMPV1_03736	CTACGCGGTTTGGGCGCTGCTACTGCAGCCGGGTTTCCGCCGTGTGCCGCTGCGGCTGCA
EMPV1_03737	GGGTGTGACCAATCCTGTTTATCCTACGGGCTGGGTAAATTGAACCTGATGGTATACTAC
EMPV1_03738	ACAGAATGCCCGGACAAGAGAGGACTTGCTGTGTGGACTGCTTTGCGTTTTCAAGCACAG
EMPV1_03740	AGGGTCCTCTTGTCTGAGCCCCAGAAAGAGGAGAGAGCGCTGGGTCACAGAAGAGGGTCT
EMPV1_03741	GGAAATGAACCTGAAGAAACAGGTCTTTGCTGAACTGTCAGCCGTGTGCAAGCCACAAGC
EMPV1_03745	GCGAGGCACTTTTGGCACTCTATGCTTGAAAGAAAAGCACTCTAATAGCCTAAGGGCAGG
EMPV1_03746	GCGAGCGCCCTGGGCAGCATCACTGAGATTGATGCTGATGACTGGCTGTCTGGAAGAGAA
EMPV1_03747	GCAATAGGGAAGCCATGGATCCTATAGCTGCAGAGCTTTTATCTCAGTTATCAGGAGTGA
EMPV1_03750	GGCATTTCCAACATTGAGGAAGTAGCTCAAGTCCAATGCATATGTGGATGCAATCAGGTG
EMPV1_03751	GAGGGCAGGGTAACTTGTCTGACAATGTGTTCAAGGCTGCATGAACTCGGGGAGCTGTGA
EMPV1_03752	GACCAGACTCTGTGCCCTACAGCAAAAGTATGATGCTAGCCAGGATGAGCAGAATGAGCT
EMPV1_03755	CCGCAGAAGAGGCCAATAACCAATCGGCCCAGGATCAACATTTCAACCGACCGTGATATT
EMPV1_03756	ACTTTGTCGCTCCAACCCCCGTAGCCCAACCTGTAGAGCACCCAAAGCCCAATACTGTTA
EMPV1_03757	GGACGTCCGCCGGCGCTTAAGTGATCACTATAATCGAATTCCCGGGGCCGCCATGGCGGC
EMPV1_03761	TTACAAAGGGAAAAAGACTCCCCTACTCCGGAAACATTCCAAATGCTCAGCCCCCCAAAG
EMPV1_03762	TGTGTACTCCCAGCTCCTAGCACTCCAGAAACATGGGCAGGGTAGTTCCTTTTGTGGTGA
EMPV1_03763	ATCAGGCATGGAAGGTTTGGAGGGGCCTGAGCTGCCTTCCTCCCTCATCTCAAATTTGGG
EMPV1_03764	TGTTGGGAGACCCCCCGACAGTTCAACCCTGGCCACTTCCTGGACAAGAATGGAAACTTT
EMPV1_03765	TGCCGCATAGCCGAATTCGTAATACCACACTGCAGATGTACTGCCTACCCTCCACGCGAT
EMPV1_03766	CACCGTGTCTTCTGTTTTTTGGGGTAGATGGCTGAGGCGTGACTGAGAAGGGCAGACATT
EMPV1_03767	CAGTAGGTCTAGGTGGGACCTGAGATTCTGCATTTCTAACAAGTTCCCAGGTAGTGCCAA
EMPV1_03768	TGGATAGGAAAAAGGCTGGGGGTGGGTTGTCTAGGCTTCCTCTAAAGGCAAAAGCAGCCT
EMPV1_03769	AAGGGCCACTCACAGAAGGCATCTTCAGAAAATCAGCCAGTATAAAAGAATGCAGAGCCC
EMPV1_03771	CTGATGGAGCGGACAGTGTAACAGCACAGAGTGGAGCTTCAGTTCAGCCTCTAGTGTCAT
EMPV1_03772	CATGTCCTTCAGTGCTGACCAGATTGCTGATCGAGTTCTCTAAGGAACAGCAGGATGAAT
EMPV1_03774	CCAGTCTTGCATTCATAATGCCCACTGATGGCATTACTCTGCACTCTAGCCATTTGCCCC

EMPV1_03775	AGCCAGAAAATGAAAGCAACCTAGACAAGGTGGGAAAGTCAGAAGATGTGGTGGATACAG
EMPV1_03777	GTTACTGGAGCGAGGCAGGCAGCTCTGCCGACTTTACCACATGCTTCTAACGTACTAAAA
EMPV1_03778	TCGCGCGGAAGCAGGACCGTGAGGTGATGATACGGATGATGCCTGAGGTTGTGAACTCTT
EMPV1_03781	AGGAGCCCATTTATCGACATGTGTTTTCTGACCTACGTGTGCACCCCCAAGGTAATGCTC
EMPV1_03782	TGATTTCTTGTGGAGGGAAGTAGGAATAAGGGTTCTAGGTCTGTGTTCTGGAAGGCAGTG
EMPV1_03783	AGGCACTGGGTGTCTGAATGTAAGGCCAAGCAGTCCTACGTGCGGGCATTGACTGCTGAT
EMPV1_03784	ACCTGCTCCCATCCCGTCAGTTTGGGTTCATTATACTTAGAACTTCAGCTGGCATCATGG
EMPV1_03786	TTGGAGCCCACCCTTCTGGGCTGTTCTTTGGGACTATAACCTCCACCACGAGGTAGTTTA
EMPV1_03787	AAGCTAGTGAGGAACCACCCCAGAGCTCCTCATCATCTGTAAGTCCAGGTACTCCTTCTG
EMPV1_03789	GTCGAACCCGCAGCCTCATGGGTACTTGTCAAATTTGTTACTGCTGAGCCACAGCGGGAA
EMPV1_03790	CTTCTCTCCCTCTTTGAAAGGAAGGGGCTTCTGTTGTTCCTACCTGGCTAGAACCATAAC
EMPV1_03792	TCATGTGTTCCCAGGGCCCAGCACAGTCCCAGGTACAGAGAAGCAAAATCAACTGCTTGT
EMPV1_03793	AAGTCCTAGTCTCTTATCTGGCATCAGAAGCCGGCCAGTGTTCCTTGTCCGACAGACTCT
EMPV1_03794	CTCGAGGACCCTGCGATCAGGAATGTCATTGTACTACAAACAGTTCTCCAAGAAGTGAGA
EMPV1_03801	TGTCTAGTTTTACCACTCAGGTCCTGGCCTACTTTTTACAGTAGTGGCTTCAAGAACTAT
EMPV1_03802	TGTCTGGATTCAAATGCCATCGGTCCTCCTTCCTAGCTGTGTGGTTTTGAGCAAGTCACT
EMPV1_03803	AGGAGACCGCATTGGAGGAGTTTAGATTGCAAGCAGAACAATGGGAAGCAGAAAGAAGAG
EMPV1_03805	TCAACCTCTCGCTCAGAGCTGGTGACCCCGCACTGCCTTCAGCTCCAACCTGTTTTCTGT
EMPV1_03809	CAGGCAAAGTGATGTCTCCATAGCCATCCATGTCTTTCAGCACCTGGGACAGCTCACATT
EMPV1_03810	ATGGAGCATGGATGTGTCAGATAGGCAGCGCATCTCTAGCTGGCTGTTACAATTTCACTT
EMPV1_03813	CTCGGAGAACTCTGGGACAACGTTAAACACACCAACTTCCGTATTATGGGGGTGCCAGAA
EMPV1_03815	AAGTAAGTAGAAGACGTGGCAAGTAGGTCTATTTCGAGTTATGTGGTCAAGCAAATTTCG
EMPV1_03817	TGCTGCAGCTGCTCAGAACTAACCACAGCCCCAGGATCTGGGAGTTGCAGCCAAAGCAAA
EMPV1_03818	CATTTACTAGCAGGATCGGGATTATGTTTACCTCACCCGTGGGCTGCTCTAGGATGTCAC
EMPV1_03819	AAATCTGCTCCAGCCCCGAAGAAGGGCTCCAAGAAAGCCGTGACCAAGGCGCAGAAAAAA
EMPV1_03821	TGCCATCAGCAGGAGCAGAGTCCAGAGCATCCTCCAGCGGGCCCTTGTCAAGTAAGAAAC
EMPV1_03823	GCCCCATTTCAGAGTGAGAGAATCTTACAGATGCAAGAGATCCTGGAAGCCATACAGCTC
EMPV1_03824	TTCTATGGGACGAGTCTAGGGGTCTACCTCACTTCTGCTGTGACCCATTCTCCCCGTAGA
EMPV1_03826	TATGGGTTGATCTGCTCACCGGACCTGCCGTTCCCACTTGACCCTGTGGCAAACATATTT
EMPV1_03832	AAGCTGCTAGCATATGTCATGGAAGAAGAGGGACAAAATCTGCCTGGGCGTGTCCTTTTT
EMPV1_03834	CCTCTATGCAGGATTCCTTGGATTTGCTTTTGGGTGGTTTGGCGCCGTGTTGTTTGAGTC
EMPV1_03835	TATCATCTCTGTTCTGAAAGGCAGGGCTGAAGTCCCTTGGCCACCATCTCAGCTTGGCTA
EMPV1_03837	CCTGGAGCCTGGCCCCTACCCCATTGCCTGTGGCATGGATGACAAGGCATCGGGTTTGGA
EMPV1_03838	ACAATGGCACCTTCACTGCATCAGAGAACCACCTCACACAATGCCTGAGCTGCTCCAAGT

EMPV1_03839	AGGAGATGGAGAATAAGCCCCGGAAACAGCAGGGCTACAGCACCGTGTCCCACTTCAACA
EMPV1_03840	CTTTTCAAATTATGTTTTTCTCTGGATGTATGCCCAGGAGTGAGGCTACAGGACCATATG
EMPV1_03841	GCCCCCAGAAAAGAATGTGAGAAGACAGATCCCATCAACTGAGCCATGGTTATAGGCCAG
EMPV1_03843	TTGGTGAAGTTTTGAAGTGGATGGAGTATCTGGGGGATAGAGAACCTTGTGTCCAGCAAG
EMPV1_03845	AAGCTCCACTGTCAGAGAAGGATGGCACCTAAACCACCAGTGCCCGAAGTTTGTGTGACA
EMPV1_03850	TCAGGTTCGTTCATGCTACACCACAGTGGGAACTCCCTATACCTACCCTCTTCTTAAAAC
EMPV1_03851	TGCTGCTTCTAGTCGCGCACCATCCCCTCCCACTCAAGTGGTCATCTCCAGGATTCTCAT
EMPV1_03852	CCTGAACACATTTATGTGTACGTCTATAACCCCACGAGCATGTTTATAGTCAGTGGATGG
EMPV1_03853	CGGCACCTAAGAATAGCTCTCAATGGTTGTCCCTCCAAGTCCCTGGATGCAGCTAGTCAA
EMPV1_03855	CGTGCCAGATTGCTAGCTTGTAGCTGACTTTCCCCTGTTCTCTCCTTTCTTACGCAAAGC
EMPV1_03857	TGGAAAGAGAACAAAACATAATGAAAGGCAAGACAAAGACAAAAGACAAAACAGGGTATA
EMPV1_03858	CCCTGAACATTGATGGGTCAAAACATTTTACTGGATTACCGAATGTTAACTCAGAACTTG
EMPV1_03859	GGATAAGGAGTTCCCATTCTGGTTCAGTGGTAATGAACCCATTATCCATGAGGATGTGGG
EMPV1_03860	TCTCATGAAGGTGAACACTTTACAAGGGAAGAAGATGGTGGAGAGCGGCCTCCAGTCTGG
EMPV1_03862	GCTTGAGAAAAGGAAGAGGTTGGAGGAAGGAGGAAACAGAAGTGGTGGAGGAGATGACCC
EMPV1_03865	TAGATGGCACTGAGAATTTGCTTTGATTTGTTCCCGTCTGGGGCTCCTGTGGCGGGATGT
EMPV1_03866	ATTTGCTGCCTGTGTGTGAGGACCCTTGATTCAGAAGGTGCTGCTTGCTGTGCCTGATGT
EMPV1_03867	TGCCTGGCACCTAGCAGAGCCTCAACTAAGATCTCATCAGGGGATGATAAACCAAGTTTA
EMPV1_03869	GCCAGGGAGCATACCCTCGTCCTCATGGATACTAGTCAGGTTGTTTCCACTGCACCACAA
EMPV1_03870	AGTACGAATGCCACACGCCCAACACCGAGGATCAGTACTTCGGGAGTTACAGCGCCAAGA
EMPV1_03871	ACGCTTGTCGATAGTCTCCACATGACGGTTGCACCGCCACAGGGGGATGCAGTGGAACAT
EMPV1_03875	CACAGTCCACCCAGAGCCTCAGCACAGTCCACCCACAGGAGGAGCCGGCGAGGTGGTGCT
EMPV1_03876	ACAAACTCCTGCCCCCTGTGCCGTCATGAGCTGCCCACAGATGATGACACTTATGAGGAG
EMPV1_03877	CCCAGCTACACCATGGAGCAGCTGTTGTTGCCTTTCACTCACAGGACCAATGAGAACACT
EMPV1_03878	GCTGCTTACATGGTCCCAATTCTGTCATTGTGGTTTGGTTTCGTGCGTGACCACCTGTTA
EMPV1_03882	ACAAAGGCCGCCCAGGCTCTCGCCAAGCTCACCATCACCTCCAACCCAGAGATGACCTTT
EMPV1_03885	TGGGTAAAGCATCCAGCAAACACGAGCTGCCACGTAGGTCATGGACACGGCTCAGATCCA
EMPV1_03886	GGGACGACTTCTGTGCTTGGTTTAACGCTCTGCTACTGCCAGCTTGACATACCGGATACT
EMPV1_03887	TCAACGCCATCCTGCAGACCAGCTTCACATTCAGCCTCCCCTTCTGCAGCTCCAACCACA
EMPV1_03890	ATCCAGGTATGCTTTGTCTTTGTCAGGGTTTCTCAGTGGTGGCACTCTTGCTATCTCGGG
EMPV1_03891	GATAAACCTCCTTCTTACACCTGTGCTCCCAGAAACCCCTAAAGAAGCCCTGTCCACACG
EMPV1_03896	AACAAACCTAACTGAAAGCATTAAAGCCATGCACATATCTGGCTCAGACCTTTTTCTCAG
EMPV1_03898	GGTGGATGAGGAGAACATCTGCGAGTCGTGCCTGTCCCCGGTCCTCCCCTCAGACGCGGC
EMPV1_03899	CCAAGGTGGTTTCTTTCCCCGTGGTTGTTTGTGGAATGCTACATGTATCGGAGGATTCAT

EMPV1_03900	GTTGTATACCACACCGGTGCAGTCTGGGATGTACAAACTGAACAACATGCTCTCGTTGGC
EMPV1_03901	TAGATGGGGCTGGGAGAAGCGGAGCAGGTTGAGTCCTTGATCTCCCCACCAGAGTAAGAT
EMPV1_03902	TCTCCTTCCACATTTTCTCTTCTTTGTGAAGTTCGTCATTCATCTTGTCCTGCTCTTGCT
EMPV1_03903	ATGACCTGGGGGTGGAGACCAGCGACTCAAAGACGGAAGGCTGGTCCAAGAACTTCAAGC
EMPV1_03904	TGGACGCTGTATTACTAAGCTGGAAAACATGGGGTTTCGAGTGGGACAAGGATTGATAGA
EMPV1_03905	CCTGCTGATAAGAGGGTCTTGAAAGGGTTTTTGGGAAGCGTTCTGAGGGGCAAACTGACA
EMPV1_03906	CACATTACTGGTTGGAAAGAGTGTTCTGGAACTAACTCACCCTTGAAGGTCAGGGTCGGC
EMPV1_03907	CAACACAAACTATCATAAATAGCAGCTTGCTTATCATTTCAGGGGCAGGGAGGAGGGTGC
EMPV1_03908	AGGGGCCCGGGGTAACAAACCCCAGTAGTTCTGATGATCTTGCTGGTCTCAAGAAAGCTT
EMPV1_03910	ACAGGCCTCCTTCTTCTGTTTCAAGAGCCCGCAATGAACAGTCTTCAGGTCCTCGGAAAT
EMPV1_03911	CACACATACAGCACAGGGAGGTTCTCAGGCAAGGGGTCCAACTGGAGCTGGAGCTGACAT
EMPV1_03912	GGTGAATCGCTGCCGTCGAGAGGGAGGTTAAGAAGGCTCTCCACTTGGCGTTGTTTAATT
EMPV1_03914	GGGTCCTGGGTTGCAGGGGTTATCAACTCTGTGGTACAAACAGCATTTGTGGTACAATTG
EMPV1_03915	CTGCTGAAACTGATCGACTGAAACCACCTGAGAAAACTGAGGATTCCGCACCTACAAGTC
EMPV1_03917	AATCCGCCGAGGAACCATAAGGTTGCAGGTTCGATTCCAGCCCTCGCTCAGTGGGTTAAG
EMPV1_03918	CCAGCAGCCAAACTTGCACCAGCGATTATCAGGTAGGCCATGCAAAGGATGTGTTTAGCT
EMPV1_03920	ATCCTTGGGTCTGTGCTCAAATGGCTGTCACTGTCTGGATCACTGGTTTTCTCTATGCCC
EMPV1_03925	GCAGGGTCAGGGCTCAAACCTGAATCCTCATAGATGCTAGTTCGTTAACTGCTGAGCTTA
EMPV1_03926	GGATTTCCGTCTGGTTAGAGCAGAAAGGCCACAGGCTTTTGATAAATGAAATCCATGCCT
EMPV1_03927	AAGCTCCATCTAGTCCTCAGTCTCTGTTTCTTGTAACCTTTCAGTCATTGGTCTGGAGGG
EMPV1_03930	GCCTGCCACAGAGTAGACTCACCTGGAGGGTGAATTGCTTGAAAGGGGGCAATCCGGAAA
EMPV1_03932	CCACACCAGGATGTCAAACAAACTCTGCCTTCAAATGATTACAGGAGCCTATATAGCCGG
EMPV1_03938	GACTTCTTCATTGCTCCTAAACACACACAGCTTTACCCAAGACAGTACAATACTACCTGC
EMPV1_03939	AGTCCTGGGCTGATGCAACAGGACCCACATCTGTCCATGTGAAGCCACCGCCTTCATCCT
EMPV1_03940	CTACACCCTGCCCCTGGAAGAAAGCATGCTACACTCGATTTCCCGCGACAGTGATATTTT
EMPV1_03941	TCCTTACCCCTACCTCTTCTCCTCAAGCCTTAGCCAGCAGGGTCTGGTTCTGGATGGTCC
EMPV1_03942	CGTGGGAAACAGTGAGACTAGAGTACATGGAAGATCCCGCCATTATTTCTTCATACAGGC
EMPV1_03943	TTGTACAGTGCTGGAGTTCTTTCTAGCTGTGCTCTCTGCTGTGGTTTGGTGGAAACAGTC
EMPV1_03946	CTTGAACTGTGCCTGTGTGATTGGGCTTGCCCTCTTGTACTCCTTCTCACTGTCATCAGA
EMPV1_03947	GTGAGAAGATGACAGAGGAAGAAGTAGAGATGCTGGTGGCAGGGCATGAGGACAGCAATG
EMPV1_03948	GGGACGATATGGTTTATCGCTAGATCCTGATTTAAACCATGGTCGAAGCAAGTCCTGCAG
EMPV1_03949	ACTGTGGATGTTCCTTTTCTGCCACTGTTAGGGAATGTATGCTTAAGCCTATTAAGGCAA
EMPV1_03953	GAACTTCTCTCTCACCAGTTCTCAACAGAGAGCATGATCCAATAGCCTGGTCCCATCAGT
EMPV1_03955	ACAAACACTAACCCGGCAGTTGCCCCACCCTGTGTAGTTGTTGAGGAGAAGCGGCCTCAA

EMPV1_03956	CACGGGCTGGTCTCTCTGGTAAAGGGACACTGGTGTATTTTCCACAAATGGTTTACTGGC
EMPV1_03957	ACTGCTTACCCAAGCTACTGATAAACAACAGTGACCTCTATCTCCTGATCCCCCTCCCTG
EMPV1_03959	CCTGGCTTTAATTATTTCCTACACTGTCATTTTGATCTCCGTCCACTGCCGGTCCTCCAG
EMPV1_03961	CTAGGTTCTAGCTCATTCACAGTAGATGTGTGGCCCCAGGCAAGACAATTTTCCCCTCTG
EMPV1_03962	TGGGGTGCTTGCTGGAAGGCAGCTAAAACCTTCTCCCAGGTGACTTTGCTAAAGGCTACA
EMPV1_03964	ATGGCGTCATTGGGCCAGACCAGTGGCAGTGGGGGGCTGGGAATGGAGTATAAATAGGAA
EMPV1_03965	GGCCCTTGTCTTTCCAATGTTTTCTTCTGTTATATGGTGCCTCCCACCTTGAGGGCGCTT
EMPV1_03966	TGTACTGAAGGCCCATGGTGTGAAGTTTCTTTTCACACTGGTTGGTGGGCACATTTCCCC
EMPV1_03968	GGGAGACAGTTTAGCAATTTCTTATAAAACCAACCACCAAACTTTTATCATGTAGGAAAC
EMPV1_03969	AAAGATGGCAGAGCTGCCCGTGGACCCCATGCTGTCTAAAATGATCCTGGCCTCGGAGAA
EMPV1_03970	CCCAGAGAGAGTCAACGCCTTGCCGAGGGTCACATGTCAGCTCAGGCTCTGAACTCAGGA
EMPV1_03972	GGCAGCCTGGACCATCTGCTACTTCTGTATCTGGAAGGGGACCAAGTCCACAGGAAAGGT
EMPV1_03973	AACGACATGCTCGATGGCTACTCTGCCTCAACACTCAACCCCAGAAGCTTATTTGGAACG
EMPV1_03974	GCTTTACCAAAGTGCCACCCCAAGACGTGGATGTCTGCTACCTGTCGAACCTGGTGAAAT
EMPV1_03976	ACGCTGATGGAGGTGGGCAGAGGCCCCGTGGTACGCGTTGGCTTTGCCGCAGCGGGAGCA
EMPV1_03977	CCTGTCGCACAACGTGAACCTGCAGCAAAAACCTTGCGCCTACCACCCGGGCGATAAGTA
EMPV1_03979	GAGGGAGGAGATGGGCCAAGTTCCCTCTGCCTGGAACGCCCTTCCCCCCCTTCTTCACCT
EMPV1_03980	CATGACTATGAAAGAAGGGAAGTGGTTTTCCCCTGACCCAATGACATATTCTAAGTTGGC
EMPV1_03982	TGCATATACACGGCATACGTTTTTAGTATAAGTATATCCTTTGCAATATTTGGGACATAC
EMPV1_03984	GCAGTAGTGGTGGAATATCACCCTGTTTTAGAGCCTGACACATTGCAGTTGTAATAGGCC
EMPV1_03985	ACTCACGAAGCGAGAACAGGACCCACATCTGGTATGTCTGCGTCTTAAAGAGCTGCTGAA
EMPV1_03986	TCTCAGAGCTGACTCGGAAAAGTGGGGGCGAGAGGAGTGGGGAACTAAACTCGTATTGAA
EMPV1_03988	GTGCTAATGAACGGAAGAAAACATTTTGTCGAAAAGAGGTACAGCGAATTCCATGCCCTG
EMPV1_03989	CAGCTAGTACTCCTACAAAACTCAACCTTAGAAGTAAGGATGGGAGTTCCCGTCGTGGCG
EMPV1_03990	GACGATGGTGTGAAATCCCTCTGTCAAGTATTGAAACACCCAGACTGCAGCTTACAGCAC
EMPV1_03991	CTGCTTCCTGCTGGCGCTGAGGATTATATCCATAGAAAAATTCAAGGGAGAAACGGCAGG
EMPV1_03993	GCTCCCAGATTTTCGACAGTGTTATGCCATAGAATCAGAAGAATAGGCACAGTGGAAGGG
EMPV1_03994	CCTGAAAATATGATTCTGGGTGCTGTGGAGGAGAGAACAACTCCTCTCTGACTCGGATAC
EMPV1_03997	ACAGAAAATTGACGCTCCACCAAGTGTCAGAGTGGAAAGGTGCCGCAAGTGTATTGGAGC
EMPV1_03998	GCAAAACACCATTCCACCCCACTCATCGTCTGTGCTCCTATGTTCAAACTTTCCCCACAG
EMPV1_03999	TGGATAACGACGGCTACATCACCAGGAACGAGATGCTGGACATTGTGGATGCCATTTACC
EMPV1_04001	AGAAGCAGCTCTGGGGGGCGGATTTCAGATGCATTATGAGAAGACCCCGTTCGATCAGTT
EMPV1_04002	TGGCTTTCATCACGGGCGGTGGCTCTGGAATCGGGTTCCGGATTGCTGAGCTCTTCATGC
EMPV1_04003	TAGCTTGAGTTCCTTCTGGCGATGGCAGCAGCCGCAATGGTGGTGTCTTGAATAGACATA

EMPV1_04004	GTGAGCTGGGTTGGGAGCTGTACCACAGACGAGAAGATTCTGCGGCACTTTATGACATTA
EMPV1_04005	GTCTTTGGTTTGAGCTTCCCTCTTACACTTCAGGGCTCCAAGATAAGCACCTCTTAGGCC
EMPV1_04007	GGGATTTCATGCGTCCAATTGTTTTGAAGCTTTTACGCCAGGAATCTGTTACAAAGCAGC
EMPV1_04008	CTCTCCCCTATTACTTATTTGAAGACAAACCTTTTGAAACTCCCCTCTCTCCTCCACACT
EMPV1_04009	AGGTGCACTCATTCACTGCTGCCTTCATCGGGGGCCTGCTGCTGTTTGGAGAAAACAATA
EMPV1_04010	AGGTCTCCGGGCTGCTGCTGAGGAGGCGCTTCCACCGCACGGCGCCGGCGGCGCTGCAGG
EMPV1_04011	CAGCATGTGAGCAAAGAACAAGCCACGGCTGCAACTTCTGGACATTTGCCATGGTCCTTG
EMPV1_04012	TCATGAGAAGAAGAAGAAGGTCCGCAAGTACTGGGACGTGCCGCCGCCAGGCTTCGAGCA
EMPV1_04015	CACTTCAGGGAAATAGTTTCAGCAGAGCCTTCAGGTATCTCCTCTGTGGGAGAAAACACC
EMPV1_04017	TGTGGAGATGATAGTGGTCTCATGGAAGTAGAGGGAGCTCATCCATCGCGGACAATGAGT
EMPV1_04018	AGCCTTCTCCCAGAGCTCCTCCCTTACTATACATATACGAGGTCATACAGGTGAGAAACC
EMPV1_04019	TGCCTGTTCTGACGCTTTTATCAATTACCTTGCCCTGTATTCTATGACTGGCCTGCTGAC
EMPV1_04021	ATCACTGTGGGGTGATAATCCTGAGCCAGAGAGATCCAGGAGTGAGCAGGGGAAGGAAGA
EMPV1_04022	AGGAGCTGTGCTTATGTCTGCTGGCTGGATGCTGGCTCCTTTCCTGTGCCTATGCCTTGA
EMPV1_04023	CAAGGTGGTTCCCAACAACGACAAGGACCGCACCTACGCTGTCTCCTACGTGCCCAAGGT
EMPV1_04024	GCCAGGAGGAGAACATGATGAGGCCAGTTGAGGAGCCTGACAAAGCCCTTAAAACTCATT
EMPV1_04025	GGAATAATCATTCCATTCAGTATTATCGTTATGATTGTTGGAGAAACCCTGTCTGTTTAC
EMPV1_04029	GAGCTCTTTAATTTTGTTGCCAATTTGGATAAGCATGGCACAAATCCAGCAGGGAGGTCC
EMPV1_04031	CCCCTCTGGGGCGTAAGTGTTGGCAGAGCCTTGTGCTTTTCCTGTTCCTGCTAAATCCCT
EMPV1_04034	AAGTAAGGAGAAAAAGTCTTCCTAATCTACCAGTTGCCTCCTCAGGTGTATCAGCTGGCG
EMPV1_04035	CACATCCCAGAAGGGGGAGAGCCCAGAGGACAAGGAGCAGGAGGAAGGACAGAACTCAGA
EMPV1_04036	TCAAGGCCAAGGTTGCTGGGCTTTCGGGCTCCCACATCAGCATTTGGTATTCCCTGGTCA
EMPV1_04037	TGTACAGGCCAAGGCCAGGAGAGTGACAGAGGACAGACAGGAGAGGGAGGAACTGACCCA
EMPV1_04038	GCTGGAGGTGAGCAGGCACTGTCCTTTGAAAATGGTATACATGAACCAGCTTGCCATGGT
EMPV1_04039	CAGACAACCGTGGTCGGCTACAACCCAGAAAAATACTCCGTGACACAGCTCATTTACAGC
EMPV1_04040	CAAGAGCCCAGATATAAAACTTCACATATACGGTCAATGATATTTGACAAGGGAACCAGG
EMPV1_04041	TCACTTTCCGCAACCCTGTCATTGAGCGAATACCCCGGCTCCGACGGCAGAAGAAGATCT
EMPV1_04042	GATATCTTATCTGAAGAACAAGGATGATGATCCCTATTTCCTAGAATTTGTGAGAGGCAC
EMPV1_04043	GTAGGTCCTCCGTCTTCTAATAGGAATGGTCATTTATTAAGCAAATTCCACCTTGTCTGG
EMPV1_04044	CCAGGAACAGCTACCCGACACTCAAAGGAAGCGACTAAAACCGCAAGCAGGGAATTACAT
EMPV1_04046	CGTCTCAGGTTTTGCCTCTTTTGGCAGCCCCAAGTCTAGAAGATGAAACCCAGTATCAAG
EMPV1_04047	CTACTTATATTCACTGTGGGTTGGGGGGAGCTCATTTTAAGCATGTTCGGTGGCAGCTCC
EMPV1_04049	AAGTGCTGCTTCCATGGTTCAAACATCCCTTGCTATGAGGCTGCCCTTCTGTGGGGACAA
EMPV1_04050	GTCACAGCAGTGAATGGCCTTTGTGTTGCAACCCCTCTTATCCTGTTAGACAGCTCCTAG

EMPV1_04051 GAAAAGACCTGAGACTGCCCTAAGCTCTCATCTCTGGTTGCCCTTGAGGCTCTGCATAAG
EMPV1_04053 TACCAAGTCATGTTCTTCACACTCTTCGCCCTGCTGGCCGGGACTGCGGTCATGATCATA
EMPV1_04054 TCCACAAGCTGCAGCATAGGTCATAGATGTGGCTCAGATCTGGCAGCGCTGTGGTTGTGG
EMPV1_04055 TGGAAAGCTGAAAGCTCCTCCTAAACCATGTGCAGGCAATCAAGGAACCCAGATCACGGT
EMPV1_04056 TGTCCCTGAGATGAGCCCTAAACCTGTGCCTATAATCTCTGTCAGTCTGCTGTTTGCAGG
EMPV1_04057 ATCATGTGCATCCCCCTGGTAGCCCTGGGGACTTTCATTAAGCATCGCCTCAAGCAGAGA
EMPV1_04058 CTTGGTCTGTTTGCTGGTTGTCCCGACAACAACCACATCCGGCAACTGTGCTGGGCGAAT
EMPV1_04059 CGATGGCTGGTCTGTGGCAGAAGCTGTAACTGTGGTTTCCAATTATTTTCGGCATCCTCC
EMPV1_04060 AGCTTAAGAATGCCCTCAAGGAGTTCCCACTGTAGCACAACAGGATTGGTGGTGTCTTGG
EMPV1_04062 AGCACAATTACCCGGTTGTGGGCCTCCTTCAGGTTGGAAGTGAACATGGACCGGAAGAAA
EMPV1_04064 GTTAATTCAAGTTGCACGGACAAGTCCATCTACAGCAGCCACGTGCGGTCTAACCTCATG
EMPV1_04065 GCTCCCTCACTACGGAGACTGAAACTGGAATTGGAGACGCAGTCACCTTGTTCAGACACT
EMPV1_04066 AGAAAGAGCTCAACCATATGCTGACGGACACGGGGAACCGAAAGGCTGCTGACAAGCTTA
EMPV1_04069 TGCATCACTACATGGAACAGAAAGCGAGAGGCTCAGTTTGGCTGAGAAACTTCAGACAAG
EMPV1_04072 GCCCCCAGATTCTTTTAACTTCAAGGAAATGAATAACAGCTTGTCAGAGACTTCCTATGG
EMPV1_04073 ATTCACGTCAGCTCACCAGCTATTTCACAAGCACCGGCTACACCCCAGGCTGTGCGAGGA
EMPV1_04074 AACCTCACAACATTCTGCAGAGACGGCTCCTGGAAACCAACCTGTCTAAGCTGCGAAGCA
EMPV1_04076 TCCAACTTCAGCTACTTTTACCACCGGGAGACAGATGACGAAGAGGCTGGGATGTACAGC
EMPV1_04079 AATCTTGATATTTTTTTTATTAAAATACTATCTTTTTTGACTAAACTAAGTGAAATTAG
EMPV1_04080 GCTCTAGGCAGGGATGCCATTTAGCTCCATCCTTTGACCCCACAATACAGACAAGGAATC
EMPV1_04082 GCACTGTGATCCTGGGGAAGGGCAGTCTTTTGTCTTCAGCTTAGTAGTTTACTGACCCAC
EMPV1_04084 TGATGCACGAGGAGTGGAAAGAAAACTAACGAAGCCCAAGCGGGTTCTGTGACCCACTAG
EMPV1_04085 CCTCCAAGGAATACAGTGACCTTATGCAGCTTTTGAAGCAGTCAATACTGGCAACGGACC
EMPV1_04089 GCGACTGTGCCTGGGAGACTGTCAGAGTGGAAATCGTGAGAGCCTTGTCTTCAACTACTA
EMPV1_04090 GTCTTGGCCTTTGGTCCTTTTATATTTGTCTATACATGGCCATCCCCCTCCATACCCCTG
EMPV1_04092 GAGAATCACATGGTGTCCAACATGCGCTGCTGCCAGAGCGACGGCTGCAACCACAATGCT
EMPV1_04094 GACATTGATGCTGCCAGTGCCATGATGCTTTTGAATACTCCCCCTGAGATACAAGCAGGT
EMPV1_04095 GGTAATTGAGAGGCTGGCGCACAGTCAGATCCACAGCATCCGGGACCTCCAGCGACTCCT
EMPV1_04096 CTACTCGTGGTGCCTGGAGTGGGACTCGCTCAAGTTCTCCGTGTGGCACAACAACACGCA
EMPV1_04097 AGCCAGTGAGAGGGCAGCGGTGAGTGGTAAGGAAACTAGTTCACCTGCAGCTACTTCTCA
EMPV1_04098 ACAACAGGCATTGTGGTCTCAGCTTTTGGACTCCCTATTGTATTTGCCAGAGCACACCTG
EMPV1_04101 GCATGCTTGCATCACATAGAGTGAGATTGGTATTCAGTTACCTCCGTTGCGCCAGTTTGT
EMPV1_04104 AGTCGCCACTTGAACCGAGTTGAACCAATCTTGTAGTGGAAATCTTGATACCATGTGTCT
EMPV1_04106 GTCCGGCTAATGGAACCGTCCCCGGATTGCTAGCCTTTACGACACTAGGGCTGAACCACT

EMPV1_04107	ACTACTCCATCAAGGATATTTCGGTTGGAGGGATGTGCATCTGCTATGGTCATGCCAGGG
EMPV1_04108	ACTGAGATGGCCTTGGTGCAGGTGAACCCGTTTTGGTCGCACACATGCCCACACATCTGT
EMPV1_04109	ATCATTAGTCTACAGCCACACCACCCTGAACGCGCCCGATGTTGTCTGATCTCGGAAGCT
EMPV1_04110	AGGTGGGAACCTCTGCAGTGACAATGCTGGATCCTTAACCACTAGGCCACAAGGGAACTC
EMPV1_04111	TGACCTGCTCAACATCCGGAGGGAGTTCATTGAGAAATACGACAAGTCTCTCCACCAAGC
EMPV1_04112	GGGAACGGAGGTAGAGTTGGATTCTCTGGGGAGTCAGAAATTTGCTTCAGTGGCCTTTGA
EMPV1_04113	GGATGGTTCCATCAAGGGAGAAATCCGAAAACAGGCATTGGACCACTTCAATGCAGATGG
EMPV1_04115	CTTGACAAGTCCTACCAACCTCTTCAGTGGCACCATCCTCGAACCTCCCACAGCACTCTC
EMPV1_04116	CAAGTGCGAGCAGCTGCAGAAGGAGAAGGAGGAGCTGGAGCGGCGCTGCGAGGACGAGGT
EMPV1_04117	AACATGAATGCCTAGCAGACCGTGTCAAAATGTGGCATTTGTCCCAAAGTACAGCATGAC
EMPV1_04119	CCAACTGGGTGGCTTAAACAACAGAAATGTATTGCCTCACAGCTCTGGAGCCTAGTAGTC
EMPV1_04120	AAGAAAGTGACGGCTGTGCACAAGGCCAACATCATGAAGCTGGGCGATGGGCTCTTCCTC
EMPV1_04123	CTTTAGCCCCTCAATAAGCTGGGGCTCCCACTCCAATAGCTGCAGGGGAATGAATTCTCA
EMPV1_04125	AGAACCTGAGTCAGGCCCTAGGTCCACAGCAGAGAATAGGAAGGAGATCATGTTTCAGGC
EMPV1_04126	CCTGTGTCCCTCACATCTTTGTGTTGACCGTCTTTCTCAGTTCCATCATCTCCGTGTACC
EMPV1_04128	GCCATGGAGGATACTAGCCCAGCCTAGCAGAAAAGTGCAATATGTATAGCATACTTTGAC
EMPV1_04129	TATTGGCCTCTTGGAGGAGAGTGCCTGACAAAAGATTCTGCAACATCAGTTGGTACCCAC
EMPV1_04139	CATGACTCAGTCGACTGCCAAAGCAATGAAGCGACCTCTCGAAGCATCTGTAGACCTGGC
EMPV1_04141	GTTTGTATCTCTGGGAGCATATTTTTGAAGGCTTACTTGAACCCACTGATGTGACAGCTC
EMPV1_04142	GCCCCTCGAGCCCGGCCGCCGCCACCGCCAGTGCCCGGGCGGGCCCTACAGTGAGCCCAC
EMPV1_04144	TCTGTGTATGCTATTTACCTACCTACCTACCTAGATGCAGTGATGTTCCACTACATGCCT
EMPV1_04146	TGCCAAAGGGAAAACGTTTGCCTTTGTTGGGCCCAATCGGTACCTTCTCTTTGACCTGGT
EMPV1_04147	ATGGCCTCCACGGCAATCATGGTACCTGTAGGGCTTGTATTTGTGGTCTTTGCCCTGCAT
EMPV1_04149	TGCCTAAGAGTGGGGCTGATGGGTCATATGGTAGTTCTATATTTAGTTCTCTGAGGTATC
EMPV1_04151	TCAGAAGGCTCCTCAGATCTCACAATCCAAGGTGTACCTGGTGCCAGAGCCACACTATGA
EMPV1_04152	CTGATACTCAGCAGTCACCCGTGTTTTTTGCCTCCTGCCTAATTGGATGTGTAGCCTGGC
EMPV1_04153	CTTTTGTACATCAGGCATTGTTCTGGGCAATAGGGATTCAGTGGCAAATCAAGGGACTGC
EMPV1_04154	AGGACCGCACCTGCAGCATATGGAGTTCCCAGGTTGAATCAGAGCTGTAGCTGCTCACCT
EMPV1_04157	CAGGCCCTTCTGAAACTGATGATGTTGATGACAAACTCCCCCTTTCGAAGTCTTTGCAAG
EMPV1_04158	AGACCTTCGTGTGATGGGTTTGGTTCTTTGAGCGTTCTGGAGTCTGGGTATGTCCCCGTT
EMPV1_04159	TCGCCAGCCGGTACGGGATCAGAGGATTTCCTACCATCAAGATCTTTCAGAAGGGCGAGT
EMPV1_04161	GCTCGGGCAAAAGCAGTGGCTAGTAAAGAAATAGGAAAACGTAAACTGGAACAAGATCGC
EMPV1_04162	CTTCATCATTGTCTCTTATGTGTGTATCACCCGTGCTGTTCTGCAGGTCCCTTCTGGGAG
EMPV1_04163	GTGGCTTAATCAGAGGTGGCTTATCAAACTTGGGGATAAAAAGTATTGTAACAGCTGAAG

EMPV1_04165	GGAAATGCATCTGAAAAGGGACCTTGGAGCTGAGGATTTTAAGTCAGCCCTGGAGTTTCC
EMPV1_04167	CGTGACTGGTTACGAGTCGTTGCTAACAGCGCTCTCTGCTGAGCTCCATTGTTGGAGGAT
EMPV1_04168	ACGTGCCGCTCTGCTCCGAGAGCTGAGGTTTAGAAGGAGGAGAGAGCGTTGGCACGTTTT
EMPV1_04171	CAGGGATAATGCTTTGACCGAGTTACATAAGGATTCCTTTGAAGGCTTGCTGTCCCTCCG
EMPV1_04173	ACCAGCAGACAGAGCAAAAGAAGAAGAAGAGGCACAGCAGCACGGGGGACAGTATGGACT
EMPV1_04174	TCAGCAATGGGACAAAAAGAATGCCGCCAAGAAGAGAGACCAGGAGCAAGTGGACCTCGA
EMPV1_04175	GGGTTCTGAGGTGTCGTGTTGACTTTGGGGGACAGCTATGTCATTCCAGCCTTCTATAGT
EMPV1_04176	GACACACATCTGTTCGGCTCATGTCCTCACAGATGAACTGGGGCTCAGCACAGAACTCCT
EMPV1_04177	CTCGTGGATGCTAGTCAGATTCATTTCCACTGAGCCACAATGGGAATTCTCATGCTGCAC
EMPV1_04178	GTTTTAGGGAGCATAGCAGATTACTAAAGTCACCTAGCACAGTTCAGGGCATTTTGGGGA
EMPV1_04179	GTGGAATTATCACAGAATAACTACTCATGACATATCACCGACTGCTTTGGGTCCACTGCT
EMPV1_04181	TTTTCTGTGCAGCAGCCGCTTGAGCCGAGACAGCAGAAAGAGCTTTGGTTCCAGGTAACA
EMPV1_04185	CACTATGAATTTAATCATCAAGGGCTGAAAGTTGGGGACTAAAATCTGGGGGGCCAGAGC
EMPV1_04187	CGGCATTCGTGCTCGCCTGGTGCGACTCCCTGACGTCGGTATTACTCATTTCAATTAGAA
EMPV1_04188	AAGCAGCCTCCCAAAGCAGCCTTCTGCACCTGGCTCACAGCCAAGCATCCATGTCTCAAA
EMPV1_04189	GAATAACTATGTTGAAAACATGACCTGAACTCTGGGGCTCTGTTCTCTTCAGGGCTCCAG
EMPV1_04190	GCGTCATCAAGGACACATCGGGAAGCATCTCCAACACTGACAGGCAGAAGAATCTGTGCA
EMPV1_04191	TAGAGTCCCAGAGCAAAGGGTTCTTGACTGGTTCTTTTGGGGTCCCTAGAACCTAGCACA
EMPV1_04192	CCCAAGCTCCTGGATTTTCACCTTCTGGGAAAACAACAGATTATGCCTTTGAGATGGCTG
EMPV1_04194	GGGACTCCGGTAGTTTCTCCAGAAGCAAAACAAGTTCCACATCATTGGCGCTGTGGGCAT
EMPV1_04197	AATGGACCTGTGGGCGTATTTGAATGGGAAGCTTTTGCCCAAGGAACCAAAGCCCTCATG
EMPV1_04198	TTAACTCTGGCTCAGGTTACTGCTGTGGCGAGGGTTTGATCCTTGGCCCGGGAACTTTCT
EMPV1_04200	GTGCTCTGCCTGGTTGTATGGGCAGTTGTATCATTTTCAGAGCTTTCAGGAAATACTCTC
EMPV1_04202	CAAGGGCGGAGACACAGAAGAAGAGGCAGGGCCCCAGGCCTCAGAGCCATGCACTCCCTT
EMPV1_04203	GTGGTGATGGAATCTGGGGGCTGGGAGGAACAGAGGCAGGTGTCTATAATGTCACTGAGT
EMPV1_04204	AGACCCAAAAACCATCATTTGGATGGAATTACCCGGTGAGAATGAAGAAGGCAGGTCTGC
EMPV1_04205	CCTGAGTTCATGCACGCCGTCGCCCACCAGATCACTCATCAGGCCATGGTGGCAGCTGTT
EMPV1_04206	GACAAATCAACACAAACCCCAAGTCCTCCTTGCCAAGCCTTCAACCATTATCTCAGTGCG
EMPV1_04207	CAGGAAAGCTGATGGTGAAAGACTTTCTGGAGCCTTTGTCTGGACTTTACACATGCACTC
EMPV1_04210	AATGAGACCCACTTTCCGGCCAGCAGGGGCATCTCTGCGGATGGTAGAGGGTTTGCCAAT
EMPV1_04212	ACAGAGCTGGGGAAATTGTTGTGCACCTGACAGGCAGGCTCGATGAGTGTGGCGTTATCA
EMPV1_04215	AGGTTTCTGAAGCAACGTGGTAGGCTTCTTACATCAGCATCTCTGTCTCGTGATCCAGCC
EMPV1_04216	CCCAAGACCTTTGCCTCCAATATCCCTCCCCCACAACGAGCCACAATCATGCCCTTTTTT
EMPV1_04218	ACTACAGCTGCCGTGATACTCTTTCCACATTTTGATATGTTAGAGGTTAGGACTTCAACA

EMPV1_04219	CATGTTAATCCTGTGTAGGCAGATTCTTCAGATAATGGTCTTTACATGGGATATCTCTGT
EMPV1_04224	GCTCGATAAATCATCCTTTACATCACCACACCAGCATATCAAGATAGGAAAATTGCTCAG
EMPV1_04226	AAACGTGGCCATTGGGTCTTGGCTCAGAGGATTGCGTGACGGTGGAGAAAGCCAAAGCTT
EMPV1_04227	CTTCTGTGATTCAGCTGAGGAGTGGCTCAAGGGCACCCAGTGTCTCCTGAAGATGCAAAA
EMPV1_04228	CTTCATCTTCATAACGTTGCTGCTCTTTGTGGGGAACACACAGTCCAACCACCTGTCGGA
EMPV1_04229	ATTCAAGCCAGGTATTTCTGAGTCCCGTGTTGTGCCTCCAAACAGCCCAAGGACACTCAG
EMPV1_04230	ATCCAGAGTGTGGAGAGCCACCACCGGCCCTGCCTCCTGCTCTTGTCACTTCCTTTGCCT
EMPV1_04232	ACAGGGTCCTCTATGAATCCCCATTGACCTTGACCCCCACCTGGAAATTATAGGGCGTGT
EMPV1_04233	ACAGCAACGGTAGAATGGCCACCCCAGTTGTCATTTTTTACCACTGTCACTTCAGTGTAG
EMPV1_04234	TGCTGTTCCAGGAAGAGAAATGACAACACCACCATTGGACAAACTGCTGCTGAAAAGATC
EMPV1_04235	AATCATCAGGCTACGAGAAGAGCACGGAGGACCAAGCACTCAGAACAGCCCAAATCTTCG
EMPV1_04236	AGGGTTTGTGCACTCTACTGTCCAGACGATTCTCACTATCCATCTGCCCTTTTGTGGGCC
EMPV1_04237	AGGAGGGTCTGGCTCAGAAGAAACAGGAATTGGTAGAACTGCACCCTGTCTATGGTGATC
EMPV1_04240	TGTGAGTAGGGTGACAGTTGGGGTAACTGGGACCCCAGGAGGGATGGAGCTTACCACTGA
EMPV1_04243	CCCTTCATTCCGCCCTGAAGATGAGCTCGAGCACCTGACCAAGAAGATGTTGTATGACAT
EMPV1_04244	TTGCTCATCAAGGATGCCCGCTTTCAGCACCTTCTCCGTCTTGTTAACAGCCAGGTGGGT
EMPV1_04247	GCTTTTGGTGATTTTAGGAACCAAACGGAATCTTTTTCTTCTTGGGAATGTCCCACCCAC
EMPV1_04248	GGCAAAGTGACTTAAGTGATCATGCATGTCCCCCATCCCTGCGGTTTATAGTTTATGGAG
EMPV1_04249	ACAAAACCTGATCTATTATGCTACCAACACAGCCTCTGGGGTCCCTGACAGGTTCAGTGG
EMPV1_04250	GGCTATGGTCCCCAAGTGTTCCCTCAAACAGTTCCTGGTGAATCTGTACCTGACATTTGC
EMPV1_04251	GCCGCTTGATACGCAGTAACCTGGGTGGAGGCAAAACACACTAATGAAAGGGCTAAAAGG
EMPV1_04252	AAGAGACAGAGTCTATAGTTCTAAGTCAGTGTTTTTCCTGAAGCATGATTTTGTCTGCCA
EMPV1_04254	GAGGAGAGCCAGAGAAGTGACAGGGAAGCTACAGGTTACATCAGAATTGTCAACTGAGCC
EMPV1_04255	GTAGACCCAGAAGAAGCTGTAAGGATTCACATTGATGTTCAAACAAAGAGATCAGTGGCG
EMPV1_04257	ACTTACATACACAGGTGCTCCTCCCCACAGAATGAGGGCCAGACTGCAGGACCAAATACT
EMPV1_04259	CGAGCCCAGACCTGGAGCCCAGAATGCTTCCCAGCAAGGACAGTTGGAAGAAAACAATAA
EMPV1_04261	CGTTCCCGTGTCTCATTTGTAAGGCTGGCCTCTGGCTTTTCTGCCATGGATCCTTCGTCT
EMPV1_04264	CTGCTGATTTGGGCACGGCTCAGCTAGTCTCTCAAGTTCACAGGAAGTGTTCTTTTCCAG
EMPV1_04265	GGAGGTTCATCTGTGCAGGCAGTTGTTTTCCGTGTGCCATTGTTTCTTGCTGCCCGATTT
EMPV1_04267	GAAGGAACAGTGCGCGTCGTACCTCCTGAATGTTACCTGGGTTGACTCGTCCAAGATCAA
EMPV1_04269	TCTAGAAGAAGCTTATGATAATCTGAAAGATGAAATGTTCAGAGTGAAAGAAGAAAGCAG
EMPV1_04270	TGGTGCTAGTTCTGCAGCGGACAAACATGGCTGCCCCGACGTTGACTGCGAGGTTGTACT
EMPV1_04271	CAAGGTTCTTTAACCCTTCTTTGGGCTTGGGTTGTTTATGGTGGCTTTGGGGGGCTATGG
EMPV1_04273	ATCCAGCAGACCAAGCCCCTTAGCCTCGTGCTCACCATAAACCTGTACCCTCTTATTAAT

EMPV1_04275	CAGCTATGCAGCAGACAGTGAGGAACTGAAACAGAAGTGGCTGAAGATCAACCTTATAGC
EMPV1_04276	CCGTGGGATGTGTTGCTGCTGTTGACAAGAATCTCACAGACTGTGTGGAAGTGTGCAGTA
EMPV1_04280	GGATATTAGGAGCTGAGATAAATAGTCCTCATCCTGTTTTTTCCATCCTCCATTCCCCCC
EMPV1_04281	GGAAGGTTAAGACAGTCACCAAACCACTTTGGGCTCCTCGGTGCTCTGAATTAACATGCA
EMPV1_04283	TTGGAGGAAAGGACTGAGGGAGAGAGTTAAGCGTTAGAGAGTTCTTAATATTCTAGGCCC
EMPV1_04284	CCTACACCGTTGTGTTCCCCCATTGGAAGTACTGAGATGAACACCATCAGGAATGTGCTC
EMPV1_04286	CCCACCGTTCTGCTTGATTTCTATATTTAAGGGGGTTCTACGGGGTCTCCATAAGGCCCT
EMPV1_04288	ATACTCGGCCCAGAATCTGGAGCTACAGAACAAAGTACAGCTCCTGGAGGAGCAGAATCT
EMPV1_04290	TCAGGCAGTCTGATTCTGAAGCCCAATCAAAATATTAGTGTGCTGGGGATGAGGGGGGTG
EMPV1_04291	TTTGCCCTCTACGTGGGCTACACCCGCGTGTCTGACCACAAGCACCACTGGAGTGATGTT
EMPV1_04292	GCTGAAATTAACCCAACAGGAGGTTTAGCTCCACCCAGAGCGGCAGACTTGTAAGCAAAT
EMPV1_04295	ATGTGCAAAGCACCTGGCGTCCGACAAGCACCAAACAGACATTGGTCCCCTTGTTCTTAT
EMPV1_04296	TATGAGTCTAAGGGCCAGTTTTGCATCTGTGCGGTCATCGCCCCTTTACGTGGCTTCTTC
EMPV1_04297	GTCCCTAAACCTGGGTCCTAAAAGATGGTTCTTAGCCTCGTCCTTCCTATATGCTCCCTG
EMPV1_04298	GCAACGAGGGCTAACAGATTTGACTGTGTGGAGGCCATCAGTAAACTTTCCAGAAGCAGG
EMPV1_04299	ACACGCCTGTGTCCATTCTCTGTGTCCCAAGTGGCAATATGAAGCCATCTGCTGTTCAAT
EMPV1_04300	GGAAGTGGAGAACCAGAGCCTGCGAGGGGTGGTACAGGATCTGCAGCAGGCCGTCTCCAA
EMPV1_04302	CGTGGCCAAAGACCTAATACTGAACCCCCGGCGTAGCTCTGACAACCTTCCCTCCATCGC
EMPV1_04304	TGCAGAACCATGCAGAGCTGTGGCACAGGACATCTCGGGCAGTGCCAATGGTCCTGGTCC
EMPV1_04312	CCTCCACGTTCTCGCGGGCCTCCTGCAGCAGCAGCTCGGGTTCCAGATCCATCGCCCTCT
EMPV1_04314	GCTCGGCGTGGGACACGGACGCAGGCTCCTCACAGACATGTACAACGCCCACCTCACCAA
EMPV1_04317	GTGGTAAGACTTATTGATCACTACAGACAGTCCAATCTAGTTGAAATAGTTAGCAGCTGG
EMPV1_04319	TTTGACCTGTAGCTCCAATTCGACTCCTAGCCTGGGGACTTCCATATGCTGTGGGTGCAG
EMPV1_04320	GTTCAGGGGACTAGCCAAGGTATTGCTGTTGTTATTATTACCCTTCCACAGGAAAGGCTT
EMPV1_04322	CAAAATTCATGTGCGCTTTCTGAGTGTTTGCAGCCTTGTGTGATTGCAAGGCATCTGGGA
EMPV1_04324	CTAAGCCCTCTCCTATTTATGGTGGACTTGGGTTGATTGTAAGTGGTGGAGTTGGTTGTG
EMPV1_04325	TCTCATGCTTTCAGCTACAGTACTTCCCATCACATTGCAGGAGGCCGCCTTGGGTCATAT
EMPV1_04326	TTCTGGAACGCTTCAAATGAACCTGGACCCCCTAGACAAGTATTCTGATGGCGAGCTGTG
EMPV1_04327	GCCTGATTCTTTAACCCACAGCACTGGGCCTGGGATTGAATTTGCATCTCTGTAGCAACC
EMPV1_04328	TACTTCAATTTCCCCACCAACTGGTTTAAACGCAGCGAGCTGGGCAACCACTTCTGTCTC
EMPV1_04329	GGTCATAGCAGCACTAACAATTCTGTTTCGGACTTGCCATCAACTACCCAACCAAAAGGA
EMPV1_04330	GGCTGATGGCATTCTGGTAGTTGAGTAAGCACAGGAACTATACTTTCACCTTTTATCACC
EMPV1_04332	TGTACGGCTTTGTCCGTGAGGCCTTGCAGAATGACTGGCTACCTTTTGAGCTGCTGGCCT
EMPV1_04333	CCATACATTTACCTTAAAGTGAGACAACACAGGGCAACTAAAAGGTCACGCTTCCTCAGC

EMPV1_04334	TGACCCATGTATCGGAGGACTGTTTCCCTTTGCTGGATGGCTGCAGAAAGAACAGGCAAA
EMPV1_04338	CTATTGAAGCCCCCTGGAGCCGTCCCTCATCCGTGGTTGCTTTATTAGCTGTAAATTCCC
EMPV1_04339	TCAGAGCTGCCCATTTCAGATGGAGAAAGATTCTGGGCTTTACTTCATTCCCACTGGGGC
EMPV1_04342	CCCCGTCTCTGTGCCCTGATGGCTTGTGCTTCATGGATCATTGGTTTTGCCAACTCCTTA
EMPV1_04343	TGTGCAGTACAGTTTTGTCCCCGAGTGGCTCGCTTTGTCACTCTTGGCAATGCAGCACAT
EMPV1_04345	CCTGAACACATATAAGTGTACGTGTATAAGCCCACCAGCATGTTAATTGTCAGTGGATGG
EMPV1_04346	GGCTCCAAGTCTGCCTGAGAGTTACGGGTTTCCAAGTACCTTATAGCATCTCCAAGACCA
EMPV1_04347	CACTGCGCCATGATGGGAATTCCCTTAGAAATAAATTTGACCAAGGAGGTGAAAGGTTTG
EMPV1_04349	GGCACGTTTTGTGACATTCCTCTTAACCTGAACAGCTTCCTGATCCCGGACAACCATGAG
EMPV1_04350	GCATGTGGTTCTACTCAGCATCCCCTTCTTCAGCATTCCTGTTGTCTGGACCCTGACCAA
EMPV1_04353	AGACACAGCAGCCACTTTGCTTAGCATGCTTCATAGCTGTTTCTCAGGTGAACAGGTGTG
EMPV1_04355	TTGCGCTGTAAATGGTAAGACGCGAGCACTGATGAGCGGGCTTTGGCTTCCTGTGTCCAG
EMPV1_04358	CAAAGACTTGTGGAGCCAAACCCAACAGCAACAGCAACTGCCTGACCATGAAAGAAGTGG
EMPV1_04359	AAGGGAAAGAAGCCAAGTACAGGCCCAATTTCAGGCCAAGTCCCATGGAGTGGATTCAGC
EMPV1_04360	CTCCTTTGATCCCGCCTCTAAGCCAAACATTGATCCGCGGGGTGAAGTTGTTCCTGCAAA
EMPV1_04362	GCATAGCCGGAAACAATAATATACCCAGGAGCAAAGATGAAGCCATCCTTCCAAGCTGGT
EMPV1_04363	TCGAGGACCGCCAGGTCCCTGCAGACCCGGTTCTCAAAAATTCGCCTTTAGACCTATTTT
EMPV1_04365	CTAAAGGACCCCTCTGTGTCAGATTTGTGTTAACCCTGAGAAGACCAGCCATGTGGTACC
EMPV1_04368	GGACTTGTGTATATACACATGCACATATTGCCCACCAGCATGTTAATTGTCAGTGGATGG
EMPV1_04370	GTCTCGTGTGTAAACATCTAGCTTCCCTTTTATGTCTTGACCTCCTAGAGTTAATGAGCC
EMPV1_04371	ATTCAGACCGGAGGAACACCTACAGGACCAACAAGCCAGTCTTCAGAGATGAAGGGCAAG
EMPV1_04372	CTGCACCATCGCTCCCTAGAGTCTCAAGAGCAAGAAAAACTGGCTTCCGTGTTTTATGGG
EMPV1_04375	CTTTGTCAAGAGATCTACAAAGGTCAGGCGATCTGCAGAGATGGAAGGGAGAAATGGCCG
EMPV1_04376	AGAGCACTCTGTCTAGTATTTGCCCAGGAAAGGGAGCTGGTACACACTCTTTGGCTGATG
EMPV1_04380	GGATTCTGGAGTCAGATTACTTGGGTTCAAATCCTACCTCTAACACTTATTGGCTACTTC
EMPV1_04381	CGCAACAGGCTTCTTTCACTCAGGAATGTTGTTGAGATGCACCCATGTTACTTCCTATGC
EMPV1_04382	TGTGGGCATAGATATGGATGTGGATGTGGATGTAGGTACAGATGTAGATTTAGATGCAGA
EMPV1_04384	GCTCGGGGCCAGAGGCCTGTCTCCTTGGTCCATTCACAGTGAGGACCCCATCAGATGACA
EMPV1_04386	TTCACCAAGAAATGGAGGCATCAGCACTGGCTACCTCTTTGGAAAAGGACCTAAGGCAAA
EMPV1_04387	GCTGAATGTAGTGAAGACAGGCCGAGTGATGCTTGGAGAGACCAACCCAGCGGATTCTAA
EMPV1_04389	TGCCAGCCGCTCTGATTTTTTTCCCGCGAAAACTTGGCGTTTGGGAGTTGCGGGGCCACT
EMPV1_04390	GTCTCTGGCCTCTGGGCAGCCTTGGGTGCGTTTCCAGTAATTTGGCCTTTCTAACGATTT
EMPV1_04391	TCCTGTCGGGTGTGGAAGAGTGAGGCACTGCTGGCAGGTCAGGCTGCACTCACTGCTGTA
EMPV1_04394	CATCTATAAAGGGGGTGATGAATATGACAATCACTGTGGCAAGGAGCAGCGCCGGGCAGT

EMPV1_04397	GGCCTGTACTATGATTTTATATGTCAGATATTGCCTGTGGCTCTAATATGCACCTCAAGA
EMPV1_04399	TTGCCAGGGCCTAATCCTTTTGAGGATGGCTCGTCTGGGCATTCTGCTGGGCCTGCTGAT
EMPV1_04402	AACTACCAGCCAATCATCGCCACGGACCTCCCGTGACCACGCCTCCTTGAACACCCATAT
EMPV1_04405	TCACAACACATTCACATGTACATATGCTAGTGCCATTCAGAAATGAGCTATGCAACAAAG
EMPV1_04406	TCCTCCAACCTTCTCCTCGTCGTAGCTCCACTGTCTATCCTCTCTTAGTTCAACCACACT
EMPV1_04407	GGAAAGCGGAATGCCACAAACACAGAATGTTCTTAAATGAAATAGGCATTGTCAGCTGGG
EMPV1_04408	CAACAATCAAATTATGGACCCATGAAGGGGGGCAGTTTTGGTGGAAGAAGCTCGGGCAGT
EMPV1_04410	GAGATGGATGAACACATGCGGAGCATGTTGCATCACAGGGAACTTGAAAACCTGAAGGGC
EMPV1_04411	TGGCTCCCAAACTTGATCAGGAGGTGCTGCTTCTGTCCTGGTGCTTGCATCGTTCAGTGA
EMPV1_04412	GACCCAATCTGTACGTTTTTGTCAGCAGGAGAGTTTTACCTGTTGCATGGAGAGATGTTC
EMPV1_04413	ATTCCCACGACTTCCCACCGTCAGGGTTAGGCCACTTCTTCCTCTGCTTTAGTGACTTTC
EMPV1_04414	GTGATCTCCCACGGCCACATCCTGAACCTTGCAAACACTGGAATCTGATCTCTTGGTTCT
EMPV1_04416	CAGCTGCAGTTTGGATTCTATCCTTTCCTCAGGAACTTACGTATGCTGCAGGTGCATCAG
EMPV1_04418	TCTAGCACCTCCTCGCCAGTCCTTAGTGATCAGGAACCATAGTGGACAGTTCCAATCAGT
EMPV1_04419	TTTTAGTACTTGTTTCCCCTTCTGGGCTCCAGCAATCCCCCATACCCACCCCTCCAATGA
EMPV1_04420	CTCCTCCAGCAGCGACCCTTTCAATTTCAACAGTCAGAACGGCGTGAACAAGGATGAGAA
EMPV1_04421	TCATCCTCCAGCTACAAGGCCAGGATCTGTATGCTATCCGCTTGTGCCAGTGCAAGGTGT
EMPV1_04422	GCCTCAGTCGATTAGAACGTGCGAACTGGCCCGCGATGCCAAGCTAGGAAAGAAAAATTA
EMPV1_04423	TATTCTTCTATCCCAAGGTTAAGGTGATTAAGTTATCATCATGAGATAGTCCTTGCATTC
EMPV1_04424	TGATGAACAACATGGGTGGTGATGAGGATGTAGATTTACCAGAAGTAGATGGAGCAGATG
EMPV1_04425	CCACCAGTTCATAAACAATGGGAATATCTGTACCGGGGACCTGGAGGTGATTGAGAAGGC
EMPV1_04426	CTTCAGGCCAAAAGTCCCTTCTGCATTACCCACCCCCAAAGTGAGTGATTTGTTCCCTGT
EMPV1_04428	TACTGTGTACCGGCTCATCTGTAAGGGCACCATTGAAGAACGAATTCTGCAGCGAGCCAA
EMPV1_04430	AGTTGGAGACTTGCGGGGGGCGAGTTCGTCACTGTCGTCACGGATAAAATGTTAGGAAGA
EMPV1_04431	TTCCAGATCTCGTTTTTGGCAAAGGGGGGTGGTTTGTGCTGAGTGATGCTGTTTTGGGGT
EMPV1_04433	CATTTTACCTTAGGACAACTGTTGCATGCCAATTTCTGTGTGTCTGTGTGAAAACACTTC
EMPV1_04434	CTTCTATGTCTATTTACAGCCTGTGTCCTCCTACACCGTCAGGGACCACATGGCAACAAT
EMPV1_04435	CAGGAAACTATCATGGATGGTACAGGACCTTGTGGTAGTTGGCTATGATTGGGAGCACTG
EMPV1_04437	GTCCATCTTGGGGAGAAACCCTTCAAATGTGTCGAGTGTGGGAAATCTTTCAGTTACAGC
EMPV1_04443	CAGTTCCAAGGCTGTGGTTGTGAAGAAGATCGAGACCCGCGATGGGAAGCTGGTGTCTGA
EMPV1_04444	AGCTGAAGCAGCCTATGGATATATTCTTATCTCATGATTGGCCAAGAAGTATATATCATT
EMPV1_04445	TAGTGCAGCATTTGTGACAGGAGAGCGCAAACAAAACCTGGTTGTCGCGGTCAGACGCTA
EMPV1_04447	CGGAGTGATCAGAGACATTGAGAGGCAAAGTCGGAAAAAAGAAAACATTCGTCTCCTGGG
EMPV1_04448	ACTGGGAAAGATAACCTTCTCAATGCCTGGAGGACGCCGTATGGAGCCAGCATATTCCAG

EMPV1_04450	TGGTGGTGCACAGGGGAACTACTCTAGCCACTTTGGGAGACCAGCCATGAAAAACCTACT
EMPV1_04452	TGCAGGATCTGTCCATCTTCATCACCAACGTCACCTACAACCACTCAGGCGACTACGAGT
EMPV1_04453	GGCAGTTCAGAGATAGACTTGTGGCAAATGTTGTGTGTTAACTGTTGTTTCTTTGCCACA
EMPV1_04454	GAGGAAGAGTTCAATGAGAAGAGTGAACATGATTCTGGTATCAACGAGGAGCCTCTACTC
EMPV1_04455	AAACATTAGCCGCCCCAGCCTCCCTCCGCCCCCACAGACCCCGCCCGCTGGACCCAGACA
EMPV1_04456	GGATCTCAGAACATGGAGAATGGCAGTTCACAGAGAGAGTCAAGAAAAGTACGTCAGAGC
EMPV1_04457	TCCAGAACTTCCAGGCCAAGCCCTCGACGCCTGGGCAGCACCATGAGCACCCTGTGTGGC
EMPV1_04458	CCACAGAGTGCACACCTGGTGAATTTTACCACAAAGGCTGAAATGGGAGGACCATTTTCC
EMPV1_04459	GGGATGAGCCTCGGGACACACCTTTCAGGGCTGCTGTTTGGAAGATGCACATTTCATCAG
EMPV1_04460	TCCCAACTGCTTTGCTTGTACCATCTGCAAGCGCCCGTTTCCACCCGGAGACCGAGTCAC
EMPV1_04464	CCTAAAGTTCGTGATGGGAATGGATTTGTAGTAAGATGTTTAAAAGTCCGTGGGTTCGTC
EMPV1_04465	CATGATTTGCACTAGTGAATTGCTGCCCAGGCTTAAACCATAACTCCAGGGAATGCAGAA
EMPV1_04467	GCCAAGAGTTGGGAGAGAAAGAAGGGACAGAAGTGCTAAGGAGGGTCTAACCACAGCAGA
EMPV1_04468	TAGGAAACATTCCCCCAGGAGATGGCATGCCAGTAGGTCCTGTACCACCAGGGTTCTTTC
EMPV1_04469	TGTTGCTGTGAGCGGTGGTGTAGGTCACAGACACGCCTCAGATTTGGCATTGCTTTGGCT
EMPV1_04470	ATATCAGGATCAAGAGAGGGATGGGAGACCTGGGTGTAGGCTTTTAAGATGGGTGGGCCA
EMPV1_04471	ACTTCAGAGGTTTTTTAATGGTGCAGGTGAAGGTGCCGGTTGCTATTTGGTATCACACCC
EMPV1_04472	CATCTTCTTCCAACTGAACAATCTGACCCTGGAGGACCGCGGCTTCTACACCTGTAGCTA
EMPV1_04473	TGCCATTGTCACCTGGTTTCTGGTCCTCTATGCGGAGTTTGTGGTCCTCTTTGTCATGCT
EMPV1_04474	AGTGAGCCTCGTGTTTGAGCATGTGGACCAAGATCTAAGGACATATCTGGTCAAGCCACC
EMPV1_04476	AAGCATCCTATCCAAAGTTGGAACTTCCAATGTCATGAACCTTTTAAGGAATGCACTTCT
EMPV1_04477	TGCCAGAGGTACCAAAGATACCTGTCTTAGAAGAAAAACCAGCTGTTCCTGTTCCCAAGA
EMPV1_04479	GTCTCCAAGTATGGACCCAAACGAAATACTTCCTAATGCTGCCAATCAGATGCCTGACCC
EMPV1_04480	TTCTGCCTGGCTCAACTGCTGCTGAAGAGGAACCCTGCCATGTTTTTCCAGCACTTCAAT
EMPV1_04481	TGGCCCTGTACTTTTCCCCCTCATTTGGCCAGTCTCATTACAAAGGCATCATTGCTTCTG
EMPV1_04482	GCAGCAGTTCGACGTCATGGTGATGCCCAACCTCTACGGCAACATCGTCAACAACGTCTG
EMPV1_04483	TGCTCGGCCCTAGTCGTCCGGGAGGCTCTGCGCCAGGAGCGGCGGAAGGTGGAGCCCGAC
EMPV1_04486	GAAACTGTAAGATCTTGAATGAGATAACTTAAATCTTTTGTGAGGATGATAAATATGGCC
EMPV1_04487	TATCCGGCTACAGATCTCAAACCCAGACCTCAAAGACCGCATGGTCGAACAGTTCAAGGA
EMPV1_04489	AGCAGAGAGAATCCCTATGGCCCTCTCAAGCCAAGGGTTGCCAGTAGGACACCAGATTAT
EMPV1_04490	CTGAACTGGAGATGTGGTTACAGGGTCATCCAGAGTTCGCTGTCGATCCCCGATTTCTAG
EMPV1_04492	AGGCTCGAAGTTTCCCGTTGGCTGAACATCCTGGGTAGAGACAGTCAGGTCCTTCTTTCA
EMPV1_04493	TGCTGGCATCAAGGGCCGGGTGTACACCAACAGCATCTATGAGCTTCTGGAGAACGGGCA
EMPV1_04494	GTTGCTGCTTTGACATTCTTCCTTGGGAAAGACGAAGAGGAGAAACAGGGCAGTGACTCA

EMPV1_04496	ACATTTGAAGCAGCCGTGCCCTCCACTCCTCGCTTGCATTGGTATTCCTCTTGGCAGCAA
EMPV1_04503	CCTCTGCTGGCTCTGTCTTTATCCACGGAGCTGGTGTCCTTTTCTCTTCAACCTGTATTC
EMPV1_04506	CCGAAGTCTGCTAATAGGATCCAAGGCGCCAGCCATGTCAGCAATCCTCAAAAGAAATTC
EMPV1_04508	GGCACTGTCTACTTATGATTTTAAGATTAAAGTTTCATGGAGTTTCTGTCATGGCACATC
EMPV1_04509	AATTTCCCCAGGGAGTGTGGGGATGGGGGCTGGAGATGAATGGAGCCTGGTGCCATATGT
EMPV1_04511	CTAGACCTCATCAAGGAGATGCGGCAGTTCTGCAAGTCGCTCTTCCCCGTGGTGGACTAT
EMPV1_04512	CACGTGTACCTTAGCCACCATAGGCTGCAGAGAAGGAGTGCAAGATAGAATTTGATGATT
EMPV1_04515	TGGAGGTTCTGCACTACCGGCTCAGTGTCTGCAGCGCCCTCCACAGCCCTGCCCAACCCA
EMPV1_04516	TCACAAGGGGCCCAAGTCAACCAGCTACCCAGAATCCTCAGTGCACCCAATCTTGCTAAA
EMPV1_04518	AGTTTCCGGGACTCCGGGCCCCGAAAATTGTCAGACTCGCACCTAGCCTGCGAAGACTGT
EMPV1_04522	GCTCCTGTGGGCCAACGCGTCGCACACAAAGGGGCTGGTGAACCCGAGCCCGAGGCTGCT
EMPV1_04523	AGTAGTTAGGTGTGCTTCTTTCCCAGCGCAGGGTGTCGTTGTTGAAGGTCAGGAGATTGT
EMPV1_04524	CTGCTTAGACTGTCATGCACTGATACTCATGCTAATAAGCTGGCCCTCATGGTCACAAGT
EMPV1_04528	GGAAAAGCCCCGCTTGCTACTGGAGAGGATGATGATGATGAAGTTCCAGATCTTGTGGAG
EMPV1_04529	CTAGACAAATTAGGAACTTCTGAACTCTTGCTGGCCAAGAATGTAGGAAACAATAGTTTT
EMPV1_04531	GGCAGCAGGCTGGATTTGGTCTTTGGATGTAATTTCTCAATACCTGGTCTCTCATGTCCT
EMPV1_04533	ATTCCAAGGAGATAGATCGCAGGAGAGCAAGAATCAAGCACAGTGAAGGGGGTGACATCC
EMPV1_04534	GACTGTTGCTAATATGCAACTCTGTTCAATACAAACTGGAATTGCACTTTAGCAATGGTG
EMPV1_04535	GGCCATCATTCATAAGTCCACAAATAACAAGTGCTGGAGGGGCTGTGGAGAAACGGGAAC
EMPV1_04537	AAACCTGCTTGTTGGCCTCATCGAAGAAGACGCAGTTGACCGGGTTCGCCTTCTCGAAGT
EMPV1_04538	AGCATTAAGTAATCGTAGAACATAGGACTGTAACCTCAGTTCGCTCTGTGACGTCAAGTG
EMPV1_04539	GACTCCACTTTGGAAAGATAAGGCATACACAGTGCCTGTTGCAGTGAATATCTCAGGCCT
EMPV1_04541	GATACTGCTCCTTGAGCCTGAGGTACTAGCAATCCAATGGCCATATCCATTTTCTGGTCC
EMPV1_04542	TAATCCAATGTCCTTTACTACCTTTCAATGTGCCAGCAACGCGGAGCCGGGCTTTTTGCC
EMPV1_04543	TTCAATCCTGAAGGGAACGCCGCCTTCTCAGCCTCTTTAGAGACACAAGAGGGACATCTA
EMPV1_04544	GAGGTCTTCCGAGGGGCCCACACCCTCTACATGCCTAATTGTGACCACCGGGGCTTCTAT
EMPV1_04546	CAGCTGCTCTGATCCTCCTCTTTTCATGGGCAAAACCAAACAAGGGCCAACTCGTTGCCA
EMPV1_04547	AATACACGTGCACATACACATCAGATGCATGCACATATGCATATATACAAACACACATGT
EMPV1_04548	CACCAAAAAGCCAAAGTCACAAAGCCACTGCTTGTCTGTGAAATGGACCGTTGTTGCCCC
EMPV1_04550	TTGACGTCTTCCTGGTTTTCTCTATCGCGGTCATGGATTCTTATCCTGGGGCCCGTTTGC
EMPV1_04552	ATAGCGCTGAGGCGTATTCTTCAGTCGTCCAGAATGCAAAATCCCAGCAGGATGGAGCTC
EMPV1_04553	TGAAGGCCTGGTTTCCCCAGAGGAGTGATTTCTGGTAGAGGTCTGAAGGTCTGATCCAAG
EMPV1_04554	AGAGTCTCCCTGCCATGTGAAGCTCCTCCGAGCACAAAAAGTCGTCTATATCAGCTGTGG
EMPV1_04556	CCTGCTCCCTCCACAAACTGGAGACATCTGGATTCTGCAGTCTTATTATGTAAATAAAAC

EMPV1_04558	TCCTTCAGTGGGCTAAGGATCCAGAGTTACCATGAGCTGTCAAACACAGGTCGGAGATGT
EMPV1_04559	GCAGAATATTAGCATGCAGCGTCAGGAAAACCTTCGCTGGGTATCAGAACTCTCTTATGC
EMPV1_04564	AGGCCTTCAGGCATTACAACATCTCTTGGGAGCTGGAGGTGCTGGAGGTGAGCAACTCAT
EMPV1_04567	AGCTTAAAATGTGGCATCTCGGTTCCCAGAGCAGGGATTGAACCTGGGCTGCAGTGGTAA
EMPV1_04568	TGGAAGCGATTGAATTAAAACCTCAAAAGGCTGGGATTCCTTTCCCCAGGAGAGACGCGG
EMPV1_04569	TGAATGTTGACCTTGGCCTTGGAGGTTGATTGGGAAGACCACTGAGGAAATGGCCCACAG
EMPV1_04570	TGCACCCCCAACCTTTCCTCTGGCTGGACTGCAGATTCCTGTTCCTGAAGATCATGGCTA
EMPV1_04573	GTCTCTCATGTCATCCCAGTGGCTGAAGGATGTGGGACTGGCAGCTGTATGAAGCACATT
EMPV1_04574	TTTGGATTCAAGTGGATTCTAAATACTTCTGCTTATCTTGAAGAGAGAAGCTTCTTAAAG
EMPV1_04575	GAGCTGAGACAGAGGATCCCTAATGGTGGTATAGTCAGATAGAGAGCTGAAATCCAGGAA
EMPV1_04576	CTCTACATCGTTGTGTAGGTCCTTAAGCTTGTCAGCATCTTCGTCCATGTCCCTCACTCG
EMPV1_04577	TGTGTGCCCAGTAGATGGGGGTGTGGCCTCTGTAAGGGTCACCAAGCAAATATCCAGAAA
EMPV1_04580	ACACAACACATTTACAAGATATTGAAAACCAAGGAACGGCCCAAGATGTCAGCTCCAAGG
EMPV1_04581	GCTGACAGTGGGGTTCTGACCATGACCTGCTTCATAGTGTTGCTGATATCCTACACGTAC
EMPV1_04583	ATGGCAGCAACTCAGGGGGCCGTGGACATGGACTAGGTGAGAAAGGGAGAAGTTTGATTT
EMPV1_04584	GGTTCCTTAATCTAGCAAAGCCAGGGATGGAATCTACATTCTCACGGATACTAGTCGGGC
EMPV1_04585	CCCACGCCTCTTGACCTGTTAGCCATTTCCTGTGTTCTAACCTACTGTCCTGGGATGAGA
EMPV1_04588	GTAACCAGGACCTTCAGGTGTGTCTTCCAATCGCGATAGCCCTTTTTGGCTGGGAATTGG
EMPV1_04589	TTCTTCAGACTAAGGAGTTTTGATAGCCTGACGCCCCAGAAGAATTGTCACCTTCCCCCG
EMPV1_04590	CCACTTGGTGGGAGGTTTCTGTACTCGTTGGAACATGCCCTTAAACATTCTGTGAACCAT
EMPV1_04591	TTACTCCCTTTGTACAGTATTCTGAGAAACGCAAATAAAGGACCGTGTGTTCCTATTTCC
EMPV1_04592	AGAGAGGGTAACAGGCGAGAGCCAGGAAGAGGAGTAAGTGATACACTGCTGGGGTTTGTT
EMPV1_04593	CTGTGTCAGCTGTCTGCCCTGCTTTCTACGTGGTCCAGCGGCATTCTCTCTATAGCATCA
EMPV1_04594	AACTGCTTCATAATACCTGACCTTCCTTTAACCTCCGTGGCAGTACCATCCCACCCCCCT
EMPV1_04599	CAAACCCAGGTCTGCTGACTCCTAGTCCAGTGCTTGTGATGGCTGGTGGGCCCTGAACTA
EMPV1_04600	ATTGGGTCTGGGAATCATGTTGGCCTCTGCTTCCTTCTCTCCGTATTTCACCCACATGTT
EMPV1_04602	TGTCATTAGCCATCCTGCACAGAAGCCCTTGGGGAAGTTTATGTCCTTCTGCTCTGTGAG
EMPV1_04606	GTCAGGGAATGGGCTTCCTTGTCAGAGATGGCAAAGGTCTCTAGTCCTGGAACCAAAAGG
EMPV1_04608	CTGTCCGTCAGAATGATGGGTTTCCTTTTAGAGATACAAACGGAAGTGTTTACGGAAGAA
EMPV1_04610	CTTCCAGGCCTTCCAGGAGCTGCGGGATCCCCTGGTGCCAAGGGTGAGCCTGGCAGCCCT
EMPV1_04611	CAGCCCTAGGATTCTAATCTTCCTATGCAGCTGTCTTCCAGACTTACTGGTACACTTAAG
EMPV1_04613	CAGATCCGTGCCTCTGACGATGGCCAGTATCGGTGTCATTTTAAGGAGGGTTACATCTCC
EMPV1_04614	CAAGCATCAGATTATGGAATTGATCATGCACAGCTTCCCCTTGGAAATTTTGTGAAGATG
EMPV1_04615	AAGCCTTTAGCACGTGTGGGTCTCATATTTGTGTCATCCTGGTTTTCTATGTTCCTGGCA

EMPV1_04617	GACTTGCTTTATAGGGTGGAGCCGAGAGAGGCCCAGGTCCAGGCTTAGAATGCCTTTAAT
EMPV1_04618	TGCAGCAGGGAGCTGTGATAAAATAATTAGAGTGTGGTGCTTGAGAACTTGCGCTCCAGT
EMPV1_04619	TGGCCAGTGCGGCCTCGGCTACTTTGAGGCAGAGCGCAACTCCAGCCATCTGGTATGTTC
EMPV1_04620	TCTCATGAAGAAAGCTGGGGTGCTTTGAAGTTAATCCCTACCCCTATGCCCCAAGTGCAG
EMPV1_04624	GAGCCCTGCTGGCTCATTCGCCTTTGCATTTCCATTGTTGTCCTACCCAAGGAGGATTCC
EMPV1_04625	CATGCAGACATCTGCTTGATGTGCTAGTTAGAATGGCTCATTGGACATGTAGGAGAATGT
EMPV1_04626	ACAAAGGAGTCCGAGTCCCAAGGTGGCTATTCCAAAGATGTCCTCCTGCGGCTTTTACAA
EMPV1_04627	GCTACTACACCATACACGTGTGGAGGATAAGCTACGACACCATACACGTGTGGAGGATAA
EMPV1_04631	GGGTTGGGGTGTGGGGCCAGGGATTAGGAACACCCTCTCTTGCTAACATATTGCTAATAG
EMPV1_04632	GTCTGCCTTCTTGGGGGGTGAGCCAGACAGACTGACAAACAAACAGACCCAACTGTGTTC
EMPV1_04633	TGTGTGTGAAGGACCAGAATTGACAACCGCTGGCAGAGAGGAGCCTAAGGGGGATGTGAG
EMPV1_04634	CCAAACCCCTATAACAAACCCGGAAAGTTTGGACAACATCATAAAGACCCGCTGGGCACG
EMPV1_04638	GCAACAGGTTTGTAATTGGTTGTCACGACTTTGTCCAGTTGTTGTGAGAGCCTTACAGGT
EMPV1_04640	CCAGGTGCAATTTATGGCAGTCTTTGGGAGTAACTTTCTTCTACTAAGGAGGGATATGGG
EMPV1_04642	CAGAGGAGGGCCACTGCGGCAGCATCTGGCTTCCTGGGAAGGCTGCGTGGGACTTGGAAA
EMPV1_04643	AACACCCCACGTCGTAAATGCACCTTCTGCCTGACTGTTTATTGTTGGCTTTGTTTCGTT
EMPV1_04644	ACAATATGTGGCTCAGGCCTGTAGTCGCCATACCATCGTGAGCTTCGCATCGAGGATGAA
EMPV1_04645	AGTAGACAATGTCCCGGATGCGAAGCTGCTTGGAGTGTTCAGGCAACCTCACAGCAAGTA
EMPV1_04646	AGAGCGATAGTGCAACTTCGGGTCGTGGCTATGAAATCATCTGGACCTCATCACCCTCTG
EMPV1_04647	GTCACACGGCCCTCTGCTGCCAGTGGGCAGACTGGAAAATGCCAAGTGTCCCCCAGTGGG
EMPV1_04648	CCACCAGAGCCAAACGGCTCACAACAATGAAGAAACACAGTCCTCTGTTCAACCTTCCTC
EMPV1_04650	TGCCCTGGAGCCTCAAACCACCGTCATTCATAACCCAGTGGACGGGATCAAGGAGTCATC
EMPV1_04651	GTGTTAACTGTTCTCTAAATGTTTGACAGATCACTTTTGAAGCTTCCTAGTCCTGGGCTT
EMPV1_04653	GCTCTGAAGTTTGAGATCGTCTAGGCTGAGACTGGCCTGTTGTCGGTAGTCAGACCTATT
EMPV1_04656	CCATACAGCCCACCAAAACTGAATCAAGAAGAAATAATTTGAACAGACCAATCACCAGAA
EMPV1_04660	CATGCTGGGCCTTGCCAGTGCTTTGGATGACACCTTCTGTGTGGATAGTATTGCAGCCTT
EMPV1_04661	CAAGGCCAATAGCCCCAACCGAAAGATCTCTAGCACGGCCTTCGGACGGCAGCTCATGCA
EMPV1_04665	GTTGGATTCGTTTCTGCTGCCCCACGATGGGAACTCTCCAGCCCATTAGTTCTTAAGCTA
EMPV1_04667	AGTGTGTGAAAGTGAGCAAGCTATTGGGAACCCGCCAAGGGTATATGGCCCGCCCAAGAA
EMPV1_04669	ACTGCTCGCGACACCTCGTGTTGAATGCACTGTTTCCCCTATTTTCTCCTTCGTCCGGAT
EMPV1_04670	CATCAGCCAAAGAGTGTGTACCAGCTCCCTTTCCTGGGCAAATACTAGACAGAGTGCTCT
EMPV1_04671	ATGCTCTATGTGGAATTTGTCTGAGGGGTAAGGAGTCCAACAAGAGAGGGAAGGCTGAGC
EMPV1_04672	GTGTTCCGAGTAGGACTATGCCATGTGCACAGCTTCTGCAGTTCCTTCAGAGGAACATCA
EMPV1_04673	TTTCCCAATAAATGTAAGACTCGAACACGGGTAACTCCAGAGGGGCCAAAGGAGGGGGTG

EMPV1_04677	AAGGAGGAGCTAAATTCCAAAGAGGCTCAAGGGGAGGAGCTGAAAAAGAGAGCGGCTGGT
EMPV1_04678	ACGGAGGGCGGTGGGGACTGGACTAGGGGTGGCAGTCGTGGGGGGTGCAGGAGGGGCGTG
EMPV1_04679	GGCAATATTGGGAGATTGGTGGGCTTGTGATACCTCATGTGGCTGAGTTTCTATAGGGGC
EMPV1_04680	AGGGCTGAGGGCACATCTTCCCCTTACATCTGGTCTCTAGAACAAACCCGGAGTGAGACT
EMPV1_04684	GGACTGACATCTCTGAGTGTTGATTTCACAGTGATCAAGTCTGCTTCTCTCTCTAAGAAG
EMPV1_04688	GGAGGTACTCAGTCCAGTATCATCCTGTTATTAAGCTCATCAGGTAAGCCTGGGCCTCAG
EMPV1_04689	ACCCAGTGCGAAATTGCTTAGTGGTGTTACAAAGCTAGGCTTGAGCCTTTGCTCCTGCTG
EMPV1_04690	AAGAGACTAAAGTTGTTCTTCCTTCCCGGCAGGCAGGTCCTGGTAGGAGGGATGGCTGGA
EMPV1_04694	CTACATTTTGTCCCTGAGAGTACTCTGTTGTCACCTAGGTTCTTGATTCCCATATATGGC
EMPV1_04695	TCTAGGCCCCTCTCAAATCCCAGCAGACTCTAACCCTCTCCTGAATGGCCACATGTCTAA
EMPV1_04697	GATCTGTAGCCACCTTGGCCTTGGGTTTCCTAGCCTCCATATCTATGAGAAATAAGCCAC
EMPV1_04701	GTAGTAAGATTCGGGAACAGGATATGGTGGGCAACAGGTGGTCAGAGGAGACTTCCAGGA
EMPV1_04702	CTACCCAGTTATCATGTCACAAAAGGTATGTTGGGTGCTGGTGGCAATCCCCTATCTCTA
EMPV1_04703	CAGCTGCCCTGCTGTGTGGTGGTCCCTTCTTTGGTATTCTTTGTTCATACTCTAAAATAG
EMPV1_04704	GAAATTGATGAAGAACCAGTCAGTAAAGCAAAACAGAGCCGGAGTGAAAAGAAGGCCCGG
EMPV1_04705	TCTCCCAACCAGCCCCACCTTCCTCAGCCTCATTTGCCTTCCCTTCCACAACAGAATGTT
EMPV1_04707	AAGAGGAAAGAGAAGACTAAGAAGTGACCAGAGTGTAACACCTCTTGTGAAAGCTCCACG
EMPV1_04710	ACCGCAAGAATGAACGAAATCTGGCTACTGACGACAACGTGGATGGACTGTGAGAGCATC
EMPV1_04711	CATCACTGTGTCCTATATTTCGATCTTTATCCCTGTCTTTCGGCTGCCCCAGAAGGAGGC
EMPV1_04713	CGTATATCCAGAACTTCGCACGTAGGAAAACGACTTGCCTCAGGGCTGGCCAAAGAAGTT
EMPV1_04714	TACATGCAGAGTCTGATTGATGAAAGTGTCCACGCTCTGTTTGCTGCGGTGGTAGAACAG
EMPV1_04723	TCTCTGTGTTGGGCGTCTGTCTGCCCGCATGCCTGCCTCTCTGTGGCTCTGAAGGAGGCA
EMPV1_04724	AGGACATCCTTCTACAGATTTCCCTGGGGCTTAAGTACATCCACAACTATGGCATGGTGC
EMPV1_04726	AAGAGGGAACCTACAGACAAGGATGACATGGCCACGGCGCTCAGGGAAGCCCAAGAGGAA
EMPV1_04729	GGCTCGACAACCGTAGGGAAAACCCTAGAATACGGCACAGAACAGTTCCCGCAGGACAGT
EMPV1_04733	CTCAGTGAAGTTGTCTGTCTACATGACCCTGTACACTCTCACCCATGTGGTGCTGCTCAT
EMPV1_04737	CATCTCACAGGGCTGCCTGCAGAATTAGATGCGTGGATACAGGTACAGCGTCTATGCAGC
EMPV1_04739	TTAACGACTTTGAGGAAGACGACACAGGTGACCACATTTCCCTCCCGGGCCTAGCTACCT
EMPV1_04740	CTTGGCTTTTGAGGGCTAGCTGGTTTCAGTAAACTGGGACGAAAGTGTGAAAGACCAGCC
EMPV1_04742	AGTCCCATCTCGGTTCCAGCTATATCACCGACAGCATGTTGGAAGACTGCTCACCTCAGT
EMPV1_04743	CAGGGACTACGACGGCAAGGTCTTCTACATCGACCACAACACCCGGAGGACCAGCTGGAT
EMPV1_04745	TCAGAAGTGCCCATGCTGGCCCCAGCCAGGGCTGGGTGCTCCGCTGGCTCAGATCCAAGA
EMPV1_04747	GCACAAAGGTAGCGTGGCACTGGGACATGAGCCTTTAATACAGATAAACCAGTCGAAATC
EMPV1_04750	AAGAGAATTCCTCGTGAGTAACTGCTGCCCTGGGTCTCGAGCAAAATGAATCCAGATGGG

EMPV1_04751	GTGTCTCACACTGGCGTTCCTGGCCTTTTCCCCTTGAGGAGATTTATGCTGACTTGCTGA
EMPV1_04752	AAGACAAACCGCCATTCTCCCAACTACCGCCTGATCAACATTGACTTCACGGATCCCGAG
EMPV1_04753	ACTCTGTTGATACCAAATGTGGCCAGGATGCAGAGCAACAGGAATTCTCATCCATTGCTG
EMPV1_04755	CCTTGTGGGCTGGATGGGTTCTTGAGTGTAAGAAAACAGGTCCTCTCTTCACAAACTTCA
EMPV1_04757	CTCTATTGGGTGTAGATTTCTGCCATCAAAACTTTGGGCCCTTGGAAAACAAGAGTTTTC
EMPV1_04759	CTCACTTCTAGGTTTGTAGGGACTTGTGGTTCTGCTGGAATTGGATAGGATCAGCTGCAT
EMPV1_04760	ATGTAGCCTGTGTTGTACAGCAGAAAGATGGCAGACACTGGTTTGGTGAGCTATCTGTGG
EMPV1_04762	GTGTGTGAGTCCGCCTTCAACCGCAAGGACAAACTGAAGAGACACATGTTGATCCATGAG
EMPV1_04764	GTTATGTCCCAGCGAGTTTGTGTGCAGCTGGTGCTAGGGCCTTATGCCATGGGTCTTATA
EMPV1_04765	ACTGCGATTCTGTCCCTCTGTTAGCATTGTCCTGCTCTGACACTTCCTTTCCAGAGACAG
EMPV1_04766	TGTAGAGTCTCATATTGTGAATACAGCCCATCAGCTGCAAGACCGGGGAGGAGTGTGGCA
EMPV1_04767	AGAAATGACCAAAGGTTTCTTCAACGCCCACTTCGGCAGCCTGTTCCGCACAGACCAGAA
EMPV1_04768	TGCAGAGTCAGTGCCCTGGGAGGAAAGAGGGTCCCACCAAACCCCTTTTTAATCACCTTC
EMPV1_04770	CAAACCTGAGCCACAGCAGTGACAATGTTGGATGCTTAAATGCTAGGCCACCAGGGAACT
EMPV1_04771	GCCCAAGCCCAGGTGCTGCTAGGTTCTAGGTGATTATGATTGAATGTGACTGGTGTTACT
EMPV1_04772	TCCAAAAGGCTCAAGACACAGCTTCCCACATCCTTTCCCATAAAGTCACCTGCCCAAACG
EMPV1_04774	TCGGAGCTTACCCGGCTCATCCAGAGAATCCGCTCAGAGATTGAGAACGTGAAGAAGCAG
EMPV1_04778	GACTTCATGTCCAGGTTTTGTTCCAACCTCTGTCTCCCTAAACAAGTACAGATGGCAGCC
EMPV1_04780	TTCCCATACAACGTGGAAGGATGAGGGCCGCCTAGCTCTTAGCTTTGGATGGACTGGCCA
EMPV1_04781	CCTGCCCTTCTGTGGCCATAATACCCTCTCGCATTCCTATTGTCTCCACCCCAACCTTAT
EMPV1_04783	CCTGCCGTGCTCAGGTGAAATCCATGCCACTCTGCTTACAGATAAAAAGTTCAGATGCTT
EMPV1_04785	AAATGCAGCTGGGCTAGAGGATCTCGTAAGTGCTCTGTCCTGTGTGGGAGGCTGTCAATT
EMPV1_04786	CTAGGGGTTTGAATGGGAGTTGTAGATGCCAGCCTATGCCACAGCCACAGCAACACAAAT
EMPV1_04787	GAACTGCTATCCCCTAGTCTGAGAACTGCTATATACCGCAGGTGCAGCCCTGAAAGTCAA
EMPV1_04788	TTGGTGCCTGGCAGCTCCTGTATTGGCTGCTTTACCTGCGAGGCTGTGTGTGGATGTCTT
EMPV1_04789	CATCGACACGCAGTACTACCTGGAGCAGCAGCTCGCCAAGCCCCTCCTGCGCATCTTTGA
EMPV1_04790	GGAACAGTAAGTGGCCCTGAGGAGGTCTATGAAATGGGACAAAAACAGAAAACTGGAGTG
EMPV1_04791	TGAAGTTGGACTGGCTACGTTTCAAGGGCTCAACAGCGCAGTTGCCAGTGACTACTGTAT
EMPV1_04793	GAGAGTGACCCTTTCTATCTGGCTTGCCTCGCCCTATGACCAGGATTGAGACACCGTTAA
EMPV1_04796	GAGCCCGAGAAGATGGCGGTGCGGAAGAAGGACGGCGGCCCCAACGTGAAGTACTACGAG
EMPV1_04798	GTATATTTGTAATGAACAATCAAAGAATGACTTTGTTATTGTTTTGATGCCACAGGGCTG
EMPV1_04799	CGGACGCGCCGCCTGCAGGGTAAGAAGTTAAGAATGGTTTATCCAAAGTCTACTTCACTG
EMPV1_04800	CTTCTGGAAAGAGCAGGGATGGTTCACCCAGGAAGGATTTTTGTGACTGAATCGGCAGTG
EMPV1_04801	CAGCTGAGAAGTTGGTGGGGCCTTGAGAACATACCCTAAAACTCTGTCTCCTTTTAGACC

EMPV1_04802	ACAAGAGCTCCTTGCTGGAACCAGGAGTTTGGTGGTGCGAGGGACTTTCTGGTCTATGAT
EMPV1_04804	TAAGACAGGAGCGTTGCAGTCCTGGGGACAGCCTTTTCTGCCCTTCTGCAACTCCTTCTT
EMPV1_04805	CAAGCTCCATGATCCAGTTTTCAAGCCCTTGCCAAATAAGCTACACAAAACACAGGAGTG
EMPV1_04806	GGGCCAGAACTTCATCTCTAGCCAGCAGGAGAGCTTTAAGCCGAAGAACATGGTGGAGAA
EMPV1_04807	ATACCCATACCAAGTTGTAAGAGCTCGTCTGCAGGATCAACACATGTTTTACAGTGGTGT
EMPV1_04814	AGCTAGACTTCAGAAAACCAATGACTTCAGATTGGTGCACTTAGTTGTGCCCAAGATCAA
EMPV1_04820	CTGGGATTTTGACCGGGGTTGCATTGGATCTACAGATCAATCTGGGGTGAACGGACATCT
EMPV1_04823	GTGCAGGCGTCATGGATATGGCTGTACAAGACTCGATGGACAAACACCGATCTCTCAAAG
EMPV1_04824	GGAATAGTTACATTATGGCCAAGCATTCATGAGTTATACAGAAACCCTTCTCTAACAGAA
EMPV1_04825	CACTGGCAGAGGAAGTAGCAGCATCACAGCCAGGGTTTACTGTTCTCATGAATGGGTTGT
EMPV1_04827	TCCAGTCATCACGCTGTTCCCACTCACCCAGTGGCTGCTTCACAAGGATTTTAGGGCACT
EMPV1_04828	GCTGGTCCCTACATTTAACTCTCTTTTAGGTTGTTCTCTCCTTGTTGCCATGAGTATTGC
EMPV1_04830	GAACGTGTTTCAGCATCTTTGCATTGGGTTCAGACAGAGGCGGATTACTATGGGTTCCCA
EMPV1_04831	TCAAGCTTCTCCCCCCTTTAATTTATTCCCCCTTAACAAGCTCTCCTCCGCCTTCCTGCC
EMPV1_04833	CCGAGGAGGAACGAAACTGGGAGAGGAAACTCAAGAAACCAAAGAATCCTTATAAACCCG
EMPV1_04834	CAGTCTGCCAATCACTGTACCTATTACTCTGGAGATAAGATTTCATATACATGTAATAAC
EMPV1_04836	TAAGACCTCCTCCCACTATACCCCACTTCAGGTGCCAATTCCAAGTTCAGGTTGCCTCCT
EMPV1_04838	GAAATGCAAATACTTAACCAAGCCAGAGCCACAGATTCGCAAGTGCAACGAGCAGCCGTG
EMPV1_04841	TGCCATGAACTACGACAAGCTGAGCCGCTCCATCCGCCAGTATTATAGGAAAGGCATCAT
EMPV1_04842	GTCTTGGCCTTTGGTCCTTTTATATTTGTCTATACATGGCCATCCCCCTCCATACCCCTG
EMPV1_04844	GAGCCAGAGCGTCGCCTTACGTTTGGACTTGCTTCTGAGAAGCCCCTGAGTGCTAGAACT
EMPV1_04845	GGGGAAAACACTTTGTGTGGGAGGAAGGTTTTCAACTTGTGTTGCACTAGGTTGTCTGAG
EMPV1_04847	GACAGAAACGCGTCGCTCCACCTTACAAGCCATGAAACTATGTGCCATGTTCAGACGGCA
EMPV1_04851	GGAAGCCCTCTCTGACCTGCAGTCACCTCTGAATCCATGTGTCATCCTCTCAGAACTTTA
EMPV1_04852	GGTGGTCAGCATTCTTTGACTTATGGTTCCTTCACTCCAATCTCTTCCTCTGTGTTTACC
EMPV1_04854	ATTTGATAGACTGCTGTTTTTCTGGTCTGACCGTCGCAACCCTCATGAAGTACAACCAGC
EMPV1_04855	GCCTCTCTGTGATAGGACTGAGAGCATGGTAATGTAATTAGACTACAGTGTTGGCTGGGG
EMPV1_04858	CCGCCCAGGCACAAAGGGCGCAAACCGAAACCTCCAGGTCGCTGGCCGGCCAACTCGGCT
EMPV1_04859	CTTTGGGGTCCTCAGGGTCTCATATCCGTTGAAAAGAGAGAGAAGTGGGGGTTGAAGGTA
EMPV1_04860	CCTGAGGTATTCTCCTGACATATTGGCTGCTTGCTTTATGTCGATAACCCATCGAGTTGG
EMPV1_04861	CGAACTCTGAACCCTCGGCTTACCCTCAGCTGTGAGGAGTTTGTCAAGGTCATGGACAAT
EMPV1_04865	GATACTCAAGTAGCTGAGATGACCTCTTTTATATGCACCTCTTTATTTGCCCTCGGCCCC
EMPV1_04866	TCTGCTTCTCGGCCATCATATAGAGGTTGTGGCAGTTGTAGATGTACTCCTGCTTGTGCC
EMPV1_04868	TGCCTGAGGCATGGATATATTGGGGAAAAGACAAATGTTCTTTTCATTAAGTGGTGTTGG

EMPV1_04870	TTGGCAGGAATACTGCAGAAGTGATATTGAGTCCTTCTCACTGAACCATATAGTGGCATG
EMPV1_04872	ATTTCGCCGTTCTTCACACCTTACCCGGCATCAGAGTGTCCATACCTCCAAAACCCCCTA
EMPV1_04875	GTTGACTGGATTATTGCTGATATTCTGGCCATCAGGCAGAATGCTCTTGGACACGTGCGC
EMPV1_04876	GCCTAGTCCCCGTACTCTATGTCTTTGTGGCCATTTCAATCTGTTTTCAACACCCCAGTC
EMPV1_04881	AACCCTTATTCAAATGATATTCTTGAATATAAAAATGCCCACGGCAAGATCATCATTCTG
EMPV1_04882	GGCCTAATGAAATTCATGTACGACTCGTTGGGCAAGACTACTTAACTGTGTGGTATTGTG
EMPV1_04883	CCCTGATGAGACTAAAAAGCCTCGGTCTTATCCACGCCGACCTCAAACCGGAGAACATCA
EMPV1_04885	TTCTGAATGGCTGACTTGATTGTGCTGTGTTGGCAGAATGCCTCTGTACCTCTGCCCCCT
EMPV1_04887	AGTCACTTCTGTGGTGTAGCTTTGATCCCTGGCCCCAGGAACTTCCACGTCTTAGACACA
EMPV1_04889	GCAGATTTAAAGGTTTTACTGTAGCGTCAATATCCGCATGTGTGTGAACTATCTGAATTC
EMPV1_04891	ACCGTCGCATAGGTCTGATTTTGCTCCTTGCTGCCTGCATCTTGGTTTCCATAGTGACTG
EMPV1_04893	AAGGCAGCCAGCAACCCCAACAACCCACCAGGTCTTCCCATCAAGAGGGATCAAAGTGTT
EMPV1_04894	GGGCAGGGATTGAATCCACATCCTCATAATATCAGGTCCTTAACCTGCTGAGCCACAATG
EMPV1_04895	CCATATTCAAGCCACGTGACCTATTCCACAGCTCTTGGTAATGCCACATCCTTAACCCAC
EMPV1_04896	TCAGTTCTCGAGGAGAAAAAAATCCTTATGGCAGATGCTGATGGAGCTACAAAGTTTCCAG
EMPV1_04898	TTCTCCACATCTCCGAACCCGCCTGTACCCACCTCAGCCCCTTCCTGTCTTGCCTTATTT
EMPV1_04899	GAATCATCCTCTCGCTCTCCATTTACGGCCTTCACCACAACCCGCAGGTGTGGCCGAACC
EMPV1_04900	CCTCTGGTGACATTTCTGCTGGGTTACACCTCCACCCAAGATTTATAGCTTCACAAAGCC
EMPV1_04901	GCACATGGAAACTGTAGAGACAGATTTTAAATTTAATTTCCTGAGGTGATAAGGTCAAAC
EMPV1_04903	TGTCTTCACGCAGCCAACCCTAGTTCTCTCCCTGGAACTGACATCCAAAACTTGAGTCTC
EMPV1_04905	TGTTTGAGAGTCTCCTGGAAAGGTGGGCATGGTTGCAGGTCACCCAGGGACATAGGTGGT
EMPV1_04906	CACGGAGGGACTTGGAGCTTGCTATTGGTGGAGTTCTTCGGGCTGAACAGCAAATTAAAG
EMPV1_04908	CCTGACACCTTCAGCAGTAAACAAGATAAAACAACTTCTTAAAGATAAGCCTGAACACGT
EMPV1_04909	ATCCTCTAGCCCCACAGGTTTGTGATAAGGAATATGGACTGGCATCTCTTCTTGGCCACG
EMPV1_04911	TCCTATGTCAAGAGACAGAACTGTTGATTTACCTTCTTCCTGACCCTGTCCTTTCCCATC
EMPV1_04912	CGTCTTACTACACAGGAAGAAAACAGAATTTGTAGCTCACCACTGCAGTCTCTACGGGAC
EMPV1_04915	GTCCCAGGGGTCAGTGTTGACCCAGCTCCCGAGTCACACCAACGTTTAAACATTACGATA
EMPV1_04916	AAAGCCGAGGAGCGGTGTCGGTTTGGCAAAGCCATAGAGAAAAGCGACTGTGAAGAATCG
EMPV1_04920	CCTAGCAAACCAGAATTTCTAGTAGTGAATGCATGCATAGCTCCACAGCTGATTCAGAAG
EMPV1_04921	GGCCTGAGTTCTGAGTGGAGACCTAGGAGCCACATCTCCCCGGGTTCAAACATTGGAAGC
EMPV1_04922	TTCAAACCCTGCAGAATCCACTTCCCCCATTCTCCCTTCCTGCCCCAGGTTTCTTGTCAA
EMPV1_04926	TCCTTCTCAGGGTCTTACGGAGGCTGTTCGTCAAGAAGTTCACCGTGTGGGTCTTCAACA
EMPV1_04927	TGGTGGCCTTCTCCTTTGCCAGCGGCTTCTCCATCTCCGTGATCAAGGTCTACTTCATCT
EMPV1_04929	GACCCAGACGGACGATGGACCAACCATTCCCCAATTTCTCGCTACTGTCCACTGGTTAAT

EMPV1_04930	CTCAGAAATGTATGTCAGGACTTGTTCACTAGGTTGGCAGCAGAGGGGCAGAAGGAAAGT
EMPV1_04931	ATTGGAATTGCCGCTGCCAGCCTACACCAAGCCCCAGCAACACCAGATCCTTAGCCCACA
EMPV1_04932	GACATGAACAAGAGCGCCACGCTGGAGGAGATAACCAGAAAGACATAATGCAGAGGCAGT
EMPV1_04934	GCTGTGAGGTTTTAACTTTTGAGCAGCATGCGTCCCATTGTTGACAGGGCCAGTAGTCCT
EMPV1_04935	AGTGAAAGACGCAAGCATGCAAGACTCAGACACATTTGAAATCTATGATCCTCGGAATCC
EMPV1_04936	TGATAATTGATTGCGTTGGCAATGACACAGATGTTCTTCGGGACACCTTCAGTTCCCTGG
EMPV1_04937	GTGAGGGATGTCCACTGTTGGAGCAATTAAACATTTCCTGGTGTGACCAAGTAACCAAGG
EMPV1_04939	GCTGCAGGAGCCAAAAAAGCAAAGAGCCCCAAAAAAGCGAGAGCCGCCAAGCCTAAGAAG
EMPV1_04940	GATGGCAGCTCTCTCCTGGGGACTTGGCTTTCTCAATTCATTGACAGAAACGATTCTTGC
EMPV1_04942	TTATGGTCGCGCCCAGCACTCCATCTCCACTGAGAAGATCAAGGCCAGGTACCCAGACTA
EMPV1_04945	AAAGGCCCGTTCTAAACAAGAAATGCAATCTGCCGGATCACAGAGAGGTCATGGAGGAGG
EMPV1_04946	GAGTTGCGAGACCCAGCTACATATCCCACGCTCCTCAGACTGTCACTCGATAAGAGAGTA
EMPV1_04949	AAGCTTCTGGAGATCCTGCTCCGTCGCCCCAGTGTTCAGACTACCTGTTCAGGACAATGC
EMPV1_04951	GATCAGTGGGGATCTTCGTTTCCATAGACACTGCTTCCCGAAGTCTGGCGACAGTCCCAG
EMPV1_04952	TCAATTCAGAGAACAGCTTTGTGAGACAGGTTATGTTGACAGAAAGATGGGTTGTGGGGG
EMPV1_04954	TGGTGCCAAGTGGTAACATGGGAGTGTTTGATCCCACGGAAATACACAATCGAGGGCAGC
EMPV1_04955	TCTTCCTGTTAAGTGGCATAGGGGTGATGACCGGAAGCATGGCCCTGATTGTGTTGGATT
EMPV1_04956	GACTTCAGTTTCATAAGTCAGTGTTTCTGAAACTGTAGTCTCTGAACATACCTTTTGGGG
EMPV1_04957	CGGCAGTGGCGGGAGCGGCCCCTCGGCCATCCTCCGTCTGCCCAGTTACCGCTTCCGCTA
EMPV1_04958	TACATAATGATGGTGATGAAGGTGACACAGGATGGGAGAGGAATGCCCAGCAGAGGAGAG
EMPV1_04959	AGTGGGGGAGCCAGGAAGTATTGATGTTTCTGCCAGGTTAGCGTCAACACTTGCTGGTTT
EMPV1_04961	ATAGTGTTGCTCCGGCTTGAGCTTGATCCCTGACTCTGGAACTTACATATGCCACAGGTG
EMPV1_04962	TTGCCTGGACTTTTGGGGCCTCTTCAGATTACATTAGGAGACATTTACCCACAACTTAGG
EMPV1_04964	ATGAGAAGAAGGCAGCCGTGGTTCAGACAGCTCGGGAATGTCTCATCAAATGCATGGACT
EMPV1_04966	GTGGTTGCCAGAACCAGGATTACAACGTAAATCGGGGAGGTCTTTACACTGTCTCATCCC
EMPV1_04967	AAATGCCAATTCAATACCATACTCCTCAGCATCTCCTGGGAACTATGTAGGTCCTCCAGG
EMPV1_04970	GGAGGTGGGAGCCTTACAGACAGAAGGGTAAATGCATATGAAGGCATAACGTGTGGAAGG
EMPV1_04971	CCCTGCCCTTACCCTACCTCTGAGTTTGTCCATTTTTATTTCCTGAAGAGAAGGGACGGG
EMPV1_04972	ACTTAGCCACAGGCTCTGGGCCACTGTTCCCCAAGCCACAGAAAGGGGGAAGCAACTGGG
EMPV1_04973	TGTCCAACGGCTATAAGCCAGCCCCTTTGGATTTGTCTGATGTGAAGCTGTTACCTTCCC
EMPV1_04975	GGCAGAGCAAAAGGTCATGGTATAAGTTGGACTATCAAAGTATTTGGTTCCAAGAAAGCA
EMPV1_04976	GGTGTGTGTGCCCCCAGGGAGGTCCTCTTTTCCAGTTTATGAGAATGACACCCATGTATA
EMPV1_04977	TTGTTATCGCTGCACTGCCACTGCTGTGTGGACTTGTACCGCTTTGAGAAATGGCACTGT
EMPV1_04978	TCATAAGAGGGGACCCGGAATTCCTATGGCCTAGCATGTCAAGAATCCAGCATTGCAGCA

EMPV1_04980	TAGTCACGACCCTGTGGTTTGTCCTTCTTCTTGTGTCTTACACAGTCATCCTCGTGATGC
EMPV1_04981	GTTTCCAGAAAAGGCTGTTTTGGAATCAAGTGGTGGGGTCTTTCGCCCTTGAGAGAAAAG
EMPV1_04983	TTTTCCTGCTGGCTCTGTCAATGTCCATGTCTTCTGTGATGGAGTCATCAAGGCCACAGC
EMPV1_04984	CCTTCCTCCTGCCCGTTTTCTCCAAGGAATCAATATTTCAGTCTGATGAGGTGTGAGTCC
EMPV1_04985	GTCTTTGAGCGTCCTTCCTACCCTGACGTCTTCCAGGCATCAGAGCACATCAAATCAGAA
EMPV1_04988	CTGACCCTGATTTGGGTAAATCATGGTTGGTAGAACTTGGAGCAGTTCACCATATGACAT
EMPV1_04989	GGGGATGGCAGTAGGATCATTTTTGATGACTTCCGAGAAGCGTACTATTGGCTTCGTCAC
EMPV1_04992	GCAGAAGTTCAAGGCGCTTTGGAGCCCCATTATTGCGTGATTGAGTAGGATTCACTCAGC
EMPV1_04996	GTGCTCTTGCTGCATCGTCAACAGCTCCAATGGCTCCCGTACCATTGTGCTCTACGACAC
EMPV1_04997	GGCCATGTTTGAATCCCAAGAAGCTGTGGACATTACCTTCGATGACAAAAGGGACTTTGC
EMPV1_04998	ACTTTGGTTTAAAACAGGCCAAGGGAGTCAGGCCTGGGGATCTAGACTCTTGCCCAAAGT
EMPV1_04999	CTCACATTCTGCTCTCCCAAGAGAATACACAGATTAGAGACTTGCAGCAGGAAAACAGAG
EMPV1_05001	AGCCTTAGACTAATAAAATCAGCTACCGTTTGTGGGGACAGGCCCTATTTGAGATGGCCC
EMPV1_05002	TCCAGATTAGATGTCACCAACAGTGAGAGCCCAGACATTCCTTTGAATCCAATTTCGGCC
EMPV1_05003	GTGGGGCCAAATTATAAGACCAGTGTAGAGCTGAAGTTCAGGCTCATAAAAATGACTCTG
EMPV1_05004	GGTGCTGCGCAAGTTGGGGCTGCGCCCTGGGATACATCACTCACTTTTTGGGGACGTGAA
EMPV1_05005	GGCGCTCAGCAAATGACCAAGTGAGATTACTGGACAAGGTTTAAGACCACAAGCTGTGAT
EMPV1_05006	GAATATGCTTTCTGTCACCTGCCGAAGTCGTAACTGTGGGTATTTATGGCCGTGTGTATC
EMPV1_05007	CAACAAGTTTGCCAGTCAGCAGGGCATGACAGCCTATGGCACCCGGCGCCACCTCTACGA
EMPV1_05008	TTTAAGGCCCTTTGTCTTGGGTGGCTTATGTGTGACAGACAACGGATTCCTGCTTCCTGG
EMPV1_05010	CTCGGGCGGCGGGAAGAGGCCGCGGGAGAATGGACGCGGGGGACTCCGGCGGTGGCGCAG
EMPV1_05011	GAGTGAAAAGGTACATTCAGGTGGAGCCGGTATTGGACTACTTGACCTTTCTGCCTGCAG
EMPV1_05012	GCTGTCTGCACTCACAGGGTTGTATTTGCCATCATGTTACGCAATGTTTAACTCACAAGG
EMPV1_05013	CAAACTGCCAAAGTACAGTGTGGGCCACAGGTAACCATAGTCACTCCAGAAACCATGGAG
EMPV1_05014	TGGACGTAGTTTTCATCCTGGGGGAAGGGGCCGTGGAGTTTGAGAGGCTGTGGAAGAGAT
EMPV1_05017	AGGTGGGAGATGGAGGCTCTCAGGACCTTTCCCACTCAGAGCACGATGACTCTTCTGAAA
EMPV1_05019	CAGGCATTTGAGCAACCAAATCTTACAGAGTTTGACCTGTCCAGTATGGTGGGCACTAGC
EMPV1_05020	TTGCCTGCTGGAAAACTGGGCCTCAGAAACTGAGATGCACTTGACCGTGTCTTCACCCCT
EMPV1_05025	TGAGGTGCTGCAGTTGGAACGTGTCTAGAAGCCTGATTTGTCCACCTTAACAGTGGCAGG
EMPV1_05026	ACTAGTCTCTTGAAGCCAGGAAAGGCATTAAGCACAAGTTACAGTGACGAGTATTTGTTG
EMPV1_05029	TACGACAGTGGCAATTCACGACGAGGAGGTCTACTGCAAGTCCTGCTACGGGAAGAAGTA
EMPV1_05030	CAGAGCATCAACAGCGACGTCAACAACCTCATGGCCGTGCTGAACATGAGCAACATGCTT
EMPV1_05033	CTTCTTAGCACTCGACCTGTAGTTCCGCACAGACTTGGACTCGATAATGGATGTTCTCCG
EMPV1_05035	CTTTGTCAAGATGGTTGGGCACCACGTCTCCTTCCTGGAAGCCCACGTGCTTCAGGCCGA

EMPV1_05038	CACCTCCAACCACCTTCAAGCCCAACAGTCCTGATCTAGCCAAGCACTACAAATCCGCTT
EMPV1_05043	CTGGCAGAGGAACGAACGCAAGACCACATACAAATCACAAGGTGGAACAGCCCATACGAT
EMPV1_05047	AGTGACCACTGCTAGCCTAATCTACCACACAAAACTGCATCCAGAAAAGCCACTGGGACC
EMPV1_05048	TGTAGGTCATGTCTGGAAAGTGATATCCCTGCTCTCCCTGCAAATCCTATTCCTGTTATC
EMPV1_05049	TTGTGCCCAAAGTGAGTGCCTTGCCCTCCTCATCCTGTTTCTAGGGTTAGAACAACACTG
EMPV1_05050	TCAGACAGTCTCCACGGCCAGTGAACTACGCAAACCAATTTACTGGATTGTGGCCGGTAA
EMPV1_05053	TGACGTGCTTTTCTACCCGCTGAGACCAGAGCTGGTGGAGTCTACTTATCTCCTATACCA
EMPV1_05054	TTGAGGTTCCCCCCCTCACACTGCCTAATTTTTGCGCTGTCTCACAAATACCTTGTAAGG
EMPV1_05056	TGATGGGGACACCAGAGCTGACTGAGCAGGAGGATACCATGCAGATGGACCAGCTGAAGA
EMPV1_05060	CTTCTGGAGCTCCACCAGCACTTCCTCTTCCACCACTTCTCCCAGTGAGCACTGTGATAG
EMPV1_05062	GCAGGGCTGGGACTGACCTCAGGGAGACCAGGTGTACCGATTCATTAGCAATATAATCAA
EMPV1_05064	AGTGGTGACGATGGTGATGAGGACAATGGTGATGACGGTGCTGAGGACAATGGTGGTGAT
EMPV1_05065	TCCCGGCCCAGGTTCTGTGATACACTCCGACTCGGGCTCTGGAGCAGTCAGTGCATGACA
EMPV1_05067	TGAGTCTGGGAGTGGGAATCTTGGTTGGCTGCCTCTGCCTTCTGCTGGCTGTTTATTTCA
EMPV1_05068	ACAACAGCATGTTTCTCTAAGGTCACCAAGATATTAGTTGCTGCTTTGACATTCTTCCTT
EMPV1_05069	CTCTGGCCTAAGCACACAGCGATATTTATACACGAAACTGTACGGAGAGCGTTGTGCCAA
EMPV1_05070	GCTTCAAAAACAAACGAGGAGTTCCCTTCGTGGCTCAGCAGTTAACGAACCCAACCAGGA
EMPV1_05071	GTCAGCCTGTTCTTTGAAGCTTTGAGCCAGGCATTGACTTCTCTCTAGCTGTGAAAGTCC
EMPV1_05072	GAATCACGGTTTAGCTTGTGTACTTGAGTGACATAACACTGTCCACAGAAACATGGTCAG
EMPV1_05073	GGTGTCCCACAGAGTGAGTACAGCTGAGATACAAGGCCAGCTCTCCTACAAGATTCGCTT
EMPV1_05074	GAGACCCTGAAGCAGGATGACCCAAATTGTAGTGTCAGGATTTTAGCTGTACCAGAGGCC
EMPV1_05078	GAAACAGCTCTTGTGAGGCTGGGCAGGAACAAGGATGAGGTTCCCAGATACAGAAGGTTG
EMPV1_05079	AGCGAGGGGGCAACTGGATACGTTGAAGGACACAGGCTGCGGAGTTTGTGCATTTTCGAA
EMPV1_05081	AACATTACCCTTGTACTTTGGTGGGGACTTGGAACACGTGGTATGGCGAGCAGGATCAAG
EMPV1_05082	CACCACCAGTGTGTGAGGGTGCACTTCTCTTTACATCCCTGCCAACATTTGCTATCTGTG
EMPV1_05083	CCACTCACAGGTCTTGGGTTCCCCGGCTACTCACACTTCTGTCCTAATGGGCTGCAAATT
EMPV1_05085	CTCTTGTGGGCAGCATATTGTAAGCTCTTGAATTTTTATCCAGCCTTTCACTCGATGTCT
EMPV1_05087	TAGGCAAGGTAAGATGAGTGGGAGCCCAGGAGACCCTTACCAGAACCTGAAACCCAGAGA
EMPV1_05089	GAAAACAGCCATTCCTACAACTTTCAGGGGGCACAGGAGGAAAAAAGGATGAAAGTATGG
EMPV1_05091	GGTTCCCGTTTCCTTTCACTGAGGAAAACCTCCATACACTGAGGCAGCACTTGTGTCAGT
EMPV1_05093	GAAGGCAGCTGTCCCAGGGACGTATTGCACAACTTGAAGCAGAGTTGGCTTTACAGAAGA
EMPV1_05095	ACCAGTCACCAAAGACGACTAAGTTCAAGGGCTGTCAGCTTCCCCAGTGGCTTCGGTCTA
EMPV1_05096	CTACATTGAAGCCCGGGAAGGCTGGCCACTCATTGACTACATCTTCTCCCAGTTTGCGAT
EMPV1_05097	GGTGGGGGGCCAAGGCATGGGGTTGAAGAATTTCACTTTTACTCACATCATACACTGGGA

EMPV1_05098	GGAGGGACCGTGCTGGCCAAGATGTACCCGCGCGGCAACCACTGGGCGGTGGGTGAGTGT
EMPV1_05102	TGTGTCCATGGCTGCACGCACTTCTCCCCTCACCTTTATGGCACATCTCTTGATACGTCT
EMPV1_05103	ACAGTTCTCAGTGCTCTGCCTGTTCATCCTTCACCTGCTCCAACCCCTGGTAATCACTGA
EMPV1_05104	ACCCAGGCCTGGTTCTGGACATCGTTGAGGACCTGAGGCTCATCAACACACAGGCCATCT
EMPV1_05105	AGGAAGAGTTTCGGCGTCTTCTGGGCGGCTAGAGCGTCCTCCCGCCTCTGTCGCGCGGCA
EMPV1_05106	CTGCTGAAAACTACAGAAAGGTGCCATCTGGTACCATGAAATGAGGTCACCCTTGAATGG
EMPV1_05108	CCTGTCCTGCATCCAAAAGGGTGCTTGGCATTGGCACTAAAATCATGTAGTTATACTGGG
EMPV1_05109	AGAGTCCTGCTCCACGTGATTCCTCCTTGAGAGAGAAGTGAGCTGGCGGCGTAGGCGTTT
EMPV1_05110	CTGCCTGTTAAATCCTCTCCACTCCTGCTTAGTCCCTTCCAGCTTCCGGCCCTTGAATCT
EMPV1_05111	CGCAGTGAAGGTTGATCACCAAGGCATTTTTAATCTGTGGCTTCCCCAGGTGGTTGGCTT
EMPV1_05112	CCTCTTCCTGGGATGGCAATGCTGGTGCTCTTTCGATGATGAGATTCACTTCCCACGTGT
EMPV1_05113	TCACTTGGCCAGAACCCATGATTAGAGCCCCAGCCGTGCCTCCTGCAGGTTTTGAGTATG
EMPV1_05117	CATGTGGGACTGATTCCGGCCAATGGGACTGCAAAGTGTATCTATTGTTTTCACGCCGTA
EMPV1_05118	AACCCAGGCTCACAGCAAGACACCCTATGGACCTCGCAGAACTGTCTACAGGACAAGTGG
EMPV1_05121	CAGCCTTGACCCTTGGGGAAGGGGAGGAGAATGGGACATGGGAGTTCCTGACGGTGGTAG
EMPV1_05122	TCCATAGAGACGGTGAACGTGGATGGCACTGGAAGGCACACCTTTCCTGAAGTCTTCTTG
EMPV1_05123	GTCCTGAGCTCTCCAGGGGAGGAGGACATCGACCTTGAGGAGGGATTCTTTTATGGATCT
EMPV1_05127	CGGGTCCTCCGCAACGAGACCAACAATCAGACCATTTGGGATGAACACGACAATAGGACT
EMPV1_05128	CTTAGAGGTTCATAAGTTCTTCAGCCCATGACAGATGAGAAGGAGCCGCGTCAAAGGGTG
EMPV1_05129	TGCATCTCTGGTCAGCTGGGAGTCTGAGATGAAGCACTGTAGCTCGGGAAGGGAGAAGCT
EMPV1_05130	TTTCCTCAGGAACCGTTTTTAACGGCTGGCGCAGCTGCTTCCCTAACTGGGGTCTGCAGT
EMPV1_05131	CCATGCTTGATATCACCAGAGATCCCTTAAGAGAAATAGCCCCAGAAACAGCAATGACCC
EMPV1_05132	TCTGCAGTAAAATCTGACACGGGAGATTCATCGGAACATGTGCCCTCGTCCGCCTCCCAT
EMPV1_05133	CACATGTGATGGTGGGGGGCAGCTGAGGGGCAGTACTAAGTAAAGTGGGACCATCTTTTC
EMPV1_05135	CGGCCTCTTCAAGGTCTTTACCCCGGAGGAAGCCGTCCACTTCATCTTGTCCTGCCTTGA
EMPV1_05136	AACAAGCTTGCCCTCCCCTATGTTTTCCAGAAATGACTTCAGTATCTGGAGCATCCTCAG
EMPV1_05138	AATACATCAACAGCCCAGAAGGTTCTTTGTTCTGGATATACCAGTCAAAAAACCCAGAAG
EMPV1_05140	GGATGAGCCACCGAAACCAACCTTAGCTTTGGAGTACACATATGGAAGGAGAGCGAAAGG
EMPV1_05141	GCTTTGGTTAAAAAATGGCTCAAGTAGAGAAGCGGTCCCATTCACATTAAGACAGTGTAC
EMPV1_05142	GAGGATGCTAGTGGGGCTATGGCGTGGAGGATGGACTGGAAGTGAAGGTACTCAGACATT
EMPV1_05143	AAGTACCCTGTCCCCTTTAGTCCCCTTACACGACCTTATGCTCTGACGTGCTATGCCTAG
EMPV1_05145	GACAGGACCCCCTGGGGATTTCAGCTCCCCCCACACGGAGCCCTCCTCCTCAGCTGGAGA
EMPV1_05146	GTCGTACCTACGCCTGTACAATGAAGGCAAACAAGGAGCATCATGGAGTTGAGAGGTGGT
EMPV1_05148	GGTTGGTTCTATGGTGAGAGGCTGCGGGATGGAGAGACGGGCTGGTTCCCCGAGGACTTT

EMPV1_05151	TTTCAGGAGCCAGCAGAGGAGGAACGAGATGGCAGAAAAAAGAAATACCCTAGTCCCCAG
EMPV1_05152	TATGACAAGCTGTGGCAAAGGTGTGGCTTGGATCAGTGTTGCCATGTCTGTGATGTAGGC
EMPV1_05154	ATGTCTGGACGTGGGAAGCAGGGAGGTAAAGCTCGCGCCAAGGCCAAGACCCGCTCCTCG
EMPV1_05155	TCATCCTGGGCGCTTATGTGTTCATTGCTCGGGCAGTCATGAGAATCCGCTCTGCTGAAA
EMPV1_05158	TGAGGAGAGCATCATGGTGCGTTTTAGTGTCAGTAACCGAGAGAAAGGTCGGCGAAAACG
EMPV1_05159	GGTTCTTCACCAATGACATCGCTTTGCTCTTGCTCAAATCCCCATTGAACCTGGGTGTCG
EMPV1_05160	TTCTGGGATTTAGGAGTGACTCTGCTGTCCCCAGACCCTCATGTAGCAGGTGCATCGGAA
EMPV1_05162	GGTCATTGTTCTGTTACCAAAAAGGCTCTGTTGAGACTGAGGCAGATTAGGGCTAAAGCC
EMPV1_05163	GCCACAAGTCATACCCAGGCAGTGCTTCAAAAAACTGTGGAACAAAAGAAATCTTTGGAG
EMPV1_05165	TTGTGTTAGTGCCTGGCCACCCAACTGCACCACCTCTCTGCTGTGTAAATTCAGTGTGTA
EMPV1_05166	CTTTGGCGGAACACAGTTCAACCCATAAACAGTTCTCCTTAGCTTCAGAACCGTATTTAG
EMPV1_05167	CAGCACCCGTCTTAAATGGTGTCTGCTTCAGATCCATTCAAGCAAACCTGAATGGGTTGT
EMPV1_05168	CTTCTGGCAGCAAAGAAATCTGTCTGGGCATTAGCTGAAGATGAACTAAAGGAACTGAGA
EMPV1_05169	GAAGAGAAAGCGGACAAGTATCGAGAACCGAGTGAGAGGCTACCTCGAGACCATGATCCT
EMPV1_05172	TTCTGTCTCTCACAACTGTCCAGGACCCAAATCAGCCATGCAAGTCCCAGACTCAGAGAA
EMPV1_05174	CCAGAAGATGATCATTGCTGCCCTGGAGTCTAAGCTGGCCCAGGCTGAGGAGCAGCTGGA
EMPV1_05175	GATGACACTGGCCGACGATGACACCCTGGACGACTTGATCTTGGCCAAAGATGACCTCTC
EMPV1_05176	GACCACACATCTCCTTCAGTTGGCTGTCTCTCTTCTGAGCCCATCATTTCGTCACCAGAG
EMPV1_05178	TCATCACTGTCCCTGAAGGAGACGCTGTGGAGCTGAGGTGCAAGTACTCCTCTTCTGTCG
EMPV1_05180	TTCCTCCGCTCTGACCTCTACCTGGACCTCATTAACCAGAAGAAGATGAGTCCCCCGCTT
EMPV1_05181	GGCTTTTGCCAAGATGGCTTCAGCTCCTGCCAGTTACGGCAACACTACCACTAAACCAAT
EMPV1_05183	ATAGGCACCACTTCTAACCATTCCTGTGCCTGTGGCAGACATTGCTAGTTGATCGCTGCA
EMPV1_05184	TCTCCAAGCGCCCATCAAACACAGAACATCCTGCTCAATGCATCAGACTTGAAAACCTGG
EMPV1_05185	GAGCAAGGCCAGGGATCAAAACCGCATCTCATGGATACTAGTCGGGTTCTCAACCTGTTG
EMPV1_05186	ATCTGTACCCGTAAGTCCGAAACCTGCTGTGATCCGGGGAGCTGTAACAGAGCCTTGAAT
EMPV1_05187	CACTGGTCTCTAATGGATACTGTTGGATAAAGATCCCGCCTGCCCTGTATCAGATTCCTC
EMPV1_05190	GCCCGTAAGCTGGGTATTCTAGATGAAGTTGTGAACTCAGACCCAGTTGAAGAAGCAATC
EMPV1_05193	AGCGTATGTAGGCAGAAACGTAATACTGACAAGTGGCTGCATCATTGGGGCTTGTTGCAA
EMPV1_05194	CTCAGCTCCTGCCCCCGTTTCTTCTGTGGTGTGAAGCAAGGAGGACTGAAAATAAAAAGG
EMPV1_05196	GCTCTACATCCATGCCACTCCTTTACTTCTCTGCTTTGACAACAAAGACCATCTGACCCA
EMPV1_05198	GCAGCCTGGTATTTTTAATCAGATGAAATGCTTACAAAATGGATTTCCTCCTCATTCTGT
EMPV1_05199	CTGCAGCTCAGGTTTGCCGAACAAGGATCTAGGATGCAGCTCAAGGATATCAGGTGGGTG
EMPV1_05200	GATCTGATTTGTATCCACTGCGATCAGCACTCACTCCCTTAGGACTTTCCAGACCAGGGA
EMPV1_05202	TCACTTCAATCAGATTGTCTTTGACTTTTCATCTGTACTTTGGGAATTCCAGGCTTTAAC

EMPV1_05203	AACAAAAAGTCGTTCCGCCGATGTAGGTTCTAGGGCCGAGGGTCGGAAATTTGTGCAGTC
EMPV1_05205	AGATGAGCTTATCCTCAAGGTTGGCTGGTGTCCAAAGGTGTTTCCACTTAACACCAACTT
EMPV1_05206	CCAGGATGGACACGGAGTCCTAATGGGTTTACTTAGGAAAGTACGGAGAGCACTTCCAGT
EMPV1_05208	AAGAAACTTCATTTCAGCAGCTTCGCCCTGAACCCGACAAGACGCAAAAGGGGGACTCAC
EMPV1_05210	TCATGTTCTCCACGGACAAGCAGATGGGCTTCCAGTGCTTCGTGACCTTCTTTGATCTCC
EMPV1_05211	CACCCCTAGGGGTCAGTGGTCAACGAGGAGATGATGTGACTCCAGTAATTCTAGAAACAT
EMPV1_05213	TTTCCGAAGGTGCGCAAGGACTGCTCTGTCGTGAGGGGCCAAGATGAGGAAGATGCATTA
EMPV1_05214	AGCGGTTCCTGAGCCCGTGGTGATCTTGCTTCCTCATCGGCTATTACTGGCTAGTTGTAT
EMPV1_05215	GGCAGCAATGACACCCACTTTCCAGTTGGCAGGGGCATCTCTGTGGATGGTAGAGCGTTT
EMPV1_05216	GTGCGCTCCCAAAGGGCTACAGGCATTGGAAGATTGATGGTGAACGTGGTTGAAGGTATT
EMPV1_05217	CGGAAGGAGGGGGAATATTTGGCCTATTGCCGCCTTATTTTCGATGTCTGAGAACTGGAG
EMPV1_05219	AGGTGGCAGCAGATAGCCCTGCCCCATGATGGTCAGCTCTGCCTCCCTATTCTGTCATTT
EMPV1_05220	AGGGACTCTCATCCTGAGTGAAGTAAGTCGGAAGGAGAAAGACAAACACAATATGACATA
EMPV1_05221	AGTGGGGCACTTTCTTCTCTGGAAACTAAGACATTGGACAGAAAGGACTGGCTTAGGGGC
EMPV1_05222	CCTGCCCTTCTGCCCCCACCGGCAGATAGATGACTTTGTATGTCAAGTCCCTTCTCTAAT
EMPV1_05224	ACACTTTACCTCTCTTGTCGAAACTCCAAGCCTGGCACAGGGTTTGTTATTGCCCACTGC
EMPV1_05225	ACTGTACACCCAGAAGGACCGAGTTGGTGGCTTTCCCAACTTCCTGAGCAACGCTTTCAT
EMPV1_05229	GTTGGAAGGGCCACAGCAAACACATGCATCTAAAAGGAGCCTGATGCCCAAGAAGAGCCA
EMPV1_05230	GATCCTGGAGGAGCAAGGACTCGGCACAGAATTCACTTTAAATGGGGCTAAAGGAGTGTC
EMPV1_05231	GACAACTCTCTTTTCATTGTGCTGATTCATGAGTTAGCAGAAGCCTTTCAGCAGGACTCT
EMPV1_05237	TTGGATCTAGTGTTGCTGTGGCTGTGACATAGGCCAGCAGCTGCAATTCCTATTCGGGCC
EMPV1_05238	TGGGGTTCAAGTAGCAGCCAAAACATTCTTTACTTAGGGGCTTGGGATGTGGCTGTTGGC
EMPV1_05239	TGGTAAACCGAGTGTTTGACAAGCTGTCCCCCCTGCATCATCGAATCTACTGTGCGCTCT
EMPV1_05240	TACTTTATAATGCAGTCCTGCTGGGCTTTTGACTCCAGGAAACGGCCCTCCTTCCCAAAC
EMPV1_05241	AAGTCCTTCTGGCACAGCAGGTTCTTCCTGCACAGGCCGTTGGCCTGCAGCTCCGTGTTC
EMPV1_05242	TCTACTTCCACCTGTGCCAGCCCTGCACCATTCGGCATTACGAGGCTGCAGTGAGTGCTT
EMPV1_05243	CTTTTATCGCATTGGAAAGACTGGCAAAAATCTTTTGGAAGGGTTGGGAAATGTGGCTGG
EMPV1_05247	CAGAAACTGGCCCGTGAGTCATCTATCATGGCCCGAGAAACCTGGGAAGTCTTACTATTG
EMPV1_05248	CACTGACTGTGATGCTGCAGGAACTGGAAGAACATTAGAGCTGAGGACCTTTATCCTAAG
EMPV1_05249	GGAGCCAAGTCAGATCTTCAAGTGAGCCTCGGGCTTTGGGAACTGAAGAGTGTGTCTGAA
EMPV1_05250	ATAAGCAGGGAGGTGTGTAACATAAAGCATTAGAGCTCCTTGGAGCACAGACTCCTGGGG
EMPV1_05253	TAAGATCCAGGAAGAGATTGACCGTGTGATTGGCGGACACCAGAGCCCCTGCACGCAGGA
EMPV1_05255	GTCCTATGATACTACCTTCCATGACCTGGTTGTGGAAGTAGCCTATGTTAGGGTGCACTG
EMPV1_05256	CACCTTCATTTATGACCCAGGCATTTTTCAAACCGTTGCCTCTGTGCTGGGACTTAGAGC

EMPV1_05257	GATCGAGGCCCCACCTCACTGCAAAACCAACATCGTCACTGCTTCGGTGGTCGCCTTTAA
EMPV1_05260	TACCAGCATCTGCTACACACCGTCCACTCCCCTCTTCACCTTTCTTTCCAAATGGTTGAC
EMPV1_05261	AATCTTTCTTGAACTTACTCAGATGAGATCGGGGTGGGCAGTGGGAGGAAAGGCCGCGTC
EMPV1_05262	GGCAAGAATACCGTATTGTCCCAAGTGGAGCATGGAAGCTTTGTGGGCTATGATAGACTG
EMPV1_05263	AGTGTCCTGGTGGTGTCAAGTCTCTCGCCCCTCTGCGTGCTACTCTGGAGGGAACTTACA
EMPV1_05264	GACAAGATGTTGTCAGCAGAAAAAACCCTACATACCTTATAGCATTTCTGTTAAGAAATC
EMPV1_05265	CCGTTCTATGGACAGTAACACTCTGATGGCCACCACCTATACAGTCTTCACCCCCTTTCT
EMPV1_05266	GGACTGAACAAATACAAACTGCGCCAGCTGGAAGCTATTCATGACCAGGAACTTCACAGA
EMPV1_05267	CCACAGAACCAGAAAATGAACATCTAGAGGATGAAAATGTCCAGTCATCTACAGCTCCTC
EMPV1_05269	CATGATTATCTGTGGCTCTAGTCAGCTGGATCTTGTTCTGTAGGTTTGGGGTAGAAACTG
EMPV1_05272	CATATATACATGTGGTAAAGCAATCTTTTGACTCCCCAGATGGCACAGTTACCATATCAG
EMPV1_05274	CGTGCAACTCAGAGATCTAGAAAAATGGCAGAATAACATGTTCCTGTCCCGTCAGTTTGG
EMPV1_05276	ACCTGAGCCTGCGGTTTGATCCGCGTGATACTCAGATACACTTTGTTGTGTCCCTGCTTG
EMPV1_05278	CAGAGAGGCATGGATAAACTACTGTATAAATTTGGAGACTAGAATCCATGAGGACGTGGG
EMPV1_05280	CAGGTTCCGGAGAGTTAGTTTGCACAGCGGGTTCTGGTGGAAGCCTGAAAATCAAAACTG
EMPV1_05281	ACCCAAGTCCCCTGACGCCACAGCTGACATGGGGCGCCATGAGTTCACCTATGCACTGAT
EMPV1_05282	TTGAAATGACTGAACTAAACCCAGGTGGCTCAGTGTGGAATCGAACAGCCAATTGTGCCA
EMPV1_05283	AAGCTCAGGGTGGCCGTGGAGCAATGGAAGCGCCAGGTCATGAGCGAGCTGCGTGAGCGG
EMPV1_05284	GGAGGGACCTTGAGCAGATTTTATCTCAGTACATTACGTTCGTCAAGCCTGCCTTTGAGG
EMPV1_05286	CTGGTCCCTGGAGACCCTTTGTCAAGCCAGACATAGGTGGATGGAGAGAGAAAGAAAGAG
EMPV1_05290	TTTGAGATTGTGACTGTGCCTGCCAGAGTGTGACTTATGCTGCCCCTGCTAAGAACAAGC
EMPV1_05291	GAATCTCGGGCTAGGCAACAGGTTCTTGGCCTTCACTCTGAAAGCATAATCCTCAAAGGA
EMPV1_05292	CTGAATGCTTTGACCCGGCTGCCTTTATTCTTACACTGGCAAATTCAGCACGTTAGAGCC
EMPV1_05293	GCTGGGAAACCCTAGGCCACGTCAGACAGGACAACAATGCTGGGTTTTACATACAGGTAT
EMPV1_05294	ATTCCCACACCAGCTCTTTAAAAGCAGAGAGTTTTCTCTGGCTGGTGGCAGAAGTCAGCG
EMPV1_05296	ACAGCGAGCCTGGTGGAAGCAGGGGAAGAAAGAGGATACGGATGGACACCGAGTATTTTT
EMPV1_05297	TCACTCTGTGTCTATGATGCTACGTCATCCACCCCCTGCTCCGCTAATGGCTGGTTTCTA
EMPV1_05300	AACAACCAGTTTACTCAAGGAAAGAGCTTACTGACTGTCCCGCTGGTGTTCCTGGAGCCG
EMPV1_05301	GCCACTGAGATGCATTTTTGTATCCCTCTGAGTCATGGAATTTCCAGCTTTCTTCTCCCT
EMPV1_05302	AAAAGCATCAAAGCTAAGGCAGAAGATTCATCACATTTTCATCATTCATTACAGGCTTGG
EMPV1_05303	CTGGTTAAAAAGACCAGTTCTACAGTATTAGAGCAGAGCATCAAAGAATCTTTAAGGGAG
EMPV1_05304	GCCCCCCTGGTATAGAACCTCATCCATCGCCACCAGATCAAGGTGATGGTCAAAACACTA
EMPV1_05306	TTGAGAGGGAGAAAAGCATGAGCGAGGAAGAAATGGAACGTTGGAGTGAGAAAGAGGAGG
EMPV1_05307	GCTTTCAGGTCAAAGGGAACTGTCATCAGCTGGAAATATGGATCTACATGAGGAATGAAG

EMPV1_05308	GGAAAATACGACTTCATAAAACATTTTGGGAAATACATTGAAAAAGAGGTTAAAGGTGAC
EMPV1_05310	GGCAAAGGGAATGGCAAGTAGGAGTTCCTTTTCCTGCTCTAGGGATTCCTGTCACTGTGA
EMPV1_05312	TCGCTGGAGTCCGACAGAGCCCACACTGGTCCCGAGATGACGGCGAGCAAGCGGGTGGCA
EMPV1_05313	CGACGGGACTTGAACTCAAAGCATGTGAAGTGGATAGTAGGTGTGTGGTGGTATGGAAAG
EMPV1_05316	GCTTGGCAGGCAGTAGGAAACGGTGGTTTCTTCTTTAGTAGTTTTCCAAAGTGGCACCCT
EMPV1_05317	TCAGTGAGGCCAGGGATGGAACCTGTGTCCCAACAGACACTAGTCGGATTTGTTTCCACT
EMPV1_05319	GTGTGGACCCTCCTGACGTGGTGAAGACCACCCCCTGTGCTCGCTTGGAGTGCTGGATTG
EMPV1_05321	AGGAATAGTGACTCTCATCTCTGCTCAGAATTGGTCTTGGCTGCTCACTGCAGGGCTGAC
EMPV1_05322	TCCAGTCTTCATTGCTTATGGACAGAACACAATTCAGACCATAGCACATGTCCACAACTG
EMPV1_05324	GAGCTGCCAGTGATGAGTTTTTCCCAGACTTGTTCTTTTCAGCCCAAAGGGGAGGGAAGA
EMPV1_05325	ATGTCGACTTCAACAAGGCCAAGTCCCCGCTACCAGGAGATGCCACACAGCACATAGGCT
EMPV1_05330	TACTCATGTGCACTCCAAAGAGACATTCTTCTTCAGGGCCGACTCTACCTCTCTGAAAAT
EMPV1_05331	CAGAAGCTGTTCCCCTATGAAGAGTGCTCGGAGATGACAATTCTGAAACTAACACTGTAG
EMPV1_05332	TGAGCAAGCGAAGGAGGAAACTGACGGAGCATCCGGTTCACCGGGAGAGGAGACCTGGAA
EMPV1_05334	TGGTCCCACTGTAATCAATGCCCCCTTCTTGCTTGAGCTGTCGCAGATAGAGCCTATTTC
EMPV1_05335	GAAGACGAGACCACCAAAGCTGAGGCGAAGAACCCGAAGCAGAAGACACCCAAGCAGCGA
EMPV1_05336	ACTTGTGTCCAACCCAGGTGCTACCTCTCCAGCTGTTGCAGGCCCCCTTGCTGCCAGTCT
EMPV1_05337	CCTAAAACCTTTGGGTGCTCATCCTGGGAGTTCAGTCTTACAGACAAGTCCAGCCTTATC
EMPV1_05339	GGCATTCTGACCCCAGAGAAGAGCAGTGGCTTCATAGGTTTTCACTGTACGCTTCATATG
EMPV1_05340	GGCCATCAGCTGTCTTGATTTGCCTGGGACTGAGGGGTTTCCTAGGACAAGAGAGTTTCA
EMPV1_05341	ATACCTTGCTCCAAACCAGATAGCTAGTAAGAACATAAGAATCCAGGTCTGTATGCCTCC
EMPV1_05343	GGAGTTGCTGCTTGTTTCTGTTTCCTCGTCTGAAAAGTCCATTTCCAGCTCGCAAAATGT
EMPV1_05344	TTTATCTCTTTAGCAACCGGAGGCTGGATCAATTCCCAAAGTTCAGTGCAATTTTCAGAG
EMPV1_05347	CCAAGATCCACGAAGAGATTGACCAGGTGATTGGACCGCATAGAATCCCACGTGTAGATG
EMPV1_05348	CAGCCCCACCCCGCCTCTTTCCCTGCCAAGGGAGGAGATCCTAAGCCCACCTGTCGCCCA
EMPV1_05349	AGACTTAGAACTAAGCAGGCAGCAGTCTCCTTGAAGAAAGAGCTTTCGGTCACTGTAGGC
EMPV1_05351	CCTATTATGCTTACTTTTGTCTCTTTTCTAGTTGTGCCTCTAATTTGTCAAATACGCAGG
EMPV1_05353	CAGTGGCTCTCAGTTCTTCAGCCGCAGTAGAGGGATTTATTACCGTAGTTTTGAAGTGTG
EMPV1_05355	TGACAGGTGCATTCTGGATTCTGTGGGGCCCTTCCAGTTTAGTCCTGCATCTCTTCCAGA
EMPV1_05356	CTGCAGTTCATGGTCACTGTCAATAGTGGGTTTATCTGTGTCGGTTCCTTCTTCACACTC
EMPV1_05357	TGTCCCCGTGGATACTAGTTAGATTCATGTCCTCTGAGCCACGACGGGAACTCCCTGACT
EMPV1_05358	CAGGCAGAAAGCCAAACTCTGCAGTATTCACCAGGAGAGGTGTATTCTGTCACTGGCCTT
EMPV1_05359	CCATTCATACGAAAGACTATCCTCACCGTTGTGAGTACTGCAAGAAAGGGTTCCGAAGAC
EMPV1_05360	TCATGGTAGCTGCATGACCACCAGTTACCTCTAACAGCTGCAGTGGCATGCCCGACAGGT

EMPV1_05361	GCTCAGGTAGCATACAACTTTCTGTCCTTCAGATTGATTTCATCCTTAGTGGCAGCAGCC
EMPV1_05362	GGAGCGTGAAGAATTGTGGAAAAAACTGGAGGATCTGGAGTTAAAGAGAGGTCTTAGACG
EMPV1_05363	TGAACCCTGGACTCTGTGGGCTCTTGGTTCTGCTGTCCTGTGTAATGATGGCCACGTATT
EMPV1_05364	GCTCTGATTAGACCTCTAGCCTGGGAACTTACATATGCTGTGGGTGTAGCCCTGAAAAAA
EMPV1_05366	CAGCTGCGTTGCTTTATCTGCTCTGGGACTCTTCAAAGTCCAGGCTCTGACGCATCTGCT
EMPV1_05368	CAAGCTGCCCAAGATCTTCCACGTCAACTGGTTCCGGAAGGACAAGGACGGCAGGTTCCT
EMPV1_05369	TGGGATGAGGTAGTAGGTTGTATAGTTTTAGGGTCACACCCACCACTGGGAGATAACTAT
EMPV1_05370	CGATCTACTAACGTCACGGGGAACAACTGCCTACCCCTCAAAATGAGAAACAGTCAGAGC
EMPV1_05372	ACAACTAGAAAAGCAGTCCTCCACCAGTTCCTGGGAGAAATAACCCAGCATAGGTCCTGG
EMPV1_05373	GGTGGTGGAGCTGGCAGGAGTGCTGCGGTGGGTTCTCCAGGAGCTGAGCCTTAACAAGTT
EMPV1_05374	GGTCCAGTCCTATATCCTACAGTTTTTAAAATCAGGAACAGAGAGAGATGACACCGGAGC
EMPV1_05376	GATTTCTTCAGGCTTGTTGTCAGCTGGAAGGGCTTCAGGAGCTGGGAGCCACATGTTGAA
EMPV1_05377	TGGGCCTAATATTCGAGGTGTAGACAAAGGCCTACAGTGGCACCACCAATGCCCAAAGTG
EMPV1_05381	AGGAATCCCTGGGGAGAAAGGATGGCACTTGGAAGAAGGGGACTTTAGAATCTTCTGGTC
EMPV1_05382	GCTTATCAGCAGTGAATGAGTTTTATTCCTACCTGGTGCTGGCCGCCGGCAGGAACAGGA
EMPV1_05383	CTGAGGGGCGAGGGAGAATCTGCAAACCAACCTGAAATTGACCAGCATTTAGCAATGATG
EMPV1_05384	CACCAAAGTTGTGTGCAGGGGTGTGGGTGTTTTTCAGTTGTCCTGCTCATGGAACATACA
EMPV1_05385	GGAGGTCATCGTGAAAGCCATGAGTGATTACTGGGTTGTTGGCAAGAAATCTGACCAGCG
EMPV1_05386	ACGTAGGCAGATGCACAGAGCCAATTTATGAGGCTCCACCTGTGTCACGAGAGCTTTAGC
EMPV1_05387	TGGATGGGATTAGAGGCAGGGCTGTGTGCCTGGGGGGTCTTTAGGCAGCTGTTTACTGAT
EMPV1_05388	AGTATTCACAGACTTGGCTGGGATGTTATCTAGACTATCTGGCTTTGGATGCCAGCTCTG
EMPV1_05389	GGGCTGAGCATTTGGGTCTCTGTTCCTCGGGTTCATCTGCTGTGTCATCATCACTTAGTT
EMPV1_05391	AGCAACAGCAACAACATCATGGGAGCTCTGGTCCCCCTCCACCCGGAGCGTATCCTCACT
EMPV1_05392	GTCAGTGACCGCCATGGATGGCAATACCTATGATGATTAAATGAGGAGGAGATGTGTATG
EMPV1_05394	GTACTTAGTTCTGTTGGCACTACGGCTTCTCGCTTTGCCTTGTTGCAAGTGGATAGTGGC
EMPV1_05397	GGTGGGGTAGGGCAAGGGAAGCTAAGACCCCTAAAGCCCTCGGGTTTTCAGTTACATGTA
EMPV1_05399	GTTGGTCTCATACTGGAAATGTCAGCTCTTTAGCAGTGAATGAAGCTTTGATGAATGAAG
EMPV1_05401	GATCTGTGCAGGAGAAAGTTCTGTTCTAGTCATAGAAAGGGTTACACCAAGCTGCTGCAG
EMPV1_05402	AGCAAGTGGTGGAAGCACAGAGCCCACAACTCCTGATAGTGAAAGACCTGCTCAAGCTCT
EMPV1_05407	TATGTGGAAGAAGGTTTGCACCCACAGATCATCATCCGAGCTTTCCGCACTGCCACTCAG
EMPV1_05408	AGCTGGGTAGATGGGTTTTCTGTGCGTGTGACTTTGGCTTAGTGCGATGTCCAGAGCAGA
EMPV1_05410	ATCAGCAGGGGTCCCTGGCTGGCAAGACCAAGCACCACGCCAGGGGGGTTGCTCAGAAAA
EMPV1_05411	CCAGACGATACTGGTACTGGGCAGTCAGAAGCTCACGGAACTAAGAGATGCAATTTGCTG
EMPV1_05413	GGCCAGAACTTGTCCAAAGCCAGCGAGTTAACACAATAGGGGAAAATGGAAACCAGTGGG

EMPV1_05414	GGCCAAGTGTAAAGCCAAGCTGCAAGTGTCAGTTATTAAGCTTGTGGAGGAGCTTGAGCA
EMPV1_05415	ACATGTGCACCGGAACATCCTCTCCAAGTTCACGGGCCAGGCGGTGGAGCCATTTGACGA
EMPV1_05417	GCTCCTGGAGATCATTAAAGAAGAAGGCCTCCTGGCACCATATCGAGGGTGGTTTCCAGT
EMPV1_05418	TTCACGAGGCCTGGAATGAAGCCACCAACGTTTACCTGGTCGTGATCCTTGTCAGCTTTG
EMPV1_05420	GGATTTTGACCACGATCTGGATGATGTGTTGGAGAAAGCCAAGAAAGCCAGTGTTATGGC
EMPV1_05422	TTACCCTGGGCTGGAAACGAGGCCAAAAATGATTCCAAAGGATCCCCCGATGAGGACATC
EMPV1_05423	TGGCCTTGCTGATTGTCATCGCCTTGTTCATCATTTGGAGGTGCCAGCTGCAGAAAGCCA
EMPV1_05424	GGGGTGGAGAGACCATGAGTGCCTCTCTGTTTGCATCAATTTGAACTTGTCTCTGATCCC
EMPV1_05425	GCCGTGTACCGACACTAAATATACTGACACTGGTTTTCCTCAAGACTAGCCCTCCATGTG
EMPV1_05426	TCCCAAACAACAGCAACTCCAGAATGGAACTCAGCCCCGTTTCTTTCCTGTGGAAGGTTC
EMPV1_05427	GACTATGAACAGATCAGAGAACTTCTTTCCTGGTTCCTCATTAGCATCCCAAGTTCATGC
EMPV1_05428	ATCAGAACATAGTCGCAACCTAAGCCACAGCTGCAGCAACACTGGGTCCCTAACTCATCA
EMPV1_05429	GCATGAGAAGCAGTTGGGTAGAGGCATCTCAAGAGTTCATGTTTCTCTCCTGGTCCTTAG
EMPV1_05431	ACGCACTGAAGGTGCTGTCCAAACTTGGTTGATCTGGGGCTTGCCTGGCCTAGTACTCAA
EMPV1_05434	TGGAGGAGGTAGTGAGTCTCATGAACGAGGACGAGAAGACCGTGGTTCGGCTTCAGGAGA
EMPV1_05435	CACACCTTTTTGAAATCTGCCCTACAAAATTTGTTTGGCTTAAACGTCAAAGCCGTGACA
EMPV1_05436	AGGTATTCATTGGTCCAAAGGAGGGACTCAGGACAACACACCAGTCCACCGCTTTTTCTC
EMPV1_05437	CGGGCCCAAGGCAATGTGTATCATCTGAAGTGTTTTACATGCTCTACCTGCCGGAATCGC
EMPV1_05439	CTGTGGAACAGCATCTTTTTGATGTGAATAGTCCTGGAGGTCAAAGTTCAGAGGACTCGG
EMPV1_05444	CTTGAACCATGAAAATTGAACACATGGTGGAGTTCTCATTGAGGCTCAGCAGAAACGACG
EMPV1_05445	AACATGCTGTGTGACTCTGGCCCACTGCGCAACCTCTCTGGCCCTAAGGAACAAGGGAAA
EMPV1_05446	TTCCTCTCCAGGCCCCCCAGCTGCTCCTTGTAGAAAGCATCACGGCGTCTCAGCTCTGCC
EMPV1_05447	ATATTTGTGGACTATGAACTGGAGAGAACCCTCAAAGGGTGGATCCCTCGGCGATTGGGA
EMPV1_05449	CTGGAGCATCGAAAGCAACAGAATGAGGGTGCAGAGGATGAACAGGAACTTTCTGAGGTC
EMPV1_05451	GATCTTGGTGTCATATGCCCTTATTTTCTCTTCCATCCTCAAGGCCCCTTCTGCCAAGGG
EMPV1_05453	GGCAAAAAGGATCTTTGGCCATCTTCTGCTGGCGTTTTCGAGAAGAACTTTGTCAGACAC
EMPV1_05454	CCGAGGTGCCTGTTGTGACACCTTCCTTAATGACATAATGGTGTTCATTGCAGCTGGGCT
EMPV1_05455	GCTCACAATCTTCAATAGCCAAGCAACCATAATAATTGGCGGGAAAGAGCAGGGCCAGCC
EMPV1_05460	TTCCTGGAAAGAATGGTACAGGAGGGCTTTTTCAGGACAAAACTCCTCTGAGAAAGGCTA
EMPV1_05461	AGAGCACCCCAGACCCTGTGAGGCCAAGACCATCTTCTAGGTGGGGAAACCGAGGTACAA
EMPV1_05462	GACAGTCTGAGCAGTTTAAGGAGACTGGTTGAAGCTCTGCCCAAACAATCGGTGGATGGA
EMPV1_05463	GTGCAGGCTACTGATTTAACACATTACCTACCTTCTTCGCCTCACCTCCCATTTTGCCCC
EMPV1_05464	GAAAGCATCCTGGCTTCTCTCAGTGGCCTTCCTATTCCTAATGAAGTGCTACTTAAATGA
EMPV1_05466	TCCCAACCTGATCATTCATTTTGGATAGGGCTTTCTCACAGCCAGACTGAAGGGCCATGG

EMPV1_05467	AAATGGGAGTATCAAGGCCGCTGGGTGCACTACACCGAGTTTTTAAGGAGGGAAGACGTG
EMPV1_05468	ACATTTGGCTTGAAGGCCAGTGGGGTTTGATTACAGGAGCTCCATGGGACTGGGGAAAAT
EMPV1_05470	TTCCTGGGAATGGTGCAGTGGTTCTCAACTTTTGGTGCACATCAGGCTCACACAAGGAAC
EMPV1_05472	GAAGTGGGCGAGTCACCATGAGATAAATGATGCCAGTCGTGGTACTTTAAGCAGCTATAG
EMPV1_05475	GACCAGAAAGCTACCATTTGGCCAAAGGATGCTTTTGAAACTCAACAGGACTTCGAGGAG
EMPV1_05476	GAGCAAAGCAGCACTAAGTCAAATGTGGCCAACTCAGTGGCCTGGTGTGGGCTGAAATGA
EMPV1_05477	AAGGAAGGGGAGGACCCTGATGAGACTGAGGCACTGCCCTCAGAAAAGCCCTTGGGATAT
EMPV1_05479	AGCCAGAAACGAGTTGATCCCTGGTCTTTGCATTTTCTCACCAACCACAGAGCTGAAGGG
EMPV1_05480	GAATGAAGGGCCTGGACGGCTAACATTTGTGGATGACTGAAGATTGCTGGAATGCGACTC
EMPV1_05481	TCTCCCATGTTTCTTCTCCTGATCACTGGGGACCCTGAGAAATCCTCCTTCTCTGCCCCT
EMPV1_05483	GTGGAGCCCAGTGACACCATCGAGAATGTGAAGGCCAAGATCCAGGATAAGGAAGGCATT
EMPV1_05485	TTAACTTCATCCTCCTGAAGTCTGTTGAGAAACATCTTTGAACTGTGTGCCAGCCCAGAG
EMPV1_05487	CTTGGCTTCTGTCGGATAAGGTCTTTACTGGGCGAGATGAACTTGATGCACCCTTCACTG
EMPV1_05489	CTGGCCTGGGCTTCCACAGCCTCATCTGTTAAATGGGTACAATGATTCCTTACTCTCCTG
EMPV1_05493	TCATGGACATTAGTCAGGTTCGCTACCACTGAGCCACGACAGGAACTCCCTCTTACATGT
EMPV1_05494	GATGGATTGACCATGTGGCAGATCCTCTCCAGAAGAAAATTATAGAAAAAGTTTGTTCAC
EMPV1_05496	CTTCGGGAGAGAGTCCCATCTGTTCTGCAGGTGGCATTCTGCTGAGTACCAGTCGGCATT
EMPV1_05498	CCTGCCAGGGTGGTCAGGCTAACAAAGCTAAACAGTCTTAGAACTGCAAGAACAACAGAG
EMPV1_05499	ACTACACTGAATTGTGTGACCTACGGTTTACTGTCCCTGGTGATGTTCTGTCACTGTGCC
EMPV1_05502	TCTGAAAGAACATTCCTCCCAAAGGGCTGAAAGCACCCTGGACAGAAGTATGAGCTTGAG
EMPV1_05503	AGTTTGTTCTAGGCACAGGTGGTGCCAACGCCACCAGACTGACAATTGTGTACTGGAAGT
EMPV1_05505	AAGGCAGAGAGCTGCTTTGCTTGACTTCAGCTGGGGACACACATGGCTTGAGATTCAAGG
EMPV1_05506	ATCTTTTTGGGAAGACAGATGAGAGGGAACCACTGACCAATGCAGTCCGAAGCGACTCCG
EMPV1_05507	TACCCCAATCTATAGAAGCCCATGATGAGGTTGTACTAGTAAGATTGTCTGTGGAATATG
EMPV1_05512	ACATCGAGCTGAAGGTTGAAGTAGAGAGCCTGAAACGAGAACTCCAGGGCAAGAAACAGC
EMPV1_05513	GAGTCTAGGTGAAGGTCCAACTATTGTTACTCTGGAAAGAAAGGAGATGCAGGAACATCC
EMPV1_05515	CTAGTGGTTCGAGTAATTTTGGAGGTTTCCCCACAGCAAGTCACTCTTCTTTTCAGCCCC
EMPV1_05516	TCCACCCAGAAGTTCCAGGACCTGGGTGTGAAGAACTCAGAGCCCACGGCCCGCCATGTA
EMPV1_05518	TTCACACCTTGGTTTTGAAGCTCTGTGGAGTAGGCAGGTGGGAGAGGACCAGAATCAAAG
EMPV1_05523	GACTGTCCTGTGCAGCATCTCTGGCCCACTGTAGTTATGACAACCAAAAATGTCCCCAGA
EMPV1_05525	CTGCCCTCCACAGAATTGGGTTCCAAGGGCTGTTCCAGACAACTGCCAATGTCACTGAGG
EMPV1_05527	AGTCTTCTCCAACCTCTGCCAGATTTTCTCGGAGGAGGATAATTACTCACAGAGTCGGGA
EMPV1_05528	GCCACGGGCAGTGGTAGGAAACCTGTCTTTGGGACTGGAACGAGACTTCGGGTCGCCTTT
EMPV1_05530	CCCTGAAACCCATGCACACGCTTTGGCTTATTCCTGCCAAAGCAGAAGCTGAGTTTCTCA

EMPV1_05531	CACAGCACTGTGGACTGCTGGTTCTGGTTTCCTGGATTATCAGTGTTGCATATTCCTTTT
EMPV1_05532	ACAACTGCACTGTTAAAGTCACGGGTATAGACCCAGAAAAGCAGGAGGAAGTGGTCAGTG
EMPV1_05534	GTTAACACTTCAGGGCAGTTGGTCCCTCATAACAGATGTGCCCCTCCGATGCCTCGTGTT
EMPV1_05536	ACGGGCACATGCAGGGCCACCTTGTAGTTCAGAGGAGCAAGTAGTGGAGTTTGACCAAAA
EMPV1_05538	CCTTGCATGTTAGGCTGCATGACTGAGACCATAGGAAATCCTTGTTGCTGCATTGGCATC
EMPV1_05539	TTAGGCTTTGTAGGCCATAAACTCTCTGCTATAACTACGTGGCTCTGCCATTGAAGTGGG
EMPV1_05540	CAGGACCAAATATTGAGACCGGGGTATGGTGGAAGTTTGGAAAGGAGAGAGGAGCAGAGG
EMPV1_05541	GGCTCTACCAACTGGCTCCACAGCAGCAACTCCAACAACCCATAATTTCACACCTTCAAC
EMPV1_05542	AGGGTGTATGCGCTTCACCTGCGTCGCTACAACGATGCGCTGCTCATCCACGACACAGTC
EMPV1_05545	TGTTCTATGGGACGAGTCTAGGGGTCTACCTCACTTCTGCTGTTACCCATTCTCCCCATA
EMPV1_05547	TGTTCCGGATGGCGTCCAAAGGCGGCGGAGACCTTCTGGAGGTAAATCTGCAGAATGGCA
EMPV1_05548	GAGCATAGAGGAATAGGCAGTATAGTCAGTAGTGGAGATTTGCTGTTGTGTTGTGGCTTT
EMPV1_05551	CTTCTCCTCATCCGTGGGGGTGACCTGCGCCACATAGGAGGTGGTGGCAGCATTCTCCTG
EMPV1_05552	TCAGCAGCTGTCACCCTCATGGTGCCCTACTACGAGATTCATCCTTACAGTCCGTTGCCA
EMPV1_05554	ACACAATGAGGTTAGTACGCTTTTGACACCAAACCCGCAGACATGGCATCGACACGCACT
EMPV1_05555	ACTCTTTTAGTATTAAGTAGTACAATTGACTTCCAATCAATCAGTTTCGGTAAACTCCGA
EMPV1_05559	TGTCGGACACGGGTAGGAGGAAGTCTCAAGGGCTCCGCACGGTGGTTCCTACCTACTTCT
EMPV1_05560	ACCACCACTCTGACACGTGCCCAACCACTGAATCTTTTCCCTCAAGAACATCCCATAGAG
EMPV1_05561	AACACCAGTGGGTCTGCCAGGGTCAAGCTGGGTCACACAGACATCTTGGTGGGAGTGAAA
EMPV1_05562	ACTAGTCTGCCATCTTCTCCATCCGCTGGCTTTTGGATTAAAGTCATTGTCCCTTGCCCC
EMPV1_05563	CTTTGCCACATGGTCATCTGCTGGGATCAGCCAACATAAGTGATCCCTCGGCATCAGAAA
EMPV1_05564	GGATGCAGCTATGAAGATAGCAAGAGAATTGAAATTGGCACCTTCTCTGAGGACATACTG
EMPV1_05565	GTGCACAGAATGAAAGGAATAGTGCAATTGGGTAGCTAAGAGGCAGCTTTATCTGGCAGC
EMPV1_05566	AGCACGGGCAGTTGCAGTGTTGGAGCTAAAGCCTCAACAAATAATGAAAGCTCTAATCAC
EMPV1_05567	AAGTTATGGTGTGAGAGTACAGGGGGTTGAGGGAAGGCAAGAAACATTCTAGTCAGGGGC
EMPV1_05568	CACCTGAGCATTCTCTTGGATGGGAAGAGCCAGAGAAGTGATGGTAATCTCTGGAGCGTT
EMPV1_05570	GGCTTGTTTTATCTTGGAACTATCAAAAAGATAAACATATTGAAACAGAGCTGCTTCATC
EMPV1_05571	AACACTAGCCTCATCAACCAGAAGAAAAAGATGGAGTCGGATCTGACGCAGCTCCAGTCA
EMPV1_05572	CCAGCTGCCCATAGCAGAAAGCTCAAAAACACATACTTGAAGTGGCTTGCCCACCTTCTC
EMPV1_05573	CAAGTTCGCTGCTAAGCTGACTTTGGGGGCCAGTGGCTGCTGATATACATTGGCTTCACT
EMPV1_05575	TGTACCTCGGGGAACAGAACCGTGAAGGCTAATTAGGACAAGTCGGAACAGGGAACGGGG
EMPV1_05576	GGGCGGAGGCGCAGTGCCCATCTGTCGGGCCTATATTCAGATATTTATGGTAGGGAGGAA
EMPV1_05577	CAGTGACAGCCAGGGGCAGAAGCACATTCCTCACACTTTGCCCTCAACTAAGAGACTGTA
EMPV1_05579	TGGCTCACTTCTGGAGTTCAGATGCGTACTGAGCTCCCCCACGGCTTAGGAGTACCCTAA

EMPV1_05583	GTACCTAGGAAGGTCAGTCAGGTGAATAGGGTACAGGTGAAGCAGGCACTAATTGAGCAG
EMPV1_05584	CCACACCTGTGACATATCCTGGGCCTACTTCCAAAAGCGTAGTGGCTTCCTCTGCTTTCA
EMPV1_05585	TTCATGTACTCTTTGCCTGTGCTTCCCAAGCCTGAGTTTGCATCAGAATCCCCTGACGGG
EMPV1_05586	GAACATATCCGCATGGCCGACAGGAAGACACTGCACACCCTAGAGATTGTCTCAGTCACT
EMPV1_05588	ACAAGGTGGCAGCCCTATGATTGAAGGAGTTGATGATGCCAAGGAGATGGCACATACCAG
EMPV1_05589	AAAATTCAGCATCACTCAGTGGAGCTGCTTCAGAGGAGACCTGTTCTGTGTTCTACCGCC
EMPV1_05590	CTCCTCTTGATAGCCCAGGAGTGGGAGGTTGATCGAAACTGACATGATTCCTTCCTGTTG
EMPV1_05591	TGAAATCCTAAGTAATGCCCTGAAGAGAGGAGAGATCATTGCAAAGCAGGGAGGAGGTGG
EMPV1_05592	GGCCCAACCCTGCTAATTTGAACTGTGTTCTGAGCCCCAACTATAGGGCAGACTGTTACA
EMPV1_05594	GGGCTACCTACCACTTGGAGGATCTCAGACTTTTGCCTTAGAAGAAAGATGGAACTCCGG
EMPV1_05596	AAATGCCCTTTCTTGGAGGGTAGTGGTGAGCTAGGCAGCAAAGCTTCAGGAGCTCTTAAA
EMPV1_05597	GTAGACCCCTGACATTCCCAAGTGACAAATCCAGAGCCTTTCAGAATCCCCAAAGTCACG
EMPV1_05598	TTTCCCCTTCACGTCGAAGCCCGGGAGGGGCTGCCCGTACTTATCCACGCACCAGCAGAA
EMPV1_05599	CCTGTGGTTCATACATATTTCAGGAGAGCTGAGATGATGCTGCTGCCATTGAGGGCCAAC
EMPV1_05600	ACCAGTCCCTCGTGTACACCCTCGACACTTCAGTGAAGGAGATACTCCTTTAGACATTGC
EMPV1_05601	GTGGAGTATATTAAGCAAGATGAACATCTCAGGAAACAGTTGTGGAAGCCCTGGTTCTGC
EMPV1_05602	GTTTACCCCGTGTTTTCTAGGCTGTTTCCTCTCACTGGTAGGGACACTGAATGGACTGTT
EMPV1_05604	TAGCTAGTGCTGCAGAGTCTCCTTCCTCCCTTCCAGATCCCCCCAAATCCTCTGAAAACT
EMPV1_05605	AGCGCAGGGCTATGAACGAACTCCTTCCTCTCCTAAGCCTCGGTTCAAGAGCTATGCCTA
EMPV1_05606	GAAGTTCTTCTGCTGAGCCGTTTCTGAGATCTTCTCCACGAGGTTCTGCAGCCTCTGCTG
EMPV1_05607	CCTACACAGCGCAGCATCTCCAAGATGTTGATTTCCTGATCTCTGCCGGAAAAGCCTCAA
EMPV1_05612	GAAAGAAGAGCAGAGAAGATGAAATCCCTTCAGATTCAAGGACTGAGCCCGAGACAGCGC
EMPV1_05613	TCACAGCGGAGGGAGAGAAATGTGCCTTCTGCATGAGAATCAATACCATATGGTGTGTGG
EMPV1_05614	AATGCCAGTGTGAATTTGCAGATGTTTTCAGCAAATCAAGTCACAATAACAATTTGCCAC
EMPV1_05615	CCGACAATGTGGTAGAAGGCAACTGTGCTGAAGAACTACTACAAAACTCCCATCGAGTTG
EMPV1_05616	TAGGTTTTCTAGTAGTTCCTCGGATCCCCAGACAGCCGGCTGCTGGGCGTCTTCCATGGC
EMPV1_05618	AATTGGTGAAAGCATAAACACTGCATCTTTGCTAGTTGAGGTCTAAGAGCCTAGAGACAA
EMPV1_05622	ATCTGGTGTTGCCATGAGCTAGAGTGTAGGTCACACGCTTGGCTCAGATCTAGCATTGCT
EMPV1_05624	GTGCACTATGAAAGAGGGAGTGCGCTTGAGAGAGCGTTTACGAAATCAAACCCGGGCAAC
EMPV1_05626	TTTAAATGTGTTCCTCAGCCACGGTGTAGCGTCATGTTCCAAAGGAGATGACCCGGCCCT
EMPV1_05627	TTACTTACAGTGGAGGGCTTGGCATGTGCTGGCCCCAGTGACTTCCCAGGTTTCTACCAT
EMPV1_05628	AGGTAACCTGGGCTCCCAGCTGGTGGAATACAAAGAGGAAATGTACATCACGTCAGACTG
EMPV1_05629	GCCTTGGAGATCATATTGTCTCTCAGTTGTCTCTCACTGTCAATAGAGGCCTATTGTCCC
EMPV1_05633	GATCTCTGTGGCAGTCATCTGAGGCTATAGGAAATTAGGTCTGTGGCTCCTTGCTGGTAG

EMPV1_05635	GCCGGGTTCCAGATCAGTGGGTAGGGATAGGGAGACGAAGCATACACAGTAAATTAAAGG
EMPV1_05637	CAGCCCCAGAGAGCCATGAAAGGGTGGCACAGTAAGAGTGGCTTGTTATCCTTGTGGAAT
EMPV1_05638	ACCTACTCAAGTTCCTCTACCTATTAGGCGTGTGGAAGAAACAGCAGGAGAGGTTTGCCC
EMPV1_05640	AAACAGCCAGATCTATAAGGTGAGTGTGGGGTAGGGAAGGTCTGGGATTCAGAGGGCACA
EMPV1_05641	CTTGATGCGAAGTGTGTTGCTGGTGATACCGTTCACTTTCCTGATCAAGCGGCTTCCCTA
EMPV1_05643	GCGGAGGTGAAAGGGGAAGGAGGTTATTTGATCTAGAAGATGAGTATTCTGATGTGGTGG
EMPV1_05644	CATGAAGTCCCAGGCTTGCACCCGAGCTTTACTGCTTACAGTCTGACCCCGAGTAAGTTA
EMPV1_05646	AGAGCCAGCTGTCCTGAAGTGGATAAAGGAGAATATCTCTGAGTGGAGGAATTGCACAAT
EMPV1_05647	CTCAGGCTGACTCTCCAGAGTGAACTGACAGGAGATGAACTTGAACGAATAGCTCAGAAG
EMPV1_05648	TGTTCGTGCAAGGGCTGGTGTGGGAACAAGCAGTGTGGGTGCAGGAGACAAAAGTCGGAC
EMPV1_05649	AAGGGCCCAAAGGAGTAGAGAGAGACCCAGAGTTACAGCTGCCACACTTCCTTTATCAGA
EMPV1_05655	GGAGGTATGACTGGGTGGTTGCTAAGCCCCTTGGCCGTGCAAATATTCTCTGATATTCTG
EMPV1_05657	TCTAGAATTCAGTGGGTGATTAGAAATGGATTTGAAAAGAGCAGTGCTCACATGGAGAAC
EMPV1_05658	ATCTATAGAAACAGGAAGTAGATTAATGATTGCCTAGGGCTGGGTGTGGGGGCAGGGGGA
EMPV1_05660	GACATCTGGCAGCTCCGTGCAGTCGTTTAATGTTGTGAGGAGTTACCTATCTTGTCATGG
EMPV1_05661	CATACACATCTCCTCCTCATTTAATCATCATAGGTATTGCCATCCATGGCGGTCACTGAC
EMPV1_05662	CAAAAGGCCAGGGAACCGACCACTCGTATTCACCCTAAGCGTACCTTTTCCGTCTTCCTT
EMPV1_05663	GCCTGGCACTACAGGACTGTTATTTTTTACAATTCACTGCCCCAGGATCTTCTGCTTCGA
EMPV1_05664	CCACGTCACACCGTAGGAGTACTTTCATCTAAAGTCATAACTTGTGGTTGGGACGGTCTC
EMPV1_05665	GATCACCAACTAATTCAAGCAGACCCCAAACATAGTCTCTTCCTCGCCTGTGCCCTCATG
EMPV1_05667	CGTGAAAATAGGTTCCTAAACTCGGCAGGGTACAAGTCTGATGCGAAGGCGGTGCTTCGA
EMPV1_05668	AAGAAGCCCGGTCCATGGTGACAGGTGGCTGAAGACTGCCTTTGGGATTGGACCTCGTTC
EMPV1_05670	CAGAACCCTTCCAATCCTCTTCAGCGTATAATTCCAGGCCCTAACTTGGAGAATGAACCC
EMPV1_05671	CTGGCCTACACCAGAACAACAGCAATGTGAGATCCAAGCCACGTCTGCGACCTATACCAC
EMPV1_05672	ATGCGAGTGGCAGTAACTCTAGGCCCCCATCCCCTGAGCAGGAGCGGAGATGCTGGTACA
EMPV1_05674	TTCAAGAGAATGCCGGTCATTTACATGGACGTTTGATGCTGCTGCACAACTTACCGTGCT
EMPV1_05675	CTTCCAGGTTGAGAAGCAAGGGAAGCATCTACACTGGGCTGAGAAAGATGGCAGGATGAA
EMPV1_05676	ACTGGCTATGAGGGAATGCAGTGGAAAACGGTGTGTGGATTGCTATGGCCTAGGACCTTG
EMPV1_05678	ACTTACTTGAAGCATGCAGCGGTGAGATTGATCCCCAGTTTGGCTACTCCAGAGGAAACC
EMPV1_05679	TTAAGCCTAAGGTGCCAGTACCTGCCCGAGTGCCTGAGGTTAAGAAGAAAGTGCCAGAGA
EMPV1_05680	CGATATCACACTGGTTGACGTGAACATAAGCCTCTGCATATACAGGGTCTGAGAAACCTG
EMPV1_05681	GTTCGGGCGACACTCCCAGGACGAAATCTCCATCCTCTCACCGCTCTCCCAGTGCTGCCG
EMPV1_05683	CGTTATACACGCGCTGAGCATCCATGAGCAATAAATCAGGGGCTTCACAAAGGTGTTGGG
EMPV1_05685	TGACCTCTTCTCTGGCTCTCCCATCCGCTCCGTGGGTTGAGATCTCACGAGTTCTCCTCA

EMPV1_05686	GGATTCTCCGTTGATGATCTTGGCTGCTGTAAAGTGATTCGTCACAGTCTCTGGGGTACC
EMPV1_05690	GCACTGCACGAGCAGAAGACCCTGCCAGGGATGAATCGTCCCATCCAAGTGAAGCCGGCT
EMPV1_05691	GGCTGGAGGTTTTTTCCTTTTATCACCTTACGTATATTGTGCCACTCCCTTCTGGCCTGC
EMPV1_05692	AGAGGCTGTTCCTGTCCCTTCCTGCTGCAACCTCTTGTGAGAACTCCGCATGGAAAAACA
EMPV1_05695	CTCAGAGAGTGGAAGTCATGCGGCATGAAGTATCTGCAGAGAAGGAATGGAGTTATTCAG
EMPV1_05696	CACTCAGGACAGACTGATGCCATTCTGTAAGAAGACTGCTCCAGTTTGCTGAATTAGTTC
EMPV1_05697	CCGACGAGTGAATTATACTTATCAAACATCTGATCGACATAGGGCACCTGGGTTCTCTGG
EMPV1_05698	TCCGGAGTTTGAGTTACAGAACGAGCCTGGTCATGCAGTGTATTTGGGATCAGCAGTTGG
EMPV1_05706	GTGTAGCAAACCTACGTCTCTTACCCAGTAGCAGTGTTTTTGCATGTTCCGGTCAGACGG
EMPV1_05707	AGAACAGTCTACACCAACAGTCACCTGAAGAAGAGACTGATGAAGGAATAACAGAGTTGG
EMPV1_05708	TTTCTGCCTGGAGAGAGGGCTCTGGGGATGGAAATGAGGAACAGCAGGCCTTCAGACAAT
EMPV1_05709	GCTAAAGACACAGAGAAGATAAGAGAATTTCTTAAAAAGGTGTGCTCCTTACCCTGTGGC
EMPV1_05712	GCCTGTCATTCTCCTGGTCATTTGTGAAGACAGCATGTTGAAGTCTGTGCTTTGCCTCTC
EMPV1_05714	CCAGCTTATTCATTGGAGCTGTGATCCTGGCCATGAATTTCAGTTCTGAGTGGTCTGTGG
EMPV1_05716	TGAAAGGGCAGAGACAAGCAAAGGATACAGTCTTCCCAGAACTGTCTTTCCTGTAGAGCC
EMPV1_05717	AGAATGTTTGTTGTTGCAAGCATAGGGCTTCCTCTCCTTTTGGCCTTGAGGCAGGGCTCA
EMPV1_05720	AATCATTCCTACCCTGCACTATGTCATCTCTGAGGGGATCCTGAATGCTGCCTCCATAGG
EMPV1_05722	CAGAAGCCCTGGGCCAAGAAGCTGAAAGAGGTAGAAGCAGCCAAGAAAGCCTACCACGCA
EMPV1_05723	TTAAAGCAGCACGTCATCGATGGAGAGAAAACCATTATCCAGAATCCCACAGACCAGCAG
EMPV1_05724	TTCAAGTGCATATTGGGGTTTTCAATTCTAATACAAGCTGTTAACAGGGAAGCTCTATAC
EMPV1_05728	GAGCTATCTTTACATGTCTTTTATCATTTTATTGTCTCCAAATCCTAAGAGATAGGCACC
EMPV1_05729	CCATATCAGCTCCCCCTTTGAGCTCTCCATACACAAAGATGACTTGACCCTGGAGTTCAC
EMPV1_05730	CTGGGGAGAAGTCCCATCCTAGCCCAGAGACCTATTGGAGCTTGAGTTGCCTTGTTGATA
EMPV1_05732	TAACCGCCCAGCCGCGCCAAAATAGTTGGTGATGGATGACTTGAGGTGGGACAAGGATGA
EMPV1_05734	ACCTGCCATTCCCACATCTCCAGTTGCCTCATCATCCCTGAGTGTGTCCAGATTTCTGAA
EMPV1_05737	AGGAGAGTGTAGGGCTGAGAGCCGAGGGCCACCCTGATAGTCTCAAGGACAACAGTAGCT
EMPV1_05738	GTCCACAGCATGGACAAACCTCAGTGTAATGGTGAGGAAATGAAACCAGGGACAAAAGAA
EMPV1_05739	GGGCCCATCGAGGTGGATGTACTAAGACCGAAAAGGCTGTAAAAGATGATGGCCTGATGG
EMPV1_05740	CGCCGACTGGTTTGGAGCCCGTCAGAATGAACAGGAAGAAAGGAGACAAGGGCTTTGAAA
EMPV1_05741	AACAAAACAGAGAATGGTCCTTGCAACAGCAAAGGGAGTGGAAGATTGCCCGTGAGGACC
EMPV1_05743	CCATGTTCCTTTCCAGGAGGTAGGGTACAGGAGGTGGGTTAGAGAGTTTATGGTAGATTC
EMPV1_05744	CTGGTGATGTGTAGAAATTCATCCCTCATTTAGGTACAGAAAGAGACCCAACCATTTCTG
EMPV1_05746	TGTGTCCCACATAGGTGCCATTTTAGCCTTCTACACACCTGCGGTCATCTCCTCGGTCAT
EMPV1_05747	TTAAGCTAAAAAGTGAAAACCATCGTTCCCAGGGGAGGGAACCGAATGAGCCAAGGCGCC

EMPV1_05748	CTGAGGTCACACCCTGCCTCTGCAGCATATTTATTTTTTGTGCTGGATGGACTCTGGTCC
EMPV1_05752	GGGACAGAGGTGTGAAGCTGTGATAAACGTTTTCCAGTTTCATTCTTGTTGGTGTTACCC
EMPV1_05753	TCCTTCGCTGCAGATGTGATTTTCCTGTGCCCGGAGTCTGCTCCCTGGTTGGCTGAGGCA
EMPV1_05754	GGGAGTCCATTAACCTGGCAAACTTCTGAATCTAAGCCAACTGCCCCAACTATACCTGTG
EMPV1_05755	GTTTTAATTTGGGATGTTCCTAATCTTTTTCATCATATTTCATCTTTAATCATCAATATT
EMPV1_05756	TTCCCAGTGCAGTCTACTGAGGTAAAGATGGTGGCCTCTTTCCATTTGATTTATGTACAC
EMPV1_05758	CACCCAAGCCCTTTTGTGAATGAGGTGCCTTTGTCCTGGTGCTTTGGATTGAGAAGCAGG
EMPV1_05759	GACAAAGTTGCCATTGAAGTTCTGGTCCCATTTCCGCTGGGGCTTTTGTCTTGTTCTCCG
EMPV1_05764	ATACATTGAGGATGATGAAGAGCAGGTGGACATTGAGACTGTGGAAGAGCTCCCTGAGGA
EMPV1_05766	TCCAGTGCAACAGCCCCAGTTAAATGAGAAAGTGCTGAAAGACAAGCGTAAAAAGCTACG
EMPV1_05769	GTGGGGGACTGTGTCTCTGTTATTCCAGATGATTCTTCAAAACCGCTCTACTTAGCCAGG
EMPV1_05770	GGACAACCTGAATTCCATCGCTTGGGCAACCCAGTCACCTGCTTCTAGCCGTTAGCTTTT
EMPV1_05771	TAGCCATGTGACCTGTGGGCTTCCCTTTGTCATCCTTTGCCCAGGGTTCTGAGTGGGGCA
EMPV1_05774	GGAGTCCTCACTCCTTCTATTTCTTTAGCGCTGCCCCTGGCACTGTGTTATTTCTACCGC
EMPV1_05775	GTGAGAAAGCCTTCAGTGACCATTCAGCCCTCACTCAACACAAGAGAATTCATACTAGAG
EMPV1_05776	GGTCTACCTTCTGCTTCCCTGGAAAGGATGAATTTACATCTTTTGTCAAGGCTATATCAA
EMPV1_05777	TACGAGGAGACGTCGACGGTACGCGTAGAGACCTCGTCCCACCGCGTGGAGACATCCTCC
EMPV1_05778	ACCACGTTGCTGAAGGGTTTGCGGACAGCAAGAGGCAGTATGAGACCAGGTTCCATGCTC
EMPV1_05782	CTCTGCAGCGGGTTCAGTTTCTGATGCCTTCACCCCATAGATGTGCATGTACCCATGTTT
EMPV1_05784	AGATTTCTGTGTCTGTGGCGACTCTGGGCCTGGGCCTCATCATCTTCTCCCTTGGTTTGC
EMPV1_05788	GGGACTTGGATCGTCTACGAAGTCTCTGCCTGTTGAGATCCCTTATCTCACATCAGCCTT
EMPV1_05789	GGATCCAGTGACATGGTCTGAAGCTTTGTGGAGTCAGAGACCTGGAGAAGCTAGGCCTCA
EMPV1_05790	GCTTGGTACCTTGTCTTTGGGTTAGCAGATGTGCCTGTCAGCTAGCGTGCATGTTATCCA
EMPV1_05792	AGAGCAGGTTCCGGATATGGCCCCAGGTCCCCATGCTTCTGGTGGACTGACTGGGATGGA
EMPV1_05793	GATCTGAGGGGATTGGGGTCTTGTATTCCATTATCTCATCAGCAGGCAGGGAAGCAACCA
EMPV1_05796	TTTCAGGGGCTCCTTCTGGGAGGGGTACAGGAGTGTGCCGCAGCCGCCGGGCGTGGAGGG
EMPV1_05798	ACACTGAAACCTTGTCGTTTGTAACCCCTTCCGCCCAAGATGGACTTCCCAGTGATAGAA
EMPV1_05799	CTGGACATTAGCTCGGTTCTATTCAGAAACAAAAAGGAGACCCTTAGCTTGGGAACCTCC
EMPV1_05800	CCGGTGGTCACCATCGTCCACGACTGCCAGGTTGTCGACATCCCTGAAGCGCTGCTCGAG
EMPV1_05801	AGCAGCCACGAACCAAAAGGTACGGGAGCAGGTACGCCTGGAACTGAGCTTCTTGAACTC
EMPV1_05803	GCAACAATGATTGCAGCCGGGGATTTGCAGGAATTTGTTCCATTTGGTCGAGACCACTGC
EMPV1_05804	CCCCAAAGTGGAATTGTTGAAAAGTGTGGCGAGTCTGTGCTCGAGACAATAAAGCTTGTG
EMPV1_05805	GACTTCTAGTTGAAGCTGTCTCTGAAGTTCAGCCACTTCTGAGGTCAACTTATTTTTCTG
EMPV1_05806	ATCTAGACAAGTTTGTGGTGAGTTCCAGCCGCCAGGGCCAAGGCTCCGGCCAGATGCTGT

EMPV1_05807	CAGATAGAGCAGCTTTTGGAGCCATCCCGAGGCCCGGAATTTGTTCGGACTCTGGCAGAG
EMPV1_05808	AAAGGCCATGTAGGACAAACCCACAGCTAACATCATCCTCAACAGTGAACAGCGGAAAGC
EMPV1_05809	TGTGCACATTGCCCTTGAGATGAGCATGTGCTGGGCAGGCTCTTGCCCTCAGCATCACAG
EMPV1_05811	GGTTGTTTCCTGATACTTAGGTCAGAAGATGTCACAGAATCCCATAGACCTGTGGCTTCC
EMPV1_05813	GACAATATTATGGAAACACTTCAGGGTCAGGTTTCAACAGCTCTAGGGGAAACACCCTCA
EMPV1_05814	GTGATTTCAAGTGGAGGCCTGATTACAAAGGGCTAGAGTCAAGTTGCAGAGCTACTCGCC
EMPV1_05816	TTGACAGTGCACACAGACCTCAGAGAAGTTGCAGGTTCAGTTCCAGGCCACTCCAATTCA
EMPV1_05819	ATCCAGGGTGAAAATGCAGACATGATCCAGAACCAGGAGGCACCAAAGCAAGAGGCTCCT
EMPV1_05820	AGGCTGCTTTGTGTGGCTGTGGGACAGCTGGATCGGCCCCCCCATCTCACCGATGACGGG
EMPV1_05821	GGCCGGGCGAACTTTAGAACTGTGAGGAGCTTCATTTCTCGTATCGTGGAAGTCTTTGAG
EMPV1_05823	CAGAAGCCCTCCGTCTAGCTGGGGAGACACACACATGCCAACAGGTGATTTTAAAGATAC
EMPV1_05824	GGGCAGGATGGTTCCGTGGCACAGTTTAAGATTAGGAGGCATACACCACTTAGTAAACTA
EMPV1_05825	AGAAGCCCCGGCTGTCATTTGCAGACAGAGCGCTGATCAACAGCATGGACCAGAACATGT
EMPV1_05827	AAGAAGCTCCTATGGAAGCAGAAACAGAGGTGCAAGATCCCTTTCCTCTACACCCAGGGA
EMPV1_05828	GGCACAGGCTCCCACCTGGACTGTTGTAAAAGCTTCCTAACTTTCCTCAATCTTGCTGCT
EMPV1_05832	TGTGGTTTGGCTACGATGTGAAGCGGGCAGTGGAGGAACCCCGGTTGCACAACCAGCTTT
EMPV1_05833	GCTTGGTCCTGAACAGAGAGCCCATCCGTAAGATCTACATCGAACTCGTCTGCGTCATCT
EMPV1_05838	GAGCCCTTGGGCCACATCTCCTTCAAGCTGTTTGCAGACAAAGTTCCAAAGACAGCAGAA
EMPV1_05839	AACCTCAAGAAGGACATCCGGGGTGTTCTTGACCAGATGGAAGACATCCAGCTGGAGATT
EMPV1_05840	TGAGGCATCACACCTGGTGCATTACAACAAATGCTCAACCATCACATCCTGGGTGATCCA
EMPV1_05841	AGATCACCCGCCTCTACAGCCGGTTCACCAGCCTGGACAAAGGAGAGAATGGGACTCTCA
EMPV1_05842	GAGTTCAAAGCCTGCCTCATCAGCTTGGGTTATGATATTGGCAACGACCCCCAGGGAGAG
EMPV1_05843	GCCATCCCAAAGTTTAAATGCTAAGCCCATACCAAGGACTCCTGACCATGAAATACAAGG
EMPV1_05844	CAGCCATTGTCCGCAATCTCCATTATGACACCTTCCTTGTGATCCGATATGTCAAGAGGC
EMPV1_05845	GAGAAAGACTGTACAAAACAGGAGGTTCCCACTTCAGGGAGAGAGAGGCCATGTGCTGCT
EMPV1_05846	TTTGAGGTCAGCGCCCTTATCTACATACCCTTGTCTCCCCAGTGCCTATCACAGAACCAG
EMPV1_05847	GGAAGCCTCAGCTGGATGAGTGTATACATTCAAAATACTTCCTAACAGAGTGAACAGGTG
EMPV1_05848	CCAACAACTCTTGTTACTGCGTTATCTTTAACGTGGGTAGGGCTGGATTGGAGGACACAG
EMPV1_05849	ATCACCCTTTGCTAAGGCAACTTCAGAAACTGATCCATTATAGGAGTTCCCATCGTGGCT
EMPV1_05850	GGAGTTTGCCAGTGAATTTCTGGTAAGTCCTATATGTGACCCGGTCGGGCTAGTGACATA
EMPV1_05851	TTTACCCCTCAACCCTCTCCTGAACAGAACTCTGAAGGCCCTCTTGATGAGGCTGGGTAA
EMPV1_05853	GCCAGGGACTGAATTTACATCCTCATAGATACCAGTAGATTTTGTAACCTGCTGAACCAC
EMPV1_05854	CTGCTCCTGAAGCATATGGAGGTTCCTAGGCTGCTAGGGGTTGAATCGAAGCTGTAGCTG
EMPV1_05855	GGGGAAGCTGAGGTTTGGAAGAAGGCCACATAGTTTTAATAATGGGCAGAGCTAAGAGTC

EMPV1_05856	GGTGCAAGTCTACAAGGTGTCAGGTAACAAAATGGGGGTAGGGAGTGTGTCTCTTCATCC
EMPV1_05857	ATTTCTCCTCCCAAGCCCCTGCCCCCCACCCACATGGCTACCAAATGTTAATAGATCCTA
EMPV1_05861	CGCTTGCTTTACCTTCGGAGCTGGAGCTCCGTCTCTGGGGGAGAATAGCCCGCGATGTGA
EMPV1_05862	GGCTGGCTGGTCCCTGAGCCAAGGGCCTCCTCCTCAGGGGAGCTCCACCCAGGAAACTGT
EMPV1_05863	GACAGGCTAACCTACTTTGAGAAGAACTTCAATCTGCGGAGGTTCATGTACACCACCCCC
EMPV1_05864	ACCAGCTGTCCAGTTTGTGCCACCTCTTCCTGAGTGGAAATCATCTGCAGACCAAAGGAT
EMPV1_05865	TTACGACACCCTGCCCAGCTATTCAAATCGAGCTATTATTACTTCTTCCAAATACCGTGG
EMPV1_05867	AGGTTCGCCTGCTGCACCTCCTCCTGGGGGCTTTGGTCCCAAAACAGGAGCCCTTCCCTA
EMPV1_05870	AAAGTCGTTCCAGGAGATGTTGCCGCCGCTGGAGCCAAGCGCGTTGAGAAGGCTCATGAC
EMPV1_05872	GGCGGGTAAATGAGGAATCTGCATTATTTTGGAGGACAAGGAGCACAAAATGCATAATGG
EMPV1_05873	GTTTCCCTTCTACTTTGAGCTGAAGATCGCCTTTGTGATATGGCTGCTGTCCCCTTACAC
EMPV1_05877	AGTGACAGGTCCCCTGGCCCCTCCACGGACACAATACCACAGAGAACTAGAAAAGGAAAT
EMPV1_05878	AGCTCCTCTGGACCTTCCCACACAAAGTTGCACTAGAGATGCAGAACTACCAGCAGCAAA
EMPV1_05880	GGCCAAAAAATAGTGTTTCCCCTTGGTACTGATGTCTTCCTCTGAAGGTGCCTGAAGCTCT
EMPV1_05882	AGTGAGTAAAAACTACCGATCAGTCATAAGAGCGTGCATGGAGGAAATGCACCAGCTTGC
EMPV1_05883	CTTCCAGGTTGTAAATTTAAAGATGTTAGAAGAAATATCCAAAAAGATACAGAAGAACTA
EMPV1_05885	TGGAGTTGTCCCAAAACCAGCTGCAAATGCTGCCCCTCAGACTACCAGCAAGGCTACAAA
EMPV1_05886	GATCAGATTACTAATAAGTATATGGAAGATTTGAAGAGCTGAAGGAAGAAATCAGTATTG
EMPV1_05888	GAGAGCCCAGAAATAAACCCATATGCCTACAGTTATCTAATCTTCAACAAAGGAGGTCAG
EMPV1_05889	CCAGCCAATGAGAATCCATTTCCAACAGGTATTAATGCCTGTTAGAAGGCACTGGAACAC
EMPV1_05892	CCAAACCAACTCATCTCTGCAGACACTTAGCCGGGTGAAGTCCATCAGCACTGGGCCAAG
EMPV1_05893	CCTTGAATAGACCTGCTTGGACAATGGGTCGGGAATAGGGTTTCCTCCTTGTAAGTCAGG
EMPV1_05895	GGGCATTGGTGGACCCTAACTGGAGTGAAGGAAATGAGTAAGAGTCAGAGAAGGTGGGGA
EMPV1_05898	TCGATGATGGGCTCTTTGTCCTCCTCCTTCAATCCGTTCAGCGGCATCGACAACGCCATG
EMPV1_05900	CCCTCACCCTGTATGACATGTGCAAGGCTGTCAGCAGGGACATCGTGCTGGGTGAGATAA
EMPV1_05901	CTGTGGGCTTCTGGTTCTGGTGTCCTGGGTGATGTGTGCCATGTATTCTTTATTACACAG
EMPV1_05902	TTTGCACTAGACATGACCCGAGAGGAATACAATGCACTGCCCGCCTGGAAGCAGGTGAAC
EMPV1_05903	TCCCCCGAGGATTGTGCTTTGAGAACTAGAGGCGATGAAGACTGTCTGGCCTTGAACCTT
EMPV1_05906	GCTCTGTCCCTTCCTACATGGTCCTTCTGGTCTGGGACTCTTTTGTATCTCCTTGAACTG
EMPV1_05907	TTCTTAAGATGTATCAGGGTCCCAGCCCTCTCTCTTGACCTCCTCTGCCCCACCAGCTGA
EMPV1_05909	ATGAAGAGCTTTGTGACCAGGGTGTCAGTTGGGGAGTTTGTAGGGGAAGGTGAAGGGAAG
EMPV1_05911	GCCTGTGCTGACACCAGGTTTAATGTTATATATGGGCTGGTTCTGATCATGGCGCTGTGG
EMPV1_05913	AGTGCATGCTCTAGTGGAGGCAGGAGCCATTCCAGCTTTGATCAACCTACTGGTTTCTGA
EMPV1_05914	CTGCACTCCCCTGTTCAAAGCAGCCACTATACACAAGAGCCAAGATGTGGAGACAAGCTA

EMPV1_05915	ACTCAGCTCGGATCCTGCATTGCTAGGGCTGTAGTGTAACCTGGCAGCTGCAGCTCTGAT
EMPV1_05918	AAGTCATTCAGAGGGAAGTCGGTCCTCAACAAGCACCAGAAAACTCACTCGGTCAAGGTG
EMPV1_05919	TCTCACCATTCGAGGAGTGAATAGGATAGATGAGAACTACAGGCTTCCCACCTGCAAAGG
EMPV1_05924	AGAGAATGGTGCCTGGGGTCTCTGTCCTCTGTGGTCCTGTCACTCGGTGGGCAGGAAAAA
EMPV1_05927	TGCCACACTCCCACCACCCATTCCAAGTCCAGAGTGAAATGTTGACATCATTATTGGAAA
EMPV1_05929	TCACCTGGAGATCCTGTTAAAATGCAAATTCCAGTTCTATGGGTCTGGGGTGGGGCCTGA
EMPV1_05930	GTCTGCTCAGGGCTGAAGAAGGGATTTGAGGTGGTGGAAAAACTGAAAGGAAAAGCCTTC
EMPV1_05931	CACTATATATATGAGGAACCTGAGGTCCAGAGTGGGGAAGTGTCTTACCCAAGGTCACAT
EMPV1_05932	GATCACTAATGACCTTTCCTCCCTGGTGGAGAACACAGATGAGAAACTGTGCACTGAAAC
EMPV1_05933	AGACAGACGAGAGATGCCATCTTTTCACACCAGTGATGTCTGTGTTTCAGGGGCAAAGTG
EMPV1_05934	TCCTCAGAATCGTCTGGAAGGACACAGCAACCCCAAGGCTCCATACCCTGGCCTAGCTCT
EMPV1_05935	CCTCTGAGAAGGACTTTGAGGACTACGTGAGGTATGACAACCACTCCACCAACGTGCTGG
EMPV1_05937	TTGTTGTTTTTATTTGGCCGTGGCTGCAACATGCAGAAGTTACCCAGCCAGGTATCATAC
EMPV1_05939	CCAGAAACATATCCATTTAATCCCCCTAAGGTCCGGTTTATCACAAAAATATGGCATCCT
EMPV1_05940	TCAGTGTTGTTCCAACTGACTCCAGAGTGCTGTCTAAGCTGGGAGGATTATACGACAGCG
EMPV1_05942	CTCATGTGTTCCGAATGCCCACAAAATAGACTCAGATCTCGAAAGGCTGCCAGGTTAGGG
EMPV1_05946	CATCCAAGTTATCAAGGTCATCGTCTTCTGAATCTTCGTCTTCCGAGTCATCCTTAGCTC
EMPV1_05948	CCTGCCGAGCTCTCGCTCGCTTCTGCGGAGGCTGCGTCGGGTTCTTGGCGAAGGGACAAA
EMPV1_05949	CACGGTGGCCCTGCGCGAGATCCGGCGCTACCAGAAGTCCACGGAGCTGCTGATCCGCAA
EMPV1_05951	TGACTCCCCAAGCCCCGAAGAAGATAAAGAAGAACACGTTTCGCTTGCGCACAGAGGAGT
EMPV1_05953	CGGGAGACCCCAGCTCTGCTGTTAGGAATTGTGTGTACGGTGGAGAATAGACCGTGATTT
EMPV1_05954	TCAGCTCTTGATTCCTCTAGTCTGAGGGTTGGGAGGTGAAGAGAGCCATGAGTGGGATTA
EMPV1_05955	GCCTCATTCAGAACTACTTCATTGTCATCCTTCTTGCTGCCCTGGCTGGATCTCAGTGGA
EMPV1_05956	AAAGCCGAGGACAATCATTTCACTCCCCTTGTGGGCCTCTAAGAAGCCTGAGCACTTGCT
EMPV1_05957	CCACCTCAGCACACAATGGTTTTGAAGGAATGACACCACGCACACACAGGCCTTCTTATG
EMPV1_05960	GATTGCACCCTGTCTTGATAAAAATTCTCCTCCGTCACCTGATATGCACCAGGACATAGC
EMPV1_05962	AGACTGAAATACAGCCATTAACCCAGAGGGAAGGTGCACAAGGATGGGTGAGTCCAGCTG
EMPV1_05963	ACTTTGGCCTTCCCAAGTGATCAAGCAACGTGGCAGGGGAATTATAGTTTTGGTACTCAG
EMPV1_05965	TCTCACTCAGAACGTAAAACTCTGTTGTTCCTGCACCATGTTGTCTCCAGCTGCCCTCTC
EMPV1_05967	TGTTCTGACCTGTTCCATCCCGATGGGTGGCTTCATGGCTCATATTCTCTGTGGCAGCTT
EMPV1_05968	CTGAAATGGAGAGATTATAACTGTTATGTGAACTTACCCTATGTCTGCAAATTCAAGGGC
EMPV1_05970	AGTGCCTCGCAATACTTCGTGCTGTTGCTGCTAACTGATGGTGCTGTGACAGATGTGGAG
EMPV1_05972	GAGGACGTACCAGCCCTGCAGTATCCCCTCATCCCACCCATGACCGAATACTTCTTCGAT
EMPV1_05974	CCAAGATGAGCACAGCTGGTTACAAATAAGACCCTTGCTAGTCACTTTCGGCCACGATGG

EMPV1_05977	ACAGCCAAAATAGCCCCCAAGCTAATCCAGGGTACCCAAAGGCCGGATTCTCTGGAGCTG
EMPV1_05979	GCTTCTAAGCAACTTCAGTCCTGGTTATTGGAGGCCGTCTCTCTGTCCGTGAAATTCTGG
EMPV1_05980	TCAGCTACCCCCTGGAGACCTTTGTGGAGAGTCTTAGTGACAAGGGCATCTCAGACATTA
EMPV1_05981	TTGACAAGGCTAAGACCAGATTCTGAGCCAGCGAACTAGGAGGCAAGAAGCCAACAGAGG
EMPV1_05984	ACTGTGCCAAGCTCTGTGAGGTAGCAGGAGTCTGAGTTGAAATCTGGGGTCTGATGGACT
EMPV1_05985	CCCCCTTAAACACCTTTGGAGAAAGCTGAAGTCCTGGAGGTCGCCCAGCAGAATTAGTTC
EMPV1_05986	TGCGACACAGCAGTGACAACATTGGATCCTTAATCTGTTGCGCGAAACGGGGACTCCTAA
EMPV1_05987	AGGGATGACCAACTGTCCTGGTTTGCCTGGGACTGAGGAGTTTCTGACAACACAGAATAA
EMPV1_05988	ACGTGTCCCAGGAGCCTTGCTTTCAGGAGCCTCTCCATAGATCTGGGACTCTCGAGCTGA
EMPV1_05989	TCCTGCTGCTGAAGCACCCAGATGTCACAGCCAAAGTGCAGAAAGAGATTGACTCCGTGA
EMPV1_05992	CATGTTCGCTGTGGGCATCGGGACCTTGCTCTTCGGATACTGGAGCATGATGAAGTGGAA
EMPV1_05994	TTTCTTTGCGGGGGGGGCTCTATCTTGAACAGTGGCTGGCACTTAATAGGCCCTCAATAA
EMPV1_05995	TTTATCTGGCGAACCATGGAACTCTCTCACACAGAGCTGGTCTTCCCCTACGTGGTCCAT
EMPV1_05998	GCTGGGCCCACCTTACCACATCCTGGTTGATACCAACTTTATCAACTTTTCCATTAAAGC
EMPV1_06000	CTTCTCTGTCTTATGGGAACTGAGGCTCTTACTTTTCCCTGAGATGGTATGGCTCTCAAG
EMPV1_06003	GTTGCTTCGTATGCATTAACGTTTGGGTGTAAAGATTGTGTGTCTATCCAACAGGGAGCC
EMPV1_06004	CACCTGCCAGCATGAGTGTAAAAATACCTTTGGGAGCTATCAGTGTATCTGCCCTCCTGG
EMPV1_06005	GTGACAGAGATCTACTCAATCTAGGGATCATTCGTGTCCTGGGTTAGATTTGGGGGCAGG
EMPV1_06006	CTGGGTGGAGTTCTCCCACGAGCGGCTGGAGGAATACAAGTGGAATTACCTAGATCAGTA
EMPV1_06008	GCAGGATGGTTCTTCGGTGCGGTTGAAGATTAAGAGGCATACACGCCTCAGTAAACGAAA
EMPV1_06009	GAGTATCTTGCGGCTGCAAATGGGCAAACTGAGGAACAGAGATGTTGAGTGACTTTTCCA
EMPV1_06011	AACTGCAACTTGGGTCTGATCCCAGAGAACTTCCATGTGCCATAGGGTAGCCAGAAAAGG
EMPV1_06012	TCGAGGATCTCTTCTAACGTGAGGGCCAGGCTGCGAAGAAGCAGAAGCTGGAACAGGAAT
EMPV1_06013	GGGTGTTTGTTAGAGGCAGAATTGCATAGATACAGCGCTACAAAATGTTTATGCCCTCCC
EMPV1_06014	AAGGCGCTGGAACACACAGCAGGACTCCACCTTGGAGAGACCCAAGACCCTGGAGTTTCT
EMPV1_06015	CCGTTCTTGTCCTTGGCTGTTGGCTGATTGGGTTAATTGTTATACTCCCACCTCTCAGCC
EMPV1_06017	TGACTCCCAGAGAGAATGGCACCATGAACTGGCATGGGTGGTTGGCCTGTGTCTTCTCGA
EMPV1_06019	CTCCTGATTCCCAGTATCTGGCCACAGGAACTCATGTGGGGAAAGTGAACATTTTTGGTG
EMPV1_06021	TGTGGGGAAGGGTATTTCAGGAAGGCCAGGGCAATATTTAGCCTGTCGGGGTGATGGAAT
EMPV1_06022	CTGTGGAGTACGCATCTGGGGGAATAGCATAATCACTTTCAGAAGCCACAGAAGAGAGAG
EMPV1_06023	GTCTACTCGCTCTTTCTGGACGAGTCACGATCCACGCAGTACATGAAGGAGTACCAGGAT
EMPV1_06026	CTCGCAGACGTTGGGGCTAAAGTCGCAGATCAACTGCTCCGAAGGCTACATCCAAAACTA
EMPV1_06028	CCTCCGTCCGCATCGGCCTCTACGACTCCGTCAAGCATTTCTACACCAAGGGCTCAGAGC
EMPV1_06029	TTTGTCGGTGGAATGCAGTTTTTACTATCCCATCGTGAGGAACGCCCCAGATGCCATGAC

EMPV1_06030	CCCAACATCCCTGATCATTAGGGAAATGCAGATCAAAACCACAGCAAGACACTACTTCAC
EMPV1_06031	TGCTGAATGAAACCCAGCATGTGCTGCTGGCGAAGGTGCTGGAGACGGGGGCCCTGTCCG
EMPV1_06032	GAGGTATGGCTAGTAGCTCCTTCTCTTACAACAGGTACAAGGGGAAGCACTCTTAGAGCC
EMPV1_06033	CCCTTTTTGGAAGCCCGAGATCTTCTGCTCGAGTTCAGTAGGTGTTCTGTGCAAATCATT
EMPV1_06034	GTGTGTGTGTGTGTGTGAGTGCTGGCTCACATCTCTGTTGCATCCACTGACACATCCAGA
EMPV1_06036	GGGGAGTCGCCTTCAACAGAATGGAAACGGAAATAACCTAGGAAACCTCGAGACTTTGCC
EMPV1_06037	AAACGTGGGAGTGGTTTTAGACCTGGGAAGTGGGCACAAGCTGGAAAAACTTTGAGGACG
EMPV1_06040	ACGGGGCTTCGTTATGTTGCCAACTTCTGCTCTGACGTTAAACCTGGAAGCCACCAGTTA
EMPV1_06041	GATTTGGCAACTCCTTTACACCATACTTGGACATATCAAGCCTTGGTGCATGATGTACTG
EMPV1_06042	TGCCAGTGTCAGTGCACAGCTTTAGTGGGAATGGGAAGTCCACCCAGCCTCTTCAAATTT
EMPV1_06044	AAAGTGCTTTGGAATGACACGATCACTCCCGTTGAGTGGGCACCCAAGTAGCCATCGGGA
EMPV1_06045	GCATAGGAAGTAACATGGGTGCATCTCAACAACATTCCTGAGTGAAAGAAGCCTGTTGCG
EMPV1_06046	AGAAATTCCGGGTAGATATGCCTGGCTCAGGCAGCGCCTTCATCCCCACCATCAACGCCA
EMPV1_06047	AGAACAGCCGTCCTGAGTGCCATCTCCTGCTGCTGCGGAGAGCTGTTGTGAGAATTAATT
EMPV1_06048	AAGGTAAGAGAGGTGATGCAGGGACCAAATGAACCTCCCTCGGTATTTCTTGAGAGGCTC
EMPV1_06052	TCTCCAACTTAGGGGTTCATTTCCCCCCTTACCGAGAGCGTGGCGAGATCTTTTGAGTGA
EMPV1_06053	TTCTGAAGATGTTCCAGTGGCAGCGAATGCTAAAAGGGCAAGGAGACGAGAGAAATCGCC
EMPV1_06055	CGATCTCGGGTCCTGGCAAAGCCCATGTGGTTAGACAAAATCAAGAAAAAGCTGACGAGG
EMPV1_06056	ATTGATGACAAGGACACCATTCAAGGTAGGAGCCATTTTCCTTGGCACCCTCCGGGCACT
EMPV1_06059	CAAGTCGAGAGCAAGGCTCAACAGTGGCTGCTGCATTAGGTTTCTTGAAAGTATCGGGAA
EMPV1_06060	GCTTCATGCAACTGTATTTATTTTTGATGATAAGACTTCCATCTAAATTTTTCTCCTGCC
EMPV1_06065	GGGATCTTGTTAAATTGCAGATTCTAGTTCACGGGTCTGGGCTGGGGCTTGAGATTCTGA
EMPV1_06066	CTGGCAGAAGCTGTTTGTCAGGATCGCCCTGGAGCTAAAGGGGAAAAATAAAGATGCTCA
EMPV1_06067	AGTGGTGGTTTGTTGGCGGCAGTTACTTTAGTGTCCGTGGTAGTTTGAGTCTGTGGTGAG
EMPV1_06069	GGAGAGGATGGGTCAGGTGCTTTGTCCCCATTGTCTTTTGAAGTGCCTGCATATGAACAA
EMPV1_06071	GTTGACATATCTGAGCTCCACTACTTTTGGTGCCCTCCTCCTTGTGAGAGCTGCGGTTTA
EMPV1_06072	TACCTTGAAGGTAATGAAGTTTGAGCATTGGGGCCCCTCACTTGCTCCGGCTTCTTCCAG
EMPV1_06074	ACGCACGGAGATCCTGCACAACATCGACCCCCTCTACAACCACGCCCGGCACGGCTCCCT
EMPV1_06077	AGCATAAAGCACTGTGAAACAAAGTAGTTGTTAGGGTTAGCTGCACTGGGGTGGGTGTTT
EMPV1_06081	CACGCTGGGGAATCAGGGCTCTAATGTCATGCACGTGGTGGTACAAGAAGGAAGATGTTG
EMPV1_06082	GACATGGAACTTCGGTGGTACAGGGACCAGCTTTCCCCAGCTGTGCATGTGTGTAGAAAT
EMPV1_06086	CCAGCTACAGAACTGTGAAAAAAATGAACGAGTGAAAGAAGAGCATAACTGTTTTTGATC
EMPV1_06089	CCTGAACTTTTTCTTCCCTTCTTGCTATGCTGTCGGGTCCTGGAAGGCAGGGACAGTGTT
EMPV1_06090	AGAACATGGTGCTGCCCACAGAAACAGAAGCAGCCCCGGCCAAGGACATACTACTATTCA

EMPV1_06092	GCCAAGGGTAAGGCAATAAGAGTTTACCTAGCACAGAGATGGCACTCAAGGTCACTGGAG
EMPV1_06093	CCTCTTCACACTGACGGTCCTTAGTAACCATGCGAATGAGAAAGTGGAAATGCTGCTGGG
EMPV1_06097	TCCTGCTATATTTGGGGGACCTGTCTGGTTTCTGGTTTGGCTGGGGTCCATTGAGGATGT
EMPV1_06098	TGGAGAGTCTTGTTTTCTGGTTACTTTGGGAAGCGTTTGACCAGTACCTCTCGAGCCACC
EMPV1_06099	AACAGAACTTTATTTCCAGATGAACTTTCTCATTCTCCCAGACAAGGGCCTGGGGCCGTG
EMPV1_06100	TACCGCACAGTAATCGCTGGTGTGGATATTATCAGGAACCATCGTCTGCAGGAAACGGCT
EMPV1_06101	GATGCCTTCACGCCATTGCAACAGGAGACATTTAGGCTGGCAGAGCAATTATCAGAGTGG
EMPV1_06102	AGGTGCCAAGAATAAGGTGTGACATCCAGAAGCATTCCAATGGGACCAAAGAGACCTGCC
EMPV1_06104	CCGCCGGCTGCTGCACGCGGGACACGCGAGAGACGCGCAACCGCGGCATCTCCTTCCACA
EMPV1_06105	ATCTCCCCTCAGATGGAGGTCACGTGTGTGCCCCCTCCCACAAGCACGGTGTCCTCCATA
EMPV1_06109	CTCCCTTTCCAGCCTCATTCTGTTCCCACAGTTTTCAAACGCCACTTCATCTGCCAAAGC
EMPV1_06112	AGCTGGAGTCAGATGGCAGCACTATGGAGGAGTATTCACAGGAGGACTGGGGAAACCACA
EMPV1_06113	GCGGCGGGTGTTGACGCGATGTGATTTCTGCCCAGTGCTCTGAATGTCAAAGTGAAGAAA
EMPV1_06114	CCTGGAGTCCACTAGGACAGAGTCTTGTAATCAATGCATGTCCTTAGTCCATTAGTTAAC
EMPV1_06115	ACTGCTACGAGGCCAGGAATGTCCAGTTAGCTGTTACCGAGGCACAGGCGGACGATGAAG
EMPV1_06116	ATACTCACCTGGCTGGGACGCCAAGTTCTTCTACCTCCCCATGAGCTACTTGCCCACCTT
EMPV1_06117	ACTGTGTGGCACATTTGGAACCTCCTTTCTAGTGCTCAGGACTTTCTTCCTTGTGTGGGC
EMPV1_06118	CCTCAAGCACCTTCTATGTACCTGGCACTGTTCTGAGACCTGAATTCAGTATGTTCAGTG
EMPV1_06119	ACGCAATAACTGTTTGGTATTTTGATGCAGATGAGAGAGCACGAGCTAAAGTAAAATATC
EMPV1_06120	GGGATATATCTGACCAACAGATATTGTCTTTGGGCATAAATGAAATGCAACACATGCACT
EMPV1_06122	TTTGCCTCTGTGGCCCGGACCTGCGTTCAAGTCCTGACGCTGCTGCGTGTCCTTAAGCAA
EMPV1_06123	CTTCGTTGACTCCGAGGGGATGCAGCAGCAATTCATGTTTTGAAGGGCTTTAAATGGTTC
EMPV1_06124	TGCGCGGCCCCAGCTGCGCTCTGTACTCCTGTGTGGCTTGGAGACACAAGCCTGTATCTT
EMPV1_06125	CCGGGTTCCCGCTTGACCGGATACTTCTGCGTTAAATGGTCTTGCTAACTACTACAGGAT
EMPV1_06127	TGAGCCACTGTCCTTGCTTTTCTCAGCTGGGGCCTCCTTCTCCTTCGAGGCGCTGGCTTC
EMPV1_06131	TACTCATCGTCATGTCCTATGCACGGATTGTCTCCTCCATCCTCAGGGTCCCTTCTACTA
EMPV1_06133	GAGCTCAAGTCACACAGCCTGGTAAATCAAGCATTCAGGCAGTACTAATACCCACAGCAA
EMPV1_06134	GTGACCTTTTCAAATAGGAGCAAACAGAAGGTTATTCTGTAGCACCAGGCCAATGGGTGG
EMPV1_06136	CCCAAAATTCCACTGAAATCAATGTGAGGCTTCCTGCCCCTGCCCTCCAATCACTATGTG
EMPV1_06137	ATTCACAAGCCTTGGTCTTGAACCCTATCCCTTCGATCGGCAAAGACCATCGTCCTCACA
EMPV1_06138	CCTGCTCTGACACCTTTTTCTTTCAAGTCATGACCTCTGTGCTCACAGTGATCTTTGGAC
EMPV1_06140	GAGTGATGTGAACACTCTGGTGGTTCAAGCAGCTGTTGTAATGGTGACTTACCCAGTTTG
EMPV1_06141	GCTCCTTGAAGTTCGTGGAAGACTCTATGAGCTTCTAACTCATTGTATTCCTCCTGAAAT
EMPV1_06145	TATCACTGCTGTGGCTTAGGTTTGATCCCTGTCCTGGGAACTTCTGCGTACCATGGGCAT

EMPV1_06147	TGCACTGTGACCTTCCGGAGATGGCGCGTGGGCAGCGGGCCATGGTCACCGCGCAGGCTT
EMPV1_06148	GGGCGGGATTATGAGTCAGAAAGATCAGCTTATGTCCCAAGCAAACGTAAATCAGCATCG
EMPV1_06149	TTCTATTAGGGCTGTTGGGCGGGAGGGACGGGCAAAGATATAAACCCCAGCCTTTAAGAC
EMPV1_06150	CCCTTGATGGGTATTTTGGTAACACTCGGCATCCTTTGTTCTTGGCAGCCTTGCTCTCCA
EMPV1_06151	AGAGTGTTTCCTACAGGATCCTTTCAACAGGGCGGCGGGGGAGCGCTACCGGCGGGAGAT
EMPV1_06152	AACTGTAAACTGCTGCTGGCCTTGCCCTTCGACTTGTCCTGCATCTGGGAAGATTGGAAC
EMPV1_06155	CACTACTGTATCCTTCATGCCTGGCACTTCCCAGATGCTCAGTAGATGTTTGGTGAGTGA
EMPV1_06156	TGTAATGAGAAAGACGCCGCCGTGTGCCAGGTGAAAAAGGCAGATTCCACGCAAGCCAAA
EMPV1_06157	GCAGAATCTAGCGCAAAAGAACCCTAAGCAGCCACCGTGACTGTTCCTGCACCTGAAGAT
EMPV1_06158	CAGACATCAGGCCACATCTCCGAAATCCTCCCAAGCCACCAGAAATTCATCTGACCCCGT
EMPV1_06159	CTACAGCCAGGAGGACACAGACAAGCTGCTCCGTCTGACGCAGTACAACATCTGCAACAA
EMPV1_06160	AGAATGAGCTAGAGGATATAGCAGCGGGAGATCAAGAAGATGCCAAGGCCCCAGCCATGT
EMPV1_06162	GCCTGACACAGCTGCATCTTGAATTTAGACTAAACGAGCCAGTTTTTAAATAATGGGGCA
EMPV1_06163	GAGACCCGTCGGCCCCGAGATGTCGTACATGCTCCCGCACCTGCACAATGGCTGGCAGGT
EMPV1_06165	GGACTGTGAGCGAAGGTTTTCTCGTTCAGACCAGCTCAAAAGACACCAAAGGAGACACAC
EMPV1_06167	TGGAAAGTCCCACGGGCCAGACACTGGACACTCATCATATTCAGCCACCTGGTAGGGGTA
EMPV1_06170	CCGTTTTAACCGTCTAAGCACCCCATGTGTTACCTGATGTGGCTGAGATTCTAAAGGGGC
EMPV1_06171	TGGGGCTCCTGGGGACTGAGTTTTCTGGACGCTCTCATTAACACCCTCATGACTTTGAAT
EMPV1_06173	GAGTCCAAGACCGCTCAGTACTCTCGGATGCTTTTATGCCCTGATGGGCTTTACAGACCC
EMPV1_06174	CTGTACCCGCTGTAGCTTCTGCAAATGTAAAGAGTGCAAGTGCACCTCCTGCAAGAGCTT
EMPV1_06175	ACAGTCATCGTTGCCGTCTTTGTCCTGCTGGCCGTCTGCATCGTGGTGACAGTTCACTTT
EMPV1_06176	CCCGCATCCTGGAGCTGGAAGAGGACATCCAGACCATCAGTGAGAAAGTGCTGACGAAGG
EMPV1_06178	AAAGCTCTACTTGCTATGTACACAAACCAGGCAGAACAATGCAGAAAAATATCTACCAGT
EMPV1_06182	ATGTCAAGAGTGAAAAGGTTTACACTCGCCATGGGGTCCCCATCTCAGTCACTGGCATTG
EMPV1_06183	AACCCTTAAGCAAATTAAGGAGTAGTCTCTTCCTAAGGCAGCCGGGCCTGTGGCAAGAAG
EMPV1_06184	TGATCCGCAAGCGGTACAACTTGACGGCACAAGATGTGGGCTCTGGCGCCACCAACAACA
EMPV1_06185	TCAATGACTTCTGGAGAATGGTCTGGGAGCAGAAGGCTGCCACCATCGTCATGCTGACAA
EMPV1_06186	GGAGGCTGGGGACAAAAGAAGCTTATAATTTTGTAAACAGAGGCTTGGGAGAGGCCTGCC
EMPV1_06187	TGACAGCTGCAGATTTTATTCCAAGACCAACACTAAGCCCCAGAAGGGACCTCACAGGGC
EMPV1_06188	GTGCCCCCACGTGCTCCGTGCTCCAGCAAGGGTCCTGGGGGAGGCTGTTCTGCCCTTCCC
EMPV1_06190	TGCTTTCCTTCCCCTCCCCCTCCCACGACCTCATGCCCTGTGAACAGCACAGATGCGGTA
EMPV1_06192	GAAGGCTTAAGGGGTGAAGGGACTGAGTAAGATCATTCAGCAATAAATGCTGCAACCAGC
EMPV1_06193	AGTACTCTGCCTGTGTGGGCACAGTCCTGGGCCAGCGCTTTTTGCTGCCAGCTGAGAACA
EMPV1_06194	GCCTTCCTCTCCCCAGTTTGGAACCAGTTTAAGCACTATTTGCAAGGAGATAGCAGGTGG

EMPV1_06197	GCAGCCTTGGGCCAGACCAACTATATTTTCTCCCTGGACTTCTGTGGCCCCTGTGAAATA
EMPV1_06198	ATGTCTCATATGGAGCTGGGGTTGGACTCGGGTGACGATGACTTGCTGGGCTTCCTGTTA
EMPV1_06199	GACCAAGACAAGAAGAGTTGGATGATTTTGATAAAGATGGTGATGAAGATGAATGTACAG
EMPV1_06200	GGTAACAGGCTATTGGCCTTTTTCACCTTTGGGCCAGAGGAATAATATCTCTTAACCCCC
EMPV1_06201	CTCTTCTTTGGCTTCCTAAACAGAGTCACACTTGCTATCTTCAGAGTGACCATGGCCAAC
EMPV1_06202	ATGGAAGCCGCTGGTCCTGAGCCCAGATTTCTCCAGAGAGGAGGCTGTGCCAATGTCACT
EMPV1_06203	AGCAGCCGAGTTAACTTTAATGAGCCAGTTTGATCAAGTAGGACTCTAGCTACATAGGCC
EMPV1_06204	CCCAGGACAGGGCACTGACCCTCCATTGTTGCTTGTTTGGGCTTTTATTAGCCTTTGTTG
EMPV1_06205	AAAGCCTGAACCTAACGCAGTCACTAAAGCAGCCGGCCCAATTGGCAGTGCACAGAAACC
EMPV1_06209	TTCTACCCGCATGATCCCTGCCTCCGAACCCCCTTCTCGGTTTGTTCATTTGTGAATGCT
EMPV1_06211	TTGTGTTGCCAGGTATCTCACTTGCACATGGTCATTTGGGGCCATGTTGTACTAGGACCC
EMPV1_06212	CAAACCCCGTCACCAAGGAGATGCTGTTTGAGTTCATCGACAGGTGTGTGGCAGCTTCTC
EMPV1_06213	CATACTAGGTACAAAACAACATGGCCCATCACCACCTAACGCCTTTATGTAAAAAGGTGG
EMPV1_06214	AGCCCATCCCCAGGAGCCTCGCCCAGCTGGAGGGCTGTACCCAAGACTTCAGACCAACAG
EMPV1_06215	AGCCCAGCCTAAATCCTAGTTGATAAGGGGTCATGGGCTTGAATTTTCCCATCTGTTCTC
EMPV1_06216	CTGGGCACAGAGCAGGCCAGAAAGGGACAAAGAATGACCAGCACATCTAAGCCTATTTGA
EMPV1_06217	GCCTTATATTTTGTCAGACTTTGGAGTGCATGGCCTTATATTTTGTCAGATTTGGGAGTG
EMPV1_06221	GGTGAAAACACTGCCTGTGTCTGACCTTACTTCACCAACTCAAAGCAATATTATGTACAG
EMPV1_06223	ACCTATGCTACAGATTCAAATAGTACTTGTCTACTTTTGTTTTACTTTATGTCTTGCTTT
EMPV1_06224	TGGGATCCTTTGACCCTTGAAGAAAGTTGGATTTGACCAAAAAAGGCCCCAGGCCAGCCT
EMPV1_06226	CTGTCCATGGCAGTAACTCTTAGATAGTTTCATATTTTCCATGCAAGGGCAGGGTGCACA
EMPV1_06227	GGAGGAAGAAAAGAGACCGAATAAGACAGTTGAGAGAAGTTCCCCGAGCTCCCAAGAACG
EMPV1_06231	CCTGTTGCTGCTTCTGAACAGTCTGAAGATGACGAGGAGGAAAGTCCTATAAATATCCCC
EMPV1_06232	TCGAAAATGAGAAGCTTTAATGCCTGACTTCAAATTTCCAGATATGTGGGTCCAAGTCCC
EMPV1_06233	AGTGTGTAGAGGGAGTCGGAGACCATTTTCAGCCCTAGGGATTGGGGTGGAGCCTTTAAA
EMPV1_06236	GACTATACGCCTGTGTGTGCCACTCTGCTTCCACAGTGTATCCTATTAACAAGCCTCACC
EMPV1_06237	GCGCTGATGATCTGAAGTGTACCCTAATTTACAGTTCCACACAGTTCTCTGGTTTCTGTG
EMPV1_06238	CTGTGAAAAACAAAAATCTTCACGTATGTTAAAGAGTGGTACACTTGAATGATCGCTGAC
EMPV1_06239	AAGGTCCTCCTTATTCCCCTGACTGGGCTGCTTACGGCAAAAAGCCTCAAAGATGTGCAA
EMPV1_06240	CATAAAGAACAAGCGGAGAAGGAGCCTGACCCCTCAAGGGGAGGAGCAGATGGAGGCTGA
EMPV1_06241	CCCTCCTCCCCATATTGAACCTGCTTCTACTCTTTTACTGTTTCCATGTCAGTTAATAGC
EMPV1_06243	AAGGAGCCTTTGTGGGAGAGAGTTGTGATGGGCTGGGGTCTCTGAGCTGACCCTTTGTGT
EMPV1_06245	CCTGCCAGCCCTGGGGCCCGGCTCCTGCTCCGGTGCTTTAGAGGAGTAAGCTGGATGGTC
EMPV1_06247	AATAGGAGGGGGTGGGGATACCGACAAATCGCATACTCATGCACAAATGCTTAGCGCCAA

EMPV1_06248	TGAACACCGTGATGGTCAGCAGTGAAAGGGAGCTGAGGAGTGTTTATCCTGCTTGTGCTG
EMPV1_06250	AAAGACTCAGTCGACCGCGGACAAGGAAAAGAGGATCCTTCTGCTCCACTTCGGCCGGAT
EMPV1_06251	TGGACGCGCACCAGTTCCCCATGGCGGGCTTCCGCATCATGTTTCTGGATCTGGTGCACT
EMPV1_06255	CAGGGCGAAGGGCCTGTGCTCATCCTGCAACTTTTCTGTAAGACTGAAAATTGTGTCACC
EMPV1_06256	CCAGATGTCCATAGCCAGTATTATTCTCCTGGTGGTGCCCTTGGTCATTATCCTTTCCTC
EMPV1_06257	CAATAGCTGCTCTTACCTCTGAGGATTTTAACTAAGGTTTTTTGAAATGTCTTTTGTTCC
EMPV1_06259	ACTGAGGCTGATGTAAATCCGAAGGCCTACCCTCTTGCAGATGCCCACCTCACCAAGAAA
EMPV1_06262	TGCTATGTTCTTATCAGCAAATTTCAGCGCACCAGTGATGAGAGGCTGAGGGGTGAGGAG
EMPV1_06263	AAATGGCACTTTAATAGGTGTGGCCAGGGTGACAGGACCAAGATGGGGCGACAGCTGAAG
EMPV1_06264	TGGCATGGGCTATTTTTGAGCTGCAGAGAAGATGCTTTAACTTGCTATAACCTGCCCCCC
EMPV1_06265	ACCGTGAACCACAACACTAAGAAGTTCCGCTTTGCCCTGCCCACCGCCCACCACGTTCTG
EMPV1_06266	TTTTGCAATACTTGCTCTCCTGTGGGGAGGTGTCCGTGTTTGTAGATGTTGGTGAGCATG
EMPV1_06267	AATGTTTTATTCCAACTAAATTCCAGTACTACAAGGGTAGTAAAACAATAATGATACACT
EMPV1_06270	CTGCTGGCTCAACTCAGAAAAGGGATTTAAATGGAGTTTCCTTGGACCAGTCTGTTCCAT
EMPV1_06271	GCCATCTCAACGAAGGCCACTTGAGCTGTTGGAGAGGAAACTTTTATTACTTTACCACCA
EMPV1_06272	AAATTCTAGTTTCACTGTCTGCCTCGTCACCCAGTTAGTATCCTGCCATGTGGCCTAATG
EMPV1_06274	AATGAGCAGCTTGGACTGCGTCCTGCCAGAGGGTCAGTATGCGCAGCGCTTGAGCTCATA
EMPV1_06276	AAGGTCACACAGCACTTGAAACCTGCCTGTTTGGTCTGACACTGGTGCCCACACCATCCA
EMPV1_06278	TAGGACACATGAGCCTGCCTAGTTTGGAGCTTCCCCCAGAGCTCTTAGAGGTGGACACCA
EMPV1_06279	AGGAAAGCACAAAGAAACAGCAAAATTACTTCAAACTTACTCATTTAAAGTTGACCATTG
EMPV1_06280	TGCGAGTCACGGCCTTCCAGCTGTGCTGGGTGATGTGGGATAGAAGGCCTCGAATGTATT
EMPV1_06283	GGCCCAGTCAAAAAGGAACATTAACAGTTTGAATATGGACCTTGAAAGGGATATGCAGAG
EMPV1_06284	GAGGGCTTCTTGTTTTGAGTAACAGGTTCTAAATTGTTGGACCCGAGGACAAGAATAGCG
EMPV1_06285	GACTGGCTGACGGTGGAAGACTACAGAGAACCTATTGAAGTGAATCTGTTTGGACTCATC
EMPV1_06286	AAGTAGCTGAGGTTTCACAACACCCAAAACGCCAGAAACACAGAGCTGGCAGGAGCACAG
EMPV1_06287	CATCCAGGTGTATGAAGGGGACATGTCCTAGCATCTGTGCTCCATAGTAGTTTATATTCC
EMPV1_06288	TAATGGGGTTCGGTCTCCAACACAGTCCAGGTGGTCCCAACGATACCTTACACTCTCAAG
EMPV1_06291	CACTCCTAGTGCCAACATGCGTCTTCTTGGTGGTCCTACTTGCACTGGGCATCGTGTACT
EMPV1_06292	TCAAAACTCCCTGTGAGCTGTCCTCCAGAGCCATTTCAGAGTCATCAGGACCAATGTGCC
EMPV1_06293	GGCCCGAAGCCTGGCTAATGCCTCCAAAACTTGATCATTTGAATCCAGATTATCTACATC
EMPV1_06294	ATCCCAAAAGCAGCTGTTGTCTCCAGAGCATTCCAGCTGCACTTGGATTTCGTTCCCTGC
EMPV1_06296	TGGACTGTGTGGTCAACAACGCCGGCTCCCATCCACCTCTGCAGTGGCCTGAGGAGACCT
EMPV1_06297	CTGCCCAGCCTGGAGCCCTGGAAGCAGCACGTGACTGAGGCTTTCCAGTTCCATTTCTAA
EMPV1_06299	TTTCAAAGTTGGACAAGTCATAGATCCGGTGACTGGCAAGCCCTGTGCAGGAACCACCTA

EMPV1_06300	TCATGAGCAGTGTGAACCAGTCCAACTGCTACTGGCGCACCTAGATGACACCCGTCTTCA
EMPV1_06302	CCTCTGTATTCAGTGCAGCTTTTGACAGGCATAGAATGAGGCGACCACTTGCTGTCTAGG
EMPV1_06303	TCAAGTTAAATTTGAATAACAATGAATAATTTTTAGTGTAAGTATATCCCTTGCAATATT
EMPV1_06305	GCTGAAAGAGTGAAACAGGAGAATCTGAACCTCATCTGTATCCCCACTTCCTTCCAGGCC
EMPV1_06308	ACATCGTGCAGTACTGTTTTGGACTTGTCTACTACGTCCTCGTCGGCCTGACTGTGCTGA
EMPV1_06309	CTCGAGTACCTAGCCCTAAAGTGGTACTTGCCAGCCAACCCGACCTGGAATGTGGATTTT
EMPV1_06310	ACCCGCAGCAAAAAGGGGAGAAAATCGCCGAAAGTTACCAAAACATTCTAGCGGCCATCG
EMPV1_06311	CGTGCAGCCATGTGTACAGGCCACCAATGCGAAAATGTGTAACTGCACCACTGTGAGATA
EMPV1_06312	ACTGGGCACCTCAGAGCCTCCCTTTCTCCCATTTGTGGGTTGTTAGCAAGATCTCTGTGT
EMPV1_06317	GCTCGGAGGGCTGTCCTGAGTACCTTGAAGGAGTCATTCCTGTCAGAAAGCTAATGACAT
EMPV1_06318	CACGTGAAGCTCATGGCCACCATGTTCCAGAACCTGTTCCCCTCCATCAATGTGCACAAG
EMPV1_06319	ACAACTATCATGAATTGGCAGGTGTGTTGGCTATTAGTTGGAGTGTCATGGGTGGGAGGC
EMPV1_06320	GATGCCATGCAAAGAGTTGGAAACAAAGAGGCGTATAAGTGGTACGAGCTTGGGCACCAG
EMPV1_06321	TAACTGCTGTTTTAGGCGGCCATCACATTGGTTCACATGCAGCTGGAGGATTATGCACGC
EMPV1_06323	TGGACAATGACACCATCACCTATTCCAACTCCCTCAGAGCGGCTGTCTCAAGTGGCATCA
EMPV1_06327	ACGGAGGAGGAAATCAAGTTGAGGCAGAGGTAGCCAGTGTTGTCTCCCTTCTAAGAGACA
EMPV1_06330	AACAGGAGAAGAGCTGAGGGCTGTGGCACCTTGTCTCTGGGTCAGAGAACCCTAGAGTCA
EMPV1_06332	AGGCTTCTGGGGCACTATCAGGCTTAAGTCTACTCTCCACCCCAAGTTCTCCATGTATAT
EMPV1_06335	GGTTTAATAGATAGGAGTTCCCTGGTGGCTTGGTGGGTTATGGATCCGGTTGTTCTTACA
EMPV1_06336	CAGCAGAAGCTGTACAGCAAGATGATCGTCGGCAACCACGAGGACAGGAGCCGGTCCTGA
EMPV1_06337	GTGCTGACAAGCTACTTATTGCTACTGGTGAGGTAGGAGGCAGATGGACCCCAAAACTCC
EMPV1_06339	GGAGTAAGCAATTTGAGCAACCACCATCTTTGTTTCTGATTCCCCTGCATTGCTTCTGTG
EMPV1_06340	TTTGGTTTCTGAGGGAAAATCCCTGGAGCTGAGGTGCAACTATTCCTATGGTGCAACCCC
EMPV1_06341	GATCGCAGTTGGGTATATAGATTTCCTTGAGGGCCACCCTTTTCTTCATAATGGATTCTT
EMPV1_06342	CCCCATGGATCGGTTGACAGCAGAAGAAGCGCTTTCCCATCCTTACATGAGCATTTATTC
EMPV1_06343	CACCTTCTAAGTTGCCATATGGGGCAGCATGTTGTTTTAAAAAGTCAGGATTCAGTCAAA
EMPV1_06344	TTTGGAGGTGGGGCCTTTGGGAGGTATCTGGGTAGGGACGTCATGAATGGGATTAGTACT
EMPV1_06346	GCAGATGAAGTTTACAACACTAAATCATCTTCTCAGATACCTGCCCAGCCACCTGTAGCA
EMPV1_06351	CCCCAGCCAGCACCACCATCAGCTTTCTGCAGCTGTGAACATACCTATTCCAGATATTTC
EMPV1_06356	TGGTCAACTGTCCTGGTTTGCCTGGGACTGAGAGATTTCCTGGGATTCAGGACTTGCAGT
EMPV1_06357	TGAGGTGAGAACACAAATATTTCCCTACAGCCAGGGAGCCAAAGACGCACAAGACCTGTG
EMPV1_06361	TTGTTAGCAAGTCGTGGTGTAAAAGGATGTGCTGTGTGGATGTAGCACCTGGCGGCGAGG
EMPV1_06362	CGTTGTTCAAGACTGAGCCCAACACTGGATTTCTTCTCTAAAGGAAAGCAGGGAGAGAAC
EMPV1_06363	AAAAGTTGCTCTGGGACTTCCCACCACCTCGCCTCTCTCTGGCTCTGCTCGAGTCTAGGA

EMPV1_06364	CCATGTGCTCTGCCTACTTCCAAAGGCTACCGAGGATTGAGGTAATTGTATGTGACTTGC
EMPV1_06366	GCAATGTCAAAGGGCTCTCAGAAGCAAAAGTGGACAAGATTAAAGAGGCAGCCAACAAGC
EMPV1_06367	AGCACCTGGGTTACGGGCCGCGCTCCGAAGGAGTGATCCTGGCGCGCTATGGGAAAGCGT
EMPV1_06369	CTCTGCGTTATTCATTCTGCTCTTCAGGGCCATTAATTCAGCTTGTGTCTCTGCAAATGA
EMPV1_06370	TTCCCCCCTCTGACAGGTATTGATTCTCCCCTGGAACCGCACAGTCTACATTCTGATCTT
EMPV1_06371	GGACTGATTTTCTCTTTGAAGAATAAAGATTGCTCTGTCTCCATAGGGATACATTTTGTC
EMPV1_06375	CACTGCGCTTATGTTTGTAGCGGGAGGAGAGAGACAACAAATCAGTGAGCAGGGTTCATG
EMPV1_06377	CAGCTACGCCTTACTCGTGGAAAACAGGATCAAGGAGGAGATGGAGGCTTACCCGCAGAA
EMPV1_06378	GGGACTTATACAGAGATGTGATGATAGAGATCTGCAGACACCTGGTCTCCTTGGAGTATG
EMPV1_06379	AAGGGAGACAGAGGCCGTGGCGAGAATCCAGCTTTGACCTTTATTCAAGAGACCAGATGG
EMPV1_06380	TCTCCAAGTCGAATCCGCCCAGGCAAACACCCTCAAGACAGCTTCCCAACAAAGATGACT
EMPV1_06381	GCCAGAGGATGACTCCCTGTCAGTTCCCATAAAGTCTTGCTCATGTCCTTGTTCTGCGAA
EMPV1_06382	TGACAGTGTATGGGCTGGAGGATGCACCAGGTGGCCATGGCGAAGTCCTGTTTGAGAAGG
EMPV1_06383	AGAGACCCATCCTTCCTACATCAGCAACAGCTGAGGATTTGTGACTGGACCATGAATCAA
EMPV1_06384	CCTGATTAGCTAGCTGGCTATAGATACAGAGCATGTTAATTTCTACAGGCACCTTTCTGA
EMPV1_06387	AGCTCCACATAATGCTTACAACAAGATACGTTTGGGAGTCAGTGGTCCAGGGCTGACTGC
EMPV1_06390	CGGAGCAATCATTTAGAGGTGATGTGCTGTTGCCATGGAGAGACTCAGAAAGAATCCTAG
EMPV1_06391	GCAGACCAAACTGGAGCATGCCCGCATTAAGGAGCTTGAACAGAGCCTGCTCTTTGAAAA
EMPV1_06393	GTAGTTGAAGTACAACGATGATTTTTCATGTCATTGGTCGTGGTTAAATTCCATGTGAGA
EMPV1_06396	TCCCTGGCTCAGGAGGAAGAGGAGGAAGACCTGGCCTTTGAAGAACCAAGCTGCAGAGGC
EMPV1_06397	TATCTGGCTCAGAGCACAAGCTTTAACTTGAAGTTTCACATAGTTATCCTCTGTAACAGG
EMPV1_06398	AGGACATCCTGTGTCGGTGATGTTTTCCACCCCATGGTGTTCATCAATAAAGAGGGGGTG
EMPV1_06399	GGTAGCTGCCGAATGTTCTACTGGTACATCGACAACTGTATGGAGCGATGGACTTACCAG
EMPV1_06400	CACTTTACATGAAGACATCATGTTGTATGTCGCATAGTTTCTAGGTTGATTTCTCTTGTG
EMPV1_06401	CCTTATTCTTTGTTGATATTGTGCCACTGCAGAATTTTAGTGCAGAGTTTATATTTAGCT
EMPV1_06402	AGCAGCGATTTATAGGAAAAGAAGAGAGAAGAATCGGGAGGGAAGGCATGGGCCTGAGTC
EMPV1_06403	TGGTCGCTTGTGATCCTTGCACCTGTCACTTTGGGTCACAGCTCCGGTCTTGTCCCGCAG
EMPV1_06405	TGTTGCCATGAGCTGTGGTATGGTTCGTGGATGCGACTCAGATCTTGCATTGCTGTGGCT
EMPV1_06407	ACCCTCCAACTCCATGTATGTTTGTGGCTGTCACCTCTGCTTTTGACCCATGGGCTATAA
EMPV1_06409	GCATTTTGCACTTCCTCCCAGGATCTATTCTTTTGGTCAAAATACGAAGTGTCGGTGCAG
EMPV1_06411	GAAAGTCAAGGACAACGCACAGACTTACGGAGTGGGCCTGAACACCCTGCGCGGCTACTA
EMPV1_06413	TTTGGTCCTGCACGCTCTGGATGATTCGCAACAGGAGGAAATATGCCATGATCAGCTCTC
EMPV1_06415	CTTCTCCTCTCGTGCTCCAGTATTTACTTCAACATCAGCCTTTGTGGGTTTAACCTGATG
EMPV1_06416	ACAGCACAATGGACAAGGAGGTGGCAAAGCCCTTGAGGATGTTGTCGGCGTACTTGACGA

EMPV1_06417	ACAGAAGGAAGCAGCCTATGAATGGGGTCTTATTTTATGATGCCCACCTGGTATGTGGTC
EMPV1_06418	GGGGTTTTTCTCTGAGACTGGCTTGGATTCCACTCCGCTTCATCCCTTGAAGGAGGTGTA
EMPV1_06422	ATAGATTCGAGTCCGAATGATCTTCTCCACCAGATACTGAGGGTTGGTGCCGTGGATGCT
EMPV1_06423	CGTTGCATTACTGAATCCAACATATTCATTACCAGCCATCTTATTCAAAAACTGCATTAC
EMPV1_06424	AAGTTCGACATGATCGTTCCCATCCTGGAGAAGATGTAGGACAAGTAGGGCTGGCAAGTC
EMPV1_06425	TATTCCACTCAGATTGACATGTGGGGTGTGGGCTGTATCTTCTATGAGATGGCCACGGGC
EMPV1_06426	GCACAGGATTACATCTCCTCCTGCGGCAAGAAGACGCTCCATGAAGTCTTGGAAAAAGTC
EMPV1_06430	ACCGAGCAATATAATGAGCAGTATGGAGCAGTGCGCACACCTTACACCATGAGCTATGGG
EMPV1_06431	TGCCAGCCCATGCCCCAGCAAAGTGAGATCCAAACCACGTCTGCAACCTACATGGAAGCT
EMPV1_06432	AGCATGGAGCCCTCCAGGAGCAGACCTACGCCCAGGTCAACCACTCCAGATTGAGAGTGA
EMPV1_06433	TACGGTCAACAAGTTCTCCCTTCGTGTGTTCGGCAGCCACAAAGCAGTGGAAATCGAGCA
EMPV1_06436	CCCAAAATTCCTTCCGAGGAGTCTCTTAGGCATCTATATATAATTACCCTCAGCTAAAAG
EMPV1_06438	ACAGCTTATTCCCCTTCCTTCTTCACTGGCAGGATCCCACACATATCTGGGTGTCCATCC
EMPV1_06439	AAGCCCCCCACGTAAGGGACGCTTTTCCCATCTGTGGCTTCAGTGGAGGGAAGAACTCAA
EMPV1_06443	TCTGAGTCTGACATCCAGCTTGAGCCCTGCCGAGTGCCCAGTTTTCTCTCTTCCAGTCAT
EMPV1_06444	TTGGAGCAGGAAGAATCACGGAGGTGATGACTGCAGGTGACATCACTGTGACAGATGGAG
EMPV1_06446	TGGGTGCCATGGTGTCTTCAGAATTTAGGTCAACCAACTCCCAGCCTTCCAGAAACTGAC
EMPV1_06447	TGGCAGAATAACCTGCTCCTGTACTGTCAGTTTGTGTTCATTGTACTGATAACGTCAGCT
EMPV1_06451	GCTCTGATCCTGGGCCTCATTCTGACCATCGGAGCCACTGTTCTTCTGGTGTTTGTATAC
EMPV1_06452	GAGGCCAGGAGATTGTGAAAGCATGTGATGGCTCAGGCTGAAGAACTGGGGTGTTGTTTT
EMPV1_06454	GCTGGTACTCATGATGGCTTTGGCTATTTTGCGTCCATCTCTGATGCCATGTTTATTGGC
EMPV1_06456	GCATGTCCTGAACAACTTTACTGCTATTACTCACCCGTCATAGTATCTGCCCTGGTTGGC
EMPV1_06460	CCAGTGATAATCTCGTTCTGAGAGGTATTCTATGGACTGCTGCACCGAGGAGGATTTGCC
EMPV1_06461	AGTCTCACTCCGGGTTTGTTCTAGAGACCAGATGTAAGGGGAAGATGTGCCCTCAGCCCT
EMPV1_06462	AGGAGGGGTCCTGGTTGTCTCCCAGCTGCAGAGCTTCCTGGAGCTGGCGTACCGTGTCCT
EMPV1_06463	GCTAGAAGGAAACGCTATGTCTGTAAACAGTGTGGTAAAGCCTTCAGTTTTCACCGTTCC
EMPV1_06464	TGTGCTGTCTTGGTGACCCAGCCTCCAGAAAGCTCCAGAGCCCATGGCTGCACAGTTCAG
EMPV1_06465	TTTGCAGAAGACGACGTACGAGGGCAAACAAATCCAGCCATAAACACTGCATGAGGGCAC
EMPV1_06466	CAATTTGCGTGGTTTTCTCTGACAGATAGTTGATCTATACTAAGCAAACTAACACGTGTG
EMPV1_06467	AGGCAAGCAGTGGACGATACCAGGGCAGTTGAGCATGATACCAGGAATTGACATGGTGTT
EMPV1_06468	GCAAATGAGGAAACTAAGGCACAGAGAATAAGCAAGGGCATGAGAACTTGAGTCCGAGCC
EMPV1_06470	AAACCCACGACTTCATGGTTCCTAGTCGGATCAACTTCTGCTGTGCCGCAACGGGCGCTT
EMPV1_06471	ACAGCTGCATTCGCCAAGCTGGACCGCAGTGGGGATGGAGTGGTGACTGTGGATGACCTC
EMPV1_06473	CTTCCGTCTCGGAGCTCGCCTGCATCTACTCTGCCCTCATACTGCACGACCATGAGGTTA

EMPV1_06476	CGCCCAGGCGTGTCATTTACTGAAACAAGGGTGCCCTCTGCTCCTTTCATTTAAGGCTCA
EMPV1_06477	GTGCCCAGCTTCTCCTGGCAGCTCGGAGGGATGGATGAGCTGAGAAAACCCTTTCAGGAA
EMPV1_06478	GGAGGATCAATTGACATTAACTTGTGATCCATGCAGCCTGTTTATCCGTTATTCCCTACG
EMPV1_06479	TCTGACATTCAAGGAACATTCTGATTATGTGAGATGTGGATGTGCTAGCAAACTGAACCC
EMPV1_06486	ATTGCGCTAAAGGAGGGATTCAAGCTCCCTAACTCTACACGCCAAGATTCCCACAGTCTG
EMPV1_06490	AGGGGCGGCCAGAAGGAGAGCGTATCTATCCGCACGCATATAAAATGTTGAAACAGATGC
EMPV1_06491	GAGTTCGCAGTCAAGTTGAATGCCCAGGAACTCGTTGATAGTCACCTGGCTGTCTTCATC
EMPV1_06493	ACCTCCACTGCAGCTACCATGGGATAGAGGTGAACTGCCCCCTCCTCCAAAACTTCCTTG
EMPV1_06494	TCTGTTTGACCGATCCTCGCTTATTCGCCACAGGAGAGCCCACACTGGAGAGACTCCTTT
EMPV1_06495	TTGTGCAGAGCCCCCTCCTGTCTGGCCCCTGTGGTTTACATGATCTTTGCTCTGTTTACA
EMPV1_06498	GTGGAGAAGGTGCAAGGATGCACAGACCTTCTGCGTCCTCACAACCATAAGAGGCGTGTT
EMPV1_06500	AAGATCAGCTCCACAAACGAGCAGGTGCCAGGCTGAGAAACCACAGAAACGGCCTCTCCT
EMPV1_06501	GATGCTGATTCCTCAATTGAAAAACAAGTGGCCCTGTTAAAGGCTCTTTATGGACATGGC
EMPV1_06502	CCTACTCTTGGTTTTTGTCACAGAAGTTGTTGTAGTGGTTTTGGGATATGTTTACAGAGC
EMPV1_06503	CGACTCATGATATCTGTTGTTCGTCGGATGTATGCCCAGACAAGTCCCCAGGGAATGCCA
EMPV1_06504	GTATCTGTGTGAAGAAATGTGAAAAGTTTATATATAGAGAGAATGGTCAGAGAAATGCCC
EMPV1_06505	CACCACAGGGTAATCCGCAAGAGATATCAACCAGGTGACAGGGGATGTTGCTTCGTAACT
EMPV1_06507	GGGGAACGATTAGAAAGACAGCTGCCATTGTACGCCCATTCGGAAAACAGCAAAACTCGC
EMPV1_06508	GCTGAGAAGCAGGTACTCGGATTCCAACTCAAAGGCCAAGCTTAACGGCACAGACTGACA
EMPV1_06510	ACTGTGGTGTAGGTTGCAGACACAGCTCAGATCTCCCGTTGCTATAGCTCTGGCACAGGT
EMPV1_06511	ATGGAGTCTGTGGGGTGTTGGTGGCTGTGGCCTGGGTGGGATCCTGTGTGCATTCTTTAA
EMPV1_06513	CTGCGGTTCACCGAGCTCATGGAACGGAGGCCTGCTGGTGGCCATTTCAATGATGGTGCA
EMPV1_06516	GCCTTTGGTAGTAATGTGACCCCACAGCCATACCTGAAGAATAGTGTAGTAGCATACAAC
EMPV1_06517	CTGAGTCGCCTCCGTCGCCAGTTTATTTCCTACACCAAAATGCATCCCAACAATGAGAAC
EMPV1_06518	CATTCCTGAGGAGCTCTACGAGATGCTGAGTGACCACTCAATCCGCTCCTTCGATGACCT
EMPV1_06519	GGTATTTGGTCTTGAATTATAGCATGACAATAGCAACTAATTATCCTCTTCTGCCTAGCT
EMPV1_06520	TAGGGAACTGTGCATCGCAGGCTGGGGAGCAGAATGACACAGCCTAGGGAACTGAGGAGA
EMPV1_06526	TCCAGAACTAAGTCTAACCAGAGAGATTGCCCTGAAACCCGGTTCTGAGGCTGAACAGAC
EMPV1_06529	GGAGGGGGCAGGCAGTGGGGGAGGCTGTCCTGAGGCCAAGACGGCCCGTCTTCCCGGCAG
EMPV1_06530	GCCTAGGAGGCCACAGTTATGATGGGGTTCAAGGTAGTAGCCCAAGAACAAAAGAAGGAA
EMPV1_06532	AGCCTAAGCCTTGGACCTCTGACATGTTGGTACGGCAATCAGCCCTCAGTTCAGCACACT
EMPV1_06533	TGTGTTCAGTGTGTTGAGGATCAGTTGGATTGGCACAGAGGAGGGTACTGCAACTGAGCA
EMPV1_06536	GACAGAAGCTGTTCAGTTGTTTAAGAAAGATTGCTCTACTAGCCCTGCAAGGTCATGGGC
EMPV1_06538	CCTAGCAGTGGCTCAATGAATTTGCCTAGTGACTGCCTGAGCCTGGTCATAGAAGTTAGC

EMPV1_06540	CCACCCCGAGCTCTCTGTGCCAGCTTTGCCGACTCTGTATCAGAAACTACATAGGAAGAG
EMPV1_06542	GTGACTGAGTACAAAGGAAACAGGAATAATAAAGGCATCACTGACTAGGAATGTCTGGGC
EMPV1_06543	CAGAAGCGGGGCGGCTGACAGTTGCATTTTCTTACTTAATCCCCATGTCCTGCCTTATAG
EMPV1_06547	CAGAGCTAGAGCAGGATTTGAGATAGCAGTCTACTTTGCTGACTCTTTCCAAGCTTCAAG
EMPV1_06548	GCTCCAGGATCTCTATTGTAATTGGCCGGGGATTCCTGAGACACTCCAAAGTTATCAGAA
EMPV1_06549	CTGAGTCGCCTCCGTCGCCAGTTTATTTCCAACACCATAATGCATGCCAACATTGAGAAC
EMPV1_06552	ACCACAGCTCACAATGATGCCAGATCCCCAACCCACCGAGGCCAGTGATCAAACCTGCAT
EMPV1_06553	GGACTTAGTCGATTATATAACCACACATTATAAGGGACCAAGAATAGTTCTTGCTGCTGC
EMPV1_06554	GAATTTCTCAATTCCAGAAGACAAGTCCCTGGGGTCCTGACAGTTTCCAGCTGCCTGACT
EMPV1_06555	GACAACTTGGTGGTTGACGTTTCCAATGAGGATCCGTCGTCCCCTCGAGGGAGCCCAGCA
EMPV1_06557	TGGCCCAGCGCCATGGGGTGGACCTAGAAGGGGCCTGTGAAGCCTCCTTGGCGTGCTCTA
EMPV1_06558	CCAGAACTTCAGCAAGAAGGAGAAAGCAGAGGAGGAAATTGAGATGGAGCCTATAGTTGG
EMPV1_06560	AAGTCTCATGTTGGGTCCCCAGAGAAGTCCTTTCCCTACGCTTCTGCCGATGGGAATGTT
EMPV1_06561	CGCCCTCCTTTAACTGTCCAGGCAAGAATTCTCAAAGTCTCTCTGATTGACTCCCTGATC
EMPV1_06562	ATCCGATTCTGCCGCCAGTACCTGGTGTTCCATGATGGGGACTCAGTGGTGTTTGCAGGG
EMPV1_06563	CCACTTGAGAATTTCATAAGCACTTTAATTAAGTCCAAAATCTGAAACTTCAAAGCTTGC
EMPV1_06564	CTGGTGGTTTCCGAGCTGGTTTAACAATGGGACTCACAGTTACCACGAAGAAGAAGACGC
EMPV1_06565	ATCTCGCCGCCTCTCCCAGCCACGGCCCTGATAACGTTCAGTCTAGGATTTTTATATTGG
EMPV1_06569	GGAGGGTTATCCAGTATATTAATGTCTTATTGATAATGGCAGAACATCCACCACTACTGG
EMPV1_06570	GGTTGAACATCCTGCATAGTGCTGCCAGGAAATCCCTATTTTATACTAAGAGGGGGCTGG
EMPV1_06573	CTCACTACTAAGCTGATGGAGCAGAAAGCTCAGTGGCTGGGCATGACAAAGAACACAGAC
EMPV1_06576	CCATCATAAGAAGAAGATTCTGGGATTATGGCCGCATTGCCCTGGTGTCAGAAGCAGACA
EMPV1_06577	GGCTTCTTAAAAACCTCATCTCTGCACTTCCTCCCTTAGGGTACTCAGGGTTCTGCAACC
EMPV1_06578	CCTCAATCCCGAGAGAGTCACACCCCAAAGCCTCTTTATTCTTTTCGGCGTATATGGTGA
EMPV1_06579	TATAGGTCACCCCTACCAAAACCGGACCCCCCCGAAACGGAAGAAGCCGCGCACGTCCTT
EMPV1_06580	TGAGCAAGGTCTGCTTCCCTCATTTTACTTCCCCTTCTGTTTTTGAAAGGCAGTTTCGCC
EMPV1_06581	GTGGTAAGAGCTTCAGTCGGAATTCACATCTTCAGTCCCATCAGAGAGTCCATACAGGAG
EMPV1_06582	TGCCACCCGCCAGGGAGCGCGGCTGGCGGCTGAGCTCCGCGAGCAGGCCGCCCTGGAGCG
EMPV1_06583	AAAAATGGCCAAAGCCCACCATATGCCAAAGATTCCCATGCGGAGAGGAAGAAACCTGGC
EMPV1_06584	ACGTTAAAATCGTGTTGTACTTCAATATAATTCTTAACTTGTTGTTGTTGATACTCATAT
EMPV1_06586	ACTTGGGAGGAGGCCCCGAGGCAGGTTCTGCTGAGTTTTTGATCCACTCTGGTTTAAACA
EMPV1_06587	CACATCTGTGCAGTGACCATCTTCTATCTGCCCATCATCAACCTTGCCATTGTCCATCGC
EMPV1_06590	TGCCATTCTGCAGATTTACCTCCAGAAGGTCTCCGCCGCCTTTGGACGCCATCCGGAACA
EMPV1_06591	GGGAATTCCTTTAGCCAAAGTTCCAACCTCATTCAACACCAGAAGATTCACAGCGGGGCG

EMPV1_06592	ACCACGTCACACAAGGACAGACCTGTTAGAAAAGTCCACCAACTCCCATCTTGCTCCCTC
EMPV1_06597	CAGAATAGGGCTCAGGTGCTTCTGGTTCCCGAAAATACATTCCTTTTGGGCTATCGCCTC
EMPV1_06598	CACGGTGGCCCTGCGCGAGATCCGGCGCTACCAGAAGTCCACGGAGCTGCTGATCCGCAA
EMPV1_06600	GTTCCCTGTCAGACCTCTTCTTGCAGTCTTGGCGGGGAGACACATACACACATGACACTT
EMPV1_06603	ATTCAGTGGCATTGAATCCAGCTCTCCAGAGGTGAAAGGCTATTGGGCAGGTTTGGATGC
EMPV1_06608	CCTTGGACAGACACAAGTCAAACACAATCCCGAGACGCTTGTGGCAAAATAGAGGTAACC
EMPV1_06613	CCTCAAACTGGCCTGTGCGGACACTTCTGCAGTTGAGATGGTGATCTTTGTCAACATTGG
EMPV1_06615	GCAGATGGGCAAAGAGTACCGTGAGAAGATCGAGGCAGAGCTGCAGGACATCTGTAACGA
EMPV1_06616	CCTACCCCATCTGGCTATTCGCCCGTCCTGAATAAATGTTGCTTGGAGTGTACCCATGCT
EMPV1_06617	GATATGTGGGAAGAAACTCCGAAAGGAAACAGGAGCGTGAATGACCAAGTGACACGAGGG
EMPV1_06618	AATTTCTGGGACATCAGCAGCTGGGCACTGCCCACAGCACTGAGGACTTTTCCTAAGACT
EMPV1_06620	TGCGGCCATCCGAAGACCAACAAGAACCTCCTCACCGTCCTAATACAGGGACAGCAACGT
EMPV1_06623	CTCCTCAAACTATAAAGCCACCTCCTAATTACTCCCAAGGGAGGGAACAGCCTTTAGGGC
EMPV1_06624	TATGAGGACACTCAAGCTGCCCTGTCAAAACACCTGTGGTGAGGAACTGAGGCCTTTTGG
EMPV1_06626	TCATTTCAGCCCACACCAGGCCACTGAGTTGGCCACATTTGACTTAGTGCTGCTTTGCTC
EMPV1_06628	GGCATACCTTTCTCCACAACCTCTGAAATGCACCTTAGTTTTCCTTACCTCCTTCCGGCC
EMPV1_06629	GGGAAAGCCTTCAGTCGTCCCAATTCCTTTCGAAGGCATGAAAGATCTCATACTGAATAG
EMPV1_06631	CCCTGGGGATCTTGTTAATATTCAGATTCTGGGGTGCTTCAGGGGATTCTGCATTTCTAG
EMPV1_06638	GAGATTTGGGGTGGGTTAGCAGGTCACTTGTTCTTCAGCAGGAAGCGGCAGCTTTTCCTT
EMPV1_06639	CTGTTTCGGAGCCTGGAGCGGGGGGGCAGGAGGGGCTCAGGGAGAAAGTGTGTGCCGCCC
EMPV1_06640	GATGAGCCAAGCTCTGGGATGGATCCTAAGTCAAAACGGCACCTCTGGAAGATCATTTCA
EMPV1_06642	CCTCGAAGTGATGCTGTTGTCTATTTATTTGGTCCTGGAGAGGGTGTTTGTGCCTCGTGG
EMPV1_06643	GGAGGAACCAAGAAAAAATAGAAAATATTTTGCAGAGACACAAACTGAGCTAAAGGCATG
EMPV1_06644	TGGACAGGACGACGAAACATGCACGAGGGGACACAGCTTCATCTCACGCGGCCCAGCCTT
EMPV1_06645	GCATCTTGAACTTGCTTCCTGAGCGGGGCAGACCCAGGTGATGATGAGTTTGTCTTTGAA
EMPV1_06646	AAATTCACCACGGCATGGATGACCTCATTGGAGGCGGGCACAGTATTCTGGAGGGACTGA
EMPV1_06647	GCCGAATACAACATAGGCAGCAAAGATAAGCTGCACATCCTGCTCACCTCAAATCAGGCC
EMPV1_06648	AAGGAAGTGGAGCAGTTGATCAAAAAACATGAGGAGGAAGAAGCTAAAGCTGAGCGTGAG
EMPV1_06652	GCATAAGAGAAAGAAGGTTAAGCTGGCTGTCCTGAAACACTATAAGGTGGACGAGGACGG
EMPV1_06654	ATGGAGGTGAACGTGGACATGCTGGAGCAGTTGGACCAGATGGACTTCTCTGTCCAGGAG
EMPV1_06655	CTGTGAATGGCGTGGGCCGAGGGGCCTTACTCAGCTCTATCCAGAATTTCCAAAAAGGAA
EMPV1_06658	GAGGAGGAAAATAGTCTCCAAGCGAAGTAAAGTGCAGACAATACTCTGACTCCCAAACTC
EMPV1_06660	GCCTAAAATCAATGGGAGAATTTGGCCTGGGTGTTGGGAGGAAAGGGCATGAATGTAGCC
EMPV1_06663	GTGGCAGGTACCATGAAACTGGAATTGGCTCAGTATCGTGAAGTTGCTGCTTTTGCCCAG

EMPV1_06664	TATTGGCAGCTCTCACCATGTCTGCCTCAAACGCCAACATACACTCCCCAACACGAGAAA
EMPV1_06665	TTTGAGGCCGGTATGGAAGGGCCCCTATACTGTAATTCTAACCACCCCCACTGCTCTTAA
EMPV1_06667	TAAAAAGTGGATAGTGGCTCTCGTGTCAAGGGATGGCACGGTCACGCTCTCGATCCCCTA
EMPV1_06668	AAGTCCTTTGGGCCAGTACTGAGGGGACCCCACGTCTCAAACGCACGACTTCCTGGGGGT
EMPV1_06669	TGGCTGGCAGCTATAGCTCTGATTCAATCCCTAGCCTGGAATCCTCCATGTGCCGCGAGT
EMPV1_06671	CACTGGGGCAGGTTCCCATGCCATCACCTCCTGATCCCCAAATTAAGGTGGTTTTTAGTT
EMPV1_06677	TTCCTGCTGTGGTGCAATGGGCTGTAGCAGCCTGGGTAGCTGCAGAGGTGCAGATTTGGT
EMPV1_06679	TCCTCTATTCTGAGAGTACCTTCATCGGGTGGGAGGTATAAAGCCTTCTCCACCTGTGGT
EMPV1_06680	ACTCCTTGCAAGAGCTCTCACTGGCCGGTTGCCCCCGAGTCTCTGAACGAGGCCTAGCCT
EMPV1_06682	AATGATGTTAGGGGTTCCCTCCTGTGGTGCAGTAGGTTAAGGATCCGCACTGCCATAGCT
EMPV1_06683	TCCCATCACGACTTGCGCTTCTACCGGGAATCGCTGCAGAGGCGTAACTCGAAGACCTGA
EMPV1_06687	AGCTTGGTTTTCAGAGTCATGTGCCTGGGGCCGAGGTGACTGTGATTTGTGAGGACTTTA
EMPV1_06688	GGGGGTGACAGAGCTGACCTGAGCCGACACGAGAATAGAGTACAAGTGACTCATAGTTTT
EMPV1_06690	GGCCACTTCTAGCCACACAAGGGCCTCTCTGAAAACCGCGCTATTGAGGTTTAATTGACA
EMPV1_06691	CTCTACAAATGGTATTTTGGTGGATGCTGGATTGTGTCTGTGCCATATTTGTGCCCACGC
EMPV1_06692	ATTCTGCTGAACCATGACCCCAACCCTCTCCTCCGGTCAGTCTCCCCCACCTTATTTATT
EMPV1_06693	TCTGATCACAGGCAGCCTCTTCTCCATATTCCCTCGTTAAACATGGGCTGTCATCTAGAA
EMPV1_06694	CCTCTGCCTTAGGACACCACAGAAGGAAGATCAAATTAAGCATAAAGCCAGAAGAAAAAA
EMPV1_06696	CCTCTTGTTGCGCCTGCCGTTCTGCCACTCCAATGTGGTTGAGCACTTCTTCTGCGAAAT
EMPV1_06698	CAGCATCGTTCTTATATGCGATTAACAGAAAAGGAAGATGAATCACTGCCAATAGATATA
EMPV1_06701	CCTTGTCTCTTATGGCTGTATTGTGGTCTCCATCCTCAGTGTCCACTCTCCTAAAGGGAT
EMPV1_06703	TCTGTAGAGGTTCAGGGTCAGCGGTTGAGCAAAAGCACTGTGATCCAGCTCCATGTCTTA
EMPV1_06704	ACTGGCCTGGAGCATTTGAAGAAGGCTTGAAAAGGCTGAAAGAAGGGGACCTGCCAGTCA
EMPV1_06705	TCGTGGTGGAGACGAGTGGCTGGATCAGAGAAGCAGGACGGACTGGACTTGGCCTCAGAT
EMPV1_06706	GACACCAAGAGAATGGAGCCTTCGGAGGATAAATATTAGCTCCAGGGCCTATGTGTATTC
EMPV1_06708	CAGGCTACCAGCAATATAATATAACAAAATCTTCAGACCCCTTGTAACCAAAAGCAGTTTC
EMPV1_06710	TGAGTTCAAGCCACTCCCACATGAAAGATGAAAACAACCCTGGAAACCATCCAAATGCCC
EMPV1_06711	GAGTTCCCATTGTGGCACGGCAGAAACGAATCCGACTAGTAACCATGACTTTGTGGGTTC
EMPV1_06712	ATGAGGAGGATGCTTTCTGGGCACTGGCCCAAATGATGGACAACAAAAAACATGCCGTGC
EMPV1_06715	TCCCTCCCCATCACTTCTACCTCAGGCTGTGGGACAGAAACTTTTAAGACAGGCCTCAAT
EMPV1_06717	AGTCTCTCAACCCATCCAGGTTCCCAGTGTAAAATAAGCAGATAAACGGGTGGATCGGAT
EMPV1_06719	GATGGTAGAGTGGTCAGAAGAATTTCAGAAGGACCCACAGTTTAGTCTAATATCTGCAAC
EMPV1_06722	TCGCTCCTGATCCTTCCAGAGGTCCTAGACCACCAGCGCTCAAGTAAGTGGGTGCTACCA
EMPV1_06724	CCCTCCGAGCTCCGCGGCGGCAAGAATATGGTGCGCCGTCTCTTGATTACTGTGCGGATT

EMPV1_06727	GAAGCAATTCCAGAGCATGCCCCCTTGCACATACACCATGCCAAGTCAGTTTCTTCCCCA
EMPV1_06729	CATTAAGAGACTGAGGTTTTGTGAAGCCAACGTGCTTTCCCACTCATACTGCCTGCACCC
EMPV1_06730	GTGCCGGAGAGGCTGCTGGTGGTTTTGGACATGGAGGAGGGAACTCTGGGCTACGCTATT
EMPV1_06733	TACAGACCTTACAGTCTCAGCCAGATGCAGCACATCAAATATCTCAACAAGTGGGCTGGC
EMPV1_06734	ATGATCATTCTTGATAAGCCGCTCATGCAGCCGCTCTGGCCTGAGGTTTGCCTTGTAAGC
EMPV1_06736	AGCCATTACGTGGCAGAGTGAGAAGCATTGCAACTTAAAAATCTGCACTCAGGGCCCAGC
EMPV1_06738	TCAGCCTCAGGAATTTACACTTGCCCCTCTGCCGTCTGCCGAACATTCCTGCCTGAAATT
EMPV1_06739	AGGGTTCGATTCCGGAGAGGGAGCCTGAGAAACGGCTACCACATCCAAGGAAGGCAGCAG
EMPV1_06741	CTGAGCCAAAAGGCCAAACCGAATTCACGCCTTGCAGTGTGGCAGTTAAACACTACCTTG
EMPV1_06743	CACACGTAAGTGATCTCACCCAGTATTTGTCTTTCTCTGCCTGACTTCTTTCACTTGGCG
EMPV1_06745	TCCACACCACCAAGAGCGTGTGCAAGGAGACTGCCATTATTTCCAGCAAGAAGCTCTGCA
EMPV1_06746	TCCAGGAATTAAGCCTCTGCCCACTTTTTGCCCTTTTATGGTCAGAACTGTCGTGGCACC
EMPV1_06748	GAGCGGCGGCTGCGAGCCAAGCTGGGGAAGAAAACAAAGAAACCCAAGGCAGCAGAGTCT
EMPV1_06749	ATTGTGCAGACGTTGCCGGTGCCACTGGAGCCCGCTCCTGAGGCCGCCACTGCCCCACAA
EMPV1_06751	GCCCATTATTGTGATCACTCTAAGCCCTGCTCCTGCCCCGTCCCAACGAGCAGCCCTGCA
EMPV1_06753	TATAAGGAAAACATCATGCGCCTCTCCAGCCTTCACAAGGACCGCCCCATTGAGCCTCTA
EMPV1_06754	AGGAGGGGTTGCGGAAGGATTGATATATGAGGTACATTTGAAGGTCCAGCCAACTTGACC
EMPV1_06756	ACGAGGACTGCCCAGCCATCGACCAGCCTGCCATGTCCCCAGAAGACAAGAGCCCCATCA
EMPV1_06757	TGTTGACGTTAAGAGCAAAACGCCTTGTTGATAGGGTGTCCGTCCCACCACCAGATCCAA
EMPV1_06758	AGGTCTACCACACTCTTTTCTGGCCTCTTGAGTGGACAGTGGCCTGCAGAGACAACAATA
EMPV1_06759	GAGCTTACCTGCTCTCTGCCAACCACCGTCCCCACAATCTGTTTACATCCATGCAGATAT
EMPV1_06760	TACCACGTCCACGAGTGTGTAATTACAGCACCAAGTCGAAGAGGGAAGACGGAGGCTATG
EMPV1_06762	TTCCACTTCAGGTTTCTGTACTGCTCCTGCTGCTGGTCCAGCTCACTGTTCCTGTCACTG
EMPV1_06763	ATATCAGGCCTTAAAGGGTGGCCTGAAGGCAGACAAACAGGTGTCCCGGACTAGGAGTCT
EMPV1_06764	ATGTCTGGTCGAGGAAAACAGGGCGGAAAGGCACGCGCTAAGGCCAAGACTCGCTCTTCG
EMPV1_06767	AAAAAGAGAGCGGCTGGTCTTCAGGCCGAGATTGGTCAGGTGAAGCAGGAATTGTCCAGG
EMPV1_06769	CTGCAGCAGCTCTGCTCATGCTTGTATCTTGTCTGCACATTCATCGAGGTGTCAGGGAGC
EMPV1_06772	GGGGACTGAGGAAGGGATGTGGTGACAGAACCACTTCCTGCCACTGCTGTGTTGAAAGTT
EMPV1_06773	CCGAAGAACCTAGGGCAGATGGGCACACAGCTGACAATGGATGAAAAGCCGGTTGATTAT
EMPV1_06774	GATGGGAACTCCTCAAATGGATGCTTCCTAAGATGCTGTATTAGTTGCCTAGTGCTGCTG
EMPV1_06777	GTTCACGACTAAGGAATGCTGTCTCTCGGGTGGGCTCTTTGTTCTGGATGGTGGTCACTT
EMPV1_06781	ACAGGCTTGTGTGTGTTGATATTTAATAAGCTCTGGGGGGTGCTGGCCTCCTTTATAGCC
EMPV1_06783	TACCCGAAATTCCACCAAAGCCCGGAGAACTCAAAACAGAGCTTTTGGGATTGAAAGAGC
EMPV1_06784	TCATTTTAGGGCTGCAATAGTGGCATAAGGAGTTTCCCAGGTTAGGGGTCTAATTGGAGC

EMPV1_06787	AGCGGAGAAGACTACTTGAGAAGAGCAATCCAGATCAAGCAGGGAAATACACCAAAGCAG
EMPV1_06788	GGCCCTAGCAAAACCTCTCATGTTACATGGTCACAACTCACAATTCTGTACAAAAGTTCG
EMPV1_06789	CATCGACACGCAGTACTACCTGGAGCAGCAGCTCGCCAAGCCCCTCCTGCGCATCTTTGA
EMPV1_06792	CGCTGGGAAGAAATGAGAGCCAGGAGATGTTGCAAGTCCTTGAATCCCTACCCAGGTTCA
EMPV1_06793	ATGCCGGTCATCATGAAGAACTCGGTGTACATACACAAGGCGGCCACTCGCCGCATCAAA
EMPV1_06796	AAGGGAGAAGCCGCTCAAGTGTCCGGACTGCGAGAAGCGCTTTCAGTACGCCTCTGACCT
EMPV1_06798	TGCAGTTCTTGGAGGATCTCCTGGGAAAACAAGGGGTGACTATGGCTGGATGTGGAGGAA
EMPV1_06801	TGGGTCAGTGCCTTAACCTCACCTTTCAAATGGGATGACGCTAACAGTGCCTCCATCACA
EMPV1_06802	ATCTCTTTCATGGCTGTTGCACACCTACACTAAGTGCAATGCTACAAATACACCTACTCC
EMPV1_06804	GGGACTTCCATATCAGGTATTGGTAGATAGAAATTTAGAAATGAAGACATGTAGAAAAGC
EMPV1_06806	AGCCAAGGACAGATGACAGAAGCAATTCAATACCTGAAAAAAGTTGTGAACATTGCAAGA
EMPV1_06807	TTCCTCTCCACAGGCCTGGGGTTGTGTGGAAAGAGGGAACGTGGAAGTGTGTTGGAGTTT
EMPV1_06808	AGAGGAGAGAGAGCTTCTCATGCACAAGAGTTCTATTGCAAGCTGAGGCGCAGGTTTCCT
EMPV1_06810	GGACTCTGATGGAAGGACCGAGGGGAAATCCCAACCAGAAAAGGAGAATGCCTGACTCTT
EMPV1_06811	CCCAGTCTTGGGGCCTTGATGCTGCATGGGATGGTTGCATTCCCAACCTGTTTTCTTATA
EMPV1_06812	CCGAAATCTGCGGAAGTTAAACAGCCCGAGGATGGAGTCGGCGGCGTTAAGTTCCTAATA
EMPV1_06814	CAGACCAGGTCATCATCAAGAGACAGATGGATCCCAGAAAACCAAGATTCTGCAGATGCT
EMPV1_06819	GGTTGTTTCCATGTCATACCTACTGTGAATAGTGCTGCTGTGAACATAGGGGTCCATGTA
EMPV1_06820	CCAGAGAGATACCCAGGAGAAAAGGGAACAGCCATTTTATTCCTGAAAGCCAGGTCCCAG
EMPV1_06821	TCATGACAGATACTGGTTCCCACATCCAGGTTGAACAGTGTGGCTCAGAGACGCTTCCGA
EMPV1_06822	GCGACTTGGATCCCGGAACGGAAGGCACCAGTAGCGGCATATGTCCGGATTAAGCGTGGT
EMPV1_06824	GCAAGTGCAACCGCTGTGAATGCTTTGCCGGCTATTATGGTGCTCTGTGTGATCAGTGCT
EMPV1_06825	GTCCAAGCAAGATTGAGAGTGTCGACATATCTACAGATGTGGTGTATGAGGAAGAGGGAC
EMPV1_06827	GTGTGTGCACCTGTACAGGCGTGCCTGTGTTTGTGCATCTGTACACAGTGCATGCACGTG
EMPV1_06828	CAGTGCATCCGTGGAAGCGAGGGGAATTCTGGTTTTGAGTGAGAAGGTGTGGACTGGTAT
EMPV1_06829	CACAGTTCGGCTTGTCCACGTTTCATGATTGTATGTTTTCCAATCTATTAATCCAAATTA
EMPV1_06830	ACTATTAATGCAGATACTACCTGTGGCAATGACTGGGTCTGTGAGCATCGATGGCGTCAG
EMPV1_06831	TTAGAATCTACTTGTGGCTGAAGACCCCAGCTCTGATGCAGATGAAGTCCAGGGAAGGGC
EMPV1_06832	TTGGCTGTCGCAAGTGTGTTACTTTGCGGCTAAGCCTTCTAATCCTATGGTCATGCCCTG
EMPV1_06834	CTTGCCTTTAAGGGCTATGGTTGTGGTGTGATTCTTGGCTAACCTGCTTTCAAAATCAGT
EMPV1_06835	AGCCCACTTGACCCTCCTCCCAAGGAGGAAATAGGGGGAGGGCTGAGTGTGTGGGGATGA
EMPV1_06836	GAGTAGGACTGTATAGGTAGATCCAGACTGTATGATGTTGTTTTAGGGGCTAAAGGGGAG
EMPV1_06837	GACCATCCTGGTGGCCCTTCGAGATGTCTCCTGGTGCATTAGCTTTTGCTATCATATGGC
EMPV1_06838	AAGCTTAGTAGCCACTGTAGTCTTCCAGCTCCTGAAACTCTTAGTTGGATGCACAGTTCC

EMPV1_06839 GCTTCTGGTCATGTTCAGTTAACGCTAAGGGATTCCTAGAAAAACAGTCCCTGGGATACC
EMPV1_06840 ACAGACTGCACTTCCGCCCTCTACTGCTGTCGCGAGATGAGCGCGCAGACTCAGTGTTTA
EMPV1_06841 AGACTCGAGAATAAGGATCAAGATATCAGAATAGGAAGACCCTGAGCTCACCTCCTCCAG
EMPV1_06843 CTACCCTTTGCTGGTGCCCCAGGCTTGCCAAAAAGGCCTTGATAAACAAATAACGCTCCA
EMPV1_06845 CTGGAAGAGGCTTCTCAAGCAGCAGCCGAGGGAGATAATCAGTGTGAGCAGAAGCTGAAA
EMPV1_06847 ACAGGTCATTAGAGAAGACAGCAGAGACCTGGGATGAAACTGGAACCTGCACATTCTGCC
EMPV1_06849 AGCAAGCCGACCCGCATTCCCGGGAAAGGTTCACGTCTCACCGTGGCCCTGATCCCACTA
EMPV1_06850 GGGTAGATTTGACTCCTTTGTGGTCAAGGTCCACACAGGCATAAAACCATTCGAGTGCGG
EMPV1_06851 CCGCCTCACAGATGACTATGGGACAATCTTCACCTATAAAGGGCCCAAAACTGAATGTGT
EMPV1_06852 CTCGAGGTCGGGGAGTTGCCTTGTTTGGCACCAGTTGGAGAGTTATAGGTCATGAAGCTT
EMPV1_06853 ATGTCGGGAGCTCAGAGATTGCCCTTTGCCCACGAGCTAAATCAGCCAGAGTTGCCAGCT
EMPV1_06855 GTTCCTGCCCCATGTCAGAGGCCAGGCCTTGGAAGCCATGTTCCTGACAGTCCCGTGGAA
EMPV1_06856 TGCAGCCGTGTCCAATGTTGCAGTGGGGGATGGTGTTGCATGACACAAATTCTCCTCCAA
EMPV1_06857 GTTTCCCTGCCCAGTACGTGAAGCTGCGGGAGCGGATGCAGATACAGAAGGAGATGGAGA
EMPV1_06861 CACTCTCTCCCTGTCAGGAGGATCTGCCATTGCCCTCATCGTGGTCACATTAGTGGTGAT
EMPV1_06867 TGTCAGCTCTGCCTTTTTCTTTGTTCCCTGAGGTGGAACCCATACAAGTTTCAGTTAAGG
EMPV1_06868 GCTGACAGTGGGGTTCTGACCATGACCTGCTTCATAGTGTTGCTGATATCCTACACGTAC
EMPV1_06869 TGGGAGTGACAGTGCTGACAAAAAGCCTCAGGGCCCCAAAGGTGGTGGCAATGCAGTAAA
EMPV1_06871 ATGGATGCATTGTCCAAGCAGTAATGAGGATCAGGTCAGCTCAAGCCTGGCATAAAGCCC
EMPV1_06872 AACCGTCCTGGTTTGCCTGGGGCTGAGGGAGTTCAGAGAGACAGGGTTTCAGTGCTAAAA
EMPV1_06873 CCGTTTTAACCGTCTAAGCAGGGGTTGTGTTACCTGATGAGCCTGAGATTCTAAAGGGGC
EMPV1_06875 TTTAGGGCACCACCCAAGGATGGCTCATGCCAAGGTATTATTCTGTGGTCTCAACCTCGG
EMPV1_06876 GAGGAGTAGGCCTCTGGGCCGTCCGTGTGGTCCAGGGAGGATGGGCCATAAGAGGCAGCT
EMPV1_06878 CAAGTGAGAGTTTTGATAAGCCCCGTATGGACCGCGTTGTGAATCACTTGCCAGTTGTAC
EMPV1_06879 AAAGCCTTAGCTCCTCAGGGAACACGGGTCCCAGGCCCCAGGGAGTGGGACAGTCTGTCT
EMPV1_06880 CCGCGTCGCCATGGCCGCGGTTCCCGAGTTGCTGCAGCAGCAGGAGGAGGATCGCAGCAA
EMPV1_06883 TCACTGTGGGATGATCAGAGCTACAATAGTGTGAGTGATAAAAGAGCTGCCTTTACTCCC
EMPV1_06885 GCCATGCTGGACTTTGTGTTCACAGTCGATGACCCTGTAGAATGGCATTCCAAGAACCTG
EMPV1_06886 GAGTAGAAGCTTGATCCTTGCAGTAGAGTGAACTTTAGTGGTTGTATTGGCTTGACTGGC
EMPV1_06888 ATATGATCTGATTATGAAAAAATTATGAACATAAAAGTAGAATACATCTTATAAAAGGTA
EMPV1_06890 AGGCTTGTCCTCTGTTGCTGGACAGCTGGAATGCTGATCATACTCCCACCACTTATCATG
EMPV1_06891 TCTTCCCTGATGTCCCTCCCACATATATCTTCAGTCGTGCTAACAGGACAAGAGGGAGAC
EMPV1_06893 ATATGCAGGCATGGAGAAGGCTGACATTTAGAACTAGCTCATGGGCGGCAACCGGCTGAA
EMPV1_06895 CAGAACAGGAAACCTCTCTGGTGCTGTTTCTCGTGCATCTGTCCACCCAGTTCCCGGTAT

EMPV1_06896	ATGCCACCGCACCTGTCCCTCCTTTCGGCTCAGGGAAGTGTCTTCTTGCCCACAGTTTCT
EMPV1_06897	GTTGAATAGATGATAAAAAGGAAAAATGCCCTATAGGACAGAGAAAAGAGACTCCATCTC
EMPV1_06898	CATTAGCCAGCACTGCCTCTTTTGCTCACTTGCTGCCTGTTCAGAGTGAGTGGCATGAAG
EMPV1_06899	ATAGTCTGCTTGGCAGTGACCTTGGCGGGGGCATTGGAGGAAGTCCGGCAGTGGGACAGT
EMPV1_06902	GTTCATAAAATTACACTGATGATAAAAAATTTGTTCTATTGAATAGAAATTGGAAAATAA
EMPV1_06905	GAGAAAGAGAGACGGAGGGAGAAGGAGATGGAAGAAGAGGGGGTCACGTGAGCCTTTCAT
EMPV1_06909	TGACTTCAGTACACTGGTCTTCTAATGGTCATATTCTAGAGTTTTAGGCAGTTGCATCGC
EMPV1_06911	CAGGGCCATCCTATACACAAATGTCCATCCTCCAAAGTCATCTGCTCCTCAGTATCTCCC
EMPV1_06912	CAGGGCCGGTGCCAATGCTCAGGTCAGATGAGAAACAACCAGCTTTGTAAGGAGAACCAA
EMPV1_06914	TCCTTCCCGCCTGCTCAATTCTTCTCAGTTCTGCCAAAAGCAGGGCCTGTGCAGCTTCTC
EMPV1_06915	CCTAGACGGCGATGGCCATGGAAGTAATTTTCAGAAGAGATCAGACTAACCCTAATTGTC
EMPV1_06916	GAAGGACACCCCGTCCTCCAAATGCTGCCACTCCACTGAGCCGGCCCTGGCGGCTGGACA
EMPV1_06919	GCTTCCTCTGGCTGTGGGAAAACTCAGGCTAGTGACCGTTTCTTTAAGGTTCTACAAGGC
EMPV1_06921	ACATGCTCATGGCAGTCACTCTGTCTTGCACTTTATCAATGGTCTGGGAAACCAAGCAAC
EMPV1_06930	GGCAGCCGTAAAGACCATTTTGGAATATGCAGAAGCTGGTGAAATCTGCCTCCCCATCCT
EMPV1_06933	CACTAACACTGAAAATAAAGACATTTACTGGAAAAATGTGAGAGAGAAGCTGTCAGCCGG
EMPV1_06934	TCTGATGACAGTGAGAAGGAGTACAAGAGGGCAAGCCCAATCACACAGGCACAGTTCAAG
EMPV1_06935	CCCGGTCCTCACCCGGGTCCCCTCAGCCGTGCTGGTCTCTGATGTCTATTTATTGAAACT
EMPV1_06936	AGTGGAGACGGTCAAGACGATGCAGGGGAACTTGACTTTAGTGGTCTCCTGAAACGTAGA
EMPV1_06939	CCACATACATAAGAAACCGTACATGTCTTTCGCTGTCCAAAATCACAAGATGCCGTGCTG
EMPV1_06942	GCAGATTCTCCAGGATGACAAGAAACAATTAGCTGGACGATACCAAACTCACAGCTTAGC
EMPV1_06943	GTGGACAGAGAAGTGCCAGTGTCGGCAGAAAAGCATGTTTTCTCAAGAATGCCCATCTGT
EMPV1_06944	GAATTACTCACCTAGACACCAGAGCCCTGCTTAGCCTGGATGCAACCTCAGGGATTCCAA
EMPV1_06945	AACCGGGGAAGTTGGGAAGAGTCGCAAAACGCGAAAGCAACTCACCACAGCCAAAGAAAA
EMPV1_06946	CTGGTTACCCTGCTTCGGTGGAGTACCATCCCTCAGTGCTCTTACAAAACTATTCATGGG
EMPV1_06948	ATCTTAGTCCCGGCTCTCTCCGTCTGCTTCGTCTTCACGGTCACCATTGGGGTGTTTCCT
EMPV1_06949	GGTCGATGGGGTACACAATGACGATGTCGAGGAAACAACTTTCTTGAAATTACCTGGTGG
EMPV1_06952	CTGTGGCTTCATCCACCCAGTGGCCTTGACTATCTTCTTGACTTTACAACTGGACTTGCC
EMPV1_06953	CCACAGCCCCTCGAAGCTCTGACAGTTGAACAAGTTCAAGATGATGTAGAAATAGACTCT
EMPV1_06954	ACCATTTCAGGCGAGCTTTATATGAGTTCCAAGGAATGTGTGGGAGCAGAGATGTCACTC
EMPV1_06956	GAGCCGGGCGGCGCTACTGCTGACGCCTCTGATGCTCCTGCTGCTGGCGACCTTTGCACA
EMPV1_06958	CAGCAAGCCCAGGGACACTTGAACGTCAGATAAACGACAAATAATTGTTTAGCATGAGTA
EMPV1_06959	AAGGCTGTGCGACACCATTGGCACTCTTCTGTGATGATGGAGATGTGAAATTTGTTCTGG
EMPV1_06960	TCCAGCTATTGCTTATTGCCAGTCCTTCTGAGCGTTCCACTCCCAAACCATCTTCTCTAG

EMPV1_06961	GGTGCCTGTGGAGCCAAGTGCAAGATATTGGTGAAGGATAAGGACCAAAGGAAACACGGT
EMPV1_06963	GAGCAGAAAGATGTGTATGGAGCTTGTTATTGTGGCCTGGTTTAGCGGGGCTCTCTATGC
EMPV1_06964	CTTCTGGTCTGAGAACCTCCTGACCAGTTGCCTCAGATTTAAAGATATGTCACTGCCTTC
EMPV1_06967	CTATGTAAATACAGTGAAGTTCCAGGACTGAGAGCATGGACTACATGAGGAGGATTGGTT
EMPV1_06970	GGGACACGATGGGGAAAAGGGCTGCGGGAACTACAGGCTGGATGGGAACATAAACAATAT
EMPV1_06971	TGGCCATTGCCTGAGCTTTGGCGTATATGTGCAGGAGCAGCTTGGATCTGGCATTCTTGT
EMPV1_06972	CCTGGCACATGTGCAGAAATAAAGACGTACGTGAGGATGTTCATCACAATATTGTTGGTG
EMPV1_06973	TTTCATTTATATACCCTGGACCCCGCAAAACTCCCCACCGCGACCAGGGCTCATGCTTGA
EMPV1_06975	TCCATACTGTTCAAATGAAGGGATGCCCTGTGGTTGCAGATCGCCCATCGTGACCTTACA
EMPV1_06976	GGCCAGTACTCTTTCTGTGAGGTGAAAGCCTTAAAATGTAGCTCCCTGGCCCTTGATTGG
EMPV1_06978	TGGCATGTTGCCAAAAAACCTTCACAGAAGAACTATGATGCAGAGGTTGCATCTTTTCCC
EMPV1_06981	GTGCTCCCGGTTCAGCCAGGCGGGTGGCTCATGGTGACACTTTTACTCATGTAAATAAAA
EMPV1_06982	GTGTTGTTTTGGACTGTGCTACCAGCTTAGTGCAGTAGCTGCTTTGTCATAAAAATGTCT
EMPV1_06985	TTGGCAGAGACCAATGGGGGAGCAGGCCATTCAGACTTGGGGAGCAGCAGCAGCAGCAAC
EMPV1_06986	AAAATGTTATCAATCACACGGACGAAGAAGGATTTACTCCTCTGATGTGGGCTGCAGCAC
EMPV1_06987	AAGGCATGGCCAGGTATGTCTGTTCCATTTGTGATCAAGGCAACTTCAGAAAACATGACC
EMPV1_06988	AAACCCCGCTCAGGCTGGCTCCTTGGCCTGCCACTTTGATGGAGGAGGTATTGTAAGCTT
EMPV1_06990	GAGGAGAGGCTAGTTCCGATGGGATCAAAGGCACGAAAAGCAAAGAACCCTATTGGCTGC
EMPV1_06991	GGGAAGCCCTGCTAATGTCACCGAGTCCAGGCTGTCTTTCTACTCTGGACATTCCTCCTT
EMPV1_06992	AGGTGCAGGTTTACCAGGAGCCCGACAGAGAAACAAGAGTGCAAATTCAAGAGTCTGTGC
EMPV1_06993	TCTTGGCCACAGGTTCCTGGCTCTCGGGCTTCACCGTCTCCATGGGAAAGGTCTTCTTCA
EMPV1_06995	TCCGGCACAGGACACACGTTCTGGACTTTGATCACTGCCACCCTGTGGGGTGGACGCTCG
EMPV1_06996	GATGAACTCACACCTCTGTGGCCTTCTGGCTCTAGTCTCTTGGTCCCTGAGTGTCTTAGT
EMPV1_06998	GTCTCACATGTACTCACAGACACACATACTCCAAACTTACATGTGCACACACCCATGTGC
EMPV1_07000	AGAGGACTAAGTGTGGGCATGGGACCTCATTTCAGTTTCTAATCTTCATGAATCAGGGTG
EMPV1_07001	TTCTCCCACTCTCCAGTAGCCCCTGTAACCACCAATCTACCTTCTCTCTCTGTGGTCCAA
EMPV1_07002	CTCCTCGTAACAAAACACGCCCCCACAGTGATTACCTGCACTGCTTCCGGAGTTCCATTT
EMPV1_07003	AAAAAGAGAAAGTTAAAGTACCAAATGTGGATCTGCCTTCAAGTACATATCAACAGCTCG
EMPV1_07004	GAAGGGGTGTTTAAGCTCAAAACTGAAAGACAGGAAGGAGCTAGCCATGATGAAGTCAAG
EMPV1_07006	TCCCCAGTTGTTACCAGTGGAAAAGGAGCGGACTGTGGGCCCTCTCTGGGATTAGCAGCA
EMPV1_07008	GACACAGCTCTGCGCATGCTCAGCACTAGCCTGACGGATAATACACACGTGCTTACCGAT
EMPV1_07011	ACGTTAAAATCGTGTTGTACTTCAATATAATTGTTAACTAGTTGTAGTTGATACTCATAT
EMPV1_07013	TGAGCACCACCCTATTCCTTGTGTTGCCTTTTATTATCATTGCATGTTCCTATGGCCGTG
EMPV1_07018	GGCATGCTCAAGGGAAAGCTAGGTGGCTCAGAATCATTGGAAGCCTGGGAAGTTTTACTG

EMPV1_07020	GCATATCCCTTCTAGTCTTCTCTCCCTTTTCTCAAGGACGTCTGCTCTTTTCTTCGCCGT
EMPV1_07021	TTCTTCCTCTTGGCCCTGGAACTGCAGGTGTTGGCTGGCCCACCTCTTTGATTCTGTATT
EMPV1_07024	GTCAGCACTCTCAACGGATGGATGAATCATGCTAAGGGGAATTCAGGTTGGAGAAGCCAG
EMPV1_07025	AGCACCTGACATTAATACGGAGCCAAACCAGAATATACTGGAAATCTTACTAGACTGTTT
EMPV1_07026	ATGGAACTCTCTTCTTCATGTATGTACGTCCTGCGTCTGGTCCCTCTGAAGGTCATGGCA
EMPV1_07027	TGATCGCTAAAACTCACCCCCACACCTCCAGATGCACCCAGCTCAGCACTTCGTATCAAT
EMPV1_07029	ACCTTTGCTCAAGCTCCTGTCACACATCTTACTCTGTCCTTTGTAGGTGCTCCTGGTGAC
EMPV1_07030	TTCCCTGGTGGTCTAGCGGTTAGAATGCATTGCTTTCACTGCTGCCCGGTTCAACCTCTG
EMPV1_07032	TGTCTTCCCAGCCCCATTTTTGTTCTGTGCCAGGCTTGTGGCATTTTGGTGCTCACAGGA
EMPV1_07035	CCTTGGCAACATGAAGGTCAAGGGAGAAAAAGCAACTCAGGGGCCAGCTGTGACTTCTTG
EMPV1_07039	CTTGTGAGATCCTGGCCCTTCTTAAACTCACCTGCTCAGATATCACCGTCAATGTGATTG
EMPV1_07040	CCACAGCAACAAGCCCCCCACATTCTCCACGACACTTTGAGACTATCCAGACTCAGAAAT
EMPV1_07041	CCGAGGCGTCGAGGGCTGGGCGTACCCAGTCGTTCTGCCGGCTGGTGGAGACGCAGGGTT
EMPV1_07042	ACTGTTCCCAATGATGGCGGGCCCATCGAAGCTGTGTCCACTATTGAGACCGTGCCATAT
EMPV1_07043	GCCCCCATTTGCTGAAAGCATCACTAATGAATTCAGACAAAGAGTACCATATTCAGTTTT
EMPV1_07045	CTGGGTTCTTCTCTGGAATTGGTAGTTGGAGAAACTCAACAGTGGGTGAACATCCATGGC
EMPV1_07046	GAGAAAAGGCCTGAGAGAGAGGTTCTGAATACGCTCAAGATTTCACTAAATGCTGCGAGG
EMPV1_07047	GGCACACTGCCAAGTTCAAGGCTACACTGTATTCCTGTTCCTCCCCAGCCACTTACTTTT
EMPV1_07048	ACAGCCAGCTCACGGCGGCGACGGCGGTGCCCTTAAATAGCATTCGGAGCCAGTGCAGGG
EMPV1_07050	AAAAGATGGTCCGGTTTTACGTCCTCAAATCCACCAAGGCATGCCGCTGCCCGTGCCATT
EMPV1_07051	TACTCGAACAACAACTGGATGATGAAGATGAAGAGGAGGAAGATTGCCCGAATCCTGAGG
EMPV1_07052	ATCCACGGTAAATGTGACAGAACATTCCTGTTGTACAAGCCAGGTGCTGGTTGAGCCCAC
EMPV1_07053	GAGAAAATGTTCAAGGGCAAACGGGGTGCACAACTTGCAAAGGATATTGCCAGGAGAAGC
EMPV1_07054	CTGTGTAAATTCTGTGATGTGCGATCATCCACCTGTGACAACCAGAAGTCCTGCCTGAGC
EMPV1_07055	CCCACTCTCCTATCTAGCTCAGAGCAAAGCCGAAGTGCTCACTGCAGGAGTTCCTGTTGT
EMPV1_07056	GACTACAGCAAAGGGAAGTGTCTGAAGACGTACACCGGCCACAAGAATGAGAAGTACTGC
EMPV1_07059	CCTTCCTAGTTTCCCCAAATTCCCAGCTTTCTCCTCTGCTATGCATTGTCTTCACTGTGC
EMPV1_07060	CAAGCACTGTCTTATCCTGTCTCAGAGTCTGATGCCCTCTGAGAATCTGGCTAAACCTTC
EMPV1_07061	GTAGAGCAGCCAGGCCCCATCAACCAGGGGCCCCAACAAAAGAAGACAAGATTCAGTCTT
EMPV1_07062	AGGGGTGGGAGGTAGGAGAGTTCATTCAGCTGGGGAAAGAAGGAAATGGTACCTGCTCTT
EMPV1_07069	GCGAAGAGGGATGGAGACTCTTCAAGATCGACAAGGAGTACTTGCTAAAAATGGCAGCAG
EMPV1_07071	CAGGGATACAAACAGGAACACCATAGGTGTGGTGTGGTGATCCATGTGATTGCAGTCTAG
EMPV1_07072	AAGAGCACAAGTAGCTTTTGGAAGAGTCCTGAAATACAAGTCCCTGCTGTCAACATAGAG
EMPV1_07074	GGGAAAAGGGAAATTGCATTAGAAGGCCAAATCATTGTGGAGTCATTCTCCTAGGGTTTG

EMPV1_07075 CCCACCCAAAGAGCCAAAGAATAGTTCCTGTGACATTCCGCCTTCCTCCATTTTAGTCCC
EMPV1_07076 GGCGCCTTTATCCCGGGAGTGCCCGTGCAGCCCGTGGTTCTGCGGTACCCGAACTCGCTG
EMPV1_07078 TGGGGCTGCCCATCCACCTGCTCTTTCTGCGTTTCTATAAGCGTGACAAGGAAGAGCTTT
EMPV1_07083 CTCTAGGATGTGCTCATTGCATGGGCTGTGTATATTTTTATTCAATACCCAGAGCGTGCA
EMPV1_07085 GATGGATGCCTCTCAAGTTGATACATGTTCTCCCTAGAATCTGCCCCATCCCTTATTCCT
EMPV1_07087 AAAAGTAAACAGGACACTCCCGCTCTGCCTCCGAAGAAACCTGCTCCTCCTCGGCCTAAA
EMPV1_07088 AGTGATATATATGTCATTTTTCATTTGCCTGAACCTATAGAATGTGTTGTACCAAGAGAG
EMPV1_07092 CTTTGTGTTGAAATCCTAACAGGAATCCTGGGTAGCTGTCCGGGGTACAGTGAAGTCCAG
EMPV1_07093 TGGACTAAATTCCAAAGTGTTGTTTAGTGATTACAAATGATCAGAATTAGCTGCCAATTG
EMPV1_07094 AGAGACAGAAGACAGAGCCTGCCCTAGAGGTACAAGCCTGCCCTTAGAACTGGAGATGAT
EMPV1_07095 GTTTGCTGGCATGGGTAATCTCATTAAGGTGCTAACCAGGGACATAGACCACAATGCAGC
EMPV1_07096 CCTCTCCTTTCACACTTAGTGTTCTTTTGGTTTTAGCCCCTTATCACTGGCCCCAGAGTC
EMPV1_07099 CTTTGCTGTCATTCTCCCCTCCAGATGTCCCCTTCATCTCTGCGCTCTAACAGAAATCTG
EMPV1_07100 TCTTCCAAGGCTTTGCATAAAGACGGCTGCCCACAGAGTGGAGTACAGAAGAGAACTGCT
EMPV1_07101 GGAATAGGACGATGCCTGCCCACTCTCCCGGGTTCTTTTAGGTTGTCATCAGGGGCTGAT
EMPV1_07104 GAATTAGCAGAATCTGGATCAAACTTGAGTGCTGGACAGAGACAGCTGGTGTCCTTTGCC
EMPV1_07110 AATCCCCACCGAGGAGGATGAGGCAGAAGACACATCAGCCAGCATCATCTCTGACCTGAT
EMPV1_07111 AAGTCTCTCTGGGCAGGTTTAGTGGTCTTGCTGCTTCTTCAGGAAGGATCTGCCTACAAA
EMPV1_07114 CAGATTCTGCGATAATGTGTGGACTTTTGTATTGAATGATGTTGAATTCAGAGAGGTGAC
EMPV1_07118 CCAAGAAGCTCAGTTCCTACTTCCACTTCCGGGAGCCTGTTGAGCTGAAGAACAAGACCT
EMPV1_07119 CTGCATGGGGATTGACAGGCAGCATATTCCTGATACATATTTATGTGAACGTTGTCAGCC
EMPV1_07120 CTCACCAGCACCCTCTGGGGCCAAGCAGGAGGGAGGCCTGGGCGCCCGGCCTCCCTCCTG
EMPV1_07121 GTAACTGGCAAAAATGGAGCTGAGCAGAGTCGCCAGTACCTGAGTGCCCAGGAAGGAGAC
EMPV1_07122 CGTCCAGGTGTTCATAGCCACGCTGCACTTCCCTCACCCCAAGTATGTGGACCAGTACAA
EMPV1_07123 TGGCAGAGCAACCCGAGGTGCAGGATGGAGGCAGCGGCCAGAAGGAGAATGGCTGTGTGT
EMPV1_07124 CAGATGCACCATAATTCATTTAACCCATCTTCTATTATGAGACATTTAGATTGTCCTTTT
EMPV1_07125 CCGAGACACAGTCCTCGGGACTCTCCTTACACTTGACACCAATGGCAAATGTGGGGGCTT
EMPV1_07127 TTCCTCTGGTCCCTTTCCACTGTCCAGCACCGGGGATGCACTGATCACCCTGCAGACGCT
EMPV1_07128 GGACCAGAAGATGACTTTGAATCTTGTTTGAGAAATATAAAGTCACAATATGAAGTTTTC
EMPV1_07129 CCTTGTAGAGCACCATGGCTGAGCAACTCCTTCCCCTGGCTTTGTATTTGAGCAGCATGC
EMPV1_07130 AGCTGCTGTTGATGTCACCCAGAAGTGCACAGATGAGTCCTGGCAGCATTTGGAAGCAGT
EMPV1_07132 GAAGAGTTTTAAGGAGAGGTGCTGCTTACTGTGAAGGCTAAGGTGCTGGGTGCATCAGTC
EMPV1_07134 GCAGAAGCCTAGGGTAAAATCATTTTCACTGCCCTAAGCATCACTGGGATTTGTGTTGGA
EMPV1_07135 ATGCCCCGCAAAATTGAGGAAGTCAAGGACTTTCTGCTTAGGGCCAGACAAAAGGGTGCC

EMPV1_07136	GTGGTTGAGACAGTGTTGTGTGTGCATTTTGTCCCTAGAGAGATACTGTTAGAGAGGGCC
EMPV1_07137	CCCTGGAGGAAAAGTGATAGACAACAATGACCACCTTTCCGGTGAAGTCTGGCTTTTTGG
EMPV1_07138	AGCAGTCTAGCAGCTTCCCACCCACAGTGGTACCGACAAGAACAAAAACCTCTTGTAGCA
EMPV1_07139	AGCTGTTGTGTCACTTCCTCATGCTGACATATTTACTAGAGGGTAAAGTTAACAACCTTC
EMPV1_07142	GTGAAGGTAAGTATAAGGCTGAACCAAGCTTTTACAGGTTGATAAATAGAGTTGTTCCTG
EMPV1_07143	TTGCACCAGTGTGGGATGTGTTCAAAACATCTACAGAGAAATTAGCAAACTGGCACTTGG
EMPV1_07144	CAGCAGGCTGCAAAGTGTGTTTCATTGGCCGGAGCTGTGTCATGTGGCTGACACAAGAAA
EMPV1_07145	TGACAGTGATGGGGAGCTGGAATCCAGATACTCCTCCGGGTATTCCTCTGCAGAGCAGGT
EMPV1_07146	CCAAAAGTTGCTAGGACCCAGTTTGAACATGTAGAATATAAATTTCTCCTTTGGCTAGAG
EMPV1_07148	CGTCCTGTCACCTCGGGTTCATCTTATTATTGTGAAACGCATCATGTCCAGGTTCGTCCC
EMPV1_07149	TTGCTTGTCCAGAAAACACCACCACGGATGGGAAAAGGCTTCCAGCCCTCTGCAAAACCA
EMPV1_07154	GATTAGGAAGTGTGGCCTCCAGCTTTGTTCTTTCTCAAGAATGCTTTGGCTATTCCAACC
EMPV1_07155	TCTGGCTTGGGAATGTCCATATGCCACAGATGTAGAAGAATGTAGAGAGGCGTCCAGGCT
EMPV1_07156	AATGGGTACCACTTGCCCTTAACCTTCTGGCTTCAAGGTCCTGCTATGACTCAGGCCCCA
EMPV1_07157	ATCTTGCCTCTGCTCTTCTACCTCCGCAAACGCAACAAGACGGGCAAGCACAACGTCCAA
EMPV1_07158	CCTTCAGCTTTATAATGTCCTCTGTACACCACCTTACATACCCATCTCGCAGCCTCCAGC
EMPV1_07159	TCTGCGGGACCGAGCCAGACGCTTTGAGGAGGCCTTGAGGAGGAACACTGAAGAGCAGCT
EMPV1_07160	CAGTGTGTGCACAGTCTAAAGATAGAACCAAGACAAGGGCAGTTGTTCCCAACAATATGA
EMPV1_07161	ACAGATCCTTTTCCAGAGCATCCAGAAGGAATGTGCCCCTGTGGTCACCTTGATTGAAGC
EMPV1_07162	CTCCTAGATGCCAACAAGTCCATCCTGCATCGTTTTGCTCCTTTGCGTTTTCCCGAACAG
EMPV1_07163	ATGTGGGCAGGTTCACGTCCTTGACGGCAGAATCCTTTCTGGTCTCTCTCATCGTAACCA
EMPV1_07164	ACAGAACTGGGAAAGAGAAAAGGAGAATCAGGGGGAAAATGGATTTGGAAAAGCTAGGGG
EMPV1_07165	ACCTGGTGAAGATCATAGGAAGGAATTACGCTGGGGTGTCCGAGAACAGATGCTTTGTCG
EMPV1_07166	CCGGCAGGGCAAGTTCAAGCCCAACAGTGTCACTTACAGGACCAAATCTGGCAACATCTT
EMPV1_07168	ACAGCCAATGGTTATGTGATCATGTCTGGTGGTAAGCCTGCCCTGAAACGTGACTTCTGC
EMPV1_07169	GCCTACGTTTGTCTCTCAAGCATTCGAAGACACATGAGACTGCACACTGAACAGAAACTG
EMPV1_07171	GAGAATGAGCCAATATCAAGTCCAGATAAAATTCCCTCCTTGGAGTTCCCACTGTGGTGC
EMPV1_07173	CAAAGGGGATCTAGCAGTTCATCAAGTGAGGAAGTTGTTGGCAATAGTGCTGAGCAGAAG
EMPV1_07174	GCCTCAGGCAAAGAAAGCACCACCTTCATTGTAGACATTACTCCAGTGAGGCAAAGTCCC
EMPV1_07176	GGGGATACTTTGGAAAATAAAAGTGTCTTGTGGAGCTTGCACTGTACTGGGGAAAATGGA
EMPV1_07178	TGTGAGAGGACAAAGGCCAGGCTGGAAGGCTGTGCAGAAGAAGGTAGGAGGCTGAAGAGC
EMPV1_07179	CTCAGATCTGGCATTGGTGTGGCTGTGACTTTAGCTGGCAGCTGTAGCTCTGATTCAAGC
EMPV1_07180	ACCAGCTGTCCAGTTTGTGCCACCTCTTCCTGAGTGGAAAACATCTGCAGTCCAAAGGAT
EMPV1_07181	TTCCAAGTTCACTGCAAAGTTCGGAGAGTCGGAGAAGTGCCCCCGATGTGGAAAGTCAGT

EMPV1_07183	GTATGGCAGAGATTAGGATGCATTAATTTCATTTGCATGTTTGCTCTCTATAAGCCAATC
EMPV1_07184	AAGCCCAGGACTAGGAAGCTTCAAAAGTGATCTGTCAAACATTTAGAGAACAGTTAACAC
EMPV1_07185	GAGTAAGGGAAGGCCTGTGGACTGTGGTTATCCTATTTATTATTGAACATGTTTTTCAAA
EMPV1_07187	AGGACATCGCCTATGCAGCTCTGTCTTTGGGGGACAATGAGAATCAAGAGGCGACCTACA
EMPV1_07190	GTCAGTGGTCTCCAACATCACACAATTACCACCACTTCAAAGGCCAATTGCTAGTCCAGG
EMPV1_07196	TGGACCATCTCATTCTTCTGGCTATCATGACCATCACCTTCGCTGTCTGCTCCTTGCCTT
EMPV1_07197	GCTGAGGAAGCGTGACCTTTTAGTTGCCCTGTGTTGTCTCACTTTAAGGTAAATGTATGG
EMPV1_07198	GGCAAAATGAGAAGTGTTCCCTCTCAGAAAAAAAGGAATAATATTGTACACCTGCAATTA
EMPV1_07201	GCCAGGTTTCTTCCTCTCCGCATGGGAATCTTTGGCATATGGTGGGCTTTGGCCATTTTT
EMPV1_07202	GTCCTATCATCCCATGGTCACAGCCTCAGAAAGGATCTTTGTTCTCAACCAACTCAGAGA
EMPV1_07203	TGGACATCGCGTCGAACAGGATCAAAAAGATTGAAAACGTCAGCCATCTAACCGAGCTGC
EMPV1_07204	TGAGGAGGAGTCGCCACTGGACGTTGCATCGTGGCTGAAGTGGGGATCAAAAGACAGCAG
EMPV1_07205	CAGCTTGGTGGTCGTATGTGTGGCGCCATCTACTTGAGCGGTTAGGAGTGACATCACATA
EMPV1_07206	TTCCAAAATCCCCACACCCCAGAGGAGGCCGCCAAAATGATCGAGGCCAGGGAAAAGGAG
EMPV1_07207	CCTATTCTCAAGAATCAAAAGAGCAATTTGCTGTTATGGAAGTGGATCCCTGAGCACACC
EMPV1_07208	CAGGAGTTGGCTTTGAGTGCTGTTCCCCAAATCACCTCTGACTGTTCAACCACTGAAACT
EMPV1_07212	CCAATATTTATGGCAGTTTACCGCGTACAAGTCTCTGGGAACAGTGGTGAACAAGGCACC
EMPV1_07213	CTATGCTGGCAAGGAGTATATCATCCAGGTGGCAGCCAAGGACAATGAGATTGGGACGTG
EMPV1_07214	GGGCAGAGGTGGAAATGAAGAAAGTACAAAGACGGGAAATGCTGGAAGTCGTTTGGCCTG
EMPV1_07216	GATCGTTGGCAATATTACTTCGACAGAATCATGGGACTCGGAAGAGAGAGGACAGAGGCC
EMPV1_07220	ATGAGCGAAGCCACAGAGATGGAGTGCGGGCGCTCGGAGATCGAGGAGCTGATCAAGGAG
EMPV1_07221	AAGCACTGCAAAGAAGACTTGGATTTCCTCTGAGGACAGATTCAGAAGATCCTGCAGAAG
EMPV1_07222	GTTGTTGGGCCTGGATTTCTGAACACAACGACATTAAACCACCCGACTCACGGCAGTTAC
EMPV1_07223	AACACCAGGACCGCAGGTCTTCTCGCTCGAACCCACAATCAAGAGGCCTCAGTATGAATT
EMPV1_07224	CAGAAGGAAGACTTTGAGGAAGCCAGGAAGAAGGCGCTGAAGCTCGGGGCCAAAAAGGTA
EMPV1_07228	ATGGAAGTGGAATTGTTTCAGCTCTGATGTGCCACGCCTTTCAAGTGACACAGAGCAGTC
EMPV1_07229	AAGATGTGGACGTTGTAGGAGGCAGTGATGGAGAAGGTGCCATTGGGCTCAGCAGTGACC
EMPV1_07231	GGCTTTGCCGACTTCAGCCAGATGTCCAAGCCCCCAACTTCTGGCCCTTTCACCTCCTCC
EMPV1_07232	TCTCTGAACTTGGAAGACTACAAAAAACGAGGGGGGCTTATTTGACCACACCCAGCCTGC
EMPV1_07233	GTGAAGGAAGTAGACCCCACCAACCACACCTATGTGCTCATCAAAACACTAGACCTCACC
EMPV1_07235	GATCTGTTGACTGAATTGCAGAAAAAACTTGATGATTCATATTCTGAAGCAGTAAGACAG
EMPV1_07236	ATACGTGACACTTCTCCTTGAGTCGAATGTGGCTGGCAAATGTGAGCTGCTTCATCATGG
EMPV1_07239	CCACCACCTCTGTCCCATCCCTGCTCCAATCCCGAGTATGAACCCTGACTTTACAGAAAA
EMPV1_07240	GTTACCGGAAGTCCTCATGAGGCGGGGCCGGAGGGGCGGCGCGGTGGGAGCGAAATGGCG

EMPV1_07242	GTGCAGCATGAGAATTCCCATTGTGGCTCAGTGGAAATGAATCTGACTAGCATCCACGAG
EMPV1_07245	CAGAGTGGCCCAGCCTTCAACAGCATCTTCCAGAAGGAGAACTTCCAGTTGCAGCTCATT
EMPV1_07247	GATCTGGATCATGGATTTTCTCGGCACCCAATTGATGATGACTGCCGTTCTGGCATCGAG
EMPV1_07248	TCCAACTCCCTTTCAAGTAGAACGTCCCTGGTCATCATTTGCATGCCTCTCCCCGAAGGA
EMPV1_07249	AAGTGAGAGATCGGAGGACGTACAATACTTCGACATGGAGGACTGCGTGCTGACGGCCAT
EMPV1_07250	ACCCCCGTCTCTGTACCCTGATGGCTTGTGCTTCATGGATCATTGGTTTTGCTGACTCCT
EMPV1_07253	TTCCTTGGACCAGGTATTGATATTGCAGGAAAAAACTGCCCCTGCCCCACATCAGGCAGC
EMPV1_07255	GCTGAGAGCAGAGTGACAAGCAGGCAACTATGCCAAGACCTGAGGAAAATGTATTTCAGG
EMPV1_07256	CAGGATTCGGAGGAGCCCGACAGCTACACGGAATGCACAGATGGCTATGAGTGGGACCCC
EMPV1_07257	ATGCCTTTGGAGGTCAGAAGGAAGGGCTTCCGGTCTGGGATATAACCACAGTCGCTTCAG
EMPV1_07258	TTGAAATGTCCTTGTCCCAAGTATGGGGTCAGTTTTGGAAGTCTTAAGCCCGGTGCATGC
EMPV1_07262	TGCCTTACCTCTACACGCTGATGCACAAAGCTCACGTTGAAGGCAGCACTGTTGTCCGAC
EMPV1_07265	GAAATAGGCTCTATCTGCGACAGCTCAAGCAAGAAGGGGGCATTGATTACAGTGGGACCA
EMPV1_07267	TCTGACTACAGCAGTTTTAGGTTGCTGAAATTCCTCTGCCCAGCAAGATCCAGTGTTGCT
EMPV1_07271	AATTGTGGATCGAGGATTGGAGACCCGCCTTTTTCTCCTGGGTGTGGCTGAAAGTCCCTT
EMPV1_07272	CTTGGGGAGTCTAGAAAAAGCTCTCTTCATTTTGACAGCAGTTTCATGGCTGACTTCTGG
EMPV1_07274	CTGGAGGAGCAAATGACCGATGGGTACGATCTGTATCATTTAGTCATGATGGACTGCATG
EMPV1_07275	TCGATCAGTGCTTATGTTTATGCTCAGAAGCCACAACTGGATATTCATAGTTTTGAAGGC
EMPV1_07276	CCCTCATCCTTTTCTCCTACTGGAACATTATCTCCACGGTGATTCAGATACACGCAGGGG
EMPV1_07278	AGTGTGAGTGTGATCTGTGATTTGCAGTCTTTAATGTTACCACTGTAATTATTTTTGGGC
EMPV1_07279	TGGACCGGAAAAGGATTGTTAAATCTGTGCAAACGATGCAGGCGATCTCCCTAGGCCTGC
EMPV1_07280	AGGCCCAGGCCTTGGACACAGCAGACAGATTTATCAATTCCAAGTGTGCAAAGTACATGC
EMPV1_07281	CACAAAGTGCCGAATGTTCCGAATCACCAGGCAAAGCTGATCGAGGGAGGAGTTGATGGA
EMPV1_07282	GGTACAGACAGTACCTCCGCTTTATCATCACGAGCCAGAAACTTGCCAGTGGAAACCTTC
EMPV1_07283	TGAAGGTCTCACTCAGCAGTTGGCATTTTAGGTGAGATATGAAAGGTGCCAAGAGCCAGC
EMPV1_07285	ACATGTGTCTGATGTGAGGGGTCTCTGGAATGAACCTGAGATTGGTACCGTTCCCTGCCC
EMPV1_07286	CACATGTATCTTCACAAAGTCCCTTCCTGGTTTCACATCAGACAATGGGAACACTGAGTT
EMPV1_07288	AAAGCCGAAAGCATGTGCTTCATCGTCTCACAGCAGGGCCTTTGCTTGGGGAAGAACTTT
EMPV1_07289	CAGAGGAGAACTACTGCGCTATCACGGTTACTTCTCTGTTTAACCATTTAAGGGACTCTC
EMPV1_07290	ATATTCCAGGAAAAAGAGAGTCTGCATTTGTTTCACGGCCTAAAGCATCTGGCTATGCAT
EMPV1_07291	CCCTTGTGACTCTACCTCTCAGTAAGAAACATGCTTTTGAGGCACACACATTTGTGTGTG
EMPV1_07292	ATGTGGGCGATGAGGTCGGGTTCTAGGCATGTGAGGTCAGTGCATACAACACAGCGGCAT
EMPV1_07293	TTTGCCCTCGCCTGTTCAAACTCATCTTCTAGTCCTGCAAAACTCGGGAAAGGTCTGCCT
EMPV1_07294	CACACAGCACACTCTTAGGTTACTCTAGGAGCCTATTAGGAAGCACTTGCCTCTTGTGAC

EMPV1_07296 TCTTTGATGAGAGGTATGTGTTGTCCTCGAGAGTCAGAACTGGCCGAAGTATCCGGGGAC
EMPV1_07299 AACCAGCTTGTCAAATGTGAAGAGTGCTGCAACGAGACTGCAGGGGTCCCTTTAGAACCC
EMPV1_07300 TCCTTAATCCACCGAGCTAGGTCAGGGATCGAACCCACATTCTTGTGGATACTAAGTCGG
EMPV1_07303 TCTGAGGCCTCTAGGAGACCGAATTTTTCCCTGCCCGGGGAGATACAGTGAAGAAAAACA
EMPV1_07305 TGCACTTTAATGAAACAGTACATTGAGTTCGTGGCAGACAGACTCAGGCTGGAGCTGGGC
EMPV1_07306 TGGGATAACTCCAAACCGCATGAAAGGGTGTAATTGCAGCATGAGTTAAATCTTGAAGCC
EMPV1_07308 CCCGTTACAAGCAGCAACGACTGTTGGCAGATCTTCCATCCCTGTAGGATAACGGGAAAC
EMPV1_07310 AAGGTCACTTCCAGGACATGCTCTACCTAAATGGTCCAAGGTCACTTCCAGGACATGCTC
EMPV1_07312 TGGTATGTCCCCAATGCCTTTCCCAAGGCTTCTTCATGTCAGATGCACCCGAGTCCTTAG
EMPV1_07314 CAAGGATCACCACAATCCAATTTTATAGGATTTCCATCCCACACCCGCAGTACATCCCCC
EMPV1_07315 GTGCACATGGACAGCCACACAACTGAATGTATTCATGTGTATACATGCATACCCATGCAC
EMPV1_07316 TGGCTGATACCTTTGTCAGGAGCGGGTTCTGTTCCCCTGAAGGACACGAGAGAAGGACTA
EMPV1_07317 GAGGGCAGCTGTTAGTTTTGAGGCTTATTGCTGCTCCACCGAGTGCCTGCAGACTTTTGA
EMPV1_07318 GGTGCTCTGTTTCATGAATTACTGCTGTCTTCAAGTCATCCTGTTTACTTATGATCTTCT
EMPV1_07319 TCCTTGCTCACCTGCGGTATGGATTGCTAGGTCTGGGTGACTCAGAGTACACGTACTTCT
EMPV1_07320 GGACCTGGAAGGAAATATAAAGGAGGATATAATGGTTATGGAAGAGGCCCTGGACTGGGG
EMPV1_07321 TTACTGAAGAGGCCGAGAGGGCTTCTAAACTGGGCCAGCTCCAGCAGAAGAAAATTCAGA
EMPV1_07323 CCACTTCTTCTCTGGATTGACCATCCCTGATGGGGGCGTGCATATTGTAGGGGGTGAACT
EMPV1_07324 GGGGCACCTTGTTCCTGCATCCTCAAAGGAGCATCTTGTTCCAGAGTTAAAAGGTTCCAG
EMPV1_07325 CTGCTTCCACCAAAACAAGCCGGATCCTAAAAACTGCCTATGGGAGCTGGTCAGAACCTT
EMPV1_07326 AGAGCCTTGAGAGGGAGAAAACGGAGCTGGAAATGAGGCTGAGGGAGCAGCAGGCAGAAA
EMPV1_07327 TGGCCCAACCCATGCCAATCCATTTTAGTTCCTCGATCCTCCACCAAACGCATTCTGAGC
EMPV1_07328 ACCCAGGCTCTTTGTCAGGATCTCTTCCAGGAATGTCTCGAGGTAAATCGATGATCAGCT
EMPV1_07330 CATGTGGAGTTCCGAAAGCAGAAGGACATCGACAACATCTTGAACTGGCAGCCTCCAGAG
EMPV1_07333 ATCCTCGGGTCTGTAATCAAATGGTCGTCACTGCCTGGATCATCGATTTTTTCCATGCCC
EMPV1_07334 CATTTATACGTGCATTCCTTCTCACACACATCAGTGTAACCGCAGCCTCTCCCGGAAGCT
EMPV1_07340 AGGGCCCTTTGCCAATAGATAGTAATTCAATCTGGTATTGATCTTTTCACAAACAGAACC
EMPV1_07341 TCTCTCGTGCTGTCAGCCAGAAGAGTGCAGGAGCTGGAAAGGGTGAAAAGAAGATGCCTT
EMPV1_07342 AGAGCCTGGCACGATCGAAAGGTCCTTGGCCACCTCCATCATCCTTGTTGCCTTCACCAT
EMPV1_07343 CTTCTATCCAATCTCTACCTGCTTGTGCCCCCGTTGCTCAATCCTGTCGTTTATGGTGTG
EMPV1_07344 GCTGTTGGCTTTTACCTGAGTCTCGTCTCTCACTGCGGGTACTCCTGCGGCTTGGATCAT
EMPV1_07345 GTCACAGGGGCCAATTTCCTGACTGGAGCCTGACATGCTTTTAATTATCTGTCTCTATTC
EMPV1_07346 GACGTCACACACTCCTGGTTTCCTTCCACTTGCTGCTACGCCTCTTTCAAACCTCAAGAT
EMPV1_07348 TCTGCAGCACATCACGTTCCCTTGACTATGGGAGCCTTGCAAAAGCAGCGACTGTTCCCA

EMPV1_07349	GGGATTCCTGTTGAGGAAAAGTCAAAGAAGAAGCAGGCAATGCAAAGCCACACTAGCCAG
EMPV1_07350	ACTGAGTCCTAAAGAGAGAACTACAGCACTCCCCTGTCTTCCCAAGCTGCACCACCACAC
EMPV1_07353	GTGACGTTTACATCAGGTCTGCAGCGTTTGGGAGAGAGAAGCACATTCCAAGGGCAGGAA
EMPV1_07355	CCTTCCCCGGCGGCCGCGTGGTGCGGCTGCACCCGGTCATCTTGGCCTCCATCGTGGACA
EMPV1_07357	TCAATGCCATCCTGCAGACCAGCTTCACATTCAGCCTCCCCTTCTGCAGCTCCAACCACA
EMPV1_07359	GGTGGAAAAGGCCACGGCTTGCAACAAGAAAATTACAAATGAGAAGGCTCACCAGTAAAG
EMPV1_07362	GCATTTCAGACCCTTCCCCATCAACCTTTTTGCCCGTAGGGCCATCAATATTCAAAAGGC
EMPV1_07363	GTGGCCTTAAGAAATCAGATCTTGGGGAGAGTCCCTTCCTTCATGACTGGCCTCCCATAA
EMPV1_07365	ACACTCTATGGCCGGTGCCAAAGCTCTAGCGTCTGCTGTAACCAGTGGAAAACATGCAAA
EMPV1_07370	AGCTTCATTGGGGCGGTGCAGGCTCTAAACAATGGGAAAGGATTTTGAAGTCCTGGTGGG
EMPV1_07372	CAAGGAGGTTGAGAGGCAGGGGGAGCGGCCAGCGGAGGGCAGGAGCGCGCCTGCTGACAT
EMPV1_07374	ATGTGACGTCCCCGGAAGCACTGCCAACAAACCAGCCTCTCCAGACCCTGATGAACTGTG
EMPV1_07375	TGCACATCAAGGAGAAGAGGGAGTCCATCGCCAGTGGCGACGACCGGGCAGAGGAAGACA
EMPV1_07379	ACTCGAGGGTAAGGATTTCTTCTAGCACACCGGTGGTGGAGGATACACAGAGCTGATCTT
EMPV1_07384	GCAGAGAAACAGGTCAACGGAACAGAATAGAGTCTAGCAAAGACGCAGGTGGATACGTGC
EMPV1_07385	CACTAATGCCAAGACTTCAGTGACAATCACATGGCAGAGTAGCGTGAGAAAGATTTGGAG
EMPV1_07386	ACCTGTTGCCTATGCACCTGTTCTCTTTGGAAAAATAGTTATTCGGGTCCTCTGCCCAAC
EMPV1_07388	AGGCCTTGTCAGAGCACAACGTCTTGGTTGTAGAGGGAGCTCGCAAGTATGCCTGCAAAA
EMPV1_07389	TCTGCTTGTCGGCCTTGATATAGACGTTGAGGCTGTTGTAGTTGAACTCCAGCTTGTGCC
EMPV1_07390	CCCAGGCACCACAGAGTTTGGCAAAGAAGAGAGCACTGCAACTGAGCACCACCTGTTGTT
EMPV1_07392	AGTGTGGGATAAGAGACTTTGCTAAAGCTGTCTTCAATCATTGTCCATTTTTGCTGCCTC
EMPV1_07393	CCTTCCAGACACAATTTAATCAGTAGTTTTAATGCACATTCGTTAGAGCTGCACTTGAAC
EMPV1_07395	CTTCCGTCTCGGAGCTCGCCTGCATCTACTCTGCCCTCATTCTGCACGACGATGAGGTTA
EMPV1_07396	CTGCCCAAGGTGGCAGTCAGGTCTCTACTGGGTCTGTGGTTCCTGATGGCAGTTTCTGCT
EMPV1_07398	TGGGGTTGACTTGTTCTGCATTGGTCTTTCGTACATCCTCATTGCACGAGCTGTCCTTCG
EMPV1_07399	CAGTAAATACATCATGCAGTATATCGCGGCCATCACCAACCCCAGTCAGAGAGCCGAGAT
EMPV1_07401	CTGGTGTCCTGGATCTTGGGTGCCCTGAACTCCCTGCTACAAACCCTAATGGTGTTACAA
EMPV1_07402	CACAGTGAAGAAGGAGTTTGGGGGCGGCCACATCAAGGATGAGCTCTTTGGGACTGTGAA
EMPV1_07406	ATAAGGTAAGTTCAGGAAACCTGGCTGGTCCAATGACGGATGGAAAAACTGCCGTCCTCA
EMPV1_07411	AGAAAAAGAGATACCAGATGTGAAAGCCTAGAAATTCCAATCAATGTGGTTCTGCCTCAG
EMPV1_07412	GGCTTCGTGGTAGGGGAGGCATCATTTCCAACCCAAAGTCATAAAGATATTCTCTGCTGC
EMPV1_07413	CTTGTTATAGTCTCGCCACAGTTGTTCAATGTTGATCATTGGATTAACACAACCTCGTTG
EMPV1_07414	GAGATGATCCAGCCGAGCTTTAGCTTCTTCAGCACAGACAGTTGAAATTCCTCACTGGGC
EMPV1_07415	AGCTGGAAGAAACCTTGCCCCTGATTTTGGGGCTCATCTTGGGCCTTGTCATTGTGGTAA

EMPV1_07416	GGCAAAGACAGATTCCAGAGCAAAGAGTGCCAGCACGTTATCAGGACTTCTTATGGCCTG
EMPV1_07417	TGCTCCCAGGGCTCCTTTCCAGGCCACCTGGGCCCGCGAGCCCCTCTTGACCAGTTAGCT
EMPV1_07418	TTTGGAGACTGAAGAACCTACCTGAGATGAAGAGGCCATGGGTAGCTGAACAATGGGCAG
EMPV1_07419	ACATTGGGAACAAAGAGCAACAGACAGACCCCAAGGAGTGTCATAAGAATGATGGCATCC
EMPV1_07420	CACCAATACCACACCTTCTTGATTATTGTGACTTCAGAGTAGGTCTTGATGTCAAGTAGG
EMPV1_07423	CATCCCAGTGCCTGTGTTGGCTGCCCAGAGACAATACTGCTCCAGAAACGAAATTGAGCA
EMPV1_07424	GAGTTGCCGTGAGCTGGGTATAAGTTGCAGACGTGGTTCGGATCTAGTGTTGCTGTGGCT
EMPV1_07425	TTTGGAAACCTGTTGTGGTGCCCCTTCCCGTTGTAGCCTCATCCTGCAGCTTCTCTCACT
EMPV1_07426	TGGGACATGCTTATACTAAAAATTGTCATTGTTTCTCTGAAATTCAAACTTAATTGGGCG
EMPV1_07427	TATCATGTCAAAACTGGACAAAAGAAGAAAGGGAGTTTTTGGTCCTCCTCTGGGCAAGAG
EMPV1_07433	GAGGAGTGGGGTGGACAGGGAGTTTAGGGTTGGTAGATGCAAACTACTATATTTAGACTG
EMPV1_07434	ACCTTCCGCTCTGTGCTGCACTTTGTTGAGGTACGCGTGAGACTGGATGCCTCCTTGCAA
EMPV1_07435	AGGACGCCGCCAAGGTCTGCAGAGAATTCACTGAGCGTGAGCAAGGCGAGGTCCGCTTCT
EMPV1_07438	CGGCATGAAGGAGAACTTAACAGATCTAATTTTCAACATGACCACTTCACTGCGGGAGAC
EMPV1_07439	CCGTGTCCCTCTTTTATGGAACTGTCTTTGTCATGTATGCCCAGCCGGGAGCGGTGGAGT
EMPV1_07440	TGTGATGTAGGTCGCGGATGCAGCCCAGCTCCCAAATTGCTGTGGTTGTGGCTTAGGCTG
EMPV1_07442	CCACCTGAGGCCTGAGTCTAAAGCTTCTCCGCCTCCCCCCGCCCCTGGGGTGCCCTGCAG
EMPV1_07445	AGGCGCGCTGAAAAGGCTGGCAGGGCTCGGGAAACTGGGGGCTTAGGGACACACATTCAA
EMPV1_07446	ATTTCTGTGACATCCGCCCAGTTATGAAACTCTCCTGCGTGGACACCACTGTACATGACA
EMPV1_07448	AGTGTCTAAGTTACGTGGCCTTGGATCAGTTGGTTCCTTGCTGTCTGTGCATGGTGCATG
EMPV1_07451	GCTTGGACAGGACCAGGCTCCGCCGAGTGTCACAGTTAAACCTCCAGAACCTGCTATGGA
EMPV1_07452	AAGCCTAAGAAGGCACCGACAGCCCGAAAGACTACAACTCGGCGGCCCAAGCCCACCCGC
EMPV1_07453	GCCAGGGAAGGACAGATCACATGATGGGCAAAAGTCACCATTCAAGGCTAAGGTGGGCTT
EMPV1_07458	TACAAAATTATTTTTGCAATATGTTCCTGAATATGTAATATAAGTATATTGGGAACATTT
EMPV1_07459	TTCCAGTTATCATCTCCCTGAAGACCGTATTGGAGAAGAATAAGATCCCGGCTCTACGGG
EMPV1_07460	GGACATGTGCTCTGGGTTCTCTCCAACCCCAGTAACATCCCAAGTTTGATTACAGGATGC
EMPV1_07462	ACTTCATCAGAGAGTGCCGAGCTTCAACTGCCTGGGCTCTCCACAGCACCTCTCCCTGAA
EMPV1_07465	AGCAGTCTTTCTCTTCGAGCCAGGGCGCATCCTCAGAAACCTACTCAACACAGCACTCTA
EMPV1_07466	CGTGCTATTCAGAAGGAGTAAATCTGATGGGAAGGCAGTTAGTTGTTTCTGCTCACGGAC
EMPV1_07467	TAACACCTACACCGAGAAGTCCTCCACCGGCTGCTTCAGCAACAAGAGCGACAGGATGAT
EMPV1_07468	GTGGATACATTTGTACCTGGGTAGGCTGGTCTTCTGGTCCTATTGTTTCACCTTCCCTCC
EMPV1_07469	CTTGGAAGAGAAACCCACCATAGCGTTTGCCACCATCCCCGCTGCCAGCCGAGTGATCCA
EMPV1_07471	CCTTAGCCCAAATCATGCACAAAATTCTTATTGTCCACTTCCTACAAACCTTCTATCACT
EMPV1_07472	CTGGGTTCAAAGGACCACTGGAGAGCCTAATGAGCACCCATGTACCAGAAGAGAGAAGCA

EMPV1_07473	TCTCAGCTTTGCAGGCATCTCCCGAAGCACCACCATCGTGACAGCATATGTGATGACTGT
EMPV1_07475	CGGCGTGTTGAGCTCCATTGGGAAGATTTTCAAAGAAGAGGGGCTGCTGGGATTCTTTGT
EMPV1_07479	GAAAACTCCTGTATATCCTGGCTTCTCCCTTAGCTCTTTGGAGCTATTTGCGAGGCTGCC
EMPV1_07480	GTTGTAGATCACTTTGGAAGACTGGACATTTTGGTCAATAATGCTGGAGTGAATAATGAG
EMPV1_07482	CAGACTCTGATTCAGTAGACCTGCGGTGGGGCCTGAGGTTCTGCATGTCTAACAAGTTCC
EMPV1_07483	GACGACTGCGTCTTCGTCCACCTCGTCTCCCAGCTGAGATACTACAACTCGGACGTGCAC
EMPV1_07485	CAATAGATGAACTGAATAGCAGCATGTTTGTTTCACAGATGTAGAAGTTACCAGTAGAAC
EMPV1_07486	GTTACTGTCATGAGGACCATTAACCCAACTTTCCAGACTTTCAAATGCACTCAAGACGTT
EMPV1_07488	CTGCGTGTGGCTTTGTCAACTTTGGGAGTAGCGGTTTCTTCGGTGGCTCTCATGTGTTCT
EMPV1_07489	GCAGAACCATATGGAAAATTCCACTAAGCGCCACCTCCAAGTTTGCGTTCTGTATCCTTC
EMPV1_07490	ATACTCAGCCTATGGACAGTGCGAAGATAGAACCACCAGATACTGGACTTTATTGTGATG
EMPV1_07491	CAGGCCCTTCCTTGATTATGTCTCACGGGTTGATACGTGGGTTGATATTGATTCTTGCTG
EMPV1_07492	TACCGTAGTCATTGTCCACTTTGGTTGTGCCTCCTTTGTCTACCTGCGACCATCAGCCAA
EMPV1_07494	TTTCCTAAGCTTAACTGGAGTGCCCCAAGGGATGCATACTGGATAGCGATGAACAGCTCT
EMPV1_07495	ATTGGTAAACAGAGGTTTGGCAGTTTGTTACAAACCTGACGGAAAACAACCCAGACTTCG
EMPV1_07497	GCTCGTAATTTAGCTTCTATTTTATGTTTCTTTCTTTCCTCATTTTTTTTGGCAAAGTAC
EMPV1_07498	CTCTTCCCAGATGGCCACTCGAACCCACAGCCGCTGGGTGAGGGTGATCCCTAGTCTGGC
EMPV1_07499	TTCTTAATTGGAGACCAAGAACCCTGGGCCTTTAGAGGTGGTCTAGCAGGAGAGTTCCAG
EMPV1_07500	GCTGAAACCAGTCTTTCCTGTGACACTGTTGGAGGATCCTACATCCAAACATGAACAATG
EMPV1_07504	ACAGAGTACGTCCTCGGAGGATGTCATCTGCTTCCTCAGAGACTGGAATGAGTCCCTCCT
EMPV1_07506	ATCAAACAGGCCATGTTCAACTATTTCCAGGTGCCAGATCGGCTAGGAATCCTCACCCAC
EMPV1_07508	GGCTTTTAGAAAATGGTTGTAAATCTGACTTCTTTGCATGTAACTGTGTATATTGTAAAT
EMPV1_07509	TGGTTTATAATCTGTCAGACGCAGAGTTCTTCCCCAGCGCGAGCTCTAAGGGCACCAAGA
EMPV1_07510	CTTCTCCACCACCTCCACTGCCAGAAAGAACCTTAGAGTCCTTCATTGTTGCTGATGAGG
EMPV1_07512	CTGTGATCCTCTATCGGGTATGAGTGGATTTTGATAGAAACTGGACAAAGAGAACGCCTC
EMPV1_07513	TTCTGCCCCAAGAAATAAAATTGCAAGGTTCCAGGCAGCGCTTTCTGCACCGGAGTACAC
EMPV1_07515	CTTCATTTTTTCCTGAACTTCTCCTCTGTCTCCTATGGCCACACCTAACTGACCATTTTA
EMPV1_07517	TGGTTCAAACAAGGTCGAGGATAAGTGCGGTGTCTGTGGTGGTGATAATTCCCATTGTCG
EMPV1_07518	GAAAGCCTAGAGAGGAATAAAGAGAGCTCCTTGGGCCCCTGTTTAGATGGATTTATTTTT
EMPV1_07519	CACAGATGGGGAAGTTGCGGCTGTGGAGGAGGCACCGGTAAATGCATGTCCCCCTGGATT
EMPV1_07520	TGATCCAAGGCTCAGATACTCTCTAAAAGCCACCGGAAGTTCACTGAAGTCGTAGTGCCC
EMPV1_07521	CCAAAACTTGACAGCAAGCAAAAGACGCGGTTTTACATCTTCGGCAGCCGTCGCCAATGT
EMPV1_07523	GAGGAAATGGGATCAATTGCTCCTGCCCAGCCTGTTGGATTTGCATTTGCCAAGGTTTTC
EMPV1_07524	GATACACATCTCAGCATTGGACATGGTGGACGGACCCGCATGGAAAAAGAGTTACAAGCA

EMPV1_07526	CCCAGAGCCACCCTAGACTGCCCACTGGCTCATAGCTACCATATGCCTTGCTTTTCTGAA
EMPV1_07527	GTGCTGGTGCAGATTGAGCTGTGGACCCATCCATACAAGTACCCTCTCAGGGTCTGCTTT
EMPV1_07528	TACTGAGGACAAGACACTAAGGCGAGTCGTTCTCTCCCGGCATATACAAGCTAGAGACCC
EMPV1_07529	ACGACGGTAAGTGCCACGGGAAAGGTGAGCATGCGCTAAAAAGGACTGGCTTTTGTCTGA
EMPV1_07530	GCATTAGTGGCCGCCTCCCATCAGACCTCCCGCAGTGCTTGTCTTGGTAAATGTATTTCT
EMPV1_07532	CATTCTCTGGATTCCTGGCTACTCTGTGTTAAGGGGCTAGGGGCTGAGGGTGCCTAGTAT
EMPV1_07535	ATACTGTAACCTATGGCATCTGCCCCGCAGCCAGACCTCTCTTGACCCCACCTCTGACTT
EMPV1_07536	GAAATACTCTCCCAGGGCCAGGCTGCGGCTTTACACCCTCGACAACTACGAGAAGTTCAT
EMPV1_07537	AAGAATGACCAGAGATTGTCAGACGAAAGGGAGGCTCTACCCATCCTCGCTACGTTTGCC
EMPV1_07538	GCCAGAAGCAGGAAGCCCTGATGGTGCTGGAGGAGGAGGACGTGCGTGAAAACATTATCA
EMPV1_07539	CAGGGAGCCAGCTCTCTGGCAGTGAAACAGGTACCAGTGAAGCAGTTTCCAACCCAGCTA
EMPV1_07540	AAGGGGCTACAGCTCTGAGGCGAAGACGGAGGACGAGCTGCGGGTGCGATATCTGGAGGA
EMPV1_07541	TTCCTTTTTATGAAGCATTGACTTTGTACATGTGCCCGCCCCCAACCCAGGTGGCCATGC
EMPV1_07542	CATTGCCCTGTTCCCTTTCTGTGCCCATCCGTGAATGCTGGTGTTTGATAAGACCTCCAG
EMPV1_07545	GGGGTAGCTATACTTATATCTGAATTTGTATAACAATATAGCAAGATAAAAAGAAGCAGT
EMPV1_07546	GAACTCCTAGGCTTGTAGCATATGTTCCTTCACACTGTCCTGTGTACCTGGGTCATCTAG
EMPV1_07547	GCTTTCCTGTGGCTGCCCAGCCTCCGCCAGAGACCCCTGGATCAGTTTGTGCTGCGGTCG
EMPV1_07548	TGCCACTGTAATTAACATGACATTTGGAGGAAGATCCCCTCGTGAGACCTTGTACTCTGT
EMPV1_07549	TGGCAGATCTGGGCTCCAAGACACTGACCGGGCTCTTCCGAGCCTGGATTCAAGCAGGCT
EMPV1_07550	CCGCTGCTTCCTATCCAACCTCCTCTTATGTAGAGGCACTGGAGAGTGCAAAAATTTGAC
EMPV1_07551	TCCGCTGAGGAGTTTGGTTATTGGGCAGTGGAAGTGCTTGCCTGAGTGGCTGTTGAAGAA
EMPV1_07553	GAACAGTCGGAGCAGCCGCTGGCAGAAGGATGACCCAAGACGGGAAGCGGGCTGCAGAGT
EMPV1_07555	AGTGGAACTGGCCTTAGGTGTGAAAGTGCTGATCCGTCGAGCCACTTGAACTCTCTGAAA
EMPV1_07556	CCTTTCCCTGGGGAGACGGTAACCATACCCTATTCCATAACCCTCATGTGAACCCACTTC
EMPV1_07558	TTCAGTGCTGGACGTTGTCAATGGTGGAAACTGGAGGAATGGGTTAGGGGAGCTTTCCCT
EMPV1_07560	AGACCGATCAGGCCAGCGGATTCCGGTGAGCGGCAGAGTCGGGGATAATGTTCTCCACTT
EMPV1_07561	CAAGCCTCCGATAAGCTCCTCCAGTATGACATCACGCATCTTGCTACGCCAGCAACTCAT
EMPV1_07562	GATGGGGGCTGGCCTGAATTATGAGTGTTACCAGTTCAGCATTTCACCTTCCACAAACGG
EMPV1_07564	CCTTGCCCCAGTGTGTGCCCGAACCTAGAAGAAAACTCCAGAGTGCAAATCTATGTACAG
EMPV1_07565	TCTATCAGGCTCCTGTTCCATCTCTCTGGATGTTGTTGAAAGAAAGCTCTAGGCCATTGG
EMPV1_07566	GGGCAGACTTGGGGGTTGGGTGTTTCTCTCTCGAACACCAGGTAGTAGTTAGTTTAAGGT
EMPV1_07567	TATTTTCTGGGTGAGAGGGCTGATCGCAGATTGTAGTACCCACTTGGATCCCTCCACATG
EMPV1_07569	GGATCCTGGAATCCGCGGGGAGGTGGGGATTCTGGGAGAAAAACTCCATTTCCTCCTTTC
EMPV1_07570	GACCAGTGGGCAGGATCCCCTGACACAAAGCCTGCTTTATAGGAAAGTGTTGGCTGTCTC

EMPV1_07571	TGGAGAAACGCCAGACCCCTCTGACCAAGAAGAAACGAGAAGCACTTACCAACGGCTTGT
EMPV1_07572	CTCGTAGTAATCTAAGACATCATCTCCATACCCACGTGTCCGGATCCCTCCCATTTGGTG
EMPV1_07573	TGCAAGATAGACATAGAAAATACGGGCAGTACTCCTGTTGGCCTCTTGATCAGAAACTTC
EMPV1_07576	CTTTACTGGCCCTCTCTTGCATCCCTGCCCCTGGAATTGAGCTCTCCTGTTCCATATTGA
EMPV1_07577	CGTCCTATCAACAAGGGGTCCTGTCCGCCACCCTCCTCTATGAGATCCTGCTGGGGAAGG
EMPV1_07578	TGGGGAAGCAGACCAGGCCCCAGGAGTGGAACCAGGAGACATTGTTCTTTTGCTACAAGA
EMPV1_07582	ACAATGTCGGCTCCTTAACCTCTAGACCAGCAGGGGACTCTGAGAAAAGTGACAGATCTT
EMPV1_07583	TCAGCTGAGGTCTCTGCCACAGCCACAGTCACAGCAATGCTGGATCCTAAATCCACCGAG
EMPV1_07584	TTTACTCGAGAACAGCTGGAGAACACGCCGAGCCGCCGCTGCGGAGTGGAGGCAGATAAA
EMPV1_07585	ACAAGGTTCATCAAATGTCGCTGTGGAAGGCGCTTTAGAGACCAACCCCGGGCAAATGGT
EMPV1_07588	TGGAAAACACGTGAGCTTACCCGACGGAACCGCTGCTGCTTTTCATGTCCACTTTCTTCG
EMPV1_07591	CATCCAGCTTTGGCTTTACACATCAAGCTGTTTGCCATGGTTCAGAAAGGGACTCTTGTC
EMPV1_07593	TGGGCACAGGCCAGGTCATCAAGGGCTGGGACCAGGGGCTGCTAGGGATGTGTGAGGGGG
EMPV1_07595	ACCCTGCAATTCCACTCCTAGGTACAGACCCAAGAGAATTGAAAGTGATGCTCGAGAAAC
EMPV1_07598	GTTTGGCTCGGCATCACGGCCACCACCCTTTAAACCCAAAGGAAGCAAGACTTGTCCTCA
EMPV1_07601	GGCCCGTGTTTGGAAACATGGACAAATTTGTGGGGCTGGGAGTATTTGTAGACACCTATC
EMPV1_07604	TGATGACCTCTATGACCAAGACGACTCAAGGCACTTTCCACAGTCCCGGGATCCCCACTA
EMPV1_07605	TATCAGAGGTACTTTGGGGCTCAAAAGCTAGGATTTCTAAGTCAGTATGTGTATTTCACA
EMPV1_07607	CCTGCCGTCACTCTGCCTGCGTGTGTTTCTAATTCAACAAACTTTCCACAGACTCCATGC
EMPV1_07608	TTTCTCATCTTAGGGGTGCTGAGAGAAAAAGGGATGAGAGTAGAGGCCAGTGTCTTCCTG
EMPV1_07609	CAAACCAAAAGATCATCCTCAGAGATAACAATCAAAAATGACAAGGAGAGGGCACAGAGG
EMPV1_07611	GGCCATTTCCCTTTCATCTGTGATGGCAACACCCTAACCCAGTTTCTGTGTCTGCTCTAC
EMPV1_07612	GTGCAGATCTCACTCCCTGAGGGTATCAACTTTGTGCGCAAGGTGGTGACAAACAACAGA
EMPV1_07613	AGGCAGAGAGGGTGCAAGGGAAGCCCAGATGAGTGGCTGGGCTACATATAGCGGATATCA
EMPV1_07614	ATGAAGCTTTTGGGAATGCTCTTGGGAGAAATAGCTGTCAGAGGGAGGGTGGCAGGAGTG
EMPV1_07616	ACGAGCCCATTCGGCCCCCGGCTCTGGGACCACTAGCCGCCCAGGCTCCATCAGGAGAAA
EMPV1_07618	CATGGCTGTGGCTATCAGGTACTTAAGAATGCTAGAAACTGTTCTTAGATAGTCAGAGGC
EMPV1_07619	TCCTCTCAAGACAGGGATATGGACCCAGAGGAAGGAAATGGGCTAAGAATGGGTGAAAGG
EMPV1_07620	CAAGGTGGTTTAGTAAAGAGTGGCCCATCTATGTGATATGGCTGCTAGTAAAGGACGAGG
EMPV1_07621	GCTCGGTGAGCACCCGCTGGGTTCCATCCCCTGTCTTTGTCCAACTATTTATGTATTATG
EMPV1_07622	GTTTCCTCACTGCCAGAGCACCAGATGCTGAGTTCCTTTCTGTGTTTGATTTCAGGGAAG
EMPV1_07624	GCTCATAATAGCACTGCAACGTGGGCTACTGCAACTCTCCCTTCACTGTGTTGACCATCC
EMPV1_07625	GACCCCATCACTATCAGTTAATTGTTGAATACCCAATTGCATGATGCTGTGCTCACGGTT
EMPV1_07626	ATGCCTATGGTTTCTTTCACAAGCCACAGATCTCGCCAGCGCCAGATTCCTTGGTACAAG

EMPV1_07627	CAGCTGCAGGCTCTGAAAGAAGGCAAGACGAACATGCACCCGCCGTATTCCTTGGGACAG
EMPV1_07628	ATCTCCCATTCACGAAGACCTCAACCTATGGAGATCAGGGCATCTGTCATGTCTGTGCAG
EMPV1_07631	TCACATGGTGAACCTGGCCATGGATTGGCTGACAGAGTCATTAACTGCAGAGAGGTTCTG
EMPV1_07632	CAGGAGCTAAGTCATCCAAATATAATTGGTCTCCTTGATGCTTTTGGACATAAATCTAAT
EMPV1_07633	GGATCGTGAAGGTGCAGCTCCCCGCGTATCTTAAGCGGCTCCCAATCCCGGAGAGCATTA
EMPV1_07638	ATGGCCCAGTGGACATGAAGAAAAGAGGCCATGAAAGGAAGGATGTAGTTCCCTGCAGAG
EMPV1_07639	AAGAGCCAAAAAGAGCCCCAGTGCTTCTGATAATGCACATCCTACAAAACAGCTGACTGC
EMPV1_07642	TCATGAGCCCAAGATTGTGCATCTTCCTTTTAGTGGCTGCCTGGATAATTGGCCTTGTCC
EMPV1_07643	CTCGCTGAAGATGGACGGTCAGCAGGTCAGTCATTTCACAGGATTAGGAAACTGACAGAG
EMPV1_07644	CTAGGAGGTTAATGGTTTGAAGCAATCAGGGGCAGGTTTCTCACAGATAAAGGGGAGGCC
EMPV1_07645	GCTTTTAAGTCCGCCAATCTTTGGATTCAATCTCCTATGGCGAGGCACAAGCATGACCTC
EMPV1_07647	AGCAGCTGTACTACAAAGAGATCACAGAAGCCTGTGTAGGGTCGTGTGAGGCCAAGAGAG
EMPV1_07649	CACATCAGGTTAAGGAACCAACGTTTTCTCTGAGGTGGCAGAGGTTTGATCCCTGGGCCT
EMPV1_07657	GGAACCCCTTAATGCGTTTAAAGAATCAAAAGGAATGATGAATGATGAATAACTGAAGTG
EMPV1_07658	TTCTCAATAAGACAACTACAAGACCTAGTAGACCGAAGCCCAGTGGGATGCCCAGAGGGA
EMPV1_07660	GAGGGGCTCTGTGTGTTGGCGGTAATCCTTTAACTCTCAGGAAAGCAAGTTACAACTCTG
EMPV1_07661	TCCGATTTCCGGACCAGCTGTATTGAGGGAACGGAAGAGGCTCCAAGGTAAAGACAAGGA
EMPV1_07662	AGGAGCTGCAGAAGCAGAGGAGGATTCAAGACGGGTCCATCCTCGGTGCTCACGGAGTCA
EMPV1_07663	AGACCCTACCACAGACCAGAGAATGAGTGCCACGGGCTTTGAAGAGACAGACCTCACCTA
EMPV1_07665	GCAGGGCCCCTACATGGGCTGGCAAATCAGGAAGTGCTTGTTTGGCTGACACAGCTGCAG
EMPV1_07666	GACCCAGTACTACGACAGAAACAGAAACGATCGCTAAATATGAAATAATGGATGGTGCAC
EMPV1_07669	GGGGGCTTATTTAACCACACAGCTCAGAGAATAGTGAGGCTGTTGGAAGATGTTAGGTGG
EMPV1_07670	CAGGTGACAATTACTCTGCCAAATATAGAAGGTTCAACTACACCAGTAACTGTTTTCAAA
EMPV1_07672	TTAGAAGTGTCTGGGGAAGCTGGCTGGGATGGAAGGGGCAGGGTGAGAGGTGTCTCAGTT
EMPV1_07673	TGGATGGAGGCTTCCGCTTCAGCCTGTCTGATGGTGAGCACACTTCCCCTGGACACTTCT
EMPV1_07674	CTGTGCCTTCAGGTTAAATTACTTCAGGATTTTCCAGCTATGTTTAAATAATGGTGTGAG
EMPV1_07675	TGAATCCGGTTCCCACTTCACTACCTCAGATCTAATCGACTTGCCTTTTCCCCTCCTTCC
EMPV1_07676	AAACCCTTCTAAGCAATGTCGGGAACGGTGTTCAGAGAGCTGCCACCGGAAGGAAAAACG
EMPV1_07678	GTCCACTCCTTGGGATTTGAAGAGAAAATGCCTGGTGTCATACCTAGTGAAAGTAATGGG
EMPV1_07679	CCGCTTTAAAACCAACATGTCACTGTTCTTACGGGAACACATTAAGATATCAAACTGTGT
EMPV1_07680	CTGTTCTCCCCAAGCAGCACAAGCTCCTGATGATTCACAATATATCTCTGGTGCCTTTGT
EMPV1_07681	TCGGATTGCACACCCTGTTGTGAAGTGCACCTACTGTAGGACTGAGTACCAGCAGGAGAG
EMPV1_07682	GTTACCAGGATTACTTTGGTGAACGATGTGGGGAAAAGTCCATGAAGACTCACACCATGG
EMPV1_07683	GGCTGAAACACAGCCGCTTCCAAGATTTGTGCCGAAAGGACCCTTACAATGCTGTACACT

EMPV1_07685	TGGTACCGGCAACATCCTGGCAAAGGACCTGAATTATTGATCGCCATACGTTCTGGTTCA
EMPV1_07686	ATGTAATTCCCTGCTTGCGGTTTTAGTCGCTTCCTTTGAGTGTCGGGTAGCTGTTCCTGG
EMPV1_07687	GTCGTCCATCAGGACCTCAGGCTCTTTTGAGTCCAGTCACCAAATCTCTCCCTTCAAGTA
EMPV1_07689	AAAGAATTACTCGTCCCACTGACGAGTTCTATGTACGTCCCTGGGAAGTTGCATGATGTG
EMPV1_07690	GCTGGAGCTATTCAGACCTTTCTGATGACGAGGATTTCCTGGCTGATGAGGCCTCCGGAG
EMPV1_07691	CAGTTCCTAGTAGGATTTGTCTCTGCTGTGCCACGACCAGAACTCCTCCATGAGTTTATT
EMPV1_07692	AAGAGCCTCTGAGAGAGGAAGACTCTGATTTCATCCTGACCGAGGGCGACCTCACCTTGA
EMPV1_07693	GTCAACTTGCCAGGAAGCTACAGGTGTCAGTGCCGGAGTGGTTACGAGTTTGTAGACGAC
EMPV1_07694	TTCGCAGTCGGCACCACGTTTCTGTTGGTGCTCTTTGATGTCTACGACGAGTTCGACTCT
EMPV1_07695	TATGCCTATGGTGTCATGGATCGGGGTTACTCCTATGACCAGGAGGTGGATCAGGCTTAT
EMPV1_07697	ATTACGTTGTCCTGTAGCCTCCGCATACTACTCACTGTCATCACACCAGCGTACAGTAGC
EMPV1_07699	TGTGTGCGGATGATTGTCACCGGAGCAGCCCCCGCATCACCAACAGTCCTGGGATTCCTC
EMPV1_07701	AAGCTGCTCAGCTGGCCATAGAATCTTTGGATGGCATCCTGGTAGAAGGTGCCTGCATCA
EMPV1_07703	GCTGCAGAAGCAGAAAAAATATTATGGATCTGCCTTTGGAAAGGAAATGACTTTGGATAA
EMPV1_07704	GCCCAGGTGTTCCTGCATCCTGAATGGCTCTTCTGCTGCCATAAGCACATATCTAAGAAG
EMPV1_07705	ACAACTTGGAGGAACAGACGCAGAGGGGTTGAGAGGAGATTAAGTTGGACTCCTACCTGA
EMPV1_07708	GAGTGGCTGGCTAACGAGACGATGCAGGAAGGACTACGTCTGTGTGCGGATCGAAATTAT
EMPV1_07714	GCTTAGTCTATTGTAGATGAGCCACCAGAGTGTTAGGTTATGACATGCCCAGAATTATGG
EMPV1_07715	CCATGGAAATATCTCTCCGACATACTTGTGGAATACAACACAGTCTCACTGTAGATCATG
EMPV1_07717	TCAGATTTCAGGGATCTGTGAGCCTCCCGGGATTGTTACTGTCCGGATGAGAGAAGGAGT
EMPV1_07718	CCTACATCTTCTACCCGCTGCCTCCGGGCATGGAGTTCTGTGACTACAGGAAGCATAATT
EMPV1_07719	CCACCTCCAGGTAAATATTTTTTTTTGAACTTAGTAAAGATACGTAGTTATCGTATTCCA
EMPV1_07720	GCACAAAGCAAGTGGCAGTAAGAGCCTTAGAAATAAGACCACGTCTGCCGTCTGTGGCAG
EMPV1_07721	TTTTGAACCTTGTGGCATGTTCGCATCCATCCCTAGAGCAAATCGGCCAAGACGCCCGCA
EMPV1_07722	TTCTTGCCGGCACTGCTCTCCTCCGTCGCCGAGGCGCCACACTTGTTGAGCAGTTTCTTG
EMPV1_07724	GAATACCAATGACTGCAGTCCTCATCCCTGCTACAACAGCGGCACCTGTGTGGATGGGGA
EMPV1_07729	GAAGGGGATGTGGATACAGGGTCAGGCTGTGCAAAGACAATAGCCACCGCATATAGTGAT
EMPV1_07733	GCCAAAGCCTCAACAATTCCACAGCATAACTGCAACTGAAGCCATCATTCCTCAAGCCGG
EMPV1_07735	GCCGCTACCCCAGCCCCGGATCTGCATATGGAAGTACCTCGACATCCATTCCATGCAGAG
EMPV1_07736	GCCTGGACCTCAAATGGAAGCTACTGACTTGTCTTTCTCTATGGGAGCTTCACAAAGGCG
EMPV1_07738	ACACGACTTGGCTATTGGAGCCAATCTGCTGTGACAACTGCCCCCCACCCTGCCACATCC
EMPV1_07740	GCCCCTGATATGGCAAGATTTCTACCTTTCATGCACAGGCATACTCTCCAAATGCTGGAG
EMPV1_07741	TCATAGTGTGGCTCTGGCACCAGGTACACCTTGGATTGTGAGATCTGAGGAGCCTTCTGA
EMPV1_07742	GACATTAGCACTTTGAGATTGAGGTAGGAGAACATAATTTGACCAGCAGATCACACTGTG

EMPV1_07749	CAAGACCATCACCCTGGAGGTGGAGCCCAGTGACACCATCGAGAATGTGAAGGCCAAGAT
EMPV1_07750	ATCCAGCCACCTGGCCCTCAGCGGCTCCACCTTTGGTGTCCTTTTGAAGCACTTCCCCAA
EMPV1_07751	GCTAGAAGAAATTGTTCAGCCAATTATCAGCAAACTCTATGGAAGCGCAGGCCCTCCCCC
EMPV1_07752	TCCGGATTCTGGGGCTGGAGGACAGATAAAGCAGAAGTTGTTAATGGTTACGAAGCAAAG
EMPV1_07754	CATTATAGTAATAGGGAGACAGTCGTCATCTTGGAACAGCTCAGAGGGCACAGAGACGGG
EMPV1_07755	GGTTTGAGAGGGAGGCTGTCCTGCGATCTAACCAACTCACACACGCACAGATAAGCACAC
EMPV1_07758	TTTAGACCTGGCTGGTGGGGACCTCTCCAAGGAAGAGTTCGCAGGCTGGGACTCTAGGTT
EMPV1_07759	GGGGATTAAGAGAAGGCAGATCCTGTACACCACGGATAAGGCATTAGAGACACAGCTAGC
EMPV1_07763	CGTGAGCCAAATGGCATTCGAGTGGCTCCAGTTCCTCTCTACAATTCTTTCCATGACGTC
EMPV1_07767	ACGTCCAGGTCCCAGTCTGCCCACTTTGTAACAGCCCAGTCCCAGTGAGAAAGGGGGAGC
EMPV1_07769	AGTTAGTTGGGAGACTGTCCACCTGGGATGATGTGGTGTGAAGTGGGTGATAATGCGGCA
EMPV1_07770	CAAACCTGCATGCCCATGATTCCTAGTCAGGTTTGTTAATGGCTGAGCCACGAAGGGAAC
EMPV1_07771	AGGCTGCTGCCACCCTGAGCTTCCCTTACTGTCATTCTCGGGAAATCAACCATTTCTTCT
EMPV1_07772	CAGCCCTATAGGTCCTGTGAACTAGTGTTTATCAAACTGAAGGTTGTAACCCATTGGTGG
EMPV1_07773	GTCCACAGCAGAAGAGGCCACAAAATGCATTAACCACCTGCACAAAACAGAGCTCCATGG
EMPV1_07774	ACAACAAGGCCAGCCAAGACCCGTACATCTTCAAGGTCAAGGAGATTCTGAAATCCCAGG
EMPV1_07776	AACGTGGAGCACCTGACGGAGAAGATGAAAACCACCATCCAGAGGGGGCTCGTGCTCAGG
EMPV1_07782	TGGCTAGAAAAAGCCGGAGGGGAAGGGGTGTCGGAGAAGAGTGGAAAGCAACCTGTTTAT
EMPV1_07783	CCCGTGTCGGAGATGTTCTCCCTGGAGGACAGCATCATAGAGAGAGAGATGATGATTCGG
EMPV1_07787	TTTAAGTTACTTCTTCAGCCCCCAAGGGGCTGTCTTCAGTCTCTCCCCTCCTCCCTAAAA
EMPV1_07788	TGTGATCCCTGTCCTGCCAGTCCCTGCTGAGAATACTGTCATCCTACCCACCATACCACA
EMPV1_07789	AAAACTATGAGTCACTTGTACTCTATTCTCGTGTCGGCTCAGGTCAGCTCTGTCACCCCC
EMPV1_07790	GGGAACTCCCACATAACAAATCTTAAAAGCAAAATTCACAAGAATCAAACAGCTTCCCCC
EMPV1_07791	CACCCCCTCTCATTTTGGTATCATTACTCTTCTGCCTGGAATTTATCTTTCAGGATTCCC
EMPV1_07794	CACCTGGGGAACTGGTTAAAACCATGAATTCTGATTCAATAGGTCTTTAGGGCCTGAGAG
EMPV1_07795	CCTGCTGGAGGACAAGTACCGGCGCGCCAGTGAGGAGCTTGACGGGCTCTTCCGGCGCTA
EMPV1_07796	GACTGGCAGAATGGCTTTTAACCCTCTGCTGTCTCCTACTGTGAGTGGCTCAGGCAGTTT
EMPV1_07797	TATTGTGGATAGAGCACCTACAGGCAAACATTTTGGGGATGTCCTGCAGCCAGTGAACCC
EMPV1_07800	TGATGGAGTACTTCGCCGCCGACTACCGCGTGGTGCAGCAGTTCGCCCGCCACTACCAGA
EMPV1_07801	CAGACATGCAGGGAGGAACACAAGAAGAAAAACCCCGAGGTCCCTGTCAATTTTGCCGAA
EMPV1_07802	CCTTCATGTTTGCATTGTTCACTGGAGCTCCCCAGGACTGTAGCTTCAGAGGTTGTTCTG
EMPV1_07803	TCGGGCCTGGCCAAGATGTCAGCCAGTCAGGTGAAGGATGTTTTTCGTTTCATAGACAAC
EMPV1_07804	AGCATTTGTCTGTTGGAAGCATGCAGCCCTGGCCCTCTTGGAGAAAGTGAAAAGGTTGTG
EMPV1_07806	TTCACCAACATGATGATGAAAGGAGGAAACAAAGTACTGGCCAGATCCCTCATGACACAG

EMPV1_07809 CTCTCGCTCTGTCTGGGACTGGAGAAAGCTGGCAAGGAGACTGATGAGAAAGAACCAGAA
EMPV1_07811 ACTGTGAAAGTCTGTGGTGAACAACTAAGGGCTGTTGACATCTATGCTGCTGGTGTGGTT
EMPV1_07812 ATACACATTATTGTGATCGAAGAAGGTTCACTAGTTTCAACGAATCTTGGCCCAACTCCC
EMPV1_07813 GCAGATAATTTGTTTGCATATCACCTGGTGTGAAAGGCGGAGGTGTCATATCATCCGTGA
EMPV1_07814 GGTCCTGTACCTACCCTTCGTGACCTACCAGGAAATCTGGTACTCCTTAGGTTCTGCTGT
EMPV1_07815 GCCTGTTCATGGGAGGCTGCTCTGGTTACAGGTATGCTGTTTGAACCTTCATCTTCCTAC
EMPV1_07818 GCTTGATCTAACCATGTGGTGGAACGATGGAAACGGAACATGGTTCTGTCAAGCACCGCG
EMPV1_07820 TCTAAGAAAGACCGAGTATCAGACCCAGAAAAGAGGACCACGGCAGCTCCAGGCACATGA
EMPV1_07821 GGGTGTTGTGAGCAGCATCGCATTTAAAGTCATCTGTTAAAACATATGTATTTGGAATTC
EMPV1_07822 TGGGCCAGTCAGGGAGAACTGAATGGAAGCTACAGCAGAGATGATAAAATCCGAGAAAAC
EMPV1_07824 TGTGGTCAAGCAGGAAGGGGGTGACAATGACCTCATAGAGTGAATGCAGGGTGATCCCTA
EMPV1_07825 AGGGTTCCTCCACGTGACCTTGATGGTTTCCGGAGCTTTCATCACTCCTGGGTATCCGGA
EMPV1_07826 CTGCAAAGAGGGAACCTGAAGGCTCGGCATCCAAAGAAAGGCAATTATGTACAATGAGCG
EMPV1_07827 TGCCATTGGGAGTTGGGGATATCGGACCTCAGCAGTGAAGAACGCTTGTGAAGTACTAGG
EMPV1_07829 TGCCACCCCCTGTAACAGCAACTCCATGTGGAAGTGCCCACTAGTTCCAGTGGGGCTGCT
EMPV1_07830 CACCTATGGCATGTTAGTTCCCAGGCTAGGGTCAAATTGGAGCTGCATCCTCTGGCCTAT
EMPV1_07832 GCTTGGCGCAAGGCTCTCGGGACCTGTGGTTCCCATCTACTGGTGGTGTTCCTCTTCTTT
EMPV1_07833 GCCTAAGCCAGCTCCCTGTTTGGATGTTCTGTATGAAGCTCAGGGGTCTTGGGCATTGTT
EMPV1_07834 TGTGGCAATGCTGGATCCTTTAACCCACTGTAGGCTGGGCCAGGGATTGAACCCATGCCT
EMPV1_07836 GGAGAAAGCCATTCAGCGCATGGAAAAAGGAGAGCATTCCATTGTGTACCTCAAACCCAG
EMPV1_07837 GAGAAAGCGCCTCCAGTTCGGGGTCTATTGATAGTTCAGCTGCAGATAATGTTGCCGTTG
EMPV1_07839 GTCCTTTGCTTCTCAGATGTAATGCACTTTAAGTTTGTTATTCAACAGTGAAAATGAGTC
EMPV1_07842 GAATCACAGCTCCCTTTTCCCAGCGACCCCCACGGCCAGAAAAGCAAGCGTTAGCTCATT
EMPV1_07845 GCTAGCCATGTAGATGCCGACTGCTTTTTGTGTGTTTTCCTGAGTCACGGCGAAGGCAAT
EMPV1_07847 GAACGTTGAGTCCAATTCAGGGGAAGGAGACCTGGACGCCAGAGACTTGGAAATGTCAAA
EMPV1_07850 TAGAATTAAGCCAGATGAGATCTCTGTCTCCTCTCTCCGAAGTGGCCAGTGTTTCGGGGC
EMPV1_07854 TAATCACGTCAGTTACAGGCTTGCGTCATCGTCATTGTGGGCAGTGAGCTACTCTGTGAG
EMPV1_07855 TAGTTCTCTGGGTTCATCCCCCAAGGCAGCAAAACTCCATTCTGGTTAGTCATTTCCAAG
EMPV1_07858 GAAAGTTGACAGCAGAAGCAGATAGCAGTAGTCCAACTGGAATATTAGCCACCTCAGAGT
EMPV1_07861 CCCACACCCTCAAAGTTATCATCAGCTGGCATCTACATCCACAATCTCTGCCTTTCCTCC
EMPV1_07862 GTCAGGAAAGGTCGTGTTAGCCCGGGTTGTACCTACTGAAACCGAAGCCCAGAAAGGGAA
EMPV1_07863 AGTGAGTCATTACCACCGGGATGTACCCTAATCTCAAAAGCTGAAGTCTCGGCCCTGAGC
EMPV1_07867 CTTCTTTCGGGAGGTGGCTGAGGAGAAGCACGGGGGAAGAAGCGTCCAGCTTTTCATGAA
EMPV1_07868 CCCAGAATTTGAGAATGACGTGGAGTTCACAGAGCGGTTGGTTGCTGAGCAATCCGTCCA

EMPV1_07869	GGTCCAGTTCTGTCATTCAGTAGTTGTGGAACCCTGAGAAAATCACTTATCATCTTTAGG
EMPV1_07870	GTACTGCTTCTGCTAAACTCTACTAAACTTAGCTCCCCCACTCCACATGAAAATAGCAGG
EMPV1_07871	GATTTGAGCTACTGCAGTGAGATTCTTTACCCTCTGTACCACAGTAGGAACTCCCTCTCC
EMPV1_07873	GGAACCTGAAATACTGCCGTCATCTGCCATAGGAAACACACCAAGAGACCTGTCATGCTC
EMPV1_07874	GATCTGTGGTTTCCAGATTGCTTGTTTCAGGGTCTGGGACTTGCGTTCAGGTGATGCTTG
EMPV1_07878	CTAAAAAGAATGAGCCTAAGATCAGAGGGAAAATGTAGGTCTTCTCTACTCCTCAGGGGC
EMPV1_07879	TTGCCCCAAAGCTCCCAAATTAGAGGAAAGGCTCAAAGTACTGACAAATAAAGCTTCTGT
EMPV1_07881	CTCCTTTGAGTGGCCATGTTCTTTATTTTAGGGAGCAGAATTAGCCCCCTGGGCAGCTGT
EMPV1_07886	GTTTCAAATGACTCTGAAGCCCTAAGTGAAAGAAGTGGAAACATCCCTAACTCTCCCAGG
EMPV1_07887	ATGTTCTGGAAGCTTTCCGTGTCCTTGCTCCTCATGGCTGCTCTGGAGAAGGTGGAGGAT
EMPV1_07888	CTGTATTCAGCCAAGGCCCACCCTCTTTTAATCTGTGACGAAGAAAGCACTTGAGGAGAG
EMPV1_07889	GGCTTCGTCAGCCACTTGCAGACAACTACAGAGAGTTGTTCCTGTGCTTTCTCAGTGTCA
EMPV1_07892	AGCCACTAAACATAGGAAAAAACTAACAGTTACATGATCCAGCAATCCCACTCCTGGGCC
EMPV1_07893	AAGATGTCGAACTACACCGGCGGCATCTACGCCGAGTACAAGGACCAGGCCTACATCAAC
EMPV1_07894	GGTGAAAATTCCAAGGCACCTCATAACCTTACACAACAATAATCTACTAGAGTTCATCCC
EMPV1_07896	GCCAGCCAGATCCTGGAGCAGTCTGACCTCATCATGAATCTCTCCCCGTCTGAGGAGACG
EMPV1_07897	GAAGGCGCTTCCGGCTGCTGCTGCTGCTCGCCCTGGTGTCCGTGGGGCTCTGGACTCTTT
EMPV1_07898	ACAGGGTCTGAAGACTCTTGGTGTTTTGGAGAAAATTCAGACTTATCCAGAAGCATTTTG
EMPV1_07900	AGCTGGAAGCCTGCAAGACTGTTAAAGAACTTCATCTGGGGACAGTGGGATGTTGCAGTG
EMPV1_07901	CTGTAGCTTTACGTCGCTTGGTGCTGCAGAGACTACCATGTACCATTTTTGTGCCAGGAC
EMPV1_07904	CTTTTTATTCTCTTTCATCACTCGGTTTCTCAACCGCGAGCCTGGACCCATGAAGGCTGT
EMPV1_07905	ACGTCAATCCAGACCCCCCACTCCCGCCCGTCCCGTACACTCTTTACTCTTGTTTCTGTT
EMPV1_07906	CTCAGCAGTAAGTTTAGCCACAAGAGTGTGTGACGAATTGGGCTGTGAAAATGGACCTGG
EMPV1_07907	GAGACTAAAAGGAGGTTGTCGTAATTGCCCTTGAACAGCACACAGTAAAACACTGAGCTC
EMPV1_07908	AGCGACATGACCCGCGAAGAGATCAACGCGCTGGTGCAGGAGCTCGGCTTCTATCGCAAG
EMPV1_07910	AAAGGACAGAAGAAAGTTGGAGCATGAGGGAAGTGGAGGAGGCAGCAGGAGCACAAGGAT
EMPV1_07911	GTCATTTCCTTCAATAAGGCCTCGTCCATCACCAAAGATGTAAACTCCCCCCTGATTTCC
EMPV1_07912	AAGAATTCCTCGGAGCAGTTTGAGAAGTGGCGCTTCAGCATGCCCTTTAAGCTGGCGCAG
EMPV1_07913	ATCCACCTTTCATTGAGGCCTCCAGCCCACAGACCAGGCTGCTGACGGGCAGATGCCATT
EMPV1_07914	GTGATCTTAGCCTTTGCAGCCTGGGTGGCACTGACAAATGAGCTTGGCGTGGCTGTGTAT
EMPV1_07915	ATGTTACCGTGATGTTTTGCTTTTAAAGTCACGTGGGAAGCCCAAGCCTCGGACCGCATC
EMPV1_07916	CCAGGGTCACAAGTGAGTGTGCTACCAAGAGCCCTCAGTGAAAATAGCAGTTTAGGGATA
EMPV1_07917	CAGAGAAGGCATCAGAAGGAGAAATCTGGCATCATACCTACCTCGCCAACACCCTACACT
EMPV1_07918	AATGATGCTTCCTCCTCCAATCCTGGAGCGCACATTTTTCCGGGAGAAGCTAGAGGTCTA

EMPV1_07920	AGCTGTGGGAGCCATGGTTTTCCCCTGCCTGGGGCTCCCCTCTCACCCTCAAGGAACGAA
EMPV1_07924	ATCTACACAACAGAAACAGACTTATTGACATGGAGAGCAGACTCGTGGTTGCTGGGGGGC
EMPV1_07926	ACACTTCAGGTCAAGGAGGAAGTACTCAAGAGAGCGTGAGCTGCTGGGGAAGGTGCAGTT
EMPV1_07927	ATGAGCACACGCAGTAAAGGCGCTGAGCAGAAGTCCACCTCAGCCCAGGGAAAACCAGCA
EMPV1_07928	GGACTTACCCCCTCCCCATATTAAAGAGGCATAACACTGGGCAACATTCGTGAGACAGCT
EMPV1_07929	TTGACAGTAAACTTTTGGAAGGGAAAGTAAAGGAGGATCCTGACCAGGGGGAATCCATAA
EMPV1_07930	TCACTGTGAAAAGTCCTAAAAGAAGAGCCAGCTCCTCATGATCAAGGCCCCTCTCCGGCA
EMPV1_07931	GCAGACGCTGGTATCCAGAAGAATATGAATTTGCACCAAAGAAGGCTGCTTCTCACAGGG
EMPV1_07933	GTGCCTGCACCCTTCCCCTCTAACAGTACCTCTGTATACTCTGCTGATTGGTGTATTCAT
EMPV1_07935	CATGTGTGGGGTGAGAACCCGAGATCTACTCTCCTGGCAAAGTTCAAGTATACAACGCAG
EMPV1_07936	CCTCTCAGTGTGATGGTATTAGGAGGTGAGGCCTTTGGGAGGTAATGAGGATTAGATGAG
EMPV1_07937	ACCACACTGATGATTGGGTCCCGCACTTCTCTTCCTAACACCAACTGCTTCATAAAGGCC
EMPV1_07942	GTCTAGAGTTTGTAGAGTGCTTTGAAATGAAGAAGTATTGCAGAAATGGGCCTACTGAGC
EMPV1_07943	CTGTGGAAAAGCCTTTAGGAGGGGTTCATACCTTACAGTGCATTGGAGAACACATACTGG
EMPV1_07944	GCCGGAGGTGCATATTAGTGATAGGATTAAAGGAGCCACAAGCAAGGAGAACCACTTCCG
EMPV1_07945	TGTGCAATTGTGGGGGTGGTCCGTGTGACAGCGTAACTGGAGAATGCTTGGAAGAAGGTT
EMPV1_07946	CCCTAGCGCAGTTACGTTGTAGTACTCGACATTCAAGAAATGGAGACGAGGTTTGGAAGG
EMPV1_07947	AGGCACTGCTTGGCTTCCTGTCCCAGCAGATTGCAGTGTCGTGACTTGTGCATCACCTTG
EMPV1_07950	GTTCTCCTTCCACCAGAAACCAGACTTCACAGCGACAGCGACGACAGCGTACCCTGACTT
EMPV1_07954	GGGTGTCTCAGAGCAAGCTTCTAGGCATCAGCATAAGGAACATTTCTCCAGGATGGTGTC
EMPV1_07955	CAGGCTGTTCAGGGGACTGGCTTAGCGTTTATCGCCTTTACAGAAGCAATGACGCATTTC
EMPV1_07957	TGAAGAACTTGGAGTATGTGACAGAACCAATCCAGGCCTACATCATCCCATCGGGAGACG
EMPV1_07958	ACTCAGAGACCCTCAGACACTGAACCAGGCCTCTTCTCAGCCTCCTCTTTGTCCAGATTT
EMPV1_07959	AGTGGGCCGAGTTCTTTGCGCAGCAGAAGCTGAGTAAGGAGCGGGCAGAGCGAGAGGCAG
EMPV1_07962	AACCCAGCCCGCAGCTCCATGCCAAGATGCGGCTAGAATCCGAAATGTCGTCGGGTAAAT
EMPV1_07964	TATACATATTTGTAATTTATGTTGCATGTATAAACGGTGTAAATCTGCTATAATGGCAAA
EMPV1_07966	ATGACATCGTGCTGTATTTTGTAACTGGCCTGCTGGGTGTTATTCCCCTGATTGGGATCC
EMPV1_07967	GAGTGGGAGATAAGGGGACTCAGCTTTCAGGAGGTCAAAAACAGAGGATTGCTATTGCCC
EMPV1_07968	TCAAGTACTGAGGGGCCTTCGACAGGATGCACTTCGAGTAACCCTGTTTTATGCACACGG
EMPV1_07970	GCACACAATTTAAGGGGGCACTCACTCTCCAAGTCACACAAGTGCTAGCTCATACCTGCA
EMPV1_07971	TCCAGCTACTTTGGGGACTTCTGGCTGCTGCTTACTCAGGGAAGTGAACAGAGGCGATTA
EMPV1_07974	CCCCCCAGACCATGGAAGGGCAGAAGAGCCCAGAACCCTCAGGGCTCCCACAAAGCCAGA
EMPV1_07976	TTGTGTCTGGTTATTTTTTCTGGAAGTCTACAAATTTAGTTAAGAGACCAGAGTCACTGG
EMPV1_07977	GCCTTCAAACTTGACTTTCAAACTGCCTTAATTTGGGTGACACGCGCTCCTGAAGCAGGC

EMPV1_07978	CCTAAACAGGATCACAGATAGTATATAAACAGTGTTATGCCCAGCACACATTGGAGCTAC
EMPV1_07982	TCAGTGATTACCAGGGGTTGGAGCAGGTGAAGGATGAACAGGCAGAGCACTGAGAACTGT
EMPV1_07983	AGGACAAATCAGTTCTCTGTGACCAGACATGAGAAGGTCGCCAGCGGGCTGATGGGTGAT
EMPV1_07985	AAAGGGCACTTGGAACCCACCGAGCTGCTGATGAACCGGGCTTACTTGCAGAGCATCACT
EMPV1_07987	ATTTAACCTTTTAATTATCACCTCACCTGAAAAGGTTGGTTGAGATACTCACGCAGCATG
EMPV1_07992	ATATTAACGCCAGCTTGGTTGACATAGCAGAGGCTCGGAGGAGTTACATCTTAACACAGG
EMPV1_07996	TTCCACCTCTTTTCTACTTCTCCGCTGCTACCCTACTATATTCAAAGACCCCAGGGCTGG
EMPV1_07998	CAATTGGCAGCTAATTCTGATCATTTGTAATCACTAAACAACACTTTGGAATTTAGTCCA
EMPV1_07999	TTCCTTGTGAATTTATCCCGATCTCCAGTCTTCTAACACACCTTCACGCAGGTCCCTGGG
EMPV1_08001	TCATGTGAAACATACCGTAGGAGAACTTCGTCTTCGCCATCCAGCTGAGTGTGGGGGCTT
EMPV1_08003	GTGGGCATGGACAAAGCCCTACTAGTTCTCCATTGTGTGAGCTAATTAGATTTTCTCCTG
EMPV1_08004	TGTGCCATCTCCCTCTCCAACTTCATCGGCCTCAAGCCTGTACACTTCTTGGCTCTACCA
EMPV1_08005	ATGTGGCAAGACGTTCCTCACCCTGGAGAAGTTCACTATCCACAATTATTCCCACTCCAG
EMPV1_08006	CAGGAAGGTTCCGTTTCATGAGCCTGCCCACGTTCGTTTACTTCTGACCACTAATTGCAC
EMPV1_08007	GTCCAGAGGCAGTGAAATGTTCCAGTTGGGCAGTCATGAGCTAAAAGTAGTATCGTCCCC
EMPV1_08010	CACTTTCCACAACCAGCATCACAGCCCAGCTCGTGCCTACCTCATGCCTATATGATAGTT
EMPV1_08012	TACGCATTACACAGCAGCAGGAGAACCCCAAGGTCCTGTCCCACGTCACGAGGAAGTTCA
EMPV1_08013	GACCAAGAAGCAGCCGATCAAAGTCCAGATCGCCATCTCCCAAAAGAAGTCGTTCCCCAT
EMPV1_08014	CACTGTCCCCCAACGAGTCACCCTGTTCCGACAGATGTCGTCGGGAGCCATTCCCCCCAA
EMPV1_08016	ATCTAGTGTCCTGCACATGCTCTGCCATTTCCACCACTAGCATTCAGCTCCCCCTAAAGA
EMPV1_08021	AGATCCAGGGCTCTCGAAGCTGAAATGATTGGAACCGTTGTGGGACAGATCCTGGGAGAC
EMPV1_08022	CCATCGTCTCTGTGCAGGAGATCGTTGATTTTGCCAAACAGCTGCCAGGCTTCTTGCAGC
EMPV1_08024	TACTCACACGCTCTTGCTCCAGCACCCGGGCTAATGAACTCACCCTGTTCTTCTTCAGTT
EMPV1_08025	GTAGGAGGCAGTGCTCGGCTTTCAAAGGAGAGTTTTTCAGCAGGAAGAGGAGATGGGTGA
EMPV1_08027	CTCAATCAGTCAAGGATCCGGGCCCGACACGTACTTTCACAGTAGTTCCCAGGGCAGCAG
EMPV1_08028	GCCTCCAGAAGATATGAAAACAATCGATGGCCTCAACCAAAATTTAAGATACTGTGATTT
EMPV1_08030	AGTTACACCAAGCTGCATGGAATCCACTTCATTCAATCGAGATACATTGCACCACTCTGC
EMPV1_08031	CCATGGTCCTGCCCTTTTTCCTGGCTGTGGAGGTGCCGTGACATCCTTGGCCATCAGGAT
EMPV1_08033	ATCAGGTGGTTCAGATGGTGTGATTGTGCTTTATGATCTTGAGAACGCCAGCAGACAACC
EMPV1_08035	AACGTTTCAGAGACCAGGATTGGCAGTGCGCTCGTAAAATTGCTGGACATAGAGGAGCGG
EMPV1_08036	GGCAGGATTCAGCATCTTCATCCCCGAGAGGTACCTGGATCAAGCACAGCTCAGCAAGGC
EMPV1_08037	CTCGAAGGCACTTTGAATGGCAAGAGGGGTCTGATTCCACAGAATTACGTCAAGTTGCTG
EMPV1_08038	TCCCCAATGTCCTCAGGCGTCCAGTCAGGGCAGAACGTCAGCTTCAAGTATACAAAGGAT
EMPV1_08039	AGCAGACACAGCCTCTGGACTCGTGTGGGGAACAGACTCTCACCACTCTCTACACAAATT

EMPV1_08041	TTATGAGTGGGCGGTGAAAAACAAGGACGATTTTCTCTGCGCTCGTGGTTATGGAGGTGC
EMPV1_08042	CCTTCCCAGAAAGGGAGGGGTCCTTCTTCTCAAAGCTTCAACCACTCGTGTATTAGGGGT
EMPV1_08043	TAGAAAGCTGGCTGCTGCCACTGGCCAGACGTGCCTTCCGTTATCGCTGGATGTCCGAGT
EMPV1_08044	GGGGGTCACATCAGAGCTACTTAATGCAACACCAGATCCGTAACCTACTGAGCAAGGCCA
EMPV1_08045	ACGTTCTCAAAACCGAGATAAGGTGGGCATGAAGGAACCATGGAACCAGGACCCCTAGCA
EMPV1_08049	TTGGGTTCATTTATTCATACAGGGATTGCATTTCTGAGGGTCTCTTCCACCAGGAGAACC
EMPV1_08051	GATATCCCCAGATACTCACAGAGTCCCCTATCACATCTCATTTGGTTCGAGGATGCCAGG
EMPV1_08052	CACAAAACCTAGAATCCACTCGTGATGAGCGCATGTCCTCCTGCCTACTCCCGGGAACAA
EMPV1_08053	TTTGGGAGGAGGCAGCTCACTGGAGAGCCTACATTCCTTACACAAGTGCCTAAAGAGAGT
EMPV1_08054	ATGGCACAGGTTCCTTGCGGCCATGTAATTGAAATCTGAAGTTGTGGGCAACTGAGGAAC
EMPV1_08056	TCTCCACTATGTGAGGACACAGCAAGAAAGTGGCTTTCTGCAAGGCAGGAGGAGAGCTCT
EMPV1_08057	AAAAGGCGCGAGAGAATGGATGATAGTAGTTACACCTCTAAGTTACTGTCTTGCAAGGTG
EMPV1_08062	AACAGGCTGCTGACGCTCTGCGAGAAGGTGTTGACTGTGCGGCGGTAGCTGTTCTTCTCG
EMPV1_08063	TAGTGGAGCTGGTGGGCGTGTTCCCGACTCTCATCGGCAGGATCGGAGCAAAGATTATGC
EMPV1_08065	TCTGACCTTAAGGCATGCTGGTTAAACTCTGTGCCTCAGTACCCTCAATGTAAATGTGGG
EMPV1_08066	CGGGACCGATGCCTGATCATATGTGCCTCGTGAAATACCCTAGGATCTGCCTTACTAATC
EMPV1_08068	GCTTTAGTGATCATCTTTGTACTCTCTACATTTGGAGGAGCAGCAATGTGGGACTATACG
EMPV1_08069	AGGACCCAGTATTGCCACAGGTGCTACACAGGTTGCAGCTAGGGCTCTGATTCAATCCTT
EMPV1_08071	AGTGAGTACGGGACTTGATTGAGATGGGCGGGGTCTCTGTGGAACTGTCCATAATGTCAC
EMPV1_08073	GAGGCTCTCAGACAGTTGGGAACTATCACAATTTCTATGAATCCATGTGTGAAGGAAGAT
EMPV1_08074	ACCAAATAATCCAGCAGTACATGGTGACCCTGGAGAACCTCCTCTACACGCCTGACTTAG
EMPV1_08076	AGAGTGACGAGGCCGTGGCCTTCAGCGTGCTCCTGGGGGACCTAAACTTCGACAACTGCT
EMPV1_08078	TCGGGCTCTTACCTGAATGGGCGGAAGCCTGTTTTGAGCAGAGTGACTGGATCTGACATT
EMPV1_08079	AGCTAAGCTGCTGAGGGGGGACAGGTGTGAGGTTCCAGCTGGGAGGCACCATCGAGATGA
EMPV1_08083	GTACGCGTGTCCTCAGGAGACTTCAGCACTTGCAAAACACCGCGCTCTACCCCGAGTACA
EMPV1_08085	GATGAAATCAACAAGGGAGTGAAATATATACTCAGAAATGCAAATACCACAGCATATGTG
EMPV1_08086	GACTTGGAGAGGAAATCTCGGCTTCTGTCCATCAGCCTCCACAGTGTGCTGTCCCTGCCA
EMPV1_08087	GGCAGTATCCTGGGAAAGGGCTTACTTCTCTGCTGTTAATTCAAGCGAACCAGGGAGAAC
EMPV1_08089	ATTACACTTTGTTGCAGGTAGTCTTGCTGTATTTTGGGAATTGCAGAGAACGTGGAGCTG
EMPV1_08090	TTCCTGAGACATTCTTGTGAATAAATGGAAAGGTGTTCCTGGCAGATATGAAACTTTTAC
EMPV1_08091	TGTCTTCGGCAGATGCTCGGCACAAAGCCTTCAGCACCTGTACATCTCACATCTGTGCTA
EMPV1_08092	GTGGTCTTTCCTTCCCAGGACCGCCAAGACAGAAACACATATTCCGAAAGCCAGCATTGC
EMPV1_08096	GGTTTGTTTGGGTTTGTTCTAGGTATGGTCCCAGGGATCCCAGATCAAACCAGGCCCCTG
EMPV1_08098	TATCCTGCACTGAAAACTCTGGAACATCTAGAGCATACCTATCTGCCTCAAGTAAGCCAC

EMPV1_08099	AGAAGTCACTGCTGACTGGGATCAGAATGGCTTGGCCTGCAAGACTTGGAAGGAACTTCT
EMPV1_08102	TTGCAACACAATCGAGAACAGCAGAAACGACTCAGTAGCCTTTCAGATCCTGCCTCAGAA
EMPV1_08103	TGACCTGCAGGCTTGGAGACGAAGAGTGGTTGTACCAGAGGTACCACTAGGCATCTTTAG
EMPV1_08108	GTACAAGAAATATGGCACTCGGTGCTCTAGCTGCTGGCTGGTGCCCAGGAAAAATGTCCA
EMPV1_08110	TCAGCAGGGAGTATTTGGGCCTGAAGAAGATAGTGTGCAAGTGTCCCCATGTTAAGGACG
EMPV1_08112	ACCTTGCCTTCTACTTCACTGGGAAAAGGGAACCAGCTGTCCACCACTCTACAAACATCC
EMPV1_08117	GAACGGCTGATGTAGGTTCAGGCTTCCACCTTGTTATGGCCACAGTTCTTTGATGTCGTC
EMPV1_08118	AACGGGCAGTTTCTAAGCCGGAAGTTTGTGTTGACGGAACGCGAGGGGGCCCTGAAATAT
EMPV1_08120	ATCACCCACGAGCATAAAATGTCATCTGCCACCAGCCTCTTGTACTAAAGTCATAGTGCC
EMPV1_08122	ACTGTGAAGCCCTGAAGACACTGGCTGAGACGCTGGTGAATCTGGATGTAAGGGACCACA
EMPV1_08123	TGCATAGACCTTTTCAATTGCTTTGACAACTGGGAGCAGGGGACGCACAAGAGACAGAAG
EMPV1_08125	AACGCTAACAAGCTCCAACTCTGCCCCAACCCCGGCGCAAAATGTCCTTTGTACACCTAA
EMPV1_08128	CTGCACAGCCACATGAGGAACCAAGAGCGCACAAAGAAGCAGACCACATACCAGAGTGAT
EMPV1_08133	CTTTGTCTCTTTTGGACTTGGAGCAAAAACTAAAAGAAATCAGAGATCTCGTTGAGCAGC
EMPV1_08134	AGAGGTAAACATGTCCGGTCGCGGAAAGCAGGGAGGCAAAGTGCGAGCAAAGGCCAAGTC
EMPV1_08136	GGTACCTCCTTGGTGGAAATTCCTGGCAGACATCACTTTAGCCAAGTTATCAAGTTGAAC
EMPV1_08137	ACCACTGTGTGAAGAATCCGTCAGACCTGTGCAGCATTAATGTGAGCGGCCTCAAGTTCT
EMPV1_08138	AAGGGGTTTCCTGCAGAATTTGCCATGTACTTTTGTGGTGGACTGCGCTTTGAGGAAGCC
EMPV1_08139	TGAAACCTCCGACTTGGGTTTGCCTGGGTTCTCACTTCATTCCCTATGACCTGTCACCTG
EMPV1_08140	ATACCTTTGACTGGACCCTAAGCCTCAGCCTTTTCACCCAGAAGTATGGGGAGGGCCTCC
EMPV1_08141	ATGCATTTCCTTCATCCTGGTTTCAGGTCTTCATCAGCACAAGCTGCATCCTCCACTGGG
EMPV1_08142	TGCTGGGACTGGCTCAAGGATGTTTTGACCTCACTATTCCATACCTTAAAGAAAGGGTAC
EMPV1_08143	AGAAATCTGGGTTTCATTCTGGTGGTGGGCTGAGTAACGCCTCTCCCCTGCCCCAAGATA
EMPV1_08144	AAGGAATGACGCTGCTTTCTGCAAGGGGTGACATGCACATGTGGGAGGTTTCAATGCTGA
EMPV1_08145	GGACTGTTCAAACTCGTCCTGCTCCACGAATTCCTCCAACCGTGGCAATAATTGAGAAGG
EMPV1_08146	TGTTCTTAGGCAGGGAAAGGAGGGACATTCTGGATCTGGCGGGTCTGAGCAAAGGGAGAA
EMPV1_08147	CACTGCTATGAATCTGGAAAATTCATTTGCCAACCCGTACACTCAGACCATGACACTGGC
EMPV1_08148	CACTCAGAGGGCTCGCTGGTCGTTCTTACATGCACAAGACCCCTTTCCACAAGCTGTCCA
EMPV1_08150	TGATTACAAAGCCTGAGGGAATTTTTGGAGGGTCAGTACTGCTGTGAATCCTGCTTGCGG
EMPV1_08152	TGCACGGTGTCCTTCTTGGGCCGATACCCATAATACTCCTCCTGGCGCCTCATGAACTTC
EMPV1_08154	GATGAGGAACGCAGTGGCAGGCTCAAGGCCCTGCTCCGCCGCTACTTTGAGGAAGAGGAA
EMPV1_08155	AGGTTGTTTCTATGCCTTGGCTATGGCGAATAGTGCTGCAAGGAACATAGGGGTGCATGC
EMPV1_08158	TTCTAGGCTCCAAAGTTCACAGGCTACCCTCTTCCCTGGTCTCTGTGTCCCCGTAAATCT
EMPV1_08159	CCTGATCCTTTGTACCCAAAGCCAGTAAGGGCCTCAGCTCTGCAGCCTAGGACAGTGGGC

EMPV1_08162	AGCTGGAAGGGTTAGGATTGAGGTCCTCAGGCCGCTCCACTGAGCAAAGTGTTCCATGGA
EMPV1_08166	CCCTGCCCCAGACCTCACCCTCTGTATCTGACTGTTCATCTGTAATCTTTACCATACCCT
EMPV1_08167	GCCAGTGTTGGCTGCTTTAGGGATGCCATAGGCTTTTGTGGCAGGTAGGCAGAAATGAAT
EMPV1_08168	GGTCCTCTGACTCTCTTCGGTGACGGGTATTCTTGGGTGGATAATACGGATTACGTTGTT
EMPV1_08170	CATGGGAGTAAAATGTTACGTTCGGAATCAAACTCTTCAATTACTACTACTCAGCCTACT
EMPV1_08171	TGCCTGGGGACCGGAAGAGACTGGAGCAAGAACTTTATCAACTGGAGCAATGGAAACTAA
EMPV1_08172	GATGTCAAGATCTGGGCACCCACAGCCGAAACTTCCACTGAGCTTACAGGGTTAAAGGAT
EMPV1_08173	TGGAGTAGTCCGTGGTCTCTTGAGTGTGTCGAGAGAAGCCGTTTGTATGTAAGGAGTGTG
EMPV1_08174	AAGAGGCCAGCAGCTCAGGCTGCTCTGAAAGAGACTCTACAGAACTGGCCAGCATCCTCT
EMPV1_08175	AGCTCTGATTTGACCCCTAATCTGGGAGCCTCCATATGCCTCTGGTGCGGCCCTAAAAAA
EMPV1_08177	GCCTTGAGTAGGAACTAGACTCACTCCCGTCCACTAGACATAGAGATGAAGCGTCCTTCT
EMPV1_08178	ACAAATCGACTGGTGGTAAAGCACGGAGGAAGCAACTGGCTACAAAAGCCGCTCGCAAGG
EMPV1_08181	AGCCACCTCAGCCATCATCTTCCACTTATGTCCCAGGCAACATGCGATCGAGCAGCTATA
EMPV1_08182	TCACTGAGCATGGAACTGCTGATGACCCCTACTGAGGGCAAAAGATGCGTGTAGAGTTAG
EMPV1_08187	AGGTCCTGAGCCCTTCTGCTGTCAAGGCAGTGGACTTTTAGTTAGGAGATTGTAGCATCC
EMPV1_08188	TTTTGGAATTCATCTTAATCTGTTCTGCACCTCAGTTCCAGGGCGCCTTTCCCCAGCAGG
EMPV1_08189	AGACTGGGCTGCCCGCTGGTACTTCATTGCCTCTGTGGGCCAGTGTAATCGGTTCTGGTA
EMPV1_08191	GTTTAATCAGGTCGCCGTCGTGTGTGGTGATGAGAATTGTGGAACATGCCATTCTCTCCC
EMPV1_08192	GGCCATACACAGAAGAGAGACCCCGGTCAACAAAAAGGTGGAAAAGAATGACGTTGTCCT
EMPV1_08193	GACGGGTTCATGACGCACTATCTGAGAGAGGGTGAACCATACCTGAACACTGCATATGGG
EMPV1_08196	GCAGGATGGAAGTAGGATATATGAGCAGTAATCCTGTCACTGCTTCTCCTAGGTGACGTT
EMPV1_08197	CGTTGTTGAACCATGAAATGCTTCCCTGCAGGTGGGGAAGAAACAACTGCTTTTAGGAGG
EMPV1_08200	GCCTGTAAGGAGTTGTCTGTAGGGGTCCAATGTTCTTCTAGAGGTCCTAAGGGACAAAGC
EMPV1_08203	CTCTGGACACTCTCTTCTGGATGTCACAATGCCAAGTCTTTTTGCTCCTCTCTCTCCTAG
EMPV1_08205	CCTCGAAGGAACCCCAAGCACGCCAAAGCCCAGGACCTGTTTCAAGCCTGTTTCTTGAAA
EMPV1_08207	GGTTTCCAGTGATAGTTCTGGATTCCCCCATGTTGCCTTGTCAGTCTCACCTGCCATGTT
EMPV1_08209	GGAGTGCCTACTTCTTACAGGAGGATCAGAGTACCTTTACTGACCTACGCTACTCACCTC
EMPV1_08210	GACCTGGATCATGGTGTTTTGGTGGTTGGCTATGGCTTTGAAGGAACTGATTCAAATAGC
EMPV1_08211	ATCGAACCTGAACCATAGCGGTGACCTGAGCCACAGCAGGGACAACATCAGGTCTTTAAC
EMPV1_08212	AAATGGCAAGTCTTGGCTTCTCTGCAATCACTCCCTGATCCACAGCATCCTCCGTGCTGC
EMPV1_08215	TACATCATGGAGAAAGTGGATCTCGTCGTAGTTGGCGCTGAAGGAGTTGAAAACGGAGGA
EMPV1_08216	TTCCAGCCCTAGCTCTTCTCCGTCTCCACAGCCCGTTTCAGAGCTTGACTTGTCCTCAGA
EMPV1_08219	TTTTAACCCTACTCCCAGGGCGGAGGTTGGAGGAGCGAAAACGGCTTGCGGGGCTGCTTT
EMPV1_08221	AGCTCCTCTATCTCCCTGATGTAGTTCTGGATCAGAGCCCCAATGGCCTCGTTGCCATCA

EMPV1_08223 CTGCCAGGGAAGGACTTTCTGAGGAGGTAGAATTTGAGCCAAGATGTGAATCAAACAAGG
EMPV1_08224 AGAAGTTTGTGGAGGGACCAGGGCGTGACTTCTATCCTGCTCTGTGGTTGGTTCTTCTGA
EMPV1_08226 CTCTGAATTGTCCATAACCAGTCCTACTTCTTCCTCAGTGGTGGTCACTGTCCTCTTGGC
EMPV1_08229 ACTGTGGTGGGGAGTCCTAACTGTGAAGCTTAATGCCTGAGTTGTAGAAACTCCTCCCTG
EMPV1_08230 GAGTTAAACAGGCCATGAAGTGCCATTTCCCCAGTGTTAGCAACTTACTCCCTGTGGCAG
EMPV1_08231 GCCTCACGGCCCATCAAGCTACAGGCTTCTGGCAGTAAGTGACGTGAACCAACATGCTGA
EMPV1_08232 CATTGAGTCTGCATATCGCACTTTTCTCAAGGGCTCAGCAAGACAACTTCAGCACTAGAG
EMPV1_08234 GACGAGAAGTTAAGAGTTCATTCGGCTGTCCAGATGTATCCAAGTACCCCGATATTTGGC
EMPV1_08237 AAGGCATAAGAAGGCTGCACAGACTAAGGGGAATAATGCAGTGACATCAAGGAAAGGGGG
EMPV1_08239 CTTCCATCCTCTCACTCTCAGTCCTTATGTGCCTCTAGATCTGAAGTGGGTTTCTTGTAG
EMPV1_08242 ACCCAGTTTAACCATTGGACCTTGCAGAGTCAGATACCCCCGGGCTTTGGGCAGTAAGAT
EMPV1_08244 TTCCAGTTGCTCAACACCCTCAGCCGTACTTGGTGCCATCAGCTTACTGGTTTTAGCCAG
EMPV1_08248 CAGGCTGGCCAATCTCTTCCTTGTGCTGCTTCCTGCTTTAGTGTTTGTCTCCAAGCCCTA
EMPV1_08249 GTAGATTTGTGCCTCTTTTAAAATTCCTTGAACTACTGCAAACAGAGGCTTTGGAATCAG
EMPV1_08251 ACATTGCGCAGTCGGGACCATCGCCGGAGCCTGAGGACACTTCTCTGTCGTCACAGTTAG
EMPV1_08254 TGGGAAATGAGAACAGTAGGATTTCTGGAAGCCCGCTAGGCCAGTCACCAAAGCCTTGCA
EMPV1_08257 ACAGGTGGCCTTGTCGGTCGCTGAATTTGGACTCATCGGCATTGGGGACGTGAACCCCTT
EMPV1_08258 CTGACCCATTCACTTAAGCGTGGAGACAGGAAAGGCCACCGAATCTCCCCTTCTGCATGT
EMPV1_08259 TGAGGATGCAGATTTGATCCCTGGCCTCAATCCGGTGGGTTAGGTATCCACCATTGCAAT
EMPV1_08260 TGATCCGATGTTGCTTCAGGCCCAGGTGCAGGAGAGCTTCGGTTCTCTGCGGCGCTGCTA
EMPV1_08262 GGCCAGGGCAGGGACTTTATCCCATATAATCTCAAACAGCACACTCAAAAGGCCACCACA
EMPV1_08264 TCTCCATCCTCTGCGTCCTGAGTTCGCTTCCCGCGGCCAGGTGATGGGAGCTGATTGGGA
EMPV1_08268 GAGAAGTGGAGAAACACACATCTCAGAAAGATTACTCTCGTGGCTTTGGTGGCCGTTATG
EMPV1_08269 TAAGGAACCAAAATCAGTCAGGAAAAATCACTGCTGATGGGCACCTCCATCGAGCCCACC
EMPV1_08271 TGATCTCTGTCTCTTCAGAGTAACATTGCCTGGCTCTTTTTGGGCTTGCCGTTCCCGCAC
EMPV1_08273 AAACTGCATCTCAATGAACCTCCAGAACATGATGTTGGAGCAGACCCCCTTCTGGCCCCC
EMPV1_08274 AAAGAAACTCTGAATCCGACCAGTGTAGGTGATTCCATTAGCCTTTGAGGTCAACACAAA
EMPV1_08276 GCTGCGCAGGAAAGTGGTGCTACTTATGGTATGTGAATTTTACCCTGAAAAGCAGTTTCC
EMPV1_08277 GAAAGTGGCAGAGCTGGGATTTGAACCCTGGCATTTTGGCTCCAGATTCATGCTCTCAAA
EMPV1_08278 TGTCTGTGTGCCCAGTTTGAGGCCGTCCTGCAGCATGGCCTGAAGAGGAGTCGAGGATTA
EMPV1_08279 AGTGATGCAACCTCGACAGAGAGTGAAAGCGAGGACAACTTCCTTACGCTGCCTCCCAGG
EMPV1_08281 CTGAGGCTGAGATGTCTGAGTGCAATTCTGTCCTCTTTCCCATCACCAGCTGCCTGACTT
EMPV1_08282 GGTAATCATCAGATTATAGGACAGCCTCAGATTGCTCTTACTGGACATGATCAGGGGCCG
EMPV1_08284 ACTCCAGGGGACAAAGATGGGCTCCTTGCTGGAGTCTGGGCCTCAGGGTCAGTCGGCTTT

EMPV1_08287	CTGAACTATTGTCTCCCCTGGACGAGTCCCGCGCCAGCATCACCTCGGTCACTAGCTTCT
EMPV1_08291	ATACTCCGAACGAGGATACCCTCCATTCCTGTGGTAGAAGATGTAATGACCTCTGGCTGC
EMPV1_08294	AGCAGGACCTTGTGATCTTGGCCCCTTGGAACTGAGATGGTTTTGCATCTTTCCAGGAGA
EMPV1_08295	TATCAAATATGCCCTCATCGGGACTGCTCTGGGCTTCATCATATCAGCTGGCTTTCTGGC
EMPV1_08297	TCTCTTCAGTTAAACTTGCTGGAGACCAGTAACCACCTTAAATGCCAGTCAGTGCTGCCT
EMPV1_08300	GAGTTGGTGTTACTCTTCATCGGGGTCTTCATTGGATGGGTTCCTTTTCTGGGTATCATC
EMPV1_08302	GCCTGGGTGGGGTCTTGTGTGCATTCTTCAGCTCAGATTTTTCTGGCCCTGAGTTTACCA
EMPV1_08305	TAAGGTGTTGCTGCTAGATGCTGTGGAGGTTGCAGACGGCTCGGCATTGCTGTGGCTGTA
EMPV1_08306	GAGATGTTTGGGAACCAGTACCGTGCCCTGTCCTCCCCTGAGAAGATTGTCCCCTTGAAA
EMPV1_08309	GACGGACAGACGGACGGACAGCCGCCCTGTCTAGCTGGATAGTTTCCAACAATTTTCCCT
EMPV1_08310	AGAACACTGACCCAATCTCTTTGAGGGAGGGCTAATCTGGTAACTAAGGCTTTTACGTCG
EMPV1_08311	CAAGGACTTCCTGTGCTGCAGACCAATCAAAACTTCCGCACTCCCCTCAAAATCAGTCAT
EMPV1_08313	GAACACTTTGAAGGCAAGTACCTAAAACGAGGGTCATAAATTTGGGGTGCCTGGGGGAAC
EMPV1_08314	GAGTTCATCAAGGCAGAGCCCCCGGACTCCCTGAGGACCCCCATTCGGAAGAAGGCCATG
EMPV1_08315	AGAGTACCCTCGCTGGGATGCTTGGAGCGGGAGTAATGCCTACCACATTGAGCGACTCAT
EMPV1_08317	CTGGTTTTCCATTCAGCTACTCCATATAACCTTGTTTTTGATACCACAATGTACTGAGGG
EMPV1_08318	AAGACTTGAAGGCTGCTCCCTGCCCAGGTCTTCTTGGAAAGAACCTTTCTCAGCCGTGGG
EMPV1_08320	TGTGGCTCCCTCCGTCTCCTTTGCCGAGTGACTGTCCCCTTCTCTGCGCTGCTTTCTAGA
EMPV1_08321	GGCTGGAAGGCTGTACAAAGTAACATGAGGTCATGGGGAAACAGATTGGTCTAACCTTGG
EMPV1_08324	AAATGCCAGCTTGGATATTCCCTTGCCACCCACTTCACGAACAGACATACCACATGGCAT
EMPV1_08327	ACCGTAATGACTTTGGATCCCTCTGAGACTTCATGTTCCACCTTTGTGAAATGCAAGAAC
EMPV1_08332	CAGCTGCAGATAATAAAGATGAAGAAATCAGAGTTGCCCCCAGGCGGTCGTATCTGGACT
EMPV1_08333	GGTTTTAGTTTAAAGCACTGTGAATGAAGGTAGTCGTCAGGGTTAGCTGCTGGGGAGTGG
EMPV1_08334	AGTCAACATCTACTATGGGGTGATTGCGGCTCTGTTTTCCGTATGCCTGGATGTGGTGCT
EMPV1_08335	TTGCGTAGTAACAGATTTCGCAGAGATGCAAAGACCTTGGAAGAGGATGAAGAAATGTGG
EMPV1_08336	GGAGGTCACAAACACAGGTTGGATCCCAAGTTTCTATTGCTGTGGTGTAGGCCCTCAGCT
EMPV1_08337	CCTTGTTAGGGTCAAGGGTGTCCATCCACCCTTTAACAGCTGCCTAGCAAAGCCGTTAAA
EMPV1_08338	TGTTACAACAGTTACCTGACCACAGAGAGGGGCGCACCCAAGTGAAAGCTGAGAAAAGCC
EMPV1_08343	ATTAGGGGTTGGGGGAGAAAGCTCTGCTGGTTGCCCCTCCCACCCCGCCTGCCCTTTCAT
EMPV1_08344	GAGCCAGCACCCCTTGTTTGACTTCGGAGACCCATCGTTTTGCGTGATTTTGCTGTTTTG
EMPV1_08346	ACATAGTACATGATGAAGTTAAGGATAAAGCTTTTGAACTAGAGCTCAGCTGGGTTGGTG
EMPV1_08348	TTTCTGCTGTATCGAGGGAAGGCAGACCTAACTGTGCTGGATGAGAACAAGAACACTGCC
EMPV1_08349	CAAGAGTCCATGTCTTTCCTCGTCGAGACTGTATCAAGGCTTTCAGGGAATTTGCAATGC
EMPV1_08353	CGGAGTTCAATTTAACTGGCACAGTAAAGAAGGAGATCCATTAGTGTTTGCAACTGTAGG

EMPV1_08357	TTTTTTTCTGGAAGCCCTGCAGCTACCCTAAAATTGAATGTATTGTTGCACAGACCTATG
EMPV1_08358	CCTTGAATTTCCCTCTTGCCCTTCCTCCCACTTCTACCATTTAGTGCTCACCCATGTGAC
EMPV1_08359	TCAGACCTAAAGCAATACTGTTCTCGCTGTAATTTATGAGTCTACTCACTTCCCCCTCCC
EMPV1_08361	ACACTGTGGGACCCGCCTGTCCAGTGTTAGCCCAAGGCATAGTGGATTATTTCCCGATAA
EMPV1_08362	CTTCCCTTTCCATTAAACTGCCATTGCCTTCCTCAGAGCCAATGCCTATTAACATTTACT
EMPV1_08363	AGCAGCCTGGTTGGAAGCACAGCCAGAGTCCTCTTCTTAAAGCTGCCCTCACATATCCTT
EMPV1_08364	CCACAGCCTCTGACATGCGATTTGAAGACACGTTTTATGGAGCAGACATAATCCAAGGGG
EMPV1_08365	AGTACATACAATTGGTGAGAGTGCTGCATTGGGGGTGGCAGGAGTAATATTGTGGGGTGG
EMPV1_08366	CCCCTGCTTTAGGCTCAGGGCAATCGGGAATGGTTTGGTGGACATTTATGGCTCATCTGT
EMPV1_08369	GGCTGACCCCTACTGTTGAATTCCTTCAGCAAATGTTTATTGGCACGTGGTGTGTGCCAG
EMPV1_08370	TCCAACAAAGGCACCTCCTGCTGTTCTCTTGGCCTGGATTTCATACTTCCCAGCAATAGG
EMPV1_08372	GGGGCTTCCAGTTCATTGGCAAAACAGGTATTTTGAAACACCTTCCTTTCAAGCTGAGCC
EMPV1_08373	CTGTGCTTAGCCAGGCTGATATAGATCATAACCCCAAAGATGCAAGTGGCTGATACAGCT
EMPV1_08379	CGGTACCTGCCTCGTTTTGTCACAGCCATCTGGAATTTACTAGTTCCAACAGGTCAAGAG
EMPV1_08380	CAGGATCTCATACGTCGACCATTTTGGTCTGGCTACAATCCAGTATTACTGCTAAGAGAC
EMPV1_08382	TTCTTATGTTTGGGCCTGGTATCTTCATCTACACCCGCCCCTTCAGAGCCTTTCCAGCAG
EMPV1_08383	AATGGACTAGACCAAGGACATAACCTTTGAACAGTGGATTGGATCTGTGCCAGTAGTGAC
EMPV1_08385	CACTTGGATTTTGGAGCAGACGGCCAGTTTGGTGAAACAGCTGGGGGTGTCAGTAGACGT
EMPV1_08386	GTGTGGTCATGGGAGGAAGTGAGCTCAGGGTCTTTCTACTCTATCATCTTTACCACACCC
EMPV1_08389	CTCCTGTGGAGAGAAGAGGGGTAATCTCTACATCACTGGAAGAAAGGAATGTGTTCCTCT
EMPV1_08390	ACTGCTGGGGATAGCTGCGCTCGTCACCGTCATCACCGTGCCCGTGGTTCTGCTGAACAA
EMPV1_08392	CATCACTGTGGCTCACCTGGATGACAAAACACACATGCTCTTTGGCTCTGACCGGATGGA
EMPV1_08394	GAGTGTGTGAAGACAAAGCACCCCCAGCTGCACATCGAGAGCAAGTTCTACAAGATGATG
EMPV1_08395	ACCTGGAATATTCTCCAGGATACATCACATCTTCGGCCACAGACCAAGCCTTGTTAAATT
EMPV1_08396	CTGTCCAAGAAGCTGGAACACTGTTAGCTAGCAAGAATGTTCGTGTCAACTGTTTGGATG
EMPV1_08397	TGAACATTCAAATTCAGCTGTCTCACTGTCACAAATCCACCCCTGACCTGGCCACTGCTG
EMPV1_08398	CCAGCTCAAATGTTACTACTTCGATTACAAGGAGCAGCTCCCGGAGTCAGCCTATATGCA
EMPV1_08400	TCTCAGAGGCATGTATCGAAGTGTTTGGTGCGGACCCGAGAGAATCAGTTCGTGCTGGGT
EMPV1_08404	GGTTTGCCACCCATTTGAGTGCAGTCCCCAGCTCCTTGGCAATATGTACTTGCATTCATG
EMPV1_08406	TAGATAAACTATGGACAATATTTTAGTATAAGTGCTTCTAAGCAATATTTGAGACATACT
EMPV1_08407	GCCTGCATGGACACTTACATGATCAACCTGCTCTTGGTGTCTAACAGTGGAGCTATTTGC
EMPV1_08408	CCGGAGTCGTTGTCATCGTTGTTCTCCTGATTCTAACGGGTGCTGGCTTTGCTGCGTATT
EMPV1_08409	CTATGAAAAACGCTCGGTGTTCGAGTCTTTGGACCTGGGCTGGAAGCTGTTGCGCATCTT
EMPV1_08412	TTCCTGGCAAATGCTCTCCCCTCTGGAAACGTGCACAGAGAACCCCTGCTTCCTTAATGT

EMPV1_08413	CTGACGAGTCTGGCAGCAACCTTGTGGACTCATGGCTTGTATTTCTGTGGGAAAGTTGAG
EMPV1_08415	TGGATAAGGGCTCAGAGTTGCACTGAGTGTGGCTGAAGCAGCGAGGCGGGAGTGGAGGTT
EMPV1_08418	CCTCACAGGTGCAGCTTCTGGAACAAACATCCCTTCCAATAGTCCAAAAGATTGTCTCCC
EMPV1_08423	CCCCACCTTTCTCCTTGGGTAAGCATAAGTTTGTTTCCTATGTCTGTGAGGATCTATTTC
EMPV1_08425	ACTAGGCGAACAATTACACTGTCAGAGGAGGAAGTTCTTTTGGGGTTGACACCGACTCCC
EMPV1_08426	TTCTGACCGCCCCAAACCCTATGATCCTCCTGGAGAGAAGGTGGTAGCAGGAAAACTAAA
EMPV1_08427	ACGTGTAGACCACCTTCCCCATCATGCCCAGGTTGGCCAGTGAGAGGGACTGCAGCTGCT
EMPV1_08428	GAGAATGCAGGCACAGGTCTCCAATCCTGATTAGCAGCCCCGTTAGCCAGATTCAGAGAT
EMPV1_08430	ATTGGGAGAAGGAGCCCCCAGAGCATTTGGGTTTGAAGTCCAGTAGGGCTTGAATGCAAA
EMPV1_08431	CTCCCCAGCCTCCAATCATTCCGGCCTCAGGCAACCTATAATCTGCTTCTATCTTAATAT
EMPV1_08432	TTGCCACTGCTGTTGACTCTTTCGGTAAATGCCATCGCTGCCCGTCAGTGGAAAGTGACA
EMPV1_08434	AGCCGTGCCTGTGACCTACACCACAGGTCAGCAATATGGGATCCCTAACCCACTGAGTGA
EMPV1_08436	GGGGCTTCAGGGAAAACGGCAGAACTGGAAGTATTCTGAATCCACCTCTCTACCTAGACA
EMPV1_08437	CTTGAGGTCAGGGACTCCTTTATACTTTCTGTATCTCAAAGCGAGGCAGCGAGCTGGATG
EMPV1_08438	GACCATGAGAATGAGAGTTTTTATGAATTCCCCTGACAGTCTCTGCATCAGTGTCCGACC
EMPV1_08439	CCATGTTGGTGCTCATGGCCCTCTCAAGTGGATACATGGTGATTCTTTTGTACAGGCACC
EMPV1_08440	ATGAAGAAACTAAAATTTGGAGATTTGGGGCCCATTTGTTACTTTAGCTACACATAACTC
EMPV1_08441	CCCATCCTGGCAAAGAGGAAGTGAATCAGAACCCATTTCTGGCGGGCACTGGTAATGTAT
EMPV1_08442	TCTTTGTGTATAGAATGAGAATGCCTGTGGGGAAACAGCCATGGGGCCTAGAAGGTGAGC
EMPV1_08443	TATATACAGTGGAGTGTGCAGGGAATCATGGGGCTCAAAGAGGCTAGCTAAGGAGACACC
EMPV1_08444	CTGGATTACTGTGCAAGCCTGAAGAACCTTGAAACCATTTCTGATAAACAAAATTACAAA
EMPV1_08445	GAACCGCCCCCAGGTGGGTGAAGAGGAGGATGATGGCTTCATCGAGGACAATTACATTCA
EMPV1_08446	TCCAACAAGGCCATTTTTTATGCTGAGTAACAGGCCACCGAGCGGCTACTGCATTACACC
EMPV1_08447	TCTTGATGCTAGGTTTCACGTCTGGACTTGAGGCTTCCAAAACAGGCAAAACCTGAGACC
EMPV1_08448	AACATCCTTGATAAGAGCTTGGGGTTGGGGAGGAGAGACAGCCTCAATCTGATGGGTCAG
EMPV1_08449	TCAAGTGGAGCCTGCAGAAAATGGACATGATGTTGCGAGCTCAGTATGGCGAGGAGCCTT
EMPV1_08450	GTGCTCCTCTTTCTGTGCTCCCCAACTTGGTAACAGATACATTATTCACTCAGCTTCCCA
EMPV1_08451	CAGCAGCACTAGGCAACTAATACAGCATCTTAGGAAGCATCCATTTGAGGAGTTCCCATC
EMPV1_08454	CTTGGGTGCATCAGCACTTAGGAGCTGAGTTACATCTTAATCTGGGGAAAGAATACTGGG
EMPV1_08458	AAGGATGCCTGGTGAACGCTTCTGGAAACCTGCCCGTGTGGGGTAATCAGAGCTGGAATA
EMPV1_08460	CAAGATCAACATCGACGACCTGGTCAAGGTGATCGAGCTGGTGGACAAGGAAGACATTCA
EMPV1_08464	CCCTGCCAACTTGGTATATTGGAAAATTCATCATCTTCAAAGCCAGAAGATCTCAATTCT
EMPV1_08465	GACTGTAAGAGGTGAGTTTAGACAACTAGGTGATGTCACGCAGGCCAGAACCTCCTCTGT
EMPV1_08466	GTCCAGGTTTGTGTGTTAGCCAGCGTTGTGTTGAATAAGGTTCTCATTCCTGTGTCAGTC

EMPV1_08467	ACTTCTGGCTGGCCTCCTGCTTCAGCCGCCATGGACCTAACAATCTCTGGGTACATTTAA
EMPV1_08468	GACACATGCTAAGCTGGAGTAAAAACGGAGTCGTGTGTATCTCTCTTCTACAGAGCGCGT
EMPV1_08469	CTTCCTTTCGCGTTGGTCATGGGTTTTTAGAAGTTTTTAACAGGAGTTTTATCCCAACAG
EMPV1_08472	TGGATATGGCAGTTTCCAAAGACTTGGCTATGGCTATGGCCATGGAGGCCATGGATATGG
EMPV1_08473	TACAAGCACACCAGGGTTCGCACTAAAGAATCATCATTGTAATAACACTATTTGGTAGCC
EMPV1_08476	GACCAGGAATTGAGGATTTGCCTTTCAGAATGACTGTCTCAAGAAGTGCTCTGCCCCCTC
EMPV1_08478	GGTTATCCCTGCTGCCACGCTGAGCCTCTGGGACGTTCTGTACACAGCCTTGGAGAAGTA
EMPV1_08480	GAATTCCAGAGGTCACTCCCTGCCTTTGTCTCCTCCACCAAAGCTCATGTCACTTGCATC
EMPV1_08482	TGAGTCCAAGCGGCTCTTCCAGCCCTGATGGGTCATTCCTAAACCATCTACTGGGAAAGA
EMPV1_08484	AACTGTACTCTAAACCCAGGGAATTGAGGCAGAGGCCGTGTTAGTGGTCTGTGTTGACAG
EMPV1_08485	AGCAATGCCGGTTCTCAGGCATATCCCGGGGCTGTGTGCCAAGCTCTTCCCAAGGCAGAA
EMPV1_08487	TTCCTTCCCCAGGCTTGTGATTAGGAGGAGAGAGGGCTTGTGAAGAGACTTGGCTGTGAA
EMPV1_08488	AGGACGACGCATCCTGTGTGTATATTTGGGACCTCCTGGAAAGCGATTTGGGTCCTGTAG
EMPV1_08489	CCACTGTGACCATTTGACTATAAGCCTGTGTGTACATTATTTTTATTTGTCTAACCATGG
EMPV1_08490	CAACAGAGCTCTTCAGAAACTGAAGGAGAAGGCGGAAATACTGCAGATGCAAGCAGTGAG
EMPV1_08491	AATGCATCATTAAGCCCAACCAATGGATCTAAATTAATGAATCGTTGAGGACAAGACGTG
EMPV1_08492	TGCAGGCCTCTTCATTTCGAACCTCTGCTGTGGACAAATCAATACTAGCAGCTGGTGTGG
EMPV1_08493	TTACCCCGCTGCCTATGGAAGGAGCTAACAAGGATTTGGCAAGCCCCACCAATGCTACTT
EMPV1_08494	TGAGGTAGTAAGTTGTATTGTTGTGGGGTAGGGATTTTAGGCCCCAATTAGAAGATAACT
EMPV1_08496	CAGGGACAGAAGGCAGAGCACAGAGGAAGCTGAAGCAGGAGCAGGGACCAAAAAGAGGAT
EMPV1_08497	AATCCTGAAGGTCACACACAGAGAGGCTGAAAGCTTTACCCACATAGGCATCCCTGTGGA
EMPV1_08498	TCTGAGGGGATGAGGGTGGGGCTAAGGAGGGCAGGCTTTAACCACAGTGACTTCGGATCA
EMPV1_08499	GTGTGTGTGTACACGCACGAGTACTCGAGCGTGCACAAGAGCACACACGTGCACACACAG
EMPV1_08500	CAACCCTGAGAGATAGCTACTATGATTCTCATTCTATACACAAGGACACTGAAGCTCTGA
EMPV1_08502	TGAAGCATCAAATGCTTCTGAAACAGAATCTGACCACAGAGACGAACTCAGTGATTGGTC
EMPV1_08503	GACTAGTTGGCGAGGAGTCAAGAGTACCACTTTCCTTCACTGTAACCTGGCATGTGATGC
EMPV1_08507	GGCCCCAAGCCCTGCCCTCAGTCAGTGAAACATTAGCCGCCCCAGCCTCCCTCCGCCCCC
EMPV1_08509	GTGAGAGACTTCGTATCAAAGTCATCCCCACACTAGCCCTGGTGAAAGATGGAAAAACAC
EMPV1_08511	ACATGCACCCAATAGAGCTACATTTCAACATATGAGGCAACTGCTAACAGCCAAAAAGTG
EMPV1_08513	TACATCAAGAGGTATGTGCCAACACAAGCAGATGTGGCGGGATTCGAAGCAGTGTCCCGC
EMPV1_08516	CAATGATGAGAGCAGCTGCCAGCTGGGCCCGCGCTGCCCAGAGCAGTGCACGTGTGTGGA
EMPV1_08517	AATGGAGAGGGCGGCGAAGAGGACCCGCAGGCTGCAAGGAGCAACAGCGATGGTGAGAAG
EMPV1_08518	GATCCTCCAGAGTTCCAGACCATTATCATCGGAGACAGTAAAACCCAATTCCACATGGGC
EMPV1_08521	GCCAACACCACATCCTTAGCCCACTGAGCAAGACCACGGATCAACCCACATTCTTATGAA

EMPV1_08523	GAGCATGTTTAAGTGGAAGATGGATGTCATTCCAGCCCAGTTACCCACTGGTACAATCAG
EMPV1_08524	TCCCAGAACAGCCAGGTTTGCTTTCCTCTGGATTCCCTGCTAGGCTGTAGCTGATCACGT
EMPV1_08525	ACTTCCGAGAGAACTTCAAGAAAGACATGAAGAAGTGCGACCACCACATCGCAGACCTCC
EMPV1_08526	GCATTGCAAATTCCCTGAAAGCCTTGATACAGTCTCGACGAGGAAAGACATGGACTCTTG
EMPV1_08527	AGATGGTGGTCATGACTGAGACCCTGGCTGTCATTGCAACCCCTTTCCTATGCATTCTCT
EMPV1_08528	GAACCCACATCTTGGGGAGTCTCCTAACTGACTTGGAGATCAGCTGAGATGGAGGGACCT
EMPV1_08529	CTGAAGCCTCTGCTGAAATTACCACAGCTCAACTTCTGCCCGTGATTGCTTGACTACACC
EMPV1_08530	CTCTTACGCCCGAGACTTTCACATCAACCTCTACAAAGTGCTGCCCTGGCTCAAGGAGAA
EMPV1_08531	GCAGGGATCTAAGCTGTGTCCAAAAGGGTTCTGAATTCTCCAGGCTCATCCGTTTGAAGC
EMPV1_08532	TCTGGTCTCCGAAGAGTGAGTTGCATCCACGTAAAACCCAGCTCCGGATATGATTTGCAC
EMPV1_08534	GCTATCTACTTACCACCAAAGGCAGAAACTGGAAAATGCCCTGCACTATACTGGCTCTCT
EMPV1_08537	TAGACCAGCCCTATGAGCCATCTCCAGTAGAGCATCTCCCTATGAGCCAACTTCGTCACT
EMPV1_08538	CTTAGTATGCATTTTCCCACAAAATATTTGGGACTTACTTATACTAAAACTTATTCACTG
EMPV1_08539	TGTCCCAGGTGCTTAGGCGGTGTGCCCTCTAGGCTGGCTGGTTACCTCACTGGACTTCCA
EMPV1_08540	TGCAGAGCCACTGTGTGCTGGGAATGGAAGGGAAACATAGTGGCCCTTGTGCATGACAGT
EMPV1_08541	GGAGAAAGAGGGGGGACTTTCTGGTACTGCTGACAAAATAATGGTCTGGACTGATTTACC
EMPV1_08542	GCATTGCAATGTTAGTCTTTCCGGCTCCAGTGGGGGCACAAATCAGCATGTTCTCATTGG
EMPV1_08543	ACCTCATGCTGCTTTGGAGGGAAGGATTACTCTGAAATGTACGTGACCTGTGCCCGGGAT
EMPV1_08544	GGGAAAGTAGAGGCAGAGTTTGAGCTGCTGACTGTGGAGGAGGCTGAGAAACGGCCAGTG
EMPV1_08549	CTTTCTGTAAGCTTCGGTAAATCTCACAAGCCCCTGCCCAGCAGATGTAAATTCAATAGC
EMPV1_08553	AAATAGCCCTGAGAAAGTACTGCAGCCCCCAGGAAACATTAAACAAAGACACAGGGGCCC
EMPV1_08554	TTCCTGTGTCCAGATGCTGGTGGGCTTCTCGGGCCTCATAGGCTTCCTCATGCGCTTCAT
EMPV1_08556	CAGAGTTCTCTTGTGGAACAAAAGGTTAAGGATCCAGCATGGTCACTGTACTGGCTTGGG
EMPV1_08559	GCCTTCAACTGGGTCTCCTTCTCTACGCCATGTCCTTTTAAAATGTTAGCATGCAAAGAG
EMPV1_08560	GCCCCCTAGCTTCCCGACTGGAAACCCAGATTTACCTCCAGGGAGAGGTGAGATAAATTT
EMPV1_08562	CTTGTGTCCACCTTTGGCATGGACCTGTTATGTATCGTCCTCTCCTACGTGCTCATTCTG
EMPV1_08564	CATCCTCACATCTCATTGGTTTCAGCTCCATTTTAGGAGCAGCAGTGGGGATAGGAGTGG
EMPV1_08566	ATATAACATTGATGTCGAGCACAGGAACATGGCCAACGGGCAGCCCCACAGTGTCAACAT
EMPV1_08570	AAGCAGCCCAAGTGGGGGCCTTTACGCTCTGCCTCTACTGATGTTTCGCTTCTGAGAGAA
EMPV1_08574	TATATGCAAAAGAGACACAGTGCCCATTACTGTCCCCCTAGCCCTTCCTCCCCTCTTGGT
EMPV1_08575	TTCTAAATGTGTTCAGGCTTCTGAAGGTAAAGGGACACTGGATCCAGAAGCTATGGAACC
EMPV1_08577	CTGTGCATTTCTCCATGTCCAATCCATCAGAGTTGGTGGTGACAAGGGCAGTGTCAGTAC
EMPV1_08578	AAGTGTTCTGGAGGAACTACTTTTACCGCGTCTCCCTGATTAAGCAGTCGGCCCAGCTCA
EMPV1_08580	CACAGATTGTTTACTACTCCTGTGAGGCCCAGAACACCCGACAAACCACACGTCAGACCT

EMPV1_08581	GGTAAAACCTCTAAGCCGCAGGGAAGATATTTTTGCCCATAAGTTTGGAGATGCTCTTTG
EMPV1_08582	CAGAATACTTAACCGAAATCCATGTCTGGGGAGACAAAGCCAGGAGTGGGTTTACATGAG
EMPV1_08583	AGAAGGTTGTACGATGGTGCCTGAGAACTGCTACGTCAACCAGCGGGAGCTGGAGCAATT
EMPV1_08584	CGGGTTTGCCTCTGCGGTCGTCATGTGTGAGTAGCCCGTATTTATCCACGAGTTCTTTGT
EMPV1_08586	AGTCGGCTTTGGACCACAATAATTTGACGCTGGGTGGTGTGGGATCAAAGGTAAACAGGC
EMPV1_08587	CTTAGCTTAATTAAAGTGTTTGATTTGCATTCAATTGATGTAGGATAAGTCCTGCAGTCC
EMPV1_08588	TATCACCCTTTGCATGGACTCTTAGATCTCAGTCGTGAATGGTCACTCCCGCAGGAACCT
EMPV1_08590	GGATAAGGATGGCTATATTTCCAATGGGGAACTCTTCCAGGTGTTGAAGATGATGGTGGG
EMPV1_08591	GGAGGAGGCCTCTTCACAGTAGTGCCATGGAGGTACAGACAAAGAAAGTTCGAAAAGTTC
EMPV1_08592	CCTAGAATCCAGGCTGCTTTCCTAGAAGTATGGAGGAACCTCTGTTGGCCCAGAGGAGTT
EMPV1_08593	GGAAAAAGCGCCAGATCGGCTGGTTCCCTGCTAATTACGTCAAACTTTTAAGCCCGGGGA
EMPV1_08594	AACATACCTCTGCCACCCACATCTAGACTTGTTTGTGAGCTGGGTTGCTATGCAGTTGTG
EMPV1_08596	GAGTGCAGGATTTAGATTTCTCAGCCCACCTCCCGTTTCAGCGGACCTGGAGAAAAAACT
EMPV1_08597	TTCAGCAGTCTCTGATGACTAGCTACCAGGGGGAAGAGAACTAATGTGAACCCACAGGGA
EMPV1_08598	AGAGAGAGGAGAGACAACTACGTGCCCGAGGTCTCAGCCCTGGATCAGGAGATCATTGAA
EMPV1_08599	GATTTATTCACCAGCCTTCATGCCCTTGCCTTTTCCTTAAAGAAGGGAGTCTACCCCGTG
EMPV1_08600	ATACTTTTCTTTGGATTTGGGTGGCTTTTTTTCATGAGACAACTGTTTAAGGACTATGAG
EMPV1_08602	GCCAGGACCAGAACTACATCACAGCAGCAATAACACCTGATCGGTAATCCATTGCACCAC
EMPV1_08604	AACCCTCAGAACATCGTGCGGGCTCGGATCTTCCAGTCTAAACCCCTGGAGGTGCGCTGT
EMPV1_08606	GTGCTTTTCTGTTATTCTGCCTCTTAGGTGGTGTTGGAGGTGTGATCTGTAGGGCATGCA
EMPV1_08607	CACTCGCTCCAATGTGATTTTCTACATTGTGACGCTCAACGGTACAGCAGACCATCTCCG
EMPV1_08612	AGGGAAGTGGTCCCTTGGCTCATTGTGGGTATTCAGGGGAGTGTTGACAGGATTATCTGA
EMPV1_08613	TATGACTAAGCATCTAGGGGGTCTAAAAGAGGTTGAACTACAGCACCCCCACACCACTCG
EMPV1_08614	CAACACAGTTTATTACTGAACTGCACTTTCACCTTCACACAGACTATTTCAAAGAGCTGG
EMPV1_08619	CGTCACGATGCAGGTCTGGCGGGAGGCAGTGGTAGGGACTCAGAGGCCGGCCTCTCCTCT
EMPV1_08621	AACCCACGATGCCCACAAGGCGAAGCAATAGGTATCATCACAGCATGAACAGCTCTTGTT
EMPV1_08622	CCGCTTCCTCGTCAACATGGTCAGCAGCGGCTCCTCCCTGACGCTGGCCGGCTTCGTCCT
EMPV1_08624	GGACCCTGCATCTGAGCATTACAATCATCTTCCACCTCATTAGCATCATAGTCCTCGGCC
EMPV1_08625	AGCCAAGGCTTCTGAAGACCAGCTGAAGCAGAGGTTGCAAGAACTGGAGGCAGAACTGGA
EMPV1_08626	CACCTCCACAAATGGCTGAGATGTCGTAACTGGAGAAGTCCGGTTGGTTCAGAATGTCCA
EMPV1_08627	GCAGCCATGGTGCTCAAAAGCTCCTTGCTGAAACTCTGGTTTGAAAAGTGCCAAACGAGG
EMPV1_08629	GTTCTCTGATACCTCCGAGTTCTCCCCGCTCGGAGTTCCAGTTGCTGCGGGTTGTGTGTT
EMPV1_08630	CAGCTTCTTGCTTTGTGAACTTCTCCAGACAACAGCACATCACAGATGTTCAAGCTGAAA
EMPV1_08631	GCAGCTGGCAGTCAGAAGCCAAAACTCCCTTTGAGTCTAGATGTGTCTCATAAAGTCAAC

EMPV1_08632	TTGCTCTGGAAACGTGCTTAATGTCTATTTCTTGCTTCTTGTCACGGGGTCTGGGGACGG
EMPV1_08634	TCTCTGCTCACCTCCCTTACGCTGGTTAAACGCGACTACCGTTTCGATCGGGTCCTTTTC
EMPV1_08635	GGTCAGACAAAATTCTTCTAAGGCCACGGGAGAAAAAGTTGGGACTAGGAACTGCATATA
EMPV1_08636	GATTAAAGGTGCTGCCGCGACGACGCCGGAGATTGTGTAGTTAGCTCTCCCAGCCTCTCT
EMPV1_08637	GACAGATGGACAAAGCTGAATGACAAACCTGTGATTGGTAAAGACAGACTTACAGTGCCA
EMPV1_08638	CCCGGGAGTCAGGATCTTAGGACTCTTCTAGTCATTAAAGCTCAGTCACCAGCCACACAA
EMPV1_08640	AGAAACGAGCACATCAAGCCATGCCCGGAGCGGTTTCAGGACTGCAGAGAAGCCTTCGAT
EMPV1_08641	CCGGAAACGGTCACGGGACTCAGAGGAAGAACAGGAATCCAAAGACTAGCCTCAAAGTAT
EMPV1_08642	TTCTTGTTCACCGTCCATGACTAAGCCCGCCCCTCTAATGTCGATGAAGTCAGAAGCCTG
EMPV1_08643	GGATGGTCAACACAGTGAAGGGAGAGTTGCAGTAGCCCACGTTGCAGTGCTATTATGAGC
EMPV1_08644	CAAATACGGTCCTGGATTCGGGGTTCATCAAGAGTACTAAGCTGGTGGTGATCTTGTACC
EMPV1_08646	ATCAGTACGACCAGGTGATGCCAGGGGTCAGCTTGCTGATTCGAGGGCTTCCTGACGTGA
EMPV1_08647	CACTGCTCCAGAACCTGCAGAGATCTTTTCTGAACTTTTCCATCCACTGGCACATCACCT
EMPV1_08651	TTACATGTGGTGAAACGCAGAGATCCTAAGTGCTTGGTTCGTGAGTTGTGCCTGTGGGAC
EMPV1_08652	GTAAACCTAGCCTTTAAGCAACCTGGAAAGAGGCTAGAGCACCAAGGAATTAGAATTGAA
EMPV1_08653	AGTATTCCCAGCATGGTCTAGATGGGAAAAAGGGGGGCTCTAACCTCATTCCTCTGGAAG
EMPV1_08654	GCTTCCTTCTCTTCTGCCGCCTTTGCATCTATCTGAACAGCGCCATCAACCCCATTGTCT
EMPV1_08656	CAATAGGTGCCACAAATGAGACGTAATATGCAATCTTCGCCCAGGTGGCAGCAAGAAGCC
EMPV1_08658	GCAACATACAAGCAGAAAGCAATTAAGCCTTGGAGCTGTGCACTGGGTGTCTACCAGCTG
EMPV1_08659	GCCACAGAGACCTTCAGATTGGTCCAAAAAGAAGAAGGAGCCCCTGGGAAAATTGGCTTC
EMPV1_08660	GCCTTCAATACCTGCAGTTCTCATGTCATTGCTATCTGTTTGTTATTTGGGTCCGGTGTC
EMPV1_08661	CCTTGGGCTATAGGAGAATAAATGACTTTGGAGAAGGGAAATACAGAGTGCCTGCTTCAG
EMPV1_08663	GGGAACAGTTGACCCTGGGATATGATGGGACTTTCAGGGACGTGTGTTTACAGAGTCAGA
EMPV1_08665	AAGAGGCATTTTTTATACTCGCACGAGATATAATGACAAAACTCAACAGAAAAATGAATG
EMPV1_08670	AGTGTCTCTTGAAAATTCACAACCAGCATGGTGGCCGCACCCTCTTCCAGGACATGCAGA
EMPV1_08674	CTGATTTAGAGAACGGCCATAAATTTCAAAGTGAGGATGACAATAAAATGGAACCAGGAG
EMPV1_08675	TGCCCAGGAACTTCCACATGCTGTGCACCTGGCCCCCAAAAGTATGATTTTAAGGACACT
EMPV1_08676	TCCAGTTACAGACACAGAGGTGGAAACTGTAGAGCCCCCTCCTGTTGAGATTGTTGCAGA
EMPV1_08679	ACTCACCCCAAACTCCCAGTACCTCCCACTCTCCCCCACTTGGCAACCACAAGTCTATAC
EMPV1_08680	CGTGACACTGAACTAATGAGCCCTGGAAAAAGCGTCAAGAAAGGCCAAACCAGCTTGACC
EMPV1_08682	AAATTTGCGGTGCTGGCTGGTGGAAGAGTTTGTGGTGGCAGAAGAGTGTGCTCCATGTTC
EMPV1_08683	TTTGTCCTCTCCTGTTGGGCTGGAGGCTTGTTCATCATAATCCCTCCACTTAGCCTAGGC
EMPV1_08686	TCAGGGTCAGAAGGAAAAAAGAAATCAGAAGTCCAGTCTGAAGGGGTCTGGGGAGCAGTC
EMPV1_08687	TGTCCTGTGGAGCAAGGAGGCTGTCCCGGAGGCCCATCAACCTCGGGAAAGAGCAGGAGC

EMPV1_08688	GTGACACTTCTCAACAGACTTGAATGTGACCAGATCAACTCTCCACATGATGTTCTGGCG
EMPV1_08689	AGTCCTGCTGGCTGCTCAGTTCTCTTGGTTGACAGGGCTTGGGTGGTCCCTCATGATGTT
EMPV1_08690	AGGCCATGGTCCAGGAGTCCGGTGGCAATTTTTCTGAGGGGGCAACTCATTACATTGTGA
EMPV1_08692	ACCCAGGACACCTGCAGACAGCACTGCCTGCCCCTCTCCCAGTGGCCAATCTGGCAATGA
EMPV1_08693	TGGGATGGTAGCCTTTTCTGTGGGGTTCGTGGACCTTGTCATAATTGGTTTCTCCTACGG
EMPV1_08695	CCACCCTCCTCCTAGTCTCCCAAGTTTCAGTTGTGTCTGACAGAGCATTAGGTTTTCCTG
EMPV1_08696	CCACCTGTGCATCAGTTAGGAGGTGCTGCAGGGTTTCTTACTATTTACATGAACATTTTA
EMPV1_08697	GCTGGCTACAAGCTGGTGGTGGCGGAGGCTGTGATTGAGAGATTTGCACCAATTAAGAGT
EMPV1_08699	ATAGACCCCAGAGCATGGCCGTGATATTTTGCATTTCCCACCAGTGAGAGGCAGCGTTGA
EMPV1_08700	ATCAAGTCTCGACCTGGAGGGTAGTAGTATCCCCATCCAAGCTAGGGATGAAGGAAGGGT
EMPV1_08701	GCCAATAGAAAGCACCTGTGTTTAAGTAACTAGACTGGAGATCTATTATGCTCCTATTCT
EMPV1_08702	CTGGCTCCTCCCACAATCTTCTTCCACTTCGTTCTATTCTCTGGCTAAGTAATCCTGCAG
EMPV1_08703	CTTACTTTTCAAAGACGAAAAGATCACCATCCACAAGGACACCAAGGCACCCAATGCCTG
EMPV1_08704	GTTCCCTGTGTATTATAGCAGGTCCCCATTGGCCAATCATTCCGTATACTTCAGTGTGCA
EMPV1_08707	GGATGGCACGATCAAGAAAGTGTTCTACAAAGAAGGCTCTCAGGCCAACAGACATGCTCC
EMPV1_08708	CTCTTTTTTTTTTTTAAATTTTAATTTTTTCCAATTCTCTTGTTCTAAATAATTTTTAGG
EMPV1_08709	GCCTTATTGGTAGTTGGAGGAAAATACACCCACTAATACAGACAGCACATGTTGAGTTTC
EMPV1_08710	TGAAAGTTTCCTTTAGAACTTTGCCATCATCAGTGCTATTCTGAAGGCAGCACCAGCCTG
EMPV1_08711	TATCTCCAGATATTCACAGATTGAGAGCAATTGCCCCACCAGCCTGGCAGGAATGGCCTT
EMPV1_08714	CCTGAGATCTTTCTAAAGTGTGGGAATGACGTGATGCTCCTCAGGTGACACTGGGGCAAT
EMPV1_08715	GGAATACAGTATTTCAGTTACTTGGAATGGAACCTTAGTGAAATTCATCGCTTGTCATGC
EMPV1_08716	CCACTAGACCACCAGAGAATTCCCATTCTGTATGCTTGGCATGAGTTCTGCTTGGCTATG
EMPV1_08718	TCTTCTCTCGAGCTTCCGCTGAGCCCTCGTCGTGAGCTGTTTATCCGTGTCGTTCGTCCA
EMPV1_08719	AGCGCTTCAGCACCGGCCCGTCTGGCTCCTCCTTGACGTGCACGTCAGGCTTCACAGGCA
EMPV1_08720	GGCACCCCTTTTAGAAATGTCCTGCAGTGTCATTTTTTGCCTATTTCAGCCATGGGACTT
EMPV1_08721	GCGACTACTTCATGTCCTGCCCGGACAGAGGCCATGCACACAGGAACGCAACCCACCGTG
EMPV1_08722	AGTCCTGAAAATAGACAGACCTGGGCGGCCACCACCCCAGTCGTTTCAACCCCTCCAACA
EMPV1_08723	TGATCGCTCACCGCCTGTCCACTATCCAGAACACAGACTTGATCGTGGTGATTCAGAACG
EMPV1_08724	GCATCAAAGAGGGTCTGGATTAAATGTAGCGATACCAAGAAGTGGGTGCTGTTGGCTTAG
EMPV1_08725	TTTATACAGCTTCCTCAAGCGAGAGAAGGCCCCCAGAGAGAAACTGCTCCGTGTGCTGAA
EMPV1_08726	AGTTCACCAACCACCCACATCCTACCAAGGCCAGTGACCAGTTATATGGTGTTCAGTAGC
EMPV1_08727	TGAACATCTCCTCTGCCAGTGGCATGTACCCAGTTCCACTGCTGACCATCTACTCTGCAA
EMPV1_08729	CCCACCGATGAGAGGAGCTGGGTGTACTCCCCGCTTCACTATAGTGCACAGGCCCACCCC
EMPV1_08732	AAGACTGCTATCTTCACAGAAGCGAAGGTGTTGAGCACAACGTTCCAGCTGAAGCGCCTC

EMPV1_08734 GGGCTACAAGCCTTTCAGATGGTGGCAAGCATCCTGTATGATGTGCAATAAGCATCCAAG
EMPV1_08737 ATTCCACTAGGCACTGTTTGCGTGCGGCAAGACCAACGGCTATGGCTTGGTCAGCTTTAA
EMPV1_08738 GGTGTTGTTGGCAGCAGCGGTCTGCACGAAAGCAGGAAAGGCTATTGTTTCTCGACAGTT
EMPV1_08739 GCGGCATCCCTACCCTCTACCCATTAGATCTCAGCAGCACTTCACGAATTTTGCAACCAA
EMPV1_08740 AGCAAAGGTAATCCAGGAATTCCAAAGGGGCGACACACGTGCAAAACAAGCAACACCGTC
EMPV1_08741 CATCTCCCCAGGAATCATCTGGACCCCGCTCACTTTTGCAGCCACGTAAGCAGGGAATAC
EMPV1_08742 AGCCCTCTGCTCAGAGGGCCAGCAAGATGCAGTTTGAGATGCATGACAACGTGAAAGGCA
EMPV1_08744 CGGGAGTGGGACTGAAAAACGCAAAGCCATGTACACCAAGGACTACAAGATGCTGGGATT
EMPV1_08745 CCTGCTCTTATACAACCGGGAAAACAAATGTAGCTGAAAGGGCAGCCTGCAAAGGCATTC
EMPV1_08749 AGATTAAATTTAGGCAGGTGGTCTAACCTCAATGAGATTATTTCCAGGGGTTACAATCCC
EMPV1_08750 AGTGACGTGTCTTGCTCAAAGTCTTCCAGCTGATAAAGGGAAATCTGACTCCTGAACCTG
EMPV1_08751 ATCTTTACTGCCCCAACCTGGGCCCACATCACCATCTCTGCTCCCCTGGCTGACTGTAAT
EMPV1_08755 GGCACGCCCAATACCAGTCTGTCCCAGTCTACGAGATGAAGTTTCCAGATCTCTGTGTGT
EMPV1_08758 GCTCCATGTGGGGGATGGGCAGGCTTTGGGGAGACCAGTGATGAGCATACTTCTTCCAAA
EMPV1_08760 GTCAAGCTTGTATAGGAGGCCATGACTTTCATGAAGGCGTTAGGGCAGTTTTAATAGATA
EMPV1_08761 CTTTGGAAGAAGTCAATTCCCAAATAGCCCTCGAATATGGCCAAGCGATGACAGTCCGAG
EMPV1_08763 GTCCTTTACGCCAGTCTTTTACCCACAACCTCCATTTTCTGAGGTCGACGTGGTAACTTC
EMPV1_08764 ACGGAACCCCAATCCCCCATGCCCCTCAAAAGCGGCAGTGCCACCATCACTCAGCAGCCA
EMPV1_08770 TCTTCCCCACTCCGAGCCAGGGGCTTCTTGGATGAAATCAGCTGCATGCCTTTACTCTCA
EMPV1_08771 GGAGAGATCTGTGGAGAAGATAGCTCACCAACTGGAAGAGTTGAATAAAGAGCTTACTGG
EMPV1_08773 GCAATATTCAATTGCGAGGCGGAGAGTGAATTACCAACAGGGAATAGGGCGTTGGCCCTA
EMPV1_08774 ATGAGGAACAACCCTGTCCCCCAGTACCTGCCTGAGATGCTGGAGAAGGTAGCTATCAGT
EMPV1_08775 GATCAAACTGGAGCCTCTACAGAGACAAGCCGGATCATTAACCGTACAGCAGGAACTCCT
EMPV1_08776 GGTGAGTGAGGTGTGCAAGGCAACTGTCTGGACTTCTCCCAACACAGCATGTGCACTTTT
EMPV1_08778 ACTACTACTTTGTGACCAGGGAGGTGATGCAGCGGGACATTGCTGCTGGAGACTTCATCG
EMPV1_08779 CATTGAGGTGGGGAGAACAAAGATGAGGAGCAAATCACACCTGCCACCCCAGAAGGGAAT
EMPV1_08780 TTGCAAGCAGAAGCCAGAAGTGATATCCTCACCCACTGGTCTCCCTCCAAACTACTCCTC
EMPV1_08783 GATGATGAGGTTGCCCAGCTCAAGAACAGTGCTGACACTCTGTGGGGCATCCAGACGGAT
EMPV1_08784 ACTTCATATGTGCCCATGGCTGCTGGGTCTTGGATTGCAGGCAGTGTCGATTCTGTGTTG
EMPV1_08785 TGGAGAGTGTAGATCATGTCTGGAGAATTCCTCTCGACCCACCAGCACTATCTGGGTTAG
EMPV1_08789 ACTGGACACGTACCTAACACGGAACGTTGAGGAGACCTGCCCATTCTGTCGTAAAGAAAT
EMPV1_08793 TGAGGAGCCAGGCACGCCCCCCCCCCCCCCGCCCCATTTGTTCTGCCCAGGGAGGAGTGA
EMPV1_08794 AATGCTTAGAACTTCGTCGGCCACACTACTCTCCTTACAGCAATCCCAGAGGGTCAGTGC
EMPV1_08796 CACGTGTATATCTTTGGAGGGATGACCCCCACTGGAGCACTCGACACAATGTACCAATAT

EMPV1_08798	TTCCACACTACCACTCTCCTCCTCTTATGCCTCTTAGTTCAATGAATGGTATCACAGTCC
EMPV1_08801	TGGTGTGAGTGAATCCGAATTGATGGAACTCTATCCTGAGATGTCCTGGGCTGTCCTGAC
EMPV1_08808	GCATTCAGCTTGTCCTCAGTTGTTGGCTTGCTGGGTCCTTTACCATCTTTGTACCTCTCC
EMPV1_08809	TAGGGAAAAGCGTGGGGCGAGTCACACCCCAAGAGGGGAGAGCAGAGAAGGAAAGGCTTT
EMPV1_08810	GTGTGAAGCCGAGGGAACCATTTTTCATCTGGACAACGGCCATCTTGGTAAAGGCAATGC
EMPV1_08811	ATAGACCCGTCTGTTCTTATATTAAACCACTTCGAGCTGGGAGGCTTCTGGATACTCCAA
EMPV1_08812	CCACGAGGATGGTGATGCCCAAGAAGCCAGTTGCAGGTGCCCTGGATGTTTCCTTTAACA
EMPV1_08813	TACTCTGGTCCTCATTCCATGGGGTGTGGGCTTCATCCATACTACCAGCCAGCTGGCATT
EMPV1_08815	AGAGAAGGTCATCAGCAGCCAAGCCAGACGTGGAGCTCCCCGAGCCAGATCACAGCAAAA
EMPV1_08816	ACAGTCTTAGTATTCACTGTAACTCTGAAGCTCGCCTTGGATACTCGTTTCTGGACATGG
EMPV1_08817	CCTAAATAGTTTGGGTGGTTTGTCTTCTGAGACCATGTGGCAAGGTTAACGTGGGGAGGC
EMPV1_08819	CTGCAGCAATGTCAGATCATTTAACCCACTGTATCAGGCCAGGGGTCAAACCCGAACCTC
EMPV1_08820	CAGTAACTAGTGTCTTGGAGTATCTGAGTAATTGGTTTGGAGAAAGAGACTTTACTCCAG
EMPV1_08822	AAAGGCACCGTTGTATTTCTTCAAAGGCCTCATGGCAGCCCTCTGTCCAAACATGGCACG
EMPV1_08823	TTTCTTCAGCAGTAAAAACAGAGGCAGGGGCAGATAAGAGCAGCACCAGAAGTAGGATCT
EMPV1_08824	GCATAAGAGAAAGAAGGTTAAGCTGGCTGTCCTGAAACACTATAAGGTGGACGAGGACGG
EMPV1_08825	CTTCAGCTGCCCTACTTCCCTGCTCATTCTTTTAGCTCCGTGTCTATCAAATGTTTTATC
EMPV1_08826	GCCAGGTCCTACAATTCCTTGGGGAGCAGAGACTGCACTTTTCTACTTGGGCTTCAGTTA
EMPV1_08828	CTGTCTGACTCCATCTGCAGGGCTGTAATACCTACTCTCCTCCGATCCATTCACCACACA
EMPV1_08830	CCCCTGGTGAATTCAATGGATGTGCCAGGAATCTTAACCAGATGTGTGGATGATGCAGCC
EMPV1_08833	GCTGAACAGCATCAGGAATAAAGCAACGTGGGATGACACAAGCAAGGAAGCGCTCATGGA
EMPV1_08834	ATTCCCTGAGTATCAAGAGCGTGCCCGAAAACGCATACGTACTTACCTCAAGTCCTGCAG
EMPV1_08836	GCTTGGTAGTAATTATTCAACTTGAAAAACTTGTGTATCTCATGAGCGACCACATAACTG
EMPV1_08837	GATGCTGCTGCCTTGTATTGACTGTGGTTTGTCTAGAAGCAGCCATGCTTACTGTGGAGG
EMPV1_08838	GTTGTGACTTATGCCACAGCTGCAGAAACACCGGATCCTTTAACCCACTCTGCTGGGCTA
EMPV1_08839	TGAATGCCATCAGAAGGGTCAACAGCTACAGCTTGAGTTGTGAGCACTATACTGCAAGAC
EMPV1_08842	GCTGAGCAGAACACCAGACAGGAGGAAGTGTGCGGAGAAAGAGCTCCATAAATGTGAAGA
EMPV1_08843	GAAGGAATGTGAGTACAACTGATGGTCTGGTGGTAGTGGAATTAGAGGAATGGACTGCAG
EMPV1_08846	GGAGCCGTGGCCTTTGCCAGGGGACTGGAGGCCAGCATCTTCTTGAGGGTCCTGGACATC
EMPV1_08847	CGTGTGTTTACTTGCAGTGCCATAAAGGCCAATATGCATAACATTAAAGCCAAGACATGG
EMPV1_08848	GACTCCTCGGATTTTCTGGAATTTGTAAACTACGTGAGGTCCCTGAGAGTGGATGGCGAG
EMPV1_08849	TGCGCAGCCCTGTGTGTCCTGGCTCTGCGCAAACCAGAGAACAGCCGGGTCATCATGGAG
EMPV1_08852	GCATTCAAGCAGAAACAGGAGGAGCAGAAGGAACGTGAGGAGCTGGAAGCGAAGGCTAGT
EMPV1_08853	AGCCAGGTTTCTTCGCCCAGCAGGTCGGCAGACTTCGATTTGGTGCTGACAGATTTCTTA

EMPV1_08854	TTTGGGGGCAACTGCGACGTGGACGTGTCCTTCATGTACCTGACCTTCTTCCTGGAGGAT
EMPV1_08855	GGCCTTTGAAGAAGAATTCGGAAGAGTCAAGGGTCACTTCGGACCTATCAACAGCGTTGC
EMPV1_08856	GAACCACTGGAGGAAGAAGATGAAAAGGAACCAAACTCCCCTTTGATGGTGCCTGCTGCA
EMPV1_08857	GTATTTATAGCCAGGACTCACCTTGCCCTGCCAAATGCCTAAGCTATGTGTTGGGTGAGC
EMPV1_08858	CAGAATAGAAGGCAACCTCCAACTAGAAACAGCTCAAATATGGAGAAAGGCTGGGGGCCC
EMPV1_08859	GGGAAGGAGGTAAGAACGCTGAACCAGAAACCCAAGAGGAATGTGGTGAATGTGGCTTGT
EMPV1_08860	TGGCTCCCATTAGACCTCTAGCCTGGGAACCTCCATATGCAACATGTGTGGCCCTAAGTT
EMPV1_08861	AAGCTGGAAGAACAACCTCACAAGACTGCCCTCATTTCAGACACCAGCCACAAGTTCAGG
EMPV1_08864	GTCCCCCAAACAATCTCTTCTTGGCCAGCACAGGGAAATCCAGACTCAGGGTTCCAAAAA
EMPV1_08867	GATGAAATTGCCACTGTGTCTGGCAGGATAGAGGAAAGCTCTACCTTTTCCAACACCTTC
EMPV1_08868	TCTCTCTAATGGCCTGTTTGGGGCATTACCACTTCCTCTTTCTACCTGTGCTGGCTGTGC
EMPV1_08870	GGCTGCCTCTGAGGGTAGAGACTGACTGGAAGAGGGTTAGCTGGGAATTTATTAGGGTAA
EMPV1_08872	TCTTGTACACATGAACTTCCTGCTTGAGCCACTACCAGTCTGGACCTCATGGTCTCAGGG
EMPV1_08874	GAAACCAAGAAAATCCAGATGCTATAGTCTCTTACTCTGCTTCTCATAAGTGCTCCATGT
EMPV1_08875	CCGGGTTTGGAAAAAAGACCGGACTACTATTATCGGAAAGTTCTGCACTGCTCCTTTGCC
EMPV1_08876	GCAGAGGTGCAGGCTGACACGAGGCCCTGGAGCGGCCCTTATGTCCTGAGGAACCAGGAT
EMPV1_08877	CCCATCATTTGGTTGAGGACTTGCATAGAGCACATACATCTATCAAACTGACTCATATTG
EMPV1_08878	GGGACCCCTGGAACACAACTACCGATTAAAGCAGTTCCATTTTCATTGGGGGGCCATCGA
EMPV1_08880	GCTCCCCAATCCCGCAGCCCGCAGCGCCAGCTAACCCTGAACCTCCACGTCAGCCCCGTT
EMPV1_08881	AACCGGTCACAGCTTGCTCCCCTAGCGACGCCCTGTAGCCTTTGGACAGCACCCAGCTAC
EMPV1_08882	ACGCCCAGCCAACTGGGCCCCGGGGCGGAAGAAGAAGGACTTCTCGTGTGCAGACCGCCT
EMPV1_08883	ACATCCGCCTGTGTGTGTTTATCCTGTGGTTGTTGCTTTAACTCCAGTCTCGCTGGTCCC
EMPV1_08885	CCCAAGAGCAAATGGAGCACTGGGAGATGGGGTGAAAGGAATGAAGGTGATAGCCAGTCT
EMPV1_08887	TGGGCAGGTTAAGAGTGTAGCGGATGAACCCAGAATTGAACAGTGGTGTGCTACTGAAGG
EMPV1_08891	ATTTGGCCAGATAAAGTAATGAAGTCCTGATATGTGCTACGGCATGGAAACACCATGCTG
EMPV1_08893	CACCTCAAATGCAATCTGCACCTAAAAGATGGCGTGAAGAGCTAAAACGAACTGGTGCTC
EMPV1_08895	TCATGGCTTTTGCTCTGACACATTGTCCCCCCAGCAGCATTGAGCTTCTTCTGGCAGCTA
EMPV1_08896	GAGCAGCAACTTCACGAGCATTTGCAAGATGCAATGTCCTTCTTAAAGGATGTCTGTGAG
EMPV1_08898	TAGAAAACGTCAGTGTGGAGATGCTGCTGAGAAAGTTTGCAGAAAGCAAGGGCACAGGCC
EMPV1_08899	AAGGCTTTCCCACACTCATTACACTGATGGCCTTTGGAGTGGCTGTGGATTCTCTGATGG
EMPV1_08900	GAGGAATATGGGCAGCCTTTAGAAGGTGGGAAAGGCAGGAAAAAGAGCTTCTCCTAGAGC
EMPV1_08902	GGGACACCTCATGGCCGCTCTATACCAGTAAGATGAAGCTACACCCCAAATAGTCTGTCA
EMPV1_08904	TCACAAGCTTGGCAAGAAATGAAGCTCCTGCATTCACAATAGAAAATAGATCAAGCTGGG
EMPV1_08905	GCTTGTGCCTAAAAGAAGGAATCAGAACTAAAATATGTGGCCATGTGGGGACCGCATTGG

EMPV1_08906	ATTGGCTGCTATCCCCACTGCAGCTTCCGTACTCGGGGAATGCCTTTGGTTCTCTGAGGT
EMPV1_08907	AGCGAAGTCCTCCATGCCAGTGGTTGGAATCATGGTCGGCTTCCTACTGCTCTTGATCGT
EMPV1_08908	CCCACATTGCATTTACCTGGTATTTCTCTTTAGTCTCAGCTGGTCTGTGACAGTTCCTCT
EMPV1_08909	AGAGCCGGTGGCAGCCTCGTAATGCGGCCTCCTCCTGTGGGACGCGGGGACTCCCGAGAA
EMPV1_08910	CTGCGTCTAAGCCAAGCTCCTGACCCCTCGTCTTTCCTTCAGCAGAACCCTGGGGCACCA
EMPV1_08911	GGAGTGGCAAAGGGTTGGTTGTTTGAGGAGCCTCAATGATGCCAGCGGCAGTGCCAGGCA
EMPV1_08912	CAAGATACAAGAAGCACAGAGGGCCCCAAACAAACTGAACCCAAACAGACCCACACCAAG
EMPV1_08913	GATCTCGTTGCACCAGAGCAGGAACTCCCCATCCTTAGATTTTGATGGGTTGCCCACTCT
EMPV1_08916	TGGTAGTCTACTATGCACCTTCTGCTTGCCAGGTCCTAAGAACACTCTGAAGGCATTATC
EMPV1_08917	CTGTCTCAGGAGGGACCATCAAGGAGATTACTGACTCCCCAGGCTTATTAATGACCACAG
EMPV1_08919	AGGAGTTCTGTGCTGAGCCCCAGTTCATCTGTGAGGACATGAGCCGAACAGATGTGTGTC
EMPV1_08921	GGAGAGCTGGCCTCCTCCACATTCCAGTGTTTTTCAATCTTGGCACTACTGGCATTTTGA
EMPV1_08922	GGCCACTTCTTCTAGGCGTGACTTTAACAAGTTAACACTTTCATTTGACAGACCTTTGGC
EMPV1_08924	TTCAAGGTGAATGGATTCTCTAACAACTACTGGGGATGGGGAGGCGAAGACGATGACCTC
EMPV1_08928	ATCTGAGAGCTCAAAGCCGTGGCCAGATGCCACCACATACAGTGCTGGTTCTACATCACG
EMPV1_08931	GGACGAGTGACCAAAGGAATGGAAGTTGTGCAAAGAATTTCCAATGTGAAAGTCAATCCC
EMPV1_08932	CTAGAATCCAGTGAGGGAAGCTTTTGGGCAGGAGCCCTTACGATTTTACTTGCAAAGGCA
EMPV1_08934	GAAAACATACCATATGTATCATGCGGAAAGCATCAGTGCAGAGAGCAAGCTGAAAGAGGC
EMPV1_08935	CTTCATCCTCCTAAGAACTTCCAGCCTCAGTCAAATGCCAAGTGCTAACTCTCTGGTGTG
EMPV1_08938	ATCCATGCTGGCCTTGAATGCTACTCCAACTTGCAGGGGACGTGTGGTATTTGGAGGTTG
EMPV1_08939	TGTCTGCAGTGCCAAATACACAACCAGTTCTGCAATATGGTAATCCTTACGCAACTCAGG
EMPV1_08940	TCTCACAAGGCGGCCCCCACTTCAGACTCCCATCACAGGTCCAGGTGGTTAACTGTAATA
EMPV1_08941	GTTATTTCCTTGGCATAAACTCTTGTAAGTGGAATTGCTAGGGTATAAGGTAGCCTGCAG
EMPV1_08942	GAGAGGCATCATCAAATTGGAGCCAGGCAAAGAGGGTGCTGCAGGAAGTCAGGGAGCTGA
EMPV1_08944	TCTTAGAGGCAGACCTTGCAACGGAATGAAGAGGGTACATGCTGGCCTTTTGTGTGCTGC
EMPV1_08945	GAAGAACTTCGGCATGAGAAGGAGATGCAAAGGGAGGAAATTCAGAAACTGATGGGGCAG
EMPV1_08947	TCACAGTTAAATGGAAGAGACAGAGGCACCTCTTTGGACTCATCAAATATGACCACCTTG
EMPV1_08948	AATTAATTCTCACAACAGCTCTCCGCAGCAGCAGGAGATGGCACTCAGGACGGCTGTTCT
EMPV1_08949	GTGGTACTGCCACAGGAGTGAGGGACGTTCAGACTCAAGGAAGACATGATTTGTTCAAGC
EMPV1_08950	CACATCTCCGTTATCCTGGAGGCGTGCAAAAAGTGCTTTAAGTGGTTGGCATGGGACTAG
EMPV1_08951	AGCTGGCATTTATGACACCAAATATGCTGCAGAGTTTCACGCCCGCTTTGTGGCCTCCTA
EMPV1_08954	ACAATGACAACTTTTTTGGATTGTACTGCATTTGCAAGAGACCTTATCCTGATCCTGAGG
EMPV1_08955	CATGGAGGAGATGGTGGAGGACAGTGTCGACTGTTACTGGATCACGCGATGCTTCGTGAA
EMPV1_08959	TGTGGCATATTTGAACTCAACGGACTTCTTGATCACTCCTGATTTCTACTGGGACCGAGA

EMPV1_08960	GGCAGAGATGATCTTTCCTTCTGTGGTGTTGGTGGTGCTTGTAATACATAAGCATGCCCT
EMPV1_08961	GTCTCTCTGGATCAATGAAAAGATGCTTACAGCCCAGGACATGTCTTATGATGAAGCCAG
EMPV1_08964	TGCGCAGCGAGCAGCTACCCAAGAAGGACATTATCAAGTTTCTGCAGGATCACGGTTCAG
EMPV1_08965	CTGTGTTGCCTATCGGGACGAGAGAGGCTCCGACTTTATCCAGACACTGGTGGAGGTTCT
EMPV1_08966	GGCTGGGACATTCCAGGCCCTCCTCATATGTCTTTGAACCAGTTGACACATTACCTGCAA
EMPV1_08967	ATGGATTGCGTGTGCAGGGCGCGGATGGCGTGTCCAGGTTCTCATGACCAAAAAAATGTG
EMPV1_08969	TTAAATGGAAGTGGCCAGCTCAAAATGTCCAGTCACTGCCTTTCCGCTCAGATGCTGGCA
EMPV1_08971	GGGATGGTCATCAGACCCACAGGGTCTTGAATGCATGACAGCCCTTTTCTAGGACAAAAC
EMPV1_08972	CAGCTGTTACCCACAGCCACACCAGATCCAAGCAGTCTGTGACCTACACCACAGCTCATA
EMPV1_08974	GGGATGACCAAGGGTACTTTTAGGCAGTCCATCTTTTCAGAACACATGCCGGTGTGTTGC
EMPV1_08975	CCTCCTGACCCCGTACCCAAGAGACTCCAGGCTGCGCTGGCTGACCGACCCAATCCCCCC
EMPV1_08976	CAGAAATCGAGAGACGATTAAGGGGTGGAAACGGAAACCCCGAGGAGGGAGTTCTCGGAA
EMPV1_08977	AGCGACCCAGCTCAAGACAACCCTGCTCACCCCCCTGACTAAAGCGTTTATAGCTGGAAG
EMPV1_08978	TTGATGCTGCTTTGCGGGCATGCAGACGCTTAAATGATTTTGCTAGTGCCGTTCGCATCC
EMPV1_08980	AGACGATTTCTTCCTCTGGGGCAAAGGTGGGGCAAGATTGCTTGCAGCTCATTGCAAACA
EMPV1_08981	CAAACGCTCAGTCGCTGATCATACATGACGCCCTTACTCCCAACACCGCAGTCAGTGAAT
EMPV1_08982	GAAGAACCGGAAAGAGAATGAAGACAGTTGGGACTGGGTCCAGAAAAACCAGTGACCTGC
EMPV1_08983	GGCAGCCATTCTGAACGACCTATTTGGTGGCGTGGTGTATGCTGGGATTGACACTGATAA
EMPV1_08984	AGCAAGTCCTGCGTGAGTGATGGGCATAACCACTTCTCTTGGTGGAGTAAGAACTGGACA
EMPV1_08985	GTCTCCTATGGCAAACTCATCTCCACCATCCTCAAATTGTCATCAGCCACAAGTCGCACC
EMPV1_08986	TCTACTTATGAGCTGAGTCAGGAAGCCTGGTGGTGCCATTTTCAACTATCGGAATCCGGG
EMPV1_08989	GTGAATTTACCCTAAAATGTCCAATCTGTATTTATGTACCTTGTCAGTGTTTTGCTGTTG
EMPV1_08990	CCGGAGCCAGATTTTACCTGTAGAGGAGCCTGCCGGGAACCACAGGCTACAAGATGGTTT
EMPV1_08991	GCAGCTGGCTGGCGAGCGCAGCAGCCTGGCCCAGGTCCTTCGCCAGGAGTTCGCTGACCG
EMPV1_08992	GTTTGCTCCTAAAGTGAAGTCTCAGTATCCAGGTTCTTGATCAAGGCAGCAGTCCCAGCA
EMPV1_08993	CCTCAGCTGTTTCTGGGAGGAGCTGTATTATCCTGGGTTGGAGAGAGGACTTCAGGTAAA
EMPV1_08996	ATCTAGAGTGTGTGGCTGGAACCGAGGGAACAAGAATGAGGTGTAGAGTGAGGAGTAGAC
EMPV1_08999	GGAGCTTATCAGAATCTCCAGGGGTACTTATAATTTGAAAAAGTCCCCCAGGTGTGATTC
EMPV1_09000	GCTGGCTTTGGAGATGGGGGTATTAATTGAACAGCGCACTTGTTAGCTTAAAAATGCCAG
EMPV1_09001	GATTCGTGTCCGCAAACCATTCAAATGCCGCTGTGGTAAGAGTTACAAGACAGCTCAGGG
EMPV1_09002	GAAAGCATGAAAAAGGAAGCTTATCAAACAAGAACTTAGAGGAGAAATTGACGGTCTCTC
EMPV1_09003	CCAGATTGTGGGTAGGAGGTGCATCACCCCAGAGCTGGTGGCCATGGCGGTCCACAACAA
EMPV1_09005	CCTGGCTAGCTTTTGGCTGGCAATGAGTTCAACCATGTACAATCCCATCATCTACTGCTG
EMPV1_09007	GACTTCCGAGCCCAGCAGTGTTCAGCCTACAATGATGTCCAGTATCAGGGGCGTTACTAT

EMPV1_09008	AACTCTCATGGACCTCGTTGGTGCTCAGAGATTTTCCAGTGCTGTGGGACTCGTCACAAT
EMPV1_09009	AGGTCCTGTTCCTGCTCCTTCCCCAGATCCAGCTCCTGCCCCAGCCCCAGCCCCTGTTCC
EMPV1_09010	TGGATTGCAGAGGAGACTCGGATCCTGTGTTGCTGTGGCTCTGGTGTAGTCCAGCAGCTA
EMPV1_09011	AAGGACGATAAGTTGGAAATGATGTGGTTCCTAGCCTTGAGGAGCTCTTGGGGGACTTCC
EMPV1_09012	ACAGGACCTCTTGGGCAGGTGGAGAGCCTCAATTTTCCTAGGTGGCAGAGAAAGTTATCT
EMPV1_09013	TGGGAGCAATATGGGAAATGGTTCAAAGGGATCGATATAACCAAATCCAAGTCTGAGAAG
EMPV1_09014	GGAAGAGGATGATAGGAGATACATACTTCTGTAGTACTCACGACTCTCATAAACACGAGG
EMPV1_09016	ACGAAGTACCCCATGAAATGCCCAGCAGGGTACAGCCAACACCAGGCTTACCTCTTCAAT
EMPV1_09018	CGTACACACTCACACATGCGCCCAGCGCAACCACTTGGGTTCTATATCAGGAATGTCTTA
EMPV1_09019	ACAGACCAGCGTTATCGTCAGGTCCAGTACATCTTGCTGGATCCTTCGTGCAGTGGCTCT
EMPV1_09021	GAGTACCCTGGTGGCACAGTGGTTAAAGATCTGGCATTGTCATTGCTGTGGCCTGGGTCA
EMPV1_09022	GAGGTCTGATTCAAAGTTCTGGGGCATTAGGCATAAGAGAGCTGGCTTCGATGCTGTTGG
EMPV1_09024	CCGAGTACTAAGTAGGGGTCCACATGTGGCTGGCTTTATTTTCCTTTGCTTTTGTTTATG
EMPV1_09027	CATGTGCCAGCATGGTCGGTGGTACTCTTTTTCCGAGCACAGAGATTAAACCAGAATTTG
EMPV1_09029	GACCTTGCTGGGCTATCAGTTACAATGCTTTTGGCCTTGCTCCTGTTTTGCTGTTTGACC
EMPV1_09031	TGAGTTGCCTTCCACCTACCTTTTGTCTGCCATGGGGCATCTCTGACTCATTTGCCCACA
EMPV1_09032	AAGCCAAACTGTGCTCCGCCAATGCCTCCCGTCAAGCCTGGGGCTGGAGGGGCCCAACCT
EMPV1_09034	CACAGCAGCTCACACCGCCTGCAGTGGCCTGTAGCCCGCTTTTCTCACTTAGTGCATCCA
EMPV1_09035	TCCTAAATTGGAAAAACGACTTAGGGCTACGGCTGAGAGAGTTAAGGCCCTGGAGGGTGC
EMPV1_09036	CTCAACCCCATGGTGGATGTGAAGGTGGACACTGAGAATATAGAAAACAAACCCGAGTCA
EMPV1_09037	CTGTTAACGGCCTTCGCTACCTAAGCCTGCTCTGTTGGGTTCTTTTCTGCCTTGATTGTC
EMPV1_09038	ACTTACAGCCCAAATCTGGCTACTCCCCAGACACCAAGAAGCTCCTGTCCTTGGCTTACT
EMPV1_09041	TAGTTCCTTTGTGGAACGACCTAACTCTAACACTTACACCGGGCCCTCCAGCTCCTAGAT
EMPV1_09043	CCCTTGAATTTCTCAACAGTATTGCCACCTCTGGCCAAATATGCACTTTCCCTAGAAAGC
EMPV1_09044	CAGGCCATGTCCATAAAGCTCTGTGCATTCTTGGTAGCAGCCTCATATCTTGGTGGCTTT
EMPV1_09045	AAAGGAAGTTTCGTGGATTAAACTGTGGATTTAATGCAGCAAGCAAATCTTACAATGTCC
EMPV1_09046	CTTTGGCCTGGCATTTGCTTCAGCAGGCAGTCTTCCTTCAAGTACCTCCCTGATTGATGT
EMPV1_09050	AAGTCACTGAGCCGATCTTAACCCCCCTGCTTTTAGATGAGAACTTTCCCATCCTCTGCC
EMPV1_09054	GCCTCAGTGTTCCTGGCATTTGGTGGGAGCCTCTAGTATCTCTCAAGGGTCTGGGTCTTT
EMPV1_09055	TACTGTATCAGAATCTTGGGTATGTGTTAGGAGTGATAGAGCAGGGCAGGAGAGGAGACC
EMPV1_09057	CCAAGGCTTTGTGGACTCTATAACGGAACTGTTGAAAGTGAGAGGAGAAGCCCAGAAACT
EMPV1_09058	CGAGGGAGTGATGCCTCTGACTTTGACCTTTTGGAGACGCAGTCAGCTTGTTCAGACACT
EMPV1_09060	TGAGTCAGGTAGAAGAGTGCAAGCTAAAAGGTCCTGGAATCTCAGAATTTGCTAAAAAAC
EMPV1_09061	GTATATAGTAGCGTGTATATGTTAATTCCAGACCCCGAACTTAACCCCCACCCTTTCCCC

EMPV1_09063	AGGTTCTGGAAGAGAATCTGCAGTGTTGTGAGAACTGCCTGCTGAGACCATGGAGGGAGA
EMPV1_09066	CAGGATGAGTTTTCACTGGGGAAGAGTTTTGTGGGTGAAGCAAATGTGGGAAAGCCTGCA
EMPV1_09067	CCAAAACTGGCCGAAGTATTGTGTGTCCCTATGTGGAGGTGGAAATATGCGGAGCCGAGT
EMPV1_09068	TTGGCCATCTGTACACCATTCTTACCACACAATTGACCAACGATGAACTTTCTGAGAAGG
EMPV1_09069	ACTAGTAGCGGTTCCATACGTGTACAGCTTTCTGGTTGCCCTGTTCCACACCATCATCAC
EMPV1_09070	GCTCAGAACTCCCAGTCGTCCACCTCCAGCTCTTCCAGGTTTGTTACCAGGCCAAAGATT
EMPV1_09075	GAGGTCTGGGGCTCTAGAAAGATGGAGAAGATCCACCCTGTCACCAGCTCAGAATAACTG
EMPV1_09076	ACAAACAGCCAGTTATTTCTCCAACAAAGACGGGTTTATCCGAGCTCAGTAGAGAATGGC
EMPV1_09078	TGCAGGACGAGGCGGACCCCAACTTCCAGCTCGCTCTGCACTTTGCCTGGTCCAACTTCA
EMPV1_09080	TGTGCTGGAGAATGTGTCGGAAGATGGCGAGGAACTTTCTGGCTGCCTGGGGAGACACCA
EMPV1_09081	CCTTCATTTTGCAGTCCTGAGTAACGGAAGTGCCTCTTGAATCTGTGTCTCCCAGGCAGT
EMPV1_09083	GTACTGCTGCTGTTGCATGACACCCCCAAGTATGTTCCATCAACACAAACCTGCCTTTGG
EMPV1_09084	GGCAGGGAGCCATGAGGTGTGGCTTTTCTCTCTAGTTGCTTTTAAGATCTTCTCTTTGTC
EMPV1_09086	AGATCTCAGCCTCTGTTTCTATAGTGGCCACCTCCAGGTGATCTGGAACAGGAGGTTCAG
EMPV1_09087	CCACTCCTGGCCAGTCTAGCGATGGGTGGAAGCACTGCAGGTCAACGCACCTACAGTGAA
EMPV1_09089	GATTTTAACACATTCATCAGGCCATCCAGGATTCTCTCACAGTCCCTTGAACTCTTAGTG
EMPV1_09091	GCAGATGGGGCAGCTCCTACAGGCCTTCTGTCTCTGTTACCGCCCATGACATCTAATTAA
EMPV1_09092	AAGCCTCCACTCGGAGACGTTTGGGGCAGAAACCTGCTGATTTACCCCTTGTTTTAACTC
EMPV1_09093	TCCTCCTCTCTATCCTCCCCATCTGCGCGCAGCTCAGCCCAGAAGCTCAAAGGCAGAACT
EMPV1_09095	CTTCAGCCAAAGCACGTTCCTTACCCAGCATCAGGTCATTCACACTGGAGAGAAACCCTA
EMPV1_09097	CATTCTGGATGCGGGTGCTCAGTATGGGAAAGTCATAGACCGAAGAGTGCGGGAACTGTT
EMPV1_09100	GCCCATGGTGGTGAAAGAAGTTATCAAATAGCCATGGTAGGAATATGAGTTCCGGCTGAG
EMPV1_09104	TGGTCTGAGACATGAGATGCAACATGGGCGCTACATTGTAAACTGTGCCGAGATGACTCG
EMPV1_09106	GCCAGAGTGCAAAAACCAAAACCCAAAACACATAAGCCCAAACTTAATGGGCAATGTGGG
EMPV1_09107	ATCCTGGGCTTCTTGCAGGATGCAGGAGTCTGGAGCTGCGTTTGTGGACTTGTTTGCTTT
EMPV1_09108	CCATATTCGCCAATAAGGAGCCCTACGAAAATGCCATCTGCGAGCAGCAGCTGATCCTGT
EMPV1_09110	GCCCGCGTTCTCACCGTCATCAACCAGACCCAGAAAGAGAACCTCAGGAAATTCTATAAG
EMPV1_09111	TGCTGAGGGCCTTTGGAGGGCAGAACATTTTCATACAGTCTATCTGGCACTATGTTTTCC
EMPV1_09112	AGCTGTATGCATTTCATTGCTGACTGCAGGCTTCTTTCAGGTTACGTTCATCGGTCTGTG
EMPV1_09119	AGGTGGAAAGGTCCCCCCTCTTGTTGTAGCAAAGGATTGGAATGGCGGATGTTTTACAAA
EMPV1_09120	AGAATTGAGATCTTCTGGCTTTGAAGATGATGAATTTTCCAATATACCAAGTTGGCAGGG
EMPV1_09121	ATGTGCCACCTCGAGTTTCCAGGAGAGAAGTGTTCCAGAGGCAGAGGAATATGTATTGCA
EMPV1_09122	GGCGGTTATTCTCGTCATCGTGTCCGCTTCCAGAAACACGGGTTAGAAGGATTGAATAAG
EMPV1_09123	GCCGGAGAATGTGTGTTGGGCTTGTGACACTTTCCTGGATTGTTGGCATCTTCCACGCTA

EMPV1_09125	GGGTTAAAGAGGAGGAAAGGGAGGGCATGTTCGGAGTTGGGTCTAGTTATTCGGTATCCA
EMPV1_09126	TGGTATTCAGCGGCACAGTAAGCAGGTTGTAAAAACGAAACAAAAGCACAGTGTTAACGC
EMPV1_09127	CCTGGCTGAGAAGGAGAGGCAGCTCATGGGTATGATCAATCAGTTAACCAGTCTGCGAGA
EMPV1_09128	TGCGCAGTCTGTGCACCCAGCCTGTCTCAGTTAACGAGCGCATCGAGAACAAGCGTCAGG
EMPV1_09130	GTATCTTGCGGTACCGAGTAGATTTCCAGGGAATGGAATACCAAGGAGGAGATGATGAAT
EMPV1_09131	TGGAGAACGATGAGGATGGAGCCCAAGCCTCTCCGGAGCCGGATGGGGGAGTCAGCACCA
EMPV1_09132	CAGGTGAGAGCCAGCCCTGGGTGAGGTGCACGTTTTTCACCAGTCTATCATTTGATCATT
EMPV1_09134	CATTCACCCCATCTAGGCCTGCCTCCCACTCTGAAACCAAGTCACCAAATGCTCTTTTGT
EMPV1_09136	ATTTTGTGTCCAGGGAAGAGGCAAGAAGTGTGACCTGCGTTTCACTTTGTGGTCAGGCTG
EMPV1_09139	GAGAAGATGGGTGTGGACCTGTGCCTGTTCTTTCCTCATGTGTGGGGAAGATGATTCAGC
EMPV1_09140	GCCCACCCCGAGTCTGCTCTGAATGCACGATTTTGTCCAACCCTTTCCACTTAGTCAGTA
EMPV1_09143	TGCCTGGTCACTTTCCCCTATCTCTGGGGATCTACAGTGGGCACCATGCAGGTAATATTG
EMPV1_09144	GGGCTGATGTTGCACGATCTCTGATGTTCATAGTTCTTTATCCGGACACTCAGAAATCCA
EMPV1_09148	TTGCCAGTACATGGAGGAGGCACAGACATCACGGCCTAGGGCAGACTGAAATGTAATCCT
EMPV1_09149	GCCCCACTGTCCCCAAAGTTTCAGTTCTCCCAGTAAAAGTAAGAAGAGGCAACCTGGAGT
EMPV1_09150	TGAACGCCCGGATGATGCACGGCCGCTCCTACTCGCTCACCACCTGCTCCGGCTGCAAGA
EMPV1_09151	CTCATTTCCTGTCACCCACAGAAATAAATGACACATTTTTCAGGCTCACTGATGAATCCC
EMPV1_09153	CCTAACAATCCACTTTCCTTCCGATTACCCTTTCAAACCACCCAAGATCGCATTCACCAC
EMPV1_09154	GGCTGTCAGGGACAAGGAGGTCGTTGCAGTATCTGTGGCTGGAGCATTTAGAAAAGGAAA
EMPV1_09155	TTGCATCATATGGACATTCCCGGTCTGGGGGTCAAATTGGAGCTGAGGCTGAGGCCTATA
EMPV1_09156	CATTCAGGAAAACATTGTTGTGATCTATACTACAGGAACCAAATGTCATGCATCATACAT
EMPV1_09161	AACGCTGTGGCCGAAACTTTTGCATCAAGCACCGTCACCCACTGGACCATGATTGCTCTG
EMPV1_09163	CTTGTCGAAGCTACTGGGGACTGGATGTTAAAAACACCAATTTGTGCGGAGGAGCTGCAG
EMPV1_09164	CATTTTTCCCCAAAGACGAGAAAATCTACTCAGACAAGGGCTGCAAGACGGTGCAGACGC
EMPV1_09165	CTTCAGCGGCAGCTGGCCCAGAGCAAGCAGGACTTCCGAGACCTCAGGGAGAAATTCCTT
EMPV1_09167	GGTGACTGCAACCTTCCACCCGATGTACCTAATGCCCAACCAGATTTGCGAGGTCTTGCA
EMPV1_09168	CTACTATCACGTATGTGCTTCTGGCTTCCTTACAGGACATCTCAGGGTTGCAGTGAAATG
EMPV1_09170	GAAGCCCTTTGCGTGCAGCGAATGTGAAAAGACGTTTACTCAGCTCATAGATCTTCACCG
EMPV1_09172	GTAGTTAAAGAATCTGTCTTGAGATCTGATTTCCCCTTCCCCCAGCTGTGTCCCCTCTGG
EMPV1_09173	TCTGGCAAGGAAACCATCTGTTGTGTGGCTGGTGGGATGAAGGTGAAGGCTGATGGAGAT
EMPV1_09174	AAAAAACTGCTCCAAGTGCGGTTGCCCCTCAGACCCTGGGCGCATGTCTAACGTGACTTT
EMPV1_09175	TCGGACAAAAAGAGGCTGAAATGGATTCATAAGGCCCTGGAGCAGCGGAAGGAGTACGAG
EMPV1_09176	CCTGCAAAGTCCTGCTTCCTTGAATCTTACCCACAAATTACCTATCACAAGCACAAATCC
EMPV1_09177	CGACATCATATGGGCAGCTTGAGATGGTTATGGTGGGAGGATTTACCCTGTGGAAATTGG

EMPV1_09179	CTCATTCTCTTTGTCATTGTCTTCACTATCTAGGCCGCTATTGTGGCTCCTCCATGCCCC
EMPV1_09181	AGAGTTACCTCATTACAGACAGGTCTTCAGTTAGCTGCCGTTATCTGCACAAATGGGAGG
EMPV1_09184	CTCGAGTCCTCCTGGATACTAGTCGGGTTTGTTAACTGCTGAGCCATGACGGGTACTCCT
EMPV1_09186	TCATCTCCAGTGGCGGCGAGCATCCCTGGCGTCCACCTGTTTGCAGAAGATTTGCTAAAA
EMPV1_09187	GGCTTAATGGTCCTCATTTCCTATGCCTTCATCCTGTCCAGTATCCTCCACATCAGCACC
EMPV1_09188	GCGGTGCTGAGTAGGAACAGCACTGGTATTTCCAAAGCAACCACACCCACAACTAGAATC
EMPV1_09189	GATGGTGTATCCAGTAGAATCTAGGTCAGGAGTCGACAAACTTTTCCTGTGAAGAGCCGG
EMPV1_09190	AGCCCTACAACACTCAGGGATTATTGTCTAGGTCTGCTGACGAGGCATTGCTAGTGACCA
EMPV1_09192	TTTTGCAGCCAGCGGTGAAAGCAGGAGGTCCTTGCCCTGAAAAGCACAGTTGCTAAAACC
EMPV1_09193	CGAACTTTCCCACAGAGCTTGCTGGGCAAGGCACCCTCTGCTGTTCTGAAGACCCAACCA
EMPV1_09194	TGTATCAGGCACCCAAGTTGTGCAGGGACAGATCCAGACGCTTGCCACCAACGCTCAACA
EMPV1_09196	TGGCCAGGTTTTATCTGCTCTTCCTAGAACCTCTAGGCAAGTTCAAATTCTGCAGAATCT
EMPV1_09198	TCCATCCTGGGCCTCTCAGGGTTTCACACTTACCTCGTCACCTCCAACCTGACAACTAAT
EMPV1_09201	GAGGCATGTACCCTGTAACATTTGTCAGCTGCCATGGCCAGTTTTTGGCGAAAATGATAA
EMPV1_09202	GAAGGAGCCCTAGCGTTTCATCCGGACCAGACCCAGCCTTCGGAATCTATTTTTTGCCTA
EMPV1_09205	TCTGCAGTTGAGCTCCCATGGGCTGTAAAATCTGAAGATAAGGCCAAATATGATGCAATT
EMPV1_09206	TGTTAGTCTGCTGCTGGATGCGTTTGATGGTTGCCCCCTTGGGGCCCACCACCAGCCCCA
EMPV1_09207	GGCACAGTTGCTTTTGTCTTTTTGTGGTCACTCCATTTCCTCTATCTTGGTGGGCGGAGG
EMPV1_09208	CAAAAGAGACCGAAGGAAGAGGAATGGGATCCAGAATATACCCCAAAGAGCAAGAAGTAC
EMPV1_09210	GACCCCTCCAGAATTTGACTTCTCCAGCTGGCTCTCTTTGGGACTTGATGGCTTACTGAT
EMPV1_09211	CCTCAGGTATCTTTGTCTATTTGAGTTCCAGCTCTGGCAGTTCCTCCAGCTTTGACAGGT
EMPV1_09214	GGATCCTTAACCACAAGGCTACCAGGGAACTCCTCAGCTTCCCAGTCTTACACCACTGAA
EMPV1_09215	ATGGCACACGTTCCTTGGGGCCATGAAATTGAAAACTGAAGTAGTGGGCAAGTGAGGAAC
EMPV1_09217	TATGCCTTGACTAGAACATCAGCACCTCACTGCCACCTGAGAGTGAGGGCGGGACATTGG
EMPV1_09218	AAAGAAGTCAACCATCCTCTCTTGGTCTGGGAAGGGTAGATGAACTCAGTTGAACAGCTG
EMPV1_09219	AGGTGTCTAGGCATGCCTTCAACCTCAAGCAGCTGGACAACCCTGCTCGAATCCCTCCCT
EMPV1_09221	ATAAGGCACAGACACACGTGGTACTAACTGGAAGTCAGTCATCTGGAGGAAAGACGACCC
EMPV1_09222	CTGCTTGTGGTGGGACTTGATTTACTGCTGATTGGCTTCTCCTATGGTCTCATCCTGCAG
EMPV1_09223	TGAGGTTTGTTATGCTGGGTCTCTTTCACCCCATCACTCACCTGTTCCTTCTCCTGGTCA
EMPV1_09226	GAACAGTTCTCAATGTGTATGGTGGCTTGTCACGGAGACGAGTGTATAGCTACCTGTTGG
EMPV1_09229	GACCAGTCACCAGAGACAAGCAGCTATGAATAGATCACCCCAAACCGATCTACATATGTG
EMPV1_09231	CATCCTGCAGATCCCTGCTCTGTCACCAGGGCGTCAGAGCCTCAGGGGATCCCTCGAAGC
EMPV1_09236	CTGCAGGGTGGGTGCCCGTGTGACCTTATTTTACAAATGGGTTCGGTGGGTTCAGAGATA
EMPV1_09238	AGCTGGTGTTGTGAATCAGGCCGTTGCCAATCAGAGAACGGCTACTTCACAACACCAGGG

EMPV1_09239	ACAAAAACACTCTCTACAAACACAAGTCCAGGACCAGAGGCTTCACAGGTGAATTCTACC
EMPV1_09241	AACTTCCTGAAGTGCTCCGAGGACAACCCGCTCTTCACTGGCACTGACTGCGAGGTCTTT
EMPV1_09242	TTTCCCTTTGGCTTTTTCACCACCGTCTTCAATACCCACGAGCCATTTCGTCGGGGGACA
EMPV1_09244	CCCCAAACCTACTCCACCCACGTCTTCTTTTCATTCCATGGCAACGCCATTCTTCCAGTT
EMPV1_09246	GTCCAGTCACTATTCCTGCCTCCCTGATTAGAGTCCATGACTTTTGAACATTAAGGCTCC
EMPV1_09247	AAGAACCCTAAGAGGTAGCTTCGGAACTTCTCCCCGACGAAAGCGTAGATGATGGGGTTG
EMPV1_09248	GAGGAACTCTATCCAGGGACCTCCTATAAGGACCAGAAGAACGCGCGTCCTGCCAAGTGA
EMPV1_09250	GCCAACCTCCTTAGAATTGCTCTCTGTGTTTCCCAGCACTAGAACACGCTTCCCAGTAAG
EMPV1_09251	CTTATTGCACCTTCCTGCTGGCTGTCGGCTTGTCCCGGGTCTTCCTCTTAGCACATTTCC
EMPV1_09252	TGTGCTGCTTTTCTGAGAGCTTCGGGTTCTCCGACCTGGCTTTCACTCGTTTCTGTGTCT
EMPV1_09254	CTCGGAAATGGACATCTAGGGTAAAGCAACTTCACCAGAGGAGTCACACGGGAGAGAGAC
EMPV1_09255	TGCTGCCGCCGAGAAATTACACTCAAGAAAAGCAGACCCAGATGATAATCAAACAAGGAG
EMPV1_09257	TCTGTCCTTGGCTGGTTAGATGGCTGCCTCCTTGCTGTGTCCTCATGTGGCCTTTTTCTT
EMPV1_09258	CGTAAGAGCAAATTCAAAGTCACCTCTTCAGAGATCACTGTCAACTAAGTGTGTATCTGG
EMPV1_09259	GGCGTAGGGAGTCAGGTAAAAAACGGAAAAGAAAAAGGTTAAAACCCACCTAACGCGGCC
EMPV1_09260	TACTACATGCTCAACATTGAGGCTGCTAAGGATCTCTTATACAGACGCACCAAAGCCCTC
EMPV1_09261	GCTTTGAGCATGTCTTACTTCACATGGACTTGGTATTGGCTCTGCTGACAGTGGACTGCC
EMPV1_09262	CCATGATGCTTGAGTGGCTAGGTCTGGGTGTCGGTTCTTTCACCCAAAATGTTACTCCAC
EMPV1_09266	ATGCTGCCCCATAAGACCAAGCGAGGGCAGGCCGCCCTGGACCGTCTCAAGGTGTTCGAT
EMPV1_09270	GAACTAAGGCCTACACAACAGCCACAGAAATACCAGATCCTTAGCCCACTAAGTAAGGCC
EMPV1_09271	TTGTCAAGGACCCCGCCCTCGCATTTGTCCCATTGGCCTCTCTCTTTGCTGTTTAATTTT
EMPV1_09273	CAAAAACCACACCAGGGAATTCCCATTGTGGCTTAGTGGTAACAAACCCGACTGGTATCC
EMPV1_09274	TGTTCTCATCCTGTTTGCCAACCTCTATGTGGCCATCCCTCCAGCTCTCAATCCTGTGAT
EMPV1_09275	GGGATGCTGTGGTGAAACGTTAGCCGAGGGAACAGGTGACAGAAGATTGGAATGTGCTTC
EMPV1_09280	CACTGAATTATTTAGGTATCACATGTAATAACACCAGCACTTAACTCTCAAGTAATTCAC
EMPV1_09281	ACATACTGCTGGATTATATCCAGTAGCATCTACCTGGCCCGAGCAGGTACTCTGTAAACA
EMPV1_09282	TTTCAGGACCAGGTCATCATCAGAGATCAGAAACACCTGTCGTCTTTTATGAGAGAGAGA
EMPV1_09283	AGCGCAAGGCCCGCTTTGCCAAGCTCAAGGTCTGTGTCTTCAAGGAGGAGATGCCCATCA
EMPV1_09284	GACGTCTTACTCTGCTGCCTCGCAAGGATGGCTCTTTATAATTTGGGGAGAGATCGGGTG
EMPV1_09285	GCAGCCACTGAGGAAAAGTTTGGGGGTTCCTCAGAAGGTTACACATAGAATTGCCATTTG
EMPV1_09286	CACCACCATCACCCAGGGCGTGGATACCTCCCAGCCCTCGAAACAGAGCAACAACAAGTA
EMPV1_09288	TAGCCTAGGGAAAGAGGCCAGAGAAAGGAGGCATTAGGATCTTGGACCTGTGACTATGGG
EMPV1_09290	CCCAGGGTCTGCTATGTGCCAAGCACTGTTTTAGGACCCAAGGATACTGTAGTTAACAAA
EMPV1_09291	CCCAACATAGTGTCCATGAGGATACACATTCAATCCCTGGCCTAGCTCAGTAAGTTAAGG

EMPV1_09293	TTGCAGTGAGCTGTGGTGTAGGTCGCAGGTGCGACTCAAATTCAGCATTGCTGTGGCTCT
EMPV1_09294	CAGCAGAGAAACCAAGGAGACTGTGTTTCCATGTGACTTGGGACAACGGGCCGTAAATGA
EMPV1_09295	GACTTCAACACTGCTTGCTTACAAGAGCCACAGTCGGTTTCCATTCTCCAGCATCTTCTC
EMPV1_09300	CTGTCATCTTCATATAAAGTCTATTATTTGTTTTGTATTAGCGTATTCCTGGTGCTGTGC
EMPV1_09303	TGATAGTGCATCAGTTTCTCAGATTCTGACCATAGGAAGTTAGATACAGAGAATACAGGG
EMPV1_09304	GGATTCCTGCTCCACTCGCAGCTCTCGAAAAAGAAGAATCAGATCGACAGCAACACCGAT
EMPV1_09305	GCCGCTGGTGTGTTTGCCTTCCGCCCTCGGCCTGCGTCCCTCGGAGGCGGCTCGGAGCCG
EMPV1_09306	ATGGCACACTAGGGTCACTGGCAGTCCTCTGTCCTTGGCACCCAGCTTCTGATGATCAAT
EMPV1_09307	TTGGTTTGGAGACGTTTGGGGGATAAGATTTGGACTAGGGCGAACTCTAAGAAACTTCCA
EMPV1_09308	ACACTGACTTCAAGTGGGGAGAAGCCAGACAAGGATTTGGAAAGCTGTGGTGATGACAAC
EMPV1_09309	GCTAGTGTTCTCCTGGTAGAATGAACTTCCAGAAATGGTTGCTCTAGTGTCTGTGTACCC
EMPV1_09310	AACCTGAGGAAAATGGTGAAAAATGACTGGCAAGGAGGTGCCATTGTGTTGACTGTGAGC
EMPV1_09313	ACATTGTCGTGTATCACAGGGGTGCCAGGTCTTGTAAGGATGCCTCTGTAGGGACAGTCA
EMPV1_09314	GCTAAGCCAAACTGCCTTCACCTTCTTTCCTTGGCCTGTAAGGTCTAGCACCAGAGGCTT
EMPV1_09315	AGAAAACCGCGACGCCCGTAAAGGACGAGTACGAGTTCGACGAGGACGACGAGCAGGACA
EMPV1_09316	AGCCTGGCTAGGATGTGATTTTTTCCAAGTCCTGACCCCTCAGAGCCAGGAACAGGCCCT
EMPV1_09317	AATCTGTTTTCCACTTTCACCATCACCTCCCTGCGGGAGACCACGCTGGCGGCCAACCAG
EMPV1_09321	ATTATCTGGGTGAAGACAAATCAACTGCAGCGCATCATTCGGACAGGCCGTACAGGTCAC
EMPV1_09322	CTACCTCCTGTTAGCCGTGTGATATAAGAATTAGCTTCTGCCAACCAGGATTCGGTTTCT
EMPV1_09324	CCTAGCATGTGGAAGTTCCCAGGGCCAGGGATCAAATCTGTGCCACATCAGTGACAATGA
EMPV1_09325	TCACTGCGGGGGAGAGGTCTGACAATTTGGGTTGCCTCAGGTTTTTTCCTAAACTTCGGG
EMPV1_09327	GGGTGAAAGCCCTGCCACAGGTGTGAAATTGTTCTTCCCTCTTGGAATTGGGTGTATGCT
EMPV1_09333	GCCAAGGGCCTGGCCAAAAATGAAATAAAAGCTTTCCTACAAACTCTTATGGTCGCGCTG
EMPV1_09337	AGGCATCGGCCGCCTTCAACTGGAATGATCAACGTTGTAAAACCCGAAACCGCTACATCT
EMPV1_09338	GGCAAAAAGCAGAAATGAAGTGTCTAGCAGTGAGCTGGAGATTGGACAACAGAAGGAGTA
EMPV1_09339	CACATTAGCCAAAATGCACATCTTTCTAACTCATCCGTCACACATTTTTGGAGAGGTGAG
EMPV1_09340	GAACTGGTTCTTACAGGGTTAAAATCAAGATGGTGGTAGGGCTGTGTTCCTCCTGGCAGC
EMPV1_09342	CTCAGAGGTTGGCACATAGAAGGGCACTGCAACCAAGCACCACCCATTATTGCTTTCACT
EMPV1_09343	GACCGAAAGACACCGAAAGTAACATGACAATGCAGCACTGATGAGGTTCTTCACAGGCCG
EMPV1_09344	CCTGCCGCGGGTGGAGCTAAACAAAACCCACCAAACTCACGTGGGCTATTCATACATCAA
EMPV1_09347	CCCAAGTCTCAAGATATCCTTGGGCAAGACAGTTTGCAAGCTAGGGAGGCGCTTATTTCT
EMPV1_09350	TCAAGGTCTTCTATGACCGTATGAAAGTGGCCCAGCAAGAGATCAAGGCCACGGTGACCG
EMPV1_09352	GAGAGTTTTATTTGGTTGGGGTGAGAAATCCAAGTCAGCTCGGGGAGAGGAAGGTCAGTG
EMPV1_09354	GGATGCCTCCAGGGTCCCCATCCCACTAAGAAGAGTTGTGTTGAAGTCCAAGAATGGTAT

EMPV1_09355	CATCCATGTCAGCAAACCCACTCCCAAGTATTTATCCAAGTGAGTCAGAAAGGAACAGTC
EMPV1_09357	AGAAACCACGCAAATCCCCCTTTGGTCTGTTTGAGGAAGCACGCCAATTCAGAATGCCTG
EMPV1_09361	TTCTCCCCCTGTTGAAGCTCTCTTGTTCCGACACCTATGCCAACCAGTGTGTGTTGACCT
EMPV1_09363	TGTCTGGGTTTCTGGTAAATTCCGCACAAGTCCCACTAGGTGTGGCAGAGTTCTCTCTCA
EMPV1_09365	CAGAAACCGATGGTGAGCCGGGTGTGTGAATGCAGAAGACCTCCTGAATGTGAAGAAATT
EMPV1_09368	GAGCTTTCCAACTGTGGATTGCATCCTAAAACCCTGGACCATCTGAGTCAAACACCAGTA
EMPV1_09369	AAACTGCACATATTGAAGTTAAACCGGACGGGGAAGTTCGCGCTGAACGTCGTGGACAAC
EMPV1_09371	AGAGGCTCGGACATTTCGTTGCTGATTTTTGTGGTGACCCTCCACCCTCTGATGGGGTAT
EMPV1_09373	TTGTGAGAGAAGCCAGCCCTAAAAAGACTCAGAGCACATTCTCCATCTCAGTGCCCACAC
EMPV1_09374	AGGAACCTACTCAGATTTGCCAGAGAGCACAGAGGTGTTGAGGTCAGGAGTCAGGAAGCT
EMPV1_09375	TCAACATCCCTCAAACTACATCCCAGCTTTCCCGTCTTTTCAGTACCCCCTTTGTGCGCA
EMPV1_09380	TAAAGGCTTGCTTTGGTGTGTCAGGCCCCAGCTTGTCCAGGGAGGAGTTTAAAGGCTCAG
EMPV1_09382	GAGAGCAGCTAGGAAGGGAAAGGGGAAGGGATGCAATGTGTGGGAGACAGTGAAAAAGGA
EMPV1_09385	AAGCCTAAGAAGGCACCGACAGCCCGAAAGACTGGCTCTGGGAGGTTTCCTCCCACCCGC
EMPV1_09386	CCAGTCCCTTGACCTCAGGCAGCCCTAGTGTGGCTTTCAACCCTGTGATATAGTTCAGAA
EMPV1_09387	GCTAAGCACTTACAACTGTTTGCAGAGGAAACTGAGACTTTGTAACTACATCTCAGTCTC
EMPV1_09388	TGGACCAGAGTCATGTGAGTTTCCGTCACATACCATTTTAGGGGTGTTCTCCCTCCTATT
EMPV1_09389	CTGGGGCTTTTCCGGCCTCCTGTGTGGTTTCTGTTTTGTAAGTTAAACTATTTGTCACGG
EMPV1_09392	TGCTCTTCGGACTGGTGGTGAGCATGGCACTGATCTGGCACCCCATCAACAAGCTGGCTG
EMPV1_09394	CTGGTGTTTGGGGCTCTCATTTTTTGGATAGTCACCAGACCGCAAAGGAAACGTCCTAAG
EMPV1_09395	CCAAATCTGTTTCCTCCATGTCGGAGTTTGAAAGTTTGCTGGACTGTTCCCCCTACCTCG
EMPV1_09397	CCTTGAAATCCTTCCTGGCTTCATTCTTTTCCCCAGAGGCCCAAACCCTTTCTCTTTGGG
EMPV1_09399	ACTCGAACCTCAACCCATTCCTGTCCCCGACCTCACCGGGTTCCACTCGTCTCCTCCGCG
EMPV1_09401	AATCGGCATGGGAGCCACGGTCGACATCCAGAGGCGGCAGAGGATGGAGCTGCTGGATCG
EMPV1_09402	TATACATCAGAAAATCCATGCTGGAGAAAATCCCTATAAATATAGCCCAGGTCGGAAGGC
EMPV1_09403	AGAGAAGCAGGACTCAAGGCTCTCTCAGATCCCTCCCAGTTCGAATGAAGTGTGGTTCTG
EMPV1_09404	CAATGCCCTCCTCAAGAAGATACAGACAGTTCCAGTTGTTTACGTACCAACTCTAGGAAC
EMPV1_09405	TACAATCCCCCCACTCATTAAGTCAGTTGTCCAAAGCGATGAGGGGCTGACCACCAACGA
EMPV1_09406	TAGTCTTTTGAGGAACCTCCATACAGTTCTCCATAGTGAGTGCCCCAACCAACAGTGTTG
EMPV1_09407	CAAGCTACTGCAATTGAACCCTTAACCCACTGTGCCGTGGTGGAAACCTCCCAATTTAGG
EMPV1_09409	TCAAAGGAGGACTGGTTGGTGGCATCAGATACGCTCTGAAAAGTCGAGTCAAGTGAGTGC
EMPV1_09411	TGAAGGACAACATTTTTATACAGAAGGCAGTCAAGACGCTGAACCCAGAGCCATGGAGGC
EMPV1_09412	CTAGGCTCTTTCCCGTCTATTCATACATGCCAACCAATGTCTTTCCCAGTGTCAGAAAGC
EMPV1_09413	AGTGAAGCCTGAAGTAGAACAGAGAGCTGAGAGGGGACAGGTGGGACACCAGAAGCACGT

EMPV1_09414 GAGGGAAGAGTAAGCATTTGTTGTCTTTTGTCTCAGATCCTGGGCCACCTGTGTTGGGAG
EMPV1_09415 TTCTCGGCCAGCGTCTCGATCACATCCTGAGTGGTGGCAGTACTAGAGACCCGAATGCAT
EMPV1_09416 TCAGTGCTTGTCGGGATGCCATTCGAAGCAACAACATCAACACGCTGTTCCGAATCATGC
EMPV1_09418 GTTATTTGGGCTTACTGGCTTGGATCTAAGGCTCGAAGGGAGTTGAAACGCTTGAAGGAA
EMPV1_09420 GTGACACTTTGGGAGGGTGCCTGATGAAACACGTAGGGTTGAGGAGTAACATCTGTGCGT
EMPV1_09421 TCTCTGTAGGTTGGGAGAGGGAGGGGGCAGGTACAATCTTAGCATTCTCCTGAATTAGAC
EMPV1_09422 GGTACCAGAAAATTTCCCCAGTTCTACCAAGCCAGTCCAGATTTCAGAGTCTCAGACTTC
EMPV1_09427 TTCCAGCTCTTCGGCTTCGACTTCATGGTGGATGAGGACCTGAAGGTGTGGCTCATCGAA
EMPV1_09428 GCCTACTGCCCCAAAGAGAACTGTACTAGCAAATGGGAATGTTAACCCTTGTACCTGTCC
EMPV1_09430 CTCCTCTGGAGAGTTTAAAGACAGAGATGGATAAGGCTCTCTGCAACCTTCTAAGGGAGG
EMPV1_09431 CCAGTGCAACGGGTTTGTCTGAAAATGCTGGAAAGTCTGGTGCTTGGAGAGGGTGCCATT
EMPV1_09432 GTATGGCTCCTACATGATCTGGAAAGAGCTGGGGGGCTTCTCGGAGGAGGCTGTGGTTCC
EMPV1_09433 TCATTGACTTTTCACATGGCCTCGCATTGTTTGAGCGTTGCCAAAGACCTGTGGAGCCTC
EMPV1_09434 CGGCCTTCTCTTTCAGTCTCGATGTTTTTACTCCTGAAGGGGGCCAGAGGTTTTTGTCAG
EMPV1_09435 GTATCCCCAGTTGTTATTATATAAGGTGTTTCATGTAAGGTACATAAAAGGTCACCGAAG
EMPV1_09439 CTGGAATTGGAGCACAGTGAGGTCTTCGACTGAATGTTTTCATCCCAGCTACCTTCCCCT
EMPV1_09440 AGTTCTGCCCTGCTTTCTATGAGCCAGGATTCCCGTTTAGCTCTCAAAGCGTTTGCAGTG
EMPV1_09443 CAAAAGAAGACTGGTTAAAATGTGTGACTGAGGAGAAAAAGATACCGGAACCCAATCAAG
EMPV1_09444 TTTACCATCTCGGGATTGCATGGCTTTCTGTACCGGGCTCCGGGGCCCCTTCGCCCAAGT
EMPV1_09445 TGGAACGAGTGGATTCCTGCGCCTCCATGTACAGCCGCTCCATCCAGGGACACCACGTCT
EMPV1_09448 CTCAATAAGCCTGTGACAGGGGTTCTGGTTTATCTGAGTTCAAGTAACCTGCCCAAGGTC
EMPV1_09449 AGCTCTTAGCCGTCCCCTCTCCAGGCACAAAAAGCATAGAGAACATTGCAAACGACATCG
EMPV1_09450 GTGGCACATTCTCTGGCTAACTTCTTGTAGCCAACCCCACCCAACCACCATCGTGTACCT
EMPV1_09452 CTGGGGATCGTTTACGGACAAACCATTTGCTGGATCGGTGCCTTCTTCTCGCCCCTCCTT
EMPV1_09453 TTCGTCGGGTTTGGTTCCCGGTCCTGTCAGACGCTCTAGTTCAGCCCGGGGGCTTGGTGG
EMPV1_09454 TGAGTTCTCAGGGAACTTGTATGGGACCAGCAAAGCAGCTGTGCGGGCCGTGCAGGCCAT
EMPV1_09457 AACACAAGATTAACTATTCTACCTGGTAAGGTTCATAAAAAAAGGCTTCTACACCTTCAG
EMPV1_09459 CATAAGCCCCCTTTAAATCTGGGCATGATCAGCTACCTCTGAACTGGACATTGGGGTGGG
EMPV1_09460 ATTTTCACAAGACACTGGCCCACGTCCCGGAAATTATCGAGAGGGACGGCTCCATTGGGG
EMPV1_09461 GTCCTCTTCTATGCCACGGCTCTGGTCCTCTGGCCGCTCTACCAGTTCGACCAGAAGTAT
EMPV1_09462 GTCTGTACAATGAAACTAATGTTAAGAAGGTGATCCCTGAGGGCCTGCAAGAACCTTGCG
EMPV1_09463 TACATATGGTTTTCATACTACCTTTATCCATTAGTTTCTTTATATTCCAATTATTTAAGA
EMPV1_09464 CCAGAATTTGTGGCAGAATCATTCAGAGATCAAAATATGTGATCCTCCTGAATTCCAGAG
EMPV1_09465 CGCTCAAGCCTGAGAAGAGAATTTTCCTAAATGTTGGTTTTTAAGGGGACATTCCTAGGT

EMPV1_09466	TGCGGTAGTAACACAAGTGTCAGCTCGCCCAGTTCAGGGTAGTGTATGGATGTGAGAATA
EMPV1_09467	GTGACCGTGTGCTTCGGTAAGACCGGCATCGTGGTGCCCCGCAAGGAGGGCCAGCTGCAC
EMPV1_09468	GAGCAGACGTCAATCGGTTACTCCTCTCTCATGAAGCAGTAGCACCAGCCAAGCATATGG
EMPV1_09469	TGTAGATAATCAGATATTCACTCGGATGGATGAGGTGGGATAATGAGCCCCCAGGTCCCC
EMPV1_09472	GCTAAGCCACCATGGCACTCTGGGCTTCGATTTCCTCACCTGAGTTATAAGGTCCCTTCT
EMPV1_09473	TTGATTAATTTGTCTTTCTTAACTGAACAGAAAGCAAATTTGAAGCAGCCAGCATCACTT
EMPV1_09474	TGGATTTATGGCAAATATTAAAGATGACAGGAAGATAAAAGGATAGAAAACAGTCGCTGC
EMPV1_09476	CTGCACCTGCAGCATATGTAAGGTTCCCAGTCTAGGGGTTGAAGCACAGCTTCAGCCGCA
EMPV1_09479	CAGCACCCATGGCAAAAATGGACGTCTACGACATAACTGAGGGGCCCTGCCTGGAACCAA
EMPV1_09483	ACGGTCGTCGAAGGTCGGTCACGGGACAGCCTCCGAACTACTCATCCCATTTTATACTAT
EMPV1_09489	GTTCAGATTTTGGAGATGCCGTCTAAGACGTCAGTGTGCTCCCTGAAGCCTGAGGCAGGG
EMPV1_09492	GGTCTTTCACATCTTTTGCCCAGTGATGAACTATGGCAGCCACCTCTCCCCAGATACCAA
EMPV1_09493	GGCCTCCATGAATGGGAAATGTTTGGGGGATTTCAGATAGTTTAGTATGGTGACTAGTGC
EMPV1_09495	GTAAATCACTTTTGACCTCGGACCCTTTCCCTGATTACCAGGCTCTAATCCTCCAAGGCC
EMPV1_09497	TGCAAAGAGGCCAACAGGAAAGAGTCACCATACTGGCAGGAGAGATTGAACTTGATTGTC
EMPV1_09498	GTGACAGAAAGCAGGACATCTGTTGTTTGGGGGATGAGAAGGTAAGAAAGGACCTGAGTG
EMPV1_09500	TCAGAAGCAGCAGCTGGTGAATGGCTACCGACGCTTCGATCCTGCCCGGGGTATGGAGTA
EMPV1_09501	CAGGGCACTGAACCGCACCACGTGTGGAATATCATTGCTGAAAAGCCCCAAGGCTCGGAA
EMPV1_09503	ATGTGAGGTCCCTAAAGGAGTCAGAGGCACAGAGACAGAATGGTGGGTGCCGTTGATGAG
EMPV1_09504	CATTTGAATGTGTCTGTGTCCCTATGAGCATCCTGAGAGGAGGTGGGGACCCTGGCCCAG
EMPV1_09505	TTTGGGGCCAATGGTGACACGGGCAAGCGGGAGCTGAAGCAGAAGATGCGCAACCTATTT
EMPV1_09506	AGGAAACACTTGCCCAGAATCCCTGGTAGTTGCCACTGTTAAGTTATAATCACTGCCTCC
EMPV1_09507	TGTTTGGCACGGCGTAGGCACTACGTGTTGACACATGAATGATCCCGGTGGGATAGTAAG
EMPV1_09509	ACTAAGGCGAGGAAACAGGTGTATCTGGCGCTGACGGGGCCTTCATATTTGTGCCATCAA
EMPV1_09511	TCCTCTATTCATCCATCATGTCACCCTCTCTCTGTGGTCGGATGGTGCTGGGGTCCTATA
EMPV1_09513	TTGTCGCCATAATCATAGAAGCTAGTGGGGGAGGAGCGTTCCTTGTGCTCCCTTTGCACA
EMPV1_09514	GATATTTTCGGAGTCACCCTGCGCACGTATGAAGTGACCAACCGCCTTAGATCTGAATCC
EMPV1_09516	TGCATGATATGTGCTCCACCTCAGTGGACAGAGGCCTACATGCACAGCTTCTAGAGTTCA
EMPV1_09518	CATGTTCAAGAAGTTATTCGTCAAACTCCAAGAAGGCAAGCATTTTGGCCAGACTTCAGG
EMPV1_09519	GAGTCACAGATAAGTGAGGACAGAAGCAGTGAGAAAATCCTGGGCTACTTCCCAGCACAG
EMPV1_09522	GGATGCCCAGTTAGATTTGAATTTCAGATAAATAACAAACAGTATTTTAGTATAAATATA
EMPV1_09524	CTTAATAACTCTTGGCCCTGAACTTGTAGGTGTGTTTGAGAGGAGTTGACAACCTTGGCC
EMPV1_09525	TCTCAAGATGAGTGGGGTAAAACCCAGGACGCTATGGAAGCCGCCCTTCACTTGGAGAAG
EMPV1_09529	CTGTGAACAACGTGCAGTGGTATCGTCAGTACCGAAATGGCAGCCTGGTCCACTTGTTTT

EMPV1_09535	CGTGTCCGGCTACGAAGAGTTCATACAGGCTGTGAAGCAGCACAATGGCAAGACCATTTT
EMPV1_09536	AGGAAGCTCACCGCTGGTTTAAGTGCAGGTTCGATGGGCTACAACTTGAGCTGACCAAAA
EMPV1_09538	GATCTCTTTCTGTAACTGATGAGCTTTGGGATTGTTTAAGCTTATTTTTCATGTTTAATA
EMPV1_09539	TTTTAGTTCTCAGTGCTGAACTTACTTCTCCCTTAAATTATTATAAACTTTTGCTGCTGT
EMPV1_09540	CTGAGTGGCCGAAGGAGAGGCGTGTTCTCATTCCCGAACCGTGATTCACTGGGAAAGCTT
EMPV1_09541	TGGCATCTCCATCGGAGACGTGGCCAAGAAGCTGGGTGAGATGTGGAATAACTTAAGTGA
EMPV1_09542	TAACTGGTATGAATCCTCTGTCTCCTTATTTAAATGTGGATCCACGATATCTTGTGCAGG
EMPV1_09543	CACTCTTATCATGAATCCTCGGGTCTGTACTCAAATGGCCGTCACTGTCTGGATCATCGG
EMPV1_09545	AAGTCACTACAGGAAATGCACAAACTGGCCACTGGTAAATATGACCCCCATTTAAGTGAC
EMPV1_09546	CGAGGAAAGGGAAAGGGGCAAAAAAGAAAGCGCAAGAAATCCCGGTATAAACCCTGGAGC
EMPV1_09547	CAGAAGCCGCGGGCCGACACCGTCATGGCCGCGAACCCCGGGCTCCAGGACCGAGGCGCC
EMPV1_09548	GTGTGGGCTCAGAAGTAGAGAAAAGAAGGTGCAACTAAGTGGTGTTCCTCGTTGTGGCCC
EMPV1_09549	GGCAAAATGGATACAAAGTATCTCATGGTCTTTGCAACTGATATTTCCCCAAATGCTAGC
EMPV1_09554	TTAAAAGGAAAGCTGGTCCAGAAGGCTGATCAGGAGCAGCAAGACCCTCTGGAGTACCTT
EMPV1_09559	ATATTAAGCAACAGCCGGGCTCCCCGCGCCGCATTTCCCAGCAGCACTCCATTTATGTTT
EMPV1_09560	GGAACCATTTCTTCACTGGTTTTGAGGGAACTAATCTTCTAGTTGTTCTCGATCACTGTC
EMPV1_09561	GGTCACTATGTCCCCCTATCTTAATGTCCTGCTGGTGTTGGCCACGTATCTTGCAGGCTT
EMPV1_09564	ACCACCCAGCCTTCTGGGTCCTCCCCCCATGGCCAATGGAAAGCCTGGTGACCCCAAGTC
EMPV1_09567	GGACTCTTTGGCTAAGGTACTGATCCCTCAGCTATTACTTACACATCTATACAAGATAAG
EMPV1_09568	AGTAGTGTTTCCTGAGCACTAGCCGTGTGCCACTAGCTCACTGAGTCCTTTCCAACGACT
EMPV1_09571	ATCCTTGGAGCAGTGCCATTTCTGGGCACATATTGGGCAGCAGTACCTGCAGTTCTAGAC
EMPV1_09572	AAAGTCCAGGTGCACCACCAGCAAGTCGGCCGCCTCCTCCAGCAGAAGTGCGGCTGCCGA
EMPV1_09573	GGGGACCTCTTTCGAGACCTGGCCTTTGAAACATCCAAAATGGGATTGGGGTTGAAAGTC
EMPV1_09574	CTCTAAATCCGGAGAACTGGGCAGCATCTTCCAGCTCACACCGTTGCCGCGGAGGACCTG
EMPV1_09575	ATCCTGAAAATACTTAACCACCTAAAGCCTGGTTCTGCCAGTTGGTCCCAGAGCACAGCG
EMPV1_09576	GGAGCCAAGCACTGTGCTATGCTTTGAAGAAAATAACAGTGAATAAGTCAGATGACAGGC
EMPV1_09577	GGCTCTGAGGAAGACTTCTTGGATTTGAATTCTGCATGGCCTCAAGAGGTTACCTAAACT
EMPV1_09579	GGAGGTTTGGAGATCCCTTTTGGTAATTTTCCCTCATTAAGTGAACAAAACGGGCCTGGC
EMPV1_09581	GCCACAGCGGCTCTGCATTTCGAGCTGCCTACTTCCAAGTATTTAAGGTGGCTTTAACAC
EMPV1_09585	TGTCATGAGGCCCTGGACAGTCTGGTGTGATGGCTGAGAACACTGCTTTATGTCAGATAG
EMPV1_09586	AGTGAGTGTTGTCACTTGGGAGTGGCATTCCAGGCAGAACCTTTCACCTTCGTCTGATCG
EMPV1_09589	AGGAGTTCCGCGACGAGCGGGGCCACGAGATGTTCGTCATCGACCGCTCTAAGCCGCTCT
EMPV1_09591	GAAGTCGTAAATATCATAAACTGTGTATTAAGTGGGTCTGTCAGAAGCTTATATCAAAGC
EMPV1_09592	TGAAGATGGACTGCAGCCAGGAGTACCAGCCTGTGGAATGTGCCATAGTGGTCAACGCCG

EMPV1_09593	GGTTCTCTGTTCTCAGCTCCGCGCCCACGGCAGGATCACAATTCCCATTCAGTGTTATAA
EMPV1_09594	AACCGATCAATGTGAGATTTAGCAACTGGTGCTGGGACAACTTGATGTCCAAATGCAAAG
EMPV1_09596	GATATGTATACAGAAGGTGTGGCAGATTTGGGTGAAATGATCTTGCTGTTGCCACTGTGC
EMPV1_09597	ACGCGCTCTTCCCGGCGGATGGCTGCGCGGCGGACTGCGAGAGCCTACGCGAATTTCGGG
EMPV1_09599	CTTTGCCAACTGAAAGTTCTCTTCTACAAGAAATTGAAGTACAGAATGAGGAAGTGGCAG
EMPV1_09600	GGCTCAAACAAAAGCAGATGGTAGCCAGGTTTGGCATGAAGGTCAAAGTTTGCCAGCTCC
EMPV1_09602	AAGCCTGTTTAACCCAGCATTTCCCAATCCATGTGACCCCAGGGCCTTTTTCTCCTTGGG
EMPV1_09603	GACTAGCCTCTGGGCACGGGTACAACAAACAGCAATGTTTGTCAAAGAATTCCACCCATG
EMPV1_09604	GACACCCTGGGTGGGATCCAGAAATCTGTGTTTTAACAAGCTCTGAGGCATGCCAGAGTT
EMPV1_09608	CCCCTTCTTCTGAGAGTATTCCCTCCTTAAACCACTTGCACAGGAATCCCCATCTCAGCC
EMPV1_09610	CTCTGAGCTTCCAGTCAAAGTGAATGTTCCCAAAGTGCCGATCCACAGCCTTGTGCTTGA
EMPV1_09612	CGTGTGACGGTCGTAGTGAGAAGGTGGTGATGTTTGTTGTTAATGTCTGCTAGGCAAGGA
EMPV1_09613	AGGTCCTCAGTCTGGTTTGGAGCTACTTGCTAGTTTTAAGGCCTTTGCAGAAGCTGTAAG
EMPV1_09614	GAGCCTTGGTTACAACCGACTTGTCCTACAAATAGGTAGAGGAAAGGTGGTACCTGAACC
EMPV1_09615	CTTCGTAGTATATAAAATACCCTGGTCTTGTAAACCAGAAAAGGAGGGCCACCCCTCCCC
EMPV1_09616	CAGAGAGAGGTTGAAAACAGAACTCTAAAGCATGCCACCACAGCTTTCGTCAGTGTGAGG
EMPV1_09617	GCTAGCCTTTCCTGGTGCTTCCATTGCATCCTATGTTTATCTCTAAAACTAGTGCTTTCC
EMPV1_09619	CTGGAGGAGAAGAGGAAGGAGAAGGCCAAGATCCATTACCGCAAGAAGAAGCAGCTCATG
EMPV1_09620	TGGCCACAGCCACCCTGATTTGATATCCTGCTTTCTTCCTGATGAGAGGTCGTGATGCAG
EMPV1_09622	GCAGTACTTCTCTGCCAACCACCAATGGAACCAGTAGTGGCAGTATCAGCAGCCTGGTAT
EMPV1_09623	GGCAAAATGAGCCCCTGAAGATGGTTCAAGAAGGCTTGGGTTCCTAATCCAGGGAGTCGG
EMPV1_09624	CCACTACATTGCGAGAAGGCTGTGGAGAGGATTATGGGTAAAAGGAGGGAGACAAGTCAC
EMPV1_09625	TATCTGGAGTCTCTGACAGACAAATCTAAGGAGCTGCCGTTGTACTGTTCTGGGGGGCTG
EMPV1_09627	CTGGTGTCTCTCCAGCAGAGTGAATGCGAGGCTGAGCTGCCAGAATTCTTATTTCTCTGT
EMPV1_09628	CAGCCCTCTTGGGCCTCATTGGGTATCACCCATTTTCCTTCTCACCGGAGTCCTACTTTA
EMPV1_09629	TTACCATTTCATTACCATTTCATGCCTATCCCAACCCGCAGCCGCTCTGCCTGCAAAGGC
EMPV1_09632	AGTCGGCCATGACAGCTGGTCGACCGCCAGGCAATTCGGACCCTCTATCCCTAGGTCTCT
EMPV1_09633	GTGCTGAACTTTGAGCTGCCACCAGATTATCCATCCTCCTCCCCACCTTCATTCACACTT
EMPV1_09634	AACCCACACAATCATCTTTAGAAGAGGGTGAGGATACCACCAAGGCTCTCCCAACCACAG
EMPV1_09637	GGGCTACAGTTTCCCTAATGGTAATTGCATTTCTTGGATTGTGGCTACGTTGCATCTTGC
EMPV1_09638	CAGCTAAGGCAGCTGTATCCAATCTGTTCCCAGAGTTACCTACCACCAGAGGGAAGGGTC
EMPV1_09639	CCATAGACAGGGCAGAAACAATGGCTGTTCCGGTTCCGACAAGCAAGATTCCAACTTACC
EMPV1_09640	AACCACGTCATGCCGTCCTGCTGTGAGGGTGAGGACCCCCTCATTGTGCCTGAGGTCCGT
EMPV1_09641	GAAGCCACAACAGCCACAGCACACAAGACCCTGCTACCATATATTCAGTGAGAGGGAAGC

EMPV1_09643	TTTCCTTGGTGAGTCCAGGAAGAACACGTCTGGGAAGAGAGTCGGGTTTGTCTGGCCTGA
EMPV1_09644	CCAGGTAAGCAAGGTCTCTCAAGGATGGGAGTACACTGGAAATGTGTGATTACTGCACCT
EMPV1_09645	CCAACAGCCTCCAGGTCCTAAAAGTCAAGGCACTTCATCAGTTTCTTTGGCACACGTGAC
EMPV1_09648	CCGAAGGAACGCATCCTTTTGGAGGGTGTGAGGGCTAAACCGTGTTGTCTATACTTAGCA
EMPV1_09653	GCACTCTGGTGGGTTGATGTTACTGTAGCATTTTCAACATAGAGCACTGGCTCTCACAGA
EMPV1_09656	GAGAGAGACAGATAATACCTCTTTTTCAGGATTGTGATCCTTTGTACCAAGCTTGGCATA
EMPV1_09658	AGTGGTGTGAGGGTCCAGAAGCCACGTCCCCTGAGTAGCAGCAGAAGACCTTGGCGACAA
EMPV1_09661	ATTCGAGTTGACCCCAGAGGATATGGAAAGCCTGGACGGCCTAAACAGAAACATGTGCTA
EMPV1_09662	ATGGTCTGTACCACAAGAGGGAAGGTGCCTGCATCTGTGTTCCCCGTGGCTGACCGGTTT
EMPV1_09664	ATTGAACGATGTTCCTCCACAGTTCCTCCACACTCTGGTCCCTTGTGGAAGTGATTGCAC
EMPV1_09665	CCCTCTCTTAACAGGTGGGCACCCAGGCCACTGCTACAGCTTATTCTTTTATCCTTACTT
EMPV1_09666	ATCCTTGTCAACACCTTTTCAGGTTTTATCACCGCACTTCTTCCTCTGCCACCGAGCGCC
EMPV1_09668	GTCTTCTATATGATTAGACTCACACATCAGCGAGACTAGGGGAGTCAGGAGTTTCAGGAT
EMPV1_09669	GGATGAGCTACAGGGCTGGGTTGAGGAGGTTATCAGAAATCTGAAGGTCTGTGCCTGTTA
EMPV1_09671	GGATTCACACGGGAGAAAGGCCCTACGAGTGTAACGAGTGCGGCAAGTCCTTCATCCGGA
EMPV1_09672	CCGTCTACTGCTGTTGTGGACAGCACACACAAACGATGTTCTAAAGAGTGTCAGCCCATG
EMPV1_09674	CTCAAGACTGTGCAAAAGGCGGCTGGATATGACCACTGACCGTGCTCCTTGAAGTTTTTC
EMPV1_09676	GAGCTCTCACCTTGCTTCTGTTAGAATGGCTACCATCCAGGAGACAAGAGATACCAAGTG
EMPV1_09679	TGGGTTTCTCTCCTGCATTCTTTGATGGCTGCTGATTGACAGCTGGTAGACTCGGTGCTC
EMPV1_09681	TGAAAGCCAAGGTGATGGACCTCAGCAGAGACCGCCCCCGCCAAGACACCATTATCATCA
EMPV1_09682	AACAGAGCTGTCTGAGCTTGGGGCAGAGAGCTGGAAGGCAAATCAATCTTGGAGTCCAGA
EMPV1_09683	CTGCAGATGGATATAAGGATTTTGTTTCTGTGTGCTGCTGCTTCTGCTCTTGCAATGATA
EMPV1_09684	AGAACACATTGGAACTTTGGGGGAAACTCCCACTGCACTTAACTCTTCCTACCCCTAAGC
EMPV1_09687	ACAGGAGGTGCTATAGAAGCCACAGCCAACTCAGCACACCCAGTGACTCAGAACACGCCA
EMPV1_09688	GCATCCTATGTTGGTGGCCTTTTGAATGCTTCTGTGCACACAGTGGCCACATTTAGCCTA
EMPV1_09689	TTTTGGAGGCAGCCAAGGAAAGCTGACCTTTGGAAAAGGCACTATGGTCTCCGTTAAACC
EMPV1_09690	GTACGAGTTCGAGCAGCAGCGATACCGTTACGCGGAGCTTTCGGAAGAGGACTTGAAGCA
EMPV1_09691	GTCAAGAAAACATCCAGACAAGAATCTCTCTTCGTTCCTGCCCAGCTCCGGGTATCCAGA
EMPV1_09692	GCACCATGTTGGCTTCTTGGGTCTTAGTGTCTTCCTCTGTGTAGCAAGGGGATGGGAATA
EMPV1_09698	ATCATTGTGATGGACCCTCTGCTCATCGTGCTCTCCTACACACTCATCCTGAGAAGCATC
EMPV1_09699	CGGAAGATGACAGAGAACTCTTTGAGGATCTGTTAAGACAAATGTCTGACCTTCGGCTCC
EMPV1_09700	AGCTAAGGCTGTGTTGCTTTTTGGGACCTCGGAATGCTAGGCTCTGTAGCCTCCAAAGTT
EMPV1_09701	GAGGGTACATCTCCTCCGACGAATGATACCTCCGATGCTGAGAGCAGAAGCAGCAAGGGA
EMPV1_09705	TCAGCAAATTCACCGGAATGCACCTATATGCCCTCTGTGCAAAGCCAAGAGTCGGTCCAG

EMPV1_09708	AGTCAGACTTGACCGAGAAGCCAGGTGACCAGAGTGAGCTGCGTGGGGCGGGTCAGACCA
EMPV1_09709	GGGCAGCCGTGGAAATGGAGTTGGAAATAGCACAGACAAACCACCACTGACAGAGCAAGA
EMPV1_09710	GAAGATCCAGGAGGTTCACTGTGAATGATGCAGCAACAAAACCGATGACCCAGTCTCAGC
EMPV1_09712	CACTCCTATCTGGACAGGGAAACCTCCCTTCTTCTGAGAAACATTGCAGGAAAACCTTCT
EMPV1_09714	TCCTCAGTCATCAGCTTGGCTCTTCAGCTGTGGTTTGTACTCATCTGTCCTCCTCTCAGT
EMPV1_09716	GTGGCTGCCTATAGAACAATCTTCAAACTGAGCCATGCTTTAGGAGAGAGAAAGGAGCTA
EMPV1_09718	TGACCTAGGCAGGGGGACTGCGTGAAGCCCTTCTTGAGGATTTTCGGACATCTCTGCTCT
EMPV1_09719	CGGGCCGTGTCTGCTGTGAAGAATATCAACCTGCCAGAGATGCCTCGGAACATCAACATT
EMPV1_09720	ATGTGCCGGCCCATCCGAGCCCTCACACAGGCACGAGGCCACGACCCCTCGGCCCATGTG
EMPV1_09721	TGGAGCCTCCCCAGGAGATCCACAGCCGCAAGATGGTTCTGATCAAGACTATTGAGACCC
EMPV1_09722	CAGATACTCTGAATAACCCACCGTGTCCTTAGAAACACATTCGTCTCTTCTGGATTGAAG
EMPV1_09723	GATCCCATTCTGGAGTCCTTAGAAGAATAGCCATGACAACACAGAACTGATTCTCCAGGC
EMPV1_09727	TTGGGAAAAGAAGGATTATGGACCAGCCAAGGGAGACAGGAAGAAGAACCCTTATGCCCC
EMPV1_09728	AGGGTGGTGGAGGGGTGGGGGTGGGACATTAGAGTATTCGGGATTTACAGTGCAGTATTT
EMPV1_09729	CAAGACTGCGATCCAGCTGTGCTGCGGAGAAGATGCAGAAGCTTCCCTGGAGAGATGACC
EMPV1_09731	GGGAGTGGTATGAATTGGGAATTTGGGATTAAGAGATGCAAACTACTGCCTTTGGAATGC
EMPV1_09732	AGCCAGACTGAGAAACTCGAGCCGGGAACAAAGAGGGGTCGGGCTGTGTGTGTTTCGGCT
EMPV1_09733	CTGATTCAGTAAATCTGGATTGGGGCCCAAGATTCTGCACTTCTAACAAGCTCCCAGATG
EMPV1_09734	ATAGACGCGAAAGAGCGAGGGCACCACAAAGTCCACCGCCAGGCTCTTTCTCTGCAAGCG
EMPV1_09737	ACCTTGCAGCATGCTCTACGCATTCAGTCCTTAAGGGGTTTATTTTACAAACTGTGCGCC
EMPV1_09739	TGTCTCATCTGCCTGTTTGGAGTTCAAGTGCAGTGGGATGCTGTATGACCAGGACCGTGT
EMPV1_09741	GCAGTGCTGGGGTGACCAAATGCTGTGAAACTTAATCTGAGGAGAGTGAGACTATGATAT
EMPV1_09746	CCAGACAAATTTCCATACTGTTTTCCATAGCGGTTGCACCAATTTACATTCCCAACAGTG
EMPV1_09748	AGAAGGAACACCGGCTTGGGGATCAAGAGACTCAGGTTCAAGGCCCTCTCTGTCACTCCG
EMPV1_09749	GTCGACCAACTGATGTTTGCAGCCATGACCCTGGTATCCCTTTCAGACGAGCAAGACAAA
EMPV1_09750	GACCCAGGCACACAGGAACTAGAGGTGCTGTTGGAACTATTGGAAAAGCAGAGGCTGTAC
EMPV1_09751	AGCTAGGATGGAGAGGGAGAGTGGGAAGAAGCAGAGGCAGGGAAATAATGGGGCAGATTT
EMPV1_09753	ACACAAGCCTATGGGCCTGGGGCTAGTTTACTTAAAGTGATCTGCTGTTCAGGGAAGGAG
EMPV1_09754	ACGGTGAACTTCATCAACCAGAACCTCCTCACCTACTTGCGCAACTTAGTCCAAAGTGAG
EMPV1_09755	GGCAAAATTGGGAGATTCGTGGGCTTGTGTTACCTCATGTGCCTGAGTTTCTAAAGGGGC
EMPV1_09757	CAGAGCTGCCGTATGATACAGGAATCCTACTTCTGGGGATATATCCAAAGGAAACTGTAA
EMPV1_09758	GTACAGTCTCCCCTGGAGCGACCCTATAGGCCAGGTAGTGAGGTGTGCACTCCCTCATCT
EMPV1_09759	ATGAGGACTTGGACCTTATCCCCCCCAAACGTATGGCCTCTTCAAGATGCTCCTGCTGCT
EMPV1_09760	TCTTCCGAGACCACTAACCAGGAGAGGCCTGTAAGTGGGGAGAGTAAGTGGACAGCACAA

EMPV1_09762	TCACTTCAGGGCGTTTATGAGAACCGACTGGTTCCTTTTTCTAGGAGATACCGTCTGGGC
EMPV1_09763	TAGGTAAGACTGTAACCCCTTCCAAATTAGCAGATATTTTTTAGATGCTTTGTTGTTGGG
EMPV1_09764	TGGGGCACCTCTTGCAAAGACTATGGGTGGCGAGAGGCCACTTGTTTGGGCTATTGGAGA
EMPV1_09765	GCTGGTTGGGGGAAGAACACTTTAGTAGCTGAGCATCGTGGCTCTGAATTTTCTGAAGGG
EMPV1_09766	TTGATGCTGATACCAAGGCTGGGCGAAAGGGCAAAGAGCTGGATGACCTGGTGCCAGAGA
EMPV1_09769	CAGAAGCTGAGCAGAGCCATGCGGGAGATTTTTTCAAAAGTGTTGATTTTCTGTCCATGA
EMPV1_09771	TTTGTCTCCGCGGGCTTGGGCATCATCCTGTTCTGTGTCTGTAGGCGTCTGCTTAGACAA
EMPV1_09775	CCCTGGGGAAAGATCAGACCCCCTCCAGATACACAAACATAAAAACAGTCTCCCAGCCTA
EMPV1_09776	CCCACGGGTGTGTTCAGTGCAGCAGAAGTTTCAGCCAACTCTCTGATCTTGTTAATCATG
EMPV1_09777	CTTTTAGTGTATTTTGGCCCCTTGGTCACACCAGACAGAGGAGGGGAGAGAGCATGTGCT
EMPV1_09779	CGCATTTCAGCATTTCTTCAATTGAAATACATTTCACAGTTGGTGAGTACTTAATATAGT
EMPV1_09781	GAAAGCCGCGGGTGGGAATATTGCCTGTGGAAGGGGAGCCTTTTACTACAATTTGCAACT
EMPV1_09782	ACTTTGGAAACTGCAAGTGCCAGGGGCTTCGCTCAGAACATACTCAAATGGGATGGCTCC
EMPV1_09783	TGGAAGGTGCTGAGTCTCCCGATGGATCTGTTTAATAGTGTGATGAATGTGGGCCGCTTC
EMPV1_09784	GATCATAAGGGTTAGTCAAAGCTTCTTAACTCTTGACAAAGAAACAGAACAATGGCAAGG
EMPV1_09785	CGACAAACTAGGGAAGAGAACCACAAAGAAGCAGGCTCACTGATACAGAGAACAAACTAG
EMPV1_09788	GAGAACAACCTGATCTTCTATGAATGCAGCGCCTTTTCTGGTCACAACGCGGAAGAGTCC
EMPV1_09789	GAGGCGTTGTGAAACCTCAAAGTGCAGTAAAGTAGGTCCAAGCTCCGTTCATGTGGAAGC
EMPV1_09791	GTATCGACCTCTGTGAAAACAGTGTGCAGAAGCATATCGGGCACATGAACCTCACCTTCG
EMPV1_09792	CTGTAAAACAAAGGTTCTCAGGTTAATAGGATCTGCCACGTCACCTGAAGGTAGTCTAGC
EMPV1_09793	TTGTACAGCTCCCTCTTCCAGCCTCTGGGGACCTCTCTACCTGACTTCAGAACTATTGCT
EMPV1_09794	AAAGGAGAACCTACAAGGCGAGGCAGAGGGGGAACATTCAGACGTGGTGGAAGGGATCCT
EMPV1_09797	CAAAGTTCACTTGAAGAATACCAAAGTGAGCATGGAGGCCCTCACAAGGTTCATGCAGCT
EMPV1_09798	TGACTGCTGCCTTCTTACCCCAAGGCAGGAAAAGTCCCTGAGCCAACCTGACTTCTCTGA
EMPV1_09801	ATGGTTAGGATTTCTCCAAAACAGGAGATTGACAAGGGCATGGCAGAGCCTTGAGCCTCA
EMPV1_09803	TTGCGTTCCTGAAAAAAGAAGTGGTGGGGAAGGACCTGCTGAAAGGGTCTCTCCTCTTCA
EMPV1_09806	TCAGAACTTGGTGGATCGCCGGTTACAAGTGAATCGAGGGAAACAACTGACCATGTACCG
EMPV1_09808	AAGTACACTTGCTCCTTCTGTGGCAAAACCAAGATGAAGAGACGAGCTGCAGGCATCTGG
EMPV1_09809	GGACTTCAACGCAGAGCCAACAGAGGAGGTCTACAAACACTTTGCTTCCTCCAGCCTCAA
EMPV1_09811	GTACCAGCAGACTTTGCCTAATGTGCTTCCTTCCATAATGCACATTCCAGCAGATCTTCC
EMPV1_09812	AGTTTTCCGCTGGGTGGCAACCCTGGAAATTAATGTGGAGAGATTGTCCTCATGGTGGCA
EMPV1_09813	ATTAATGAGGAAAAATAGTTATCATTTCTTAATATACATGTCAAAAATACGCAAACAGAA
EMPV1_09814	GAAGCCCAAACAGAACACCTCTTAGTAAACAGAACACCTCTTAGTATTACCATCTTAAGT
EMPV1_09817	GACAGTAGGTCGGATGCTAGCATCCATATAACACTTACTGCTATCATTATCAAACCAGTC

EMPV1_09820	ATGTCTGGACGGGGTAAGCAAGGTGGTAAAACTCGCGCCAAGGCTAAAACGCGCTCTTCC
EMPV1_09823	AAGTAGATCCGGCTCACAGAATCACAGCTAAGGAACTGCTAGATAACGAGTGGTTAACGG
EMPV1_09824	TAAATGAGGATGCTGAACTAAAACCACTTGCCCAGGGTCCCGAAGCTCAGAAGTAGTGGG
EMPV1_09825	TGGAGTTACGCACTTTTGAAGAACTCAAGAAACATGAAGAGTCTGCTATTGCCCGGAGGC
EMPV1_09826	CAACACTGTTGGTTGGGGCACTCACTATGGAGAACTGTATGGAGGTTCCTCAAAAGACTA
EMPV1_09829	GCCAGCGGGTCAAAGAAACCAAATACACAGAGTATCAGATGGACGGAGCAGCCTCCTTAT
EMPV1_09830	CAGAAAGGAGCTTTGCGGGGTAAGGAGGGGGTGCTGAGGATTGACATGGTTCTGTTATGA
EMPV1_09833	AGGAAGGCTCCTTGGTCGTGGGTGTGGATAGCATCTTTGTCCATGAGAAGTGGAACTCCT
EMPV1_09840	TCCTCTATTCATCCATCATGTCACCCTCTCTCTGTGGTCGGATGGTGCTGGGGTCCTATA
EMPV1_09841	TGGAAGAAGAAAAAGTCCAAGTCACCAAAGTACCTGAAGTTTCAAAGAAGATTGTTCCAC
EMPV1_09842	ATCGCCCTCCTAAAGACCTCTGCGATTGAGGTGATGCTTCTGGAGACATCTCGGAGGTAC
EMPV1_09843	TGTCTCGAGTGCTCAAGGAACCGCATGTCCGCCCAAGACTTGCTCTCATTGTCCCACAAG
EMPV1_09844	AGGGGGATCCTGGACCCCTGGCACCCAAAGGGGGCAAGTCCCGCAAGAAGAAATCCAAGG
EMPV1_09845	CTGGAAACTGGTCAGTCTGGGCATAGCAGAAGCCCGAGGAGTTCTACGTGAGACTTAACT
EMPV1_09847	CTCACAGGCTTCCTTTCTCGAAGCAAGTCTTCTAGACTCTGCTCACAGTGCTCAGCCACC
EMPV1_09848	CGCTTATTAAATGCACCGGGTTGTAGAGAGTAAAAGATGGTTTACCCACGTGACCTTGAC
EMPV1_09849	CCATGGGAAGACTGGGCAAGGAAATGCTTATGGATTAGAAGTAGGTAAACAGCGTGCCCA
EMPV1_09850	AGGCTCTGCCCTTGGCCCTGGACCAAAGTCCCACAGATCTTGTTGGAAGCTTGACAAGTA
EMPV1_09851	CTAGGAACTCTATGCCACAACATGGACAAAGCGCTTATGATTAAGTGAAATGTAGGATCC
EMPV1_09852	CAGAACACTTACCCAGGTTGTGGTTTTAGGACAGCCAAAGCTCACTGATGTTGATTAGTC
EMPV1_09853	ACACTGGCGTTGCACTGACTGGTCCGTCCTGTTTTGTTACCCCAGATCTTGCCAGAGATT
EMPV1_09856	TTACAAGCACCACACTTACACTTTTGCATACACACATGCATACACACACGTGGGGGCCGG
EMPV1_09858	AACGAATCTCCTTCCTGCATCCAGTCTAATGCACACCATCTTCCTGGGCAAAAGGAAGGC
EMPV1_09861	GCCCAAATCAGTACCCCAATGTTCTTGCCTTCTGCCCAAGTTTCATATCCTGGGAGGTCT
EMPV1_09862	AGAGGCGGAGCGAGTCCAGAGCATGTTTGGCCCTTTGTTCTGCGCTCCTGCGTGTGTCTG
EMPV1_09864	CCTGCTTCGTCTCTCTGAGCCTCAACTTCCTCATCAGTAAAATGGACATAATATCCTCCC
EMPV1_09869	TCCAGTAAAGCAGGAATTTCAGGAAAGCTCCCACTAATCTCCGCACACTCTCTACAGGCC
EMPV1_09870	GAGTGTGGAGGAAAGGGAACCTTTGTATATTGTTGGCAGGAACATAAACTGGTGCAACCA
EMPV1_09871	TGTCCTCTGAAGAAGGGAAGCTCTTTGTGGGAGGGCTCAACTTCAACACATATGAGCAGG
EMPV1_09876	GCGAACTCATACAGGGGAAAACCCTTATAAATGCAGTCAGTGTGGTAAATCCTTCAGTGG
EMPV1_09878	ATTTAGAAGAGGCCCCCAGTGGAGCTCAGAATGTCCTAGCATCTGAAGCAGCCCTGTCGT
EMPV1_09879	CCAAAGATTGGCATCAATGACATATTCACTACCAAGGCCGTCACCTGGAACACCACCAGG
EMPV1_09880	CCGATCGTGAAAGTCGCTATGAGGAGGAAGAAGAACAGAGTCGAAGTATGAGCCGGAAGA
EMPV1_09881	TAAAGGTTGTTGACCCCACACCAGAGCAGTTGCAGGCCTTTAGGAATGAAGTGGCTGTCC

EMPV1_09882	CCATAGTGATGTATTTTGCAGCTGTCCTGCTGGGGGGTGGTCCCTTTGCTGGTATCATTT
EMPV1_09883	GTTGTTTTCATGTGGCTGTTGTTGGGAGTTTCTATAACCATGGAAGGGGTGTAGGAAGAG
EMPV1_09885	TTCACCTCGTTTTAGTGCACATTTCCGCCCGTTCCCATCGCCCCCAAAATGGACTGTTTT
EMPV1_09888	TGTGGGATGATGGGATTATTGATCCAGCAGACACTAGGCTGGTCCTGGGTCTCAGTCTTA
EMPV1_09889	TCTAGAACAGGAGGCTAAGCGACAGAGGTTGCCAATGCCAAGTCCAGAGCGGTTAGAAAA
EMPV1_09890	GCAGAGAGACTCACAGCAACTCTCCATTATAGCTTTTACGCGGTGCGGATGTACAGCACA
EMPV1_09892	TGCCCAGGAGGCACTGTGATGAGCCAGATGCAGTCCTTCAAAATGGGATATTCATCTGGA
EMPV1_09893	AACAACTCTTGGGCAGAAGTGAACTGGGCATGAGCGGCAACTGGGAACTTTTACATTGGC
EMPV1_09894	ATCTGCATGAAGCTGGAGGAGGGCAAGTACGTCATCCTCAAAGACCCCAACAAGCAGGTC
EMPV1_09895	ATACATGGCCCTGCTACCTGCTCTTCCTTTGAAAACCCAGACTTTGCTTCTAGCCCTGTG
EMPV1_09903	TACTCACAATGGCAAAAAACCTACGAATCCCTCTCGGACATGGTAGTCCCTCGGTCAACC
EMPV1_09905	GTAGGACAAGCAAATTGAACCAAAGCTTGCCTGTTGCAAAGTGGTAGAAAATGCACATGC
EMPV1_09906	GTATCTGCTCCCCCACCACCCACAGCATTAGCCTGCTACGCCTCAACTTTCACCATACAT
EMPV1_09907	CATGTTGTGCTGGATGAGCCATTTCTTCCGGAGGCTGGTGGGCATCCTCTATGTGAATGA
EMPV1_09908	TTTGACTCCAGTCCCTTCGCTCGCCATGACGGACGGGATCCTAGGGAAGGCAGCCACAAT
EMPV1_09909	CACCAGGGCTGGAAAGGTTACTTCAAAATGAATGTGGCCAGGCAGAATAATGACAGTGAC
EMPV1_09910	CTTAGAGAATTTCATTGATTAATGGACTTTGGGGTCACTTCAGTCTTTCAGTGGGCCCAC
EMPV1_09914	ACACACACACAAGAAGAATATCCGGAGCTACCGTCACGCCGTACACCTCAGTGTGCATAT
EMPV1_09916	AGCCACTGCTGCTTTGGGATCTGGAACCTGACCCAAGACCGGGCACAGGTGGAGATGCAA
EMPV1_09918	GGCGGGTTTTAGGAACGTTCACCATGTTTGCGAGAGCACTATCGGCACGGAAAAAAAGGT
EMPV1_09919	GTAACACAGGTCACTGTTTTGTGTGTATATGAGGTGAGAATGCCGAGGATGTTATGCACA
EMPV1_09920	GGTGGCAGAAAAGGAGACAGTAAACAAGATGTCCCTGCACAACCTCGCCACGGTGTTTGG
EMPV1_09922	CAGTGGGACGGACTCCATTTTCACTGCTGCAGAAGACAGGAACTCGGGTCATTATAGTAG
EMPV1_09928	GAGCCCCAAATTCCTTAGGCCACACGAGTGAACTCTAGTCAATTGCAGGGTCAGAGCGTG
EMPV1_09930	TGGCAGTCCAGCCATCTTCCTCTTGTCCTCTGCAAAAATGGCACCTTGCTTCTCCTGTGG
EMPV1_09931	GCCTCGCTGAGTGGGTTAAGGATCCAGTATTTTGCCCTGAGTTGTGGTGTAGGTCACAGA
EMPV1_09932	GGGCTTAAAACCCAGGTCTTCTGTCCAGTGGGCTTTCCACTAAACATACTGACTATGATC
EMPV1_09934	CCAGAACTCAGACCCCACCTCTGAAAACCCTCCTCTCCCCACTCGGATTGAAAAGTTTGA
EMPV1_09938	CTGTGGCAGTATTCAGGAACGGGCTGTCCTGCACACAGACTCCTCTCTCCCTTTCAGCTT
EMPV1_09939	GCAGAGTACATGTACAGTAATGAAGTTCACTGTGTTTATAAATTGAAAAGGTACCGGGTC
EMPV1_09940	TGAGTTGGATTACGGAGCCGGCGAGGGCCACCACCTGCAGCACATCAGCGACCGGGAGAT
EMPV1_09941	GTCTTCAGAGCTACTGGCAGATAATTTTGGGGGACTTCATATTCAGAGGTCTATAAAAAG
EMPV1_09942	TACCTGATGAGGAAGTGGAGAAGGAAGAGGGCAAGTCAGCAGAGTATGAAAAGCCTGACC
EMPV1_09944	AGTTCTGATCGTTCTCGTTCTGCTACTCCTCCAGCAACAAGGAATCACTCTGGTTCTCGC

EMPV1_09945	TCAGGCTACAGGTCACGGTTGAAGACGCTGCATCCTCTGCCCATGTCTCGCTGCAGGTCC
EMPV1_09947	TACATGAGTTCAACCTGAAGAGCCGGGACAGGAATGCCTACAAAGTGGTGCGGGTCCTCA
EMPV1_09949	GGCAGCCCTTTTCTGAACATTTACTGTGACTATGAGCCTGGCTCTGAGTACAACCTTGAC
EMPV1_09953	ACAGGAACAGCGGTAGGAGCCAAGTGTGTTCTCGCAGGTGTGCTGGCACAGGCGGCTGGG
EMPV1_09955	TCTGGTAGGGAGTGTGTTCTCAGCCAGATGAAACTGCTGATGGGAAAGAAGGGGATTGGT
EMPV1_09956	TATGGGAGAAGGCACAAGCCCTAACAGCCATCTCAGACTTCAGAGCTGTGCACAGCCCAC
EMPV1_09958	CCATGCAGCTGTCCCCAGAAATCAAGCCGTGTGAGAAGAGACCCAAAGTATACCTGTGCA
EMPV1_09960	AGCTTTATTAATTCTCACGCTAGCACCTCGCAAGGCGATGGATGTGGCCGCTAATTGCTC
EMPV1_09961	TGGTAATTCTGGAGACCTGTTCCCAAGATCCAGGCCAAAACGTCCAGTCAGCTGGTGAGG
EMPV1_09965	CACCAGGTGCTTGAAATTAGAAGACAAAGATCAGTCCTCAGGAAGTATTAATATCTTCAC
EMPV1_09966	GTGGAGTTTGACATGAAGCCAGTCTGCAAGAAGTGCTATGAGAAGTTTCCGTTGGAGCTG
EMPV1_09969	AACTGATGAGCAGCCCAATTCTACGGCCCACTGAGGCCCCGTCCCCAGAAGCTGAAGCCA
EMPV1_09971	TCGATCTCTACCTGGCCTACTTCGTGCGCGAGCGAGGTGAGGCGGGGCGTGCACGTGGAC
EMPV1_09973	AATCCAGGCTTCTCACACCCTTCCTGTTAGTCCCATTAGCCTTTCAACCAGCCCCCATGA
EMPV1_09976	TACGTGGTAGTTGTAAGTTCTTGCTGTCGTTGTAAACAAGCTCCCGCCGAAGGGAACGCC
EMPV1_09977	GGCTATCAATGACCCAGACTGCTCCTTTTACACCATTGGAAATACTGTTGCTGATCGGGG
EMPV1_09978	AAATCTGCTCCAGCCCCCAAAAAGGGCTCCAAGAAAGCCGTGACCAAGGCGCAGAAGAAA
EMPV1_09979	GTGCAGAACAGATCCCTCCTTGCCTGACAAGTGAATCTTCTCTCCCCTCTCCTTGGGGTA
EMPV1_09980	CACACAACAATGAAAACCCTAACGGTGATGATATATTTCGGAAGAAGTTGCTGGTGGATG
EMPV1_09981	GAGCGCTTTGCTTCTCTTCAGCAAGCTCAGGATCTTGTTTCCTGCCTGGGGGGCCCTGTC
EMPV1_09983	CGTAGTCGCCACATTCTGCCGGCGAGGAGGTCACACAGACGCCGAGGCGGCCAGGCGGGA
EMPV1_09985	GGAGACAGGTTTACTTTTTACCAGTTTTGAGCATGTGCGCCTAACCGACTGTCTTTGCCC
EMPV1_09986	CATGTTACACCAGATAGCTACATTCCATGCCTTGCTGACCTTTGCAAAGCACTGTGGGAG
EMPV1_09987	TGTACACCCACAGGCTCAACTCACTAACTCAGGCCTTTCCAGAGCTTCTCTGCCCCTAGT
EMPV1_09990	CGAGGTTGTTTATTTGGTGCCCGAAAAGGACATGTGGACTGAGAGGGAGAAGTGGCGATT
EMPV1_09991	AGATGAAGATTCTCCTGCTGAGTCTGATACTTGGTCTGGTTTGGGCCACGGAACCTCAAC
EMPV1_09992	TGAGGATTTCCACCAGCCTCCCGAATTTCCCTGCCTGAAGATGCTCTTGGCTTACCTGGA
EMPV1_09994	TTGTGCTTTCAGCTTCTTTACAGTGCTGCCTTGTAGCATTCAGGTCAAGCAGCATTGTAC
EMPV1_09997	ATTGGGGAGTACTTGCAGATAAAAGGTGCCACGCCTAGGGACTCCGGCCTCTATGCTTGT
EMPV1_09998	GGACCCAGGGATCAGTCATCCAATAGGGCCATTACTGCAATGATCAGCCACAGCTATTGT
EMPV1_09999	TTGAGAAAATGAAACAGCTCGACAAGAACAAACTACAGGCGGTTGCCAATCAGACTTATC
EMPV1_10003	GCTTTCCTGGGCCCTACAAGGATCAGGTCCCTGACTTGGTTTCTGCACATCCATTTGAAA
EMPV1_10011	AAGATTAAGTCGGGAGAGGAGGACTTTGAATCTCTGGCCTCACAGTTCAGCGACTGCAAC
EMPV1_10013	CTGGATTCTGGACAGACCCAAGATGGTCCCCTTGTTGTGAGTTCTAATCAGGGATGCAGA

EMPV1_10014	ATGACGAAGTCACCTTCTGACTCTACTTAATTGCAAGGTCCCCTCAAGATCCAGTCGGGG
EMPV1_10015	GTGTAAACAAGACACCAAGCCCAATCAGTATCTATGGGAGTAGGAAAGTCCACGCCGCGG
EMPV1_10016	TTCAACATGCTCCTGCTGCTGGTGCTGTCCTGGGAACTCTTCCTCCTGTACCCTCCAGTA
EMPV1_10018	GTTGGGCAATGTGGAGGAATGTTACCGCTACTTCACCAGCGTTCTATTTTGCCACGGAGT
EMPV1_10022	GAGCTGACAGCTATTTTGAAATTTGGAGCAGAAGATCTCTTCAAAGAACTTGAAGGGGAG
EMPV1_10023	CATTTCCTCGTGTGAGTGATCAGTTTTACTGGGAACCATTTAGCCCAGTGACGTACAGTG
EMPV1_10024	ACTTAAAAAGAACCTGAGCTGGAACCGTGCCTGGCCTTTTCAGAATTAAACACGGTGGCC
EMPV1_10025	CACTCAGATGCCCCTGTTCAGAGTTTTGGGCACCCCTCCTCCCATCCTCAGGCTACGATT
EMPV1_10026	AGGGGAGAGAGAGGAAGCGGTGAGCAAAGCCACCAAGAGACAGGAGATAAAGATGGATGT
EMPV1_10027	GACCCTTCTCTACTCCTCAAGCTTGCCTGTTCTGACACTCTCACCCATAACTTAATCATG
EMPV1_10028	GTTTTGGTACCCCAGGTGGCTCCTGGAACCAATCCCTCATGGATACCAAGGGGTAACTAT
EMPV1_10030	TCTCCGAAGAGACAGGCTGTGTTGAAGGAAGCAGCCACCGGACCAATTTATTGTTTATTT
EMPV1_10031	GGCACACTTTGTCCCCATTCCAGCATATCTGTCCAACTGCACACCCGTTGAGAAGACCTG
EMPV1_10036	TCGCTATGAACAACAGTAAGCCCTGGGGAGAAGGGAATACCTGATTTCTGGAGTTGTGAC
EMPV1_10038	AACTGCTTCCAGACTGAGCTGGTGGTGAGAAAGGAACTGAAACTGACTTGCCCATGGTCT
EMPV1_10040	GAAGACCGGATCCCCAACCCACTTCTCAAGCCCACTTTGTCCATGTCTCCACGGCAACAG
EMPV1_10041	CATATTACCCATGCAGTCCTGAAGATAAAGTCAGCCCAGGGGAGGCGCAAGGCTTTTGGA
EMPV1_10044	GTGGGCTCCGAGGAGGAGCTCTATGACTGCCCGGATTACTACTACCTGCGCGACTTTCCA
EMPV1_10045	GAGACTGAACATTAGCCAGAGACTGCACTACCATCACCTCGATGTAGACACAGCACACAC
EMPV1_10046	GCAAGGATGTCATTGAGGAGCAGGGCAAGAGAAACTTTTCCAAGGCCATGTCGGTGGCTA
EMPV1_10050	GATTCTAGTTGTGGGTGTGGTTGCTTTGGAAATACCAGTGCTGTTCCTACTCAGCACCGC
EMPV1_10052	TTCGAAGCTGTTCGTTTCGCTCTCCCGTCAGGAAAAACCAAAATACTGCCCCCAGCAAAG
EMPV1_10054	AAGATAGTCTTTTTAGGGAGTTCCATGAGGATGCAGGTTTGACCCCCCTGGCCTCACTCA
EMPV1_10055	ACCAGCAACCACGTCAGCTACAATGGCCTGTATGGACTGGCAGTGTTCAGTCAGAAGGAT
EMPV1_10058	ACAAGTACAGACCGGAGACAAAGCAAGAGAAGAAGCAGAGGTTGCTGGCCCGAGCTGAGA
EMPV1_10063	TTGCAAATGACAGAGCCAGGATTCACACTGAGGCAGTCAGGTCCCAGAGCCTAAACTCTC
EMPV1_10064	CTGTAGCCTGAAAAGAACAAGATAGTTGGCAAACAGCTGAGGTCTCATGGATGCCGTGCC
EMPV1_10065	CATGAGACATTTTCGGGACTGCGGTCGTAATCTGGTCTGTGTAGGTTCCTCTGCTTTCTG
EMPV1_10066	AACTTTAACACACAGCACAAGGGAGAAAAGGGAGTATCAGTCATGTGGCCTGGGAGACCC
EMPV1_10067	GAACTGCTTAACGATGTGGAAAGACTGAAACAGGCGCTCATTGGCGTTTCGCAGCACACC
EMPV1_10069	CAGGTGCATATACCCACTTAGGGCTTTCCAAGTCAATTCACACACATTCTGTTAAACGAG
EMPV1_10072	CAGGAACACAACAGTGTACGAATGAGGAATGAGTAAGCCAATGCCAGCAACCTGAGGGTC
EMPV1_10073	GGGCCTGCCTTGAATATGCCAAGAAAGAATCACCACTTTGTTCTACAGCATTGGATCCCA
EMPV1_10074	GTAGATGGCATGGACCTGGGCAAAAAAGTCAGCATTCCCAGGGACATCATGTTGGAAGAA

EMPV1_10076	TGGAACAAGAAACTCCTGTATGAGAAGATGAAGGGAGGACAGAGACGCAAGCGGAGGAGT
EMPV1_10080	GCAGCTGAAAGTAGAGCAGGGCTTTCAAGTGAGTAGTAGGATGGGAGAGTGTTCTCAGAG
EMPV1_10082	TTGTCCTGGGAAGAGTAATTGTTTCTGGGAAGCGCAACATTAATTACGACCCAGTTTCTC
EMPV1_10083	GCTCACAGTGCACTGTTAGCACACACTGCCTTTCTGCCCCAAGTTCTCTGTACATGAAAT
EMPV1_10084	TCCCTCCCAGAGCAGCAGCAGCGAGAGGAGATGAATAACTTAGGGAGGCCCCTTCGCGCC
EMPV1_10087	TCTCCGTCTTTCAGCGTTACAAAGTCTCCTGTGGCCCGCATTGTGCCAAGTGGCACTGTC
EMPV1_10092	CATGTAATCATGGGGTCACTACTCTTTGGGAAGCCACCAGTGTTAAAGGTGGGGAGTACC
EMPV1_10094	CAGGGCACTCCTGTGGTATGGGTTTGATCCCTTCCCCTCAAATATTCACAGTGGCCTAAA
EMPV1_10096	CAGCCCCTGTGCCTGGGAGATTGTCAGAGTAGAAATTGAAAGGTGCTTTTCCCTTACCTA
EMPV1_10097	AATGTGGCTCAAGGACCTGCAAACTAAGAATCACCTGGGAGGAGTTGCCGTTGTGGCTCA
EMPV1_10099	AAAAATATCCTCGGAGTTCCCGTTGTGGCGCAGCTGAAACACATCTGACTGGGAACCATG
EMPV1_10101	CCTGACTGTCGGATGCTCCAGCGGCTCCAGCACGAAGGAGCACTTGGAGGACATCAGGGA
EMPV1_10102	TGTGATCATGCTGGCCCCGTGACCCGCGGAGCTACTCGGGAACTCCTCGAGTTTGCTTTT
EMPV1_10104	CCAGGGCACCTGAAAGGCCGCGAAATCGGCTTGTGGTACGCGAAGAAACAAGGACAGAAA
EMPV1_10105	TGTGGACCAGGAGCTGTTTAACGAGTACCAGTTCAGCGTGGACCAACTAATGGAGCTGGC
EMPV1_10106	GAGAGAAGGACAAGCCAGCTCCATACCCTTTGAGACATCTGCTCCCAGAGCTAGAATACT
EMPV1_10107	GTGTGGCACTGTGACTTCTGAATGAGGGACACGGGAAAAGGCTTTGTAAAGAGCAAAGGG
EMPV1_10108	CTCTCTTGAAGTTGGCTTGTGGAAATGAGACATCTTCTGTCACCTTGGGTGTGATCCTAG
EMPV1_10111	TCATCGTCAGGCGCTGTCGACCAGTCGTCCTGCACAGGGACGCCTCTGTCATCCACAATT
EMPV1_10112	AGAAGATCTCAGAAAACATGAAGATAAATTGAACCATCATCAGCGAATTGGGCTAAAATA
EMPV1_10114	CACAAAGTTACCACTATAACAACGATTAATAGGAATTTTTCTGGAATTTAAAGAATGGCG
EMPV1_10117	CAAACTGCCAAAGTACAGTGTGGGCCACAGGTAACCATAGTCACTCCAGAAACCATGGAG
EMPV1_10120	CACCATTTACTGAAGAAACTATCCTTTCCCCGCTGTATATTCTTGCTTCCTTTGTCATAG
EMPV1_10121	GAAGAAAGGCGTGGGTGGCATCGGGCCGCCAGGCTGCCTCCTTAAACCCATGACCTCTAA
EMPV1_10123	GATACAGGGACTTCATGGAGTCACTCGGATACTTCTAATGCAGGGGCTGTGATCAAGAGG
EMPV1_10125	CTCTGACCTGAGACCTCTGGGTTCTGAGCTGTGATGTTGCTTCCAAGCTGGGATCTCTGG
EMPV1_10126	TCTTGAGATGTTTATATGAAGAGAAACAAAAAGTTCATGTCCATATTGGGGAGATAAAGC
EMPV1_10128	TCCCGTGTTCCTTTCCCTACTTTCTCTTGTGCCAAACTGACAGAAACCATCACCAGACTG
EMPV1_10130	GCAGGTACATAATCACTTCAGGAGGTACGAGGTCGAATACCTACAGTTTGCTTTCCGTTG
EMPV1_10131	ATGTCACCCAGACTCAGGAAGACCTGGAAACTTAGGGGCCATGTGAGCCACGACCATGGT
EMPV1_10133	AGGCGGTTTCCCAGACCTGCCGAATCAGAATTACTGGGGTAAGAACCAGAAAGTTCGTTT
EMPV1_10134	AGGTTGAAGTTCCGACTAATACCTGCGAAGTCAACTAGTGTGGACGGGGTGTTTGCTGCA
EMPV1_10137	CGGGTCCTGTTTTCCTACTTTGCCTTTTGGTTTCTTCTAGTTAATACTGGGAGTTGCCAG
EMPV1_10139	GAGTTCCAGACCAGTGTTGTCTCCCAGGACATTGACACAGCGGCCAAGTTTATTGGTGCT

EMPV1_10140	CAGGTCAACCATCTTCTCAACCATCCTCTCAATTTAGTAATCCACAGTGTCCTGCTCTGT
EMPV1_10142	GCTGTTGGTGGTGTCGCTTCCCTGGTACACACATCCTTGGCAATTCAGCTTCCTTTCTGC
EMPV1_10144	GAGAGGCTGTGACCCGGTTCCTAATGAGCTGCACAGAGTGTCAGAAGAGGATGCACTTTA
EMPV1_10145	CTCCTGATGTTAAATGACCAAAACGAAGAGTGCCTGAGCAGGGTAAACTACAGCCACAGT
EMPV1_10146	CAGATGAATCCTTGTAGTGGAGAGATCCACCAAGATAAACCAGAAGTTATGGGCAAGGGC
EMPV1_10147	CTTTCTCGCTTGGAGCCCTCAGACCTCTCGGAAGACTCTGACTATGACAGTGTATGGACA
EMPV1_10150	GTGGGACACTACATAATTTCCAATGTTTTTGAAGCAGCACAGGTTGGAGAACCCCCAAAA
EMPV1_10153	AGTTTAGAATTGTATCAAGGGAGATAACTGTACAGCCTCCTAGCTTTCCTTGGGTCTTGC
EMPV1_10154	GTGGTGGAGGTCTTTTGCATCTTGGCTTCCAGTGCTGGGTTACTGGGGTACATCTTTGTT
EMPV1_10162	GCTGGTTTTGCAAGCCGTGAACATTAATCCACTACCTAGTCCTCAAATGCTCTCACTTCC
EMPV1_10164	CCCAAACAACTGAATTTGCCTGGTCAGTCTTTATCATGCCCCCCACCAAGTGTGTGATCT
EMPV1_10165	CCCTTCTTACCTCAAATTCTCTCCCACCTTACCTCCATCAGGCTATGCTCCTGTAAAAGA
EMPV1_10166	TGCCTTCACGAAGCATCATTCTAACAGTCAACAGCCAGTTCCCAAGATGGACACATGGAA
EMPV1_10167	TTCCTGTCTCCTCTTTTATTCCCCTCTACTGATCCACACCCAAGAACTTCTACCTGGACC
EMPV1_10169	CAGTAGTGGACACAATCGTCTGTATTTCCATAGTGATACTTGTTTACCTCTCCGTCCACA
EMPV1_10170	CTTGGAGACCTTCTATGTGACCAAAAGCCGGGATGCCCTGACAGAAACCGCCTTGGAGAT
EMPV1_10171	AAGAAGAAAGGGGTCAGAAGCGAAAACGAGAACCTGAAGACGAGGGAGAAGATGATGACT
EMPV1_10173	CTCAGAATCAGGACGATGGGTATCTGTCTCTCAGTGCCCGGACAAACTTGCAGTACCGAA
EMPV1_10175	GTGCAGGTGTGGTGGATTAGAGGATTCTGCATTGCCACAGCTGTTTCTTGGGTCACAGTT
EMPV1_10176	TAGCTCCCCGAATGGGCTTAAGAAGAGGTGGTGTTCGAGGTCGTGGAGGTCCTGGGAGAG
EMPV1_10177	AGGCCTTAGCGGTCCACAGTTGACTGAGTAGCCACAATTTTAAATCTCATTCCCAAAGCT
EMPV1_10179	GATTTATGCAGATAGTCACAGCTGATCTGGGTAGTTTAGGCCCCAAGTGGAGGGATCCAG
EMPV1_10180	AATTGCATCGTCCTGTTTGCTGCCCTGTTCGCCGTAATCTCCCGGCACAACCTCAGTGCT
EMPV1_10181	TGTTAGTGCTCTGGTTTCTCCACATCCTGACCATCCTCTGTCTTCGTTACAGCCACCCTG
EMPV1_10183	TGCTGTTTGTGTCATCATCTTTACTGGAATTTTGTTCAGAATCTTAAGGAGAAGGCAGGC
EMPV1_10185	GATCCTAGCCTCATTCCTGAAGCAACCCAAGGAGGTACTTACAATTTTGACCCAACAGCC
EMPV1_10187	CCTACCTCTACCCCACCCACTCCTCCATGTTTCACTACATCTATTTGGCAGCAGTTTCTC
EMPV1_10188	TCATCGACAAGCTGGAAGAAGATATCAACAGCTCCATGACCAACAGCACGGCGGCCAGCA
EMPV1_10189	CCTATTAGAAGGTCCACATCATCAGGGCAGTACACAGGCTTCAATGACCACAAGCCACTG
EMPV1_10190	CTATGATCTTTCAGCTGAGGGCAAGGGCCCCAGCAGGACGCTAAATCGCATTGGCCGTCT
EMPV1_10191	GGAACCTGCGGTGTGGTGGTCTAGTTGCTGGAACACAGCAGGTCACTCTCCCACGAACGA
EMPV1_10192	GGAAACTGCTTTTCAGGGTAAAATTCACATACCATAAGTAGCACCACTTTCCTGCGCAGC
EMPV1_10193	GGAAAGTTCGGGCTAGTAGGGAAAGTTCTACAAGGTGCTTGGTGAGGATGTCCTTGAGAC
EMPV1_10196	GGAGGTTCAGCACAAAGCTGACATTTTCAAGCGGCAGATCATCCAGAGGAGACAAAACCT

EMPV1_10197	TGTCCTTCCTCTCACACGCTGGCTCTCTTGAGCACACGTACAGCCCTCACCTTTATCCTT
EMPV1_10200	AGGAGGAGCAGCAGGGTGAAACTGACACAGTTCTGGTGAGTTTCACTTTGCTGCTCCTCC
EMPV1_10201	ACCATTAGATCTGCTAGGTCCTGTGGTGCAAGTGACAGAGAAGTTTGTACAGCAGCCTGG
EMPV1_10202	CTGGCAGCTCTTCCTCTGATGAGTCTCCATTTCCTCTGTGGATCTTCACCCAAATAGTAA
EMPV1_10205	CATTGCAGTGAGGGAGACCACATATCATGAAGAACCCCAGGCAGCTCAGCAAGAGACAAA
EMPV1_10208	AATCATCTCAGAAGGACAAGGTCTGCCAGTTCATGGCATTTACTCGGGCTGGCAAGAGGA
EMPV1_10211	TGTTCAGAAAAGGGTCAAGGGGAGCAAGGGAGAGATGAGCCATGTCCCTAAGCCTATCTT
EMPV1_10213	TAAAGGCAGATCCAGTCACTGGTCACCAGTAGTCGGCCCAGCAGCACGGACTCCAGCTCA
EMPV1_10214	TTTCTTCCTGTTCCCGTCCTTTCTTCGCGACTCAGATACTGTGCAGAGAACGTCATCCAG
EMPV1_10217	CGTAGGGAATAACGGATAAACAGGCTGCATGGATCACAAGTTAATGTCAATTGATCCTCC
EMPV1_10219	AAGCCATTGCAGGGCCAAACTTTACAAGGTCACATTTTGCAGAGGCAGATGATGCCAGGC
EMPV1_10220	AAAGCCATCCACTTTCTAGATGTGGAGCAGGGCCGACTGGAAAGACGCATTTCCAAGGCT
EMPV1_10223	CACCCTTTCTGGTACTCCAGCTTGGGCTTGAAGGACTCTCTGGGCAGGTAAGAGTGATAA
EMPV1_10224	ATGTTCGCTTCCTTCCACCCCTCTGCCAGCACCGCAGGGACCTCTGGGGACGGTGAACCC
EMPV1_10227	TCAGAGGTAAATTTTCTGCCAACTTCCTGGTTTACTCCGGTACCCACCACAGTCCCTAGC
EMPV1_10228	CCCTTATGCTGAATTTCCTGTTTTGTGGCTGCCAGGTCTTCCTCATGCCTGGTTTACACT
EMPV1_10230	CTTGAACCTGCGTCCCAGCACTCCCAAGATACCACTAATTCTGTTGTGTCACAGTGGGAA
EMPV1_10231	TTCCTCATGAAAAGACTTGGGAGGGGGCTTGTTGTGCTCGCTGAGAAATAACTGCACGTG
EMPV1_10232	GTTATCCAGTCCATGGCTCGCTTCATGGCCGCCTATTTCACCAATCAGCCGCTTTGCATT
EMPV1_10233	GCACGTCCATCCTCCTTCTATCCTCTTACAGAGCAACAGGCAGCGGATTCTATTTACTAC
EMPV1_10235	CATAGCAATAAGAAAGAGGCAGAGGAAATCGTCAGAAAAGCCCTCCTTGCAGGGGACAGG
EMPV1_10238	AATAAAAAACCCATGAGCCTTGCTTCAGGCTCAGTACCAGCTGCCCCACCCAAACGCCCT
EMPV1_10239	GCTCACATCAGGTGAAAGCTCACGTTTTCCAGGAACTTTGGAGATTGAGGCTTGTGGCTC
EMPV1_10240	GTCTATTGCTCAGAAACACAGGTCAAGTCACTGCATTTTCCCCTTTCTGTGCCTGCCACA
EMPV1_10241	CCTCTCTCAGCCAGTGTATGAACACGTAGCATCTTTCCTGTCAGTCTTCAAGCTAGTATC
EMPV1_10242	GATGCCTTGATTGACACGTATTTGCCTGGAATTGATGAAGAAACCAGTGAGGAGTCCCTC
EMPV1_10243	AACTACACCTTCTGGCAGTGGTTTGACGGGGTCATGGAGGTGCTGAAGAAGCATCACAAG
EMPV1_10244	ACGGGAAGAAATGGCAAAAGGGAACGAAGAAAGACCTGAGTGAACTTTGTCTGCAAGGGC
EMPV1_10245	ATCACATTAGGAAGGAAAGACCTCCCAGAGATCCCGCCGTGGCACAGTGGGCTGTTGTTT
EMPV1_10246	TTCCTTGACTCTGTCTCGAAGGGGAGTTAGGAGTCCCTTACAGGTTTTCTCCTGGTAGCG
EMPV1_10250	TCGAGCAGAAGCAACCCAAGGCGCGGGGCCCCACATCCCAGGATTCTATCCGGAACCAGG
EMPV1_10251	CACAGGGATAAGGAAGATCTGTCAAGAATTATGCTCTTGGAGAGTTGACTGCTAGGAGGG
EMPV1_10253	TGGCTATGACACCAAGGAGGTCACCTTCTATGGGGAAAATGGGGGTGATGCTCGGCAGCG
EMPV1_10254	CAATTATGACCAGAAGTCACAGGAAGTGGAGGACAAGACCAGGGCCAATGAGCAGCTGAC

EMPV1_10256	AGTTTTGTGGTTCTTTTCCTCAGTCACTCCTGACTCCAGGCTAACTGGAAGGCAGCACCT
EMPV1_10259	AACATCTGCCCTTTCTTCCACTACTCCAAATGGAACAGACTCAGCCCAGACTGCAGGGTG
EMPV1_10260	TTTTGTGGGTGAAACTTGCTGATCCCCTGGCTAAACTCCAGACCGTGCATGTGCTCGACT
EMPV1_10262	TGGAATTATGCTCACTCCCATCCCCTGTTTCACTCTGGACCCTGCTGCCCTATGGTATCA
EMPV1_10263	GAATTCTGGGGGTGGAGTCCAGTAACCTGCATTTTGACAAGCCCTCCATGTGACTCTGAT
EMPV1_10264	TGTGATGGGAGCTGAGACAGCCCTTTCTCATTATTGTTTTTATTGACGTTGCTGCTTTTC
EMPV1_10265	GGGCCCTCGGAAAGCAGATACTGTGGCAAGAGAGAAGGTCACTGAATTGATCCAAGTCCA
EMPV1_10266	TGTGGTGGCCCCTTCTGGACAAGGTCTGCTCCCTTCAATCAAGACAGACATGAAGGAGTT
EMPV1_10268	TTTATTAATACCCCACGCCAACACCAACAATCTTTCCATGCGTGAGGTCCTGGGGTGAGG
EMPV1_10269	GAAGTGTAGGTCCTCCAAGTTCTATACATAGTGCAATCATACATCAGAGTCCCTTCTTTC
EMPV1_10270	TGCAGCCCGCAAGGGGCATTGATTCCTGTAGGAGTTGCCAGTTTATTATGGGGGCCTAAT
EMPV1_10272	CCAGCTGCGGCGTCCTGGCGTCTACAAGCCCTTCGTCATCGGCGTCTCACTGATGATCTT
EMPV1_10273	ACACATAGAGGGACAGCCTCACCGCGCAACCAGGAGCGTCAGCTGCACCTGAGGCGATCT
EMPV1_10274	ATCAACCACTTCTACTGTGATGATGTTCCCTTGGTTGCTCTGGCCTGCTCGGACACTCGA
EMPV1_10275	TCAGTCCATGGACATCTCCTTGAAGGAGAATTTGCGGGAGATGATTGAAGAGTCTGCGAA
EMPV1_10280	GCTGGCAAAGCTAGGAATATGCATTCACTGTACTACCTCAGAGTCTATCAACTGTCCAGG
EMPV1_10281	CCTGAGGAAGTCAAATTGGTATTTGTGACACAGAGAAAGGAACACATGGAGACACATGGC
EMPV1_10282	CCACTTCTTCTCTGGATTGACCATCCCTGATGGGGGCGTGCATATTGTAGGGGGTGAACT
EMPV1_10285	CCCGCAGACAGCTGGAGTTTACACCTCGCTGATGCCACAGGTTCATTGGGAGAAAAGTAT
EMPV1_10286	CTGTTGGCCTCCAGCCTGGGGCAGCCTCCTGCTCATCCTCTGGTGCTTGTGAATCCGAGT
EMPV1_10287	GCGAGGACTGGAGGTTTCAGAAGACGAGGCAGACCTGGCTGCTGGTGCACATGTACGACA
EMPV1_10288	GATGAATCTGCCAAAAAGGATGATGAGGTGTTTCGCACAGCAGTTGGCGACCTCAACCAG
EMPV1_10289	TGTTTCCTTTGGGCTGCTTGTCGGAGGCTGAAGAGCTAGTGGTGGGTTCTGCAGCCTTCA
EMPV1_10290	TTTCCAAAATCACATTCTGTCCTCTAGGGAAAACGGGGCTCTCCACCTGCACACCGGGCC
EMPV1_10292	CAGTGCTGTGTTGCCCTTTGTCTCCTGTTCCCCACTGTCTCCCCTTCTTCTAAAGAGTAT
EMPV1_10294	TTGTACAGAGATATCCATACCCAGCTCATCCTCAAGACAGATTATTTTTGCTCACTGGTG
EMPV1_10297	TAGGAGCTAAGCCGTAAAGGTGGATGGTGCCCTGTTGTGGAGGCAGAGGAGTTTTTACCT
EMPV1_10301	TCCTCCAAGGGGTTGGTCAGAAGAGCTGGATGAACGTGGGCATACCTTATATACCAGTGA
EMPV1_10303	CAGTGTTCAGTTAGCAGTGAAGATATCGAAAAAAGCTTCAAAACAAACCCTTGTGGCTCC
EMPV1_10305	TTGAAGGAAATTGGGGCAGGTGCAAGGCTGAAAGCCAGGGAATTGTAAAGGTCAAAGCCC
EMPV1_10306	TGTAGTCCATGTAATTGGTGGTGGAGGGAGAGAGTCGTGTAGTTTGAGTCCCTGGATTTG
EMPV1_10307	TGATGGGTCCACCTGTAAAAATTGTGTTTCTCAGCAGGAGACTCTGATGCGTGCTCAGGC
EMPV1_10309	AGTAACGGCACCTAAATTCCCCTCAGCCCGGCCTGAGAGTCTCCACAGTCCCAACTCCAT
EMPV1_10310	AAGTGCTCAAGATATCCCACGGTATGAGTCAGGGTCCCACTGCTTTGCCATCAGGGTTCT

EMPV1_10312	AGAGATAAGCCTGAACTCGTAGGTGTGAAAGTTGGTGTCCGAACCAGGGGTTGTAATGGC
EMPV1_10313	TGGACAACTCGGCATAAGCACGACTGGTTCCGCATCTCTGAGGCTGCATCAGATCCATGA
EMPV1_10315	TTTGTGAGATATAGCAAAATCAATTCCAAGAGGGAATTTCAGAGGGACAAATGTCTAATG
EMPV1_10317	GGACGACCAACTATTCTGGTTTGTCTGGGACTGAGAGGTTTCCTAGAATGTGGGAATTTC
EMPV1_10318	GCTTTGTGGGGCATGCTGATTAGATAAACTGGGCAGACCACAGATTTGGGCAGCAATCAG
EMPV1_10320	TCCACCTATGAGGAGGCCTTAGGCTACTTTCACAGGGCAGAGCAAGTGGATCCAAACTTC
EMPV1_10321	CAGTCAGAACTGCCCTGGACTAACCGCCAAATACTGTGTGCATTCTGTGGGAGGCTCATT
EMPV1_10322	TCCAGCCTCTCATGGCTTCTCTCCAAAGGTCTACGAATCTGTCTCTGGTGTCAGAACTTC
EMPV1_10323	TGGGATTTTCAGTTAAAGCAGTTGCATTCCAGAGGACCTCAGAGCACCCTCAGAACCCCT
EMPV1_10324	GCTCTTTGGTTTAGGTATCCTCATGGCAGGTGCACCTGTGATTCTCATTGTCACCTCCTA
EMPV1_10326	CTATTCTTAATAGTGCACCACCTGATTGTGACCCAGACACAATTCACCCTGGCAGCTCGG
EMPV1_10327	GACGACAAGTCTTCCTACAACCCAACCAGACAACTATACCAGCTTCTTCCCCGAGTACTG
EMPV1_10328	AGTGGTCGAGAGGGCAGCTTGATGGCTCATTGCTCCCACTCCTCCAGCAGAGCACGAGCT
EMPV1_10329	CAGAAAGACTTCCCAAGGGGTGGTTCCAAGTTTGTGCTGAAGCAGAGAAGGGCCCACATT
EMPV1_10330	CAGAAATTCAAGGGAAAGCTGAGCAAACTCCCCAGGCTGCTGATGCAGAGACCACAGGAT
EMPV1_10331	ATCCTCATGCTCACCAACTCGCTGGAGGAGATGAAGCGACTGGTGAGCGAGATCTACGGC
EMPV1_10334	TGTGTACAGAATGAAGGGAAACCAACAAGGAAGCCCCCCTATGAAGCTGGCAGTAGTGAG
EMPV1_10335	GGAGAAGGAATAAATGAAACAGAGACAAAGAGAAGAATAGAAAAGATCAATGAAACTAAA
EMPV1_10337	TGCCTTTGGGCTGAGTGAGTCTCCGGTGAAGGGGGAGTTTGTATTCTGCCTTTAGGATGT
EMPV1_10338	ATTTCCTTTAAATGCCCCTGGCATACACAGATCCTGCCCATGACCCACCACTCCAAGTTC
EMPV1_10339	TCCAACTACAAGGCTGTTTATGGAGGCTTCTCATCTAGTGTCGAGGGGGTCTTTGCTGCA
EMPV1_10341	AACTTGCTCTTTAGCGTCTGTGGGCTCACCGTTTGCGCTGCCATAATCTGTACCCTGTCT
EMPV1_10345	TGGCATTACCCAGCTGACTCTCTACTCTGATTGTTTCCCAGATCCCACAGGCCGAGAAGA
EMPV1_10347	GTGAATGGTGTTCCTGGGCCTTGGGTAACATGAACTGCAGTCACTGCGAATGGAATCGAG
EMPV1_10349	GATCAAGAACAAGGGCTTGATGGCCATCCCGGTCTCCATCTTCTTTAGCCTGCCGCATCA
EMPV1_10350	GTGGCGGCATTTAGAAAGAATCTAATTGAAATGTCAGAACTGGAAATAAAGCATGCCAGG
EMPV1_10351	ACTCCTGCACCTGGAAGGACGGACAGGGACCGCAGAGAAGAAGCTGGCTAGCTGTGAAAA
EMPV1_10353	ACTCCTTGACCCTCTCCTTGGCACTCTGAGGGCCCCGCTCGCCATTAAGCTGATGGTGCT
EMPV1_10354	TACCACTCACAGAAAGGCTGAGACTCAAGTTACACACCGCAATATGCTAGAATGTGCACC
EMPV1_10355	CCAGCATGATTGCGGGGTGCAGGAAAAATTCATCTTGGGGCCATTTGCCTTCAGGAGACA
EMPV1_10356	TTTCTCCGAAGGGAGGAAGCCTGCATGGGAGACCAGACTTTGAGGGGCAGGGAGAAGAGG
EMPV1_10358	ATCGGTCAGAGATGGGGTGGAAGTTAATCTTGGGATGCTTTGCCCCAGACATGCACCCGA
EMPV1_10359	TCCTTGTTCTGATCTTTGTCCAACCAACAATGGCCTTCGAAGTTCACACATATCAAACCT
EMPV1_10360	CTTCATCACCCACCTGTTCAACGCCATGCTGCCTTTCCACCACCGTGACCCAGGCATCGT

EMPV1_10362	TCATAGGGCTTCCGCTGCCTAAATACCATGAACCAAGTCTTTTGGAGGCATCTCTGGAGC
EMPV1_10363	AGACGCCTTCCTAGCTAGCACACAGCTCAACAGAGGCCATCACTTCAGCCACCCACTCCA
EMPV1_10365	CATCATATCACAGAGCCAATGGTGACTTCTGATCATACTGTACCTGGGGACACTGCCCGA
EMPV1_10366	TTAGAAATGCACCCAGGTTTCTTGATTCCACCACAAGGCTGCCCCCTGCCACACGAGTAT
EMPV1_10367	TCACGTTCAGGTGTGAAGCAGACTACGTGTTCAGCCTGGACAGCAAGTACACCAACAACC
EMPV1_10369	CGAGGCCCCGGATTATCTGGCCACTGTGGATGTTGACCCTAAGTCTCCCCAGTATTGCCA
EMPV1_10371	GAGAAGGACCATGGCTTGAACCCACATAGCACCAAGTACAAGGCAACATGAAGCTGGATC
EMPV1_10372	GGAACTGCTCCTGAGAGAGGAACCCCAATCCCAAGAGGTCACTTACAAAGAGGCACTAAA
EMPV1_10373	ATTAACGTGGTGGATGACTTTGGCTTTGAAGCAGACGAGGCCCTACACTCTAGCTCGGTT
EMPV1_10376	TACAGCCTATGCACCTTCTCTCCTTTGCCGTGCTCATCGTCGTCTTCTCCCTCTTCATGT
EMPV1_10377	AACTGGGTAGACCAAAGGGAGAAAAAAGAGGAGAGAAAGGAATCAGGACAGGGCCAGCAC
EMPV1_10378	ACGTGCACCATCTCAATGGCAGTACCCTTTGGCCTCCCCCAAATAACCCTCAGAAGTAGT
EMPV1_10381	GGCTCTCATTGCCCAAACACATCTGGAAGACAACTGCTGGGGATAGTGAAAAAATCCACT
EMPV1_10382	CGAGTCACCATGGCTATTCCTTCATCCTCGCCTGGATCTGCTTCTGCTTCAGCTTCATCA
EMPV1_10386	GTGAAGTGGTTTGAGGCTGTCCAGACCGGCCTGCCCATGTGCGTCCTGAGTGCGCTCTTT
EMPV1_10388	GGTTCCTTGGATGCCACCTAACCAGGAGATCTGAGCAGTTTGGAAAGACATCGAAAGGAG
EMPV1_10389	GTCTCCCGAGTGGGGAAAGCCATTGACAGGAACTTTGACTCTGAGATTTGCGGTGTAGTC
EMPV1_10391	CTGCCATGGATCTACAGAAAAGAATTGAGAGCTCAGAAATAAACACACACATCTTCAGCC
EMPV1_10392	CCTAAGTGTTCTGCATGTCCATTCAATGTGCTTTAAACTATAGCTTATTGCATTAGAGTG
EMPV1_10394	GAGGGTAGACTCTCTTTTGGTCGCCTTACTTCAAGAAGCTAACCTCCAATCGTCAGACTT
EMPV1_10396	GGTGGGGGGTAACTGCCTGAAAAACATATTACTATACAGGCCTTAGCATCATGAATGGCT
EMPV1_10398	GCTCTGGATTAGGCTTTGGCTTAAGGGAATGTTGTGACTGGTTTGATCTTTTATCCAGAC
EMPV1_10399	GATAAAGTAAAATTCACTGTTCACACAAAGAGTTCATTACCAAATTTTAAACAAAATGAG
EMPV1_10400	ACGACATGGCCGGCCTGCAGTACAGCCCCATGATGCCGCCCGGCGCCCAGAGCTACATGA
EMPV1_10401	TGCTCCTAATGAAACACCCTGAGGTTCAAGCCAAAGTTCATGAAGAAATTGACCGTGTGA
EMPV1_10402	CCCACCCAGATTAAACCATTACCAAACTAAGTGCCTGGTTTTATCTCCTGTTCAGATGAG
EMPV1_10405	GGGACTTTGTTTTGCTACAATAGCCTTTTGGGTCTGGTGCCTTCTTTCTGTCCCCCCAAT
EMPV1_10406	GCTGTGGTGCAGGTTCGATCTGTGGCTTAGGAACTTCACATACCATGGGTGTAGCCAAAA
EMPV1_10407	GGTTAAGAAGGACTTCCAGGCACGAATGAAACTTTTGGTGAGACTCTGGGAGGGAGACAC
EMPV1_10410	AAGAACTTTCTATAATACTGATATTAGTTCCAGAGACTACTTTATTTCTATGCTTGTGGC
EMPV1_10412	CAGGTGGCTTTGGGGAACATTGCCAGCAAGAGGGAGCGGGTGAAGATTCTATACAAAAAG
EMPV1_10414	ATGCATTCTGGTGAGCACAGGGACCCAGCACCTGACCCCCAAAATTGAGGATGGAAATGA
EMPV1_10416	CTAGCCCTCGTTCATGATATGGCAGAATGCATCGTTGGGGACATAGCACCTGCAGATAAC
EMPV1_10417	GAGGGTTCAGGCCTCAGACTGGGCGAAGCATGTGGGAAAGAATTGAGATAGGCTGTCTGA

EMPV1_10419	AACGGTTAGTAGTTCCAGCTTAACTTGCCCTCGTGCTGATCTTGTGCTGATGGAGACAAG
EMPV1_10422	CTCCTAGATGAAAGGACATTAAAATGGAATTGCCTGCCCAGGAAGGGGAGCTTGCATCCC
EMPV1_10423	TGTGTTTCGTGGCTTAGGACTCATGACCCCACTCACCTGCTTACTGGCAAGACTGAGCTA
EMPV1_10424	GTGCTGAGGTTGAGACACTGTTAAGAGAAGTGTTTTGTGCGCCCTTAGTGGACGTTTTCC
EMPV1_10425	ACCCAAGCTGCAGCGCACCTCTAGCCAAGGTGGTCTTCTGGAACCCGTACTTTTTAAGAA
EMPV1_10426	GGGAGAGGCTGCTCTCTCGGTGGCAAGAAGAGCCCTGGCTGCCCAGTTGCCAGTCCTACC
EMPV1_10427	TGCCTGGATATGTAAGCAGGCAACTAAAACTATCAGCAAACACCATATTGCATCTTCCAC
EMPV1_10428	GGTACATTTGCTGGAGTTGGCAGTAACTTTCATTGAGAGATTAGAAAGCCATCTGGAAAC
EMPV1_10429	CCAGCCATGCGGAGATCTTTATCAGTGGAACATTTAGAAACCAAGAGTCTACCTTCTCGG
EMPV1_10431	AAGAAACTGGTGAAGAAACAAAAATCAAAGCGCGTCAGCTGACTGTTCAGATGATGCAAA
EMPV1_10432	AGAACAGATCGGCCATATCCCTTTTGGCTGCAGAGGAGGAAATAAATCAGCTAAGAAAGC
EMPV1_10433	TAGGGCAGGGATCGAACAAACTCTTGCCACAGCAGCAATAATGCCAGGTCCTTAACCCTG
EMPV1_10434	ACTTCCACTGGGGCAACCCACCCAAGGACAAGTGCTACTGTGGCTTCATGACCTCCAAGA
EMPV1_10435	AGGAAAAATCAGAAACATCCCAGGAACAGTACATGGAGGGGGAGGAGCAGTCAGTGGCCT
EMPV1_10439	GGGGTCCCCAACTACCTGCCAACAGAAGACTGGGAAGCATTTAGAATATATATCTACGTC
EMPV1_10440	ATCCCTAAACCCCTAGCAAAATAGTGCCAGAAGCTGAGATTACCGTGTAGAACTGCATGG
EMPV1_10441	CCATGGCTGATGAAAACATCATCGTACGCAAGCAGGGTACCATTTTCTTGGGAGGACCTC
EMPV1_10442	GAAGAAGACAGCGATGATGAGAAGGTAAAACAGCTTCATTGTGCTGATTCGGAAGGGACC
EMPV1_10444	ACGGGCTAGTGAAATGAAGAGAGATGAGGAACGTAGCCATGATGACAAGGACATTCCCCC
EMPV1_10446	GTTCGAATGCTTTTTGTTCTATTTTTTTACCAGTTCCAATTTCAAATGTCTCAATGGTGC
EMPV1_10447	TCTCCCGGGCGCGGGGTGGGGGGCGACTAGGAAAACCAGGATCCGGGCTTTGTCTGACTA
EMPV1_10448	TTCAGGAATCAGTCGTGGTTGGTCAGGACGAAAGTTGGGGTAAGTTTGGTTGGTCAGAGG
EMPV1_10449	GGACGTCCGCCGGCGCTTAAGTGATGTGATTAATCCAATTCCCGCGGGCGCCATGGCGGC
EMPV1_10450	GACCCATTGGAATTGGGTCAAGTTTCAGCTTTCCAAAGACTTCACACTGCTCTTACTCGA
EMPV1_10451	AATCTTTAGGGACGGTCCGGGAGTGAACTGTGAACTTTGACACTCTAGGCAATCAGGTCC
EMPV1_10452	GTGACAGGGTTTCCACCATTTCTCTATCAGTCTACCCAGAATGTCTCCATGGATGGCACC
EMPV1_10455	CCTTAGCTTTGCTTCTCTCTCTTGCTCTGCTGCCTTCTCTTTCCTTCTGCAGGTACTGGT
EMPV1_10456	CCATGCAGCTAACTCCCAAGGACAGGCTTCAGCATCAGCAAAAATTACAGTGGTTGACAC
EMPV1_10458	CCTGCACGATGATGAAGTGATGGTCACAGGGGATAAGATCAAGTCCCTCATTCATACAGC
EMPV1_10459	GGTCGTCTGCATGGCGGTAAAATAAGCGTTTTCCTCTTGCCTACATCATTCGGAAGGTGG
EMPV1_10460	CACCATTCTGAAGCCCCTCACCATGCCCAACATTCAGATCTCAGAGGTGGATGCCCTGCC
EMPV1_10461	AAGGGCATGCATGCCTTCATTGTGCCCATCAGGAGCCTTGAGGACCACAGCCCTCTGCCA
EMPV1_10462	GGGGAGAAGTGTCTGCCATTAGAGTAGAAGGTTTCTTTCGGGGGTGATGAAAACGTTCTA
EMPV1_10463	GACGACGCTTGGAGACCCTCGACAACGCAAATACGCCAGATTTTGAGGCTCTTAGTAAAG

EMPV1_10465	GACCATCAGTGGACCAAGTGTTAGAAGACCTGAATTCCAGCCTTGTGTTCTTAAATATGG
EMPV1_10467	CCAGAGCATGTGTAGGAAGACATTTTGGTAATCATTAACTCTGCCCTGAAATGATGGACA
EMPV1_10468	GGCCCCATGTCTGCAGCTGGAACTTCGAGAACGTTAGTAAAACCATGGACTACTTCAGCA
EMPV1_10469	CCCATACCACACAACTGTTAAGTAGTAGAAACAGGATTCAGACCTGGTGGTGTGGGATCC
EMPV1_10470	CAGCTAAAATTAAATTAAATGTAGTTACAACAGCAATGATAATAATTAAGACAAAACAAA
EMPV1_10471	GGGTTTCCTCCTCGACCACCAGGGTTTCTGCCATTCATAGAACTTGCCATCTGGAATCTT
EMPV1_10472	CGAACCCTGTCCTGGTCAGAGGAATTGGCAGATATTCCCATGTGGCTGTCTATTCCAGAA
EMPV1_10473	ATTCCTAGGTAATGTGTATAATGTTTGTAAGTCATTCCTAGAAATTGTTCACAATGCCTG
EMPV1_10477	CCCGCTAACTCTGCAGTCTGATGCTATCTGCCTCCTCCAATACTGATCACTCACAGCCAT
EMPV1_10478	AGGACGAGGAGCTCTCCTCCAGCACCGAGAGTATTGATAATTCATTCAGTTCCCCCGTCA
EMPV1_10479	ATCACCATATCTGAGGATGAAGCCACCAGTTCACACAGCCACAGTGACAGCTCCGAGGGC
EMPV1_10483	AGGCAAGGGGCAAGACCTAAGAATATTTCCAAGTTGCCAAACTGCCCGTTCTCTCTGCTG
EMPV1_10484	TGATGTCAACACGGGAGAGGAACAACTTTGCCCCCTCCTTCCACCCGATGCTCAGATATA
EMPV1_10489	TGGAGACGCCGCCAATGTCCTCTGTTAATCTTCTGGAAGGGTTGAGCCGAACTGTGGTTT
EMPV1_10490	GACTGAGCAGAAGAGCTCACGTCAGACCATGCTGAGCTTAAGAGTAACATTAAAGGGTTC
EMPV1_10491	CACAGCCCTCCTGAAACAGGAACGGGTGGAGCATCAGTGTGACATCAGAGACATCGTGGA
EMPV1_10493	TGATCTCGGGTGCCAGTGTGAAGGAGTACAGGCATCTGTGGACTGGGAGGAAACCTTAAA
EMPV1_10494	TGTGCCTGTCTGCAGGGTACAAGAGCTTCCTTGTCAATTTCCTCCATCTGAGCAAGGCTG
EMPV1_10496	TATAACAACTCAACTGGGATCAGCTACGAGACCCTGGGGCCGGAGGAGCTGCGTAGCCTG
EMPV1_10499	TGGGAACAGGAACGGCTGCCCTAATCACAGGACCACAACAGCTGGAGAAAGGACTTAGTA
EMPV1_10500	CTGCTCCTGGGCAAGAGAGAGGGAAGAGTTCTGGAGCACCTTAAAACAGCAGTAAAGCAC
EMPV1_10502	GAGTCCTTGGTCTTCCCTTTTTCCTGCCACCCTGCTCATTCTTCTTTTTGACTCACCAAC
EMPV1_10507	CCGCAGACTGTGTGAGGCTCTTGTGGGGAAAAATGGACTTTTCAGATTCCTAAGAGGCCT
EMPV1_10508	CTGAGCAGCCACCTGCTAAAGGAAGAGAATGTGTCACCTGTACCTGCCCCTGGAAGATCT
EMPV1_10510	ACGTGCTTTCAGACTTGGTCTATACACTGGCTTTCTCTTGTGGCTTCTGTAGAAAGGAAA
EMPV1_10511	GCCGCTGTGGATTACAACCCCAGGGACAACCTTCTCTACGTCTGGAATAACTATCATGTC
EMPV1_10512	GATGCGCTCTGGAGGTTTGCTGTTATGACCACGACATACCCTCTCTCTTTTGTGCCTCCA
EMPV1_10513	TCTTCACATTGGCGAGTGTCTGAAATGCACGTGGTTCAGGATATTAACAGGCTCCTGAGG
EMPV1_10514	CTCCTGGCCCTATTCTCTCCCGCTACCCTCCACTCTTTGTAGGAACACAAGACATCTTTG
EMPV1_10516	TGCCAGTAAGGAGATGGGAACTAACTACCCGGGGGGCTTCCTTTAGTGCTGGCAGGAACT
EMPV1_10517	AGTCAACCTGCCCAGACATTTGGGGCTGGGATCACTCTGTTGTAGGGCCTGTCTTGTGCA
EMPV1_10518	AAGGGGAAGAGCCCAGGGAGGTGACATACACAGAGGTGGATCACTGGATTTTCACACAGA
EMPV1_10524	AAAGACCCAGACCCTTGAGAGATACTAGAGGCTCCCACCAAATGCCAGGAACACTGAGGC
EMPV1_10525	TGTGCCTAGTGTAGCCATAGGACAGAACATGACAGTGACAGAAAGAGTGCTAAGCCCCGC

EMPV1_10526	GCAATTGGTTAAGGACTTCCAGTAGTAAGTTAAAATCTCAGGAGAGGAATGGATAGTACT
EMPV1_10527	GCTCTTTGATTATGAGGTCGAGAAGGAGCCCCTGCTCTTCCACGTCTTCAGCAACGCCGG
EMPV1_10530	CTTCGCTTCTTTCCCCAAACTTAGCATCTCCTTCAAAGAAACCCAGAGCCACGGCCAGCA
EMPV1_10531	CACAGGAACAAAGCTTTCTGGGTAGTTGAACATTTTTGCAAGTTTCAGGCAGTATACCCT
EMPV1_10532	GATGTCCTTGCTTATGTGTTTGATGATGTGTGGAGCAAGGTCCTAAGCTGTATGGAGCAG
EMPV1_10533	GGCTTCTCCTTTCTTCACCACTGCTGATTTTGTCCTGCTGTTAGACTACTCCTGATCGAT
EMPV1_10535	GTTAACGAACCTGACTAGCGTCGACTTGGGTTTGATCCCTGGCATTGCTTAGTGGGGTTA
EMPV1_10536	CAATGAAACCTCTAACCTGATGGGAGTGTGACTTGAGGGGATGGAAGTAGAATGCTTCTG
EMPV1_10537	TCTTCCACGAAGCACTGGTGGTTCAGCTGGATGGTCAACGCGCTCTTGCCTACACCACTT
EMPV1_10539	CAGGAGGTCATGCCTGATCGCTGCTTCTTGGGAGATCTTGAAGAGGTTAAAAAGGAGGAG
EMPV1_10542	GGCCTGTTTGCTCTTGTCTCTTCTACTGCTCTCTTTATCCTTATGGGATTATGATGGTCA
EMPV1_10544	GCGCAGATGAGAGCAGCGATTAACCAAAAGTTGATAGAAACTGGAGAAAGAGAACGCCTC
EMPV1_10547	GGTTCACTTCCAACTAACGGACGGCTGCCAGATCCCATGGACGTCTTCGCCGGTCAATTC
EMPV1_10549	CTGACATTGGGGCGTTCCAGAAGATGATAAAGAAATGATAGCAGCTCCAGAAATACCAAC
EMPV1_10550	CGGGAAAGAAGCAGGAAGAGAAGACCGTCCTCTCTTAGGAATCCAGCACTGGAGAAACGA
EMPV1_10551	AGCCATTCTCCAGTGCCCAGGATCCAGAGAAAAATTCAAAGAAAGTCACACACCTGATTC
EMPV1_10552	TCATTCCTCTTCAAGTACCTGATGCACCAGAAAAACTAATTGAAGGACTCAAATCTCCTG
EMPV1_10555	AATAAAGTTGTCCCGGTGCTTCCAATGGGGTGTCTCAGTTCTGCTCGGGGCTCCCCTTTC
EMPV1_10556	ATGTGGATCCCACTTAGGAGAGGAATGAGCAAACCAGACGGAGAAGAGTAGGGGCAGCTC
EMPV1_10557	TACTGCCACCATGACCTGTGCAACTCCACTTGGCTGTAAGCAAATCAAAGGAATTATGCC
EMPV1_10559	TTAAAGAGCAGAAAAGCTGCCATTAGATGCCCATCCCGAAGTTCCTCTAAGAGCGAGGCG
EMPV1_10560	TGAGAGTCATTTATCCAGCAGTTGAAGGTCGGATCCAATTTTCTGCAGGAAGTCAATGCC
EMPV1_10561	GAAGTGATCAAAGTCCGAACAGAAACCAAATTAGACTTCAATCTAGAAAAGAGCAGAGTC
EMPV1_10563	GGTGGGCTCAGCAGGGGCAACCTCACGGTTTATTCAGAGCACATAAAGAAATCTGAACAG
EMPV1_10564	ACTCGACATGGAGGAGGGCAAGGAAGGAGGCACGTGGCTGGGCATCAGCACTCGGGGGAA
EMPV1_10565	GCCACCACCGCCACCGCCCCTGCCATCAGGGGTAGAGCTCAAGAAGACAGAGGGAAGAGA
EMPV1_10570	CCAGTGGGTGCTGGCCAAAGCTCGGGGGGACTTGGAAGAAGCTGTGCAGATGCTGGTAGA
EMPV1_10571	TTTAAGCTGGACTATGCTGGGCTCTGACCTCATCCTCATCTTCGCCTCCTACACCCTCAT
EMPV1_10572	GTCCTCTCTCACAGTTTGTATGTTGAAGTCCTAACCCCTCAGTACCTCAGAAAGTGACCC
EMPV1_10573	GTCAGCCCATCCCTCAGTGAGTGCCTTACTCTAACTGAAACCAAGCACATGTAAGGTACA
EMPV1_10574	GGACACCATCTCCCTTGTTGTCCTGGCTGAAAAGTGATGCATTCGGTCTCTCAGTTTTAG
EMPV1_10575	GCTTCTCAACCATAGGGAAAACATTCTGTGTGGCAGGGTGATTTTCTTCTTTGTGGCCAC
EMPV1_10578	CAGATGACAGCTTGTTCAGAAAGATAATAGCAACAATGTATTCAAGTAGTTATGCTTATG
EMPV1_10580	CAGCATGCAGATCAATCACTACCAGTATCTGCTCCTGCTTTTCCTCCACGAGTCACTTGT

EMPV1_10581	GCCCCAGCTGTGCTCTTCTAACCTGCTGCTCAGACTAGGTGTCACTGTTAGGGATGAATA
EMPV1_10583	TTGTACAAATAACCTGCTACGAATGGGTTGAATGTTCCTGTCGGTGGTGCAGACCGAGGC
EMPV1_10584	TTTCCTTCCAGACCCTGAGCAAGTGCAGCGAGACACACCACAGCATCTCTGACACTCTCT
EMPV1_10585	AGAAAGAGTGCATTGTCTGGCTGCGGAGATACTTGGAGTTTGCAGGGGAGAGCTTGAACC
EMPV1_10586	TAGGCTTTGGCTTATGGGAATGTTGCGGCTGATTTGATCTTCTATCCAGACGATGGCAGC
EMPV1_10587	GGGTGTGTATTTCCCTCACAGGTCTCTCAGCCAGATGCTAATGTGGAGCCAGTACTCAGT
EMPV1_10588	CCTCCTGGTCGACTCTACTTTTACCACAAAAATAGGCGTAGCCTGTAGAAGATGCCAGCC
EMPV1_10589	ACAGGTGATACTGCCCAAAATCAGAGAAGTTAAGCTAGTGGCCAAGGGACACTCAGGTCA
EMPV1_10592	GTGCCATCATCATGCAGCTCATCTATTTCTACATTTGTCTCTCTGCTGGTGATTTTGTCC
EMPV1_10593	GAGGAGGCACCTATGACTCTGAACTGCACTTACCAGACCACTTATTCGGACTATCTTTTC
EMPV1_10594	CCTGCTGTACTGCCTTGAATTCCATCTGTAGTTTGAGGGGAGCAAACAGACCCTGGATGT
EMPV1_10595	GGAAAATTGTATGAAACTGCATAGTGTCTGAGCAGACTGACAGGGCACCAGCACTGCAGC
EMPV1_10597	CGCTCGGCCTCATCATTGGTAGATGTGCCTTTTATTTATCTGCCTTCAGCCTATCTTACC
EMPV1_10599	CACAAAAAGAGAAAATGGGCTCAGAAATGCAGTGGGTAAATAAATTATGTACGGCGAAAG
EMPV1_10600	GCTGCTAAAGTCACATGTGTGGTAAAGTTTGGTAGTCTTAAGCCTGTGAAGCACATATCA
EMPV1_10601	AGGGATTGTCTGGTTGTCATTTCCTCCCATTAGCCAGCTTGGGACGTGGAGTCTGCTTCT
EMPV1_10603	TGTCATCTGTGGCTTCTGTGCCCGCGTGTCCGTCTGTCTCCTTTCCCTGGTCTGTTGTGC
EMPV1_10605	TTGCATATCTGGTGTGCCCAGTAAAAGTGGGAAGTACATCGACCACTACGTGAACTGTGG
EMPV1_10606	GCAGCAGATTCTGTGCAGCAGAGACGCCAATACAGACGACAGAACCAGCAATCTTCATCT
EMPV1_10608	AAGTCAAGAGCTTTACCTCTGGGAAGTGGCGGAGAGACCAGGCTCCAGGCACAGCCTCCT
EMPV1_10609	CCATTCGTTGTGTAAGAGAAATGTTCTCCCATTCTTGAATGCAGTTAGACCCTGCCAGTC
EMPV1_10613	ACCTTGCATCTAAAGAGCAGCAAAGTGGCTTTCGCCTGAATCTGCATCCTAGCCATTCTG
EMPV1_10615	GTATCACCCTACCCGGAGCCTGCAACATCCCTGCCACTGTGAGCAGCGGCAACTGGTTCT
EMPV1_10617	TGAACAGTGGGGCAAAGGAAACTCTGGAACCTCTCATTCAGGCTGCTCAGCTTTTGCAAG
EMPV1_10618	TAGTCTACAACCACCAGCCAGGCAACAAGATCCACGTCACCTACTGGGTTGCCCTGAAAA
EMPV1_10619	AGCCCCGACTGCATCCAGAGAGGGAACCTGCAAACAAACTTCACAGCAAACTTTTTGTTC
EMPV1_10620	GACGCTCCTACCGAGGTGACAGCCCAACAGATTCCCAAACGGACATGATTGAAATCCCTT
EMPV1_10622	TACAGGGACTAGAATTCAAGGGACAATCCAAATTGGGCCTGGACCCTCCAAGATAGCAGC
EMPV1_10623	ACTCCAGTGGGACTGTGTTCATTCGTCTTTTCTGTGACTATCCCTTAGGCTCTTGGCTGG
EMPV1_10625	GTGGCGTATGTGTTCGTGTTTTATTGTATTCAGAGCCATTGTGCAATAAGCAGTCCAGAA
EMPV1_10627	GTGGGTTGCAGGGCACAAAGCATCCACACAGCCAGGAATCATTTTACTTTCCAAAGAGCC
EMPV1_10628	GAGACATTGACGCTGCTCAGCAGGGGAATGATCACCTCTGCACACTTGACCGCCTTGTTG
EMPV1_10629	CCCTTCTCAAACAGATCCAGGAGGCCTATGGCAAGTGCAAGGAATTTGGTGACGACAAGG
EMPV1_10630	AAGGGAACGGGCTTGGTGGAATCAGCGGGGAAAGAAGACCCTGTTGAGCTTGACTCTAGT

EMPV1_10632	CTGATTAGTCCCCTGGCCTATGAACCTCCATATGTTGAAGAGTGTGGCCATTAAAAGACC
EMPV1_10633	CATGCAGTGTTGTGATTGATCCCCAGTTTGGCTACTCCAGAGGAAACCCTTGTATTCTTG
EMPV1_10634	AGCAGGTGCTGCAGATGTCTGTTGGAGAAATGGATGAATAAACGAACGAGGGGACCAGGC
EMPV1_10636	CCCGGAATATTTGCTGGGCAAGTGCAAATGCTAGTGTCCACCTTAGACTATGTTGCTGAA
EMPV1_10637	GCTGTGGGCCTTCAGTACTCAGAGAGGGGGAACAGATCCTGTCTACAACATCGGGGCTTT
EMPV1_10639	CCCTTTAACATCTGACCCCTTGCTAGAGCCCATGAATTGATTCTAGAGCTCTGAACTTGC
EMPV1_10640	GCTCAGCAGTAGAAGGAGGTTTCTCACCTACTTGTCCCAGATGTGCCTTTAGTGTCATGT
EMPV1_10641	CTATAGGCTTATTGTGCCTGAGAGGGAGGGCGAGGTAGTCATTGAAACTGCCATGAATTA
EMPV1_10643	CCCCTGGCTCGGCCTCGCTCTCTCGGCCGCCAGCTCTCTATGGTACTGGCGACGACATGT
EMPV1_10645	AAGGGAACGGGCTTGGCGGAATCAGCGGAGAAATAAGACCCTGTTGAGCTTGACTCTAGT
EMPV1_10646	AACCTCCCTTGACTATTGTCTAACTACTTCCCCGTCCTTCCCGCAGTCCTTTTCTCCTCC
EMPV1_10647	GGGCTCATGGCCCTTGGGTTTCTACCCTGCCTGCATGTCCCTTTGCCATCATTTTGAAAA
EMPV1_10648	GGGGACACCTGTGCGGGGCCCTCTTCCATCAACTTGTGCTCTTTAAAAAATGGGTGTGAA
EMPV1_10652	CATGGGAGAGATTAAAGTCTCTCCTGATTATAACTGGTTTAGAAGTACAGTCCCCCTTAA
EMPV1_10654	AGTGGAATATTGGTGAACAATACTAAGTTAGATGGACCTTTATTACAGATTCTATTTATG
EMPV1_10656	TGATCGAGTTCTCTAAGGAACAGCAGGATGAATTCAAGGAGGCATTTCTCCTCTTTGACA
EMPV1_10657	TCTGTGGAGTACAGCTTGTCGTCCCCAGTTCACAGATGCAGACAGCGACGCGCCCTGTGA
EMPV1_10658	CCAGGAACTTTATGTCTGCACAGTCAAGGATGACGTGAACTTGGATACAGTGCTTCTCCC
EMPV1_10660	GGAAAGGATGACATTTACCTGTGTTCACTGCCTCGTTCCGTGTGAAGCAACCGCTAGTTG
EMPV1_10661	CACGTAGCATAAGAACGTCTAGGTCAGAAAGTTCCTCCAGAGATAACAGCTATCAGGTTG
EMPV1_10665	TGCTAGGTTTGCCAGGTTAAGTCATTTCTCTCTACAGCTATGCATTTCTCGTCATGCTCC
EMPV1_10666	CCACCTTTTTACATAAAGGCGTTAGGTGGTGATGGGCCATGTTGTTTTGTACCTAGTATG
EMPV1_10667	CGTGGCTGGATTGCTAAAGTGCCTGTGAATTCCGACATGGAAAAAGCTTGAGATGAAAGC
EMPV1_10668	GCTCTAGGGCACACCCTGTATACCATGCACTATCCCTTCTGTAGGTCCCGAAAAATCAGC
EMPV1_10670	CCCTCGTGGGTGGTGGTTCTTTATAGAAAAAATCGAAGCTTAGCAGCTCCTCGAGGCCCG
EMPV1_10671	CATAGATGAACTGCACCGGCACCTTTATATCAAGTCCACGAGCCGAGTTGTGCAGCAGAA
EMPV1_10674	TAGGGTGGTGGGGTTGGCTGGCAGGAGAAGGGAGAGGTTGGACCTAAAATTGCATATGTA
EMPV1_10677	AGCAGCTTTGATTAAAGATGACATCCCTGTTTGCTGAGGAAATTTGCCTTTCTAAAAGAC
EMPV1_10678	AAGATTTTCTGCTGTGTCTCTCCCAGTGGAATGGGGGCAGGGTTTGCAGGTTTCTGGGTT
EMPV1_10680	CATGTGGTGTGAAAGACACCTACCAGGACATGCTCTACTTCCTCCTGGCCTCCAATGTCA
EMPV1_10681	TGTGTGCCCTTGAGCATACTTCCTTCTAGTCCCCACTCTCACCTGCCCTTCAGGAAAGAT
EMPV1_10687	GATGGCAAAACTGGGTTCAGAGATCAACTAGCTTCCATTTTCTTGGCCTGCACATCCCAC
EMPV1_10688	CAGGGTTAAGGACCTGGCATTATTGCTGCTGTGGCAAGAGTTTGTTCGATCCCTGCCCTA
EMPV1_10689	ACTGTCACACCAACCAATAGGAAGAGACAGCTCAGGACGCCTCTGGGGAAGGTGGTCGTA

EMPV1_10692	CCAGGTGTCTCGCTGGTTTGTTCAGTGCCTTCGGGAACAGTCCATGCTGCTAGAAATTAT
EMPV1_10695	GGAGTGCAGAGCATGGCTTCTGTCATCTGCCTACAGTGTTTTTCTCTGAAAGGAAGGTAA
EMPV1_10696	ACTTGGTTTACATTTGGATGCTTTGAAGACAGAGGTTGGTACAAATACCCTCTAGGGAGG
EMPV1_10699	TCAGAAATCAGGGTCAGGTTAATAGGCCAAGGTCAGAAACATGGAGCTACAGTCCTGGGG
EMPV1_10700	TTTGCACTACATGACCATCATGAATCGGCGAGTGTGTGGCCTGCTGTTGGGAGTGTCGTG
EMPV1_10701	TTCGGGCGATTCTTCCTGGACGTATGTGCCGGTCAGGTACCGGTGCACCAGGGCCGACTT
EMPV1_10704	TGCTCAGACGTCTGAAATCAGTCATTTCACAGCCCCGTGTTGTGGGTTTGTTGGACTCCA
EMPV1_10706	AGTCTTGGCCTTGGTCTGTGAAAAGAGACTGATGCCTTGGGCCCTGCATTTCTGCTCCTG
EMPV1_10707	CAGGAGGTGGAAGATTTGTGTAAACTGCACTGTCTCAGCGTGGCCTTTCTCCTCTGCTTA
EMPV1_10708	GTAACGACGACCTCCACATTTGGACGTTAATACTGCTCTAGAAGAGGTGCTGAAGACCGC
EMPV1_10712	CTGGAATTGGGTGTCTCTGGGATAAGGAAGTTAAACCTATGGTGATCTGAGACTGGTCCC
EMPV1_10713	AGGCCGTGGTCTCAGAAAAGGGAATTAAGGGGAAAATCGAAGGTCGGCCTCACTCCACCT
EMPV1_10717	GCTCTGGGAACTTGCGCTGACGTTTTGCTCTTCGACATGGATCAGGAAATGCTTGAGATC
EMPV1_10719	TGAACTTGCTGATCGCGCTCGTCGCCTTCGTGCTCTTCAGTTTCTCCTGCTTCTGCATCT
EMPV1_10721	GCTGGCCAGCCGGAGCTGTAGCGCTGCAATGACTTAAGGGCAGTTTTGTGCACTCTTCCT
EMPV1_10722	AGTCACTCATATCCGTGCGACGACCTCCAGCCTCGCAGTACTCCTTTCAGGTATCTACTT
EMPV1_10723	CGGAGTGAAAAATCACCCGCCGACCACGCATCAGATGTAGCCATGACTGGTGAAATCACA
EMPV1_10725	TGACAGGTCTTTGAAAAATGAGGGCCCCAGTAACAGCCTCTCTCCCTCCAGTGTTTCCCT
EMPV1_10726	CGCCGTGCCCCACCCAGACAGCCAGCCTGACACCATCGACCATGATCTCCTCCTGCTGAA
EMPV1_10727	CTGCCTTTAGAGACCATGAGAAGCGGTTTTGTGCTTCACTGGGGAGTTGTTAGATTTTGG
EMPV1_10728	ATCTGATCCCAACCAGTAGCAAGGAGCCAAGACCACCTAGAACCACCACTTGAATCTGAG
EMPV1_10730	CTGATTCAGCAGATTTGGGTTAGAGCCAGAGAATCTGCATTTCTAGCAAGTTCCCAGTTG
EMPV1_10731	CATGCAAAGCCGTGTTGAGTGTGGACTGCCTCTTACTTGCTTTCCTTAACAGTGGTGCTG
EMPV1_10732	CAGCCTGATACAGGAGTGTTGGGGTCAGTGCTGGGGATCAAGGAAGACCTGAGAGTGACT
EMPV1_10733	CACTTTGATTTTAAGACCCACATGTTGTTGGATGTGCCTGCAGTGCCTCGGCCACGTTTT
EMPV1_10734	GCTGGCCATCAAACAGCCACCCAGGGTCTAGCAGTGTGTCTGTTGAAATAGCATTGACTT
EMPV1_10735	ATATCAACCAAAATGGGTGTTGATGTCTGTTCCTAAGTCCCACCTCACTGGGGCTGAGTG
EMPV1_10736	CAGGTAGACTTCATTCTTGGCTTTAGTGTCATCCTGGTTCCGCTCTCCCTGATCCTTGTC
EMPV1_10737	GAGAATGCTATGAGGTTATCAAGGATCCACCACGATCAACCTGTAAAGCCCCTGGATCGC
EMPV1_10738	CCCCCCGGGTACTGAGTCCTTGGCCTTGGATTTCATCAGAGCAATGGAGACATTTGTTTC
EMPV1_10739	GAGTAAGACTTCTGGGTGAACTGCTTCCACGAGTGAACTTTCTTCTGACAGGGTCTGGCA
EMPV1_10741	CCAGGCAGCGCTTTCTGCACCGGAGTACACTGTGACACCATCGCCACAACAGGTTCGGGT
EMPV1_10742	CAAGTTTGTGGAGCTTGGCCTCTCCAACTATGCTGCCTGGGAGGTGGCCGAGATCTGTAC
EMPV1_10743	TTGGACTCTAAGCCAAGTAATCTCCCCGGCAATGGTTTCCCTGAAGGTATGAGCCCACAG

EMPV1_10744	TTCTCTACTGGCTGCTGATCTGTGGCATGGTCTGCAGTAGGACTTCTCTTGAGCCTCAGA
EMPV1_10746	AAGGCTGTCCACTCCCTGTCCCGTATCGGGGACGAACTCTACCTGGAGCCCCTGGAAGAT
EMPV1_10747	AGGCTAGGGTGGGATTCTGTACCAGGGACCGATATGGAGAGACACCTGCAGACTGGGATA
EMPV1_10750	TCTCGCTCTAGCACTCGAGCTCTGTAACTTTTGAAGCAAGTTGCTCATATGAAGCCCTAG
EMPV1_10752	TCAGAGTGAGGTAGTAGATTGTATAGTTGTGGGGTAGTGATTTTACCCTGTTCAGGAGAT
EMPV1_10754	GTCCTGGAGGAAATAGATCAAAATGATGGGGAATAGTGCCTCCTTAGCTATGTTGTTGTG
EMPV1_10757	TTCTTGTGCCAGTCTTGAACAGCTCGCCTTCTAAAAGTCTCTGTGGATGTGACCGTGCAC
EMPV1_10759	GGAAAGAGAGGCTTGTCTGCTCCTCCAGGGCTTCCGTTTTTCTCTTGCCAATGTATTTTC
EMPV1_10761	CACGGGGAATCCTGGGCACAGGGAGGCAGGGGACAGGAAACCCACTGATTGTGGCTTTGA
EMPV1_10762	ATCGTGCCCCTTACATTGGTTAGGAAGTCACTCGCTCTTCCCTCCTGCTTCTCTTCAGGA
EMPV1_10764	GCCATTTTGTTAAACTTCCAAAACATGGCCTCGTTCATGAGCTGTTTATAGCCTTTCAAG
EMPV1_10765	GGTTAAAAGATCCAGCATTGCCATAGGTGCGGCTTAGGGTACAACTCCGCCTCAAATCTG
EMPV1_10767	CCATGGCTACAGGGCAGGTGTTGTTCCAGCCGTTCTTATATACCATGTCCTTAGTGAAGC
EMPV1_10769	AGAAGTCACTATGGGGTTTGTTCGTTTGGTAGCATCTGGATGGGGAGGTTACCTCTGCAG
EMPV1_10770	GCCACAAAAAAGACATCGGGGAGATAAGAGTACTGGGAGTGGACAGAGTTCTTGAGAAGC
EMPV1_10771	GCACAAGGAAGGGGGCATGAGGCTATTTTTTGTAGGATGGTCTCTCTGATAGCTGTATGG
EMPV1_10772	GGTTATTACCCCTGTGATCTGCATTAAGTTCCACCCTGCTTCTGTGGCTTATTCCTTGGA
EMPV1_10773	GCTAGCCAGCTGGATTGCAATTTCTTAAAGGTTGTGTCTAGTTCTATTGTAGACAAGTAC
EMPV1_10775	TAGCTGCAGAAAGAAATCGGGATGCCGCGGAGCACAGAGTGATTTGGAACTCCATTCCAC
EMPV1_10777	AGTGGACAGAAAGGCCGCTTGGGGCAGAAAACTCTGAGCCCGCTGGGAAGGCCTTTGAGA
EMPV1_10779	AGAGCTCGCCAAGGCTGAGAATCTCTCTCTGGAGGAGGGCGGGAAGGATGCCCGGGGCTT
EMPV1_10781	GCTTGAATCCCAGCCATAGCTGCGACCTACGCGCAGCTGAGGCAATGCCGGCTCCTTCAA
EMPV1_10783	CTCCAGTTACCCCATCTGATGGGTTCAGTGATAGAGCTTTCCTTGAATACAGAGTAACTC
EMPV1_10785	AAACCCGTTCTCCGTTTGCAGCGGGGCCTCGACCTGCCCATCACCAGTTTTTCCAGAGGC
EMPV1_10786	TCCGTCTTGGAGCTCGCCTGCATCTATTATGCCCTTATCCTGCACTACGATGAGGTGAAG
EMPV1_10789	GTATATAGTCAGCCCTCCACACCCCAAAAAGGGAAACGTGCCCCCTCGGAAATGGTTCTT
EMPV1_10790	GATGGATGGAAAAAAGTCTATGATAGTTTGGAACCACATCATGAGGTTTTTCCTGATGAT
EMPV1_10791	GCAGAACTGGACCAGCATGAAAAGGACCAGGAAAATACATCTCGGGTGGTACAGGAACAC
EMPV1_10795	GGGTTCTTTAATACGAGAAAGTCATCCTCCAGCTCTCTCCCCCAGCAGATCTAGTGCTGA
EMPV1_10796	GTGGTCATCTTTGCTGCTTGTATGTTTATCTTAGTTGGGCCCCTCTGCTTGGTGCTGGTC
EMPV1_10799	CAGAAAGGTGTCCCCCTGAACTAGAGTAGATCATGCCTGGTCTTGCGTTTGACAACTGTG
EMPV1_10800	AGCCCTTGACACCTCCCTTAGCATCTTCGTGCTCCTTGGCCACTTCTGATGTCTTAGCAT
EMPV1_10801	GGTGGCTGAAGCCATTTCCTACGTGGATCCTCAGTTCCTCACATACATGGCACTTGAAGA
EMPV1_10804	ACACTGACTTTTCAGAAGGAAGTGGCAGAGAGACTTATAGCCAGTAAGGGAAGCAAGCAG

EMPV1_10806 TGTGACCTCACTACCCTCCTGAAGTTGAGCTCTTCAGATACCTCCATCAATGAGCTAGTC
EMPV1_10807 TGGGGCACTGGATCAGTAAAGTAGGAGGTTCATACCGAGGGCTGCAAACAGGAATCCCCC
EMPV1_10809 GGATCCAGCATCACCATGAGCTGTGGTATAGGTCACAGACTTGGCTCAGATCCATTTGTG
EMPV1_10810 ACACAACCGGCACAAGATAACTCATGTCCAGAACTTTCACACTTCAGCTCTCCTGGTTTG
EMPV1_10811 GGGAACTCCAAATGCGACTGTCTGGAATGTGCTCCAAAATAACTTAGAGGTTGGGTCTGA
EMPV1_10813 CACAAGGGGGCGCGCGTAGCTCTCCTTGTATATCCATTCTTGCAACAAGGGGCTTTTTTA
EMPV1_10816 CAGGATGTGGTGCATTGCTGAGCTTACGGAAATGCTGTGGAGTGACTTCCAAGAGATGCT
EMPV1_10817 CCTCAATTGCCAGAAGTCTATTATATATACTGTGAACTTTCTCATACTTCATGCGACTCT
EMPV1_10818 ACATTCGAACACCGTCCTCTTCCTCGAAAAATGCCAGTTGCCTTGAGCAAGTTCAAGCCC
EMPV1_10819 ACCCATGAGGCAGAGCTCTGGTGTCATATGAGACTCCGGTACCAATTCTTTCTATGGCCT
EMPV1_10820 CATAGGTCTGGTGGTCTGGACGGGAAATGAGGGCAGATCCTTAGGGGGTGATTGTGTCCT
EMPV1_10821 CCACGAACCAAGGGCGCCATGACAGCGCAGGTCGTTATAGAAATAGCTCCTGGTATTCAT
EMPV1_10822 CGGCAGCTATATCTCCATTTTGACCCCTAGCCCAGGAACTTCCATATACTGATGGTTCGG
EMPV1_10823 GGCCCATCTTCAGGATCCACCACCAGAAAACCCGCTATATCTTTGACCTCTTCTATAAGC
EMPV1_10824 CGTGTCAACACGAGTGTGGTGGAATAAGTGCTAGCATTGGACATGATCCGCATCTTTTAT
EMPV1_10825 CTAAGGTCCACCTCGCACAAGGACTCTAACCCAGACCACAGCTTGCCACGTTCACTCTGA
EMPV1_10827 TCAGAAATGCTTTAACAGTTAATGATGAACTTAATATCTGAACTACTCACCCTGAAGGCG
EMPV1_10828 AACCCAGGGCACCATTAGCCACCGAGAGACTCAAACTGCAACTCCAAGGCTCTCATTTTG
EMPV1_10829 GTTAGGCTTTTTTAGGACAAAGTCAGCCAGACACCTTGTACTGGGCACGCCTCAGTCTAC
EMPV1_10830 AACCTCAAACGGGGCAACTGGGGCAACCAGATCGAGTTTGTACTGACGAGCGTGGGCTAT
EMPV1_10832 CAATGATGGCTGCGTAAGTGTCTCAGAGTGGGTTTACGGATTGTCGGTGTTTCTTCGAGG
EMPV1_10835 ATTTGACATTGAAATAGACACGCTGGAAACCACCTGCCATGTCCTGGACCCCACTCCCCT
EMPV1_10836 AGAACGTGAAAGGCCACCCGCCCCGGAGGGTCACTCTGAACACGCTGCAGGCCTGGAGCA
EMPV1_10837 TCGTGCATCCTCCTCCAGAGTGTCTGTGCCAGGTGCTGGATGCACAGTTCTCACCACAGG
EMPV1_10838 TCAGGTGCCTCTTCAAGGTTTTGTTGGTTTGATGGAGGTGCAGGTGTGGTGCGGTCATGA
EMPV1_10843 GAGACTGAAGAGGCAGTTTCCCCAAACCACCCACTTTCCACACAGGCTTGCGAGGCCTTT
EMPV1_10844 CAGGGAAGGTCAAAAACACATGGCCAACATGAAACCCATTTAAGAGGGACGCTGGGACAC
EMPV1_10845 TCCCAGCTCTCACTATGTGATTACCCTGAAAGCGTTTAACAATGTGGGCGAAGGCATCCC
EMPV1_10847 CTTGAAGCCAAAGTAGTTGAACCTTTGAAAGCTTATGGAACCATTGTAAAAATGAAACGA
EMPV1_10848 GAGGCCTTGAAACTCAAAGAGAGGGAGACGGCCCTGGATATTCTGCACAACGAGAACTCT
EMPV1_10850 CTCTCCTCACTCGATATTCTCTGGGTACTCCTATTTACCCACCGCAGGAAGACAGTGCAT
EMPV1_10851 TTCGGGAAAAGAAGGAAAGCAAACCTGTGAGGCCAACGAGGGCCAGAAGCAGCTCCCTTT
EMPV1_10852 TACTGACAGCCTTTCAAAACTTCATTACCTCATCTGGCATGGCTTTCATAGGCAGGGCCC
EMPV1_10853 GGCGCTGCTGGCCACCGAGTGTCGTTTGATTTGTGACCTTGACCTAATAGATAGGCTTGT

EMPV1_10855	AGCTTTCCCACACTGAGTGCATGTAAAAGGTTTCACTCCAGTATGAGTTCTTTGATGTAC
EMPV1_10856	AGAAACAAGCTCCTGCTGGATTCAACATTAAGCCCCTGATCCTGCTCAAGGCCCTGCTTT
EMPV1_10857	GCAGTTCCATGACCCAACACAAGCATTTTTTTCTTCCCTACATATTGCTCTGTCCTACAT
EMPV1_10860	GCAATTTTTCCAAGAATGTGAACAGCGTGCAGTGGTATCTTCAGTACCGAGGTGGCAGCC
EMPV1_10861	GCCAAAAGCTATTTTTAGACTCATGGGGACGGACAAGAAAGAAGCTGGAAGGTGTCCCCG
EMPV1_10862	GGAAGAAGGAGGGGATTGATTAGGGGAACATGGTAGTAAGAGCCCACTTTTTGTCAGTGG
EMPV1_10863	GTTGACTAAGCCAGGAGAAGTGAAGAAGCTGAACAGATTGACTGCTTGGTTTGGAGACAC
EMPV1_10865	CATCACGCAGTTGGTGATTTTCAGCCTGTGTAGGTGTTCTCGGCTGGCATGCCTGGCTTT
EMPV1_10866	TGCTGTTGTGCTTTTAGAGAAAAGTCTGGACTCAGCCACAAACTCTGATAAGACCTGTAC
EMPV1_10868	TGAGCGAAGTGCTCTGCGACATCCAAGGCTCTTTCCAAGACAGGTGAGCAGGTGCTAAGT
EMPV1_10869	GTGTGGACACCAAAGTGGGTAGGATGGTATGATTTAGTAGACATCACAGAAATGCAGGGG
EMPV1_10871	GAGGTAGTGGTGAACATGCTGAACTCGCTGTCACGGAACCAGCCGCTGCCGCAGATCACA
EMPV1_10873	GTGCAGGTATAAACGCGTCCAGCAGAAGCACCACCTGGAGAATGAGAAGACACAGCTCAT
EMPV1_10874	GATCTAGGCACTGATCCACGAACCGCGTACTGACACCTTCCTTAAATAGAGAGTGGTACC
EMPV1_10876	TCCTGAGTCGCTGTGAGCTGTGGTGTAGGTTGCAGAAGCTCCTCATATCCCCAGGTGCTA
EMPV1_10877	GATACATTGACCTGATCCCCATCAGCGTGCCTCACGCAGTGACCAGGGACATTAAATTCA
EMPV1_10878	CTCAGAAGGGCGTCTACAGAGAAGTGATGCTGGCCAATTACAGAAACATGGTCTCCTTGG
EMPV1_10879	TCCTCAACCACCATGTTCTTTGCCAACGCTGAGCTCTACGGTGACGCGCTCAAGCAGAGG
EMPV1_10880	GTCCACACATGGTAAAACAAATGATTTAGAGGAGGGTACTGCAAACAAGTGCATCCTCCA
EMPV1_10881	CCAGCAGATGTGGAAGAGAGAGTGGATCACTACATAGGAATTTTGATGTACCCAGCCTGC
EMPV1_10884	ATGATACTGCTAAGTGCCTCTTCAGACCATCCACCAATTCCCTTTCAGGACAACCCACCC
EMPV1_10885	ACAGGAGCTGGGATTCTAGCACTGATGGATGCTCTGAGAGATAATGAAACCCTGGCTGAG
EMPV1_10889	CTGAAATTTATTTTGCCAATGTTCTCTGAATGAACTGAATAAAGCTTCTGTTGTAGCATG
EMPV1_10890	TTAAAATCAACTCACGTCTCCCGAAAATATACAAACGTGGTGAAATTTCACAGGGCTGTG
EMPV1_10891	GGAAGCCAGTTTGCAGAGGCGGAAGAATAACACTTCTATATCTTTATAACATCTGGGCTA
EMPV1_10892	CCAAGCGAGAACAATGTAGTTAATCCTGTACTCATCACCCAGCCTCCACAAGGATCACCC
EMPV1_10894	CGTAAGGCACTTAGAACAGTGCTAGGAACAATGAAATACTCCACCAGTGTGACCTATCAC
EMPV1_10895	GTGAGCATGACCGCCGAGCTCTACCCCTCCATGCCTCTCTTCATCTACGGCGCTGTCCCT
EMPV1_10897	TTCGCCTGTTTCAGGTCCAACCCGCCAGACCTGGGAGCAGCAGCTTCGTCTGGGGATTCG
EMPV1_10898	TAAAGACAAGGAAAAGAAGCTGGATGATGAGAGTAATAGCCCGACGGTCCCCCAGTCTGC
EMPV1_10900	AAGCAGCCCAATCGTAAGCTGATGAGCTAAGACAACCGACACCAAGACACTACAGGGACT
EMPV1_10903	ATGGGCTCTGTGCTGCTGTCATGGGCTGTTGGTTTTGTGCATACGATGAGCCAGATGGTT
EMPV1_10904	ACCCAGGAGACAAACATGGCAGAGGTGAGGACTCAGAAAACATCCCTGGAGCTGCCCTTT
EMPV1_10905	CTTTGGGTCTCTCTGAGTGTAAGCTTGGGGAAAGGAGATGGTAGTGAGTAAAACTGTGGA

EMPV1_10906 TTGGGTATCACTGGCTTTCCTCGACCTTCAGCTTCTGCGGCTGTTTTCATGGTGGACACA
EMPV1_10907 AGGTGCCAGACCCGCCTGGGTTTTGGAGACTTGGAAATTAAAGCAAGAGACTTGGGGATT
EMPV1_10908 AGAGTGGAGGGCAGGACAGTACCTTTGAGTGCAACATCTGCCTGGACACAGCCAAGGATG
EMPV1_10910 CCAGCGGGTAGAAGAGCTACAAAGTCAGCTTAATGACGCTAAGCAATCGTCCATACCGAG
EMPV1_10911 CTCCCTTTGAGGAGCCTAGTGGAGGAACCTAAGAAAAGAGGAGCAGTACCTAATCACCTC
EMPV1_10912 ATTAACAACCTTGGATGGAGAGCTAAACATTCCCTCTTTTTTCCTGACCGGCAATGGCCC
EMPV1_10913 AATGCTTTTGCACCCTTGACTGATGCCAAGACTAGGAGCCATGCTGATGATGTGGGTCTC
EMPV1_10914 GTAGGACATTGACCCCCGGCTCCCTCCACCCCGAAATCATTGTGAGCAAAAACCTATTTG
EMPV1_10915 CCTCCCTGGACTTTGGACCTGCCCAGATGCCTTAGATTGGGGTATGAATAAAATGAGGTG
EMPV1_10917 GCGTCTCGGGCTCAGAGCCGCTGGCCGCCGGGCATGGACCCTCTCACCGCTGGGGCCCAA
EMPV1_10918 GCAGCAGGCAGTAGAATTTATGTCCACCAGTGCTGTGGCTTCCAGGTCTCAAAGGCAGAA
EMPV1_10919 TGGAGGGGCTGTGGAGAAAAGGGAACCCTCCTGTACTGTTGGTGGGAATGTAAACTGGTA
EMPV1_10921 GGGGAGTTGGGTACCGACCTTTGCTCTTGGTCGGAGGCGGTTTTCACCCTTTCGCTCTTT
EMPV1_10922 ATTTCCATGGTCCGTTGCAAGCAAACCTCCTAACAGCCTTTGATACACGTCCGTCGCCAG
EMPV1_10923 CAGTGAAACCATCAATGGACAGGGAATCACGGCCAGGTTGAAGGAGGGGTAAACATTTGG
EMPV1_10924 TGTGAAGCTGAACGAGCACTTCCTGAACACCTCGGACTTCCTGGACACCATCAAAAGCAA
EMPV1_10925 GGGGACCTGGAAAAGAACAGGACTATGTATGTGTATAACTAAATCCCTTTGCTGTACCCC
EMPV1_10926 CAGCACTTCTATAACAGCACCTTCATCTCCAAAAGCTGCATGGTGAACAGCTCTGTCTCC
EMPV1_10927 AAGGCAGATGGGTGAAGTGTGGGAATCTCTTGAGACCAGGCTGACCAGTAAGTCATGGAC
EMPV1_10928 GTGGAGCTGTGATTTCTTCTGGCCATTTTTCAGGAGGAGCAAATGATCTACCATGTTGGT
EMPV1_10929 TTCTCGCTGCTCCATCTCGCCGCCATTTTCCGCGCCCAGCCGTCGGGACACGAGAACTAC
EMPV1_10930 GATCTTCCTTCACTCACAAATGGCTACCCACCATCAATCAGCATGTACGAAACTCAGACC
EMPV1_10933 AGATCTGCCTCCCCACAGTCTGGAACAGCCAGTCCTGATTAGGTTGCCAGGAGAAGCACT
EMPV1_10936 CCCGCGCTGTGTGTGGTGTTCAGCAGCTTTTTGACTGGCGTGGTTTTCATGCTGATGTAT
EMPV1_10940 ACGTGGTACTCACACAGTGGCTGCTGTTCACCGACAGATGAGGACAGATGCACCAAAGAG
EMPV1_10941 GGGAAGATGAAGAAGGAATGAATAGAATGCTACCCACGAGAGAACGGTCCAAAACAGAGG
EMPV1_10942 AGCATTCGTAGGTTTCCCAGGACTGGAAGGCCGAGCTCCTACCTGGACAGAGGCCCTTCT
EMPV1_10943 AGCAAGGCCCTCACTGAAACCCTCAGAACAGCAAAAACCCATCAGGAAAGTTCCCCAGGG
EMPV1_10945 ACGCCCACTCACCACTGCAGTGTACTTGCTTTGAAAGGACACATACAGTCGGAGTGGTGA
EMPV1_10946 TCTTTGGACCTGCCATCTTCCTCTACATGCGGCCCCCCTCCACCTTCACTGAGGACAAAC
EMPV1_10948 CCCCAGCTCTAGTTACTGGAACCCTGCTCCCCTTTGGGTGTGTGACTGTGATTTATTTTT
EMPV1_10949 CCAGAAAATCTAGACGAACAGATCAAGAAAGTGTCCCAGCAGGTCCTAGAGAAGCGAGCC
EMPV1_10951 TGATCTCTTCCACCTCCTTCAGCGCCAGCAGTGCGTAGCCGACCGCGTGCTCGAACAGCA
EMPV1_10952 GCTGTAGAGCTAAGATGACTGAGAACCCCAAGAGAGCATGTATGGATATATAACGTGTGC

EMPV1_10953	GTCCCACAGGGATTTACGGTTTTTCCCTCATCCCTAAGGGGTAGCCTTTGTAGCATCAAC
EMPV1_10954	GTTAAAGCCTGGAATTCCCAAAGTACAGATGAAAAGTCAAAGACAATCTGATTGAAGTGA
EMPV1_10956	GCCAGTGGTGTGCAAGCACAGATGGCAAAGCAACAAGAGCAGGACCCCACAAATCTATAC
EMPV1_10957	GCTATAATGAAAGACAAAAATGATCATTATGTAATGATCAAAGGAACAACACAAGATAGT
EMPV1_10959	GCACCCCACTAGCTGCTTTTGATTCTGTTGGTGTCATGTTGAGAGAAGGTAGAATAAGCC
EMPV1_10960	ACCAACTAAATCCCCTGGTGAGAAGGAACAGAGACAACCTTCCTGTCTTCTGGCTGTGCT
EMPV1_10961	TGTGTCTTCCTTCCGGCAAGCACATGGGTACATTGATGAGGAACATCTAATAGGGGCTGG
EMPV1_10962	AACCCAGAAAGCCCCAGGGGAAGGTGGGTTTGGGTTTGATTTGCTGGTCTGAAATCACAA
EMPV1_10963	GCACTTGATGCTATAGACAAGGCTCTCAAGCTGAAACCAAAGGACCCAAAAGTTATATCT
EMPV1_10964	CTTCAGTGGAGTCTACGGACTGGGATTCAGCCATCCAGACTGGCTTTACCCGGCTGAATA
EMPV1_10970	CTGCTACTGCAGAGTTCAGTCAAATTGTTCAAGAATACAAACTGCATCGCGCCCGTCAGT
EMPV1_10978	TTTATGCCCTTAAGACGTTTTATTGAATATGCTTGGGGAGGGGGGCAGTGAAGGGGAAGG
EMPV1_10979	GACCCATTGTTTCAGACATCTGCTCAGTGTGAATGGGAAAACTTAAGGTGATCCAGAGGG
EMPV1_10983	GCTGGTACACAACAATCAAACTGCATGTTCAAGTAGCCAGTTACAACTCCTTTCCACCCC
EMPV1_10984	ATGGTCCAGGTCCTCACTCAAATAACAGCTCCAAGACGATGTGGGAACACGGACGCTATG
EMPV1_10985	AGGACCGCACCTTTGCCCGAGTCATTGTTGGGGGTTCCTCTGAGTACAAGATCAACAACA
EMPV1_10986	GTCGAAAACTAAAGGACACTTGGATGCTGAGTTGGATGCCTACATGGCACAGACAGATCC
EMPV1_10989	ACGGCCTTAACACTAATGGTCGTCCTCCGAGACAACAGCTGCATCAACGTCAACAAAATC
EMPV1_10990	CACAGCGAACATCACACTTATCACCAACATCCTTGGGCAGTCTCATCTCGTGTGTGTGCA
EMPV1_10991	ATTGTGACTCCAGAGCAGATTGAGGAGGCTGTGGAGGCTGCCATAAATCATCACCGATCC
EMPV1_10992	CAGACCCAGGCTGCACAGGTATAATTTGAAGAACCAACAACTGACCCTTCCAGACACCAC
EMPV1_10994	TTGCTATCCCGTGCAACAACAAGAGAGCTTGCTCAGTGGGTCTGATATGGTGGGTGCTTG
EMPV1_10996	CGGCGTAAAGCATCCAAGGTCTGAACATACATGCCACGGACTATAATGAAGCACAGATTC
EMPV1_10997	GATGAGATCGAGCGCAAGTTCGACAAGTGGCAGGAGCCGCCGCCCGTGAAGCAGGTGAAG
EMPV1_11002	GTACGTCCCATGCAATATTTAGGACATACTTATGCTAAAAAACTATGCATTGTTCATCTG
EMPV1_11004	GCTGCCGCCGCTGCACCCGGTCTAGAAGAGCTGCCGCCTGCAGGACACCAATAACTTCTT
EMPV1_11005	GGAAGGGACAGTGATTTTGTTTGGGTAGCTAATGCAAGCTGGACTTTTATGGGAAACGTG
EMPV1_11006	GACTTGAGGATCCCGCGTTGCCATGAGATGTGGTGTAGGTCACAGATGTAATTTGGATCC
EMPV1_11007	ATCAGCAACCTCTTCAACACAGAGGAGAGCCGAGCTGCGTGGAACAACACTCTCCTTGAT
EMPV1_11008	AAATTGAAATACAGTTCGCACAGGGGGATCGGAAGACTCCAAATCAGATGAAAGCAAAGG
EMPV1_11009	AAGTTGGCTTAGTTGGCTTACCTGTGGCTCAGCGGGCTCCCTGGTCCCTCCCTTATTTCT
EMPV1_11011	CACTCTTGGCATACTTGCCTTTCTGTGTCTGCCAGTTTCCACTTCTGACCAGGTTGATCC
EMPV1_11015	CCTCCAGAGCCTCACTGTTATTTTCTCCAGGCCCTTTCTGTTCGCCGTAATCGACAAAGA
EMPV1_11016	TATGAAGCAGAAAGGCTTTGGTAAGGACTTCAAGAAGCTGGAGTCCTTCTACATCCAGAC

EMPV1_11018	GTGACTTGGAAAGGGGGGAGGATGTGAAAATGTCCTGCTCTAACTTTTGGCTTTATGTCC
EMPV1_11019	CAGTGATTGCTCAGATGTAAGGAAATGGCCCAATGAACATGGTTGTGGGAGGGGAAAGAG
EMPV1_11020	CAGACGTGAATTCCTAGACGCCTTTCATTGACTTCCATGTGTAACAATGCCGGTTAAGCC
EMPV1_11021	TCTTACCCAGAGAGTCACCTTGCTGGAAGGAGGTCTGGTTGCTATGAGCGAGGTAGAAAG
EMPV1_11022	TGACTTGCCCCAGTCAGTCGCCTTTTTCAGTGCAGTTGATATTGACCAGTGCCTCAGGAA
EMPV1_11023	CTCTTGGTGTCCAACAGTGGAGCCATCTGTACAGTGAGTTTTGTCATGCTGATGGTCTCC
EMPV1_11024	CCTCCCCCCCACTTGCTCCCTGCTATTGAACATTAAGCCTATAAATGCAGAGGATGTGGG
EMPV1_11025	GCCTCCGGATGGTCTCGAGAGTGCAGCAGATAATAATGTCTTCCACTTGGTGGCCACTTT
EMPV1_11028	GAAGCCTCCACCTTTGTGACCTAATTACTGCCTAAAATTCCTACCTCCAGGAGTTGCTGC
EMPV1_11029	GGAGCAAATGGAGACGCCTGAAAAACAACATTTGTGTTGTCTATGCCCCTGCCCGAAGAT
EMPV1_11031	TCCCCCCTGCCCTCTTCTTTTGACTCCCTGTATGAAGTCCTCTGGTGTGGCATAAAAGTG
EMPV1_11036	TGATCCCAAAACCCACTCTTACATTATCGTCAACAAGGGGCAGAAGGGTGACTCAGCAGC
EMPV1_11039	GCACTCCAGAGATGCTGCTGATCCCGTTGTGCCACAGCAGAAATTCCCAAAATCTTATTT
EMPV1_11040	CCTTCTGTCCATAACTGAGCCCCACCCAGCAAAGTACAAACAGGATGCAAATGCAGTGGC
EMPV1_11041	CTTCATCACCCACCTGTTCAACGCCATGCTGCCTTTCCACCACCGTGACCCAGGCATCGT
EMPV1_11042	ATAGTTCTTGAAGCCACTACTGTAAAAAGTAGGCCAGGACCTGAGTGGTAAAACTAGACA
EMPV1_11045	AGGAAACGCAGTGTCGAGCAGCTGTCCTGGGCATCTCACGCTTGAGTCAGTGGGCCCCCC
EMPV1_11048	CTGGTCCAGCTGTCAGGATTGGATATCTTCTACTGCAATGATGAGACAGCACCTACTCAT
EMPV1_11050	CCAGCCTTCCCTCGAACCAGTCAGGCAATTAAGGGTCAGGAAAGAAAATGCTTTGGTCTC
EMPV1_11051	CCAGACCCCAGTTTGTAAGAGAACCTTTGTCACGCTGGATAAACACTATTCCCCAGGACC
EMPV1_11052	GGAAACGGATGACTCTGACTCTGAACCGAAGTCTTCTCCTGAAACTCCGAAACCTAGCAC
EMPV1_11056	CTTCGTGCCTCTGTGTCCCTGGGCTCCATAGTTTGGCATCTGGATGGTTTTATCAGTCAT
EMPV1_11057	GTCCTTTATTCTGTGTTCAATATGGTACAACAGTGGGGGCACCTACACGAGCACAGGCAC
EMPV1_11059	ATAATGTCAATATGGCCATATCTCCAAAAGATTGATCAGACAGCCGATGCAAGTTTTTTG
EMPV1_11061	ACGTCCGGAAACTGGTTCGGACCGTCATGGTTGCCCATGAATGTCCCAACTACTTCCGCT
EMPV1_11063	CCCGGACACAGCCTAACTTATTCTGCCTTCAGGGGGGTGGAGGGATCTTTTTATCGGTAA
EMPV1_11064	ATATAGAGTCCCGCAGCTGTAGCTGCTGCGGATGCGAAACCAGTTCTGAATTTCCATTCT
EMPV1_11065	CACGACGGTCCATAGTTTTATACGTGTGGTTCCTGCCCAGCCTCAATAGTGTAGATTTTG
EMPV1_11066	GCTCCCGCAATCCTGCTCGGCGGAGGGGCCACTCTGCTCCTGTCGCTTCTTTGGATGCCA
EMPV1_11067	AATGTGTCAGATGTCTCAGGCGGGTGCCAACATCACCAACATAACCCCCTCTCTGGAACA
EMPV1_11069	GCTTTAACATGGGGGTACCTGCTGTCTAAAACTAAAGTAAGTGCTTCCATGTTTCAGTGG
EMPV1_11070	GCCTCTGGATTAGGGACTTGTCCATTCAGGGCTTTGACCCGTTTTATTAGATGGGCTTGG
EMPV1_11072	GGCGTTTTCCTTCTCAGTTCAGGGGAGGGTTTTGCTACATAGAGTTGCCGTGTAGAACAG
EMPV1_11073	TACTAGGCTACAGCCACCATACCGGCAAAGATGAAAGCTGGCAATAGAGAGCAAGCGATG

EMPV1_11076	ATCTACCAGATGCCAGGTTCCACCACCGCAGCATCCCAGGACACAACACGCCCAGTGGCT
EMPV1_11077	CCGTGTTACACTTGACACAACGCGTAGGTTTTGACAGTCGCATAACCATCCGGTATAGAC
EMPV1_11078	GTGCTCAAGTCCTGCAGCTACAACTGTGTCACCATCTCCCCTGATGGCAAAATCATCTTT
EMPV1_11079	TTTCTCCCTGGTACTGATGTAGCAAACACCCTCTGCACGGGAGCTGCCAGTCTTGAGTTT
EMPV1_11080	GTCCTTGGCTTGTTTCTTGGTGCCCACAGTTTCTGCCTCTAAGCTTTAGTCCAAATGGTT
EMPV1_11081	ACCTCCTCCTGCTTCCAGCTGGAGTGTAAGCTTCCTGCACACCAAGACATCATGTGTTTT
EMPV1_11084	GCGAATAGAAGACTATGGCTTGTGTATACGTCCCGGGCAGAGAAACTTCTTGAGCTGGGT
EMPV1_11085	TGCTGAGAATGAGGCTTGCCCTGTCTGCATCACCTTCACCACCAGCATCTGTGCCGGCTA
EMPV1_11086	AGTGGATGCCAAACCATCAAACATAAATGCCCCTGCTGTGTCCCTGACTGTGTAATGAGC
EMPV1_11087	CGAAATCTGCTACTACTCTACTTGCTTGTCTGCTAATCTGCTCTACTTTTGTTTCTTTCC
EMPV1_11088	TTTTACCAGTTGAAACGACAGGACGGCTGTGAAGGCAGTGGTGACAGGCTCCGAAGCAAA
EMPV1_11090	GGCTTTGGCAACAACATACCTTTCATCCACCAACAACATTAGGAGGCTGAAGTAGAGCTG
EMPV1_11091	AAAATGAATATTCCAGGCCCCAGACCTCCTGATTGAGAAGCTCAGAGTGGCATGTGCTGG
EMPV1_11094	TAAGCGCTCCTGGTCCTCACTCTCTTAATCACCGCCCTTTACCCGCGTGAGGCTCTAGCC
EMPV1_11097	TGCGTGTGCCGGGAGGCGTGGGAGTTATTAGAAAAGTTCGGGCTGGCAGGTTAATATCAT
EMPV1_11098	GTGACTGTGGCAAAGCCTTCTCTCGGAAATCACACCTCATACCTCACCAGAGGACTCACA
EMPV1_11099	TGACACCTGCACCCCCAGCTCTTTGCACTCTCGGTGCGTGGTTTTGACTCTCTTTGCTTT
EMPV1_11101	TTTTTGTCTTTGAATTTGATCCATATGTGCATGTGGCTTCATGTAGAAATCAAGAGCTCG
EMPV1_11103	TTGGTTCAGCGTTTGTGCCTCCTACACGCGCATTGGAATTGCCATTTTGAGAATCCGCTC
EMPV1_11108	AGGAGAACGAACTGAGGAAAAAACAGGAACAGGCACTGATGGAGAAACTCCTGGAACAGG
EMPV1_11109	GCCTACATGGGGTGTGACCAGGAGTACAAATTCAGTGTGGATGTGAAAGAAGCTGAGACA
EMPV1_11110	AGGGAGTCGCACAGAAAGGGTTTTCAGGCTTCCAGCCTCTGCGGAATTCAAGATCTCTTA
EMPV1_11111	TAATATCTCTGGTGCTGAACGAACTGCCCACATTTCAGGGCTCCGCCCCGGTAATGATTT
EMPV1_11115	GTTTTTGCTTTCTGATCAGAACCGTGAAGGCTGAGGCCGGGCTCCTGCTCTTTACAGGTA
EMPV1_11117	TGCAATTATAGCACAAGATGTTCATTATAATTGATAACTTGATGTAGTTGTTACTCATAT
EMPV1_11118	TACGACCACCACTGCCCCTGGATGGAGAACTGCGTGGGGGAGCGCAACCACCCGCTCTTT
EMPV1_11120	CTAGATGGAATCCCACAACAAAACTTTAATGATTAGAACCCACCAAGGTTTTTGCAGGGG
EMPV1_11122	TTGAATATCAGTGGGGCCAGGCTCTCCTTTGCTGCCAGGAGCACTCAGGGTCAATGCTGT
EMPV1_11125	TTTACAACCTCGAAGGAGCATAGCATTTAGATTACGTTTGGCCTCTTTTGATAGCAGTGG
EMPV1_11126	ATAGCCTTCAGAGCCCAAGTTTGAGGCGGAGGGTTCAACTCCATAGGTCATTTCCTCCCT
EMPV1_11127	GGGGTATTATTGGTCATCTATGAGATCAGAGGTACACATGCCAGTGAAGAGACAAGTCAC
EMPV1_11128	AACAAACCAATAAGTGCTGGTAACGGAGGATGTGGAGACACGGGCAGACCTGTGCACTGT
EMPV1_11130	GCTTGTACAGTCTGGCCCTTGCCTATCTCTCAAACTCTTCCTTTTACACTGTGCTCCAGC
EMPV1_11131	CGTCCACGCAGCCCCTGCTCACAGAAGGATGACATTTCATGTGTGAGTAGTCTCAGAAAA

EMPV1_11135 GCACCAGAAGTAAAGAAACTTTTACAGCTTTTGTTGATTTGGCTGTTAACGCTGATCCCC
EMPV1_11137 CTGCTCTACATTAGAAGGAACGAGAAGCACCAGAGCCCCGAGAGAGGGCAAAGGAAAATT
EMPV1_11138 GGGCTTCTTCTTGGACTTCTTTGTTGTCTCGACACCACAGAAGACACAGAGCAAGACGAC
EMPV1_11139 ATGGGCATTGGGGAGAAGCAGGGGTGCCTCCCCATCACAGGCTACATGCTTAAGGTAGGT
EMPV1_11141 AGTGTTGTTTCCAGGGAGCAGGTAGCAGTAACTGTGGAGGCGTTTGTAAGAGCAGAGACA
EMPV1_11142 CAGGGTCTAGACGTTGTTCGCGCTTCGCGGGCGCTCATCGGGGCCACGAACCCGGTCGAT
EMPV1_11143 CCCAGAAGCCCCACAGGGCCCAGCTTTGTTTCACTAGTCCTGATCTTAGGAAGAAAGTAC
EMPV1_11145 AATTTCCAGCTCTCACCCCAGAACAGAGGAAGGAGCTCTCAGAAATTGCCCAGCGCATTG
EMPV1_11146 CCGTGAGACCTGCTAGCCCTTCAACACCACTGCAGGATAACCGAACTCAAGGCTTAACTA
EMPV1_11147 CTGTAAGCAAAGCGTTAGAATTCATTGGCCTAACTCCTATCTTTTCTCTGCCAAAAGGGG
EMPV1_11150 CCTCATACTCACAGATACTCAACACATGGTTTTCTTCTTTCCCCTGCAAGAGTAATGTAC
EMPV1_11153 ACATTGGGCTCGGAATTTTAGTTAGGAGTTTCATGTTCATCGTGGCTCCCATAATCCGCC
EMPV1_11157 GGAGGAAACAAACCACAGTGGATGCTGGGCCTTGTAAATATGGAGATGTAGAAAAGGAGG
EMPV1_11158 ATTGGCCACGACTGGGGAGGCGTGCTGGTGTGGAACATGGCTCTCTTCTACCCTGAGAGA
EMPV1_11160 ACAAAAGATTCACGTTTAGGATTGCTGAAGCTTAGACACATGGGTCAGGGAAGGCCTACT
EMPV1_11162 GGCTCTGAATCTACATGACCAATGGCTGTTCTCTGCCGAGGCTCACAGTTCTGGCTTAGT
EMPV1_11165 CAGTGGTCACTCACCTGGCCTCACATTTAAGGATTATAGTCACTCTTTTGGACCCATAGC
EMPV1_11166 TGAACACGCCCGATCTCGTTGGATCTCAGAAGCTAAGCAGGGTTGGGCCTGGTTAGTACT
EMPV1_11168 ACTCGGGTTTGTTACCCGCTAAGCCAAGATGGGAACTCCAAGGAGGTATATTTAAATGGT
EMPV1_11169 CAACACTGGTGGTGATGGAGTTGATGGCTCACGGAGACTTAAAGAGTTACCTGCGTTCCC
EMPV1_11173 GCTGCTTTGGATCAGTTCAGCAGCCACGACTACCCTGCCACTTGCTTCTGGATAAATTCT
EMPV1_11174 TCACGTCTCTGCTTTGGACATGAGAACATCCTGGGCTGGCTTGCTGAATGTCTATGAGAG
EMPV1_11176 TTTTTGTGGCCACATCTGTGGCATATGGTAGTTCCCAGGCTACGGCTGAATCAGAGCTGC
EMPV1_11178 GCAACTTTGCTTCCTTTATTAGATGTGGTAATGGTTGTTTGCCTGTGGCTTCTTTGGGGG
EMPV1_11180 TCTGACGCCCCCTGGTGGACATCTAGAGTTGACATCGGTAACGGACAAACACGACCCTGT
EMPV1_11182 GCAGAATGAGAATCACTGTGTGGCATGTATCCAGTTTATCTTGTTATGGTCTTATGGTCA
EMPV1_11184 GTAACAGAGAAATGGTTTTGTGCATGTTCTTGGAAGTACTTATGTACAGAAGGGAGGGAC
EMPV1_11185 TTCTGGGCCAGCAGGAGCGGAGGAGTGCTGGGGAGCCAGGAGCTGAAACCACTGGGTCCT
EMPV1_11187 CACTGGACTGCTTTATTATAATCAGGGCAAGATGGATTCTTTTTATACTGGGGATTTGCA
EMPV1_11188 TTTAAACTGCGCAGAATTGTCTAGTGTCAGCATTCATGTTACAGACTCAGCACCTTGTTC
EMPV1_11189 GTTTCTCAGTAGGATTTCAACCTCCGAAAGGTATCTCTCTTTCTAAATGCATACGGGTGT
EMPV1_11190 ACTCAGAATTATCCAAGTTCACTGGGGCTCAGTCTCATTTTGAGGAGCTGCAAGCGACCC
EMPV1_11192 GTATCTGGTACATCAACTTCTTTGGGTGTGAGACGCACGCCATGCTGCCATATGACCAGT
EMPV1_11195 TGCAGCTCATTGGCACTGCAGGCAAGCTTCAACTATTCATCAATGCTGTCCTCTGGCTGT

EMPV1_11197	CCTTTTCCTCCTTGTTACCACGGGAGAGCAGAAAGCAGGATCCGCCTGGAGTTGATGGAT
EMPV1_11198	GGTTCAAGATACAAGAAGCACAGAGGGCCCCAAACAAACTGAACCCAAATAGACCCACAC
EMPV1_11200	GGTGATTACTATGACCTGTATGGAGGAGAGAAGTTTGCCACTTTGGCTGAGTTGGTCCAG
EMPV1_11203	GAAGCTTTTGCACGAGAACATGGACTTATCTTCATGGAAACTTCTGCTAAGACTGCTTCG
EMPV1_11204	AATGTTTTCGTCCCTATTACTGCTCTGCATCTGGTGGTGAGAGGCCCCACTTGCACCATG
EMPV1_11205	AACAAATAGCAGAACTTAAGATGACACAATCCATTATTTCCAGCTGCGTGCATAGCTCGC
EMPV1_11206	TCACATGAAGGCCACTGTGAGGCACGAGATGACGGAAGGTGTAACTGCCTATGAGGAGAA
EMPV1_11211	TGTGGTACTCAAACTGTGGGGGCACTTTCTGGTCTAGGGAGGGAAGTGCCGCCATTTTTT
EMPV1_11214	GGTGCACACTCCAGAACTTTTAACTACTGTTTTGTAAGCCTCCAAGGACGGCATTGCGGG
EMPV1_11215	CATCCCCTTGGGAAGCAGAATAGATGGCCTTGGGGTGGCCTTATTTTTCTAGCAACTTGT
EMPV1_11218	ATCACCGTCAAGGAGGTGCTCAAGGTGGAAAAGGATGTGGCGACCGAGAGGGAGGTGGGC
EMPV1_11219	CCCAGTAAAGTACCAAGGTCTAGGTCGTCTCACCTTCAGTGGACTGCTTAACGCCTTAGA
EMPV1_11222	AGTGCTGTTGGCATCTTGTGGTAAACCTACAGTGTCTGCGACCTTCGTGGTAGAAGAACC
EMPV1_11223	GTTTCATGCAGCATTTGAGACCTACTTATACTAAAAATGTTGGTTGTTTATCTGGAACTC
EMPV1_11224	GCTTTAAGCAGGAAGTCTACCCACAAACTTCTTGGAGTATCCCACTGAAGGATCCCCCCA
EMPV1_11225	CTGGAGATCCCAAGTGACAGTGAGGAGAATGCAATTGAGAGTGGATCCTCAGCCTTACAG
EMPV1_11226	CCACAGGTGACATCCACTCAACAAAAAGCCTGGACAGAGAGCAGAAGACTCACTATGTGC
EMPV1_11228	GAGGGTCATCGTTGCATCTTAACATAAAGGAACAAGCCCAGAGAGGTGAAGTGACTCCTC
EMPV1_11229	GGTTCCCCAGATCGTTCCTTGCTGGATCAGACCCATCCTACTGACCAAGAATAAATGAGG
EMPV1_11230	GCATAACTTGCTCCCAGATCACAGCAAATAGCTGGGGGAGTTAAGATTCAAGTCCAGTTC
EMPV1_11232	TGATGCTCCTACTCAGAAATGGCCCACAACCTGTAATTTGAAATGTAGCAGAAAATGCCC
EMPV1_11234	GCCCCACCTTGAGACAAACTAGACTAATAAGATGCCTTCTCCTTAACCTGATCCCGTCTT
EMPV1_11235	CACTCAACAGGGGCCACATCTCCACCTGCAGCGACTTGACCGCTTCCGTGTCCTTTTCGT
EMPV1_11237	GAAACCACTTTTGAAGGGCCTAAGATGTTGGACTTAGCAGACAGAGATTTCAGAACAGCC
EMPV1_11238	GTGAGGAGTCGGAAACTCAGCCACTGGTTGCCTTGGTGGGCAATAAAATTGATTTGGAGC
EMPV1_11239	CACTGGAGAGGCTAATGCAAATGGAAAGGACTGGGGAAGGAGTTGGAGTGACCGTTCAAT
EMPV1_11241	TTTCAGTCCGAAATCAAACGCTTGAAAATCTCATCCCCGGACCGCGTTTGTCAGAACTGC
EMPV1_11243	CGGTGTCTTGTTTTCTTCCTCCTCCGTGCATCTTGGCAGGCTCGCACTTCTGTGGCCGTC
EMPV1_11244	GAGGGACAAGGAATAGAAAGGTAGAGAGAATCACTGAGGAAGCCCTTTTAGCCCACAGCC
EMPV1_11246	TCTTCATCCTCTCCTACCTCCTGTCAAGGGCAAGGACGTGAGCCAGTGGGAACTGGTTTC
EMPV1_11248	CCCTGTAAACCCTTTGGACCCCGCAAAGTTGCTGTCCAGAAATTAAGTGCCTAGTTTTCA
EMPV1_11251	CAGGGGTCCTGATTCCCAGATTTCTAAAAAAGAACTGAGGTAAAACAGATGCCTTCCCTG
EMPV1_11252	TCACCTGAAGATTTTGTTAACGTGTAGATTCTGATTCATTAATCTGGGATGGAATCTGAG
EMPV1_11254	TAGGACCAAACGGGAAGCCTTCTTGGGATGACAAATGCAAAATGCCTTATACGGAGGCAG

EMPV1_11257 GTCCTCTGGATATTTCACTAATAACCAGATTTGGGGGAACATGACCTGTTTGTAAAGTGG
EMPV1_11258 GCCCAATCGTGGGCAGAACGGTACACATTTTGTGCGTATCAGAGGAAAGAGATGCCTTAG
EMPV1_11259 TGGCGGGGGCACCCTTTTTATGACTGCCAGACTGATTTCCGATTCTACTGGATGCATTCA
EMPV1_11263 CCTCTTTAGATAATTTGTCTTATGTTGCTTGGCATCTGCCTGATTACTGGGGGTGTTTGA
EMPV1_11265 GGGGATGATATACCCAGGAAGGCCAAAAGAAAAACCAACAAGGGGCTGGCTCAGTGTCTC
EMPV1_11267 AACAGGCATGAGACAGACGCCCTGGACGCCCACTCTTGTTCGTATTCATTTTATGCTGCC
EMPV1_11269 TTTCAGCTGTCCATACCTGGCCCTGGTTGATGGCCCCGTGCTGAGCGGTGCACGTGAAGA
EMPV1_11270 GGCGGACCGTTTCTTCCACTTTGGGCCTCATCTGGATATACACCATTCTTCTTTGTAGAG
EMPV1_11271 GCGGACGCAGAGCCTGTGACCCCTTTCTTTTATTTAACGCAAAACTGGAGTGTCCACATG
EMPV1_11272 GCGAAGGAGCAGAAGGCATCTTCTCACTGTGCTGCAAATCTTTATAGCCTTTACAATACG
EMPV1_11273 GCAGCGACTGTTTTCTATCCTTTTATCTTCCTGTCATCTTTAATATTTGCCATAAATCCA
EMPV1_11274 CCTGGCAGCATCCAACTAAGCGAGGAACAAAAATCAGCTCTGGCTTTTCTCAACAGAGGC
EMPV1_11275 AAAAGAGGACGCCCATTTGTCACCCGTGAGAGTGTCGGCCTTACTAGGTGCCCCAAAAAT
EMPV1_11276 GGACTGGGAGTTAGGAGCCCCGTCTTGGATTCTGGTTCTTAAGCTACTGGTTCATTCATC
EMPV1_11281 GGTCTTGGCTGTGCGCTCCCCAATAAGCCTGGTGTGTATGTCCGTGTTTCAAGGTTTGTT
EMPV1_11282 CGACTTCCGGCAGGTAAACAGGGTGAGTTTTGCCTTGAGGAAAGCATAGGTCTGCCATAT
EMPV1_11283 TCATGACCTGCTGGGCTTGTGGGTTTCTGTGGTTCCTGATCCCCATTGTTCTCATCTCTC
EMPV1_11284 GTCCAGTTGGTTTATGGTTGATGATGCAGGTTCACCAGTGCCTTGTTCTCCCCAGTTAGG
EMPV1_11285 GATGTGGTAACAAGAGAATTTATCAAAGTCTCTGGCGGCCAGCTGATGCCAATGTTATGC
EMPV1_11287 CCACAATAAATACATAATTACAGGCTGTGAGGAGTGTTCTGTGCTGCAGTAGGGCCCGGC
EMPV1_11290 GCCGACTGCACAATCACGATGTCCGACTCAGACTTAGTGGCTTTGATGACTGGTAAAATG
EMPV1_11291 TTTCTGGAGATATGTTTTAACAATCCTTTTACTTTCAGACTTCTTGTTTCAGTCTTAGCT
EMPV1_11293 TTGTGGGAGGACAAGGTATGTGTGATGGAACACCACTGTGCCCAGGAATAAAGCTCCCAG
EMPV1_11295 CTACCAGTGGCTCAAAGACAGGAAAGTGCAGAGTGAGGACGGCAAGAAGCAACAAGCCAA
EMPV1_11297 ATACCGGTGAGAAGCCCTTCTTCTGCGGCGCCTGTGGCAAGGCCTTTAGCTGCCACTCAT
EMPV1_11298 GGATCAACGTGTACTACAATGAAGCCACTGGTAACAAGTATGTGCCCCGGGCCATCCTGG
EMPV1_11301 GGAGATGACACACCTGGAGGTTCAGCACAAAGCTGACATTTTCAAGCGGCAGATCATCCA
EMPV1_11303 ACACTGCCACGTGGCTGCTGCGTTTGGGAGTGCTGCTTCCTATATTTGTACCCAACAGAT
EMPV1_11305 TTGGCACACTACAGCAAGCGTTCCACTATCACTTCCAGAGAGATCCAAACGGCTGTGCGT
EMPV1_11307 CATGGGGTTGAAGCTCCTTGGAAAGAAACCCCTGAGAACACTGTTGGTCCTCAGAGTATT
EMPV1_11308 AGGGAACGCAAAACCATTTGGTCTCTGTCTCTTAGTGTCCAGAATGACGGAAAGCACAGA
EMPV1_11309 TAGAGAGGGCCACAAGGTGAAGAACAGCAGGGGCAAAGGCCCTGGGGAGACAGAAAGCCT
EMPV1_11310 GGCAGGGATCATGTCTATCTTGTTCATGAGTACAAACCCAGCACTGTCCGCTTGCTTCTC
EMPV1_11311 ATCTGTAAGAGCGGTGCCTCTGTGACCATCCAGTGCCGTACAGTGGACTTTCAAACCACA

EMPV1_11312	GGAATGGATCGTGATTACGGCCCTGGATCTTATGGAGGTCTGACATTCAAGGATATTTAT
EMPV1_11314	CTCGTTCTAGGCCAGGAGCAAGATAGTGTGGGGGGTGGATTTGACAGCTCCGAGGCCTTT
EMPV1_11315	CCACTGGCGAAGACCCTGTACAAAAGCAAATATTTCACCTGCCAGTGTGGCTCTGAGAAG
EMPV1_11316	TGGAAATGTGGAACTGGTGGAAATCCTGCTGAAGGCGGGCTGTGACCTGAAGGTTGTTGA
EMPV1_11317	AACTGCGTGGTGCTGAAGCCCTCGGAGATCAGCAAGAGCACTGAGAAGGTCCTGGCCGAG
EMPV1_11319	TCATTCATGGCATCACCCCTGTCACTTGCACTGATCCCTGATGCCGGTTCCTAGTAGAGA
EMPV1_11321	TTTCCCTGAGTCTGCTAAAGGGATTTCTGGGCACAACCACCATGCCACCTTCCGGATGCT
EMPV1_11322	GATAAGGTTCTGGCACATTCCTGTTAATCTTTCTGAGTATCTCCCTTGCCCAAATGTGCC
EMPV1_11323	ATTCCTGGGATGAAATGATGGGAAGCGTTCCTACTTCAGGCGCTGCGGTTGCTGCTACAT
EMPV1_11326	ACCAGGTAGACCCTAATAGCCATAAAACCGCCCAACCAAGTGCTGCATGCTAGGCAGTGT
EMPV1_11329	GCTGCCATGGGAAGATTGAGTAGAATGAAAATTGGAAACAAACCCATGAAGATTTACCAG
EMPV1_11330	CATCTAATCCTTTTAGTAACTCTAGGAGAGAAGGCGAGCTCCTCTGAAGTGGGAAGCTGC
EMPV1_11331	ATGAGCCAGATTCTGTTCCAGCAACTCGTCCCGTTGCAGGTGAAATGTAAAGACTGCGAG
EMPV1_11332	GGAGCAGTCCTTTGAAGTGGGCATCCTCGCTGAAATACACAGCCAGAAAGAAGCAGCAGA
EMPV1_11333	CTGTCTCAGGAGGGAAGGGCAGCAGTGCGGGGTCTACACTCCTAACTGCGCTCCAGGACT
EMPV1_11335	TAAGGTCTTGCGGGATAACATCCAGGGTATTACCAAGCCCGCCATTCGTCGTTTGGCTCG
EMPV1_11339	AATGCTGGCTGTGTCCTTGCCTGACCCATGCTCACCGACTCAGACTCATTTTAATGGCCT
EMPV1_11341	GCGGGGATAAGGAACTCTGAGGCACTTCCTGTTGTATGGCTTTCTGGGCAAGTGCTTTGT
EMPV1_11342	TATCCAGCAAGCACTGGCGAATCGCTGGCTTAATGAGAATTACATGGGCAAGGTGCCATG
EMPV1_11343	GATACTGCAGCGATTGTGTGTTTAAGCCCTCACAATTAAACAGACAATGTGCGTCTTCTC
EMPV1_11347	CTCCTACTTAAAGGTCCATGTTAAAACCCACCACGGTGTTCCCCTTCCCCAGGTCTCCAG
EMPV1_11348	GTGCACCTTTTGTTACAAAGGCCACTCAACACAGGCCAACAGTCAAGGTGTCAGATGGAC
EMPV1_11353	GGTGTGTTGCTTGTCATTCCCTTGATTTTCTTCACCAAGAGACTACCCTACTGCAGAGGC
EMPV1_11355	ATGCCACCAAGATTCTCCCCCAGATCCTACTTCAGACCCTCCAGCACAACCTTGGACTGA
EMPV1_11358	ATTATGATTTTGGAGATGTGTTTCCAGCAGTGCCGTCTGTACCCAGCACAGACTGGGAAG
EMPV1_11359	AGGAAGATGGCAGAAAGTGCGGCAAGACAGTCAGGACTGAGAATGTGCTCCAGTCTCAAT
EMPV1_11363	CTGAGCCTCTTTTTCTCCTCCGAAATAACTGCCTCGGCTGTGACCTTTGATCACCAGGGA
EMPV1_11366	AGATGGTGGGGTTCGTTAGTGTGGGTCTCATGCCCCTCAGCTGCTTCCTTCTTATCATCA
EMPV1_11367	GTGGCTCAGGAGGTTAAAGACCAGATGTAGTCTCTGTGAGGATATAGGTTTGATCCCTGG
EMPV1_11370	AGGATGTCGACCTTTATGATGCAGAGCATGGCGTTTCTCCAGGCCTCCTTCGCGGTTCCG
EMPV1_11371	AGAATTTGAGGTGATGGTGAGGGAAGTGGGGTACTTTGATGTTGTCCTGTATTGTGACAC
EMPV1_11372	CCCTGGCAGCTGTGCACGGCTCTGAATTCAGTCAAACGACTATTTGCCGATTTGAAAACC
EMPV1_11374	GTCGGGACCTCGGGGACCGTGTGCATCTAAAAGACAATGATCTCAAGATGCCTCTGGTTT
EMPV1_11375	AGGGAGCACAGCCTTTGATTCATCCCTGATACGGAAAACTGGCACACTCTAGGGGTCTGT

EMPV1_11376	GATGAAGCTCACCAGAGGAAAGCCAAGAAGAAAAAGGCCCCAGAAGTGATTAAAGATCCC
EMPV1_11377	TACCACGAGGGGCTCCCCACCACCAAAGGCTCCCGCTTCATCGCCCTCTCCTACGTCGAT
EMPV1_11380	GTGCACATACCCTGCCTTATGCACCTCTTCCGGCTGGCTGTTCCTGAGTTGTTTCATTTT
EMPV1_11382	GCAGTGACAATGCTGAATCCTTAACTGCTAGGATCCGCCTGAGACATCCTTTTGCTTTGC
EMPV1_11383	TAGGGATAAAGTAAACCCATCTTCCGAGTTGTTGGACATGGGGAGGGAGAGGGCAGTGTG
EMPV1_11385	GCGGGGGAATGGATGGTCTCAAAGCACATGATTTCCTCATATTTCATCAGTTTCCAGGCT
EMPV1_11388	GACTATGGCCTCTAAGCCAGAACCATGCCCCTGTCTGAATAATATGCTTTCTTCTCCCCG
EMPV1_11395	CGGCAGGCAGACTTGGGCGGGCTTTCTTCAGACGTCGAGAAATAAAACCGTTCCTCTGGC
EMPV1_11396	TGTCCCCATCATCCGTGACAACCTGGCTTCCACACGGAAATACTTCACTCAGTTCTGCAT
EMPV1_11397	AAAAATAGGAAGGGGCTTCAGCAGCAAGGACCCCGATTTCCACGATGACTACGGCTCTCT
EMPV1_11400	ATCCTCTCCAGCATCCTTCACATCAGCTCCAGGAAAGGCAGGTTTAAAGCGTTTAGCACC
EMPV1_11401	CTCATCCAGTGACGTAAACTTCAAGGACATAAGCAACAAAACCACGAAAAAGGACCCTTG
EMPV1_11403	GAGCTGAAGAAAGCCATTTTGGAAGATATGGTGAGGTTAGGAAAAGAAAGCGGACTCCAT
EMPV1_11404	AGCTGCTACTCTGTCCTTCTCCTACTGTGGCTCCCGAGAAATATCCCAGTTCTTCTGTGA
EMPV1_11408	ACCCTTGCTGTTGTCTAGGCCAGGAATGCTTTTCATCTTACTCATCCTCCAAGATGCAGC
EMPV1_11409	TTCCCAGTAAGCTTCAAGCAAGCTATCACCAACAGAAGTACTTTGGGTGTGATCTGTTAC
EMPV1_11410	GTTCCAAGAACACTTTATTTTTAGAGCATCTTGGCTCCCGGTGCTGTGGATTCGGGCTCC
EMPV1_11411	ATGTGACTTCACAGTGCAGGTCCAGTTAAGGTTTTGTTTATCAGAAACCAGTTGTCCACA
EMPV1_11413	GGAGACAGTTTGGGGAGTGGGGGATGTGCTTGAGTTATGGGATGGAAATCCTGTGAAGTT
EMPV1_11414	ATACTGAGTGAGATCAGTAAGTCGGTCAAACCGCGGCTAGCATCCTTCCGCAGCCTTCGA
EMPV1_11415	GTCCTTCTCCATAAGGGAGCATTCTTAACTTCCATTACAGTGCCTGGCATAGTTCTGAGC
EMPV1_11416	TTTGTTACCAAATTTAACAAACAAAAACCACTTTTGGATGATGTAATTTTATCTAATATT
EMPV1_11419	CGTGTGGGTGGAAGAGTTGCTACGTTTCGCTAAGGAAACTATGTACCTGATCTTGGAGCC
EMPV1_11420	TGCCTGGAAACAAAACCTGAACAAAGTGAAAAACAGCCTTTTGAGTCTTTCTGATACCTG
EMPV1_11421	GAACCAGGCTCAGAATTGGGAGAGGGAGAAGAGGAAGTGGGCCTCTCATATTTAATGAAA
EMPV1_11422	TCATACCCCGAAACCAGAAGGCCATTGCTAACTCCCTGAAATCCTGGAATGAGACCCTCT
EMPV1_11425	GAGATTATTAAATTCTACCGGACACAAACATTGGACTTCCGTGTTCCTCCGCCAAAGAGC
EMPV1_11426	GACTGCTGGGAAAGATCCTGATGGCTATCCGAGATGCAGGTTTTGAAATCTCAGCTATGC
EMPV1_11431	TTTCACAGGGGCGACCTTCTGTTCCTAACAAATTTCCGGGAAGACCCGATTAGAGCTGGT
EMPV1_11433	GAATTTATTGTTATTCATAATGGAATCATCACCAACTATAAAGACTTGAAAAAGTTTTTG
EMPV1_11435	GTCACGGAGAACGGATGTAGAAGACCTGACTCCAAATCCCAAAAAACTACTCCAAATAGG
EMPV1_11439	GCGAGCACATCCGCTACGCCACGGACACTTTCGCCGGGCTCTGCCACCAGCTGACCAATG
EMPV1_11440	GTCCTATGATACTACCTTCCATGACGACCATGTGGAAGTTGGCTATGTTTGGGTGCACTG
EMPV1_11442	TGTCTAGGGGCATCTCCCACGCCAGCTCCAGCATTGTTTCCCTGGCCCGGTCCCATGTCT

EMPV1_11443	GGCTCCATGTGAAGAATAAAGGTGATTCAGAGGGATATGAGAGTAGCACACCCTGAAAGC
EMPV1_11446	GGGGTAAATATCCCATCATACGCCCACCGACCCATAATTTACTGACTTGACCCTTTCCCT
EMPV1_11447	GCTAGGAGGAGTAAGAAACAGATTCTCCCTTAGAGCCTCTGGAAGAAATCAGCTCTGCCG
EMPV1_11448	CCAACCTGCCCGCATGCCCTATATTTCGGACAAACACCCTCGACAAACCTTGGAAGTGAT
EMPV1_11449	ATCACTTTCAGTAGCAGGTGTGTGATTTACACTGCTGTCGCCCTGGTAGAGTACAGGCCC
EMPV1_11450	TGCCTGTTCTCAGCTCTCTTACCTGTAGACATCAACCTAATGGCTCACCTCCAGGAGTAG
EMPV1_11451	CTGCCAGCCTGCTGCTGAGGGGTCTGCTGCAAAGGCATCTGGGTTCCGTACATCGTATAG
EMPV1_11452	AGCAGGAGTTATTATAGGTCAGTGGTGTGGGATGGACTGAGGAAAGCATGGTGATGATGG
EMPV1_11453	GTACCCTCAAGCCTAGCACAGTGATGACACACATTAGGAACTCAAGAAATGGTTGTTAGG
EMPV1_11460	CTCCTGTCACACAAAGGTGGTGGGTTGTAAAGGTAAGGCAGAAACTGTTTCCAGGAGGTC
EMPV1_11462	CTTGTAACCCAGGCTACATGTTGGTGGGAAAGGCCTTCATCTTCTGTACACACCAGGGAA
EMPV1_11463	TACTCACGCCACCCAGCGGAAAACGGAAAGCCAAATTACCTGAACTGCTATGTATCTGGG
EMPV1_11464	ATTGGAGCCTCCCCACAGCCCTGGGAGCTTGATATTGCTATTGTCACCCATGCTTCAGAT
EMPV1_11465	TGTCCGTTCACGGTGGCCAGGACCGGCGGCAGCGCGTTCTTAGTCCACGGGTTCACCTTG
EMPV1_11467	TCATCCCAAGTTAGAAGGCTCCTTGACTCATCTCTATTGCTATTCATGGATATCAGCAGC
EMPV1_11468	ACTCCTGTCTTTTGCAGTTTAGGAAGGTGGGACTGGGATAGGGAAGAAGCAAGGGCCCAT
EMPV1_11469	GGGTTGATCCGTACGGGTTTGAAAGGCCTGAAGACTTTGATTATGCAGCTTATGAAGAGT
EMPV1_11470	TGTGAGCTAGGCTGCTTGCACCTTCCCTCCACCTTTATCACGTGTATCTTGGTATTTCCC
EMPV1_11471	CCATCGTCACTGGCTATACAGACCAGCAGCCTGACATCATCTCCAACGGAAGCATTTTGT
EMPV1_11473	TCATCTGCTGTGTCCTCTTCTTTGTGTTCATTCTGGGTTACATTGCTGTGGGGCTTGTGG
EMPV1_11474	TTTACAATACCTCCGGCAGCGAGAAGTCCCATAGCCACATATCCTGTCTGCATCAAGGTC
EMPV1_11475	CCACCTGTGACAAGGAACATCTACATCACCACCAGGCCAAACTTGTCGGATCGCTCTTTT
EMPV1_11477	GATGTGAAATTTGTTGCATCCTGAATCCAAAGGCTGTCTGCAGGTACTGTGCTTCCTTCT
EMPV1_11478	AAAACGTCTGTCGAACGAGCCAATTCCCTTCTTAGCTCCCGGTTCAACAAACCCTTCCTG
EMPV1_11479	TTTTTTGGCTTTTCATGGCCACAACTGGGGCATATGCAAGTACCCAGGCTAGGGGTCAAA
EMPV1_11480	GGTCTGTATGCAGCTTGTAACCGGCCCCTACTTATACAGCTTTTCTGTTGCTTTACTCCC
EMPV1_11483	GGGGCATAAAATTGCCAAGCTCCCTAGGAAAGAAGATGGACCGATGTTTCTGGTGGCATC
EMPV1_11486	AGTTGAAGCTTAAAGTAATTTGTCCATGGACACCCAGCTAGGCACCAGCTGAGCCAGGAG
EMPV1_11489	CCAGGACGTGAGATCTGGATCATGGAAGAAAGACCTGCTGTTGAGATAGAATGACCCACG
EMPV1_11490	GTTTATAAGAAAGGTATCAGAAGCTGTTAGCTCCGAAATAAACCCTCATTGCAGACAATG
EMPV1_11492	GGCAGACACATCAGCAGCCAAATACCATGGAGGTGTAACATTTCAGACTTACTACAGGCA
EMPV1_11495	TGCTGAGAATGAGGCTTGCCCTGTCTGCATCACCTTCACCACCAGCATCTGTGCCGGCTA
EMPV1_11496	AACAGCCAGATGCTAGTGTTGATCCAGCCTTGGTGAAGGTTAGCTGGCTCATCTGTTGCT
EMPV1_11497	AAGGCGGTCTGCTGGGCCAACCTTGGTATGCAGGGAACTGTGACCGCCATGCTGTTGAGA

EMPV1_11500	GAAAAGTAGACAAAGATTTAAAACCAGCAGCCCCCACCTCCCTCCACCATATGCGTCTGG
EMPV1_11501	CGAGAGGAACTCTGTACTGCCTCCCATGACACCATTACAGTCCACTGGATCTCGGATGAT
EMPV1_11504	CCATGGCCCTGAGAGGCTGGTATGATTATCCCCATGTTAGAGATGCCAAAAGAAGCTCAG
EMPV1_11505	TCTAGTAATGTGCAAGGAGAGCCCTTAGCCTGGTAATCATTTGGGAGCACCAGGCTGGTT
EMPV1_11506	TATCAGTCAGACAGGTGGACAGCTTAATAGTGTGGTCCGCTGGGCCTGTGATCGAGGATT
EMPV1_11507	TGAGCTCCCAGCTGCTTTTACCTGCCTCAAACCCATTGTCAAGTGGTAGTCAAGCTCCAA
EMPV1_11508	CAGGAGCAGTGGTTCAGGTTTTCTGTCGAAGAGGAAGACCCCAAAATGCACACTTACGGT
EMPV1_11509	ACAGTGGTGGCCAGAGCTTCCTTGTGCACCGTCACCGGGTTCATCCTTCCAGAGAAGATA
EMPV1_11512	ATCCCTAAGGAACATGCTTTGGGAAACAGTCATCTAAGATGGCATTCCTGCAGCCAGGGC
EMPV1_11513	GATCCCCAAAGCAGAGAAGCTGACCCTACAGGGACTATTTTGTCCACTCAACTCCATCTC
EMPV1_11514	ATTGCTAAAATGGGTTATTCTGCCCAGGAACACGCTTGGTGGTGTTAGTAAAGCGTCTCC
EMPV1_11516	CTAAGGCTTAGTGATTCTTTCATTTGTGAAGTCAAGAGTCTCCTTAACTTGGGGAAAGGT
EMPV1_11517	CACTGGTGACTTGGGGATATCCTCAGCTCCTCTGTTAATGAAATTAGCAAGATGATGGTC
EMPV1_11520	AGGAGGTAAGGGAAGTGACCGCTTTATATGAACCTCCCAGGATTTGCTCATTGTCTGTCC
EMPV1_11522	TGTGGATGGCTCACCCAAAAATGACGACGGGCACAAGACTGAACAGCCTGATGAAGAGTA
EMPV1_11523	CCACATGGGGAAACTCAGACTTAACAGCATTCTTTCCGCTTTACCCCAGCACCGTGATAG
EMPV1_11525	TACACACTGGCCACCTAGCACTTTGTAGTGTTTTCTAACGCTGTCACCTACCCGCTCCCC
EMPV1_11526	AGGTCACACTCATTGGCCTCACCCTGATCTTCTGGAGTCAGTTGGCTGCCTCTGTCCTCT
EMPV1_11529	CGTCTTGGTTATTCCACCCCTAGATTCTTTCCTCCCCAAATTCTATGGTGGGTCAAACCG
EMPV1_11533	TGAGGGGGAGATGTGTGTGAATCCATGTGTTCCTGACCCCTGCAAAGCATTTGTAGAGTT
EMPV1_11534	GGTTGTATAGTTGAGGAGGACACCCACGGAGATCACTATACGGCCTCCTAGCTTTCCCCA
EMPV1_11537	AATGAAGGAATAGCCCGGGGACTGGGCCGTATTTAAGCTTTTCTGTCTGGGCTTAGGGAG
EMPV1_11538	GGGTTGTCTGTTTAGATCTTTAACGGGTATTTAGAGGGTTAGGTTGCTTGATTCCTTCCC
EMPV1_11540	CCTGGAGGGAGATGTGGTGAGTTGAAGTGGGAGCAGAGTTTCGCTTTTCTCAGAGCTGAT
EMPV1_11541	GTGCAGGAGTTTGGTGGCACCATCCTGGAATGTTTTTGGCAAGGCACCAAGTAACATGGT
EMPV1_11543	GAGTAAATCTCTTGAAGCCGATGAGTGTCAAAGAGAACTTAAAAAACTGAAGTTTGAATC
EMPV1_11544	GACCACGCTGGACAAGTTAGATGAATGTGTATGTCTCAACAAGGATTGCTCTGTGTCCTC
EMPV1_11545	ACAGCTCAGGGTAATGCTGGATCCCTCACCCACTGAATGAGGCCAGGGATCTAACCCACA
EMPV1_11546	AGGACTCAGGCCTGTTGTGTGGGGAGCCCTCCTCATTTGTGGTTTGTGTGTCTTAATCTT
EMPV1_11547	TATGAGATCTGTTGCCTCTAGACTATAACTCTACTTCATGACAAGACGGTTCTGTGAGAT
EMPV1_11551	GGAAGAGAACCCAGAAGCTTGCTCTTCAGAAGGCTATGACAGATGCGTTCCAGAAACTGC
EMPV1_11556	CAATGGGCATGAGTTTGAACTTAGACGGAGTGTGGCATTATCCGTTGAGGCCGGATTCAG
EMPV1_11557	TTGCGGCAACTTTGTGGGGAGAGACGGCTGGGTTTCTTAAAGCCACGTATTACTGACACT
EMPV1_11558	TGATGTGGGAACTGGTAAACGGAAATCGGGACGCTCACGGAACTCTCTTCGCCCTCAGAT

EMPV1_11560   AGTACGACTTCCAGCCTACGAGTTGAGTGGGTCGAAGCGGATACCATTGCTGCTGGGACA
EMPV1_11561   AAGGCCCCTTCTCAGGATTTGCTGCCGAGGACTCAACAAAACGTGCCTGTTGTCGGGTTT
EMPV1_11562   GGAATCCTGTTCTACGTGCAGTTTTATACCTTGGTTTTATTAACCTTGTACTTTATGGTG
EMPV1_11563   GACAGAACTGTGGAGTCCTGGGTCTTGTGGCTCTCTGATCTGTTTTCATCAAATCCTGGT
EMPV1_11564   GGGGGCAATTATGTACCTCCGACCATCATCTTCTGGGTCTATGGATAAGGGGAAAATCTC
EMPV1_11568   GGCAGACTGGAAAATCTCTGCAGGCAAATGTGATGTTACTAAAGAAATCACACACTTACC
EMPV1_11569   GATGCAGTCAGACTCTTAAGCCACTGCACCATAGCGGAAACTCTGGTACTGCTTTTCACA
EMPV1_11570   TTCTCTGCCACAGCTGGCCATAGGGCCAATATTCCTAGAAGGAGTTGAGCCCACATCTTT
EMPV1_11571   AATTGAAGTCATGGAGAACCCGTCCTGTAGACGGAACACAAACACCAGCAATGGGGATGC
EMPV1_11573   GTGTCTCTGCACTGCGCCCTGCAAGCAACTGAAACCCGGCCAAGCACAAAGGAAAAGAAA
EMPV1_11576   CCTCACTCTTTGGAAGACACTACACCTCTCCTGTCCCAGGTAGTTCTGGTAGGGATGGTT
EMPV1_11577   CACTGATGTTTGAAGGCAGTGAGGCAAGAAGTTTATCATCAGGCTTATCAGAGACCAGGC
EMPV1_11578   TTGAGCCAGGAAGAGTCTAGAGCCTGGTAGTTAGGTTCAAGTGAAAAGGCCACTTGGGTC
EMPV1_11579   CTCCTACAGGCGGCTTGCATCAACACTCATTTCCTTGAGATGATAAGCTTCCTCTTGTCT
EMPV1_11580   GGTACCTCCAAACTCCTATCTCTGTGCTTAGTCTCCCCTTTCATTCAGTCCTACATTTCT
EMPV1_11583   ATATGTTTCCACGCCGGATCATTTCCTGGAAGACACCACCTCTGACCCCAATGCACAGTA
EMPV1_11585   GTACTCGGACACTGAGCGCTACCTGTACTGTCGTGCCATGGACCGCACCTCTTACGCTGT
EMPV1_11586   CTGTCGTGTAGAATACATATCCTCTCCCCCTTCGGAATCCTCTCTCTGCTAATAACCGGG
EMPV1_11587   CCTCGCCGCCAGGTGCTACATCCACACAGCACATCCTTTTACACCACGACTTGCTAACAA
EMPV1_11588   GAAAGAGGGTAAGGTTGTCAATACCTGCGTGCAAAGCTTTATATTGGCTGGTCTGCAAGC
EMPV1_11589   TTGGTTCCAGGCAGGGCCCCTCAGTTATGTCGTAGACGTCCATTTTTGCCATGGGTGCTG
EMPV1_11590   CCATGGCCACATCCATGGATGCCTTCCTCTGACACTGGTCTGCTCCACCTTTCCTGAAAT
EMPV1_11591   ACCAGAAGTTTGGTTACGGTGGGAAGAAGACAGATTCAAGGACTGAGCCCGAGACAGCGC
EMPV1_11592   TGTTCAGGAATGCAGCGGCCATCTGTTTGCACCTGGTGGGGCCCCTCTGGGGACAGAAGT
EMPV1_11598   ACCTGAAGCTGCTGCCACACTACGTACGGGCCGGGTCGGCCCCGGGACACGTTCCTGAGG
EMPV1_11599   GATGGTGGTTCACATAGGAAACTCATCCACACGGATCCACAGTTCTTGGTTAGCCTGGTA
EMPV1_11600   TTGTGAAGTCACTGATTTTGGCTAAACCACACAGCCACTTACTAATCCAGGGACATGGGC
EMPV1_11601   TTTGTCTTTTCTATGGACCCGTAGGCTTCACATACACTCGTCCTGCCTCAGCCACCTCCA
EMPV1_11602   TTGAATTGCAAAGGAGGACTGAGGTCTTCACAGTCTAGAGAGCGCCCCTAAGATGAGCCA
EMPV1_11603   AGACTGTCCCCGCATCCCATCTTCAACAGGGAACAGCCTCTGGTTCCTCCAAAGCGGTCT
EMPV1_11604   AAAAGAGATTCTGATTGCATGGTCTAGAGCAGTTCTTACTCCTAAGAAAACTCAGAGAAA
EMPV1_11607   AGCAAAGGACAGAACCCCATAGAAGAGTGGCTGAAGCAGAATGCTGTGCTCATGCCTCCT
EMPV1_11608   GATCCATTTACTGTGATGCTTCATGTTGAGTTTTCTGGTTTGAACTACTACTTGGGTGGG
EMPV1_11610   GTATAGCAATCCCTTTAAAATGAATGGGAGCCGTCTGTTGTTTTAATGTACGAACAAGCA

EMPV1_11611   CGGCTTCCCGAGAGACACCTGGTATGAATGCCTGATCGAAAGAGAGGTCGAGTGGTTTCA
EMPV1_11614   AGGAAACAGATAGCAGTGAAGAAGAGGACATTGTTGGACCGATGCCTGCCAAAGGACCAG
EMPV1_11615   TGCCAAGGAGATCGATGTGTCCTGTGTCAAGATCGAGGAGGTGATTGGAGCTGGTGAGTC
EMPV1_11617   ATGCAGAGGCTCTCCTTTGCCCGGCTCTTCTACCTGCAGCCCAAGTACGCAGTGCTTGAT
EMPV1_11619   CGAAGATGTCATAGCAAACGTTGGCGCATCCCGGCTGCAGCGTGTTGCACACGAACCTCT
EMPV1_11621   TTCCTTGCTTAGACGTTCCACTTCTCCACATCAGCAACCAGAGACACCCTGAGCTTTGAG
EMPV1_11622   TGTGCCTGGACAGATTACACACTCCACACTTCTGCTCATCATCAACCCTCCCAAGGGATA
EMPV1_11625   AGGTGCACATGTTCTTTGCCGATTTCCAAGGCTTAGGTCACGCTGTTTTCCTTCTCGCCG
EMPV1_11626   CCCTTAATCGTCTCAATGAAAGGTTGTACTATATACAAGAAACTATTTGCAAAAATCCAT
EMPV1_11627   TTCATTAACCACTGAGCCACAATGGGAACTCCTACCTGCTCCAGCTTCTACCATCTCGGG
EMPV1_11628   GAAGTTCTCACTAGAGAAGCTGCATCAAGGCATCGCCGTCTCAGACCCTCCCTTCGATTC
EMPV1_11629   GCTGCACTTCCAAGAGGCCTGTGTCTCTCTACCCAGGAGCACGTGGACACACTGCGCCAG
EMPV1_11633   TGCTGCAGAACTCGCCAGTACAATCTGCTTCCCGAATCATCTTACTTTCACAGCCCGCTT
EMPV1_11637   AATCCGCTAGGCTCTTCCACGATGTTCTTCCGGGCTGCGGCCGTGTCAGATTTCTATGTT
EMPV1_11640   AACTCTTTGCCTAGTATGAATCCGTTTCTAGCTGCCCACGGGCCTGCATTTCACAAAGGC
EMPV1_11642   TTCCTGGCTCCTCATCTTCAGCATCCTTCTCGTCTTTGACTACGCAGAACTCATGGGCCT
EMPV1_11643   GCTGAAGGTCTCCAAGGTTTGGCTGATTGGTTGAAGCAAGGCCTCCAGACTGTCAGGTGA
EMPV1_11646   AGGCCTATCTATCGCCTTTCTTTTTGAGGAGGAAACTGCAGGAGGAAGGGTTCGATCTGG
EMPV1_11648   GAAGGATAAGTATTAATGTCCGCAGTGGCCAAGCTTGTTTGCGGCAGTGAGAAGCTCAGG
EMPV1_11649   TAGATGATTCTCTGGGACCCCAGCTCTGTGAGCATCTCCCTAACCATGCCTGTTTTCCTA
EMPV1_11650   GTCGCAGAGTCTTATACTGTAACCCCATCTCTATCCCTACCACCCAGAATACAGGAAGAG
EMPV1_11652   GTGGTTCTCCAGTTCCTCAATCTGCTGCCTGTACTGCTGGAGCTGCACTTCAAACTGCTG
EMPV1_11653   GGAGCTCTCAAACTGAAACCAAATACTAAAATCATGATGATGGGAACTCGTGAGGAGAGC
EMPV1_11654   TGATGGGCAATTTTCTAGTGTTATAGGGAGATAACAACGCCCACCACACAGGGACAGGGG
EMPV1_11657   TCCTAACAAGATAACCTTATTGCCTGTCTTCCCCCAACCTGCCCTCCACCCAGATTAGCT
EMPV1_11658   GCAATTTCCCAATCTCCTGATAGGCAATCTGAACCAGCCACACATCCTGGTCTGTTCCAG
EMPV1_11660   ATGGCTCGTACTAAGCAGACCGCTCGCAAGTCCACCGGCGGCAAGGCGCCGCGCAAGCAG
EMPV1_11661   CAATTGTGCACTTTGGGGTGAGGGGGACATTGGGCAAGCCTGTAAACTGGGTGGTGGCAA
EMPV1_11664   CTTCCTTTGCTCACACTGCTTTCACCTTGCATTTTCACATCTGCAGCCCCAGAGAGATCC
EMPV1_11665   GCTGGAGAGGGTGTGGAAAAAAGGGAACCCTATTACACTGTGGGAATGTAAAATTGGTGC
EMPV1_11667   CAAGTTCACGCAGGTGCAGGTGCAGGAGCTGGAGCGCATTTTCCGTCGCATTCAGTACCC
EMPV1_11669   TGGAAGTATGTATGACAGAATGCGACGAGGAGGTGATGGATATGATGGTGGTTATGGAGG
EMPV1_11671   ACCTGTTGGTGTGATGGATTACATTAGTTTCAAATGTTGAACTGGCCTTTCATACCTGAG
EMPV1_11677   GGAAGTGACCGAATCAGGATTTAAACAGTCTCAAAAGCCTGGCAGTCCACTGCTTACCAT

EMPV1_11680	CTGCAGGCATTTAGTGCCAGATTTCCCTTCTGGCTTTGACTACATCCCACATGTTGTGCC
EMPV1_11682	ATGGTGCAGGTCCTCACACAAATAACAGCACCAAGACGATGAGGGAACACGGAGGCTATG
EMPV1_11684	CTCAATACAGTCTGACAAGAGCACAGCAGTCCTACAAGTCCTTGGTGCAGATCCACGAAA
EMPV1_11685	CAGATGTTTGGTCCCTTTCCATCGTCATCTGCCACTGCAGCCTGTGATGCTACTAATCGA
EMPV1_11686	AAGTGTCAGTCTTAGATATTAACAGTGGTCTGGGCATAGAGGAGGGAGGAATTTCATTCC
EMPV1_11690	CCTTGAACCATATTTCTCCAAAGAAGATAGACCATAGGCCAATAAGCACATGAGAAGATG
EMPV1_11692	CCAACAGATTACCCCTTCAAACCACCTAAGGTCGCATTTACAACAAGAATTTATCATCCA
EMPV1_11694	GGCTCCTGTAACTGGTAATGACGCTGGAATCCGTAGAGCAGAAATTAAGCAAGGGATTCG
EMPV1_11695	CTTGCCTCGTGTTTCCCATCTGCATCCCTTCTCTCCTCCCCGAATCAATACACTAGTTGT
EMPV1_11696	TATCAGGTAAAGTTCTATGAACCTGGCTTTTCTTCTGTGATGTGGTCCCATTGAGATGAC
EMPV1_11698	GAGATCTAGGACTCCACCAGTAACCAGAAGGCGATCTCGAAGCAGAACTTCACCTATCCC
EMPV1_11700	CCGGAGGTCTCAGAGAAGTGCTTCTGGAGAATCTGGAGAAATGATGGAAGGTGTGGATCA
EMPV1_11701	TAGCCCATGCCCAGTTATAGCAGATCGAAAACAGGAGGTGCTACAAGGTCTCAGGAGTGG
EMPV1_11702	CTTCTAGACTCTAGGTTCTGATCACGGTCGGGAACCCCCGGAGGTGGTCATTCCTGAAGT
EMPV1_11703	AAGGAGCCTCTGTCCAGGTCAACTGCACCTACCAGACATTTGGGTTCAACGGGCAGTATT
EMPV1_11705	ATTTTGAGAGTTTCGACCTGCCTGAAGAGCACCAGATCGCACACCTGCCCTTAAATGGAG
EMPV1_11708	CCAGTGGAATTAGAGTTTAGAGTGTGGAGGGGAAAGAGAGCACGTTGTACTGGTTGGTGA
EMPV1_11709	TAGTTCCTTTAACGGTATGACGAGACCTGGCCCAATGTAGCACTGGTGCTCGGGGCAGGA
EMPV1_11715	TGAGATCCCTGCCTGCTGCCTTCCTCCTGGACCCCTGTTCTAACCTTCGCTGTATCTGCA
EMPV1_11718	TCGCCAAACTTGGAATTAGTGTGTTGAGTAAACCCCATCCGTAGTAGTCACGGCCCCGCT
EMPV1_11721	GTTAGTGGGGCTTTGGAAAGGTGGGATCCGCTCCCATCCAGGCCTCTGGGCCCCAGTGCA
EMPV1_11723	ATCTCAAAATCAGCATCCCCCAGATCAAGCTGAGTGTGTTGACCTGCAACTAGCTTCCCC
EMPV1_11729	TGGAGCCGTGGAGTTGGTTTTAAGAGTCAGATCTAACCGTGTTCGTAACTGGTCCCTGGA
EMPV1_11730	ACACACTGGTAGGTGCTTCGAGACTAGACCGTGATTGACTCCCAGACGCATTACCGGCTC
EMPV1_11731	CGGGAAGCAGAGACCTAGGCACCCTTGTGGGGGCAAACGCCCAGGCAGCTTCTAAAATTT
EMPV1_11733	TCCAGGTCTTGGAGTAGGTCATTGGGTGGATCCTTTATTTCCCTATGTGGGCCACTGGAT
EMPV1_11734	TGCTGGGGCCTGCGTCCAGACGGCATTTTCCTGCCAACTCCTGCTTCTCCACGAAGAAGA
EMPV1_11739	CTCCCCCAATCCAAGTTGACTTTTAAACCTTTGGCAAACCTGCTATTTCAATGACTGGAC
EMPV1_11741	CTCCAGTGCATGACAAGTGCCAATCCAGATGGACTTACCTGGCAAATCAGAAGGTCACTA
EMPV1_11742	TCCTCATAGCCAGAGAGGAAGGGCGTTCAGGAATGGAGTTTGGCTTGGTTAAGGGCAAAA
EMPV1_11747	GAGTCTAGTCATGTGGTTTCTTTGCACCACCATTGTTCGCTTGGATGGGAGTGAGTCTCC
EMPV1_11749	TGGTAGCCGCACCATCCTCCATGCCCACAGCTTCCTGGTGGCTGACTTCGGGCAGCGGGG
EMPV1_11750	TGTGCATCAAGGAGGCCCTGAGACTCTATCCGCCAGTACCAGGAGTTAGCAGAGAGCTCA
EMPV1_11751	GACAGAAGCATGCAGCACTTCCGCACCAATGGGGAGTTCGACTACCTGGATTTGGACTAT

EMPV1_11754	GTCCTTCTCAAAGTACCAGTATTTCGACTCGAGGGGGATGTTCGTGTCCCTGGTCTTCTC
EMPV1_11755	AGAAGCACAGGCTCGAGATGACGGCGACGAGGAGGGGCTGCTAACACACAGCGAGGAAGA
EMPV1_11756	AGTGACTTCTCTGGAGCAGGATAATTTAGAAGTTAGTGGTGAGACCTTAACATGGAGAAG
EMPV1_11759	GGACGCTAGTCGGCTTCATCACCACTGAGCCACAGCAGGAACTCCATACATGCTTATTTT
EMPV1_11760	GGTGGCTTGGCAGTTTTAGACATAAGGGCTCCTTCCAATTTGTTGCTCTTCCATCCCTAG
EMPV1_11761	GGACGTCCGCCGGCGCTTAACACTAGTGATTAATCGAATACCCGCGGCCGCCATGGCGGC
EMPV1_11762	CCAGGAGATTTGGGTAGGAATGGGGTTGATGGGATTCATCGTCTATAAAATCAGGAGTGC
EMPV1_11763	TTTTGACAGTTTTGGCTGGGCCTTATTAGCCCTGTTTCGGTTAATGACGCAGGATTACCC
EMPV1_11764	TTTGTCATCTTCCTGGCCCTCTACCTGCTGACCCTCCTGGAGAACACACTCATCGTCTTC
EMPV1_11765	CCAAGTGGAACACAGGACAGAGGAAGCAAGAGAAATTGTCCTTTAGCCCAAGTGATGGAG
EMPV1_11766	ACTTATGGTTTCATCCTTTCCAACATACTCCGCATCAGTTCCACGGGAGGCAGGTCCAAG
EMPV1_11768	TATGTCAGCTTCCTGGCCTCTTTTGTTATGCTCAGCACATCGCCAGCATCGATGGGAAGC
EMPV1_11769	CTGCAGGGAAGTTGTTTTCCTCTGTGTCTCTTCTTTGACCTTAATTGATTGAAAATAGCA
EMPV1_11770	TTAGTTCCTCCTGCTCCTGTGGACAGTGATGGGGAGAGTGATAACTCAGACCGTGTTCCT
EMPV1_11774	ATGCTGCCTGACTTCCCCACAGCAGAGGGTCTAACCAACAGGGACTCGTGGAAAATCACA
EMPV1_11775	AGGACTCCATCCTGGCTGTGAGGAAATACTTCCACAGACTCACCCTCTATCTGCAAGAGA
EMPV1_11777	GGTGAAATCACGAAAGGTTCAGAATCGGCTGCAGAAGAAAAAAGGAGTGACCCCCAACGG
EMPV1_11778	GTTTAGAACACAGGATGGAAGAAGAATGGCATGAGGTGGGCTGGAGATATAAGCCCGGAC
EMPV1_11779	CCTCTAAATCACTAAGTTCTAGAATATATCATCCAAAGAGTTCCTGTCGAAGCTCAGCGG
EMPV1_11780	TCTTTAAGGAGAAAGCTAAGGCTAAGGGCAGGGACGCGGAGATAAAAAGTGAGGACAGGG
EMPV1_11781	CCATTTCCTGGTGGTAGTGGCCTGGATTGGAGGGGTAATACACGCTACTGTGCAGATTCT
EMPV1_11782	TGTCTCCTGGAGGGACCGTCACACTCACCTGTGCCTTTAGCTCTGGGTCAGTCACTACTA
EMPV1_11783	TCTCGGATCCTTGAGGTTTGCTCCGCCGCAGCCTGCAGAACCATGGAGCTTCGTGAAGAA
EMPV1_11788	ACACCCGCCTGCCTGCCAAGGTGAACACAGAGCCAGCTTACCCCTCTTAAGTGCCAAAAC
EMPV1_11789	CCTGCAGGCGGGCGCGAATTCACAAGTGATTAATCCAATTCCCGCGGGCGCCATGGCGGC
EMPV1_11790	CATCAGGGTGTTTGATGATGTGAGAAGTTTGAAGTACAGGGATGACGGCCTCTACGTGTC
EMPV1_11791	TTTAAAGAGCTGGGGAGGTTCATGCTTCGCTACAGTGGTTCTACCATAGCTGCTGGTGTA
EMPV1_11793	ATTTCCCTTTTCACAGTGGAGATCCTGGTGCGCATCCCACAAGTTCACAGACAGGCAGGA
EMPV1_11794	TTCAAGGACGTGGCGGGAATGCACGAGGCCAAGCTGGAGGTCAAGGAGTTCGTGGATTAT
EMPV1_11795	TGAGCTCTTCACTCGCATCTTCATCCCCACCTCCTTTTTGCTGGTGTGTATCCTGCACCT
EMPV1_11796	GAAGATGAGGCTGTTTTGCCACTCTCTGATAAGTGTGAGTCCTCAGCTGTGACAACAAAC
EMPV1_11798	ACTTCTACTGTGACATTGCCTCTTTGCTCAAGCTGTCCTGTTCTGACATCCACTTCAATG
EMPV1_11799	CTTCAGAGCATGAAGGCAAGCAAAGAGAAGCCAAGGAAGCAGGGCAAATTGACACCCAGG
EMPV1_11800	TTTTTCTCCAGCCACGACAAGGCCCACCTCTTAGAGCAGTCCAACGGCTGCTTCCACTGT

EMPV1_11801	GTACATATCAGACCACACTGAATTGTCACGCACCCTTTCCATACTTGGTTACTCACTGTG
EMPV1_11804	ATTTCCTGGCGTCACACTGGATTACTTGGTGGCTGGTTCCTGTTGGGGTCACTTTCAGGA
EMPV1_11805	GGGCCTGCTGTTGTCATTTTTCCATTGAGTGGCATCCTGGTCTCTTACGGCCATATTGGG
EMPV1_11806	GGTCCTGCTGGATGGTGATGAAGGCAAGTTACTGCTTCAGGTCTTGACCAAGTCCCTTTT
EMPV1_11807	TCTCAGGTGAAGAATGTGAAGACTCCCAACTTGCGTCTTACTTGATGAGCTGCAAGGAGG
EMPV1_11808	AGAACAGTACTTCTCAGCCTCGTTACCTTTCTGTTGGCTATGGGAGCTGAACTGAGGTGG
EMPV1_11810	GTGTTTCTTCTGTAGTGGCTGCCTGGATGGTGGGAATAGTCCATGCTGTATCTCAGTTTG
EMPV1_11811	AGGCATGGATTTCATTTATCAAAAGCCTGTGGCCTTTCTGCTCTAACCAGACGGAAATCC
EMPV1_11814	CTTGGCTGACTAGCAAAACCCTTCTCTCCATGTGCTTTCTACTGGTTCCCTGGCTTCTCC
EMPV1_11815	CTTTAGAGCTAATGTCCTTCTATTTGTATATGGCAGCTGGGGGTCCATGTAGGATGGAGC
EMPV1_11816	TGCCGCCTGGTACATCCTCACCATCTTTGGCATCTACGGAGTGATCTTCCTCTTCCAGTT
EMPV1_11819	AACAGACCTCTCTGCATTGTGACTGTGAGCATAGCCTGGGCAGTGGGATTTCTGATTTCC
EMPV1_11825	CGACCAAAGGTTTCTTTGTCAGTCCCCATGACAACAAATGGCTTGCCTGAGAGCACAGAC
EMPV1_11826	TGCCAGTGTTTTCCGCACCCCACTGCAGCTTGACCCAAAGCAGGAGCAGCAACTATTCCG
EMPV1_11830	TATCATCCGCCTCCAGCACTTGCACTTTTTTACCATCTCTTTCTGCACATCCGACATCAG
EMPV1_11832	TGCTTTGGAATCCATTAACTGGGCAATGTCACAAGCAGTTTGACCCATTTTGTCCCTTAC
EMPV1_11834	CAAGCCGTTCCTGGATTACTACGAGCAGATTGATGGCGTGTCCTACCGGAAAGCTATCTT
EMPV1_11835	GTTGAGAGGAATCGTAATAGACTGCAGCACGGTGTAAACATAGCATTTCCATGCACTCGG
EMPV1_11838	GATTCGGGGCAACGTGAGCGCCTGTGCCAACTCTGTGTTCATCTTTGATGAGATGGATAA
EMPV1_11840	CCTCACTCAGTGTAGGTCACAGATGGGGCTTGGATCTGGCGTTGCCATGGCTATGACATA
EMPV1_11842	AGTTCTTGAGGAGGTAGACCAACGCAGCAAAATACTTACCACCAGGCTCCACTCTCTGGA
EMPV1_11843	CATTGGAAAGCATGGAGAGCTGTATTTCATAATTTGATGAAAAATGCTAACGCATGTCAG
EMPV1_11847	TTGAGCTCTCTTGCTCCCTCTCAGCCACTCACACAAACTGCCAACGGAGCACTTGTCACA
EMPV1_11848	CCAGACTGACAACACCCATCTCAGCTTACTCGAGGGCATCTGGTCCTTCAATAGGTGGTT
EMPV1_11850	ATCCTTGGGTCTGTGCTCAAATGGCTGTCACTGTCTGGATCACTGGTTTTCTCTATGCCC
EMPV1_11851	ACATAGGGAAGGAGACGTCTTGTAGGAAGCGACCTCAGCTCTTTGTGGATCAGAGTGCAT
EMPV1_11854	GACGACGGCTTGTTAGTTCTCGCTTTTGCTGCCCTCGCCGTTGGTGCTTCAATAATGAAT
EMPV1_11856	GTATATTCCTAGATATGTTCCATGAGGGCTAAAGGAGTTTCTTGTCAATGTACCCGTGAC
EMPV1_11857	TAAGGATCCAGTGTTACCGTGTAGGTTGAAGATGCAGTTCGGATCTAGCGTGGCTGTGGC
EMPV1_11859	GCACTCTAGAAGCAATGAAGAGCCAACTCAATGACACAGAAGAATGCACAAGTGATCTGG
EMPV1_11861	CTTCTTTCAACTATTGCCAAACCTCACGTGGCTGGACCTTCGCTACAACAGGATCACAGC
EMPV1_11862	ATGCAAATTCGGTCCCCGGCCCCACTCACCGGGTTAAAGATCCAGTGTTGGAGTAAGCTG
EMPV1_11864	GTAAGTACTTTGTATTCATTCCCTTGCTTTACAAATTTATAGAATCCTAAGTTAAGGACC
EMPV1_11865	GTCCTAGAGTATGCAGCTGGTAAAACCAGAGGCTACAATCCAGTATTACTGCTAAGAGAC

EMPV1_11867	TCCTCAGCCCCTCAGTCTCGAGCCACCAACTCTCCTGCTTGCATATCAGGGTCTGACTCA
EMPV1_11868	GGAGCAGAGACAAGGCGTAGAAAAGCACGACGGAAAGAGCTAGAGATGGAGCAGCAGAAT
EMPV1_11869	GGATGGATCTAAAGGTGTCCCATTCAAGACCTCTCCACTAACCTCTTCTGATCTTGCCAT
EMPV1_11872	ACCTTGACGTATTTTGGCAGTGAAAAATGTGCAGGTTGCTTTGTTTAAATCTGGCCCAGT
EMPV1_11874	CGGGGCCACCGTAAAACTGCTTCATTTGGCAACATTCTGGATGTCCCTGAGATCGTCATA
EMPV1_11875	ATGGAAACGGGTAGTATCATGAGCTCTGGACTTTCACCTCTGCTTAGAACAGTCCCCTGG
EMPV1_11876	TCGACACTTGAGAAAGCTGGATCAGGAACTGGCTAAGTTTAAAATGGAGCTGGAAGCTGA
EMPV1_11879	TCCCATTGTGGCTCAGTGGTAATGAACCCAGTAGTATCCATGAGAACACAGGTTCGATCC
EMPV1_11881	CCAGCAGTCTGGGTGCTCTACAGGAAAAGGACACTGGCATCTCAGCTTTCAGATATGACA
EMPV1_11882	TTTCCTCTGGAGCTGGATATGACACCATTCATGGCCTCGAGCAAAGAGAGCAGGATGAAT
EMPV1_11883	CTATGACCCCTATGCAAACCCACCCAACTACGGCAACCCTGACCCCATCGTTGTCAACAA
EMPV1_11885	TAATAGAGAGGAAAGAGAGGCCCAAAGTGGCAGTGACTCGCACAGTCGTATAACTGGCTG
EMPV1_11886	GATGCCCTTTGCCAAGCAAGAGAATGAGAACACAAAAGTGCTCGCCATGGTATTTTTGTC
EMPV1_11888	CGGGACTGTCTCCGTCGCCGAGAGTCCACATGGGTAGGAAAGGTTTTTATGGAATTTGAT
EMPV1_11892	GCCAGTCTCAGAAGAACTATGCTCCTCCTCTCAAGGTCCCCAGAAGCACAGCCAAAGACA
EMPV1_11893	GGGAGGAGGTCAACTCGGATCTGTTCCCACCCAAAAAGAAATCAAGGGCAGAGCAACAAA
EMPV1_11894	CTGGTTTTTACACCATCCTAAAGGAAAACTTATGAGGTTCTGTTGAAGTAGAGAAGTTGG
EMPV1_11895	GTCATCATACTGAAACTGTGTTCAACCGGGTTTTGCCAGGGCCTATTGCACCAGACAGCA
EMPV1_11897	GAAGTGCTCCCGCTCCCCCACTTACCATCTGTAACTGGAGGTTGGGGCTCCCAGCCTTCT
EMPV1_11898	GTGTGTTACTCTTGTACTCATCTCCTGGTGTGTGGGCTTCATCCATACTACCAGCCAGCT
EMPV1_11899	TCCCGCCCTGGGGACATGCCCCTTGCATATCTTATCAGCAATAACCCCATGGCTCTGGAA
EMPV1_11902	GAAAGGGGAAGAAGGGATTGTTTTGCTCTCCCCGGGTACATTCAGCCATGTAGAGAGACC
EMPV1_11904	TCACAGGCTGTACACTGCAGAAGAATAGACTGTAGAATTAGTTGAGCCAAGTCACTAAAC
EMPV1_11905	GCAGAACGACCTGGCCAGTCTCATCCAGGCGGGCCAGCAGTTCGCTCAGCAGATGCAGCA
EMPV1_11906	GCATAACATTGGCATCAGCTGGCCGCCAGAGACTTTGATAAATTCTCTTGTTACCACATC
EMPV1_11908	CATGCATCAGTTTACAAAGACAGTCTTATCTTCTGAGAATGCGCCATCTTTTCCTCTAGA
EMPV1_11910	TGCACATAGGAACCATCTATGGCAGCCTGAAAGTGGGGGAACAGGATCGGGTGTTAATTG
EMPV1_11912	AGTACGAGCCGGGCCACATCCACCTGCTACTCCCACATAACCATTGTGGTGCTAATGTTT
EMPV1_11916	GATGTTGTCAACTCTCTGGATCTGTGTGTGAACATCAGTCTGGAAAACCCTGGCACCAGC
EMPV1_11917	TGTTAGTTCAAGCAATTCTAGACGCACATGCCTGTCCACTCTGCTCGGAGGTTCAAGGTG
EMPV1_11918	GATGGAGTGTCATGCCCATGCAGCTGTGATGACAGAAGGCCCAAGTATGTTGTTAAAGAG
EMPV1_11919	TTTGAGGCTCTAGCTGCCCATGATGCTTTGGTTGAACTCAGTGGAGCCATGAACACGACT
EMPV1_11921	TTTTCACCGGGAGATCATCTCAGAGATGGGGGAGCTTGGTGTGCTGGGCCCCACCATCAA
EMPV1_11922	TGAAGTGCTGTGGATTTCTTTGTGAATCACCATATCTAAGCTAATGTGGTGGTGGTTTAC

EMPV1_11924 GTGGCCCTCTCCTGCTCATTGCCTCACCTCCACTCACTGCTACACACACTTCTGCTGAAT
EMPV1_11925 AAGAAGTGGTACCAGTGATACCAGTCAAAGTGCCTGAAGTACCCAAAAAACCTGTCCCAG
EMPV1_11927 TCCGTAGGGTAGCCTTGGTCCTTCGTTACTCGGTAAGCATGAAGATGCTACGGGAGATGT
EMPV1_11929 CTGCGCCTGCAGCACAGAGACTTTCTTCTGGAGATCCTTCTACAAGCCCATCTTTGTCGC
EMPV1_11930 TAGTCCTTGCTGGGGCACAAGGAGTGTCTGCTTCTTGGCAGTGAGACCTCCCAGTGGAGA
EMPV1_11931 TGGGAAAGTTCTCAGATGTGGTGTTAAATCAGACTGTATCCACCAGCCTGCACCGCAGAC
EMPV1_11934 GATGAGGCCGAAAGGATTCTTTTTTCCTATGGCTATGGTGCTAATGTTCCCACAACAGCT
EMPV1_11935 GGGCTGTGTCTTTGGTAACAGATAGCACCTCTACCTTTCTCTCTCAGACCACATATGCAT
EMPV1_11936 TGGGTTAAGGATTCAGCATTGCCACAAACGGAGGCGGAGGTCACAGACATGGCTCGGATC
EMPV1_11938 ATCTGAGCCTCACCTGCAACCTATACTGCAGCCTACAGCAATGCCTGATCTTTCATCTGC
EMPV1_11940 ACTTCTCACGGTGTTGGGATCCTTTGGGGACACCATGTGCTACCATAAGAACAGCATCAG
EMPV1_11942 AGCTTCATGAACAACTGGGAGGTATACAAGCTTCTGGCTCACGTCAGACCCCCAGTGACT
EMPV1_11943 TTTCTGAGAGCCTTCCTCACAGAGATCCGCCGATCACGACTGGAGGAACCAACATTTCAA
EMPV1_11944 GGGAAGGTTGTGGCTGGTTTGATCTTCTATTCAGACCATGAGAACTTTCTCCATACCAGC
EMPV1_11946 TCGTAAAATGAACTGTCCATCCTCCATGGGACAAACCCAGTGCTGCTACTCATCCCTCCA
EMPV1_11950 AGACAAAGCTCGTGGTGAAAGGCTGCTCCAACATCAGCAACTCCACCTGTCAGTTCCTCT
EMPV1_11952 CAGTTCCTGCCCCCCATCTATCCAGCTTCTACCTCTGACCCCCGGCCCCAGGTGACCCGG
EMPV1_11953 AGGCCAGCTGCCCATCGATGCCCAGCTCCAGTTCCTCTGGTCCCTTTCCACTGTCCAGCA
EMPV1_11956 TGCTATAGTTGCTAAAGAAAGACACCCAGGGCAGCAGGTCATCTGTGTAGAAGGAGACGG
EMPV1_11959 ATCCTCGCTTCCCTCCTTATGACTCCTTCCCACAGGAGCCGCTCCAAAGAGGTAGAGTTA
EMPV1_11961 AGCAGCCAGCTCTGTGTAAATCTGGTATGGGTTTCATGGGGCCTGGGTTTTCTGGATGCA
EMPV1_11963 CAGGAGCCTGGAGGTGCTACCTGCTACCCCCTAACTACTTGTAGCTGAACAAACTCTAAA
EMPV1_11964 TGTCAAACCAAAGAAGCGGAGAAAGAGCTACCGGAGCTTCTTGCCTGAGAAGAGTGGCTA
EMPV1_11966 CCCTCCTCTCACCTTCTCTGAGGTTTATTAGGGCTTCTTCTGCCACACCTCGAGTAACTT
EMPV1_11967 AGGCAAATGCCAGGTTCTTATTACGCCGTGCTCCAAAGTCATCGTCAGGTCTCTAACGGT
EMPV1_11968 ATGGCTGCATCGCCTGCTGGCTGGAAACGGAAGTTCTTCATGGCCACTGTTAGGGTTTTA
EMPV1_11969 CACTGCTTTTCATGTCCAAACCAGCCATTTTGTTGTACTTTGGTAAAGGACCTCTTCCCC
EMPV1_11970 AATCAAAGAGCGTGTGGAGGAGAAAGAGGGAATTCCCCCACAGCAGCAGAGGCTCATCTA
EMPV1_11975 TGTAGATCCTATGGCTCTGACAGTGAAAGTGACCGAAGTTACTCTCATCACCGGAGTCCC
EMPV1_11976 ATCTACCGGGGCCTCATCAGCCACAATGAGATGTTTTCCGTCGTATACCAGATCCGGGAG
EMPV1_11977 GCTATCCTTCCATCAATGTCTAGAACAAATGGCTACAGGGCACGGTGATGTGAATTGCGG
EMPV1_11978 ACTAACCGCAGGGTCTGTCACACCTCAGTGTCCTACTTGCTGCTACTGTTTGTACTGTGT
EMPV1_11979 AGGAGCCTCAGCATATACACTGGAATAGGAGCCGGGCAGGCCTCTCTAGCTGCCCTGGTT
EMPV1_11983 GGCTTCTACTTGTGGTAAAGGCAAACCAGGTCATTTGCACTACCCCCTTTGTTGAAAACC

EMPV1_11984   ATCAGCTAAAGACAGGGTGTCTCTGAGCAAGGGAGTGGCCGTGCTAAACACCTCAGTAGC
EMPV1_11985   TTCTTGGTCATGTCTTCCTCCTACCTGGCATTTCGCATCTCCCAGCTGGAACAGCAGTTA
EMPV1_11986   CAGCAAAACCGGATCCTTAACCCACTGAGCAAGGCAGGGATCAAACCTATTCCTTATGGA
EMPV1_11988   TCTGTTTCTCAAGAGCAGTCACATCAGCGACGTGTGAAGAAAGGAAACTCACAGATATGC
EMPV1_11990   TATCCCAGTCTGCAGGTGTCTCTCCATATCGGTCCCTGGTACAGAATCCCACCCTAGCCT
EMPV1_11992   CTCAAGTCTGAGTGGCAGAAGAGCGCCTACCTCAAGGACAAGATGCGCAAAGCAGTGGCA
EMPV1_11993   CTGTGATCCTCTATGGGGTATGAGTGGATTGCATTTAGAGCCCTGTTTTCATTGAGCCCC
EMPV1_11994   AACACTGGATCTTTATCTCACTGTGTTGGGCTGGGGAATCGAACCTGCTTCCCAGTTGCA
EMPV1_11995   GTGTGGGTTAGCCTTGCAAAGGAAGGAGCCAGAGCAGAAGTAGAGAGTGGTAGAAAACTT
EMPV1_11997   GTTAACCAGGAGGTAGCTCATCACTGTCGTTGTTACTGACCACCAAGTCGGTCTCATTGG
EMPV1_11998   AGAAGACAGCCCTCTACAGTTCTCCAGAGTTGTCCCTCTATACAAATCTCTCTTCCTTGG
EMPV1_11999   TGTGCGGCGGCTCAGGCCCCTTCACCTTCCTGCACTTCGACAAGAACTTTGTGCAGCTGT
EMPV1_12000   TGTGACAACAGATGCATGATAACGCTCTTGTGCTCTTCCACCGACCCTGCCTCTCCTTCA
EMPV1_12002   TAGAGCAGGAAGAAGAGTAGAGAACATAAATAAATAAAACAATAAATGAAAGAGGAAATG
EMPV1_12003   TCAGTTCCGCAATACCACGCTCTTCATAGACCAGGTGGAGGCCAAGTGGGTGGAGGTCAA
EMPV1_12005   TCACGTTATAGTCCTCTTTTGAGATTTATCAGGAGTGCCCCTCCCCCAACTTTTGGTTGG
EMPV1_12006   CTGTGGCCTATCGCCCAAAGCCGGCTGCCATCCGCCCGCAACGATGCCAATCCCTGCATC
EMPV1_12007   AACGTGAAATACCCTAGAAACGAGAAGAGCATTCGCATGGACCAGCAGTGAACCTGTGGG
EMPV1_12008   AAACCGTTTTGTTGGAGGTGGTGTAACCAGTGCAGGACTTGTGCAAGAAGAAATCCGCTT
EMPV1_12009   CGGAATCCTGTCTGTAACACACTTCATGGCGTTCCCATAGGCTTTGCTGTCTAGTCCTTA
EMPV1_12011   GACAGTGACAGGGAGGCGGGGACCGAGACTGGAGGAGAGAATAATGACAAGGAAGAGGAA
EMPV1_12012   AGGCAAGAAGGAATTCGAAGCGACAACCCCAGAGTGCTGGGAGGTCGGATTTCGGTGCCA
EMPV1_12013   ACTGATTTATGTCAGTACATGGACAAGCATCCTGGGGGGCTGCACCCAGAGAATGTGAAG
EMPV1_12016   AAAGTCCAGCCCAGATCCTGAAGTCGGTAGAAAGGGAAGAACGAGGAGAACTGGAACGGA
EMPV1_12018   CTTATGGCCATGTCCCTGAACCTGAGGATTATGGCTGCTCTCCTGATGAATGGCAGAGCT
EMPV1_12020   GTGGAAAGAATTTTCGGCGCAGCTCTCACCTTAATCGACACCAGAGAATTCACAGTCAGG
EMPV1_12021   GATGAAGGAGGCAGTGATGGAGGAGGTGGTGGTGGTGGCAGTGTTATGGATGGAGGAAGT
EMPV1_12022   GAGTTCCGATTCTTGGAGTCCTGCTGTGCTATAATGGGTTAAAAATCTGACTGCCTTGGC
EMPV1_12023   AAGTCCCAACTGACCCGGTGCCAAAACCAGCCTCACAGGTCTTAACACCAACTGTCAAGT
EMPV1_12025   GGATTAAATGCAAGTGTGACCCTCACCCCTTAGCCAGAGAAAGGAGATCTCTTGGCCTCT
EMPV1_12029   GGAGCATGGCTTACCTTCAGGGACAATCACCCAGGAAACTAATAACTCACCACTTGTTGG
EMPV1_12030   TGAAAACTGGGCGGTGCCTCCTTCCATGTCAGCAGCGAGACCCGCAGCAAGATGACAGCT
EMPV1_12032   CAGAAAAGAAGCCCACGAGGGGACGCCTGCCTTCCAAAACCAAGAAAGAGTTCGTATGCA
EMPV1_12033   CAGTTTCTCTCCAAGAAGCATAGCCAACAACCTACCCTTGTGACGCCTGCCACCGACATT

EMPV1_12034	CCACTGCTATGGAAAGATACCCACCATCACATGGTAACGAAAATGGTGCGTTTGGAGCTC
EMPV1_12035	ATCTTCCGGCTACCTCCGTAAACTCTCGGCAACTCTCGGTAAACCAGTCTTCCCACTCCT
EMPV1_12036	CATGTTTCCTGAGCTTTCCCTCAGCCTCCTCTTGGGGCTTCCTTGGATAGAGCATTAGTA
EMPV1_12037	TCTTGTGTGGGCTTTTATTCCTGAATCTTGGCTAAACTCTCTAGGCTTAACCTATTGGCC
EMPV1_12038	TGGACGTGATGCCACTTCCTGGAGCAATCCATTTTACTTGAGGAATCAGAAGGGAAGCGC
EMPV1_12040	ATTAGGCTTGGGCACCTGCTGTGGCCTGGAATGTCCTCCAGCTCCAGGTTTAGGTCTAGA
EMPV1_12043	TCCTGCATCCAAACGTGGGGAAGAAATCCAAATATAAGACCAGTGTTCGGAAGAAGACCC
EMPV1_12045	AATATCATGCTATGGATATTCGCTGTTACCACTCAGGAGGACCTTTACATTTGGGAGACA
EMPV1_12046	ACCTCCAAGTGTCGGCTCTATGACTGATGAGCACGGCCATCAAAGACCAGTAGCTTTCTT
EMPV1_12052	GTGCTGGGTTAAAATGTAAAGTACATTATGTTCCCAACTCAGGAAAGTACTGTGAACCAG
EMPV1_12053	CCCATATTGTGGTGCTCGGGAAATACCCCATTTTTTCTGTGATGTCCCTGCCCTTCTCAC
EMPV1_12054	CTTAACAGGGGCAGTGAAGACTCTCGAATGGCAAGGGAATGACTTAGGGAGTCCAGATCC
EMPV1_12058	TTCCCCATGCAAATGAGGTATCCAGCACAGGGGTACAAAGGCAACTGTAAAGTGGGGCAG
EMPV1_12062	CCAGCCAAAAGCCAGGAAGAAAAAGTCTCAGAATACCCGAACTAAAGATCAAAGCCTATG
EMPV1_12063	ACACAGCGCCCTTCCTCACTCCCGGACCGGGCAGGTACTTCCCGGAGCGAGCGGGGAACG
EMPV1_12065	GCTGTAATGTCACCAGGAAGCCCACAGTCTTCACCAGGGTCTCTGCTTACATCTCTTGGA
EMPV1_12066	TATTCTGCCTCTCATGGGCTCTCTGGCTGCTGACAGTCCTTTGTCCCCCACCGTAACCAT
EMPV1_12067	GAACTGACAATGACCATATGAGCCAGCTGCCATAGTGCTTGGTGCCTCACACTGACCTTT
EMPV1_12069	TGTTTTCACTAGACGACACTGGGCAGGAGCTGCAACTGGACGTGGGATCCTGTTACGCAT
EMPV1_12070	CTCTCTGTCCTCAGATGGGCTTTTCCCCGTGTGTGCCCGTGTCATATGGGATTACGGTCA
EMPV1_12071	TCAGCAGGGTGATGTCGTTGCGCACGGTGAGCAGGCTGAAATTGGGGTTCTTGAAAACCT
EMPV1_12075	CTCTTGCTATTTTCACCACCTCTGCAGTTCCTTCCTCCACTGAAGTCTTGAACCTCTCAG
EMPV1_12078	CCACGGAGGGGGTTTTGTGCCAGGATTCCTGCCCTTGAAGTTTCCACATTTCAGCTTAAA
EMPV1_12079	TCATCTGGCAAATCTAGCAGTGGCAGCAGTAAAACAGGCAAAAGTGGGGCTGGCAAAAGG
EMPV1_12080	GTAGAACTTGGTGCTCCAGGATTTCCCCCTCAGGTCCTAAGTGCCTTCATCAAACAGCAG
EMPV1_12084	CTGGGCATCAATATCACTATTCACTAGAGGAGACCGAGCCTGGGGCCAAACCATTGTCAT
EMPV1_12085	ACTCCTCCCTCGTCTCCCATCAGGAGATCCACCACAAAGAAAAATGCTATCAGTGTAAGG
EMPV1_12087	CTGTACCATCTGCTGTATTCATTTCTCCAATCTCATGTCCATCTTGGTGTGGGAGTGGGG
EMPV1_12088	CCTCCCTAACACTGCTTGTAAGCACATTGTCCTGGCGAAAATTTCCTGTAACGATATCCG
EMPV1_12089	CCTCGTTAGCTGGCAGGGATTTTCTCCAGATCACTGTAAAAGACTGAAAAAGGGCTCCTC
EMPV1_12090	CAAGCTCCTTTTACAGAATATAAGCAACAGTAGTGCCTGTGGTTTAGCCCACCAATCTTG
EMPV1_12092	GCAAGAAGTGTAGAGAAATTTTGTTTTAAGTCTTCAAATATCTTGAAAAAAAGTACACTG
EMPV1_12093	CATGTAAGCCATGGGGAGGGAGTATGTGCAAGAACAGCAGTGGAAACCCTATACCTGTGT
EMPV1_12095	TCGGGTTTCATCCATACTCAGGACCCCGCGCTGCGCCATGTCCAAGAAACTGAAGCAAAA

EMPV1_12097	AACAGGCTCCAGTGGCATTGTCTCCCTCCCAGCAGGGGGTCAGCTGGAGCTTGAGGAGAT
EMPV1_12099	GTCACATGCTTGGTTCATTCACTTGAAATAGGGAAGAAAGTTTCAGTGATTCCATAGAGG
EMPV1_12100	GTCAGAAAGCCCAGCAAGACAAGGCCACACTGGAGGAACACATTAAGAGTTTAGAGGAGG
EMPV1_12103	ATCATCGTCTCCGACTACACAGACCAGAACCTCCGCGAGGTGGAGAAGTGGTTGAAGGAG
EMPV1_12105	TGTGTGTGTGACTTGGCAAGAGCCAACCTCTGCCCCACTTCCCACAACCCCACAAGTCAG
EMPV1_12106	AAACCAGAAAACCCACAGCTGAAACAAATCGAGGGGCTGGTGAAAGAGCTGCTGGAGAGG
EMPV1_12107	CTCAGCTTCATTGCATGGGATTTGTATTGGGGAACCTCTTAACCTCAATTTCCACATGAG
EMPV1_12108	AAGGAAACCTTTGGGAATGTACAACGGAACAGCCACTACCGAGAGCAGTGTGGAGATGCC
EMPV1_12109	ATATTGCTGTGAGCTGTGGTGTAGGCCAACAGCTGTGCCTCTGATTTGATCCCTAGTCTG
EMPV1_12110	GAAAGAGGGCATGAATATTGTGGAAGCCATGGAGCGCTTTGGGTCCAGGAATGGCAAGAC
EMPV1_12112	CTGGCACAGTGGATTAAAGGATCTGATCAGGCATTGTGGCGGCAGCAGCACAGGTCACAA
EMPV1_12113	CAAGACTGACCTCGCAGCCTGGTCTGCTGCCATGGATGCCGGCCTGGAGGCCATGCAGAA
EMPV1_12114	GAGGGCCTCAGTAGGAGATAAGCCTCTGCTGTGTGTTTATAACATGATATTGTTGGTGAT
EMPV1_12118	GGTCTCAGTGCGCATCAGTGCAGCTAGAATGAAGTGTCTTTGTTAGAGGTTAGGTGGCTG
EMPV1_12120	CCAGAAGAAACAAGGAAAATGGAGGCAAAATAGGAGTAAAAGAGTAACCCCAGGACTCCC
EMPV1_12122	GATCCCCAGTTTGTCCTCCGAGGCACCCAGTCAGCGGTGCATGCACTGCATTTCTGCGGA
EMPV1_12126	TTAATATACCTTTGGGTTCACACAGTGCAACAGGGGTGGAGACAGGGTGCAGAGAGTTCC
EMPV1_12131	GATACAGTGGTGCTCAGGACAGTGGAATCGGCAGCGACAGTGTTAAAATCAGAATAGTGC
EMPV1_12132	CCAGACACTTGGACTGCAGAGAAAAAAGCCAAAGTATACCATGTAGAACAGGAAGATGTG
EMPV1_12133	ATCTTCATCTTCCGTGAAGCCTACCATCCACCCTACTCCAGCCCCGTCTGCTACGAGGAT
EMPV1_12137	TATTATAGATGTTATTCCTTCCAAGGTCGTCCCCCAACCCGGTACTGGTGCCCAGAGAAG
EMPV1_12138	AGGTGAACTTTCAAAGGCTGTCCAACTACAACTTCCACCATCGTGCAGGCCACGGTGCCC
EMPV1_12141	GAGTAACCTGCCAAAAGCCCGAGCTGCCTTAACCTCTGCTCGAACCACAGCAAATGCCAT
EMPV1_12144	CCGAGTAAGCATGAGAAGAGACAGGGAAAGCTACTATCTCAGCCAAGCTCTGATGTCTGC
EMPV1_12146	ACAAGCTGTGTGCCAGCCAGGTAATCACGAGCCTTTAGAGAGACAGGCCACCACTAATGA
EMPV1_12147	TTTGGATTGTCAAGAGATTGCTGGACCCTTTTTGCCATCGGAGGAGGAAACACACGCGCG
EMPV1_12151	CTCTGGACACTTTATGTGCAACCTGCTGTCAGCATTATAAGAGAAGATGGCAGCTTTCTC
EMPV1_12152	AAGGGAGAAGGAAAGGCTGGGGACCAGCAGATTTCTCTTGGCAATGTGCTTCTGAGTGCT
EMPV1_12155	GTATCGCAAACCCAAGACCATCTCAAGAAGGATGGAGCTAGAACATCTGGTCAACCTGGC
EMPV1_12158	TCGTCCCCTTGAGGGGTATGCTGCACTGGGTGCCCCAGGTTACAGATTCAGTGCCTGACT
EMPV1_12159	CTACCATGGATATGAGTGATAGTGTTGGAGGTTTCATAATTTTACCTTTCTTCTCCTTCC
EMPV1_12160	TACCTGTTAGCTTTCCCCTGCCTTCCAGCCCATTGACTCATCCTCACTACAGGGTGACAA
EMPV1_12161	TCTTTATAAATTACCAGCGCTGAGATACTTAGACCTGGGAACAACACAAGTGTCACTTAC
EMPV1_12165	GAGGCCGCCATTGCTGCTTTGAACACCTCAGAAGGGGAGAATGGATTTATCAGGAGTGAA

EMPV1_12166 GGACCAGTCTGTGGTGGACGCCTCGGAGAACTACTTCGTGACAGACAACGTCACCAAGCA
EMPV1_12173 TTCCTAAATTGCCTTAGAATAACGATGGAACTACCCCATGCCAGGCAGCCACAGCTACCG
EMPV1_12175 CTGGCACCTCGCTCTTCAACTTCGAGCCTCCAGAACTAGGAGAAAGAAATGTGTGTTGTT
EMPV1_12178 AGAGTGTGACCAGTTCCAGTAGCAACAGCAGTGACAGTTCAGCCAGTGATTCTTCATCAG
EMPV1_12180 AAGTAACTTCGACTTCAAGAATCCCCTAAAAGGGCTTCAGAGATTCCCAGGAGTCCGTGG
EMPV1_12181 AAACAGGATGAAGCCATGGACAGCCTGCCCCACATGGCCCCTGGCCTGTCTCTAGTCAGC
EMPV1_12183 AATACAAGTTACTTCACCCTCCCTGGCTCCACCAGCTCTGAATCCAAATGTCTGGGGTGG
EMPV1_12184 TGGCAGCATTGGGGATATGAAGGCGGAAGGAGTCGAAGGAAAAAGTGAAGAAAAGCGTGG
EMPV1_12186 CTTAGAACACACATGAGAAAACACTACAGAGGAGCACTTACCTAAGGAATGTGGAAAACC
EMPV1_12187 GTCCTATTCCCTGAACAGTAAGACATGGAACAGATAAGCGCGTGATGAAAGCGGCCCTCA
EMPV1_12188 CAGGGAATAATTTAGTTAGAATTTCAGCTTTGGGTGCTGATGGCGGAGTTAGTCTCCTTC
EMPV1_12189 CCCCATCAGGTTCTTCTCCATCCCTCCAGATGAGACTGAGCATATTTTGTAGAGCTAGCC
EMPV1_12190 CAAAAGTGCGGCCACAAGGAGGCGGTGTTCTTCCAGTCACACAGTGCCCGGGCCGAGGAC
EMPV1_12191 ATCAGTGTGTGCGATGCTGTCCTGGACCACAACCCCCCAGGCTGTACCTTCACAGTCTTA
EMPV1_12192 CCATCGACGGCTTAGAAGATGACCATGTACGACAACTGTATGGAGCGATGGACTTACCAG
EMPV1_12193 TGAACACGTCTCTGGCCACCCAGATTTCTTCCCACTTTCTGCTGGCTCTGTGTCCCTGTA
EMPV1_12194 ATGCACAGTATTAAGCAAGGGAGCAGGATGGTCGAGAAGGGTTGCCGGCCCAGCTATTCA
EMPV1_12196 TGCCTATTGTCCTGCTGCCGGTAGAGGCTGGTAGCTGAAGAGCCGTGTGCATCCTCGGTT
EMPV1_12197 ACTAGTCTGTTGTTGTCTGAAGTGGGACTGAGAACACAGTGAAGTGTGCCTAGATACAAC
EMPV1_12198 CCAGGAAGGGAATCCAGTCAACAAGATTGAAGGCTGGCTGCAGGATTGTGGATATTCTGA
EMPV1_12199 CTTGGACTTGGGTGAGGAGCCGACGGGGTTTAATGCTTATTGTGGAAAATGCAGCCATGA
EMPV1_12200 ACGTTAATGGCTGTAGCACTGCTGAAGAAAACATATCTCAAAATGCAGAAGGGACCCAGC
EMPV1_12201 TGCTGAGGGAGGAGCCCAAGAGGAGACTGCCAGCTAAACCTGCTCCCGCAAAAGTGGAAA
EMPV1_12202 TCACCACTCATGCCCCCTTTGGCTTGGGTGCCCATTTCACTGTTCCCTTGAACCAAGTAT
EMPV1_12203 AGATGAGGTTTGAGTCCAGGGTCATGAATATCTGGCACCAGTGAGGAGCCAAGGCTGGCA
EMPV1_12204 AGCAGGAGCGAACACAGTGGGCACAGTATCGAGAATCTGCAGAGAGGGAAATAGCTGATT
EMPV1_12205 TTTGGAGAAAGAGACTTTACTCCAGAATTGGGAAGATGGCTTTATGCTTTATTGGCTTGT
EMPV1_12206 GAAGAAGGGCTCCAAGAAGGCGGTTACCAAGGCCCAGAAAAAGGACGGTAAGAAGCGAAA
EMPV1_12207 CTGTGCGCTCATACTGTATCAACAAAAGCTAAAGGAAGGTGAATCCATGTATTACTCTGG
EMPV1_12209 AACAAATGATAGGAAACAAGAAGAAAGGCGACCAGAGCCACGGCACCAGGAAACGAGGAC
EMPV1_12210 TCAGCTGCACGGCCCCGCTGAAGGGCGACGCCATGGCGCCACACTGCCGTCGGGTCACCG
EMPV1_12213 TGATTTATCGGGCAAGCATGTTATGGAGTTTTCTATGTACTTGGTCTTGGGACAGGGGAG
EMPV1_12216 TCTAGCTTGAAAAGTAAGAAAGTAGCAGCCCTGTTCCAGGCACCCCCTGTGGGTACTGAA
EMPV1_12217 AGCAGCTGTTGAACCTGCTACACCCGTGCACAAAGATTTTAATGTGGGGGGTGGGAGACT

EMPV1_12219	AAACTCAGTGCACGGCCCTGGTGTAGAGACCACTCTGCTTGAGGACAGGCCCCTGGACAT
EMPV1_12221	GGGCACTAGAGGATAGACCAAGGAAAAATCGTGACTCGAGAGAATGGACAAATCTGGCAG
EMPV1_12223	GTAGAGACAAGCCTAGTCACAAGTTGACTGTGACCCCGAACACAGCTTCTCCACATGACT
EMPV1_12224	CCAGGAGTCTATATGTCTAAGGAGCTTCCCAGATTTGCTGTGATGCTTGTCCATGGGGGT
EMPV1_12226	ACTCTCTCATTCACACAGCAATCTTGATGCACTTCCCTTTCTGTGGGCACCGGAACATCC
EMPV1_12228	TCATGGACCTCGCCCGCACATCCTCTAGCTTTCAGTCGGACCTGGATTTCTGTCCGGATT
EMPV1_12229	GCCTCTTATCCTGGTGACTCAAAACTGTGTTCTTAACTGCCGTGATTGCTTTTGAGGGCC
EMPV1_12230	CACATTCAGAGCGAGGAATGAGGAAACCTCCATAGCAGAGGCACAGCCTGGTTGGTGGGT
EMPV1_12231	CTCCAGCTTGTCAGCCTATAAAACTGCAACCCTGATGTCTCCTGAGGGGTAGTAGCATGC
EMPV1_12232	GTAGACTGTAAGCTGTGTGGGTGTAGCTGGTTTCTGTTTTCGGCTCCCATCCTTTCAAGC
EMPV1_12233	AATAGAATTTTTAGAGTATACCCACTTGTAATACTGTTTATGACAAATATGAGTTAGAAA
EMPV1_12234	AGAACATTCTCATCGACCGAACGGGACACATCAAGCTGGTGGACTTTGGATCAGCTGCTA
EMPV1_12235	TGGATACCTTGGCTGGTTTGGAAAGTTCATTGTTCGACACCATGGATCCCCAGGTGGGTC
EMPV1_12236	AGGCAAGGTGGTTCTGGAAGGTATAGACTCCTTGTTCTGGGAGCTGATGGAGGAGAAGCT
EMPV1_12237	GGAAGGCTTGTCCTCTGTTGCTGGACAGCTGGAATGCTGATCATACTCCCACCACTTATC
EMPV1_12238	CTCAGGAACTGATCACATCCCGGGGCACAAAGCAAACCTCAACAAATTTCAGAGTAGAGA
EMPV1_12239	TGATGTCCGATCTCTTCAGCAAGCTGGCCATGAGGCGCAAAGGTATCTCTGGAAAAGGAC
EMPV1_12240	ACACCTGCCCCAGTAGCTTCAGGCAGAGTTCAGCAGGCAAAGGAGAAGCTGTATTTACAC
EMPV1_12244	GATCCATACCTACATAGTTACAATGCCATTTAAAAAATTCCCAGTGGTGGCCTGCCTAAG
EMPV1_12245	TGATCAAGTGGGACTTATTCCCAGGATGCAAGAATAGTTCAATTGATGTGGTATACCACG
EMPV1_12246	CCCGGTGGCTTGCAGCGGCATCTAGAGGTTGGAACTTGGCATTGCAGCTAATAGATTTAA
EMPV1_12248	CCATCGACGGCTTAGAAGATGACCAACATGGACAAGTGTATGGACCGTTGGACTTACCAG
EMPV1_12250	AGCAGTGGTTTTAATGGAGTTCCTGTCGTGGCTCAGCAGAAACAAATCTGACTGGGATCC
EMPV1_12252	GAAGGACAGAAAACTTTACACCAACGAGGGAAGGAGATAGGTGGAGTGCTAGAGAGAGGG
EMPV1_12254	ATGGGTCCTGTCGTCTGCACATCTTTCTGAGGATTCTAGCATATTGGACTGAAGGGGTCC
EMPV1_12256	CAGACACTACATTCTAACTTGTCCAAGAAAGAACCCAAATCCAACCCCCACCTTTCTGTA
EMPV1_12258	ATCTGAGATATGAAATTCCTAGTTGGTAGCACTTTGGAGCTCCCGCTATGGTGCAGTGGG
EMPV1_12262	TCAACGCCATCCTGCAGACCAGCTTCACATTCAGCCTCCCCTTCTGCAGCTCCAACCACA
EMPV1_12263	GAGTACAGTTCCATCTCTCTTCCACCCCATCCCTGCCTATTTTCTTGCTAATCTCAGAGC
EMPV1_12264	GAATGACGCTCTATACAGGAAGCTGTGTTTGCAGCCAGAGAGCTGTCAAGAAGATTGTTC
EMPV1_12266	CATTTCGGTTGGGCTTTTCAACTTGCCACCACCCTTCAAGCTGTGCCTCATCCCTAGATA
EMPV1_12268	TGCTGACTGAAGCCAGAGAAAAAAATAGACCTGAATTTCTTCAGGGTTGGCAGACTGCTG
EMPV1_12269	AGCCTCCATTCTGAACACCATAGGTAGGATGTAAAGCTTGTCTGATCATTCAAAGCATGG
EMPV1_12270	GGTGGAATCGGGAGAGCCTAGAGGTCCTCTGAGTCATCTTCACAGAGAACTGAAATTTCT

EMPV1_12276	GGAGCATGAGGTCATGTGGCGGTGTGATGCCCAAAAGGCAAAAGAAAACTGCCTTTCCTT
EMPV1_12278	GGTTAGGGATAACTCCCGCTCGCATTTTCTCTCATTAAACATCTTTTTCTCCAGGGAGCC
EMPV1_12281	CTCCTGGTCACTGCCTGTTATGCTTGTGCAGACTCTTGTAATCATTTTTAAGCCGGCCTC
EMPV1_12282	GGGGCCATTGATTACACAGGGCCCAGGAGAAAGCATTTCCTACAGAAAACAGTGCTTTGT
EMPV1_12284	AGGAGCCGATGGTTTCAGAGGAAGACCTGGGGAGAGTGGCCGAGAATCTGTCAGTCACCT
EMPV1_12286	GCAGGCTTTGGTTGGGAGAATGGATTAACTGAAGGTGTCCTTCACCAGCAAGAGTCAACC
EMPV1_12287	GTGACCAGTTCTGCTGGTTTGCCTAGGACTGAGGTTTCTCAGGATATGGAACTTGCCATG
EMPV1_12288	GTGGGAAAACCTTTAGGCAAAGCTCATCACGTATTGCCCATCAGAGAATTCATACTGGAG
EMPV1_12289	GAGGGCCTTCTCCTTCATGCCCCAGTTGAAGATGTAACAGTTGCCATAATCCGGGTGGAA
EMPV1_12290	GTTCTTCATCCATAGTTTTACAGCTCTGGAGTCGGGCTTCTTTCTGGCCATGGCCGTTGA
EMPV1_12293	CAGGACAGGTGGGCTATCTGATTGCTGGGATGAAAGATGTCACTGAAGCACAAATAGGAG
EMPV1_12294	CGTCTGTCACTCGTTTTGTTGCCTTGATCAGTGTTTGGTTGGGGGTGGATCCCAAAAGGA
EMPV1_12296	CTGTTCCTCTCGTAGGTCGCGGTCCTGCAGGCTCTGGATAAAGCCATTGGCTACTGCTTC
EMPV1_12299	ATGAGGACTTGGACCTTATCCCCCCCAAACCTATGGCCTCTTCAACATGCTCCTGCTGCT
EMPV1_12305	TCCACACCCAGTAGTATGAACCCTCTTCCCGTAAATCCCCAAGTGGCTGTCCTGGCATAT
EMPV1_12307	GAGCATCAGGTATGGAATTACTGACTTAGTGCAATATCTGTCTGATTATCCAGAGGTATC
EMPV1_12308	TGGCTGATGAGCTTGCCCTTGTTGATGTTGCAGTGGACAAACTGAAGGGAGAAACAATGG
EMPV1_12310	CCTGAACTCATATATGAGTACGTGAATAACCCCTCCAGCATCTTTATTGTGAGTGGATGG
EMPV1_12312	AAGGCACCAAGAAGGACCTGGATGACATCAGCACCAAAACAGGCATCACCCTCAAGAGCT
EMPV1_12313	CTGCTACTGCATGACGGATGACAAGGTGGACAAGACTCTGGAGCAGCACGAGAACTTCGT
EMPV1_12314	CTGGCCTCAGCAGACACCTACAGTGCCGAGATGGCTCTCTTTTCAATGGGTGTGGTTATT
EMPV1_12315	CCGAGTTCACCACTAAAGTCCAAGAAGCTATCAATTCCCTGGGGGGCAGTGTGTTTCCTA
EMPV1_12316	TGAGAAAAAGGTGAACCTGGCAGAGCTGTTCAAGGGCAAGAAGGGGGTGCTGTTTGGAGT
EMPV1_12318	GGGAGTGCAACTTGGCAACAGCATTTCTCTTCCAAAATGCGTTTCTTATTTCCAGCCTTC
EMPV1_12321	GAAACAAAGTCAGATTCAGAACCAGCAAGGCACAGGGCCCCACTCCGACCTGGAAGATTT
EMPV1_12322	GGACTCTGATAGGCCTCTTAGAGAATCCGTGAATGTGAACACATAAACACGGCTAGTCAT
EMPV1_12325	CTTCTCGTGTGGACTTGGGTCTCCCAGCTGCGGGCCGAGGTCGTGAGTACACGGTTGGAA
EMPV1_12327	ACTGGGCTATAAAGATTTCCGGTTAGACGCCGCCTGGGCTGGAGGGAATGAATGGATAGT
EMPV1_12328	TCCTCTGCCTGAATGGTTAAATCTTTGCGTGGTTAATTTTAGCAGAACCAGTAAGTGATC
EMPV1_12331	GCTGTAAAATCCCAAAATACTTGAAGGTTAAACAGCACACTTCTAAATAACAAAGAGGTC
EMPV1_12332	CACATTAGAAGAACACCTTAGCACTGAGATACAAGCCAAAGAGGAACTAGAGCAGAAGTG
EMPV1_12334	TATAAGTTTGGACCGCTGTTCATGAGGCTGTGCTACGAGCTGGATCTGGAGGAGTCTGCA
EMPV1_12335	CGAACTTGTACAGCCACACATAGCCAGGCTCTACGCCTCTATGCTAGATGATATGTACAA
EMPV1_12336	GCATCTCTTCTAATCACACAAAGATGTTGTACAGCAGAAGCCCTGAACCAGAACTACTGG

EMPV1_12337	GGCCAGAAATTTCATTTTCACACTGCCCATCCCCAGTCTTTCAGGACGTCTCTATTATTG
EMPV1_12338	CTAAGGACACCATTGGAATGGATATTTTCATGCATATCATCTGTGTTGAGTATGTGCAAG
EMPV1_12341	ATGACAAAGAATATCTTCTGAAAGCAAGGGCAAACACAGAATACTATATACTTGCCATGC
EMPV1_12344	CCCTGAGTCATATTGACCCGCCAGTGCCTCTTCATCTTGCCTGTCTTTGGTTTTACAGGA
EMPV1_12345	TAAACTGCTGTTATTACCCACCTCTAGGATCAAACTAGGAGCCCGGCGTTTGGTTGGGTG
EMPV1_12346	CACCGTGTCTGCCTCGTACGCGCTGGAATACGGGAAGGCTGAGCTGGAGATCCAGAGAGA
EMPV1_12347	ACCAAGGGGGCGTCACCCACCTCTGCTTTCACCCTGATGGCAATCGCTTCTTCTCAGGAG
EMPV1_12348	GCGAGTTTCGGAAGTTATGTGCCATGGTGGCTGAAAATCCTAGCTACAACACGAAGACCC
EMPV1_12349	TGAACTCCTGGGATCACCCTCAAGATGAGCATGCACCGATTTGATCCTAAACACTGTACC
EMPV1_12353	AGTCCACATCATTCCTGCAGTCTGCTCTCTACACCAGAGTCCAAATGACCCTTCAAACAC
EMPV1_12355	CTTCTCACAAGAAACACACTGAAGCTCTAGGAAGGAAGTACCTTTGGAATTTGGCACTAT
EMPV1_12357	GGCTTTGAAGCCAGGCATTGACTTCTCTCTAGCTATGAATACCCTAGAGATCTCTTTTCC
EMPV1_12358	GTCCAGCTTGAACAACTTGCTGGTGACGATAGTTGTGGCTGTGGTTGCTTGGATTGTTGC
EMPV1_12359	TGACTCCGCGGCCTCGCAACCCTACGTCCCTGCAGCATGTCTGTCAAGAAGATTGCGATC
EMPV1_12360	GAGGAGAAGGCCAGTAACATCGTCATGCTGAGGATGCTGCCACAGGCAGCCACTGAGGAT
EMPV1_12362	GGACTTGTGTATATACACATGCACAATAACCCCACCAGCATCTTTATTGTCAGTGGATGG
EMPV1_12363	CTGTCCCAGGTAGCAGTTCCCAAACACCTTCAAAGGGCAGAAGCCACAGAGTTGCAGGAT
EMPV1_12364	GGACCTGGTGAAGGACAACGGCCACAAGTACTTCCTCTCCGTCCTGGCAGATCCGTACAT
EMPV1_12365	GGACCTGGGGCCTTTCTCTGCAGTGCTTGCACTTGGACAAATATCTCCTCCAACAACAAA
EMPV1_12366	CAGCAGTGGCCAGGTCAGGGGTGGATTTGTGACAGTGAGACAGCTGAATTTGAATGTTCA
EMPV1_12367	AGGAGGGCATGGATATCATTACCCACATCCTGGCCTTGGCACCTCGACTCCTGAAGGACT
EMPV1_12370	ACAAGAGGAGTCCCCAGTTAAGGATTGGAGCCCCTATAATATCTTCCCGTCAGGAGCCTG
EMPV1_12372	GGGATGATATAAGACTACATCTCCGACGTTTCTTAGTGAGCAGATTACAAAGCCACAATG
EMPV1_12373	ACAGTAATCATTTACTCTGTTAGGCACCGCACTGCTAGTGGCTCCTCTAAGGCCTTCTCC
EMPV1_12374	CGGCCAGTTCGAGTCTCTGGCTGATGGTTTCACGGACCTAAGGAAAGACCATCTAACTCA
EMPV1_12377	CCCTATTCAGCAGGCCAGTTTTCCTCGAGCTTTGGAAGTTTCACTCAGCCGTGCACTCAA
EMPV1_12379	GCCTCATGGGGCTGGAGCGAGTCAAAGAGCAAAACATACATTTTCAGCTTCTGTAAAAAA
EMPV1_12382	GAAGGCTTCAGATCTAGAATACAAAATACAGCGAAGAACTCTTTTTAAGGAAGACTTTAT
EMPV1_12385	TCTAGGAGCACTGGTATGAGTTAGACATTGAGACGCCTCTGAAAGATTCAAGAAGAGTTG
EMPV1_12386	GGCCAGACCCTGGATGAGAGCTGCCAGGAGCTACGGGACAAGTTCTGGGAATTCTACAAG
EMPV1_12387	AAAGTGTCATGCTTCCAGTCTACTGTAAAGTGTTTAGCTGTTCTTCAGAGACTAGATGTT
EMPV1_12388	ACTATGATCCGCCCACTGCCCACGAAGCTCAAGCTCAAGGCTAGGGCAAAGCAGAGAGTG
EMPV1_12392	GCCTTTGTTCTGAGGTCATTTTGTGGAAGACAGCGACATCCGGTGGCTTAATGTGTAAGT
EMPV1_12395	CTAACAGGTTTTAGTAGCATATAGCCTCTGTGTTCTCTCCTCCTGCTTGCTGGCATGTAG

EMPV1_12397	TTTCTGTGAAGGCTGAGCACTTCCTATCTCTTGCTCAGAGACATCGCACGTCCCTGACTT
EMPV1_12398	TGGAAGACAGGTCAGGCTCCTGGATTCACTTACACAGATGCCAACAAGAGCAAAGGTGTC
EMPV1_12402	GCCCCAGCACAAGGTGCGCGGCTACCGCTACCTGGAGGAGGACAACTCGGACGAGAGCGA
EMPV1_12403	TGTGCTTGCCGCATAGGGAAGTTACTAGGCCAGGGATCCAACCTGCACCACAGCAGCAAT
EMPV1_12406	TGACCCAGCTGTCCTATGATCCTACCCCACTGTGAACTTTTGTGTAGCCATGTAGCTGCT
EMPV1_12411	GATGACGGTATTTTCTAGGTTACGGTTGCGGGTAGAGAAGTGGCGGAAGGTAGTGAACTG
EMPV1_12412	GGTGCCTTGCCCTCTGCAGCTGCAATCTCTGTCTCTTAAGTAAGTCAGATGTCTCTTCTG
EMPV1_12413	ATGCAGAATGCTGGACACTCCCACACCCTTAGCCCCCTGGACACCAAGGAGAATCTGGAA
EMPV1_12414	ACCAGGAACCCAGGACCGGTCACAAAAACTATGCAAGAGGGGTGAGAGCCCTTCCGGCTA
EMPV1_12415	TTGGTGACGGAGACTTCTTCTGCGGAGTTCCAGATATTCCGGAGCAGATACAAGCGGGTT
EMPV1_12416	TTGGACTGTATCGGCTGTGCCTTCCAACGCCAACAAGTCGCTTGAGTCTCTTTCAAGACA
EMPV1_12417	CTCCACATAATCAGTATTAAGCCCTCCACTATGTGGTTTGGAGAAAGAACTAGAAAAACC
EMPV1_12418	GGCCTCTAGTGACTCTTCAGAATTGAGTGATGAAGGTGCATCTCGGAAACCATACACTTC
EMPV1_12419	CATCCACTGTTTCTATGGAAAGTGGTTTTTGGAGTGGATTCTTGATGAGTCTGTACATTT
EMPV1_12420	AAATGAAATCCTCAGTCCCTGGCGTGGTTCACACAGACTTGACTGGAGCCTACTCGAGAC
EMPV1_12421	ATACGGCCAGTTGTACGTTCTCTTCCTGCGGCATCACTCTCGGACGTCTAACAGTACAGG
EMPV1_12424	TTCATCACCAACGTGCAGGTGTCTGGAGGGGGACCTAGCATCTCCATGGTGATGAAGACT
EMPV1_12429	CTAAAGCCAGGCTCAATTTTAGCAACTCATGGCATCCAGCGCAGCAGGGACCAAATGCTT
EMPV1_12430	TAATGGCTCTCCTCCCACAGGTGTTGGCCGAACTCAATGCCATGCTTGGTGCAACTAGTA
EMPV1_12431	GAATTGTGGTTGATGCTAAGACCACTCAACTTAGCTCCATGGACTGTGCTGAAGGAGAAG
EMPV1_12432	GATGCGCAACCAGATCCTCCCCGACGCGCACAAGGACCACACTCTGAAGTGGCGTTTGGA
EMPV1_12434	CTGGTTGCTAACCTGGAGAAATTGTGTTCTGAAAGACCTGATGGAACACTTTGCCTTCCG
EMPV1_12435	GGGCTGGGACAAAGAAAATTATATTGACCCTTGGCTATCTTCTTGCAGTGCCAGGGAGTT
EMPV1_12436	AGACACCAGAAAGACTCCGAACGAGAAGCAACACGCACAGATTCAGAGCAGGATAGAGCT
EMPV1_12437	CGTTTGTTTGATTTCATCACTGCTGGGTGGCTGACTTTCCTATTTGTGCTCCTGGCTGGG
EMPV1_12438	CCCCATTTTTATCCAACAGGATGTCAACAGTGGCAGGAACCTCCTCAAAAGGGCCCAGAG
EMPV1_12439	AACGCTCCAGAGATTACCATCACTTCTCTGGCTCTTCCCATCCAAGAGAATGCTCAGGTG
EMPV1_12440	AATCTGTTTGGATTTCTGTAAATTGGAGTCCGACAAGCCTGCAGTTCCGTCGCCAACCTG
EMPV1_12441	AATGGTGCCCAGCCCTGCTGCTATGTTGTCTTAGTTGTATGCAATTCTCGGTGCAGAGTG
EMPV1_12442	CGCTGGCTCAGCCTTTTGACTGGCAAGACCCTAAACCCGTTGCCCATATGAAATCATCAG
EMPV1_12446	GTCATGGCAGCTACTCAAGCTGAGGGACCTAAACGAGTGTCAGATAGTGCTATTATCCAC
EMPV1_12448	AGGAGGAAATCGCCTCACTGAAGGAGCGGAACGTGCAACTGAAGGAACTCGCCAGTAGGA
EMPV1_12452	CACCCAGAAACCACCAGCCCAAAGATTCTCACAAATCATATCTTCTCCCAAGACACCAAC
EMPV1_12454	CTGGTTTTCTTAGCTGTGCATATGCTAGTAGTTTGATTAGGTCTTATTTGTTTATTTTCC

EMPV1_12455 GGTTTACTTCCAGGCACCTTATTCAGACCTCCATTATAAACTTGGGATCAGATATGGGTG

EMPV1_12456 CAGATTTTCCAGAGACTGATAGCCCTAGATAACCAGAATAACCTCGAATAACGAAGCTCT

EMPV1_12458 GTTGGGTCATTGGTCATTCTTTCTTAGAGTGGATTCCCTGAGCATCACCTATCCCCTGTC

EMPV1_12460 TATGACAGCCTGCTTCGGCAAATGTGGGAGAGGATGGACGAGGGATGCGGAGAGACCATA

EMPV1_12461 GGAATTGAGGGCATTACAGAACATTTGCCCAACACACAGCCAAACGCTGAGACACCAAGG

EMPV1_12464 TCTAGTAAAGCAACAAGTCTGGGGAGATGGGTGGCAAGGAGGGGCCATGTTCCAGTTAAG

EMPV1_12466 TTTGAGCAGAGAAAAATCACACAAGGCCCTCTCCAAAGAGGAAAACCGGAGGCTGCCCTC

EMPV1_12468 GAGCACACATGGAACATTCTCCAGGATAGATCACACTCTGTTCCACGAATTAAGTCTGAG

EMPV1_12469 ACCATTTCCTGTGTGTGAAAGTAGGAGAGCTGTGCTGGTGGCTGGTGTCCTTGCAGAGAA

EMPV1_12470 TGAGTCTTCAGGGTGGTTGGATCTGGTCCACTGGGCACTGGCATCACCTGTGGTTTGTGT

EMPV1_12471 GAGTGTTTTAAGATGCCACAGTCTTTAGGGGCATTAGGAGGAAAACCAAATAACGCCTAT

EMPV1_12472 TCTACTTGATGCCATGAAGCATTCAGGTTGCGCTGTGAGCAAAGAAAGACACTTTTCCTG

EMPV1_12473 TTATCAGAGGAGGGCTACCTGGCTAAAGTCAAATCCCTTTTAAATAAAGATTTGTCCTTG

EMPV1_12474 ACTGGGCCATTTTAACATCCCGACCCTCTGCGACCTAGCCAACCTCCAGTGGTTTATTCT

EMPV1_12477 AGGAGGACCTCTGCCAGAGCGGCATCTGCACCAACACGGACGGCTCTTTCGAGTGCGTCT

EMPV1_12478 TCAAGAATACGATACCATGGAGAGATCCTAGAACACAGAAGTGAGGCTGGAGCACCCCCT

EMPV1_12479 AAGGGCTGACAGTTCTCATAAACGAAGACAAAGAGCTGGCTGAACTTCGGGGTCTGGCAG

EMPV1_12481 CCCCCATGTGTTTTGGAAGTTAACACCTCTCTCCCCCTTGGTTGCAGAGTGTGGTGACAA

EMPV1_12483 TGTCCTCGTCAAATGAGAAATCACCCTGAGTTTGGGGATAGTCCTCCTTCCCAACGCCTA

EMPV1_12484 ACTAAAAAACGCAATAATCAGATTTGGAAGACCTCCTATGCCCAGCACCAACAGGTCTGC

EMPV1_12487 GAGCCAGTTGTGCCTGGGGAGCTCTGCAAGAAGCCCTTTGTAGCCTTGGCAAGCGGTAAA

EMPV1_12488 TCTCTTTCTCTATGTCGTGAGCAGGTAAGAGGAAAAGCGGAAACGGCTGCACAAAGGACG

EMPV1_12489 AAAGATCATCCCCATCTTAGCCCAGCCAGGGGGATTTGCTTCCTAGACATCGTTGGTCAG

EMPV1_12490 TGGAAGAATTTCATGCGTAGACTTCCCAGTTTGACCCGTGTTTGACCCCTACTTCACACC

EMPV1_12491 AGTTCTGACCTTCAGTGGATGAGAAATTTTATCAGATTGATGCCCCTGGCAGTCTTGCCC

EMPV1_12492 CGAGGAGCCGGAGAAGAAAAGAAGGAGAATAGAGGAGCTGCTGGCTGAGAAAATGGCTGT

EMPV1_12493 TACTGGAGGGCCTTGCGGAGGGGCAGAGAAGGCAGGACCATGGCATCTAGGGCCTCTGAA

EMPV1_12502 CCACGCCAACCTGGAGCTGCACGCCTCCTACGCCTACCTGCTCATGGCCTTCTCCTTCGA

EMPV1_12503 GTGGAACCAGAGTTATCTTTCCAAAGCACGAGGCCAAACATCAGAATGAACCTACAGACC

EMPV1_12504 GACATGAGTTCAGTCCCCAGCCAGCACAATGGGATAGAGATCCAATGTCGCCACAGCTGT

EMPV1_12506 TGAACACGCCCGATCTCGTTGGATCTCAGAAGCTAAGCAGGGTTGGGCCTGGTTAGTACT

EMPV1_12509 GGCTGCAGTTTTCATTTTTCCACCGAGTGGCATCTTGGTCTCCTATGGCCGCATTGGGTT

EMPV1_12512 TGCAGGTGCCTATGGCTGACACATCTATCTTGCATCTCTGTGACTTCTTTGACCCCAATG

EMPV1_12513 ACATCTTGCTGATCAAGCTCTCCTCACCTGCGGTCCTCAATTCCCGGGTGTCCACCTTAG

EMPV1_12514	GGCAGCCCCGAAAAATGCTGGAATCTGCATGGCTCCTGCCGTGACAAATGTTCCAAGAAT
EMPV1_12515	GAAGGGAAATAATTCAAAGAACCAGCAGAGGAAATTCAATACTGCCTTGAAAGAGGAGGA
EMPV1_12517	ATCTGCAGCAGGCAGATAGGCAGACGGTTTAAAAGCTGTTTGAAAAGCAGACGCCTTTGG
EMPV1_12518	ATGAATTTCCCACAGTCCAGGCAGAACCCCGTTAAGGAGGCACCGAGAAGGCTGATGGCT
EMPV1_12521	TCAGCCATTAACGGAATGATGAGGTAACCCAGATGGAACTAGAGACTCTCGTACTGAGTG
EMPV1_12523	GGTCACACAACTAGTTAATGGCAGAGTCAAGATTTGAATCCAAGAAACCTAGCTCCCATG
EMPV1_12524	CAGCCTGGCTAAACTGGAAACCTAAGCTCCCCACTTATCCTTTGTTAGTATGGGAGCAGG
EMPV1_12525	GCAGTTAGGGCTCTTTGGGTCACAAGGGAGAGAACTCAGTACAACCCAGCCTAAGCCAAA
EMPV1_12526	AACCGAGGGCCCCATGATTTCCTTATTCATCTGCATCCTCATTTGCCTGTGCCCAACTCA
EMPV1_12529	ACAAAAGAGCTCCCGTTGACATCTCATTTACCGCCTCCCCCAGGACACAGACAGAACCAT
EMPV1_12530	TGACTACACTCTGGGTGATGCTAAAGCCGGTGGCATGGAAATCCTGAGATGTATTGCCTG
EMPV1_12531	GGGGCGCCAAGGCTGCCATCGTCTGCTATGACCTGACAGACAGCAGCAGCTTTGAACGGG
EMPV1_12536	TGTGTGTACGCGTGTGCAAGACCAGCCTCGCCTCCACACGCAGCCCCACACGCCAGCAAG
EMPV1_12537	GCCGAACTTGATGGTTTAGCCACTGACCCATTTCACAGGTAAGACTCACAAACCCTCAGG
EMPV1_12538	GCTGGTGGTCGTGACCCTATTCAATTTCCTGGTCTGTTTTGGGCCGTACAACGTGTCTCA
EMPV1_12540	TATTGAAAAATTGTTTTTAACCCACAGAGCCAAGGGCTGGAGGATACCTTGGGACAGTCC
EMPV1_12542	TGCGCACGTATAAGCCTTTAATGGATCGGCCTAAATGCCAAGGATTAGAAGCCCGGATCC
EMPV1_12543	CCCAAACGCCTCTCACGTTTTGTCTCTGGAACGTAATCTGGTTATTTAGGCGTGGAGTGG
EMPV1_12545	TCCACACATGCATCTCTAGGATTCACCCAGAGGTGTGGCCGTGGCGACAGTCAGTGCCCA
EMPV1_12546	TATAGACTTGGAACAACTAGCATCAGATCCCATTCTAAACCAAAAGGCCTAAACTGAGGG
EMPV1_12547	TGCTGCTGAGACGTGATCATGGAGAACATGACCTCTTGAGAAACCTCATTCACAAAAGCC
EMPV1_12548	GACTCACAGACATAGAGAATAGATTTGTGGTTACCAAGGGGGAAGCTGGGTAGGAGAGGG
EMPV1_12550	CTGACTGTATATATAAGAGCTATCTACCAGTGCTAAACAGCCCGAAGAACAAGAAGTGGG
EMPV1_12551	TCGCCAGTATGATAGCAGAGGTAGCAAAGGCAGAACAACAGCATAACCAAAGTAGGATGG
EMPV1_12552	CTGTGTGTGATGGGTTGGCAGTGTATTGTTAGCTGGTTGAGTATATGAGTAGCATCAGCT
EMPV1_12553	GCTATACGGCCAATTATAGAGCTCTAATTATGTCCTGTTGTTGATAGACTGGGACTCCCC
EMPV1_12554	AGAACCCAACCACTATTCATGAGGATGCGGTTTGATCCCTGGCCTTGCTAAGGATCTGAC
EMPV1_12555	AGGGTGTTGGGAAGACTGGATAGCCACATGTAAAAGAATGAAACTCAACCACTATCTTAC
EMPV1_12557	GTCGACACACTCCTGACCTTTGTTCCACTGTTCGGCTCCCAGAGAATTCCAGAAACGAGA
EMPV1_12559	TCTTGTGTGGCTTTTGGATATAACAGAAGTAATGTAGGCAGCCCAGTCCAGCCCATCTCC
EMPV1_12561	AAATAAGACAGCGTGTCAAAATCAGTATTTTCTCTATCAAACACTGTATGGAAGGTCACG
EMPV1_12562	GGATGCCTCATCATTTACGTTGACATTTTTTGGTCAAGGATACAGCCAAGGTCTTAGTCC
EMPV1_12565	TGAACCGTGGTGTGGGCTGCAGATGGGGCTCAGATCTCATGTTGCTATGGCTGTGGTGAA
EMPV1_12566	CTTGGACTACAAGACCGAGAAGTACGTCATCGCCAAGAACAAGAAGGTGGGCCTGCTCTA

EMPV1_12567 CGCTGCTGTCTCCTAAGATCTCATTTTCTGAAGCCTTGGTTAGACGCTTATAGATGTCCC
EMPV1_12568 AATTGTCCTTCCCCTGGGATTGGTCTATGCCTCCTTCCCCGTGTCCTGTCTCCAACTAAT
EMPV1_12569 AGAGTGTGGGAGAGACACTGGGTGGATCATGGAAAAAGAGGAATTCATCATCAAGTTGTC
EMPV1_12570 CATTTGGCAATGGTTTCTTGGGGAGTGGGATGCATGGTAGCCTTGGGCCAGACCAACTAT
EMPV1_12571 ACTCATCAGAAGTGGGGGACCAGAGCAACAGCAAGGCAGCTAAGCAGATGGTAGAGACAA
EMPV1_12572 ATTTTCACCACCAGCACTCAGCAGCCCAGGCAGAGGCCAGCCAAAGACAGATGTTGGAGT
EMPV1_12574 GAAAGGCAAGGTGTTTCTGGAGTAAGCATCATTGTAGCCAAAGGACCTCCTCCCTGACAC
EMPV1_12575 AAAAAGGACAGCCGTGCTGCTTGGAACACCACCCTCCTCGATCAACTACTCTCTAGCCTT
EMPV1_12577 CATTCTATGGATAAGAAGGCTGAGTTCCAGAGGTATTAAGTAACTTGTCTGCAGTGACAC
EMPV1_12578 TCCCTTATCCTTCAGAGTTTCGCACATGGACAACCCGGCCAGACACAGGAAGATTAAAGC
EMPV1_12579 GGTCTGTTGTAGGACTTGATTTTGTGCTGTTGTGAGGCTACTGGACTTAGAATCAGGAGC
EMPV1_12580 GTCTCTGGAAAAACAGAGCCATGAGACAATGAGATCCCTAGAGCAGGAGAATCCAGAATC
EMPV1_12582 CAGCACTGCGATCTCCTCTCAAGTCTGTATCATCAGTCCCACAGGGAAACGTCACCACTG
EMPV1_12583 CTTGAGTCAGAGGCTGTATCTACTCCACATACATTCTGCTTAAACAGTGGCATTCTAGCC
EMPV1_12584 CTAAAATACAGACTCCAATGGGATATGAGGGGTAGGCATGGACAAGACCAGGCAGAGCCT
EMPV1_12585 CTTTGGCTTCGGGCTGCCCACGTCGCGGGCTTATGCAGAATACCTCGGTGGTTCCCTTCG
EMPV1_12586 GAGGTGAAAGCTCCTGTTCCTTCTGCCTGTTTCAGGAATATTTGTAAGCAAATGACAAAA
EMPV1_12587 GGTGTAATGGGTGGAGACCCTGCTGTGGTTTCTTTTGAGGGATAATAAGAAGGGGGATGG
EMPV1_12588 GCAGCCTCAGGAAGCATCTTCCAGGAGCGTGATTGGGAATAAACATGCCTCTCCTTTGTA
EMPV1_12589 TCTCTCAGAAACCCACGATAGCAGCAACATGGCAGAGAAGAAGGTGGCATCTGAGCTCCC
EMPV1_12590 AAATGCTGGGAGCACCTCAAGTCCCACGGAGTCCCCCCACTGCCTCTTAACTGACTTACT
EMPV1_12591 GACTGTAGTGATGTGCTACACTGATTTATGGTGGGTTTAGTCATGGTTCAGGTTTAGAGT
EMPV1_12593 ATTCCAGTGACCTTGAGACTTCAGAAGTTCTCCCTGTTCAGGCATCAGAGGCTGCAGGCA
EMPV1_12594 GAGCAGCAGAATGAGGCTCCAGAAGAGGATAACAACCAGCAACCAGAACAAATTCCTGAG
EMPV1_12597 TGTGGTACTCAAACTGTGGGGGCACTTTCTGGTCTAGGGAGGGAAGTGCCGCCATTTTTT
EMPV1_12599 AGGGATCAAACCCACACCAGGGCCATGACCTGAGCCACAACAGTGACAAAACTGGATCTT
EMPV1_12600 CACACGTACACACTGAGACACCTCCCCACATACCATATACACTCTCACATATATACATAC
EMPV1_12601 GTTGAAAGCACCACTTATGATCACCAGACCAGCTGCAAATGCCAGGGTGTATTGGTCATG
EMPV1_12602 GTTTGGTAGCCAACACTGTCACCCCCTGAATCCCGAGGAGCACTACGGTTAGGATGAGAT
EMPV1_12603 TGCCTGATTCAGCACCCTGTGCGCTGTCCACGCTCCGGGCCTCTTCTGGACCAGACACCA
EMPV1_12606 CCATATATGTAGGTCTGTGTATCCACCCCACAGAATAGTTCCATCACTACAAGGGTCCCT
EMPV1_12608 GCCCATATTGTCAGTCCCTGAGTCACACATTCTGCCCAGAGGCATTACTATTCCCAGTAG
EMPV1_12609 GCAGCATGGTCGAATTGGAAATTTTGTTGTGAAAAAGCCAATGGTGCTGGGGCATGAAGC
EMPV1_12610 ATCTTGTCCCGAGCTGCTGACACGGAGGAGTTGGACATTGAGATGGACAGTGGAGATGAA

EMPV1_12611 CAGGTTCTTTGTGAACTTCCCGTCGGCCAAGCAGTACTTCAGCCAGTTCAAGCACATGGA
EMPV1_12615 TGTAGCTGCCGGCATACACCAGAGCCATAGCAACGCCATATCTGAGCTGTCTGAGACCCA
EMPV1_12617 AGGAGTAACTTCTTCTGGGTTTGGTACGGCCCTCTTGGCTCGCAAAGAATGTCTGAAATT
EMPV1_12618 TGCATTTTCCATGGTGCTACCAATAGTCCAGACTACTAGGCCTGGTGGATATCAAAGCTG
EMPV1_12619 CTTTAAGACTCCGTCTTGGGTCCACTGGAGTCAGTTGGTTGAAGCAGCAAGATGGTAACC
EMPV1_12624 AAACTGTGCTGCCCCAGAATCTTCTTCTCAGCATGTGCTCTGACAGTGAGGACACACTCG
EMPV1_12625 CAGCTCTTGCAGGGAATCAACCCTAGAACTAAGAAGGCTGATAACGTGGTGAACATTGCC
EMPV1_12627 ACATCTGAGTGATGAGTGGAGGTGTGCACTGGATTTCTCATTAAACTATAGACTGTTAAC
EMPV1_12628 GCTGACGGTTAATACTTATATAGAATCTGCAGATCTCACCAGTCTGGACTACCTGCTGTC
EMPV1_12629 GTTGGAGCCATCTTTCATGTGGACTTTGGCATAAAGAACATCCACCATGCCAGGATCATC
EMPV1_12630 GAACAGGCCTACCTCAGTTGCAGAAATTATCAACCATGCTGCCTCCAGTCCTATGTAAGG
EMPV1_12631 ACCTGCTGGCATTGGGGGAAATGAAATGAAACAAGTTTGGCATAGAGGAGAAAAATCGAG
EMPV1_12632 TGTGTCTGTGCTGTCCTTGTGTACGTGCTTTGTGGACCCCGACGTTGTCTGACTGTGTTA
EMPV1_12633 GACAGTTCTGTTTGACTTATCAGGAAGAGGGACAGTGTGGGAGACCTTGGCTGCTTTTGG
EMPV1_12634 GTCCTATGATACTACCTTCCTACTGGACCATGTGGAAGTAGCCTATGTTTGGGTGCACTG
EMPV1_12635 ATAGCCATTCTCGGTTGTGCTGCTTACGGAGATGGTGTAGATGTTGTTGGTGTAGCCGTC
EMPV1_12636 GAAATACTCAAAAAGCTCGTTGGGCATGAGAGCTACTCATTACCCTTTGCCACTGTACAG
EMPV1_12637 CTGGACAACAGTGGGGAGTTCCACCTTAATAAGCAACTTGAATCTATAGGCATCCCACAG
EMPV1_12638 ACATGTCTGTCTTTGTGTCATGGTTGTGCAGCCGGGAGAACCAAGGTCACAGCCGCACCA
EMPV1_12641 CCCCAGTGGTTAATTTCACATAGAGATTACCATGACCTACAGAGGGGGGAGAGGAAATCT
EMPV1_12642 GATTGGGTGTGGTCTTGACCGTCTGCAATGGGAACATGTATCTGCGATGATTGAGGAGGT
EMPV1_12644 GGCCCTCTCTGCCATTGTCTTTCATTTTCACAGTGAAATTAGACCCCTTTACTTCACCAT
EMPV1_12645 GTTCCTCATCAATGTACCCATGTGCTTGCCGGAAGGAAGACACATTCACTACTCGAGCTG
EMPV1_12646 AGGCCTTCACCCGAGTGCAGGACTTCCGCTATTTACAGCTCATCAGCAGCATCGAGGAGA
EMPV1_12647 CTGCAGACACTTTTGGGTAAAACAAGCTTACATTTGGACTGCCTGCTGTCTACAAGACTA
EMPV1_12648 GACTGGGAGGGAATGTCAGTGAATTGGCTTGAATGTTCTGTAGCAATCCACAGGCCTAGC
EMPV1_12650 AAGTGAATAAAGTGGAAGTTAAGCACCCCAGTGGGTCTGAGATTCTGAGGGAGTCTGGGC
EMPV1_12653 CTGCTTTTCTATGGCACCGTCATCTTCATCTACATGAAGCCCAAGAGCAAGGAGGCCCGT
EMPV1_12655 TTTGGTCCGAAGATCCAAGCAATATACCTCTGGGCTGGAAAGCCACACATCAACCAGGAG
EMPV1_12656 CACATTCCTTCAGCAAGTTTCTTCTGGCAATGAAGCTGCAGTTCTAAGTTCGCTAGAGCG
EMPV1_12657 GGCCAGGGATTGAACCCGCAACCCCATGGATACTAGTTAGATTCTCCATAACAGGAATTC
EMPV1_12658 CAGAGGATGGAGAAGAATTTCGGGCTTTCACCGCTATTTGTCCCGTCTCTTTCACTCCAG
EMPV1_12659 GCAGGAAACTTCCCAGAGCCCCAAGTAGTTTTCAGTCTGCTTGCTTCACCTTGTTCTCAG
EMPV1_12660 CCCCTCTTTGAAGCTGAACTTTGAACCTTGCTCGTGTGATCCACCGACTGACCAAACAGA

EMPV1_12661	CTGTGAAGCCTCCTTGGCGTGCTCTACCTGCCATGTGTATGTGAGTGAGGACCACCTGGA
EMPV1_12662	TGTCATGATAACCAGAGAGGTAGAAGGTGGAGTCAGCAAATGCCACCATTATTGGCCCGT
EMPV1_12663	TGTGGGAGAAACGGCAGCCTCTGCTGGAGATGGAAGCAAGAAAACATCCACCTTTGGGAG
EMPV1_12664	CAGTCCCCTGTCTGTCCTCCCCACCTTCTTGAATAAAGTAATTTAAAGAGAGTGTGACTG
EMPV1_12666	CATGCATACACATAACATTGGCAGAGGTGACGTAGATTAGCTGGAGGAAGTGAGAGTGAC
EMPV1_12667	GGATGTGCTCCTAATATTAGATATGCTCAGCTCCTTCCATGGGACTTCAAGAGGGGTAGG
EMPV1_12669	GCTTTCTCCTGATCCTCCCATCCATCATTCTTAAGGCAAGACTGCCATATTGTGGCCCCA
EMPV1_12670	AGGAATAAATGAATGGACGTGAGCTTGGCTCTCTCTCAGGAGGCAATGGGGAGGTTCAGA
EMPV1_12671	CAAGTCTGGTTCTTGAATGAGGTCCACAGAAGGCAAGAAATCGGTCTGGTATGATCCAAT
EMPV1_12672	CATACGGTTCAGAACGCGGACCAGATCCTGGTGCTCAAGCAGGGAGAGCTGCAGGAGCAT
EMPV1_12673	TATCAGCTTCCTGCCCCAGACAGACTTCATCATAGTGCTAGCGGACGGACAGGTGTCTGA
EMPV1_12676	TCCTCTACCTGCCGAGCTTCTTCACCTACGCCAAGTACATTGTGCAAGTGGATGGGAAAA
EMPV1_12679	GGCAGGGTAGGAAGCCCTAGCTCTCTTTCCTACACAGGCATGGATTTAACAATGGTATTA
EMPV1_12682	TCGAGCTCAGGAACGGGCTGCTGAGAGCTAATAAACCAGGCCATAGTTTTTCTATGAAGA
EMPV1_12684	ACACAGTTGGCTGAGGAGCCCCAGGTATCTTGGCGTTGGTTTCAGCTCATTGGCACATAG
EMPV1_12689	CTATTGGCAGGGATGTCAATTTTGTAGAGAACAACATCAGAGGCCCCCTGTGCCTTTTCA
EMPV1_12690	TGCATCTTGGACTTTTACATCCATGAGTCACTTCAACGCCACGGCCATGGACGAGAACTC
EMPV1_12693	CAAGAAGTACAGAGGCACCACTAATAATCAGACCTGATTCTGGAAATCCTCTTGACACTG
EMPV1_12694	TCAGGATTTCAGTCACTTCTTCCTTCTTTGTCCAGAACACATTGACCAAGCTCCCGAGAG
EMPV1_12695	TGGTCCCCCTTGCCTGGTCTTAGGGCAGAATCTGTGGAGACTGCTTCCAGAAAGCCTCTA
EMPV1_12696	GTTCAGACGATGGAGAAGTACCCCAGGAAGCCCACTACATTGTTTGGAAACATCGCTCTG
EMPV1_12698	AACTAATGAGTGATGAACTGGGCTTGAACACTGGCCGTTGAGGAACTCGCCACCTGTGTC
EMPV1_12701	ACTTTTACGATGGCTCTGAGATTGTGGTGGCTGGGCGCCTGGCAGATGAGGACATGAACA
EMPV1_12702	GGCCTGAAGGGGCTCTTTTTGAACATTCAGTGGAGACACCCCTCGTGAGACCTGACCCCT
EMPV1_12704	CCTTCTGGGGATGGTGGGCCATATGATGTATTCCCAAGTCTTCCAGGCAACGGCCAACTT
EMPV1_12706	GGCTCCGGCCTCCGTGCTCAAAGTCTAATTGTCTTTGCAGTAAAGTGGATGAAGACATCT
EMPV1_12707	AATATGTCTTGGTGTGGGCCTGTTTGTGTTCAACTTGGGGCCCTCTGTGCTTTCTGTATC
EMPV1_12708	ACCCTTACATGGACTCACCACAATCAATAGGGTTCCAGGCAACAATCAGTGCTCCACACA
EMPV1_12710	CTGGTGTACTTCTGGAACAGCCTCACCAGAGAGCAGAAATTAGGTTTCAGGGCCGGGTAT
EMPV1_12711	GGAGTAGAAAAAGTTATTCTTATCCAAGAGCAGCTGTCTAAGAACAGGATCATCGTGGAG
EMPV1_12712	CTCCCAGGTTTGGTATGTCGCTCAAGGATGACAAAGTCAGCCAAGAAAGACAAAGACCCC
EMPV1_12713	TCCGCTATCTTAACACCTGCTGTCCTTATAGGCTTGGGGTTGTTCATCGTCTTCAGAGGC
EMPV1_12714	GGATGTAGAAGACTTGAACAACGCTCTCAACCAACCTGACATGAAAGTGTATCTCATCAG
EMPV1_12715	CAACGACGTGGTACAGAACTTCTACAACCCGCTGCTGCCCAGCGGCATGAAGTTCGAGAT

EMPV1_12716 CTAGTCTCTGCTGCCCAGGACCTTTTAGGCTTGTCTTGGCGCCTTTGAAGTTCAGGAAGT
EMPV1_12717 TTCAGAATGCCTAATTTGGTCCCACAGGAACTCCTTCCGCGAAGCTGCGGGCTCTTCTAT
EMPV1_12720 TTCCTAGGCTGCTCCCTGGTCCTCTGTCCTGTTAGCTTCCAGTCAAACTCCATTTGCCAA
EMPV1_12721 AGAGACCAGGTGGAGGCTGTGTTCCTGACACTTTCCAGTTGGGCTGCATCAAGGGTCCAA
EMPV1_12722 CATAGAAGGTCCTGGTTATGCCCAAAGGGAGCCTAAAGATGACTTGGACAGAGTGAGCTG
EMPV1_12724 ATCATCTAAACTTCTACTGCACCGGCAGCAGTCTGGGGAACTTGCTGCTGTCCATCAAGT
EMPV1_12725 CAGAAGAAAAGGATGATTAAGAAGCCTTTGGAAAGGGAGAGCATTTGTACAACTCCAAAA
EMPV1_12726 CGCGCGACGTAGTATTGTACCCACCTGAGGATTGTACCGAGCCTTCTCTGTATTTTAACA
EMPV1_12729 AATCGAACCATCAACCTCATGGCTACTGGTCGGATTCATTTCCACTGAGCCACAATGGGA
EMPV1_12732 GGGGCCTAATGTAAGGGAGGGGACCTCAAATTCTTGTTTGGATGGGTGTCATGAGTGTTC
EMPV1_12733 GATCTTTGCAGAGACTTTGCTACACATGAGGAAACTGTATCAGAGGAAGAATTGTGGGCC
EMPV1_12734 ACAACTCTCTACTACTTGGTTTGGTAAAAGTAACTAGTACTAGTAACTGACATCGCAAAG
EMPV1_12735 CATGGCTCAGAATCTACAGGTCAAATTAATTTGAACAGTTCTTGTCAATCTGAATTGTTG
EMPV1_12736 GTGAATGTGTATAAAGCACTTAGAATGATATTTGGCACACAGCTATGATGATAACGATAG
EMPV1_12737 TATAGTATCTGGGAACCAGTCACCTCTGCCTCTCTCTCGCCACAGGAACCTGAACAGGAA
EMPV1_12739 GAACCTTCTGCATCATTTCACTGTGCACCTGTGTGGCCGGGATCAACTTTGAGCTGTCAC
EMPV1_12741 GAATCTTGGCTCGTAATATCCCAGAAGACAGTGCTGACATGGCCAGACTTGATTTCAGTC
EMPV1_12742 TTATTGGGCTGTGTTTAAGGGTAAAACTTCAGCGGTGTTTACCATTTAAACATAAGTTGG
EMPV1_12743 GCTATGGTCTTGCTGATCCTTGAACATACAGTAACTCAGACCCTGGCCCCATTCCTGGTA
EMPV1_12744 AGAAGTGCTGTTTTCAGCTCCCGGAAAGCTCTTGATCCACTCATATCTCCACTCCCGCTG
EMPV1_12745 TCTGGAGGGTGTGAGCCACTTTTTCTATGAATTGGTCGAGGAGAAGCGCAAGGGCTTGGA
EMPV1_12746 GCAAAAGCTTTGGAGTGGAATACCACCCAGGAAATTCGGGCAAGAAGTTCTGGAAAATAT
EMPV1_12748 TTCAGAGACTTGTATCTGAGTCCTGCACCCCGACTCTAGCCTTTTGAAAGGCATCCCACA
EMPV1_12749 TGGCCAACTTGATGCCTTGGCGCATTTGACCTCACAAGCTACCCGTTGGAGATGATGAAG
EMPV1_12752 AATGAGCTGTGGTGTAGGTCACAAATGTGGCTTGGATCCCGAGTATCTGTGGCTGTGGCT
EMPV1_12755 TAGGATTTCGACCTGACCCTCACACTGAATGATTAGACAGGATGGTGAGCTGGGGAGCCA
EMPV1_12756 TGCTGACCTGTTGCAGTTCTGCATGGAGCAGGCCAAGAGCGACCCCTTCCTCGTGGGTAT
EMPV1_12757 GTGCTGGGAAGAACTTTAAATGTGTTTGTGGAAAAACTTTCCACTCTCCTGCCTAGGTTC
EMPV1_12758 GATCTTTGCCAAACCCAAGTTCACTGAGAATTTCTCCCAAGTTTTCTTCTAAAATTCTGT
EMPV1_12759 GACTCCAAGTCTCCCAAGAGCGCCAAGGCTAATAAGAGAGCTAAGAAGTCGAGAACAACC
EMPV1_12760 TGAAGTTACTTTAACACATAAGACTGACCCCTCAGTCAGGCCTATGCAGGAAAGAATGCC
EMPV1_12762 TCTGAAAATGGTGAAACTAAAGCTGAAGAGATCCACATCTCTCGCTCAGCTGTTAATGTG
EMPV1_12763 TGGGGTATGGGCCAGGCCCCAGGAGCGGGGCCTGGGCAGCCCCGTGTGTTGAGGAAGGAA
EMPV1_12765 GTGGACAGGAAGCAACTCTTTCTTTGTGAAAGGAGACTTGGATTTACTGATGATAGGCTG

EMPV1_12766 GAGGAGGATCTGATGGATATGGAAGTGGTCGTGGATTTGGGGATGGCTATAATGGGTATG
EMPV1_12767 CCTCTGCTCTAGAGGTGTTCCACCATAGGCTGGACATAAATATCACCTGGAGTGCTTTTC
EMPV1_12768 GGAGGGTAGCTTTTAGTGCAGATGGTAATATAATTGTATTATGGCTATATTGTAGGGCAC
EMPV1_12769 TTTCAATTATGCTCATCTTCGCCCTCACCACCTAAGCTCTCCAGAGGACTTTTCCGCCCC
EMPV1_12770 AACAACACCCTGAGCAATAGAGACCGCCTGTCCTCTCCAGTCTCTGATCAGGAGGTAGAA
EMPV1_12771 GCCCAGAACTGTTAGGTGCCGGGAGTCGCAAACGTTGAGCAGGAGTTCGTTGAGGATCCC
EMPV1_12773 GGAAGGGCTTAGGTGTAAAGAAAATGGTCTCTCTCTGCAACATTAATATGATTCAGATGC
EMPV1_12774 CAGGCATGGTCAGCCCTCCTGAGTCAAATTTCAGCATAAAGGCAAGTGTGGTCCAAGGGT
EMPV1_12777 CCAGCTCCCAGCCCCTCCAAAGTTTAACTTTCACATACGGGACTACTGGAATTCAGGCGT
EMPV1_12780 ATCGGTCCTTGTTCCTCCTGTTGGGCATTTTCCACGCTCCATCATGTTGAGGGCTAAGAC
EMPV1_12784 CTGTGTTTTCTTCAGTCTCTCCTCCTGCCAGAGCCCTCATTGCCCTCAGAAGAGTAAAAC
EMPV1_12785 CACCACCTCCCATAAACACTCACAGGAGGTTATGCCTCATCTCTTCTCCAGAGATCCAGA
EMPV1_12786 CGGCCAAGACCAACCGAGAAAGACCCAGGGGAACCTACCGTGGGAAGAGAAACTTTTGAT
EMPV1_12787 AGCCTGGGAGTATATCAGCCATAATGTAAGGAAACAAGGATCGTTACTAGTGACAGTGCC
EMPV1_12792 CATCACTGATCTAGAGAAGGGAGCATCATTGATGGAGATAATTTTAGGGAGAGATGCAGC
EMPV1_12793 GCCATCTGATCACTCCAGAGCCACAGGGCCAGCAATTATCCAAGTCATCTATACTGACAC
EMPV1_12797 GCAGTAGTTGACTGTGACTCTCCTGAAACAGCCGCTAAAATTTATGAGGATTGTGATGGC
EMPV1_12798 CTTCTTTGGCCTGATAAAGGGCTCTGTGAAAACCCACAGCTCACAAGACGCTTCGTGGTG
EMPV1_12800 GGCACCACAGTGAAAAAGAAGATGAGGTTCAGGTTGTTGCCACCTTCATGAGAAGCAGGC
EMPV1_12802 AATAATAACGCAGGCAAGGCGTTTACATTTGGAGGGGGAACAAGGTTAATGGTCAAACCT
EMPV1_12803 CAGTTCAAGTCTCTGTCCCCACAAGCGCTGCAGGCCTTCAAGAGGGCCAAGGATGCCTTT
EMPV1_12805 CGCGAGAGTGTGTGGCCTCTATGCTGAAGTCAAGTGTGTGTTGGAGGACTACTTATCTCT
EMPV1_12806 TGACTTACATAGTGGTGAGGAAGAGAGAGGTTTGAGGGAAAGAGAGACTGAGAAAGATTT
EMPV1_12809 GAAAATGAGCACCAGTTATCTTTAAGAACGGTCAGTTTAGGGGCTGGCGCAAAGGATGAG
EMPV1_12810 TTGGTGCCCATCATGACTATCTTGCTGGAGGAGCTAAATGCTTCAGGCCGGTGCACTTTG
EMPV1_12811 TCCTCCATGCTTAGAAAATACAGGGAGGAGGCCAGAGTGGCAACTGTCACTACGATTGAT
EMPV1_12812 TTCCCAAAGGCTGCTTTGGTCCTGCTGTACGCTACTCACCCCGTCACCCAGCAGACCAAA
EMPV1_12813 TAAGGTCTGTGGAAATACTCTTCCCTCACCATTGCTGACAGAGGCCAATGAGGCCACAGT
EMPV1_12814 CTATGGAACGAGTCTAGGGGTCTACTTCACCTCTGCTGTGACCCATTCTCCCCATAGAAT
EMPV1_12815 AAACCTGAAGCCCAAAGGAATGAGAAGGATTGAGAGAGGAGACAGGAAGGGAACAAAGGA
EMPV1_12818 TTGTTCACTGTGTTACCCATCTTGGAAAAAGGCGTGAGTGTCCGCTCGCTCTCTCTGAGG
EMPV1_12823 ATTCTTTGACCTGGGTTTCATCGGAGTGGAATTTGTCCTTACCGTGGATTGTCGCGTGGC
EMPV1_12826 CCAGAAAAGCTATGCTTGGACTGACACACAACTGGGGCACTGAAGATGATGCCAGCCAGA
EMPV1_12827 TGGAGAATACCAAGAAAGCTCCTATTTTTCTCGGTTTTTACAGTGGAAATGGCTGGAAAG

EMPV1_12828 TATACGGACAGGTTTTAGTCTGTGGCAGGGGCTTCAACATTGGCTTCTCTTTTCTCTCTC

EMPV1_12829 GAATGATTGTTCTCTTCAAGGCATAGCACGCACCCAACAGTCTTGTTTGAGCACCCCTCC

EMPV1_12834 AAAACCTCTAATCTGGACCACCGACTGGTTGATGGCAACGACAGACCATGAACGTGACAC

EMPV1_12835 GAGCACTGGTTCGAGTGCATCAACAGCACAAGGGACTGTTGACATTTGCATTTCTGTTCT

EMPV1_12836 AGTTGAAGTCAGAGATCCTGATGCCTCCAGCTCCATTTCTTTTCCTCAGGATGTCTTTGG

EMPV1_12837 GGGAGTAGGGGGATAGGAAAGATATTGTTTACCATGCAGCAGGTAGCAAATAATCCCTAG

EMPV1_12838 TTCCTCAGAAACCTTACCAAAGGATGTTACCACCTGCCCCAAAGAGGGAGAACTGAGGTG

EMPV1_12839 CTGATTACAGGAGTTTTCAGGGGTGTCCCTACCCATGGCATAGTGGGTTAGGAATCTGAC

EMPV1_12840 GGAGAGGGTATGCACTCAGCTCATGAGACCTAAGATAAAACAAAGACTGGATTGTCAGGC

EMPV1_12841 GTCACACATCTGATGAAGCGGATTCAGAGAGGACCAGTGAGGGGTATCTCCATTAAGCTG

EMPV1_12842 TCAGTGGTTTAAGGATCCAGCATTGCGGTGAGCCATGGGGTAGACTGCAGACACGGCTTG

EMPV1_12845 TGACCTGGGAGACAGGACATGGGTATTATCCTCAGAAAGCTATGGTTCAGAGTCCAACTC

EMPV1_12846 AGACCGAGGAGGGTTCAAAAAGAGGTGGAGGGATATACGGGCCACAGTCGGAACTACTTT

EMPV1_12847 TGGCCACACCAAGTGCGTCTTATTTAGGGTCCTGTTATCCATCTTTCCCGTTTAGTGGGC

EMPV1_12848 GGCCAGTCAGAATGTTCAGTATAAGTGACTCCTTTCAGGATAAAGGCTCCCTGTGCTGCC

EMPV1_12849 TGCACCCTGAGTTATCTGAAATGACTGGCCAACGGGGACCTTTTGTATAGCACAGAGAAC

EMPV1_12850 TCTCCATCCTCTGCGTCCTGAGTTCGCTTCCCGCGGCCAGGTGATGGGAGCTGATTGGGA

EMPV1_12852 GAAATACTTCCACAGACTCACCCTCTATCTGCAGGAGAAGAACTACAGCCTCTGTGCCTG

EMPV1_12853 AGGGTGCTAGCAGATGCCCTGGCTGCCATGGGCCGTGAAGATGTGGTCCAGGTCCTGGAT

EMPV1_12854 GTCAGTGGAGGAGTCGTATATCACCAGCGAGCACTGCTACCAGAAGCCCCGCACCTATTA

EMPV1_12855 AATCTTCGCATACCTCTGTGACACCCCTGCCCAATTCTCCCTTTATGACTTAACCACGGC

EMPV1_12857 TGCCGACAGCAGAACCAGACGTAGAACTCAGACGTATCTCCTTGCGAAGTATGTGGTACC

EMPV1_12858 GAGGGGACACAAAGGCCATCTTCCCAAGGTGCAAATAACTGATGGCAGAGTGTTTCCTTT

EMPV1_12861 GCAGGCTTGTTGAGATTTGTGATTATATTGCTAAAGCTAGTTCCATGCGGGAAGATCTTG

EMPV1_12862 CAAGAAGAGGATAGCCCAGGCACCTCACAAGCTGCACCAGACACCGAGAATGTGCTCATA

EMPV1_12866 CTGGACCCTCCTTCCTCCATAACCGGGCCTTCCTTGGTGATAGAACCTAACACGTTTATC

EMPV1_12867 GATTCTGATTCCTTCCTGGGGTAGGGCCTGAGATTCTGCATTTCTAACAAGTTCCCAGGG

EMPV1_12868 GGGCCCACACCGGTCTATGCTAACAATGGATTTTATGCTGGAGACTTTAGGCCACAGCTC

EMPV1_12869 ACTCCAGGACATTCTTGGGAGATGTCTCTCTGAGCCAGGCTCAAGTAGGGAGAAGGCAGT

EMPV1_12870 CAGCTAATGCTGTTGCCAATGCAGCTATCCAGCACAATGCATCTCTTCCAGTGCCTGCAG

EMPV1_12872 CAGGACCCAGGATAAAAGCCGTAATTTGACAGGATACTGAGCCAGGCTTACCTGCTAGTC

EMPV1_12873 CTCCTCACCACGGGCTTGGACTTTGTCCTCATCTCCCTGTCGTACTGGTTAATTCTGAGA

EMPV1_12876 TATAATTAGACCGGTTAGACTAAGCAGCAAATGAGTCCCGACAGGAGGAAGCAGATCAGC

EMPV1_12877 GACCGCCTCTTGAAGGAGAGAATGTTTGATCCCCGTCATTGTGAGCAGGTTTATTCTGGG

EMPV1_12878 AGATCATAAACCTTGACGTACTTGGAGAAGGCTATGCAGATCTGATGGGGAAGAACCAAC
EMPV1_12879 ACAACTGCACGCCAGCAGAGAGCCCCATTAGGAACAGAATGGATGTTACCCTGATCTTGG
EMPV1_12881 CATATGGAAATTCCCAGGCTAGAGGTCGAATTGCAGCTAGAGCTGCTGAGTCTACACCAC
EMPV1_12882 ACCCGTGGGTCCTAGAGGGACGAGAGAGAGGAAATTGCAGAGAGAATGTGTGAAGGAGTA
EMPV1_12884 AAGGTGACAATACTACAGTTGAGGGAGTGAGACCATATGGAGAAAGGAGAGACTATGGGC
EMPV1_12885 GATGGAAGCACGGCTTGCTGCACTGCAGGATAGAGTTCCATCTTCTCAGACCCCTCAGTG
EMPV1_12886 CCAGTAGAGAAATCTCAGCCTTTTGAACAAAACATACTGGGTGGGAGTTGTGCTTCGACA
EMPV1_12889 CGGCCGCCAGGCACTGTTCTGAGCCCTCGAGTTGCATCAAGTCATTTAATCTTCAGGAAA
EMPV1_12890 GTCAAAGTTCAGGTGTTAAGGAAGCTCCAGTAAAGGATGGAGGGGACCTTAGCGACTCTG
EMPV1_12895 TTTTTCCTCACACTGAAGAGGGCAGCAATAACCTGTCACTCCGGACCCAGCCTCCTTCCA
EMPV1_12896 CCTTCACGTCTGGGCATGTCTGGATCCCATCCTCTCTAATATTCGCTGGGTTTTTCTACC
EMPV1_12897 CCTCTGCATCCTAGATAAAAAACCTCTTCATGAAATAAAACCCCAAAGTATTGAGAACGC
EMPV1_12905 CCTCGACGATGGGGTCTTTTGGGTCAAATAGAGCTGGTTCGCCTAGAAATGGCAGAAATG
EMPV1_12908 CCAGAGCCCTGCCATTGTGGCAGCTGTTCAGGGCAACCTCCTGCCCAGTGCCAGCCCACT
EMPV1_12909 GGCAGAGAATAAAGGATTCACTGTCATTTGCTGACTGTGTCAGGGTGGTGAATATCCCTT
EMPV1_12910 CTCTATCTGGGACTTCCAGTTCCATGTTGAAAAGAAGTGGCAAGTGTTAGAGTCATTGTC
EMPV1_12913 AAACTGGGATGTTTGAGTACAGGGCAGGTGGAGGACAGGAGGCTGGAGTAGAAGCAGAAA
EMPV1_12915 CTCTGGGTCGAAGGGGCCGGGCACAATGACATAGAGCTTTATGCACAATACCTAGAAAGA
EMPV1_12916 CAGCAGAAGGAGATCTATCAGGTTCAAAAGAAATACTATGGGCTGGATTCGAAATGGGGT
EMPV1_12917 GGCATGAATGGGAAGGAGACAGTCACTAAGAATGAACACCGTGAGCCAACCTGGAAGTAG
EMPV1_12920 TTTGCTTTGAGTTTCTGGTTTTCGGGAGTGACATGGCCAATTGTTATCATCTTTCCTACG
EMPV1_12921 CATCGTGTCTTTCCTATGCTCTGAAGATGCGAGCTACATCACTGGGGAGACAGTGGTAGT
EMPV1_12922 GAGGATTCCCACGGTGGCACTGCACATGACGGAAAGGTTTGACACAGCAGTTGAGCAACT
EMPV1_12923 AGGAAGTGTAAGCCCCCGCCACTCAGTGCCCCTCTCCCTGCCAGCTTCCTTGGGAGGGGA
EMPV1_12924 AGGATAAGGAGGCCATTCAGGCCTATTCTGAATCCCTGATGACACCAGCTCCCAAGGGAA
EMPV1_12925 TTTCTTCCGAACCCTCATAGCAGCCATCCTCTACCTGATCACCTCCATCCTTGTGCTCGT
EMPV1_12929 GCGGCCATCAACCGCCAGATCAACCTGGAGCTCTATGCCTCCTACGTCTACCTGTCCATG
EMPV1_12930 AAATGATCAGACAAGGCAAAGCGAAACTGGTCATCCTCGCCAACAACTGCCCAGCCTTGA
EMPV1_12933 GAACCTGTTTATAGGACAGAGACATTCTTACACAGAGACTACTGTCTGTCCCAGGGCACC
EMPV1_12934 TTGATTCAGCACCAGGAAGTTCTTCTGGCCTCTCAGGTGGTGCTATTGCTGGCATCGTGA
EMPV1_12938 GGACATTCTATTAGAGTCCCATCAGTGTATTCATTTCTTCGGCATCAGTGGTATTATATG
EMPV1_12939 CAAGGCTTCTCCCCTGGGAGCTCCCCACAACACTCTTCTACCCTGGGCGGCGTGTTTGGG
EMPV1_12940 TGAGTTGGGTTGTGGCCTTACCCCAGGTGAAGCAAGAGAAGGCGGATGCCCAGGAAGACT
EMPV1_12941 ACTTCATGTCGAGGGAGACCACAGACACCGACACAGCCGACCAGGTCATCGCCTCCTTCA

EMPV1_12942	GGTGTGAGCAGTCACCACTACCTCCCGCTTTTAAGTTGTTGCTCCCTTACTTAGCTGAGC
EMPV1_12943	AAGTAAATCTGGAGCCAACAGGAGAGTGGGCTGAGGTATGGTGCTCCATGGACAAAGATG
EMPV1_12945	GCCTTTGAGAATATCTAGACTGACGCCCTTATTTTACAGATGTGGAGAACACAGCCCAGG
EMPV1_12946	AGGTTGTCAAGTTCATAAAGACTTTCAAGGAGGTGGGTGAAAGCCAAACCCCAGGGAGGG
EMPV1_12947	GAACCAGTAACTTGTCCCTCCATACCCCCAGCCTGTGAGGTGTAGTTGGAGAGTTGATGG
EMPV1_12949	ACCTGCTTTCAGCTTTCCCTACCTGGGGACCAGTGCCTATTCTAGAGCTTTTCATGGGCT
EMPV1_12950	GTCTCCACGCTGGGGTTCAAGTTCTTCACGCCCATCCTTGAGTCAAAATTCAAAATCCAA
EMPV1_12952	CGACAGGACACCATCAAACTCCTAGAAGAAAACATAGGCAAAACACTCTCTGACATCAAC
EMPV1_12953	GTCTATTCTTCAGAAAGGTGGGAAAAGACCAGAAGGGACAGAGTACAAACAGGCTACTGG
EMPV1_12955	ACCTCCCGCTTTGAGGAAATTCTCACAGAAGGATTTTGCACAATTTCTTGCCATGTCATC
EMPV1_12956	TAGGTCACAGTTGCCACTCGAATGTGGCGTTGCTATGGCTATGGCTGTGGCTGGCAGCTA
EMPV1_12957	CTCATCAGAATCACCAATGTCACCACATCCCCGTCATCACTGACAAAGCATTCTCCTGGC
EMPV1_12958	TCTCCTAAGTGCCCCTTTCTCACCACCTGACCAGAGACCTGTAGCAGCAAGTCAGTGAAT
EMPV1_12960	ATGTGGCTCTGGTCAGCGTGGGCCGCTTATTCCGCAAATTGACCGAGGAGAATCACAAGA
EMPV1_12961	AGCGATGGATCGAGCTCTGATGACGGCAGAGAAGCCCCCGACGTGAAAACCAAGAGGAAT
EMPV1_12965	GACTGATCTTTGCTGGCAAGCAACTGGAAGATGGACGTACTTTGTCTGACTACAACATTC
EMPV1_12966	CAGGAAGTAGTTTTGATACATTGTGACCCCCTAGTCCTTGGACCTTCATTCCACTGAAAG
EMPV1_12968	GGGCAGGACCTCGGGGGTGGCTGAGCTCTTACCTGGTTTTCAGGCGGGGCCCATGGAAGA
EMPV1_12969	ATGCCGATTTGTCACGGATCTCATCAGTCTCATCCGCCAGCGCTTCGGCTTCAGTTCTGG
EMPV1_12971	TCGCAGTAACAACAAGGGGAAATAAGTATGTAGTTCTTTTGAAATATGTGGTGAAGAACT
EMPV1_12972	AAAGTGAAGGCTCCTGTGTAGGCCAGGGAAAGGAGGTGCACACCAGACAGAAGTCACTTT
EMPV1_12974	ATGCCTGGGTCTGCATGGGACAGAGCTCCTCAATACATGCACAGGGGAGTGAAAAATTGT
EMPV1_12976	AAGATGGCGTCGCGGGAGTCCCTGCTCTAGGCTGTCTGAGGGAACGTCGAGTGACTACCT
EMPV1_12978	CAGCACTACAGCCCCCTCCCATGATCTCTCTTAATTGTTGCCTTTAACCACTACTTAGTC
EMPV1_12979	AGGTCCTTTTAGAGAGCGGGCTGGAGCGTTTGTGATCGGCCAAGAAGAGTTTTGACTCTG
EMPV1_12980	ATTCTAGAGCTGCAGGAGGGGCCTGGTGCCAGACTGCCCATCCTAGAGCTCTCATTTTAA
EMPV1_12981	ACAGCAAGAGAGGATTGTGCCATCTGCTTCCACCAGGCTGCCAACACCTGCCTTGTGCCT
EMPV1_12982	TGACCCTGTCGCGTGGTTATTTCCGACCAGGGCAGTTTCCGAGCGATGTGTATTTAAGAG
EMPV1_12983	CTGGTGAAGACGCAGATTCTGACTCAGCAGGTCTGGAGTGGGACCCAAGATTCTGCATTT
EMPV1_12984	ATCACTGCTTCCTCTGTACTGGGGGCCTATCGAGCAGATGGGGTAGAAGCTTAACACACA
EMPV1_12985	TGTGGAAAATGTGCTTGATACATTTTTCAAAGATGAGAAAGCAGAATCACAAGAACATGT
EMPV1_12988	AATCCCATAACCCTGTATATACCCTTGAATTTCTGTCCTCTCCTTCCTTTAGGCATCCAG
EMPV1_12990	AGCGCTAAGTGCGAACTCCAACCACCCCGGGTCTTTACACATTCTGCGTCTACAGACTCT
EMPV1_12991	CGGAAGAATATTGGGACAGGGGAGCTTCGGAATGGTCATCGGAGCCATAAACAAGGAAAC

EMPV1_12994 CCTCCCTTTCGTGCTTCCTTGCTTTTGCTCTTGATGTTCTCTTAGCTGTGAGTGTCCTGC
EMPV1_12995 TGACATGATGTTCTTCCTGCAGGGCCAGTGGATGCGCCTGTGCACCAGTGCCACAGAGAG
EMPV1_12996 AGCTGCTTTGCACGACGAATGATGTGGGGTTAAAACGACTCCAGAAAGAACCTGCCAGAG
EMPV1_12997 TCAGATTCCACTTCCTCTGTGTCTGTACTTGATCCCAGTGAACCACGAACACCTGTAGGG
EMPV1_12998 ACCTACTCTGCTTAGGACTTTTCAGAGCTAACTGTGCCCCCAAGTGCATGGAAACTAGGC
EMPV1_12999 CTTTGAGCAACATTGGAATTGACTCTGCACGTTTGGCGGTAGTACCAGTCTGTCTAGTCC
EMPV1_13001 TGTTAAAGTAAACGTCTTCCTACAGCGCCGACCTTAACGAAGGATCAGGAGGGGCCTTGC
EMPV1_13003 TTGGGATATTAAGTCTATGAGCAATTATTAACAGGTATGTGTTTATTGCCACATTAAACC
EMPV1_13006 ACATCACAACTGGAACAAACATGCTTACCCAAGGCCACTCTCCACCACCCACTCCGTGTA
EMPV1_13007 ACAGTTCTGGAGACCTGATGTCTGAAAACCGGTGTCAGTAGAGTTGTTCTCCCTCCAGAG
EMPV1_13009 TCAGATCCGACGGGACATCCGCCACCTGCTGTCCTCGTGGCCTGAGCAGCAGTTCTCAGG
EMPV1_13011 AAACACTACCATCCTGGCTGAGTTTGCCACCGACGACGAAGTTAGCCGCTTTCTGGCACA
EMPV1_13015 CAACAGAGAAGCAGTCATAGACATAAACTCACAATTCTAGATACAAATCAAGCACAGGCT
EMPV1_13016 CCCTAGATACATTATCATGGCATCCCTTCAATGCCTCCCCATCCTCACTCTCAGCCAGAG
EMPV1_13017 GGGTGGAGCCAGTGTTGGCTACACTGCAAATACATTGACAAAGGAAGTGGACTGGATTGT
EMPV1_13020 AGGCCACCTGCCTTCCTGCTAAATTGTATAAAGTGCTGTACCCTTAAAGAGTCCCATGTG
EMPV1_13022 CACCAGCCCAGGAGAGCTTGTCTTTCCAGGTTGTTAGCTCTCGCTCTATTTCATGTGACA
EMPV1_13023 GAGCTCCTGTAATAAGAACTTGATTGGAAAGTGCAAACTGTGGATGGTCCTCACCTCTGT
EMPV1_13024 GCCCAACTATAGAGACAGTTCCTATTTCTCCAGCAATCCCGCTGCAAATTCCAGTCCTTT
EMPV1_13028 TGGTGTAGGTCACTGATGTGGTTTGGTCTTGTATTGCTATGGCCGTGGCTGTGGCAGCAG
EMPV1_13029 GCTCAATGTCAAATGGGACCGGATTGCTCCTGCTAGCAATGTGTCCCACACCGTGGTCCT
EMPV1_13030 CCATTCCAAATCCAATAGTTTGCTTCTATTAACCCCATATTCCCACTCCATCCCACTCCC
EMPV1_13033 GAGTGCCTGTAAGTAGCCGCGTATCAGCAAAGATCCAACAGCTTGTCAACACCCTCAAAC
EMPV1_13034 CAGTAAAACTGGGCTTGGGTTAGGATTCATCGCCTGTGTGGCCTTTGCCTAATCTCACCC
EMPV1_13035 CATCTCCAACATCCTGCAGAGCGGCATCCGCCAGACTTTTGGCCCCTCAGGAACTGACAA
EMPV1_13036 ACATCCCATAGTGGCCTAAGTAAGTCCTCCAAGTCAGGCCTGTAAAGATGCACACAGCAG
EMPV1_13037 TGTGGGGAAAAGTAGAATGACGAGTTAGTGATGATCTATAATTTCATTCCTGGGAAATGG
EMPV1_13038 GCTTTTAAATCAACTCACTGCACTAAACCCTTGCTTCAGGCTCTGCTTCTGGTGGAGACC
EMPV1_13039 GCTCATAGTTTCACAGTTCAGGAAAATGGTTAAGTGACAAACTTCCATGGAACTCAACCC
EMPV1_13040 GCGGGTGACACTGGCTTCACGTTGTTCCATGAGCCGATGCTACTGTTACTTGCTTTGAGT
EMPV1_13041 AGAAGAACAATGTGGCTCTCTGGAAAATGGCTGTGGAAACACTGGCATCAGCTCCAGGGA
EMPV1_13042 ACACCCTAAGCATCAACGTCTACATAAGTTTCTCTCCAGAGGGTTAGATTTGGGTAGATG
EMPV1_13043 CAAAGCTTGGAACCCAACCATGTTCATAATCCCAAGGCCTTTACTTCTCACCCAGCTGTC
EMPV1_13046 GGAGCCTGACAAAGCCACAGACATAGTTCAGGAACCAAGAGGTCTTTCAAGATCAAGCAC

EMPV1_13048	AGCCTTGCCAAGAACAGCCAGCCCAACCGCTACACCAACCGTGTGGTGACGCTCTGGTAC
EMPV1_13050	GAATTCCTGTTATGGAGAATCTAACTAGTATCCATGGGGTTGCGGGTTCAATCCCTGGCC
EMPV1_13052	GTTGAATCTTATCAGGAGTGAACTGCAGAATGCGGGAATCGATAACCACGAGGGGAGACG
EMPV1_13056	GTGTCAATTTCTGCTGTACAGTGTAGTGTGTTTATGTATATATATAGATACAGACACACA
EMPV1_13058	GACTCCAGAAGGAAGACAAATGAAACAGCAGTGGAATCTGTTCTCCGAGCTGATGTGGCC
EMPV1_13062	GAACCCTTAATGAATCACAGAGGCTGAATTAACCAACCGTACTGCCCTCCCTAACTTGGG
EMPV1_13067	AGGGTGAGGACTTTGGAGACGTAAATGGGGAGCGAGGGATAACTAACCTCAGTGGGACAT
EMPV1_13068	GAGAGAGGATGACTGAAGAGGAGGTGGAGACAGTTCTGGCAGGACATGAGGACAGCAACG
EMPV1_13069	ACTACCAACTGCTGACCTTGGCCCTGCTGGCCGTCAACGCGCTGCTGGTACTCCTGGCCT
EMPV1_13070	ATTGTAGCCCACCTAATCCTTGGGGCAGAGTCTTCTTACCTGCCTGCTTGCATCTCTCAT
EMPV1_13071	GAGATGGATGAGTTGAAGGAGCTGAAGAACAATCCTCACCGGGACTTTTACAATTGCAGG
EMPV1_13074	CATCATGGAGGAGATCATGAACCTGCTACGGCCCATCAACATCACACAGGTCTTTGAGGC
EMPV1_13075	AACGCTACCCAAGAGCTAATGGATTTTTTGAGGAAATAAGACAGGGCAACATTGAGCGTG
EMPV1_13076	TGAGTCACCTGTTCGACAAGTTGTGCTGGATCATCTGCACGGTCACCAACGCCGTGGGCA
EMPV1_13077	AAGGGGCAAATACATACGATGTTTAAAGCTGGAGTCGATGGATAAGCAGCTAGTGGGGAG
EMPV1_13078	CGTATCAACAACAAGAGCCGAGTCAGGGAAGAAGGAGCTCCAGACATGAAAAGGGAAGAC
EMPV1_13079	AAGCTCCTCTTTGGAGCCTCGGGGTACCAGGGCTTGGGAACAGTTAATAGTGGAAGATTA
EMPV1_13082	GTCTCCGTAAAGGAGCTGACGTCCACTTTCCCTTCTCTCCGGCCCTCTTTTTACTTGAAT
EMPV1_13084	TGAAGCCAAGAACCCGCATTTCCGCCCCGCAAGGCGTTTGACCAATATCCCCGCAAGTCA
EMPV1_13085	TGCAATTTTAGGACAACATGTTCTATATAATTGTTTACTTGTTGTAGATGATACTCATAT
EMPV1_13086	GAGGGCAGGAAGGACCTGTACTATCATTAGGGTAGTACAAATACTATTAAAAATGCTGGA
EMPV1_13087	CTATAGTTTCTCGTGGAGGTGTATCTCAAGCGGGACATAGATGACTTAGTGCAGCGAAAC
EMPV1_13089	CACGATTTTACATATCTCCTCTGCATATCACTAGAACTTTTCTTTGAGAGAGAGCTAGCA
EMPV1_13090	TGTTGGCGTCCACCACCCCCGCCGTGTGCATCCACGAGCGCACCGGGTTCATGCCGCTGC
EMPV1_13091	GAAGTTCAAGGACAAACACGGAGACCCCTTGGAACGCCTTCTCTCTGCTTACCGCAACAA
EMPV1_13094	GGACAGGGTGCAAGCGGATGACCTGGTGAAGATGCCTTTCAGCCCAGAGGAGACGGACAT
EMPV1_13095	CAGAGGAGATCAGTGGTCCCTTGACCTCGGCCAAGAAGATTACACTGGTGTCCAGTGGAA
EMPV1_13096	ACATGGTGATGGTGAGATCGCTAGAGCCCCAAGAGATTGCAGATGTCAGCGTCCAGATGT
EMPV1_13097	CAGTCAATGATGAGGGAGTTCCAGCAGGGTCCAGCTGGCAATATGTAAGGCATGATGGGA
EMPV1_13099	TGACACCCAACATTGCCTGCTGGTCCAGTACCTAGAGAAGGGGATCCTGCCACAGCAGCT
EMPV1_13100	GGGCAGAACCAGAAGAACCTTGTAGAGGATTTGTCTGAAAATGGGTTTCAAGGATGAACT
EMPV1_13101	ATCTTTTCGGGGCTGTTGGTGAATCTCAAAACGGTCGTGCCTTGGCTCTCGTGGTTACAG
EMPV1_13103	CTTATAGTTCAAAAGCTCCTCCAACCTTACCCTTTAAGGACACCTGCCACTGACCTGCAG
EMPV1_13105	ACCTGCAGAAGTGGAATGGACAAGAGACAACGCTTGTTCGGGAACTAGTTGATGGGAAAC

EMPV1_13106	GCTGGCCGAAACACAGCACGAGAAATCACAGAAGGTTCCTGACATGCATTTCTTTGGGGA
EMPV1_13107	TTCAGGGAGAGGTGCTGTGGAGAGCCCAGGCAGTTGAAGCTCGGCACTCTCTGATGAAGT
EMPV1_13110	GTACTTCCTCACCAGCTTCTACACCAAGTACGACCAGATCCACTTCATCCTCAACACTGT
EMPV1_13111	GTATTTTTCAACTGGGCTTCTGGCTGGTGGTCCCCTTGCTGGGATACTTTACTCTTACTC
EMPV1_13115	AAAGATGGCAGAGCTGCCCGTGGACCCCATGCTGTCTAAAATGATCCTGGCCTCGGAGAA
EMPV1_13116	AGTATTTCTCAACTATGCTGTAGTCTGGCATTTCCCTAAGGGAGGAAAGCAGATTTCATC
EMPV1_13118	CTTCACCTACATCAAGGAGCGATACGGCCCATACGTCGCAGGCGCCTATTTCGTCCTGAA
EMPV1_13119	GAGTGGATGCCGCACGCAGGAGTTGGGAGGGATGTTCAGCCTACAAGCTGGGGTCTGGGT
EMPV1_13120	TTTGAGAGGCAGCACTACTCAGACATCTTCACCACCGCGGAGCCCATCAAGCCCGAGCAG
EMPV1_13121	AAGGAGTTCAGTGTCAAGCTTTCCAAGCACCACTGCCGGGCCTGCGGACAGGGCTTCTGT
EMPV1_13122	GATACACTCAAATGCAGTGCTTTACCGATTATCTGGGCATTCCTTAGCCCAGTCAAGTTG
EMPV1_13123	AAACCTGCCGCGTGGCCTGTACTCTCCCACAAGGATGTTTGATGGCTCATAAGTTCACAT
EMPV1_13127	GTGTTAAGTGCTGCCAATTTAAAGTGTTACAAGGGGAGCTTCCACTGTAGCGGCAGCGTC
EMPV1_13128	GGTCCAGACCAGAGCCATTCTCAACTATATCGCTACCAAATATGATCTCTGTGGGAAAGA
EMPV1_13129	GAAGATTTTGGAGGAGGTGCGGTACATTGCCAACCGCTTCCGCTGCCAGGACGAGGGAGA
EMPV1_13130	GGCTCCTGTGGGTAGACTGAGATCGCGAGGAGGTGTCATTTGCGGTTACTTTATTCCTGT
EMPV1_13133	CCATCGACGGCTTAGAAGATGACCATGTACGACAACTGTTTGGACCGATCGACTTACCAG
EMPV1_13137	CAGGGGAAAGGAACAGAGGTCTCCTTCACTCTTGGAACCTTGAAATTTTTAGGGTAGACG
EMPV1_13141	GCCCTCGGGGAAAGCCTGCACAGTTATCACGGAGAGAAAGCTTATTCGGGAGATGAACAT
EMPV1_13142	CACCGACTCAGCCCATCTTTGCCCAGGTAGGAGGCTTCCCAGGAGGTGAGTTCCTTTTCT
EMPV1_13144	GAACAAGAAGAGGAAGAGGAGAAAGAGAAAGTTCAAGGAGACCCCATTTACGCCGTGGCG
EMPV1_13145	TCATATGGCTAGGATTTGAAGTCCCATGTTGAATAAAAGTGGCTAGACTGGGCATCCTTG
EMPV1_13149	TCACAATGACCACAGCTACCCCTTTGGGGGGTACCCCCTTCTTCTCAGTGAACATGACCA
EMPV1_13151	GCCAGAGCCAGCTGATCAACACTCTGACTAAGAAGCAGAAGAAGCATCTCCGCGATTTCG
EMPV1_13152	TGCCTGGATTTCTCTCATTTTTATAAACAGTTTCATACATCACGATGATTTGTCCTCTGC
EMPV1_13153	ATCTCACATTCCTTACATCCGTGACTTGAGTTTGCAGCCAGTCCCTCTTCGCGGGCTTCA
EMPV1_13154	AGGAGTTCCTGGCTCTGATACCAGTGGACCAAATACTCAACAGCTTCCTTGCCCACTTCA
EMPV1_13155	ACTGGTCCCATAAATAGACACACACTTGGCCTTGGAGTATTAGAACACGGTTGTCACCCC
EMPV1_13157	TTCTGTTTGTCCAGGACCCTGCGCCTTCTTGACACATAGTCACTTGACTGCAGCAGGAGA
EMPV1_13158	CTGCTCCACAGCCAATGGAGAGAAAGACAGCATCACCCTCATCTCCATGAAAAATATCAA
EMPV1_13162	CTGAGGCTGTTGTTTCATGGGGGTGGTTCACTTTCCTTATGCATCACTTGACTGTCCTGC
EMPV1_13164	ACTTGATTGGGCTTGGTTGAGTCTGGGCAAGGCCTCTTTCCACTGCATGTATAAGCCCCA
EMPV1_13166	TGATAACATTGGTATGCTGCTGCCTCGCCCAGTGTTTGCAGTATCATTGGAGAGGACTGC
EMPV1_13169	GAACCACACAGACCTGTTTTCCTGAATCATAATTAGGGGTAGCTCTCTTTCACTCATACA

EMPV1_13172 GCCATTTACTGTCTTGTGACCCTGGTCATCTTCTACTCATCTGCCTCGTTTTGGGCCTGG
EMPV1_13175 TCTGAAGTTGCCAGCATGATCTGTGGATACCACAGCTTAAAAAATACATGCTGATCTGTG
EMPV1_13176 AAACACGTGGTTCAAAATGGAGACGAGAATGATCAAGAACGTCTGTGCCCCCGCCGTGGT
EMPV1_13177 ACCTGGAGGACCCGAAACGGCGGAACTGGAGCACCAGCTGCGGGCAACTGAGCGCAGCCT
EMPV1_13180 GGAGAGGCATCTGGAACCATGTTGCAAACTAAAGCTTCCAAGATTTGAACATCCCTTCCC
EMPV1_13181 GATGTGATGGTAAACAGGATTGCTTTCCCAGTTTCTCTTTCTGATCTTTCATTGTCAGTA
EMPV1_13183 GCATGTGAGACTGGTTCCGTGGCCTCTTTGGATCTATAAATTAACCATATCACCACAGAC
EMPV1_13185 ATGTAGCTGCTGTCCTACGCCACAGCCACAGCAACTCCAGATCTGAGTTGTGTCTGTCTT
EMPV1_13189 TCCCCTTTCTCAAAATTTCCAATATGTGCTGTGAATAACTCATGACCAGCTGCTTCTGGG
EMPV1_13192 CTTTAGTGACAATGCCAGATCCTTAACCCACTGCACCATAGGAGAACTCTGAAGTCCCCC
EMPV1_13193 CTTTGCAGTTCGGAGTTCATTGCCAACTCAGGTTAAGTGTCTCCAGAGAGAAATGTTGTG
EMPV1_13194 CCGCGCCCGGCCAACAGGGCGGTACACCCAGCGAGGCCACCTCTCCACTGGACATCAGCT
EMPV1_13196 TGAGAATGAAGGTGACTCAATTGCCGTGATCCTGTTCAGTGGGGTGCATTTTTATACTGG
EMPV1_13197 CCCTCCTACCTAGCACCCCACTGGTGACCTGTCGTAGGGAAAAATAACCTAGTTATCTGT
EMPV1_13198 GCCGGTCCCAATCAGTAGTCTCCTTGTGTCGTCCATCTGGGCTTCTTGAAAGGATATCTC
EMPV1_13199 AAGACCACGCCTCCAACGTCAAGTTCACCATGCACAGGGTCTTCCAGTTCCTCAGCCTCG
EMPV1_13203 TGAAGCTGGGCCTCGGAGACTTCATCTTCTACAGCGTGCTGGTGGGCAAGGCGGCGGCCA
EMPV1_13204 GCATAACCTTCCTTTCATTGTCCTTGCCATGAGTTCCCACCATAGACCCACGCCATGGAG
EMPV1_13206 CTGGCTGAGGTGTTGTGAGGTGCATCGAAGTGTTCCAGCCTGTGGCTTACCTTAACATGT
EMPV1_13207 TCGACACCGAAACGTTACCGACCTTTACCTCCCAACCTTTATTCTGCACCTGAGACCCCA
EMPV1_13208 CGTCTCTACCGTGGAGGTCCAGTGGATTCCCGTTCTGCTGCCTTCTTACTGCCAATTTGA
EMPV1_13209 CAAGGCAAAGGTCCTGCAGAGGGACATCCTGAGGCAGTGCAAAGAGGAAGTTAGCATCCA
EMPV1_13211 TTAAAGTGTAATTGTTGACAGTGTTTGCCAATTTATGCTGTACAGCAAAGTGACCCAGTC
EMPV1_13212 TATGGTGCCGGGCATTTTCTTCGTTGAGCAATCCTGATATAAATCCTCTCCCTGGCAGCC
EMPV1_13214 GGACCATACAAACATAACAAAGAACAGATGGAAAGCCACAAGGTAAACAACGGCCTTTCC
EMPV1_13216 TCTGGTGGAACAGAAAAATGGCCGTTTAGTTTTGCCTTCCAACTACAAACACAAGAGGGG
EMPV1_13219 CTTATCGCGCTGAAGGAGAAGCCCGCCCCAGAGCTGAGTTTGTCTAATCAGGACCTGGAG
EMPV1_13220 AGCGGCCAGCTTCATTGAGGAGCTGACGTCCATTTTTAGAGAAGCCGCAAAGCCAAGAAA
EMPV1_13223 GTGGAACGGGGTGGGGACCGTGTATAGCTTGACAACCCTTAATAAAGAAGGGACTTTGAC
EMPV1_13224 GCCGGCAGATCCGGGTCTCGTGGCTAAGAGTGACGTGGTCAATCGAATCAAAACAGAGGA
EMPV1_13225 ATCCTGATGGATGTGTTCACGGATGTGGAGATCTTCTGTGACATTCTGGAGGCGGCCAAC
EMPV1_13228 GTGGTAGCCTGAGATGTTGAGAATTCTGCCTGTCTTCAGCCACTATGTTCTCCAGAGACG
EMPV1_13230 GTTACTGGAGAAGGGCATCACAAGAGACTACCATCACATTCAGTTGGGAGCAATCAGAAG
EMPV1_13232 ACCCCTAGGACCTGCGGAGCTGGTGCCAAGCAGAGAGGTGGTCATGGTTGGAGTGATGAT

EMPV1_13233	AATGGGCTTCCGTGTTTGGCTTTCTACCTATTCCAAGGCTAGATCAGAACTGGTGGGCTG
EMPV1_13236	TTTCTGCAGTCACAGCCTTGGCCTAAAACCATCGCCGAAGCACTTAAACCCCAGGAGCAT
EMPV1_13238	GGACGGCGTACCTTTCTGGGAATTAACCTGCTCCTGCTTTACTGCTAACCTTTTCCTGCT
EMPV1_13239	CTGAGCAGTTTAGCAAGGACAGGCCGGAGAATAGCATTTGTCGCTGCTGACTTTTGGCGT
EMPV1_13240	ATGTTTCTGTCCCTGGTGTTCCTGGCTGAGCTCGTCGCTGGCATTTCAGGCTTTGTGTTT
EMPV1_13241	CTGTCTTCACCTGTGCTGAAAGCTGTGGATTGGGCGCTGGCTACACAGAGGAATTTGTCT
EMPV1_13243	GCCAGTCTGAGAGGGTCCAACAACGTTGCTTTGGGCTATGATGAAGGGAGCATCATTGTT
EMPV1_13244	GTCACTGTGGTCGACGTTCCATGTTTTATATCCTGTCTTCACAACAAGCTCAGCAGCTCA
EMPV1_13245	CCTGTTGATGATTGCAATGTTTGGTGCAAGCTGTGTGGCAACTGAAGGGTGAACAGAGGT
EMPV1_13246	GCTGCTCCTTTCCCAGTCTGAATTCAATAAATCTGTCCAACCCCCTTTTGTGGGGGTGGA
EMPV1_13247	TTGGGAGAGACAGTGCCGGAGAGCACTTGAACTGAAAACCAAGTGTCCTAAGCGTCAGTA
EMPV1_13248	CAGGCTTCATCAATGCGGGGCCATTTTCTTCTCCGATCTCAAGTACCAAGTGGCAGAGCT
EMPV1_13249	CAGGCTGCCGTGATGCAGCTCTGTCGTGGTGTTGGACCAGTATTGGAGTTCTGTAGTGTC
EMPV1_13250	TAAGAAAGAGATGGCGGAGTTCTCACTGTGGTGCAACGAGATTGACGGTGTCTCTGCAGT
EMPV1_13251	GATTATCCTGAATTATCAAGATGGGCCCCCAAAGGAATCGCATGCTGCCTCACTTCCTGG
EMPV1_13253	GCTGAGTTCACATGGGCAGGAGATGGAAAGGACCAAAGCCAAGGCCAAACCCCTTCTAAT
EMPV1_13254	AGTTTTCGACGCTTTGGCCCTTTGATTGTGGATTGGCCGCATAAAGCAGAGAGCAAATCC
EMPV1_13256	TCTTCAGCTACACCACAGTTGGTCTACACCACAGCCAATGCAACACCATATCTGAGCTGC
EMPV1_13257	CAGCAGAAGCGGATTCGGTGAAGCAGGCACAGAACGTGCCTCTGAAGGAAAACTCTTTAT
EMPV1_13258	GGAGCGAATGGCTTCTGAGAATTAGGTAAAAATGTATCCAACTCAAAAGGCTCTTTTGGG
EMPV1_13259	AAGAGGGCTTTGGTGTTAACCACTTAGGGGCCTCCCAGCACAAGACTCCATTTCTGAAGG
EMPV1_13260	AACCCAGTTGGCTACTAAATACCAAAGCTCCAAGGCTGAGGGAGCCAAATGCAAGGTCTG
EMPV1_13261	AGTCAAGGAAGGCCCACACCCTGATGCGTTCTAACTGGCCCTTTCATTAATTGGGTCATC
EMPV1_13263	TGCACCACATCTCATGGGAACGCCAGATCCCTAGCCCACTGGGCGAGGCCAGGGATTGAA
EMPV1_13264	CATGGAGGTGGAGGTGCTGGGCATGGCCATCCAGAAGCATCTGGAGCGACAGATCGAGGA
EMPV1_13270	TAAACCGCAGCTCTCACAAGGAGGAGGGCACCAAAAGTAGTGGAGCTCAGATATGTCAAC
EMPV1_13274	GGAAAACTGATGAGGCAAAAGGTTTCCTAGTACAGGACGACGTGCCAAGCATCCAGCTAC
EMPV1_13279	GGTTTCCTTTCTGACCATCTTCTCTCCGGCAGGATTAGGCAGCTCATTTCCAGACTCTAC
EMPV1_13283	CCAATACCTGCCCCAGCGATAAGGAGGTTGAAATAGCATATAGTGATGTAGCCAAAAGAC
EMPV1_13285	GTTACTGTTGATGACTTGGTGGCTGAAATCACACCAAAAGGCAGAGCCCTGGTACCTGAC
EMPV1_13287	GCGAGCCTTTTTACTGATGTGGAAGTTTGCGAAGAGGTGAATTGACTGCCTTTGGGCTTG
EMPV1_13288	CAGGACAGGCAGTAGTCTCTTTTAAAATTTATTCACCAAGAACCATTAACTGCACAGTTG
EMPV1_13292	AGATGAGGTTCAGGCACCTTCTTCCCCTCCTGGTGAAAGTGGGCTTTTGCTGCCAGCGGA
EMPV1_13293	CACCACGTCCATACCCTTTGTTCCTGCTCACTCAGGCATCTCATGGAAAAGCCAAATCAC

EMPV1_13295	TCAGCAGCATCTCTGCAACACTGAGACAGGTTTGATCCCCAGCCGGCACAGTGGGTTACA
EMPV1_13297	GCCTGGGTGGGGTCTTGTGTGCATTCTTCAGCTCAGATTTTTCTGGCCCTGAGTTTACCA
EMPV1_13298	TCAGACTAACCCCACGCCAGGCAGTCTTTCTGCTCAGGGCTTCACTTGGATTACTGCATT
EMPV1_13299	CTCCAGTTCAGCAGTTCTTCCCCTCCTAAACCTCAGTTAGTGTCAAATAATATACCGGAG
EMPV1_13301	CCACACTAGGAAGTCGGACCTCGCCCCAATGCCGAAATGCTCTTTACTTAAGATGCTTCT
EMPV1_13303	AATGCTCTTCCCTTCGCCAGGCCTCTCGCCCTGGGGTCACTGCCTTGAAAACGAACCATT
EMPV1_13304	GTGAATGTACAGACATCTCTTTGCCTGGTCTGAGTCAGGATCCCTTAGCAACGCGGTGTT
EMPV1_13305	GGTCCTTGGCTTTGCAGAAGTCTATTGTATGTGAGCCATACAAAAGCTTAATTTATCTTG
EMPV1_13306	TGACAAGCTTGGGTACCCCTGTGGTGCCTGATGTGTGGAGAACATAGGCCAAGCAGTTCT
EMPV1_13309	GGGAGTTTTCCTTGGTCCAGTTATGAAATGTGACTCTGGGAGATAAGCGTGTTCGGCGCC
EMPV1_13310	AGCCCCTCCCACTCCATAGACTTAATTTAAAGGTTTCTTTTTAGATCACAGTATCCATGT
EMPV1_13311	AAAATGGACTTCCAAAATAGTCACCTACAGGATTGTGTCATATACTCGAGACCTACCACA
EMPV1_13312	GAACTTCCCGTATATGAGTGATGTGAACTGGGATGCAGTTTTGCAGAAGAGGCTCACCCC
EMPV1_13314	TACGGCACATACGCGGCGCTGCTTCGGGTCAAGCTGAAGGCGGGCAGCGAAGAGCTGGAT
EMPV1_13317	TCTCAGGCAAAGAGCCTATTCATACTCATAACCTCATACGCATGTCCTTACATGGATAAG
EMPV1_13319	ACTAGACTGAGTTCTGCAAGGGTAAGGACTTTCGTTGTTCCCCGAAGAGCACATGGACCA
EMPV1_13320	TGCTTATACTTATCTACGCCCAAAATTTCTTCGTGCTCCAACAGAGGACAAGGCTCTGGC
EMPV1_13323	ACTCACTGTCCAGCTCAACCAGGATAAAAATCAAACTCCTTCCTAGAGCTGACGGGAAAG
EMPV1_13324	CCCGTAGCTGCCCCTTTATCTGAGACCTATAGCCTTCTTGGTTGATGTGATAGTGCACCA
EMPV1_13325	GGAAGACCATGCCAAAGACAGGACCAGAACTCAGCTTGTTCAATACAGTTACTCAAGTAT
EMPV1_13327	GAAACATACCTCCTTTAAGCAACAAGGAACTCTGCAGACGTATGATCCAGAAAGCAGTCC
EMPV1_13329	GCTCCCAGTGGACAAAGACGCCCAAGGCTTGTGATTGAGGAATGTCCTTTAACTTATGAG
EMPV1_13330	TGCTGCTGCGGAAGCCGCTCAGGTTAAAATGTCATCCTTTAGCAAATTACCACTATGCCG
EMPV1_13334	AAAAGACATCCATCACTAGACAGAGCATCTGCTGGCATCATGGTCTTGGACTTGCCAGCC
EMPV1_13336	TCAAGGCGGCCAAGCACTGTCATGTCTGAGTTCATCCTAGCCCATCCCTGCATCTACAAA
EMPV1_13337	TGTAGAGAAACGCGAGGAAGCAGTGCCATTGCCATGAATGTGTGTGACTCGAGAACTCAC
EMPV1_13339	CAGTTATCCAAGCGGAGATTTACAAGCTGATCTTAGGGATTAGACCATGTGTCAGTCCAC
EMPV1_13340	CAAGACCCAAATGCTGGACCATGTCTCTTGCTGTGCTTGGTTATCTTGGGCTGGTGACAC
EMPV1_13343	TAGCCCCGCTCACTTCCCCTTTAAGGAACTTCCCCGTGACGCACCAGGAACTGGCCCATT
EMPV1_13345	TATTCAGAGTGGTCACTGATGAGAGAGGATAGAACCAAGGGTGGGGAGGTGTGCTCCACA
EMPV1_13347	AGGTTCACACGGAGCCAGGCTTTGGCTCACAGCTTCCCGAGCCTTGGGCTCAAGAGGATA
EMPV1_13348	CTGCAGGATGCCATCTCTTTGTGGTAGAGAAGGTTCATCCTGACTTGTTTGAATACCTTC
EMPV1_13349	ACCCCCGTAAAAGCAGTAACTCAGACAACTTCACCTAGGCGCCTGATCTGCTGTGGATTT
EMPV1_13350	CGTGGCCTTGATGGAGCAGCGAGTGAAGGAATTACAGCGTCAGAGGAAGGAGCTGAGGAT

EMPV1_13351 CGTCAAAGTGCTGTGAGCAGATAATGCAGAGAGCCGTCCAAGCGGAGTCGCACCTTTTAA
EMPV1_13353 ATTCTCGCCCTGGAGCTGTGCCAGGAGATCGTGGTCTATGGAATGGTTAGCGACAGTTAC
EMPV1_13354 TACCTGACTGGAGGAGTCTGGAGTCTGAATGGTGCAGAGAGCATGCAAGAGACTATGCAG
EMPV1_13356 GAAAATCAGTACCTTAGCCATCTCTCAATGTCCACCAGTCATTCGAGGAGCCTCCAACCA
EMPV1_13357 AGGCCCCTCCCCAGGGATTCGGAGTTTGTTAAGCCTGGGAAGTTTTACTTTTCAACAGCA
EMPV1_13359 GATGACTTTCACTGCTTCTCCCAAACAGCAGCCTGGCCCCAGAGTCAGAGATATTTGAAC
EMPV1_13361 CAATGGTGAATGTTGGGAGTTACAGGGAATCCGAATGAACCAATGGGAGCTCAGCACGAA
EMPV1_13362 CTTTTGGGGGAAATGAGTACCGGGTGTCATGAATATCCAATATGCCTTGGGTCTCAGGCC
EMPV1_13366 GTGAGCCCCGTCACCACACCCACGGATGAAGATGTGGGCCACAGGATCAAACATGTGGCA
EMPV1_13368 AGGCTGTTCCTGCTAATCCCCCGTGGCATCTTTAGCTTTCCTCCAGAAGCTCACTGCTTT
EMPV1_13370 CCACCCTGCTGGAAGAGATGCACTTGAAGAATGAAGACCTGGAAAGCTTGACCCTCCCAT
EMPV1_13372 GCTCGCCGTTCCGCCTTCCTATGCATATACTTCTTTGAGGGTCTGGCCTGAAGAGGTGTA
EMPV1_13373 AAGTAGACGAACACAGGACAGAGCCCCTGTAAGGGACCCAGTGATGCTGGAGACAGGAGT
EMPV1_13374 CCTCCAATAGCATCCATCAATCCTGTTAGCTTTCAAGCACCAACTATGATTCCAGGCATG
EMPV1_13375 GATCATCAGTGGCCTTAGCAACGCTGAGAAGCAAGCAGTAGGAAAATGAGGAAATCAGTG
EMPV1_13376 GATGGAGGTCACCCTTACATACACACCGTACTCCGCCGTACGACAGCTCTTGTCGAAGCT
EMPV1_13377 GAGATGCCCTGAGTCAGACCACTGAGGTCAAGGAGGAAGAAGCAGAAGTGGCAATTTCGA
EMPV1_13378 CTCCGGACCAAAATTTAACAGTGCCATCCGAGGAAAGATTGGGTTGCCTCATAGCATCAA
EMPV1_13380 CACACAACATGCATAGCTCTTTCCTGGGTCCTCACTCCACACTAGAATAAAAGGTTTCGG
EMPV1_13382 GCATATCTAGTCTAAACCGAGTGGCACTGGGTGGTAACTCGCTTCCAATATTCTCCGCTG
EMPV1_13383 CGATTGTGAATTCCTACCCATTGCCTCCCTCGGTGAATATGGAAATGGCCACCTGGGTTT
EMPV1_13384 GTGCCAATAGAAAAGTTTGTTGCTGAGAAGGGTCAGCGCTATGGTAGTACTCCTGGCTGG
EMPV1_13387 TCTCCACCTTCCCGGCGGCGTCCACCTTCCCTGCCGTGTCCACTTTCCCGGTGGCGTCCA
EMPV1_13388 GGGTGTTGGGGATGTTTGCTACCTCCTGCTATCTTTGTGGTTATGGTCCTCTCCTTATGG
EMPV1_13391 TGGCATAAACCAGGGGGCACAGAGACTTAGGCCAGCTCCTCTCATATTTCAGAAGCGTAG
EMPV1_13392 TTCAGTGCCACGTTGAAGGGCTGCCAGCACCCGTCATTACTTGGGAGAAGGACCAGGTGA
EMPV1_13393 ATGATATTAACCTGCCAGCCCGAACTTTTCTAATAACTCCCACGCCTCCCGGCACACGCA
EMPV1_13395 TGTCATTGCTGCAAGCTTTGGTGTAGGTTGAAGATGTGCCTCAGATCCAGTGTGGCTGTG
EMPV1_13397 CTGACCTATGTAGACAAGTTGATAGATGATGTGCACCGGCTGTTTCGGGACAAGTACCGC
EMPV1_13398 AATTAGGTTTGATGGCTCGCCCAGCAGCCACCTCATCTGAGATCCCTGTGTTTCTCCAAA
EMPV1_13399 TGATTGAAGTGATGTTTGCTGTACGGAAGGATGGATTCAAGGACCACCCTGTTATCCTAG
EMPV1_13401 AAGGTAGACCAGGAGATATGGAACAGTGAAGAGATAGTACCCAATGTGAAGGTACAAAGG
EMPV1_13402 CCTCCTATGCACTCGCTTTGCCTTTTCTGTCTGTGTTACTAACTTGGGGGTATATGTGCT
EMPV1_13403 AACTATACCCACTGGCGGCGTGTGTGCGATGCTCTCTTCATCATCTTCTCCTTGGTCTTC

EMPV1_13406	CCAATTTCCATTCACCTAGCAAAAGGAAGCAGCTGCTGTTTAGGGCTTTATTTGACCCAC
EMPV1_13409	ACGGCCCATGGCAACTTGGAGCTGTTCACTCTGCAGCAGGGACAGTGGCGAATGCAGCCT
EMPV1_13411	TTTCCTTCATCTGGCGTCTGGGAAGAGTCAGAAGGCCAAGGTGATCTCTGGAGTGGGTCC
EMPV1_13412	GCAAATGGTACATGCAGGTGATTGTAAGGACAGCAAATAACAGAAATCTTCGCATCTGGC
EMPV1_13413	TTTGGGATCCTTGGCACCTCTGTCTTCACCCTGTTAACTCCCCTGGCTGCAGATTTCGGA
EMPV1_13414	CAGGATGAGTTATGATGCCCTCCAAGCACATAGTGCCCAGTGCCTTTGCCCTCCATTTAA
EMPV1_13415	TGGGTTCTTCTCTTTGGTTTCTCTAAAGTCAGTTCTGTCACTCCATTATTGGGAGTTGTG
EMPV1_13417	GCCATTGTCCAGGTAACATCCGTGTTTGGCCTGCCCTTCTGTGATGCTTTTGTCCTCTCT
EMPV1_13418	CAGGGCCCAGCCCCTTCCTAAGTGGAAAGCCCTTTAAATGCCCTGAATGCAAAAAAAGCT
EMPV1_13419	GGTCCAGCTTCAGCAGAAGGTCTGTGACACTCTACAGGGGGAAAACAAAGAACTTTTATC
EMPV1_13420	TGTTCAGGCTGTGCTGGTGCAATCCTTTGTGCCAGTCTCCCTTTCCTCGACTTAACAAAG
EMPV1_13421	TCAGTGGGGGCAGTATGGGGGATGCCACACCCAATCTAGAGATGGGAAAACTGAGACCCA
EMPV1_13423	AGGTCGCTGGGGAGGGGGTCGACCAGCCCCTAGGGAGCAGTGGCTTCACGAAGTCAGAAA
EMPV1_13424	TTGCTTTTGAAGCTTGTAATGAAGTCCCTCCGGCTCCTAAAGAGTCCTCAGCCAGTGAGG
EMPV1_13426	GTTCTTACTCCTGTGCTGGGTTTCTTTCTCCAGATGTTGCCTTCATCACGACCGTGACCT
EMPV1_13427	CTGGATTTCCTGAAATGTTAGGAGGGCGTGTGAAAACCTTGCATCCTGCAGTCCATGCTG
EMPV1_13428	CAGACAGCCATGGTGTTGGCTATTGCTGTGTCCTACAGGTTCATCGCTGGTGCTGTGATG
EMPV1_13429	GCCCCTCGGGCACTCAAAGAAATCCGTAAATTTGCCATGAAGGAGATGGGAACTCCGGAT
EMPV1_13432	CCCACGCAATGAGCCAAAGAATAGTTCCTGTGACATTCCGCCTTCCTCCAATTTACTCCC
EMPV1_13434	GGAGGCGAGCTTGGCAGCAAGAAACCCGTTCATCCCAACGATCATGTTAATAAAAGCCAG
EMPV1_13435	GGAATAAATAGGCAGAAGCTGACTTCCTTTAGGCTTTGGACCCCCCAGGAGGTAGCCCTT
EMPV1_13436	GGGGCAGCACTTGGGTCTCTCTCTAAACCTAGACTGATAAATTGCCAGGGAAGATGCTAG
EMPV1_13440	TCCCGGGCTCCTCTTCTTCTAGTGCAGGGTAAACAGACGTGAAGGCATGAGGCACAATTA
EMPV1_13441	CTAACATGCTATAAAGGAAAAGGCCAACATTCATCTTCCCCGTGGCTCTGCTGCTCGACC
EMPV1_13446	AGACACTTGGATGGTCCTCTGTGGCCCAACTGCTGTGATGTGTCTGCTTTTGGGAGCAGA
EMPV1_13448	AGATTCTGTCGCCACTGGTTCAAACCTCAACCCCGATGCCACTGGTCCTTTAGGAGGAAG
EMPV1_13449	CGAGGACCTTGGAGAACCATTTTACACTGACCAGTATGACCAGGAGCACATCAAGCCTCC
EMPV1_13451	GCGTTGGAACATAGAACCAAATGGGTTTAGAAGGACGGAGCTCTCAGGTTACTGCTCTGG
EMPV1_13453	ACTACCCAGGTGGACATTTTATTGTGTTTCTCAGCAGGAGTCTCTGATGCGTGCTCAGGC
EMPV1_13455	CTGTAACTGCTGAATTGGCATTCTGATGTTCAGAAAAAAAGCCACATTAAAAGCAGCTGA
EMPV1_13456	CAGCTCCATGCACGAGATCATGAGTCCTAGTCCAAGGGGGCCTGTTTCCCTAGTTAAACA
EMPV1_13457	GCCAAGCCTTCGGAAACTCTTATTACCACTGTTGAATCTAATTCCAGCTGGTGGACCAAC
EMPV1_13460	TACCCTTCATCACAAGCCAAAGCCCAGCCTCTCAGGAACCTGAGGAAACAACAGAGACAA
EMPV1_13462	TTTACAACCACGAGAGGACAAGAAATCCCACAGACCCAGACTGCAACGTCGAGCAGCTAG

EMPV1_13464	GCAGCAAATTGTGTGAGGTTTTAATGCAGAATTTTGTCAGAAGACAATGGCGCTGCATGT
EMPV1_13465	CCTCCTAAACCAATGTGGCCAGACTACTGGACTTCTCTGAATGGGACTCTGCGCAATGCT
EMPV1_13466	TACGAATGCTTCGAGTGTGGAAAGGCTTTTAGGCGGACCTCGCACCTTATTGTCCACCAG
EMPV1_13469	GAATCATTACAACTACGGCTGCTTGGACAACTGGAAGGTATTCCTGGGTGTGGACACAGG
EMPV1_13471	CACACCTACCGAAGCAATTGAACAGCTGAAAAGTAAAAATGTAGAACTTGCCAACGAAGA
EMPV1_13473	GATACAGAGGATTAGATCAGCAAGCTGGAAGACAGAGAAGAGGAAATCACTGAAATTGAA
EMPV1_13474	GGCGTAAGGCCCACTTCATCCTTTTTTTCTCCCCTGGAACTCTGGGTGGAACTTCAGTTA
EMPV1_13476	TTCGTCTGTCGCTCCCGTCGGAGTCATTGCCGTCTGTTCCAGTTTCCTAATACTTTCCTA
EMPV1_13478	CCACAGCCCCAGGTTCACGGGCTTGCTGTCCACCATCACATTGGCTGAATAGTTGTCAAA
EMPV1_13480	CTGCTTCAATTTTTTGAAGCATATCATCTCTGAGGAGTTCTCTGCTCTGAAGACACAGAG
EMPV1_13481	GTAAAGGAAAATGATCTAAGCTAGGGAAAGGAAGAGTGGTTCCTAGTTCTAACAGGGATC
EMPV1_13483	GCTAACAGCAGTCTTACTGAAGGTTTCCTGGAAACCACGCACATGCTGTTGCCACTAACC
EMPV1_13486	TTGAGGTCAAGTCCGGTGACGCCATCAAGAAAGTCAAAGCCAACGTCCAAGACAAGGAGG
EMPV1_13489	AGGACACACACAGCTAGGTGCAGCCTGGCCTCGTCCGGTCGGAGTGTTGAGGTTCGCCGC
EMPV1_13491	AGTGCTTGAGCGCCTTCACTGTCCATTTCTCGGGCCAATTCACCGGCACAGCTGGAGCCT
EMPV1_13492	GTGACCAGATTTCTCTCCAGAGAGCTTACCAATGATTACTCCGCCACTCCTTCCCTAGTG
EMPV1_13493	CAGCCAGGTGAATACCCGGGGTTGGGCTGTGCCCCGTGGGGTCCCCGGGGAGGGCAGGAC
EMPV1_13494	GAAACCTATGACTGATGTAATGAAGAATTTCATGTAATACTCTTCAGAATGTTTAAACTG
EMPV1_13495	TGGAGGAGATGCCTGCTGTGGTTCCTTCCAGTCCCAGTACCTGCTGTTTCTTCTGCCATA
EMPV1_13496	CTTTCACGTGTCACTCAGCTATCTCCCAATCTGGGGAAGTTTTTCTTACAACAAGATGCC
EMPV1_13500	CTGACAGATGTGGCCATTACACAATCACTTAGCCCCTCTCGCTGCCCCTTCCAGACCTTT
EMPV1_13502	GGTGCCTTCAAGCTACAGCACGACTTGTCCTGGACTCCGTATGAGGACATCGAGAAGCAA
EMPV1_13503	ATCCACTCCTTGTATCGCCGCACGGGTGCCAGCTTTCAGAAGGCCCAGCAAGAATTTGCT
EMPV1_13504	TGGCCTCTATGGACGGCTTGAGTGGGATGGCTTTTTCAGCACAACTGTCACCAACCCTGA
EMPV1_13505	TGCTGGGGATGTCCACACAGTACGAAAGATGTTAGAACAAGGCTATTCCCCCAATGGCCG
EMPV1_13506	AGCCCTCAGATAACCAGCAGTTACCAGCATGGATCATCCCCCGCTCAGGCCTAACTGTCA
EMPV1_13507	CACTCACCAATCAGCTGGCTTATCCGCACCACCCTGAGTGAATCAGAAAGCTTCGAAGCT
EMPV1_13508	CTCTGGCATCAGGATCAGCTGCTTTTGGTCACTCACCTTGACCTGACACAGAATCCTCTC
EMPV1_13511	CAATTGGGAGTGGAAAACATGCTTACCATTGCTATCTGCATAGATCTGGAGATGTTCACC
EMPV1_13513	TGATGGGGGCGCATTGGACCTGGATTCATGAATGAAGTGACAAACAGGCGCTCTTGGGGA
EMPV1_13515	ATTACCTAGAGCAAAGTTTCAGCTGTTTCGCCCTGGCTACTGAGTCTGATCCATTCTCAA
EMPV1_13518	AGAAGAAATGAAGAGGAATGATCTTCCGGAGACAGCGGTGATTGGACTCCTATGGACCTG
EMPV1_13521	GAAACGACCGAGAAAAATGAGGACCAAATACATAGGCGCGTTTGCTGACCGAGTCCCATT
EMPV1_13522	GCTCCAGTAAAGGTGAAAAATGCTCAGGGATGGAGCCCGCTGGCAGAAGCCATCAGTTAT

EMPV1_13523 TTCGAAGATAAGAAGCGAGAGAACTTTGAGCGTGGCAACCTGGAACTGGAGAAGCGGAGA
EMPV1_13525 ATTGCGTCTTCTGAGTCTAACTTTGAGAACTTCACTGGCCAGTCCTGCCTGGATTCCCTC
EMPV1_13526 TGGGACCACAAAGTAGGAGACTTGGTCCCTGCAGAAGTGCCTGGTTCTTAGAGGAGCTCA
EMPV1_13527 AGTGCTCTCATCTCCCCTCTGGCTGCCAATTCCATAGGTCACAGGTATGTTCGCCTCAAT
EMPV1_13530 GACCACTGTGCCTGTCTGGGTACACATTCACTAGCCAAATAACTTTTAACATGCCCAAGG
EMPV1_13531 CAGAAAGAGGAGCTCAGTGTGTTTATACCATCGCCCACTAATGTTAATAGCAAACGCGTG
EMPV1_13532 AGGTCACCCACCACATTCCATGACAGACAGCTGGCAGTGACTATGTTTAGCAATAGAGCA
EMPV1_13534 CAGAGCAGATTTCAAGGCCAGATACAGTGTGGGTGACGGTATCTCCAGTTATGACTTGGA
EMPV1_13536 GTCAGTCAAACTTGAGGTTCGGTCCTGATTAAGCTACCTACCAGCTACGTGATCCAAAGC
EMPV1_13539 TCCTCTACCTCTTATTCGGGCGGAAACAGCAGGGCTACAGCACCGTGTCCCACTTCAACA
EMPV1_13541 AAACAAGCAAGGCTAGATGCACGGTAGGGCTGGTCACAGCAACCAAAGGCATTGCAACCA
EMPV1_13543 GGATGAATGTCAGAGACAGTTTTCTCTGGGGTCTCTAAATGAATTCTACACAGATTTAAG
EMPV1_13544 CCCACACCTCCCCAAAGAATCGAAATGTCAGCTCAGGATATGGGGCCAGTCTGTGTGAAT
EMPV1_13545 CTATTCCATTGAGAAGAAACCTCTTTCCCCTGATGCCTGAAAAACAGAAGAAACCCAAGC
EMPV1_13548 AGCAGCATAGGGTATAAGAGCCGCCCCATCGACCCCAGTGTGCCCTTTGACCTCAAACTT
EMPV1_13549 TGCCACGATGTGGGAGATGCAGGGTGAAACACTTCCTATGTGAGATGCCTGCTCTGATAA
EMPV1_13552 TTTCTCCTGATCCTCCCATCCACTGTCCTTAAGGCAGGACTACCATACTGTGGCCCCAAT
EMPV1_13553 TGTTGAAAGGACACCACAGGGACCACTGGCTGTACCTATGTGGAAAAAGCACGGATGCTA
EMPV1_13556 GGCTCTAGGATAGTGCCTCATGCCACAGGATAGTATGCTCATAAGTATTTCGGGACAGCA
EMPV1_13557 GCTGGAGAAGGTAGAGGAGATGGCGCAGGGGACACGGGCCAAGACTAGGGGTTGCTGGGA
EMPV1_13558 AGAATCACTTCTGCTCTTGGGCTCCGGCGAGGCCTGACTGGATAATGGGAGACTGCCTGA
EMPV1_13559 AAACCCAGGGCCGCTGTACGCAACGCTCTGCCCGGCGCCATCAAACCACCTAACACATCA
EMPV1_13561 TGGTGGGCCTCTTCCAAGAAAACAGAATGCCCAGAATGCCGAGACAAATGGGAAGGTTTC
EMPV1_13563 GCTGCCTACTTGGTGGACAGTTTATGGGAGAGCTCTCAAGAACTGTTGAAAGACTGGGAA
EMPV1_13564 CATCTACTCTGCCCTCATCCTGCACAACAATGAGGTGACGGTCCAGGAGGATAAGATCAA
EMPV1_13567 TAGTGCTTTGCTTGTGGCTTCTGGAGTTTACCGCGGCATCCTTCGACCTACAGGGTGCGA
EMPV1_13568 ATCTGACATTACTGGGGTTGTGGTGCAAGCCAGCAACTCAGCTCCGATTAGATCCCTAGC
EMPV1_13569 GTTAATGATCCGGCTTGTCTCTATGGAGGTGCTGGTTCAATCCCCTGCTCTGCTCAGTGG
EMPV1_13570 GATAGACTCGGCTGAGAGCAAGAAGTGGTAAAAGGTGAAACTAGAAAGCTCCCCAGGTGC
EMPV1_13571 CACTGGTTTTCCACCTGAATAGTGCTACCTTCACGCTCGCTGGAACACACCCTTCCTGAA
EMPV1_13572 AACAGGGACCTGAGCAAGGAGAGCAAAGCCGGGAAACCGTCACAGCGTGGGTTGCAGGAA
EMPV1_13575 TTTCGATTTAAAGTGTTAATACTTGCATACGCGGTGTGCAGACTGCGCCACTGGTGGGCC
EMPV1_13577 GCTCCAGAACTCATTCATCTCACTTAACTGACCTACATCTCCTCATTTCCTCCTCCCCTC
EMPV1_13580 CCGAATTTGGGTCCAAGAGGAACTGAAGCATTTGGAACAAGAGGAAGAGGTGGTGGCTGG

EMPV1_13581	GGCAGGTCCAGAAACGGATGCCCAATTCCAGTTCACTGGTATCAAAAAATATTTCAACTC
EMPV1_13583	GAGGCAGCCCTTCTGGCTCTGGAAGAAGACATTCAAGCCAACTGCATTATGAAAAGACAC
EMPV1_13584	ACACACTGAGATGGGAGCAAGGAGCCACCTGAACTGAGAGGAGGAAACATCCTGGAAGAA
EMPV1_13585	TCTACCAGCCATTTAGGTGCCTGACTTTGGATACAGGAGGTTCTCGCTGCGGCTTCTACT
EMPV1_13586	CCGTTTTAACCGTCTAAGCACCCCATGTGTTACCTCATGTGCCTGAGTTTCTAAAGGGGC
EMPV1_13589	ATCCCATGGCTGTCCCACTCCCTTTGAGTCCTGTGCACTCATGCTCACCACATCATGAAT
EMPV1_13595	TGACTCAGAGGTTTGGACGCCACATCCCAGCTCATATCCACATCTTGCTGGCCAATGTCT
EMPV1_13596	CCCAGGACGAGCCCGAGGAGCCACTGAGCGAGTTCAAGCCCTTCTTTGGGAATATAATTA
EMPV1_13597	ATAGGCAGACTCCAAGTCACTCAAGGATGTTAAAAGATGGTGAAGAAAGCCCCAGAGTGC
EMPV1_13598	TTTCAAGAGTTGGGTTTTTCAGGGTCGAGGTGGCATCTTGTTTCGTCAGAGTGGCACAGC
EMPV1_13599	CCACTGCGCCACGACGGGAACTCCAGTTTAGCCATTCTTAAGTATTTTCATTAAGAGAAT
EMPV1_13601	CCTGAGGCTGAGATTGGCATGGTATTTCTGACAGTGATTGCTGCATTAGAAGCATATGTC
EMPV1_13603	CCATCTCACTTCCAGTGTCAGACTTAGGAGTCGTCAGGAAAGGAGGAGTGAAGTCTCAAT
EMPV1_13604	TTCTCAGCCAGAAGACCCAGACCCCAACTCTGTCCATACTGTCCCAAGGGGTGACCCTGA
EMPV1_13605	CGGTTCCTAAAGGAGTACGCATCCCTTCGCCCCACGCCTGGGAGGAGTGTGAACTTTTTG
EMPV1_13608	GGATCTTGTCGTGTATCATGTGCTGCGCTTCCAGACAGTTAGCTTTATTGGCTACCCCAG
EMPV1_13610	ATGATCCTGCTAAACAGTGATGGTTGTGGCATAAAACCACAGAAAATGTATTCGTGTCTT
EMPV1_13611	GTGTACGACCGAGATATGCTCATGTGGTGTTGAGGAAAGCAGACATCGACGTCACGAAGA
EMPV1_13613	TACACCACAGCCACCGGTCTACACTGCAGCCACAGCAATGGCAGATCCAAGCCGCATCTT
EMPV1_13615	GGCATCTGCTACTCAGCTTCCCTTAAAGTTTCCATGTGGGAATGTGGAGAGTGTTGCCAT
EMPV1_13617	ACCCTGGCCCCCTTGCCACGTGCCGAATTCATCTATTACTGTGTAGACGAGGACGAGCTA
EMPV1_13618	GAAGGAGTTAAGTACTTTGCAGCCTTACTGAAAGAGGAGACCTCACGAATTGATGTTACT
EMPV1_13620	CTCGATCAGTGTTCCTATTTATAGCCGGGCGCTTTCTCCTCCGTGAGACGGCATCGAGCT
EMPV1_13621	AGGGTCTATATTTCCCAGTGAAAATGTCTCTAGGTCCACTTGTAGAGCTGGGGGCCCTCT
EMPV1_13622	CAGAGCTGTGGATAGAAACATAGGAACCCTTGACATCTTTCTTGGGGGGAGCTGGAGCAT
EMPV1_13623	GGTACCTCCTTAAATTTTGTTCCCTAAGTGCCTCACTTGCTTCATCTTAGTGCTGACCCT
EMPV1_13624	CCTGATGAAGGTGACCATTGATGACCGTAACTACATTGTTGATGCTGGATATCCTGGCTC
EMPV1_13625	CGTCCAGAAGGCAAAGGGATAGCTAAGTCCAAAGTGAGTTCTGGGATGCAAATGGAGCTG
EMPV1_13627	CGCTGCCAGGGAGTCAACCAGCAGTGGAAGGTCCTGAATTACGATTAGACGAAGGTCATA
EMPV1_13628	AGCAGGGTCGGGCCTGGTTAGGACTTGGATAGGAAAATATAAGGTGAGGTAAAGCACAGT
EMPV1_13631	GGCATTGCCATCAGCTGGCACACAGATGAGGCTTGGACTCGATGTTGCTATTGCTGTAGT
EMPV1_13633	GGGAGTTTGCTGCCCGTGGGGGCTTGAAGGGTCAGGTGTACCCCTGGGGAAACCAGTTCC
EMPV1_13637	TACAGCCATATGCAGATGTCACAGCCACGAAGACCCCTCTGGGCACAGTTCTCAAAGATG
EMPV1_13639	GGGGTGGCCAAAAAAATAAGAAATGTAGTTTTTCAGCTGTCAACCCAGCCTCTTGAGTCT

EMPV1_13640	CTCCAAGAAGGGCAGAGGCTTCCACTGTTAATAAATACCGTTTCATTCCTCTCTCTCCTG
EMPV1_13641	TAGGGCATAGAGTGCTCTTATACATCTCCTGTAACAGCCACAAACCTTAAGCCATGTAGC
EMPV1_13642	CTTCTTCTCGTTTGCCTTTTTCTGCTTCTGCTGCATGATCTCCGAGTCCCTGGGGGTAGA
EMPV1_13643	CCCTGTCCAGAGATCTGTCGCTTTGTCTTCCGGACACTTTTGCTTGGTTAACATTAGGAT
EMPV1_13644	TGGAATAGCTACCTTCTCTTCCAGGCCACAGCTCATCAGAGGGGGAAAGGCAATGATGTG
EMPV1_13646	GCTCCTACTGCTTCGACCAGGATGACATTGACGAATCCCTGGATCCCTTTAAACCCACTC
EMPV1_13647	GAAGTCTGCCCTCACGCTCAGAATCGCCATAAATGTGCAACGTATATTCCAACGAAAAAC
EMPV1_13648	AAGGTCCACGTCACACAGGAGATCCCCTTGCCTCTTGGGAAGCTTCTGCCTACAGAAAGA
EMPV1_13649	GTTCAGACACAGTTAAGTTCTGTGTAGCCACTGTATACCCTGTATACCTGGGAGAGTCAG
EMPV1_13652	GACATTTCAGTATGCCGTCTTGTGTTTTCCAAGGCAAGTCCACATCACAAGTAACCTGCC
EMPV1_13653	CCTGGGGGTTTTGTTCCCCTCCTCTTCTCGCCGTTAAAATACACGAACACAGTAAGAAGC
EMPV1_13654	GAACAACGTAGACTGGATGGATGTTTGATCCCTGTTGGTCATGAAGGTGTTAAAAACAGC
EMPV1_13656	CTATGGTGACCAGACCAACCTGATGCTCAGAGGCTGCGAGCCGCTCCTGGTGACATTCGT
EMPV1_13657	GTGCCATTTTCCCTACAGCCTCTGCTCACTTTGTGTCTCTGTCGCACGTTGTCCCAATAT
EMPV1_13659	GTGGAGGGAACAGTTCAACTTACTGATTAATCACAGCTTCACTGCAGGACTTTGCTACTG
EMPV1_13660	ACAGTTAGTTTTGCAGGTTTGCATTTCAGCGTATGTATGTGTATATGGCTGTGCAAATCC
EMPV1_13661	AGGGCAAGCCTTCTGCTGGAGGAAGAGTGGGATTAACTTGACCTCAAGACCAAAGACCGA
EMPV1_13662	CGCCTTGTAGTCCTCATGGGTCTCGGCCACGAATTCCGACACGGAGATCTGGTCCGGCAT
EMPV1_13664	ATCGATTCTGCGAACCTTTGACCGCATCCCTCTTGAGGACATCCATGCACCCCCACTTCG
EMPV1_13667	TTCCCATCCCGAGCTGCTAGAGCGGAAGGTGTTCGGTGTAGAGGGAGGTAGCGCCTTCCT
EMPV1_13669	GGAAGGTGACAGTCCTTTGGGTGACCATGTGGGTTCCTTGTCAGAGAAATTAGCAGCAGT
EMPV1_13670	CGCAGATCCATTGCCTTAACTGGGTTCTCTTAATGAGATGTATTAAGAGTTGTGTCTGCT
EMPV1_13671	ACTGAACGATGGGGCTTCCTGAGGGAAACAGCTCCCTTTCGGTTAGTCCAGCAAGAATGA
EMPV1_13674	GCAGTGCAAACAATAGACAAATCCAACAGAGGAAACCAGGCCAAACCTACAGGAGCCCAG
EMPV1_13676	CTAACTCAGCAAGCTCCTTATGCTCACTCTCGCCCCCTCAGTGTCTAAGTCTGTAAGAGT
EMPV1_13677	GGTGGAGAAGAGACTTCAAGAATGTACCAGGCTCAGCACTTACCCTGGACTTACCGTTTT
EMPV1_13678	CCGTATCCAGAGGGGGCGCCAAAAGCCCAGAAACTGTCTAACTCACAAAAACTGCAGAAA
EMPV1_13680	GATCTGAACCTCTTCTCACCTCAAAAACAGTGACGCAGAATCAGAGGAATGGCGGAGCAG
EMPV1_13681	TCTTTTCTAGCTGTCCCCTGCCCACGCAGGGCGGGAGCACCGGTGTATATGTGTTTTGTA
EMPV1_13682	CCAGCTACTCCAGCCAGCCTTATCAGAGCACCCACCCTTCTACCAATCCTACTCTTGTAG
EMPV1_13683	CTCCCCCTCGGCCCGCACGATGTCCTCCTCAGCCGGCCCGATGTCCTCCTCAGCGCTCCC
EMPV1_13686	CACAGCAACTGCTCCTGGGAGATTGTTAGAGTAGAAATCATGAGAGCCTTGTCTTTCTCA
EMPV1_13687	CGTACTTGCACCACTCGCTCTTCACAAAGTTCTCGGAAAGCACGAAGATGGTTTTCTGGC
EMPV1_13689	TCAGCTGGGGATGTTGATAAAGTGCAGATTCTGATTCAGTAGGTCTGGGATGGGTTTGAG

EMPV1_13690	CCTTTGTTTTTCTCGGGGCACAACTGACCTTGGCCTTGGGTGTTCTTCTGTGTCTGAATC
EMPV1_13692	ACATTGACCAGTGTAGAAATTCCAGGAGTTCCCTGGTGGTTCAGGGGGGTAAGGATCTGG
EMPV1_13693	TTGCCTATGGAACATACCCAGATTACATAGCCAACAAGGAGAGCCTGCCAGAACTGAGCA
EMPV1_13694	TCTTCCCCAAGCTCTCCCCAACCTCCCCCAAAGGCTTGATAACTGTGGGACCATATTCAT
EMPV1_13695	GCCTAGAGATCAGCCGCAGCGTCCACCTCACGGACAGACAAGTTAAAATCTGGTTTCAGA
EMPV1_13697	CAGCAGCAGTCTGTGTGGCAAAACTCCATGACATCAATGCCCAGATGGTTGAAGATCAGG
EMPV1_13698	GTATCAGCTGGAACTCAAAAAGAACTACCACAAGCTGAGAGAGAGCCTCCGGCCAATGAT
EMPV1_13700	AAAACAGTCCATTTTGGGGGCGATGGGAACGGGCGGAAATGTGCACTAAAACGAGGTGAA
EMPV1_13702	TGCCACAGGAGCTGAAGGCAACATTGTCTGAGAGGCAACCATCACTGAGAGAGCTACAAC
EMPV1_13705	TTTGCAAGGCCACATTCTGAGAGCATAAAGCTCGTGTGGGGCTGTGATGGGGCTGCTTTT
EMPV1_13707	CTGGCTTCATCATAAGACATGTCCTGGGCTGTAAGCATCTTTTCATTGATCCAGAGAGAC
EMPV1_13708	TACCTGCGTCCAAGGTCCAGCACCTCAGAGGATGAAGACCGCCAAATCGCACTGGTCTAT
EMPV1_13709	TTTTGTCAGCCTCTTCCTCTCTTGAAAGGACCGGTTTGTTGCCAACACGAAGCACATGGC
EMPV1_13710	AGAGCAGCCCTCCTGGTAGCTCCATGTCTCATCCTCATCAAATGCCGCCTGAAACACTAT
EMPV1_13715	GGCAGCACCATCTTCACATATGGACGACCCATCTCAACATATTCACTGGAGAAAGACAGG
EMPV1_13716	AGAGCAGCAAGATCAATGGGCTGGCGGAGGGCAAGGCCTCCGAGGACTTCCTCGGCTCCT
EMPV1_13719	TTCTTGCTCTGGGGTCACAGGCCCTCTTTTTTGTCTTCGGTTCTGGGTGGGAGGTTGTGG
EMPV1_13722	GTGGTGTTCTGCATGAGCTTTGTGCAAGGAGATGACAGCGATGACAAGATACCTGTGACC
EMPV1_13725	GAGAACCTATAGCATCTTCTCATTCCCACGTGGAACAGGATGCCCACATACTGTCTAATT
EMPV1_13726	GTGAATATCATTTTTGACGGCTGCATGGCTTTATCTTAGGTAAACACCCCATGGTTTACC
EMPV1_13728	TGTCACTGCTGTCCAGCTGCAGACTGAGGCCCAGGTGGCATCTGCCTCAGGCCAGCAAGT
EMPV1_13730	AGTCAGCTGCAGGGGCCTTTCACCAAATGTTGCCTTTTTAAAATGTTGTCATCATGGGTC
EMPV1_13731	TCTAGGGTCCAAAGGCTAACAAAACCATTAGCAGTAGGAGTGGGCCATGTTCATTAAGCC
EMPV1_13734	GCCCAGACACCTAGCCTATTTTAACCCCATTCCTTTGACTGATGCAGGCCATGAGAGGCA
EMPV1_13735	CTGTTTGAGGACATTGACACCTCCATGTATGACAGTGAACTTTGGGCACCAGCCTCTGAG
EMPV1_13736	TGAAGGATCCGGCGTTGCTGAGAGCTGTGGTGCAGGCGGGTTCACTGGTTTAAGAATCCA
EMPV1_13737	TTACCAGTTGCTAACCCAGTACAAGGAAAGTGAAGATGCAGAGTTACTGTGGCGTTTGGC
EMPV1_13738	AATCTCTCGGTTTTGATAACTGGATTGGGATGCCCCCTGCCACACTGGGATGTGGTTTGG
EMPV1_13739	GGAGTTCTTGGCTGTTCCATTCACCTACTACAAACAACTGCAGGAGAAGCTCAAGTCGGC
EMPV1_13741	TCTCGCAGATTCCAACAGCGAGATACTGAGGGTGGGGCCATGTGGTTGGAAAATGACCTG
EMPV1_13742	AAACAGGAGTGAAATTGGACACAGCCCTCCTCCTGCCTACACCCCCATGTCAGGAAACCA
EMPV1_13743	AGAACTCTAAATCCCTGCTGCCCCTTAACCTGTTCATTGACGGCTCCCAACCTTGCCCTT
EMPV1_13744	ATAAAAAGAGCTGCCCCGAGTCCACACAGCCAATTGTAAGATGCAGGTGTGCTGGCTCCA
EMPV1_13745	ACCAAAGCCAGGATCTCCAAGACACTGCAGCGGACCCTGCAGAAGCAGAGCGTGGTGTAT

EMPV1_13746	CCTATTGTTCCAGCTTTAGCCATGAGGAGCTCTTTTGGTTGGCTCCTGTGAGGCTTTGGC
EMPV1_13747	AGGTGCAGGGGAAGTACGTGAAGAAGGAGACGTCGCCTCTGCTGCGGAATCTCATGCCTT
EMPV1_13749	GACTGAAGTCAGAATGCCAGTCCTTTTGAGGAGTAAAACACCACTCCAAAACTGGAGACT
EMPV1_13752	AAGAACCGCTACGCCAACGTCATCGCCTACGACCACTCTCGAGTCATCCTGACCTCCATT
EMPV1_13753	AGGTCATTGCAGAATATGAGGCGCTGGACCGAGAGCTCCCGTGCATCCGCAAGTTTCCCA
EMPV1_13755	TCTTTATTCCGCTTCCGGTCCGACGCCGGCGTCTTTGCTGAGGGTCACATTGAGCTTCCA
EMPV1_13757	GCATGCACAGTGAGACACAGTCGCTGTCCCTGTCTGAGGGCGTGTACACGCAGCACCTGT
EMPV1_13758	AGATCGAGGCGGTGGAGGTGGAGGTCGGCCGCTTCCGCGACCAACAGTACGAGATGCTCA
EMPV1_13760	ATGGCATTCTCTCAATTCCAGGAGCCACACATGCTCTCTGCTTCCAAGCCTTTACACACG
EMPV1_13761	CAGAGTAAACAAACAGGAGATGCAGATAAGAAGAGAGCCACCTTCCCGCAGATGTGGTCC
EMPV1_13764	TCATATTAGTTACACCCATTTCCCTGGTGCTGGTCTCCTATGTGCGCATACTCTGGAGTG
EMPV1_13767	CCTGTAGAAGCAGCAGATGAGCTGAGATCCTGGCTAAACACTTGGTAACAGATGAGGGGA
EMPV1_13770	GGTGCCAAGTGCCAAGGCCCCTGCGAGAAATGCTCTGGACTCTGTTGTTTTGCTCATTAA
EMPV1_13771	GCCCAAGGTTGTCACCAAGCCAGGCCATATCATTAAGCCTATAAAAGCAGAGGATGTGGG
EMPV1_13772	ACCAAACCTTACAAGGACAGTGGAAAATATACAGATTAATGAAGAGGATAATGAGATAAG
EMPV1_13774	AAAAGCGTCTTCCCCCCCAGGACAGAGCTTAGACGAGGAGACTGGAAAACAGACAGTACC
EMPV1_13775	ACCAGGAGGGCCACTTCTTCGACCTGGACTTCCTGGCCAACGACCCCATGATCCCTGCTT
EMPV1_13776	TCTACCCCACAGTGTGGCATTTAGGGTCTTTGTACCTCCAGACGGGCAGCCACGTCCATA
EMPV1_13777	CCAGCATGTGGGGGTATTTCTCTGCTTCATTATTTTCTTGAGTGACTGCCCTGGGTTGGC
EMPV1_13779	TGAGAATTCCTTGCATACTGACTACATCACCGAGAAGCTATGGAGAAACGCCCTGCTGGC
EMPV1_13780	CAGTTCCTAACCTGACTGAGCCACAACAGGAACACCTACATTAGGATATTAGCTCAAACT
EMPV1_13781	GGAACAAAGGTACAATCAGTTGAGAGGCCATGAGGGCCCTGGCTGATTGAGGAAGAGGAA
EMPV1_13783	TAGCAACTACCACGCTCACCTTTCTTCCTCCTCTCTCAGTATCCAAACCCTCGGCTTGAG
EMPV1_13785	GGATTCTCCTACCACCTGAAGACGTTCGCCTTTTTGATTGGCTGACCAGGGCGTCCGTTA
EMPV1_13786	TCATATCCGAGAGGCCACCTTTAGTGTCTTACCATGGGGGTGTCCACAACACAGCTCCTT
EMPV1_13787	GTGAGCACTAAGGGCAAACAAAAGTCGGTATTTAAGAACCATCCAAGTAAAACCCTGGCC
EMPV1_13788	CTCAGTGCTGAAGCAGTATCAGAATTTATGGGCCTTTTTAACAGGAAGGAGACAAATGAC
EMPV1_13790	GGACAGCTGCCCTTGTGCTCCTGAGCTACCAGAGAATGACCGGCAGGTACAGCTTCAAGA
EMPV1_13791	CAGTTTTTATAAGGAAGCTGGTAGATCCGAGGGTGGGATGAGTAGCTGTGTGTTGCTGGC
EMPV1_13792	TAGCATCTCCTTGAGCTCCTGGCCCTTCCTCCCACACCTGTCATCCCAGCTGCCTAGATT
EMPV1_13793	TCCCGAGCCCTCCACCTGGACTCCCAGATGATAGAGTTCTATGCCTTAAGAGCCGAGGCC
EMPV1_13794	CAAGACTGACTCTGGTAATGAGGATTTAGTCATAATTTGGTTAAGAGACATGCGACTCAT
EMPV1_13795	TCAGCTCTCGGTGCCTCCTTCTTGCCTTGGGAGTCTGACATGAACAGGTGAAGTTTCTTC
EMPV1_13798	GAAACTCTCTCAAAACTTAGAACTGAACAGAGTGAAAGACAGAAAGTAGCAGGTGTCTTG

EMPV1_13800 CCCATGGTTCTCAAATAGTCATCATCAACCCGAGAGGAATGAATTCTGGTTCCTACCACC
EMPV1_13804 TCCTCGGCTCAGCTGCTTCTCTTTTCGAAGGTTTCCAGACATCCCTTCTGAGCACGCATT
EMPV1_13806 AGTCGGGTTTCTTCTAATAGTTGTCCAGCAGTGTGGCTCTGGGGGTCGTGGGAATCGCAA
EMPV1_13807 GGACCCCCCTGGCTGTTACAAATATCTCTCCATGTCACAAATAAGATAATCCAAAAGGAC
EMPV1_13810 TGTCAGCGTGGTGGTTGGAGTAGCTGCTTCGGGCGAAGAAATCGCCACTCGTCCATTCCA
EMPV1_13812 CTTGGAGCACGGCCAGAGATGCCAGCAGCTTCTATTGTCCTCAGTCCTGCAAACCAAAAT
EMPV1_13813 CCATGTGGTTGCCAGGTATGAGTAAAACATGGTTCTGTCAAGCACCATGGAACGTCACGC
EMPV1_13814 GCTTCTGAGGTCACCATTACTGGTGAATAAGAACACGTACGAGGATGTGAAAAGACAAGG
EMPV1_13815 ATTAACCAGATCATGCTGGACGAGGGGGAAGTCATCACTGTCGGGTCAGATGGATGCGTT
EMPV1_13816 TCTGTGTCTGTAGGCGTCTGCTTAGACAAGGCAACAAATGGGCAGTTGCCAAGCCCTTGA
EMPV1_13818 TAGCGAGGGATAAATGAAGTGTTGTCTAGGTTCTAAGTAAAAGAAGATCACAGTGGATGG
EMPV1_13819 GCTCAGGATTTCTGGGACATGTATGTAGACTGCTTGCTTTCAAGATCTCATGGAATGGGC
EMPV1_13820 CCTTTGCAGAACAACAGACGGCTTCAACAGATCCTTTTGGAGGGGATCCTTTCAAAGAAA
EMPV1_13825 CCATCCCTCGTCCACTCCTATCCTCGCCTTTATTTCTCTCGGAGGGCTGAGCGCGTGCTT
EMPV1_13829 CCCCACTGGCTCCCCAGTGCGTCTGTGTGGGATTGAGTTCATTTATGTAGCAGAGATTAA
EMPV1_13830 GCTCCTGTATATGTTGTTCGAAGTGGTGGCCTTGTGAAAACTAGGTCAAGGAACATTCGG
EMPV1_13831 TGAATGGCTTCCCCAATGAATACTGGGGCTGGGGTGGTGAGGATGATGACATTGCTACCA
EMPV1_13832 AAAGTGGACCTGGTATTTTCCAGCCAGAGGAGAAAGACTCTAATCCAGCGAGGCATGGCG
EMPV1_13834 TCACACTACCTTTTGAGACTGAGAGTGATCCTGGCGGAGTTCACCTTGACCTATGTGGTC
EMPV1_13835 TTGGGCAGGATGAAAGGTGCTGGGAGGAGCTTGGGCGTGAAAGTTCTCAGCCAAGAAATA
EMPV1_13838 CCAGAAAGCATGTGTGGCCGATAAGCACAGGCCTCTGGAAATGTCAGCCAAGGTCCGTGT
EMPV1_13839 TACGTTTCCCAACCTGCAGGCCAGGCTTCAGTTCTCGGCCTAATGACCCTTCACGAGACA
EMPV1_13841 GTCAGAAGCTTATCAAATCCTAGACCAGAAATTTAAGTTGATTCAGCCCCATGTTCAAGC
EMPV1_13843 GAACGGTTACCCTGCTGAATAAGGCAGCCCTCGAACGTCCCCACTGTCTTTAACGTTCTA
EMPV1_13845 CCAGCATAGGCTGCATGAGGGGGGTGCGGGCTCCCGCAATCCTGCTCGGCGGAGGGGCCA
EMPV1_13847 GGTCGGAATGTTGTTTCTGAGGATTAAAGAGAAGATCTACCTGCTCCTTCGTCGGCAGGC
EMPV1_13849 CTGGCAGTGATGGTGAGAGCATAGGAAACTGCCCCTTTTCTCAGAGGCTCTTCATGATTC
EMPV1_13850 CTCCATTGTATGAGTCACGTTCTATCAGTGGCATTTACTTCAGCCGGTGAGAACAGGGAG
EMPV1_13851 TGCAACTTAGAAATGCAGGTCACCTAGATATTTTCCTTCCGCAGCCAATGCCCCTCTATG
EMPV1_13853 CGCCAAGTAGAAAAATGAGCAAAGGCTGTTAGGAAATCTGTAGAGGAGGAAAAATGCTAG
EMPV1_13854 ACCTGGCATTGTATCGTGGGCCGAAATTTTGGCAGCTATGTTACACACGAGACAAAGCAC
EMPV1_13856 CCTACCTCCATCCGTGTGGCCCCATCGTCCAGGCTGTGTACCAGGCTCTGACTTCGTGCA
EMPV1_13857 GCTTCTTTCTGGTCATGGTGTACTAGAAACTAGATCTGGGTTAAAATCTCAACATCCCAC
EMPV1_13858 CCCACACTCAGCAGGGTCTTAGTAAGTGGCTCACGATTGCAGTGGGAATCTGGACAGCTT

EMPV1_13859	CCATCTGAGACTCCACTCTCGGGAGAAATCCCACCAGTGTCACGAATGTGGAGAAATCTT
EMPV1_13863	AGGCATTCAAGCAGAAACAGGAGCAGAAGAAATTCGAGGAGCTAAAAGCAAAGGTCATGG
EMPV1_13864	CCTGCTGGAAGAGCAGATCGAAGGCGCCCGGCGGCGAGTCACTCAGTTACAGCTGAAGAT
EMPV1_13865	GTTTCGGAGACGGCCTTTAGCTTGGTTGATTTTGGACAGCTGCTTTTTACCAAGAAAGGG
EMPV1_13866	AGCAGGAAGCTCAGTCGGGGATGGGGAAATGTGATATGCTGCTGTTGAAGATGGTGGGTA
EMPV1_13869	CAACGCCTGCTCTGGGATGCTGTGACATGGGCACGTGAAAGTGGAAAAGCCATCGTTATA
EMPV1_13870	GCAAGGTCCCACTTGCAAAGTACGTCATATGCTAAGAAGACTCAGTAAAAAGGCACAACT
EMPV1_13872	TGGAGACCATTACCATTCCGGATCTGCGGGGCAGGGAAGGCCACTTCTACTACAACATCT
EMPV1_13874	CCTGATCACATTTATGTCTACGTCTATAAGCCCACGAGCATCTTTATAGTCAGAGGATGG
EMPV1_13875	CTCTTTAACAACATACTTGGGCCTTCTGTCATCACAGCTGCATGGGCATGACACTCCATC
EMPV1_13877	GAGCATAATGAACCCTGGACTCTGTGGACTGTTGGTTCTGATCTCCTGGATAATGATGGC
EMPV1_13878	CAAGGAAATTCTGTGTCATCTTTGGTACGACACCTCCCAACCCCTACCCCCGTGTCTAGA
EMPV1_13880	TTCTGATCCCAGTCTATCTTCCATCTCAACTGACACGTACTCCAGGCAGCATCCCAGGCA
EMPV1_13881	TTTCTGACTGCCCAAGCTTTCCCAGCAGGCAAAGGCAAACTCTCCAAGCAGAGCAGGAAG
EMPV1_13882	AACTTAGGGCCACACATGTTGCATATGGAGGTTCCCAGGCTAGAGGTCTAATGGGAGCCA
EMPV1_13883	GATGAATCTAGTTGCTCTAGTGAAGAAGATGAGGAAGATGATTCAGAATCAGAAGCTGAG
EMPV1_13884	GAGGAGGTGAGAAAACAACTGCAAAGTCTCCTCCATGAAGACAAAGAGCCCCGTGAACTA
EMPV1_13885	GCCTCCCTCTGAAGTTAAAGGTGGGTTTGTGATTTGGCCAGTAAAACAGGAGCGGAAGTG
EMPV1_13887	CTGTGTTAACTCGTCTTCCGTGGTAGTTCTTCGCCCCAAGGATGGAGATGCAGTCCAAGA
EMPV1_13888	CCTGGACCTGAGCGATCAAACGTCCGGAACCATCACTGATGACAGTGACATCTGGCTGTT
EMPV1_13891	CAGCTCTGCCCAATCACTAACTGACTACCAAACTATATGGCCAAAGGGCAACGCCCAGGA
EMPV1_13893	GCATATTTCTAGAGTTCCTGAGAAAGTAGGCAAGGACCAGGGGCAGAAGTTATGCTTGTC
EMPV1_13894	CAAAGGGGGCGCCAAGCGCCACCGCAAGGTGCTGCGAGACAACATCCAGGGCATCACCAA
EMPV1_13896	GGCCTTCGTGCAGTTTGCTTCACAGGAAATAGCTGAAAAGGCTCTAAAGAAACACAAGGA
EMPV1_13900	AGACAGACCCAGTGAGTGAATCAGGCAAAGTGCTTATAATGTGTGTCGTGTGAGCGTGGC
EMPV1_13901	TCTTTGGAGAAGGGCAGCGAGTGGTGGGGAGCGGAGCTGGGCTGGGTTTGAGCATCATGG
EMPV1_13902	GCTGGAACTGGTACCAACGGAGGTAGTCTTGTCCTGGGTGGAATTGAACCAACTTTGTAC
EMPV1_13903	CGGAGCCCTGCTGGTGTACGACATCACCAGCCGGGAGACTTACAACTCGCTGGCTGCCTG
EMPV1_13909	CGGCTGTGACCTTCCTGGGACACATGATGCCCCTTTTCTCCTGTAAATCTGTTTTGGCAT
EMPV1_13910	TTGAACAGTTTCTTGAAAACCTGGATAGATCCCGGAAAGGAGAACTTCTCAAGAAGAGTG
EMPV1_13911	ACGGCGCTGTGGGGCCTGGACTCTCTGGGTATTTTTGTGTCTTATGTTTTCATCCTTCGT
EMPV1_13912	GGTGTGAAGGGATATTAGCTTGGACGCAGAGCAAGAAAATTTAAACTGGGAGCTGCCCGG
EMPV1_13913	TAGCGGGCAGCAAGAATGCTACCTTGTGTTCATCGGCTGCTCCCTGAAGGAAGAAAGTGT
EMPV1_13915	CAGCACTTTCAAGCTTATGATGAAGGTTTTGAGTATGGGTCCCAGCTGCCAGAACGTCTC

EMPV1_13917 TGCTGTGCACCCTGACCTTCTGGCTCCTGCTGCGCCAGTTTGTGAAGGAGAAGCTGCTGA
EMPV1_13918 CCTGGAGTTGATTGTCCTGCTTTAGCCTTGTGTTGAAGAGGCTGAAACTGTCTGTGGCAC
EMPV1_13921 GCTAGATGGGTCACATTGGAGCCAGTGGCAGAGAAGGCACCTATGAGTTTGACCCTTTTT
EMPV1_13922 AGCCATGCGGGAGATGAATGACATGCAGGACACTGAGGTCTTCATCTGCACCAGCCCGCT
EMPV1_13923 GGTCTCTGGGGAGATGAAAGTTGAAGTGGAGAAGTTTTGTATGAAATTGAAGCTAAGTTG
EMPV1_13924 GGCCAGTCTGAGAGGTCCATCTGCATACCTCTTCCCCCAGATTCTTTGTCATCAGTTTAC
EMPV1_13925 GGTAGACAATGTAACAAAAAGGTGTCGGATATATGCTTCCGCATTTGCACAGGTGCTGGG
EMPV1_13927 TTCTGCAAAGTGGGGCCAAGGATTCTCATTTGTCAGCACTCTTACTACCGAGCGCCGAAT
EMPV1_13928 TTCCTCGGAGCATGGGTAAATGCACACACCTTGTGGAAGTTCCTGGGTTTTGTGGCTTTG
EMPV1_13930 CACTTAGAATGGGTGTTGAGGGGGGATACTAAATACATAACATTATCTTCCTGCATGCCA
EMPV1_13932 ATCTTCTCCGTGGCCAACATGAGGAACAGCAAGCTCAAGGACATCCGCAACGCCTGGAAG
EMPV1_13933 CCTTCCAAAAAAGCTCCTGTGCAATGGATGCCCCCACCAGACAAACGCAGAAACAGTGAA
EMPV1_13934 TGTCTCTCCTCCCCGCCTCACCTCCAAAAGAAAACCCTGCAGACAATGCTTCTTCCAATC
EMPV1_13936 TAGGGTCATAGGTGCAGCAAATGGAAGTTCTCTGGCTGGTGATGGAATTGGAGTTACAGC
EMPV1_13937 GAGCCAGAACTGTGTCCTCCTTCCAGTGTCCTTAGATTGGCCCATAGAGTAAGGAATACC
EMPV1_13938 TGCCATTTCTGCGGTATCAGGAGCAAAAGCACATGAGCCCGACACACAGCACTACCAAGA
EMPV1_13939 AGCCAGCAGATCGAGAACATCCGGAGCCCCGAAGGCAGCCGCAAGAACCCCGCCCGCACC
EMPV1_13940 GCACGTAATGGACAAAGTTGTGAATCTGGTTCCGCCTACTGTTATGGAGGACGATGTCGG
EMPV1_13941 CCAGGTACTGAATAGGAAGAGAACGGGTGAGAGCTGGGAAGGAATCTCTCTTTCACCTTC
EMPV1_13942 CCTTACACTAGAGACAAATGTAATGAATCTGTTTAGCCACTTTTCCCTAGCTCTTAATGT
EMPV1_13945 GGTCTCAGCCCCGGCCCGAATTCTCTGTGGGCAACTTTCTGTTATTGATTACTTGAATGC
EMPV1_13950 TTCTCTTCCTATGTCTGTCCTCCATGCCATAAGCACTCCTGAACCAACCTGTCTTCAGAG
EMPV1_13953 GACTGCACTCTGAAGTTAGATCCTATCACAGGGCGATCAAGGGGGTTTGGCTTTGTGCTA
EMPV1_13955 TATGGTGTAGGTCTCAGATGTGGCTCCGATGTGGGGTTGCTATGGCTGGTTATGGTGTAG
EMPV1_13956 GCGATTTTCTGTTTGTATTGCTGTGCACCTGAATGCTTTTGCAGGCAGCACGTGTATGCG
EMPV1_13958 CTGGGACAAAAGCTTCTTTTCTAGAGACAATCTGATCCATGCTCGAGCTCGGCCGACATC
EMPV1_13963 AGGCTACTTTGGACTCTGCACCCTCGGGTGTGTACGTCCTGTTTCCTGACGGGCTGTTTA
EMPV1_13967 GGCGGCTCCTCTTTGCAGAGGGGGAAAACTCCGAGGGATAAGAGCCGGAATAATGCGGTA
EMPV1_13968 TTCTCAAAGTTGGTGCCCAGACCAGCCACAGCAGCATCTCCTGGGAACTGATACACAATG
EMPV1_13970 AGTGGGTGAGAAGCTAAGTGAAGGAGACTTATTGGCAGAGATAGAGACTGACAAGGCCAC
EMPV1_13971 TTGTTTTATTCTCTCTGTGCCCACATCATCCCACAGCCCTCCCCAACTCAAGCAGGTGCA
EMPV1_13972 AGCATAACATGAAAGAAAGGGAAGTCAGGAGTTATCTAGTGGCCCCATAACAATCCTCTC
EMPV1_13973 GGGAACCCTCCCACACTGTTGATGGAAATGTAAATTGGTGCAGCTACTATGGGAAACAGT
EMPV1_13974 GAGAACCAAATGATCCAGGAACGGTTAGCTGATGAAGGATTGCAGAACAAAGCAGAAAGT

EMPV1_13975	GGAAGAAGGAAATTGCCCAGAAGGCAGCAGAGGAAAATGAGAGATACCGGAAGGAGATGG
EMPV1_13976	TTAGAGACGCAATACTGAGCCATGGAGGCAGGCTGGCCCCTGAAAGCGGGCAATTTCTAT
EMPV1_13978	GCATTTGTGAACAGCAAAATCCAGCCTGGGAAGGTGGTAGTTTTCATCAAGCCCACCTGC
EMPV1_13980	TCAGGACAGAAGCCTCGGGCCCTTGATGTCTATTCAATGCGCTTCCAAGTTCTCCCTGGC
EMPV1_13981	TCACGGAGAACGGAGCGCGCGCGAAGCGGGGGCACAAACAAGATGGCGGCCACGCCGTTT
EMPV1_13982	GTACCCGATGAGTGTGAGGATGGTGATAGCGAGTGACGAAAGTGAGTCCGAGAAGGAGAA
EMPV1_13983	ACTTGTAAAAGAGTCTACTTAATACGTTTAAGAATAAGTAGCTATGAATTAAGTATTCCT
EMPV1_13985	GTGATGCACATGGAAGAGCCTTGTTTTGACTTCCTTCGAACCAAGCAGACCCTTGGGTAT
EMPV1_13988	CCTGACCATCTTCTGCTCACCCCACTCCTGGATTCAGTGCTAAGGCCTATCAGGAACGAA
EMPV1_13989	CAATGAACACAGCGAATGTTTCCTAAATCCTTGCCTTTCACTTCCTCCGATTACAGACCT
EMPV1_13991	GTCCTCCGTGGTTCAGAGCATCAGGAAGCCTGGCAGTAAATGTAGAAGACGATTTTCAGC
EMPV1_13992	TGAGAAAAGGCCATCAGCAAGCAGTTTATTGTCCCATGTTTTCTTCAAACAGATGAAAGA
EMPV1_13994	AGAAGTATTCTCAAGACCCTTTTCCAGGTCATGGCCACCAAGCCCGAAACAGACGACAAG
EMPV1_13995	GTAGGAGGGAGTATTAGCCCTCGGAGGTGCGATCATTATTAGCTGGAAGAAAGAACTGGC
EMPV1_13999	TCAGAACGGTGCCTGCTCTCGGAGGGCGTGCTTACTGCCTCCCAAATAAAAAGTCTCTCT
EMPV1_14000	TCTCCCCTCTCTATTAAATGGTGCAGCAGCTGTGAACAGCTAGTAGCCTTGCAGTCAGTG
EMPV1_14002	TTCTCCAACGAGTACCTCAACAGGTGCTTTTATCTCACCCTGGAAGACGTGCTCTACGCG
EMPV1_14004	CCTGTAATCGGAAGAGCAACCAGCAGAACCTGGGAACAATCAAATGCAGCAACCTGTGCA
EMPV1_14005	GGGAGAGAACACCGTCTGAGTGGTTTTGTGATGTATCTTGTGTTGTCTGTGGTAGTGTTA
EMPV1_14006	ATCCTTCAGAGACCTCATTCATGACCAAGACGAGGACGAGGAGGAAGAGGAGGGTCAGAG
EMPV1_14014	GAGGATATTCGTGGGCCTTGATGATGTCACCGTGGTAGCCTTTTTCTACAGCTCTTTTGC
EMPV1_14016	ACTCGGGTGCAGGTTGAAGAGACCCCTCCTGCCAAAACCTGCTGTGAAACATTAGAAGAA
EMPV1_14017	ATCTTATCCTTCAAAGCACAAACAAGACCTGCAGCCATTACGAGTGTGCGTTTCTAGGTG
EMPV1_14018	GAGATCCTAGTTAAAAGCTGCTGCCAGTTGTCCAAGAAGATACCCACCAAACTGCTTTGT
EMPV1_14020	GGGAAAGAAAACAACCCCTGCATCTTATACTCTTTTGGGTTTTTCAGAGAGTGGGATGCA
EMPV1_14022	TCATGATAAAGAGTCAGCAACTCACACCAGAAGCCATGGAAGCATGAGGGTCCCCATCAC
EMPV1_14025	ATGGCTTTACTCCCAATGGTGCTGTTTCTGGCTGCTGTGCTGCTACCATCTTTTCCCACT
EMPV1_14026	GGCACGCGGGGCACCACCAACCCACCCAAAGCTGAAGAAGAGCAGGCATGCCCAGTGCCT
EMPV1_14029	CTCTTCATGTATGTGCTGCCTGGATCTGCAGAAGAAAATGATCAAGATATGATGGACTCT
EMPV1_14032	CCCAAAGAAGGCACACCCACCGCCCTTATGGAGCATGCCTGTTTTATTCCCCAAGGTCGT
EMPV1_14033	GCTCTTTACTCTAAAACTGTTGCCATAGCATTGGCCTCTGTGTGCATTGGACAGTGAGTC
EMPV1_14034	AGGACCGAGCCGACGCCTTTCTCCAAACACAGTCTCTCTAAAAGAAGCCTCCGAGAATCT
EMPV1_14035	AGCTGTGGCAACCCGATCCTTAGTGCCAGTGGGGCATCAAACCTACATCCCAGCACTCTA
EMPV1_14036	GAAGTTGTCTATGAGTTCCTGTGGAGAGAGCCTTGTGGAAAGCAAAAACGGCGGTGATGC

EMPV1_14038	AAGACTGTAACTTCCATCCTGCTGGCGGACTCTCAGCCCTTGTCGGCTCTGATAAGAAAG
EMPV1_14039	GCATTTTGAATGCCGTAAGTGTGGCAAATTCTTTAGCGGATGGCAGCTCCTCCGTGACCA
EMPV1_14040	GGTGGCACCGTAGTGAGTGTTTCCTGGTCACTCAAAGGAGAGGCCCAGGTGGACACCTTC
EMPV1_14041	TTTGGGGACTCATACTATGAGCAGCAGATGGCAGCCAGGCAGGCCAATGCTCTGTCCCAT
EMPV1_14042	TCCTCCCCTCTGGCGATCACCTGTTTGTTCTTTGCATCTAAGAGTCTGTTTCATTTTGGT
EMPV1_14043	GAAAGTGCTGTTTGAAAAATGGCTCTGATAGACTTGCTCAAGACAGGGTTGCCACAAACC
EMPV1_14044	TCGGTCCCTCTGGTGGTGGGGCTTTGTCTCTGGGTGAGATTAGATATTCCTGTTTGCCTA
EMPV1_14046	ATTATCTCGACTCTAAGAAAGACGTGGACCGTCACCTGAAGGCAGCCTGCGAGCAGTTTA
EMPV1_14048	GACGGTGCTATCCAAGTCAAAAAATACATGCCTGCCTCGTAGTGAAGTTGTAGCTCTCCG
EMPV1_14050	GCAAAGCTGTGGAATTACTAGAGAGGGAAGACAGTGGTTGATAGCAGTAGTTGGGTCAAG
EMPV1_14051	TAAGTCCGCTCCTGCCCCCAAAAAGGGCTCCAAGAAAGCAGTGACCAAGGCGCAGAAGAA
EMPV1_14054	AGCTTGAGACCTCAGGTGTCCTGGACAATGTCACTCACCTGGAAGGAGAGCTGCCAGCCT
EMPV1_14057	CAACAAGGAGAAGGTGGAGAGGGACGAAGACTGCCGCCGGAGAGAGCTGATCCAAGCCAT
EMPV1_14058	TTTAATGACAGTCTGAGCCTAGAGGAGAGCAGAAGAGGAAGGTCACGGAGCCGAGAGAGA
EMPV1_14060	CGCGTCATTCTTGCTTTCTGTCTTACAGTCGTTCTTCTCGCTTGCTAAAGAGGCACCGTC
EMPV1_14062	CTACCTGGTGGGGCTGTTTGAAGACACGAACCTGTGTGCCATCCACGCCAAGCGCGTGAC
EMPV1_14063	GCTCCCTCTATGGTTGATTTCAAGGGTGGTGATGTTTAGCAACCAGCTCACAAAACCTAG
EMPV1_14066	CAGAGCAGGCTGTTATTCCAGGATCAGTAATATACCACAAAAAGTCACAAATACTGCTGG
EMPV1_14067	ACTCTCTCATTCACACAGCAATCTTGATGCACTTCCCTTTCTGTGGGCACCGGAACATCC
EMPV1_14068	CATGATGTATCACTCTCGGCAAGCCCATGGCCCTCTCGGAACCTCAGTTTGATCTTCTGT
EMPV1_14069	CGATCAGGGGGCAGGACACTTTTCTTAGCCTTTCCCTGTGCCTCATTCCCTAAAAAACAG
EMPV1_14071	AATTTGCCCAGAGATCAGAGCAATGGCCTAGAGGTTATCAAGGAGGACGCTGCTGAGGGG
EMPV1_14074	CCTCTGATCAGTGTCACAAACTCAAAAACACATGTATATTTTTATATGTCCTGACCTGTA
EMPV1_14076	AGTTTGTGTATGTGGGTGTGTTAAGCCTTTGAAGCACGTTCTATAAACACACACTTACAC
EMPV1_14077	ACTTCACATCCAGGCGCCATTGGCATCGATTCTTCCGTGTTCCTGGTGCCTGAGCATGAA
EMPV1_14079	TATTTACGTTTATTTCCGGCCCCTGTCCACCTACACCATCCAGGACCAGGTGGCAACGGT
EMPV1_14083	AAAAAGAGAAAGTTAAAGTAGGTTTACACCATGTGCCTTGAAGTAGATAACAACAGCTCG
EMPV1_14084	CCTGTGACATTCAGACACACATGACGAATAATAAGGGTAGTGCTGCTTGGATGGCTCCTG
EMPV1_14086	GTAGATATAGTTTAATAAAAACCCTAGATTGTGAATCTAGTAATAGAAAATTAAATATTC
EMPV1_14089	CACAGACAGCACACACCAAAGGCAACAACACAGAAAATACATGCTCCCACACACACATCC
EMPV1_14090	AGAAGCCCTTTGTGTGTGCCCAGTGTGGCAAAACCTTCAGTAACACCTCCAACCTGAGAA
EMPV1_14091	GGCCCGGGGGCTGCAAGACGTGTTGAGGACCAACCTGGGGCCTAAGGGCACCATGAAGAT
EMPV1_14093	AAAAGGACTGACCTGTGGCCCATCTGCGACTTCATGCAGCCACTCTCACTTGATGTCCTG
EMPV1_14095	AAGATGCCAACTCTGTTACCTCCTCTTTGAGAAAAGCCAACTTAGACAAGAGGCTGCTTG

EMPV1_14096 CTTGGGTGGTTCCTAAGGGAAAAGAGTGCACACACGATCATGGGAATGATAGCCCAGAAC
EMPV1_14097 AACCGCAGAGCCGATGACAATGCCACCATCCGTGTCACCAACCTGTCTGAGGACACTCGT
EMPV1_14098 AAACTGTGCCTTCGTCACCTATGAAAAGATGGAGTCAGCAGATCAGGCCGTTGCTGAGCT
EMPV1_14099 TTGCAAAAAGACACCTTCCAATAAGCTGGAGAACGGAGGCACGCACCGGGAGAAAAAGGA
EMPV1_14100 CTTCTGGCAAAGCCACGGCCGGAGGAACCAGCAGACACCTCAGGAAATGAAACTTAAAAA
EMPV1_14102 CTGTGTCGACGTGAACAAGGCAAAGTGAAGGAATACATGGGCAAGCAGGAGCGCTTGGAG
EMPV1_14103 AGAGAGAGGACCACTGCCTTAGTTAAGCCCTAGCATCTTTAGCTTGTTACCTCCCTAAGG
EMPV1_14105 GATAACACCAAACCCTGGCAAGGATGTAGAGCAGAAGCAACTCCCATTCACTGATAGTGG
EMPV1_14106 ACAAGCCCAAGGGCACCTTCAAGACTATGTGCGTGACCGCGCTGACCTCAACAAGGACAA
EMPV1_14108 AACTTCACAGGATTTCCATCCCATAACTCAAGCACATCCCCCACTCCCCAAACTGTCTCC
EMPV1_14109 AGAATCTACGCATCAGACTAAGCGGCTGAATCAAAGCACAGAGACAGAAGTGCCTGGGTG
EMPV1_14112 CGTGTCTTACGGTATTCTGTTGCAAGGCATGGTCCTTCATAGGCCACAGAAAAATCCTAG
EMPV1_14113 GAAGACCAGGATATCGGCGTTCTAGACCCCTTTCTGCCACCGATTAGACATGTGTCAGTG
EMPV1_14114 CTCAGGCCTCCCTGTGCCCACTGTTCTGGATGGAGTTCAAAGGCCACTGCTATAGATTTT
EMPV1_14116 TCACTACACAGTAATAATGCACTCTCAGCTCTGCCAGAAGATGGTGCTGACCGCCTGGTT
EMPV1_14117 CCTATGGAAATCGCCACAGCAGAGCCACAGGTTTCTGAGGCAGTATATGATTGTGTTATC
EMPV1_14118 GGGGGTGCAACAGGAGGTAAGGCCGGCTGGAAGGAAGCCCTGGTAAGGCACGGGGCTGGT
EMPV1_14121 TCTTCAACATTTTCCTGTACACCTGCTGCTACACGCCGCTCAAGAGGGTCAGCATCGCCA
EMPV1_14123 CCAAAGTGGAAGCCAAGTTCATCAATTATGTGAAGAATTGTTTCCGGATGACTGACCAGG
EMPV1_14125 ACTTGTACAGAAGGAAGGTGAACCAGTTAGTCAGCATCTTCTCTGCCACCGACTCAGTCC
EMPV1_14126 GGCAAAAGGGATTTCATGTACCTGGCAAATAAAAACAGAGATTTGACAATTCTGGGACAC
EMPV1_14127 TCAAACTGCACTGCTTCCAAACACTGACAGGGATAAAGTTTGTGGTGCTGGCAGATCCTA
EMPV1_14128 GAGGTCATCGTGAAAGCCATGAGTGATTACTGGGTTGTTGGCAAGAAATCTGACCAGCGG
EMPV1_14129 ATCTGCCCTTGAGGTCTATAGCACTGGAAACCAAGATTTTGGTTTAAAATGTTGAGAGGG
EMPV1_14130 AGAGCTCATGTGAGAGGCGGGCAGGTCTCGAGCAGAACAATTCCGATCCCTTCAATCTTT
EMPV1_14131 CCAATAATAGTGGTCTTTTATTCTTCATCCATGGACTAGTGTAGGGAAGGACCTGTGGCA
EMPV1_14134 CAGCTCACGTTTATAGCCTTACCCCAGCCCATGTCTATTGTGAATTGTGTGGAGGTCCCT
EMPV1_14135 GGGGCTGGGGCGAGGCCTGGTGAGATTTATTTTAAGGGGAAAGACAGGAGAAAAAGCCTT
EMPV1_14136 TAGCTTCAGTTACACAGAAGATTGCATTGCACAATAAATGCTGAGTAGAGCACTGCAGCT
EMPV1_14137 CTTCTAGAAGCTGGCCGCAGGTTGCCAAGAGGTGACATGAAAGAGGAGCAAACTCATTGA
EMPV1_14138 CCCAACAAGGAAACAGAGTTCCTGCTGATTCAGATATTTGGACGCAAAAGCTACCCCATG
EMPV1_14140 TCAAGGACCACTCCGTGCAGCACCACCCTGACGGCTGCGTGACTGTGGTGTCCATCCTCA
EMPV1_14141 GATGACTTCTGCTACCGGCAATGTTAGGCTGACTCCCAAACCTATAGCTTGAATCGCAGC
EMPV1_14142 ACCTCACAACCATGCACGCACCTGCTGGGCCCGCTGTCCTTGGAAACAGGTGACTAATGA

EMPV1_14143   AGTGAGCATGTGGGAACCACTGCCACCTTCTCCATTGGCTCTAGTGGCAATGTGGTCTAT
EMPV1_14144   AAGTGCACTCTAAACATATCAGAACCTTATTTCTTCCTGTGAACTTAAGCTGCCTGCGCA
EMPV1_14145   TGGCCGCAGGTGCTCTGACGAGGTTGCACTACTGTGCTTTGAGGAGCAGTGCAATGATAT
EMPV1_14148   ATCTCACTTTCCCCCAGGAATGATGCAATGGCCTCGGTGATGTACACTGTGGTCACCCCA
EMPV1_14149   TAAGGAGAACAATCTTGGTGGGGCCATGGTTTGGGCCATTGATCTGGATGACTTCTCAGG
EMPV1_14150   CTCATTCAGGTTACATCAAGTGACACTGGGAAGGATGTTCAGGAAAGCAGCCCTTGGTGG
EMPV1_14151   AAATCTGCCTCTGACTTAGCCCTGGAGAAAAACAATGCTGAGGTTCTGGTCAGCCTCCAG
EMPV1_14152   GACTGCTGCAAAATCAATGAGGAAGCAGGAGGCCATGCTCATCACAGTTCTACAAGCTGG
EMPV1_14154   AGAGACTGGCATGATGTTGAAAGTGCCCTATTGTGAGCCCCTCATCAGTCATTACTTCTG
EMPV1_14155   GACCCCTGAATTCACTGCTCTTCAATCAGTACCTCACTGCTGGGCTCTGCCAAGTTATCA
EMPV1_14157   TGGTGGGCACGTGGCTTCCTGATTTGCACCCTGGGCCTCTGGTCTGAAAGAGATTAATTT
EMPV1_14161   CCGGTCCTCTTTCCAGACTTAGCTTCCTCCTACCCTCAGCCTTCACCCACTAATAAAACA
EMPV1_14162   TGGCAATAGGACCCTGCTTCCCAGTACTTTGGGTTTTGTGAATTCGATGCTTTGATTGAG
EMPV1_14164   ACGCGTGACAAGCACAAGCGCATGGTATTCCTCATTTTATACTTCCTCTACCGGCACCGG
EMPV1_14165   AGGTGACCATTTTCTTTAGGCCTGGCTTGTCCCCTGACTCACTGTTAGGATTTGCACTTC
EMPV1_14166   GCCTTCAGCTTTTCTCCATCAAGTGCGATGTTAGCTGTGGGCTCGTCAGATATGCTCTTC
EMPV1_14167   TTGATGCAGGTTCTGGTCTCTGGTTCTTGTTAGGCATCCCCAGCTGTGAGTGTGCAGAGT
EMPV1_14168   CACCAAGACTCAGAGGCCTGGGTCCATTCAGGAACTGAGGATGCGCTCAACTGAAAGCAT
EMPV1_14170   GTGGTCACACTCATGGTCACGCTAACACTAGTTATTCTCTCCTACACATATATCGTCCGG
EMPV1_14173   TTCTCATCTTAATATCCTCTGTGAAAGGAGAACACAAGTACAGACCCTCCCCAGGGCCCC
EMPV1_14175   TTCCTCTTCTCGAATGCAGCAACCGCAGATTTCTGTCTATAGTGGCTCAGACCGACATGC
EMPV1_14176   TGATGTCCGATCTCTTCAGCAAGCTGGCCATGAGGCGCAAAGGTATCTCTGGAAAAGGAC
EMPV1_14179   GTTACTACCGCTATTACAACAAGTACGTCAACGTGAAGAAAGGCAGCGTCGCCGGGCTGT
EMPV1_14180   GGCCGGGAGCCGCTGCTCCGTCTCTCTTCTCGTCCGAGCTCCGGGCCCCCTCCGAACTGG
EMPV1_14181   CATTTAAGGAATTTTTAGGAACAATTTATTTTCAAGCCGAAAAAATTGTCACGTGCTTTA
EMPV1_14183   ACGTGAATAGCATGGAGCCCTTCCTTGTGCTTCTCCCTTGTTGAAGTGGACTCTAAACCC
EMPV1_14185   TAACCTCTACCGGATCTGCTATAGCCCCTGGGGCCAGGAATTCTCCATCTGCAATTACTG
EMPV1_14190   GTCTTTGGGACCTGAAAATCTCTTGTTGAAAGGAGCTACGCTAAAAAATACCAAGAAGAT
EMPV1_14192   TTCCGGCCCATGTCTGAGTCCCCTCACATGTTTGACTCTCCCCCCAGCCCTCAGGATTCT
EMPV1_14196   AAAGGACATTTGCTGGGGCCCTGGGATGCTGGGGGACGAAGCCCAAGCTTGGTTTCCATT
EMPV1_14197   GGCAAAAACTTCTCCTGTTAAGTCCAATGCCCCCGCAGCTCATCTTGAAATAAAGCCAGA
EMPV1_14198   TGTTGCCACCACTCGGGTCTGTCAGGAGCGGAACTTCCTCCTCGGCGGCCACTAAGAGGC
EMPV1_14199   TTAAAGTTTGTCTAAACTGTTTTCTTATAATTGCAATATGTGAATATTTATTGTAAATAC
EMPV1_14200   TGGGTCCCATACGATTGTATTTGCCTTTGTTAATGAGGTTTTTCTGTTCAGCGGCTTCAG

EMPV1_14201	ATGATGGAGTCCGTGCTGGTGACCCTCTTCTGCTGCCTGCTCACCGGGCTCCTCGCCATT
EMPV1_14202	TGATGGATCACCTCCATCCAAAAGTGAACCAGCTAACTCCCACTCCAAAGCTCATGGATT
EMPV1_14203	CCTGCTATTTTCATGTGGAGTGGGGGAGCTAAGTTTAGTAGAGTTTAGCAGAAGCAGTAC
EMPV1_14207	AGTTTTATTCCATTGCTGGGCTTCTAGAGTGGACAGTGTGGACCGTGGACAGAGAGGGTG
EMPV1_14210	AGGAGGACTTTGCTGCCTTCCGAGCCTGGCTGCGGTGTTATGGCACACCGGGCATGAACT
EMPV1_14211	AAATAAGATTCACAGGGAGACAGAGCCTTGAAGAGCCTTTGCTGAGTTAGAGCGGGAGGC
EMPV1_14213	CCACTTTGCTTCAGAGAGGATATAGGTTGGCTTTCAAATTGCACTTGGTATAAATGGAGT
EMPV1_14217	CAGAATAGCCTACATTTTACCAGCAAATGAAAGCTTTGGACCTCTGCAGATATCTCTTGG
EMPV1_14218	GGCATTAGGAATGTTCCTGCTTTGAGTGAATTAAGTGTACCAAAAATAAACCTGGAGCTA
EMPV1_14225	GTTGTGTGATTCCACTCCTATGATGTATCTAAAGTGGTCAAGATCGTAGACACCAAAAAC
EMPV1_14229	CTCCAAAAGAAATAGCTCCAACCAGATGACCATTTCCATGACACTGGGACTACACCCATG
EMPV1_14230	CCTCCAGATCGTCTTCTTTCTGGTCAGCGCCTACTTCTTCTCCTGCCCAGTTCCGGAGAA
EMPV1_14231	GACCCTCATGATTTCTCCATTTGCTGGGATTGGGTCTGAATTATGCCCATGGATAGACAT
EMPV1_14233	ATTCACTTTATTGCCGTGGCCTGGAACTGAGCCCACAATACCCCCAAGGTATGTCTGTAC
EMPV1_14234	AAATGCTCAGAGACGTTGTGATGATGCGACTGAGGATTATGCAACGTGGTCCAACCGGAG
EMPV1_14236	ATCAGCAGCATCAAGGATGAGATGGAGCCTCGCTTCCGGGAGGTCTCCTTCTACTACAGT
EMPV1_14237	CGCCTTCAGGGTGAGTAGTTCAGATATTAAGTTCATCATTAACTGTTAAAGCATTTCTGA
EMPV1_14238	TGACAGTGACACAATACGTCTGAATTTAGCGGAGCAGGGCAGCAGACCAGCAGGCAGATT
EMPV1_14239	GCGGGTGGTTTACTGCACCTGTCACTCCCCTAATGCCAGGACCATCTTTCTCCAGCTTCT
EMPV1_14240	TAGCAATTAACTTTCCCGTTTAACTGGTTGTGTGCGGTTTTGCATATACAATCAACCCTA
EMPV1_14241	CCTCGTGGAAGAGGGCTTTAACCCTGCGGTTATCAAAGATGCCTTGTATTTCTTGGATGA
EMPV1_14242	TGTAGATGATGCAAGTTGGCTGCCAAGGGCAACTGGAACCGGATGCTTTCTTGGCCTCAA
EMPV1_14243	TTGGAATGCGACTATTTTCAAAAACCTAGCTCATGAAGTGCCCAGTGTGGCTTAGCAGGC
EMPV1_14244	CCACTCAAGCTTCACCTCCGCCCTTGAACCCTCTCTCACATGAATCCATTGTCTTCTCTT
EMPV1_14245	AGCAGACACTTAGCTGGCTCTCTCCACCAGGGATTGGCACACCTCTCAGTCAGGAGCACA
EMPV1_14246	CCACCGAGCCACAGTGGGAACTCTATCAATCTTTCTTGTGACTTGCTTTCCTCACTTGCA
EMPV1_14248	GCATTTGGGGACTGGGTACAAAACTATTGCATTTGGAATGGATGGGCAATGGGGTCCTGC
EMPV1_14251	CAAAAGTAGGTAGACCTACTAACCAGTAAAATTGTTGAAAGTTGACCTATCAGATTGTCC
EMPV1_14253	GGGGTATACCTCTTGGGCTATTGGACTGTCTGTGACAGATCTGGTAGGATCAATTTTGAA
EMPV1_14254	CAGTATGTCCTCAGAGAAGGTGCCAATTTCAATTCTCTTGCTATCTTCATAGCTGCATCC
EMPV1_14256	CCTATGTGATTACTGTTCCAAACTCATGTAGGGCTAAAAGCCATGGGCTACAGTGAGGGG
EMPV1_14258	CGTGGTTTGCGACAGTTCTCCCAACCTCATTGTCTCCTAGTACCCTGTCATGGAAATTTG
EMPV1_14260	TTACTGCGATGCTGTCCCTCTGTTAGCATTGTCCTGCTCTGACACTTCCTTTCCAGAGAC
EMPV1_14261	GACGCCGTCAATGAGTTCGAGGGTGGGATGATGCTGGTCAGCCATGACTTCAGACTCATT

EMPV1_14262	GGCCAGTCAGAATGTTCAGTATAAGTGACTCCTTTCAGGATAAAGGCTCCCTGTGCTGCC
EMPV1_14267	ATGCCTGTCTGTCCCTGTTCAAAATGACGCCTCACATCCTCTGATCATGAATTACACAGG
EMPV1_14269	TTTCCTGGTTGGTCCCTGTTGTTTTCGACGTTACTGCCTCCTGTGCATCTGAACTCATCC
EMPV1_14270	AGTTTCTCGAACGATGGGCAAGACTTAAACCCTGAAGAAGCGAGTGGGGGTTGTTCTAAG
EMPV1_14271	GGAGACGGGGGCAGACGAGGAGAGTCTGTTTGTTTTTCGTCATCTGCTCACAGGATTATT
EMPV1_14274	CCTTCAACGGGAAGGACACCATTCTGGGAACTTTGAGTAACTTATTGCTGTGTAGGAGGC
EMPV1_14275	TACCACTCCAGTCTCATAAAACACCAGAGAGTTCATTCTGGATCAAGGCCCTATGAGTGC
EMPV1_14276	AAGAGGCCGGGGCCGGAGGCGGGGCCGAGAGCTGCCGCGATCTGCCAGGGGCGGCGGGGC
EMPV1_14277	GCTGCCAGCAGGGAGTGAGAGGGATGGTCTGGGAATTTGGGCTTAGTAGATGCAACTATT
EMPV1_14282	AGCAGCAGCTCAGGTGTTTCCACTTTCCCTCTTCTTCTCAACCTCAGAATCACAGCATGC
EMPV1_14285	GGCGGGCGGCCGTTAAGACTTGCAGTGATGTTTAACTCCTCTCCACGTGAACATCACAGC
EMPV1_14286	CTCAGCTTTTAAGGCATGAGCCAGCTGTGGCCTCCTTTACCCGGCAAAGCAATAAATGAT
EMPV1_14289	GCACATTCTTTGTACTGGCTTCTTTCACAGCGATGAACATCCTCTCTCCTCCCGGGTTGT
EMPV1_14291	GGCAGAATTCTCTTTTGTTATAGATTTGCAAACAAGACTTTCCCAACAGACTGAAGCTTC
EMPV1_14296	ATGCCTGTGCCTCACCGACTGTGTCCCCCTGTTCCACAGCCACCTGGCCTTGTCTGTCAT
EMPV1_14297	GACTGTTTTCAAAGTTAAAGGAAATGAAAGACAAGGCTGCGACGCTTCCTCACGAGCAAA
EMPV1_14298	GAGTAAGAAGGGCGATTACCTCAAGTACCACTACAATGCCTCGCTTCTGGATGGCACCTT
EMPV1_14303	AACGTAGGCTGGTGTTGTATCATGCGCGACTAAAATTCCTCAAGCAAAGCTTTCCCAAGC
EMPV1_14304	GAACAGGGCTGACACCGTTCAGCTACCGCTCCTTCTCCAAGCTATGGAAGCACCGCAAGA
EMPV1_14307	CCAACCGTGATTTCCCCCCTTATTTACTATACCTCCACTCACAGTTTGCTCCCTCCAATC
EMPV1_14308	GTATTAACCAGGAAAGGAGAGAAAGTAACATGATCTGGTGGCTGGGAGTGGAGGGGTCAG
EMPV1_14309	TTTAGCCTTCTCACCAGGAATGCACCCCAGACAGACGAGTGTCCATCAACCTCTTCCCTT
EMPV1_14310	ACCTGGAAAAGGTCTCCGGTTACTTACATACATTCGAGCAAATGGGGTCAAGCAGGAAGA
EMPV1_14313	TGCTGTCGGTGGGTTGAACTGTGTAGACAAGCTCACTGAACAATGAATGCAACTGTGGCC
EMPV1_14316	ATAAATATCCCAGTCCTAATCATATATGCAATAGAAATGTATGGATATTAATACAATGGA
EMPV1_14317	GTGTCTGGGTTCGAGACCCTAGGATTCAGAAGGAGGACTTCTGGCATTCTTATATTGACT
EMPV1_14320	CAAGAGGAAGCAAGGCAACAGCAGGAGAGAGCAGCAATGAGCTATGTTAAACTGCGAACT
EMPV1_14321	CACTTTACAGCATCACGCTTCTCAACCCTCATCTGACTGAAAGAACAGCCTCTTCAGGAG
EMPV1_14322	TGTACATCTCCTTCTGCATCTACCCCTGCACCTGCCTCTGCCTCTCCTTCCGCATCTACT
EMPV1_14324	GCTCTTTCAGGGCTGGCTTAAGTTTGGAGAAGAGGAAGCATACTCCTGAAGATGTCCCAG
EMPV1_14325	TCACCCACCGTTTTGGAGAACACAGTATCCCTCACCACATACACATCATTGTGGCCAACC
EMPV1_14326	TTGGGTGCATGCACATACACACCACAAACAGAATCCTTCCCTTCCTGGCTTGCTCAGCCT
EMPV1_14327	GCTGGGGGCCCGGACTCTTCTTGCTCTACTTCTGGACATCTCTGTGTTGTAATTATGTAC
EMPV1_14329	GAAGAGAGCTGGCATTAGCATTTGGAAGCAAAGCCCCCAGACAGGAGTAAGTTGGAGTCA

EMPV1_14330	CCGCAGGCCGTTTATTGTAAGAACGTCTTTGACATTGAGCAGTTCTCCTCGGTGAAAGGC
EMPV1_14332	TCTGCTGGTTTTGCCTGTTGATTTTGCCCTCCTAAGCAAGCCTGTCCACACACAAGTCCG
EMPV1_14333	CTAGAAAGAAGTAAAAATCCACTCTGTTATTGCTGTGCATGCTTGCATTCTCACCATGGG
EMPV1_14334	GATGGAAGCAGAAACCAAAACTCTTCCCCTGGAGAACGCATCCATCCTTTCAGAGGGTTC
EMPV1_14335	AGAGTCTGCAGATCTCCACCAACCTGACACTGCATCTGCTAGAGCTGCTAGCCTCTGCCC
EMPV1_14337	GCCAGAGACTGTCGTGAACACTTTGCTTGAGATCCGTGCCCTGTAAAATGAATATGATGT
EMPV1_14338	GTCTTACTCAGGCCTCAATTCCCGTCTCAAGGCCCCACCTTCCACCTACACTGGACTTTT
EMPV1_14339	CCTCAGTATGGACAAGAGGATTAAGGGGAGGGATGTTTGGGCTGGTATCATTCTCATCAG
EMPV1_14340	TGGAGCCCCCTGCCTGAGATACTCCGGCTCACATGGATAACCTCTAACAACAAATCCTAA
EMPV1_14342	CTCTAACTATGATTTCATTGTTTTTCCTGGTCATCTATATGGGATCCTTTATGCTGATGC
EMPV1_14343	AATCCCAAGCAGCACGTCCCGCTGGCGGAGTTCGCGGCCTACCTGAAGAGCATGGTGCAG
EMPV1_14344	GCCACTACCGCCAACAGCAGGTCTTTTCACTGTCTCTGCAGTTTTGTCTTTTCCAGAACG
EMPV1_14345	AAGCTGGAGGAATTGATCTTTTTGTTGGAGGAATTGGTCCAGATGGTCATATTGCTTTCA
EMPV1_14346	CCCGATTTCCTGAAGCAAGGCTTGAGCTCGCCAGGAAGTACGGTTATGTTTTGCCTGATA
EMPV1_14348	AACTCTAGTGTGGAAACCCAGACGGAGATGCTTGGATCCAGTCCGGAGCAGGAGTGTCAA
EMPV1_14349	CGATCTACATCGTCATGGAGCTCGTGCAGGGGGGCGACTTCCTGACCTTCCTTCGGACAG
EMPV1_14350	GAGGGCCCCAGAGGGTAAACACTTTGAGGCAAGCAGGAGGCTCTGCCTAGTATGTATTTA
EMPV1_14352	TGTAAGAAATGGGCCTCCACAGCACCAAAAGCCACCTTCCCCAGAGAGCAAACCCATTCC
EMPV1_14353	CGTGACTGGTTACGTGTCGTTGCTAACAGCAAAGAGAGCTATGAATTACGCTACTTCCAG
EMPV1_14355	AGTGCCAGGTGCCAGCAACAGAGGGGGCTGGCTCATTTTACCCTAAATCTTAGCTGATAG
EMPV1_14356	AATGAATCACAGAGGAATCAGAGTGATGCAGGCAGACAAGCTGGAGGAGGCTGCAGTGGT
EMPV1_14358	CAGCAGGGTTTGGAAGGTGCGTTCCCGGTAGGTCTGGTTGGTGACATAAGGCAGGTAGAC
EMPV1_14360	TTGGTACCTTGTTGGCATAGTGAGCTGGGGAGAAGAATGTGGTGAAGTCAATAAGCCGGG
EMPV1_14361	GCCTCCCAGGAGAAGCTAGTTCAGACACACTCATTAGCATCTTTGGTTACAAGGGCAATA
EMPV1_14362	GCTTTGTGTAGCCAAGGTCCTGCTTATATCTGCAACATCACAGCTCTGCAGCCTTATACC
EMPV1_14365	GGAGCAGGCTTTGCAGGATCTACAACAGCAAAGACAACTGAACACGGATTTAGAGCTTAG
EMPV1_14366	GTTGAAGAGCCATGGACAAGACTACCTTGTGGGCAACAAGCTGAGCAGGGCTGACATCCA
EMPV1_14370	AACATGGAGCAAATCAAACTCTTAGGAGCCTCAAATCTCAAAATCATCTATTTACTATAG
EMPV1_14371	ACATGTAAGAGGGAGTTCCTGTCGTGGCTCAGTGGTAGCGAACCTGACTAATGTCCATGA
EMPV1_14372	AAGGACATCGATAAAGTATGGAATAACCTAATCGGCTTCATGTCACTCGCCACACTCACG
EMPV1_14374	GGTCAGTCATATGGGCTGGAAGATGGGTCCTGCAGTTATAAAGACTTCAGTGAATCGAGG
EMPV1_14375	GCAGTTCTCAGAGGGCAGTGCTTTCAGTGGTGTTCTAGTGCCATCCCTAAATAGGTGCTG
EMPV1_14377	GGCCTGTAGAGTGCTCAGCCTTCCTCACGATGCGTTTGTAACTGACTACGGTGTTTTCAA
EMPV1_14378	AAAAAGTGCAACCCGTACGTAAAGACCTACCTGCTGCCTGACAGGTCGTCCCAGGGGAAA

EMPV1_14386	CAGGGACAGGGGAATCATGGTTGTTGGAAACGGTAAAGAAGAGTATGAAAGCACCAGCCT
EMPV1_14391	CCACAATAGGAGCATATAGAACCTGAGACCAAGAGATCTGCTCTTCACGGCACCTGCTTA
EMPV1_14392	AAGCTTGGATTTAATGATGGAGGACAAAAGGGGCAGAGGGGGTGGGCCTGAGGTTAGAAG
EMPV1_14393	CAATTTATAATAAAACGTTTTGATGGAAAGAAAATTGCCCAGGAACGAGAAAAATTTGCT
EMPV1_14394	GCCAAGTGTATCTGTGGACTATAACACCTCTGACCCCCTCATCCGCTGGGATTCCTATGA
EMPV1_14395	GAAGAAGCGCTCAGGTGAAAAAGGAGGGAGCTACACTCAGGCTGCAGGCAGTGACAGTGC
EMPV1_14396	GAGAGGGGCTATGTTCCTTACTAGTAAGTGTATCTTGGATTTTCTCCTTGGCCAATGCCC
EMPV1_14397	TGTGAGCACCACTGTCTTCCTCTTGTTACCTTTCTTTGGTATTGCATGTTCCTATGGTCA
EMPV1_14398	TTGGCAAACTCTTTATGGCGAAGCTGTTCCACAGTCGTGCCTCCAACCTCTGGGGTGAAC
EMPV1_14401	GTGTTGTCATCATGTTTATGAAGCAGGGTGTGTTGGGGTTACTCTGTCATTCCCCCCAGC
EMPV1_14405	GCCCTTGACCCTGCCCTTGCCCTTGCCCCTCAGGCCTTCTGGAAAATAAATCCTCTACAA
EMPV1_14406	TTAATAGTGTGCATTGAACGAGCCACCCGCTTGGTGAAGTCCCAGCAGAGCGCAGGCAAA
EMPV1_14407	CTTGAACCATAAGTGTGAGCAACGACGCCGAGGAGCAGGCTACCGCTGTCACCGAAAGAA
EMPV1_14409	GGTAAAGTGCAAGTTAAGAGTTCAGACATACAAGTTGGAGACCTCATCATAGTGGAAAAG
EMPV1_14411	GGGTCAGATGGCCTTCCTCTTGATGTATACCCAGAAAAATCTGGACATCAGTGTTTTGGC
EMPV1_14412	TTTGGAGGTGGTGGAGGCAACTTTGGCCGTGGCGGAAACTTTGGTGGAAGAGGAGGTTAT
EMPV1_14414	GCTTCTCAAAAGGAAACATATCTCTTCTTTCCCTCTTTAGAGGCACTTGTTGAACCAAGA
EMPV1_14416	CTCCTCAGCCACGTATCTCAATATCGTATTTGTCATCGTATCTCTATGGTGGGGGCGGAC
EMPV1_14417	AGGAGGCAGCGCCGCTTCGGCAGAGGGTCTGAACGCCGGAGGCCTCTGGGATGGTGTGGG
EMPV1_14418	TTGGGAAAAGAAGGATTATGGACCAGCCAAGGGAGACAGGAAGAAGAACCCTTATGCCCC
EMPV1_14419	CCACATCTGTGACTTGGGCCATCGCTTTTGGCAACGCTGGATCCTTAATCCACTGATCGA
EMPV1_14420	CAGAGGTATAAGACTATAGGCCAGTTTTCCTCACTGGTCCTTACGAAGGCATTGAAGCGG
EMPV1_14424	AAAATTAAACAGCAAAGAGAGAGGCCAATGGGACAAATGCGAGGGCGGGGTCCTTGACAA
EMPV1_14426	CTTTCCCTCAGCACCTCAATATCATCACCCCGCCTCCCCCACCAGATGAAGTGTTTGATA
EMPV1_14431	TCTCAGACTTGTCCACACTGAACCCCCAGCAGTTTGTCAAGAGTTCAGGTTTTACTGCCC
EMPV1_14432	CCTGAGGCAGTGACGTGATGTCACAACGTGAAATGTCACATCCACGCTCCACACCTTAAT
EMPV1_14433	TGTGAGCTGTAGGTTACAGATAAGGCTCGGTTTCATGTTGCTGTGGCAGTTGTATAGACC
EMPV1_14434	GGACATTCTATTTCTAAACCAGATGTGATCTCCTTCTTGGAGCAGGGGAAGGAGCCCTGG
EMPV1_14435	CAGCTGCCCAATTACTTTTGTCTCTTTGTATAAGGAATCACCCATCTGGCCCCCAGGAGG
EMPV1_14436	AATGTCCCCCAGTCTAGGTCGTCTGCTGTTTCTCTCAGGATCACCTCAGAGGGACTTTAT
EMPV1_14437	GCATCTTGAAAAACAAAGTCACCTACCCGCCGCCACTGACCGAGAAGACGCTCAAGTCCC
EMPV1_14438	AAGGTTAGAGAAGGTGGTGGCCAAGCAGCATGGAAAGGTGGTGATGGCCAAGGTGGATAT
EMPV1_14439	GAGCAGCAGCTTCCAGGCGCACTTCCTCCTGAATGTACCCCAAACATAGATGGACCAAAT
EMPV1_14440	CACTGGTGCTGTACAAAATGGATGTTAAACTAGGTGTCAATTAAATCGTATGTTTGGCTC

EMPV1_14442	CAGATGTGACTGGGATGTTTCTGGTCCTCCAGCCCAAGGTCTTTTGTTGGCTTTAAGGAC
EMPV1_14443	CCGATCTCTGGTTATTTTCCCAGGAGAGATTCCTAGAAGTGGCATTGCAGGGTCACAAGC
EMPV1_14445	ATTCTTGATCTTGGCTGGGGATGTGAGAGCAAAAGGGATGCATTCAACGTCCGGGTGGTG
EMPV1_14447	CTGAATCACCCAGTAGGCTGGGGGGTCCTTATGTTCCAACTCAAGAACTCAAGTTCACTG
EMPV1_14450	TCCAACTTCAACGCTGATTATGGTCGCTTCTCAACAAGTGTGGAGGGCGTGTTTGCTGCA
EMPV1_14451	TCTGTTCACCTGATATCCTTTTTCCGTGTGCTTTCCTTTCCAGGGGCCAAAGACATGTGG
EMPV1_14452	ACTTTGCTATCTAAGGACAGTTGGGGCTGCAGGGATGTGGGGTGTGGGTGCTAGGAACTA
EMPV1_14453	TCTCTGAGGTTTGTAGGAGTCTGTTTAGGGACCGCGATCCTTGGCCCCAGCACACTTTGA
EMPV1_14454	GGTTCTGGTCTCTGTCATAGGGCAAGTGGAGAAGGGCAAAGGAAATTGGACTCATGGTTG
EMPV1_14455	CAGGGAGCCAGTTCCTCTATTCGCAGCCTAAACTTCCCTAACTGGGAGGATCCCTGTAGA
EMPV1_14457	GGTTAATCCCTAAGAACTACAGGGGCATCTGTTCAAGTCAAACAAAATGACTCAAAGCCC
EMPV1_14458	GCAGGTGATACCAGGTCTGGAGCAGAGCCTTCTAGACATGTGTGTGGGAGAGAAGCGAAG
EMPV1_14461	TTGTCAAGCTTCCCATGTGACAGGCACGGCATTGTGTGTGGGAACAGAGGGACTCTAAGA
EMPV1_14464	CTGCAGCAGAAGTGGCCCCGCTTCATCCGGCTCTCCGCCTTCGCCATCCTGGTCTTCAGA
EMPV1_14466	TGGTTGCACCAGTTTATGTTCCTGCCAACAATATACAAAGGTTCCCTTTCCTCCACACTC
EMPV1_14467	ATATTTGTTATTCATAAATCTTTTTTTTTTACTTAAAATACACATCCTATTCTTATATAT
EMPV1_14471	ATCACTGCTATCAGCGTCACACTGTCCATACTGTGCCATTCTACAGACCTCGGCCACTTA
EMPV1_14474	CACCTTCCTGGACCAACTCATCAGAGGCATCAACTACCTCGACAGATCCACCAATGCCTT
EMPV1_14477	TACCACCTCTGATGCCTGGTATGCCTCCAGGTATGCCCCCACCTGTTCCACGTCCTGGAA
EMPV1_14478	TGTTTATCCTAAAATATTAACAGATAGGTGTAGGTGAAGAGTAACATTGGAAGCAGTAGC
EMPV1_14480	TTCATTGATTTCCTCACTCATTCATACATTCACTCATTTCCCTTAGCAGTCATAATTCAT
EMPV1_14481	TCATCCCAGAGCCCATATTTCAGCGTTGCTACCTTTCCACTCAGGAATGCTCTCTCTTCC
EMPV1_14482	CTGCAGGGGCTATGCCTGGATCGCCCCCGGCATGAGGAACCTGCAGTTCTGTGTCTACTG
EMPV1_14483	CACACTCTCAGGCCTCATCCTGTTGGCTGGTTACATTGCCTTTGAGAGCTTCACCTCCAA
EMPV1_14484	TATGGGCTGGAAAGAGGGCAGTGGCCTGGGCCGCAAAAAACAGGGCATCGTGACTCCCAT
EMPV1_14485	CTTACCGTGAAAAAATAGAGAAGGAGTTGGAGGCCGTATGTCAGGATGTGCTGAGCCTGC
EMPV1_14489	ACAAATGCCAGAACCCCAATGTCCAGTCGTGGCCACAAGGCGTGGTGTCAGCGTGTGTGA
EMPV1_14490	GTCACTGGAACATTTGATCATCTTTCCTTTGGCAATTCCAGCCTTCTCTGAAAGGTCAGT
EMPV1_14492	CTGCTGATGCTGCTAATGGAGCAAATAACCCAAATTATAGTAGCATGATTGGCTTGGTGG
EMPV1_14493	AATGTAGCTTAAATTATCAAAGCAAGGCACTGAAAATGCCTAGATGAGCCTCACAGCTCC
EMPV1_14495	TGATGACAGATACAACCAGTTTCATATAAGGAGATTCGAAGCATTACTTCAAACTGGAAA
EMPV1_14496	AGTTTAAAAGCTGCCCAACTGGTGGATATTGAGCTCTCCCCTGTCAGTGCTCTGAGAATG
EMPV1_14497	ACTTGCTGTTCCGGTCCTGGTTGTACCCACCTTTGACCAGTCACATAGCTACTCAAGAGA
EMPV1_14498	CTTTTAATTGCTTGTGATGGGACTGATCTTTGTGGGTCCCTGGAATGAGAGAGTGGCTAG

EMPV1_14499	GAAGTGGAGTTCTTGTCCACGTCCATTGCCGAGCTCAAGGTGGTACAGACCAAGTATGTG
EMPV1_14500	TCAGCACGAGGAGAAGTCAGGCCAGATGAGATCGAGGACGTTGGGAAGTGTGACTGCAAA
EMPV1_14503	ACTACTGATTTTGGAGCCTGGTCATTGCATGTCTTCTGGACAGGTTCTGGACCATCCCTG
EMPV1_14504	TGCCCCCCACTATGAAGTGTTTGTGGCACTGAAGGGGCTGAGGAACTTGTCAGAGGAAAA
EMPV1_14505	ATTTGGGTGGTGTAGGCGTCCCTGTTACCTGCGACAAAGCAATGTTCCCAAAGCACCGTT
EMPV1_14506	TCTACACCTACGGCTTCCTAAACGCGGTGATACAGACAGTTCTGACCTTCCAGCTGTCCT
EMPV1_14507	CATTCAGAAAGTGTTCACCCACGAGGCTGACCTGTCAGGAGTCACAGGGGACAACAAACT
EMPV1_14508	GTTCAAGGGAAGAAGGCAAATCTCCCCGTTTCACAGTCCTCAGAATCTCTCACTTCACCC
EMPV1_14509	CCAGCAGGAGGCCATCTCAGCACCATCTCTTTCCTGCTTTCAGGGATCCAGCCATCTCTA
EMPV1_14511	GTTAAGGATCTGGTGTTGCTGGGGCTGTGGTGTAGGCCTGTGGCTGTAGCTCTGATTTAA
EMPV1_14512	TGTGGCGGAATCTGTCCCGCATGCAGAGCCGCTTTGGCAAGAAGGAGTTCAGTTTCTTCC
EMPV1_14513	ACTTTGCCATCCTGCTCCGGGCCGGCTTTGACCGATGGAGCGCAGCCAAGCTGCAGCTCT
EMPV1_14515	TGTGGCCGTCAAAACCCAGCACTGTGAACAACCCCATTTGGGGCATTGTTAGGCAGTTCT
EMPV1_14517	TAAAGGAGGATTTGAACAGAAAATGAGTAGGCGAGAAGCTAGTCTTATTTTGGGTGTAAG
EMPV1_14518	ACCCCCCCCAAAGTGATCAAATCAGGAGATCTAATAGCAAGGGTTCATCACTCTTCAACC
EMPV1_14524	ATAGCATGTGCCTTACAGTGATTGTCCCACTGCTGTGGTCAGACTGTTCTCTTCCTGGAG
EMPV1_14525	TCTGCTCTGTGCTTCGAACCCTCCCCGCTCGGTGCCTCATGCCCTCCGTTTGATTCTCGG
EMPV1_14527	AACAAGAGGTTCTACCCGATGGTGTGCTCCACGGCGGCCAAGAGCAGCATGAAAGTGATC
EMPV1_14528	CCTCAGAGGAACAGGTCACTGCTAATTCTCACCTGGGTGGCATTTCTCAAAGGGAATTTT
EMPV1_14529	GGATTGGTTGGGAGCAGGAGCGCCATTACAAGGACTCTGAACTTGGGGACGACATATACT
EMPV1_14531	TGTGTGTGTGTGTGTGTGTGCGCGGGCATGCGCGCGCATGCACAGCACACCCATGCACAG
EMPV1_14532	CCGCCAGTAGGGGACAGCGGCGAGGATCTTAATGATTCAACTGCTTGCCTTGCTGTTTTT
EMPV1_14533	CTAGTCCTCTCCTGCTCTGAAACCTTTTTCCTACAAGTCATGAAATTTGCGATAGCAGTG
EMPV1_14534	GTGGAGTAGGGCGGTGGCCACAGCTCTCATTAGGCCCGTGTGACCTGGGAATGTCCAAAT
EMPV1_14535	GGGACACCAAGTTTGGATCTGGAACGAGTGCTCTGTCAGTTTATTAATTCTTCTCCCTGC
EMPV1_14537	GCTCTGACCTGGGGGTCACCTTGAGGTGCTGGGCTCTGGGCTTCCACCCTAAGGAGATCT
EMPV1_14538	CAGGTGCTTCCATAGGATAGACATAGGTGAGGATCACACAGAAACTGGCCAAGACCAGGA
EMPV1_14540	GCCAGGAAAATCTGAGAAAGCAAAAGAAGTGGGAAATCTGTGAAGCTGGAAAAGTTACCC
EMPV1_14543	GGGAGCCAGTTGGTTCTCTTAAGTAGTCTTGTGGGTTTTCTGTTTGGAAGGAGTCACAGC
EMPV1_14545	TGACCCATCCCTGCCCGGTCATGTACCTCGGTCACAATATCCTTGTCTACAAAGTGGAGA
EMPV1_14546	GGCGCATTTAGCCCGTTCCAGACCTGCGACCTTCAGTTTGTCAGTTGCTGTGTAATAAAG
EMPV1_14548	AGGGCCAGTACTCAGAGGATGAGGACACAGACACAGACGAGTACAAGGAAGCCAAGGCCA
EMPV1_14549	ACTCAATTCTCCAGTTTCCCCTGCTTCTCTGATCATGCTGTTCTTCTAGTCTTTCCCTGT
EMPV1_14550	GTGCCCCCACCTTCCCTTTGTTCCATCCTCGATTTAAGACCCATTTGTTCTCACTGGCTT

EMPV1_14551	GGAGGCAGGAAACACCTCCAGGAAGCAGTGCCGCCGCCCCAGGTGGTAAGACGCCTTTTA
EMPV1_14552	CCTTCAGGAATTAGCGACAATTTCAGGTGTCTTCTCTCTTTGACTTCCAGCTCATGCTGG
EMPV1_14553	GCGATTTCCTTTCAGCTCTGTAGGGCACTGGTGGGGCCAAATAAAAAGCCTTTCTTTTTT
EMPV1_14554	TTTATGACCGGAGTCGACCTGACCGGAAGTGGAGATTTCACCCGGGCCATAGGCCATTGA
EMPV1_14555	GACAGCATGCCTTCGCTGCGATGCCTGTATAACCCAGGGACTGGCGCACTCACAGCTTTC
EMPV1_14556	TGATGCCAAGGGAGGAGGGACAGGCAAAGACAAGGAGTCAGGGAAGGCTTTTTACAGGAA
EMPV1_14557	CACTTGGTATCTCTTGTCTCCTGGATGGTAGCCATTCTAACAGAAGCAAGGTGAGAGCTC
EMPV1_14558	GAGAGAAGTATTAAGAGAGGTATCAGTTTCCACATACTAGAATGTATACTTGTACCTGAG
EMPV1_14559	CTAGCAAACTCTGTTTCCAGATGAGTCTGTGAGGTCGTGTTACAGTATAGCAAGGCCAGC
EMPV1_14561	ACAGCGAATGTTTGAAGTGTGCCCTGAACTTTGAGCGATCCGTGAGCTGCGCTTCCTGAA
EMPV1_14562	GACTATGAATGGTGTGAAGATATTTCATCAAGAGGTGTGGCTACTCCACAAACACCACAG
EMPV1_14563	CTAGGGATTATTTGCTACCTGCTGCATGGTAAACAATATCTTTCCTATCCCCCTACTCCC
EMPV1_14564	GGTGACAAAGGCCTGAGTGGAGGTTCAGGAAAGAATGAGGAATTAGAGACAGCAATAGGC
EMPV1_14565	AGCCTCCGAGAGCCCCATGCCCACCCTGGCCAGATCCACCTCCAGTGACACCAGTGAGGA
EMPV1_14566	GCAGGATTTTGGTGTGCCTGTTGTGTTTGAAGACAGGTGGATTGACAGGGAGCCTGGGAA
EMPV1_14567	ACTCCTCATTTACAAACTTCTTGAGGACGCCATTCGTTCTGCTGGAAGCCTAGGGCCCAG
EMPV1_14568	AGAGGAAGACCAGGCTCCAGAACAGGAAGAAGAGGAAGACCAGGCTCCAGAGCAGGAAGA
EMPV1_14571	TATGACTGCAAAGCTCTGTGGAATGCTGGTGTCTCTGTGCTGGCTCATTGGATTCCTTGG
EMPV1_14572	GGAAGGCTTTCTATTTCTTGCTGGCTGCCTTTTTCTTTCTCCTTCCATCTCATGCCAGAG
EMPV1_14574	AGAGGAACAGCAAGAAAACCATCACAAGAAACTTTTGCCCTTATCAGGAACACTTGAGGC
EMPV1_14575	CCTGGCTGCACTCTTTGCATTCACAATGTGGGTTATGCTTCAGACCCTGAACGATGAGGT
EMPV1_14577	AAGATGCGGCTTGGATCTGCCATTGCTGTGGCTGCAGTGTAGACCGGTGGCTGTGGTGTA
EMPV1_14579	ATTGCCTTTGGAGAGCAGCTTTGATTCTGCCACTCTCACAGAGGAGGAAGTGTCCCTTCT
EMPV1_14580	TGTCTTTCCCCAAAAGAGGGGGAACAGGTTACAGCCACACCTCAGATCACCACATCAGAA
EMPV1_14581	AAAGCATCGTGGGAGCCTTAGCGTGGGTTGCATAGAACATCAAAGAGGCTGCATTGTCAG
EMPV1_14583	GAGGTGGAGGTATGGATTGGAATGACTGTTGAGGAGTTGGCCAGAGCAATGGAGAAAGAC
EMPV1_14586	TGTGTGCTTTTCTACCAGGTTCCACCTCCACCGTCAGCCTAGAAAATGTGCCTTCAGGGA
EMPV1_14587	GACATGAGGGCTGCTTTTGCTTTGGATGCTTGTGGTCTCTTTCCTCAGTGTGTGCAGGCT
EMPV1_14591	GACACCTACCTGAAGGAATTGAATGAGGACTTAAAGCTAAGGAAGCAGGAACTTCTAGAG
EMPV1_14592	CTTTCTGCTGGGGCTACTGAGCTGTGCACTATAGAATATAAACACAGAGCTGGCAGGAGC
EMPV1_14593	GTGTAGTGCATGGTTAATTCAAGCTTCTGTACACTACAGTATATTCCATTTTCGTTCAGT
EMPV1_14596	TCAAGTTGTGTTGTCATGTTGTATTGTTTTTTCCAAGTGCTGCCAGTTTAAAAGGGAAGC
EMPV1_14597	GAAACAAGCTCTGTGTATTTTCTGTGTACCATATCCTTGGGAGGTAATCAGTCCCCTTCC
EMPV1_14598	CCAGGGTCAGAGGGGCAGGTGCACAGAAGTGTGTCCAGAGACGACCAATGAGAGGGAAGA

EMPV1_14599 GCTTCGGAACGACCCCGTTGGCCATGCTGGCGGCGACCTGCAACAAGATCGGCAACACGA
EMPV1_14600 GTACAGCACAGGCAGCGACAGTGCGAGCTTTCCCCACACCACCCCGTCCATGTGCCTCAA
EMPV1_14603 CCCTCATCTATGACAACGAAGGGGACCAAGTCAAGAACGGCACCAACATGAAGTCTCTGA
EMPV1_14604 GCTGTGATGCTGCTCCCATCCTGAAGATTACTTGCTCAGACACAGCCTTCATAGAGCGAT
EMPV1_14605 CACTCTTATCATGAATCCTCGGGTCTGTGCTCAAATGGCTGTCACTGTCTGGATCACTGG
EMPV1_14606 CAGCCAACAATGGATCATAAATGTAGTATACAATCTGTGGTTGATTGGATCCATGGATGT
EMPV1_14610 TGGTTCATGGAATGACTCCTCAGTTTACACCGTTGCCTCCCCCAGCAAAAAGATTAGTGC
EMPV1_14611 CGACGTGAACTACGAGGAGCTGGCCCGCTGTACAGATGACTTCAACGGGGCCCAGTGCAA
EMPV1_14612 CTGATCCCTTTGCAGCAACCACAGCGGGAACTCTACCCATGACTTGTTTTTAACCCAGGA
EMPV1_14613 CACGAGAGACCACTGTGCACTTGTGACAGATTGATAACTGAAAGGTCTGGGAGCCGCTCA
EMPV1_14614 TTTGCAAACTAACTTCCAGTTCTATTCAGCCAATGGAAGATCTCAGTCAATGACTGGGGG
EMPV1_14618 GGAAATCTTGGGTGGTCTGACCTGATATTTGAGTTTGGTGGCCACAGATATTTTAGTCCC
EMPV1_14622 TCAGCTTCGACACGGACGGGAAGTAGTAGAAGTCCTGCTTGATGAGGTCGGCCATGAACT
EMPV1_14624 TCGTGAATGGACCTATTGAAGCCATCATCTTCCGTAAGGATCAACAATATTATAAGGGCC
EMPV1_14625 GCCAAATGCCAACAGGATATTCTGACAGCGTAATAATGGTGGGGAGAAGAAATGCATGTG
EMPV1_14626 TCCCACGGCGTCAGAGCCCACCAGACGGTCAACAAAATAATTCGTACTACTCTTCCATAG
EMPV1_14628 AGAAATAAGGCGAAGGCCAGGCTGCCCGGCGTCTTTGCTGAGGGTCACATTGAGCTTCCA
EMPV1_14629 GAAACACTTGGAGAGCACTGAGTTTAGTAATAGGTGACTGCCGGAAAAAAGGGAACTTTG
EMPV1_14630 GGGGATATGCCGGAGTATAACTGTTGTCTCACCTTTAATCTTCTTGCTGAGGCGACTCCC
EMPV1_14632 GGGGCCAGTAGCTCTTTGTAGCTCACTCCCCACCTGTGATTCCAGTCTTTGTGCATTTGT
EMPV1_14634 CGGAAGAGAGAGCTTCTATTGATACAGTATGCTCTGAAGGAGAGAACAGCAATTCAGCTG
EMPV1_14635 TCAATCTAACATTTGGAGCTTCCACAGGCCCTGGCTACTGGGACCAACACTACCTCAGCT
EMPV1_14636 GTGAACAAGGAGGAAGAGGCGGATGAGAACGAAACCGAGTGGCTGATGGAGGAGATCCAG
EMPV1_14637 CGTGTGCCTTCTTGTGGGAGACTGAGATCTTCAGTGTTTTGCGCTCTAGGTCCTGTTTTC
EMPV1_14638 TTGCCAGATCGAGAGCTAGAGCGAGAGGCTGTTGTGAGACGACGGCTCCCCAAACCGACC
EMPV1_14640 AACCATTTGGACTAAAGCTTAGAGGCAGAAACTGTGGGCACCAAGAAACAAGCCAAGGAC
EMPV1_14641 AGGAGCTACAGAACGAAGTAGAGAAGCAGATGAATATGAGTCCACCACCAGGCAATGCTG
EMPV1_14642 GCGCTTTTCAACGTCTTAAGCCCCTTTTCTGTTTCTTCCCTCCCGATAGCCTTTCCAAAT
EMPV1_14643 CTAGGACACATGCTTCTGCTGGTTGTTAATATGTGATCCAAAGTCCTCTCACTTGTGGCC
EMPV1_14646 GGCTAGGGATCAAATAGGAGCTGCGGCTGCCAGCCTACTCCACAGTCACAGCAACACCAA
EMPV1_14649 AACCTTACGCATGGGAGATGCATGCCTTACAGGGAAGAACCCAGAAGGACAAATCTGCAG
EMPV1_14653 CTCCTGCAGCCTGAAAGTTTTTGAAAGAAAAAAGATATCAACTGCCTGCTGCTACCACCC
EMPV1_14654 GCCTAGTCCAGAGCAAGACCCTAACTCTCTTCAATGTTATGAATGCTGAGCGAGGTGAGG
EMPV1_14656 GCTAATGGAGCATCAACTTTATCAAAACTGCCTACACCCACATCTTCTGTCCCTGCACAG

EMPV1_14657 AGAAACAAGTGCTCTAGCTCCATAAAGTAGCCAGGGTAAGGCGGCATCTGCTACCAGGTA
EMPV1_14658 AGAAGAGGCTGACGCAAGACCATCAAGCCCCAGCTGTCCTTCCTAAGTTCTCAATAAGCT
EMPV1_14659 TCCCCTGCACCACAACGAGAACTCCCAATGGTGATAGTTTTGATAACAGAGTGTAGTATA
EMPV1_14660 AGTCCCTCGAGCCAGTGCCAGGAAGATAACATGTGGAACCCTCCTTTGGCCATATGTACA
EMPV1_14663 GGGAATGCCCATAAAGCTATCAGCCGATTTCTCTACAGAAAACTACAGGTCAGAAGGGAG
EMPV1_14664 GGGCTTGAAGCCACTTTGATCCCTGTTCTGTCCTTTGAGTTTCTGTCTCTTCCCAGTTTC
EMPV1_14665 AAGTCGGCCCTGGTGCACCGGTACCTGACCGGCACATACGTCCAGGAAGAATCGCCCGAA
EMPV1_14666 GATTGAGTTCAAGGAGACCCAAGCACTAGTATTGGCCCCCACCAGAGAACTGGCTCAACA
EMPV1_14667 TTGGGGGCTTCGTCCTGGGTCTGCTCTTTGTTGCTGTGGGGCTGTTCATCTACTTCAAGA
EMPV1_14669 ACAAGTCTATTGAGATAGGAAGACAGTAGGTGTGTGGGGACACCACGTAAGAGCCACAGG
EMPV1_14674 ACCAAAGTGGAGCCGTACGAAGTGCATAACCCTCTCCCACTCATCGTGGGCAGCTCGATA
EMPV1_14675 GGGAGTGATATAGGAGAAATAAGATATAGCTCTTGAGTAACCTTGGTAGAAACAGACTGG
EMPV1_14676 CAGTCCCGCGTGGCCGCAGCCCATGGCTTGATCCTGAAGTCAGTGGTTTATGGGGACCTT
EMPV1_14677 ACCCCCGTCTCTGTACCCTGATGGCTTGTGCTTCATGGATCATTGGTTTTGCTGACTCCT
EMPV1_14681 GGACCCACATCATCACAGTGACCAAGGCCGTCCCAGGCACCCTTACTTGGATTCTGTGCA
EMPV1_14683 GATGTCAGAGGCAGAGTGAGCATGCCAGTGAGATGAGCAACAGTGAGAAGCATGTGACTT
EMPV1_14684 ATCCACAGGAAAGGGGTAGGGGTGGTTCTGATAGTGGTTCTCCGGCTGTGGCTGTGAGAG
EMPV1_14686 GTATCATTCCAAGTGTTTGTCTGATGAGTACAGGCTTAGAGACAGCAGTAGTCACTGGCA
EMPV1_14687 TGGTCTTCCGTGGGTGTGGGTGTGTCTCAGCTTCTTGTATGGACACCCATCATATTGGAT
EMPV1_14688 TGTGCTGGCCGGGGATGGAACTTGTGTTCTGGTGTGCAGAGACGCCACGGATTCTGTTGA
EMPV1_14690 CAGCATGTATGAAAACTAGTGGCCTAAGGAAAGCTTATACCTGTTTGCCCAGAGGCTTGA
EMPV1_14692 TGCGCCACAAGGGAACCTCTCACCCTTCCTTTTGACTGGCACAACTTTATTGAAAGGATG
EMPV1_14693 TGCAGTTTTTAGTGCAACATCGCACCCTACTGGGAGATGACAGAACTGCAGGTCCTTGGA
EMPV1_14694 GATGAGATTACTGTCAAGTGGGAAGAGGGCAAACAGAAGAGAATCCCCCAGGAACTGTGG
EMPV1_14697 TTGTTGTTAGCCATTTGGGAGGGGCAGGCTGAGGCAAATTCCTATTGAGCTTTGGCTTCT
EMPV1_14698 GGGAACTCCCACATGCCCCAGGTGTGGCCAAAAAGCGAACAAACAAAAACCACAACTTCT
EMPV1_14699 TTTGAGTAGGCTAGGTTGCTCCTAATTGCTCCTTCCCATTACCGATTTCCTCACCTGCCT
EMPV1_14700 TCCATTGACGAGCAACTGTAGGGTTATTCTTGCTGACTGGGTTTGGGGAAGGTATCAGCG
EMPV1_14702 GTGTGGTTCCTGGAGGGGGATTAAAGGCCACCATGAAAGATGATCTCGCAGATTATGGTG
EMPV1_14705 CGGCCCCACCCTCACTTACAATATGTGGATGCCTTGGGCTATGTCAGCCTTTTTCCCTTT
EMPV1_14707 GAGGAAGAGACATCCCCTGCTCTGATGATTACTCGCTTATGAATATCTTGGGAGAAGCAG
EMPV1_14709 GAAGAGAGGTGCAGTCGATTATCATTCACGAAAACTATGCTGCCCATAAGCATGACGACG
EMPV1_14711 GTGTAGATAGCTAGTTGTCCCAGAACACCATTTAATGAATTTCTTCTTCATGCCTCATTG
EMPV1_14713 ATGGGATCCTCGCGCGGAGAAACTCCCTCAACATCCATCTCCATCTTCCCATTCACTGGA

EMPV1_14714	CACCTGATGCTCCATGAAACAACCTAGGAAACAGCCTGCAACAGCTGCATCAGCTCAGTT
EMPV1_14715	AGATTCAGAAAAATGAAGGGCAAATGTCCTCCTCTGCGGGTCGGTATGTGTGCCTGTTGC
EMPV1_14716	CTTCAGTGCATTCAGGTTATGACACTTGGGCAGAACACCTTTGAAGAATAGGCTTTCCCC
EMPV1_14718	CTTCCAGCTCAAGTGCCCACATCATCAGGGTCTGTAATCCTGTGACCCCGGTGTTTAGAA
EMPV1_14719	GTGACAATGATAACGAGGAGGATGGTGACAATGAGGATGGTGAAGACCATGACGACCAGG
EMPV1_14722	AGACTGTCTACTTCATGGTCAATGTCACAGATGCCATCTCGTCCGGAGATGACGAGGACG
EMPV1_14723	ATCAGGTCAGAGATGATGCTGGCTGATGTGTCTTCTGCCTCATCCTCCTCGGTGGGGATT
EMPV1_14728	TGGCCGACAGCATGAGCATCCCTATGGAGAACAGCATCTCGCTTTAAAATCACCTGGAAG
EMPV1_14729	GTATAGTAAATTTGGTTCGTTATATTGTGAAGGCGCTGGAATTACATGAACATACCACCT
EMPV1_14730	CTATCCTGGAGGGCGACTTCTCCACCAAATCTGATGTCTGGGCCTTCGGTGTGCTGATGT
EMPV1_14733	GATGTTCCCACTTTCGGGCTTCTTAGAAACAACCTGTGGAGCTATCCACTGCATTTGAAC
EMPV1_14734	TTCCACATATCCACCTGCTGAGCTTCTTGAGGGGTATTCTGAGGAGGTACTGAAGGAGCA
EMPV1_14741	CTTAGCCTGCCTGAGAAGGACAGAATAGTGGGGATCCTGGAGGAAACACTCCTCAGAAGA
EMPV1_14744	TGCTGGTTGGACTTAACCCTCTTGTCACAGGTAGCTTTATTTGGAAGAGTGTCTGTACAA
EMPV1_14745	CCCAGCAAAGGAAAAACTCTTATTTTCAGATGTCAGTGAAAGCATGTGTAAGAAAACTGC
EMPV1_14746	TTCATTGACTTAGATCGTGTGGGCATCCATGGCTGGTCCTATGGAGGATACCTCTCTCTG
EMPV1_14747	CCTTTGTGGAATTTGAAGATCCTAGAGATGCAGAAGATGCAGTGCGAGGCCTGGATGGCA
EMPV1_14748	AGGATGATACTGTCCATACCCAGCTGCTCAGGGTGGTTTAAGGATTCAGAGAGTGAGTGC
EMPV1_14749	ACAGCATCTGCCTGCAGGACCCCATCTACCTCCCATGTCAATGTCTGAGCAACTGAATAT
EMPV1_14750	CTGCTCTACCTGGCTGGTGTGCTGGCAGGCTCCCTGACTGTCTCCATTACTGATATGCGG
EMPV1_14751	TCCCCAGCCCTTCAAGGCAGCATACATAGGATGACAGGATGACCTTCTAACAGTTCCAGA
EMPV1_14752	TTTGTATTCTAAAGCAGTATTTCTACTTTCTACTAAAATCAGTTTTAATTTAGCTTAATT
EMPV1_14753	TTCGAGGTTCTGCTTTGATCCTACAGCTTGCTCGAAATCCTGATAACTTGGAAGAGCTAC
EMPV1_14756	TCATGTCCCCCCAAGATGCGGCCCATTCAGGATACAGAGTTCTACATGCGGACCTTCGGT
EMPV1_14757	TTATCGGCAGGTGTCTGTCCCTGTCTGAGCACATTCCGCAGGGCTGAACGACAAAGGTCA
EMPV1_14759	TCATTGCTGAGGGCACCGTGGGGAACCAGTTTGTGCTGTTTGATGATGTCCCCCTGTACT
EMPV1_14761	AAAGTCATTAACCCAACTGAGCACAGCGATGCCGACTTTGAGTTCCGAGAGTCTTCCTCC
EMPV1_14763	TTCCCGCGTGAGGAATGTCTTCCAGCAGAATGATGTCATCTACAGGCTGAGCCCTGAAGG
EMPV1_14766	GAGAGGATGGCAATGGGAGACCCAGTCCTCATGACTAAACAGCTTTTCAATCCCTTTCTC
EMPV1_14768	GGAAGCAAGATAGGAGAAAAGTCCAGTGTGAACCCATTTAACTGTAAAACTGCACGGTGC
EMPV1_14770	TGCAAGCTCCCACTCTCTGAAGGAGAGAGACACTCTTCCATGTGAACACAAAGAGACCTG
EMPV1_14771	CTGACGGGACTCATTACTGAAGGACAACTTTTTGCTTTTTCTAATTGGTGCCATGAGGAG
EMPV1_14773	AGGGAGACAGGACATTGGAGAGAGAGGACATCAGAGAGACCCCCTTATTTGTGTCATCAG
EMPV1_14774	AGGAGAAATAATGGATCTGCAGCACGGAAGCTTGTTCCTTCAGACACCCAAAATTGTGGC

EMPV1_14775	GGGACTGAATAGTGATGATGAGTCCTCTTGGAAGGATTTGAGTGAGCTTGATGCCCAGGG
EMPV1_14776	TTCCCTAATCCTGCTTTGGAATGAGCCAGTACGGGGAAAGGCACACCATGTCGGAGAGAA
EMPV1_14777	TGGGTCTTCCTTCCAAATCACCTGGTGCAAAAGCAAGTATCCAGGAGAACTAGTGGCAGG
EMPV1_14780	CATCTGTGAAGCTGGGAGAGGAAGGCAGTAACAAAATATCTAGCACCCTACCTGGCACAC
EMPV1_14781	GTTCATGTTCCAAGCAGTAGGATGGAAGAAAGAGACAAGAAAGGGCAAAGACACTCCCAG
EMPV1_14782	CTGGAGTGCAGAGACTTCATGGTGCAAGTTCCAAACTACAGAAATGATTCGTTCTCAAAG
EMPV1_14783	ACAAGAGGAGGTATCTGCGGTTATTATTCAGCGTGCTTACAGGCGCCACCTTTTAAAGCG
EMPV1_14790	CTCAATCAAAGCATCGAATTCACAAAACCCAAAGTACTGGGAAGCAGGGTCCTATTGCCA
EMPV1_14792	TTCCAGCTGCAAGATGCGTCCTCAGGACCAGGAGCCAACAGGGGCAAAGAGCCACATGAG
EMPV1_14793	TATGAGCTGTGGTGTAGGTCACAGACTGCTTGGATCTGGTGTGGCTGTGGGTAACAGCTG
EMPV1_14794	TGCATCTCTCCAGCTCACATTCCAGTCTTCTCTCCTGCATTCCACCTCTGCAGGGTCTCA
EMPV1_14795	CAGACCATGATAAGGACGTCTCCCAACTCCTTGGTCACGATTCTCAATAATTCCCGCAGC
EMPV1_14797	CTTCTGGAGAAAATACCTGCACCTGAACTTCCACGCCCTGATGCACCTGGCCACAGAGGA
EMPV1_14800	GACACATTACCATTTCACTCAAGGCATGACCCCTGTCCACAATTCAGTTGCACCTCAGGG
EMPV1_14801	GACGTTCATTGTTCCGGCCATCAAGCCCTTCGACCACTATGACTTCTCCAGGGCCAAAAT
EMPV1_14802	TTGTCTCTCAGTGCCCTAAGCATGGACTCGTGGTGGGGTCAGCTTCTCTGGTGTGATGGA
EMPV1_14803	ACAGCTTGGTGGAAAGAGCTGATGTAAGACTCGATAACATTGGACTAGATTGTAATGCAT
EMPV1_14805	ATATATGGGTCCCTGTGCCTCTAACCCTCCGTGTGGGGTGTTTTCTTTCCTTGATGGCAG
EMPV1_14806	CAAGAAGGCTCTGGAAGGCAAGGAGGGCAGCATGGAGAATGAGATCGCTGTCCTACACAA
EMPV1_14807	CAGTCTTGGAGTCCATGTCCACATTCTTCTTGTGACAATTCCTTGTCTTCTAGCCATTGC
EMPV1_14810	GTCTTTTAGGCCTTCCATCAATACGGCACTTGCTCCTGGGCTCAGAACAGCACTCCTAAA
EMPV1_14811	TGCAGAGAGCTCCACCGTGAGCAGGAGGTGCTGTGATGGGTCAGCATTCTTTTTATGGAG
EMPV1_14812	TCATTTTCCATCTAGCCTGCGGGTCAAGTTCCCTCCAGTCGGTCTTAGCCACGGTGAAAT
EMPV1_14814	ACCACAAACCCTTCCAAACAGTTTGACAAAAATGCTTACGCCTAAGAAGAGCACTGCCGC
EMPV1_14816	GTAACTTGGATGAGGAGGATGAGAATTCTGCGCCGGCTGCTCAGAAGATACCGTGAATCT
EMPV1_14817	GGTGCCATATTGCAGAAAAGGGAGGCAAGCACAAGACTGGTCCAAATCTCTCTGGTCTGT
EMPV1_14819	CTGTGTGAGATTCCTTGCAGTTTCCTCCTGTATAAGCTGAGCACGCCCACTTTCAAAAGA
EMPV1_14820	TAGTTCGTACCTGTTGGCTTGGACGAGCCACATTGAAGGACAGAGTATTGGCGGAAGTCA
EMPV1_14825	GCTTTTTCCAAGGAGGAGTTGGTTAGTAGTGATCTTGAGGAGAGAGCTGGCAGTGGAAGT
EMPV1_14826	GATGCATAGGATCCGCGAGCGTGGTCTGTGGGGGAAGCAGTTTGACGGGCTCACTGACAA
EMPV1_14827	ATCTCCGACAGATAAGTTTCAGAGACCCTGATCCCCCTTGGGCCTCCAGTCCGTACGACA
EMPV1_14828	TCATTTTTGCTACCACCGCCGCCTCCAGTGGCCAGACCTGTGCCCCTTCCTATGCCTGAT
EMPV1_14829	TGAACAGTCCGGACGTCTAGGAGCGACGAGTGGCTACTAACTGGCCCACACTATACGAAA
EMPV1_14830	CTTACAGCTTCTGCAAAGGCCTTAAAACTAGCAAGTAGCTCCAAACCAGACTGAGGACCT

EMPV1_14832 CCAATACCAGTGTGACCTTAAAAACAGTAAAGGAAAAATTACAGTTGTTCAAGACACGCC
EMPV1_14834 GGCTTTTATCTTCCTGTCCACAAACTGATTTATAGCTATCATTTGTAGACTGTCCTGGCA
EMPV1_14835 CAAATACTCAGTATGAGGCACTTTTCCCGGGACTTCCTGGTAGATCTCTGGATCCCTCAC
EMPV1_14836 GGGGGCGTTTGCAGGCGTTAAGAGAAAGCCACGTCTTCAAATTCTTGTGGGTTCGAAACT
EMPV1_14840 ACATTCTGAAGGTGCTGGTGTCCGACCTTAACCGCTCCAATGGGGTAGCGATCCCCCCAT
EMPV1_14841 GATCTTGGATGTGCTGGATGAAAATCGCAGACCTGTGTTGCGTGGTGGGTCTGCTGCCGC
EMPV1_14846 GCGGCAAGTGAAGCGCAGAAAGTACAACGAGGACCTGGACTTCAAAGTGGTGGATGATGA
EMPV1_14848 GTTACTTCCCCAGTCCTGATGTGGGTCCAGGCTTTGGATATCATCTTGGAGAAGATGAAG
EMPV1_14851 CAGGCTAGAGTGGAAATACCCCTGTTTCCCTTCAGCATCGTGCAGTTCTCTTTTGAACCC
EMPV1_14852 TGAGTGGAAAATGGCCCGGACCGTGGCGGCTGTGGCGGCTTGGGTTGTGGTGGAGGCAGA
EMPV1_14853 CAACACGATCAGTTTCACTTGCAACCGGGGTTTTTCTCTGAAAGGAGCTAATTCGGCTAA
EMPV1_14854 GGTAGAATTCACCTGTGAAGCCTCTGGTCCTGGACTTGTGTTTGTAGAGAGTGTTTTTGT
EMPV1_14856 GGTGCTTCGAGTCCAGAAACAGCCGCACGTCCTCTACGTTGTTCTTGATGGCCGACTCCA
EMPV1_14858 ATGAGCTATCATCAAGCCAGTTCATGTAATAGTGCCAGAAGTCTGAGGTTCATGCTTCCC
EMPV1_14859 AGGTCCAGAATGACATACAAAAACTACTTTTGGGGGAAACAACTTTGTAGCAAGAAATAA
EMPV1_14864 GTGGGTATGGGTCAGAAGGATTCCTACGTGGGTGACGAGGCCCAGAGCAAGCGAGGTATT
EMPV1_14867 ATGACAGTGACAGTGAGAAAAAGAAGCCAGACGATCCAGAGGAGCCAGGACCCAAGTCTG
EMPV1_14868 GAACAGGATCTTTTGGGGATCGACCTGCAAGACCTACTCTTTTAGAGCAAGTGTTAAATC
EMPV1_14871 GTGCCCCGCCCAGCACTGACAAGAAAGAGGACCTGATAAATCTTAGTTCCGGTTGTCACA
EMPV1_14872 CCTGCAGGAACACCTGGAGGCAGCCAGCCTCGGACCCATGCAGATAGTGATAGAAGACCA
EMPV1_14876 CCATGAGAAAGATACATCTCTAGTGGGAGTCCATGAATATGGTGGGACGAGGGACAGCTC
EMPV1_14877 CAAACCAAGACAGGTGACTGTAAAACTAAAGTGTAAAGAATCAGATTCTCCACATGCTGT
EMPV1_14878 TGCGGTTGACTAAGCCTACTTTGTTCACCAATATTCCAGTAACATGTGAAGAGAAAGACT
EMPV1_14879 ACGCATCTCGCCCTAAGCATCTCTCCCATCTGGCTGTTCGTGAGTTCCGTCCTTACATAA
EMPV1_14880 TTTATGGCATGGGAATGGCGGATGGTGCTGGAAAAGAGGCTCAGGTAGGAGAGCCAGATT
EMPV1_14882 TTTGAAAGTTGAAAGGTTACCAAGCGGGCCAGTTCTTGGAGGAGGACTCTCCTGTTGCTG
EMPV1_14883 GGGATACTTCTTACAGGCCTCACTAAAGATGGAGTGGACTTAATGGAGAGTTATGTCGAC
EMPV1_14886 ATCCTGTCATATATCTTCATCCTTCGAGCAGTTCTGCAGCTTACCTCTCAGGAGGCTCGC
EMPV1_14887 GGAACCAAAACCTTCAAAGCTAATAGCCTAGGCCTAGAAGATGACCTTGACCTTATAGCC
EMPV1_14888 ACCATACTCTTAGTTTTCCCCTACCAGATTTTCCAGCAGTTTCCTATGAGGGCACGTCCC
EMPV1_14892 TTCTCCCAATAATATGAGCCTGAGTAATCAACCGGGCACCCCGAGGGATGATGGCGAAAT
EMPV1_14893 TTGAGCTTGTGCCTGCAGGAGAGAAGGCTGGTGGACACGGCAGGATTCAGCATCTTCATC
EMPV1_14894 CCATCTGGATTTTTAAGAAAGTTTGTAGTCCTCTGTTTTGCAGGTCATAGGGAAATTACA
EMPV1_14895 TCAGACACAAGTACCCACCATTCCAAATGGACACAACTCCTCACCCCCCATGGCGTTCCA

EMPV1_14896	CTTTGTCTACATGAGGCCCTGTGTCACCCTCCCGGCAGACAAGATAGTGGCCGTGTTTTA
EMPV1_14897	TAGGTCTGGGCCAAGGGCGAGCCAGGGGCGCAGCACAGCCCAGGGGTGCTGGGTCTCCAG
EMPV1_14899	GGCCCAGAGGAGTCTCTGCAACCTTGGCAGTAAAGTCCTGAGAACAGTTTCTGCTTCAAA
EMPV1_14901	GCATGGCTTTGGGAAGAAGACAAGAACCTGCGATCTGGTGTGAGGAAATATGGAGAGGGA
EMPV1_14902	ACCCGTCAAGGAACAGAACTGCTGATTCAGTCTGATAATGACACTGTTATTAATGATTGG
EMPV1_14903	CTGAGTTTCTCAGAGAGTCCCCAGAGGGACCAGATCCCAATTGCCTACAGAAGTAACTCT
EMPV1_14904	TGTGGCCCTCAGCTGCTCTCCTTCTCAATCCTCAAAATGACATCCCAGCCTCTTGCAACA
EMPV1_14906	GGCTGCTAAGTGGATAGATGAGAAGCTTAGTGGTCTTTGGTCATTTCCTGACTTGTGACA
EMPV1_14907	CATCTCCCTATGACTGGCCTGCTGGAATGCTTATTAGAGATGCTCAAGATGGGTTTCTGC
EMPV1_14909	GTTCTCTCTCTATGATTGATTACCTCATCTGGCCCTGGTTTGAAAGGCTGGAAGCCCTGG
EMPV1_14911	TGCGGGGGTCAGGCTCAAAACAGGATACAATGAAAGCTCTGGCTTCAGCGGAAAGGGTTT
EMPV1_14913	CCTGATGAGTTGGATAGAGGAGAGAAAAGGTTTGGTACACCTGTGCTGTAAGAAACTGAG
EMPV1_14914	ATGCCAAGTGCTTTCACCAAAGAGGATGGATACAGCAATGAACAATCCAGGCAAGACTGC
EMPV1_14915	CTGATGAAGAGACTGTCGGTGAGAGGAGTTCTAAGACAAGGCAGGTGTCAAAGAGGTTGT
EMPV1_14916	TAAAGGATGTCCTGGAGGACGCCGAAACTGCAAAATGAAGTGCGACAATGACGAATATGC
EMPV1_14917	ATCCTCAATTTGGGGCTCTTTGCTGCAGGCGTTTGGCTGGCCAGGAATTTGAGTGACATT
EMPV1_14918	AGAATTGGCCATCACAGGAAGCCACACCCAGAACTTGTTTCTCCCGGTTTGGGAGAGGCA
EMPV1_14922	CAGTGGCAATGCCTGATACTTAACCTGTGGCACTGCAAGAGAACTCCAAGATTTAGCGTA
EMPV1_14923	TGAGTCCGTGGACAGGAGTCCCTGGGCGGGGTGGTGGGAGGAGAGAAAGAATTCATCAGT
EMPV1_14924	TCCAACTGACTCTGGTCTTGGGAGGCCAGGCTTCCCAGCCACCGAAGACTACTTTAAATA
EMPV1_14925	AATCTCCCACCCGAGCTGCATCCACGCAAACATGGTATGGAGTCCCAAAGGAAAAGCGAT
EMPV1_14927	CCAGGACTCCAACCTCAGGGACTTGGGTGGCCAGTGTTAACTTCTTTTGATACTAAGACC
EMPV1_14930	CATCTTCTGTGAGATGTATGTCCTGCTGAGGCTGGCCTGTTCCAACACCCAAGTCAATCA
EMPV1_14932	TAAAATACAATGCAGATGAGGCTAGAAGTCTGAAGGCATATGGCGAACTTCCAGAACATG
EMPV1_14935	GCACTGGGTCTGGCACAGATGGGGCTCAATAAATGTAGAGTAAACTTCCAACTGGAGGGA
EMPV1_14936	GCTTTCACTGAGTTCACACCCCATCAACTTGCGGACTATTCACAGCAGCTCTTCCAGCCA
EMPV1_14937	GCTGTAGAGAGAATTCTGCAGCTGCCTTTTGGGTTGATTCTATCATCTGACGTCATCTTA
EMPV1_14940	AGTTTGGCAACTGGATGCCCCAAATATCAGAAGATGTGTGGAGAAAGGACAGGGACAGGG
EMPV1_14941	CCTTTCACACCGTCTAGGTGCCTATGATGAGATCAAACTTTCTGACCTTCCAAGTTCCCA
EMPV1_14942	AAAATATGCAAATTCGTACAAAGGTAGCAGGAATCTATAGAGAGCTCTGCTTAGAATCTG
EMPV1_14943	TAACCTTGAATGCGTTGGTGTCTGTTCCCAGGCAAGTGGGTGAGAGGAGCTGTCTGCTGA
EMPV1_14945	AACACGTGCGGGTTGTGGCAAAATCTTTGAATAACAGGGGCAGTATAAGTGAGGCGGGGG
EMPV1_14946	GCAAGCAGGATTGCATCTTAGGCAAGTCCAATAGAGGGAGGATGCAAAGGAGTTGGTAGC
EMPV1_14947	CCCCCAGACGGACAGATGGACGGACAGACATGCAAACACCAGACTGAAGCACATGTAATA

EMPV1_14949	AAAGAAGCAGGCATGGGCTGGGACTGTTCTCAGCTGCCATCGTGGGCTTTCTGCACATAT
EMPV1_14950	GCCCGACAGGACATTTGAAATCAAAATTGGTCAACCCACTGTTTCCTACTTCCTGAAGGC
EMPV1_14952	ACGATCAAGAGAGAGGGACCTTAGTAGATCACGAGAAAAGTGTCGACGTCATAAATCCCG
EMPV1_14955	CTCTAGTTGCATCCATGCTGCTGCAAATGGAATTATTTTGTTCTTTTTGGGGCTGAGTAA
EMPV1_14958	GGGAGTTCCCAGGCTAGAGATCAAACACACATCACAGCATTGACCCAAGTTGCTACAGAA
EMPV1_14959	GTCAGAAAGGGTCCCAGGAAACCTGTGAATGTTGAAGAAAATTCGTCTGTGAATTTGTGG
EMPV1_14960	CACATCCAGCAGCTGATTGCTTGTGGCGCGCTGCCTCCCTTGGTGGCTCTGCTGAAAAAC
EMPV1_14962	GGGCTCCCCTCTTAGTCCCCAGTCATCCATAGACAGTGAGCTGAGTACTTCTGAATTGGA
EMPV1_14963	CTGATCCAAGTTTACAGGACGACAGCCTTGCTTTAAGCCATGTCCTATGGTGAGCAAGGT
EMPV1_14964	CATGGCCCAGAGAAGGTGATATTCGAGTTGCTGTGGAAAGATTTGGATAGGAGACAAGGG
EMPV1_14965	CTTGACTTATAGATGCATGGCTCACCTCTGCCTTTCTCTTCACATGGTATACTCTCCAAG
EMPV1_14966	ATTTAGCAAGCAGACCAGAAGAGGAGGCCGGATGGCACTTTTAGCCTCCATCCCTGTTTT
EMPV1_14967	CCAGCAGCTTCAAAGTCAAGAATCTGGCCGGGAAGCTGTCAGCCGGTACTTCTACTGCAA
EMPV1_14970	CCGGAGAGATATGTCCTGATTCTAGAGTGCTAGCCATGGTGACAAGCATAATGTGTGGGG
EMPV1_14971	AGTCCTCAATTGCACAATCCATTCACAGGGGCTTTGAGCGGGAGTCATGGGAACCAGTCA
EMPV1_14972	AGATTGACCAAGAAAGACAGATGCTGAAGCGAGAAGTATCCAGCAAGTCCAGCCAGAGCC
EMPV1_14974	GGTGACAGCGGATTTCATCAACCGGGGAGAATATGAGATTGACATCGCGGGACACCGGTT
EMPV1_14975	GCTCTTACTTGCTGACCTTTTACTGAGTGCCCAGTTGTGTGCTAAGTCCGTTACATGCCC
EMPV1_14976	GCATCCCGTGGCCGACTCATCCCCGGCGGGTGCCAAGTGGACGGAGACGGAGATAGAGAT
EMPV1_14977	TGGGCCACTTCTGGAGCAGGGGGATATCACAGGTAGAGAGATTTGAAGTCAAGAAGCCCT
EMPV1_14979	GGTAATTATTAGCATCAAGACCTCTCGGCTCCACTCTAAGCCAGGCCTATAAGCTGGTCC
EMPV1_14980	ATGGAGGAGCTTATCTGCAAGAAAACACAGGGATACCATCAGTGGAAGAACTGATTTCAA
EMPV1_14981	GGTAGGTGGGGCTGAGTCTTGTCCCTCTGATGGAAGGGGCTTTGTCTCTGAGTGTGATTA
EMPV1_14983	TTAAGCAGCTCTGTAATCAGTCCCGGAATAGAGCCATGAGCCAGGAGATCCGCCGTGTTT
EMPV1_14985	GCGAGTATGTGTAGGATGCCCCTCATAAGCTCAGATCCTCCAGGGATCAGGAATGTCACA
EMPV1_14987	TTCACTGTGGGCCTGGTGGTCCTGGTCATTGGCACCTCCATGGGCTTCAACTCTGGCTAT
EMPV1_14988	ACCCAACGCTCCTCTGTCACCATCAAGTCAGGGGGCTCTCGGAACTTCAGTGCTTCCTCT
EMPV1_14989	CCCTGGACCTTTCTGGAAGGGCAGTGTATGAAAACATCAGTCCCAAGGAGAGACAGTAAT
EMPV1_14990	CACCAGTCAAGGGCACAGGCCTTAGCAAGAGACGCTGAAGCAGACAGGAAGAAGGACTTT
EMPV1_14991	TGAAAAAGAAATGATGCTGGTCCATTGGGGAAGAAGATGCCTTCCTCGGCGACTGAGAGC
EMPV1_14992	GGATAAGGATCGTTGGATCAGGCTCTACTCCCAGTTCTGTAATAAAACAGCCAATGAGGA
EMPV1_14993	CTTTGTCCTCATGACTACCTTCCCGAACAAAGAGCTGGCCGATGAGAGCCAGACCCTGAA
EMPV1_14998	CAGCTGTCTACTTCTGGTGTTGTCTTCTCATCCTGGTGTAGTTTCCCCTCTATGTGAGCG
EMPV1_15001	CCTTATTTCTCCAGCTTGTCAAACATACCATGCCTCGCCCTCCACCTCAGACCTAAGCTT

EMPV1_15003	ATGCTGGTGTTTGGCTTTGCCACGTGCGCCACCCTCTTCTTCATCCTCCGGAAGCAGTAT
EMPV1_15005	ATCAAGAAAACTTTCAGCAGGTGTGTCAGCAGGAGGAGGTCAAGCAGGAGATCACTGCCA
EMPV1_15006	TAACGTATTGTCCCCGTGACGTACATCATCATAGTGGTGATCAACCAGAAGAGAGAATGG
EMPV1_15007	CAAGATCGATTGCTACAGTGTCATCCAACAGGAGGGAAAGTGCCAATGGAAGCTGAAGTG
EMPV1_15009	CCCACGGAAGCCTCATACGAAGATGGCTATTCTGTGTCTCACCAGATCTCAGCCCGTTTT
EMPV1_15011	CTTGTTAGACTTGGCTCAGACCTCAGCTTCACAAGCTGATGGCCTGAGTCAGGAGGCCAA
EMPV1_15015	CTGGGGTAGGAGTACAGCAGCCATAACAAGAGGAGCAATAAACTCCATCTGGACTCTTCT
EMPV1_15019	TTGGCCAGTTCTACTTCTTAATCCGGAAGAGGATCCACCTGAGACCTGAGGACGCCTTAT
EMPV1_15020	TAAACTGACCACCTCTGGGACCCCGTACCCTGTAGGAGTAGCTCATCACTTCTTAGCAGA
EMPV1_15022	AAGAGAAGAGCCTGTCCATCAATCTCCTGTGCTCTGAACTCATCTGCGATATCCTGGCAG
EMPV1_15024	CCCTGATGTGTATTTTTGGTTTTGTGCCAGTGCCATGCTGTTTGGATGACTGTAGCTTTG
EMPV1_15025	AGCGTTTCCGGGGTCACGGTCTGCGCGCGAACCTGTGGCGTGTGCCAGCCCTGGAGACGA
EMPV1_15027	TTGGACAGAGCAAAGCCAGGGTCTGGAGCGTCCTGGGACCTTGGGCTCCGGGTATTAAAA
EMPV1_15028	GGTCTCGTGACCCTGAATGACATGGAGGCCAAGCAGGAGGCACTGGTGAAGGAGCGGGAG
EMPV1_15029	GGCCTGTCCTTTTCATGGTTGTATTCCCAGCACCCAGCACTGTACTTGACACAGATATTC
EMPV1_15031	CTTGGCCTCCCCAAATGGACAAGAAACTGGTGGGTTGGCAACAACATTCTCTGGCGGCCA
EMPV1_15035	GATGAACTCGCCTTTAGATTCTCCTAAGCATTTGCCCTCGCCCTTCACCGGTTTTGAACC
EMPV1_15039	CGTGATCATATTTTTCACGGTGTTGTCCAGCCCCACGAAGGCGTCCCAGCTCTTCATCTC
EMPV1_15042	GTGCCTTCTCCACAGTGCCATTCAGACTTCCGTGATCTCCAGTGGTCAGAAGACATTTAC
EMPV1_15043	GAGATACCTGGACCTCAGAAATACCGGCTTGCAGACCTTGGACCATGCTGCTTTCCAAAA
EMPV1_15049	CCACTCTTACTAACACCTGACGGTACGATGCCCTGTGCGCGTTACTCAGCATGAATCACT
EMPV1_15050	TGAATCTTTGCTTGTGCAGACTCCAGCTGTGCCTCCGGGCCCATGCCTCCCTCCCAGATT
EMPV1_15053	CTGCAGGTCCAGAGCTTGGTGGTAGAGACGCTGTAGTCAAGTCAGGTTTACTCAGTGCAC
EMPV1_15054	TCTCGCAGTGACCCCAATGCAAAATACCACGGGATCCTACAGGCCGGGAGACAGATTTTG
EMPV1_15055	TTAATGAGCAGCAGCCCAACTGGCAGCCCTACCTCCCCACCTAACACATACACACAAAAG
EMPV1_15056	TCAGTCAGGCCTGGATCAAGCAAGTGGCTCAGATCCTGGAAAGCCAGAGGGATTTTCTCA
EMPV1_15057	CACCTGCGGCGCTTTCTACCTCCCGCTCGGTGTGGTGCTATTTGTCTACTGGAAGATATA
EMPV1_15058	TCTCTGCTCATCATAGTAGTTTTCTAACCTGTCTGCCTCCCTGTAGTCTCACTTTTGTGC
EMPV1_15059	GGGAGGAAAGCAGAACCCAGAGAAGGCTGATCCATTCCAATGTTTTGTCCTGACCTAGAC
EMPV1_15060	GGACTCCAGGGAGATAGTTGGTAGCCCTAGTTCTTGATGACATTCCAAAAAGTGTGGTAC
EMPV1_15064	AGCTGCTCGAGTTTGTGGCTTTCTGTGTCCCCACGCTTGTGATTATGGAACAGTTTGCCG
EMPV1_15066	CACTAGCTTGCTCAGATCCCTGGGTCACGCAAATTGTGGTTGTCAGCTGTGGAGGATTCA
EMPV1_15067	TTTGGCCAAGATAATGAAAAGAGTTAATGAGGTCTTGTGCCCAGAGGGGCGCTGGAGCCT
EMPV1_15070	GCATCTTTAGTTGCTTCCTGGACATCGGGTCCTTTCCCCTAACCATATACACGTACATAG

EMPV1_15071	CTGCATGTAAGGGCTTCAGACATTTCTCTCATCAGTCTAAAAGTTAGAATTTCCCTCTCC
EMPV1_15073	GGAAATGGCTGCCGAAAACAAGCCGGAAGAGCGTTTCCCAAAGTGTATTCTGCGGAGCTA
EMPV1_15074	ATCTACTCTGCCCTCATTCTGCACGACGATGAGGTGACGGTCACGGAGGATAAGATCAAT
EMPV1_15077	CTCTTGCCCACAATGGTGGAAACCAGTAAGGGCAAAGATGACGTTTATGATCGTATGCTG
EMPV1_15079	TGCACAAACTGCACTTCAGCCTGGGCGCACCGACCATCAGCTTCTTCCTGGAGCATTTCA
EMPV1_15081	GCGTTTAATGAAAGAAGCAGCAGAATTGAAAGATCCAACAGATCATTACCATGCACAGCC
EMPV1_15082	TGCTCTTCAGTGCTCAGCGAAAAGCAGTCTGAAGGATTCTTCACGAAGTTTTCAGTGCTC
EMPV1_15083	TGCTGGATGAACTGGTGGCTCTTCGGGAGAGGGTGATCAAGGAACTCAAGAAGGCTTTAA
EMPV1_15085	GTCAGCCCTCTATATCTGTGAGTTCCACATCTGCAGTCAGCCAACCTCCACTGGAAAGTA
EMPV1_15086	TCCAAGCTGCCCTGATGTCCAGGACTTTAGAAACTGTAAGGTCACCTCGGAGCCTCTGAA
EMPV1_15091	TTTTTGTATGATTTGATGATTTTTCTAGTGTTATGCTTTAATTCCTTTATCTTTATTTTT
EMPV1_15094	CAATCTAAAGGTTCTCCTGGCAACAGGGCTCTCACCTTACACGTTACCTAGATTCAGCCG
EMPV1_15095	CGGTCACCGTGGCCTTGATCTCTTGCTGGGCCACTTTCATACGGTCATAGAAGACCTTGA
EMPV1_15096	GAAGGAGGGCTGCTTATCCTTTTGCTTTTTAGGGTCACAGGCGTGGCCTATGGAAGTTCC
EMPV1_15097	GCCTGGTATCTGAGCTCTCCCTGTGAACCTTTATGAGCATAGGTAGTGGATTACAAGGCC
EMPV1_15100	CTGACAATCAGTTAAAATGTGGCAATTGTGGAGAGAAGCTGTTCTTCATGTTGAACACAT
EMPV1_15102	CTTCGCCCTCTTCTCTGTCTTCGCCTTCGCCTCTATCTCCAGCGTGCCTCTTTGCCAGCT
EMPV1_15103	CTTGCGGTAGCAGAGAAACTGTACAGAACCAGGATGAGCAAAGCAGAATCATACTTTCCC
EMPV1_15104	ATGTTCTGGAAGCTTTCCGTGTCCTTACTCCTCATGGCTGCGCTGGAGAAGGTGGAGGAT
EMPV1_15107	GGGCCAGGAAATTGCAAGGAATGTATATCAGGAGCAGCAGGCTTATTACAGGGATCTGGG
EMPV1_15108	AAAAAAGGAGCCTAGAGTTCCCATTGTGGCTCCGCAGGTTAAGCACCCAGTTAGAGTCTC
EMPV1_15109	GATCAGCTTTGATAAGTGGGAGTTCATCTTTTGGGTTCATTCATAGCCTTTAATTTTGCC
EMPV1_15111	GTGAGAGCCAAGTGGAGGAAGAAGCGAATGTGCAGGCTGAAGCGCAAAAGAAGAAAGATG
EMPV1_15112	GGGTGCTGACTCCTCTAATCCCGTACCCCTCTATCCATTTGGTACTGACACCTACTCTGA
EMPV1_15113	ACAAGTGATATGCCCTTATAACTGTATACAAGCTGGTATGTGCACCAGTGTGGGAATTCC
EMPV1_15114	CATCGACACGCAGTACTACCTGGAGCAGCAGCTCGCCAAGCCCCTCCTGCGCATCTTTGA
EMPV1_15116	TGGCTAGAGAGCCTTATTTGTGAGTTGCAAACATAAGAAATAGGTGATATTTGTCAGGGG
EMPV1_15119	AATGCCATCCTAAATCAGTTCCAGCCTGCGGCAGGGGGCTACTTTGTGCCAGCAGTTCCA
EMPV1_15120	CCATAGTAGCTGTTCAGGCCATTATTGCTGTTCCAAAGACAGACCATAGACTGGGGAAAG
EMPV1_15121	GGGACCATTGAGGAATGATAGTTTGGTATTGGGATTTTCCTTCTTGGCCTTGTGTGGCAG
EMPV1_15125	GATGAGCTGAGGTTCCCAGAATTTACTCATTAGCACAGTCCAACAAGACCGCTAACGCGC
EMPV1_15128	CACAAGCCTCACAGAACATCCGCATCGTGGGGGCAGAAGTGGCGTATTTTATTGAAGTTC
EMPV1_15129	GGTGTGTTGCTTGTCATTCCCTTGATTTTCTTCACCAAGAGACTACCCTACTGCAGAGGC
EMPV1_15130	GCTGTGTTCACTTAAAACTCCAGAGCTTACTTCCTGTGGTGCCAGTGTATTTGTTGCAAT

EMPV1_15131	TTTAATGGGAGTTCCTACTCTGGTGCAGTGGGTTAATGATCCAGCTTGTCTCCGTGGCAC
EMPV1_15132	ATTCTTCCAGTGGCAGCATATGGTTCCTTGGCAAGCTTGTGCAGCATCTGTACAAAAAGC
EMPV1_15134	AACCTTCCAGAACCTGAGCTCCACCCACAGACGCAGAGCTGACCACCCACAACCGACTCA
EMPV1_15135	GATCTCTCCTGGAGTCATGAGAAACCTCAAAGGCCACCCTCAAAAACCTCTGCTGCAAAG
EMPV1_15137	AGACATAAGCATACAGACAAGCCAGTTGGGATGGAAGTGGGGAGGGGGCTGCAGGATGGT
EMPV1_15140	GAGTCAGGACCCCTATTCCATGGGAGGTTTTGCTTCCTTCACTCCCTACCAATATGTGGA
EMPV1_15141	TTTCAAGATGGCGGACGTGGCAGGCCCCTCCCGCTCGGGCGCGGCGGCGTTCTGGAGCCG
EMPV1_15143	CTTTGGGTTGCAGGGTGAACTGAGGGACTTGCTTGAATTTCTTATTTGGGAGATCAGGGC
EMPV1_15144	ACGGCCGAATGAAATTGAACCACCACCCCCTGAAATGCCCCCTTGGCAAAAGAGACAAGA
EMPV1_15145	AATCATTGGGAATAAATACGGCGATCAGCATAGCGCGGCCGGAAGAAATGGGAAGCCGAA
EMPV1_15148	GTCTTTGGCCAGAGTGAGTACCTGCGCTATCAGGAAGCCCTGAGTGAGCTGGCCACAGTG
EMPV1_15149	AGGCCTTCTCGGCTCTGCACTCCGGTGTCTTATATGCTCCAGCCTTGCCAATGACAGTGA
EMPV1_15153	AAGACACTGAGGTTCAGAGATGTTCAGCGATTTCCCCCGAGTCACAGGGAGCAGGTTTCT
EMPV1_15154	TGAGCTGAAGGAAGTCAACCGCAGCAATCACACCTACACCCTCGTCAGCAAGCTGAGCCT
EMPV1_15155	ATGGTGAGTTATTTGAGCATTGGAAAATGTCTCTGGAAGCTAAAGGATTGTCCCTTGACC
EMPV1_15157	AGCCCCCTGAACCGTGTGCTGAGCCGGCTGCCTTTGGAGACACGCTGCACATACACTACT
EMPV1_15158	GTCAGTTTCTGTTCTTGCTAAGGCCAGTTTCAAGCAACCAGGGTGTATCCTTGGTCCCAG
EMPV1_15159	CCATGGACCTGCGCTTTTACCTGGGTGTGGTGGTGCGGTGACTTCCTTCGCCATGAGGAT
EMPV1_15160	ATCTGTCCACAAGCCCTGCGAACCCAAAGTTGGCTCTTTCACAGCTCACTTCTTTGGTCT
EMPV1_15161	TCAACAACCGCAACTGCCTCGGTCTGAAGGACAAACCTAAGGTCATCATTGTCCAGGCCT
EMPV1_15162	TAAGTTTCGTGAAGATCCTGGTTGTTGTATTGCTGTTCATTGTGTTGCAGGACTTGGGAG
EMPV1_15163	TGGAGAACTACAACCCACGGCAGCGCAAGCTCCGGAACCTGGTCATTGAGGACGAGAAGA
EMPV1_15165	TGCCCGGAGCGCCACGATCAGCTGCGGGTGCAACGCACGAGCTTCCTACTTGAGACCAAG
EMPV1_15166	AGAGCTTCAGGCACCTTCAGAGGAAGACATCAGTACCAAGGGGAAACACTATTTTTGGCC
EMPV1_15167	TGTGAGAAAGACCAAGGATCATGCAGAAAGAAATGCAGAACAGAGGAAGTTGAATTACAC
EMPV1_15168	ACTACGCAGCCCGACCTGGATGGGATTTGCTTCTGGGTACAAAACGGGACACTGATCAAA
EMPV1_15170	CTCAGCATCACTAACCACCAGGGAAATGCACATCAAAACCACAATGACATAGCACCGCAC
EMPV1_15171	CTCAATCTGAGCTGCTCCACAGTTCCCACCTTTGTTCTGTCCATCACAGCTACGACACAG
EMPV1_15173	TCAAAGTCGCTGTGCTGAACCCACTGACCCTGCTCGCTTCCATCCATTCCGCTGTGTTCT
EMPV1_15174	ACCTCTTCATGTGCTCGTTGGCCACTCCTTGGATGTAAACGGACATTCCGACCCGGAGAC
EMPV1_15176	TTCCTGATGCACCTACACTTGAGACTTGGGGAAATGGGACGTGGAAGATATGGGTACTAG
EMPV1_15177	TGTGGAAACTTGGACACCTCATCCCTAGGTCTCGCCATGACTCGATTTCCCAACTTGCAA
EMPV1_15179	GCCAGCTCAGGAGTAGCACAGTCATTGCAGCATCAACAAGTGGACCAGCAGCCAAGTGAT
EMPV1_15180	GATTGCCCACATCCCACTGTACTTTGTCAAAGCCCAGTTTCCACGCTTTAGCCCAGTGTT

EMPV1_15181	CCCTTGGTGCCTCCCGGCCACAATCTCTGCTTATTCTTCCGCCTTTAGGTAGAAACACTT
EMPV1_15182	TAGTGCGAGAGAGTGCTTCCCAGGTGGCCTCCCTTTCCAATGCCGTACGTAATAAAGTGT
EMPV1_15183	TTCAGCTTGGTGAAGAGTTACTAGCTCAAGGTGAATATGAGAAGGGTGTAGACCATCTGA
EMPV1_15184	ACAGGTGTAGTGTGCGACGAGAAAAGAGCAGACACGGTGCATTTTCCTGAGTTTGTCTAG
EMPV1_15185	GCCTCACAGATCTAAACAACAGTCTCAACATATGTTGCCAGCTTGGACTGAAGGAGACAG
EMPV1_15186	AGGGCTTGTGGGACCCAGCATCCGAGTACATCTACTTCTGCTCCAAACACTTCGAGGAGA
EMPV1_15187	GTGGAAGGGGACAGGTACAACTGGGAACATGAAGATGACTCTAGGAGTTAGGGTCTAGCT
EMPV1_15188	CTTCTTTGATGGGGTGTCCTGCGATGCCAGGAACTTCACGCTTTTCCACCACCCAATGTA
EMPV1_15189	TCCCTGGCTCTCCTCAGCAAGGACCACTTCTGCCTGTGCTACCTGCAGGCGGAGGTAGGG
EMPV1_15190	TGGGAAATGTGGCTTCTGTTCATTTCGTTTAATTAAAGCTACCTCCTAAACTATAGTGGC
EMPV1_15191	CTCTACTTCGTTCTGTAGCTCCTTTAGCTTCTCAGCTTCTTCCTCCATCTCCCTGACTCG
EMPV1_15192	TCGATTAAGGCACGGAATGAATATCTCCTAACCCTTGAAGCCACCAATGCCTCTGTTTTC
EMPV1_15193	TGTGGCCCCAATACCATTGACCACTTTGTCTGTGACTCTGGTCCTTTACTGGCCCTCTCT
EMPV1_15194	AAGGTTCAGAAGGACTGGTGGACGTGAAAAGGGCATTGGAAGGAGGTGTCTACAGAGTGT
EMPV1_15196	GGGCTGCTGGTCTTCCCTTACACACACCAGAACTGGGAAGTGCAGTACAGTCGCGATGTG
EMPV1_15197	GTGTGCATTCGCCTTCTCAGTGTTGAGTGACAGGATGAGGAGAAATTACTTGACTGACAT
EMPV1_15198	GGTAAGAAGGAAGACTGCGTGAAGGGTGATCCTGTCGAGAAGGAAGCCAGAGAAAGTTCT
EMPV1_15199	GCTACTTTATGGGGAGTGACATCACCCTGTTGCAGCTGTATGGACCTGTGAACTTTACTG
EMPV1_15200	AGGGGCCCGGACTCTCGCATCTTCCCATACCTAGAAAAGCGCTAAATTCCCTAACTTGAG
EMPV1_15202	GAGTGGGCCGAGAGCTCAGGATGGTCCTGCATGTGGTCAACCTGGGGACAGAGAAGACCA
EMPV1_15204	TGGGAGAGGAGCAGGACAGGCAGTGAGGAACCGGTATGTGACCTGGGAATGGTTCAGAAA
EMPV1_15205	GTTCAATCCCTGGTCTCACTCATTGTCACGAGCTGTGGTATAGGTTTCAGGTGCTGCTCA
EMPV1_15206	CTCTCAGTTTGGAGGTGGGAGTCAGTATGCCTACTTCCACGAGGAGGACGAAACTAGCTT
EMPV1_15207	TCAATAGCAAAGAGCTGAAAAACGTCAGGTCCCACAAAGGAGCAGAACCTGACCCAAGAC
EMPV1_15208	GAGGTCTAAGTCGTCCACTACCCCCAGACAGGACACTCTTGCTTTTGTCTTTAGGACTCT
EMPV1_15209	CACGTGCAAAATTCCACGTACTTTTTCTGTTCTTTGTGTCTACAATGTTCTTCATCAGCG
EMPV1_15212	TAGCGGCACTGAGATTGACCAGTGGGTTTATAGGGGGACAGAAGCAGCGGGTTTTTTGGA
EMPV1_15213	TAGTTCAAGAGCACAGTAATTCTGTAGGAGGCCAGGACACAGGAGCTACCTGGAGGACCA
EMPV1_15214	AACGCAAACCCAAACTACAATAAGGTAGCACACCTCACATCACTCAGAATGGCCAGCCAG
EMPV1_15215	GCTGTGCGCGGACATCATCGAGGGGCTCTGCTGCGTGGACGCCACCACCTTCCTCTACTT
EMPV1_15216	CCCTTCCCTCTCTCTTCATCCTGCAGGCCCCATGGGCCAGGCAGGCTGTCGCACACTGCT
EMPV1_15217	TGCAGGTATAAACGCGTCCAGCAGAAGCACCACCTGGAGAATGAGAAGACACAGCTCACT
EMPV1_15218	TGTGTCTTGAGGGCTGTTAAAAATGTCACTGATGATGGCTCTGTAGTTCCACGTGCTGGT
EMPV1_15221	CAAACAGAAGTGATGGAGTTTGAAGGATGCCTCCACTGACATCCTGACCAAAGGCGCCCA

EMPV1_15222	CCATCTTCTACATGCGCAAGAAGGTGCGGAAGGACCTGGACGACCAGGAGGAGCTTCTGA
EMPV1_15223	AGCCTGTAGGGCCTTAGCCAGCTTTGACCTCCATTGCCCAGCAAAGGCATAAAACCTTTT
EMPV1_15224	AGATATAGCAGGTTCTGGTGATGGTACACAAGAAGTATCTAAACCTCTTCCTTCAGAAGG
EMPV1_15225	CATCGGGTTCTGCCTGTCTATGCGGGGCCGAAAGGCTTACATGAAGAAAGAGGGAGATTT
EMPV1_15226	TGCTGAGAAGGTGTCTGAGGTCCACAAGCAAAAGAACCAAAGGCTGAAGCAGGCCAAAGC
EMPV1_15228	AAGGAGCCCTCTGTGTTTCTGCATTGCCCATTACTGCAGAGATGACGGTGCTGGCATTCA
EMPV1_15229	ACCGTCCAATGCCCAAGGCAAGAGCTTGTCCAGAGGCGGGAGGTCAGTGGTGGGCAGCAG
EMPV1_15230	GAGATGATGCTATATGCGAATGGAAAAAGCTTCTTGGACCTGCAAACTCTGGACTGGCAC
EMPV1_15231	AGGTATAGCTCCATCCTGACAACCGTCAGAGTTGCCCAAATAGGAATGGTGTGCCTTTCC
EMPV1_15235	GCAGTACATGCTTTTGACATGGAGGATTGGGGAGATGAGCTGTGTATTGCCATTGGGGGT
EMPV1_15237	AAGAGAGGCACTAAACTATTTTTGCGATGTGTTCCTAATACGTAATCTAAGTGTATTGGG
EMPV1_15238	ACGCGGAGACCAGCAGGTTGCAAGTCCTGGAGCATCTGTTTGGCGTTCGTGGGAGGGGTT
EMPV1_15239	CACCTCCTGGAAGAAAGGGCACATGTCAACCCAGGTGTTTCTCTCTTCAAAGGCACTGTT
EMPV1_15240	GACCTGCTGCTCGGTTTGCTTCATTGGCAGGAGTACTGAAGGTAGAAATATCTCCAGGTG
EMPV1_15241	GAACACTAACAAGTTATGAATAGGGTAAGCTGTCAAAGGCCACCTCTTCATCCTCTAGAG
EMPV1_15243	CCCGGGGCTGGAGAGGGGCAGATTTTAGAGGAAGAAGGGAGGGAGGAACAGAGGGAGAGC
EMPV1_15244	AGAGCTGAGAAAAGAGCTCATGAAACGCAGGAAAGAGGAGCTTGCACAAAGCCAGCATGC
EMPV1_15247	TTGTACCTCGGTTTCCCCACCTAGAAGATGGTCTTGGCCTCACAGGGTCTGGGGTGCTCT
EMPV1_15248	TGTTCATACTATCTACTTTGCCCCTGGAAAGCTGAAGTGTGTGAAACACTAGCTGATGGC
EMPV1_15249	ACAGTTATTTATTGGCCCAAGATGCAAAGTCACACATCTGACCCACCTTAGGGGCCTCCG
EMPV1_15250	GCGCAAGGTGAGGGTGCTATCTGTGACTGAGGGACATGGCGCATAGAACTGTCTCACACA
EMPV1_15251	AACCAAGTGCAAATCAGGCTCTGGGCTGGAGTTTCAGATGAGGAGACAAAGGGAGTCCCA
EMPV1_15252	CAGTCTGAGGGGTATAGCCTCGGGCAAGGTTCCATGGCAAGAGAATTCAGTTTAGCAAAT
EMPV1_15255	ACTGCCCAACATTTCCTGGTGCTCTGTCTGTGTGACCTCCTAACCGTAGCGGCACTAAAA
EMPV1_15257	GTGGTTCCCTGAGCACCAGCGAGCCACAGCCAACATGATTGGCACCATGTCAAACCCTCT
EMPV1_15258	CTTATCTCATTATCCTCTTCATTAATCTGTATATTTTCCACTGTCCTTGTAAGGTTTGGT
EMPV1_15259	TCCGTTGACGGGTACCCTGTGCTGTCAGCTGAGTGGCTGTCAGAGGAGTAGCATTGCTTT
EMPV1_15262	TAAGTCGGAGGTACCTGACCCCACCCAAAAGGCTTGGTCTAGATCAGACAGTGACGAGGT
EMPV1_15263	CGTCCAGTTCCTCTGGAAGTTCACATTCAAGGCTTCCCAGGTCAACATTACTGTCCTCGT
EMPV1_15264	CTTTGGGCTGGCATCACCCATAGGCCATCAGCATCTACTTGGGAAAAAGCAGAAGGTGCA
EMPV1_15265	TTGTCTCCTGGACCTTCACCAGACCTACCTCAGAGCACCACAGCATGCACAACAGTCAAT
EMPV1_15266	ATGCCGCCTAAGTTCGACCCCAAAGAGATCAAAGTCATGTACCTGAGATGTACCGGTGGA
EMPV1_15267	AAGAGGACGACTCAGCCGGAGGAGTGTGTCTTCATGTGGACAAGGATAAGGTGTCGGTGG
EMPV1_15268	GGTGAGCTATTGGAGGTGAAGCCAGTAAATCAGTCACTAAAGGAGACAGAACGCCAACCC

EMPV1_15269	TTCAAAAACTGCTGGTGTGTGAATTTCCTCCTTCCCTCAGCCAGGCCAGAAAACCACCGT
EMPV1_15270	AGGGCCTTTTCCAAAGAACGCAAGCACGAGGGCAGATTATGTCTCAAGAAGGGACGAAGC
EMPV1_15271	CAGACCTCAGGCAAGTCTGCATAGCTCATGGCACAGCCAGATCCTTAACCCACTAAACAA
EMPV1_15272	TGCAAATTTAGGACAACTTGTTCTATATATTTGTTTACTTGATGTAGATGATAGTCATAT
EMPV1_15274	AAAGAGCTAAGCACACACATCCTCTCAGCGAGCTTGGTAAAGTCCCCTAGGCAAGGAGGT
EMPV1_15277	GTAAAAGATGGAGGGCCACAAGGATTTACTCAACAACCCTTACGGCCCAATTTAGTGGAG
EMPV1_15280	TTACATCTGTAATGTCACCCTTAGACTCCCAGCCACTCCATGTCCATATGCCACTGCCTG
EMPV1_15282	TTACTTGGTTCCAAAGAAGAGACTGAGGGTCACAGAGGTTGGGATAGGAGGTTGTCAGGC
EMPV1_15283	TGAGTTGAATATTAAGACATGGTGTCACATGACCTCTCCTGTGAGGCAAGCATTGGCCTC
EMPV1_15284	TGACGATGAGGATGTGGCCCCGCTGTCCGCCAAATTCGCTGATATTTACCCGCTGAGTAA
EMPV1_15285	GGGAAATGTGATGGGGTTTGCTCTCCCTCAGTCTGTTTGCCCCGAGACTCTTGTAACTAT
EMPV1_15286	TCAGCTCGGATCTGGGACCAAATGGGCCGGGATAGCTGGTCTATCACGTGTCAGAAAGGT
EMPV1_15287	ATGTCCTGCCTGCTACAGTGTGGATGACATTTCCTGTGAGCCAGATTTCCTCACCTGCAC
EMPV1_15288	CTGTGGTTCCCTCCTTTATGAAATATCTCCCCTCACTTTCCAGCTGTTCTGGCAGCTCTG
EMPV1_15290	GGACCCACTGAGGACTGGCTCAACCAAAGTCTTCATTTTACCAGGGAAGAATATGATGGG
EMPV1_15291	AGGAAGCGACCGGCGGCTGAAGGCTGGAAGACTTGCTACTGGATAATCGTAGCTTTTAAT
EMPV1_15293	CCGAGACTTCCAGCAACCAAACTGCTCTCTCAACTGAATGAAGCAACCAAAACTGCTCTC
EMPV1_15295	TGAGTGACCCAGCAGGTGACACGGGTTTTAATGGGGCTACTTCCTCCCTAGAACCAATAC
EMPV1_15296	GAGTGTACAATGGTGCTTCTGTGCTTCTTTTCCCTTGTAACCCATATGCCAGGTGCGTGG
EMPV1_15298	GAGCTGAGTTTTGATGGACAAACAGGAGTTCTTCAGGGAACCAAGGAGTATTCTGGAAAC
EMPV1_15299	AAACTTGAATGGGTAGGCAACTGGCCAAGGGACCTGTTAAGGCAGCTTCTGATCCAGCAG
EMPV1_15302	GAAGTACTCTTTGAAATGGCTTTCTGGGTAGTAATGGGATTGGAAGGTAAGTTGAGAGGA
EMPV1_15303	CTATTCAGTATGAGATCTTTCATGCCTTCGAAAGGAATTGGGACGACTGAAGGCTTTCCC
EMPV1_15305	GCCATGCACGAACTTTGGGAGTGCGGGAAGTGTTCTGGGGAGATACTCAGGAATACACAA
EMPV1_15306	CTAATAATTGCAGCATCACTTTGCAGACTTTATGCCACGTGTTCTCTGTGTCCCTTCTCC
EMPV1_15307	CTGAGACAAAAGGGATGTCTTCACATAAGCTCCCAGGAAGGTCTGCCCTGTATTTGTCAC
EMPV1_15308	GAGGGCTACATATGATAGGTTTACTTAAACTAAGGTACCAGGAACCCAGTCTGCCTAGAC
EMPV1_15310	TTTCTCCTCCTCGTCGCCTGAGAATGTGGAGGACTCTGGCCTGGACTCACCGTCCCACGT
EMPV1_15311	TGGCCAGTGAACTCACCAAGATCAAGCCATAAACTGTCAGCATGCAGGGTGGTGGTATTC
EMPV1_15313	AGAACACAGGGAACCCAGAAAGGCAGGAGTGGACAAAGGGCAGAGGGTGTGGGAGACACT
EMPV1_15314	CCAACAAGGAAGAGTTTACCACCCTCCAAAAGATAGTGCTGCATGAAGTGGAAGCCGACG
EMPV1_15316	ACATCAACAACACCTTACATGTTGAGGACTCAAAGCCCACGACCACCAAGCAAAGGAAGC
EMPV1_15317	CTTTTGGATTCCTGTCACACCCAGTAAATTTTACCTCAAGGCCCTTACTTCAGAGGCAAG
EMPV1_15319	GAATGCAGACCACCTCACTAAACCGTGAAAGGAAAAGATGGTTGAGCTGGTTTACTTGGG

EMPV1_15321	AGCAGCTCTATCTCCAGCCCTCACTTCCCTTCGGAGTATGAGAACAATGCAGACTGTACG
EMPV1_15326	GTTCTGTTCCCTGCAAAGGCAATGGGAAACTTACTCCTACATCAGAAATGCTTGCCTCTC
EMPV1_15327	CAAGCTCTGTAGCTGTGCCCCGAGCGGTCTTCAGAAATGACCTGGACGATCTTGGTATTA
EMPV1_15329	GCTGGAAATAGATTAGTGGTTGCCAGGAGTTGAGTGGAGGGAAAAATGGAGAGTGACTGC
EMPV1_15332	TTTCCCAGCCAGGCGTCCAACCTGAACTGCTGAAGTGATGAAGCATCCTTAACCTGCTAT
EMPV1_15336	CAACAGTGGGGAAAGTCCTTAAAAGGGTGGCTCAGTTCTGCACTACAAGTGACCTAGCCC
EMPV1_15337	CCATTGAAGAGGTGACAGGACTGGCCTTTTCTCGGATGCAGAGCCCTTCAGATTTCACGT
EMPV1_15338	TCATCGCTTCTTGTTTTCAAGTGCTTCTGAGTACCATGTGCTTTGGGGGCCTGACAGCCG
EMPV1_15341	GGTCCTGGAATTGCGCTACCGATCACCAAGTAGCCATAAGATATAATAAACCTTCAGCAC
EMPV1_15342	CAAATATCGCCTTGGTACCTTGCCACCTAGCCCCCTGCTTCTGAGTATGAATGAAGTGAC
EMPV1_15343	GTGCGGGAAACTAGAATCTCAAGAGGAGGATGGAGATATAGAGGTTGAAGAGGCAGAAGG
EMPV1_15344	TCCTGCTATTTCTCGTCTGTGGGTCTTTGCACCTGCCATGGCCTTGACCTGAAACACTCT
EMPV1_15345	AGAGGTCCCAGCGTGATGGAAACAGAGGTGGAGGGAATGCCAGCTAGTGAGGCTAGAAAT
EMPV1_15346	GCGACGCAGGACATACAGAGCACAAGGTCAGATCAAGACTTACATGAGATCTCCCTGCCA
EMPV1_15349	TCTCCGTCTGCACCTTGAGCATAGACTTGACTCCCTTAATCCCAGAGAGAGCTACAGGGA
EMPV1_15350	GGCGGACTGACGCCTCCGCCGGGCTCGCAGTGGAGGCGCCTGGGGTCACTGAGAACCCCT
EMPV1_15351	CTCTTAGAGTATCTGCCCACAGGGTCACCTTGCCCTTGCCTATAACTGACTAGACCAAGG
EMPV1_15352	AGTATATGAAATATTTTGAATACACATCTGTCAGTGTGAAGACTCAGTGGGAGTGTGTTC
EMPV1_15354	TCCATAGATGAGGCTCTGTGGCTGGGCCAGACCTTTCAGTACACAGATGAACATAGGGAA
EMPV1_15355	TCTGGTAAAAACGGCAGCATTGAGAACTGTGTCGGTAAACCCACAGCAAAAAGAGGGCCC
EMPV1_15356	TGCAACAACATGCTTTACCCCAAGGAAGACAAGGAGAACCGCATTCTGCTTTACGCGTGC
EMPV1_15357	TCAGCATAGATGATTTGATAGTGATGGGGGGGAGGTAGTACGGAAAGCAGAGAGAAAGGA
EMPV1_15361	TTTGTCCCCTCATCCAGTGCCTCCAGTCCCAAGGACACAATTTCCTCTGTGATGTACACA
EMPV1_15362	TTTTGTGTGTTGGAGCCCTTAAGATGTCACCTCCGCCTTCTCTGTCCTTGTCATGCAGCC
EMPV1_15363	CTCATTCACACGCCATTCTTCCAGGTCTTGCTCAATCTCATTATCTCTGTGAGGCAGCCC
EMPV1_15365	TCAACTTCAAGCTGAAAGCAGGACAGAAAATCCGATTTGGGGAGGCTCTGGGCTCCCTCT
EMPV1_15369	CCAGAGTTTAGAATGTAGACATGAAATGCTTAGCTTTGCCTTCCCTTGGGGGTGCGGACC
EMPV1_15370	ATCTCCCTTATAACCCGGACATGAGTCTTCAGAGCAAAGCCCACACAGCCGCCAAGCTTT
EMPV1_15371	GGTTCGTGTGAGCCAGGATGGGCAGTTTCTCCACTACATCTTTCCTTACCAGTTCATGGA
EMPV1_15372	GTAGATTCTGATTCAGTAGGTATAGTGTGAGATTCTGCATTTCTAAGAAGCTCCCAGGTG
EMPV1_15373	TTTCTAACTCATATTTGTCATAAACAGTATTACAAGTGGGTATACTCTAAAAATTCTATT
EMPV1_15376	GTAGAAAACCTTCACGCAGTACTTACTTGCTCTACCTGTCCATAGCAGTTCCTATCAGCC
EMPV1_15377	GTCATCAGCTGTGTTACGATTGTCCTTATTTCCTACCTGTGCATCCTCATTGCCATCTTG
EMPV1_15379	TTTTGCTGTGCGTGACATGAGGCAAACAGTGGCGGTTGGCGTCATCAAGGCAGTGGAGAA

EMPV1_15381	CAGCATGGACGAGCTCTTGTGGACGCGTGCAATGAATTTTACACCAGAATCCCACATGAC
EMPV1_15382	TCCAAGTCTGCCTCTTAATCTTCCACAGATTCCTAGCTTTTCTACTCCTTCGTGGATGGC
EMPV1_15384	GTCTCAACATTACTCCCTAAAATGGGAGGAGAGCTAACAGTCAAGGAAGGTACCACTCCC
EMPV1_15385	CTCGGTTTAATTTAAAACGGGCACAAGTGCAAACAAACTTCACAAAAGTTTCTACGGTAG
EMPV1_15387	ATGGTGGTCTGAAGCGGGAAAAGGTGAAGGACACATTCAAGGAGGAGCAGCAGAAGCTGT
EMPV1_15388	CAGCTCAACAGACACACAGTCTCCCCAGGATAGTCAGTTTGAGACCTCTTTGGACTTGAG
EMPV1_15390	TGGGAATCTCAGTTGGAAAACATGTGGATGCTCAAGGCAGAATATACTTGCCCTATCTCC
EMPV1_15391	GATATCTCTCTGGCTGAGCTGGCTGACTTTGTCTCCTCTGCTGTGTTACTGTTGGGTTCC
EMPV1_15392	TCCTGCTGGGTCTTTGGATGAAGAGAAAGTTTCCACAGTTTTTTACACCATTGTGGGGCC
EMPV1_15396	TGTTCCCTCTGCCAGTAACTCTCTGGACAGGGTTCTTGTACAATATTTAGAGAAACTGCC
EMPV1_15397	GCACAGTGGACCTGAAGAAGCTCCGAGTTAAAGAGCTAAAGAAGATCCTAGACGACTGGG
EMPV1_15401	AAACGGCAGCTTTTAAAGAAACGAATGAAGCTTTCCTCTTTGGATGTGTTCATCATTGGT
EMPV1_15402	GGAGCCCTACAGCTCTTCCAAATATGCTATAGACCTTCTGAGTGTGGCTTTGAACCGGAA
EMPV1_15403	CATGTCCGACGACTGTATTTGCTTGAAGTTCTTCCATATGGTCATCTCAGGAAAGGCATC
EMPV1_15405	AGATGGTAAGAAGGAAGACTGCGTGAAGGGTGATCCTGTCGAGAAGGAAGCCAGAGAAAG
EMPV1_15406	CCACAAGAAAGTGGCCCGGATCTTTGGAATCTCGGTGGATCAGTGGCAGAAGATGCACAC
EMPV1_15407	AGAGCTACTGGCTTGTGGCTGACCTGAAAACCTAGCTTGCATTTATCACCTCCTGTTACC
EMPV1_15408	TCTAGCTCGAGTTCTCAAACCAGTCAACCCTTACACAGGCCTCCCGGTTAAGACCAAAGA
EMPV1_15410	TACCAGTAAGACCAGTTTGCCTCGGTGGGCTGTGCAGACGCTGTTATTTCCCCTGTGTAT
EMPV1_15412	TGGTTTAGAAGTACAGTCCCCCTTAAAAAGATTATTGTGGATGATGACGATAGTAAGATC
EMPV1_15414	TGTTCACTGTCACTCTGTCCCAGATTACCTTTTCCTCGGGCAGCATGGTCCTGGATGTGA
EMPV1_15415	ATAAACAAGACGCTGTTTTCTGAGTAACTGTGCCGTCACCACCGGGGGACTAAATGGCCC
EMPV1_15416	GGCTTTCTCATCCACTAGAATGGTCTCTGTCTAAGGAGGTCCTTTGCCCCTCACCCAAAT
EMPV1_15417	TACTGTTCCAGCTTCGACAAGCACAGACTGAACAACAAAAAGTCACAGTAGACCCTGCTC
EMPV1_15421	CGAATGAATCGAAGAACTTCAAAGAGAGTCAACATTCCTGCAAGGAAATGGGCTCCACAC
EMPV1_15422	GTTTGGGCTGTGAGCTTGATATCCTTGTTGGGCGGGCCCTTCCTGCTGCTTCCCTTAACT
EMPV1_15423	TGTACAGTGGGCCCTGAGGTTTCCTCAGCCCCAAAGGGAAACTCAGCAGCCTTAGGAAAT
EMPV1_15424	CCAAAAGGAACCAGAGCTGATGTGGCTGGAGCAAAAGAGCATTACATTCCCACTGCCCGT
EMPV1_15426	CAGTCATGCTCGATGTCATCTGCTATGCAGTTACCAGACAGCGTGGCAACCAGTAATTCC
EMPV1_15428	AATGGCATGCCTATTTGTGGCAACCCTGCATGCCTAGGTTTGCATCTTGGTTCTCTTTGT
EMPV1_15430	TCTGAGTCTGACGAGATGGCTTGCCAGTCCTACTACCAGAGTATTTGTCGCATTTGTCCC
EMPV1_15431	GGCCCTCAACACCTGTGTCTCTCATATCCTGGCTGTTCTGGTCTTCTACATCCCAGTCAT
EMPV1_15435	TACAGTGACTTTGTAAGAAGGGCTGGTAAAGGCAGCACGTTCAGATCCATCCCGTTGGTG
EMPV1_15436	CCCCCTGCCCCAGGAGTCATTCTGTAATTACCAAGATATGTCAGGAGTAGGATTATGATT

EMPV1_15437	GCAGGTGCCTGCCCACTGCAGCACACAGGCCACACCACGGTGCTGGCTGGAAGACTCTGA
EMPV1_15440	AGGTACACCCACCGTGGGCATCCTTTCTCCAGGGAGGCATCCTGATAAACAGAAATGTAC
EMPV1_15443	TCCATTTCCCCTTCTCCTTCTCCTTGTCTTTTTTGGTTCCGGGCGCAGTGGTCGAGTCGT
EMPV1_15444	GTGTTAATAATGGAATCGACGAAAGGGAAGTGGAGTCATGCGGAAGAAAGGGGACCGCTG
EMPV1_15446	ATCTAGAGGGGGATGGATAAGCCTCATTGGGGGAACATCCCTTTCCTGTCCATGGGATAG
EMPV1_15449	GTTTGCTTGCTCACTTGCTCCCTGAAGAAGCAGCACTATGGTTTCCCAAATGAGGTGAGC
EMPV1_15451	ACTGTCAGGAGTCCCTGGGCTGCCAGGGAAGCCAGGCCACGTCAAAGGAGCCAAAGGGGA
EMPV1_15452	AGAGGCTGCTTCCTGGGGCCACACAGACTGACTCTCTTATGGCTACTAACAAGTGTCGAG
EMPV1_15453	CATTGTCACACTGGGAAAGCTAAAGAGACAAAATGGTTTTGGTTTCGCTGAAGACTGGCC
EMPV1_15454	GCTGACTGCCTCTCTTAGCTAGTATAATTTCAGTAGTACACAGCAATTACCTATTGTGGG
EMPV1_15455	TGCTGCTTGGGGTCAAGGGACACGCCTTCTGAGCGCCCCCCTATTCCCGAACGGACATTT
EMPV1_15456	GTACTGAAGACGACCTGGAATTCTACATCAGGAAGTGTGGTGACATTCTTGGAGTCACTA
EMPV1_15457	GGAATTGCTCTTAAGAAACAGTTTGGATGGAAAGTAGATTTGAGGAATCCAAATTTAGAG
EMPV1_15460	GACACAGGATTAAGAACATGAAGTGCCGTCTAATCAGGGAGTTAAAACGATTCAATGCGG
EMPV1_15461	CGGGTTAGCTCTTCACGAAGATTATTTGGTTCTGAGGAATAAAACTTAACTCGTAGATGC
EMPV1_15462	GATCAGCAAGGACAACGCACAGACTCTCCGAGGGAAACTGAACACCCTGCGCGGCTACTA
EMPV1_15464	CGTTGCACCGTGGTTCTGGTTTACTCTTTTACACGTCCTTTCCTGAGATGTGAAGTCCAG
EMPV1_15465	GGGAGATTTGAGACACCAGGTTCTAAGCTGCAGACATCTCAATTAAGCACAATTCCTGCC
EMPV1_15470	CTGCAGGATACTCACCGTTCACACCTCAGAGAATACGAAAAATACGTGGAAGATATCAAG
EMPV1_15472	GCTATGAAGCAGCTAACCCAGCTCCTTCCAGAGGATCTGAGAAAGGAGCTCTACGAACTT
EMPV1_15474	CCTCAGGAGGAACAGAAGAGAGGAATCGAGGCACATAGTTGTTTTGGGCCTACTTCAGGA
EMPV1_15479	GCTGAGCAACGCGCTGCTGGATACGCCATGGAAGAAGCTGTGCTTCGGGAAGCAGCTCTT
EMPV1_15482	GAGAGCCGACTAATCTGTTTCGCCGTGATTTGACTCTCCTACACACTACAGTAATCGCCA
EMPV1_15484	GGAATGGGTTTGCTCTCTGGGGAAGGTGGCTTTTGGTGCTGTGGAGGCCCATTTCTTACA
EMPV1_15485	GAGCAATCTAAGAACTAACTTTCTGCACATTAGGAAGCAAACTCTCCCAGCCCAAGTGGG
EMPV1_15487	CTAGGCTCTTGGACCCCAAATGAACCCCTTTAACGTTGTGAAGAGGCAGCAGATCCTGGA
EMPV1_15490	TTGACCATGAGCTGCCCAAGCACTAACTGTGCTGCGTGTCTGCTTTTGCCTCTGGATGAC
EMPV1_15491	TGGTGGAGCCTAGATGACTCAAAGCACACTGAAGTTTTAGAACCTCTGTCATTTAAGAAC
EMPV1_15493	GGGAGGGGATCCTTGAGTTTCCCGAGAATTCTCTTTCAGATACCCAGCATTGTTACTGAA
EMPV1_15494	ATGGCTCCATCTTTACAATACCTGATGACTACAAGGAAGAGCCAAGCCGTATTCCTGATC
EMPV1_15497	TTTGGGCCAAAGAAGAAATTCAGACCTGTGGTCCAGAGGCCAGCCCCGAAAGACACATCC
EMPV1_15498	TCGACACCTTACTACTCGAAGTCAGTTCTTAAAAGAGGAACGATCCTTTCTGTCCTGACA
EMPV1_15500	GCATGATGGCACACTGTTCCCAAAGTACCAATGCTTCTTAAAGCTTAAGCACAGAAGTCC
EMPV1_15501	AAGAAAGCCCGGGAGGAACGCCGCCGAGAGCGCCGGATTGAGATGGAGGAGAACAAGAAA

EMPV1_15502	GGTATATGATCCTTCCCTCAAAAAATTTGCAATCTAGTTGTGGAGACATAAGAGACACAG
EMPV1_15504	GCCAAACAAAGGGAAGAAGCGTATACACACTTCTAGAGGACATAAGAGACCTAGCTCTAC
EMPV1_15506	TGTCCTCCAGGAGCTCACTTACCTTGGGCCGTGAGCTCCTCGAGGCCAGAGCCCGTGTCT
EMPV1_15507	CTTCCAGAGGGCAGTTTCAATTCTCCGTACTTCTTACTTGGATTCTGCATCTGAGCATGG
EMPV1_15512	ACCGGCTGTCAGGGATTGGGGTGAATAAATGGAACACAGAGGATTTTAGGGCAGTGACAC
EMPV1_15514	TGGGATGCACACCCATCTCATCCAGCCAAGTGTTGCCTTCACCCAGCTTTGCTGAGGCTA
EMPV1_15515	AGTGAGGAAACCGCGCCCTAGCAAGGTTTGTCCCTTACCCAGGTCGTCTGAATAACAAAG
EMPV1_15516	TTAGCTGATTGTACTCTGGCGCTTACAATCACTAATTCCACTGCCATCAAAACAAGGCAC
EMPV1_15517	TACCTCTCGGAGAATTCACGCCTGAAGAACGCGTGATAACAGAGCGTCCTCTTTGACAAC
EMPV1_15518	CCATGGAGAAAGAGAGGACTAAGTTAATTTTGTGTGAGATGCCAGCAGCTAATTGGGTTA
EMPV1_15520	TTGAGAAAAACTGGACTTGGGAGCACAAGCAAGGCCAAGAGCTGAGTTGTCGGGGGAGAA
EMPV1_15522	GATAGAGGCAAGGTCTCAAAGAGCGAGCCCAACACCAACCCACACAATCCCGTGTATGTG
EMPV1_15523	TGGGAAATGACCCTTCATCTGTCAAGTGCACTTGTCATTTTGCAACATGGTAGCAACCCC
EMPV1_15525	GTTGGAGTGGTATCTGCCCTTGTGCTCCTCCTCTCTTATGGCTACATTGTTGCTACTGTC
EMPV1_15526	CGCTAAAATCAAAAATATCATTTCCACGGAGGATGCCAAGGCCCGCCTGCTGGCAGAGCA
EMPV1_15529	AGCAGCAGAATCTCCAGACTTGAAAGGCATAGACGATGGAACCAAAGAGGATAAGCTCTG
EMPV1_15530	CTAGCTATGAGCTCGGTCTGCTACAACCCCTTCATCTATGCCTGGTTGCACGACAGCTTT
EMPV1_15531	TTGGAAGCAGAAACTATGATGCTTTTGGAGGACCATCAACAGGCAGAGGCCGAGGCCGAG
EMPV1_15534	CGGGCTTGGTTAGGAATGGCGTTGGCTTTGCTAAAAGCATGTGGTGGCACCGTAGCGCTT
EMPV1_15535	CAGGAGATAAGGGAAAAGCAGGCAAGAGGTCTCATTGATTATGCTACTGGTGCAGTTGGA
EMPV1_15538	CATCTGAAGTTTTTGCAAAGATCAGACTGGTACTGGAAGTGGTCCAGAAACAACTTCTAG
EMPV1_15540	CCAGTGGGCTGCCTGAAGAATACTCCTTTCTAACCACTTTTCGGATGACTGGAAGCACAC
EMPV1_15543	CAGCCCAAGCCTGCCACTGCTGCCACCGCTGACATCTGGGGAGACTTTACCAAATCCACA
EMPV1_15544	TCCATGATGTGGCTGTGCTGAGTCATGCTTAGTGTCTGAGGATCCGTATGTCACCTTTCG
EMPV1_15546	CTTTCCCAGTTCATTTTCAGTCCTATTGTTGGCACAGTTCTGAAGGGTATGTTCTTGCCG
EMPV1_15548	CCCCGCCCTTTGCTGTTTATTTCAGGACTTTCCCAGTTTCTGCAGCCGGAAAATAAGGGT
EMPV1_15553	GGCTTGTCATAAAAGGGTCATTTCTATAGGCTTAATTGTCCTAAAAGGGTCATTTCTCTG
EMPV1_15558	GAACCCAAATATTGTGAACTACTTGGACAGTTACCTCGTGGGAGATGAACTGTGGGTTGT
EMPV1_15560	AGGCCAACAGCGTTTATGCTTAGGTTCCAACAGTTGCCGGATGTTTTTGTCTACTCACCC
EMPV1_15562	GCAAGCTGACCCTCAAGACCACAGAGATGGAGACCATATATGACCTGGGTACCAAGATGA
EMPV1_15563	TGCAGAACAAGGACCAATACGACTGGCTCCTGAGGGAGCGCAGTGACACCAGCGACAAGA
EMPV1_15564	CGTGACGCCGACTCTAATGGCTCTTACTATCCTTCAGTCACAGCAGAGTTACTTCTGAGT
EMPV1_15566	GCAGGGCAAGGAGGTGACCGAGTTCTGCAACAAATACTGGCTCATGCTGGATGAGAAGGA
EMPV1_15567	CCCAAGCCAGTACAGTGACCATGCTGGATCCTTAACCTTTTGTTCCACAAGAGAACTCTG

EMPV1_15568	AGGAAGGAGAGGCTTTTCCAGAAACAACAAGTCTATAGGAGGGTCTGACTTGCTCGAGTC
EMPV1_15570	AGCCCTCCTTATAGCCTGGAAAAAATGACAGATCTCGTAGCCGTTTGGGATGTTGCTTTA
EMPV1_15571	GGACAAACTGCCACCCTGAAAACCGAAGAGATAGATGGTTGTGGAACATATGCAGACCGG
EMPV1_15572	GAGGCTACCCTACGACACCTTGGATGCCAAATGCACTGATGAAGGAGAATGGACCACAGA
EMPV1_15573	CCTCTACACTATATGACCATAATGAAACCCCAACTCTGTGGAGGCCTGGTGGCCATGACC
EMPV1_15574	TGCATTGCTGTGAGTGTGCTTGTGGTCACTGTGCTTGGAACTGTGATCTGGAGAAAGAGG
EMPV1_15577	GCAGTCAGTGTGCAAGAGGTTGGGTACTTCCATTTCATTAGGTTCGCAGTTTACTGGGCG
EMPV1_15578	CTTTATCTCTTATGGTCTCATCCTCACCACCATCCTCCGCATTAGCACCATGGAGGGAAG
EMPV1_15580	CTTCTCCCTTTATGTGTTTTTGTGTGCACCCTGACGTTTTGCTCGAACCGTTCTTGTGGA
EMPV1_15584	GAGCCTCTGGGTGAGGACAAAAAGAAAGGAAAGCCAGAGAAATTGAAGCGCTGCATTCGC
EMPV1_15585	GGTTTGGAAGTGCATCTTATTTGGCAGATCACCTTCCTCTGACACCTCTATTGGGTCCCC
EMPV1_15586	CCGCATCTGCTGCCCAGCAACGAGTGCGTGGAGCTCTTCAGAACCACCTACTTCTGCAGA
EMPV1_15587	GATGTTCAGGGCAAAAACTGCTTGACCACTTTCCACGGCATGGATCTTACCCATGACAAG
EMPV1_15588	TGTGGCTCAGTGGGTTAAGAGCCTGACACAGTTTCTGTAAGGATGCAGGTTCAATCCCTG
EMPV1_15589	AGGTCACAGTTGTGGCATAGATCCAGTGTTGCTGTGGCTGTGGCATAGACTACAACTGCA
EMPV1_15590	TAGAGGTTATCAAGGAGGACGCTGCTGAGGGGCTTTCTTCACCCACAGGCTCCTCGAGGG
EMPV1_15591	ATGGAGGCAAGTGGAAGGATTTGCCCAAGATGGAGCTTATCGAAGAGGCGTATAGCAACC
EMPV1_15593	TCATGAATGCCTCTCGCCTCCATCACTTGTTTCCACCACGCAGGGCTTCCTTTACCCACA
EMPV1_15594	GACTTATTTGACGTTTTATTTTGGTTTCCCTACCCCCTCAATCTGTCGGGGAGCCCCTGC
EMPV1_15596	AAGCACAAAGAGGAGTGGGGACTCCTACAGGTGCCTTGTGGCCAAGATACTCAACAACTA
EMPV1_15600	ACTACCTGAGGCTGGCCGGTGAGATCATCACACTCTTCACTGGGGTCCTGTTCTTTTTCA
EMPV1_15601	GGTTGAGAGCTGATTCCTGCCTTCAGGTGGCAACATAAGGATCAAATAATTCTACTAGGC
EMPV1_15604	GCTCGAAAGGAAGAAGAGCGAATAATGCTTAGAGACGCAAGACAGTGGCTGAATAGCGGC
EMPV1_15606	CCGAACACAATGCCACCTCTCAAAACAGACATAATTGGCCCCTCGGACTAATCCTGGCAG
EMPV1_15608	ACCCTGGGATTTTAACTTTTAATGTGGCCAGCCCACTGGGATCACTTATTAGGAAAGCTT
EMPV1_15611	TGTACAGTGGGTTGAGAAATTGCCGTGGTTGGGAAAACAGTGAAGAAAACCTCCCAGAAC
EMPV1_15612	AAGAAGACAGCCACCACGACCAAGGTCACCAAGGCGGCAGTGAAGGCCAGCAAGAAGTGA
EMPV1_15614	GCTTCTGGGGCTTTAACACACTGAGCATGGCCTTCAGCTAGGAAAGAAATCCTTAGAGGG
EMPV1_15615	ACCTGGATCAGTTTGGGACTTACTTCCATTTGCCATCTCTCAGACATCACTGGCCTCCAG
EMPV1_15616	GGCTGCATTTACTCCCTCCAACAACCTTTAATGTACAAGACCATTGTACGCCTCACCCGC
EMPV1_15620	CTCCACGACCATCAACAGGAACCGCATGGGCCGGGACAAGAAGAGGACATTCCCACTCTG
EMPV1_15621	GGCATCACGTTTGCCGTCTTTCGGGTCCCATTTCTAGGTTGTCATAAAGCTCTGTCTCAG
EMPV1_15622	AAGACCAGCCAGATGAAGAGACACAACGGACAAGGTCTGAGAGGGTTTCGAACTCAGAGC
EMPV1_15623	CTCCCCCTTGACCACCACCCTGGAGGTGGCCGTTTTGGATTTGTTGTCTTTAACTGGTGA

EMPV1_15624	CAGACTTAGACTCAGATCCCTTCGGGGAAGACGGCAGCCTCTGGTCCTTCAACTACTTCT
EMPV1_15625	CTCGGAAAGGATATGACTTAGAATTTGGAAAATGCCCACCGCTGCTCCATGGGGTTGCAG
EMPV1_15627	GGCAAACCCTTTCTTCCGAGATGCCTGGGCTTCAGCTATCATCAACATCATGGAATTGCT
EMPV1_15628	CCATACACTCACCCACCTTACTGGCTTGCTTGGTCTGCCTTTCTGTGGACCCAATGTCAT
EMPV1_15631	AGCTGCCTCTGTACAACCTCCGTGAGTCTTGCCATTTTTCTTAGTGTGACCAACCACACC
EMPV1_15632	CCCGCGGGTTCCCTGGGTCGCCGCGCTGCGCGCTTCAAAGACACCTTCACTCAGGAGCTA
EMPV1_15633	AAGGATCTTTGTTTTCTGAGAGTTTGTTCCTTCCCACCTGTCCTGCGGCTGTCTGATCCT
EMPV1_15635	ATGAGGAAGGCAGCAAGTGTAGCATCCTCTCCAAGCACCAGAACTTTGTGGAGCACTGCT
EMPV1_15636	ATTTACGAGGGAATATGAAGACCAGGGAGTGGAAATGTGCTGACTTTCCCATTCGAGGAG
EMPV1_15637	GAGTAGATTGACCCATTAGACTTGGCCCTTCCCTCATTGAACTCTGCCGTATAGCCTCTG
EMPV1_15638	TACGAAAGAGCTGGAGGACCTGGAGAGGTGGAAGGAGCAACAGAGGGCAAAGCCCATTCA
EMPV1_15643	AAGGCCTGCTGAGGGATCATCAGCAACTGGCCATTCTCGCTTCGCACGAGGACCATACCT
EMPV1_15644	AGGGATGATTGAGGCAGCATTTGATTATAGTCGACGCTGTTACAGGGACACATCGAGTCT
EMPV1_15645	GTGGATCTGAGCTATTGCAACCCAGAGAGAATTAGGCAAACCATCCCAGACCACCAACTG
EMPV1_15646	GACCGGCTAGGAATCTTTCAACAGTGCCAAATCAGAGTAACGACAGCCGGCATCTTTTCC
EMPV1_15649	GGAAGCACTGGGACAGGTTTCCGGGGTGATTCAGGCTTTGAAATTGGGAGCAGAGCTTGC
EMPV1_15651	GCTTGGGTTCTAATGTCCATGTTTCCCCAAGATGCTCTTTACTCTGTGTCCTCAGATCAC
EMPV1_15653	CCATGGAGCAGGCTTTGGTGCTCAAATTACATCCAGACCACATCTGCGTGAGTGCAGAAA
EMPV1_15654	TGGTCACTAGCAATGCCTCGTCAGCAGACCTAGACAATAATCCCTGAGTGTTGTAGGGCT
EMPV1_15655	ATCTCCCTACCTCTCTCCCCACCAGTGCAGCCATGCTGAAGATCGCCGAGATGGAGTACA
EMPV1_15656	TTTCAAGGTTCTGACAAGATGACAAACCAGCAAAGGCGCCTCATTTCCTCCACCAAAATC
EMPV1_15657	GGTGGTGAACTTCAACAGCAACAGGCTGAAGGCCGTGAAGGGCTTTAGGAACGTCATCGT
EMPV1_15658	TGGAGCCAAGACACTCTGGTGGGGGAGCTTGGATGCCTGGCTTTCTTTGAGGACATCTTT
EMPV1_15661	GTGGCTAGTGTCTTATTCCTGCTGGTACCCTTGTCACTCATCCTGATTTCTTATGGGCAC
EMPV1_15662	TTTTCCAAGGGGGAGGACAAGATCGGGAATCGGACTGGCCTTCTCTCTTTGCACCCGAAA
EMPV1_15664	TTTGACGGAGCAAGTATTACCTAGTCTGGATCTGTCTTGATGTGGACGGCCTGAGGAAGG
EMPV1_15665	GTCCTGTAATGAGGACTAAGAGGAAGTGTGCTCTCCAGCTAGGTCATGGCTTTCCAAACC
EMPV1_15666	GGCTCGCAAAGAGGGCAGAGTCCAGACTAGGAGTGTCCTGGAAGACTTTCTTGTATAAAG
EMPV1_15667	ACAAAGGTGAAAAGAAACCTGAAATATTTGTTGATGGCTGGAATATTTATTTTTTTGATC
EMPV1_15671	TGCCAACAGCCACTGAATTTGCCATATTTCACATCAGTGCTTGTAATTTGCTGATGCCTC
EMPV1_15672	CCTATCAGTGGGAGAAAAATTCTTGGGCACCACATGTTTCTAACTGCCAGGTCAAGGCCT
EMPV1_15674	AGCCCAATGGTTTATTCAAGGTGGTGTTGGGAGTTGAGTGCAGCCAGGATAAAGCCCTTT
EMPV1_15675	TTTGAGAGCCAGTCCTTCCTGTACAGACAGGTGCGGAGGATGACCGCTGTGCTGGTAGCC
EMPV1_15676	CCCTGTATCAGCAAACAGTGTCAGAAACCCCTCAGTGACCAATCATACCTTAATCAGCAT

EMPV1_15677	CTCCCCGGTGCTCGGATCTCGAGGGTGCTTATTGTTCGGTCCGAGCCTGGGTCTCCCTCT
EMPV1_15679	GACCCAGGAGCTGGAGAAGGAAGCCCATAAAAGGAGCCAGGAGTTAATACAGCTTAAGAG
EMPV1_15681	GTTCATGCCTTTGGAACCCGTACACTTGCACAGCGTTGTCCACGACCCTCTCGCCTTTAT
EMPV1_15682	GACGTGGAGAAGTAGGAAGAGGCCAGATCACAGAGGGCCTGGCAAATCACAAAAGCGATT
EMPV1_15683	GACTATCACACAATGGTTACCAAGTATTTGTCTGGCTTTCTCTCCTTATTAGCCACTGGC
EMPV1_15685	GACTGGGCTTTGGCTCTTTTGTGGAAAAACCCATCTCCCCATTTATGAAAACAACGCCAG
EMPV1_15687	TGCATTTCAAGTGCTATGCCTGCTTCTATTGCTTCAGCTCCCCTCTTCATACTGAAGCAG
EMPV1_15688	GCTCTTGATTCCTGGCCAAGGTGACTATGTGAAAGTGGCTCCTGGATTTCAGTTCTTTGC
EMPV1_15689	ACTGGTCGCGCGTTCTTTGTCATTTTGCCCGAGGAGACTTCTTTCACTGTGCCTTGATGT
EMPV1_15691	AGAGGCTCTGTTGAGTGAACTGTCCCTAGCAGATGGGCCTACAGTATCTGTTGGGACTTA
EMPV1_15693	CTCAGTCTCAGCGCTACTGACATTCTGAGCTACACCATTCTCTCTTGATGGTCTGTCCTG
EMPV1_15694	CCTTCTCCCCTGAGTGAGAATGTTAGGGAACTAAAGGAAGCGGCCATTCTCGGAACTGAA
EMPV1_15696	CTGAGTTGCATCTTTTATTTTCTTGTCCCCATGTGGAGAAGAGTACTGCAAAAAGGGACC
EMPV1_15698	ACGATTCTTACCTTCCCCTGGCATCCTTTCCCCAGCTTGGAGCTATTTGGGCTTTCTGGT
EMPV1_15699	AGTTCCTGTCATGGCTCAGTGGAAAGAAATCCGACTAGGAACCACAAGGTCGTGGGTTCA
EMPV1_15700	CACAGCCTCCAGATGCTTGCAGAATTCATGGTCATCTGTATGTCAATAAAGTAGCAGGGA
EMPV1_15701	TGAAACCCGAATTCCTCACGAGTCGCACCTCCGTTTTGGACCCATCTCTGTGTGGGAATA
EMPV1_15703	CTGAATTCTGCCTGTTCTGCTCTACCCTCTCCATGAAACTGTCCCAGACGGTACTGAATG
EMPV1_15705	GGTGCCATCAAACGTGCCTACATCCCCAACTTTGAATCTGGCAGCAATAATCCTGTGAAG
EMPV1_15706	AGCAGCTCTGTTAACCTTTAGGCTCAGCTTGTGGCATTATCTCTAGTCATTTGTTCTCTC
EMPV1_15711	CCAACCTCACCATGGTGGATGCCTTGGAAGCTAATGGACAGCAAAAATTTTGATGACTAC
EMPV1_15712	GTCATTACAACCAGGGATGTCCAGAAGGCTCTATGTGCAGACTTCAAGATGAAGATGAAG
EMPV1_15713	TTTGAATTAATAAACCACCATCCTGCAACTGGGCAGCCACTGAACCGCTCTGAGTCCCAG
EMPV1_15715	CTGCTGTTTTTTCACTAGATTGTGTGAAGATCATAAACTGACGAACTTTATCCTTCTGCG
EMPV1_15716	TACCAGAGAATTCCTGCTGGAAAGGAGACCTTTGAATGCCAAGAGAGTGGAAAACCCTTC
EMPV1_15717	GACTGGACACGGATGATTTGGACGAGATTGAGAAAATAGCCAACTGAGAAGCTCCAGCCG
EMPV1_15720	TGAGGGAAGCAGTTGATGAATTTCGGCGTTTTCAGCGGGACAAGGAAGTGAACTCTCAGC
EMPV1_15721	AGAGGGAGGCTGGCTTTATTCGGGAGGCCAAATGAGAATCAGGGACCCAAAGACTGATCA
EMPV1_15723	TGCTGTGCCAGGGCTTCGCCTTCGACTTCCCGGGCTTCTACGACGACGGCTTCCTCAAGG
EMPV1_15724	CCAGGGCCTGTAGAGATAGGGGCTAGACAGATGGGTCCAAGGGTGAGAAATAGATTTTAC
EMPV1_15725	TCTTTGGACCTGCCATCTTCCTCTACATGAGACCCTCCTCCACCTTCACTGAGGACAAAC
EMPV1_15726	GGCTAGAACTTGACATACGATATCCTTAATATTAAGGCTTATCTCTGCTTGGCAGCTTCT
EMPV1_15727	AAAGTCCGTGCGTGCTGGTGCCTCCTACAAACGGCAGAATGAAGGAAAGCCAGAAAGAGG
EMPV1_15729	GGAGCCGGACTATTTGCTGTTTTGTGGATTTTGTGCTTATCAGGGATCTTAAAGGTATTT

EMPV1_15731	TGCTCAAGCTCCTCCACAAGCTTAATAACTGACACTTGCAGCTTGGCTTTACACTTGGCC
EMPV1_15735	GCTCAGAGTCCTCTTTCAAGCTCATTCAGGTGGTTGGCAGAATCCAGTTCTTTGCCTCTG
EMPV1_15736	CCAGAGAGGAGTGCAGTTCTCCGCCCCACCAGGCGGCAGCGGCTAGGACGACTCTGAGAA
EMPV1_15737	CTGTGTGCCAAGATTTTGAGTCATTAAAGTGTGGGATCTTAGGAGTTTCCGTTGTGGCTC
EMPV1_15739	GAGCATCAGAGATCTCATACTGGAGAGAAGCCTTACGAATTTGGCAAAGCCTTTAGTTGG
EMPV1_15740	GGTGTTCTTCAGCCAAAACTGTAAGATATGTTTGATTTGTGTACTGAGTAGCTTCGGCAG
EMPV1_15741	GGATGAAGAGTTTGCATCTAGGAGCACAGGGCCCAGCCCTGACGCTGCCCGGTAGAAATA
EMPV1_15742	TGTCCCAGTGTGCACACGAGTCCGTGTGTTCCCGTGTATGTGCACATGCGTATGTGGAGA
EMPV1_15744	TGCTCTGTGTCACACCCTCCTTCTGACCCAGCTGTCTTTTTGTGCTGACAACACCATTCC
EMPV1_15746	AAGGTGTGGCCCCCTTTGGCCTGGAGGGTACCAGCTTTTGAGACTCTAAAGGTTCAGAGA
EMPV1_15747	GAGCACCACACCTCTGGCATATGGAAGTTCTCGGGTTAGGGGTTAAATTGGAGCTGCAGC
EMPV1_15749	TCATGCTTATGCTGCTGGTGCCAGTCATTTGAAGAAAAACAGTCCTTGGAGGAAAAGCAG
EMPV1_15750	GGCCTTAGTAGAGATGGACAGCCGGATTCCTTACGATGACTACCCGGTGGTTTTCCTGCC
EMPV1_15752	TGTGTCGGGAAGGAAACCAGTGCCTGTTCTCTCATGACTTGGCAAACAGCAAGCCATCTA
EMPV1_15753	AAGGAGCACTTGGAGGACATCAGGGAGAAGAATGAGGCGTTGCTGGGGGAGCTCTTCCTG
EMPV1_15755	TCATGAACGCTCACAGCATGGGCATCGGGGGTGGCCTGTTCCTTACCATCTACAACAGCA
EMPV1_15759	TCCCCTCACTGGAAGCTACAGGTTACGACAGTTTCACCTTCACTGGGGGTCTGCTGATGA
EMPV1_15760	AGCTGCGGTGTAATGAGTGGCAGAGGCGGCTCAGATCTGGCGTTGCTGTGACCGTGGCGT
EMPV1_15766	GCCTAAGCCTCCAGTAATAAAGCAGGACACTAAATACACCAGATTTTTCCCTGTGTGATA
EMPV1_15770	ATGCTCGCCATCCTGCAGCACTCCACCCAGGACTTCAGGGTTGGCCTCCAGAGTCCATCG
EMPV1_15772	CCTGTGTCTTCTGAAAATTAAGGGCAACTGTGACCGTGTTGGGACTTCCTTTTGCGGCCG
EMPV1_15775	TTTCACTAGCTCCTCAAGCCCACGATCCCTTGGCTAGTAGATGTGGCATGGCCTTCCCCT
EMPV1_15776	CACCTGTTCACGTGGGAAGAGCGATATGAGCACGAACATCATAAAACCAGGCTCCTCAAC
EMPV1_15777	CGTTTCCCCTGCGCCACAACAGGAACTCCTGGAGGAATAATCTTAATTGACACCATCATG
EMPV1_15779	AGCCAAGTTGAATATCACCCAGAACCTCCCCTAAAGGGTGGATGTTTCATGTTATGCCTG
EMPV1_15780	GAAGGAGATGACAAATGCCGACAGCCAGAACGAAGGGCTACTAACCGTAGAGCAGTTAAG
EMPV1_15781	GCTAGCATTTGTAGTTTTGATAAATCCTGTTGCTGGCAATGGAGTTGTGCCAGAGAAATC
EMPV1_15784	GAATAGCAGTCAATAAAGAAATGGTGAATAATGCCATATCTGTTATATCATACACCTGCC
EMPV1_15785	GTAGGCCGGGCACAAAAGAAAGCAGAGCGACAGGCTGAGTTAAAGCAAATGTTTGAGCAG
EMPV1_15787	TCAATTGAACTCTCCTGCCAGACTTGGCATCCTGAGCTCAGAAGAAATGGCGGGAGGAAG
EMPV1_15790	CCCCGGGCTAGTTCTGACCTTCTGGAAACTGATGTCTTCACTCATTTGTCCCTGGACTGT
EMPV1_15791	AACGGGAAGGGAGTTGGGGTGGAGGGGGCTGACACCAAGGAAGGGAGCAGGGGCAGCTCT
EMPV1_15793	ATAAGATCATTGAACTCCATGTCCAGAAACTCTCTGTTTCCAAGCCCACGGTGACAACTG
EMPV1_15794	CGTGGAGAAAGAAACGCAAGAGAAGAAGAGATTGAGCCGAACCAACGAAGAGCTGCTTTG

EMPV1_15795	GCCCAGAGCTGGGAGGACAGGTGGGAAAGAGGACATGTGCTCTCATCACTTATTTGAAAA
EMPV1_15796	TTCTCTGCGGGAAGTGTAAGAAGCAGTTCAACTCACTGCCAGCGTTTATGACCCACAAGC
EMPV1_15797	CAGCTCGGACCTCTGACCTCCCTGACGGGTCCTGTACTGAGCTGCCCCGAGGCCCTCACT
EMPV1_15799	GAGTTTGAACCATCTTGCCAAACCACATCTCAAAGAAGCATTTGGACTACGAGGGTCACG
EMPV1_15802	TCCTTTGGCCACTTTGAATAAGTAACACCCGCCTGCGAAGAAGGGAAGCCATGTCACCTG
EMPV1_15803	CTGAGGACATCCAAGCCGTGGCTAGCGGAAAGTGTTGGCAGGCTCGGAACAGCCAAAGAA
EMPV1_15804	GTCTTATCACCACTGTTTTCCCATTGCCTGGATCAGTGCTGGACAGGGCATGACTCAATG
EMPV1_15807	CGGAAGGTCCTCGTCGACCAAGTAGTGGCCTCTCCCATGCTGGGCGTCTGGTATTTCTTG
EMPV1_15808	GGTCCTAGAGAGAGCAAATTATTAAAGATGGGGTTTCACGAAGGTCCTCAGTGGAGCTGC
EMPV1_15809	GCTCTGACAATCTCGCAGCCCCCATCGTTTCAGACGCATTAATCACCTGGTTTCTCCCCT
EMPV1_15811	GTTCTTTGGTGCCAGAAAGTCACTAATGGATCGGTTGTTACTCAAACTGCATAGGCTCCC
EMPV1_15813	CGGAAGAGTAGCTGAGCCGCGGCTGGTGCGGGACTGGGTTTGCTCAGCCTGGGCCCGCTC
EMPV1_15815	TGCCTACGCTCCGCTGTGTTACTTGGACCACAGTGTCTGCCACAGTGTCTGCCACCCTTT
EMPV1_15819	CTCAGCTCCTGGGCAACTGGATTCCTGCTCATCTTTCCTCCAGTAATCTTAGGACTCAAG
EMPV1_15821	CCTGAACAAGATTTGGTCTGGGACATAAACCAATAGAATCCAAGGACCAAGATGGCCTTG
EMPV1_15822	ATGTTACACATGCAAGCCCTTTAGGTAGGTGCATCCCCACTTTACTGTTGGCTGGGAGGC
EMPV1_15823	ATCAAGACCAAAATCAAGAACTATCAGATTTCTCCCTTTGACAGCCGCTTCCCCAGTGAG
EMPV1_15825	GTCTGGGAGCCTTGGCTTTCTCTTCACTCATTGACCCGTCAGCTCCTTGTTCTTGAATTG
EMPV1_15829	GGTCCAATCAACTGTGACTGCGTGCATGTGGACAAGAGGGGCTGTTCGGGTAACAATTTT
EMPV1_15831	TGAAGCCAGACCCGACACCTCTGCTGACATCTCGTCACAACGTCTTCCAGAATGATGAGT
EMPV1_15832	AATTCCAGAATCCTGTCAACAGCCATGTGATGAGCCCTCTTGGAAGAAGACCCTCTTGCC
EMPV1_15833	CAGCAGGGTCTGCTTCCCTGACTTAATAACGATAGCTGCCTGCTGAGTTGTCAAGAATCT
EMPV1_15836	CTGTTGGGGGATTACTACCAGGCCATCAAGGTGCTGGAGAACATCGAACTCAACAAGAAG
EMPV1_15837	ACGTTCTTGAAGGCTGGTCTCTCTCCCTCTTAGGACTGACCTGTAAGTGGCTTCATTTTC
EMPV1_15838	CTTGATGTGCCTGAGCCCTAAAAATACCTCCCTGGTTGAACCTCCCTAATAAGCCTTCGG
EMPV1_15839	ATGGAGCATGGATGTGTCAGATAGGCAGCGCATCTCTAGCTGGCTGTTACAATTTCACTT
EMPV1_15840	TGCCAGTCGAGGATCCGAACCCTGCCAAAAAGCTACTCTTTCTCCTGCTTACCATCGTCT
EMPV1_15841	TGTTGAATAGTAGTGGTGTGAGTGAGCATCCCTGTCTTGTCCCAGATTTGAGTGAGAAGG
EMPV1_15844	TTCTTCTCCCTACATCAGACGCCTCGTAATCAGATGGAGCACAGGGATGTCCCAAACCAC
EMPV1_15845	CTGGCTACCAAAAAGTCCCTGGAGAGTGTGGCAAGCAGGTTTGAGAACGGCAGCTGAAAT
EMPV1_15846	TGGATTTTGACAACTTTATCAGCTGCCTGGTCAGACTGGATGCCATGTTCCGTGCCTTCA
EMPV1_15849	CACTACAGCCCATTCCAGTCTCGACAACAGCGCATTTCCCTTATATGACGCACCCTTCAG
EMPV1_15850	CTATGTGCAAAGAGCCCCAAGGGCTGAATGACTCCCTTTGTACCTGAGAAATGATTCTTG
EMPV1_15857	GGCCTCCCAAGTTCTTGGGGACACATACAATATTCCTTTAGACCCCCGAGAGACCAGCTC

EMPV1_15860	TTTATGGTGTTCCTAATCTCGGGGACCACTCCTCTGGTTCAGTCACTTGCCACTTCCCGT
EMPV1_15862	GTGAGTGCAGAGAATGAGCTAGAGGATATAGCAGCGGGAGATCAAGAAGATGCCAAGGCC
EMPV1_15863	GGAAGGGTCTTCAGATGAGGAAGATAAAGAAAGTGAAGAGGAGCCACCAAAGAAGACACC
EMPV1_15864	TTCAAGGTGGCTGTCCCCCTCCCCCATATGTACAGACAATAATAGGGTGTGGAATGTCGT
EMPV1_15866	TGCAAGCGGAACTGCTCTGATACAAAATGAGTTCGAGGATGCAGGTGCTACTTCCACTCA
EMPV1_15869	CACCCACGGGCTCGGTGTTTACGAGGCAGATGGAAAAAGACCCGTTTCTCAAGATAATTC
EMPV1_15870	CTAGCCAACATGCCTGAAGCCCTCACCTGAAATAGAAGGCCACACCTTAAATCCATCCTC
EMPV1_15871	TGCAGACAGAAGACTGGTGGTGGAAAGACCCGGACACTCCTTGCAGGTTTGGGGTCAGAT
EMPV1_15873	TGAACACTCAGCTCTGCGTGGGGCTGGTGGTGGCTAGCTGGGTGGGAGGCTTTGTCCATT
EMPV1_15875	ACATCAGCCTTCTCCCCGAAGGAGAACATGTTTGGAATGGGTTTCACATCACGGAGACCA
EMPV1_15877	GTTTTAGAGTTTTCCTGGCTCGATAAGTTATACCCCGACCTTGGCCTATTGGTGAAATGG
EMPV1_15878	GCCAAACAGAGACACTCTAACCTGCATCTTCTTGACTATTTGTATCGGTGTCAGTAGCCG
EMPV1_15879	TCAGAGTAGGTGTCAGTACCAAATGGATAGAGGGGTACGGGATTAGAGGAGTCAGCACCC
EMPV1_15880	AGAGTTTCTCTGGTCAGTTCTATGACCGCCCCGTTGGCTTCAGTCAGCAAGACACGAAAA
EMPV1_15881	ATTTTGTACCCCCAAAGGGATTCCTTTGAAGTAGCACACTCATTTTGGTTATGCATCCTG
EMPV1_15882	TCTTGTTATGTGAAGATCTTCTCTATGGAAGAATGTCATTCAGAGGATTTAATCCCGAGG
EMPV1_15884	GGGTGGGTGTGGGGCAAAAGGTACATGGAAAGCCTTTTAAACTACAACAATGCTACTAGC
EMPV1_15885	TGGTGTCACTGCAGGCTGAGTAGTAGGGAGCCAGGACTTTGACTTCTGCTGGGTTTTATT
EMPV1_15886	GCTATTTTTAAGATGAAGCCCACAATTAGCTGTCCTTTAACGCCGCAGGGCCCCCACCCA
EMPV1_15887	GAGGAGAGCCGTCGAGGGGTCATTACTTCGTATTCTCCAGAATCCTATGTCGGCTGGTTT
EMPV1_15888	TGCAGGAAGTGATGAGAAAGGAGCCAGGAGAGAGCACTAGCAGAAAGGCCTGTGAGGTGT
EMPV1_15890	CTGGTCTCCTCTTCAGAACTGGCCATTTAAGATGCTTTCTGTGAATTCAGGTCGTAATGC
EMPV1_15891	CAGATCTTGGCGACCTACATGTTGCATAAGCTAAAGCAACAGACACTCCTAGGCAAAGTT
EMPV1_15892	CCTTCAGTGCAGGCTGCCATCCCCCAGACCCAGCTTATGTTGGCTGGAGGACAGATAACT
EMPV1_15893	GGAGAAGAGGTGTCAGACCTGCAAAGCCAATCTCTGCCACTTTTGCTGCCAGGCTCATAG
EMPV1_15894	AACTCAATCCAAGCCTGAATCATCTATTTTTATAAACAGCTGTAGATAACCAAATATTTT
EMPV1_15895	AAAGGCAGTGTGCCTCCATTGCTCCTTTCCGTCAGAGGTTCTGACTTGGGCAAAGGAAAC
EMPV1_15897	AAAGCAGCCCCGCAAGCCGTTTTCGCTCCTCCAACCTCCGCCCTGGGAGTAGGGTTAAAA
EMPV1_15898	CCTCTTCTTCCTCCTCATCCTCCTCCTCAGCAGACAACAGTGATGCGGATCAGCACCCCC
EMPV1_15899	AGCCACCTAGATGTCAACGTTGCTCAAGCAGAAGCTACTTCCGCACCAGCTGTATGGGGT
EMPV1_15900	GTGTACCTGATCAAATGGATAGTACTTCATCTGTCTGTAGCAGTCCCCTCATTAGGACTA
EMPV1_15902	TGTTAAAATTCAGATCCTCAGGTCCTGCTCTCCCCCAGTTCTGATTCAATAGATCTGAGA
EMPV1_15904	TGAAGAAGCTCAAGTTCAGAGAGAAGATGCTTCTGCCCGTGGGGAGGGCGCTAAGGAGAA
EMPV1_15905	CAAAGGATTGTCTTCTGCTGTTGGATGGCCACCTTGTTGATCATCTCCCCACCGTTTAGC

EMPV1_15906 GAGCTTGTAGGAGGAGGGAAAGAAGAGGATGCTGTGAAGAAAAGTGTAGACTGGGTGTCC
EMPV1_15907 TGTCCGAAGTCCTCTATTCAGACATTTAGTTGGGCAGCCATTCGGTCAGTCAGCACGGAC
EMPV1_15909 TTCTCTCCTGGCCATTTGCTGGTCAAGAAACCTACTTGAAGCCTCAATGCTCCGCACTCT
EMPV1_15910 ATATACAACAAGCAGTCACATATTGACTACCCAGATATATAAAACATCCGCAGTACACAG
EMPV1_15914 GTGGAAAGTGTCACAGGAGAGGGGACCCTGGGCAGAGATCTGGGGGCTGAGGGGAAGACA
EMPV1_15915 ATGGTAATGCGAGGGGTGCAGAAAATGAACTGTAGAAGCGTGACCTGCACAAAGTTGGGG
EMPV1_15917 GGCAATCCCGATGATGAAGAGCCTGGTCCTTAGTTTCTTCAGATATGCTGGGGGTGCTAA
EMPV1_15918 TTTCAGTCTTGCTCACACCACATGGTATCACCTCCCCTGTTTATCCTGCCGTTGCCACAC
EMPV1_15920 AGCATCATGGCTGACTGGTCTGTGTGGCGCCCTGAGTATGTGCTCCTTTGCTTTTACGCT
EMPV1_15921 CGTAGAACTCTGGGGTGGATGGTATTTGATGGTTTGACATCTCCGAACTGCTAAAGTCGC
EMPV1_15922 TGCTTCTGAACTGCATTTCTTTCCTTATTACTCAGTGTCCACACCCAGGGCGGTTGCCTG
EMPV1_15924 TGTATCTTCGTTTCCGTAGTTTGCCAAGAAACAGACCTCCCTTCGACTACACTGCAATTG
EMPV1_15927 CTGCGCAGGGCCAAGGGCATGTTCACGGCTGAAGACCTACGCTAGGGGGACTCCCCGCAG
EMPV1_15928 GAAGAGTGAGACTGGAATACCACAAGGACAGGGGTCCCAAGTAAAGAAGAGTGAGACTGG
EMPV1_15930 AAGCAGACAGCGAGGGCAGCCTCAGTAACGCGTCTGTTCCTGTTTCCTAAAACTTTCCTA
EMPV1_15931 GTCGACTCACCACTTGTTAGGAATGTTGGTACTTGAAGAAAGCCAGGGGTTAGCTACTAG
EMPV1_15934 GGAGAATATGGGAAGTGAGGAGAAAAAGGTTGTCGAAGGCTACTTTTGGGATGAAGGATC
EMPV1_15935 TTATTTTTGGTAAATAGTCATGTTTTCCATTGTATCATTTCCTTATTTAACTTGATGATG
EMPV1_15937 CTTTACTACTTCACCTATCATGCCAGCTCTCGAGGGAACCTGTGCGTGAACCTCATGCGT
EMPV1_15938 TCAGAGGAAGAGATTTACATGCTGATGGAGAAGGACATGCGGTCGTTAGCGGGGCTTCTG
EMPV1_15939 AGGCCGAAAAAAGCCCTTGCAAAGAAGGCAGAGAAGCTGGCGAAAGGGAGAAAGGGGAAA
EMPV1_15944 AAGGGAAGAGGTTGATGGACACTCGTCTGTCTGGGGTGCATTCCTGGTGAGAAGGCTAAA
EMPV1_15946 GTCTTTTAATAGAGCCTTGTGAAGCCTTTCTGGAAGCTGGAGACTAACACTCTGGAGACC
EMPV1_15947 GTCTAATCCTGACACAGATGGTTAGCCAGGAAGCCCAAGCCTTTTGTTGTCCCCTATAGT
EMPV1_15949 CCTGCCGTTGCCCTTCTCACATTACAAATAAGATCCTAGTCCAGGATGACTGTCAGCATG
EMPV1_15950 GCATCTTTTTTGTGTGTTTTGCCTTCCACAGTTCCTCTTCGGTGACCTCTTTCACCCATT
EMPV1_15952 GGAATGGACAAGAGACAACGCTTGTTCGGGAACTAGTTGATGGGAAACTTATCCTGACAC
EMPV1_15955 GCAGTGCACACACATACAGTAGGCCATACTTACCCAATGAACAGGACATTTTGCTTTAGC
EMPV1_15956 ACACGGTATACTGTTGTTTCAGAAACCTAAATATTCCATCTGTTTGGTACTGTATTGTGG
EMPV1_15957 GCAGATCTCTCTGCTTTAGTACGGGAGGCTTCTATCTGTGCACTGAAGCAAGAAATGGCA
EMPV1_15959 CACCTCAAATCAGGCTTAAGACAGCGCCGAATGAGGGGGAAAGGGAAATGCCGACCAATT
EMPV1_15960 CAGAACTTTGCGCCACCTGACTCTAAGCAGCCTAATGAAATCCAAAAGGAAAACTGATCA
EMPV1_15961 CTACATTATCACACCTTCTGGTCCCTATTCTTCTCTTGATTGGCTGGATTGTGGGATGCA
EMPV1_15962 GAACCTGACCACTTCAAGGTTGCGGTCAATGACTTCCTCCGAAAACAGAGCCAGCTGCTT

EMPV1_15967	GTCTGTAGGCAAGAGCTGGGTCATATGGGATTATACTCAATTGTGGAAGAGACCACGCTG
EMPV1_15971	CCTACACCTGTGGCTTTCTAGCTGGCCTCATCCATTTAGGGCAGTGGTTCTCTAGCCTGT
EMPV1_15972	ATGAGATCAGGTCCCTGCGAAGAAGACAGCCTCCCTACATGCAAATGCAGCCTGGACATT
EMPV1_15973	AAATTGGATTTTGGATCAGATAAACAATAAATTTTTTTGGTATAAGGATATCCCAAATAC
EMPV1_15975	CTGTGAAAGGCAACCCCAAACCAGCGCTTCAGTGGTTCTATAACGGGGCAATTTTGAATG
EMPV1_15976	TATGTGATATTCCTACTCCCTTACAAACAGCAGATGAAAAGGAAGTAAACCAAGAGAAGG
EMPV1_15977	TAAAGCGGATTTCCAAGGAATGCGCCCGAAAGCCCAACAAGAAAATTTGTAGGAAAGGAG
EMPV1_15979	GTAGTTGCCCACAACCCTTCGTGGTCCGTCACATCAAGAATCTGGGCCTGAAAGCTGACA
EMPV1_15980	TACATCGTGGCAGGGGTTGCATTTTTCATGAGTCCGGGGTGAACAGGTGCGAAGTGCGCT
EMPV1_15983	GTGGTTCCTATTCATGTTCCACCAACCACTGAAAACAAGCCAAAGGTGCAGTGCTGTCAG
EMPV1_15984	GAGCAAGTAGAGATACCTAGCCCCAGAGTTCTTCCCCGACGGGTGTTCAGCAACAACTCA
EMPV1_15985	GATATTTTGGACCTGAGACTTCAGAAGAAAACTCTCACCCTCAATGCTCCCGTCCCCTCC
EMPV1_15987	CAGTACGTGGAGGTGCTGCTGCCGCCTTCGCCCTCGGTGCCATCTCGTCGCATATACACC
EMPV1_15988	ACACCACTGTAAAACATGTGTTGATCCTGCAGACGAGCTCTTACAACTTGGTATGGGTAT
EMPV1_15989	CATTGGGTGGTCTGCTTTTCCGCTGCCGAGTTGTATATGTGGTGTGTACGTCCTCGAGAT
EMPV1_15990	ATACGGATACCTTGCAGACAGCAATCATGCTGGTGGGGTCTTTTATCCTAACTGGGTTTG
EMPV1_15991	ATCAGGGAGCAGCAGAAGCTTCAGATTGCTGTGGCCACTGGAGGCCGAGTATTTGAGCAA
EMPV1_15993	TACCACCCACCCCAGGACAGCTGAGATATGTATTCATCCACAATGCGATACCTTTCATAG
EMPV1_15994	GCGGTTCCCCAAGAAACTAAAAATAGAACTACCATATGATCCAGCAATCCTACTCCTGGG
EMPV1_15995	AAGTCTGACATTGAGGTCATTTCTGAGCCACCTGAAGAAAAGGTGACAGCCAGAGCGGGG
EMPV1_15996	TCACTCAGTGGGTTAGGGATCCCATATTGCTGACCTGTGGTGTAGGTCACAGGCACGGCT
EMPV1_15997	TGATGGAGAATGCAGAAGGCAAGGAGCAGAGCTCTACAGAGAAATTGGGAGGAGAATCCG
EMPV1_15998	TCCATAGCTTCTGACCCCAGGGAGAGTAAAACAAAGCAGAGTGGAGATAGAGTGTGCACC
EMPV1_15999	CTCTACACTATATGACCATAATGAACCCCCAACTCTGTGGAGGCCTGGTGGCCATGACCT
EMPV1_16000	TGCTGATAAAAGCGGCAGCTCCCTCCTCGGTCTCTGCTTTAAAGATTGATGAGCACCTCT
EMPV1_16001	TACCTTGGTGCCACCAACCCATTTTATGCCACATGCAAGTTTTGAATAAGGATGGTATAG
EMPV1_16002	ATTCGTCTTCCTCATCAACCAGGACCTCATTGCCCTTATTCGGGCCGAGGCTGCCAAGAT
EMPV1_16004	ATGCTGGATGAGCTGGCCATGGAGACGCTGCAGGAGAAGTCCCAGCACAAGGAGGAACTG
EMPV1_16005	GGCATTGGTGAAAACCAAAAAGCACATTTAACTTGTTCAGGCATCGCTTAGTCCCAGGTG
EMPV1_16008	ATTCGAGGGAAAGGCTTCGACTGGCCTCTGGTGGTGAAGGATTTTAACCTGATGAAGTGG
EMPV1_16011	CTCCCCCTTGGCAACCACAAGTCTGTTCTCTACGTCGGGGAGTCTTGTTTATGTTTCACA
EMPV1_16012	AGGACAAGCTGGAGGCTGAGGTCTATAACGACCCTTGAACTGCAAGCTCCTGAGAGCTTT
EMPV1_16014	GTGAGCACTAAGGGCAAACAAAAGTCGGTATTTAAGAACCATCCAAGTAAAACCCTGGCC
EMPV1_16015	GTCATTCGGTTGTACCATGAGGCCACGACAGACACTTACAAAGCCATCACTTACAGTGTT

EMPV1_16016	GTGGGAAATCCTACACCGCTATCTCCCCTATAATGCACATGCCGCCAGCTACACATGGAA
EMPV1_16019	TGAAGAGATTATTTGTAGAACGTTTAGAAGATGAAACTCTTTTAATTAATATGATATGTG
EMPV1_16021	CAGGATATGGAGCCTCACCTGAAAGATATGGAGCCCCCTCTGCAGGATATGGACACACAA
EMPV1_16022	GTCTATACAGGCCGTGAAGTTGATACACACGCCATTTCAGTGGACAGAGGAGAAGGACTC
EMPV1_16023	CTGATGAATGTGCAACTTGGAATGCTAAAGAGCTGAAATTCTTGGCCCAGCTGGTGGGAT
EMPV1_16024	ATCATCCCTCAGGAAACATGCCAGGACCCACAGTGGCAAGAAGCCCTATGAGTGTCAGGA
EMPV1_16025	CTTGGAGCTCCCATCGTGGCTCAACAGTAATAAATCAACTGGTATCCATAAGGAATGGGG
EMPV1_16026	TGACGACCCAAAGGCAAGAAATTGAGAGCATAAACAAATCTAGAGAGTTGAGAATTGCCG
EMPV1_16029	CAGAAGGCGAAGCTGACGTGCGAATTGACGGGGCTTGTGCTTTCAATGATGTGACTCAGA
EMPV1_16030	ACTCTGCAGTGGAGCTGTGGGAGTCAGATGCACAAGATGCAAGCAGCCAGTCTCTGGGAA
EMPV1_16031	TGAAGACATGTTCCCACCCTTGCTGCCTCCCACTGAACAGGACCTCACTAAGCTTCTCTT
EMPV1_16032	TTTAGGAAGCCAAGCAGCGTCGGGCCTGGTTAGTACTTGGATGGGAGAAGCCTGGCTGTG
EMPV1_16033	GACAATGCTGGATCCTTAACCCACTGCACCACAAGGGAACTCCTAAATGCATCTCTTGAG
EMPV1_16034	TACCAGCCTGTACCACTGCATACTTCATTCTGCTCCCTGGTCAAGCATAGCAGCGTAGTC
EMPV1_16035	CACGGAAGACAGATAATAGCATGACTGTGACTTGTACGCCTCCTACCGACCTTAATGGCC
EMPV1_16036	CGTAACAGCAACACCACAGCAGCACTTGCGGCCAGATCCTGGGCAACAAGGAAACTCCAA
EMPV1_16040	GTCTATGCCAACATTGAACAAATTAAAGCTTTACAATGCAAGTCGTGGCGACAACAATGG
EMPV1_16041	AGCTGGGAGCGTTACCACTCATGGTTTGCCGGCTCTGATTGAAGAATTTGGAGGCAAGGA
EMPV1_16042	GTGACTTCTCACCAGAAACCACCCTGAGAACACAGACAGAAAACGACGAAAGCTCAAGCT
EMPV1_16045	CTAACTCATAACCACACCTGTGTTCCCCCCTAGGCATGGCAGCTTTTAATAACCATTATC
EMPV1_16046	CAAAGCTGTGTAGATCAGAAAGAATGAGGGCAGTTGGGCGGCTTTCACCGCCTCCTTCCC
EMPV1_16047	CGTGCACCCCCATGTTCACTGCCGCATTATTTACAGTAGCCAAGATATGGAAACAGCCTA
EMPV1_16048	CTCTATCACAAGACCTTGCACCCGCTGTGTCCCGTCTTCAACCTCGGCTACGTGGTACAA
EMPV1_16049	GGCTGCCACTGTCTGGATCATTGGTTTTTTCCATGCCCTGATGCACTCCATAATGACCTC
EMPV1_16051	TGTCTCCCACACTCGCAGGTGACCGTCAGTAGAAGCTAAGGCGAAATAGGCCTGGTTTTG
EMPV1_16053	GTTCTTTGTACGACCGCTCGCTCTAAAGAAGCTGGCCGCCAAGAAAAAGACGGACAAGAA
EMPV1_16054	CAGTAACATGTAAGACAGAGCTGTTACCACTTCAGACATTCCTCTTGATGTCCAGTCCCC
EMPV1_16058	TGTAGTCTCGGTTGGTGGCTTCGATGTCCATGTCCCAAGCCTCGACCAGATAGGTCAGGA
EMPV1_16059	GGTATCCTCATCACCATCACCAGCAATGTTATCCTCATCACCATCATTGCCGTTGTCCTC
EMPV1_16060	TAGAACCTGGGGTGGGGGAGGGTCTAGGGAAAGAGGACAGCTGATACATTTAGGAAACAT
EMPV1_16063	GGCTGAGTTGCTTAAGTCTGCCTGCTCTGATGCTCTCACCAATTACATTGCCTTATACTC
EMPV1_16064	TCGTGTTAGCTCACGCACAGAATCGGCATCTGCTCACCCAGCTCTCTCTCCAGTGCACAG
EMPV1_16065	GGAGGAGGTATTAGAGACTAAGAAGAGACAAAGTTGCTAGTTACGAGGGAGGGAGCAGAG
EMPV1_16066	AAACATCTCCGACCCCTCCAAGAAGAGCTACGTGGTTCAGCGGCTGTCCCGACCGGACCC

EMPV1_16068	AACCAATGCAGCACTATCTGATAACACTGAAAAACTGCATACCCTGCAACCCAGCAATTC
EMPV1_16074	TCTCAGTGTGTCCAGGATGTTCTGCCATTGTGGGGGGAGGCCCACGAATTTGCCTTCTTT
EMPV1_16077	AGTGATTCTAGGGAAACTTCTCAGATTGGTTGGGGATGTGGTCACAGTGGGAGAGGCTCC
EMPV1_16078	GCCTGTCCTTTTTGGTTAGTCAAGTGGTGTGTATTTGGAAGTAGAAGCTGAACCCAGGAC
EMPV1_16080	AGCCTTCGCACCCTTTCATCCGTGGGTGGGGATTTGTTGCATTACTTGTGGTGTGTATAA
EMPV1_16082	TCGAGGCTGCCTGGAGTCTCCTCAGGCCTCTCGGAGTGGCCCTGGACCTCACTGGCTGAT
EMPV1_16083	CCGGAATGCTGTACTTGGTCCATTTCTTAAGCCCGGTTTCCCCACAGCAAGACTGTGACT
EMPV1_16084	GGCTGGAGAGGCCCCAGGCTGACATTTGTGTTCTCTTTAGTTCAAAACCATTACTATGGC
EMPV1_16087	CCTCCAGCGCCAGCTTGATGCAGCTCACAAGGAACTCGATGAAGTAGGAAGAGCTAAAGA
EMPV1_16088	GGCTGCTGGGTAGCATGTCACACCGGACCAGATTCCCTGATCATTGTCGAATAAAGGAAT
EMPV1_16090	TAGGAGGTCAAGGGTTCCATCCACTCCAGCGACTGGCAGCCCAAGAATCAGTCTCCATGA
EMPV1_16091	TCTAAATCCTTCGACAGACAGTGCAGGGACAGCCTCTGGGGACGAGTGAGAAATGCAGGA
EMPV1_16093	AATGGCTCCTCAGACATCCGTTTCTACCTCACCGTGTATGCGACCATTGCTGGTGTCAAC
EMPV1_16094	CATGGTGAGCCAAGTACTTTCCTAGTTCACACTTCCTGGAGACTGTCTCATCTACCCTGC
EMPV1_16095	GACTCCCCAATCCTCAAACTTCTGAATTCTCCACACTGCCTTATCTCTGCCATCCCATGG
EMPV1_16097	TGCAGACAGATATCCCGGCTTTGCAAGTGTCTGGTCGGATCGTGGGCAATCTCTCCTTTT
EMPV1_16098	ATCAAAATAGAGCACACCATGGCCACCCCACTGGAGGATGTTGGCAAGCAGGTGTGGCGG
EMPV1_16100	ATTTCTCATCGACAAAACCGGAACCAGCTAGCACCCCTCCACAAGGCTCCATGGCCAATA
EMPV1_16101	ACAGAAACTCCTGCTCCTTAAGTGGCCAACCTGGTGTGGATGGATCAGGGAGAGGGTACT
EMPV1_16102	CCACACTGGGGTAGACTCGGTATGTTACTTCTCACCTTTGGCTCGGAGACTGGACAAAGT
EMPV1_16103	TTTCTTGTGGGAACTCGGTGGCCGACCAAGCCTTCCTCGACTCCCTCTCGGCAAGTACAG
EMPV1_16104	CTACGAACACCTCTTCAAAGTGAATGACAAATCCGTGGGTGGCTCCTTCTACCTGCAGTC
EMPV1_16105	AATTTTTTCTTCCGGCTGTATGCCTCAATCCCGCGTTCATGTCACCGCGATGTGGAGATG
EMPV1_16108	TCTTGTCCTACCTTCAGACTCAGACTGGAAGCCCACCATCAACTTCCCTGCATCTGCAGA
EMPV1_16109	GGAGAGGCACTGAGGTTTGTTCTGACAAAGGGCTGGGAAATATTGGTTCAGGTGTTTCCT
EMPV1_16110	TTACAGGCAACACATTGCTTTCTTTTGAAATGCAGTAGTAATCCTTTCAGGGTGTGATTG
EMPV1_16111	CAGACTTACTAGAGCTGGGTTCCTGGCCTCTGCACACTGTATGACTTCCTTGGAGGGTAA
EMPV1_16112	CTCAGCCTTTCTCTTCGGTGCTGCCCGAAAATATGGAAACCAGGTTGGGGAGCACATCCT
EMPV1_16115	ATTGACGAGGACATTGTGAGCAAAGTGGCCGAGGAGATGACGTTTTTCCCCAAAGAGGAG
EMPV1_16116	AGAAGCGATGAAGTACAGGGGTGAAACCAGAGGTGTGTTTGGCAGTAGCTATGTTGGTGG
EMPV1_16119	GCAGGGTTTGATTTAGGTATGGACTAGGGCCTAAAACTCTGCATTTCTAACAAGCTCCCA
EMPV1_16122	TATATGTTCAGGAGAAGAGGGATGGATTGAAAAGAAGGCAGCAGCTAGAGGAAGAGCTGC
EMPV1_16123	GAAGAGTATCTCTCCCATCTGAAGGTTCGTATGTGGAAGTCCTAATCCTCAGTACCTTAG
EMPV1_16124	CCCCTTAAACAAAGAAGGTAGGAAACCTAGGACCGAAACACCCAAGATTCAGCCTCGGGT

EMPV1_16126	GCAGAAACCCATCTTGAGCATCTCTAATAAGCATTCCAGCAGGCCAGTCATAGGGAGATG
EMPV1_16128	ATGGCTCCGAGCAGGAAGACGTTTTACTTTGCCTTTGCCTTTTTCTTCATTTTGGCTCAA
EMPV1_16129	GTTTCTCAGGATGCACTTCCTCCAAAACAGAATCCTCAGACGTTGCAGTTGATATCTAAA
EMPV1_16131	CAGCTCAAAATCCCCTCAGACACGCTGTCCAAGTTCCCAGCCTTCGACATCTACTACGTG
EMPV1_16132	GACTTCAAAAAGGAGCGATCAGACCGAGAAAGACTTAATCAAGAAAAAGTGGAACTACAG
EMPV1_16133	GGTGCCCACCTATGGGTAATTTCACCTGACACATGCGAGTTTCCAGTTCAGAACCAGCAT
EMPV1_16134	TGGCCCCACAGGGCCTTATCCTACACCAAATATGCCCTTTGCAGACCTTCGAAGAGCATA
EMPV1_16135	TGTCCCTGTCTCGTACCTTTGTTGTCAGCAGAACAGAGTTGTTAGCAGCAGGTTCTCCAG
EMPV1_16136	GCATGTTGGTGGCATCGTCTAGTGAAGCTGAACCTGTGGAACAGTTTTCAAAGTTGTCAC
EMPV1_16137	GCAAAGAAAACTCATCCAGTGTGACTGTAGCAAGCCCTGAGACGGAAAATAAGGCAGGCC
EMPV1_16138	AGGGACAGCGCCGTAGATGAAGAGAGGCATGGAGGGGTAGAGCTCGGCGGTCATGCTCAC
EMPV1_16139	CAATCTTACTCCCTGTCAGAAGGCAGTCACCAGGTGCCCAATGGGCATGCCCAACCCTCG
EMPV1_16140	CGCAGAAGGAGGTGGTGCTGTACGCGCACTTCCGCCGCCTCGACTACTTCTCCGAGGAGT
EMPV1_16142	CAGTGGCTGTGCCTTTATGGTTCACGTCTGCCAGGATGAGCATCAGCTTTACAATGAATT
EMPV1_16143	AATGGAGAGGGCGGCGAAGAGGACCCGCAGGCTGCAAGGAGCAACAGCGATGGTGAGAAG
EMPV1_16145	AGCAGCAGGAGGGGGACAGCAAGGACAAGAAGGACGAGGAGGAAGACATGAGCCTCGACT
EMPV1_16146	CTGAGTTCTTTGAAAAACCGGAGAAGAAGAACTAAGAGGATAGTACACAAGGACTTTCCA
EMPV1_16147	CAGAGCTGCTAGAGGAGGTATGTGACCGGAAGAAGGAGTATGGGGAACAGATTGATCCTT
EMPV1_16148	ATTACAGTCAGCCAGGGGAGCAGAGATGGTGATGTGGGCCCAGGTTGGGGCAGTAAAGAT
EMPV1_16149	ACTGACCCTGACATCCTGACTTGGATAAACCAGTATAAGCTTCCCTTTGATGACTGCCAG
EMPV1_16150	GATGGCTTCTGTGTCATGGTTCTCTGGTGGAGTCAACTCAATTGTGCAAACAGCTCTTGC
EMPV1_16153	AAAACCCAACATGGTGCAATATGGGCTACAGGTAAGGCAGTGGGTGCATTTTGGACGCCA
EMPV1_16154	GAGGCATGGACAGAAAATATACCCCTCACATCATCGGATTGAGATGGCAGTCGAAATAGC
EMPV1_16157	AGCAGCTTGAATCCATAGGCATCCCGCAGTTTCACAGTCCAGTTGGGTCACCGCTTAAGT
EMPV1_16158	TTTAGAAATCAGCCCCCAGAACGTGGATGTCAACGTGCACCCCACGAAGCACGAGGTTCA
EMPV1_16159	GTTGGAGAAGATCGCCGTGTTGGAGAAAAGAACCGTCTCAGGAAAGAGATCCGTCTCAGA
EMPV1_16160	TCTTCATCTCTTCCTTTACTCACTTGGCAAACACTACCACGGAAAGGTCCTCTCTTATCT
EMPV1_16163	TGAGCTCTGCTTGGAGAAAAATGAGTGCTCCAGGGACGACCAGTGTAGGGGCAACAAGAA
EMPV1_16165	ATTCTTGATCCTATACACTTGTCTACAAGCTCTAGAGGTAGCAGCTGCGACGGGTTAGTC
EMPV1_16166	CACTAATCTATGTTCCAGCCTATGCATGAAGGGTAAATCACGTCCCACATAGACTCCTCG
EMPV1_16168	CAGAGTGCTGATCACCCTCGGGGACCAGGACATAGATCTGTCACCGTCCTTTGTCATCTT
EMPV1_16171	AATTCTCCCAGAGACAGCAGGAAGGTGGCCTTGGTACAGGGCGGAGTGACGAAGTGTAAG
EMPV1_16173	ATGTATGCTAGTTGAATACCAGCATGCTAATTATCTAACCTTTTCTACTGCAAAGAGCTC
EMPV1_16174	GGGGGCAGTGTCAACCTTGAAAATATCTTGCTCAAGTAGACCCAGTTATTCCACTCTTGT

EMPV1_16177 CTGGGGGAGAGTACTTTGTAGAAGGAGAAACGTGGCACATCGACAGCTGTACTCAGTGTA
EMPV1_16178 TCGAGAAACCCATACTTTACCGGCTATTCAAGGTGAGCATCAGGTACAAGTGGGATGCGC
EMPV1_16181 GGATTTCCCCTTCCTTCGGAAAAGTCAACAAGGCAAGCACCAAGAGGCACAAACAAAACC
EMPV1_16185 CTGTGCCTAAATAGTTCTTTGTAAGCTGGCAAAGTTAAAGTAACCGCACCCAACCTTCTG
EMPV1_16186 ACCAGGCCTCACTGTCTCTCTCCTTCCTCCCAGGGTCCCACTGGTGTGACTGGTCCTAAA
EMPV1_16188 ACTTCAGGAAGCATAGGGTCAGGTGGAGTTTGATCCACTTGGGAGGTTTGGGATGGAATG
EMPV1_16189 CCAGTTACAAGAACCTCACGCTCAAATTCCACAAGCTGATCAACGTCACCATCCACTTCC
EMPV1_16190 AGTGCACGTGCTGGACCTGGAACCTCGGGGCTACATCAAGCCACACGTAGACAGCATCAA
EMPV1_16191 TCATGGTCCCCTCCACTTGGACCCACAAGTTTTCTCTCTGTTCCCGGTCAGAACATACAG
EMPV1_16193 GAAGAGAAAGGGAAGCTTCGATGCCTTTCCAAAGATTTTCCAGTTGTTCATACTGTGACC
EMPV1_16194 TGCTTCATCAAAATCAGCGCGGCCTATGGCTCCACGAAGGACATCTCAGTGTACAGTGTG
EMPV1_16198 TATATTTCGCCTCAGATAAGGAAACGGTCACTCGTCACGTCCCTGTGTTTGAAGATCTGC
EMPV1_16200 TTTGAGCCACTCCAGTGCCCTTAGATTACTGCGGTCTTTGTCACACGTGGGGAAACTGAG
EMPV1_16204 TCAAAGTGATGGATACACAGGGCCTCTGCTGGCGGGGTTTTCACGGAACCCGAGACTGTA
EMPV1_16205 AGTGTGACCCACAGGCACGTACCATTGAAGAAAGTTGTGAGTCTGTTGCACTGGATTCGG
EMPV1_16206 AGCTCGGCGACCTCCTTGGTGGAGCTGGATATGCCGCGGGCTCCGGTCCTCTCCAGGCCT
EMPV1_16207 TAGGGAAAGCACTGTCATTGATCCGGTTCCCACTGATGACCATTGAGGAGTTTGCAGCTG
EMPV1_16208 ATGTCAGATTAACTTACGGAACTAAAAGACAAAGTGGGTCTAATTCAATGTGAACCTGAC
EMPV1_16209 TTCTCATGTCTGTCTTCCCTGTCCTTATCAGCCACTACACAGTTCTCTAGTCTGCAGGTT
EMPV1_16210 TTGTCACCCTTCCTGGATCTGCTGGGGTCTGGGGGCTTTGAAAACCAGCCAGTGCAGCTG
EMPV1_16211 GCTATTAACTGACATGGAAACAGTAAAAGAGTAGAAGCAGGTTCAATATGGGGAGGAGGG
EMPV1_16216 GGACTTGGGGCCACAGGGACTTCTCACCAGGAAGTTCATCCCAACAAGATTTTCTTTCCC
EMPV1_16217 AGTTGAAATAAAAGAATCTGTTCGTGGCCAAGATATTTTCATTATACAGACGATCCCCAG
EMPV1_16218 GTCGCTGTTACTGCCCCAAGCCCATAGTTTATTCGGATTTCCTGTTTTCCCTCCTGTCCT
EMPV1_16219 GTCGTTCTATTAGCTTCTGTTTAGGCTTGGTGCTCACGTGTCTGGATGTGGGACAGTTTC
EMPV1_16220 AGATGTAACAAAAGCAGTGGCATAAATCTGCCCGTACACACAGACCCGGGGTGCGGAGTT
EMPV1_16221 GTACCCTTTTGGACAAGGATGGTCTCTACGTTGAGGAAGTGTGCACCCCCTGACAATCTGC
EMPV1_16223 TTCTTCCAGAACAAATATGCAATGTTGAAGAAGATGGTGATTCAACCCTGGAACACCTTC
EMPV1_16224 GTATTGCTAATGGGAGAAGTGGCAGCTGGACAGAGTTCAGTGTTGGGAACTTTATGCCGC
EMPV1_16226 GGTGAGATTTTCTGTTATGGTACCCAAACTCACCATTTGGTCCTCTTTAATCTTTGAGGG
EMPV1_16229 TTCCTGGTCCTTTTGGTCTCCTACGCGGTCATCTTGCTCCATCTGAGGACTCAAAGCTCT
EMPV1_16236 CTGGAGGCCCCTGGTGTCTACGTGTTTGGGGAACTGCTGGATATGCCTAATGTTAGAGAG
EMPV1_16237 CTGGCTCTTCAGGGTCTGTCTGCGCTGCCTTTTTCTTGGACTGCATTTTCTTCTTTGCAG
EMPV1_16242 AGAGCCAGCTGGAGTCTGTGCTTGGCTGCTTAGGGGAGTGGGGGGCACAGCTTAGAAGGA

EMPV1_16244	GGAGAAAATGTGAGACGCAGAATGCACTCATCCTCCTGAAACTGACACAGGATTTGGGCC
EMPV1_16247	CTCATCAAAATGGCTTGTGTGGATGCTCGTGCAGTGGAAATGCTGGCTTTCACCTTTGCC
EMPV1_16249	TTTCAGGGAAGGGGAATGATGTCACTTACACCCTGGGACTACTTGTCTGGATAGGCTGGG
EMPV1_16252	AAGCACGTGGTGGAAGGTCTGGAACCACGGACCCTGTACAGGTTTCGACTGAAGGTCACC
EMPV1_16253	GAACACCCGGCGCTGCGGAGTCTGCGGCTGCTGACCTTGGAGCAGCCGCAGGGGGATTCC
EMPV1_16254	GCCTATCCAGGTGTGGTTTACCAGGACGGATTTTACGGTGCTGACCTCTATGGTGGATAT
EMPV1_16258	GAAGAGGGGTGGAAAATGCCACACGTTACAACCTCTTATTCCTATGATGCACCAATGGAC
EMPV1_16259	TGTCTGTCCTGATTCCTGATGGGACAAATGTTGGGGGCCAAATAACAGAAGCCCATCCTC
EMPV1_16260	GGTGGTGCCGTGATGCTGCTGGTTTATGAATACACCTATTCATGGCTTCAGGAGAACTGG
EMPV1_16261	CTGCCTAGAAAAGCCTAGACAATATCCAACAACTAAAAAAGTGGGCCCAGAAGTCTGCCC
EMPV1_16262	TCAGTCTGGCGGCTGCACCTGGGCTGATGGTGTGAAGTAGGGACCCCGTTTGGGGTTTTT
EMPV1_16264	CTGGCTCCCTGAGGCAGCGAGTAGCCAATTTCTGTTCTCTTCCTGTGGAGAAAATCGAAA
EMPV1_16267	CCGGAGGAAATTCCTCATTTATGCGGGAAATGACTACCAGGGGCCCGGAGGCCTGCTGGA
EMPV1_16270	TTCAGTAAGTCTGGGTTGGGCCCAAGATTCTGCATTTTTAACAAGTTCCCAGGTGATGCC
EMPV1_16271	TTAACACTGGTGGTCCAAAAGCTGCCCCATGGGGATCCAACCCCAGTCCAAGTACAGAAT
EMPV1_16273	AAGAGTTTGTATCATGGCAGCAGGATTTGGAGGACTCCGTAAAACCCACACAGCAGGCTC
EMPV1_16274	GTTCACTCTTTTATACAAATGAAGAAGTTGCAAAGAATACTCATTGCAGCAGCCTTCCTC
EMPV1_16275	GTTTACAATATCTAAAGTAAACGTTCTATTTCTCAAACAATTAGCACTGATCACAATACC
EMPV1_16278	CCCCTGTAAGCCCTGCCTATGTTGTGTTCCCGTAAACCTTTCCATCAGGAACTCTGTGTT
EMPV1_16280	GGCACCATGAGCTCCCTCCTCATTTCTGCCACCTTTTTCTACATTACTGGATCCTACACC
EMPV1_16282	CCCTGTCACCTTCTCCGACGTCATTTTCCTGTCCCACTCTTCTCCAGCTATTCCCTGAAT
EMPV1_16284	ATCGAGTGCGTGTGTTTCTCTGGCAGACAATGAAGCCGCTGGCCGTGCTGGCTTTCCACA
EMPV1_16285	CTGGACAAGATGCAATTTCCTAGTCTTAGCTTCTTGGGCTGGTGGAGCCGTTCACGCCTT
EMPV1_16288	AGTGGTCGCAGAAGGTGGGGCTTCCATACGTGTGGATCTTAAACTTGTGCTTGCTCCGGG
EMPV1_16290	GTCCTTGGGACAGTGAGGATTTTACCAGGAGGAACAGAAATGTGAGACCCAGCAGAAGGC
EMPV1_16291	GGCACTAATTTATCGCCATGTTCCTTCTCCATCACATTCTAGGATCCAGACCTGCCCCTG
EMPV1_16296	GCTGAGCTTTATTTCTGGTTCCTTTGACGTTTCCATCGTGGGTTGCCTGCAAGTCTGTGC
EMPV1_16300	TCAGAAGTGACCTTTGAGCGCCTGTGAGGCCTGGAATCCGAAAGCCAGGGAGGGTTTTGT
EMPV1_16301	GCCCGGATGGTGGTTGGCTCCCAGCGTGTCTGGAGAAATCACACAGTTCCAATGAATTTT
EMPV1_16302	TTCCCTGTGACCTTGTGGTGGAGGCAGAGAGGGAAATTGTCACTTGAGCAGGACAGGCCT
EMPV1_16303	CTTATCTACGCCCAAGATCCCTTCGATCTCCAGTAGAAGACAAGGTTCTGGCTGTGTTCT
EMPV1_16304	AGACAGAGAAAGTCCAGCCAACCCTAGAAGAGCTACTATTTCCAGCTGCCTCAGTCCTGG
EMPV1_16306	CTGTTTACAGCATCTCTACAGTTTCTAGATCTCCGACCTCGCGGGCTTTATCTCCGGGAT
EMPV1_16308	TGCAGCTGAAGATCCGTGAAGTGCGCCAAAAGATCATGCAGGCGGCCACTCCCACAGAAC

EMPV1_16309	CTGTAGGCTCAGAAGATCTCGCGGTTGATTCTCCTGCCTTTGACTCTTTACCAACACCCA
EMPV1_16310	TGCTACCCAGTATTGAAGGGCCTGAAAAAGAAGTCATTGTGGAGACAGTCAGCAATCCTG
EMPV1_16311	AGCGTGCAGAGGGCTGCCCAGTATCTGAAGGTGGAGCAGCTATTTGGGTTGGGTCTGCGG
EMPV1_16312	TTCTGAGCAGAAAAGCACCCCGAGCTGGAAGAAGGGATCTGAGCTCATCTTGCCTGAGTC
EMPV1_16313	AATGATGGCGGTGCAGGCATTTCCTGAGGATTAATGACCAGCTGGGAGGAACCAGTGGCC
EMPV1_16315	AAGGATCGGGTGGGTAGGGGAACTCCTAAAATAATACGTGGCTACTTCCTCCTTTCTGGG
EMPV1_16317	CATGGTCTTCATCCCAAGCATTTACCTCTATGCCCGGCCCTTCACCTCCTTCCCCATGGA
EMPV1_16320	ACAGGTGAGCGAGGGTGAGACTCCCTAACTGCTCCACTCCTGCAGCAACAAGCTTGCCCA
EMPV1_16322	CCAGAAAGGACTGGGTAGGGTGTGGGGAATTTCTACTGAAGTCTGGTTCTTGCCTGGGAA
EMPV1_16325	GGCAAAAAGATCACCACCAAGCGAATCATTGAGAATGGCCGAGAGAGGGTAGAAGTGGAC
EMPV1_16326	TGTGAAACAGCACCAGCAGCATCACTTGGGGACTTGTTATAAATGCACACTCTCACTGTG
EMPV1_16330	TAGCGTTAAGGGGAGAAGACGTTCCCCTGACGGAGCAGACCGTGTCTCAGGTGCTGCAAT
EMPV1_16332	TCCAAAGGACTCTCCCACTGACTGTTACCTTTGCCCCAGGCCACCCTAGACTTTTATGCT
EMPV1_16333	GGCGTGTTTGGTGTATGGTTATGCAATTGTGTGTCCCGCGAGGTGATGTGCTACCAACTA
EMPV1_16334	ACTCGGGTTATTCGCACAGTGCCGCCTGGGCTGTCTCTTCCCGTAACGCGGTGGTTCTCG
EMPV1_16336	GCCTTCTAGAACAGTAGACACAAAACAGGCTCAGGACTTAGCAAGAAGTTATGGAATTCC
EMPV1_16338	TGGCTCTTGCATCATTGTCTTCCTTATGCTACTGCCAGAGATTGTGTGGATTGCCACGCT
EMPV1_16339	GCATCCGAGAACTTCACCTTCACCCCAGAGTGGGGTGCTGATCTACACACGGAGCATGAA
EMPV1_16340	TGAGTGTCTTCCACTGCTGCCTTCCTCTCTGTCTACACTGCCTGGTCACTCTTGGTGAAA
EMPV1_16341	GTGGCAGTGTGGGATAGCGATACAGGCGAAAGGGTTAAAACACTGAAGGGACATACTTCC
EMPV1_16342	AAACCGAGTTGGCTCTGAGCTCCCTGTTTGGCTTTTGGGCGGTGGCAGCCTGAGGAGGAA
EMPV1_16343	CTAGTGGTGGACTGGCTGGTTCAGGTGCACGAGTACCTGGGCCTGGCGGGGGACACGCTC
EMPV1_16347	ATCCAAGGCTTCCGGCAGCTGCTGGAGCTGAACCTGCTGGGGATGTACACGCTGACCAAG
EMPV1_16348	GCCCTGAGGTGATCAGCGGTGAGGGCTACGGAAGGAAGGCAGACGTGTGGAGCCTGGGCT
EMPV1_16349	CATGGACCTTAGTCACTGGGGAGAAAACACGCAGACTCTAGGCCTTCAGGAATCTTCGAT
EMPV1_16352	TGGATCGACCATACCCATTGCCCAGATATTCCAGGCCACCATCCAGAAGAGTGTGATGAT
EMPV1_16354	CACCTTCCCGGAGGAGTTAGTGATGATCTTTAGCAGCGGCTTGATCTCCGTGGTGTGTTT
EMPV1_16356	CTACAGGAACAATTCCAGATCCTTTACCCACTGTGCCATAGCAGGAACTCCATGAGCTCT
EMPV1_16357	GATGGAGTCCTGGTGGATGAATTTGGATTGCCACAGATCCCTGCTTCGTAGACATGCACC
EMPV1_16363	GCAGCTATTTAACGTCATTTGAGGGTCACTGTTGACTTATATAAAGTGACCTCAGTGTTC
EMPV1_16368	CAAGGTGTCCGTGTTCCACGTGAAGCTCACCGACAGTGCTCTGAGGGCCTTCGAGAATTA
EMPV1_16369	CCGGCCCGGGCAAGTACAAAGTGATGAAACTATATAAAGATAAGAGCTGCTCCCCATCAG
EMPV1_16370	GTCAACTCTGAATATCCATCTCTGAATGCCTCCCCACCGACCTTTAAACACTGGAGTGCC
EMPV1_16373	CTTCCAAAGTGTCCCTCCCCCCTATCCCTGCAGTAAGCAATATCAAGTCTCTGTCGTTCC

EMPV1_16376	TTCAGTAAGCCGAATAGCTTCCAGGGATCTAATGTAAGCATGGTGATTACCACCACATCG
EMPV1_16378	AAGAGGTCTCTCTGGATGAACTCAGTGCTGATGAGCTCCTGGGGAAACTGGCTGAGATCC
EMPV1_16382	TAGGGCTTGGAGACAAACACTAAAGCAGGAAGCAGCACAAGGAAGAGATTGGCCAGCCTG
EMPV1_16383	GCTGGGTACAGAGCATACTGTTCCTTCATCGAGTCCAGAGCACCCTATTCCTTCATCGAG
EMPV1_16385	TCGTTCCTGAGTCCAAGGACTCGCGCGGTCGAACAGCTGTCCCAGGTTACTCGGGCCCAT
EMPV1_16386	AAATGGAAGTTCCTTAGGGCAGATAAGCATTTATCAGAAACTTTAAGATCGCTCATGAAG
EMPV1_16387	ACACCTTAGTTCCCTCCTGGGTAGCGGGGAGGAAGCAGAGATTCTAGGACCTGTTTTGCT
EMPV1_16389	AGCTTGGATCTCGTGTTGCTGTGGCTGTTGTGTAGGTCGGCAGCTGCACATCCGATTCAG
EMPV1_16392	ACAGCAGCATCGGGAGTACTATCGAGACAAGACAGGCACGGTGCCCCGGGTCCCCTACTT
EMPV1_16393	TGTCACTGAAAATGAGTGTCAGAACTACAAACGCTGTTTGGAGATCTTAGAGCAAATGCA
EMPV1_16394	CTACAGTCCCTTTTGTTAACCTATGATCCCGTTCCCATCTAACACCCTTTGCATGACTGC
EMPV1_16396	GGGTGAAGACACCTCATCGTTACAGGCCTGGTATCGTGGCCCTCTGTGTAATTAAACGTT
EMPV1_16397	AGAAGTTCCAGGACCGCCTGGCTGGGTGCACTATGCATTGCAGTGACAAAGCCAAAGATG
EMPV1_16398	TATTGCGAGAGTTGTGCCATTCACAGCAAGGTAATCAGGAATCGTTCTCGGGAAGCCCAG
EMPV1_16399	GGGTGTGGGGGCAGGGGGAGGGATCATTCAACTGTGTTATACTGTAATAGTTGCTAGAGT
EMPV1_16400	TTGTGGTCTGTGTTTCATTCGAGACCTGGGGACGGTGATCCGCTCCTGAATTTGGGAACT
EMPV1_16401	CGGATCTCATTATTTACTGTTTAGCCACTGGACAAGTCACCTGTAAGCCATAAAGGAGTG
EMPV1_16407	CTGATGTGTTTTGGCAGATAAGCTTTCAGCTGAGGCCTGATGGAAGTTGAGATAATCTGC
EMPV1_16408	AGGCATGTACAAGGTAGTGAAGACGGGACCTTCCGGTCACAACATCAGAAGCTGCCCTAA
EMPV1_16412	GCTTCCATGCTTACCAGTCCTGATGGACCTTTTATAAATTTATCTAGGCTGAATTTAGCA
EMPV1_16414	ATTTCTCTTGCCGCACAGAATTGGACCTGCGGTCTCAAGGGCTGGGACTGTTCCAGAACA
EMPV1_16415	TGGATGGGTGAGCTGTTCCTGAAAAGGGAATAGAGGGGGGCGGATGTGTTGGTTGGAAAT
EMPV1_16416	TGTTATCCCGCCCCCTCTTTCGTCACTTCCTCTCTGGCCCCTGTGCAGTCGATAAGGAAA
EMPV1_16417	CTTATGATCTCAGACAGTGGGTTGCTGTCGATGAGCTGTTTTCTGCTCCTCCTGATCTCC
EMPV1_16420	TTTGCCAGGTCGGATGATGAGCAGAGCTCTGCGGATAAAGAGAGACTCGCCAGGGAAAAT
EMPV1_16422	ATCAGGAAGCCGTGAGTCACCGCGTGAAGGCTTGCGCTACGTGAAACTCTGATAAAACCT
EMPV1_16424	GCTATTTAGTATCAAAGGAACTTCCCAAGCATTGCCTCTATACCAGACTCAGCTCCCTGC
EMPV1_16425	AGTAATCATCCATCATCTTGCCCCCACTCAGTCCCTTCCAGCTACAGAGGCCCCATTAAT
EMPV1_16427	CTGTACTGAAAAGTGTCCACGGCCCTTACAAATAGCAGGTGTAAAAGGTTTCAGATGGTC
EMPV1_16429	ATGATTACGGTTATCAGCAACCGTCGTATCCTGAACAAGGCTACGATAGGCCTTATGAGG
EMPV1_16431	GTGGCTTATCTGGTCCACTACGTGTGGCAGAACCGAGATGAGAGCCGCCGTCCTCTCAAG
EMPV1_16432	GAAGTTCTTTGCTGGTGGGGTTTGTAGGACAAACATGCAGCTTCCCGGGAAGGTGGTGGT
EMPV1_16433	GGCTTTTGTACAGTCCGTAGGCTCAGCTTTTGTCCCGCGTTCCGCTTTTCCGTGTTCGCG
EMPV1_16436	CAATCGCCCCCTTCTTGCCCTGGGGGCCCAGGATGTAGATATTGTACAAAGGTTTCTAAA

EMPV1_16437 CTGCAGGGTGAGACTGCTCCTCCTTTCGACACCTTCCGGTTCTGCCCCGCTCCTGGAGAT
EMPV1_16439 CTGGAACATAGTAGGAGGTCTCTGAATATCAAGTTCCTTCCACATCCACCCTCTCAGTCC
EMPV1_16440 AGTGAAGCGGCGGGAGGGTTGGGGAGAAGGAGCATTCGCCTGAGGCTGGAGGAGGCTGAA
EMPV1_16441 GCTCACACCATGGCCCTGAAGTCGCTGGCCTTCTTCCTCGTCTTCCACACGTCGTATTTT
EMPV1_16444 CCTTGTAGCCAAGGTGACAGAAGAGATGGTTTCCTGCCAATGATACTGCTCCCTCCAGGA
EMPV1_16445 TGCCTGGTCACTTTCCCCTACTTCTGGGGATCTACAGTGGGCACCATGCAGGTAATATTG
EMPV1_16451 TCTGGATTTCCATGGTGGATGCGTTCTACCAGAGCCTTGTCTGTTTCTTTATCCCCTACC
EMPV1_16452 CTCCACATGTAAACTTAGGAAGGAATAATCTTTGGAGAAAAGTACATGTTCGTAGACTGC
EMPV1_16455 GAGACCACACAGGAGAATCCAAGCCTGAGCATCATCTGCAAGGACCTACCTCATTCTCTA
EMPV1_16456 CAGTAGTGGATGTCACCATTTCACCTTTCAATGGTCCCTTTTCTCCTTCAGGTGTCTTTC
EMPV1_16461 TATCTGTACCGAGTGTTCTGCCGGCTACGTCCCGGTATTTCTATCCTTGCTCTGCATGGT
EMPV1_16462 GATGATCTTCTCTCCAGGAGCCATCATATACGGGCCAGGATCTAAAGTTCTGCCTCATCA
EMPV1_16463 GTGCTGGCGACTCTGAGGATATCACCCTGGCCGACGTGGAAAGGAAGGAGAGAGAATATT
EMPV1_16464 CCTAGCCGTTTGCTGAAAGCCCCCAGCCCTTTTTCCCATTGATATTTTGTCTCTTGCACC
EMPV1_16466 CAAGCAAAGACAGCAGGTGCTCCGAGAGGTATGTGCACTGCAGGGTTAGGGAAGCAGGAA
EMPV1_16468 TAAATGAAATCAGCTGTGGCGCCCGTTCCACTCAGTGTATCCCAGTGTCCTGGAGATGTG
EMPV1_16469 ATCAAAGACAAGCAAGAAGGCATCGAGTGAGGGGCCAGAAGGAGTCCTGCTTAGCCAGCT
EMPV1_16470 GGAGTTAGCTATCAGGTTACACTGGTCATTTTCTGTGTCACGGTGGTTATTTCCGTGTGT
EMPV1_16471 GTCTTTCTCTGGCCTCAATTCCCGTCTCAAGGCCCCACCTTCCACCTACAGTGGAGTTTT
EMPV1_16473 AAGGCCACACTCAGAGACATCCTGCAGAAACCCATTAGTACACTCGACAGCACTTGTAGG
EMPV1_16474 GGAACTCGGTCACTTGGCATTTTTCAAGCCTCCGTTTCCCCAGATCAGTAACGAAGACAA
EMPV1_16478 AGGCCTTGATGACTCTACCTACCGCCAAGGTTTTGATTTCCCTCCCACCTAACCCTGAGC
EMPV1_16479 GCCTGCTAAGCCACACTGGGCACTTCATGAGCTAGGTTTTTGAAAATAGTCGCATTCCAA
EMPV1_16482 AGACTCGAACCAGTCTGGGGAAGCCGACTAGCTTCAAGGCTGAGTGTCCTCTGAGATCTC
EMPV1_16484 GCTCCGTCTTACACAATTCCAAGAACTGGCCTCAAGGAAATAGGAACATACTGACTCTGG
EMPV1_16485 GAGGAAAGGCAGGGATGGTTGAACCCTCATGAACTTTCAAATTGGCTGTTGTGCTATCTC
EMPV1_16486 AGAAGCTGGGCCTTGTTTGTGGACTATTTAGCCCCACCCCTCTTCCCTACGACTTTGATT
EMPV1_16487 TTTTAGTACGTTAGAAGCATGTGGTAAAGTCGGCAGAGCTGCCTGCCTCGCTCCAGTAAC
EMPV1_16488 GTCTGGCCGGCTTCACCCTACTCCTCAGTCTCCTGGCCTTTGCCATGTACCGCCCGTAGT
EMPV1_16489 CAAACCCACTCCAACCGCATCTATCACATGACCAAACGAGGCCTGGGCCCTGACGAAAAT
EMPV1_16491 CCATCGAATCAGGATTTAACCAGATCAACGTGAAAAACCAGCGAGTCCTGGCGAGCCCGA
EMPV1_16492 CAAAAAATCCTGCCAGCATGGGGCTTCTGTTGCAGTGGGAAGAGCAGACAAAACCCAACA
EMPV1_16493 GACCATGAGCCGAGCTTTCATTCTTACATTCCTGCTTAAGAGCCAGTTTCGTAGTGAAGG
EMPV1_16494 CCTTTCAGGAGAAAAATCTATTACCTGCCCCGCCGGTACAAGTTCATGAGCAGGATCGAT

EMPV1_16497 AGGGTGGTGGAGGGGACACGTTGAGAGTCCATTATTCCTAGGGCATTTTCAGAAGGTCTG
EMPV1_16502 AGCTGCTCCCAGACTAGCTGCTGCCCATCAACCTGTAGCCAGACCAGCTGCTGCCAGCCA
EMPV1_16503 GTGGAAGAGCTTTTCATGATGGCTGGCAATGTCTGGAGACGCTTAATGCCATTCTAGACC
EMPV1_16504 GGTCTTGGAACAGGTGAATTCTCTAGGACTTTAGAAGAGAAAAAGACAAGAAATGGAACA
EMPV1_16505 ACGTTGTTCACGCGATGTAGGAAATTTCTAAACGACACCATGCATCACTGGCGGGCCTGT
EMPV1_16507 AACCTGACTAGTATCCATGAGGTTCGCTCTGTGGGTTAAAGGATCTGGTGTTGCTGTGGC
EMPV1_16510 GCTGTCCGAACCTTGAGAGCAGAACGTGGGATATTGAGCTATGGATGCAAAGCAGAAAGT
EMPV1_16513 CTTACAACTGTTACTCCTGTCATTGAGTCATGGGTCTGCGTCCCTCACCTTGAAATTGGG
EMPV1_16515 TCTTTTCTGAGACCATCCGCCGCTTCGGCCGCCTGGACTGTGTGGTCAACAACGCCGGCT
EMPV1_16516 CTGGCTTCAGATGTTGACAGGAAGACATGGACTCTCTTCAGTCTGAAAACATGCTAACTG
EMPV1_16517 CACATTTCCTTTAGCCTACCTTTTGATCCTTCCTGATCACATCTGGGAGTCCGCTGACAG
EMPV1_16518 GGCATGTGGCCTCAGCACCCAAAGAACAAAACCTGACCACTTTATTCAATGACGGGGAGA
EMPV1_16519 CAGGATACTATGATCGAAGGTACTGGACCTTGTGGAAGTTGCCTTTGTTTGGGTGCACTG
EMPV1_16521 ACGAAAGGCTTGGCGAAAAGCTCGAGACAAGGGAAGAACCTAAGCCATTACACTTCTTAC
EMPV1_16522 AAACTGCAGAAGAAGGAACACTCACAAGCACATTCTATGAGACACCATAACCCTGTTACC
EMPV1_16523 GAAAGGCAAGAGTAATAGCTCCCCTCCACTGTTGAGAATCTGAGATTTTCCCTCTGCATC
EMPV1_16524 GTGGCGGCATCGTCGCTTCTATCCATCCCCATCGTCTCTCTCACAATTGCACCTATTAAA
EMPV1_16525 CTGTTGTTTGCCTATTCCTTGTCTTCAGGGGAACTCCCTTTTCCTATTGTGTGCTTCTCA
EMPV1_16526 TCTTCTTTGGAGATGCCCCCGCCTCCCTGGACGCCTTTGTCTTCAGCTACTTGGCCCTGC
EMPV1_16527 AACTGACCACCACGTCCCTTACCCAGGATGCGTCCACTATGAGCACCAGAGCTCACATGT
EMPV1_16531 CCTGATGGGCGACCACACTGGCTGTGAACTTCTCAAGGAGGCCTGACTAAAGAGGCAGGA
EMPV1_16534 CAGAATTACCAGAATAGCGAGAGGGGGGCAAAGAATGAGGGATCAGAGAGTGCTCCCAAA
EMPV1_16539 TTAATGGGGAAATGGCCCCCAGGGGTGGGGCTGACCATAAGAGCCCCTTGGGGAGGTAAT
EMPV1_16543 GTGGAGTTTGTGTGTTGCCCACTGGCCGAGGAAAGTGACAATATCGACTCAGCAGATGCA
EMPV1_16544 GGTCTTGGAGGGTCTCCTGGGGATGTAGGTGTTAGCTGTGTCTCACTGTGGGGACAAGAT
EMPV1_16545 TCCAGTAAAAAACCAGGGGATCCTAACAGACACCTGCAAAGAACTGAGACCCCTCCTGAG
EMPV1_16548 TTCTTGGTGTTTGACGATGGTAGGCAGATCCCATATAAGAACTATCGACAGGCGGGAGGG
EMPV1_16554 CAGGGACAAATCCAGGTCCTTAGCCTGCTGAATGCAAGGGAACTCCATGGCTTTTCTCAT
EMPV1_16555 AGTCAGCTGCAGGGGCCTTTCACCAAATGTTGCCTTTTTATAATGTTGTCTTCATGGGTC
EMPV1_16557 GTGGACGGGCTCCCAGTTACCGAGTTATTCAGGACGTTGTCTCGGGGTAACGAATTTTGA
EMPV1_16558 CAGACTTACTCACACCTGAGCATCGCCGGGCTGGTGGGCTCCATCGACAACGACTTCTGC
EMPV1_16561 CTTCAGTGTGTAACTAAGCACAAAAACAGTATCAATGTTGAATCTGTGAATGGTTTTCCG
EMPV1_16562 CTCGCCATGTAAATACAGATTTAATTAACACTTGCAACCTGTGAAGATGCAAAAACTTAA
EMPV1_16565 GTAAATGTACATCAGTTTTTATATTTGTGAATTCATCTGTGGGAAGAGGAGCAAAGAAAA

EMPV1_16567 CCACTGGTTATCCTGGCAAAATGGCAAGCCAGTTTCAAATTCATCATTTAGGGCACCCTC
EMPV1_16569 TAGACTTCCTCTACCAGGTCCTGCAGAGCGACGAGATGGACAATAAGATGACCAGCTGCA
EMPV1_16570 AAAGACTTCTCTCCTTACCATGACAATGTTGCAGGATGTCAAACTGGAGTTTTTGGGGGG
EMPV1_16571 TTACAAGGGTCCTGGCTCTCGTGGTGCTGCCGTGAAGGGAGAGTCTGATAATAAGGAATC
EMPV1_16573 TTAGTTGTCAAAGCACCAGCAGGACCATTTGGGGAATCTGAAAGGTGATCTTGGAACCTG
EMPV1_16574 CCTGAGCATCTTGCCCTGAACCCCAAAAAGTCCACCAATCCAGACTCAGATTCACTCAGT
EMPV1_16576 GCAAATGGACTTTTTTGACCCAACTTTTGACTATGAGATGATCTTCAGGGGAACAGGAGC
EMPV1_16577 GATACTAACGAGGAGATTTCCGAAGGCTTTGTTGTAGGAGGTGGAGATGAACTTACTAAC
EMPV1_16579 TGGGTTCCTTCCAGGTGGCCCAGGGCTGAGGTCCTTCAGAGCAGTGAAAGTCCACTTAAA
EMPV1_16580 TAAAAAGTATGCAAATATATACTGAGGGGCTGCAAGTGTAAGCCCTACTGGCCACCAGAG
EMPV1_16581 TGCCAGAAGAGCATGACATCCAGAGTGCGCAGAGAAGAAAACAGGTAAATGAGAGGGAAC
EMPV1_16582 GTCCATGAGTGGAACCTATCCTCCCATTACTCAGTCTGTAGTTTCTTGACCTTGCCCTGC
EMPV1_16584 TGGCTCGAATAGAGGAATTGAAAAAGGAGGAAATGAGGAAGTTACAAAAGGAACGCAAAG
EMPV1_16587 GCCATGACCCAGTCTTCTTCCTCAAACCCCTAGGAAGCATGGATGACCTGAGCTTGATGT
EMPV1_16588 GTGTATGTGCGTGATGAATAAGTTTGGGTCTGGCTAGAATGGCATACCTTACTCTTCTAG
EMPV1_16591 AGGTCTGTATCTAAAGCTGGGGAACCTTAGGCCAGTGCTGAAACCTCTCTGCTCCTCAGT
EMPV1_16593 GTTGTCATAATGGTGCTGCAGGGCTATATTTCAAAGTCCTCGTCTGATAAAGAAACATTC
EMPV1_16595 CGTCTGCACATTCTCGTCATTTCACAGGGTCCAGGGACACCCCCAGAGGCTTTTAGTCGT
EMPV1_16596 GTGCAGGCGGGCGCGGAGCCGGCAAAGAAGGCCAGCAGAGCCAGCCGCAGAGCAGCCAGC
EMPV1_16597 CACCGGCTAGGCGAGTTTATTGGCTGTTTGCAGAGCCCACTTGTTGCCTTTCAAGCTTCT
EMPV1_16598 AGATGAGGAAACCGAGCGTCTCCTCGACCTCAGCAGGGAGATGCTGGCGGCGGGCTCAGA
EMPV1_16599 CAGCAGGCACGGAGAAGAGGGAGGAGCTGGTGGTAAATAAAACCCGGACAACTGTGTTTT
EMPV1_16600 CACTGATTGTAAGATCTTTGGCATCTCAGAGATGGAGTACAGGTCCTTGACTTGTGACTT
EMPV1_16602 AAAAGATGACAGCAACCTCTGGTTCACGTCCTCCCCCCAGCAGAGCTGTACCCACTGTCT
EMPV1_16603 GTCAATTTCGGACTGTTTCTCCCACTTCCGTAGTACAAGCTCCCTGAGGACAGGAACATT
EMPV1_16604 TTGTGTCCTGGTCCTGGGCTGGAGGTTCAGCTGTGGGGATGATGGTGACATTGATAGTTT
EMPV1_16605 ACCTCCTAAAGCTGTTCACTATAATAAAGTTCAACCAAAAAGGCAGCTAGGGCAGAAGGG
EMPV1_16607 AGGGCTCCTGACTCCAGGTCCTGTGTGTTGCCTGGAAATAGCACTGGACTTGGAGTCAGA
EMPV1_16612 GGGATCGAGTGTTTCCGTGGGAAGATGTGATTCAGACTGGTTTGTACGATGAACCTGCTG
EMPV1_16616 ATCAAGTCGGAGCCAAAGTTGTGACCGAAGCTGTACCTGCAGCAATGCTGCCAGATCCTT
EMPV1_16617 AGTGTTAGGCTCGTGCTGTATTTGTGTCGGGAGGAAGGTTCGACTCCCGTGCAGATGACA
EMPV1_16618 GTGCAAATTCTTCAGCCTGACGGAGACCCCTGAGGATTACACGCTCATGGTGGACGAGGA
EMPV1_16620 CTGCAATTCCCGACCCCTCTTATAAAAAGCTCACCAACTTTTGAAGAAACTGGAATAGGC
EMPV1_16621 TGCTACCCCCAGCTCACAATCAAATGCAACAGTCTCGGTTATATACACTGTGGTCACACC

EMPV1_16623 GTGGTTTGTTATTCCTGAAACCTGGGGCACCTGTGACGTAAAGACCTAGAGAGAAGGACC
EMPV1_16624 GTAGATACTGCCTTCTACCAAGGCCTTCCTGTCCCTGTTCCCACTCCCACCTTTAAATGA
EMPV1_16625 ATCCATCACGGCTACTAACTTTCTATGACAAAGCTCTTCAGCCTAATCAAGCTAGAGCCC
EMPV1_16626 CTGCTGTTTTTACTGTACTCTCTACTGTACTGCCTTTTTGTGGCTACAACTGTTTTACAG
EMPV1_16627 AGGTTGTCACACCTTCGTCCCCTCGAACGCCTCTGTCGCCTGCAGAGCAAGGCTGGGTCG
EMPV1_16628 TTTAGCTGACCACCATTTCGTGGCTGGAAGCAACTGGCTACAAAAGCCGCTCGCAAGAGT
EMPV1_16632 CTCGATGATGGGCTCTTTGTCCTCCTCCTTCAATCCGTTCAGCGGCATCGACAACGCCAT
EMPV1_16633 GTAGGTACAGTGTTCTTCTGGACATAAGGCTATTGCACACTTCCTAGACTACAGCGTAGG
EMPV1_16635 GCAAAAGGTTTGTGTACTTTACACTCAGAGGTTTTGCCAGTGGCCTGCACCCCCTGAATG
EMPV1_16636 TGGATGGGGCTGGCATGTTTTATTTGTTTTCTGGCAATACGACATGTGAGAATTTTGAAG
EMPV1_16637 TTGTGGGTCGATTGAGTTCATCTAGTTTGTAGTGAGGTGTGCCTCCCGACTCCCTTTTGC
EMPV1_16639 TCAGCCAGCTTGCTCACACAATCCTGATGATCCCTCTCTGGATAAAAGTCGCTAATATTA
EMPV1_16645 CAGGGAGATAAAGCAAGGCGGAGCATGGGACACGGTTTGTGAAAAAGGATAAAGTGCCTG
EMPV1_16647 TCGTGTAGGGGAAGGGTGAGTGCTGGGAGAGGATGTCTTAAGTTTCAAGGCTGGTGGATA
EMPV1_16648 ATGACGAGGACAGCGATTACCACCAGGAATCCTACAAGGAGTCCTACAAGGACCGGCGGC
EMPV1_16650 GCGAGGCTTTGGCGTGTGTATCCAGGTTTCCAGTAAGAGACGTTACCCAGGTGGTTGACT
EMPV1_16651 CATCCCAGCAGTGGCACAGACCTCCCACATGGCTTTTGCCAGCACTGTTTACATCTACCA
EMPV1_16653 GAGAGCTACCAGATATACAGACAGGCTCGTTCCACAGTCACTACTGCATTTTCAAGCGCC
EMPV1_16659 CTGCAGTGCCCCTAACATAGAAAATAGTGTGTCCTTGCAAGCTTTCTCTGCTGTCTTTAG
EMPV1_16660 CACTGCCCCTCCCATTCACCTTCATCCTCATTTCCTACTTTTGCATTGTGGTTACTGTGC
EMPV1_16661 GGGATAACCAGGCCTCTGTCCACATCCTGCTTTACCCAAAACTGAACATCTATGACCTCT
EMPV1_16664 GCTGGAGAAGGTAGAGGAGATGGCGCAGGGGACACGGGCCAAGACTAGGGGTTGCTGGGA
EMPV1_16666 CCTGGCATCATCATTTCTAGGATGGTTCTAGCCTCTCTCCCCACCAAGATAATTTCTAGC
EMPV1_16668 AGTTTGTCACCAGGAGTTGTTACGAATGCCGCAGTGAAGCCCTAGGTGTGACAAGCCTGG
EMPV1_16669 TCCTAAACATCTATCTGTCTCAAAGTATTTCATCCTACTTGGAAGCAGAACTCTTATTGC
EMPV1_16670 GTGAGATCATGTGATTATTCAAGGGTGGCTGTTGCTGAGGCGTGGGTGGGAGAGCAGGAA
EMPV1_16671 CAGGGAGGAGAGGCAAAAGCCTGATATTGAAGGATTTCACAGAAGGCTGAATGACCTCCC
EMPV1_16672 CTGCTCTACCAAGAAGATTTTATACCTAGAAGTATTATATTTTGGAAGGTCAGATTTATC
EMPV1_16673 GCGTGGTGTTCTCCAGACTCTGCCCTTTTCAAGGAAGAAGAGGATATGACCAGTTCTCTT
EMPV1_16674 TGCCGGCTAGGCCCTTGGGGAGATGCCTTCCTCCTGCCGTCAGGGGATCTAGGGTTAGCA
EMPV1_16675 CTTCAAGGCCCTCAACGCGCACTGGAGCGCGGACACCGTCTACCAGGAGGCGCGCAAGGT
EMPV1_16676 TACGTTGCCAGCGAACCCAGGATGTCCAGAACTCATATGGATAGTGGGGAATCAGGTCTG
EMPV1_16678 TAGCTGCAGAAGTCCCAGTCCAGGCCTCTTGTCATCTTACCCTGCCTCCTCGTATTCATA
EMPV1_16679 ATGCCTCCTAAGTTGTCATTTTGTGACTTAGTCATTGGGGAAATGTTTGGGGTTTGTTCC

EMPV1_16680 AAAAGTCGAGAGTGGCAGCTGTGTCTGGGGATAACGTCTCTCATTTCAACACCAAAGCCC
EMPV1_16681 TCCAGAAGCATTTACCACATTAGGCACAATCCAACGGCTCTGCTATCATTTGTACTCAGG
EMPV1_16682 TGCTCTGTGTCACACCCTCCTTCTGACCCAGCTGTCTTTTTGTGCTGACAACACCATTCC
EMPV1_16685 TGGGATTACCAGCCTGACATTTGCAAGGACTACAAGGAGACTGGCTTTTGCGGCTTCGGA
EMPV1_16687 AACCTATGAGAGTGGACAGGTATATGTAAATGACTTCCCTGTAAATAGAGGTGTAACCCG
EMPV1_16688 GCAATGGAGGTTTACAGGTTGCTTTCTTTGGCATCCGGCTTCTTTCTCTCGGCCAAATGC
EMPV1_16689 TCAGAACGAGTTAGAGAAAGGTGAGCGAGAAAATACGGAGCTGCACGAGTTTGCCAACGC
EMPV1_16690 AGGGTGTCCCTTATACAATATTTCACCCTGAGGAGCTGTGTCTCTTGTGGCAGTGGCAGG
EMPV1_16691 GCACATGGAACATTCTCTAAGATTGATTACATCCTGAGTTGCACATCCAACCTTGCTAAC
EMPV1_16694 CGGTTAGGATTAGCCCAGGGTATGAGGAGTGCCTTCATTTCCCATGTTTGTTCTTGTGAC
EMPV1_16695 CATCACGGACAAGGGCCTGGAGCTGATCGCGGAGCACCTGAGCCAGCTCACGGGCATAGA
EMPV1_16698 CCCCTGAGTGAATCAGAACTCATTGATGAACTTTCGGAAGATTTTGACCAGTCTAAGCGT
EMPV1_16702 CTGTCCCAAATCTGGGTTCACCCGACACCGGCTGCCTCCACCATGCAGCCTAAGTTTGAT
EMPV1_16703 CTCGGCGGAGGGGCCACTCTGCTCCTGTCGCTTCTTTGGATGCCAGCACTGCTGCCCATA
EMPV1_16704 AAAGGGGGATAAGTCTGCCCGCTCTCCGTCATCTCTGTCCGATTATCCAGAGTTTCCTAA
EMPV1_16705 GGGGTAAACAATATCTCTGGTATTGAAGAGGTGAATATGTTCACAAACCAAGGAACAGTG
EMPV1_16706 GGCTCCGAAGAAGGAGCCTGAACTTACAGAGACACATAGATTTGGCGTTGACTTCCAGAT
EMPV1_16713 CAGTGTGGTATTGGCACAAAAGCTGACACACAGACAAATGGAACAAAATAGAGAACTCAG
EMPV1_16714 GTTGTGTTGGACGTTGAACTAAAGCAGCCTCTGGAACCACTGTGCCCTCTGAAGCCACGA
EMPV1_16717 GGATTTCATATTGAATATATGTACACAGTCTTAACTATAGTGGTGGTAAACATACTACTA
EMPV1_16719 CTGCTGCCCGTGGGGCCCATCGTGGACGTGCTGCAGTACAGCCAGAGGGACCTGGACGCA
EMPV1_16720 TACAAGAAAGTCAAAAAGGACTCGGCTCTGCTTTTGTCCGCCTGCTCCCACTTACTGCAC
EMPV1_16721 AGGATGCCAGCTATGGGCTGGGTGGGATCTCTAGGAAAAGCCAGTAAGTGAGAGAAACCC
EMPV1_16723 AGAAGGCTGGCGGAGAGTGAGGTGGACCCCGACAGGCGGGCGTACGCTGTGTGGGTGTCA
EMPV1_16724 GTGTTTTTCTGGGCTCCAATTATGAAATGGGGGTTGGTGTGTGCTGGCTTGGCTGACATG
EMPV1_16726 CTTTCCCGTCAGCTCTAGGAAGGAGTTTGATTTTTATCCTGGTTGAGCTGGACAGTGAGT
EMPV1_16727 CTCTACCCACTGGAGAATAGCCCACAATTATAATGGAGACTAATAAAACAGGCAATCCAG
EMPV1_16728 ACATTGCTTTGACTTAGAGGAAACCGAGCTTTGGGACGGCAGGTGAGCGGCTGCATGAAA
EMPV1_16729 CTGCATGTGCATTTGTACGTGTGAGTGCGTGTGCATGTGTGCATTTATGTACGGACATGT
EMPV1_16731 CTTTAGGCCTTGGGCAATCTGCTTGCCTTCCAGACCTTGTAGACTTCGTCACTCGGACAT
EMPV1_16735 CGCCACACGGACATGCCCAAGGCTCAGAAGTATCAGCCCCCATCTACCAACAAGAAAACG
EMPV1_16737 CCTTGTGATTACGTTGGGTCCAACCAGATAAACCAGGGTGTCATAGCTAGGATTCTCCAG
EMPV1_16738 ATGTAAATCCAGGACCCTGGGCTTGCGGAATACGAAGACAAGGACACGTGTTACAGCCAT
EMPV1_16740 GCAAGCCAGGAGCTCATTCCCATTGAGGATTTTATCACGCCAGTCAAGTTCTTGAATAAA

EMPV1_16741	AGAGACTCTGCATCCAACTGGTGGTTGGACCCTATACTGTGGGGTTTCTGAACACCATGA
EMPV1_16742	GTAACTGCTCAACATCCCCATCTAGACTTGAGGTTACAGAAGTGGACCTCCCTGTCCTAC
EMPV1_16744	GGCTCTGGGACGAGGGAAGAATAAAGTCACTACTGTCAGGCAGAACCTCGGACTATGTAC
EMPV1_16747	TGGACCAGCACGAGGTGCGGTACCTGCAGTTTGCCTTCCGCTGGATGAACAACCTGCTGA
EMPV1_16748	TTCGGCCGGGGCGCCCACTCTAAGAAGGGCAAGATGAGGAAGAAGATGTAAGGAAGGTGA
EMPV1_16749	CCTCTGCCAAGGGACATCACCCCTGACGTTCAGCGTGTCGAAGAAAAGTCTCTACCTTTA
EMPV1_16750	GACGCTGCGCCAGGGTATTATCGACATGGTCTTAGCCACCGAAATGACAAAGCACTTCGA
EMPV1_16751	AGATTCTCTTTTCTCAGAGCCTTGCATGTCGGTAAACAGGGAGGATGAGGCCATCTGCTC
EMPV1_16752	ACTTGATGCCTGTACCCGCCGACTTTGCAGCCAGGGAGGGCCTGACAGCCCCACCCCGGA
EMPV1_16753	AACATCGAGCTGAAGGTTGAAGTAGAGAGCCTGAAACGAGAACTCCAGGGCAAGAAACAG
EMPV1_16754	GTTGCCGCGTGGAATCCATCTACCTGAATGTGGAGTCGGTGAGCACACACCGGGAGAGGT
EMPV1_16757	ACATCTCCACAGAAAAGGAAAACATGGCCTTCAGAGTATAGACTTTTGGCTGTCCGTGGG
EMPV1_16759	GAAAATATTGGTAAAAATCTTTGATAAACATGGGGTGAGGAAGGCCTTCCTAAGTAGGAC
EMPV1_16760	CAGGAAAGTTCCAGTTCATTTTCATCCATGGATCATCACGTTTTTCGCTCGTAAACATCC
EMPV1_16764	AACCACTTCTTCGGTTCATGAAAAGCATCAGACTTCTATTTGATGGTTTACAGCAGCCAG
EMPV1_16767	GTGGCACTTTGATCTCTACCCAACAGTGAAGGCTTTGGAAAACAGTCTTTTTAGAAGCTG
EMPV1_16769	TTCATCTGAATAGGCGTTTGGAGTGAAGCAACCTTAGAAAGTGGGGCTGGTGTCCAGCAG
EMPV1_16770	GGGGTTTGTAGTTATACGCCATGAATTTCCAATAATCTCTCCAGAGCCATATTCTCTCAT
EMPV1_16773	GAATGTGCATTTCTCTTTCGGCTATTGCTTGGACCATTGGACTCATCCACTCTGTGGCTC
EMPV1_16774	TGTTTTGATTGAATGAGACGTCTTTCCGGCTTGACCGCTCTTCACCCACCTCTGCTGTTC
EMPV1_16775	GTGAAGGTGGAGTTATTGTTGATTACCATGGTTGTGATTTTTTCCCTGAACGTTGGTTTC
EMPV1_16776	ACTCAAGTAAATTGTCTGAGAGCTGGTATGTGAACTCAGGCAGTGGGTCCCCAGGGCTCT
EMPV1_16777	TGTGCAAACCTGGGGCTAGAACTGGGAAGGAATCTGCATCTAGCCATCAACTGTGCTGCA
EMPV1_16778	CAACCTTGGCTTATATGAACGAATGGGTCAGAGTGATTTTGGGGCTGGGCACTACCCACA
EMPV1_16779	CACTGTGCAGAACAGAGAACTTGAGTGTATTCGAGAGATCAAAGAGAGAGTGGGCACATC
EMPV1_16782	TTCCTATCCCACCCTCAGTTCTTTAGGGGATGGTCATAGTGGCTCATAACTGGACAGTGC
EMPV1_16785	GTTTAACCACAGGCTTCTGCTGCCTCTCCGCCTCTATCTTAACAGTGCTTTTGGAGTCGA
EMPV1_16787	GGCTTGAGTCTTCGCCACTGTCTGGTAGTGACCTTACTTCTAAGGAGCCTGTCTTGTGAT
EMPV1_16790	GGAGCCGGTGGTAGGGAAATTGGAATAGGGCAAGTTAAAACCCCACAAACCTCACTGTTC
EMPV1_16791	AAACTCAGGTCATCTTCCGTCCGGGCTTCGTAATCGTAAAGGGCCACAAACAGCGTCACT
EMPV1_16793	GGACTTTCTGTAAGAAGTGCGGCAAGCACCAACCCCACAAAGTGACACAGTACAAGAAGG
EMPV1_16794	GCCCCTCTTCCCGTCAAAACGTCGCAAACACGAGCTGCTTTCAGGTTCCATTAAGACTCT
EMPV1_16795	ACTGTCTGCATCACAGGATTCCAGTTCTTCTAGTATTATTTTGTGTTTAGTGTGGAGGCC
EMPV1_16797	TAAACCAAAGCACAAGCAAGAGTGAGTCCCTGTAACCAAAGATGAGCAGCATCACCCTGG

EMPV1_16798	TGAATGGCCTCTTGCCCTTCCTGAGCCTGTGGCCTGCTCATCACAAAAGCACAAACCTAC
EMPV1_16800	CTGGTCAGATTCAGAGGCATTCGTGATCACAAATTTGGGGGAAAGTGACAAAATAAACTC
EMPV1_16801	CCGCAGTCAAGTGATTTGTCTAGGAACATGCAATGACAAGAGAGCCCCATCCTACAGCCT
EMPV1_16802	TGGTGATCTTTATCAACATTGGGGTGGTGGCCTCGGGTTGCTTTCTCCTGATTGTGCTGT
EMPV1_16803	TCCCGTCTGCCCCAGAGCTTTCTTCTCCTCTTTTGTTGCTTCTGCCAAGACTGACTTCCA
EMPV1_16804	CGCTGAGTTCTCCGGCAGCCTGAGCCTCTCCGAGACGCAGGTCAAGATCTGGTTTCAGAA
EMPV1_16806	ACAGGCAACTTCCGCACTGTCCTCCAGTTTGTGGCCAACCTCAGCAAGGAGTTTGACATT
EMPV1_16808	ACATCCAGAAAAACCTGGAAGCAAGGAAAGAGGCAGTAGCACATCACACCCTCCTTCTCC
EMPV1_16809	TTTTCAGCAACTAGAGCATAAGTTCGGGCGGATGCGTCGACACTACCTGGAGCGGAGAAA
EMPV1_16810	AGCCTCTCAGCCCCTGAACCGCAGGGGGAAGAAGGAAAACAGATGACAGCGAAGCCTCCT
EMPV1_16811	GGCTTCAGAGAAGTGGTGGAGATCCTAGACGGGAAGACGACTATAATCGAAGGCCGAATC
EMPV1_16812	GGGGTACCAGCATGGATATCAAAGGAAGGGAGGCATTTATCCCTGTATTTAGAGTGGACA
EMPV1_16813	CAAGTTGGTAGAACACGGATTGAATGAAACCCTCCACATCAGAAGTGGTGTCTTCAGTTG
EMPV1_16814	TCCTCGCCCTCATCGTCTCTCTCCCGGGCCATCCCTTCAGCCCTGGGGCTGGTCCCTGGC
EMPV1_16816	TAGCAGCAGCGACGGCAAAGGGGCGGCTGAGGCCGAGACACCTCGTGTGCTTGTGTCCAT
EMPV1_16817	AGGTCTCTTGATCAGTTTCTACATTTTCCTACCTTTGCTCATTCTAATTGCCCTCATTAC
EMPV1_16818	CCGGCTGAAGGCGGAGGCTAGGAATAAACATGCTATTGCAGTTATTTGGGCTTACTGGCT
EMPV1_16819	TTTCAACGTCATCAACGGTGGCTCCCACGCCGGCAACAAGCTGGCCATGCAGGAGTTCAT
EMPV1_16820	AGGTGGAGGAAAAGCTTCCCTAAAAAGATAACACTGGAAGAGTTCAGATTGATGAGAACC
EMPV1_16821	TGTTCCGGACACACGCACTACAGTCGACCGACCCCTTTGAGGAGAAGATTGTCAGCAGTT
EMPV1_16822	GCAAAGAAGACTTTATGAACATCTGCATGGAACCCAACACCATCAGCAAAGGAGACTTCA
EMPV1_16823	CCATCAAAAATGTTTCATCCCTCTACTGTTCGGGCCTCTAGGGAACGCTTTCTAATCCTG
EMPV1_16826	TACTCCACGGTCTCCGGGTCGGGCTGGAAAGTGAAGTGAGCCACCTGCCGCTTCTGGGCC
EMPV1_16829	GACTGAGATCACTCATGTCTCACAAGCCCTCTCAGAAGTGGAACAACTTCTCAATGCTCC
EMPV1_16830	CATTATGAGGAAGAAGATCAGGATCTGGCCCAGTGTGCTGGTCATTCTCCATTCGCCCTC
EMPV1_16831	GATTTTTGAAATGAGTATCCTTACTCAGTTAATGTCTCATTGGGCCAGTTCAGTTTTGCC
EMPV1_16833	TGGTGATCTTTATCAACATTGGGGTGGTGGCCTCGGGTTGCTTTCTCCTGATTGTGCTGT
EMPV1_16834	AGGCCTCCCCTGAAGCAGATGTTCCTGAAAAAGCCAAGGGAGTTAACAGTGCATCTTCCA
EMPV1_16835	TGCAGAGGAACCAGATGCTCATGACCCCGACCTCGACCCCGCACAGCAGCATCATGAAAT
EMPV1_16836	AATCTCACGGTTCAGTTGTCCTTCCATTAGGGTGGTGCTTTGTGCAGTCAAGGACTTGCC
EMPV1_16838	GGCCTATGTAGTGATTGTATGCCAGAAATGCAGTTATACTCAGATCAGTGATGTTCTCTC
EMPV1_16839	GAGCTGCATTCATATCATATTGTGCCTTATGCTTCGGAGAATGCCATTTATGGAATGGGC
EMPV1_16842	GTGAGGTATGAGACACATGAGGCTGCCCAAAAGGCTGTGTTAGACCTACATGGAAAGTCC
EMPV1_16843	GCAGCTGCTGTCCTGTGCCATGGTCACTGCAATGCTGGATGCAAGCCTCATCTATGACCT

EMPV1_16846	TGCAGGATATCAAAGCTCGCGAGAAAGATGGCAAGGGGCGGACGGCGTCTCCCGTCAACT
EMPV1_16847	AGATAACCAGCAGTCTGTACCTTGGCAATGGTGCGGCAGCCAACAGCAGACTCATGCTAT
EMPV1_16849	TGGCCCAAATCACATCTGACAAGGGGTCGTTAGTTGGCTTTAAGGATGGCTATGGTTGTG
EMPV1_16851	GGAGTTGTTGTTCTTCAAGATGCGGGAACAGTCTTACAAAAAGAGGGATTAGGCACTCAC
EMPV1_16853	CTCGGGAGCTGTCACCACGTGGGACGGGAGCAGCTGGAGCCCTGGGGACAGCCTGGAAGA
EMPV1_16854	GTGTTCTAACTCATCCCTGATGGTCTCTGAGATACATAGACTACTCCACCAGACTCCATG
EMPV1_16855	GCCACGGTGTGCCTGCTACAACCAGTTGGGTGCTCAAGCATTTTCCAAACTAATAAATCC
EMPV1_16856	AGTAAGGAAGGGACTTGAGGGCTACGGTGAAAGAGTCCTGGTGCTGAGGAGTCAGATTCA
EMPV1_16860	CAGGAAAGGTGGTTTAAAGGTACCAAAATACTGAGCAGATAATTGCTACGGTAATATACC
EMPV1_16861	CAGCTGCCTGTTGCCATGCAAGAATCCTAGGTCAGGGTTACCAGCTCCACTTTTCTATGA
EMPV1_16862	CGGAAAGCTCCCCCAGATGGCTGGGAGTTGATTGAGCCAACACTGGATGAATTAGATCAA
EMPV1_16868	AGCTTGCCGGCGAAAAGAGCCAAGGAGAGGACAGCAACGACGCAGAAGAGGACAGAGACA
EMPV1_16870	GGAGCTCCCTGTCTCACAACTTCTTCATTCATCTGTTGATGGACACTTAGGCTGTTTTCA
EMPV1_16871	GAAATGGAACTAGGATTTGAAACTAGACTTCCGAGGACTGTGCCCCCAGTGGCAATGACA
EMPV1_16873	GCCTTGCTCAGTGGGTTAAGGATCCACATTGCCATGAGCTGTGGTGTAGGTCGCATTCAT
EMPV1_16875	GTATCCTGGAAAGCAAGAAAGAGGATGAGGGCTGGTACTTTTTGAGCCTGGAGGAAAACG
EMPV1_16876	CTTCGACTTTGGCACCCAGAGCACCACGGAGAAGATCCACAACCTGATCCCCATCATGCT
EMPV1_16878	CGATTCAGGGTCAGGAGAGATTACAATCAAAATGGAAAACCTACTCTGTAGGGCGCTGGA
EMPV1_16880	CTCCATCCCCACCATGAACAGCTGGAACCCCTTTTTAGCATATTTCATTCTGCCAGGTGG
EMPV1_16881	GGGTAGATGTTGCCGCAAGCAGAGGAAGAAGGAAAAGTCTCAAATTTGTTCACACGTTTT
EMPV1_16882	CACAGACTCAGGAGGACTCCAGGCTTTATTTAGGACCTCACTTCCGATACTCTGCACTCA
EMPV1_16883	GTGGTTTGAGTCCGAGCCTCTAAAAGCCACTCTAGCAACGGATGCTGTAATTGGAGCCAT
EMPV1_16884	GAGGCTTCCATTCTGTGTGATAATGCTTGGAAAGAGACTGGGTTTGTATGATGGGGTTGC
EMPV1_16885	AGAAGCGTATCTTCGGGGCTGTGCTGCTCTTCTCCTGGACAGTGTATCTTTGGGAGACCT
EMPV1_16886	AGGGCAAGGAGAGCACAAAACCCCTTGGAAGGAAATCATTCAAACTTCTGTTATGACCAC
EMPV1_16890	TGAGAAGGTATTTTTGGCCTAAGCCATTTTGTGATCTAAGCCTGGCCATGATGCTTGTCC
EMPV1_16891	TTGTCTGAGAGTAATCTGTAAGTCCCCAGGAGACTCTCGCAGGCCAGCAAGTTGAGAAAG
EMPV1_16892	TTCATCTTAGTGGTTTTGGGGTCTGCAACAGTTTTTCCCACATAGCATCCCTCGGCCCCC
EMPV1_16893	AGAGTTCTACTCACCCCATTAACCCAGGACGCTATGGAAGGCGCCCTTCACTTGGAGAAG
EMPV1_16896	CTTGTAGTAACACTCTTCTATGGCTCAGCATCTGTCACCTACTTGAGACCCAAGGCTAGC
EMPV1_16898	TCTGAGCGCCACCCAGATCCAAGTGAAGCGAGAGGAGCTGATTGCCAACTGGGAGCAGAT
EMPV1_16899	GGAACTGTCTTTCATGCCACAAACTGTGCGTCATAATCCCACCCAAACAACTGAGATGTG
EMPV1_16900	GCATGGTTTAAAAAACAAGCATTTGTTTCTCACAGTCCTGGAGGCTGGGAAGTCCAGGAC
EMPV1_16903	GATGACTGTCCTGTTAAATATGGAGAAAAGAAAACTAATGAGAAATGGAATCTCACCGTG

EMPV1_16904	GGTTCTCTTATAAAGCTCACATCCAAGCAGCCCGTAGGTTTTGTCAGTTTTGACAGTCGC
EMPV1_16906	TGGGGATAAGCTGCGCGGCCGTGACAGCGTCCTGGTGCCCCTCGATCTTGCTCAGCAGCA
EMPV1_16908	CCAAATGCAACAGTTTGCATCCACTAACTCCAAACTCCCTGTCCATCCCACTCCTTGCCC
EMPV1_16911	AGTCTCTGCTGTGCCCACCGCTCACAGCAAGTCCATCTTGGGCCTGTAGTGCAGCAGGAG
EMPV1_16914	CATCACGTCCCTGCACGAGGCCCATGAGTTCTCCAACACCTACGGCTTCCCCATTATCTT
EMPV1_16915	TGCGTGAGAACACCTGTCGCCTCTACAGTCTTTGAACAGAGAGCGTGCCATCAGGAGTCT
EMPV1_16916	AAGAGCCAATGAGAACTAAGCTCAAATGCCTTCCCTGCTCTTGCCAACCCGATGGGGAGA
EMPV1_16921	CAGCCAGAGCAGTCATGCATTCTTTCCCAAACGTCTCTTCTTCTAGGCTCTGCTCATCAC
EMPV1_16922	GGAAAGATTCAGATGAGGCAGACTTGGTGCTGGCAAAAGAGGCAAATATGACGAGTCCTC
EMPV1_16923	TTTAACAAAATGAACAGCTCCAACCTGGCCTGCGTCTTTGGGCTGAACTTGATCTGGCCG
EMPV1_16924	ACTGGGAACGTCTGGATGCTGACAAGGGAGAGCTAAATACTGTTAATGCTATCAGTGCCC
EMPV1_16926	GGCCTCGCCCGCAGGGGCCAGGGCAGCGTGATGGGGCGGGAAAGCCCAGGTCCTCAGCCC
EMPV1_16927	AGCACTGTGATGAGACCAAAACATGTGGACACGGAGGCGCTGAAGAACTCAACAAGAATA
EMPV1_16928	AAGAAAATGTCATGGGAGGCCTCTGCAAGAAGGCAGCCAGGAAGAGAGCTCTCACCAGAA
EMPV1_16932	TGAGCAGTCAGACCGAGTCCAGCTCCAGTGAGAAACAAACCAAGAAGCAGAAGGTGGCCA
EMPV1_16933	CAATTGGCTGCTTCGCTCAGTTGGTTAGAGTGCGGTGCTGAGAAACTCTATTCTGCCGGA
EMPV1_16934	GCACTGAACGGAGAGTCTCAACAAAGGTATTCCTTACAAATATATACTGAATGCGGGCTG
EMPV1_16935	TGTCACTTCTGGGAAGCTGTGCAGCAGAGGAACCTGCTTCAGATTTCTGACTGGATGGAT
EMPV1_16937	CACACACTTTAGATCCTTTACCCAGGAAATCTCAGAGAAGCAAGCAGGCATAGTCCTGTC
EMPV1_16938	ACTTGGTCTTCTTTTTCAAATTGAGGCGCCGAGTCGTTGCTTGGTTTCCTGGGCTTAGGC
EMPV1_16939	TCATACCGTTTTCCACCTCAAATCCCAAGATGCACGTCACAAGACACTGAGCACTTGCGG
EMPV1_16940	CTGTGGTGAGTGTCCCTCGGAGCTGCCAGGGGATTCCCAGCCCCGGGAATATCGTTACAT
EMPV1_16941	AGCTAACTGGTCAAGAGGGGCTCGCGGGCCCAGGTGGCCTGGAAAGGAGCCCTGGGAGCA
EMPV1_16942	AGAGGTCAAAACCTGGGCGAAGTCAATATCTTTGTGAAGGAGGTGCAGCCGGGGAGTATC
EMPV1_16943	GGCTTGGCGCTCCCTTTCAGAACCAAGGGAAGCAAAGACCGTTTCTCATAAACCAAGCTT
EMPV1_16946	CATCAGCTTCCCTACTACTTGATGTGACCATGACCGAAAAGCTAGAAAATGGCATCAGTG
EMPV1_16947	CGTTCTCCAATATCATCTCTGTAGTTCCTCTATGCAGGGCGGTGAACAAATTCGCGGGAC
EMPV1_16948	GTGGGGCGCGGGCTTCCTTCCGTGGCCGTGGACGGGGGCGTGGTCGCGGCCGGGGACGCG
EMPV1_16949	GGTCATCAGCATTGTCAACTGTCTCAGCCACGTCAGCTTCGGTCACTTCAATTACCTCAG
EMPV1_16951	TCTCCATAACTCACAATGGCCTGGTATTTTGGTCAGTAAGCTGCTGTGGAATGTGAACAT
EMPV1_16954	ACGTGTGGACTTTTGGATGCCTGGCCTTAGGCTGTGGCCCAGAAGATGGAGGTTTGGGGA
EMPV1_16955	AGGAGCCTACATAGCTGGAAACCTGCATTCCATGATTCATGTTGGGCTTCTGTTTAGATT
EMPV1_16956	TTTTGCTCCTGGGCCTACTTCCAGCCGCTTATTTGAATCCTTGCTCTGCGATGGACTACT
EMPV1_16957	CCTTGGCAGATAAGAAGAGCTGGTATTACTCCGGGGCAGTGTTAAGCCTTTGTATAGCGA

EMPV1_16959	TGCTGCTTTACCTGCGTTCCTGCAGTACTTTATGTAATCTTCTATGCACTGAGCATCACG
EMPV1_16961	GGTAAACTTGAAGCTCACAGAAATGCCAGAATTTAAGAAGGATGGTGGGCCATTAGGGTC
EMPV1_16963	AGGATATGGTGATAGAGGAGCTGGATACAGCAACCTGAGTAGCGGCTTTGGCGACGGTTA
EMPV1_16968	GTCCTGTTGGCTAGTAGCTTCGTGCCCATTTACGCGGGACTGAGACCAGTGGCGTACCGC
EMPV1_16969	ACTGACCAGGACCTAGTCAAGTTGTGTCAGTCATATGGCAAGACTGTCTCCACTAAGGCC
EMPV1_16970	AAACAGATCCTGATGTGTTCATAACCATCAAGTGAATGGCTCAAAACGTTTCTCAGAAGG
EMPV1_16971	AGAGCAGTCACTCCCAGACACATCCTCTGTGCCAAGCTTTCGGTGTATGTTCTCTGGTTC
EMPV1_16972	CCGGGGCCTACATAGCTGGAAACATGCATTCCATGATTCATACAGGCTTTCTCTTTAGGT
EMPV1_16973	CGTGTGTGTGGTTCTATCCTACGTACACATCCTCAAGACCATTTTGCAATTCCCTTCTGC
EMPV1_16974	GTTGGCAGCTCTGTGGGCATATGACTTTAGGTTCTTTAGGGTTGGGATTCCTGTTGGCAC
EMPV1_16976	CTAGACCTGGGCTGCTGAAGCAATTAGAGCTCACTTATCCTCATAGGGAGACAACAGACC
EMPV1_16977	TTCTATAACAAGTCTCTGTACCCTCCCACCCTTACCCCCTACTGTACCCAAGGTATCGGT
EMPV1_16978	TGCGAGAGCTGAGGAGATTGTCCACCTGCAGATCATGACACCGGGGGCAGGACTCCATCT
EMPV1_16979	GGGTGCAGTGTGGAAGCATAGAGAATGAGATGTTGCTGTTGAGAGGGATCTGTTCCTTTT
EMPV1_16980	GAGCATGCAGACACACGTGGCTCGACTGAGAACCTTTATGTATTGGCCATCTACTGTACC
EMPV1_16988	GATTACTGTTCGGAAACACTCTTCAGGGAGCTCATCTAAGGCCCTCTCCACCTTGTCAAC
EMPV1_16989	GTAATGCAGTTTTTGAATAAGATGGCTGGTAATGAATATGTTGGATTCAGTAATGCAACG
EMPV1_16990	CACAGTGACTAGCAGGAGGAGGGGGCTTTCTTTGTGATATTAGCCATACTCACGTCTTCA
EMPV1_16991	AGCTGCACCTGAGGCATATGGAAGTTCTTGGAGTAGGGATCAAATTGGAGCTGCAGGTAC
EMPV1_16992	TAGCTCCCAAAGTCAAGGAAGGCTTGGGGGCAAAGTCTGTAAAAGAGGTGATGGTGAAGG
EMPV1_16994	GCCCGGGGCCATCTAGCTGCAACGCATCCAGCTCCCCTGTTCATTGATGTTCATAATTTT
EMPV1_16995	CTGGGAGTCATCCTTCTAACTCCAAATCCAGTATGCTAAAAGCTACATCAGGGAGTTCCC
EMPV1_16996	GGACAATATGTTTCACGGATTGGGGACTTATACATTCCCAACTGGGGCAAAGTACACTGG
EMPV1_16997	CTTCTGCTGGTTTCCCTCACCTACGTCTACAGCTTTTCTACATCGGTTGTGGCTTCATCT
EMPV1_16998	GCGCTGGTTCTGGTTGGTGAGAGTTCTCCTCAGCCTGTTCATAGGCGCAGAAATTGTGGC
EMPV1_16999	AGGGGACATCCCACATCTGAGAGAAGAAAGGGCCTGAAAGCAAGGCCTAGAGTTGCTATT
EMPV1_17000	AGGACAGAATGATAAGGAAAAGGAATTGGAGGAGCAAGAAGAGGAGAAACAAAAGGAAAG
EMPV1_17002	GCAAGCTCAAGAATGTGCTTTCACCAGCCATCCACCTGCTGGCACATAGATCGTGCACTT
EMPV1_17003	ATTCACCCGAATCAGAAACATCCACCTGAGAAGGTATTCCCAAGAGACAGTTCCCCTCGC
EMPV1_17004	GTTTCCATTCTCCGAGCGTTGACACACTTCAAATCAGTGAGTGAATCCATAAGGTGGTCA
EMPV1_17005	ATGCAGAATCAAAGAATTCAAGAGTTGATGGATAAAACTGATCAAAAAGAGGATACTATC
EMPV1_17006	TTCCACCTATAAAAAGCTATTTGGAGTCAGCCTGCAGGAGCTTCACCAGCAGGGTCTCAC
EMPV1_17012	TTCTCTCCTTGGCAGGGCTCCTTCCAAGGGATCCCACGGACTGTTCCGCCGCACCGCAGA
EMPV1_17013	GTCCCCTCATCCACTCTCCACTCCCACTTGCCTGAAGGTCTTGAAGCTTAACAGGATGTT

EMPV1_17014	CAGGGAACCAATCTTCCGCCTTCAAGGCAAATTGTACGAGCTAAGTTCTGTAAGCTTTAC
EMPV1_17016	AAGTCCGCTCCTGCCCCGAAGAAGGGCTCTAAGAAGGCGGTGACCAAAGCCCAGAAAAAG
EMPV1_17019	CACAACCCCTTTGAGATGCCGACTTGAAGACAAGGCTCCTTCTGGGCGATGCACCAAATT
EMPV1_17021	ACGTTAAAATCGTGTTGTACAAGTTTATAATTGTTTACTTGTTGTAGATGATACTCATAT
EMPV1_17024	GAGCCTATCAACCAAATGAGCAGTTAGCTACGGAGTCCATTGGCCTTAATCATAGTATGC
EMPV1_17027	CCAGGTCCATCCCGAAGTCGATGAACGTCCACTCAATGCCTTCCTTCTTGTACTCCTCCT
EMPV1_17030	CTCTGTGACACCCTCTTTGTGGTCTTCAGTGCTGTTTTTGTGGTGACCCGTCTAGGAATC
EMPV1_17032	CAGTGTTACCTGAAGAAGTACACAGTCGTTCCTTACGAATTGGAGCTAACCGGCGTACAG
EMPV1_17033	CATTCCTGCTTAAGGGAACAGGGTGAGCGAAAGAGGGATGTGGCAAGTCAAAGACAACAC
EMPV1_17034	TGGATCCTCTGTGAAAGCCACTGCTTCAAGGTGGCCGTGGATGGTCAGCACCTATTTGAA
EMPV1_17035	ATAACCACGAGGACCACCCCTGTGATAGATGAATGGAGTGAGTAGGGTTATCTCAACCGC
EMPV1_17036	TCATTAATAAAGTTATGGCTTCTTGTTGGATGAATTCCGTGGGTGCTTAATCTCTTCCCC
EMPV1_17037	GAGCCACATTCTTTACTTGATAGAACTCAAGATTTGTCCTTAGCTAGCCAATTGTCATTG
EMPV1_17040	CCTAGGTTCCTACTGAAAGCCTCATCTCCCATAGCTGACCATCTTTTTCTGACCTTTGAA
EMPV1_17048	AGCAGATCCGGAAGCTGATCAAAGATGGGCTGATCATCCGGAAGCCTGTGACTGTCCATT
EMPV1_17050	AAACCTAGAGATGGGTAAATGCCTCCTGCTGCCACTGCTGCTGGTAGTCCTGTCTTCACT
EMPV1_17052	AATAGAAGACTTTGGTGTACACTGCAAACAATACTATGCCTTAGAAGTCTCATATTTTAA
EMPV1_17053	GATCTTCAGATGCTCCTGGGTTTTGTCGGCCGGAGTAAGAGCGGACTCAAACACGAACTT
EMPV1_17055	AGGCTGCCTGCGGTGGCTCCGGCCCAAACGGGTCTCCAAGAAGCTTTTCAAGCAGCTGGA
EMPV1_17056	TCCATTTGAATTGGATACTTTTAACCCCCTGCCCCAGGCCAAGGATTGAACCCTAGCCTC
EMPV1_17057	GAGCTGCAGAAATTCGCTGAGGAGGTTGGCATCTTCTTCACTGCCTCTGGCATGGATGAG
EMPV1_17058	GAGGAGCTGGGACCAATAGCTCATGTCTGTGTCTTGTTTCTTACCAGCCTCTCAGGGCCC
EMPV1_17060	ATATAGTTCATGGCCTTCTAGTCTGCAGGAGTTGCTCCGCCCCACCTTTCCTGGTGTCAT
EMPV1_17061	CTGCCAAAGTGGTTTAACTCCCAGGAAGGTTAGGAATTTGAAGTCTTGGCTGTCTAGAGT
EMPV1_17063	CAGGAGGGACACATCACAAAATGGTGCTGGGGCTGGCAGGTAAGTTTCTGGTGATTTGGT
EMPV1_17064	AAGAAGTCTTACACACCTCTCCATGCAGCAGCCTCCAGCGGGATGATCAGCGTAGTCAAA
EMPV1_17065	CTGAGGGTGAAGAACTTCGGCATCTGGCTGTGTTATGATTCCCTTGTTAGCACCCACCAG
EMPV1_17066	AAAGAGTGGAAAAGTGTATAGCTTTCAGCAATTGGTGTTTGCCCATTCTAAGCAAGGTGC
EMPV1_17068	GGGAATATTGTGGCTGGTTTGAGCTTCTGTCCAGACCATGAAAACTTTCTCCATAACAGC
EMPV1_17071	CAGAAGAGATGTGGAAAACTCGAGGAGAATAAAAAATTGGAACAGGCAGTAGTAAACCTC
EMPV1_17072	TCTTCTATGCGGTCCACCATTGTCATCTTGGCTAGGTTGGCTGCTCTGAGGGAATGCATG
EMPV1_17074	TTCTCTATGAGCACATGGGGCTTCAGTGACATTAGGGTGAGATGGAAGGCAGAGGGCAGA
EMPV1_17076	CAGGGACGAGATAAAGTCCGAGAAATGTTTATGAAGAATGCCCACGTCACAGACCCCAGG
EMPV1_17079	ACGGGAACATTGTGGGCACAGATTCAGAGGAGGACGCTGTAACTAAAGGTGAAGCAGGCA

EMPV1_17081 GGCTATGTGGGATTTGCAAATCTCCCAAATCAAGTATACAGAAAATCAGTGAAGAGAGGG
EMPV1_17082 TTAGGCTTTGTGGGCCATGTACAGATTCTGCTGCCTATTCTTTTGTGAGTGTGTGTTGTT
EMPV1_17084 TGCCTCTATGGGAGACATTTGTGGCATTGTACCACGTCTTGCGGATGTTGCAGGAGCGAT
EMPV1_17086 TTGCCCCCTGCCTCTTCGAGATCTGCATGTGGGTCTAACCCTTGTTCATTTCAAGTCAAA
EMPV1_17087 AGTTGGTGATGGTGGAGAGCGGGGCGATGATGAGGAAAGGGCCGTGGATTCCCCTGAGGA
EMPV1_17088 TTTAAAGTGCATTTCCTCGCTGCAAACCGCTGATGACATTCCTCGCTCAGAGGATCCCAG
EMPV1_17089 TGCTGTGGCATGGAGCTCCTGGAACCGGCAGAGGGGAAGGAGCCTACTGTCTCTGATCTC
EMPV1_17091 CGAGCGGACATTCCCGCCGCGCCCAGGCAGGACGCCGCTCCCCGTCATGCTGTTCAGAAA
EMPV1_17092 GGCAGAAAAAGCAGGTGTAGAAACCAGCAATGTATTACAGTATATGTGCCTAATTCTGGC
EMPV1_17093 CTGAGAATAAAATGCAGGAGCCTGAGATTCAGCATTTCTAACAAGCTCCCAGGTGCTGCC
EMPV1_17094 GAGATTTCAAAAGGGAGATGCACAGCAGTGATTTGTCACCTGTCCATGAGATGCCCTAAG
EMPV1_17096 ACACTTCTTCCTGGGGCAGTGCCAGTTGGAGATGGAGAGCTATGATGAGGCTATTGCCAA
EMPV1_17099 ATCGTCATCGATCTGCATTACGGCTCTGGGACAATGACCGGACTCCTTGGCTATGACACC
EMPV1_17101 TTGCAGTTCTCATGTCATCGATCATTTCTTCTGTGAGGCCCCAATGCTGGTGCGTTTGGC
EMPV1_17102 TTAAGGGCATTCAGCCCGAAGATGTTAATCTGTTGGTGACCTGCAAAGAGGAGGGCAACC
EMPV1_17104 TTTTATTTTCTTATCATATTTTTGGTTCAGCTTATGATTGATACACCTACCAGTCCAATT
EMPV1_17106 ACCTGCACGAACCACCTAACTGCTGCGGCAATTTTCCAGGGAACGATACTCTTCGTGTAT
EMPV1_17108 CTCAAGTATAGCTGTGCTTTTGGTGTGTGTGTATATATATATATGCATGTATACACACAC
EMPV1_17109 CATTAACACAGATGAACTTGGGAGGGATTGCCTGATTAATGCTGCTAAGACATCAATGTC
EMPV1_17110 GCACGTGAAAGGAGTTTTCTGTTTCTCTAACTGGACTCTGATGGAAATAGCCTGAGAGGA
EMPV1_17114 TGTGAATGGAAAGCTGACAAACATGGGGACCGACAAAACAGATCCTCCTCATGAGACCCC
EMPV1_17116 GTTAGTGGTTTCATCAGTTGTAGCAGGGGAATCCCAGAAACCTGAACTCAAGGCTTGGCA
EMPV1_17118 TGGCTGGGGCATCGGCATCGACCGGGTCACTATGTTACTCACGGATTCGAATAACATCAA
EMPV1_17121 GGTATGATGCAAAGCAAGCAAATGGTTATCACATAAGTCAGAATACGGTTACCTCGAGGG
EMPV1_17123 GAATGAAAACTTTGCCTGTCCTACAAGGTACACAGTCATCTCCCAGAAGAAACAGGCTGA
EMPV1_17124 ACCTGGACACCAAAAGGAATCGGAACCGGGAGGGCCTGAGAGCCCTGCAGAAGGATCTCA
EMPV1_17125 GTGCAGAGCACCCCTGGACAGATGTTCCCTAATCCCATGATCCCACCTATGGCATATCCT
EMPV1_17127 TTCAGCTCTTAGCCCAGTATCAATTACCTATTCCCCACGCACAGCTGCATGGCAGGAAAC
EMPV1_17130 GACGCTGTGGTTATTTCACTGCCCTCTTCTGCATTTCTTCCTGGCGGGGATTGACTCTAT
EMPV1_17131 CGGAAGAAAATAGCCCAAAGATGAGAGTGATTCGTGTGGGTACCCGCAAAAGCCAGCTGG
EMPV1_17132 AGGACTGCAGATTATTTCACAGGCACTTACTTTGTGGATAAAATCTGATATGAGCAGATC
EMPV1_17133 CACATGAAGAGCGGGTATCCAACGCCTTACATTCCGTGGAGGTGGAGCTGCAGAAACTAA
EMPV1_17134 ATCCACAATGCATACTTCTGGGGATTTTCCACATTGATGGAAGAAAGTATTCAGGATACC
EMPV1_17136 GGAAGCCAGGAGAGTGGTGGAACATGCAAGCTATTCACAGGGACCCCAAACAGATTCATG

EMPV1_17137	ACATGGATTTAACCAACTGCAGATTAAACCACAGATTCCACTATGGTAGTGTAGAATGTC
EMPV1_17139	CTGGGCCTAGCTATGGTACCTGGACACCACATCGTTTCTATTGAGGTGCAAAGAGAGAGC
EMPV1_17141	GTGGTAGATAGCAATGATCGTGAAAGAATCCAGGAAGGAGCAGATGAGCTGCAGAAGATG
EMPV1_17142	TGGCCCAGGCCTATGAGATGACAGGAGCCGTGTGATGGGAAGAGAAACTAGAGTTAGGTT
EMPV1_17143	CGGAAGAAGGAGGGATAGTATCCATTAGGCTTTGTCTTTACCTACCAGTATGACCTGGGC
EMPV1_17144	TGAGCAGCACCTGAAACCTATACCACAGCTCGTGACAATGAGTGAGACCAGGGATTGAAC
EMPV1_17147	AAGCAACAGGAAATGCCAACAAACCCTGGTGGAACTGGAGAGTGTCCTCAGCCACTTGGA
EMPV1_17148	TATGATACCAAGGGCGAAGGCATCCTCATCACTTCACATGGGGAGCTGCAATACTACCTG
EMPV1_17149	CAACAGTTGCAGATGGAGAAGGAGAGACTGCGACTGAAGCAGCAAGAACTGCTTCGGCAG
EMPV1_17150	AACATTGATGACTCGCTCATTAGTGGAAATGCATCCCCTGAAGGTCCCGAAGGTGAAGGT
EMPV1_17152	CTCACCAAGGGAGGAGGTTCCTGTCGAGACTAATACTACTAAGGAAACGGAGTCAGGCTT
EMPV1_17153	AGCCACCTCCCTGACTTGGGTGGGAAATCTCCTTTTTTGGAGGAAGGCGGTCTCTTTGCT
EMPV1_17157	GAAGCTGGACCTCTGAAAAGAAATCAGGCCCTCCTAAAGCTCTGTCTGAAGCTCCTCATG
EMPV1_17158	TCGGCACCTGAGTTCTCATGCTGAAGAGAAGCCTTACCGATGTACAGAATGTGGCAAAGG
EMPV1_17160	TTACAAGAAGCTGGTCCAGGACCTGGAGGCCAAAGTGGCCACATCAGGGGATTCCTTCTA
EMPV1_17161	GAACAGAAGCAAGGAGGCTGTAGTGCTATAGCCTGCAAGAAGATCATTCATATTATTAAC
EMPV1_17162	AAGGTGGGTCAGATGCAGTATGAAAAGCAGCGGATGGAACAAAAGTGGGAGTCACTAAAG
EMPV1_17164	AAGCTGGGTAGCTGCACGTAAATCAATGAAGTTAGAACACACTCTCACACCATATCCAAA
EMPV1_17165	GCAGAGCTTTGGACCCTCTAGATGAATCCCTTTGTGAGTGAAGAGGAAGGCTGGGTTTTG
EMPV1_17168	AAACTCAAGACCGGGTATAAGCGGGAGCACGTCAACCTGGGCTGCAACGTGGATTTCAAT
EMPV1_17169	GCTGCCTGCTGGAGGAATCACCATAAATTATCTGCTGTCTACATGGTACAAGGCTCACTG
EMPV1_17170	TAGACAAGGATGCTAGGAAGTGGACCTGCCCAAGGTGGGTTTCCATGGCTTTATTTTCCC
EMPV1_17171	TGGGCAGTGAAGGGCAGAAACTAACATAACTTGAGCATCGGACCATGGGCTTGGCCTTCT
EMPV1_17173	CAGCAGCAGAACTGTCCCTACATTATAAGTAAATGTACTTATTCTGAATTCACTCACCGA
EMPV1_17175	CAACCGGTCTGTTATCCTGGCTCTGAATGCACCTGCTACTCATCGTGTTTCCTTTAGGGC
EMPV1_17176	AGCAGTACTACACAGCCAAAGGAGGAGTACAGACTCAGGCATCCCCTGGAATGAGGGGCA
EMPV1_17177	CTCTAAGGGTTCAAACGGGCAGATTGTCCCGAGGAGGGGCACCATTTGCACAAGAAGTGC
EMPV1_17181	CTAAGCTGGTGTTGTCCTATCGACCCAGCTGGATCGTGTAACCAGTCTATGGGAAGTGAG
EMPV1_17182	CCTCAGAGAAGGCTCAATAGCACACAAAATGAGGAGCCATTATAAAATCATATTGCTAAG
EMPV1_17183	ATAGGCTGCAGCTCAGATTCAATTCCCAGCCTGGGAACTTCCTTGTGCCATGGGCATGGC
EMPV1_17184	GAACAGCCACAGCAGATGACAAAAAACTTCAGTTCTCCTTAAAGAAGTTGGGGGTAAACA
EMPV1_17185	AGAAGTTTCTGACTGGAGGAGTTGAAGGCTCACAGAAGGGAAACTGCCCCCAAAGGATTG
EMPV1_17186	AAGGGTTTTTCTGAAGCTGCCTTCACCACTGCCTACCTGTTCACATCAGAGCTGTACCCT
EMPV1_17187	TTGCAAAACCCAAGCACTTAGTTGTGATTTCGGGCGCTGGTGATAGTGCCGTGAGTGGGA

EMPV1_17188	TACTCTCAGAGGTGCCAACTTTGCAGGATACAGACACATCAACAGCTACACACAGCTATG
EMPV1_17190	CCCTGCTGATTTAACTTAGGAAGTAAGGGTAGTGATACAGCTTCTGCCTCACTTTCTGCC
EMPV1_17191	GCCATCCTCAACTACATCGCCACCAAGTACAACCTCTACGGGAAGGACGCCAAGGAGAGA
EMPV1_17192	GGCGTAAGGACCCCTTGGAATTGAGATAGTTGGCTCTTTCCTAAAAGTAATTTGCAAGAG
EMPV1_17193	TTCCCTTTATCTGAGCCCTCAGCCCGGGAATGCGGCGCGGGGTGTGCAAGACTCCACTCA
EMPV1_17195	TTGATGAAGTTGGCGGTCATGCTGAGGAAGCCCAATTGTCAAGGAACTGTGGGTGGCATC
EMPV1_17197	TCCTCATGGCTGGAGGATCCTGGCTTATTGGGACAGTCAATTCCATTGTTCACACAGTTT
EMPV1_17198	AGAGTTGTTCCTAAAGGAAAAGGAGAACAGATTGCAAGAGAGCATGGTATTAGATTTTTT
EMPV1_17202	CACATGTAGCAGTTTACCGTACTTTTGGTTTAATCTCAATTAGTCTTTCTCCCCCTGGAC
EMPV1_17203	TGCAACAAAGCCTATTACAAGTCATCGCATCTGAAGTCCCACCAGCGCACCCACACGGGT
EMPV1_17204	CTCCTTCATTAGACAGACAAGGAAATTGGGGTTCTGGGTGATCGTGACTTCCCTGAGAGG
EMPV1_17207	GCACACCTGGCTATCTTTCTCCGGAAGTTTTACGTAAAGATCCTTATGGAAAGCCAGTGG
EMPV1_17208	AGTGGTGACTCGGAGAGATGGAATTTGAGACCTCTTGGTGCTCCCTGTCTAGAGGCAGTT
EMPV1_17209	AGAGTTTGCGAAGTGCTTTTCTAGTCACTGTTTTTCTCTCGGTCATCGGCCCCTGTCCTG
EMPV1_17211	AGATGCCTCATGTCTGCTTTCTGAGTCAGTAATTTCTTCGTGAGATTTCATTAAAGAAGT
EMPV1_17214	TTGCAGGTTGAACTTTTCGTGAAGGCTGGCAGTGATGGGGCTAAAATTGGGACCTGTTCC
EMPV1_17215	ACTGGCTCAAAGGTCCCCAGCTAGTAAATGCAGAACAGAGACAGAAACTCAGGACTCCAG
EMPV1_17217	GGCCATTGAAAGACTCTCTTAGGTTGCCTGTAAGGTCTGCTCTTGGGTCTGCTGATAGCA
EMPV1_17218	AGATGGGTCTCATTTGCAGGAAAGGCTACTGATATGGATCATCGTCACCCAGATTCTCTC
EMPV1_17219	AGGCTACATGTGCCATAAATTTGCCTGCAATCAAATGTACTATGCTAATTATGAACAATG
EMPV1_17220	ACAAGCTCAGGTTCAACTTCTACACCTCCCAGTACCCCAACTCTCTGAACCCGTTCTGCA
EMPV1_17223	TGGACACTCAATGACAGCCACGGGGAGGCCTTGGAAAAAGGACCAGACTGAAAACTGCTT
EMPV1_17224	GGTCCAGAGTTGAAGTTCCCCTAATTGCTTCCTCTACCATCTGGGAGATAAAATTATTAC
EMPV1_17225	GCGCTCTGAGATTGTCTGCTTTCATCACTGCCACTCTTCTCAACAAACAGCACACCTCGA
EMPV1_17228	CCTCTATACCCTGCCGTAAAGAAGCAAGTTAAAGCATGTCGGGGGCATTTTTGGAGGCTG
EMPV1_17229	GCCCTCAGTAGCTCGAAGAGGTAGATCATGTTGTTATCACCTTCATTTTACAGATGAGGA
EMPV1_17239	GGCGGCCAGCAGCCACATGCTGAAGGAGGTGCTGGACGGCCCCTTCGTGGATCCGCTCAA
EMPV1_17242	TAATTTGAACGGGGATGCTGAACGGCTGCATGAGACCAGGGGGCTGGGCAAAATGGACTC
EMPV1_17246	AACACCGATGGCTTCCTCCCCACGTCCCAAGATGGATGCAATCCTAACTGAGGCCATTAA
EMPV1_17247	GTTTTGTACAGAGCCATACCAGGAACCCAGCCCCAAAAACCTGCACCACATCTTCATGCA
EMPV1_17248	CTGTGGCACAGTCTCTGGCACAGATGGCTGCCGTGAGCTCAGTGACAGGGGACCCATGGC
EMPV1_17252	AAAGCTTCTTCTCCCTCTGACCCGCCCCCCTTGAGATGAATGTAGCCAGAGACGATGAAG
EMPV1_17253	CTCAAGTGGAAAGGAATGCTACGGCTCTTTATCATGTAGAAGCCTTCAAAACTGGACCAG
EMPV1_17256	ATATTTTCCAGAACTTCTTGCCCGAATTTCCTGGGTGGTATTCCACTCCAAAGCTTTTGC

EMPV1_17259	GCCTCCTCTTTTGAAAGTGTGCGTTGTACCTTTTGTGTGGATGCTGGGGTGTGGTACTAC
EMPV1_17260	TTCCTGCTACAAAGGGGCACCGTTAGGGGAAAGCAGTTAGGTTTCTGCTCATGGGATGGT
EMPV1_17262	CAGCTGCATTTGTCAGGACTCACTCCGTCTTACTCTGTTCTCTCTTGTGGGTACAGCACC
EMPV1_17263	TGCACAGCCACAGGCTCCTCAGCACAGAGTGGGGACTGCCTTCCATAGTGAAATCTATTA
EMPV1_17264	AAAAGGCGCTGGCCCCCTTGGGTTGCAGTAGAAAGGTGACAACATAGTGGAAACCAGGAT
EMPV1_17265	GAATAAACTTCAATAGAACATACTGTATTCTCCTAGAATCTTCACTTTTAGAAAGGGACC
EMPV1_17266	TGGCCGCTCAGCATCCACGATCCCACGCGTCGCGCCCGCGTCAAGGAGTACTTCGTGTTC
EMPV1_17268	TGGTAAGACCACATGGGCCATCAAACATGCAGCCTCCAACCCCTCCAAGAAGTACAACAT
EMPV1_17269	GTAGAATACTCTTCACTGCCACTGCCACCGCCACTACCAAATGGGGTTTTTAGAGCTTTG
EMPV1_17270	CAACCCTCTTGGTCTGACCTGTGGAATGGTATGTCCAACCTCTGATCTTTGTGTAGGAGG
EMPV1_17272	AAGTAATCTAAACACAGAAGCCATACGGAGTTCCCACTGCGGCTCAGCAGGTTAAGGACC
EMPV1_17273	CGAGCCAATGGACCCAGAGGAAATAGCACGACGGGAGTTGGAGCGTTTGGAGAAGAAAAA
EMPV1_17274	GTCCCGCTGGATCTGGCCAGGGGTCTAACATTGTGACTTGTTGAGATCACATTCAAACAG
EMPV1_17275	CCTCCGCCCTGGCACCTGGCGGGAACTCAGGCAACCCCGTTTACGACTTTTTCCTGGGAC
EMPV1_17277	AAGTTGCGCCAACCACTAAAAATGCCGACTGAAGGACTGGACAGGGCCTCACTGCCCTCT
EMPV1_17280	AACCAGGGCAAGTTAAAGTATTCAGAGCTCTTTATACATTTGAACCCAGAACTCCAGATG
EMPV1_17282	ATGAGGTCATTGGGCAGGTGCTGAGCACACTCAAGTCCGAAGAGGTCCCGTACACGGCAG
EMPV1_17285	GCAGTTCTGACGATGAGGTCCAGACGCTGCTGAACATTTACTTCCGTCATCAAACGCTGG
EMPV1_17288	CCTCTGAGCACCATTAATAACTGCGAACTTCCTTAAATTTAGTTCCATGATTGTAATGGG
EMPV1_17289	CCTCAGGAAACATGCAAAAACCCACAGTGGTAAGAAGCCCTACACATGTCAGGAGTGTGG
EMPV1_17294	CTGGGTCCTCTGTCTCTTGAAGTCAGTGGCTGAGACAATCATCACCATGAGGCTGCCCTT
EMPV1_17295	AGTGGGTGTGGAAGGTAGTGTGTTTGGGGGAGGATTACCAGAAGAAGGAAGGCTGTTGGT
EMPV1_17297	GGTCCTCCACCCCGCAGCCCACGCAGCCCGCGCCACCGCTCACCTGCACGAAGCCCCAGA
EMPV1_17299	GCCCTGTCTTTCGATTCAAAAATGCTGTATCTGTACTTGGAGGTTAACAGAGTCCTGTGG
EMPV1_17301	CGGCGCCTAGATGTAAATCCAACAGAAAAGTGTGCTTGTGAACTCGAAAGACAGGAACAG
EMPV1_17302	GTGATCCTGAGTAATCCCATCTGCGGCGTGAGCTATGCCCTGACTGTGTGCCGGTACTTC
EMPV1_17304	TTGCATCCAGCTGTGTGATATTCTGCTGTGCAAATCCATGCAAAACTGACTGTGGTAGTG
EMPV1_17308	AGATGCCCCACCGTGTGCAGAAGAAGACAGGAAGATGGACAGGACCTGGCGGCGGGCTTG
EMPV1_17309	ATGGACACCTCATACTCCATGTCATCGCTGGTCTCCTCAATGGCTCGGAAGAGGTCATTG
EMPV1_17310	TCCCTTTGCTTTTGTATGACCACATGTTTTCTGTACCAGTCACTTGGGAAAGAAGTGAGC
EMPV1_17311	GTGGCATCAGGCTACATTATGCAGGTTCCATTTTTCATCGAGTAGTACGGAATGGTTGGG
EMPV1_17312	AGCCACGCTGAAGAAAGGGCATAAAATTTGCTTGGATCCACAGAACCTCCTGTACAAGAA
EMPV1_17314	TAGAGCCAGAATAGGACAGACTCATATACCATTCTGATCTGGGTGCATTTCTTCAGAAGG
EMPV1_17317	ACGAGGAAAAGGCTAACTTTCAAACCACAGAACCCGGGAGGTGGCAGCCTTTCCGCTACT

EMPV1_17319 CCTCCTGAGGACTCTGATCTTTAGTGACTATGGACTCTCTCTGGCTTTTCTCAGGGCAAC
EMPV1_17321 AATAAACAGCTTCTGGCCCTCAAGGGATGTATGATCTAATATGGGCATGAGATGAACTAC
EMPV1_17323 GATACCCTCCCCCACCAAAGAGAATCATTTAAGGGTTTAGGAGTTGCTGTTGTGGCCCAG
EMPV1_17324 TGTTCCCAGATGGGAAGGAAATGGCGGAAGAATATGATGAGAAGACAAGTGAACTACTCG
EMPV1_17327 AGGCTCTTACAAACATGGGTGAAATTCTGAAAGCTGCGGGCTGTGACTTCACGAATGTGG
EMPV1_17329 GTTTTCCCTCTTCAAGGTAAAGCGACCAACTTGAACTTTCTCTGGCAACACGATTCACGG
EMPV1_17331 AAGACATCGAGGATTACATCAGCCTCTTCCCCCTGGACGACATGCAGCCCTCGAAGCTGA
EMPV1_17333 ACCATCACAGGCGACATTGGCACTTGAGCAGCCCATTGAGTGCTGATTCCTACGGAGAAA
EMPV1_17334 CTTGGGTGAGAGAGTATTGTTGCATTTCCCATTGTTAGTCTTTAGTCCTGACCTGTTGGG
EMPV1_17335 GTTGGCTATTTTCTCTCTCCCAAAGGGTAGCCTAGAGACCTGTGCAAGGATGTGGTAGTG
EMPV1_17338 CGTTGGTAGGGGAAAAAGGAGCTCAAATGAGTGGAGGACAAAAACAGAGAATTGCGATTG
EMPV1_17339 ACCATCTGTTTCCTCCATGCCACCGTGGTCCTTGTGCTGAACCTGGGGGACTGTGAATAC
EMPV1_17340 CTCTGCGCTACGGGAGATGTCAAAGAATCAAAAATGCAGATAACACCTGAAACTCCAGGA
EMPV1_17342 TTTGACAGGAGGAAATACTCTTTTCCCAGGATTTAGGGATCGGGTTTACTCCGAAGTTCG
EMPV1_17343 CAGCACCAGTGAAGATGATGGCTATCCCGAAGACATGGATCAAGATAAGCATGACGACAG
EMPV1_17344 CTCTCCTTTCACTTTCATTCTGCGTCTAAAGACTCCTCTCTCTTTTCTGTGTGTGTATCT
EMPV1_17345 GAAAGGATTTCCAAGCTGTGGGGCTGCTTCTCCCCAGTACCTCCCTAACAATTCATGGTG
EMPV1_17346 GTGGACTCCTGTGGCAAATATGAATGGGAGGAGGCTACAGTTTGGTGTGGCGGTCTTAGA
EMPV1_17347 GGCCTGCACGCTCTGAAGATGTCCCTAGAGGGCGCCGCCCGCGGAGAGGCCAAGTACAAG
EMPV1_17348 TGTGGGAGGAGATTCTGCACATCTCAGGAGCCATCTTTCCTGGCCTGAGCTATACCTCTT
EMPV1_17349 CGCTCTTCAAGCTCACAAACTCTGACCCAATTTGATTCTAACATTGCACCAGCTGATCCT
EMPV1_17353 CTCATCAAGTTTTTAGCTTTGGGAGACTCTGGAGTAGGGAAGACCAGTGTACTTTACCAG
EMPV1_17356 CCTTGATGAGTTAGAGGGAAGTACTAATCCTGGTGCTAGTTGCAAAGACGCAAGTGGGGA
EMPV1_17360 AAGCTGGCTTCCCGTGAATGCTTATTTCTCGTTTCCTCACCCTTTTAGCCCCATCCTCCC
EMPV1_17362 GGGAGGTGAACATAAAATTTTAAGTAGATGTAATGGAATAATATTGTTGTCTGTGAAGGC
EMPV1_17363 AAACACCGTGGCGAGGTTGTGCAGGGACATCTTGTTTACTGTCTCCTTTTCTGCCACCCT
EMPV1_17366 CAGAGGTCATTGCCATGCTCACAGCGTTGAGACAGTAGTCTATCCATCAGTGTGTGATTA
EMPV1_17368 GCATAGGCTTTATGCTGCGTCGCCTTTTGCAGTCTTCTTTTACCGTTGAGGGTCTTTAGA
EMPV1_17369 TACACATACCAACAAGAAGCGATCTGAGTGGCTGGAGCCCACTGGGGCCAAAGGTTAAAG
EMPV1_17371 TGAGCCTCATAAGCAGCGCCTCTAATATCCCTATTGCTACCGAGTCTGTGCAGGCTGATT
EMPV1_17372 TTGGGTCCACCCCAAATTCCACAGCCAGCCGTGTCAGAAAAAGCAAGCCAATACTAGACC
EMPV1_17375 GATTTTCCGCCCTTTCTACAGACATTGGGCCATTTACGTTGGCGATGGATATGTGGTCCA
EMPV1_17378 GTCATCCTGGACGTGGGAACAGAAAATGAGGAGTTACTTAAAGATCCACTGTACATTGGA
EMPV1_17382 TCATGAAGCTCTGGACACCTCTACTCATGACATTGCTCTGTGTGGTGCTACTGTCTGCGC

EMPV1_17383 GGGAATGTCGTTTTCCTTTAGCAGGGTAGTGATGTCACAGGAGTGCCAAGATGGCAGAGT
EMPV1_17384 GGTGGATTGGGGCAGAGGTTTCCGTCTGGGATCTGGGGACATTTGGTTTAGCTTTAGTAA
EMPV1_17385 TCTCTCTTCGGAGACCAACTTCACCTGTTCCCACAGCCCTGCCTTTCCAGGTCATAGCCA
EMPV1_17388 GCCAAACAGCCAACAGGGCTGGAACACAGACCTCACCATCATCAGATAGGCTTCTTAAAG
EMPV1_17389 TGTGGCAAGATGTTGAGCTCGGCTTATATTTCGGACCACATGAAGGTTCACAGCCAGGGC
EMPV1_17390 AGGGTCTTAGGAGTGCTGCCACCTTCCACTGTATAACCGGGATCTCTCTGTTGACATGAG
EMPV1_17392 GAGCAAGACCTGAAGACATGGAACCCGGCGCAAAAGTTCCCCCCTTTACCTCTTCTAAAT
EMPV1_17393 TCTTTAATTCACGCTGGAGCAAGAAGGATGGATGATCAAACTCAGCACTGCCCTCAGTGA
EMPV1_17394 AGTGCTGTAGCCCTGCTGGTGGGAGAGAAGGTACAAGAAGAGACCACTCTAGTGGATGAT
EMPV1_17395 TGGGCACAAATCCCACCAATGCAGAGGTCAAGAAGGTTCTGGGAAACCCCAGCAATGAAG
EMPV1_17396 GGTGCATGAAGTCAACACCAGTGAACACATCAGCCAAAGCCAAAGGAAGTGAGAGATGCG
EMPV1_17397 ATGCTTCAGGCCGGTGCACTTTGCCCATCGATGAATCCAACACCATCCACTTGAAGGTCA
EMPV1_17399 CTGAATACCGTGCACAAATACGACCCTATCCAGGAGATGCTGCTGTGCACTATCAAGTGT
EMPV1_17400 GCTAGTGGCTCTCTTCCTGTGTTTTTCTGGAATGAGTCAAGCAGAGCTCTCAAAGTCCGG
EMPV1_17401 CAGAACAGGTTTTGAACACATTTGGATTTATCTTCACTGAGATTATGTTGTGACCACACT
EMPV1_17402 CCCTAGTGAATACCTAGGGTTTTCTTCCCTAGTGTGGCATTTTTTTCATTTTAAAATAGG
EMPV1_17403 TGTTGCTGTCACTTAGGAGTCAGGGGGCCTGTGAGTTCATCTTCCGGGAGTGGGAATAAT
EMPV1_17404 CCAGGTGGGCATGTGGAACCTGATGAAGAGCTGTTGGAAGGAGGGCTTCGAGAGCTTTGG
EMPV1_17406 GGCGTGCCTGGGGTGGTGACAGTGAACAAGGTGTATAGAATGTGCCCAGTGGTAGCAATG
EMPV1_17407 AAGCAATAGCAATGCAGGCTGATAAGAACTATGGCAGAGCAAGCAGTTTGGCTGGGGTGG
EMPV1_17409 GGCGGGTCATTTGGCCATTCCCTCTAGGTATCCGCCCTTCCTCTTAGGATAGTGACATTA
EMPV1_17410 GCCGGTGCCCAGAGACACAGCCCCACCGGAAGAACCCCCCCCCAGATATCATCTACCTAG
EMPV1_17412 GAAGCCGTTCCTCCTATAACTGAAAGGTATTGTGTGATGCCAAATGTACCATAAACCTTC
EMPV1_17415 CCAAGTCATTTGAGGATCTCACGGACCATCCGGTCACGAGAAGTGAAAAGGCCTCGTCCT
EMPV1_17416 TTTCCCTGGAAGCTGGAGGCAGGGATGAGACCAAAGGACACACACGTTGGCGTCAGTCTT
EMPV1_17417 CCATCATGACTACTAAGTTCTGTAGCAGCCTGGTCGCCTTTTGCTGGGTGTATGGTTTCC
EMPV1_17419 CACTCATGAAGCCAAAGGAGCCGATGATGCAGCAGACGCAGACACAGCTATAATCAATGC
EMPV1_17420 GCATCGCCTGTGTGCAGGTGTTCGGCATGATCTTCACGTGCTGCCTGTACAAGAGCCTGA
EMPV1_17421 TGCCTGTGCAGGACAGACGCACTGTGACTCACAGGCACTGCTGAGGGTCTCCGAGCAAAG
EMPV1_17422 CGGGATTGGTGACGTACTGGGCAAGAAGCTGGAGGAAAGGGGCTTTGACAAGGCCTATGT
EMPV1_17423 GAGAAGACGCACATTGTCTGTTTAATTGTGAGGGCTTAAACACACAATCGCTGCAGTATC
EMPV1_17424 TTGCCAAGGGCTATTGTCCACTCTTATTTGTGATAGATGGAGGCACCGAGGGGGCCAGTT
EMPV1_17425 GTGAGACTTGTAACTGTGCTCAGAGGTCACACTGCTTGCCAGGTGCTCCCATTTGGATAA
EMPV1_17426 TTATAAGTTCTCCAAATCCCAGTCAGAGGAACAGGACACCTCCTCCTACCACGAGGCCAA

EMPV1_17427	CTGCCAGCAGCGTGGGGTTCTGCTGGGCCTTCTCGGGGGTGGGCTGGGAGGCCTGCTCGT
EMPV1_17429	GCAAAAGAGACAAGATAATAAGCAGTTAAACCCCAGTCAGTCTCATAGAGGCGACCCAAA
EMPV1_17430	TAGAATCCTTATCCTCATGGAGACTGGGGTTTCGGGGAGATTCGAGTGAGCACAGAGAGA
EMPV1_17432	TCCGGGAACAGAATGCCTGTCTGACTCACCCTGACAGATCAGCTATGGCAGGTCTGCTGT
EMPV1_17434	CTGATGGCTTGATCCTGTCTTAAAGAGCTGTTCTGAAACTCCCAGTTACCTGGCTGCCTC
EMPV1_17435	AGAAAGAAAAGGACGGTCCCCACAAGGCTAATGACCGTCAGGGCAATAAACACTGTTCGC
EMPV1_17436	GTCCGAGACCATGAATTTTACTTACATATCCTTAATGCTTTCAGTCTGTGAATCCACATC
EMPV1_17437	ACGTGTCGAGGCATCATCAACGTGAAGGGAAAGGGGGACCTGAAGACGTACTTTGTGAAC
EMPV1_17438	CCTGCAACCATCTGCATTTACATAGGACTGCATCTGTATCTGTATCTGTCATTCCATCCT
EMPV1_17439	GGTTAGCATAAAGGAATCATCCGTAGCAAAACTAGGATCTGTATGCCGTAGGATCTACAG
EMPV1_17441	TGTCAATTAAACCTCAATAGCGCGGTTTTCAGAGAGGCCCTTGTGTGGCTAGAAGTGGCC
EMPV1_17442	TCAGGAGACATGAATCTTCCTCATTTGATGTCACTGGCATCCCATCTTGCATCTCATGGG
EMPV1_17443	CCTCTCTCCTTCATTCCTGACCTCCTATCAGCTCTTCTTTCCCTGCAGCCTAGCAATTCA
EMPV1_17444	TGCGTGAGGCGAGCGGAGCCGGAGACAAGACCAGAGGCCGTACTCGGGATTTGACAAGAT
EMPV1_17445	CCCAAGTTTAATTCCACATCTTACTCTGACAATCCCCAGGCTTTCGATGCTTCCTCCACC
EMPV1_17447	GAGGCTCATCTTCTCCACAGGTTTCGCCAGCTTGGGTTTCTTGATAAAGGTTCCACCAGG
EMPV1_17448	TCATGATCAGAGATGCAAGGTGGGAGGTTGTGGTGGCAAACTGGAGGTAGGAAGTAGGAG
EMPV1_17449	TGGTGGCCTTCTCCTTTGCCAGCGGCTTCTCCGTCTCCGTGATCAAGGTCTACTTCATCT
EMPV1_17450	CCCCAGGATTTTTAGTACCAAATGACTCTGGCGCGACCCGGAAACGCAGTTACAAATGAG
EMPV1_17451	TTTCCAATGCAACACATACAGTTCTGCTCCAGGACCATGAGAAATATGCCAACTTACTTG
EMPV1_17452	ATCTTCGCCAAGCGCTCTGGGATGATCATCCTGGGTGGTGGCATGGTCAAGCACCACATT
EMPV1_17453	GTGGGACCCTCGGCCCTCCTCTGCCTTAAATCCCAATGCTTCGAGAAAGAAGTTTGTGAA
EMPV1_17454	TTTCAGGCCAGACAGTAAATCCGATGGTGCTAACTTACTACAGAGGAGAGCTCAGCCAGC
EMPV1_17457	TGGTCTCCTGCTTCCAACAGCTGATGGTGGACACCTGCTCCTGCGGCTACTACCTCTACC
EMPV1_17460	AAGACCCCCAGTCCCATATCCACAGGAACCGAGAAGGCAACACTATCCCCAGTTTCTTCA
EMPV1_17462	ACGTGTTTGCGCAGATCATGGGGGCCACCGGTTTCTCGGAAAGTAGCCTCATCTGGAAGT
EMPV1_17464	CTACAGAGAGACTCCCGTTGGAAGATTCTTCAATGGACATGGCTTTGACATGTGTTCCAC
EMPV1_17465	CCCTGACACCCTGTAGTCTTCTTATAGGATCTCTGAAGGCAGAGTGAAAGCAGAAACCTC
EMPV1_17466	GTAGTTCTGGAAAAGCTACTGGGGACGAGACAGGTGCTAAAGTTGAACGAGCTGATGGCT
EMPV1_17467	TGTCAGAGGGGAGCTTCAGCGCACAGGATCCGGCCCTCCCAGACCAGGACTTGTTCAACT
EMPV1_17468	TGAGTGAGGCCAGGGGGGTCAAACCTGCATCCTCATGGAACTCCCTAAAAAGACTATCTT
EMPV1_17471	GTCGGAAGGCTAAGGCAGATCACTTTGAGCAAGTGCCCATGATGGTTCTTCTCTTTGGGT
EMPV1_17472	AAGTGGTAATACTGTGAACTGGTCCCAGCAGCTCCCAGGAAAGGCCCCCAGAACCATCAT
EMPV1_17473	CTGGAAGAGGATTCGCAGCCAGCAGGGGTTTCAGGAAAATTCCAACGGACAGAGCTTCTT

EMPV1_17474	GGTACGCTGTGTCCTCTTATTCTTCGACCTCTATGAAGCTATTGATAGTGAGGGACACAG
EMPV1_17475	GGTTGTCACGTATGTTGGTCGCTTCTCCCTTATTGATGAGCACTGTTCTGCTGTGTGGAC
EMPV1_17478	TGCAGCACAGAGCATGGTAACAAGAGACTGGATGCTGCTTATCGCTCCATGAACGGGAAG
EMPV1_17481	GCTGTTCTCCATAGTGGTTGCATCAATCTACATTCCCACCAATAGTGTAGGAGGGTTCCT
EMPV1_17482	AGTGCTTCTCCTGGCAACCTAATCAGGACTGGCTGTTCCAGACTGTGGGGAGGCAGATCT
EMPV1_17484	AGGGGAGTGAAGGGTGTGGGCTCAGATGATCATTTTGAAATGGCCCCACTGAACATCCAA
EMPV1_17485	TGACGTCTTCATCCGCCAGTACTACACCGTCTTTGACAGGGCCAACAACAAGGTCGGCCT
EMPV1_17488	TGGAAAGGAGCATCGAAACCAACAGCACGCTGCAGAGCAAGCTGGAAGAGCAGCTGGCAA
EMPV1_17490	GGCTGAGCTAGAGCCCTGTGAGTGAAAGTATCTCGGATTTGAGTTAGCATGGTGTGATGC
EMPV1_17492	GAAGGTCCAGCAACTCCACATATTGAAAAACTCCTTCAAGCTGCCCCCTCACATCAGCCT
EMPV1_17493	ACAGGGAGGAGGATGGCGGAGTCATCGGACAAGCTCTATCGGGTCGAGTATGCTAAGAGT
EMPV1_17496	GCTGCTCGCTGTTTCTGGGATGCCGACACTGACAACTGCACGCATGATGTTTTCATGAAT
EMPV1_17498	TCAGAGCTGAATGTGCGGACTGCGGGCGCCAACGGCACGTTGGGCTTCTTCTGTGTGGAT
EMPV1_17500	TATTTTCCTGCACTGCTGATGCCACCACAGACTCTGCTACTTATGGGTGAAGTCGCCTTG
EMPV1_17501	TAACTGCCCTCAAGGAGCTTACAATCTAGCTGGGGGTGAATGACTTGCACATGAACGCAA
EMPV1_17502	ATACAAAGTATGGAAGAGTGGGCAGAACCTTCGGGTAGACACCACGCTCCTGGGCTTTGA
EMPV1_17503	GGGTGAGGCATTGGACCTATAACAAGGAGAGTATGTGAAGGCCATCTGCTAATCGGTGAG
EMPV1_17504	CCTTAATGGCAGCAACGTGGTGTTCTGTAGTGGTAATAATACATGGGAGCCTGAGATACC
EMPV1_17506	CCAGGAAGTCAAAGAGAGTAAAAAAGTGGTCTGCCATTTCTACAGAGACTCCACATTCAG
EMPV1_17507	TGAATGTGTGAAGACTGACGAAGGGGCAATGATTCTGAACTACAGTGTGGTCTGTCCCCC
EMPV1_17509	GTTTTTAGGACCTGTCATACTATTTTCCAAAGTGGCTGTACCATTTTCATTCCCATCAGC
EMPV1_17512	GAGTTGCTGAAATCGTTTATTCCTGGCCACCGCTGGAAGAGCTCCAACATCAGGGAGGAA
EMPV1_17513	TAGGTGTCACAGAACTATGCTGCCTAAAGTGATCTTGGCTCCTTAATGCTTTTTGGCCCC
EMPV1_17514	GACTTAGCCAACAGAGGGAAGTGGTTACAGGATCTTCAAGAAGAAAACCAGTCTTTAAAA
EMPV1_17516	ACTGATGCTGCAAGTAGACCTTATGCAGATGTGCGAGTTATTGACTGTGGGGTGCTTGCC
EMPV1_17517	TGGTTAGGATTTGCTGCTTTCACCGCTCCTGTGGCTGGAGTTCAATCCCTGGTCCTAGAA
EMPV1_17520	ATGCACAGCTTTGGAATTTTAGGGCCGTAAAAACAGAGTGAGGCTCGGGCCGTGGTCTCA
EMPV1_17522	AAGCTGCCACAAGGTCTTCCGACAGAGGAGGACATGGCCAACACGTGCCTCAAAAGCAAT
EMPV1_17524	GACATATGAGTTCAGGTTTAGCTCCTGATTTGCTGAGTGAGCTTGGGCAGGCCTTCCTCC
EMPV1_17525	TGGAAGGCCTGGCTTTGTGGCGTGGATTGGACTGTGCAGACCCGGCCTTGGCAGACTCTT
EMPV1_17526	GCTGAATTCTTTCCCTCTATTTCCAGAGTACAGTAATGAGTTGGTGGCCTAGCAATCTCC
EMPV1_17527	CATGATGTCACCCGATTCTCCGACAGCCCCGAAAAGCTGGAGGGTTAGCCCATTTTATAG
EMPV1_17528	GTCCTGTACAGTCAAAGCAGTAATTTACAAGAGTCAGCAATTCCACAGGGCAGGTAGGCC
EMPV1_17530	CGAATTGGAGAAGAGGCCGCCGTAGTCGCTGCTGAAAGCGGACGACGTGGGGGACGTGGA

EMPV1_17531	CTACCAGGACTGCTCAGCCTTGACGCAGAAGAACTTGAAGGAGGTGTTTGTCTCTCCCAT
EMPV1_17532	CGTTGTTTTCATGTGATGGATTCTACGTTCTGTGCTACACACCCCTTGAGGGCAGCGCAT
EMPV1_17534	TGTAACTGAGGTGGAGGAGGTTGAGCAGTGACAGGTGCAGAAGTTCCAGGCAATACCACA
EMPV1_17537	GGAGCTGCAGTTCATGGTAACTGTCAACAGTGGGCTCCTTTCTATGGGCTCCTTTGTCTT
EMPV1_17538	GGCCAGACGAAGAATTTGTTCCCCAGATTTGTATTTTCCACTTAACACCATTTCTTCCCT
EMPV1_17539	ACTGTCATTCCTAATCAGGTGTACCAGCCCCTTGGTCCCTGTCCTTGCAATTTAACAGCC
EMPV1_17540	GAGCGAGAAGGATGGAAGTGGAGGAGCGACATAACCACCTTTGTAGACAGTTTAGTGGCA
EMPV1_17541	CCTTCACAAAACCAATGTTCTCTTAAATCATGCTGTGATAATGTCTTCCCATAAGGACAG
EMPV1_17542	ATTGGTCCGAAGGTGTGCAGGTGCCTTCTGTGCCCATCCAGCAATTCCCTGCTGAAGATT
EMPV1_17546	AGAGAGGAGGCCAGCCTGAGAGGGGCTGTTCTGGCAGATGTCCACACGAGGGCGCCAGAG
EMPV1_17548	GATTGACTTGCTCCAGATTCATTTTGCCCACAGCAAGAGGACTGACTTGAACCTGTTCCC
EMPV1_17549	AATCGGATCATCAAGCAGGAACCTACCCTGGAGAACTGCAGCCAAGACCTCCTCCAGCAT
EMPV1_17550	CAACGCCAAGCCCCCGTACTTCTACACATCTCACTCTGCCCAAGAACACCAGGAACTTCT
EMPV1_17551	CAAGCCTTAGGTAAACTATCTTAACAGTGGTACAGGCGATCCATGATTTAGCCCCAGTCC
EMPV1_17552	CTACGTGGAGAGCTACAGCGTCATCGAGAAGCTGTCCAAGAAGTTCCAGGAGGCCTTCTG
EMPV1_17556	AGTCTCAAGTTTAACACTCCGCCAGGCACATTCCAGGCTCCAACTTACTGGCTGGGGAAT
EMPV1_17558	CTGGCGCTTCAGAGATGCTGCCAATCCTGTTAGGTCACAGTGGGAACTCCAAAAAGGAAT
EMPV1_17559	GGCTGCTATTAGAACCTACCCCCTAGGTAATCTTGACACTGTAACCGAGCTAGTGCCCTG
EMPV1_17563	ACTGCTCAGATCCTGCGTTGCAGTGGCTGTGACCTAGGCTGGCGGCTACAGCTCTGATTC
EMPV1_17564	TGTGGGGAAGACAGTGACCCTGCAGGGCAACCTGGACCCCTGTGCCCTGTATGCATCTGA
EMPV1_17565	CAGGAGAGACTTCCCTCCAATGCTTTTCCTTCAAGTAGCTGAATCTTTGCATGTCTAAGC
EMPV1_17566	GACTACATGCAGCCTCTTAAAAATGTATTTGTCCTAACTTGGTGGGGGAGGGTTCAAAGC
EMPV1_17567	CCTTGAGCAAGACCCTTTGGGGTTCATCAGAACATGGATTATAACACTCCCCATACTGGG
EMPV1_17568	TTCTCTCAGGAAAAGGGCTGTGCTGGAGTGAACCAGGGTCCCCAAGCAACTGGATGTTGG
EMPV1_17569	TAGAAATGCAGACCCTCAGGCCCCAGCCCGGAGCTGCTGAACCAGAAATCTCGGAGTGGA
EMPV1_17570	GATAGCTACTTAAGGTGTAACAGAGAAAGGAAGCTGTTACTTGGAGAGAGAACTTGAAAC
EMPV1_17572	CAAACAGCCGTCTTTCCGCCAGTACTCTGGCTACCTCCGAGGCTCCGGCTCCAAGCACCT
EMPV1_17573	TGCCTGGCTCCTCATCCGTTCTGTGGTTTTGCAGATGAGTCAGCACACATTCAGGAACCT
EMPV1_17574	GGGGAACCCAACTGCAATAAGCTGAAAAACTTACTGTGTAATGGCAGCAATGTTCAGCTC
EMPV1_17575	AGATGAAGGAGGAGAGCCGGCAGATGATGCGGGAGAAGAAGGTCACCATCCTGGAGCTGT
EMPV1_17576	TCCCCTCCACCCACCTCAGCAGACAATGCTTGGGAAAAGCTGAGTGGGGAACTCCATTCA
EMPV1_17577	CTGCAAATTCAAGCTGGAAAAAGCATATCATCTGAGGGACATATTTGGGGACAGCAGATC
EMPV1_17578	GGTCTATTCATCAGGATAAAATCCTAGCACTTAGACAGCAGACACAATTCTTGTGGGAGG
EMPV1_17582	AAGAAGCAAATAGCCACTTACTCATTTAAAGACAAACCGCGATCAAGCCCCAGCACGCTG

EMPV1_17583 ACGACTAAAAGCCTCTGGGGGTGTCCCTGGACCCTGTGAAATGACGAGAATGTGCAGACG
EMPV1_17587 ATTCGCGATGGAGGGGATTCATATTCTCTATGGGCCTGTGGGAGCACTAGCTGGAGGGCT
EMPV1_17588 GGGTCCTGGATCATAGGAGGTGTCAACTCTGCTGTACAAACAGCATTTGTGGTACAATTG
EMPV1_17589 CTTTGAGTCTGTCAGCAATGTCACCTCCTTCTTCCCCAAGGACACGGCCTACGTGCTCTT
EMPV1_17592 CTCCCCGCCTCCCGCCACCCACCAGTCAAACAAAAACACACAACTATTTACAACTGAGAA
EMPV1_17594 TGTACAAGCATGTCTTCCCCTTCTCTTTGATTCGCTATGATGTCACCACAGGGGAGCCGG
EMPV1_17596 GCTGCCTAGCCTGGTGAGCAGTTACCATTTTCTCACTTAGCTTTCTGGCCTCTGGATATG
EMPV1_17597 CTCCTACTCTTCCCTATCTGTGACTTCATCCAGACGTTCATGGTGTTGGATATCACTGTG
EMPV1_17598 GGGACTGCTTCGGAGCCCTCTCATCTCTGGCCAATTTACTCACCACATAGAGTTGTAATT
EMPV1_17600 TTCAGAAATGGGGCACAGAGGAAGTTGCTGCTTGGCTGGATCTGCTCAATTTGGGAGAGT
EMPV1_17603 TGCATAGGGCGAGGAGATGAACGCAGAAAGAGGGCTTGACAGCCTTTCTCCAGGATACAA
EMPV1_17604 ATGTTCCTCCTAATGGGCAACTCATGGGCTTTTGACTCTGAATAGAAGTACTTGTGGGGG
EMPV1_17605 GTTAGCTCAACCAAGCTGTGTTGGAAAGAATGTCACCGAAGGCCAGGTGCCTATCGAGGA
EMPV1_17606 TTACTTCCGTGTAGGAACGCTGCCTCTGAACCCCAGTCTGTTGCTTCCGGCCTGGATCAT
EMPV1_17607 CACCACCAGTCATACCTCATGCAACCTTCAGGTTCAGTTCTGACACCAGGGATGGACCAT
EMPV1_17609 AGGGTGCTTTGCATTTTCCTATGAATTTTAGGATCACTTGTCAGTGTCTCAAGAGAAGCC
EMPV1_17611 TATAGCTGCTGGCCTAAGCCACAGCCACAGCAATGCGGGATCCCAGCCGCATCTGCTACC
EMPV1_17613 TGGACCAATGTCTGTATCAGCAAATGGGAGCAATCTTTATGATGCTGTAAGGATGGGAGC
EMPV1_17614 TGATCTGTGTACCAGCTGTGTGTCCCTGGACATAGGTGTCACTTGACCCTCCCTGAGTTT
EMPV1_17616 CTGAAACACTTGAAAGACACGAACAAGAATTGAGCCTCTCACCCCTAAACTTGCATGCAG
EMPV1_17617 ACAGAACGTTTGGCGAGAGGGAGAGGGAAGGTAACCAACCATCCATGCCTGAAAGATTCT
EMPV1_17619 ATATCTGGTTTGCTTAGGGTTCCACATGAGATCAATTATCAAAGGCTACATTTAAACCCC
EMPV1_17620 AAAGTGGAAGAGGGAGGTAAAAGCAGAGGTCAGAGTGATGTGGTGTAAGGACTTGACCCA
EMPV1_17624 GGGGATTTCTGCGGTTGGTGGTAGTAGTGATTAGGTTATTGGGATGGAAACTTTGTTAAT
EMPV1_17625 CCTGGATAACAAGATCTTCTGTAGAAGTGAGAAACTGAAGGTTGAGATGGAGGAGACAGG
EMPV1_17626 ATCATCAGTGAATCAAAGACTGGATTGAACAAGTGACCGCTGCTCTCCCGGTCCCTCCTG
EMPV1_17628 GCTCCAGTCCTCCTGTTCTGATGCTCTCAGCAATTACATTGTGCTCTTTTCTGTGACTGG
EMPV1_17630 CCTCACTATAATGTGACTACAACCAGATTAGTGAATCCAGATCAACTCTTGAATGAAATA
EMPV1_17631 CAAGCCTTTGCACTACATGACTATCATGAATCGGCGAGTGTGTGGCCTGCTGGTGGGAGT
EMPV1_17634 GCTTCACAGATATGTGTACTCTTTCTTCTGAAACTGCCAGATTTCATCATGACAAGATGC
EMPV1_17636 GTGCTTGGAGCCCGGAGAGATTTGAGGATTGGCTGTGTCGATGGTGAGAGAAGGGACTTC
EMPV1_17637 CTGATATGGTAACTGTGCCATCTGGGGAGTCAAAAGATTGCTTTACCACATGTATATATG
EMPV1_17640 TACAGGGCATTCTCATCACCCGCAACATGAATGAGCGCAAGTGTTTGGAGGAGGCCTACA
EMPV1_17641 CTGAGCATCTGTGTTCCAGGTCCGCGTGGCGAAGGCAGATTAAAACAGCGATGGCATTAA

EMPV1_17642	TGGGTTTGATCCCCTGCCTTAGAACTTCTGTGTGCTGTGAGTGTGACCGAAAAAAGAATT
EMPV1_17643	ACAGGGATCAGCCCAATGTCTCATCTGCCTGTTTGGAGTTCAAGTGCAGTGGGATGCTGT
EMPV1_17644	GCCCTGTCTGCTGCGACCCGTGCAGCCTGCAGGAGGGATGCTGCCGCCCCATCACCTGCT
EMPV1_17645	GCCTACATCACAGACATGGCAACACTGATCCAAGCCACACCTTTGCCACAGCTTGTCATA
EMPV1_17647	GCCCCCCGCAGGAAGAGGGGTGTTTTGTACCTTGGTGGGAAAAATAACTACGCGTTTAAA
EMPV1_17649	GCCATTTCCAGATTGTGTGAGGACAAATACAAAGACTTGTGCAGAGCTGCCATGAGACGG
EMPV1_17651	GCTGGAAGAGTGATCAGCTTCTCAGCATGGCTCCTTTTGTCCTCACTTTGGGGAATCTTT
EMPV1_17652	TTCAGTACCCACAGGGGGTGCCTGGAACAGGGCTGCTACTTTCTTACTTTTCAAGCTAGA
EMPV1_17653	CTCAGACGATCTAGGTCTGGTTCTATAAAAGGATCGAGGTATTTCCAATCCCGGTCCAGG
EMPV1_17656	GTCCCAGGACACCCAGGACCAATCTTCTGAAAGTTGCTATCAATCAACTGCTTGACCTAA
EMPV1_17657	GACATGTACCATATACGGAGAGTCCAGACTTTCGGATTGAAGTTGTCTCTGTAAGACTAG
EMPV1_17658	GTGCATGTGTGTCCAATACGTATATATTTATACACACACACACACACACACACACGTGTG
EMPV1_17659	CCCAGGGACTGCTAGTTTCAACTTCTGATGCAGACTATGATGATAGATTCTCTTTCCATG
EMPV1_17661	AGTGAGGAAGTTGTTGGCAATAGTGCTGAGCAGAAGCACGTTCAAAAAGAAGAAGATGTG
EMPV1_17662	AATTTGCTCTACACACAGCCAGCAGATGGCAGAGTCAGGATTCGAGTCCAGGCATCCATG
EMPV1_17663	GGAGAGCAACCTGGCTGCCACCGCTGAAGCCCTGGTGTGTCCCCGGGGTGGTACCCAGCA
EMPV1_17664	TTATATACCAGTGACTATACTAATGAAAAGTGGCTCAAGCATGTTGACGATCAAGGTAGA
EMPV1_17666	CCAATATAACAACCACTAATAATTTGGAACCACGGCGATGACCTGACTGGTGCTAGGGCA
EMPV1_17667	AGGCCTCCAACATAGAAACCAAGCAGGAATGGATCAAGAACATACGCGAGGTGATTCAGG
EMPV1_17668	CCGTAGGTCACACTGAGTGCAAGGAAAAAGAAACCAAAGCAAAACTAAACAAATGAGTCC
EMPV1_17670	TTAGGGTCTCACTCTGAAGTGTAAGGACATAGCAGTTGGGATTAGCTGGGTCCATCTGAC
EMPV1_17671	AGCTGCCAGCCTATACCACAGCCATAGCAATATTGTGACCTACACCACAGCTCACGGCAA
EMPV1_17674	ATCTACATCTATACCTACATTTACATCCATATCTATAGCTACATCTGTACCTATGCCTAC
EMPV1_17675	CCTTTCAGCTGTTGGGGCATTGGTGGGTCTCAGTAATGCCCGTCTTGGTTCTATCAAAAC
EMPV1_17676	TTTACAAATGCTATTAACTCACCCCAACTGCTACAGCGTCTCCCACTCAGTCTCCCTGCC
EMPV1_17677	AAAAATAAAGATAAAGGAATTAAAGCATAACACTAGAAAAATCATCAAATCATACAAAAA
EMPV1_17678	CTAATCCAGCTTCCCATGAGAGACAACGTGTTGCACCCACAGGAGTTCTGAAGTGGGCAT
EMPV1_17679	GGTTGCCAGCTTTGAGACTGATGAACGCTAATGGATCTCATGAAACCTGTACCAGGTGCT
EMPV1_17681	CCCGTTTCCACGGAGGAGCCGGCTTTGCACGGAGCCTCTTCTTCCGCATCCCCGCCTTCC
EMPV1_17682	AGACCCAAAGGGGTTGGGGGGTACCCCGGAATATTCTTTTATCTGCTCAACCTTGAGTCA
EMPV1_17683	TGTGTGCAGTGCTGTGGCTCATGCAGTGGCCCAGGTTAACCATGAAGAGTCTGACCTCAC
EMPV1_17685	TGGTGGGCCTTTGCGTTAATTCTGGTTTTGAGCGTTATACATGGCGCTGTGGTGGACACC
EMPV1_17687	CCAATGAGCTGAAGAGAAAGTGCCGTGTCTGAAGCACAGGTGCATGGGAACCAGGTCTGT
EMPV1_17688	AGACGATCATAGGGACATCTGGAGTGAGTTAGTTTTGAAGTTACTGGTGTGAGGCGCTGG

EMPV1_17690 CTCTGTTGCACTATGATGCGGTTATAATGTCACGGGGAGAAAGCAGAAGCAGAGTCCAGC
EMPV1_17692 ATGCCGCCTGCCTGGGAAACAGACCAGCCAACAAGAAGAGGCTTGCCTGATGTCACTTGG
EMPV1_17694 GAACAGATGATTCAGTGAAGGCTTTTCCACATACATGACATTCGTATGGCTTCTCCCCTG
EMPV1_17696 TGGAAGTGCCCCACCTGGGGGCTTGGCACAGCCATTGTGCTCTCAATAAATGTGTTTCTT
EMPV1_17697 GGAGCAGAAGAAGCAGGACATCTCCAAAACCAAGCCAACTCCTAGCCAGTGGAAACAAGA
EMPV1_17698 ATTGGTTAAGTGCAGGCAGCCACACCTTTGACTTCCATTTCAACTTACCTCCCAGGCTCC
EMPV1_17699 CTGCCATGCAGGAACCCCGTGAAGTGTGCATTTTCTATGATGTGTGCCTCTTCATGCAGT
EMPV1_17700 GAGGAGTGGGGGAATTGGGTAGAGGAAGAAAGAGCTTCACTTCTAAAGTCCCAGGAACCA
EMPV1_17704 ATTTACATCCAAGCCGGTCTGTAGTTTGTGCAATACTGCCATAGCATCGCTCATATTCTG
EMPV1_17705 TCTCCATAGAAACAGCAGCAACCTCCTGGTTCTCTTCCTCATCAACACCAAGGATCTGCA
EMPV1_17709 CACGTCGGCTGCTGATGTCTAGATGAGTTCCCTGTAGGGTCAGAGACAATATCACACTGT
EMPV1_17711 CCATCTGGTTTTTATGTCTGCTCAGGGACTTGTATGCACAGAGTTGTCTAGGATCTGCTC
EMPV1_17713 TGTAGTATCTGGCTCAGGATCTAGTACCCCTGTCTCCAGTTCTCATTTACCTCAGCAGTC
EMPV1_17716 TGATGGCAGAAAGTTGTGTGGCTCAGGATGTCCATCTGCAGAAGAGGGAAGGGCTGACTT
EMPV1_17719 ATGGGGTGGAGCAGACGGCCATCAAGGTGTCCCTCAAGTGCCCCATCACCTTCCGCAGGA
EMPV1_17721 ACCATCTCGGCCAATAAGTGTCTCTGGAGGTCCTGGATCAGCTAGAGGCGTTCGCGGTGC
EMPV1_17722 TTAAGAGCAGTCCTAGGGAGCCGGCACCCCTGCCCCCAGCTCCGGTCGGGGTCGCCCAGG
EMPV1_17723 ACAAAGTTGTATCTGCCCTCATACTGTCACTCATGCTATCTCTGTCACCTAGAGCCAGGC
EMPV1_17727 CCAGGACCTATGCTGGGTTATTTCTCCCAGGAACTGGTGGAGGACTGCTTTTCTAGTTGT
EMPV1_17730 ATATAAAGTGGAACCGGTGGACAGAAGGCAAGGGCTGCAGGACTGAAGAGACTGGGAACT
EMPV1_17732 GGAGCCATGAGCCCCACACATGTCACAGTTCACCAACTTCCCCAGATTATTCACTTGGTT
EMPV1_17733 CTTTAGCTGCGCACACTGCGGCAAGAGCTTCATCCGAAAGCACCACCTCATGAAGCACCA
EMPV1_17734 CGTCGCAGCCTGGAGTGATGTTTCTGACCTGGGTGAAGGATGGTGTCATCTCAAGAAATG
EMPV1_17739 CTTTGAAAGGGTGCCCTCGTCGAGTATTTTTGAAGGGATGACTGGCTCTTCACGGATTCC
EMPV1_17740 CAGACAAGTGGCTGCTCCGTGGACTGCATGACACTAAGATGCTTGTCCCCAGGGGCCCAA
EMPV1_17741 GCTTGCTTTGGCATTGACTGGGCTCATCACCATTATCACTGCTGTCCTTCTAGTGTTGCC
EMPV1_17742 AGAGCTGGTGTGTTCTCTGATGTGCCTGGAGAAACAAGACCTGTGCAACAAGTTCAAGGG
EMPV1_17744 AACAATAGGTACAGAAAGAGAGAAATGCCCCCAAATGGCAAGAGCGGGCAAGCTCCTGGA
EMPV1_17745 GGGCACAGGGAGAACTTAGGTATGTTCAGCTTTCACAGGGTCTACCTCTCTTTCCAAAGC
EMPV1_17746 TGAATATACAAACCATCTCACTCTTCGCCAGATTTTCCAGCCAGGCACAGGCAGAGGAGG
EMPV1_17747 TTCTGAGCCTTCCAGAGCTTCAGCCTCTCTATAATCATCCTACCCCACAGAGACCACGGT
EMPV1_17749 GCATCATCATTACAAACCATATTTAATCTTCATCACTGCCTAAGGCACCTTGAGGTAGCT
EMPV1_17751 CCAAGAAGGTGAGATCTTTATTGCTCTTGCCATCCCACATCCCTACCTGAAACTTACTGC
EMPV1_17752 TTGAATTCCAGCCCCTGAAACCTGGAGAAACCTTCGGGAGACTAACACTGCACAACAGTG

EMPV1_17753	TACGATAGGACCCAGCCACCTATGCAGTATTACAGTAGCCAAGGGGATGCCACTCATAGA
EMPV1_17756	CCTCCTGACAAAGTGGTATAGACAAGCGATTCTGAGCCTCCAGGGATCAACGTAGTCTTC
EMPV1_17757	CGGTCGAGGCAAGTTGGTTTACAGAATGTTTGTAGGGAAAAAAAAAAAAATTTGACTGAT
EMPV1_17759	GATGTATTTTGCAGCTGTCCTGCTGGGGGGTGGTCCCTTTGCTGGTATCATTTACTCTTA
EMPV1_17761	TACAGGTGGTACCCAGGCCGTGCTTTGGTGAGAAAATTCAACAGAACACTATTCCCACAG
EMPV1_17762	TACAGGTGAGGAAACAGGCTCAGGGGAGGTGAAATGCTGAAAGTCCCATGCTAAACCATG
EMPV1_17763	AACAGCGGGGACCAGGACTACAAGTACGACAGTACCTCAGATGACAGCAACTTCCTCAAC
EMPV1_17764	CAACACTAGGTACTGAAAGCTCCAAAGAGTGCAGTGGATACAGGGAAGGGTGACTACACC
EMPV1_17765	CAGCTTGGGACTAACCCCAACAGGCGTCCTTGTTTTTGAAGGAGAGACCAAAATTGGCTT
EMPV1_17767	CTCAGGCAACTTCCTTACTCCTTCTCTTTGGGATCAACCTCCTCCTACACTGACTGGGTA
EMPV1_17768	GAGTGGCCAGATAGGGAGCAATTGGAATTCTCATTCATTGTTAGTGGGAGTGCAAAAAGA
EMPV1_17769	TGTCTTTTACCAGCACAAGAGCATGAATGAGCCAAAACACGGCTTGGCACTCTGGGAAGC
EMPV1_17770	AACTCAAGTGGCATGAGCTTTCACGGAGTTTACACATGGAAAAGTGGCATCTGGGAATCC
EMPV1_17771	AACCTCAGGATTCAGTCTCTCTGCTAGGATGGGTGGCGGTGCGGATACAGGAAATGTTTT
EMPV1_17772	GCTCGCCATGTCAGAGATAAGGAAGAAGAGGTGGATCTGGTGATGCAAAAAGTTGAAAGC
EMPV1_17774	GCCAGAACACAAAGCGTGAATCTGGAAGAAAAGTTCAGTCTGGAAACATCAATGCTGCCA
EMPV1_17775	ATCTTCATCGGTGGGGTCTTGGTGCTCGTCGTGCCCTTGATCTGCATCTGCATCTCTTAT
EMPV1_17776	GTTCGATTGCAGTTAAAGATTGAGGGAGAGGGCAGATGTCTGCAAGTCAGGAAGAGAGCC
EMPV1_17777	GCTGGTGGTGAAGAATAAGCTGGAGACGGTGATGTCTGCCTCCTCTCTGCATCTCACTTT
EMPV1_17778	CAGTGGGGTAACTAAAAGGATTGCGAAAGAAGAAAACTCTAGCCAGGACTGAAAATTAAG
EMPV1_17779	GGCTCCGTGTACCAGAAAATGGGTGCTAATTATGACTTACACTTGGCTAGTTCAGCCGGC
EMPV1_17780	AGAAACCACGGCCATGATCAAGCAAACACGCACATCCATCACCTCGACAACTGCACTGAG
EMPV1_17783	CAACATGACGGAGCGAGAGGCCGCGAATTTCATCATGAAGGTCATTCAGAGCTGCTTCCT
EMPV1_17788	GACATCATCACGCTGCTCGTCCCGGAGGAGAAGGACGGCTGGCTCTACGGCGAGCACGAC
EMPV1_17789	CCTCGAGTTCGTGGTGATGTTGGAATGGCTGGCGTTGCTATTGACACTGTGGAAGATACC
EMPV1_17790	ACGTTAAAATCGTGTTGTACAAGTTATATTTTGTTAACTAGTTGTAGTTCATACTCATAT
EMPV1_17791	ACTCATTAGAGTCAGATGGTTGTATATGTTTCTGACTATCAGCTATCCCTGCTTTCTCCC
EMPV1_17792	TGCATCGGATTTAAGCGAACTAGGTCAAAATGGAGTCACAGCGGCCGAAGCCAACTGAGT
EMPV1_17793	AGAGGGGCAGGAGGAGTTGGCACCCCTTAGTTTGATTGGTCACCCCGACCTGTTGATCACT
EMPV1_17796	CAGGATGAACAAGAGGAGAAGACCTAGTTACCGTCCAGAAGATCAAGAAGCATTCTACCG
EMPV1_17797	CCAAGCTCGGTTTGGACATGACCCTTACCCTGAAAAAGCAACCAGGAAACAATGGGCCAA
EMPV1_17798	TACTGTTGCCAGAATTTGCCAGTTCCTTGGAGGGGAAGGCCCTGGAGCTCAGAAGGGAGC
EMPV1_17799	AAGCAGTGAAAGTGCAGTGTTTCTGAGAAGTCCCCAGTGTTGGGGTATCTAGGCAAAGAT
EMPV1_17800	CTATCGGCACTAGTTTTCCAGCAGCATTTGCTCACATCTTGTCTCTGTCTTATTTTGGTG

EMPV1_17802	ACCCCACCGTGTCAGTTCAAGCATCAAAATACCTTGCATCTGGGGCAGACTTCCTACATC
EMPV1_17803	AGACTGTGCAAGATCAAGGGAGGCTATTTGTTGCCTAAATCAAAGGCGGGAACCTCAGGG
EMPV1_17804	TCATACTGTCGAAGTGCGTGGCCCGTGAGGTCAAGGCCGTCTATCAGTATTGCTTCAGTA
EMPV1_17805	TGATCCCTGGGGGCATGAGAAAAGCACATGGTGGATGTGGATTGAGAGGTGATACTTTTC
EMPV1_17807	CCCTGTGGACCCTGTTGCTCACCAACTTGGATGGACACCTGCTGGAACCAGATGCCGAGT
EMPV1_17809	CCGAGAAACCTACAATGCGCTTACTAATTGGTTAACAGATGCCAGAATGCTAGCGAGTCA
EMPV1_17812	GGACTCCATCTGCTTTTTGCTGGGCATCTCCAATTTGTCTCAGGTTCGGGCTTCTAATTT
EMPV1_17813	TGGGGTCACTCATTTGGTAGTCGCTATGTAGTCATTTTTTGTGTCAGGCCCTGGATGCAG
EMPV1_17815	TCTTTGGACCTGCCATCTTCCTCTACATGCGGCCCCCCTCCACCTTCACTGAGGACAAAC
EMPV1_17819	AGCCACAGCAATGCAAGATCTGAGTCGCATCCACGAACCATACCACAGCTCATGGCAACA
EMPV1_17820	GCGAGGCAGAAAAACAAGGGAGTGGGGAGTGCGATTAAGAAAAAAGGTATTCTCGCTTCC
EMPV1_17821	TTCCATGCACTTAAATCCTTGTCTTAGGTTGCTTTAGGGTTGGGGGTGGGGCAGGAATCC
EMPV1_17822	GACAATGAGATACTGCCATTTCAGAGGTGCTGCGTGAAAGCAGGGAAAGGGTGCATGACC
EMPV1_17823	GAACCGCACGTTCCTCTACTCCGTGCTCGGCTCCATCCTGGGGCAGCTGGCGGTGATTTA
EMPV1_17825	GGCATCATTGATCCAACTAAGGTTGTAAGAACTGCATTACTGGATGCTGCTGGAGTGGCC
EMPV1_17827	AACTGGTCGGTGATTTAGGTAGTTTCCTGTTGTTGGGATCCACCTTTCTCTCGACAGCAC
EMPV1_17828	CTTGAGAGTGTAAGGTATCGTTGGGACCACCTGGACTGTGTTGGAGACCGAACCCCATTA
EMPV1_17829	AGCTCTACTACACGGGTCAGGGTGACCAGCTAGACGTGGATTATCAGTACAAACTGGACT
EMPV1_17831	CATCTGTTTCCCTCCTAAAGAAGTGCAAGATGCAAACATTGTCACTGCGACCAGACTCCC
EMPV1_17835	TCAGACTATGACATGATAGCATCGACATGTTACACCATTGTAATTCCCATGCTGAATCCC
EMPV1_17836	GATTAAATGTGCCGGAGCAGTTCTTCCCATTCATGTAAACCAAAGCGACAGGGAGCTCAG
EMPV1_17837	AGCAGAGTCAGAGCAGGAGTACGAGGAAGAGGAAGAACCTCCCAAAGTTGAAAGCAAACC
EMPV1_17838	AGCTACATGAAGGTGAGCATCTGCCTGCCCATGGATATTGACAGCCCCTTGTCACAGCTG
EMPV1_17839	TCAGCGTCCAGCCAAAGACCTGGGCCTTCCCCAAACCAATGTGGAATACATCCAGACTGA
EMPV1_17840	GGAGCTCAAGTCTAAGCTGATTCTTGCTTCTCTGGAACATGGAAGGTTTAGAAGAAGATG
EMPV1_17843	TGACCTGATAGGCTCAGAAATCATAGAAGAGCTGGGAGAGCAGCGTGACCTGTTGCAACG
EMPV1_17845	CATCTTCTTCTGCCTATCCGGGGTGGGCTCACTTTTGGGCTCCGGCCTGGTGGCACTATT
EMPV1_17846	GCCCAGCCCTCATGCAGGTTCCAGAGTGTGTCTGCTGCTGGCCTGAGAGCAGAAGGTCAA
EMPV1_17847	CCTGGGTTTCCAGTGTTCTTTTCCCATCAAAATCCATAGCAGAAACTCTGGGGTGCAGGG
EMPV1_17848	TCCCAAGTCGTAGAAAGCCAGAGTGAGAAGGAGTTACTGTGCCGGCTCCAGAAGCTGCAG
EMPV1_17850	ATTATGGAACCCAGGCTCTGCTTGCTGCTCACAGTCATCCCGTGGGTCTTCAGCCATATT
EMPV1_17851	ACACTGCCTTAGAGAGAGTGGAAAAGTGGAGCAGCCATGCTGATGCTTGACCCTGGATTT
EMPV1_17854	AGCTTTCCTACCTGTTTCTGTAGCCAGCAGAGAGCATCCGGTAAATACTTGGACATCCCC
EMPV1_17857	CCTAGTGCACGCAGCCACCAGGGAAGTGGATATCAAGGGCGTGTTTCGATACTGCAATAC

EMPV1_17859	ATACAGGTAACATCTGTGTTTGGCCTCCCCTTCTGGGATACCTTTGTTGTCTCTCGCTCC
EMPV1_17860	GCCTGGTTTCGTGGCAATGGTAGCTGGTGGTAGTTATTTCAGGGACCTTCCTCGGTAAAA
EMPV1_17861	TTCCTGGCTTTCTGTCCAGAGCATTTCACTCAGCCCTGGGCACAGGCAGGATCTTTGCCT
EMPV1_17862	TAACCACGAACCCAGCTTCAGATCCTTTTAATTGGTCCAAGGGCTTGCCAGTTGCTGCAG
EMPV1_17863	ACACTGCAGACATTGCAGGAGCACAGCAGGGAGCAAAGCCTGCAAGGGAGAAACTGTTCC
EMPV1_17864	TGAACTCTAGAAGCTGTGCATGTAGGCCTCTGTCCACTGAGGTGGAGCACATATCATGCA
EMPV1_17867	CCAAAAGCAGCCAAGGTCTCCCACACTGTCCCTCTTCCTGATAAGTCAAACAGAACTGTC
EMPV1_17873	TAGCTTAAAAATAATCATCCTCTGCCACCTCAGACCGCTCTCCCAACACCTACCTTCGCC
EMPV1_17874	GGGCCCTGCACAGCCACCTTCTGTCCAGCACCTTAAGTTTTACTTACCAACTAAACTGGA
EMPV1_17875	GGTATAGGTCATAGACATGGTGTGGATCCTGCAATGCTGTAACTGTAGCTTAGGCTGGCA
EMPV1_17876	CCCCGCTGGAAGACAAGGATTGTTTTTTCCGGGAAGTCCCGATTAGAGGTGGTGAAATTG
EMPV1_17877	GCACCATAATTTTCCTTTGGAGACCCATTCTTGCACACAGCCCACATGGTTTGGGAGACC
EMPV1_17880	CTTTGTTACTGCAGCGTCATTTAATCCAACCTGACTCGCCCACCAAGGTCCAGTCCTAGG
EMPV1_17881	CCATTGTTCTCTGTTCATTCATCCCTACCCGTGTGTTCGCTCGTTCGCCCATTCATTCTC
EMPV1_17882	CTCACATGAGACAGATGAGGAGTTTGATGCTCGCTGGGTGACATACTTCAATAAGCCAGA
EMPV1_17884	CAGTTCCCCAGCTTTAACCCGAGGCAGAAGGTGATTGTCTTGTGTCCCTTATGGATGTTG
EMPV1_17886	GCGCCAGGCGGCCCTAGGGAGGCGCTGCGGGCCGGTGGCCCGGGCTCGGCTGGCGGCTGC
EMPV1_17889	CTGGATGATGTGTTGGAGAAAGCCAAGAAAGCCAGTGTTATGGCTCTTGTGGTGGTTGCT
EMPV1_17891	CTCCCATGCTGGGCGTCTGGTATTTCTTGGGCCTTGGATACCTGGAGGGCCAGACCCTGG
EMPV1_17892	CCCTTACATCCCGCATTTGGTGAGACTGTCTAAAGAGAGGAAGGCCCCCGAAATTAACAG
EMPV1_17893	CCCAGCGTTTCTTCATTTCAGTGGGCTGTTTTCTGGAGTTCCCTTGTGGTGCAGTAGGTT
EMPV1_17897	CCTACACGCATAGGATTCACACATAACTTGAAGAGATCTGGCCATAAGACAAACCCTAGT
EMPV1_17898	AGAGCAGCGAGGATGAGGCCAGCGACCACACGGCCAACCGCTTCGGGGACGCGGAGTCCA
EMPV1_17901	GGGTTATTTCCAGTTCAGAGGAATGTGGAAAAAGGTATGCAATTGTGAAGTGTTTTGTTG
EMPV1_17905	AGCTGCTGTCCTCAAAGCTGCCACTATTCCGGCAAGAATGAAAATACATCGGGCTCATGG
EMPV1_17908	CTACTTAGATCACAGGGAAGAGTGGCAGAACACGCCACCTCCAAATACCCCACTTTGGTA
EMPV1_17909	CAACACCATCATCCGGGACTTCTACAATCCCTTGGTGCCCGACGCGCAAAAGCGCGAGAT
EMPV1_17910	CCATCACCTTTGAGCAGTTTCTGGCTTCCCTTCTCACTGAGCCAGCCCTGGTGAAGTACT
EMPV1_17911	ATCAGCCTCGTTTGCCTTTGAGTTCCCCAAGGACAGAAGTGGGATTGAGACATTCTCACC
EMPV1_17913	GTCTACATCTAGTCTGTGCTGGGATATCACCTGTTCTTCACCTTGACTTCCAGAGGCGCC
EMPV1_17914	CTAGGTTTCACCAGTCAGTTGCTCATAAGGTCAGTGGAAACCAGGGAGAGAAGCTACTAG
EMPV1_17916	GTAGGTCCTCTGACCACAATGGAATGCAATCAGAAGTAAACAGCAGAAGGAAATGTGGGA
EMPV1_17918	AGCTGGGCACAGTCAAAACTCATTGCCCAGTAAGCATCTTCCCAGAGCAGCAGAGGCCCT
EMPV1_17920	CCACGTCATGCAGTTGCACCGAAGGTCTCTGCTCCCATTATTTCTGCTAATAGTCTGCTG

EMPV1_17922	CCCAGCCTTCTTGGCACAAATATCCCCTTTCTTCCTGCATTAACCATGGAGGTGTGTATA
EMPV1_17923	CACTCAGCTGGTTGCTGGATCTTATTTCTGTGGTTGGGTCAGCTCCATCCTCCAAGTCAG
EMPV1_17924	CCGACAGTTCCTGGTCGAAGACAAAGGACTTTATGGAGGATCATCTTATGTGGATTTCCT
EMPV1_17925	GCCTGACAATGAAGGAAAGTCGGATGAGGAAGAAAAACCAGAAGTGGAGGGGAAGTCAGC
EMPV1_17930	ATCTGGTCCCAGGTGAGAAATTGGCCCAGCAGATTAGTTATTTTCCCCAAGGCCACGCTC
EMPV1_17931	CACAAATTTCCCAATTGCCTTTCACCAAAAGCTCCTCAGTTCTAGAGAGTCCCCTTAGTC
EMPV1_17932	GGAGACTTTGGAAATATCTCTGACTGGGCAGCCATGTATGCAGTCTAAAAGGGAAAGGGG
EMPV1_17933	CAGCTAACAGTGCTGTGTGTTTACAGAGTGCTGCCCTATAAATGTGAGCTTTAAGATGTC
EMPV1_17934	CCTGCAGGCGGCCGGGAATTCACTAGTGAATAATCGAATTCCCGGGGCCGCCATGGCGGC
EMPV1_17936	CTTCAGAGCTCAGTCTGGAAACAGTTACAGCTGGTGATTGTGATTGAGGGACGAGGTGGA
EMPV1_17938	ACAGTTGGTGATGAGAGCAGCTCTGCGCCAGGCACTCCGTGGGGCATTTTACATATATAA
EMPV1_17939	CAGGTCCATTCTTCAGCCTTGCTGGCACATAACCCTTCAGGCACAATACTCTTGGACATG
EMPV1_17940	AGAGATTGGGATCCGTCCCCCTCAGGTCTTGAAGCAAATAAGAGAGAGGCCCAGGAGAGA
EMPV1_17941	CCTCCATCACTGTACAAGTACTCGGAAGGTCAGTTCTGATACTCTGGAGACCATTGCTCC
EMPV1_17942	ATCGAGGATGTTAAAGACTCCCAGCTCCGCGAGCTGCTGCTGCCTCCGCCAGACACCAAA
EMPV1_17943	GTGGTGCCGGTAAAGCTGACCCAAGAACTGGGGAGAAGAATGGATGCACAGTGTCAGCAA
EMPV1_17944	GCTGCAGTCTTTGTTGCAGCCACCCTTTGCATTTCCAGCCCATTTTTACTCATCATTGCT
EMPV1_17946	TGACAGCTGAGGACCTCAACAACTACCGTGCAGAGCTGATTGAGCAGCCGCTCCGCATCA
EMPV1_17947	GTTCTCTGGTAACAAGTACCTCTGCTTTCTTGCTAAGATTACTACGTCAGAAACCACTGC
EMPV1_17948	TGGAAGTTTCTTAGAGTTCGCCCTAGTCCAAATCTTATCCCCCAAACGTCTCCAAACCAA
EMPV1_17949	TAGGGCAAGGGCAGAGAAAGGGCATTGGAGTTGGAGTGTTGGAGGAGGAATGGTAGTGAA
EMPV1_17950	TTGTGGAAACTGGCAAGAACTTAAGGGGAATTGATATTGTGACTTTGGGGAAAGAGCAGG
EMPV1_17951	CCTGTCATCGTGAAGGCCAAATTTTTCAGCAGACGAGCTGAAGAGAAGATTAAGGGTGTT
EMPV1_17952	GGCTTCATTTTGGAATAACTGATAACCCTGTGGGGAGCCCCTCTGTCTACACATGTCCTC
EMPV1_17953	AGCTCTAATTTGACCCCTAGCCTGGGAACCTCCATATGCCGCCAGTGTGGCTGTAAAAGA
EMPV1_17956	AGCTAACCCTCCTTTTAAGCTAACCCTCTTTTTAAGCCATACTCAAGAAATCCCGCCCCC
EMPV1_17957	AAGTTAAAATATGGGCATTATGTCAGCAAACTTAAGGGCATTATGTCAGCAAGCTAAAAC
EMPV1_17959	TTTCTTCCCCATCACAGCAACTTTGTTGCTCAATGGAAAGTATTGGAATTAGCCTTAGAC
EMPV1_17964	ACCGCAAGGTTCTTCGCGACAACATTCAGGGCATCACTAAACCTGCCATCCGGCGTTTGG
EMPV1_17965	CAGCTACAATTACTCCATCAGAAAATACCCATTTCCGGGTAATTCCCAGTGAGGACAGCC
EMPV1_17967	AGATCACTGTCAATGATTTGCCTGTGGGACGCTCTGTGGATGAGGCTCTGAGGCTGGTCC
EMPV1_17969	AAGCCAAAGGACTCACCAGCCAGGAGTATCCACTGTTGGCCGCCTCTTCATCCTCTGTGA
EMPV1_17972	TAATTGCAGGTGTACAATATTATTCCTTTTTTTCTGAGAGGGAACACTTCTCATTTTGCC
EMPV1_17974	CGTCACGGAGAAGGAGATGGAGCTGCAGCGCAAGGAGTATTACAGGAAAGGGAGGTACCT

EMPV1_17976	CGATGGGGGCCGACAAGAACAACCTCAAGTACACAGGGAATGTCTTCACTCCACGCTTTA
EMPV1_17978	TAACGTGAACCTGCCCAAACCTGTGGAAGCCTTTAAGGAGTGGAAAGAGTGGCTAACCCA
EMPV1_17979	GGCACCGCTGGAAATGAAGAGAAAAGCCTGAACTCCAATGACCTTGAACTCCACAATCTA
EMPV1_17980	GAAGGATGAATCCGTGCTTCCGGCAGGCCGACAGGGGTGTGAGGAGAGGGTTCGAGAAGT
EMPV1_17982	CAGTCTCTGGAATGGGGTGTGACCTCTAGCAGGTTCAGGATAAAGATTTACTGCAGAGGA
EMPV1_17983	AAGCGAGCCGACATCCAGCTGAACAGCTTTGGCTTCTACACCAACGGCTCTCTGGAGGTA
EMPV1_17984	GCTGCATCGCTTTTTCTCTGACAAGCAGAGGTTTATCGATGAAATCAACAGCGTGACCGC
EMPV1_17987	GTCCTTTCTGAAGCACCTGCTAGCCTTTACAATGAATTTGCACTCTCCTGAAGCCAGTGG
EMPV1_17988	ATTGCGCTGGCCCCCCTCTCCCTCATCATCCTGGTTTTGGGAGGAATATGGATACACAGA
EMPV1_17990	ATTTGATTAATCTTCAGGACTGTTTTGTAAAGTGGTGGGTGGCTTGGGCGCCGCGAGGAG
EMPV1_17991	GTAGGGTGAGGGTCCTGATAAAGTGTTTGTTCTGGTGTGAGCCCAGGGAGGGGAACAGTA
EMPV1_17993	GCTGAGCATTATCGGTGCAAAACAGAAGGCCCACTTCTCCCAAGGAACCCACAGTCTAGA
EMPV1_17994	GGCCGTATATAAGGTAAGCCCACAGATGAACATCATACTCAATGTTGAGAAGCTGAAAGC
EMPV1_17996	GGACACACCACCGCAAAGCGTCTATAAACTGTACAACCAAGTGGGATACAACTGCTTCAT
EMPV1_17997	CTACCCACTCCTGTGTCACTGCTTCTTGTGCCACCACTTCTTCCTGGCCTGACAGGGTAA
EMPV1_17998	CAAGCATCTCTGTCGATTAATAAGAAGATTTGGAAGTTCTAATTGTGCCACCAGGGAATC
EMPV1_17999	TACTCTTGTTCTCAGTGAAAGTGGGGCACCAACCCGCATCACCTCATTGCCTCTGAGTAG
EMPV1_18004	AGGTTGACTGAGACCAGAACCCTCCCCAGGCTTCCCTGCCACCGAAGAGTACTTTAAATA
EMPV1_18005	GGAAACAAACACCCAGGCGAGGACTAGAGCCAGCACATGAGTCATATCTTTGGCATCCTA
EMPV1_18006	AAGTACCTTGTAGAAAGCGCCATTGGAGCCCCGCACTTCCACCACCAGCTCCTCCATCTT
EMPV1_18007	TCATCCACTTAGCTACTCATCATCCAGCAAATGCATGCAGAAGCTCTGCTCCGTGGCAGT
EMPV1_18008	TCATCTCCATCACCCTCGTCATCTCCATCACCCTCAACTCCATCACCCTCAACTCCATCA
EMPV1_18009	GAAGGGCTGTGTCGCGTAGTACTGCACGCTGGGTGGTTTTAAACGTCAGAAATCGATTCT
EMPV1_18011	AAGTGCACGTGTGTTTTAACGGTGCTGCCCCTTCCCATTAAAAGTTCCTCTGTGCGCTCA
EMPV1_18012	CACATAGAAGATGCAGCAAAATGAACGTCCCTACGACACGCCACTGCCTCATAGAGGATC
EMPV1_18014	TAACTCAACACGTGCCATTGAGCTAGTCCCATCCCCTTTTTGGGACCTCAGTTTCCCCAC
EMPV1_18015	AGAACCACATGTTCTCTTCTCTTCCACAACCACAGTAAGATCATCAGCATCAGGGGATGG
EMPV1_18016	TGTTCCTGCTAGGTCTCTGTGCTGTTGCTTTCTGCATCCCTGATGAGCATCTCTTCCCAG
EMPV1_18019	TGTCGGAATTAGGTACCGCACCTCCCCTTCAGCTAACTCGTGGCAAGCCAGGTGGGTGAT
EMPV1_18020	ATCAAAATGAGAAAGGAGCCCAATACCCGGCAAATGCCCTGCAAAGGCAGAGTGGACCAA
EMPV1_18021	CCGGAGGTCTTTGCAGTGGATGATGAGTGTCTCAGGGTATTTCTTCAGCTGTCCAAAGAG
EMPV1_18024	TAGCTGCCAAAGGCGCTTCTCCTTCTGAACAGAGCGCTTTGCTCAGCCAGTGTAGACATG
EMPV1_18026	AGATCTCCAAGGTCATTGTGGTGGGGGACCTGTCGGTGGGGAAGACTTGTCTCATTAATA
EMPV1_18027	AGTTTCCAGCTAGTTACCCATATTAGAGAAGAAATCAAGAGTCTTAGAAGACGACAGGAG

EMPV1_18028	AGCTCATGTGGAGACAGTGGTTTTATTTTTCCCGGATGTTTGGCATTGCCTTCCCACCCG
EMPV1_18030	TGGCGAGTCACACGTGAGTACTGCATAAAAGTTCTAGCCAGCGTTTACCCGAGACCCAAA
EMPV1_18031	AGAAGCAAGTCTGGATCTGCCTTGATCTTCAGTCATGTACTTCAGGGCCTAGAGGTGGGG
EMPV1_18033	CAGCCAGTTATTCCCAAACCATTTATCGACTGTCTGTCACCGTCACCAGCACTTGGTCAC
EMPV1_18038	GGTATAATGTCAGCCTCCCCGTATGCAGGAGCTGGTTTCCCTCCCACCTTTGCAATTCCT
EMPV1_18041	GAAACGGACATCGTCATTGACCACTACAAGGAGGAGAAGTTTGAGGACAGCTCCCCAGCT
EMPV1_18042	AGCACGCCACAAACGTCGGAGTCATGTTCAGGGGCAAGGAGACTGCACTGATGCCCAATT
EMPV1_18043	TCCCATGGAGAATGTTGCGACAATTGCTGATTGTGCCAGTGTGATTGAAGGGGTCAGTCG
EMPV1_18044	ATGGTGCAGAAACCAGGATTTGGAGTGGGAAGGGGCCTTACTCCAAGGCAGTGAATTGCT
EMPV1_18047	AGTAACTTACCTGAAATTTCTGAATATGAGGCAGGATCCACAGCACCATTATTCATAGAC
EMPV1_18050	AGAAAGGCCAGTGAGCCTGTCCTTCAGCGGGTTGGATGATAGAGCTGCCACTCTGAAGAG
EMPV1_18051	GATTCTCGTTCTATATCCCATCACAGCTCCAGCCCTGTTTTTAGCCAGAAAGGATTCAGG
EMPV1_18052	TGCAACTCCAATTCGACCCCTAGCCTGGGAACGTCTGTATGCTTCTGGGGTAGCCCTTAA
EMPV1_18053	ATGTTCCTCCCCAGAGGGGTCCTAGACCAGTCCGAGAGGCTGGTGAGCAAGGGGATGTTG
EMPV1_18056	CTCCTTATCCATGTTTAAGTTGGCATTAAACCTATTCATTGGGTTCTTAATTTCATGTAT
EMPV1_18057	GCAACGATCTACCAAAGAAGTTCAGAGTCTTAGAGAAGAAGGGACCGGACTACAGGGCTC
EMPV1_18058	CATTCTGCGTACGTAATAGAGCCAGAAGAGTTGTGAAACCCAGGAGGTTCTGCTAGGCTG
EMPV1_18062	AAAGATAAGGAACCCGAAGTCGTCTTCATCGGGGACTCCTTGGTTCAGCTCATGCACCAG
EMPV1_18063	CAACTTTGTTGGAGGGTCCACTCAGACTCTCACCTTTGCTTCCAGGGGGAGGAGGAAGAG
EMPV1_18066	TCCTTTGCCGCTGCTGTCGTGGTGCCCTGCCTGTCCTCCGTGGTCGCGGGCTACGGCTCC
EMPV1_18068	GTCGATGCTGTTTTCCTAGCCGCCGATTCCTTTACATCGTCAATCTGGATGCCCCTTTGA
EMPV1_18071	GATGTCGGCCGAGCTCGAGCATGGATCAGATTGTCTCTAGAAAAGAAGCTTTTGTCCCAG
EMPV1_18072	GTATCAGAAAGTCAGGCAGAAAAACAACGATCTGCAACTGCAAACGTGACGTCACTGATT
EMPV1_18073	AGGTGGTCAGCAATGAGGGCCCAGGCACCCTTACCCTCCAACATGATTAGACAGCATTTT
EMPV1_18074	GCCAGCTGTCCTTTGCTATGGAGGTGCCAAGAAAGTGTGGAGTTTAGAAGCAGATCCTCC
EMPV1_18075	CAATCTCAAATTCAAGAATATTATCAATGAGGAGGATGCCGATACCATGCGTCTCCAGCC
EMPV1_18076	TGCGCATTTTAAGAGAGAAATCTGCAGCGAGCACCGCTTACTCCCCAGGAAAGTTTAGCT
EMPV1_18077	GGACTCATCAAGACTAACTTCAGCCAGGTGTTGTGGATGGATAAGGCAAGAAAGGAGTAC
EMPV1_18078	GGTTCAAAGAGTCAGATATGCTGCATTCCTGAGAAATCTCAATCAGCTTCTCATTCTGGG
EMPV1_18079	AGGCGAGATTTAAACTGCAGAAGCCCGAGGCAGCTAAGATGGAAACCTGGACCCACCAAA
EMPV1_18081	ATATGAATCTCTGCCTGCAACACATATCAGGGGGAAATGCAGTGTTGCCCTTCTGAATGA
EMPV1_18083	CTCTTCCTTGGCTGGAGGAGGCAAAAACTACGTGTAGCGACTTTAGAAATCACGAGGGCT
EMPV1_18084	CACAAGCTTTGGTGTGGTCACAGGTGCCGCTTGGATATGGCATTGCTGTGGTTGTCATGT
EMPV1_18085	CACAACCACAGGGATCTGAGCCAGGATTGTGACCTACACACCATAGGTCATGGCCACACT

EMPV1_18086	AATGATCTGGAGGAAGCAGCAGATGGCTTCCTCCAGGTGCTCTCCATCAGGAAAGGCAAA
EMPV1_18087	TCCCCACTCTCCCCTTGGTCAACAGTCTCCTTCAGTTGTTTATTTTTAGCCGGCTTAGCC
EMPV1_18091	GCTTTTGAATACGTTGGTGGTGTTGTCATGTGTTCTGGACCATCAATGGAGCCTACAATT
EMPV1_18092	AGAGCTGCGTCTATTTGTTTTGACCCAACCGACCTGCCATCCAGTCCTGCCTGAGCAAGA
EMPV1_18094	ATTGGTTTTCCACCAACTTCACTGGCCACCACTCCAGCTTCCCTTGGCTTGATTCTCCTT
EMPV1_18096	CCTCCGCCCACAGAAGTGGCCGTCGTCAAAGAAATAATCCACTGCAAGAGTTGTACCCTT
EMPV1_18097	AAAGGCAGGCTCGCAGTAACACCAGGGCCTGGGCCCCGAGCTTCCGAGAAGCACACGCTG
EMPV1_18098	CCTTGTGCTGCATCCCCTTATTGGCGACCCATTACTGTGACTTTGGGAATTTTATGCTTG
EMPV1_18099	AGGCAGTTCCATTGGTGCCAGTGACGGAGGAAAATGAGGATGCCATGGAAAGTGAACAGT
EMPV1_18100	GTCCGCAAATGGCTCTGAAGTTAGCTTTAACAGAATGGTTGCAGGAGTTTGGTGTTCCTC
EMPV1_18101	GAAGCTGAGACTTGTGGAGCCCCAGGCTTGTAGGCACTTCACTGCTTTTGACTACAGTCT
EMPV1_18102	AGCACTTGCTGGCCGTGCCTGGGACCCTGTGGATGGCAGGTTTTGTGCTCTCCTACAAGC
EMPV1_18103	GACCTGGACTGCTTCGTCATTGATGGCAATGGGTTTATCCTGATCTCGGAGAGGGCCCAG
EMPV1_18104	CCCATCTCTTGTTAACCCATTCATCTGTCGGTGGACGTTTAGGTGGTTTCCACGTCTTGG
EMPV1_18108	TGTAACGGGAGGAAGGATGACCTACAGCAACTGGAAGAGTAATGAGCCGAATGACCATGG
EMPV1_18109	CACTCCCCCTGAAAAGGGGAAGGGGGAGTGTGACATTTTCTTTCCATGTTCAAGTGAAAA
EMPV1_18110	TGCAGCAGCCAAACCACTTTCCATGTGCCCCCTACAGCATCCCTGGCTTTATACAACTAA
EMPV1_18111	TGCATGTTTCACACAATGCAGTCACTGCGGCATAAGGGCATAATACCCCCCAGGGCTAGG
EMPV1_18113	TTCTGCCCACTGTGAAGTCTCCTCCTGAAGCAGTGACAGTCCTGGCAGAGCATTGCTATC
EMPV1_18115	TGGACGACATCGACGCCATGCCAGACCCCGCGCTCTTCCTCTACGCCCGGAGACCCCCGC
EMPV1_18116	GACCACGCTGGCCTGCATAGACACTTACGTTATCAGCCTACTAATAGTTTTAGACAGTGG
EMPV1_18117	AGTATTGGGAACCATGACTGTTACATAAAAGCCGTCAGTAGTGGGAAGCGGAAAGAAGGG
EMPV1_18118	TGATTGGGCGGCAAGGGCCCCAGAACAAGCAGCCCTTCATGGTGGCCTTCTTCAAGGCCA
EMPV1_18120	TCTGTACCTTTGCCTACATCTCCAAGGACCTGCAGACCAGGCACCACTATTGCCATGTCT
EMPV1_18121	GGCTCACTGACATTTCCATATGGGGCTCTTGTAAACTCTAGGAGCTAAAGTCAGACTCAG
EMPV1_18122	GTGTTCCAGTGCCTTCTAACAGGCATTAATACCTGTTGGAAATGGATTCTCATTGGCTGG
EMPV1_18124	AACAACACGCACACACACACACACTTCCAGATTGCCACCCCATCTTTCTAACTCATACAT
EMPV1_18129	GAGCTGTATCATGAACTGTAATGCTAATGGTGAGGGTTCTGTGCTTTTAGGGGCGCTCTG
EMPV1_18130	ACAGGTTTTACCTGGCATTCTGCAGAAGCATTGCTGTATCTTACCGGACAGGAACACAGG
EMPV1_18131	AGTCTGCTGCCTCCTATTGTCAAGGACAGTAGTGGAAAGAGTCTGGGGCAAGGCACGTGG
EMPV1_18136	CCAGAGCTGCAGATAGGAGGTACAAGTTTCAGGATGAAGTACCTACAGCCATTGTGCTTG
EMPV1_18138	TGATGGCGTGGGCCAATTAGAGGAGTTAGTTTTCCTGATTATCCCCCATGTTTATGGGCG
EMPV1_18139	AGAGGCAGCCTTCAGGATGGACCCGCCGGCAGGTGGGAGAAACAGTCCTGCAGCCAGATT
EMPV1_18140	GGAACTGCTGATCATGCTTTGCTCTTTCCTCTACAGTTATGGCCCAAATATCCAGGCTAC

EMPV1_18141	CAAAGGGCAAGAAGCCCTTGACGTCACCGGGTGGAACACAGGAAAACCAGCGAGAGAAAA
EMPV1_18142	GTCTTTTTGCATGGGCTCTTTGGAAGCAAAGCCAACTTCAGCTCCATCGCCAAGGCCCTA
EMPV1_18144	GCTCCTAGTGACCTGGAAACGGGCGTACTTTCATTTACTGATAACAAATGTTGAGGCCTG
EMPV1_18145	TGACCTTGTGGTGGAAGTGGCCCATCCCAAAATAATCCACGAATCTGGGGCAGAGATCCT
EMPV1_18146	TGGGAGACCTCCTAGACGCTTATTTATCTCGCTTGGGTTTACAATGGGGCACGTAGAGGG
EMPV1_18151	TGATGCTACAAATTGGGGATGTGACAGATAAAATTGCCTTGGAGAGAAATAGATGGCGAG
EMPV1_18153	GGCTGCCATCGTGGAGGTTGTCGTCATCATGGGGAAATCTCACCAGCTCAGCTATCCAGA
EMPV1_18155	AGGCAAGTTCTAACCAAGTAGGGAAGAATTAGGCATCAGTCGTGCTGTCTAGTAGCGCTC
EMPV1_18158	TGTGGGGAAAAGTAGAATGACGAGTTAGTGATGATCTGTAATTTCATTCCTGGGAAATGG
EMPV1_18160	TGACTTAGGCTTTCTCATCCCCCAGGACCTCCACTATCCAACCATAGCCAGCCTGCATTT
EMPV1_18161	CCTAGCTGTACAATAGGATTTTACTGTCGAAATGTTCAGGACTTCAAACGAGCTTCTGAA
EMPV1_18162	CCAGCCTGCTGAAGCTCAGAGGGCTCTGATTCAGAAAGATCATCGGATCCGTCTGAGCTT
EMPV1_18163	TCCTACAGTATCCGAACAGTGCAATCTACCAGGAGAACGTCAGGAGAGACCTACCTTCAC
EMPV1_18164	CCCCAAGATGAAGCTCAATGAAACTTCCTTCTGAACACAGCTGCCATGGTGCCTTGGGAT
EMPV1_18165	TGGAGGCCACCTGGTTGACTTTCGCCTTGGAGTCGGTCCAGGAAGGCATTATTTACCCTT
EMPV1_18167	AAAGACCCTGGGGTGGTGCTGGGTGACAGACTAGCCGCTTTAATTCTACTGCTCAGGTAA
EMPV1_18169	CATCTTCTTGGCCATCGTCCTGTTCCCCTTTGGGTTCATCTGCTGTTTCGCCTTGAGGAA
EMPV1_18170	GCAGAAGGTGGGGATTTGTGGTCCCGTGGGAACTACTGGATCTTTTTCTTCTTAGGGAGA
EMPV1_18176	CAGATTACCGGCTCATGCAAGCAGGGGAATAGGTGAGCCAGGCAATTCTCAGGGAGACTT
EMPV1_18177	CGGAGGTGCAAAAGCAATTCAGTAGAAGAGGGATAGCCTTTCAACAAATGATGCTACAGC
EMPV1_18178	TAGCACCTGGGGATATGAGGAGCTTCTGCAACCTACACCACAGCTCACAGCGACTCAGGA
EMPV1_18180	GGGTAGAGACCTGGTTATTGAAAGCCTTTGTCATTGTCCCCAGAAGCTCCACTCAGGTTG
EMPV1_18183	AGGTGCCTGCTCCCTCTACAGGCTCGACTTTGCTAGGAGAGGTGTTGACAAAGTTTCTTT
EMPV1_18185	CACGGGATGAGGGCGACCAATCAGTTTTATCCAGCAGAGTCAGTAACAAAGCATTGCCTG
EMPV1_18186	CAATCCTCAGCGCCCCCATAAGACAGAGGGGGACCTGGGCCGCCTGTTTCCTCAGTCCTT
EMPV1_18188	TCAGTTCTGCTCAGGAAGGGGATAGGCGGGTAAAGGCTCCGTGGCCTCTTAGATTTATAA
EMPV1_18189	GTACAGTCCCTGTGGCTCGGAGTTGACCAAACGTAGTGAATGCAGTTAGCACCAAAGGCC
EMPV1_18190	GCTTTCTGGGCTTTTAAATAAAGCAACAGCAGAAATCTCTTCCGGGCTTGATGAAGAACC
EMPV1_18191	TCACATGAGTGTAACAACCTTGAACAGCTTGGGTAGCTGCCATGCCCTCCCAACCCTCCA
EMPV1_18192	TTCCTGGGCACCCCAGCTCTATAGGGTGGAAACCTGGGATGTGGGCAATACTCAGGAACT
EMPV1_18195	TGCAGCTCCAGTACGAGGGTGTGGCTGTCATGAAACTCTTCAATAAGGCCAAGGTCAATG
EMPV1_18196	TCCGTCATTAAGGGTATTAAGAACCAGCTGATAGAGCAGTTCCCAGGTATCGAGCCGTGG
EMPV1_18199	TTGACCAACACCAAGGAATGGAGCTGTGCTACCTACATGACCTTTGCTGATGAAAGCAAC
EMPV1_18200	TGGAGCAGATACCTGGTTTCCAGTGTTTGGCTAAAAACATGCCTGACCCAATCACAGCCC

EMPV1_18201   GGTACCATTACTACCACTAGCACCACTACCGCTATTACCATCACCACCATGACTAGTATT
EMPV1_18202   AGAAGGCTTTTCTAAGGATACCACCAAGAGGAGAAGCATAATAGGCAGATTTGTTCATAC
EMPV1_18203   TGGTGTGTTTCCAGGGTCCTGTTGGCTTTTACTGAGCTGCTGCCACCCAAAAATAGTGGT
EMPV1_18204   GTCATCCAGAAAGCCCTTTTAGGGAAACTGCAGTCCCAGTGTCATTACCATAGAACTTCA
EMPV1_18205   GAAAGCAGAAAAAGTCCCAGGCCTCCCTAACTTGGTCTGAAATAATCCCGGCGCAATCGA
EMPV1_18206   CCCCTTCATTCTACTCCTATAGAGGCCACTCACTTACCTTCTGCCTCCAGAAATCACTGC
EMPV1_18208   CGCCTGGGCCCGGACCCGGGGAGTGGCCAGGAGAACACGTTTCACTGTAGGAGAAACCCC
EMPV1_18209   CTATCAGTGTTTTTCCTGCCTTATGTGGAGGCATTGGCAACCTGCGTGTTAATCCTCAAG
EMPV1_18210   CTCTCTAGCTGTGAAAGTCCTAGATGGCCTCTTCTTCCAATGAAAGGCAGTTTTGTTTCC
EMPV1_18211   TTCTACTCCTCGCCGCTGCTACAGCAACTTTAGTGGAGGCACAGAAAGCTTGGGCAAGGA
EMPV1_18216   GGCGGATTCACCACTCTACGGAGGCCGGTCTCCATCATTTCCAAGTAATAAAAGAGCAGA
EMPV1_18218   CTACAGAAAAAACCCACACGACGCATCCCTGGTCAGCAGGATTGGGCAAGCCTATGTGAA
EMPV1_18219   TGGAGAGCAACAGTATGTTTATGTGAAAGATGGAGATGTGATTAAGACTGAGGGCGCCAC
EMPV1_18222   GGGCCCTAACCTGAGAAGACAGTTCTCTGATCAATCACCTTGCTACCCACTTAAGCCTTA
EMPV1_18224   AGAACTGGCTGATACTGCATATTCCAGATGCTCATATTTGGGTCAAAAACTGCCGGGATC
EMPV1_18226   TCTCCCACAGAACCCCTGCACATAGCCATGCTGCATGTAGAGAGCACAATCCATCTTGTT
EMPV1_18233   ATCACTAACAAACAGTAACATTTTACAAAGGCTTGCCCCTACTTCATTCTCAACTAAACA
EMPV1_18235   ACCTCAGAGAACCAAAGGCATTCCCCGAGTACGGAAGCTGCAGTGGGGATAGCAGCCAAT
EMPV1_18236   GAAGTGAATCCCTGGTGCGTGAACAATCTAACACAAGCAGACTTCTCGGATCTTGGAAAG
EMPV1_18240   AAGTGTCCCACCAGGTCCAGCTCAGTATCCTAACTATCCAGAGGGACAAGGTCAGCAGTA
EMPV1_18243   GGTTTGTGAAGCTTTAAACGAGTCTTTATGGGAGGTGCTGAGAAGTGTCCCAGATGCGGG
EMPV1_18245   GGGAGAGAGGGGGACAGTCACTCCCCTACATGATAACTGACGTTTAAGTCAACTTTTTTG
EMPV1_18247   TAAGGTTGTATATAAGGAAGTCTGTCCTATGAAAAGAGGGACAAATAAATACCTTCAGCA
EMPV1_18248   CGTTTTAAAGCAAGCCCTTTCCCTCATAAGGGTCAGAGCTTCGGGAGAGAGTCCCATCTG
EMPV1_18250   CAGTTTGACTTTCTCATCGATATTGTCCCAAGAGATGAGCTGAAACCTCCAAAGCGTCAG
EMPV1_18252   ATGATCTTGGTGACGGGGCTGCGGTGCACGGTGAAGGTCTGGAAGAGCTGAGGCCCCGAG
EMPV1_18253   AGCTCAGTAAGGAGACTCAGACCTATACCTTCACCATCAGCCAAAGTGCGTTGGTCACCA
EMPV1_18254   CTGGATTGCAGAGCTATGTCGTGCGTGTGGTGTCTACTAACTATAGCCAGTTCGCCATAG
EMPV1_18255   AGCAGGATCTCCTACAACCAGCTTGACCCTCGGCCATGAACGGGCAAATTTCAGTGTGTT
EMPV1_18256   CATGCTCGGTATCTATTAGGGGGCTTTTATTTTGGCCGTGCCGGAGGCATGTGGAAGTTC
EMPV1_18257   ACTGTTGGTCCCAAGTTGATGGTGCACAGGCTTTTCGTGGGAGCCGAGCTACAGTATACA
EMPV1_18258   CATCATGTTTCTGGATCTGGTGCACTCCCTGCTGCACCGCATCTACTACAACGACCACAT
EMPV1_18259   AAAGTCTGGGAAGTGCTGAGTATGATGGGGGTCTATGCCGGGCGGGAGGATTTCATCTAC
EMPV1_18260   CGTGGTGAGGCTGCACGCCGCGCCCTGCAGAACCTCAAGATCATGCCGGGCAGCAAGTGA

EMPV1_18263	CTCTTGACCTTCAGCGTGGATGACCGGCAGAGCTTTGAGAACCTCGGTAATTGGCAGAAA
EMPV1_18265	AGCCCGTCAGAGAGAACAAAAAGAACAAGAACTTACTCAGGCAACAAAGAAAAAAGAGAG
EMPV1_18266	TAGGTCAGCGTTGTCTTGAAGAACTCAAGCATTTTATTGCAGAATTTGACTCCTCAAGTC
EMPV1_18267	TCAAGGGTCAAAATTCAAAGACCGTCGGCTACAAATATCATGGCACAAGGCCAAGGTGCC
EMPV1_18268	TGATCCGAAGTCACTGTGGTTAAAGCCTGCCCTCCTTAGCCCCACCCTCATCCCCTCAGA
EMPV1_18269	GCCCACAGGCTGTAGCATCTCCCATCTAAATTACTGCAACAACACTCTTAACTGGTCTCC
EMPV1_18270	CGGATGATGCAATACAGATTCCTCAGTCACTGGGAAATTTTAAGATGAGAAATGAACTGG
EMPV1_18271	TTGCTTGTGTCTCTCCGCTCTGAGCAATTATGTGCAGTGCCAATATGGGAAAGCAGGATG
EMPV1_18274	ATGTTTGGTAGAATTCACTTGTGAAGCAATTGGTCCTGGACTTTTGTTTGCAGGGAGTGC
EMPV1_18275	TGGGACCAAGGCTCTGTGTGTGCCTTCAGGGAAGTGAGGGGTGTTGGAAAGGGTATTAAA
EMPV1_18278	GATAGAACTGTTTTCTGGAACCCTTCAGCTGGTGTCATTCTCTCATCTGCCAATGCGCTC
EMPV1_18279	TTTCTCGCCCTCTGGGGGCCACCAGCCTGTGTCGGACTGGGGTGAAGAGATGGAGCTGAA
EMPV1_18281	GAAACGTCAGAGACTGGAAGGGTTACAGGAGGCTGAGTTACGTGATCTAGTGAAAGAGGC
EMPV1_18282	AAGAGAGCAGCCAAGTTACCCAGAAACGTGGAAACCCGGCATGTGTTTCACTGCTGGTAT
EMPV1_18283	TAGGTCTGTCGGAGTCAGAGGAGAAGAAGGACCTGAAGCCGGACGACAGAACGAGCCTTG
EMPV1_18284	ACTTTCTTCAGGGACCACCACAGATGCAGAACACTGCCGCCTACGCTCACCTTTCCTTAA
EMPV1_18285	AGGGAACACAGACAGCACCGAGGCCTTGTCCCTCTCCTATCTTGTGGAACTAAGTGTTGT
EMPV1_18286	AAGGAAGAAACGTGGGGCATGCTACAACTTGGGTGGACCTCAGGGGAAATATGATGGCCT
EMPV1_18287	GGTCCTTCAGGAAAAAGAGGAATTCGAAGACTAATTAGGGGTCCAGCGGAAACCGAAGCC
EMPV1_18288	CGGCTGAAGGTATCCTTTCTGCTCTGAGGCCAACCAGACCCGCCTTCCTTTCTGTGCTTG
EMPV1_18290	TCCACCAGCTGAATTTGCACTTTACTGTTTGTATCAGGAGTTAATCCATGCTCGGTTTTC
EMPV1_18291	TGTGGCTCTAAGGAAGCCCTTGGTGATCCACTGCCGAGATGCTGATGAAGATCTGCTGAA
EMPV1_18294	TCTCACATCCCGCCCTGTGTTCACATCCTCTTATCCAACCTTTATTTGCTAGTCCCACCT
EMPV1_18295	AGGTCCAGAGATGCCACTTGAGGATACAGTTCTACAGAACCTGGAGCATCTGCTAGACAG
EMPV1_18297	ATTTTGATCTTCGTTCTCTGGCGGAAGCCAACAACCTGAGCTTCCCCTTGGAGCCTCTGA
EMPV1_18298	TTTGTCTCTTTCCCGGAGATACTGAAAATGGACCCTCACGCGGTGGAGATCAGCCGGGAG
EMPV1_18299	GTTAACAGTATCAAGCCCATACGAGCCAGAGCTGTCGGGAGCATTTCACATGGATTCACG
EMPV1_18300	GATACCTGTACACCTTGGTCATCACAGACAAAGAGAAGGCAGAGAAGCTGAAGCAGTCCC
EMPV1_18301	TGTCTATGGCTGCTTGTGCTCCACTGTCCTGAGCCTGTGTGCACATAAAGCATCTTGCAT
EMPV1_18307	CAGCCTACAGTGTGTGTCCTTGGGGAATGGCGGAAATTGCTCTGCGCCTCTATGACAATA
EMPV1_18308	TCCCACATTTCCTTCTGCTGTTTACTTCTGATTGCATTCCATTGTGGTCAGAGGACCTAC
EMPV1_18309	TTTTAAATTTGGATGAGATGGTTTGCTTTGTTTTCTTTCTGACACTTTTGAGTACTTTCC
EMPV1_18310	GCTCCATCTATCACGTTTTATACCTTTCCACCTTCTAGTCTTCCTGCCCACCCTCCAAGG
EMPV1_18312	TGGGAGAGTAAGCTGACCAAAGTGGAGATCACTGGCAGCAGTGGTTAAAAATACACAGTG

EMPV1_18314	TTCAAGTGGTAAAAGTGGGGGCCAATGGAGAGGTGGAGACACTAGAACAAGGGGAACTTC
EMPV1_18315	AGATGGCTCTGTGAAGAATGGCGGCTCCTCCCGGGTTAGAATCTTTTCTAGGTTTCCGGA
EMPV1_18316	AATCTGAGAGCAAGAAAAATAAGTGGGACTGACCATCAGCCAAAAACGAAGATACTTCAC
EMPV1_18317	CCAAGCTGAAGAAGATGAAGAGCCAGACGGGACAGGTGGGCGAGAAACAATCGCTCAAGT
EMPV1_18321	AGCATTTCTCATTCTGCCCACACAACATTTCTTTAGAGTCTGGAGCGAAACCAGCACTGC
EMPV1_18322	CCCGCTGGGCAGCGCCACTGTCCCCTTCCTGATTCTAAAATGTCTCAAGTGCAATGATGA
EMPV1_18325	GCTGAACTGCTACAGCTGGCAAATAGTGCTCATGGTTATGTTGGAGCAGACTTGAAAGCC
EMPV1_18326	GAATTGCACCTCTGCAGCAACCTGAGCTGTTGCAGTTGGATTCTTAACCCACTGTACCAC
EMPV1_18327	CCATTCTGACCGGCTGCACTTGCGTTGATGGCAGCTCTGTTTCTCTTAGCTCCACTGAAT
EMPV1_18328	TACAGTGACTTCTGGGAGGGAGAGGGAGCAGGAGCAGTTGGCCTTGGGTTTGCACAGAGT
EMPV1_18330	CTGAGGAAGAATTGGACTCCATGGCAAAGCATGAATCTAGAGAAGATAGAATTTTCCAGA
EMPV1_18331	TAAACATTTAGTGGCTTTGACTGTGACCACCCTTCTTGGCGTGGCTGTTGGGGGGTTTGT
EMPV1_18332	AATGCTTGGAGCTTTATGCAGAAGACACGAGACAGAGCCTATCAATGCTGGCGTGGGTCA
EMPV1_18333	CTGCAGTCTTGCCCTGTTGACTTACATAAGAGATTCCCCCTCTTCCAGTTGCAAAGGACA
EMPV1_18334	TGTTCACCTTCACCTCTTCACACCTCATGGCACACCAGGATGCCCGCACCTTCACATGCA
EMPV1_18337	GTGGATGCAACTCGAGAGACAAATCGCCTAGGAAGACTGATCAATCACAGTAAATGTGGG
EMPV1_18339	GCCTTCTCGACAGCCTTTTACTGTGAACAGTATGTCTGGATTTGGAATGAACAGGAATCA
EMPV1_18340	AGCCCCTGTGGACTCTAATCATCCTCTTTCCCAGACACTCAGGAGGAATTTGGGCAATTG
EMPV1_18341	ATTGAGGCCCAGGACCACGACTGCAATGAACAGGAGGCTCGAAGAGCTGGAGCAGCTGAA
EMPV1_18342	ATTCCCTAGCCATGCCTGCGACCTACACTACCGTTCATGGCAACACCTGTATCTTTAACC
EMPV1_18343	GGTTTATAGAACAGGCGTTCACACAGTTTCCTGGAGGCCAAGCTGACCATGAACAAGAGG
EMPV1_18344	TGGGTGTACTTATGATCTCAGACAGTGGGTTGCTGTCAATGAGCTGTTTTCTGCTCCTCC
EMPV1_18347	TGTGCACAGGCAGAGATCCACCCAGAGTTGAGAAGAACCTTTTCATCCCACTTGGTGTAC
EMPV1_18349	CCTAGGTGGGGTTAGATACCGTTACAAGAGGAAGAAAAGGAGCCCCATTGTGTGACAGTC
EMPV1_18352	TTTGCGCAGAAGAAAGTTGTCCAGTGATGTCGGCTGGCCCCAAATGTGAACATCGTTGGG
EMPV1_18353	CCGAGATTAGGCCCACTGCCTTCTGATGCTAGAATGAGAGAAAATCAGCTTGTCAGAAGG
EMPV1_18354	AGCCGCTTTGTGGCAAGAACTGTGGATCATAGCCCCCCAAATGTGTTTCCACCCCAGCAT
EMPV1_18356	GGGTGATTGCCCAGTAATTCTATCCTAACTTGGATGCTGCTTGTACCCATCAGCTGTGAA
EMPV1_18357	TTTCTGCAAATGCACTTTCGTTTACTGAGGGGGAGGGTCATGGCTGCATGCTCTGGCCTT
EMPV1_18358	AGGTCATCGAAGGAGCGGATTGAGTGGTCACTCAGCATCTCGTAGAGCTCCTCAGGAATG
EMPV1_18360	GACTGGTTTGCTTTAAGTCTTGCACTAAGCCTCGGGTAGAAAGTGCTGAGGAGTCAAGGT
EMPV1_18361	GCAGGTTCTCAAGTGTTGTTGGCTCATCACGATAGCAAAAGAGGAGATTCCTGCCCTGAT
EMPV1_18362	TATCAAAGTTCTTAAGGACCAGCCACCTAATAGCGTAGAAGGTCTTCTCAATGCTCTCAG
EMPV1_18363	AAAAAAACACAGCTCTGGGAGTTTCCATTGTGGCTCCGTGGATACGAACCCGGCTAGTAT

EMPV1_18364	TAAGCTACAGAAGACGGACAGAAGACCAGACACACGTGGCCAAGCACAGACACATCCCGG
EMPV1_18368	GGAAGAGAGAGCATTCCTGAGTGGAAAGGTAGCAACGCTGAAATATGGGCTCTGGGATGA
EMPV1_18370	TCCTGTACCCACCAGAATGGCTGGATTCCTTAATTCTGGCTGTTCTCAGTGTCAGACCAG
EMPV1_18373	GTACACGTACTTCTGTAATGGGGGGAAAATAATCGATAAACGACTGCAGGAGCTCGGGGC
EMPV1_18375	GTTGGGTGCTGGTCGCAATCCCCTATCTCTATTGCACATTCGTGTCTCTTCTAGTCACTG
EMPV1_18377	AAGGCAGGGAGACCCAACTGCAGAAGGGACATTGTTCCAATTTAGAACTGGACATGGGTG
EMPV1_18378	CTTCCCTGAGGGGTTGCTCTGTCCACCTCGGTGACTCTCCCAGGGGAGATAAGGGAGGAA
EMPV1_18379	GCAGGGAAGCACCACGTGACCAAGTAAAGCCAATCATATACCCCATGACCCTTTCCATGA
EMPV1_18381	CAAAGGCTGCTCGGAAGAGCGCACCGGCCACCGGTGGCGTGAAAAAACCCCATCGCTATA
EMPV1_18382	TGACTGTCCAGTGATCAGCGTGGCTCAAATCCCCTTTAGTCTGGATCTGTGAAATGTGCA
EMPV1_18383	GCTATATGGCTCCTTTGGGGCCTGGTGTTCAACAACTCCGTGCCTGTCCAGAATCCTAAA
EMPV1_18384	GGATGACATGCAGGTGGCTCTGGAATGTGAGCAGAGGGAGTTTATCCATGTGTCTGAAAA
EMPV1_18388	AAGCCCTTCGTCTGGCTTACTAGAAAACTGATTAGAGATCCTAACGTGGAGTTCATCGCC
EMPV1_18389	GTGAGTCTCCTATTGAAAATGAACAGGAGACTGATAAGTTCCCGGGAACACCCACAAATC
EMPV1_18391	GCCAGGAATTCGGGTGGATGAGGTCTCATTTCACAGCTCAGTGCATCTGGATGAGTTTGA
EMPV1_18394	CCCAGTTTTTCCGATTCTCGATTGGGTAGAGGTTGAATAAGGGTAAAGGTAGGCTGCTCC
EMPV1_18397	TCCGAAGGTTTGAGCATTCCGGGAGCCAGCATTCATGTAGTTTCCCATTAGCAGCACAAG
EMPV1_18398	CTGCATTCGAGTCCATGTTGACAGGAGGTAACATGGGAAAGCTGATAGTTTCCATCTCAA
EMPV1_18399	GCGGCCATCAACCGCCAGATCAACCTGGAGCTCTATGCCTCCTACGTCTACCTGTCCATG
EMPV1_18405	AGGAAGTTACTGTGCTGACTGGAGTAATGGATCCTGAGTACCGCAGGGAAACTAGACTGC
EMPV1_18407	GTTCTCATGAACAGAATGGTGGGACAGATGATGGCTGTTATGTCTTGGTTCGGCGCAGCA
EMPV1_18409	TGATTCCGACTAGAGCCTGCAGCGCCCACAGCTTATGGCTCTGGACACATGAGACCGAGT
EMPV1_18410	GCAGGAATAAGTTTGACGACTACATGAAGGAGCATTTCTGTGAGCAGCTGGCCAAGGTCA
EMPV1_18411	GAGATTCTGGGGGTCCCTTCGTCTGCAATCTGAATAACGTCTGGTTTCTGATCGGGCTGT
EMPV1_18412	TGGAGATGTGCCGGTATGACCCTCTACAAGACAACACTGGGGAAGTGGTGGCTGTGAAAA
EMPV1_18414	GTACTGGCAGTGATGGAACACGCAGAGTCCAAGATGTATGCAACCAGCCCTTACTCGGAC
EMPV1_18417	CACTGGGAAGTGAAGAAGTGGATAAGTTGACCACGGGGTTTCCAGGAATAGTCCTTCAAG
EMPV1_18418	TCCAAACCCATGTGGAGTCTTTCCGACCAGATTACTTCTCTCCTACCCTCGCCCCAATGA
EMPV1_18419	TGCCGATGCTGTCCAAGTGGAAACAACTCCACTGTAACTAGGTTGATCTATGCCCTTTTT
EMPV1_18420	ACCGTGAGTCCTGTGGCTGCTGTAGGAAATTACCCGTTAGGGGAAAGGATTAGGAATCCA
EMPV1_18421	GCTCTGTTCCGCAGTGGCCTTTCGGTATGTGCCTCCTGAGAAATTCGTCTCTTTTTGTAC
EMPV1_18422	GTCACCATTACAGCTGCTGGTGTGGACCATCGAGTAATCTTGGGCAATGAGCTGCCAAAA
EMPV1_18423	ATTCCCTCAGCCGGGACAGCATCCCCGACCCCTGCTCACCCTGTACCTTTACGGCAGGCG
EMPV1_18425	GGGTTACCCAGACCGTCCTGGATGGCTTCGATATATCCAAAGGACACCTTATAGTGATGG

EMPV1_18426	CTTCTACTGGTCCCCTTGCTCTTGAACCTGATGCAGTGGGAGCTGGTAAGGGTATTGAAT
EMPV1_18429	CGTCACTGGGAAGCAAGGTCACTGTAGGTGGGGATACATGAAGGCTTTTCTTATCGTGTG
EMPV1_18432	GGGCAAATGTGAATGTGAAAGATTTCGCAGGTTGGACACCACTGCATGAGGCTTGCAACG
EMPV1_18435	CAGAGAGTTTAGGTAACATGTCCAGGGTCGCCCAGCTAGAAGGTAGCGGAGCTGAGATAA
EMPV1_18436	ACCACCACCCGTGTCATGGAATTTTATCTGATTGTGTAGATCAGATTTCTTTATTCTGGG
EMPV1_18438	TACGAGATACACCTTAAACAGACTCGGGTAAGGAGTCCAACAAGAGAGGGAAGGCTGAGC
EMPV1_18439	AGTGTGCAGGATGCAGCTGGAAACTACAGTGTATAAAGTCAACATTGCTGTCTGTGACAG
EMPV1_18440	ACATATCAGGAGGAAGTGGAAAAGGCATCTGGGCTTGAGGATGAGCCAGTGTCTGGGCTT
EMPV1_18442	GAGAACTGGTTTGGTGAACTGCTGGGACTGCTGCTATGGGAGGGGGACAGGTATAAAATT
EMPV1_18443	GTCACGTCCCAGTTACTGGATGCTCAGCACCTTTCTGAACTCCACATCTCATCCTTGAAC
EMPV1_18445	ACCAGCTTGTGTTGTTTATCGTGGGCTTATCAGTCATCACCTTCCCCTTTGCCCTCATCC
EMPV1_18450	CCAGATCCAGATGGGAGAGCACTGGAATCATCTAGAGGTGGAGGTGGGGGTGGAGGAAAA
EMPV1_18451	AGGAAACCTGCGCCTCAGCTTGCAATAGAACTCTTGTGCATGAGAAGCTCTCTCTCCTCT
EMPV1_18452	GTGTGGTTCTATCCTACGTACACATCCTCAAGACCATTTTGCAATTCCCTTCTGCCCAGC
EMPV1_18454	GACAACGTGCTGAACATCATTAACCAGATCATGGACGTGTGCATCCCCCAGGACCGGGCC
EMPV1_18455	AAAGTTGATCGGTTTTCCGCACAGGTTGTACTAGAAAAGCGTAACCATGACAATGTAAAT
EMPV1_18457	CTTGTAGTTCCAGTTCGACCTCCGACTCTACAACACCTACCGCCGGCTCCTGGAAGATGG
EMPV1_18458	GGTACCCTTCTGTAGCTAACACCCCTGGCTGATGGTAAATCATGGGAGAGTGATTAATTT
EMPV1_18460	TCATCGGAGCCTATTTGTCATAGACTTTTGAGTTTATTGTTGGGACCACGTAATAGGACC
EMPV1_18461	GCCTAGGAGAGACAGCAGGCAAATGCTCGGATTAGCGCTTAGCTGTACATACATGTGTGA
EMPV1_18462	ACGGTTTGGGGTAGGAGTTGGGAAGGGAGTCCTTCTTGGTGTTTAGGAACGAGGCCAACT
EMPV1_18464	TTCTGTTCTCATCTGGAAATAGTGCTAATCCATGGTTCTGCCTCCACCTGAGCTCCTGCC
EMPV1_18468	AACTCTCTTCCATTCTGTGAAGGCTGAAGGAGGTGAGGAAGCTGCAGACGAAACAACTGC
EMPV1_18469	CTAGCCTCCTCCCAGGGTCATTTACCAGGAAGAGATAGTCCCAGTGCAGTTATTGAACAG
EMPV1_18470	CACGGTTAGAATAAGGGAAGAGGGTTCACGGGTCTCTGTTCGTGGTCAGCTTCAATCCCT
EMPV1_18471	TTCAACAGGAGGCCAGGGCGCATGAGCTCGAGGCCCATCAACTTCGCTGTGATCCTGAAG
EMPV1_18472	TCGCCCCAGAGATCATCACAGCCACCCAGTATAAAAAGTGTGACGAGTTCCAGACAGGCA
EMPV1_18473	GAAGTTCATCCTGTGGCGGCGTTTCCGGTGCGCCATCATCCTCTTCATCATTCTCTTCAT
EMPV1_18474	TGCTCAATGTGAAGCAATCGAGAATGCATGCCAGCAGGTTCTGTGGCTCTATGGAGAGGA
EMPV1_18479	GAATCCCTGAATATTAGGGACTTCTACTTTTAATATATTCTTTGGAATAGGTAAGAAGGG
EMPV1_18480	GAAGCACCTTGTCTGCCTCGCTCATTCAGATTTGATTCTCGTCATAGGAACAGGAGAGGG
EMPV1_18481	ACAGCAAACTTGCATGCTCGCAGTACCCAGACACTATAGCTCAAGTCCCGTGTGCACCCA
EMPV1_18484	CGAGAGACTCTGGGCAGGTGTGTCTCTGGCTAAACCCCTTCAGTCAACACTTACGGAGTT
EMPV1_18485	TCTACTTTTTAAGGAAGGATAACGAGGGAGGACTCATTTCTCTGCTGGCCCTGACTGCGG

EMPV1_18487	GACATGTTCCACTGGCAGATCATGGATGGGTACCTGACTGGGAACTTCCGGGAGTTCCTG
EMPV1_18488	AGATGAGTAGGTACGTATGTAGGTAGATAATTAAATAGCCTTTGGATGATTGAGGCATGC
EMPV1_18489	TGCAGTACCGCCCCGAGGAGTTCACCAAGGTCAAGGGCCTGCCTGGTGACAGCTTCTACA
EMPV1_18490	GCTGTAATACACATTGTCCCACAAAGCTGAGCTGTCACATTTAGCTAACGGATGGAAGCA
EMPV1_18492	TAACCTGCTGTGCCTGGCCGGGATCCAATCTGCTTCTGGTGCTTCAGAGATGCTGTTGAT
EMPV1_18493	CCTATGATGCCTATTCCACCTTCTACCTGAATTCCAATGGCCTCATTTGTCGCCATCACC
EMPV1_18494	TCCCCGAGTCACAAGATTCCACATTAACTGCGAAGATTACACAGACAAACTGCAAGATCC
EMPV1_18495	CCTCCCTGGGCTTCTGCTTCAGCCATGTGCAACAAAGGCAGAGACGCTTGATCTTGCTTT
EMPV1_18496	CGGGAAAAAAGAAGAGCCCAGCAACAGAAGGCTGAGGGACGAGGCGCCCAGTTGTGGGAT
EMPV1_18497	TCATTATGTCCCAGCGAGTTTGTGTGCAGCTGGTGGTAGGGCCTTATGCCATGGGTCTTA
EMPV1_18499	TGGCTCGTTGGACAGACTGTCGCTATTACCCTGCCAAGATTGAGGCGATTAACAAAGAAG
EMPV1_18500	AAAGGGCCAAGAGCCTGAAAAGGAGGGAGGAGAGGTAGAAGTGAGTTTGTAGGGCTTGGA
EMPV1_18503	GTTCTGGGGCACAGCAGACATCTGGGACAGGAAATCCACTTGTTCAATTTGCCTGATCTC
EMPV1_18506	TGTCGTTATGGACACCGGGGGAGTTTTCTGCCTGTGTGGTACATTAGACATCCTCATCAC
EMPV1_18508	AGGATATCTAGAAGACATGTTTAGAATGGGATTTGTTTCGATATGTAGCTGTGTGGTCCG
EMPV1_18509	TTAGAACAGCTTGCTCTTCAAGGCCAGCGAAGCAGAGCGGCTCCCGTTGAGACCGGCATC
EMPV1_18511	TCACCAAGGGGCTTGCTTTCTTCAGCACCATCGTGTGGGCCCCGCTGAACCCCTTCATCT
EMPV1_18512	TGGACCTTGGAGGTAAGGTGATAACGGGAGATGGAGGATAGCTCAGAATCCAAATAGAGG
EMPV1_18513	AAGGCTGCCCAGATCCATGAGAAGCTGCTGGATGACTCGCTGACAGAGCTCATCGAGGCA
EMPV1_18514	TCATGTTCACGAGCAACCAGTCAGTTTGCAGTATTTTCTCTTCCATTGCTGGATCATCCT
EMPV1_18515	CCTGGTACCTACACATCTGCTCTTACATTCTGCTTTACCCTTAGGGATTACCTTCTAAGG
EMPV1_18516	ATGCAGGGGCCTGTTGTGTGAAGCTTGTGCCCTGACTCTGTCTTAAGTGCATTCGTGCAC
EMPV1_18517	AAATTTGACACAAACTTGCGCAGTGATGACGCGCTGCTTAAGAACTACGGGCTGCTCTCC
EMPV1_18521	TCACAGCTGAAGGAAAACTGACAGCAACGAGAGAGGAGCTCAGATACATGAAATCCACGC
EMPV1_18525	GGCATCAGTAGTTTAGCAGAATATCCATTTATTGCAAGGTCTGATGTACAGTGACATGCC
EMPV1_18527	ATGATGCACTAGAAAGGCAACAAGCAAATGAACACGATGCCCAAGAGTCTGAACCAGCAG
EMPV1_18533	ATCGCAGAGACGTTTCCCGAGGATGCGGGGGTCTTCACCTGCTCTGCCAGCAATGACTAT
EMPV1_18534	CTCTGCGCTACCCTGTTCTCATGAATGCTAAGCTGAGTTCCTTGCTTGTGGCTGGAGCTT
EMPV1_18537	GGGCATAGATGTAGAGTCTCTGATCATTGAGCATATCAAGGTGAACAGAGCCCCCAGGAG
EMPV1_18538	ATCCTGATTCCTCACCGGCACAGAGAGAAGCACCTACTGTACCTGCTGGAACACCTTCAC
EMPV1_18539	CTTCAGCTCTGGGTCTTTGCTGTTGCTTGGACCCCTCATTTGGAGCAGGGTTAATGGGAA
EMPV1_18540	AGGTCATGTGACCTGTCTAGCAGGCATCATCACCCTGGCGTATCCACAGAAGAAGGTGAA
EMPV1_18542	CTACCAGGGCTGGAAGAACTCCGTGCGCCACAACCTCTCGCTCAACGAGTGCTTCATCAA
EMPV1_18543	CTGCTGTGAACTCCTCAAGCATCCATAGACAGATCCTTAACCGCCCAAACGTACTGCTTT

EMPV1_18544 CTCAGTGACAGCAACAGTAATAGCTAACTGCCAGTCTCATTCCTTCGTAGGGGGACCTAC
EMPV1_18545 GTGCCGGAGAGGCTCCTGGTGGTTTTGGACAAAGAGATGGTGCGATTCCTGAAGGGCATT
EMPV1_18546 TATAGGCATGCACCAGCTACCTTGGAATAGTTGGCTCCATAGTCTCTTTGGGGTCCTTGG
EMPV1_18547 TACGGCAAGGACGTGCCTCGGCTGGACTACTGGCTGGCCTATGAGACCATCATGAAGAAG
EMPV1_18548 AGCTAAGCCAGAAATATAAGACCTTCAGCGACGGTCTGCACAGGGGCCCAGAGTAGTCTG
EMPV1_18549 GCAACTTCCTGTAGTGAGCACTTTGTATGAATCGTGGACTTCCTGTTCTCAAGGCGCAGG
EMPV1_18550 GAAAAGGTGTTCCCTGAAGCAGTAGATCCTGGAGATGTGGTTGCACAGCTTGAATATGAT
EMPV1_18553 TCACTGGAGCGAAGGGTGGAGGACAAGGATGGCTACCGGATGAGGTTCGCCTGCTATTAT
EMPV1_18557 GTCTGCAAACCTGCCTGTTTTTACCAAGCTTTAATCAATGTTTCTACTGAGCCGGAAGCC
EMPV1_18561 CTGCATTGCTATAAACTACTCTTTCAGTGCCACTTTTTGCCGCATTCCATAGATTTTGGA
EMPV1_18565 TTCCTGTTGCCCTCACTTCCCCCACAACAGCCTTGTTAAGATACTGTTACCTCTACTGGG
EMPV1_18569 CATATACACCAGGGAACACGAAGCACTTGCCAAACAAAGAAAACCACAGCGTGAGCAGGG
EMPV1_18570 AGGGACGATGCCACAGCCCCTACACCGGATCTCACTGCATTCTTCTCGCTCATGGTCTCT
EMPV1_18571 TCATTGGCCCTGTGACCCATGAAATCTTCGAGAACAACGTCGTTCACTTGATGTGGCAGG
EMPV1_18576 AAGCAGGCGGAAAAGAACGTAGAGAAGAAGTTCGACAGGTTCACGGAGGTCCTCAAGACT
EMPV1_18578 CTCTGTGAATGAGGGACACCCTCCCAGCCATGTCTCCCTTTATCCTGAAGAAATGTCTTG
EMPV1_18580 ACCAGAAGTTTGGTTACGGTGGGAAGAAGAAAGGCTCCAAGTGGAATACTCGCGAGAGCT
EMPV1_18581 GGACACCTGGATCTATGAATACACAGTGTTCGTCAGTACTATCCTCTTCCTTTTGTTGCC
EMPV1_18582 CGCCTGAAGCTCCTCTGCTTGCAGTTTTTGCCATTATTTATCTGGATTTTTGTCCTTGGG
EMPV1_18583 GAGTCCAGAACCCCTCAGCAGCATCCCCTGAACAACCTGCCCAACCTGCAGCTAAGAATA
EMPV1_18584 CTCTTGCGGCGCAGTGTCCAGGGTTCGGCGCTGCTCGGTATGACGTTCTGGAGCTGAGCA
EMPV1_18587 CGTGTGCCTTCTTGTGGGAGACTGAGATCTTCAGTGTTTTGCGCTCTAGGTCCTGTTTTC
EMPV1_18589 TGTCGGGTTTATTTAACAACTTGGAGTCTCAGTGCCCAGATATGGAATGCATGAATGCTA
EMPV1_18591 GGCTCTTCTAGTCCTCCTTCCATTGCTCCAGTGGTGCAGACCTTAGAGTTCTCATACACT
EMPV1_18593 ACTGGCCATGTAGGATTAGAGACCACCCCACTCCAGTACGACCGACCTCATCTTCACTAT
EMPV1_18595 GGTATTTAGTCAACCAAGAGACATAAAACACACTCTTGGCTGGTCCTTTGCAAGTCCTGG
EMPV1_18596 TGTATAAAGAGAGTGGTGCTTGCCAGGGTGCAGTGGTTGTGAGTTCAGCGAGACACGACA
EMPV1_18597 GGTACAGTCTCGACGAAGAGGTGTCGACATTGCTGGAGGCTAAACTTCTTCACTTGCTTG
EMPV1_18598 CAGCATCAAGGAAGAGCGAGGCGATGAAGTGATGGTGGAGTTGGCGGAGAATGGCAAGAA
EMPV1_18602 GTACTCCAACGAGGACACACTCTCTGTGGCGCTGCCGTACTTCTGGGAGCACTTTGATAA
EMPV1_18606 GTGGATTGGCTTTCCCGCCTTCCTTCATTCTTTTCAGTCTCCAATTCCTGCTCCAGAGGA
EMPV1_18607 CATCTATTCCCACTTCACATGTGCTACAGACACAGAGAATATCCGCTTCGTGTTTGCTGC
EMPV1_18608 ACATGAGTGGCATGTCCTTCACCAGGTTCTACTCACTCTCCAAGTTCTACTCGGTCTGCT
EMPV1_18610 TTTCTTTGGGGGGGGGGGTCTATCTAGAACAGTGCCTGGCACATAATAGGCGCTCAATAA

EMPV1_18614	TGAACACACGGCCACGCTTCTCTTTCTCTTTCCTCCTTCAGCAGAACTCCGCACCTCCAG
EMPV1_18616	CACAGAGATACTCCTGAGAATAACCCAGATACTCCATTTGATTTCACACCAGAAAACTAC
EMPV1_18617	GCATGCTCCTGGAGTTCACTGATACATCCTATGAAGAGAAACGGTACATCTGCGGGGAAG
EMPV1_18618	ACAGTTAGGTTGGGGTGTGAGAAAACCGGGAGGGTTAGGAACCCCACCTCCCCAGACCAT
EMPV1_18619	ACCACCCACATCGTCGTGGTGTCCATGGTCTTTATTCCAAGCATTTACCTCTATGCCCGG
EMPV1_18620	TACGGAGCTGATGAACCCTATCACTGAACCCAGAGTCACTTAACTTGAACGCCTGTCCAC
EMPV1_18624	GTTCATCACAGTGCTGTTCATAATGTCAAAAAACTGCAAGTGATATAAATGAACACTGGG
EMPV1_18625	TCCCACCGTCGACCTGAACAGCGTGCTCAACGAGACCAGGAGCCAGTACGAGGCCCTCGT
EMPV1_18626	CTTGTCCTTAAGTGTGATAACATCTCTCCTACATCTGACCACCACATCCAGTGGGGATGC
EMPV1_18627	GCAAGTGCAGATCTCTGGGTATTTATTGAGTTCTGTGGGATTGGTGGAGGCAAGCACCAG
EMPV1_18628	AGACCGTCCACTGCAGGAAGCCATTCTTTTCCTCCTGGTAGCAGACGTCCGAGTAAGAGA
EMPV1_18629	GATGTCTAAATCCAGAGTAAAATCTCTCAGAAAGAGTGAAAAGGAAAGGATGAGAGAATA
EMPV1_18630	CTGATCTGGAGGCACAGGAAGCCCCCAGAGGAGGGGCGTTGAGTCAAAAGGAAAAGGTGA
EMPV1_18631	GCATTGATCATAGAACTTCTGGGGAAGGTGCCTCGCAAGCTCATTGTGGCAGGAAAATAT
EMPV1_18632	GCTGGTGAATTCTTAAATAGGCAAGGTACCCAGTCATAACACCGTCCAGACCACCCCTTC
EMPV1_18634	CAGATCCCACTGCAGAGAAGCCTGTAGGCGCTATGTCAAGGGCACCCCTATTCCTCTTTT
EMPV1_18635	ACTTCTGCACCATTTCAGACGCGTTAGCGTAGATGTGGGTTTATGACTGCCGAGTTTGCC
EMPV1_18636	TTCCACAGAGGGGGTGGCAAATGAGGAGCCCAAGAGGAGATCGGAGAGGTTGTCGGCTAA
EMPV1_18638	GCTCAGTGGCTTCTACAGCATGCAAAGACAGAACCACCTACAGGCAGACAACTTCTACCA
EMPV1_18639	CACAGGGAAACCCAGGGGAGCTTGCGGCATCTATGGTTAGGCAGGTGCATCGAGTCAAAA
EMPV1_18641	AATGGAACTCCGTCTCCGGGATTTCTCCCAAGGGAAGGCTCTACTGCTGGAAGAGCTAGA
EMPV1_18642	ATGGTATCCAGCCATCTCAGACCATGAGAGCAACCATAGGATGGCAGAGCAGCAAGACAG
EMPV1_18644	GAAGAGACCAAGCTCAAAGGCGTCATCTACTTCCAGGCCATCGAGGAAGTCTACTATGAC
EMPV1_18645	AAACAAGTCCGTACAAAAGTAGGGAGAACACTCTCTTGAACCGCCCCACTTGCTCCCTCT
EMPV1_18646	CTGGCCACCAGCCAGATTCCTGTTCAGCAAATGCACCTGTTTGACATCCACAACTATCCG
EMPV1_18648	CCTGGAGCAACCAGTACTGTGTACACAGGAATAGTGTGCTGACCTTCTTCAAGGATGCCA
EMPV1_18649	GCCTGTAAGTGTGCATGTTGAGTGGCCCTGAGGCTGTGGAACTAACTTGGAATTTGAGTG
EMPV1_18650	GGCAAACCTGGGTTTTCAGAGGTTCTGTGATGAAAAGCAAGGATCTACATGGGCCATCTG
EMPV1_18656	CAGTGGAAATGAATCCGGATAGTCTCCCTGAGGAAGCAGGTTCGATCTCTGGCCTTGTTC
EMPV1_18657	GCCGCGATCCCAAACAAAACCCAAGAATTGGCGTGTGACTCATCTGCTTGGATACCTCCA
EMPV1_18658	GAGGCGATCACCCAAGACCACCTTCTGATTTTGTGACAGGTATTTGCTCATCTCTCCAGG
EMPV1_18659	CCCAAAGTCCCTTATGACGAGGTCCCTGTTTCTAGCATTTTTTTGGCCAGGAGTTTGTGC
EMPV1_18660	AGGGCTGGTGGTTCTGACTGTGTTGTTGGTGGCAGTGTTCTCAGAATCCTTTCCCTCAAA
EMPV1_18661	TCTTGTGGTGAAATGAAGTGCCTTTCCTTGCCTAATCCCTCCCTGGTGTGTATCAACATG

EMPV1_18663 CCAAGGCTTGATTTGTGGTCTAAGATGTGCTCTATCCTGGAGGATGTTCCATATGAACTT
EMPV1_18666 CAAGCTGCGGTATTGTGCCTGTGCTTCGGGAAGGGGACTTAGGAGTGACTGAGAAATCTT
EMPV1_18667 CATCATGGAGAAAGTGGATCTTGTCATAGTTGGCGCTGAAGGAGTTGTTGAAAACGGAGG
EMPV1_18669 GGAGGAGGAAACATACCAAGCTCTCAGAGATGTAAGCACACTGCATGTTCAGGGAACTGG
EMPV1_18670 CCCTAGAGTTGGTTCTACTTCCAGGTAACTTAAGAGCAGAAGTGGAGTTGGTGGTGTTAC
EMPV1_18671 AGCTCCCCTGTAGGATTTTCACCTCCTGTATTCTGTCTCCACTCACCTGTGCAAACATAC
EMPV1_18673 CTATTCTGGATGCCTCTCGGTCCTCCATGGGCATGGACATATCTGCCATTGACTTGATAA
EMPV1_18674 GTCCCCTCCTCTGGACCATGGCTATCAAAAACTGACCCACTGACTAACACCCAAAGCTGT
EMPV1_18680 GGCCCTGTTCTAAACACTGGACACAGTATGAGGCTATCAAGGAACGTACAGTCTAGTGGG
EMPV1_18681 TCAGGAGTTCTTAGTGTCAGCTTCCATCTCCACTAGAAGTTTTCCACTCATCCCTGCCCC
EMPV1_18682 TACAGGATTGCATGTGCGTGAGCGTGTGTCTCTGTTGGGGGCGGCTGTGCCCTTGAATCT
EMPV1_18684 ATGTGGGTCAGCCTTCTGAACCTGCTCAGCTTGCTTTGTCATTTATTGTGGACTTCCTAG
EMPV1_18685 TAATTTTTCTTTCCTAGCTTGGCATCGCGGGCCAGTTCGCACGTTCTAATCGACTGAGGC
EMPV1_18686 ATTTCCGACCCGTAGTGCCTCCAGCAAATGGGGTTGAAGGAGTCAGAGTGGAACAGGATG
EMPV1_18687 TGAAATAAATGTAGGTCACTTGGACAATGGGTTGGCAGGGACCGGAGGCGCAGCGGTATT
EMPV1_18688 TACCAGGACCTAGCAACCTCTGACAGTGGGCGGGCCTCCAGCAAAAGCGGGTCATCCTCA
EMPV1_18689 GATGTCTTTGGGCCTAATATTCGAGGTGTAGACAAAGGCCTACAGTGGCACCACCAATGC
EMPV1_18690 CGGTCTTCTCAGATGTGGCTGAAGAAGCTCTGAGGCTAAAGTAGGAATACAGTCCCTTGC
EMPV1_18692 ATTATCTTGGGTTAATCTTCTTATAAGATGGGATTCTTTGGGGTCGCAGCAACACGTCCT
EMPV1_18694 CCTCTGAGAAGTTTAGAGTGTTGATGGAGAAGCTTTGTAAAGAGGGGCCAGCGGCCACCA
EMPV1_18695 TGTTCCATTTCTTGTCTTTTTCTCTTCTAAAGTCCTAGAGAATTCACCTGTTCCAAGACC
EMPV1_18696 GCAGCCCAAGAGCCTTCTGGCTCTGAGCCATCGGCTTTCGCGTTCTTGAACGTGTGACCT
EMPV1_18699 TAAGGCACCCCTTGGACTAAGGCCACTGGCTTAACAGGACCTGGAAGCAAATTTGAGCCT
EMPV1_18703 AGGAGGAAGTGGCAGCAGAGGACCCCTTGACATGCTAGTCACCTGAGTTTCCTAATCACA
EMPV1_18704 GTCCAGCAAATAAAACTATATACAAGATCCATGCAAGGAATCCAGTTACACACAAGACAC
EMPV1_18705 GGCCAAAGAACTGGTTAGCAATCCTCGAGTCCATCCAGACATTTTACAGGAAGGGGAACG
EMPV1_18706 ATGCCTAAAGTCAAAAGAAGCCGGAAAGCTCCCCCAGATGGCTGGGAGTTGATTGAGCCA
EMPV1_18707 GGCTGGAGTGCTCAGTTCATATGGTTTTTCTTGGTATCGCTCGTGGCTTTGGCAAGGAGA
EMPV1_18708 GAGCATTCAGATGGAGTTTGGTTAGCTCCAGGCTTTCGCCAGCAATGTAGGTCATAGAGG
EMPV1_18711 GCAACAGGATTACTTTTTGGTATGGGAGGTAGAAGGTATGGTCTGTAAGGTTCCAACTGC
EMPV1_18714 ACATGTGCCTCCTGATTGAAGGACTCGGCGGGATTGATAAGATTGAGGCTTTGCAACTGC
EMPV1_18715 CACCTCACTGGTAGTTTATTTTGGTGTTACTCTAACAAGTACATCCTCACTGTCATTTTG
EMPV1_18716 GCACGCCCGCTCAAGGAACAGTTGGTATTTCTGTTGTTTTTATTCATGGGAAGTAGCAGG
EMPV1_18717 ACATGTGGTTTCACTTTCCATCTTGGGTCAGGTTCAGCGCACAGAAGTGTGGACCCCCTC

EMPV1_18718 AGGAAAACTGGAGTTCCTGTCATGGATCAGGGGAAACCAATCTGACGAGCATCCATGAGG
EMPV1_18721 ACTCCCTGCAAAACGTGAACATCTCTGTCATCTCCAACACCGAGTGCCGTGCTGCCTATA
EMPV1_18722 GGGCATGGTTGGACATATCATTGGCATGGAAATCTGTTGTTTATTGCACACATGTGCTCC
EMPV1_18723 CGGGCCGTGTCTGCTGTGAAGAATATCAACCTGCCAGAGATGCCTCGGAACATCAACATT
EMPV1_18726 TCCCTGACCTTGCTCAGTGGGTTAGTGGGTTAAGGATCTGGTATTGTTGCAAGCTGTAGT
EMPV1_18727 TCTGTCTTGAAGGATGACCTCATCCGTTTGCCTCTCTGGCTTCAAGGACCGCCTGTGGAT
EMPV1_18729 AAGACAGCACCTCGGGTTAGCAGTTCCACAGACGCTAAAGAAGAGGGCGGGAGTAAAGCT
EMPV1_18730 CTCCGTGTACCACGAACGGCAACGCCTGGAACTCTGTGCTGTCCACGCCCTCAACAACGT
EMPV1_18731 TCATCTCTAAGATAGCCTTGGAGTTCAAAGAGAGAAGATCCCCCTGAGTTCCTGGAGCCT
EMPV1_18732 ATCAGGAAAGGGCTGGGTATGATGGTAAGAAACAACCCCCGAATCTCAGTAGCTTAAGAC
EMPV1_18733 CTCTGCCAACACACACCCCCTTAGAAACACACACCCTTCAGCTACATGAAACACCGGACT
EMPV1_18735 ACTAGTAGCTGCGCTGGGAATTCCCGTTGTGACTCAGCAGGTTAGGAACCCGAAGGGTAT
EMPV1_18736 GGCATTTTCTTGAGTCCCTGGAAGCCATCTCAATTGTTGTTTGAGTGGTTCATCTTCAGA
EMPV1_18737 TACTAAGCATCCTTCCGCGAGTTGAAGGCACCGCCTGGATGAACAGGCAAGAATCCTCAG
EMPV1_18739 CAGGAGGTTGGGTGTTTGGGGCGGAGGTATTGATGTTGTTCTGATTTGGGTTGATTCAGA
EMPV1_18740 AACCATCTCCATTGCCAAGGCAGGCATTCTCACAACACTTAATGCCCGCTGCTCTATCCT
EMPV1_18742 TTGGCAAAGCCAACGAGAAGCACTACTACTGGGGAGGCTCTGGGCCTGGAATTCAGAAAT
EMPV1_18743 AGGTGTCAGAAGTCAAGTTCAAAAAGAGCACCATGAAGATGGCGCACGCACTGTATGGCG
EMPV1_18745 AACGGAGTACTCATGATGATGCCTTTGAGAAAAATCAGAACATCATATAGGGTGACCTAG
EMPV1_18747 ACAGCGTTTCTAAGGGTGTTAGCCACAAGGCGACTTTCACATGAGCCCCTTGTCACGTAG
EMPV1_18749 CATGGATAACAGGGGCTTTCAGCAGGGAAGTTTCAATAGCTTCCAGAGCAGCTCCAGTGA
EMPV1_18750 GCTTGCAATTCTTAGGAATGGTGGTTTTCAACCCTGGCTGCACTTGAGAACCCTAGCAGC
EMPV1_18753 TCCAAGCCTGTGGATATCCCACACATGGACATTGAGGCACTCAAGAAACACAACGAGATT
EMPV1_18755 ATAGGCTGAAAGTCATGAAAGGCGATTTTCCGGGTAAGAAACAACCCGTTCTCAGGCCCC
EMPV1_18756 TCCAACCTATTTCGTAGCGACAGTTTAGGGTGACTGCCTCCCCCACTTGACTGAACACAT
EMPV1_18757 ATCATAAGTGCTGTAGCGCTTGACGTGGATGCGGCGGACGCGGTCCCGCAGTTCAAAGGT
EMPV1_18758 GCTTGACAGCTTAATTCCAGATGTGCTTGCTGAGGACAGAGCCTGGGAGCGTCTTGTGGA
EMPV1_18760 GTTCTCCCACCACTCATCTCCAAGATCGTCTGCCATCTTCACGCAGAACTCAACGTGGAA
EMPV1_18761 TCCTGCCATCTGTCCATTCTTCTTACTGAGGCCCCAAGCAACCAAGTGCCATCTGACTCT
EMPV1_18763 CCAATTTAAGCCGCTGCAATCTTGCCCATGCTAATCTCTGCTGTGCAAATCTTGAACGAG
EMPV1_18764 CAGGGTTTGACTCTTCCCAAGGTAAGTCCCAGGAGAATATGCAGGTAACCACAGACGAGC
EMPV1_18770 CGGTCAAAGTAACCTCCTTAACAGGAACAGTTGGGTGTGCAGGCTGTAATTTTCTTTCTC
EMPV1_18771 GCAGGGATGGAAGGGGAATAGGGAAGTTATGAATTACTCCTTCCTGTAGTAGCTCTGAAG
EMPV1_18775 CATCATTTTCCACGCTTCATCAGAACATCCACGGAACCTCTGCTGTGTACACTAGACATG

EMPV1_18776	CCATAAGAAATCTTGTCTACTGTTGACATCTCAACCTCCTAGGCTGGTGCAGGCTCTCAG
EMPV1_18777	GACATGCAGATTAAGCTAACAGGAAGAAACAGTCTGGTGCGGGCCTACCGTCTTGCTGAT
EMPV1_18778	GCCTAGTAGAATTATTTGATCCTTATGTTGCCACCTGAAGGCAGGAATCAGCTCTCAACC
EMPV1_18779	AGACTGCTACTAAAGGTCTGAGTTTTCAAGCTTTTGGTGCTGCTGGATTTCTTGTTCCTG
EMPV1_18780	AGGAAGGGAAACTGGTGGACGTAAGAAATTATGACCCCTTAAAGTCAGTGGGCATCGCGC
EMPV1_18781	CCACTGCTGATGAACCAGAGGGTCTGTGAATGCCTTGTGTCTGGATGCTGGTTTCTAGGA
EMPV1_18782	CAATAGAATCTGTGCAGTAATTCATCGATCACCCCTGCGTGACGCTATATGCTCGTCTGG
EMPV1_18784	CTTAGAGGTTTGTCTGGACTTGGGGCACACATTTGGTGTCGAAATCAGAGTCGTTGCGTC
EMPV1_18788	GAGTTTATCCCAAAGTGGACTGAAAGTTTGTGGAATCTGGTATATTGGAGGCTGTGCTGC
EMPV1_18789	TGCTCTTGGGTGCTGGGGAGTCAGGGAAGAGCACCATTGTCAAGCAGATGAAGATCATCC
EMPV1_18790	GGGATTATCAGAATGAACTTATAAAACTACTGTGGTTAGAAATTAGGAAAGCCACATTTT
EMPV1_18791	ATGGGGACAGGGTGTTCCCCGGCATCTCCCCAGCCCTGTTAACCTTTGATTCACCAAGGA
EMPV1_18794	GGAGTCTTTCCTGATTTAAAACACATGCACACACAGATCTTAATCTCAGGCACTACAAAA
EMPV1_18796	GGACGTCCGCCGGCGCTTAACACTAGTGATTATTCGAATTCCCGCCGCCGCCATGGCGGC
EMPV1_18800	TGCGTCTTCAGACCGCGAAGCCTTGTTTTAATGCTGAGGTCGCTCCTGACGTCTTATTTG
EMPV1_18804	GAATCTTCTTTTGGCCTTAGACAAGCCTCATTTACCCCCTGCCTCAGTTTCACAACCAAG
EMPV1_18805	GCTCCAGGGTGATGATTGCTGCACTTTAATGCCGTTTTCTTGAAACGTTGCGCAGCTCTG
EMPV1_18806	GACGAATAGGACCTTGTCGCTGGATGAGGTGTACCTCATTGACTCGGGTGCACAGTACAA
EMPV1_18807	ACCAAGGAAGAAGTACCCGGAGACGCAGACTGAAAGCCAAGAGATTGGATGGGACTCAGA
EMPV1_18809	TGTTCAGGTCTCCAACGACTACATGAAACAAAAGACATCAGCTGCCCTGTCCACCATGGG
EMPV1_18815	GCCCTTCAAGTTCTCCTTCAGTTTGATAAAGCTATAAATTCAGCATTGGCACAGAGGGTC
EMPV1_18817	CCTGTTCCTTGTTCTCTGAGTCATGCCTTCCCCAACTGTGTTTTTGCCAAGACTGTGCAC
EMPV1_18818	GACGGACCCGATTACCTATACCTAGAGGATGAACGTGTTGTTAGGCCTAGAGATTCACTC
EMPV1_18819	TGAATGGCGAGGAGTTTATGAAGTTCGACACCAAGCTGGGCACCTGGGATGGGGAGTGGC
EMPV1_18820	TCTACAACCTGGACGAGAGTGAAGGTGTCTGTGACCTCTTAGATGAGCCTCTTCTGAACC
EMPV1_18823	CAGGTTATTTACATAAGAAACACAAAATACCAATAACACGTTTTAAAAGCCCTCAGTCTC
EMPV1_18824	TGGAGGTGTGCATGTGTGAAAGGAGAAGGGGAGAGAGAAGGGTGCCGCAGAGGTGATAGT
EMPV1_18825	AGCAGGCTCACTTTTGGAAGACGACAAATCGGTAAGGCTTATTCATGGCTCAGTGCCAGA
EMPV1_18827	GGGAGTCGGTGTCAACCCCAAAAGAACTTCCTCCTCTGACAGTGTAATTGTTCGCCTAGT
EMPV1_18828	TGTATGCAGATCCTGATTGCCTGCCCCGAACACTGGACTTTGGCCTCAACGTGAAGCTTT
EMPV1_18829	CCTCCTGGGGGCCAGATGGGTGATTCCTTATACAAAGAGACAAAAGTAATTGGGCAGCTG
EMPV1_18831	CAGTCAGCGTCCAGGCCACAGGAACACAGTCATCTTATCCATACAAAAAAGATCTGCCCC
EMPV1_18832	TGGGCCTCTACTGTGCCGATTCCTATTTCCTCCTGTGTTTTAAGTGCAAACTAACAGAAG
EMPV1_18835	TCCTCTCCCTTAGGCCACAGCGAAGGTCACAATCAACATTCATTGTTGTCGGTGGGTTGT

EMPV1_18837 TAGAGCTTATACACATCAGGTTGTAACCAGATGGTATCGGGCTCCTGAGCTACTATTTGG
EMPV1_18838 GTGTAAACCTTCACTGATAGTCAGAAGCTGGGGAAAGGGAGCTGGATCTTGTCTATGCTG
EMPV1_18843 GGATCAGTGCTTTGATTGAAATACCTAAGAACTGTGTTGTGGCCGCAGTTGGCAAAGAAC
EMPV1_18846 ACTGCCACAAATGTTTTCATGGTTTTGGATTCTTCACGTCCTATAGATCTGGCCCACGAC
EMPV1_18849 CGTGGAGCGTGAACCCTATAAGGGAGAAGTCGTCACACTGAAATCCAGAGGGACAGAAGA
EMPV1_18850 GTTCCGTCCTGTCTGCGCTGCCACACGCACATCCTCACGTGTTCTTGAATGATGAGAAAA
EMPV1_18852 CCTCAAATGGGCTTTGGATTCCTTTGGAATGAAGCATGAAAGAAGAAAGCTGTTGTGGCC
EMPV1_18853 AGTTTCACTTGTTAAGACTTCTCCTACTGCCAAGACCTCCCTGCGACACACACCTTCCCA
EMPV1_18857 TGGACACCAATGGGTTATGTTGTTCGGCAGACATTATCTACAGAACTGTCACCAGCCCCT
EMPV1_18859 TCTTCCTCCAGCAAAGCCACCTGTGCCCCTGCTGCTGTCCTCGACGACATAAGAGGACCT
EMPV1_18860 GCAGGGAGAGAGGACTTCAGTGAGATGAGTAGTAGGTAGGAAGACGAATAACTTGGACCC
EMPV1_18861 TGAAGTGCTGTGGATTTCTTTGTGAATCACCATATCTAAGCTAATGTGGTGGTGGTTTAC
EMPV1_18863 GTGGTGGAGGTCTTTTGCATCTTGGCTTCCAGTGCTGGGTTACTGGGGTACATCTTTGTT
EMPV1_18864 CCGGGGGCCTTGGTCTGCACCTGGCTCTCAATCCGTTCGAGTATTACATGTTCTTCTTTG
EMPV1_18866 GCAGTGGTTTCTGACGTAGTAATCTTAGCAAGAAAGCAGAGGTACTTGTTACCAGAGAAC
EMPV1_18867 CATTTGCCCAACAGTTAACAAGTAAGGTGTCTTTCCCTGATACGCCCTGCACTGAGATGC
EMPV1_18868 CTTCCACATACTAAGACCTTGTCTCTGTAAAGAAGGACATGACTGCTGAAAAGATGTCTC
EMPV1_18871 TAGCTGCTGGACTACACCAGAGCCACAGCAACACAGGATCCGAGTCTCCTCTGCAATCCA
EMPV1_18873 ACCTGAACTGGACGCTGAGCACGGGCGCCTCTGCGAAGTCGGACAGGCACTCTATGTTGA
EMPV1_18874 CACGGAGGAGAAGACGCAATGGGAACATCCGAAAACTGGGAAAAGAAAGCGAATAGCAGG
EMPV1_18875 TTGCAGCTTGAAGTTTTCCTGAAGCCTGGCTGTGATCGGGCTTAAATTCGGACCAGTTCC
EMPV1_18877 GGAGATTCACTGTCCACTCTGCTCACCAAGAAGGAACAAGAAGACCAGGAGCAGGAGAAG
EMPV1_18878 TGTACCACCTCGGTGAGGGCGTGTACGGAAAAACTGCACTGGGATCTCTACACTGGGACA
EMPV1_18879 ACGAATAAGTCTGCTTTGAAACTTTATGAAAATCTTGGTTTTGTTCGAGATAAGAGGCTG
EMPV1_18884 TCAGCACACCCTGGAGATGTCCGTGAGCCAGATTGTCATGCACCCAGACTTTGAGAAGCT
EMPV1_18886 ACCTGCTGGCACTACGTGGGGGAGTTTGGAGAAGACCAGATCTATGAAGCCCACCGACAA
EMPV1_18887 GGCAGCATCTACAACCCTGAAGTGCTTGACATCACCAAGGAAACTCTGCATTCTCGCTTC
EMPV1_18888 ACGAACGTCGTGGAACAGACTTTAAGCCAACTCACCAAGCAGGGGAAACCTTTTAAGTAC
EMPV1_18889 ACCAACATGGTAGATCATTTGCTCCTCCTGAAAAATGGCCAGAAGAAATCACAGCTCCAC
EMPV1_18890 GAGCTCTGGAGAGCATGGTATTGTGGTGTTTTCTTTGGGGTCAATGATCAGTAACATGAC
EMPV1_18891 GAAAAAGGCAGAGAATGTCCGCCATCTACCCTCCGCTCCTGGGCGCGCTCTCATTCATAG
EMPV1_18892 GAGCCTTTTGGGGACTGGTCATAGGATGTATGATTGGGCTGGCCCGTATGATTACCGAGT
EMPV1_18894 AGGTCCTTTACAAAGAGTAGTTACTTCAGTCAATAGGTGAGGAGGCTCCACAAATGAACT
EMPV1_18898 ACTGGTTGGTTCTTTGACCAGGTAGAGATGCCAGTGGAAGAGGCAGTCGTTGCTGGTTCT

EMPV1_18901	GAGGACGGCCACAGGCCCAATTCTCACCGCACTCTGCCTGAGAGGTCTGGAGTGGTCTGC
EMPV1_18902	TCAGGGATCCATCTCTCAGTCCCAAGTCCTCTTGCAGTGCTTTCATCCTCAGACAGCTCT
EMPV1_18904	CAGTTGGATGGTTGATTCATTCGGAAGGTAAAAGTGGGGGGAGAATGGGGCAAGGTAGCT
EMPV1_18905	GATGAATCCCACTTGGTCGTTGTATATGATCTTTTGTATATGTTGTTGGATTCGGCTGGC
EMPV1_18906	CTCTGGTAGTTGTCATTATTTCTCCTAGGTGATAATGACTTATACCCATAGATGGAGCTG
EMPV1_18907	GGAGGGAAAGAGGTCCAGAGAGACAGTGAGATGTCCCACTGAAACCCGAATATCTGACAA
EMPV1_18908	GTGTTTTGTGGCTGTCCATTGGGGCAGAGGTCCTGAATATCCTTCTGACGTTGACAAGCA
EMPV1_18909	GGGCTTTAGGAGAAGCTGATGTGATTGAATGTCTCTCTCTGGAAAAACTGGTCAATGAGC
EMPV1_18910	GTCGGGGAGTTTGTAGCATTTTTCATTGGCTGGAACCTGATCCTGGAGTACCTGATTGGC
EMPV1_18913	AGGTATAACTGGGTCACTTTGTACAGCAGAAATCGACAGGACATTGTAAATCAACTATAG
EMPV1_18914	GCCCATGAAGCCAGTCCATTTCTGGAAAAGTGAAAATGCCTGGAAGTGCATCTGTCGTGC
EMPV1_18915	TAAGAAGAGTTCCAGGGTCAAGCGGTCACCTTCATAAAACCGAAGACGGTGACTGGGAGT
EMPV1_18919	ACAGGACGCCCCAGACTGCTGATCACAGCAGCTGTATCTGCTGGAAAAGGAACCATTGAT
EMPV1_18921	TTAAATATGACCACACCAAACAAGACACCTCCTGGCGCTGACCCTAAGCAGTTGGAGAGG
EMPV1_18922	CTCGATGGCACCGCAGCTTTCTGCACTTCATGGGGACACATCTGAGAGAAAGTAGCCAAA
EMPV1_18925	CACAGTGTGATCTGCTGGTCAAATTATGTTCTCCTACCTCAATCTCAAAGTGCTAATGTC
EMPV1_18926	ACTCTCCCACCCTCTCAGTCACCCTAAGACCTTTGGATTTTCTCTCTGGGGAAAGACTGC
EMPV1_18927	CAAAGCATAGCTTAAACTAAAGCACCTAGTTTACACCTAGAAGATCCCACAATGTATGGG
EMPV1_18929	TGGGAAAAGAGAAGCGTACCTAGACCTTCGCCGAAGCAGAACCTCAGCCGGAGAATGTTT
EMPV1_18930	AACAGATGTTGCCTTACATGAGGTTGTGGACCTGTTTAGAGCTCATGATGCGACCCTCGC
EMPV1_18931	GATGACTCAAAGGTATGGCCTCTGTTCTTGGTTAGGGATGAAGCAGCTGTGGGGTCAAGA
EMPV1_18936	GGGCTGATGATTCACACAAGCAGCGGCTACACACAGCACGTCAGAAGCCAAGACTGTTTT
EMPV1_18937	TCTGCATAAATTAGGTACAAAGCAAGAACCTATGGTGCGACCTGGAGACAGGGTGAGTGC
EMPV1_18938	TGAGGCACCACCATCCCAGACTCCTAATCCATCTACACATGTATCCAACCCCCCTCTGAT
EMPV1_18939	GACCGATGTGAGTAGGGGGCCACCTTCCCTGACCTGGCTCCGTCCCCAGGGTGGGATGTA
EMPV1_18945	GAAGCTGGTGTTTGAGGCTTTGGGAAAACAGCTGATGGAAGAATGACTGGCGATGCGGCA
EMPV1_18950	AACAGCAGGCAGAGTCCTACCCTCAAACATGTGATCAGTCCACCCAGAACTTACATCTGC
EMPV1_18951	GTGTCCCAGAATTGTCAAATCTCTGTTTTTATTTGCCAGGTACATGAAATCCCTTTTGCC
EMPV1_18954	TGAGTGATTCAGGTTCTAGGATTTCTCACCTCTGAGACCTCTGCAAGGCATCTCTAGGCC
EMPV1_18956	AAAAAGTATACCTCCAACGAAGACGCGGCCGCTCAGTCAGGGCTCCAACAAGCCGCTGCT
EMPV1_18958	AGAGCATAGCCGGGGTGTGCATGAGAAAGCCAGAGGAGTTTCAGTCTGTTAGGTCAGAAG
EMPV1_18959	GACATAGAGCTAAAGGTCCCAGCGTGCCATTTTCGCTAAGCCATTCCAGAAAGAAACATT
EMPV1_18960	TGCTGTGCAGATCGCGGCTAGAGAGAGGAGTGGTAGGAACGGGTCCCTGGAGTTGTGAAT
EMPV1_18963	GAAGAACCTGTGTCACCTCTGCATTCTAGACAGAACTCACTCCCCAGTCTCTAATAGCTG

EMPV1_18967	CCTCTCTGACGAGGTGCAGAGCTTAGCTGATTGGTGAACAGTGACTGGTTTCCGCTTTGT
EMPV1_18968	TTGCCATTAGAATTCGGAGCAGGTTCAGTGTCTCTGGCTTGGCCTTGGACCAGGAGGAAA
EMPV1_18970	GTAGGAGTATAGTTGACTTACAATGTTGTGTTAGTTTCATGTGTACAGCAAAGTGAATGT
EMPV1_18974	CAGTGACAATGCTGGATCATTAACCACTAGGCCACCAGGGCACACCCATCCCGATTTTCT
EMPV1_18976	AAGAACCATGAAACAAGACACATGAAGGAGCTGCCCACACCCTCCATCTGGCCTGTCTGT
EMPV1_18977	GCGGTCTGGGCTTGCTGTTCCTCTCTACAGTAGTCAGGAAGCCCTTACCCCAAAAAGTAT
EMPV1_18982	AAGGAACTAAAGAAGTCATCGTGCTAGACTCCACTTCACACCCGTCTCTGTGCGTGCAGA
EMPV1_18983	CTCAGTGGGTTAAGGACCTGATGTTTTCTCTGTGAGGATGCAGAATCGATCCCTAGCCTC
EMPV1_18984	TCTCCCTCTTCACCAAAGCACCAAGATACAGCCAGCAGTCCAAAGGGCCAAGAGAAATAT
EMPV1_18985	CTCTGGATACATTTCCTTCAGAAAATGAGAACTTTCTATGTGATCTCATGGACACAGCAG
EMPV1_18991	TTTCTGTGGGCACGTTCCTGTTCTTCTTGCACTGTTATTTGATCCCAGAATGGCACACTG
EMPV1_18993	CACGTAGACACAGAAGCTATGTCCTCTCTTAGTATTTCTTCCCTGATCTTGGGCTTGGGC
EMPV1_18994	GAACAGGTTGTTAATGATGTCACTCAGAATGTTCTTACTGCTGCATCCCCTGCAGCCGCC
EMPV1_19000	CATGTGGAAGTTCCCAGACTGGGGATTGAATCCGAGCCATAGCTGTGGTGAAGACCAGAT
EMPV1_19002	TCTTCCGAGGGCGCATGTCCATGAAGGAGGTGGACGAGCAGATGCTGGCCATCCAGAGCA
EMPV1_19003	GCGAAAAAGAAATGAAATCTTAGCAGAAGCTGGAGCTCCTTATACTTATAAAAATCACCT
EMPV1_19007	CGCTAGCAAACTCGGTGTGTCTGCATCGACACTAGGTACGCAGCAAGTAATCTGACAGCT
EMPV1_19008	CACATGACCCTCCTGGCTGACCTGAAGACCATGGTGGAAACCAAAAAAGTGACCAGCTCA
EMPV1_19011	GGTCACCAGGATGGTATCTAGTCATTTGCATTTTAGGTCCATAGGGCTCAAGAGAAAGGG
EMPV1_19012	TCCCTTCTTTCTTGAACTGTTTTAACAGATTGGTGTTTTCAGTTATACATGAAGGCCGAC
EMPV1_19013	CTGGAGGGTGGACTACACCGTGAGCTCTAGCCTGCTGTACTCCGTGGAAGAGCCCATGGT
EMPV1_19014	CTGGCCAAGCAAACAGCACCATTGGCTGGATTTCGTTGTCTAATTCTGATCAAGCCCTGA
EMPV1_19015	TAGTAAAATATTACATAACTTTGTCGAAGTTATATTATAGGTGAAAGCCCTATATGCCTC
EMPV1_19016	TTGACTCTGATGTGACCATTCACCTGGCCTTCCTGCCCCACCTCTCTATCTGATCTTCTG
EMPV1_19018	CGTTTGATTGGCTTGAACACTGTAAAGGGCTTGACCAACATCCCAAGAGTCCTGTCCACC
EMPV1_19020	AAGGACCAAAGAAGTCGTTCAGGTGACATGGTTCTCAGTGCCTAGGCACGTGCCCCTTTG
EMPV1_19021	CAACATCCAACACAGGCACTTCAGCAAACATCCCTACCGTTCTGACTTCAGGCTCTATCC
EMPV1_19025	AGCTTAGCCACTGGAAATAAGCACCAAGAGTTGAGCATAGTGACCTAGCTCTGAACTGAC
EMPV1_19027	AGATGCTGTCCTTTGTCATGGACGACCCTGACTTTGAGAGCGACTCAGATACTCAGCGGA
EMPV1_19029	AGGACCAGAGGATTGAGTTTTCTGAGTTTCTGTCCTTGCTGGGGGACATAGCCACGGGCT
EMPV1_19030	AAGGAGGGGAGCTGGTCAGAGAGAACCCTGGGCTGTGCTCAGCCCGCAAGCTGGGTCCCT
EMPV1_19034	TTATTACTGGTGTTTGGGGTCTGCCTCTTCACCCCATCTCTAGGCAAACAGGCTTGTGTC
EMPV1_19036	AGCCGCACCAAGGAGGAAGTCGTCCTGTTCCATGTGAATTAGATGATATTTACTGCGGAT
EMPV1_19037	TTCCTTCTGTCACCCACTGGCAGCTTCCTTGCATCTCATTTGTCCTCAAGCCATGTCCAG

EMPV1_19039	TGATGTAGCAGAATCTCCAGAGCAGAAAGGCATAGAAGGAAGCCCCAAAGAGGAGGAGAG
EMPV1_19040	GATACCAATATAGTTTCTCTCTTAAATACCATGTTCCCCAGACAGAGCACAGAGGCGGGC
EMPV1_19042	GTGCAGCTGAAGGAGCAGAAGTGAATCTACGTGTGGTCAACATTATCATAGCAGCACCAC
EMPV1_19044	TTCCTGGATAAGGAAGATTGGGGCCCCCAGCAGACCTCGAAGGAAATGAGCCATTTGCAT
EMPV1_19046	ACAACACTGTTTAGTCCTAAAACTGAATGGTGGGTGAGGGGTTAGGCTGGCCGGGCACCC
EMPV1_19047	ACACAGTCATTACATACAGTCATGAAGTAGTTGGTCCATGCGACTGAGACCCAGACAGTG
EMPV1_19048	CCTCATCAACAAGCGGTGGGCTCCATTTAACACCCACGCCGGAGGAACACAGCCCGGTCA
EMPV1_19049	CAGAGACTACTACCACTCACACGGGAATGTTCTGTCAAATAACCAGCTACACTCGATCCG
EMPV1_19056	CTTAGGGCTTTAGAAAGGATTGGGGTGTCCTGAGCTGATTTGTCTTGGAACATGGGTGGG
EMPV1_19057	TGCATCCTAGGCGCCCGAATAAAACCGATTTAGAAGAGCAGAGACCTGAGTGTCTTCCGT
EMPV1_19058	CGAAAACCATCAAGGACGACATGCTGTGTGCCGGCTTCGCGGAGGGCAAGAAGGACGCCT
EMPV1_19060	AGCCTCTAAAGCAGCCTCTAAACAGAGGGAGATGCTTATGGAGGATGTGGGCAGCGAAGA
EMPV1_19061	CTTTGGAAACAGCCACCGGCAGCTTTGTCTTATTGTGTGAGTGGGTCCTTCAATGGCAGA
EMPV1_19062	AGGCAGATCAGATTCCGATTTGACGGGCAGCCAATCAATGAAACAGACACACCTGCACAG
EMPV1_19065	TAAAAGGGGATTCTCAGTACACGCCTTGAGTTGTTGTGGTGATTGATGGGCGAGCGAGGG
EMPV1_19067	TGTATTATTCCCCTTTAGGTGAGAATTGGTAAGGGGTTGGTACAGTCAGGTCCGAAGCCC
EMPV1_19070	CTCTGTACCCAGGTGGATATGTTGTGGGATTTGGGGCTGTTTTGAGAGCAGTGGAAATGC
EMPV1_19072	CTTGGGGATCTGCGCCAAGGGTGTCATAGTATACGAAGTGAGAAACAACAGCAGAGTTGC
EMPV1_19073	GGGTGTACTACCAAGAATGCTGGAAGAGATTTTGAAGACTAGACTCATGGTGAAACAGTC
EMPV1_19075	CAGCAATGGATGAGAATTCCTGTTGCTCTGCATCCTGGCCACATTTGGTATCAACAGAGT
EMPV1_19076	GCTAGCAGCCTTTGTGGGGTTTAACTTGACGTGCACTGTGTCGGTCGTCATCTTTTCCTA
EMPV1_19080	CCAAGGGCTTTTCATCCGTGAACCGAAATCATCCCCACAACAGCTCCATGTTATTCATCC
EMPV1_19081	TTTAGGTACCCAGAGGTCAAGGCCCTCAAGTCCACAGCATTTTGAGACTCCACTCAACCC
EMPV1_19084	TATGAGCCAAAGCATGTGTGTGGGGCTTGTGGTAGCTTCTTGGACAGTGGGCTTTCTGCA
EMPV1_19085	AGAAGTCTGAGGAGTAGCGAGTTCTGGGGACCTTTTCACCCCCGGCATTAGCATCTTCAA
EMPV1_19086	ACCTTGCATCTAAAGAGCAGCAAAGTGGCTTTCGCCTGAATCTGCATCCTAGCCATTCTG
EMPV1_19087	GTGGGTGAAGCAGCCCTAAGGATCTTCCTGTAATTCCTCTGGAGTAATACTGTACCATAC
EMPV1_19088	CGTATTTATATTCACATGTGCCTGTTGTGGGAGCAGGGCCAGCAGATCACCATGATCAGG
EMPV1_19090	TGAGCGGTTCAGCTTTAGTCTGCAGGTCTTCACGAAAAGGGCAGCTTCAGTTCTCAATGA
EMPV1_19093	CCCCCTCCTTAAATGGAGCTATAAGGTGGCCAAATCTGAGAACCATTAAATCCACTCTAG
EMPV1_19095	TCTCAGCAGCGGCACAAAACTCACTTCGGGAAGCTGCATTCGGCAAACCTAAACTGTGCT
EMPV1_19097	TTGCTCTGGGATGACCCCTCCTAGGGAAGTGGGAAGACGTACCCAGGCCCTGCTGTGAAT
EMPV1_19098	CCTGGATGACCTGGTGAAGGTCTGGAAATGGCGTGATGAGAAGCTGGATCTACAGTGGAG
EMPV1_19099	GGATGGAAGCCTATTTTATTCCCCCAGGGTCTGTGTCTGTCCCCTTGTTTGCACAAATGC

EMPV1_19100	TCTGACAAGCCAGAAAGATGTTGTGGGCATGAACTGAGCCTCCCACTGCTACTGTCCAGT
EMPV1_19101	GCAGAAACTTGAATGATGGCTTGTGGCACTTACTTAGCATCAATGCCCGGCGAAACCGCA
EMPV1_19102	CCATTGAGTAAAGAGAAGTCAAAGATTCTTTGCAGCTTTCTGACTTCAGTGTCTGGTGGC
EMPV1_19103	GGAACTCTTGTGCTGCATTTTATGTCCTGTAACCCTTAGAATGGTCTGGGGAGCAGGTGG
EMPV1_19104	CCACCTGATAGGTGTGAGGTGCGATCGATCATATTTTGGTTTTAAGTTTTGTGCAGGCGG
EMPV1_19106	GCGAGTTCATGGTGACGTTTCCCTATGGCTACCACTCGGGCTTCAACCACGGCTTCAACT
EMPV1_19108	GCACATGTTGATGCTTTAGGGTGAGGCTGTAGTACAGAATACATAAACTTTTCCCCCAGG
EMPV1_19109	TGTAGGCCGGCTGCTGTGGCTCCGATTCAGCCCCGAGGCTGGGAACTTCCATTTGCTACA
EMPV1_19110	TACGAGAACACAGTGGTTTTTTCTCTGTCCAGCTTTCAGTACCTCATCCTGGCCGTGGCC
EMPV1_19112	TGTCTAGGAATGTCTCAAAAGTCATCTGGAGCCTTCAGCCGGATGGATTCTGGTAGAGGG
EMPV1_19113	CTCAATTTTCCCAGAGAAACAAGAGCCTAAGGGAAAGAACCCAGGGAAGGGTGGTGACCA
EMPV1_19115	TTAACAAAGAACAACAACCACCTCTCTGGAATCCCTCATACTACGGAGAGGGGGTGCAGC
EMPV1_19117	ATCAACCCCTTCCTCGGGTTGCTTCTGCGATCCCTTTTAGGCACAGCTGGAGAAGTCACT
EMPV1_19119	TACATGACTTTTGGCCTGCTGTGATGGCCTCCATAGTCCATGTGGCTCCCTCAGAACGAA
EMPV1_19120	CCCAGCCTGTCAGCCCTAATTGGGGAAGCGGTAGGTGCACGTCTCATTGCTCATGCTGGC
EMPV1_19121	AGGTCCCGAAAACCAAAGAAGAAGAACCCCAACAAAGAGGACAAGCGGCCCCGCACAGCT
EMPV1_19125	AGGTGAAAGCTGTTTAATGTTTTCCACTTGGGAACAACGCCTGGCCTCGAACGCTAGCTG
EMPV1_19127	GCTCTGGATTAGGCTTTGGCTTAAGGAAGTGTTGTGGCTGGCTTGATCTTCTATCCAGAC
EMPV1_19131	GTTGCTGTCTTCTCCCACGTGGTTAGTACATGTTGGTACCTAGAAGGACAGTGGCATTGG
EMPV1_19132	CAGACAGTTGGGAAGTGTTTCAGTCGCTCTTGTATGTAGTAGCTGACATTGTGTTGGTGC
EMPV1_19133	GGGGAATGAGCAGGGGGAAGGTTATTAGACTTCATCGGCGGAAGCAGGACAACATATCAG
EMPV1_19135	CCTGCGTACATACAATAATACGCACACATGGTTGCAAACATGGTATGTACCTTCATTTAC
EMPV1_19138	ATTTGAAGCTGACGTCCTGTGTCTGTACACTGCTGCCACTGTTGTGTCCTCGCTCTGCTT
EMPV1_19140	GTGAAGCAGCAGCCAGCCTTAAAATAGTCTTTTCTGGCTGAGGGTTAGAACAATGGGTAC
EMPV1_19142	TCAAGGCTATCTGCTGTGTGATATAACTCTATGGTCCTCTTACGGTACAGGGGTTCCAAC
EMPV1_19144	CTGTGCTCAAATGGCTGTCACTGTCTGGATCACTGGTTTTCTCTATGCCCTTCTGCACTC
EMPV1_19146	TCTTTCCAGTAGGCAGGGCCCATTACCAAACCTCCTTCAGTGGTGTCTAAAGCAAGTGGG
EMPV1_19147	GAGCCTCTTCATTTTTTATTAAGTGTTATGAGCCTGGCACTGTGTCTGCTTTTGGGTATC
EMPV1_19148	GCTGCCCTTATGTTATCTGCTCTGCCAGGTTCTGTGTTCTGTTTCTGTGAAGGCTTTTGG
EMPV1_19149	CTGCCACCTGCTTCAAATGAAGCCCAACCCTTTCAGCTATAACCCAGATCACACCCTTTG
EMPV1_19150	TCCGGCACAGATGAAGATGACTATAGTTGCAGTGTATTTACTCAAGAGGACACCTACGAG
EMPV1_19152	TTGTGCCATCTGCCAGACGTGCTCTTCTCTCGTAAGAGTGAGCATAGGAGTTCCCCTTGT
EMPV1_19153	TCTGCATGAGGTCTTGCTGGAACAAGCCAACGTTGTCAGGCAGTTGCGTGGCTAAGAAGA
EMPV1_19154	CCAGCACTATAGAGAGCCATTGATCCTGTTGCACCACAACAGGAGCTCCTGAGAGGCAAA

EMPV1_19158 CCTGCCCTTCTGCCCCCACCGGCAGATAGATGACTTTGTATGTCAAGTCCCTTCTCTAAT
EMPV1_19159 TTAAAGGATCCAGCCTTGTCACTGCGGCGGCTCAGGTTGTTGCCATAGCACAGGTTCAAT
EMPV1_19160 TACTCCCGCAGATCCAGAAGAAACACATCGGCTTCGGATGAAGACGAGCGCATGTCCGTG
EMPV1_19161 CACTAGATACAGAGCAGACAAACCAAGTGATTGAACAAGCATCTGGCACAGAGGAACCAG
EMPV1_19166 TGACTTGAGTTTCTCCTCAGGGAACTGCAGGCAAGGCCGGGGACCCTGGGAGAGATGTAA
EMPV1_19168 CATCACTTTCTCTGTGACTTCAGCCCTCTAGTGAAATTGTCCTGTTCACCCACATCTGTC
EMPV1_19169 AAGCCCTGGACTCCCTCCTTCTCCGTCTTCCGCCTCTCTCTTCTGATTAGTTCCTATCCA
EMPV1_19171 TAGTCAAACTAGTGCTTTTCCTGTCAGTTCCTGGGTTCTGCTGCCCTCTAGCAGCCATCA
EMPV1_19172 ATGGCAATGTGGGATGTGAGCTACATCTGTGACCTACATCTGGTGAGCTTGCAGCAACAC
EMPV1_19173 TTTTCCAGGAGGATCTGGGACCAACTTTCCCTCTCACCACCCCCAACACAGTTCCATCAT
EMPV1_19175 GATCAATATACTCTCTCCACCAACTCCCCACCCTTAATTCAGTACGTGCAAAGGCCCCTG
EMPV1_19176 CGAAGGGGTGTTTTCACCTAGGATGTGCTTGGGTTTGTCTGTCTCTGGCTTTAAGAGCAG
EMPV1_19177 GTGACTTCGTATCCCGATCTTTATTTCTAGCTTCTGGCAAATTGGCTGGGGCCACTGGAG
EMPV1_19178 CACCAGTGACTCAAGAGTACAGTCTGGATGGAGCAAAGCATTGCATAGAATTTTCACCCC
EMPV1_19179 TCCTCAGCTCTCTGAGATCCTGGGGAAACTCTGGGACTGGTCTCAGCCCAAGATTCCTCT
EMPV1_19180 GAGTGGAACCCAGACCTCCCCCACAGCACCACCAAGAAGGAGAAACAGCCCAAGAAGGCA
EMPV1_19181 TGGCAGTTCTGCTGAAATTCGTTTCAGAAGGAGACAATATCCCAGATGCGTTAGGTCTCG
EMPV1_19184 CACTACCATGAGTGGTCTCTTCACAGTTCTTCTACGGTCCAGAATGTCCTGACGCAGTCT
EMPV1_19186 ATCCTCATGACATCCTCCATCATGTCTGTAGTAGGCTCTTTGTACCTGGCCTACATTCTG
EMPV1_19189 GGGACTTAGACACAGTGAGTGGAAGAAGATCTGGTGACGATACTACAGTCAAGGAAGGCA
EMPV1_19190 CCCAGGAAGAAAAAGTTCATGTTGCTGTAACTAAAAAAGTGGAACCTCCAAGACCAAGAG
EMPV1_19192 CGCTCTGAAGACGTCGCTGGATCTCCAGTGAAAAAATCTGTAATTAATCTGTACTGTGGG
EMPV1_19195 GCGATTTTCTAAAGAGACAGAAAACAGGAAGATTTGCAACCGCAGAAGAAGTAGCCCTGC
EMPV1_19196 ATCATCCTGTCGCTCTTGTTGCTGAAGCAGCCGGTGGAGGACTTCTCGGTGTAGGTGTTA
EMPV1_19197 ACAAAGATCAGTGAACAAGGTCAGAGCCAGGGTTGAGGGAGGAGAAGGGCTGGGAACAAT
EMPV1_19200 AGTGGCCAACTTCTACTGCGATGCTGTCCCTTTGGTCAAGCTGTCCTGTTCTGACACCCA
EMPV1_19201 GCTAAGAACAGGGCTTTTTAAACTACACCTCCTCCAAGCACCACCAGGAAGAGCACTACC
EMPV1_19203 GTGTCCACATTGGGCTAAACCTGAAATAGAAGTAAAGGAAGAGCTTGGGCTTGTGGGCCC
EMPV1_19204 AGGTCGCCCACCCCTAAAAGAACCTGATACATGCTGGTGAGGTCTGCTTCTACAACGTGA
EMPV1_19205 GCTGACCAGAACAGCTCCAGGAATGGGTGCTACTCAATAATAGCGATAACTTGGCGTGGA
EMPV1_19207 CGGGGGTGATGGCAGTCAAATGTGCAGTAGTTATATAATTGTATATTTGTCAAAGCTTAC
EMPV1_19210 GCATCTATGGTTGATACAGACCACTCATCTTCTGGCTTCGTGTTTCCCCACTTTTAGCCC
EMPV1_19212 CGCCCCCTTAAACATCTCAGTCTCACAACCCTTATATTGTGCTACGCAGCTGACTCACTC
EMPV1_19213 TTAGAAGAGACCCCACATGTAAGCCAAGTCCAGCCACTGCCATGTTTATGGAACGGTCTC

EMPV1_19214	ATGCTCCAGTTCCTGCTTGGATTTAATCTCGGCAACGTGGTTGGAATGTATCTGGCTCAG
EMPV1_19215	CACTGGTCCTGGCTCCAAAATAACTCAGCAGAAAGGAGTTCCCTACTAAAAATCCCCTTG
EMPV1_19217	TAGACTTTTACGTCTCATTCCACAAGCCTCTAGCTGTAACCTTCCTCTCAACACCAATTC
EMPV1_19218	GGGGCTGTTGAATCTGTAAGGCCGGATGGAAGGAGGTTGAGCCATGTTTAACTTCATCTC
EMPV1_19219	CAGCTCACCTAGCTGTGTGCGTCAGAAGGTGCTCAAGTGCTTTTCCAGCTGGGTGCAGCT
EMPV1_19220	GGGGGAAAGAAAACAGAACCAGGACCAGTAGGTGGAAAAAGGAAGGAACACATGCACTAA
EMPV1_19221	ATGCATTACATGCCACCTCCGCACACAGACAGATCGCTGTAGAAGAGGCTTCGGTGTCTG
EMPV1_19224	CATGGACACAGTGCAGAATGGACAAAGCCCTAACTTAAGAAGCAGTGATGCTCACGGCCT
EMPV1_19225	TGAAGTCAGATGAGAGGCTCCCAGGCTTGTATTTCTGTGCATACAGGGCTCATCCAGTCA
EMPV1_19226	CTCCACTGCGTTCTCCTGTATTGCTTTTGCCGCTTATGCCATGGAAGTGGCCTGGAACAG
EMPV1_19228	AGTGAGCGAGAGGTTAGAAAGAGGGGTCTGGGGCTCAGGAGTGCCTCCAGATTAAAAAGT
EMPV1_19230	CTTCAAGGCCTGCTTTTTTGACAGTCTGAGGAATGGCTATGATACTTGGTATTTACATGG
EMPV1_19231	GATCGGGGGCTTCGTCCTGGGTCTGCTCTTTGTTGCTGTGGGGCTGTTCATCTACTTCAA
EMPV1_19232	CCATGCCCCAGTATAGCATTTACACACCTCTGGCCACCGTTTCTTCTGAATATCAGCTGC
EMPV1_19234	GAGGCCATTTGAGGATAGTCAGACAGCTTTGGTGTGATTTGCTGGTCTCCTAATATGTGC
EMPV1_19236	GGGGGTTGTAGGAGGGATAGAGTGGAAGGTTGGGGTTAGCAGATGGAAGCTATTACATAT
EMPV1_19237	GAACTGGTCAGCAATGGTGTTCAGATATACCAGTTCCCAACAGATGATGAAACTATTGCC
EMPV1_19242	GTACAAGATTGAACACAATCCACAGTCCCTGAGCTGGTAATATTATGAAAATCACCTATC
EMPV1_19245	TGCACAGCCGCCTGCACCAGCTCTTTGTCCCCACCGCCGCACTCAATTCCTTCCTGTGCA
EMPV1_19246	TACTAGATATGTTACTTCATAATCTTAGAACACTGCGTATGTCCTTAGAAGACAGCATAA
EMPV1_19249	AGTCATCATTCTCACTGTTAAGAAACAGTCTTCAGCGGGTTCATCCAAGGCACTGTCCAC
EMPV1_19251	AAACAGGAGCCCAGACCACTGTACCAGGGAGCCAGCTCTCTGGCAGTGAAACAGGTACCA
EMPV1_19254	TACCAACCATCCCAATTCCAACATTTACAACACTTTGTCTGGCCTGGTGGAGTTTGATGG
EMPV1_19255	TCCCATTGTGTATGAATTGGACAAGAACTTGAAGCCCATCAAGCCCATGCAGTTCCTGGG
EMPV1_19257	TGGCTGGTCAAACGGAACCAAGTCCGTCTTCCTGAGAGGTTTGGTCCCCTTCAACCAGCT
EMPV1_19258	ACTGCAGGACCTCGGTCATCTAGAGAGAACACCCAGAAGTCATTCCCTACAACCTCACCA
EMPV1_19259	AAGTACTGTGTGTGCCTTGTTGGTCACTGGCTCCTACTTCTGTGGACTCTCCCAGTCTTC
EMPV1_19260	GCAGAGAGAATGCAGCAAACGTGAAAGATTACATAAATACATCAAATGAAGCTGTGCCGC
EMPV1_19261	CCTTTGATATTTCTCTATACATGGCCATCTCCCTCCATACCCCTGGATAAGTTTCTGGCC
EMPV1_19262	CACTGGCGGTGTCTCTTACCATAACAGTAGTGTCCTATGTGTCTATCCTGTCTACTATCC
EMPV1_19263	TCACTACGGGTCGCTCGGCAAACATGACTACCTGCCCAGGACCGAGCAGTACTGGGTGAA
EMPV1_19265	AGTCCCTCATCCCCAACAATGACGTGAGGGGGCTCATTTCTCATGTTATCCGGACGTTGA
EMPV1_19266	ATTGGTCTTTGCCTTGATAGGACGTTAGAATTCAGGCACTGGCCCCTCTGCCCTAGAAGC
EMPV1_19268	GAGAGGATTGTTATGGGGCCACTAGATAACTCCTGACTTCCCTTTCTTTCATGTTATGCT

EMPV1_19270 ACGGAAGGTGCGGACCAACGCAATGAACAAGCTGAAGAAGTCAAAGGACAAAGCCTCGGA
EMPV1_19271 AGCCACCACTACCTAAATCCCTCAGCTTTAAGTGGCAGTAGTTGGATGTAGCTGCCTGCA
EMPV1_19272 CAGCTGACGGATGAGACATGAGCCAACCTGCCTGGAAACACATGTCTGAGGTGTGAGAAA
EMPV1_19273 GAATCTTGTACTGTATCTTACTATGTCTATAACAGATGTTAAGAATTGGAACAGTTTTAT
EMPV1_19275 TTTGCATTTGACCCCTGACCCTATAGCCCATTCCCATGGCCTCCTGTCTCTGTAAGGAGA
EMPV1_19276 GGGAAAGGGAAGAACTAAACCTGACTGCAAACCGTCTGATGGGACGAACACTGACCGTTG
EMPV1_19277 TCCCCTCTCTGGGCAGCCATGCGGAGATGATCAGAATGCCTCACCTTCAAAACTGTCAAA
EMPV1_19278 AGCTTGGGGATGCCGTCCTGCCTTGCCCATTTTCATGGGACATACTCGGCTGATCATTTC
EMPV1_19279 TCAAATAGGCTTAGATGTGCTGGTCATTCTTTGTCCCTTTCTGGCCTGCTCTGTGCCCAG
EMPV1_19281 TCACAGACTCCGTAATCCACTATACTAGAAGGCAGCTAGAACCCCAAGCCGCATTACAGG
EMPV1_19283 CAGGTACCTGCAATGATGTATCCCAGGTGAGGTCACTGGTGATCTGGTTTCATCCAGAGA
EMPV1_19288 TTCATATTGGTCTTGTGTCCACTGGGGTTTTTACTGAGCCGGGCTGGGTGGATGCGGATA
EMPV1_19289 CTCTTGCCCTTGTTTCTTCCTCCTGACGGCCAAGTTCTACTGGATCCTCACCATGATGCA
EMPV1_19290 ATGAGAGGAAAAGGAGTCAGAGGGATCAAGTGAGAGCAAAGTCAAGAGTGAGTCGGTGAG
EMPV1_19291 TGTGCCAAGACGCAGGCAGCAGAGAGGGAACAGGTGCTGCTAAGAGAGAAACAGAAAGAA
EMPV1_19293 AGCAGTTTTAATAAAATGTCAGTTGGCTGGTTACACAGAGACACACTTTCACATTTTGTT
EMPV1_19294 GGGAGTACTGATTTTTAAGAAATGCACCCCATGCCGTATGCTCAGTCCTAGCATCAGATA
EMPV1_19296 ACACCAGCACCTTCGTAGTAGAATATCAACCTTTTCCTGCGCCCCATCTTGAAACCTCTA
EMPV1_19298 GGATCAGAAGATATAAACCTAAGTATTAACAGCTCTTACGTCTGGGTGGTAGCATTAAGG
EMPV1_19300 GGCAGGGTGTAGGCACGAGGCACGGGAATTAGCAATCTACGGTCTTCTTACATAGCTTTC
EMPV1_19301 CAGTGCATTCAGAGCTTATGCCTGTAGCCCCGACTCCTGAATTCTATGGCAAAGACAAAA
EMPV1_19303 AGGGAGCGCTGAAGTTGGCATCTCCTTTACGTTTCCTTGGGACCTGGGAAAGCTTTCAGG
EMPV1_19305 ACCTGTTCATGCCGTCACCCCCGGGCCACATATTCATTCTCAAACTGCTGGACACCATTT
EMPV1_19307 CCTTTATGTGAATAGCTTAACCCGGGAACCTGACCGCCTCTTCAACATTGGCTCCATGTG
EMPV1_19308 CTACATAGGCCGAGGCCTTACCTTTTTTGCCCTTCTGTATCTCTAGTGACCAGCACACAG
EMPV1_19310 AATGCAGGAGGCGAGAAGGACAAACTCACGGCTACAACCTGTTTGAAACCTAATTCTGGC
EMPV1_19311 GAAGAGACCTTTCACTCTGTCATACCACCAACCTACCAATGCACCAGTATTTGGGTCCAC
EMPV1_19312 CCGCTGAGCCTGTGGACCATGCTCTGCTGCTCCCTGAATGAGCATCTCTAAGAGACTGTA
EMPV1_19313 TGGCGCTCCTGGGCGTGGGCAGCGTCTTCGTACTTTCCAGTTTCCTGGCGCTGGGCTTCA
EMPV1_19314 ATGACGTTCATGTCAATTCTGAGCAGCCCCAGGAGAGTCGTTGAGCTTTCTCCTATCGTG
EMPV1_19315 GGCAAGTACCAACCCCACAAAGTGACAAAGTACAAGAAGGGCAAGAACTCTCTGTATGCC
EMPV1_19317 AGCTGGAGCAATAAAAAGGAAGAAGCCAGCCTTCTTCCTCCCTATCATTCCATTAGGCTT
EMPV1_19321 GGCTTTTGCCAAGATGGCTTCAGCTCCTGCCAGTTACGGCTACACTACCAGTAAACCAAT
EMPV1_19326 TAGCAGTGTTTACTTGCATCTTTGTACCTGTTTTTAAGCTCTCCTAGCTCCTAGCTCTGC

EMPV1_19327 GGGGTGGCGATCCTATGTAGGAGTTCCTTACTCATACCTCCAGTTATCATTCGCCTCAAG
EMPV1_19328 TTCCGTCATCAAGTAGCGAAGCCCGAAGGAAGAGCAGCAAGACATCCTATTCTGGCCACA
EMPV1_19329 GTGCATTTCCTCAGTAGGCTGTTAATGCTTGGTGTCATATGGCATCTAATGTGAATGACC
EMPV1_19331 AAGAGCGTGCGTCGTTAGCTGCACACTCAGAAGAGAGCTTTGAGAATTTTGCAGACCCCT
EMPV1_19332 GGCAGAAAATTGCCTTTGCTACCACTGCGATTAAGGGTGTAGGACGGAGATATGCTCACG
EMPV1_19333 GTGTGTTGATATACATACATATGCACAGATACACACATATGCACATAAACCTCAACAAAT
EMPV1_19334 AGGTCCTCTTATGTCGTCGAGGACAGCAGCAGGGGCACAGGTGGCTTTGCTGGAGGAAGA
EMPV1_19335 GAAGGCAGGAAAACACAGGCAGCTCTTCTTTGGGCTGATTTCTATGGGACCTCACAACTC
EMPV1_19336 AGAAATGTAGGACTGAATGAAAGGGGAGACTAAGCACAGAGATAGGAGTTTGGAGGTACC
EMPV1_19338 ACGTGTGTGTGCTTGTCTGTGTGTGCCATGAGTGTCCATAATGACACACACACACACACA
EMPV1_19341 TCTTCAAGGGCATGAGGACAGGATGCTCTGCGCCTGGGCAGAGGCAGGTGGACAGTAGGA
EMPV1_19343 CGGGTAACTCGAGCCATAAATGCCAAAGACAAGGAGGTGGACAAATAAGGAAAATGTAAA
EMPV1_19345 TGATTGGAATGAATATCACCGAAGCATCTTCTCCCAGGCAGCTACGGTCCTGTGCAAGAC
EMPV1_19346 AAAACAGGGGCAGCGGGAATCGGGGCGAGAAGGAAAGGTGAGGGAGAGGGGACTTACTTT
EMPV1_19347 ACTGGCAGTGTGTCTCTGTGGTCTCTAGAATAAAGCACAATCTTCCCAGCCTGGCCCTTG
EMPV1_19352 CCAGTCTCACCTGGAGACTCTCCAAGACCAGCAGTTAGTCTGGCCCAGGTTCCTATAAAA
EMPV1_19356 TCTGTAGACTGTCCTTTAGGGTGGGTTCGTCTGCTGCTCTCCTGTGATCGATTGAGGTGT
EMPV1_19357 GATGGTAGTACATTACCTTACAGATACAGGCACATCACCGCGTGCTCTTGCTTAGATCCG
EMPV1_19358 TCACCAATCCGTGGTTGCAGTATTGGGACGCTTAGAGCTAGGAGGTTTTGGTAGAACTGC
EMPV1_19359 GCTGGTCAGTTTATGAGATAGATGGCACTACTGAGGAGACATCATTTAAAGCAAGAAATC
EMPV1_19362 CTTGCCCTTTTCTGTCAAACTCTTCTTTGTCTTGGCTACCTGGGCCTAGCAACCATAAAC
EMPV1_19366 AATGACGTGTTATTATCCTAAGGAACGATCATGTGCCGGGGGCAGCGGAAACAAAAGCTT
EMPV1_19367 GGTCGCCACCGCCATCTTGAGCCTGTTCAGGAAGCTCCACTGCACTCGGAACTACATCCA
EMPV1_19370 GCTGGGGCTCATCTTTATGAAAGGCAACAGGATCAAGCAGACGGACGTCTGGCACTTTCT
EMPV1_19371 TCCCTACTGGGAGCTGATGATGGGAACAGCAAAAACAAGGGTGAAAAAAGTGGGAGCCCC
EMPV1_19372 GGCCACATACCGATCCAGAGACATTACTGCTATGAGAGGGAAACCTCCTACTCCCAGAAA
EMPV1_19373 AACTCACATGTCCCACTAGCCTTTTAGTGATTGGTACCCTCTCCAGTCTCATCACAGGGC
EMPV1_19374 TCCTCTAAGAGAATAAGCACTTCCTGTTCTTCCAGCTCCAGGTTCACCTCCTGTTCGGAG
EMPV1_19375 GGGGTGGAAAGGAGTTGTAACTGGCTACTTGAACATGCAGTTTGATTGTTGTGTACCAGC
EMPV1_19377 TGCCAGCAGCAACTGTAGACCATAGCCAAAGAATTTGTGAAGTTTGGGCTTGCAACCTGG
EMPV1_19378 CATACGAGTAGCTGTATAAGAAGTCATGGTCCTGTTATGCGCAGCAGCCTTTTGGAAGCC
EMPV1_19379 CCACTGTCTGGATGCTATTCCCTGCAGCCCCAGAATGTCAGTTAACCTATTCCTTTGTCC
EMPV1_19380 GCATGCACAGACGGAGCCAAGTTTGAGTTTATTGTTGCTGCCAACAGTGGCTTCATGAGT
EMPV1_19381 CGTATAATTCACTCTGGGGGATGAGTGTGTTTTGCGTCTCATTAGGCGTCTGTCTGGAGG

EMPV1_19385	ATGATGAATGCCAGCGGGAAGAGGGCCCGAGCTCTGGCTTCACCGAAAGCTTTTTCTTCT
EMPV1_19386	GAAAGGATAGTAACGGTAGGGATCAGGTGAATGAGGTGATGAGCACTTGAGGCTTGCTGC
EMPV1_19388	CGCCAGGGTTACCAAGGTTCTGGGCAGGACAGGTTCGCAGGGACAGTGCACGCAGGTGCG
EMPV1_19389	GGCAAGCTTTATGTCCTGACCCAGCTAAAGCTGCCAGTTGAAGAACTGTTGCCCTCTGCC
EMPV1_19393	ACCTGCCCTTTGTCACCCACACCTTGTCGTCGGAGTTCACCATCCTGCGGGTCGTCAATG
EMPV1_19394	TTGTCTGCACCCCCAAAAGAAACCTAGACTCAGCCCTGCAAGAACCCCCTGGAAGAACTT
EMPV1_19396	GGTGAGGGCCAGAGCTGTCTTTCTAATGATACTGCCCTTTAAGAATCCTGTTGGGTGTGC
EMPV1_19398	GTAGATCAAGGTTGCTCTTCTGTGATATACGGGATATTATGTTTTAAAGACCATCTTGGA
EMPV1_19399	CATTTAACATGTCCTGCTGTAAAGCTCACACACTTAGAGAAAGGTCAAGGCCGGACTGAC
EMPV1_19400	GCAGCCTATGGCAAAGGCATCTACCTGAGCCCCATCTCCAGTATTTCCTTTGGATACTCA
EMPV1_19401	ATGAGAAATATCTTCAAGAGGAACCAGGAGCCCATTGTGGCTCCTGCCACCACCACCATG
EMPV1_19403	TGTAAGTGATCGTGGTGAAGGCCCTGCCACTAACTACACATCTCTGCTCATGGCCACAGT
EMPV1_19406	TATGCTCCGCTGCGGACAGATGATGCTGGCTCAAGCCCTTATCTGCAGACACTTGGGAAG
EMPV1_19408	CACACACACACACGAACACTCATACGCACACTCACTTTCTGATTAGCGAGTCTTCAGGGT
EMPV1_19413	GGACAGCCGTGCAAAAAGAGCTGCTAGAGTCAACTATCGTGAGATTGCGAGATGAACTGG
EMPV1_19414	TTAACCTCAGAACTGCATCCTTGGTGGCCTCTGGGTTGGCTCTGTCATCCTGCTCTACTT
EMPV1_19415	ATCTGGCATTGCCGTGAGCTGTGGTATAGGTCACAGAAGCAGCTCGGATCTGGTGTGTCT
EMPV1_19417	CACACTGATGCCTATACCCTGAGTGGGGCTGATCCTGAAGGGAGTTTTCCAGTGATCCTG
EMPV1_19419	TTCAGTCTGTCTGGCCTTTTGCCTTGGGAACCTGTAAGGAAGCTCTGGAACAAAGCCCCT
EMPV1_19420	CAGTGGAACAGCATAAAGAGCCCAGAAATAGACCCACATAACCTACACTCAGTCTTCTAC
EMPV1_19422	AGGTTACTGTGGATACCTCTTGAGCAGTGGTGATGGCAGAATTGCCTTTTAGTAGTGAGG
EMPV1_19423	CAGAGAGAGAGCTGTTATTGACGATTCCTGGGACAGGGCCCTGTATAGTGAGAGGCCTCC
EMPV1_19424	TTCCAGAAAAGTGATCTCCCCGGTGCTGCCGTCGCTCTTGCCCGCCGCCATCTTCCTCCT
EMPV1_19425	TCTACGGCACAAGCCTTGGGGTCTACCTCAGTTCGGCAGCAACTTCATCCTCTAGGACAA
EMPV1_19427	CAGGATACATGCTGCGACTGTATGGTGTAGCTCTCAGCAAAGCCGGATCCTTAACCCATT
EMPV1_19428	TGTCTGGGAGCAATGCCATATACAGTAGGCTTTACCAGTTCTCTGATGTCCATCTGTGTG
EMPV1_19433	AGCCCATCAGAAAGTGGCCATAATGAGGAACATTGAGAAGATGCTTGGAGAGGCCTTGGG
EMPV1_19434	GTAACGTTGTCTATATATACCCTGTAGAACCGAATTTGTGTGGTATCCACATAGTCACAG
EMPV1_19438	TTCATATGGGAGCTTCCACAGTCCTCAGGAGCCCAGGCATTTGCTGCACATGGAACCCTA
EMPV1_19439	TTTCCGGAGAAGCGCTATTCATTTGTCAGATTTTCCACCCATGGAAGTGCAGCCCATGCC
EMPV1_19440	ATTCTAAGACCGATGAGTCCGGGGCCTGTGGTTTAACTGCTTCAGGATACATACGGTGTC
EMPV1_19441	CTGAGGATTGAACAAAGAGGATTCTGCCCTTCTATGTCCTGGAACTACAGGAGTACAGAG
EMPV1_19444	CTCTTAGTGCCTGTAAGTAACATTTGTGTCAGGGAATGAACTTGGACTTTCTGGCCCATG
EMPV1_19446	GTTCTCTGTCGCTGCTGGAGACTAACAACTGGAAATACTAAGGTTGGAGCACTTGGCTGC

EMPV1_19447	TCTGGCCCCGTGTCACCTCTCTCCAGGTGTCTGATGCCTTTCAGTCCTGTCTTTATCTCT
EMPV1_19449	AGGCCTGCCAGGTGCCCGACCCGCTCTTCAAATTCTTCTTCTGGATCGGCTACTGCAACA
EMPV1_19450	GTTGGTGGCTGTTTTCAGGAAGATGTTTTTCTTCCCTCACTTGCCCTGTGACCAGAGTGG
EMPV1_19452	AAGGCAGGAGGTAGAGGCGGCCATCAGAAACCTGCGGCTCTGCTTGGACTCTGGCCCCCT
EMPV1_19455	GAAAGCACAGCCAGCCCTGAAACAACCAGACACAGAAGCACCACTCAAAGAACCACCACT
EMPV1_19456	CCAAAGGCCCCACCTCCCAATAATACTGCCTTAGGGGTTAGGATTTCAGCATATGACATT
EMPV1_19457	GGGTAAAGGACTTACTGAACTGTGCGTTTTCTGAGTCTGGGACTAAGAAAGTGAAATAGG
EMPV1_19458	CACTCATTCAGAAATCTGTTACTGTGCACCTATGCCAGGCACTGTGTGAGATACTGGGCA
EMPV1_19460	CTGGGAGCTGTGTGATGCCAGGCAATTGACTTCACCTCTCTAGGTCTCTTTCTTGTCTAC
EMPV1_19463	CCTGGACTCACCGATATTACTATACCTTGTCACCTGGGGCCTAAAAGAGTTAGCAGAATC
EMPV1_19464	GGTCCACGAGATCCAGTCTCACATGGGACGCCTGGAGACGGCAGACAAGCAGTCTGTGCA
EMPV1_19467	GACTGTTTATGTACTTTCCTATCCAGGTCCGTCTCGGGCTCAGGGCAAGTTTTGCAGGAA
EMPV1_19468	ATCTTGCACCAAATGCAGATTCTGGTTCGGGAGGTCTGGGTGGGGCCTGAGATGCTGCAT
EMPV1_19469	ATTTGGACACACAAGGAGACACCAGGGACACGTGCACAAAGAAAGGGCCACACGAGGACA
EMPV1_19471	CCAGGCACATGGTGAGGAGCAGGGTAGGAGGGATGAGGGCTATTGACAGAATTTTCACAA
EMPV1_19473	GGCCGCGCTTCCCCTCCCGGCAAAGGGTGACGGCGGCCCCTACCGGCCGCGCGCAGCATC
EMPV1_19474	TGGTGGTTCACAGCTCTTGGCCCGGTTTCCTTCGTGTCCGATCCAACTCAGGAGCGGGGT
EMPV1_19475	TCCCGATGGGACATCTGGGGGACCCTGAGGATGTGGCCGATGTGGTCGCGTTCTTGGCAT
EMPV1_19476	CAATCATATTGCACCTTGGAGAATGGATGGAAGAAGACAGGGAGGGGGGCAGTTCTAGTG
EMPV1_19477	GGACTCACAAAACTCAGGGAAGCTGTTACACTCACGGTTATGGTCTACTACACTGAAAGC
EMPV1_19478	GAGCAATGATGTTCCAAGTTCTCCATACCTTACCCTCCATGCTTAACCTGCCTCAGCTGC
EMPV1_19479	CCAGGAGTAAGGACATGGGGACCAATTTAAAAGAGTGGTTAAGGAAGGCCTCTTTGGAGA
EMPV1_19480	ACCAAAAAGAATTCTTGGGAGTTCCCACTGTGGCACCACAGGTTGAGCAACTGATGTTGC
EMPV1_19481	CCCAAGATCCACCTGACTTGAGTCTCAGGACTTGATCGTAGTCTTTGTGACAATCCCGAG
EMPV1_19482	CTAAGTTATCTGTGAAGGACAGGTTGGGTTTTGTATCAAAGCCATCTGTTTCAGCAACTG
EMPV1_19483	CACACCTGCTTACTTCTAAAACAGGGTGAGAGAGCAGACTAATTTACACAGGTGGCTGCC
EMPV1_19484	ACTGTGATGCAATGGGATCAGCAATGTCTCTGCACACCAGGACAGGGTTCGATCCTGGGT
EMPV1_19485	CAGAGGTAGCGAGTGGAGGTCGTGGGCCAACGATCGAATCCTCAAGTATAACCTGATCAA
EMPV1_19486	ATTCCTTGTGGAATGCAGGTGTGCGTGGCTGATTGTTGGCAGTCGTGCTCTGACTTGACA
EMPV1_19487	TGTGTGACCATCCACGAAGCTCTGAAGACCGTGATTGACTCCCAGACGCATTACCGGCTC
EMPV1_19488	TTTGTCTCCTTCGGGCGACCCTTACAGCCGTCTCAAGAACTTTCACTCCTCTGCGCACAA
EMPV1_19493	CAGGTGTAACGTGGAGAAGGTGCCCAGTAATTCTCAGTTGGAAATAGAAGGAAACAGTTC
EMPV1_19497	CCCAGCAAGGAGATCTAGGCTGAAGGCAGAGAAGATGACTGTGACCATTTACCGAGCTTT
EMPV1_19502	TGTTCTATGATGCCTTCGTGGTCTTTGACAAAGCTCAGAGTGCTGTGGCCGACTGGGTGT

EMPV1_19503	TTTCGTTAGTCACATGGCCAAAGGATCTGGAGCCTCTCTCGAAAGCCCCTTGCACTCACT
EMPV1_19505	CGTTAAGAGAGTTCTCCCACGCTTGCCAAAACCATTCTGCGCTTCTATGCTTCCAGAGTG
EMPV1_19506	ACTAACAACCAGAGAGAATAATAACGCATCCCGCAAGTCCCGCTTGTCAGGCACCACCTG
EMPV1_19512	CTTTGCCAGCTTCACCAACGGCACCGTCAGGATCTGGGACCTTCGGAACCAAAGCGTGGT
EMPV1_19513	CAACCAGCCTCTCTCTCAGGCTTGTAGGTATCTTGGTAGGAGGGCTGAAAGGGAACAACA
EMPV1_19514	GGAAGACACTGGTCTTTGGATTTAGGGCCAACCTGATCATCCAAAATGATCTCCTCACTG
EMPV1_19515	TGCTCTGGATTAGGCTTTGGCTTAAGGGAATGTTGTGGCTGGTTTGATCTTCTAGCCAGG
EMPV1_19516	ATGCATACCTCAGGATCCAGCCACACCCACACACATAGACCGTTCCCAGCCCATCACGCA
EMPV1_19517	TACATCCAGTTTGTAGGTAGACTCTGAAATGGGGAAGAGCCGGGTCGTACGCTGTGACAC
EMPV1_19518	GCCTTTTGTCACAGCGCTATTGCCCTTAAAAATTGTGCCCAAAGAATGAAAGTGTGGAAG
EMPV1_19519	GAGTGGCTCCAGGAAGCAGGAGTGTCCACGTTTAGACGGCACATTATGAGGACTTTAATC
EMPV1_19520	CCACCTCTAGTCTCTTCTTGCTGTGGCTCCAGGTCTGGGCATGGGCGCTGCTACAGTTCT
EMPV1_19522	CTCATTTCCTTGCTTACATGCACGCAGCAAGATATAGCAGAAAATAAATTCTTGCAGGAG
EMPV1_19524	CCCAGATTCTCATCAGAGTTCCACAGAGTTGATAAACCCACCTTACTGGACTTCGATTTC
EMPV1_19525	TCTTTGGAGTCTCTTTGCTCTCATGCTTAGATGTTTGGAATCTGGAAGTCACAGAGGAAC
EMPV1_19528	CAGCTGCAGAGACAAACCAGATGCCTAAACTATTGTGCAGAGCCAGGAATCGAACATGCA
EMPV1_19529	GATTTGAAGGAAGTGGATCCTACCAGGCACTGCTATGCCCTTGTCAGCAAACTAGACCTC
EMPV1_19530	TGTGCCTCTGCTCCCAGCCTTACAACTGCTAACAAGAAAAGCTCAGCACCAGGAGAGACT
EMPV1_19531	ATGAATGAAATCTGGCTTCGTTGTGACAATGAAAAACAATATGCGCACTGGATGGCAGCC
EMPV1_19532	TCGTGACGACTGGGATTGAACCTGAGATTCTGCCTTTTTAACAAGTTCTCAGGTGATGCC
EMPV1_19533	TGAAAAGGGCAAAATCCTCCAGTTCCACTTGTCTGAGCCAGTCCTCTGGGTCCATCTGTG
EMPV1_19534	TACTACAATGCTGACGACAAGCAGTCCGTGTGGGAGAAGCCCAGCGTGCTCAAGTCCAAG
EMPV1_19535	TTCGCCTTCAGTTCAATGGAAAGTATCCCCAAACCACGATCACACTTCCCACCGGGCTAG
EMPV1_19537	CTCTGTCCTTCTCCTACTGTGGCTCCCGAGAAATATCCCAATTCTTCTGTGATGTCCCAG
EMPV1_19540	CTCAGAATTGGAACAATAACAAACCTAACACAGTGGTGCCCAACAGGGGTGACTGGACTC
EMPV1_19545	TTAGATCTGGATTGTGAAGCCAAAGTGAGAGGGTTCCAGAGCCTACCATTTCTCATCAGG
EMPV1_19546	TCTTGGCTCAACATCCAAGATGTATAACCTACTCAGGATCAGCCTAGGAGACCAAGGGTA
EMPV1_19548	TGGCATCTGCAAGCACAACTTTGACCCTCGAGTCTGTGGAAAGCATGCTACAATGTTTGG
EMPV1_19549	TTTCTGCTGTGCCTAGAGGCCATCTACCGGAAGGTGACTGCCAAGGAGCTCCGATATGAA
EMPV1_19551	TTTGAGACGGACACACAGGACCTGGCAGGGATCGAGTGGGCCCCCAACGGCTGCGTGCTG
EMPV1_19553	CAGACCTCACGACACACAGCATCAGCAAGTCCACGTGCGAGAGACACCTTTAAATGATGA
EMPV1_19555	ACCCCTGGGAATGTGGATCAGGTTAGCACACCTCCGGCGGCAAAAAAGTCACGAATCTAA
EMPV1_19556	AATGGCTGAGTAAGCATTAGACTGTAAATCTAAAGACAGAGGTTAAGCCCTCTTTTTGCC
EMPV1_19559	GACTCTTCCCAGTACAGGTCACCTTCAGACCGCGACTCAGCCAGCATTGTCACTATTGAT

EMPV1_19561 CCTCTGGATTGCAGCTCTACTTGTAACAGTGGTCGTTGTGTGTGGGATGGTGTCCTTTGT
EMPV1_19564 AGCCGAGCCTGCTGGGGTAGAAGGGGCAAGAAGGGTCGTTTTTACAGCTCAGAGATAGAA
EMPV1_19566 TGTGCCACAAGAAATAAAAGCATTGCAGCTGTGGAATGAGATAGAAACTCGCCATCCTGG
EMPV1_19570 GCATTCACTCCTACCTCCCTTACCTCTAAGAACAGCTTCACTCAGGACTGGGCAGCAGAT
EMPV1_19571 CTGCAGTGTGTGTGGCGGCAGCCCATGTGCCTTTACCTATTGTCAGACTTGCAAACATTT
EMPV1_19573 CCCTTGAAGACACAACCTTGTAAGAATGAACTCATCTTTCAAGAAACACTTTTGTTCTGG
EMPV1_19575 AGACTCCTTCAAGAAGCAGGAGGCTGTGCAGGAGGTGAGCTTTGATGAGCCACCTGAGGA
EMPV1_19576 GAGGGGGTGTTTTTTATATCCCAGAATATCTCAATCGTTCTGTTGCCACTCTCCTGATGC
EMPV1_19577 CAATATGTGCACTCATTTGCGGGCGTCCAATTGTAAAGCCAGAATATTTTACTGAATTTC
EMPV1_19579 CCAAATCTCCATGTGTGCCAACTGGTTCGTTTTATCCTCACAACGGTCCCATAGGCTTCG
EMPV1_19584 GTAGTGTGGGATGTTAATAAGTGAGGAAACGGCATGGTGGAACAAAAACTATTTCAGCAT
EMPV1_19585 CTTTTGCCAACCCCTGTCCTGGTGTTTTTATCAGACCTTTCTCGATTCAGCCTGACAGTC
EMPV1_19586 AGCGAGAGAGCGCGAGGGCACGCGCAGGGCTGGCCACGGCCGACGAGGACGCGACGGCGC
EMPV1_19588 CCATCCGCTGACGGACCCCAGCAAGGACCAGCCCCATCCGGTGACAAACCAAAGAAGAAA
EMPV1_19589 GGAGGAGGCAGAAGCCTTTGAGCACATTAAACAAGGAAGTGAGCTGTATGACGCGCAGAC
EMPV1_19590 CCCAGGACTGGCACATAGCTAGGTACTCAGTAAATGTTTCTTGCATGCATGAAAGAGGCA
EMPV1_19594 TCCCACGCTCCCCAGGTAACCCCCATCAGCGTTAAGAAGAACGGAAAGGCTGTTCATTTA
EMPV1_19595 GCCAAAGAGGAATCCTTCACTGCGATAGGAGAATATTCAAGTGTTCTCTTGGACTGTATA
EMPV1_19596 CAGGTGGCTTGTTCTACACGATCTTCAGAGAACTTTTTTCCTCATCTAGTCCTAATAAGA
EMPV1_19597 CTCCGTGAGGAGCTAAACAAGAAGATGGCCGAATTGGTGCAGTTCCTGACCGTCAAATAC
EMPV1_19601 CAATGCCATATATCAGGAGACCCAGGAGGAGTCTCACCAACCCATGCATCAGATAGTAGG
EMPV1_19602 AAAGAGCAGGAGCTCAAAGAACGAGAAAGGCGTCTGAAGATGTGGGAGCAAAAGCTGACG
EMPV1_19603 AAGCAGCTCTGGATCTTTGCCTGTGCAGGTGTCACGTTCATTTCTTCCCTTCTGGTTGTC
EMPV1_19604 AAGAAGCAGAAACGTCACTCTTGGGTGGAAAAGCTCCTTCCCGACACTCATCAAGTGGAG
EMPV1_19606 CCTGCTCAGACTCACCTGTAAAGCGGAAAAGGTAGGGATCGTGCTGTGGAACGTCTTCAA
EMPV1_19609 GTGTTCAGCACACAAGGAATTTGCTCTTCAGAGACAGCAGTTAAGAGCCTCAGTGGAGGG
EMPV1_19610 CTTGAAGCTGGAGGTCTTCCTTTGAGGCTTGAAAATACTGGGAGAAGCTCTGTGGTGCAG
EMPV1_19612 CAATCAAGATAAGCACAGGGAACAAAGCCGTGGGAGGTGTTTGTTTAAATGGCATGGGGA
EMPV1_19613 CCTACAGCAGAAGGACGCTGGGAAAGGTAGCAGCTGACATGCGCTAGACTTAAGGATAAA
EMPV1_19617 GGATGACAGCAAAGGCCTTCAAACTTCAGTCTCTCTTTCTGGCCACACAATAAACTTTGG
EMPV1_19624 TAGCCCCAAGAAAACAGCGATTTTGCATATTTAACCATCACCTCCCTGCCAGCACCCAGC
EMPV1_19627 CTTCCCTTCACCTTCCCCTACAAACTCCCCAACTGACACCCTGGTCACAAAGCTCTTCAT
EMPV1_19629 CACTGTGGTTTGTACGGCACTGTGGCCACGTCCAAACCACACTGTGGTGTTAGAGCGAGG
EMPV1_19630 CTGCAGCTCCTTTCACTTGTCACTGGCAGCTATGTCTCCGGCACTTACTCCATCGTCTTT

EMPV1_19631	TGGAGTCCCAGGCACTTCAGGTCACCAAGCACCTCCAGGGAGAAGGCGGTGAGGCACACT
EMPV1_19632	TCTGCTAGAAACGGATGTACTCTGAATGGAGCTAGAGTATAGCCAGCATTGTGTCGGGGC
EMPV1_19633	GCTTGGTTTTTGTTCTTCAGAATTGTGTTGGCAATTGTGGGTCTTTTGTGGTTCCATATA
EMPV1_19634	CCTCAGGAGGACAGATGCTTGGAGGTGGATAAAGAGGGCTTGTTCTGTTTGGGGATGAAT
EMPV1_19637	GCTTCTGAGCCGGCAGTTGCTGCAGTGACAACACTTCTACGCCCACTAGCATTATCTTGA
EMPV1_19638	AGGCCTTGAGAAGTTATTCTGATCTTGGGGAGTTTCTCCTCTTTGGGGGGCATGTGAGCT
EMPV1_19639	AGCTTTATACGCTGCACAGCGTGAAGCAGCCAGGGCAGCAAAGCAGCGAAAGCTCTTGGA
EMPV1_19641	CACCTGGTTTGACTTTCACAGAATGTTTGAGTGATAAATGTAAACTTTGGCAGGAGGGTC
EMPV1_19643	TTGGCTCGTAATTTCGTGTTTGCTGGGTCTCATCCTCTAAAGTCCATGTGCAGCGTTCGG
EMPV1_19645	CGCGCGCCTTCAGGGTGGCCTGTACCTTGATGTTATTGGAATCCGTGAGAAACATAGTGA
EMPV1_19646	CATTTGATTCCATTGCCAAGGCACAGAGGATGTGAAGGCACGTTCTGAGTTCCCAAACTT
EMPV1_19648	TAGAAAATATATCAAAATCCCAGGCCCCTGAGCCAGGTCCAGAAGAGGGAGCTACTCCAG
EMPV1_19649	AACCTGCAGATGCGGCTGGGTGGACCTGAGGGCCCCGGATCCCCACTTTTGGCTACTTAA
EMPV1_19650	CTGAGTGGCTCAGATGTCATCCCCTGAAATAACTTTTCCTCTTCACCTCCGACTCGCAAT
EMPV1_19652	CTTGAGCAGGAATTGGAGCTATCTCGAAGGTTATTGAATCAGTCAGAAGGCAGCAGAGAA
EMPV1_19653	TTGACCACACAAGCGTGGCCCTTCCTCCAGACCCCCGCTCCGGACGCGCCCACCTAGCGT
EMPV1_19654	TCACCCAGGATTATCTGTGTGGCCCTCAGCCCTGTGACCGTATCCTTCTGGGCGAGGAGA
EMPV1_19656	CTGCTTTTGGTGTCACGTCCAAAAAACTGCTTGCAAGACCAATAACATCTTCCTTACATT
EMPV1_19657	AAGCAGTCAAGAAACAACAGTTCGGTCATAAAACAGTCACAGGACTCCTAGCTCCTCCTG
EMPV1_19658	GGCCTCCGTCTTGGTAGATGTGGCATTCATTGCTGTTTCCTACACTTTGATCCTCCAGGC
EMPV1_19660	TTGGATCCTATGGGGTCATCCTTAACTCCTTAAGGAAGCAATGCTTGGAGGCCAGACGCA
EMPV1_19661	CTGGGTCGCAGAGTAGCTAGAGGGGTTGTAAGGAGTTTCTGGAGTTCTGATAAGGTCTGT
EMPV1_19664	GAAATCTGTCATTTGGTTGTAGGGCTTACCTGGCACAACTTAGTAAAGAGCTTCTTCAAG
EMPV1_19665	ATGTCTGACCAGGAAGCAAAACCTTCAACTGAGGACTTGGGGGATAAGAGGAAAGGAGAG
EMPV1_19666	TGTCAAAAGTGACCTGTGGCTCTGCCGTGGCTGTGAGATGAGTACATTCCAAAGGAGCAG
EMPV1_19667	CCATGATTTAGCTGGTTTTGCCTAGTGCTCATGGTGGGAAGCTTGGAAGAGATGCTGGAG
EMPV1_19668	TTGGCTAAACGCCTCAAGAAGGAGAAAGTAAATGTTGACATTATCAATTTTGGGGAAGAG
EMPV1_19670	CCTGGTGCCACTTGGTGTCTGCTGGTTCAGGGTTTGCTGTCTGCCTTCTTACAATGTCTA
EMPV1_19674	ATGAGTGAGGCGGAGGTGGGGGTCTTTGTCCAGCGGCTCCGAGGCCTGATGAATGAGATT
EMPV1_19675	GTGCCCAGCGTCCAGGTCCAGCTGCTGCAGGACGACCCTGCGGGTGAAGCAGGCCTGCCG
EMPV1_19676	GTGAGGTTCCTTCCATCCTGAGATTGGCTTGCACTGACACCTCATTTACGGAGATGGTTG
EMPV1_19681	CTTGAACAATCTCAATCTCTGAAGTGTTGGGTGGGAAGGTGAAGGGGTGTCTAGGCAGGA
EMPV1_19682	AAAGTTCTGAGCTGGCCACGGAGGAGTCTGTCCACATGCCTGGCAAGTAGTCCAGAGAAG
EMPV1_19683	ACACGAGAACTTTGCTCAGTCCCTCGGGGAGAAATAACCGAAGTGAGGGCACTCTTGACT

EMPV1_19684	ATGAACCTGTTGGAGTTACCATCGTGGCTCAGTGGTTAACGAATCTGACTAGAAGCCACG
EMPV1_19687	TCAGTAGGTCTGGGGTGAAGCCCAAGATTTTGCATTTCTAACAAGCTCTCAAGTGATGCC
EMPV1_19690	ATTCTGTTTCGGACTTGCCATCAACTACCCAACCAAAAGGACGACAGATCTATGTAAGAG
EMPV1_19691	GTTCGTGGTATTCAGTTATCGCGGTTGGTGGGTCGTGACTGAGCCAAGATGCTGCGGAAT
EMPV1_19692	ACGGGCTCAAAAGAGCAGCTGGCAGAGTCCAGAGTAGCCCCCTGTGACCACCTACCAGCT
EMPV1_19695	CAACCAAGGCGTGGCTTACAAAGATATTTTCGAGGGGGTTTACTTTCCGGCCATCTCACT
EMPV1_19697	GACATATTCACTACCAAGGCTGTCACCTGGAACACCACCAAGACGTCAACTATCCTGGAG
EMPV1_19698	AACTGGGAAACCAGAGACACACCACCACTGTGGCTCCACGCTCGGCAGTGCCTGGTTCTA
EMPV1_19700	CCTCTGTCATGTTCCATCACAAATGACTGAGCATATAGTTCCCTGTGCTGTACAGCAGGG
EMPV1_19702	AGACGTCGAGGGAGACGTGTACGTGCTGGTGGAGCATCCCTTCGAGTACACGGGCAAAGA
EMPV1_19705	ACATGAGGAGTTTGGGTTAAGGGGCTCCCTTTGTGGGAATTTGTTGGGGCAGAGTCAGAA
EMPV1_19708	GGAAAACAAGATAGCAGAATTTGCAGGTAAAGAGTTAGGGCTGCTTGTTCTCTGGGTGCC
EMPV1_19709	AGTCCAACATGCTTTCATTCAGCAGGTGTTTATTGCCCCACTATGTGCCAGGCTCTGTGC
EMPV1_19710	GGACTAGGCCGCCATCCAGTCTACGTTACAAGAGAACAGAGTACTAGGAGACAGAGTAGT
EMPV1_19711	CTCACCTTAGTTGCAATTTGGAACGAAAGACATTACCTCCGGGTGGCGCCTGTGACGCCA
EMPV1_19712	TCCTGCTCCGAGTCCAGCCTCTACCCCGTGTCCTTCTTTGTGCCCCTGCTGGTGGCCCGA
EMPV1_19714	CCCCAACTTCCTCCTCAAACCTGAACCATGTCCCTGTCCATATGCTCCATTAACAGCCTG
EMPV1_19715	ATCTGGCAGGCAGTCCTTGGCAGCAGCATTTCCATCTGGCTGGGGCAGCTTTCCCTGCAA
EMPV1_19718	GCAGCATATAGAAAGTATACAGAACAGATTACAAATGAGAAGCTAGGTATGGTTAAAGCG
EMPV1_19720	ATTCCCACCTGGATTAAGATGGATCCGATGGGGTCATGGTTTTCTGGCGCCTGGGCCATG
EMPV1_19721	CACGCTCCTGACCAACCACTGGCTTTTCTACGGTAGTCCTTCTTTTCACAGTCCTATTGC
EMPV1_19724	GGTATGGGAGAGAGCAGGAAGCCTTCTCAAGTAGAAGCATTTTAAGTAAGGCAGGATTGC
EMPV1_19726	TCACTGTAAAGATTGTAAGACCAGATGGGAGAGTGCTTATGTCTGGTGCATTTCTGGAAC
EMPV1_19728	GCTCTGGGAAGCTCTGCCCTGTCTCTAAGGATTTACTGGGAAGCATCATTTTAATGTGGC
EMPV1_19731	TCCTCGGACATACCGTGGCCTTAGAGGTCAAGCCGGTTGCCAGAAGGGCTGGGGTTGATC
EMPV1_19732	CCCGGGGAACACAACTTTCCTCAGCGAACTCTGTCTAAGAAGCTGTAATGAATGTTTGAG
EMPV1_19733	CAAGCTCTCAATATCCCACATGGCCCCAGAAAAGTTGGTCTCACCATGGGAAGTTTATCT
EMPV1_19734	CTTTGGGACAAGAGACGAGAAGGTAAACAAGTCAGTAGCCCACCTAATCTCATATCATGG
EMPV1_19738	ATGGGGCCACCTGCATCTGTGCCCACAATAACCCCCTTCTCCTTCAAGCAAACAGCTCCT
EMPV1_19740	TGCCTGACTTTTAAATGAGCATAAAAGGCCCTCTAACCTATGCAGGTCTCCTCATTACGC
EMPV1_19741	AGGACACAGTGAGCGGACATTCGGAATTTCACTGGACTAGGGAGGAGCTTGGCCCGACCA
EMPV1_19742	GTGGTGTTTGATATTCGAACTAAAGCCATAAAGGAATTAAAATTAATGAAGGAACTAGCT
EMPV1_19743	TGTTCGACTTCTGGCAAGAGCATTCACATTGAGAACTGTGGGATTTAACCATCTGACCCT
EMPV1_19745	GATTCTGCATTTTGAAGCCACATCAGTCCCATCTCCGCCATGACCTCTCTCCTGTGGAAT

EMPV1_19746 GCTATTGGCCTCCTTCACACAGTGGTCCAGGTAGCTTTTGTGGTCCATCTGCCGTTTTGT
EMPV1_19747 GGACTTGTGTATATACACATCGTGTATAACCCCACCAGCTTGTTTATTGTCAGTGGATGG
EMPV1_19748 CATAGGCCAAACAGACCAAAGCCCTGTAATACTAAGCTGTAATACTAATAATAATCCAAG
EMPV1_19750 AGAGACTGCACTGATATTCAGGGGCTCTTTCTGTGGCCCCAACCTTGTCAAACACTTCTG
EMPV1_19752 CTGGAGGGAGGACTACAGCGTGAGCACTAGCCTGGTGTACTCGGTGGAAGACCCCATGGT
EMPV1_19753 TCTTGACATTACAGAACTAAACTGAAGTTTATTAACATTCCACTCTTACATTTCTTATCG
EMPV1_19754 AGCATGTGAGCAAGCGTGTAATAGGTGCACAGAGCGAGGGAGCTGAAGGGAGGACTTTTA
EMPV1_19755 TTGATCCGTAAAGCTGGTTAGCACTGGAGTAGGCACGGAGAGTGCTGGTGAAGACTGGTA
EMPV1_19756 AGGACCCAACGCCTGCTTCTGCCTCAGGACATTAGAGTATTCACTCCAAATGGGACAGAA
EMPV1_19757 GGTAAGCTTTTCAGAGCTCTGTCTCCCCTTCCTTCTTCATGGCAGGTGCCCGCCTATCCC
EMPV1_19758 CTGTGAGCCTTGAAGATTTACTCCAACCCTTGCTCATTAGAATCCGGTCTCATCATTGCC
EMPV1_19760 CCCTGGCCTTCTACCCAGAGGCTGAAGTGATGTGTGATGAGAATGTAAAACAGGTGTACG
EMPV1_19762 GTCTAAAAGACAAATTTTTATGCTGTTAAGGCCTTTGAAAGTATGTTTCCCCATTAAATA
EMPV1_19763 GTCACTGAGTGCCCTTCTTTGACCCAGGCATTTTAGTGATGTTGCTGACCACTTCTGTTC
EMPV1_19766 CTCTGTTCTGAACGCCCTGAACACTGCCAATCGTTGAATCCTCATGACAACCCATGAGGT
EMPV1_19767 AGCTAGCATGATGTCTGTTACACTCAGCTGTGATCATCGGGTTGTGGATGGAGCAGTTGG
EMPV1_19768 TCAGCAGGATGTAATTGCCCTTGTGTGTAGTTAAAATGAGTCATCATCTGGTCCTGTGTG
EMPV1_19771 CACTCCTGGGGACTGGAAAAACTACTTCACGGTGGCCCAAGCCGAAGCCTTTGATAAACT
EMPV1_19772 ATGTATTAAGGCGTGGTTGCCCGTCCCATGTCAGGCTTGATGTTGAAAAGACGACACTGC
EMPV1_19773 TTTTGTCTTTGCCATAGAATTCAGGAGTCGGGGCTACAGGCATAAGCTCTGAATGCACTG
EMPV1_19778 TCCTTTCTTCTTCCTTTGTTCCCAAACCACATACTCCAGGCCCAAGAGCCTTGCTGCCAC
EMPV1_19780 TAACCCTTGCACTTCTGGGTGTTGGGTTTCTCAGTTCCCTGTCTTCCCACTAAGCCATGG
EMPV1_19781 TCACCTTTTCTCCCTACCAAGTGCAGTCCCAGTTGCTCGTGCTGCTACCCCCACCGAATT
EMPV1_19786 TCTCTGTCCCAAGGGCTGCACAAAGATGTTCAGGGATAACTCTGCCATGAGGAAGCACCT
EMPV1_19787 GGTTCAATTCAATCGTTTACCTTTGGTGGTGAGTTTCATAGCCAGCAGCAATGCCAATAC
EMPV1_19788 GGAAGGTTCTGCCGGGCTTTTTGATGCTAATGGCAATGGTTGATCTGTCTTCACCGCAAC
EMPV1_19789 TGGTCTGTCCAGCTACCTGTCTACTCACCATGGCCTGTGGTACCTGCTCTCTTCAAGCCC
EMPV1_19791 GATCATTTGGCTCCCAACTTGAATGTATAACATGACTCAGTGGGGTTGGTATCCTTCTGG
EMPV1_19792 GGAGGCTCGCCCGGTCTAAATTGGATCAGAGACCATGTCTTTCTCAACCTCGTACTTGAA
EMPV1_19794 GGTGACATAATGAAGTAAGCGAATAAGGACTTCAGGAGTGGTGTGGACAGTCGGGACTCC
EMPV1_19795 AAGGCCAAAGCCTTCAAGAAAGAGAAACAGAAGGAGAAGAAAAGGAGGGCCGAGGAGGAC
EMPV1_19796 CAAGGGAACTGAATGCAGAGCCAAGCCAGATGCAGATTCCAAAAGAAACGAAAGCAGAAG
EMPV1_19797 GGGAGATACGGAGTAGATCCTTCCTTGCCAGACCACAGAGGAAACCATCTGGAAATCTTT
EMPV1_19799 GACCTACAGAGACAGAAACCGCATGATCTGAAAGAAACACTTACTGGGACGGCTGCGGAT

EMPV1_19800	CCTGGGGCACTCTGTAGGGGGACTGGAGCAGCCTAGTTCTCTTCCTATTTGTACCAAAGA
EMPV1_19802	TGCCCAGGGGAGGGAGTATGGACAGTATCTGCAAGTTCTGGGATTCAGGTTGTTATTAAA
EMPV1_19803	AATCTGCATCGCAGGCCTAATGGAGAGCCTTCCTCCTGTGGCTGAGTTATCTGCTTGTCA
EMPV1_19808	GAATGGATCGGCTATGCCTTGGCCACTTGGTCCCTTCCAGCACTTGCATTTGCATTTTTC
EMPV1_19810	ACCACCTATAGAGCTGTGTCCACGGCCGTCTTCAGGCATGAACGCTCCATCCCCATGCCT
EMPV1_19813	ACGCACATACAAACAGGATACATTTCAAACATGCCAAAGGGAGGGAGGGCAGTGGGGAAC
EMPV1_19815	GTAACTTTTGATCATTTTCAGTTTCTTCCTTTAAGTTCATTCTACCCCCCATGTCCACTC
EMPV1_19816	TGGAGCAGCAGGATCAAAGGCTCAAGAGGGATGGCTCCAAGAAAAAAGAATGGAATGTGC
EMPV1_19817	CCAAGAATCCCTTAATGCTGTCCATTCTTCTCAGGGCCACGACATAAGGAGACGTACACA
EMPV1_19818	GAGATGGACACTGAGATGCACTGATTATGCAGCCCAAACAGGTGGTGTTATAAAGGTTTC
EMPV1_19819	TCTGAAAATGAACACCTTCAGAACTAACCCCGGCGAGTGCAGCACTTCAACGATACCACG
EMPV1_19820	CGTCTGCTGATGGACGCTCGGTTCTTTCCACCTTTGGACTCTTACGAATAATGTTCCTGC
EMPV1_19823	ATGCCCGTGTGGTGACAGTGTGCCCAAGTTCATGATTGGTAAGCAACAAAGCCAGCCTTT
EMPV1_19825	TGCTCACCCAGAAGCCTCTGTTCCCGGGCAAGTCGGACATCGACCAGATCAACAAAACTG
EMPV1_19826	ATGTCAAGAGTGAAAAGGTTTACACTCGCCATGGGGTCCCCATCTCAGTCACTGGCATTG
EMPV1_19827	TCCCTGGCTACCCCGGCTCTGACCTTGGAGTTTTCATTCTTACGTTTCTCTCTTTTCCCT
EMPV1_19829	TGATGACTCTCTCAGACGTGATGCTGGCGGAAGGAGGGTGCCTTCCATGTGATTCCATCT
EMPV1_19832	AGACTGCATTCGAATCGAGCCCGACACCACTGCCCCTAGCACCAGCCATGAGACCATTGA
EMPV1_19833	CATTACATGTTCTTAGGTTCAAATTGTGCAGTATTTTCTCTATGGAAGCCACTTCAAGGC
EMPV1_19834	TTTGCCGCATAGTGTGGTCATGGGGCTGTCTGAGCAGTTTCCAGTTTTCCAGCTTTCAGA
EMPV1_19835	CTTCTGGGCTCTGACCTCGTCCTTGTTGTCCTCTCTTACTCCTTCATCCTGAAAGCTGTG
EMPV1_19836	ATTTGTCTCAGAGGGCAGAGATGAGGGCAAGTGCCAGAGACAGCACCTGTCAAGCAGAGA
EMPV1_19838	TGTATGAGTTGCTTGTATATTCTGGAGTTTAAGCCCTTGTCAGTTGCATCATTTGCAATG
EMPV1_19839	TTAAAAAGAAAATCCCCAATTAATAAAATGGAAAATTTCAATGGGCAGAAGGGGCAGCTG
EMPV1_19840	CTAGGGGCAGGCTTACCTATCGGATCACCCACAGTAGTCAGCCATCAAGTGTACTAACAT
EMPV1_19843	TCTGGGTATGTCACATCCAGTGCCGCAGCTTTAATGACTCCAGTCTGAAGGGCTTCCACC
EMPV1_19844	CGCTGGCTCTTTCCCAGCACACCTTGTTCCAGGATAACTTGAATAAACATACACATGTGA
EMPV1_19846	CCTGTCCAGCCTCTCAGCCTGCTGCTGGCACTTGCTCTGCGAAACGCCTTGATGATACAA
EMPV1_19850	GAGATGACAGCTCTGACGATGAGGACAAGGAGAGGGTCGCGCTGGTGGTACACCCGGGCA
EMPV1_19852	GACGGATTCTACAGCACTGCTTTCATTTAATCGGGCACTTGGCACTCCGTTTGAATCCAG
EMPV1_19853	ATTTCTCAGTGGGTACGTCAAGGCAGGGAGCATTACAAGGTAAGCCCAAGGTAGAACCAG
EMPV1_19856	ATCCCATCTTGGTGTTCTTGGGATGGGTAGCAAAAAGGCAGGATTGAGGGAGTTGAGGGC
EMPV1_19857	AAGTCTGATGAAAGTGATTCCTCTCTGTCGGAGGTACACAGTAGATCCTCCAGAAGGGGC
EMPV1_19858	GGAAAGCAAACCACCTGGAAGAGAAGTTGAGAATCCTTTGGAAACTCACCAAAGATGTTA

EMPV1_19859	ATGGCTAGGCGAGTTAGTGCTGATGAACAGCTCTTTTCTGTGAGCCAGGCAACGTCGAGC
EMPV1_19861	TGTGAACCCCTCGAATATCCTAGTTAACACTAGGGGTCAGGTGAAGCTCTGTGAGTTTGG
EMPV1_19862	CCAGGGTGAGGATTGAACTTGGTTGCTTAACCCACTGTACCACAACAGGAACTCTGTACC
EMPV1_19863	AGGGGAATATGTGTGACCTCAGGGCTGGCGTAAAAAGAGCTGTCACTCAGAGGTCATCTA
EMPV1_19864	AGATAGGACTGCTGGTGAAGGGGCTCTGCAGTCTGGCGGAGGAGCTGTGCCACGTCCACT
EMPV1_19865	GGTCCATAATTTCCACTTCTGACTTGAGCTCACTATCGATAGGCACAGTGATCCAACCAT
EMPV1_19866	GCTCAGGTCCTAAGTGCTATGTGTGCAGTAGATGCTTGCACTGGACTTAACTACCTTGGA
EMPV1_19867	CGAGAAAAAGGCCAAGAAGCCCGCCCTGGTGGCCAAGTCCTCCATCCTGCTGGATGTCAA
EMPV1_19868	TGATGTCAAGGACTCTGCTAACTACACATGTGTGGCCATGTCCAACCTGGGCGTCATTGA
EMPV1_19870	ATGTTTACACGCGAGATGCCAGCGTATCAGTGGCCGACCAGGTTTTCTCTAAGCTCAAGA
EMPV1_19871	AAGATGAGTCTTTGTGTTGGGGAGTGGGGGTGAGGTTGGAGAATGTGTGTTGGGATCAGG
EMPV1_19873	GATGGATTCCAGCCCAACACCCAAGTGAAGGTGATTGCAGCCACTAACAGGGTGGACATC
EMPV1_19876	CGCATTTGATCAGCCATAGAAATAGGACAATCTGTAAAGCTTTCGGGAGGGCGGAGGGAG
EMPV1_19878	AGAGCGGGTTATAGGATCTGCGTACGCGGTGTACAAGCAGAAGATGCAGAGCAGAAGGAT
EMPV1_19879	TACAAGGCAGCCAACTGGTATGAGAGGGAGATCTGGGACATGTTTGGAGTCTTCTTTGCT
EMPV1_19880	CACTGGGCAGGATGTGGGTCTCATTCCACTATCGTTTATTGTTTGGGGGCATGGCTCATG
EMPV1_19881	GTGATTTGGCTTTTCCATGAGATGCCTGAGTGAGCAGGAACAAAGGGTATGGACGTGGTG
EMPV1_19883	AGGTGCTGGGCTCTAGGCTGCTGCATCACATGCTTTGCTATGGTGCTGTTTATGCCCTAA
EMPV1_19884	GAGCTGGTCATATTTACTGTAGGGTCTGTGGGCCTTGTTTTTCCATTGAGTGGCATCCTG
EMPV1_19885	CAGGTCGGTGAATTCTGGCCTTACATCAGGTTGTTGTCTAGCTCTATCCTCAGTGCTCAG
EMPV1_19887	TTTAGGAGTTCCTACTGCAGCCTAGTGGGCTAATGATCCGGCTTTTCTCTCTGGGGGCCC
EMPV1_19888	GATACTGTTACCGATAAACACGTTGTACCCGTGACATCATTAGGACGCAGAACCAAGAGG
EMPV1_19889	GGCGTTACTGTCCAAACAGCAAGCACCCCAGAACAAATGCCACCCATGGATCTTTACGTA
EMPV1_19891	CTTGATAGTGATCGGCTTCCTCTCCGGCTACCAGACTCCTACTGGCCTTTTACCTGTTGA
EMPV1_19893	CTAAGTGGGCATGCTTGGTGGTTGGTCTGGGGCTTAGGAGAGCATGTTTAGATGGAGTTA
EMPV1_19894	AACTGAATCAAAGCTACAGCTGCCAGTCTACACCACAGCCACGCCAGATCCAAGCCGCAT
EMPV1_19896	AAGGTTGCCCCAAGGGAGCCGCTTTAAGCTGAGATACTGGTAGGATGTCTGAGGCCATCA
EMPV1_19898	AATATGCATTTCTGAGATAATTTCTGTCTCCCTGGGGTTGAGGGCAAGGACGCGGCACTG
EMPV1_19900	CTCGTGATAACGCGTGCAACGACATGGGTAAGATGCTGCAATTCGTGCTGCCCGTGGCCA
EMPV1_19901	ATGGTGGCCATCCTTACCCCTTTTCTGGAATATTTGAAATTTCCCCAGGAAATGCTTCTG
EMPV1_19902	CAGTTAGCAGAAGAACAAACTACAACCCAGACCCAGCCAGAGAGCCCAGGATCCCGAAAA
EMPV1_19903	TCTGCCTCCACCACAACCCAAGCCGCCACAGCCGCCACGGTCCGGGCCATTTTCCACTCA
EMPV1_19905	CCACAGTGTTCTCTCATCACCCTGCTGGCCAAGATCCCTAACACGCTCAAATGTGTATAC
EMPV1_19906	CACTGTGATGTCTTCGGAGGGAGCCGTGGCCGAAATCTCCTGCAATCACTCTATATCCAA

EMPV1_19908 CAACCAGTCTTCCTGGAGGGGCAGCAACAGTTGTAGCACAGTAAGGATGTTTGAATGTAT
EMPV1_19910 AGCCTGATGACATGGTCTAATTTCACAGCCGTTGAGTGAAGAGGTTGGTGTTTGCGGAGC
EMPV1_19911 GAACATTTTTCTCTTTGTTGCATTTTGTCTTAGCTGAGTTACCTAGTTCTGTCCTAAGTC
EMPV1_19913 GTGAGTTAAGAACCTGATGTTGTCTCTCTGAGGTTTGTGGTTCAATCCCCGGCTTTGCTA
EMPV1_19914 CTCCTCCCCTTTGGCCTCACTTGATCCTAGGTTCTCCAGGTTTCAGCACAACCTCAGTAT
EMPV1_19915 GGAACTCCTTCACACACGTTATCTTCTTTGGACCTTACTACCTTTATGAGGCAGACCAGC
EMPV1_19916 GCACAGCCTATGCCTGTGGAGGTGTCCTTAGAGACAAGAGGGTCTTAGTGACACAAACAC
EMPV1_19919 CCCTTCCATGTCCAATCAGTCACTAAGCATGTTGCTACTTACCTTTGCAAGAACCCTCAC
EMPV1_19920 CAGCTGGAGGAGAAAACAGCAATAGATGGTAAACCCTGTCCCAAGATTCACAAACCAGTG
EMPV1_19922 TCCCCCTGCACGGCTAGTACAGAGCAATGCTTGGAAGACAGGCAGCTCTTACTGCCTCCA
EMPV1_19923 ATACTCAGTCGGAAGATGGTGAGAGAACCCCTCTCCCTCCAAGAAGCCTAGTGGATTTCA
EMPV1_19924 TCCCAAGGGGATCTGCAGTCAGGGCTGCTGTGTGCTTTCTTTCAAGCCAATAATCTTACC
EMPV1_19926 ACGCCCCTGGACACCCTTGTTTATCTTGGCTCTATTCTGGGCACAACTTGCTACCACCTT
EMPV1_19927 CAGAAGACTCTGCTTTTCCCGAGGCTCAGAGAGGGGCAGTGATTTGTCCATGGCCACACA
EMPV1_19928 GCTCCTGGACATGCTCAACAATGAGGAGGCGCAGCTGCCGGACCCGGCTATTGAGGACCA
EMPV1_19929 TCACCGCCAGACAAGCCCGGGAGATAGTTTAGATAGAGTTTCTTTCAGCCCAGAGAAGCG
EMPV1_19930 GTAACCCACTCGTTTCTGGTCATCCCTGAGTGCCCAATACCCCTACTAGGTAGAGACTTA
EMPV1_19931 CTTAACACTTTTAAACCTGGAGTTTGAGAGGGATTTCCAGGTGGGCGTTCCAGGCTAGGG
EMPV1_19932 CCAAACCCAAGTCCTCCCGATTCTGTTTGTTCTTCCCACAGCAATCAGCTCTGTGTTCAC
EMPV1_19935 AGTGCCTCTTGCATGATCTACAGACGGGGGTGAACCATGGCAGTCACCTCAGATACCAGA
EMPV1_19936 TATTTAGAGGCTTTTGGGATCGGTGCTGATGAGAGGGTTAAGAAACGCATGGCTCCTCCC
EMPV1_19938 CACATGGACAGCAGCATGTGCTAGAGCTGCGTGTAACTAGAAGGGTCCTAGAGAGGAAAA
EMPV1_19939 TCACTCCCCAGCCTCCCAATTCTCACAGCCCTAAGCAACCACGCATCTACTGCCTATATT
EMPV1_19940 GCAAGCGTTGAAGGTGCACCGAAAAAACCGGTCCTTCACCTACATCAAGGAGCGATACGG
EMPV1_19941 GTGACCAAGAGACCAAGAGTTCAGAAGAGTGCGATGAGTGAGAGAGGGGAAAAGACCAAC
EMPV1_19943 AGGAAGACGACGACAGCGAGGAGGAAGATGAGGATTTTGTGGACCAAGAGGAGGTGGAGA
EMPV1_19944 CCCTCTCCCTCATTCTGGCTTCCTACATCAGCATTGTCCAGGCCATTCTCAAGATCAAGT
EMPV1_19946 CTGCCTTTTGCTGTGCAATGCGAGGCATCACTCCAGGACCCTTTGTTTTAGACCTGTAAG
EMPV1_19947 CTGCTTAACTGGGTAAGAAATCACACAACTCTGATGGAAAACCTGAATGCTTCATCCAGA
EMPV1_19950 TTGGTATTGATCCTGTAAAGGAACCAGAGCTGATGTGGCTGGCGCGGGAGGGCATTGTGG
EMPV1_19951 ACCTTCACAGGGGAGACCGAGGTGGGGAGGCTCTCTAGGCTCACATAATCAAGTCTGTGT
EMPV1_19954 AGGGACTTCCTTTAGTCACCTACTCGCATGAAGGACTGACTGTAGACGCATGACCTGCAG
EMPV1_19955 TACTCCCTAGAAGCCTGATATGGGTGGATTAGCAGTAAGGCTTGGGTAAAGCTGAGGGAC
EMPV1_19956 AGCAGATGGTGTCAAGATCACCTGCAGAGTGGAGCACGAGAGCTTCAAGGAGCCTGTCCT

EMPV1_19958	GACTCATTGTCACCACCCGTCACCTAAAATCTACTCAGCGTCGGCAAAGGAGCCATGGAT
EMPV1_19959	TCAACCCCAAGGCTTTGAAAATGGGGCAAGGAAGAAGGGTGGGGGGAGGCTAAACGTAAT
EMPV1_19960	GAGGTGTCCAGTGGTGAGGAGAACGAGCAGGAGGTTTTTAGCCACAGAGCCGATCTTTAT
EMPV1_19966	AGCAAATGAAGAGCAGCAAGTCCAGTAACCTGAAGACCCACAACTCCTTCTGCTACCACG
EMPV1_19969	CCCTCATTTTCCTTTTTGGTCCCCTTATAACCTGAACCCCAGCTGAAGGGCTGCAACAAC
EMPV1_19970	AACAAGATGGATTGTGCTCTCTACATGCAGCATGGCTATGTGCAGGGGTTCTGTGGGAGA
EMPV1_19971	CATCCCACGTTCCCAAGTTCACTGTTCTGTACGCCGCTCCCGCTGTAACCAGATAACATT
EMPV1_19972	TAACATGAAAGGCTATGCACCCGTGAGCCCCTGTGGCCTACAAATGGAGAACAACAGTGC
EMPV1_19975	TGACGTGTGCCTTTTGTCCAACACAGTAACCCCAGCACTCTGCCCAGCTCTTCTACACTA
EMPV1_19976	TGGAAGGGATGCCTGGGAAACTCATCTGGCATGTGGGCTCAAGCTGTGCCTGACTCTGAT
EMPV1_19977	CAGAGCACACAGCCGACAGTTTTAACTCTTTTGTGGAAAGGCAGAGCTTGGAACATAGGC
EMPV1_19980	GCTGTATCCACTACCACATGTTGCTGTTGATTCCATGAAATATGGCTAGTGTGGATGAGG
EMPV1_19982	GCCCAAGGACTATAATTTGAGTAACAGTGGCCAAGAACAGTAAGTAGGACCAAGGGTGCC
EMPV1_19983	GTGATATTGGGTGCTTTGCCATCTACTTCTTGAACCTGCTTACCCTGAAGCCAGGGGAGG
EMPV1_19984	TATATGCCGGTTCTGGATTGGCTGGGCTCTTCCCTGCAACATCACAGTCTTCATTCTAGG
EMPV1_19985	GTCTTCTCTTTGTATCCTATCTCATGAGTCTGTTTCATGTGCAACTTGCAAGCCCTTGGG
EMPV1_19986	TCTGGACATGTGCTACACCACAAGCATTGTCCCTCAGATGCTGTTTCACCTGGGACGATT
EMPV1_19988	GGAACGGCTGCAGAGAACTAGAGACCACCCACCCCACTTTCACCAACTTCTAGGATTATA
EMPV1_19989	GATCGACCAGTGAGAAAGAAAAAAACCAAGCCCATCATCTTCGTTTCAGACAGAGCAAAC
EMPV1_19991	CCAACTCTGCAACTGCCTTGCACATGGACTCTGACTTAATAAACCCTTCATTGTTCTAGC
EMPV1_19997	CACCGCGGGCTGATGCTCTGATGGAAAGGAGAGAGAGTTGGAGGAGAGGGCAGAAGGAAA
EMPV1_19999	CCTGCTGGGAGGTGGAGGTTGGGGACATGCAATCTTGGGACTTGGGAATTTGTAGAGATA
EMPV1_20000	TGTGGGTGCATCCACCAGGCTCTGAAGGCCGATGACAGTGGCACCAGGAGGCACTCCCCA
EMPV1_20001	AGATCGCCTCCTTAGCCTGGTACACGACTGTGATGCCCTTGATGGTGGTGCTGTCCATAA
EMPV1_20002	CCTGTCCTGCTCAAGTTCCAGCCTGTGCCCCATCCCCAAGATGACTCAGAGAATGACCCT
EMPV1_20003	CAGTCTCCAAGTCGTTGGACTTATTACCCGGGGCTGGCTTCTTCTCTTGGGGGGCAGTCA
EMPV1_20004	GAGGTGATGAAGCTGTGAGCTCACCTTGAGGGTGCAGATTGACCCCTACACACTGCTCAT
EMPV1_20005	CAAAGAAGGTAGTGAGGTGTGTTGGTTCCAGAGGTTTCTTAAAACTGCTGAGGAAAAGGG
EMPV1_20006	ACCAGCCACTGCCAGGAGAGGCCCTTCCCACCTAGAAAGGAAATTGGTCTTTTCACTGAA
EMPV1_20007	TAAGTTCGGGCGGATGCGTCGACACTACCTGGAGCGGAGAAACTACATCATTCAGAACAT
EMPV1_20008	AGACTCTGTGGATTGAGAACAAGTCAGACTGCACAGCCCACTTCCAGTTCGCCATCGACT
EMPV1_20009	ATGCTGGGTGAAGATATTGAAGCCGGCCTCTGTGGTGCGCCCCGCCAGGTCCCGCCTGCG
EMPV1_20012	CGTTGGTGTCATCCCTGCTTTGGTTCTGAAGCCTGTACCATTAGCTTAACCTGAGCTTCA
EMPV1_20013	CAGGAAAATAGCTGTTAAAGAAGAAAAGGTAGCCATCGAAGTTCCAAAAAGAGAACCTCC

EMPV1_20014	GGACTAAATTGCTGAAGCTCACGATCCATCTCTTCACCATCCAGAATTCCCAAGCATGCC
EMPV1_20015	TACAAAGGAGTCTGCAAGTGCTTCTGTCGATCCAAGGGTCATGGCTTCATTACCCCGGCT
EMPV1_20016	TACAGACCACATCCTTATGGAAACAAAGCTGCTGACTAGTGCCTACTCTCTGGACTGGGC
EMPV1_20017	ACAAATGGTCTCAAAGTGCCGTGTGCTCTGGAAGGAAGTCATTGCGTATTTCCTGGTGGG
EMPV1_20018	AGTATTTGGTTTAAGAGTCTGCTCTGTGGGACTGTTGATGGCTCAGGTGGTGAGTAGGGC
EMPV1_20025	GATGAGACAGAAGTGTTCAAACAGCGATTTACAGCCGAGTGCCTCAAGAACTCCTGCAGC
EMPV1_20026	CTTCTTACCCGGTCATCCATGCAGCCGGCAAATCTATGAGCACGCAGCCTCCGTTTCCAT
EMPV1_20028	TGTCGCCTTTAAGTAACACCTATGGGTAAGACATTAGAAGAGAGCAGAGGTGTATGCACC
EMPV1_20030	ATCTGATCGGCCGCCTCACCAACCTTCGAATGCAACACCTGTTTATGATCCTGGCTTCAC
EMPV1_20032	CATACAGCCGAAAGAAAGGTGGAAGGAAATCAAGGTCAAAGTCAAGATCTTGGTCCAGAG
EMPV1_20034	CAGACGGTTCCCAAAACAGATGCTGCTAACCCTGTGCCTCACATGCTGGATTTTTCAGGC
EMPV1_20035	ATGTTTCTCTCACACATCCTGCCAAGAAAGGCTGAACTCCAGAATACCTTTTGCTGAGGC
EMPV1_20040	TTGAAGCCCCTATGGAAGGGCCCCTATACTGTAATTCTAACCACCCCCACCGCTCTTAAA
EMPV1_20042	GAAACCGTGTCTTTTCATCTTCCCCTTCCTCTGTCTTCTCTCTTGGTGCTGGTGCCTGGC
EMPV1_20043	CTGCACCGAGTGTGAGAAGAGCTTTGTCCAGAAGCAGCACCTCCTGCAGCACCAGAAGAT
EMPV1_20044	TTTGAGGTGTCACCACCCTGAGCTTGCCAGGGATTCGGGGAGGGGTATTAGAATGATATT
EMPV1_20047	CACAAAGAGCGGGCAGGAAGGAACTCCCCTGTATCATGTTCCCACAGGGTCGTCTTAAAT
EMPV1_20048	AACCTCTATTTCTACATACGACTAGCCTACTCCTCCTCACTGACTATGTTCCCATCCACC
EMPV1_20051	GGTCCAAGCTTTCTTCATGCAATGGAGACTGTGGAGCAGTTAGGACCAGAAAGCGCACTA
EMPV1_20053	TGACACCCTATATACCTGTGATTAGAGATGTGTATATATATGAGGGCTAGGCATGCTGTG
EMPV1_20055	GCATGAGGAAAACGAGGCGAAACGTACCATGCAGATGACCGCATTAAACGAAAACCTGGG
EMPV1_20057	CTTCTCTGAGACTCAGATTTAACGAAGTGGCCTGTGTTTTCTCTGCCGAATCCAGGCAGA
EMPV1_20058	CTCTCTTTTGTCAGTCTTCCAACCAATGGTGATAGTTGAGACCCTGCTGGTGTCCTGTGG
EMPV1_20059	GGACCACTCCTGTCTGTCAGTCTATTCCTGGGAAGCTCATTGCTAAAGGCTCTGGAACCT
EMPV1_20061	TACTGTCATTGAGGTGAACGTGAGTGAGTTGGGCCTTGTGACACAAGGAGGCAAGGTCAT
EMPV1_20062	AGGGTAACACCCTCACTTCATGTGGTGCAATACCATTAACGAAAGTTAGGAGTCCGTGCA
EMPV1_20063	AGTGCCTTTACGAAGAGACGGCCTCCGCAGCAGGTTTCGTCTGCCCAGTCTGCTGCTTAC
EMPV1_20064	GTGTTGGAGGTGTGATCTGTAGGGCATGCAACCTCTCAATCCCTTTCCATGGATGTCTAT
EMPV1_20065	GTCTCATCGTCGTCATCAACATTGATGCTATTGAGTGCTAGCTTTGTGCCAGGCATTGAG
EMPV1_20066	AAGGCATGGCGCTGCCCCTGGAGGTGCGGGCCCGGCTGGCCGAGCTGGAGCTGGAGCTGT
EMPV1_20068	TCCATTACCATCAACCTGAAATTTTAAATGTTTTAAAGCCTGTGTTTTCCATGTCTGAAC
EMPV1_20070	ACCCCGGCTGTCGATGCACTGTGATCACTACTAGGAAAAACTCATCCGCACCTTTTCCTT
EMPV1_20071	TTCTCATCTCTTTGGTGGCCTCACTCTTCATGGGCTTTGGAGTCCTGTTCCTGCTGCTGT
EMPV1_20074	TCGGCGAGTACCCGGTGGAGCAGATGACAACCATCGCCCAGTGCCAGTGCATCTTCTGTA

EMPV1_20075	ACTCTATAATCAACAAGCGGCTCTCAAAGTCCTCTGCCACGCTCTGGAACTCCCCCAGTA
EMPV1_20076	CTTCTCTTTGCCTGTGGTAGTTTTAATGTGTCTGTGTCCCTGGCAATAGTCCTGGCCTCC
EMPV1_20077	GCGAACTAGGTAGCCCAGGGGCCGCTTACCATTGTACCATATTCCCTTCTGTATCTTTCA
EMPV1_20078	GGCAGAACTGTGAGTTCAAGGCCAGCCTGGTCAACAAAGTAAGTCCAGGATAATCAGTGA
EMPV1_20082	GCATGGACATCGAGCGGGTGAACATTGCCTTCAGGAAACTACAGTAAAGGTGGACCAGAT
EMPV1_20085	GTTCAGGAAAATTAAATGTGGGCACCAAGAAAGAGGATGGTGAGAGTACAGCCCCCACCC
EMPV1_20087	TAAATCTCAGAGGTCTTAAGAAAGCAGCTGGAATGTCATATGGGCATAGCCTTAGGTAAG
EMPV1_20088	GGGTGGAGGAAATGCGCCAGTCCCTTCGAATCATCTCACAGTGTCTGAACAAAATGCCTC
EMPV1_20090	ATGGTGCTGGTGCTCAGACAAATTTCAGCACCATGTCGATGTGGGAAGACCGAGGCTATG
EMPV1_20091	TCTCCGATGTCCCAGTGGGCGTGTCCTGAGGAGAAGGTTTTTTAAGTCTTGCAGTTCACA
EMPV1_20092	TGTGCCAGACGATCTGGAAGCCGGCCCCATGGATCTGTACTTGAGTGCTGCTGAGCTTCA
EMPV1_20093	TTTTCTTGGGGTGTTTCTTGCCCTTCCGTGGTGCTGTGCCTGCAGGCCTTGTCTTAATCA
EMPV1_20095	CCGTTTACGCTGGTCCTAGCCTCTACCCTTTTCTCCTCACCCAAGATTTGAGTAGCTGTT
EMPV1_20096	TCAGATCATCCGGCGCAAGTGGGAGGCGGAGGGAGTAGCTGAACAGTATCGGGCATACTT
EMPV1_20097	GAACTTAGAGCCACATCTTGCACCCCAGGAACATATGCAGAATTTGCCCTTTGAGCCTCG
EMPV1_20098	AGTCATATGGGTTTGCCACCATAAACTGGCCTCCCTATCCCCAGACTAAAAGGGAATGGG
EMPV1_20100	CCTGCAGATACAGCGCAATGACTACGTCCACGCCCTGGTCACCTACTTCAACATCGAATT
EMPV1_20102	TGAGGACTCAGGTTAACTTCTTGACCCATCTATTGAGATCCACAAACACCAGCTTCCTTC
EMPV1_20103	CAGCCTAGGGTTTAATGCACTTACAGTTCCTTATGTTTGTCTTCAGCCAAGATTTGGTCA
EMPV1_20104	ATGGTGCTGTCCTTTATTACGAAAGAGAGAAGGCAGACTGAGCATAGGCAGAGATAAGGA
EMPV1_20105	TGGAGAATCCCAAGAAGTACATCCCTGGAACGAAAATGGTCTTCGCTGGCATTAAGACGG
EMPV1_20109	TAGGGTCTATTAGTTTAAGTCTTCAGCTTACTTCTACAAGACTGGTTTAATATGGACCAG
EMPV1_20114	GCTGGAATTGTTGATGCTGTGTGATGGATCCATAGGGGTTTGCGATACTGCTCTCCACAT
EMPV1_20116	AACCGGGATTAACTCGACTGACGGGAGATAGATGTGTTGGCTGTTGAGCTCAAACAACAG
EMPV1_20117	ATCCAAATGGCCATGGGCACAATTATAAAGTTGTGGTGACAGTACATGGAGAGATTGATC
EMPV1_20118	GTGTGATCAGGTCTGCTTTGGGGGGTTATTTAGGACATCCTGAGTTCTCGATTGTTGGCC
EMPV1_20119	GGGAGGGCAAACCCCAAATAATGGAATGCCTGAAGGGGAGACTGAGACCAGAAATGGGAA
EMPV1_20122	CCCCCAAAGAAGCCACAGGCAAACAACCATTACCACATCTAATAAAGGAAGCAAAGTTGC
EMPV1_20123	ATCACCGTCCTGATTCCTGGTTTCTACCGCGGCATGGACCTGGCCTGTGACGGCTCCTTA
EMPV1_20124	CTTGGAGACTTTTTCAGAGTCAACAATCCAAAGCAAAGTACTTGGTCTTTTGAACAACAT
EMPV1_20125	AAGCCTGAGCTTGAAGTGAGCAGTTGAGCTTCTGGAGGGTCTAATGGGATTGTCACTCAG
EMPV1_20127	GTTGACAAGATACTCAATAGCTGTACTGAGGGCAACTACAAGGATGGGGCCAAGCTGTGT
EMPV1_20129	AGAGTCCCCTGACCCCAAGCCCGGTGCTCATTCTACTACCTCTCACACTTCACAACATTT
EMPV1_20130	TGAGATCCCTGAGACCACCCAGACGTAGGTGCTCTAACAGCTCCTGATGCTCCAGTCCCT

EMPV1_20131	GGCCGATTATGTGGTCAACACCTACTTCCGCCACTTCAAGCTCTACAAGTATGTCTTCAC
EMPV1_20134	GCTATTGGTGGTTACCTTGGATCAGCATTGTCGCCATCTCCCAAGAAGGATGTGGAAACC
EMPV1_20136	GAGGGGATTACTGAGACAGGGAAGAGAAGTGCAAGAATTGGTTGCTGGTGCTATGTGGAG
EMPV1_20137	TTATGTCTTGTGCCAGCATGGAGATGGAGGCCACAGCCACTGAGGAAACTTGTGGAAGAC
EMPV1_20141	CTGGCCCATGTGGTGATGGCTCCTGTGTTGGGTCATTTGCTTTGTTGATGGCCTTTAATT
EMPV1_20142	CTTTAGAAGGGAAGCTCCACATTCAGATGATCCTGTTTATTCTCCTTCCACTTAATAGAG
EMPV1_20144	GACATGGCTGGCAGTGAGGACTGGCTCCTGTGTCAAGCCCTGAACAACTTCACCAGACAA
EMPV1_20145	ACAGGAGTCTGTCCATTTACAATGATCCGTCAACTTTCAGCAAAGTGACCCAGTTATACG
EMPV1_20147	CTGCAGCTCTGATTTGAGCCCTAGCCTCGGAGCTTCCATATGCTGCAGGTACGACTCTAA
EMPV1_20149	GTCCCTCTAGCCACAGCCTGCACCGCGATGTTGACATCACTCTTCCAGTAGTGAATAAAA
EMPV1_20152	TGCCTATACTGCTAGAATTTCTGTGAATAGCAACAATGTTCCGTCTCTGTTAGATGCAGC
EMPV1_20153	GGGATACCGGGGAACAGGTGTGGAAGGGACAACTACTTTCTATGATGTGCATTTATAGAT
EMPV1_20156	GGAGCATCCTGCTTTATTCTGCCTCTGCCAAAGTCAAGCCCCAAACTTGATCCCATATGC
EMPV1_20157	AGAGGCAGCGTGGTCTGCTGCCATGAGGTCAGTCTTTGCTTGTTCACATACACCGTCAGA
EMPV1_20158	GCTTATCATGCCCATTTCTGATTACTATCTGCTTTCCCATGCATTTGGGCCCCTGGATGG
EMPV1_20160	CACGGCGGAAAAACCAAATAGCCCCATGATGTACCCCCAAGCCTTTAACAACCAAAACCC
EMPV1_20162	CCGCCTCCCGGGACCTCGGGCAGGTGCAAGTTCATTGTAAAATAAATTCTGCGTGTATTT
EMPV1_20164	CAGAAGCTGCCTGGCATCACTGCTCTCAGTTTCTGGTTCCCACTGCAAGCCTCATTTTCA
EMPV1_20167	GCACAGCTACAAGGAAATCATCATCGAAGCCACTAAGCATCGCAACTCGGAGGAGATGTG
EMPV1_20168	TTGCTGGTCAGAGAGGAATCTAGGCCAGGGCAGTTTAAGAGGAACCTAAGAGCTCGTTTC
EMPV1_20170	AAGCCCCCAAAACTGGGAGTTCCCTTTGTGGCTCAGCAGGACTAATATCCATGAGAATGC
EMPV1_20171	CTGCAACCCACGCTCACCTACTGGAGCGTCTTCTTCAGCTTCGGCCTGTGCATCGCCTTT
EMPV1_20172	CCACTCTTACTAACACCTGACGGTACGATGCCCTGTGCGGGTTAGTCAGGATGAATCACT
EMPV1_20174	TCCAGCTCCTGGCCCCTTCTGGTACTAAACCTACTGCTGCTTCTACTGGCACTGCCACTT
EMPV1_20175	CAATCTTGCAGCCTGAGTTTGTGTTGGAAGCATGTGACTGAGTGTCAGGGTCCTCAAGCA
EMPV1_20176	CGATGCCCTGAAGCCATTTTCCAGCCTTCCTTTCTGGGCATTGAGTCCAGCGGGATCCAT
EMPV1_20177	ATGAGTCAGTGAGACACGCACAGGTCAGAAGAGCCAGTGCAGGGGTTTATTACACGTGGA
EMPV1_20178	TGTCAGTCATCATCTTGCTCGAGTTCTGCTGTTGTGGAAGTGTGTCTTTCCCGTGTCTCC
EMPV1_20180	GTACAACAATTTTCTTTCACACACCGTTTTTGTGGGTGTGTATATATCTGACTTGGGGAG
EMPV1_20181	TCGGCTCTGTGTAAACATCTTCCCTGACCGAGTGTGTTCTGTCACTGGCTTTGCTCTTCC
EMPV1_20182	TGTGTCCTGGTTGGGCCCCACAATGAACCACAATCTGCTGGCTTCCAGATTGAAACAACT
EMPV1_20185	AAGCCTTCAGTCAAGAGGACCATTAAACCCCCAGACATCCCAGCATGAGCAAGAGGTGGG
EMPV1_20187	TAACTCCTTGGGCTTTCGTACCTGTGATGATGATCTGTCATTTGGATCACATCTTCATCC
EMPV1_20188	GTCTTAAATGAGTATTTCCACAATGTCTGTGAACTGGACCTGGTGTTCAACTTCTACAAG

EMPV1_20189	CAAAGGAGTTCCCTTTGTGGCCAGTGGGTTAAGAACCCGACTAGTATCCATGAACACCTG
EMPV1_20190	ATCCTCGGCAGATCAACTGGACTGTTCTCTACAGAAGGAAGCCCAAAAAGGGACAGTTGG
EMPV1_20191	TTCATGCTGCTGTATGCCTGGTATTCTTGGCCCAATGTAATTTTGTGTTTCTTTGGTGGC
EMPV1_20196	GAGCAATGACTCTGGAGAAGACCCTAGTGAATATAACACAGACACATCTGCCCTGTCCTC
EMPV1_20197	TGGCGGTGCCCCTCCGTCTTGCCGGTCCTCAAGCCTAAGAAGCTGTTAATTATTACTCCT
EMPV1_20198	TGACCGCACAAGCATTCTTCACGTTATGGTCAAAAAAGGTCAAGTCACTGGTGTGGAGAC
EMPV1_20199	AACCCCTATCAGACGGCCATGTATCCCATCAGAAGTGCCTACCCCCAGCAGAACCTGTAT
EMPV1_20200	GTTCTCCACATATTTATGGACTCCTTTATCCCACGTCACATTTGCTTTGGTCTTATATGT
EMPV1_20201	AGTGAGTGCCCAGGTCTGAGATGTTCCTCCTGTGCTTGGAAGAAGTGTCATGCTGCAGAT
EMPV1_20203	GAACCTGTTCTGCAACCCGTTACCTTTAGAATTGATCTACCAGAGTCGACAATAGCTCCT
EMPV1_20206	AGCTCTAGGGAGGAGGGTCAGTCTTAAGTGTGTTGCATGGCTGGAGCTGGAGCTAAAACA
EMPV1_20207	CGCAGTATTTTCTCAAATTCATAACATCAAACCACAGATGCAAGCAGCTAGAGAACACCA
EMPV1_20209	AATCGGTGGCCGACGTCAAGTCTGTATCCCTTGTCGAAAACCTTATTCTGTGACATCGCG
EMPV1_20210	CCTCTGAACCTTGAACCTGTGCATTTCTAGGAACACTTAGGCGGTCACGTCCTCCATTCA
EMPV1_20212	GCTGGGACCTGTTTCCCTTTCTTGGGGAGAGGAAGTGACAGCAGATTAAAAGCAGAGAAG
EMPV1_20216	CCACCTTGATGCGTGCCCACAGTATTTGATGCGTTTTATACCTATGGAGACCTTAGGCGT
EMPV1_20217	TGACCCCCAACTCTGAAGCCTGTTTGAATGGTCACCTCTTCCTTGAGGTCTACGCGCTCT
EMPV1_20219	CCCCTTCTCATACGCTCCCTCCTTCTCTGCACTCTGGACACTGGTTTTCTTTGCCAATTG
EMPV1_20220	TTATTCCCAAGAACTTTGCCTCCACTGTCCTTGCCTCACAATGAGCCACAGTCACCCCTT
EMPV1_20221	CATACAGGGCAAAGGAGTGAATGAAGACGTGCGAAGGCTCAGAATTTGACACCTGGAAAT
EMPV1_20223	TTTTCCTCATTATCTGCCCCCAAGAGCTGGGACCTTGAAACTGAAAGCCACTGATCCCTT
EMPV1_20224	TCCCGTAGGGATGTGAAGAGAGAAGAGGGAGAGGCCTTTGCTCGGGAACATGGACTTATA
EMPV1_20225	TCGGCATCTCCCAGGGTCTGTCCTCATGGAGAATGTGCAGGAGTGGCGGCCTCCCCTCTG
EMPV1_20226	GCCCATAAATTGCTAAAGCACTCCTGAGGAACAAAACAAAGCAGGAGGCATAACTCTCCC
EMPV1_20229	GATGGAGTGATGAATGGCGAATACTACCAGGAGAGTAATGGGCCGACAGACAGTTATGCA
EMPV1_20231	ACCTCATCAGCTAACTACAGTGCCCTCTCGTCTCATCCCAGACGGACCACCAGCTCATTT
EMPV1_20233	CGGTGCTGCGGACCTCTATTTTGAAGGAAATATTCATCAGTCTCTGCAGAACATCCCTGA
EMPV1_20234	CAACCACAGCCCCTCACAAAGAGAGGTATTTCGACAGGGTGGATGAGAACAATCCCGAGT
EMPV1_20237	CCCCTCAACCTGCCACTGCTCAAGTAGCTCTTTGGTGCCTTTAAAACAGGTGATTTTTAG
EMPV1_20238	GTTTTGTCTTTTTCCTTTAGCCTTAGCAGTGGTGGTTATGAGGTGTGCAGGGGGAGCTGG
EMPV1_20239	TCCGTCCTAGGTCCTGATTTGCATCAGGAACAACCCCTATTTGAGGGTAATCAACATTTA
EMPV1_20242	CTGGCTGTGCCAGGGAGTGGCAGCGACGAGGTTCAGATTTTGGAGATGCCGTCTAAGACG
EMPV1_20243	GTGGGGCAGGGGCTGGGCAAAGGCGTTCCAGGCCAGCGTGGTATCCGAAAGCGTGAAGAG
EMPV1_20245	CCAGATCACGGGTCTTGACCAAACCCTGCATTTGGTTCCCGCACCCTGGCCTGTCCTCTT

EMPV1_20247	GATGGACAGCTGGATGGGCATTTGCTGTTTCTTACAAAACCTCCCATTGCTCTGAAGCGC
EMPV1_20248	ACGAGCCAAGGACAAGGAAGCGACTCAGAGGAAACTGCAAGAGGAGGAAAACGAAGGCAA
EMPV1_20249	TCGTTACCTGTCCTTACATGACAACAAATACATCAGATACTTTCCTGGACATAGCAAAAG
EMPV1_20251	GCTCACCAATATTTATGTAATAAGCGAACACTAAATTTACAAGTTAAAATGAAATCAGGC
EMPV1_20252	CCAGCTTGGGACAGTGTCAGTAACCATGACAGCAAGAAGCAGAGAGATTTCCACCTCCAA
EMPV1_20254	CAGTAACGGAGGTAATAATAAAAACCAATGAGCAGAGCTGTGACCCTTAGGCCGAGCGGG
EMPV1_20255	GTCTGGTGGATCCAGTGTCTGGAAGTGTGAAGACAGTAAGCGTTCCTTTCCATTTAGCCC
EMPV1_20257	GTGGGCATGTAAGAACAGAATCCAAATCTGCAGGAAGAAGAGATGATGGAGAGTCCTTCC
EMPV1_20260	GTGACAGGAACTAGTGGTTTATGCCAAGTGGAGTGAAATGAGCCAAAGAATTGTCTTGGT
EMPV1_20264	GTGCAGGTTTTGCAGCATTTGGACAAACAAAGCCAGTGGTAACTCCTTTTGGTCAAGTTG
EMPV1_20267	CAGCTGTGCAGCCCAACAGTGGGTCATCTTGTCAGTGGGCAGTCAGAAGGTTTGAGTCAA
EMPV1_20268	AGCGTGCCCCCTTGGGCACTCAAAGAAATCCGAAATTTGCCATAAAGGAGATGGGGACTC
EMPV1_20269	CCCTGTTGGGTTCTACTTTATTCACAAGGCCTGAGAGCTCCCCATTCTGGTTTCCTGACT
EMPV1_20270	AGTACGAGATGGTGAAAGTCAGCCTGGGCTTCGGGCTGGCCCTGGTCCTGCAGCTGGCGT
EMPV1_20271	CTCCTGCCACACCTGCATCTTCTGTCTCACCTGCATCTTCTGTCTCACCTGCAGCTCCTG
EMPV1_20272	AACGAAACAAAGCCAACAATAAACAGTCAGGCAGAAGGTGCATTTACGACGTGGGGTGTT
EMPV1_20273	ACGGCAGGAACAGCAGGGTGGTGACTGGGCCAAGAGGAGACACCCCGTTTCGGCGTCAGT
EMPV1_20274	TCGAGTGCTTATCCGGCAGCGTGTCGGGTGAAGATACCCGCGGCCCTGCCTGTGGCAGCT
EMPV1_20275	ACACGCAGTGGGCCCTCAGGAAAGAGCCTTCTTCATCCGACTAGTGTTGGTGTGTTTCTT
EMPV1_20276	GCCAAGGAAGAGCAGCAGGACGACACTGTATACATGGGCAAAGTGACCTTCTCGTGTGCA
EMPV1_20277	GGTCCGCGCTTCTCACAGGCTTACCTAGCAGCAGCCTTCGCCGCCCTGTGATCTCTGGCC
EMPV1_20278	CATGGCAGCATTTCACCAGAACTCTACTGACTTCATCCTCTTGGGCCTCATTATGCATCA
EMPV1_20279	CACCACTGCCACAGTGCAGACATCCCTAGCAATGCAACTGCCTTTCTGTGGGGACAACAT
EMPV1_20282	TACTGTTCCCCTCCCTGGGCTCACACCAGAACAAACACTTTATCAGGACCCTCACCCTAC
EMPV1_20286	GGCCTACAACACAGCCACAGCAACACTGGATCCTGCCAAATCTGTGATCTATACCATAGC
EMPV1_20290	ATTCAATGTGGGATCACGGTGCCAACTTTCAGTCACACGTACATAACAGTCGCGTCGGTC
EMPV1_20293	GTGAAGATGGCACTCCGAACATCAGGACATCTCTTACTGGGAGTAGTTCGAATCTATCAC
EMPV1_20294	ATGGCAGAACGTGTACTGTGTCCTGCGGCGCGGGAGCCTTGGCTTTTACAAGGACGCGAA
EMPV1_20295	TGGACCACTGGCTGTAGAGCATCAAACTGAGGGAATGATCCGTGCTTCTAAATGATCCAC
EMPV1_20296	GCAAGATGCCATTAAGAGAGGGGACTGTAGGAAGTGCTTGACGGAGCTGAACAATGGACA
EMPV1_20297	TGGTTTCCTGGAGGCCAATCTGATGATGTCAACATTCCTCTGGGCAGAAAGGCAGTGGAA
EMPV1_20298	GTGCTGTCATCTTTGGAGTTGTGGTCAGATAAAAATAAGATCTCTACAGTTGGTGAGGCC
EMPV1_20303	TCTCTTGCTTCTGCCCCCAGCCCTCAGATACCAATGCTTCAAACCCTCCTCAGCTCATCT
EMPV1_20304	GTGACACCTAGTTTCCAGAAAGAGACACAGGCAGTAGGAAAGTTCAAAGGCACATGCAGC

EMPV1_20305	AACACGGCTACTTCCGAGCCCCTGAGCGTGGGGTCTATTTCTTTGCAGTGAGCATTGAAT
EMPV1_20306	CACTCCGACCTCGAACCCAGAAGACGCAGGTTGACAGAACTCATAGCCAATTTCAATCCC
EMPV1_20308	ACAGGTAAGAGTTAGAGGAACACGGTCCCTGCAAAAGGTCACAGACAGTGGAGCCAGCAG
EMPV1_20311	CAGCTCAGGACCATCAGACTTTTTTCACCTGTGACACGGACTTTTTACGTCCATCAGACA
EMPV1_20312	CACGTGCTCTGAACGGGTGATGCCGTGTTCCTTGACAGAAAGCAGAGGAGGGACAGCCCA
EMPV1_20316	TCTCCTTCTTGGCCTTCTTTTTGTCGCCATGCTCTGACACGGCTGCGGGCTCATATTTAT
EMPV1_20318	AGCCTGCTGGGGAGGTGTGGCTTGGGGAAACCTCAGCTATAGGAGTTTACCTTCTTTTCT
EMPV1_20319	CTACTGCAGCCTTGGGGAACATCTCTTAGTGTTGTGTTGTTGCTTCTCTACAAAGTACAC
EMPV1_20320	GGTTTGGGGGATGTTCGTTATTACAGCCTAATTTCATCTATCCTGACTGATAGAGGCAGC
EMPV1_20321	TTTTTTACAGAAGCTGAAAATGTATGTTTTGCTCTTTGACTCGCTCCAGCCCCATGAGGC
EMPV1_20322	GATTTCCTACCGTGGGTCATAACACTGACTCCTCAGGTCTTGTAGGCTTCTCCTGTCACA
EMPV1_20323	GTGGAAGCCTCAGTTGAGATCTATAACAGGATGAGCATTGATCTTTTGCCAACACCGGCC
EMPV1_20324	AACCAGCGCTAATTTATGCTTGGCAACTGGTGTGCGGGGCGGTGTAGACTGGATGAGAAA
EMPV1_20325	CACGGACATAGCTGTCTTCTGTCTGCTGTGCGGGAAGCGTTTCCAGGCCCAGAGCGCGCT
EMPV1_20326	ATACTACACTTGGCTCCGGCCGTGTCCGGGCGCGGTTGCTTTTTCTTGGGACCTTGAGCG
EMPV1_20327	CAGGGAAGCTGATACAAAGCTGTCTCTGCCCTATACCCAGTGCTACCCATGTTACTCCAC
EMPV1_20328	CCCCGTGTTTACATGTCCATGGGAAGCTTACCAGCTGGAATTTTGGCCCTATTTCTGCAT
EMPV1_20333	AGCAATCTCCTGGATGTACTCCAAGCTGTCAGGAAAAGAGCTTCTCAGCGGAGCTGTCGT
EMPV1_20335	GCCTACACGCCCAGACACACACATACCCAACCAAAAAAATCTCCCAAGCTCCTCTCTTAG
EMPV1_20338	GGGGGTACCCGATTTCCATAGATCCTAGCTCCTCAGTGACCTGAGAGACAAAGGCTTTTT
EMPV1_20339	AGACTCTGGGCAGGTTCTCTTTGAGGCATGAGCTTGTCCTTTTCAGTATGTTTACACGTT
EMPV1_20340	GGGCCTCGGGCCATGACCTCAAACGACAGTAGACTTTTAATTTTTCTGACACTAGACTCG
EMPV1_20341	CTCAGATATCGGTTGGTACGTCCTCTGCTGTTCTCCAGGTTGGCCTCACTTATTTGAAAC
EMPV1_20344	TTGCCATTCTCACCACTCTATCACCAGTGCCTTCCCCTTTACTTGCTAAACGAAGAGCAC
EMPV1_20346	ATCAGTAACCGCTGCTGCTCTAAGAGACCAACTAACTCGGGCACTAACTATGATTGGAGC
EMPV1_20347	TTACTGCGATGCTGTCCCTCTGTTAGCATTGTCCTGCTCTGACACTTCCTTTCCAGAGAC
EMPV1_20349	CCTTTGTCACACTCTCCTCCTGGCCCAGCTGTCATTTTGTTCTGAAAACACCATCCCCAA
EMPV1_20350	GCTGGGTGCTGGCAGGGAGGTGATGGTTAAATATGCAAAATCGCTGTTTTCTTGGGGCTA
EMPV1_20351	GGAACCCGAGAATGAGAAAGCAATGGAGACTCGGTGTGGTCTAAGGCTACGCAGTAATTC
EMPV1_20352	CCTGGGAGTTGTCTTCATCCAGGAGTATTACAGCGTCTTTGACAGAGCCAACAACCGCGT
EMPV1_20353	GACCCTCAACGGCTCGCTTTGTGGGACCGGGAAGAAAATGATTATCCAAAGGTTGTCCTT
EMPV1_20354	CCTACTGGGAGGTGGAGGTTGGGGACATGCAATCTTGGGACCTGGGAATTTGTAGAGATA
EMPV1_20355	GATGCCACCTCCCTCCGAAATCCAACGGCTCTATGAACTGCTGGCTGCCCACGGTACCCT
EMPV1_20356	TTCCAAGAGATGTGTTCCTTCCCCATAAACCAGACCCTGAAGTCTCTCAGAGCGAACCTG

EMPV1_20359	CCCGAAGAAGCCATAGTAGCTTATGAGCAAGCATTAAACCAGAACCCAAAGGATGGGACC
EMPV1_20360	AGCGCTCTGCATCCCACCGCAAAGTGTGGCTCTCATCATACACATCACCTTCACACACAA
EMPV1_20362	GTCACTATGTTCATGCTGGCAACATCCTTGCGACTCAGCGCCACTTCCGCTGGCACCCAG
EMPV1_20363	GTGGAACCTTGGGAGAACCGAGATGATATTTGGGAAGAGTTGCTGCCTTCTCTGGAAACA
EMPV1_20364	TTCCTGTCCATGTGCCCACGCCCCGCACCAGCTGTTGCCCGCTACCGCAACCTGAACTGT
EMPV1_20365	GAAGAGAGGTCAATCAATTTCTTGTTCATAGACCACTCTTCATCAGATTCCATTATGGCT
EMPV1_20366	GTCAGAGGCAGAAATGTGAACTCTTCAATGGCCCCGACGCGCTTCTTCATCTTGTAGCCT
EMPV1_20367	GCGTTTTGTTGAAACTGAAGATTACAAAGACCGACTAAAACATGGGGAAAAGTGGGAATA
EMPV1_20369	CTGGAGGGGGAGTCGCTGCCCCACCCGGCCCTGGCGCCCGCCTGGCCGGCGTTGGCCCAC
EMPV1_20370	TGCTGTTTGGTGGAGAAACTCTGACCAGAGCCAGAGACACCATCTGCAATGACCTCTACA
EMPV1_20371	TTCCCGCCTCACCAGTTGGTACCTTTTGTTTTTTGAAAGAGGTGGATCCAGAAGAGCTGT
EMPV1_20373	ACTTTGGGAAAAGTTTCTCTGATGGTGGCAGTTGGGGTAGGAAGCTCCAGGGGTGCAGCT
EMPV1_20376	TCATGAAGCAAACCATCAGCAATGCCTGTGGAACAATTGGGCTAATCCATGCTATTGCCA
EMPV1_20378	ACGGTGCCTGACTTTCAGGAGACTAGACCGCGTAATTACATTCTCTCGGCCAGCGCCTCG
EMPV1_20379	CAGCTGCTACCTATGCCACAACCACAGCAACACCGGATCTGAGCTGCTTCTGAGACCTAT
EMPV1_20380	CTAGCAAAACCACTGCGAAGTTATCCACCAGTGCTAAAAGAATTCAGAAGGATCTGGCTG
EMPV1_20381	TCCAAGGGGCACATAGTACCTTAAAGGATTTTGGGAGTTGATGACGTTTATTGCGTCAGC
EMPV1_20383	GAGGTTAGTTTTGTACCCCAGTAAGTATATGGTCAATCCTTGAGAATGTTCCATGTGCAC
EMPV1_20384	AGGGTATACTGCCTGAAACTTGCAAAAATGTTCAACTACCCAGAAAGCTTTGTTCCTGTG
EMPV1_20385	CTGAGGAAACTGCTAGTATTTCTGGGAGTCCTGCACAGTCTAGTTGCCAAGTGGAACATT
EMPV1_20387	CCCACTGAACCACAGCAGGAACTCCCATTCTTGAGAATTTTATGAAAGCTTCTCCCATAC
EMPV1_20389	TGCTCTCCCCCTGCACCAGATTACAAAAACAACATCCTCAAGGAGAGGGCCGAGCTGGTC
EMPV1_20393	TAGGGTCATTTCCACTAGGATAGGGTATATAGGACATAAGTCAGAAACATTGGGAGCTCC
EMPV1_20395	CTGGAGTCCACTCAAGCTGACATTTAAATTACACAATCTAGAAGGGAAGACCAAAGGGGT
EMPV1_20397	TGATAATTAGTTTAAAACAAAACAAATGATTTCGACTCATTAGACTATGATTTACTTCAT
EMPV1_20401	GGAAGCGTTGGGTTTCCCTTTGTTTAAGAATGCTGGCTTCACACCACAGGAACGTCAAGG
EMPV1_20402	ATTCTCTGCAGGACTGTGTGTTCAGCAAGGAAGCAGGACGCATGTGCTACGCCATCATTC
EMPV1_20406	GGGACCATCCTAATTACATAGGAGCAACATATCATGGAATGGCATGCAATCCCAAGTTTG
EMPV1_20408	TCAGTGTAGGACTAGACCGTGCTTATGCCTGTGTTCTTCTTTGCTGGCGTAGAACTTCAG
EMPV1_20409	GAGCTCCTGTCAAGTGGTTCTGCTTGGTTTGCCATAATCCTCATGGTTGTTACATGTCTG
EMPV1_20410	TATTTGTTTGGATATCCTGAAAGATCAATGGGCAGCAGCAATGACTCTGCGCACAGTATT
EMPV1_20411	CCAGCGCCCCGGCCCCGCTGCTGCCTCTGTGCCCCCAGGTCCCGCAGCGCCCTTCCCACT
EMPV1_20413	AGACTGCTCATCCTAACCTAGTCCTGTCGGAGGACCGTAAGAGCGTCAAGTTTGTGGAGA
EMPV1_20415	GGATTTTACTAGACTTCCCTCCCTGTGTTGTCTTTCCACCAGCAGTGTACAAAGTGGCTC

EMPV1_20416	CAGCCACCAGATAGAAGGAAAACCAGCCAGGGATGCTGTGGGAGAAAAAATCCAATGTCC
EMPV1_20419	CCACAGCAGTTGAATAGACTCCAAAGAAATGAGAGGGAAACTGCCAACAATGCAGGGCCC
EMPV1_20420	CACTTAAAACCCACCTCATGCTTCCTTAGGCTCCTCTGGTCCTTACCCTCGTCTCTGCTT
EMPV1_20421	ATTTTAGGAATTGCCTTTATGTTCATGTTCACCGCCTTTCAAACTTGTGGAAATGTAGCG
EMPV1_20422	TGTGCGGCCCTTCAATTCTCGAGAGACCAGCAAAGAGTCCAAGTGCATCATTCAGATGCA
EMPV1_20423	CCCGTACAGTGATTTTCCAGTAGGGCTTCTTCCACTTTATCTTTGTGTCTTGGCTTCTGC
EMPV1_20425	AGTCAGGATGTCTTACTGGAAGCAGTACAGACTTGGCTCCCCAGATTAGAGGTAGCGTTG
EMPV1_20426	GATGTTGAGATACAGGGGAAAACATTAAATTGGTTGGGTGAACTTGTGACAGCAGTAGCA
EMPV1_20427	ATGGCCAATTGAGTCTTTCTCGCACCATATTGCATCCCGTCACTCTGCCACTGACTCTTC
EMPV1_20428	GAGGTGATTATGAGGCCCATGATGCAATGGATTTTGTCATTAGTTTTTGATGGGAAGGGC
EMPV1_20429	GAACACCGAACACTTTTAGCTGGAGTTGTCTTTCACTACAAGAGGCCAAGGGACCCAGGG
EMPV1_20430	AAAGCCACCAAGAATCGGAAGGATGTCACTTTTGAGAAGCCGTGTGCACCATTCTGTCGC
EMPV1_20434	GCATTTCCTGGCAGAAAGGGAGCATCCGTGCACGTTCCCCTCTGCCTCTGTCTGAATGCA
EMPV1_20435	CTCCGAGGGACAGGTTCAAGGAAGGGATACCAGAAAAGTCCAGAAACTTGGTCCTACTGG
EMPV1_20437	TGAAGTGGCAGTTACAACAAGTTACGTGGGAAAAGATCCAGCCTCATCAGATCGACCTCC
EMPV1_20438	TGGTCAGTTCTGGTCGTCCTCCTCCTTGGTCGTCCTGGTCCCAGGGGTCGCCGCCCGTGG
EMPV1_20439	AATAACAGAAAGCTGCTCTACTCATCGGCTGGAGCATGCCTTGTATAAACCTCAGAAAGG
EMPV1_20440	TTGTGTCTGGTTTCTGGGTATCCTGGCTCTGACTTCTCAATGGGTACACTGGTTTCGGGG
EMPV1_20441	GAGGACTTACTAGCTGAGCCGGATGAAAAACCTCCTCCTCCTCCTGGAAGACCAGACTCT
EMPV1_20442	CATCATGACGTTAGTATGAGCATGAGATGCACACGTATACGCTTAAGATTCGGAAACTCC
EMPV1_20443	CCAAGCTTCTCTAGGTCTGTTTCATTATCTCTATGGCAACGCCTAGCAACAGTGCCTGGC
EMPV1_20444	ATGTAGTTCTGGCCCTGGTCGGCCTGACACTTGCTGTAAGATGGGGGAGATCTTGTCATT
EMPV1_20445	CATAATGACTACTGTTCTCCATTCTGCCCACCTCATTCCATGGTCACCACATTCTATGAA
EMPV1_20448	ATTATGAGGAAGCCGTGCTCTTGCTGTTAGAAGGAGCTGCCTGGGAAGAAGCTCTGAGAC
EMPV1_20450	CCTTGAGAGCAATGCAGCCAAGAAGCTGGAGAAGCTGGAAGAATGGACCCTGAGGATCAT
EMPV1_20452	CCGTGGTGAATATCATGTTGAATTGCAAATTCAAAGAAGCAGCTTCAATTCTGCCGGGAC
EMPV1_20455	GCCAGCTTCCCAGTTTGGCTGAGAGCAAGGTCCTCAAACATCAGTGGCCAATATTCTGTG
EMPV1_20456	AGGGATGGAGTAGGCACAGGTAGCTGGAGAAGTTCCAATAACGGACCATAGATAGAGAGG
EMPV1_20459	GGACGCAGAAGAGAATGCATTTCAACCCGACTCTGTATGTTCCAACAAAGAGCTGCTGAC
EMPV1_20461	AAATTGGGAACGTCACGTGAGTCCAGGGTGTCCATGTGGTAGTAGAGGCAACTGAGGCTC
EMPV1_20462	CTCCCAGCGGCTTCATGCTGTCGAGGAAAAGAGGAACCAAGAGTCAGTCTGAGAAGGAAA
EMPV1_20463	TAGTCTAGACAGGGAAACCAGGGAGGGTGTTTCCAGCCCAGTTTAAGAGCTGCATTGGGC
EMPV1_20464	AGCATCACCATGACAGGTGTGGTTCTTTTTGATTTTGCTGCTGACTTCATTGATGGGCCC
EMPV1_20465	AAGTTACTTCCGCTAGCAGTTCACTGGCAAAAACCAGTCATGCGGCCCCCTCCACTGATA

EMPV1_20466 GAGCTTCCCTGAGTCCGAGGCTATAAATATCTGTTAGTCTTCGTGTGTACATTCTCAGGC
EMPV1_20467 AATGTCCTGGAGAAGAATAGCAAGCGCTACCAGGCCCTTGGCCATTTGCTTGAACAATAC
EMPV1_20469 TGACCAAGTACGAGCGAGCCCGCGTGCTGGGCACCCGAGCCCTTCAGATCGCGATGTGTG
EMPV1_20475 CAGATTGGCCCATGCTTCTATGAGGTGAAACAGTTTTTGGAGTGTGCCCAGAACCAGGGT
EMPV1_20476 GTTATACATATTATGTCTGGCTTGTCATCTTTCTTGCTAGTGTTGGAGGCCTCTACACTT
EMPV1_20477 GCAAGGAGAAGCCCTCTGATCCTGTGGAATGGACGGTGACGGATGTGGTAGAGTACTTCA
EMPV1_20478 GACAACAACCAGATCCAGCCTTTGGTCGAGTGTCTAGCCCTTCCAATGCGATGATGTCAT
EMPV1_20479 TCACATGCGTGGAGGTGGGGGAGCGGCATTGTGTGCAGTTTCTTTGGTGATAGAGAAGTA
EMPV1_20481 GGTCAGCATGGCTGGCAATGTCAGTTGGATTTAGTAATTACAGAAAGAGGTAACAAGTAT
EMPV1_20483 AGAATGTCCAGGATGTGCTGCCCACCCTGCCAAATCCAGATGATCATTTTCTCCTGCGCT
EMPV1_20485 ATAACCAATATCCATGGTGGAACTAAACTCATTGTTAATGACCCGAGTTTTGCTTCGCTG
EMPV1_20488 GTACAATCATGAGCTTGGCAAATGCTCACCAATCCAATTTTTCTATTTCTGCCAATATCA
EMPV1_20489 AAGGAGGAGATCAACGAGCTGAACCGCATGATCCAGAGGCTGACGGCCGAGATCGAGAAT
EMPV1_20490 CAACTCTCTAGTTTTTCTCAAAGGAGCATTACAAGAATATTAGAGCACTTGCTTGGCTTC
EMPV1_20491 CTTGTAGATGAGAATTTTACAGAATTAAGAGGAGAAATAGCAGGACCTCCAGACACACCA
EMPV1_20493 GAGTTCAAAGCCTGCCTCATCAGCTTGGGTTATGATATTGGCAACGACCCCCAGGGAGAG
EMPV1_20494 GCCTGTCCGTTGCTCTGGATGGAGACTCTATCTCTGGTTTCCAAGGCTATGTGCTATACA
EMPV1_20496 ACAAACCCACAGCCTAGTTTGTGTACTGGGAAGGGTAGCAATTTCTGATGTGGATGTAGG
EMPV1_20498 ATGGCATAAAAGCACCTTTCTCTGAAGGCATTGTGATGTTTGGGCAGTGAAATACATATG
EMPV1_20499 TAGTAATGGAGGCCACACTAGCGACTACTCTTCATCCCTCCCATCCTCACCCAATGTTGG
EMPV1_20500 CAGTGGCCTGTGTGGTCGCAGCTGACTGGCATTTTCTTCCCAATGTGTTGCACTGAATTT
EMPV1_20502 TTCGCCGAGCACCTCTCCGCGCACATCACTCCTGAGTGGAGGAAGCAATACATCCAGTAT
EMPV1_20503 AGCTGTTTAGAAAGCCCAGGAACCAGCTTGAATGTAGAAGGGGAAGACGGATGAATGCTC
EMPV1_20504 TTTGGATGCTTGCTCTCTCTTTTCTCAGTGGTGAAAACCAATGGGGAGATGGGGGCTGCA
EMPV1_20505 TGCCTGCTTATCCACTGGGAGAGCTGGGGTCATCACCTCTCATTTCCTGGTACTTGGGTG
EMPV1_20506 TTCTTTCTCTCTGGTCATTCAAGTGATGGATGGCTTCTACTTGGATGCCTGTCTGTGCAG
EMPV1_20507 GGTAGTCCAAAATGCTATCGATCAGTTAGCCCTGAACGGACAGCGAGGAGATCAGTGAGA
EMPV1_20508 TTTATTGTTTATCTTGGAGTTCCTGTCATAGCACAGCAGAAATGAATCCGACTAGGAACC
EMPV1_20510 TGTGAGACACAACAATCAGCCTTCAATCAGTAGCTCTCAGTCAGGGGAGGTGGGGAGCAT
EMPV1_20511 TTCCTGAGCATGCATTCCCACGTCTGATTCTTCAAACACCCTGAAGATTTGCAAATTCCC
EMPV1_20512 GGCAATATGAAGATGAAGGGGGCTTTTTGAAGGAGGTGGAATCAAGGAGAGTGGTCTCCG
EMPV1_20513 TTTCTCTCTTTGAGAAGATGTCCCCCAGCTAGGATGGGTCACGGTGCAGGAGTGTGGGAA
EMPV1_20514 TGCAGCTTCTTTGAGTCCCCAAGTCCTAAATCTACTTGGCCACCACTCAGGCCCTCACCC
EMPV1_20515 CTGCTGTGGGATTACTTAAGCTGCCCAGGTACAGGGAACTGGTGATGCCAGGGACAGTTT

EMPV1_20517	GATATGTACAGACCTCTCAACGCCACACGCAAGAAAAACATGGCCCAGAAGCTACACGAC
EMPV1_20520	CTAGCTTAGTGGATAACTAAACTTTTTTTAAACAAGGCCAAGGTCTAATGCTGTTTTGAG
EMPV1_20521	TCTGCAGGAAAATGCACAGCATGCCACCAGGTTCATAGCTCTGGCAAATGTTGGAAATCC
EMPV1_20522	ATCCTTCTGTTCAAGGCCTCCATGCGCCATAAATGCAGCCTAGCACCGTCCGAGTACAAT
EMPV1_20524	TCTTCCTCTGAAGACCCGGAGCCCTGTTCTGCTTTCTCAACGTAGCCCCACATCACTTCT
EMPV1_20525	CCCTTTGCCTAGAGGACACAAAACTTAAGAACTTGAACCAAAGGTAGGAGCAGTAATTGG
EMPV1_20528	TGGTGGCTGTGGTGGTCGTGATAAGCTTTGTCCTCTTGGGAAGAAGCATCCAGGCAAACA
EMPV1_20529	TGGTGTTCTCTCCTCTACCACCTACCTCTTCCCTGCCCTGGTGCGGGCAAGGAGAGGGTG
EMPV1_20530	ACCATCTCATTCCTTTTGGTAACTCAGTCTGTCTCCTAGTGGTTGTTACTGACCCGCCTC
EMPV1_20535	CAAAAGGCTATACTGGATCCTGACACACAGGGAGAGAAGACAATGTGAAGAGACCTGGGG
EMPV1_20536	TGTCTCCAGCTGACCATCCCTAGTTCCAGGCTCTCTTCCTCCTGCTTCAATCTTTGACTC
EMPV1_20538	CCCAATTGCTATGTCCCCTCAGGTGTGTTCTTTGCTGATGTCTGGTTCATATGTGCTGGG
EMPV1_20539	AGCTAGGCAGAAGAGGATAATTAGTTGCTATTGTCATGCTATAATTCAAGACCAAATACC
EMPV1_20544	CGGCAATGTGCCTTTCAAGTATGAGGACTCAGAGACTGTGGAGCAGGAAGAGCTTGTATA
EMPV1_20546	GAGTTTCAGTTGAGGCTAATGGCTCGTTGGATGCAGTGAATCTGGCTCTTCTTATGGCGC
EMPV1_20547	TATGGCGGCTGCGGAGGCAACAGGAACAATTTTGAGTCTGAGGATTATTGTATGGCTGTG
EMPV1_20548	CCTCACCAACTTCAACATATCTCCTGGGGACATAATTCAACCCCAAACAGATGGCACGGC
EMPV1_20549	AGGCGTTGGGTACCTGTGGGTCTCATGTCTGTGTTATTCTTGTCTTCTACTCCACAGCTG
EMPV1_20550	CAGGTTCATTCCACTGAGCCATGACGAGAAATCCAGGAAAGACAGGCTTTATTGAAACAG
EMPV1_20551	AGGCCTGCTTTTCCCATCCAGGGCTTCTGCAGAGCAGAACTTCAAAGTGCTCATTACCAA
EMPV1_20553	CGCTCCCCTTCTCGCACATACACCTATATCTCCAGATAGATAATCATACACTGACCTCAG
EMPV1_20556	CCCAGGCCCAGGAATAACTCCACTTAAACAGTTGTGACCATCCATCGTCCTCCACTAGAG
EMPV1_20557	GGGATAAACCTGAAGGGCCCTAAGAGCAAAATTAACTACAGGGAGTTCCCCTGTGGCACA
EMPV1_20558	ACATTCTGCCTCTGAGGCGCTTCCTGGAGAATTGCTTGGACACGGATCTGCGAAACTTCT
EMPV1_20559	CTGTGCGAGAAGCCGGGCAGCCTCGTGCTGTGCGAGGGTCCCTGCTGTGGCGCCTTCCAC
EMPV1_20562	ACTTGCAGCCTATTTTTGATGACATGCTCCCCATATATCCATCCACATCCAAAAGATTGG
EMPV1_20563	ACAAAGCAAAAGACTTCATTGGGAAGGGGTGCCTGGGCAGAGAGCAGCAGGGTCAGGGAA
EMPV1_20564	AGCCTTTCCACTCCAAGTCCAGCAGCTCCCGATTCCTAGAAGTATGATGGATCACAGAGA
EMPV1_20565	CATTAAGGGACGTGATTTGTAATTCTTCATTCTGTCAGTCAATAGAGCATTCGTGGCGGA
EMPV1_20566	AGAGCCCTTTCCAACCTCTGCGCCGAGACTCTCAACAAGTCTTACGTACGTTCTGACCGA
EMPV1_20568	TGTCTCGTACTTGAAAGAGTAACCTCCATCCGAGGTGGTGCGAACGCGTAGGGACCTCAT
EMPV1_20570	CAAGGAGACCAGTTAGGACGCAATTATAGTAGTCTAGGCAAGGGTGGTAAGGGCCTGAAC
EMPV1_20571	ATTCAAGCATCGGCCTGAGGTGGACCCCGATTAACTCTTCCACCATTATCGGGTACCGCA
EMPV1_20572	GGAATCATGCAAAAGAGCAATTGAGGCTTCTGAGAGAAAATGACCCCTCCAGATTTTAAA

EMPV1_20573 TATAATTTCTCAGAACCCCAATGTTCGATGGGATGATATTGCTGATTTAGTAGAAGCTAA
EMPV1_20574 GCAAGAACGAGGACCTGCCCTGGCCTCCACTCAGGAATGACTACAAATGTTTCCAAAGAA
EMPV1_20576 GGCCACCATCCTTTATTTCTCTCTGTATGCATGCCCTTTACAATGTGACTTGGTAGCTCC
EMPV1_20577 CAGATAGGAGACAGGAGGAAATCCTGATGTTTGTGACATCATGGGTGGACCTTAAAGACA
EMPV1_20578 GTGAGCATCATGTCCAGTGGCTCCTTCATCCTCCTCTACACGGACTTTGGGCTTCAAGTT
EMPV1_20579 TGGTCCTGGTGGTGATTTTCCTGCAGGGCGACTGCTCCCTCGAGGAGGAGATCTGGAGAG
EMPV1_20580 AGCAAGGCCCAGGATCAAACCACATTTGCATGGACACTATGTCAGTTAACCCACTGAGCC
EMPV1_20582 TGTTTACTACATGAAGCCTTATGTTAGTGTATAACAGAATTGATGTTTGTTTTCTGTTCG
EMPV1_20583 TGGGCAAGATGGCCAGGAACCTGCAAGGTCTACTCTGCCTATGACAAAAGACTGTCAAGG
EMPV1_20585 GTAGGAAGGTGGGGCTTATCACAGTTCCTTCAGAGCTACTGCTTCATTTGTTCTTAGGCC
EMPV1_20586 AGAAAGGTGTGGTTCCTGGAGGGGGATTAAAGGCCACCATGAAAGATGATCTCGCAGATT
EMPV1_20589 TGGGGTCCTCAGCAAACACGAGGCATCACAGCTCATTTCTCCATCTTCAGGGAGTGCAGA
EMPV1_20590 GCACTATGAGAGTGGCTTTGACACCTCCTTTGTGAACCACAATCCTGATGGCAGCAGTGA
EMPV1_20591 GACATAGAAACCAAATCAGCAGATAAGAAAGTGCAGGTTCTGGAGTTCCCACTGTGGTGC
EMPV1_20592 TGTCCGAGACCTTGGGCGAGGGCATGACGCTGAAGGTGTTCATGATGCGGTCGGGGTACT
EMPV1_20593 GCTGACCTTGACCTCTGAGGGCAATAACACTAACTTCACTTGCTGTGTAAGACCTTGTGC
EMPV1_20594 AACTATCATATAGGCATGAGGTAGGCACGAGCTGGGCTGTGATGCTGGTTGTGGAAAGTG
EMPV1_20595 TACCATCACTATAACACGTACCACCCTGACTGCAGAGCGCCGGCCCTGACATTCCCGAAA
EMPV1_20597 GAAGTGTGGTTCATAGAAATGAGACCTCCTTAAGTGATGCACTGGGGGGCAATTCAGCTG
EMPV1_20598 CCATCAGGGCATTTTGCTTTGATGGAACAAAGGCCCTCAAACTTGATATAGACACAGAAG
EMPV1_20599 AACTGCAGCTGGTTTTGTTGAAAGTGGCATTACTGCTGGGGGTGGAAATTCACTGGGGTG
EMPV1_20600 TGACATGGCCGAAATTGACGCCCGCCTGAACGCTTTGCAGGAATAGATGAACCGACTGGA
EMPV1_20601 CAATCTGGATACATGGGAGTGGAATGAATTAATTCCACAAGGCATATGCCCAGTTGGTCG
EMPV1_20602 GGAAGTCACATGATATTGATGCCGACGGGCTCTCCCTGCACTGGACATCGTCTGTTTTCA
EMPV1_20603 GGATCATCGCAGAGCTGGCTTGCTACACATACTCTATGGTGGTGGTCCCTCTCTATGACA
EMPV1_20604 AGATGATCAAGGTACAGCACAAGCAGTGCCTGGAGGAAGCCCAGCTGGAGAATGAAACAT
EMPV1_20606 TATTCTCTCTAAAGCAATAAGGGGGTGATGGGCCAGGGGGAAATGTCTTGCTGCTACTGG
EMPV1_20607 GAGATCAACCATTTCTACTGTGCAGATCCTCCTCTCATCAAAATCGCCTGTGCTGGGACC
EMPV1_20610 CCCTAGTGAAAGGCCTGAGTTCACAGAAACTTTGGGGAAACAGAATAAATGCTGCTCGGG
EMPV1_20611 ACTGTAAGGCCTCATGTTCTGAAAAGGGCTGTAGTTCAAGGAGACAGAGGTAAGGTTGTG
EMPV1_20617 TGTGTGTGTATGTGTGTATATACACACACACACATACATATCTATTCTCTTTCAGATTTT
EMPV1_20618 CCTAGAATCATTCCCGGACATGCAGAGAGCAGCGGAAAGTGTTCCAATTTGCATGTGGCA
EMPV1_20621 GTGGTGGCTGGCACAACCCTGGGCACTGTAATGGTAGTGGGCGATGTGTTTGAACGTGAT
EMPV1_20626 GTTCAAATGCAGTGGATAGCTCCACAGGTTGTTTCTAAGAAGCCCGAAAGTGGGAACATC

EMPV1_20627	GGCAGGGCTGTTCTATGAAATATACTGCGGGAGAACTTGCTATGCTGGAATGCCTGTCCA
EMPV1_20629	CAGGTTTTCAGGTTATGCGACCATTTCCTTACGAAGGCTGAACCTAAGAGGTTTTTACTC
EMPV1_20630	AGAACTGGGATTGCTGTGAAGGGCAGGGGCTGGTAAAGGTCAGTAGATGTAAGGAGCGAA
EMPV1_20631	AGCTCTCCGGAACCTGGGTTGTGGTGAGGTTGAGAAGCCCCCAGGGGGCCCGTCCTCCGA
EMPV1_20632	CATGAGAAAGCAACAGCTCTGAAAGGGCTACAGCTTATGGAGTCTCTCCTTGACTTGGCC
EMPV1_20634	TTTGTAGCTGGCTAGGGAGGAAGGTAGAGAGAGGTCCCAGAGCTGGTGTCTCATTACTGA
EMPV1_20635	GCCTCCCAGATAGTGAGAGTTGACTCCAGGAAAGTGACCATAGTTTCTGAACACGAAAGC
EMPV1_20636	TAGTAGAAGCTGAACCTCCAGATGTTGTGAAGTTTGCCATACCATCAGGTGCTGTGAATC
EMPV1_20637	CCCATGCTCTACAGTGATGAAAGCCATACTGAAAAAGGTCCTGCAAATCTCCTTCCCAGA
EMPV1_20638	GGATGATTCTGATCCCTCTGGTTTGTTACAGTGAACCGGCTGGGGTAAATACATAGGGCG
EMPV1_20640	TCTCAGGCGCTCGGTCCCGTGTTCTCCTCCTCACATAGAGAAGATAGTCCCAGCTTTCAA
EMPV1_20641	ATTGAGCAGGAGCGAATCGACAAGATCTGGCCAAAGCTGAGGGTGCTGGCTCGCTCCTCC
EMPV1_20644	GCTTGTAAAGCTCTACCAATAAAACCCCTTCGAAATTGTGAATGGAGCCCAACCTAATCT
EMPV1_20646	GCCCCCTGCTACCTCTCTGAGGGCAACTCGGCGCTCTCGTGCTGCTGCGGTCGACCCTGT
EMPV1_20647	CCTGCACGACAATGAGGTGACAGTCATGGAGGATATCAATGCTCTCATTAAAGCAGCAGG
EMPV1_20648	GTTCCTATGAGACCTTACCACTCCAAGAGGAACCACAACTTCTGCCCAACTGCCAACCTT
EMPV1_20649	CTTGTAGGTTTCAGTGAAAGCATTGCTAAAAGGGAATTTTATACCTGTAAATGCATACAC
EMPV1_20652	AGTGATGATCGGCATCTTCCTGGGCTTCATAGCGGCGTTTACCACTCGCTTCACACACAA
EMPV1_20653	CTTCCATCTTTCCATCCACCCAATCATTCATCCTTCCACCTATCCATCACTCCATCTATC
EMPV1_20654	GGTGTCTCCGACTTCGCCTGGTCCCTCTCCAATGACATCCTCGTGTCCACCTCTCTCGAT
EMPV1_20655	ACCACTCCAAGCCCTCCCCACCTTCAGCAGAGCATCCTTCTTCGACTTCATTGGAAAAAA
EMPV1_20657	AAAGAGAATGAAGAACCGGTCAAAAAGATGGTTGTAGAAGCTACCGGGGAGTTTGAGGAG
EMPV1_20659	CTACAGTTCTGATTCAACCCTTAGCCTGGGAACCTCCATAAGCAGCAGGTGTGGCCCTAA
EMPV1_20661	GTGGCATTGAGAAAAAACCTCTGAAATCCTTCAGGATATTGACTCTCTTCTATCAAACTCC
EMPV1_20662	ACCTAAGCAGTCCACCCAGGCAGCTTCACTAGAACAAAGGTATTCACAGCAGCACCAGCT
EMPV1_20664	TATTCTAGGCACTTGGGGGATGTTTCGTCACCACTGCATGACTGCAAAGTTCTGCGAGGC
EMPV1_20665	ACCATCTACCTCACCTTCCCTGTGGCTATGTTCTGGATTGCGAATCAGGCCGAGTGGTTT
EMPV1_20666	ACGTTAAAATCGTGTTGTACAAGTTTATAATTGTTAACTTGATGTAGTTGATACTCATAT
EMPV1_20667	CTCAGAGGCTTTTGGGCGTTCAGGGTGTTGCTTTTCTAGCTCTCTCCCGCTTGCTCGCTT
EMPV1_20668	CTAGGTACCTCACAAAAATGTTTCTTAACGGTGCCATGGCTGTTAGTTTTTAGTGAGCGC
EMPV1_20670	TGGGCGTCCCCTCCATTTCTATTATGCTCCATGAACTCATTAAATTACTGTACCATGCCC
EMPV1_20672	CTTTTCATTGCTTTTGGTGCTGCCTGTTGTTGAAGCAGTAGAAGCTGGAGATGCAATCGC
EMPV1_20673	CTGCTCAGCCCTTGTAGAATCCAAAAAGACGCCACCATGAAATTCTCCCACTCAATACAC
EMPV1_20674	ACACTACCTAGGGCGTTTCTAGAGAGTGATTGCTAAACCTCACTTAACCGGATCCCGGAT

EMPV1_20675	CTGTGCTCAAATGGCTGTCACTGTCTGGATCATCGGTTTTCTCTATGCCCTGATGCACTC
EMPV1_20676	AGGGCATGAGCTCCCTTTTCCAAGAATTGTCTGAGGAGAAGCACGTAGGCGCCAAGAATT
EMPV1_20677	CTTTCAGTCACGTCTCCTTTGAGTTTCGTTGTTTCAGGAAGAGTCTGTATCCAGTGCCTC
EMPV1_20678	ATGGAGCTGCGCTGTTTGGACCTGCTCAGCATCTCAGGAGCTTCTCAGGGTGTAAGAGGA
EMPV1_20680	TCACCTGGAGATCCTGTTAAAATGCAAATTCCAGTTCTATGGGTCTGGGGTGGGGCCTGA
EMPV1_20681	GGGTGGCTTATCTGATGTTGCTGAGAAGTTCTCCAAGAAAGAACAGAGTGATGAATCCTC
EMPV1_20682	TGGCAGCTACCCTGAAATTCCTGTGGAAGAAATGCCAGATGCAGATGGAGTAACCAGCAC
EMPV1_20684	GTGCAGCCAAATGGAAAAATTTTTAAAAAGGTTATTCTTGGGAACTATAATTGGCTGTCC
EMPV1_20685	TTCCTGAGTCTCTTAGGCTCCAGAGGAAGGTAGGCTGAAGGGGAAATAAGACTGCAGCTC
EMPV1_20686	AGGAACGCACGAATGAATTCATTAAGTATTTTATTTCTGCCTACCCCTGCCCGGGTTGTG
EMPV1_20687	TGTGATTGGGGTGCTTGTGGCTGATTTCAGCCTCAAGGACATGCACGTGGTGGATGTGGA
EMPV1_20688	ACTAGGCTGATAGAGAAGGCAAACTGAGATTTAGGTCCAGCGGCTTCCACCAACCCCACA
EMPV1_20690	TTTCCCTTGTGCTCTTCATGTGTCTGGGCTGCGCCCGGGGCTTCACAAAATAAAGTCGTG
EMPV1_20691	AGCAGCAGCCTCACCACCTGGCCACCTCCGTGCCCCTCACCCCGCGCGTCAACATCAACA
EMPV1_20693	ACATCTTCAGGCTCTTTGACTTGGTCCTCCTGATGACGTCTGGCACCACCATCTACTTGG
EMPV1_20696	TGCAATTGAGTGTTTTATTTGCTGCCCCAAGGAGGGCAATGGCCAGGAGGAAGATCTCAG
EMPV1_20697	TGGATCACTGCAAAATACCTTTGACTTGTCAATGTGTTGATAATGCAAAGCACGTTGGAC
EMPV1_20698	GATGTGAAACATACTGGTCCTGGCTTACTGTCAATGGCCAATCGAGGCCAGAATACCAAC
EMPV1_20701	ATCATGGCTCCTGGCCTCTGTGTGAAGATGATTGCTGGGGCTTTTGGGAGTGGTTTCCTT
EMPV1_20702	GGCCCAGAGGTTCTTTAAGCAGAACATTTTACAGTTTATCCAGGGGGCTGAGTACGAAGG
EMPV1_20704	TTGTGGAGCTTCATGAGGTGCGGGCAGGCGCAGGAGACGGTCCGGTTATAGTTGATGAGA
EMPV1_20706	CCAGGGAGTGACCTTTATGAGACCCAGTCTGAGGAAAACAGGGGTGCAAAGGCACCAGAT
EMPV1_20710	CTGCCAGGAACAGCCAGCCCTGCCACTGGGCAGGCTCAGGAAGGGTGGTTCAGTGAGTCT
EMPV1_20711	AGCTAGACCCTAACTCCTAGAGTCATCTTCATGTTCCCAGTTGTACCTGTCCCCTTCCAC
EMPV1_20714	GGATAACGGCTCTGTACTCTGGACTGAAACCTACCATGATTCGTGCGTTCCCTGCCAATG
EMPV1_20715	ATTCTCCCTCAGTGGCACATTTGGAACGAGTTATGTTTTCCCACAGCTCGTCCTTAGCGG
EMPV1_20717	GGCTGTGAGACTGACCACCAAAATAATTGCCATAGTTGCAGTCACTTGAGCAGGATTGCT
EMPV1_20718	TCTGGTGTCAGCCCGTTCGTCCAGAGTCAGTTTAAGCACAGCGCACGGGCCGGGAGACTG
EMPV1_20723	GAAGCAGCAGGTCATCCAGTCTCACAAGCAGTAAACAGACCACCACGTATCCAGGATGTA
EMPV1_20724	GCCGCTGCTCTTGGCTCTGTTTGGTTTCTATTTTAACACTGAAGGAGTGAGAGTATTTCC
EMPV1_20726	CTCTAACTGTCGATGTGGTGATAGACTGAAACCATTAGAGCGGAGAGCTGCACGCCAGCA
EMPV1_20727	TTTGCTGTGTCACTTGGAAACACACTCGTGGGCATATTCTCACATTCGGATCAGCAGGCG
EMPV1_20728	GCTGACGCAATAAACGTCATCAACTCCCAAAATCCTTTAAGGTACTATGTGCCCCTTGGA
EMPV1_20729	TGGACTGCTGAGAGCCGATGTCGACAACTTCCGAGCAACACATCCTTTGAATGGGCGAAT

EMPV1_20730	TCAGTGAAAACACGAATGCAAAAGCTGGCTGAGCAACGGCGCCACTGGGATAGCGATGAT
EMPV1_20731	TTGATAACCGTACCCAGCAAGTGGTTGCTATTAAAATCATAGACCTCGAGGAGGCCGAGG
EMPV1_20735	AGAGCAGCTCACCATCTTCCAGTTCTGGAGCTATGTCGAAGCCCTGGACAGCTCCTCCAT
EMPV1_20736	AGGCTACGATTCCAGGATAGGCAGTGGCAGAAGAGCGTTTGGTAGTGGGTACCGTAGGGA
EMPV1_20740	CACTGCATCCTCAGTAAAATGTGCCCTATAATGGCTGCACTCCTTTTGAGCAACCCCTCT
EMPV1_20743	ACTCTTGCCCAGGGACGCGGTGGAATGAAAATGGATGAGAAGGCACTGTCTCGGGGACTT
EMPV1_20744	CCCTGATGGAGAAGCTTTACAAAGTGAACTCCGTCCCGAAGCATCCCGATTAAAAAGGAG
EMPV1_20746	GCTGCAATACAGAGCATATACAATTTGCTATCCTGAGCTGATTGACAAGACAGTTCTTCA
EMPV1_20749	AGCTCTGTGGCCTTCATGTTCCGAAAATTCAAGATCCTCCGTCCAGCACCCTTGCCTGCA
EMPV1_20751	ACATTGACAGCCCCAAAAACCTGGTGACCACCCAGGTGACAGAGAACACAGCCACCGTCT
EMPV1_20752	TCTCTGCCACATTTTCCTCCTTTACTGAACCCAGGTCAGCCTCTGTTGATGGGGTGCCTA
EMPV1_20753	AGTGGAAACAAATCCGACTAGTGTCTGTTGGGACACAGGTTCCATCCCTGGCCTCACTGA
EMPV1_20754	ATTCCTCCCTGCTCTTGCAGGATGATTGTTGGCCTGTCTGGCTCTTAGTCCTTGTTTCCC
EMPV1_20758	AGACGAACAGCCGGTACTATATCTGCAACAGGCTGTTGTTGTTGTACTCGAGCTTGTGCC
EMPV1_20760	GCCTGTTACTTGCTGTGTGGTCCTAGCCAGTGACTTCTCCTATCTGAGATCATGGTTTAC
EMPV1_20761	ATCCTCTGAGGTGGCCCAGCCATCTCCTCTGAGGGTTTCAGACCAGCTCCACAACAAGGG
EMPV1_20762	CAGCAAGGAGCATAATCCAACCCAGTATGAGGAACGCATGAGAATACAAAAGGCTGGCGG
EMPV1_20765	GCATTTGAGCTAGATGGTGGAGCCAAAATGCTATTGCCCTGTCTTCATCAACAGTTAATC
EMPV1_20767	AAGGTGAGGCAGCTTCACCTACAGCAACCACCTACACAGCCTCAGGCACATCTCAAGCCA
EMPV1_20768	CCCCTGGGCTTCCAATGCACCTTCATTAACGACTTTGACTGCTACCTGACGGAAATCGGA
EMPV1_20774	AGGACGGCCTGAAGCACGAGTATTTCCGCGTGACACCCCTCCCCAACGACCCCTCCATGT
EMPV1_20775	TTGGCCCTAATTGAGCTGACTGCCCTCTATGATGTGTTGGGTGTTGAGCTGAAACAACAG
EMPV1_20777	CCACCTTGAGACCAATAAAATGAACAATCACACTCTGACCCTACACCTTGTTCCCACTCC
EMPV1_20778	GTGACCCATCATTCTGTTTTGCCTGGGACTGAGGATTTCCCAGGATGTGGGGCTTTCAGT
EMPV1_20779	TCCCGCAACAAGGCCGCCTCCTGCAGCCTCCGACAAAGCTTCTCAGCAGGTCATCGCGAA
EMPV1_20781	AGGCAGAAGCTCCCTATCAGCCTTCAAGAGCAATCCTTCCACTCCTGCAAGTAAACTCAG
EMPV1_20784	ACTGCCATATTGGCATTCCACTCCTTGTTGCCATCTTCACTGTTACCTGTTTTGGGTTTC
EMPV1_20785	GCTGCATGGAAGTAAGATTCGTGTTACCTTGGCTTTTTGTCTCCGTTGACACGGTTGCAC
EMPV1_20786	CGAGGAGGAGACATCGTCTGTGGAGAAGCCAAGCAGAGCCTTGCCCAGGAAGTCCAGAGA
EMPV1_20788	GGCCTTGGAGGATTAGAGCCTGGTAATCAGGGAAAGGGTCCGAGGTCAAAAGTGATTTAC
EMPV1_20789	CCAAAGAACCTGAGTTACGATGGCGTGAAAGAGCTCATCTTAGCGGTGGATGGAGTGGTG
EMPV1_20790	CACCCTAAAGAGCTCTGAAAGCCGCAAAAGCCTGGGCTCTACGCCAAAGGATTTTAATTC
EMPV1_20791	GCGGTGGCCTTTCGGAGTTTTAACAGTCATATTAATGCATCCACCAGCTCAGAACCGTCC
EMPV1_20794	TTGACTCCTACGATCCAACTATAGAAAACACTTTCACAAAATTGATCACAGTAAATGGAC

EMPV1_20796	CATCATCCCCGTCCTGCAGAGGACCCTGCACTACGAGTGTGTCGTGCTGGTCAAGCAGTT
EMPV1_20797	CAGGATGCCCAGTTAAGTTTGAATTTCAGAGAAGCAACAAATAAATTTTTTCAGTGTAAG
EMPV1_20799	GGCATGATACTTACATTTGTTGTTCTGGTGCTGATGAAAAAGGGGCAACCTGCTCTCCTC
EMPV1_20800	TAGTAATGTCTTCTCGGGTGTGGAACCACGGTCATAGCTGACATGTTGGCCGGACTAGGA
EMPV1_20801	GCTCACCTTCCAGGCGGGGGACCCCTACTACATCAGCAAGCGCAAGCGGGACGAGTGGCT
EMPV1_20802	GGTGGTAGGCTAAGTTGTTTATTTGAGAGTTTTTTTTTAATTTCTTGAGGAAGGCTTACA
EMPV1_20803	ATCTGCAGGAAAGAAACAAACTCATGGGCTTGAAGAACAGACTCGTGGTTGCTGAGGGGG
EMPV1_20804	GATTTCCCATATACCATCCTCCAGATTGCCCCAATTCAGCATTCCACATAGCCACAGTGT
EMPV1_20805	CAATTGTTCTAAGGGGGGACCATCTCAATTGCTCATAAAGTTTGCATCTGGAAATGAAGG
EMPV1_20806	AGGGCAACTTTCGGCTCTGCTTCGACGACTTCGTGACCATGAGAGCCTCTCCGATGCTAT
EMPV1_20807	CGGAAATTCTTTGGGGTTGTACCAAGTGGAAAGAAACTTGTAAGTGAAACAGTAAAGAAG
EMPV1_20808	TCCCGGTGAAGATGCACAGCCAGGGGAGGAGCCCATCAAATACGGTGAACTCATCGTTCT
EMPV1_20809	TGCTCTGACGTCTCCACCAACCTCCTCGTCCTGTTGTGTTCCAGCCTGCTGCATGGGTTT
EMPV1_20810	GCTTCTCTCACCACTTCCCCATCAGCCCCACCCATGGAACACTTACCACTGATTCTCTAC
EMPV1_20811	ACTGGAGCGCAAGTTCTCAAGCACCACACTCTAATTATTTTATCACAGCTCCCTGCTGCA
EMPV1_20812	AGCTGAACCCAGGCCAATGTCTTCATTAATGCTGCAGCTCCTTTCAGGCTCACACATTCC
EMPV1_20813	GCCACTAGTTCAACCATCTGTCTGCCCCAGTTTCATGTTAGAGTCTTAAAGCTGCATGCC
EMPV1_20816	GGAAGAAAAGTATGAAATTCCTGAAGGGCCAAGGAGAAGCAGGCTCAACAGAGAACAGCT
EMPV1_20820	GCTGCCTGACTTGCTCAAAGCCCTGTGATCGTGGCTGCAGTTCTTTGTCTTGTTATCTCA
EMPV1_20822	GGTCAGAGGGATCCTTGTTCAGTTGTTGGCAGTGGCTGGTATAAAGCAGAAGGAAGTAAG
EMPV1_20823	CGTCTCCCTCTTGGTGGCTGGCTTCTTTTCCATGTTGTCTGGATTCTACTTCGCTGAGTT
EMPV1_20825	TTGCTCCCCGCTCCTGTATCCTACGCACATGTCTCAGAAAGTCTGTGTTCAGTTGGTAAC
EMPV1_20828	TCATCTCAAGGGTCACAGACGTGGACGGGCTGCTGGATGCCCTGTACGGGAAGGTGCTGA
EMPV1_20829	AAGTTTGCCATCCGGAAGTCCAGACGCTACAGGCGGGCATGAGTGGCCCAGACTGGACTT
EMPV1_20830	CGAACTTGTTTGTCATAGTTCGCTTTGCGGCCTTCGCCTAGACGGTCAAGTACGTGCTGA
EMPV1_20831	CAGAGAGGAGAAGCTGGTAATTGGGGCGTGAACTGGCTTCAGAGGTGGGCAAGGTAGAAA
EMPV1_20834	ACGCTGCATCTTTCTCGGTACCCTTGGTCCTGCATCTGCTATCTTTTCCAACTCCTAACC
EMPV1_20835	GGATCAACCTGGTCAGAAGTGGAAACTGGCAGACACAGAAAGGCAAGTATGCCAAGAGTG
EMPV1_20836	ACTGAGTGAGTGAGGCCAGGGATCTAACCCACATCCTCACAGAGACCACATCAGGTTCTT
EMPV1_20838	GAGGTCTGGGCAGCTCTGGACAGGATGGGTGTGTGTGCTGGGAAAGAGCATTGCATCTAT
EMPV1_20839	CTACCCCACAGCACTGTCCTGAGAATTAATCAGCTGATATGTGAGATGCACTCCAGTGTC
EMPV1_20840	GTGATAACTTGACTTTTACATTGTATGGGAATGTGCCAGTACCCCCGCCCCTACCCCAAG
EMPV1_20841	CCAGTGTGCAGCTGGCGAAGGAGTAAGGCAAACCCATTACTGATAACGTACTCTTGAGTG
EMPV1_20842	CAGTTTAAACATTCTGAAGAGTATTACATGAAATTCTTCATTACATCAGTCATAGGTTTC

EMPV1_20844	TGCAGGAGGGATGCTGCCGCCCCATCACCTGCTGCCCCTCGTCTTGCCAGGCCGTGGTCT
EMPV1_20847	CGTGCTGTGACCTGTGCTGTTCTGTGTTGCACTGTTTACACTCCCAAGCCTCAGTTCTGA
EMPV1_20849	ATCCTGACCTGGGACCTTCAGCCTCCATAACTGTGAGATACCCATGCTGTTGTTAAAGCG
EMPV1_20850	TCCACAAACTTCCTGCACTTGGAATCTGCTGGAGGCTGGACATGGGATTAGGGCATGAAA
EMPV1_20851	CAGGACGGTGAGCCTGTCGTTGTTCAGGGAGGCCAGGTCCTCTGGGCTGAGAAGGATTAT
EMPV1_20852	ACAAAACAGCCTGGAATTTTGGACCTCAAAGGTAAGGCCAAGTGGGATGCCTGGAATGGG
EMPV1_20853	GCCAGGGAACAGCCTGGACAAAGGGCTCAAGCTAAAACGTTCAAGGCCAGTTTGGAGAAT
EMPV1_20854	AGTCTCTGGATGGTGTGCCTGCACCCATGCTTGCAGATCCTTAGGAAGAATGCTCAGAAA
EMPV1_20855	TCCTCAGGAGGGGAGTCCCAGAGACCAGGCGATGGCGTCTTCACTGCTCACGGCAGATTC
EMPV1_20857	ACCTCAGGGCTGACCATACCAGTCCTTTGTGAATAGTGGGTAAAGAATGAGAGCATTTTC
EMPV1_20858	GAAAACCAGTGCTATAGGAGGCATGAACCATTTTTTGGAAGAGTCAGGATAGGGTGACGC
EMPV1_20859	GTCTTTGACAGCGGCGTCATGGTCATTGAACTTCAGTCCCACAAGGAGGAGGAAATGGTG
EMPV1_20862	AAGAGGAAGAGAAGGCCAAGCGGCTGCTCTACTGTGCTCTGTGCAAGGTGGCAGTGAACT
EMPV1_20863	ATTTCACTGCTTTGTGCCCCGAGCTGACGCCAGGGTCTCCACCAGCCTCTGGATAAAACT
EMPV1_20866	GGCTGGGAGCAGAGGAAGTTGGCTGCAGATGGAAATTTGCAGAGTAACCGAAAAAGGACA
EMPV1_20868	CGCCCATGTGGATTCAACCTCCCAATGCCTCCATCAACGCCCTTGTATTTAGCGAACTTA
EMPV1_20869	GGTTCCTGTAACCCCTTCCTCACGCTCGATAATTTGTTAGCACAGCTCACAGGACTCAAG
EMPV1_20870	GGGCCCCAAGATGCTCCTGAGTGACCTGTAAGGACTTTTAAGAGTAAGAGTAATACGATG
EMPV1_20871	GGGGAGGCCACTGCAACGGTGCTAAGGCTAACAGGGAATCTTCTGTTGAGGGCCTTAAGC
EMPV1_20872	TCTGTGGACTGCCTAAGAGGGCATAAACACCATGTCTTTGAAAAGACAGTGGTCAAAGTC
EMPV1_20873	GTGTGCAGGATTTTTGTTCCAGAAAGTTGGAAAACTTGCAGCAACTGCAGTAGGTGGTGG
EMPV1_20874	CTGGAGCGTCAGGGGACAGCAGGCTCACCTCCGCCGCGCTCCAGCCATGTTGGGCGACTG
EMPV1_20876	CAGCACTCAGCATATTTGGCATGGTTGGTGGACCGCTTATGGGCTTGTTTGCTTTGGGGA
EMPV1_20877	AATCAAAGAGAAGGCATTTAGAATTGTGGTGCTTGGGGGCGGGGGAGGCTTCAATTTGGA
EMPV1_20879	CAACCAATTCTTTTTCACTTGTTAGGTTGAGAAGTTTCATTTAGTGGCAGCTGAAGGGCC
EMPV1_20881	GACTGGTATCAAACAGAATCTCAAGTAATCATTACACTTATGATCAAGAATGTTCAGAAG
EMPV1_20882	GCAGTTCCTTCTTACCAACCTGGTGGAGGTGGATGGGCACTTCGTGTGGAGAGTGAACTT
EMPV1_20885	CCCCTTTGACCTTCTCTGCCACCAGCCACCACGACATTTACCATGAATTTACTTCCTCTC
EMPV1_20887	TTTTTGCATTGGCTGCACTGGGTCTCCCACCACGGGCCCACCTCCTTTATTAATGCCTGA
EMPV1_20890	CGGCTACCGCACAGTCAAACAGCAGCAGACAGTGTTCATTCATCCCAACTCTTCCCTCTT
EMPV1_20892	TCATCTCCTATGGCTTCATAGCTCAAGCAGTATTGAGAATCAGGTCAGCCGAAGGACGGC
EMPV1_20893	AAAGGGCAGAGAAGGAACGAATCATGAATGAGGAAGATCCTGAGAAACAGCGCAGGCTGG
EMPV1_20896	TGCGAATACATGCTCAACTCGATGCCCAAGAGACTGTGTTTAGTGTGTGGCGACATCGCG
EMPV1_20898	GTTAGAGATGTTGTCAAAGCTGTGTGGGCTGGTGACGTCGAAGCAGAGGAGCAGGACGCT

EMPV1_20901	CCTAGCTGCCATCAAGGTGCTACAAGAGTCGTATGAAGATATTTTTGGCATTTCTAGGAA
EMPV1_20902	CTGGTGGTGAGAACCCCGTGAGGACGGAGACAGGTGGGGGTTGGGGTGTTTGAAGACAGA
EMPV1_20903	CGGGGCCAGAGAACCCTTTCCCGGGGGTGTTAGAAATGTGTCTGATGAGAGGATGAATAA
EMPV1_20906	TCCTGCTTCAGCACGGTGACACAAAATATGATGGCTTTGGCCTCTGGGGTCTGCCTTCTA
EMPV1_20908	AGGAGGAGATCCAGAGGCAGGTGCGAGACATCGAGAGGCAGCTGGACGTCCTGGAGCTCA
EMPV1_20912	GGTCAAGGAGCTTCCTGCCTATTTCTGTAGGAACCGCATGGTTTCAGGTCTCACATCAAG
EMPV1_20913	TGATTCAGCAGGTCTGCAGTGGGGCCTGAGATTCTGCATTTCTAACCAGTTCCCAGTTAG
EMPV1_20915	GAGGAGGATCTTATGGATATGGAAGTGGTCGTGGATTTGGGGATGGCTATAATGGGTATG
EMPV1_20916	AGTATTGCAGGTCTCTGATTGTTTTGGATCCGCTTCTGATACCTGTGGACTTAGTTCAAG
EMPV1_20917	TACGGTCATGTTAACCGAGAATGTGACGAGGGCCGCAGTTTTTCTCATGATCGAAGAAGT
EMPV1_20918	ACTGCTATCGAAGGAACGGCATCTTACACCTGCTGGGGAGAAACGAGCGCATCAAGCCAT
EMPV1_20919	ATGATGAGCCCTCCCAGCCTGAGCCCTCACCCTCCACACTCATCTACTTAAACAGGCAGT
EMPV1_20920	GCCTGCAAATTTCAAGACATCTTCATCTAAGTATCTTCAAGACTTCATGTCATCAAGCAC
EMPV1_20921	GAAGAACAAACAGACCTACAACATGGAACCCAACAACCTGAAGGCCCGATACTCCTTCTG
EMPV1_20923	AAGACTACAAGACCACTCTACAATGGGGCACCTCTGGCTTTCTCAAGGCTACGTGACCAC
EMPV1_20925	GCTTCAGCAAGAAACTTCAAGCACAGAGTTGAAAACTGAATGTGCAGTGGTATTGGGAAG
EMPV1_20928	GACCCAGGGCTGGCTTCTAAGTTTCCGTCCAGTCTACAGCCAAGTTCTGTGTTAGTCACA
EMPV1_20929	ATTCTGTTCAACTTGGACCCAGTCATGTTCTTCAGATGTCCTTCCCCAGACTCTGCCAGC
EMPV1_20930	CATCTCTGCCATATTCTATTATTGTGTATCGAGACGGAGTGGGAGATGGACAGCTTCAAG
EMPV1_20931	ACAGCTATCCAAGGTGATGAGCCTGTGGGTCAAAGACCATACCAACCTCTCCCAGAAACA
EMPV1_20932	GCTCTTCAGAGTGGAATCTGCCACTTCTCCCCGACAAGAAGCACGTACAGGATGTATGAT
EMPV1_20934	GGGCCGTCGAAGCAGTACATACTCACTTACACATTAAAGTGCTGGTAATTCACAGTCTTC
EMPV1_20937	TTTGTAGATTTCAGAGGCAATAGAAATGCCCTCACAGCAGGGACTTATCTGGCTGTTGGG
EMPV1_20940	TTGCAAAGTGGGGTCCCATGCGGGTTCAAGGGCAGTGGATCTGGCACTGATTTCACCCTC
EMPV1_20943	AGGAATTGTGACAAGGTTATTCAGCCAGCAGGGATATTTCCTGCAGATGCACCCAGATGG
EMPV1_20944	TTCAAGGGGATTCAGGTGAAGGAACCCCCATCTGTGGTGGAGAGCTGGACGCTTCTTTAA
EMPV1_20945	AGGTTAACACAGACCAAGAGCCTTCAAAGCTCTAAGTAAGTACAAAGTACTTAACTCCTG
EMPV1_20946	ATAAAGAAGATCCAGCTGAAATGCTCTACCCTCTAGGAAGCCTGCGTTCACTTCCTCCAG
EMPV1_20947	GAGGAGACAAAAGGTTGTTTTGAAGACCTGGGGGCTCACTCTCACTAGGCACTGCTTCTG
EMPV1_20949	TAAAGTTTTATGAATGTGAGAGTCTTTGCTTTTGAAAATTGCTTGTAATTCCTGGCATCC
EMPV1_20952	GGAACTCTCAAAGCTGCGAAACGAAGGAAGTTCATTAGATACCCACGAGAGCAACTTTTG
EMPV1_20953	CATTAGTGGCCTCGTGGCTGCCAAGGTGATCCCCTCACCTTTTGAGCATGCGGATGTTGT
EMPV1_20955	GCAGCTTATAATTCAGCCTTGAAATTCTGCATTTTTAACAAGCTCCCTAGCAATGGCTAC
EMPV1_20956	ACTTCAAAGTATATGTGCAAAGAGCCATACCAGAACCACCCCTGCCAAGGACTGGGTGCG

EMPV1_20957 AAAGCTGGTCTCTTATCAAGAATACCTGCCCACCCAAGGATGGACCCCAGGTTATGGAAG
EMPV1_20959 GCTTTAAGAATCGATGGGGGATGCTTCATGTGAACGTGGGAGTTTAGCTGCTTCTCTTGC
EMPV1_20962 ACGCTTGAGGCAAAGTTTACAGAGAAGGAAAATGAGGGCAAAGAGATGGACCAAAATTCC
EMPV1_20964 AGAGGATCTCTTGTCTTTGAACCTCATCGGTTACCTCACTTAGGCTTTGCAGCCCTGGCA
EMPV1_20965 GGAATGACAAGCCTGTGGTCTTGGAGCCAGGGACCTACGGGCTGAGCAACGCGCTGCTGG
EMPV1_20966 CTCTGTGGCTGCCAGACCGCAGAATGGAGGGACACGCAGAGTGCTGGAAATCTGGTAACA
EMPV1_20967 TGGAGCCACACTTCTTGGCTTCAGATCCCAGATCCCCTAATCAGTTGCATATGGCCTTGG
EMPV1_20969 GTTAAGCTGGTTTACACTTCTAGGGTTTTGTGTGACTCATGCTCCTCTCAGTCCTTGACT
EMPV1_20970 CCCTTCCAGCACTCCTCTCATCTCCTTGAATGCTGAGGTGTATTCGCCAATGAAGTCATG
EMPV1_20971 CCTCTAAGAAGCATAGACCCCAGTTCACTCCCCAGGAATTCTTATTTTCCTTTTTGGAAG
EMPV1_20972 TATACAACCATCATGAATCGCCGAGTGTGTCGTCTTCTCATGGGAGTGTCGTGGGTGGGA
EMPV1_20973 CCCAGGGATGCAAGGGTTCTTCAACATCCACAAATCAATCAGTGTGATACACCACAAATC
EMPV1_20974 CTTCACCCCTCCTGCCGGTATCCAGCATGTTTCCTATTTCCCAGAGCTTCTGAAAACTTG
EMPV1_20975 GGAAGCTATCCTTTCAGCCATAAAGATGGTAATAAACCTTTGCCTCCCACAGTTCAAACC
EMPV1_20976 CTGAGCAGGATCCCTTTGTTCTGCTCCAGGCTTTGCCACAGAAAAACATGTCTGCACTGG
EMPV1_20977 TGGTTCCTCATATTTCCCAGCTTCTTCTGCCTTTGCCCACCTACAAGCCACCCTCCACAT
EMPV1_20979 ATTGTCACCCTGCTGGGGCACCTGTGCCCGGTGCGCTGTATGTACCACACTTGTCTCTCT
EMPV1_20981 GGCCAGAGTTGATGGTAGGAGAGGCCATTCTGGATCTGGGCTTGTTAATATCCATGGTCA
EMPV1_20984 CTGTCGAGCAGACAGTTGGATTCAAGGATCTGGGGAGAGATTTCGTTTGCAGATAGAAAT
EMPV1_20985 ACGAGAAATCGTGCGAGACATCAAGGAGAAGCTGTGCTATGTGGCCCTGGACTTTGAGAA
EMPV1_20986 TCACGCTGGCGGGCACTTTGCCAGCGTTGCTTCGCAGAGACAGCACCTTCAGCTGCTTCA
EMPV1_20987 TAGACCGGCAGGAGTCATCTGAGCCTTGTTCCCTCCTTTTCCCTGGCTGACTCCTGGTCC
EMPV1_20988 TCCCCATCTGCACCTTTGTTGAACTGTGGTAGCTAGGAGGATCTTGGGGTGAAGGGTGAT
EMPV1_20989 AGTGGGAGTTCCAGGTGACCGTCTTCTACCGGGGCTGCCAAGTCTTCCAGCAGACTGTCT
EMPV1_20990 ATTTAAACCCTGGACTCTGCTCTCTCACCACAGTTGTTCCCACTTGCCCACTGACCTCCT
EMPV1_20993 TGATATCCGAAAGACAAATGTGGCCCAAATCCGCATGGCCTATCGCTATGAAACCTGGTG
EMPV1_20996 GGAGGATTGTAACGAAGAAGGGTTTGATGATGAAGAGGATGAAGTCAGGGGGGAGGAAGA
EMPV1_20999 GCGCCCTTCCTCTCAGGCCTGATCCAGTCTGGCTTAATTATTACAGTTTGTCAGTCTCTG
EMPV1_21000 GACAGTGCAGTCACCCATAAAGTAGAAAGCACTACTAACAGCACTGGAGGGTGTAGTGTT
EMPV1_21002 CTGGAAGCCAACATGTGATGTATTTTACAAACATGAAGACTATATGCAGGACCAGTTTAC
EMPV1_21004 CTGGACAGCAACTAGCTGCAGGACAGTCTTCTGAGTGTCCCCTCGGAGGAAGACACTCTT
EMPV1_21007 CACCATTACCGAAGCACTCCCCCCCCGGGTCTAAGTTCTTTTCCGCCGTAACTAGGTGGT
EMPV1_21008 ACCTGTCCGGGAAGCTGACCCATACACGTCAGCAGTGAACATCTTCTGCTTTTCTCTAAG
EMPV1_21010 TGGACAAGGTCAACAGTTCATTAGCAGAAAGAGAAGCCAGTCGCCTCATGTCCACCAGCT

EMPV1_21012	TCCAAGTCATTGTTTACCACTCTACAAAAGCCTTCAGGAGAACCACAAGATGAAGATGAT
EMPV1_21014	ACTATTGAAGACAAGGACTGTTTCAGATAAATTTTCTCCAGAAACAGCCTCCAGAAGAGG
EMPV1_21015	AGCAAGTAAGAGTCAGCAAGACTTAGGTGGTGGGGAGAGAGGCTTAGATGGTTGAGAACC
EMPV1_21017	TCCAGCCTAAAGTATCTCACGTCCATCCAGTGAGCTCTCAGTCGGGGAGGGTTTGGTGGA
EMPV1_21018	TCTTTCACATAGGGAAGAATGGTAAATGGATTTGGCAGGCAGGCTCTGTCAAAATTCTGC
EMPV1_21019	CCTTAAAGGATTACCTTATCAAGCACATGGTGACACACACAGGGGTGAGGGCGTACCAGT
EMPV1_21022	TTCAGCTCACAGTTCAGCAGAGTGGCAATTTGGGCAGGGCTTAGCTATGTGGTTCTTCTG
EMPV1_21023	CTGTGTGTGATGGGTTGGCAGTGTATTGTTAGCTGGTTGAGTATATGAGTAGCATCAGCT
EMPV1_21026	AATCAGGCTTCTCACCTGGATCTTTGAGGTGCCTCCTTCCACCGCACGACTGGCCAAAAA
EMPV1_21028	TGGAAGCAAATCAGTGGACTCTGGAATATCCTTGGATGTCAGTTACAAAATGGATTATCC
EMPV1_21029	AGATGACAATGAAACAAATGGCCAGAGAGAGATGTTTGCCCCAGCTAAGGAGTGGCAGGG
EMPV1_21030	CTACAACAGTGGGGACCCCAGTACGAAGACCTGTCAAATTCCCGGAAACTCCCAGCTTTT
EMPV1_21031	AAATGTAGCTCCCATGATAGAGCAAACTGGTCAGCCCGAGGCACCACCTCCTTAGCATCT
EMPV1_21034	CCCATGGACTAGTTTACCTGTGTCATGAGAAGGACTTTGTGAAACCTGTCTGTAATTTGG
EMPV1_21038	TCGCGATTGCCGGCTGCCTGGAAAAATCCAACCATTGTTAAGGAGGGACCATCTGTACAA
EMPV1_21039	GGTTGTCGTGAGGAATACACCTGGCACTTGCTAAGTTCTCAACCCACAGTTGCCCTACTG
EMPV1_21043	CAATGTTAGATAAACAGTCAATCATTTTTGAGTATAAGTATGTCCCATGCAGTATTTGGG
EMPV1_21045	CCCTTGAAAAGGAGGCACAGACATCTTTCCTGCATCTTGGCTACCTTCCTAACCAGCTGT
EMPV1_21046	GCTTCAACGAGTTCATGACCTCCAAACCCAAAATCCACTGCTTCAGGAGTCTAAAGCGTG
EMPV1_21048	AACGAGTTTCGCTGTTACGTGAATTATGCTTCTGGGCTGGACCGCGAACTGTGAGTGCGT
EMPV1_21049	GTTCTGTGACTCTGGACAACGGCATAATCCGGGTGAGGCTGGACCCAATCGGCCGTCTGA
EMPV1_21050	CAGAACCGTTTTGTCTCATTACTCCTGTTTAAGTTGAAAGAATTGTATTCCATTTGGAGA
EMPV1_21052	ACTAGTTGACAGTCTAGCCAAGGTGCTTTACGCATCTGCTGCCTTAGACGGCTTCTAGGA
EMPV1_21053	ACAAGTGCGGGAAGGGCTTTACTCAGAGCTCCAAACTGCACATCCACCAGCGTGTGCATA
EMPV1_21054	GACAGTCAGAAGGTGAAATTGACTTGGGTCCGAGGTTGCGAAGAGTCCCAGAGTGGGTGA
EMPV1_21055	AAAAGGCAGCTATGACAAACAAGCATGTGCCCAGGAGGGCCCTTGGCCTCAAGTGGGCTC
EMPV1_21057	TCACTGCCAGGCCCCCATGTCTGACAAATGGTGGATGCCCAGTAAATGTTTGTTGAGCGA
EMPV1_21058	CCAGGTGGTCAGGATGTAGGACTTTGAAGAATGACTATGGTTTTACTATGGCAAGAACAC
EMPV1_21060	ATCTGGCTTCTCTCCTCAGACCTCAGCAGCCAGGGAGGGCAACCAGCACACCAGTCTTCT
EMPV1_21061	AGTCAGGGACTCTTATCCCAGCTGCAAGGACAGTCGAAGGATATGCCACCTCGGTTTTCT
EMPV1_21063	ATGGGTTAGATGGATAGGTGGTGGGCAGCAAACTGCCAGGGTACTAGAAAACAGAGGGAG
EMPV1_21064	AACCACCTCATCTGGCTCATCTTCTTCTACTGGCTCTTCCACTCCTGCCTGAACGCTGTG
EMPV1_21065	TTGCCAGACCAGCAGGCTGTAGCTGAGAAAAGCACCAAATCCTAACCACTAGACCACCAG
EMPV1_21067	GCACACCTGGAATGCCATTATACCTTAACTCCATATATCCATCCACATCCAAAAGATTGG

EMPV1_21070	CGGTGATGCATAAATGTGCAGTGACGTAGAGAAGACATTGGAACAGATGCAGAGAAACAG
EMPV1_21071	AGCTTCCTTGCCCTCCTTCTCTATCTGTCTGAACCCTGGGGTCTGGGGAAGGCGTCTTTT
EMPV1_21072	CTTGCAGATTGCATGTTCTTACAGTGGCCTGCTATTCAGGATAGGTATTCTTCCATGCAC
EMPV1_21073	GGTCCTGCTCTCACAGTAGTTCCTTTCTAGTGAGAGGAAAGTCCCCATGAACAGTGAGGA
EMPV1_21074	AGGAACTGAAAAAGGGGCCTTTCGCAGCTCCTTGTCACTGGTTCAGATGATGGCTACATC
EMPV1_21076	GTAAGGACAAAGCTTGAGGCAGGGAGGTTTCCATTTGTTTCAGCTTAGGAACTCTCACTG
EMPV1_21078	GCAGTGGTACCTTGAGTACCCCCATACCTTCCTTTTACAGATATATAGAGTGGTCTGGCT
EMPV1_21081	CCGCCTAAAACTCAGATCACCTCCACAGACAGATTTCAAGGTCCAACCTAAAGGGCACCT
EMPV1_21083	CTATGCCCAGCCAAGGAAGCCAGATGTATTTTCCAGGGTGAGTGTCTTCTTGGAGTGGAT
EMPV1_21084	AGAGAGCTGAGAAGGATTGCACCTGTCGTTATTGTAGGTGACAGCCGGCCAACCTGTACA
EMPV1_21085	CCCCTTCATCAAGGAGTACCATGCCAAGGAAAACGACTTTGACAGACTGGTGCTGCAGTA
EMPV1_21086	AGAACTGTGAAGTCCATGCTCATCCAGACGTAACCAAAAACGACGTCACCCCACACGTCT
EMPV1_21088	ATATGCTAAAATGCAAATTCTCAGGCTCTACCCCAGACCTACTGAAGAAGATACCGGAGG
EMPV1_21089	CTGCAGGGCTGCCTCTCTGGATGGGTTCTACAGCGGCCGCAGCCTGGATGTTGCCAAGGC
EMPV1_21091	GGATCCTATAGATACCTCAGGAGAATTCAGGAGTCAGGCCCCCTTGCCAACTGTATATTC
EMPV1_21093	GCTGCCTCTGTTTAGCAAATTTAGAAGTTTTGACCCACAGGGTGCACTGAGGAACATAGC
EMPV1_21094	GGGGAATTTTTCTCCTTGGATCAGTGGAGGCAGGGCTCAAAGAATATGGAGTCTAACCTT
EMPV1_21095	TCACCGTGCAGGGTCTGATCCATCTGCTTGCAGACCTCATAAACTCCCTGCCGCTGTACT
EMPV1_21098	CTGGTTTCAGTTTGTCTAAAGAATACAATGAGAGGTTAATTTCTGCTCAAGTGGTACCAC
EMPV1_21099	GAGAGCGTTCCACAACTTCAGCTACTTTCTGTTCTTCTATAACGTGCTGCTGGGCCTGGG
EMPV1_21103	CAAAGAAATGTCTCTGTCCCACGGTACAGTTCCTGTAGTAAACGTGCAGACATGTGCAAG
EMPV1_21106	CCTCTGCTCATGTCTGCGCCTTCTGATAGTGATAAATCTTCTTCAAGTTCTAGCTGACAT
EMPV1_21113	GGAAGCCATCATCGTAAAATGCACTTGACCCACAGTTAATTCCTTCCCAGAGCCACCCAT
EMPV1_21114	AGGGAATGGAAGTTGTTGAAGGGATGTGGACAGTCGTAGGACAGAGGAGGACACAGCTGA
EMPV1_21115	GTCCATCCTGGCATTCAATTAGTAGGAAAGAGCAGGCTAGCGTGATGACTTTCTGAGGGC
EMPV1_21120	ACGATCAAGACCGACACCAATTGCTGGGCCAGTTTGTGGTACGCAGATTCTTCAGGTCAT
EMPV1_21121	TGGGAAGTACCAGACAGAGACCTGCTCCACTGTCCCTGTGTAGGAGACACAAGTACCAAT
EMPV1_21122	CCACAAAAGGAAGGAGACTTCAGAAAGGAGATTAACAAGTGGTGGAATAACCTAAGTGAT
EMPV1_21123	ATCCCGCCATCGTGCCGCCACCCACCTCAGGTCATCCCAATTCCCGGGCCCGCTTCATAA
EMPV1_21126	AAGGCGTGGAGTGCATCACCTTGGGTGCCAATAAAATCCGATTCAAAGCGGGCAATCGAG
EMPV1_21127	TGCTCTTCCGCATGTCTGAGGACAAACCACAGGACTATAAGAAGCGTCTGTCAGTGGAGC
EMPV1_21128	GGCAAGCAGGAGGACTTCAAGACGACAGTTCTGGAGGGTATGGAGACGGCCAAGCATCAG
EMPV1_21130	GAGTATCCTCATCTCCTACGTGTACATCCTCGTCGCCATCCTGAAGATCCGCTCTGCCGA
EMPV1_21131	AACTGACTGCCCTGCACTTCTGTAACCCCGCACTTCCCTGATGTATTCTCGGTATAGCTG

EMPV1_21132	GTTCCAGAGGACCACGTACAAGTACGAGATGATTAACAAGCAGAACGAGCAGATGCACGC
EMPV1_21136	GCCGTTATATGAAAGAGCATTAAAGATTTACGAAGATAGCCTGGGTCGAATGCATCCTCG
EMPV1_21138	ATGGACCTGGTACCTGAGGACGAATGGAACATTTACAAGTGTGAGGATCAGCCCAGGCCT
EMPV1_21139	GCTGTCTCCTGAACAGGGCAGCTCAGGCATTGAGATGCTGGAGACAGACACATTCAAACT
EMPV1_21140	ACTTCCTGAGTGTGGGTGGGACTGGCAGGTGTACACTGTGGTGGAAGTACTCTTCCTGTG
EMPV1_21143	TTCTACAAGATTTTCCAGCTCGAGATAGGGGAGGCGTATAAGAACCCCCATGCCTCCAAG
EMPV1_21145	TAATTGGGGAGCATTGCTTGGATGGTCTGCTATCAAGGGCTCCTGTGAGCCATCTGTTTG
EMPV1_21146	TGAGACTGAGCCCCAAGCTCTGCCTGTCTCACCTGTCACTTACACCTTCACTTCCATTGT
EMPV1_21147	CCACTCTTTGTTCTTGACCTCTTGAAGTCTCACAGCGGGTAGTGTTGCCTGGCATTTAGG
EMPV1_21148	GGAAGCCACAGATATAACAGCAGATTCCTCAAGTGAGTTCTCAGAAGAAACTGGGGCTTC
EMPV1_21150	CTACCCTCTGCTCCCACAAGCGAAACATTGCCTATTGCGCCGTTTATTACGCTGCCTCTT
EMPV1_21152	CACCCCCTCAGGACCACACTTCATTCACCTTTCCCCTAAAAGGATCACGTAACTTGGGAA
EMPV1_21154	TCTAGCCTTTCTCAGGCTGCACCAAAGATTCCATCGTCAGGGCCGACGGGGAGTGAGGAA
EMPV1_21156	CGCGCGCCATCAGGGTGCCCTGTACGTTGATGTTTTTGGAATGCGTGAGAATCATAGTGA
EMPV1_21157	TGATTTATCTCAACCCTAGCAGAAACCAACCGAGCCCCGTTCGACCTTACAGAAGGAGAG
EMPV1_21158	AGAAGATGAAGCGTAGGGAAGTCATGGACACCACTGACCGTGCCGTTATGCCATAATTGC
EMPV1_21160	GAGTCCCAGCCTGAGCCAGCCCCAACCGCACTCGGAATCTTTATTTATTTTAACTATTTC
EMPV1_21161	CAGTTTTCCAAGAGCAGGGAGGAAACAGCGAAGGCTGAGACTCTGATACCCCTAAACGTT
EMPV1_21163	GTCTAGTTACTAGGCAGTGTAGTTAGCTGATTGCTGATAAAACCAATCACTAACCACACG
EMPV1_21165	CAACAGGGTCCGATACCGGTGCGTCATGATAGGAAGACTGTTCCACGATAAACCTGTCAA
EMPV1_21168	CTCGACTGCGGTGCACGTCTGCTAAACTCCCAGAATCATACGCTGAAAGAGGCACATTTC
EMPV1_21170	AGTGAATGGTCCGAATGAGCGTCTATCGCAAAAGCACATGATATGGCTCGATGACCTGCG
EMPV1_21177	CTTACCGTCAGACCTGAAGTAGCAAAGGGAGGCAGTGCAGTTTGAGGAGCCCAATGAAGA
EMPV1_21178	TGAACCGGCAGACTTGTATCAGGCTGGTGTCGGTTTCATGGGTCATTGGATTTGTGCACT
EMPV1_21179	GCACAGTTCATATCCATGTTGCTCAAGGGTTGGTTGTGTCACTAGCTTCAAGTGTCCGGC
EMPV1_21182	GTACAAGAACACCACGATCTCCCCTTTATTGCACTGCATGAGATTAGTGTGGCCCATTCA
EMPV1_21183	CTACAATGGCTGTATATTTCACAGAAATATCAAGGGCTTCATGGTTCAAACAGGAGATCC
EMPV1_21185	GTTGGTGATAGAAGACAAGCCTGGGAAGATAGGCAGTGGAGGCAGAATCATGGAGGGTCT
EMPV1_21186	GTGAAATCTCCTGTTTCCTCCCTTGCCAAATAGCAAAGATAGCAAGATGACTCAGCAGCT
EMPV1_21191	GCTGCATTTGCATGGCTGAATGGGTTTGGACTGGAAGCTAAGTAAGACTCTGGTGTGGCT
EMPV1_21192	GATGGTCCCTTCTAACTTCAGGCTTCACCGATCGTGCTCACTGCCAAAAGTACTGATCTG
EMPV1_21194	TGTTGACACATGAGGGACAAGCTACCCAAGTGCCACATAATATGGAAATGGAGCCTCGAG
EMPV1_21196	TCACTCACCAAACATCTACTGAGCATCTGGGAAGTGCCAGGCATGAAGGATACAGTAGTG
EMPV1_21199	AGGTTGTGGAGAAGTGGCTGGATTACTTGAGGAATGAGCTGCCAACCGTGGCTTTCAAGG

EMPV1_21200	GATGACACCAGAGGAGAAGGAAGCATACACAGCAATGGGACAGCGCATGTTTGAGGACCT
EMPV1_21201	CAAGGAGACCCATCATTTCTAAACTTCTAGCCTTCACTGGCCCCATCTGCAGGGAAGTGA
EMPV1_21203	CCTTATCACAACCCCAATCCCTCCCACCTGAAGAGACACTTGCATGGCCAATTGTTGCCA
EMPV1_21205	CAAAGTCAGGATGCTCCTGGAAGAAATACTCTTACCCCCGTCATTGCTGCACAGGTCACA
EMPV1_21207	TCGCCACTGATACTGCCCCTTAGTAAGAGTGTGAGGCTGATCTGAGGGCACTGCAAGATG
EMPV1_21208	CATGGGGAAGATGTACGAATGGACGTAACTCATGGATTGGAAGGTGCTCACGTGGCTTCG
EMPV1_21209	GAGTGCGCCACCAACATGCCACCAGGCCTCGCCTCCCTTGCGTCTGCAGAAGTGACCTCT
EMPV1_21210	TTCTGGAGAGCGGCCGCAGGCCAACCAGAAGCGAATCACCACGCCGTATATGACCAAGTA
EMPV1_21211	GGGGGGGTGGAGCGTCGTCAGCCTCCACCCGTTGAGGAAGCAGCCGTCCTCCTCCCCAGG
EMPV1_21214	TGCACTGGATGTAGTGTACGCAAAGCCGTGGTCAGCTGTAAGGATCGCACAAATCGAATC
EMPV1_21215	GGTGCGCAAGGGCAAGCAGGCAGAGTGCGAGCAGCGCGGGATCCTTTCCTTCCTTTGGGC
EMPV1_21217	CTCTGGGGGTTCTTCAGTGTTCTGTGGATCATTTTGAATTTGGCATTTGTCCCTTACCGC
EMPV1_21218	TGGATGGGGCATGGACTACACCTTTGCCAGTGTGCGCTGTCAACCTGTGCCATTGGCATA
EMPV1_21219	CCACCCTCCTTACCGAGCTCCAAAGGGCAACAGCAGTCCTGCCCACTGCTCCTCTTCTCA
EMPV1_21223	GATCCCCTCTGCTCTTCACTCAAGGGATAACCCCAGGGTGATAATTTTCTATTTCCCTCT
EMPV1_21224	TTACAACATATGAAAGTTCCTGGGCCAGTGGCTGAATCCGAGCTGAAGCTGAGACCTACG
EMPV1_21225	CTTCCTTTGTTCATAAGTCTACAAGATTGACCAACTAGTGGCATGGCCATGGACTTCCCA
EMPV1_21227	CCCCTTAACCTCCCAGCTGCAACACCCTACTTCATCCCAAACAACCTGGAAGACACAGTA
EMPV1_21228	GCCACTGCAATTAAGTTGCCTTCGTCAGTGTTTGCATCAGAGTTTGAGGAAGATGTTGGA
EMPV1_21230	TTCCAGCCAGTGGGGGTGTGTAAATCTTTATGGTTATCCTGGCCTAAAGTCTGAACAGTC
EMPV1_21231	CTACAGAAGTGGTAAACAAATCCAGTGTTAAGGATTCAGTACTACGTGAAGATGGGAAGA
EMPV1_21232	GATTCTTGGGTTAAAAGGCATGTGTGGATTCACGGTCTACACAAAAATGAAATTGGTTGC
EMPV1_21233	CGCAGAAGACGTCGACCACGAGCAGGGCCAGGAGCTTCCGGGCCTGGAGAGGCCTCTTGT
EMPV1_21234	GGAGTGGCTATATATCAAAGGCATAAGAAAATTGCCAGCATAGTAAGGAACAACTTCAGT
EMPV1_21235	CAGAGAGGGGGAGAACTCACATGAATGCAAGGTTAACAAAAGAGGGCCTGACTCACTCTG
EMPV1_21236	CTTATTAGCCTGGAAGGACTGGACTCAGCAGGATGGGTCTGGAGGGGAAGGAGAGAAAGT
EMPV1_21237	CATTGTTGTGTCTATGATCTTCATTCCCTGTATCTACATCTATGCCCGGCCTTTCACCTC
EMPV1_21238	CTCAGTAGAAGAGCCAAGAATTGCATAGATAAAATCCCTTTGGGGCAGTGTTAAGGTACG
EMPV1_21241	TCCAGTGGCTGTTTAGTGTCTCCATCCAGCCTCAACCTTCCAGGAGTGGATCTCATCACT
EMPV1_21242	GATCGAATGGGCTCTTCACTGATGATTGAAACAGCAAGAAACCCCACATGTCCTAAGGTA
EMPV1_21243	ATCTCACGCCTTTGCAGACAGCCCCCCACCCTACAAGCTGAAGCTGGACCTGACCAAGCT
EMPV1_21244	TGATGTGAGCACTGTTAAAGAGCTGTGCAGACGCTGGTATCCAGAAGAATATGAATTTGC
EMPV1_21245	GGTGGCTCTTAATAAAGGAGTTTCCCTGCTCATTTCTTCTATTTCACCCACGTTGAACCC
EMPV1_21246	GCCATTAAAAGGAAAGGTCAAAGAGTAAACAACAGGAAGAGGAAGTGGAGGACTGCAAAG

EMPV1_21248	GAGTGCAGTATCTTTGACCTGCTAGGCATCCCTCTGTACCATGGCTGGCTTGTTGATCCA
EMPV1_21249	AGGAATCGATCCTGCATCTCTGCAGTGAACCAAGCTGCCACAGTCAGGTTCTCAACCCAC
EMPV1_21252	AGTGCGAGGGAAGCCGAAGATGCAGAAAAGGTGGTCGAGGACATCGAGTACCTGAAGTGC
EMPV1_21254	TTGTCTGTTAGAGTCCTTGCCATTTGTCAGCGTCTGCCTCGCCACCTCTGTGCTTGCTAA
EMPV1_21255	CCTAGCATTCTAATGGAAAGATCGCTCCTGGCTTGCTAATTACCAACAGAGGTCCAGGCA
EMPV1_21257	GGACACAACCTCTCAAAATCCATGGCATGCTGCAAAAGAAGTGGTCATAGTAATACAGGC
EMPV1_21259	CTGCATGTGCATTTGTACGTGTGAGTGCGTGTGCATGTGTGCATTTATGTACGGACATGT
EMPV1_21260	CCTGCTGTCTTTCCAAAGCTCCAGAGACTCCAGGTAGGGTCAGTTCTGCTTTTCTGCTTT
EMPV1_21262	CTTTTTGGCCTTGCTTCTGAGCTCGTTTAGTGCAGACACCTTGCAGCCACTGATGATGAC
EMPV1_21264	GCATTGCTGTGTGCTGTGGTATAGGTCATAGATGTGGCTTGATCTGGTGTTGCTGTGAGC
EMPV1_21265	ACCCAGGGCTTTGTTAGAACAGGGCCTTCCTTTTCCAAGGGTGCAGGATCCAAGCCCCCA
EMPV1_21266	AGATGGATACTACGATGCTGAATTTGCGGAATCTGTTTGAGCAGCTTGTTCGCCGGGTGG
EMPV1_21267	GGGCTCTTGGATGACAGTTGCTGGGTAATTTTTGCCCAATAGTTCAACTTCCACTTGTTG
EMPV1_21270	GAAGACTACGTTGATCCCTGGAGGCTCAGAATCGCTTGTCTATACCACTTTGTCAGGAGG
EMPV1_21272	CCTGAGCCTCTGCATCTTCAGGTTCTGCACAATAGGGTGTTTTAAGTTCCCAGCCTCTGG
EMPV1_21273	GGGATGTGGAGATGTAATTTGTGCTCCTCCCTATTTACAAATAAGTGGCCCGTTGGGGGG
EMPV1_21274	TGCAGTATGTCTTTTGTGCAGGCCTCCTACTGCTTCTGGCGATCCAGCAGTTTCTCAGCT
EMPV1_21275	TCCCTAGAATACTTAGGTCAATCCTTGCTGCCTGTTCTTAGCGCGCCATCATCTGGAGAG
EMPV1_21276	AGTGTGCAGGTGGAGAGTAGAATGTCTTCAGGTGGACAGTTGCAGGGGCTTGCAGTAGAA
EMPV1_21279	CTTCAAGCATCCATTTGCCATTGAAAACATCATCGGCCGGGACTACAAGGGCGTGCTGCA
EMPV1_21280	CCAGTCCTTGGCTGGATTCTGTGGTTCCTGTGTTCAATAATATCGTGACCCCTTCCCTGA
EMPV1_21281	CGCCACCTTACATAGAACTCTGAAAATGGTATGACTGAAAACAGATTAATGCAACATGGT
EMPV1_21283	TTCCACCATACGAGAAATCACATCAGTGCAGGGGAAGTATAAGGCATTTTCCACCTGTGC
EMPV1_21285	GGGAAGTTGAGGTAAAACTCCCTCAGGAAGTAAAACAGGTGGAAAATGACAGGCCTGAGG
EMPV1_21286	CTGCCTGCATTCTGATAGAGAAGGGGTGCAGAAGATACACAAGACAAAGCCCCTGTCCAC
EMPV1_21289	CTCACCTCAAGAAATCTCTATGTATGCACAAGTAACTCATCAAAAGGCCGAGGAGATCTA
EMPV1_21290	CGCATATGTGCATGTGTCTCCGTGCCATCACCTCAACACCTGTCCATCTAGTTCAGCCTA
EMPV1_21296	TGTTCCTTAAGACAGGAAGCAAAAATATACATACACGGAGCCCCAGTTGTGCCACAGTGG
EMPV1_21299	TGGATCAGTGGGATCGATCCAGCATTGCCTTGAGCTGAGGTGTAGATCACAGGCGTGGCT
EMPV1_21301	GCACACTGGATTACATGCTAACCTTGCTGCCTTCCATGACAGAGCAAACCATTTGGCTGG
EMPV1_21302	CCACTCCTACCTGCCACAGCCCACCTTCAAGGATCCCTACAGTAAGTTCGACATCAAGAT
EMPV1_21304	GGTGTTAGTGTCCCTAGGCTTTCCTCCTACTCTTCCACGGGTGTGTGACTTAACAGTTTC
EMPV1_21307	TATTCCCACTTTTCCCCATGTTTTAGTCGGTCTTTGTAATCTTCAGTTTCAACAAAACGC
EMPV1_21309	GTTTTTGCCCTCCCAACCTTGAGGTACTCATCACGCAGGGTGTTCGAAGCCTCTCTGGAT

EMPV1_21311 CGATTGCCTCTCTTAGGAGAAAAACAGGAGTCTAAGGAGAATGGAACAAATCTTACCGTT
EMPV1_21313 CATTCCCACCACAGCCTCCTCTTCCAAGGTGGCCAGTAAGAAGGCTGAGGCCAAGAACTA
EMPV1_21314 ATCCGGAGTCAGCATCTCTACCTTGGTGCTGATAGTGGTTGGCTCTGTAGTAATCGTGAG
EMPV1_21315 GTCACGGATGACGACTACGCGGGTTTCATCTTTGGCTACCAAGACAGTTCCAGCTTCTAT
EMPV1_21316 GAAGTTCTGTGCCATCCGAGAAAAGCTGGACCCCACAGGGATGTTCCTTAACGCCTATCT
EMPV1_21320 CCCCAGCCAACACTGTAGTCTAATTACATTACCATGCTCTCAGTCCTATCACAGAGAGGC
EMPV1_21323 GGTCGTGACAGATTTGTGCTGTTCTTAGTACATGCGATACCCTTGACGTTCCTGTTTTAC
EMPV1_21324 CTTGTTAGCACTTCCATCAGAAGAGAGGAAAAGGGGATGAAACTATAACGTGTCAATTTG
EMPV1_21325 ATGAGCTGTGGTGCAGATCGCAGATGCGACTCAGATCCCAAGTGGCTATAGCTGTGGCAA
EMPV1_21326 TCCTCATGGCAACCCTCAGAATTAGGTGTTATTGTTACGGGAGTTGGACAGATGGGGAGG
EMPV1_21327 CTACAGAACATCACTGAAAGGAATGAAATCACAGGTATGAAAGCACCCAGTTTGTAAGCC
EMPV1_21328 GGTGCCATCCTGAACTTTGAGCTCCAGATACTTTGTTTTCTTATACAGGGAGCCAGGGCC
EMPV1_21330 CCCGAGCACTGAATGTCTGGCTCCTAAGTGGCTTGTTGGTTTTCGTATATAACACCTGGC
EMPV1_21332 ATGGATGTAGCTGGGGAACAGCAGCTGGATGTAGAACACAACCTGTTCAAGCAGCGGCTA
EMPV1_21333 GGTACCAAGATGATACCCTGTGACTTCCTTATCCCGGTCCTGACAGAGCAGCCGAAACGG
EMPV1_21334 ACCCAGTGGTTTTTAGTCTATTCACAAGGCTGTGCAGTCAGTACCACTATCTAATTCAGA
EMPV1_21335 GGGCCCTGTTCTGACATGGTTTCCAGAATATCCAGAAGCCCAAAAGAGTCACTGAGGACT
EMPV1_21336 GAGGCCTGGCCTGGGACGGGTTGTCCCTGGATCATCCATCCTTTTCCTGTGTGACATGCA
EMPV1_21337 GGTCCTTGATATGGGGCATGTGTGCAGTCCTGGAATTAACCATGAAGAAAACGCTGACTT
EMPV1_21338 TCTCAGTTCCTTTAGTTCATCTTCAGCTAATGCCCAGACAGATTTCTTTGCTGCCAGAAG
EMPV1_21339 GATCAAAGCAAGGCAGGACTGTGTCCACACTAGGATTGTGACTGATGTTTTAGCAACTCG
EMPV1_21340 AGCGTACTCAATGGATTCTGCCAAAAAAGGTGAAGGAGTCACCCATCATTGACGCCCCTG
EMPV1_21345 CCTGTTAATGTACGTTGGCTACTTTGTGGCTGCCTTTGTTGCTGCTGCACATTAGGTTGC
EMPV1_21346 ACTGCATCGGGACCTGACTATCCAGTGTGAGAAGATGGACATCCCCTTCCTGTCCTATCT
EMPV1_21347 CTATGCCCAGGGAAAGAGGTGCTATGATAGGAAACAGAGTGGCTATGGTGGGCAAACAAA
EMPV1_21348 AAAGGTTGGGACCCGGGGATGGCCAGGGACGGGCCCGGGATTAAACCCATGGCTTTTTAT
EMPV1_21350 GGTTGCAAACTCAGCTTGGATCTGGCATTGATGTGACTGTTGTGAAGGCCAGAAGCTGCA
EMPV1_21351 ATGATACCTTCTTCAGTTGTGCACCAGCCTTTTCAAGTCACACCAACACCACCCGTGGGG
EMPV1_21352 AGCATGTCCTTTGCCACCTGAAGACACAGATGACGAAAAAGAATAGAACTTTCTTAGTCA
EMPV1_21353 AATTCCCTTGGTGGAGCAGAGGGTTAAGGGTCCAGCGTTGTTGCTGCAGTAGCTTGGATT
EMPV1_21354 AGGCATCCCTGGACCTCACCAGGCTGCCACTCTAGATACTTTTGGCAGATGAGGAAAACA
EMPV1_21357 TGGCCAATTACAGAAACATGGTCTCCTTGGGATTTCAGTTCCCCAAACCTGAGGTGATCT
EMPV1_21359 ACTGAGTGAATCTGAGTCTGGATTGGTGGACTTTTTGGGGTTCAGGGCAAGATGCTCAGG
EMPV1_21360 TGGCCATCACGGTTTCGAGCATTGTCCTGCTGATGACACCTTTCTGCTTGGTTCTCGTGT

EMPV1_21363	CTTAGCAACGTCCAGTCCAAGTGTGGCTCAAAGGATAATATCAAACACGTGCCAGGAGGC
EMPV1_21364	CCCACCACAGCTCTGACATTATTCTCGGTTCAGACCTCATCTGCAGCTTTTTCCTCCTCA
EMPV1_21365	AGACTCTCGGTAATGGGCGAATATGACCTGATGGCTTTCCTCCGTTTAGGATGGTGGAAC
EMPV1_21366	CCATCCCCATGGACCCCCTCCTCATGGACACCATCCCCATGGGCCCCCTCCTCATGGACA
EMPV1_21367	CCAGGGAGCTGTTTGTCTGGGTCAACTTGTTTGGTGAGGCTCCCAGATCTTCCATATGAA
EMPV1_21370	TTGCAACTGTGGCTTGGATTCAGTGCCCTGGCCCAGGAACTTCTATATGCTGGGAATGTG
EMPV1_21372	ATTAATGAGGAAAAATAGTTTTCATTTCTTAATATACATGACAAAAATACGCAAACAGAA
EMPV1_21373	ATGCCATGTCAATTTGTAGCAGAATAGGCTCAGACAAGTACGTGCAGTTCCCCCGTTGCC
EMPV1_21377	CCTCCCTCTGGACTCTGAGATAATTGCTGTGGTCTGTCCCTGCCACCAATAGATCATGCA
EMPV1_21378	AGCACTTGCTTTCAAAGAGTGGCGGGGACTGCACTTTTATTCATCAATTCATAGAATGCC
EMPV1_21379	AGGCTACAACATTGAACAAATGGCAAAGCGAGGCAAGAAGCTAGTTGAGCTGCCATACAC
EMPV1_21380	GCCGAGGTGGGATCCCGAGGCCTCTCCGAGTCCGCCGAGGGCGCACCACCGGCCCGTCTC
EMPV1_21381	GCTGCAGAAACCTGCTTAACTACTGATTTACAGTTTGGGCCAAGGTATTTAACTCTGTCA
EMPV1_21384	TGGCCCCTACCGTGAATATCTACCCTCTCAGACTCAAGAGACCTGCACTTCATCTCTGGT
EMPV1_21385	GGGACTATTTAGCCAAGTCAGAGCTTCTCCTGGATGTGTGATTCTAGGGAACCTTCTTCC
EMPV1_21386	ACATGTTGCTGGCTACATCGTCTCGCTTCCGTATGATGAATCTCCAGGGAGAGGAGTTTG
EMPV1_21389	ACAGAAGGCGGGCACAGAGGGGTCACAGCCTCAATGGGGCATGCCCACACTCCAGGTGAT
EMPV1_21390	TGACAGACCCAATGTTGGCTACAGCCTGGACATGGACATCCTAATCTCTGTGCTCTTCTG
EMPV1_21391	TGGCTTGAGACCCTAATTTGTGAGTTGCAAACATAAGAAATAGGTCATATATGTCTGGGG
EMPV1_21393	TCGAAAGCCAGTTGGAAAGTATCATATTCAAGTCTGCACCACTACGCCTTGCATGCTTCG
EMPV1_21396	CCTTGTCTTGAGTTTGGCCCTGAGCCCTTTGTGAATCAGTGACCAGCACGCATGGCAGGT
EMPV1_21397	TCTGCCAAGTGGAGATGATCACAGAGAAATACGGTGAACAGAAGTGGCAGTCCATCCAGG
EMPV1_21398	CGGGCTAGCGGTAGCCCGGGATGATGGGCTCTTTTCCGGGGATCCCAACTGGTTTCCCAA
EMPV1_21399	GAACCGTAGGAACCATATCTGGTGCTGAAGCATCTCCTGGACTGACACCTATCATCCATC
EMPV1_21400	AGTCCCAACAGCTCCTGTGATTCGCCCTATCATTGCGACCAACACGTACCAGCAGGTCCA
EMPV1_21404	GAATATGGTGGAACGCAGTCAGGTAGAGCACTGTGAACAGGAGACTGAAGAAGGAGCAAG
EMPV1_21407	CACAAAGAGAGCACTACAGAGATCACAGTGGGCGTGGACCAGATGGATACGTGTGAACAA
EMPV1_21408	GCAGTGGAAGAGGGATTCTGCTCTATATTGGAGATTAAAGGAATTTTGTTAAGCTATAAA
EMPV1_21411	ACTGACCCGATTTGCTGTCTGTGGGATCCTTATGGGTGGTGGGAGGCTCTCATAAACTCT
EMPV1_21413	AGTTTTCTATCTAAACTAATCTAAACTAATGTCACCTAGATTTTCACCTGTGTTTTCTCC
EMPV1_21414	TGCTGGAGCAGCACCTGGAAAAAATGCGCCTGGAGCCTGCAAGGACGTCAACCTCTCAGA
EMPV1_21415	GTGACTCGCAAGCGGAACTTCTCGCCCCTCTTTCGGTCCAGCTGAACTTCTATTTAATTG
EMPV1_21416	GCAGAAGATTCAGAAGTTTCAGATAGCCCAGGTGGTGAGAGAATCCAGCGCCATGCTTAG
EMPV1_21417	CCCTCATCTGCCTCAGTTCGTTGTGGCTCAACAGGTGTAGCTATAGGAATGGTTAGGGTT

EMPV1_21418	CCAGTGTTTCTGGGTCGAGAGAAGATGGGGAGGGGCCTGGACAAACTTGAATTTATTTTG
EMPV1_21419	GTGCATGCAGCTGTCTAGTTTTGCCAGTGCCACTTGCTGAAAAGACATTGTTTTCCCCAT
EMPV1_21420	CAGTAGCAAGATGTCCAAGAGGGTCTGTGTCCGCCTGGTCATTATCCCTTATGTCTATGG
EMPV1_21421	CCAAGGAAGAGAGATTTGTATAGAGGGACAACTCTGGAGAACTGTAGAGGGCTGTCTTCT
EMPV1_21423	GCAAAGTCCTGAGTGAGTATTAAATCGGGGTGGTGGTACTCCCTGCGAGGCTAAATTCAG
EMPV1_21424	GCCCGACTAGTATCCGTGAGAATGTAGATTCCATCCCTGGCTTTGCTAGATTAAGGAACC
EMPV1_21425	CTGGCCTAGGACTGATGGGAATAAAAGATGAAGAGGAAAGCTCCACCCCAAACATGTGTT
EMPV1_21426	TCCTCCGAAGTAACTGCCTGTTGCCTCTGCTGCCTTTTGAACCATGTACAGTCACCAGAT
EMPV1_21427	TAACGGTATAAACTGAGCCAGCCCTCTGGTAGACACAGGATAGATAACTCTTTGGACAGC
EMPV1_21428	CGAAATGGATATTCGACTGGAGACCTGACACAGCGCGCCTGCTTTGATGGGAACTGGTAT
EMPV1_21432	CCCCTACAGCCACTGCCCTTTACATCAAAGGCTATTTGGTTCCACTCTAGGGATTGGCAA
EMPV1_21436	ACAAAAGAAGAATCGTGACTGAGAGGGTGGCAGTTGAGGGAGGAAAGAGGTGGCTGCAGA
EMPV1_21438	ACTTTCTGTAACTGGAGAGGTAGAGCCTAGAAGTTACCTGGGACCACCTCATCAGGGTCC
EMPV1_21441	CTGTAAGTGCTAGAACAAAACCCAAATCAGAGGCTGGGAGGGTGAGTGACGGGGTGTTTT
EMPV1_21442	GCAAGAGTGGGTGAAACTGAAGAGCGATTGGACGTTGCCGTTGGCCCCAGACGTACCTAT
EMPV1_21444	AGCAGTTCATCTGTGGCTAGAACTATAGGGAGGAGCCCCTTGCTGAGCCTTCTCAGTCCC
EMPV1_21446	GTTGGGGTTCTAATAGAGCTGAAGTCACAGCCACAGCCATGCCAGATCCAAGTGGCATCT
EMPV1_21449	GAGCTCATTTAATCCTCACGGCAAAGGGACAGAAAACAGTAGCCCCGTTTCCCCAGTGGG
EMPV1_21450	CCTTCCATGACACAAGCACGGAACAGCTCTAATCCTGACCAAATGGCAACAGCAGCTTGT
EMPV1_21451	ATAATCTGGCCCTGTCAGCCCAGCTGCATTCCTCTGGATGAAAAACTCCTGCCCCAGCTT
EMPV1_21454	GATGTGGGAAGAGGTAGAGAAAGGTGAGGAATTAGAGGGAACCAGAATTAGGGATGTGGG
EMPV1_21457	GGCTGGCAGGTGGTAGGCACCTGAGGCTGGGGAGGAAGCACCTCTGAGGGGATAACTGAA
EMPV1_21458	TGGGCCAGATACCATCAGTACATTCTGAACAGCAACCGTGCCAACAGGAGAGCCACTTGG
EMPV1_21460	CCTGTGTAACAGTGGGCAGGCACCTAACCGTTGTCTGTGCTTTTAACTCTCTTCCACCTG
EMPV1_21461	AAGACAGAACTTTGATTGCTGGCCTAGTGCCCTCCTATATGGCTCTCTCTCCCTCCCACA
EMPV1_21462	GCCTGGTTGCAGTATGCTTGAAATTTGGGATGTAGAAGACCCTTCCAACTCAGCTAACCC
EMPV1_21463	TCAGTCATGTTCCCTCAAGCGGTGATGCGCGCCTTCCGCAAAAACAAGACCCTACGTTAT
EMPV1_21466	TGTCTTTAAGGTCCACCCATGATGTCACAAACATCAGGATTTCCTCCTGTCTCCTATCTG
EMPV1_21467	CATGTTGGACCAGATCAACTCCTGTCTGGACCACCTGGAGGAGAAGAATGACCACCTCCA
EMPV1_21468	GCCAGTGTTGGAAGTGGAGATGTCCATAAGATGGTGGTGAGCATAGGATGGAACCCATAC
EMPV1_21470	CATTGTACGCCCTTTGGCCAGTCTTGTATGTGCCTTGATCCAACGCTACATGTATTCAGC
EMPV1_21471	TATCTGTCTATATTCCATCTCTGCTTGATGCCGCTGTTACCTGGGAGCTCTACGGGAGTG
EMPV1_21472	AGGTTGAAGTTCCGACTAATACCTGGCTTCTCAACTAGTGTCGAGGGGGTGTTTGCTGCA
EMPV1_21473	GTACCATATCCCAGACACTTTGCAAGAGACAGAGAGGATATCAAGCGAGATCTAGACGTG

EMPV1_21474	GAAGGCATGGGGTCTCCCAAAGTAGACGAGCCTCAAGACCTTTCCAACCATGGAGATGTT
EMPV1_21475	GAGGAGGACTCCATCCTGGCTGTGAGGAAATACTTCCACAGACTCATCCTCTATCTGCAA
EMPV1_21476	AAAACTTCGGGGTAATTGGAAAATTCAGAGCTTAAAAGATGAAATCACATCTGAGAAGCT
EMPV1_21478	CCTCATAAACTTCTCTTGTATGGAGACGTGTGTGACCTGAACCACCAACACGGAGGACAG
EMPV1_21479	AATACAGCCCCGCCCATACCGGAATTACACGGCATCAACCCTGTGTTCCTGTCCAGCAAT
EMPV1_21480	GGCATTCTACACTCATCCAGCCATCTAGCATTGACAGTTGACCTTCCTTTCTGTGGACCC
EMPV1_21482	ACATTCTCTCCTCCATGCCTCTGCCCAAGCTGTTCTCCCTGCCTGAAGCACCTTTCACAT
EMPV1_21485	CCCCGAAAACAAGAAGAGGGTTCACCGCTGTCAGTTTAACGGGTGCCGGAAAGTTTATAC
EMPV1_21486	CTGGGCGAACATATTGCCAGTAATTAAACATGAATGAAGATAGACATAAAGTGAAAACTG
EMPV1_21488	GAACACAATGCACTTTGCACGCTGTCCTGAGTCCCCACGAGCGTCTGGCTGGCCTGGGTT
EMPV1_21489	GTCTCTCTGGGCTACCTGAGATTCTGTCTCCAGGGCTTAAGTCCTAATTTCACCCCAAAC
EMPV1_21491	CGGGCCGCGGCTCTTGATTGTCCAAACGCAATTCTCGAGTCTATAGCTCTGGCCGAGAGT
EMPV1_21492	CAGGAAGTGGTGGGAGTGAACACTTAGAAGATCAGACCACTGCTCGTAACACATTCCAAG
EMPV1_21493	ATCAGGCGGCTGCTGAAGAGTGAACTTGGATCATTCATTACAGACTATTTCCAGAACCAG
EMPV1_21494	CTGTACCATGTGCAAAATAAAGTGATCAACGCATAACCCATTTATCATCATTAGGGTGAC
EMPV1_21502	GCTGGCCACATCTACCCTGGGATGTTCTTAGTTGGCCTGCAGGGTTTGGAAATATGGATT
EMPV1_21507	TCGACAGAACAATTGGCCTCCTCTACCTTCGTTTTGCCCAGTTCAGCCCTGCTTCTTCCA
EMPV1_21512	GTGGTCTGGGAAACGCTGGGTGTCATGGGGGTGTGTGCCGGGATGGTGCACTGCATCTAT
EMPV1_21513	GGTGGAGGGTGGGTGGGGGTATATGTGAAAACCGGGATAAAAGCAGAATTCCTTATCTGA
EMPV1_21514	TGGGCTGTTTTGGTTGTGAGCTGGTGTGTGGCAGAGGACAGAGCCCATAGCTTTTGGATT
EMPV1_21515	GTCGTCATGCTAAGGAAGGGGAATGTTCACTTGCAACCACAGACACAGCTCTGAGGTCTA
EMPV1_21518	TCCTTGGCGCGTTTACTGTTGGAGGCCTTGACTTCCTTTTGATCACTCTCTCCTACACAC
EMPV1_21521	AGAGCCCCCCACCTCTGGTTGCACCCTCTGTGTTTCTTTCGGCATTATTTCTAAGTTGGA
EMPV1_21522	TGCATCTTGGACTTTTACATCCATGAGTCACTTCAACGCCACGGCCATGGACGAGAACTC
EMPV1_21524	CTAGATGCCATCTCTGAGGAAATCACCTGGTAGCCAAGGCTTAGATAAGGATTAAGAATG
EMPV1_21525	GATGATGACGATGATCCTGACTACATTCCTGCTGTCCCGGAGTGCGACTTGTCCGATTAA
EMPV1_21526	GCACTCTTATTTCCTCTCACTCTCCTCCACCACCACCCGGCCCATAAAGCGCTCCTGTTT
EMPV1_21531	AGAAACACCTGCTGACTTGCAGTTTAAGGGAAATTTATTCTCCCCATTTCCAAACCATGA
EMPV1_21532	GCTGGGCCTTAAATTTGTGTGAGGTTAGCGAGCCACTGCCCTTTATCAAAGTGATGCATG
EMPV1_21533	TACACCGTGCTTGAGTACAACTGTGAGCCAGCAGAAATGTGTAATGGGAAAGGAGTTTGC
EMPV1_21534	TGAGTTGGGCACAGGCAAATGAGGATGCAGATGAATAAGGAAATCATGGGGCCCTCGGTT
EMPV1_21535	GCAGAATCAGATGTTTCTTTTACAACCAAACCAGATGCTCCAAATGCTACAAAATATGAG
EMPV1_21537	AACATTTGAGAAAGCCTTAGGCCCCTGTGCACTGCCATCGCGGAGAACCAGACACATCCT
EMPV1_21539	AGCATTACCACCCTGAGGTGTCCAAGGCTGCCAGCGTCATCAACCAGGCACTGTCTGTGC

EMPV1_21542 GGCTGGCACCTCCGTCAGACTTCCAACCACCAGAGCTATGAGAAATAAGTGCTCGTTTAA
EMPV1_21543 CATCAAGGACCGCTTGGATGAACCTCACTTTGGGGCCAAGAGCATAGTTTTACAGTCATT
EMPV1_21544 TCTTGCCTCTTGGCATTGTTCCTGAGGCACTGATTTCTTCATCCTGAGCCCTAGATACCC
EMPV1_21546 GGTACAGAGTTCCTGTTGTGGTACAGTGGGTTAAGCAACCAAGTTCAATCCTCACCCTGG
EMPV1_21549 ATCCTGACAGTGCGGTTGAGAAGAGGAAGCAGTATGTGCAGTCTCTCTCCAGGGCGTTGA
EMPV1_21550 GTCAGGGAAAAAGTATTGGGGGACGTGGCCACAAAATTAGCGACTATTTTGAATACCAAG
EMPV1_21551 TGGCCTCAGGCTTGCTTCTCGTGGCTCTGCTGACCTGCCTGACCATAATGGTCTTGATGT
EMPV1_21552 GTTCCCAAGATGTACTAAGCCATAAAGCTGACTTACCTTGCTGCTGGACTTCAGAAATTC
EMPV1_21554 ACTTCACCCTGATGAAATCGACACCGACTCTGCCTACATTCTTTTCTACGAGCAGCAGGG
EMPV1_21555 GAGGTTCTTTCAACGGAAGGCAAAGCAGTGTGTACCTCCCAAGGGCTTGGACCCAGAAAT
EMPV1_21557 GGAGCGGATGCTATTTATAGTCCAAGTTTTGGCATCACGTCCATTGATCTGTTGATCCCT
EMPV1_21559 CCTCGGGACGGCCAGAGCCGCAGGAGGACGAGGAGGATGAGGAAGAGGCACTGCCGCACT
EMPV1_21561 GGCCGGCCACGCATCCAGAGAGGACGGCAGGATGCAATTCTTCCTGGAGAAGAGGCGGCG
EMPV1_21562 ATCACTGTGTATGAAATCGCCGTGTACATTGGTGACATCGTCCTGCTCCTTCTCCCCATC
EMPV1_21563 CCCTGTAGATAGAGACATTTTACCCTTGGAAGGAGCTGTGTCCCACAACGTGGTTGCCAT
EMPV1_21564 CCGTGGTGGCTGAGGCCTTTTCCCTAAAAATGTCCCTTTCCCTGCACTGATGTTGCATAT
EMPV1_21566 GGACAGAAAGGCTGCCCGAATCATGGCCTTACTTCTACATGTGCGGAGAGAGTTCCAGAT
EMPV1_21567 TCCACCCTGGAGTCAACATCTTCCGAGCCCTCCCTTGGGGGAAATAACGCAGCTCCAGTT
EMPV1_21568 CTGCAGTTACCATGTTAGACATTAGATCCCAGGACCTTCTTCAGCTTATACCTTGCATCT
EMPV1_21569 AAGGCAGCCAAGCACAAGGCAGCTGAGGTGGCCCTCAAACACCTCAAAGGGGGGAGCATG
EMPV1_21570 CAAAATGTATCAGGAAGAAGGCTTAAAGGCATTCTACAAGGGGGTTGCTCCTCTCTGGAT
EMPV1_21573 TGATGTATGCACCCACCTCGGGCCTGGCTGATGGCAGCCTCACCGTGCTCAATGCCTTCT
EMPV1_21576 CAGGAGAACCCAAACCAGAAATTACATGGTCGTTTGAAGGAGAACTATTGCAGGATGGAG
EMPV1_21577 CCCGAAAGGAAGGATCGTCTTATGCTTACATTTCCTGGAAGTTTGAATGTGGGTCAGTTG
EMPV1_21578 TTGACTTAGCTGGGTAGTGGGGAACCCTTCCATGAGGAGTAGAACACTCCTTATGCAAGA
EMPV1_21581 CCAAGGAGGTAAAAGCACTCTACACTGAAAACTACAAGACTGTTGAAAGGAACTGAAGAC
EMPV1_21582 GACTATGATTGATGCTCAGACCACCCAGCCAAACTCCAAGGACTGTGCTGAAGGGGAAGA
EMPV1_21583 GGCACCAGAGGGCATCAGTGATTGTTAGGATGAACCCTTTTCCTCCCTCCATAGGTTCTT
EMPV1_21585 TTGCTGTACGAGCTTCCGATGAGACCCTCTACACTCAACCCGAGTAGCTTATTTGGAACG
EMPV1_21586 ACTTAAGAGCCAGGGTTCAGTTTGCTTCCACATGCCCTGGAGCCTCTTAGAAGGAGAGCA
EMPV1_21588 CTTCCGTAACATCCAGGTTGATGAAGCTAATTTATTGACTTGGCAAGGGCTTATTGTTCC
EMPV1_21589 TCTTCATCCTCATCTTCCTCTTCCCCATCCTCTTCTCCTATGGGTGGGAAGGTCTCCAGT
EMPV1_21590 CTGCAGGCACTTTCCCCAATGAATACGGTAGTGGTAGACACACTCAGAGGAACCAGATTC
EMPV1_21592 GGGGGTATAACATGTATTCTTTCACTGGTCTGTGCCTTGGCCCTTGCTTACTTGGATCAG

EMPV1_21593	CCAGATGCAGGTGGTAATGATGTGCGAGCAGAGAGGAGACGGTACGTGTAATAATAAGGG
EMPV1_21594	CTTCATCAAGAAAAAGTGGAGCCAGCTGTCCCTCCAGCAAAAACAGCAGCCCTAACAGCA
EMPV1_21601	ACAAACTCCTGTCATAGTTTAAGGTCTAACCTCTTTATTTTGGGTCGGGACGAGGTTAGG
EMPV1_21602	GCTTAGTATCACTACCCACTTGCCGAGGCTGAGCGTTCCTTCCAGAGTATCAGCTTACTC
EMPV1_21604	CAGCCTGGTGCCAAGACCTCTAGCCGCAATGACTACATCCACTCTGGCTTATATTCTTCA
EMPV1_21605	CATTAGATTGTGTCATCACTCTGCCTTGTATATTGTCAAATAGCCCATTGCAAAGACTGT
EMPV1_21609	GTACAGGATAAACACACAGACTTCCTTGGGGAGATCCTAGCCAGATTACTAGAAATTTCG
EMPV1_21611	ATCCACAGCACCACATGGGAACTTCCCCCTTTCTGTCATTCTTAAAATAGCATCTTACTG
EMPV1_21613	ACATGCTGGACTGTGAGGGCCGCCATGTCTCAAGCCACAAGATACACTGTGGTCACTTTT
EMPV1_21618	GTGCCTTCTCCTGGTGGTGCTCACCTTCCTGTTCTGCAAAGCCATTCACAATACAAGCAC
EMPV1_21621	ACTAAAAATTCAAATAATTGGCCTGGGTACCCACGTATCTCAGCGACCAGGAGCACAAGC
EMPV1_21623	TTACAACAGGCTATGATGCAGAAGGCTCCCAGAGCCACATCCACTTTGATGAGAAGCTGC
EMPV1_21624	GGGCTTTGACCTTTACAATTCCCTGGCTTTCAGCCTTGCACCTGCCCCAATTTCCTTCAA
EMPV1_21625	TTCCTAATGAAGTCCTGTTCGTATCGTGGTAGGGGCTGGGCAAGAAGGCTCCCAGTCCTT
EMPV1_21626	AAGATCCTGAAGCTGAGTGCGGAGATACTGCGACTGGAAAAGGCAGTGCAGGAGGAGAAG
EMPV1_21629	GCAGTGCCTGGCACTTGGTACTTAATTGAACATATGAATGACCATCAAAGGAAGAACCAT
EMPV1_21631	CCCAAGCAGTTCTCAAGGGTGTGATGCATTTCTTCGCCACAAGATGACACTGATCTCTCC
EMPV1_21632	TGTGTGTGTGTGTGTGTCACCCTGCGGCAAACACTCAGGCTGGGACACCGAGGTCTCCAA
EMPV1_21633	GCATCCTCATGGCTGGAGGGTCCTGGCTTATTGCGACAGTCAATTCCATTGTTCACACAG
EMPV1_21634	GAGCTTCATATGGCTGAGAAGACTAAACTTATCACTCAGTTGCGTGATGCAAAGAACTTA
EMPV1_21635	GTCCGGAGGTCTAATTTACCATGGAGTTTCTCTCCCTTCTTCCCACTGTCATAGCTCTGC
EMPV1_21636	ATTACGACTAATTGATGACCGGGGATTCTGCTGGAGTCCTCTCGTGAAAGCTGTGCCAGC
EMPV1_21637	AAAGCTCCACCAGGCCGTGGCTGCGGGGGACTACAATTCAGTGAAAAGGATTTTGAAGAA
EMPV1_21638	GGCAGAAGTCCAGGATAAGCTGAGGAATATTGACAGCATGGAACACACAGGAAAAGGCCC
EMPV1_21639	AAACATACAGCCCTAGGTCCCGACTCGGACGACTTAAATTTCAGGGTACGTGCCTTCGTT
EMPV1_21640	AAACGTCTACCACACATGGACCAAGTGCTCATGGGTCTGCTGCAAGACTACTTCGTACCC
EMPV1_21641	AGTCTGAATGGCTTTTACATCCCCAAGGGGCGTTGTGTCTTTGTGAACCAGTGGCAGATC
EMPV1_21643	GCATTCTTAATTTCCTTTCACCCTTGTACAGAGCCTGTGATGTGTGGTCTCCAGAAGCTC
EMPV1_21644	TTGGTTTAGGCACTTGGCCCTCTGCCGTCTCCTATAAAAAGAACAAGCCCTGGGGAACTA
EMPV1_21648	GAGAAATCCCAGCCGGAGAAGCCAGTTTGAATTATGTGTCCCAGTTGGCTTCACCCATGT
EMPV1_21652	AAGCCTCTAGACCAGCCCCACCCACTATGAGGCAGAAACCAGAAGGAAGAAGGCTACAAT
EMPV1_21653	GTGGCTTAGTGGATACAGAATAAAGTTCTTAACAGTGAGAGCTACCATTCCCTGAGAGCT
EMPV1_21655	ATCACGTTTTCTGCAGTTGTGGGCTGCCTTCTTCCTTGTTGAGTTGTTGCAAGAGGTCCC
EMPV1_21656	GACAGAGGCCAGGTGTTTAGCTTAGAGACAGCCACTGGAGAGAGCAATTAAGTTCTGCTG

EMPV1_21657 TTGATCTGCCTGGCCCTACAGCTGGCCTGCATCACCCTCACCAACTTCTCTCTGGGCTTT
EMPV1_21658 GTGCTTCTCGGGACACCAGGCAGTCACAGGTCATTTTGCCTGAATGGAGGGATTTGTTAT
EMPV1_21659 CTCCAACCTGCTGGTGAACCCCCTAGAGCCCCAAAATGCAGACAAGATCAGGATCAAGAT
EMPV1_21660 TATACCGGCTACTTTGGAGGTGTGTCGGGCCTGAGTAAAGCCCAGTTCCTGAGGATTAAC
EMPV1_21661 CAGAGTCCATCCGCACCTGCACTTTCACCTTGCTGCTCCCCCATCTGGAAAACCTTTTAT
EMPV1_21662 TGTACAGATCCTGCTGCTAACGAGTTCCTTCTGTCAGGGACCAGTGCATTTCTGGGACTT
EMPV1_21664 GCATCCAGAAGCCAAAGAACACCTTGAACCGCGAAAAGCCACAGGAGGCAAAGAAGATTC
EMPV1_21665 CTGCCCTTGGGCTTATTAACATGGCCAGTTTTATTACCAAGAAACGAGTGAGTTTGCCTG
EMPV1_21667 TGTGACCTTACTGAGGTCTGTAGCCCAGGAGAGAAACTCTCACAACAGCTTCTCAGACTG
EMPV1_21671 ATAGAGTAGCAAGCTCTTCGAAAACCAGAAAGCACAGTTCTCAGAGTCACGACCGATGAC
EMPV1_21672 GATCTTAGAATATGTTGGAGGCTCCTCCTTTCATTTAGGACCCCCTTCCTGCCGGCCCTT
EMPV1_21673 TGAGCCGCACACAAAATAAACATCCAAATGAAGAGGTCCTAGAAGCTGAGGGGCATGAGG
EMPV1_21676 ATGGAGCAGGCCGAGGACCATTTACACCTGAGAAAGACTACAACAGACCGGTACATACCA
EMPV1_21677 GCTCTGGATTAGGCTTTGGCTTAAGGAAGTGTTGTGGCTGGCTTGATCTTCTATCCAGAC
EMPV1_21679 TGCTGTGGATTAGGCTTTGGCTTAAGGGAATGTTGTGGCTGGTTTGATCTTCTATCCAGA
EMPV1_21680 GAGTGGAAGAAGGTAGCAACTGCATTAAAAGATGTGGTGAAGGTTGGCGCGGTCGACGCA
EMPV1_21682 AGAGGAAGGATAGTCGCTCACCCCATTACGCAGGTTCTCCTCTTGTCCCTTGTATCTTAC
EMPV1_21685 GAGACTACCAATGGGAAAAACAGCCAAGCTTCCAAAGATCCTGTAGCATCCTGTTCCCTG
EMPV1_21688 AGTTCAACTTCAGCAAAGTCGTGCTTGTCAATTACCCACTGCTGGATGTGGCAGCAGCCA
EMPV1_21689 GATGGTCATTAAAAGTTTGGAAATGAGTGGTGTTTGGTATTAACCAGCTGTGGCCTTGAG
EMPV1_21690 GCAGAACCGGTGTGAGACGAAGAGACAATCCTTCCCAGCTGCCCGGATAATCAAGAGTTT
EMPV1_21691 AATTATTAAAACAGAGCTTCCTGCAGCTATTTCAGAATTTTTGTCTGGAGACCATAGTGG
EMPV1_21693 TTCAGCATGGGGAACAACTCGTACGGGCAGTGCGGAAGAAAGGTGGTGGAAAACGAAATT
EMPV1_21694 CCCCCCTTCACCGCGCTGTATCGCATATTGCTGAGTTTTCTATTTTTGCAAAATAAAGTG
EMPV1_21695 GCCGCTGTAGGGACGTGCTTTGCTGTTTTAGAATAAACCTTGCGAGAGTGAGGGTTCTTG
EMPV1_21696 CTGGAAGTTCTGTAGGAAACTCTGAGAACTCACATATGATCCTTGGCTCTACAGGAGTGG
EMPV1_21697 TGGAGAAGACATTTCTACTGCAGAGGGTTAGAGGCACCTGAGCAAGGGCCCCACTCCCTC
EMPV1_21698 GTCAACATCTACTATGGGGTGACTGCGGCTCTGTTTTCCGTATGCCTGGATGTGGTGCTT
EMPV1_21699 CTGGAGAGGAGCAGAGCTATATCCACACTATACACCCTTGTCCCCATCTTCCTTAGCATC
EMPV1_21703 TTCAGATCCAGGACATACAAGTGAAAATTGGGGGGAGAGACTTATATCTTCTTACAGGAC
EMPV1_21704 GTATCCCTGAGTCCTTACTGTGTATTTGTCTAGTATAATTAGCACTTCCCCACTTTCAGG
EMPV1_21705 CTGGAGTGAAAGAGACGGGACAAATAGCGGTGAAAGCCCGAAATTCTTCTCCATCCTCTG
EMPV1_21708 AGTACTGTATTCCTTGTTTGCAGCAGCCAGCCAACTAAAAGGGCAAACCGTTAGCAAATG
EMPV1_21709 GACCCACACAGCCAAATTTAAGGAATGTACCGATCCCAGGTTTCTACACTTACTCCAACG

EMPV1_21711	CTGCTGGTATGTTACTGTGAGGACTAGAGGTAAGCCTTGGAATGGCTCTGAGCTACCTTG
EMPV1_21712	GGACTTGAACCAGTTGATTTCCAGCCTCAGGCTGGATTTGCTGCTGAGGGTGTGGAATCA
EMPV1_21713	GACAGACACAAGTGCATCTGGCTAGTTTTGACATCGTGGATTGTTGGCTTTCTGCACGCC
EMPV1_21714	TGCGAAAAGCCAGAGCCAGAAGGAGGAAGGCCGGCAGAAGCACAGACCGCATCCGTACAA
EMPV1_21715	AAATGTACACATCGTATTTGAAGGAGAGAAGGAATTTCATCAAAATGTGAAATTATGGGG
EMPV1_21716	GAGCTCCAAACGCACCATTTTCGTTACCATGTGATGGTGGGTATCTTTCCATAGCAGTGG
EMPV1_21717	CAGGCTCCTATGTTGCACCCAACTTCATTCTCTTTTCTCTTGTGCAGCCAGTTCGCCCAT
EMPV1_21718	ATGGACCCTGTCCCGGAGATCCCCTCTGCCTTCAATAACCTCCAATGGACACTCAACTAT
EMPV1_21719	GAGAGTCAGTCCAGGCTCCTTGATGGCCTTCCATCAGGGACATGAATGGATGGGAGAAAA
EMPV1_21721	TCTGTAAGCCTAGATCGGAGGAAAACAAGAGAATGAACCCTTTGCTGCTCCCAGAGGTGG
EMPV1_21722	GGGCCTGAAGCTAGGACCTGCACTGAAACTCTGCTACCACATTGATAAACTGAAGCAAGC
EMPV1_21724	TGCCAGGAAGAGAGTGACATTGCAGCCGAGACGGGAAGAAGGTGTGGGTGATGGCTTCAA
EMPV1_21729	ATTGAGCCCTTAGTGTACCTGTGCCAAACACACACAGAGGCTGTGTGGGAAGCTGCTCGG
EMPV1_21732	AGGACGACTGCTCACATATTTGCATGTCCCGTTTAAAAATCTTTGTCAAGCCCACAGACG
EMPV1_21733	CTTTGCAGCCCAACACGGCATCTGGGCAGTTCAAAGTAGATAGCAAGTACCTTTCAAGTA
EMPV1_21735	TGGCACAAGATCTCTGCAAAGTAGCAGGTGTAGCGAAAGTTCTGGTGGCTCAGCATGATG
EMPV1_21738	CCTGGTGGATTCTTAAGTGACTTGAACCTGGCATCACTACAAGTGGTGGAAGCAGCATTA
EMPV1_21740	TGTGGCTGGTTGAGAAATGTCAACTAGGAAATGGATCATTCAAGGAAAATTCTGAGTATC
EMPV1_21743	CGGTCGCGTAATCGCACCCAAGAAGGCACGCATCGTGCAGCAGCAGAAGCTTAAGAAGAA
EMPV1_21744	ATGGCTGAAGATAAAAGCAAGAGAGGCTCCATTGGAATGAGTCTGAAGGGCGGCCAGACA
EMPV1_21745	GGTGAGACAGGAGGGAAGAAGCTGTCCAGTGCCTTTGGGGCTGCGCCTGCAATAAAGAAA
EMPV1_21747	GTCCCTACTCCGCAGCATCCACTTCCTGCTCCTGGTTTTCCTGAAGATGCCTCTGTTCCT
EMPV1_21748	GGGCTTGGAGGGACCGGTGGTCATTGGGACCTGGTTCCAGCCAGTTTGGCCTCTCAAGAT
EMPV1_21749	ACTTGTGCATAGAACTCAAAGACGAGGAGGAAAAGTAAGGAACAGCATCACAGCGTCCGG
EMPV1_21750	TCTCCTTTGGACATCAGAGACTTCACGTTGGAGAGAATGTGAGTGCTTAACTGGACAGTC
EMPV1_21752	AGGAACCAGGAACCATTAGACCGGAGGGAGTGAAGCAGGAGGGCATCTTGAAGTTTCTGA
EMPV1_21753	ACTGACTGCTGAAGCTCTGGGCTTGGGAGAGACAATGCCAAGATCACTGTCTGGTTTGGT
EMPV1_21754	CCTGTTACCAGATAAATGGAAGGAGTATGGGGGAGACAACATTGACAGGCATCCACAGTG
EMPV1_21755	AAGCTCTTTATGCCTCTTCATAGGTCCATGACCTGAAGTGGAACCTCCCAGTCACCTTGG
EMPV1_21756	CAGTAAAGCTAGAAATGCAGAGTGATGAAGAGTGTGACGGGAAACCCCTGAGCCGAGAAG
EMPV1_21757	TGGAATTTTGATGCTAAGTGTGTCTGCCTTTAAAATATCAAGGGAAAACCATCAGGTAAC
EMPV1_21758	GTGCTGGCTCCTGCACATGCAAAGACTGCAAAGGCACCTCCTGCAAGAAGAGCTGCTCCT
EMPV1_21760	GGACACACAGTATCCTCACCTTTCTCACCTGTGCATCAGTATCAGCTCTCCATCTGCTTC
EMPV1_21761	CCTCCCACCGACTGACTAGCTAAAGGAGCCTTGACCGATCACCTAATTTCTCTGAGCTTT

EMPV1_21762	CGCAACGGGGAGTGGCAATTTTAAATCAATTTTGCTTCTCAGTTTGGCCTAATTGCTTGA
EMPV1_21764	CAGACCCTTATACGTGAATGTCAAGTTATCAGAGAGACCAAAGATAACCAGATTGCAGAG
EMPV1_21765	TGAGCGTGTTTAGGGCTGTGCGTCGGTGGTTATCATCAGAAAGGGGTGGTCTTGAGTAAC
EMPV1_21767	GTACCGCTATTACCAGAACGTGTGCACACAGAGTTACTCCTTCGTGTGGTGGGACTGGGC
EMPV1_21769	TCCTCTTGTCTGACTCATTCTTGGCCTCATATCCTGATGATAGAAGTGATGACCTGAAGG
EMPV1_21770	TGCCTGCAGATCTATGGTAGGTGACGGTGGCTTTAGGAACAGAAAACAAAGATCAGTACA
EMPV1_21771	ACTGGTGGACGCTGCAGTTGTAGAAGACGAAGTCCACGGACGCAAACTTCTTGCCCGTCT
EMPV1_21774	AGCACCGGGCTCCTGATGGACTTGGCAGCCAATGAAAAGGCAGTTCATGCAGACTTTTTT
EMPV1_21776	CAACACTAACCCTTTGACTGCTAATGTATGTTTCTGCTTGCCGTGCCTTGTTATGGCTGC
EMPV1_21778	GAGATTCCATCAGACAGCCAGTTTATCAGAGGCGATTGGGCTCATTCAGGGTGCTATGGC
EMPV1_21779	GACCTTTGTCAACGACGTGATGACCATCACCGCGGATGTGTTCCTCTCGGGCATGAACTT
EMPV1_21780	GTCAGGTCGGGTAGTGAGTTCGTGGGGTTGTCTAGTGTCTGGTGTTAAGTCAGTGCTTGA
EMPV1_21781	CGAAAAGGGAAAGCTGATTCGTGTTGGTTTTTATAGGCAACTGCAGCACCATAGTGTCAG
EMPV1_21782	TTCTCCAGTGCCTGAGTAGCACACTCCACCGAATCCTGTTGCATCTCCTCCGACATATCG
EMPV1_21784	CCTCACTTCTTTTCCTGTGGTTGATTTCTAGCTTCACGCCGCATAACCATAAGTCACAGA
EMPV1_21785	GAGTTGCCAACAGTTGTATTCAAAGCCTCAACTGATCTGAAGGACAAAGGGAAGAGAATC
EMPV1_21786	AGCTCCTGCAGCAGCGAGGTCTCTTCTACGGCATGGCCAAAGATGCCGGCTTGGTGTGAG
EMPV1_21789	TAAGAACCGTCCCGCGTCCAGCTGAACTGGTCCTTCGCTGGATTCTCTCGCCGGCCCTGG
EMPV1_21791	CCTCGGGCGCCTCGGCGCCGCCAGCAGAGTTCCCCGCCTGGCGGCCGCAGCCCACGGCCC
EMPV1_21793	GTCAGCAGACATTTTCGGTTTTCAGGACAGAAGTGGGTTGCATCCAGTGTGGAGAAGGAC
EMPV1_21794	GTGACCACGTGTGACTTTGTGTGAGCCACGTATTTGAGTGTCACGGCCTGTATTTGGCAC
EMPV1_21795	AGCAGCATAGGGTATAAGAGCCGCCCCATCGACCCCAGTGTGCCCTTTGACCTCAAACTT
EMPV1_21798	CAGGGGTAGGCACAGGATATTTACTGCAGACTTGCTTGCAGGAGCAAAGGTCAGAAGACT
EMPV1_21799	CTCTGAGGCCAGTCAGAACTGAGAAGATGCCTAAGTAAAGCCGACAAACCACGTGAGCTC
EMPV1_21800	CGTCATGCAGGCCACACTGACGGTAACGTTGCAGGTCGTCTTGCAGGGCTTCTCGCAAGA
EMPV1_21803	ATGCAGAGGATAAGATCCCTTCTGAACGGGACACGTGCTGTGAGACATCCACAGGGTGTG
EMPV1_21804	CAGGGAAGGGGTGGGGAGTGGCATCAGGGGCAGCATTCTTTGTCCCTCCTGTGTTACTAA
EMPV1_21805	TCTTGGGGGCAACGATGGGGGGACAGCGGAAGCCCTAGGCCAAAATAAGGAAACCCCTCA
EMPV1_21808	AGCCTTGGGCAGGTAGGGAAACTTTTGTATCAGCATCATTTGACCTGTTGGGGACATGAG
EMPV1_21810	CATCACTAGTCCACAGTTTGCCTGCTGGTAGTTTGTGTTCCGTGGCTCCTGAGCTCTCCA
EMPV1_21812	CAGCTGGGGGTAGTCCAACACCAACGATGAGGTGGCTGAAAAACGGGAAGGAATTTAAGC
EMPV1_21814	ACAAGTAGCCTTCAGGTACCCATTTATTCGTTTGCCCTTCCAGTTTCCAGCGGGCACTAC
EMPV1_21817	CACAGAGCGCTGCCAGTCTCCGCCGATTGTTTTATTTTTGAATTAACTATTTCGGCCGGC
EMPV1_21820	TTTTGCTGCCAAGGTGCTGACGGGCCAGGTGCGAGAGCTGAAGGACGTGAGGGAATACGA

EMPV1_21822 GTCGGATATGCTCCGGTCACTATCAAGCACAAAACCCACAGTCAATGAACACGACTTGTT
EMPV1_21823 ATGCTCACTTGAAGACGAGGACTTTTCTGGTGGGCGAACGTGTGACGCTGGCTGACATCA
EMPV1_21825 GGGAACTCTTGGATTAGCTTATGTTGGTTCTCCCAGAGCAAACAGTCATGGAGGTGTTTG
EMPV1_21827 ATGATATCGCTCGGGGCTTTGAGAGAGGATTGGAACCAGATAAGATCATTCGGGCGACAG
EMPV1_21829 GATCGCAGAACACTCCTCAACTGCAATCTATGTGGTGTTATTCTCATGATTAGACTGGGC
EMPV1_21830 CCATAGAATATGAGCAAAACAAGCCGAGTGACCTTGGGGAACTACACTGGGGGGAGTGGA
EMPV1_21831 ACTGACTAAGTATTGCTCCGCTTTTGCCCCGCACCATTCTCGGAACTTGCTGAAACTTGG
EMPV1_21832 GCGTGTCCTAGCCGATGGAGCATAACAGTGAGGAAGGACTGGTGGTACTGGTCTGTAATG
EMPV1_21833 CTGTCCCAGCCAACGGGGAGATCGGCTTCCAGCTCACAGACAGACAAGTGGGTGGCAGAA
EMPV1_21834 CGGATTTAAAAAGTACCCAAGCATCTACTCCAGAACTGACAGAGATAAGCCAAGAAAATG
EMPV1_21835 GCACAGACCCTGGCTTTTCTTAAATTTTATATATTGGAAAGCCCATGTTTGTATTGGACC
EMPV1_21836 GTGTTACCAGTGGAGCTTGTCTTCTGAGCTAATCCTATTGTCCTTTTATTGAAGGATGGC
EMPV1_21839 CCATGAACAAGAACCTGAACTCTAGTAACATCTTTCCAGTATCAAGGCGACAGCCTCTGC
EMPV1_21845 TGAGCCACTCTTTTTTATCTTTATAACTTTCCCTTGAAGACAGACTTTTGGGGTTGGTGC
EMPV1_21846 CAGGGAGACATCCCATTTGGTGTCTAAGAGAGATGAGCATGTTTGCAACTTCCTAGAAGG
EMPV1_21847 TTCCGTCCGATAGAGAAACTGGTGGTGCTTTCAACTAAAGTCCGGTTCCTGTTAGCCGCG
EMPV1_21848 GAGTCACTTGCTCATTGAAAGCCACCCTTCATGCCCAACAGCTATTTGTGCCAATGCCTG
EMPV1_21850 GTCCCACAATACTCTTCCCCGCTTCAGAGGCCAATAGCAAGTCTAGCGAGCCTAGGTTAT
EMPV1_21852 TTTTTTGGGCCACATCTGCAGCTTATCAAAATTCCCAGGCTGGGGGTGGAATTGGAGCGG
EMPV1_21854 CGCAGAAGAAGAAGCAGCGAATCCACCGAGGAAATCGAGAGGCCTTGTGAAATTTCAGAA
EMPV1_21855 AGCCTCAAGAGACTGGGCCATATTTCCAAAGTCCTGAAACCTACCCCTACTCAGCTACAG
EMPV1_21856 ACCGGCTGGTGATGCACAACAAAGCAGACTATTCCTACAGCGGGACGCCCTGACCCCAAA
EMPV1_21857 CCATGAGGTCATTCTGAAGGCCAAGGACTCTCAGCCTGTAGACCCCACCCTAAAGCAGAA
EMPV1_21858 GCCGTAGAAAGAGGCAGCTGTAGAATTTGATCTTCTAAGTACCCCACGTACCTTTACTGA
EMPV1_21860 AGAAACATGGGAATAGTTGGAGGGAGGACTTGGGGGTCAAAGGGATTTTTTCTAGTGGAA
EMPV1_21861 ACCTTACGTGTTGCTCCTCAGCACCCTGAATTCCTGCGTCGACCCCCTCATCTACTACTT
EMPV1_21862 CAAGGGGGTCACCTGCATCTGCCTGCGTGATGGCTCCTGGGAGCATTGCATCCAGTGACA
EMPV1_21865 GTTAATAATATGAATGATCTTCTTGCAGGCTATAGCACTACAGCCTCCTTGCTTCTGTTC
EMPV1_21868 CACCCAAGGAAACCACAGCTCTGGCTGAAAACCAAGATGAAGATTCACTAGAAGATCCAA
EMPV1_21871 GCTTTTACAAGCGCAAGGTGCAAAAATATATACGGTAGTCTCATTGATGACTGTAAAGTG
EMPV1_21873 CCACTATCTGACCTGCACCTCTGCACACAGAGACAATTCAGTGTCTTAACTACCACAGGC
EMPV1_21875 TCACTGTAGGGTCTGTGGTTCTTATTTTTCCCTTGAGTGGCATCCTGGTCTCTTATGGTC
EMPV1_21876 ACTCAAGCCTTCCAAATCTCCAGAAGAGGAGTCGCTGATACGGACACACAGTGGGGAGTT
EMPV1_21877 GGGTGGCAGAGACCGGTAATTGCGTAAAACTTAAACCTTTATTACCAGAGGTTCAACTCC

EMPV1_21881	AAGCTGAAGAATGAAGCCTGGGAGTGAGCAGTTTGGGTAGACAGGAGGAACGAGTCCCAT
EMPV1_21886	AAGGCGCTGTGTCTGAGGAATGACACCGTTTTAAAGCAAGCCCTTTCCCTCATAAGGGTC
EMPV1_21887	GGAGCTGAGTGCGTTTCTTTGTGGAGAATGCGAGTGCTTTTCTAAGTGGCCGTGCTCAGA
EMPV1_21888	ATCTCCAGGTGTGGTCCCAGGATAGCTTTGGCCGCTGCCAGCTTGCAGGCTACGGCTTTT
EMPV1_21889	CTTACCAAGCAGTATGAAAAGTCTGAAAATGATCTGAAGGCCCTACAAAGTGTCGGACAG
EMPV1_21890	CATGTTGGGGTCTTACAGTGTGAATGATGGTGCACAACTCTACATGATTGACCCATCGGG
EMPV1_21891	TCACCCGTGAGACCTGGTCAAACCCGCAGATCATTGGATCCGGGCCCCAGTCGCCAAAGA
EMPV1_21892	CTGGAAGACCGTGAAGTAGATGATACTTATATTGAAGATGCTGCTGATGTGGATGCACGG
EMPV1_21894	TGTGACTTACAATCTTCTGGTGCCAGTCTACACACTTGTTACCTACTGGGCTCTGGGCTC
EMPV1_21895	CTGAGCCAAACATCCAAGACCTTTTATTGATTTCAGCCCCACCTAGCCAAGAGCTCCAAG
EMPV1_21898	TTTCATCCTGCTTTTGGTCCCACTCTCCTGCATCCTGGCCTCTTACATCTGCATCTTTGC
EMPV1_21901	TTCATCGAATCCACCCTCTCTGGACACAGCCTGCCCTTGCTCAGATACGACAGCTCTTTT
EMPV1_21902	GTCCTGATCCCAGCATGTGATACCCAGAGACAGGTTTTGATGTCATCTCCTCCTTGTCCA
EMPV1_21904	ACCAGTAGCCTCAAGTAGCAGGCTCACGGCGACCAGCCCAGTAACAGTGACTTTGCCGCA
EMPV1_21905	ATTTTGTGTGGTCCCTGGGTAAGAGATTGGAGTGTAGGGAAGAAGGGGTGGACATCTCTG
EMPV1_21906	TAGATGAACAGTGAATAATTTTTTAGTATAAGCGTGTCCCATACAATATTTGGGACATAC
EMPV1_21907	TAACGATGGTGCTCACAAGCAGTTTGACCACTACCTCGAAGAGCTGATCCTGCCCATCAT
EMPV1_21908	GTGCGAGACTTAGAATCAAATGGCTTAAGGTCTGTAAAGATGTTTCATGTGTGTTACCCA
EMPV1_21909	GTTGGGGAGCACATGGGGTGATGGCCTGGAAAAAAGATGCCAAAAGTTGCCTCTGTGTTA
EMPV1_21910	TCTGTGGCTATTGTTATGAGAAGGTGCGCAAGGAGACAGCGGAAATCAGACGACAGGTGG
EMPV1_21911	ATTCCACTGTGAGATGGTTTAGCTAGGTCGTTAAGATGATGGTCACTTGGCTTCAACTGC
EMPV1_21912	CATATGAGCATGTGGAAGTTCCTAGTGTAGGGGATGAATCAGAGCAGCAGCTGTCGGCCT
EMPV1_21913	TAGGAAGCCTGGCAGCCCATCTCTCAAAGAGTCTACTCTTCCTCCTTGTCCTTGGTGAAA
EMPV1_21914	GTGTGTGTTATAATTGCTGTGAGGCTGAAACCCCTGGGTCCTAGAGAGAAATTGCCCCAG
EMPV1_21916	ATCCTGTCCCTGCTGCTAATGGAGCAATACGTTTCCATTGTGATCCCACCTTCTGGGCAA
EMPV1_21917	CCGCTGGAGGACCTGGACCCCTACTATATCAATAAGAAAGTGAGTGTTGTTTATCAGTCA
EMPV1_21918	CTAGTGGGTCACACAGATGGGTCCTTGTCAGCCAAGCATTTGGCTCTGGCTCTATCTCTA
EMPV1_21920	GGTCATTCTTCCTGCTAGATAAATGGACAGCCAAGCTCCCTACTTGGTGTTCTGCCTCCT
EMPV1_21921	TAGTGTTACATCTGCCTGAAGACAATGCCAGGCTGGGCAAACACAGGTCAGAGTATCTCC
EMPV1_21922	CTCTCTCGTTACTGAACCTAGGACTGTGTATGTTTAGACATATGTGCGTATGCAGTCTTT
EMPV1_21923	CGAGACTGTCAGATTGCCCATGGAGCAGCCCAGTTCTTAAGGGAAAGACTATTTGAGGCA
EMPV1_21924	GGATGAAGTAGAGCCATTCCCAGTACCAAATATGCCATCGAGAACACAGGAGAGACCCTC
EMPV1_21925	CACAGGGTATATATTTATATCTATTGTCCAGTGAATTGAGTTTTGAATATATCTTTCACC
EMPV1_21927	CCTTCCAAACACCAGCCTCCACCCATCAGGAACCTCCACCAGTCCAACTTCAGTTTATCA

EMPV1_21930 GACCTAGTTTGTGGTTGAGAGTAGTGATTTCCAAACTAGCTAAATCGCTGGGGTACCCTG
EMPV1_21931 CTTTGGGGACAACAACTTCACCGAATGGGCATCACTCTTTCAACACTATTCCCCACCTCC
EMPV1_21932 GGCCACTGCCGGGTCTGTCTGGTATTTCAAAGGAATATTGGGTGCTGCAAATAGGAACTG
EMPV1_21933 TGTGAGCTCAGACCACACTTCGACTTCCTGATTGAGTCATGCAGGGTCGGAATGGCCAAG
EMPV1_21935 GAGAAGGAGGAGTCCAGGAATATGGCAAAATAAGAAGTACCAGGAATTCATCTTTCCAGC
EMPV1_21937 TCTTTCGATGCCAGATGCCGGGATCTTTTGAATTCCTCCATCACCTGCACGACCACCACT
EMPV1_21941 AAGCTGGGGGCCGTGCCCTTCTTCTCCCTTCTGCAGTTCGAGTGACCCTGCCTCTGCCCA
EMPV1_21942 GATTTTCAGGCCCCTTCCCCGGAGATTGAGTCAGCAGTTGTAAGGGACCAAGTGATTCTT
EMPV1_21943 CCTGCCTGTCCCAAGATACCCCTGCCCACCCTGACCGTGCTAACTGTGTGTACATATATA
EMPV1_21944 CACAGGGAAAATCCAGCGGGCAAAACTTCGAGACAAAGAATGGAAGAGGTCTGAGCAAGC
EMPV1_21945 CTTGATAGTGATCGGCTTCCTCTCCGGCTACCAAGCACAGCTCCAGAGGAAGAAGGACAA
EMPV1_21946 CTGCAAGGAGCTTGGCGCGAACTCATGATGGGCCATCTTCTTTTCTCTGAAAAATGAGCC
EMPV1_21947 TGTGTGAGCAGAAGCTCGGCTACGCCGCCTACAAGTGCGTGGGCATCGACAGAGACATGA
EMPV1_21948 CCCTGGTCTTTAACGTCTTGGTAGATGAGTCCTATTGCTGTATCTGAGGGCCAGAGGATG
EMPV1_21952 TGACTGAGGAATAGGTTGATGGAGAGTGTGGGAAGCCTGGGCGGGTCCGCACTGATATTA
EMPV1_21954 AAGCCATCCTCCATAGCATAGACTGTTGTTCATCTGATGATACCAAAAAGAAGATGTACA
EMPV1_21955 AAGCTTGCCAGATGCCCCCCTTTGCTTAAGTTGTATTCTTCCTGCTACGTGCGACCACAG
EMPV1_21957 GGCAGGCTATGATGTTAATATTAAAGATTATGATGGCTGGACACCTCTTCATGCTGCAGC
EMPV1_21958 ACCTCGTGGCTGAGGTGAGAACCTGTTTATCGTGGAAGAGAAATCCGTTGGCTGGTAGAG
EMPV1_21960 GGAAGCAGAAGCCAGAGGACCGAGCCTGGACTGGTAATAGGGCTGTCCCCTTTGGCTTTT
EMPV1_21961 ACTGGTAGGGAAGGACTCTGCATCTGTTGTTTTAGGGAAGAAATGGTTCAGCCCCACGTG
EMPV1_21962 GGGCTACTGGGTCAGACCTTCTCATTTCTGCAACTGGCTCAGAGCACCTCTATGAAATCT
EMPV1_21963 TGAACTGGCAGGCCAGGAAGAGGAGGAAGCCCTGGAGGGGCTGGAGGTGATGGATGTTTT
EMPV1_21965 CATTTCCATACACTTTTCCGCTTTCTGTGATTCAAATACCCCAAAGAATTGTGTACAGTG
EMPV1_21966 CTGTTCTTCACCAGTTTAACAGGCGTTAATGGACGAGCACGTGTGGCTTCCAAACCTGGT
EMPV1_21967 AGCAAGAGGGGATGTGGGGCAAGATATGCTCAGAGGGGCCCAATTCCTAGTCTTTGAGTT
EMPV1_21968 ATCATCCCTGTGGGCGGCACGTACCTCATTCAGTCTGTTCCAGCCGAGCTCAATATGGAT
EMPV1_21969 CATCACTTTCTCTGTGACTTCAGCCCTCTAGTGAAATTGTCCTGTTCACCCACATCTGTC
EMPV1_21970 GAACAGAGATGAACACCTTAAACAGACATACACAGACTCTTTATCAGTTAGTAGGGTAGT
EMPV1_21972 ATCAAGCTGTGGGACCTGAGGAACAATAAGTGTGTAAGGCAGTACGAAGGGCACGTGAAC
EMPV1_21973 ATATGCTGTAGAATCATTAAAATGAGTCCCTAAAGTGCTGAATGATTATGCCCTCACCCC
EMPV1_21974 GAAGAAGGGAAAACAAAGTCTTAGCAGATGCCCTAAAAGAAAAGGGGAATGAAGCATTTG
EMPV1_21975 GAAAGGAGAGGAAGGATAGAAAGGCAAGATTTGAGCTGCTATCACCCATGTTCTAATGTC
EMPV1_21976 CTCTGGTCGTATTCATTAAAGGGTACTGAGAGGATAAGCCTCCTCATTAGTGATCCTCGG

EMPV1_21977	ATCTTCCGAGTGGTCCCTAAGCCTTTGCCGTGAGCGTGTGCACTGAGAATGGTAGTGGTT
EMPV1_21978	CGTGGTGACGAGGACAGAGATACTGCTGTGGGAGCGTTGAGAAGGTCTTTATGTTTCACA
EMPV1_21979	GTCCGTACTATCCCTTCCCAGTCTCGGTTCGAAGACTCCATTTTCCTCGGTGGGGGCTTA
EMPV1_21980	TGTACAGCTTCCAGGCCACGGAGAGCGACGAGCTGGCCTTCAACAAGGGGGACACACTCA
EMPV1_21984	GACAATGCCCGATCCCCAAGCCCCTGCGCCATAAAAGAACTCCAAAACCTGACCTTTTTT
EMPV1_21987	TGTTCTCCAGCAGAAATGTGAAGATGATGCTCTCCCAGCCAGCTTTAGGTTGGGGGAAGA
EMPV1_21990	TGCCTGTGAAGCCTGACGTGCACGTCAAGGAGGAGCCAGACGGGCCGGTGCTGAAGCGCT
EMPV1_21991	ACCAGGACAGCTCAAATACCTTCTGACAAAAAGCTCCCTCATGGCCTGGGAAGAAGTGAC
EMPV1_21992	GGCAGAGATTTTGCCCACAAGGAGGTAACTATACGACCTGCTGCCTTTCTTAGGGCCTTA
EMPV1_21993	CTATGGCACTCTCTTCCTCATGTATGTGCTTCCTGGGTCTGGACCATGTGAAAATCAGGA
EMPV1_21995	AGACTCTGGTACAGTAGGTCTGATGTGGGACCTGGGACTCTCCATTTCTAACAAGTTCCC
EMPV1_21998	AAGAGGCAGGCCCTTCTGATCCTTACGCCCCTTGAATGATAAATCTGTAGCCCACCTGAT
EMPV1_22001	GCGCTTGGCTGAGCTTGGTGGCTGACCTCTGATGTCTATGTGACAGTTTTTCCAGAATCT
EMPV1_22002	AGCATCAGGTTGGCGCATTCTTGTAACTCTCTTACAGACAATCGAGCGGAAGGGATGCCA
EMPV1_22003	GTAACTCAACTAACGGAGAAACTGAAGAATCAGTCAGAAAGCCATAAACAGGCCCAGGAG
EMPV1_22004	TTTGTGAACTCAACTGGCTATTTGACAGAGGCTGAGAAGTTTGGTGACTCCTTTGTCTTT
EMPV1_22008	GCACAAACAAGAATGCACACATGTCCACGCCGTTCTCAAGAGGAACCATTGGAAGCTGCT
EMPV1_22009	TCTATGGCTGAACGTCGGGGGCAAAGAGGCAGCTGCCCTGTCCATGTTCCACGTCTCTGT
EMPV1_22012	TGAAAATGGATGATTTTGGGGCCGTGCCCTTTACAGAGCTGGTGGTGCAGGGCCTCGGAT
EMPV1_22013	CTGGGCCTACTCGTGTAGGTGCCTTCTGGTTCTCTATTCTGTTCCAATGATCTCTGTGTC
EMPV1_22014	GAGAAGCTTGCTTTCCACCAGAGGCTTACTTGACCTTGTTTTCATGCCCAGAATACTCAC
EMPV1_22016	CCCTGAGATCACTCACATTGTCATCAAGGAGTCTGTGGTCAGCATTAACAAGCAGGACAA
EMPV1_22017	GCTGGCAGGGAGGAGAGAGAAATGACTTTGCAGCACGAGGGCGATTCGTCATTAGAACAA
EMPV1_22020	CCCTCCAAAGGCAGTATTCACTCATCTCTGGCCCCATCCTGACCCCCACAGTCACACTCA
EMPV1_22022	AATGGGTCCAGGTCCTTCACGAGCTTGGGTGTTTATACTCAGGAGAGTAGGAAGCCTCTC
EMPV1_22024	AACCATAAAGTGATAAAAGGGGTGGCAGGGGCAGGGGGAGCAGCTTTGACAGATCTAAGG
EMPV1_22025	GGGACACTCAGCTCCACATTGCCCACAAACAGAATCCTCCTGGTTTCTTTACTTTCAGCC
EMPV1_22028	CTGCAGACACACTCGGATGTCAAGAAGTACCAGTGCAAAAACTGCTCCAAAACCTTCTCC
EMPV1_22036	TCAGCCCAAAGAAGATTATCAACAGCAGCAGTGTGGCTCTGGGGGTCGTGGGAATCGCAA
EMPV1_22038	TTGCTCCCTTCTCAATAGAGGTTTGCATGAGGGCAGAGGCTTGTTTGCCACAGTTATCAG
EMPV1_22042	ATGCAGGTTTGATCACTGGCCTCGGTGGGTTGGGGATCTGGCATCATTGTGAGCTGTGGT
EMPV1_22045	GTCTCTGAGGATCAGATGGAAGCTTCTGGAGCTGTAGTGGGACTGAGGCTCGAGGGCCAA
EMPV1_22050	GTGCTCTGGGCAGCAAACTTTACCTTCTCAGCCTTTGTTTTCCTCTCAGCCACCATTGTG
EMPV1_22052	GGATGCCTCAGAAATTCGGCTCTGGGTCTGTGGGGAGCGAAATGCAACCCAAACCCCATT

EMPV1_22053   TCACCTTGTTCTCCACCAGGGCGGCAATCTGCTGCAGGCGGCAGGCCAGTTGCATCTTCT
EMPV1_22055   GGTTCCATCCCTGGCTCCTGCGGTGAGTTAAGGACCTTGCTGTGAGCTGTGGTGTAGTTT
EMPV1_22057   AAAGAGCCCGGCACACTGTTGGCCTCCAGCCTCCTCCTCAGGCTGTGCTCCCATCTCCGG
EMPV1_22059   CACAGGCTAAGCTGCTCTAATAAGGAGAACCTAAAGTACAGTGGCTCTTAGAAGTTGGTG
EMPV1_22060   CCATGGGGCCAATCTAAGGAAAATAATTCTCTGAGTCAGCTTGGAAACAGTGTTAGCTTC
EMPV1_22063   GCCTCGATGAGGGGGAAGTCAACAGAAAAGGCCTCAAAGGAGCTACAAGCTGTGGGAATA
EMPV1_22065   TTTGGATTAAGGGAGTGCTGCCTGCAGAGGACAAGGGGACCGTCTCTGGAGCAAAGTAAA
EMPV1_22067   TGACTCAGCCAGAAGATCGGGTTCCTGTCACCGAAGGGGATCCAGTGACTGTGAAATGTA
EMPV1_22068   TGGATTACATGACACTGGGAAACCTCACCGTGTGGCGAAAAACCCTTGACTTTCAGGCCA
EMPV1_22069   CGGGACCCCAGCCGGGGAGAGCAAAGAGATCGTGCGCGGCTACAAGTGGGCCGAGTACCA
EMPV1_22070   CAGGGCTGCCACTTGAGAATTTCCTCTGAAACTGCTGTGAAGAGAGCACCATTTGTCCTA
EMPV1_22073   TGACCACCTTATGGATTCACTCACTGATTTGAAGTGTGTCAACGCTCGGAGAATGGAAAC
EMPV1_22074   CAGCCCTAGAGAAAATATCCACGAGTCCTCAACGTAGAATTGCACCGGGACAGGGTAGTT
EMPV1_22075   AGGCAAGAAAGAGTTCACCAGCAATTCAATGTCCCACAGGGATGGAGACGCAACTTCTGG
EMPV1_22076   TCACTGAAAGTCCCCCAAATGAGCAAAGTCTAGGTCACACTCAGCTAAGCCACCCTGAGA
EMPV1_22077   TTCCAGCTTAGGACCAATCCAAAAAAGCCAAATTGTGCCCCCCTCGCCAATCATAGTGGC
EMPV1_22080   TGAATGGCCTGGCTCGGCACCAGCCTGTTACTTCCTGGCACTTTCCCCAAATGGATGGGA
EMPV1_22081   GGGAGGTGTGATGAATAGGTTCCAAGGCCTGCACAGATCATTGGGGATCCAAATAAGGCA
EMPV1_22082   TTGAACAGTGGCTTCGAAAGCACCACCCTCTTCTTGAAGTGTATCCGGAAGCCAATGCAC
EMPV1_22083   GACCGACAGGAGAATGACAAGGAGAACCTCAACCTGGAGAACTGCAGGGACCAGGAACCT
EMPV1_22086   CCTTCCGGACAGTGATGATGAAGAGGATGAGGAGACAGCCATCCAGAGAGTCCTGCGGCA
EMPV1_22090   TTCTCTGATCCATCGGCAGGTACTGGACGTCAGCGTCAGTGGCCAGTAGATCACCCATTT
EMPV1_22091   CACCCCTGTCACTTAGGACATTGCTGGGCATTTAGGAGCTCTGTCAGGAACCAGGACAGA
EMPV1_22092   TTGCAACTTTCCTGTTCAGACACCAAATTCCTAGAGATGGTGGGTTTTTCCTTTGCTGTG
EMPV1_22093   GCAGGTTCAATATAATTGTAGTTAAGGAACTTCATGTTAAAATTGTCAGTTATCACAGCC
EMPV1_22095   AGGATGGGGAGGCAGATTTCACCAGCTTCTGCATATTCCAAAATGGTCTTTACGGCTGCC
EMPV1_22097   CCCTTGATGCTCCTCTTACTAGCAGTTCATCTCACTCACCCTCTCCCCTAAAAACAAGCC
EMPV1_22098   CTTCACTGCTGATGGTGCCCATTGGTATTAACCACAAAGTGAGAGCAATATTTGTCCACC
EMPV1_22100   GCAGTGTAGGATTTTCTATTGTGATAATGTCAATATGGCCATATCTCCAAAAGATTGATC
EMPV1_22101   TCTCACCAGGCAGAAGAAATAGCCCAGGATATATGTAGGCCAGAATGGTCTCTGGAGTGC
EMPV1_22102   CTCCATGTCCCTTTGTCACCCTCCCTCTACACTGGCCCTGTACTCCACATCTATTGGGTA
EMPV1_22103   ACTCTCACGGTAAGTGAGCCAGAGTCTCTCATATGCTGTCTTAGCAGCTCAACTGCCTCA
EMPV1_22104   CCTGCAACTATCCTGCTCTGACACATGGCTCCTGGAAGTGATTGGTTTCTACTCTGCCAT
EMPV1_22105   AGCTAGCTAACTTCCGGGAGATGGGCAACTACCTTAAGGCCAAGTATGACCAGGCACTAA

EMPV1_22108	TGTGGTGACAACCCCCAAGCTTCGCTTTTCTCCCGCTTCCGAGTGTGTATTTATCTTTGC
EMPV1_22109	GACAAGAAGGAGTTCCTAGGACGTTTAAAGAAATATGTGCCGTATCACGAATTTCAAAGA
EMPV1_22110	CAGGATAGTATGATGGTAGGTACTCGACCATGTCGAAGTTGGCTATGTTTCGGTGCACTG
EMPV1_22112	CCCTTTAACACACCTGTTTTCTTTATTTGCATGCATTAAAGAAAGACAATATCCAGCATC
EMPV1_22114	GACAGCAGTTGGTTCAAGATGTACTGGAAGGACTCAGGGGACTCCTCCATAGTTTACAGG
EMPV1_22115	TGACTACTGTTGTGGCTTGTATTCAGTCCCTGGTCTGGGAACTTCTGTACGCTGCAGGCA
EMPV1_22117	AAATGTTGACCCAGAAGTCCACTGTGGGTGAGGAGGTTTTCTCCTGAAGAGCCTCTGAGT
EMPV1_22118	AGTGTCCATTCATTTAAAGAGATTTGGCCTAAGTACAAGAGTATCATCATTTAAGCCTCC
EMPV1_22120	ACATTCCACCTTATGAGTCGTATGAGAAGCTATACGAGAAGCTGCTGACGGCGGTGGAGG
EMPV1_22121	GTCCCAGGCCCCCTCCAGATCCCTTCAAGGCTGGACAGAACCACTATGGTAAACTCTTTA
EMPV1_22122	GACGTTGCAGTCTTCGGTTTCAGTAACTTCACAGGTCCGTCGTTTTCGGAGAGGTTTTTA
EMPV1_22123	GCTGCCTGGTTCTGTATGGGCTGTGGGCTAATGAGAGGTTTTGCATTTTCACATGGCTGA
EMPV1_22124	GACAGCAGCCACCATGTCGGATACGAGGCGGCGAGTGAAGGTCTATACCCTGAACGAAGA
EMPV1_22125	AGACATGCCCAGTTTGGTGCTTCCCTGGCTGCTGCTTCCCTTAGGTCCTAGGGCCTGAGA
EMPV1_22127	GCATGATTTTAAAATGTGCAGCTTGGTGGGTCTTAGCACGTTCACAAGACTGTGCCACCA
EMPV1_22128	CCCCAGCAAGGGGTGCTTTCAATGGACTGAACAGAAATTCTTAGCTAGTAGAATGGCAGC
EMPV1_22130	CATGTTCTGACTACCATTCTCTGCCAGCCCATTAAGTATGGGTTTTGATTTCCCTGCAGT
EMPV1_22133	GCTGGAAACAACGTGGAGGTAGATGAGTCTTTGTTCCGGGAAATGGATGACTTGGAGCTG
EMPV1_22134	TGTCACAGGCTCATAGGCATTTGATAGTGAGGTCTGAGAAGTTAGGGAAATTTCTCCAGC
EMPV1_22135	ATGAATTACTTTCTAAGTGCACTACCCTTCCTGGCTGCAGTTGATTCTGGCATAATGGGG
EMPV1_22136	CTGGGCCAGTGCCGTGTCTCTGCTGCCCTGTGTCGAGGGTCTGACATGTCAGCTCAGGCA
EMPV1_22138	AACGCACTTGTCATCTATGAAGGCCACCTGCTTTCAGGGCCAAGGCTCACTAAGGGACAT
EMPV1_22139	GGTACCTGTAACACTGGTCTCACAAGCTGGCCCTCCTCTGTTGGCTGGAAACACTTGTCA
EMPV1_22140	TGGGCCAGCAAGTACCTCACATTCCTTTGGGAAAAGATGGCCTTAATGTGGGCAAGGAAC
EMPV1_22141	CTTATGACAGTCTGCCTACCTCACAGACCTGCTTCTTCCACCTGACGCTGGCCCCATATT
EMPV1_22142	CTCGATGGCCGAGGCCATCTCCTGGGCCGCCTGGCGGCCATTGTGGCCAAGCAGGTACTT
EMPV1_22143	CTGCACAGATGAGGAAACAGAGACATCTAAGGTAAAGTCGCTGGCACAACAATGCACAGC
EMPV1_22145	TTCAGCAAGGTTATGTGCCAGCCTGTCCCTGAATAGAATCTGATACACAGGGTTGCCAAC
EMPV1_22146	CGAGTTGGCAGAAAACAGGAGATTGGCCCGAGAACTTTCAAAGCGGGAGGAAGAGAAACT
EMPV1_22150	CTTCTTAGAGAGAGAAGCAGACTTGATCACTGTGAAATCAACACTCAGAGATGTCAGTCC
EMPV1_22151	TCTTCTTCAGCTGTCCTTGTGTACAGTGGCATATGGGCATATCTTCTTGGGAAGGGTGAC
EMPV1_22152	GTCAGTGGAAAAACCAGTGGATCGTCTTCCAAGTCAGGAGAACAGAAAGGATCAGATGAG
EMPV1_22153	TGTACAAGATCCGCAGACGTGTAAATGTTCCTGCAAAAACACAGACTCGCGTTGCAAGGC
EMPV1_22154	GAAGATCCAAAGGGATTTCTGTAGGAAGTGTGGAAGGCATCGGCCTCACAAAGCGACCCA

EMPV1_22155   AAACCACAGCCCCAATGCGCCTTTGCATAGCAAAGCAACGCTTTCCTTTTTCTCCCCTAG
EMPV1_22156   TGGTGACAGCCGCCTACAACTATTTCCATGTGACCAACTTCTTCTGGATGTTCGGCGAGG
EMPV1_22157   GTTGTCAAAGAGGACGCTCTGTTATCACGCGTTCTTCAGGCGTGAATTCTCCGAGAGGTA
EMPV1_22159   CTCAGCGGATCCTGAGAGCTCATCGCACTGGACCTCAGTGAACTTTGACTTCTTAGTGAA
EMPV1_22160   AGCTATTCATTGCATCCCTGGTCTTGCTCCTCTTGCCCTTAGCACTGATACTGACGTCCT
EMPV1_22163   TCACCACCAAAGGGGAGGCCATCTGCATGGCTGTCGCGTTAATGACCACGGCGGTGATCT
EMPV1_22165   GACTTTAGGCGTGTGCGGCGCTTGGGTTTAGTGTCCTCACTACTGCTTTTGTGATGGTTT
EMPV1_22168   TGCATTTGCAGGCCCATCTCGTACATCGCTTAGAGCGCATTTTGTAAAGGAGGAAGCTGG
EMPV1_22170   GGATCTGGGCGAAGCCGGTATTCAGGGACCTGGATTTTTGACCAAGCGTTGAGATATGCA
EMPV1_22171   CCAGATGATCCTGATAAAAAACCACAAGCAAAACAGTTACAGACACGTGCAGACTACCTC
EMPV1_22172   GCTCCAAAATACAGCTCTTCATGAAGCAACCCTGCTTGGCCGGGCAGGAATGGAGTGCAT
EMPV1_22174   GATCCTGAGTTGCTGCGGTTGTGATGCAGGCCAGCAGCTGAAGCTCTGATTTGACTCCTA
EMPV1_22175   GGGGAAACCCCACTGAAATGCATCTGGGACTGGGTTGACATTGCTCTTGAACTTGGAACT
EMPV1_22178   TGCTGGGTGGAGGCATCAGCGGCCTGGCCGCCAGTTACCATCTGAGCCGGGCCCCTTGTC
EMPV1_22180   GATCCTGGATGGCCTTCTCGAGACATGCAGCCTGCTTCCACTCTGCCAGGACAGCTGCAA
EMPV1_22181   AGCAAGGAGGCACAAGGGAGCTGATGGTATATGCATGTGAACTATTATAACATTGTAGCC
EMPV1_22182   GTCGAGCTAAACTGCAGACAACCTTGCACTGAGATAGTCAACTCACACCCACCTCTGAGG
EMPV1_22183   AAATTTGCAGAGCAGTCGAGAGAGTGGAAAGGCAGAACTATTCAAGGGTGGGATGCTCCC
EMPV1_22184   CCCAAAGTATTCTTCTTTCCTTAGTTCCAGGAGTGGCATCACACCCAGGTGACCCAGTGT
EMPV1_22186   TCCAGGAGCAGGTCTTCTCAGAGTTTGGCTTCCCTCCGGCCGTGCAGCGCTGGGTCATTG
EMPV1_22187   TCAGTGGCAGAAGATCCTTCTGTAGTATATTTTCAGTGCTGCCATATCGCGACAGTGCCC
EMPV1_22188   GTGAAAGCCCTTGGCACTTTGGAGACCTTGAGAGAAAGGCTCACTGTTTTGATATGTGGG
EMPV1_22189   TACAATGTCACGCTCCTACCAGCAGCGCCTATGAGTTTGTCACCTGGCTGGATGGCATTA
EMPV1_22190   GGTAGCAAGAACTGGTTGAAGATTGTAAGACGCATGGATTGTCTGTTGTTTGGGACAACG
EMPV1_22192   CTACCTGCTCGTGACACCGCTGCTCAACCCTCTTGTCTACAGTCTCAAATCCCAGCAGAT
EMPV1_22198   AATGCTTCCATTCAGACAGCGCTAACCCTGCAGTTCCCCTACTGTGGCTCACGGAAGATT
EMPV1_22201   TCCTCGAGTTTTACACCCTTGCCTTCTTCTCCTTCTTCAGCCATCGGCTTGGACACACTG
EMPV1_22205   CAGGTGCAGAAGGAAACAGGAACACACAGACTGATCCACAGACCCAGCCAGACAGATGTA
EMPV1_22208   TCTTGGGATGCTTGTTAAGCTACAAATTCAGATTCAGTTGGTCTGGGATTCTGCATTTCC
EMPV1_22209   AGAGGGAGCCAGCCTTCGAGAGGAAGAGGAGGATGAGGCTGAGTATTTCCGACAAGCTGT
EMPV1_22215   TCTATCTGCCGCCAGTCCCCCTTCGATGTGACAGCTACCAGGCAGAGGAAAGTGATGATT
EMPV1_22217   TGCTGCCTCAGTGGAATGTGGGCTCTTAGGTGAGCTGCTGCTTATTTCCGTAGGACAACT
EMPV1_22218   TGCTGTCTCCAGCCCATACCATTATCTTCTTCTCAGCTGCTTGTCCCTCTGATCTTTGCC
EMPV1_22220   GAGAGGTTCCAGGCCATGTCCAGGGAATTCGTTGAGAATTTCCATGGCTGTAACACAACC

EMPV1_22221	TCCGACTGGGACATAGGACATTCATGGAACCACATAAAGCAGGTCTTTTTGGACTCTGTC
EMPV1_22222	TGAATTCTGCTTGGATGAACACGAGTCTGCGACATGGGCTCATGGAACTCTCCAGACCTG
EMPV1_22226	TCTGATTCAGTAGATCTGATATGGGACTGAGATTCTGCATGTCTAATAAGTTCCCAGGTG
EMPV1_22227	AGTGACAGGAAACCAGAGTCAGAGCAGGAGACACCCATGTAGGGTGAGCCTGAGGCATAA
EMPV1_22229	TGACCCAATCCAAGGCTTTAGCTCTTCTGACCAAATTCTGTCCCACGATACCTCAGTGCC
EMPV1_22231	CAAGGAAGTGAGGTGTGCAATAGGTGGCAAGGACACGGTGGTGTTTTGATGTCATACTAG
EMPV1_22232	GCATCTCCCCGCTGGCACTGTTATCTGTGACTCCCCTGGTGTTTCATGTGTCTTTTCTAT
EMPV1_22233	GGCCTTCTTTGGTGCTGATGATGTTCTGTTTCTCAATCTGTGTGCTGGTTACACTAGTGC
EMPV1_22234	CCGAGAAATGTTTATGAAGAATGCCCACGTCACAGACCCCAGGGTGGTTGATCTTCTGGT
EMPV1_22236	AAGCTATCAGACAGAAGGAAATAAGGTAAGAACAGTTATCAGTGAACTTGATCACAGAAA
EMPV1_22238	GGGCCCTTCTAGGTGTCTGTAGTATTCTATCTCTTTACCTGGATGGTGGCTGCTGACAAG
EMPV1_22240	CCGACGGTACTAGCACAGGTACATAGATATCTGCAACCTGAAATATGTCTGCCTCCGCCT
EMPV1_22242	CCTCAAAATCAGGGTGAAAAAGTTCTGAGGTCCGTGGATGAGAAGAAACGTGATGGCGTG
EMPV1_22243	CAAACTCTGCAAAACAATGGTTGCCTGCTGTTGGCTGGCAGCACTTCTGATTATCCTCCC
EMPV1_22244	GGGTTTCAACTGGGAATCCTGCCAGAGGAACAGGGCTTTATTTTTTCCTTCTATCCTGGA
EMPV1_22245	CACAGATACTAGTCGGGCTTGTTACCCCTGAGCCAGATGATAGGGAGACTAGATTTTCAG
EMPV1_22248	GCCCAGACTCCCTCAGAATCTCAGACCCACTGGGGTGCTTAACTTCCACTTTATTCACTT
EMPV1_22249	AGTGTCTATCACTGTACCTGAGATAATCTTCTACTGGTTGAATCTTTGCCCCTCTGATCT
EMPV1_22250	AAACGGTGGTAGTGGAACTGCTTCATTGACAAGAACGGAGAGACGGAGCTGTGCATGGAA
EMPV1_22254	GAGGAAGTCAAACTATTTCGTATAAATTCAGCAATGGCACAGAGGGACAGGAATAGAGTC
EMPV1_22258	ACCAGTGAATTCAGGAGGGTGGTGGACAGTCCATGTTTCTGTCCTCCAAGAAGCCACTCT
EMPV1_22261	CACTCTTTCCTCCCCCAACCGAACCCAAATGAAAGTCAAAGGACTGAGGAATCCAGCTGA
EMPV1_22263	CCACTCTTACTAACACCTGACGGTAGCTACCCCTGTGCGGGATACTCAGGATGAATCACT
EMPV1_22264	AGAACGGCCAAGAGGAAACAAGAAAATCAGTGTCTTTACATCCAAAATGCAAAGCCGACA
EMPV1_22269	CACCGAGACCGTGTCTTGACATTTGAGACCTGCGATGGACCCAGGTAAAGTTGTAAGAAG
EMPV1_22270	AGCTTCTTCTGAAGAGGTTGTCTCTGAATGTCCTAAAAATCAACAAATCATTTCTTCTAC
EMPV1_22272	CATGACTTCATTGGCGAATACACCTCAGCATTCAAGGAGATGAGAGGAGTGCTGGAAGGG
EMPV1_22275	CTGCCTCCTAAATCGCCACATTCAACCAGGTCGTTCATTAACAGCATTGGCACTAATAGC
EMPV1_22276	AGAAGCGGGCCGAGGTGGACACGGTGTGCAAACACAACTACCAGATAGAGGAAGGCACGA
EMPV1_22277	ATGTCTCATTCACTCCCACGCTCCCCTCTAGGCCAAGGTCGTGCCTGCCACCCCCAGAAT
EMPV1_22278	TTGGGCTCGTTTATTGCATTTATTGAACTGGGCCTGTGAAAAGCTGATCGGCCTACGGCC
EMPV1_22280	CACCCAACACTAGCGTCTTCATGTGCCAAACCACGACCATTGCCCGCGTCTCAGTTTAAA
EMPV1_22281	TGGAGTCTGCAGGAAGAACCACTAAGCCACTGGGTCACACTTTTCTCCCACTGTATTCCT
EMPV1_22282	ACGTTCTGTGGTCGTCGATGGCCAAGATCCTGACCTGCCCCAAAACCAAGCTGAAAATCT

EMPV1_22284	GATGTTTGAAGGACATCAAGGACCAATTACTGGCATCCATTGTCATGCAGCTGTTGGAGC
EMPV1_22286	GCCCTCGTTTTCAAGAACTTGAGAGTGAGACTTTGAAATCGGAGGAATTCCAGAAGAGGC
EMPV1_22289	CTTGCTCCCCAAAGTCATATTAACACAAACAATTATAAATTTCAGATAGACGAATAGGCC
EMPV1_22290	CAATGAGGCATGAAGAAGAAATTCATTAAATGGTGTTCTGGGACAACTAGCTATCTACAC
EMPV1_22291	GACTGGAAATTCTGCTCCATACTGGTGGTGTCCTGTTGGCTAGGAGGGTTTGTGCATACC
EMPV1_22292	TGGGGCTCATTTTAGATCAAAGGCCATGAAAAAAGAAACTGGCCACAACCTTCCTGTGCT
EMPV1_22293	TGGTGTTACAGCGGTGGCATTTAATAAGGAGCTCGATCCTGTGCAGAAGCTCTTCGTGGA
EMPV1_22295	CTCAGCTAAGAGAAGTGTTCACGAAATGGCTAAAGCATGGATGTCAGCTCGGGTTCGGTG
EMPV1_22297	TCCAGGAAAGCGGCGAGGCGGAGGAAGAGGAAGAGGGGAGCGAGAAGGGGGCGGAGCCTA
EMPV1_22298	CCAGTTCGGAGGGGGCCCGGAGCTCGGACGAGAAGAGAGACGGAGCAGCGGCTCCCGGCC
EMPV1_22299	CTTGATCGCGCCGGGACTGACCCTCCTGTGCTGGCGATTAGGAAGTGGAATGAGCGGATA
EMPV1_22300	ACATGCGGCCAAACGCCAGGACCACAAAAGAGCAGGATAAGACGATCTCTGTGTTCTACA
EMPV1_22301	GTAAAAACACGATAGGTCAGTGGACATTTCTCAGCAAGAGCATTTAGTGCCTCCTGGCAG
EMPV1_22303	GTCCACAAGAAGCTGCTGCTCCCTAGTTTTCGGATCTTATTTCATTGGCTTCATGGACTC
EMPV1_22304	ACACACAGGCTGAAAATGGCTCACGGTATGACCCTGTGGTAGATTTGAAGAGCAAGGGCC
EMPV1_22305	CCTAGATAATCCTCTCCCTGGAAGCCTCATTAAGTATTGGCTACTTGGCCTAATGTTACA
EMPV1_22307	GTACCAAAGATCTGCATTGTTTCTGTCCAGTTCTAGTGCTTTAAGATGTGCTTCCTTTGC
EMPV1_22309	CATAAAGCTCTGTGCATTCTTGGTAGCAGCCTCATACCTTGGTGGCTTTATTAACTCCTC
EMPV1_22310	CTATAATTCTGCCCTCCCTCAGCCCCTACACCAACTACGCCATCAGGATCAACCGGGCCA
EMPV1_22314	TGATCTCCGTCTAAGTGGCCGGCATGTTTCAAATCGTCGTCCTCGGGAACAGGGCACTCA
EMPV1_22315	AGATCTTTTCCAAATTCCCGAGAGGCTGCCTAGGGCTGGTGCTGCGGCACCAGATCAACC
EMPV1_22317	CCCTCTCCTGTTGTCTCATTGAACCTTAAGACTGAGACTGTAAATTGCCTCTGGTGCTTT
EMPV1_22318	GACAAGAAACACTGTAAGATAAGCAACCAACACAGGCCTCAGCGAAGAGTATAGTGTGTG
EMPV1_22319	CCTTCTCCTTTGCAGAGAAACTTGTTAGCTGAAATGGTGCCAGACGCTAATGCAGGAGCC
EMPV1_22320	CAAGCTAGCCTGTTCTGATACCCTCATCAATACCATTATCGCATATTTTGTTGGTGCCAG
EMPV1_22322	CAGTCAAAAACCAGGGTTGTGGTTGGACCAGCCCTTGTGGTTCATGCTATGACTGCAAGA
EMPV1_22324	AGCCTCCAGGGGCAGCATGTGTGGCTATTGATTATTCTCCATGGTTCCAGCCACTTGCAA
EMPV1_22325	GCCGCTCTCTCCCGCTTGGCTCTTGCAGGTTCTGTGTGGGAGACAAGTTCTTCCTGAAGA
EMPV1_22326	GGTTGCTCTCGCCTAATGAACACATCTTCGCTGATGACAGAGGCTTGTACAGAATCCCTG
EMPV1_22327	CAAGTGCCCTCCAGTCTCTTCCTGCTGCAGTGTGAGCTCTGGGGGCTGCTGTGGCTCCAG
EMPV1_22331	TTGCTCGCCGGGATGATTTCGGACTCGTTAACCAAGGAGGACTTGATGTCGGCTAAGTCA
EMPV1_22333	CCGGCTTTCTGATTCCAAGTCCAGAGGTCTTCCACCAGCTCCCCCAGATTTAGTGACTCA
EMPV1_22334	TGAGCTGCCATATCTCAAGTGCCCTCTGCACACAGTGCTCAAACTTACGCCTGTGGCTTA
EMPV1_22335	CAAAGCAGGCATTAAATCCAAGGACAATAAATGGTGAAATGGTGAGACGAGCGTTCCAGG

EMPV1_22338	GCTTTGAACCCTCCCCCACCAAGTTAGGACAAATACATTTTTAAGAGGCTGCATGTAGTC
EMPV1_22341	CGTTTTGATTTTTAGACCACCACATGGCATGCTTGACTCTTTTCTTATTTCGAATGTCTG
EMPV1_22342	TCTCTCTCTGCAGCCCTTCGCCTACCCGGTCTGCACTCCTGAAGGCATCGTCTTTGACTT
EMPV1_22343	TTTTCGAAGCGGGAGGACAAGATCGGGAATCGGACTGGCCTTCTCTCTTTCCACCGGAAA
EMPV1_22346	GGGTTCGGCATTTCTTGTATTTATTAGGGCGATCTCCTCCGAGTAGGTCGCTTTGACGTC
EMPV1_22347	AGGAGCAGAGCTAGGATTGACACCGGGCGGTCCGATTGCGGCTGTATACTCTTTATCATT
EMPV1_22350	CGCCAAGTGAAAGAAGTCAGGCAGAGAAAGACAAATACTGGGTGAGATCACTTACGTGTG
EMPV1_22351	TTTCCTCGCTGCTCCTGCTCACAAAAGCCCAATGGCCCAAGTTCAGAGGAAGAGACAAGA
EMPV1_22352	GCAGAAGCCAAGACACAAAGATAAAGTGGAAGAAGCCCTACTGGAAAATCACTGTACGGG
EMPV1_22354	ACGGCTGTGATAAAAACAGACCCCAGTGTTATTAAGCTACTGAAATGGCTATCCAGGAGC
EMPV1_22358	AGGACTACCTCAAGAAAACTTCCCAGGCTGTAAAAATGCTTCGAGATAAAATTGCACAGA
EMPV1_22359	GTGGCAAATGAGCAAGGGCCTGAAGGATCTGACAAAATCGACAAGCAGTGAAAGTGGAGG
EMPV1_22360	CACAGAATTGGTGACACACTTGGAGAGTTTTGTCCCATGTTGACATACCGATGTTCTATG
EMPV1_22361	GCATGGCCTGGCTGCCAAAAATGTATCTCCTGATACTTCCAACCTTTGAGAGTAGAGCCC
EMPV1_22362	CCTGCTGAGCTTGGCTGGATTTCTGGGATGAGAGCTCAGGAAATTGTGTGCATTCCACTT
EMPV1_22364	AGATTCTCGGAGGCTTCTTTTAGAGAGACTGTGTTTGGAGAAAGGCGTCGGCTCGGTCCT
EMPV1_22366	TGTTTCAGCTAAACCAGAGGCCACACAATTTGCATCACTGAAATGGTCCTGGGCAGGTCC
EMPV1_22367	GTGTGTGTGTGTGTGTCTGCTCTAGGCTATCACTGAGCACCACACGGACGTGTCACAGAC
EMPV1_22369	TCTTCCATCATGTCTATCCCAAGAGACTGGATATACTTCTCTGTACTATAAAGTAGGACC
EMPV1_22370	AGGCAGTCATGGCAGAGCTGAGTCTGAACCACGTTGCAGATCGACTGATTGGCAACTACA
EMPV1_22371	TGCGACGAGGAGGTGATGGATATGATGGTGGTTATGGAGGTTTTGATGACTATGGTGGCT
EMPV1_22372	CTCACCGTGCCCCTTCCTACTAACACCCACTGACCACACAGTAAATCGGATGTAAACAGC
EMPV1_22375	GGTTTTATGCTGAGGGTGAGGAGAAGCCACCGAAGAGTTCCGGTCAGTGTAGGATGTAAT
EMPV1_22376	GGGGTCTGCAAACTATTACACAGGAAAACATTTTGGGTATGCTGTGGTAGTGACAAACAG
EMPV1_22379	AGAGATAGATGTTCCAGCAGAGTCCTTCTGAAGTAGTAGGAGCAGAGGAATCCGGAGACC
EMPV1_22380	TGGTAAGAAGATAAAACTAAATGGAATGCTCTTTACGTTCATTAACTCTGCCCCCACTGC
EMPV1_22381	CCTTCTGAATGGAGTGGCAAACAGGGATACATTTCACCAGCTCTGCTTTCTGAATTCTTG
EMPV1_22382	ACTGCAGAGAACCGGAAGAAAACCATGGACAAGATCATCAGAGGGAAGCTGGTGCTGCCA
EMPV1_22383	CCCGTGTGGAAAGTAATATGTATGTTGCTCAAACACAAGAAGATGAAGTCGGTGACATGG
EMPV1_22385	ATTTCAAGTATTATTCCAGATGACATGGCAAACCCAACGCAGGAGGAAGGGGCAGGGAGG
EMPV1_22387	GGGTGTTTATTATACAGCAGGGGTACAACGGTAGTGCTAATGCCAACAGGGCACTATGGA
EMPV1_22393	ATGGACCTGAGACCGCCCATCCATTTACACTTGATAAGAGTCTCCTGTATTCCAAACCCC
EMPV1_22398	TTCCCAGGTTAAAATGTCTCTCCTGCACTCAGATGAACTCGCCTGCCTGGACACAGACTC
EMPV1_22400	GAACTGGCTATGTCAAATTGAAGGAGGGGATCTCGAGGTCTGCGTTCATAGACTGACAAC

EMPV1_22402 TTCCTGCCACCTGCCCCAGCTCTGTGGCCTTGGACAAGTTACCCCCTCGCAACTGAACTT
EMPV1_22403 CCAGTGTCAGCGTTGGATTATTCATTCAGTGTGTCTGATCCAGACTCCTATAAAGGGTAC
EMPV1_22404 GCGTGGGTCAGCAGTTTGTAAAGGAAATCTAATGTGCTAAGTTGTCTGCCTGACGTTCTG
EMPV1_22405 CATCCTCGTGAATGTGTCGCTGGAGCCTCTCCCCTTGTTTGTGATCACGATGATGAAGAT
EMPV1_22406 AGCCCTTTGAATATGTCTGTCTTGTGTGGCAGCAGATGTGGCAGGATGTATCGAGACCCA
EMPV1_22408 GGATTCAGTGGCATTGAATCCAGCTCTCCAGAGGTGAAAGGCTATTGGGCAGGTTTGGAT
EMPV1_22411 CATCGTGTCTTTCCTATGCTCTGAAGATGCCAGCTACATCACTGGGGAGACAGTGGTAGT
EMPV1_22415 TTCCACTGCCTTTCTGCCCAGAGGAATGTTGACATCATCAGATTGGCCTCCAGGAAACCA
EMPV1_22417 CCAGGTGTTCAAGACAGACCACAAAGGCTGGGGACTTCGTACCCTGGACTTCATACCAAA
EMPV1_22419 ACGGCAAATCTGATGGCCAGTACCCTTCTCATCTAAGGGCAAACCCTACCATTACCCTGC
EMPV1_22421 CTATGAGGAAACCAAGGCACAAAGATATGACCCGACCTGCGTAGATGGTCCAGGAAGGCA
EMPV1_22422 CTGACAGCTGAAGACAGAGCGTTTTACCTTTTCCCGAGATGCCGTGATCCACCGTGTCGA
EMPV1_22424 GGAGTCCCTTGCCCTATCAGACACCTCTGAGACTGTGTGTCTGACCATTCACAAATAGGA
EMPV1_22427 CCAAGGCCCTGGACCTGCTCATGGAGAACAACGCCATCGTCCAGTCTATGGCAGCCAAGA
EMPV1_22428 GCGATCCGTATGTATTAACCACAGTAGTACTACATAATAAAAGAATCACCTGAAGAAAGC
EMPV1_22429 GAGTAGGCCTCTGGGCCGTCCGTGTGGTCCAGGGAGGATGGGCCATAAGAGGCAGCTGGG
EMPV1_22430 TGTTTTTCAGAGGCATCACGGTCAACGCCGTGCGGGGCTTCCCCATGAGCGCAGCCATGT
EMPV1_22431 GACCAGAAAAGCTATTGGCTATTTATTCCCTGCCCACCTTGTTTTGGCTACTCAAGGGCT
EMPV1_22433 TCTGTTTGTATGTGGCCACTGCCCTGCTGTGTATGCTTCCTCTCACTGGGGTCCTCTTTT
EMPV1_22436 ACTATCTGGAGTCAGAGGTAATCAGGCCCAGGCTGCGTTGAAGGCTGAACAAGACATGAA
EMPV1_22438 GGCAGAGCTGGAATCTGAAATCAGGGCTCCTGCCACCTGTGGATTCTGAGATACTCACAA
EMPV1_22442 ACAACAGGATGGGACGTTGTGCGGCCTAATTAGCCTTGCTTATTGATATCTGGCACATCG
EMPV1_22446 GCATTGGGGGTGTACTGCAAGCCACTGAGCAGCACCCTTGAAAATCGCTAAAATGGTGAA
EMPV1_22447 TGGCCTTCAGTGCAAAGACTTCAGACCAATTCTTTGTATTACACTTGGTTGCCTCTTGGC
EMPV1_22449 GATACATTACTTTTTCCTGGGTCACTTTGCTCTTCAGAGGCTGTGGCAGTGAAGGGGTGC
EMPV1_22451 ACCCATACAGACTTTTGACTGGTGTTGAGTACATTGTTGGAAGGAAAAATTGTGACATTT
EMPV1_22452 GAGGGAACTTGGCCAAGAACCAGGAGATCAAGTCCAAGACCTACCAGGTCATGCGGGAGT
EMPV1_22454 CAAGCCAGACGACAAGGAAGGCGAGAAAGATGCAGAGGCGGCCAAGGAAGATGCGCCGAA
EMPV1_22456 ATAAGAACAAGGGGACCAATGTCTGTTTGGTGCTTGTCGGACGCCAGGTGCTTTGCACAT
EMPV1_22457 CGACATGGAGGACGTGCGCAACCGCTTGGTGCTCTACCGCAGCGAGGTGCACAACATGTT
EMPV1_22459 GACGCTGGGCACCTGTGGTTCTCACATCTGTGCTATCCTGATCTTCTATGTTCCCATTGC
EMPV1_22460 AGAGTCCTGGTCACCGAGAGTGACATGAAGAAAGGCCTCCAGCACTCTCCCACCCTTTGT
EMPV1_22461 TTATTGCCCCCTTTGTTTACTGCCCCTGCCTCCTCTCTGGACCACAACCTGGTTTACAAA
EMPV1_22462 AGAGGAAGAAGAGGAACCACCTCCGGCCTGCACATCTAGGGCCTGGCGGCGAAGCTGTGT

EMPV1_22463	TGCGGCAGCACATATCTGAACGTGTACTGTGGGACTTAGGGAGAGATTTTCGATGCACTG
EMPV1_22464	ACGTACCAGTGTGCCTTTCTAGTTTGCATGAGGGCATATTGCAGGTGTGGGTCTTTGACC
EMPV1_22465	GAATGTGGTGTATTTTGGTAGCACGGAGAATTTTGAATTCTCAGGTTTAGGTTCGAGTCC
EMPV1_22466	AGCCCCACGGTCCCCTTGGACCTTAACAGGTAGACAGATTAAGAGAGGAGAAAGATGGGA
EMPV1_22468	TGAGATTAGGCGGCTCTTCCCTCGGGCTCAGATGCAGACCTTGCCTAATGCTGGCCACTT
EMPV1_22471	AGCAAGTGCTGCTGTCAGTGGCCTTCTAATCCAGGACTGCCATATTGCAAGAACACATGG
EMPV1_22472	GATATCATGTGTGGAATGCTGCACTGTGCTAATGTGCAGAAAATTCCTGGTGGCGGAGAG
EMPV1_22473	AGAGGATATTCCAGAGGACGTGGAAACACCAAGAAAGGGTCTTCAGACTAAAGCAGAAGA
EMPV1_22474	TCTATGGTCATTCCGCTGTGTATCACGAGGCCACAGACTCCCTCTACGTGTTTGGGGGTT
EMPV1_22475	GGTTCCTGTGAGTGCTTGGGTGTCGAGAGCCTGTTTTTGGCGTAGGGAACCAACTTTTGA
EMPV1_22476	CAGTGCCTTTCGACAATGGCCAGGGTTTCCCCTATTTTCCCACCATTCTTTCCCAAAGGA
EMPV1_22477	ACCAAAACCTGCACCAGAAGATCCAGTATGTGGTGCGGCTAAAGAAGGAAAACGTCAGGC
EMPV1_22478	GCTCATTGGCACCGGCACTGTCATCATTCTATTGGGCACCTTCGGTTGTTTTGCTACCTG
EMPV1_22479	GCCTGCCAAGACCTTCTCTTACAAGCGAGGCTGAAGAGGTACTATTAGAGAGACGATCCA
EMPV1_22481	CAGCAGAACTGGTGGAAATTCAGAGACACTGACAACAGGATGTATAGAAAGTTCAAGGGC
EMPV1_22482	TACCCTGTTCTCATGAATGCTAAGCTGAGTTCCTTGCTTGTGGCTGGAGCTTGGGTGGCA
EMPV1_22483	GATGAAGGCTACTGGAATATTGGCCCTGATGGGCTGCCTGATCACAGTCATTGAGCCTAA
EMPV1_22485	TTCCTCGACGAAGCCGTTCCTAGGTCGCCCGCGCCCTCTCGAGCCCTCCTTTTTCCCACG
EMPV1_22486	GATGAGGATACTGGGGTCCAAGAATAATGTTAAGAGGCTTCATTCTTCAGAGGGACAGGG
EMPV1_22489	ACAGCCTTCATTCACTCTGGGGTGATCCTGGGTCTTGTGTGTTCAGCTCACTGGCCCCTT
EMPV1_22490	TGAACTTGGAGGCAGGTGCGCTTGAGTCTGCCCTGTATCAACCAGTTGTCTGAACTTAGG
EMPV1_22493	GTGGCACTGTGGACATTGTGGAGAGTGAGCCTGAGAATGATCATGGAGTTGAATTACTTG
EMPV1_22494	AGAGCTCATGGAGTTCCTGCTATGGCACAGTGGGTAAAGGATCTGGAATTGTTCCTGTAG
EMPV1_22496	GATGAAACAAGTGAAGATGCTAACTGTCTTGCTTTGAGTGGACATGATAAAACAGAAGCA
EMPV1_22497	GGTGAGATTGATTGTGCACTGCCAAGCTACCCCAGTGCGGGATACTCAGGTTGAATCACT
EMPV1_22500	TGTTTCCCATCACGCAACACTCCTGCTCGCAACCCGACAACTGGTAGATGAGTCTATGTC
EMPV1_22501	AGTCCTTCATTCCGAAGCTGAGCAAAGTGTGAAAGGATTCCGTAACTCAACGCCTGCCAC
EMPV1_22502	CATGCAGAAAATCTTTGCCCGCGAAATCCTGGACTCCAGGGGCAACCCCACAGTGGAGGT
EMPV1_22503	ACATGTTCGGCTGGTAGTGTAGAAGAGCTAAGAACTCACTGCAAAGGAGAAGGCTGCTAC
EMPV1_22506	GTGCTCCATATGGTCACAGGACTTTTCTACAACTGTATCTGTCTGCTTGAAGTAGACTGG
EMPV1_22508	CACTGTGCACCCCCTGGCTTCTCGCAGACTTACAGGGAAGCTCTACAAATGCATCAAGAA
EMPV1_22510	CTTAGAACATTTGCATCTCCTCTTGCCTGTGGGCATCTTTACCAGGTGTCCTCTGATGCC
EMPV1_22511	ATCTGGAAAGGAAAAACAGGATGCTTCGGAAGGAAATGGAGATGTTATGGAACAAGACAT
EMPV1_22513	TCACCCCGCTTTGCCGTGGTGAAACTTGATCCACTTCCCAGTCCTGCTTTGAAATCACAT

EMPV1_22514	CAAATGAACCAACGTAACTCAGCAGAAGCGGAGGGGTGGACTCAGAGAACAAATGGTTGG
EMPV1_22518	ATTGTCCATCTCTTTAACTTTAGCCGTGTGAGTGGCTGTATGATGACATTTCATGTGTGG
EMPV1_22523	ATAGAGTTCTATGCCTTAAGAGCCGAGGCCTACATCCAGCTCTGCGACTTCTCCTCGGCC
EMPV1_22528	CGGTTCAGTTCCATTCAAATCCAAAGCTTGAAAGACGTGAACCAGGGGACGTATCCAGGT
EMPV1_22529	ATTAATGTTTTGGTTTTAGAAGCCCGGGACAGAGTTGGAGGAAGAACATACACAGTGAGG
EMPV1_22530	AAAGTGAGGTCCGTGGCCCAGCAGCTTTAGCATCACCTGGGAGCTTGGTAGAAGCACAGA
EMPV1_22531	GCTGTCTTCCTGTTTGGTTACTCAGAGTTTTATAAAGCAGGGGAATAGAGTGGATAGGGA
EMPV1_22532	TTGCCTTTATTAGTGAGGTGGAAAGCGATGGGGCCGTCACAAGTGCTGGGACCGAAATCA
EMPV1_22534	GACTCGCGATGCCAACCATAGCCACCTAAGCAGTCAACCAAAATACCCCGCCTTTCAACA
EMPV1_22536	CAACTGGGGTTTTAAAGCCCTAGCGGATCCGCGGAAGGGCCGAGCGCGACGGCTTTGGTA
EMPV1_22537	GGGGAGGCTGTCCAGTCCCAAGAGGAGGACGGAGTGGGGTGCGGGAGCTGGAAGGCAAGT
EMPV1_22539	CGCAAGAGAGCTGTAATGATTGAGCAAGCTGCCATGGAAGACGGAAGAACATTTTCATTA
EMPV1_22540	ATCATCAAAAACTGGAAAGGGAAGCTAGAATCTGCCGTCTTTTGAAGCACCCCAATATTG
EMPV1_22542	ACTGCGATTCTGTCCCTCTGTTAGCATTGTCCTGCTCTGACACTTCCTTTCCAGAGACAG
EMPV1_22543	CCCTGGGGATCTTGTTAATATTCAGATTCTGGGGTGCTTCAGGGGATTCTGCATTTCTAG
EMPV1_22544	ATTCTGCGTCGCATCGGCTGCCAGCATTGCGCAGACCTTCGGCCTCGCGTTTGGGAGAAA
EMPV1_22548	ATTAATCTGACTGCACTGCACAGGGACCCCGCAGAGTGGGCCACCCCAGACACATTCAAT
EMPV1_22550	GGTTAGATTCATATTGTCCACTATGGGTGGAGCCACACTTCTCACTAACTGTTTGGCTAT
EMPV1_22551	CCGATGCGCTGGCAGATTTGCAACAGATGATCAGGAGCCACATGCTTATTGGACAAGACC
EMPV1_22554	ATGTGTTTGTGGGAGAAGGTGAGCATGATGTCTTAGGCCCCTGCCGTCTTGATCCTTCCT
EMPV1_22555	CTTCTACCCTAAGGAGATCTCCCTGACCTGGCAGCGGGAGGGCCAGGACCAGAGCCAGGA
EMPV1_22557	GGTGGAGCAATAATTTCCTGTTCGTATTATTTCCATCTTATCCTCCATCGCGGTGTGGGC
EMPV1_22559	AAAAAGAGGAAGCGGCGCATGTATAAGGTGCCTACCTCTGGGACCTCGGACCGGGAGCCT
EMPV1_22561	TAACAATGAAAACAGAACTAACTCAGATGGACCAACTGCAAAACGGCCAATACCAGCATC
EMPV1_22563	CTCGTAGTAACACTCTTCTATGGCTCAGCATCTGTCACCTACTTGAGACCCAAGGCTAGC
EMPV1_22564	GAAGACAAACACCCTGGGGAAACAGCCTAAGAGCAGAGGATTCACCAAGGACAAGACTCT
EMPV1_22565	TCCACTTTAGTTATTTTTCCTTTGGGGGTGTTCGCTGTTTCCACCGGCTCCCTCAGCCTG
EMPV1_22566	CTAGCCCTTATGTGTAAAACTGGTATCCCACACTAATGAATTTTAGCTCTGATCTTGCAG
EMPV1_22570	AAGTCAAGGCTAAAGGCCACTGAAACACAGGCAACAAATGCATTGGATGAGAAGTCAAAG
EMPV1_22571	GTTTTCGGTCATGCGGAGGGGGCAACAAATGAAGAAATACATCACTGATAGCGGGTGCTG
EMPV1_22576	GTTGCGACTGTTAAGTACTCCAGAGACTCCCACCACCCAGCCTCCTCCTTCAATGTCTAT
EMPV1_22577	GTAGACCAGTTCTTTACTGACAGTTTCTTCCCCAGCAGACCATTTACGACCTGTCCCGCC
EMPV1_22578	ACGTGCGGGGAGAGCTTTGTGCAAAACTGCAGGGCTATCTGAAATCCTGGACCAGCTTCA
EMPV1_22579	CAAGCAGCACAGGAAGGTGGCCTGGATCCAGGATTTCCTCATGCTGCAGTTCTGCGAGCA

EMPV1_22580	TTTTGGGCTGTATTCTCAGGGATACGGAAAGACCGGAGAGGAGGGAGAATGTTGAAGCAC
EMPV1_22582	GTGGGGGATCCACAGGGTCCCTGACAGGTTTTCCCAGAGGAGGGTCAGGATGCTCCAGGG
EMPV1_22584	GGCAAAAGCCATCCTGAGAGGGAAGTTTACAATGACATAAGTCTACCTCAGAAAACAAGG
EMPV1_22587	GCAGAAACTTCTGACAACCCGGGCTAAGGCTTATGACTCACATCAAAGTCTAGCTCAGAT
EMPV1_22588	CCTGGGTTTGGGATAACAGTTCTTACAGGTAGACACCCTCGTGCAGGGTGGTCGCGCAGC
EMPV1_22589	AGGAGAAATTGCTGGTGTACAGCTTCTACAAACAAGCCACTCAGGGCGACTGCAACATCC
EMPV1_22593	CAGCTGGTATTTCAGATGCAGCTTAGGCAGCTGGACCTTGGTGGGCCTCTCCCGCAGCAA
EMPV1_22597	AGGTGTGGTTGAATACAAGGAGAGCTTCGGGGTGGATCCTGTCACCAGCCAAAATGTTCA
EMPV1_22598	ATGACGGCCTCTTGGGTGGCTGGGTTCCTGGTTTCTCTGTGCATTGTCGTCATCATATTC
EMPV1_22599	GGGTGAGTAACTCGAATTTAAAACCGTGGGTTCAAGTCCCAATCTAGCTTTAGTCCAGGC
EMPV1_22603	GGAATACGTAAACTACTGAAATACATTAAGGATACATATAATAACCCTGTAATTTACATC
EMPV1_22604	TTCTTCTCGAAAGCCTTGTTGGAGGGCTTCAGGGTAGCCAGGTTCTGGAACTCGACGCTC
EMPV1_22607	GACGCTCCAGAATGGGAGACAAGCCAATTTGGGAGCAGATTGGATCCAGCTTCATTCAAC
EMPV1_22608	TATGGTCCTGGCAGTGAGAACGGCTGAAACTAAGCAGGAGCTGGGAATTAGGGACGTGAA
EMPV1_22609	GCCTCCCCTGCTCGGGCAGCACACGACGCACATCCTGAAGGAGGTCCTTAGATACGACGA
EMPV1_22611	ATACTCAGACCCCAATCTTGAGTCCTCAGGAGGTTGTGTCATGCAGCCAGTATGCTCAAG
EMPV1_22615	AATACTCTTCTCCAGCGAGACCTATTCGGTAGATGAGACGGGGAATGGTCAGAGGTGCCT
EMPV1_22616	ATAGTCTGCTAGAGCACAGAAGAGCAGGAAGAAGTTCCCTAAGTCCAGACGTCCCAGGAG
EMPV1_22617	ATTCCATCAAAATTGCTTGGGAAAGCCCACAGGGGCAAGTTTCCAGGTACAGGGTGACCT
EMPV1_22618	AAGGAATAACACTTAGCACTTACGTAGCACTTCATCTATGCCAGACAGCATCCTAAGGGC
EMPV1_22619	TCCAGGAGCTCATCCAGGAATACGAAGACAGTGGTGGATCAAAAACGTTCAGCGCAAAGG
EMPV1_22620	TGCTGCCAGGTTGGTTAGATGAGTTCTATGGAGTAGTCAGCACTCCTTGGGCTTCTTGAG
EMPV1_22621	CCTTCTCTAGCCTCCCTTTCTTAGCTGGTTCATTGGTTCTCACCAGAAGGAACTGCATGC
EMPV1_22622	AGGTAGTAACTGCGAGTCTGGGGATCTTGTGTGTGGCCTTGATGACAACAGTGGGTGTCT
EMPV1_22625	AAAAACCGCAAACGTCATTTCAATGCCCCCTCCCACGTGCGCAGGAAGATCATGTCGTCT
EMPV1_22626	CGTGACCACCTGGGAGGCAGCTCTCAAGCAACGGAATCGGGTCGTGATGGATAACACGAA
EMPV1_22627	TGTAGGTCGCAGACATGGCTCAGGTGTAGGCCAGCTGTGGTAAAGGCTGCAGATTTAGCT
EMPV1_22628	GCCGGCTCTGCGTCTGGCCTGTCCTGCTCTGACCATGGCGTTTGAATGTTTTGGGTTTGG
EMPV1_22631	GTATAACGGGTTATCGCTATCATTGCAATCAGTCGACATTCCATTAGCCCTTATCGCTAG
EMPV1_22632	AAATTGTTTTGAACGGAAGAGGGTTCAGCTTGGGCAGTCGGAAGGGCAGCGTCCTCTGTA
EMPV1_22635	GGAGCATCCTCACTGTCACCTTTATCTGCCTTCGGGATTCAAGACGGAGCAGGACTGAAA
EMPV1_22636	GGACGTCCGCCGGCGCTTAAGTGATGTGATTAATCGAATTCGCGCGGCCGCCATGGCGGC
EMPV1_22637	AAGGCATTTTCTGGTTCATACTGGAGAGAAGCCATTTCCCTGCACTTTTGAAGGATGCGG
EMPV1_22638	TCTCCCAGTAAATGCAGTAGACCCCGTATTATGTCAATCATGTCATCCCTGCAGTAACGA

EMPV1_22641 AAAACTCCTCTCTGACCTGCAATTTTTCTGTCTCCATGACCAGCGTGCAATGGTTCCAGC
EMPV1_22642 CCGGTGATGCCCTGGATGAAAGCCACGTTACCATGCGTGAGGGTACCGAGAAGATTATAT
EMPV1_22649 CCTCCTCTCTCAGACTAAGTACCCTCCACCTGGTCAAGAATCACATGGCTGTTCACTATA
EMPV1_22651 GGATTAGGCTTTGGCTTAAGGGGATGATGTGGCTGGTTTGATCTTCTTTCCAGATCACTG
EMPV1_22652 CTCTAATGATCAAAGTCCACAAATTCGAGCATCACCTTCTCCCCAACCCTCTTCACAGCC
EMPV1_22653 CAGGCCAGGGAGGCCGAGCTGCAGGCTGTGCTCCGCCCTGAACTTCACGGCCAGTTTCTA
EMPV1_22654 TGTCCAAGGGAAAGGCTGCACCAGACGCTCAAAACTTCCTCCCAGGGCCTTACTTGTATA
EMPV1_22655 GTTCCCTCCAGAAACATGGGCATAATTCAAGTCAGCATTGGAGGACCAAAGACTCCTGGC
EMPV1_22656 GCCCTTTTAGACTCGCCCACAGCCTTAAGTATGGAGGAAAAACTTCAGCAGAATGAAGCA
EMPV1_22661 GTGCCACTGGCGATTGCATAATTCTGCGTTCAGAAGTGCTGTAGGCTTTCTGGATGATAC
EMPV1_22662 CTGACAATGATAACTGCATAGATATACAAACCTTCACCATCTGTGCCACTGTCCATTTGC
EMPV1_22663 GCGCGGTACTTATATAGCAGTGCAAGCGGAGCAATGCCGAGGTTGTGAGTTCGATCCTCA
EMPV1_22664 TGTCTCGGCTTCCGTCTGGTTGCTGATCAGAGCGTTAATTCCTTCACACACAGCACTGCT
EMPV1_22666 GCCAGAAAATTGCACAGTTCCAAACAAATCCTCTGTACAAACACAAGGGCTCATTTACCA
EMPV1_22667 GCCACTCCTTTCCGACTTCCAGAGTTTCTGCTGAAAAATCAGCCTATATCCTTACAGGGG
EMPV1_22668 TGATGCATGACAGTCCTCAAGGGGGGAGTTGTGAGAGAGCAGGATGCGAAGAGTTCCTCT
EMPV1_22669 TCTGGGCAAGATCCGAGATGTTCTCCGTAGAAGCAGTGAGCTCCTGGTGAGGAAGCTCCA
EMPV1_22671 CACCCCTGAGCTCCTTGAGCCCCAGTCATCTCCTGCCTCGGAATGCAAATAGTTCTTATT
EMPV1_22673 GAGTTCAAGTTCTAGGTAGCATCTTAGTCCTTAGCACCACTGGTAATTTCAAAGCCGGCC
EMPV1_22674 TCAGCAAGAGCACCGGGAAGGTTGTGCTGAAGACGTCCAGCTTGTACCGGAAGCTGCTGA
EMPV1_22675 TGACCTGCCCACGCGGCCCTCGCTTGGGGTCAGGCAGCAGGGACTCAGGAGAGAGGGAGC
EMPV1_22676 AAAGCATCTTTCACCACTGACTCATGCCCTCATGGCTAATGCCTACCTCCTTGTACCCCC
EMPV1_22677 GCGGTGTATTTTAAGGGTTTGTGGAAATCGAGGTTTCAGCCAGAGAACACAAAGAAGCGC
EMPV1_22678 GACCACTGCAGAAAAGTAATAGAGCCCAAAATCTCAGAGCTCAACCATCGATTTGCAGCC
EMPV1_22679 TGACTTTCAGTGGGACCACGTCCAGTCTGTGTGAAGTGTGATTGGTCCTGTGTGCTGCAT
EMPV1_22680 AAGGCCAACCTTCCCCGACACTCTCATGGTGCAACATGTAAAGACATAACGACAGGGTGG
EMPV1_22681 TGTTTGTAAAGAGAGGTCGGATTCCATAACCCATGCTACTAAATCATTGTCAGTGTGTGG
EMPV1_22685 CGCCCCCGCCTGTGGGCTCGGTGGGGGCCCTGACCGCGGCCTTCCCCTTCGCGGCGCTGC
EMPV1_22686 AAAAGGCCAAGGTGCAGCAAGCTGAGGCTCTGCCTGGACCTTCGCTGGACCAGTGGCACA
EMPV1_22687 CTGGTCCAGAAGCCCTTCACCCTGACGAAAAGCATCCTCCTGGACCTGGCCTATGCCTAT
EMPV1_22689 AGCAGCTTCAATCCATACGCATCCCCCAGTTTCAGAGTCCAGATGGGTCACGGCTTAAGT
EMPV1_22693 ACATCTGATCTCCCCTCTTGTGACCCCATGACGCCTTCTCTAACTCCCCTCCTCTACACT
EMPV1_22694 TGTCACTGCTGTCCAGCTGCAGACTGAGGCCCAGGTGGCATCTGCCTCAGGCCAGCAAGT
EMPV1_22695 GGCCAATTGGTCCATATACCTCAGTGTGCATATGCCAGTCCTAAACCACTAGTCCATCCC

EMPV1_22696	GTCGGGGAAAAGATCATGTACAAATTGAATAAAGACTTAGGGGTTTATTACACTAGAGCT
EMPV1_22697	CCGAAGTGCCAAGAGAGTTGAAGTCACGACACTTCTACCCAGGAACAAACCGTGCCTGAA
EMPV1_22699	CTACGTGGACTTCGCAGAGTTCTACCGCCTGTGGAGCGTGGACCACGGCGAGCAGAGTGT
EMPV1_22700	TTGGCTCTGTCTGCTCTTGGAGGTGGGCCTGTCCCAATGCTGAGGGGGAACAGCAGCTCT
EMPV1_22705	GTACCCTTTTCCTTTTCGTGTCCCCAGCAGCAGGGAAGAACCTAGTGGCTTTGAGTGAAT
EMPV1_22711	GCAACTGACACCTATATATCACTGAGCCTTAACCTCCCAGGACATGACAAGGGGGCACTA
EMPV1_22712	TCCTGCCGGCTTGGTTCCCCACTGGCCTGAGTTTCGTAGCCACCCTAGATAAGAACCACA
EMPV1_22713	CCTGAAGTCATCTATAGATTCAATGTAACCCTCATTCAAAATACCATTGGCATTCTTCAC
EMPV1_22714	GCGAGGAAGAGGGGCGCGAGGAAGAGGATGTGAAGGGGCACAAGAAAGAGGAGGAAGACG
EMPV1_22715	GCAAGCTTCCTTGGACTTTTCTGGCAACCATGTCAACACAAGGGAGAAGCCAGCCTGAGA
EMPV1_22716	TTGGTGCCTGCTCACCCAGCCCTGGTAAATGCCATTGTCTTGGTTCTGCACTCAGTAGCA
EMPV1_22719	CACTGAGAGGATACTGCCCCCATCTCAAGCAAGAATGCCGTTGGTCTCTTCCGTTTCAAA
EMPV1_22720	CGCTTCCCCGTTCAGAGCTGGTTGAGGCTTTTGTTTCCCCTCTATTGCTAATTGGTGCTA
EMPV1_22721	GCCCTGAGGTACTGGAATGTTTCAAGGCGGGGATGAAAAAGAAACAGAAAGGAGCCAGAG
EMPV1_22722	ATCTGCGGTGACCACTCTACGCTGTCAGGCTCTGAACTTCTACCCCCAGAACATCACCAT
EMPV1_22725	CAAGGATTAGAAGCCCGGATCCAGCATTTTGTACGTAGGGGACGCATTGAGCACCCACAT
EMPV1_22728	GTGCATCTGGCCAATTATGCAATGCTCTATCACAAGGGGAAATTCCTTCCTCACCCCAGC
EMPV1_22730	AGCATCCAGGAGGGGGAGAATTCCACCATGTACTGCAACTCCTCAAGCATCTTCACCAAC
EMPV1_22731	CCAAATGTTCACTCGAGGGTAAGGATTTCTTCTAGCACACCGGTGGTGGAGGATACACAG
EMPV1_22734	CACTAGCTGGTTTACACACTAGTAGGTAAGCCAGAACCACACAATGAGGAGGAAGCGAGG
EMPV1_22735	CACGCTCATCCTCACCGTTTCAGATGTTCCTTATAACGGTGTCCTGATGACAAGGTTGAT
EMPV1_22738	TGTAAGCTTGTGGCAGGAGAAGGAGCTTTTATGCCAGTTGCCTTGAGCAAGTTCAAGCCC
EMPV1_22741	AGCCTAGAAAAGTAGAATTATCCGAGTCGGAAGAAGATAAAGGTGGCAAAATGGCTGCAG
EMPV1_22742	ACTGTGCTACTTTTCGACAGGATGGTAGACTGCTTATAGCTGGCAGTGAAGATGGTGGCG
EMPV1_22743	TGCATCTATCCAAGCCACGAAATCTCCCGACAGTGTTAATGGAAGTGAACCCACAACTCC
EMPV1_22744	GTGCAGTAGGTTATGTGTTTCTTTTTGGTGAGGGAGGGAGAGAACAAGTCTCCAAATTCC
EMPV1_22746	AGAGCACGGACCAAAACTCTTGCCTGCGAAAGGACTCTGGCAAGACCAACCTCTTGACTA
EMPV1_22747	TCCCTGACTACCCCGCCACTCTCTGAGTCTGTCACCTGGATCATTAAGAAGCAGCCCATA
EMPV1_22749	AGCTAAAAATGAAATCCTCGATGAAGTTATCAGTCTGAGCCAGGTTACACCAAAACATTG
EMPV1_22750	AGGAAAGTGTGGGAATTGCAAGCAGCTCACCTATTTCTAGAGCAGTGCAGGAGAGGCCCT
EMPV1_22751	GACAGACACAAGTGCATCTGGCTAGTTTTGACATCGTGGATTGTTGGCTTTCTGCACGCC
EMPV1_22754	ACAATGCACATGCCTCTGACCACTGGGGTTTGGTTCTTATGTCACCAAAAGTCAGGCTTG
EMPV1_22757	ATTGCCAGCCCATGGGCTGTGGAGCCGCCACATCCTGCAGCAGACACTGATGGATGAGGG
EMPV1_22759	CCTGGAACAAACCTTTAATCCCTCGGATCCTGACTGCGTGGATAGGCTCTTACAGTGCAC

EMPV1_22760	ACCACCTCCCTGTTTTTGCTCCAGTATTGGGTTCCTCTGGGCTTCATCCTTATCTGCTAC
EMPV1_22761	GACAAGTTGGGGCTGTGAAGGTCAAGGAGGAACCAGTGGACAGTGATGAAGATGCTCAGA
EMPV1_22762	TCTCCTTCTGCGACGGCCTCACCCACGACCCGCTGCGCCAGAAGGCAATGTTTCTGCAGA
EMPV1_22765	TTGGCAAAGCAAGTGCCTTCAAGAGGTGGGAAAAGCAAGAAGTCAGCAATGCCTCCCTCC
EMPV1_22771	GGTTGCTACAGAGATGCAAATTCAATCCCAGGCCCAGTGCTGTGGGTTAAAGAATCAGGC
EMPV1_22772	GGATGCTGACAGTGAGAAGCGATGACTCGGAGCACAAGTACAGCTCCACGCCGCTGGACT
EMPV1_22773	TTTCTACATCAACTTGCGCTCGGGGAGTGACATCGCCTTCCACCTGAATCCCCGCTTCAA
EMPV1_22775	CCCCACCACCCATAACATTCAAACCATAGCGGACATACCAACAGAATGGTACCTCTGGCT
EMPV1_22776	GACCTTTTATGCCACAAATGTTTTTATTATGTTTTGCCAGTTGGGTACACAATGAACGAC
EMPV1_22777	AAGATACCACAGCAAAAGCTTCTCTTCCTCCAAAGTGTGGAGAGGCAGCCATTGTCCTGG
EMPV1_22778	GAGTAGGATTTCTAGTCCTGCAGCTTTCTCCATTGTTCCTAGGAGAGCCTCTAAAGGGAA
EMPV1_22780	GGTCATTCACTGAGAGCAGCAGATTGGTTTGGGATAAGGGGCCAGGGACAAAGGTTATGG
EMPV1_22781	GGCTGTCCGCCAAGACCCGGACTATCCGCATCATTAACACGCTCACGTCGCAGGAACACA
EMPV1_22782	AACACCCTAAACATCCTCTATGACCATCCCAACCCTTACTCCTGGGGCAGCATCAGCTCA
EMPV1_22783	ATCCTGTCATATATCTTCATCCTTCGAGCAGTTCTGCAGCTTACCTCTCAGGAGGCTCGC
EMPV1_22784	CAGAATTATGTACCGTTGTGTTATGAGGACCAGAAAGAAATGTAAGCACTTGCCCCTCTG
EMPV1_22785	ACCATTGCTTCCCTGAATTTCTGACCCACTGTCTCCAGGCACTGAAGGAGGATGAAGCTT
EMPV1_22786	ATCATCGTGTGCATCAAGGGCTCTGTCACTGCCTTCTATTGTGTCTTGGGATACCTGGGC
EMPV1_22790	CCTCAAATCGTGTATGGGCTGAGGATAGTACTGAGAAAGACAAGGACAGTGTTCCCACCG
EMPV1_22793	TAGAAGGACCACCGCAACCACCGTGTGGTGGATGTGTACAACTATGGCGCCATCATCCTA
EMPV1_22794	TCTTCCCTGATGACCCTCCCACATTTATCTTCAGTCCTGCTAACAGGAGAAGAGGGAGAC
EMPV1_22796	ATCCTAAATCACTTTGGCTGTGAGTATTCTGCCATCATCTCTGCCTCCTGCTCTGACACT
EMPV1_22798	GGCATCGAACAATCAATGTAGGAGCGTGGCAGTGGGTAAAATGATGGACAGGCACCAAAG
EMPV1_22800	TTCCTTGAAGCAAATGAGTAAACACAGCCCCAGAGGATGAGGATGTGGACCCGGCGGAGA
EMPV1_22801	GAGGTTACAGAAGCACCAAGGAAGGTCAGAGACTTGGGATCAACGTCACAAGCTGATGAG
EMPV1_22802	CCGCCTCTTCTGTGTTCAGGTCCATCATGAGTGAGAAATGGGACTCCAACTCGTCAGAAA
EMPV1_22804	GGTGACCAGTCATTCTGAGCCTCCATCTCCCTCTTTAATACCCATGTGGATGCTGGATTC
EMPV1_22805	CCTTTCGAAACTGTACCTGAAGGGATAATCTCGAGGACTCTAAAGTGTCTCTGTATCCCT
EMPV1_22806	AACTGGGTGTTCGTGATAGACACACTCAACTTCTCCTTCTGGTCAGAGCGCGACGAGCAT
EMPV1_22810	GGGTGAGCAGCCAAAAGATGGAACAGAATCCCCCATCTCTCAGCATTCAAGAAGGTGGAA
EMPV1_22815	TTGCTGCGTCTGCTCAAACTGGCACGGTCTGATCCGGAAATACAGCCTCGATATGTGCCA
EMPV1_22816	ACTGCAGACCCCAGTTAGGGAAGCAGCTGCGCCAGCCGTTAAAAACGGTTCCTGAGGAAA
EMPV1_22819	GAAAACACCCACAGACCACCAATCACATTCTCGCCCATAGTAACCATCACCCCCTTGTGA
EMPV1_22820	TAGAGCCCTACAATGGGGAGGAAGTGGGGCAAGTTACTCAACGAGATGGAAGAAGCTTTC

EMPV1_22821 TCTCATCAACACAATGCAAAAGAGACATGCAGGAAGAGTCCTGGTTTTCACATTACGTTG
EMPV1_22822 TGTGCCGCCAGGGCTACCAGAGCGCCTTCCCGTCGGATCTGCTGCTGTGCAGCCTGCAGA
EMPV1_22823 GTTGCAAAGGTCGCTGCTTTGAGAGAACATTTGGGAACTGTCGCTGTGATGTTGCTTGCG
EMPV1_22824 TAAAGCACCTGAATCCACGTGGGAGTGTTCCACAGAGCCTGCCGTTCCTACTTTCTACAC
EMPV1_22825 GAAAGGTCTGGGCAAAGGAGGCGCTAAGCGCCACCGCAAGGTGCTGCGGGACAACATCCA
EMPV1_22826 CGCCTGACGCTTTTCCAAATGTGAATGCCGCCGTCCCTTAGAATGTGTGCTGATTGGAGA
EMPV1_22828 GGGGTTTAGAAGATGAAGCAACTCGTGTGAGGTCCTACAGTTAGTCAGGGTAGAGCTAGG
EMPV1_22831 ATTTGAAAGCCCAAAGCCGAGCAGATAAAAGGCAATCATGTTCCCACAGCCTGGCCCCCT
EMPV1_22832 GGAGGGAACCTGTCTTCAACTGAAATGGGAAGAGGAAGAATTATGACCAGAGAGAGGAGC
EMPV1_22833 CGGAAGTATGACTATAACCCCAGCTTTGCCATCCGTGGCCTCCACTACGACATTCAAAAG
EMPV1_22835 GGCTGCCTTCAAATAAGAGATGTGGACAATGGGGGTTATTGTATGAGATTTGGGGGTTAA
EMPV1_22838 TCACACAGACAATTAACTCTCCAAAGGGGGTGTAAGGAGTGGGGACAGTGATGGTGAGGG
EMPV1_22839 GGCTTCTCCATGACACCATACTTCTGCCCAAAACTGCCTCTGGGAGGAGAAAATCTACAC
EMPV1_22840 CAATTGACTTGTCCTACTTTCCTCCATCAGGGATTCCTCCTTTTCTTCCTCAACAGCTCC
EMPV1_22842 CTTGAAGCTTGGAAAGAGTCAGCAAAGTAGACTGCTATCTCAAATCCTGCTCTAGCTCTG
EMPV1_22847 GCGATGTCCAGTGAGTTTGGGAAAAGCCGCATCTATACACCCCTCTTGAAGCAGCAGAAT
EMPV1_22848 CGTATATATGGCTTTTTTATCAACACTGGGACAGAGGTGAGCAGAAGTGACCGCGGGGCA
EMPV1_22850 GGATTGTGTGTCTGTGGCATCTGTCTCACGGGGTTGCCAAGGGGGATAAAAATCAGTCTA
EMPV1_22851 GGACCCTCTTTCCTTTTGTTCTTTCTCAAAACCTGATACATGGTTCACCCAAAGTACCGG
EMPV1_22853 GTCCTGCACTGAATACGTATTAGATATTTTCTTTGAATTAATGCTTTTGAGATGATTTCC
EMPV1_22854 AGAAGCTGCACTGTTCTCCTTTTTCAGGAAAGACTCACGCACCGTGTCTCCAGTCCAACC
EMPV1_22855 AGAGGTCCAAAGTGTGAAGATCATTTCTTCCTAACGCCCACCACAATGCATCTACCATGG
EMPV1_22856 CAGGCTGAGTCAGGTCTCTGCTCCAGATGTAGTTTCCCTCTATCATTAAACATCCTCCAC
EMPV1_22857 AGACCAATCTGACTTTCGTTGGCTGTGTGGGCATGCTGGACCCTCCAAGAATCGAAGTGG
EMPV1_22858 GACAGGACTATGCCTGCTTGCTTCTCTGAGATTTCCTGGGTAAAGGATCTAAAGTGTGTG
EMPV1_22860 ATGCTTCAATTAGTTTTAGGAGCAGGCTGACATGGGAGTGGGGAGCAGGAGCTCAAAAGG
EMPV1_22863 TTTTGCCAAAGACCTTGAAATGTTTGCCAGACACGCAAAAAGGAGCACAATTAACACTGA
EMPV1_22864 CCACCGTGCGTTCCCAGGAGGTGGAGGATGAAGAAGCTCCAGATTACGAGAATCTGCAGC
EMPV1_22866 ATTTCCAGGTGCTTGAAATCGCCCGGAAGTTGGAAATGTACGGCCTCCGATTTCACGTGG
EMPV1_22869 GCAGTGCAGCCGAGGTGGCCTGTCCGCCTTGTCGTCGACCGTCTCTGTTTCCTCATTACA
EMPV1_22872 CCTGTCCCCAGAAGAGCCACTGGTTCTGCCCGCCGGTGATCTACAGCTCCTGCACTTCTA
EMPV1_22873 TCTCGGGGGGCTGCTCTTCCACAGTCTCATACTCGAGGCCACTTTATTGACTCCCTTTTA
EMPV1_22875 GTGGCCAGCGGGATTGTCTTCTGTGGAATCGTTGGACACTCTGTGAGGCATGAGTACACA
EMPV1_22876 AGATCCTTGTCCTTCAAGACCTTCCTTGTCTGGGTTTTGATCAGCGTTTACCAAGGCGGC

EMPV1_22877	ATTGAACCTGCATCCTCATGGAGACTGTGTTGGGTTCTTAACCTGCTGAACCTCCATGGT
EMPV1_22878	CTGGGACGGTGGCAGAGAGAACATGAGGCTTCAGTAAAACAAGAAAAGGAAGGGATGTGG
EMPV1_22879	GAGAAAACGCAGGACCCACACCCATGAACATCAATCCTCAGCAGACCCGTTTCCCTGATT
EMPV1_22882	AGCCAGTTGCCTCCACTGCGACCACCTTTTGATCGAATCGGCGCTCTTCCACGACCTAAT
EMPV1_22883	GCCCTTGCATTAGTACAGTGTAATTTCTATTGCCCTTGCCCCTCCCCCCTTTCCTATTTT
EMPV1_22886	AGGAGCTCATGTTAGAGGGGGATCTGCTTGAGGTGACCCTGGATGAGAACCACAGCATCT
EMPV1_22887	TCTCAGATCTATTCATGGGCCTGCGGACCTCCCTGCGAAGGACCCCCAAGCGGAAAGAGG
EMPV1_22888	CTCTCCAATCTAACTTAGGACTCCAGTGTAGTTTTTCTCCCAGAACTCTGGTCTTCCCTC
EMPV1_22889	GTCGGAAGAAGTCGGTGAAAATAAAAACTACAAGTCCAACACCATGCTGTTATGTCACAG
EMPV1_22890	ATCAATTCTTCCCATTAGTCAAGAGCTGGCGTCGCCCTCACCCCTCCAAGCGGCTTTCTC
EMPV1_22891	GCCACGACTCTGCTGGCATTTCTTCTATAGTCACTGGAATCTGATCCTCATCGTCTTCCA
EMPV1_22893	GTAACCGGGGCAGCCGGGCCTACTGGATCCGACCGGAGGACCCCTTCTGCGACCTCCCTT
EMPV1_22894	GGAGGTGGCAATTTTGGAGGTAGCCCTGGTTATGGAGGAGGAAGAGGAGGATATGGTGGT
EMPV1_22895	AAGTGCCTCATGTGCCTCAGCTCAATGAACCATCACCATACCCTACAGGGAGATGTTATC
EMPV1_22899	GAAAGGAACCTTATCCAGAAATCCATTGCCTTAACTGGTGCCCAAGGGTGCCCTGGAGAG
EMPV1_22900	TAGGGCTCAGCAGGTTAAGGATCTAGTGTTACTGCTGCGGTCCAGGTCACTGTAGTGGCA
EMPV1_22907	AAAAAAGCCACTGGTGGCAACCGCTTCTCCGAACCTACTCGCAGCCAGTGCCTCCAAGGA
EMPV1_22908	TGGCACGAAGCCCACATGCTGTCCACCTCAAGAAGTTGGACCTGAGTGGCAACGATTTAT
EMPV1_22909	GAGTCTATATACACCCTGATTCTCTAGCTTCTGGAGATACTTGGATGAGACAGGTGGTCA
EMPV1_22910	GATGTCGTCTGTGTCAGGAACAGCACACCAAGCCAGTTCAAGAGGAAAACCAGGCCAATA
EMPV1_22915	CAGGAACTCCTGTTGTAGGGGGACTAACCTATCGAGAAGGCATGTATATTACTGAGGAAA
EMPV1_22917	CTACTGGAGTCTTGGGTTCCAGCTTAGTCGTAGGGATTATGGTGGCATCGACGGGTTGAA
EMPV1_22918	AACCACGAAGAGCACGTCGACAACTTGCTGGCTTTGATCCCAATGACAAGCAAGTTGGAC
EMPV1_22920	CATGAGGAATAGGTTTTTATCAGTTTACAAACAGTGTGAATGCTGGACCTGCTCTTTACA
EMPV1_22924	GGACTATTGAGTGTGCCTAAATGAATTCTGCAGTAACTGGTATCCTTGGTTTTGTGCTAC
EMPV1_22926	GCAGACACTCTCTCCAGGGAACCTGTTAGTCTGGTGACGATCTGCTTAAGACAAGAAGCC
EMPV1_22928	TTTGCAGTAGTTCAGAGGATGAAGTCTCTACCGAGAATTTGTCTCCTCCAAAAAGGAGGC
EMPV1_22929	GATATAGAGTTCAAGATTACTTATACCCGGTCTCCAGATGGTGATGGCGTTGGAAACAGC
EMPV1_22931	TCTAAACCTATGTGGAGGAACACCTGATGTCCAGGAAAATCCCCTGAGATGGGCGCTGGT
EMPV1_22932	GCGCTCCCGGCGCCTGCTCTCCGGCCGGCAGCCGGCTCGGCGGTCTCCGGCTCCGGCCCC
EMPV1_22933	TTTTGGAGACGGTGGAGCTTCAGATCAGCCTGAAGAACTATGACCCACAGAAGGACAAAC
EMPV1_22936	GTTCTGCGGTAGACTGGTGGGCACTTGGAGTTTGCTTGTTTGAGTTTCTAACCGGAATTC
EMPV1_22937	CTGTGGCCATTCTGAAACGCCAGCTGGAAGAGAAGGAGAAGCTGCTGGCCACGGAGCAGG
EMPV1_22939	GCTCTTCTTCCAGCACTTGCCTGGGCATGGAATTTTCTCTTAGGAATATAGGAGATTTTC

EMPV1_22940   CGACTATAAAGTTGCAGACCCTCTATTTCCATGTACAGCACACTGCATTACAGTAAACCG
EMPV1_22941   GTTCTCTTTCTATGATTGATTACCTCATCTGGCCCTGGTTTGAAAGGCTGGAAGCCCTGG
EMPV1_22945   ACCACTGCCTCTCGTGTCTACAAGTCCACGAACGCACTTTCAACGGCATCTTCCTCGTGG
EMPV1_22946   CCAAAAACCTGAAGGTGTAATAACTTTGGACAAGCCGGTGGTATCCATGATTTCAGGCCC
EMPV1_22947   ACTTACTGACCTAAGCAAGGTCCACCAGCATGTGCCTGTCCTCATGGATTCTGTGAATGC
EMPV1_22948   GTGTTCGTGTGCGACGAGGACGCCACCCTGGAGCTGAAAGTGAAAACCGTCAAGTATTTC
EMPV1_22950   CCGTTTTAACCGTCTAAGCACCCCAACACATACCTGATGAGCCTGAGATACTAAAGGGGC
EMPV1_22952   TGACGCAACCCACGTGTAACTGTCAGCCGGGCCCTGAGTAATCGCTTAAAGATGTTCCTA
EMPV1_22954   CCAACCCTGTTTCTTTCTACTTCCCAGTCTGGCCTGTCCCCACAGTGCTTGAATGTCACA
EMPV1_22955   GCGTAGAGAAAATGGAAGGGACGCCTGATGCTGAAGATTAAAGATGTCTGTGGAGACAAC
EMPV1_22956   CTCTGCTTGTGGCGGAAACAGGGCCAGAGGACAATGGGAACAACAGCTCGTTTATTAAGC
EMPV1_22957   TACCTCAACATGCTCTACATGCTATAGTTCACACCTGCTCCCTTGTATTTGCTCCTGGCC
EMPV1_22958   CTCACCGGCATTATTCAAGCCATGGTGGATGGTCAGCCAAGCCTGCAGCAAGTGCTGGAG
EMPV1_22962   AGGAGGTGGTTTTGGTGGAAATGACAACTTTGGTTGTGGGGGAAACTTCAGTGGTCCAGG
EMPV1_22965   CTGCAGGACAAAGTGAACAAGACTTCCAAGGAAAGACAACAACATGTGGAAGCTATTGAG
EMPV1_22967   CATTGAGAATGAGGATGGCACCTATGACATTTTCTACACGGCCGCCAAGCCAGGCACCTA
EMPV1_22968   CCCAGATTGCAAATGCATCAGTCAGTAACATCACTGTCTGTATGTGGAAGACATGTTCCC
EMPV1_22970   GCTATTGCCGCTTTATTTTCAATATGATGTGCCTGTAAACCATGGATTTTTCTCCCTTTG
EMPV1_22971   TTGGTGTTGCTGTGACTGTGGAGTAGGCTGGCAGCCGCAGCTCCTATTTGATCCCTAGCC
EMPV1_22973   ACTGGGAGTTCTTTAGATGCCTGAGGAGCAGTGGACCCCAAGGGACATGTGACTGAAACT
EMPV1_22975   ATCACTTAATCGAATCGGCGTGGCGCTTGTTGAGCAGCAACCGGCGTACGCAAGGAGCTT
EMPV1_22977   CAGCCACCACCTATTTTTTGGCCTTTTATGGTTGGAATTGTCATGGCACCTGTGGGCATG
EMPV1_22978   CTGGGAGGACACCAAGAGGAAAAGGGAAATTATATGTGGGGAGGCACTTGCCCTTGGGAT
EMPV1_22980   GTTGCTGCTGATGGTTGCTCTGCTGGCTTCTTACTCAGTTCATCTCCTGCTTAGTATGTG
EMPV1_22982   ACAGTAAGACTAACCATCTTAGGACATACATCTACATCTCTTGAGGGTTCCGCAACATCG
EMPV1_22983   AGCCCAGAAGAAACACGGGAGGTCTGTCAGCAACAGCGAGCAAGGCTTGCCCTGGCCTTG
EMPV1_22984   AGGCCAGTCTCTACAAAACCTGATCTATTATGCTACCAACACAGCCTCTGGGGTCCCTGA
EMPV1_22985   TCACAGTCAGGCCCTAGTTGAGGATAACAGGATGCTCTGTGATGACTCTTCCTACTGCTG
EMPV1_22986   CTGCAGTGACAGTCGCCTGGTCCATAGAGTGGGCAGAGCACCAAAGGACAGCATTCCATT
EMPV1_22987   TCCTTAGTCCTCACCCCTTTCTGCTTCGGTGGCTGCCCTACTGCAGAACCAATGTCATTT
EMPV1_22988   CCTACGTGGAAAGCAGGCGTCTCTGTGGGTGATTCTGGGACCAAGTTCTGGTCTTATTTT
EMPV1_22989   AGACGTGAGTTGTCACATGGTTTGGCTGCTGCCAGGCTGTCCGGAGGCCTCTGCAGGCGG
EMPV1_22991   CCCCAGGCTCTTGGGTACTCTTAGCATGTTGTTATTGGTTTTCAAGGGTGTTAAATGGCA
EMPV1_22994   CAGACCTGTCTGGGGATGAGCCCAAGTTCCCAGCTGTTGGCCCCTTGATGGTATGATGGGT

EMPV1_22996	AGAACACTGGGCTCCTGGTCCTCTAGGACACTGTAAAGCTATTTGAATGAGTGGGCTTGC
EMPV1_22997	AGTACTTGTTGTCCTCCTCCCTCAGCAGCTTGGACAAGATGAGCTGGTGCTGCTCGTTCA
EMPV1_23002	CCTTGCTCAGACTCACGCTCTGGCCCATCATCTGGTTTAAACAGCTGGCTGATGGCCACA
EMPV1_23003	TACACCACAGCCACAGCAATGCCAGATCCGAGCCATGTCTTCAACCTACACAACTGGTGG
EMPV1_23004	AAGTCTGGGGACAATGGGTGAGCCCTTTCGCATCTGTTCTCTCCCTTTCTAATATTTGGA
EMPV1_23006	CGCTCCCCCAACTGGCCTGAGGAGAAGCCACCCAGCTACCGCTCTCTGGATGTCACCTCA
EMPV1_23008	CAGATGACACTGGGGGATCCCCTCTCCCCCCACCTCGAGCACTTCATACCTGAGTCCAGT
EMPV1_23010	CAAGAGCCATTCTGGTACAGATGTTACTGATACTTGAGCTCTGGTTCCTCACCTAGCTGG
EMPV1_23012	TCTGCCAGCCCTGCAGTACACCCTCAAGCACTACCTGCTGTACCTGAGCCGGGTGCAGGA
EMPV1_23013	TTTCCTCTTCGCTGATGGTGTGTTCATCTTAGTTGGGCCCCTTTCCCTGATGCTGGTCTC
EMPV1_23014	AAAGCTCGGAGAGTTCCTGCACATGGACATCCTGTCATAGAAAGGCTTGTTACCTATCGA
EMPV1_23015	CAACGGAAGCAACTTGGAAGAAAAAGTCTCACAAGAGCTCCACTTGAGTCCTTTCTTCAG
EMPV1_23016	AGCAACATGGCCTATGGGACCTTCAACTTCCTCGGGGGCCGGCTGATGATCCCTAATACA
EMPV1_23018	TGTGTGTGTGTGTGCGCGCGCGCGCGCAGGCATGTGCGCGCACCCGCACGTTCCTGCCCA
EMPV1_23020	CCAGCGTGCTACCCAGTTTCTCCTGGGTTTTCTGCTGGGAAGCGATGAAGACTAATGATA
EMPV1_23021	CTGAAGATACTGAACCACGCAACAGTGGGAGGAGACCTGGAGGAGAAGGTGGATCTCATA
EMPV1_23023	TATTGGAACATAGCAATCCTTCATGTAGATCTGGCCCGTGGCTGCTTTCCTGCCACTGTG
EMPV1_23024	GACCACATGTCCGTGGCCAAGTTATCCTGTGGCAACATCAAAGTCAATGTTATCTATGGC
EMPV1_23025	GAATTCCCTAATCACCCAGGTTTGAAGGGGCTGGGCCACAGGATAAGGTTGACACTCTAG
EMPV1_23027	ATCCTCAGGTCTGTGCTCAAATGGCTGTCACTGTCTGGATCATCGGTTTTCTCTATGCCC
EMPV1_23029	CAGGTAACGCAGGAAGAAGGACAGCAGTTGGCACGGCAGCTTAAGGTAACATACATGGAG
EMPV1_23030	GTCCACAAGAAGCTGCTGTTCCCTAGTTTTTGGATCTTATTTCACTGGCTTCTTGGACTC
EMPV1_23031	TATTGCTGTAATTTATTTGAGACTAGGTGGCTGGTGGGCAGTGCTGGGGTCAGGGTAGGG
EMPV1_23033	TCCAACTTCAAGGCAGATTATGGACGCTTGTCAACTAGTGTGGACGGGGTGTTTGCTGCA
EMPV1_23034	ATCCAGGCCTATGAGGACATGGTGACACAGGTCCAGCGCTCACAGGCCATCGTGGTGGTG
EMPV1_23035	GAATTACTGATCATCACGCAGATGAAGGAAGTGAAGCGCAGAGAACGTCAGGGACTTGGC
EMPV1_23036	CTTGATAATACTGCTGATGCTGGTGGTGATACCAGTGGTGTGAGCTGGAAATGGGGTGCT
EMPV1_23041	TACACAGAGAAGCAGCCAGAATCATTTTTTGTGACGGTAGCCACCATTCAAGTGAATTTG
EMPV1_23044	CAACATCAGGAAGCCTGGCGACTGTATCAAATCATCCCAAATAGCACCATCAAGATCAAG
EMPV1_23045	GGGCAATACTTCTCCTAGCGCCAAGGACATCAAGAAGATCCTGGACAGCGTGGGCATCGA
EMPV1_23046	TATCTGCAGAGAAGGAATGGAGTTATTCAGAAGAAGAGGAAAGAGTATCTGTTTCAGTTT
EMPV1_23047	CTCAACAATGTCTTCAGGAATTCAGGCTCCTCCCCTCTCTCTGCTCAGCCATTCTTCGAA
EMPV1_23048	TCCTGGAGCAGGCGCTGGTGATCGAGGAGCAGCTGCGGCGGGCGGCCTACCTGAACCTAT
EMPV1_23050	GACCCTAAAACTGGCAACATTACTTTGGAGGAGGCGTTGCTGCAGACAGATTATGGGCTC

EMPV1_23051	CCTCTTTACCTGTTCCCTGAGAGAACTACTATTCATGGATATGAGGTCATGGAAGATCAG
EMPV1_23052	GGCTCACAGAGACTCACCTACAGGAATGACAGTTTTGAGACCCACATCAAACCCTCATAT
EMPV1_23056	CGATGCCTGATGAAAATGGCCACATTCCTGAAATGATAGGTTGGGTCCCAGTGGAGAAAA
EMPV1_23057	ATCAGGTAACAGGTAGATGGACCTCAGTATTACTGTGGACCTTGCAGTAGCCCATGACGC
EMPV1_23059	TGCCGTGTGAGCAACTGGGGAGAGATGATTTTGGAGAGAGGCAATGTCAGGTTCTTAACC
EMPV1_23060	AAATGTGAAAGGAGACGCGGCTAGCAGAGGGGGTCCTAGGCAATGAGTCGGCGAAGAAAA
EMPV1_23063	CACCCAAGTCTGAGCAATGGAAATGGAATCCACCACGGGGCCAAACATGTATCTGCCGAT
EMPV1_23064	CTGATGCTGCCTCATTGATTGGAGAGGAACTTCAAGTAGATTTCCTGGATCACGTTCCCC
EMPV1_23067	GACAGCGAGCTCATCATCGAGTCGTGTGATGCCTCCTCTGCCTCGGAAGAGAGTCCCAAA
EMPV1_23068	CCCACATTGCCCATTAAGTTTGGGCTTATGTGTTTTGGGTTTTGGTTTTTGCACTCTGGC
EMPV1_23069	TAGCAGGAAAACATAGAATAGTGGTTTCTATGGGATGAGGGAAGTGGGAAATGGGGAGTT
EMPV1_23070	TTATCTTTCTATCCATTGAGAGTTTTTCTTCTATGTGTTGAATGTTTCCAAAGGCCATTT
EMPV1_23072	TCATTGAAGGGTCCAGGAGATGTACTGACTTCTGTCTTGGCTGGTTCGGGTTGCTCAAAT
EMPV1_23076	ACCACCCTCCCGCGGGCTTGAACGACCCCTCGCTGGGCACCCACTGCTACGTGCGCATCA
EMPV1_23078	GGTTTTTGACAAATGCTGACATTTTACAGTGCAAACAGGACACTCCGCTTAGGGGCCAGG
EMPV1_23079	CAGAGCGGCAGTCCGCGGGCAGGGATGGGCTCCGACCAGGAGGACAGCAAGCCCATCACA
EMPV1_23080	ATGCACCGTTACTGACCCAGAACCCTTGGATGGCTTCCTGCAGAACCTAAGCCAGATCCC
EMPV1_23081	GAGGCTGTGCGCACACTCAACACCCTGCAGACCAATGCCAACTACCTGGAGCAGGTGAAG
EMPV1_23087	TGTCATCCTGTGCTCATGCAGAAGCTGTGGCTCAAAGAATGAGGTGCCCCCAAAGTCTCA
EMPV1_23089	GGGCAATACTAGCCCAGGGAATTTTTGCTGACTCTTTCTTCTGCTTGAAAAACTCTGCCC
EMPV1_23090	GCTGGCTTTCTGCTTACCTTTCCACCAGTGGTCTTGGGATTGAAACTGGAATTTTGTGCT
EMPV1_23093	GGGATGTAAGCTTCTTTATGTATTCTGACCGTGAGTCCTGTGGCAGATATATCGATTGCC
EMPV1_23094	ATGGCCACCAATACTGCTGAAGGCCCAGTCTGCCCATAGCACAGAGTAATGCTGAACAAT
EMPV1_23095	GGTGATCCTCTTGGATGGTGGTTCTCAAACTGGGTTGTTCATCAGTATCAGCTGGAGGGA
EMPV1_23097	TGGTGGCAGAGGTAGCGGTGGGAGGGGTGACCCTGATTATAGAGAACATCTCTCCTAGTA
EMPV1_23099	GAAGCAGTTCATCCTGGACTTCAATCAAACGCTGCCGTTTCTTTTCGATATCTGAAATCA
EMPV1_23101	ATAAACAGTGCTGCACCAGCCCCATTTCTTCCAGTCTACATGGTCAAATGGAATCGCCGG
EMPV1_23103	TTCTGCTTCAAAAGAGAGAGCAGCACATCACCGCCGGACCATGTCTGATGGACGTGCTTT
EMPV1_23109	GCACAGCCAGCAACCCTGTCAGCAACAACTCCTGGCAATTTCTTTCTGGAAACATTTGTC
EMPV1_23110	GGTTTGTGGCAACCCTGTGTTGAGCAAGTCTAGTGGCGCCAATTTTTCCAACAGCAGTTG
EMPV1_23112	TTATTCTCATTCAGCAGTGACTTGTGACTGTCTCCTTGTACTGGGCACCATGGGAGCTGC
EMPV1_23113	CATGATGAGTACAATCGCTACGAGATGCTCAAGGAACATGAGAGAGGGCGATATCAGGAG
EMPV1_23114	GGAAGAGAGGCAGGGAATGCAAGGTGAGTGTACAAAAATGCAATATGTTGTCCAATGTGC
EMPV1_23118	GCATCTGGATGCTGTAGCTAATAGAGTAGGATTAATATGCTTAGACGCCTCACCTCCTAT

EMPV1_23125	CTCTTACCCACTTGGTTCTTTGGACAAGCCTTTGCTGATCCTGGTCCCCTGCCAAGTGCC
EMPV1_23131	CGGCCCTGTTTTATCCTCCTTCATTGTAGGATGAAGCATTTCACAGTGTGTGAACTTCTC
EMPV1_23133	TCCAACACAAATCCCAGTGATGCTTAGGGCAGTGAAAATGATTTTACCCTAGGCTTCTGC
EMPV1_23134	GTGCACCTGGCAATAATTTGTATGTAGGGTGTGTGTGATGGGTATGTCATGACATGATCC
EMPV1_23136	GAATGAGAGCACCACAAATTCTGATCAAGGGAAAGCTCGTCCAAAGCTCAGCGTGACTGC
EMPV1_23137	GCCGCAGATTGATAATATTGGCTCCGTCTCTCCTTGGAAGCCTGTCTGGACTCGTGATTT
EMPV1_23139	ACGTGTGAGGGATCTGGCAGAGCCAGCAATAGGGGAACGGAAACCAGTGCCAGTAGAAGA
EMPV1_23140	GCTGGGCTAGGCCCCAAAGGTGAACAGCTGGATAATGAGGTAACTTTGACCTGCTCACAT
EMPV1_23142	GATTATAACCTTGTTAACTATGCATAGGCCTAAGAAAGGTGGCACATAAACTGTGCATGT
EMPV1_23147	GAAGTTCTACCCCAAGGGCATATGGCTGACCTGGCTGAAGAATGGAAATGTGTCTCGGAT
EMPV1_23148	TTCTCAACATTCCCGAGGGGATGAGCAGGGAAGGCCTACATAGCAATCTTTAAGTCCAGG
EMPV1_23150	CTCTACCTGAAAGAGAAGAAATACAGCGACTGTGCCTGGGAGACTGTCAGAGTGGAAATC
EMPV1_23151	GGCGAAGTAACGTTCCGGGCTGTTGAAGGAGCGTTCATCCTTAAGAAAACTCCAGCCTGT
EMPV1_23154	GGACATTTTGCACAACATTGACCCCATTTATACCAAGGCCAAGAATGGCTCCTGGGCAGC
EMPV1_23156	TCCAAGCCATGCTCTCAGGAAGACAGTTTAGCATAGACCCAGATCTCCTGGTTGATGTAG
EMPV1_23157	ATGTGGCATACCTCTACGAATCCCTAAGTGAGAAGCAAGGCTTGACAAGGGACTCCTCTG
EMPV1_23159	GACTAGCAATGTGTGCAAGAAAATTAGGAAGAATAAGAGAAGCAGTAAAAATAATGAGAG
EMPV1_23160	GAGATTGTCCTCTGAGGAAAAGAGGCAACCCTAATGCATTGCACAGGTTTGAGGGGGCAT
EMPV1_23161	TTCTGGGACCTGAGCTACAGATACAGCTCTTCTTGTGGTGATGGCAGAGGCATAAGAGAC
EMPV1_23162	GCAGCAATGACAACAAGACCTTTGACTCTTCCTGCCACTTCTTTGCCACCAAGTGCACAC
EMPV1_23165	ATTCCCATTAATATTTCCTTTAATGCCTGTTATATTTGTTGTATATATTTGGTCTGTTCC
EMPV1_23167	CATGTTGCAGTTTCCTCAAGCTGATTTCGCAGTGGTCATCAAAGTCATTCTCAGGGCAAG
EMPV1_23168	CCGGCAACTCACAGAGACAAGACGGTGTTTCCAAGCCGACCACCTTGTAACTCAATCCTT
EMPV1_23170	ACGTCGTCCAGAGAGAACTGCGAGCCTTGGGCGAAGATGCTGCCGTTCATGGCTATCAGG
EMPV1_23171	ACACCTCGTTGCTAGCTCAGTAGGCCTGGACACAAGGAATTGGACTTCCAGAGTACGCAA
EMPV1_23174	CATGTGCACAGAGTTCTCATAGAAAGAAGACCTAAGAAGTGGCCTGAGCAGACAGCTTTT
EMPV1_23175	AATTCCAGGGCAGGACTCCCAGCACTTCTACCCACTGCATCTTGGTATCAGCTTCACCAA
EMPV1_23176	CTGTAAGCTCCTTATGGGCAGGATCTGTGTGTGAGTCATCTTTGTATCTCCCATAGCCGC
EMPV1_23177	TGCCTTTCAGGCTGACGTGTCCGAGGCAGAGACCGCCAGGCGCCTGCTGGAGCAAGTGCA
EMPV1_23178	CAGGCGCTCGCCGGGAGTTAATGTTGTTATGTACTTCTTTTTGTGTGAAGAGGAGCTGGC
EMPV1_23181	GGTCACCTCACATTCCAGGCACTGTCTTTCCATTTAACTCCTGATCCTTGGTTTCCTCCT
EMPV1_23184	GCAGCAAGCAGCACAGGATACAATTCGCCAACAAGAAGTGACAGAAAAGGAACAGCAGCA
EMPV1_23187	CATGGACTCATCTCTGCTGACCGCTATTCCCTGTTCCTCGTCTGTGAAGACAGCTCCAAT
EMPV1_23188	GGGATATCATTTTCAATGCCCAATATCCAGAGCTGCCTCCCGATTTTATCTTCGGAGAAG

EMPV1_23190	GGGAATTAATAGCCAAGGAGAAAGCCCAAGGGGGCAGGGAGATGAACTTGTAATTTCCAG
EMPV1_23192	CACATTTCCATCAGCTCCACGTTCACTTGCTCTCACTGTGGTTTTAACTTGTACTTCACC
EMPV1_23193	CCCAAATAAACTTTAGGGGTATTGGGTGGTCTTTGGGAAAGGTGAATTGTCATGCCGGAG
EMPV1_23194	GTAGCAGGTTTGTTGGCAGATGCTCGTTCTTTAGCAGACATTGCAAGAGAAGAGGCTTCC
EMPV1_23196	GAACTTTGAGGTGGCCTTCTCATCTGCCGAGACCCATGCGGACTGCCCGCAGCTCCTGGA
EMPV1_23197	CGCAGGAAATTTCAGTCTGCGGAGGAGACGGGGACAGAACACACAGTCACACTGACAGTT
EMPV1_23200	TTGAGTGGCACCCCCAGGACAGCGGGGTCTTCGCGGCCTCGGGAGGAGACTACCACATCA
EMPV1_23201	CTGAGTAGCAGAGAGTAGCTGAAGGACAAGATAGTTTTCTTCTAAGCATCAAAGCTTCAG
EMPV1_23204	TTACTGAGCCTCTAGAACACACCAGGCCCCAAACCGAGTCCAGCCAGGTCCATAGGTATA
EMPV1_23207	CTCTTAAATTTTGCCCTCGATCAGGTGCCTCCCAGTGCAGTCTCTTCACAGCTGTTTGCT
EMPV1_23208	AAAAAACACAGTGGAGGAGTTCCTGCCGTGGCACTTTGTGGTTGAGGACTGGTGTTACGG
EMPV1_23209	ATCCAGGACAAGGCCAGGCTATAAGAAATGGAGTCAACAGGAACTCGGCCATCATTGGAG
EMPV1_23211	GCTGTGGTTCTTCCCATGTGGATGCCTGACTTTTTCAAAGGGATTAGAAGGCCGTGGAAG
EMPV1_23213	TGAAGAAAGTGCTGATTGTGGGGGCACCCATTTGGTTCCGAGTGCCCTACTCCATCATCA
EMPV1_23214	TACATCATGGAGCTGACCTTCAATCAAGCCGCCAAGGGCGTCAACAAGGAGTTCACGGTG
EMPV1_23216	AGATCAAGACCTTGATTTTGCCAGATATTTTCCATAATGCGAATAATCTGAAGTGTTAGC
EMPV1_23217	ATCCTCAGGTCTGTGCTCAAATGGCTGTCACTGTCTGGATCATCGGTTTTCTCTATGCCC
EMPV1_23218	AAACTCCAAGCAACACGGAGCTGGTGGCGGACAAGCCCAATGATCTATACGAGGAACACA
EMPV1_23219	TTCCTCTGAAAGAGCCAGGTGTCCCCTCCTGTCACCTGAATCCGTCCCACCCCATCAGAT
EMPV1_23221	AGTTCGAGGACTCCCGCGACGCCGACGACGCCGTTTACGAGCTGAACGGCAAGGAGCTCT
EMPV1_23223	ACTGTGGCACAATGGGATCTACAGCATCTCTGTAGTACTAGGGCACAGGCTGATCCCCCA
EMPV1_23224	ATCTCAACACCTCCCTGCCTGTCACTGCCCCCATCCAGTTCCTCCCCAAGCCCTGACAAT
EMPV1_23228	ACTTTACAGGCCCTGGTGGAGTTGGAGTGCAAGGCAGTAAATACGCAGCAGACTATAACT
EMPV1_23229	GTCCATGTACATCTTCCTTCATAGTATATTCAGAACAACAAATGGTGCTCCTCACCAATG
EMPV1_23230	TGCTCTTGTAGGAAAGCATAGGCACCCTTGGGTCTATTTGTGTGTGGAACCAGTTCTGCT
EMPV1_23231	TATCCTCGCACTTGTGGAAAACTTTCAGCCATGGCCCTGAAGCGACTGGACTATGCTCGT
EMPV1_23232	CTGTTAATCAGGACATTGCTATATTGCCCCATGGAGTCACATTCCAGGGCTGTATGCCCA
EMPV1_23233	GAAGAAGAAATGATTAAAAAAATGGATGCGCTGGAACTACAGCTGGCCAAGAAGGAGGAG
EMPV1_23235	ATTAAGGTGATCCGCCATATGTTATCACTCTTGAACTTTATCATCTGTGAAAATGGGAGG
EMPV1_23236	TACAGGTGTGTGGTAGTAGTTTAATTTGCAGCATTAGCTCTGTGGTGTCAGGCTCTAGAG
EMPV1_23237	CTCACACTTGCTCAGACACACAGAAAAGCCGAGTCTGGGCATCGGAAGAACTGCCACTCT
EMPV1_23238	AATCTGTTTTCCTCACTTCTTTCCGGTCCTGGCCACCCTCGCAGTCAGGTGTGTCTGTGA
EMPV1_23239	GGCTGACAACTTGGTTTCCTTTGAATTTCCTTCCCCACCCTGACGCTTTGGTAGCCATGA
EMPV1_23240	TTCAGATTCTTATTCTGTAGGCTTGAGATTCTGCATTTCTAATAAGCTCCCAGCAGATGC

EMPV1_23243	TGTGAAGTCAGTAGTGAGGTACCTAATTGTACACCAGAGAGTTCATAATGGAGCAAGGCC
EMPV1_23246	GAAGAACCGGAGTCTATTTCTGGTTGGCATAAAGCACCTGCAACTGTACTTGAGTCTGCC
EMPV1_23247	GGATTCCGCTATAAAACCAAAACATCACTAGCCCACTTTCTACCACAAGGAACACCCGCC
EMPV1_23251	TTCTTTCCTGTGACCTTCTTCCCTGTTGTCTTGAACCATAGCAAAAGGATACTGCATCTC
EMPV1_23252	CACTGAGAGAGGACAACTGTAAGGACGCAATTAGTGGCAGAAGTAGACTTGGGCTGTAGC
EMPV1_23254	GAAACATGCCTTAGTTCTGTGTCATCTCTTACAGGCAAGCTGTTGGGACACCTGCTTTGG
EMPV1_23255	AACCCAAACTACAACAGTGACTACACTGGATCCTTAACGTGCTGAGCCACTGGGAACTCC
EMPV1_23256	TCACGGCACCCTGTCGAAGTCCGATGCCAAAAAGGCTGCCTCCAAGACACTGCCGGAGAA
EMPV1_23258	CAGTGATCCACTAAATCCTCTCAATCCCAGCACTAACTCTACGGGCATGAAAGACGATCC
EMPV1_23260	AAACAAGAGTGCAAATTCAAGAGTCTGTGCGGGGGAAGGATGTTTTCATCATCCAGACTG
EMPV1_23261	GTCACGGGTATTGGTGCTGTGTAACAAACAGTTGTAACTAGCATGCGGTTCAGAAACACA
EMPV1_23263	CTGGCTCAGTCCTTTATCTTGTTGAAACCTGTGTTTCACTGCCTGAATGAAATCTTAGAC
EMPV1_23264	GGCAAGTTCCCTTCCTTGCTGACCCACAATGAGAATATTGTGGCTAGAGTTGATGAAGTC
EMPV1_23270	GCTCAAGAGAAATATGATGCCCCAGGCACTGGAGGAGAAAAGACAAGTCCACTTAGCCAG
EMPV1_23271	GAAGCCAAGGCCTCATGGAGCTTCGTTGTGTGTCACCTTGGCTCCACGTTACAGTTTTTG
EMPV1_23272	TTTCATCCTGGTGACGGTATGGAAACGCTCCTCAGGCAGTTTGTGCAAGGCTCTCTCTAC
EMPV1_23276	CAACATGGCTACTCTAAATAGAACCGCTGCTGGCTTGATGCACACGTTTAATGCCCACGC
EMPV1_23278	AAAGGAGAGTCTGAGTGGAAATTCAAAAACCTCGATGGTTGCCACCATCAGCCCGGCTGC
EMPV1_23279	AATCACCATCCCCGTTAGATATTGAGCACCTGTCTTCCCGTGGCTTCTGCTGCACTAGTC
EMPV1_23280	GTCCAGGCCACTGCTGCTACTCTGTTTTTAGATCGGCCAAGAATGACCTTTGAACTTTGC
EMPV1_23282	GTGACTTCCTCTGGGGATAGGAATTTGGGAATTAGGGTTTGGAAATGAGAGGCTCCCAGG
EMPV1_23283	GATCCAGTATCACCACACGCAGTGGCCAGACATGGGAGTTCCTGAGTATGCCCTCCCGGT
EMPV1_23284	TGACACCCCCCTGAGAACATCCCGGTGGGAGGGAAAGAGAAATCCCGTTTATTCCTCAGG
EMPV1_23287	TAGACCAACAAGAAGGAAACCACCCTGCTCCCTCTCTCTGTTCCCCCCCAAGGTAGAGCT
EMPV1_23288	AAGGGATATGGGGAAATAGGACAGTTGTGTCGGTGCTTGTTTTTCCTGCCGCTCTCCCAC
EMPV1_23290	ATCCTGCCTGGATGCTGCAAAGCTGAGGCTAGGAACCCAGAGACACGTGTGCGCTGGGCA
EMPV1_23292	TTCCCGTCTCCGAGTCTCCTTTTCCTCTCTGTTTGTCTCTGAAGATGTGGCACTTAGCAG
EMPV1_23294	GAGTCTGAGGACAGTAAGCTTCGATTTGAAACTTATCAGTCGATATGGCAGCAGATGAAA
EMPV1_23296	TTTGAAGTGGTCCCCACTGAGGGGCACTCTCCACCAGAGAGCGGTGGCCACACCAGAAAT
EMPV1_23297	GAGACCTCATGATCTTATCCCGTGCCACATCTCCAATACAAAGACATTGCCTCAGCAGTC
EMPV1_23298	GAAACCACCTGGAGAGAATGATTTTGAGACCATCAAGCTCATAAGCAATGGTGCCTACGG
EMPV1_23300	ACCTATGCTGAAGCTGTGGCAACGCTGGATCCTTAACCCACTGTACTGGTCTGGGATTGA
EMPV1_23302	AGTAGCCGCATGGCTGGAGGAGGCCAGAAGGTGTGTTACATTGAGGGTCACAGGGTAATT
EMPV1_23307	GAAGAACTGGGGCTTGCAATAGAGAAATTGAAGGATGGATTCACCCTACAGGGACTTTGG

EMPV1_23309	TCCTTGGATTTCTAGGGAGGTTGGCTGAGCTGCCTTTTGATGCTTGAGAGCACATAACCC
EMPV1_23310	GTTTGTCTTCCCCATCAGACTGTGGGCTGAGAACAGAGATCTGTTTTGCTGGAGTTCTGC
EMPV1_23311	CCTATGCTCTGAGCTAAGGGCTGGAGAATAGCAGGTAAATAAAACTGCTTGAGCTCAACC
EMPV1_23313	AATACAGAATGCCCAGTTAAACTTTAATTTCAAACAGGCAACAATAATTTTTAATATAAG
EMPV1_23314	CTGGTACCTGCGATATAAATTGCTGCTGCGACAAGGACTGCTATCTTCTCCATCCGAGGA
EMPV1_23315	CCGTCGGGTGGTGGCATATACAAACCTCACTTCCGAATGTTAAAGGTTCACACGCCTTTG
EMPV1_23316	AGAGAGCTTTCTGGCAAGAGTAAGGCCAAGTGACCAGTGGGCTCTGACGACTCAGTCTCT
EMPV1_23318	ATTCTGGATGTACTGGACAAACACCTCGTTCCAGCAGCTAACACTGGCGAGTCCAAGGCT
EMPV1_23320	GAGTGTTAAACATCGGAAGTCTGAAGCAAAGAAGGAGTCTGTTTCCAGGAGAAGAAGGCC
EMPV1_23322	TAACCTGGCTCATCAGTGCTCTGGGGGCCCTCCCTGAGAGTTTAACCACATTGCAACTTT
EMPV1_23323	CTTCTCTGGACTACTTGCCAGGCATGTGGACAGACTCCTCCGTGGCCAGCTCAGAACTTT
EMPV1_23324	CAGCCACCTATCCCAAAACAGTCACGAGCTGTCCACCGGCTTCCTGAACTTGGCTGTGTT
EMPV1_23325	TGGGGAGGCAGGAAACCAGCCAGCAGCCGCGTGTACCCAGGGACCTATTCCGTCACCGTG
EMPV1_23326	AAGAAATAAGAGCAGGGCTCCTGGGCACATAGGGTGATGTGACTTGGGTGACCAGCCTGT
EMPV1_23327	CGGCATAGCTTCCAGCTGTGTAAACTGTATTATGAAAGAGTATGTAAAGAGAACCTACAG
EMPV1_23329	TAAGGACCTGAGTAGGGGATAGGGAGGAACCATGAAGGTGTGACCAATGGGGAGAAGCAA
EMPV1_23334	TTACAACAAGTACCCCATAACTTTGAACACTACTGATGTGGACAAGCCCCCTGAGCTGGC
EMPV1_23336	TGAAATGGTAGAAGGCGCAAGTGGTAGGGAAGTAAGGTCTGATGTCAAAGCAGGGTGGAG
EMPV1_23345	AAAATCAAGCTCTCTGTCCTGGTGAAGGAGTAGAGTCTGGAGTTCGTTTTCCTCCAGCCT
EMPV1_23346	GAAATTGGCCTATACCAGGAGAGCGAATCCCAGACGTGGCTGCATTATCCATGGGCTTCT
EMPV1_23348	TCTAGAGCTGAGTCTCAGAGCAGACAAAGAAACCTCCCAAGGGAAACTTTGGCTAAAAAC
EMPV1_23349	CTCCCATGTAGAGGAGGCTGGAATGATACCTCCGATGCTGTGAGCAGAAGCAGCAAGGGA
EMPV1_23350	GGCAGTTGTCGGATCGCGGCGGGTGCGGATGCACTGGCCCTACAGGACTGAACTTATACA
EMPV1_23353	GGACATGTTCCCAAGCCTTTACCTCGACTTTGGGTTCAGTGTAGCAGACGGCTTGTTGGA
EMPV1_23355	TGCTAGATGTGGAGGGCAGGGGGACAAATCAAAGGGCATCAGAGTGGATCATTTCACAGC
EMPV1_23356	TACTTTCATGGTGGGTCCAGAAGATGAAGGAATAAGGCTGAAGTGCACCGGCAAGGGATG
EMPV1_23357	GGAAACCAGAAACATTCTTTTGAGTTTGTTGTTTGTCATTTCCGTCAACGGTCATCAAGC
EMPV1_23361	CACTGTGTGTATATTTACCACCTATCTGTTTTCTGTCCTTCTGAAAGGTAAAATGGAGTG
EMPV1_23362	TTGAAGAACCTCTTTGGCAAATATGGAAAGGTTCTGAGTGCAAAAGTAGTTACAAATGCT
EMPV1_23368	TGAACAGCCACAGGGTTGGGTGTCATGGGCCTGGTCATTTGTGCCTGCAATTGTGAGTTA
EMPV1_23369	GTGTGTCCCACATAGGTGCCATTTTAGCCTTCTACACACCTGTGGTCATCTCCTCAGTCA
EMPV1_23372	GCCTATCATATTTACTGCCTGAATCCACCTTTGGATAAGGTCCCTGAAGAGGAGTACTGG
EMPV1_23373	GAACGCGAGACTATGTCATGTGACGTGGCCCAAGAGACGTTGCAGGTGCCATGAAATTAA
EMPV1_23375	TTGAACATGGGCAAGCAGTGTCCTTGCTAAAAACTTTCCAGAACACAGTAGAACTCATTG

EMPV1_23377	GCCGGGTTTGGTCTGGGTACACATAGTAATTATAGACTGTCATGTAGCCTACGGTCGCAA
EMPV1_23378	CAACACAGGTCATGAGTGCAGCTTGGGTCTGATCCCAGGCCCAGGAACTCCATATGCCAA
EMPV1_23381	ATCCGAGGTCGCAGATACAGCCTGGATCCAATGTTGCTGTGGCTGTGGTGTAGGCCAGCA
EMPV1_23386	GAACTCTTTCAAATGAAGCAGCAACATGGAGAGGCAAACAAAGAAAACATACGGCAAGAC
EMPV1_23387	ATCGATGAACCAATCTTTGCAGGTCCTCTCTCCGACATGCTCCATGAGGCTTTCTCACGG
EMPV1_23389	GGTTTTCTGAATGAAGCTTTCAAGAAAGGAGGCCAGGATGGCTCCGTTGCTCGCGTGATG
EMPV1_23390	CAGAAATTGAGCGTGAGCTCAAGATGTAATGGAGCCTTCTTAGGGGATACCAAAGTTGGC
EMPV1_23397	AGCTCATTTCCGAGCTGTTCAATGACTGTGGCCTTCTGGACTCCTCCAAGCTCTGCGATT
EMPV1_23399	CCCTATAATTCCCATAGAACTCTGGCAATCTCACCCATTTCCCTTCAGCAGAATAAAGAG
EMPV1_23400	CGGTCTCCATCTTCTTTAGCCTGCCGCATCAGAAGCTCTTCGACCACTATATCCGCTTCT
EMPV1_23401	CTTTCCAGTCAGCTACAATGACAAGTTCAACAAGGATGTGCTGGAGGTTGGCGAGCTAGC
EMPV1_23402	CGCAACGTAACTTGTACCCAATTTTGCCGTGGTCCTTCCATTCACTGTCACAGCTTGGAG
EMPV1_23403	GTACCTGCAGCTCCAATTTGATCCCTACTCCAAGAACTTCCATATGCCTCAGGTGCAGCT
EMPV1_23405	CTGGTGCTGGACCTTTCCCACCAATCAGAAACAATAGTCCCTACTCAGTGATACCTCAGC
EMPV1_23407	GCTTAGCAAATCATGGCCCACCTCACAAAGAATCTCTTGCAGTAGCTGGAAAGGCTGATT
EMPV1_23408	TTTAACAACCGCTGGTATCAAATGGGCATCGTCTCATGGGGTGAAGGCTGTGACAGGGAT
EMPV1_23411	GGGGTGAAGGGCATCAGGGCCTGGGGCTTGAGAGAATTGGCTGAATAAAGATTTAAGCAT
EMPV1_23412	GTGGTTTGATTGCAGCTACAGACCCTGCTGTTAGACTAGCATCCATTCTCCTCCTAGATG
EMPV1_23413	AGGAGGTCCTGACCCAGTCACCACTGCTGCCCCAAGAGAGGATAAGAAAGTAGAAGCCAA
EMPV1_23418	ACCCGCTCTGTGTCGATGGACTGTTGGCATCTTTTTAATACCAGGAACCCAGCGGCTCCA
EMPV1_23419	CTGCCAATACTGGAATGGCTCGTGGCACTGTTGATACTAGAAACGCTTTCTGAAGAATGC
EMPV1_23421	AGCTTTAGGACACTAGAAAGGACAGGAAAGGTGAGAGAGAGCCTCTAAGGGGCAGAATGC
EMPV1_23422	TTCCATTCATCATGTAGGTTTGTATTTTACTCAGTAGGATTAGGGAAGAGAAAAGCACTC
EMPV1_23424	TCAGACACTTTTAAATGGTAGGAGATTGTAGCATTCTAGTTGCTGTGAGATAAATGACAG
EMPV1_23425	ATATTGACGAGTGCTCGCTGGAAGAAAAGCGCTGTTCCCGGCAGATTGAGAAGTGCTACA
EMPV1_23428	TGCCATAGGCAAAGGGTGAAATTGTTTCAAATATCCACGAGTAGATGAATGGCTAGAGAA
EMPV1_23431	ACTATATTTGTAATTCGGTTGTGCGCAGCTGGGTAAGTTATGCGCGTCCACGTTGTGCGG
EMPV1_23433	CGGAGATAGAGATGCTGAGGGCTGCTGTGAAGCGATTTGGGGACGATCTTAATCACATCA
EMPV1_23434	GCTTTGTCTTTCTTACGTGAGCAGGTCTAGTGGTGAAGTTTAAATCAGGATGGCACCACC
EMPV1_23435	ACAATCTGGTATGAGAATCTCTCAGGCCTGCGGGAACAGACGGTAGCTATCAAGTGTCTG
EMPV1_23436	CCATAACACACTCAACATTCATCATGTGTAGGTTCTCCAACACTTTCCCCGAAGCAGAGA
EMPV1_23437	AGACACCTGGGCAGAATTGAAGAGATGGGGACGAAGATAGAAAGGGTTACGTTGGGGGTC
EMPV1_23438	GGATTTCCTTAAGGCCTGGACACACAAGGAGCTCTTGCCTGGACATAAGTTTAGGCTGAG
EMPV1_23439	CACCGTTAAGGAGTGAGATGCAATCAAGACAAGGAGCTGTCATCTGAGCCCATCCGAATG

EMPV1_23440	GGCCAAGTTTGTACTATATCAGGAGGCCCTTTCTAGGAGTTCTCTTATAGCACAGCAGGT
EMPV1_23444	CCCTCATGTACCTGCGTTTCTCTGCCACTTTCCCACCAATTTTGGACACAATTATTGCAC
EMPV1_23445	AGCTCCAGGTAGAGAGGCTGCAACATACATATGTGGAGGCCCTGCATGCCTACGTCTCCA
EMPV1_23450	TGGAAGGCCATGAGCAAAGGGGATCTAGCAGTTCATCAAGTGAGGAAGTTGTTGGCAATA
EMPV1_23451	ACTTGGCATAGGTTCTGGGAGGGGTGCTTTGAGGACCTTCAGAGTGGGGAGCATATCTTA
EMPV1_23452	CAAGTAAAACTACACTACCTCATACCTACATGATTTTCAAGTTGTAATCCAGAGGGACAG
EMPV1_23453	TTCCTGCCACACGGTTTTCCTAAGGCTCTGAAAGCATTCTGTCTCTCAGCATCTCGCTCT
EMPV1_23454	AAAGGACTGTGGCTTGCCTTGGTGCTGAGGACAGAGCAGCTAACTCAGCAAAATCTTGGC
EMPV1_23455	TAATTGCTCCCTGCGGGTGAAGAGACCTCTGTTGGATCCCCGCTTCGAGGGTTACAAGCT
EMPV1_23456	ATTTTCTTTTGCAGCATTCCTGAGTATCTTATTTTCTTGATGTTTGTGTGTTATTTGTAT
EMPV1_23457	GCAACATGAAAACTAGAGGTGGAAGCATTGCTACTTGCTTGTGGAGGTGGTGGACATAAG
EMPV1_23459	AGGTTCCAAAATAGGTTCCAGTAACAGCACTTCCCCCACTGTACCATTGAAACCACCGCC
EMPV1_23460	TTGCTGCAAAATGGTATTCAGTGAAATGTCTCCCTTCCACAGGTCAGTTTTCTTCCCTGA
EMPV1_23461	GGGCTTCAGGAGAATAAAGGGGTGAAAGAGTAGCTAAGATGAAGGGCATCATCTAAGAAT
EMPV1_23464	ACCAGTAAATGTACACAGGAACCCTATGAATTCAGTTAACCCTGTGAAAGTTCTCAGTGG
EMPV1_23465	ACTGATGGGTGGCCAGGTGTACTTCTGCTATGACAACTCACCCTACAAGAGCTACGAGGA
EMPV1_23466	GTCTAGAGAAAGCCTTGTCTGCCCACACACACAGACCCCCATCGCAGTTTCCAGACACTG
EMPV1_23467	CCATTCTGCGTGACTGGCATTTCGACACCCGCCACGTGTACATAGGTTCCATATTGTCAC
EMPV1_23468	CTGCAGCTTTTGGCCTACACGACAGCCACAGCAATATAGGATTCAAGACTCATCTGCGAC
EMPV1_23469	CTTTTGGCCCTACAAGATTGACGTGAAGAAAGGCCTGATGGAAGACCCTGGAAGCCATGG
EMPV1_23472	TAGAAATGCCCAGAACACATCTAGAGAAACAGAAAGTAGATCAGTGGTTGTCCAGCCTGG
EMPV1_23474	TGATGGTTGGCCTGCGCTGGTGGAACCACATTGATGAAGATGGAAAAAGCCATTGGGTGT
EMPV1_23476	ATTCAGCACACGGAGGACATGGAGAACGAGATCGACGAGCTGCTGCAGGAGTTCGAGGAG
EMPV1_23477	GCTTGGATCTATGTTAAATGCATGGAATGTGTTGGGAACTCTGATCTGGCGTTTCTGTGG
EMPV1_23478	ATAAGACTCAGTACTAGAAGACCCCCAGCTCACCTTCCAATCCATGCCTCCTCCGCTTCA
EMPV1_23479	GAATGAACGCTGGGCACGGCGCCTGAGTCGGACTGAGAGTGTTCCTTCTGAGAACAGCTT
EMPV1_23480	ATTCTCTTCCTCTGCATGTGAGCACCGAGGATTGGCTATGTGAGGGTAGAACCAGGAAGG
EMPV1_23487	CACCTTGACCTATGCAGATAGCCGACAACCGCACATGGATTTGGAGGTCAGGAATTCAGA
EMPV1_23488	TGCCAATGAAGGGCAATGTTTACCAGGAATGTGGCACTGCTATGAGGCAAAGTCATGAGC
EMPV1_23489	GGGAGAAGAGCACATTAAACCTGTAAATCCTGTTTTATGCATGCCTGATGAAGTACTTCG
EMPV1_23492	AGCTCCCTGTCCCAGACTCAGCCTGTGGGAGTCCTTCAAGGGAGCTCACACACCTGCCCA
EMPV1_23493	ACCCTCATCAATGACATCTTCTTGCTTGTGACAACTGTCCTGCTGGGTGTGTTTCCCCTC
EMPV1_23497	TGCTGGCCTACACCACAGCCACAGCAACATTGGATCCAGGCTGTATCTGCGACCTCGGAT
EMPV1_23498	GGTTAAGAACCTGACATTGCCTCTCTCCAAAATCATCTCTCCCCAGTTGCTCACACGGCA

EMPV1_23501	TATCAATCACCTTCTCACACGAAGAGTCACAGAAAAGGCCCAGGACCGACGTCTGGGTGA
EMPV1_23503	TGGTGTCTCCCCACTAGGACACTACTGGCTTCCTTGCGCCCTGGATCATCTCTGAATTAA
EMPV1_23504	GTTCAGATTTTGGAGATGCCGTCTAAGACGTCAGTGTGCTCCCTGAAGCCTGAGGCAGGG
EMPV1_23505	TGAACGACGAGCTGTCCCACATCAACGCGCGGCTGAACACGGGCATCCTAGGATCCTATG
EMPV1_23506	TGCCGAAAGCCAGAAGCCACCTGAGGTTAAAACAAGCTTACCTGACCCCACAAAATTAGG
EMPV1_23508	TGGGCATAAACTCCTTCGGCTTCGGCGGCTCCAACGTGCACGTCATCCTTCAGCCCAACT
EMPV1_23509	CTTCAAGGTTGGTATCAACTACCAGCCCCCCACCGTAGTGCCCAGGGGTGACCTGGCCAA
EMPV1_23510	TCACTTCAGAAGGGGACAGAGTTCCCCAGGGACTTGCTTTAATAGGTCAGAAGGGGCGCT
EMPV1_23511	AGCACAGTTTAGGTTTGCCGAATGCAGCTTCCCGAAGTGAGTTTTGTGCCGCTGCTGAGA
EMPV1_23514	CAAGGCTCCCTGTATGTCTTGCATTTAGCACACTTGAGTTCGTGTAGTAGGTTGCCAGCG
EMPV1_23515	GGTTCCGCTTTGGAAAGTTCCTGTAAATGCCTTCCTTATATTCTCCGCCCGAGTCACCTG
EMPV1_23516	GCTAAGGAAGCAGGGTCAGGGATCGTTTCCCATCAGTTCCATGTCTCAGTTACTACAGTC
EMPV1_23517	GAACAGCTGTGCCAAAGACCTTACCTCGTTCGCAGCCGAGGCCATTGCCATGAACCGGCA
EMPV1_23519	GGTCTCAGAGCGCATCACTGCAGCTTGAATGAAGAGTCTTTGATAGAGGTTTGGTGGCTG
EMPV1_23520	AGCAACAGTTGTTCCCATTGTGAGCGTGGTAGCCCCAGAGAAGCTGTCAGCCAGCACTAG
EMPV1_23523	CTATGCCCAGGGAAAGAGGTGCTATGATAGGAAACAGAGTGGCTATGGTGGGCAAACAAA
EMPV1_23524	TCACAGAGGAACATATTCGAAATAACCAAGGGAGAGATGCCCGAAGCTCCATCACAGGCG
EMPV1_23525	GGCTCATTGACCTACATATAACAGGGTTAGAAGATACATGGGAGTAAGTCTTGATCTTGG
EMPV1_23526	AAGTGTTCGCATCTCCCAGTAAACACCCCATGGACAGCAAAGGGGAGGAGTCCAAGATCA
EMPV1_23530	CAGATCCAGCCTGACCTTTATACTAATGATGATTGTGTGGCTACATACAAAGGCGCTCCC
EMPV1_23533	CTACTTACCCTCAATTAAATACAGATCTCAGAGTTTCCACTGTGGCACACCAGGATGGGC
EMPV1_23534	GGATGACTGCGGCTCGGATTCGGGGGCTTTGGGGATGTGAGAAGCTGCTGGAAGCTGGAT
EMPV1_23536	AGCTCTTTGATTGTGACACCACGCTGGTCATTATAAACCTTGATGGGCTGGCCCTGGCCT
EMPV1_23537	TTGACCAAAAGAGGAAGGACAGGGAAGAGGAAGATGGAGAGAGAAGCTTGTGTAGCAGCC
EMPV1_23538	AGTGAGATTGCCTTCTGCCCAAAGCTGACCAGGATGGCTACGAGATAAAGAAGACGGGCT
EMPV1_23543	TTACCCCGGAGGCCCCACAGCCCCGCTTCTGGAGGAGAAAAGTGGTGCTCCACCCACGCC
EMPV1_23545	GAAGGTGTATGATTCTGGAACAGAGACATTAGAAGGACATGACTGGTGAAAAGATGTCTC
EMPV1_23546	CTCTCACTCCCACAGGGGCCAGCTCTGTGACAACTGGATCAGAACTTTTGTCCCAAGTCT
EMPV1_23547	CTACGAGGCTCTGGAGAAGTACCCACTGGCTTATGTTGACTACGTGACTGTGCTGCAGAT
EMPV1_23550	GGTTCACATGTACAGGTAAGTATATTGTGTATTTCTGTTCATTTTCTGTTTATAGAGTTG
EMPV1_23551	CTGCTGGGTGAGTGTGACGTGGTGCTCCCTCTTTTGCGAGTGGAACTCTTGGAATTTCTA
EMPV1_23553	GAGGCAGTACGGCCCAAAACACCACCTGTCGTAATCAAATCTCAGCTTAAAACCCAAGAG
EMPV1_23554	AGGCCTGAATGCAGTTCTTGGTGGATCTCCTGGGAAAACGGGGTGAATGCGGCTTGTTGT
EMPV1_23555	GGCCGAGTGCGAGGACGGATGTGTACCTGTGTTAAAAGTGACGACCACAACAATAAAGCC

EMPV1_23556	AGACCCCAGAAGAAAGTTGGGTGGTTTTTAGACGGTACACTGACTTCTCTAGGCTTAATG
EMPV1_23557	TGCCCGCGTGCGTCTAGTGTGCTCATGTCTCAATGAAGCCTCCAAACGATTTCAGTGGAA
EMPV1_23561	GGTTGTGTCCCTGTTTCTCATTCCAGGAATGTTTATATATCTCCAGCCCCCATCAGGAGG
EMPV1_23563	TGAACTTCCCATCTGCTCTAAAGGAGGAGGTACCCAGGCTCTTTCATCAGCTTGCCCAGA
EMPV1_23564	AAAGAGATGCCTAGAAAGTGGGTGTGGTGGCAAGGGATGGTGGTGGGATTGACCTCCTAG
EMPV1_23565	ATCCGGCCAGGACCCCGTCCCCTCGTGCACTTTACGGGCAAAGAAGTCACCGGATGCTTT
EMPV1_23566	ATTGAACCGAGAGACCTTCGAGCTTTCAATGACCTCTACTTGCAGAACAGCCCAGAGGCC
EMPV1_23567	GGCTTGTTGATCATACTGCCACCACTGACTCTGTTCCTAGATTTGAAATTCTGTGACTCA
EMPV1_23568	AGAGAGAGGGCTCCCAATTACGCTTCTACATAAACAGCCAACCCGGAGTTCCGATTGTGG
EMPV1_23569	CCTGCGGCGGATCAGCTGGATATTCTTACTACCAGAATTCCAAAGGAACTGATCAAATCA
EMPV1_23570	AATTCCTACAACTTAACGGAAACTTGGTGTCTGCCTCTTTGTTACGCAGGGCTCGGCCAG
EMPV1_23571	AGATTATAGTATATGGCCCTAAAGGGCAGTAGCCTAAAGATAACAGTACACAAGATGGGG
EMPV1_23572	GATGCCTTTGTCCACACTACCTGGATGAAGTGCCTCTTCTCTCTTGGGAAGCATAACTTG
EMPV1_23574	TGAGGGCAGTGGTGGGCCCTCTACCTCCCGGGTGACAGATCCACAGGGTAAATCAGATAT
EMPV1_23575	TCCTGCTGCCCTCCACCTTATCGCCCACTACAAAGAGAACAAAGACAGGGAAGACAAGAA
EMPV1_23577	TAGGCAGACTCAGCCCTAAAGGTCTTTCCCGTCTTCAACACCTAAAGCACTTCCATCGCC
EMPV1_23578	ATGATGGAAGGCCTGGACGACGGCCCCGACTTCCTCTCCGACGAGGACCGCGGACTTAAA
EMPV1_23579	GCAGGACATTGCCTTCGGCCTTCTTTCTGCCTTCTAAATCTTGCCTGAGTGCAGCCGCTT
EMPV1_23581	TCTGGCTATATACAAAGCTCCAGATAACGTAGAACAATTCATGGAAAAAATTCTAGATAG
EMPV1_23582	TGTCTGGCTAGGTGATATGGACTTTATCGCAAAGGTCAGGTGACAGAAGGGCCCAAGGGC
EMPV1_23584	TCCTGATAATGGGTCCCCTCTCCATGATTCTTACCTCTTACAGTTATATTGCCCAAGCTG
EMPV1_23585	GGACTGGGGTATTTCATGCAGAAGAACAAACTGGTCAAGGTCATGGTATACAAGCAGCAG
EMPV1_23586	GACTCACCAGCTCCAATCACCTCCTCGATCTTGACACAGGACACATCGATCTCCTTGGCA
EMPV1_23587	TACATCCTTATCATCTCCACTATCCTTAAAATTGCCTCTGCTGAGGGCCGGAAGAAGGCC
EMPV1_23588	CTACCTGCACTTTCTCACCCCTCCAGTGCTCAACCCCGTTGTCTACACCGTTAAGACCAA
EMPV1_23589	CCAAAACCGTATTTTGCCTGGTCCTTGTCTTTGCCCTGTGTTGGCTTCCCCTTCATCTCA
EMPV1_23590	CCCTTTGCCCCGAAGACCCTTCACTCGTCTCAGCTTTCGTTTCTTTGTAAAACAAGGCCA
EMPV1_23592	GAACTTCTAGACAATGCTCATAATGATGCTCCTGCCAATACTGTATATGAAGAGGAGGAC
EMPV1_23594	TGGAGGCGTGTGAGCTGACCTCGGGTTGCTGTGAGGCCCTCTCCTCCGCCCTCTCTGGCA
EMPV1_23597	TGTAATGAATGTTCCGGTAGCAGAGGCTGACCATAATAAGCACTTCTGCAGGCTCAAGGC
EMPV1_23598	TCAGGCTAAAGCAAACAGATACGGGAAGGAGTTAAGATACTTAAAAAACAGAGCAACCAC
EMPV1_23599	TCAGGACTCGCATAATTAGCAGAATGCCGTCCTAAGGTTGTTGAGTTCTGCGTTTCTGGG
EMPV1_23601	CCTCCAAGTATCTAAGACTCTTCTCCAATAAAATTAAAGTCCTCATCTTCTGACACAAGC
EMPV1_23602	ACCATGTGACTGGCATACGATGGTGTCTTGAAACATGACTTTCCCTAACCCGAGATGCAT

EMPV1_23604	GTCTCTGAGGATGTTGTTACTACTGAAAAGTACACTTTCCACTGAAGATGCAAACATATG
EMPV1_23605	CCCCAAAGAGAGCACGTAGTTCCAGCCTCACCCAAGCACAAGAGCACAATGATCAAAGCA
EMPV1_23606	TTTAGCATCGAGAGTATCATGCAAGGAGTCCGGGGAGCTAGTACTGGCGCTGTGCAGAAT
EMPV1_23609	GGGGAAAGAAGGGCCGAGCTACGCAACTGAATAAAGACTACGATTCCCAGCATTCACTAC
EMPV1_23611	AACTGGCAGAACTGAAACAACAGACTATGCCTTTGAAGAAAAAATTGGAGTCCTATTTAG
EMPV1_23612	CTACAAAGTCACCACTGAGCAGTACCAGAAAGCCGCTGAAGAGGTGGAAGCAAAGTTCAA
EMPV1_23614	TTTCACTGAGTTGCATCCTTCTCTCTTATGGCCAAATTGTGGTCTCTATCCGGAGGGTCC
EMPV1_23618	AGGGAACAGAGGTTGCTTTTCCCCTTCTAGTCAGCCATCGCTCCCAGCCCCAACAGCCCT
EMPV1_23619	ACTTAAGCTCAGGAGTCCCATTCTTCTTGGATGGAGAGGGGGTGCAGGGATGGAGGCATA
EMPV1_23621	AGAAGCAATACTGAATGCTGACGGCGGGTGCCGAGACGAGCAGGTTATAAATGCGGCTTT
EMPV1_23622	TCGTGGTGGAAGCTGGGAAGGCAGCAGTTTACCAGCTGGGTTCTGGAGGAGCCAAGTGGA
EMPV1_23624	CATCAGTTATGAGCCAAAGCATGTGTGTGGGGCTTGTGGTAGCTTCTTGGACAGTGGGCT
EMPV1_23625	AATGTTCATAGCAGCATTGTTCACAAGAGCCAAAAGGTGGAAGCCATCCAAGTGTCCGTC
EMPV1_23626	TGGGAAGGACAGACACATGCTCTAGAAACTTGCCTGGATGTCCGTGAACGGGAACTCAAG
EMPV1_23627	GCGGCCCTGCTTTTATGACATAGATCTGGACACGGAAACAGAGCAAGTTCAAGGCCTGTT
EMPV1_23629	ATGTACGTGACGCTGGTGTTCCGCGTGAAGGGCTCCCGCCTGGTCAAACCCTCACTCTGC
EMPV1_23630	TTCAATAAATAAGAGAAACGGATGGCGGCGGGCCCGTGCCCGCACGGCCCCACATAAATA
EMPV1_23632	GCAGGCCTTCTGGAGCACCCCCTGTGGTTTAAGATCATGAGAGAAAGAATAAGCAATGGG
EMPV1_23637	CCTTTCAAATGGTTTGGAGGAGGTGGTGAGCCCTGAGAATGTCTGGAACGGCATACCCAA
EMPV1_23638	GGGCAACCTGGAAGAAATATGTTCTTTCCAGCAAATGCTTGTACAGTCTTTAGAAGAATG
EMPV1_23640	ACGACGTCTAGGTCCGTAGGGTCCTAAAGGAGCAGAACGAGATCCTGGGGCAGGCTCTGA
EMPV1_23642	CAGTCAGATATTACATACCATCTATATGAATTTGACAAATGTTAGAATGTCTGGTTGTGT
EMPV1_23644	CTGGAATGTCAGGGAGTAGAGGCACAGAAACTATTACTTGGGCAATCTGCGGAGGGAGAT
EMPV1_23647	GTATGAAACGCTCAGAACAGCTGATGGACTATCACCGCAACATGGGCTACCTCAGCTCTT
EMPV1_23649	ATGTCCCGAAATGTCTGTGTGGAGCTGGTGTCTTGTTGCTACCTGTGTGGGACTGTGTGT
EMPV1_23652	ACCCTGAGACACTGCCAGCCATCGCCACCACCCTCATCGATGTCCTCTTCTACTCGCACA
EMPV1_23653	CTTCAGCTCACGGCACACTCCTCAACTCTGACCATGCACGCTGCTATCCTGTCTCCAATT
EMPV1_23654	TCCCCCTAATCTTACCTACGGGAAGAGTGCAAGAGCCTGCAGCTCACCTCCATTTTTCTT
EMPV1_23655	CCTCCGGTATCTTCTTCAGTAGGTCTGGGGTAGAGCCTGAGAATTTGCATTTTAGCATAT
EMPV1_23657	AAATGTGTTTAGTATAAGAGGTGGAAGAACCTCTGTCCCTCCGTTCCTCTGGCAGCAGCC
EMPV1_23658	AGAGGGTTACTCTGGCTCAGCATATGGACTGGAGTGTGCAATACCAGAGTCAGCAAAGTC
EMPV1_23659	TTTCTGAACCTCCTCTCCTCAGGTTTCCAGACCCTGGAAAAACAGGACTTTCAAACCCAC
EMPV1_23662	TTTGCTGAGCTCTGGCTCCTTCGGAAACCTGGAGAACCTCAGTTTGGCCTTCACCAATGT
EMPV1_23663	ACCCTGAGCGGACACCCTCGAGACTTTCTCCTGCTCCCCAGAAAAGGTACTTAGAACAAA

EMPV1_23664 TGAGGATGTGATCACTGGAGCCTTCAAGGTTCTGGACCCAGAGGGGAAGGGTACCATCAA
EMPV1_23667 CCAGGAGGAAGGTGTTCGCCTGCAGTTTCACACAACTCTTCATCACGCTTTCTCATTACC
EMPV1_23668 TTCAACACTCCCTCTAAGCACCCAGACCTCTAGTGGCAGCTCCACCCTGTCCAAGAAGAG
EMPV1_23669 TTCAAGCTCGTAGCGGCGTCTGGAGCCGGAGGTTGGCGCCTGGGGTCGGGAACTGAGTCT
EMPV1_23670 CGTGCTGAGCAGCAGCCTGACCCATTGGAAGAAGCTGCCGCCGCTGCCGTCCCTCACCAG
EMPV1_23671 CAGTTATAGAAGAAGTTCCTCGACTACTACAAAACCTACTCCGAGTCCGCCTCTGTCACG
EMPV1_23673 CTGCAGTGATGCCAGATCCTTAACCCACTGCACCACAAGACAACTCCTAGATTGCTTTTA
EMPV1_23674 CCGGCTAATCTAAGCGTGCAATGGTCAGTTCGGGATGGTGGCGCCTCCGAGCACCAGTTC
EMPV1_23676 TCAGTCACTCATGCCCACCTTGGTGCCCAGGAGCCTGCCTTACTCCATGCTGGGAGTAAA
EMPV1_23678 CTCCAGTGACCTTGAGTGCCATCTCTGTGCTAGGTAAACTCTTATTGCCTTACCCTTGGC
EMPV1_23680 GAAACCTTCTCTGTATTGTGTGCTCCTCTGATCCCATTTCGACCACGGCCCTGATGTTAT
EMPV1_23681 GACCATTCCTCGTGGGCCTCTTCCTGAGCATCTTCATGTACCAAAACCAGCAGGCAAACA
EMPV1_23682 ACTTAGGTTGCCCCTGCTTGGGGGACTCAAGCTGTAATGCTACCCTTTACCAGTAGGTGC
EMPV1_23684 GGACATGCTGAAGTTCCATAAATATACCATTGGTCATGCCTGGACCACCGATTATGGGTG
EMPV1_23685 CTCGTAGTACTTCACGTTGGGGCCGCCGTCCTTCTTCCGCACCGCCATCTTCTCGGGCTC
EMPV1_23686 GATCCCTGAAAGTCCTGAGGCCTATATTAAACTCACAGAAAGTGCCATCAGAAATACCTC
EMPV1_23687 GCACTGTTTACACCTCTGCCACGACACAGCACGCGCATCGTCTCACCTGTGCACGTGTCT
EMPV1_23688 TTCGGTCATGCAGAAAGGCCTGAACTTTGGAGAAGAGCTCCCACGAACGTGCTGTCGACT
EMPV1_23689 AAGAGATAAAGACACCTGCCTGGCTTTGCTAGGGAATAGTGTGCAAGGAGAGGGTTCTGG
EMPV1_23690 CTTTCCCCAGAATATAACACGGAGTGTTTTTCAAAAATCCTAAAATAGAGGGCTTAGGCT
EMPV1_23692 GTGGTGAGGAGGGGGGCTTTATTCCACAGAGAGTTGGAGGCCTTCGAAAGATTCTGAACA
EMPV1_23693 GAGACTCTAACTGGGTGCTTAACCTGCGGAGCCACAATGGGAACTCTAGGCTCCTTTTTT
EMPV1_23694 TGAAGAATCATCTGAGGGGGAGGGAGATGGATGCTCAGTTGTCACATCAAAGGAAGCAGC
EMPV1_23696 TCCATTTAGAGAATGATGTGGTTTCACCCAACGCTCTCACTGGAGGGCCTCCTGAGGGTG
EMPV1_23697 GACGGGAGATTCGGGCAGTTTTCAGGGCAGCTACGTGCTGAAGGTGGTGGGCGGTGGGCC
EMPV1_23698 CACAATTCTTGTGAACTCTTCAGGGTGGACCACGGTGTCATAGCATCCTGACCATGGAAC
EMPV1_23700 GGTGGAACCTCTTATGGCACCTTCAAAAAAGCAAGAATCATTACCCTTGCATCAAGAGAC
EMPV1_23701 TTCCTCTACATCCGCCAGCTCGCTATCCACCTGCGCAATGCCATGACCACACGCAAGAAG
EMPV1_23703 GAGTTCACTGTAGTGGCTTTCATTTAATATGGCTGTCTGGGAGGAACAGGGTTCCCTGGC
EMPV1_23704 AAAGTGCAATGCAGTAACCCTCTGGGGCTGGGATATTGTGGTTGATAACTGTGCCAGCTG
EMPV1_23705 GGGCCTCCTCGGAAATGACGTTCTACCAGAAAAAGCATGATATTAAACTCTTTAAACCTC
EMPV1_23708 ATGACCTTGCTCTCGGAGGCTGAGTAGCTAATCACCATGTGCCCCTGTTCAATGCAAGTT
EMPV1_23709 CTTGACAAGGAGGGGGCCTCCAATCTGGTCATCGACCTCATAATGAATGCGTCCAGTGAC
EMPV1_23712 AACTGGCTTGGACAAAGACTGGGGATAAGGCTGGAGATGGTCCTGACGGCAAGGAGGAGA

EMPV1_23713	TAGCGAGGACGAGGAGCAGACGGAGGAGGCAAAAGAGGAGAGGCAAGGGCCGAGTCCGGA
EMPV1_23717	TCTAGAGGAGAGAAATGGGAAGAATGCCCCACTACTCAACTGGATGGCAGGCTGAGCTGT
EMPV1_23718	AGAGAAAAGCAGCGGCACTGTGAATATCCGCACCAGAGATAATAAGGTGCATGGAGAACG
EMPV1_23721	TTTCCTGGTGGTGAAGTGGGAGTAAGGACATTACAATAGGGCCGCATACACGCTGTGTGT
EMPV1_23722	GGACTCTAGCTGGGGTTCAAGTAGGGCATGTGTATCCAGGTAGGAGAGAATGGATTTGAG
EMPV1_23725	TGTGATTGTCCCTCTTCCCAGGCACACCTCTCTGACCATATCTTTCCTCGCACCTGCTCA
EMPV1_23726	GCGATGGCAAATCGTGCTGCAGGGAAAGGCTATGAGAATGAAGACAATTATTCCAATATC
EMPV1_23727	AGCACCGCAATGAGCACAGCCCACGTTTTCTGATAAGACCCCAGAGTAGGGCTAGTAACA
EMPV1_23728	GAGAGGAACTCTGCTCCCTCTTGCTGGCATGTTGGTGGATTTTGTGGTGGATGGTGAGTT
EMPV1_23729	AACATGAACTTTCCCAGCCAACCCTTCAACCAGCCTCTGGGTCAGAACTTTAGCCCACCG
EMPV1_23730	CAATTTAAATTACCGCACAGAAAATGGTCTGTCTCTACTTCATTTATGTTGCATTTGTGG
EMPV1_23731	CCACAGCTTGGCCCTTGCAAGAGAAAGTTTTTGCTGTTGAACTGTGATTTCTGCCCAAGT
EMPV1_23732	ATGGCAAAAACGATGGGGCTGAGAATGAAGCTGCAGAGACCGAAGAGACCAAGGAGGAGA
EMPV1_23733	CCCTGGCCATGGGTACCTCGGGGAGCATGGCTCCCTCTTCCAGCAGCCCCTGGATGTTTG
EMPV1_23734	CAGATTTGAACCCATGTTGTCTCTTTTCAGAACCTGAGCTTCTAAACCCTAGGAAACAGG
EMPV1_23735	TCTTCCACTTTTGATGCTGGGGCTGGCATTGCCCTCAACGACCACTTCGTCAAGCTCATT
EMPV1_23736	TTCACGTGTAAACAAAGCTCCCTGCCTCCCACAGAGGGGGTCCTAAAAGTGCAGAGAAGG
EMPV1_23737	ACAGACAGTGTCAGCCAGCACCCGAGGGGCTACTTGAATCCACGTGGCTTGGGAAGAGCT
EMPV1_23739	AACGCTTCGTGCTCATGGCCACCCCCAACCTGTCCAGGAAGAACAACAACCGGGGCAACA
EMPV1_23740	TTCAGGTCTTCATCACCATCATGGGCACAAGGGTCACCAAAGACAGGGTCACTCTGAGAA
EMPV1_23741	GGCCGCTGTCAATGCAAAAGTAGAAGAATGGAAGGTGCTTCATTCAGATACGACAATAGC
EMPV1_23742	GTCGGGGGCACAGGCCCGGAAGGCGTTGTTTGAGAAATGGGAGCAGGATACAGCGGGCAA
EMPV1_23743	GCCGAAAACCAGATGTGGAGCCAAAACTCAAGGACAAGTTTGTGGAGATTTGCCAACAAT
EMPV1_23745	GAGGAGAGACTGGACACATTCTGTACCCTGGACTGAAGAGGAGACCCAGTTGGCTTCACT
EMPV1_23747	GCTCCAGCTAGAACTTCCACAGCCCGGAAAAGAGATGGCGTATAAGGGCCTTGAATTTAA
EMPV1_23749	GTACCTCGGGTGTCATTTGTGTCTTGGATGGCCTCCCTTTCTTAGAACTAACGTGGGAAC
EMPV1_23752	AAGGACTTGAGGATAGTGAGTTTTCAGAAGAGCTTAGAGGCCAGTAGGCAGGTGGGGAGA
EMPV1_23753	GAGAGGCAACCTGCAGTGACATAACCACCATGTGATGAAGGTCTGAGTTTCTGTGCCTCT
EMPV1_23755	AGGAGAAGATGCACTTGCTAATGTCAGCATCGAAAAGCCAATTCAGCAGGGACCAGAGGC
EMPV1_23756	AGCAAATCAGGTGGTTCGTCCTAAAACTTCAAGTAAATGGTCTGCTCCTGGTTCAGGTCC
EMPV1_23757	AGCGCCCTGAACCGGGCGGACTTACTCCAGAGACAAGGCCACTATGCCCCACCCCCAGGA
EMPV1_23758	CCAGCTGTTAAGTACTAATGTGATGATAGGTTTTCAAAGTATCCCCTTAGTGGACGCTGC
EMPV1_23759	GGGAAATACTGGAAGGGATTCCATTGGGTTCATTTCTTCTCAGATGCCTGAAGACCCTTC
EMPV1_23760	CTACCGCACATGGCCTATACTTACCCAACTGGAGCAGCTACTTTCGCTGATATGCAGCAA

EMPV1_23761 CCATGTGATACAAGACGTTTCTGCCTTATTTCTACGTGCAGAGTAGCTGGCTGTCCCCTG
EMPV1_23762 CATGAAGCTGACGCGGAAGATGGTCCTGTCCCGGGCCAAGGCCTCGGAGCTGCACAACGT
EMPV1_23763 TTCCAACACTGAATCTGTCCATCTTGGGACCGAGCCCCCATTAGTACCTAGAAAGGTCCC
EMPV1_23765 TCGTCACCCGGCCGCCACGGCCCAGCTGGAGACAGCAGTGCGGGGCCTCAGTTACCTGCT
EMPV1_23766 CGGTGAGTGACGCTCTTGATCTCCCGAGACCCAGGCACATCCTTGATTGAATAGGATTTT
EMPV1_23767 AAATTTTAATCTATGGACAGCTCAGCACCTCCTGCCCACTCCACCTCCCTGGACAGGCAA
EMPV1_23768 TGGGAGCACATGAAAGGCTCCTTACTGCAACAGTCACTCTGAAGATTCTGCTAGTTTATG
EMPV1_23771 AAGCAGCTCAAAATCATGTTCGTGGGAGGCCCCAACACCAGGAAGGACTACCACATCGAG
EMPV1_23772 AAAAAAACCTGCGTTCTTAGTTGTCGGGGGTGACATCGGGGACGAAACCGAACACGTTGT
EMPV1_23773 CCCGTCAAAAAGAAAGACCTGTTCCTGGACTTGATGGGGGATGTGGTGTTTGGTGTCCCA
EMPV1_23774 CCCTCAAAGCAAGAGGTAAGCATGTTTATAAAATCCCACCAAGATTTGGCTGGGCTCCAT
EMPV1_23777 AAAATGATCAAGTTTGACTGTGGCAGAGCCCCAGGGCTGGAAAAGAGGGTCAGGAGAGAA
EMPV1_23778 TTATTTCAGTTCCAAGACTGGCTTCCTGAGAGCCCAGAGGACAGATCGGGGCAGAGATGA
EMPV1_23780 GTCTACGGCCATACCACCCTGAACGTGCCTGGTCTCATCTGATCTCAGAAGCTAAGCAGG
EMPV1_23781 TCCCTTTCTCTGCTCCCTCCAACACAAATGGCCTTGGCAGAAGGACAGTACATAGGGCAA
EMPV1_23782 CAACCAGCAGGGATAGCGCAGTCTCCCAAACACCACCTCATCTTGCCTCTGTGTCTTAAA
EMPV1_23783 CATGGTTCCGAGGAGCTCGGCACACACGCGCACCCCTCACAGCTCGCGATCCGGTCCAGA
EMPV1_23785 CCGTAGTGACTGCTTCATTGCCATGGTTACATACTAATTGCCGTGGCTCTGTGGCTCAGC
EMPV1_23786 ACTACATCAACCTGGATGAGAGTGAAGCCGCAGCATTTGACGATGAGTGGCGGAGGGAGA
EMPV1_23787 GCATCCCCAGTAACCAGCTCTCCTTCATGGCCATTCTGTCACTGCCAATGTTCTGAGATA
EMPV1_23789 CTAGAGCTTTTATGGTGTGGTTACCCAAATAATGAAATAGAGAACCTGATCCCTCAGCTG
EMPV1_23790 CAGCCCATCCCCATCTGTGATATCCAAAGCCACTGAGAAAAGGTGGACTTCGACTGACAG
EMPV1_23791 ACGAGTTTGGTCAGGCCCACAACACATGCGAACATCGGGGACAACCCTTCTAGCTTCTCA
EMPV1_23793 GCACTCTGAGATTAAACCCTGGCCAGACAACATCCCATGTGGCAAGTGGCACCCAAATAA
EMPV1_23794 TGAACAGTGGGGGAAAGGAAACTCAGGAACCTCTCAATCAGGCTGCTCTGCTTTTGCAAG
EMPV1_23795 TCGTAACCTCCGGCTCTAAACTGGGCCTGGCGCTGGAAGAGAACGTTGACAGTCAGTTCT
EMPV1_23798 TATTTCATCATTCAGCCCCTTGCCCAAATTACAGCCTAGTGACAGGGTCTGCCTCACTGC
EMPV1_23800 CTGAGTTGTTGAGATGTGGGTTTCCACTGTTGAAGTAAACATCTTCTCCGTCAGTGTCTA
EMPV1_23802 AGTTTTCACTGGCGGCAGGTCGGCATTCTGCTCTTTGTACCACTATTCCTTCCAGGGACA
EMPV1_23803 GTGTTCTGTGATCATGCTATTTGATTTGGAAAAAAAAAACAAGAATATATCAGACTTGAA
EMPV1_23804 AGTCGCATGATGGGTTTTCATGTCCAAACACAGGCTCATTCAGTGTTCAGTATTGCTTCC
EMPV1_23805 CTTGTCTAGCTTCAATCTCTTTCTGCCCCGTTGGAAATCTTTACGCTGTTTTCCTGTGGA
EMPV1_23807 CTGAGGATGATCAAAGTATTGATGATTCCACTCCTTGCATATCAGTTGTTGCACTTCCAC
EMPV1_23808 AGGAACAGTCCAATCCTGCTGTGGCAACAAAACGGAAGAGAACAGAGGCCTTGGAACAAG

EMPV1_23809 CTTATGGGACACCTGGAGAAAGCACTGCCTCAGATCAGGGAATTCTGACCATTTCTGAAC
EMPV1_23810 CGGTCTTTCAGTTGAAACGTCTCACCCGGGGTCAGGACTTACTGAGGCTCAGGTTCTTTT
EMPV1_23811 AGGTTGAGCTTGGGGAGGCTGAATACGCAGCGGAAAACCTTAGACGAGCCCTACCTTGGA
EMPV1_23812 TCTCAGCTGTGAGCTCGAGGTGGCTGGGAGAGGGTTGTTTACTCCTTCTGCCATGGAAAA
EMPV1_23813 GCTTTGTCTTTCCTAAACAGCTCTCTCTCATTCTTTCTCCCCCAGTCTCTCCCTGACCCC
EMPV1_23814 TCATGGCTCCGTGGTAACGACCCTGACCAGTATCCACAAGGACCCAGGTTTGATCCCGCA
EMPV1_23815 ATGGCTGCCTTCAGGCTCTGTCCTCACATGGCCTTTCCTCTAAGTCTCCACGTAATAAGC
EMPV1_23816 ATTGGGGCTGGCGTGGACTCTTACTTTGAGTACCTGGTGAAAGGAGCCATCCTTCTTCAG
EMPV1_23817 GAGGACATGCTGGAGTTGGTGGCCGAGGTCCGAATTGGGGACAGGGATCCAATCCCTCTT
EMPV1_23820 AGAAACGCTCCTTCACCGCAGCCTGTTTCTCATGCCATTCAGCAATCAAACCATTGCGGC
EMPV1_23821 GTGGGTGTGTATTGTACTCTTGATGGTGTCTTGGGTTGGGGGTTTTCTCCACTCAATCAT
EMPV1_23822 AGCAGGCATTCCCATCGTGCCTCAGCAGAAACGCATCTGACCGGCATCCAGAGGTTCGAT
EMPV1_23823 CGTATGTTGCCAACACGGAGTGTCCCATTTTTTGTGGCAGATGTGGGATAGAGAAAAGGG
EMPV1_23827 AATTTGCAGTCCCTGACTACCGTTCTTCTCATCTTGAAGTCAGTCAGGCGTCACAGCTCT
EMPV1_23829 TAGGACCTGTGACTGCTGTGTGGAGAAAGGGCCGACACCCCGCAGGCCCTGCTTCTGATT
EMPV1_23831 GGAGCTGTTGGAATTGCAGAGCCAAGTGGGGACTAAGACATACAAACAATTGGTAACTGG
EMPV1_23833 CGCCTCCCACTTTGGCTGCACCGTTGGCCTCAAGGACTCTGTCAATGCCGTAGTCTTCGT
EMPV1_23835 GGAGAACATATTTTGGGGACTTGCTAAAATTGATGAATATGGTAATAGGGAGTTTGAGTC
EMPV1_23836 CCAGGTGTGTGAAGTACAAAAGCAATGGTCCTCCGGAAAAGATGGGCAGCTAGGCTGCAA
EMPV1_23838 AAAACTGGATGGACTTTTCCCACCAGGAAGCCATGGGAAAGACGAAGGATGTGCCATCTG
EMPV1_23839 TGTTTTTCTTTGGGGGGGGGGTCTATCTAGAACAGTGCCTGGCACATAATAGGCGCTCAA
EMPV1_23843 CTGGGATATTTATCCTCCAGATACTTATGTACACAGTTGCCCTGACATTCTCTCCTCAGG
EMPV1_23844 CACTGGTTGCCGTGTGTTGTGGGAGACAGAAAAGATGAATAAGACCCATCTCACAGTCAC
EMPV1_23845 TTGCTGTACGACCTACCGATGAGTCCCTCAACACTGAACCCGAGAAGGTTATTTGGAACG
EMPV1_23846 GTGATCCCTAAACGAACGAACAGACGAGGCATCACCACAACACACCGGGGTATTCCACGA
EMPV1_23847 TTGACATCCCAGACTCCCTGGCTATTTAAACAGAGATGGGTGCCCCCATCGGCACACTGT
EMPV1_23850 TCTGGCTCCGTGTCTTCACTCCCGTGTGTGTCCGAGGAGGGAGGGAGGGACGGGGGCTGT
EMPV1_23851 AGCTGCTACTCTGTCCTTCTCCTACTGTGGCTCCCGAGAAATACCTCAATTCTTCTGTGA
EMPV1_23852 CTGTAGTCTTGGTGGGCTACCTCCATGCAAGAGCACCTCTTTGAATTTTCAGGGGGTTAC
EMPV1_23853 ATGAGGAGGCCTTGAGAGATGAGGCAGAGAGAGCCCGAGCTAGAGTTGCCACTGAGGCTA
EMPV1_23854 CAGTAATAAAGAGGTCTACGCCAAACTAAGCTGGAGGGAACAGCAAGCCTTACAGGTTCG
EMPV1_23855 ATGTCGCATGTTTGGGACAGTGGTCCCTGCTCTCTTGTGCTCGAGAAGTCGACGTTACTT
EMPV1_23856 GACTCGCTGGCCAAGAAGAAGCAGGTACACTTTGAGGACAGCGTGGTCAGAATCATTCCA
EMPV1_23857 GAAGAGATCCTGCACTTCTTTGAAGAATTTCAGTCAAAAAAGAGATGAAGTGTGGTTGGG

EMPV1_23858	TTAGGTGTGTGGTTTGCAAATATTTTCTTTCTCCCATTCCATAAGTTGGGAAAGGCTGAC
EMPV1_23859	CACGAAAAGGACACGGAAGCGGTCAAGTCGCTGCAGGTGGAGATGTGGCCCCTGTTGAGT
EMPV1_23860	TCGAAGCAACTCATGATCTTTTGGTGGAGCGCATATTGAACCCTGGCCATGAGCTTCAGG
EMPV1_23866	GTGTCTGACCTCAGCCTTTTTGGGGGTCTCCCAGCCAACCACGTCCTTGTGAACCAGTAT
EMPV1_23867	CAATCTAGACGATACTGCTTATGTGGGAAAGCCATTAGTAGATATAGAAACGGAAGCCTT
EMPV1_23868	GATGCAGCCGATCTGGTGGTCCAAGGCAGAGGGAAATTCGAGGAGCTGATGGTGTGTTCA
EMPV1_23869	TGCGGAATTTGAAGCCAGTGAGGCGTCCTGGAACTCGTCTCCTAGTAACGTGTGGTACGA
EMPV1_23870	TGTGCCTCCTTTGAGGTTGTACTGAACAGAGCATGGGGGTGAGGGTGCCAAGGACAATTT
EMPV1_23872	TCCTTGCTTCCAGCACCATAACTGTAAGTGCACACACTCCGTGCCTGGAGAAGCACAGTT
EMPV1_23873	TGTGTACCAGGCACTACTCTGTGTGGCGAACACACGACTGCTTATTTGATCCTCACAATC
EMPV1_23874	ATCTTTTCCGGGCACAGTTCAGCTCCAGTATGGTTGGGTCCCCAGATTGCAGGATTTCCA
EMPV1_23875	ACTGACCAGGAAAAAGGTTGAGCTGATTCTGGGTGATTCTATCAAGGGAGCGCCAGATAG
EMPV1_23877	GAAAACAGCTACCCGCTGCAACAGGACTTCTCCCTCCTGGATTTTTATCCCAACGATGTG
EMPV1_23878	CTTCGACTACGTCTGGCTCCGAGATCACTGCCGCTCGGCGTCTTGCTACAACTCTAAGAC
EMPV1_23880	GCATTAGCAAGATTTCCCAGGTGTTGCAAACTGAAGACTGAGGGGCACTGTGGTTGAGAG
EMPV1_23881	TCTAGGGGTGAGGTGGGGCCTTGCAAGCAGTACTAAGAGCAGACTCTTTAAAACCCCTAA
EMPV1_23883	ATTCTGCCAAAAGCACAGCTGAAAGCCTGAGTTTAGAGCCAGCACGGCCCGAATGTAGCT
EMPV1_23886	GCAGAGCTCTCATAGAAAACTAAAATTAAGACGTGTTATGATGGCCAAAACTCTGGCAGA
EMPV1_23887	CGGCTCTGGACCCTCGGTCTACTTTAATAGAAGTTTTGTGTGCTCTCCCTCTCTTTGCGT
EMPV1_23889	GCCTTCCCTTCGGTTTTCTTAACTCTGGAAGAAACTGTTTCTACTGATAAAAACATAGGC
EMPV1_23891	ATTACCCACTGCTGATGAACCTGAGAGTCTGTGTATGCCTGGTTTCTGGATGCTGGTTCC
EMPV1_23895	AATCCATATAAGTCCACCTGGCTTTTCCAAAGAGCTGCAGGGGTGGGATGGTTGGGTCCA
EMPV1_23900	GGTTCTGCCCTGGAGACTATCACTATATTACGTGGCCCTCTTTTGAACCCTTCTCAGTTC
EMPV1_23902	TGTGGCCGCAGAAATTACTCACTCTATGTAAACAGGGTCCTAAAGGAAGTGGTCCCCCAG
EMPV1_23906	CTGGAGAGCCTGGCTTTACCTCTTGTCAGAAAACACCAAGTTTGTGTACCCGGCTAGTAG
EMPV1_23907	GGTCTGTTTGTGGACTGTCTAATGCCTACTGTGGCTTTAGCTTTCAGGCAGATTTGGGAC
EMPV1_23909	TTACACATGGATTGAGCCCCGTTACACTGAAAGAAGCAAAACCTGCTGCTGTGTGGCAGC
EMPV1_23911	AAATATCCAGGAGCCAGCCCAAATATCCTTGGAAGAGCACCCCTAGGAGAAACCAGCTTG
EMPV1_23912	GGAGCATGCCCTTTTCCGCAGATTTCGAGGACTTTGACTACAGCTCGTGGGATGCCATGT
EMPV1_23913	GGTGACAATGAATGTGGTCTGCCTTGCTAGCCCTGAGCGGGATACTCTGGATGTATCACT
EMPV1_23914	TCCTGGTGAAGGTAAAGATATGCCAGAAGACAGTGAAGCGCTATGATGTGGGCGCACCAT
EMPV1_23915	ACCACAGTTCCAAATGCAGACAGGTGCCCTTAGAGAGGAAAAACCATAGCAGGTGGAATG
EMPV1_23918	GACCAAGTGGCACAGGAGGACAGGAAGAGGCACTTGCCTTCAGACAGTGAATCTGGTATA
EMPV1_23920	TTCAATACGCAACCCACCATAACAGCTTTCAAACTTAGGTTAGACCAAGGCGGGGAGGGG

EMPV1_23921	AGGAGATCATGTGTTGTTACCAAAGTGAAAGAGAAGATAAAGTCCAGGCCATTGCCACCC
EMPV1_23922	AGGGTCTAAGGATTCTGAGCGTGCAGCCCCTGACCTTGTCTCCCAGCAGGCTGAGCAGCT
EMPV1_23923	GTGGATCTCACGATGTACTATGGTGCCCCTAGGCCTGCTAAGGTCCTGTAACCCAAGACT
EMPV1_23930	TGGCTATGGCGGCTATGATTATACTGGGTATAACTATGGGAACTATGGATATGGACAGGG
EMPV1_23931	GTTCAGCCCATCCTTGGCATGAGGAGCAGAAAGGCTCTGAAACGGCCCCAAGGCTATGGG
EMPV1_23932	CAGCTACCCATCCTAAGAAGTCAAGTACAAGCATGCAAGTAAATTCACAGGCACAGAGGA
EMPV1_23933	TGCGACCCGGCCAGTCAGCAACCTGGACCGTTAAATGGAAGTGGCCAGCTCAAAATGTCC
EMPV1_23934	CCGACCAGCAGAGAACACTCAATTATCAGCAGTACAAGCAAAAAGCTGCACAACCATGAG
EMPV1_23935	GAGGCCTGGCTGCTGCTGCCCTTCTTCAAGAGAGGCACGCTGTGGAATGAGATAGAAAAG
EMPV1_23936	TCAACAGCATGTATGGCCTGTTCATTGTCATCTCTGCCTTTGGTGTGGACGCTGTGCTCA
EMPV1_23939	AGGGTCCTCTTGTCTGAGCCCCAGAAAGAGGAGAGAGCGCTGGGTCACAGAAGAGGGTCT
EMPV1_23940	CCCGTGTGAACTCACCCCTGTCCTTTGGGGCTGGGTCATGCAGTTTACGTTTTTAAAGCT
EMPV1_23941	TAAAAGCAGTCCGTATGACATTACTCCTCCCAGGATGTGTGGTTCTCCTGTAAGGACCAC
EMPV1_23942	TAAGACAAAGGTCAGTGAGAGATTGAGAGGGAGCAGAGGGTAAAGCCTTGGAACCTTGGG
EMPV1_23944	GTACAGGACCTTCCTTCAGCACGTGACTCTGATCACCAACCAAGGGCGCTCAGCCTCATT
EMPV1_23945	GCAAGCTGCAGCGTAGGTTGCATGTGCGGCTTGGATCTTGCGTGGCTGTGTCTTGGCATA
EMPV1_23946	ATAAGAGGAAACACAAGACACCCCTGGCCACATCTTTGCACAAACAGTCACGGCTCCGTC
EMPV1_23948	AGGAACCAGCTGCCTGTTAGCTGTGTGACTTTGGAAAAGTGGCATCCCCTCTCTGAGCCT
EMPV1_23949	GAGTCCAGTCACCCCTGTTGGGCACCACTGTGTTAGGTTTGTTATTGTTCCAATTCTGAG
EMPV1_23950	AATTACCTGGGAGCCATTGAGAGCTGGGTGAAATTACAGGATGAGCATGGCTCCGTGCTT
EMPV1_23955	GGTGCATTGTCCTTGTGCCTGGGGGGTGGGTCAGACTCGAAGTACCCTCTTTATTTAACT
EMPV1_23957	AGGTGCAGTGTACTTCGACAGCTTGGGTGTAAAGATGGGAAAAACTGGAACAGCAACCAA
EMPV1_23960	TGACAGAACTTAGGGATGACAAACACGGGAGAAGGCTGTGGGGAGTACAGGACTTGGAGA
EMPV1_23963	CTGCCTCTCCCCCAAATTAACCCTCCCCATTTGCTCGACAAATCTATGTAAAGAAGTTTG
EMPV1_23966	CCGCTGTGTTCAGATGTTGGAGTGGTTTGAGCATCATGGTCACATTTGCATTGTGTTTGA
EMPV1_23969	GAGCTTTCATCACAACAGTATCCCCAAGAGTTCAGGTGTTCCAGGGGAGGAAGAGGGATA
EMPV1_23970	GTTCTCCGTGTCCTGCTGTCTGCTGACCTGAAGGGCGTCCTGTACTTCTCCACCCTGGAA
EMPV1_23971	ATAAGAACCTGCCAATAGCTCTCTGAAATGTGTGCCTAACTCTGGTCAATAGAAAACAGT
EMPV1_23972	CTGTCAGGAGTAGCTGACTGTTGTAGCACACGCCCCATCCACATGATGCTTTAACTGTGT
EMPV1_23973	AACAACACCCTGAGCAATAGAGACGGCCTGTCCTCTCCAGTCTCTGATCAGGAGGTAGAA
EMPV1_23974	GATGGCCTTCTGTGGTGGAGACAGCATCTGAGTGGGAGAACAAATCAAGATCTATTCCCC
EMPV1_23975	TTGGCCTCCGTCTCGGAGCTCGCCTGCATCTACTCTGCCCTCATTCTGCACAACTATGAG
EMPV1_23976	TCACATTCACCAGGCGACCTCTACTCCTTCTCTGAGGTTCTGGCTCCTGCTCGCTTAATA
EMPV1_23977	TTAGGGACGGTTTTTGGCCCTCCGCATTTGCCCAACTCGAAATTCTGACTCTAGGAGGAG

EMPV1_23980	TTGCCTTTTTAACATCTAGCGGCTGCCTGTGTTCCTTCCTCCAGCTTCAAAGCCAGCGAC
EMPV1_23981	CGAGTGTCTGGATGCCTACAACAGCCTGACGGAGGCCGTGCTGCCCAAGCTGCACCTGGA
EMPV1_23982	GCAGCCAAGATTGCTAAGACTGCACACAAAAATGGGTCAACCTTAAAGGCAACTGCTGTT
EMPV1_23983	CTTCAAATCCTAAAAAAGAGAATGCCCAAAGGCTGGACTTATTATATAAAGGCTCAAGCC
EMPV1_23984	GAAGCTGGAAGATGGGACGGAGTTTGACAGCAGCCTGCCCCAGAACCAGCCCTTTGTCTT
EMPV1_23986	TCAGGGTCACCCCTTGGGACAGTATGGACAGAGTTGGGGTCTGGGTCTTCTGGCTGAGAA
EMPV1_23987	CATTCGCTATGACAGAGATGAAAGTGGTCCTGGCTCTCACACTACTGCGCTTCCGTGTTC
EMPV1_23988	ATGGAAGACAGTGGTCTGGACACCTCACTGGGCAGTAGGCTGAGTTTTGAGGCCAATGAA
EMPV1_23989	CCTCAGTCAGTGAGAGGGTCTGTGAAAGTCATAAATATGATTCAGCTCTGTGGCATCTTG
EMPV1_23992	CCCGAGAGCTTCCTAACGGAGGAGGTTGATGAGACTGTGTCACTAAAGCAAGGGAGTCGA
EMPV1_23996	TTGTACAGATGAAGGGACGAAGAACATGGGTGGCTGTACCATGAGTTAGAAGACCAGGCC
EMPV1_23997	AGTTGGGTATGTGGCCTCTTTACGTGATGGGACATGAGTGAAGAGATTTCAAACTGCCTG
EMPV1_23998	CAAGCCAGGCTGGAGTGTGATGAGGAGTGTTCAGCCTTGGAAAGGAAGAAGCGATTAGCA
EMPV1_23999	CGTCACCGACGTCACCGATGTTACAGATGTTCCAGAAAATGAGATCACAGAGGCCCCAGA
EMPV1_24001	CTCTCTGTCCCGACTCCGCATCCTTCTCCTCCTTCCTGTGAACTCCCGTTTTAGAACTAA
EMPV1_24003	TGATGGGCTGGTGCTGTTTTCATTAACCTTCAGCTCCTGGTTGGTGGCGTGTGCCCATGA
EMPV1_24004	TGATGGCTCAGTTCCAGTGGTCCGCCCTTGCCCTGGGAGAATCAGACACTTCAAGATCTA
EMPV1_24008	GTCCACCTTAGCTGCCTTATCCTCATGATCACAATACCAGCCCTTGCCCTAGCATGTGCT
EMPV1_24009	AAGGTGAAGGACGAACCACAGAGAAGATCTGCGAGGTTATCTGCTAAACCTGCTCCTCCA
EMPV1_24011	CTGTATTCAGCTCCCAAGGCCAGTCAGAGCTGATCCTCCTCCAGAAGGTTCAAGAATACT
EMPV1_24012	GGGAGGAAGGCATGGTTGTTATTCCCAATTTATACGGGTCTAAGCTGAGGCTGGGGGTTA
EMPV1_24013	CTGAGTCAGGGTGGGATGCTTTGGGGAATCTTGACACTTGTGCCGCATAGTAAAGTTATA
EMPV1_24014	TTGACGTTCCTGACCAAGCAGGAGATTCTCCTAGCCCACAGGAGGTTTTGTGAGCTGCTT
EMPV1_24016	AGACCCTCCTGGACACACTCTGCTCCGCGGGGCTGTTCACAAACAGCTGCTCGAATGTGT
EMPV1_24017	ACCCTGAGCGGACACCCTCGAGACTTTCTCCTGCTCCCCAGAAAAGGTACTTAGAACAAA
EMPV1_24018	AAACGCGCGTTCTCTTCGCCTTTTGACGCGGGTGATCGGTCTGCGCGTGTGCAGTGTGGG
EMPV1_24020	TGCAAACAGAGTTACTGCTCTCAGTTCGCCCAGATCTACCCCCTTCCTTTCCCAGAAAAG
EMPV1_24024	GTTTGGAACGACTGTAGCTGTCGGCCTGACTATCTTTGTGCTGTCTGTTGTCACCATCAT
EMPV1_24025	TGAAATAGCTAACTCTCTGCCTGCCCTGCCCTACCCAGAGGATCCCAGGACTGGTTGTTT
EMPV1_24027	TTTAGGGCCGCACTTGTGGCATATGGAAGCTCCCAGGCTGGGGGTCGAATGGGAACTGTA
EMPV1_24028	ACCAGCAGTTTTGTGATCATCCTTGTTACCCTTCAGCATACTCTGGCAGCTGTGTCCTCG
EMPV1_24030	TCGTTGACCTTGTGTTCTTCCGACACCTTTAACTTATTGATGAGCGTCGCTACCAGTTCC
EMPV1_24032	GTCATCAGGATCAGAAGTATCAATGGTGTGAGTTCAAAAGTCCAAAAAGTGTTGCAGCCT
EMPV1_24033	CAACGCTACCTCTAATCTGGGGAGCCAAGTCTGTACTGCTTCCAGTAAGACATCCTGACT

EMPV1_24035	GGTCATCATTCATAGGCAGAGAAGCTGATAGAGCCCCGTTTTATTTGCAGGAGAGCAGGC
EMPV1_24036	GATTACAAGGGCCAGAAGCTAGCTGAACAGATGTTTCAGGGAATTATTCTTTTTTCTGCA
EMPV1_24037	CTGGACTGGCTGTGTTCTAGTCTGTCGCGCTGTTACAATCTGCATTGGTGCTAGTCAGAA
EMPV1_24038	CTGATGATCTGTACCATCTAATTGTCCAACTTCCAGACGCCAACCGCTCCATCTTGGGTC
EMPV1_24040	CACAAATCGTCATTTTCCTCCACCCAGCACCACTGGCAATGAATGGCCTTCTCAGAAACC
EMPV1_24042	ACAAGGAGGTCCGGAGCCAGAGGCCGTGGAGCGGGGCCCCTGAGACCAGTCACCGGCCTG
EMPV1_24043	TCCTGCTGTCTGCCTTATTTCCGCCCCAGCCCACCCTTACCTGCTTTTGGAGTCAGAATT
EMPV1_24044	AATCATGAGTGGAGTATTTTCCTTTGTGAATACCCTGTCTGACTCCTTGGGGCCGGGCAC
EMPV1_24045	GCCTTAGTCATCTTCCTGCAGTAGCATCTGGACATCAAAAGTCCTGCTATAAATCGTTGA
EMPV1_24046	CCCAGGAAAAGTAACCAGTTTAACCTGTGGAGACATGGACACAACCTTCCAAGCATAGGA
EMPV1_24047	GTACACTAGTTTCTTAGGAACCAATTGATTTTTACCAAGTACAGAGAGAAATGTCAACCT
EMPV1_24048	GGGTCACCGTTGGCGACACCAGCTGCACTGGTCAGGGCCCCAGCAAGAAGGCAGCCAAGC
EMPV1_24050	TTAGAGGAAAACGAGGAGCCTTTCATTGCCCCTTTAGGATTGAACGTGCCGCCTGATGTG
EMPV1_24051	ATCTTCAGAATAACTTCATTGGAAAAATTGAAAATGTTAGCAAACTCAAGAAACTTGAAT
EMPV1_24053	CCTGTAGATGATGGATAAAAGAATAGGAATAAAAGTAGGTGAGTAATAGAATGGGAGGGG
EMPV1_24056	CCTAGTGACGTGTGCCCTGCGTCCCACTGGCATCTATGGTGAAGGCCACCAGATCATGAA
EMPV1_24058	CTGGAGCCTCCTTCGCCCGCACTGAGCAGGGGAGCAGAGAGCAGAGGCTGCGAGCTCAAA
EMPV1_24059	CCGATTCAACCCCTAGCCTGAGAGTCTCCATATACTGCGAGAGTGGCCTAAAAAGACAAA
EMPV1_24060	GGTGAAAGTCCTGGAAGCCATGGGTATGAGTGAAAAGAACAAAGAGGAGAAGCAGTGCGG
EMPV1_24062	GCTGTTGAGATCCTGAAGACAGCTCGTGAAATCAGCATGCGTGTCCGCTTTTTTCCCTAC
EMPV1_24065	GTTCTCTATTTCCTTATTGATCTCCTGTTTATGGGTTGTTGTTCTATTCATTACTGACAG
EMPV1_24067	CTTGCGGATCCTAAGTTTGCTCAATTTTCACAAGAAATAGGCCTGGCATCTCTGGGAGCA
EMPV1_24069	ATTACTCCCTTTCCCTTTTGTTTCGGCATGTCCCTCTGGGAATTTCCGGTATTGCGGGCT
EMPV1_24073	ATCTTACGTGGCCATTTCACCCTGGAACTGAACATCTTCCACATACGAACCATTGCCGAA
EMPV1_24074	GCGCTACTATGCTCCTGAGGGAGAAAGGTGCAGTTCTGTTCCCTGGGCGATCTGGACATT
EMPV1_24076	TCTTCCAGAAGCACCTACAGGAGGGCCGGAGCCTGCACTTCGGGGAGTTTCTCAACACCA
EMPV1_24077	TCAGGCTGGTGGAGAGGCGATAAGAAATCTGGCTATGGGATCCAGTTTTTCGGACCCAAG
EMPV1_24081	AGGACAGACGGCCGGACAGCACAGATGGGTGGAACAACTGTCTGAGGACACAAATAAAGT
EMPV1_24082	CCGTCACCGTGGGAAATAATCCTGGTTTGGTCTCCAAAAGAATTGGCCCCCTTTCATGGA
EMPV1_24083	CATTGATGAACGGATGAAGAAGCTGGGAAAGTGAGCACCTTTGGGAGATGGAGAACAGGG
EMPV1_24085	TCCGAGCGCGCCGCCCATAGGGAAGCCCTCGGAGAAGAAGACCAAGTGGGGCAAGAGGAA
EMPV1_24087	AGATCTGGAGGACGATACCCAAGAAGCCAGTGAGAACCAGCCAGAGAAGCCTCGCTTTAA
EMPV1_24088	AAATGTACAGACTCATCAAGAATCCACTCCAAAAACCACTTTCCATAGAAACAGTGGATG
EMPV1_24089	TGGCACAAATGAGGAGAATCTCTCCCTTCTCCTGCCTGGACCACAGAAAGGACTTTGGAT

EMPV1_24090	ACCAAGATGGAGTATGAGTGGAAACCTGACGAGCAAGGACTTCAGCAAATCCTGCAGCTG
EMPV1_24092	TGAACATCTACTCCAGGAATCTGTTCTGATCCACCACATGCTAGGGCCTGGCTCATCTGG
EMPV1_24093	TGCTAAACCTGCTCCTCCAAAGCCAGAGCCCAAGCCTAAAAAGGCCCCTGCAAAGAAGGG
EMPV1_24097	AAGGAAGCCAAAATGGAAGAAAAGTTGTTTAACCCACCCCCAGCCAGCGCTCCCGGCAAA
EMPV1_24099	CCTTCATCTTCTTCACTTGTCTGATGCTCAAGGTTTCCCAGTTATGGTTCTGTGGCCCTA
EMPV1_24100	GGGGCTGAAATGCTCAAGTTTCCAAGCTCCAGCGCTTTGACCCCGTTTGGTTCTACAAAA
EMPV1_24101	AGGTTTTATCAGAGTTTCACGTAGCGCAAGCCTTCACGCGGTGACTCACGGCTTCCTGAT
EMPV1_24102	ACGCCCGATCTCATCTGATCTGGGAAGCTAAGCAGGGTCGGGCCTAGTTAGTACTTGGAT
EMPV1_24103	GATGATCTTAGGAGTGTGGAGGAGACTCGCAAAGTAGAGAGAGACCAGCTCAAGGAAAAC
EMPV1_24104	GGAGTATAGTTTGAAGCCAGGGATTGTGATTCTCCCAGATATGCTCTTTCTCAAGGTTGC
EMPV1_24105	CCTGGGCGTCTGTGTGGACTTGACAGAAGGGACCAAAAGCCTTGGCACATACATGTCTTT
EMPV1_24108	TCCCAGGATAGTCCCAGGAAATTGAGTTGAAAGAGACTGCAGAATAGTGCATGGCAAAAA
EMPV1_24109	GATTAAACAGCCTTTCTTATCCCTGCAGTCAAACCTTAAGTCCACCCGGGGACCCATCCT
EMPV1_24111	TGACTGTTCCCATGTTCCCGTGTCTGCCATAAATCATACGTTTTCATCCTCAGAAGCAGG
EMPV1_24112	TGTTCAAGAGGCAGGATATGCCCAGAGAATGTCTCCCCTTTAGATCCCAGGGATGGAGGT
EMPV1_24114	TGGCAGGCACCTGGACGACATTCATTGATGTCTGCTTCACAGCGCAGGCCAGTGTATCCT
EMPV1_24116	AGAAGCAGGTTCTCAAGTTCACTCTTGATTGCCCGCACCTGGTGGAAGATGGAATCATGG
EMPV1_24119	CCATTTCACCAAAAGTTACAAAAGTTTCCACAGTTCCCCTTAGCAAACTGAAGCTCACTC
EMPV1_24120	GTTAACAAGGCAAATTTCCAAGTACCTCCCCATGACCTGCTGAATCAAAACCTTCAGCGG
EMPV1_24121	TGTGGACTCACATTCACAGCAGGCAGTTCCCATGAAGTGCTGAGGTTGTTTCCGGGACTT
EMPV1_24122	CCTGCAACAACACTAACGCCCTTTGGAAGAGTGAAGCATATAAAGACTGCCTTGGGTCGC
EMPV1_24123	CATCATCAGCGACAAGCACTTCTACTTCAACCAGACCAAGGGCTTCCCCTGCCTCAAGAA
EMPV1_24125	AGAGGCCATGAACGCAGTGCTGATGCCAATTCGAGACGCTCTGACCCCCGAATCTGATGT
EMPV1_24126	CTGTATTGAGACATTAGTGACCACAGGGGCCAGTGTTAATGAAACAGATGACTGGGGCCG
EMPV1_24127	TGGGCCATTAATCTTCTTCTACACCTGGCCCTTCCCCTCATCACAGCTGGACAAGTTTTT
EMPV1_24130	AGACACGGAGGAAGCGGGAACAAAGGAGTTAACAGCAGCAAGCTGGAACTGGGTGTGATT
EMPV1_24131	AGCCTTTCAATAACTCACTGCCTGCTAACGGAAGAAGACGTGATGCATCTGTCCCAGTGC
EMPV1_24132	CACAGTGGACTTGATTATAAAACTCAGAACTGGCATTCCCATCCTGGCTCAGTGGTCAAC
EMPV1_24133	TGGTCGGCATTGGTGTTCGGCACACCCTTGGGCGTGATTTTTTACCGACTCAACCAAAAA
EMPV1_24134	GCCACTCAGCCCTTGATAGCAGCACTTGCTTTCCTATGCGGTCAGTCTTTTGTTCAGTGA
EMPV1_24139	TTAGTATATAAGGCGCGGAGGCCGCGGTCAGGGTTCAGTTCTGGGCGTTAGCATCGTCCG
EMPV1_24141	CGCATTCCTTCTTCTCCCTGGAAAGATCTCAGCCCCCACCAATCAGCTTAAACAACCTAG
EMPV1_24143	AGGGCCAGCCAGAGAATGTACTTGTGGGGTGTGAGGTAGAAGTGATTGATACTGAAGTTC
EMPV1_24144	AGATCCCACAGTGCAACTCTCAACTGCCGCCTGATGGTAAAACAATAGTACACGTGCAAG

EMPV1_24148	GTCAGGGCAACCACATCTGGCACAGGGGATTTGGAAAACTCTCGTCGGATTGGTGTTTTT
EMPV1_24149	GGCAACACCAAAGGAGATGATGCTCTAGAGAAAAGGTTTCTTGATAAAGCTCTTGAGCTG
EMPV1_24150	TGTCCCATGCAGTATTTGGGATATACTTATACTAAAACATTACTCCTCGTCTATCTGAGG
EMPV1_24156	TAGCCTGCAGTTCGATCTGGTCAAGAATGGCGAGTCCATCGCCTCTTTCTTCCAGTTCTT
EMPV1_24157	GCTGTGTATTTTAACAAGATCCTGAGGTGGTTCATATGCATCATAAAGTTTGATACTGGC
EMPV1_24160	ACAGACCTTAAAGAACGCTTATGCAGAATCCATGCTGAGTCTCTGCTCCTCGAGCCCCCT
EMPV1_24161	AGGTCGACACTTCGTTCTCGGTGGAGACCCTGAGGACGACAAGGAGGACAACAAGGATGA
EMPV1_24162	TCTGTGACTCGGGTCCTTTTGGACGAGTTGAAATTGGTGTGGGGGTGACCAGAAGCTGCT
EMPV1_24165	CTTATTCTTGGGCTGTAGCAGTGGACAATTTAAGAAGAAGTATACCCACTCTAAAGGGAC
EMPV1_24166	CCATCATCACCCAAAATCATTGACACAGAGACTTGCCCATGCGATCTGTACCAGTTCCCG
EMPV1_24167	ACTGCGCCCGGACTTTTACGCACCCATCGAGACTCACCTACCACCTGCGGGTCCACAACA
EMPV1_24168	AAGCAGCTCGTTCCAGGTGATGTATTTTCTTCTCAGGAAGCACTGATGAATTATTTCACC
EMPV1_24171	TTCATTGTTCACATATTTGTCTCTGTGAATACTTTGGAAAGCAAGGCATGAACTGCCAGG
EMPV1_24175	ACTCATTTCCTCTCCCTCCGCGAAGCCGAGATCTGGAGCTTGGAAGAAGCGGCGTCTGGG
EMPV1_24176	CAAACTCTGCAAAACAATGGTTGCCTGCTGTTGGCTGGCAGCACTTCTGATTATCCTCCC
EMPV1_24177	CAGATGGTAGATGTGGCCCTGTGTCCTGGCATCAGCATAGCTTCCGTTAGGCGGTTCCAA
EMPV1_24178	TGCAGACCATGCTCGAACTGGAGATGTCAGGGAGGCTGGCAAAAAGCCCTAAGACGGTTT
EMPV1_24181	AAGTCAGGGACGTGCGATGTCTCTGAGCAAGAGATAGGAAGTGCTCAGCCTTCACAGAAA
EMPV1_24182	GAGTTTGGAGTCCAACCAAATACGACACAATTCCAAAATAGGCAGCCAATGAATTTCAGC
EMPV1_24183	GTTCGATTCTGTATCCAGTCCTCTCTCTACCGCTCTATTGCTTGTCACATATGTTTTGGG
EMPV1_24184	TCCATCTCTAGGGACCAGCCCAAGTTCATGGCTCTCTTCTATGGAGTGGTGACTCCTACA
EMPV1_24185	TCCCTTAGGGTGTTTTGATCTTCCTTGCATCATCAGTTTACAGTTTCCATCAACTTTGGG
EMPV1_24186	TCTCCATCCGTACCTTCCTCCCAAGAAATTCCTCCTCCACAAGGCGAGCCTTGCTGCTCA
EMPV1_24187	TTACACGGAATACAGATACGGTAACGTGATATATTGGAGGGGAAGCCAGAGCTCACCCCG
EMPV1_24188	ACCTCAGTTTGACACAACAGCAGAAAGCTGGCTCCCAGACAATCAGAAACGTCGTCACCG
EMPV1_24189	GGACTTGGAGAACAGACTTGTGGTTACCAAGAGGAGGGTAAGTGTGATAGACTGGGAGTC
EMPV1_24190	GATTCCTACCTAGAGGCTGTCTGACCTTAAACAAGTCACTGACCTCCTCTCTGAGCCTCA
EMPV1_24191	AAACAACCAGTCCTGGGATCCTCTGGGTAGGGCAGGGCAGGCAGAGAGTTAGCTATTTCA
EMPV1_24192	ACTCTTGGACTGTAAGCGCGTGGTGCAGATTGGCATCCGGGGCTCTTCCCTGACCTTGGA
EMPV1_24194	TGAAGAAACCGCCAGCGTCCTGATGGGAAACATCAATAGCACAGCCCTTCTTCCACACAC
EMPV1_24196	AAGACACTCAATTTTGGCCATGACCCCTCCTGGGGGGCTTGTATGGGGTCTGGGAAACAA
EMPV1_24197	CTCCTTTGGGAATAGAGAAAGAAGGAAAAGGGGAAGGGGTACTCCAGCATGAGGAGCAAG
EMPV1_24198	TGCTGGACCGCTTTGAGAAGACCAATGAGATGCTATTGAACTTCAACAACTTGTCAAGTG
EMPV1_24199	GGTGGGTCCTGAATATATCAAAAGTCCAGTCTTCATTGTCTAGCACTTAAGGAAGAAAGG

EMPV1_24201	ACTCTATGATCCTGAAAAAGATAACAGAGATCAGGCTTGGGTTGATGCACAGAGAAGAGG
EMPV1_24202	TATACCAGGATGAAAAGGCAAGACCTGCTTGGCTTTCCTCCTTGCAGGCGGTGTTAGTGA
EMPV1_24203	GTATCTTTAGGTGGGGTTGATCTCACAGTCCGGGTGCTCACAACAGGATATTGGCCCACT
EMPV1_24205	TGGCCTTTGCAAGCAAGAAGGGCAACTGCGGGCATATGGAGCTGGACTGCTTTCCTCCAT
EMPV1_24206	TCTCTCTTGAGTCCGGCATAGGCCTTCACATAAATCAGCCTTATGCAGTTCTTGCTTGCC
EMPV1_24208	CTTCTAGATGCCAACCAGGGTGTCCTGCATCAGTTCGCTCCTTTGCCTTTTCCCAGAGAG
EMPV1_24209	TACATGGACCCCTATGTTCACAGCAGCACTATTCACAGTAGGTATGACATGGAAACAACC
EMPV1_24211	CTGTGTTGAATGTGAATGGTGGTTGTGGGCACTCTTGTCTCGTGCCTGATCATAGAGGAG
EMPV1_24214	GGGTGCACTGTCTTTGCACACACATTTGGGTATCTTCTGAACACAGGACTCTTTCACCTT
EMPV1_24215	GTCCTTCTCCACACTGGATGCAACCCACTTCTGTCCTGAAAACCGAAAATGTCTGCTGAC
EMPV1_24216	CTCCTGAAACTGTTTCTGCTCAGATCACCAGAGAACCTCCTTGTTGCTAAATCCAGTGGT
EMPV1_24218	CAGTCTGCATCAATACAGTTCTGTTTCGGGGATCACTTCTCAATCACTCCAGTTTTCTCC
EMPV1_24219	TATATCCCATCACGGGAGGATTCAGGCAACCCTACCCATCCTCACTGTCAGTCGACACTT
EMPV1_24221	CCGAGGGTCAAACACCTCACCCGGACAGCAGGAAACCTCAAGCCGGTGTGGGTGAACAGA
EMPV1_24222	CCTCACTCGGTGGGTTAAGGATACGGCGTTGCCACAAGCTGCAGTATAGATCTCAGATGA
EMPV1_24224	TCTCTCCACCCACCAGCTCCTACGCCCCATCTTCAGTGAGGACTCATTCATTTCCTCTTT
EMPV1_24226	GAGAGTGACCATGGACTACCACAGCCATGATTGCAGAAGCTGTAGCAGAGTGGCTCTAGT
EMPV1_24227	AAGGGTACAACTCTTGCAGTGGATTATTTCTTTGACGACGGCCACTTCTGTGGGCGGAGG
EMPV1_24229	CCACTTTAAAGACTCTATCTCCAGGTCAAGTCACATTCGAAGGCACCAAGGGCTAGAACT
EMPV1_24230	CCCGACAGTGATGATTTTTTGGACAGTTCAGAAGAAATATACTACACTGCAAGATCTAAT
EMPV1_24231	GCTGGAGAACCAGGGAGATCTGATGTTGCAGATCAAATGTGAAGATGGTCAGCTGGCAGA
EMPV1_24232	GCAGAAATTTTCTTAACATTAATGCCCTAGGCCACCCCAGACATGGGATGATCACAGCAG
EMPV1_24236	CCTGCTCAGATGTCTCTGTTCTTGAAATTATCCCTTCCATCTCCTCTGGGTCCATCATCG
EMPV1_24237	GAGCTCCTTTCTGTTACTCAGATAAAGTTACTGCATTGGGAATCCGGAGAGGCGTGAAGG
EMPV1_24238	CGAGACTCTAGGCTGCTTCCGGCTGGAGCTGTCTGTGATTGGGTTCGAGGAGGGCCCATT
EMPV1_24240	TCTGCTTTCCAGCCAACACTCCGGTCTCTGCTGTCCCTCTGCCACATGAATCCCTGCTGG
EMPV1_24242	ATGTGGGGCTCACTTTGGGGGTTCTCTTTGGGAAGGTGTTCAGCCAAACGACCATCTGCC
EMPV1_24245	GTCCCAAAGTGTCTGCCTGGCTCTTGTTGCTACTCCTTACATTTACGGCTTTGCCAATGG
EMPV1_24248	AGAGTTCCTTTTGTGGCTCAGTGGTAAGAAACCCAGCTAGTATCCATGAGGCTGCGTGTT
EMPV1_24249	AGATGTGGCTCAGATCCTGCATTGCTGTGACTGTGGCTGGCAGCTGAAGCTCTGATCCTA
EMPV1_24250	CCCACCATTAGTGCTCATTTCTCTTTGTGAAGGTGGTGTGAAGGGTACTGGGTCCTAGAG
EMPV1_24252	CATGGCCAGACTTGATTTCAGTCTTATAAGAGTTGTCGTTTGTAATCTGTATCCCTTTGT
EMPV1_24256	ACAATCCTTATGTCACGGGCCCATATTCTGAACCCCTTCACTGGCACATAGTAGATGCTC
EMPV1_24257	CAGGGTGCTACCCTTGTTACATCTCTTTTTCAGATTCATGTTGAAGCTTCCAGGACCCAG

EMPV1_24258 ATGGGCACGCGCCCTCCCTGGGCCCCACTTACAGAGTGGTCCTCCTCGGTTTCTGCCTCT
EMPV1_24259 GTTGTATTCAGCATGACGAACGAGACCCACAAGTTCCACGTGCCTGTGCTCCTGACCCCG
EMPV1_24260 TCAATAATGGCACTTTTCCGAGTGGATTTCAGTGGTCGTGGGGAATTGGCTGAACGGCAG
EMPV1_24261 AGATTGGAGATGGGGGGTGTTCTGTGAAGGCACCTAGGCTTGCTGGGGAGATGTCGTTTT
EMPV1_24262 TCTGCTGCTAACAATTAAATTCAGAAGTCTTCTTCCTTCCACTGTAGTGCCAGTGAGCAA
EMPV1_24266 TGGATTCTTAAACCAGTGAACCCGCCTGCACCACAGCTCTCAGCAACGCCGGATCCTTCA
EMPV1_24268 ACATTTATTGACCAAAATGGCAGACATTGAGTACAGACTTTCAGTTGGCACCAATGAGAA
EMPV1_24269 GACCCAAGTTTGATCTTTGCTGGAGTGCTGATTATGCAGAGAGTTGTTGCAGAGAAAACC
EMPV1_24271 TGTCAATTCCTTCAGCTCCCCCCTTGCCAACATGCAGTCGGTTCTCAATCCATAACAGAG
EMPV1_24273 CCACACATGAAATGTCATCATACAGCCACTCACACGGCTAAAGTTAAAGAGATGGACAAT
EMPV1_24274 CTCCAGATGGCCAAGTCTGCAGCAACAGAGGGATTTGCGTGTGTGGTGAATGTTCTTGCC
EMPV1_24276 TCCACAATCACCACGTATCAGGAAACAACTCTCTGGGGGCACAAGTGAGCAGAGGCTGAT
EMPV1_24278 GGGCAGAAATGGTAGCCCAGCTGTGTATGAATGACAGGACACATAGTTCATTACTGGACC
EMPV1_24279 TGGAATGTGACCATCATGGAGGAGACTATAAACACTGGAGAGAGTGGGGTTCTCATGCCC
EMPV1_24281 CCCCTCCCCCTAAATGGTTGCGCCCCTTGAACCTGTGCAATATGAGGCCAAATTCATTCT
EMPV1_24282 GGGAGGGGGAAGTGAGTAGACTCATAAATTACAGCGAGAACAGTATTGCTTTAGGTCTGA
EMPV1_24284 GATTATCGCAGAAGATGGGAATTACCGCGTACACCAGAACAGCCACACAAGGTACCACGA
EMPV1_24285 GCAGCTCGGTTAGATGGCTGACGTTTTCAATCTTTTTGATCCTGTTCGACGCGATGTCCA
EMPV1_24286 TGTGACACCATGGAGCAGTGGAGACCTTGCCCCAGCTTCTGTCCTTGGGTCCACCTTAAA
EMPV1_24287 ATGTGCCTCATCTTCCTCTTCTACTATCCCCGAAACAACATCTCCAGCTGCATGGGCTAC
EMPV1_24291 TTTCTGGGGCAGGGATCAAGTCTGTACCACAGCAACAACCCGATCCGTTTCAGTGACAAT
EMPV1_24292 TTCCCCTGCTCAAACTTGCCTGCTCTGACACTTTCCTGATTGGTCTGCTGATGGTTGCCA
EMPV1_24294 CGAGGTGAATATATTATGTATGGGCAGCACTATCACTATCCTCTACTCCGCAAAGACCAC
EMPV1_24295 CAAATCCACTCCTATGTCCTTGTCAACTCGCTAGACCTCACCCATGATGAGATGCTTAGT
EMPV1_24296 AGGTCCCAGTCCAGAAACATCCAGCTAAAGACCAGGAGCTGCCTCTTCGTGTTGGTTCTT
EMPV1_24299 TTTCCCATGCCAGGACTGTCATTCAGCCCAAAACCCTCAAGGAGGAGAAATGCTGCCAGT
EMPV1_24301 TAATCGCAAGGGTCGTTAAAAGTGGAAGAGGGAGGAGCTCCCTGGTGGCCTTGCGGTTAA
EMPV1_24302 CTGAGTTCTCTATTTTGTTCCATTTGTCTGTGTGTCAGCTTTTGTGCCAATACCACACTG
EMPV1_24305 ACGCCCCGCTGTAACCTGGAGGAGAATCTGCTGAACGATCACTGTAACCCGAAGTACATT
EMPV1_24307 AGACCTCGAAGCATGGATTCCTCCAGCACGTCCTGCACATCCAGCGGTAGGTTGACCTCT
EMPV1_24308 TTTGACCCAGGTGCTAAGCCAAGCTAACACTGGAGGTGTCCAGAGCACCCTGCTGCTGAA
EMPV1_24309 GCATGATGTATGGTGGACTCCCTCTGCCGACTTGTACGGTGATGCTGAAAAAATGAGTGA
EMPV1_24310 CTGTTAATTCGCCTAAGAGAGCGAGGCAATAGAGTTCTTATTTTTTCTCAAATGGTGCGG
EMPV1_24311 CCATAGCCATTATCCCACCTCCACAAAGTACCAAACCAGGAAGAGGCAGAAGGACTGCTA

EMPV1_24312	GGACAAACTTGTTGGCATCTGTCCATCCGTTCCTGATAAGCCACCAGGCTGAGAAACCTC
EMPV1_24315	GTGTATCATCTTTGGAGACTGGATCAAGATGGCGGAATAGAAGGACTGGAGCTCAACTTC
EMPV1_24316	GCCTGTGCACCTTGTGTGGCAACTTGGAAAGAAATACAAATTAAGAGAGCCAGTATCCCT
EMPV1_24318	CAGAGTCTCCATAGAACTATGCTTCGTGGTGTTCTGGGAAAAACCTTTCGACTTGTTGGC
EMPV1_24319	TGCTTCCTGCGGGCTGAGCTTGGGCTTCGCGCTGCCCGTCAGCGCGCAGATTAAGGCTGA
EMPV1_24321	CAGATGTGCTAAACTCAACCTTTCATAGTACATGACCTGCACTCCGCATCCCAGTCTAAC
EMPV1_24325	GCTCGAGGCAACACTGGATCCTTTTCCCATTGAGCCAGGATAGGAATTCCACCTTTTTAT
EMPV1_24328	AGGAGAAGTTGAGTCACAGCCTGCCTTTCTACCCGAACCATCACATGGTAACCTGTTAGC
EMPV1_24329	ACTAGTGAACCTGTTCCAGGCGAGCGGTCTGGGTGATAAAAGATCTCGGGACTGGAGCCA
EMPV1_24332	CCCATCGTGAAAGTCAGCGTGGAGGTGCAAAACCAAATTCAAGTGAAGAAGTGAAAGGGG
EMPV1_24335	CCACTGGGCAAGTAGTATCTAATTGGCAGCATCATCATTAGCAATAAGTTTCATTAAGTC
EMPV1_24336	GACTTGTAAATACCTCCACCTTTACCCTGTAACACGCTAAATGGATTTTTGCTTCACGGT
EMPV1_24337	GCATTCAGCTTGTCCTCAGTTGTTGGATCGCTGGGTTCTTTACCATCTTTGTACCTCTCC
EMPV1_24338	TGACGATTAAAATGGCACAATAGTTACAGCTACACAAAAATATATACAAGGATTCGAGAC
EMPV1_24340	GAACAGTCAGATACACAGGCAATCATCAGCTAACACCGCTGACCTAAGAATGACCCTAAG
EMPV1_24341	TTCTTCAAAGGTGCCTGGTCAAACGTATTGAGAGGCATGGGCGGTGCTTTTGTATTGGTA
EMPV1_24343	GCACTCCCTGCAAACAAAAGTCCAGGACCAATTGCTTCACAAGTGAATTCTACCAAACAT
EMPV1_24345	ATCAAGAATCGCTGAGACAGACACAAAGTGGGACTTCTGAGGTGTCAGAAAGCCAGCCCT
EMPV1_24349	GCTTCTTGGGCCTTAATGCTATAACCCATATCCCCACCCTACTTCCCATCTTCCTGTCAG
EMPV1_24350	AAGGGAAGGCTCGGGCCATTGTTTAAGCACAGATTCCTCAAGTGCCCTCTCTCCTCTGTG
EMPV1_24351	CTCAATTACTCACCCAGCAGTTAGGGACAACTGGACAACTGCAGAACTCCCCAAGGAGGT
EMPV1_24353	TTCACCTCTATCTGACCAAACCGCTCTACACATCAGCACCACCCCTGCAGCATCTCTGTA
EMPV1_24356	AGTCGGAGCCGGGGCAGGAGTCACCGTCGCGGCTGGAGCGGAGTGAGCGGCGTGTTGAGA
EMPV1_24358	CTGTATGAAAACATGGAAGTTCACTAGATATGACCTAAACCCTGTTGTACAGGGATAGAG
EMPV1_24359	CGGTTTGCCATGAAAGAAATGAAGGTGGTCGCAGCCCTCAGCTTGCTCCGTTTTGAGTTC
EMPV1_24361	GAGTCCATTTTGCCCAGCCCCCTGGTCTCATGCAGCCGTTCAGCATCCCCGTTCAAATTA
EMPV1_24362	TGAGAGAGACCCCACAGAGCTCCCTAGCCCCTTCTGCCAAATGAGGAAATCTAGAATCCA
EMPV1_24364	TAATTCTCATCTCCTCCCCTAAACCTGCCTAGCCCCACAAAATTCCCCAACTCAATTAAC
EMPV1_24365	ATGGCTAGAATACGGTTCCCTTGGGGTCACCTGAAAGTGTGGGGGCACAGTTCTGATCAA
EMPV1_24366	GCTGGAGAAGGTAGAGGAGATGGCGCAGGGGACACGGGCCAAGACTAGGGGTTGCTGGGA
EMPV1_24367	CCAGAAACACTGCGGACCTGAGAACACTGGCATGGTTGGTTACTTTTCCAAGACTCTGTG
EMPV1_24368	GAACTGCATTCTCTCAATGCCATACACACAGGTCCCGCCATACAAGCGGAGATTCAGGAA
EMPV1_24369	CAGATCTGGCATTACTATGTCTGTGGTGTAGGTCTGCAGCTACAGCTCCAATTAGACCCC
EMPV1_24373	GTTGGCCCTAACTCCACGCGAAACCATTCTTCCTAACTAGCTCAGAAAACCAAGCATTTC

EMPV1_24375 GGAACTGACATTGAAGACCAAGATTTGGAGAAGTTTAAAGAGGTGACAAGAGAGAACGGG
EMPV1_24376 CCCCGTGCTGTATGGGGAGTAGGACCTTGTTTAGGTGACACCGTTGATTTACACGACTTT
EMPV1_24378 GGTTATAAAGTCATACCCACTTCTTCCACAGTGACCACAGCAAATGACCAAGCATGAACT
EMPV1_24379 TAGGACTGGGGGCCTGAAGGTCATCCAAAGAACACTCACTCCTCAGGCCCACTGTGGCCT
EMPV1_24380 TGCCTCTTCTGTCGTCTTTGGAAAGGAGTCTAAACTGAGAGCTGGAAACCTGAACCTGGC
EMPV1_24381 CAAGATCTTCCCGCATGGAACTAGCTTTAGCAATATAATCACAAATCTCAACAAGCCTGC
EMPV1_24382 TACATTTGGTGGCTGCAATTTCCTCTCCACTGTTCCCTTAACCCAGAGGTGGTTCGCCAT
EMPV1_24383 AGTATGACAATTCTGTGGATGTCTACGCTTTTGGCATTCTTTTCTGGTATATCTGCTCAG
EMPV1_24384 GAGGATGCCGTGGGACCTTGTCCAGCGTCTGTACAGATGTGCGGTCTGACCACTGCCAGT
EMPV1_24385 AAAAGAGCGATCCGACAAGTTTGGCCTGGTGGTGATGTAGATGTTCCTTGTCACAGGTGG
EMPV1_24386 GGTAGCTGGTCTTCTTAAGGTAGGAAAACAATAGTCAAGGAAAGACACCTGGTGATGAGC
EMPV1_24387 TGAAGATCTCCACGATGTCATTGGACGTGGTGCGCACAGGGTCCACGTCCTTCTCGCTCA
EMPV1_24388 ATTGCCTTCCCCATTGGCTCTTCCATGGCTCCTTCCACCTCTATCTTGGCTATTCTGGTC
EMPV1_24390 CCATGGAGAGCAGCGACGAGGAGTCGGTGGACAGGCGCCGCGTGCACCTGCGCCCTTTTA
EMPV1_24392 TAGGATCCTATGTGGAGGAGACAAGCCCCTTGGCTGGTTGGAGAACCACTGGGCTAGAAA
EMPV1_24393 CACACAGACACACCCCTTCCTTTTACTTGCAAAGGAGAGTCAACTTGTATTTGCTGAATG
EMPV1_24394 GACACTGTTTAGATGGAGTTTTCAGCCCAGTGAATCTGAGGGCCATCTGTGACCCATAAA
EMPV1_24395 TGCTAAGAAACATCAACGACGGGCTATCCAGCCTCTTCAGCGTGGTGGAGCACTCGGGGA
EMPV1_24396 CTGTGCCACAGCAGGGACTCCTCAGCTTTCATTGTTGAATATGATAGTGGTGAGCTTTTC
EMPV1_24399 CAGTGCATTCCGATCAACAAGATACCGCACTGAGAGCAAACAGCCCAAACTGCAAAGGAG
EMPV1_24403 GGCAGTGTCCAGACAAGAAGAAATGTTGCCGAGATACTTGCGGAATCAAATGCCTGAACC
EMPV1_24406 GATGCGTCCCACCCAGGTGTCTCCTCGCCTGTGTTTTCTCAAGTGTAATCCATGCACTTT
EMPV1_24408 TAAAACAGGCTTGTCCTTAGAGCTAGAAAGGTGGCGTTACCCTTGCATCCTGCCCAGAGC
EMPV1_24409 ACCTCCGGGACTGACAGGGCAGCCACTTGCAGAGGTCTCAGAGTTATAGTATTATATTTT
EMPV1_24411 TTCGTTCTGAGGGAGCCACATGGACTATGGAGGCCATCACAGCAGGCCAAAAGTCATGTA
EMPV1_24412 CACGGTGGGCACTTAACGCCCTTGGATTTCAGGCTTGACTTTCCTCTGTTGTTGAGGATA
EMPV1_24413 GGCAGGTCAGTGGGTTTATGCTCATTTTAAAGGCCAATTGGGAAAGCGGAACAGAGGAGC
EMPV1_24415 CACGGATTGAGCATTTCTTCTGTGATGTTCGTCCTGTGTTAGACTTGGCCTGTGCTACCC
EMPV1_24418 CTTTCCGCCACCTTTTGTTGGTCAGGGAGGCCCTTAAATGGAGAATATGACAAAGCAGAA
EMPV1_24419 AGAAATCGCATTCGCCGCACGCCTCTCCTCAGGGCACAAACCACCTGATAGAAGACTTTG
EMPV1_24420 ACACGGCTATATGTAAAATAGACAACAAGGTCCTACTGTATAGCGCAAAGAACTAAATTC
EMPV1_24425 GACTTTGCCTTAACTTTTGTATAAGACAGCTTAGAGTTCGTGAGCCCGGCCCCGGTTGTC
EMPV1_24426 AGAAACAAGGTATCCATGACGATGAAGTTCCACGGAGAGCCCAAGAAGCCCCCGATGCAT
EMPV1_24433 AGCTGCCTGTCTGGCCTACTGGGTTCTTACTCCTTTGTCACAGCCAGCACCCCCTCCTAT

EMPV1_24434	GTGATGGATGCTGACAAGGGGCGCAATGCAGAGATGAGCCTGTACATAGAGGAGAACAGT
EMPV1_24435	TTGGAACCACAAATGCAAATATGGCCAATGAAATCACGCGGTGCCTCACCCAGAGACCAC
EMPV1_24438	CACATTGTTATGTGAGAAATGTTACTGGGGAAATAGATCAGCCACTTTTGAGGTGCTGTC
EMPV1_24439	GCTGTTTGCACAGACACTTGGAGCCGCAACCGAAGCATTTGAAAACTGAGGGAAAGCGAT
EMPV1_24440	AGAGCCTTTGGCCCTCCCTGTGGAAGCTGGTGATAATTGCGTACCTAGAGGAAATCGTAG
EMPV1_24442	CATAAATAGAATCTGTAATAAAGGTCCATCTAACTTAGTATTGTTCACCAATATTCCACT
EMPV1_24446	GGTTTCGCTGACCCACCACCTTGGCCTTAATCCCTATCTCTCTTCCTCCTATTTTGTCAC
EMPV1_24447	TGACCCCAGGCCCAGTTGTCTCTACAGCCTGATTGGTTCCTCCATTATTGTGGGTTCTGA
EMPV1_24449	TTCAGAGCCCCCGTTACCTCCTTGCATTTCCTCCTCTGGGGTTAAAGTTCCCTGTATGGG
EMPV1_24452	GTGGAGGAGTCCAAAATAGTTGCAGTCTTTTATACATTTCTGAGCCCAATGCTGAACCCG
EMPV1_24454	CTTCTGGGCTCTGACCTCGTCCTTATTGTCCTCTCTTACTCCTTCATCCTGAAAGCTGTG
EMPV1_24455	ACTGCTGGACAAGCGCTGATTATTCTTCTGCGGCATCACACACTTGCAGTCTCTACGTTG
EMPV1_24456	CCCCTCCTAAGATGGACTTTCCTAGAGATCCGAGAACCGCATGTTTGGTCTGTTTCAGTG
EMPV1_24460	ATACTTTCATTGCAGAGAGATGTAGCCACAAGCCAACGAATGCCAACAGCCACTAGAAGC
EMPV1_24461	AAACTTGAACCCTGCTCACCAAGCATGTCTAACTCCTGTCCCTCCAGGCAGTACCAGAAG
EMPV1_24462	GGTAACCACAGGGGGAGAAGGTCTTGATGCCTGCTGGTCAGTGGGAGCATATAACCTTTT
EMPV1_24463	GAGTGTCCCCGCTGGTCTGATGGCGGGACTGCAGATACTAATGAGAGCTCTGGGAATTCC
EMPV1_24464	GTCATACCTGTTGGAAGGTTTCTTTTTCTCTGGTCCTGCATCTTCATTGGAAGTTCTTAT
EMPV1_24465	GGGCCTGTCTGTGTTCATGACTTCAGTACTAAAGGTAAAATCTCTTTATTGCAATATCTA
EMPV1_24468	GGAGGCACTGAACTCTGACTCCTTTTCAGGCTATGGAAGAACGAAGTCCTTACCTGTTGG
EMPV1_24472	CCCAGAGAAAAGGAGTTGTCGGTCATAACACCCATCAGACATTTGGAAAACAACAGTGGC
EMPV1_24475	AGCACCCCAATCCACTCCGTGAGAAAAACCGACGGGTGAATGAACTGGTACGAGCGGCAT
EMPV1_24476	TGTGGTTCCCATCTACTGGTGGTGTTCCTCTTCTTTGGGCCCAGCTCAGCCATATACATT
EMPV1_24477	GTGTGGGAGAAAGGGAAGGAAGAGTAGCCTCTGCATTAGTTGCTCGGCGACATTACAGGT
EMPV1_24478	TCCTCTCTGTCTCATCTGTGGAACAAAAACCTTCATCTGTGGAGTGCAGGGCATCTCTGC
EMPV1_24481	CTGTTTGTGATTAGTTGGAATGACATGTGTGGGCTCAGAGACTGCTGGTTGCCTGTGAAT
EMPV1_24485	TTACCAGGTTCTGCCCACTACATCTTGTCTCATCTCTGCTACTAACACCCTAGGCCAAGC
EMPV1_24490	AAGTTGCTGCCCAGGAGCAGTGGACACTGGGATTCTGAAACCTCACCTTGGGTCTTTTGA
EMPV1_24491	GCACTGCACACATGTAACTTGCCCACACACACCCTCTCCGACTAATGTAAAGGTCTCAAC
EMPV1_24492	TGTACACACATGAACATATATACACACGCAGGGAAACACCTGCCCCGCCGACGGAGAGAA
EMPV1_24493	AAATTTGAAGGCGCCTTTAAATATAAAATGGGTCAAATAGAAATTGACGATCAGGTGGAA
EMPV1_24495	GAGTTTGAATAAAGAAAACCATGACGACGCAAGCCCGACTGGACTGCTTGTGTCTCCTGA
EMPV1_24496	CTAATAACTCTAAAAATGCCAACTGAACAAAATCTCTTTGGCACAATCAACTTGGTCTCC
EMPV1_24499	TCCCAGTTTCTGGCCTGTAGCCTAGAGCTTCATTCCTTGAATTTACACTCCCTTTTCTCC

EMPV1_24500	TAAGAAACCGGCCACCAAGAAGCAGACTAAGAAGGGAGCAACCGTCACCAACCAGAACCA
EMPV1_24501	CTACCAGTAGCCTCTGTATTAGTCCCCAGAGGTTCCCTAACAAAATAACCACAGCTTCGG
EMPV1_24503	GAAGACGTGAAGGCTCTAGCTTTGATTTTCTTCCTCAAGTTTGCTTTGGCTCTTTGGGTT
EMPV1_24504	AGAAGCAGAAGCTAACCAAGCTGCTGGCAGCCCGTGGAGCCAACATCCAGGAGCTGAACA
EMPV1_24507	GCTGAACATCGCAGTGCTGCTGGGTCACAGCCACGACGTGACGGAGCGTGAGCTACGAAA
EMPV1_24508	ATCTTCGACTCGCTCTTCTCACAGCCTCTCAACCCGTTACCGTCAGCCGGCCCCGCCGCT
EMPV1_24509	TATTTCAAAGGTAATAGGGGCCGCATATTCTATTTAGTTGCTCCGGGTCCAGGTCCGGGC
EMPV1_24510	CTGAGGGATCCAAGTGGTGAAAAGAGGCGGACATTCTCTCAGATGGAGACAACCACCCTT
EMPV1_24512	ATGTCTGTAAGCATCCTGTTGATGGGCATCGTGGGACCAATTACTGCCGGAGTCTTGACA
EMPV1_24514	CAGCCTAACCCCAGAGAAATGTGGGCGTCTGAAAAATCCATCAGTTGTGAACCTGAACCC
EMPV1_24515	CTTGGTGTCAGCTTTTCTACTCTTTGGCGATGCTGTTCCTCATAGTTCTGGACTTGGTAG
EMPV1_24517	ACTGACCCGACTGACCTTGTTGAGGTGCAGCTGCTGCTGCTGAAACTGCTGCTTGTGCTT
EMPV1_24518	ATTAAGGCACAGGGCTGGGACTGTCTGGAGATGGAGTAACCTAGTTTGGCCTGGCTTCAA
EMPV1_24524	ACGCACTGTGCCTGTGGTCATTTCTAGAACTTTCTGTGTTCTCGGCCTGCGTTGTTACTG
EMPV1_24531	ACAAACTCTGCAAAACGATGGTTGTCTGCTGTTGGGGGGCAGCACTTCTGATTTTCCTCC
EMPV1_24532	AAGGATGCAGAAAGTGAGAACAGCCCGAGTTTGCCGGATGGAGAACAAGGGCATCAGTTC
EMPV1_24533	TAATCTGTGAAGATACTGTAGTGCCAGCGTACACCTCCCTCCACCCCTTACCCCTTTCTC
EMPV1_24534	CAGGCTGGATATGTAGGTGAAGATATTGAATCTGTGATTGCCAAACTGCTCCAGGATGCC
EMPV1_24536	AGCCTGGGCTCCCAGGCCCTGCGCCTGCCTCAGGTCTTTTCTCGCTGCAGCCTGCTCCAG
EMPV1_24537	TGAGGAGAGTGAGCCCATGGAGAAGCTGGAGAGGGCCAATAGCCAACTGAAAAGACAGAA
EMPV1_24538	CACATTTGTACAGCTCGTGGTGCTCATTTGTATCCCGTGTTAGGACTTCGCAATTGGGCG
EMPV1_24539	TGAGTGGCCTGCAAGATCCGATTGTGGCGCGGATGTCCATTTGCTCCGAAGACAAGAAAA
EMPV1_24541	GCCTCTGTTATCAGGGCTTTCAGGCCCCACTGAATGATTTGGATAATTGCTCAAATGTGA
EMPV1_24542	CTGGATGCATTCAAAATTACCAGAAGAAGAAGGACTGGGAGAGAAAACTAAGCTCAATCC
EMPV1_24543	CTGACAGCGAGGAGGGCAAGGCCCCGCAGAAGTCCTACAGCTCCAGCGAGACCCTCAAGG
EMPV1_24548	ACTCTGTGCCCTGCTGGTGACTGGCTGCTGGATTATCTCCTGTTCCAGTGGCCTTCTTCA
EMPV1_24552	ACACTTCCCATGTTTCCAACCTCAGCTTTTGTGATGCCAAAGGAAGCCACAGTCACCTCC
EMPV1_24555	CTGTATGCCTGGTGTGGCAAAAGGAAGATGACTCCATCCTATGAAATTAGAGCAGTGGGG
EMPV1_24556	TTCTGATCCTAGATGAAGCCACAGCTGCCATGGACACGGAGACAGACTTACTGATCCAAG
EMPV1_24557	GGGTTTCAGTGTTGCCCGAGCACCTAGCTCATTAATTTCTGCTGGAAAAGGTGTATTCGT
EMPV1_24558	AGACGAACAGCCGGTACTATATCTGGTTGTGGCTGATGTAGTTGTACACCAGCTTGTGCC
EMPV1_24560	GGGAACTGAAGTCAAAGCAATAACATATTCAGTAATGCAGGTTTATAATGAAGAGAAGCC
EMPV1_24561	CCAAATCCACATCTCATTCATTCAGCAGTTTTCGGTACCTACTGTTTCCCAGACACTGCC
EMPV1_24562	ATGACAGAGCGCTACACGATCCACACCCAGTTAGTGCATCTGGAGTCCAAAAACATCGGC

EMPV1_24565	TGACTCAGAGGTTTGGACGCCACATCCCAGCTCATATCCACATCTTGTTGGCCAATGTCT
EMPV1_24566	GAAGGTGTAGACAAACTTGGTTTGCTCTGCTTATGTTGATGTGTGGTAATGTTTCTACTC
EMPV1_24567	CTGCTCCTCCCTGTCAGAATTAGCCACTGCTCCTTTCTCAATGTCATCCCTGCAAATAGC
EMPV1_24568	AACCAGATAGCCTGTCCACGAATGGTTATGTATCTTCACCTTCTCAGAGGCCCTGTACCC
EMPV1_24569	AGCTGTAGCTTTTGAGTGGAGGAGACTCGAGCTGACTGTAGCAGGATCGCTTTGACTGCA
EMPV1_24571	CGGCAGAGCTGATTTCTTCCAGAGGCTCTAAGGGAGAATCTGTTTCTTACTCCTCCTAGC
EMPV1_24575	CTGCAGGGTGAGACTGCTCCTCCTTTCGACACCTTCCGGTTCTGCCCCGCTCCTGGAGAT
EMPV1_24576	TTCATGCAAAATATGGCAGCCTAGACAAGTGCGCTAGGTATGATCTGTCAGCTGTTCACC
EMPV1_24577	AGCTATTCATTGCATCCCTGGTCTTGCTCCTCTTGCCCTTAGCACTGATACTGCCGTCCT
EMPV1_24578	CAGCTGAGAGCAGAGAAGTTATCCCAGAAGGACTGAGTCACTAGCCAAAAACTCAGCTTC
EMPV1_24579	AGGAGCCTCTCCTCATCCTCTGGAGCCGACATTCAGCAGCAGCACGGGAGGACGATACGC
EMPV1_24580	GCTTTAATCCTTCTGAGACCAAGATTCGAACACCTGATGGGAAAGTGTGGCAGGAGGCTG
EMPV1_24582	ATGAAGCAAAATGTGAATAGGTACATGCGTAAGGGTGTGTGGGGGCCAAGGGGTGAATCA
EMPV1_24585	GCCCAGAACTGTTAGGTGCCGGGAGTCGCAAACGTTGAGCAGGAGTTCGTTGAGGATCCC
EMPV1_24588	CCTTAGAGCAGCCTTTCCAGTTTTCCCAAGAAGACATGCCCCGAGTGCGGAAGAGGATTT
EMPV1_24591	AGGTACCCTGTCCCAGTAACTATGTGCTTTCTCGCCCTTGAATGTTAGGCAGTCGTTGTG
EMPV1_24593	CTTGGTCAAGTAAGTCTCTATGCTGTCATTTGAAGGGCGTGGACTTGAGGTAGAAATCAC
EMPV1_24594	TGGGCTGAAGAAGGCAGTACAGGGAAAGGGGAAAGCAAAGAGATGGACTGGGAGGCTTTT
EMPV1_24595	GATCCAATAATGCCCTTGTCAGTGTGTCTTCTCCAGTATGGTATCTGGTAGAGGGCCAGG
EMPV1_24596	ATTGTAGAAGCACTAGCACTGTGTTTTTGGTTCAGTAATGATGATAGTATGAAAAATGGG
EMPV1_24599	AGCAGACATGCTGGGCTCCTATGGTTCCTTGGCTCGTTTCCGCAACATCTCTCGCATTAT
EMPV1_24601	CTCTTGTACCAGGGGCCTGATCAGGCCTGTGTCTGCCTCCTTCCTGAGTAGGCCAGAAAT
EMPV1_24603	AGTCATGCAACACTAACCTTTCACTGGTCAGGACCCTTGCTATCAAATGCCCGCTGCCCA
EMPV1_24604	GGATTCCTTTAGCAGAAGAGCCAAGATGAGTGTCTGGAACCCTCTGAGACTGCGGGACCT
EMPV1_24606	GGATAAGTGTCTTTTTGTAGAAGACCTCCTAAGTGTCCCAGCAGCATACTACCCCTAGTC
EMPV1_24607	TCTATGACTCTCCTTGTCTTCCTGAAGCTGAAGCTATGTTTGCCATCAATGCGGATGGAG
EMPV1_24608	TTCATACTGACACAAATCCTCACTAGTTGACAGCTCCATTTAAGTGAGTTTCAAGATCAG
EMPV1_24609	ATCGAGAGCTACTCCTGTAAGATGGCGGGAGACGACAAGCACATGTTCAAGCAGTTCTGC
EMPV1_24612	TGATGAGTTAGGGACCCAGTGTTGCTGCAGCTGTGGCTTAGGTTGCGACTATGTTCTGAT
EMPV1_24613	GGGAGTTGAGACTGTAGATGCCTGGTTCTGTTCAGACACTATTGTAAACCTATGCTGCCT
EMPV1_24614	GGGAAGCATTCCTTACGTTTCTATATTTGTTTGGTTTTCCTCCATCAGTAGATCTGCTCC
EMPV1_24616	AAATACAGGGAGAGGGATCAAGCGCAGCGTGGCGATGGGCAGGAGTAGAACCCCGCCAGA
EMPV1_24620	AAAGCAGGGCCTTGAGCAGGATCAGGGGCTTAATGTTGAATCCAGCAGGAGCTTGTTTCT
EMPV1_24621	TACCAGCTGTCCCCTCCCTCCCCCAAATAAGTGCAGTCTACACAGTGTCAATATATTTTT

EMPV1_24625	ATGAACTCTACTGTACGGGTGCCTGTGTCTTTCTCAAGCCGAGCTTTGTCCGGATACATG
EMPV1_24626	GTCTTAGTGATGTTGCTGAGAGTACATGCTTTGTTGTATAATTGCTTGAAAGCCCAACTG
EMPV1_24627	TTCGAGGCTGAAAAAGTGCCAAGAATTAAGCACAAGAAGAAAGGCACTGTGTCCATGGTG
EMPV1_24628	TCATTGACTGTGTGCAGGAGCTGAGCCACTCTGTGAACAAGCTCTTCGGCCTGGCGTCTG
EMPV1_24630	GGCTGCTCATGCTGGTACTTGAAGCAGTCAAGGTGGAATTCTATGGAAAGCATCTCTACT
EMPV1_24632	ACTTCTACCGGAAGCTCTACAGCCTCCTTGACCCATCTGTCTTCCATGTCAAGTATCGGG
EMPV1_24634	GTGGTTTAGTGTCATTGCTGTCGAAGAAGGAGGCGGAGTAACCTCCGGTCAGCCAAGAAA
EMPV1_24635	TCAGGACTCGCATAATTAGCAGAATGCCGTCCTAAGGTTGTTGAGTTCTGCGTTTCTGGG
EMPV1_24636	TATAATTCAGACCCCAAAGGGATAGGGCCAGAATCCCCACGCCTCTCCTGTCTGTTCCTT
EMPV1_24637	GGACTGATTCCATAACCTGCCCATTAGCAGCTGTCTCTCTATGACTTAATTGGTGGGCCC
EMPV1_24638	CTGCCATTGACTGCAGGAGGCTTTAACTATGTAGGAACGGTTACGGTCTTGGCTGAGCTG
EMPV1_24640	CTTGGCTCTAGACTGCTTACTGCCCGGGCCGCCCTCAGTAACAGTCTCCAGTCACGGCCA
EMPV1_24643	CAAAGGGGGCGCCAAGCGCCACCGCAAGGTGCTGCGGGACAACATCCAGGGCATCACCAA
EMPV1_24644	ATGCGACTCCCAGGCCAGACTGCTCATTGCTTTCTCCTGGACCTAAGTCTGTCAGCAAAT
EMPV1_24645	TTCGAATGCTAAAATTAAAGCCCACAAGTCACTAAAGGGGCAAATAGAGTGGTTTGCACC
EMPV1_24647	GCGGCTGGGCGTTCGCACTGCTTGTCAAAAAGGTTGGAATCTGGAGACGGAAAATAGGAG
EMPV1_24648	ACAAGCGACCTCTGATTGGGGTCACATAATCCCCAGACTCCTCCAAGTTCAGCCACCTGT
EMPV1_24652	CCATGATCCTTGCAGAAGCCATCAGGAGAACTCACAATGGGGAATCTGTCTCCTACCTGT
EMPV1_24653	ACTGGCGAAAAGCCTTACAAATGCGAGGAGTGTGGGAAAGGCTTCGGGAGGAGCTTGAAT
EMPV1_24654	CAGTAAGTGTTTCCAGTTGTGCAAAAGCAAATTCTTAGCTTTCATTAGAAACTCTGGTTC
EMPV1_24655	AAGAAGCAGCAGGCCATCCACCTGACAGGAGTCTTTTTTGATTTCTTCATGGGCGTCCTC
EMPV1_24656	ACGGACCACGCTAGATATGAACGGGGAGAGCATTGGAGAAATTCGGTTCAAGGCCACTTC
EMPV1_24657	CCAGGCTGCTGTACAAACTTCTCTGTCACTTGCACCACAGGACCTAGCAGATCTAATGGT
EMPV1_24658	CTGAGGATGATCATGAGCGGCCACTACCAGTTCGGCTCACCGGAATGGGATGATTACTCG
EMPV1_24659	TCTGGAATAGAATGCACCTTTGCTTCCCATCCTGTCACTTAGCATCAGCTCCCCACAGTG
EMPV1_24662	CTGCCTCCTCTGGCCTGGGTGATGATCTCATACCTTGACGATGATCTCAGAAGACAGGTA
EMPV1_24664	CTTGTTGGCAGCAGAGATAGAAAGAATGGGGTGGATCCAAGAGTTTCTTAGAAGACCAGA
EMPV1_24665	AGCCCTGGCCCCAGTCTACCTTACACCACAGTCTTGCACTAGATCTTGGATGAAGACAAT
EMPV1_24666	CTAAGCCACCTGGGAATTCTCAAAACCTTTTTCCATTAGCATCCTCATCTAATTCCAGGC
EMPV1_24668	TGCAGGCCTCTGTGTGATATGTTTGATATATTAGGTTGTTATTTAATCCAACTATATATC
EMPV1_24671	GCCAAACAGCAAGTGTAACTACACCAAGTGCTGTAACTGTAGATACGAGAGGAACTATGG
EMPV1_24673	CTGCATTGCTGTGGTGTAGGTCACAGATGCTGCTCAAATCTGGTATTGCTATGGCAGCTG
EMPV1_24674	TGGACGACTTGGACACGGAGTTCACGGAAGAGACCATCAAGAACCCGGACGAGAGCTACT
EMPV1_24678	AACTCATCCAGCTCGGCCTCCTCCGAAGCCTCGGAAACCTGCCAGTCAGTGAGCGAGTGC

EMPV1_24679	GTAGCAGGTGATTTTTGCAAGGGGTTTCCCTCTCACACGGCCTCAGTGCTCACAAGAACT
EMPV1_24680	GAGAAGGAGATAGAAGAGTGGGAGGACTACAGGAAGGAGGGAGAAGAAGAGTCCGAGGAA
EMPV1_24683	TGAGTGCCATCCACCCCGCCTGTAGTCCTGCCAGCCACAGAGCCCCTCACTAGACCGGAA
EMPV1_24684	CCCGACTCCACCGTTGCCTGCGTGTCAAGTCCCCAGAAACAAAGATTTTCGCTTAAACTA
EMPV1_24685	TGACCCCAGTGCTCCTGAATACTTGGAATATGATACTGAATGGCTTGCTATTCTCAGGGC
EMPV1_24686	TGAATGCCAGCACCGTCATCTCTGCAGTAATGGGCAATGCAGAAACACAGAGGGCTCCTT
EMPV1_24687	GCCCCTATCTGTAGTGAAACGATTGGGCCAGGTAGTAATTCCTTCTACAGTCAAGATTTG
EMPV1_24688	CGAAACTCGTACAGAATGAAGTGTAGGACTTGGTATTGGCTCTGCTGACAGTGGACTGCC
EMPV1_24689	GGGTGTGAACACTTTTTCTCCTGAAGGAAGATTATTTCAAGTGGAATATGCCATTGAGGC
EMPV1_24690	CCATGGCCAGACTCAACTTCTCGTGGGGACCCTGACTTTGGATGACAAGGCTTTATCTGT
EMPV1_24692	TGCCGACAACTGGAACCCCCAAATCTCTGTAAGAGTAGGGACCTGTGTTACATCATGCTG
EMPV1_24693	TGGAGTCTGTCCCCGCCATCGTAATTGATCGCGCACGGAATGCTTTGGAATTTTAACGAC
EMPV1_24696	CATGAGTATGTGGACCCAAAGCTCTTTTTCAATGTTCTTCGCATATACTTGTCTGGTTGG
EMPV1_24697	CTTGATCCAAGTAAAATCCGAATCTTGCAGTAGGGTCTTTGGAATGAAGACCGATACCAA
EMPV1_24698	TGTTGAAGTCCTAACCTCTAACATATCAAAATGTGGAAAGAGTATCACGGCAGCTGTAGT
EMPV1_24700	TTATGTGTGTAGGTGCATGTGCATGTGTGTGCGTGCACACACATGCACACACACACACAC
EMPV1_24703	TCGAAATGGGGTGACATCGAGCAATGGATGGAAGACAGTGAGCGCTACTCCCGCAGATCC
EMPV1_24704	GGATGTTAATAGTCAGAGTCCCTACCACAGCTTTGACAGTGCCCATAAAGCTCCCGCCTC
EMPV1_24705	GTTACCACAGGGATAACTGGCTTGTGGTGGCCAAGACTGCATAGCGACGTCACTTTTTGA
EMPV1_24707	TGTCCTGCAGGCCCGAGAGGCGCAGGCCTGGGCGCAGCTCAAGGAGCAGGAAGTGCTGCA
EMPV1_24710	TCCTCTCAACTTACCTTCCAATCCCATTACTACCCAGAAAGCCATTTCAAAGAGTACTTC
EMPV1_24712	TGGAGCGAGAAATAGAAGAGCTCCGTCAGAGATACACTGCGAAAAGACAACCCATTCTGG
EMPV1_24714	AGGGTTTCTAGGATCTCTTGGACTCACTCCAAAAGCCTGGTACTGGAGCAGTGGACACAG
EMPV1_24717	GGCAAGTGCCAGGACATGGGGTTCTAACTTATTTTGCCATCAGTGAGGTCTGTGGTTGTG
EMPV1_24719	GCACTTCTTCACCGAGTAGTTTGCATTGGATATGTACCCATTCATTTTGAAGGCGAGGGT
EMPV1_24721	GTACCAAAGAGATCATATTCCAAGTAACAGCCAAATTGAGCAAACTCCAGTAGTTCCTTC
EMPV1_24722	GCATATTTTTCTCCCCTCTTCTTCTGAGAGGGCCTTCCCGCTTCACTATGGCATGTCCTA
EMPV1_24723	TCTGTACCTATTAACCTACGCTACCCTCTGCATTACAAGCCTCTGGTCCCGTTACGTGCC
EMPV1_24727	TGGCTCGGCTTCAATCCTGGCTGAGGAACTTCCATATGCCCTGGGTATGGCCATAAAAAA
EMPV1_24728	TGGTCTTTCGCATGGCCTGTCACCTCTGCTTGCTTCCCCTGGGTTACCTTGCCATATTTT
EMPV1_24729	CCATAATCTGATCTCCTTTGCCCAGAACTCTATTATTCTTCGGGTCCCCACTGTCGCACC
EMPV1_24730	ACCTTTGGTCACCCCACAGTAGAAAGCCTCCAAAAGCAGCTAAGCCAGGACTCCTACTTT
EMPV1_24732	TAAGCTCAGCAGTTAACGAACTAGCATCTATGAGGATTCAGGTTTGAGCCCTGACCCTGC
EMPV1_24734	CCCTCTCAACATTTTAAACCAAAATCTTGGTTTCCAGTGCTATAGACCTCAAGGGCAGAT

EMPV1_24737 TTAGCTGATTGTACTCTGGCGCTTACAATCACTAATTCCACTGCCATCAAAACAAGGCAC
EMPV1_24739 CTGCCTGACACCTGCCACATGAATGGGATCTGCAATAATAAGCATCACTGCCACTGCAGC
EMPV1_24742 GAGCTACTCTTGTGTCTGCAAGTCCGGATTTGAATCCAGCACGGGCAAGATGGGCATCCA
EMPV1_24743 TCCAGGTCACACCCAAGGATAAAGAACCAGGGCCTGCTTCCAGCTGTGAGCATGGGTGTA
EMPV1_24744 GCTATTGTGCACCTCGAGTCCTGGTCTGTTTGTCCCTTTCTTATGCACGAGCACAGGCAT
EMPV1_24745 TGAGGAGGTGGTCTGGTTAGAGCTGGGCAAAATGGGGCTGTATCCTGGGAAGGTGCACTG
EMPV1_24746 GTTATAAGTAACTGTTACATTGCTTTTTTCTTCGCTTTCTCCTAGGTCTTCCTTTGGCTG
EMPV1_24747 AAAAAGTTTCACCTCTTCGCCAATGACAGTGCAGCATGGCCAATTCTCCCAGAAACCCCC
EMPV1_24750 CTACGACAGGATAAGTGGGATTTATATACCCTGCGTATAGAAGCAGAGCTTTTAGGACAA
EMPV1_24751 GGATCGAACCTGTGTTCTCATGGATACTACTGGGTTCATTACCACTGAGCCACAATGGGA
EMPV1_24753 CTAAAGGGTGAGAAGAGTAGAGATAAGAAAGCCCATGAGAAGGTGACAGAGAGGCGCCCT
EMPV1_24754 CCCTCGTTTCCTTAAAGCACATTCTTCTGACATAAACTCATGACTAATCTTTGTGCAATC
EMPV1_24756 ATCGCTCTTGAAGCAAATCATAGCCTGGTTATATAAATGTCCATAATTAGCTACTCTTGT
EMPV1_24757 GGAGAGATGTGGGATAGTCCTCCTAGGCAAAGCTTGGCACCTCGCTGAGACAGAGACACT
EMPV1_24758 GTGTTTCCCAAGTTCGAGTTTGACAGGGCTCATTCCCCTAAAACAAGCTCAGGTGAGAGG
EMPV1_24759 GCTCATCTTCTCCTGCCCTTGGACTCCTGGTTTTCCGGCCTTTGGACTTGAACTAGAACT
EMPV1_24760 CTGCTGCTCATCAGGACATCCCTGCCATGAAGCAGCTATCTGACTGGCTAATGAAAAACT
EMPV1_24762 TAAAGCATTGTGGGGCTGAGGGCACTGGTCAAACACCCAGACCCTCCTCTGTACTTTGTT
EMPV1_24763 AAGGTGGCTCATTGTAGGAAATCCTGTGCCTTCCCCTTTGAGCACAAGTGTTGCATGAAC
EMPV1_24764 CAAGGCATTAGAGGCAGATATATACAGTGCAATCCCAACAGAAAAAGTGGATGGAACATG
EMPV1_24765 CCCCTGCCTCCACCCCTACTATTGCACCATTAAAGGATTATTTACGGCAGTTCCATTCCC
EMPV1_24766 CTTCTGGGCTCTGACCTCGTCCTTGTTGTCCTCTCTTACTCCTTCATCCTGAAAGCTGTG
EMPV1_24768 GTAGAGGGTAGCCCCAGAGCCGCCAGTGCCCGTTCAAGCTCTCCAACCGGAAGCGAGTTA
EMPV1_24769 CTTCGTTCATTTTGTTTCACAATTTGACTAGATGGACTTTTTGGTAAATACTTTAGTGGC
EMPV1_24770 CCTCGTTCCATGTTTGGAGTAGCGGTCCATAAAGGCAAAATTGTGATTGCTGGGGGTGTC
EMPV1_24772 CAAAAGATGCGGTTGTATCTATGTATCCTGGTACAGTATATCAGAAATATGAGCCAGTCT
EMPV1_24773 GAGAAGCAGAGGAGCTGATAGAAATTGAGATCGATGGAACAGAGAAACCAGAGTGCACAG
EMPV1_24774 GAGAATGGGACAAGCAGGAGATACTATATCAGACTCATCTGGTTTCTGTAGATGCTCAAC
EMPV1_24775 GAGAGGACCAGAGGGCTTCCATTTACATTCTGGCTCTTTCATGTCCTGGCTGTGTGATCA
EMPV1_24776 GGTTAAGAATCCGACGACAATAGCTCGGGTCCCAGCCCTGCACACAGGGTTCAAGAATCT
EMPV1_24778 TAGCATTCATGCATTCCATATCTGGGCACTGAGACTCCAAGTTGTTAAATAAACCCGACA
EMPV1_24779 TATTGTCGCCAAGCGTGCTTTAAAATTGTCGGTGCCTGGGTCCCCCTCAGAGATCCTTAT
EMPV1_24780 TGGCAATACTACAGACTACTCATCTTGAAAGAGAGGCTCCGGGTTATGGAAACATGAGGC
EMPV1_24781 GAAGATGCTGCAGAGCAGGCAGAGACCAATGTGGAAGAAATCTATCACCCAGTCATGTGC

EMPV1_24782 GTAAGACTTCTGATGCAAGCCTCCTATAACTATCAAGGAAAAGGGTGTGTGGCTGGCTCG
EMPV1_24784 CGTGGAGGAGGTGCTGAAAATTGCTCTGAGTGATGGCTTCTGCATTGATTCTTCTCACCC
EMPV1_24785 CTGGCTTGCAAAGATTTAGTTATTAGGTAAAGCCAGGGCCAAGGCAGAGAACAAGGGCTC
EMPV1_24789 CCCAGAAGCTGAAGAAGTTGAAAGCTGACACCAGGAAAAGTAACAACTCAGACTCGCGTG
EMPV1_24790 TACTTCTTGACGGAGGCCCACGACACCCAGCCCACACCCTCCACAGCTGGTGAAGCTGAA
EMPV1_24791 CCTGCTGTGAAGTCCTGTACGAAAGATATTGATTACTACCCTAGTTGTTCAGGAGGGGAT
EMPV1_24793 TCTGCTGAAGTCCCTGCAGGCAGCGCTCTCTGGCAACCTCGAGAGGATTGTGGTGGCTCT
EMPV1_24794 GTCTTGGATATGGAGACACATTACGAAGGAAGCCAGTGTTTGTGGAGAAGGCAGCGACTC
EMPV1_24795 TGGCCAAGAGGAAAGCCAAGGCCTGGAGCGACTGCCCACAGATTATTAAGTAACGAGTGA
EMPV1_24796 GCAGTAGGCTTCCTCTGTAGGGTTCTGGACAAGCTGACTAGAGAGAGGTCGTGTATGTTT
EMPV1_24797 TCAGGCCAGGATCAGCAGCAAGGAACTGATGAAGAAGATCGCCAACTGCGGTCAGATACA
EMPV1_24799 TCAGGCAGAGGTGAGGAAGCTAGAAAAAAGGCAAGGGGGGCACAGACAAGAAGGGTAACT
EMPV1_24802 ACTCTACGAGTACGAAAGCTTCTGCTCTTGATACCCACTGATCCAGCCATTCAGGAAGCC
EMPV1_24806 CCAGAGTTACTTGGACCAGCATTTAGGAAACTAAAATTCAGCGGATGTAAAAACGACGCT
EMPV1_24810 GCTCTGGATTAGGCTTTGGCTTAAGGGAATGTTGTGGCTGGTTTGATCTTCTATCCAGAC
EMPV1_24811 AAGGACCTAGTCCAGACCATTTGGCGCTCTTGGATCGGAGCAATTAACTAGACAGATGGC
EMPV1_24813 CCTTCGCCGGCTCTATCAGGGCCGTCTTCGAGAAGAAAGTGGCCCTCCACCTGAGTTAAT
EMPV1_24814 TGTGAAGGAGACGACACAGGCCGTAAAGGGCGATGCCCCTGGGAAGTTCAAGTTTCCTAA
EMPV1_24815 TGTACCTGCTTAGGAGTCTACTTCAGCTCTGCTGCTACCAAAAGCTCACAGTCAAGTGCG
EMPV1_24817 CCTCGACTACTACCGCAAGGGGGACTACGGCTACTTCGGGCTGACCCTCTTCTTCGTGCT
EMPV1_24818 TTCCACAGGATTGTCTGACATGCTGCCTGGAGGCACATACACCAACTTGCAGGAGTAAAG
EMPV1_24820 ATTGATAATAATGAGCATCACCACCATGGAATCACCACCATCCGCGTCACCCCGGTAGAG
EMPV1_24822 CAGGCACTGTTGAAAGCCCTTCACGTGCATTAATTCATTTAACTCAGGACAACCCTGTGG
EMPV1_24823 CCAAACTTCGGTGCCTCTTTGCATATTCTAAAAGTTGAAATAGGTGGTGATGGGCAGACA
EMPV1_24824 TGGTAGGGGTTGTGTTGGGAGCCGTGGTCCTCTCTCTGCTCATTGCACTTGCTGCCAAAT
EMPV1_24826 TGGACCTGGTAATCCTGAGTTACATGGGTAGTCTACTGTGGCAACCAGATGTCTCACTCC
EMPV1_24828 CTCCCAACACAACCACCCCTTCCTTTCTGACAAACTACCCCTGGTATTCATCCACGAACT
EMPV1_24830 ACCTGTCTCCCCACTTTTGTCCCCCAGCCGAGCTCTGAGCCCATGTAGCCGCTACGATTT
EMPV1_24832 ATGTTTGGCATCTGCGGGCTCATCATCGTGGGCACCACACTCGGGGTCCTCATCTCCTAT
EMPV1_24835 TGCTGCTCCAGAGTGTTGCATCTTGGTGTTACTTAAACCCTGACAACCAGAGAAGAGCCG
EMPV1_24836 CTGCTGGCAGGCAAGAGATGTAAGCTGTGATAAATTAGAGGCAGGCCAAACAGGCTCACT
EMPV1_24838 GCCCACCTAGGGAGGGAAGGAGGGAGGCTGGGTTGCTGGTGGGAGGGACTGCCCACCAGT
EMPV1_24840 CGTCAGTGTGAATTGTTCTCTCGTGGGCCTCGTCCAACATGATGATCGCATACTGGGTGA
EMPV1_24843 CTTCTATGGCCATTCCATCTATGCCACACTGTACATGGACGCCTGGCGCAAGGACTCAGT

EMPV1_24845	CCACTGAGCTGCTGAACCTGCTCAAGATGCCCAGCGTGGCCCAGTTCGTGCTCACGCCCA
EMPV1_24846	GGGAGGGGGTGGGAGAGAAGGGAAAAGCGGGAAAGTAAAATGAGGAAGTTGCTAAGAGCT
EMPV1_24847	TCAGTGTCCTTGCACGAGGCTTCTAGCCAAAGATGACAAAGGGGAGAGGGTTTCCATGCA
EMPV1_24849	GTCACTGTTGTTGTACTTACTGCATTTCCAAAAATGTAACTCTGTCCAAAACACAGCCAA
EMPV1_24851	GACCTCAGCCAAGTGGCCCCAACCTCTCTGTGCCTTGGTTTTCTCAACTATAAAATGGTC
EMPV1_24855	AAGAGGACATGCACTTCATACTGCAGACGCTGGAGGTCAGGTTTAAATCCCTCGGGCTTC
EMPV1_24856	CCCAAGGTCAAAGAATGATGTCACTCTGTAATGGTACACTTAGGAGAGCCACTGAAGACT
EMPV1_24857	CAGGAGACAGACACCTGAAGCAAGAGGAACTACAATCAGGCAGACTGGAGGAGATGACAA
EMPV1_24858	CTTTGGCAGCATCTGGTGACATTTTGGGTTATCACAGCTAGGGGTGGTGGTACTACTGCC
EMPV1_24859	TCATAAATACGGCCACGGCCACCGAGTCCCTGAACAGTCCTGAGGAGCAGAGAGTCCTGA
EMPV1_24863	GTCCTTCAGTCTCTTTCTTGCACTTTTCTCCCCGTGTCTCCCTTTCTCTATTCCCCTTCC
EMPV1_24865	TGATTGTAGTCTAATTATGTGTGATCCCAGTAGGGAACCTCGAACAGTTCCTATCCCCTC
EMPV1_24866	ACTGTGGGACTTCTCTGGCTCCAAATGTGCCGCTGTTTTGATAAGCCTCTGTAGGAACAG
EMPV1_24867	GCAACTCCTTGGTGCAAACAGTGTTAACTTTCTTGTTACCTCGCTGTGGTCAGTATCAGG
EMPV1_24869	GGGGAGGAGGGAATAAAGGGTGTAGGAAGGCAAAGCTTGCAAAAGGGTCTTTGATCATGT
EMPV1_24870	TTCAGTGTCTTATCTGTCAAACGAAGCTCCTGACAGAACCTCCCTCACAAGGCTCTGGTG
EMPV1_24871	AACAGAGATCAAGTCTGTCCTATTCACCACTCCTGGCACATAGCAGGTGCTCAGTTAATC
EMPV1_24874	GCTCTCTGTAAGGAAGACAGTACAACAAATTGCTACTCTGCAACAGGGCCATTCCAGCCC
EMPV1_24875	AACTGCAGCTGAGGAAGCATGAGCACAAAAGCAACAAAAAAGACGGACTGGCCACGGAAG
EMPV1_24879	CGTTCTAGTTTAAGAAGCTGGACGGGTCGTAATCTGTCATCTCCCTGGTGTTTGACCTGG
EMPV1_24880	AATTTTACCTTATAATAATTATTCCAGTAAGTGAGTTTATGAAGTTGATGATTTGGAGAA
EMPV1_24881	GGCTGTGGATTTGGCTATGGATCTGGCTGTGGCTCTGGCTGTAGATCTGGCTGTGAGCCT
EMPV1_24882	ATAAAAATAACCAGCTCCCCAAATTCAGTGTGAGCCCCAGCAGGAGAGGGTGGTGGGGTT
EMPV1_24883	AGTTGGAGTGGAAACTGTCCGAGTCTGGAGCGATCAAGACGAGCCTGGAGGAGAACCCTA
EMPV1_24884	GAGGTGCTGGAGTCGGACCTGCCAAATGCTGTGGCACTTTTGAAAAACCTTCAGGAACAG
EMPV1_24887	TATTGTCTGCAACTTTGGCAGCGAGCTCAGCCTGGTGTATGTGCCCTCTGTGCTGGAAAA
EMPV1_24888	AACTGGGATGGGGTCCCTGGGTTTTTCCTACCCTTCCCCTTTCTGTTTGTTGTCGCTCCA
EMPV1_24889	AAAACCATCAAAGTTACTACCGCAGCCGCTGCCGCAGCCACATCTCAGGATCCCGAGCAA
EMPV1_24890	GCAGCTTAAAAGAAGTCGTCTAATTGCATGGTTTCTGAGGGTCAAGGCTCCACTCAGGAC
EMPV1_24898	AAACCGACATCATGTGTTTCCTGATATGGTGCGCTGATAAGGATAAGGCAGCGCTTGTGC
EMPV1_24899	GTGTGTGTCTTTTTAGGGCAGCATGCTTGTCATATGGAAGTTTCCAGGTCAAGTTGGAGC
EMPV1_24902	TGCCTATGGGAGCAACATCTTCATGTATGTGAGGCCCAGCCAGAGTCATTCCCTGGAATT
EMPV1_24903	CAACCCAGCCCACTTCCCAAAGAACTGCCAGGTGCCCAGTGGCTTCATGAGTTTCATGGA
EMPV1_24904	AAATCCTGTTAGGACCTTTCGCCCATTGATGCGTGATCTGCGTGGGCACCTCTCTAGGAA

EMPV1_24905	TCAGTTCCAGGAATTCTCCCTCCACCACCCGCTCCACGGGAAGCTCTTCAGCACCTATCT
EMPV1_24907	AGAGACTGAGTCGTCAGAGCCCACTGGTCACTTGGCCTTACTCTTGCCAGAAAGCTCTCT
EMPV1_24909	TGGAAGTGCCCCAGGGGAATCCAACTTTTCCACCTGGTGGGCCTTGTTATACTATGCCAT
EMPV1_24911	TCAACCAGATGGACCAGGACAAGGTGGCCAAGATGTACAGTGACCTGAGGAAAGAGTCCA
EMPV1_24913	CATGATTTGACTCCTAGCTATAAGCAGTTCAACACCATCTAGGCTTCTAATTTAGCTGAC
EMPV1_24914	CATCCCACAGCACTGATCATCTTTTAATGTGCCACCCTAAGCGAACGGTAAGAAGGCCTC
EMPV1_24916	GGCAGTGCCCGCTGCTTCTGCCCCAGAACCGTGCGAAAACCGTGTATGAAGGATTCATTT
EMPV1_24917	TGGCAACAAGTGCGGGGCCTGTGGGAGGACCGTGTACCACGCTGAGGAGGTGCAGTGCGA
EMPV1_24918	AAGTTTATGCTGAGTGGTTACAACTTCTCGGTAATGGAGAACATGCCGGCGCTGAGTCCG
EMPV1_24919	TCCTCCTCTGAGGGGAAGACCAAAGCCATGTCCACATGCACCACCCATATCATTGTTATA
EMPV1_24920	GAGCATGCTTGTATTCACGTAACTTGTGTACCTCTGTCTGATCATGTCCACGTGCAGAGG
EMPV1_24921	ACAACGAGTCAGAGCTGCAGGTGTTCTGGGTGGATCGCGCCTACTCACTCAAAATGTTGT
EMPV1_24922	ATCTGGTTCTGGGTGCACAAAGGCGAGACCCAGCGATGCCCCAGCTGTGGAACCCATTAT
EMPV1_24925	CTCCCGTCCACAGTCTGCTCCTCAAACTCTTCTCCGATCTTGAAGTTGATCTCCGTGGTA
EMPV1_24926	AAACTCACTGTGCCTGACGACACGGCCGCAAACTGTGTGTATCTCAATATCCCCGGCAAA
EMPV1_24927	CCAAATAGTAGGTAGCATCCCTCTTGTCTCCCAAACATGAGCTCCAAGTGTGACTGTATC
EMPV1_24928	GTTGTTTTCACCATCACGAACGAGACCCACAAGTTCCACGTGCCTCTGCTGCTGAGCCCG
EMPV1_24930	AGCTGTGGCATAGGTCTCAGATGTGGCTTGTGTCTGGTGGTATTGTAGCTGTGGCTTAGG
EMPV1_24931	GACAAAGCAGGACCTCATAAGGAAATCTACCCCTATGTCATCCAGGAGCTTAGACCAACT
EMPV1_24934	GAAATGAACCGGAATGAGAGAAATAGCAGAGAGCTCACCGCATAGAGTGCCGTTGCTCTG
EMPV1_24935	AATCACCTGGCCCAGAACATAATGGATCCCGCTGTCCAGAAATCCAGCATCACGGCCAAT
EMPV1_24936	CCAATGAAGAGAAGTCCGAAACCGTCTGGACAAAATGGGCATGAAATATGTAGCCATATG
EMPV1_24937	GAAATAAACTTTAACAATAATCACCATTTGGAATGGGAATTGGAGGAGGAGGAAAAAGAC
EMPV1_24938	TTGTACATTGAAATGAGGGGCTCGGTTTAAAAAGAAACCTCCCCCACCCCTACCCCAGAC
EMPV1_24939	TGGTGACCTGTGATGAATCAAGAGTATTCCAGGAGAAAAATACTGTAGCACCAGCAGACC
EMPV1_24940	CCCCACGTGATTAACTTCTAGGCTTCTTCACAGCATGGTGGTCTCAGGGAGGTCAGATTT
EMPV1_24941	CCAACCCCTCTTCTGATAACAAGGAGCCTGCTGTAAATCCATCAGCTGACAAAGCTCTGC
EMPV1_24942	ACTTTCAGTGGCAGACTCCGGACTACCTGGTGCCACTTCCCTTCCAGCCTGCCTTTCCCA
EMPV1_24943	TTGCCTTTCATTTCTGCTGCGGCTGCTGTGTGTAGTCTGTCTCGTGAGCGCGTGCGGAAG
EMPV1_24944	CCAGTTCCCTGAACATGCAGTGTGCTTACATCTCTGAGAGCTTGGTATGTTTCCTCCTCC
EMPV1_24951	CCTGTTGCACGAAATCTATCTGCCCGTGAAGATTTGGCTTGGGTGTTTGGTTAGTGATGC
EMPV1_24952	AAGGACTGATTTTTTGCCACGTCAATCTCACGAGTGATTACAAGATTTCCAGAAGTCCTG
EMPV1_24953	GGAAGCAGTAATGAGAATCAGTATCATAGCCCAACTTCTGGAACAGTCAGCGCGGGCCCG
EMPV1_24954	CCCAATGGGGCCAACATCTTGGTAACAGAGGAGAATAAGAAGGAGTATGTACACCTGGTG

EMPV1_24957 GATGAGGAATCATGACATCACTCAGCAGCCCATGTCCTACGACAACCTCACTCAGAGGCT
EMPV1_24959 GGGGTTGGATGTTTGATTATGGGCCTAGGGTGTTTCTTACAATCACTACCTCATTTCCTC
EMPV1_24960 GCAAGGAGCCCGGCTTGTTTGACCTGATCATCATCAACGACAGTCTGGACAAGGCCTACT
EMPV1_24961 GCATCTCTTAATCCCATATTCCCATTTTGCCCCTCCCACCTCTTCTCCCTACTTGTAACC
EMPV1_24966 CCAGTTCTAATATCATGAAATATCAAGTAGGGTTAGGGCAAGGAAGGCTAATCAGATATT
EMPV1_24967 TCATGCGAGGCTGTTCCTATCGCAAATATCCTTTCCTGCCCTCATGACCTACATGGGCCA
EMPV1_24968 TAAGTATGCAGAGACTGTCAAATGTAGAAGAAGCAGTCCAGGAAACCGCCCTCCGGTAGG
EMPV1_24969 TCATCAGGACGCTGTCCAGGAGCTGCCCCACGGGCATGCACTGGACGAGGCTGGACCTTT
EMPV1_24970 AGCCAGAGTGGAAAGAAGGTATGGAAGGCTATGTCCAAACTGGGTCTTCGGCAGGTTACA
EMPV1_24972 GGACCCAGCCATCTGTGTTTGAACAACTATCCAGGTGATTCCGACAAATGCTCACACGTG
EMPV1_24973 AAAATAGCAGAGAAGGAGGCCAAGCAGAAGGAGCTCAGTGAAAAACAGCTAAGCCAGGCC
EMPV1_24975 AGTTTCACAATCAGCCACGCAGCAGCCTGTAACTGCTGACAAACAGCAAGGTCATGAACC
EMPV1_24976 TTTGACATGCAGAGGATAACTCTGGAAGAGCTGAAGCACATCCTCTATCACGCCTTCCGG
EMPV1_24978 TCCTGTTATTCCAGACCTAGAAGAGGTACAGGAGGAAGACTTCGTTTTGCAGGTGGCAGC
EMPV1_24979 TTGAGCTCCAAAGTCATAAAGCCAGCATCCTATGCACCATCTCCATTGGGGTGTCTCGCG
EMPV1_24980 TCCTGGTGCCCCTAGTTTTCATCTCTGCTTTTCTGTACCTGGCTGCCTCAGTAGTCTCCT
EMPV1_24982 TCACCCCAACATCCTTCGCCTTGTGGCTTATTGTCTGAGGGAGCGAGGCACTAAACATGA
EMPV1_24983 GGACGCACCTAATCCCCGAGGCATCCGTCCACCCAAGTTTTCAGAAGGAAATCTTTGTTT
EMPV1_24987 AGATACTGGTTGGTTTCTTAACCAGCTGAGGCACGATGGGATGACATATTTTGACTTGGC
EMPV1_24988 AGCCGGCGCTGGTGAGGTGCATCCCCGCCGCCTCCCGGTGGTATCTTCCATCTTATACGT
EMPV1_24990 CAAGAGCAGCTTAGCACCGAGAGCTTTCAAGGGAATCATTCAGCAGCTTCTCAGCCAACA
EMPV1_24993 GACAACAGCATCATCATGTGGGACATCGGAGGAAGGAAAGGCCGCACGCTCTTGCTCCAG
EMPV1_24994 AAGGGCCCTGAAGTTGACCTCAAGGGCCCCAAGGTGGACATTGATGTCCCAGATGTGGAC
EMPV1_24995 TCTTTCACCGGATGACGCCAATTTTGTGGACGCCATTCACACCTTTACCCGGGAGCACAT
EMPV1_24996 AAAACACAGTACAAAGACTTCACAAATGTGATTCAGGGCCCCCAGCACCCCTGTGTCTGC
EMPV1_24997 AACTCACCATCCAACCGTCTCTCAACGCCATCCTCATCCCTCCTCACTTGAGATCCCCGG
EMPV1_24998 GGAGGTTCAGCAGTCCTGGCCGTCCTCGGCGCTCAGCACCCTCTACTCCCTGTGGTTCTC
EMPV1_24999 GACATATGGCACTCTAGCTTTATTATTCTCCCCTTTGCGGACCTGGCTGACACTGGTGAC
EMPV1_25000 GTCAGAAAAACGCACAAAGTGTAAAACGACGGCCAGTCAATTGGATTTCGTTAAAACGCG
EMPV1_25001 AGAGATCCTCTTTGAGGCTGGATTTGGGGTAGGGGGCAGAGGCAGCATTGAAATGAGAAC
EMPV1_25003 TTCACAGGTTCTGGGTGGGTATCTCATTTGGAGGGCCATCATTTCAATTCACTACAAGGC
EMPV1_25004 GTTATGATTGTCCTCATTTCCTACCTGTGCATCCTCATTGCCATCTTGAGGATTCCCTCC
EMPV1_25005 CTCTGCCTAGAGACTTGTAACAAAGGAAGCGGGACGCATTTCCACAGACCCTGCTTAGAA
EMPV1_25006 GTGCTTTCCAACGTTTGACATCTTCTTCTAGTAACTTCTTCTCTGCTTGCAACATACCAC

EMPV1_25007	ATCTGGCACGAGTGGGAAATAGCCAACAACACCTTCAGGGGCATGTGGATGAGAGACGGC
EMPV1_25009	AAGAAGAAAACCAAACATTTGGAGGTAGAAGGAAAGAAAAGAATGAGAGAAGGAAAAAAA
EMPV1_25010	TACACGTGAGGTCAGGGGGAGACACACACGTATGAAACCGAATCTGTGTATGAGCGAGGA
EMPV1_25011	CGCCACCACCTTTCTGTGGGTCCACTGGTGGGAAACCTGTATCTTTTTCACACTTTCCTA
EMPV1_25012	ATGGAAATTCCTTGGTCAGGGATCAAGTTTGAGCAGCAACTGCAATCTATGCCACAGCTG
EMPV1_25014	GGAGCTGACCCCTATTTTATAGTTGGGTTTTCAAAGGGAGAATGAGTCATGTCCAAAATG
EMPV1_25015	CGAGAAAGTGATCCCAATGACCCAAATAAAGACATGGTAGTACAGCTAATTGATGACTTC
EMPV1_25017	GCATTCCAGGAAAAGCGCCCTTTTGACTTCGATTTTTTTGCTCATTTGCTTCAGAAAGTG
EMPV1_25018	CTGAGCAGGATGTATTGAGCAGAAAGTCAAGGTCAGCAATGTCCTTAGTCCTGAGGGAGG
EMPV1_25021	AAGTTGGTGTTAAGTGGAAACACCTTTGGACACCAGCCAACCTTGAGGATAAGCTCATCT
EMPV1_25022	CCGACTGTGTTGACGGACTTGATCTGTGGCAAGAGCATGAAGATTTTTGACTCTCAGTAG
EMPV1_25023	TGCAACATAGATTTCTGCAACAGGAAAGTCAGGTGCCCGGGAGGAAGATGCCCAAGGAAG
EMPV1_25025	CATCCTCCCGGGCCTTTTTCAAGTGCTTTGGCTGTGACTTTCGTACTCTGCTCTTAGTCT
EMPV1_25026	GAGCCCCTAACTGTTGCTTCCAGCTGAGACGTGACCAAACACAACAAACTGTCTGAGGGT
EMPV1_25028	GGGAGTTCCCATTGTGGTGCAATAGAAACAAACCTGACTAGCCTCCATGAGGATGCGGGT
EMPV1_25029	CAGGACTTCTGGAAATCTTGTAATCACTCGTGAGATTGACGTGGCAAAAAATCAGTCCTT
EMPV1_25030	AAACACTGTGCCCAAAATAACCTCGCCAGCAAAGCCCTGCTCATCTTAGACAGTGCACCG
EMPV1_25036	ACAGTTAGTTTTGCAGGTTTGCATTTCAGCGTATGTATGTGTATATGGCTGTGCAAATCC
EMPV1_25038	AGCATTGGGAGCTATTTACTACTGTAATGGGAGACCTGAGCAGAGGTACGATGAGGAGGG
EMPV1_25039	ATGTCGGGCCGCGGAAAACAAGGTGGCAAGGCTCGTGCCAAAGCCAAGACTCGGTCTTCG
EMPV1_25041	CTTGTGAGGGTGTGTGTGGGCGGCGTGTGCCATTTGTCTAGCATGTCTTCCCTATGTGCA
EMPV1_25042	GTCACACCCAACCCCCAGTCCCTCCTAGACAACAATTGAAGCAGAGCAATATCTGTCATG
EMPV1_25044	ACGCATGGGACACTACAAGCAAGGTGATACTGTGGACATCCAGGGAATGGGCACTGCTTA
EMPV1_25046	CTTCTCCCTTTGCTCTGTGTCCTCCCATGTTGGAAGGGGTTAGACACATCTGAAAAACAT
EMPV1_25051	TCGAGCCCAGGAGCAAGGCATGGCCGAAGCCTGTCCCAAGCTTGGACTTGATGCCTACAT
EMPV1_25052	AAGCTGATTGATGAGAACGACCTGCTTGACTGTCTGGTGCACGTGTCCTACCTGGAAGTG
EMPV1_25053	CACACATCCATCTGTGTATCTACTGGCCTGAAAGGACGTTACATTCTTGTATGTCAAGTT
EMPV1_25054	GGGGAGGAGAGCATCATCACTTTCCATTAGCTGTGTTGGCTTGCAGGTCACATTTTTACT
EMPV1_25055	TGTAGAATGAATGGTAAATGCAGCGGTTTGCATTCAGCCACAGCCGCCGCCTCTTGGCAG
EMPV1_25056	TAGATATTTGTTTTATCCTCGTCGCTGTGCCAGAGTCATTGCCTGAGAAGATGCGGCCCG
EMPV1_25058	ACCTGAAAATCCAGGTGATTGAAGCCAACTTCTACAGCTCTGCTGAGGCCCAGAAGCAAG
EMPV1_25059	TGGTAAAGGTTATAGCAGCAATTATAGAAGGTCACCAGAAAGACCTACAGGGGATCTTAG
EMPV1_25060	GGGAAGCACTCTCGCTTATTAAAGATCTTGGGCTGCGTCCAAAGAGGACTCTGCGTTTGG
EMPV1_25061	GAGACTCCAAGAACTACAGAACCTATTTCTGTGTCTCAATACGTAGGCCGGCTCTCCGAC

EMPV1_25062	AGGGGCGCCGCTTCATCTCCAACCAGAGCCGGAGGAAAGACCTCTCCGACCGGCCGCCGC
EMPV1_25064	GCATGATGCTCCAACACAGAAAATTGTGACTCTGTTCTATTCTGTTGTGACCCCACTTCT
EMPV1_25067	TGTGCCTGCCAACCTCAAACCCAGCAAAATCAAACAGCGGGCTGATAGCAAGGAAAGCCT
EMPV1_25069	TGTTCCGGGCTGGGACCCTGCCCCCGGAGCACCCTCCCCAGCTGACATCACCCATCCCAC
EMPV1_25072	TCGTTCGTGGGAGAGTGACCTGCTGTGTTCCAGCAACTAGACCACCACACCGCAGGTTCC
EMPV1_25073	CACACGAGGTATTTGGATGCCAGGTTTTCCCCACATGGGAGGCAGGGTTCCTGTGATTTT
EMPV1_25076	CTAGCCATCACAGAGAATCCAAGGTTCTGAAATGTGGACTCTTTCCAAGGACCCTGCTGG
EMPV1_25077	CTTAAGTCTACTCCCCGCCCCAAGTTCTCCGTGTGTGTCCTGGGAGACCAGCAGCATTGT
EMPV1_25078	GGGTGTCAAAAGCAATTTTAGAAGGTGCTGGACCAGCTGTGGAAAATGAATGTGCTTGGC
EMPV1_25079	AGCTCAGAACGTTTCCGCAATCTTTTAACTACTCCCTCCTAAACCTGGGGTCCCGGCAGC
EMPV1_25080	TGTTAGAATCATTAGGTTGACTTGCATACTTGTCGCTCACTCTGGCCACGTATCATGGAG
EMPV1_25081	TTCTATAGTGAACTCTGTGGGCGTCCTCCAAGTATTACCCCTGGACAGCAGCGATTGGCT
EMPV1_25083	CAAAGTGGAACTAAGCCTGCTCCAGTGTTTCCTCATCATTGATTATTGGGCTAGCTGTGG
EMPV1_25084	TAGCAGGACCTCCAGACACACCATATGAAGGAGGAAGATATCAACTAGAGATTAAAATAC
EMPV1_25085	TCAGTCAGTAACTGAACAGAGCTCCCTGGATGACTTCCTTGCAACTGCAGAACTTGCAGG
EMPV1_25086	CAGTAAATCTCAGGCTGGTCCGCACCAGCCTCAGAATCGCGCAATAAAGGTCACCAACAA
EMPV1_25088	GATCTCTTCCCTCCTGGTCATCCTAGTGTCCTACCTATTGATCCTGATAGCCATATTTCG
EMPV1_25090	GGGTCTACCCTTTAAGCAGGTCTCGGATGCCCCCACCATCATCCAGTTTCCCAGTATTTT
EMPV1_25091	GCACCTGCTATAACACGGCAGATATTTACGCTCTCGTGGACGAATTTAGTCTCTGTGGAC
EMPV1_25094	TAAATAACTTAATCTGTAATGAACCGCGGAGGCTGGCGCGCTCTGGTGTGAGGCTCTGGG
EMPV1_25095	CTGCAGTTGCTACGTGGTTACTCGTCTGATTACCTCTTTGGCGCTGGCATAAGCTGCCTG
EMPV1_25096	GGGTCACTGCAGAACAGGACACAAGGACAACAGTGCGCTCAAAGGAAAAACAGAAAGTGG
EMPV1_25097	AGGCAGAGGAAGCTGAAGAGACAGGTGGTGAAGTACAAGCTTTTCGAAGACTATCTGATC
EMPV1_25098	GCAAAGGGTTATATCAAAAATTATTAGTATAGAATCACAGGAAATCTGGTTTGGAACCAG
EMPV1_25099	GCTACCACTTCTCCCAGAGGAGCAAAGCCCTCAGTGAAATCAGAATTAAGCCCTGTTACC
EMPV1_25100	GTATGACATTCTCGAGCCACTTTCAGACACTATTAGCTGAGACTGCTGCATGGCAAAAAC
EMPV1_25101	ATTTCAAATCCTTCCTGCTCAAAGACCCAGAAACGTGCCGACGGCTGGCTCACCTCAGGC
EMPV1_25103	GTCAATCTCGCCCTGAAAAATGGAGCTGTCTCTCTGGTCATTAATTTGGGATCAGGGGCC
EMPV1_25104	AGCAGCCTCTGAGTGAAACGCTTCTTCAGGAGTAAGTCGATGTGGTCATTGTTCACAATG
EMPV1_25105	AACAACAAGGTTCTCCTCTAGAGCACAAGGAACAATACTCAGTATCTGGAAAAGAATCTG
EMPV1_25107	ATCAAGGACTTTTTGGTCAGTCAAGACAGGAGTGCCGATGTCACGCTGGAGGGACAGGTA
EMPV1_25109	GCACTTCTTGTGCAAATGGTGCCCCTCCTCGGGACAATCTGCCCGTTTGAACCCTTAGAG
EMPV1_25112	GAACGGTATGATGGAGCCCGACGACTTCCGGGCCTGCCTCATTTCCATGGGCTATGACCT
EMPV1_25114	GTCTCTCGTTGTATTCCTTCCGCATGGAGACCAGAAGAACCTGAACCTCTGTGAATCCAG

EMPV1_25116	ATTGCAGCGCTCCAGAAAGTTATAGATAATAGCGTTTCTCTGTCTGAACTAGAATTGGCC
EMPV1_25118	GGACCTTACCCTCTGCATCACATAGAACTGTCCACTCTCTGTTTCACCCGATGAGTCTAC
EMPV1_25120	GCCATCGAGGTGGCTACTGATAATTACGAATGGCAATTTAAAAGCATGTTTCCAGGGGAG
EMPV1_25121	CTTTCTCCCAGCAGGAAGCCAGAGGCTCACCGAAGTCAAGGCGTTTATTAGCTTAAGGTC
EMPV1_25126	GGAAGGACTAGGGTAATGGTGATTCTAAGCGTCACGGTAAGGTGCTGTGTGACATCCAGA
EMPV1_25128	TAAGTCACGTCTCTCCAACCAAATCCGTCGTTGCAGTGAGCAGGGCCCGCGTCTCAGGGA
EMPV1_25131	GCTGCCTTGAGATTCCCTTGTAAGTTTTGTGGCAAGCGTTTTGAGAAGCCAGACAGTGTT
EMPV1_25133	AACTGTTTTCCAGCTTCTCTGCTTCAGGGCCAGGGACGCAAAGTGTACAGAGAGGTAGGT
EMPV1_25135	GCTGCTAGCGCCATGCTACAAAGACTCCATCATGGGAGCAAACAGCTAAATCACAGATAG
EMPV1_25138	GGGAGGCTATGGATCGGTTGGAAGAATGGGAATGGGGAACAATTATAGTGGAGGATATGG
EMPV1_25139	TCTCTGTGGCATGTTCTCTGTGGGAGTGGTTTCACTGTTTAACTGCACGTCAGATTCATG
EMPV1_25140	GCCTTGCTGAGAGTGGCTGCTTTACGAGTAGTATTTGGTAGGTGAACAGGGCTCAGAACT
EMPV1_25142	GGCATTAGAGGACGACAGAAACAAATTGTTGATACGCTGTGTAATGTTGTCTGATAGACA
EMPV1_25143	CGTTCTCCCTCAATCCCCATTTTCTGTCGGCCTCCTTTTCAGAAACGTTTAGTCTAACCT
EMPV1_25145	ACAGTGCCATTTCTCAAAGCGGTACAAGTCCACACAGCAGTGGCAGTGCAGCGATAACAA
EMPV1_25146	GGGCAGACTTTGAGTATCTTCATTACTGCCCAGTCTTCTTAGGCTTGTTTTAGAGCCCTG
EMPV1_25147	TGCTCCGAATCCTCCCAGGAGAAGAAAACAGGGAGTGGCAAATAGAGAACAGGCCAAGGA
EMPV1_25148	TTATGCACTGTCATTAACTTTGACCGGTACTAGAACTTGGGGCTGAAGTGTGAGCCAGTG
EMPV1_25150	CACTCCCTAGGTACTTGCTACCATTTTAGTGTTCCCTGGGTGCTTGGGACAACTTCATCC
EMPV1_25151	CCGCCATCTCTACTACACGTCGCCCTTCTACGGCAACTACACAAACTACGGGAACTTGAA
EMPV1_25153	TTGTCTCAGCTTGGCTCTGCCGAGAACAGACCTGAGCAAAGCCTTCCTCAGCAGAGATTC
EMPV1_25156	ACATCATGCAGGGTTCTTGCACACATTCTGATCTTCTCTGTGACAGGCATCTCCAGCACG
EMPV1_25158	TTACTCATTCCTTACCAGCCCCCCTCAAATCCCCTTGGCTTTCTCTCTGCTGTGTGGACA
EMPV1_25161	CCAGTTGGCCATTTGCAGAAATGGAAGGTAGGGATGACTTTTAGAGATGCCTCTTCATCT
EMPV1_25162	GGCAAGACCAACATGGGCTAAGTTGTTGAGATGTTAGAACCTGGAAGCACACATCTGATT
EMPV1_25163	TTTTACCCATGCGAGGAGTGTGCTGAAGACATAAGGAGGAGGATATGCCGGAACCAGCCA
EMPV1_25165	GACCAGCGGCTCTGGAAACCCTAATATGAACCAAAATGTGACGCTGGAAGTTTGGCGAGA
EMPV1_25166	AGGGGCAGAGAGGTAGGCGGCGTAGCAGATGGGGCTTTATGCTTGTAAATACTGATAGCA
EMPV1_25168	GTGGGTTAAGGATGTGGCATTACCAAGAGCTGTGGAATAGGTCACGTGGCTTGAATATGG
EMPV1_25171	TGTGCGTCCGCTGCTGGATACGCTTGATGGTGGCGCCCTTGGGCCCCACCACCAGCCCCA
EMPV1_25172	TGGCTACCAGTCCAACCTCTACGAGCATTTCCCCAGTTCTGGTTACCTGGTCTCTGAAGT
EMPV1_25173	GGGGACCCTTGTGCACTGTTGGTGGGAATGTAAATTGGTTTAGTCACCACGGAAAACAGC
EMPV1_25174	AAGAGAGGAACAGTGGACTTGGTGGTGGCTTTGTCTTAGATTCCTCCTTCTCCGTCACGG
EMPV1_25175	TTCTTGTGACATCGTGGTAGACGTGGGTGGAGAGTACGACCCTCAGAGACACCGATATGA

EMPV1_25177	TCTATATTTACAAAATGGGCAGGAAGCCCCAGACAGGGTGGCGGTGACAATCACTGGAGG
EMPV1_25178	AAACTAAGTATTCCCCTCCCGCCACGTCTCCCCTGGTCTCTGAAAAGCAGGCCGAAGTCA
EMPV1_25179	TGGATTACTATGCCATTTGCCTAATTTCAGTGGCTACCCAGCTTTATGGATGGTGCACCC
EMPV1_25180	CGCAGTGCTGATTTCAACCCCGACTTTGTTTTCACTGAGAAGGAGGGGATGTATGATGGC
EMPV1_25181	ACTCCAACCAAATCAGAATTGCGTTGCCAGGGTTTAAACTTCAGTGGTGCTGATCTTTCT
EMPV1_25183	GAGGCCATCCAGGCCCTAATTTCTACCATTCTAATTGAGGCTGCCATGAAAGTCCGATGG
EMPV1_25184	GACCACCCCCAGTGCTGGATGCTTTGGGACGAATTATCTCAGAATCTCCTCCAGATCAGT
EMPV1_25186	AGGAAAGATTATGAAGAGTCCATTAAAGGCAGAAACCTGACTGGCCTGGAGGTCACGCCA
EMPV1_25189	AGAAGTGTCCATAGTGTTTATTCCGTGTGGTCATCTGGTAGTCTGCAAAGATTGTGCCCC
EMPV1_25190	GATGTGTCCCTTTTCCTAAATAGTCTAAATACTTGTGAGATGGCTCTGTCAAGTTGAAGG
EMPV1_25191	CAACACATGATGGAGACCTATATATACTTCACACTTTATGTCAGGAAAATTCCTAGAAAT
EMPV1_25192	TGGAATAAAAAGTTTATGGCTGCCAGTATAGCCTCAAGCAACCGGGATGAAGCTCTGGAC
EMPV1_25193	TGTCTTTAGAAAAAGATAGGACCTGTGCCGCTGCCTTGCGGGATACTGACAGACTGGAGC
EMPV1_25195	CGGCAAGCAAACCCAGACGTGTTTCTGTCCTCATTTACCAGACCGGATCTTATCCCTTTC
EMPV1_25199	TTTATGGATCACAACCGAGGCATATGGAAGTTCCCAGGCAAGGGACTGAATTGGAGCTGC
EMPV1_25204	ATAGCTATTTGGAACTGGGTGTGGGGGTGGAGGGAGGATGTCCTCTGTAACCAATAGTGG
EMPV1_25205	AGCTGCTGAGGGGCCAAGGCTTTTCCAGTCCTGCCTAGTGGAAGGAACCTCCCTACAAAG
EMPV1_25206	TACCAAAGACAGCAGGATTTCTTTGTGAACAGTAACTCCAAGCAGAAAGAAGCAGATGCC
EMPV1_25207	GACTAGCAGAGGACGGCATTCACAGGACATGGGCTTTCATTAAGATTTCAACCTGCAGCA
EMPV1_25209	ACATGAGGCTGAAGTGGGACCAGTACACAGTTGGGCGATCTGGGAATGATTTAGCCCTGT
EMPV1_25213	GTTTTAAGAAGAGGGTAGGTATAGGGAGTTCCCACTGTGGTGTAGCATGAACGAACCTGA
EMPV1_25215	AAATCGAAACTACTGGGGAAACAGAGCACCCCTGCACATCCCCTCTCCAGACCACAGCTT
EMPV1_25216	CTTTCTTTTCCAATGGTCACGTCAGGTCCAAATCCTCCAGAGCTGTTAGAATTGTGCTTT
EMPV1_25217	CTCAGCATCATCAGGTTCGCCGTGGCCAAAACGAGAGATCACACCCTCAACGACCTCTGT
EMPV1_25218	CTGCAAATACTACTCTACAGCCGTGGACATCTGGAGCCTGGGCTGCATCTTTGCTGAGAT
EMPV1_25223	GTAACTCTTCAGAGAAATTATACTGATCGTTGCTAGGCTCTGGGAGGAGGGTTAGTAGGG
EMPV1_25224	CATCACCCACGACCCCAGCGAGGAGTGGCTGTTAGTGGGCTTGAAAATGAGTGAGATCAT
EMPV1_25225	GTCCTGCGTTTCAGCCGGAGAGTGCGGTTTATTTACACTTACTATGATCACAATGTTGAG
EMPV1_25227	GGCCCGAGCCAAGCTAATTGTGTGGAATGGACCTTTAGGGGTCTTTGAATGGGATGCCTT
EMPV1_25228	CCCTTTCCTGGTGACTTCAGTTTTGGTAGGGAGACTTAGTTTTGCTGCTCTGACTGGCGT
EMPV1_25229	ATATACACAGTAAGTTCAACCTAAGCAACCCAGGGGGCTGTGCTCGTAGGCGTTTCAGTG
EMPV1_25231	GGAAAGATCGCAGGAGTGGACTGGTGAGAGGCTGGGAGCAGAGAAGTGGTTTCTGTATTT
EMPV1_25234	AAGAACTTCCATGTTTCACAGCTGCTTAACCCCACCCACAGCCCACACCCCATCTCTCTT
EMPV1_25236	GCTGGTCTCCAAGAGTTTAAAGCAGCCATAGAAGATTTAAATGCAGTTCTGTTTCAACTG

EMPV1_25238	GTAGTGAATGCTGGGAATCGTAGTCTTTATTCAGTTGCGTAGCTCGGCCCTTCTTTCCCC
EMPV1_25239	GCTAAAGAAGTGTTCTTTCTCCTTCCCTACCCACCACTCCCAGTTTTAATGTATTAAATC
EMPV1_25240	CAGACTCTTTCCCAAAGCCTGGGCATCTGGGTGGGACCAGATATGGACTCAAATCAACTT
EMPV1_25241	TCTGAAGGACCTACATGCCTGGTTGTGGCATCCTGGAGGGAAGGTGAGGCTTAGCCAGCA
EMPV1_25242	CAACCTCACCGTCATCCGGGGCTCACGCCTCTTCTTTAACTATGCGCTGGTCATCTTTGA
EMPV1_25243	GCCACGGAAACACCATCGCCTTTTTCTTTCGCTCGCTGTTGCCAAATTACACCATGGAGG
EMPV1_25245	TTAAATACAGTTCAGGTGGAGCCCCAAGTGTGCCAACACCCTCCCTCTGCTTTCCAGATG
EMPV1_25249	GGAGTTCTTTCCTTCGGAAATGGTATGATTTAATGATACAAAATAAGGATGACCTTGCCA
EMPV1_25252	GGTCTATACAACTGCCACAGCAACATGAAACCGAGCCTTATCTGTAACCTACAGCTCACA
EMPV1_25254	ACAGCTCATGGACAATGCCAGATCCTTAACCCACTGAGCGAGGAGGGATTGAACCCACAT
EMPV1_25257	GCCACCTTACTTAGGTACGTTGGGGATCGCAAAAACCCAGTCTGTCGAGATATGTGCATG
EMPV1_25259	AGACAGCTAGGTGAGTGGCTTAGTGCAGGATGTCCGGAGTTGCCTTGACCTTGAGTAGAA
EMPV1_25260	AACGGTCTGGGGGGTAATCTTTCACTCCTAGAAGATGAAGAGGGACCTACAGCATTGAGC
EMPV1_25264	ATATGTGCAGAAAGCCCACGATGGCAGCTGAGAACAGTCCCAGCCCATGCCTGCTTCTTT
EMPV1_25265	TTCGCTGTTACCACTCAGGAGGACCTTTACATTTGGGAGACATAGAGGAATTTGATGGAC
EMPV1_25266	TCCCTTTTCTGTTGATACTTGGGTCCTACAGTCAGATCGCCTCCACCATCCTGAAGTTGC
EMPV1_25268	TGTGGGGGATGACCAGCTGGGGGAGGAGTCTGAAGAAAGGGATGAGCATTTATTACCGAT
EMPV1_25269	CATTGATGATGACTGTAACCAGACAGGCCAGATGACAGCCCGATTACTGGTCTGGGCACA
EMPV1_25270	GCATACTTTTTTAGTGACAGGGGAAAATATCTATATGCTAAATCTAAGTTAAAAGTATAC
EMPV1_25271	AGCAAAGCTCTTCTAGCAATATCAGACTCTGCTGAGACTCACCCTGGCATCTGGACTCTG
EMPV1_25273	TTGCCGAGCATCCTCCGAATGCCCAAGTGGCTTATGACCCTGTACACCAGGAAGTACTAA
EMPV1_25274	CAGCTGGGAACACCAGGAAGGGGCCACACCCACACTCTCGTTTGTAAAATAACCTATGAT
EMPV1_25276	CATTCCCCACTCCAGGGTGGTACTTATTCTTCCCTTCATGACACCTCATCCTCATCCACA
EMPV1_25277	TTCACCACAGCCATAGCAACATGAGATCTGAGCCCCATCTGCAGCCCACACCACGGTTCA
EMPV1_25278	TTAGGACACTCTCAGTATTAGTCTGCACTAAGGTCCGGCTCTGCCACACACTGCTTGTGT
EMPV1_25283	CTAAGTCTTCGAGACCGTGGAGCACAGTATCACAGAGGAATTTGGACATGCACAGGAAGC
EMPV1_25288	CACTGTTGCTCAGTCTCTGCTTTTTCCAGAAGAATAGCATCCAGACGTTCTGTCAATGCC
EMPV1_25289	GAGGAAACAGCACAGAGCTGTTGCAGAGCTGAGACTGGTTAGTCTAGCAGTTAGAAGTGC
EMPV1_25290	AGCCCTTAGTCAGGCATTTCCTTGTTAGACTAGGTGTTCAGAATTGGATTTTATCTTTCG
EMPV1_25291	AAAGCACCTAGTAAAGTGACCGACGCCCTCCCAGAGCCTGAACCTCCAGGAGCCATGGCT
EMPV1_25297	TTTCCGAGTCAGCGCTCCTAGGAGACGGCGGGCTTTGGGGCTTCAGGTTGCATTTCTTTG
EMPV1_25298	GCCACTTGGCTTTTACAATGGATCTGCCCTTCTCTGGCCCCAATGAGGTAGACAGCTTCT
EMPV1_25302	CAAAGTACCCAGATGGTTCATGAGATATTCTGCCCTACCATGATATTCTGTCCTTGTACC
EMPV1_25303	GGATTGAGCAGTGGGTCAAAGATCATGGCTCTTGACTTACAAGAATGGACACTTATACTC

EMPV1_25305	ACACCTTTGCATCGTTCTCATCTTTTACTTGCCCAAACTGGGGCTGTCTGTGTTGCACCG
EMPV1_25306	GTGCCAGTAAAGGAATCCTGCTTCACTTTTTGCCTGCTACAGTAAGAGTTCAGCGCTGGT
EMPV1_25307	GCCAGGCACAGTCCATGAACAGAAATCAGAACCACATAGAAGGAAGAGTCCTGAGCAACC
EMPV1_25309	ACACCAGCCACGGGTAAAACAGGGCCAAAATCACACGGTGCACATCCAGGAAAACGAGAA
EMPV1_25310	ACCGTGGGGTACTGCTGCATGTACCTGTGTCAGATCTGCACTTGACCGATAGAGCAATTT
EMPV1_25311	ATGGCTAAGTGGTTTGGTCAACTCCTTGCTTCAGTCATCCCTCACCATCCAGCTGCCACT
EMPV1_25312	ACAGGGGCACGTTCAGAAGATTGTTGAAGCTGGTGGTCAGCAGCCTGCAGGGAGAAGACT
EMPV1_25313	GCTGTGACGGAGACCTGCTCCCACCCCCAGTGAATTTTCCATCTAGTCTGAGAAACAACT
EMPV1_25315	AGAGGGATTATGGAGGTGTTCGGGGTGGTTCGGCCTGTGTTAGCGTTCTCTCTCTTCCTC
EMPV1_25316	CTATGTTCCCGGCTCGGTCTCTGCAGCCTTTGTCACCTGCCCCAACGAGAAGGTCGCCAA
EMPV1_25317	AAACTTGAACAGGCAGCAGTAGAGCCAGGAGAGTCACCACATCTTGGTCCTGTGTCCTCT
EMPV1_25320	CTCTCACAGCCACAGCCGGAGAACCACTATCAGAACCACCCCTACCCCTTTCCTGTGGAT
EMPV1_25322	GTGCTTTACTGCTGTTTTAAGGTGCTCCAGAACTCTTCCCTCTCTCTTGCCCAGGAGCAG
EMPV1_25323	GACTGCACTGGAGATGAATTCTGCCAACAGCCTGAAGGAGCTCGAAAGCAGATCTTTCTC
EMPV1_25326	ACGGAGAGACACCAGAGACTTTGCCTTCCTTCCTTCCTTCCTGAAGTTTATGGACAGTAG
EMPV1_25327	TGGCATCTCTGTCACCTGCCGCCGTGAGCTTTGCAGAGGAAAGTGCCCTTCTGGTTCACA
EMPV1_25328	TTATTCCTCATAACTCTGAGAAACAGGGCTCACTCGTCCGCCTCATGGATGAGATGCCAG
EMPV1_25330	ATTTTTCTGACCATCGGAGCCGTCGCTCTTCTGGTGTTTGCATGCATAGCAGTCTACGAG
EMPV1_25331	GCCAGGTATCAAACCCATGCCACAGCAATGACCCAAGCCACAGCAGTGAAAATACAAGAT
EMPV1_25333	TGGAGGAGCAACTGCAGCACGCCAAGTTTGAGATCACCGAATGTTTCATCTCCGACAGCT
EMPV1_25335	TCTAGATGCACAGCCCAAGAAGGTCCGAAAGGTGCCACCAGGTCTCCCATCCTCGGTGTA
EMPV1_25336	TGACTCCTTCCTGAAGCTCTGCCCCCCTGGAAAATACTACAAGGAGGCCACGCTGACCAT
EMPV1_25337	TTGACAAGGATGGAGGGTTGCCACCTGGAAGTGGGAGGGCTCTAATGAGAATCAGATGAT
EMPV1_25338	CTGCAGGGGCCTGACAAATAGCTGTAGTATGCAGAGGATACAAAATTACCCTGGCAGTGC
EMPV1_25340	CGAAGCCTATGGGACCGTTGTGAGGATAAAACGAACCAGTTGGCACACATGGAGATTTGG
EMPV1_25341	CCAGATTAAAGTCAGTTATTCAGGAAGTTGCTAGAGATTATAGAGACCAGTTCCACAGGG
EMPV1_25342	CTCTAAGGAATGTCTGCCTGGTAGTTGAGTCTCAAGCAGTATTCTGGTACCCTTTTATCC
EMPV1_25345	TTGCTTCCACGACATCAGGAAACCTGACCAAGACAACTGGGAGAGCGGCCTCCAGGCCAT
EMPV1_25346	GCACAACAGGTGCTAGTTTTGAGAAGGCCCAGCAAGAGTTTGCAACAGGCGTGATGTCCA
EMPV1_25347	AATTATTGCTCACTTAGGAAGAATAATGACCATTCTCTGGTTAGGGATGCTAAGATTCAC
EMPV1_25348	TGAATCGGACATCTGAGGACCCCGTATCAATGGATGAGAGTGGATTACCACAGCTGACCA
EMPV1_25349	TCAGGCACTGGCTCAGGTTCTGGCAGGTGGTGAGAACTTAACTAGAAGGAACTGGAGTGA
EMPV1_25350	TTGCACACATGCATCTCATCATGCCTGTTGGACAACCGTGATCTCCCGAGGCCTGTCTCT
EMPV1_25354	GTGCGCACCTGCAAACTCCGCCTTGTGCACCTGCGGCCCTTGAGCCAGAGCTATGGCCAA

EMPV1_25355 AAGGGCCAGCAGAGTTAGCAGGAGTGTAGAGAAACAGAAGCACAGATGTACCGGGGTTTA
EMPV1_25356 ATTTTCCCCTAACCTCTGTGCTGAACTCTTGGCCCTTTGTGCCATCAGCATGTATTTACC
EMPV1_25358 AAAGTCAGAGCAGCCAACTGGGAGAGATGAGACTAGGAAGGCAGCAGGAGCCCAGATCCC
EMPV1_25359 AAACCTGATATCTTTAGTGCCTCATTTGAGCGAGGGACTCGCCTGAGAGTCGGAAGAAGG
EMPV1_25360 GTAGTTTGAAGAGCCCTACAGAATGACTCCTGACAAAGTGCAACCTACAGCTGCCTGGAG
EMPV1_25361 TCCAGGGCGAATGTTCCAAATGGCTGGGCTTAGGATGCCCCTTTGAAGTAGGAGAAATTC
EMPV1_25362 ATGAGGACGACCTTTCCAGCTCCATCAATGAACTCCCGGCCACTTTCGAATGCAGCGATA
EMPV1_25363 ATTTTACTTTTGCTAGAGGCGTCGAGATTCAACAGAAGAAACGATTCCCACAGCACTGGG
EMPV1_25367 CTCCGGGACGAGTTTCAAACCGCTAACAGCAAAGTGACCTGTTAATACTCGCTCATCAAC
EMPV1_25369 GGCCGACGGGCAGGACGCTGGGGAGCAACGCGGCCCCCGCCGCCCGCGAGCCCGAGCTCG
EMPV1_25372 GCACATGGAGCGTAAATGGTGCAACTTAAAGATAGAACCGTGTGTGATATTTTAAGTTAC
EMPV1_25374 CACGCTCTGACCCTGCAATTGACTAGAGTTCACTCGTGTGGCCTAAGGAATTTGGGGCTC
EMPV1_25376 CCCTGGCAAGGAGCTATAATAAACAAGGGAAGTACAGTCTCACGCGTCACTGAAACCACT
EMPV1_25378 CTTCAACTGCTCCTATTTAATCCTCTCGTCAGAACTCAAGGGATCTGCAGGAACCGTGTG
EMPV1_25380 AGTGTTGTGAGCCTTCCCAAACTCCTGTACATGCCTGGCTAGGGCACTTAGTTCCTACTA
EMPV1_25382 GACTTGATCAGGCTCTGCCCTGTCTGACAGAACTCATTCTCACCAACAATAGTCTTGTGG
EMPV1_25383 TTCTCTCTGTGGGAAAAGCCCAGGACCCCAACCAGCAGTAATTCCAAATGGGGACTGATG
EMPV1_25385 TGTCTCCAATCCTGGAATAGGGTCAGAGTGTGCCCTGAGGAGAAGCAGCAGAGAATGAGA
EMPV1_25386 ACCTGCTTTCAGCCCCCCGAGAAGCCCCAGGACGCAATCCTCAGCGCCCCCATAAGACAG
EMPV1_25387 TTCTATGACAGGCGGTTTTTGAAATTAATGAGATTCTATATTTTGTTGACTGGAATTCCA
EMPV1_25388 AGCCATGCACCGACAATTCCGACTCCCTCATTGCCAAGATCAACCAGCGTTTGGACATGA
EMPV1_25389 GCAGGTAAGGGAAGAAAAACCATATGGCGGTGAGGTCGCTCTGTTACAGGACACTTGTAC
EMPV1_25390 TCACTGCTTTGACCCTGGCTTCACAGTTCTGAGCTTCCTCACCTGCTGAGAGGAGAAGGT
EMPV1_25397 ATTGGTTTATTTGTTAATAAATTGGCAACACAAGGAGGGGCCAGGGGTGTGCCCCCAGGC
EMPV1_25398 TCATATCAAGATTCTTTTTCTTGTTTCCGGCTGCACCCACAGCGTGCTGAAGTCCCTGGG
EMPV1_25399 ATCATTGTCATGGTGCTAGGAGTCCCCTCTGTAGCTGGGAAGTGGATGGTCTTCTATACC
EMPV1_25400 TGCTTTGGTGACACAGCACATGCACCAAGCTAACTGTCACCCAGAGGATAATATGGCCTC
EMPV1_25401 ATAACTTTTAAAGCGGGACCATCTGTCAGTGCACGTATGCAGGCGGTGTGGAAAGAGACG
EMPV1_25402 ACCCCAACAGAAAAACAAGGGAAATGGGGTCCAGGGGGTGTAAGTCCATCCACAGATACT
EMPV1_25404 TCACCAAGAAGGGGCAGCGTGTCTGCACCAACCCTGATGACGAGCAAGTTCAGAAATGCA
EMPV1_25405 TCTCCTGCTTTCAGGCTCCTCCCTCCCACCCCTCCCCGACTTCCCCCAGCAGCTGGGGGA
EMPV1_25407 CGAGCCTACTGGGTGGAGCAGCAGAACAGGCTGCCCCTGCCCCTGACTGTACTCATGGAG
EMPV1_25408 ATTGGAACATGGAGGTTTATTTTAGGTTTGCTTAATATCTCAGTAAAAAGTTTTCATAAA
EMPV1_25410 CATCATCGCCAGCAACACCCGGGACAATAACACTGGGGCCACCTACCCCCACCTGAACTT

EMPV1_25412 CTGACTTTTCCTTCCATTATGGGGTTGGGGTACGCCTGTTCCAGTGCCTCTAAGAGAAGT
EMPV1_25413 TCCTAATTGTAGTTCTTGGGTAAAGGACTCTCAGTAAGACCCGAGTAAGACCCGAGTGCC
EMPV1_25414 AACCTGGTGTGTGAGGACGACTGGAAGGCCCCGCTCACGATCTCCTTGTTTTTCGGGGGT
EMPV1_25416 GACTCATTGATTCTGAGCCGGTGCCAGCCTGACCTCTTTTCCTGCGCCGTCTCACTTAGT
EMPV1_25419 GACAAGCTGCAAGATGGCAGTGTCAAAATGAAACCTCTGATGGGAGTGATATACGTGCCT
EMPV1_25420 AAATACTTACTGAGTGCTGGGGATGCCAACAAGGCCATGTCAGAAGGGATCTCTCTCCTC
EMPV1_25425 GTGGCTACAGCTCCAATAAGACTCTTAGCCTGGGAACCTCTATATACAGTGGGTGCAGCT
EMPV1_25426 TGAACCTGGGTAGGGATTCAAGGACTTGCAACATCTCCTGGCTCTCATTTCTTCCCAGCG
EMPV1_25427 TATATGCCCAAATACAGGGCAACCCCCAGAGAACACGACTCTCCCATTTTTCTAGTGAGG
EMPV1_25428 TGTCTTAGGGAAGACGTGGGGACAGGAGTGCGACAGACGTTTCTCTCTTTAGAGGCCAAT
EMPV1_25431 AACACAGCAAGTGTGGCCCCCGGAACTGTCTCAGGAGGCCCTGGCTGGTGCCTCAAAGAT
EMPV1_25432 TTGCTCATATCCACATGACGTAATAGCCCGCAGTCAGTTTCCAGATCTTTCCCCTGCTCC
EMPV1_25433 TACATGGACATGTAACTCATTAGTTTAACATTTTGAGATGATTGCCATTAGCAAATTGCT
EMPV1_25434 TGGACACCACGCAGAAAGGGACTCGGCTTGCGGTTTCTGCTTTTTCAATTCTGTGGCAGT
EMPV1_25437 ACGTGCATCCCTGATGTCTCCCTCTTTTCGTAGGGACCCTAGTCAGATTGCATTAGGGCT
EMPV1_25438 GCAGCATCCCCGACGACCTGACAGAAGCGCTAAGAATGAAGAGGTCAAATCTCAGGCGTT
EMPV1_25439 TCACTCTCCTCCCAGGCCTCAGCCTCCTTATCTTGAACATGAGGGGACAGGGCGATTTAA
EMPV1_25441 GAAACTTGAGTCATACCTTTGACCCTCTGCTCCAGGTGGCTTTCAGTCTCACCCAGAGTA
EMPV1_25442 GTACAGCTGCCGGTGCAGCCTCCCTCACCAAACGGATACAGAAAAATCCCAATTTACTGA
EMPV1_25443 ACTCTAAGGAGAAGGCAGTCTGAGTGAAACTCGGCGTCGCAGCTTCCTTGCGTTCCAGTG
EMPV1_25446 CTGTCTACCTGACTTCACTAACAGCAATATTTGTAGGTACCCCAGCCACAGGAAGGTCAG
EMPV1_25447 AGGTACTGTCGGTAGGAGTGGATATTTGTGCAGCCACTGTGGGAAACATATGGAGGATCC
EMPV1_25448 ACCAGCAGTGGGACCAGTAGCTCTGTGTTTGGCAGCACGACGCCATCTCCCTTCACATTT
EMPV1_25452 TCAAGCCTTGCCTGTCCTGGAGTGGACTCTGAAGCTTTATCTCAAAGTAAAGAAGGACCG
EMPV1_25454 CTCCTCTAGCAGTTTGGTAGCCCGGTAAAGGTTGTTTAAATAAGGTGGGAAGGAGGAGTG
EMPV1_25455 CTGCACAAACTAGATTCTGGATACCAGTGTGCGGAAATGCTTCTGCTACATTTTTAGGGC
EMPV1_25456 AACGCGCTTAACATGGGCAAGGTTCGTAAAAGAGAGGGGGAAGCCAAGAAGACGCCCCTGT
EMPV1_25457 GCAGAAGCTGAGGGCCAACGACGCTGCTAAAAGCAGACAAGCAGCTCTGAATCTTGTTTT
EMPV1_25458 TGCTCCTGTTCAAGGCAGCCTCACATCTAGCATCTCGTTTCATTCTCCCAATAACTAGGA
EMPV1_25462 TTGTCTGGACATGGGAAGCAGAGAGGTAAGTCCTGTGCTGAGGCCAGATCCCGCTCCTTC
EMPV1_25463 CCATCGGAATGACATGGAGACCATTTACCCCTTCCTGTTCCTGGGCCTCGTCTACTCCTT
EMPV1_25464 GGGAGGCCACTCATTCCATCACCTAGTGCTTCTTTCCCAAATGTGAGCAGCCATCCAGGA
EMPV1_25466 AGGCCCTTTTGTCTACTTGTCTTTGGTAGGAAACATCACAGATTCTGATGCCTTGATTGC
EMPV1_25467 CGACGTGGTCGCGCTGCAGGTTTTGACCCAACTCAGTAATTGCACGTTCAAATTGTATGG

EMPV1_25469	GATGACAACCACAAGCCCATCTGGATGCATGCAGAGGAGCGGGAGGCAATGAGCAAAAGC
EMPV1_25471	CCCGAAGAAGAACCTGAAGAAACAACAGAGGACACCACAGAAGACACAGAGCAAGACGAC
EMPV1_25472	TCCTTACGCCCAACTGGCTCAACACCGCCCCGAAACCAACTTTACAGACTGCACTACTAG
EMPV1_25474	TTGATCGGACTGCCGTGTCAAGGTTTCCGTGGGTTCCTAAGCAAGGGAAATGAGCCTACC
EMPV1_25477	CCAAGCCACCCTGGTTGAACAAGTAAAAAGAGTCAAAGAAATTGAAGCCATTGAAGGCGA
EMPV1_25478	TGGAATCATGTCGTCCGATTCTGCAGATCCTTTCTACTGGATGAGAGTTATTCTCGCATC
EMPV1_25479	GCCCCGCTGACCACCAGGCCGAAGGATTCTACACAAATTATTGAGTTTACCCCAAAACTC
EMPV1_25480	CCCCTGGATTGGGAGCTACCACTTTCCGAGGCATTGAGCGATTGGGACGTAGAGGATTTC
EMPV1_25482	TGTCATTCCTCTCTCCCTGGAGGAGACGGTGGTAGTAGCCGGGGTTCCGGTCCATCTGGG
EMPV1_25483	CTGCAGCGTGAAGTAATGTGTGCATTTGTGGACACTCAGTCTCTCACTTGAGTTACAGGG
EMPV1_25486	CATGGTCTTTTTCGGGTCCTCATCGGAGCATGGGAGCCCATCATGCTAGGAATAGCGATT
EMPV1_25488	AGGATAGCAGAGATGGGCAAGCCGGTTCTTGGGGAAAACTGCCGGCTGGAGGTCATCATT
EMPV1_25489	GCTCAATAGCAAGGTGGTAGCTCTGCTTTCATTTTAAGGAAGTGGAGACTGAGGGCATTG
EMPV1_25491	AGGCAAAGATCCTGCCTGTGCCCAGGGCTGAGTGAAATGCTCTGGACAGAAAGCCAGGAA
EMPV1_25493	GTTCCTCCAAGAACTAAAAATAGAGTTTCCATATGATCCAGCAATCCAGCTCTGGGCAAA
EMPV1_25494	TTTTGTGACGTTTCACCACTGCTCTCCCTTGCATGTGCAGACACCTGGGCCAATAAGGTG
EMPV1_25495	TGGCCCCTTACCGTATCCTGCACCAGACCCAGGACTCTCAGGTCTACTGGACAGTGGCAT
EMPV1_25496	ACAGATGAACATGAGATAAGAAACAGGGACCCCCAGAGGTAAAGGCTGAGCATCCGCGTG
EMPV1_25497	GGTTGTGTCTTGCCGCCCCAGGGGCTCTGGTAGAAAGACTGAAAGCAGAGAAGCCAAGAA
EMPV1_25498	GGTCAAGGATGATAAGAAGAACCATCCCCTTGAGAAGAAGGTCACACCTCTAACTGGAAG
EMPV1_25499	GAAAGCACGACTTGAAGCTGCTATAAAGGGGAATCAACAAAGTGTGAAAGTCTAGATACC
EMPV1_25501	TCCTGGACATTTCCCACTCTGCCCAGGCCCTGAGTGTCCACATTAAATGGGTCTCCAAAA
EMPV1_25502	GAAAGCTCTGGAAACCATCGGTGCAAATTTACAGAAGCAGTACGAGAACTGGCAGCCACG
EMPV1_25504	CAGCAAGTAGCCAAGAGATGTAGCACCAGGAACCCTTAAACCATTGCACAGCATCTCAGC
EMPV1_25506	GCCTGGCCCCCATCCAAGCCACATAGAAACCTGTGCAAATTGAGCTTTGAGTTTGATTCT
EMPV1_25508	CCTCTGGCCATAAACTCGCTCTAACTGCCCTGGTTTGTAAGCACCATTAAGTGGCTAACC
EMPV1_25509	CAATCGACAGGGCCTGAAAAAGGCCGACTGCTCCTTCTGGTCCAAGTACATCTTGTCTCT
EMPV1_25510	CCTGTATTATATGGAAGTTCACAGGCTATAGGTCGAATTGGAACCGCAACTGCCAGCCTA
EMPV1_25511	CAGAGGGTGTCTCCCAGGAACTCATTTCTGCTGGCCTCGTCGATGGAAGGGATTTAGTAA
EMPV1_25512	CAGGGATCGAACACATGCCCTCATGAATACTAGTCGGGTTCTTTACTGCTGAGCCACAAC
EMPV1_25513	AAATCGGAGGTCCTTGCTCCTTGCTTCGCTGAAGGGCGTTTGTTGCCAGGTTTCGGCCTT
EMPV1_25514	ACTTCCACTGGGGCAACCCTTCCAAGGACAAGTGCTACTGCGGCTTCATGACCTCCAAGA
EMPV1_25517	TGTCCCATGCAGTATTTGGGATATACTTATACTAAAACATTACTCCTCGTCTATCTGAGG
EMPV1_25519	GTTCCTCTGCATTAATCCTTCTGCTCTTCAGTGCCTTTTAATCAGCTTGCATCTCAGAAA

EMPV1_25520	TCTCTTCCACATTCACTTTGCCCCACAGGCCAGTGATCACAGACTTCTCCTCAGCAGTAA
EMPV1_25521	TGAATCTACGGATGCAGAACTTGTGGGTATGGAGGACATGGAAGATCGCCAAGGCATTCC
EMPV1_25523	TCCTTGATGACCTGTCCTTTCTTAATGGGATGAGTGGTCTGGATCCTAGCTATTGCTGGC
EMPV1_25526	AGCCAACACTGCACGCAGAAAAAGAAACCGAGCGAGTCCTCCCAAGTGCGCGTTGCTTTG
EMPV1_25527	TCCACATGGGAGTCACCCTTTAGAGATGTTCCTGGGGCCTTAGAGATGGGATTATGGCCT
EMPV1_25528	TCCAGCAGCCGGCTCAGGCACCAGTACGTGTCGGCCTCCACGTTGCGCAGCACGTCCTCG
EMPV1_25529	CATTTCCAGTTACACCCTCTCGCCATTGCCCATTTGTCTCTAGTTTCCTTGTCCAGGTTC
EMPV1_25530	AAGAGCCATTTTGGGAGACAGGGGGAGAGGAAGAAAGTGTGCGAGACAAATGAAGAGAAG
EMPV1_25531	GCTGACCCTGACAGCACAGGTGAGAAAAGTAGGGGAAGTGAATGAGTTCTAACAGGACAT
EMPV1_25532	GGGAACTGTGAAGGGCTGGGCGGAGGGCTCAGGCAGGGTTTTGTAAGCAGTGATCTAGTT
EMPV1_25533	CAGTAATATATTCGGTGGTGTCACATGGGTAATGGCTAAATACGTTCTGCATATGAACTG
EMPV1_25534	AGGAGGGAACTCCGATCTGCCCTCTTTGTCCTTTTGTCCTGCAGCTCCAGAGTTTATAAA
EMPV1_25535	GCAGGCTGAACATTGAAGGGTTTTCAGAAGGTGGTAACATATGCCATATATTTTGAAAGG
EMPV1_25536	CTAACGGAAGTAGCTTCCTTCGTCTGCGAGACCACGCTACACCCTCTGGTTCTGCATGGG
EMPV1_25538	TGAGCACAGAAGTGACCGGATTCTGAAAAACAAAGTGTGCTGCTCCACAGAGCTGCCTCT
EMPV1_25540	TAGCCGGAAGGGCTCTCACCCCTCTTGCATAGTTTTTGTGACCGGTCCTGGGTTCCTGGT
EMPV1_25541	ATGGCCTCTGTCTCTGAGCTCGTCTGCATCTACTCTGCCCTCATCCTGCATGACAGTGAG
EMPV1_25542	ATGATCCTGGCATTCCCATAAATGGACGGCGTTTTGGTGACAGGTTCCTGCTTGGCAGTT
EMPV1_25544	ACACTCACCCCAGCAGTTCAGCAAAGCTAGCTGCTTGGTTTTGGGACTCGGGAAACTACA
EMPV1_25545	TGGCTATACCTAGTACACTTCTCAATTCGTCAAGAGGCATGTAGCTGTCTTGCACTGCAC
EMPV1_25553	CAGGGAGCTGGGGCAGGGGCAGTAAGTGGAAGCTCCTTATAGGTAGAATTTGTCATGCTT
EMPV1_25555	TCCTTCTTCCACAGCGAGGAGAACAAAGCACCCGAGAGCGAGGAGCTGGAGATGGAGTTT
EMPV1_25557	CGTCATGGATTCCAGGTTCGGGAGACTTTGTATCTCTTCGGTTGGGAATACTAGCTCTTC
EMPV1_25558	ATTGTGAACAGGACAAAATGCAATCTTATAGCCTTGGCTGCTTGGGGTGGTGGAGCGGTG
EMPV1_25559	AAGAAAGATGCCGGAAAGTTGGCCAAGAAAGACAAAGACCCAGTGAACAAATCTGGGGGC
EMPV1_25561	CTGACTGAAGGAGAAGGGCACTGTCATGGCTGAGACTTTAGCACCTTATCTCCCAGAATC
EMPV1_25562	TGTGCACCTGAAGTTATTTCACTAATAAACACGAGAATGCTCTTCTGGGCATGCTCCACA
EMPV1_25563	TGGTTGAGTGGCTTTGGCAATTCTCTCATAATGGCACCTCAGACATTGATGCTACCCCGC
EMPV1_25564	TATGACATAACAGGTGGCACCAGCAATCCGGGCTCACGGCAGCCTCTCCAGTGACTATAT
EMPV1_25565	ATCTTCCAAAGAGGTGGACCCGTCTACAGGTGAACTCCAGTCTCTACAGATGCCTGAATC
EMPV1_25567	GCATGAAGTCAAAAGGATCATGAAGCTAAGTTTAAAAACTGCTTTAGTAACTATGAAGCT
EMPV1_25571	AGGAAGTTCTGCCGGGTGCTCTCCGCTGGTGCCTCCCTCGCTATATTGTTGAACAGGAAA
EMPV1_25573	ATCACCTGAACCTCCATCACCTGAACCTCCTCCATCACCTGAACCTCCCCCATCGCCTGA
EMPV1_25574	AGCGGAGAAGACTACTTGAGAAGAGCAATCGAGATCAAGCAGGGATATACACCAAAGCAG

EMPV1_25576 GTGACTTGTCTCCCTCCTTTTACCCATAATCCTCTCCACAGCCTTCTCGCAATGTAGTGG
EMPV1_25577 GTAATGGAAGAAATGCACTACCAAGTTAAGAGGACAGATTTTGCTTAGAGCAGGAGGCCC
EMPV1_25578 GACTGTCCTCCTGATTTGCCTGGGACTGAGTGGCTTTCTAGGTTTGCAGGACTTTCAGTG
EMPV1_25579 TGATCCTGGTGCACGCGTCACTGCGTCTACGCAACCTCAAGAACAAGATTGAGAACAAGA
EMPV1_25580 AGTGAATGGTGTGGGCTCCTCAGGCCCCAGTGAGTACATGGAGGTCCCTCTGGGGTCCCT
EMPV1_25582 GAGGTTTGGCCTGCCGGCGCAAGGCGGATCCGAGAGAGTGGCCCATTTGAGCCGGGCCAA
EMPV1_25583 GTGGATTTCACACCTAAAGATAATGTACACGGACTGGCTTACAAGGGCCTGGATCCCCAC
EMPV1_25584 TCATGACCGCACCACACCTGCACCTCCATCAAACCAACAAAACCTTGAAGAGGCACCTGA
EMPV1_25585 GCTGCCGTGACCGGGTAGCCAATCTTCATGAGAAGCCATGGATCTGGACTTTTATGTGAA
EMPV1_25586 GAGATCATTTGGCACTGAGCTGTGATGTTGCCAAGGAACAGGATGTTCAGAGTACCTTTG
EMPV1_25590 CAGCTAGGACTTGAAGGGCAAATTCCTAAAGCAGAGAGAGAGCATTGATTGTGTTCTGTG
EMPV1_25592 CTCGATCAATGATGTGTATGGCCATCGTCATGAGGGTCCAGCACTTGGAACATATGTCTG
EMPV1_25593 CCTTCCATAGATGAGATAGCGCGTCAGATCGAGGATGAGGAAATTAACCTGCGGGAGAAG
EMPV1_25594 TCTATGGGAGGAGAAAACAGGGTGGCCCTGACTAAGATCAGACAAATGCCTGCAAGGGAA
EMPV1_25595 CAAGCAGGGTTTAACGTTCCTGCCTACACTCTCAGGTGGCCTCCCTATGTTGGTGCAAAA
EMPV1_25597 TCCATTTGACTTGGAAAACATAATCTTTCGGATGGTGGATGTTGGTGGCCAGCGATCTGA
EMPV1_25599 CAATGGAAGGAAGCAATGCAGCAATTTAGACTGTTTGGGAACCAAGGTATCACCCTGCTG
EMPV1_25600 AACGGCCCTCCAGTGCTGGTGAAGAAGGAGAAGGAAATGATGGGGGCGTTGGTGTCAGAC
EMPV1_25601 ATTTTAGAAAACTCACTCTGGTGTTGAAGGGTAGGTTGGCGGGGGAAGCCAGATTCGGAG
EMPV1_25602 ACAGCTGGGTCTCCAAGTGGCAGCGAGTCAGTAACTTTAAGCCGGGGGTATATGCAGTGT
EMPV1_25603 CAAGCATTTTCCCACCTGTTCAGTCCAGCTGCCTTTCTAACCTCAGATGTTCCTGCCTGA
EMPV1_25605 GACAGTGCAAAAAGCTGATGAAGTTGTTTGGGTGCGTGCAAGGGTTCATACAAGCAGAGC
EMPV1_25606 GAAGCTGCCCTGGCTGTTGGGCCAAATCAAAGAGGAGAACCGAGGCTAATCAGAATTTCT
EMPV1_25607 GCCGGGGCCATAACCACTCATCCTCTTTAACAGAACTTCACTGAGTTCTGCTGTGTTTAG
EMPV1_25610 CTACAGCACTGAAACAAGGCCAAATTACTCCAACTGAGCTCTGTCAAAAGTGTCTCTCCC
EMPV1_25612 TGTATAGTTCTGTGAGCTGTGCTTTCTGGAAATTTGTGGGGCTGGAGGAGACCCCGGTGT
EMPV1_25614 CATCTCTCCTGTATGGACACCTCGCTAACTGAGATGGTTGCCTATTCCACCTCTTCTGCA
EMPV1_25615 GATGCCCTGAAATAACCTGCACGCCGCCCCAGCTTTGCACGCACCCAGTCAATAATTAAT
EMPV1_25616 TGGATGAGGACCGCCAATTTGCCCTCGTTTACACTTTTGTCACCCCCTTACTCAACCCTT
EMPV1_25618 TATCACCCTAGCCACATGCTTCCTGGAAGTCCGATATTATCCCCCGCACTACGATCTACT
EMPV1_25619 GTACTCCCTGCAGCAAACAAGAGATGGCAATGTATTCCTTGTTCCCAAAAGTAAGAAGCC
EMPV1_25620 AGGCAGGCCTTTTGGCGCTCTCGGCTTTCACCTTTGCTGGGCTTTGTTATTTCAACTATC
EMPV1_25621 ACCTGGGAAGGACACCAAAAGAAAATTAAAGGCGTGGGGAAAATATACAGACATGACTGG
EMPV1_25622 GGCCAGGGTTTTACTTGGATGGTTCCTAAATACCGACTTTTGTTTGCCCTTAGTGCTCAC

EMPV1_25625	AGAAAGAGCCAGCAGAGCCGCATGGATCCGGAAGATCAAAGGCCTGCCCATAGATGATTT
EMPV1_25626	GCCCCCAGCCTGCCTATGCCCTATAAAGACAAGAGAAGCTGAGACTACGGTATTTCCTTA
EMPV1_25627	AGATGAATGGAGCCTGGTGCCATATGTGAGGAACCTCGGTGTCCACAGCAAAGCCTGATG
EMPV1_25628	TCTCCTCTACATTACGTGGACATTGCCAGTCCATGCAGTAAGGGAGCTCACTCAGTGGGT
EMPV1_25630	TGACTTCATGCTGCCTAATGGCTCCTTCACCCAGAACCTCCTTGTCTTTGGCAACAGCTG
EMPV1_25631	CTGCAGGATTTGATAAGCACGGGGAAGCTGTGGATGGCCTTTACAAGATGGGCTTCGGTT
EMPV1_25632	AAGCGGTCTTCGTCCGAGGAGTCGGAAAACGCCGAGAAATGGAACAAGACGTCGTCGAAA
EMPV1_25634	AGCCTGTCCCTTCTATGCCCTTGGCCCTTCAGAATATTTCCATCAAGCACCTGGGAACTG
EMPV1_25635	CCGCGCCGGGCTTCGGCAACTTGGGCAAGAGTTTTCTTATCGAGAACTTGCTGCGTGCTG
EMPV1_25637	GAGGTTCCCTTCCACATTGACTGATTATATACCTCCTTTGGCAGCAGTTGGGTTACTCCG
EMPV1_25640	TTCCTCCCTGCCCCATGACCAAACTGTCCCGAGCCACTCTAACATGACTTCAGTTAGCTT
EMPV1_25641	GCTCCCGAATCCGAAGTTGTTCATGGGGGGCAAGGAGCAGAATTCGCCCATCTACATCTC
EMPV1_25642	CAGGCCCCGACAGAGAAGAAGGAGGCCAACCCCAAGCTGAACATGGTGAAGTTCCTACAA
EMPV1_25643	AGGAGTTGAAACCTGATATAGTAACTAAATCTGCCCTTGGTGATGATATCAACTTTGAAA
EMPV1_25644	ACTGTACAGACCTGCCTAACATGCCAGCGCAGCCAGCGGTTCCTCAACGATCCTAGGCAT
EMPV1_25646	TTGGTAGCAATTCACACTGCCTCCACTTAACTCTGAACAAGCGTGTCTCTGGAGCCGCTG
EMPV1_25648	TGTAGAGTAATCGTCAGTGTCCCTAATGCAGGCTTGAAGCATCAGTGCCCACGTGACAGT
EMPV1_25649	ATTCTGACCATAGAATCGGATGTGGAAGACCTGGGGGAGGAGACTGACACGGCACCTGAG
EMPV1_25650	CTTGCCTTAGCGTCAGGCTCATGTTGGTTACCCGCTTTGTGACTTGCGGAGCTTTGAATT
EMPV1_25652	AAACGAGAGCATTTCAGAGTGCGGGAGGTGTTCCCTGTCTCCATTCTGTCAGTTTGAGGA
EMPV1_25656	AACTGAAAAGGAAACAAGCCACCTGCCACACTTGAGGCTGCATGACCACCCGGAGAAATG
EMPV1_25658	CTAGAAATGGCGAGGTGGAGGTATACAGACAAAAGGCCAAGTACCAGAGTGAAAGACAAG
EMPV1_25660	GTCGGAGCACTGTCTGTGTGGCTTACCAGCTGATACACTTTGCTTTTTAGGCAGCACTCA
EMPV1_25662	GAAAGGGCTTACCTGGCAGCACAGTTCCGAGGGCAAGTTCTAAAAATAATGAGACTCACC
EMPV1_25664	TTATGAAAGCGCCCTTTCCTCAGGGAGCCCACAGACCCTCCCCTGAGACTTGGAATGAGA
EMPV1_25665	TGTCAACAGTCTTGGAGAAGCCACCTGCACAGCAGAGCTCATTGTGGAAACCATGGAGGA
EMPV1_25666	TAACCAAAGCTGTCGTATACAGAGAAACAGACCCATCCCCGGAGGAGAGGGACAAGAAGC
EMPV1_25668	CAAGAAACTATTTGCAAAAATCCATCCTTCAAAAGTAATTTACAAATAATGCCACTCCTG
EMPV1_25669	GCTGGACTACCCCAGCGAGAATCTTGCCTTCATCGACGAGTCCTCGGACACGCAGAGCGA
EMPV1_25670	TGCCTCCTGTATTAAGGAACTAGACACTTTCTGGGTAGCAGGCTGCAGCGGGATAAAAGG
EMPV1_25673	ACTCCCCTTGGTTTGCCCTCCTTATGGGAACCCCCACAGTGCAGGAGAGAGAACCAAGTT
EMPV1_25674	CAGTTTACTGTAATGCAGTGTGCCGTACATGGAAATAGAGGGTCTGCAACTTTATAGTCG
EMPV1_25677	CAATCCAGCACTCCAAGCGAGCACAGGGGGTGGTCTTCACCACGTCAGGAGGGTCCACAC
EMPV1_25678	ATAATCAAACTAATTGCCAGCCAAGTCAGTCACCCTCCTGGGAGTATATAGCGTCCCCAG

EMPV1_25683	TTAGATGTCTCTAGAAGGTTCGGGTGTAGGAGGCCAGTCAAGGTAATATGCAGCCCCTTG
EMPV1_25684	CAGGGCAGTATTAAGCAACATATGCACGATGGGAGAACCAGATGTAAAGGAGAGAGTGAA
EMPV1_25687	TGGTTCAAAGAGCCAAGATGACACCTACTGCTTTGCTCTAATCCTGCCTTCAGTGTATAC
EMPV1_25688	GATGGATAAGGCCGCAGCAGCACTCAAAGCCAAATCTGGTGAGAAGGGCGTTCCAGACAA
EMPV1_25690	TTCACACCTTGGTTTTGAAGCTCTGTGGAGAAGGCAGGTGGGAGACGACCAGAATCAAAG
EMPV1_25692	GAAGAGGCTGGTCTTCAAAGTAACTCCGCCAATAAAACACAAAGCTGGAGGCGAACTGGG
EMPV1_25694	CCTAGCCTTGTCTTTACGGAGGGATGCTAGTGAGTCCATGTGGTGGAAAATACAGACTGC
EMPV1_25695	CCCAAATCCAAGGCTAAGGTCATTCCTGGGAAGGTCAGAGGTGGTGTTTCTGTTGAGTAG
EMPV1_25698	ATCAATCAGAGAGTGAGCTTCCTCCAGCTCCAGGGCACACAAGCACCATCAGAGGACTTT
EMPV1_25700	AAGCTGCTGGATGCGGGGAAAGCCACTGCCAGGCCTGCCGAAGAGGATGCTCACCAGGAT
EMPV1_25704	CATCTCTCCTGCTGGACCAGAAACGGTAGATGCAAATCTGCAGAAACTCACACAACTTGT
EMPV1_25705	GCAGCAGCCAAGTTCTCAGTCATGGTTTGCCTTGATTCATCTTTCAGACTTCGTTTCGCC
EMPV1_25706	TGCTCAACATCCGGTCCCTGGAGTTCTGGTTTAATCACCTCTATAATCATGAAGATATCA
EMPV1_25707	CACGTGGAAACTCAAGGTCTCAAATGGCAAAGTCATCGGCTGAGTGTAGGACATGGCTAC
EMPV1_25709	CATCATTGGCTTTTAACCCAGTATTGGACTGGTGGTTGGTGGAGTAGCTGTCAGGCTGCA
EMPV1_25710	GAAGTCCAAGGAGTTATGACCTTCCACAGTAGCCTTCTCAGCCTCTGCCATTACCTCTGA
EMPV1_25712	TATATTTCGCCTCAGATAAGGAAACAGTCACTCGTCACGTCCCTGTGTTTGAAGATCTGC
EMPV1_25715	CATGTTTGCTTGCCTGCTGCCTCACACAGCTTGGGCACTCCTTAAAATCCTAATTATGCG
EMPV1_25716	GGAGCTCAACAAGTACAAGTACAAGTCCGGCAGCCACGAAAGCTCACGGCACCATGACAA
EMPV1_25717	CGTCTTTGCGCGGCTCCCTCGTGGAGACACCCGCATCGCTGGCCACCCCCTCAGTGTCTC
EMPV1_25718	CAGGTAGATTTGTATGATTTGGGGGTGGGGAGCTGTTGTATCTCTACGCTTGGCCTTAGG
EMPV1_25719	TTTTAGGATGGTACCCGAAGCATATGGAGATTCCCAGGGTGGGGGTCTAATCGGAGCTAC
EMPV1_25720	TTTCGCTTCCTGGTTCTTTCATCTATGTTTTCACTGATGCACGGTCTAAGGATTATCGGC
EMPV1_25722	GGTTGGGATCGGTTGCAATGCTGTGTTTCTGTATGGTATTGCACTTGTCCCGGCCTGTTG
EMPV1_25725	GTGCTCTGCTGGCTGCCCAACCAAGCACTCACACTCTGGGGAGTGCTGATCAAGCTGAAT
EMPV1_25727	CTCCAGTCCTCCTGTTCTGATGCTCTCAGCAATTACATTGTGCTCTTTTCTGTGACTGGC
EMPV1_25729	GGGCCCCTATACTGTAATTCTCACCACCCCCACTGCTCTCAAAGTAACAGGAATAGATGC
EMPV1_25732	CAAAGGGAAAGAGGAGGGGCACCTGCTATGTGTTCTCTAGGCCTTTTGGAAAACATGGAG
EMPV1_25733	CACTCGCTGTAGTTCAAGTATGCAATATAAACCCTGTCCCTTCCAGCTGTGTCTGCCTCC
EMPV1_25736	TCAACCTTAAACAGCAAGACCTACGAAACTATCCGGAACCGTCTCACTGTGGAGGAGGCC
EMPV1_25737	CCAATAGCTAACAGTTCATTCCTCTCTGTTAAAATTAGGGATAGGATTAGGGTTAGTGAT
EMPV1_25738	GCTGACAGTGGGGTTCTGACCATGACCTGCTTCATAGTGTTGCTGATATCCTACACGTAC
EMPV1_25739	TGCGGGCATGTCTCCCTCACTAGACTGAGGCTCTGTGAGGTTCACGGGTTCTGCCCTATT
EMPV1_25744	GCCCCGACTGCCTCCCTTTCCTGATCTCTTTATCACGATGCCTAATGTAGTATCAACAGC

EMPV1_25745 CAAATGGGACCGAGAGCCCCAGGTTCCTGATCTGAGTTTTAATGAGGGTTTTTAGGGCTC
EMPV1_25746 GCATATACCTTCAGCCCCCATCAGGAAGTTCTCAAGATGGAAGCAAGTTCACTGCCCTCT
EMPV1_25747 AGCATAGGCTGAAAATGTGGCTCGGATCTGTGGGCTGTGGTATAGGTTGCCGATTCGATC
EMPV1_25749 GTGAAGCCGCTGCCTGCGCCCCTGGACGGGCAGCGCAAGAAGCGAGGCGGCCGAAGGTAC
EMPV1_25750 TGGAGATAGCCATAGCCATGAGGCAGAAGATCTCTATAAGGAAGGTTTGTCTGGCAGCGG
EMPV1_25757 TTCATACAAGCTCTTGTCAGACGTGTCCGAGGCCTACTGAGGATATCACGGAACTGAGCA
EMPV1_25758 ACTCCAAAGAGGCCTCATTCTAGTACTATCCGTCATGGACAATCCAGTTCTAGGAACCAG
EMPV1_25760 GGCACCTTGATTACAGCCTTGTGAAACTGAGCAAAGGACCCAGCCATACAACATGGGACT
EMPV1_25762 CCCGGCAGCACCACCAGACATCGGAGCTGTGAGAGGATTGGTTTTAAAATGCTACTTTGT
EMPV1_25763 GTGGCACCACTGCAGGAGGACTCCAATTGAGGCTTGAACAAATGGAACTTAATGGCAAGC
EMPV1_25766 GTGGCCGATTATGACTTCTGGTGAGGAGGGGTGAGCAGGGAGTGTTGATTTTTCTGATGT
EMPV1_25771 ATGGAATAGCTTCCCCTCAAGGGCTGATTGATGTGGTGTTCTTGACCCAGGCCGCCAAAA
EMPV1_25773 GCGCACCTTGGCAGTCATTATTGCACAGTTTGCTGGAAGCTTTTTCTCGAAGAGCACATC
EMPV1_25774 TGCAGATACAGCGAAGGTTAGAACAGGGGTCCAGGAGGAAGGCAGCAGGCAGGGATCTCA
EMPV1_25775 TGTGATGTCACCCTCGTCACCCCTCCCACTCCTGAACAGGGATCCAAGAATGTGCCAAGA
EMPV1_25777 CCTCGAGCAGACAGTACTCCTCCCTAAGATACTGAGCAAGAGAGGTTACAGAAGCAGAAC
EMPV1_25778 CTGTCCATGTCCAGGGAGTAATTCCGACACCACAGCCATCGATTATTCAGTCACCACAAA
EMPV1_25781 AGGTTTGAGCCAAAGTTGTCTCAGAGCCCCTCAAGAACACACCCACGCCTATGACGGGCT
EMPV1_25782 ACTTACTGTGACTAAGGTGATCCATTGAAACTATGTACTCAGTTGCCAGTCAGTGTCTGC
EMPV1_25785 GCTTCCCGTCTACCTGAACTTCACCCGAGCGGACCTCATCTTCACGGTGGACTTCGAAAT
EMPV1_25786 GGCTGTGACGGTCAAACATAACTCGGAAAACTCAAGAAGAGCCTGTCGTGCTGTACTTTT
EMPV1_25787 GTTCCTTATCAGCCATAGCACTGAGGAGAGAAAGCAGGGACCCAGAATTCCCAGAAAGGA
EMPV1_25790 CACTGATCCTTAACCTACTGAGCCACAGAGAACTCTAGGAGAGGGAAGTTCAGAGTGGTG
EMPV1_25792 GGTCTTGGGCAAACAACAAACTCTGAATGGAGCCACTGTCCCTGAACCAAAAAGCCTCTG
EMPV1_25794 CCAAGAAAAGCAGTTTTGGAAAGGCAAGAGGAAGAGAAGGAGAGATGCAGGGGTGCAAGG
EMPV1_25796 TACCTGCTGTTACTGGACCTGCTGGCAGAGCCTTCTTAAACTTGGCCCTTACTGAGTGTC
EMPV1_25798 ATGCAGAATTTCAGGACGTGTCGGAGGAGGCCCGGGAGTTCATCTCCAAGCTTCTGATCA
EMPV1_25799 TCTAAAGCTAAAGTCCTGAAAGTTGAAAGCAGGGTCTGCCAAAGCCCCCACTACTCTCCC
EMPV1_25800 GACTGGAACCAGCACAAGGAGCTGGAGAAGTGCCGGGGTGTCTTCCCTGAGAACTTCACA
EMPV1_25801 CCCACACCATTTCAACTGCAGTGTCACATTGAAAGTACACACACACCCCACGAATTTTCT
EMPV1_25802 GGATGTCAGTTGACCCAATGGATTGCTAATGGATTAAAAACAATTTATGGTGTTGCAATG
EMPV1_25803 TGTGATGCCAGGCTGGAGACACTGCACTCCTGAATGACCACGAAGAGTGTATTGCAATCT
EMPV1_25806 CTGCACTGACGTCTCTTCCCCATTGGTCGCCAGACACCAGCAGTCCAGCCTCTTTAAGAA
EMPV1_25807 AATGCTGGGTCCAAATGAGTCTCCTCTGTGGGGACCTACATCCAGGCCAGTCACACAGCA

EMPV1_25809	GGTGGGTCATCAGACTGGGCAATCAGGTTATCTTTGTTTTGGTCCTGCTGTAGGTCTTCC
EMPV1_25810	GCTAGTTGCCTAAAGACAGGTGGTTTGCTAAATGGCACTGAAGAAAATCCTGACCCAGTG
EMPV1_25811	CTTCCAACCTCTTTCTGAACTCCTTATCCCATCTAAGTCCCGGGATGAGGCCTAAGACCT
EMPV1_25816	GGAAGGGATCACTCAGCCATCTTTCAGGAAGAATTCAGGAAAATATGAAGTCCCTGGAAG
EMPV1_25818	TACGATGGGACATATCACTCGTTCAGCCCTAAAAAGCATGGACAACAAGTTTTTGTTCTG
EMPV1_25823	CTTCCAGTTAGAGGTGTGACCTTCTTCTCAAGGGGATGGTTCTTCTTATCATCCTTGACC
EMPV1_25824	GAGGGGAGAGCATGATTTATATGTGTTCTAGAGTGCTGGAGTGACGTCTGAACTTCTGAG
EMPV1_25826	CAGGCATAACATGAAACATCCACCCTTTAGGGGAGGTTCTGGGTGATATTCAACTTGGCT
EMPV1_25827	GTGCAGAGCTGCTGTCCACCGCGCACCCAGCGTCCGGGAGCCTCCAGATAGGCTTGGTGG
EMPV1_25828	CATGGGGATCGGGCTCCGCTAGAATATACCGATAGCCGTCTTTGTTAAATGGGTGTTCCA
EMPV1_25831	GTTACGACAACACGGAGAGCAGTGTGCGCAAGGCCAGTGTGTTTTGCTTGGTGGCAATTT
EMPV1_25832	CATTCCGCATTTTAACCTGAGCCTTGCAGACTTTTCATTTGGAGTTTGAAGCTTTTTTGG
EMPV1_25833	TGCATCTCTCTCCTGCTTGTTAACATGTCATTATCCCACTCCTCTCAGGTGGAGTCCAGC
EMPV1_25834	CCTCACACTGTTTGCAGTGAGAACCACAGCTCTGACCCCAGGGAAAGGTCCGGGCGCAGT
EMPV1_25835	GTCAAGGTTCCGACACCAACACTTCAAGATCGAGACCCGCGATGGGAAGCTGGTGTCTGA
EMPV1_25836	TGTCCTGTATAGCACCATTATTTTAACCTTTCCTGTTTATAGAGCTTTAAAATTCTGTC
EMPV1_25837	TCCTGCCTGTGACTTTGCTGCTCCTCCTTCTCATCGCCTATTTCAGGATCAAACTGATGG
EMPV1_25838	ACTAGAGGAGTTTCAGAATATTGGGGCACCACAGGAGCATGATGAAATGGAACCAGCGGC
EMPV1_25839	TGCGTTCACCCCCTGTCTGGCGCCTGCCAGTGTACATAAATGCTGTTCAGAAAAAAACTCC
EMPV1_25841	CTGAGGCTTAACTGTAATGTTGAGAGAAATGGGAACTACTGATGCCTTTAGACCAACTAG
EMPV1_25842	AAATGACTCCAAGTCAGCACGGATGGTATCCGAGGCCTCGTCGTGTTGCCAACACAGGGT
EMPV1_25843	GAGTTATATGCCGAGATAGCGTCACAACGGTTACAGCCCCATGGAAAGCGAAAAGCCCAG
EMPV1_25848	GCTGTCCACCCAGGGCCCAGCCCAGGGCCCACTCATCTCCAGCACCCTCTCAGGGCCCAG
EMPV1_25849	CGCTCTCAGCTTCTTTGCATCTCTAGGTACTGACTCGAAGTCTGGTGTGTGCACTTCTGA
EMPV1_25850	TCTGAGAATTGGCGTACAAAAGGCAGAGGGGGCAAGGAGAGGGAAGGAAAGAGCGAGCAT
EMPV1_25852	ACCCACAGACATAGAAAACAAAATTACGGTTATCAAAGGGGAGGAGAGAAGGGGAGGGAT
EMPV1_25854	GAGATCTCAGAGGACACTCAGCCTTGAAGCTAGTCGGCTTCCCCAGACTGGTTCGAGTCT
EMPV1_25855	CCAAATGGGAAAGAAAACTTCTCAGGAAGCACCTCTTTGTGGCAAAACATCTAAGGCCTG
EMPV1_25856	TGCAGGCAGTGACAGTGACCAGGGCTCCGATGTGTCCCTTACCAAGGGTCCTAGAGTGTG
EMPV1_25857	GTGGAATCACAGCAGAGGCATGAAATACTGGACAGTTGCCTTAGGTCTTCACTCGACTCC
EMPV1_25858	TTCTGGGAGGAAAGCCAGGGCTTGTGGGCTCTGAAGGCATCCCCATTTCTGGCAGTTTCT
EMPV1_25863	AGGTTCCTTGGCTAGATATCACGGGTTAACTTCCGCGCACACTCCTCTAGCATTCCGAAG
EMPV1_25864	CAGGCGCTGGGGGTGCCGGAAATGGGGAGATGTTGTTAAAGGTACAAACTTACAACTAGA
EMPV1_25866	GAAGAAGACCACCACAACTGCTACAGCTACACCCTCATGTTCATTAATTGTGTCCTGACC

EMPV1_25867	CTCTTAGCTATCACCCTCCAATCTCTGTAAACCTACTCCTTTCAGCCTTAGGCAACCATT
EMPV1_25868	CTCGAGGAGAGGGTCCAGGAGGAAGCCCAGTTTCTGGTGGAGGAGCTCAGGAAAACAGAG
EMPV1_25869	GAGAAGGGGGCATGTACGGCCCTGGTTTCTGGGACAACTTCCAGGGTGAAACTGATTATT
EMPV1_25872	GAATGCTGACAGTGGGGTTCCAGCCATGACCTGCTTCATAGTGTTGCTGATATCCTACAC
EMPV1_25874	GCCCCCTAACAAAAGACAGAATGCCAAACTTCGTGTTGACCAGTTAAAGTATGACGTCCA
EMPV1_25877	GACAAAAGTACACTCTAATCCCAGTCTTTTGGATGGAAGAAAGGCTGCATTGTTTACAGG
EMPV1_25878	CAGTTGAAGAGATTGTGACCTTTCTGACGAAGGTGCCCGAGTTCCAGTTTCTAATTGGAG
EMPV1_25879	TCGCACAGGACCCTGACTGTCGTGCGGTGGTGATCTCTGGTGCAGGAAAAATGTTCACTT
EMPV1_25880	TCAGGGATGTTCTGCAGAGACTGATGAATATTTCCTTCAAAATAGAGGTCCGCAGCACCG
EMPV1_25882	CGGCTGCACCCTCATTAAGTTGAGAAGAGTTCACTGATTTCCGCCATGATCTATCTGCTC
EMPV1_25884	AGCAGTGCTGTGTGTGAAGGAATTAACGCTCTGATCAGCAACCAGACGGAAGCCGAGACA
EMPV1_25885	CCACTAGGAAGAGGAAGGCTTGGGAGCACAGGTGCAAAAATGCAGGGAGTGCCTTTAAAA
EMPV1_25889	GACCAACCCCAGATTCTGATGGAGACCTGACAGAGATCCAATACCTTGCCACCTACGATA
EMPV1_25890	AACAGTTTCCTCCACAAATCATCCTCAACCCACCTTTCCAGACTCATCATCAGTAATACG
EMPV1_25893	ACGGTTAAGGACCTGGCATTGTTACTTCTGTGACTTGGGTTTGATCCCTGGCTGCAGGTG
EMPV1_25895	CTGTGAAAAGTCCCGGAGTCTAAACAACATAGCAGGAGCGGCTGGAACCAGTCTGGGGCT
EMPV1_25896	TTTTATTTTCTCATCATTTTCCTTGTACAGGTCACAGTAGACACACCAACTAGCCCAGTT
EMPV1_25897	AGTCCGAGTGAGCAAGGGGGATAAGCCAGTGACCTATGAGGAGGCGCACGCGCCTCACTA
EMPV1_25901	TACGGCCAGAGGCCCACAGTGAAGGGCGTCTGAGGAGCCTTGGCCAAGGTGATTAAAGCT
EMPV1_25902	TGCAGTCGGAGGAATGCAGGAACCTGTAAATGTGGAGGTGGAACCGAAAGCCAGTTTTGG
EMPV1_25903	ACGCAGGCCGTGTGCAGGGGCTTCCTCATGCGCGTGGAATTCCAGAAGATGGTGCAGAGA
EMPV1_25906	ACAAAGGTGGGATCCCTGGCCCAGAACAGTGGGTTTAAAGGATTCAGCGTTGCTGCAGCT
EMPV1_25907	CGTCACCAGCATCCTGGCCGGTTTTGCCATCTTCTCCGTGCTGGGCTACATGTCTCAGGA
EMPV1_25908	TGCTTGTTCCAGTTCGTCCCATTCCGAGACTATGTCGACCGGTCAGGGAACCAGGTGCTA
EMPV1_25909	AGTGAGGAAGTTGTGTGTCATGTGGCTAGAAACCTGGCTGTGGAGCAGTTAGCGCTGAGT
EMPV1_25910	CTACAGCACCATCATTTGTATGTATATGCAGCCTGGGAATGTGGCAAGCTAGGACCAGGG
EMPV1_25911	GTAAGAATCATCAGGGAAACAGTCCTTTTCCAGTGTGCATGTGGCCGGCTCTGAAGAGTC
EMPV1_25912	CTTGCCAACAGGTCCTTCCTCTCCACCTCCTAAGAAACCAGGGCATCATCTGTTAGACAG
EMPV1_25914	GCTGAAGGGCCTGAAAGCTCTGGTGGAGAAACTTGAATCCCTCCCTGAGAATAAGAAGTG
EMPV1_25918	TACTGCAACACCCCTAGCAAGCACCACGTGACCTGGAACTCAGTGTGAACCAGTAATTCT
EMPV1_25920	GACACCTCTGTCATTGGTATCTTAGTGGTGGTTAATGGAGGACTGATCTGCAGTATCGTG
EMPV1_25921	TTTAGTTTCATTGATCTTTTCTATTCTTCTCTTTGTCTCTGTTTCATTTATTCCTTCTCC
EMPV1_25923	AACATGGATGATACTTCAGGTTCCAGCTTCGAGGATATGGGTGAGCTGCATCAGCGCCTG
EMPV1_25926	CCCTTTAACAGTCAGTATGTTGGAGGCACATTGTAGGCACTTGCTTATTACCCAAGAGTC

EMPV1_25929	CCCCCTGCTAGACTAATAAGTGGAGAGGAACATTTTTCAAGCAAGAAGTGCCTGGCTTGG
EMPV1_25930	AGAATTGTAAATATTGGTGTTTGTTTCTGACATTTTAACTTGAAAGCGATATGCTGCAAG
EMPV1_25933	GCACAGATGCAGGTAAGAACCTAGATGGAGTGCTGCCATTTGCTAAGGTGTCTGGATGCA
EMPV1_25934	GATGGAAAGATTGGCAGAGACAGACACAAAACAGGCAAATAAGCTGCTCCCCATCAGACC
EMPV1_25936	TTTCCCTCCTGCCAAGTGCGCCATTCTCAGAAAGCCCAGATTGCTGCCTTCCATCTTGGT
EMPV1_25937	ATTTAACCAGCAGCCCCAGGTGAAATGCCTGCAGCTGCTTTCAAAGCCGAAGCCGAATTC
EMPV1_25939	TCGTGGCAACGTGATTCGGCGGAGCCGCCGGGCCGTGTTGGAGCAGGACTTGTGTTTGGA
EMPV1_25941	CCCCAGAAGGGTCAAGCCACCAGCATTTCTTACGCCCTCTGGATCCATATTGCAGAAATT
EMPV1_25944	CCAGTTTCATAAATGAGGCTTGAGAGGCTGATCCAAGGTGTCCTGGCAAGTTGTTTAGCG
EMPV1_25946	TTTTCTGCAACAAATTCTGGGAGTACAGGCAGGTTCCCACGCAGCTCTACCCTTCACAGC
EMPV1_25949	GTTGATTGTTTCTCTGCAGTTGAATAGCTGGAGTCAGCCACGTCTGAATACTTAGATTTG
EMPV1_25950	CAGGCAAAGACAGAACAGAACCTATAAAAGGCTTTCACAAAGCAATGGTCAAGACCATGT
EMPV1_25955	TGCGGAGCCAGGAGCTAATGGTGGGGTGGCTGCGGGCCTTCTGCAGGTCCCCCACGTTCT
EMPV1_25960	GAAGAGGAAGGAGGCGATCCAGATAATATTGAATTAACTGTTTCAACTGATACTCCAAAC
EMPV1_25963	GGCTTCCTGAATGGCTGGATCAGTGGGTATCAAGAGCAGAAGCTTTCGTACTCGTAGAGT
EMPV1_25964	CAGCTTCCAGCAGGCCCAAGAGGAGTTTTCGCAGGGCTTCTTCAGGAGCAGGTCCTTCCA
EMPV1_25966	TGCACTCTATGCATCGCTACCTGCCAAGGCTTCATTTGGTGCCTGCAGAAAAGGCTACTG
EMPV1_25968	AAGCACAAGCAGCAGTTTCAGCAGCAGCAGCTGCACCTCAACAAGGTCAGTCGGGTCAGT
EMPV1_25970	AAGGAGGAGGTCAAGATTATCGAGCACCAAACACAGAACATGCGGCTGATGCCTCACCTG
EMPV1_25971	GGTAGCTGCCGTATCTTCTACTGCTACATGGACAAGTGTATGGACCGTTGGACTTACCAG
EMPV1_25973	TGTGCTCAGGACTTGGATTCCAGATGGGTTGGGAGCCCTGAAGGGTTCTGAGCAGAGGAA
EMPV1_25974	CCATTTCTCCTTCCCGAATGCCTCAGCCTCCAAACATGATGGGCACACATGCCAACAACA
EMPV1_25979	GTGCTCAATGTCTGTTCCCTGTTGTCATCCTCACAGCATTCTCCTTAAAACCCTCTCCTC
EMPV1_25980	CATTTGGTGGGAACAGAAAAGGCAGTGGCTGTTGCAGACCCACTGGACACTGGACAAATA
EMPV1_25981	ATTTGGAAATCATAGCTTCCTCCCTTCTCTATCCCATGAGTCCGGGACACGAAACGCTCC
EMPV1_25982	TCCCACAGGACTCAAAGTCACCGAATCTCCTCTGCCCAGCACTTTCTGCTCCTTGGATAC
EMPV1_25983	TCGTATGCGTGTCTGGAGGGGCTGCCCAGCGGGACACGGGCCTCTGCCTTCTGAGGCCGT
EMPV1_25984	CTCCATTCTCTGGTCTCCCCACTACAAGGAGCTCATTTCACGCCAAGGCTATGCCGAGAA
EMPV1_25986	TTTAAAACAGAAGCCGCTCAACTCACAGCTTGGAAGTGGGTGGATGGAGGTTGTCCCCAG
EMPV1_25988	TCAAAACACAGCACACACTAAAATCCATTCTTTTCTTTCCACAACCCAAGCCCCACCTCC
EMPV1_25991	CTTTCTAAGGTGAGTCCAACCCACAGACACTCCCTTCTCTCTCCCAGAATGGCTTGTTTC
EMPV1_25993	GGTCTTGTTGAACTGCAGATTGGGTCAGAAGTTTGGGGCTGGGGCCTGAGATTGTGCATT
EMPV1_25995	GACCTAGAGACCATGCAAGCATTTGCTCAGGTTTTTAACAAGTTAATCAGGCGCTACAAA
EMPV1_25998	ATTAGTTTCAAGCATACAACACGGTGACTCAATATTTTTATGCATGATGACATGATCACC

EMPV1_25999	TGTCCTTAGAAGACAGCATAAAATGGCTTGGAGAAGTTATGGCTGAAATAGGGCCATCCC
EMPV1_26002	GAGAAGAAGCTGGAAGGAAGCCCAAGTGGATTAACTGAAGCTCTGGACAAGGACAGGATC
EMPV1_26003	GGCTCATATTGTTCAGTCAATAGCATTCGGAGGAAAAGTAGACGCCTCATGGGATCCTAG
EMPV1_26006	GGATCCTGGATTTGGTTCCATCTTACCAAGGCCCCCATTTGCTGAAAGCATCACTAATGA
EMPV1_26009	CGCTGGGGTCTAGTAGCCTTTCCAGCCCCTACGAAATGAACTGCAGACTTGAAATCAATG
EMPV1_26014	TGAGCTGCAGAACAGCTATTTATCCCTCTTAGCTGGTGGAGTGAAAAGGCCAGAGTCGTG
EMPV1_26017	AGATTGAGGAGAGCACCTCAGTGGTCACCTCACAGTCGGCTGAGATTGGAGCTGCTGAGA
EMPV1_26018	CCCATGAACACGCAGCAGAGCTGGTCAGTATGGATGAGGAAAAGTTTGTGGATGCCATTA
EMPV1_26020	GGGGCAGAGGGTGTGTCTGGAGTGTGTTTGCTTTTCTTGGGTAATTGTGGTTGCAGGTTG
EMPV1_26021	TTTGATTATCTGCAAGGCGGTAGCCTTCTCAGGGTCTATGACTGAGAATGGTTCACTACC
EMPV1_26023	GCATACGCCACACTCCGTCTCTGCCTGCATCCTTTTCAGCATTCCTGTATCCTTTACCCA
EMPV1_26024	GTCTGACAAACCCGATATGGCTGAGATTGAGAAATTCGATAAGTCGAAATTGAAGAAGAC
EMPV1_26026	AAAGAGTCGCTCCTTATTGGTGGCCATGGCAGCTGCAGTGTGGGAGGCTGAATCCCCTGG
EMPV1_26027	GGGTCGTCTTTCCTCCAGATGACTGACTTCCAGTTAGTACCACGTGTGTCTGTGCCTTAT
EMPV1_26028	GACGGGGTTTCCCCAAGAAATTTCTAACACAGAGATGAGCCCTGCAAATCTAATTTCTCG
EMPV1_26029	GCGGTATTAAGTCCTCCGTGTAAATGTGCCCGCTTCTCTCCACGCTGCCATTAAGCCAAA
EMPV1_26031	CCTCACACAGGGTGCTACGCTATGCACTGTCTCTTGGAACAACAAATTCCTTCATTTCAG
EMPV1_26033	GGGATCTTGTTAAATTGCAGATTCTAGTTCACGGGTCTGGGCTGGGGCTTGAGATTCTGA
EMPV1_26034	TATATAATGCCTTCCCCCCATTAAGAGAGCCGGTTTTCCGAAGGCCTCGCTTGCGATACG
EMPV1_26035	TCATCTTCCCAGGACATGTGCAGTTCAGGGGTCATCCCAGTTTCTGAGGGGATCTTATAA
EMPV1_26037	GTACAGACAGAACTTACTGACCCCCCAATCCTTGGCCCTGCCTTCAGCTTCCTTCCCTGA
EMPV1_26038	CTACCTGGACAAGTATGAGTGGTTTATGCGCGCCGACGACGATGTCTACATTAAAGGTGA
EMPV1_26039	TGGTGCCAGACGCCACATTTAAAGAAAGGAGAGAGCATTTCCAAAGAGGCCCGTCGTGTC
EMPV1_26040	CCACTCTGTGAAGCCAGCATCTAGGGTCTAAAGATGAACAGGAAAGCAGCATGCATTGTC
EMPV1_26041	AACAACCATCTACTCAGAGTGACACTTACTCTATGCTGGCCCTTGGCAATGTCTGGCTCC
EMPV1_26046	TCTCATTCATTTCGCCGGTTAACATGAGAGATCATGGCCGCCTTCGGGCTTCTCAGCTAT
EMPV1_26047	GCCTAGAAGTAGCAGTGGTCACAACTGAGAGGGCTAAACATTTCTACAACCCCCAGGATG
EMPV1_26048	AAGGAGAAGAGCAAATACAAGTTCCCTCCTGCTGCTTTGCCTTGTGAATTCAGCACCTTC
EMPV1_26050	GTCAGCGTCACCGTAGGGGGCAAGCAGTACCTCCTGGGACTCTATGACACAGCCGGACAG
EMPV1_26051	TTTATGGATTTTCCATCCAGCAAAGGTTTCTGTTCCACGGCTGGGCAGATCCGACCACAG
EMPV1_26053	AAAGCATCACGGTGTCAGCCAGTCAGTGCAATAACCAGGTGTAGTTTTACAGTCGCTCCC
EMPV1_26054	GGGGAGTTCCCAGCTGTAGTTTGCATCCTCTTTAGGACTGCGTGTGTTATGTAGACGCGA
EMPV1_26056	CCATCTTCTCCTTGCCTCTTGTAGGCCTGGTGGTCATATCGGCTTTGCTCTGGTGCTGGT
EMPV1_26059	ACACAGCAACGTCCCATGCCATATGTTAAGATGCGATGAGGAGAATCAAGCATCACTTCC

EMPV1_26060	CTGGGCACAACGACATCGAGCTCTACAGCCAGTACCTGGAGCGCCTACGCCGCTTCATCT
EMPV1_26061	GGGTGTTCAGCAGCCATCACTCCTTGGAGCATCTCCTACCATTTATACACAGCAAACTGC
EMPV1_26063	CCAGCCTAATCTTAGAACCACAATAAAATGTGTGACCTTAGCAATATATTACCACATAAA
EMPV1_26068	AGCCAGCAAGAAAGGCGGATGGCTGCGGATAAAAGTAGTGGCGAAGACAGCACTTACTGT
EMPV1_26069	CTGTGTCTTGTGAAAAGGTGTGTGTCCTTGGGTCTGTGGGAGCAAGTGTTCGGGTGAACA
EMPV1_26070	ACCCACGGAGCGAAATATCTGGAGTAAAAGTCAAATGCTGGAATCTGCCCACCAGGCTCC
EMPV1_26071	CTCACCTGTAAGTATTGATACAGAAGATTCATCTAGTGACTCATCTATAGAACCAAGACC
EMPV1_26074	ATAAATAAAAATGACTACTTGCAGTACTTGGATATGAAAAAACATGTGAGCGAAGAAGTT
EMPV1_26076	CTTTTCTCTTGTGTGTTCACTTTGTTGGTTAGTCAGCTGCCCCAGAATCTTGACGATCTG
EMPV1_26077	GTGCTTGTCTGTTTAGTTCTGTGTATAAATGGAATGATGTGGAGATGACCCCTCCCTGCG
EMPV1_26079	GAAGTTAGGCCGGCTCCTGGGCATTTTCGAGCAGAACCAAGATCGCAAGCACAGAACGTA
EMPV1_26080	GTTCATTCAACATCAGTCACCTCCACCGTGCCGCCTAAGTTCGACCCCAACGAGATCAAA
EMPV1_26082	GGGGTGTAAAGACAACCAGCCTGCCCTCAGATCACGCTCCTGTAAATAGACGGCCTTTTT
EMPV1_26084	AGAGGCCCTTCAGATACGGACCGAGAACTCCTGTGTCTGATGACGCAGAGAGCACCAGTA
EMPV1_26087	ACTGAGGAGCACAGGTGGCCTTGGAGAGGCTGAGAGAATGTTGTTAGGTTTCCCTCCCTT
EMPV1_26090	ACTGGTGGGATTGTGAATCATAAGAAATGGAGAACACCAGAAGCTCCTGGGCAGCGGAAG
EMPV1_26091	GTTCTCCATGGTGGATGTCGGATACGTGGATCTCTCCTTTCCCGTGCTCAACGTCGTCTT
EMPV1_26094	CACAGAGGCTGCAAGTGTTTTGAGGCTGCGAGTATTTTGAGGGACTGGTAGGAAACCGTT
EMPV1_26096	GGGTGGCTTGGAATGCATAACTCCAACTTTTTTAGAGTTGGTAGGATCAGGACTCCGTGG
EMPV1_26097	ATCATTTATTGCTTTGCCGGGTAAAGGAGGCCACAGCTGGCTCATGCCTTAAAAGCTGAG
EMPV1_26099	CAGGCACGGTTAGATCTGGTTCTTCTGTCTAATACTGTCTGGTAATGCCGTCCATTCCCG
EMPV1_26100	TCACTACTGGAATTGTTGTGTCTGCCTTTGGATTTCCCGTTATTCTTGCTCGTGTGGCTG
EMPV1_26101	CTTGGAGCAGGAGCGTGTGTGTTTTGAGATCCTCATCTGTGAAAGGTGTGCCAGAAATGG
EMPV1_26103	ACGAATGACTTTTGACTCTTGAAGAAGCAGCTGGTGTTGGCCAGTTGACTCCAGACACGG
EMPV1_26107	CCTCTCTGATCACCTTACTAGAACATTCCTTCAGAAAGCAAACACTTAATCCTTCCCGAT
EMPV1_26108	GGCCCTCGGGGAAAGCCTGCACAGTTATCATGGAGAGAAAGCTTATTCGGGAGATGAACA
EMPV1_26109	CTGACTCTTTGTGCCCAGGGCTGTGCTGCCATTCTGGACACAGGCACATCTCTCATCACA
EMPV1_26110	AGGCTACTGATTGCTGAGTTGGCAGTTTCAGCCAGTGAATTCATGTTTGAAGAAACTAGG
EMPV1_26111	TGAGTTCCACCTCCTTTGCTCAGAACTCTAGGTCGTCATTGCCATCAGACTTCCGGACTA
EMPV1_26112	CAACTGCTTTCTACCAAGCGTTCCATTTGAACACGATTCAGGAATCAAAGAACCTCTGGG
EMPV1_26113	AGGAGGGGCAGGACTGTTGGGATCAGCAGACGCGGAATGTCAAGGAAGAGGCACAGATTT
EMPV1_26115	ATGCTTGTAAATCGCCTAACAGAAAAGGGCCGGGTGTCCAGGCCGTGCAAAGAACTCATA
EMPV1_26116	GGGTCCCTTCAAGTTCCTATCCCATGTGCTGTGGTGAATGTGGTCTTCATCACTGTTTTA
EMPV1_26118	CTCTCCTTTTCTCCTTTAGTATCAGAAGTCCCAAATTTCAGCGAGACACTGGCCACCGGA

EMPV1_26119	GATGGTCGCTGCTCAGAGAGGATACACCAGGCTCGTGAAGATCCTCGTTTCCCACGGCAC
EMPV1_26120	GTCTGTTTGTGCCTGGGCCTCCCACCATGACTGTTTCTGTTGAAAATCTCCTAGGTTTGC
EMPV1_26124	AAGAACCTCAGACGGATCTCAGCCCACAGGGTGAGCCGCTCGTAGAAAGGTTCCCTGACA
EMPV1_26125	TGGTCTTCGCTGTCGCCGTAACCGCCACGAACGCCGCCGAGGCCGGGCTCGAAAACATCT
EMPV1_26126	TAGAGGAGAGAAGGGGTGTCAGTTCCAGTGACTCTTCAGAAATCCCAGTAATATTGAGTG
EMPV1_26128	TTGGTGTAACAACCTGAGGAAGCCACCCTGGTACCCATCCCACAGAGCCCTGCTGCTGGT
EMPV1_26129	AAGCAGACGGTGTTTTACATCGAGGACTACCTGACGGTGATGACGGGCGAGGAGATCTTT
EMPV1_26130	TGAACATTGCCAGGAACTTTTAGACTTTTCCTCAGCACAAGGCCCAATGACTTGCAGTGC
EMPV1_26133	ACCATCACCTCCTCCATCCCTAACACCTCCGCCACCATCACTTCCTCCATCCATAACACT
EMPV1_26134	TCCGCGGGTGTGGCCAAAAAGAACTCTCAAAACCAACCTGTTTTTACCATGTGTGTACTG
EMPV1_26135	GGAAATCAACGAGAACTTGCCCGCCAGAAAAACATGAAGAAATCGCAGGAAATTAGTAAG
EMPV1_26138	AGAGTGGATCAGTTACGTGAAGACTCTGCATCAGGACAGGGTGTCTGGCAACAGGCCGTG
EMPV1_26140	AGGTTGAAGTTCCGACTAATACCTGGCTTCTCAACAAGTGTGGAGGGCGTGTTTGCTGCA
EMPV1_26142	TCGCCTCAGTCCTGTGTTGAGCCACCATGCAAGAAAATGTTCAACCTCAGGCAGAGGCTT
EMPV1_26143	GGAGCTGCATTCAGAGCTACACAGGGACTAAGACACTGGGATAATGTTAGGTTCCTGTTT
EMPV1_26144	CTCATGTCCCCAACAGGTCAAATGATGCTGATACAAAAGTTTCCCTACCTGCCCAAGGCT
EMPV1_26145	ATGGAGGTGCAGCCCCCCGTGTCCCCTCAGCAGTCTGAGTGCAACCCTGTTGGCGCTCTG
EMPV1_26147	CAAAGCCATTTTCGAGAACACACTGAGCACCTACCCAAAGCGCACCGATGTCTGGTCAGT
EMPV1_26151	TGCACCAGACCTAGGTAACTTTCCTCCCGTGTCCCCTAGTTTGGGTATCCTCCCAGGTTA
EMPV1_26153	GAAGAGGAATGATACTGTAGGAGACCAACCAGCCCAGATGCAGCAGAAACCATCTAAGCG
EMPV1_26155	TCACAGAGACTTGGCGGCCAGAAATATCCTCCTTACTCATGGTCGAATCACAAAGATTTG
EMPV1_26157	TACAGTGACCAACCATTCTGGTTTCCCTGGGACTGAGGGGCTTCCTGGATATGGGACTTT
EMPV1_26159	CACTTTATCAACCTGGTCAGCTTGGTGGTGTTGGGAGTTGAGAAGGCATCAGCATGTCCA
EMPV1_26160	GAACTCACATGCCAATTCACAACATCTAGGGATCTGAATTCAATAAATGTGACTTGGAAA
EMPV1_26162	CCTGCCCTGGGTGGTCTGGGCAGCGAGGAACGTACATACTTGCGACAACTTTTATCTCAT
EMPV1_26163	TGCATGCCAGCTTTTCAGGCAGTTCCCTTACCAGGTGATGTCTGGGCCCCATGAAACCCT
EMPV1_26166	TCAAGCCCTCCTCGGCACCTGAGCTCCAGGCCATCCGCATGTTTGCTGAGTACCTGGCTA
EMPV1_26167	CACATTATCTGGCTCCTTGATTGACCTTGGCCATGTTGACACCAAGACAGGGATCCATCC
EMPV1_26169	TCTTGATTTCTATCCCACACATGTTCACTGGGGCGTGGTGGCTTCCTCAGCATGCATAAG
EMPV1_26170	CCCTGTCCGTTTTCTGTGCTTGTGATTACCTGTGAGTTGTACGCCGTCTCGTGTTTGCAT
EMPV1_26172	TGGGGAGACCCTGCCCCAGATGTAAATAGAGCAACTTGGACAAACTTGTATTTTGCTATT
EMPV1_26173	AAGACGGCCCCAATGAGGATGGGGAGATGTTCATGCGGCCAGGAAAGCTGTCTGACTACT
EMPV1_26175	CTCCACCTGGCACTCCTCATGCCATGTTTCTGCCTTGCCAGCTGCTCCCTGTCCGTGTAT
EMPV1_26176	AGCTCTCACTACTCGAAGCTGGAGAAGGCGGACATCCTGGAAATGACCGTGCGCTTCCTG

EMPV1_26179 GAGTCGCTGCCTTCTCCACAAACACTGGCTTCCTTCGTAATGTGTCTCCATATCCAAGAC
EMPV1_26181 TTCAAGCACACTCGGGTATTGGGACGCGTTAGATGAATTTCCGGCTGGGGTGCAGGCCAT
EMPV1_26182 ATTGCCTCTGCTATGGCTTGGATCCCTCCTGTGTCTTGGGTCTGATCCCTGGTCTAGGAA
EMPV1_26184 TTTAGGAAAACTAACCACAGCTCCCCCGAAGCCCAGTCTAAGCAGACAGGCAGAGTATTG
EMPV1_26186 CCCCAGTCCACGAGTTTAACTCTGGCCGCTGCATGGCACATTCGTTGCGCTTTTTTCATA
EMPV1_26189 GGAGGCCCGTTTCTCTCTTTGGTTATCTAGCTGTATGAGTGCCACAGAGCCGTCATAAAG
EMPV1_26191 TCTCCAAAGCCATAGAACAATGTGGGAGCGTCCATCCTTCTCTAACCTTCCCACTGTGAC
EMPV1_26193 GAGGCCATGCTCAGAAGGGCTGGAGACAGAGATGAGGAACTAGGCACATCGATGATTTAA
EMPV1_26197 AGACGTTGAAAATATGCACGATCCAGTTTCAGGTCTCCCAAACTTGTTCTTAGACCATGC
EMPV1_26200 CTGAGGAGGCCCGCAATGCTTGTACCAAAGCAGAATGTTTCACCTCCAGATATGTCATGC
EMPV1_26201 GGACTGTGGTGGATCTTGAAGTCATATTTATACTTTGGCCCACAAGGCTGTTTTTGTTGC
EMPV1_26202 CCAGGCAGGGCTACTATTTTTCCCCTTCACACTCCCTTTCCCAGTCGGGCCTCTGTGTAA
EMPV1_26203 GTACTACCTTCCCTAACGATGCCCTGTTGTGTGGCTGTTTTCCCTCTGCATTATGGCTAC
EMPV1_26205 GCTTGGGTCCCTTCTGAGGTGCTATGTGTATCTGTTCACTGACGCTCTTTCAAGTGGCAT
EMPV1_26206 GGTCACTGCCTTTTACCGTGGCTGCATGACGCTGGAGGTCAACGGCAAGGCGCTGGACCT
EMPV1_26207 CCAGTGTCACGTTGGGTTTCAACTTGTGACATCGGCAAGCCACATTCCTGGCCTTTTCTT
EMPV1_26210 GTAGACAGGCAGAGACTTCCACCTGGCTCTTTGGTCCAGGGTTCCTGCTGTGAAATAGCT
EMPV1_26213 TCTTCACTGTGTTGAGAAACTTGACCCTGGCCCACAAGGCATAGAACACCGCCTTTCGGA
EMPV1_26215 TCCTGGAGTTCACCCCTCAAATCCCGGGGTGCATCCCCCAACTCCCATGCCTCCGATGTT
EMPV1_26216 CTGGACCTGCTGGACAAGGGGCAGGTGATGACCGGTTACTACCGTTGGCTGCAGCGCGCT
EMPV1_26217 GGGAACTAAGGGACCTTCTTGCCAAATAAGGAAACTTGGCAGGCTGCACTGTGTATTTTA
EMPV1_26218 CTACTCAGCCATAAAACAAGAATGAAATAATGCCATTTGCAGCAGCACAAAAGGACCTAC
EMPV1_26219 GGTGGACTGTTTTCATTTCGAAGCAGAATGTCTGTTGTGAATGCTGAAACCCATTTCAGG
EMPV1_26221 CAGCAGCTCAAAAAGGAGAGTAAGGTGAACAAGCTGTGCAAATGGTACTCAATGAGGAGA
EMPV1_26222 GCAAACCCACTTCATCCCCTTGGCAGGTACACAAAAGGGCTGTCTTCAATGCTTTAATGG
EMPV1_26225 AAGGCAGAAGGCTCGGTGGCAAGGGAAGAATGGCACGGGCACGTCACAGCTCTTTCTGTT
EMPV1_26227 AGTAGGCCTTCCCTGACCCATGTGTCTAAGCTTCAGCAATCCTAAACGTGAATCTTTTGT
EMPV1_26228 AATTGGATTCAAGTACGTGGCTCTGGGAGACCTCGTCATCCTGATCACTTTCGGCCCACT
EMPV1_26229 GTGGCCTCTATTGAAGCCTTAAATCTAGTTTCTTTACCCCTGAGATGTTAAATGTGGGGT
EMPV1_26230 GGTTGAGAAGACAGTATCATTTTGTGTGTGGTCGCCGTGGACTGTTAATGTTTACTGTCC
EMPV1_26231 GGCATTAGGAAAGAACTGAGCAGTAAAGCAGAAGTGAGAAAATAGTTGTGTGGTAGCAGC
EMPV1_26232 TCCATTTCATGGAGGGGGAAACTCTCGGAGAAGAAGCACTTTCATGTGGCCCTGAGGCTG
EMPV1_26233 GAAGGAGATCTGAAGAGTAGTTTTTGCCACCTGATGCCGTTATCTCTTCAGGCAAGTTCC
EMPV1_26234 ATTGGCATCATAATCTCGATGTTCGTTACCTTCCCCTTCATACGGAGACGCCGACGCATC

EMPV1_26235	TTAGCAAGATGGCCTGTGTTCTGACCAAGATGGATAAAGCTGTCTTCCCAACTCTGGATG
EMPV1_26238	GCTGGAGTGCCAAACTGTATCTTACACCAAGTAACATTGTTCTCCTCACGGCCATAGCTC
EMPV1_26239	GCAAAGTCATATTATTAAAAGAAATGTGCTCTGTGAACTCTCTAAGAATTCTTCCCAGGG
EMPV1_26242	ATCTTGATGCTAAGTAAACAGGGTCCTCCTTGTAGAAAGGAGAAAAGGTAATAAAAGACC
EMPV1_26243	GGAAGAACAAGTAGTATTGCCTGGAAAAGGTCCGCTAGAAGATGGTTTCAGAGGGCACCG
EMPV1_26244	AATCAGTCGCAAGCATCATTTTCTTAGATGCCCAGGTCGCCCATCTTTGCCCAGCAGAGC
EMPV1_26245	CGAGGAGGGAATAGGGAAGTCAAGGATGGAGGCTGAAAGACCTGGTCAGAGAGCTAGGAA
EMPV1_26246	AGAAGCCACACAAAGGGACTCATAGCATCAGGAGCTGGAAGGGAATTCCTCACCCCTGCT
EMPV1_26248	TTGGGAATCCATTTACTCTGACCCTGGGAGGTAGGCGGAGTGGAGAAACACCATGAAGTT
EMPV1_26250	CCTCAGAAAGCTGGCGCCGGCGGCGATGGAGTCCTTGGTGCGGATGATGGCGTCGGGCAT
EMPV1_26251	ACCAGAGCCCCCAGAAGATCCTGGCTCACAGAGTGGATTTCGACAGTAGGATGTCCTGTT
EMPV1_26252	GCCCTATACACCGAGAAGTTTGAAGAGTTCCAGAACACTCATTCCTAAAGGAGTGTGGTG
EMPV1_26254	TTCATCGCCAGGGGTACCTTCATTGAATTCCGAAACGGGATGTTGAACGTGTCTCCCATC
EMPV1_26255	CTGTTTGGAAAAGATCTTCAGGTGGTGTATCCAAGGCCTTCTCCACTCTGTCAGCTCATG
EMPV1_26257	CTGTGCCAAAGATTATCGAGGTCATTCTTGTCCAGAAGTAACATGAGCGTTGCCAGAAGC
EMPV1_26260	CCCAGTTCCTCTTTTAGCACTCACTTGTACCACAGCCCCAGTAATGGAGGTTTTTTGGTC
EMPV1_26261	TATTTCGTAGGAGGAAGAAATCAGGGCAACCATGAGACCTGGCTGGAGGGTGATTGATGG
EMPV1_26263	AGACCCCTTTGGACAACCTCATAATAACTCACTGCCTGCTAATAGAATCAGACCTGACCC
EMPV1_26265	CATGGTAGAGATGGTGCATGCAACTCAAACATTAGTTTCTCTACTTACCTCCTCTAACAA
EMPV1_26266	GGTGTCCTGCGTTTCACTTCGAGGATTGGGAGCTCCGGGGTCGTGTGCCAACATGTATGA
EMPV1_26267	ATCCAAGTTTCTATTTGTATGCAGTCAGCTTACAATGAAGGAACACTAATGAAGTTTCAG
EMPV1_26268	TGGGAGGCTTTGAGGGGCATCTGTACCCAGGACTGTCTCTCTTCTTCTATGGACTTTATC
EMPV1_26270	GACTGCCCTTTTTGAGGAACAGACCGAAGGATAAAGACAAAATGAAGGCCTGCTACCGTC
EMPV1_26273	TTCCATGAATTAACTCGGGACCATTGTAGACCCTTGAAAAGCGGCATCAGGCTCTGGTGC
EMPV1_26274	GTCACTTACAGGGATGAATGCAGCTGTCCGATCACTATATTTGCTCGCCTATCTGTGGGC
EMPV1_26276	TTAATAAATCCACAAATAACAAGTGCTGGAGGGGCTGTGGAGAAAAGGGAACCCTCCTGC
EMPV1_26278	ATCAGATTAGCAGAAGTGCCGAATGAGGGCACTGTGTCACCTATAGAACTCGGGCCTGGA
EMPV1_26281	GCTGGAGAAGCTTTACACTAGCAATCTCTTCTTGGAACACTCATCTACATGGCTTAGTCC
EMPV1_26282	TGACCCTCTAAGGAAACACCATTTTCTTGGGAGGTTGACCTGGAGGCACCAAGGGACAGC
EMPV1_26283	CACCTTGTAATGTTCAGAGAGCGCTTGGATTATGGGTCTGAATAGGGAAATGCTTTCAGA
EMPV1_26284	CCAGGGGTACCTGCCAACCTGGGATAGGTAATCTGAGGGCAGAAAACATGGTGTTCCTTT
EMPV1_26286	CCCACACCTCATGAACCTCTGGGTAGCTTGATTCTGCCTCCCTTTCAGGAAAACTTATGC
EMPV1_26287	AAGCTTAATGAGGAAGGCACGCCAATAGCCAAGACAGGCTGAAAGCAAGGCCTCTTGCAC
EMPV1_26290	GGGACCACAAATGACCCTGAGGAGGCAGAGTAAATTTGGAAAACAAGAACAAAGCTGGAG

EMPV1_26292	GCACAGCCAGAAGCTTTCATGTTGGATGGTAACAATAACCTTGAGGATGACTCCAGTGTG
EMPV1_26294	AAGGCTCTCAGCGATTTGGGAAGATACAAAGAGGCTTACGACGCTGTAGCGAAGTGCTCC
EMPV1_26297	CTCCCACTCCACCAGTGCTGCTAATGGAACAAACAGTACAAACAGTAAATCTGTAGTGGC
EMPV1_26298	TGAGGTGGAGGCAGACATAGATTGCACTGGAACAAGGTGGCATCTGCTGGGGCAAGGACA
EMPV1_26299	TCTATGAAACTAGTCCAGCCCCCCGCACGAGTTTCTGGAGCTCTGGGCCCATGACGAGTC
EMPV1_26300	TTGTTCTGCCAGCCCTTTTGTTGCAGACCAGAATACAGTGCTGTTGCATCTCCATCCCAG
EMPV1_26305	TCTTCCCTCCCAAATTCATAGACTGGAGTCCTACTCTCCACCACCTCCGAATATGACCTT
EMPV1_26307	ATGTGAGCAATGTAGACAGACAAATAGGATAACTGTGACCCAGAGGTCTACTCCGGGGCC
EMPV1_26308	CTAATTCACCTGGCTCCTGGAGCTAGCCTTGCTGCTGAGAAGGCAGGGCAACGACTGTGT
EMPV1_26309	AAATGACTGTGCTGCCCCTTTTCACATCAAAGAATGGCGAACTACTGACACGAAGGCCGC
EMPV1_26310	TGACCATGGATATTAACAAGCCCAGATCCAGAATGGCCTCTCCTACCATCAACTCTGGCC
EMPV1_26311	CGCGGTGTCTCAGATTCATTCTTAAGGAACTGAGAACTTAATCTTCCAAAATGTCAAAAA
EMPV1_26312	GACAGTAAAATTCTTAAAAAGGCCCGGAAAGATGGGTTTTTGCATGAAACGCTTCTGGAC
EMPV1_26313	GTGACGGCCTGAGGGTTTGGGCTGGATCTCTGAGGACCTCCAGCCAGGATCCTTCTTTCA
EMPV1_26314	TCCCAAATGTTGGACAAGGAGGCTGAGTCTGTGGTTGCCAGGGTGTGAGATGTAGTTTTA
EMPV1_26315	TTGTGCTCGGAACGGGCCGACCCTCGTTTCTCATCGCTGCCGCTCCAGCAACTGGACTTG
EMPV1_26318	TTTGTTAACTACAGTATCCTTGGGTCCTAAAACAGTGCTTGGCACATAGCAGACCCTGGG
EMPV1_26319	AGGCCCTGTTCCTATCTTTGAATTCCGATTGGGCTAACATGGAACCTACCTGTGCCTGGA
EMPV1_26320	CAGAACTCTGAAACTCTTGATTCTACCCTGCTGTGTGCCTGGGACTTGACCTGCCTCTCA
EMPV1_26322	AAGGTCATATTCGGAGGTGGTGGAGAGTAGGACTCCAGTCTATGAATTTGGGAGGGAAGA
EMPV1_26324	GTATATGATCAAAACAAAGTTCTATGAATGGAAGAGAGGGCTGCATGAGGGAGTAATAAG
EMPV1_26325	ATGAACTTCATCGACAGCGTCTTCTGCCTACTCCTCTAGTTCGGCAGCGAGCTCTTCTCC
EMPV1_26326	AGGCCTTGGGGATGGAGCCCAGGAATCTGAATTTTAACAGACTCTTGCGGTGATTCTGAT
EMPV1_26327	CTTCTGCTACTATATGCTGACATCAAACTCCTAGGGGTGCAGAACCTAGGCTTCCAAGTC
EMPV1_26328	GAAGTGGGTAAGTTGGTAATACACAGCAAAGCATCTGTTGGCTGGAGCTGGACTATTAAG
EMPV1_26329	CCCAGCCCCACGCAGCTTCCTTCTCACAGGTATTGATTGGTCTGGGTGTTCTTGTAACTA
EMPV1_26331	CTACCACTTCTGTTTCTAGCATCCTTTGATGAGAGTAGGAATTTCCCCAGGTATCTGGAC
EMPV1_26332	AGCAACGCCAGATCCAAGCAGAATCCATGCCCCATGCCCCAGCTTAAGACAATGCCAGAT
EMPV1_26335	GATCAGCTCTTGTGGTACACCAGGGAACTCAGATAAGTGAAAAATCCTATACATACCTGG
EMPV1_26336	TAAAGAAGAGATTCGGTTACGGAGGCTGGGTGGAGCTCCAACACCACAGCGAGGGAGAAG
EMPV1_26338	GACTAAAAGAAGGTATCCTGGAGGCCACATCAGTAGGACAAACAGACCTCCCTCTCTCCT
EMPV1_26340	ACCAAGGGTGGGCCTCTCGGGGCCTGCGGAAATAAAGATGGAATTGGTCAAAGAACGTTT
EMPV1_26341	GTCTCCATGGTCAGCCCTCTACCACTCCTTTCCATGCACCTGCTACCTCACTGCTTTAAA
EMPV1_26342	TAGAGAGCTAAGCCCCTGTACTGTCTGTGACTCGGGGAAATGCCTGCTCTTTATTTCTCC

EMPV1_26343	GTGAGGGATCCAGAGATCTACCAGGAAGTCCCGGGAAAAGTGCCTCATACTGAGTATTTG
EMPV1_26345	ACCATCAGATCTGCCTGCACTCCAACTCTACCAGGATGACACTCTTTGCTGGTGGCAGAC
EMPV1_26347	CTACAAGCTGAAGCTGGACCTGACCAAGCTGTAGGCCTCCATTGCCAAGTACAAGGGCTC
EMPV1_26348	GCAGGTGGACAACTTCTTTTGTGATGTTCCCCCTGTCATCAAACTTGCCTGTGCAGACAC
EMPV1_26349	CTTACCCCACGAAAGCAGGATAACTTGCTTATCCCCAAACACTGGCCCAGAATAGATACG
EMPV1_26351	CCGCCCTCAAGAAGGCATTTAGCGGAGGAAAACAGCCACAGACAACTGCATTTCAGAACA
EMPV1_26352	CAGTGAGTTCAGTTGGGACAGCAAAGTCCAAGAGGAAAGGGAGGAAACTGATAGTCCAGC
EMPV1_26353	GAGTAGAAGGGATCTAACCAGAACAGATGCCTGGATAGAAAGGCTATCCCACCTCCACCT
EMPV1_26354	GTCTGTGCAATTTGCTTTCTCTGCGTATCTGAAGTCCGCAATCCACAGCGCGACTAGAAG
EMPV1_26355	CCCAGCACTTCCGGGGGCACACGGGGGCGGTGTTTAGCGTCGACTACAACGATGAACTGG
EMPV1_26356	GAGCCAGTAAAAGAAGAAGGTTCCGAGCTGGAGCAGCCATTTGCACAGGATACAAGTAGC
EMPV1_26358	GCAACATTAATATGATTCAGATGCTGGAGGAAGATTCTAACCAGGGGCCTCTGATTGGCG
EMPV1_26359	TCGCTCATCTCTGAGTGCCAGTCACCCAATGGTGGACAAGTGGCTGGAGAAGCAAGAACA
EMPV1_26362	TCTTCTTCGTGGAGAAGCAGGAGTTGGCAGGAAAATGCCGTCTGGACGCAGGCCCCAGCA
EMPV1_26364	GTGTGGCAACGGCTGCATTTGCCCTCACAAGGGGTTGTCTGTTTTGTTGCCGGAAGTTGG
EMPV1_26365	CAACCTTATCCAACAAGTGGGGGAGGGGTTGGAATTTAACCAAGTGTCAGCATCCCTGGG
EMPV1_26366	CTATGGATGCATTTGCAGATGAGGACGTAGAGATCCCAGAGGAGCAGGAAGAAGAAGCTG
EMPV1_26368	CCAACCAGGGCAAGAAGGCAGAAGGAACCCAGAATCAGGGCAAGAAGACAGAAGGGTCCT
EMPV1_26371	AGCTGGCTTCACAAACCAAGTGATTATCTTGAACCGTCCCAGCCAACTCGGTGCTGGAGG
EMPV1_26373	ATGGGCACAGCTGAACTGTGTGGCAGGGCCTCACTGTGAACTGCAGGGAAACAACTGTGG
EMPV1_26374	GTGGGCTTTTCTTTGTTATGGGGAGGGGCTGGGGGGATAGTTTTAATTTCTATTTTCTAG
EMPV1_26380	TGTAGGTGGGCTCTTCGCTGCGGGACCCGCGCTAGGTTGGAGCCTAGAGTTGAAGATCTT
EMPV1_26381	GGATCTCACTCAGTTTCTAGGCATCAGACCAATCATCAGCACCACCATGATCCATGTCCC
EMPV1_26382	AGAGTTCATCGAGGGGACCATGGCCAATAAGGAGATTCTGCGACTGATCCAATTTGAGCC
EMPV1_26383	AGCAAACTTGGTGGATCTCCAGAAGAAGCTAGAGGAGCTAGAACTCGACGAGCAGCAGAA
EMPV1_26385	AACCCCAGTGGGCCGTCGTTGGAGACACCTTCCCTGTGGGCTGCCGTCCGCAGGCCTCTG
EMPV1_26387	AGCCACAAAATTTATAAAGCACCCTGGCCACTGCCTCCAAGATGGGAAAAGCTTCCTGGC
EMPV1_26388	AACAACACACACTCACAAAAGAATAGGCAGCAGAATCTGTACATGGCCCACAAAGCCTAA
EMPV1_26389	AAGATTACCAGGAAGGAGGGGAACGTGGTAGGCACAACGCTACTGGAAGCCCTGGACTCC
EMPV1_26390	CTCTGTATGACCTTCGTATTGACCAAAAGATTTACGGAGAACTATGGAAGACAGGAATGT
EMPV1_26391	AGAGAGCAAGTGCAAATAACTCAGGAAAAGATGAGAAAGGAAGCTGAGAAAACTGCCTTG
EMPV1_26392	CCTCTGGGGCTTTGGGTTTGACATTGTGTTTGACTGTTTATGTAGCAATGTGCCAGGGCA
EMPV1_26396	TCTCAGTTCAGGACTGTGTGGTGGGAAGTGGGGGCAGGCTGTGTCTTGGGTTTGGTCCAT
EMPV1_26397	GAGAAGCAGATCCTCAGCCCCCTTAAGCCTTCAAATGACTATAGCCCTGGAATACACCTG

EMPV1_26399 TACTCACGCCACCCAGCGGAAAACGGAAAGCCAAATTACCTGAACTGCTATGTATCTGGG
EMPV1_26400 TATCACAGATTTATGTAAGCAGATACTCTGCTAAGAGCCATGGCCTGTACTCTGAGTGTC
EMPV1_26401 GAGACCCAGCTTCCCCGGCCCGTGTTACTGATAGAGCTTTATGCACAATACCTAGAAAGA
EMPV1_26402 GTCTGCGGCCCAGTGAAAGTAGCTGGTGCGCCAGGCACCCTGACTGCTCCCGAGTACTAC
EMPV1_26403 GAGAAAGGCTGCAGTTTCACTGAGTTCTCTGACTTGTTCAGAGGTAGCAGCTTTTGACAT
EMPV1_26404 GTAAGACGCTGCCAAATGGGGAGGGGGCTCTGACCACTCCCCCAGGCTCCGCTCCCCTTA
EMPV1_26406 TGCGGCTGCGTACCAACCGCCACTTCGAAGTCCTGAAGAATCACACAGATGTGTGGTTCA
EMPV1_26407 TGGCATCTGCAGAGATGTCTGTATTTGCATCTGGTCAACAATTTTGAAGTTCGTCCTTGC
EMPV1_26408 AGTGCACAGCACGATATGTCATGGTAAAATAAGAGGAAGAGGAGCCTCATGGCCCCACGG
EMPV1_26409 CCATGGTGCAGATGGTGCCAGCAGGCCATGTGTGTGGAGACTTCTCCAGTACCACTCTAG
EMPV1_26410 CTGATTCGTATGAGGAGGCAGGCTCTGGCTCTGGAATTGAAGTTTTCTGAGGAGCAGTAA
EMPV1_26411 TACTATACACACACCTCCTGATGCACCAAAAATCATTCACAATTGAGCTGGGCCTCGGTG
EMPV1_26413 CCACCTCAGAGGAAGCACTCAAGTGGGGCGAGTCCTTGGAGAAGCTGCTGGTCCACAAAT
EMPV1_26414 TGAGCTGCATCGCCCCTTGATCGGTGGTATGCCATCTGTCATCCCTTGATGTTTAAGAGCA
EMPV1_26415 CACAGTGAGTAGCAGGACGAGGGGGGTTTCTTTGAGATATTACCCATACTCTCGTCTTCA
EMPV1_26416 GACCATTGAAAGCCTCTCAAATGGTGAATTCCTGCATCACCAGCCCTTCTACACCTTCAA
EMPV1_26417 GTGGGTGTGTATTGTACTCTTGATGGTGTCTTGGGTTGGGGGTTTTCTCCACTCAATAAT
EMPV1_26419 GACATTAAGAAATATGCAAGAGTGGAAAAGATACCTGGGGGCATCATTGAAGATTCATGT
EMPV1_26421 CACAGGGAACAGTCGTGGTTGGCTCAGCAGTCAGTATTTGCACCAGTTTCCACTTTGCAA
EMPV1_26424 CAGGGAGGCATTCAGTATATGTACAGGTTGGGAGTGCGAAGTTGGTCTGGGGACGAGATT
EMPV1_26425 TGGCCAGCTTCTACACCAAGTATGATGCTGCTCACTTCCTCATCAATACAGTCTCATTGC
EMPV1_26426 ACTGAGTCCTGCTTTGGCCTCATAGTGGGGAGAGATGTGGTAGATGACAAGGTCTTGAGT
EMPV1_26427 CCATCACTTCCCGAATAGCTAAGCAGGGCTGGAGGGAGGAATGAGGTGGGTTTTATTTTC
EMPV1_26428 AAGGGTCTCGGGTGCGAAGAGGGCCCCTGGTTCCTGCAGACGGTGAAGACCCCACAGAAT
EMPV1_26430 GGACCCCATGGTTGATAACACCACTGATTACTACAATTATGGCTGGACCCTGCTCCCTGG
EMPV1_26434 GGATGCCAACGGGAATATCCAGCAGAGGACAGTGTTGCCAAAACTTGTTAGCTGAGCTGA
EMPV1_26436 CGAACGCTGTACTAGTGCTCAGAATTCTCATTCTATGCATGTGGTTCTTCTGAACCTCCA
EMPV1_26438 GGTAGCCAAGAACTAAGGAAGGTCAGAGTAGAGTAGAAACATGTGCGTATCAAGAAAGTG
EMPV1_26443 GAGTTGTGAGGTCCCATAGAAATCAGCCCAAAGAAGAGCTGCCTGTGTTTTCCTGCCTTC
EMPV1_26446 ACAGACTCCTGGGGATAGTGTTGGATGTGGAATATCTCTTTACCTGTGTCCACAAGGAGG
EMPV1_26449 ATGCTGTGTGCGGTGCTCCACTTCAAGAAGTGCCATCCATGTGGCTAGAAATATGATTGG
EMPV1_26450 GTCTGTATCCAAATGACACTTGTCTCCCTGCTCAGTGGGCTTGTGTATTCAGGTGTGCAC
EMPV1_26452 GTGGGCCGTAAACATGGCTTCTTCACACCCATTGTTAATGACTCAGAATTACAGTCCTAC
EMPV1_26453 ATCCCCCCACAACTATCCCTCCTCCCACAACAACCACCACCACCACCACCACCACCACCA

EMPV1_26455	GTGGACGATGAGGTTCTGAGGCACTGTGGAGTTTTTCCCGAGGAGTGAGTGTGACTTAAT
EMPV1_26462	GCTTGATTCAGATGTCTTAGGCCTTGAGTCGGCTTTAGATTACTTATTTTGAGAATCCAT
EMPV1_26463	CCAAAATTTGACAAGTCTACATGAGCGACTTAGTGACCGCATGTGTGTGTTGCTCTGTCC
EMPV1_26466	ACACTCTGGAAAGGAGGGGATGGGAGAAAAGAGTGAAGGTGTTTCTGATGATCCAGTAGG
EMPV1_26470	GAACCCGGACTGCGCGCTGGAGACACTGCTGTTGATGCATTCTTGCCTCACCTCTGTGGC
EMPV1_26471	GACACAACCTGTTCTGCATGGAGTGTGCAGTACGCATCTGCGAGAGGACGGACCCGGAGT
EMPV1_26472	TTCTGAGGAGGTTTCTACAGTTCCCTCTGAACCCCAGAAGACATCTGCTGACCCCAAAGT
EMPV1_26473	AATACAGATAGGAAAGTATGGCATCGTCGTCAGCCCTTGACCCCACCCTCATCCTGGGAA
EMPV1_26474	ATCTCAGACAGAGGTCATGTTAGATCAATTGTGGATAACTGGCCAGAAAATCATGTTAAG
EMPV1_26475	AACAATCTCGGAGGGACGACCTGGAATCTCTGGGCTACGTGCTCATGTACTTCAACCTGG
EMPV1_26477	CTGCCGGGTAGCGAACCTCCTTCCACAGCTCCGGGCTGCACAGGCCCTTCAGATACTCCT
EMPV1_26478	TTTCCCTTCCTCTGATAATCATCCCAGAGTCGTTAGACGGTCTCCAGTGTTGGTCCAGAC
EMPV1_26479	AGAGCAGAGCTGCAATTCCCGGTGAGCCGCGTGGACCGCTACCTGCGAGAGGGTCGCTAT
EMPV1_26480	ACCCACAAAGCAGGATCTGAATTTATCAGCCAAGGGCCCATAGAGCCGCAGGGACCTGTC
EMPV1_26482	GCCATGGCTTTGCCTCCTGCTCTGCATTCTGAATGTCTCTCCCTATATGTTGCACAGGAT
EMPV1_26483	GAGAAGCGAGCAGCAGGGCAGAATGGAGTGCCTGGCATAGGGCGCCAGTGGGTGAAGAGA
EMPV1_26486	AAGCTGCCTAGACAAACTACCTCTTGGTCATCCACCAGCAGACCACAGCTCCATAAGGAC
EMPV1_26487	ATTGGTAACCTAAGTAGGCAGAACCAGGACTACATCTGAGCCTGCTCCACACCTGGGCCT
EMPV1_26488	CATTTAACTCAGGGAATTTGTACTACTTGGAAACACAACTGTAATGCAACATGCTTTGGG
EMPV1_26489	TGCACACTGTTCTGTCTAACCGACCTGCCGAAGCACGTCGTGTTCGGGGAGGTCACAGAA
EMPV1_26490	GTCAAAACTACTGGCCTTGGGAGTAGAAGGAAGACCACAAGAGAAAAATAGGCGGCAGGG
EMPV1_26492	CCCCCCTGGGAAAAAGAGGAACCTGCAGCTTTTTGACAAAGGTCGTGATGAATTTGCTGC
EMPV1_26493	GGCTTTGACCACACGTTCCGTCTGAAGGGATCTAAAGAAAAGCAATTTCGTGTACGGGTC
EMPV1_26494	TTTTCTTTGGGATTTCTCTGGGGGAAGGGCTGCGGACCCTGTGGGTGCCCTGCTGGGTCA
EMPV1_26496	CCCCGCAGTGCTACCAATGGAAAGTCAGAGACCATCACAGCTGATGTCAATCACAACTTG
EMPV1_26497	TTAGCTGGTAAGCTAAAGGGGTGGCTTCTATCAGTGTTTTCCTACTAGTCTCAGAGTCTG
EMPV1_26500	GATGCTGACACCCTCCACCGAATCCTAAGTGGAATAATGTCAGGAAGAGACACCATCCTC
EMPV1_26501	TTCCCACTCTAGCTCTCCTACCAGCCTAGTTCCATTTTTTGCCTCTGCTCTCGGATCCTA
EMPV1_26505	TTTATGGCAATGGCCCGGGATACATGCTCAGCGAAGGCGCAAGGCCTGATGTTGATGACA
EMPV1_26506	GATCCCCTAGAGTTTGGCGAGTAGAGAAATCATCTCCATCCAGCTCTGTGGTGAGCAGAG
EMPV1_26509	GTGCTGTACAATATGACTTCTACTGTTAATCTGCTCCTATGTTGCCCCTTCCCCCTTCCC
EMPV1_26510	ATCATGAGCCCAAGAATGTGTATCTCCTTTCTAGTGGCTGCCTGGATGACTGGCCTTATC
EMPV1_26513	CAGAGGTGATCACTGGTCGCTTGAGCTCGGGCAAGATGATTAGACTGGAGTCCACTGGAA
EMPV1_26515	GTGGGGAATCCCACTGCTCTTGGCCTTGTGGGGTTTTCTTCTCATCTTGCTTTTCTCCTT

EMPV1_26518	AAGTTGGGTGCGCTGGGGTTACCAAGGCAACCATCCTTCTGTGAGGCTTTTCCAGAACTC
EMPV1_26519	AATTAAACTGCCACCATGTCGAGCAAAAAGGCAAAGACCAAGACCACCAAGAAGCGCCCC
EMPV1_26522	TCCCCTTTGGGGCCTCTGCCTTCGAGAGGAAATGTCGAGGCTATGTAGTAACTCGAGAAT
EMPV1_26523	AGCTGACATGTTTCCAGAAATGGTGGGTGGATTTGATTGGAAAAGTCGGGCTCCTGCAGC
EMPV1_26524	CAGTATTTATTAGCTGCCCTTCTGGGTCAGGCACTATGCTCTGGGCAGGGGTACCACACA
EMPV1_26525	TAACCCTTCTCTCCCTTCTCCCTCCATCTGCACCCCTGAGATCCTGAGAAGAGCTGTACA
EMPV1_26526	AGACTAAACAAACACCAGGAGGTGGGGGCAAATTGACAGCTGGATAGCCCAACAGCAGGT
EMPV1_26527	ATGAGGATGCTGGCAGAGGATGAGCTGCGGGATGCTGTGCACCTTCTCTTAGCGATCAAA
EMPV1_26530	TTCACATGCTTCGCGGTATTTATCTCGTGTAGAGAAGCACAAAAGAGAAGGTGCTCCACT
EMPV1_26531	CCATCTTTAGAGGAACGCCAGCAAGAAGCCGTGCCAGAGAGAAAAATGAAAAATGTGCTG
EMPV1_26532	TGCCTGCGGACACTGCTTAGGAAACGTGAAGGAATGGCTTCGGATGTACCAACTGAACAA
EMPV1_26534	CCACCCAGCTGTGCTTGACAGTTCACCCAGTGTTTATGAAGATAGCCGAATGGAGCAGAG
EMPV1_26535	AAGTACAATCCCCTCCAAAATTCATGAGATGGCTGTTCCCCAGACATAGGGAGGGCCTTC
EMPV1_26538	CGAGGAGATGATGTGACTCCAGTAATTCTAGAAACATTCACGGAAGACCCTAATCTGGTC
EMPV1_26545	TGGCAGTGACCAAGAGCTACCCTCGGGAACGGCTGCCCATCAAGTTTGACAAGATCCTGA
EMPV1_26547	GCTGTTGAGAAGTGTGAGAATTTAGGAGAGAAACACCTTCAAGATACCCTCAGCGAAGAC
EMPV1_26549	AATATCACTGCTTGCTGCAATTGCTGTGCCCGGATTTCCCGCTGGAGCTCACCCAGAAAG
EMPV1_26550	GCATTCAGTGGCAATAGATTCTGGTTTCAGGAACTCTGGCATAACAGTGGGAAAGAGAGG
EMPV1_26553	GAAGACTCGGCCGGCACATCCTCGTTGCGTGGACCCACCGGAGCAGCACATCCGCGACAA
EMPV1_26554	ACTACCGCAGCACCCGGTACAGCATCTAGGACACAGAACCGCACAGTATAAAAGTGGTAT
EMPV1_26555	CCCTTTGAAGAAACTTTATGCCCTGGATGTCAGAGTCAATTCATTAGATCCTGCTTGGCC
EMPV1_26556	GTCACTGTCTCAATCCATGATCCTGTAAGTTGTCATCACAATATGATTTAGAAATACTGG
EMPV1_26558	TCTTCTACTCGGCGGAGATCGTGTGTGCCATCGAGTACCTGCACTCCAAAGAGATTGTCT
EMPV1_26560	AAACCGAGTCCTGCAGTGAACCTCCGAACTGGAAAAGCAGAGTTTACCCAACATCCTGAG
EMPV1_26561	GGAACAAATCATTACAAGGGAAGTGTTTGACTTCTTGCTTCAGTCATCAGCCAGCAAGGG
EMPV1_26562	AACAACCCTCGGGAGTTGAAAGCTTTCCTAGAGCACATGAGTGAGGTACAGCCTGACTCC
EMPV1_26563	CAACAGTTATGCTTCATTCACCATAGTCATCCACCCATCACCCTTTCAGCCACCTGTCCA
EMPV1_26566	GGTGTAACTCGAACTTCAACTCCGGAGTACCCATCTTCAGCCAGCTCCCGAGTAAGAAAT
EMPV1_26567	ATGCTGATTGCTGTGTGGATGACTACATGGTATCACAGTCTACAAGGCTGCGCGGAAGAC
EMPV1_26568	TTGTGACATAAGGGATTACGTGTTGCAGAGACCCAGCCAGGAGGCCAGCAGCGAGGCTTT
EMPV1_26569	ATGTCACAATCCACCTCCTCTTATCTCCATCTTTATCACAGATCTGACTCTTTCTTTAAG
EMPV1_26570	CTGGAGCTCCGGGCTGGTGGATCCTCCGGGGAAGGACAAGCAGTGGGAGCAGCTGCTCCA
EMPV1_26571	AAAAGAGGCACCAAAGAGCAAAGCAGGTTAGACAGGGATGCTCAGTGCTGGGTCAATTCC
EMPV1_26573	GATGTCTATGCGTACATGCAAGTTGGAGTTCCGGACTGCACAATATTCACGGTGAACCCC

EMPV1_26574	TCCTGGCACCAAAGTCAAAGACACCACAAGAAATGAAAGCCACCCAGCAATACCCCCTAG
EMPV1_26575	TGGCTGTGGCTGGCAGCTGTAGCTCTGATTCAGTCCCTAGCCAGGGCTCTTCTAAATGCT
EMPV1_26576	AAACATGGTGGCAGAAAAGCGGCTTATCCCCGATGGCTGCGGGGTCAAATACATCCCTAA
EMPV1_26577	GTTGTGGAACCAAAAATTAAAGTGATTGAAGGCAGTCTTCAGCCTATTATCAAAACGGAA
EMPV1_26579	TACTGGCAGATTTATTTGAGAGAGGCCCAGGGTTGGAGAGGAGGATGGAAAATGTGGCTC
EMPV1_26580	TAAGGTGGGTATAGGTGTTGTAGCTGAGGCAGATGCGGCTGATGCTGTTGGGTTCCCTGT
EMPV1_26581	GTAAGGCATTCTGGAAAAGGCTAAACTATGGAGATTTTAAACAGCAGTGGTTGCTAGGGG
EMPV1_26584	CGGAGGTCATCCTGCTGGACTCAGACCTGGACGAGCCCATAGACTTGCGCTGCGTCAAGA
EMPV1_26585	GGGCCAAGTCCTTCACCAGGGACACAAACTCATGGTGGTTCCCACGGTCATAGTAGAGTT
EMPV1_26586	AAGGAATTGACAGTGACCTCTTCTCTGGATGGGGTTGCAGAGTAGTCATGCGCAGAGCGA
EMPV1_26587	CTGGCAGCCCGTCATCATTGGCCTGGTGCAAGGCATCAACTTCGTCCTGGGCCTGGAATA
EMPV1_26588	GGCTGGGAGGTAGAACACCATCCATCAGCTGTCACACCCTAATTCCAGTCTTTTCCCACA
EMPV1_26589	CATTTCTAGGCAAAATTGTGAAGCTAACGACCAACCTATTTCTACCTATGTGCAGTCTCT
EMPV1_26591	CTGTGGTAGACTGATCAGTGGGAAGGCAAGTTTGCCCCTGCCTTCACTTGTGCCTGGACT
EMPV1_26592	CAAGCATGAAGATGACACACTGAGGATAGCAGGATGCACAGATTGAAGGCACCTGAACTG
EMPV1_26593	TGCAGTTTGGTCAGCTTCTGACACACTTGGATACCACACAGCAGATGATCGCTTGTTCCC
EMPV1_26595	AGCACCCCCTTGCACTGGCTGACACAAAAAGAAACTTGCTGAGCACCCCAAGAGTATCTT
EMPV1_26596	CAGTAACTTGCACATCCTCGATGCCAAAGTCAAGCTGATGGCTGACGGCAAAGGCATTAG
EMPV1_26598	CACACAACTCTCAGATTCAAACACTCTCTCCCGCACACTTGTACACATACACACTCACGC
EMPV1_26600	AGCCACTGTGCTGAAGCAGGGATCCAACCAGGCCATCCGCTTCTTCGTTATGACCGCCTT
EMPV1_26602	ATGTTAGTGATTTTCCCAACTTCTCCAATCCGTTGTTTGTGTTGGTCGGGGTGGGGGGCG
EMPV1_26604	TACACAGGCTCATCACAGCAAGTAAATGATTTCAGGTTATAATAGCTGTAGGGAATTCCC
EMPV1_26606	GAAGATCAGGTCCCGGCAGTACCAAAGTGCTCACAGTGCAGGTAGGTTTTGCATTACTCT
EMPV1_26607	TGGCAACGAACCCGACTAGTATCCATGAGGACGTGGGTTTGATCTCTGGCCTTACCCTTA
EMPV1_26609	CTGTTGCTGTGGCTGTGGTCAGCAGTTGCAGGTCCTATTCAACCACTAGCCTAGGAAATT
EMPV1_26610	ACGGGAAGCTTAACACGGCGTTATTTTTAGCCAGTGATTTGGTGAGCTAATGGTATGAGT
EMPV1_26612	TTTCTTTGTCTGCACACGGTGCACCTCAGATCCAGTATTACCGCAGCCCCTGGTTTAAGA
EMPV1_26613	CGAGGAATAGATATTCCAGAAGTGAACTGGGTTTTGCAGTACGACCCTCCCAGCAGTGCA
EMPV1_26614	GGAACTTTAGGCCTGTGAAAATCTAGGAAAGAGACAGCTAAGAAGAGCCCTCCTGGGGTC
EMPV1_26616	CTGGTAAAGAAGTGGCAGAGTATCCAGCGTGAGTTAGATGAAGAGGAGAACTCCAGCTCC
EMPV1_26617	TTCTATCTGACTTCCTTCTGTGAAGTTGGGGTGCACTTGCTGGCTGCCGTTTGCTCAGGA
EMPV1_26618	GCCAACCCCTATACTGAATGCCCCTAGTGAGAGACTTCTCATCCGCTATAATGACGGTGA
EMPV1_26620	GCATAAGAAACACAGTAAGAAGAAGAAAAAGAAGGCTGCTAGTTCAAGTCCTGACTCACC
EMPV1_26621	CAGGGATCAAACCCATGCCACAGCCACAGCAACACCAAATCCTTAACCTGCTGCACCATA

EMPV1_26622 GCTTCCCCCAGCACTACAAGAGCCTCTACCGGCTGGCCTTTCTCTACACCTACAGCAAGA
EMPV1_26623 AGCAGCTTATTGCTGAACGCGACCAATACGGTTGCACGTCCTGTGTTCACCGAGTATCAG
EMPV1_26624 TGCTTACCCTGGGCTCGGTCACCATCTTCCTGGAGTCTGCTGTCTACCTGTACAAGAACA
EMPV1_26625 GATTCCAAGTGATCTGTCCTTAGAGCCCCTCTTCTTATCCATGGTGCTTTGTTCCCTACA
EMPV1_26629 TGTCGATAGTGACGACTCAGGAGCAACGGGAGGATTAAAAACCGCAGCCAGGAGGCTCTT
EMPV1_26631 GAGGAAATGGCAGGCTGCATCAGGGCAGATGGAAGACACCGTGAAAGAGAAACAGTACAT
EMPV1_26632 GTCGGGTTCTTGGTCTGTTATGGGCTTAAAGGTACATCTCAAAGGGATTCCAGGCAGGGA
EMPV1_26633 GCGAATGCACTTCTCCAAAACGAAACACAGCAAGTTTAATGAATCTGGTCAGCTTAGTGC
EMPV1_26634 TGGTCACTCAAGAGAACTGAGCACCCCCTGCACTTTGACTAGTGCAGCCCTGGGCCTCAG
EMPV1_26636 GGTCCCAACGGCAGGCAGAGGGGCAAGTGTAGAAGTGGGGAGACCAGCATCTTCGGGAAT
EMPV1_26638 AGTGGGGCCTGTGGTCGATGTTATTCTGTACAGGGTTCCATACATGAGGACCAGATGTTC
EMPV1_26641 GTACCTTGTTCCTGGCTTTGTAGTAGACAGTTATCCTCCACCACAGCTGCATATAAGAGG
EMPV1_26645 ATTGTTATGGATCCTCAAGTCTGAGTGGTCTAAACACCTGGTAACCATGCTTTTCTTTGG
EMPV1_26646 GGTGGGGCTTATGTCGGACCGACCCAGAATAGGATCTTAAGGTTATCTAAGGAACTGGGT
EMPV1_26648 GACAAGACAGCCCTGATCTTTGAGGGCACAGACACACGTTGGACCTTCCGCCAGCTGGAT
EMPV1_26649 GGCCCAAAGAATCAAGTCTACAAGCTCCATAAGCGCCTCCCCGGCATTTACCTACGGAAT
EMPV1_26650 TTGCAGGAGGAGGCTGAAATCCCAGCCAGCGCCTTCCAGAAAGCGCCGGAGCCCTTAGGC
EMPV1_26651 GAGGGATCCCAAGAGACCTACACACTGGCCCACGAGGAGAACGTCCGATTTGTGTCCGAA
EMPV1_26655 CCGTCCCCACCGCCTCAGTCATGGCTCCTCGCCGCGGCCGCTGTTTCTGCACCTCCAACT
EMPV1_26656 CGGGAAAGTGGTGAATCCAAATTTAATTCTGTATCTGCATGACTCCAGTGCCTTTGCTTA
EMPV1_26657 TCACATTCTGAAGGCCAGTGGGATGGTTGTCACATGAAACCTGAAATGTCGGCAGAGAGG
EMPV1_26660 GCATCATAAAACATTCAGAAGGAAAGAATGTTTTGTGGACCACTTGTTTGTGTGTGTGGC
EMPV1_26662 GCAGCTCCTTGGACTGGAAAGAATTCAACTTCGGGACCACACACTTTGAATTCAACAAGG
EMPV1_26663 CTGTGCACCCAATGCGGTTCTACATCAAGGACATTCTGGGCAGCAGGCCCGCCAAGTCCA
EMPV1_26664 ATCAAGAGCCACAGCCTTAGAGGGAGAGGGTTGATGCAAGAGGATCAGGGGAGAAGTCCA
EMPV1_26666 AGTCTGGGAAATGGCCCCAGCAGCCCTGAAGACGGATCTGGCTGTGGATGTGCTTCCCGG
EMPV1_26667 CCCTTCTGCTGTCCTCAATATCTAGGAAGATAGCTGTAGTCTTGGCTTCTCTGGGTAGTG
EMPV1_26668 CATCTAGGTCTAATTCTTCAGATATTATGATGAGAAGGGGATTTATTACCATTAGATCAG
EMPV1_26670 GGCTTTTGCTCCACACCTGTCTTCAATCCTTGTCTTCAGGATGCTCTTGGCAGGGAAGTT
EMPV1_26672 CTGATCTCTAACTCCAAGTACACAAGGTGGGTTTAGCTTTCCTTTGGAGCTTCCCTGGAG
EMPV1_26673 TCTCCCAGCGACGCCGAAGAACCTACTCAGATACAGACTCCTGCAATGATGTTCCTCTTG
EMPV1_26675 GTTTACCATCCCATACCAATGCTCACCTCCGACAAATAATGGGATAACACAGAGTAGCAG
EMPV1_26677 TCACTGACTGTCGGGGGTTTAGTTATGTGGAGAGAGAGGCTGGTGCTGATGAGAGAATGG
EMPV1_26678 AGAGCCGTAAACAGTTGCTCTGTTACTGTAGAACCAGCGATCTTTCAGCATCTACATGCA

EMPV1_26679	GGTGTCACATCTGCCATCACCCCGTTGGCCAGAGCAAATTCCACCGTCTGTAGTGTCAAG
EMPV1_26680	TGGCACTGGGCAGCAACGGGCGAGACAAAGACACGGATCCTACAAAAATCTGCACCGGGA
EMPV1_26682	GCAGGGATTACCACTTCGTTTCACGGCAGGGATTTGACGCAGACAAAGCAGCAGGAAAGT
EMPV1_26683	CTAGTGGATCTCCAAGAAAAGTCTGGGGAAAAAGCCCTACAGATTCTGTTCTAAAGGTAC
EMPV1_26684	AACGATGTCATAGAGGAAGACCTTGCGAAGGAGGTTCAGAATGGAAGACTGTTTAGGCTT
EMPV1_26686	AACATGACGTTCTTCAATGGTGTGTTTAAAAATGTGGAAGGTGTGGCTGAAATTTTTGAC
EMPV1_26687	GCTGGGAAATGAGGAGCAACAGGCTGAGGTTCCAATTCTTTATCATGATGTTACCTCCCT
EMPV1_26688	GTGATCATTGTTTTTAAAGCACCAGTGGATGGAGGGAATGGATAGAGGGAAGCAGTAGAA
EMPV1_26690	TGGGTTAAGTATCCGGCATTGTCGTGAGCTATGGTTTAGGTCGAAGATGCTGCTCAGATC
EMPV1_26691	CAGCTCTACGACGAAGCTGCATGCCCGGAAGGGCCTGCGATGGAGCCAGACCCCTCCTCT
EMPV1_26692	CCCCATAACGGACTGCTTATTTTGTCCCCATCATTCAAGCTCCCTGGTGAAGAATGTGGC
EMPV1_26693	GGAGGGAGCCGCTGAATTTCATTATCACTAAGTCCAAGCTTTCGTAATTTGACCAGCTGG
EMPV1_26694	TCTCTTGGTTCTGCCACCCTCCTCCAGAGGATGGCTTGCCGTGAACAGCAGGGATAACCA
EMPV1_26695	GTGAAAAGATGTGCTGAGTTTTTGTCTCTCTCAAAGGCCAGGATTGAGGCATATGAGAAA
EMPV1_26696	GGCGCCGGCACGGGCGGCGACGGCTCGGCTGTGATGGGAGCTGGAGCCGGGGCTGGGGCC
EMPV1_26697	TAAGGGAAGTCGAGATAACATGAGTATTGTACTAGTTTGCTTTTCAAATGCCCCCGTCTC
EMPV1_26699	GCGTACACCCTGCTGACCTAAAAATACCACTTTCAAATATGAGCACAAGATCACACCCAG
EMPV1_26700	TGCCTGCCATTTCCTCAGAAATCTTGGTGTTGTGGTCACCCTACACGCAGTACAGTTACC
EMPV1_26702	CTGTCTCTCTTAGAAGCCCAGACAGGTTACAGGTAGAATATCGTTTCCTGCAGGTGCCAC
EMPV1_26703	AAAGGACCACCGTTTTACCACCTTCCATGCACGACCCTCAGGCACTTTCTCCTCATACCA
EMPV1_26707	ATGGCTTCGCTGTATCAGAGGTTCACCGGCAAGATCAACACCTCGCGGTCCTTCCCGGCG
EMPV1_26710	CGCCATCTGTTTTCATGGATCAAGGAGATACACCAACACTGGAGGAAGACCTGAAGAAAC
EMPV1_26711	GCTCTAGGAGAAGCTCTTTTTCCTGCCTTTCCCACCTTCTAAAGGCTGCCCATATTCCTC
EMPV1_26713	CCTGCAAAGATGAGGGGAGTAGTATCTCAGGCAATGGTAATGTGTGCTAGTTCACCAGAG
EMPV1_26716	CTGTCTGCTAGGGAGGCAGGTGAGTGGGAAATGTCAGATACACTGAAGTCATGATTGTGG
EMPV1_26717	GCAAGATATCCCTTTGAAGACCAGCCTGCCACCACCATTCAGAAATTATGAATATGATAA
EMPV1_26718	GGCAACTAGCAATGACTTCACCCTGCAGCTACCCAGACTGCACCTGGAGAGCTTTCCAAT
EMPV1_26719	CATTTCCATTAAATTAGTCACTGCTGTGTAGGACCCTTGAGACCCTCTTCATGACCCTCC
EMPV1_26723	TTTACACAGCTCTTCCCAGCCCGCGTCTGAAGTCTGGGATCCAGAGGGATCAGTGAACCA
EMPV1_26724	GGCAGCTGTCCAGGTGACATCCATATTTACTTTACCCTTTTGTCACAGGGTGGTTGGTCA
EMPV1_26725	ACTGATGCACCTGTTCGCCATGCTCTGAGGTAGACGGGACACCCAAAATGAGGAGTTTTG
EMPV1_26726	GGCCTCTTTATCACAGACACCACACTTACAGACTGGGAACAGCTGGAATGCTATTGATGT
EMPV1_26728	GGAAGAGTCCTTCGAGAGCTGGCTGGACCACGCCAACGACATGCTGTACCTGTGGCGCCA
EMPV1_26730	CGTGGATTTCAGTGACCCCAGAAGCGGTTTTCGCCACAGCGAGGTAGTCATGTTCATCAA

EMPV1_26731	GTGACGGGCCTCCTCTCCGGGAACTGCCAGAAAAAGAATACAAGGGAAATGAACTACTTA
EMPV1_26734	TCCAAGTTCAACGCTGAATATGGTCGCTTGTCAACAAGTGTCGAGGGCGTGTTAGCTGCA
EMPV1_26740	GAGGCACTGTCCGAGAGTGAGTAGCAGGTCTCACAGTGAACCGGTCTCTTTCCCTACTGT
EMPV1_26741	GATCTCTTCGCTCTCTGGCATGTCCTGGATGGCAGCGTTGATCTTCTCTCGGATGGTCAA
EMPV1_26742	CCATAGCCATTTTCAACCCTACTTACGGTGTGATTGTGAGAAAGCCCTATGCTGACAGGG
EMPV1_26743	CACGGAGCCTATGCCAAAGCAGGTTGTTTCCCTAGTCTATACCCAGTTTCCCCAGGTATA
EMPV1_26744	GGGACCGGAACTTTTAGCGACCCGCCTAACCAGAATTCTTTCCTGTCGCAGTACTCATTA
EMPV1_26746	CCGTACTTACTTCAGTCGGGAACAGCTTCGGGTGCTAACTGATACCTTTGAAAAGACCAG
EMPV1_26747	TCTGTTTAACTTTTATTGGGATTGCATTGAATCTGCACTTTGGTTGGTAATAACATCTTC
EMPV1_26749	GGGGGAAATGATGTGACCCTCTAAGGACGTGTTTCTAAGACGCTGTAAGTGCTGCTTCTC
EMPV1_26751	TCACACTGGTGAGATAATGGGATCCGGTGGTTCTAGACTTGCCAACTATGGTGCGAGGGC
EMPV1_26752	AACTCCAAGTAGGGGTACCACTGAATCTGTACAGAGCCGTACAAACCGAAGTTCTGCCTC
EMPV1_26755	ACAGCCCAGATACTCAGAGAAGCAAATCTCATGCTCCGTGGGAAGAAAATGGCCCCCAGA
EMPV1_26756	GGCAAAACTGTGATTCAGGAATGAGGGAGAAACATAGCCAGACAAAAGTTGAAGGAGACC
EMPV1_26759	AAGCTGTACTGTGGGGTTCTATGCTGGAGACAGGAAGGAGTTTGAGACGCTCTGCTCAGA
EMPV1_26761	GGATGGGTGGGAGCCCACTAGTTGGAGAGAGAGGAAATAACATGTTCAAATATCCAGAGC
EMPV1_26764	CTTGGTCTGCTCTGACATCATAACCTCAAATCTAAACCTAAAATCCCAACCCCCTCCCTC
EMPV1_26768	GGCCCTTAGAACATTTCAGCCAGTGTCATTCAAATCTGGACTATTTTTAAGCTTGACCTC
EMPV1_26769	GACCAAGACAGATGCAGCTACGGAAATGTCGCGTTTGTTAATATATGGGTTGACGTCCGG
EMPV1_26771	TTGACAGCCTCTGCTGATAAAACAGTTATTGTATGGGATTGTGATATTGGAAGACAAGTT
EMPV1_26772	GGAATCCGGATGAGATAGGCTCCTTCCTACGCTTATGCCTCATGGTTCAGTATTTGCCAG
EMPV1_26773	GAAGAACTTGGGACCAAAGCCACTGTTATGTATAGAAAAGGGCAAAATCAGTAAACTCAA
EMPV1_26775	GAGGCTTGTCAGTAAAATCTGGGAATGACCGGATATGGAAACATCTGCCCTTGTGTACCG
EMPV1_26776	ACTTTTCCTACGCTGACTTCGGGCCACAGTTTACAGCGCACTTCTTCCATCCTGACAGCT
EMPV1_26777	GTGTTTTTCCTAGAAAATGACGAGCAGCACAGTTGCCTGAGTGACCCTGCAGATCACAGC
EMPV1_26778	TGTTCCTCTTTGTAGAAGTTTTCTCTCTCGCAGTGGTGTGAGATTTCAGCGTCAAGAAAG
EMPV1_26779	TCAACTGCATCACAGAGCTGGTCCTCCGAGCAAAGAAAGACAACTTAGCAAAACAACAGC
EMPV1_26781	ATGGAGTTGGGACTGTGGAGACTCTCAGGCCGGGCTGAGGGGAATTTAGGTGCCGTTACT
EMPV1_26783	TAACTGGTTTTGTACACCCTGGTTTGGTTGAGAAACTCTTATAGGCAAATATAGGGTAGC
EMPV1_26785	CCTAGCAGCAGGTGCAAAGGGGGTCATAGATTCAGGAAAGTGAGTAGAGGCACTGATTAG
EMPV1_26787	GAGGACTTTGTAACCACGAAATACTCTGGGAGATTCAAAAGGCCACAGAGCTCCAGATAA
EMPV1_26788	TTCTATTTTAGACATTTTACAGAACAACAGTTAAATCCACTGGGAGCATTTTTGAGTGAG
EMPV1_26790	TTCACACCCATTTTTTAAAGAGCACAAGTTGATGGAAGAGGGCCCCGCACAGGTGTCCCC
EMPV1_26791	ATCCGTTTTACACACGAAGAAAGCCCCAGAAAGGTGAAGTGCCTGGTCCTTAGGTCAGAG

EMPV1_26792   GTGCGCTTGTGACCTCCTCTTCATCTCTGCCTTCCTTCCCCTCCACACTTTCCTGGTCCA
EMPV1_26796   GACCCTCACTTCCGCCCTGTGGTTTTAATTCGATGCCACGGATCGTCGGCCAAATTTTAA
EMPV1_26797   CGCCAGAAGAGATACAGGGATAAGAGCTGTGGCAGGCTAGCTTATGGACCAAGAGTTTGG
EMPV1_26800   TAGTGGCCACTCCCCCGGGCTGGGCCTCTCCAGGAGTCACAGAAGAGGCCCCCGCCAAGA
EMPV1_26801   TGACATCATCATTGGGGCTCTCTTTTCCGTCCACCACCAGCCCACGGTGGATAAAGTCCA
EMPV1_26802   ATCAACTTCCCAATGAAGGGACGGAAGGGGATGGTTGACTGGGCAAGGAACTCAGAAGAT
EMPV1_26803   ACAAGCACTGGAAAAATATTCAGCGTGATTGCTTCGTTTGAAAATGATGTGGAAATAATA
EMPV1_26804   TAGAATGGTTAAGCAATGAAGTCCTACTGTAGAGCACAGGAAACTATCCAATCTCCTAGG
EMPV1_26807   CCATTGTCACTGACGGCTAATCATGTCATCAGCTATTCAAGTACTTGCTGATTTCAGGGC
EMPV1_26808   GGTTTGCCCAGCGTACGGCTCGGTGCTGTCTCTGTACACCACCTTTTCCTCCATCCTCCT
EMPV1_26810   TCTGAATGGCAGGTGCTGGTAAGATAAATCAGTGCACCACATCTGCTGTACCAAAGGGCC
EMPV1_26811   GAGAGAAATGGAATAGGTATTCATACCACTCCTAATGGGATTGTCTACACAGGAAGCTGG
EMPV1_26813   GTCCTTGTTCGCCCTTTTCTGCAAACTGTGGAAAAGCCATGTGCAAAAACTGCAGTAGGA
EMPV1_26815   ATGGCACAGGACTCTGAATTTCTGGATGAGGCCAAAGGGAAGACCCAGAAGAGAGGCTCA
EMPV1_26816   ACCCATACTGGGGAGGGCCTTCTTTACTTAGTCCAGCAATACAAACGCTGGTCCCTTCCT
EMPV1_26818   CGTGCTGCCCACAATTCATGACCATATTAACTACAACACCTACAAACCAGCACACGGGGC
EMPV1_26820   TAAGCTGGCAACATTAGGTGAGGGTTTTTCTGCTCCAGTGGTGTGGATACCGCCTTGGAT
EMPV1_26827   TCAGTGAAGGTTCGTGCTGGTTTAGTGCCCATGACCCTGGTAGTGTCTACTGTGTTCACT
EMPV1_26828   AGCAGGTGCAGTGTGTTTTAACTTATTGGCTGACAGGCTTCCCCGCCTTCGCCTCCTTAA
EMPV1_26829   GCGCGGCGCAGCTGTCGGGAGGGCGCGTCCCCGTCCCCCGCCGGCCCGCCCTGCCCCTCC
EMPV1_26830   ATCTGGCAGGATGAGGAAGGGGCCAGCACCTCTCAAGCTTGAAGTACCGACATATGAGTT
EMPV1_26832   TGTACCTAAACACCTCTGCTTTCGGGTCAAGCCCCAAACCATGTCTCTGTCCCAGGCTGG
EMPV1_26833   ATTCACGATCACCATGAAGACAAGTTTCACTAGGCAGCCGCAAATATGAGCCTGGGGCTG
EMPV1_26834   AAAGCCCTCCGGTCTATTAATTGGGTGCAGTCACCTATCTGCAGTCTCCATGGCTAGCGA
EMPV1_26835   GCCGGGGATCATGAGAGCTGTTTTATGGATTTGCAGTGAGGATTACACGAGACCACATAG
EMPV1_26836   TCAAGGCCCGGGAATAGGTGAAACGTTGGGAGTCATTTCTTTTTTGTGTCATCCTAGGGC
EMPV1_26837   TGGGACAGGACGGCGAACCTGTACGACGTGGAGACTTCGGAGCTCGTCCACTGCCTGACA
EMPV1_26839   ATGATGCCGTTCACTTTCCTGATCAAGCGGCTTCCCTACTGCAGGGGCAATCTCGTTCAA
EMPV1_26840   ATGGAGGCAGGGGAATGAATAAGTGAAATGGAAGACAGAGTGGTGGAAATCACTGCTGCA
EMPV1_26841   CGTCCACAAAATCCAGAATGGCTGCAAATGCTTCGCATTTGTGGATCTGGGCTCCATGCA
EMPV1_26842   GAAATGAACTTCACATTCTAATTCATGAGGTACCGGGGCAGGTTTTCTGGGGATTTTCTG
EMPV1_26843   ACCACCTGTGCTCCTCCGGGTGCAGAAGGCGTCCATGCCCGGCGTGGCGCTCCGGGGGGT
EMPV1_26846   AGTGGTCACAACTGAGAGGGCTAAACATTTCTACAACCCCCAGGATGTTCCTGTCACCCT
EMPV1_26847   GGTGGATATGCAGCCAACAGATATGCACAGGCTGCTACTGCAACCCCAGCCACCGCTGCT

EMPV1_26848 CACCTGCTCTCCAAGCTGCCTATCCCTGACAGCCAGGTGATCACCATTAACCCTGAGCTG
EMPV1_26853 ATTTCTGCCTTCCCTGAGTCAGACAACCTTTTCAAATGGGTGGGGACCATCCATGGAGCA
EMPV1_26856 GTCACATAGAGAGCGAACAGCAGGGCAGCCGGCTTTGAGCCTAGGCACATCTTAATTCTA
EMPV1_26857 CAGAGATTAACCAAAGTGCTGACAGTCTACACGTGTTTCTCCTGGTCTGTGGACTCAGAC
EMPV1_26858 GTAGAGATGAGCAGTGTGTGTTATGCCACAGTCATGGGTGAGACGATTAGAAAGATGACC
EMPV1_26859 CTCTGGGGAGCTGGTTGCTGCTGTCATGTATACCCTGGTAACTCCCACTCTCAATCCTTT
EMPV1_26861 GTCACTGTAATAAGTTATTCCTTATTGATTTTGGTTTGGCCAAAAAGTACAGAGACAACA
EMPV1_26864 AGGTAATATGCAAGACACTTCGTTTTGTTGCACATTATTACGGAGCATCGTTAATGTTTA
EMPV1_26865 GATTATCATGGACGATGTAGAGGTGCCAGAGGAGAATGTGCTGCCCAAGGCGTCTAGTCT
EMPV1_26867 CACCCCTCAGGATGATAAACGGGAGCCAGCACCAGATAACTAACCTCTCCTAGCCTCTTT
EMPV1_26868 AAATCCAGGATGCAGGGCTCACACCAAAAGGAGCATCAGGAAGTATCTGGCCAGGTCACA
EMPV1_26869 AATTCCACTGGGCACCAGCATTACCTGGAAACTGGTTAAGCACGAATTTCTGGGTCAGCC
EMPV1_26871 AAGAACTTCCTGCACAAAAAGTGGAAGGAGCAGGCTCGGGAAATGGCAGATACCGCATGT
EMPV1_26872 AGCCCTCTGCACACCTAGAGCTGGTCTTCGGGGTAGATGTGAAGGAAGTCGATCCCACTA
EMPV1_26873 GAAGGAGGGCTGCTTATCCTTTTGCTTTTTAGGGTCACAGGCGTGGCCTATGGAAGTTCC
EMPV1_26874 GTGAGAAAGGTATTCAGCAATTATATGGAACACCCGTGGTGAATCGCTCATGGAGCCCAT
EMPV1_26875 TACAAAGGCCACCTTTATCCTTTTTAATGCCATATTTTCAGTGTTACACCTTTATGGCTT
EMPV1_26876 CCTTCATCGTGGTCACTAGTAAATGAAATTGTCTTCACAGTTCTATCAAGGGAGCCAGAC
EMPV1_26877 AGCGTAAAGGGACACATCGTCTGCGAATTAGACCCTGAGCCAGAAGCTGAAAATACCTCC
EMPV1_26880 TAGCTACGGACTTTCCTCAGAGTTTACCAAAGGTAAAAGGATTGGGATGGGGGAGGGTAC
EMPV1_26883 CATGGGCCAGAAAGTCCAAGTTCATTCCCTGACACAAATGTTACTTACAGGCACTAAGAG
EMPV1_26884 GGAGGACTTAGAGAACAATCATGATAATCTTCTTACTGGTGCACACAATGAACTTAGAAA
EMPV1_26886 TGTTGGTAAGGGTGCAAGTTAGCAGGTGCCCTCCTATGCCAGGTATCAGGTGTTTCTTGT
EMPV1_26887 TGAAACAGCCAATCTTAATGAGCACGCTGAAGGCCAGAGCAACCTAGAGTCTGAGCCCAT
EMPV1_26888 CTTGGTGAGCTCTATCCTGCACCCCCGGCCCATCAACTTCAAGTTCTATAAACACAGCAT
EMPV1_26889 AGGAGGCAGAGTTATGCAGGCCTGAATCCACATTCAGCTACTTCTGGGCCACGTGACTGT
EMPV1_26890 CTTTGCCTGTCGTCCATCAAAGCTCATCGGTGCTTTATCTTCATTCCGAGTTGGCTGCTC
EMPV1_26892 AGCAGATAAGTGCAATCGGACGGGGCATCTGTGTGCTGCTGGGTATTTCTCTGGAGGATA
EMPV1_26893 ATCAATGAAGGGAGGCTGACCCTGAAGATAAGCAATGCCAGGACTTCAGATGATGGGCAG
EMPV1_26895 GCTAATTGGGGTGAAACTGTGGATTACAGCGGGGCCAAGGGAAAAATTTACTGCAGCTGA
EMPV1_26899 GAGATAATCCATGGGCTCCTGGACAGAATCATGCAGCTATTTGCTGTGCCTCCTGGTGAA
EMPV1_26902 CAGCAGTGGGAGGACAGGGCCCTGCGCTCCCGGCAGAGGCAGACCTACTTGCGCCTGACA
EMPV1_26904 TACAGCAGGGCCCGGTCGTTTCTCATCCCTATAGACGAGTTTGCCTCTGCTCACCCCAGA
EMPV1_26910 CGTGGTCAGGAGCGCCGTCATATGCTTCCCATTCATCTTCCTGGTACATCGACTTGCATA

EMPV1_26911	TCCAGTGTTGAAGGGTGCACACAAGGTCTCTCATGCACAGGGATGCAAGGCAAAGCAGTA
EMPV1_26912	GTGCAACTCGTGGAAGGTTTCGGAAGGTGCTCTGGCCGCATCGAGGTGTATTTTGAAGGC
EMPV1_26913	GGTGCGGCGCTTTATTCAGGGATGAAAGCAAGTGCACGAGGCGTTTATAGTAGGGTCGTC
EMPV1_26915	ACTCAGGTCTTGGTGTTGGAGTGGGTGTCAGGGTTATCTGTGGACATTGCAAGAATACGT
EMPV1_26916	GTTAACATCAAGATTCTGCTTCAGTGGGCCTGGGATTGGGGCCTAAGAGTCTGCATTTCT
EMPV1_26919	GCTCTACACATGCGCTTAGGGAGTCTGGTCCATTGAGTAAGTGGCTATAAATTCCTCCCC
EMPV1_26921	AGCATTAGCGAGACTCAAAGGCCATGACTGTGCATGAATCAGACAAGTGGACAACCGTCC
EMPV1_26925	GCATCCTAATATTAGTTCCGTCACAGGGGCTATTTGTTTGGATATCCTGAAAGATCAATG
EMPV1_26926	CCCTTCAAACGCTCCAATCCTTGGAAACTAAACCTCCTCTCCAAGCCTCCACGTCTAGAA
EMPV1_26927	GAATACACAGATGGAGGGATCCTAGATCCAGATGATGTCCTGGCAGATGTCGTTGAAGAC
EMPV1_26929	ATCCTACGAAGAGATGCTGCGATTCTACAGCTACTACAAGCAGGCTACCGTGGGTCCCTG
EMPV1_26930	GACTCCTGTCGCAGCCCCTCCTCCAGCTTCAGAAAGTCAAGGTAACAGAATTTGGCAGAA
EMPV1_26931	GCTGAACTTGACATGGCCACCTGCTCAAGAACCTTGACTAAAACAGATCCTTCTTTGGAG
EMPV1_26934	AGTTCCTCCTGGTTGCCAAAAAGCGGGTGGTAGCGGCGGTACTTGCCCTCTCTGTATTTG
EMPV1_26935	CTTAATGTTACACACAGCAGCTCTCCAGCAACAGCACTTAAGCAGCTCCCAGCTTCAGAG
EMPV1_26936	CTCTATCTCTTAGGTGTGGTCCTGACCCTGCTCTCAGTTTTCGTTAGACTGATGGAGACC
EMPV1_26937	CTCGGGTCTCCCAACACTGATTGGTCAGCAGAGGAGTAAGAAAGGTCTAAGAGAAAGAAT
EMPV1_26939	GATGTGCTACCCGGTGTCTTGAGTCAGTGCGCCAAGTTCTGAGCTGATTCTTTGGACATT
EMPV1_26940	AGAGGGAACAGCCTTCACCCAGACCTGATAGAGGGCAGGAAGGGGGCACAGATAGTGAAG
EMPV1_26941	CAATCCTCTCCTTGATTCCCTAGCTGGATATTAGTTTTCATACTCTAAATTCTCCTGATC
EMPV1_26943	AACCTGGAAGAAAACCATGAACCACATCAAGTTCTGAATAGCCTGCAATCTGCCATGACG
EMPV1_26944	CAATGTCAGCTACTTCTCCCATTGGATTGACGAGATCCAGAAGTGCACACCCCTCCCTGA
EMPV1_26945	ATGACTCAGTCGGCTGTCAAATCACTGAAGCGACCCCTCGAGGCAACCTTTGACCTGGGA
EMPV1_26947	CCATGGCCTCTGGAGGGAACATCCTCCGTTTTATGGGAGCAGCAACACATATCAGAAGCC
EMPV1_26950	TGTGACACTCAACTCTCTGGTGTCTGAAGCATTTGTCAGGTTCTTTGTGGAGCTGGTGGG
EMPV1_26951	TTGCAGGGGCGCTCATTGCTGACTTCTTGTCTGGCCTGGTGCACTGGGGAGCCGACACCT
EMPV1_26952	AATACCCCCGCCCGTAATTCATTCTGGCATCAAGTCCCTCTCAGTGTTCTGAAGTTCTGC
EMPV1_26954	GCTGGAGAAGATGCTTGTTTTGGACTCAGATAAGAGAATTTCAGCAGCCCTAGCCCTTGC
EMPV1_26956	CTTTGTCCCTGCCCAGTCCTTTCCTGTGGCTAATGGGATGGCCTATCCAGCCCCTAATGT
EMPV1_26957	TGAGGCTTTATGTCCCTGAAGAATTTGTAACCTTTCCCAAGGAGACCGCCTCTAACATCC
EMPV1_26958	CCAAATTAGGGTTCTCCTCCACGCCGACCAAGAAAAGCAAAAGCTCCGAGGACACAGTGA
EMPV1_26959	GTTAAGAAAATACATCTCTTGTTTTATCAGGTGTGTGTGGTTTCAGCGCAGCATGGCTGT
EMPV1_26961	TATGTCTAGCAATGTATGTGTTGGCATTGTTGCTGCTGCCTGGGGAACTGGCTTCCTCCA
EMPV1_26962	GGATCCCCTGGCACCTGCAGAAGCTGAAAAATGAGAGGAGAAATAAGAGCAGGAAGAGGC

EMPV1_26963	AGACTTCTGTGCCCAGGAGCAGTGTGGACAGGGCCATGTGGGGCTGAGGAACTTGATGAT
EMPV1_26966	TTTTCTCCCCTAATGAAAAACTGGGAGGTGGTTGTCTCCTGCGGGCCATTCTGAGTCTGG
EMPV1_26967	GAGAGACTCCAGACCACATTAACTGGCAAACAGGCCCAGGAGTAGTGATATAACCAATTG
EMPV1_26968	AACGGGCGGAGGCTCCCGGTTTCGTAACCGTCGCTCCACCTCGCTGACTCGCGGGCTGCT
EMPV1_26970	TGGCGGGGACACTGACCGCTGGGTAGAGGAACAGTTTGATCTTGCTCAGTATGAGGAGCA
EMPV1_26971	CTTTACAAACCCTTAGATCCTGCTGTGGTTTCTTTCCACCGCCTTCTCTGTACATGCAAG
EMPV1_26972	AGTGCCCTCGGCATTCCACCATCAGCGTGCAGTCGGCATTTTATGGGCAAGATTACCAAA
EMPV1_26973	GTGTTGGATTTAACTGAGTATGCCAGAAGACACCACTGGTGGAATCGAGTGTTTGGCCAC
EMPV1_26974	CGTCGCTGGCTTCTCCAACTGGACCTCGAATTTCATTGTGGGCATGTGCTTCCAGTATGT
EMPV1_26975	GACAGTTGCTTTCTCGAGCGAACCCTTCAGGACCAAAGAGCCGAAGTCTAGAGCGCACGA
EMPV1_26976	ATGGCCAGCTTAGCCCTATTGCAGAGGCAGTTTGATGTGGACATTCTTATTTCGGGACAC
EMPV1_26979	AGCGCTTCAGTTTGAGCTAGAGCCTGTGAATAAGCAAGCTAGCCAGGCCTTCTCGCGGCT
EMPV1_26982	GATTTCCTCAAGAAAAGAAACTGTACCAGGGAGTCTTGGCATTCCTTTATTCCATGAAAG
EMPV1_26983	CTCATGCTTTGAAGAATTAATGCTGACATCTACATCTGTTTCATGGGAAGGATCCATGAT
EMPV1_26986	TTTTACCCATGCGAGGAGTGTGCTGAAGACATAAGGAGGAGGATATGCCGGAACCAGCCA
EMPV1_26987	ACCCAGGAATGTGATGAGGCAGGTGGGGAGGAGTCCAGGGAAACTCTTGGAAACAACATT
EMPV1_26988	TTGCAAATCAAAGGAAACGTCAGTTGCTGATTGGACTTCTGAAATCTCTGAGAACTATAA
EMPV1_26989	CTGTGGCACCGCCTTTGACTTCCACAAAGAGATAGACTACATGTTCCTAGTGGGCACAGA
EMPV1_26991	TGCATGTTAATTCAGAGCACCGAGGAGCCCAAAGTGGTTTGGTGACTGTCTTAACCTTCC
EMPV1_26992	ATGATGACCTGTTGTATATAACTTTCCACATCCATCATATCCACATCGAAAGGCCTTCTC
EMPV1_26993	CACAGCAGGAACCTGTATCTTGACTCTTCAGACACTGATTTTTGTGCCTCCCTCATCCAC
EMPV1_26995	TTTGAACATGCACTGGCTGTGGAAACTCCAGGGACTCTGCCTCCGATGGGGGACTTGGAT
EMPV1_26996	GACAGACACAAGTGCATCTGGCTAGTTTTGACATCGTGGATTATTGGCTTTCTGCACGCC
EMPV1_26997	ATGGTTCAAGGGAGGATTTCCCGATGCCCTGGGATGTCAGAGACAGAGCTGCAGGAAGAT
EMPV1_26998	GTCTGCTGGAGGAGCCTGGAAGAGAGTTCCACCTTCTTTTAGACTGCAGCCTTTTGCAAA
EMPV1_26999	CAGGACTGTGAACTACTTTTCCTAAAACCCACACTGAGCTCGGCAATCGGATGGAAGGAG
EMPV1_27000	AAAGTAGTGTTACTTCTATTTTCGGTGCGCTTCTCGCGGGAAATCCGGCCACCGTAAGCT
EMPV1_27001	ATGGTGTGCAGGGCTGCGGCTCCTGCGTCCCTTCTCGCGGCGAGTGAGCTGCACTGATTT
EMPV1_27004	CTGTCAGTGTCGTAAGCAGAGGATCCCAGCACCTAGATTCAGGCTTATTGAAAGCCAGGC
EMPV1_27006	TGGGCCTGGGAAGCTATGAATCCAGAGTTGCCTCCTTTAAGAAAACCAGTGAACACAGGG
EMPV1_27009	TTCCACGTTGAGTTCTGCGTGAAGATGGCAGACGATCTTGGAGATGAGTGGTGGGAGAAC
EMPV1_27011	AATGATCAGGCCACGTCTGTGCTACTCTCTTCCACGGTAAAATTGCCCATGTCTGGAGCA
EMPV1_27012	CCACGAACCAAGGGCGCCATGACAGCGCAGGTCGTTATAGAAATAGCTCCTGGTATTCAT
EMPV1_27013	CAGCTGTCTGTGGGTCTGAAGCTATTCCAGGGCTATTGTAGATACACTGGTTGGTGAGGC

EMPV1_27014	TTGTCCATCAGAATGTGGGCGAAGTTACAGCTAAAGACCAAACCATGGAAGGACGGAGGC
EMPV1_27015	ATCTTTGAGGCACCAGCCAGGGCCTCCTGAGACAGTTCCGGGGGCCACACTTGCTGTGTT
EMPV1_27018	AAAATTAAATCTGTATCTCACCCCTGGGCTCTTCCCGCCCAAGGGGCTGGCAGCTGTGAG
EMPV1_27019	GGAGACGAATGTGCCCAGAAAATGAGAGCGTTAGTTTATCACCATGACGCGGACGTGAAG
EMPV1_27020	AAGCATCTGGCACAGAGAAAAATGACAAAAGTCAGCAAGGAATGTCTCCCAAAGCTCTGA
EMPV1_27021	AAATTGAGGTGAAGGTGAGTTCCTGCTGTGGCGCAATGGGTTAAGAATGCAACTCTGGCG
EMPV1_27023	AGGCTGGAGCCCGTGTGCTCGGGGCTGCAGGACCAGATCCTCCGCTGCTACCGCGACCAC
EMPV1_27024	ATCCAGTCCCGTGAGAACTCAGAGCAGCAGTGGAGAAGGCAGTGAAAACAGGATGCAGAA
EMPV1_27026	TGTGTCCTCTCCCCTCGCCCTGACCTGCTGGCATTAGACTCACCCCGTGTACCTAGTAAA
EMPV1_27027	ATTACTGAGTTGCCTGTAAATTCTCACAAGATAGTACTAGAATGGGCTTCTCCACGGAAT
EMPV1_27028	TTGGGTGCCAAGGCAATCTCCAATTAGAAGCTGGTAACACAGAACCCACCTGACTCATGG
EMPV1_27031	GTTCGAAGGTCCAGCCCAGTGACCCAGGTGAAAGAGATGATCCAGATGAAGACCGCTATA
EMPV1_27032	ATTGATATGGTCCTCCACGTTCCAGACGGAGCTGAGGGTTTCCTTTAGGTATGGCGTGCA
EMPV1_27033	AGAGTTGAAACCTCAATGAATGTTGAGCTGTTTCTTTTGTTGGTGTTTCCTCTTTGTTGT
EMPV1_27034	ATGTCCTGTGGTTGGAGAGTGGCGATACTATGGTGGAAGCACCTTCAGGGTGGCCTTTGA
EMPV1_27036	ATGGAGGTGGACCCGTCTGTGCTCCAGCACATGTTGGAATGATGGTGCACCCATTAACAC
EMPV1_27038	TGGGGAGGGTGCCTGGGCCACCCCGGAGGAGCCACCGGTCGACTACAGCTACTACCAACA
EMPV1_27039	CAGCAGAATACACATCAAGTGCACATGAAACATTCCCAAGGATAGACCATATGCTGGAAC
EMPV1_27040	AGAATCAATCGAGAGTGAGATTAGGCGAGAACGGGAGAGGCAGACCAACCCATCGGCCAT
EMPV1_27041	CGTGGCTCTAATAACTCCGTAGAATAACAGACGTCATCACCACGGGATCAGTGCACGCAG
EMPV1_27042	GCGCCTGTCACATCGCTGTCATTTGATCTTTCTCCAGAGATTTTAGCTGGCTCTAAATGG
EMPV1_27043	AATAGGCCACCACCTGCTGATCTGTGCTGAGCTACTTTACATCGAAGCAGCTTTGTCTGC
EMPV1_27044	TCTGAGGACAAGTCAAGCTCTGAAACGGGCTGTGGAGACGGAGAAGAGCTAGGGCTGGAA
EMPV1_27045	GTCTCAGAAACCCTTTCCACTAGCTGCCTTGTGCCTTTCCCTGTCTTTGCTGATCTGTGT
EMPV1_27046	GATAGAGGCACAGATGCAGGTAGGTGGGTAGATGTGGATGGTAGCGGCATTTGGAAATTC
EMPV1_27048	GAGGATCAGACGACTGGAGAAGGCAAGCCAGGAGCTCATTCCCATTGAGGATTTTATCAC
EMPV1_27049	GGACGACAAGGTGAGCTTCGTCCTGGAGGTTCTTCTGCCCGAAGCAATCATCTGCTCCAT
EMPV1_27051	CACCAACAACCTGGACATTGAGTCTATCGACGCCCTAGGGGAGGCCATCAACGAGTACAA
EMPV1_27054	CTGCTCTAAGAGCACCAGCCAAACGGACCAGGGCTCCAGGAAACCAGAGGGAGGCAAGAA
EMPV1_27056	GAAAACAAGTCCCCCAAACCTGTTTCCAAACTTGGCCCACCCCGGAGAAGCAAGTCAGAT
EMPV1_27057	GTAAATTTGTTGGTGGTCTTTATTGTTGTGGCCACTGGTATTGGGGTGTCCATTGTGACC
EMPV1_27058	TGAACCCGACGCACTTCCCCTAAAAGGCGGCTGTGGCTGCTATCTCTGAGATGTAACAAC
EMPV1_27060	GCCCTGGTAACATATGACCTTGAGACAGCAATCTCTTCATCAACTCGCCAGGCAGGAATC
EMPV1_27061	AACTGAGGAGCAGTGCAAGATTACAGGCATTTTGGTAAGCAGTCGTTAACCCAGCCCTGG

EMPV1_27062	GCACAGGCATTGACTTGCTGCGTTGTAAAGCTTAAGGAGCATTTAACATAAGCGGCTGCC
EMPV1_27065	AATCTACATACGAAGAGTTTGTGGAAGGCACGGGCAGCCTGGATGAGGACACATCACTCC
EMPV1_27066	CATCACAGGTCATCAACAGCCTCAGTCAAGACCTTCATAAGAGAGAAAGTGGGAGGGACC
EMPV1_27068	AGAAAGCAAGTAGTTATGAGATTATTACAGGTAATGAAGAAGGATATTTCACAGCTATAA
EMPV1_27069	GGAATCTGCAAGGCCAGCTGGTCCTGGGGCAGGATGTGAGGGACTCGGATTTCCTCCTCC
EMPV1_27070	AAGAGTCTGTTCCAAATCACCAGCACGTGGGCGTAAACACTGGCACCGTGGACGGGGAAA
EMPV1_27072	GACCAAGCTGGCCTGCACAGACACTTACGTTATCAGCCTACTAATAGTTGCAGACAGTGG
EMPV1_27075	CGAACCTATGGGCAGACACTGATCTGCGTATCTTTCCCTCTCACCCAGTTCCTGAATATC
EMPV1_27077	AATGGGTCTTGCATGTTGAGCAACAATTTCATCCATTGCTCCATGGAAGTGGTCGGCCGG
EMPV1_27079	ATGGATGCCTTCATGCGGCGCAAGTGCCAGCAGTGCCGGCTGAGAAAGTGCAAGGAGGCT
EMPV1_27081	AGACGTACGAGTTCTCGGGTAAGAATTTATCATTCGCAAGCAACAGCGCCAGTCCCCTAC
EMPV1_27082	AGCGAGGACTTCTCAGCTGGGCCGACTGCTGTGCTCTTCAAAACCGAGCTGCAAATCGTC
EMPV1_27083	CTTCTTATTTTACTGCACAGGATTAGGAGTGTACATTAGCTCTGCTGCTACTCACAGCTC
EMPV1_27084	GGGAGGTGGTGAATGAAGAGGAATCTGCTGATGTTTTATTAGAGGATGAGGCTGAATTGG
EMPV1_27085	AGGGCTGTGGGGGCCGTGTCCCAGCTGAAATCTTTTTAGTGTATGACTCTGAATGGCACT
EMPV1_27088	TCCTGTTGATGCTAGGAAGATTGGCAGCCGGTCACCATGAGTGGGAATGGAGTAGGGTTT
EMPV1_27090	ACTGTTTTCCATAGTGGTTGCACCAATGTTTCCCACCAACAGTGCTAGACACCTCCCCCC
EMPV1_27092	TTGTGTTGGTGCGGATGGTGTAATATGTGAAGACGTTAAAGTTAACACCATAAGCAGAGC
EMPV1_27093	AAAAGTTACCACAGGGATAACTGGCTTGTGGCGGCCAAGCGTTCATAGCGACGTCGCTTT
EMPV1_27095	GAACTGAGTTTGTACAGGCAAGTCCTGGGCTAGGTAAGGGGTTACAGTAGTTTTGATATC
EMPV1_27097	CAGACTTTATCAGGATCCCAGAGGAAGCTAAGGCAGTGATGCCGAAGAGGAAGAATACCG
EMPV1_27098	AAGAAGTCTCCGGGGTGTCATGGTTATATCCGCCTGCCTGGTTTAGACAATGTTCTGCAC
EMPV1_27100	TGTGAGTGTGTGGAGCTAGCGTGGGACTTGAGAGCTGCAAAACAGCTGCAGAGCAAAGCA
EMPV1_27103	GCATCCTCCAGCCAAAGGGAAACTTCTATTTGTCCCTAAGAGCTCTGAGGTCAGTGGACA
EMPV1_27104	CCTTAGAACTAGTCAGGTGCTCCCCCCACCCAATACCATTTCTTACCCTCTAATCCTACC
EMPV1_27105	TGTGTGGTATGATTCAAAGTTCAGAAGATAGTGCTGACTGGCAGCGGGTCTCCCAGGTTC
EMPV1_27106	TTGCATTTGAAGAGAGCTAGGCAGAATTTCTGCAGGGCGGAGCAGTCGCAGAAACACCTC
EMPV1_27108	TCACGGCTCGGGTCCGTCTTGGGGCCTGGCCTGCCAGACTGTCTGGTTTTTCATCTGGAT
EMPV1_27110	GTAGAGGGGGTCGACAGTGACTACCATGACGAAAACATGTACTACAGCCAGTCTTCTATG
EMPV1_27111	CCATCTTCTCTTCGAAGAGTGACCATTGCCTCTTTGCCCAGAAATATTGGAAATGCAGGA
EMPV1_27112	ACGTAGGACCTTGTTTATTTAGTATTTACCATAGACATCAAAATGATCATGGCTAAAACC
EMPV1_27113	TTAAAGCCAGCATTTTGGACTGGTAGTCGAAGGCTACCACCTCGCCCTGCAGCCGCTGCT
EMPV1_27114	TGTTAGCCTCCATAACTGGCAGTGGGTTCTGCTGAGGAGCATGGTGTGCAAGCTGAATGA
EMPV1_27116	TGCAGTGGTAGATCAGATTGGGGGTATGTAGCAAACTGGACTTTAAGAACTGGAAAGAGG

EMPV1_27117	ATGCCATTTTCCCCCACAAAGCAGTTCAACAAGAAGAAAAGCCCTGATTGAAATTGGGGC
EMPV1_27119	CCTTTGCCCTTGAGGTCTTGATGATTTCCAACAATGGCTTGATCTCTACACTGTGGTTCC
EMPV1_27120	TTACCTACCACCACGCCTTGACGAGGTCGATCCTCATCAAGAGGATCAAGCAGACGTACA
EMPV1_27121	ACGTCCCAGGCTACTGCCTCTACTTCATCCCCTATGTGTTTCTGAGTGACTGGATCACAC
EMPV1_27122	CTACTCCTGCCGTGCTTGAGAAGGAAGATCCTGAAGATTAGACACGGTTGGGAAGATGTC
EMPV1_27125	GCTGCAATCTTCGTTCCCGAGTGGTAAAAGAGTAACATAATACCTTCTATTTATGCATGG
EMPV1_27126	TAGCGCCTGAACTGATTAGGTAACAACAGAGCACCTTCCCTCCCTGTTTCCTCTCAATGC
EMPV1_27133	GGACCAGAAAATGGATATGGCCATTGGATTGCTAGTACCTCAGGCTCAAGGAGCAGTATC
EMPV1_27134	GCATTCGCCTGCCTCTTCAAGCTCACAGAATCAAAATCGAGTACTTGGCATCCAATAACG
EMPV1_27135	GTCCTTGTGTCCTATGTTTTTATCCTTGCCACCATCCTCCAGATGAATTCTGCGGAAAGC
EMPV1_27138	GTGTGCCAACCCCACTGCACACCTACCAGAATGACTAGAATGAAGAAGGCCCAAAATACC
EMPV1_27139	TCGAACGCCTTTCCGACTCAGCCAAGCAGGAGCTCATGGACTTCAAGTCCCGCCGCGTCT
EMPV1_27140	ACTCACGTGCTGACATCGGAGGGTTTTATTTACGCCTGTTCATGTCGGAGTGTCCTCGGA
EMPV1_27141	CCTGTTTAACCTTGCTAGTGTCCTCATCCGTAAAATGGGAATATCCGTACCCAGCACTCG
EMPV1_27145	TGCCATCAGCTTCGTGTTGGGTGAGAAAACCAGCAACCTACGGGTAAAGACCCTGAGAGA
EMPV1_27146	GGCAGTGACGAGGTCATTACAGAGCAGGTCTTGTTAGGTAGGAGGGCTTTTTTATGTGGG
EMPV1_27147	TATTTTTGTATCAGCCAGTCTCCTTTACCCACATCCCACGCTTCACCTTGGAGGGCTCAG
EMPV1_27148	TGAGCATGAGTCCTCCTCAGGGCCCTGCACTTGCTATTCTATCTGCCTGGAATGCTCTTT
EMPV1_27149	TGACCCTTGGGATCCTCTCAACTCTATGCCTCACAAACTAAGCTCCAGGCTTCCTCTGAG
EMPV1_27151	AGTCTGCTTCCTTCAGTCCCTGCTCTCAAAGGGGAAATCTCACCACCTCAGCTATCCAGA
EMPV1_27152	CACAGGTTTTCACTCTGTCTAAGCCCAGAACCGGTAGAAGTGAGCAAAGAACAGCAGGAG
EMPV1_27154	GATGGGGTTAGTAGCTCAGGAGAGCCAGGTTTTCCCTGTCGGCAGCACATACCAAAGAAA
EMPV1_27155	TGTTCGCGCTCTCGGGCAGCTGTTTCACATCACCTGCTTTACCTGCCACCAGTGTGAGCA
EMPV1_27156	CATTGAGGTCCATGTCTTCTTGATCAGAGTGAGGCACCAAGCTCTCAAACTTCAGCCTCT
EMPV1_27157	CCCATTGGCATAAACCCGTAGATCCGATCTTGTGGTGAAGTGGACCGCACAAGCTCGCTT
EMPV1_27158	GTTTGTCTACGGATTTTGAGCATCTGAAGCTGCCCTGCGAATCTAAGCACCGCCCGCTGC
EMPV1_27159	GCAGTCAATAGAGACCCTCCCTGAGCTGGTCAGATTCAGGAAATAAAGTCTTGAAAGCAG
EMPV1_27160	AAGGTTGGTGATGACATTGCCAAGGCAACTGGTGATTGGAAGGGTCTGAGAATTACAGTG
EMPV1_27161	GCTTCCAGAAAAATCTCAAGGAAAAGTATTGCAAGCAACCGTAGTAGCTGTTGGATCAGG
EMPV1_27165	GGCAGTGCTTGGAGTCCTAAATCTGATTCCTCCTCACAGAGGTCTTTATTTCACCTGAGC
EMPV1_27166	CCAGGTTTTCAAGTCTGATGCATTGAGCAGGATGTTCTGTGTTTGATGGGCGCTTGGAGA
EMPV1_27168	GGAATCAGGGACACACAGTGTGGGTTCAAATTATTAACTCGAGAAGCAGCTTCACGGACG
EMPV1_27169	TCAATGGTGTGGACGTGAATGGTAAATATACGGCCGATGGGAAAGAGGTGCTGGAGTACC
EMPV1_27170	AACCCTTGGGTTGCAGACTCGGGAAGGAGAATCTGGAACCACGGAAGCTTCAAGTCCTGC

EMPV1_27172	GCTACAAGCAGACATTCTGTATATCCCAGTAGTGAAGCCCTCAATGCCAGAAACAACCGC
EMPV1_27174	GAATCTACAGGTCATGTAAACACCTGATTGTTATAATCAGCACCTTACATTTCCAAAGGT
EMPV1_27175	CTTCCAGAACATCCACGATGTCCTCAAGAGCCAGGCTGGTCCCTTCATCTCTACGGGCAA
EMPV1_27178	GATGTGCCTTGTCACGGGAGCACCGAGCATCAGAGATGTTATAGCCTTCCCCAAATCCTT
EMPV1_27179	CTCCTGCAGAGACATCCTTGGCATCACCACCACTCTGCTTTGGTCGTTTTATAAGCTGTG
EMPV1_27180	AGCCTGCTCCAGTCACCGTGGGCTGTTTTCACTGTTTGTCCGCTGTCTCCTCTATAGTAA
EMPV1_27181	TCTGCCCAGTTCGAGCACACAGTTCTCATTACGTCCAGGGGCGTGCGGATACTGACCAAA
EMPV1_27184	GTTTGAAACTTGTCCCTGAAACCAGCACCTTTGTTACATTTATGTTGAACTCGTCAAAAG
EMPV1_27185	GGTTGCATCTATGTCTCAACTATTGTAAATGGTGCTGCTACGAACATTGGAGTGCATTGC
EMPV1_27187	GGCATCATCTCTTGCTGTCATCCACAGCCTTCATAGGAGAGTCACCATACAAGTTTAGGA
EMPV1_27188	GTCCTGCAGTAAGAGAAACAAGGATGGAAGTTGGAAAAGGATGTGATTTAAATATTTCAG
EMPV1_27190	GAAGAAGACTATCTGGTTATCATCAGGTTCACGCCAACAGTACCTCATTGCTCTTTGGCG
EMPV1_27191	CATCTTTGACGTGGGCACACGTTTGGGACTACACTATGATACCTTGGCAACTGGAATAAT
EMPV1_27193	TTCCTGTGGCTGTACTGTTTCCCTAACTGCACCCTCTGTCAACTCAAGCGGGATATTGAG
EMPV1_27194	ATACCTCGAGTGCTCCGCCAAGTACAATTGGCACGTGCTGCGTCTCTTCCGCGAGCTACT
EMPV1_27195	GGCATTTCGGGCTTGGTTCCAGAAGAAGTGAAAACCTAAACCAAGCGAATCAGGCAGAGT
EMPV1_27197	GCACACACAAGGCTGTAACTTTAGGAACCCTGCCGACAACTTGAATCTCAGCCTCTAACC
EMPV1_27201	GCCTCAGTGTCAGGTGTGCGCAGAGTGGAAACGGGAAATTTTGAAACATCACACCAACAG
EMPV1_27202	AAAGAGCCCGGCACACTGTCGGCCTCCAGCCTCCTCCTCAGGCTGTGCTCCCATCTCTGG
EMPV1_27204	GGATGAAGATGTGATCCAAATGACAGATCATCATCACAGGTACGAAAGCCCAAGGAGTTA
EMPV1_27206	GCCAGAACAGGAGAGAATGTGTATCACTCCAAGGATGAGGAGGGTGGCTTTTGCCAGAGA
EMPV1_27207	CAGCTCTATGATTGAGGCTTCTGTTGGTCCTCTTCAGACTAGTAAATCCAACTCAGATGA
EMPV1_27208	GAGAGAAGGAATTCGGGTGCTGGCTTTTTAATTTGTCCCTTTCTGCAAAACATGAGCGCC
EMPV1_27209	ACAGTACGCAGCTTCAAACATACCCAATCTGGGGAAGCGGGTTTTCTCCTTCCCTCCCTG
EMPV1_27210	TAGACTGGTTTCCATGGAGATAGGGCATCAAGGCTCCAGTGAGGTTGGAACTGACTCCAC
EMPV1_27211	CAGTTTCTACCCCAAACCTACAGAACAAGATCCAGCTGACTAGAGCCACAGATAATCATG
EMPV1_27212	GTGGGAAAACCTTCCGTGTGAAGTCAAATCTTACTCAACATCAGAGGACCTACACAGGAG
EMPV1_27214	CTCTTTCTCAGTTGGGTAAGCAAGGATGGGGAGAAGGAAGCAGATTGGTGCTTCAGCTCT
EMPV1_27215	TTTTTATTGTGGAACCTAGGAATTGCCCCACTCTTCCAGGCCTCAGCCTCCCTGCTTGAG
EMPV1_27216	AATGGAGTGGCTGTGGGTGCTGCTGACAACTCTATAAACCTCGGCTCCCCTGAGCAGACA
EMPV1_27217	CCAGAACTGTGGCATCCTGGGAGCTCTTCTGACAATAAGGGTTAATGGAGAGCAGAAGCA
EMPV1_27218	ACCTGAGGATACTGAAGACATTATTGGTGAACTCGAGGTGGGCAAAGTCCCCCAACCTGG
EMPV1_27219	CCTCTCATAGCCTCTCCTTCCTAACCTCTGCATCCCAGCGTCACATCCTGGCCATGCCTA
EMPV1_27222	TCCTTTGCGTTGTCTGGTGTTGGTTGCCCAGGTTGTTGCTGAAATGTGGCGAAGAAATGG

EMPV1_27224	CGGTCTGCTTGCTTATGTGGCCTTCAAGTGCTGGCGCTCACATAAACAAAGGCAGCAACT
EMPV1_27225	CCAGGTCTAACATTGACTGCCTCTGCCTGTCGCATGAGAACATTAACAAAAGCGATTGTA
EMPV1_27226	TCAGCCAAGTCCAGCCCCCTCCACCCAGTCTGTTCTTGTATGACGTCTTCTGCTTTATTT
EMPV1_27227	ATGGCATGAAAATCTTGGGGGCCCATTTTCCAGTCTACCAAAGTCAGTGTTACTTCTGAG
EMPV1_27229	TGGATCCTATGCTTGCAGTTGCGTGGAAGGCTACCTAATGCAGCCAGACAACAGATCTTG
EMPV1_27231	AGAACATCTACACCATTAGGGATGTGGTTAGCGAGATTCGAGACAAGGCCACGCGCAGGA
EMPV1_27232	ACTTACACTACCCAGACTTCCCAGCCTACTAATTACACTGTGGCCCCTGCCTCACAACCT
EMPV1_27233	TACAGCTGAATGAGGAGGTAATGGTTGCTGATGATCTGAAAAGTGAGAAAGAAAAGCTGA
EMPV1_27235	CACGTCACAAGAAGAGACAAGGCCTCTCCGGCAATCAGTGAGTCTTTGGTGGACACAGAC
EMPV1_27236	GTGACTGGTGGCTGAAGGTCTTTGTGTCACTACTGCTGGGCTTATTCAGAGGAGTCTTTC
EMPV1_27238	GAGAAAAACCCCTTGTGGTGAAGGTTCCAAGACTTGGGATCATTTTCAGATGAGGAGCCA
EMPV1_27240	TACACTTGGTTGTATGAGGAAGTGATACCAGGAAGCTGATGCAGGGATGCTGGATGCTGC
EMPV1_27242	GGTGCCATAATCTGGGGCCACGTGGTGTTTGCCACCCAGGAGACATCTCCCTATGACATA
EMPV1_27243	TTGACACACCAGACCCCTACGTGGAACTTTTCATCTCCTCAACCCCTGACAGCAGGAAGA
EMPV1_27245	CTGCTTTTGGTTTGACGTGTGCCATCTCTCCTCAGCGGATGCAGGCTCTTGGCCCCCGAT
EMPV1_27247	AGATCAGTGAGGGAGTGGGGTACGTGATGCTTACAGATGGGAACCAGCGATGCTGATGGT
EMPV1_27248	ACACCTTCAAGGGTGACTACAGCACCAAGAAGCACGTATACGGCAATGGCTACAGCAAGG
EMPV1_27250	TGAGTACACTGTGGAGAGGACTTCCGAATGTGGTTACTTCAGCTATATCACTGCCCAACA
EMPV1_27251	ACTGAGATGAAGATGGACTCAAATGCCGCAGCCCAGAAACGGAGCTCCTGAAATGTCCTT
EMPV1_27253	CGAGTGCCAACTACGGCCAGGGAACGGGTACCAGCCACAAAGACAGTGCACCTACAGTCA
EMPV1_27254	GGAGCTGGCAAACTTTGACCTTCATGCCAAACCTGGCTACCATCTGCTTTTGTTTGAGCC
EMPV1_27258	GAAGGCACCAATTGACAACGGTTCAAAAATCACCAACTACCTTTTAGAATGGGATGAGGG
EMPV1_27259	AGTTTCAAGTTTCTACTCACAAGCTTCAGCTATTCAGCAAGAAGATAGCAACCTGAAAGG
EMPV1_27261	TACAGAGGCAATGGGGAGAGTTACCGAGGCACGTCTTCCACAACCATCACGGGAAGAAAA
EMPV1_27262	TGGTGGCCACTTATGTCTTTGTGGAGTTCTTTGTCAACCCCATGCGGCTGCGTACCAACC
EMPV1_27264	TGCGTATCGGACTCCTGATGGCATGCCTGTGAAGGACTTGCAGTTGAAGGAGTTTAATAC
EMPV1_27266	GTGTTGGCAACGTAGCCAGGGTGCAGCAGATTCTGTTGCTCAGAAAAAATGGTTTGAACG
EMPV1_27267	GTGGCCAGGGATGTCTGGGACACTGTTGGTGTAGGGGATGAGAAGATTGAAGACATGATG
EMPV1_27268	ACAGTAATGTGGAGGTAACGTGCTGACCTGAGTTGGTGTTGCTCTCTGTTGGCTCTCCTC
EMPV1_27269	TGAAAGTCAGTACATTTGAGCAGCAGAGATCAGGGGCATGGAGGGGTGGAGTCAGGTTCT
EMPV1_27270	GGCAGGTTGTCAGTATGGCCATCGAGAAGCGCATTGGCATTTTCAACGTTTTGAGTTCTG
EMPV1_27271	CTGGAGGGTTCCACAGTCATCCTATGGAATCAGGATTTTTCGCAAGTTTGCTTGTGGCTT
EMPV1_27272	GTGGATTGCCGTCAACTATGAACACTTCTGTTTATCAGCATTTATTGACCACCTACTGTG
EMPV1_27273	ACAAGGTCTGCATAGGATTTGACCCTTGGCCTAGTGTGTTGTCACTGGACAGAACGATAC

EMPV1_27277	TAGTAAAGGCACAGTCGCTCCCATAAGTGGTACACGTAGCTGGAGTGTGGCATGTTTCTG
EMPV1_27279	CCAGGCAGTTACCCCAACTACGTCCTCTTTCAATTGTAGTAGCAAGTTTTGGTGCTGTGG
EMPV1_27280	GCCAGAATGACTGGCAGTGTCCAAAGAGGAAAAAATGCTGCCGTGACACTTGTGGCATCA
EMPV1_27281	AACATTCAACGCTGTCGGTGAGTTTGGGATTTGAAAAAACCACCGACCGTTGACTGTACC
EMPV1_27282	AGTTGGAACCCCAATTGGATCCAGAACCAGATTCGCCCCCATGGCAACAGTGGGGAGCAT
EMPV1_27284	TGATGCCGAGTTTGAAGGGGAGGGCGACTACGAGGAGAGCATCTATGCCGACTGGTGGCA
EMPV1_27287	GCAGAGACTGGACTAGGAACGATGCTATGGGGGAAATGGGACACAGATTGTGCCTTCAAG
EMPV1_27288	CCTTTACCCTGTCCGTATGTCCCAGCAGGCTTGTGTTCGTTTGGTGATTGGCTCTTACAT
EMPV1_27291	CGGAAGGACTTAGTGGCCTTCTTGTTCAAGCAGTGCGGCTATGGCCTCACGATTGGCTGA
EMPV1_27296	GAAGAATGTGGTCCCCTCCCAAGACGGTTCTGGATGTGCTTTTTACACTGTGGAGTCCCA
EMPV1_27297	GGTACTTCATCGAACAGCATTCTTCCTGGCATTAGTTTAGGCTAGAGAGGCAGAGTGATC
EMPV1_27298	CACGACGGAGAACAAGGACGCGCTGGGGAAGGAAGTTCTGAAACTCTGCCGCTCTTTATA
EMPV1_27299	GATTCTGGAAGGAAGTGGTGTAATGAATCTCAACCCCAGCAACAACCTCCTCCACCAGCC
EMPV1_27301	TTGGCAAAGCAAGTGCCTTCACGAGGTGGGAAAAGCAAGAAGTCAGCAACGCCTCCCTCC
EMPV1_27302	ATTTGGAAATTAGGTTCTACTCACTTGGACATCCCAGCACCACGGACTCTTGCCGCTCCC
EMPV1_27303	CTAAAAGGGTAATTTTCGCCAGACGTAGCCCTGGAACGTTCGAAACCAGGGTGAACTAGC
EMPV1_27307	ACCCCTGCGGACTGCGCCGCCATGCTGCACCTGCTGGCTCTTTTCCTGCACTGCCTCCCA
EMPV1_27308	CAATGACTGTCATCCATGGTTGGCGGTAAATCATACCTAGACTGGTTGCTTTTTTTGGTC
EMPV1_27309	TTGAAGGAGCCGGCATTGCCTCAGCTGCGCGTAGGTCGCAGCTATGGCTGGGATTCAAGC
EMPV1_27313	AAAGGGGTTTGGGGACCCGTTTAGTGGAAAAGATCCATTTGCTCCCTCCTCTTCAGCTAA
EMPV1_27314	GAAGAACCGCGGAGTCCCTGGCGAGACTTCCAACCAGCTTCTCGAGTCCGCACTACATCA
EMPV1_27316	ATCCAGACATCATCAAGCTCTCCTGCTCCAACACCAGGGTCAACAGCATCTTGGGACTGT
EMPV1_27317	CGCCGGGACGAAAATACTGTATCTACTGGACACGGATACAGAGCAAGTACAAGGCCTGTT
EMPV1_27319	TCTTGGCCAACTTTATCTCCAGTTTTATGGCCCTGAACAGGCTACCGGTAATTGGTGGGC
EMPV1_27320	CTCGTAGAGGAGCGCTCTTCTGGTTGAGGCGAATTCAGGCAGCTGCGCCACCTCGGTCTG
EMPV1_27325	AGCACATATACCCCCAAGTTAGTAACACAGACAGAAAAGGCAAAGCGAGTGCATAGGAGG
EMPV1_27326	CATCTCCCAGACTTCAGACACTGAGTGAGAAAGAGATGCTTTGTGCAGAGCTGGCCCAGA
EMPV1_27330	GACTGCCCTGAGTAATGACTTCCTGTTGAATTTGCTTTTTCTAATTGGTGCCATGAGGAG
EMPV1_27332	GGGCCATGCTTCTACTGGATGAAAGGCAGGATGCCCACCTGCTAATTACCAACAGCATCA
EMPV1_27335	GCAGGCCTTCATGATCCTGTCTATCATCTTCTCTGTCGTCTCCCTCGTGGTCTTTGTGTT
EMPV1_27338	TGGGAACCATCTGATCCAACTCCCAGACCCAGGGGAACCGAGAGAGGCCTTTTGAAATTA
EMPV1_27340	TACACATGATGAGGGCCATAGACCTTGGTCCCAGGAAAATACACAGGAACCTCCAGTGCT
EMPV1_27341	AGATGCCAAGGAGCTGGAGCTGATGTTTGGATGCCAGGTGGAGGGAGATGCAGCTGAGAC
EMPV1_27342	GTCCTGGGACAGTTGAGAGCAATGGAAACTTTGTTCATATCCGAGTACGCTGCGATCTAA

EMPV1_27343	TTGACCAGCCACACTCCAGTGTTCTGTTTGGCGCCCGTGAGGTCGAGTTCCCCGCGCTCA
EMPV1_27345	AGTCCTGATAGTCTAGTGTAGTTTTACAGCATTGCCTCATCCAGAGCTCGACCAGCATTG
EMPV1_27346	CAGATGTGTGGAACAGTCATTGGTCCTGGGCTGAATGGTGCCTTCGTGTGTATCTGCCTA
EMPV1_27347	CTGGTCTCCAGCTCCACCAATTTCATGGTTTGGGTTTTGTGAGACAAGCTCCTTGGTGAG
EMPV1_27349	ATTCTGGTGACAGTATGGAAACGCTCCTCAGGCAGTTTGTGCAAGGCTCTCTCTACTCTG
EMPV1_27351	TCTGGACATGTGCTACACCACAAGCATTGTCCCTCAGATGCTGTTTCACCTGGGACGATT
EMPV1_27352	CCAAGGGTTCTCCCTGCTCATCACATGAACTATAAACTCTGCCTGGTCCACCTTAGCTTG
EMPV1_27353	TCTCGTGATCTTTGTCCTTCCAAGGTTAACAGCAGCCCAACTTTGCCTTTGACCCCACTG
EMPV1_27354	GCTTCTCGGCCGTGGCGCTCTGCAAGGCGGCCTAATGCCCAGTGCGGGCCCTTCGCTTTA
EMPV1_27355	GTTCAGAGCCATGTCCACGAATCTGCGCACCGCGCTCATTTTTGGTGGCTTCATCACCCT
EMPV1_27357	CACACAGTGGCTAATTCCTTCCTGTTCCTGCCTAACCGTCACCGCCTCTTCCTGACCTAT
EMPV1_27358	AATACAGCAGGTCAACTCAAAGAAGCCGTGCAGCACTAGACGTGATGTGGCTGTCAAGAG
EMPV1_27359	GGAGTACCTGAAGCAGTTCAAAAGCGAATAGGAAAGGCCAGAGACCATCAGGAAGTTGCC
EMPV1_27360	AGAGTGCCTGAATGACACTTCTTCCTGGAATGCTACTTGGAGGGAGGTGGATGCTTCAGA
EMPV1_27361	ACCAGCCATCCTGGTTTGCCTGGGACTGTGGGGGTTCCCAAGATGGGAACCGGGATTAAT
EMPV1_27363	TGAGGATTTCCACCAGCCTCCCGAATTTCCCTGCCTGAAGATGCTCTTGGCTTACCTCGA
EMPV1_27367	TGTGAGGCGTTCTGCTGCGTCCTCTGATGTGGCTCCTCCAGAAACTGACATCTCCAAACT
EMPV1_27368	GGGCGGCCAAATACATCAGGAACCGCATCATCCAGTTTAACCCAGGGCCAGACAAGTACT
EMPV1_27369	GAGAGAACTACAGTGGTTATGAAACAGGCAGTACTCAGTGAACACCAAGGTCAGTTCCCC
EMPV1_27372	GTTATAACAAGCTCCAAAAACGAGAGAAGACATGCAGGGCTTACCTTAAGGCCATTGTTG
EMPV1_27376	GAGCGGGTTTCACTTTTAGGCATGTATTGGCTCTCTGCATGGCCTTTTTCATGTTACCAC
EMPV1_27377	TGGGCGGTGGGTCTGCAAGGAAAACTAGACACTACGATGAGTTCCTCACCTGTCTTCTCA
EMPV1_27380	TCCCACGCCCGCACAGTTCAGTGACAGGGTTCAGGATTTTGTGGGTCTGTTTTCCTTTCT
EMPV1_27381	ATTTCCATGGCAGGCCTTAACATCTTTTACTCCTAGGGTCAGGTTGTAACCCACCGGGAC
EMPV1_27382	GTTTATGCAGAACCAGCCCCCCGTTGTTGCTGCCCCTTTACCAACTGATGAAAACATCGC
EMPV1_27384	GCACTTCATGAGATGCTAAAGCTGACCCACAGGTTGAAAATGTTGACCCTATCCTGTTAT
EMPV1_27386	CTCGGGCATCTCAACCTCTTTGTAAACTTTGGCCTCCTGCAGAAACAGACTCACACAGAC
EMPV1_27387	AGCTGCAGTTCTACCTGCTGTGTTTGCTGCCCCTCCTTCCTGTACCATGTCAGCCGTCAT
EMPV1_27389	GCACAACAGGCATATGAAAAGAGGCTCAACACCACTAATCAGGGAAAGCAAACCCAGGCC
EMPV1_27390	TGGAACCAGTACTATCAGAACCAGGGCCAGTGGACGCCATACTACGGGAACTACGACTAT
EMPV1_27391	ATAGCCAACCTCCATTTTTAAAGAATCCAGTTATTCAGTTAAAGCAACAATGTGCAACTG
EMPV1_27392	CAGCTCCTAAAACTCGCCTGTTCTGATGAGTATGTCCGTGAGCTTGGGGTCACAGGCTTT
EMPV1_27393	ACATGTAAAAAACGAACATTACAATTCCTGTGGTAGCGTTTTGGCAGGAACACACTGCCC
EMPV1_27394	TGCTCTTGCAGAGATGTTAGAATTGAAAGCAGACCCTACTGGTCCAGTTGAAGGAACAGT

EMPV1_27395	CTGAAGCATAAACATCACATGGCATGTTATGTCATTGTTTTCAGGTACTGGGTTCTTGAG
EMPV1_27398	GTCATTTCACATATGAGCCCTGCGATTGTCATGGAGAGTGACACGACAGGGATGCCGTAG
EMPV1_27399	TGGGATCAAAGTTGTCATGAATACTGACCTGGGCGTGGGACCCATCCGAGATGTGCTGCA
EMPV1_27401	AATATCTACCTGGGATGGGGTGATAGTGACACCTTCAGAAAAGGCTTATGAAAAGCCACC
EMPV1_27403	CAAAACCTTGAGGAAGATGATGAAGAGAGTGAAGAAGACAGCAGCAGTGAGAGTGAAGGG
EMPV1_27405	GGTCCTCTACTCAAGACCAGGATCAAAAATGTCAGCCACACCAGGGTCTTATCTGGAAGC
EMPV1_27408	AATGGCCACATTGCCAACAGGTCCGAAATGGACAGCAACACAAGCGCTGAGAGCAGTAAC
EMPV1_27410	ACCGTGTAGCTAAGAATGTGAGGAAAAACCTGTCCTTCAAGCCAGTAAATGGGGAGGAGG
EMPV1_27412	TAATACCTGGTTCCAGCCGCAAATGTCCCAGTAACTGGCCAATCGAAGCTACGCTGAGAC
EMPV1_27414	CTGGATTGCTGTGGCTTCCTGGTGGCATAAAGCTTTTTGCAGTATTTTATACCTTTGGAA
EMPV1_27417	GTCAGCCTGTCTGTTGATGCTCTGAAGCCAGGCATTGACTTCTCTCTAGCTTTGAAAGTC
EMPV1_27420	AAATTCGCACCTTGCTTATAGAGTGGGAAGGGGGAGAGCGACCTATTTTGGCTTCCTCTC
EMPV1_27421	TCTCCGGGGCCGCTTCCTGGAAGAAGTCCTTCTGGTCTTTGACGTTGTGGCCGGGGCGTT
EMPV1_27422	AACCACAAATGGGTCTGCAGCAAGGTTGGGGGCAAGGTGCTAGCCCTGATGGACTTAATT
EMPV1_27423	CTTGGGAAACAGAAATGTGCACCTGACCATTGGGAAAGGGACAGAGGTAGAGCCTTAGGG
EMPV1_27425	CGATTTTTATTGAATGGGGTGCCTGCCAGGGCCACAAGACAGGGGGGGAGGTCCTCGCCA
EMPV1_27429	GTTGTGCAGAGACTCTCCCAGCCTGCTTCCATTCCCCTTGCCCTGGCACCAAGATTATTG
EMPV1_27430	GCTGTTTTCCGTAACCGGCACACGAAGCTCACGTGGACCGCAGGAACAAGGTGGTCACCA
EMPV1_27431	GGCCATCTACGACACGATGCAGTACATCCTGAACCCCATCTGCACGTGGTGTGTGGGACA
EMPV1_27432	CCTGGTCAAGTTTGCATAAGTTCGCTGGGCCGAATGCTTGGTTGAGTTTGGTCTTCGGTA
EMPV1_27434	TATGGGCATTCTCATCAAGGGGCTGGCCAAACTGAAGAAGATTCCAGAGACAGTGAAGGC
EMPV1_27437	CCAACATTCTGTGGGTGGGGCTAAGGAGGAGATTCTCTATCATGAGGACTTGGGAAGAGA
EMPV1_27438	CCACCATTTTGCCTGCCATGTTCTTGCTCTCTTGAAGTTGGCCTGTGGGAATGAGACATC
EMPV1_27439	TGTCCACACCGTGTTTCTGAAAGAAAAAGTTCCTGAAGCGAAAGTTAAGGCTGGCCCCCC
EMPV1_27440	GAAGTATCAGCCCCCATCTACCAACAAGAAAACGAAGTCTCAGAGGCGAAGGAAAGGAAG
EMPV1_27442	CTGTGGTTGGCCCCTGGGCTCGGCACATGCTGTGCCTTCTGCCCGAGACACCGTCCCTGT
EMPV1_27443	GTCACAACCTGCCATTTGAGATTTGCTTCTCTAACGATGGCTTGATGCTGGCTCCACCAC
EMPV1_27445	CCCCCGGGTCAGTCAGTACCGGTTTTGTCAGTTAATGATAGAAAATCTTCCTAAACTCCG
EMPV1_27450	CACTGCTGCGACTGTCTTGTGTTGATACCCGTGCTAATGAGCTGACCCTCATGGTCATGA
EMPV1_27453	AACCGTTTCTGAGTCTAGTGCGTTGGAGTGAGAGGAATGAGCACGAGCGTTGCATGAAGC
EMPV1_27454	CCTAGTTCGATGGGCAACCTGCCTTTGCTCTGGATTGGTCTTAAAAGTATTACTACCTCC
EMPV1_27459	TGAAGTGGTGGTGCTAGCTAGATCACTGAGCTAGGTCAGGTGCAGAATACACTTGGGTTC
EMPV1_27462	GACTAGAGTTCCCATTGTGGCTCAGCAATTCCAAACCCGACTAGTACACATGAGGATTCC
EMPV1_27463	AGCTGTTAGGCCAGGAGGGAAAGGGCAGCCGGCCCCGCATGGAGGAGAATGAACTGTACA

EMPV1_27465	AACAAAAGGAAGACATGAAATTGTTCCAAAAGATATAAGGGAAGAGGCAGAGAAATACGC
EMPV1_27466	GGGGAAGGCCAGCTTGGTTCTCTTCTTTGGGGGAGAACGTAGAGTTTCATGCTAGATGTT
EMPV1_27468	GCCTTGTGGAGGAGTTGAGAAAAACCAATGCTTCACCCTGTGATCCCACTTTCCTCCTGG
EMPV1_27469	TTATTTAGGCCCAAGCGGAGTGGTTTCCTTGCGGGCTTGTGGTTACATGGATCCCCTCTA
EMPV1_27470	AAAAAGGTACCGTCATGGACAGTCTAGAGACAGCAGCGCCTGCGCCTGAGCTCTATACCT
EMPV1_27473	ACCTGTGTACTGCCACTGCTGGAGGCCCAAAAAGTTCCCATTCTATGATGGATCACACAT
EMPV1_27474	CTGCACGTCAACTTCAAGGACATGGGCTGGGATGACTGGATTATCGCTCCCCTTGAGTAT
EMPV1_27475	GGTGGAGGAGGTGAAGCTGGCCATCAAGCCGTATTACCAGAAGAAGGATATCACCAAGGA
EMPV1_27479	GGAGTCTAGATATGGCTCAACTCGATCCTCTGCTTGGGGTCTAAGGAGTCTGCTGTCCAA
EMPV1_27481	ACCTACCAGTTGGAAGCAGAGTTCGTTGGGGTGTCTCACCTAAAACAAGAACCAGAAAAG
EMPV1_27483	CGCTGCTGACGCAAGTGTGGAAGCGTTTGAATCTGATAGAATGTGACTACTTTGGCTTGG
EMPV1_27485	CTGCTGTTTTGCCTTGTCTGTCAGTGCAGTGCCTGTGCTAGAGGTAGAGAGGTGAGTTCT
EMPV1_27486	TGTGACACTCTCCGGAAGAGACCACATGTTAGGCCACAAAGCAAGTCTCAATAAAAGTCT
EMPV1_27488	GAGTCTCTGAGTGGAGAGTGTTACATCTCTGACATTAAGGGGAGCAAGTTAGACATCAGC
EMPV1_27489	TGATCCCTAACCCTCACCCCCATACCCTTTTACGGAAATTGAAGAAGTCCTTGGGAGTGG
EMPV1_27490	ATTTCACCGTGGAAGTGGAGATGCTGAAAAAGCCAGGCGCTCCTGACAGGACTTTCCTTC
EMPV1_27491	AGCCAGTGAAGTAAGAGATGACCCGTTTTTGGAGACGGGGTCTGTGCAGCTACTTCTGAA
EMPV1_27492	GTATTTCTACAAAGGGGGCCTGGTTTGGGATGAGAACAGCACTGAGGGCCCGTATCTGAA
EMPV1_27493	ACCTGGTCCCAAGTCTTATTCCAAAGCCAGTCCTTTCCAGACTTGTACATAGCCTTAGGC
EMPV1_27496	TTTGTGTGGCTGCTAGATTTCAGCAGCCCTGCTGTATTGATTCAGACCTGTAGCCCTGAG
EMPV1_27498	GGGCATACGAACAGCTGCAAAAATTGTCCCTAAAGTCCAGAACACTTTCCTCGTTTTGAC
EMPV1_27500	AGAACTGTTAAATCCCCCAGTGTCTCCTGAGTCCCGTAGGTAAAAAGAAAGCTGGAGCCG
EMPV1_27501	GCTGAGGAGCCCAGCCTGCCATCTGTGTGTGAAAGCCTGCTGAGATGATGCCTGAAAGAA
EMPV1_27502	ATCATGCCTCTCTGCTCTGGGGTGTTTTGTCTCTAGGCAACTGCCATTGACCCCTCAAAT
EMPV1_27504	CTAGAACTGTGTCCCCCAAACTTGAGAAACTCGCAGCTCGCTACTGCCCGTCACTATAAG
EMPV1_27505	CCAGTACAGGACCTCCTGAGGAGAGAGCCATTGTTCAATTCCAATTCAGTGTGGGTGACA
EMPV1_27507	CTTATCTTACTAAGACTACTTGAGACTGTAAGCTCTGTGAGGGCAGAGACCATGGCAGTC
EMPV1_27508	AATGCACCTCTGCCTGGGAGGTCCAGTCGCCATATGATTTCTAGCTGCCTCCTCAGTTAG
EMPV1_27509	TCCTGCCCTCCCTGCTTCTTGTTTCCCTGTGTGTTCTGTGTCCCCACTGAAGTGATTACA
EMPV1_27510	AGGGGCCACTGGGCGTGACAGAGGAACTACACATAATCAGCTTCACGGTCAAATACACCT
EMPV1_27511	GGCTAGCTTAGCCTTGAGCCAAGGGAAAATGAACCTCAGCCCATTTGGAAGGAAACGGTA
EMPV1_27514	TTTGCAGCCAAGGCAAGAAGCAAGCAAAGGGCTTGAGAGGCTTCAGGGAGCCCAAGAGCT
EMPV1_27517	AAAATGCCGGACCCTTAACCCACCCAGCAATCTCATGGTTCCTAGTGGGATTCCTTTCTG
EMPV1_27519	CGATTGCTAGACAGCGAATTCCATATCGACTTGGTCGAGTAGTTGATGAATGGCTACTCG

EMPV1_27521	CTACACGTAATCACATGTGTGCACACATACTTTCATTCAGGCATGCATGCTCACACCCAT
EMPV1_27523	GTGTTGTCTCACCCCCTGTTCCATTTTCCTTTCGCTACTCTCCTCTTCAATCACGTTCCC
EMPV1_27526	CTATTACAAATGTAATTCTGTTACAGAGTTGTCTGCCTTCTCAAGGATGATCTAGGGAAC
EMPV1_27527	GAGTGTGGGGTGTTAGACAGGAGAAGGTCTTTGGAGAAGCAGTTAGGAGGGGCAAGATCA
EMPV1_27529	CCAGCGAAAGAGTATTAAGACAGTGACCTGGGACACATTGAGAATCAAGGCCAAGACTGT
EMPV1_27530	TGTCAGGAGTGGGGGGGTCTGTGCAGCCAGGTAATAACCCAGCTGCTTTCTGCTCCTCTT
EMPV1_27531	GATGTGCATATGAGGGTAATGAGGTGTTAACGCTAGGAAAGACTGGGTGAAGGATACAAG
EMPV1_27532	GGGTAAGGAATCCAGCATTGTCTTGAGCTGTAGTATAAATTGCAGATGCAGTTCAGATGC
EMPV1_27534	GTTTGGGTCACGAATAGTTCCTCCTTGTGAGTAACAACATCGATGGGATCCTGGAGGCTG
EMPV1_27535	GCAGCTGTGTGTGGGCGATGGGCTGGGTGATGGGTTCAGAGTTCCTTTTTTACCAAAAGT
EMPV1_27538	TAATTCGTCATGTAAACGGTGCGTTCAGCGTGGATGGCGAGGGCGAGTATGGCCAGGCAT
EMPV1_27539	TAATTCATCTCTATTGGCTTCCATCCTTCTCCTCCCATTCTCCCAGGGGTCTTTCCTAGG
EMPV1_27540	AAATGAATCCGACTAATATCCATGAGAATGCACTTTCCATCCTGGGCCTTGCTCAGTGGG
EMPV1_27542	TTTGCTTTTCTAGGCATTGATCAGGGAAAGGGATGCTGGTTGGGTAGGAGAAACCAGAAG
EMPV1_27543	CAGCTGTGGACGAGCCAACGGAATGGGTGTGATTGGACAAGATGGTCTGCTTTTTAAAGT
EMPV1_27545	CTGTTGGGATAAAACTCCTGTTAAAAACTTCTAAAAACCCATGACCAACGCGAAAGGAAG
EMPV1_27546	TGAGAAAACTGGAGACCTGAAATGCTATAAAATACCCTGAGAGTGGACTTCCCTGGTGGC
EMPV1_27547	GACCAATGTGAAAGGAGCTGGGAATGGGTAGTTTGGGCACAGAAGGAAAAGCCAGTTTCC
EMPV1_27551	CCTCTAAGATGAGGATTTCACTCTCCAGTTTTATCCAACCTAGTATTAGAATCCCCAGCC
EMPV1_27552	GCATGGTTGGTGCCGAAAGAGACAAAGCAGGATGAGAAGGCAGGTAAGCGTCTAGTAAAG
EMPV1_27553	CAGCATGAAGAAAATGCAGCAGAACGATGTCTGCCATCACGGGCACTAACCAGACAGCAA
EMPV1_27554	TTGGAGGCATGAATGTGTTTCTGGAGCAGAGAGTGGACTGGGGAAGGTCCTGAGTGACAT
EMPV1_27557	GCGCTGTCCTCCGCCAGGTCATTTCAGGAAGATTCCGCAGTTACTTTGATTGATATGTCC
EMPV1_27558	TACTAGGCCCTTGGTTCACAGCAGAGAGTGTGAATTTGTTAGTGCCGTGTGCTGGAGGGC
EMPV1_27560	TGGTGGTGATGTCCTATGTAGGGGGATCTCTGAATGCCTGGATATTTACTGGCTGTGTGC
EMPV1_27563	TACCACGTCCACGAGTGTGTTTAATGTCGTCCAAGTCGAAGAGGGAAGAGGGAGGCTATG
EMPV1_27565	AAGCTGGAGGGCATGTTCAAGGACATGGAGCTGTCCAAGGACATCATGGTCCACTTCAAG
EMPV1_27566	TTCTTCCAGTCGTCCTGCTCCACACACTTATCCTAGACACGACAACATGCGGACTGACAG
EMPV1_27567	TGGGCAGCAAGGCTGGCACCGGTGGGTCTTCAGGTTACCAGTATCTGCGCTCAACAGTGA
EMPV1_27570	GAGCCGGGGTCTTACCCTGCAAGCGCTGTATAGTCACGTCTCCTAGTCCTTAATTGCTAA
EMPV1_27571	CAGTAGCTCTAAAACAAGCCACCTGAATGCTTATGACCAAGAGGGAAGTCAGACTGTATT
EMPV1_27572	CAGAGACATCATCGACACGCCAATGGATTTTGCTACCGTTTGAGACACGTAAGAGCCTGG
EMPV1_27574	GACTGACACTGAAAGTGGCGTTCTTCCCTACCCTCTACCTGCCTGAGTGTTGTTACTTAG
EMPV1_27576	TTAGCCCTGCGGCCCCACGCACCAGGGTAAGAGAGACTCTCGCTTCCTGCCCTGGCCCGA

EMPV1_27577	AAGCTGGTGTCATGGCTTTGGCTAACCTTCTTCAGATTCAGCGTCATGATGATTACCTGG
EMPV1_27578	CAGTTCATGAGCAACATAAGCAAATGGACCTCAGCAAGGACCAGGGGTGACCAGCCACTT
EMPV1_27579	AAGAAGGATCAGGTGGCCCAGCTGGATGACATTGTGGACATTTCTGATGAGATCAGCCCT
EMPV1_27580	CAACCATCCTTTCTAACAATGTGCTTGTGAAGATAGCCCTAGCCGTCCTAGCCAGGGCAG
EMPV1_27582	CTAGAATCTTACAGCCTGCCAAAACTGAATCAAGTAGAAACAGACCAACTGAACAGACCG
EMPV1_27583	CTTGAAGTCTACTGAAAGTCACAGAGGAAGAAGTGGGCAGGAACCATCTCGTGATTCCGC
EMPV1_27584	AGCTTCCTATTACCTGGGCTTAGCACACTTGGCTGCTGGGGAGTATGAAACAGCATTAGC
EMPV1_27585	AGCTCCAAGAACCTCGGTGGAAAGTCACCAGGCAAACGCTTTGGCATCAAGAAAATGGAG
EMPV1_27586	TGGAGACTGAAGCAGCAGTTCCTGTTAAACAAGAACCAGAGACGGTATCTACTCCAGCAT
EMPV1_27587	TTTTTCTGTTTCAATTACATCCAATTGTGGATGAGCCTTCATGTAGATATGAACAGCTCG
EMPV1_27588	TCACGCGTCCTTGCCTCTTTGTCCCATGTCCCCTGCAGTCAGCTCGTTGAGCTATCTAAT
EMPV1_27591	AACCTAAATGTTCTGCCCACCCTGTTGGTATAAAGGTATTTTGAGCAGATTGTGAACAAG
EMPV1_27592	CTGGCTGGCACTTTTCCGCCTTCCAGTTGCTAAACTGTATTTATCTGATGGATTCCCGCC
EMPV1_27596	GTGATGAAAATAGTACCGGACTTCTGTGGCCTTCCTCCTCCAAAATCCATAACCCCAGGC
EMPV1_27597	CTTAGGGAGTATTTTAGTTTCCTTCTCGAGCGTCACCTCACACTCCCCACGTACCAAGAG
EMPV1_27600	CCCCACATCCAGGGGCCATGCATCTCCCCAAGGTCCCCACCAAGGGCGTCTCTAAGAAAT
EMPV1_27601	GATCAGGACACTGAAAGGGAAGCTGGCCAGGCAGCACCCGGAGGCCTTCAGCCACATCCC
EMPV1_27603	TGCTTAGAGCTGACAGAAGGGAAAGACAGAAGTATACGCATGGGAAGTGGGATCTAGGGC
EMPV1_27604	CTCATGCAGATGACATCGAGGTGTGGCTTCTTAACCTTCGCTGTTGACGACTAGAGTTGT
EMPV1_27606	GCTGATGCCAGCAACCTCACTACAATTGACATGAGCCACAATCAGATCTCAGTTTGTCCC
EMPV1_27607	TCAGTGAGGATCAGAAAATGCCACAGCTCGGAAACAGAAGACACCCGCTCCTGAGTGGTT
EMPV1_27610	AGCCCCACAGAGTGTCTGAGGCAAGCACAGTGTGGACAGGATTTCCAGTGTAAGGAGACA
EMPV1_27612	GTCGACTGGAGCCATGTCAACAAACCTCCTTATCCAGCTCTTGGAGGGAACATGAAGAAG
EMPV1_27613	GGAATTCAGGAGTTCCTGTTGTGGCTCAGTGCATTAAGATCTGACACAGTGCCCATGAGG
EMPV1_27614	GCTGTGTCTCTCAGGAAGTTCTGTACATTTTCATGCCCAGAAGGCATTCAGGATAGCAGC
EMPV1_27616	GGGGGGACAAAGCAGGAGACATAACTCTCTCAGACTTCAGACAATACTAGAAAACTACAG
EMPV1_27619	CCCACATCCTCACAGAGACAGTGTTGGGTACTTAACCTCTGAGCCACAATAGGAACTCCA
EMPV1_27622	ACTCACTGGAAGGTGTACATTATAGCCAGACACCAGAGATAATAACGTGCATGGAGAACG
EMPV1_27624	AAAGCTGGTCTCTTATCAAGAATACCTGCCCACCCAAGGATGGACCCCAGGTTATGGAAG
EMPV1_27627	TTGGCAGCTTAGAGTGAGAAACAGCAGTTTTTGGAGTATATCAGCCCAGTCTGACGGCCC
EMPV1_27630	TCTGAAATTCTTCCCCGATATACTCAGGATCGCTCTGGAGGTGGGTCTCGTGGTTGTACA
EMPV1_27632	TGTCTGCCAGGGGTGAAAACAGGGACAGCCGATACCTTTGTACCATGAACTTGCTTTCCC
EMPV1_27633	ACAGAGAGGGTAGCAGGGAGGTACTCAGTGACCAGGACTTTCCCCTGAAGCCCAGAAGAA
EMPV1_27636	AGATGCTTCTGTAGACATGAGAGACCCTAGGTACTGCCTTCTCACTCCTGTATCCTCAGG

EMPV1_27637	CCCGCTTCCACTGGAGAATGATGATGGTAGTCGGGTGCCAATTCAAGGTTCTGGCATATT
EMPV1_27639	GATGCCCTGGCTGGGTGGTAGGTTTTGAAGTTTCTAGTCTCTCTGGCTCACCAGCTACAA
EMPV1_27640	ACAGGTGTTACTTCTCTGACTTTCAATCCTACTACAGAAATCTTGGCCATTGCTTCAGAT
EMPV1_27642	GCATCTATCACCAAGCAGCATCTCTTTGCTTCAGTCCACGATGCTGTCATCTTTGCCCTC
EMPV1_27643	CCCCAGGGGACAAGCTACCACCCCCCACTGCCGATGCTTTTCTTAAAATTTGTGTTTTTT
EMPV1_27644	CCCAAAAAGGGGTCTAAAAAGGCAGTGACTAAGGCCCAGAAGAAAGATGGTAAGAAGCGC
EMPV1_27645	TCCAAATAGGTAGGACTTGAGGGTATCTTTTCTGAGAGGTTGCCTCACATTTTCACCCCC
EMPV1_27646	GTGTTGCCAAAGGATCAGTTCTGAACATAGCCAAGGCTCTATATCCCCCTGGTGATTTGC
EMPV1_27647	AAGACCTGAGGCCTTTAACGCCCAGTTCCTGAACGTTGACAAGTTGCGATCTGTCCTGAA
EMPV1_27648	GAATGGGCTCTTTCCACGCCATGATAAACTCCTCTGTGCACGTCGTCATGTACCTGTACT
EMPV1_27650	CCCACTGGTTGCCAAGAGATTGCAGAAGTAGAAGTTCGAATGATAGACTTTGCTCATGTG
EMPV1_27653	AAAGAAGCATGTCGGGGGAATCAGGAGCTTAAGACCTACAATAACAAACAACCTTTTCAG
EMPV1_27655	AGGCGATGGCAGGCTCTCTCATGCCGCCTTCGAATGTCGTCTGCTTCCCGCACAGAAAGG
EMPV1_27658	GCAAAAGAATGAGCTGCTGGCTGGGATGCAGGGCTGTGTTCTAGACCTCTCTGGATTCTA
EMPV1_27663	AGAGTCTGAATTTACCATGGACAACTTGATCACCCTCAGATGGGATGTGTTTAGTGCAGG
EMPV1_27664	TGCGTACCCACCAGGAACTCAAGTCGTCTATGCTGCAAATGGGCAGGCTTATGCTATACC
EMPV1_27666	TCACCAGGCCACAAGCCTGGTAGAATTTGCAACCTCTATGTTTGAGTTATGATCAACCTA
EMPV1_27667	AGGGGTGACAACATCTGGTTTCTCCAAGCACCTGTATCTCCCACTCATCACCTACCAGCA
EMPV1_27668	AGTGAGCATGTGGGAACCACTGCCACCTTCTCCATTGGCTCTAGTGGCAATGTGGTCTAT
EMPV1_27670	CTGAGAGATTTCAGGTGGGCCAGAGGCTGAGCTCCTTTGGGAACAACTTTTAAGGGAATG
EMPV1_27671	GAGCTTTTTAGAGACTGAGTAATCAACTTCCATGATTTTCCCATGCAATTCCACTTTACC
EMPV1_27672	AGCCTCATCGCTGACATTTTCTTGCTGGTGCCCCCTCTGATGAACCCCATTGTCTACTGT
EMPV1_27676	GATGGAGGAGCTGAGGGACCGATTTCCCTACCTGAGTGAAAGCTACTTAATCACCACCGA
EMPV1_27677	GGCCAGCTGACTACCGATGTTACAGCTTCTGAAATGGGGCTCAAGTCCATAAATTCCAGC
EMPV1_27678	TCTCAGGCTGGCTTCTCCCTTGTGTTGACATGGTTGCCAGAAAAGTCCAAGGAAGCTTGC
EMPV1_27679	ACTTCCGCATCGAGCGCGGTGCCATCGAGTGCGACAACGAGAGCTTCGAGCTGGGCGTCT
EMPV1_27680	GTCGGCTGTCAGAGGATCAGTGACGATTTCTGCTTCAACTACACTATTATGAATGTGTTC
EMPV1_27681	AGTCTCCAAACCCCGGTCAGTCCCAACACCGGAGCGCAGATTGCCCAGTACGAACGTAAA
EMPV1_27682	TCCTGTGTTGTGACCTCATGGTTTAAGTGGGAATAAAGATGAGTATAAGCAGTGATGAGG
EMPV1_27684	ACCCGAAGTCGCCACCACGTGCTCTGAGGTACGCATCCTTGGCATTGCTTGTTTATGATT
EMPV1_27685	TAGCTCCCCTATACGAAGACGAAAAGATACAGAGTGCCCTTGCTGATATTGCGCAGTCAC
EMPV1_27686	GAGGACTGCTGAGTAAGCGCCGCCTCTGCGTTCCGACCGAGGCACGAGGCCAGTCAAGCA
EMPV1_27688	CAGATCCCGATCAGTTTGTTTATAAAACACAGCCTCCCAGGGAGCAGCCCGACACATTTC
EMPV1_27689	CTCAGTAGCCATGCAGAAGCGAATATTGGTAAACCTCAGGATCGTGGAATGCAAGCTGGC

EMPV1_27690	CAGAGAGGATGGAGTGTAACATGAGCAGTACAGAGTCTTAGGCAATTCATGAGGACTGCT
EMPV1_27693	GTGCACTTTGTCGGAAGTCAAATAAACCAGGGGCGTGCAGTGAGGTCAGCCCTTCTTTGT
EMPV1_27694	TACGAAAGAGCTGGAGGACCTGGAGAGGTGGAAGGAGCAACAGAGGGCAAAGCCCATTCA
EMPV1_27695	TGCCCTGGACCTCAAGGTGTTTGATGGGAAGCCACCGCCCAATGACAAGAAAAAGCGGAT
EMPV1_27696	CCAATCCCCCAGCTTCAGGTAACCACTAGTCTACTTTCTGTCTCTATTTGCCTATTCTGG
EMPV1_27700	AGTTGAGCCCTGTTCTCACTGGTGCCAATGTTGCACTGATGCCTCTCTCTATGATGAGGG
EMPV1_27701	CTAACGTTTTGCACAGTGATGGAACCAGGTAGTAGTGGGGCTGTTGCCCAAATGCTGCTC
EMPV1_27703	TAGTGCAGTAGGTTAAAAATCTGACTGCTGCAGTTCAGTTTGCTGCAGAGGTGAGGGTTT
EMPV1_27704	GAGTAACACAGAACCACACTGCTGGCCGAGGAGAATGACATGATTACCTCAATGCTATTC
EMPV1_27705	GTTCCTCCTCTCAGGCCTAAGGTCAAGGAGGCGCTTGGTACATGATATATAGGTCATTGG
EMPV1_27707	GATGGAGACCCAGACCCAGCAACCTACGGGAATGGATGTGCTTCTAACAACAGATCTGGG
EMPV1_27708	CCCCGTCTCTGTGCCCTGATGGCTTCTGCTTCATGGATCATTGGTTTTGCCAACTCCTTA
EMPV1_27710	CAAATATCTGAACTCACCAGTCTGACCAGGAAAAACTAGAGGGCTCTCTCCAGTCTGCAC
EMPV1_27712	GTCCTCATGTACTCTGTAATCCACCTTTCCCTTCATTCCTGTTCTCAGGCAGCCATTGAT
EMPV1_27713	AAGGAGGCTGCTTCTGAAGTCCGTGCAGCTCAGTGACTCTGGAAACTATTCATGCTACCA
EMPV1_27714	CCACAGGGTTGTCCTGAGTTAAATGAATTAATGCACGTGAAGGGCTTTCAACAGTGCCTG
EMPV1_27715	GTTGAATTGATACTTAGAACATCTGTTATACAAGGTATTCACACAGACCACAATACGCTG
EMPV1_27716	TCACACATAAGAGCTGATACCTGACACCTGAGAAAAGAAGACACGATCCCAGTGGACCCG
EMPV1_27717	GTCTCCCGGGCATTGAGGCAGAAGAAAATTTTGAAGAAGACTCACACAGGCCATTAATGA
EMPV1_27718	GAAGAGAAAGCGGACAAGTATCGAGAACCGAGTGAGAGGCAACCTGGAGAGCATGTTCCT
EMPV1_27720	AGGACTCCATCCTGGCTGTGAGGAAATACTTCCACAGACTCACCCTCTATCTGCAAGAGA
EMPV1_27723	AGTTATGCTCGAGATGAAGGCTCTGGAATCTTCGGACCTCACCGAAGTCATGGTTTACGG
EMPV1_27724	TTTTCCGAAGCAGAACCCACTTTGACCCCATTAAGAGGCAAGCCTAGCACATGTATCCCC
EMPV1_27727	GAAGATCTGGATCATGGCGTTCTGGTGGTCGGCTATGGCGTTGAAGGAGCACACTCAAAT
EMPV1_27733	CAAGTTACCCAACTGCTAATGAGAAACTGGAACAGGATGTGGGAGGCAGCTCAGTGTCTC
EMPV1_27735	CTGTCTTCAACTAGACCACTGAGAGCCCTTGGAGAATATTACACTGGTCAATACTACTGC
EMPV1_27737	ATCAAGGATCCGACTTTGCCAGCTTCCAGAAGAATTCATGGTTGCCCTCTCCAAAAGGGG
EMPV1_27738	GATCCCTTCTTCTCCTGGGGCTGGGAAATCCAAGATTGCAAAGGCTGTAAGATAGATTGG
EMPV1_27740	AAGTCTGGTGAGAGTTGGGGTGGGTGTGTGGTGACTCAGAATGTGTAAACCATCGGGTAG
EMPV1_27742	GACTCTAATCAAAGTTTCGCTTGCTGATGATCTTTTACGCGGAACGCTGCCAACTGCCAG
EMPV1_27745	CAGAATGAGAAGGGGGCTAATCAGCAGATTATTGTGGGGACAGACTTTTTTCTTTCGCTG
EMPV1_27746	CCACTCTTACTAACACCTGAGCCATGCTACCCGTGTGCGGGTTACACAGGATGAATCACT
EMPV1_27747	ACAGAGAAGGCATTTTTGTGGTTGATGAGCGGTCACTCTTGTCTCCCAGTCTGTCCCCAG
EMPV1_27749	TTCTGGGTCAAGGAGTCTTGGATGGGCCTGAGATTCTGCATTTCTAAAAAGTTCCCAGGG

EMPV1_27750	GCAGATCACATCCATCAGTATTGAACCAGGAGTCGAGGTTGAAGTCACCATTGCAGATGC
EMPV1_27752	CCTCATCACACTCCCCTGTGCTCTGTTTGGAAAGCCACGGATGATTAAAGAAGTCACTGC
EMPV1_27755	TCCAGATGTGCATCTCAGCCCCGCCTCTGCCCAGAACTCCAGGCTGGATCACCTTCAAAA
EMPV1_27758	GAGGTGGACCAATCTCTAGAACCAGGCAGGGACCATAGGGCAGATGAGACGAAACTGCTT
EMPV1_27759	ATGACGCAGAAGGGCACAGCACTGCAGAACTACAACAAGCTGGTCAAATGCATCAAGGAG
EMPV1_27761	AAATGCTATCTGAGTACCTAAGGGGGCAGATGCGTGTTCATGTCAAGGAACTGGGAGTGG
EMPV1_27762	TGCCTTAATCTCTTCACTAAACAAGAAGGGGTGAGGAATTTTAAATTGGAACAGGAGCAA
EMPV1_27763	GTTCTCAGATGGTTTCCATGGGGAATAACTAAAATGAGAGACACATTCTCTTCTAGAGGC
EMPV1_27764	ATCTTCACCGATGAACAGCTCGAAGCCCTGGAGAACCTCTTCCAGGAGACCAAGTACCCA
EMPV1_27765	CATTTCATTTCATCAAGAACGAGGACATCAGCTGCTAAAGAAGCATTTGGATACTTTTCT
EMPV1_27771	CTCCTTAGCAATCCTGACTCATCAAGGAAGGCTGCCTGGAGGAGGAATGTGGTACAAGTT
EMPV1_27772	GAGAGCAGCTACATTCGGCTCTGCAGGTGGGTGGATGAAGGTTTACCCTGGAGACTTAAA
EMPV1_27773	TACAACGGCCGGCCTACACCACAGCCACAGCAATGGTTGGATCCTTGACCCACTGAGCGA
EMPV1_27774	TGTCTGTAGAACTCCGACCAGCCACGTTTACATGTTTGAGAATGGCAGTGGGGACTCGGG
EMPV1_27776	CACATTGCCTAGAAGCCATTGATAGTTCAGTGGAAGTTCTGTAAGATGTGCATGTGCTAC
EMPV1_27780	TCAACTCGGCTCAGGGCTTCGTCTCCTCTACCTGGACATACTACCTGCAGACTGACAATG
EMPV1_27784	AGGTTGCAAGACGCCATGCGAGAGACACAGGGACTGCGCAGAGTGTAAGGCCTTTGGGAC
EMPV1_27786	GGAGGTGGATCACAGCACTAGATGGAGAGTTTTGTGACATATGATGGGTGGGGATGTTGC
EMPV1_27787	CATTCTGCACCTCCATCCCCTGGAGACTGTGATTGAGTTGATATGTTGTGGAGACTGAAA
EMPV1_27788	GATGTCTGGAGGTGTGGCCTTTGGGAGGTGATTGGGTCATGAGAATGGAACCTTTTTGCA
EMPV1_27789	GTCTCAGACAATGTTTGGGACATGCTTATGATAAATATTTCTTGTCTATCTGAAATTCAA
EMPV1_27790	AGAAAGGCGCACGGCCACTGACATGCATTCACTTGTCTCTTCGCATCCTAGGACATGACA
EMPV1_27791	TTTGGGCTCCATTCTCTTCCCCAGTCCGGAGACCTGCTTCAGATGCAGCGAGAGCCACGA
EMPV1_27793	GGACATCATTACTTAGTCCTCCACCTGGCCCAATACAGGGTTTCCTCTCAGCAAAAGATT
EMPV1_27798	ACCTCACTGATGAAAAGGCCTCCAGGTTTTTCACCTTTCCGATCAGTACAAGTGGAGAAA
EMPV1_27800	GAGAGTGGAGGGCAGGACAGTACCTTTGAGTGCAACATCTGCCTGGACACAGCCAAGGAT
EMPV1_27801	GAGAGGCGGAGTTGTGGGCAGGGAGGTAGAGAGAAACGAAGCTCGACTGTAAGCGCGTTT
EMPV1_27802	GCCCTTAACTAACAGGGCATGAGTCTAAACCAAGGGATAAAGAGTGAGGGTGAGAATCCA
EMPV1_27803	AGGCCTGGACTAAGAACCAACTCTGAGTATGTGAAATGCCACCGGCACCAAGTAGGTAAC
EMPV1_27804	AAGTCCCCTCCTCACTGGCTGCGTTCTCACTCCAGTAATGCGTAAAGGACAATTTCGCTG
EMPV1_27805	AAGAGGATCAAGCACTGCCACAGTTGTGGGTAGTTGTGGTATAGGTTGTAGGGGCAGCTC
EMPV1_27806	GGTACCTCGGAAAAATAAGTATAGAACTGCCATATGACCCAACAATCCCACTCCTGGGCG
EMPV1_27808	AGAAATGTGGACCCCTGAGAAATGCATAGTGCAAACCAGAGCACTCTGGAAGGAGGCCAA
EMPV1_27812	AGGATGTTTGTTCACTGTCAAATCCCAGCTGACCTGTGAGCCTTGCCACTCTGAGGGGAT

EMPV1_27813	TTAATGGGAGAGATGTAGGCCAGCGTCCCCACGGAGAACGTGCTCCAGTTCCACTACATG
EMPV1_27814	TGTCCAGGACCGCGCAGAACTCGCCCAGGAGCGTGGGCGGCATGTCGAACAGCAGCGTGT
EMPV1_27817	ACCCTACCTGACCTTATATGACTATAAAAACAGCCAGTGGGTCCAACTCCTGGGCTGGCA
EMPV1_27818	GGCATTATGGAGGAATGTGAGAATGAGGTCCTGTGAGAGCACAGATAAGGGAGTGACTGG
EMPV1_27821	TGCTCTACACGTTCCAGGCCGACTTCCAGGGCAAGCTCTACCCCATCCTGGACACATGCT
EMPV1_27822	TCAGCCACTACAGCCTGGCTGGGTCCTGGAGCCACAGGATGGGCACAGCTCATGTGTCTG
EMPV1_27823	GGCACAGTGGTTTATAACCATTAATACAGATCCATTCCTTCTTTGTGGCTGAGATAGGCC
EMPV1_27824	TCGCCACAGCTGAAGGTGGATTGAACAGTGATTCCAGTGAGGAGGAAAATAGCAGAAATG
EMPV1_27826	TGGCCTCAGTGATTTGTGGGGAACCTGCGTTGGAACAGGTATGCCAGTGATGGCTAGTAT
EMPV1_27830	TGCATGTCCTAAATTACCATACAACAAGGAGACAAGCCCTTCTTTCTACTTTGCCATTTC
EMPV1_27832	ACAGAGGGTGGAAAGAATGGTGAAACTTTCTGACACCGGCACCCGACTGTCACGTAGCTT
EMPV1_27833	AGGAATGCGTCAAGACCTCTGGCCCCTGTGCTCCCTTTCTTGGCTTCTAGACTTGGAGTC
EMPV1_27834	CAGCCTACAGGGGGAAATGTTCTTACTATTCCTGCAAAGGTGTGGAATTGTCTGTCCACT
EMPV1_27835	TGTTCAGAATGATGCTCCCAGTTTTTGCCCTTCAGCGGCCCTGCCTCCCGTCCCAGAGAA
EMPV1_27837	GGGCTGCATGACCATGTGTGCACGGTTTTATTCTGTGAATGATAGCAGCATCTGTCTGTG
EMPV1_27838	GTCGATATAACTGAACTTCACAGTTGGATTACTCGTTCAGAAGCTGTGTTACAGAGTCCT
EMPV1_27843	GGTCCTAGTGTCATTTTTCAGCAGCTTTTTGGACTCCCAGATCCACTGCTTGATGGTGTC
EMPV1_27847	AGTCAGTCCTCATTTCCCCCTTATCGTGGGCGCCTCTAGGATCATACATGGGATCATGTT
EMPV1_27850	TGCCTTCCTTGACTGTAGTATCGTCACCAGATCTTCTTCCACTCACTGTGTCTAAGTCCC
EMPV1_27854	CGCTCTTACTGTGATTTGTACCTGAAGGACACCCTATTCTCTTCATTGTTAAATACTTCC
EMPV1_27855	TGCAGGACACACCCAATGCCTGTGCCACTTTCAACTTCCTGTGTCATGAAGGCCGGGTGA
EMPV1_27859	TTGAAATAGCCGGTATGAAAGTAACCACGGGTAAGGTGCTTGACTATGAACACCAGGCCC
EMPV1_27862	TGAGTTGGAGGCAGAGCCCAAGCCAGACCAAAACCATCTGAAGCTCCTTCCCTTGTTCTA
EMPV1_27866	ATCACCAACAACTACGGGAAGCTGCTGTTCTGCACGGCCAGGACCATCTTCTTACAGTGT
EMPV1_27867	GGGTAAAACCCCCAATGGACTAACACCTGCTTGTCTTTTCTCATCACAGGTCTCAACCAC
EMPV1_27869	GGGTCAAAGAATTCGGCGAGGAAGCATTGGTGTGTGGAATTACATAAGCCCTAAACTGGG
EMPV1_27870	TATAACTTTACTATGCGGCACAAGTGTCAAGATTCCCCAAAGCATCCCACCCTGACTCAG
EMPV1_27871	GAGTGCAGGTTTTTCCACACTTCAAGCAACTCTATGCAATACAAGCTGGGGGTCCTACAA
EMPV1_27874	GCATCCTCATGGACACTGTTGGGTTCTTAACCCATGGAGCCACAACAGGAACTCCAGAAC
EMPV1_27876	AGAAACACAAAGTGTGCTGGCCTCAAACAGATGAAGAACCCAGGTGGCATGTCTCCCACG
EMPV1_27879	CTGCTCTTCCAGGAGCTGCAGTCGATGAACTTTATCCGCAGGTTATAACTGTGCAAATAC
EMPV1_27881	GTATGCCTTCTTGCCAGCAATAGTTCATTATAAAGTAAGCCAGGTTTTTGCCAAGATCAG
EMPV1_27882	TACATTTTGGTAGTGAACCAACCTTGCATATAAGCTGACCTCGCCTTTTGGCTTTTGGAG
EMPV1_27885	CTAATATGAGTGATGGACCTTCACTATCTTCCAGATCTACGGGCCACGTTGGGGTGCCCT

EMPV1_27886	GTCCCATTTCATGCCTGTCATGTTCTCCCTGACTTGGGACATGGCCTGTCGGATCATCTT
EMPV1_27888	CATTAGGCAGGGAAAACATACCAAGACTTAAGCAGTAAACGGCAAAGAAGCTACAGTCAC
EMPV1_27890	TGTGCAGATTAGCAGTGTTCAGACTGACGGCATTGGTGATGGTCCCTGGCAATCCAACTT
EMPV1_27892	GCCTGTGGAGCCAAGTGCAAGATATTGGTGAAGGATGAGGACCAAAGGAAACACGGTACT
EMPV1_27895	TCCGCATTTCCTTCTCGAGGGATTGTTGCTCTCCTTTGGCTTCGTGATCTCTGCGCCAGA
EMPV1_27896	AGTTCATGACTCCCCGGAAGCTGTCCTACGACTTCGAGTAACCCTCTTTTATGCACACGG
EMPV1_27899	CATATGGTCCTGTAGCCTCACTCCTGGGCATACATCCAGAGAAAAACATAATTTGAAAAG
EMPV1_27900	GAGGAGGATGAAGGCTTGGGAGTTGATCTTTCTTTTATTGGCTCTCATGCTTTTGCTCGG
EMPV1_27901	GCCTGTATATGGGGGGAGGGCAGAGATGGAACAATGAGGGTTGTGATGATAGTGAATAGC
EMPV1_27902	GTCCTCCGGTGAGCAACATCCAGGACAAAATAAGCTAAGTCACTCTGAGAATGGTGTTGA
EMPV1_27903	ATATGCGTCCCCTGCTCGGTCTCCTCCTGGTCTTCGCCGGCTGCACCTTTGCCTTGTACT
EMPV1_27904	GTGGAGCCACTAGGGGGAACTTGAGGACCGAGAGATGCTTCTGCTTCCTGTTTTCATCAC
EMPV1_27905	GGATACCAGTCGGGTTTGTTACCACTAAGCCACAATGGGAATTCCCTGGTGTGGTTTTTG
EMPV1_27906	TAGGAGTTCCTTCTGTGGAGCAGCGGGTTAAGGATCTGGTGTCATCTTTACAGCAACACG
EMPV1_27907	CCTCTGGGTTAGTGGCTAGAATCACAGTCCTCGGGAAAAGGGTGTCTTGATCAGCCATGA
EMPV1_27909	TGGGGAGTGCCTCCTGGTGCCACTGTCATCGGCCTTCAGAGCCTGGTGGATGCACCCACA
EMPV1_27910	ACCCACTACCAGCTGATGCGCAAGAGTCAGATTCTCATTTCCTCTTGGTTTCGGGGCAAT
EMPV1_27913	CCTACCTAGAGGAGCTAAATGCACAAGTGGATGTGACCCAGACAGAGTATCACCTTCACC
EMPV1_27915	GGATCCTGGCTGGTCTGGGCTTCGACCCTGAAATGCAGAATCGGCCCACACAGAAGTTTT
EMPV1_27916	TCTCCAGTAGTTTCCCACCTACTTCCGGTTAAAGTCCCTGTGTGGGATATGAGGTCCTTG
EMPV1_27918	TTCCTGGTGGACATCAAGCCCTCCTTGAGTTTTGATCTCATCCCCCTGCTGGACCCCTAT
EMPV1_27920	GTCGAGGACCAGAAGTTTGTGTTTTTGAAGAACCTGGTGTGGACCAGCGGCATCTCCGCC
EMPV1_27921	AGTATGCCCTGTGCAATATTTGGGACATACTTATACCAAAAGGTATTTGTTGTTTATCTG
EMPV1_27922	TTTCCTATACTGTCATTTTGATCACTGTCCACCGCTGGTCCTCCAGTGGGTCATCCAAGG
EMPV1_27923	GACTTGGTTATCTTAGGGTGGCACCGTGTATGTAGATGACTCACACTTTCTAGAGTCACC
EMPV1_27924	CTATTGGCAGCTTGGTTAATTAGTGTCCCGGGGGTTAAAGGGCGGGGCAACGCCCCCGGA
EMPV1_27925	AGCACATGCCAGGTGACTCCTCACAGCTCCACAAGCCAGTATCGTCATTGCCGTGCAACA
EMPV1_27926	ACGGGGATCAAGCTTAGAGGAGCTTCTCTGGAACCTCCCAGCTGGCCAGAGGAAGGAGTT
EMPV1_27928	GTGGCAAAACATATACCACCCATAGTGCTTCACAAATGCACTTCTATTTAGCCAGCGTTT
EMPV1_27929	AATTTAGGGCCACTACAAGAGGCTGTGGGATGCGTCTCCCCTCCTTGAGAATCCACACTC
EMPV1_27930	GTGATCATCTCTTACTTACGGATTCTCACGGCTGTTCTTAAGATCCCTTCAGCAGCTGGG
EMPV1_27932	ATCCCCAGGATGTTCAGACCTCTGTGCAGATCCCTTTAGCTCCATAAGACTCTCTGCTGG
EMPV1_27933	GCTTGCAGAGTCATATCTTTTTCGTACAATGGAAATCCTCAAGTCCACTTTGTGTGCCCC
EMPV1_27934	AAACTCTGTACCTGTTAAGCAATAACTCCTCTTCCCTCTCTCCCCTTGGCCCCTGGCAGA

EMPV1_27935 CATCGTCTGCCCTTAGAAAATGACACGATTTCTGTTACTGCTCTTGCCACCATTGGGACG
EMPV1_27936 TGGGTGTCCTCCCTAGAGAAGAAGGCAGAGCAACTCAGTTCTCAGTACATTCTGCTGAGT
EMPV1_27938 GAGGTGCAGATCGTGTAAAGAGAGGAGGGGAAATTTATTACCTTGTAAAGGCCGGCAGAC
EMPV1_27939 TGCTTGGGAAATCCGAGACAGATTACTGTCCGAGGGGGTCTGTACCAACGATAACATACC
EMPV1_27940 AAAAAGTGGAGCCATTTGCCAGTGTTCTTAGTTTGCCATACCCATTCACTTCAGAAATCA
EMPV1_27941 ATGTTATAATAATTTCTGCAGACATGCAGACGGGACCTGCAAGGGGGACTCAGTTGAGAG
EMPV1_27942 ATGCAGACCATGAGTTCATCAGTAGTAAGGATTTCAGAGTTATGCTTTTGCCCAATGAAG
EMPV1_27943 CATGTATATGAATGAAGAAGATGCCTTCTGGGCCCTGGTCAAACTGTTCTCGGGCCCCAA
EMPV1_27944 TTTGTTGCTCTGCCCTTGATTTCTTTTTGGGTGGGAACAGATCCGAGTTGACCTCCTCCC
EMPV1_27946 TGGTCCTCTCACAGTTTGGAGGCTAGAAGTCCAGAATCAAGACAGACATTGGCAGAGCTG
EMPV1_27947 GCTGTGATTGTCTGCTATTATTTACCATCTAATAACTCTCCCATGTGGTCCAGAAGTGTT
EMPV1_27949 CCTTCCTCCGGTACGTGGTATGGTTTTCAAGGTTGGTGTGCTGTACTTTGGTATGACATG
EMPV1_27950 TCACCCAGCACTAGAAAGAGGTGTTCCCGCCACACTCACAGGAGAGACGGAGCAAGAGAA
EMPV1_27951 CAGGGCTACTGGCTTCAAACCAAAGGCTTCCCAGGTTGGACCATGCTAGAGCTAAGCTTT
EMPV1_27954 ACGAATCATAGTTCTTACCTCTCTCATTAATAGTCTGATTTAAGGGTTCAATCTAAGGTG
EMPV1_27956 AGAGGTTCGGGTGGTCTCTGCGGCAGCCTGTCCCGTCGTTGGATCTGTGAGCCAATAAAG
EMPV1_27957 CCCGCCTCATGTTTGAATACCAGACATTACTGGAAGAGCCGCAGTATGGAGAGAACATGG
EMPV1_27958 TGGCTTTAGCTATTTTGGAGTGTGCAGTTAAGCCGGTTGTGATGCTGCCAATATGGCGAG
EMPV1_27959 AGAGTTACACGTTGATTGTCACAGCTGCACAGACCTCTGGGCTGCGATCGTGTGTCAGAT
EMPV1_27964 AAAATAGCTGACTTCAGCAGTCGCTCATGTGACCACTGCAGCTCCAAGTCCTGTCCCTGT
EMPV1_27966 AGGAGGGGTTGTAACAATCGAGAGAAGCAAAAGCAAGATCACTGTAACTTCCGAGGTGCC
EMPV1_27967 AGGTCAAACTGAATCTGGGGGCCTCCTCCACCTACCTCTCTTTAGGCTTCCATTTCAACC
EMPV1_27972 CCAGTATGCGTGTTATTTCTGACAGGAGTTCTCTCAAACAGGTTTTGCACTTTGCCCAGC
EMPV1_27974 TGGAGCCATGGTTGTATGCAATTGAAGCTGTGAGAATGAGGAAAACCCTGGTGGAAACCG
EMPV1_27975 AGTTTGAGCTAATATCCTAATGTAGGTGTTCCTGTTGTGGCTCAGTCAGGTTAGGAACTG
EMPV1_27976 CATGAAGCAGAACAGGCGAAAGTGGAATTAACTGAAACACAAAAGCGGCTGCAGGAACTG
EMPV1_27977 GTGGTGACACGGGAGCATTTGGATCGCATGAAAAACAGTTGTATCGTGTGCAATATGGGC
EMPV1_27978 GGGCACTCACTCTCCCATTCTTTCTGGTTCCGGTATCCCACTAGCTTCTAAAGACCTAAG
EMPV1_27979 AGAGCCACACCATCAGTACATGGGCGTTCCCAGATTAGGGGCCATATCAGAGCCACAGCT
EMPV1_27982 TTCCAGAGACTCCTGGACGCCAGCACCTACAAGGAGTCCTATCGGAAGGACATGATCCGC
EMPV1_27983 ACCCCGATGATTTCAACCCCTCCGTTCACGCCTCTCTGGACAAGTTCCTGGCCAACGTGA
EMPV1_27987 GCCCGGATCGTGGTTGGGTCCCAGCCTGTCTGGACAAATCACTCAGTTCCATTGAATTTT
EMPV1_27988 GCCCTTAAAGATACTGGCAAGACTCCCATGGAGAGTGAGGTCTCGATTCACCGGATTAGA
EMPV1_27990 GGACACCAAACTGTTCTGCTTGTTACCTTCCCTCCATCTTCTCCCTAACTTTCACAGTCC

EMPV1_27991	ACGTCGTGGGGCTTCTGAGTTTTTCCACTGGCCTCGGGGCACAACCTCCCTTGGACAGCT
EMPV1_27993	GCACTCTAAATAGAAAGCACATATCCCCTGCTTTCCAGCCGCCACTCCCGCCCACAGATG
EMPV1_27994	CAGTTTGTGGAAACCCATGGAGAAGTCTGCCCAGCCAACTGGACACCGGATTCTCCTACA
EMPV1_27995	ACCTCCATGGCTCTGCTGGGTAAAGGCAGAATTAGGAGTGGCATCCCTGACATGCATCAT
EMPV1_27996	ACAAGATTTGCTTCAAACGACAACAAGAGAGACAGAAAGTATCTGGTTCAACCGCTGCCC
EMPV1_27997	TTTAAACTGTGACAACCCATCCGAGGCAGAAGATAATTACACCCGTCCCCTACACACGCC
EMPV1_27998	CCTGTCTGTTTGCTAACTTTGCCTTTACTGCTTGCACACTGATTTCCTCATGACCAATGG
EMPV1_28001	GTACTTTACAGGTTCCTTATTGTAAGTTGAGGAAATGAGTGAACTCAAGACGAGGCCTCC
EMPV1_28004	GACCTGGAGACCCGAAACGCGGAACTGGAGCACCAGCTGCGGGCAACTGAGCGCAGCCTG
EMPV1_28005	AAGATCTCCGTTTCGTGCATCAAAGCTCTCTGGTGGTGTTGGAGCACAGTTACCGCCGTT
EMPV1_28007	TGTTGTTGGTGGGAGAGACTGGGACTGGCAAAACCTCCACCGTCCAGTACTTGGCTCATA
EMPV1_28008	TATCGAGACACCGTCAGGCTCTGGAGGCTTTTCCGAGAGATTCTGGATGGACTGGCTTAT
EMPV1_28009	CATCGCAGCTATGGATGGGTTTGCCTTGGGCGGAGGCCTGGAACTTGCCCTGGCCTGTGA
EMPV1_28010	GACATTCAACCTCAAGTTCATCTCCCCCTTCACGCAGTTCAACATCACACGGGCTTCCAA
EMPV1_28013	TACAAGGAAATAGCAAACCAACTGCCAAGTAAATCAATTCATGACAGAAGACAGCCTGTC
EMPV1_28015	TAGCTTCGAATCATGGCCATCTGCACCATTTTCGAGCCAGACAAGATCTATGGCGCCTCT
EMPV1_28018	GTGGTTGTCTACTCTGACTCTCCAACTGCCACTCTGTGGCCCATATATACTAGATCACTT
EMPV1_28019	GATGAGCACTTTGGTGGTTCTCGAGGGAAAGATGACCCTGCGGAGATGGTCATTTCCTTG
EMPV1_28020	GCCAGTCCCACAAGGAAACCGGAGCCCCAGGTGCTGGCTGCTCTGCTGCATGCCCTGCCA
EMPV1_28021	AGGTTCCAGGTGTGGGACTACGATGAGGCAGAGGTGGAGGCCGTGCTGGATCGGTACTTT
EMPV1_28022	GCCGTGGGCATGCTGGGGGCGTCCTACGCCGCCGTGCCCCTTTACCGGCTCTATTGCCAG
EMPV1_28023	TTCAGCCAGCAAACCTCCATCGCTCCTTGGTGGCGTAAAGGATGAAGATGCTGCTACACC
EMPV1_28024	GCTGATGGCAGCATTAGCTGCTGTTGGGCCTCCTAATCCACGTGCAGATCCAGAATGCTG
EMPV1_28026	TGGCACTTGCCTTAAGGATGTTTAGCAAGCTGTTTCCAGGATCAGAAACCACAGAGAAGT
EMPV1_28027	GGGAATCATTAAAGTGCTTTCAGAAAATGAGGAAATGGTCCCTGCCCCTGGAGTGGCTGG
EMPV1_28028	ATAAGAGGGAGGCAAAGGGAGATCTGACCACAGGAGATGTAATGACAGAAGAAGCTGGCG
EMPV1_28030	AGCTGCGGCTCCTTCAACTCCGAATTTAGCAAACAAAGCGGCCTGTCCTTGAACAGAGAG
EMPV1_28032	ATGACCAAACACCCGGGCAAACGACTGGGTTGCGGACCTGAAGGTGAACGTGACATCAAA
EMPV1_28033	CCGAAGCAGTGCTCATTTTTACAGTCCTTGGAACACAGAAGTGAAAACTTACTAACCAAG
EMPV1_28034	AAGGAACATTAGGGTGAACTGCCTGGCGCCTGGACTCATCAAGACTAACTTCAGCCAGGT
EMPV1_28035	TTTGAGCTGCGCGACGTCTCAGGGGCGCACTCGGGCAACTACAGCTGCGTCTACACGGAC
EMPV1_28038	TCTGGGATTTATCTAACAGCCACATGAAAAGAAACAGTGCCTTCTGCCCTCCACCCAGGC
EMPV1_28039	TCCGTGGAAAGCACTGGTGGCGGGAAGCCTCAGAAATCAACAAGGCAAAGTACTCACGTT
EMPV1_28041	CGACCTAGGAACAGAATAGTGCGAGGTCTTGCTGTTTTAGTTTAAGAAATCCAGTCTGTG

EMPV1_28042	GGCATGCTACGGTCGGTTTCTGTCCTAAAGGCTTTGCCTCTTTCTTGGCAAAGTTTCTGG
EMPV1_28044	CCACTGGAGATGCTCCCTATCTAATACTTTGGTTGGCTTTGTCTTGATTTCAGATCTCAG
EMPV1_28045	GCTGGTGCTGGTGTCCTGGATCTTGAGTGTCCTGAACTCCTTATTACAAAGCCTAATTGT
EMPV1_28046	GGGCCGTAACAGCAAATTTTATCAGCTCTGCCCCATCAGCTTGTGCTGAAAGCAAATTTC
EMPV1_28047	CCGATCGTGAAAGTCGCTATGAGGAGGAAGAAGAACAGAGTCGAAGTATGAGCCGGAAGA
EMPV1_28050	GCCTGTCATCATGCCCTACTCCTCTTCAGAAGCCCAAAGTTCAGCCTTTTTCAAGTAGCC
EMPV1_28051	CCCAACAGGTCCATGCATCGTTTTGTATGAGAGATGTAGGTTAACTAGCAGACGCCATGC
EMPV1_28054	TTACTGTGACCTCTTCTGCAACCGCACCGTCTCCGACTGCTGCCCTGACTTCTGGGACTT
EMPV1_28055	TGTCACATAACATGGACATGGTGTCAGGCCGCCATCAGTGCCTGGAGCATCATCAGTTAC
EMPV1_28061	CAGGGCAGCCTCTTCCATGGCTGTGATCACAACGTAGGGGTCATTCATGAACTCTTTGAT
EMPV1_28064	CTTCCAGTATGAAACATGGGGTGGTCAGAACTGCATGGAAGAGGTTAACGCTGGACATGC
EMPV1_28065	AGCCAAGTTAGTCAGACAGTGAGACACAAATGTCATATGCTATCACTTATATGCGGCAGG
EMPV1_28066	CTGCAGGATGCCATCTCTTTGTGGTAGAGAAGGTTCATCCTGACTTGTTTGAATACCTTC
EMPV1_28067	GAAGCCATCCCGTTATGGACGACTTCCACTCAAAACACAACGCTCTGGACTTCAAATGCC
EMPV1_28068	CTGTGACAACTTTGGCCGCAATGATTCTACCTCTCAGATTTCTCCCAGTCCTGCTGCTGT
EMPV1_28069	AGCCTGAAAGTGTTACCATCGGCTTTTTACCGAAGAGGTTTGTGGACTCTGCTCGTCTTC
EMPV1_28072	CCGGAGCCTAACCATCAAACATCGGAAACGGAAGCCAGAGTAAAAGGTAGAATGGACGAG
EMPV1_28073	TGTCCCTCTTTTATGGGACCCTTTTTGTCATGTATGCCCAGCCGGGAGCGGTGGAGTCCA
EMPV1_28076	CAGGTCAGGAACTGTTACGGCTGCTCCTCTCAGATGTCATACTCAAAGACACATATCCCC
EMPV1_28078	ATCATTGTCATGGTGCTAGGAGTCCCCTCTGTAGCTGGGAAGTGGATGGTCTTCTATACC
EMPV1_28079	GATGACCTTGGCCATCTGTACATCATATGAAACCTTCAGTTCTCTCTATGGAAACTGCCT
EMPV1_28083	GGTGCACAGGATTACCCCTCTTCAGCCTTCTGTCTTGTCACCAACCATTTCTACTTGGTG
EMPV1_28085	TTGTGTCCAACTTACTTCACTGGACCAGCCACACAACTTTCATGAGCCTAAGAGGGAAGG
EMPV1_28087	CTTGGGTCCTGACCTACGAGAGTAGCATCAACATCTGCCTATCAACCATCACCATCGTGC
EMPV1_28088	AATGGTTGTGTGTGAAGCAGGGTTGGCCTTGTGCTTCTGTGTATGACCTCAGGCTTATGG
EMPV1_28090	GTGCGAGCGGCAAACTCCGCAAGAAGAAAAGGAAACAGAAAAACAAGAAGAACGCTGCCG
EMPV1_28091	CTGACAAGAATGAGTTTCTTTGCAGGACAACAGGTCAGTGGATGCAGTCCTGTCCCGAAA
EMPV1_28094	TGATTTGCAGCGGCCATTGCAGTCATTTGGACAGACAGGAAAAAGGTCTAAGTCAAGCTC
EMPV1_28096	CTGGATGGTGGCCTACGTCTTCTACTACCCCGTCTTCCACAACGGGCCCATCCTCAGCTT
EMPV1_28097	TCCTCAGTTCTCTGTGTCCATTAACCACTGTCCTTTGCATCCCAGTGTCCTTAAGATGTC
EMPV1_28100	GATGAGAATATCGAGGTGGTGTATTCGAAGACTGCCAGCGTACCAAAAGTTGAAGCTGTG
EMPV1_28102	AAGGAGCTAAAGCCGGAGCAGGAAACCACGCCGAGACACAAGCAGGAGTTCTTAGACAAG
EMPV1_28104	TGAAGGAGAGGCAGGGTCGGTGGAAGAGCACAAGAGCGTTATCATGCATCTGTTGTCACA
EMPV1_28108	GGCCATTCTCTGATGGACGGGTGAAGCAGGCAAGGTGACTATATTTTGACTAAACGGTAG

EMPV1_28109 GCACTCGATGCAAACAGGGTAACTAAACAAGCCACTTCAGTCCTTGAAGGTGTATCCAGG
EMPV1_28110 GAGGGCAGGATCAGCCTCTTTAGAATCACATAGATCCTTAAATGGAAACTGGGAGTGGGC
EMPV1_28114 CCAGGTTTGAAGATGAGAGAAAGAGGCTACCTGCCCTGAAAGCGTAATACACATGAGCAA
EMPV1_28117 CTACAGCTCTCACCAATCGATATTCCCTGGGCCTCTGCCACCAGTGTCCTTGCCCTTCTA
EMPV1_28119 ACTGATGCACAGAGAAGGGGAGTAGTTTGTCTAAGTAGCTAGAAGATCAATGACACAGTC
EMPV1_28120 AGAAGAACTGGTACATCAGCAAGAACCCCAAGGACAAGAGGCACGTCTGGTACGGCGAGA
EMPV1_28121 GTCTGCGCTGCGGCCGACATGCAGCTCCAGGTCTTCTACGCGGCTCTGGATCAGCTCTAC
EMPV1_28122 CCGTTATGTCGATTGACTTCTTGAGTTTCGCAGTGATGTACTTGCCAGGTTTCTCAGCCA
EMPV1_28123 TCAAGAAATGTTTAATTATAATGAACATGAAGTTAGTAAGAGGTGGACATTTGAAGAAGG
EMPV1_28125 AATGCCCGGGAGGACTAGTGGAGAATTAACATCCTTCAACAACGGCACCAGCTACCCGTT
EMPV1_28127 TTCCGCTTAATGGTTTGCTTTGTTGGCATCTGAGTTTACTTACCTGTATGCCACATGCGG
EMPV1_28128 TCTTCTATGGGACCATTGTCTTTATGTATGCCAAGCCCAAGTCTAAGGACCCGCTGGGGA
EMPV1_28131 AGCCTTCAGTCCGCTCCCTTCTTCCTCGAATCTCCATGTCCATATGTCCACCTGCAGTAA
EMPV1_28132 TTTACGACACAGAGAAGGCAGTGATGCACCTTTAGGGACCAGCACCAGCCTGACTGTCAT
EMPV1_28133 GTAGTAAGTTTCAGCTCTGCCAGTTATAAAAGCCACGTCCATCTTGGAGTTCCCTGCTGG
EMPV1_28134 TCCACCATGGCTCTTACATCTTTCTCGTGGCTGCTCCGCTCGGCTTCATTCTGCCATCCT
EMPV1_28135 CCTAGTTCCCTGTGGTCTCACCCTAGTCTTACATGTCTTTTGTCCATTTCCATGACACTC
EMPV1_28137 TTGGCGCTAGGGAGGGATTGGGCTGTGGTGATGGGGTTCAGGTGTGACACGCACCGGAGG
EMPV1_28141 AGGTGTGTGCATTTATCTGGGGCCCCGGGAAGGGGGCACTGTCACTCGGAAACATTTGAT
EMPV1_28142 CATTACCAGCCTGAGTCCCCATGTTCCCAGCCCTGGTGCAGGTCAGATTCCAGACTAGAT
EMPV1_28146 GGACCAGCATTAGGACATGAAACCCACCTGGCACTTTGCTTTTGACTAGACGCACAAACG
EMPV1_28147 GGGTTTGCATTAGGGCATCTGCTCATTTTACCATTTCTTCCCAGTGACTTGTGCGTGGGA
EMPV1_28148 GCGTATGTGTGTGTGTGTGTGTGTGTGTACGCTCATGTCAGTGAATTATGTCACATACAC
EMPV1_28149 TGCAAACTTATGCCCCCACAAAGCCTGCATACCTGCACGTGAATGTCATAGGAGTTTGAG
EMPV1_28151 CAGGGTCCTTCAGAGGAGCTGAGACCACGCAATTCAGAATAATAAGTGAGGCGAGCAGAT
EMPV1_28155 CTGACGTCCCTCTGCCCCACCCTCCCCCATGACCAGGGGATTCTGGTTGCAATAAAACAT
EMPV1_28156 TGCTGACCGGTGCAGAGTTCGGCACACACAGAAAAGTGAAAGTCGTCTTACTCAAGTCGA
EMPV1_28158 ATATCCGACTATCCAGGGTTCAGGATATCAAGTATGAACCTCAGCTCCTTGCAGATGATG
EMPV1_28162 AGGGCAGCTAGAGGCTGGCGGCGGGAGGGACTCGTTGTCCCAGCATGCCCTGCGCATCAG
EMPV1_28166 GTCACGGAATCAGGAAATTGAGGTTATTCAGGATGTGCTGTGGAACATGTGAAAGGCAGG
EMPV1_28168 AGGAAGACTCACAACTTGTCCTTCCAGGACTGCGAGTCCCTGCAGGCCGTCTTCGACCCA
EMPV1_28170 GTAATGGAAACTGATTTTGCCAAGCTCTTACGAGGTGGTTCATCTATCGATGGCATCCGC
EMPV1_28171 CCAAAGGATCTGTCTAGGTAAGTATCTCCAGAAGTCCAGGAAGTTCCCTCGTGTTGGAGG
EMPV1_28172 GCTGCGCAGATGCTGCGGTCCGAGAAGAGCGTCCACCTCTTCATTACTGGGGAGAGATGA

EMPV1_28178	TGAGAACATGGCACAGGCTCTTAGTCCCGACATGCTGGCCACTGACCTCGCCTACTACCT
EMPV1_28179	AACTCCCCAAGCCACAACAGGAACTGCACCTAGATCTTGAAAGGGAGAGGACTGTCTAGG
EMPV1_28180	AATGGAGAGACCATATACAAACAGACAATGGCCAGACCATGGATGGTGACAGACCTCTGA
EMPV1_28181	GAGTGAAGAAATGACAAGCCGCCTTGAGCTCAGCTTTAAAACCACATCCTCCCTCTGACA
EMPV1_28182	GGAGGCTTAAATGATGATACTCTTGTACTTAGGCCAAATCTCTTTAAATGAATGGACACT
EMPV1_28183	CAGAGCAAGCGGGAAGACGTGCGTTGTCCCTGGGGACGGAACGCGTTGAGGGTTCTAGTA
EMPV1_28186	ACAAACTCTGCAAAACGATGGTTGTCTGCTGTTGGGGGGCAGCACTTCTGATTTTCCTCC
EMPV1_28187	CTGCTGGATGTACTGGAGGGCAGAGAGCGCCGCCTGCTGCTTGGCCTCCAGCTTACTATT
EMPV1_28189	CCCAGTGACTCCACCAAGAAGGCCTATTCCAAATCTGATGATCCCTTAATTCTAGGCACC
EMPV1_28190	TCAGAGAGACCCCCTCCTACAACGCCCAGAGAGCACGTGTTAGAGTCATTGCAAGGCTGA
EMPV1_28191	GGTCCATCTAAATCCATATCCCAAGATCCAAAACCCTAGCCTGTCTTCATGCCCAGGCAG
EMPV1_28193	CAACAGGTGCAGGTGCAGGGAGGAATTCACCCTACTTGCGCTCTCCTCCCTTTATAAATT
EMPV1_28194	GCCAAAAGGTATGGTTCAGACTAGTCTGGAGTGGATTTTACCTGATGCCTGCAAGCAAAG
EMPV1_28199	AAGTAGGTGAGTAAATAAATCTCTGGCATCTGTCGGCAGGGCTTCTCCGGGCTGGTGTGT
EMPV1_28204	CACATTTGATGACAATGTCAGCTCCACATTAGAAATTTCCAGTAATCAGGTAAGGAGAAA
EMPV1_28211	TCCGAAAAATGTGGAGTCCTTCGCATCTATGCTGAGACATTCCCCTCTTACACAGATGGG
EMPV1_28213	GAAGGAAGCTCATCTACACTTGAATCTGTAAGCAAGATAACACGCAAGTGGTACCAAATG
EMPV1_28215	AATCTTACCTAGAAAACCAGCCTGTTGCTGAGGTTCAAGTCTAGTTCCTCCCCCTTGGGG
EMPV1_28216	GCTTGACTGTGGGCACTTCTAGAGCAAGGATTGTGTCATTCTCATGTTTGCATCCCAGGC
EMPV1_28217	TTCCTCTTCTACTGCTTCTACTACTGTGGGGGGGATTCGAGTGGTGAATGCATTTCGTAG
EMPV1_28219	CTGTACATCTTCAACGGACTAGCGGTACTCTGTACTGACAAAGTCAGAGCACTGGACAAC
EMPV1_28221	TGACCCTCATGTACCCAAGATCAGGCCCAGCCACAGCCACAGCCATTCACTACTCTTATA
EMPV1_28223	CTTCATTTAAAAACCCATTCCTGGAGTGTTGTGGTGACTTAGTGGGTTAAGGATCTGGTG
EMPV1_28224	ACCATGTGTCCAGTGGCTCAAGTTTCGTGGAGTGAGACCACATATCCTTTGGAAGGCTGA
EMPV1_28225	TGCCTAAGTCAAAGGAACTTGTTTCTTCAAGCTCTTCTGGCAGCGATTCTGACAGTGAAG
EMPV1_28226	AACGGGTGTTTTATCCAGGTACTCGTACACTATTTTTTTGTACTGCTGGTCCTGTACCAG
EMPV1_28227	TTGTGCCTCCAGGACCCCAGGTGGGCCCTTACCTATTCTCAGTTTTACTCTGAAGCAGAT
EMPV1_28228	TCCGACTCCACCTATGCCCACGGCAATGGTTACCAGGAGAGGGGCGGCGGACACCATAGA
EMPV1_28229	GACTGAAGAGGAAGAAGAAACCAGAACCCCTAAACAGAAAATCACCCAGACAAGGCGCCC
EMPV1_28231	CATGACTTCATCGAGACCAATGGGGACAACTGCCTGGTGACGCTCCTGCCACTGTTAAAC
EMPV1_28235	TACTTGAAAGGTACTTGCTATCTACTTTGAACTGCCCAGATGCCGTGTTGGGCTGCAAAG
EMPV1_28238	ATGGACACTAGTCAGGTTCTTTACTGCTGAGCCGCGATGGGAACTCCAGGATGGTCACTT
EMPV1_28245	GAGTTATTTGGTAGAAGAGGTGACAGAGGAAGAGGGAGAGAGTAGTTTTGGGCCTGGTGC
EMPV1_28246	ATTTCCTTGGAGTGTGAGGTACAGGGTATCCCCCAACCGACAGTGACCTGGATGAAGGAT

EMPV1_28247	AGGACTCCATCCTGGCTGTGAGGAAATACTTCCACAGACTCACCCTCTATCTGCAAGAGA
EMPV1_28248	GTCATGTATTTTGTTGGCGCCGTTTATGGTGGTACTCAGGTCTCAGGGATCATTTTCTCT
EMPV1_28254	TCCAGCCAGTCCTGTACCATTCTCTCCAAAGGGGCTGCAACCTGGCCTTTTGTCCTTGAT
EMPV1_28255	ATGCCTTTGCCAGTGTCATTCTCTTTGGAACCAACAACAGCAGCTCCATTTCTGGAGTCT
EMPV1_28257	GAAGTGAAAATGGACACAGTCACTCCGATCAGTGGAAGGAGTTAAGGCTGTTCCCACAGC
EMPV1_28258	AGGTCTTGACTCAGATGAAGAACTGGGGCTGCTCTGTCACCACGCGTCCTATCTAGACCA
EMPV1_28259	CGACCTGTTGAAGAAGATGCTGACCATAGATGCCGACAAGAGAATCACGCCCATTGAGAC
EMPV1_28261	ATCCCAATAGAGCCACTGATCATAGCATTTAACCCTAGTCGTGAAAGCCAAGTGACTCAC
EMPV1_28262	AGTAAAGGAATCCTTTCAAGTCATCCCAGATATGGTTCTATACCTAAACTTATATTTGCT
EMPV1_28263	CTCAAATCTTGCCTGGCAAAACAATCATTTGGAAGGATTTGAGATACTGTGAAGGGTGCA
EMPV1_28264	TAGCCAGGAAAGCACCTTTGAGGGGCAGGCTGGGGACACGCCAAGGGGCCTAATTGTAAT
EMPV1_28265	TCACAGCAACATTCATTTGTTTACATGCTATCTGAGGCTGCTTTTGCCCTACAGTGGCCC
EMPV1_28268	ACATAGCCAGCCGGATGGACGCGTTGGATGGGCTGTGCCCTTCCTCTCTTAACTCTGACT
EMPV1_28269	TCAAGAAATATGGGTAGGCTAAAGCTGTCTTCAGGGGCTAGAGCTGAGGGATGGCCAGTG
EMPV1_28270	ACAAGTTCATCCAAACTCCCATGAGGGGCCTGAGCCAGCCACTGAGACACTTAGCACCGC
EMPV1_28271	CCAGTCCCTGGCGGCCACTAATTTACTCTCTGTCTATATATTTTTCTGTTCTAGACATTT
EMPV1_28272	GAGAAGAAGGATAGTGTGGATGGGAAGCCAGGTCTGGAGAGGAAATGGGCAGTGGGCTGA
EMPV1_28273	ATCTGTACTGTGGGAAGAAAGGATAGTTGGGTTGCTGGTCTTCCTCCTTGAAGGACACCA
EMPV1_28276	GAAAAGAAAGTCCAAAACGACACACATGGTGCTCCTGGAGATCATTAAAGAAGAAGGCCT
EMPV1_28277	GAAGTTCTACCTGGAGGGCGAGGTCCTGTTTGTGAGTGTGGGCAGCCTGGTGGAACACTA
EMPV1_28279	AGGAGACACTCCCTGGCCATGTGGTTTGCGTTCCCGGCCTTAGCACCTCCTCTGTATGAT
EMPV1_28280	AGGATCCTCCACGGAAGGGCAGCTGAGTGGACTCCAGAGCAGCCTTAATTCTGCAGCCTT
EMPV1_28282	TGGGGAGAAGCAGCGGGTGGCCATCGCTCGCACCATTCTCAAGGCTCCGCACATCGTTCT
EMPV1_28283	GTTCCGTCAAGGCCTCATAGGATCCCTACCACCAAAACTGATGGAAAAGAAGCAGCCAAC
EMPV1_28284	GATATCTATGTGCTCCCATTCACTTCCCAGCCCCTTGTAGTTAGGCAATGCCATTTGACT
EMPV1_28288	GAGTTCTGTGCCTGCACCTCAGGGTGGACATCCAAGAACAGCTTCATCCATTGTCCCACT
EMPV1_28289	GTAGACAGGTATATGGATGAACAGGACGACGGAGATGGATGTGTGATGTCCTCGAAGAGC
EMPV1_28290	TCCTCCTGGGGCTGAAGAACAACTTGCCATCTACCAATGTCTTGGACAAAAAGTGGCCAG
EMPV1_28291	AATGCCATCCCGTCTGTGCCGACCTGCAGGCCCAGATTCTCCAGTGTTACCGCCAGAACA
EMPV1_28292	AGGATGAGCTTTTTCGGGGGGAAAAGGAAAAGACAGTGAGTCTTCAGTATAGAAGGAGCC
EMPV1_28295	AGCTTGTGTGTGACATTCCATCAGACCACTGAACCAAACAGCTCAAGCCAAGATCGTTGC
EMPV1_28296	TCTCTTGATGTGCTGTGATTTTTGTATCAGCCAGGAACCCATCCCTGCGTTTTGCCTCCC
EMPV1_28298	CAGCTTCGGCATCGGCTGTTCTGAAGGTCCAAGATGCTTCACTATCAAATCTGTGCCTTC
EMPV1_28301	TTGCATCCAAAGGGTGCTCTGAGGTCAATAAAATAGCCAAGGCCACCCAGCTGCCTCTTT

EMPV1_28302	AGAGTTCTTGGGGACAGTAGCTCTAACCTTGGAGTAAGTTTCTGCCTTTGACCTGCATGG
EMPV1_28303	GCAAAAGTGCCTTGGGCTTGTGGTTATTTCCTGGACCATTGGCTTTGTGCATGCCATGAG
EMPV1_28304	CTCTTTACCCAGTTTATGGGGGTTGTGATGCTGATCTCACTCCTCGGGTGATCGCTGAAG
EMPV1_28305	AAAAAGAATACCACAGCCTCCTTGAAGCAGTGGAAGGTTGGTGACAGATGTTCTGCCATT
EMPV1_28307	GAGCAGCGGGAGCTCTACAAGGAAGTCATGCAGGAAAACTACGGGATCCTGGTGTCCTTG
EMPV1_28308	CCTTGGGACGGGGGGCCTGGTGGAGGTAGACTATAATATGTTAGCTTTCCAGCCTGCCTA
EMPV1_28312	AAGTGCGCTAAGGGATTTATCTTCTCCCTAGGAACCAGAGGGGAAACTGAGGCTCGGGTT
EMPV1_28314	CGGGCGCTTCATATACAACAACGCAGTTACCAAAATGGCTCAGTCTTCCAGCAAATCCCC
EMPV1_28316	AAGAGTCACCACACTGCTCACCCTGGATCTCTGCCATCTACCTGCTTCAGGGCCAAGCTT
EMPV1_28317	ATGAGGCTTACTCTATCCAGACTTTCCTCCTTGAGGTCTGTAGTGAGCTTGCCCCCTTGG
EMPV1_28318	TGTGTGTGTGTGTGTGTCACCCTGCGGCAAACACTCAGGCTGGGACACCGAGGTCTCCAA
EMPV1_28321	CTCTTCTTGCAGCTAATGTTTCTCACCGTGTTGGTGGTCTTTGTTTGAAGTGTGCTCAGC
EMPV1_28322	GCCTCCGGCCGTGCCAGTCTCCTTTTAGGCCCGATAATTAAAAGTACAGGATTTGGTTGA
EMPV1_28323	ACTGAGCAGTGGAGGTGGTAAGGGGTGAGGGAGCCATGCTGCTGAATTCTGGTTGGCATT
EMPV1_28327	CTGTTCTCTTTCTGAACTCTGATTCGGCCCTTATCTCAGCCAGCTTTGCCCTGCTTGGCT
EMPV1_28328	ACCAGCTATACCCTAAGCTCCCTCATCATCCTGCTCAGCTTCCTTTTCATCCTCATCTCC
EMPV1_28331	TCACCACTTCAATGCAGCGCTGCTGCAGCTGGGCCTCAAGTACCTCTTTGTCCTTGGTAT
EMPV1_28334	CTGTCACAGAAACTCTTTATCTCCGTCTCCGTCCTCTCCAGCCTGGGCATTGTCCTAGCT
EMPV1_28337	GGACCTGTGCAGACTTCCAGCTAACTGAGAGTAACGTGAATGTGGAGGAAATCAGGTGAC
EMPV1_28339	GTCTGACAAACCCGATATGGCTGAGATTGAGAAATTCGATAAGTCGAAATTGAAGAAGAC
EMPV1_28341	TGTTGATTCGTGAGGTGGTCGAGAAGAGATAGAGTCTCTATGAGCGTAAGGACGACCGGC
EMPV1_28343	AGTCCCAGAAAGCCTATAGTCCCTAGCAAGCTGGTCCCTGGAATGAACACCTCTTACTGC
EMPV1_28344	TGGACAAAGTTACCGACTTCCTCTTCCTGTTGGGCAAACTTCTGATTGTGGGTAGCGTGG
EMPV1_28345	GCTGGTCAGTTGAACCCAGGAGACCTGCAGTGGATGACCGCCGGCCGGGGCATCGTGCAC
EMPV1_28346	CTGCCAGTTTTTCAACACCTGCTCTAAGTGTATTATTGTCTGCTTGATCTTCATCAGCAT
EMPV1_28347	GTGTAGTGTCCAGAGCTAATAGCATCGGTTCCACCAGTGCCTCTTCTGTCCCCAACACAG
EMPV1_28351	ACCCAGAGAGGAAGTACCACTTTGAGTGCTGCAGCGAGGAGCAGTGTCAGGAGTGGATGG
EMPV1_28353	GACGCTGGATGTCTCTGGCTGAAGATTTGATGTGGTTCCTCCTTAAACTGTGCGTCCTGT
EMPV1_28356	GTATTTGTATAAAGAGGGAAGAGAATTACAGCTCCTTTTGTGATGGTTTCCATCAGCTAC
EMPV1_28357	TTGCAGTTCATGGTAACTGTCAACAGTGGGCTCCTTTCTATGGGCTCCTTTGTCTTGCTC
EMPV1_28358	CATGTTCTTTCCACATCTGGCCTCTGGGTTCCTTAGACTGTTCCAAGCAGGGTTTTTGCC
EMPV1_28359	CCAAGTCTGTGAGCGAAGATGTTCACGTGCACTCCCTGCACTGCTACTTTGTTCGGACAG
EMPV1_28360	CCTGCTTCTAAGCCACCCAGTCTTTCTCGGAAGTCATCTGTGGATCTCAATCAAGTTAGC
EMPV1_28361	CAGGATGCCTACTACATCTTCCAAGAGATGGCTGACAAGTGCTCGTCTACCCTGCTGCTA

EMPV1_28363	TAACGTCTGAAGACTCCGGCCTCCTGCAAATCTCCACTGCCTCCTTGCTCAGCAGGCTTT
EMPV1_28364	TTACCAAGGATAAAAGGCCCTACACACTCCTGTCCTCTATTACTGCTCCCAGCTCACCTC
EMPV1_28367	GAGCCTCTGAGCACATGGAGCTGGCCTTTGGTGTTGATTTGAAGGAAGTGGATCCTACCA
EMPV1_28371	GGGTGGATTTTGCTTCTGGGAGCTTAACTGGGAGCCTAACCGCTATTTAGAACAGGGCTT
EMPV1_28372	TCATTCTTAATGAGATATTCTCATTCTGCATCTCTCTTTTCCAAAGAGAAAATGCATCAC
EMPV1_28373	GGGGCCGTATTACTGCCACTTCAGAGAGAAGGAAACAGACACCAAGGTGATGTTGCTGAT
EMPV1_28374	CCGTTTCCTGAGAATTATTCATGACCTGGGCAAGTTGACAAAGACTCGGCATCTCAGGCG
EMPV1_28375	GGGACAATTATGGCACTATATCTTTCCTCCTCATCAAGTCAGTCCCAAGACAAAAGCATC
EMPV1_28376	ATCATTCAGAGGACTGTGAGGAAAGGCTGTGGTACCTGACTTGTTGGAATCAAGGCCCAG
EMPV1_28377	GCTCTCGATGTGGAGCTGAGGGTGCTTAGTTTTGACACATTCAAGCTTGATGGCAACTTC
EMPV1_28378	TCACGCCTGATGCTATGACTAGCTTGTTCAGAACGGCATTAGGAGGGCCATTCCTGTCAT
EMPV1_28385	GAGGAAGAACCAATGCAGAGAAAGCCTGGATATTTACATCAAGTTCCTTGGAAGAACGAC
EMPV1_28386	TCAATGTCAACCACTTGCTTGGGGACCCAATGGCCAACGTGGCTATGGCCTATGGCAGCT
EMPV1_28388	TTCATGGCCCTCGGCTCACTCCGTCAGCAACATCCTGGGCATCCGGACGTTTATGGAGCA
EMPV1_28389	GTTTGGGGTGAAATTAGGACTTAAGCCCTGGAGACAGAATCTCAGGTAGCCCAGAGAGAC
EMPV1_28391	CCCTTGCCACAGTGCCCAGTTGATGATGACTATAATACCCTATGGATTGATGGTGGTCCC
EMPV1_28393	ACCGGTTACCTGGAGGTGGAGGGTGCACCTGTGCCGTTTCACCTGCTCCACCAAGAGGAT
EMPV1_28394	CCTCTCTTACAAGGACTTGGAGGCGGCGCTGGCCAAGGTGGGCCCTAGGGCTTCGCCCAA
EMPV1_28396	CTGCTGATCAGAACTGGACCGACTCCTGTCTGCACTGAACTCCGGGTGGATAACCTTCTA
EMPV1_28397	TTTAGTAAGCCTGACTCAGACCGATTTGTCTTTTTGCTATGCACCCGGGCCGGTGGACTT
EMPV1_28398	TCACAGAATCAGCCAGCACCGGCACTTTACAGAGAAGCAAGCCAGCCAAGTAACAAAGCA
EMPV1_28401	TACATAACGATCACAAGAAAGGAGACTATTGCCGTGTGGCTGATTGTAACTGCGCTGCAG
EMPV1_28403	CATTAAACCCTCAGCAAAAGACAGGGTGTCTCTGAGCAAGGGAGTGGCCGTGCTAAACAC
EMPV1_28404	TCGTAAGGTCTTCGACCCCTTGGAATCTAGACTAGTGAGCGGCAGTCGTACTTGCAGAGA
EMPV1_28405	ATCCTCAATCCCGATGGGTTCGCTGCATACCCCTGCGCTTCCTACAGGGCCTTTGAGTCT
EMPV1_28406	GCACTGTGTACATAGACTGATGATTCAGCTGCGTTTTAGCCTCTAGTGTTTGAGAGCTAG
EMPV1_28407	GGCTGCAGTCTTTGCTGTGGCCATCCTTTGCATTTCCAGCCCATTTTTACTCATCATTGC
EMPV1_28408	CCTGCATTTTGTTGTACCAGTGCATGCAGAGAAATGTCATCCTTCCATCACCTGTGAATC
EMPV1_28410	ACGTGAACGACAATCCCCCGGTGTTTTCTTCCGACCACTATAACGCCTGTGTCTATGAGA
EMPV1_28412	CTTCCACTTTATTCTGCCCTTCATCATCGCTGCCCTCGCAACCGTACATCTCCTATTCCT
EMPV1_28413	TCAGCAACATCGCTATGCAAGTGCGGAAGCTGGTGGGAACTCAGCACCCTCTGCTTACCA
EMPV1_28419	GCCAAGAAAGAAGACAGTGATGAGGAAGACGAAGACGACAGCGAAGAGGATGAGGAGGAC
EMPV1_28421	CCTGTCTTCACAGAGCTCAAAGTCTAGTGGGTGAGGTAGCCATGTAAACAGTGAAGGCCT
EMPV1_28422	GATTCTGGGTCTTTTCCCTTGATTTCTGGTCTCAGTCACAAACTCCTGGGCTTTCTGGGG

EMPV1_28423	GTGAAGTTAAAATGTCTGATCAGGAGTTCCCATCATGGCCCAGCAGTTAATGAACCCGAC
EMPV1_28424	AAGGCCTAAATCCAGTCACTCTGCAGGAATTGACAAACTGCTCTCTCTTTTCTACACCAC
EMPV1_28425	GGTCTATACAGCAAGAAAACTGGCCAGAGATGACACTCTAATCCCGCAACTCATCAGGCC
EMPV1_28426	CCGTGTCTACAGCATCAAAATCGGGGACAGCCTCCAGCAAAATCATAATTGAACGCACCC
EMPV1_28428	GGCTGTCCTACAGCTTGGAAGACCCCTCAGGTGGCTTCCAGCAGCTTCTCCTGCAGCCCA
EMPV1_28429	GTCCCTTCTTTCTTCACCTATGTATGTCCCGTGTCTACTTACCATACAGGCAAGTTGGTG
EMPV1_28430	TGAGTCGTGTAGTTAGGGTTCTCTTGCCAAGGTGGCCAGTGCATCGCAGGCTAAAGCTAC
EMPV1_28435	CTAAGGCCAAAACTCGTGCCAAGAAAGCCACACCCTCACCCTCAGACATCAAGAAGCCTA
EMPV1_28436	GTTCCTGCAATCACACGTGGGCTGTGCTCCTGTCTTGCTCACAGTTGAAAACTGGACTTT
EMPV1_28437	ACAGACCTGTAAACAGCTGCCCTCCGCTCAGCTCTGACTAGTTTCCATTTCCTTTTTTGT
EMPV1_28440	GGTTCGAATCCTCACCCTGACTTAGGGGTTGGAGCAACAGAGGCTCAAGATTGTGAAGAA
EMPV1_28441	ATCAATTTCGTGTGCATGTGTCCACAACCTCAGAGCAGCTAGGCTCTTCAAGTGTCCACC
EMPV1_28442	AGCACGGTCCAGCAGGCTGTATGTGGAACTCTTTTACTATGGCCCTTCCCAAAGAACTTC
EMPV1_28444	TAAGCTGGCCCCAAATCAGACCAAGGAACTTGAAGAGAAGGTCATGGAACTGCATAAGTC
EMPV1_28448	TTAATGTAACTGTTGCAGTGAAAGTGCAATATCTAACCCCCTGCTCCCAGCGGGACTCCC
EMPV1_28449	GCTACATTGCCAAAGCTGTGCTGAGAATGAAGTCAACGGCAGGCCAGCGGAAAGCAATCA
EMPV1_28450	CATGTCAGCCTGGAGGAAGATTTTTGGAATGCAGGATAGGGCTTGGTTTCTGGGTCATCA
EMPV1_28451	CACCCGTGCACCCGTCCAGGTCAGATCGGACCCTGGCCCCCAGGACACACGGAATGCCCC
EMPV1_28453	TCCACACCTGCACCGCACACATCTCTGTCATGCTGCTCTCTTACACCCCTTCACTGTTTT
EMPV1_28454	GTAGATGATCTGCTAACACCCTGTTCTCCAGGTGACCCTGGTGCCATTGAAATGACATGG
EMPV1_28456	ACGGATGCCTTCGTGCTTGTCTATGACATCTGCAGCCCAGACAGCTTCGACTACGTGAAG
EMPV1_28457	GCAGTCATTCCATGACTCCAAACGTGTGCCTCGATTTCTTTCGCCAAATGTGCCTAATAT
EMPV1_28460	GGTCCCTTTCTAAAAGTGAAGATTCTAGGAGAATACAGTATGTTCTATTGAAGTTTATTC
EMPV1_28463	CGGTTCATCAAACTTGCCTGCGTAGACACAGATCAACTAGAGTTCATGGTCACGGCCAAC
EMPV1_28465	ACCAGATCTGCTTTGAGGAGTTCCTCTACATCCTGGGCAAGCTGGTGAAGGACTACCACC
EMPV1_28466	GCTCGAAACTGGGCCAATCAAAAATCAGCTTTGGGAAGTGCCCTTGATCCACTTGCTGAT
EMPV1_28468	AGGAAGGCTCAGAGAAATAACGAGGACGTTTCCGTCATCCCGCCTCTGTTCACAGTCAGC
EMPV1_28470	GGCCAGCATGCAGGAATTCAGGAAATAATCAGGGAGCAAATGGGCAAATCATCTCTTCTG
EMPV1_28476	GGGAAGACAGTTGCTACTGCAAGCCAAGAGATAATAATTGCCAGCACAAAGGTTCTGGAA
EMPV1_28477	GGTAAATACATGCTGATGGCACAAAGGGCCAAGAGTTCAGCACAGAGGTTAGGGGAAAAT
EMPV1_28479	GACAAAAGAACTCCCATAAGCATACCATCAGACCTCTCAGCAGAAATCTTGAAGGCCAGG
EMPV1_28481	CTGGCTTCTCCAACCTGAATTCCCCTTAGCATGATTCATCCATCCGTTGAGAGTGCTGAC
EMPV1_28482	GTGCACATTTGCATTAGTATTCTTGCCATGATTGTAAGACCCAGAACACCTAGAATAGAG
EMPV1_28483	TCTGTGGACAGCAGAGTGCAGAGACCAAAAGAAGAGAGTTCAGAGGACGAGAACGAAGTG

EMPV1_28484	ACGAATATTTGATACTTCATCAGGGCATTTAATCCAGGAGCTGCGGAGAGGGTCTCAAGC
EMPV1_28487	TAAGGTCATGCAATGAGTATGTGATAAAGTCAAACCCTAGACTCTGGAGTCCTCTTTCTG
EMPV1_28488	CGTGGAGAAGCAGAGGCTCCTGGAGGAGAAGGACCCCATTCCTCTGTTCAAGATCTTCGT
EMPV1_28491	CCCACATTCTCATGGATACTTGTTGGGTTCTTAATCCTCTGAGCCACAACTGGAGCTCTG
EMPV1_28497	GGGCCGTCAGACTGGGCTGATATACTCCAAAAACTGCTGTTTCTCACTCTAAGCTGCCAA
EMPV1_28498	GTTTGGGACTTCAAAGGGCAGGAAGGCAATTCACATGGAGACAGCAAAGCACATGTTCGG
EMPV1_28499	CAACCAGCGTGAGCAGTATGACTGGCCCATCAACCACCGCATGGACGAGATGGTGAGCTT
EMPV1_28501	CTAAGCCAGCCCATACTGCTCTAGCACCCGTAGCAATGCCTGTGCGTGAAACCAACCCTT
EMPV1_28502	TTACCCCTTCCTAAACTTCATTATAGATTGAATTAGTGAGATTAGGTAGATTTAGTGAGA
EMPV1_28503	GGCACTCGGGTCTTACTCGGGTCTTACTGAGAGTCCTTTACCCAAGAACTACAATTAGGA
EMPV1_28505	CGAGAGCGCTTACGGCGACTTCTACGTCGGGGATGCCTACCTGGTGCTACACACGACCCA
EMPV1_28506	TCCGGAACAAGGTGATCGTGAAGGAGTATGAGTGCAGATCCTCTGGCCTTGTGGTATCAA
EMPV1_28507	CAATGCAGGAGGAGTAGGGGAATTTAATGAAAATCTGCAGCCCAAGAACCATATGGTCTA
EMPV1_28509	TACAGTGTGTTATGGGCTGATGATCTTACGCCTCAAGAGTGTTCGCATGCTCTCTGGCTC
EMPV1_28511	GTGCGTACACCTGGAATGGAACCCAGGAATCTGCATTTTAACAAGAGCTCAGTTGATTCC
EMPV1_28512	TCCTTAGTCCTCACCCCTTCCTGCTTCGGTGGCTGCCCTACTGCAGAACCAATGTCCTTT
EMPV1_28514	ACATACGTACGGCCCATCTCATCTTACTCACTGGAGAAAGACAGACTCATCTCAGTATTG
EMPV1_28515	TACACAGGGTGAGGTACATGGAAGGGTTGGGAGGGTTGGGATAAGGGCTGCACATTTAAA
EMPV1_28519	GGAAAAGGGTGGCTCCCCTTCCACTACAATTGGCGTGTCATGCTGTAGTAACAGTGAATA
EMPV1_28520	AGCTTGTCATATACACTTCAGGGGCTGCAGTTTTCATTTTTCCATTGAGTGGCATCTTGG
EMPV1_28522	GGCTTTAGCCTCAGCTTCCCGAACAGGCCCCTGAGTGGGACCGGAAGCTGGGGCGGTCCT
EMPV1_28523	CCAAGGCTGGGGATAAATTCTGAGGTGTCCAAGCAATCATGTGGTTCCACTGTCCTCATC
EMPV1_28524	CCCAACGTTCGGGGCCGCCGGGCGACTCGCTCCCCGCCGTAGTATTTATAGTTAAATTAA
EMPV1_28525	CTGAAATGATGACACCCAAGTGATAGGATCGAAATAGGTAGCATGTACTGAGGACAATGG
EMPV1_28526	ATGTCGGGAAGGTTGTGGTCCAAGGCCATTTTTGCTGGTTATAAGCGGGGTCTCCGGAAC
EMPV1_28529	TAAAGACTCAAGTATTCATAACCCACTAGGCCCTGGCTTCTCTTGCCTCAGAAAGGGGCG
EMPV1_28530	TTTCCTCTTCCCCAGGAGGACGCAGCGAGGATGCCTGCAGCTGGTCACCACTACTCCTAT
EMPV1_28532	GAACAGTTTGCTCATGGTCTTCTTAGGGTATCAAATGGTATTCAACTATCTTTGGAGGGG
EMPV1_28535	CCCCTTCAGCTCCTGTATTTAACTCACCAGCAACCTACAGAGAGACAAGACATTCACTGA
EMPV1_28536	GGCCTACATTGGCATTTTTACCACCATGGTGGGAATGGCTATGACTGCTTTTGCCAAGAC
EMPV1_28538	ACTTCTTCTCTGGATTGACCATCCCTGATGGGGGCGTGCATATTGTAGGGGGTGAACTGG
EMPV1_28540	ATATTTGAAGCACCTTCCTGAAAAATTGTCTATGGGCCAGGGAGGTCTGCCTCCCAGAGG
EMPV1_28541	CTAACTGTGCTTCTCGGAGGCCATCTTACCATTTAGCTACTCCACAAAAAGCCAGCAGCA
EMPV1_28542	AAAATGGAATGAGTCTTTGAGCTCCCCATTTACTATGGATTTGTCATGAAGGCACATTGG

EMPV1_28543	CTGAAAAGGAAACCCTGGCTGTTCTGGCAACTCCATTTGCTACCAGTGATGAAATCTCAG
EMPV1_28544	AACATTTCTTCCTTATGTCTGGACAATGTTGTTTTATACTTTGATGGTGGTTTGTTATGC
EMPV1_28546	CATGAATGTTCAGGGAGACTACGAGCCAGTCGATGCCACTGGCTTCATCAACATCAACTC
EMPV1_28547	TGCAGGCTCTGTGCTCTATCACCTCTTCATGTGCCACAAAGGGGGCAGCCCTGTGTACAC
EMPV1_28549	TCCTGGCTGGTGAGAAGTGGACCTTGGTGTCCAGTAGCTGAAACTCAATCTAGGGTCTAG
EMPV1_28551	TCAGGACGTGCCGGGACATGCCAGGGCCAAAGCCGTGTGTCTACCCCACGCGGATGGTAT
EMPV1_28553	GCGCCTTCTATGTTCCTGGGGTCGCACCAATCAACTTCCACCAGAATGACCCTGTAGAAA
EMPV1_28555	TGCGGCAGAACACGCTGGACTCAGAGGTCATGGTGAGGACTGTTTGCCTGAATTCAGAGT
EMPV1_28557	CTAAAGGACTACGCAGTCCAGCAGTATGTACGGGACTCCAGGGTCCACCAGATCCTTGAA
EMPV1_28559	CCACAGCAAGGCTGGGTGCTTTCTCCTCTCTTGGGATTATTAGAGGCTATTCTAAGGCTC
EMPV1_28560	CTGTGAAATGGGAGGATGCCCTGGTGCGGCAGACAGAAACAATGCCCCCAAATACCAAAA
EMPV1_28564	GGGATGTATGTCTTCTTGGCTTTGCTGTGCTTTCTTACAACCTTTGTGGTTCACCAACTG
EMPV1_28566	GAGATCAAGGATGGAGATGGATGCGAGTGGGCTCTGGGGGTCTGTAGGAAAGATGTGAAA
EMPV1_28569	TAGACAGGTGCCCCAGAGCTGTTTATGGCCTCAGCTGCCTCACTTCCTGCAAGGAGATCA
EMPV1_28570	GTGTATCTTATGTATTTCATGGTTATGTTGCCTTGGGTCAGGTGGAAGCAGAAGGTCAAG
EMPV1_28571	CTGCTCTCCTGGGTGACCACAGACTTGGCAGCAGAAACACTACAGATCATTCTCTTTCAG
EMPV1_28572	CAGTGCAATGATTGGAGGGTTTGCAGGAAGAATAGGAGCTTCTAACATGGGTCCAGTAGG
EMPV1_28573	CCGGAATGATATGATTGAAGGTTAGTTGTAACAGAGAATTTGCCTTCAGGAAAACAGTCA
EMPV1_28574	TGTTTAACATTTTGTCTTTCAGTGTAAATGAAAAACAACGTTTTTTGTGTGTGTGTCTTC
EMPV1_28575	GGCCAAAGACGTTTTAAGAGGAGGCGGAGGGTCGTGGCTCTGGAAGACGAAATGAAGATT
EMPV1_28576	CCCAGCTCTGAGGACTAGGTTACTCGGAAACTGCCTGCATCTTCGGTCCCTTTGAACTTT
EMPV1_28577	ACTGGTACACGGGCGAGGGCATGGACGAGATGGAGTTCACCGAGGCCGAGAGCAACATGA
EMPV1_28581	GCCCTTTTACGGCTGAGATTCCAGGAGGCTTTGGGGATTGCAAACTTGAATGTCTTTAGG
EMPV1_28582	AGCCAACTCACACTTCGAGCACCGGGGGCTGCCAGGTACCAAATAAAGCCTTTGTGACAT
EMPV1_28583	CAGGTCAGTTGTCATCTTCGGCATTCAACACGGAGAAAAGCGAGAGTAGGAGAAATCTGG
EMPV1_28584	AGTTCCTGGCAAGCCTGGAAGAGTTGTGACTCTTGTTGAAGATCCTGGGGGCTGTGTATG
EMPV1_28585	TTGAAAACTCTTCTGAAAACTGGGAGCAGGAAGGTACAGAGCTTGGAGGCCGTGATGCTC
EMPV1_28586	AGCGTTAGGCTACAAGAACGTAAGGAAGCAGCCCACGGGCCTGAGCAAGGAGGACTTTGT
EMPV1_28587	CCCAACATGGGGAAGGTCAGCATTGAAAGGGTTACGCAGCAAGCTTTAGTTCATGGTTTT
EMPV1_28588	TCTGGGCCACATGTAGGTTTAATGACCACGCGCCACCGGGAGAGTCTTTTTCTTCCTTGA
EMPV1_28589	CGCAGCGTGCGCATCGAAGTGACCTGTAACCACTGTACCAAGAACCTGGTGTTTTACAAC
EMPV1_28590	AGCCTTGGGGGCAGTGTTGTATATAAGCTCGTGCAGCGTCCTCTTGTTACTGGTGAGGTT
EMPV1_28591	GTACGTGCCAGGCACTGGTGTGAACTCTTGTATTTAACTCTGATCCACACTATAAGGTGG
EMPV1_28592	AATGACAGGGCAGCTGGTCCCGGGTTTACTCCACAACAGCCCTAATAAGCACTCTTCGAT

EMPV1_28593	TTTTTCTCTTTCAAATTCATCCAAATGTGCATGTGCCTTCAAGTTGATATCAACAGCTCG
EMPV1_28594	TAAGCCAAAGGACGTGGACCTGACTCTGCCTGGCTGGGGCGAGTGGGGTGGTGTGGGCCT
EMPV1_28596	GTCCTATGATACTACCTTCCATGACGACCATGTGGTAGTTGCCTATGATTGGGTGCACTG
EMPV1_28597	CAGGAGGATGTGATACAGACCATTTGGAGAGTAGGATGGCCACTTTCTATAAGGCGATTG
EMPV1_28599	CAGACCATCTGGCCCACAATTTACTCCACAGTGAATTGTAGAAGTTAGGCTTCTAAACCC
EMPV1_28601	GGCTGTGTCTTTGTCCAGGGGATCAGCTTCTCAGCGCAACCATATTCTTCGACGGCATTA
EMPV1_28604	TACAGCTCCAGCGAGACTCTGAAGGCCTATGACCACGACAGCAGGATGCATTATGGAAAC
EMPV1_28605	CCAGATCTATGTTGCACTTTGCAGCAATGCCAGATCCTTTAACCCATTGAGCAAGGCCAG
EMPV1_28606	AATCCCAGCATCTTGTAGTCCTTGGTGTACATGGCTTTGCGTTTTTCAGTCCCACTCCCG
EMPV1_28607	GGAGAGAGAAGAAAGGGAGAAGCTGAGGAGGTCTCGATCCCATAGCAAGAATCCCAAAAG
EMPV1_28608	CATGTGTGGGAATAAGGGCTGTATGAGAGACCTCTGTGTCTTCCCCTCAGTCTTACTGTG
EMPV1_28609	AATGGAAACGGGAGCCCTCAGAAGATGAACAGGAACAAGGCACCAGTGCAGAAAGTGAAC
EMPV1_28610	TGGTTACTGCAGAGTGAGTGCTAAAGGGTGACCCAAGCTCCAGGCAGTAAACGGATAACT
EMPV1_28612	TCATCACTCTCATCGATTCCTACGAGTCTTCTAGCTTCATGTTCCTGGTGTTTGACCTGA
EMPV1_28614	CCAGACAGCCATGGTGTTGGCTATTGCTGTGTCCTACAGGTTCATCGATGGTGCTGTGAT
EMPV1_28616	ACGAGCTGTACGCTCAGAAACTGAAGTACAAAGCCATCAGCGAGGAGCTGGACCACGCTC
EMPV1_28617	CACTCATCTAACAGTACTGCGGTAGGAATCCCTCTGTATTGCATTCAGGCTAAGAGGATG
EMPV1_28618	GCTGCAGTCTTTGTTGCAGCCACCCTTTGCATTTCCAGCCCATTTTTACTCATCATTGCT
EMPV1_28619	AAAGTGGTCCATCTACTGGTCTTGTCAGGGGCCTGGGGCATGCAAGTGTGGGTGACCTTC
EMPV1_28622	CAGATGGCTGACAGACTTAGATCTCGGCAAAAGTGCATGTCTCAGAGCCTGGACCTGTCC
EMPV1_28623	CCAGAACTGTCCTTTGGCAAATGTCATCTGTGAATATTGCAATACGGTGCTCATCAGAGA
EMPV1_28625	ACGTACACTCGCTTTCAGCACTACCTTGACAGCCTGCAGAAGAGAAACATGGATTACTTC
EMPV1_28626	ATAGGAATGGTTACTGTGAGTTTCCGCCCTGGAATCATTTGACAGCTCTGGTGGGTCTGG
EMPV1_28627	TCCCCATACCACCCATCATCCTGGCCGAACTGGGAAACAATCCTGAGAAACCCACAGTTT
EMPV1_28628	GAGAAAAAGAGCTTGCCGTGTGGCAGGCCGGTAAAGAGGTGAGCTGTTGGGGCAAGAAAT
EMPV1_28630	GCCTAGACCAGAGGCCTTCCTCCTTGGCTATATCTCATTAACCGTATGTGAAACCTTAGC
EMPV1_28633	CCCGCTCCCTCACAGCAGAGCTACCTTTCTATGGAGAAAATCATTCAAGTGGCCAAGATC
EMPV1_28634	GGTCACAGAGGTTAAAGATTGTTGGTTTGCATTGGTCTCCCCAGCCTTGCTCAAACATGT
EMPV1_28635	GCTGTCCAGGCATGGCCACTGATAGAAAAGAGGACATGTTGGCATGGTCATGCAGGAGGA
EMPV1_28637	AGTATGTTATCTCTATGCCAGACCTGGTTCGCCTGTTAGAGCAAAAGAAAGATCCCTGGG
EMPV1_28641	AGGAGGAATGGAGCCTGAGGAGGAGCCTGGCGGTGCTGCCGTGAGGGAGGTTTATGAAGA
EMPV1_28642	CTCCCACAAATAAGCTTTCACCACAAACATTTTGTACAGAATCTCACAAGTTCACAGCCC
EMPV1_28644	TTCGGACCTCCAATAAAACGAGCCCCAGCTCAGCCAGATCCTGACGACCAGTTCATCATT
EMPV1_28646	GGGGACCCGAAGGTTACGTGCTTCATTAACGACTTTGACTCCTACCTGACGGAAATCGGA

EMPV1_28647	GGCATTTTCCTGATCAGAAAATCACCCCACACTCACAGGTTCAGCAAAGGTACGGGAGAC
EMPV1_28648	CATGTCACAGACACTGGTGTGGGGATGACCCGGGAAGAGTTGGTTAAAAACCTTGGTACC
EMPV1_28649	GATGGCCGACCAGACCTTCATTTCCATCGTGGACTGGGCGCGCAGGTGCATGGTCTTCAA
EMPV1_28650	CCGACAGCTACAGCAGAAACAGGAGAGTTATATCAGGGAGATTTCTGATCTTCAGGAAAC
EMPV1_28651	GCCACTGGAATGGCAGCTGCTCTCAGCTCTCTCTGATTCTGTGAAGGATGATGAAGATAG
EMPV1_28652	GACTGGAATGAGGCAATGGATGGGGAGTGGGAAGGGCCTCTGATACCAAACCTGAAGTAT
EMPV1_28653	CTTAAATACAACAGACCCTAGCAGTACATTCCGCTGTGTTCAGATGTTGGAGTGGTTTGA
EMPV1_28656	ACAGCTAGCAGACGTGCAGAAAGACCTGCAAAGTGCTATGGCTGCTGCCGAGGTCCTGAA
EMPV1_28659	CAGCAGTTGCTTCAAAGTTGGTGTAGAGGAGGAGAAGCGCCCAGTGGATGTGTCGTGAAT
EMPV1_28660	GGCATGGACCTGAATCTCTACTCCCAAATATGCACTTTTCACTGTTCTTTTCCTGGCTCC
EMPV1_28661	CCAAGCTTATTAGGAGTTGGAGGTTGGGGGAGGTGATTAGGTCATTAGGTCATGAGTGGG
EMPV1_28662	GGTTAATTGTGCTTTAGTAGTAATGCTCCTGATAGGCACCTTGAGGGACCCATTAATTGC
EMPV1_28663	CTTGAGACCAACTTTTCAATGTGATTCTTCTAAAGCACTGGTTTCAAGTGTAGAGACTGG
EMPV1_28665	CATCAGCAAACAGGATCAGTTAACTATTGCATTTATACCTACCGTAGGCTTTTTATTCCA
EMPV1_28666	TGAACTAGAAGTGTGGCAGCTTCAAAAACCAGATGTGGTGGGTATGAGGAGGCAGAGGCT
EMPV1_28667	AGGAGAGCCGGTGACTAAACATTCTTGCAGCTTCCGAGGGCCACTGGGCTGGCATTTTGT
EMPV1_28668	TCAAGTCCCAGGTGGTGGACCTCAAGGCCGAAGGGTATTGGGAGGAGCTGATGGACACCA
EMPV1_28673	GAGCTCTCCTACTTTGGGGTGAAGGTGGCTATGATTGAGCCCGGTTTCTTCAAGACCAAT
EMPV1_28674	TCCTGCGCGAGTACCAGGAGGAGATCAAGAAGCTCAAGGCGATTCTAGCCCAGCAGATGA
EMPV1_28675	CTTGAAATTCCCTGATGGCTCAGTGGTTAAGGATCCATGTTGTCATTGCTGTGGTGTGAG
EMPV1_28683	GGGCTTGAGGCCCTCGGTCTGGAGCCTCCTCCCCCCGAGGCTCCCGTGCTACCCGGCGCC
EMPV1_28684	GGACTTTCTGTAAGAAGTGCGGCAAGCACCAACCCCACAAAGTGACACAGTACAAGAAGG
EMPV1_28688	AGTGGTTTCTGGAGAAGTTAAAGCCTGAAGGTCTTCACCAGCTCCTGGCAGGGTTCCAGG
EMPV1_28689	TTCGGTGCTTGATGGGGCTGTCTGTTGGTGGATCAGATTTTGCAGCGCCTCTTAGGAGCG
EMPV1_28694	ACCCGACCTTGCTGCACCAGAGAAAGAAGTAGCGATTCCTCAGTAAACACATCTCTCACC
EMPV1_28703	GGTTGTCTGAACATGCAGCATGCTTGTCTGCTTATATATACCCCATGGTTCTTGAGCAGG
EMPV1_28707	GTCTTCCCTTTTCATGTCTGGAGCTCCTTCTTCCCTGACTCGGCTCTTGTTGTTGATACG
EMPV1_28714	TCGTCCCCGCTCCGCGCGCGGGCTCTCACCCACCTGCAGGTGTCTGTGCGTCTGTCCATA
EMPV1_28715	CCCCACTCACATCAATGGACAGATCCTCTAGACATAAATACGTTAAGGCAACAGAGATAG
EMPV1_28719	TTCCCATCTCCGACTCCTCCCACAGCAAACAGCCCTTCCTATGCTGTGCCAGCCTCTAGA
EMPV1_28721	CGGTACTGAGCCTGTGCGAGAAAGGGGATGCCATGATAATGGAAGAGACAGGGAAAATTT
EMPV1_28722	CCCCTGGCTGTTTGGGGGACTGGACCGAATAATGTCAATAAAAGCTGCCCCATTGGTTGT
EMPV1_28724	TCTGGACATTTTCCTGCTATATTTTCTCCTTCAGCCAGGTCTCCACTCAACTGTCACCCC
EMPV1_28725	AAGTTGGGGCGTTGAGAGCTGAAGGGTCAACTCAGCATGAACTGGTCACCCGGGAGGTAT

EMPV1_28726	GATGTGCCCTCTCTCCATTCTGTTGCGCAGACCTCTCATCTGCCCTTAGAAGCAACCAGA
EMPV1_28727	GTATTTTGCACAAGCGTACTGTACTAATGGACATCCAGCACCTAACATAGTGCCTGGCAT
EMPV1_28730	CATGGAAATCCTTACTCGTTCCAGGGGTCAAGTCCACGCCCCTTGTGGGAGAACTGATAA
EMPV1_28731	ATCCCCACGCTGGTGGAGGAGCTGATTGCAGTGGAGATGTGGAAGCAGAAGGTGTTCCCT
EMPV1_28732	CCCATATCACTGGTTGGGGGGCGGTAGTAGTTTAAAACCTTGTTTTATATACATCTTGCC
EMPV1_28733	CTGTATCTGTTCCGATTTCTGTTTTCTGGAAACTTGGCCACTCTATGAGGATAGTCGTGG
EMPV1_28734	TATCCTCCTGCTGTGGTGCCTGCGAGAGAGCTGGGACTTTCATGCCCATTGTGCTAATGA
EMPV1_28737	TTGAGTCTTAGGAAACTAATGTTGGGCCACAATCACGTGCAGAACCTCCCAGCACTGGTG
EMPV1_28738	CCTCTGTTTCCTTGTAAGTATCATGCACTCATTCTCCTTCCCTGGGCTGTTGCAGAGGGA
EMPV1_28740	GCTTCGAGCAGCCTGGGGGGAAGCAGCCAGGGACAGCCTCAGAGTCTTCTCCAACCTCTG
EMPV1_28743	TACATCGGCCATTATAATACAACCTGATAAGTGTTACAGCACTTATCAGGTTGTATTGTA
EMPV1_28744	AAAGTCAAGGTCCACAAAAGGGAGATTTTCTTCACGTCCGCAATGGCCTCTGGCCCAAGA
EMPV1_28746	GGCTCAAAGTACTGACAAATAAAGCTTCTGTGATGCTCTTTATGAAGGGAAACAAACAGG
EMPV1_28747	CAGATGGAGGGGCTCCTGACATAGCCAGCTCCTGTGAAATAATGGTGGAGCTTGAAATCT
EMPV1_28748	GATGCAGCAGTTCCAAAAGTACCATGTGGTCGTGTTATGCATTAGTATTTGGATGGTGGG
EMPV1_28752	GCAAGGACTTCAGGGTGGAGATCAGGAATGAGGCACTCTAGGAAAAACTGACAGAACAGG
EMPV1_28754	CCAAAGTTTATTGTGTGGCCAGAAAGAGAGACTGAAGTTTGAAGGCCTTTGCTGTCATCC
EMPV1_28755	TAATGTGACCGCGCCTGTTCCCGCCCCTTCTCTGGACGCGGTGGTGCTCTCAAAAGAAAT
EMPV1_28758	TCGAGCTGGACAAGTCGGGATATTACACGCTCTACCACTCCCTCCACCACTATAAGTACC
EMPV1_28759	CAAAGTCGCATGACCCAGCTCCTGAGAAACACCTTCTCTTCAGTGCGGCAAGAGCTAGAA
EMPV1_28765	CTACAACGAGAACCTCAAGATCCAACTCTGCCAAGAACTCTGTGAATAGCAGTGTGAGAG
EMPV1_28766	TGGACCCAATATGATCCACCATTTCTTCTGCCACATGCCACCTCTGGTGAAGTTGGCCTG
EMPV1_28768	CACAGGGAGCCTATTGAAAATACAAAAATGAAGCATCTGACTGAAGGACCAGCTACACTG
EMPV1_28769	TTTACCAAAGAAATGATTCATTCAATGCAGGTTCTCCAGCAAGTGGATAACAAGTTTATT
EMPV1_28770	GCAGAACAATTCATGAGATCCTGTGTATGCTTCATTTGTAACCCATGGCCAGTGCACAGC
EMPV1_28772	GGAGCGCAAAGTGAAGAGGAGGAGTCCTAAGTCTTTTAGTGCCCACTCTACTCAGGTTGT
EMPV1_28773	CGCAGAGATGGGGGGCAGCGCCCGGAACCCGGGAGCCTTGCAAAGGGACGTCGCCCCCAG
EMPV1_28774	TTTTCTCTCTCTTGAACTCTGCCCCAATTCCACCTTGGTCCACACCCCAAAGGAGCCCAT
EMPV1_28775	GAAAATCAGCTGCTGTCTGTAAGATGGACCAGGGGAAGCCTTTTGCCACATTCTCAGGAC
EMPV1_28776	GAGACACTGAGGGGGTGGCCAGCGATGCGGGTGTCTCCACGAGGGAGCCGCGCAAAGACG
EMPV1_28777	TGTCCATTCCTTTTCTCAGTTCTCAATGGTCATCCGTTTGCCCTTCTGTGGCCCCCGTGA
EMPV1_28779	TGTCATGTCCTGCTTGCCTTCTGTCTGCACACAGTGGGGTGTTGCTCCAGGACCTTACTT
EMPV1_28780	CAAGCGCAAGGCGGAGCTGGCCAAGATCAAGCAGAAGAACCGGCGCTTCAAGGAGAAAGT
EMPV1_28783	ATCCCATACTGGTTGGGTGGAGCTTCAGACCTGGAGCATGTGATTGGGCCAGTGGTAACA

EMPV1_28784	CTTGGACAAGCCAACATCTACAGAACAGTGTCTCACCATCCTGGCCAAGTCAGACATCCT
EMPV1_28785	TCCCAAAATAAACCGATCAAGTCCGCCAGAAGGCGAGTCCTAGAGCTAGAGAGCGATCCT
EMPV1_28787	CCTGCTACTTTGCTAAATTTGTGGATCAGTTCAGGTAGTTTTTGGGTTGTGTCCTCAGGG
EMPV1_28788	GAAATGGGAGACAGATGATAGTACGCAGTGAGCGTGGCCAGTCCCATAGGAGACAAGCAT
EMPV1_28790	AACGACATGCTCGATGGCTACTCTGGGAGTACACTCAACGCGAGAAGCTAATTTGGAACG
EMPV1_28791	AGCCAACCGGTGAGCCCTCTCCTAAGAGACCAAGGGGAAGACCCAAAGGCAGCAAAAACA
EMPV1_28792	CGAATGGCTGAGGACCCAGCAACATTCCAGCCGCCCAAGATGGACATCCCAGTGATAGAA
EMPV1_28793	CCTGTTTCTTAGGCCAGGATCAGGTGCTTATGCATAATTCAATGCCTCATTCCTTTCCCG
EMPV1_28794	AAGAGAGAGAAATGAAGAGGCCCATGTGGTTTGTTCTCCTTTAGACAGCCATAGGGGGAC
EMPV1_28796	AGATCTTCCCTGGGGTACAGGAATTAAAGGGGCCAGCTCTGTGGGTAGTGAGATGGCTGT
EMPV1_28797	TCATGGAGACTGATTCTTGGGCTGCCAGTCGCTGGAGTGGATGGAACCCTTGACCTCCTA
EMPV1_28801	CATCTAGGAAGCCAAGGAAAACCAACTGAACATATTTCATTGGCTGCATCCTTCAATTCA
EMPV1_28802	CCATTATTTTCACTCCCCAACCATCTGCAGCCCAGCGATCTGCTACTGCCTTTTCTGAAC
EMPV1_28803	AATGTGAATAGACCTGCAGGAGAGCCTAGCACGAGGGCGAACAAACGCAAGTTGAGTGAG
EMPV1_28804	TCTTTTGTCGATTGTTCCAAAAGCAAATTGCTAGAGGCAGCTATTACACCAGAAACCAAG
EMPV1_28807	ACACAGCCTGATTTAGATGGAGCACCAGAGTAAGTTCCACATACCACTCATGGCCTCACC
EMPV1_28809	GCAAAGCAGAGAGCAGAGGTTCTTCAGTCCACGCAACGGTTCTTCTCTGAACAGCAACAG
EMPV1_28813	ATCTAGTGGGTAGAGGCCAAGGATGCTGCTAAATATCCTGTAATGCGCAGGAGGGTCCCA
EMPV1_28815	GGGCAGGGAGTGTGTGTTTGTCATCTTTGTAATCTTATCACTTATCAAGGTCCAGGTCAC
EMPV1_28816	TAAATAGGTGTTCCTGCTATGGCGCAGTTGGTTAAGAACCTGATTGCAGTGGCTGGGATC
EMPV1_28817	TCACCCTCTGGGTTGAGGCAAATTGGAGTGGATGGAATGAGGAACCTGACTCACTTGGCT
EMPV1_28818	ATGCCTCCACACACCTTGCCATCTTCCGTCATCCTCTCCCCTGTACCCTGAACTCCTTTT
EMPV1_28820	TCTCGGTGTCCACCTTGCGCAACTTTGGCCTGGGCAAGAAGTCGTTGGAGGAGTGGGTGA
EMPV1_28822	CCAGGCCTCTAAGAATCTCGAGCAAGCCGAGAGGATGGAAAGCCTAGTCCAGGAGGCTTC
EMPV1_28823	CTGGCAGATTTTGGTCTTGCCAGAGCTTTTGGGATACCTATTAGAGTATATACACATGAG
EMPV1_28825	GGTGTCGGCGTTGGAGTTGTGAATTCGCTGCGTTTCCATGAAATCCTGCGGAGTGTCGCT
EMPV1_28828	ATTGCAGCAGGCAGAGTATGAATTTCTGTCCTTTGTACGACAGCAGACTCCTCCGGGGCT
EMPV1_28829	GGAAAAGAGTAATAGCTTGGTCATGGAAATGCAAACTGGTGCAGCTTTTTGGGGAGTGGG
EMPV1_28830	CGTCTACGAGGGGGAAGATCTAACGCAAAGCCCACTTTACGGCCATACATTTGCAAGACT
EMPV1_28831	GCCATAGCAGTGACAATACCAGATTCTTAATCTGCTCAGCCACCAGTGAACTCCTACCAC
EMPV1_28832	TCGCAGATGTGGCTCAGATCTGGTATCGCTGTGATTGTGGTATAGCTCTGTAGCTGTAGC
EMPV1_28833	TTCTGGGTGGAAAGTTGTGATTGGATCGGGTGTTAAGGATTAGAAAGGAGCCTCTAAAAG
EMPV1_28834	GCGCCATAAGAAGAGCCAGATTCACTGCATCCAACGAGCCATTAGCCTCAACTGAAACTC
EMPV1_28835	CAGCAGGCTGGGTGACCGTAGTTGGACTGCCACAAAGACCTCATCTTAACTTCAGTACCT

EMPV1_28839 AGGAAGCTTTTGGCAACACAGAACTACATGCCATCAGAATTGTTTACATGGCGTCTGGGG
EMPV1_28840 TTCCACACCCACGAAGGACCCAAGAAATCCTATTTTTAACACATGAGGGGCGATCCCCAC
EMPV1_28841 GCTTATCAATACCTGTTGTGTCTCCCTTTTATGTACAAACACTAGACTGAGTTTACAGGC
EMPV1_28842 TTGGTGCCAGGGCTAAGCCTGCAGGCCACTTCCAGTCAGGAGGTCTATCAGGTGAATCTC
EMPV1_28844 GTGTGCTAGGCTGAATTCTGCCAGGATTTGGGAATATTTTCCTAAGTTTTGGGCTAGGTT
EMPV1_28845 ATAGAGCTGCTTGTTGGACAGGGTTTTGGTGAAGCCCGATTTGTTGCTGGGCAGTCACAG
EMPV1_28846 TAGAAGGGGTAACCCATGAAGAAGCCGTTGCCATCCTTAAGCGGACAAAAGGCACTGTCA
EMPV1_28847 GACTGTGGTCCTTGCGTTGGACCTAAATTACCACGAATCCCTCTTCTTCCTCCCCAGTTT
EMPV1_28848 GTCTAGTTACTAGGCAGTGTAGTTAGCTGATTGCTGATAAAACCAATCACTAACCACACG
EMPV1_28849 GAAAAAGCATCAGTCATTCCCAGTCTTACTGAATATGACTGGAGAAACTATCAACCCAAC
EMPV1_28850 GATATTTTGAAAGCACTCCAGACTCTTCCCACTGCTGCCTTCTCTCCATTCATCTGGGTT
EMPV1_28851 CTGCGCTGGCTGACCGACCCAATCCCCCCACCCGCCCTCTGCCCGCTGACCCGGTGTTGA
EMPV1_28852 CTGACCTGGAAAGGACATCCAAACCAAGACTGGGCCAGGATTTGCATGTGATAGAAAAAA
EMPV1_28853 CAGTGCTAACTACAAATGAATTCTATCCACTCATCTTGACAATACTGGTTATTAGTTTGG
EMPV1_28855 CTATTTATATCATCCAGAGAAGGTTAAAAAAGGAAGGGAGGTCGGAAAGGAAGACAGGCG
EMPV1_28858 ATGAAAGCTGCCCTGTGCAATGCGGGTGTCCAGCTGAGCCCACAGGAGATGTGTGAGGCT
EMPV1_28859 CCTGTGGGACAAATTTACTGTACCTTACTGTTCCTGACAAGACTTGGAAAAGCAGGAGAG
EMPV1_28861 AGCCAACAGGGCATACACATGCGGAGAGCCAAAGGTCTATTGGAAGTTGAGATTCTGCTC
EMPV1_28863 CCACCACCCCCCTCCACCTTGTTAGTTGCTTTGCTGGCTCTTACTTTATGATATAAAGGT
EMPV1_28865 TGTGACGTGACTCAAGAGGATGATGTGAAGACCCTCTTTTCTGAGACCATCCGCCGCTTC
EMPV1_28867 GTCCCCCACCACCAGAGGGGATGCTGGAGAGGAGGAGGAAAAGAGGGAGCGGTGGTGCCA
EMPV1_28868 ATCGATTGGAACAAGCTCCAGAGCCTCTCGGCATTCCAGCCTGCTTTGCTCTTTAGTGCA
EMPV1_28869 GGTTTGTTGGGTTTTGGATCTTTCTTCATTTTGTCAACCTTTCAAGTAAAGCCCTCCATG
EMPV1_28870 AGATGGCCACACAGACAACCACTTACCTCTTTTAGAAAATAACACACATTAGCGCCTCCC
EMPV1_28871 ACTGCTGCGACTGTCTTGTGTTGATACCCGTGCTAATGAGCTGACCCTCATGGTGTTGAG
EMPV1_28872 TTGCATCGGGGAGATCAAAAGATGAATTAGGCCCTGTCCCTTTGATGTTAAGTGGAGGGT
EMPV1_28875 GTCCGAACCCGAAATAACAACAGTACCTCCAGCCGGGAGAGGCACAGGCCCTCCCCACGG
EMPV1_28877 AAGACAAGCGGGCGGAAGTCTGGCGCCAAGCAAGCCAGCAGTGATGCTAGTCAGAGCGAC
EMPV1_28878 TTCTACACCCCATCGACCTGCACCTGCTCTTCAACCTCATAAGTTCTTCCTCGTCCTTCC
EMPV1_28880 AGAACATAGAGTGGCATGGCCGTGCTGGAATGGCAAATAACTGGCGTGCTTTTTTGTGTC
EMPV1_28881 TGCTTCCTCAGGGCTCGTTGCACAGAGGCCTGATGGGGAAAGAGGAGCTGACCTGCCGAA
EMPV1_28882 GTGTTTGGCAGATGCGGAGGGTTCTGTGGCAAATGTATTCCTCACATGACTACTGGTCTC
EMPV1_28883 AGTTCAGGAGTTCCTATTGCAGCTCATCAGGGTAAGAACCCGACATAGCGTCTGTGAGGA
EMPV1_28885 AAAGACTAGGAACTTGAATACGATCTAACCTCTGTTTCCGAGGGGGCAACTTCCACGGGG

EMPV1_28887   ATGATGGTCGGCAAGAAATGTGCCGCTGACCTGGGCCTGAAAGAAGGGTTATCGGATGGA
EMPV1_28888   CTTTCTCTTGTCCCACATGTTCAACCTGACAGGAAATCCTGTTGGCTCTGCCATCAAAAC
EMPV1_28889   CCTTGACGGTACAGATCGGCGAGGCTGCTTTCTTGCTTGCCAGCTCAGGAAATTCTCCCC
EMPV1_28890   GTTGGCCCTCACCTGCACTAGAGTACCTGTAATAGAGTTAACGAGCTCTACTTTAAGTTC
EMPV1_28891   GGACCTGGCCGCCTTTGAGAAATCCCATGATCTAGCCATCTGTACCTACCTAGAGCACAA
EMPV1_28892   GCTGGAGTAAGGCTCCTGGCTCTCAGAGGTGCACCAAACGTAAGTTTCCATATTCCCAGT
EMPV1_28893   TATGGCACCACCCTGTGTCCACACTCGGAACCACAGAAGCGGTTGAAAGAGAGGCACTGA
EMPV1_28894   AAAGATAATCCTGTGAGAGCCACAGAGAGCACGAGGCCAGTGGTGGCGGCTGTGTATTTA
EMPV1_28896   TGAGCAAACTCAGCAAGCTCCTCACCTTATGCCTCCACCATTCTTAGTAGATGTGGATGG
EMPV1_28897   TTACGACAACTACAAAAAGCAAGCCAATGGTGACTCCTGCAGTGCCTCTGGGCCCGCCAA
EMPV1_28899   CTCTGTAGGGACTTTGGGAACCCCGAGGAGTCGAGGCTCTTTAAAGCGCTCCTCTTTTGT
EMPV1_28900   GTGATGCATTTTCTCTTTGGAAAAGAGAGATGCAGAATGAGAATATCTCATTAAGAATGA
EMPV1_28901   CCCTCAGATAACCAGCAGTTACCAGCATGGATCATCCCCCGCTCAGGCCTAACTGTCATT
EMPV1_28903   TTTTCAGAAGGGGCATGCGGCCTCTCGGGTGACTTGGAAACTCCTGGATGAAATTCCATT
EMPV1_28905   GCCTTCTTTGGTCCTCATACCCAATGATTTCTGCTGGTAGATGGCTTCCTTTGAAGCAGC
EMPV1_28907   GTTGATTTCACAATGGTTGAAGAGGTGGCTGAGGTAGATGCTGTAGTGGTCACAGAGGGG
EMPV1_28909   TGTGCGTGTGTATGAGAGGGCCATGTAGCAAGCATATACATACACACACACACACACACA
EMPV1_28910   CTAATAACAGGAGTTTGGCTTTAGTAGCCCTGGATGTTGCTGTGTGGTCTCGGGCAAGTC
EMPV1_28911   TATTTGAACCAGTCTTGCCTCAAGCCTAAAAACACTGTTGCCACCCTGGATGTCCCTCCC
EMPV1_28912   TCAAAGAAGTCCAAGTGGAAATCAGGTGATCGTGGAACCAATGGGGACTTGGGCGAGGGT
EMPV1_28913   GGAGCCACCACGTCTATGAGCTACAACAGACGCTAGATAAATTTCACACCATGTCCAACC
EMPV1_28917   CAGAAAACCACCTCAACCAATCACAGACGACCTGATGTCCACGGTGTGCTGGAGAATCAA
EMPV1_28918   GGAAACGATGGTTCTATCGCATAGGTCGGGGATTTGCTGTGTTGTCGGCTGATTTATCCG
EMPV1_28919   TTAAGCTTATTCCAGTTTTCAGCCTTCAGCTGGTTTCCACCTCCATGACTCCTCCTTCTC
EMPV1_28920   TGCAGCATCACCCTGGACCACTCATGGCCCAACACACTGGTGGCACACTCTAACCCATTT
EMPV1_28921   TCCCACAGAATATAGTAGGAAGGGTGATTGTGAAAACACTGCAGTAGAATGCTGTAGTGG
EMPV1_28923   TGGGTGCGGCGACAGCCAGTATTGGACTTTGAGAGTGGCCACAAAAGCAATGTCTTCCAG
EMPV1_28925   GAAGCATGAGGACAAACGTGGAGGTAGCGGATCTCACAACTGGGGAACTGTCAAAGATGA
EMPV1_28931   TTCGTTTTCTCGGCTTTCGCCCGCAGTCTGCTGCACCTCTATCTTGGCCTGGGCGTCCTT
EMPV1_28932   CTATCTTTCTTTAGCATCCGCACAGTTTGCTTCTCCACAATTGCCTCTCTTTGCTCAGTC
EMPV1_28933   GAGGGATGCACCGGGGGTTTGGGATGGAAATGTAAAATTGGGTTGTGATGATGGCTATAC
EMPV1_28935   CAAATTACACAAACTAAGTCCTCTTCCTCATGGCTTCCTTTGCTCGCGGTGCTCCTCCCA
EMPV1_28937   GCAATTTAACCTACATACCTCTCAGGAGTTTGAATATTATCCAGATCTTCTATGTCCAAG
EMPV1_28938   TACCACAAAATTGATGGGAAACAATTCCTCATATGTAGCATATTCAGATTTACCAATTGC

EMPV1_28939	TCGATCATTTCTTTTGTGAGGCTCCCTCTCTGGTGCATTTGGCTTGTGCTGACACGTCTC
EMPV1_28940	AGGAGAGTGGCAAACATAGCACGGACTAATGCCACGAACAACATGAATCTAAGCCGAAGC
EMPV1_28941	TGAAACCCTTGCTGCAGTCTCTCTCGTCCGGTGTCTGCGTGCCAAGCTGTATTACCAACT
EMPV1_28942	ATACGGCTCCTGAAGGCTGGAGGAGACTCTTTTCCTCTGATGGGAGGTTCCGGCGCTGTC
EMPV1_28943	CCATTGGGAGGTGGAGGTTGGGGACATGCAATCTTGGGACTTGGGAATTTGTAGAGAAAA
EMPV1_28944	CAGTGTGTAAGGACTGAGGTATTGAGCAATACCAACCATAAAAATCTCACCTAATTGGCA
EMPV1_28945	TGCTGGACATGGTAGAAAATTACCCCACAGCTTTGCGGAGTCTGGTGTTGGCTGAGAACA
EMPV1_28946	GAGGGTTTCTGGGGCCGTGTGAACATCCAGTGAGACTCGTCCTCCTAGAGCAAGTGGTGT
EMPV1_28947	ATATGGAACGAAATCGTTTGTCTCGGGACTATTTAGACCCAAGAAGTGGCCCCTCCGGAG
EMPV1_28948	GTTCAATTCGGGGTGACAAGTACAACTTGGGGTACACCTGAGTGGCTTCAGTGTCCTCGA
EMPV1_28949	GGCCACAACAGCTTTCTTCTTTCATGCTTCATTCCAAAGGAATCCAAAGCCCATTTGAGG
EMPV1_28951	GTCCAACTCAGTGGAGCAGCTTATCTGTAAGAGTAGTAATTACTGTTTATTGCTCTAAGG
EMPV1_28954	AGCCACGATGGGAACTCCTATAATGGATCAGTTTCTGAAGTTGCCTTAGCAAAGGGTGAT
EMPV1_28955	CTCCTCTGAGAAATACTTGTTTTTGATACATCTCCATTAAGTGCTTTTGTCATGCGTGGA
EMPV1_28956	TGGAGGGATACTCCATGCCATTGTGCAGATTCACTTAGTCCTTGACTTGTCCTTCTGTGG
EMPV1_28958	TTGTGTTTCGATCTTCTTCCAACATCGCCACCATCAAGTACCTCAGGACATCAGGCCAGC
EMPV1_28960	TAGGAAGATGCCCCTGATTTTGTTCTCTGCATGGGACTCTTCAGGCCTCCTGCACCCATT
EMPV1_28961	TTAATTAGCCACCTGCACTTCTCTGCTTTCCTCTGCGGACTAGTGTTGTAGAAGCCAGGG
EMPV1_28964	TGCACCAGTTGATAAAGAATGCCAAAAGCCACGGCATCGACCTCTCCAAACCTGTAGACT
EMPV1_28968	ACCTGCTGCTTTGTGGGTCACACTCTTAGAGCCAGGGTTGATTTTTGGTCACCATCTGCA
EMPV1_28969	CTGGTGACCCAGCAGTGAAGGATCTGGCGTTGTCACTGCTATTGGGCAGGTTTTGTCTCT
EMPV1_28972	CTCATTTTACAAAGACCTCCTTTGGCTGCTGAGAAACCAGTGGTGTAGAGAGTGACGTTG
EMPV1_28973	CATCCTGGGCTCGAGGGGGATGAAGCTGCCTTCTAGTTTTACCTGAAAAGGTCATTCATG
EMPV1_28974	GAAGCCGGCTCTGAAGATATTGACATACTTCCCAGTGGTCTGGCTTTCTTTAGTGTGGGT
EMPV1_28975	TGCCGATCACCGGACACAATCCTGCAAAAGGACACCCGACTCTATTTCTTACAGTGCGAA
EMPV1_28976	CCTATGTGCCTTCCTGACTTCCTAACTGCCTCCTTTGTTTAAATTGTGCAACAGTGAATT
EMPV1_28977	CACATAATCTTCCTGTCACTACTACCACAGCTGCCTCGTGAAACTGATGAAGCCCGTCAC
EMPV1_28978	GCAGCATCTTGGTGTTGGCATCAGTTAGTAGTCTTTCCTCACTCAACGGGTGTTTTTCCC
EMPV1_28980	CCGCCCTGTTTGGGTGATTTCTATTATTCTATGTTCCAAGTCACTAATTCGTTCCTCTGC
EMPV1_28982	GACATGGTGATACGTAAGAGACTACGTGGAGATGGAGGTAGAGTTTGCAGTGCTAGAGTG
EMPV1_28983	GGCTCAGGTCACTATATAGGTGTGGGTTCAATCCCCAGACTGACACAGTAGCATGCCACA
EMPV1_28984	TGAGGACCAGCTCATGCGCTGGCACCCCCGCTTCAATGTGGACGAAGTGCCTGACATCGA
EMPV1_28985	AGACTGGCTTTTGGGAAGCCCCAACCTCAGCACCAACAACTGGCTCTACTTTTGGGTCTA
EMPV1_28988	TCTGGTTGCCTGGCGGGCCCTGGGACCACAGCTCCTCCGCTCCTCCAATGAGCTGGTGAC

EMPV1_28990 CTCGGCCAGGCGGCTATGAGCTAGCTTAGGAACGACGTCCTACTGCGTGTATTTATTTGA
EMPV1_28992 TAACTTACGTCAGGAGAGCTCATGCCCATGTGGGCCGGTTTTTGGAATTCGTGGGTGCTC
EMPV1_28993 CCATCAAGGGAGAAATCCGAAAACAGGCATTGGACCACTTCAATGCAGATGGGTCTGAGG
EMPV1_28994 CTCCCTGCCTCATTCTCTTCAGTTCGGTCCAATTGTCCTGCGTGATTTTCCTAGAATGGG
EMPV1_28995 TAGGGCATCAGAACCTGGAAACCAGAAAGGTCGACCCGGGAGTGAGAAGCCTTAATGGAA
EMPV1_28999 TGCAGGCCTTTGACCAAGCCTGGCACATGGTTGCCCATGAATGTCCCAACTACTTCCGCT
EMPV1_29000 TCACTCCCTTTACCACTTCCCCGGAGTGTGTTCCCAGGATGACTAATCAAGTGAGAGACT
EMPV1_29001 TGGTGGGTTCCACCCTCTTCCATATACTGGGGAGCTAAAAGTGGCACTGCCTAGTGGAAA
EMPV1_29002 TCTGGACCTGTCACTCTGGGCGTCGATGGTGGAGAAGATGATCTACGACCAGAGACAGAA
EMPV1_29003 AAGGCTTCATGGCTCCCAAGGCGTGGACCGTGCTGATGGAATACTACAAATCCTTGGAGA
EMPV1_29004 TTCCACCCTCTTCTCACCCCTCCTCTGCCGTCTGGGACTGTTTGAAGTCAGCTTTGATAA
EMPV1_29005 TGCCGAGGAGCGGGCTGAACTCTCAGAAGGCAAATGTGCCGAGCTTGAAGAAGAGTTGAA
EMPV1_29006 GCAGTGGGACGTGCTCACGGAGAAGCAGAAAGCACGCTACAACACCAAGTTTGCCCTGGT
EMPV1_29007 GCCAACTCCTGTGGAGGAGAATGTAACTCAGAAACTCAGTCACCTGGAAATCTGTCCTGC
EMPV1_29008 GGGGCTGCTGAATCTGTAAGGCCGGATGGAAGGAGGTTGAGCCATGTTTAACTTCATCTC
EMPV1_29009 GGCCAAGTAGCTACTCCTTCAACAATATTTATCAGTGGATCGAGAACTGAAGTTTAAGAT
EMPV1_29010 TTAATGAAGCAGGGGCTGTGTTTTCACAAAGCACTGGTGACTGCTGTTGGCTAAGCTGCG
EMPV1_29014 GATTCCGGCCAAGGGAGTTCGTTCCCAGTTTGAAGAAAGAATTTTGCCCATGGAGACGTC
EMPV1_29015 CAGCCTAACGGTGAGGAGATGACCCTCAAACAGTTCCTTGACTACTTCAAGACAGAGCAT
EMPV1_29018 CCTTTGTTCCAATGATAACGGCGCCCGTGGCTGAGCATGAGCAACATGCCTGGAGTTTTT
EMPV1_29020 GTCCTTGCACCTCCTCAGCTCCCCGCACAAACTCACAGACATAGAAGACAAACTTATGGT
EMPV1_29021 ACGATGAGGTGATGAGCCAGAACCTGGCCTACTACACAGCCATGCTCGGAGACGAACCAG
EMPV1_29022 CCGCGTAAGCAGAGAGCAGAGTGTTTTTCAAAATGCTGAACGCCGTGAGTGTACAACAGC
EMPV1_29023 AAAACGTCTGAAGTCTCGGAGGCCCGTCTGGTGACTCTAAACAAGGGACTTGGATTTGGG
EMPV1_29024 CTGGACCCTGAGGTGACAAAGCGAGATGGAAGAGAGTCTGAGCTGACCTTGTCGTTATAA
EMPV1_29025 TGTGCAGCAAAACTACTTATTCAGGCCAAGTGGGAAATGAATGTGTGCAGTGTGTTCCTG
EMPV1_29029 GATGCTATAGACACCTTCTGTAGACTCTGAGGGTTACTGCAACAATGGATGTCCCAAAAC
EMPV1_29030 AGCTGCTAAAACACTGGGTATTGCCGTGGCAGCTTTTCTGGTCTCTTGGCTCCCATACAT
EMPV1_29031 CTTGCCCTATGGGTTCACGAAAGCTTAACGGCCATGGTGATGCATTCATGGGTAGAAGAC
EMPV1_29032 GAATGTTCAAGTTGTATTGAAGCACCCAGTGACCTGGCATCAGAAAGTTGGACAATGGGG
EMPV1_29033 GTGTCCAGCCCAACATCCAAACAACCAAAGTAGAACAAGAGTCACAAGGCCGATGGCAAG
EMPV1_29034 TAAACTACCCGGCCATGCTGGCTCCATCAATGAAGTGGCTTTCCACCCTGACGAACCAAT
EMPV1_29040 GCTATCAACAAAACATAGGGAACAGGCGTGGGACGGATTCTTCCTCATAGCCCTCAGAAG
EMPV1_29041 GTACATCAGTAGTGTGAATCCACTGGCACAGTGTGTGTAAATGCCAGATGTGGTGAGATT

EMPV1_29044	CCATCATGACCCGCCTGCACAACCTCGCCCAGTTTGAGGGCGGGGAGAGCAAAGTGAACA
EMPV1_29045	GGATAGGAAAATGGTCCGTATTGTTCTAACAAAGACAAAGAGAGATGCAGCGAATTGTTG
EMPV1_29048	CGGCATTCCACATATAGTCAACAATGCTTATGGAGTGCAGTCTTCAAAGTGTATGCATCT
EMPV1_29050	ATATATCTATTACTCACAGGCCAATCACAAGGTCCCCCCGCCCCAAACCCCTCCTGGTAG
EMPV1_29052	ATGTCATGCCCAAGGCAGGCCTGGCCCCTCTCAATGGACACACATGGCCAGACCCCATAT
EMPV1_29053	CCTTGGAGCTGAGTACAGGGCACAACTGTGGCTGAGGGTCTCAGAGGTTCCACTTTCTTT
EMPV1_29054	CTTCTGGCGCATGTTCGGGGAAGCCCTTGAAGACCTGGAGTCATGTGGTCAGTCAGAGCT
EMPV1_29056	GCCCTGATCTCCCAAATAAGAAATTCAAGCAAGTCCCTCAGTTCACCCTGCAACCCAAAG
EMPV1_29057	GATCTCCCAGGACACTACTATGAGACCCTCAAATTCCTCGTGGGCCATCTGAAGACGATT
EMPV1_29059	CAGCTTCCACTCAGCCAGGACCACTGGAATCATACACTGTTGACCACTGACCCGTCTGTA
EMPV1_29061	ACGCTTCAATCGGCTTTGCCTCTGGACTTGCGGTGAAAAGCCTCTCTTCGGACGCTGACT
EMPV1_29062	GCAGAAGCAAAGAAAAACAACTAATAAGACAGGGACTGAGGCAGGTGGCATGAGATCGGC
EMPV1_29063	GTACGTCCCATGCAATATTTAGGACATACTTATGCTAAAAAACTATGCATTGTTCATCTG
EMPV1_29065	AGCTCTGGCCACCTCCTACACGTCCTGACTTCCAGACGGTTCCTTCGCTATGAACTGTTA
EMPV1_29066	GCTGCACCAGCGCGACGTCCTTTTGACCCAACTGAGTAATTGCACGATCAAATTGTATGG
EMPV1_29067	AAGCATAGGCCTTGGTGCTGGTTGGGGATGGGGCCTGCAGAGTCTTAGTTACTGATTTCA
EMPV1_29068	GCTAGCGGCTGTGATCAAAAATTCAACACAAAATCAAACTTGAAGAAGCATTTTGAACGG
EMPV1_29069	TGAGCTTAGTAAAGAGCAGCAGAGGTTGAGCTCCTGTTCCCTGCAAGTGCCACATTCCCT
EMPV1_29070	AGGAAGTCTGCACCGAAGAGTTCTGAATGTCTTGCGAGTTCCCTCTGTGGCGCAGCGGAA
EMPV1_29071	TGTCCACCATTATTATCATCCTGTGTTCGCCTTCTGTTCCAAAACGTTCAGGGAGTAGGC
EMPV1_29072	GCCAGCCGAATCCAACAACATATACAAAAGATCATATACAACGACCAAGTGGGATTCATC
EMPV1_29073	CAGAATGGTTTCCTGTGTTTAGAACTGGGCCCCACCCTGGATTTCCTGATTTAGTGGGTC
EMPV1_29075	GAGACTTCTCCTCATACTGAGACCTCAACTAGAAGAAGGATGGCCACGTCAAGCAGATGC
EMPV1_29076	CAGCATTTGTTCTTTGCCAGTCTCATTTTCATCCTCTCCCATGGTTCCAAAGAAGCCTTG
EMPV1_29078	ACCTGGACAGACAGGATTTCTCAGCTATGGCACAAGCTTTGGTACCCCTCAACCTGGACA
EMPV1_29081	GTGCACGCACACTAACACTTGTGTGCTAACACTTGTGTGCTTTTCCCACGGTTACAAAGA
EMPV1_29083	GATGGTGGCCAACAAGAAAAGTCAGGACCAAATGACAGAGGACCTGTCCCTATTTCTAGG
EMPV1_29084	AGGATCCAAGGTGTGTCCACGGCCTGCACCACAGCTCACAGCAATGCCAGATCCTTAACT
EMPV1_29088	AGCCAGTTGATTAGGGTGTTTAGCTGTTAACTAAAAGTTTCGTGGGTATGTAATCCCACC
EMPV1_29089	CAGAGGGTTCTGTGGGGTCACGGAGCTGGTACAGATGGGGGAATTGTGTTCCTGTCCATA
EMPV1_29091	CGCCCTTGCTAAAGCTGGCCTGCGCGGATACCTCTGGATATGAGTTCATGGTATATGTGT
EMPV1_29093	GGACCAGCAGATGGCAGTATTTTCCTTCCTAACACCTCAACAGCAAAGGGAGTCCTGTTC
EMPV1_29094	CTGCTCACAAAGGGAGGCTCTGGAACCTGTAGAAGAGGAACTGGCATACAGACCAAATTC
EMPV1_29095	GTCTGACACCTAGCCTGTGAGTTGTCCTGACCCTCAGCCCCAGTTTGGTCAGATAATTTC

EMPV1_29096 CTGAAGGAAGGCTGAGGAATTGGTAATAGTTCATCCCCTGTTTTGAGTACATACTTCTAC
EMPV1_29097 ACGAACTTAAATACATTGAGTGTCTACTGTGTGCGAGGAGTTGTATCACTTTAAAGCACC
EMPV1_29101 GAAGTGGAAGCTTCACAGCTCAGCCTGGTGCCTCAGGTACCCATGAGAATGCAAAGCGCT
EMPV1_29104 GGGTATGTCAAAGAGACGCAAGAGCCACCTGAAAGAGCTCCCAATGGTCAAAGCTGGAAT
EMPV1_29105 AATACATGATCATCAAGAGGTAAGAACCTTCCTCTGCACCTCAGCCAGTCCTGCCAACAG
EMPV1_29106 AGCTTGACAACCGTATGGAGTCTTTCTTCCTGGCCGAGACTGTGAAATACCTCTACCTGC
EMPV1_29107 GCCGTACTTCCTCTTGAATTCAGATCTAATTTTCAACATGTCCACTTCACTGCGGGAGAC
EMPV1_29110 AGGTTTTGCTCTGGGAGCATCAGAAAAGCACTTGAACGATGAACAAGGAAGCCCTGCGGA
EMPV1_29115 TCATGCACCCTAGCTCCATTCTTAGAGGAAACCCATACCCTAAGCCATCCTTTGCCACCT
EMPV1_29116 GAGTCAGCATCTGTTCTGCAAAGTTCATTGTGTCCTTGTTTCGGATTCCACATGTCAGCG
EMPV1_29117 ACCGTGCTTCTCACGGTCCAGCAAAGAAGAGAAAATGCTATCGACTGTGGCATAGAGTGA
EMPV1_29118 ATCTGTCTGTTAAACTTCCTACTCTTGCTTGGCTCCTACGGGGTGATCCTTCACACCCTT
EMPV1_29119 ATGCCAGTGTCACATGTCTATCCACTGCCTCACTGCCTTTAGAAAAACCACGTCCCCGTG
EMPV1_29121 TCATACGAGCTATTTCCTTGGTTCAGATGTCACATAGCATATTAAGCCGTGCAAAACTGG
EMPV1_29122 GCCAGGTGCCTTTCAAAGTATCTCTGGATGTCGGTCTGCTCTATGATGAAGTGCCCATGA
EMPV1_29123 AAGATCATGTACGTCGGGGACGTCCGCAGCGTCACGCAGAAGCATGTATGTCACTCTCTA
EMPV1_29125 CAGACATTGACTTCTCTCTAGCTGTGAAATCTCTAGCTGGCACCTCTTCCAATAGAAGGC
EMPV1_29126 GACACGGCTGGCCAAGAGCGATTCAAGACGATAACTACCGCCTATTACCGTGGAGCCATG
EMPV1_29127 ACCTGCAGCAATCCATTGCAAGCACTGTGCTGTCCTGTGACAAGCAACCCCACAATGTCA
EMPV1_29128 AGGAAAAGTCCAGCTTGCTGAAGGGGGCCTCAGTGATGGTGATGGTGAAACTAGGCCATA
EMPV1_29131 ACGGTGCCAGGACCGCTCTTGGATTGTTAATTGCCCACCTTGAACGAGTAAGAAGGTAGT
EMPV1_29133 GAGGAAACCACGAGAGCCGACAAATTACCCAAGTATATGGCTTTTATGACGAATGTCTGC
EMPV1_29135 TGATTCAAAGTACATTTCTCGGTCATCTCAGGGCACTCTCGGTCTCGGGGTCAGCAGAGC
EMPV1_29136 CCTAGGGCTGTCTATTACTGTTTACGTGTGTGGGTGTGGAGAAAGGTTTTGGAAACAAGG
EMPV1_29137 CTGAAAGTGAAAGAAATTTGGCTCTCCAACGTTAGGATGGCATCCATTTCAGCTCTGAGT
EMPV1_29138 ACATTTCACACGCCAGCGACTGTTCAATCATCACTTGACCCTAAAGACACAGGCGGGAGG
EMPV1_29139 TGTTGATTCTGCTACGACTGCTGGTAATGGTAGTATGGGAGGAGGTGGAGATGTCACTGC
EMPV1_29140 GACAGATTGCTATTCCTTTTCTCGGTTTGGAGACCCTCCTCCCACTCCCTGTCTTCACTT
EMPV1_29141 CTTACCAAACAGACTCTACTGCCCCAACAACTGAAGGGATGGAGCTGCCATTAACTGAGA
EMPV1_29143 GAATTTGCATTTTTCATCTTTCTTTGTCTTGCTCGTGCGTGTGCGCTTGCACCGCTGGCG
EMPV1_29144 ACTACATCATGGACTTCGAGGTTGGAAAGGAGTTTGAGGAGGATCTGACCGGCATAGACG
EMPV1_29145 TGAATAGTCAGACAAAGAGATATATAAGGAGGGGTCTGGAAGGGTCCCAGGTACAGGAGC
EMPV1_29146 AAGGTTCGGAATCCTGCCTCCCAGGAAAAGTTAAGTGGCCCTTTGCAAACTCCGGTCTTC
EMPV1_29148 AGAGTACAAATGACTCAGTCCTGGCCGTTGGCTTCAGCCCTCGTGACAGCAGCTGCATTG

EMPV1_29149	CGGACTTCCTTTCGGGATACTAATGTCTGGCTGAAAGTGAGCACCTGGCTGACATCCTTG
EMPV1_29150	TCTGCGACCTGGCCTCCGCAAGAGTTATTGAACAAGATGCGCTAATACCTTCAAAATGGC
EMPV1_29151	ACTGCCTGGGAGACACAGATTCAAGAGGCACTTCCGTTACTCAGGACTGCAAAATGAAGG
EMPV1_29153	ATTCCACAGGAGAGAGGTCATGGCGGAGATGGGACTGATGTGGCTTCAAAATGCAGAATC
EMPV1_29154	GCAGTCGGCCCTGGATATCAGAAAGAAACTTCAGAGACTGGAAGGGTTACAGGAGGCTGA
EMPV1_29156	AGTTGAGTGTCAGCTGATGTCTGTCCCTTGATGTCGCCCTGATCTGTCACTCCTCCTCTT
EMPV1_29158	GAGCTTTTCTCCTGCGTCTGCTGTGCCACAGGCGCTGAGCTTTTCTCCGGAAAAGAGTGA
EMPV1_29159	TTCTCATGCAGTGTAAGTCATGGTCACTATCCTGGGTGGGTTAACTCTCCTACCAGGGCT
EMPV1_29163	GAAAAGGCGAAGAAAACCTACCTGCAAGCCTGTAAGAGATCACCTTCATGCCTCACCTGG
EMPV1_29165	TGGGCCCTAATGCTGGACTACTAGGCTGCATCTTTGTCCCAAAGTGCTATATTATTCTCC
EMPV1_29167	CTACGTGGCCCCGCCAGCGAAGAGATGCCTCAGTCGTCGGTACGGTATCAAACATGTATC
EMPV1_29168	CCAGTATGGCCCAGAAAGTTACTCAAGACCAGCCAGTTGTATCTAGGCAAGTTGGAGAGG
EMPV1_29169	GAAAGTACCCACTCTCCCAGGGTTCTGATTGGGAAGTCACGGTCCAAAACGTCAGCTTTT
EMPV1_29170	TCTCTGAGCTGAACCGGGTGATACAGAGGCTGCGCTCTGAGATTGACAACCTGAAGAAGC
EMPV1_29171	CCACTCCTCACTCCTCCCAACAACCCAGTGAACTTCTTTTATTGTGAAGACACTCTTGGT
EMPV1_29172	GATATCCCAAAGCCACTGCCTGATCGTCCTACCCTGGCCACATTCTCACCTGTTACCAAG
EMPV1_29174	TGGCTTAAAGACTCATAAATATGACAAGACACTATCAGACTCCTAGAAGAGAACATAGTC
EMPV1_29175	GGCTCTGGAGAAAGATTACGAAGAGGTGGGCGTGGACTCGGTGGAAGCGGAGGCCGAGGA
EMPV1_29177	TTTCGTACATTTTGCTTCTTGCCCGGTGGGGACAGGCAGGACTGGGTAATCAGTTTGATG
EMPV1_29181	AGGGTGTTCACTGGTGGTTCAGTGGGTCAAGGATGTGACGTTATCACTGCTGTGGCTCTA
EMPV1_29182	CTTCTGTGCCTCAGCACTTCTCACCATTAGGACTCTTCTGTTAGACTTCCACACAGCCAG
EMPV1_29184	AAGTCAGTCCAGGAAGCCGGAGGGTAAACTGCTGAGCCAAAGGGTCATCCTTGTACTAGT
EMPV1_29188	AGTGGGTTAAGAATCCTGCGTTGCCGCCACTGTAGCATGGGTAGTAACTGTGGCTCAAGT
EMPV1_29190	CCAGAAGCTAAAACAAACAGTGGCCTGGGGAGCAGGGATAAAAATAACGCTGCAGTGCCT
EMPV1_29191	CCCACTGCACATCCCCGTAACTCTGTGATCTTTTGGGGGTGACTTTGCTTCTCCAGACTT
EMPV1_29192	AGAAACAGATTGCTCGTCCTTCTCACCTTCATCCAGGGAAGGCAGGCTCTAGTGAGACAG
EMPV1_29193	GAAGGGAGACTTAGGCAGTTGTGGGGGGTTAGATTTTATGGAATGGTAGGGAGAGCAGAG
EMPV1_29198	TGGCTCATACAAAGCAGACCACCCACAAATCAACCTGTGGTAAAACACTGGGGAAGCAAC
EMPV1_29200	TTGACACAGCTCTTTGCAGCATCCAGAGGGTCCTCATAAAACCTCTTTTCAGCGTCCCAG
EMPV1_29201	TGATCCCTGGAGGCAGCAGCCACCAGCCTGCTGAGTAGCAGCGACAGCATGAAGCCTCCA
EMPV1_29202	GTCCACACCGGCGAGAATCCCTACAAGTGTGCGGACTGCGAAAAAAGCTTCAATAACTGC
EMPV1_29203	GCCAAAGTCATTCAGACTAAATCAACCCAGCGCCTCTCCTTTTCACTCACCATTGCCACC
EMPV1_29205	GTTGCTGACATGTGGAAGTTTCCTGCTAATCGTGCTTTCTTCATCTGGGCTGCATCTGGG
EMPV1_29206	CAGGGTTAGAGGAATACAGGATTATAGTTCTCTGTTAGGTTTAGGGTTGAGGAGAGGTGG

EMPV1_29207	CCTTCTTGGAGACCCCACAATGGGTAAGAAAGAGTAAGCAGAACCTGGAGGGCCTTGAAC
EMPV1_29208	AGAGGTTGTCCCATTACTTGAAATAAATGAAGATCAGATACCTCAGCCACATGATGTGCC
EMPV1_29210	AAGCAGGTGGTGAACATCCCGTCCTTCATTGTCCGCCTGGACTCCCAGCAGCACCTCTCC
EMPV1_29215	GAAAGGTGTGTGCTATTCTGGTGGGGGTTTCTTGGGCCATTGGCATTCTACACTCATCCA
EMPV1_29216	CCCTCAGACCTGTTTCATACCCCAAAGTTCTCTGTTTGAGGGATGGTCCGCCCTTCATTT
EMPV1_29217	GGTCCGTTTTACTATATGAAGCAGCCACTCACCACAGACCCTGTTGATGTTGTACCGCAG
EMPV1_29218	CTCTTTATTCTGCTGAAGGGAAATGGGTGAGATTGCCAGAGTTCTATGGGAATTATAGGG
EMPV1_29219	CAGTGGGGCTAATCGGGCACAGATCTTTCTGCTTGTGATGATTAAACAAGGTCGGCGTGA
EMPV1_29220	AACAACCTGTGAGGAGGAACAAAATGGCCTCTAGCATCTTCGGGACACCTGAGGACAATC
EMPV1_29221	AGAGTCTTTGCTGAATCCCTGACTACACCGTTTTCCGGGCCAAGGTCGGCTCAACTCCAT
EMPV1_29222	GGGTAAGAATGGCGCCGGAAGGGAAGAACAAGCGGAAGAAAAAGCAGAGCGCGCAGGAGG
EMPV1_29223	CCCTTGTGATTTGATGATTATTTTTAGTGTTTGGTGCTTGGATTCCTTTCTCTTTTTTTC
EMPV1_29224	GTGAGCACGAGAAAGCGCTTGCACAACAGGAAACACGACACATCCCTAGAACATGGCAAA
EMPV1_29225	GAGTTCAAGAAGCTCAGTTCCAGGCGTACCTGCTCCCGTACCTTTTGGTTCGTGGCTGCT
EMPV1_29226	GATCATGAACAGGACACGATGCAACCTCCTACTCTTGGCTGCTTGGGCTGGTGGCGCAGT
EMPV1_29227	GCATCGTAGCCGCACCTGCCTCATTATGAACGGGGTCAACGACCCCCCTCTTTTTATAAA
EMPV1_29228	GTTTCAGACAAGCTTATTTCCCTCTTGTATGGAGTGCTGACCCCCATGCTCAACCCCCTC
EMPV1_29232	ACTCGGTCTCATGTGTCCAGAGCCATAAGCTGTGGGCGCTGCAGGCTCTAGTCGGAATCA
EMPV1_29237	TGGATACCTTGGAGTCATGGATTGCAAACTGAAAGGAACTTAGACGTTATTGCCTTCGAC
EMPV1_29238	CGTCATCAGTGGGGACCAAGCTTTCTCCTTTCCCAGCATGTTCCATTACGCCTTCTTTTG
EMPV1_29239	CCAAGGACCTCAGTGGAACAGGCAGTCTCCCAAGTGGTACAAGGGAGCAGAGCTAGAGAA
EMPV1_29240	TCGGAGAGGAAGAGATTAACGGCAGAGGAGGCCAAGAAAAGATGAACAAAGACGCCCGCA
EMPV1_29241	ACTGACAGACATGAAAAAGTTGGCCAAGTGGGCAGCAGAGTCCAAGCTTGACCCCAATGA
EMPV1_29242	GGTGGGTGGTTTCTCCGTTTGCCTGTTTTGCTGACGTTCATTTGACTCATTCTCAGCAAA
EMPV1_29243	ACAAAAGCAACTCAGCCAGACCCCCAGGCAGCTGAGAGCACGTTTTCTTCCTCTTTTTCT
EMPV1_29245	GCATTGCCCGGCTGGCCTGTGCGAACATAAGAGTCAACATCATCTACGGCTTATTCACCA
EMPV1_29248	GCATATATCAGACTAGCACGGGGCAATACATTGCTATAGCCCAAGGTGGAACAATCCAGA
EMPV1_29250	TCTACGTGGGATCCGATGCTGTCGCCAGACCTTCAGCCAAAATCAGCCTTCAAATTAACC
EMPV1_29251	AGACTGGCTATCACCTTCATTCCTTTCACCCCATCTCCCAGTGCTCCATTTGCTCTTGGG
EMPV1_29252	AGTGGACACACTTGGCACTGGTGTGCTTTCCAGGATAGGCTTAAAACCCGATGATCCGAA
EMPV1_29253	TGAAATTCAGAAGCTTGGATGCATCCGCGCGAATCAACAGACGGTGCTGGGAAGGAGCAA
EMPV1_29256	AGTGCCTCATCTGCGTGTCACCCTTCGACAGCGTGTTCAAGCTGCCCAAGCGCCTGGACT
EMPV1_29257	GGAGGCAGCTTCAGACTACTTGATTGGTGGTAATTACTCACTGAGCTCTTTTAATAGGTA
EMPV1_29258	CACTGCTCTGGTTCTCATCGTTTCTTCCCAGATTCCAAGCTTCGTTTTCAAGGGGAGGGT

EMPV1_29260	CGAGAGCGTGTGCCATTTGTTTTGACACAAGATTTCTTAATAGTGATTAGTAAAGGAGCC
EMPV1_29261	AGAACCAGAGCTGACCAAAACATATCAGAACGATGGAATATCAAAGCAGAGGCGGGGCCG
EMPV1_29263	AGCACAGATTGTTCAGACGGGAGCCAACACCAACACAACCAGCAGTGGGCAAGGAACTGT
EMPV1_29265	CCTGCAGGAAACACTAGTAAAAGTTCAGACTGAGCATTCAGAATCAAAACAACTTGTTGG
EMPV1_29268	GTCTGTGAAAACAGATCAACCCGCAGAACAAAGCAGATTTCCAAGGCATCACCCCTGAGC
EMPV1_29269	ACTCCAACGTGGTGTATTGTTCATGCCGGGGGGAGTAAAGGATGGGTGCCTTGGACATAT
EMPV1_29272	TGTGTCCCGTGAGAGCAAGGGCTATTCGGAGTGACCTAACTGAGCCCCTTTGGACTCAAA
EMPV1_29274	TTCCTGGGGCCGTCCAAGTCAGGCCAGGGGAGGAAGTCCATGGGAGATGCCACTTTCTGC
EMPV1_29276	AACTGAGGGCATCTCAGCATGTTTTCCTCCACACTTGTTAGCTGCCTACTCTCCTCTGCG
EMPV1_29277	CTACACCTCACCACCCATGACCAGCTGCACTTTTATGCCCATCTGGGCTACCATCTGGGT
EMPV1_29278	TCTTGGGAACCTGGACAAAGCAACTGCTGAGCTCAAGAAAGTGCTGGCAGTAGACCCCAA
EMPV1_29281	GTCTCCAGGGCAACCGTGGCTTTCGATTGTTACTGTGGGAACCGGAGGTAACAGTCTACA
EMPV1_29282	GCGAGCATGTCTGCCCAAAGCATGTCCTCCATGAAAGAGTCCTTTGTAGAAATGAGTTCC
EMPV1_29283	TACTGGGCAGATTGGACTGACCGAGCATACATTGGGAGAGTAGGCATGGACGGAAGGAAT
EMPV1_29284	CAGGATCAACCGAGCCACACCCTACAAGTACCCAGCGCCTCTCCAAGACAATGGGAACAT
EMPV1_29285	GCTCTGGATTAGGCTTTGGCTTAAGAGATTGTTCTGGCTGGTTTGATCTTCTATCTGGAC
EMPV1_29286	CACTTTCCCGAGCTCGTGAAGATCATTAATGATAACGCTACATACTGCCGCCTTGCTCAG
EMPV1_29288	TGGATCTCTGCAGCATCTTTCTCATGCTCAATGCTGTACAAGTAGCTTGGGATGATGGGG
EMPV1_29291	TCCCTATGCCCTCCTGTATCTATATGCAACCTTCGCGGATGTGACCTCCATCTCCCCCAA
EMPV1_29292	CTGGCTGTGCCAGGGAGTGGCAGCGACGAGGTTCAGATTTTGGAGATGCCGTCTAAGACG
EMPV1_29293	GGATTTTTATCTTGAGAGCACAATGCAGCGAGTCTCTTGGAAATCATCGTCCAGGTCTTG
EMPV1_29295	AGACAAGAGCTGCCAGAGAGCGAGAAACCCGAGGTGGTGGCGATGATCGCAGGGATATTA
EMPV1_29296	CCAAATGGTTTGGTGATCGCATCTCTCGAAAACTATGCTCCTGCATCAAGAATTGGTTTG
EMPV1_29298	GTCCCTGACCAAGGACAAGGTCCAGGCTGGGGACGTGATCACCATTGACAAAGCCACGGG
EMPV1_29299	CAGGAGTTCGTATCCAAGGTGAAGCAAGTGCCCTTTACTGAAATGTCAGAAGAAAACATC
EMPV1_29301	GGTACAATTCCTGGGAAACGACAGAAACAACTGCACCCTCCTCATCAGCCCAGTGAACGT
EMPV1_29302	AATTCCCACGGGACACTGTCACCCAGACTCCTCGCAGTCAACCGTGCACCTCCCGACAAT
EMPV1_29303	CCCAGGAGCCATCATCGCTGAGACCCTTCATCAGATAAATCCCTGCGACTGCTGTTATTA
EMPV1_29304	CCATGGGTTACCACCACTGAGCAACTCTCTCACGAAGAGCATTTTTGCTTCACAGCTCAG
EMPV1_29306	TTGAGGGAGAGGGGGCAGAAATCAAGACATAACTGACACTTATTTTGGCAAGATAGCAAC
EMPV1_29309	CAGATGCCCGATCTCTGAAACGCCACGTCAGGACACATACAGGTGAACGGCCCTATGTCT
EMPV1_29311	CTCGTCTGAGGCTGATGCAGACCGTTCATCTTTTGGCTTTATGTTTCCCCACTTTGAGCC
EMPV1_29317	GAGATTAGAGAAGGTGGCTTCATGGAAGAGGATGATGAGTTCGGACAAGCAAAGGCAGAG
EMPV1_29318	CTGTGGCTACAATGGGGACACTGGAGGAAGGGGACAGATGCCTGCAATGACTAGGTGCCA

EMPV1_29320 TCCTTAGGTTACTATTGTCACCCCCCTACATCCTGGACTCTTTCTTCACATTTTAATGAA
EMPV1_29321 AAGTTCATCAACGTGATCCTGGCGCTCATGGCCGTGCTGCTGGTGTTCGTGTCCACCATC
EMPV1_29322 AGGACGTTCTGTCAACATGAAAGCAGCAAAAACAGCTTGTCGGGGCTGGGAGCTGACCCT
EMPV1_29324 AAGTCCGTCCCCTTCCCCTCATGTCCCTGAGTCTCACTTGGTGCTATTTTCTCTGTAACA
EMPV1_29331 CTGATAGCAGTGGCAGATCAAAAACAGAACCAAGGACTCTGGGGGAAAAAAGGTCCACTT
EMPV1_29332 GGTCTCCAGTTCCTTACATACATTCGAGCAAATGGGGTCAAGCAGGAAGAAGAAAGACTC
EMPV1_29333 ATGCCAGACTGAGAGCAGTCATCGTCACCGAACCCCGAGACCCTTGCCTCATTTCAAACA
EMPV1_29334 GAGCACCAGATCTGTCTTCACTCCAATTCCACAAAGTTCTCCCTTTTTGCTGGAGGCATG
EMPV1_29335 CAGATTCCGGGACCAAGGTTCAAAGGCCAATACAAGTGTCTTAACTGTGTGACAACCGGC
EMPV1_29336 TCATTACCCTCTTTATCTCCAACATTGGGGGGAATGCGCCCTCCTTCATCAACCGGCTGA
EMPV1_29337 ACTGTCCTTTCAGCTGTGAAACACAAGCCTGGTCAATTGCCACTATTCTTGAAACACTCC
EMPV1_29338 TCTTGTCAATCCTAATAGTCTGGCACTTAATGCTCCCCAAGAGTCCACGGAACTGGTCGC
EMPV1_29339 ACAGTAATCATTTACTCTGTTAGGCACCGCACTGCTAGTGGCTCCTCTAAGGCCTTCTCC
EMPV1_29340 CGTGGAGAAAGAAACGCAAGAGAAGAAGAGATTGAGCCGAACCAACGAAGAGCTGCTTTG
EMPV1_29342 CCTCGACATACCTTCGGCCTCGTACAGAGCAAACTCTTCCCTTTCTACTTTCACATCTCC
EMPV1_29343 GCGTCTTGATTTTCCCTCTCAAAGTACCTCTGTGCTGACCAGCAGTGTTACAATTACCTG
EMPV1_29344 ACCTGTACTCCAAGCGGCTGGACCGAGGCGTCTCCTACCAGCTGAACCCCCCGGAGGGCA
EMPV1_29345 GCTGGAGAGAAAAGGAAATTTGTTATGAAACCTCCACAGGTTGTCCGAGTGGGAACCAAG
EMPV1_29346 CAGTGCATTGGATCCCCATTCTCAGGTAGCAGGTTTTATTTCCCTTCTTACCAGCTCTTG
EMPV1_29347 CCAACACTTTGAACTGTAGTCTGGATGTGGTGCTCAAGGAGGGCGGTGAGATGACCACTT
EMPV1_29349 CCCAGGCTGCACTCATCCGATGCTTGCATTTGAGATTTTGCATTGAAAGCTAGTGACACA
EMPV1_29350 TCGGGTGCATGTCTGGGGCAAAGCATCCCAAGATTAACTTCCACCCCATCTCTGACCGAT
EMPV1_29351 AAACTGTGGCATAACAAAATCGTGACCATCCCGCCCTCCATCACCCACGTCAAAAACCTG
EMPV1_29352 GGTTATGGAGGACCAGGAGGATATAGCAGTACAGGACATGAATATGGCAACCAGGATGGG
EMPV1_29353 AGACACAGGAAACCACACGCCTTGGCTCTGGAATTGTGAAACAAACATATCAGGCCATGA
EMPV1_29354 TCCACCCCAGTGCTGCATAAGGCTGCTAGTAAAACAGAAGATAATGGCCAAAGGGAGGAG
EMPV1_29355 GATCTGATCCTTGCCCCCATCCCACTCCTATGGCTCCATGGTTTGAGCCAAGAAAAGGAA
EMPV1_29356 ACAAGCTGCTGGAACCTGTTCTGCTTTTGGGCAAAGAACAATTTGCTGGTGTGGACATCC
EMPV1_29360 TGCCCAAGAGGGTGGGCAGCATGGTGGGTGCCTACAACAGTAAGACATTCAACCAGGTGG
EMPV1_29361 CTCACCCAAGAGTCCATTGTGTTGCTCCACACTTGTTTACGACCAAATTAGACAAGAAAA
EMPV1_29362 CCTTCTGCAGTGTTAACGTCATCAACCATTACTTGTGTGACATCCTCCCACTGCTCCAAC
EMPV1_29367 AGAAGTTAATCGAATACTGCAAGTCCAAAGGCATCGTGGTGACTGCCTACAGCCCCCTCG
EMPV1_29368 GAAATACCTGGAGCCACGAGAGCGACGTGCCAAGCCTGCAATCATCCAGCCTAGAGCAAA
EMPV1_29370 ACTGGGCGTGGCCCACATGACATCTCGTGAGATTGAAGTGCAGGGTTTCCTCATCCCCAA

EMPV1_29371	TAGACACCGACTACTAAAAGGTACATCTAAAACGATTCAGGATCACTTGTTCCATTTCTG
EMPV1_29372	CTGATTCCAAGAGGGAAAGGAATCTAGAAGCCCCACTTTGTATTTCGTCACCCTGCTTTC
EMPV1_29376	TCCTCAGAGAGAGAAGACTGTAATAATGGCGAACCCCCTAGGAAGATAATACCAGAGAAG
EMPV1_29377	CAGCAACATGAAGCGGGCAGCCTCCTTGAAGTATCTGAACCAACCCAATGCAACGCCCCT
EMPV1_29382	CTTTGAGGCTGGTATGGAAGGGCCCCTATACTGTAATTCTAACCACCCCCACTGCTCTTA
EMPV1_29383	CCACTCTTACTAACACCTGAGCCATGCTACCCCTGTGCGCGTTACTCAGGATGAATCACT
EMPV1_29384	CCCGGCGGCATTGGCGTCTATCTGGATTATGAACTGGGAGTGATTTCCTTCTACAGTTTC
EMPV1_29385	CTTGAAAGCAAGAGCACCATTGGCGTAGAGATTGCAACAAGAAGCATCCAGGTTGATGGG
EMPV1_29387	GTGTGGAGAACGGGAAGCCGGGGGCTGACGTGGTGGACCTCACGCTGGACAGCTCATCTT
EMPV1_29390	AGATATGCCACCGTTCTAACGCACACAAGGATTGCACAGATTGGTTTGGCAGTCGTCACC
EMPV1_29392	GCCCAGTCTAATCATGAGAATAACACCACATAGATTGCAGTTGAGGAGTGTTCTGCGATC
EMPV1_29393	GCTGCTCCATGAACTACTGCACCTTCCACTCTATATCTGATGCAGCTGAAAATATGTTGC
EMPV1_29394	CCTTCACCAGGCCAAGAATCACAAACTCCAGGTGGATAAGGGAACATTAGAGGCCAACTG
EMPV1_29395	CCCCTTCATCAAGGAGTACCATGCCAAGGAAAACGACTTTGACAGACTGGTGCTGCAGTA
EMPV1_29398	GATTGGAAAGGAAGAGCAGGAGGCTGCTGAGAAAGTGATGACTAAGGAGGAATTTCAGGG
EMPV1_29399	TGAACCTCACAGAGAAGCTGGTGTCCTGTCTCAGTTCAAATATTGCCGTAGCTTTGGGAA
EMPV1_29400	TTGCCGCGCAGCCTTCCCCCGCTGGTACTTTAATGCCGAGAAGAACTCTTGTGATAAGTT
EMPV1_29402	CAGTGACCAATCACATGTTCAACAGCTTCTATCAGCTGCCTCCTCGCTTCTCCTAGCTGT
EMPV1_29405	CACTGGGTCAAAGAATCTGACATGGCTGTGAACTGTGGTGTAGGTTGTAGATGCGGTTCT
EMPV1_29406	TCTTTCCTCCTCTCTTCGTAGGGCTTATTTTCCAAACGCTGCCCTTTCCCCAGTCTTAGG
EMPV1_29407	TTCATCGTGTGGATGCGGACAGCTGCCTTTCCCACTTTCAAAAAACTATACCGCCGACTC
EMPV1_29409	GAGAGATGCCAGTGGAGCAGAATTGTCCAATAAGCCCTGCCCAAATTTCTCACCCACACA
EMPV1_29410	GTACGAATCCCTTTCGGAGTTCTTGGCGATCTCTGAAGAGACAACAGTGACAGCGTTGCA
EMPV1_29411	GGGCCAGGGGATGATGTATGGTTTACAGGATCCAGAAAAAGAACACGATAAAGCCCAGTG
EMPV1_29413	GGAGCATTTGCCAAAACTTCCAAAATTGATATGAAAGGTTGTATCAAAGTTCTTAAGGAC
EMPV1_29416	CCCTCTCTAAGCCGGCAGCCTACAAACAGTGCCACCAAGAAGTCTGCAATGACAAGATCA
EMPV1_29418	AGCTGCAGATGTGAAACAGTTCTGTCTGCAAAATGCTCAACATGAGCCCCTGCTGACTGG
EMPV1_29419	GCCTGTTGCCACAAACCCGTAGATCCGAACTTGTGCTGATAGTGCACACAAGCTTGTGTC
EMPV1_29420	GACCCTCCTAAGGCTGGAGATAAGTAAAACAAAGGAGGTTTGTACCTCACGCCGCTGTAC
EMPV1_29421	CGGAGAGGGGTTTTCTTGGCTGTTAGTCTAGAAAACAACTTAAAACACCCTGGGGGTTCC
EMPV1_29422	TCCTCAACACAAATCTCACCCTCCTAATCACTGCAATTTGGGGAATGGGTAGCCGGGCAC
EMPV1_29423	TTGACCTTCTGCTCTCAGGCCAGCAGCAGACACACTCTGGAACCTGCATGAGGGCTGGGC
EMPV1_29424	TCCGGCCCAGCTCACCTTGAGGAAGAGCCAGGACACTTAACATGGTTTTGCACATCTCTT
EMPV1_29426	TGTAGACTGAGTTAGAAACTTGACCAACTGAAGAAAAGGCATAACCAGGATGGCCCCTTC

EMPV1_29427 ATCCTGGGCTCCTATCGCAAGAAGAGGAAGAACTCCACAGGTAGGCGAGCGCAAGGCACT
EMPV1_29428 CCCACCTCCCAGGATTTGGCGGGGTTCTGGGACATGCTGCAGTTGTCCATAGAAAATATT
EMPV1_29430 TGCAGGTCTTTCGCGGGTGAGGGGCAGGAAGCAAGTAGCAAATCTAGCCCGACTACTCGT
EMPV1_29431 AACAGCGTCAAGGACACACAGGGCTGCAGTAGGCTTCTCCTCTCCCTCTCTCACCAACTT
EMPV1_29433 AGACTCACCATAATTTCTTTCAAAGTGGACTTGATCAAAAGTCTTCTCTGCCCCCAAGGC
EMPV1_29435 CCTGTACCTGTAAATTGTGCAATAACGATACGTGACTATTGCCATGGAGAAATCTGTGAC
EMPV1_29437 CCGACCGCTTTCCTGGTACATCTCTGAGTTATCTCGTTTTGTCTGGCTTCGTTGTCAGTA
EMPV1_29438 GAGGTGTTCGACACAGAAGACTAATGATGGCTCGAGGACCTAATGAATGTTTTCCTCGTG
EMPV1_29439 GAGGCTGTTTCTCAAATGCCAGGGTGCAGATACTCCGGGGAAATCTATCCTGATACTCTC
EMPV1_29440 GGTCCACATCAACAGAAGTCTAACTCCCTGAAACCTCTCTCCATGTCACAGCTGAAGCAG
EMPV1_29442 GTGATCCCAGCTTATTATTTTCCGGACTGCTCTAGGGTTTGGAAAGCACCACATGTTATC
EMPV1_29443 GAGAAACATAGTATTGTCAATATCCCCACAGTTGTCCAAAGTCACAGGACCCATGTGCCC
EMPV1_29444 CCTCCCCAGGTCTCAAGGGGCAGAGGCTATTTTTAACATGTCCGTTTGTATTGATGTATG
EMPV1_29445 GTGATCTTGATGCACTTCCCTTTCTGTGGGCACCGGAACATCCACCACTTCTACTGTGAG
EMPV1_29446 CCTGCCCATGTAATAACATGCAGGGTGATAAGGGGACACAGGCATAGAGAAAGGTCACTG
EMPV1_29447 GAAGATCTGCTGTCTCATGGCTGTTCCCTCCTGGATCATTGGCCTTCTTGATGGTGTAAT
EMPV1_29448 TGGAACTGTGAGAAGAGTAAATGTGGTATCTGTGATCCTAGGCCCCTGGTTGGTGCAGCT
EMPV1_29451 TCCACAGTGAACTCATCCTGGGTCTGGACCACTGAAATGGATGGCTACATTGAGAAGACC
EMPV1_29452 CCCGAGAGGGGCAAATGGACTTTGGAGTTAAGATGCAGGGAGGTGAACCACTGTCAACAA
EMPV1_29453 TATCTTGGGCAAGTTTGCAGTTGTGATGGCTGACCCACCCTGGGATATTCACATGGAGCT
EMPV1_29454 TGGCCCATGTAGGTCATGAGGGCAGGAAAGGATATTTGCGATAGGAACAGCCTCGCATGA
EMPV1_29455 GTGGACTTCAGGAACAAGGCAACTGCTCTGATTGAAGATATATTTGCCCGAGATAAAATT
EMPV1_29456 GAACTGTTATGACACAAGTTAAGGAAATGGCTTAACAGAATGCTTTGGAGGAAAGACTGC
EMPV1_29457 TGTTACCTGGAAGGCCAGGTCATGGTACTTCTGCTTCTCCTTGGGCCCCAGAGCGTACCA
EMPV1_29464 CAATGGTCTGTCAGACTGCAGGGTTCTGTTTGATTGTTGACTGCTTTAGGTGAACTCTCC
EMPV1_29465 GGTCACCGGTTAATTCCAGAAAAGGTCTCTTTCCCACTGTCTGGCCTTCCTGTTGAAAGG
EMPV1_29468 TGAGCGTCCCTTCTACAACCACCTGGGCGGGAACCAAGTGTCCAAGGAGATGAAGCGGAT
EMPV1_29469 CTCAAGGCCATGCAGTGCGCCTTGCACCTGGAAAAGGGCGTGAACCAGAGTGTGCTAGAT
EMPV1_29470 ATGGTGCCAGCATCCTCAGTCGCTACATCACCAGAGTGCGCAACAGCTTCCAGAACCTGG
EMPV1_29471 TCTTTGTAGCAGCAGCCTTCATGGGTGTGGCCCCCTTGGTCCTCATCACTGTGTCCTATG
EMPV1_29472 GAGCCTGTACCTGCCTTTGCCCCTGTGCTGGGCAGTTTACACACATTTTCTCATTTTATC
EMPV1_29474 GCCAGCTTCACACAGCTTTACTCTAGCTCAGCCTCTATGAATCAACTCAGACGAAGCCTC
EMPV1_29475 GTCACAGTGGGTCCGAAACTGGGCTGTTTGGCGCTACTTTCGAGACTACTTTCCCATCCA
EMPV1_29477 CGAGATTGAAAGTAGAGAGAAACCCTTCCATACAACAAGTCAGGGGAAGAACCTTCTAAG

EMPV1_29478	CAGCAAACAGAACAACTGAGGCAGGAATTAAACTTATTTGGCCAGCCAGGAGAAAGGAAG
EMPV1_29479	TAACCTCAGGGTTTCTGGCACACGTGGAAAGCTGAGAGATTTTTCCAGCCCTCAGCGAGA
EMPV1_29480	ATCAGGTACCAAGACGCCGGAGTTCTGCCCAGTGCCCTAAACCCCTAGGTCAACTAATTT
EMPV1_29483	CGTAGTTCTGGGGAGAAGAAGTCCAAAAAGCGCAACCGGCGCAAAGAAACCTACTCAATG
EMPV1_29484	CCTTTAACTGACAAGCCATGTTGAGTGGTCTTGGACCACTGCTTTTCCCCTATGTGAGTC
EMPV1_29492	AGGGCACGGTGTTCTCCGAGGAAGACTTCCGAGAAGAACTTCCGAATCCTGGGGAAATGT
EMPV1_29494	GAAAAAGAATAGGGTCCCCCTCTCCCTCCAGAGCCACTGTCACGCTGGAAGCAGAAGGAA
EMPV1_29495	GGAATCCCTATCAAGAGTGTTATGGGTTGCAGTCTCTCGTGAAAGCCGTGATCATCCATG
EMPV1_29496	CCCACCTCAATGTCACTACAACAAATGTCAAGTTTAAGCTCTAAGAAGCCCTTATTGACC
EMPV1_29499	GTCAGTGCACAGTCACTGGCTCATTTCATCGCCGGGAAATAATCAGCTGTGAAGAGGAAA
EMPV1_29501	TCCCCAAAATGCCCTGAACTGTGCTAGGTGACTTTAGTAATCTGCTATGCTCCCTAAAAC
EMPV1_29502	AGGCATCCCATCGGAACCAGCTTCGTTACTCGGAAAGCATGAAGATCCTACGGGAGATGT
EMPV1_29504	ATACAGCCAGAAGCTTCAGACAGGTCTGTCAATGGCGGGTCCCGTACTTACCACTAACTA
EMPV1_29505	GATCGCAATCATTACCACCACAGGGGGGCTGTCCATTTTGACCTTTGTGGCCTTGGTCAT
EMPV1_29506	GCACATCTTGTGCTTTGTGTGGGGGTGATGACTTAGAACTGATCAGATGTTACTGCCTTG
EMPV1_29507	ACAGCCCTGCTGGTGGCCTTACTGTTGATTCTGATTCACCGCAGAAGAAGAAGCAGCGAA
EMPV1_29509	ATGCTTTCAGTTGAGCGCATCCTCAGTTCCTGAATGGACCCAGGCCTCTGAGTCTTGGTG
EMPV1_29512	CCATATCAGCTCCCCCTTTGAGCTCTCCATTCACAAACATGACTTCACCCTGCAGTTCAC
EMPV1_29513	GGTAGCGGTAGTGGACCCAATATGCTGGCCAATGCCAGATACTTACGTGAACTCAAAACC
EMPV1_29515	GATTCCTCATAGCATGGAGGTCTCAGGGCAGCTGAACTTCTTACATGGCAGCTGGTTTCC
EMPV1_29517	CCGACAGCATCAGTGCTCAAAGCAAGCTAAAGGAAGCGGAGAAGCAGGAGGAGAAGCAAA
EMPV1_29518	GAAGGAAAGATAGATGACATCATTGATAAGACAATTATAGGAGATTTTCAGAAGGAACAG
EMPV1_29519	TGCCCCGCCCACTCCTGACTCCAGCTTAAGACATATCTTCTCAAAGCCGGTGACGGCTGT
EMPV1_29520	GCCAGTGGCTTCTCAGAAGCACTGTCTCCTTGGGGACAATTTTTATCATGACTGATTTTG
EMPV1_29523	GGCTCACCCTCCCAGTACAACAAAATCTTTGGACCTAGCCATCCTGAATCTACCTGGAAC
EMPV1_29524	GATTCCCTTCTTGTCCAAGCCAGATAAATTTAAGCAAAGGGAGCTTAGGGCCAGTGACTG
EMPV1_29526	CTCTCCCACCAGAGAAGGGAAAAGGCCAACGCCGCACCCTGAGGAATAATAATGACCATT
EMPV1_29527	GCCGTCGGACTGATTGCACTCACTGTCGCACTCTTCACTATTTACCTCTTTTGGACCCTA
EMPV1_29528	CTAGAATCTTACAGCCTGCCAAAACTGAATCAAGTAGAAACAGACCAACTGAACAGACCG
EMPV1_29529	CATGAACCAGATAACAAATTCCAGTTTATATGGTGCTTTGTCTGTGCCAAGCACTGTGCC
EMPV1_29531	GAAGAAGGAGACAACAGCAGCCCACAGAGATGGAGCAAATACCCTTTGGATGTGCAGAAG
EMPV1_29532	CATCAAAGAACTCATACTGGGGAGAAACCTTATGTCTGTGATGAATGTGGGAGAGTCTTC
EMPV1_29534	CAGCACCTTTGGCTCCATCTTGTTTCCAGCTTTGTCCACCGCAGCCTGTCATACGCAGGT
EMPV1_29535	GACTGCTTGGAGTTCAATAGGACAGCAAAGTTAAAGAAAGTACAGACAATAACCACCAAG

EMPV1_29537	CTCCGAAACGTGGATTGGAATAAACTGCTAAGGAGAGCAATCGAGGGACTTGAGGAGCCC
EMPV1_29540	AGGACTCCATCCTGGCTGTGAGGAAATACTTCCACAGACTCACCCTCTATCTGCAAGAGA
EMPV1_29541	ACCGTATCTACTTCTTGTTCCGGGAGTCCAGTATAAGTGTGATCCCGCAGGTTACTACTG
EMPV1_29542	GGCCACAGCCCTAATCAACACATAAGTGAGACCTTAAGCAAAGGACCTAGTTACACTGAG
EMPV1_29545	GCCATTTCCCTTCTTTGAAGGACTCCGAGACAACAACTTCATCACCGATACACTATACAG
EMPV1_29548	GAATGGACAACGAGGATCATGCAACTGAGATGGTTTGGTGGGCTTTGTGGTCTTGGGCCA
EMPV1_29549	CGGAATGTAACTTTTGAGGATGCTGGGGAATATACGTGCTTGGCGGGTAATTCTATTGGG
EMPV1_29550	TACAGTTCCCATTCGACCCCCAGCCTGGGAGCTTCCATATGCCACAAGTGCGGCCCTAAA
EMPV1_29551	TGACCTCAGGTCTGAAGGGAAGGGGCTTCTCTGTGCTCCACACCTACCAGGACCACTTGT
EMPV1_29552	ATGGCTGGTACCCATTGGCAACTGTCTATGCAATGCTGGGCATGAAGAGCGGAATGGAGA
EMPV1_29553	CTCCTATCCCCTTATCCCGCAAACCCCGGTACCCCCAGTTTACTCTCTGTTTTTACAAGT
EMPV1_29554	GAGGCTCCTCTCAGTAGGTACAGTGGGCACAGCTGCAGACTTGGAAGCTCATGTCCTTTT
EMPV1_29556	GCGTAGGAGTATATTTGCTTTCTTAACCCTGCTACCCTCATGTCTTTGGACTGATTATCT
EMPV1_29558	CGGCTGACTAAGTAGGCTTTGTCTCCTAAGCCCCTAATCTCCAAAACAGGCCCTCCACTT
EMPV1_29559	GTGCCTCTGCTGATAATTTGGTTTGCTGGCTTAACAAGGGACGCTGTGGTCAGTTGAACC
EMPV1_29565	GCCTGTATGAAGAAATAATGGCGGGATCTTCCATGTACTCTAGTCTCACTGTTTCCCACG
EMPV1_29566	CTGTTTGCTCTTCCGGAGCCTCCCTTAGCGCCCTTTGAAAATAAGAGCTAGTAGTACACC
EMPV1_29568	GTCCAGCTCAGGACTCCTGCTCTTTGTTGGCTGCTGCTTTTAACTAGAAATGCTGTCACA
EMPV1_29569	GGAGTATGGTGGACAACCTCCAAGGCATAAATTAGCAAGTCTTGGTGACACTGCTTCTCC
EMPV1_29573	CTGAGGTGGGCAGGAAAGAAATTTGAGGAGATCCCCATCGCACACATTAAAGCATCCTAC
EMPV1_29574	AGTGAAACTCAGAGTGCGGCCTTCTTCATCCCAGTGCCTACAGCCTCTGCCAGCTCTGTA
EMPV1_29575	TTACTCTTTCCAAATGTTACTTATTTTAACTTTTTCCATGCTCCCCTCTCTTCGGGATGG
EMPV1_29581	GACCTGCCACCCTCCTACGATGAGGCCATCAGGATGGCCAAGACCAGACAGGAAGAGCCA
EMPV1_29582	TGTTAGAGGCCAGACTTGCAGGTATCACATGCTTTCTCCCCTAGTCCACTGCCCAGAGCT
EMPV1_29583	GGTTGCATTCAGCCACTTCATTTCTAGGAATTTATCCCCAAGACGGTCCCCAACTTACGA
EMPV1_29584	ACGTCTTATACGTTTAGCTTACATAAAATTACAAAATCAAAAATTGTGCATAAAGTACTA
EMPV1_29586	GGAAACTGCCCTACATAAGGAGCAGCTCTAAGTGTCTGGTGGTACTCCCATGCTGTGTGG
EMPV1_29587	AGAAAACTGACACGGCTGGTGCCCAGCCTCCCCTCTTGTCAGTGGCACACATGCCAAGAA
EMPV1_29588	CTCTCCCAACGCAGACTCAGGCCGCGGCGACGGGAGGAATCAAGTTGTGTGGTCGGTGAT
EMPV1_29591	GATTGAAGGGTTGCTGGGCCCCAAGGGCCTGAACTGCCTCCCTTGGTTCCAAAAGCCTTT
EMPV1_29593	TTGTTGAATGAAAAAATGAATCATCAACAGACATTAATTGGGCGCCTGCTCTGTGATCTC
EMPV1_29594	TGTCCCGTCTACACAATTGACGTAACAATTTCCCCACGTGGAACCTGCAAACAAGTGACC
EMPV1_29595	ACTAGCAGCTGGCCTTGCACAGGGAGAGCCCACGTTCCTCAGTAAAGTCCCCCATGTTAT
EMPV1_29598	ATGCCAGGCACTATGTTAGGTGCTGGATGTCCATTAGTACAGTACGCTTGTGCAAAATAC

EMPV1_29599	CCTGCTGGGAGGTGGAGGTTGGGGACATGCAATCTTGGGACTTGGGAATTTGTAGAGATA
EMPV1_29600	CGGCCGTCGAGGGTGAGGCTGGGCAGGGGCCGAGGCGGGGGCGAGGGCCAAGGTTTCTCT
EMPV1_29602	ATTTGCTGGGAAAAAAGTGTTGGACCTTGGTTGTGGTTCAGGGTTGCTGGGTATAATGGC
EMPV1_29603	GTGGACTGCTCTCATTTATCTGTTTCCTCCTGCTGCTTGTCTCCTATACTGTCATCCTGC
EMPV1_29604	TTTGGGGATTATTAGTCACAGCCGCATCTCCTGTTCTGGCTCTTACATGCAGGTGGTGCC
EMPV1_29605	AGATTTCAAGCGATTGCAAGAGGACCCACCTGTGGGTGTCAGTGACGCACCATCTGAAAA
EMPV1_29607	CTCCTGAAGAGGCTGTTCTTTCAGTCAGATGAGGGTTGAGAAGCGTGATGCTGTAAAGTG
EMPV1_29609	GAGCTCTACCTGGACCCTGCATACAAGCAGTTAAACCACAACATCCTGTCCACCAGCACA
EMPV1_29610	GATCCAGTATTGCCCAGAAGGTAACTCAAGCCCAACCACCAAAGTTCACACAGGAGAAGG
EMPV1_29612	GAAGCCAGCGCCTAAGAAAGATGACCGCAGAACACCTGCCATCAAAGCCCTTAATGCCAC
EMPV1_29613	CAATGTTAACTTGTACGATCGGCTCGATCTCGAGGTGGAAGAGGTAGAGGACGCTACTCA
EMPV1_29614	TCGAGAAAGACATAGTGGTTATGACGTTGGCTTGAAACCAGTGGGAGAGGGTTCGATTCC
EMPV1_29616	GGTGAGGAGATTCTTCTGCTGAGGTGGTGCTGAGGTCCCACCAACAATACCCTTGGTTTA
EMPV1_29617	GGGTCAGAGCGTTGGAAAACGGCAAATTTAGGACTGTTGGGAAGCAAAACCAGCTACCCG
EMPV1_29619	GGGAGTCTGGTCATGCACAGCATGGCCATGTTCGGCCGGGAGTTTTGCTATGCGGTGGAG
EMPV1_29621	AGCTATTCCTCCAAATCTGGCAAGAGGAGCCCGCCTAGCAGAAGCTCGAGATCCCGAAGA
EMPV1_29624	TGGGTAGGATCCTGGAACTCAGAGGCATACAGTGGGCCTCAGCCCAAGCAGCCAAGAATC
EMPV1_29625	CAGTGTTCCAGAGGACACAGACCAATGGGATCCAAAGCCAGACATGACAAAGGCGAGTCA
EMPV1_29626	ACATTGTTCGGGAGTACATGGAGACAGACTTGGCTAATGTGCTGGAACAGGGCCCTTTAC
EMPV1_29627	AGCCCTACACCGACTGCAACTTACGGCCGCCAGAAGGTCTTTCCAACGCCGCACACCCCA
EMPV1_29629	AGAGCCTGTTTCCAAGGGGCGAGAGGAGAACTATGACTTAACTCAGGTCTCAGAGAGCAT
EMPV1_29631	GTTGCGCTCCAAAGGGGTGACCTTCCCATTTCATCTCTTAGATTTATCAGGGTTGAGCTT
EMPV1_29632	TGCCTCGCAGACTGAGGGGGAGCGCAAAGTGACCCAAATTCTCAAAGAAAAATTTCCTCG
EMPV1_29634	TGGGACCGACCTGAGTCACATCTCGTACACCACAGCTTCGGACCCTGGATGCAGGCCCAT
EMPV1_29636	CCAAGGAAAGCTCTAAAACTGAAGACTGCACCTCCTTACATAGGACCTCAGGGTCTCCTC
EMPV1_29637	GTGGGTTCACCATTGAGACCTTGTATTGTCCTCTTCCTGCTAATACAGACATCAACCTGC
EMPV1_29642	AAGCATTCCATTTGTGAAAAGACATGAAGAGCTGACAGGTCCAGGGTAAGTACGCAAGGG
EMPV1_29645	TTGTGGTGGGGACATCAGCTGAAATGATGCTGACACCCTTTGGAGCTCTGGCAGCGGGCT
EMPV1_29646	GATAAATGATTTGGCGTTCTCACTCTGGACACTGGGTTAAGGATCCGGCTTTGCTTCAGC
EMPV1_29650	CATGCACATGTTTGACTTTCGACATTCAGCTTGTAGAATAATGGAGTCTATCCAGCAGGC
EMPV1_29651	GAGGCAGAGTTTACAAGAAGATTTACTTCGTCGATTTCCAGATCTTAACCGACTTGCCAA
EMPV1_29652	CTCTGAAAATTGCACTGAACTTTGGGAATTGATCCAGCCTCCGGTTGCTAAAGAGATAAA
EMPV1_29654	CTCCTGACCTCAGGGATGGCAGGAAGTGGAATGCTGACTGGCCTCAACTAGCACTGCTTA
EMPV1_29655	TTCCTCTTGAGTCAAGTCTCCTCTGCACAGGCAGGGACCTACAGATGCTGCGGTGTCTTC

EMPV1_29656	CCATTTGCTCTGCCCTGTCCTTACCTTCATTAGGCTTTAAGCACCTTGCTGTTTCCTAAC
EMPV1_29657	TCCACCCCCAAAAGGAGGCCGAAAATCAGCACAATAAGATCCTTTACCGTCAGTTCAAGC
EMPV1_29658	CAGCACTGAGTTATGCGAAGCCTCACAGGACAGTAGTGAAACACCTAACAGGAAGAGAGC
EMPV1_29659	AGTGGGGTCGTAGGGATTTGCCGAGTGACCTGCTTTTGGCCTGGATAGGGTTGTAATAGA
EMPV1_29660	TCGCTCATCACTGTACCAGGAGAAGGCGAACCTGTACCCGCCGAGCAACACGCCGGGCGA
EMPV1_29663	AAGGCCCCGAAGAGCCCGAAAGACCTGTTAGGAGGAGTTTTCAGATCCCCAGGAAGAGCA
EMPV1_29665	AGATCGATGTCATCAACTCCCGCACCCAGGGCTTCCTGGCCCTCTTCTCAGGTGACACGG
EMPV1_29666	ACCGGAGTTCCAGTCAGGAATCCTAAGCTGTGTGCCTCTTAACAAGGCCAAGTCCTTTTC
EMPV1_29672	CTTCTAATAGCCATGGCCTTCCATGTTTACCTAATCAGCACATTTTGTCTGAATACTTGA
EMPV1_29673	GGGCCTCATCCGTCACCTCTACTGCATCCTCATCATCGAAATACTCCTGGTAGATGTCAA
EMPV1_29675	GAAACCACCACCTCATCTAGGCACAAAAGTAAAAGCAGAAGTTGGAGGTGGAAAAAGGAG
EMPV1_29678	GAGCACCTCAAGCCTCCCATGCTTTCTGCAGCACCCTGCACAGTTTGTCCTGCTCCTGCT
EMPV1_29680	GGGGACCAGACTTCTACTGGGAATGTGAATGGGGCTGATCTGGCTAGGACTGATGAATCA
EMPV1_29682	TACGGACCCAACTTCATTCTTCAGGTGTACTCGGCTCAGAGGAAGTCCTGGTACCCAGTG
EMPV1_29684	GTAGCTTCAGGTCATATCTTCAGCCTCCTCAAAGTACAGGTTCCTCTGCTGCTGGGGATA
EMPV1_29685	AACCTTCTGACCGAGAAGCAGAAGCTGCGAGTGAAGAAGATCCACGAGAATGAGAAGCGC
EMPV1_29686	TAATAAAGAGCTGGCAGAAAGATAAATTAAGAAAATCTGAATTACAATGGCATCAAAAAG
EMPV1_29688	CTAAACTGTGTGGGCGTCTAACTGTTGGCACTGAGGTTTCCTTTCTCTTGAAAGTGTCAG
EMPV1_29690	CTTGACCCAAAGATGTCACCATTCCTAGTTATTTGTCACCACATAATTGGTGTTGATTGG
EMPV1_29691	TTGGCACCACCCCGATGAAATGTGTCCCCCTCTTTGACTCTCCCCGAGAGCTCCTCAATT
EMPV1_29693	CTTCCCTTGACTTTTTGGCATGTATTTTTACTTTGTTGTTGTTAAAATCTAGATGTGTTG
EMPV1_29695	AGATAGCTGCATCATCAGCATGGTTGTGGGCCAGCTCAAGGCCAATGAAGACCCCATCAT
EMPV1_29698	GAGCAAAGGGGATCTAGCAGTTCATCAAGTGAGGAAGTTGTTGGCAATAGTGCTGAGCAG
EMPV1_29699	CCCTGAACTCTCAAAACAGCTAGGTGAAAGCTGGAAAATCTGGTGGAACGGGGAAGGGAG
EMPV1_29700	ATGCAGGTGCTTCTGAATGAAGAGAAAAGCAGTGGTCCCAGATGGATGACCAGGTGGCTG
EMPV1_29705	ATCCACAGGATGAGAGGGTCATTGCATTCTAGGTGTGGCTCCAGTCAACCAAACATCACA
EMPV1_29706	CCAGAGTGAACAGCCTTTGCAGGTGGCAAGATGTACAAAGATAATCAACGCTGATTCGGA
EMPV1_29708	GATAGGGCCAAGCATCCTCTGCTAACTCATACAATTGACAACATAAGTGGCCTCCTGTGG
EMPV1_29709	AAGTACAAGTTGCTCAGTGTGGCCTTCACAGATCCTCATGGTTCACCCTCGCCTTTCCTC
EMPV1_29711	CCTGCCACCAAACTTGTGGGTTCAGCCTCAAAATACTTGGTTAGAGTTATCCTACAGTCT
EMPV1_29712	GCATCTTCAAAATGCAGATGGACACAATGCAACCACTGCCTTCTGGCATTGTTATGGGAT
EMPV1_29714	ATCTCTTCCACTGGGCCTGCCTCAATGAACGTGCCGCCCAGCTACCCCGAAACACAGCGC
EMPV1_29719	GTGAAAGAGGGGAAGAGAAAGTGGTTACTACCCAGGCTTACCACCTAGCATAAGTCTGGG
EMPV1_29720	ACAACTTGGAGGAACAGACGCAGAGGGGTTGAGAGGAGATTAAGTTGGACTCCTACCTGA

EMPV1_29722	TGCAAAGATGACCTAGATTTGAAAAGAATGTGACCAGAGGGGAGAAGTGGTGTGGGGCGC
EMPV1_29723	GAAGATGTCTCCTGAGAAACGGGTCCTCATCCGGCAGATCCCTAACACCGCTATTGTGTT
EMPV1_29724	TGTGGCGTAGGCCGTAAGCTGGCAGCTACAGCTCCAATTCAATCCACAGTCTGCTAGTCT
EMPV1_29725	ATCCAGCCAAGGCGGACTTTATGCGCCAGTTCCAGAATCCAGTCAGGGTGGACTTTATGT
EMPV1_29726	GTGGACTGCTCTCATTTATCTGTTTCCTCCTGCTGCTTGTCTCCTATACTGTCATCCTGC
EMPV1_29729	ATTACCATGGGTCACCGGCCCATGTACTGTTCCAATGCCGATTTGGATGACTGTACCTGG
EMPV1_29730	ATCTTTGCTAAGCATGGACATTTTCCCCTGGAGGAAGTGGCCCAGGCTCGCTACGAGGTG
EMPV1_29731	CATCTACTCATGCTCTGGAAGATTCAGAAATTACCAATTTCGTTCTACAGTATATTCTGC
EMPV1_29732	GTGTTTTAAGGACTTGAGTGAGGTGTCATTCTCCAGTTTAAGCCTTGTCAAATGGTGTGC
EMPV1_29733	GAGCTTGAGGTCGGGCCCAGCCCCACCGGGTCGGTGTTTCCCACCTCGCGGATCACGTCT
EMPV1_29734	AAAGAGGTTATTGTGTGTCCGAACTCTTCACACAAGAATTGGAGGAGATTTAACACCAGG
EMPV1_29735	CAACCATAATTAACTTGAGTTTAAGGCATGTTCAGATGTGGTCTTTGTTCTTCATAGGCA
EMPV1_29736	CGATACTTGGAGCACAAATATGAAGGTGCCATAACATTATGGCCTGCCATTGTTACCTCC
EMPV1_29737	AACATGGTCGCGCTGGAGTTCCAATGGAAGTTATGGGTCTGATGCTTGGAGAATTTGTTG
EMPV1_29738	TTGTCGGCTCACCGTGTGTTAGAAGCCCCGGATACAGAGAACAGTGTCATGCAAAGCCTT
EMPV1_29741	CGTGCCGTTTAAACCACTGGATCTATCTAAATGCCGATTTGAGTTCGCGACACTATGTAC
EMPV1_29742	AGACAGACTGCTTCTGCCGTCATCTGGGCTAAACGACAAGGAATTACAAAGCCTGCACCT
EMPV1_29743	TTCTAAATTCAGTTTGCCTACATGAAGTGCCTCTCAGCCTGCTGTGGCATCCACTGATGC
EMPV1_29745	GTAGCGGCTGGCCGAGGGCCGCACCCGCTTCGGCATCGTACTTGGCGGTGGCTGCTGCCA
EMPV1_29746	AGAAGGCGATGCTCTACAGAAGCCTAAAATTAAGAAGGTTAAGTGAAAAGAGGACGGGGC
EMPV1_29747	ACTCTACTACCCGACCGTCAGCACCATCAGCTTGGGCTCCCACACCATGCTGGACCTCTA
EMPV1_29748	ATGCAGATTCTGAATCAGCAGGTCTGAGATTCTGCATTTCTAACAAGTTCCCAGGTGATG
EMPV1_29749	TTAATGCCATCGCTGTTTTAATCTGCCTTCGCCACGCGGACCTGGAACACAGATGCTCAG
EMPV1_29750	TGAGGCTCTGAGCCGGGAAGGGCAACACCCTCTCCTCGGGCTCCTTCAGCCCCAAATCCA
EMPV1_29752	TAATGCTGTGCCACCACCGCTGGCCAAAGCCATTGGCTTAGAGATCAAGCGCTGTATGTT
EMPV1_29753	CCCCGACATGCCTGTCCTTGTGAGGCAAAAACTTCAATGGGATCCTTCCTCTCCCTAAAG
EMPV1_29755	GTCCACAGCTTCTCTCACATGAAGCTGACTTACCACGTGTTCGGCCTGGCCCTGGACGAA
EMPV1_29756	AGAAACTCAAAGAACTAATGAGCGCCACCGAAAAAGTCCGCCGGGAGAAGTGGATCAACG
EMPV1_29757	GGAATGGACAAGAGACAACGCTTGTTCGGGAGATGTACTGAGCCCTCTGGAACGAGCATT
EMPV1_29758	CTCAAGTTAAGGTCCAAAAGGAGAACCGCTCTAGTCCCACTTTCCAGTCCTTTTTCCATG
EMPV1_29760	CATCCAGATGTTGGCACAGCGCCTAGGCTGGTCACAAGACAGTCACGAATTACATTATGC
EMPV1_29761	GCTTCAACTTTCTGGAAAACCATGAAAAGAGGCAGTAATGGTAATAAACCCGTAGGCAGC
EMPV1_29762	AGAAAACTCTTAAAGAATGCAGCAGTTTAGAGCACTTGCTTTCAAAGAGTGGCGGGGACT
EMPV1_29769	CCGAATCTGAAGAACGAGACGGCGAAGCCGAGAGAGGTCGTACAATGGAAATAACTCCAA

EMPV1_29771	CAAATGAGACATTTCAGAGTGAGTATCCTGGCAACATGACTACCTGTGTGTAGCCTAGGC
EMPV1_29772	CATGCAGCCCTAAAAAAGGAAACTGGCTGACTTGGCGGTGAAATGAGTGTCACCTTATTG
EMPV1_29775	GGACTTGGGGAAGAGGTTGCTAGAAGCAGCAAGAAAAGGCCAAGATGATGAAGTCAGAAC
EMPV1_29776	GCTGCTCTAAAGCCAGTAGATGTTAAACCGAAGACACATTATTCCTGCCATGAGTCCGGC
EMPV1_29777	GACCATGGGCTCAGTGTCTCTCTTTATATTTTTCGTCACATTGCTTGTTCTGGGCCGACA
EMPV1_29778	AAGAACGTGACCCTGGGTGTGCCCCGCCTTAAGGAGCTCATCAACATTTCCAAGAAGCCA
EMPV1_29779	GGCCCCATAGCTTGATGTAAAGACAGCGAGGAAAGAAGCACCAGTCAAGTTGTGTCAAAG
EMPV1_29782	CCGTAAATTGATGCCCACTTTGGAAGGTTTGCGACTCTTGATCCATTCTGTCCCCAGGTC
EMPV1_29784	CGTTTTTCAGCCCCTCTGGCCTGTCGTACAGTCTCAAGCATATTCAGGAATTATCTGGTG
EMPV1_29786	GTGTGTGAGGTTTTCACTTACCACACATTCATCATTCACTGAGGGGGTTCATCTGGGAAA
EMPV1_29788	TTGTTGCAGAAGTGGGAGAGCGTGAAGCTCTTGTACGTGAAGACGGAGAGGTCGGTCTCC
EMPV1_29789	CACTCCGGTGCTGTACTTCAGCATCTTTTCAATGTTCTGCCTCATTGGACAAGCCTCCTG
EMPV1_29793	AGCTCTTCCTTGCTGTTTTAATGTGGTTCACGCTGTAGCTTCACAAGCCTGTTACTCCAG
EMPV1_29795	CCAGGCCAGTCGAGCTGACAATTTTAACTGAATTATGTATGGCAATTTCCCCCTTATCCG
EMPV1_29796	AAGTATCAGCTAGCAGCAGGACAGAAAGGTGTCCAAGTACCTGTATCGCAGCCAACTACG
EMPV1_29797	CCATTGGAGCCCTACTTCTCCAGGTCAGGATACTGTCTGTTTGGCACCCAACTACCAATT
EMPV1_29798	TTCTACATCATCCGCTTCCACTCCTTCTACGCGTGGCACACGGGCGGCGACTACCGGCAA
EMPV1_29800	AGGCCGTGGTGCTGACGCTGCTGGCTGTCTATGCAGCTGGCCTGGCGCTTCCACACAACA
EMPV1_29801	AATGTCTTCAGTGCCTCTGCGTCTGTCTCCACTACTCATCTTAGGACCCCCATCAGCTAC
EMPV1_29802	GCTGTGTCTGCTTTCTGACTACAGCTGCAGAGAGTAGATTTAGCTAATGGCCACAAACCC
EMPV1_29806	ATCCTCTTTTTGGTCCCTGCTCCTGCTTCAGCTTCCTCTGTGCTCTGCCTTCTGTCCCTG
EMPV1_29808	CAATGAAAGCTGTGGAAGCATAAGGCCCAAGGTGGAGTTAACTGAAGAGGAGCTGAAGGC
EMPV1_29809	GTGGCACACTTACAACATCATATTAGTCAGGGTTCTCCAGAGAAATAGAACCAGTAGGAG
EMPV1_29810	GACTGTCCTTTCCCCATGTCCCCTCTCTCACTGAACCCTCAAATCTGCATTATACCTTAT
EMPV1_29814	ACAAAGCTCAGGACGCACAAGGGCTGGAGCAGCACAGGGCAATCACTTCAAATTAGATAG
EMPV1_29815	GATCAGCCAGAGCAAGCCAGCACTCACTTACGGCACACGAGGCAACAGCTACTTCATGGT
EMPV1_29816	ATGTCCAGTGGGTAGAAGGACTAAAGGACGCCCAGATGCGGGATCTTCTGTCTCCACCTA
EMPV1_29817	TATAGCCTGGAGGAGTTAGACAGGGAAACCAAAATACACACCACCCTTCCTTCTGCGGGG
EMPV1_29818	GGGGCGGCGGCCGAACGATGTGCGAGAACTGCGCAGACCTGGTGGAGGTGTTAAATGAAA
EMPV1_29820	GAAAAAGATGACTGCTTTCACCCTGTCTATTCAACACGTAGTAAAAGTTCTAACCAGTGC
EMPV1_29821	TGAGCTTTTGCCAAATAAGACGGGATAGTTTGTGGAGTCTTTTGAGGAGCATCAGGACTC
EMPV1_29822	CCAAGAGTCGCCAGGTTTGGGGAACACATGGGTCTTTTATCTAGCCCAACTCCACAAGTT
EMPV1_29823	CTGCAGGTTGTCGACGTCCTGGCAGATGACAGGAGGCAGACTCTCATCCTTGAGCGCTTT
EMPV1_29824	TTACAACTGTGAGCCCTACAACAACCCCGCAGACTTCTTCCTGGACGTCATTAACGGCGA

EMPV1_29827 CAAGCCTGACTTTTGGTGACATAAGAACCAAACCCCAGTGGTCAGAGGCATGTGCATTGT
EMPV1_29829 GACTTGTAACTCCTAAGGTCTGAGATCATGGTTTGGGTGAGAAGGAGAACGCTTTGGGGG
EMPV1_29831 GGCACCAGTCTCATCCCCGTTTAGTAGAGACAGTGAGTAGAGCTCATTAAGGGCAGTACA
EMPV1_29834 CCTTGTTTTTAACTCCTTCTCCCTCCATTTTCCAGAGTAACTTCCCCAGAGGAGCCTGCC
EMPV1_29836 GAGTTGGTGTTACTCTTCATCGGGGTCTTCATTGGATGGGCTCCTTTTCTGGGTATCATC
EMPV1_29837 TTATTACAAGCGGTGGCTGAAAGTGATGCCAGCAGAGGAAACCCCCCACCCGTGGCAGAA
EMPV1_29838 CAACCTTCATGACGATCTTCATGGTGCGTGTGCGGCACACGCCTCCCTCTCGGTTCTCCA
EMPV1_29840 GTTTGAGGGTGAGCATGGTTCTAAGGATGAATGGAGAGGAATGGTCTTAGCCCAAGCACC
EMPV1_29841 GGACTGTAGGACTGAGTGCCTAAAAGTTTATGTAACACATCTCCTTACCTACTTCTGAGG
EMPV1_29846 CCCCCAAAGCGTTCTCCTTCTCACCCAAACCATGATCTCAGACCTTAGGAGTTACAAGTC
EMPV1_29847 CCCCATTCCCACTTTCTGTCCCTCCTTTTTTCACTTCTGCCAGGAACTTTGATGACCTGC
EMPV1_29849 GGAAGAGTGTCTTTGAAGGAATGAAGAGTCCCAATATGACAGATCACCAACTTTCACCTC
EMPV1_29852 AAGGCACGACAGGACATTGAACCAGAATGGGCTTATGGAAAGAAACGACACCCTGTTGCC
EMPV1_29853 TTATTTGGGTGCCACTTGCCACATGGGATGTTGTCTGGCCAGGGTTTAATCTCAGAGTGC
EMPV1_29854 AAGGCAATTCTACCACAGAGACTGAGAATGCAGGAAACTTGGCAGGAGATCCGTGTTCCC
EMPV1_29857 GTGCTTGGGTCCAGGGAAGTTCAAGTGCAGGAGCGGGGAATGCATAGACATCAGTAAAGT
EMPV1_29858 CTATTACTTGACTGAGGAGATGGAACTGTTACTACTTGATTGAGGTTGATGGTTACATGG
EMPV1_29859 ACTTCTGCCAAGACCCTGAAAGCTCCTTTTAAAACACAACTCCTTTGCCTAGTTCTTGTC
EMPV1_29865 ATGGGAAGCATGCCCAACCTGTCCATTCCTCAGTCATTGCCTCCAGTTGCACCTATAGCA
EMPV1_29869 TGTCCCCAAATTACAGAGGAAGGGTTCTGGCCTAGGAGGGTGGGTTCATGTTTTACAAAA
EMPV1_29870 GAGGCCTTTGAATGGGTGACAGTGATTCGTCAAATCTTATTTCCTAGAACATCTTCAGAG
EMPV1_29875 CTGAAGTCACGGCGAGACTAAGGGCTTCCATTCAGTCCCGTGTATTGCAAGAATCCATGA
EMPV1_29877 ATATTCCTGGACTCACCGATACTACTGTGCCTCGCTGCCTGGGGCCTAAAAGAGCTAGCA
EMPV1_29883 TCTTCCACAGGCTACAATTTAATTAAGCAGTGACACTGGAGATGAAGAATGATTTGGAGA
EMPV1_29884 ACCCTTAAGGCTTTACTGTCTGGTACAGTTACGAGCCGTGTAGATTGCATCAACTTCAGA
EMPV1_29886 CCATCTTTGCCTTGAAGTGGAACAAGAAGGGGAATTACATTTTGAGTGCTGGCGTCGACA
EMPV1_29888 TCCTGCTAAGGATGTTCAGCAACAAGCTGGAACTGTGTTAGGTGTTTCCTTGGAGGCATG
EMPV1_29889 TAACCCAGGAGTTGGGAGTTAAAGAGTTGAGGAGAAACTAAAGCCGAGGTTTTGGCCCCC
EMPV1_29894 GATGAACATTTCCTTATGTACAGCTGCTGCCAGGTTCAGGTCTAGCTTCTCTGGACAGGC
EMPV1_29896 CCCCCTAACCATCCCCTTACGACTCAGGCATCATTTAACGGAAAAGGTGAGCTGTGTGTT
EMPV1_29897 CTGGAGGGGGTGGCTTCTAAAGACAGGCCATGAGAGGAAATCACTAAGAGTCGAAATCAC
EMPV1_29900 CTGGATCATTCAAGGTGATGCTTTTTCTGGTTGCTTTTTGAGATTAATGCTTTGAGGCTG
EMPV1_29902 AATCGTTCAAAGGAAACGCAGGGGCTTGAGCAGGGCCGGGGCTTGCGAAGGGCTTCGTGT
EMPV1_29903 CAGGAAACACAACAGGATGACAGCACGTTTAATTCGAGCTACTCCCTCTTCAGCGAACCC

EMPV1_29905 CAAGACGCAGTCAAGAACGAGACAATCTTGGCATGTTGGTCTGGTCGCCTAATCAGAATC
EMPV1_29906 AAACGCAATCGACTCTACCTCCTATCCACCGCCAACACACTGGGCAACTGTCAATGCGAT
EMPV1_29913 ATCTGCAGTTCACAGTCCCGCATGGAGTCGGCATACTACAACATGAGCTACGATACTTTT
EMPV1_29915 CAGCTCCAAGGGATCTTATCCTGGTCAGAAGGGTGTATTATGAGAGGTGATGTTGGCTAC
EMPV1_29919 AAGAAGAAGCCCAGCGTCGATTAGAGGAGAACCGACTGCGAATGGAAGAGGAGGCAGCCA
EMPV1_29920 TGTTTTTGGATACCTTCCTCACACCAAGCATAAAAGATAACTCAGAATGGTTCGAAGACC
EMPV1_29921 AATCTGCCTTTGTGTACGCCATCTCCTCAGCTGGAGTTGTATTTGCCATCACCAGGGCCT
EMPV1_29923 GAAAAGATCTGGATGTCACCTACCTTCCAACAGCTTAAAACACTGTGGCTCAGGAGGCAG
EMPV1_29924 CACAGAGCTTGGCATGTACCAGCCCCACTGTGGGCTCGAGAATGTCCTGATGTCCTGGGG
EMPV1_29926 CCTCCAACAACTACAAGCTTAAGGTGGGTGTCCCGCTCTCTCAGGACTCCATCTACATCT
EMPV1_29927 CCATGGGCTGATGACATAGTGATACGTGTGTTCGGTCGGGGTGCCTTTAAATCTCATGTG
EMPV1_29928 ATACATTTTCTGAACGTTGTTTTTCTTTCATCTTGTATACTGTTGCTTTATTTTTATAA
EMPV1_29929 GTGATATATCAAGGAGTAAATACCCCTGGACAACCAGTCTTCCTGGAGGGGCAGCAACAG
EMPV1_29933 GCCAGGGAGAACTTGGAAGCGCATTGAATAGACATCAAGGGCCCGAGGCTTCTGTCCTGA
EMPV1_29934 CTCCTCTGAGCTTACCTCCATTGTCTTTGCACAGTTCTGCTTAGCCATGCCTTGATTTGC
EMPV1_29936 AGCCCTATTTTTCAGTTGAATCAGAAACAGGTATCATCAAGACAGCCTTGCTCAACATGG
EMPV1_29938 GCCCCCTCCACGGCCTCCTCCCTGTCTCCGGTCCGGAGCCGGTCGCTAAGCTTCAGCGAG
EMPV1_29939 ACAGGTTCAAGGGCAGGAAGGAGAAGACGTTGTACTTAGCCGTGTGGATGGCATTCCTCT
EMPV1_29942 GGCACCTAGAAAAACAAGGGTGTTTGCTGCTAACAGATCTCAAACATCGGCAACTGTGGC
EMPV1_29943 CTACTCACTTAAGGTCAGTGTCTAGTCAAGTAACCCTGAGCTGGCTGCCATCTTTGAAAG
EMPV1_29944 GGACATTCTATTTCTAAACCAGATGTGATCACCTTCTTGGAGCAGGGGAAGGAGCCCTGG
EMPV1_29946 TGGATCCCTCAGTTGAATCCAAAAACTATGTAACAGGAGGCTTATCTTGTCACAAAGTCT
EMPV1_29947 TCTCTACTGCTGGCTTCTTTGCTCACTCAGTTTTTGCTCCTCTGTGACTCTGGCCCACAC
EMPV1_29950 CCGAGTCATCTTCAGACGCTTCACCGTCTACAGGCATTTTCAGTTGCTGACATTCAGAAC
EMPV1_29951 AAATGGAGTTGCCGCACTGCCTGGGCCTCTCGTGTATGTGGAGGTGGTGTTCCTACTGAT
EMPV1_29952 GTGGTTATGGCCAGCACAGCTACAGCACAGGTTATGACGACCTGACCCAGGGGACAGCAG
EMPV1_29953 CCGAACTTGTGTCTGAGTTCAGGTTTGTGCCCATTCAGACTGAAGAGATGGAGCTGGCTA
EMPV1_29956 TAAAGATCTGCTGCCGCGTGAATGGGAATGTGGTCCAGAGCAGCAATACTAACCAGATGG
EMPV1_29959 GTCCATGCCAGAAACCTGGAAGTCGTTCTTGATTTTCCTTCATCTTTACCAACCAGGTAC
EMPV1_29961 GTCATCAGGCTATGTTCTCCTGTACTGTGAACTTCCAGCTGCCCAAGGAAGAGATCACCT
EMPV1_29962 GCTTTTCTCCGTGGATGATCAGCTCTGCCTGCTATACCTTTGGCTCAAACATTGCTGGGC
EMPV1_29964 CACGCTGGCAGCAAGGTGGGAGACCTCAAGGCTTCCAAGGAATGCCAAGTGCTATACGCC
EMPV1_29965 GGCTGGAAGAGATCCGGCAAGAAGTCGTTAGAGCCAACCAGGACACAAAAACAGCTCAGA
EMPV1_29967 ATGGAGACTGAGACCCCCACGGCCGAGGACATTCAGCTTCTCAAGAGGACAGTGGAGACA

EMPV1_29968	AATATCACGGTCGGTTGAAATGTTGATCCTGGGCCGATTGGTTATTGGCCTCTTCTGCGG
EMPV1_29970	ACGTCTTTAATTTGGCAGAGCTAGCGGAACTTGTCCCCATGGAATATGTTGGCATACCAG
EMPV1_29973	TGTGCTCACGGGTAAATTCCACACAGAGTAATGCACTTGCTGTCCCAGCAGACCGTCTCT
EMPV1_29974	GTTGTGACAAGAAGAACGTAATAACCTTGTGACCGCTGTCGTCACTGTATGTTTGGTAGC
EMPV1_29975	TGGGACAACAGTAGACGTACTGCAGGAGAAGATTGGCATCCTCCAGAAATCCAGGATGAG
EMPV1_29976	TGAATTTCTGCATGGGAACTGACCCGGGCTTTGAGCACCTCTTGGCCTCAGCAGCCCCCT
EMPV1_29977	GGAGCATTGGATTTACTGAAGGAGCTTAAGAATATTCCCATGACGCTGGAATTATTGCAG
EMPV1_29979	GTGTACTCCTGGCTCCTTTGTCATAAACAAACTGACCATGTATGTGTGGATTTATCTCTG
EMPV1_29981	GTTTTAACCGCCTGGACCTGCCACCCTACAAGAGTTATGAGCAGCTGAAAGAAAAGCTAT
EMPV1_29984	CTCCATTGGTATCCTTCTCTCTGACTCTTCCTGGTCACATGTATAGCTAATTGCAGAGCC
EMPV1_29987	CATTGAGTCATGCCCTGTCCAGCACTGATCCAGGCAATGGGAAAACAGTGGTGATAAGAC
EMPV1_29990	GTGGCCCAGATCCTCGACTTCATCTCCTCCCAGACCCACGTGCCTATCCTCAGCATCAGT
EMPV1_29992	AGGCATGTCTATCCTACGTGACCAAGCAAGGGCACAAGAGGGCCAGTCTTCGGGATGTGT
EMPV1_29995	CTACAGCCTAAGGAACAGGGACATCAAGCACGCCCTATGGAGGATTATCACCAGAGTAGG
EMPV1_30000	TTATGTCACCGAAGTGGTCTGGATCTGGGCTTGTTACCTGCCTGGTGAGTTCACTGGCTT
EMPV1_30001	ATGAACTCCGTCCGAGCCGCCAACCGGAGACCCAGGCGAGTGTCGCGGCCGCGCCCGGTG
EMPV1_30003	ACAACCTTGCCAGAAAACCTTGCTCAGAAGCTTCCAAACCTTGTGGAACTGTATCTGCAC
EMPV1_30006	AGAATATGGAACTATCGATGACGTGGACATTGACCTCCACATCAACATCAGCTTCCTCGA
EMPV1_30011	GCCTGAAACATGATCTCCTTCCTATTCTCTGCTGTGGACCTAGGGCCTGACTCAGGTTCT
EMPV1_30012	TCAGTCTCTACCCTGCTGACTTTCTACCTGAACTTAATCATCGGCTCTTCTGGCCTCAGG
EMPV1_30013	GGTGACCTTCCTCAAAGTCTGGATACCTCACTCTTTTTTGGAACAACAGCCGCCGGTTTT
EMPV1_30015	TAACAGCTGATACCGAGTCTACAACTAGACATTCCAAAGCAGATCAGCACCCAGCTGCTG
EMPV1_30016	GGTAATGATACTTCTACCTGGACTTCCCTGGTATGGTGTAACCTGATGGAGACCTCACTC
EMPV1_30018	TGCCAAAACCAGGGGTTAGAGGAAAGCCACCGAGCCAGTCTGTTTCCCTTGCAGACAAAA
EMPV1_30020	GGCTGTGGTTCCACCGACTCTGCTTGGTCAAGATCGCTTCTTACATTCTGATGAAGTGTC
EMPV1_30021	GCTCTGGATTAGGCTTTGGCTTAAGAGAAGGTTGTGGCAGGTTTGATCCTCTATCCAGAC
EMPV1_30024	AGGATGTCTGACTTGGCCAGGATGGTGAGACACTGTTCTGTAGATGTTGGCTTGTCCAAG
EMPV1_30027	TAAAAAGGTTGCAACCAGATGAAAAACCTGAAGGCAAAAATCAAGATATCAACAATCCTA
EMPV1_30028	AGTATGCTTTCCCCTGTTACTGTTGGAGAAAACTCAAACCTTCAAGCCCTAGGTGGAGCC
EMPV1_30030	TCTCCAGCGTCAACACTGTGGCCACCAAGACTGTGGAGGAGGCAGAGAACATCGCGGTCA
EMPV1_30031	GACCGACGCGACTGTTATGTACGTGTGACTGAAAGTTGGCACCGTGATCCCACATTGAAT
EMPV1_30032	GAACGTTGTCATGACAGGGGAAAGAGTAGTGGTAGGAGTGATGACTACAGTGAGACCTTG
EMPV1_30033	GCTTCAGAACTTGTATTTCACTCTTGATTGCTGCCTGGACCATTGGATTCATCCACTCGC
EMPV1_30034	AGCATTTAGAGATTCGTTGGGCCACATACTAAGAAACTCAAACCCTCCTCCGTGGGTCTA

EMPV1_30037	GCAGTCTGGCACTGACACTGGCTTTCTCAACTTCAAGATTCATTCCTTGGTCTTAAAGGA
EMPV1_30038	ACTATTTCAGCTTCTGGGTGGGGGGCGTCCTGTTCCTCGTGCTCAACTCCCAGTTCCTGT
EMPV1_30042	CTCCCGATGTATGCTTCCGAAGAGATGAAGATATGTGTGTACACATTTCTTATAGGTGAT
EMPV1_30043	CGTGGAACCCAACTTTGTAGCAGTGGGGCTTTATCACCTGGCTGTGGGAATGAATAATCG
EMPV1_30044	CTGGCTTCTGGAAACTGCCCATTCTCAGGGGAAAGGACAGAAAGAGAACATGGCTACTGA
EMPV1_30045	TTTCAGGCTAGTGCGGTCAGCTTCTCCACTGTCCCTCACCAATCTGGCTTGTCAATTCGA
EMPV1_30046	CGAGCTCCGCCACAGTAACTACCACCTCCAAATCCTAGGGGCTTAAAAATGACCATGGTA
EMPV1_30047	AGAAAGCCCCGGGTGAGGACGAGCCCCTCTCCTGCATGATCTCCAGAGTGAAGAAGCCAT
EMPV1_30048	AGGAAGTCCTTCGAGAATTTCATTGTGCGATTTGACAGCCCCTTTCTGTTCGCCACGGTC
EMPV1_30049	ATTCTTAGAGAGCCGAGCTTGGGCGGTTGGAGGAATGGGTGCTGGCTTTTCAATTCTGGA
EMPV1_30050	CTAGGGGAGAAAAAGGAAAGCGTTGCTTTGCTATGCAAAGGCGCATTGGGGCTGTGGTTT
EMPV1_30051	GCACTACCCATGAAGTGGCATTGGACTAGTGTAAATATGTATGGCAAACTCCAGGGCAAC
EMPV1_30052	GGCCTTCAGCAGCAAATCCTACCTTGTTATACACAGGAGAACACACACAGGAGAAAGACC
EMPV1_30056	GAACACTGCAATTTATTCTACCTAATGTTCAGCTTGTTCTTCTGGTAGTTCTTCCTAATC
EMPV1_30058	CAGGTCTAGGGATCAAACTTGAACCACAGTAGCAACCGAAGCCATCACAGTGACAATGCG
EMPV1_30062	CATTATGTGATTGTTTACTATAAATGTGTCAATTTCACAAAGCCCCTAAAGCTCTTCTTT
EMPV1_30063	GTTTAAGCTGTATTGAACTAAATCTGTGGAATGCATTGTGAACTGTAAAAGCAAAGTACC
EMPV1_30064	ACAACAAAGGAATTGGGAACTGTAATGAGGTCTCTTGGGCAGAATCCCACAGAAGCAGAG
EMPV1_30065	AGTTCAAAGAGCTGCCTTGCAAGGGTTAACTACCTCCCCAGCCTTCAGTTCTGGAATCCT
EMPV1_30066	TCTGGGTGCTGGGAGCCCTGTTCCTGGCCATTGGCCTCTGGGCCTGGGGTGAGAAGGGCG
EMPV1_30070	ACCCATGGCCTTTCTAGGAGCCACGACCTCCGGGAAGTTCTGGAATCCCACCGCGGAGTT
EMPV1_30072	TGGCGCACATCTGGAGGACGCAGCACAAGACCATCTCAGACTCGGGCTTTGTCCTCAAAG
EMPV1_30074	AACCTGTACCGACTGGAAGGAGACGGTTTTCCCAGCATCCCCTTGCTCATCGACCACCTG
EMPV1_30075	TGACTGTGTCCTTAAGCCTGAGTGCGTCGGTGTCCCTCGGCATGCTCTATGTACCCAAAA
EMPV1_30076	CTCCCATCTTCCACAGAGATGCTATGTCCGAGACCTCATTAGTGACCTTTCTTAGCCTCC
EMPV1_30078	CAGAGGTGCATGACTGGGTGTGTAGATAATCACTCGCTTACTAGATCCAGATGTCTCCAA
EMPV1_30080	CCTCCCTGGTGATTACCTGCAAAACTTGGTCCTGTTCCCACCTTGTGTCATCCCAGCATT
EMPV1_30081	AAAGCAGCATGGGGACCTGATCCATTGACTTTTGTTTGGGAGCGTCTGTTTAAGCGGAAG
EMPV1_30082	GCACCTTAGATGCAAAGTGCCCAGTTGAGAACTGACTGCACGACAGTCCAGCCTCTGGAT
EMPV1_30083	CTACCAGTAGGATATGGAGGCCAACCAGCAGAATATAGAGCCCCACCTCCAGGATACAGA
EMPV1_30084	TTGTTGTCTCCGGTTGATTATGACAGATCGAGTCACTGTAGATTACCAGACTACCTGCTC
EMPV1_30085	CATGAGCCGTACATACCATCATGTGCCCGCCCTGCATCTCTTTGCATGTCTGTGGTGCTG
EMPV1_30086	CCCTCAGAAATGGCCTGACTTGTTGACAGAAATGGTGAATCGCTTTCAGAGTGGAGATTT
EMPV1_30089	GAAGCAGAAGCAGGGAAACAAGTGGTTCATTTAGAGAGGCCATTTCAGCCTTCTGGGGAG

EMPV1_30090	TTTTCGGGCTGCACCTGAGGCATTTAGAAGTTCCCAGGCTGGGGGTTGAATGGGAGCTGA
EMPV1_30091	AAACTACTCTCCCAGGACTTTCCTCAGAGTCCTTGCTTTGGAGCCACCTCTGATTTGGAG
EMPV1_30092	CGCTGCAGAGACTCCTCTGATCCTATTGCACCACAGTGGGAACTCCTCTCTGAGATTATT
EMPV1_30094	GACACCTCTGTCATTGGCATCTTAGTGGTGGCTAATGGAGGCTTGATCTGCAGTATTGTG
EMPV1_30096	CAGTCATAATCCTAGAGCTGAAAGCAATCCTCACTCTCTGTAAGTGGGTCGCCCTGGTTG
EMPV1_30097	GTTTCTCGATTCAGAATACGCAGGACACCGGCCCGTTCTCAGTTAAACCTTACGCCCATG
EMPV1_30099	CCCCCTGCACTACTCAGTTATCATGAATCCCAGACTCTGTGGGCTGTTGGTTCTGCTGTG
EMPV1_30101	GAGCACCGAGTCTACCAGCTGTCAATCAGCAGCCATCAAAGAATGCAGGAGAGAAACCCA
EMPV1_30103	CACTGAGTTTATGCGCTATGCAGTGGGTGTAGCCCTGCACAAGGTACAGCAGCTAAAGGA
EMPV1_30105	AGGTTGAAGTTCCGACTAATTGGACGCTTCTCTACTAGTGTGGAGCGGGTGTTTGCTGCA
EMPV1_30107	GCAAAACCTCCGGGTCACCGAACAGCATTTTAACCTCTTGAACGAGATTCCTCCCGCTTT
EMPV1_30108	GGTTGCTTGTTCGCTTCCTCTTAGGTCGCTCTTGTACATGGAGCCCCAACTAGACACTTT
EMPV1_30109	TGGTCTGAAGTATTGCCCACCACCCTTGGGGTACATAGAGGGGAGCAGAAACCAACTAAA
EMPV1_30110	TATTCCAAGAGCAGAACAAACATCAAGAGAAAAAGCGCTCAGGTAAACCCCTCCCGCCCC
EMPV1_30112	GTCAATATCTTCTTCAAGGAGCTGAACTACAAAACCAACTCCGAGTCCCCCTCTGTCACG
EMPV1_30113	GGGGACTTTCGTCGTAGATGCATGTTAAGCCATGTGTCATAAACAGGAAATGGAAACCAC
EMPV1_30115	AGAGGGTCAGAGGGGGAGGATGGACCGGGGGACCTCCCTGCCCTGTGTGCTGGAGCAGAA
EMPV1_30117	CACAGCTTGGTGTGGGCGAGTGGGGATGGGAAGATTCAAGAGACTTCATTTTTAGCTTGT
EMPV1_30118	GAACCATCCTCTTCACATATGTGAGACCAAGTTCCAGCTATGCCTTGGATCATGACATGA
EMPV1_30120	AGCAAAGAAGCCAGAGAAGGAGACATCCAACAGTACCAGAGAAATAGAGTCCCCTCCAAA
EMPV1_30122	CAAATCCCGCCTCTAAAGTACGACGGCCACTTTCATTGGTAGCTGCTCCTTTCACTCCAA
EMPV1_30123	CTCAACTAATGCAAGTACCCCACAGTCAGTGAGCAACGTGGTCCATTATCTTGCAATGGC
EMPV1_30124	GAAGAGAGATCAGAAAAGGACTTTGAGAAGGGCTCCCGTAGCCTGCCCAGCCTGTCGGCA
EMPV1_30125	AGCTCTATGCCGAGTGGAAGCACGTCAACGATGACTCGCAGGAGAAGAAGATCCTGCTGA
EMPV1_30126	CCAGAAAAGGTTATTCCAAAATTTATATCTTTACTACAACACAACAGGAAATGCTGCATT
EMPV1_30128	CACTCTGAAGATCCGCTTAGTGAGACATTTTCCCGAAACCTAGATATGGCCTCCCCGGCC
EMPV1_30129	AACACCTCTCTTCTCCTTAATGATCTCACCCTCATATTGCACATGAGAGACTGGGCCCCG
EMPV1_30130	AGATGTGGTTATCACAAGTCTGTTCAAGGGAAAAACTACTGCATAAAATAACTAACTTGG
EMPV1_30131	GTGGCCTGTGGTGGGAAGGATTGGTCTGGCCATTGTCACCCGAAGTTTCTGCATCATCTT
EMPV1_30132	ACTCTGGGGCCAAATGCCCCTGTTCCTGGTCTCTGCTACCTTCCTGCTCTTTGTTTACTA
EMPV1_30133	CGGCACCCCCAGAGGGAGATCCGACTGATGGAGCCAGGCCACTCAGCACTGGTCACTGAA
EMPV1_30135	GCTCCTGGAGGCGCTGACGTTTGTTTTCTTTGGCTAGTACTGTATATGAGGGAAGCGTGT
EMPV1_30136	TGCTGCATGTCCAAATGACCAACGTTCAAGCAGAAGACTCAGGACTGTACCGATGTGTGA
EMPV1_30137	CCAGGCCGGAGCAAAGCGCTCATCAACTGTGACCATGTGAATTGTTATATGTTGTCTAAT

EMPV1_30139	CTTATGAGTGTTCACAACGAACCACAGGATGGGGCCAAAAGGCCGTTGAAGTCCGCTCTT
EMPV1_30141	CCATCGACGGCTTAGAAGATGACCAACATGGACAACTGTATCGACCGATGGACTTACCAG
EMPV1_30142	TCAGGGATGTGGTTTATCAGTAGACAATAGGCTGTGCTTTGACATGGTCCAGGCTCTCAG
EMPV1_30143	CACCTAGAGAGTTAGAAATAAGAAGACGGGAGGATGAGTACAGATTTACAAAATTGCTTC
EMPV1_30144	CAGTACCTGGGTGTGGCCTTCCGTGGCCTCAAGGGCAAGAAGCTGTATCCGGTGGTGAGT
EMPV1_30148	CAGGAGGCCCAGGTCCGAGTCTGTGAGTGTGAATAACTTCCTGAGCAGTGTCGACGGGCA
EMPV1_30150	ATCAAGCCCCGAGAGAGATGTGGAAAGGGATGTCTTCCTGTACAGAGCATACCTCGCACA
EMPV1_30151	TTAAAGGAAACCAAAGGCAACTGTGCTGCAGGCGCTTTGACAGAATCCACATCGCATCCG
EMPV1_30152	GTGCAGACAACGATGCGTGAAAACCTGTCCACAGTTGAGGCGAATATTGCGAACAATGAT
EMPV1_30155	TCGACTTTCCAGACAGTACAGCTGACAGCAGCTGCAGAAATCGACACCTATAACCCTCAG
EMPV1_30156	CCCCAATCTCCCCCTGGGGTGGTTCTGATTGTGGCTGACGCATGGTTACAAATTGGTTTT
EMPV1_30159	GGCTTCCTCACCACCTGGTTCCTGGGTGAGTTCCTGGCCCAGAGCGAGGAGAAACCACAT
EMPV1_30161	TTCTCAAAGATGTTCGCGGGAAGCGTAACTGCAGGGCGGCGGGCGGCTGCGCTCACACAA
EMPV1_30163	CCTTCATTCCTCACCCAAAGGTCACGGCTCTCCTTGCCTAAAGCAGCGATGGAATTTCTG
EMPV1_30164	AGCAATGCAGGATCTGAGCCGTGTCTTCAACCTTCACCCCAACTCACAGCAATGCCAGAT
EMPV1_30165	CAGCCCCAAACAGCCGGGACTTGATTAATTACTGACAGAGCCCTTAATTTCTGCCCCAGA
EMPV1_30167	ATGAAGTTGAAAAGGTCCTGCGCCGGTTCAGGTCGCTCGAGTACCAGCAGGAAGCCGTCA
EMPV1_30168	ACTTCTGTGGAGGCTCCCTAGTGAAGGAGCAATGGGTACTGACTGCCCGGCAGTGCTTCT
EMPV1_30169	TGAGCGACTCCCTGTTTGTTATCTGCGCGCTCTCTCTCGCCGCTTGCCTCTGCCTTGTGG
EMPV1_30170	TTAAGAACATAGACTCAGGTCCCAGCCCATCCTGGTTTGAATCCCAGCTCTGCCACTTCC
EMPV1_30172	AGCCCTTGTTGGTCTGCTGGCTGGATTTGCCATTTCTTTGTGGGTCGGAATTGGAGCTCA
EMPV1_30173	TGTCAGACAGAAGGCGAGGGATGTACTGGACTGAAGGCAGGGAGGTGGTTCCTTCATTCA
EMPV1_30174	TGGCTCTGCAGGCGGAGGGATGCACGGACAATTTCTCAAGGAGGGACAAAAATGGACAAA
EMPV1_30176	AACTGGGCTTGGAATTGACTCGAGAGAGGGCTCATCGCTGTTGGTAGTGACAGAGGTTGT
EMPV1_30178	AACTGTGAGGCCGGGACTAGGAAATGAGCCCTGAGTCCGCCAGCTGGTGCCTGGGAGTCA
EMPV1_30179	CCGAGGACTTGGATCTCAGCTAAAGGACAGTATTCCAGTTACTGAACTTTCAGCAAGTGG
EMPV1_30180	AGCTAAGCGCCAGGACACGCAGCACCTGTTTCTAACGTTGCCTAATAGCCTCTGGCATTT
EMPV1_30181	CGGTGGTGCTTATGGTAGCCAAATGATGGGAGGCATGGGCTTGTCAAACCAGTCCAGTTA
EMPV1_30183	CAGCGCCTCAGCTCCGGAGGAGAAAGGCTGGGCCAAGTTTGCTGACTTCCAGCCTTTCTG
EMPV1_30185	GGCCTGTGGAATGGATGGAAGGAAACACAGAATGAAGTTCCATAATACTTGAAGAAATGT
EMPV1_30186	TCAAACTGGAGAGAGACCAGAGCACAGCGTAACTGCCAGCTCCCAGCCCCATCTCATTTT
EMPV1_30187	GATGCACGTGAAGCATGAGCAAGAACTGCCTCTGTCTTGTAAATACCAGCTCTGGGGTTC
EMPV1_30191	CTGCCATTCTGTGGCCCAGATATACTAGACAGCTTTTACTGTGACCTTCCTTGGTTCATC
EMPV1_30194	CCAAAACGAGGAAGCCTGCCATGCTGATAGGGTGTATTTGAGTGATGGTTGGCAACAAGA

EMPV1_30195	TAAGCACCTGCTGCCTCCTGTGCCCCGCTTCATGTTTCTGTTTCAGTAGACTTGCCTCCT
EMPV1_30196	GGGACTCCAGTGCCTGCTCAAGTGCTACTGTTTTTTCATCCTCATGATTTTTGCTCTGTT
EMPV1_30197	GACTCAACTGGGTGACCTGTCCCCAAAGATGATGCTCTGATGTTCCACTGTGATTAGCTC
EMPV1_30200	CTGCAGAAAGGTCTCCTAATAGCCACATAGGGCTTACCTCTGTTTTTCCCCACTTCCTTT
EMPV1_30202	AATGAGAACTTGAAGAGCTGGCCTCTCCGCAAGATGAGCTCCTTGCTGTAGAACCTGTCT
EMPV1_30203	CAGCAACCTGGAGCTCTGGGGATTTCTGTTGTGCTGCTACTGACGTACGAACATCCAAAG
EMPV1_30205	CGTGCGCTTGGGTCGCTACCCGCGCGCGCAGCTTGAAGTGTCGGCGCTGCTGCTCGAGGA
EMPV1_30206	TTATCTATGTGGACTACCGAGAATCTCCCGGAGAGATGGTAACTCCCTGCTGAGGGGTCT
EMPV1_30207	GCGCTCTGGCAAGAAGCTGGAGGACAACCCCAAAGCCCTGAAGTCGGGGGACGCGGCCAT
EMPV1_30210	GACACAGGAGGAGGCTGTGTAGGTGTGTGTTTGCATCTTTTACTGACACCTGACCAGCTT
EMPV1_30212	GCTCCCTCCTCTCAATTACTCTGTTTCACAAATTCTAGCTGCCTTGATCTCCTGGAATGC
EMPV1_30213	CTAAACTGCACAAATGCCGAAACAATAGTCATATGCCAGGAATCCCCTGGGCAAAGTGGG
EMPV1_30216	CATGAGCTACCGGCATCCTTCTAAATCTAGGGGCGCCAGGGAGATACACTTTTTGGATTG
EMPV1_30218	TGTTGGAAACTTCTTTTGAGGGGAATAGGGGGAAATAATCTCAAATGAGAGGCTCTGGAC
EMPV1_30220	TTGCTCCCCGAGTTCATTCTCTGAAGTCTGATCCCCTCCCTAAAAGTGGCTAGAAGGGTC
EMPV1_30221	GGGTTTTGTAACTTTTCTTTGTCTCTTGCTGTGACAACTCATCACTGAGTCCATACTGGC
EMPV1_30223	GTGTGCTGATGATTCAAACCCTGTCACATCGACTCCAGCTAAACCTAGTAGTCCACCTCC
EMPV1_30226	GTACAAGAAGCTGGAAATGAAGCAAGCTATTGAGATGGTGGAGACTGGGGTACTAAGTTC
EMPV1_30228	TTTGCCTACGCTGGAGCATGCGGACCCCCAGACCTTTGTCAGACCGTTCCTGTCATCCTT
EMPV1_30229	ACACGAGGCAACAGCTACTTCATGGTGGAGGTGAAATGCAGAGATCAAGATTTTCACTCG
EMPV1_30231	GTGTTTATGGCATCCAGACCCACACTTGTGCAGCGGTGAAGTTTGATGAGCAGAAGCTTG
EMPV1_30232	CTAGTGACTTGTATGTGTCATCTGCACGGTGGAAACTGCGCCTCATTGGTTCTTATCACC
EMPV1_30234	CGTCGTAGGAAGCCAAATCCTCCAAAAGACAGATGATACTGAGATTTTCAGAAATCAGAG
EMPV1_30236	GGGGACAACACCAAAGAAATCCCCACGGAAAAAGTCATAGAAGCTGCAAAGCAGGCTCAG
EMPV1_30238	GCTAGGGCCAACTAGTCAAACGGCATGGGAAAATAGCCTTGCTTTAGACATGATGCTGGC
EMPV1_30239	GGGGAGTGGGTGTGTTAAAATCCAGATTCCAGTGTTTTCTTGTGGTGCAGGGGCCTAAAG
EMPV1_30240	TTTCTGACTTCGTGAAGCCACTGCTCCCTAGGGGCTGGTCGACCCCCTCCCCAGCGACCT
EMPV1_30241	AACTTCCCGCTCCTGGAACTCATATGTGCAGACACAAGTCTGATAGAACTCCTGGGTTTT
EMPV1_30244	ATCACGGCGCCATTTCGTGGGCCTGCCCGGCACGCGAGCTGGCTCTCAATAAATAGCTTC
EMPV1_30245	AGGAGGATTGGGCAAAGTGTTGGTTGTGTTCTTTCCTGTGGGTAATAGGTCTGGCCACAG
EMPV1_30246	TTGATACCCAAATAATACATTTGACTCTACTGGGGTTTAGGGGTCTTTTTGGCCAGGCCC
EMPV1_30248	GTTTCCAGAGAAAGGCGGCTCTTAGTCGCAAGCTCCCGTCGCGAAAGTAAAGGGATCCAT
EMPV1_30250	GCATGGGGAGGGGGAAGCTTTTTGGAATCACTTTTTGGTCACTGAGTCTGCCATTTTCAG
EMPV1_30252	CATAAGCATAACTACTTGAATACATTGTTGCTATTATCTTTCTGAACAAGCTGTCATCTG

EMPV1_30253	AAACGCGGAGGGGGGTTAGCGAAGGATGAAAATCGGTGGATTGTCGAGTGAGAACTGCCT
EMPV1_30254	GAGGAATGGCGGGGAGCCTTCAAAAGTGTGGAAACTTCCATCGTTGCTCACAGATGCATT
EMPV1_30255	CTTCCTTGGTGAAGTAAACCAGTATGGGCCAGAAAGATGTGAGAAGCCAATGTCAGGAGG
EMPV1_30256	CATCCCTAGTGGCAAGTACGTGGTGGACAACTCTAAGCCGTCTACAGACCTGGAATACGA
EMPV1_30257	GAAGAGTTTGGCTTGGGTTTGATTGGATTGTTGTTTGGAGTCGGGGAAAGGAGGCACAGG
EMPV1_30258	CAGACCAAAACCCAGAAAGAGCTGAGGCGTGCCCTTGTCTCCAAGAAGGTACAGAAGCCT
EMPV1_30259	ACTGACACAAATGGGCATGTGGTGGATGCTCAATGGTACAGGGTCACGTTCCTCAGGTGT
EMPV1_30260	TACCGGAACAGCCACCCCAAAGAAGACCGCCAAGAAGACCCCCAAGAAAGCCAAGAAGCC
EMPV1_30261	TTGATGGAATCTCACTGGTAGAGGGTCCACATGTCTACTTACTCAGCCTCCCACTGGACA
EMPV1_30262	ATGCTGTGATTGGTTCCTGGCCACAGCCTCCGTAGATCAAACAGTGAAAATCTGGGACCT
EMPV1_30263	CACAATTGGAGTGGAATGAATTCTTTCTGCTTCTGGTCAACCCATGCATTTGTGCTACCC
EMPV1_30264	CACTACGGCCTATGGCACCGAGTGGACATTTGATAAAACATGCTAGTGAGAAACGTGGCT
EMPV1_30266	TTTACAGCTCTGCGAAGCAGGTGCCACAGTTTACATCACTGGCCGCCATCTTGACACGCT
EMPV1_30269	AAGCGAGCTCTAGTTGGAAGCTTGGTGCCTGCTGAAAAGAGGCCCTTCCCTGGAGCTGAA
EMPV1_30271	CCTGTGCAGAGTTAACATAAGCCAGATTTCCTCAGCTTCGGCACTACTGACATTGGGACC
EMPV1_30272	GACACTCCATATTAGAACAAGGCAGCCGCCTCGAAATCCCTGCCTGGCTGGTAGGTTTCT
EMPV1_30273	GGCCGTGGTACAGTTAGCCAAAGTTGTCTGTGCAATTCTTTATGCTTGAGAAGAGACCAG
EMPV1_30275	TAGGGATGAAGGCTCTGTTGTTGCCGTGAGCTACGGTGTAGGTTGCTGACACAGCTTGGA
EMPV1_30276	GGGGAAGTGACCCCCCTGTGGTACAGATTGTGCTTCTCCACCCAAGACTTAGTATATTAT
EMPV1_30279	CTCCCGACACTGTTCTACCCAGGCTTTCTCTCCCATTTTCCTGACACCGGGAATAACTAA
EMPV1_30282	TTGTGCAACCACGAACTCTGTCTAGTTCCAGAATGTTCTCAGCACCCCAAAGGAAACCCC
EMPV1_30283	CCTTCAGTCGAATGAGCCTGCGCAAAATGGATCTCTAATATAAAGGCACTTGTTTTACTC
EMPV1_30286	CCTCGGCAGGAGAGACGGTTGGTTGCTCTCATCTCGTTTTCGAATATGGTGCGGACTAAA
EMPV1_30292	AAGTTATCTGCAAAGGAAAACTGTTTCTTTATTCTTAGGCACTATCAAAAGCCAGATTTG
EMPV1_30294	GATGACTGATTTCTTTTGGTGTTCAGAGTCAATATAATTTTCTAGCACCATCTGAAATCG
EMPV1_30295	GTCATGCACAGGCCACAGTGTCACCCGAAGTTCTCCTGCAAGAATCTCTTTTATCCTTGG
EMPV1_30297	CAGGATTGGGTCGTTTCCATCAGTACTTGCTCTGATTGGCTGTTCTGGTTCTGCTCTAGC
EMPV1_30298	TTTGACCCCTAGCCTGGGTACTTGCATATGCCCCAGTTGTGGCCATGAAAAGCCAAAAAA
EMPV1_30299	TGTACAGTGGCACCAAGGGGAAGGTCATGGTAGTGGTGGAGGTCTTTTGCTTCTTGGCTT
EMPV1_30300	GGTTAAAGATGCAGACTTGTCACTGCTATGGCTCTGGTTACTGCTGTGATATGGGTTGGA
EMPV1_30301	GGTCAGAGATCAAATGTGTAACCTCATGCTTCCTAGTTAGGTTTGGCAACTCCTGGCCAA
EMPV1_30302	CTGATGACTTTGTGCGTCCGGTGCCTCCCCGTTCCTATTTTGCCAGGTTTTACTCGTGTA
EMPV1_30304	TGAGAGCTTCCTGTCAGCAGAACACAGTGTGGTGGGAGAAATCGCGGATGACCTGGCCCA
EMPV1_30307	CACCATGTTCAGTTTCACGGGGATGTGGAGGTATTTTGATGTTGGACTGCATGATTTCCT

EMPV1_30308	AAGAGATAGGAGTAATCCTGGACCACGGTGGCAGTGGCAAGAGAAATAACCCAGGCCGCT
EMPV1_30309	CCGCAGCCACAGGGTCACAGGCCAGAAGAATATTGGTGCTTATGTCAACTCCTCAAATAC
EMPV1_30313	TACTCCTAATCCGGGATGGAGCAGCAGTCCCCTCAAGAAGAGGCCGAAGCCACAGACCTC
EMPV1_30314	GCAACAAGATGGGAACGCGAGTCCTTTTGCACCTCTGAATGATCGCTTTGCTGAGATTCT
EMPV1_30316	GGTTTGAAGGCCCTAAATTAAGCTGATAACTACTCATAGGGACAAACCGAGGGGGTTCAG
EMPV1_30318	CCTGGGGACCTATGAGCTTCATGAACTTTGATGTCCAAATCTTTCTCCGGATTTGTAGAG
EMPV1_30319	GGTGGAACAATTCTAACAGGACACCTACTGAACAGCAGCCGAAGACCTCAAAGGGCTGAA
EMPV1_30321	TCCCCAAATTGAAGTACTGATGCTATTGTAAACAAAAGTGTGTATAGTGCCTAAAAACTG
EMPV1_30322	GCCCCACTTGGGCTTTTCAGCTGTGTCAAACATGAATCTAAATCCCCACAGAGTACGGAC
EMPV1_30326	GGATTTCGAGGCCAAAACCCAGCACACCCTGTATGTTGAAGTCACCAACGAAGCTCCCTT
EMPV1_30327	TGAACAACCACATGAAACTCCACACAGGGGAAAAGCCGTTCAAGTGTACCTGGCCCACCT
EMPV1_30330	CCACGTCCTGGAGCATTACGACAAGACCCTGTCTGCCAAGATCCCCTACAACGACTTCCT
EMPV1_30332	AAAGGTGCTGTGGAAACAGGTGCAGCCAAAGGGTCACAGGGCATCTCCCCTATACATTGT
EMPV1_30333	GAATAGGCAAATCCATAGAGACAGAAAGCAGATGAGTGGTTACCGGGGAGATGGAGGTGC
EMPV1_30334	CTTTATCAAGTCCAAGGACCAGGATCGCACTACGTGTCTCCATAGCCTATTCCATGTCAT
EMPV1_30336	GGCAGTGAAAGCCAGTTTAGCACACAGTTGTCACAGAGTGAACAAGAGGAACATAGATGC
EMPV1_30337	AGAAAGGTATCCTGTGCTGCTGTCCTCCAGGTAACCCCACCCGGAAGAGGGACTGATATT
EMPV1_30338	CAAACAAACCAATAAGGAAGCAGCTAAAGAGAGGATCTCCTTACAGTTTGAACTTTAGAG
EMPV1_30339	TCTCCGAGATTTGCCAAGGTTCTAGGCCACAGCAACCCAAACACCGTGGTTCATTGCATA
EMPV1_30340	AGGACAAGAAGACAGAAGAGTTCTTCTCTGTGGTGACTACAGACTAGAGGAATGCTCTAC
EMPV1_30343	ACCCCTCATTAATTAAGGCTTCCTAATCAGACTCCTGTCTTGTTTGCTTTGTCCACCGGT
EMPV1_30347	CCACTATGGGGTCAAGATGAGCCAAACCCTGTGTGTACTTTTGGCAGTCAGCTCTGCCTT
EMPV1_30348	GTTTCTGCTGAGAGTGAGTCTGCTTTTCAGGGCCACAAAGACTCCTGGGCAAATGAAGCC
EMPV1_30351	CAGCAGACAATTTCTTCTAGATAGTTCACAGGTCTGATCCATCCTGGGCCAGCATCAACC
EMPV1_30353	GGAGGGACTGGCATCCTTAAAAGAAGAACGTGAGATGATAAACATGAACAGTTGGCCCCA
EMPV1_30354	GACTCTCAAATGGGTTGGGAAATCATGTTAAGGAAGTGAGTCTGAGCATCACCAAGAGTG
EMPV1_30356	GCAGCTTTAATGATCCCAATGCTAAGGAGATGCATTTAAAAGGGCGAAGACACAGACTTC
EMPV1_30357	ATAGCCTGAAAATAGAATGCGGACCTAAATACCCAGAAGCTCCTCCATCAGTTAGATTTG
EMPV1_30358	TAGCAGAAATCAGACCTTAGGAAATCTGCCCAATTCCCCCTGTGTGGTCCTGTGGAACAG
EMPV1_30360	TCTCTCAGAAACCCACGATAGCAGCAACATGGCAGAGAAGAAGGTGGCATCTGAGCTCCC
EMPV1_30361	GATGAGACTCTGCCTGTGTTCCTCAGCAACCACTCTCACTACTTTAGGAGACACATTCAC
EMPV1_30362	GGCAGTTCGTTTGTGGCCTTTTTGCATCTTAATGTTCGTAATTGCCCCCACTGCACATGC
EMPV1_30364	CCTGGTCTTCTCCAGGAAAGGGGATGCTGACAAATGAGCATTCTCACCAAAGTCGAGTGT
EMPV1_30365	GGGAAAAACACCCGTTGAGTGAGGAAAGACTACTAACTGATGCCAACACCAAGATGCTGC

EMPV1_30366	TCCTGGTGAGAAGAGGCAGATCCCTGAACAGCTAAATTTGGAATGGAGAGGAAGAGTCAG
EMPV1_30370	CAGACATCCAGTGAAGTGTTTCCGATGCAATATGACAGTGCCAGCCGTGACGTTAATTGC
EMPV1_30371	CCTAAAGACATTCAAGTTTGCAATCCCCAAAGCCTCCTGGAATCTCAGCCGTAAAAGGGC
EMPV1_30372	CCCACACCATCCCAGAGGCCTCCGGCGTTCAGACCCTCTGCCGAAGCGGCGCTGCCTCCT
EMPV1_30374	CTTTCCATTCAACCCTTAACTGAGCCCAGCTTTTGTCAGGGTCCCGGTGTCTATCAGGAT
EMPV1_30375	GGCGGAGAACCCGGGGAAGAGAAGGAAGAAAACTGTGAATAAAACCAGGATATTGGAACC
EMPV1_30376	CCCAGGAGCTGGATGAACAAATATACCAAGAAGATGAGTGTTGAAGGAACCAAGTCCCCG
EMPV1_30377	GCCCTGGAAATGGCCGTGAAGAGGTATTTTGAGGGAATCCATCTTTACCTGAAAGAGAAG
EMPV1_30378	TTAGGCCCGCACTTGCATTCCCTTGACAGCTGGTGAGGTTGGCTCTGTTGTCGTATGTTT
EMPV1_30379	TGTCACTGGCGATTTTTGTGCTTTAGGCAAACTCTGAAAGCTTACAAAGTACTCTAGGAC
EMPV1_30380	CTCTGGAGAACAAGGTAAAGAGGCTAGAGACCATGGAGCGTAGAGAAAACAGACTGAAGG
EMPV1_30381	ACATTCTGGAAAACTGCACAGAGAATTCCATCATGGTCCTGACCCAACTGATACAGCCCC
EMPV1_30384	GACCTCGAGATTGGGAATCTGTAGTCTGGAGCTACCTTGCCTCTGATGTTTTGCTTTATT
EMPV1_30386	AAAGGATCCACAAGAGTTCATGCCCTGAACAATGTCAACAAAGCACTGCAGGTCTTGCAG
EMPV1_30389	ATCGAGGGGTTTGATGGGAACTGGTACCTGCAGCGAATGGATGTGAAGCTGACGTGCAAA
EMPV1_30391	TCCATCAGTGACTGTCACCAGCCTTCTGCAACAGCTAATGAACCTACCTCAGAGTGCAGC
EMPV1_30392	TTCCTCCAGGACCAGAATGCACCACTTGTATCCCGTGCCCCTCAGACCTTCAAGATGGAT
EMPV1_30394	ATAGGTGGAGACCACACAGAAAGTTCTCACCAAAGACCTCCTGCACGCCAGGACAGTTCT
EMPV1_30396	AGGCCATTCTGGGCTTCTCTGCACCCTTATTTGGAGATGTCAGTGGGATTCTCTCTTCCA
EMPV1_30397	GACTCGGCCCTCTTCTCCCAGGACTACATGGACCCCAGCCAGGACTCCAGCATGCCTTAT
EMPV1_30398	AACTTTCAAAAATCAGATGTCCACCATCGCCGGGGACTGTCTCTTGTCCGCCGCGTTCAT
EMPV1_30399	TTGTGTCCACTGCCCTTGTTTTTCTCGTCGCTCTGGGAGTGGTGGTTTCTGATGAGGTGT
EMPV1_30400	TGATGTTCAAGTTCAAGGGGGTCCGTTACGGGACAGAAACCAGTCTCGGGGCCAAAGTAT
EMPV1_30402	CCCAGTGCTCATGAACCAAAGGGTTTTCGTGAAGTTAGTAGGGTCCTCTTGGGTCTCAGG
EMPV1_30403	ACTACATCTGAAGGCAACACAGCAAGTCTTCCCACACAGCAGGTCCCAAAGTCAGCTACT
EMPV1_30404	TTACACTTCAGGCTGTGCCCCTTCTATGCCAGCGAGTCCTGTCTTAACTAAAGCTGGAGT
EMPV1_30405	GTGCAGACAACGATGCGTGAAAACCTGTCCACAGTTGAGGGGAATTTTGCCAACATTGAT
EMPV1_30407	TTGCTGGACGCCAGTGTGGTGTCGGCCACCTCTCGCTACATTGAAGAGCAGCAGGCTACA
EMPV1_30409	CCATCACTTCCCGGAAAGATCTAGATTGTTACTGCCGTGTTTGACCTGTCTTGGTGGGAG
EMPV1_30410	TCTTTGGACCTGCCATCTTCCTCTACATGAGACCCTCCTCCACCTTCACTGAGGACAAAC
EMPV1_30411	GGGGACACATACATGCCCCAAACGATGCAAGTAATCTCTCATAAGAAAAAGCCAGACACC
EMPV1_30413	CCTGAGGCTTTGCGGATCAAGTTGGAATTGGATGGTGAGAGTCATATCCTGGAGCTGCTA
EMPV1_30414	AATACTCAGTGGCCATCGATACAGCTGACTCAGTTGGTGGAGAAACTACAATATGATGCT
EMPV1_30415	AGTGAACTACGTGCACCAGTTCTGTGTGGGGGCAGCCAAGGGGGTGCTGAGCCCGTTTGT

EMPV1_30416	TAGTACTTTGAGTGTGGTCGGTGCATAAGAACTCCTGGCTGTGAGGGAGAGCTGGTTTTG
EMPV1_30417	GTCCTGTGTTGAGGGATTTGGAACATCCTGTGTGTCCCTTAATTTCAACTCTTACGCTGT
EMPV1_30418	ATGCAGCCTGGATCTCATGATGCTGTGGCTGTGGCATCCTCCATATGCTGCTGGTGCAGC
EMPV1_30420	GGTTGTGTCCCTGTTTCTCATTCCAGGAATGTTTATATATCTCCAGCCCCCATCAGGAGG
EMPV1_30421	CTTCCAGCTATAAACGCTTTAGTCAGGGGGGTGAGCAGGGTTGTCTTGAGCTGGGTCGCT
EMPV1_30425	GTGTCACGAAATCTTCTGCTGCCCGCTGAAGCAAGTGCATCACAAAGAAAACACAGAGCC
EMPV1_30426	CCCTACGTACTCACGACCTGCTAAGAGGCTTGCCCTCTAGTTTTATTATCATCTTATCCT
EMPV1_30428	GAGATTCACTCGGAGAGTCAGGATTTGAACCAGCCAGAAATTTGGGCATCTTTCTCTCTT
EMPV1_30429	GTTGTTCTGTCTCTCCTCTGGTTGGTGACAGTTTCGCAGTCTTGTGTTTTCATGGCCTTG
EMPV1_30431	CTGGCAGTCAAGCTTTTGGTGTGCTGTATTGCACCTGGTTAACTAACCCATGAGATTTTG
EMPV1_30433	GTGATGAGACGGATGAAGATGTGTGTAGCCTGGAGTGTAAAGCAAAACACCTTCTGCAGG
EMPV1_30436	AACGACATGCTCGATGGCTACTCTGGGAGTACACTCAACCCGAGTAGCTTATTTGGAACG
EMPV1_30440	GATGATGAACTTGGGTATGTAGCTGAAGAGCAAAATGTTGAAGACGTGGCCTGGTTTCTC
EMPV1_30441	ATCTATATCACTTAGCGGTGTCATTTTCCTGCCACCCTGTCCGAGGCCTTTGGTTCTCAC
EMPV1_30442	CAGGCGACCCGTGGTAAAGGGAGGAAGTAAATGGGAGAAGTCCGCAGTCAGATAGTTGTT
EMPV1_30443	GGTCATTTAGCTCTTCTTTTCTCGCATATTCTGTGATCGGTGCCCAAGGCATGAACAGCC
EMPV1_30444	CTCAAGGGCCAGTAGAGGCAAACTCAAAACCTCTAAACCATCTGGGGGACATAATTTTCC
EMPV1_30445	TTATTGTACACTCTTAGGCACATATCTTAGCTCTGCTGCTACCCACAGCTCGCACTCAAG
EMPV1_30448	TCCATGGTGAAAAACAATCTCTCCAGGATTCATCTGGGGTCAGCGATGGCGAAGGGGATG
EMPV1_30449	CACCTATGTGGGACAGCTTACCTTCTCATACTCTTGGTGTCATCTGGACTGGTCTTGCCT
EMPV1_30451	CTCCGAAATCAGCCTGCCCTTTGTTGCCGACTGCCTGTCTGGACCTTGGTTTTTCTTACA
EMPV1_30452	GAAGTGCCTTGTGCCAGAAGTCACCTTCAGGGCAGGTGACATTACGGAACTAAAAATTCT
EMPV1_30456	TGTGTACATCTCATGACTGAGAGGAAGAAACTAGTCATGGGTCAGAGGCCAAGATACTTC
EMPV1_30458	TAACCAAATAGATGCGAATCCAGGACTAGAGAACCCAGAGGAGGTGACCTTCAGGACAGG
EMPV1_30459	TACCACTAGAGCCACAGACTCATATCCACTCTTGGGCAGCTCATCCCCAGATACAAACTC
EMPV1_30462	GAAGGATTGTCCAAATGTGGAAGATGCAAGCAGGCATTTTACTGCAATGTGGAGTGTCAG
EMPV1_30465	GTTGGGAGGCCAGTGGTGTACAAATACCTAACAGAGAAAGCAGTGAAATGGTCACAGCAG
EMPV1_30466	GGGCCTGCGCCGGCTCCGCCGCCTGTGGGCTGGCCCGCGCGTTCAGCGGGGCCGGGGCCG
EMPV1_30467	AGCCCATTCGGCTGACACATGTCAATACTGGCCGAAACCTCCATAGTCATCACTTCACCT
EMPV1_30468	TGTGGCCGGATCTGTAGCTGTAGCTGTGGTGTAGGCATATAACTGCAGCTCCAAATTGAC
EMPV1_30469	GAGTCTCTACCACCCGTCCAGTTTGACTGGAGTAGCAGTGGCCTTACTAACCCTTTAGAT
EMPV1_30473	CGTTCATCACCTGAGCATCCGGGAATGCCAAAGAGCGGGCCTCCAGAGAACAAATAAAAA
EMPV1_30475	AGATAGTCCTTTAGACAGTGGACAGGGCAGCTCTGAAACAATTGGGCCCTTGAGTGAAGG
EMPV1_30476	ACTGAAGAAAGTCATCAAAACCCAAGAGGAAGAAAGTAGGAGAAGGGGAAAGGACACAG

EMPV1_30477	TTCAAACCGGCTTACAACCCCTACACCGAGCCCAGCATGGAGGTGTTCAGCTACCACCAA
EMPV1_30478	AGAAAAGGCCAGATTCCGGCTCCCACTCAGAGAGAAACGGCAGTGCCAATCAGACTTCAA
EMPV1_30479	GTTTGCTCAGACTCTGGAGAAGGTGTGTGTTGAGACAGTAGAGAGCGGAGCCATGACCAA
EMPV1_30480	CAGTGCTCATGAACCAGAGGGTTTTCGTGAAGTTAGTAGGGTCCTCTTGGGTCTCAGGGA
EMPV1_30481	GGAATTGAGGAAATCGACTATTTCTACAGGTCAGTGTTGGCTTCCTTTTTCATCGCTCCC
EMPV1_30484	CCCACTTCAATGGCAGAGCCACGTAGTTATAGCAGAGAGTTTATGGCCTACAAAGCCTAA
EMPV1_30485	GCCAAAGTCTTGAGGAAGATAACATATGCTGCTAAGATATGATTCAGACCAGGAGAGCTC
EMPV1_30486	GGACCACTCTGAGTGCTGAACACTGAGACAAGGGAATATCATCTGGGTTGAATGCAAGAG
EMPV1_30488	GGCAATGGAAGGGCAGTTATGGAAAGTTTACTTTGTTTAGAGACTCATTTTTCCCTAGAG
EMPV1_30491	GCCACTTATAATCTTCTGCTACATTGGTGGCATTTTGCATGGTGCTTTACACACAGTGGC
EMPV1_30494	AAGACCCTGGACCAAATAATTCCAGCCAAAGGCTCTAACAGGGCAAGCATCTTGGGAAGG
EMPV1_30496	TCGAAGTATGATCGAGTGGTGAGGAGGGAGATCTGTAGGGTCATTACCAAGGGCACACAG
EMPV1_30497	GGAAGAGTAACCGGGCCGCCTTGCACCTCTACTCCAACACCCTCAACTTTCAGATCAGCG
EMPV1_30499	AGTCACCCATGGTAGGAACTTCCAGGTTCTGTTTCACATTTGCTTTTTACTTACCTCAGG
EMPV1_30500	CAATCTTTGATCTAGGAATTGAATTCCCACATTAAGCCACTGCACAGCTTGGCCAAAAAC
EMPV1_30501	GGCAAGACAGTACATCTTCATACTGAGTTAGCCACAGCGTCTCACGTGCACAGATGCTCC
EMPV1_30503	ACTGGTGACTCAGGTATTGAGCTCTGCCCTTGTCCTGACTCCAGTGAAGGTGAGCCAGCC
EMPV1_30504	GGATTTGAATGTCGAGTTCAGCTTGGAGGCAGAAAGAATAATTCATGGCTATTACCTCGC
EMPV1_30505	AGAACACGGGAAAGAAGAAACCAATGGTCCGAGCAACCAGAGGCCTGTCAAGTCCCCAGA
EMPV1_30506	TAAGCTTGTATTCTTCTTCAGAGCTGGTGTTCAGTGGTCTGGGACTGGTACTGGTGGTCC
EMPV1_30507	CAAGGGTGGCCTTCTTTGAGGTTTGCAGGAATGACCACTAGATGTCACTAGCACATTTCC
EMPV1_30508	CTTGAAGCTCTGGATTGCATTCTACCACCAACTCGTCCAACTGACAAGCCCTTGCGCCTG
EMPV1_30510	TTACACTGTTGCATCACAAGGGACTCGCCCAGGGACCATGACCTGCTGGTGTGTGTATAT
EMPV1_30511	TCCTGGTTTGCCTAGGACTGAGGGGGTTTCCTGGGATGCAGGACTTTCAGGGCTAATAAT
EMPV1_30512	TGGCACAACCCTCTGTCTGCCAAAGAGCCCCCAGGTGCTGAAGATGCTATGGATGTGTTT
EMPV1_30514	CAGTACTATTTCTGCTACAACGCAATCCTGGAGTTTGCAGTAAGGGAGGGCATGGTGCCC
EMPV1_30524	TCACTCATCAGGTTAAGGATCCAGCGTTGCCATGAGTTGAGGTGTAGGTCGCAGACTCAG
EMPV1_30526	ATCAGTGGTTTCGTGGAGGCCACAGTTCAGACCATCCTGGTTTTCCAGTTGTCTCTCTGC
EMPV1_30527	ACAGGTGAAACAGCAAACATATTTCCCTCTCCAAACACTTCTGCCCAATTCCTTTGAGAC
EMPV1_30528	ATCATGAGAGAGGGAGTCTGTGCTCTGCTGGTGGCTGGCTCATGGATTGTCTCCTGTGCC
EMPV1_30529	CTGGGCCTGGGGAGCCCTGTGGGCCATGGGGAAGTCTTGGGTCTTTTTCTGGAGTGCGCT
EMPV1_30530	TCTCCCAGCCTGATGGTGGGCGTCCACTCTCTCCTGGCCACGTCGTAGGAGCAACCATAA
EMPV1_30531	TCCGGTTGGCAGTCTCATCACTCTGACCTGTGCTTCTGTTGTCATGGGCCTTTCCTGACT
EMPV1_30533	CGAGCCTTCATTGATAACAATCATTCCCATATTCTTCCTCAGAAGTTTGCAGGAGTGTCC

EMPV1_30534	GGCGACAGCTCCCGCCGCACGCCATCGTCGGGAGGAGAGCGCGGCCCGCGGGGAAGCACC
EMPV1_30535	CGGAAAACCAGCTTCAGTCAGTGCTCCTAGAGAACATCCATTTGTCTTGCAAAGTGTTGG
EMPV1_30536	GCATTTTGGTCCCTGCTCCTGCCCATAGTGAAACAGCCTGAAAAATCCCATGCAAGAGAG
EMPV1_30537	AAGAAACACATACCACCATGCTTCTTTAGTTCCCTCTGCTTGCCCAAGTCTGGACCCTGG
EMPV1_30539	GAAACAGCTTTGCATCCTCTACCTAGGCAACAACAAACTCTGCGACCTCCCCAGTGAGCT
EMPV1_30540	CCAGGACTTGCAAAGGACCAGCCAAGAGTGTGTTTTATGTCTCTTGGTTGACTAAATACC
EMPV1_30542	TGTGCGCGCTCTACAGAAGGAAGGGCTCTGGCCACAGGCCTTCTCCGAAGTGGCTGAGAA
EMPV1_30543	TGGAACCAGCTCCAGCTACAGGAGAGGAGCTGCCAGAACTGACCCTGCTGACCACCTTGT
EMPV1_30544	TGCCAGAAGAGCATGACATCCAGAGTGCACAGAGAAGAAAACAGGTAAATGAGAGGGAAC
EMPV1_30545	ATGAATGGATAAAGATGATGTGATGTGTATGTGTGTATATATATATGTACTATACACACA
EMPV1_30546	TTCATTCTAAGCCGTGCAAGTCCTGCTTCCGTTGTAACACCCACTCATCTGCAGCGTTTC
EMPV1_30548	AACAGATAAAGTCTATAGATGCAGGACCTGTGGATGCCTGGACTTTGGCCTTTTCTCCTG
EMPV1_30550	AACTAGAGGCACTGGAATGTAGCAATAATGAGAAGTTAGAACCTGGCCCTGATGTGGAGG
EMPV1_30551	AAAGCCAGGCATGCCAGCCGAGAACACCTACACAGGCTGAAAATCACCAACTGCGTGATG
EMPV1_30554	ACGTCGGCCGCCACCACGATGCATGAGTAAGAGTAGAGAGAGAATCAGAGGACCTTAGTT
EMPV1_30556	TGTCAGGGAACCTTTCTACGAGCGGCTCACCCTGTGGGCTGAGATCCGTCTGAGGTTCTT
EMPV1_30557	CTCCCCTGATGACTCAGTGGCCTCCTGGCTCTGCTTCACCCAAATACTAGGTTCCTCAGA
EMPV1_30558	AAAGACTGTGCCCTTCTTTGAGAGAGAAAAGGATGAGGTTAGGGAGTGGGAAACAGGGCC
EMPV1_30560	CCAGCCCAGTGTTAGAGGGAGTCCCCCCTCGACACCTAATCAAGTCCCCTTTGTACCTTC
EMPV1_30561	TAGAAAGGTGTTCTCCTTCCCTTGATGTGTGAGGACTTGCACGACCCATGGTTGCAGACC
EMPV1_30563	ACTTCTGTCAGCCCCCCGACTTTCCCTGCCTCAAGCAGCTGGTGGCCTATGTGCAGCTCA
EMPV1_30564	TTGCACAGGTGGATTTTCTCACCACGTTTGCCTGGTTTGTATTCAGCGATTCAGCTGGAG
EMPV1_30565	TTTCACAACAGCCTCTCTCTGCTACAAGTGTTCTTTCCTTCATCCCTGGGGAAGTCGGGA
EMPV1_30568	CAACTTCCTCCCTAAGTTTAATAAACAGGAGAACAGAAGCAAGGATTCAGATATTCACGG
EMPV1_30569	AGGAAGGACCAGAGCCGGGCCCCGCCCTGTTGTCCATTTTCCAGCTTTTCCGCCCTGCAA
EMPV1_30570	TATGCCACAACCCTGCACGAAATCTGGGCCGACCACAACAGGGGAGAAGATGCAGCCTTA
EMPV1_30571	GGCTACGCAGACAAAAACCTGATCGCAAAATGGAAAAAGCAGGGATATGAGAACCTCTGC
EMPV1_30572	CAGAAGTTCATGCACGTATTTATTTCCTTGGTTTTTCCTAAAAGTGTGATACATATTTTG
EMPV1_30573	CCTGATTCAAACCTGGAAAGCTGGAGGTCAGGACAAGATGGTGGAGAAGCAGGACATGGA
EMPV1_30575	TCTGACAGCTGCACCAGATCCTTAACCCACTTCCATATGTTTCCAGGGATCAAACTGCCG
EMPV1_30576	CTTCAGTTGAGTGTGGTGCCCTACTCCAAAGGGTGTCATTCATACAGACTAATACATGAC
EMPV1_30577	CAACAACCCAGCAAAGTACAAAGCTCTCCAAAGTGCAAAGTTGGCAGGTATCCGAATCTG
EMPV1_30578	AGCAGCAAGGAAGTCGGCATGTTCTATTGTGATGTCCTTCCCGTCATGAGGTTGGCCTGT
EMPV1_30582	GCAATAACTGATGAAATTAGTTTCTACTCCCCAAGCAAGAGTATAGCAAACATGGTTCAG

EMPV1_30584	TGATTGGTTCGTTCACTTTTCTCCCAAATGCATATTGAGCATAACATGCATTAGTCATTT
EMPV1_30585	CTATGAAAAGAAATGGAAGCGAGTGCCAGGGTTTCTCAGTTGTAATAAAAGGAAATGATC
EMPV1_30587	GTCCCACATTTGCCTTAAGATAGGACTCACATGGCAGGAGGATTACTACCCAACAAGAGC
EMPV1_30588	CCTGAATCACCTCGCGTATGTTCTTGATCCATTCCTGCTTGGTTTCTATGTTGGAGGCCT
EMPV1_30589	CATTTGGCAATGGTTTCTTGGGGAGTGGGATGCATGGTAGCCTTGGGCCAGACCAACTAT
EMPV1_30590	TGGACTGCTGGTTCTGGTGCCCTGGGTCATGAGTGTCCTGAATTCCCTGTTACAAACCTT
EMPV1_30594	TCACCCGGGGGACCTCCAGGCCCCTCGCCGGTTCCTGCGTGGCCGAAGCCGGGGGGGCTG
EMPV1_30595	ATGCAGGGAGGCTTTGCAGTGCTTTTCAGAGACTGAGAGTGAGTGCGTGTGTGGACACGT
EMPV1_30596	TTTAGGAATCTCCATCCCGCAGTCCTGTCCTCTGAGTTACGCCTGAGTGGCCCTTGGAGA
EMPV1_30598	ACCAAACTGTATGTGGGTCCCCGAATGCATCGTGCATTGCTGTGTTTTGCCTCTACCTGA
EMPV1_30599	TGAGTCAACAGGGGTGTTGCCGCCAGATGTGCTGGTGAGCGAAGCCATCAAGGTACTGAT
EMPV1_30601	TACAACTGGAGGATTTTCCTACATCTCCTACAAGCACAGCCAAACAAGAGTTTCTAACTG
EMPV1_30602	ATTCTACTCTCCAACTTCCCGCAACCCTTTGGGTTCTGGAGATAGCAAGGGGGAGTCGAT
EMPV1_30606	AGCTGGGCTGTATCCAGAGGATCATTTAGAAGGTAGATGCTGTCATGTGGGCCAGCAGCT
EMPV1_30607	GCGGCCGAGCGGGGGAGCGAGCGCGCGAGTGTGTGCGAGGGGCCGCTTCACTTTCCCCCT
EMPV1_30608	CCACTTGGCAGTACATATTTTCTGTTCGACCTTCTCATCCATGTCATTTGTCTCTCTGGT
EMPV1_30609	CAGCTAGGCCCTCTGCAGGCCTCGGCTTTGAAGAACAGAGTCTTTGGAATATTGTACGTA
EMPV1_30611	TGATAGTAGTTACACCTCTAAGTTACTGTCTTGCAAGGTGACTTCCGAGGTCCTTGAGGC
EMPV1_30615	TTTGCACTACATGACCATCATGAATCGGCGAGTGTGTGGCCTGCTGGTGGGAGTGTCGTG
EMPV1_30616	TGGGCACAGACACTGGTCTCCCACAGACCCTGCTCTGTCTGCATGTGGTGTCCCTCACTT
EMPV1_30617	ATCACTTTAGCAGCTTACCTAGCTCTCCAACTCATTGTCATTCTCTCTTAGCTGGTCCCC
EMPV1_30618	GGGTCCTGGATCATAGGAGGTGTCAACTCTGCTGTACAAACAGCATTTGTGGTACAATTG
EMPV1_30619	CTACACCAAGTCTGGGGCCACGCCCCAACCGATGACCTGTGCCAGGAATGTGAGGACATC
EMPV1_30620	GATGGTGGAGAGCTTTCCTTTGGGTTTTGGTCCCCAGTCAATCATCTCACCAGGGTGAAA
EMPV1_30621	CACCAGTGCTGGGAGGTTCTGCACGTGATTGTGGCCCAACATTAGTTTCCTAAGACTCAA
EMPV1_30622	GCCGCCGCAGCAGCTCCCCCAGCCCCTGTAGCAGCGGCACTTGATGCATGTTCAGGAAAT
EMPV1_30623	CTTGCTGCCAGTGAAGCCAGAGATGACAACGCACCAGAGATATCACTGTCCCCAAATGTC
EMPV1_30624	GAGCCAGCCCGGATCATTTGTTGGCAGTACATATTGGCTGAAGTCAGTTTTCATTTCAAG
EMPV1_30626	CTGGATCGTGAATGCCCTCCACTCCCTCCTACACACACTCTTGATGAACAACCTGTCCTT
EMPV1_30628	TTGGGAGTGTTGTTCTGCCTGAGCCCATGAACGTCTCACTTATATATTTCACAGTCCGTG
EMPV1_30629	TTCACCGTTGAGAAGCTGGAGAAAGTTCCACAGGCTGCTGAGATCCTCTTGGATAACCGT
EMPV1_30630	CAGAGTCCTGGCGTGGCTGACCCCCTGCAGCAGGCCTACGCTGGGATGCACCACTACGCA
EMPV1_30633	ACATCTTTTGAAAACTTTTCACAGAATGTGGGCAGAAACATCCCTATTTTCCCATGCCAT
EMPV1_30634	GAAGAGGGTTGTCATTGCAAATATAGCTTACCCCATTAGGCCAGTGGATTACACCCGACC

EMPV1_30637	GATCATTTGCTGTGAGGACAAAGGTCAGTGCCAGTCCACAGTAAGAGAACACACTGGCCC
EMPV1_30639	GAGCAGGCCTGTCTAAAGTGATAAATGGAGCCCTGTGTGGAAGATCAATGATCAGGGAAA
EMPV1_30640	GATGCCATTCCAAGCTTAAATCCTTTCCTCACAAAAAGTAGTGGTGATGTTCACCTTCCC
EMPV1_30641	CTGTAGCAACTGGTGAGAAGCCACCCGTGGGCTCCCCTCTGCCCGCAGGTCTTAGCGGAC
EMPV1_30643	CCTCTCCCGGGCTCATAGGGAGCTCAGAACATCCAGCTCTGGGACCGAGGACAGACACCA
EMPV1_30644	TTGCAGTAGTCTGCGGGGTCCTCCTGCTCCTCATCGTCTGACCCCAGGATCTCCTCCTCT
EMPV1_30645	AATCCATCAGAGGAACGCGAATGGTACAAATGCTATCAACAGGAACACAGCCCCACCAGC
EMPV1_30646	CCCTTCCTGTCAAGACTGTTTGCGTGTTTCAGTTATCCTTATGAATTTGGTTATATCAGC
EMPV1_30647	CCAGCCACACCTGACCTCCAGTCCATTCTCAGTGCCTGAAATAATAAATCCCCTTTCTAG
EMPV1_30648	TGCTCCGCTTTGAGCTGGCACCAGATCCCTCCAGGATTCCCGTTCCTATTCAAGGAATTG
EMPV1_30649	CTGAATGCTGGGATCATGCCTAATCCACAGCTAAATATAAAAGCACTTTTCTGTAGCCTG
EMPV1_30650	TAGTACTTTATGCACAATTTTTGATTTTGTAATTTTATGTAAGCTAAACGTATAAGACGT
EMPV1_30651	CTTACCTTGTTGGAAAAACTAAATGAAAACTTCAGGATTCTGAGCTCCCCGTGGATCCAG
EMPV1_30652	GAAGAGCTCAAAGCTATCCGCCTTTCCACCGAAGCCAGTAAAAGTATCGTCCCAGTGTCC
EMPV1_30654	GCGACTCTCTTCTAGAGCCATATAGTTCCTTGGGATTAGCTCTTGGCCACAAAGGCTGAG
EMPV1_30657	AGAGACAGCCACAACGAGCATCTCGAAGCATCATGGCACGAAATCCGGAAGCTGTCCTTC
EMPV1_30659	TTCTGCTACCATAACACCTTTCTGCTGGCCGACCGGACTGAGGCCTGGGTGCTGGAGACG
EMPV1_30660	ATCAATTGCATTTTCCCTCAGGTCAAAACAAGCCATGGAGAACCTGCCAAGGGAGCGGGC
EMPV1_30661	GAGGGAGGAGATGGGCCAAGTTCCCTCTGCCTGGAACGCCCTTCCCCCCCTTCTTCACCT
EMPV1_30662	AGCACCTCTGGCCTCCCATCTGCCTCCTCCGGGAGTCAGACGACCTGCTAACCTCTGAAA
EMPV1_30663	TCAAGGAAGCTCCAGGTCCAATTAACTTTACTGTGTTCCTAACGATGTTTGGGGAGAAAC
EMPV1_30664	GCAGCAATTTTCTAGCATATCCTATTCCATTAATGAGGGAGTGTACAACTTCTTTAACCC
EMPV1_30665	TCTCATGAAGAAAGCTGGGGTGCTTTGAAGATAATCCCTACCCCTTTGCCCCAAGTGCAG
EMPV1_30666	CACATGCTGCCACCTAATGCCTTTCTAGGGAGTGAAGTCAATCAGACTCAGAACCCAGAG
EMPV1_30667	AGATGCTGGCCTCTCTTCCACCTCAGGAATCTCACTGACCTCCAGAACATGTATCCTTAC
EMPV1_30668	TGTATCTGCGCCATGTGGAGGTTCCCAGGCTAGGGGTCAAATTGAATCAGACTTGCAACT
EMPV1_30671	GGGGCACCCAAAGTATTAGCTTCCATAGAGGATTAGTGTGCCAAGATCCCAGAGTGAGCT
EMPV1_30673	AGTTTCTGCACAGCAAAGGAAACCCTAATCGACACAAAAAGACAACCCACAGAATGGGAG
EMPV1_30677	TCTGCTGTTCAAAGCCCTAGTGGCCTAGTGGGGGTGTATAATGGAGAGACTCTGGTGTAT
EMPV1_30678	GAAGGTACAGGATGCACTGCCATGGATCAGGTCTTCCACGTAGATTGTTTTACCTGCATC
EMPV1_30679	TGCAGAAACAATGCCAGGTCTTTAACCCATTGCACCAGAAGGGAACTCCGCAATTGAACT
EMPV1_30680	TGGACCAAGCTGTTAGGCTCCAAAAAATGAGTGGAGCGGTAATGATGTGAGTGATGTTGT
EMPV1_30681	CGGATGGCTTTCCGAAGTGGCCGCATCATATTGCATTCCCATCATCACCATGTGAGAGTG
EMPV1_30682	TAAATATGCAGCAGATGGTAACCATTACAGATGCTCTGCCCATCGCAGGGGTCCTCCACG

EMPV1_30690	TGTGAGAAATAGGTGTGTGTCGCCCAGTGCCCCAGTCTGTGGTATTTGTTACAGCTGCTC
EMPV1_30691	TCATCGCCCCAAACAACCGGGAGCTGCTGCAAGACTATTATGTGACCCACAAGATGCTGT
EMPV1_30693	GCCTCTGGTTCATCCCCGGCTCCCACACAGGTGGGGTGTCGAGAAGGATGGTCCGGGCCC
EMPV1_30694	AAGGGCAGGGATTTTGTAAGCAGAGGGCTGAAGTCAAGGTGGTAACATCTGAATGTGGGG
EMPV1_30695	GGCCACAGTGGGAACTCCCTGAGTATGATTTTTGCTAATAGTGTGTCATTTCCCAGGTCC
EMPV1_30698	TTCCCCTCTCTCCGGGGAAGCAACTGAAATGAAGGAAGCTCTAGCAGAAGCTCCAGGACT
EMPV1_30700	GCAACCTTGAGTGATGGAGTAGTTGTCCAGGTCATGGGTTTGCTGTCTAATAGCGGACAG
EMPV1_30701	GTGGCTTCAGCAGATTCCAGGCCGAATGCCCTCACCTGTGTGAGACCAGCCTCAACAGCC
EMPV1_30702	TGCATCTGGTGCCTCCTTCTCAGGCCGGACTTTGTGTCATCCGTCATTCTGGCCCCTGTA
EMPV1_30703	GTACCAATGGTTCTTTTTCTCCCAACTTATTTACCCTACATTAGAATGTACGACTATTTG
EMPV1_30704	AGAATTAGAGAAGGCCAGGCGGTATGCCAGATTGCCCCAGATCACTTTTACTCTCTGCAG
EMPV1_30705	TCTGGCGGCTCATCTCCTTTACTGGACAAGTTATCAAAAGGCAGGAGGCATAAAGTGCAC
EMPV1_30706	CGCTGTTGCTAGCGGCCTGCGGCTGCGGCCTGGCGGGGGGCTCGGCTGCGGTGCTTTTCT
EMPV1_30707	GAAGTTCCTCTTCTCGCATCTGAACTAGCACCTCATCGCTGTGGCTGAACACATTCCAGC
EMPV1_30710	GCTTAGTTTTGGGCATTGAAAACACTTCTCCCCGCCCCACAATTCTGCTCATGTCCCACA
EMPV1_30711	CCTTGTTTGATTCACATCTTGGCTCAAATTCTACCTCCTCAGAAAGTCCTTCCCTGGCAG
EMPV1_30713	CAAGGAGTTTCTGTTGTGGTACAGCTGAAAGGAATCCAGCTGGTGTCCATGAGAATGCAG
EMPV1_30714	GCATTTGCTAGAGGTGACTGCTCAGTATCCTGCAGAAGAGTATTATAAGGGCTGTCTTAT
EMPV1_30716	TAGAAGTCAGTTGGTCTCTTCCCTTGGACCTGTGAAAAGGATAGGCTGTTGGAGGGAGGA
EMPV1_30717	AAGCATCATGCCAGGCACCTCAAGCTATAAATGTGTGTGATGTAGACTGGAAAAGTCCTG
EMPV1_30718	CTGGAACTTTGGTTTTTGCTGTCCTCACTGCCGTGCTGGGTTCCTTCCAGTTTGGATACG
EMPV1_30719	GGGGATTTTGGATTCCAGCTCCCAGTAATAACATAGGACTTTCCATCTGAACTTTTACCC
EMPV1_30720	GAAGTATTTCTCCAGCATGGCCGAGTTCCTCAAGGTCAAGGGCGAGAAAAGCGGCATCAT
EMPV1_30721	GAAAAAGGTGGAAAAAGAAGAGGCAGTGAGACACAGTCTTAAAGGCAAGAGCCCTGTGGA
EMPV1_30724	TTTGACATCAAACGAAGTCCCTTGGAGGGCAGTTTAGGCTCAGCTCCACGGAAGAGTCTG
EMPV1_30725	GAGGTGTGCAGATGTTGAGAGGCCCTTTGCTTGGCTCTGGGACAGGAGGGAAATAATGTA
EMPV1_30726	TTTTACCGAGGAAGGTCCCTGAAATAACTACCACCAGCTACCATTGCCACGAAACCAGGC
EMPV1_30727	CTTCCCAGAAGAGAGAGACCTTGCGTGGAGCAGATAGTGCTTACTACCAGGTGGGGCAGA
EMPV1_30728	CAGAGAGTCACCAAGCACTCCACGGAGAACAGGCAGCTTGTAAATGGGACCAGGGCGGAA
EMPV1_30729	GGGACTTCCCCATGGTGTGTGCGTCTAGGTCTTGCTTTAAAAAGTGTACAGAAATGGCAC
EMPV1_30731	AATCACGGGCAGCCAGGGTCCTCTGCTCAGGTGCCTGGAGTCTGCTCAATCTGTCCACTT
EMPV1_30733	AACGACATGCTCGATGGCTACTCTGCCTCAACACTGAACCCGAGAAGGTTATTTGGAACG
EMPV1_30735	TGTGTGAGCAAAACTGCGGACTCTCCCAGAAGCCTGCTTCCTAGATTGCTCTGAGAAACT
EMPV1_30736	TAGGTTTAAAGCCGGCAGCAAACTGGCAGAAAACTTTTATGTTAGACAAGATGGAACCAG

EMPV1_30738	GCCTGAGGGCTGAAATCAAAGCTAGTCTCTCCTTTTAGTGTTGCAAACAAGGAGGGATTC
EMPV1_30739	CTGTGTCACCGTCTGGTGGCTGGTTCCTTTCTTGGGGGCTTTGTTCATTCTATCATTCAG
EMPV1_30740	GGAGGGGCAACCTCATTCCCACAAAACATTGTCGTCTGTCACATAGGGTTTGGGTAGTCA
EMPV1_30742	TGTCTAGAAAGTCTGTTGTTGTAAACATCCCCGACTGGAAGCTGTAAGACATAGCTAAGC
EMPV1_30744	GGAAATCAAGTTTAGGTGAAAACTTCAGGGATGAAGATCATCTGGTGTAGCAGCATCCAA
EMPV1_30746	GGCTCTCAGGCCGAGCTCAAGTGCCCCGTGTGTCTTTTGGAATTTGAGGAGGAGGAGACT
EMPV1_30749	TTCTGAAGCTGGCAGACAAGTACAAAAAGAAAATGTGGGGGTGGCTGTGGACAGAGGCCG
EMPV1_30750	CCCCTGTCCAGTCGTCTTGAATTGTATTATGGTCATTATAAATGGATCCAGTGTAAAGGC
EMPV1_30751	AAGACCATGTGGGTTGACCTGCTGGAGACAGAGGGAAAGGTTGTGGGCATACTTACACCT
EMPV1_30752	TCCTTGAGCCACAGCTAGCTGCCAACCGGGTCTCCAAACACCCTCCAGTGCCTGCCTCAA
EMPV1_30753	GGCTCTGAATGATGTTTAATGACATTTGGGCGAATCACTGAAAGTATCCGTGGTGAGACC
EMPV1_30756	GTCAAGTAAATATTTTGGCAGCCCAACAAAGGTAAGAAAGGGAGAATGCACCTAATGGGC
EMPV1_30758	TTCAGCCGTTTGGCACAGTTGTAAAGCAGCTGACCAGGTTCCCACGTACGTCGCCCTGTA
EMPV1_30760	CACTGGTGCTGACTACATAGAATGGGGTTGAGAGAAGATCAGCTGGGACTTCACATCGTC
EMPV1_30761	CTCCATGTTGATAACCATATCGGTATCTCAATTGCGGGACTTACTGCCGATGCTAGACTG
EMPV1_30762	TCCCTTCTGCAGGTGAGTGTTGTTCCAGATTGCACTTTGCTAATGCAGGACTCTGTGGGC
EMPV1_30763	AAAGCTTCTCATCTACTTGGAGTTGCTGCTGTACAGACATGGGGCCTCCTGGGAACCAAG
EMPV1_30764	ATGTGCAATATATGAGGATGAGAAACAGAAGGGAAGGTTCCCTGAGTGTTTTGCTGGGGG
EMPV1_30766	GGCCACCGTGTTTAATTCTGAAAAGGCCAGGCACGGTTCCAGCTCAGGTTCTTTTTAAGT
EMPV1_30767	AAGTTCGTCCAGGAGTCAGAGCTGAGCCAGGGCATCAGGGACTGGCAAGAGACGGTCCAG
EMPV1_30768	CCCTGCTGGTTATTCCGGTGGTTGTCCTGCTGGCCATTGCCTTATTTATTCGGTGGAAAA
EMPV1_30769	CTACATGCTCGTGGACGAGACCCTGTTCTCCAACGACAGCCTGAAGGTGACGTTCCTCAT
EMPV1_30770	CTACTACTATGAGCTCTGGTGGTTCTGGCTCCTCTGGACTGTCCTCATTCTCTTTAGCTG
EMPV1_30771	AGAGTCTCCATTGTGGCTCAGAGGATGAAGAACCTGACATAGTGTCCTTGAGGATGTAGG
EMPV1_30772	CGCTACGTCGCCGAGTTTCGTCGGTACATTCCAGAGCAGAACTCCATTGGCTTCATGTTT
EMPV1_30773	AACATTAGCTCCCTTAGTCTTTACTCCATTCATCGAGATCCCAACTACTGTCATCACCCC
EMPV1_30774	CGGCAAGCAGAAGCGGGAGGAGGGCAGATCTCATTAGAATCTACTTTGAATTCCCGAAGA
EMPV1_30775	GTGAGAGAGTGACTGAACACTTGGTTCCATCCATTTAGGGGCCTTGCCATCGGGGGCATT
EMPV1_30776	AATCTCCTGCCAAAGGGAACCACTGGAGGCCATGGAGAGTCGCTGTAGATAAGACTCTGA
EMPV1_30777	TGAAAGTCCCTTTGGCCTTGCAGACCTCCATAGAGGAGCGTGTGGCCCATCTACGAAGAG
EMPV1_30779	GGCCTCCACACTAAACACAAAATAATACTAGAAGAACTGGAATCCTGTGATGCAGACAGT
EMPV1_30780	CTGGTGTTGACCTCGGTGTCTACCTGATGCGCCTGGAGCTGTGCGATCCAACCCAGAGGC
EMPV1_30782	GGTGGAGAGCTCCGAGAGGGTGTCTTACTACTTCTCCTACCAGCACGTCACCAAGGTGGA
EMPV1_30783	AGCTGCAAGTCACTCTGGTTTCAAAATCATCACTGCTCCCCTTGTTCCCCCTGCTGCTAG

EMPV1_30784   CCTCCTCCACACCAGGAGTTTTTCTCTGAACTCCGAGAGTGTGCTAAAGCAAAGGGTTTC
EMPV1_30785   GGGAAATTGCTCCTGCTTCTCCCATCTTTGAGGTTTCTCACTTCCGAACGTGTTGAGCTC
EMPV1_30787   GAGGGCTGTGTGACTGGAAGGATATGAGCGAAGGGGAGAGGCGTAAGGAACGGAATTAAA
EMPV1_30788   GTGGTCCAGGTGGAAGTGATTTTATCTTCTGCTCTTTCTTCTTCCACACATGCAAGGGAC
EMPV1_30789   GGAGGGATGTTACTTTCCGAAACATTGGCATAATAACCCATTACAAGGCTCAGAAGACGC
EMPV1_30793   CTCCCGCCCACTGCTGCCAGAGCCCAGAGGTCTCCGTTCCCTCTGCCTGCGGGCAGCTTC
EMPV1_30794   ACATTGTTTCAGGCTATTGTAGAGGGGCAGCTGGGGGCAGCCTTCCAATGTATAGGACAG
EMPV1_30796   CTTATCCTTCATTATTTTTGCCCCTTGGGAGGTTAATAAGGTTCGTGGGCTCCCTCTGGG
EMPV1_30799   GCCTCAACAAATAATGAAAGCTCTAATCACAGTTTTGGAAGCTTGGGATCTTTAAGTGAC
EMPV1_30800   AACTTCAAAAGAGCTAACCCCCTTCCCAACACCCCAGCGACCCACATTCCCTCTGTGAAA
EMPV1_30801   GGCTCTCATCTTGGCTACACAGAGAATCAGCAGGCCTATTGTCAACCTCTTTGTTTCCCG
EMPV1_30802   AAGGGGGGAAAGTGCTACCTGAAGTGTGACTGTTGTAACCAGCAGAACAGCATACATGCC
EMPV1_30803   GAGATGAAGAGCTGGGACGCCTTCGTGGGGCTGGACAACACCGTGAAAAATATGATCACG
EMPV1_30804   CATCGTGAAAACACCCCTGTTGCAAAGATGGTCAACGTACCTAAAACCCGAAGGACTTTC
EMPV1_30808   CCCAGGCCTCAGGATAGAACAACCTGGGCTACTTCATTAAGGTATAATTGATCTGGGCCC
EMPV1_30810   GTTCCTAGAACAAGTTCCACCCAATGCAGTTCTGTGCCTGAACCAAGGTTTGGAAGAAGG
EMPV1_30811   CCCATGTCTGTGGATGAGAATTGATGTGTACTGAGGCCCAGGCCCCTAAATAAGCTATCC
EMPV1_30812   CTTGCTACTCTTCTGTCGTCCTGATTACAAGATACTGGGTCGCTCCGTCCTCCACCTGGT
EMPV1_30813   GCCCAGACAAATATGGGATTTGCAGACAAGTGGGATCGCTAGATAATATGGCATCTCTAT
EMPV1_30815   ATCGAACCTCTGTGTTGCCTCCTTCCAGGAGACGGGAGCACGTTGTTCATCGTTCTGGGG
EMPV1_30817   TGTACGCCATCCGAAACCTCACCGAGGACAACAGCCGGAACCAGGATTTGATCGCGAAGA
EMPV1_30818   GTTGTCCATCTCCTGTTTTAATCAGGCTGTTCCTCAAGCCTGGAACTTTGGTACCACCAC
EMPV1_30819   ATGGATACTGGTCGGATTCTTAATCCACTGAGCCACATCGGCAACCACCTAGACACTGTG
EMPV1_30820   ATGAAGCCCAGCAGCCTTTTCATCTTCCTGGTGATATTTGCCTCTGGATTCCTGACGTCC
EMPV1_30821   AAGCAGAACAGGAAGTAGAACAGCTGAAGACCCAGGTGGCACGCCTTCAAGCTGAAAAGG
EMPV1_30823   CTCCTCCATTGCATATTCTTACCTCCTTTGTCATAGATTAGTAGACCATAGGTGTGTAGG
EMPV1_30824   GGAAGTAGAGGGGATAACACTGATGACACTAAGGGCTGAGATGTGAAGGGCAGCTGTCTT
EMPV1_30827   CCAGTCCTTTACCCTAGTTTGTATGCTTTGGACGTTTTGGAATTTGCCTTCTCGAACGTC
EMPV1_30829   AAGCAGGAGATTGGCTTCTTAGCAGTCCCTTTGCCTTTGGACAGATGTGGGGCACAATTG
EMPV1_30830   GTACCTACTATGTATAAGAACATTGATGGGATTTTAAGTGATATTTTAGGTCTCAGTACC
EMPV1_30831   ATCGCTGATCTGAACCAGAAGATCTTTGACCTTCGGGGCAAGTTTAAGCGGCCCACCCTG
EMPV1_30832   TAATTCTCCTTGTCTCCTTGTGTGTCCCTTCTACCACCCCAGGTGAAGCTGTGAAACTTT
EMPV1_30834   GCTGGCCCAGGCACAGGTCAAGAGCTATTTGCAGATGCTGCTCAAAGGCGTTGCCTTCTG
EMPV1_30836   GGGTCTGACACATAGTGTGCAAGCTCATGACATATTCTGTAGCATCGTGGATGAGGCACA

EMPV1_30837	TAAAAGTGCACCTGCGTGATAGAAACTGCAAGAACCTGGAAGCAGCCCACTGAGCCGGAT
EMPV1_30840	GCAGCTACCACGAGGGACAAATAGCCAGAAAAGTTCAAATCAACATCCCATGACTTCCCC
EMPV1_30841	CTTATGATCTCAGACAGTGGGTTGCTGTCAATGAGCTGTTTTCTGCTCCTCCTGATCTCC
EMPV1_30845	GATCTGTCTTCCAGAACCGTCACCTGCCCTTTCTCCTGTGGTCTCTTAATCCAATAATTG
EMPV1_30847	AAACCAGAGCCCGCCACAACCCATCCAACAGGAGCTACACCCTCAATGGGGCCAAGACCT
EMPV1_30848	GTGGTTACTCAGGTTACTTCCAAGTTTTAGCTGTTGTGTGTAGTACTGCTGTGAACATGG
EMPV1_30849	AGCCTTGATGTGGCCTTGAGGGAGGCCGGTCTGCGCGCCCGAGTTTCCTTGCCTGAGCAG
EMPV1_30851	TAGGTGCCATAGCCTGATTTGGCGTAAGGGGCATCTGTGTAGCGGGCTGATTCTGTGTAG
EMPV1_30852	CCTAGTGGTGAAGGACACTCGTCACTGCTGTGGCTGCAGTTGTTCCTATGGCTCCAGTTT
EMPV1_30853	GCCTGGGCCGGAGGAGTCTGGAGGGCGGCGGTGCAGGGGCGGCGGAGCCCGGGCCGACCG
EMPV1_30855	GAGCATCAGGTATGGAATTACTGACTTAGTGCAATATCTGTCTGATTATCCAGAGGTATC
EMPV1_30856	AGAACGTCTACACAGGCGACATCATCAGTGGCCTCCATGTTACAACCAACTTCACGGTGA
EMPV1_30858	ATAGCACGGACTCGAATGGTCGTTTGTTGTCATAAAGCCATGGGCTGAAGCCTAGAGCTG
EMPV1_30860	TGGTCTTCTATCACTATCTGCATGGGTCCGAGGCTGGCTGCCTCCAGGTGTTCCTGCAGG
EMPV1_30865	GTCACCTCACTCTTTGTTAGAAAACAGGGTGCTAGCTAGGGTAGACAGATGTCTCCAGGG
EMPV1_30868	TTAGGAACAGGGATGTTCTTTGCTGTCATCCTGGTCATCGGAGCCATCGCTTCAGCTGCA
EMPV1_30869	TGATCAGACGCCAGGATTACCTAAAGGCTTGGATGTACCTAATACGACATTTGGGACAGC
EMPV1_30870	GGGGTCCCCAAACAGTGCAAATTAATGATATTCTACTGTATAATTGGCACGTCTCCAATA
EMPV1_30871	AAAGTGGACAGGACCCCGTTGCATATGGCTGCAGCTGATGGACATGCACACATCGTGGAA
EMPV1_30872	CACTGTGGATGCCTGTCAATGTTGTCTCCCCCATACTCCTTCCATTTATCTGGTAACAGG
EMPV1_30873	ATGATTTCTGACAAGCACGGCAGTAACAGGCTGCGGACCAGACCGGGCAGCAACAGCATT
EMPV1_30874	AGTACAGCAGCCTGGAGCACGAGAGCGTGGCAGGAGTGGTGGAGAGCCTCAAGATCATCA
EMPV1_30875	CAGGATCAATCACTGGCTAGGCTTTCCGCGACATAGTACAGCCATCTCCGTTTTTAATAT
EMPV1_30876	GGGAATATTCACAGCCTAAAGAACCAGGTTCATGTTCTGACAAGTGAGGTGGAAGAAGAG
EMPV1_30878	AGTCATTAGTTGGGCTCTACCGTTCTTTCTAGCTATTGTACGGCATACATATCTGCCTGG
EMPV1_30879	GGCTGCTACACAAGCTTCTCTCTCCATCTTCCTCTTCCCTGTCCTTCCTCTTTTGGTCAA
EMPV1_30880	CCATGTTAAGTTCTGGCTACGCAGCATAATGTCCACAGTGTGCTTGTTAGTACTACTGGT
EMPV1_30881	GGCAAGAGGAAGCAGAAAGGGTATTTGCCACCTGAATCTGTGAAGGTCTTCCATGACTGG
EMPV1_30882	GGAACTCTCAACCCGGTAAGTTTGACCTCTGGAAAAAAGCCATATCCACCTATGGGCTAT
EMPV1_30884	CATATGTGTGTATTTGTGGGTGTATGTGTACATGTGTGTATATATGTGTACACACACACA
EMPV1_30885	TAGTACAAGGTCCCATCGCGCACCACGTAGCAGGCGGCTGCCTTACGGATTTTACGCTTA
EMPV1_30886	CCTATGTATTTAACCTGGTTGTAGGCATCTCATTTCAAGTTCTGCCATTGGAGAGGAAAC
EMPV1_30887	TGGTCAAAAGCAAGTCACAGAATCAGCCCAGATTCAAGGGGAGGGAAAACAGACGCAACG
EMPV1_30889	ACCCCAGGGACATCAGGAAAAGGGGGTCTCTTGATTTCAGGTGTGCTGGGTTCTGAAAAT

EMPV1_30890	AGCAGCACATGGCCTCTCTCTCCCTGAAGTGGGAACCTCCTGTTTTGTACAGTCTTTCTC
EMPV1_30892	ACCATGTTACTTGGTGCCTTGCCAAAAACATTCCAGGATGGTGCCACCAAACTCCTGCAC
EMPV1_30893	CGCGCTGATTTCCAAGGTGTCCTTCCTCCGATGGTGCTTTGAAGGGTTGATGAAGATCCA
EMPV1_30897	CTGAGTGGTAGTTTTCTGACAGGTAGGCCATGTCTTCTCTTGTTTCAAAATAAATATTCC
EMPV1_30899	GTAGTGTTTTCCTCAAGCACAAAGCTCTCAGGCATATGAGACCTCTGGTAACGGAGGGTC
EMPV1_30900	CACCACCAAGAAATGAACTTAGAGAAACAGACAGGCGAGAAGTCCGAGGGGAACGAGATG
EMPV1_30901	TCCCGGACAGTTCAACTTCTCTTGGATCTGGGGAGTTTACCGGTGTGAAGGAACTTGATG
EMPV1_30902	GAAGCACTTCGATGCCACCTCCCAAACCTACTACAGGGACTCCATCTTCTTCCTCACAAT
EMPV1_30903	TACATCATGGAGACAGACTACACAGAGGCCTTGACCCCCATCTGCATCAGCCTCACAAAC
EMPV1_30907	CCATGTTCCTGGATTGGAAAAATCAATATTGTGAAAATGGCCATCCTACCCAAAGCAATC
EMPV1_30908	TGGATGATGTCGGAACAGGACCTGGCGGACGTGGTGCAGATCACCGTGGAGGAGCTGAGT
EMPV1_30909	CACAGCGGGCGCGATGCCGTTCTCCGTGCTGACGCTCCCGGAGCTGTTGTTGGGCGAGCT
EMPV1_30910	TTTGGCCGAAAGGACTGGAACAGTTCTCTACGACCCGTTGGTGACGCAGGCAGAAGAGCT
EMPV1_30911	GTGCCTAGACTAAAGCCTGAGGGGCAGGGCCCACGTGTACAGAATCTGCATATGAACTTT
EMPV1_30912	CCGCACCCCAGTTCATGTTCACCAAGCAGAAGGAAAACGAATGTCATGGTGACTACCCTG
EMPV1_30913	TGCAAGTTACAATGAAAAGATCGAGGTGCAGGCAGTAGCAGGGAGGCTTTGGAGGATTCC
EMPV1_30914	CTACTCATACCCAGCAAGCCCAGAATCAATTGAACTCAAAACTCAAAAACATAGCACCCC
EMPV1_30915	TGGCGAGGCTGGAGAGCCAGACGCTGGTCATTTGCCTATCAGCAAGCTCACTCTGGAGTT
EMPV1_30919	CTGAAGTTGTGACGAACGAATGTTCTCGGTCAGTCGGTTTCCATTGTCCAGCATCTTCTC
EMPV1_30921	CATCCGTATGAGCTGATGGCCAAGGTGTGCCACTTGTGTGTTGAGCACCAGCGACTCAGT
EMPV1_30923	AGATAACATGGCATGCAAATAGGCATTTCTTTAGAACTCAGCAGTAGGATAGTTGGGATC
EMPV1_30927	CCACTTTGTGCTGCCCATACCTGTTGCCTTTGAGTTTCTTTGCCTTGCAGCTGTTCTTGG
EMPV1_30928	GGTCACATTCCACCAGGGTGACATCTCAGCATTTCCCAATAGAACTGTCTGCAGTGATGG
EMPV1_30929	ACTATGCAGAGGTCGAGATCCCCGCTGCTGAAGTTGATTGAGCAGCCTGCTGGGAACAAA
EMPV1_30930	AAAAAGAGAAAGTTAAAGTAGGTTTACACCATGTGCCTTCAAGTTGATATCAACAGCTCG
EMPV1_30931	CAGAACTTGTGGGGTCAGAGATGGGCGGTTTTGCCTTTTTGGGCCTCAGTTACTTCATGA
EMPV1_30934	GCCTCTGGAGAGATTAACAACTGGTACTTACATTATGTCCTTACATTCCTACACTATGCT
EMPV1_30938	CCAGCTAGTGGGAAGGTGAATAATACGTAAAGGAGCTGGGATCTACACATACGTTGGATG
EMPV1_30939	GGAGACACCAGTAGGAAGCAGCATATAGGATTAGGCATCCAGATCCAGGCATTACTAACC
EMPV1_30940	TTCAGCGGGAAAACCGCGTCTCCAGGAGGCGAGAATCTGTGATCTCGCTTCCCGGCCCCG
EMPV1_30941	CAGCTTGCCAGGTTCGCCCCACAGGTCACAGATGTTGCCTCTGCATTTTCAGATTGTATT
EMPV1_30942	TACCTTAACTCGGAGCAGGAGTTGGAGCTGGCGCGAAGCCAGGGAAAGTACCAGGTCAGT
EMPV1_30943	GGCTCACTGAAGAGAGCAAAGTTCAGAGCAGAATGATAGTGAATACACAGAAGCTGCAGT
EMPV1_30944	TGAGGAATTGGAGCAGAAGGTATGGGAGCTGGCACAGCTGGTCTGGCAGTCCTCCAACGT

EMPV1_30945	GCTTCTGTGTGATATGTTTGATATTGGGTTGTTTAATTAGGAACCAACTAAATGTCAAAC
EMPV1_30946	CCAGCCATGTGCATATATCTCCTGCCATCATCCGGAAGTTCTCAAGACCAAGGAAAGTTC
EMPV1_30949	TATTTATCGGGTGTTGGGACAAGACATTGTCCTGCCCCCAAAGACTTCGAGGTCAGGTAG
EMPV1_30950	GAAGGTAACTCTGGAAGGAGAATTGGCCTGAGAGTTGAGAGACCTATTTAGACGCTTCTG
EMPV1_30952	AATGGATGGTGGTGCGGAACTGCTCCAGCTTCCTCATCAAGAGGAACAAGCAGACGTACA
EMPV1_30955	TGCAATTTTAGCACTACATGTTCAATATATTTGTTAACTTGTTGAAGTTGATACTCATAT
EMPV1_30957	AGTGCTTATTCACTTTGGAAGCGCACTCACAGGAGCAGAAGAAGAGGGTGTGCTGGTGCC
EMPV1_30959	GTGGTCTGGGAAACGCTGGGTGTCATGGGGGTGTGTGCCGGGAAGGTGCACTGCATCTAT
EMPV1_30960	GATTCTGAGTATAATCCCTGGAGAAAGCTTACACAGTTGGTAGAAGAGATGAATTCGCAG
EMPV1_30961	CACGTGGAGTCAGCAGGCAGGTCGCGTTGCTCATTTGTGACCCGGTGGTTTTGGTTTGGT
EMPV1_30963	GTCAACTGTACTTACAAAGGTATGAGTGAGGTAAGGGAGGCACAAGGACTGTGCAGAGCC
EMPV1_30965	GTATGCTGCTCCAGGGGCTCTTATTGGAGTTGGAACAAAAATTGACCCCACTTTGTGCCG
EMPV1_30966	GGCTACAACTTATGCCCCGCAGAGGCAGGAACTGCTGGAGGTAAACAATGCATATTCTTC
EMPV1_30967	GGCAGTTGTTCCATGCACTGAAGAGGGTCGAGCAATCCTTCTCTCCTAAATCACAGTGGT
EMPV1_30970	TGCTGAAAGGAGAGGAAGATGCCAACTGAATCCATATTCCTTGTGACCGAGTCGGCTGCA
EMPV1_30972	TCAACCAAAAATTCTCCTTCCGCGAGGGTTATGAGGAACCTTACCCAGGCAGCTCGAGAG
EMPV1_30973	TGGGACCACTTCTTCACTGAGACCCTCCCCACTCTGCAGGCCATATTCTACCCAGTTCAG
EMPV1_30974	TCTGCCAGTAGCTTCTAATTAGACCGGTCGGCCAATTTGTGAGCAGGGGGATGTGTCTCC
EMPV1_30976	GTGGCTCCCACTGTACAAACGTCTGATTGACTTCTTCATCTAAAGTTCACGCTGGTGTGG
EMPV1_30977	GGGTTCAAGGTGATCTGTGCAGTCTGACCAAAGTTCTTACAGTCCTGTGTAGACCCCTCA
EMPV1_30979	GGGGGAACACTGATGGGAGTAGACCTTGCTTCCTCAAGGCTTCTAGGTACCAACGATTTC
EMPV1_30981	CAGTGTGATCTAGTTTCCTTTATTTACATACAGCAGTCCAGTTTTCCCAGCACCACCTGT
EMPV1_30983	GGTCATCGGTTATTTGGTACCTCAGTGGTCCGGGTTGCATGTATTGTGCAGGTGTTTCTC
EMPV1_30988	CCGCACAGCAGCGAGTGGGTCCAAGAAACCAAGTCGGACAGGACCCTCTGTGTTCTTTCT
EMPV1_30991	CAGCGGGACTCCGTCGACGCCATAAGTATGTTTCTGGGTGGCCTGGTGGTCACCATCTTC
EMPV1_30992	GATGGAAGAAGAAAAGGGGGAGAGGAAAGTTGGTGACATGGTGAGTAAAAGCCTGGAGCT
EMPV1_30995	CCGGCCACCCTGCCTGGTCGCCTAATGGCCCTGAAAAATAAATCAATGCGATTCAGTTTA
EMPV1_30998	GAAAGAATTGAGGCAAGACCTCATGGCAAGGAGTGTGGAATCCCAGGGGCATGATGAAGG
EMPV1_30999	CTGCAGGGCCTACCCATGGCTGAGGTGGCCGCAGGTGGCTGGCATTCTGTGTGTGTGAGT
EMPV1_31001	GTGATCCGGAAGGGAGAAGTCAGCTGTTGTTGGACCTGCACTCCGTGTAAAGAGAATGAG
EMPV1_31004	CACTGCTCCTCTACTGGTAAGTCCTGAACCCATGCTAGTTTGTGTCCTGATGCTGTCATC
EMPV1_31005	TGAGCCTCAAGTGATCATGTGATCTAAGCTAGCTGTCTGTTGGGCTGCAGTCTTCTCAGG
EMPV1_31007	TACTCAGACACCGGCTATGCACTCAGCTTTGGCAAAGGCACCACACTGCTGGTCACACCC
EMPV1_31008	CTGTGGATCAATGCTTAACTTCATCAAAACACTTGGATTCTTGGCAGTAGGTGCGTGGGT

EMPV1_31009	TGACCTCTGCCAATATCACAGTCACAGTCCTTAGAGAACAGCAAGCACCCCACAACCTCC
EMPV1_31010	CCGTTTCACTGGGACAAGTGTGCAGCCTCCTACACAAAGTTAAGGGCCAAGACAAAACAG
EMPV1_31012	CCCCTGGGGTATGAACAAAGAAGATTAACTGACTCGGTATTTATAATATCGCCAGAAGGA
EMPV1_31013	TCATGGATGAAGCACAGGTCATAGGCCACATGAGGTTGGATTTGCGGGCGGTAGAGGAAT
EMPV1_31014	GTCAAGGATCTAGACCAAAGAGAAGTAGACAAGGTCATGGAGGAGTTTGACCTCACCACC
EMPV1_31015	AGAACCGCTATGTGGTCCGGCTAAGCGAGAGCACCCTGGTCATCTGAGGGGCCGACTCAT
EMPV1_31016	CTCAGCTGCAGGAGCTGAACAGGGAGACCCAGGGTTCTTCTCTTGCTTTTCCTCCTCAGG
EMPV1_31017	TTCAATCCCTGGCCTCGCCCAGTGGGCTAGGGATCTGGCGTTCCCATGAGATGTGGTGCA
EMPV1_31021	CCCAAATGTATATGCCTCTGGCCCCACAGAACCTGGATCTAATCCTAGAGTCAAGGATGG
EMPV1_31023	TCCTGCGTAATGTCTTTGCTTGCTGCCTTGTGAAGAGCATCCGAAGTGGCAACGACGTGA
EMPV1_31024	CAAAGGAAACTACTTGCACCCCAGTGTGTATGTGTGCCTGCAGGCTAGCAGAGGGCAGAA
EMPV1_31027	GTAGCCACTGTGGAAAAGCACTAAGTAGTCAGGCATCTCTTAAGACACACCTCCGGATTC
EMPV1_31028	TGGCTATTTTCATTCAGCGCCTGACCACGAGCATCTGTGGGTTCCTGCTGGGATTTTATC
EMPV1_31029	CTCCAACAAATTCTCACGAGTTCCGCCAGAGTAATGGATCCCGATTCAGCCTGCTGGACT
EMPV1_31032	CACCCCAAAGGTCAGCAAGCAGGGCCGGAGCGAGGAGATCTCAGAGAGTGAAAGTGAGGA
EMPV1_31034	TGACCCACAGTCAAAATCAACGAAGCGAAACACTCATCCAAGAAGCATCTCCTGGTCCCG
EMPV1_31035	AGGCATTACTGATGAATCAGATGGGGGCTCTCGTTGTTAACAGCACCTCGCTGTGTCAGT
EMPV1_31037	CCTGCTGAGATAAGCAATTCCCTTGACACAATCTACTCCACCCCAAGAAAGTAGCCCAGC
EMPV1_31041	GGTTTGAAATGAATCATATGAGGCATGTAATGTTACGCCAGCTGAGGAGAAACCAGTAGA
EMPV1_31042	TCACGTAGGTCTTCTCCCGCGACAGGCATTTGTCGATCTCTTTGGACTTGCGCTGCTGCT
EMPV1_31043	CTGATTATAACAAATGCATCACTCTGTAGGGGACGTAGATATCGGAGGACACTGTGCAAG
EMPV1_31045	GGACTTCCAAGAGTTTCAGACCCAGAATTTCGATCGCCTGGATAACTAAACCGAGGAAGC
EMPV1_31046	CGTTGGAAATTAGGGTGGGGTTTCAGTCTCCAAATTGTGCCGATGTTGTGGGGCAGACTT
EMPV1_31048	TGCTCCATAAGAGGGACAGTGACAAGGTGGATGCCCAGGAGGAGAACTTCCTGCCCAAGT
EMPV1_31049	AAGCTTTGATGACAGAGAAGTGTTTTACTGCACACTAGAGTCCTGGGTAGGGGCCACAGA
EMPV1_31054	GCAGATGATTGGGATCCTTGCAGGAGACAGGATTGAGATGGCAGAATAGAAGGACTAGAC
EMPV1_31058	GGGTTTCTCTCACTTACTGGCTTTTCCTAGAGATCCCACCCAGCCCATAGCTGGCATCCT
EMPV1_31061	TGCGGGGGGAGCTGTGGCTCCTCTTCTCTGGGGCCTGGAATGAGATGGTGACTCATCCTG
EMPV1_31062	CCTCTTTATTCTGAGCCCAAGCTGTGAGAAAGAAGGACCCATCCTGATTCCCCTCTAGTC
EMPV1_31063	GACAGATTTGGACCTGAGGACCCATGTTTCTTTTGTGATGTTTGCATCCGTATGCACCAC
EMPV1_31066	CAGAGTGGACTGTGGAGGATCTTTGTTCTTATAACATTACTGCAGCCAGGTCTGGAGAAG
EMPV1_31070	CACTGATGAGCTTTTGTGCAGAAGTTTGTACCATAAACGTCAATATAAAAATCCTGTGTA
EMPV1_31071	AGGTCACTCACTTGTAGCTTAGGTGGCAGCAGGAAGAACTTCAGACACAGGAACACAGTG
EMPV1_31072	AAATCCAGAGCGGGTGAGGCCTGTCTGACTGTTTCTAGGCGACCCACTCTTGGTTTCCAT

EMPV1_31075 GAAGTCTCAGAGAAGTTAAGCAGCTCATTCAGATTCCTTAGCTGACAAGGGGTGGAGGGC
EMPV1_31081 ACACAGAGCAACGGTCGCAGGCCCTTTGGGATCTCCGCCCTCATCGTGGGTTTTGACTTT
EMPV1_31087 ATCTGGCACTTTACTCCGTTCCGCCTGATGAATTGAGAGTTTGACTGTGCAGAGCTGTCG
EMPV1_31088 ACCACTAGGTCACCAGGGTTTTTCAAACTACATGCTGTAGAGTTCCCTGGTGGTGCAGCA
EMPV1_31089 CTTGCACATACTCAACACAGATGATATGCATGAAAATATCCATTCCAATGGTGTCCTTAG
EMPV1_31090 AGATACAACGCATACCGCACACCAACGTTTCCACAGTTTCGGACGCAGTGTATCCGGCGG
EMPV1_31092 CCAATCCAGGAAGCACAACAAGTACCATACAACATAAACCCAAGGAGGACTACACCGAGG
EMPV1_31094 ATACAAGTGCAGCCACCATTGAGAAGAAAGCCACAGCAACCATCAGTGCCAAGCCACAGA
EMPV1_31095 TCTTGGAGTCCTCCTTGCCCAGGAAGGCCTCGTGGTCGTACTGGAAGCTCTGATTGTCCT
EMPV1_31096 CTCGCTCACACGGAAGCTGGGGGAACAACCTCACGTTCGGCCTTCTCATCTTGTGAAAAT
EMPV1_31099 TCTGTGCTTCTCATCACCCAGCGTCTTAGTTCCGTGGAGCAGGCCAATCACATCCTCTTT
EMPV1_31100 ATCCTGGCAGCCTCCACAGGGTCCGCTGGTGCCTTCATGCTGCCCGCTGCCCCTTCTGGG
EMPV1_31104 TTCTTGGGCAAGTGGCTTTACCACCTCAGCACTTCACTCCTCATTTACCTCCTGGGTACC
EMPV1_31105 TAGGGACTCGTGATAGGATGGCTGTAATGTGTAGTGGTGGCTGGCAGCACAGAACAATCT
EMPV1_31106 GCCATAATGTGCCATTGTAATTTGATTATCCCAAAGATACCAGTTGTGATACTGTAAGTG
EMPV1_31108 ATGGTTGGTAGTGCCCTCCTCCTCCTCGCGGTGCTGGTGCTGCTCGGACTTGTAGGGCGA
EMPV1_31109 GCATCCTCAGTAGACAGCTAGCATTCCCTGCCAGAAGGAGGATTTAGAGGAGTATAGCTG
EMPV1_31110 CTCATGTATGAGACGACGCCCATCCCAGAGTCCAAGCTGCCACCTGTGTATGTGAGGAAG
EMPV1_31112 AAATCCTGCTACAACCTGCCTTGCATTGTGACCTCCTAGAATGAGTGTGTCTGGACAGAC
EMPV1_31119 CAACTGGGTTGATCTGGGACTTCTGGCCTTTAGCGCTCTGACCCATTACATTTCTGTGGT
EMPV1_31121 CCCATTGAGCATCACTCTGCTTTTGAGGGTATTCTGGGATCAGTCGCATTGTTTCAATCT
EMPV1_31123 CTTTCTGACTCACTAGTCGTATGCATATTGCTCTGACTCTAGTTGTATCCATATTGCTGC
EMPV1_31124 CTCGGCCTCGGCCTCTGCCTGTTGATGGTCCTCCAAAAGCATCATAGTTTCTGCTTCCAA
EMPV1_31127 GGACGGAGGCGGTTTATTCTCCCCCGGCTCTGCAATCCGAGTGGCATTTTGTAATCTTAT
EMPV1_31128 CTGCTGTTGGGCTCTTCCACAACCAATCAGGAATACCTAGGGCTCTCTTCACACTTAGAG
EMPV1_31129 GACGCACGTGGGGCAGCAAGGCACGCAGGGCTTGGAAAGGATGTCTTGTTATTATTCTTT
EMPV1_31130 CGCCGTTCCTGTGCTATATGGCATCTTCGTGATCCCACCTTCTAAGCTGTGTGGTCCATT
EMPV1_31131 GGGTGCAGTCTGAGGAGGGGGAAGTTCATTTCCATGAGGTGTGGAAAATAAAGGTGTTGG
EMPV1_31133 TGAAAAGGTACAACAGAATAACCCATGATGAACTTACCTGGATTATGAGACGGGAGAAGC
EMPV1_31134 AGTTTTGTTGGCTCTACCTCCGAAATCTTTCCAGAATCTCCTCGGTGCCCACAACCTGGT
EMPV1_31135 ACCAGATTCATGGGACGCTCCATGCTTGTCTGTGTATATATCCAGTGCTTGGGCACATGC
EMPV1_31136 GAACTCAGTTCTCAATCCCCTGGTCAGCAATCTGCCCACTACTGCATCTTGTTCTTTGGC
EMPV1_31137 ATGACCAAGATCTATGGGGGTCGTCAAAGAAACGGTGTCATGCCCAGCCACTTCAGCAGA
EMPV1_31138 TGGGGAATGGAACAGGCATAAAGTATCTAGATTGTCCTAGATTCTGTTTCCGACTTTGCC

EMPV1_31139	ACCTACTTCCTCTCCTATGCCATCCTCTGTGAGTGGCTCACTCCCGCTGGCCGCAGCCAA
EMPV1_31141	ACCGTGTTTGGACATACCTCAAAGTAGAACCAGTATCGATTAGCAGGGGACCAGCCATGG
EMPV1_31142	ATGGTCGTCCCTCTGAAACCCTACTGTCCCCGTGTGTACTCTGAGAACCAGCCAATGCTT
EMPV1_31143	TTTAAGGGCAAATTCCAGCGTGTCCTCAAGGCTCACCACCAGCGCGTCATCCAGGTTTAG
EMPV1_31146	CGTCTGGTCCGATCTCTTCTTGATCTGTTTCTTGATATGGAAGCAGCTACAGGGCAGGAG
EMPV1_31147	CGTAGGAACTGGTTTTTACCAACTCTCAGTATTGACGTCTTTCCTGTGTCAAGCCCGGAG
EMPV1_31148	GACAGGCAAGGGCTTTTCGGCTATATCATGTTGTCTCTCTAAGGGAGGAAAAGTAGCTGC
EMPV1_31151	CAAGTTGAGGTAGCTGTGCATTCAGTGTGGTGGGAGTGGAGCTATGACGACATGAAGGTC
EMPV1_31152	TCTCACCAGCCCGAGACCGTGACGTGTGAAGACTTGCTGAACTGAGCAGACACGCAATTG
EMPV1_31154	AAGGCATCATTCCGTCAGAGGGCAGCTCTAGTTGCTCAGAGCTCCGGGATATGGAATCTT
EMPV1_31155	AGTCAACTAGTCACCAGCAATCCCCTGGAGGCCATAGGCATGGAAGCTCAAGGACTTGTT
EMPV1_31158	CCAAGGAACATCAATCCACTGTTGTTGGAGACATGACAATCTTTTTCATCCCAGGAACAC
EMPV1_31160	TTTAGACCAGTCCTCGGAGTTGGCGCAGCGCCCTACTGATAGCGCTGGGACAGCACTTAT
EMPV1_31161	GTGTGAAAGCAGACTGGATCAGACCCCAGGAGACTAGAGTATCTGGAATGGAGGGAAAGA
EMPV1_31162	CCTCATTCACATGCTCACCCACGTGGTCGAGGCGCTGCACCAGGCCCGGCTGCTGGCCAT
EMPV1_31165	GCAGAGCTCACTAGAACTTGCTTCGCCTTTTACTCTGGGGGGAGAGAAGCAGAGGATGAG
EMPV1_31166	GATGAAGCAACTGAAAATAAGCAGCCCCAATCCACTGAGAACCAGGGCTTTAGCTGCAGG
EMPV1_31167	AGGGAAGTTTGCCTTCTCTTGCTTTTACCCTCCCTACTCTAGCAAACCTGGGAGAAAGAA
EMPV1_31168	TGGGAACCCCACCACCATCACCATGGGCTGCATTTCCTCTCAGTATAAGGTCTCTGTTTA
EMPV1_31169	GCCAGGATCTTCACAGAAAAGCCTGTGTCCTGAATGACAATGACTTCTTGTCCAGTGGTC
EMPV1_31171	TTTGGTGCTGTACTATGTCACCTCACTGAAGGCGTGTCAGATTGACCCAGTTGGGGAGGA
EMPV1_31172	AAAAGTTACCACAGGGATAACCGGCTTGTGGCGGCCAAGCGTTCATAGCGACGTCGCGTT
EMPV1_31174	TACTTTGTACATCAGGAGTTCCCATTGTGGCTCAGTGGGTTAAGAATCCAACTAGTATCC
EMPV1_31176	CCCTGACCTGGCCAAACCAAGGCTTTCTTCTTTGAGTCCGTGGGAAAATCAAGAAGTTCG
EMPV1_31177	CCTGGGCCTCACCCTGCTCACCTTTTACTACTCCTTCGCCATCGTGGGCATGGAATTCTT
EMPV1_31179	CCCCTGGTTACTCTCTCAGACTAATACTAAGTCTTGATGGGAAATCACTGAGTTAGGTTC
EMPV1_31180	GTCAAGACAATGGATTCGGTCTCCACTAACCATGGGAAGATCCATGGCAATGAGCTGTAC
EMPV1_31182	GGCATGCCCATGTGCCTGCAGCTCTGGCAGGCAAACTCCAGCCCCCGCCCCCTCCTCCTG
EMPV1_31184	GCAGAAGGGGATCCTGGATTTAAAAGAACCAAGGGTCTGATGAACCGGACTTCTACTCTG
EMPV1_31186	GCCTCCCTCCTTCATGATCACAATTCCAAGTGCTAAAATTTACTGGTGGTCAGAATATGA
EMPV1_31188	TTCTCTGCAGGGCTTGGTCCCAACACCCCCGCTTGGTTTAGTTCAGACTCCTTTTCACAT
EMPV1_31189	GGTCTGGTTCCAGAACCGCCGCACCAAGCAGAAGAAAGACCAGAGCAGAGACCTGGAGAA
EMPV1_31192	GGGGCAATGCATTATTGTGTAGCCACGGTTTTCTGGAGAAGTTGATATTTTAGGAATTGT
EMPV1_31193	GGGACTGGGCAGAAGCCACTCTGTAAAGACTAACTGTCACCACTGCTGTCCTCATGGTTA

EMPV1_31197	TTTGGGGGAATCCATGAGTTTTTCCCTTACTTCTGGTTTACAGAGGCAGTCACAAGGCAC
EMPV1_31198	CGGAGGAAGATCTGGAAAGGCAGCTACAGGACAAGCTCAATGAATCAGATGAACAGCACC
EMPV1_31199	TTACTCAGCACATGAAATAAACAAGGGGCACCCAAATCTTGCGGCAACGCCCCCGGGACA
EMPV1_31200	CCGTGGAGGACGACAGAGGAAAAAACATCTGGAAACACGACCTTGCACACACCATCACCA
EMPV1_31201	ATACTAACGGACCAGATTACCAAGGACCAGTCCACCTGAACCACACGACTCTAAAAGAAA
EMPV1_31203	GGATCTCAAAACACGTCTTTTCAGGATTCTTGTTCAGATCAAGACCCAAAGCAAGTCCCC
EMPV1_31204	TCCGCACGTTACTTCCCATTCTCCACATTGGGCGACACTGCTGACATGCAATACTCCAGA
EMPV1_31205	GGAAGAAATACTGTAAACTACACTCGGCCACTCCTTTCTTGGGGTCTTGGAGACTGTGCC
EMPV1_31206	GGGGTATCACAGCGCAACAAATGTCCAAATGGGTAGCTGAGAGAACTAAGAGAAGGGTGG
EMPV1_31207	GGTCCAACCTGTGAGATTTCCAAGGAGACAGAGAGCAAAGGCAGTGTAAAGGAGAATTCG
EMPV1_31208	CAAGGCTCAGGACATATGGGCTTAATTCCCTTGAGTTCTTGGGGTGATCGTGTGATGCCA
EMPV1_31209	CTGTTGTCGTCACATCTTTCACTGTATGTGCTAAGGGCTAAGTGTTGGCTGAGACCCAGC
EMPV1_31210	AAGTTCTTCCTCCTCCCTGGTCCCCAAACCCCCATCCAGTCACAAACAAGTCCTCAGAAA
EMPV1_31211	TCTCCAGGGCGTTGAACGTGAAAGGCTTCTTTCTAATCACCTCCTGTAATTGGACCAAGG
EMPV1_31215	CTAAGCGACTCTCGCAGCCTGCTGGAGGCCTTCTGGACTCTATCACTAATATCTTTGGGA
EMPV1_31216	CTCAAGGTTACCAGGTGAGTATTTGGGGCTCAGTTGGTTTAGGCTCACACTCAGAATCAG
EMPV1_31217	GCAGTCTACGATGGAAGCAGCTCTATTGAAAATCTCAAGGCCAAGTTCTGCAGCACAGTG
EMPV1_31218	ATGGGTTTCTTCAGCTCTGTCCTCCTCTTGGTCCTACACCTGCTGCAAGGGTCCAGAACT
EMPV1_31222	CTTCATGCTCGCTTTACAACCTCATCGCTTTACTTCCCATGCACCTTTTACTGCAGCCCG
EMPV1_31224	CTGGTTTTGGTTCTGTGCCTCGGCCACATAAGATGGAACCCCTGGGGAGGTCTGGCTGAA
EMPV1_31225	GACAAGTTTAGGCAGAAATTAGAGGAGGCCTTAAGAAAATCCCAGGGCAAACCTGGCTAG
EMPV1_31226	AAGGGAAATAAAGGTGAACCTGGACTCCAGGGACTGCCGGGGGCTTCTGGGCTCAAGGGA
EMPV1_31228	GGAGCCATGCACATAATTGGACAGAGATGTGAAAATGGGTGGCTTCCTTCTGGTAAGAGG
EMPV1_31230	AGGGCTCCATCCTGGCTGTGAGGAAATACTTCCACAGACTCACCCTCTATCTGCAAGAGA
EMPV1_31231	AGGGGAACCCTCAACGCCGGGGGCACAGACTCACCTCCGCCGCGGACAATGGCGGCTACT
EMPV1_31232	AGTCTTACGAGAGACACTGCCACTGAAATAGCCATCAATGTGAAGGCCCTATATAATGAA
EMPV1_31233	TATTTTGAAACTTTGCTTCTTTGGGGTTGGGGCACACTGGTCACCCCATCTGGCTGTCAG
EMPV1_31235	AGCAATGGAAGAGTTACGATGCCGAGGACGCTACACTGCAAATCTGAACATGGCCTTCTG
EMPV1_31236	CCTAGAGGTGCTCTTTGACGTAAATCGCAGCAACATCTTGTTTGATCCACCTCTAGAATA
EMPV1_31238	CCTGGGCCCAGTGGAGCCCAGGGTGCAGCCGGACTGACCGAGCCCCTGTGCCGCCCCCAG
EMPV1_31239	GTCTAGATCATGGTGCTGGGGCTCTGGAGCACATACGCCTTACCCCTTGAACCCTTTGCT
EMPV1_31240	TGAACCTTCTGCCCCATCCTCGGATCTTCTCGACCTAAGCCCCAGCCCTCTGATGCATAG
EMPV1_31241	TACGGGAAAAGGATACAACCACCCTCAACTGTCAATCTTCTGGAAGCAAACCTGCAGCCC
EMPV1_31243	GAAAGATGAGGACAGCCAAACTTCCTACAAGCTGTTACGTGTTCTTTGCTATGGAAAGCC

EMPV1_31244	AACTCCTTTTTGTCAGGTAATATAGCCAAGGGGATGACGACAGGGGTAAAGGTCACGGGG
EMPV1_31245	TCACAAGAAGTCTCGGGTGATGGATTTCTTCCGGCGAATTGACAAGGACCAGGATGGGAA
EMPV1_31247	GCCACCACAGGCCCCATCTCTTCCACCGGCGCCGGCACCAGAACCCTGACTGCTGTGAAT
EMPV1_31248	TTCATGTGCCAAGGCTGTGGAAGGTTCATGTGCCAAGGCTGTGGAAGGTTCATGTGCCAA
EMPV1_31249	ACATAGCCTATTTTTGTGCTTAAGATACTGAATGGAAAACTCCATTGTGTGTTGCTGGAC
EMPV1_31250	TTCCAGACGAAGGTCACAGCATACGATCTCTATAGCCAAGCATCAGTTTGGTTGCTGCGG
EMPV1_31255	TCCAATCCCGGTCCAGGTCGAGATCAAGATCCAGGTCTCTTTCACGACCAAGAAGCAGCC
EMPV1_31257	GCTCCACCGTCATCTTCAGCTTATGCACTGCCTCCTGGAACAACAGGTACACTGCCTGCT
EMPV1_31258	AAAAACTATACCCTGGAGGTTGTCACTCACCAGAAGGAGCAGGGTGCAGATGCAGTCCTT
EMPV1_31259	ATCCTGTGGCATGGGTGAATACGATGGTTTTTGACTTTAAAGGACAGTTGCGATCTGGAG
EMPV1_31260	TGTCCTCTTGAGACCTGCATTGGTGCTCCCAAGCATAATCTCACCCGTGAGCTTGCGTTT
EMPV1_31261	TTGCCCAAAGAGCAACCAGTGAGTGACCCAGAAGAGTCAGAAAACAAGAGAATTCCCAAG
EMPV1_31262	AAGTAGGTGCTCCAGCTTTTCTCCTTTGGGATTTAGGCTGCGTGCTGGTAGAGGAAGGAG
EMPV1_31263	CTGCCAGAGAAAGCAGAAGGAGAGCCTAAGGGGAGTGGGCTGGTTATCTTGGTATCTGTT
EMPV1_31265	GGGTTTCCCCAGAAGCCCTAATGAAGCCTGAGTCCTTAAGAGGTTGGAATACATCTCACA
EMPV1_31266	CCCCACTGAAGATGTTGCGGAGTCTTTACAGTCTTTTCTTGGCTGGATGTTGTGAAAGGG
EMPV1_31267	GGCGCCAAAGAAAGACAAGAAGCCTAAGAAGTCAACCTGGAAGTTTAATTTAGACCTGAC
EMPV1_31268	GTGCTCACGTGCAGTCGTGCCTAACAATCGGAAAGTAAGCGCGGCCCAAAATCGAGAAAT
EMPV1_31269	GCAGATGATCAGGTTAAGACAGGCATTCGGGTGTGTTGTAATCCTGTACGACTGGTGTCC
EMPV1_31273	GTTTTCAGGGGTTAAGGTAAAGGGGTCCATTCTGAAATTCCACGCCTAGATGTCGGCTGC
EMPV1_31274	ACTGTCACTCAAAGAGAGATATTAAGCCAGAGAACCTGCTCCTTGGATCCGCTGGAGAGC
EMPV1_31275	TCATCCCTGCTCCCAAGGGCACTGGCATTAGCTCAGCCTTTGTTCCCAAGAAGATACTGA
EMPV1_31277	CATAGTGGGGACGTGAAACTGTATTCTGTGCCTTCCATCATGATTTCCACATGAAAGCAC
EMPV1_31278	CAGAACCTGCGAGGTCCAGGTGGATGGAGGTGGTTTAAATTTAGTAGATGTGTTGGATGC
EMPV1_31279	CCAACTACACATGCACAGAAAGTCTCCTGGGGTCAAAGAGGAGCAGGCACCAGGTCATAA
EMPV1_31280	TTACGTGTGGTGAAGACGGCGGTTCACCTCTGAGTTGCGTCAAAGTGCCCCTAGTATGAA
EMPV1_31282	CATGTGTATGCAAGGAAGCCAGAACACAGGTTTTCTTGATAATTTGGGTTTTAATTTTGT
EMPV1_31287	CAGGTATAATTCTGACTGGGGAGAGACAGGCACTGAACAAGATGAGCAGGAGGATAGTGA
EMPV1_31288	GCCGCCGATTCCTTTACATCGTCAATCTGGATGCCCCTTTTGAAGGTCACCGAAAGATCT
EMPV1_31291	CTTTCCCTCTTGAGTGGGCTCCCCAAAATCTTACCAGGTGGACCTTTGAAAAAGCTTGCC
EMPV1_31292	CTACAAAGCAGAAGTTGTGTTCGAATGTAACGAGGGTTTTATCCTTCTTGGCAACAACAC
EMPV1_31294	CCAGATCGAACCCTTCCTCCTGCAGTTTCCTCCTCAAAAAGAAAGGCGATAGATAGGCCT
EMPV1_31295	ACGCGGAGACCAGCAAGTTGCAAGTCCTGGAACATATGCTCAGGGTCAGTGAGAGGGATC
EMPV1_31297	ACCTCACTGCCGTGGCGATTTTCCAAGGAACAATGCTCTTCATGTACTTCAGGCCAAGCT

EMPV1_31298	AATGGCATTGTGGTGTCATGGAGTCTCCTTGAGGTACATAGAAGCTGCCCCACTGTTGAC
EMPV1_31301	TCATCGGGATTTCCAGTTCAGGACTTCAGTCAGTGATGAGCAGCCTCTTCCTCTTGTGTG
EMPV1_31302	GCTATCTGGGACATCTTCAAGGACTGAACAAATACAAACTGCGCCAGCTGGAAGCTATTC
EMPV1_31304	GGGGGCAATTATGTACCTCCGACCATCATCTTCTGGGTCTATGGATAAGGGGAAAATCTC
EMPV1_31306	CAGAGCAACGGCACTCTATGCGGTGAGCTCTCTGCTATTTCTCTCATTCCGGTTCATTTC
EMPV1_31308	CACAACCTGCACCACCACAGTTGCTATTTGGAATTACTGGGAGGAGTAAAGGAGAGAGGG
EMPV1_31309	CAAGGCCGGCTTCACCGGTGGTGTGGTGGTGGACTACCCCAACAGTGCCAAAGCCAAGAA
EMPV1_31310	TGTGCGTTAGCCATCTTCATCTCTTATGCCTTCATTCTTTCCAGCATCTTGTGCATCCGC
EMPV1_31311	CTGTAACCTGTTCATTTGCCATCTGCCCCAGGAGTTTGGGGACGCTGAGCTGATGCAGAT
EMPV1_31314	ATATTGGCCAACTGATGGGCGCCCCAAAACCACCTCGCAAGAAGGGTTAAGGGAAACAAA
EMPV1_31315	ACTGCCACCTCCTCATCCTGGAGACTTTTCTGTTAACCGTGCTGACACGAATGTACTACC
EMPV1_31317	AGAAGTGCGTGCATAATACAACTAGGCATTGGTGGTGCCCTGGTACAGTCTGAAGAATGC
EMPV1_31318	CAGCTCCATTTGCATCCCAGAACTCACTTTGGACTTAGCTATCCCTTTGCCTTCTGGACG
EMPV1_31319	CTGCGCCGGTGGTCTCCAGCCTGATGTCTTAGTGTTCTCTTTCCTTAGTTCCGGTTGTTT
EMPV1_31320	AAAGATGTTCTTCCAGACAGCTTGATGAAAATGCCTTTGAGTAATGGACAGATGGGCCAG
EMPV1_31323	GAGCCTATGCTGAGGTCTCACTATTGACACTTTTTACGTAACAACTCTCCATGCATCTGT
EMPV1_31324	GTGGGCACACAACCTCATCGCCATTGCTCGTCTTCGTGGCTCTGATCTAAAAGTACTCGA
EMPV1_31326	GGCCAAGGAGTTGGGTGAGAACCTCAATGATGGAGAGCTGCAAGATATGATAGATGAAGC
EMPV1_31329	TAGCCAAGCTGAGATGGTGGCCAAGGGACTTCAGCCCTGCCTTTCAGAACAGAGATGATA
EMPV1_31331	TAGGGCATTGGTGTGAAGCTGGAGACCCACTGCCCCAGGTGCTGCTGGGGGTTGTAGTCT
EMPV1_31332	CTGACTTGCTAGGTAAGATTTTGTTTTATGATGATTGCTTTAGGGCTCACTAGATACAAC
EMPV1_31335	CTGCTCCTCCTCGGCCTAAACCGCCCAGCGGTAAAAGTACACCCGTAAGCCAGCTTGGTT
EMPV1_31337	CCTCATGCAGAAATGGTTCACCTGTGATGCCCAGAGGACAGTCAGGATGCGTTTAATGCT
EMPV1_31338	CATGCCGAACTCGGAGGCAATGTTACACAGTCCAAATTAGCAGCGTTCAAATTAATGAGC
EMPV1_31339	TCTGCTGAGCAAGAGGCAAAATGGGACAAAGAGAGGGCCCATCAGATGTTGGAAACCGTC
EMPV1_31342	ATCCTAGGTTAAAGATGCTCACTCCCCATCCCACCCACTCTGGTCTGCTGATGAGTCAGT
EMPV1_31346	TTTCGGCAAGAGAAGGCCAATGGCGAAACTGGAGATCTTGGGAATGCTGTTCACGCTGAA
EMPV1_31350	CTCTCCACCTGAATTTCCAAACGTGTGGCGGAGGCCATTCTGAATGACATTTGAGATGCC
EMPV1_31351	CAACCTTGGATCCTTTGGATTGCTGGGAAGGGCTGTAAGGACAAGAAATATCAGCAGGGC
EMPV1_31352	GGTTATTTGCCTTGGAGAACTCTCCCTGGTCTGGCTTTTGCCAACTCCATTACTGTGATG
EMPV1_31353	CTGACTCATTATCCACCAGACCTTCAGTCAGACCTCTTCTTTTACGAACATTCTCAGACG
EMPV1_31354	CTCCCCGAGTCCAGCTCCCTCTTAAGCCCGGACCCTGAGGTGGTCGTCGCCGTGGGATTT
EMPV1_31355	TGCAGACTCCCAGTCAATGTTTGAGTTTCCCAGATGGAGGAGGTGAGGCTTTGCAGTCCT
EMPV1_31359	GGACATACTTATGCTAAAAATTATTCACTATCTGAAACTCAGATTTAACTGGGAATCCTG

EMPV1_31361 TCTTCTGTCGAACACAGTGGTATCTGAGGATTGACAAACGAGGCAAAGTCAAAGGGACTC
EMPV1_31362 GGTTTTACTGAGACACAGCCATGCTTATTTACATATTCGGCTGCTTTTCTGCCACAGTGG
EMPV1_31363 GACATATGCTTCTAATGCAGCAATCACTGTCAGAAATACCATGCCAATCTCAGCCTCAGG
EMPV1_31364 GGTGAGAAGTGTTTAAGTGTGAGGAAGCCCATCGTTTTTGCGCTTCATGCTTTGGGTGCC
EMPV1_31365 TTATACCACAATGCAACAAGCTTTAGAACTAGCTTTGGATCGTGCAGAGTATGTCATTGA
EMPV1_31366 AGGACTCCATCCTGGCTGTGAGGAAATACTTCCACAGACTCACCCTCTATCTGCAAGAGA
EMPV1_31368 GTCTGTGACTCAAACCAGATCCTAAGCCACAACAAGGGTTCTCAGAGTGCCGTGGCAGTT
EMPV1_31369 GCTCTCAAAGGAAACCACTTTGCCTGTCACTTTAACTGAATGGAAGCTTTTCCGAGCTGG
EMPV1_31371 AGCGCTCTGACAAAAGCAGCTGATTTTGTGAAAGCCTTTATTCTTGGATTTCAGGTGGAG
EMPV1_31372 AAATCCTGCCCAGCCAACTTCTTTGCGTTTAAAATCGTCAGTGGGGCAGCCAACGTCGTG
EMPV1_31373 GGGGTGGGAAAGGCCTTCCTGCTAGGACTCTGCCCAGTTTCCATCATCAGAAAGTAGTAT
EMPV1_31374 TTCTCCCTCCCATCCGTCAGCCAGTCCCCAAGCCCGGGCTCTGGGTATGAAATGCGTGCT
EMPV1_31376 GCTAGCTGTTAGAGGGTGGTCGGGGTTATGCTACTCTTAGGATTTCAGAATGTCTTCAAA
EMPV1_31377 CTCTCGGCCCTATCTCCCAACCCTTGTACCAGTGCGTGCCTCAGTCCCTGGTACAGGAAT
EMPV1_31379 GGGTTCCTGGCATGCTGATTTGTGACTTAAGATTAAAATCACATTGCCAGGGATTACCAC
EMPV1_31380 CTTCCCTCATACCAGAGAGTAATCTGAGAGCCCAGGCACCCAAAGCCCACGATGTCAAAA
EMPV1_31381 AGATAATTGATGACCTCGCCAACCTGGTGGAGAACACCAACGAGAAGCTCCGCACCGAAA
EMPV1_31383 ACGACATGGACGTGGACACGGGTTCTGGGGGGGTGCTGAGACGGAACCTGCGGAACCAAT
EMPV1_31385 GAATGAGGGAGCAGGCCAGTCTCTACTCTCTGGATATCAAATTTATCAACATGGATGTGA
EMPV1_31386 GAGAATCCACTAGCTGCTCAACCCCGGGAATCTCTGAAACTACCAAGCCAGGTTCCCTGG
EMPV1_31387 GTCATCTAGAGTACTGTCGCTTTCCAGGTGGTAGTGTGCCGCCTGTATAAAACCTAAGGC
EMPV1_31390 TTTCTGAGTATTTGAACCGGCAGCACACGGACCCCAGCGACCTGCATTCCCGAAAGAATA
EMPV1_31392 TAGTTCAGACCAACCAATGCGGAGGAGGGATTTAGGCCTTGGGGGAGATTTTGCGGTGCA
EMPV1_31393 CAAAGTGGGCAAAGGCCAAGAAAGGCGAGGAAGCTTTATTTACAACGAGGGAGTCTGTGG
EMPV1_31394 GGGGATGAGAAGAATCCTGGGAGTGCCTGGGGGTAAGGTGGAAAATGGAAGTCTTTCTTA
EMPV1_31396 GATGGTCTTTGCATCAAAAAGGCCAAGTAAACAGCCCCCTCCCTTCCCCAAGTGTGAGGT
EMPV1_31397 ACTGGTCAGACGCCTGGGTTTGCAGGAAAGAGCAGGAGCCGAGGGATGCGGGATGAGCAT
EMPV1_31400 TGCGGCGGGTGTTGACGCGATGTGATTTCTGCCCAGTGCTCTGAATGTCAAAGTGAAGAA
EMPV1_31401 CATGCTTATGAGCAGTGTGCTCTGGAATTGGGGGAGCTGTGTATAAAGACATTTGAAAGG
EMPV1_31402 CTGCAGATGTGGAACCCACAGATATAGAGAGCCCACTCCCCAGTTCAGTTATTCTAAATG
EMPV1_31403 CTCCCAGGATCTGGCTTATCTTCCATTACTGAAAATGATGAAACTGCAGTCTATAGCCAG
EMPV1_31404 CATGTACATGAGCCCTGGCTCTGACTCCCTGTCAAACAGTGGCAAGAAAATGGCCCTTTT
EMPV1_31406 GGACAACGACGAAGACTTGTGTCTGAAGATGACGAGGAGGAAAGTCCTATAAATATCCCC
EMPV1_31408 ATTACCACCAGACAAACGTCTATGTCCCTCCAGTTCCTGCAGAGTGAGACCACCTACTGG

EMPV1_31410	CATGGTCGGTAGCGAGTCTTTTTTCGCTCGTGAGTATTGGTGAGGAGATTGAAGAAGACC
EMPV1_31412	CCAATACCAGGCCTTGGAGCGCAAGTACAGCAAGGCCAAGCGCCTCATCAAGGACTACCA
EMPV1_31418	GAAAGCAGTGCATGAGTTTCTGGCCACGCTAAATCACAAAGAGGAAACAGGTGGCAGCAC
EMPV1_31422	ACATCCATGTAAGAATTCAACCGAGAAAGGGCAGGAAGACCCTTCCTACTGTCCAAGGGG
EMPV1_31425	CTCGACCTAGTCTTCCCTCTTCGTCTCACACAACAGGCCTTGAATCTGTTTGTCTTCACC
EMPV1_31426	TGGTAGAAGATGACGCGAAAGCACCCCCTGAAACATCCAGTCGTACGGATTACGAGAGCA
EMPV1_31427	GATCCATTGAGACAAGTTCTAGACAGAGGCATGCAGTCCAACAAATCGTACCAGCGTCCC
EMPV1_31428	TCCTTTTACATGACCTGAACGAAACTTGGGTTGTATGGCCCAGGCAGGTTGCTGTGGACA
EMPV1_31429	GCTAGGATTTCAGTGTCCAACTAACCTCTCCCATATTGCGTTAGCACTGGAAAGTAGCCC
EMPV1_31431	AGCCGCCCCCTGGCAGGATCGGAGCAGGAGCTGAGTAGGCATGTGGAGCGCTGCCTTTCT
EMPV1_31432	TACGACCACAAACCGAAACGGTGCACGCGGACCCTCTTCTACATCCTCCGCAAGCAGTAT
EMPV1_31434	ATTAATGATCAGCTCCTGGGTGATGCACTTGATCTTCAGAAATGGGCCCACAAGGTGTGG
EMPV1_31435	GTTTAAGAATCCGAATGCAATGGCTTGGGTCAATATGGAGGCACAGGTCCAATTCCTGGC
EMPV1_31438	CAGGCAACTAGAACAGGCTTGTCCGTCTCCAGGCAAAACTCCAAAGAGGTAGAACATTCC
EMPV1_31439	CCAGCAAGTGTATACCAGACAGAGTATGGCGAAGTCCAGATACATGGAACTCTACACGTA
EMPV1_31440	GCGGAGCCGCTGCGCTCCGTGCTGTGGGTGAAGCAGCAGCGCTGCGCCGTGAGCCTCGAC
EMPV1_31443	TGGCCGAGGGATTGCTGTCCTCCTCGAGTCTCTTCAGGTGGAAGGTTAAACAGAAGTTAA
EMPV1_31444	GTGGAGTGTGCCAACAATGCCCTGAAAGCAGACTGGGAAAGATGGAGACAAAACATGCAG
EMPV1_31446	AGAAGGTGAATGGGTTAGACGGATGGAAATGCACAGGCAGGGACATGGATCATGGTGGTG
EMPV1_31448	GGGACTGAATCCGAGCCACAGCTGTGACTTACGCCACAACTGCAGCAATGCCAGATCTTT
EMPV1_31449	CCGCAAGCAGGATTCACAGCAGTACTGACCCTCCAAAAATTCCCTCAGGCTTTGTAATCA
EMPV1_31450	CAGCTTCCTTCGTCGCAAAACAGACTTTTTTGTTGGAGGAGAAGAGGGGATGGCAGAGAA
EMPV1_31451	ACAACTCTTCTGGGCCATCCAATAAACACGTGGACCCTCAGCACCGCTCGGCTGGCGCCT
EMPV1_31453	CTGGAGGCCTTGGTTGAGAGTGGAAAAGGGCTAACAGAGCAAAAGAAAGGACACTCAGTC
EMPV1_31460	GAGTGTACCATTTCAACTTTGATTTTCAAGTGCTGGCTGGTATTGGTTCCAGCGCTAACC
EMPV1_31461	TAGCTCGGGTCCTATGTTGCTATGGCTGCAGTGTAGGCTGGTGGCTGTAGTTCCAGTTAA
EMPV1_31464	ATATTGTCCAATATAGAGAATCATTTGAAGAAAATGGCTCTCTCTACATAGTGATGGATT
EMPV1_31465	TGGCTCCCCCAAATGTGAATTCAAAGGGTTCGGGTTGGGTGCAGGAAGGGTCCTTTAAAA
EMPV1_31466	AACCAAGTGAATAATCTGGGGAAGTTGGTGAACTGTGACATGTGTGGGGCTCATGGCTCC
EMPV1_31470	GGTAGAAGAGGCAGGCTGCAGCAGAGAGATGCAGAGTTCCCTTGCACATCTTGACTTAAA
EMPV1_31472	GACTGCCTTCTTCGGAACTATAGCTGTGGCAGAAGTTAATCCTCAGCATGGCCATTATTT
EMPV1_31473	AAACTGGACACATCCTACCTGTCCATCCAGAGTAGAATGGATAAATCCAGTGTGGTTTTC
EMPV1_31475	ACCCTTCATCTGGAGCCTGGAAAGCCACAGCCACAGCAGGATGAGGAAGAACAGCAGAAA
EMPV1_31477	TTTGCCATGTTCTAGGGATGTGTCGTGTTTCCTGTTGTGCAAGCGCTTTCTCGTGCTCAC

EMPV1_31482 GGAATCCAAGGATAAAGCCAGCGTGGGGAAGAGTAGCCTGATGAATTGAGACGGTCAGTG
EMPV1_31483 AGGTGGGGTTTGGGGGTCAGATTCCTTAATTTTCATTCCGGTTCCACCTTCCCAAAGGGA
EMPV1_31484 GCAAGCCGACCACTTCCAGGCTCCAGGAACACCACAAAGCAATATGAAACCTGTTCATGA
EMPV1_31485 AAGCACACACAGGAAGTTTGCAGTGGAGACAGGGTCTTTACCTCTGACCCCCAAGGGTTT
EMPV1_31489 CTATCACTTAGGGGAGCCAGAGAGGGAAGGGGCACACACCAGCAAAATCTTCCTAGACAA
EMPV1_31491 ATTGCCTGGGGATCTAGTTAAAATGCAGATTCTGATTCAGCAGGTCTGGGTCCAGCTGGA
EMPV1_31497 AACTTGATTCTTTAATTGCTGTCCCTGACAGTAGATAAGAGCCACCTCTCCCTATCTTGG
EMPV1_31499 CCCCCATTTCAAGAATGCAGTGAGTGGCTCAGAATGTCTGCTTTCCATGGTTGAAGACGG
EMPV1_31500 CCCTGAGAGCAGACGGCTTCGGAGAGAATCCTTTCTTTCACTGTTTGGTAGCAGAGATTC
EMPV1_31501 CCTAGAGGCATCAGGGAAGAGAAGTCAGAACACACAGAGGGACTCCTTTACTTTCAGTTT
EMPV1_31505 CGATACGGCGAATTGGGAACAAACACACTTACCTCCTGGCCCTCAAAAGATCCGCTAGGT
EMPV1_31509 CTAGAACGGCATACAATTTGCATAAAACACACTGTGGCCTTCCTGGTGTACCTTCCAGTC
EMPV1_31510 ACACACCCAGCCGGCTGCCTGCTGAACAAGATGCTGCTTAGTCTGCACCTTTGTTTCTGT
EMPV1_31514 GGCCATTCGAAGCAAGATTGCAGATGGCACTGTGAAGAGAGAAGACATATTCTACACGTC
EMPV1_31515 AATATCCATTTTCTTCTCTGGACACCATGGGCTGTCCGATTCGGCTCCACCAGCAGTACT
EMPV1_31518 TGAAGAAGGCTCGGTACAATGCTCAGTGCCAGGAGACCATCCGTGTGACCAAGCCCTGCA
EMPV1_31520 CATCGGAAACAAACCCAGCTTGTACCCATGAGGATGCAAGTTCGATCCCTGGCTCTACTC
EMPV1_31521 AGGTTCCAACAAACCAAGGCAGTACCATCTGGAGCAAGGGCAGGTGGAGCTGGGCTTTTA
EMPV1_31523 AAAAAGAAGATGGAGAATGAGTCTGCCGTGGAGGGGGGAGGATTCGGCGATGACAGACATA
EMPV1_31524 TCTGAGACTGTGATCAGGGGAGGTGGAAGGGGTGGTGAGAGGAAGAAAGAGGAAATGTCT
EMPV1_31525 GTCCTCAGAGACAGGAGCACTAGGATCAATGTTAGGAGATGCCGCAGGATGCAGGAAAAT
EMPV1_31527 TAGTGGATAGCTGGCACTGGACTGTTGGACCGCTCTTAAAACACTCTTGGTGAGTTGGCC
EMPV1_31528 CACTGTACCTAAATAGAGAATGCACCTGTACTCTCAGCAGTAACTACAAATAAATGGAAA
EMPV1_31529 AAATGCGACCTCTGGAAACCAGCAAGGAAACTCTGAGAACTCCTATGAGCCACCAAGAGG
EMPV1_31531 ATGCACAAGATGCCCGAGAACGAATACTGCAAAGTAAGACAGAAGTCGCGAGAGTGCCAC
EMPV1_31533 CTTCCAGGTTGTAAATTTAAAGATGTTAGAAGAAATATCCAAAAAGATACAGAAGAACTA
EMPV1_31535 TTTCTGACTCCCAGTCCCAGACCTCAGCCAGATACTCTGGTCTTCATACACCCTCACCAT
EMPV1_31536 GGGGTCATCATCACAGCCACCGTCATCCTGGTGTCGGCCGGGGAGGCCTACCTGGTGTAC
EMPV1_31542 TGGTTCACATTGAGTCCAGGAAATCCCGGCGAAGAAGTTCGGAGGTTGAAATCTTCGTGG
EMPV1_31543 AACCCTCAGGTTCATCTGCACACTCAAGTTTGAGAACCACTGTCTTGGCCCGATGAGTAG
EMPV1_31544 CTGCAGGTGGAACTAGAAGAAAAGTACCAAGATACCCTTGTGAAGGAGAAGGGCAAGTGG
EMPV1_31547 TTCTCCGCAGGAGTTAAAGATGAGGTTGCGGCCGAGGGGCGCTCGCAGGCAGTAGCGTGC
EMPV1_31548 TACGTGATGGGGCTGGAGAGCCGAGTCCGGGGTCTGGCAGCCGAGAACCAGGAGCTGCGG
EMPV1_31549 GTGTTGAATACCAGCTCTCCAGCCCAACAAGCAGAAAACGAAGCCAAAGCCAGCTCTTCC

EMPV1_31551	TAGTTGCTGGACTAATGCTCACTATCATTGTCATTGTTGGAGCCATTCTTTTCATCCCAG
EMPV1_31552	CTCACTCTTTCTGTTGCTTTGCTACTTCAGCACTCGGGAAAGTTTGAATTTCCCAGTAGT
EMPV1_31553	AGGAGGCGTTGCTCAAGAACCTGGATGCCCAGTACAGCGACCAAAACAGATTGAACCTCT
EMPV1_31555	GAGGGCTTGGGTTTGATGCCACGTGTTCTCTGGTTGTTTTGGATAAGGAGTTTCGCCCTT
EMPV1_31556	AAGCGTCGCGGTGGAGTTGGGTCGCTCGAGGTCCTGGCGATTCTGAATCGAGGATCCCAA
EMPV1_31557	GGACTTGTGTATATACACATCGTGTATAACCCGACCAGCATGTTTTTTGTCAGTGGATGG
EMPV1_31559	CCTGACTGCAGATATCCAGGAAATGGTGGTAGTGTAGACAGTGGTCCACCTGGCAAATCA
EMPV1_31562	AGGTGGGGTGCGCGATGTGTCAGCAGAGCCTGCCGAGGCACTTGCTGGAGATTCACGAGA
EMPV1_31564	GCTAGGGATCTAATCGGAGCTGAGTTGCTGGCTTGCACCACAACCCCAGTAATGTCAGAT
EMPV1_31567	AAGCCTCCATCTGGGCAAGGCACGTAATCATACCCACGCTGTGCAAAACCTGTCCAAAGT
EMPV1_31568	ACATTTATCTGGAGGCATGCATGGAAAGAGCCAGGACGGTGGTTCCAAAGTCCCAGCATG
EMPV1_31571	GCAAGAATAAAATTTAATTGTGCTCAGGTGTGGGCAGTCCATTCTGTTCTGCCTTGGGAG
EMPV1_31572	GCCTAGGTGGGCATGGGTCGGATAGTACAGTGTGACCATTGAGGAGATGTTGAGCTTCAA
EMPV1_31574	AGTCATGTACTTTGTTGGTGCCATTTTTGGTTTTCTCCCTATCTCAGGGATCCTTTTCTC
EMPV1_31577	AACTGGTCGGTGATTTAGGTAGTTTCCTGTTGTTGGGATCCACCTTTCTCTCGACAGCAC
EMPV1_31578	TGCAGCCCTATGCCAGCTACACGGAGAAGAATAATCCACTTTACGATACCACAAACAGAG
EMPV1_31579	CCTTTGTGAAGTTACTCACGAAAACTTACCCAGAGTGGGACGTAGCTACAGAACCTGTAG
EMPV1_31581	CTTATATCCTGCTAATTGTCTGCCATCAGTCTAAGGATGGGGCATCCAAGGCTCTCTCCA
EMPV1_31582	GTATACCTGGACCCGAAGTGGTAAATATTCTTCACAGAGATCATCCCACAGCTCCTGCAG
EMPV1_31586	GATCGGGCAGCACATTTCCTACATGGCTGCAATAGCAAGAAAGCCTATTTCCTGTACATG
EMPV1_31589	GAGGCAATCATGAAGCCCCTTGATCATAAGAATCTGGATCTGGATGTGCCATACTTTGCA
EMPV1_31591	TTTTTGTCCTCGTACACATGACATTAAGCTGGCCTCTGGGCCTTCTCCTCTCTACCTCCC
EMPV1_31595	CATGGTAGGCTTTCATTCATTCGTTTGCACATGCGGTGAAGGTCTACTGTGTGCCAGGCC
EMPV1_31597	CCGAGTCCCCATAATCAGCAATGATGTCTGCAATGGCCCCGACTTCTACGGAAACCAGAT
EMPV1_31599	GCTCAGTGGTAACAAACCCAACCAGTATCCATGAGCATGAAGGTTCTATTCCTGGCTTTG
EMPV1_31601	GGCAGAGTGTGTAACAGTTGCCTTTATTTCCTGATACCTTCTGCTGGCTTCAGATCATAT
EMPV1_31603	AATAACCCCCATGCTGCTTCCTCCCAAAGCTTAACTTCCACATGCAAACGCCAGACTGTC
EMPV1_31604	TGAGTGGTACCTCCACCGGAGTGCCAGGATGGTGGGCAGCTACCGTGCAGCCATGAGCCT
EMPV1_31605	AGGCACATTCTATAAACAGATACTGCAGAGTGCTTGGTCAATCAATCGGTCACTCAAAAT
EMPV1_31607	AATACCAGAGGAGGGAAGAACCGAAAACCTGAGCGGAAGGGGCTGGAAGGCAACCTCCTT
EMPV1_31608	GCTTCACTGCTGCAGATTGCTGATGGTTTTATCAAGAGCGTGGTGACATCATTCTGCCAG
EMPV1_31609	CATCATTGATGCTTTGGTTGTTTCACTCTGTTATTTGATCGAAGAATTTTGTCTATTTAC
EMPV1_31617	AGCAGACGGTTCCCAAAACAGATGCTGCTAACCCTGCGCCTCACATGCTGGATTTTTCAG
EMPV1_31618	CGAGCCCAGACGCATCCATCGCCCCACCCAGAGAAAAATGCTCAGTGTTTTCACGTGAGA

EMPV1_31619	TGCTAAGGGTCAGCTGATTTCTGTCATTTTGCTTAGGAGAGTCAAAACGAGTTGGAGATG
EMPV1_31620	CCTTTCTCCCTCTCTCCATCTGCTCATTCAGTGCATTCCATCCCTAGTGCTCTTAGTCCT
EMPV1_31622	CAAGGTTACTCCTTTTTTAATGATGCTGAAGAGACCAAGGCTGCCATCAAAGACTATGCT
EMPV1_31623	TCAACTACTGTGAAACACCTCAATTCAGGAATGTGAGCAAGATCTCAAAATTCACCAGTG
EMPV1_31628	TTTACGCCAGCCACCCTGCTTTTGCCCAGGTTCTCACGGTATTCCAAGGAAAGGTGTTGA
EMPV1_31629	ACCAAAATGATCTGTCCATCTATTTCTGACCTAGGGACCCGTCATCAATTGCCCCCTCCC
EMPV1_31631	CTGGTGCCGTCGTGATGCCTCAGGCTGCCTCATTGCACATGGTGGTCGTGGAATTCAAGG
EMPV1_31635	TACAGCTACCCGAGAGGGGCAATGCCTAAGTCAAAGGAACTTGTTTCTTCAAGCTCTTTT
EMPV1_31636	TGTAAAACATCTGCACATTTACTCCAATGACGACAGGATGCCTTGCAGGGGCCAGCAGAG
EMPV1_31637	GTCTTAGCAGTGGGCCGACGCATTTTCTACATTAATCAGTGTTTCTGACCAAGACCATCG
EMPV1_31639	CTCCAGGAACTACCTTCAGGAAGGCTACATGGAGAAGACGGGGCCCAAGGTACGTCCACT
EMPV1_31641	CTGCAGGGGAGGAATAAAGAGTGCATCACTGAAGGATTTATGTCTTGAGGACAAAAGGCG
EMPV1_31644	CTTGTGCTGTTGGGACCATCCAAGGTGACTGTTAGAAATCCCTCCCCCACTGGCCTCTTC
EMPV1_31645	TCCCTGCCAAGAACCTCCCTGAGCACAGGCTTCTGTCTGTGGCGGATGCAACCTGTAAGA
EMPV1_31646	GGGCCCTCGGGTCATTTACGATGAGAAGAAGAGCACCGTCTACACCTATTCCTATTTCCA
EMPV1_31647	CTGTGATGCTTATGACCCACAGAGTAAGAGGTACTGTAAGCGACTCCAGGTATTATGCCC
EMPV1_31649	ACAACCAAAAAGGTCACACAGCTGGACCTGGACGGGCCCAAGGAACTTTCCCGGAGAGAA
EMPV1_31652	AGTTTTGGAGAATTTAATTCAGATACTCAGGAAAAACTGATAGACTCTCTTAGTAAAAAA
EMPV1_31653	AGTTTTTTCCGTAGACGTGCTCTCTGCAACCCCCACAACCCTCTGGGCACCTCTCTTCTT
EMPV1_31654	CGAGGTCTTTGAAGTCTACGAGATTGGGAACCATGAGCATTTCAACAGCTGGAACCAAGG
EMPV1_31655	GATACGACTGGCTGTGGATGAGTTATTCTGTTCCAGGATTGAACTGTGTGAAGAGCGTGG
EMPV1_31656	TTGCCTGCACCATATCATGTGTTTGCCCACTGTGCCACTCGCTGTGTGGTAGTTTGTCAC
EMPV1_31657	AGGTCACAGCTGTGGCTCAGGTTTGATTCCTGGCCTGGAACTTCCATATGCTCTAGGTGT
EMPV1_31658	CTGTTTTGTCTTGGCCCTTAACTTTGTGTAGGAGGCTGCACACTTGTCCCAGTGAAACGG
EMPV1_31660	TCCCAAAGATCCTAGAGAACCTGAGACTCCAAAAGCGTGGTACTGGAGGAGTGGACACAG
EMPV1_31661	CGTGAGCGTGGACCCCTTCTATGAGATGTTAATAGCTGAGGAGCCAGAAGTCCAGATGGT
EMPV1_31663	GATCCACCATGCTATGGATCTAGCCTCATGCGGACACACTTTTGAGATACTTTTCCTCTT
EMPV1_31666	CCGAGGAAGATCTGGAAAGGCAGCTACAGGACAAGCTCAATGAATCAGATGAACAGCACC
EMPV1_31667	CGTAAGTGCCTGCTGAGACCCCTTTGTTTTGGGGTTTGGGAGAGGCAAAGTGGCAGGCAT
EMPV1_31668	ATCCTCAGGGGATACAGTATCCTTATTGAGGTCTCACAAAAATCTCTTCCTATCTAAGCC
EMPV1_31669	TACCATGAACAGCTTTCTGTAGCAGAGATCACCAATGCTTGCTTTGAGCCAGCCAACCAG
EMPV1_31670	AATTTCCTCCACCATTCGTCATGCAGTGGAGGAGAGTGCGGGGCCGGACAGAAGGCTGGG
EMPV1_31671	AACATGCATTCTGATTGTAGTGGTTGCCTGGATCATTGGTCTAACCCACTCATTGGCCCA
EMPV1_31672	TTCATTGCTGGGCCCTGGACAGCCATGCCAACAAAAGCGGCCACTCATGGACCCAGAACA

EMPV1_31678	AGTGAAGAAGCTAAGAGGCTAAGAGAAGAACGCCTTGCACAGTATGAGTCAAAGAAAGCC
EMPV1_31679	TGTTGAAAGGACACCACAGGGACCACTGGCAGTACCTATGTGGAATAAGCACGGATGCTA
EMPV1_31680	AACAAAAAATGAATAATTTTATAGAAAAGGTGAAAATCAAAAGCAGAAAGTTTTCCAAGA
EMPV1_31681	GCTTCAGTTAGGAAGTTTGAAGGGCTGTGGGGGAAGGGTGAAACATGCGGAGGCTACTTT
EMPV1_31682	ATACCAGGGGCTGGATGGCTTCAACATCAGGCATTTGTTTGTCCACACTTCCGGAGATGA
EMPV1_31684	TCTGTGACCTACACCACAACTCAGGGCAAAATACTGGATCCTTAACCCACTCAGCGAGGC
EMPV1_31685	AAGGTTAGCTCATGGTGGGCAGGTGAACTTGGACATGGAGGATCATCGGGACGAGGACTT
EMPV1_31686	ACGACCCAGAATAGCAAAGAAGACAGAAGCTCAGCACACCCAGAGCTGCTTTTAGCTGGG
EMPV1_31687	ACTGCGGCAATCCCAGCTCCTTAACCCATTGTGTGAGGCCAGCAATCAAACCCACATCCT
EMPV1_31688	TGGAGGAGCAGAACCGCGTGGACTTGACGCTGATCCACTGGCAGCGGGCGGAGCTGGCAT
EMPV1_31689	TGGAATATCTTTGCTCTACACTCTGCTGAGTCATGGGGAGCAGCTGGTATCCCTGGATTC
EMPV1_31690	CCAGTTCTGTAAGGAGTTCAACGAAAAGACAAAGGACATCAAAGAAGGCATTCCTCTGCC
EMPV1_31695	GGGTCTCTAACCACTGCGTGACATGGCTTGTTCTTAGTTCTCTTAATCCTCTCATGGTAC
EMPV1_31696	ACTGTGTCAGATGCCATGCGGGGGGTGATGTCCAAGAAAGCAGGCCCACAGCTCAGGCCA
EMPV1_31698	TGGATCCTGAAGCAGAGGGACCAGCAATTATCACGGTGACACCTCTTCAACAAATGCTTG
EMPV1_31700	TATGGTGTAGGTCTCAGGTGTGGCTCCGATGTGGGGTTGCTATGGCTGGTTATGGTGTAG
EMPV1_31702	TGTGCGCTACGGTGGTCTTGGACAGTGTGTGGAAACAAGCTCCGTGACAGTAGAAGACTT
EMPV1_31703	ACAACTGCTCCTGCACCTGGGCCTTACATCTTATCGAGACTTCCTGGGCACCAACTGGGC
EMPV1_31705	AGGATGAAGACCGGGCCGAATTCAAGGCCCTCTTTGACCTGGACAGTGATGAGGATGACT
EMPV1_31706	CCCCCCATTCATCCATAATAACTTCAAGAACCGGCTCCTCCTGAACTGGGAAGAGGGTAT
EMPV1_31707	TACAATTAAAGTTGGTGGTTAAGAAAGGAAATCAAACTGAAAATTTATACATAGAATTAA
EMPV1_31711	GAAGCACTTTATGCCTTTTTGAGCCCTTCTCCTGACTACCTCAAGGTTATTGACGTGCAG
EMPV1_31712	TGGGGAACCCAAAGTGTGCTTAGCAAAAGGGGCTTTCCCAAGGAAAGCCTTTGTAATTGG
EMPV1_31713	CCCAGCCTGGCACATTAGTAGGCCTCAGTAAATGTTTATTGGATGAATAAATGAATGACT
EMPV1_31714	CTCGTGGCGGGGCAGCTGGTTCACCCATTCAAACGGAAGGATGAGATCAGTTCATAGGAA
EMPV1_31715	TTCATGTGATGCTGCTATGAACTTTGACCCGAGATGGGGCGAGGACGGCAGAGACTTCAA
EMPV1_31720	CTGTCACCAGGTAGCCTACGCTCCAGCCTTGGTTGCATCATTAATCCCTTTATCCTGCAT
EMPV1_31721	TTTGGGATGTACTGCATGGTGTTCTTGGCGCTCTACGTGCAGGCCCGGCTCTGCTGGAAG
EMPV1_31722	AGGGCTCATACTGGGCCACCCTGGGAAAAGCACCTGGAGAGACAGGTTTACATTCTAAGA
EMPV1_31723	TTTCTGCCCACACGAATGTGCTTCTATGTCAGTGGGAGGTGTTCTCATTGGTGCCTCTGG
EMPV1_31724	CTGCTGCCTGCTGGACAGGAAGATCTACATTGTTGGGGGCTACAACTGGCGTCTCAACAA
EMPV1_31725	AAATAAAGTGAACGAGGTGCTGAACAGACTGCACCTGGCGGTTCCCAGCAGAAGAGATGA
EMPV1_31726	CATCTTTGTCTACTCCATGGTACCAGGTAGTTTACTGTGTGTGCAGTAGCTACTACGCAG
EMPV1_31728	ATAGCCAGGAGAAGGCTGAGTGGCTAGGGGGTGCAGTGGAGGAGTATTTCTTCCGCAATG

EMPV1_31729	CTCACCAGGTACAAATTACTGGTATGTTTTATAAGCCGCAGCTACTGTACACAGCCTATC
EMPV1_31733	CCCCACCTCACTGCTGTGCTCCAGAACAAGTTTGGCCTGTCACTGCTCCTCGTCATCCTG
EMPV1_31734	GTGAGCACTAAGGGCAAACAAAAGTCGGTATTTAGGAACCATCCAAGTAAAACCCTGGCC
EMPV1_31737	TGAGCGCTCGCTAAGTGAATGTTGATAGTGGGCTAGGAAAGGACAGTCAAGGAACATGAC
EMPV1_31738	TCACACCGAATCACCTCGACCGCAACAGCGAAACCGCCAACAATGCCAGTCGCTTCTGAA
EMPV1_31739	CGGAGAATGAGGGAAGTCAAGATTAACTCCAAGGTTTCTTCCTTGTGCCCCTGAATCGGG
EMPV1_31740	TACTCTGTCACAGAATGGCTAGTCGCTGTGAAGTCAAATGCCTTTCCTTTGAAGATAAAC
EMPV1_31741	GCTGAGTACTATAGACAACAAGCAGCCTACTATGCCCAGACAAGTCCCCAGGGAATGCCA
EMPV1_31743	ACGTGCTTCTGGTGTCCCACCTGTCCCCTCTCAGCTCTCTGTTCTACTTCAGGCTTCACT
EMPV1_31744	GCCTCCAGGGGAAACGGACACGTCAGGAGCCTTGAAGTTGAACAATAAGCCAGAGCCTAT
EMPV1_31747	TAATGGCACCCATTTACTAAGCAACAGGCTCAGCAACTGATCTCAAACCATAGTCAGACT
EMPV1_31748	AGGCCATAGAAAGAATTGGTACCGGAGTCTCATATGACACCAGAGCTCTGCCTCATGGGT
EMPV1_31751	AGACCTTGCAGCTGGATGTGAGGGATGCAGATTCAATAGCGGATGCCCGGGCACACGTGA
EMPV1_31754	CTACGGTCTAGTTGGTACCCACACATCGAAACCCTCAAGCTGGATTCGGTCACTCCGTCT
EMPV1_31755	TATCACCCCAGCTGTCAGGGCCACACAGCTATGCTTGGAGTTATGGGCACCTTCCAGGGT
EMPV1_31756	CAGCCTTGGCTGCAGTGACCTTTTTTGCCACATCCAACAGTAAGCCCCCCAGGCTTCACT
EMPV1_31758	ATGGATATCACCTCCTTAAAGATACTGACTCCCCACTCTGGACGAGGTTGCCCACCCCAT
EMPV1_31759	CCCCGTGCCGTCTCTCCCCCAAATTCTCTAACTTCTTGCAGATACCTCACACAGTTTGTT
EMPV1_31761	CCACAACCTCATGGCTCCTAGTCAGATACGTTTCCGCTGCACCACGATGAGAACTCCTAT
EMPV1_31762	CAATCCACACCACAGAAAGGACGAGGAAGACCATCAAAAACGCCATCACCATCACAACCC
EMPV1_31763	GGCAGATGAGCTTCAGAAACAAAAAGAAGAGCTACAACGTCAGCATGACCAACTTGAGGC
EMPV1_31764	CATGCCAGGGTAGTTCCCACTGATATAGACGGTCCAATGTAATCTCATTCAGGAGGCCTG
EMPV1_31765	CCCCAGGCAGTTGCTGGCCTGAATATGCTGCTAACTTCTAGGGCATCCTGACATATTTGT
EMPV1_31766	GCTACCCTGAAGGAAAGCCAGCGAAAATAGTAGATAGAGCCATCCCCTCCAAAAGACTGC
EMPV1_31767	CATTCTTTAGTGGCAGACTGAAGGCCAGAGGGAACATCATGCTGAGCCAGAAGCTTCAGA
EMPV1_31768	CATACGTGGAAGTCATAACCCTTAGCCCCTCAGAATGTGACTGTGTTTCGAGATGGGCTC
EMPV1_31769	ACTGCAGCTGGAGCAGCAGCTCAGCAAGTGGTGGATCAGGCCACAGAAGCAGGGCAGAAA
EMPV1_31770	ACCACGGATGTCACTATATACCAGAACGCAGGAACTAGCCTGTCCCAGGTGCCAAACTAT
EMPV1_31772	GAGGAAAGAAGCAGTTCCATCAAACAAAGCAGAGAGCCTGGTGCAGAGGAAAGACTCAAA
EMPV1_31773	ATGGTGAGAGTGGGGATGTCTCAGGTGGAACACCCTGGTAGCACCTCCAAGTGCTTATCA
EMPV1_31774	AGGAAACAGTGTGCTGCCACACACAGGCCTTGCCACCCTTGTCAGAGAAGAGGCAAACCA
EMPV1_31775	TCCCAAATCACAGGCCGAAGCTTCCTGAAGGATTTGTTCCAAAGAGCAAACCCCGACTCA
EMPV1_31776	ATCCAACGATGAGCTTCTCAGCTGGCGCCCCGTTGTCCTTCCAGTAGTTCATGGCATAAT
EMPV1_31780	CTCTGAGGAAGAACTGAAGCCACAGCCCATGATTAAGAAAGCTCGCAAAGTCTTCATCCC

EMPV1_31782	AGGTGGCGCCAGAGGTCAGAGCTTACCTGTTTTGGATTAGATAAGCATATTTGGCTGTGG
EMPV1_31783	TGCTTGAGGATGGACCGCCGCGAAGCAGGAGGATATCGTGAATTAGTACATTCTGTCTCT
EMPV1_31784	GCTAGCTGGCCATTTCAGCTTGCATCTCCATTTCTGGGGGTTGCCATAATTAGAGCGATC
EMPV1_31785	GACCGTGGTCCCCTTGTATTTTATGACTGTGTGCAAAACAGGGCTAACCGTTTGGACTGA
EMPV1_31788	CGGTCTGAGAGCGAGCGTAGTTGCAGGCGGAGAAGAGAAACTCGGGAGGCCACTGTGGCT
EMPV1_31790	CTGTTCATCTATCCATCTGTTCCTTTATGTGTTTTTCTGTCATACTTACACCAGTACGGG
EMPV1_31792	CATATCAACACACATGTGTATGTATTCCTATTCCATGCACATGTATGTATGTGTATATAT
EMPV1_31793	TCAGTTAAAGGGAAAATATATTCCATACTGAAGCAGATAGGAAGTGGAGGTTCGAGTAAG
EMPV1_31794	TGGGTTCCCACCCAGTCAGGTGAGGCTCCAGGGTACATTCTCTTAAGGACTACATTGTGT
EMPV1_31796	TCCTCTGGCAGTCCTCCAAGGACTACGCTGTTGGGGACCATATTTTCTCCTGTCTTCAAC
EMPV1_31798	AGGGCCTCAGCCACACCTACAGTTGTTTTCAACAGTCATCAGCTTGGTCAGGTTACACAC
EMPV1_31799	AAGCTGCAGTGTTTACTTTTGGGGAAAAATAGCCTGATGAGTCTGTCCCCTCACGTGGGC
EMPV1_31800	AAGTCGAGCCTGTTGACGCCCGCCCTGCAGCCGACCGAGGACTGACCACTCGACCAGGTT
EMPV1_31801	TCAAATACTCCTGCCGCTCGCCACCGTTCTAGGGCTTTTCAGCAAAGCTGCCATCATCTT
EMPV1_31802	AGAAACAAGCCCTTTAAGTTTGTGCTAGGCAAGCAGGAGGTGATCCGAGGCTGGGAAGAA
EMPV1_31804	TTACATAACCTCTACGTGGGAGTGCCCGTCCGGGTGCAGCAGAAAGGAATCTGTTGAGAA
EMPV1_31805	TCCATTGCACAGATGAGGTAAGTGGGGCACAGAGATACTAAGATGCAGAACCAAGGTCTG
EMPV1_31807	ATGATGATCATGGAGTATTACCGGCAGAGCAAGGCCAAGAAGCTGCAGGCCATGCGTGAG
EMPV1_31809	CCCAAGTCCAGTTGTTCTGGGAAGAGGATGAAGACCGTGGGCAGTAACATAGATCAGGTC
EMPV1_31810	GACAGGCACCTTGGGGCTGGTGGAATTCAAGACGCTCTGGTGGAAGATTGAGAAGTATCT
EMPV1_31812	ACACCTAAAAATGTGCATCAGAGTCGAAATGCAGTCCCGAGGCTACGAGGTTGACGAGGA
EMPV1_31813	ATCACTGTGGCTGCAGTATAGGCTGGAGCCTGCAGCTCTGATTTGACTCCTAACTCAGAA
EMPV1_31815	ACATTCACAATGACCTCATTAGAGACAACAAGAACCTAGGGGAGCCCATCCAGAGGGCTG
EMPV1_31816	TTTCGACGCATCTTACATGTGACACAGGCTCGTATAGCTGCTGGCTTACCAATGGCAGTG
EMPV1_31817	CCTGGACAGTGATCTCTCCCTGCTCCTGCTTGCCACACCAGTACAATTCACGAACTTCAA
EMPV1_31819	TAAGCGACTGTTCTGGTTTGCCTGGGACTGAGGGGCTTTCCAGGATGGGGGACTTTTAGT
EMPV1_31820	GGGTAGGATGTTGCTTCTGAATCACTGTTGAGTTCTGATTGTGTCGAAGAAGCGTTATCC
EMPV1_31822	CTAGAGGAGACTAGAAAAGCCACAAAGACATCTATCACTCAGCACTCGAAAGGGGCACTC
EMPV1_31826	AACAGAAAGCTGTGAAAATCAAAACAAAAGATTCTGGCCTTTTTTGTGTTCCATTGACGG
EMPV1_31827	CCAATTACACTGCCCTAATGCCTATTACAACAAGTTTTGAAAGAAGAGTTAAAGCATTAG
EMPV1_31828	ATCCTTCTTTAGCAGAGACTGTTAGCCCGTGCCGGGGTCCCGATTCACAAGAGAAAGCTA
EMPV1_31829	CAACTACCCACCAGGCAAGAACATTGGCTCTTTGCTACGCTCTCACTATGAACGCATTGT
EMPV1_31830	TCAGCAGGGAGCCGTAATTGGAGCCTCTCTGAAGGGAGGGAGCACAGGAGGGCCCGTCAG
EMPV1_31832	TATTAAACATGAAAAATAAGCTTAAACAATCCCAAAGCTCATCAGTTACAGAAAGAGATC

EMPV1_31834 GAGAACTTTTCCTTTCAGGGTTTAAGCAAAGGCACGCCAATCATGTAACTCCTGTCATTG
EMPV1_31835 TCTAGAGCTCCCCCAAGACTGACTGTAGTGTCCCCTAACCGAGCTACTTCAACGGAAACT
EMPV1_31836 GCAGTCTTTGCCACTGTAAACATCCTTGACTGGAAGCTGTGAGGCGTTCCCAGGAGCTTT
EMPV1_31837 GAGTGGCTGATGAGCAAGTATCTGTTGTACTCGTGACAGGGATTCTAGGAGCCATCCTGG
EMPV1_31839 GAATCCTGAACCTGTTTGGGGTGTGATATTACTTCAGAACATGTCCGGCACAAGATTTCC
EMPV1_31840 CCTTGGTAGTTTATTTGTGGGTGGCAGTCTTCTCTAGTTCTTGGGACTGTGACAGCCTTG
EMPV1_31841 CCCATAGCCTTGGGGCCGTTTCAGAGCCTTTCTGCTCCTCATGCCAAGGATGGGCTGAAC
EMPV1_31850 AAGGTTTCTCAGAGCACAGAGGCCTGGGGAAGATGGTCAAGGGCAGGGAGGCTTTACTAG
EMPV1_31852 CCCAGCACTGAACTTGGCAATATTTCATCAGCCTGAGTCCTTCCTGGGTTTCTTCCTAGG
EMPV1_31854 GTCCATCAGATGCGCCTTGACATTGGGAAGCTCAACAAAATCCGAGAACAAATCCATAAG
EMPV1_31855 CAATCCCCCGTCAAAAGAGTCAAGGCTACATGCCCCCCTAGCACATTTGGGGTCTTCAAA
EMPV1_31856 GGTGAATGTGGCAGGTGTGTCTCTCCAGGAACTGAATCCAGAGATGGGAACGGACAATGA
EMPV1_31857 ATGCCATCCTGGAGGACCTGGTTTTCCCAAGTGAGATTGTGGGCAAGAGAATCCGCCTGA
EMPV1_31858 GTTTCATTCTAGCAGGTTACATCATTCAGCCGTACACCTAGTCAAGGAAGGTGAACTGGC
EMPV1_31859 GTATCCAAGGAGCAGAAAGTGCTGGGCAGAGGAGATTCGGTGACTTTGAGTCCTGTGGGA
EMPV1_31860 GTAGTTTCCAGGGACTCTTGTCAGTGGTTCTGCGGTGGATTCTGAGAACTATGCTCATGG
EMPV1_31862 TGGCTGGCACTCATGGCATCTGGCTGGACCAAGTTAAAAGAAACACGAACCATTGTGCTC
EMPV1_31864 TAGCCATGTGACCTGTGGGCTTCCCTTTGTCATCCTTTGCCCAGGGTTCTGAGTGGGGCA
EMPV1_31866 CCGTTTTAACCGTCTAAGCAGGGGTTGTGTTAGCTGATGTGCCTGTGATTCTAAAGGGGC
EMPV1_31867 TCACATGAGACTGCACCAGGAAGAGACGTTCTATGACCAAGACGAGTGTAGAGAAGACTT
EMPV1_31869 CTTGGAGCAGCTGGCACCAAATCCAGGATCTGAATTTCCTGCTGCAGAAAGGCAGGCTGA
EMPV1_31871 CATCCGCTGTGGAGCCCTGCTGCTGCTGTCCTGCTACTTCTACTTCTCCATCCGAGACAA
EMPV1_31873 CCGCCTTACACTGGAATAGTCTCACACTCTATTTTAGGGCTTTTGTGATGTGTGTTGTGG
EMPV1_31874 GTCACCTTGCCCTTTTGTGGCCCTAATGTCTTAGCTTTTACTGTGATCTGCCTCGGCTCA
EMPV1_31875 TAGTGTCAGGTTCTTAGTCACCCCTGTAGCTCCTACTCTTGATTCCCATCTTCTCCTGGC
EMPV1_31878 CAACGTGGTCATCCGCCAGAGCCTGGGCGACCTGTTCCTGGAGACCTTCGCCTCCCTGGT
EMPV1_31881 CAGCTGAGTGGCGATTTTATGCAATGGCTTCAAGCCACAGTTCTTCACCAGTGCCTCAAG
EMPV1_31882 AATCTAAGGGACAACAGCCAAATGCTGGTGAGGTGGGAGGTGCCGCATTCATCCCCTCGT
EMPV1_31883 CAAGTCCCAACCCACGTTCTTGGGGCCTCGTGGCAAACATGGCTCAAATCTGGCTCTAGG
EMPV1_31884 TGAAAAAGCACTGAGCAAGGAGCCAAGACCCACCTCTTCACTGACTTGACATCGTACAAG
EMPV1_31885 GTATGACGTTTTCAAGCCACCTGGTTATGACATTCTTCAACATCCCTAATCCTACTAGCT
EMPV1_31886 GAACCAGCTTCTAGCAAAAGGACTGCTGTTTGTGGAGGAGAAGATTAAGCTGTGCGAAGG
EMPV1_31887 CTTGAATCCCGGAGTGGTATCTGCCTTTATACTGTGATTTCTTGTTGAACCTTGGGTGGG
EMPV1_31889 TGCTGGAAGTCTTTTTGACTTGGGAAGTGCGACGGGAACCCAGGTGATGTGGCCTAGTGA

EMPV1_31890 GTCAGCACGGACCTTCAGCCCTACAGGGAGGACCCCAGCCGCAATGACTCCAGCTACTGT
EMPV1_31894 TGTGTGTGTGTGTGTGTGTTCATGCATGTGCACGCGCGTGCAGACAAGCACATCCTGCTC
EMPV1_31895 CCAGCATGTTTGCTCTTGTGATTGTTTGATGAGGAAGTGCTTTGGACAGGCACGACGGTC
EMPV1_31896 GAGGGAAGATGTCTCAGTACCTGAATAGCCTGAAGCTTGGGGATGTGGTGGAGTTTCGAG
EMPV1_31897 CCCCCCCAATCCTGTTGTGCTGGATTCAGAGGGTTGGGCGGAGGCTTTCCTGAAGGCTCT
EMPV1_31899 ACCTGCCATGCGTGCTGGTCACTGATTCACAAAGGGCTCAGGGCCAAACTCAAGACAAGG
EMPV1_31900 TACTCCTACCAGTCCACCCGGATGCCAACATTAATACTCAGGACCCCAAGATACAGAAGG
EMPV1_31902 CGAGACCTGGATCCTGGCAGTAGCCACAGGCCTCTTTCTCTACGTGGCGCTCTGTGACAT
EMPV1_31905 AAGATGCTTCAGAAAGAGCTCAACCATATGCTGACGGACACGGGGAACCGAAAGGCTGCT
EMPV1_31906 GTGATATACACCTACTTAGAACAGACTGGCAACAACTACAGGTGCCCTGCTCTCCAACAT
EMPV1_31908 CTCAATAGAGATTTATCCGGGAGATGGATCTGTTTTCAAATCAGAAGGAGCTTATTTAGG
EMPV1_31909 ATGTTCGTTGGTGGCCTGAGCTGGGATACCAGCAAAAAGGACCTAAAGGATTATTTTACC
EMPV1_31911 CTTTGAATTTGTCACCTTATTCTCAGGGCGTGACCCCAAATTTCCCACCTCCTGCACAGG
EMPV1_31912 CTTCTTATAGGACGTTTGATCAGAGTGAACGGAACATTGTGAATCAGCCGCCAGGGCCTG
EMPV1_31917 TTTTTAAACTGGAGACTTTGTGTGCCCTCTGGGCTCTTGAGTTCCCCTGCTGTGGGCTTG
EMPV1_31920 GCTATGAATAGCTCTCATTTAAGCATACTTGGGCGGAAGTGGGAAGACAGGTCTGATTTA
EMPV1_31921 TCATACTGTCAAAGACAGGGAGTTCCAATGATTTCACTCAGGTTTCTCTTTGAAGGTCAG
EMPV1_31926 TTGTTTCATCTGCAGAATAGCCAGCCGCCGACAGGTCGTGGCACAGTGAGAATGGGCCAA
EMPV1_31928 AAGATCTGTCACTTTTCTCAGAGTCCCCTGCTGGTCTAGAGGTTAAGGAGCCGACATTGT
EMPV1_31929 CAGTCAGCACATTATCAGGGGCCCTGGCAGTTTCCAAGTACTCAGGAAAGACAGATGGTT
EMPV1_31930 AGACCACCTGAGGCACCTCAAGCACTACGACCGCAGGATTGTCGACTAGAAGCCCAACCT
EMPV1_31932 ACAGTGTGGGAACAGTTAGCTAAAAATCTGGTGAAGATTGTACTGACAACAGAGAAACAG
EMPV1_31933 TTGGGCACAGCAGTGACAGCCCAAATGGGGGTGGAGGAAACCTGTAATTCCCTTCTTATA
EMPV1_31935 TACGAGGCTCTGGAGAAGTACCCACTGGCTTATGTTGACTACGTGACTGTGCTGCAGATC
EMPV1_31936 CAGGCACTTGACCCTTGGTGTAGCTTATGTTCAACTTTATATCACGGGAACGGATTGGTC
EMPV1_31937 GGAGTGCGAGCTCTGATGCAGGAAGGAAAGGCGATACGTAAAATAAATGATCAAGTGGAA
EMPV1_31939 TCCCAGAGAAAGGAAGTGGGAGAAGTGCCAGGACAATGTGGGCTAGGAGAGACTAAAGGA
EMPV1_31942 TCACTTGTGCCAATACGGCCATCAATGAGCTGGTCATGTTTAGCATCTGCGGGCTCATCA
EMPV1_31943 CACCTCCACCTCCATCATTTCCACCACCTCCTTTACCTCTACCACCTCTACAGCCTCCAC
EMPV1_31944 CTGAAAGACAACAAGAGTCCCCTGCACCTCGTCCAGATGCCACCTGTGATTGTCGAGACG
EMPV1_31945 TGACCCAGCAGGGCACCCACAGGGTCCGCAGCCCTTCCCCCAGAGAAATCCCAAAGAAAA
EMPV1_31946 AAGCCTACCTCTTAATCTTGTGTGAGAAGAGGAAATAAGCCCAACAGCAACAAGGGCTGG
EMPV1_31947 TGGTAGTGTGAAAGGCATCGCTACAAACGACGTAGGGATACAGAAGGATGGGGCACCAAA
EMPV1_31948 GAATCCGCATCCTCATGGGTACTGGTCAGATTCTTAACCCGCTGAGTCACAACAGGAGGT

EMPV1_31950	TGTCTATCAGACAACATTACACAGCGTATCAACAATTTGTTTCTGTCGTCCTCTAATGCC
EMPV1_31952	CCTAATTTGTCTCCTGTTCTTCAGGATCACTGTCCTCCACTGCCTGATAACCAATGTCTC
EMPV1_31953	ATCTGTCCCTCCTGCTCAGGACAGACTGACACTGGGGGGTCAGACCAACCTCCACTCTAA
EMPV1_31955	TGGTGGCAAGATCATAGGAGCCGTTGGGAGCCAAGAGCATTCATTTGTGGGTATTGGGGA
EMPV1_31960	TCACTCACCGGACGGATGAAAATATCCGGAAGGTCAAGGACTTGGTTTGTTCCAACAGGC
EMPV1_31962	CCATAGTGATGTATTTTGCAGCTGTCCTGCTGGGGGGTGGTCCCTTTGCTGGTATCATTT
EMPV1_31963	TTACCGAGGAAGTCTCAAAGGGTCTCTGCTAAACTGTCACTGCCAAGTACACAGGCTGAG
EMPV1_31966	GCAGGCTGGGGAACGTGGTTGGCGCAGGGTTTGACAGAGGGAAGACAAATAAATACTGTA
EMPV1_31967	CTTTAAACCATTTCCTGAGAAGCTACAATAGCAAGAGAGCTGATAGGTATCCTTCACGTC
EMPV1_31968	AGCTGTTATAGAAATGCGACTGCAGGAAGACCCCAACTACAGTCCGCAGCCCTTCGCTAA
EMPV1_31970	ATCCAAGATGAAGTCCTAACTGGAGGCTGCTGTCTGGGTCCAGCCGAAGAGCTCATGTAA
EMPV1_31971	CAGCCACAGCAACACGGGATCCTAGCTACAACTGCAACCTACACCACAGCTTTCAGCAAT
EMPV1_31975	ACCATCGGTGTATACTGACTTGGCATCACCTAGAACCACTGCCTTCGGCACAAAGTACAG
EMPV1_31977	TCATATTCTGACCACCAGTAAATTTTAGCACTTGGAATTGTGATCATGAAGGAGGGAGGC
EMPV1_31978	TTTCCCAGAATTTCCAGGGCTTCTTTCAGTCCAGTTCTCTCCCCAGCCTTCGTGGGGGAT
EMPV1_31979	GTGTTCTGTATGGCACTCTGGGTCATAGGAAAGGGTCATGGCACATCTCACAGTTGTCTC
EMPV1_31981	AGGGAGACAGTCTTGGGCCTGGATGGCCATTAGTACTTGGAGTTATGGGAGCAATCAGAA
EMPV1_31983	GGGATTCATCAGTGAGCCTGAAAAATGTGTCATTTATTTCTGTGGGTGACAGGAAATGAG
EMPV1_31987	GGATGTCCCTTGTTTATGAAACCTGCAGCCACCAATCCTGGGCAATCTGCCTCTTTCTTC
EMPV1_31988	TTGACACGGAGGTAGCATACGTGGCCTCGCCTTGCTCGAAGGCGAAATGGTGACGCTAAA
EMPV1_31990	TGATGTCCTGGACTAAGATAAACTTCCACCACACGGCGTGGACTTCTGACAATGCCTAGC
EMPV1_31991	TGCTGCTTTCCCATCATTACAAAGTGGGGGTGTTCTACCCACTCTTATGCCTCGTCCCAT
EMPV1_31993	CCTGCTTGCCTTCGTGTCTCTGCTGGCTCCCGCCCTGGCCCACGGCAACACCGTGGTCTT
EMPV1_31994	GGAGAGCCTTCCCACTTTCTCCTGTCTCTGTTAATGATTGCGCCACCAGTTTCCGTGCCT
EMPV1_31995	CCAGTTGCCAATGGATTTGCTTCTGTGCCTCCCTTCTACCAGATCTGCTTCATTCCACTG
EMPV1_31996	TTTAAACTGTCTGAGCAGGTAGATGTAGAGGGGGTGCTGGTAGCCTAGAGATGTGGAGGA
EMPV1_31998	CAGGAGGTGGACATCACACTTCCAGAAAACTCTGCTTGGTATGAAAGGTACAAATTTGAC
EMPV1_31999	ACGGCGAGGGCTTCCGCTGGCTCTCCCAGTATATTGACTGATGTTTGGACAGTGAAAATA
EMPV1_32000	AAGTCCCTGTAACCTCACACCTCCAAACTGTCCAGAAGTCATGCCTCTCCATGGGAAGAG
EMPV1_32001	TACAGACATGCCTCTGTCTTGTACTTCTCCCTTCCCCTTTCCGACATACACCTGAGATCC
EMPV1_32002	TTACCACCTTCTCGTCAGACCTTGAAACCCTCCTACCTGCTATGTCATGGTTCTGGGTTC
EMPV1_32003	CTATAGTGACAGTGCCTCCGATTATTTCATGTGGTACAGGCAGTATCCTGGGAAAGGCCC
EMPV1_32004	GAATCTCACAAGGAAAGCACGAATTCCGCTAACGACCACATTGCGAGCACACTGAGCACT
EMPV1_32005	AAGGCTTGGTTCATACACATGGGCGTCAGGATTGGACATAGGCTTTGGGGCCACCATTCA

EMPV1_32006	GCAGGTTTTTTCCTTTCATTGCTTTAAGCATATTGTGCGACTCCCTTCTAGCCTGCAGAG
EMPV1_32007	CCTGTACCAAGACTGTATTTCTCTTTCCTCCACTGTACCTGCCCTCCTACCTGTACATGC
EMPV1_32008	GCACAACTGCAAGAGCGGAATGATCCTCAGCAACTGCTGTTAGACTTCAAGCACATGTTT
EMPV1_32011	TATTCACTACAGTAGAGACCCCTGAGCTGCCTCATTCCCACCTCCACAGGGTCAGGCTTC
EMPV1_32013	AGCACATTGTTCCCTCGATGTGCCTGTGCTGGATGCTCCAAATGCTGGTCAATGTCGTTT
EMPV1_32016	CCATGCGACTGAGAGAAGGAAAGAACCAGCTTTGCTGCTGGAGGAGGATGAGGGATAGTT
EMPV1_32017	ATCTTAGATGGCGGGAGTAGGACGAGAACGAGCGTGAGACGCGAAGGCTTTCTGCCGCTC
EMPV1_32018	ACTGCAGGGAAAACAACATTGACCATATAACCAAAGTCATCAAATCGAAAAATGTGGATG
EMPV1_32022	CCTGCTGCACATTGGACTCAAAAGGAAAACTGGACCAACAGTAATCGAGGAAATAGACTC
EMPV1_32023	CCCTGTGGAGTCGGTACCTCTTGGCCATGGAAAGGCATGGAGTTGATCATCGAGGGATTT
EMPV1_32025	TCTGTGTCCAGAGGCAGTGCTATGTCACCTGCAAAGACAGTCGAATTGTTATCAGACCCC
EMPV1_32026	TTTTTATTGTGGGGGAGGGGTGAGTAGGGACTGAGGGCGTGGTGATGACAGATCCTTTCT
EMPV1_32027	ACACTTCAAAGTCTGGCTGGAATATCTTGCTGGGTAGGGCCTTGAACTCTGGCAAGGATC
EMPV1_32028	GGGAGCGATGAAAAAGGAAGCCAACACTGACCTGTAGAAATAGTCGATTTCCTCAATTCC
EMPV1_32030	ACAGCCCCCCTGGAAGAGGTGATCTGAAGAGAGGGAACAGGAAGTCCCCTTTAACAGAAA
EMPV1_32033	CCATCTTCTCCTTGCCTCTTGTAGGCCTGGTGGTCATATCGGCTTTGCTCTGGTGCTGGT
EMPV1_32037	TTTCAAGCCAAAGCCTTAGTAGAAGAACAACGCCTTTTGTTCCTAGGGTTCAGATAAAAC
EMPV1_32039	CCAGTGGTGAGCACGTCTTCTCTAAATCCTCACTCCGGTACATGACCACAATCTGCAGCT
EMPV1_32042	GGGGAGGTGAATGCAACCAAGCACACATTTTACAGAAGGTCACTGCCAGTCTTATGAAGG
EMPV1_32045	GAGAAAAAGCGGTGGACTGGTGTGTTGTCCTGAGTCCCTCCTTTGGACCAATGAATACCT
EMPV1_32049	TAACTTGGGCAAATATCTTATTGTTTGGGCTCCCTGCCCCTTGAAGAACAGGTAAGGACC
EMPV1_32050	CTCCCATTTATTCAGAAAATCCTTCCGTGGGAGCTGGAGAACCTGAATGTCACAGACTTG
EMPV1_32051	TGACCAACCGTCCTAGTTTGCCTGGGACTGAGGTTGTCCCCAGGACCTGGTACTTTTAGA
EMPV1_32052	ATCCCCGTGATGACTACAGTTCCTTCCAAGTCCTGCGGCTCCATCCCAAGCCAAATACAT
EMPV1_32053	GACATTATCAAACTCTCCTGTTCAGACCACCGCATCAGCAGCATCTATGGCCTCGTCATG
EMPV1_32055	GGTCACCAAGGTCACCAAATTCATTGAAGGTGGCGATGGTCATTTATTTGAAGATGAAGA
EMPV1_32057	ATAAGAGTGTAGCCAACTTAATAGAAAGGAGGCAGTTTGAAACCAAAGAAAACAAGCGTC
EMPV1_32060	GGTGTAGGCAACATGGGGAATCCAATGGCAAAAAATCTCATGAAACATGGCTATCCACTC
EMPV1_32062	CTGAGACCTCCTGCTTCTCCACCCTGCATGATTGGTATGGCCAGGAGATTATGGAGCTGA
EMPV1_32063	GTTTGTGTCTCCCCATCCCCACCGCCAAATTCATACGTTGAAGTCCCCCAATATGATGAC
EMPV1_32068	GTCCGTAGGGCATCAGAACCTGGAAACCAGAAAGGTCGACCCGGGAGTGAGAAGCCTTAA
EMPV1_32069	ATCAGGTATTGGTTCCATGTCCCATGGGCTATGTTTTTCAATTTCAGTATTGTCCCATCT
EMPV1_32070	ATCTAGTTTGCAGTTGCCAGCTTGGGGCTCAGATTCACTCCAAGATAGTTGGTGCACTGC
EMPV1_32072	CACCATGAAATACCAAGCCCTACAGTGGGTTTTAAGTTCCAGTCTTAATTTTGGCGCATG

EMPV1_32073	TCTTTCGTTGTTTCCCCTTTCAAGTGAGCAGCACCACGACGACTGTCTAATGCATCCCTT
EMPV1_32074	TGTCACTGGAGGACGAGGTCTCCCATAGTATGAAGGAGATGATTGGAGGCTGTTGCGTTT
EMPV1_32075	TCTGTTAGCAGCTCCTCCCTTCAAGCTGGAAAAGGCAGCCCTGCCCCTAGAGATGCCCCT
EMPV1_32076	GAGTTGTTCCAAAAGTGTGTCTCCACTCTCCGCCTCTCAGGAAGGAACCGAAACTGCGTT
EMPV1_32079	GAAGCATCCATGGATCAGATATTTAGGTCCTAGATCAGAAGAGCTAAAGAGAGAACTGAC
EMPV1_32080	GGGATACACGCGCTCAGCCAGGTCGTGAAGGTCTCAGTGGACACAGACGTGTTACAATGT
EMPV1_32081	TCTGTACGAGAAGGGATAACATTTAAGCAACAGAAAGTGCTGAAGCGGGACGGTGTGGGC
EMPV1_32083	TCATACTCAGCCAGGATCCGAGCAAACCTTGTTTGCAGGAGGTCTACTGACCCCTCCATT
EMPV1_32085	TAGAAGGTGTGGGAAGAGCAGCAGTCCTCGAAGAGCACATACAGGAAGATACAACCCAAG
EMPV1_32088	CCCTGTTGCACTACTGTGGGCCACTGGGAAGCAGTGCAATGATGAAAGGGCATCAGTCAG
EMPV1_32089	TCAAGCCCAGGGATCAAACTTACATCCTCACAGAGACTATGTTGGGTCCTCAACTGGCTG
EMPV1_32091	AAAGCATCCTTGGAGGGTAGCCTGGCAGAGACAGAGAACCGCTACTGCGTGCAGCTGTCC
EMPV1_32092	GTGTCACAGCGTACGACCCGGCTCTTCCCCATTTCAGAGTCTACCTACAAACTGGATGTA
EMPV1_32094	AGACAGGTTCTACAGCAAGGAGCTCATCTTGCGGAGAGGCCAGCTCTTCAAGTTCTCATT
EMPV1_32095	GCCACACACCATGTTTTCTATAACGTTAACTGGCTGTAACCATAACTTGAACTTCATCAG
EMPV1_32096	GAAGCCTTTTCTCCAGTTTACAAATATGCCCAAGACTTCCTGGTGGCCATTGCAGAGCCG
EMPV1_32097	TCCTAGCACTAGCGTGCTTCTAGTTTTAGATAACTCCCACCTTTAGTCCCTGGCCCCTTG
EMPV1_32098	CCGGATTGCCTCACTAAAATCGAACTCTTTACCTTCAAACCCGTTCACGGCTGCACCTCA
EMPV1_32099	CCAAGATGAGCACAGCTGGTCACAAATAAGACCCTTGCTAGTCACTTTCGGCCACGATGG
EMPV1_32104	GATCCCTGTGCCCAATTTTGGAGTTGAGAGGGTTGTTGGTAATAAATGTATGATGTACAC
EMPV1_32105	AAGACTGGCTTCTCCACACTGATCTTCGCCCCATGTCCAAGCCACTTTCCTGTTCCAGCA
EMPV1_32106	ACTTAGTGACGTGCGTTTAAGGTTCCTCCGGGCCTCTTCATGGTCTGATGGCTCACTTCT
EMPV1_32107	CAAGCAGCTCTGTTTCGGCCCTCAAGCCCTCTTATGGCCTTATTGCCTACTTGCCCTTAT
EMPV1_32108	GGCACTGGCGTCAAGTCCACCTATGGGCAAAAATTTGATAAGAATGTCATCCCGAAGCAT
EMPV1_32111	GGTCTCAGCTACTGGGCCCGGGAAGATGGTTCTAGGTGGTTGCCCGGTGAGTTACTTACT
EMPV1_32114	TTGGATAACGGCAAGGTGGGGGTTGGTGGGTATGGTGTGTGCTTGGATGGAGAAGAAAAG
EMPV1_32115	AGGTGTTTCACCCCTAAGTTTGTCTTCCAATTTGCCTACGAACAATAAAATGGAACCCAG
EMPV1_32120	GGGCTGTCTGGTCTTCGAGGAAGGGACTTTGAGCTGTCTGACGTGTTTTATTTCTCCAAG
EMPV1_32122	AACAGCATGAAGGATCTTGGAACCTGACCCCTTTTCAGGTGAACATCCAGATGAGAGCTC
EMPV1_32124	AACCAGGAGGTCTGGGTTGACATACTGGGCACATGCACTTGACCCTACACAGAGGACCAA
EMPV1_32127	CTTCAACCTGGACACCCAATTCCTGGTGGTGAAGGAGGCTAAGAACCCGGGCAGCCTCTT
EMPV1_32129	GAGGGCGCCTCCTCGGTCCCTGGCGCTTCAGTATATTCACGGATATGAATGTAAAATATA
EMPV1_32132	CTCCATGTATGAGCTGGAATACTTCTACGAGGAACAGTGTGAACGGATGGAAGCCATGGG
EMPV1_32133	GACAGAGCTGAGCAAATCTGCCCGCCAAAATATCACCCCTGTTCCATGCTCCATTCACAA

EMPV1_32134	GTAGTGTCTCAGATGTAGAATATGCAAAGAGAAGGGAAACATGGTCCTCGCTCTCCAGGA
EMPV1_32135	ACCCAGGAGCAGCTGGAGCAGATCCGCCTGGTTCAGAAACAGCAAGTCCAGGAGAAGCTG
EMPV1_32136	TCTTGTGTGTGGAGGATCTTGGAGACTGGCCCCATGTCCACGTCAGAGCTTTGGGTATTT
EMPV1_32138	GGTAGCATGGATTTGATCACCAGCCTGATGCAGTGGATTAAGGATCTGGTGGTGTCACAG
EMPV1_32139	TCGCCTCCGGGCCAGATGGTAGTTATCAGCAGGGTTAGTGGACTGTTTACTTCCTGATCT
EMPV1_32140	TGTACAAATGCACTGACTGCGCTTACAGCACCAAGAACCGGCAGAAGATCACCTGGCACA
EMPV1_32141	TAATTCACTGTCTCTTGCACTTGAAACTTCTGATCTACTTGTCCTGAAGACCAGCTGCTG
EMPV1_32142	GGGTGAGTTTCCCACTGACGATGTAGTTTCAGTCCTTTTTATCAATGGTTGTTCAGCAGT
EMPV1_32143	CGTGAGGTCCCCTCCGGTTCAGGATGAGAAGACGAAGTTTGGTTCTGGAAGACTGAATTT
EMPV1_32144	TCGGCTCAGGAACCAAGTGGGTTGAGAAGATGACTGGGTGGTTCCACATCAAGAAATCCT
EMPV1_32146	ACCTTGATCCTGCCCCGGGTGACACTGAGAGACACCGCTGTGTACTACTGCATCCTAAGA
EMPV1_32147	AGTGATAACCACCTTGCTGGGGTGAATGCCCACATGGACAGTTGTGCCATTAGCCTTCTC
EMPV1_32149	TAATGGGTTAAATCTGTTGCCATAAATTCTAGCAGGTTGGGACAATGGGACAAGCACTGG
EMPV1_32150	AAGTGAAATCCTGGAAGCTATTAGTCAAAATCCAGTTGTCATTGTCCGAGGCGCTACTGG
EMPV1_32151	AACTTGACATACAAGAATGTAACGTCCTTTCAGGCCAGTAGATACACAGATGGATGTGTG
EMPV1_32153	CTGCTTTGAAGAATTGAGGTGTACGGTATGAAGCAGCCGTGTACTTTCGTTTTCCGGTCT
EMPV1_32154	CCCTGAGACCCTAACTTGTGATGTTTACCGTTTAAATCCACGGGTTAGGCTCTTGGGAGC
EMPV1_32155	GGATCAGGTATCAGAACAGACTTCCCACGAGATGCAGAAGGAGAGTCCAGACACACACAA
EMPV1_32157	AGTGCTGCTGTTTTAAACAATAACTACTGTGTTCCTGGCAATAGTCTGTTGTGATAAGCG
EMPV1_32159	GCTACCACAAGTTTCAGTGTAGGTTACAGATTTGGCTCAGATCCAGTGTGGCAGTGGCTG
EMPV1_32161	TCATCGGGCGGCTCGGTAGCCAGAAGTAGCAGGAACAGCACCACCAATGAAGCTCTAATT
EMPV1_32166	GTCACACGAAGGTGGTCCTTGTAAATCGTGTAAGCGCCTTGGAGATTAGCTGAGGCAACT
EMPV1_32167	AATATACTGTCCCATCCTGGCCTGTGTGCTTGTGACCTTCCGAGTGTTACCCCTGCTTGA
EMPV1_32168	GAGAAAGTCTATAGAGGAGCTGGCAGCAAAAAATTTGGTCAGTGTTGCACCATGCAGCAA
EMPV1_32170	CCGTGTGATGTTTCTTATAGAACACAGCCTCATCAATGCCCACTCTCTGGACCCAGCCAC
EMPV1_32171	ACCACATCTCCAGCCCTCCTTGTGGTTCAGTGGAGCAAGGTCATGGCAACAATCAGGATT
EMPV1_32172	CTCATTCTGCAGGATCCTCAAAGCTTTCGCAGCAGCATCGTGTTTCGCAGCCTGTCTCGT
EMPV1_32173	CTTCCAGGTACCACTGGGATTTTCCTCTGGGGTATGAGTAAAGATGCTTCAAAGACCTCT
EMPV1_32174	CCGGGCTTCTGCTTTCTGCCTTCCTTGGTATATCAGGTGTCACTCACAACCCCACTTACT
EMPV1_32175	CTCCATGCCATTGTGCAGATTCACTTAGTCCTTGACTTGCCCTTCTGTGGTCCCAATGTC
EMPV1_32178	AATCAGAGTTACAGCTGCCAGCCTATGCCACAGCCACACAGGATCCCTGACCTTCTGAAC
EMPV1_32180	GGAGAGCAGGACGGGGGGCTGATTGGTGCCGAGGAGAAAGTGATTAACAGTAAGAAGAAA
EMPV1_32181	ATAATGCTGGCTCAGGAAGAGAGGATGTGAGGGACAGAAACAGGCTCCCGGGGCTCACCT
EMPV1_32182	TGCCACTACAGTGACCTGGACCGCAGCAGTAACACTAGATCCTTAACCTGCTGAGCCCTA

EMPV1_32183	GAAGGCTCCTTTATACTTTACCCCAGCCCTGTATTTCCATGGTGAACTTTTCTCTGAAAC
EMPV1_32185	CAACAACGCCTTCGCCTTCGGCCCGGAGCTGAGCAGCCTCATCACGCCGCTGGCCATCCA
EMPV1_32188	GAGTGGCTGAGATGAAGTCTGTTGTGGACTATAATACATATGCTGGTGTCTGGTCTCAGG
EMPV1_32189	GTGCCTGATGATAGCAGAATAGCAGAAACATTTGTACTGAACTGTGCAAGCCTTGGGGAC
EMPV1_32190	CCGAAGTCTCCTCATGCCTGTCAGCTTCCGATCTGCACCCATAAAATTCAAGCTCTGTGT
EMPV1_32191	TTAAGGATCTGGGGTTGTCACTGCTGCGGCTGAGGTCACTGCTTTGATGCAGGTTCGATC
EMPV1_32194	GCCTAGTTCAGGGTCCTTAGGGAGACAGTTGTACCTCAGCTTAGAGCAGCTTAACTTCAG
EMPV1_32197	ACATTCTTGGCACTTACTACTCCATCCTAAGTGTGGTGCTACAGATGCCCTCAGGCTCAG
EMPV1_32198	AGACCCGATTCTTGGCTCAGGTTTCCAGAAGCCATCGGCAAACATACACGCGCTTGCTGT
EMPV1_32200	CTTTAATTTCATCACTGTCTTGGAGGTCCAGTGCGCAAAGGGCTGATAGGGGACAACTCC
EMPV1_32201	TACCCTGTTCTCATGAATGCTAAGCTGAGTTCCTTGCTTGTGGCTGGAGCTTGGGTGGCA
EMPV1_32202	ACGGAAGTACCTGTGCCCCGACTGTGGCTACAAGTGCAAGTGGGTCAACCAGCTCAAGTA
EMPV1_32203	AAGTGCCCTTCAGTGGCCACTCCTGGTCAGGTCAAAGAGCTAGTGGACGCCGAGTTATTC
EMPV1_32205	AGGCCGCAAGAAGAGAAGTATCCTGTAGGACCATGGCTCTTGGCCCTGTTCGTTTTTGTT
EMPV1_32206	AGGACCTCCTGCTCCCCCAGAACCAGTTCGCCATGTTCCTGTACTGCTTCATCTTTATAC
EMPV1_32207	CTGTGCCTGGATGCCATCTGTGCCGTCTTTGTCATTGGTGTTGCCTTTGGGTCCCTATTT
EMPV1_32212	CAATAGTAAGACTTCCCAGGTTTCTCGGGCCATGATAGATGACTCACGGGCCAGTTTCTG
EMPV1_32213	GTTCTGGAACCTTGGGGAATCTGCCCTCAGAAGGTGTTTATGAGGACATTGGAGTTGTCC
EMPV1_32214	TAATTCACAACTCTCTTACCCACTTGGGGGTGGTAGGCAGGGATTCAGGAGGGACCAGAT
EMPV1_32218	TTGACGCTTGTGTAGCTGGTCTGGGTCTCGGCCTCCGCGCTGCTGCAGCAGTGGCGTCTC
EMPV1_32220	CAATCAGTGGTAGGTATATGTCATCATCTATTCTACAGCTGAGGAAGGAAAGACTGGAGG
EMPV1_32223	GGAGTGGTGCCTCCTTCGGCTATTACCTGGCCCTTATCCAGTAAAAAAGTCGACAGTTAG
EMPV1_32224	CTAGGAACACACTTGATAGGAAATACTCGTGTACTTGGCTTCAACCAGCAAAATGAAGGG
EMPV1_32227	CGCCATTGCAAATGTTGCCAAAATCCAGACGATGGATGCCCTGAATGACACGCTGGAGAA
EMPV1_32229	TTGTCACTTGGACAGGTCACTTTATATGTCCCAAACCACTGTCCTCTTAGTCTTCCATGG
EMPV1_32231	CTAAGATAGAAGAACCACCTCCCACCAAAGTGACTGAAAGGCACATGCAAATAACCCAGG
EMPV1_32234	TTCTTGGGAAAGGACCAGGTTGGTTGTCAGGTGTGTTGGGTGTGAGAGTCGTCTTGCAGA
EMPV1_32235	GGCTGCCTTGGAGGAAGAGGAAGAGCTGATTTTTTCTAACCCTCCTGATCTTTACCCAGC
EMPV1_32236	GCTGGACTCCATCTCGGGAAGCCTTCAGAATATTAAGCAAGGGCTGAATAAGGACGTCGG
EMPV1_32237	AAGCGGTGGACCCGGATTTCCAGTGCAGGCTGTGCGGCCAGGTGCTGGAGGAGCCCCTGT
EMPV1_32238	ATAGAGATCCTCACACCGGAAGTTAACAAGGGGTTGAGGAATGATCTGAAAGCAGCTTTG
EMPV1_32239	CAGAGCTGGACATGAGATCTCAAGTATCTCTTCCGCACATGGTGTGTCTGTTTTGGTGGG
EMPV1_32242	CTATGGGCTGCTTCATACAGCTGCTGCCAGTGGCCAGATTGAAGTGGTGAAGTACCTGCT
EMPV1_32244	ACTTGGTCTGTCACCTCCTTAAACAGGGGGAGCTGAATTTCGGACTGGTCGAGGTCGTGT

EMPV1_32245 AGCGGCAGAGTCGGGGATAATGTTCTCCACTTGGCCCAGCGCCATGGGGTGGACCTAGAA
EMPV1_32246 ATCTTCCTTCACACATGGATTCATAGAAATTGTGATAGTTCCCAACTGTCTGAGAGCCTC
EMPV1_32247 ACCCATTTACCCAATATCAAAGTCCACGCCTACTTCGCACCTGTCACTCCACCCCCATCA
EMPV1_32248 TCAACAAAGACATCAGCGCCATTGAAGAGGCCATGAGTGCCAGCCTCCAGCAACACAAGT
EMPV1_32249 GCGAACCTGCACGTGGACTACGCCATCCAAGAAAAGGAACGATTGCTGAAGAAGCACAAG
EMPV1_32250 TTTTTCTTTGGAGCTGCCATTTTGGGGAAACGGAGGGTAGGGACCCTTTAACCCGGTTCA
EMPV1_32251 ATAAAGACACAGAAAGATAAAGGGATGCAGAAAGGCAAAGTGGCACGGGATGGACGGGGG
EMPV1_32253 ACAGCACACTACCAGGGTTCCCAGTGTTTACAGGTCTCTGTCCCAGCTGCACCTCAGTAT
EMPV1_32254 CATGTATCTCATGGAACATTTGTGACATATTTATGCTAAACATTATTTGTTATCTCTCTG
EMPV1_32255 GGAGTCTTCTTCCATACTGCTAACCTAAATCTCTGTATCAACCCAAAGCCCAGACCTCAG
EMPV1_32256 TATCTGTTCGCGCGGCATTTACATGCTTGTGCCGGATCTGCGTGCAGATGTTTACACACC
EMPV1_32257 CTGTGTGTTACAGAGAAGTACAGAAAAGTTCGGGATAGTTGGGGGCTGCTTTTCTCCCTC
EMPV1_32258 CCAGCTGTTGAGACTTTAAACTTTGATGAAGTGTCTGCCACTGGACTCTGGGGTGTGTGG
EMPV1_32259 AGGATTGGGCAAAGTTCTCCAGGAGGTACTCCGGCAACAGAATCTGCACCACCAAGTATA
EMPV1_32262 AACTCCGGCAGACAGAGCCGCAGCCTGAAACCCTACTCATCGTCCCCCTCCCAGATGAAT
EMPV1_32264 GTGTTCAGGTGTGTATGCAGTTTTCAGCTCTATCTTGGATTGGGGGTTTCATTGCCCCCG
EMPV1_32267 TCCTCTGTACTGTTTCCCGAAGGGTGTGCAGTAAAGCTGTTATATGTGTATCATCTACTC
EMPV1_32268 TAGTGGAGATGTCGCGCCCACGGCCTCTTAACACTTGGAAGATATTCGTGGGCAATGTGT
EMPV1_32269 AGCTTCAGGAACCAGGGGACCCCAAAGTACTAGCAGCCCTGTCTCAGGAAACTGAAAATA
EMPV1_32272 CCTCCTACCCCAACTTCCAGTCCCATTTTCCTCCCGTCTCAAAGAAGAGCAGTGTATCTT
EMPV1_32273 ATTGCCACCCCTGCCCTCTCCATGTTCATGCCCCCTCTGTTTTAAAGTTGGCTGAAGTGT
EMPV1_32276 GCAATTTCAGTTTCCTTCAACCACTTCCAGGGCCCATCCAGTTCCGATGCTGAAAGAATC
EMPV1_32277 AAAAAGAGAAAGTTAAAGTAGGTTTTGTGGATGTGCCTTCATGTAGATATCAACAGCTCG
EMPV1_32278 CCGCCATTACAAAGGTCGTCCTCCTTGATCCAGTTAACGACTCAGAACTCTTCTCCCAAT
EMPV1_32280 ACTCCCCACCACCAACAGAAGATGTAGCCACCATACCAAGAATTTGGCCACAAGCAAAGG
EMPV1_32281 ACTGGGATTTGACTCTGAACTCCAAAGCAGGCGGCCACACTGTCTCTCGGTGGATTATGT
EMPV1_32282 GGCAGTTCATGATGCTGGTAATGATTCAAGTCAGGGAGGAGAAGCACTTGCTCTGAAACG
EMPV1_32283 GGTTCAAGGGACTTTTTGAGCCTCTATGGAGTGTGACATTGGGATGCATGTAGAGCCTTC
EMPV1_32286 CTTGATAAGGCCAGTAAGGGGAGGACGTGCCTAATGGTGGCTCACAGACTCTCCACAATA
EMPV1_32287 CTGATGGCAGCCTCACCGTGCTCAATGCCTTCTCCCAGTTTCCATCCACCATGCAGGTGT
EMPV1_32288 GGTAATAAAGTAGCTCTGGATCATCAAAGACAACGCAGGAAGGGACAGCAAGTGGCACGG
EMPV1_32289 ATAAATCAGGGCAATGTGTTACATGACAAGGCAACTATTTCCCAGTCCCTCGCAAGGGGC
EMPV1_32290 AGCGCAGCGAGCCTCTTCGTGACGAGGACGACGATCTGCTACAGTTTGCCATCCAGCAGA
EMPV1_32291 TGGCTATGATGACTTCAACTGCAACATCTGGGATGCCATGAAGGGCGACCGTGCAGGTGT

EMPV1_32293	GAAGTAACCTAATTTGGCACAAGGATCAGTTACTGTTGGAGAACTAGCAGAGATGGGGGG
EMPV1_32295	AGAGGTGGAGGAAACCCTAAGGAGGATCCAGAGCCATAAAGGGGTGATAGGAACGATGGT
EMPV1_32296	GACACTTACTTTCATGTACCTGCGTCCCAGCTCCAGCTACGCCCTGGACAGCGACAAGAT
EMPV1_32297	AACACCGCCCCCTCCTGCCATCTTCACCAGGAAACAAGAGCCTAATTCGCAATCCATTTT
EMPV1_32298	AAGGAATTAGCGTATTCTCCTGGCTCATCCCTCTTGGGTACTTTCTGTTCTAATACACAG
EMPV1_32301	TTTGGTTGTGCAGGTTTCTCTCTAGTGCAGTGTGACTGATTGGCATCCTGGCTCCAGCAG
EMPV1_32304	TCCGCCCTCCAGCCTTGCTTCCTGGTGATGTCATATACGGTGATGTGAAAATACGTGACT
EMPV1_32305	CAACGGGCCTCTGGACTCGGTCCCTTTCACCCAGCTGCACGAGTGGTACGGACAGGAGGT
EMPV1_32306	GTACAAGGTCACAGAGCTAACGTGTAGGGGAGCTGGAATTCAAAACCACATCTGCCAGTC
EMPV1_32307	CAAGGGGTGGTAAAACGAATCATTCCTGCAGTAGCTTCCACAAATGCAGTCATTGCAGCT
EMPV1_32308	CAAGGTAACGCGAATGATCAGCATCGTGGCCGCGCTCTTCTGCCTGTGTTGGTTGCCTCA
EMPV1_32311	TGCAAAAATGCATAATTCGTGGCTTCCCACCAAGGGCAGACAATAACCCCGGTGATAGAC
EMPV1_32312	TTCCACCTGCTTCAGCTCATTGATTTCGCAGTGGCCAGCCTCATTCTCCACAGTGATGCC
EMPV1_32313	AGCCACTGGGCTAAAACCCACCTTGTGAAGACAGACTTGGTGCAGGAAGTGCACCAGAAC
EMPV1_32315	TGCCGGCAATGGGCAAGCCTAGTAGTACTGGCACAGAGCATTTAACTCTACACCTTATTA
EMPV1_32318	TTCCATCATCCTCAGCCTCGCCTGTTCCTCCACACGTATCTCAAGCAGCTCACCTGTCTC
EMPV1_32319	AGAGCTCGCTGTGAATTTTCCTTCCGTTGCTGCTCCTTTGAGTGGCCCGTATGTGCAGAA
EMPV1_32323	TCTGTTTCTCCTGGGTCTGGGTTCCTTGAGAGACTTCTGCTTCATCGGCTCTGTTGTAGG
EMPV1_32324	GGCCTCTGTGGAAGAGCCCATGAACATCTCCATCATTGTCACAGACCAGAACGACCACAA
EMPV1_32325	CTGGCTCCCAGGGGCACTTCCGAGTCCCCACGCTGGGCTACCTGGACGTGCGCGTCGTGG
EMPV1_32326	CCCGAAGTTTAGGAAAAAACCTGAGGCAACCCAAATTGTCAGACCTCTCCCCCGCAGTGA
EMPV1_32327	GGGGGACAAAGATCCATTGGAGACCCTACAAACTGACCGTGACATTGACCGCTACACAGA
EMPV1_32328	AAACCAGGACCCCAGAACTCAGTCTATATGGGTGACTGAAAAGTCCTCAGAAGTGGACCC
EMPV1_32329	CTTCTTGTGTGAACCCTGGCAATCCTGTGTTTTCGTGTATGTTGGACCCAAGGTCACTCC
EMPV1_32331	AATGAGAAAGTGCCATTACGTGACGTAACCAGTAGCCAGGTAGGATGCATCCTCCGCTGT
EMPV1_32333	CTTAAGACCCAGTGGGCTACCCGTGAAGGGCAAGCATGGAGCTGATAGGTGGTCTGACAT
EMPV1_32334	ACTGAGGGCTGTAATCCTCTAAAGCTTCTTGCACTCCCATGTGTGCTAGCTGATGTTGGC
EMPV1_32337	GGCAACATTCCTGAGAGTCCTTCTTGCTGATGGGTGAGCATACTGGGGAAATATCCAACA
EMPV1_32338	TGCCCACTGCAAACAGCAGGCAATGTGGCAAAGCGAGAAACAGACGGGAGCATCAAGCTT
EMPV1_32340	CACACTCTGCAAAACAATGGTTGTCTGCTGTTGGGGGGCAGCACTTCTGATTATCCTCCC
EMPV1_32341	TGCGTGACAAGTTTCAGTGTTTACGCTTGTGTACCGAGGTGGATTCACACGAGACCAGCT
EMPV1_32343	CGTTTTCACCCTCCGAAGTTCCATCCCTCTTTCTTTAGGACACACTGTACAAGCCATCCC
EMPV1_32344	CTGATCAAAACCACCCAGACAGCTGCAATCGTGTCAAATATAGACCACAAAAGAATTGCT
EMPV1_32345	GAATATCTGTGTCAAGTACAGTGCTGGGTGCTGGGAATACAACCATGAAAAGGACAGGCC

EMPV1_32356	TAAGAGCACCTTAAATGGAACAGATGCACACATCTGCAAAATCTCCGTACTCCCAGTATG
EMPV1_32359	TCAACACCAGGGTGCAGCAGTTAGAGGAAGAAAATGCCGAGCTCAGAACGACAGTGACTC
EMPV1_32362	GGCAGATAGGGAGAAAGTGAGAAGGAAACACAATGAGGAGGCAGGAAGAGAGAGAAGGAG
EMPV1_32364	GGCACCCTTGGTCCTACTTACTGTTCTTGGCCACTGTTACTCAAATTATAGTCCTTGGGC
EMPV1_32366	CGTTGCAGTGTAAAGCCACACAAGAGCTGGAAGCCGAGACGTGGGAAAACGGAAGCGGAG
EMPV1_32367	AAGCCCACAAAGTTGAAGAAGACATTGCTGACTTGAGGGAACAGGTCCATGATGCGGTGG
EMPV1_32368	CGCTTCACAAAGGATTTCTCAGGAGGACAACAGAGGAATCTCCACACATGTATACCCAGG
EMPV1_32369	TGTTCATCCTGGCCCTGCTCTGGGTGTCAGTGCTGCTGCTGTGTGTGCTGCTGTCCAGAG
EMPV1_32370	GTCTGGATTGGCCTGCATGACCCGCAGAAGAGGCAGAAGTGGCAGTGGATTGATGGGGCC
EMPV1_32372	ACTAACAGACTTTGGTCCACCATGTCCTTTTCACAGCCCACTTCTGGAACAGTCACCTTG
EMPV1_32374	GGAGGAAGGGCGTTTGCATCGCCCCACGTGAGACACGTGAAGGAAGAAGCGGATAATTAT
EMPV1_32375	TCTAGATGGTTCACAGGTAAGTCAACATGTCAGAGCATCTGGAGAGATGGTACTGCCCCT
EMPV1_32376	GCTCAGGCTTGTGCCTTTGGGTGATCCTTAAACCAGGGGAATAAGACAAGCCAGGTTCAA
EMPV1_32377	AGTGGGCCCGAGAGATCGGGGCCCAGCTGCGGCGGATGGCTGACGATCTCAACGCGCTGT
EMPV1_32379	GAGGCAGTGAGGATGATAAATGAGTCAAGTAGCCCATGTGTCATAATGGCAGTAAGTGCC
EMPV1_32380	TGAGCCTAGTGAGAGAGACAGCGATGAATCGAAGGCTTCCCTTGAAACTGACACTGAAAG
EMPV1_32381	GTCCTATGATACTACCTTCCTACTGGACCATGAGGAAGTTGCCTAAGTTTGGGTGCACTG
EMPV1_32382	GAGTCTACATGGTGCCTTTAGATCCCCAAACCTTCTTCAGCCCTTCCAAGTCCCAGACAC
EMPV1_32383	CTTGGCTTCCTGGGTCGGAGGAGATCTTGTAATGGAGTGGTTCTTCGTCTCACACTAACA
EMPV1_32384	GCCCGGCACATGGTCGGTGCTCAGTAAATGTAGCTTCTCAAACTTCTTTCAGCAGGTCAC
EMPV1_32387	TCCAGGCTATCTAGCACCGGAGATCCTCAAGTGCTCCATGGATGAAACCCACCCAGGCTA
EMPV1_32388	TATCATTCAGGAGCTTCCCTGAGAGATTATAAAGCTGCAGCCCAGAGAGGAGCAGGTGAG
EMPV1_32389	CTGTGTATGTGCCCGTGAGCTGCAAGCCCACCGTGTGTGTGGCCCCCTCCTGCCAATCCT
EMPV1_32392	TCTCTCCACACTCAAGCTGCAGAGGCTTTTCCCTCGCCCTCCCTCTGGCTTTTCCTATTA
EMPV1_32393	TAACATGGGAAGGATGCTCACCAGAGGAGGGTCGTTGTGAGGAGTAAGCCAAACCGCAAA
EMPV1_32396	ACTGCAAAACAGAAAGGTGCACACAGACGACAATCAGCAGGACTCACCATACTTGACACC
EMPV1_32398	CTGAAGAGCTCAAAGTTCCACCAGCTAAAGTGCCCGAGGCTCCCAAGGAAGTTGTCCCTG
EMPV1_32399	GCTCCACCATGAGTGGTGGGAAGGCCATAGAACATTTGTCCAAACAGGGAAATAAGTTGC
EMPV1_32400	CGTATGCCAATATCCTCATCACCAGCAATAGTCCAGCCCGCAGAACAGCCAGGCCACCTT
EMPV1_32402	CTCCTTCAGTTTGATAAAGCTATAAATTCAGCATTGGCACAGAGGGTCAGGAATAGAGTC
EMPV1_32403	AAATCGCCAGAAAAGTATTTCCTTTGTTCCCTTGAATACGCTGTGAACCTAGACTGTGGC
EMPV1_32405	CATCTGCTACTTTCTCCATCCACTTCTGTGGTGCCAACGTCATCCACCAGTTCTTCTGTG
EMPV1_32406	AGTCCAAGAGATGGGACTCCCTCCAACCCACATGTCCCTGCATTCCTTCTGACTTCTACT
EMPV1_32407	ACCCCCACAGGCAAGCAAACTGACAAAACCAAGAGTAACATGAAAGGTTTCTAAGCATGA

EMPV1_32410 GATCTTCGTGGTGTTTGATGCCAACCAGATCTACCCGGAGTACTTAATAGACTTCCACTG
EMPV1_32411 AAAATGAAGGGCACAGCCTCTGGCAACTCCCGAAGCAAGAGCAAGGACCACAAGTACACT
EMPV1_32412 TCTCACACACACTCTCCAAGCTCCAAGCTGCAGAAACACAGACATGAGGAATCGAGGTGT
EMPV1_32413 CGTGGAGTGGCTTAACAGAAGTTCATTGCTTCGCATTCTGGAGGTTGGGAGTCTGAGATG
EMPV1_32415 GGTAGCTCCCGAATCTACTACTGGAACATGGACTACTGTATCGACCGATGCACTTACCAG
EMPV1_32416 ATCCGTCCAACCAGGAGCTCTCCGTCTGCCACAAGGGGCATTTTAATCTTGGGACCTTTA
EMPV1_32421 CCGTACTGAGTGTCTGGACGAAAGTAGAGGGTGTTCTAGCATCTGTCAGTGGCACAAATG
EMPV1_32423 GTCCACCGTTTATATCAGAGAGCCTTACAGAAGTTACCTCTTTGTGCATCACTGTGGAAA
EMPV1_32424 GCTATAACATGGAAGAACCCTGAAAACTTACTAGATGAAAGAAGCCACTCCAAAGGACTG
EMPV1_32426 GCTCTGGGCAGGATGCAAGGGTAACGCCACCTTTCTAGCTCTAAGGACAAGCCTGTTTTA
EMPV1_32428 TTTGGTGTAGGTGCCCTGGAAGAGGAAGACGATGACATCTATGCCACAGAAACTCTGTCC
EMPV1_32431 TGGTAGGATAGGTGAAACTGCCATCCGAGCTTTTAGACCAACCACCTTCCCCTTGCAGAC
EMPV1_32433 TTAAACGAGCACTTATTTCTCATAGCTCTGGTGGTTGGAAGTCTGACGGAGGTGCCAGCC
EMPV1_32434 CTGGGACAAGACTATCCACATCTGGAAACCCTCAACCAGAAGCCTGCTTGTCCAGCTCAA
EMPV1_32440 CTGCTTTTGGAGCTTGGTGCAGACCTGTATTCACTCCTCCAGGTATGGTCTGATGAATGC
EMPV1_32442 CTGACAGGAGCCATTGCTTATTTGAAACCAGTGCCTGAGTCTCCCTCTCTCCTGGACTTG
EMPV1_32443 ACTGGCTGTCCTGCCTTCCAGCTCTCCTGGACCTAAGACGCGGATGACCATATTTTGGGG
EMPV1_32445 AACGTCGCCGTATTGCTCTGAAGAAACAACGTACCAAGAAAAATAAGGAAGAGGCTGCAG
EMPV1_32446 CCAGTCCGTGCCAATATTAGGTATGTAGGCGGACAAGAACATACACTTACGGTTGTTTCC
EMPV1_32448 CAGTCAGGGAAATGGGAGAATTAACTTCATTTAGTGGGGAAGACTGGGAGGTGAGACTCT
EMPV1_32449 TGGCGTGTTGGTCATGGATAATCTGCAGCACCTGTATAACTTTGTGCACCTGGACCTGTC
EMPV1_32450 GGCCTACGCCACAGCTACAGCAACATTGGATCTGAGGTATGTCTTCAACCTACACCACAG
EMPV1_32451 GGCACAGTGGCCCCAAATCCCAGAACCTTTGTGAAGCTGGTTCTCCTTGGTAAGAAACTC
EMPV1_32455 ACATTCTCCCTCCGAAGTCTGAGAAGAGCTCAGCCCCCAACAGCCTGGACGTCTGGTCCT
EMPV1_32458 GCTGCTGGTAGGTTTAAGAAAATCATGACCTCAGCTGCCTCACTCTGACATTTATCATGC
EMPV1_32459 AGATCTGAACCACCTCTGTGACCTACATACACTGCCGCTCACGGCAATGCTGGATCCTTA
EMPV1_32461 GTACTAAAGCACAATGCCTTTCCTCACGTGTGAACATTACAGTGAAAGCCACAGAGAAGT
EMPV1_32462 AGTCAAAGCACAAAACCCTAAGGACCGTGAACTCTGCCCCACAGAGGCTGCAGTACCAAT
EMPV1_32463 ATGCAGGCCACGCCCGAGTGCTTCGCCCACCTCACGCAGCTGCTGCAGGTGCTGGCTGGT
EMPV1_32465 CCTGCCCTCTGTACTTACTTCAGGGTTTAGGGAGTGGCTGGGAAAGGGATATTAGGAGCT
EMPV1_32466 TGTTTAGGGAGTTAGATCAAGAGCCTCAGAACCAAAATTCTTACAATCAAAATCTTACAG
EMPV1_32467 ACCGGTGGAAGGATCTTGAGACGCATTGAAGGCAGAGTGGAGTATGGTGGTAGTTACAGT
EMPV1_32468 TATCTTTTCTGCACCATGTGGAAGTTGTGGGGCCAGGGATCAAATCCATGCCACGGCTGT
EMPV1_32469 CCATTACAGATCCCCAGACACTTGGGTTTGATGACCGTATCTCTCTTATATGACCCCAGT

EMPV1_32471	GTGTATCACAAGGGGATGCTACACACACGAGGGTATTGAGCCACACCTCTTCTGAGACTG
EMPV1_32476	CACTGTAGAAAGATAAAGAGTATTAGCCCTGTGGGTCTTGCTCCTCCAAAGGCAGGGACC
EMPV1_32477	CTTCTCTTCCCTCCCTGGGCTTTTGCTTTTACTGGTTTGATTCACTGGAGCCTGCTGGGA
EMPV1_32478	CAAAATTGGAGAAGAACAGTCGGCAGAAGACGCAGAAGATGGGCCTCCAGAACTCCTGTT
EMPV1_32481	TCTCTACAGGCATCTCAAGAATGACTTCAACAATCTCATGTGGCTTGGGTGACTCCGGGC
EMPV1_32486	GCGTTCATTTATCCGTCATTTTTGACTCTCCACGGTCTTTGGTGAGAAGCCCCGCTTCAG
EMPV1_32487	TTCGAGAGCTGTGCCCAGGAGTGAACAACCAGCCCTACCTCTGTGAGAGTGGTCACTGCT
EMPV1_32489	TACAGTGGCTACAGCTTTGACTATGATTACTACCGGGACGACTTCTACGACAGGCTCTTC
EMPV1_32490	AAGCTTATTTCAACCACTGCGGGGAGATCCACCGGGTCACCATCCTGTGCGACAAGTTCT
EMPV1_32491	TGTAAACAACACCTAATTTTTCCCTCTTCCTCACCAAAAAGGAAAGACTAAACTTGCTGC
EMPV1_32493	AATTGTCGCAGAACGGGTCAGTTGGAGCCTTTTTGAGTTGGCGTTAGAGTTCCCTTCACC
EMPV1_32494	GACGCGCGTAACTAAGGAGGCCGGCAGCGTGTCCCTGCGTATGAAACAGGTGGAAGAACT
EMPV1_32495	GATCGCTGTCAGAATAAACCACCAGTCAAGTGCAAGTCCTTGCTTCCAGTCTATGGGGTC
EMPV1_32496	AAAAAAAAAGAAAAGAAAAAAAATGAAATTGTTGTGGCAGAGGCAGCAGTTCCCTAGGTAG
EMPV1_32499	ACTGGAAAGTGACTCCTACAGTCCACCACATGTCCGCCGGAAACAGAAAATCACCGACAT
EMPV1_32500	GGCATCTCTGGTTAGCCCAACAGGACTTCCCATGTATAGGCAACTCAAATCTTAAAGAAC
EMPV1_32501	CCAAGAAGGAAAGTCAGGGTTCAGTGTCAGAGAGTGTGTCAAATTCTGGAATAGGTTCTG
EMPV1_32504	ACTGTATGGAGCGACCTGAGCTGTAACCTGGGGATTCTCTCAACTCTCTTGTCCCACACA
EMPV1_32507	CTGAAGACACGCACCCTCACCCTTGTCCAGCCTCTGAACTCTGTGCCATTTTGGCTTAAA
EMPV1_32508	TCAGAATCTGCTAATCTTAGAAATGAAGTAAACTAGGGCTTCTTGTTGTGCCTGCTCTGA
EMPV1_32509	CTTGAGAAACCTTGTGAATATAGGGAATGTTCGAAAGCCTTACTTACCTGTGTGTGGACA
EMPV1_32510	TCTCTGCCTTCAAATGACCACAGGCGCCTACATAGTTGCAACTGTGCATCCCATGATTCA
EMPV1_32513	GCGCACCATTAGTAAGGAATGCAGCCTGTCCCAATTCTCTTGGACGAACCTTTGAAAACT
EMPV1_32515	GTTGTGAATTGTCCCCTTTGCCCTAGGAAAGTTTGGATGTTTCATTAGCTCTCTTGCTCC
EMPV1_32516	GCTTTCGTCGGACACGGGAGGCATCGGGAGGAGCTACAACAAAGCAGAATCATCGATAAT
EMPV1_32521	GTTAGAGCCTAAAGCTGACAAAGAGAAGGTGGTAATGAAGCTCAAGAAACTGGAGGCCAG
EMPV1_32522	GTTCCAGTATTAGCCTTGGCACTTTAGAACTCTGGAAGTCTGCAGAGAGATGTGGCGACC
EMPV1_32523	TGCCATCAAAGCCCTTAATGCCACAGGCCAAGCCAACCTCACCCTGGAGACGCTCTTCCA
EMPV1_32524	GGCTGACATTCGTCTTGCTAAGCTTTATGGTGCTGATGGTTTGGTATTTGGGGCATTGAC
EMPV1_32525	GGGAATGCTGGAAAACTGCATCCTGCTTAGCCTCTTTGCTAAGGAGCATCTGCACCAGAT
EMPV1_32527	TCCTCATTGTCCTTTAGGGAGATCACCAGCTTTGGCATCTTAACGACAAACGGTGGACGG
EMPV1_32530	GCCACACTGGCTGCCTTTGGTTTCCATAAATGCACAAACCTCTTTCCTTCCTCAGGCCTC
EMPV1_32531	TGCTTGCGCTCCAGGCCCGAAAGAAAAGGACCAAGGCCAAGAAGGACAAAGCCCAAAGGA
EMPV1_32534	AATTGTACCATGACTCTTACGAACACTACCTCTCTGCTCCCACCAGCCAACACTGTGTGC

EMPV1_32535 CCTGATGAGCTCCTGGATAACCCACGAAGCCCTTTGCATCCCCCCAAACAGTTTTCAATT
EMPV1_32536 TGGTTGGAAAGCATTGGGCTACCTCAGTACGAGAACCACCTGATGGCTAATGGATTTGAC
EMPV1_32537 GTACAGTGTGAGGGGGCCACGATCAGGGCTTCAACGTACGAATTTAGGAAGGATGCTAGT
EMPV1_32538 ATGATGAGTTCCGGGGAAGGCGACAGGAGGCGGCCAAGGTGGAGGAAGCGCTAAGGGAAG
EMPV1_32540 ACCAGTGGGCAGCTGCATCTTGCCCCTCTGAAGACTTAACCAATGGCTCATATGATGATA
EMPV1_32541 ACACTTGTGTAGTTAGTCCTGAGGTGTCCCTGTCAGACGTTCGTTCTGTGCCTGACGTGT
EMPV1_32542 CGCTCCAACCTGCTGGCCCTGGTGGGCGGTGGTAGCAGCCCCAAGTTCTCAGAGATCTCA
EMPV1_32543 GGATCAGCCTGAAGAGTATATCCACATGTAGCAGGACCTGGAGAAGGCACAGAATGAGGA
EMPV1_32546 GCAAATAAACAGGACATGGAACAGGCCATGACTCCGTCAGAGATGGCAAATTCGCTTGGC
EMPV1_32547 TTAAATCGCCCTGTCCCCTCATGTTCAAGATAAGGAGGCTGAGGCCTGGGAGGAGAGTGA
EMPV1_32549 CACAGTCACCTTGCCCTTCCTCCCACCACCTAAGAAAAGAGGGTCAGACTAACAGGTGAA
EMPV1_32551 ATTGATACGAAGTGCTGAGCTGGGTGCATCTGGAGGTGTGGGGGTGAGTTTTAGCGATCA
EMPV1_32552 AGCCAAAGTTTTACTTGTATTTGTTTCGCTGCCTCTTCGCGCTGGCTGGAGCAGGAGTTG
EMPV1_32553 TTCCTTTTCTTCCCCCCACGTGTAGAGGTGAGAAGTCAACCAGAAAATGTCAGACATTCT
EMPV1_32554 AGCAGAGGACACTGTCTTGGACCTTGTGGACTACTGCCACCGCAAACTAACTTTGTTGGT
EMPV1_32555 CTCGCTCTGTTGATTTCTACGATGCATGTGTTGTGGTGTTATCCTGTTCCATCTCCTAAG
EMPV1_32556 GAGAGCCTGGCACGATCGAAAGGTCCTTGGCCACCTCCATCATCCTTGTGCCTTTCACAT
EMPV1_32558 ACTAACAGATCCTAGAAGGTGGGGTGAGGAGCAATATGCCAGGTTTGGCAGGGCTGTCCT
EMPV1_32559 TCTGTCACCGCTGGAAGAGGCTGGTGGACGACCGGTGGCTGTGGCGACATGTCGACCTGA
EMPV1_32560 TATCCCTGGTTATGGAGGAGGAAGAGGAGGATATGGTGGTGGAGGACCTGGATATGGCAA
EMPV1_32562 ATTCTATAGGGTGTATGTGTGTAAATGAGGTGGAAAGCTTAGAGGGAATTGGGGTGAAAG
EMPV1_32565 TGGAGAGGAGCAAAGCCTGGTGTCTGCCAGCCTGGTGTCCCATCAGTGCCCCTCAACTGT
EMPV1_32566 TGAGGACAGAAGTTCACGGCCCCTGCTTAGCCCCAATTCCTGTTGCTTCATTTCCTAAGG
EMPV1_32569 GAGCTGGGACTTGAGCTGGGGAGAGGGCAAAGGAAGCTCTAGAGAGCCTGCGCAGGGTAT
EMPV1_32572 AAGAGTACCATCCTTCTTCCAGGCCCCTGACTGCTCCTTTGCATTGGGCCATGGTTTGTA
EMPV1_32574 TTCATTTGGAGGTTGGGGAGGAGGTGAGGAGAAAATGCTCTTGTTCAGTGGTTTGGCTCC
EMPV1_32575 GGCTGGGGATCTGCGAGAAAAAGGCCAAGAGTTGTGATTCAAAGGATGTTGCAAAACGAG
EMPV1_32577 TGGCTAAGGTGCAGGTCTCGGGGCTCACAGTAGAGGCAGGGAGGAAGCCTTGAAATTGCA
EMPV1_32579 CCTTTGCCCCTGGTGGATGGTACCTTGCCACCTACTCAAACACTGACAGCCACATTTCTT
EMPV1_32582 GGCGGTGGACAGTTTGATGACCATAGGGCAGTCTCTCCTGCAGGGGGCTCTTCAGCCTCC
EMPV1_32584 GAGCATTGTGCAGAAGCCCACCGAGGTGTCTGCTTGAGATGAAGGCTCTCTGGATTTCCA
EMPV1_32586 CCAAAGACCTGCTATGAATTCCTTTCCCAGTAAGAGGTGAGGCCCGGAAAATGGTGCATC
EMPV1_32588 AGCTCCAGGACTTCCCAGCTGCTGCCGCCCTGGCGGCCAGGAGTAAGCCCCGAGCACCTT
EMPV1_32589 GTGGGAGAATAACACAGGGACTGAGTGCTAATGGGTATGGAGTTTCTTTCCGGGGTGATG

EMPV1_32591   ATGTGAGTGCTTACAAGAATGCAGGCACCTATGATGCCAACGTGAAGAAGAGAGGCCTGG
EMPV1_32592   ATTCACTGGCTTGCCTTGACCGCCCCTCCCAACTACAGTTCTCGTCATGCCCATTTACAA
EMPV1_32593   TTTAAAGCTCACTGGAAAGGGATGAGTCCAGGTGGGTTCGGGGAAAACCGTGCTCAGCCT
EMPV1_32594   GACTTCAAAACCAGCAAATATGTCGGGAAGGTTGTGGTCCAAGGCCATTTTTGCTGGTTA
EMPV1_32595   GATCAGAGCGGCATCTTCTACCTACACTGCAACTCTCGGCAACACCGGATCCTTAACCCA
EMPV1_32596   CAATACTGATTTCTTCCTTCAGCTCTTCAAATCTTCCATATACTTATTAGTAATCTGATC
EMPV1_32597   CAGAAAGAGCTCGAAAGAGTGTGCAACCCCATCATCAGCAAACTTTACCAAGGCGGCCCT
EMPV1_32598   GCATGTTTTATTTGCTTAGGGGGTTCCTTGTGATTCTTGAATGCACGGCTTTACAGCTTC
EMPV1_32601   CTTAGACAAAATGAAATGGCAGAGGAACAAGTTCTACATGAAGGAACAAAATAAAACCCC
EMPV1_32603   CATATACACTCTTCCGTAAGAGCCAGAGCATTGGGCTGATACAGCATTTGAGAACTCATG
EMPV1_32605   CAGACCCTGCAAAGTGCCACAGTTGAGGCTATTGAGGCTGAAAAAGCAATACGAGGATTC
EMPV1_32606   GAGCTCATCAACACAGCCTACACCAACGTGCTGCTTCTGCGGCAGCTTTTCTCACAAGCT
EMPV1_32610   AGCATCACCCAGCCCCCCATCCCTGCCGCCGACACGGGGTGTACAAATCAAGAGCCATTT
EMPV1_32611   CTCCAGTTGTAGAAAAGCAGATGTACTTCCCTCTTCAGAATTATCCAGTGAACAACATGG
EMPV1_32614   CTTCTTGGAAGTCCCACGTCTTAGGAAACGAAGGTATCTTTTCGAGCTAAGTCAGACCTG
EMPV1_32616   AGGAAAACTGGGTTCCACACAAGTGAAGAGCTGCAAGGACCCTTAGGGGCAACAGAACCT
EMPV1_32618   TGTCTACATCGAAGCCCCTACGTTTGATCTCCAGGGGTCCTACAGTGATTCTAGTTGCTG
EMPV1_32619   GTGCTGACAGTAAAGCAAGCTCAAAAAAACAGGACTAAGCTGTGGCTCCTCACCTCTGCC
EMPV1_32620   GTGGGAAAGCCTTCAGTCATAGGTCATCTCTCCTTCAACATCACAGAATTCATACTGGAG
EMPV1_32621   CAGACTGAAAGTGCCAGGTGCACAAACGTCATCAATGTCATCCCACTTCTCTACCTCCAG
EMPV1_32622   TGGAAGAGCAACTGGTTTATCTTCGTGATTCTTTTGGGTTGAAGACTCTCTTGGCCCGGG
EMPV1_32624   ACCACGATGGACCCCATCTGTCTGGTAAAGAACCAGAACAATCATCTGACAGTAAATCCC
EMPV1_32626   AGTGGACATGCTGGGTTTGGTGCAGGCCAAGAAGTGCCAGATCCTTGTAAGCGACATAGA
EMPV1_32627   GGGGCTCTACTTCAGAACCCACAACCAAGTCAATAAAAAGGAACTGCTGTGTCCCATCCC
EMPV1_32630   TACTTTTGGAAAGAATTCAAATAAGACCTAAAAGCAGAAGACACTAGACACAGCATTCCC
EMPV1_32631   TGCAGTACCTGTCTCAGCCACAGCCACAGCCCTATGCTGTGCACAGCCACTTTCAGCCCA
EMPV1_32632   TCTAGTTGTAAGTTTGTACCTTTAACAACATCTCCCCATTTCCGGCACCCCCAGCGCCTG
EMPV1_32634   TTGAGCTCTGGACAAAAGTGGACCAAGGATTCAGCATTACCTGGCCTTGTACCATCTTCC
EMPV1_32638   ATACCACATGACTGGTTGGGTATGGTTGGGAGGTTGGTTGTTCTGTCTGTTTAGGGGGAG
EMPV1_32639   CCTTCATTTGTTTGGCAAAAAGGAGGACGGGCGCATTTTCCAGTGGAGAGGTAATGCTGG
EMPV1_32640   TGCTGGCTAAGCCTCACCTTCCCTCCAGGATGCCACAACCAGGCATGTAGGTCCTTCAGA
EMPV1_32641   CATATGGGCCACACCCAGAGGAGCCAAAGAATGTCTGATGAACCTCCGTCAACCTCTACA
EMPV1_32642   TCTCGTCCTTTCCCCAGAGTTCCTTTAATTCTGCTGGAGTCTTCGTTTGTGTCTGACCCC
EMPV1_32647   CCACTCTGGAACCCAATCTCAGAGTTTGTAGTATGGTACAAGCCACACAAAACTAAGCAC

EMPV1_32651	AGATATCCACTGCTTATGACCAACGTGGTATGTGGGCAACTTGTGGCCTCTGCTTGGGTT
EMPV1_32653	AAAGGGGATGGAGGGATCACGCCCTCGGAAGACATAAATGGGACGGACGCCCGCTACCAA
EMPV1_32654	TGTGTCGGAGATAAGGGTCTGGAGATTATTTACAGTGAGGAAGGTGGAGAGTGGTGCCTG
EMPV1_32655	TGAGACGTCCAGTCCTCTGTTGAATAGAAATGGCAAAAGTAGGCATCCTCACCCAGATAG
EMPV1_32656	TGAAGAGAAGGCACTGACCATGGAAGTGGTCCGCCAAGTGAAGGGAGATCTGGCTTTTCT
EMPV1_32657	ACAGGGAGATACCACCTGACACCCATTAGGATGGTTTTTATCAAAAGGACAAGAGATAAA
EMPV1_32658	TCAAACTCTGTGGCTACCAGCCAACCATCCATCACCCCAAAAGGCCTGACAAGCCACTAT
EMPV1_32659	GTGTAGTCATGTCCTATACCTACATCATCAAGACCATCATAAAGTTCCCTTCTGCCCAGC
EMPV1_32661	CTGCTTTGGATGAATACCAAGGAATGCGACTGCTGGATCACATGGGTAGATTACGTTTGG
EMPV1_32664	AATGTTCCTGTCCTCAGGGAGCTTGTACTACGGAAGTGGGAGAAACAGTCCGAAATTGAC
EMPV1_32665	CAGCCACTTTGTACAGCTCTTCACTTTTTAGCACTTCGGTCTGTTTACTGAGGCCCTGGC
EMPV1_32666	ACAAGGTCTTGGGACCGTGTCTGCCTCCTCCCTACACCCGGACCACACTGCACCTGTTCA
EMPV1_32667	CCTTGGTTTCCCTGGTCTAAGTAGGAGGACAGGATGGCCTCAAACATTAAGGACTTTCTT
EMPV1_32670	TCTTCATCATTGTCTCTTATGTGTGTATCACCCGTGCTGTTCTGCGGGTCCCTTCTGGGA
EMPV1_32672	GAGATGGGCTTTCTCTGCTGGCTTCAACCTGTCCTCTTTAGGGCATTGTGCATCTCACTG
EMPV1_32673	ACTTCCTGAGGAGAACGAGGACCTCGAGGCACTTCCTGAGGAGAACGAGGGCCTCGAGGA
EMPV1_32674	ATCTCCAGGAGCGGGGCAGAACCGGAAGGTGTCGAAAGGAGGAGCAGTCTCACCCTGCAG
EMPV1_32675	TATACCCAACTCTAGCCCCTAATAGCCATCCTGTCTCATTAAGGGGATGTGGGGAGGACA
EMPV1_32679	GAGCTCTACACATCCGGAAGGTCCTCCTGGTCCTGCCCAAAACCACTCTGATTATCATGA
EMPV1_32680	TCTCCAAACCCGCTGAGGTGTTTCCCACCGACTTTCTTTCCGTATTGCACTAGCAAACAC
EMPV1_32682	GGAAATCAGGGGGGAGTTTACATCTTTGGTGATGGACGAGGCCTTATTGAAGGAAATGAC
EMPV1_32683	GCTGAGCTTCGCAGTGGCATCATTAGAAACAACAGCCTGTGGGATAAACTGATCTTCCAC
EMPV1_32685	TGTGGCCCCCTTCCGCTTCAACGATGTCTAAGCCTGCTTCAGGAAAAGTGCCCACCCTGA
EMPV1_32686	CCTACTTTGTCAAGACAGGGATAGAACCTGAGTCCTAGCACTATAGAGATGCCACTGATC
EMPV1_32688	TTAAACCCACGGAGGACGCCATATTGGAGAGGATTCGAGAAATGGGCAGCCAGGAAGAGA
EMPV1_32689	CCACTCCTCAAAGAGGCCAAATTTCCAATTCTGAGTGCAAACATTAAAGCCAAGGGGCCG
EMPV1_32691	GATTATTAACCATGATACTGGGGAATGTCGACTAGAAAAACTCAGCAACAACATCACTGT
EMPV1_32692	CCTGGATCCCCAACAAACATTATTCTGGGATTTATGGCCTGATGAAGCTTGTCCTCACCA
EMPV1_32693	TGCTCCTGTAACTTGGAACTGGAGAGGAGGCAAGATGCTGGCATAGCTGTTAAACTGAGA
EMPV1_32694	GCATGATCAGCATTTCCACGTTCTTGCTCCTTTGCCTTATTGTGGCCTTTCACGCCAGAG
EMPV1_32695	GAACAAGGAGCATAGCTTTTTCATCACAGACTCTGACACCTCCGGAGGAGATTTTTGGAG
EMPV1_32696	AGATATGGGCTGTTCTATAACACCCCTCTGATATCTGGCTGTCAAAATACCGGATTATTC
EMPV1_32697	CTGAGCTTCCATCAGTCCCCCTGCTTTGTTCTGCCAGCCCTGAATGTTGCAAACCAGAAT
EMPV1_32700	CAAACTGTTACATTATGGTTCAGGGAAACACGGTTTCAGCCATTGGACCTTTTAGTGGCT

EMPV1_32701 AGAGACAAGTGGTGTTTCTTGCGGACATGGAGTCTTTCTTAAGGACGTGGAACTCTTGCA
EMPV1_32702 AGAATAGAATTGTTTTGCACAGAATATCAGAATAATAGAAATTCCCTAGAAATCCTACTG
EMPV1_32703 CCTTGTTTGGAAATCCCTTTCTTAAGGCCCCGAACGTTCTCTGAACCGTTGATCGCTTCG
EMPV1_32704 CATCACAGACCGTGGGGACGTGCGTGTGCAGTTCAGCCACAAGATCCGCTGGACCTTCCA
EMPV1_32706 GCGGCGCTGTTTGGCGTCGATCCTCTTTTCCGATTGTAGCTTACAGATAGTTTTCTTCTG
EMPV1_32707 AAGAAAGAGGAGGATCGGCTGTTCATTCGTTACCCACCCATGTACTCGACACCCCCAGCA
EMPV1_32709 CTTCCTTTCTTTGCCATGCTCCAAATGCTAGAAGGCTGATTTTGGGGAACTGCATCATCC
EMPV1_32710 CTGGTGACAGGCGAGTGTAATCGTCCTCAGGTAGGAGCGCCAGCACATTTTCCACAAATA
EMPV1_32711 ATCATGTGGAGAAGAAACCCCTGGTGGCACTGGTGCAGACCGTTATAGAATATGCTACCC
EMPV1_32712 AAGCTTCGTGCGCCTCTACCTATGTTGCCTGACGTTACAGGACTCCACCTTCAGCTTTTC
EMPV1_32714 CAGGAAGCGCTGAGCCAGCAACTGTGTGGAAGGAACTACTGGGGATGAAAACCATAAGCT
EMPV1_32716 AACCAGAAGGCACCACTTCACGCTTTTAGAATGGCCAGAATCCAGAATACTGACAATATC
EMPV1_32717 TTGCTCTCATCAATGGTTCCTTCCGGCTTCCCAATTCTCCCACACTTCCGTCGGGTCCCT
EMPV1_32718 GCTTTAACATCCGGTAGGAAGACTCTGTCACGTGGGTTTGGAATCTTTGATTTTCCCCTT
EMPV1_32719 CTTAGGACAGGAAGTGGCTGCTGTTGGACAGGGATTCTGAAAGGAGGGGACAGTTGGTTT
EMPV1_32720 CACAGTGGTAATGACAGTTAAGCCTCTGGATGAGCCTCTACGCGAAGCAATCCCCAAAGT
EMPV1_32721 CAGAGAGGGGCGACACTCGTGAGTGGGTGATCTCAAAGGTAAACGAGATTTTGTTTGGCT
EMPV1_32723 TGCCTGAGCTGACATGTCAGACCCTCGACACAGGGCAGCAGAGACACGGCACTGGCCCAG
EMPV1_32724 GAATGGTTTATTATTGCCAGTTTTGGCCTCCTCAGTGCTCTCACACTCTGCTACATGATC
EMPV1_32731 AAGAGTTGGAAAGTTTCCCTGTGGTACTGTGGGTAAAGGACCCAGCACTGCTACGGCTGT
EMPV1_32733 GAAGAACTGAGCCCTCCCTTTGATGCCCAGAAGATTTTGTCTGGGGGCCACACTGTAGAC
EMPV1_32734 TGATGGAGTTGGGGGCCATCCACAGCGGAATCCACTGGAGCAGCCAGGTCCAGTAGAGCT
EMPV1_32737 GCCCCCACCTGCTGGCCAATCAGACTACACTAAGGCCTGGGAAGAGTATTACAAAAAGAT
EMPV1_32738 TGGCCAGAGCCTCGGGGAACTATGCTACCGTCATCTCCCACAACCCCGAGACAAAGAAGA
EMPV1_32739 GTCTCTGTGATACAGTTACTTTCTAGTATGTGGGCTTCTCACCTAGCAGGTATGGGGTTG
EMPV1_32740 CTTAATAGTCCAGCTCCAGCCAACAGATGCTTTGCTGTGTATTACCAACTTACCCACTTC
EMPV1_32742 ACTTTATAGAGACTGAGCAGGGGGAGAAAAATCAGGAAAGCCCAAAGCCTCAGCTGTCGC
EMPV1_32743 ACAGGCCTGATCCCTAATGGAGACTGGACCTTCCAGACCATGGTGATGCTTGAAACGGTT
EMPV1_32745 CTCGCCTAGCTCTCAATACAGTTCTCTGCATTTTCCCAGGAGTCAGGAATAAAATGGGGG
EMPV1_32747 CCAGGAGATTTGGGTAGGAATGGGGTTGATGGGACTCATTGTCTATAAAATCAGGAGTGC
EMPV1_32748 GTGTCCTGTTTTCGCCAATTTCATGTATTTACTGCCACCCTAAAAACTGCCAGGAGGGGG
EMPV1_32750 CGAACAGATAACATGGATGGACTTTGGTGGCCATTGAGTATCCCCTGAACGGAGTGTGGA
EMPV1_32752 GCAGCCAGTTGCTATTGATGGGATATCTTATTTTTGGATCTCTTTGAAAGCAGAGCCTGG
EMPV1_32753 CCCAGCCCTGGTACAAGTCTACTCTCATCCACATGACTGTTGGAGGCTTCCTGCCTTTCA

EMPV1_32754	CTGGCGGTTGCTATTCTCTATGATCCTCTTTGTCATCATGGTTCTGTGGCGACCATCTGC
EMPV1_32755	AAAGTCTTCACTGGGAGGGTCTCCAAAAGGCCACTATGAAGTGAGTAGGGCTGTCGCTGA
EMPV1_32757	GTAGGGCCACAAAGATCTGGAAAGAACCCTCTTTTAGGGCCCAGCTCCCTTCTTGCAACA
EMPV1_32758	CCCAGCCAAGCAGGGTACTTCCACTCTTAATACTTTACCATTGATCCCTCCAACCTCTGA
EMPV1_32759	GACCTTGATGCAGACAGGATATGTGGCTATGGGACTTCTCGCTGCCGGAGGTATTGTAAA
EMPV1_32761	ACCCAAAGCCAGAGAGTGAGGGAGCCTATTGCAAGTGTCTGCTGATGTCAGTGTCCAGGA
EMPV1_32762	GGATCTGGTGGACAAAGGCACTGAGGACAAGATAGTGGACGTGGCGAGAAACCTGGTCTT
EMPV1_32766	CAACCAGGCCTTTACCATCTACCAGCCAAAAAGCAATAGAGAATCAAGAGCACAGGCGAG
EMPV1_32767	AGGACACGAGATCAGCAGATCAGAAAACAACACTACTTCATTTTCTTGTGGAAATATGTG
EMPV1_32769	CGTTCAACCTCTACAAGAAAGAGTTCTAATGACAGAGATGGGAAAGAAAAGGCGGAGAAG
EMPV1_32773	TAGTGGTTAGGTTATATGATGCCAAGTGTCTGCTACATGAAGAACGTGGGTGTGACGACC
EMPV1_32774	TCAGGGACCATTGACCTTGGAGGACGTGGCCATCAAGTTCTCTCGGGAGGAGTAGGCCTG
EMPV1_32775	GACATAGACTCATCTACCAGTTGTCGGGTTGCGAGCAGGAGTGTTGCGTGATGGGAAACA
EMPV1_32778	CAAGGCACTCAAAGCACCTGACAGCATTTCACCTTACAGAAGCTGATTAAGCAAAGCCCG
EMPV1_32780	GCTGTCGAGAGAGCCAGCTGTGTAATCTAAATCTCTCTGATTATCCACCATGCGCCCGAA
EMPV1_32781	CTTCATGACGGTGCTGGCATTCATCTGCTTCATCTCCATTGCCCACTCCTACATCCACAT
EMPV1_32782	TGGTCCAACCTGCAATGCCACGCTCCGTGTATTTGACAAGCTGAGTTGGACACTCCATGT
EMPV1_32783	CCGTGCCTGCCCACTTGCATCTCTTATAAGCGTCCTATCTTGGTAAGTCTCTTTCTTGCC
EMPV1_32786	CACTCGGTGAAGCCCTTGGCGGGTTAACCTTCGAGAAACTGGGCCTTAATCTTTTTGGGA
EMPV1_32788	AGCAACCCGGGAGCCAAGCCGCATCTTGAGACTACACCACAGATCACAGCAACACCAGAT
EMPV1_32789	GAACCACGTATTGTTAGTAGAATTTTTCAGTGCTGCGCTTGGAATGGCGGAGTATTCTGG
EMPV1_32790	CCAGAGCGCAAGTATCAGCACCTCGCCAAGGTGGAGGAAGGGGATGCCAGCGCCTCCTCC
EMPV1_32793	AACACGTGGAAAGAGACTAGCACGGGAAAAAAGGTGAAGCTGCAGGGGCCTGATTGCTAT
EMPV1_32794	AGCAGCCTTCATACCCTTTCCCTCAGCCCCCACAGCAGTCTTACTATCCACAGCAGTAAT
EMPV1_32795	GAGGGCTGGAAGTGCACAGGCTGAGAGAGAAGTCGATACTTAACACAAAAGGCAATAGGG
EMPV1_32799	CTTATGAAGTTCTGGCCCTGAAGAAGTCTTTGACATTAGATACTCAAGTGGTAGAAAGAG
EMPV1_32802	GGCCTACCCACCACCCTTTGTCCCAAGGAGGCTGATACATACTTAAGCACAAATCTAGAC
EMPV1_32803	TCTAAGAGAAAAGAGTCTCGGAACGTACAGGGTAAACAGGGAGGATGAGGCCATCTGCTC
EMPV1_32804	GTGGGAAGTCTTTTACATTTAGTTCCAGCCTCCGTTATCATCAGAGAGCTCACACTGGAG
EMPV1_32806	ACTGTTCTTGCTCACCCTCGCTATTTGCAGCACCACATTAAATATCAGCATTTGCTGAAG
EMPV1_32807	AACCTACTGCACCACAAGGGAACTCCAGAAAACAGCCCACTGAAATGAAGAAACGCTGGG
EMPV1_32809	TCAAAAGCCCAGCTGACCTAAAAATTGCCTTTGCTCAAGAAGTTACTCTGGGGAGTTCCC
EMPV1_32811	CTAGTTACATTGGAAACTGGTCCTTTCTCTAAGACTAAAGGAGAAGCCCACGAAGGAACG
EMPV1_32817	AGAGGATCTGAACGGCTGAAGACTGGCCCTCAGGCCTGGCGCACAGCCAGTCCCTAAAGC

EMPV1_32818	CAAGCCGTGTCTGCAGTCTACCCCATGGCTCACCGCAATGCTGGATCCTTAAACCACTGA
EMPV1_32820	TTGCTCCTCAGTCGAGTTCGTACACAATGATCCACCCAGCCCCCGAACTGCCCTTCCAAA
EMPV1_32821	AAAGAAGCTCAGAAAGGATCTCTGCTCTTTGACGGCCTCCCTCCAGCCAGCAGTGCTGAC
EMPV1_32822	ATCCTACAAAAATCTGCACCGGGAAGGGAGCCGTGACTCTCCGGGCCTCGTCCTCTTACC
EMPV1_32823	CGGGTGATAACCACTGAGGATGATGAAGATGAGACGACTGTGGAGTTGGAAGGGCAGGAT
EMPV1_32825	ACATCACAGGTGAGACCAAGGACCAGGTGGCCAACTCGGCCTTCGTGGAACGTCTGCGGA
EMPV1_32827	TAGGTCTGGATTCTCAGCTCTGCTTCATCGACCTGTGCCTTTTCACCACCACAGCGTTGA
EMPV1_32829	TTACAGACTTCCCCTCTCTGTTCACTTGATACCAGTTACTGATGGTTACATGTCCTGGGG
EMPV1_32831	CGCTTGGGTACAGGTGTGTGCCTGCTTATGTGTGCAGGTGAATGTGTATGTTTGCACGCA
EMPV1_32832	TCAGACACCAAGGGTGCTGGAACAGCTCTAGGGCATATATTTCTCTTACGCAGGAGAAAG
EMPV1_32833	CAACCTCCATAGGTCAGTTTCCGTAAAAGAAGAGGTGAAATTGGGATGAAGTCCTCAAAG
EMPV1_32834	TGACCAGTGTTCCTGCCCTCGGAACAGACCCCAGCCTCTGCCCAGGCACATTTGGATTTT
EMPV1_32838	AAGAATGCTTTAGAGGGCTTGGGCCAGAAGAGTGAGAAGGCAGGGTTAGGTAACTGCTGT
EMPV1_32839	AGCCTGAGCCTGGATATGGTTATGCCCCGAACCAAGGACGCTATTATGAAGCCTATTGCC
EMPV1_32842	AACTACCTGAGTCATTTAGACAGCAATAGCTTCTCCAGCTTTCCAGAACTGCAGGTGCTG
EMPV1_32843	AAGCCCTTCACATGACTCGCTTCACTGTTTCAAGGTGTGGGCAGAAGGCACCTGATCTAC
EMPV1_32846	GCCAAGTCTACCTGCGCCATCAACAACACCCTCATTGCTTTCTTCATTTTGACCACGATG
EMPV1_32847	AATAACATTACTCCCTCCGTGGAACCTGTGGATTGGAAGATGGCTCCTGCCTGCTCACAG
EMPV1_32848	GATTGCCTTCATCGAAGGAAGCAGACGCTCACCACGCTACACCCTCTGGTTCTGCATGGG
EMPV1_32849	GGGACACCTGTACCCTCGCTGCTGCTTTGGATATGTATTTCAGCTTTACTTCTGCCTTAG
EMPV1_32850	CTGTGGTGGAGAAAAATGACCACAGGATAGCTGGACTCTGAAGCCAGAATGGGCTTTGCT
EMPV1_32852	GTAAAGAAGGTGAGACTGAACACTGGGGCGAGGCACCGTCAGGAAGGTGACTGCAGCTTA
EMPV1_32854	GAGCCACCAGTCAGCTCTTGGTAGCGCAGCAGTTAAAGAAAATGTATCTGCTCTGGACTA
EMPV1_32855	TTGCTGTTCGAGCTACGGATGAGAGCCTCAACAGTCAACCCCAGAAGCTTTTTTGGAACG
EMPV1_32857	TGCCTGACTGCTGCTCGAGGAATAAGTGTCCTGACCCCCTGAACCAGCCCAAAAGAATCA
EMPV1_32858	TCCTGTGGGAGACTCTTCAGGCTCCAGAAGCAACTTCTACGACATGCCAAACATCACACA
EMPV1_32859	GTCCTGCTAAGTAACTGAGATGGCCAAATGCCAGGGTGAACCACTGCAGCCTCTTTATAA
EMPV1_32860	TCAGTGCTGTATTTTATATGCCAAGAACATTTGGGAGGAGGGGGAGGCTACTGTTCGCCC
EMPV1_32863	AATTTGAAGTGGTCCCCACTGAGGGGCACTCTCCACCAGAGAGCGGTGGCCACACCAGAA
EMPV1_32865	CTGCCAGGTGCTTTCTCAATGAGCATAACCGCCCATCATCCCAACAACCCTGTGAGATAA
EMPV1_32866	GGGTGTTAATCTTATTCTCTGAGATCCAGTGCAGCACGTGTCACGCATTTCGTCTCCACG
EMPV1_32867	CTTGAAGGCATCTCGAAAATTGCAAGGTGATCCAGATTTGCCAATGTCTTTTACTCTGGC
EMPV1_32869	CTACAGGTGGTGGTCGAGCATGTACTCGGGAGAGGGGTACCAGAGCCTCCAGTCCATGAT
EMPV1_32872	TATACGAGCCGTAGAATGGTTTGCCTTGGGAAGTGTGATTTTACCCTAAAGAGCTGGAGG

EMPV1_32873	TCCTTACTGACAAGGACACTCCTGACTGGCACTGGGGAAGAAGAATGAGCAGTTTTAGGG
EMPV1_32874	AGACTGCTCATCGTAACCTAGTCCAGTCGGAGGACCCTAAGAGCGTCATGTTTGTGGAGA
EMPV1_32875	TGAGTCCTTTTCTTCTGTCACCTGATGAGGGGGAGTTTATAGGACCCTGCCCACCACCAA
EMPV1_32876	GTTGGCAAAGGACTTTGAAGATTTCCGTAAAAAGTGGCAGAGAACAGACCATGAGCTGGG
EMPV1_32877	TTCTGCAGCATGCCGCAGGACTCTTACATGGAATGTGCACTCTCTGCTTTGCTGTCACCG
EMPV1_32878	TAGAAGGCCACTTCCAGACCATCAACCTGAGCCCCTTCCTTTTCACCCGCTTTTACTTGG
EMPV1_32880	GTGGTACATGCAGTTTGATGATGACGAGAAACAGAAGCTGATTGAGGAAGTGCACGCCGT
EMPV1_32883	CATGGGAAGGAAGATGGAACTGAAAGATGAACGGACCTTAGACCCTGAGCACATCAAGGA
EMPV1_32884	AAGTCCAGCATCGTGTGTGCCATTTGCCTTGGATTAGCGGGCAAACCTGCTTTCATGGGA
EMPV1_32885	GGCTTTGGCCTGGATTATGGTTGGGAGTGACATGGCTTTGGTCTTTGCTTCCTATGCTTG
EMPV1_32886	TTGAGCGGATCCACGAAGGGGCCGTCCAGCACCTCCTTCAGCATGTGGCTGCTGGCCGCC
EMPV1_32887	GGGAACTTCCTTCTCTATGCACACCTGACCTACGTCACTTTGCCGCTGCATCGGATTTTA
EMPV1_32888	CATATGTGAGGAACCTCGGTGTCCACAGCAAAGCCTGATGGGGACCTGAGGATGCTCCGA
EMPV1_32889	ACGTGCCTTCCAGAGTCGGAACCTTCCCGGCGCCATCACAGGACGGAAGTTCTAGAGAAT
EMPV1_32892	GTCTCATCTAGTCCCATGAGGAGATTTTCCTAAGACAGAGACTAGTGTTGCTGGTGGGGC
EMPV1_32893	TCAAGAAGGTGGACGGCTTCAGGTTGTCTTCTCCAAAGAGCATTTCCCACGGTTTGCCAA
EMPV1_32894	CTCATTGCTTACCCACTCCGCATGCAATAGTTTGCATCTATTAACCCTAACTCCCAGTCC
EMPV1_32896	GGGTAAAATCGACACTCCAGGAAGATGGCTAGCATTCCCAGTTTGAGAAGCAGGAACTGG
EMPV1_32897	TGAAGTGGGGACATTGAAAGCCAGCTTGAACCCCATCCTTAACTGTACTTTGTATTCGCC
EMPV1_32901	GTTGAACTGGATAGTGTGAGTGAGGGGAAGTTACATATTCTCCCTGGCCTCTAGCAGGTA
EMPV1_32902	GGTGACGCCTCTCTCTCATTTCAAAATTTGCCAACTTAGGGCCTGGCTCTGGAAAGCAGT
EMPV1_32904	GGAATATGAGACTTCTCAAGGTTTCGGGCATGGAGTCCTCTTTGCTCACTGCGCTCTGAA
EMPV1_32905	CTCTTGGTCTCTTTATTAGTTCCTTCTGCTAACCTTTGAGCATAGTCCCTGTGCTCTGCT
EMPV1_32907	CTGCTGCCCCAGTTTTACGTCTGATGCCTGTTTACGTCTGGTGCCCTGGGTTTTACGTTT
EMPV1_32910	AATGTAAATGAACATCAATGTGCCCACAGGCTACTGAGTGGGAGGCACAGAAGGCAGAAG
EMPV1_32911	CCGCGCCCACCTCCAGGAATATTTCTACACCAGCAGCAAGTGCTCCATGGCAGCAGTCGT
EMPV1_32913	ACCCTTCGCTTAGAAAAGCCAGACCCAGCCCTTGTTTCTGTTTCCCTGGAGTCCCATGAT
EMPV1_32914	AGACCTGCTAGAGACCTGCCACTTCCAGGCCTTCTGGCAAGCCCTGGATGAAAACATGGA
EMPV1_32916	CTGATAGTCTCTTCCTTCATTTCAGAATCACCTCTCGGGCTGTGCCTGAGGAAAAGATTG
EMPV1_32918	GCCAAGGAACCAAGTTTCGTTAATTTCTCACTTCTACCCCTGAAGCCACCCCATGTATAT
EMPV1_32921	TGCTCAGAGGACCCTGTATCGGGATGTTATGCTGGAGAACTACAGCCTTTTGGTGTCACT
EMPV1_32923	CACATTCTTCCATCAGGTGGCTGGATTCTAAGAGGAATGGAAATTATGATCTCATGTGAA
EMPV1_32924	CCTCCATGAGCACGGCGGAATGGACGTTGAAAAAGATTCCAAGAATTATCGACATGATCA
EMPV1_32927	GGAAAAGAAGTTAGCGCTGCATCCACTACTTACCAGAATAAACCAACCACTCCAGCAGAG

EMPV1_32928	CATTGCGGTGGTTTGAGTTGCTGCTTTGACTCGGGTTCAACCCCTAATTGCCACTTAAGC
EMPV1_32931	GGACTCTACTTATAATATCCCTAACAGCTGGATTCTCCTGCTGCAGCTTTTCTTGGACAG
EMPV1_32932	TCGGAAAGGGAGTGACCCAGACAAAGAGAAGAAAGGCCTGGAGAGTCGGGCAGACAGCAT
EMPV1_32933	ATTGCGCGGCAGCGTAGTTCGGCGAAGTCACCGGGCCGTCTTGGAGCAGGACTTGTGTTT
EMPV1_32934	CAGGGGTCCTTCAAGTAAGAGCAAGTTGATGAGTTGTTAGAGGTAAGCCTTTTGCCTGAC
EMPV1_32936	AACAAGTTAATGCCTTTGCTCTCTCACTGGCGTCCCTAACTGTGGGTAGGAGATAGGACC
EMPV1_32937	GGCACAGATGGCCAGAAAACCCTAGACCACAAGATGGCAAAGGGAAAAAAGTAGCCAATC
EMPV1_32938	ATGCAAGAGTTTTCATTCACTAACCCCAGACTCCCAATCCATCCCACACCCTCCTGCTCC
EMPV1_32940	GCCTTATCCAGAAAAAGGTCCAAACTCCCTTCAATTCCGTGAAGCCTGAGAGAGGTAAGA
EMPV1_32941	GTTTTCGTTGGCATCAACCACGCCAGTGCTAAATTGGACTTTGCCAATAACGTTCAGCTC
EMPV1_32944	TCAGGGGAGGTTCCGCAGTCCAAGTCTAACCACAGAGCCAACGTCCCCCAACACTGGAGA
EMPV1_32945	CCTCATGTCCCATTTTGCTGGGCTTCTGATAGTTTCAGTAAACTGATTCCATTCTGAGAT
EMPV1_32946	CTCAGCTTCCATTACCTGTCCTTCCTCATAGATGGTAGGTCCCAGTGTAGTTAAGCATTC
EMPV1_32948	ACCTCCATCCCTGGGATCTAAAGGGGAGACATTCTCTGGGCATATCCTGCCTCTTGAACA
EMPV1_32949	AGAGGATCATTACAGAGACAGACTCTCCCGAGACACGGGCAACACTGATAGAATAGAGAA
EMPV1_32950	GCTAATTTCATGGGACTCATTCCAGTGTCGATTTGGCTTACAATCCTCCTGGGTGGTTTA
EMPV1_32952	GGCCAAGATGCCCTACACGGAGGCCGTGATCCACGAGATCCAGAGATTCGGAGACATGAT
EMPV1_32954	GAACTCTTCCAAGTTACCATGTCTGCAAGTGGGACGAACGGGAATGAAGTTACATCTACC
EMPV1_32955	ACAAGGCTACTTACTCATAGATCCACGCGCATAGGGAGTCGGAGTGTACCTGTCGGACGT
EMPV1_32956	CTGGGACATCTTCAGGAGTATGCTTCCTCTTCTCCAAACTTAAGCCAGCCCTGAAAGAGC
EMPV1_32958	AGGTTCCAAATCGTGAAGTGCTTGCTTCGACCATGTATGTTGTAGGCATGTGCTGGCTGG
EMPV1_32962	GCATGGCCAGAGCCTCACACATAAGTAAAGGGAGTCTAAAAGAAAATGCTCGTTCCCTGG
EMPV1_32963	AGGATCACCATCTGGACGGAAGAGTCCAGCCCGGGAAAGGCCACCTGCTGAAGTCCAGGA
EMPV1_32964	CTGGACAAGGTGGTTGGAATGATCCCGATGGGACATCTGGGGGACCCTGAGGATGTGGCC
EMPV1_32965	CCTCGCCATGTTTATACTAGTATCTGATGTGTTAAGTCTTAAATGGTAAAGGTAATACCG
EMPV1_32966	GTCATTTATGTCTATTTTAGACCTCTGTCCATGTACTCAGTGGTGAAGGACCGGGTGGCC
EMPV1_32967	AAGGTCACCATGTCCCGAAATGTCTGTGTGGAGCTAGTGTCTTGTTGCTACCTGTGTAGG
EMPV1_32968	CGTACGTGCCGCTCTTCTGCAGCGTCCTCACCAAGCTGGGCTGCGGCAGCCTCGACTACA
EMPV1_32969	TGTTCCTCTTGCCTGAAGAGATACCCCACAAAACACAGCAGACCCTGGAACAACTCGGGG
EMPV1_32970	ATAGCCCACGCTCGTCAGTACAAACTCGATCTGGTTGCCCCAGTTGCCCCGTTTGAGGTT
EMPV1_32971	CCGGCTTGTGATCCTGAAACCAAACGGGGAAGTCATCACTGACAAAGGACGGAAGCAGAT
EMPV1_32972	TTCAAGTACGAGACGGTGCCCAACCTTCTCACCCAGCACTGTGCCTGCATCTAAGGGTGT
EMPV1_32975	TAGCAGTCCCCTGGAGGTCCCCAAGCCCAGATCAGTGGACCATCCCCTGCCCGGATCCTC
EMPV1_32978	GTAAGGAAGGGGCAAGCTTCGCATCATCTACTTGTAAAGCTCCGTCCTCCAGCACCTGTT

EMPV1_32979	GCAATCCCTAATACTGAGAGGTGACTGTACTGGAAGGAGTCTGTGAGGGAAACCTACACC
EMPV1_32980	TTGATCCACAACGGGAACTCCTGGCCTCCTTTGTCTTGGCCTAATGAGAGTCTTGGCTTC
EMPV1_32981	TTCTGCACTTCTTGCCACCCGGGCATCCGGTGTTCTTGACCTGGGAAAGATGGTTATAAT
EMPV1_32982	TCAGATATTTCACATCCAATCCAGCCTCAAAGGTCCTTTGCGCTCCAATGTTGCTGTCAA
EMPV1_32983	AAAAACCCCAAACGGGGTCCCTACTTCACACCATCAGCCCAGGTGCAGCCGCCAGACTGA
EMPV1_32984	CCCAGGGGAAGCAACACAAATGCCAGAATTGGGGAGAGAAGAGTTTACTGGTTGGCTACT
EMPV1_32987	TGTCACTTGTCACTTGAGCCTCCATCCACTCTGTTCGCCACTTCATGCCTTCCTTCTGGG
EMPV1_32989	CCGCGTGTGATAACGTGATGACTATGTCAATTACGTAGGAAGACTCCGTTAATCGTGGTG
EMPV1_32990	AGATTCAAGGTTGCCTCTACCCTTTGCAACTATGTAACCATTTTAGACCCCTACCCATCC
EMPV1_32991	AAAAACAAAGGCAAACTTCTGGCAGATCTGACCTTGTACCTGGGGCACCTGCTGGGCAGA
EMPV1_32993	TCATGATCGGGGATGACATCGTGGGCGACGTTGGCGGTGCCCAGCGGTGTGGCATCAGAG
EMPV1_32995	GGCACTCAAAGAAATCCGTAAATTCGCCATGACAGAGATGGGAACTCCGGATGTGCGCAA
EMPV1_32997	ATCCATTCAGGAGTTTGGGGCACACGTCCAAAGTGACTACGTAGCCAGAAATGGGATAAC
EMPV1_33002	GCACTTATCACAATTCCCACAATAGATAAGCAGTTAATACAGAATGGTTATTTTCCTTCT
EMPV1_33003	TTCAGAGTCTAATTGAGGGTTTGCTCCAGTATTCGTGGCCCAGTGACAAAGATCCCCTGG
EMPV1_33004	GAATTCGGCTTCGGCTTTGAAAGCAGCTGCAGGCATTTCACCTGGGGCTGCTGGTTAAAT
EMPV1_33005	CCAGTCAGTTTCTTCCCTCCAATCTCCACACTGTTTTCCATCCTGGCTAGAAGCTGCATC
EMPV1_33006	GTGTTAGGGTTGTAAAGAATCTCAGTTTGGTTAGTGCAGAGTTCAACATGGACACTACTG
EMPV1_33007	TCTCCTTTGCTGACTACAACCTGCTGGACCTTCTGCTGAGCCACCAGGTCCTGGTCCCCA
EMPV1_33009	GAGAAGGACAAAGGGAAGCTGAAGCCCAACCTTGGCAATGGGGCGGACCTGCCCAATTAC
EMPV1_33012	CTATAAAGTGTAAGTGTGACAAGCCACCTCCACCCCATCCCAGACAAAAACTGCTTTCTG
EMPV1_33014	GAGTCCAGAACCCCTCAGCACCATCCCCTGAACAACCTGCGCAACCTGCAGCTAAGAATA
EMPV1_33015	CTGACTGTACAGGGAGACACCTTTCTTACACATGACACCTACTCCTCACCTTTGGTTGCA
EMPV1_33016	TTTCACTTCTGACTTGAGAGGTTGTTCAAGGAAGAGTTCAACGGGAGGAGAATCTAGGCC
EMPV1_33017	CCAGTCCAAACAAAGAATGGTCTACACGCTTGCTCTCATTTCCTGTTAAGTTACTACTAG
EMPV1_33020	TTCTCGTTCTTGCTTCTTAGCCTGGGGGACTTTCTCTGGACTTGAGGTCCTTAGGCCGAT
EMPV1_33022	GGAGTCAGCGTCCTGTTAAGTGTTCCTCATGAAATAACCATAGCTGTCATACGGAATGCA
EMPV1_33024	GGCTGTGCCATATATTCTGATATGGGTTGCAATACTTACCAAACAAGGTGGATGGGTGAG
EMPV1_33025	GCAAGGCTTTCTCTCCCAAAGCAGCTTGTTCTCCGCCAGTCAATTGTGGGAGCAGAAGTT
EMPV1_33028	CCCATTATTCTATTAGTATTTGCAGCTTGCGAGGCCGCACTCGGTCTATCGTTACTAGTG
EMPV1_33029	AGAGATATAATGTTGATTGCTGCTGTTTTTTATGTCAGATATCGAACTCTTCCAACACCG
EMPV1_33030	CTCCCTCTACAACCTGGACGTGTTCTACTCCTCCAACATTCCGGCCGCCGCCAGGCCCTA
EMPV1_33031	CCATGGGAGGTCAGACAGCCAAAATCCCACCTCACTGACACCAGAGGGGAAGAATCATTT
EMPV1_33032	CTCTGAATGTCTCATCCATTAACAGAAAGATAAGGAACCATCTCCCTTGGAGCTGCCCAG

EMPV1_33035	CTCTGGGCTTCCTGAGCCATTACCAGATGAAAAACCCAAATGCACTGTACCTTTACACCC
EMPV1_33038	AGGTTTCTTGGCGGTGCTGGCCCTAGGGGCCGTGCTGGCGATAGCGCTGCTGAAGATTGT
EMPV1_33045	ACCCCTGGCAATGTGGAACAGGTTACCACACCTCGGGCGGCATAAAAGTCAGGAATCTAA
EMPV1_33047	GTAAGAAGAGAATTGCCCTTTGAGGTCCAGCAAGGTCTTTCAACTCCTGTACCTTACCAG
EMPV1_33049	CACCTTTCTCTACATTCTTGGGTCCTACACCTTGGTTTTGCGAGCTGTACTTCGGGTTCC
EMPV1_33050	CCTTCACGCTCTTCTCTCCTTGTTCGTTCTGGAGTTCGTTGCTTTGTGGGAACTAAGACC
EMPV1_33051	ATTCCACAAGGATAACAAGCCACTCTTACTGTGCCACCCTTTCATGGCTCTCTGGGGCTG
EMPV1_33052	AGCTCAGTAGCCGAGCACTGTCTCCCTTCCTTACTTCGCACCTTGTTCGACTGGTACAGA
EMPV1_33055	TCAGACTGGGTGCTGGTGATAGTAGAAGAGGCTCCCAAACCTCAGAGAGGAGACCTAGAT
EMPV1_33057	TCACACATGTATGTACAGCTAAGCGCTAATCCGAGCATTTGCCTGCTGTCTCTCCTAGGC
EMPV1_33058	TCCCTGGGCCGTGCGGCGCTGGACCTTGCTATCATTTTCCTTTCCTGGGTGATGAAGAGC
EMPV1_33059	TTTATTGAATCTATTCAGTCGATTCCAGAGGCTTTAAAAGCTGGGAAGAAAGTGAAACTA
EMPV1_33060	GCTCACACCAGGGTTACCTGAAGCTCATTGCCTTTTTCATGCTGTTTATCCATGTAGAGC
EMPV1_33061	TGCATCCCCTTTTTCTACCGGGCTGACGACAACGACGAGGTGAAGATCATGGTGGTCTAA
EMPV1_33062	AGGGCAGGAGGAGCACATGACCTGAACTTGGTGTTCTGCTTCTTTGGCTGTCTTGCTTCT
EMPV1_33063	ATCACAGAGTTTCAGCTGGTTGGCCCTGCAACCCCCTCAGTTTGCGGTTGAGACAGATAT
EMPV1_33064	CCCTCACCATCACTGTCCCCACTCCTTACACCCCCTTTGGAGAGTTAATTGTCTGTGTGA
EMPV1_33066	TCCCCATGAGCATATGGTGTTCAGTCCTGGCTGTGAAGGCACTTGTGGACTAGAGGTAGA
EMPV1_33067	ACACTGCGAAAAGCACTGCCTGGTGCGGAGCCCTTTGTGGCTTTTGTCTTACTCACTCAA
EMPV1_33068	CATGTGAATATGAGGAGAAGAGCACTCTGAGCAGATGGAAGCGCAAGTGTACCCTGTGGC
EMPV1_33069	GGAAGGTGCAAATGGCAAATTGCAGGCATAAGGAGGTAATTTTAGGCACAAAACTGTGCC
EMPV1_33070	AATTAGACGTATGGGACGGGCCTGATAACGAGCCTGTGATTTACACGGGCCACACCATGA
EMPV1_33071	GGGACCCTCTCTTCTTATCCACATATCTGCTTGGACAGGCACGAAAGATGCCTGGCTCAT
EMPV1_33072	GGGCTCTGGTCTTTGAAGTTTGAAATATTTCCCCAGAATTGCCACATTCTTCTTTCTCCC
EMPV1_33074	GCACAGTGGGTTAAGGATCCAGCATTGCTGCAAGCTGAGACATAGATCACAGATATGGCT
EMPV1_33075	CTTCTTCTGGCCTCCTCGTGTCAGTCTAGGGGCCTGTTGCCTCTCCGTGTTATTTACATT
EMPV1_33077	CGGATAAGCAGAGCGAAAGCCAAATCACTTCATGCCTGCGGTAGCCTTCTGACTGAAATG
EMPV1_33080	AGCACATACTTTGTCAGCAAGCGGAAGCCTTGCAGACCCTGGGAGTGGGCCCAGAGTTAA
EMPV1_33082	TCACCATGCCGTCTCCCATGCCTGAGTACCTGAACGTGCACTACATCGGGGAGTCCGCCT
EMPV1_33084	CAAAGGTGAAACCTTGACTCTATGTTGAGCTGTTTGTCCCTTGAGGAGTGGGGACCTGTC
EMPV1_33085	AGTAACAGTATCTTGGCTTCTCTCCTGTGGCGATGCACTTTGTCAAACCCTCCTCCTGAC
EMPV1_33086	GATGTACGATGACATCGCCAACTCTGAAGACAATCCTACCCCAGGAATCGTGATCAACAG
EMPV1_33087	CCTGTGGTGAACCGTGGCACCTGCATTGCTCTGGTCTAGGCATTATTTGCAACACTCAGT
EMPV1_33088	GACCATCGTCATCCGCCGAGACTACCTCCACTACATCCGAAAGTACAACCGCTTTGAGAA

EMPV1_33089	CCTGCTATGGATTGCCATGAATTGAATGAGGATGGAGAGCTGTGGCTGGTTTATGAAGGG
EMPV1_33090	GTATCAATCTGTTCCCATCTCCACACACATCTGTTCACCTATTTCTTCATTTGTGTGCAC
EMPV1_33094	CCCCCAGTCATTGACTGAGATCTTCCATTGGCTGAATAGAACTGGAAGTTAGTTTGCAAA
EMPV1_33095	TCACGATTTCCTTTAACTCATAGATGCTGGGCCTCCTGGCACCTTCTTCCTCTTGGGCCT
EMPV1_33096	CAGACATCCTGGGCCCTGCTCTCTTTCCTCTGCAGTAAATTACATATTAAAGTAGCCTGG
EMPV1_33097	GGACACTAACAAGTGTTGGCAAGGATGTGGAAAAACTGGAATCCTCATACAGTGCTGGTG
EMPV1_33098	GAGCGTGGACGTGACCAACACCACCTTCCTGCTCATGGCTGCCTCCATCTACTTCCATGA
EMPV1_33099	GGAGATCAAGATCATCCGGCGCCTAGACCATGACAACATCGTGAAGGTGTACGAGGTGCT
EMPV1_33102	GTGACACTTCCCAGTCAGATGATTCTGCATCCAGCAGTTCGGAGGAAAGTGAGGAAAGAG
EMPV1_33103	GACAAACATTGCTGGGAGGAAGAGATCAGGACACTTCATAGCCACCTAGAAGCAGGAGAG
EMPV1_33107	CAACACCTCGGCCAACTCCAAGCACACCATCCTGAGCCCGTGGATGAGGAGCAGCAGTGA
EMPV1_33109	CTACTTCTTCTGCGACATCCCCCCTGTGCTGAAGCTCGCCTGTGCAGACACCACCATTAA
EMPV1_33112	ACTTCGCCCTAAGGACAGAGGAAGGGACAGGATAGGCTGAACCCCAGGCAAGGAAAAGGA
EMPV1_33115	GACAGTGACATCTTTACCTTCTCTGTGTCTTTAGAAATAAAAGAAGATGACGGTAAAGGT
EMPV1_33118	GCTTTACCTGGGCTGTGCGTAAACACATTCAGAGAAACCAAACTGTGCACGTCCTCTGGC
EMPV1_33119	CTGAAATGGAGAGATTATAACTGCTATGTGAATTTACCCTATGTCTGCAAGTTCAAGGAC
EMPV1_33120	CGAGTGTGGAAAAGTCTTCGCTCAGCTCTCAACCCTCATTAAACATCAGAGAACCCATAC
EMPV1_33122	GGTAACCACGTAAATCATTTGATTCACCTCATGTATGATCTTGATGTGGCTCCTTACATA
EMPV1_33126	ACCCGATGGCCTTATCAATGAAGCTTCTAGGCAAGTTGCTTTGGCAGATATCATCATCAT
EMPV1_33127	GGGGCAACATATACCCCCAACCAACAAGCAAGTATATCCAAAGTCCCCATCCTTCCCCAA
EMPV1_33128	ACGAGTAGAAGACCTGTGGGAGCTTCACCCATCTTTCAACATTGTCGTCAACTGCTCAGG
EMPV1_33129	GACACTGTTTGTGTTCCAGCTGGTGTATATCCAAAGGTCCCAGTGTAATTCCAGGACCGG
EMPV1_33130	GGGGCTGCAACAGACACTGGCCTGCAAAGTGTGAGTATTTCCTATCTGGCCCATTAAAGA
EMPV1_33131	GTCTGTTCAGTTTGTTTTTATTTCATTGACAAAATCAAACCAGCATTCCCCCTTGTGTAA
EMPV1_33132	GGCTGTGCTCTTCCCCCTCCCTGCTGAGAATCAGCCATCAAAATGCTGAGCTAGAGCTAA
EMPV1_33133	CCGTCTTCAGTTACCAAGTATAGCCACTAGGCATCACTTCTTCGTGTTAAAGTGCCTTTG
EMPV1_33135	GAGGAGGAGACAGAGGCCCAGCAGCCACTTGATGATTCCTAAGAGGATGACAAACACACA
EMPV1_33136	CTGTATTTCCAGTTTTGAAATTAGTATATGGATCTACATTTTAAAATCTCAGACAATCAC
EMPV1_33139	GCAACAGCTGTACCAGCTGCAGATGCTACCGGGTGGGCTGCTGCTCCGGCTGCTGCCCCT
EMPV1_33140	CCATCCTTCGGCATCCAGGAAGAACTCAAGGTCACCCTCAAGGGCAATCTCTCATTTGAC
EMPV1_33143	AGCTTGGAGCCCTTCTAGCACCTCACCAGCCCAGAGTGGGTTCGCTCTTTCCGTGTGACT
EMPV1_33146	TATCAAATTCCATCCCAGGAAGACCCAGCAGGAGCAAGAATCTGGGGAGCCTCAAAGGAA
EMPV1_33147	AGCAGACCGCAGCTCACCTTTTCAGAGGAAACATTTCAGTCCCTAAGAGATGCCTGTTGG
EMPV1_33150	AAAGAAGAAGCCCAGGCTGAAATCGAACAGTACCGCCTGCAGAGGGAGAAGGAGTTCAAG

EMPV1_33151	TTACCACGCCCAGCACAAAGGCCGCCTCAAGCACAAAGGCTCCAGCCTGCATCCTTTCAG
EMPV1_33152	GCATGAGGGTGGATTTTCTCTGTCAGTTTGGCAGGCTGCTCTCACTAATACCATGTCCCT
EMPV1_33153	TACTTGAAACTAACTCCTCTTGGACCGATCACGTCTGAGCCAACAATTACCCCCGGTGTC
EMPV1_33156	CAGACAATGTCGGACACTACTTTCAAAGCCTTACGTCGACAGTTGCCAGTTACACGCACT
EMPV1_33158	GAATGGCAACCTGTGGGTTGGATAATCAGACTTAGGGGGAACTGACCTGCTTAAACTCCT
EMPV1_33159	CTCCTGGGTGATAGGTGCTCTGAATTCCTTATTACAAAGCTTACTGGCATTGCGGCTGTC
EMPV1_33160	TCCAATGCCCTCCTTCATCTAACTCTTATGCACCAAGCTAGGATCTCCCCAGGGCTAGGT
EMPV1_33161	CTCTTTGGGAAAAAAGAGATGCGGATTCTGATGGTGGGTTTGGATGCCGCTGGGAAAACC
EMPV1_33163	ACTAGCAGTAACCTCTACTTCTCTGCCCCACTGCCTCCCAAATATACCATGGAGCTGACT
EMPV1_33165	TTGATGTCTCCTATCTGCCAAAGCAACTCTCTGAAAGGAGCAGTTGTGAATAATCCCAGC
EMPV1_33166	CTGCTAGTTTTCAGTCGCACTTTCTGGAGGCTCATCGTCCCTTTGTGGGATCTGATACTG
EMPV1_33167	CCGACAGAAAGATCATGTCGCCATCCTGGGTCACTACGGCAATGAAACCCTCCAAGGCTT
EMPV1_33168	TTCCCCTGCTCAAACTTGCCTGCTCTGACACTTTCCTGATTGGTCTGCTGATGGTTGCCA
EMPV1_33169	ATGACTCTTCACAAGGCAAGATGCTGTGTGGAATCGGGGCCTCAGTTCTGGGCCTGCTCT
EMPV1_33171	CCTGCACAGTGAGATCCATGCTGAGAACAGCCAGGACCGTAAGATCCTCCACTGCTGGTT
EMPV1_33172	TGTACTTTAAGAGTCACTTTATTGTGAGCACCTGTAACAGGTGTGGGCTTTGTGAACCAG
EMPV1_33175	TTGAAGGCAAAGGAGAGGGGACGTTACGGGGAAGTTGCTGACAGGCGGTCTCTTCCACAC
EMPV1_33177	CCATCGTGCCTCAGCGGTTAACGAATATGACTAACATTCACAAGGTCGCAGGTTCGATCC
EMPV1_33178	GTTCTTAGAATCAGAAATCAACAAGCATTGGGCTGTGGTAGCCAAAAACCATAGGTTAGC
EMPV1_33179	TATGACCAAGTTTTACAGGAAAACTCCAGTGATTTTCAATCAAACATTGCATAGGCAGCC
EMPV1_33181	CTCTCAGTCTTTATGTTGTCTTTTCATTTTGTATATAGTTTTCTTTGCTGTGCAAAAGCT
EMPV1_33184	CCATCAACCTGAAACAAAGACAGAAGTGCCGGCTGCTCGCTCCCGAGTGGATGGACGTAG
EMPV1_33186	TTCCTTCTCTTGGCTTCCTCTGCTCTCTGGCTGTAAACTTGGGCATAGCTGCCCTCCCCA
EMPV1_33187	GGCCACCATCAAGGAAAAGGTTAAGAAAGCAAAGAGTAAGTAACTGGGAGGTCTTACAGC
EMPV1_33188	GAGGCGCTGCGCGGCGGCGCCGGGCTCGTGGCTGTGGCTGGGAGGGAGAGGCGGTGAGGG
EMPV1_33189	CTGTTAATTTTCCTAATCTTGGTTGGCTCCTATGGGGTCATCCTTCACACCCTTAGCACC
EMPV1_33191	CCTGGTAAAATTCGATTCCTTGATCTGTGATGAAAACAATTTCAGTAGAACTGGTCCAGC
EMPV1_33192	AGCTTGTGGCCTCATTCCATCCTGAGAGTCAAGAAGACTCTGGAAAGGTCACCAGGACAA
EMPV1_33194	TAAGCTTTGCTCGTGTGGCTAAACTGGCTTTGTCTGCTCAGGGAGTTTTCAAGGCTGGTC
EMPV1_33195	GTTTGCCAACCTGGGAAGGATTTTCAAGCCCTGGAAGTGGAGGAAGAAGAAAAGCGAAAA
EMPV1_33198	AGTGAGAGTAGAAGTCGAAGTAGGGGCCGGGGACATAACAGAGATAGAAAACACAGGAGG
EMPV1_33202	ATGGCAGCCAACGTGCAGGCCAGTAAGTCTACCCCAAACCACGTCATTTTCAAGAAGACC
EMPV1_33203	CCCAGCAACACCTAAAAATCACTAATCTACTTTCTGCCCCTATGGATTTGCCTGTTCTGG
EMPV1_33206	CGGTCCTCAATGAAGGTACGCTGATTATAAACCCAGTTTCCCTAAGAGGGCCAGGCAAGA

EMPV1_33207	TGTTACATGGAGCAAGAAATGAACTACTGGGGAGTTCCCATCGTGGCGCAGTGGTTAACG
EMPV1_33210	GGTACTACTATCCAATAGGTACCACTGTAGGTTTTATACAGTGTGGAGCTGACTCAGTTG
EMPV1_33211	AGCTCATGGTTTGGGTAAAACTCCAGCTGTGAAACAGAATGCAGAAGCAGCAGGTGGCAC
EMPV1_33212	AACAAGTGAGGGTGCCCCAAAGGATCAGCCAAATACAGTGCCTGCCCAAAGGAGTGTACA
EMPV1_33213	TCGGGAACCTCTCGATCACACTGCAAGAAATAGCCACCAAATTGTGTCATCAGCTATCTA
EMPV1_33214	GAATGAATATGCTGCGCCAGAAGTTTCCCTCTGTTCCAGTGATGGCGCTTACAGCCACTG
EMPV1_33215	ATCCGGAAGCAGCTCGTGCTCCTATACCCCAAGGAGGCCCAGCTCGAGGAGTGGTTCTAC
EMPV1_33219	AGGCCTTGGATTTCCGAGCACGGTTTGGTCCCCTCGTTTGAAATGTTGCTGTTTTCCCAT
EMPV1_33220	TGCCAGTTTGACAAGTGAAAAGAATTTCCTTGTAGTTCAATTGTGTAATTCTCTTTGATG
EMPV1_33222	GGTCTTAAGGCCACTGACTTGTACAACCCTTGGGGCATAATTCACATGGGACACAACTCA
EMPV1_33223	AGCGATTTCTGCTTTGGTTGACAGGAAATTGCTTTCCCAAGCAGGTCCCAATGCCACCTC
EMPV1_33224	AGGCTCCAGGAACTCAGGGGGATCTTCTCTCTTTGAACTCCAAGGCTATCTTAGAGATGA
EMPV1_33226	AGCTTCAGTTCCAACAGCATGTGATAGAGTCTGTTTCCCCTTTTTACACACCTACGCTGC
EMPV1_33227	GCAGGCCTTCTTTCTTCCTCTTGGTGAGTTAAGTCCGTCTCTGCTTTGTTTGACGCTGCC
EMPV1_33231	GCAGCAGGAGAAGCTGGAGGGGGATTTCAGGTTCAAGCACATGCAGATCCGGGTGAATGC
EMPV1_33233	ATGATGACTCTCAGGAATGTTGGAGACGTTGCCTCACAAGGATCACCAAGGGAGCTTGGA
EMPV1_33234	CCTGGTAATCGCTCCACATGGTTGCATCCATGTGCAAGTCCAAAATAAAGTCCTTGAGGG
EMPV1_33236	TGGACCTGCTAAAGCTGAACGTGGCGCCCCTCGCCGTATTCCAGATGCTGAAGTCCATGT
EMPV1_33240	GTACTCTCCACCGGCCTGGGACCATATCCAAATTAACTGTGTCTGGCACACTTGGTGTAT
EMPV1_33243	TTCAGAAAGCAACCTTGGGCGAGAGTCTCTGGGTGAGAGAAGACTGATCACGAATCAGGA
EMPV1_33245	GATGACCCCACCATCTATTCTGTCTGACTGCCTCTCTGGTACGGGTTTTAGAAAGGTGAC
EMPV1_33246	GAATTAAAAGCACAGCTAAGAACACAATCATGATCTACAGGCACCCTTGTCCCTCATGTG
EMPV1_33247	GCATGGACTTAGCATTCTTCTGGTAGTTGCCCCAGATAATTTAAACCAAGACTGCACAGG
EMPV1_33251	GAGCTGTAGGACAGGTGGCCAGTCTCTGAGTTTGAGGTACAAATTCCATGAAGTTCCAGG
EMPV1_33252	CCTGATGTGACCAGGATGCCGCTTTGATCGCTGCCTTTTCTCTGAAAGACTTGGCTGTTC
EMPV1_33253	GCTTGCCATTCTTATCATGCGGTTCCTCCTTAGGCCTGCCTGGATTTCCTTGAAAGAGTG
EMPV1_33254	TCACTGGAAGGGCTTGTTAAAACATAGACTGCTAAATCAGAGGTGGCACTGTGAATTTGC
EMPV1_33255	TAGGGAGAGAGGCTGCTGTGATTTGCCGAAAGGCCACACTACCAGAGACATCGTGCACAA
EMPV1_33256	AACCTCTGCTTGGTGCCTGGACAAAAAGGCTACCAATGCTGCCTGTGCCTCTGAGGAAGG
EMPV1_33260	AGCTTTGAAGGTCCCAATAAACGCCGTCGATACTAAGCGTTCTTGCCCTTCCTGCCCACT
EMPV1_33261	TCACCTGTGCTCCGCTAAACTCAACGATGGGTGTCTGCCCAGGGGGAGGCCCAGGGGAAC
EMPV1_33262	ACAGTTTCAGTACAGTGCCTCCTTGAGAGCACACCTTATTCGACATACCAGGAAGGATGC
EMPV1_33263	CCTTGGACACGGCTCGACTTCGACTTCAAGTTGATGAGAAAAGAAAGTCCAAAACGACAC
EMPV1_33264	TTGAGGGAAGATTCCATAGCTTTTATATTTCATAATTTGTGGAAGCATTTTGCCCTTTAG

EMPV1_33267	CAGCCTCGGTACAGGCGGCACACACAGTCCTCTGGGCAGGATGGCCGGCCCCAGGAGGAG
EMPV1_33268	AGGGCTATCGCCCAACGAGAGAACAAGCCGGATCGGGTGGATTATCACACAACACTAGCA
EMPV1_33269	CCTAGACAGTCCTCTCCCTTTCAAGATCTAGGTGCAGTTCCTGTTGTGGCTTGGGGAGTT
EMPV1_33271	GGAAGCAAATTGTAGAATCTTAGGCATCCTGTTTGTTCATGCCATGGATGTTTGCTCTGG
EMPV1_33272	ACATCATCCACACCATCCTCAGGGTCCCCTCTCCTGGGGGAAAGCACAAAGTCTTCTCTA
EMPV1_33273	AGATTCACACAAGGGATTGGAATCCTAAACCACTGGACATGTTTCTAGAAGAACTAGCAG
EMPV1_33274	CTCCTCTGTCCTGCACATTTAGCTTGTCCTATTTTGTAAACCAGGCTTCCCCTACTCCTG
EMPV1_33276	CTCGTACTCCTTCCGCTGCTCCAGGGCCTTATGAATCCATTTCAGCCTCTTTTTGTCCGA
EMPV1_33278	TTTAGCTGAAATGGCCTAATGCATATTGTGAGTTTTTATATTTTGTTTTAGAGAATTTTT
EMPV1_33279	GCTGTGATTTCACCTGTCGTATTTCTTGGCACATTGAATAACTGCATAACCCTGCCTCTC
EMPV1_33280	AACCAGAGTAATCAGGACAATTATTGAGGAGGTGGCGCCTGATGGTAGAGTCCTTTCGTC
EMPV1_33281	TTTGGCTTAGGCTGGGTTGTACTGAGTTCTCTCCCTTGTGACCCAAAGAGCCCTAACTGC
EMPV1_33282	GCGAAGGGAACTGAGCCGGCTGAAGGCGGAGGCTAGGAATAAACATGCTATTGCAGTTAT
EMPV1_33283	CACCTCTTCATTACAGGAAGGGCTTTCAGTCAAGGGGAAAAGGGAGCGACTACAGAGAAG
EMPV1_33284	GTGTTTGTGTCACTAAGACCCTCTTGTCTCTAAGGACACCTCCACAGGCATAGGCTGTGC
EMPV1_33285	CTGGGACTGTGTTAACCATGGCTTTTATTTTCTGGATGCTGGTGCTTTGGCATCTGGGCC
EMPV1_33286	AGTACTATGACCGAATTCCTGAGCTTCGGCACATCATCGATCAGCTGAGCAGTGGCTTCT
EMPV1_33287	TAAATCCCAGGTTAAGAGTGATGTCCAGCAGCCTGTGCATCCCAAGCCACTAAGTCCAGT
EMPV1_33292	CTGTAGATGAAGCTCTCCAGCACCCTTACATTAATGTCTGGTATGATCCTTCTGAAGCTG
EMPV1_33294	GAGATGTATGTCCTGCTGAGGCTGGCCTGTTCCAACACCGAGATCATTCACACATCACTG
EMPV1_33296	GGGAAAAAGCGGACCTCTCCAAAACTCGACTCCAGGATTCTTGCAACACAAACAGGAAGG
EMPV1_33297	AATGGTGTAAAATTGGTTGACCCTTTGGGCGAAATGTTGGCGCCCTCCTGGGAGGAACAT
EMPV1_33298	ACTGACGCCGAAACGGGGTGTCTCCTCTTGGCCCAGTCACCACCCTGCTGTTCCTGCCGT
EMPV1_33300	ATTCCTGGACTCACCGATACTATTGTGCCTCGCTGCCTGGGGTCTAAAAGAGCTAGCAGA
EMPV1_33301	GGCAGCTGTCTTCACAGGTCAAGTGATTATATTGAGCCATCCAGGTCAAATCAGTGCTGG
EMPV1_33304	CCTTCTGCACAGCTAAGGATGAAGATGGGGGCAATGGAGGCTGGAGAAATCGAACACTTT
EMPV1_33306	TTTTCTGTACCATTTCGATGCAATAGTTTGCATCTACGAATTTTGTAATTTGGAAAAAAC
EMPV1_33307	GCTACCTTAGCTGGGGCAACAGCAGTTTCTGAGTGGACCGAATATAAAACAGCAGATGGG
EMPV1_33308	TTTCCCCAGAGCGGAGAGGTATCTGCTGCGCCTGAGATGAGTAAACTGTCGTTTCGGGCG
EMPV1_33309	GCTTAAGAGTAGTGTTGCTAGGTTGTATGAGTGTGTGATTATTGTCATATAGTGAATGCC
EMPV1_33311	GCCACTCTGAGGAAGAGGTCCTGGGCATCTCTGATTTGTGGGCAAGTTGGACATCAATTA
EMPV1_33313	AAAGTGTGTGATGCTCAGAGAGACTGTCGGGACTGGTCGGATGAGCCTCTGAAAGAGTGT
EMPV1_33315	CTGTTTAGGCCATTCTGGCTGTGGTATTTTTCAACAGGTCATTGTCCCTAAATCTGTCAG
EMPV1_33316	TTTCCCTGGCGGAGGGGGCTTCTTCCGTAGCACAATATGTTCAAGTGAATGGCCGAATTT

EMPV1_33318	GCAAGAGGCAGAGAACAAAAAACGCTCCCAAGACGATAAGAGCCGACAACTCAAGAGCCT
EMPV1_33319	GGAAGTCTCATATTATGGCCGGGACCTGGACCCGGGCCTGGGCCTGGGCCCGGACCTGGA
EMPV1_33321	CCAAGAGCCTCTGGACAGGTACTTCTGGGTGGCAACTCTGTGTTGATTCTTCTCAAGAAC
EMPV1_33322	GGTAGCTTGCCAGAAATTACGTCATAGGCCACCTTATTTTTTCCCAGCATAGATTGACCC
EMPV1_33324	TGCAGAAGTTACCACTTGAGGGATGATGACAAACCTGGGGTCAGGCTGCCAGGACAGGCA
EMPV1_33325	GATGTCAGATGTGCCTAGTGAAGAGTATGGCAAAGTCAACCCAATTTCCCAGAGCAAGCC
EMPV1_33326	GGAGAGAAGCAAAGAGAATTCCTGGGTGACAATCTACAGCAGGAGAGGAGGACAGAGATC
EMPV1_33329	GAGCACCTGGGAAACTCAGTTTTGTACCACTGACTTTGTGCCACCGTGTAGAGAAAGCAG
EMPV1_33330	GAAGATGTTACTCTGCACAACAGTAGCCTCCCACCCCTGGCTATCAAGCACCCAGAATGT
EMPV1_33331	GTGGCCCCACACTTGGCAAACGATAATCCTCTTTGGAAGAACTCAGCTTTATTGCAGACC
EMPV1_33332	TTGTGGCTCAGCAGGTTGAGAACCTGATACAGTCTCCCTGAGGACGTGGGTTCAATCCCT
EMPV1_33334	CTGCCAGATTGCAGCAGAAAAACTAGGGCCTGCCTAGGAAATGGGAGTTTGACATCCTAA
EMPV1_33336	TTGTGCAAAACCACTTCTCCCATAAACCCCTCCCTGCATCGGTGTGTCAGTATCTGTCCC
EMPV1_33337	GATGCTCAATAGGTTAAGGATTCAGCGTTGCTGCAAGATGCGGCATAGATCGAAGATGCG
EMPV1_33338	TGTGGTGCTTAAGATGAAGCATGACCTAAAAGCATGAGCTTGATGCAGTGTAGCAATTAC
EMPV1_33340	GCACAGGGTTTCCTAAACTTGTTTCTTTCCCTCTCCAGCACTTAAATGGCTAACAAGCTG
EMPV1_33341	CTGCCATACACTGTAAAGGGCATGGACGTCTCATTCTCGGGGATCTTGTCTTTCATTGAG
EMPV1_33342	TTTAAAGGACTGCATCTGTGGCATATGGAAATTCTCAGGCTAGGGGCCGAATCCGAGCTG
EMPV1_33344	TGGTGGTGCCATGGTCAATTCCATGATTCGCACTGGGTGTACCTTCTCCATCTCCTTCTG
EMPV1_33345	GTTCCACTGGCAATGAGACAAGTCTTTCCCCTGATTTTCTCTTTCAGCCTCAACAGCTTG
EMPV1_33347	GCATTTGAGGTGAAGTTCTGTTATACACTCAGGCTGTGGCTCTCTGAAAGTCAGTGCATC
EMPV1_33350	TGGGCAAAGTCTGTCCCTCCTTCTCAGGGCCAGAGCAGCTGTAAGGAAGTGAAGAGACCA
EMPV1_33351	AAGTGGCACCTTTGTCATGTACAACTGCGCACGTCTTGCCACACTCTTTGAGGGTTACAG
EMPV1_33352	CAAAGAGCAAGGTTCGGAAATGAAGTCGGGAGCCGGGCTGGAAGCAGAGTCCGTGCCGAT
EMPV1_33353	CCAACAAGATTCAAGTTTATCTGCATCATCTCATGGGAGCCAGAACAGCACCTCGGGTCT
EMPV1_33355	CTGTGGAGGGTACCTGTAGAAACCTCGAACCCAAGAAATGCCCTCAGAGTTTGGGAGAGT
EMPV1_33356	CCGGGGTTACTCTTATGATGACATTCCAGACTTTGATGACTGACATCCACCTCAGCCCTG
EMPV1_33357	CTACCTGCAACCTACGCACAGCTACGATCAGGGTCGGGGCAAGTTCGTTTCGCTTTTCTA
EMPV1_33358	TATATCATCCAACATGGTGACACCACCCAGTGCCTGCCCATGTGCTCTAGCACCCAGTTC
EMPV1_33360	TTATCAGTGTCTTTGGCGAGCCGGTCAAAGGCTATGGGGAGACAACGAGACGTGGTCGAC
EMPV1_33361	ATCTGGTTATAGTGACAGCAGAGGCACGCAGCTTCCAGGCTACAGGGTAACACTCCTGGT
EMPV1_33362	CCTCTATGACCTACATTGCTGGCGAAAGCCTGGAGCTAACCAAACTCCATCTGAATGCTC
EMPV1_33363	GCCCAGATTAGTATTACAGTTGAGATTATATCTAATTGACACTCCCCATAATACCCTGAA
EMPV1_33364	TCATCTGTTGTCTGCTCCGAAGAGTCAGAGATGATCTTTCTAAAATGAACACGTCAATAG

EMPV1_33366	ATGCTGAAATAGTTTTTGTTCCACCATGCCGTTTCCTCACTTATTAACATCCCACACTAC
EMPV1_33367	GACTCGGCACAGGTTTTAGAGGAAAGAGTTGAACATGAACTAGGAAGCTGGGCGATGCTC
EMPV1_33368	GACTACGTCATCGACCTGGAGGAAGGCTCCCAGCACTTGATCCGATACAAAACCATCGCT
EMPV1_33375	CAGTTTCCAGCAGCTCCTATCCCAACTTTAAGTGGCTTTCCTATGACCTTGCCTTCTGCG
EMPV1_33376	GCGTCTTCACTGCTCACGGCAGATTCCCAGGCAATGGTGAAGATCGAGGACATGGCCGTG
EMPV1_33378	GGCCAAGGGGCTGTCAGATGTGAAGATGGCCACGGACCCAGAGAACATTATCAATGAGAT
EMPV1_33379	GTTTCACCTGAACCAGTATATGTTTCAAAAGTACATTATGGTAGGAAAGTCCTTTATGAT
EMPV1_33381	GTTACTGCTGCAGACCCAGCAGAGGGAGCCCACGTTAACAAACGAAATTTTCTCTGGAAC
EMPV1_33382	GGGAGAGCTATTGGTTCTTGACAGAACATATGCTGTGACCAGGAACTTGGAATATTTTCA
EMPV1_33385	CTTATTCCCTCATCCCCAACCTCCCCCCACCCAAAGCCAGCATTAACACTCCATCGTAAA
EMPV1_33386	TGCTGATTTTTTCGATGGATTCCACAGCATCTCTTCCGAGTTCTAAGAGGGTGGGGTCCC
EMPV1_33387	AACTGGCACGTGTGTTGGCTACTAGTGGGAGTGGTTTCGGTTGGGGGTTTTATCCATTCA
EMPV1_33389	ACAGATGAGGAAAGGCTTACAGGGGTTGAACAAGTGGCCCGAGGAAACACTGCTAGTCAG
EMPV1_33391	CAGATCTCCTCTCTAAAAATGTCACTGTAAAAAGAATTTTCTCTGTGGGAATAAATGACC
EMPV1_33393	TCTTCTCCGTAGTTGGGTCAGTGAGAGATCAGGAAGCAGAGAGGGACTTGGTCACCCTTT
EMPV1_33397	CTCCTTCCGGATTTGTCTTCGTCCTCTGTGTTTGCTAAACTCTTAATTAGGTCTTCTCCC
EMPV1_33398	ACCTGTCCACAGTCACAGCTGCATTCACACCTCACGCGTTTAGTGGGCTCCTATTTAATC
EMPV1_33399	GAGCTACAACTGCAACCTATTCCATAGCCACAGCAACACCAGATCCAAGCTGCCTCTGTG
EMPV1_33401	TAGGCTTCTCTCACCAGGAGTGGGAATGTCTTGATCTTGTTCAGAAGACTTTGTACCGGG
EMPV1_33404	GTCCGACTCTAGAAAGAGCAGCATACCAAACAGTAAGGAGGTCCCTTCACACCATCCAAC
EMPV1_33406	ATGGTATGGGCAGTCGTGGTGACAGAACTGGCTTTGGCAAATTTGAACGCAGTGGACACA
EMPV1_33408	AGGCCTTGAAGCCAAACTGCTACAGACAGTGCTCACTCCTGCTCTGATGTTCGTCGTTTA
EMPV1_33409	CCCAGTGTCAATCCCACATAAGGTGAGTTCAGTGTACGTGTCTCTTTCCCCTTGTTGGCC
EMPV1_33411	AGAATCCAAAAATCTCAACATCCAAGGCAGCTTATTCGAACTCTGCGGCAGCGGCAACGG
EMPV1_33412	GATCCGCACGTCTTTCTTCATGTCGGCGGCTCGCAGGGGCCGAGCTCGGCCCGCACGCAT
EMPV1_33416	GGGTCCTGCTATCTCTCATGTACGTTGTGATGGATGTTTCCACCTCTTTCTGCAGTCTCC
EMPV1_33417	ATTTTCACAGCCCACCCACAAGGAGTGGTGGTACAGCCAACTGCGGCCGTGACTACAATA
EMPV1_33418	GTTCGAGATCAACTTCTTGTCGGAGGCGGGGGACATTGTCTTCCACATCAAGCCCCGCTT
EMPV1_33421	AGCTGGCTGTTCAGATAGATGAGGTCCTGTCGCATTTCACGAAGCCCTTCCCACAGTTCA
EMPV1_33422	GGCTCCTGTCTTTCATTGCTGGCGGCCTGGCATCGGGAGCTACTTCCACGGAAGTGGGTA
EMPV1_33423	GATATCATGTGTGGAATGCTGCACTGTGCTAATGTGCAGAAAATTCCTGGTGGCGGAGAG
EMPV1_33426	GGTCCGGGGTCTACGTGAGGATGTGGAAGAGACAGCAGCAGAAGTGGGAACGAAAAATTA
EMPV1_33428	GCTTACAATAGTGTATACAGCACATACTCATTCAGGCTACTCCTTAGTAGCTGGGTAAAG
EMPV1_33429	TTACTCAGCTGCCAGAGGGTTTTGGCTTCAACTCATTCATGGCTGGAAGGAGTGCCAATC

EMPV1_33432	TTTGACTCAGCCTATCAGGGCTTCGCATCTGGCAACCTAGAAAAAGACGCCTGGGCCATT
EMPV1_33433	CTAACTTCCTACACTGGCATTCTATGCCTTCCAGCTCTTTGCTGACTTCAAATGCTGTTG
EMPV1_33434	AAGGCCCACCTGCACCCAGGAAGGGAGGAGGGGCTAATAAGTGAGAAAAGGAAGAATATT
EMPV1_33436	CATGTTACCCACAATTCCACTCTCTGCCTTCTCTGACCCCAAGAAGACCAAACCGTCCCA
EMPV1_33437	AAAATAGCAGCCCCGGGGTTGAGCAGCGAGAAACCTGAGCCAGTGAGAGGCCAGCTCAGG
EMPV1_33438	AGGAAATTCTCTCTGGCTTTTTTGCTACAGGAGTGTTCTGCCTTGGCATTGGCATCATCC
EMPV1_33439	ATTACAAAGGTAATTGAAAGAGGGGAGAGACTAGATGAACTACAGGACAAATCAGAAAGC
EMPV1_33440	CTCCACCACATTGGAGAAACAGATCTCAGTGGATGCAGATGGCAACTTTGACCCCAAACC
EMPV1_33443	CCTCTGAAAAAATGTATCTTGTGTGAAAAACATGTAGATTATAAGAATGTGCAGCTTTTA
EMPV1_33444	TACACTGGGCAGAGGACGACGATGAAGGCAAGAACCCACGGAGCTGAGCAAAGGAGAAAA
EMPV1_33446	GGAGCCAGGAAAAGAACAGTGAAAAGTGCATATTTGGGAGTAGAGATTCAGGTCCATGCC
EMPV1_33449	GCTCCCAAGGGTGTTGATCTCTGGGATGGTGCCTCTTCCAATTATCCAGTGATGTCAGCA
EMPV1_33450	TGTCTGTTAAATTTCCTCCTCTTGATGGGTTCCTATGTGGTCATCCTGAACTCACTAAGG
EMPV1_33451	GAAGAGGAGGAGCAGCAGGCCCTTCAGCAGTCCATCGTGAAAACATATGAAGACATGACT
EMPV1_33452	TGCACCACAGCAACAGCAATGCCAGATCTGAGCCCCATCTCACTTGTGACCTATACCACA
EMPV1_33455	CAAGAAGAACTAGAACGCCAGCGTCGGGAAAGAAGATACATTTTACCTGATGAGCCAGCC
EMPV1_33456	TACTGAAGCTTGGATAACTGGTCAATTGTATTAAAAGTCTGTGTGCTTCAAGCTCAACCC
EMPV1_33457	AGACCGACTACCGTCTGTTCGTGACCTTCCGTCTCCAGAACATCAGAAACGGGACAAAGA
EMPV1_33458	ATTGCACATTTATTACAAAAGGAGCCTGGGAGGTCATTGGGATAGAAGCTCTTACCAGTG
EMPV1_33459	GTACTGGGGCATCCTGCATCTATCCTTTACTCGGAACAACCTTAAACGGCTGGTATTTCC
EMPV1_33460	CTGCCCAGAAACCAGAAAGAGCAGAGACACAGTATTGAGCGAGAAAGTGCATATGATCTG
EMPV1_33463	ATGTTGCAGGACAACCTGGAATCCTCCATATTCCCGATGCAGAAGGGCTTCCTGAACCTC
EMPV1_33464	GTCTAGACAGCTCAATATTTTTTACTTATCATCCAATTAGGAAGACAGTTTTGAATAGCT
EMPV1_33467	AAAGCGGGTGCGAGCCGCAGTGGACACGTTCTGCAGAAGCAACTATGAATTTTTTGAGAG
EMPV1_33468	TTCTGTGTGTTGGATAAGGATGGCAATGGCTATATTAGTGCCGGGGAGCTTCGCCATGTG
EMPV1_33469	TCATGAGACACCAAAGGATCCATACAGGAGATAAATACTATGGATGCAGTGAATGTGGGG
EMPV1_33472	CCAACGCCTGGAGGCATCTGTATTTATCAGTGACCGCCTGCGGGAAAGGCTGGAATATGT
EMPV1_33474	GGCTCAGAACCCAAGAGCGTCATTCTCGGAAGATGCCACATACTTTTTCACGCAAATTGG
EMPV1_33476	TTTACTCCCTGATTTTGGGCCAAGATAATGCTGCTGACCAATCACGGATGATGCAGGAGC
EMPV1_33478	AATTCGAGTGCTCACTTGGTTTCTCACCGGGTAAAAGCTGATCCACTGCAGCCACAGAGC
EMPV1_33479	CAGACCTACTATGTTTTGGACGTGATGTGTTGGCGGGGGCACCCTTTTTATGACTGCCAG
EMPV1_33480	TAAACCACAGTGTTGCCAAGGAAACAGCCCGAAGGAGGGCAATGCTGGATTCAGGCGTTT
EMPV1_33481	CAAGTTAGTAGTTTAAGATCACACAACTTGAGAAGGGGGAAGCCACAATTCACAGCTATC
EMPV1_33483	CAACCTGCACCACTACAAGCTGCACGGGGTGAACATCAACGTGGAAGCCAGCAAGAATAA

EMPV1_33484	CACTTTGCTCCATAACACTGATTGAGTTTCAGTTGAGGGGTAAGAAACCTTTCCAGCGAG
EMPV1_33485	GCCAGCCTTGTCCCAATTGTTGTTGGTTCGCTCTAAGTAGGACCCGTGGTTGTATTGGTT
EMPV1_33486	TTGCTGGTATCTTGGGATTTGGCCTTGGAAAGGCATCATACATAAGAGTGTGCCAGAGTA
EMPV1_33487	TACCAGAAAATATAAGTGGTGGGCTTGGGTATTAGGTGAGCAAAGGGGCCTGGGCAGCCA
EMPV1_33490	GCGTCATGGGCTACTGCAAGGTCCACAGTAATACTGAGGTCCATCTACCTGTTACCTGAT
EMPV1_33492	ACTACAGACTGCTCTCCAAGGAGCTGCAACCCCTCTACACCAATCTCACTGTGGACATCA
EMPV1_33494	TTGTCCCTGAGTGGCAGGACTATGATCAGGAGATCAAACAGCTACAGACTCGCTTTCAGG
EMPV1_33495	GGCTGTGTTAGCATAGGGACTGACAGACTTAGTATAGGACGGACAGCTGCGATGGAATCA
EMPV1_33497	CTAGTTTCCCTATCATCTGGCTCAGGGGTAACAAGCCCGACTAGTATCTGTGAGGACTTG
EMPV1_33498	AGCCCGAGTCCTGCTCTTCCTGGTGGTCCCTGGCCATGTGATCTTGTTCTACTTCATTTA
EMPV1_33499	TTGGTCGTGGGGGAAACTTTAGTGGTCGAGGTGGCTTTGGTGGCAGTCGTGGTGGTGGTG
EMPV1_33500	TCAACGCCATCCTGCAGACCAGCTTCACATTCAGCCTCCCCTTCTGCAGCTCCAACCACA
EMPV1_33501	CGACAGCATTTGAATCCCTTGTGCTTAGAGTAGAAAATTGGTAGGTTAAAAAGCCCATGT
EMPV1_33505	AGAGAAACGTGGTTACAAGACAGCCAAGGCGGCCCGGAACGATGTGTACAGAGCGGCCAA
EMPV1_33508	CTAGAAATCTTCGAGGGCTGCAACTTCACGGGCCAGTGTCTGGAGTTCAAGGACGACTGT
EMPV1_33509	CAGGTCGATTGTGTCTGGACTTGGGTTCTGTCTCGAAGGTGGATGCTGATTGAGTCATTG
EMPV1_33510	AATACACATGTTGTGTGTTTGTTTTATGCGGGATCCCATATAACATGGGGCCCAGGGCAG
EMPV1_33511	GACTGAGTTACCAGAGGCATAAGCTGCAGGTCCAGAGGCCTTTGTCAGAACATTGAAGAA
EMPV1_33513	ACAACAAAGAGGAAACACCAACAAAAGAAACAGCTCAACATTCATTGAGGTTTCAACTCT
EMPV1_33517	TTTCGGAGGCAGCACTACCTTCTGGAGCACGCCATCACCGCGGATTGTGCTTTGGTGAAA
EMPV1_33518	GAAAACGTCTGGAAACTTCACCATTGATCCTGATGGCAGTGGCCCCCTGAAGCCATTTGT
EMPV1_33519	GCTGCAGGTGAACACTCTCCGACGTTATAAACGACACTACAAATTGCAGACCAGACCAGG
EMPV1_33521	AGTGAAAGTGACCGACAGGTTTGGGATCTGGACCGGGGAGTACAAGTGCGAGATCGAGCT
EMPV1_33525	CCTCCAAACCCAAATAGGTAGGAACTGCGTCTTTCTGGCTTACCACTCCTTCCCAGCTCT
EMPV1_33526	GTTGCTATCTTGCCAAAATAAGTGTCAGTTATGTCTTGATTTCTGCCCCCTCTCCCTCAA
EMPV1_33528	GATCCCAGCCTGTAGCCACTGTGGTGTTCGAACACTTCCTACACAGATACAGGATTATGG
EMPV1_33529	GGGGCAATGTCGCTGCATTTCTGCAAGTGGGGTCAGAGGGCTTCTCTCTAGTTCTTGTTT
EMPV1_33530	TACCTGATGAGGAAGAGGAGAAGGAAGAGGCCAAGTCAGCAGAGTTTGAAAAGCCTGACC
EMPV1_33532	TTTCGGACATCCCAGGCGAGGCCATGGTGAAGCTCTACTGCCCCAAGTGCATGGACGTGT
EMPV1_33533	TCCATTTTCTGAGTGCCGCTCCCTGGGCGTGAGAGCTGAAGAGTGCATGGAGCTGGAATA
EMPV1_33534	ACCAACAGAAACGATAGGAGATTTGTCAGTTTGCCTTGATGGGCTACAGTTAGAGTCTGA
EMPV1_33535	ATTTTTCATCTGAGTGTTGGAGCCTTGGAAACCTTCAGCCCTGCTCCCTCTCGCAGGCTT
EMPV1_33536	GACCATGGCACAGAATGTCCGTGTTTCCTCTGCCCTCCTGTTCTCCTTGAAAAATCATGC
EMPV1_33538	AGAGCTCATGTGCTTTGATCCTGATACTGACAAATGGATCCAGAAGGCGCCGATGACCAC

EMPV1_33539 AGTAGGTCTGCATCGTGGCCCTGGCGATGGAGCCCAGCCAGTTGTCAGCCATGTTGTCCA
EMPV1_33540 TGAGAGGGAAACTCACAAGTAAACAGGGCAGCTGTGGAGCCTGGTCTGCAGGGAGTGCTG
EMPV1_33543 CCTGATAGCCATGAACGGCAGCATCTTCGCCCAAGGCTCGCAGTTCTCTCTGGACGACGT
EMPV1_33545 GCAGCCCCTCAGCATACTGGGAACTTCTTCCTAAACCAAGAATGTAAATTTGGTCAGGTC
EMPV1_33548 TGATGGAAGCAGATGAGAAGGAAGGCTTGCGATGCCAGATCCCAGACTCCACAGCAGAAA
EMPV1_33550 ACGGGGGTCGCTACCGGACTGTGCACACTGAGTGGACCCAGAGGGACCTGGAACGCATGG
EMPV1_33552 ACAAGGCTGCTGTGTCTGTGAGGGATGGGGCTGAACTTCCCGTTACAAATGTTGGACTGT
EMPV1_33554 TCCGGCCAATACTACATCTACACAGATCAACAGAGAAATGTGTTGGAGGTGGAGGTGGGG
EMPV1_33555 TGGAGGCACAGGCCTTTTATTGACTTTGCCCCTTACCCTGCATCCTCTTGTAGGACTCAG
EMPV1_33556 ACTTTAGAAATAAGACCAAGCACACTCAGGCCAACAGGAGAGCAGGGGTTTGAAGCCAGC
EMPV1_33557 AATTACAGACTCTGAGGGGAGACCCCAGCCATCGGTGTTTCAAGCCCTCTGAGCTATTTT
EMPV1_33561 CCAAAGACATCCTGAGGAAAAGAAATGGAGCTGGAGGCATCAGGATCTCTGACTTCAACT
EMPV1_33562 CCTGCTGGCGGGCGCGATTTCACAAGTGAATAATCCAATTCGCGCGGGCGCCAAGGCGGC
EMPV1_33563 CATTGATGGCCGGGAATACATCGTAGATGCGGGGTTTGGACGTTCTTACCAGATGTGGCA
EMPV1_33565 CTTGGGGGTGGCGAGCAAACCGTTACCATTACTGAGTTTAGTAATGGTAACGGTTCTCTT
EMPV1_33567 TTCTGATATAGTAGGTCTCGGGCGGGACCCAAGAATCTGCATTTCTAGCAAGTTCCCAGG
EMPV1_33569 CTTCTCCCCTGACAGACGTAGTGTAGGAACAGCCCTGTTCAGGCCCAAGAGCTGCCGAAA
EMPV1_33571 GGCAGCACTTGGAGAGCAAGTCTAAACGGCCCCGCGTGTAATCTGCTATGAAAAACCATT
EMPV1_33572 GACAAGAAGGACGAAGTCCCACAAGGCCAGCAGCTCGGATTCTACAACGCGGTAGCTATC
EMPV1_33573 GTTGACTGCGAGGTTGTACTCCCTGTTGTTTCGCAGGACCTCTACCTTCGCCCTCACGAT
EMPV1_33575 ACACTGAGGGGTCGTCTGAAGGGCTTCGCCTGCAGAAGTTCTTGACCTTACATTGGATGA
EMPV1_33576 CAATGCTCATCCCCCAACCGGAATGTACAAGAGAGGTGCCATTGTGCCATGTGGTCCCAA
EMPV1_33577 TGACTCCAGCATCCGGTACTTTGAGATCACAGACGAGTCCCCCTACATCCACTTCCTGAA
EMPV1_33581 GTCCTATTGCGAAGCTTTCAGCGGGAACCCTAAGCGGAATCGTCCTTCTTCTCACGTGTT
EMPV1_33582 CCACTGGAGCAGGGGTCCTAGTTTTGATGTCTTCTGTACTTTTACTGAGTGGCATTTCGA
EMPV1_33583 CATGGAAAAACAATGGAAATGCACAGTAACTGAATATCTATCTCATCAAATGCCACACCC
EMPV1_33586 GCACACAACCTCTTCGAGCTGTTGAACCTTCAGTCCCTCTTCGTGACATCTCGGGGCCGA
EMPV1_33587 GAGGAAGAGCCAAATGCTCATCCACAAAGGCCAGGCACAGAAATCATGAATTGTGGTGGA
EMPV1_33588 ATGGATTCGCCTGCGTTGGACCACCAGCCAACAGCAATGATGCAAGCTTGGCCTCTGTCT
EMPV1_33589 AAGCCATCCCAGGCCACAGACACCCCAGGAACTCCCCCAAAGGATTCATCAACATCCATA
EMPV1_33591 GTTTGGAGTTCCCTGGTGACCTATCAGTTTAAGAATCCAGCGTTGTCATTGCTGTGGCAC
EMPV1_33592 CTGGAGTGGGAGGGTCAATATGAATCAGGTAGTTCTGGATGAATGAGGCCATCTGAGCTG
EMPV1_33595 TTGGGACTGAGAGAGACAAGGGGCCCAAATGCTCACTCTCCAAGAGGAAACTGGAACTTT
EMPV1_33597 CAAGGAAAGCAGCAAGTTTCCATTCGGCATCAACCCAGCACAGAGCCACCGGAACATCAA

EMPV1_33598	AAGGCGGCCTCCTTGAGTGCCTCACTAGACAATGACCCGAGTGACAGTTCAGAGGAAGAG
EMPV1_33602	ATATGAAATGTGTTGCTTCTGATCACAAGACTATACAACCTCCTAGGAATAACTGAAGTG
EMPV1_33605	GCTGGGAGAAAGCGGACATTCATCTCTCCCTCACAAACAGACATCCTTCGGCAAGCCTTT
EMPV1_33607	AAAATTGACCCTGAGGTGGCTGCCTTCCTGCAGAAGCTGCGCAGTAGGGTGCAGATCGGC
EMPV1_33608	CTGAGCGGTGTTCCATTGTCTGTCTAGACCACATCGTCTTCATTCATCCATCTCTCGATG
EMPV1_33610	GCTGAATACATTCTTGGATGATCCAGAATTTGCTGATACTATATTGAAAGCAGAGCAGGC
EMPV1_33611	GGCAGTGACTCTGGCCTCTCGTCTGATCTTTGCCTTTTGACTTTGTGCCTATTTCGATGC
EMPV1_33612	GAGATATGGTGGATGGAGGGCAGGTAAGTAGCAACCTTGTCATGGGGAGCGGTCAAATAT
EMPV1_33614	CGTCGATACCAGTAATACGCTAGAGAGATCTCAGAAAAGTCCATCGTAACTCTTCACACG
EMPV1_33616	TTAGCTTTGGCCATTAGAGAATGAACTTCTGAATGCAACAATCTGAAATGATTGTTAGAG
EMPV1_33619	TCTGCTTGTCGCCCATGATATAGTCGTTGTGGCTGATGTAGTTGTACACCAGCTTGTGCC
EMPV1_33620	CTTCTCCCTTTGCGGCGCCTTCCGATTAAATGAGTGGATGCGAGTCCTTGACCAAACACA
EMPV1_33622	CGCACGAGCCCAGGTTCCAGCTAAACAGATAGGACCCAAAGTATCTGGATAAAAGACTGC
EMPV1_33624	AGAAGAGGAACAGCTCCTTCATGCTGCCCAAGCTCGTCAAGTCCCAGCTGCGCAAGATGA
EMPV1_33626	TGGTTGAAGGCCTCTCACTGTTGGACTTGGGAGTGTCTCCGTATTCTGGAGCAGTATTTC
EMPV1_33627	AGGGTGTGAAACACATAAGGTTGTTAATACAAGTTCTTTTCACACCACTCCGAACACATC
EMPV1_33628	ACGCTGTGATTAGAGCAGATATCCTTAGTGGTCACATTTGAGGTCAGGAGCTTCAGAGGG
EMPV1_33632	CCAATGTGCCTTCATGACAAATCCATAGTAAATGGGGAGCTCAAAGACTCATTCCATTTT
EMPV1_33634	CCTTCCCCTGCTGACTGCGGGTGCTGTGGCCTCTGGTTTTTCGGCTAAGAATGAGCATTA
EMPV1_33636	GACCTGGAAACCAGCATCCCAGCGATGCCTTGCAGATGCTGAGTCCTAAGGTTTTCGAAG
EMPV1_33640	TGCTCTTCGACGTGGGCATCATGGCTGTGACCATGGACCTGGCTGAGCATCACATGTTCT
EMPV1_33641	AATACATGGTAAAATCCTGCCAAGTGGGGTCAGGAGGAGAACCTGCCGCCCAGCTGACTT
EMPV1_33643	GCAGTGTCTGTTGGAAATGTTGGTCAGCTTGCAATAGATCTAATTATTTCTACACTGAAT
EMPV1_33644	GCAGAATCTGGAGACAAAGAAAAGGATACTTTGAAGAAAGGGCCCTCGTCTACTGGGGCC
EMPV1_33645	ACAGCTGCCCAGGGGGCTAATTCTGCTCCCTAAAATAAAGAACATGGCCACTCAAAGGAG
EMPV1_33647	GAGATAGGCCTTAGTTGGTAGTATTCTCACCTGCCCCAGCCCCAAGCACTCCTTTCCTGT
EMPV1_33649	CCTTGCCCTTCAGCCCCCTGCTTGGCTGTATGCACTGTCTGTATTGCACTGAGAAAGGAA
EMPV1_33651	CCAGATATTCTTGAATCTGAATCCTGCACGCTTTCCACTACAGCGCACTGCTGGCCTGGA
EMPV1_33653	CCAAGAGCTCTCAGTGCTTACCTACCTCACAGGAAATTCGAGGTTGGATTAGATAAGGTG
EMPV1_33654	CCAGGGACTGGCGCACTCACAGCTTTCCAGAATTCCTCAGAGAGAGAAGACTGTAATAAT
EMPV1_33656	AAACTTCGGAGGTGTGGCCTACTTTCACAAACAATTGCAGCCTAAGAGGAGTCTGGGCTG
EMPV1_33658	TATATACAGCGGAAGGAGTGAATGCAGGGGTGTAGGATAGAGTGTCTACGTCATGGCTCC
EMPV1_33659	CTGGGAGTCACCAGAATAGCCAAGTAGAATGGCTCTCATTCCCATCTCAGGTAGTAGCTG
EMPV1_33660	CAGGGTACCCCAGCGATTTAGCTAGTTTGGAAATCACTACTCTCAACCACAAACTAGGTC

EMPV1_33665	GTGACTTGTGTTCATGTCAAATCCAGCTGTCCCACCAAAGCCACTGGGGATACTACAAAG
EMPV1_33667	CAAGGCAGATGGCGTTCAAGACTGTAGCTTGCTGGATTTGGTGTTAGCAGTGGGAATCAC
EMPV1_33669	TGCACCAGACAAGGAGGGCCAACTACAGAGATAAACCAGAAACGCCGTGCTATGGAAGAA
EMPV1_33670	ACTCTCCTCACCACACTTATTAAAAAGGACCAACAGTTGCCCACTAAGGGTCAGTTGTGG
EMPV1_33672	TTGCCTCTATCAACTTCTCAGAGACCCAGAGTCCCCCAGCCTTACCTCTCAATCTGTTCT
EMPV1_33673	CCACCACAACTGCTACAGCTACACCCTCATGTTCATTAATTGTGTCCTGACCACAGAGGG
EMPV1_33674	AAGCGATGGATCTGTCGGAGGAGTTCACCTGCTGAGTTAGTGGCAGACCTCGGAACTGAA
EMPV1_33676	TTCGTGACCGACACGTGTGTGGCAGGTGCGCTGCTGTGCGGGGCCGGACTGCTCTTCCAT
EMPV1_33679	TTCCTGGAGCATTTTGAGGCTGCCTCTTTTGGGGACCATCTCTTTGGGGCTCTGGTTCTC
EMPV1_33680	GACCCAACGTTACTGTGGATTTTAGATGCTGCCATGACTCACCTTTCTGCAACAAGTTCT
EMPV1_33682	CCTCTGCTGTGTCTGCTTTCCCTTCTTTTCCCATATTGAGTTTAGGGCCCCATGTAATCC
EMPV1_33683	AGCCAGATCCCTTCTCCCAGGGGACAGCATTGCCCAAACAGGTTTCTTAGGAGGAAAGGT
EMPV1_33684	CGGGAAAGATTTAAGGAGTGAGGGACATTTTAGGAGTGATTCTTGCTTTCAGAAGGGGGG
EMPV1_33686	AAATTCACCCCATGCCACCCCACTCCTTCCATGAGGAAAACCAACCATGAGCCATGACCT
EMPV1_33688	TCAGCCCTCCACCATCTCAGATCAAACGTATTTGCACCGATGTTCCATCCCCTGCAACAT
EMPV1_33690	TGGGCTGAGAGCAAATAGAGACTGGCTTGGAGGAGACATTGGGGACCTCCTGTGATAACA
EMPV1_33691	ATGAAGACGTTCACTGGGGAGACCTCTCTGTGCCCTGGTTGTGAGGAACCCGTCTATTTT
EMPV1_33692	TTTTCCTTGCTGTTGGGGTCTACTTCATTGCCGGACAGGATGGAGTTCGCCAGTCAAGAG
EMPV1_33694	GACTACATGGACCCCATGAACGAGTTCAGTCCCCTCAACGAGGCCAAGCAGATGATCGCC
EMPV1_33695	GTTCGCATTCTTAACCCTCGTGCTGACTGTAGAGATGTCTATAAGCTCAGGAGGTCCCAG
EMPV1_33696	AATTTCCAAAACATACTGCGCCCTTCCTTGTCGTAACCAGGTCACTCCCATCACCAGCTG
EMPV1_33699	TCTCTTTACACACAGTTCCCACCCCAAAGGGGAATGAGGGGCCTAGATAAGAGTAAGGGC
EMPV1_33702	ACACAGCGGAGATGCGCCCAGGGGCTTTCATTAAGCAAGGTCGAAAGCTGGATATTGACT
EMPV1_33703	TGGTATGATAAGAGTCAGGACTTTGGCAAGGGACAAGGTGGAATGGCCTTCAATCCGCTG
EMPV1_33705	AATTAAAAGAATTCCAGCATAAGAAGGTGGCTGTTGCATGTAATCTTCCTGGCACCAAAG
EMPV1_33706	GCCAATGAACTTCCGGCAGAACTAACCAAAAATGAACGTATATTGCGTCTCCTCCGAAGT
EMPV1_33710	GTGCTTATTAACTATGGTCACTGAGACTAAGAGACCTGGGATATGGCTTGAAGGAGTAGC
EMPV1_33711	TGGAGGTGATTGAGTTCACCAGCAAGGAGGAGATACTATGCCGGGACACCAAGGGCAAGT
EMPV1_33712	TGTGGACGTGAAGTCGCGAGCAAAGCGTTATGAAAAGCTGGACTTCCTCGGGGAGGGACA
EMPV1_33713	CTCAGTTGGCCATGCAAGGGCTTCCCCAAAGCCCAGAGGAGTTCTCTTGTTCAACAGCAA
EMPV1_33715	CCCGCGGATTGTGGAGCACCCTTCTGATGTCATTGTTTCCAAGGGAGAGCCTACCACTTT
EMPV1_33716	TTCGCTAGCTTCCCCTTATCTGCAGGGTCTTTGGGTGAACCCTTCCCAGGTAGCAAAACA
EMPV1_33718	CTGACCAAAGTTCCCCCGAGGTCATCACAGGATGTGGAAATTAACTAGTCTCTCCCATAC
EMPV1_33719	CCTTCAACGCTGTTTACTGCGCCAGCAAGTTTGCAATCGAAGGCTTATGCGAGAGTCTGG

EMPV1_33721	CCTCTGCTTCCAGTTGGGAACAAACCTTTAATATGGTACCCATTGAACCTGCTGGAACGC
EMPV1_33722	GGACTTGGCTAAAGCTCTTGCTGTACAGTGTGTGGTATTTAACTGTTCAGATGGGCTAGA
EMPV1_33723	AGTAGTGCTGCTTCTGCTGGTGGCAAAGTTGTTGCTCCAACTCCAAGCCCACTTCCAGTG
EMPV1_33729	GGGTTCTCTCTTAAAACATCTTTGGCAAAGCACCACAAAGGAAGCCAAGGGGCCTTCCTC
EMPV1_33730	CATGTGGCTGCACCACCCCTACGTGGACAGGGACAGCCAGCTGCTGCTGGAGAGCATGCT
EMPV1_33731	AGTCCCACTTCTACCTTAGGCCAAACCCAGATTAGAAGAGCCCGAGAAACTCCCACGCTA
EMPV1_33733	AGTCATTCACAGCGAAAACCTATTCGGTACTCAGGAATGTACCCATGATTTAAGGAGGGG
EMPV1_33736	GAGGATAAAATGGTGGTAGAAAATGGCACTCAGCATCTGAAGAGAGATAGAGGCAGTGGC
EMPV1_33737	CTCTGTCCTTCTCCTACTGTGGCTCCCGAGAAATACCTCAATTCTTCTGTGATGTCCCAG
EMPV1_33739	GGTGGCCAAAGGAGTGAGGTTACACTGAGGTTGGTTGTTTACGGAGCAGAAAGCAGAATG
EMPV1_33741	TCACCCAGACGTCGGTCCTGGGCCTTTTCTGTGACTCTTCGTGTGAGAAGGTGATTGATA
EMPV1_33750	TTTGCCCAGAGCTGGATTGCTGGATCATATGGAAACTCTATTTTTAGTTCTTGGAGGAAC
EMPV1_33751	TATGGCCACATCTCCAGCATCGACATCAACAGCTCCCGGAAGTTCCTGCAGAGGTTTCTG
EMPV1_33752	TGGGCCCTATGACTTGGTGACAGAAGGTCTGGTTACTGAAAATAAATGACCCTTCTCCCT
EMPV1_33754	GTGCTGGGTCACTGGAGACCTCTATACAGATGGAGGACCCTTGCATTATTCCTTCTGACA
EMPV1_33756	GCAATTATTCTTGATGAGACACTTGAAAATGTACTACTGGTTCAGGGGTACCTAGCAAAA
EMPV1_33757	GAGAAGAGTAACACGTGGTCCTGGGGGATTTTGAAGATGATAAAGGGAAAAGATGACAGA
EMPV1_33758	AGCGCGACAAGCGCGAGCCCCCTACACGTGGATTTCCTTTACAGCACCTGCAGGCCATCT
EMPV1_33760	CAGCTTGGGCTGCTTACTATGCTCACTATTATCAACAACAAGCACAGCCACCACCTGCAG
EMPV1_33764	GTGACGTCGCAAGGAGACGCCATGGAAGTCTGTTCTGAACACCTGGAGCCCTCTCCAGAA
EMPV1_33766	AGAAGCCTCTGTATCCAGTTGGTGGCTGGTTCCTATCTCTGTGGCTGGGTCAGTTCCATT
EMPV1_33768	AAAAGACCGAGACGCCTTCTCTTGACGCCTGAGAACGTATCACTCCTGGTTCCTCAAGGA
EMPV1_33770	AGGGAGGAATGTAATACAACAGCAGTGGCCTAAGCGGTAATGAAAGGAAGACTAGGCGGG
EMPV1_33772	ATCAGGGCCAAGTTTGTGGGAGCCCCAGAGTTCAACCAGACCGCCTCGTACAAGCGTTAT
EMPV1_33773	CTTTGGGTCAGTCAGGTTCCCTCAGACTTGGGGCCTTTTCACAAGATTCCCTTGAATTGG
EMPV1_33774	GAGTTCTGATTCTGCCTGCTCTCATGCCTCTGTGATCAGAATTGGGCCTCCTCGGCACCT
EMPV1_33777	GATCGGTTTGGAGAGCGGCAAGATTAGTGATTTCATCAAGATTGACACAGGGACTGGAGG
EMPV1_33778	AATGACCTGCTGCCACCAAGCTGCCTGTGACCTCTATCAGTTCATCCTCGAGCTACAGTA
EMPV1_33781	CCATCACCTGCAGGGTGGTTAAAAGTAATCTTTTGGGAATGTACATTTAATCTAGGGCCC
EMPV1_33782	AAAAACGAGACAGCCAGATCATCATTGACACTTTGCGGGACACGCTGGAAGAGCGCAACG
EMPV1_33784	AAAGCAGGGTGTGTTGCCATGAGGACTGGGGTTGCAGGGGGCTTGTGGGATGTTCTGAGA
EMPV1_33785	CTCAGGCTCACTATTCCCTAAGTTCTGCTTCCACATCATCCAGATACTCCCAGCAACACC
EMPV1_33787	CTTCCTGGATTTCCTCCTGGGCTTTGCTGCTGGCACTGAGTACCTGGACCTGCAGAATGA
EMPV1_33788	TCAGTAGATCTGAATGGCTCCTGAGATTCTGCATTTCTAACAAGTTCTCAGGTTGGTACC

EMPV1_33790	GACTGTGTTGCTGAAGAGGAATTAGATGAGATTTCTGATTCCCAGGTTGCTCCTGACACG
EMPV1_33791	TGACGAGCCCCGAGAGCCTGAGCCGGGACCTGGAGGCACCAGAGGTTCAGGAGAGCTACA
EMPV1_33792	AGGCTCATGAAGGATCTGGGCTACGGCCAGGGCTACAAGTACAACCCCGCGTACAGTGAG
EMPV1_33793	GCCAAAAAGCCTTTAGCAGTATGGCAATGCTCAGCAGACATCAGTTGATTCATACAGGAG
EMPV1_33794	AGCTCGGCCATATACATTCAACCAAAGAGATCCTACGGCCGAAGTCAGGGCAAGTTCCTT
EMPV1_33795	TTATACCTGGTGTAAATGTATAAACGTAGAGACGGCTCTATGGCCCCGGACCACCTGGCA
EMPV1_33796	GCCACAGCAGTGACAACATCAGATCTTTAACCTCTAGGCTACCAGCGGACTCCAAGAACT
EMPV1_33797	CAGAGGATGTGAACTTGGCCTATGAAATACTTGAACCAGAACCCACAGAAATACCAGCCT
EMPV1_33801	CACGCTTAACTTTAATCATGGTTCAGTTCTTACTGCACCCTTGGGCGGGCTTTCGCAAGG
EMPV1_33806	GATTTGGAGAATGGGGAAGACGTGGCAACGTGCCCTAGCTGCTCACTCATTATTAGAGTG
EMPV1_33807	ATGGGCTCTGGCATCCAGTACGGTGACGCCACCCTTCCCCAGCTGGGGCAGCGTCGGAGT
EMPV1_33809	TTGTCCTAAAAGCTCTGCTTCTATACGCAGGGTATATAAATCCCACTTATCCTGTCGTAG
EMPV1_33810	TTTACAATTGCCTATGATGCAGCAGGTACAATGCCAGGTGCTGGGGGGACATCACTGAAC
EMPV1_33812	GACCCTCTCCCATTTCCCTTCTCAGCCCATCCTCCAGCAAAGAGGGATCAAAGTTTTTCA
EMPV1_33813	AGGCATCTAGTTGTATGACAGATACCAAAGAAACATTAACCCCTTTACCTTTACAGGGGC
EMPV1_33815	CATCCCTCTGATGGCAAGAGAAATATGTACGAGACAGGACATCAGGCTGGCCCTGGAGGG
EMPV1_33817	TTTAAAAGGAGGAAGAGCCCCCACAAACTCCTCCGACCCATCACTGACTCCCAATCTGGG
EMPV1_33821	GTTTGCATCCACACGCTCACCAAACACCAGGAACCCGTCTATAGTGTAGCTTTTAGCCCC
EMPV1_33825	GCATGGATAGAGTCCAGTGTTCTAGAGATCTAGAATTGGCTCAACAGCTTCAACAAGAAG
EMPV1_33826	CCCCCACCCAGTGTTTCTGGTTCATGCGTTTAGGGTAGGATGCAAGAATTTGCAGTTCAA
EMPV1_33828	TCCATCATTTCATCTGTGGTTGGCAGAGAGCAGGAGTCTAAAAAGGAAATGAAGAGAGGC
EMPV1_33831	AGCTTATGCTCAAAATTATAGTGTTTCTGGTTCATCATAGCATAGAATGGTCTTTGGCTG
EMPV1_33832	CTGAAGAAGAGCGACCAGGTGTACGCCATGAAGGTGGTGAAGAAGGAGCTGGTCCACGAC
EMPV1_33833	CAGGGAGATCGAGTTATTGGCTTAAGGAGGCTTAATTGTATGGCTGGAGAATGCATCATT
EMPV1_33834	GGTGGTAGGGACCACAGGTGAAGCGAGATTTACGGGACCAGGGACTTAACATGGTGAAAA
EMPV1_33835	GTAGTCCTCCTGGAAGCCCATAACAAATGGAAGAGTCTACCCAAAAGTGGTCTCCTCTGG
EMPV1_33836	GACTCAGTGGCTTACAGTCCATGCTTGGGAAATATAGCTGTAGGGAGAGTGGACACATCA
EMPV1_33840	ATCAACAGTGGGTCTTTTCCACAGGGCCATCCGCCTGACAATGCCCCAGCTGACACTGCA
EMPV1_33845	GCGCTAAGGACCCAGTTCTCTTTGGCTTGCTTATTCCAATCGGATGTACCTCTTGTGCCT
EMPV1_33846	CAGGCACATTGCTTCACATCAGCCCAGACCTCAGGCTGAACAACGCTTGTTTGTGTGTAT
EMPV1_33849	TGGCTGTGCTGGCTGGAGTTGTCGCCTTCGTGTATGAGAAACGCGGTGGCATCTACTGGG
EMPV1_33850	GCATTAATGTCTGCTCTTATACAGTTAAGCACAGTGATGCATTCTACGGCCAGTTCTACA
EMPV1_33851	GCTACTCGTGTGACGTCGTCAGAAACATCCCCAACGGCTTGCAGGAGTTTTTGGATCCTT
EMPV1_33852	CCTGATGCTATTAGTTGCAACGCCGTAATTCCTACTAAGCCTCAATTTCCACTCGTTAGG

EMPV1_33854 AACGACATGCTCGATGGCTAGAGACCCTCAACACTCAACGCGAGAAGCTTATTTGGAACG
EMPV1_33855 CACCTGCTGTGTGCCAGGTACCATTTGAGGCACTCAGTAACATCAGTGAACAACTCACAG
EMPV1_33857 AGCCTACATCATAGCCACAGCAAAGCCAGATCCAAGCAGCATCTAAGACCTACACTGCAG
EMPV1_33859 ACCTCTGAGCATAGAGAAATGGAAGCCCCAGGGGGTCCTTCTGAGGACCGAGGAGGCAAA
EMPV1_33861 TCCGAGACGATGATTATTTCCGCACTTGGAGTCCAGGAAGAGCCTTCGATCAGGCTTTAG
EMPV1_33862 TTTGGCGCCTGAGCCTACACAGACTGATTACTTGCTAACCCAAAAATCTCAGCATTTCCC
EMPV1_33864 CGAGCAGGCAGCTTCGCAAGGCCTGATTTGTCATAGAGAAGAAGGTCACTCTTTGCAAGG
EMPV1_33865 ATTCGACACACCAGGTAAGGGGGGGCAGTTGTCCATCTGGACCGAGATCCCTAAGTCTAT
EMPV1_33866 AGCGCAGGAAGGAAAGGCTGTGACCATGGACTGCACGTATGAAACCTCAAGTACGGCTTA
EMPV1_33867 CTGGGACCAGAAGGTATAACCAGAGGGAAGCGCGGATTTAAATTCCAGGGCATGAGACAA
EMPV1_33868 AGAGGTCAGGACTCATGGAGCATCAGAGAAGTCACACTGGAGAGAAGCCCTATACGTGTA
EMPV1_33869 GCTGCTGAATCAGGAATAATAAAAGTTAAAACAATAGCTGCACGAAACACCGAAATTTTG
EMPV1_33871 TGTCTGAGAGTGAGGCAGAGCCAGATGGCGAGCACAACATCACAGAGCTGCCCCAGGCTG
EMPV1_33875 TGAAACTTAAAAAGGTTAACATCGTGAATGCCAAATTCAGGCCCTGAGCTGGCGTGAGGC
EMPV1_33876 GGTTAGCCATGGAAGAGAAACTGGACCACGAGTATATCTTTGCCGAGGCCTACAGACCCT
EMPV1_33878 AACGGTCGGAGTCATTGTATCAGTCGAATCTGACACCAGCACCACTGACTTCTACACCTC
EMPV1_33879 CCTCCCCCAGAAGTAGTTCAAACAGTAACTCCTGTGGAGGTTCACTTAGGCACCTGGTTT
EMPV1_33880 CTTGGACTAAGGATTCGTCAGGAACAGGACATTTCACCTCAGGAGTGCGGGTCTTCCGGC
EMPV1_33882 GATGACGATGAATTTGGGGAATTTGGTGGGTTTTCTGAAGTTAGCCCTTCTGGTGTAGGG
EMPV1_33883 GGGCTCTGTGTTGCTTTCAGATATTTTTATCCAAGTTGTCAGCTTCTTATTGCTGTGGTG
EMPV1_33884 CCAAGGTAGGGTCCATTGTCTTATTCTACCACTGTCCCCAGGACCTCACAGAGATGAGTG
EMPV1_33885 GATGCAGAACATGAGTATCCACACCAAGACCACCAGCGGCTACTCAGGTGGGGTGACCTA
EMPV1_33887 TGGCAGCATCTACAACCCTGAAGTGCTTGACATCACCGAGGAAACTCTGCATTCTCGCTT
EMPV1_33888 CAGGTCCCTTGATGGTCGTCTTCCGGTATCTCATCGAAAAGGACTGCCACACGTAATCTA
EMPV1_33890 GCACACACCCAGTGGAAGATGGAATCATGGATGCGGCCAATTTTGAACAGTTTCTTCAGG
EMPV1_33893 GCCGGAAAGGAAGAAACGCCCAAGCCTGGTGGGTCCCTGTCTGTGGACATCTACCACCCA
EMPV1_33895 AGAAACTGCGTGGTAGCGGTGTCGCTGTTGAAACAGGAACAGTCTCTTCAAGCGATAGCT
EMPV1_33900 GGCCAGCCCACTTGGAAGTCCCTAACTTTCTCTAGTCTCCAAATGACACAATTAGGTCCC
EMPV1_33901 GAGGCAGCTACAGGAGATACTGGGCAGCCTGAGTCTACAGGAGGAAAAGACACGAGTGTC
EMPV1_33902 ATCGAATTCATTTTTGTAGCTCCCTCCGCCCCCAAAGCCCAGAAAGTCACAGCTGCAGGA
EMPV1_33903 GCTACATGGCAGTGGGAGGCAAAAATAGGAAGTTTGCTGGTCCCTAGATGTATCACAGGC
EMPV1_33904 TTTCTCAGTACCTGGAATAAAAAGGTTGCAACCAGATGAAAAACCTGAAGGCAAAAATCA
EMPV1_33905 GCCATGTCTGTTGGGAATCCACAATCTCCCAACAGGCATCGTGGTCTCTGCTGATGCTCT
EMPV1_33907 AGGTACGAACAGCACAAACACAAATACCTGCCAGCTCTGCCCATAAGCTAGAGGAAGACC

EMPV1_33908	ATCAGCAACCTCTTCAACACAGAGGAGAGCCGAGCTGCGTGGAACAACACCCTCCTTGAT
EMPV1_33909	AGACTGACCACATTTCGAGTTCTCAGTAGCCGCATTTGTGTAGACAGTGTAGTTTCTAGA
EMPV1_33910	AAATTCTTCTCAGGGAGCCAGGAGCCAACACCCTCCTTTCCTGTGCCGTATGATTTGCCT
EMPV1_33912	GTATCACCCTACCCGGAGCCTGCAACATCCCTGCCACTGTGAGCAGCGGCAACTGGTTCT
EMPV1_33913	ATGATCAGATGGCAAAAAGTGGAGTCCGTCTGCTCTCAACTCAGGGAGGACCACTCAGCA
EMPV1_33915	GCAGGGAATACCACAGTCATTAGTCTTCTTTTGGAAGCTGGAGCTAATGTTGATGCCCAG
EMPV1_33918	GGTCAACCTTAGCTGCAAGTACACCTACAACCTCTTCTCAAAGGAGTTCCGGGCATCCCT
EMPV1_33919	CTAGAAAATTATTGTATTCCTTTAGTGGCATGACGTTTCCTTGATTTGTCATGTTCCGTG
EMPV1_33920	CCTGGAGGGATCTTCTGTTCCTGACTCATCTTGGGTGGCCTCACAGAGCAGCAAAAAGCA
EMPV1_33921	AACTATTTCTACTTCTATGACCTCCCTCTCTTGGCTCTGTCATGCTCAGACACACACATG
EMPV1_33922	TGGCCTGCGACAGCCAAACAGCCACAGCCACGTTAATATAGAAAACGGTGGTTTAAAAAC
EMPV1_33925	TTCCTAAACTTGGAGCCCATCCCTGGAGCCTTGGAAGCCATGCGGGAGATGAATGACATG
EMPV1_33928	TCCTTCTGTTTGGCTTCCAGTGGAATATGCTGGGGCATGAGGCAGTGGATGCAGCAAGAA
EMPV1_33930	TTAGAAATTCATGCTCTAAGGTCATCCAAGGCCCTCAGCCAAGAAGGGACTGTGCTGTCG
EMPV1_33931	TGCTCGTCTCTCTTCTGCACTACGGCTGCGCCATATTCATCTACATCCGCCCCAAGGCAT
EMPV1_33932	GGAGAGAAGAGGAATGGAGACCTGTGCATTGGAAACCAGAGGAATGGAAGCAAGAGGCAT
EMPV1_33936	GTTTTCTTTGCCTGGGCCCGATTGAGTTCAGATTTCTGTTACTTGCAGCCCAGGGTGCTG
EMPV1_33937	GGCGGCGCTGAGTACAGAACCTACGTCTCTAACTATAATACTCTGCCAACTGGCCTTTAG
EMPV1_33938	TACCGAAACATTGATAGTGAAAGCTGGAAGACGATCATTACTAAGAATCTACATTACAAA
EMPV1_33939	TTTAAGCTGGACTATGCTGGGCTCTGACCTCATCCTCATCTTCGCCTCCTACACCCTCAT
EMPV1_33940	TCGCGAGTCACCACTGGGTTAATGATGTGTTTGGAAGCTCACTGACAGTACAAGCAGCTT
EMPV1_33942	GTTTTGGAATGTTATTAAGCCCAGGTCGAAACGTGAAGAACAGTGACATGCATTTACTGG
EMPV1_33944	TTCGTCTTCAAACGGGCAGCCAAAATACCCGGCCAACTGCACTTACTACTTTGAGAATTG
EMPV1_33945	ACAAAAGAGGAGCGCTTTAAAGAGCCTCGACTCCTCGGGGTTCCCAAAGTCCCTACAGAG
EMPV1_33946	GGGGCCAGAAATGAAAAGTAAACTGAGAACCAAAGCTAGAAGCACATGGCCTTGCCCTGC
EMPV1_33948	TGTAAACCTGTCATTCCTTAAAAGCAGAAAGGGCGCTGCTTATGTCATTATCAAGGGGCC
EMPV1_33949	TGTATCACTGGAGACATCTGAAAAGCACACACCTCAGACAGAGTCGGCCACCAGGCCTTT
EMPV1_33951	TGCAAGTCCTCAGAAAAAAGCCAGTCACCTCGGACATCTCAAGTGAGTTCTTATCTGCCG
EMPV1_33952	GACTCTGCCAGTGTACATAAATGCCTGTTAAAATGACCTGTTTGTGATGGTGCAGTTTTC
EMPV1_33953	CAAAAGCTGAATGAGTTTCTCTTGAGTGATGAGATTGGGGAGGACAGCTGGAGGAGTGGG
EMPV1_33954	GTGCTGAGGGACACTATGAAATCTTGGAGTGATAGCCAGTCAGATCTCTGTAGCAGTGAC
EMPV1_33955	TCTGCAGCAGGACATCTGAAGGTGGTTCAGTCTTTGTTAAATCATGGAGCGTCTGTCAAC
EMPV1_33958	CATGTTCACCTGTGGCTGGATGGAGTATTAAGCTGGTAGAGAGCAAACTAGAAGAGAGTG
EMPV1_33962	TGCAGCTGTGCAAAAAGCGTGCTGGGAGCCTGGGACTCACCAAAGGGGACTCTGTAAACA

EMPV1_33963 CTACCTGGCCAATGACATGGAAGAGGACAACCAGGCCCCCAAACAGGCCATCTTTTCATT
EMPV1_33965 CCTGGACGAGCTGACGGCAGCCCACTCGCTCTGCTTCTCCCCCGATGGCTCCCAGCTCTT
EMPV1_33966 CTGAGGTGGCCCTTAGCAGCCGCAGTCGCAACTTCTGGAGGCGGATTACCCGGAGCTTTT
EMPV1_33968 GGGCTAGGAAGGACAAAAGGTAGAGGCTAGGGTTCCGCAGATCCTAAAAAATCAGAGGAG
EMPV1_33969 GAGGTTCCCTTCCACATTGACTGATTATATACCTCCTTTGGCAGCAGTTGGGTTACTCCG
EMPV1_33973 CCTTAGTGCTGGTTCATGGAGGACTACTGATAAAACTTAGCTTCTGGTTTTGCTATTTTG
EMPV1_33975 CAAGACAGGAGAAGGCCCGGTTTGGTAGAGGAGGTGAGTTCAGTTGTGGACAGGTGTGTT
EMPV1_33976 GGTTTGATCTCTGGGGCTCTGTGTTTGCAACAGGAATTGTTTGCACATTCTATTGTACCC
EMPV1_33977 AGGCTGTGCTACCCATTCCTCCAACAGGAAAGTAAAGCCCACTCTTGCACCTACGTGTAA
EMPV1_33979 GTCTAGGTGTCCATACCTCTATCTCTGTTCTCTTCTAGGCAGCTAGACTATGCCAGACCC
EMPV1_33980 CTGGTGTCTAATACCTCCAACTTCTGATTACTACCTGGAAGAGTCTATCCGGCCCCCTGT
EMPV1_33981 CCTACCAGGTATCCTGCACTGAAAGCTCTCATGGAAAAGAAAGCATATTTGGAATGGCCC
EMPV1_33982 GCCGGAGAATGTGTGTTGGGCTTGTGACACTTTCCTGGATTGTTGGCATCTTCCATGCTA
EMPV1_33984 TTGGGGGGTAAAAGCCATTGTCTGCTCCTTGTCACCTTCCACCTCAGACAGCAGCTATGT
EMPV1_33986 GACTGGGATATGTAAAATTAAGTATCAACAAAGACCATCGCACAAGTCTACCAACGCATG
EMPV1_33987 TCAGCCTTTCAACTTGGTGGGGAGGGTTGGTGTTTGTTTTAATGAGCTGTTGAGAAAACC
EMPV1_33988 GTTTCTGGCTGCAGCTGAGTACAGCACGTCACCAGGCGCAGACCTCAAGGGCCTTCTCCA
EMPV1_33990 TGGAGAGTACTGGCCTTTGGCTCTTGAGAAAATCCCTCCGCCCGTTGATACTTTCCCATG
EMPV1_33992 TCTGGATCAGATCATCTCTCAGATTTGATTTCAAGCCTCCGAATTTCCCCTCCCCTGCCC
EMPV1_33999 ACATAACTTCCCGCATTCAAGTCTACCTGACAAAGAAGCCCAAAGAATCTAAAATTGGGG
EMPV1_34000 CACATTTTATAAAAACCTAGCATGCCTAAAATATTCTGAAGAGGTTTGAAGATGAAAGGC
EMPV1_34001 GATGAAAGTTTTGTCCGGATGTTTGCCCAGGAGTGGGATTTCTGGGTCATATAGTAGTTC
EMPV1_34002 GGTGTCAAGTATGGTGAGTCCTGCTGATTGTCGTCTGTGTGCACCTTTCTGTTTTGCAGT
EMPV1_34004 GTTACTTGTATTTTGAGAGGAAAAAATTGTTTGTGGATAAAACAGAGAAGAGTCTAAAGC
EMPV1_34005 CCTAGAGAGCATGCAAGGCCTGATGGACTTGAGGCGGCAGCTCGAGCCACACTGGAAGAT
EMPV1_34006 TCCTCCGATCCCAAGGCTTCCAAGATAAAACCAGAGCAGGGAAAATGATGCAAGCGACAG
EMPV1_34007 CACCTTTTGGCTACATAGGAGGCTGAGGACCTTGAGACGGTCAAAGAGCGAGATGCTTAT
EMPV1_34008 ATTGCTGCAAGCTGCTGAAGATGCGACTTGGATCTGGTGTTGCTGTGGTATAGGCTGGCA
EMPV1_34010 CTTTCTCCCTTGATCCGTCCGGAACCCGTTAAAAGGGACTCCTTGTAGGGCGTGATGACG
EMPV1_34011 AAAGCAATCAGGTAAGGAGCCATATTTACTTGGGGCTGGCTGGGTCACTGTCAATCTCCC
EMPV1_34012 TCACCTCCTTCCAGGGCTTATGTGGCTTCCGGCCAGTTGAAGAGATTGTGACCTTTCTGA
EMPV1_34014 CTTCATGTACGTTCGCCCTGGGTCTGAACAAGCAGGTGAACAAGATATGATTGACTCTCT
EMPV1_34019 GTGTAGGTATTTCTGCTGCCAGGTGCTATCTTGTTACTAGGAATCACCAAAGTGATGCAA
EMPV1_34020 ACAGTCACTGAGCTAGAGCAGACAGGCTGTGAAATTCGCCTTGACGTGTGATCTCTGGCA

EMPV1_34021 CTGCCAAAGTGGGAACTCTCAAAAGGAAACAGCAGATGAGGGATTTTATGGAACAGATGC
EMPV1_34023 AGCAGCTGCAGTCCCTCTCACTGGCCAACCTGGGCATGATGGGGAAGGTGGTCTACACGT
EMPV1_34025 GGAACAGGCCAAAACCATTGTCAGCCAACAGAGTTCCCAGATGACCCACGTGAAAAGCAT
EMPV1_34026 GACTTGGGGTGAGCAGACTATTCCATAAATGAGTCCTTGGCGCTTTGTGAATCATGCTAG
EMPV1_34027 TGGAAGACTGGCCCCAGGACGACCATATTTGTAAAATCCCTGGAAGACCCATATTCCCAG
EMPV1_34028 GTGCGGGGAAGGGACTTCGGTGACAGGCTTTGGCTCCAGCTGATTTATAGAAGGAAAGTT
EMPV1_34029 CTTTCCTCTCTAGGATTGTTCATTGTGGCTACTGCTCTGTTCCCCTGTGTGTCAGTGAAG
EMPV1_34030 TTGCTAGTCCTACAGCACTTGACACCCTGCCATTGGTAGTTACTTGCATATCTGCTTCCC
EMPV1_34031 GCTCTTAACATGATTTACAGAACTCCTGAGGACTAACCCCAGGCTTCCACCAACCTCGTC
EMPV1_34033 GCTGGAATCTGGTGTAATGCTTCTTACCCAACACAAGGAAAACCACCCCCATGGTGTGAG
EMPV1_34036 GGTTCAAGTGAACATTAGGGTTTCGGAAAGCTTGTGTCCGCCATGAGCTGGATAGCTTTC
EMPV1_34038 AATCCACATGAAGGTGGCCAGGACCTTTGGAATCTCGGCGGATCCCTGGCGGCTGAACAA
EMPV1_34040 CTGGCAAACCCAGTCTCAGGTGTAATGCTGCAGTGTGTGCCCTTGCTCTTTTCCCGACGA
EMPV1_34043 TCCAGGTGTGGTTTACCAGGACGGATTTTACGGTGCTGACCTCTATGGTGGATATGCAGC
EMPV1_34045 CATCGAACCCACGTTAAAACAACCCGAGCCACTGCAGCGACCACGCTGGATACTCAGCCT
EMPV1_34046 AAGCTGACGCAGGTGTAATTCTGAGGCCCCCATTGCAAACCTGAACCGGAAACTCCAGGG
EMPV1_34047 GGAGGTGGTAAGCTATTGCTATGTCCCTTGCAGTTTTAAAAACCACCGCTGTCCACTGTC
EMPV1_34048 ACTTCACATATTCCCTCTCAGTCGTCCTCATCTCCTACACCCTTATCGTAGTAGCTGTGC
EMPV1_34049 ACGTTAAAATCGTGTTGTACAAGTTATATTTTGTTAACTTGTTGAAGTTGATACTCATAT
EMPV1_34051 GAGAACACCAAGAAAGTCATTCAGTACCTGGCTCACGTGGCCTCTTCACACAAGAGCAAG
EMPV1_34052 CTGGAAACAGCATCGCACAGCATCACAAAGAGACAGAAATTACAGAAGCCTTTCAAAGCC
EMPV1_34054 CCACTGTAAGTAAGACTTGAGTGTTGGGTATTTACAGTGTGCAGCTCTGGGGATATTGCC
EMPV1_34055 TGCGGCAAAGTCACTGTTACTGGGCTGGTCGCCGTGAGCCTGCTACTTGAGGCTACTGGT
EMPV1_34057 TCTCCTTTCACCTCTAGTGCTCCTGCCATAGTTCTGTGCCCTTACACTCTGCAGAGAGCT
EMPV1_34058 GTGTTCCAGGGTTAAGAGGACATCACTAGAGTTTGTCTGGGATGACTCAGCAACCAACCC
EMPV1_34059 GTGTCAATTTCTGCTGTACAGTGTAGTGTGTTTATGTATATATATAGATACAGACACACA
EMPV1_34060 CACAGACACATTGGACCGTGGGATCTTGAAGCGCAGGCGGTAGTAAGGGCTGGGGAAGTT
EMPV1_34061 TCAGCTGCGAGGGAACCAACAAAATCCCCTTCCTACCGGCCGCCCATGCTGAGGATAGAG
EMPV1_34062 GACAATGCCTGGTCCCTCCAAAATTCTTACGGTGAAACCTCACCCCACTCTGATGATATT
EMPV1_34063 CCATCAATGGGAAGGCACGGAAGCATGGTGAAAACTACAGTGTGGACTACCTGACAGATG
EMPV1_34066 TACAGAAGCAATGTGTGCCATTCAGTCATTACAGTGGTGGAGTTGGTAGGTGTGCCCAGG
EMPV1_34068 TTATCTTCAAGCCAGGATGAAGGGAGACTGGGCAAGACTCTTACGCCCCACACTGCAATT
EMPV1_34069 AGAAGCAGCAGGATGTGGTGAGATTTCTGGAAGCCAACAACATAGAGTTTCAGGAAGTCG
EMPV1_34072 GAACTGTGATCTGGAGAAAGAGGAACTCAGGTGGAAATAGAGGGAACTATGTTCAGGCTG

EMPV1_34073	TGAGCACTAAAGGCGAGCTTGTCCATTCCTAGTTCCTGTTGGCTTCTTAAACAGACGGCC
EMPV1_34075	CACAGAATATTGGCCACTGATGTTTGAGGACCTTGCTCTCAGCCAAACTGGGAAGCTGGC
EMPV1_34076	GTGAACATCTCAAAACTTGAAAAACAGCTCCAGAAACTTGAGCAGCAAGGTGCCGAAGGC
EMPV1_34079	GGTGGATCTGAGGGAGTTCTTTGATTTTTGCCTTCCCATACCTGTGCCCTTGCTGAAGTG
EMPV1_34080	CAGGGGCCCAGAACCTACCCGATATGGAGATTGGGAGCGAAAAGGACGCTGTATTGATTT
EMPV1_34082	ATGAAATCGGAGGGCGGCTTCATCTGGGCCTGTAAAAACTATGATGGTGACGTGCAGTCG
EMPV1_34085	CTGATTATCAAAAATCGGCTCAGTCGTCTTCCCTCCCATGAACGACATTTGATGGGTTTT
EMPV1_34088	AGCCTTCCTGGTACACGATGGACTTCAGGGCTTTACTGGTGTGACTCTTGTCTCACTGAT
EMPV1_34089	TTACAGAGAAAGAAAAGCCCTGCTCTGGCCGCATGCATCACTCGGGATAAAGCTTGGCTG
EMPV1_34090	TTTGATTTCCGTTTTAAGGTAGTGTCTGTGCAGCTCTTGCCCTGGTTCTGGCTGTCCCTC
EMPV1_34093	CTCTCGGCCCTATCTCCCAACCCTTGTACCAGTGCGTGCCTCAGTCCCTGGTACAGGAAT
EMPV1_34096	ACAGCTGAGGAAGGTGTCTTGCTGCAGGTAACTAATGAAGAAGTGGTCAACCACAGAGCC
EMPV1_34098	ACTTTGAGGGGAAGGTTGTGAAGACCTCAGTTGTGTTCCAGAAAGTGGGGACAGCCATGC
EMPV1_34099	GTGGTGGAAATTTGTTTGATGCCATATCCATTGCTGTAAAGGCTGCGCTCTTCAATACCA
EMPV1_34101	GTGGCGTCAAGAAAAATCTCTAGTTTGTTGAGTCTCCCCAAGTCACATCTTTGGTGTGGC
EMPV1_34103	TATCCACCTTATTCGGACAGTTATCCCCCAGCTGGAGACCCCATGTGGACATACTTCACT
EMPV1_34106	TCTCCAGGAGGCTCTCCAAGATCAGCAGGTGAGTCTAACCCAGGGTTCTCTCAAATTACC
EMPV1_34108	GGCCCCTTCTTGTTCAGGATGTGGTTTTCACTGATGAAATGGCTCACTTTGACCGGGAGA
EMPV1_34110	CTTGGCTTCCTGGATAGCCAAACAAGTGACTATTCTTCTACACCACCCAATTCCCATTTT
EMPV1_34111	AGCCAAGTCATACTACAGAAGAACACATTCAGATGCAAGTGATGATGAGGCTTTCCCCAC
EMPV1_34112	GTGCAATTTAAGTTCAGGGAACCCTCTGCTGTCATTTCTATGGCTATAACCTTACTTTTC
EMPV1_34114	TTTGGCCGCAGTGCCATCGAGCTCACCAAAATGCTCTGTGAAATCCTACAGGTTGGGGAA
EMPV1_34117	GGCAGGAGCTGGAGTCGGAGAACAAGAAGCTGAAGAATGAGCTGAATGAACTGAGGAAAG
EMPV1_34118	ACATCGACTACAGGATGAGGTGCATCCTGCAAGACGGCCGTATCTTCATTGGCACCTTTA
EMPV1_34119	GTGCAATGAGGGACCAGTACATGAGGACTGGGGAGGGCTTTCTTTGTGTATTTGCCATAA
EMPV1_34120	GAAGGAGAAACACCCAGCACTATGCCTGTTTTCCGGAGTGTATATAGCCTACTGACCAGG
EMPV1_34121	CTGGGCCCTCTCCGAGATCAGGATAAACCCATTGCCATCAATGACGAAGCAGTCCAGGTC
EMPV1_34122	TTCACCTGGCCCCAGTACATCAAGACTGTTCATCAAAAGCAGAAAGCCATGAAGCCATGG
EMPV1_34125	TGCCTGAGCTGACCCCCTCTGAACGTGATGATCTAGCCTCCATCATGAAGAAGCTCTTGA
EMPV1_34127	GACCCCAGGCCTCTCCTGGGGCCTGTCGCTGGCTGGGTCACTTCCTCTGGACGCGCCATT
EMPV1_34128	GAAATATGAATGAAGGGATGTGAAAGTGATGGGCTCTGTCCTTACACCCAGCCCCAGTGG
EMPV1_34129	ATTCCGAAATGCTGCAATTTAGCTGGTTTCTTCACTCAGACAGCTGGCTTTGTGGGTGGC
EMPV1_34130	TCATCTCCAACAGTGGGCTGATCTCTGTGGTCTGTTTTGTGGTCCTCGTGGTGTCCTATG
EMPV1_34131	GAGGGAAGACCAGAGTTCTGGGAGAAAAACTACACTGGAGTCCTAAGTTAGATTGGAGAG

EMPV1_34132	CACTCCCAAATCTGACAAAATATAAGGCCATGCACTCCAAAGTCTGACAAAATATAAGGC
EMPV1_34134	GGTCTGATTGCTGTTCCAGGGGCTTCCAATACTATGTTGAATAAAAGTGGTGAGAGTGGC
EMPV1_34136	CCGACTACCCTAAGGAAGAAGAAGGAGGTTCAGCTGTTATTTTTTCCAACAAGACCCCAC
EMPV1_34138	TGTCATGTGAACGCTTGTTCTGGATCATTTGGGGACCCGGAGGAAAGCCTTGTAGGCGAA
EMPV1_34140	AGAGACCATCACAGCTGATGTCAATCACAACTTGAAGGATGCCAATGATGTACCAATCCA
EMPV1_34141	TGTGGTGAGGAAGATGTGGTTATTGACCCTTTGGAGCTGCTGGGCAAGAGGATGGATTTC
EMPV1_34142	GACTGAAATAGATAGCAGGCAGGAGATAGGTATAGAATCTGGGTTAATTGAAATTTTGTG
EMPV1_34143	AGAGAACTCAGCCTCCACCTCCACAAACAGCGAGGTCAGCTTCCAGTTCAACCATCAATC
EMPV1_34144	TTGGGGTAATGCCTGGGTTTGTGTAACAAGTGGTCCATGGGATGCTTGGGTCTGAACAAG
EMPV1_34146	TCAAGATCTCCTTCAGCGAGACAGCCCTGGAGACCACGTATCAGTACCCCTCCGAGAGTT
EMPV1_34149	ACAGCATCAGTAGCCACCTCTGGAAATCCTAGTCTCCCGGGAAGGAGTAAAGGTAAGCTT
EMPV1_34151	GCTGACAGTGGACTGCTCTCATTTATCTGTTTCCTCCTGCTGCTTGTCTCCTATACTGTC
EMPV1_34155	CAGCTTCTCTAATCTGCTGAGAAGTAGGTCTGGTGTGGTGAATAGAGTTTGGGTGCTCTG
EMPV1_34156	AACCCACAGTGTCTTTGGGGAGCCCTCACAATGGGACTCATTCACCTCCAAACATCTTAC
EMPV1_34157	AAGTATCAGCTAGCAGCAGGACAGAAAGGTGTCCAAGTACCTGTATCGCAGCCAACTACG
EMPV1_34158	TGTCTGAAGCATCAATGATTGTTCCTGGTGCAGAGAGATACACTGTGAGGAGTCCAATAC
EMPV1_34159	GGTTGACCCAACAGCATGACCAAATACAGACACAGTCACTTGGGAAAGGTGCAGTGGCAC
EMPV1_34160	TCCTCGACAAAGGTACACAATCAATTGGGGGTATAAGGGAGGCATCCTGCTGCCCCGATT
EMPV1_34162	TTACATGAGGGAGAGACACTCTCCAATTTTGACCCTGACAACCTCCCAGGCACAGAGGCT
EMPV1_34163	AGCCGCTAAAATGGCTCCTTCGTCAATGTTCATGCCTGAGCTCTCTTCACTGCCTGGGAA
EMPV1_34164	GGGGGCAATTATGTACCTCCGACCATCATCTTCTGGGTCTATGGATAAGGGGAAAATCTC
EMPV1_34165	TTTGTGCTGCTGTTAAGTGATGGGAGGACCCTTCCTGGGCAGCGCGTCCCCGCACTGCGT
EMPV1_34166	AACCTGGAGAAGTACCGTCTGCCCTACCCGGAGGCCATCTTTGAGATTGGCTACTTCAAG
EMPV1_34173	CGGTGGGCGGCTCCATCTGCGTCATGCTCGTGGTCATCTGCCTGCTGGTGGCCTACATCA
EMPV1_34176	CTGAGCCCTGCAGTGCTGCAGCGCATCCAGCTGCAGCCCACGTCCATTGAGAAGGAGATC
EMPV1_34179	GACGCAGCCTTTCTGTGCCCATTCTTCAGGCAACTTTGGATCCCTCACTGTAGCTAATTC
EMPV1_34181	ACTGCCATCTGTCATCCCACCCAAGATTGTGCCATACCAGCTGAGGCTTTCTCTCTGTCT
EMPV1_34185	CCCCATTCCTTATGGATACCAGTTGATTTATTACTGTTGAGCCACGATGGGAGCTCCAAG
EMPV1_34188	ACAGAGAAATAAGAATTCTGGCAGCTCAGCCTCGCATTCACTCTGCTGGAGAGACACCAG
EMPV1_34189	AACACCCTGGTTTTCAGTCATAGAAGCCATCCACTCCTCCCACAGTAGATCTGAGCCCTT
EMPV1_34193	GAGCCTTACTTGCATCCTAACTTTGTTTCTACTTTCACTCCTCCCTCAGCACCTCATCCT
EMPV1_34194	CCAGTATTATGATTTCTATAATAAAGCTCATGTTTTCCTATTCAACTGAGGCTGACTGAA
EMPV1_34196	TGTGTGTGTGTGTGTGTGTGTGTGTGTGTGCGCGCGCACGCATGCTCAAGCACACACTGGGG
EMPV1_34197	GCCCCCTGCTACCTCTCTGAGGGCAACTCGGCGCTCTCGTGCTGCTGCGGTCGACCCTGT

EMPV1_34199	AAGGGCCTGCAGAGCTGCTCCCTCTGGGACTCTGCCTACCAGCTTCTTGTTATGACAAAA
EMPV1_34200	TTTCCCAGTCTTCACGGGGACAGTCGGCTCTAGGAGAAGAGGTGGAGCAGGATCCTTTGT
EMPV1_34201	AATCCATGCAGAGTTGTGGCGGACAAATAATTCCTCCAGCAGGCTACTTCCAGAAAGTGG
EMPV1_34204	GCTCTGATATAGCATTGCTGTGGCTGTGGTGTAGGCAGGCAGCTGTAGCACCGATTCAAC
EMPV1_34205	ACTTCCCGGTTGCCTGACGGCACCCCGCCACCTACCCTGACCAGGACTGACTCGCCAGGG
EMPV1_34206	TCCTCAGCCCCTCAGTCTCGAGCCACCAACTCTCCTGCTTCCATAACAGGCTCTGTCTCA
EMPV1_34207	CTCTTTCTCCAAGGGGTCAGCCTGGGTAGAGTCCAAAGGGCAGGAAAGGAGTAGAACAAA
EMPV1_34209	GCAATCTGTATTAGCGATGTTGGAGATTATGAAGGAATCAAGGCTAAAATTGCAAATGCC
EMPV1_34210	TGTGTTGCCGATGGATCACTTGGGCCTGTACACATACCAATTAGCGTGACCACTTCCATC
EMPV1_34211	TTACTGGTCCCAGATCTGACCACAAGCTTCTCACAGAGGCTGCTCATGTTAACCCTCCTA
EMPV1_34212	CCGGCTGAAATGAACAGGTCCCATGAAGGTTCTGCCTCCAGCCAACTATGGAAATTCAAC
EMPV1_34213	ACCAGCATCCTTGGGGGGGCTGCAAGGAGCCCCCGGCCCTTTGGTTTGTCCTTCTCTGTT
EMPV1_34215	AGCTGTGAGCTGTCTTCTCTCTTCCCTCTGTCTTGCTCTGACACCGCCACCAATTTCACA
EMPV1_34216	TTATCCGGTCCCATATATCAACAGTGCCAAGGCTGAGAAACCCTGCAGTAGACAGAGGTC
EMPV1_34217	ATAAAAGCATGTGAATGTCCAGCCTGAGGACAAGAGGGGCCGTTTCCTTTGCTACCGGCT
EMPV1_34218	AGGAAACCCCAGAAGGGTACAAAGTCATGTACACCCCCATGGCTCCTGGAAACTACCTGA
EMPV1_34220	CCCTGGCAGCTTCTGAAATTCTCCAGAGGTTCCTGTTCCAGAGACACACTCATAACTGTC
EMPV1_34223	CTTCGCCGCAGAACAGATCTTAGAGAGGCTATTGGCCTCTTGGCCCCTCCTGAGTCCATT
EMPV1_34225	GAGGAAGTGCTAGGCTTATTCTTGGGAACAACAGCACACTTCAACCAGAGGATGGGGAAG
EMPV1_34227	TGGAGAAGGAGTTTCACTACAGTCGCTACATCACCATTCGGAGAAAGGCCGAGCTGGCAG
EMPV1_34231	GCAATGAGCCTACTCTGGTTAAGGTGTTCTTTTCCTCAAAGGTGCCCTAGTGCCATGAGC
EMPV1_34232	CTCTCTTGGTACAACACATTCTATAGGTTCAGGCAAATGAAAAATGACATATATATCACT
EMPV1_34233	CCATCGCCCGGTTTGGCTCCGGCCCGTGCAAAACCCGAGAGGACGAGCCTGCTTGTGGGA
EMPV1_34237	CCATGTTCACAGCAGTACTACACACAACAGCTAAAACTTGGAAGTAACCTGAGTAACCAC
EMPV1_34239	GAGCTCTCAGGCCACCCACCTGAGGGTATTGAATCTCAGTAACAACCAGATCCCAGAAGT
EMPV1_34241	AATCCAGGGGAAGCATCTACAATAAGAAGAGAAGATGGACAGTTTCAGTGCATTACAGGT
EMPV1_34244	GCCAGCTGTTCCTCCCGACCCAGTAAAGTCTGAGGCAGATTCCTCACTTTGTTGTTAGAA
EMPV1_34245	TTATCTATGTGGACTACCGAGAATCTCCCGGAGAGATGGTAACTCCCTGCTGAGGGGTCT
EMPV1_34247	CTTCTACCAGGACCCCAGTGTGCTCTACATCTCCCTTCATCGCCATGACGATGGCAACTT
EMPV1_34248	GTTCAGAGAGCTTGGGCTGGCTATTACGACTACAACACCTTTGACCAGAATGGCGTGGTC
EMPV1_34249	CTTCGGGTAGAAGGACGCCTCCTGGAGGATTCAGCCTTGTCCAAATATGATGCCACCAAG
EMPV1_34250	GGGAGAGTAATCATTAAAGGACTGTCTATACTCAGTTTTTGCTGTTTCTTACCAGGAGCT
EMPV1_34251	ATGTGCTGTTTTTGAGATGCCTGGGAACATGTGGCGTTTAGGAAAGAGTTCTTCACTGGG
EMPV1_34254	AAGAACTCCGAGATGACACAAACAGATGGGAAGATGACACCATACTCTTGGATTGGAAGC

EMPV1_34256 TCCCACTGTCGACCTGAACAGCGTGCTCAGCGAGACCAGGAGCCAGTACGAGGCCCTGGT
EMPV1_34257 GGGGCAAGAAGATAGTAAGGTCTGTGTTGGAGTGGGGTGCAAGAAGCCACAGTGTTTGTA
EMPV1_34258 AGGCTCAGAGAGGGGATGCCACTTTTCCAAAGTCACACAGCTAACAGGCAGCTGGTTCCT
EMPV1_34259 TTCACTAACCGCCTCTTCTCTCGATGGTACGAAGTACGGGAATGCTGCTAGCCGAGATGA
EMPV1_34260 GAAAGACACTAATGATAGTCATCAACTTACAAGGGAAGAGAACAGAATAAGAAGAAAGGA
EMPV1_34261 GCAAAGCTAGGGATAGGAGAATCAGGTAGGAAGCCAAGGGTAGGACTGCACAGCAAAGAG
EMPV1_34263 TCTAGAGGACAGAAGACAGAGCCACAAACACTGATAGGAACCATAAATGCCAGCCGGGGC
EMPV1_34264 CTTAAAGATCTATGTGGCTTTTATGTGAGGGGACGGAAGGCAGACACACCATGGCCCCTT
EMPV1_34266 AGTGATACCACGTCAAGCCGTTCAAATCAGGAAGCCTGTTGCAGGGGTTCTGTAGAAGAC
EMPV1_34268 TCCACCTTGGAAAAGTCAGGAGACACCTGGGAAATGAAGGCAATGACCTCCTCCAGCTTT
EMPV1_34270 TTCCTGCCACGGTGCAATGGAATCAGTGGTGTCCCTGCAGTGCCAGAACACAGGTTTGAT
EMPV1_34279 GTCTTTCTCTGCCTGACCTCTCCCTTAGCATAATACCTCTAGGTCCATCCATGTTGTCAC
EMPV1_34280 TTTCTGTTTGATAAGCCAGTATCCCCTTTACTAACTTGTGCTGGGATGGCCCGTGACTGG
EMPV1_34281 GTGGACTGCTCTCATTTACCTGTTTCCTCCTGCTGCTTGTCTCCTATACTGTCATCCTGG
EMPV1_34282 AATAAATACCGGTTCTTCCTCGGCAATCAGTTTGGGGACTCCCAGCAGCTGCGGCTGGTC
EMPV1_34284 CGCTGCATCTATTCATAGGCTGGTAGACCCTGATCTGCTTTAAGATGGGTTTGGGCTTTC
EMPV1_34285 GAAAATGATAAGGGCAAGCGCATGGAGGTGTTTATGTTTTCAGCTCCAGCCAATTGCTTG
EMPV1_34286 CAGGTCTGGAAGAATGGCCGTTGGCTTCGTCTCTGTCTGGGGCTCTGACCAGGGCGTTGT
EMPV1_34289 AGTACGAAGTACCCTGACTCCTTCAGGTCTGTATTGGCGTCCAACCATCAGGAAAACAGG
EMPV1_34291 GCAGTGCCAATTCCTCGTTCTCTACTCACCTGCTGGACTGGCTAACATGCTTCTTAAGCA
EMPV1_34293 CAGATTTCACCAGCTTTACATACACTCGTTCTCTCTTGGTGTATGTGTGTTTGGTTCTAC
EMPV1_34294 TTAGCTGGGACTGCATACAGGATGCAGAGAAATGGACTCCAGCAGGAATACAGCCCTTAG
EMPV1_34295 TGTCTGGGAACAGAGGTACTAAGGTGGCTTTTGTAGGCTTAAGAATTTATCCTAATGGCC
EMPV1_34298 GAGAGCAAAAAGTATGAATGGGGGCAGAGCTGAGAAGTCTGAGTAGTTAACAAAATGGGC
EMPV1_34301 TTGCTCAGAGGTTTGGACGCCACATCCCAGCTCACATCCACATCTTGCTGGCCAATGTCT
EMPV1_34302 GCGTCCTCATACCTTCCTAAGTGTGCTGTGTTCTGGTAGCCTGTATTTGAGGTTTGAAGT
EMPV1_34303 CCTGTCCCTCACTAAGTCTTTATTGTCACAGGCTCGCTTCCATTAAAACCACAGCAGTGC
EMPV1_34305 GGGCTCCATGGTTATGAAATAAGGGCAGCAGCTGTGTGCTTGCAGGTAAAGTACTTTAAC
EMPV1_34306 GGAATTTCACACAACTAAACTGATGTAAGCGAGGGAGGGCAGGAAACACCACAAGAAGAG
EMPV1_34310 CGTTTCCATTACACAAATTCTGAGTGTAGCCAGGCGACTTTCCGGTCCTTAACTAAGAGG
EMPV1_34311 CCAGGCCCCAGAGACACTGCTACTTGGGAGGAGCCACTTAACCTTTGTACCAGTTATTAA
EMPV1_34312 TAGAAGCTTGTGAACACATGACAGCAGATCTAAGAAAACAGATCTACTCCAGATTGGCAG
EMPV1_34313 TCTGTTTTGATCTGCCAAGGACTTCCTCTCAGAGCTGTTGCACAAATAGAGGCTGTGCTT
EMPV1_34314 CCAACTACTCTGGCACCATTGCTTTGGCCTTGTTGGTGTCACTTGTTGGTGGTTTGCTCT

EMPV1_34315 GCATGTCCTTTTAAGCTTTGTCCATGCTGGTTATTGCACTGAATGATATATTATGGCATG
EMPV1_34316 CCTGGTGTCTGAGGCGTCTGAGTCCCTCTTTCCTTTACAAGTTTGTGTTTGAAGGTGAGC
EMPV1_34317 GAACCACCAGAGTAACAAGCTCAGCCTCCCAGGGCCAAGTCCAAGCTGTGATGAAATTTA
EMPV1_34319 GAAAGTCCTGGGAATACTATGTAGGGGAACAAATTCCAATCTCAGAAAGCTTCCATGATC
EMPV1_34320 GTTAAGCTTGATCAAGATGACAACTTCCCAAAAGCACCGAGATTTCGTGGCAGAGCCCAT
EMPV1_34321 TGCAGATGGGGTGGGACTACTGGAACTGTAGGAGAGGCTCCTTTTGGCATTCCTTGTAAT
EMPV1_34322 AGGCTGAGGGGCAGAGAGCGAGACTTTTCTATTTTCCAAAAGCTCGGTCTGAGGCCCCTC
EMPV1_34323 TTTTGCGCTCTAGGTCCTGTTTTCTGTCAGGCTGGCTACAGTAAACATCATTCAAGATGT
EMPV1_34325 CAGGTGTAGCGTCTGGAGCATTAGGTACACTCACAATGATGTGTGGCCATTATTAGCATC
EMPV1_34326 GGATGGTTTTTAACTGAAGAATTAGATGTGAACCCCAGTTTCTGTGCCGTTCAGTCACCC
EMPV1_34327 GTTAAAGTACTTTACCTGCAAGCACACAGCTGCTGCCCTTATTTCATAACCATGGAGCCC
EMPV1_34329 GGTTTGAACTAACCTGGCAGTGGAGATGGAGAGAAGTCAGTGGAGAGACGGGTTATAGAG
EMPV1_34331 ACCCAGCAGGTCCCAGAACAGTGTTTTTCTGGGCTCCAATTATGAAATGGGGGTTGGTGT
EMPV1_34333 CATGATGACCTTTGACCCCCAGGACATCCTACTGGCTGGCAACATGATGAAGGAGGCGCA
EMPV1_34334 GACCATGGAATCCATCTTTCAACCTGGACAACAATTATACTGGCAGAAACAGTCTGAGGG
EMPV1_34335 TGTCATTGCCACTGGGGGCACAGTCCTCGGAAGTCTAGTTTCAAATCCTAGTTCCATTTC
EMPV1_34336 CCAGACTGTAAACCATACTCCCATGTTCAAGCCCTTTCCCTTGTCTAAACTGTTTGTGGG
EMPV1_34337 AAAGTTTTCGTCCTGAAAGCCTCACAGTGTTCAGAAGGGGGAGTGGAAGACCAGAAGACC
EMPV1_34339 GTGCCAACATTGCCAGCACATCCTTTCAGGAATGGGGCAGATAGCTCCCACTCGTATTAC
EMPV1_34345 AAATATTTATAAGAAGGGCTAGCAGCTGTTATTGCGGGACATGGGCGACCGCGCCAAAAG
EMPV1_34346 GGCAGAGTATCTCAAACTGTTCACTAGCAAAGAGGCAGGACCTTGATTTGTCAACTGTTC
EMPV1_34347 GAATTGGTGGCATCTTAGGAGCAATGGGACAAGGGTTCAATCCCCCACCTGGCACAGTGT
EMPV1_34348 ACGGGCTGCCAGCTTGTGGTCTACAGCCAAAGGTGGTCACAAGCATTGTTTGTATCTTCA
EMPV1_34349 TTAGCAACACTCCCTCAGCCAGTGCCCGGTCCACAGCCTGCCTTGCCAGCTCCTCCAGCT
EMPV1_34351 ACTGAGTGTTAAACATCGGAAGTCTGAAGCAAAGAAGGAGTCTGTTTCCAGGAGAAGAAG
EMPV1_34352 ACTGGACAGTGGAGAATATTCCTGTGAAGCCCGTAATTCTGTCGGACATCGCAAGTGTCC
EMPV1_34353 TCACTTTGTCCACATGCTGCTACAGCCTGAACCGGTCAAGGACACGGGAAGCAGAAGGAG
EMPV1_34357 GGAGGACTGCTATACAGTCTTGTCTGTGCCTTTTGGTGAGGTTATGGATACCTCCTGTTG
EMPV1_34358 TTTCATCATCAGTTTGTTGGCCTCCCGGGGGTTGCTTGAGTCGCTCAGTTCTTTGAGGTG
EMPV1_34361 CTTCTGAAGCTGGTGTAAGGTTCTTCCTTTGGTCAGAGCATCACAGGCCACAAATGAAGC
EMPV1_34363 AAAAAGAGAAAGTTAAAGTAGGTTTTGTGGATGTGGCTTCAAGTAGAAATCAACAGCTCG
EMPV1_34364 AGTTTGGCTTTCTGATAGCGAGGAAACCATGTGATTACACCCAGGCAAGCCTCAAGCCAG
EMPV1_34367 CGGAGGACGCCGAGGGCGGAGAAATCGCAGAAGGTCAACAGGGGAGCGGCTGGCGCCCCA
EMPV1_34369 GGCCCAGTGACCTGTGCTGCTCTGTATGGTGCCGTGTGTAAGAATAAACCCGTTGGAATA

EMPV1_34370	CCTCACCACTGGGTGCCTGCTGGCTATGCTTGCATGGGCGCCATCTTTGCTGGCCACCTC
EMPV1_34372	GTAAGCCTTGTTGTTCAATCAGGGCATTTCTGCAAAAGTGAACTCCTGCGCCTGCCTCTC
EMPV1_34373	GAGGCATACCAAGATCCACTTGCGGCAGAAGGACAAAAAAGCAGACAAAAGTGTGGCCTC
EMPV1_34376	TCAGTAACTTTAAGCCGGGGGTATATGCAGTGTCAGTCACAGGTCGCCTTCCCCAAGGAA
EMPV1_34377	GGGCCTTGAATCTCTCGAGGTCTTCTCTACAAACAGTATGAAAAAACAGCCATGGTGGGG
EMPV1_34378	AGATGTGCTTCATCTCCAGCGGCGAAAACACAGAGCCACTCCCCGCCAACTCGTACATCG
EMPV1_34380	CAGCTCCAGCTGGACAGGTTGATTATACCAAGGCTTGGGAAGAGTACTACAAGAAAATGG
EMPV1_34381	TCTATGAGATGTTCTACTCGGTGATGAAACACCTGCCAGGGCCGCAGCAACAGGCATTTA
EMPV1_34383	CTTCTTGCTTCCAAATGAAGCTCCAAAATATTAGTGGCCTTGGGAATTCAGAGCCTGCTG
EMPV1_34387	TGGGTTGGGGGTGGGGGTTGTGTCCCTAAGGATTACCATAGGACAGCTGTACTTCAAAAT
EMPV1_34389	AGTGGGGCTTGGTCTTGATAAGATGGGTATTGGTCTCGATAAGATGGGTATTGGCCTTGA
EMPV1_34390	GATCACAAAGCTAAGGAGAGGCGGAGCAGGAACTAAATCCTCATTCTGGATGCCATTCTG
EMPV1_34391	TGCTGATAGACAAGATTCCATTAATCTCTTCCTAGGAGTTTTCCATCCCACTGAGGGGAA
EMPV1_34392	TGCGAGATGGTAACGCTCCCTCCCCAACCCTTCTCTCCCAACTGTTTTTCTCCAAATGGA
EMPV1_34394	GTCACACACCTCATATTGATTCCAGTGTGATTGATTCAGATGAAAAATGGCTCTGTCGAC
EMPV1_34396	AGCTGCAGGTTTAAATGGCGTCCAGTGGTTAGAGAGGCAGCTGAAACTCCACGAACTTGA
EMPV1_34397	CCTGGTGGTGAGCTAACCCAGGAGAAGATGAAGGGGCTAAGACGCTTAATGACAGAGATA
EMPV1_34398	TCCCGGACAGGTTTCCTTTCTCCTCCAGCCCGGCCCCGTTATCCACCGCGGTGGGAGCGC
EMPV1_34399	CAACCCTCACCCCCAAGAATGCTTCACCTTGCAGAACTGAAACTCTGCACGCATCAAACA
EMPV1_34400	ACAAAACAGAAACAGACTCACGGACATAAACAACAGACTTGTGGTTGCCAAGCGAGAGGG
EMPV1_34402	AGAGATTCCTAAATGGCATACACAAAACTTCACCTGCTCCAAGACCCAGGGCAAAAGCAG
EMPV1_34403	CATTGACACACTTGCCTTTTGTATACGTCCTGGGTATTGAGAGGTGGCTTTTGCGGCCAG
EMPV1_34404	CCCTGTAGGGTGTTGCATTGTCCCTGCCTGAACCACAAAGGCCAGATAAAATCCCTTCTA
EMPV1_34405	CTGAGACTTGTGGGCATTGGACTTTCTACTACTAAACCTTGGATTCAGGGAAACTGACCA
EMPV1_34406	AGAAAGCATGAGCCATGGATGCTAAGGGTGAACTTCTGATGGGAGACTGAGGCCAGGTCA
EMPV1_34409	GAAGTCTTGCTCACCTCTGCCTGCTCTTCCTGAAAGGCTGCTTTAGCCACAGATACCCTT
EMPV1_34410	CTCTATTCATGGATCGTGGTTCTGACGGCAAACTCCAGGAAGTGGGACAAGTCTCAGTGT
EMPV1_34412	CTAGGAACGCCGGAACGAGGGACCTTTCCCCAAGAACCCTGAGGACACCTGTCTCTAGAA
EMPV1_34414	TCCTTCCTGCACCCCGAGCACAGCCGTGGCATGCTCCAGTTCATGGCCAAGGAGATCATC
EMPV1_34416	AGCCCTTGACCTCACAAGGGGTGGAAGGAGTACAAGATGCTGTTGCTCTTATGGACACAT
EMPV1_34417	TCCTGCCGGTCACCCTTGGTTGCATCAGCCTCCTCTACCTGCAGCTGCCAGGCACACTGT
EMPV1_34418	GGGGAAGGCATCTGGCAACTCATGAAAGAACAGCATCTGAATAGCTGGGAAGTTAAGGCC
EMPV1_34420	AGTGTTTAGGATGCTGTGGGAAGTCAGAGATGGTGACCTTGGAAAGTGGGCGCTTGAAGG
EMPV1_34423	TCCAGGAGAACTCCTATTCATCCTTCAAAACCCCAGTGGGTTTCACAATCTATCTTGCCC

EMPV1_34424	AAGCTTCCAGGACCAGCACCCGACAAGCCGAACGTTTATGATTTCAGAACCACGTATGAC
EMPV1_34426	TGCCCGCAGCATTCCCGCCATGCCTCTGGTAATTCTCTCTGTTTCCTCTATGCCTTCATT
EMPV1_34427	GGCAGCAGCTTGATAATTCAATTGCCTCTGGGCAAAGGGCCCCTAGAAAAAGAGCCTGTA
EMPV1_34429	ACCCCCCATGGAAGGGATTCCTTTAGGAGTAATACATATGGAAAGGGACAACCCTCACAG
EMPV1_34432	CTGGAAGCCTCAAGTACAGTTGGTTCCTTGCCCTCGGGAAGTGTGATTTCATCAGAGAAT
EMPV1_34433	TAGCATGGCTCAGTGGCCTCATCTCTTCCCTTATTCTATGCTCCCTGACTGTGCAGCTTC
EMPV1_34434	CTTGGCCCCCGTGGGATCCCGGTAACCACTTGCTAAACGTGATAAAGAGAAAAGATAGGA
EMPV1_34436	GGACGTGTTGCACGAGAGGAACGAGCTCAAGTCCAAGGTGTTTTTGTTGCAGGAGGAACT
EMPV1_34438	GCAATAACAGGTTGCTTTTATTCTGGATTGCAAACATTATTTTACAGAGTAGCCTAACCC
EMPV1_34439	CCCCCCAGTGGAGGTCCTGCATGGAATTCAGTGGATCATGACAACATTATGTTTCTCAGC
EMPV1_34442	ACTTACCAGCTTCCGCAGGGCTCCCTCATCCAGCCCAGCTAAGGTTTCGTCCGCCATCTT
EMPV1_34444	TGAACCTTCAGCTGGGCTTCTGGGTTTTGTGACGCACAGCGGGTGGAACGGTACAGAAGA
EMPV1_34446	ATAGTTGCTATTGGAGTTCTCCTGAGGTGCAGTGGTATAAGGATTTGGTGGTGTCACTGC
EMPV1_34447	CCACGACCCCGCAGCTCCATCCACGGACCCCCCCGGGGAGCCCGCAGACCCCGCACTGGG
EMPV1_34449	GATCTAGGCATGAACCACCTGGGAGACATGACCAGTGAAGAAGTGATATCATTGATGAGT
EMPV1_34451	CCCGCAATTAGAGTCTCCAACTCTAGAAGAGGGGATTGAGGCAGAATACTAAGTCAGGTC
EMPV1_34453	TGACGGATGAGTTCCCGTACTACCTGCGGCCCAAGAAGGCTGTGTTCTCGGGGCTCGTCT
EMPV1_34454	GATCTAAGCCTTTCCCACGTTATGGATATAAACCCTCCCCACCGAATGGATGTGGCTCCC
EMPV1_34456	ATACCACCTCCTCAAAGAGCCCTCGCTGACACCACTAACTAAAAGGATGCTGCCGTCTGT
EMPV1_34457	GACTGCAGCATGAGGGAATTTTCCGGGTGTCAGGATCCCAGGTGGAAGTGAATGACATCA
EMPV1_34459	GGGCGGTGTCATACAATTAAAGAGATTCCATGCAAAGTGATGATAGGTATTTACCTGGTG
EMPV1_34460	AGAATGCAGCCTGGGTGTCTGTCCTCTACGAACTGAGTGCAACCAAAGTGTAATGTTACT
EMPV1_34461	CCAAGCACAACTGCTCCTGGGTCTTGAGATTTTGCCTCCAGCTCTTGAACTTCATCTACT
EMPV1_34462	AGAAGTAGGGCTGCTTTTGCTGTACCTCTCTGTGGGGATTTCCATCTTCTCTGTAGTGGC
EMPV1_34463	GGAAGTTCATACTAAGCGTATTCAGCAATTGACTGAAGAAATTGGAAGACTTAAAGCTGA
EMPV1_34464	ACAGCTTCTGGTGGCTTGTCCTTCTTGCCATGGTCCTTCACATTCTCCTTCCCTGGAACA
EMPV1_34467	TAATGGGCATCCGCCGCGAAGAAGAGATGGAGATGTCGGATGATGAGAATTGTGACAGCC
EMPV1_34469	GTAGACAGGACACTGGGGACTCTAAAGGGTCAGTGGTCTCAGGGCCAGAAATAGATTCCT
EMPV1_34471	ACTACTGCTCAGCCCTCAAACTTGTGGTATCCTGGCTGGGGTCTGCTGGTTTATGGGATT
EMPV1_34473	GATGAGGTTGACCCTGAGCCGCCTTTGGACTTTCTTTGGAGCTAGGCATGGAAAGCAAAA
EMPV1_34474	CATTCCAGCAGAGCTTTTCCTGACTGTCTCATCCATAATTTATGTGATCTCTACTGCTGG
EMPV1_34475	CTTATGCCCTCCCCCAGCATCCCTGCTTATTCCTTAAGTTCCTAATGGTATTTAGGGCTG
EMPV1_34477	TGTGCACTGTCATCAGGGACACCGTCATGTGCTCCTGCTTCCCTGGCTACGCCATCATGG
EMPV1_34478	TGTACGTGTGTGGGCATGTACACCAATGCACGCGCACGCACACACGCACACACACACACA

EMPV1_34479	TCGACGGCTACGTAGCAATCTCTTACTGGGTGCCCCAGAATGCGCTGCCCTCAGACCTCC
EMPV1_34480	ATATTTAACTTGCCCGCGTTCGTGGCTGCTGTGAATAAAGCTGCTGTGAACTCAGAGGGG
EMPV1_34481	TGTGCGTGCGCGTGTGCACACGTGCTCATGCTCACACGCACACACGTGCACATGCTCAGG
EMPV1_34482	GCAGTGGGAAATCCGCCTGCTTCAGAGCATCGAGGAGGCCACCCAGCATGAGCTCACCGT
EMPV1_34483	GGGGAACACACCTGAAACTAGAGGAACAGCTTATGTGGTCTATGAAGACATATTTGATGC
EMPV1_34488	AACTAGCCTGACGGCACAGTTCCAAAGGCAGGGTTCCTAACCAAGGGAAAAGAGCCTACC
EMPV1_34491	GTACCTCGGGTGACATTTGTGTCTAGGATGGCCTCCGTTTCTTAGAACAAACGTGGGAAC
EMPV1_34492	CGTGCATCACGGCGGTTTTAAATACAAGACGCCAGGCCCCAGCTCATCTAAGTGAATCAG
EMPV1_34495	GCAAACCAGAGTCATAGTGGCGAGTACCACTGCACAGGATTTATCGGGAAGATGTCGTAC
EMPV1_34496	GAGGTGGAGAGGGTGTTCTGGAATGTTCTGAGGGTAGTTCGACTATTAGGAACTCTCATG
EMPV1_34498	AGGCCCATCAGAGGAGAACTTCGACATGGAGGCCTTCACTGAGATGATGGAGGCCTACGT
EMPV1_34500	CTACCTGAACCGGACACCAACTCCTGCTTCATCAGTTGATGGGCTCAGGGAAATGATGCT
EMPV1_34501	ACACTCAAAGTCAAAAGAAAGTGATGAACCTCACCGTTCTAAGAATGACAGGCCAGCACG
EMPV1_34502	CACCTTCCCAGTGTGGATGTTTTCATTAACCTCGAACTCTCCAAGCTGCACACCACTTGG
EMPV1_34504	GGAAATTCAGCCAACAGATGAGTCAAGTAAACCTTTGGAGGGTGAACAGCCCATAGCTGA
EMPV1_34505	GATGACTACTGGTGGGGGATGGCAAAGAAGATTTGGCTTGGGTGATTGGTTAGTGATGC
EMPV1_34506	ATCGTTGCCAAATGCTGGGTGAATGTGAATTGATTGGAAGAGGCTCTGTTTCCTGTGAAC
EMPV1_34508	ACAGACCCCTCCCTCTGCAGGCTCTGGGAGAGAATCCTATCTGATTGTGCTTGCTTATCT
EMPV1_34509	AGAATCTCAGTCGGGAAGAGCTGGTTGATGTACTGAGGGAGGCTGTGGTGGACCAGAAAG
EMPV1_34510	GAATGATAGAGTACCAAGTGCCAGCAGAGATACTGTGAGCCTATTCAGAACTGTAGAGCA
EMPV1_34511	CACTACTCACAAACGTAACCCTAGCCTCCCTACTCGTACTAATCGCATTCTGACTACCCC
EMPV1_34515	AAATTTTACAGATTTAGATATAAGAGAGAAGATCATGTGTTTTGGGGGTATTGTGCATGT
EMPV1_34516	CCTAGACATAAGCCACTGCTTTTCTTACGGCTGCGATGGGATATGACTCTTGCGTTTCCC
EMPV1_34517	GGTCTGGCAATATTCATGTATTAGCATCACTCAGCAGGAACACAGTGGCATTCAGCTTCC
EMPV1_34519	TACCATCAGGATTCTCAATGAGATGCTTTGGCAGTTTTAAGACATATCATGGTCTCTACC
EMPV1_34520	CCCTTCCAAAATGGTTATGGTTCTGGCAGCTACTAATTTTCCCTGGGATATTGATGAGGC
EMPV1_34521	ACTGTGGACAGGCTGTCTGATCCCTGTTGGGGAAACTTGGGTCTTGGTTCAGGGCAGGCT
EMPV1_34522	CAGGGGAAGTGATTGACAGAAAAGCAACGGAAACGCAGTGCTGTGGTCCTTAACACCTCA
EMPV1_34524	GTCTTTCTGTTGGGAAAATAGTTTTTGAAGGTGGGATAATTGTGCGCTACCTAGGAAAGC
EMPV1_34527	ACTAACAGACGGCAGCGGTAGGTAGGGACGGTGGGTGGTGCAGGTGTGACAGTGACAACA
EMPV1_34530	CAGCTACCCTTCGCTCCTGCAATGCCTGAAGGCTCCCGTCCTAAGTGACAGTTCTTGCAA
EMPV1_34531	CAATGTTTGGGCACTGACGGATGTGCCGGGGTGCAGATGGTGACGTAATGATGATATCCT
EMPV1_34532	AGCTGCAGATTGTGGTCATGTACCGGAGTGAGGATTTAGAGAAGACGTGCTCACCACTGG
EMPV1_34533	CAGACAGAGAAGGTGTATCAGTCGAGAGGGGACGAGGACCCTGGTGACATGGATGAAGAT

EMPV1_34534	ACCAAGACACAGAAAAAGCAGTGACTTCATGGGAGCCTGGGCCAGACCTACCTGCTGTTT
EMPV1_34536	AGCTGTTGGACCAGCACATTTCTATGCTACCAATGACCACTATTTCTCTGATCCTTTCTT
EMPV1_34537	GGCCCCTGCTAATGGGAATGCTAATGAGGAAAATGGGGAGCAGGAGACTGACAATGAGGT
EMPV1_34538	CACAGAGCGACCAAAACAGTGTAAGAGCATTACCCACGCATCAGACAAGATCTGTCTAGC
EMPV1_34540	CAGGTACATGGCCCCGAATTTTGAGCATTGACCAAAGGGACGCCCTAGTTGAAAATGACC
EMPV1_34542	TATCTTCCACTCCAGAGAATTCCTTTTGTTTGCGGAGAGAGGGGGACACGGAACTCGCTG
EMPV1_34543	TGGTCACTTAGCCAAATAGAACCACTGTCATTAAATGTGACCCAGAGTGGGAGGTCGGGG
EMPV1_34544	AGTTGTGGTTTTTGAGAATAGAGTCGGTCTGCTGTGCTTTCACTTCTGTCATCCCAAAGG
EMPV1_34545	ATTGACCACATGAAGACGACAGATGAGGAGCTTGTGTCCAACATGCACTTGGCTGTCAGC
EMPV1_34551	TTGGGTTCTGCTTTGGTGCAGGTTGCTGCTGTGGAGAGGCTTTGATCTCTGGCCCTGGAA
EMPV1_34553	CGTGTTGCCTATATGACCACAAGTAGCCGAGGGTTCTGGAATTGATTTGGTCCCTCCCTT
EMPV1_34554	TTGTTTGAATTGAGGGGCCTCGCAGGGTACCTAGTCCCTGCCACTGTCCAGAGCAGGAGC
EMPV1_34555	TTAGCGCTCTTCATGGGGATTCTGTGTCTGGAGTGTCTGTAGGTCCCTCACTCTCTGAAC
EMPV1_34556	GCTGCCATGTCTGCCCAGGAATACTACGTTGATTACAAACCGAACCACATTGAGGGAGCC
EMPV1_34557	ACCTCAGGGGACTCAAGAGGGAGACACAATGGAATCTGGCTTCACCTCCAAGGATGCTTA
EMPV1_34558	GAAAACAGGGCACAGTACCATTTCAACCTTCCCTGAGTTACCTGGCCAGATGATACTGTC
EMPV1_34559	CCTTTGGAGACTCGTCTTATTTCAGAGACCACATCTGTTTGCAAACCAGAGCAAGTGGCC
EMPV1_34560	GGACACACCAAGAGAAGCAAAGGCTCTGGGGCCCCCAAACCACTCCAAATGGACAGTCAA
EMPV1_34561	AGCAAGGAGAAGCAGGAGAAGGAGGCTCCCGGCTCCCAGGGGGCCAAAGAGAAGCTGGAA
EMPV1_34562	GCAAATACAAATCATGTGTTCTAAAACAGGTGTCACCGTTACCCACTTAAAGTTGTAGTC
EMPV1_34566	TGTTGTTATGCCTGGGCTGGTCAGGAATGGCGACTAGGAGAGAGTTTGGTTAGCTCAGCA
EMPV1_34568	GCCCTGTGAGGCCTTCCTATCTCCAAGTCTATGTATTTTCTGGAGACCAAACCAGATACC
EMPV1_34569	CCCTTCCCCTTTCTATGAATAAAGACACCATTCACACTGGATTTCGGGCCCACCCTAGTC
EMPV1_34570	ATAATTAACGTCTGTAACCATGGCAGGCAGTATGTTGAACCAGGCAAAGGTGGCCCTTGG
EMPV1_34572	CCCTGATGGACCTACACCCTACTCACATGAATGTAATTGTCCCCTGCCCCAGCTAGTAAT
EMPV1_34574	GGAGCATCAGTGAATGAAAACCATGAAATGTACATGAAGGATTCTGTGTCCGCTGCAGAG
EMPV1_34576	GATTTATCTCTGTTTATGTATATACATGCATATTTGATGATTGGGAGAGGGAAAATCAGT
EMPV1_34577	ACAGTAGCAGCTCCGGGTAACCGAACTCCACGCAGCTCTCGTCGGTGGGATCCTTGAGCA
EMPV1_34580	GGTGGCTGACCAAGAAAAATGTGTACAGTACTACGAAGGCAGCACCGTGCCAGAGAAGAA
EMPV1_34581	CCAAGCTTCCTCAGTACCTGGAACGATTTGAGGCTTTACATTCTAAGCTGCAGGCTCTGG
EMPV1_34582	AGCTGAGAAACTGCTGGATCGCCAGAAGCAGTAGGAGGCCTGCACAAAAGACATACTGCA
EMPV1_34583	TTAGCGTCAGAATGAAGGATGACTTGGACTTCCTGACACATGCTTGGGGGGTGTAGCCCA
EMPV1_34584	GAGAAGTTCACACACTGTGAAATGCTTCATCCTACAATGAAGGAGGATAAAACAGGGCCG
EMPV1_34585	CCTCCCACGCCATCCTTGTCTAGTTGCCTGGCTTTCTCCTCTAGTCTCTACTTTTAGCCA

EMPV1_34589	AATGACAGAGGTTGCGTCAGAGGACTGGGATTTGCAGGCCATGTTAGGTGAGTCTTCTAG
EMPV1_34592	TCCTGGGGGGCTGGTGCTGCTGACCCTTTTAAGACCTGTTAATCACGTGGAAATTGAGGT
EMPV1_34594	CTCTAGAATTAGTGGGCCTCTTTCAATTTACTGAGGGTCTGAACACAACAAAAGGCAGAC
EMPV1_34596	ATCGAAGGAAGCAGACGCTCACCACGCTACACCCTCTGGTTCTGCATGGGGCAGTCGTGG
EMPV1_34598	CTGGAGGAGAAGGTGGCGCTTCACCACGGACCTCATGCCTCAAGGTTAAATTTGTACCAA
EMPV1_34599	ACAAACATTTCTCTCCAGCCTAAGGTATTGCCATGCTACTAAATCATTGTCAGTGTGTGG
EMPV1_34600	GTGTAATGAGGCAGACTTGCTGTATGGAAAGTTCTTAACCTGGATCACTGCCAGCTCCTG
EMPV1_34601	ACCTGCTGTTGGGCTATCCAGCTGTCAATTTGCCCCCACCTCCTGGTGTTTGTTTAGTCT
EMPV1_34602	TTTATTCTTTTCCAGAAATGCAGGCTCCGTGACATCAGAGACGGTGTGTGCGCTTGCTCC
EMPV1_34603	CCTACCCTTCTTTAGGGCTTACCTCCCAGCCTCAAGACACAGGAGCGAGTCAATCAGAAT
EMPV1_34604	TTGAGTGGACATGATAAAACAGAAGCAAAGGAACAACTTGATACAGAGACAAGTACAACT
EMPV1_34605	CTACTGTAACCCGCAACCTTGCTTCATCTACTTGATTGTCATCTGTGTGCTGGGCATCGC
EMPV1_34606	CAGGGGCAGGTCTGGATCCTAGAATGTGATGGAGAAGGAACATGGCGATAAATTAGTGCC
EMPV1_34610	TGACCTTGGGCAAGTTGCTTAATCTGTGTATGCCTGTTTCCTCATCATAGTACCTACCTC
EMPV1_34611	CTTTGGGGTTATGAAGCAGGGAGGAATGAAGTGCTGAAGGGCCACCTCAAGAGCCACTGG
EMPV1_34613	CTCCTAAACGCAGTTCTTTGGAATCACCTTCCTGTGTTGGGCATGGAAGGGAGCCATCAG
EMPV1_34615	CCCACATTCTGCAGAACTATAAGGCTCTGCCCACCTGTGCTGGCTGTGGTGTTGCTCCCT
EMPV1_34616	CCAACCCTCCACACTCAGAGCCTTTAATTTCCCCCATAGCTGTTATACAACCTCTGCAGC
EMPV1_34617	GTTCAATCTACCTATGACCAGTAATACATATTTACAAGATGGTGGGCTAGAGCTGGGCAG
EMPV1_34618	ATCCCTTACTCCCGCGTCAACCACAACAAATACATGGTGACTGAACGTGCCACCTACATT
EMPV1_34619	CAAAGGATTCTGACACCAGGAATTTTCAACAACGAACCACACCCCTCCATCACCTTGCCT
EMPV1_34621	TTATAGATCTAACAGAAGCTTCAAGGCAATCGAGTCTGTGGATGTGCATGTACGGTCCCG
EMPV1_34625	TGCCAGAAAAGCCTTACCGATTTGGGTGTCCCTCTTCAGACAGCTGCTTTGCACTGATTC
EMPV1_34626	TTGAAGGCTTGTCTGGAGTTCCCGGGTGGCTCAGTGGATTAGGCATCCATAACTGTCACT
EMPV1_34627	TATGGTTGCCCAGAGAAAAACATGGGGGCCTCACCCCTATAACCTTTCTGCCTGGTTTCT
EMPV1_34628	CAGTGCCTAGAGCTCATTTAATAATATTATCCTCACTCATTCCCCTCCCTTAGTTCCCCA
EMPV1_34629	CCCAAGGCACGCAAAAGTTCCTGAGCCAGGGATGGAACCTGTACCACAGCAGTGACAATG
EMPV1_34631	ATACGTGATTTGGGCTAAGCTCCCTGAAGACATGGGACAGGAACATGAGCAACAGACACA
EMPV1_34635	GACTTCTCCAGGATCTCTTAGAGGGAATTTCCAGATGGTCATGGTGACCGCCAACACAAC
EMPV1_34638	ACACTTCGTCCAGGGTTAGGGTCTTCGTTTATTCCAATTGTGCTTCCTTTGGCAGACGGC
EMPV1_34639	TGGAGCCGCCTGCCCCTGAGAGCCCCATGGCTTTGCTGGCTGACCCAGCTCCAGCTGCTG
EMPV1_34640	AAGAGAAAAGCCAACAGAAGGAATGTGCCCACTCTCCATGCCCTGCTGGGATTTGACCAT
EMPV1_34641	CGTCGGGGGAGCTGGCATTGCTGGCCTGCTCAGTCTCTGTTCCTGCCTCATCTTCTTCAT
EMPV1_34642	TCATAGGGGCCACAGCTCTCATAATTGCCCACGGACTAACATCCTCCATACTATTCTGCC

EMPV1_34643	GATAATTCTAGGAAGGTAGGTGGGAGGAAGGAAATGGGTTCCACTCTTCTGACCATGGGC
EMPV1_34645	CAGCCTGTCCTCATCAAATCCACTTAGTTCTTAGTTGGGAATGGTGCTTAGTTCTATTCC
EMPV1_34647	AGTCGGCATCCTTGTCTTGCTCCAGAATTCAGCGGGAAGGCTTTTGGCTTTTTACTGCCA
EMPV1_34648	GCAAGGTCACTGTGGTGCCCGTGTTGGCTGGTGTGGGCCCACTGGATCCCCAGAGCCTCA
EMPV1_34649	CTTTGACTGGGCTGCCATCAATAACCCGCAGCCATGAGTTCCGACCAGGAAATGGCTATT
EMPV1_34652	AAGTGATTTGGCTTCTTAGCTGATTGGGGGCAGTCATTTTGGTACCTGAGGGTTGGACGG
EMPV1_34655	TCATCAACCCACGACAAGTGAACTGGCTTTCTCTGGAGGGTCTCGTTTGAGGCTGCACAT
EMPV1_34657	CCTTCTGCAGGGAGACATTACTGGGAGGTAGAAGTAAAGGCAAAGACAGACGATGGATCC
EMPV1_34658	TAGATTATTTGTGATCAGATGCAGTCACCTGGGTTTTCTGTGGGTGGACAGCCCCTCACC
EMPV1_34659	AGAAAATACACAAGTCTCCTCCATCATTGAAAAGTGGTCCATGGAACGGCTACAGGCACC
EMPV1_34660	AACCCATTGTAATCTCACCATGCAGAATTAACTATTGTGTCCCTGTGTAGCGGTCCATAA
EMPV1_34661	GTCAGAGTCAGGGAGTGAGCCACGGTTGTAAATGTGAATGATACCTGTGCAAAGTCTCTG
EMPV1_34662	GTCAGAGTTGTGCTAGGCTTTATGACAGGGTTTTGTCATTGTTGATGCTGTTGTTGCTTC
EMPV1_34663	CCAGAGGTCATAACAGTAGGTACTCCCTCCATGTCCCAGGCTTTGCTCCGAGTACTTGAA
EMPV1_34664	AGGATCTCTCTTCCATGCTGGGGGTCAACCAGACCCTGACAGAGCTTTATCTGACCAACA
EMPV1_34665	ATCTTACTGCCCAAAGCCCGGGGGTATCTGACTCTGCAAGGTCCAATGGTTAAACTGGGT
EMPV1_34666	TGATATCATGTGTGGAATGCTGCACTGTGCTAATGTGCAGAAAATTCCTGCCGGCGGGGC
EMPV1_34669	CTTCGGGCTCCTGGTTGCTCTAAGCTGTTTTCAGATGAGTAGGTTTGCTGGGGAGTAAAT
EMPV1_34670	AGGAGGGCCTATCGAAGATGAGCAACACCACAGCGAAGCCAGAACTCTCCCTGATTTTCA
EMPV1_34672	ACGAGAAAGACGGCAAGGCCTACTGCCGGAAGGATTACTTTGACATGTTCGCGCCCAAAT
EMPV1_34674	CTCTTGGTCTAGGTTGAGCCTGAGCCAAAACCTGGGCTACTACTTCGCCAAAGACAAGCA
EMPV1_34675	ATTCCATGGGTTGTGTCATTGTCAGACCTGTGAAGTTTAGTTCTTCAGCTGGGATATCTC
EMPV1_34676	AGATATAAAATCCCTCTACTGTGTCCCAGCCAGCATGACCCTGCTCTTCCAGGAGTCTGG
EMPV1_34677	CCCACTGGTTCACTTTCTTGTTGGAATCCTGAAGCCTTGGGGTGAAAGCATCGAAGACCT
EMPV1_34681	TTCCAGGAACTGAGAAAAGGCCAGTGTGGCACTGAGTCAGGGGCCGAAAAATCAGGGTGG
EMPV1_34684	GTGCACTTATTAACTTAGTGATTGCCCAAGTTCAAGCGCTCCGGGACCAGCTTTGTAAAC
EMPV1_34686	CCCCACATGAGGAGAATGACGTTGCACTTGAACGGGAGCCTAGAATTCCTCTAAGATTGC
EMPV1_34687	TGGAAACTCCGTTTTCTACCGCCCAGGCCCATGCGGAGCCAGTCAAGCTGTTGTTGCTTT
EMPV1_34689	GCAAACTACCTGAGGACGTTAATGAAGAGGCAGTACCTGCATGTGATCCAGCGCAGCTCA
EMPV1_34690	AAGGTCGGAAGTACTGCGAACATGACTTCCAGATGCTGTTTGCTCCGTGCTGTGGGTCAT
EMPV1_34695	TGAAAAAGCTGGTGTTCTCTGGCCGTCCTCGACTCTAGCGTGATTTTCAGACAGACCCTC
EMPV1_34696	AAGACCATTGTGGCTCCAGTGGCATACAGACAAGTGATGGCTCAAAGACAAGGCTGTGGC
EMPV1_34698	TGATCACCTCTGCGCTGCATCCTCGCTTCATTCAGCTCTTCCAGGGTCAGTACACTGCGT
EMPV1_34701	GTGAGGGTGTGGAGAAAAGGGAATTCTTGTGCACTATTGATGGGAATGTAAACTGGGGCA

EMPV1_34703 GCAAGAAGCTGTGCTTTCCTTCCCTGATTGAAACACTAATTTCAGGCAATGCCACTCACC
EMPV1_34704 CCTCTGAGAAGAGGAGGTCGGACTGGAGCAGACGTTGTAAAGGTTGGAATGTATATTTGT
EMPV1_34705 GTATCTCATCTGTGCAAACAATTTAGATATGAGAGCAACGTAATCCCTGCTTAGCTAGGC
EMPV1_34706 CACATGGAGCTCTAATCCCCACGCCCGGGATGAGTGCAGAATATGCCCCGCAGGGTATTT
EMPV1_34707 GCTTCCACCTCAACAACCAGCTCTATGACATCATCACCATGCGGTACGCAGACAAGTACA
EMPV1_34709 ACTGGCAAGAAGTTTGATAGCTGTAGGGCTGGGTATTGCAGCTCTTGGATTTGCAGGTCG
EMPV1_34710 CCCCCATCAGGAAGTTCTCAAGATGGAGGCAAGTTCACTGCCCTCCTTTATACTGTTGTC
EMPV1_34711 TTTATTTCACCCTCTGCAAATCCCTCCTTGGCTGTCCCATCCAACAGGTGGCCTGTGGCT
EMPV1_34712 AGCTGCTGCAGCCAAATTCATAACCCACTGCGCCGCAGCAGGAACTCTAAATGCCACTTT
EMPV1_34715 ACGGTGACAGTTGCTGCCTCAGCCCTTCCCAGTTTAACACTATACCTGGGCTGCATGATA
EMPV1_34718 CCAGTGCTGGTTTTGGCAGCACACTTGGCACAAGGGCTAACTTTTGTAGTAGCCTCAGCA
EMPV1_34720 CTTTCAGCACAAGAACCCTGAGGATCCTGCTGAGGTGCCTGGTGGATTCTTAAGTGACTT
EMPV1_34721 CAGTATTATCTAAGCAGAAAATTGTGGCTGGAATATGGACAGACTCCTGGGCAACAGGAG
EMPV1_34722 CCCCTGTCATCTCAGGTAGAAGGTTGACGAGGTTGTAGGGAGCGAAGACCTGTGTGAAAA
EMPV1_34723 CAAGAATCAATGAATGAAATCTTATTTGAAGAATACCAGTTCCAAGCAGTATTAAGAGTA
EMPV1_34726 GAAGCCAGAGTGATCCTGTCCAATGCAGGTCAGATCATGTCAGTTCTCCACACAAAACCC
EMPV1_34727 TTCAAAACCATGCACGTGTGCCCCCACTCTTGGACAGAAGCACACCCCTTCTCGCGTGAT
EMPV1_34728 GTGTATATCGTTATACCCTTTATCTGGATCCACAATTTACTGACTTCTTGCCACATTGGC
EMPV1_34729 GAACTTTTTAAGCCACAAAGAGGGGGAAAACCATCAATATTCAGAATGGGTTGCCTCTCC
EMPV1_34730 TCATTTGGACAGACAGGAAAAAGGTCTAAGTCAAGCTCAAAGTTAAAGCTAGTTCGGAGC
EMPV1_34731 CCTTGTTAGTTATACTAAACTCCTAAAGGCAAATGGCAGAGCGCTAATGCAGCCTGAGCC
EMPV1_34732 GCTGCTTTGGATCAGTTCAGCAGCCACGACTACCCTGCCACTTGCTTCTGGATAAATTCT
EMPV1_34736 GTGCACGCTGGTTAAATCCTGGTTACGCCGGCAATCTGGACGCGTTGTTTCTTGAAGCTT
EMPV1_34738 AAACCCAAGCCACAGCAGTGACAATGCCACATCCTTATCTGTGAGGCAACCAGGGAACTC
EMPV1_34739 GGAGCGTGAAGAATTGTGGATAAAACTGGAGGATCTGGAGATAAAGAGAGGTCTTAGACG
EMPV1_34740 GTTGCAACGAGAGACCAGAGGACCCATATGGTAGACAGACTAATGGCCTCTAAAGAGGTC
EMPV1_34741 AAGAAGAAACAAAGGCACTGGCGGCCCTGTCTCAGTTCTGCCTTTTTCAGCGGGAATCCC
EMPV1_34742 GAACTAATGAGTAGATACAACATCTACGTCCAAGCGATCAATTACCCCACGGTGCCCCGG
EMPV1_34745 TCGTCAGATACCAGCTGTTGCTGGAAGGCGAGGGAGGAGCAGAATAGAGTGAGCACAGCA
EMPV1_34748 GGAAGAAAACCTTTCCAGAACTCACCAGTAGACTTTCTCTTACAGCTCATAGGCCAGAGC
EMPV1_34749 AAGTGGAAGACGCCCTTCAATCCCAACTTAACCACGGAGGCAGATTTCCACGTGAGCAAG
EMPV1_34750 CACAACCTCATCATAAAGCAGGCCCGCCTCTCAGATACTGCAAACTATACTTGCGTTGCC
EMPV1_34751 GTGGGGACTCGGAGCCCTCGGTTACCGTGTGACCTTGAGTGAGTCAGCAGCCTTCATTTT
EMPV1_34753 TGTCATGGCCAACAAAACGTGAGTGGGGTGTATGGTTCTGAGGAGAGGTTTGAGAGCCTC

EMPV1_34754	TGAGGAGCCTGCAGGATTCGCAGCTCGTTGCTCGCTTCCAGCGCCGCTGCGGGCTCTTCC
EMPV1_34758	AGGTGGAACTAGAAGGGGCCCAGGTGTTACGCAGTCGGCTAGAAGAAGTCCTTGGAAGAA
EMPV1_34760	AACGAAACACAGAGCGAAGCCAGGAGCTTCTTAAAACACAACTCAGAGGCTGATCGCTGC
EMPV1_34761	GTCTGATGCTGATCACTCTGGCTCTGACCCTGCTTCTACTCCTGCTTTACATACCTGTTT
EMPV1_34762	AATGACATCGAACAGCTTTGCTGTGTGCTTCGCATCTTGGGCACCCCCAGCCCTCAAGTC
EMPV1_34764	CTATGGAACGAGTCTAGGGGTCTACTTCACCTCTGCTGTGACCCATTCTCCCCATAGAAT
EMPV1_34766	AGCTCCAGACGCAGGAGATGTTGGCTCTTGAGTTGTAGAAGCACGTGAGCTTGGAAGCGG
EMPV1_34768	TGCTGATGGTGCGCTTTGCGTCGCTGTTCAACGTGAAGGACCAGACGGTGATGTTCCTGA
EMPV1_34772	ATGTGTCCCTGCCTGTGGGTTCCTCAGTGACAATCAGAGGGAAACCTGCTGTCTGTTTCA
EMPV1_34773	GGTAAGGTTACCAAACACTACAGGTTTCCTAAAAGCCTGGAAGAACTACCCAAATATCCC
EMPV1_34775	AAGTTCGATCTCTGGCCTCTCTCAGTGGGTTAAGGCTGTGGTGTAGGCTGGCAGCTGTAG
EMPV1_34776	GGAATCTTGAACTTCCAACACCACATGGCTTGATACAGTGACTTTGGGGTCCTTGGATGA
EMPV1_34777	ACACAGCTGACACGATGAAGAAAGTACGTGAAGGAGACTCCGAGATCCATGTCAGTGCAC
EMPV1_34778	GCTTTTTGGCACATTCCCAGAATGATCTGATGGAGAAAGGTCTGGACAGATGGAGGGCTG
EMPV1_34779	GGAGGGGGAGGTTGATACTCTATGGGATGGAAAGTGCATACTTTAACTCCGGTGGATTGG
EMPV1_34780	CACGTGTAAGCCAACTGAGATACCGTGATGGTGTTGATTTCTTTCAATGATGCTTACCAT
EMPV1_34781	GCGTCTACTACAATCTGGATTCCAAGCTGCGGGCGCCCGAGGTCCTGGGGAACGAGGACA
EMPV1_34784	TGAAGGCAAGAGAGAGAAACCATCCTGTCCGTGGAGAAGGAGAGCCAAGCCTATGCCTGA
EMPV1_34785	AAGCAGGCGGAAAAGAACGTAGAGAAGAAGATCGACAGGTTCACGGAGGTCCTCAAGACT
EMPV1_34786	AACTACCTGACTGAGGATCTAAATTCCTATGTGCCCCCACACGGAGAACCACCGCAGATG
EMPV1_34788	TCTCAGGCCATGTCCATAAAGCTCTGTGCATTCTTTGTTGCAGCCTCATATCTTGGTGGC
EMPV1_34789	TCAGCCCATGACAAATGATAAGAACTGGCTTCCTGGAGTTCCCGTCGTGGTGAAGGGGAA
EMPV1_34790	CATTGACGCAGTTAGAAGAAACAAATACCCAGAGGACAGACCTCCTGAAAGTCATGACCC
EMPV1_34791	CTTGTGCCTTACGAAGGATGAATCCAAAAGGCCAAAGTATAAAGAACTTCTGAAACACCC
EMPV1_34793	AGATTGGGATACAGAGCAGCAGAGTGGTAAGATATGCTATGAAAGAGGCAGGAAGGGGAC
EMPV1_34797	GTCTGACCACGCTCTGTGGTGGGAAAAGAAGAGAACATGGCTTCTCAAGACACATTGGAC
EMPV1_34798	GTCTACACCAACAGCAAAGGTTCGACGGCCAATCACACTGGCACCAAAATACTACCTACC
EMPV1_34801	CGGCAGCCCCTTAGAAGAGCCAGAGATAGACACCGAACCCTTGGAGAGAAACTTTGGTCT
EMPV1_34804	GGAATCTCAGTCTGGTTGTAGGGATCCAGTGATCAGGTTACGATGGATTTCTCAAGTGGC
EMPV1_34805	CTTGAAACTCTGCACAAACTTCCTATCCCTGACCCAGGAGTGTCTGTTCATCTCAGCGTG
EMPV1_34806	CCCCATTTATGGTGCAGGTGTTAGGTCAGCAGGGGCAATATCAGCCCTTTGATGTATTCT
EMPV1_34809	GAAGTTCATCTTATGGGCCAGGCTTCCCCTGATGGACGAAGACCACAAGGATTGGAGTGG
EMPV1_34811	AACCCATGTTTTCACATCTTTCCTTTGACTGTGTCCTGCCCTTGCTGCTACTCCTCACAA
EMPV1_34812	CCTCCTGGATGTGTACGAAAACGAAGTGGTCAAGTTCTCGGCTTCACCCAATCCGGTTCT

EMPV1_34816	ACGCGTCCAACATCTCAAACTTGATCTCCATCTTTGGCTCGGGCTTCTCGGGGCTGGTGA
EMPV1_34817	TTATTCTCCTTCCTAGCCCGGCCCCACTTGTATGTGATCTCAGAACTGTGCCAAGCTGAC
EMPV1_34818	GAAATCGCTGCTCCAAGCCCTGAACGAGGTGAAGGGACGCATCTTGGATGACGACACCAT
EMPV1_34819	GGGGATTTGGGCCATCTGTTGCTCACAGTCTATGCAGTATTGGTTATTCCTAGCTGTTAC
EMPV1_34820	GGGTCACAGCGGCATCAGGTGGAAATTGGTGATATAACAAGTGGGGTAGACAAGGCAGAA
EMPV1_34821	TTTGACTGCGGCAGCATCGACCATCCTGACGTGCTTGACGTCACAGAGGAGACTCTGGAT
EMPV1_34822	TGATGACATCCAACCTGAGCCCTGCCTGAAGCACCAAACCACCTCAAACATCTTCACTGA
EMPV1_34823	CGGTGCAGGATTATAGCAGGGAAGTGTTTACTTGGAAGGACTTGGGGGTGTGAGGGATGA
EMPV1_34824	CAAAACTGAAGTAGGCTCACAGACATAGAAAACAGACTTGTGGATGCCATGGCGGAGGGG
EMPV1_34825	ATAATACCGCAAACTTCTGCAGCCCCTAAGCCTGACAATGGGCCCTCAAAAGGGACTTTC
EMPV1_34826	TTCTTGCGTGTCCGCGTGGTCCTTCTGCATTGATGGACTCGGACCATGTATAAACCCTAA
EMPV1_34829	GCCGGGAAGTATCGTTGTGAAATTAGTGCTCCATCCGAACAAGGTCAAAACCTGGCAGAG
EMPV1_34830	CAGAATTCTCGGGTCCGCCAGAGCCCACTTGCAGCCAGGAGAGCATTCCTTAAGAAGAAA
EMPV1_34833	CTTAAGGACACATCCCTCCAGCTCTCCCCAGAAGCCGTGAGTTGTAAATGGAAAAAAGGC
EMPV1_34836	CAGACCTTTGTCTACGGTGGGTGCCGAGGCAAGCAGAACAACTTCATGGATGAGAAGGAA
EMPV1_34837	AAGTCTCGCGCCGTCTCGCGAGAGTCCAAGTTGAAGGAACATGGCGACGTCTAATCTGTT
EMPV1_34838	CAGAGGTTCTTCTTACTGGTGTGAGGAATTGCACAAGCCTTCATTTTGTTCACACAGACC
EMPV1_34841	GTATTTCCCTTGCCTGGGGCTAAAGGCCCTGATTATGTTCGGCGTAAATGGATGGCTAGC
EMPV1_34842	AGTAGATATTCATGAGGTCACTGGGAAACAAGTCATTTACTATTCAACAGGTTAGTCATG
EMPV1_34844	GCAGAGGGGATCATGGATGCTGGGTAGTCTGTATACTTAACCTGAAACTGTGAAGTGTCC
EMPV1_34845	TCTTTGTTAATTCTCACTCTGTTCATCCAGTCTTCTCCTGAGTCCTTTGACTGTCTTTAC
EMPV1_34847	CAGGGCAAGACAGGGAGGTGATGGGGAGGCTAACAGAACGTTTGTGAAAAGGAGGTGACT
EMPV1_34849	CTCTCTCCATTGCTTTCCGATAAATACATACCTATCAACATGTCTCCACCAGGGCTCAAC
EMPV1_34850	CTACAACCACTTTGGCCAGTGGGTGCAGGAAATCTTTGAGCTGAACAACTTGCCTGTGGA
EMPV1_34851	GAGGGCAGCTCTGGGGAGTGGACATCCTGAAGAGATGGTAAAGGTGGAACAAAACCCTAG
EMPV1_34852	TTGAATAAAGGGATCAAAACCTTCTTCACCAGCCCCTGCACGGAAGAGCCTCGGATAAGG
EMPV1_34854	GGGAAGCCACAGCTCTCCAAGTTCCACCAGTCCTACAGTGAAGATACACAACAACAAATC
EMPV1_34855	CGAACACACCTGCAGTCTCGGTCAACAGTGTTTTGGCAGCAGTTGGAATTTGCAGATAGC
EMPV1_34857	AATAGTCCTTCTGAGACTGCCATCCAAGAAACTCAGGGAGAGGAGCTGACAGTCTCTGCA
EMPV1_34858	ACCTACAGGCTATACCTCCGGTACTTGGTCCTATACACAGACTTTGATCGCTGGGCCACA
EMPV1_34859	TCGAGAACTTGCCTCCGTGCTCCCTGTCAATCCAAACCTGAGAAATCTGCAACTCGGGTA
EMPV1_34860	AGATTCTCACATGAATGACACCCCCCTGTAGTTGTGCAATGTCCTGGCCCTGGTGCTAAT
EMPV1_34861	GGAGAAAGTCACTGTTTCTGGAAATGGGTATGACACTAGGAAAGGGATAAAGAGATGACC
EMPV1_34865	TTCTGCCTGGAGATGCCCTGGAATTGTCTGCAGGTGTGAACTGGGTTTTGAAAGGCACAC

EMPV1_34866	CCGAAGGTCGAAAGCCACCTAACTACTTGGGCAAAGACCAGAGAGATGCAGCCTTCAGAA
EMPV1_34867	ATCAAACCTGTGTTCTGGCACTGCAGGGACACCACTGATTCCATTGCACCGTGGCAGGAA
EMPV1_34870	ATCGCCAGGATGTCCGAGTAGTACGGTTCTAAGAAGCTGCGCAGAAGGTGCGCGCTGTTG
EMPV1_34872	GGGAGGCAGAGCGCAAAGCCCAGAAAGAAAAGGAAGAGTGGGAACGAAAACAGAGAGAGT
EMPV1_34874	GAGGTCCCTGTGACTACATCTCAAAAATCGAGAGTGGGCGCCTTTCTCGTCCACAGCATT
EMPV1_34875	GACGTTTCGCCACTGCTCTCCCTTGCATGTGCAGACACCTCAGTGATTAAATTGGTGCTT
EMPV1_34877	ACGCAGGCCCCTCGAGTAGAGAAGACACCTTCTTTGCCAGATGACCTCAGTGATAGCACC
EMPV1_34881	CTCTTGGGTTTAAGTCACTAATGCGTTGGGTGGGCAAATGTAAGAACCTCAACGTTAGAC
EMPV1_34883	GATCTGAAGACAAGAGACTTGGGGGTTATCTAGAGGAAGAGTGTTTGGGGCACAGGGTAC
EMPV1_34884	GGTCACCTACACCTTCTGTTCACCTTGGTATAAATTAGAGGCTCCCATGACTCTCCATCC
EMPV1_34887	AAAACCTGTGCCCGGTGGCTTTTGTGGTCAGTTTCTCTGATACCGATTCCCGGAGGAGAA
EMPV1_34889	ACATACAGGCCACCCAAATGGCCAAGGACCCAGCAACGTTCAAGCCGTCAAAGATGGACA
EMPV1_34890	CTGGCAGCTCTGCACATGGATTCACCTGACTTTAAGGGCCTTGAGGGCCTGGGATGAGAA
EMPV1_34897	GAGGCAAAGGGTATGTGTTGCACTACTGTTGCTATCCTGTGTTGGAGGTTTTCTGCACTC
EMPV1_34900	GGCTTCCCACACTCCGAGGAATACTTGATGTCTCTCTTCCCTTCTTTGCCCAAACTTCCA
EMPV1_34902	AAGCAGCTCTCAGTTTTGGCCTGTGCTGGTGTTACTTTCATCTGCTCTGTGCTGGTTGTC
EMPV1_34904	GACAGGTGTGTAACCTTCTTATAGGAGTGGCTTGGGCTGGAGCCTTTATCCATGCGACCA
EMPV1_34906	TTTAAGAACTACCATAGAACACGGCTTGATGAACTGAGAATGTTTCTAGAGAATGAGACC
EMPV1_34908	TGCTTAAGGCGGTCAACGCGACCCAGAAACAGATAGACCTGGTAAAACTCCATGAACAAG
EMPV1_34911	TTTAAAATCTGGAGGGGTCATTTTCTCTCAGAAGCCTCAATTGCTCTTTTGCATGATTCC
EMPV1_34914	TGGATAAAGATCTCGGGTTGGTGCCGGGATTCGAAGATGCAAGTAAAGCTGAGCGAACTG
EMPV1_34915	GGACCTGGGTGATACCCTATGGAACTTTAGACAGAACTGGGTTTTAGAGTCACATACATC
EMPV1_34916	GCAAGAAATTCACAGGAGGTCAGCTCGGCTCCGAAACACTAAGTACAAATCTGCTCCAGC
EMPV1_34917	CAGACACAACACCAGATATTTCCAATTTTCCACAGTCCTTACCCTGCCCTATTGCCTCGC
EMPV1_34919	GACAGAGTAATTTTTCAGAAGTTCCAGTGTCTGGTGAAATACAGGTTGCCTCAGTTTCAC
EMPV1_34920	TGAACTTGCAAAAGTGCCTTGGGCTTGTGGCGATTTCCTGGACCATTGGCTTTGTGCATG
EMPV1_34922	ACTTCAACCAGTTCGGAAAACATCAAAGTCTAAGCACAGTGGAGTTTCTGCTGTTGCGCC
EMPV1_34923	AGCTCCAGATGCTGCTGTGGCGCAGGCTGGATCCCTGGCCTAGGAATTTCCAAATGCTGT
EMPV1_34924	AAGTTCATCCCCCCGCCTCCCCAGCCTACCTGTATTTCTGTTAATGACTTCTGGATCCTG
EMPV1_34925	AAGAGTCTGAGGAGCCAAAGGCAGAGGAGAAGAAGCAGCAGCGCCGCTGCAAGCCAAAGA
EMPV1_34928	TTTCCAGGCTGAGATTGTGCCTGTGACCACCACGGTCTACGATGACAAGGGCAATGAGAA
EMPV1_34929	ACATTGGCGACGGCTGCCGAAGATGTAAAACCGCGTCTTTTGCTTGCTGTCAAGTTTTGG
EMPV1_34931	ACTTAAGCTACCCAACACAGGAAAAGATGTCGAACCCCACACCAAGTGTCATGTGGCAGG
EMPV1_34933	TCCCCATGTAATAGTCAATTTACTCTCTCTGCTTGGTGGTTAGGAGGGAGCCAGAAGCTG

EMPV1_34934 TCTCTACGCCTTCACGGGACTGAGCCTCACACCGCAGCTCGAGGCCTGGATCCACAATAT
EMPV1_34935 ATCATCCACGCGCAGTACAACACGCCCATCAGCATGTACTCCCAGGACGCCATCATGGAT
EMPV1_34936 CACAGGTGACAAAGGCACACAGCAACAAAGGGGCACCCCACTGCAACACACAAATAGTAT
EMPV1_34939 CCGAAACAGACCCTTAAATATCAGGCCCCAAGTGGGAAGCTCAAAGAAGTGCTCCAGGGA
EMPV1_34940 GAGTAGTTGCAATAGCGACTCTATGACCCACAAAACCTAGAAAGTTTACTTCGCATGGTC
EMPV1_34941 TACGGCCAATTGAGACAGTTCCTAGACCATTCTTCCACCACTGATTAAGACTAGGGTGGC
EMPV1_34942 CTGTGCAAGATACAGTCTCTGGGCAAACCACCCAGGTTCATCAGACTATATGCTCAGGTG
EMPV1_34943 GATCCAGGTACAGTGAGAGCAATTTCAGCGTAGACGACCAAGACCTCTCCAGGACGGAAT
EMPV1_34945 CACTTCAAAGCCTCACGCCTGTCTACAGGACCTACCTGCCGTTGGTGTTTCAGTTTAGAT
EMPV1_34946 GCAGCTAATCCTTGATGGTCAGTGGGATGAAGTTCTTCAGTTCATTCAGCCTCTAGAATG
EMPV1_34947 CGCTTAAGATGGATGATTTCGAGTGTGTCACTCCTAAGTTAGAGCACCTCGGCGTCTCTG
EMPV1_34949 AGGAATGTGTGCTGGGTTGGTATGTGGGTCCTTTGGCACCGGTCTGGTTATGGCAGTTCT
EMPV1_34951 TAGGATCTAAGCTACTGGCTCTGCCACCCAAACCTGTCTTCTGTTGGAATCCTGGGCTGA
EMPV1_34952 GCAGCAACAGCACCAGATCCTTAACCAGCTGAGCCAACAGAGAACTCCCTACCATTTAAT
EMPV1_34953 GGAAATGTCCAAGGCCCCATTCTCTGCCTCCTGCTTTATCAGATCCAGATCCAAGACTTC
EMPV1_34954 GGGCACTCAAAGCACAATCTTTTAATAATTCACTTACAAGGAAGTTCAGGCTGCAGAAAG
EMPV1_34956 AGCTCTGCCCTTTACCCTGTTTCTGATTCACCTTCTGTTGGGCACTGCTCTGTTCACCAG
EMPV1_34957 AAACGTGATGAGGTTATCAACCATTCAACATGATCAGCCTGTAAAGCCCCTGGACCGGGC
EMPV1_34958 GATGGTGAGACACCCACCCCCAAAGACAGTCCTCTTGCTGCTGCCAACCAAATCAGTATT
EMPV1_34959 CTTCAGTGTGGAGGTCATTATCCTCATCGTGATTGGGGTCCTGGTTAGGGTGGCTCTGGT
EMPV1_34961 GAGAAGCTGCACAGGCCCTGCTTTTGGCAGAACTGAGAAGAATTGAGCAGGCGGGAAGGA
EMPV1_34962 GCAACAGGCACACATACCGACCCGCAGAGGAGGACATTTGCCCTTCATTTTTCTGAATCT
EMPV1_34963 CAAGGCGGATTAGTAAGTGGTTGAAGCCCTGTTCCCAGAGGATGGTGATTAACGTTCACC
EMPV1_34967 ACATCCATCTATGAGTGGAAAGGCAAGATTGAGGAGGACAGTGAAGTGCTGATGATGATT
EMPV1_34968 CCTTCTGAAGCTCATCCAGGAAAGAGAAGGCCAGAACATTTGGGTAATTTTCAGTGCACA
EMPV1_34969 AATCCTCGATCACAGGCCCAGCGGACCACACTATTAAGCTGTCCACCTGTCTGACTGATA
EMPV1_34970 CAAAACCAGCTGTGAAAACTCCTCAGACCAAGGCCTCGCCCAGGAAGGGCGGCCCTGTTA
EMPV1_34971 GCACTGGAGTTATCAACTGCATCTGCTGGAAAAAGTTGAATAATACCGACTCCTCATCAG
EMPV1_34972 AGAGATTTCCTTGCCTCTGAGAAGTCCGGTTCTTCACTCCACAGCCTGGCCGGCGCCTGT
EMPV1_34973 TCTTAACTCTTAATGCTTTCCTCTTTATCCGGCCCAAATCAGCTCCCACTGCCAGCAATG
EMPV1_34975 TTTGCTTGTGAGGAACATCCTGAAACATGTCAAGCCAGTCTCCAAAGTCAGCACCAACCC
EMPV1_34976 GATTCTGATACTCAAATTGGGAATGTTATCTGCTTGGTTGTTGCTGACTTGGTATGATTC
EMPV1_34978 ATGTGAGCTTCCTCAGACCCGCCTTCCCGCTACATCTTAATCTAACAAGACCACTTTGCG
EMPV1_34980 GGCAAAATTGGCAGTTTCGTGGGGTTGTGATACCTGATGTGCCTCAGATTCTAAAGGGGC

EMPV1_34981	GTCAATGGTATCAATGTCTCCCGATGGAGGGGGACTCCTAAATCTGAAAGATGCTTCTAG
EMPV1_34983	CTTCTCAAAACAGAAGCAGATGGAACCCAGGACAAGAAGACAGAAGAGTTCTTCTCTGTG
EMPV1_34985	ACTGAAGACTTAAAAAACATTGGAAATACTTTTTTCAAATCCCAGAAGTGGGAGATGGCC
EMPV1_34986	AGCGTGGGAGTGGCTCATTCCCTCCTGGGATTATCCCTTTAACGAAGGCCCCCCGGGAGG
EMPV1_34987	GTGGATCTCCACTTGTGTGGGCAAATCCAAGGTCATACAGTGTTTTCAGGAAGTATACTC
EMPV1_34989	TGCCCCTCATTCCAGGGGATGCCTGAGTCTGTACTCCTCCTTTGGCTGTGTAGTACTGCT
EMPV1_34991	GTGATTTACACTCGTTGGCTAGTTGAATCTTCAGGACACGGCTGTGAAGTATCATCACGG
EMPV1_34992	TTTACCAGCCGGGACTCACGTGCCTTCTCTAGTTACAGTTCAGCCAAAGACCAGCCTTGG
EMPV1_34993	GGATTGACCTGTGCCATAGCAGCAGCCTGAGCCACTGCAGTGACAATGCCGGATCCTTAA
EMPV1_34996	TGATTTAACCATGTCACAGAGACGCTACCTAGGCAAGTCATTTCAGCCTCGCGTGGGGTC
EMPV1_34997	ATCCTGCAGAAGTTTCATATGGGGATGCCAAGGACACACCTCAAAGGACCAGGCTGCACA
EMPV1_35000	TGGATTACGGCTTTGTTTCTGCCCTGGTTTTCCTGGTGAGCGGCATCCTGCTGGTGGTGA
EMPV1_35001	ATGTTGACCAGTTTGTTAACAGGGTCCAAGAGGTACATTGCCATTGGCGAACTGGGGAAA
EMPV1_35003	CAGGAAGTGGAGGGAACTAGGGAAGAGAGGGCAGAGGACAGTGCTGGTAAGGATTAGTTT
EMPV1_35008	GGTGAGCACCACCTCCTTCCTAGAGAGCTTGACCAAGTTACATATACTGAACATCCCGAG
EMPV1_35011	GGGTCATCTCTTGCATGTTGGTGGATACTCAAAGATAGGCAGAGGAGGGAGAAAGCTTTG
EMPV1_35012	TGGAGACGCCTCACCCAGAAGAACAGGTGAAATCCATCCCAGAATTGGGTGACCACGTTA
EMPV1_35014	TTAGTTCTAAAACGGGAGTTCACAGGAAGGAGGAGAAGGATGCGGAGTCGGGACAGAGAG
EMPV1_35015	GGTGGGGAGGGATTGCCCTTGGTTGAGACTACTGCGCTAAATCTATCTCATTGCTTCTCT
EMPV1_35016	CATTTGGGCACAGCCCTAAAGGCCAACCCTTTTGGAGGTTCTTCTCATGCCAAGGGAATT
EMPV1_35017	GATTCGTACCAAAAATCTTAATCTTTAGTAGATTGTAAAGAACCTAGTACTGTGCCTGGC
EMPV1_35018	ATTGCTCGAATCTACCACTGCTTCTGTGACCACCTGGCTCTAGTTCAGGCCTCCTGCTCT
EMPV1_35021	AAATTCAAGTGTCCTGAGTGCACAGCCATTGATGGGCTCTCGCTCGACCAGACACACATG
EMPV1_35025	GAGATGGACAGTTTACTGTCAGTGGCTATTAAAATAATACTCTTGCTCCAGGACCAGAGG
EMPV1_35026	TGGAGTCTGGGTGACAACTGGCAGCACTCCAGTACTGAGAAGAAACAGGATACACAGTGA
EMPV1_35029	CCGGTCATGCTGGTCTGAAACAGTGACACCGCCAACTCAAGACACAGCCAGCCTTTGAAA
EMPV1_35030	AGAGTCTTCGAGCAAATCAACGCACAGCCTCGTTTTCTGTATGGAGACATTCCACAGGCT
EMPV1_35035	TTCTGCAGGGCTGCTTGGGTCATCTAGAGCAAGACAGAGTGAGACTGATATTCAGCACTC
EMPV1_35039	TAATACAAAGAGCAACCTCTCCGCGGCTGCCCACCTCATAAATCATCAGCTACCAAGGGC
EMPV1_35040	ACACAATCCCTCCATCGTGTCACATCATCGTGGCCAATATCTACCTGCTCTTGCCTCCAA
EMPV1_35041	GGCACAGGAAGGAACTCCAGAAGAGAGAGTACAGCTGGGCTTGGTGAGAGAAAAACCAGA
EMPV1_35043	GTGGACTCCCCATCTCGACCTTCCTCTCCAAACATCAGCTCATACAGTGAAAGTGGAGTT
EMPV1_35045	ATCTTAGTGTCCTATGGCCACATTACCCATGCAGTCCTGAAGATAAAGTCAGCCCAGGGG
EMPV1_35047	TGGCTGTTCTTCGAGTAGCTGGGCCCTGGGGGCCCGGCCTGGTCCAGCGATTCCGACCGG

EMPV1_35048	CCCCTGTAGGGACTTAGGCTTCTACTCTGAGGAAAAAGTCATTGGAGGTTTTGAGGAGAG
EMPV1_35050	TGTGAGATGTATTCCAACCTCTTAAGGACTCAGGCTTCATTAGGGCTTCTGGGGAAACCC
EMPV1_35052	CACTATCTGAAGGGCCGCGGAGCACCGAAAAAATTAAGGCCTGTGACTACAAATCCAACC
EMPV1_35054	CAGGTGGAGCAGCTAGAGGTATTTCAGAGGGAGCAGACTAAGCAGGTGGAAGAACTGAGT
EMPV1_35055	AACAAGAACAGAGGACGAGCCCAGAAGTGGGAAATGGGCTTTTGACGTATCTGACGTCTC
EMPV1_35056	GTACAGAGAAACCCATCACATTGAACCACGTGTCATTAGCCGGGATGAGATCCTTGACCG
EMPV1_35061	CTAACTCAGAATAGCAAGGGCTGAAACTGGGGCCTAGGTGGAAAGCTGTTGCCAGAGACA
EMPV1_35063	TTCTAGGGACACTTATGCGCTCGAACAGTCGCTCACTTTCGTCCAGGCGCTCATCCAGAA
EMPV1_35072	ACTTAAGAACCTTTCCCTGCCTGTGTTAGCTGAGAGCAAAGGGCACCTCCTATTGGACCA
EMPV1_35073	TTGTAAACTGAGCTAAAAAGGGCTGCTTTTTGGTGGGGGCAGATGAAGGCTCACAGGAGC
EMPV1_35078	CCAAGAACCACGTTAGCGAGCATAGATTTGTCCATGGGGTTAAGTTCTCTATCGGGCACT
EMPV1_35079	AGAGGAATTAGTTGCTATAGTCTGTGCATGGGCCTCAGCAAGGTGGATGTGCAGATGAGG
EMPV1_35080	CTTAGGTCCTGTGACAGTTGATCCTAAAGGTTGTGTCACCATAGCCATCCATGCCAAACC
EMPV1_35082	GTTCCAAAAGAGAGCAGTCGGGGAAGGAAACTCCTTTGAGTCTTGATCGAGTATCTGGGA
EMPV1_35084	AAGAACGTTTATTTAAACAAACTGCTCCCTGGTGGCAAGAGGCGCTTGCGGGGACGGCGA
EMPV1_35086	TGTATCAGCGTTTTTCCAGTTCTCCTTTGTCCCCCGCCGGGCCCACGAGTCCACCAAGAA
EMPV1_35089	GTGCAGCTGGCCCTCCTTGCGGGGCACCACGATGCCGGTCTTACCGAAGCACACGGTCAC
EMPV1_35090	GAATCTGGAGAAGTACCAGTGCACAAGCTACAATCCCTCAAAAAAGTGCTTCAGAGTGAG
EMPV1_35091	GTTGAACAGCAGTCCCCTGAGTCATGAGGAGGCCAAGAATCTTCCCTATTTGCAAGGCCT
EMPV1_35093	GACTCACAGCTTCGCCGGGGGCAGGACTGCGCGGGCTCACTCATGGAGGAGGTGGCCAAA
EMPV1_35094	GTCCTGGACCACAACATCATCCCAGGACTGTCCATCCTGAATCTCATCTTCTTTATCTTG
EMPV1_35095	AGGTCTCCCCGAGTTGTGGATAGGCTTCTGCTGAATGTATGCCAGCCCACCTGCATAATA
EMPV1_35096	AGCCTCGCCAGGTTTTCACACCTCTGGGCATCATTTTCCGTGTACTCTACTTCGAACAGG
EMPV1_35097	GAGGAACGTATTTCTTCTACTTGCTCCCTGTCCAGCTTCTCGCCACCAATTTTCTCCTCC
EMPV1_35098	TTTCCTCAAATGGTGGATAATCATCAGAAGCAAGTGGTCCGACACCTGAGCAGCAAGCGG
EMPV1_35099	ATAATGACCACTAGGCAACAGTGGCTCCATTGAATACATGAGCAGACAGGTGGGCAGAGG
EMPV1_35102	CCACCTGCTGGATTTAAACCTACTCCTTCAATTGAGTGCCATCATTTGGAATTTCGTGTC
EMPV1_35103	TGTCCTCCTGGTTTGTTAGGTTCAGGCTAGGGGTGCTGCTGGAACACTGGTACTCCTGGA
EMPV1_35104	CTGAAGCCCTGGCGTTTGGTTTCTTTGCCGCCATGCTGTTTGCACTTGACTTCCATCTGA
EMPV1_35105	TCCTGCATCGATATTTATGTGATGAACCTACTCGTGGTATTTAATAGTGGGGCCATTTGC
EMPV1_35106	TGTCTCGTCCCCCAGAATGTGCGCATCGACCCCAGCAGCCTGTCCTTCAACATGTGGAAG
EMPV1_35108	TCTGGGGAAGCAATCACATCTGGTGACCAGGCTGCTTCATTCAACACTGTGTAAACACTA
EMPV1_35110	AAAGACGTGGCTCGTGTTAGGGTCGGGAGGCCCATACAAGCCGTGCTGGAAGGGGTTGAT
EMPV1_35111	TTTAGAGTTTGTTCAGCTACAAGTAGTTAGGGGGTAGCAGGTAGCACCTCCAGGCTCCTG

EMPV1_35114	GCTGCTTCTGCCACCCACCTTTGGTATACATTGGCTAATAAAAATCAGTTGCGAGGAACT
EMPV1_35116	TGGGACTACCACCTTGCATCTCTTCCCCTGGCTAACCTTTAACCCTTGAGCCCCACTTTA
EMPV1_35117	CCAGTGGGAATAGGTAATGTTGTACGCCCTGTGTTGAGGGAAGAATACTCCTATGCATTG
EMPV1_35118	TCAGATCTCAGTAGGAAGTGGGACCGGTGCTTGGAGGATGAGGTCGTGAAGATAGGTACT
EMPV1_35119	TGAAACCTGTACTAATTGCAGTTAGATTTATGAGCATTACCCGCAATTCAGTCTCGGGAA
EMPV1_35121	GCAAACCGAAACCTCCAGGTCGCTGGCCGGCCAACTCGGCTCCCATTACTCAAACTGTCA
EMPV1_35122	CCATGGGCTCCCAGAGTCACCAACCTGGCAGCATTTCAAGAAGATTAACGAAGCTCAGAT
EMPV1_35123	TCTTCTTTGACCAGATGACATGAAGGTGCGATCCTTTTCCACCATGACTTACCTGTGAGG
EMPV1_35124	ACTTCCCACAAGGAAAGTGGCCCCAATGACTGGAGAGAATTCCAAGCCTGTGCAAGCTGG
EMPV1_35127	CCTTACAAGGCAAACATCCGTGAGCAAAAAGGGAATCGGGGTGAAATGAGCCTGGTGGAT
EMPV1_35128	CTCCGAGCATGAACAACTTGTAGTGACTTTGGAAGACCACAAGAGTGCACTAGCTGCTGC
EMPV1_35129	CACACGTGCTGGAAACGCAGCTCAAGTATTGATTCGGGGCTTACCAAGTGCATTATGGAG
EMPV1_35130	ACATACATGAAACCATCAGCGAAGGAAAGGGTGACCATAACTAAAGGTGTAGCCGTGCTC
EMPV1_35131	GAAAGTGCACCAAACACCGAGGAAGTGGATTTGCTGAACTTCTTCCCTGTACCTGAGAGG
EMPV1_35132	AGGCCGTGGTGCTGACGCTGCTGGCTGTCTATGCAGCTGGCCTGGCGCTTCCACACAACA
EMPV1_35133	ACAAGCCCTTCAAATGCCGGCTGTGCAGCTATGTGACCCTGCGGGAGGAATCCCTGCTGA
EMPV1_35135	CCTTCTTCCCCAGAAGAGAGCACGTCTCAAAGCCCTCAACACTTGCACCTCCCATATTGT
EMPV1_35137	CAAGTACGGTTAGAACATCTTCAAGAGAAAACTCTTAAAGAATGCAGCAGTTTAGAGCAC
EMPV1_35139	GCCTGGTGCATAGTAGGTGTTCAGTAAGTGTTAGGTATTAGCTATTATTACCGCATAGCC
EMPV1_35140	GTGAGCGAGGCCAGGAATCAAATGCATATCCTCACAGAGACAACATTGGGTCCTTAACCC
EMPV1_35142	GGAGCTGTAGAAATCAGGCTGGATGGGCAGAAGTAGAGAGAAAAGTCCTAAAGGATGATG
EMPV1_35143	ATTTGACAAATCCATTATGGACATTTCTTGGACGCTGAACGGGCTGGGCATCCTGGTATG
EMPV1_35146	ACTGAGGTGAAGTGTGTTGGCCCTGAAGCCCTGTCTGCCTTGGTGATTGCATCTTCGAAA
EMPV1_35148	GTACCCAGAGACATCGCTGCTGCACTCCCTCCTGTTAGGCTTCCCTCCACCGTGCTCAAT
EMPV1_35149	AGCAGGAGCGAACACAGTGGGCACAGTATCGAGAATCTGCAGAGAGGGAAATAGCTGATT
EMPV1_35152	GGAGGTGCACAGCGTCCGGGAATTTGTCGAGAAGTCGTTTATGCAGATCGCCAAGTCCAT
EMPV1_35153	CATGGAGAAAACGACTTCAGTGTCATGTATTCCACAAGGAAAAACTGTGCTCAGCTCTGG
EMPV1_35154	GTTTGTGCCCATATGACTATCAGTCTAAAGCAAGCACATATAAGAAGTGGTTAACATACT
EMPV1_35157	TGATTCAGTGCCTGATGGTGCTGTCTTGGGGACAGGGACCTGCTCTATTCGTCTTTGTTT
EMPV1_35159	TTTGTACACATACACACAGACATACAGCGATCCTCCCACCAGGAGGTGAAGGACCTGGTG
EMPV1_35160	CCTTCTAGGATCAGAGCTGGGGCAGACAAGCACCTCGATCACACACAAAAATCCCCATCA
EMPV1_35161	AGAAAAATAAAGCCACTATTTACATATTAACAAACAGGAAATTAAGTAGTAATGCTCTTA
EMPV1_35162	TTTATTTCAGGTTTTCTCGAGGCTGCGGCTGGGACGGGGCCCCCAGAGAGAGAATTGCAT
EMPV1_35163	TGCTGGCTGTGCCAGGGAGTGGCAGCGACGAGGTTCAGATTTTGGAGATGCCGTCTAAGA

EMPV1_35164	GGCTCTGAAGTTGGTGTTGGAGGGGCACAAGTAACTGGCTCTAATACGCGGAAGCATATA
EMPV1_35165	ATGGTAATATTTTGGTACTTGTGTTTGCCGCTGCCCTGCTCCTCGCAGTCCCATGGACAG
EMPV1_35166	TTCAGCTGGCCCTGAAGGGAGGAAAGTGATGAGAAGGTGTGAAGGACTGATTGACTCACT
EMPV1_35169	TATTGGGAGGGTTGTGGTACAAGGCCATTTTTGCTGGGTTTAAGCAGGGTCTCCAGAACC
EMPV1_35170	GGCCGTGGTGGTCAGGCCTTCTACTAGACATGGATTAACTGAAAGGTGATGGAGCTTAAG
EMPV1_35171	CCTCTGTCCCTAGGTGTAGGATACTTAAGGAGAAGATGCAGCATGGAGGTGGAGAGTGTA
EMPV1_35172	GTTGAGGTGGGTTACTTGGCCTGGTAAAAAGCCGGATATCATGTAACCTCATCCTGGCCT
EMPV1_35173	GAACTGGACAGTTTTCCCCCTGGAGAGATGAAAAGCTTTTTGGCTCTTAAGTCTTTGATA
EMPV1_35174	CAAGAAATCACTGCCAAGGCCAACATCCACAAGGATTTTCCCAATGTTATCTTCTAGGAG
EMPV1_35175	CCTGGCTCTAGCAGCACAGAAATATTGGCACCGGGCGGAGAGTCTGCCAGTATTGGCTGT
EMPV1_35176	ATAATCAACCGGCTTTTCATCCATTGTCAGCTGTGTGCCCATCTGCCCTAGGTTCTTCGG
EMPV1_35178	GCTGTAGGAGAAATTGGGGGAAGCTTCACCTATGGTGCATATGGAGATGTCAGATGAGGG
EMPV1_35179	CCAGTTCATCGTCCAGTGCCTGAACCCTTACCGGAAACCTGACTGCAAAGTGGGAAGAAT
EMPV1_35180	ACTCGTGGCTAGAGTTAGGAAGTCGGGATGCGGCTAAGCTCAGCTTAAACCGTCGGCTCC
EMPV1_35181	TTGGTCCAACCAGCCTTGGAAAAGTCCCTGAAGAATCTCCAACTGGACTATGTGGATCTC
EMPV1_35182	CTTCACCCTCATGACCTAATCACCTCCCAAAGACCCTGCCTCCAAATGCCATCACACTTG
EMPV1_35185	CCGAGGTGCCTGTTGTGACACCTTCCTTAATGACATAATGGTGTTCATTGCAGCTGGGCT
EMPV1_35187	ACTTCCATCCGGCATGACTTCGGGGAGGTGGCTGCTGGATTGTTTCTGACATTGGATGGA
EMPV1_35189	AAACTCCTCGGCTCATGAAACTTGGGGAGGACATCACACTTTGGGGCATGGAGCTGGGCC
EMPV1_35192	GGCGCAACAGCAGAAACTCCACTGTGCTTAGACTTTGATGTTTTCCGAACTGGTTGAAGT
EMPV1_35193	GGGATCAAGTGGCAGTGGTTGATATTTGCCCCTAGCTAAACCAGGTGTTTAATTGGCCTA
EMPV1_35194	TGCTCTATTAGTGAAGAAAACGGACTCAGTGCTGCATTTACCATAGCCCACGAGCTTGGG
EMPV1_35195	CATGCAGATTCAGGTTAAGCAGATCTGGGGTGAGGCCTGAGCTTCTGCATTTTTAGCAAG
EMPV1_35196	GCACGTTTCCAGGGGCGAGCATTGTCGGTCAAGAGATTTGCAATGATTTCCTTCCTGCTA
EMPV1_35199	CAGGTTTCTCAGACCCTGTATATGCAGAGGCTTATGTTCACGTCAACCAGTGTGATATCG
EMPV1_35200	GGGTTTCTGGACTTCACTGTACCTTTCATCATATTCATTTAGTCTCTAGGGTACCAGTCC
EMPV1_35201	GGCAGCGAGAGAGGGTGAAATTCTCAGCTAATTCGGGTCATTTTGTGAATAGCGTGGGTC
EMPV1_35202	GGGAAGAAAAACACCCTGTTTCCTCTCGGGCCCCCACCGCGGATCATGTACCAGGATTAT
EMPV1_35203	ATTCGCTCTTTTCTCCCTTCCCCAGCCAAAGCCTCCCTCTCTTCACCCCAAGATATCAGA
EMPV1_35206	CTCTCGGATGCAGTCCACCTATAACTACAACATGAGAGGAGGTGCTTATCCCCCAAGGTA
EMPV1_35209	TTCGAGTACCTGCAGATCCGGAAGAAGATCGAGGAGAAGCGCAAGATCCTGTGTTACATC
EMPV1_35210	GGTTTTGTCCCTGTAAATGCGATAGCTGAGTACCCACAAGTGAGCGTTTGTTCTGTGCCC
EMPV1_35218	ATATGCATGTGTGTATGCATATATGTTTATATGTGTACATACACACACACACACATACAC
EMPV1_35220	GGGTTCAAAGAACTCCCTTGCTGATGAACACGTGTGGGTGCTGGGAGAGTAATGAGGGCA

EMPV1_35221	CGGCAGTGGCGGGAGCGGCCCCTCGGCCATCCTCCGTCTGCCCAGTTACCGCTTCCGCTA
EMPV1_35224	CTGCAGCCCTAGGCAGCCTCCAAGACCCCAGGCTTCCAGCGGGCCCCGCGCAGCCAAACT
EMPV1_35225	GTTGGTTCTTGACATTTGCATACCAAGTGGCAGCATCCAGTGTAGCAACACCAGCCAGAG
EMPV1_35227	GTGCTTCCTGAGGATCTGGGCAGGAACTGTGCCAGGCTGCTGCTCGAAGAAATCTATAGG
EMPV1_35228	TCCTGCCAAATGGCCCCCAACTCCACAGGCACCAGGCGAGTTGACTTTTCCAAATTACAT
EMPV1_35230	GCATGAACTTGGGATGCTAATGAGGAACCAGGAAAGAAGTTCTCTGATCTGTTCATAGTC
EMPV1_35232	GGGGGCATTGGGTTAATGGACTTACTTGCCTAGGTACAAGCAGGACTTTGGGACGTTATT
EMPV1_35235	AAAGGTTCAGCGAGAGCTGGAGCTGGATGAAGGTGCTCTCCGTGCATTAAAAAAGGATCG
EMPV1_35236	GCACAGTAGTGCCTTATGAGTGTAATAATTGTGGGAGAAGTTTTGGATGCAACTCCAGCC
EMPV1_35237	GAACATCTGGTCCTCATGTATGGAACCCTGTACAGAATAACATCGACCACAGGCCCCACT
EMPV1_35240	CGGAACACTCTCACCTGGATTCTTTTCTCCCTCAAAATAGCCTTGAGAAGGAAGCAGGAT
EMPV1_35241	ACAGTCCAACATTTGTAACGGGAAGTTCAGCCCCATCCCTCACAGACACAGCAGCCTTGT
EMPV1_35242	ATTCTTCAGGTGTGATAGATCTTTTTGGGGATGCATTTGGAAGTAGTGCTTCTGAACCCC
EMPV1_35243	GTATCTCCATAATTTGTTGTTTCCTTCCAGTCTTATTCCTTCTCCACCCCTCCGCATCCT
EMPV1_35244	ACATCCCCAACTCCATCATGACCATTCTGGTAGCCAGCGGCAAGCAGGAGCCAGAGACTA
EMPV1_35246	CTTTATGAAGCAGACCATTGGCAACTCCTGTGGCACCATCGGGCTTATACATGCAGTAGC
EMPV1_35250	AGAAATGCTGTCAACACATAGCAGTCCATACAAAACTCTGGAGAGGCGGCCCCAGGGAGG
EMPV1_35251	TTCCACCCATGGTGAACCCAATGATCTACGGCATACGAACCAGCAGGATCAGAAAACGAT
EMPV1_35252	TGTAGAGGATGATGAGGAGGGTCACCTGATCTGTCAGAGTGGAGACGTACTAAGTGCAAG
EMPV1_35254	ACGGCTTCTCTGTCTTGATGCGGGCCATGCAGCAGCAAGTGCAAAAGCTCAAGGTCAAGT
EMPV1_35255	GGCGCCATATCTCTCAAGTAAGGCATCTCAAACACACACCATGGCCTCCAACATTGCTTC
EMPV1_35256	TGAGCTTGACCCCGAGCGGATGGGCTCTGGCATCCAGTACGGTGACGCCTCCCTTCGCCA
EMPV1_35258	CAGCGGGTGTTTCCGAATGAAGCTGACAGAAAACTGTGATTAGCTGCAGGAGAAATTTTG
EMPV1_35259	CTACCCAGATGATGGCCTGGGGTATGGTGACTACCTGAAGATCCTTGACTGCTCACAGCA
EMPV1_35262	GCTGATTTGAGTTTTTCTTCTTCTTTAAGAATGCCCTGTATTGCTATGGATTTCTGTCTG
EMPV1_35263	GGCATACCCCCTTCCAACGAGGATTATTCTGTAATGATGAGTCCATCAAGTACCCTTACA
EMPV1_35264	GGAGCCACAGAGGGTAGAAACTTAACCAAAGTGCTCTTCTATAAATCCTTGCACATCCTG
EMPV1_35266	GCCGTGATGCAATAACCTTCCCCCCAGTTCCAGACCTAAAGCAAGTATTCACCTCGGGAA
EMPV1_35267	TCTGGATTCACAGGCTTACGGTGTGGTTCCTGGCAGCTTGGGTTTGCTCCTTGGTGGTTT
EMPV1_35268	AACGCGTGTTGTACCGAATGCGACCGAGGAAACTTCCAGATCCAGTGCATTGGGTTTAAG
EMPV1_35271	AATGGAGAGGGCGGCGAAGAGGACCCGCAGGCTGCAAGGAGCAACAGCGATGGTGAGAAG
EMPV1_35272	TAGGGGCAAGGCGGGGCTGAGAGTCTGGTCACTAGTAGGAGATGCCTGGACCTTGGGGTA
EMPV1_35274	CACGCAGAGGACGTTGGAGGCTGGGAGGAGGCATGTCTGTGTTATTTATACGATGGAATT
EMPV1_35275	TGGAACCGCACTAGGGTTGAAACTGATGGCTGTGGAGTTAATTGCGTTTTTGAGCTTGAA

EMPV1_35277	TATGCTGTGGTTCCTCTGCTGGGGTGTGAAGTACAGGCAACTGTGGGAGAAGTTGTTACA
EMPV1_35281	GACCTGCAGACCAAACATCTGGAGCTTAAGGAAGAATCTGTGAAGCTATTCCGAGGGGTG
EMPV1_35282	TGCTTTGTGTTAGCATGCTGCAAATCAAAATGGCGCTTAATATAAAAAGCTGGTTTAGGG
EMPV1_35283	ACCTGGAGCAGATCCTGACGCAGTACACCACCTTCGTCAAGCCGGCCTTTGAGGAGTTCT
EMPV1_35284	CCCTCTGTGGGACACCTCGACTGGTTAAATGCCACAGTCCTTCCCTAAGAAGAGAAATGG
EMPV1_35286	GCACCACAGACATAGGAATGAGCTGTGTTCCTCACATTTACATCCAGTTGGTTACAGTAC
EMPV1_35287	TGGACTGCAGAGCAATAGTAGTATAACTGTACCTGGTAGGCCTGTAGAAGTCAGTTGTGC
EMPV1_35288	ACCAGAAATGTGCTGGCTTGTGAGTGTGTAGGACGACTGCTGGCAGAGGGATGCTTGGAA
EMPV1_35290	AGAAGGTTCTGACCTTTGATTTTCGGCAGCCAGTCCCCTCACAGCCTGTCCGTGCTGAGG
EMPV1_35297	GGGTCTCACAAAATGCCTGCTGAATGGGGAGGCCCGGGTGATCTTTTCGTTTCTGTGATT
EMPV1_35299	CTCGAAGTGGAACACGCCAGGGTATGAAGTACTAAGCATGGTGTCCTGTGCTTGTGCAAT
EMPV1_35302	GGCGCTCTCTCCGATCTTGGCCACTCCGTCGTAGTAGGCTTTTCCCGCCAGGATCATAGC
EMPV1_35303	CTGTTAATTCTGCTCTCCTATGCCAACATAGTCCATGCCATCCTGCGGATTCGCACTGCT
EMPV1_35304	TCAGTTTTACAAGACAGAAAGGATGGCGAGAGGGGTGGGTAGTGGTGATGGTCGTCCCAT
EMPV1_35306	AGTGGCCCAGCTTTGACCACGTTACTGTCCCCTTAGTTCTCAGTCTTAGCCTTGGATGGG
EMPV1_35307	GGATGGAGATGCGGCTGAGGTAGAAGCGGTCCAGGAAGTACTGGATGTTCTGGTTAGAGA
EMPV1_35308	CTTGAGACCCCTTCTTCACCTTCACCATAGACCATCTAAACCCCATATCCTTTACACCCA
EMPV1_35309	GCAGTACAGGGATAGAGAGAGATGAACTAAGGGTACAGATGGCAGAAATGGCTTAAGGCC
EMPV1_35315	AGTCTTAGGAAAAGTCCTCAGTGCTGTGGGCAGTGCCCAGCTGCTGATGTCCCAGAAATT
EMPV1_35317	GCAGCTCTGATTCAGCCGTAGCCTGGGAACTACCATATGCCACAGATGTGGCCACAAAAA
EMPV1_35318	GTGGCAGGGACTCACAACTCCGAATTCTGTATAGAAAAGCACCTGGATCCAAGTCTTACA
EMPV1_35320	TCGCCGCAATTTGGCAGCCTTGACTTCCGTGGAAAATTCTATCTCGATCACCGCCTTCTT
EMPV1_35322	CCCCTCTAACCATATTTGGAAGACTTTGTGGAAGGGAGAGTCTTGTCATCCCTGTGTTGG
EMPV1_35323	CAAAGGAGGAAACAAAACCGAGCTCATAAATCCAAGGCCTTTACTTCTCACCCAGCTGTC
EMPV1_35324	CAGCATTGATATCCCTTGGGAGCTTGTCAGACTTGTAGAATTTCAGGCTTCACGTAGACC
EMPV1_35325	CCACCTTCTGCCAGTCTCTGCTCCACAGAAAGTAACCAACTTTTCAACACGGTTTCCCTG
EMPV1_35327	CTTGAAGATACTAAAGATTAAAACATTTTCCCTTCAATGTTGTCATGTATTCTCTAAGGG
EMPV1_35328	TCCGACAGGAGTTTGGTCAAGATGAGGTAAACTATATATGAATCAGACCCAGCCTAGAGG
EMPV1_35329	CTGCACCTACTCTGCCCTCAGCCTGTACGATGATGACATGATGCTCACGGAGGATAAGAT
EMPV1_35330	GCAGCGCCATCTTCACATATGGACGACCCATCTCAACATATTCACTGGAAAAAGACAGGC
EMPV1_35331	CAGTGGTCACCGTGTTCTATGGGACTTTGATATTTATGCATGTGCAACCAGAGTCCAGCC
EMPV1_35333	GCAGTCAGAAAACTGGCTGACTCTTAGAAGCTAGAGACTAAGTTGGACAACAAGCCTGTG
EMPV1_35336	GCCCTTGTAATTGTCTCCTCATTTGTAAGGACACATCTCGGCGAATAGGATGTCCGAGAC
EMPV1_35337	TGGGTGCAACGACAGCTGGTCTATCCTTCCAATGGGAAAACTGTGAGTTACCTTCTATGC

EMPV1_35340	TGCCCCCGGAAGCTGAAGGGGAGGAAGATGGTTTCCCTCCGCGCTTTGCGGCGCATGCTC
EMPV1_35342	AGTGGCAGCCCTCAGAAAGGTCACCAGTGGAAGGAGAAGGGATGAAAGAAGAGCTTGGTT
EMPV1_35343	TGGCCTCAGTGGAAGCGGATCGTGGAGTAAAGGAGCAGTTACATCTTTATGACACCAGAG
EMPV1_35345	CTACCCATCACAGCCTGTTTTGAGGGCTTGTTTCCACAAGCTCCGGCTTCTGCAGGGGAA
EMPV1_35346	ATTGCTTGTTGGGGTGTAATCATCCTGTCAGACCCCAAAGGGAAGGCCTTCCGTGCTGGT
EMPV1_35348	AGGGTCCAGTGCTTTCAAGCTTCCACACACAGCGCCGCCCATCTACCTAAACTCTGATTT
EMPV1_35349	CACTGCCAGTCAGGGCAGTCCTTCATTCTGCGTTGGGAGTCTGGAAGAAGACTCTCCCTT
EMPV1_35352	AGGTGTGGAGGAGTCCCTGCAGGTGCGAGTGTCCTTGGAGCAGATCCTCAGCCTTCCAGA
EMPV1_35353	GTGGTCCTGCCTCTCCTTGTAGTCTGGTGAATGAGACCACTTTGATTAAATACTCCAGGC
EMPV1_35354	GGGGCCTTCATCATCTGCTGGTTCCCCTACTTCTCTGTGTTTGTTTACCGTGGGCTGAAA
EMPV1_35355	CTGGAAGCCTCTGGGCCAATCATCCTCCATGCACTGTCTGCTAGTCACTGTAACACCTGC
EMPV1_35356	CCAGCAGACCACTACCTTCACTGGGCATTCTGGAGATGTGATGAGTCTTTCTCTAAGTCC
EMPV1_35357	GAAGAACGAAAACCAAGGAGGAGGCATCTCTCTCCCAGACTTCAGGCGACATTACAAAGC
EMPV1_35359	GAAGCAGAGCTTTCCCTTGGCTCTGAGGAGAGAGGACATTAAAGAGATGTGGAAAAGTGG
EMPV1_35360	GACGGAGTGGAGAATGTAGAAAAGTGAAGGTGTATGATTCTGGAACAGAGACATTAGAAG
EMPV1_35363	CTTGGAGCCTGGGAGGCCCAGGAGGATGAGTCCAGATCAAAGACTTGGAAGCATCAGAAT
EMPV1_35364	GACTTTGGTTTTCCTTGCTGTCGTCAAATACGTGAAAGAAGTCACATTGGGGAGAGGCCC
EMPV1_35366	CGTGGGAGCTTAGGGCTGCCAGAATTCCAGAGAAACCAGTTGTGTCCAGTATGCTAGTGA
EMPV1_35368	TTTGGCAGAGAGACAAGCGAGACACCTCCAATTTCGACAAAGAGTTCACCAGACAGCCTG
EMPV1_35369	CTAGAGTTCAGCCCTCCATGGTGATGCCTGGAGCTAGTGTAAGGAGTTTCACACCAGTCT
EMPV1_35370	GGGGGAAGCTGTTTGATTCTTGTGAATTTTGCTTTTAAGATTTGTTATGTGGGAGTTCCC
EMPV1_35371	CAGTTCCAAGAACCATTCGTAGCAGTGGTAATTGACCCAACAAGAACAATATCTGCAGGG
EMPV1_35374	GTCCATCCTAATTGTCTTTGTTTTCATCTAGTCATGGAAATTCAGTAAGTGCCTTGGAAC
EMPV1_35375	TTGCCTCATTCCCCGACTACCTGGTCATCCAGATCAAGAAGTTCACCTTCGGCTTAGACT
EMPV1_35376	ATGCGTTGATGTGTTCTGTATGGGTTCCGAAATCAGCTTCTCTCTTCAAGTAAGGGTTGG
EMPV1_35378	CTTCGAGACACTCTCCCTATGTGGGGAAGCAGGGACACCTTAGTTCTGAGAGAAGCAGCT
EMPV1_35379	CGAGCCATGGAAACTGAGGTCTTAGAGACACGAGTAATGGAGAGAAGAGGAATGGAGACC
EMPV1_35382	TCCTCTATTCATCCATCATGTCACCCTCTCTCTGTGGTCGGATGGTGCTGGGGTCCTATA
EMPV1_35384	GAGTCTGCCTATTTCATATAGAACTCCTGTGGCCATTTCCTGTCCCCTGTCATCCTGTCT
EMPV1_35385	CATTTCGAATGGCCTCATTGTTGGGATCCACGCCTGGAAGGTTCTCAAGGACACTCTGAA
EMPV1_35386	GAATGAGGCTACTGTGGATCGTCTCCACCTCCCCACATCCTAAGTCCTAGATAATGAAGG
EMPV1_35388	TGTGGTGCAGTGGGATTGGTGGCATATTGGGAGCGCTGGAACACAGGTCTGATTCCCGGT
EMPV1_35389	CTACAGGAAAGTGTGGTTTAGTTGCTGATTTTTACAGGTAAGTGCAAAGGAGAAAAGGCC
EMPV1_35390	GGAAGTATAATGTGAAACATCACTGAGCAAAAGGGAGATGGGGAAAAGTGTTTAGGTTAT

EMPV1_35393	AGGTGAGCCAGCACACATAAAATGCTCGGCAGCACAACAGAGGCATTCGGGTTAACTGTT
EMPV1_35394	CTTCTCCCTACCCCACAAGTCTTTTGGTACTACCATTTTGGGAAGCCAAGCAAGGATAGG
EMPV1_35395	GATGCTGAAACGGACAGAGTAATATTTAATCTACTCAGCTGTCCCCCTCTGTACGATGAG
EMPV1_35396	AACCTTGCATGATTATACCACTCAGCCTGTCAGCCAAGAGCAGCCAGCCCAACCTGTCTT
EMPV1_35398	CAAGGACTTGAGCGTCACAGAGGCCAAGGCCACAGAGAGAGACACGGTGGCTAACGTCTT
EMPV1_35403	AACGAGAGCTGGAAGAGGCGCGGAAGAAGCTGGCGCAGATCCGGCAGCAACAGTACAAAT
EMPV1_35404	GGACGTGCCAGTTCACCTGTCGCATTGGCTTCACACGAACATAGAAATCTCACCTGGCAT
EMPV1_35408	AGTGCTGAGACTTTTCGAGATCTGTGTAAAAAGACATAGGTTGCCTCCTATGACCTTTGC
EMPV1_35409	GCAGAATATACTGTAGAACGAAATTGGTAATTTCTGAATCTTCCAGAGCATGAGTAGATG
EMPV1_35410	TAGGAAAGGCAGGCATGACTCCAGGGGTGTTGCTCATGTGACCGTTTTCAGCAAGCAAAT
EMPV1_35411	GACTTGCCCCTTCCAGGTCTAAATGTTGACTCCTTTGAGCCTCCTTCTAGATTTCACTAG
EMPV1_35412	CACATTGCTCTCGCCTTTTATATTTCCTGCAAATGGGCAGCCAGATCCAGAGGTTCGCTC
EMPV1_35415	CGGGCACTCAAACAAATCCGGAAATTTGCCATGAAAGAGATGGGAACTCTGGATGTGGGC
EMPV1_35416	GAGAACAGATGGGAAAATTCAAGCCCATGACCCCTTATCAACTAGGATTTAGGCTGGGCT
EMPV1_35419	GGTGGAGTAAGACACCGTGGGCTCTTGAGAAGAAATATGGGCAAACCACTTAGGAGATTG
EMPV1_35420	GCCACCAAGGCCCCAAGTAGGAAAATGGTCATCAGTACCCCCATACCCTGGACAAAAACA
EMPV1_35421	TCGCCACTGATTGTCCAAACGCAATTCTTGTACGAGTCTGCGGCCAACCGAGAATTGTGG
EMPV1_35423	ACCCTAGCACCTCTAGTTCAGAGGCAGATTCCACAACCTCCCACCCTGCTTCAGATTCAT
EMPV1_35424	TTTATCCCAGGGTGGCAGAATCCCGGCATGCACACAACGGTTTTGCCCTTTATATTAATC
EMPV1_35425	GTGTGGACAGATGTGGAATAACATAGGAGGGTTCCTTTCAGACTAGGTACATACCCCTGG
EMPV1_35427	CTCTTTGGACAGCTGAAGAAATACCCTGAGACACTCATCATCCACTGCAAAGACCTCCGG
EMPV1_35428	CTGCCCTCGGCCGTGAGTCCTGTCCAAGATCACTAATGATTACCTGGCCTTTTTGCGTCA
EMPV1_35433	TATACTACACTCTGTTATCAAAACTATCACCATTGGGAGTTCTCGTTGTGGTGCAGGGGA
EMPV1_35435	CTTTGTGGGCTGGAGACCAGACTGAGAGAGTGAGAGAACCCTGGCGCAATTCATAGTCTT
EMPV1_35438	ATGACAGCTCTGCTTGGAGAGTGTCTCTAAATCAACTAGAAATCACAGCTGATGCCCCTC
EMPV1_35439	CAGCTGCACTGGGCTGGAGTCCTTTACTGGAAGCTGACAGATGATCTAGAATTAGAAAAG
EMPV1_35440	ACTCCGGCGAGTACTTGTGTGAAATCGATGATGAGTCGGCCTCTTTCACCGTCACTGTCA
EMPV1_35441	AGTCAGAGCATGCTCCTCAGGCCCCGAGGACCCCTGGCCGGGCTTGCTTAGAGAAGACAA
EMPV1_35442	ACTATGCCTTCTCTTTTAAAAGCCTCTGGTGTTTGCCGCCAGCTCGTGTGCTTTCCTCCA
EMPV1_35450	CAGCTAGGCTTCGACTTGGACGCACAAGGAATTATCTTCACCGAGGACTACAGGACCAGA
EMPV1_35451	ACAGCTGGAACTGGATACTGAACGGAGCCGGGCAGAACAGGAGCGGGATGCTGCAACCAA
EMPV1_35452	CCTGGCTCCATCTCCTTTAGGAAAACCTTCTGTGGGGAGTGGGGTTTTGACCCTAACCCA
EMPV1_35453	GGCAGCTGGGCCAAGGGTTAATTATGCTTCCTGAAGTCAGAGATCTGAGAGGAGTCTTCT
EMPV1_35454	AGAGCGTGCCACCGACCTGCTCCTGGACCACATCCGTGAGCGAAGCCAGGGGCCTACATT

EMPV1_35456	CAGATTTGAGGAGGGTGTGTTAATGTGTATTTCTCTTTCTGAGACTGGCCCGTGTGGGAC
EMPV1_35458	AACTGTCTGGACACAGCAGTAAGCAGAGATGCACAAGCAGTGCTGCACCCAGCCAAATCT
EMPV1_35460	AAGCATTGGATAAGGCCCGGTTAAAAAGCAGAGACATCAAGTTGGCTGAGGCACACCAGC
EMPV1_35461	AGCCTCAAGTTCAGAATGGGCCTGACCCTCTCCCCAGTCACCTACCGCATCTGTGCAGTT
EMPV1_35464	ATAAGGCCAGGATTTCAGTCTGTTGTCTTCACTGCTGGTCCCTGGTGCCTAACTCAGTGC
EMPV1_35466	AGAGAAGAAGAGTTCATCGGGGATGATCACAGAGTTAGTACCTCCAGAGTAAAGACGCAG
EMPV1_35467	TCGTGGATGACAGGGTTGAAGTTCAGCTAGTTTGCACCAGGATGGCCTTACCAACTATTT
EMPV1_35470	ATATCCAGACCCAAACTGAAGAGAGTGAGCTTAGCACCATGACCACTGAGCCAGTCTTGG
EMPV1_35471	AGCATATCTCAAGTAGAATAACCTGCACCCCGCCTCCAAATTCATACTAGGACACATCAC
EMPV1_35473	ACTACCGCTACTGAAACTGCTACACGAGGAGGACGATGGGCTAGATGAGTTGGAAAATGC
EMPV1_35474	AGAACAAGGAGCTCAAGGTCAAGCTGCAGGAGATGGAAGGCACCGTCAAGTCCAAGTACA
EMPV1_35477	TTCTCCTGTCCACGGAGGGGTTGGTGCTCCCCGCAAAGTTAAAAACCACAAGTCCTAGTT
EMPV1_35478	CTGATGCGGGAGATGACAGTGAGTGGGAAGATGTGGATGATGAGAAGGAAAACGGTAGTG
EMPV1_35479	AAAGATGCCCCGATCCGGGTGCTGGTACAGCGCATCCACCAGTTGCAGGCTGAGCGCGCG
EMPV1_35482	GCAAACAGAGACTGTTGTTGCTCTAGCCATGAGAATAAAATGCAGGCAGAGACATGAAGG
EMPV1_35485	CCACCAACAACACGGCCATAAACTTCTACGAAAACCGAGACTTCAAGCAGCATCACTACC
EMPV1_35486	TGTGGATGAGGATGAGGTGTCATGAAGGGAAGAATAAGTACCACCCTGGAGTGGGGAATG
EMPV1_35488	TCATCTACTGGGACCTCATCAGCCATGATGAGATGTCCTTCAACATCTACAAGATCAGGG
EMPV1_35489	GAGGCTCTCAAACCTCTCCTCAGAACCATACACCCCACTCACGTTTTGTTGGCCATGACA
EMPV1_35491	ACTGCCTTTCAGCGGTCTGAGGATGGAGTAGATGCTCCGTAGCTGTTGGAGTGACTTCTT
EMPV1_35493	GGGGGCTTCTTGGACACTTAGGCCCCTTTTAATCTTTGCTACGTACTTGTTAAGGCACTG
EMPV1_35495	AGGGCACTAATCCTTCCATGGGGACTCTATCATCTTCCAAAGGTGCCCATCTCCAGATAC
EMPV1_35497	GCTCTCTCTTGTCACTGTGTTACACTTATGCATTGCCAAAGTTTTGTTAGTCTTGCATGC
EMPV1_35498	GAGGAGACCGTGCGTGTGGTCGCGCTGGACAAGAACTTCCACATGAAGTGCTACAAGTGT
EMPV1_35499	AGTGATCGGGAAGATGGGTCTGGCATGTGTGATCCGTGGCTCTCTCTTTGTTTTCCCCTT
EMPV1_35500	GGAAAGAAAGTGATTGTCACAGGGGCCAGCAAGGGGATCGGAAGAGAGATGGCCTATCAT
EMPV1_35502	GCAGTGGGCGCTGCATGCCTGTCTCTCTGAACAGCCAATTCTTACCTGATTCTCAGATCA
EMPV1_35503	AGTTTCTGGGCAGATTCCATGTGTCGTCGCTCCCTCCCCCGGTTTTGGCATGAATCAAAT
EMPV1_35504	CTTGATTTACCAAAACCAGACAAGGGGAGAGTGAAGTATGACGCAAGAAAATTACAGGCC
EMPV1_35505	GAACAGCCCAGTTGGAAATCAGCTTGACCCAATTCAGAGAGAACCTGTGTGCTCAGCTCT
EMPV1_35506	AGAAAGTTTTTCATGGACTCAACAACATGGGGAACCAGGCCGGCAAGGAGCTGGACAAGG
EMPV1_35507	CTTACTGCAGTCTATCAACTATTTGCCTAGACATCTCCCTAAGCTCAACTCTCCACTGTG
EMPV1_35508	TTTACCGCCAGGAGTAAATAATATGGATGTAGCAGCAGGTGCAACATCAGGCGCCAAATC
EMPV1_35509	GACGCTACCACGTGCCACTGCTACTGATAGAAAAAGCACAAGAAAAGACTCCAACACCCC

EMPV1_35512	CAGCTTTACAGGCAGTTTCCTGCATAAATTGAGACATTAATTTGGTTAGTGAAATGGGAA
EMPV1_35514	CAGAATGGGAGGCAGAGACGGTGATGGGGCTGCTCCACAGCACCTTTCAGAACTTTGGTT
EMPV1_35517	TTAACATCGATGACTGCTGGATTGGTGGACGTGATGCCAAAGGCAACCTGATGCCTGATC
EMPV1_35520	ATGCAGGCGCATGTCCGAGGGTGGCTCGAACGCAAAAGATTCCAAAGAATAATGATCAAG
EMPV1_35521	CCTCATGGACTTCGTCAAGTTTTCGGAATAGGAAAGGCCAGAGACCATCAGGAAGTTGCC
EMPV1_35522	AGGGTACTCCAGTAGCTTGGACCCCACTCCACCTTTTGAAAGAATCCCCATTTCATAGGA
EMPV1_35524	TCGCCTTCCCGTGTTCTCCATCTTCGAAAAATTCCAAGTGATGTCACCGAAGCAGAGGTC
EMPV1_35525	TTGCTGCATATCAGCGAAAGTAGCTGCTCCAGTTGGGTAAGTATAGGCCATGTGCGGTAG
EMPV1_35526	GCATCAAATTCTGCCTAGACAACGGAGCCAAGTCAGTTGTTCTTATGAGCCACCTAGGCC
EMPV1_35528	CAATCACAAGTCATTTCTGTGCGAGTTGCGTTCATTCCAGAAAATGGCATCATCCTCCGC
EMPV1_35529	CCTGATGACCCCAGGCTGAAGAACAGGACTCGGGGGTACCATCAAAATCAGGGTTGTTCT
EMPV1_35531	CCTGCCTGCTGTTTTACCCCCAAGGTTTTGCACAGGTTAAGACCAGTTATGTCCTCTCTG
EMPV1_35532	TACCAAAGAAGACTTTGCCACTTTTGATTACATACTGTGTATGGATGAAAGCAACCTGAG
EMPV1_35534	TCGACTACACCATAGAGGGAGTTAGGAACTTGGAGCTGGGTGTTGAGCTGAGTGTCCATG
EMPV1_35538	GCCACAGCACCTGTCCTCAAAAAGTTACGTGGTTCCTCCCCAAAAGAATCAAATGGCTCA
EMPV1_35539	ATACCCTTCCTCACCTGGGGCAGATGTTGTCAGGTGCCAGAGAGCCAGTAAGAAGATTCA
EMPV1_35541	AACAAAACACCCTGGAGTTCCCATCATGGCTCGGCAGAAATTAATCCGACTTGGAGCCGT
EMPV1_35542	TATTCCATCGCTGTCAACATACCCGTGTTGCAATTCCTTCTAGCATTTGACGCAGTCCTC
EMPV1_35543	GAAGTTAAATATTTGAATACCTAAATTTTAATATTTGCAGAAAGTTTAGGAAATAAGGAC
EMPV1_35544	TACCAGTTCCTGGGCTGCTGGTATTGCCAACTCTGTAGTTCAGACATCACAGGCCATGCA
EMPV1_35548	TGTAAATGAAAGGAACCCTGGTCCTACACTTGGCTAAGCCCCAAAGTAGCAGTCTCTGGG
EMPV1_35549	GCTCCTCTCTCTGGTCATAATTCTTCCTCTTCCCATTTCAGTTGAAGACAGGTTCCCTCC
EMPV1_35550	AATTAAAAGTTTCATGCAATTAAAGCACATATATAACCTAAACATCCTAAAATTCAGGGG
EMPV1_35551	GGCACGGCTGAAGAGCAACAATAGACAAGTGTGCATTGACCCAAAATTGAAGTGGATTCA
EMPV1_35552	ACCCACTGAGTTTGTGCCTCGTAGCAACATACAGGGAAAGTGGGAACGAAAAAGCACTCA
EMPV1_35553	GAAAACCTGCTTATTGCTGTACACATCTAGTCCAATGAGGCATAAGCCCAGTACTTCGAA
EMPV1_35554	CCATCTTGCTTAGTGAAAGAGCTTCGGTTGAAATTGTCTAAAGTAGCAGGTACAATGAAT
EMPV1_35555	TGGCCACCTATGTGGCTCCTGCCGCGCATGCAGCTCTGGAACCTCGCGTTGTCCTGCACT
EMPV1_35557	CACTTCTCAGCTGGTAACTCAAACAATGAGTTGGTTTCAGAGGCTATTTACAAACTCTGG
EMPV1_35559	TGATGCATAATGAGGCCCAAGAGGATGAAGTCAGTAGAGTTCTGGTGAAATGCTGCCATG
EMPV1_35561	CCTTTATGAAGACTTTTTAGCTACAGAACCCCAAGCAGGAGTGAATCTACTCCTGAGCTC
EMPV1_35563	CGGCATCGTGCTGGCACTCTGCATCCAGGGGTGCCGGCTCTACAGCGGCTCTGCAGACTG
EMPV1_35564	AGGTCTTTTGCCATTTTCCCCTTGGGTTTGGGGTTTTGTTGGGCTGTTGGCTGGTGTCTG
EMPV1_35565	CAACAGCAGAAACCATACATCTGAGAGTGAAGACATAGCACCTGATCCGGCATCTGCGAC

EMPV1_35566	ACCTTCCTGCTATCAATGGCATTGTATTTCTGGTGGATTGTGCAGACCATGAAAGGCTGT
EMPV1_35567	TCCCTAGCTTTTTCGGCCTTTACTCAGTGTCGGAGACCACTTCCAACATCAACCAATGTG
EMPV1_35568	CCGAGGAACCCTCCACCCAGTACGTGCACATCACGGAAGCTGAAGAGGACGTTCAGGGCA
EMPV1_35571	GTAAACTGATGACAAAGAATCTGGGGGAAGAGGTATGCAGATGGACCTCTCAGACTGGCC
EMPV1_35573	GGTCATTATCGTTATTGTGCTGCCCTTTCTATGCTGGGGGAATATGACTGGGCCCTGCAA
EMPV1_35576	AGGTCGTCCACCCTGGTCAAAGCTGGCAGCCCCTCGGGGACTCATACCCTGGCCTTATCA
EMPV1_35578	GAAGGAAAGCTTCAAGGACAGCTTAACAAGTCTGATTCTAACCAGTATATTAGGGAACTG
EMPV1_35580	GTATAGAGCTTCCCTGTTAACAGCTTGTATTAGAATTGAAAACCCCAATATGCACTTGAA
EMPV1_35583	CCCATATATTTAATGGGACATGCTTATGCTAAAAAATTATTTGTGCTTTATCTGAAGTTC
EMPV1_35585	TCTGCACAGAGCCAGTTGGAACCCCTACCCACCTCCTTCAAATCCTTCCAGCTTCCACTC
EMPV1_35586	CAAGACGACTGCATGTTCAGCCGAGCTTTGACCCTGGACCTTTCTCAGTATCCAGAAACC
EMPV1_35587	GCCTTCAGGTAGGCACCATTCAAATTCCCAGTACATTCCGTGGGAGAAGAGGCTAAAACC
EMPV1_35588	CATCCCCATTTTCAGGGGGAAATAATGTGTAGTCTTAAAGGTGGGGTCCTCATTCCCTTC
EMPV1_35591	CCCAAACCCCCACAGGCAAGCAAACTGACAAAACCAAGAGTAACATGAAAGGTTTCTAAG
EMPV1_35593	TCTCAGACCTGAATTCCGCCAAAGGACCCAGTGCTGAAAGCCGTTTGTCATGACTTCTGA
EMPV1_35594	ATACGAGTCGTTCCATCATCCGAAACGTGAAAGGCCCTGTTCGTGAGGGTGATGTGCTCA
EMPV1_35595	AATGCTCAGAGGCAGAGGGGCTGTGGCTTCCCAAGCTCGTGTGATTCCTTCTCAGCTTCA
EMPV1_35596	TCATCAACAGCGGGACCAGCTTCTTTGCTGGCTTCGTGGTCTTCTCCATCCTGGGCTTCA
EMPV1_35597	CCGTATGCTTCGCAGAGGGTCCTTACATGCTCTAGTGTGGGTGTCTATGTGAATGTGTTG
EMPV1_35598	CAGGGCTCCTGCTCCACCTTCTGCTTCCTGCAACTGTGTTCCTGCTGGTGCTGCTGCCAG
EMPV1_35600	GGGCCATTGACCACTTTTACTGTGATGACCGTCCCCTTCAGAGGATTTCTTGTTCTGATC
EMPV1_35601	CCAAAGGCTTGGGTGGTGAAGGTGCTTATCCTTTCTGTTATTTTGTGGCGGGAAAAGCTT
EMPV1_35602	GCAATGGAACACTATGGCCATGTCACTTAAACTCAAAACTCAGCAGCAGGGGTGCTCACT
EMPV1_35604	AAGCTGCTTTCAGCTACTGTGGACATAGGAACCTGGAAGTGAAACTGTCAGACCATATGG
EMPV1_35606	CCTCAAAAGTCAGACAATTACTGGATACCACTGACATCCAAGGAGGAGAATTTGCCAGAG
EMPV1_35607	CAACCAGAGCCAGTGCTGAATGAAGACAAAGATGATTTTAAAGGGCCTGAATTTAGAAGC
EMPV1_35608	ATAGTTTCACCCCCCTAAAAAGCCCCTGTGCTCACCTTCTCATCCCTTCCTCCTTTCTCC
EMPV1_35610	CTGTGACCTTCAAAGCTGCACTGGGTTTTTTAGTATTGGTCCAGGAGAGTTTAAAACACC
EMPV1_35611	TGAGACATCGGCTCGTCTGCTCTTCATGGCTGTCAAGTGGGCCAAGAACCTGCCTGTGTT
EMPV1_35612	AGTTGTGACCCTGAGCAGGCCACCTTCCGGTGCCTCAGTTTTCCTTTCTGTCAAATGAGA
EMPV1_35614	TGAGCTGGACCCAGAAAGGATGGGCTCAGGAATTCAGTACGGAGATGCTGCCTTGAGGCA
EMPV1_35615	GCTTCATGTACTGATCTGTCCCGGCGCACATGCCAAATCTATCTTTCCACTTGTCTTTGC
EMPV1_35616	AAGGATAGTTACAGGTCCTGAGGGACAGCCAGACCTGCCAGGTCCCCCAGAAGCCCTTGG
EMPV1_35617	CTGGTGGAGCACTGAGGTTGGAAATGTAGGATGAGGGGTTGGTTTCGGGAAAGATGTGAA

EMPV1_35618 GCCCTCATATAAAATCAAACATACTCTCAGGAAGCATTCGGAGCCTGTGGCGGGCAGTGA
EMPV1_35619 CCCGGAACTCGGGGGATGGGGACAGGCATGTGCGAACCAGCCGCGGTGCCACTCCACATT
EMPV1_35621 AGGTTCGGGGTGCTGAGTCAAAGTCCTCAGTTCATGGGAGGTATGGCCTTCTGGGTTCTT
EMPV1_35625 TTATAATGCGCCCGGTTCTGCTCATGTTGCCCATTCTCTACCTAACCCATCGTCTGCCCT
EMPV1_35631 CCTGGTGGCACAAAAGAAAAAGTGAACACATCGTCACCAAGAAGCACAACACAGTAAGAG
EMPV1_35632 TTTTCAAAAGAGGGAACTGGGTCCAGGCCAGAAGATGTGTCTGCTTACACAGCCAGGAAG
EMPV1_35635 AGCAGACGCCGTGTGCATGTGTGTGCGTGCCTGTCCAATGTATATTGTGTCTTAGCTTCC
EMPV1_35636 AGAAAGCCGGAGGCAGCTCTTCAGTTCCCATAAACTAATTGAATGTGATACCCTGTCCAG
EMPV1_35637 CTCTGCCTTTGCTTGTCCGAACTCATCATCCTCTTCCATGAAGCCACCTTCTCTAATCTC
EMPV1_35638 GCACGCAAAGTTCGGTTGTGCGAGAACTCCTCCGTTTCTCTATGTAAACACGCAACTCGT
EMPV1_35640 ACAGCATTTGGAAATTCCTAGGCCAGGGATCCAGCCTGCGCCACAGCAGCATCTGGAGCT
EMPV1_35641 AAGGACAAGTGACAATTTTGAGAGGAGCTTGGAGAATATATATCAAGATGGATTCTCACA
EMPV1_35642 AGACAACCTAATAGGAGTTACTACTGTGGTGCAGTGGGTTCAGACTTTGCCTGCAGCAGC
EMPV1_35643 TAGCTGGGTAACAAGTCTGTCGAGCGAACCAGGCAGGAGAGAAGTCCTGATGGACCTTTT
EMPV1_35645 GATTGCCTAGCCATTGGCTTCAAGGGGGCCAGCACAAGACTGTAAGAATTGGATCAATGC
EMPV1_35646 GGCATCAGAGGACACCTGGTAAAGATGCCCACAGGCAAGAGGAGATGCAAATGTTCTAAG
EMPV1_35647 AAAGCATTTTCCCATACTTCCCTTCCGTCTTGGAGCATCTCCAGGTCTGCCCAGTGTTTC
EMPV1_35648 TTCGACACAGACACTTTAATGCAGAACAAGAAGGCCCGGGAGACCTGTAAATCAGGTAGC
EMPV1_35650 AGGAATAAACATGAGCTCCTCCTCCTCCTGGTCTGGACTCACAGAACCACTCCAGCCTTC
EMPV1_35655 AGGGTTCTGGGTATCAATACTTCAGTCAGATGTAGCACTTTGAGTGTGAAGTCATAGGTG
EMPV1_35657 TTCTCTCATCCGTGCGCGGAGCCCCGCATGGCCACGGCGAGCCGTCAGCTGCAGCCTGGC
EMPV1_35659 TGTCCAGTGCATTGCAGGATGTTTAGCAGAATCCCTGGCCTCAACCCACTAGATGCCAGT
EMPV1_35660 GGACACTTACTGAGACAACATCTGCATCAGCCACCAACTTTACCACCCACAACATCCCTC
EMPV1_35661 GCCTGCCCATATGCTGCCTCATATAACACCACACTCACACCTGCTCACATGTGCACTCAT
EMPV1_35662 GTCCCCTCAGGTCTGTTCTCTGCTGATGTTTGGTTCATATGTGATGGGATTTGCTGGTGC
EMPV1_35664 TGAGCCATGATAGGAACTCCCAGAGACCAACCTTTACTCCTGACCAGCCACTCACTAGCT
EMPV1_35666 AGTGGCCAATACCAAAAATGCCTCCCTGGAAAGGGCTAGGCTCCGACTGCAACTTGAGCT
EMPV1_35667 ACTAAGGAGGGCAAAGCAGAGTGAGAGCCACACAAATCATCTGCACCACTGCCCTAGACA
EMPV1_35670 CAAAGGCTGCGAGGTCGTGGTGTCTGGGAAACTTCGAGGACAGAGAGCTAAATCCATGAA
EMPV1_35673 CCATGGGAACCATAGGTGGTGGTATCTTTCAAGCCATCAAAGGTTTCCGCAATTCTCCGG
EMPV1_35675 CTTGGTGACCCTCATCATGTACTCCATCGCCACGGGAGGCTTGATTTTGATGGCAGTGTT
EMPV1_35676 TTGGACCCTTGATGCAGCCCAACTGGAAAGTGTCAGGAACACAGCCTCCACCTGGTCTCT
EMPV1_35677 AGGAACTGGGGGGAAGGGAGGACAATCTCTCAACTCATCAGGGTCTGCTACATTCTGAGA
EMPV1_35680 TGATTATCACCGTGCAGCCCTCTGGTTCCTGCATTGAACATTCCAAGTCTCTGGATCTGG

EMPV1_35681	CCCTTCGGTAGGCCTCTTATAGTGACGTGGACCACACCATGAGTTGTTTCTATATCATGT
EMPV1_35684	CCAGTGAACTGAAGTAAGGATTGAGGGACAGAACCTTCAGGATACCCAACTTTTAGCAGT
EMPV1_35686	TGTCTTTGCTGATCCCAACATGCTTGATACGTTGGTGCCTGCTCACCCAGCCCTGGTAAA
EMPV1_35687	TTCCAGGTCCGCGTGTGTGATTCTTGCTACGACTCCATCAAGGACGAGGAGTGAGTCTTT
EMPV1_35689	TATTCCCAGTGTGGCTTTAATTTATTTCATTACATTCTGTTATTTCTGATTGCATTTCTC
EMPV1_35690	AAGGCACCTGACACTGATAAACTTCACAGCCACTTCTCCTAGCACATAGGACTCAGCTGG
EMPV1_35691	GGGTTTATTTCTGGGCTCTCTATTCTGTTTCATTGATCTATACACCTGGTTTCATGCCAC
EMPV1_35692	TGCTGTCTTCTTGGGGAGCCTCTTCTTGGTTCTAGTCCATCACATCCTGGAGCCAGTCTA
EMPV1_35693	CGTGTTCGATGGTTCTTCCCGTGTTCCCCTGGCATCTTTGTCATACCTCTACATAACAGC
EMPV1_35694	ACAGACGTCCTTCTCTCCTAATTCATGAGCCAGCAGGATGCGGTCGCCGCGCTTTCAGAA
EMPV1_35695	CTTCCTGCCATAGCGAAAGCAGAAGGGCCGCAGCTCCACAGCAAGACTAGAGAAGTGGAA
EMPV1_35699	AGAGACAGGCCTCGGGAGATCACGGTTGTCCAACAGGCATGATGAGATGCATGTGTGCAA
EMPV1_35700	ATGGAATACCTGGGCCGTCACATCGCCCGCAAGCTCAACATTAACTATTTCGACTGTCTG
EMPV1_35701	CAGGATACTATCATGGAAGGTACAGGACCATGTGGTAGTTGCCTATGATTGGGTGCACTG
EMPV1_35703	GAAAATCCACCTGGAGGCCTATCACACTCAGACAACCCCACTGGCGGAGTACTACAGTAA
EMPV1_35704	AGAAGGTGTCCCAGAAGGCAACATTTTAAAATCAGGCAATCAGTAATCACAACTAAATAC
EMPV1_35705	ATCTATGTGTCTCTGAGTGTGTGCTTGCCTGCATATCTGAATGCGTGTGTATCTGTGTAT
EMPV1_35706	CAGGTCACTTCCCTCCACATCCCCTCAAACCGCCCATCTGGAAAATGGGGATAAATACTA
EMPV1_35707	ATGTGTGCCCCGACCTTGTGTAACCATGGGAACTTCTGAAAGGAGAAAAATGCAAGGGGC
EMPV1_35708	AGAAGCCTGAAGTGCTGCCCATGTGCAACGAATCTTACTGTGCTCAGATTGCTCACGATG
EMPV1_35710	GATCTCATCACATTCCTTTCTGCCTGCTCAGCTCTGGCTCTTTACTCCCTTGCACCAATA
EMPV1_35711	AAGATGTAGTGGGGAGATAGGCCCTGGTCACTGGGCTCCATTCGTATCTTACAAAACACG
EMPV1_35712	GCCTGAGAATGTACACACGAAGACTAACAGATATTTATAGCCTCGGACTCAGGGAAGCTC
EMPV1_35714	CCTGATTTCTCTCTCCACCCAGCCCCTGGAAAGTACCTCTCCACTCTTTGATTCTATGAG
EMPV1_35715	TGCCCCACTTGTCACCATCAAGCCATGTCATCATCAGGCCCTTTGTCCCCTGTTGGTCTT
EMPV1_35716	TATATCATCAGCCTCACCTGAGAACAGGCAGGGAGCGTATCTGAGTTCCCAGCTCCTAAG
EMPV1_35720	GCTCATGGTTCGGCTGGCCAAGAGCCGGGACCTGCTGGTCTCCACAAGTTGGAAAACCAT
EMPV1_35721	CGAAGGATTGTCTTCTGCTGTTGGATGGCCACCTTGTTGATCATCTCCCCACCGTTTAGC
EMPV1_35724	TTTGGTGGTTGAAACGGGAAATGGAGAGCAGTCTCCAGGGTGCAGCGCCTCCTGCTTCCT
EMPV1_35729	TATTAGACCGCTAGCCGGGGAACCTCCAAATGCCTCGGGTGTGGCCCTAAAAAGACAAAA
EMPV1_35730	GTAAATAGACAAAATTCTTCGATCAAATAACAGAGTGAAACAACCAAAGCATCAATGATG
EMPV1_35731	TGTGGATAAGTGTGTCGATGGGAACCAGCTGTCCCCAGTGGTAGAACCCAAGGACAGCCC
EMPV1_35732	ACAACCTCATTGTCGTGGGCCGATACCCAGATCCTAATTTCAAAAGTTGTACCCCTCACG
EMPV1_35733	ACACCAATATCGTCACCCTAACCCGCTTCGTCATGGAGGAGGGCAGGAAGGCCCGCGGCA

EMPV1_35737	CACTCCATCATCCCGAGAGGATCTCTGTCTTTGAAAGTGTTGGCAGAACTGCCCATTATT
EMPV1_35738	AAGACCGATCTGCGCAAGGACAAGTCACTGGTGAAGAAGCTGCGGAAAAAATGGGTTGGAA
EMPV1_35739	GGAGGGCTTGGGGGCCTTCTACCGGAGTTACACCACGCAGCTGACCATGAACATTCCCTT
EMPV1_35741	AATTTGAGCTAGAGCTGCAACCTAGGCCACAGCGGTGGCAATGCCGATCTTTAACCCACT
EMPV1_35742	TGTCTGACTATGGTTTTATTTCAGAGACGGGAAGTGGCCAAAACCGTATTTTGCCTGGTC
EMPV1_35743	TCCTACTGATATTTGTCCTGGAAAGTCAGTCGTGGCAACAAGTACCTAGAGGACTCTTTG
EMPV1_35744	CACCGTGCGCAGTGACCTGAGCACCTTCCACCTGCTGGCCTACAGTGGCTACAAATATGT
EMPV1_35747	GCTAGCCATCGAGACTTGGCATTCGTACAACATGGCAGACATCGACAACAAAGAACAGTC
EMPV1_35750	GAGGAATCAGACCATTTCTTGTCACCCTGCTCTCACTGCAGGTGGGGCTGGTACTTAGTA
EMPV1_35752	GATGATTTCCTACAACGGCAAGCTTTATGTCTTTGGTGGTGTCTGTGTGATCTTGAGGGC
EMPV1_35753	CAGTCCATCAAATACTTCTACTTCCCATAGTCAAGGAGGTGTTCCTCCCCCAGCTGGAAT
EMPV1_35754	AATAATCAAGCCCTGGACTCTGTGAGAAGACCTGTGAGCTTGTGTTGGAACAGCTCTGCC
EMPV1_35755	GCAAGATCTCATTGCTTCATTCCTGGAGATGTTGAACAGGATTTGGCCACCCTGAGCGTC
EMPV1_35756	AATCATTTACCGTCTTATTGAAGATGTGCAGGAGGAACTGAGCAGCAGATTGCCCTGTGC
EMPV1_35758	GTGAGTGCTGTGAATGGCGTGAAAGAGAAGTCACCAACTTCTCGTGTGCTGTCTTGATCT
EMPV1_35759	AAACTTAATATGCCAGCTTTCTGTGCTCTCCAGCTAAGGGGGGCACACAGAGGGTTTGGG
EMPV1_35760	TGGTCTCGTTGGTGATGGCATGTTCGCCGTAATCCTAAGGACCCGTCCCACCCCTGTGAT
EMPV1_35761	TTTTCCCAGAGAGAACGACACGCTCCTCGTCATCATCAGAAACGAAGACATCGCGTCCCA
EMPV1_35763	TGAAAAGCGTAAACAAAAGGCAGTAGATCGCTGAAAATGCAACATAGCTCAGAGTAACAG
EMPV1_35764	AAGAATTGAAAACTGGAACAGAGAAAGATGTTACCCTGCTGGGATCCGTCAGCAGCTCCT
EMPV1_35765	GGGCTTACTTTCTGTGGATCACCTCCTATACCATGTTAAGTGTGTTGCCCTCTGCCCACT
EMPV1_35767	TCTCCTACCTGCACGTTCTCACCCCTCCAGTGCTCAACCCCGTTGTCTACACCGTTAAGA
EMPV1_35770	GTATTTCCAGGGTAATTTTCCTGCAAGAGCTGCTTACCAGGTTGCTGCTTTGCCCAAAGG
EMPV1_35775	GTGGTTTCGCCTTCGTGGAGTTTTATCACTTGCAAGATGCTACCAGCTGGATGGAAGCCA
EMPV1_35777	AGGTGGAAGCAATATGGCAGTGTTACTGGGAATTCCTGGGGCTTGGACAAGGCTTCAGAA
EMPV1_35778	GAGGAAGCCCTGGACCATATGCTGGGAATGCGGGCCACAGATCTCCAAATCCTTTATACT
EMPV1_35779	TTGGAGGCTCAGACTGTTTCCTTAACCTGCACTGGGAAGGCTGTCCACCAATTCCTGAGC
EMPV1_35780	GGTCTCGGGTAAAATATTGTGGCTGTGCCTTCAATTGCATTGTGTGCTGCAATGTGACTG
EMPV1_35781	CTGGGAGAAGACGAGTCCTTGCTAATGAAATTGTATAGCTTCCTCCTAAACGATCCCCCC
EMPV1_35782	TCAAAGGCCACTGAAGCAAATTTCTGACTCCCCAGAGAATCCAACTCTACCTCCGTTCCC
EMPV1_35783	GTTCTGCCCAGGGAATTTGTGGCACCATGGGATCTGGAGTGAAAAATGGAGGACAGGAGA
EMPV1_35784	GCTGTCCCTTGCTCAGGGTTCGCTCAAGACTAGTGAAGTTAGAAGACCACAGATAGGAAG
EMPV1_35786	CCGTTTGCTTCTCTCTCGTCCCCTCTGGACCATAAGTTCTTGAAGGTGTATCTGTGCCTT
EMPV1_35787	GGCTGAAGTACGAAGACCAAAGAGGATCTGAAGTGATACATGGGAGGTCTCTCACACTGC

EMPV1_35788	AGAGCTCCCGCCAGGAGGTGGGTCTGAAGTACTTGTGCTTTGCTGGCCCTTCCTAGGATG
EMPV1_35790	ATTCTTGGTGGTTTTTAGCCACTCCCTGCGGTGTCAGACGAGAACCACGGCTCCTAACAA
EMPV1_35791	TTATTACAGTGGTGGCTACTATCCCGCACAGGACCCGGCTCTGGTCCCCCCCCAGGAAAT
EMPV1_35793	GTGAAGAATTATTTCCAGATGACAGACCAGGAGTGTATCCAAAATCCCTGGCCTTGGAGG
EMPV1_35794	GTATGAGCACTATAGTGGTGGTGAAAGTCAGGATTCCACTTCATCTTCCGAGTCTTTGGG
EMPV1_35795	TAATCTCAGACTCAAAGCGCTCTTCTGACTCTCTCCTCGGCGCCCACCAGGCCGAGCTCG
EMPV1_35796	TGTGGTGACAGCTTTTGTGGGAAGACATATTCAATGGCACGTGGTAAAGAGAAGGGCGCC
EMPV1_35797	TATTTCTACCCCTACACATGTAGTGTTTTCCTCTTCTACTGCTTCTACTACTGTGGGGGG
EMPV1_35798	TGGGAAAGCCTTCAGCCGGAGCACCTATTTCATCAAGCATCAGAAGATCCACACAGGCGA
EMPV1_35799	TATTTGCAAGCAAAGGACGATAAAGGACTGGCAGGAGAGCTGTGCTGAAGAAGGAGACCC
EMPV1_35800	CGTAGGCTGTCTATCTCAACTGAGAGTCAGCCCTATGGTTGTCTTCCCCTGGGTATAGAT
EMPV1_35802	TCACCCAGTATGCGATCATCATGTTGGACGAGGCCCACGAGAGAACAATTCACACTGACG
EMPV1_35804	CCCCAGTGTATGCCAACTGCAACAAAATGAAATCTGTGCCAGTATGTTTATTGCACTTTC
EMPV1_35807	CTGTTCAGATTTCTTTATGTGCTCTGAATAAACCGTGAGGTTGCCCCTGCTGAGCCCACC
EMPV1_35809	GTGGAAATCACCAAATGGCACCATCCGAAATATCCTGGGTGGCACTGTCTTTAGGGAAGC
EMPV1_35810	GCAAATGCCAAGATACATACTTTAGGGGGCAGAAAGAGGAGCCATGTTTCACGTGGGTGC
EMPV1_35814	AGTGGTCTCCAGCGCTGACCGTGTCCAAAGTTCTCTTATCCATCTGTTCTCTGCTCTGTG
EMPV1_35817	CGAGGAACCCCATAGAAGCTTGGCACGCAGAGGCCCTGGCAGGCCGCGGAAACGGACCCA
EMPV1_35819	AGGGGCCTGGCTTCCTGAACTATTTGCTGAAACCCTAGTTGAGAGGCTTTCCCATATTGC
EMPV1_35820	TAACTAGAGAGGCGGTCTTTGCTCTGGGCTGGATCTGATGACATTGCGCTTGGAATTCTG
EMPV1_35822	GATATCACAGAAGTACCTGACTTCAACAAAATGTATGAATTATATGATCCGTGTACTGTC
EMPV1_35823	ATGATGCACGGTGCTACACCTACCACTGTGTCTGCAGCAACAACACCTGCCACCAGCGTT
EMPV1_35824	ATGTCTAGCAATGTATGTGTTGGCATTGTTGCTGCTGCATGGGGAACTGGCTTCCTCCAC
EMPV1_35828	TTTTCCTAAGCCGATGGGAGGTTTCATACAGAAGGAATCAGGGACGTGAGTTGTGAGCCC
EMPV1_35832	TCTAGAGTCTGGGTGCTGGGGAACAGAACTTCCAAGAACAAGCAGCCTTCGCAGAGGACG
EMPV1_35833	CTCCCACTGCCCTAAGCCAGACTCATTCTATTAAGCAGAGTCAGAGTAGAGCCCTTCCAA
EMPV1_35834	CCTTGATCTGCGGAGCCACTAAGGAACTCCAGATTCTTATGTTTGGACATAACTCTGGTG
EMPV1_35838	GGAAATAAAGCTTCTGCACATCACTTGAATGGTTAGATGAGTCCAGTAGACTATGACAAG
EMPV1_35840	TTGGTGCTGGATGCCCTATGTCTATCTGAATTCAGGGAATGTTGACTCAGGCATCCTCCC
EMPV1_35844	TGTCTTTGGACTGGAAGAAGAAAGAAGCAGAGTTTGGCGGTGAATGTCCTACTGACCCCC
EMPV1_35847	ATGCGCTGCCCTCAAGGGGTGTGTAGCACAGAACGTAGAATCCATCACATGAAAACAACG
EMPV1_35849	CTCTGACAAGGGGCCACAGTACAAATATCCAATGCTCCTGATGAAGGGCAAGTCCACATG
EMPV1_35850	AATTCACTTTCACTGCCGGAGACGGAGATGGAGACGGTGCGATGCGGATGCTTTTGACAA
EMPV1_35851	TCTGTGAACTGATCAACAAGAGCGGGGAGCTCCTTGCGAAGAACTTATCCCACCTGGACA

EMPV1_35852	TCTCCAATCCAGCCTACCGTCTCCTTCTGGCCACTTACGCCCGCCCCCCTCGAGGCCCGG
EMPV1_35855	AGACTGGAGGGAAGAAAAAGTCACAGGAAAACCGGCTCTTGAGAAAGCTCATCAAACCAG
EMPV1_35858	AAATGTAAAATCCCACCCAACTCGATCCCCTGCCTTCCTCTCCCTCCCACCTTAGATGAG
EMPV1_35859	ACCAACTGCTATAAAAAATTTGTATCAAAATGAGAAGCCACAGAAATGGAGAGTGGAAAT
EMPV1_35862	GCAACTGCTGCTACAAAAAAGATAACTGAATCAGTTGCTGAAACAGCACAAACAATAAAG
EMPV1_35863	TGTCATAGGTTCTGAGAAGTAGGATATGGATGTATCTTTGGGGAGCCACCATGCAGCTCA
EMPV1_35864	CCGTCCTGGTCACAATCAACCCTTACCAAGGTACAAAGGGGTATGTAGGAGGGAATCTAG
EMPV1_35865	GGACAGAAAGGTCTTTGGATTTCTCCCTCGGCTATCCATGGCTCTTCTCCTTGCTCCAAT
EMPV1_35867	AGAAGGCCTGTCCCTGAGCATCTCAGAGCGCTGCCAGGTCGCAGAGGAGTGGGTGACAAA
EMPV1_35868	TGCCACCAGAGTGGTGTCTGACAGCCCCAACAGGCTGCAAGTTTTAGCAATATTTGAACC
EMPV1_35874	CTACGAGAACTCAATTTCTTGCATCTGCTGAATCTCAAAAACCTCAGCTGAAAAAAGGGA
EMPV1_35876	AGGATAACAGCTATCCGTTGGTCTTAGGAACCAAAAAATTGGTGCAACTCCAAATAAAAG
EMPV1_35877	ATGCGGCTTGGATCTGGCGTGGCTGTGGTGTAGACTGGCAGCTGTAGCTTTGATTCAGTT
EMPV1_35878	GGGAAATTGCAGGAACTGAAGGGATTTTCTGGGTCCATGTCCCTTTTTCTACATCCGACC
EMPV1_35880	TGACGTGAAGAAGAAAGGGCGGCTCGACCCTTATGCGTACATCCCCCTCAACAGAACCAA
EMPV1_35881	AAAAGGCCAAGGTGCAGCAAGCTGAGGCTCTGCCTGGACCTTCGCTGGACCAGTGGCACA
EMPV1_35882	GCAGTGCTGGGCCGGGGTCACTTTGGGAAGGTGCTGCTCTCCGAATTCCGGCCCAGCGGG
EMPV1_35885	CAAGCTGCCCAAGATCTTCCACGTCAACTGGTTCCGGAAGGACAAGGACGGCAGGTTCCT
EMPV1_35886	GAAACTCTTTAAGCATGTGGCCGATTACCTGCCCAATTATGCAAGGCCCCGGTTTCTAAG
EMPV1_35890	CTAGCACAGCAGGAGTCGGTCACCACATTCATCCCAGTCTAGAAATCAGTCTCCTCAGAG
EMPV1_35891	AGGTCTTTGCTGAGAGTGCAGGGCAGCTCCTTTCCTACAGGTGTTGATCCAAAGAGCACT
EMPV1_35892	TCAGTAAACTCCCTCATTCACACAGCAATCTTGATGCACTTCCCTTTCTGTGGGCACCGG
EMPV1_35893	AGGTTCCTGAATGTGTGCTGACTCATCTGCAAAACCACAGAACGGATGAGGAGCCAGGCA
EMPV1_35894	GGGGGCAAAATCGCCCTGATTGGGTACCACTGAATTAGAATATGGTGGGTGAATCCAGAG
EMPV1_35895	TCGAGTGCCACCGCCTTCTGGAGCTGAGTGAGGAGCTGGTGGAGAGCTGGTGGTTCCACA
EMPV1_35897	CACAGGAATTAGAAGATGGAGAAGTTATGAATGGTGTGCAGACAGAACTACTGACATCGC
EMPV1_35901	TCCATCCTGCTCCTTCCCCCTCCACCTTGGCAACCATACATCTGATCTCCATGTCCATGA
EMPV1_35903	CCGGCAATGGATGTCTCTGCGGTGATGATCCTTCAGAAAAGGATTCCTGCGTTTAAAAAC
EMPV1_35904	CTCTCTGTAGTGCAACAATGACTATCCTCAGCAGCCTGTCACGTGCAAGAAATCAGCCTG
EMPV1_35906	GACCCTTTGTTTAGCCAAATCGAAATCACAGTTCTTAACACCCTTGGTGTGGTTGTTCTC
EMPV1_35907	CCACTGTTCATTTCCCTCCCCTACCCACACTTTCGTACATAGGACCCTCATTAAATCCCC
EMPV1_35908	GATTTTCTTTGTGCTTTATTCGTGCGGGAGGGTTGGGGTTGCAGTGGGTTCGGCGGGTGT
EMPV1_35909	ACCCCGAAACCAGACGAGCTACCCATGAGCAGTTTAAAAGAACCAACTCATCTATGTGGC
EMPV1_35910	ACAGATTGCCTCCGAGAAAGAAGACATTGAGCGCCTGAGAGCCGAGATAGCCGAGATTCA

EMPV1_35911	GACCTGAGCAGAGAAACCCAGCTTGTAGTTGAGGAGTTGCTGAAGCCTCTGTTGTGAGAT
EMPV1_35912	CTTTTGAAACCTTGTGTGGGATTGATGGTGGTGCCGAGGCATGAAAGGCTAGTATGAGCG
EMPV1_35913	CACTCTAATGCAATAAGCTATAGTTTAAAGCACATTGAATGGACATGCAGAACACTTAGG
EMPV1_35914	GCATCCAGGACCCTCTGATTGAATTTGCCCAGGTGTGCAGCTCTGATGAAGACATCCTGT
EMPV1_35916	CAGGCAGCACCCCTGCTGCTTCTGTGCCACTCGCTCGGCCACCTTGCGGAGCCTCTCCTC
EMPV1_35918	TGGGAGATGTAGACAGGCTCAATGACCCAGGGAATCTCATTGACAAAGGAAATGGCTCCG
EMPV1_35922	ACTCCTCTATTCATCAACCATGTCACCCTCTCTCTGTGGTCGGATGATGCTGGGGTCCTA
EMPV1_35924	TGAGACTCAGACTCTGGAAATCAGTCCCAGGGAGAGAACTGGGGATGCATGAGGGGCTAA
EMPV1_35925	CCAGCTCCTGAAAGCAACATCTGCTGTCATTATTGCAAACACATGATCCCAGGAAATAAG
EMPV1_35926	ACTCATCCTGGCCGACGCGCTCTGCTACGCGCACACTTTTAACCCAAAGGTCATCATCAA
EMPV1_35927	AACCAAAGGAAGAAGTTGTTCTCAAAAGCGTTCTAAGAAAGAGACCTGAAGAAGAAGAAC
EMPV1_35929	TGTCTATAGCCATCCTACCCTGAATGTGCCTGATCTCATCTGATCTCAGAAGCCCAGCAG
EMPV1_35932	AATCGTTTTCCGTCCCTCCTCGAAAATGCTATCAGTCTGCGTGAGGTGGAACTGGCTGAT
EMPV1_35933	GGGAAGTTTGGTGTCATTTGCTTGGAAGACCTCATTCATGAAATTGCCTTCCCTGGGAAG
EMPV1_35934	GGCGCAAACCGGGAAAGGCGGAAAGACCCAGGGCAGATCGGCCAAGTCCTCACGGCCTGA
EMPV1_35935	TGCTCTCCTCACCACGGGCTTGGACTTTGTCCTCATCTCCCTGTCGTACTGGTTAATTCT
EMPV1_35939	ATTTCTGGTCATCCCTGAGTGCCCAGCACCCCTCTTAGGTAGAGACTTACTGACCAAGAT
EMPV1_35940	CCAGACGCAAGTGCCAGGAAACTTCAGCACACGCTCACAAGGTGACAGTACAAACAGTGT
EMPV1_35943	CTCATTGGTAAACAGAAGGGAATAGATGGCAGAGGGCAGTGAGTTGTAGAAATGTGGGAG
EMPV1_35944	CAGCTTGCCTTTGGAGCCTTTTTGGGAGGCACCGTGAGCTCGGTTATTCACACCACTAAT
EMPV1_35946	CGACCAGGCAGGAATCCACCAAAACAGGAGGGCCAACGACATACAAACATAGGATTCAGA
EMPV1_35947	AAGCAAGCAAGCAAGCAAACGAGACTGCCAATACCAAATGGGAGGAGGGAGAGAGATGGC
EMPV1_35948	GGCTTCACCCAGAAATTCACATTTAACTGGTCCGGGGTACAGCCTGGGTACTGGGACTTT
EMPV1_35949	CCAAAAAGAAGGAAGCCCCACCAGCAAAAGTGCCTGAGATTCCTAAGAAGCCAGAAGAGA
EMPV1_35950	TCTTCCCAGAGGGGTCACAACTCCCCGCCCCCGCCACCTGGAATTATTTTTGTAATAGAT
EMPV1_35951	TGGCCTGTCCCCACCGCTGATGTGAACTCTCCTGCAGCCTCTGTCTACCAGTTTCTATGT
EMPV1_35952	CTCCCAAAGGCCTGCTCCCTTACATGCTCTCTCCCACAACTGAACCCTACCTATCTAAAA
EMPV1_35953	GGGCAGCAGCAAGTGGGTAGCCAGGATGGGGGTGTTCCTGGCTTCAGCCTTCTTCCACGA
EMPV1_35954	TGGCTAACCAAGGTTTCTGCCGTTCCTATTTCTCTGGGCTTAAGTCAGGGCGGCCATTGT
EMPV1_35955	CAAAGAGAGAAATGTGCCGAGCTGTGGAAGACGAGGGCTATTGGGTTCATGAAAGTGTGC
EMPV1_35956	AGAACACACCTCCCCCTACACCAACAGCTCCATCATCTGGTTCACTGCACACAGGCAAAA
EMPV1_35958	AGCATCCCCGTGCACATCAACAACATCTGCCAGCGCAACTACGTGGTCGTGGAGAGTGGG
EMPV1_35960	GGAGACAGGGGGATGAAAGGAGAAAAAGGAGATACTTACGAAGGCTTCTCTGTGTCAGGG
EMPV1_35962	AGAGGCAGCTCTGTGGAGCAGCACACTTTGTTTTTCAGAATCCGGTCACTTCTGTGCTCA

EMPV1_35963 CATTCTTTGCTCTTTCCACTCATCCACTTTCCTCCAAGGGCATCTGAGTTCAAGGTCATC
EMPV1_35964 AGCCCAGAGACCCAGCAACCTGAGACAAAACGCATTAAACGTGCCCCTGCGAGCAGTGCA
EMPV1_35965 CAGTATGGTACTGGAAAACAATAGGAACATAGACCAATGGAACAGAATAGAGAGCCCAGA
EMPV1_35968 CATTTCTCTGCAGCTTCTTCGTGGAGACATGGAACATATTAGGAGAGAAAATCCCATGAT
EMPV1_35969 TTGCAGCATCAAGCAAGTGGCTGGAGGGTATACGAAAATGGTATTATAATGCCGCCGGGT
EMPV1_35971 GCCTGCCCCTCTCTGCACTCACTTTTTAACTGATCTTTTTACTCTGCTGTCTGCTTGCTC
EMPV1_35972 CAAGATAAAGAAGATGAGATTCAGGATGGACAGTCCTGGGATGATGTTGTGGTCCAGGAC
EMPV1_35975 CACATCTCAAGAGAGAGCATCAGAGGTTTCACAGCTCACCTCGACAAGAAAGAGGCATCC
EMPV1_35976 CACACAGGACCAGCGGGTGGTTTCCTAACATGCTCTAAAAGACCAGAAGGGAGGCAAATT
EMPV1_35977 CACGGTGAGTAGAGTTATAGGTAAAAGCGGCCATTTTCTCTCGTTACCACTTTCCTGTGC
EMPV1_35978 ATGAGCAGGGCACATGTAATTAAATATACTAGGGGTTCCCTGGTGGCCTAGCAGTTAAGG
EMPV1_35979 TGCTGTATGGGCAGCCTCTGTTGGTTTCTGTCCCAAAGCACAAGCTCACCCTCGAATTTT
EMPV1_35981 CCCTCAGCCCAGGGAGTTCAGCTTTTTCAGGTTTTATCCTTGCCAGAGAAGAGGTGAAGT
EMPV1_35983 AGCCTTCGCACCCTTTCATCCGTGGGTGGGGATTTGTTGCATTACTTGTGGTGTGTATAA
EMPV1_35985 GATGACATGGCAAGAAATTGTGCAAAATCCTTCTGTGAGAATTTCCTCAAAGCGGGAGGT
EMPV1_35992 CCCAGGCATTTGAATTCTTTCAGTGGGATGGTTGGCCCAGATATTTAGCTCCCCATATTA
EMPV1_35993 TTGAGGTCACTGCTTCGGTTCCGTCTTGGGGGCCTGGCCTGCCAGACTGTCTTGGTTTTT
EMPV1_35994 GTTTACAGATATTAACGACTCTTCAGGAAAGCTCGACTTCAGCCGCCTCTCCCCGTCGCA
EMPV1_35995 CATGTAGCAGTCACATAGACCAGGATATTTTAGAAAGCATTCATCTGCCCCCAGGAGAGC
EMPV1_35997 AACGGCAGAATGAAGGAAAGCCAGAAAGAGGCAAAGAGAGACCTGTTCAGTCTTTGAAAA
EMPV1_35999 CCTAGGCCAGACCCAGCAGTCTGTGAACTTTCTGTCACGCAGCTCAGATATCTTGTTGTA
EMPV1_36000 TCAGAAGGACAAACACATCTTGGGCACCATCAGGCTCAAGTCTACTCCCTGCCCCAAGTT
EMPV1_36001 ACAGACTCATAGACATGGAGAACAGATTTACAGTTGCCAAGGGGGAGATGGCAGGAGGAG
EMPV1_36002 AAGTTGGTGAGGTAACATACGTGGAGCTCTTAATGGACGCTGAAGGAAAGTCAAGGGGAT
EMPV1_36003 TTCCTGTGAACAAGTATAAGAGCCTTGATGAAGATTTTTCCTCTTTGCCCTTGTTCTTAA
EMPV1_36005 TCCGGGAAGGAGAGTGGTTTCACCAAGTTTATGATGCATAGACAGCTCCTCCTCTCCCTT
EMPV1_36007 ACGCTCTGGAGCAGATCCTGCAGAGCACAGCGGGGAAGTACTGCATGGGAGATGAGGTGT
EMPV1_36011 ATCCCGTCTGAGGATCCCCGAGTTCATCACGGGTTTCTGTGTGTGGCCAAGTTGAAGCTT
EMPV1_36012 AGAGAAGCTGAGGGGGCGGTAGCGGCAGCGGCGATTCGCGTGCCTGTGCTCAGCTCCTCA
EMPV1_36014 GGAAGCCTCCACCTCCAACCCCTACATCTACTACAACATGCAGCTCCAAGCCATCATCAA
EMPV1_36018 CGCTTCTGCTGCGCCACAAAGGGAAGTCCCAAACTACTTATTTACTTTTATCTTGATCAC
EMPV1_36019 GGATCAACCAAAGAGAAACACGTCTGCACAGAATCCAAGAAGAATCACAGAGCCGGCAGG
EMPV1_36020 AAGATGCCTTCTGGAGAACCCTGAGGCACGAATAAGAAACTGATGGACTCACCGCAAGTC
EMPV1_36022 GGGGATGTTTCTGTTGTTTTCCTAACCAAGCGGTCTCACCCCCTACTGTGTATATGCAGC

EMPV1_36023	ATTCCCAGAATAGCACAGAATCCTCAGAAAGTTGCATATGAAAAGAAGCAGAATGAACAG
EMPV1_36024	AAGAAAGCCAGTTCCATCAGCCTCACCATCACAGCCTCACAACTCGAGGACTCAGCAGTA
EMPV1_36025	TGCATTCAGACAGAGGCAGAGGGGAACGTGCACGGATGCTCCCTTTCTGCCAGGAAATGC
EMPV1_36026	CCTGGTCTCCTGCTTCCAACAGCTGATGGTGGACACCTGCTCCTGCGGCTACTACCTCTA
EMPV1_36027	AGGCTGTGGGGGGTCCCTGAGCCAGACATTTCCATGTATGTGGAAGCATTTTTGCATTCA
EMPV1_36028	GGCATCTCAAATGTCATTCAGAATGGCCTCCGCCACACGTTTGGAAATTCAGGTGGAGAG
EMPV1_36029	CGCGTGTGCACACTCACCTGTACACAATCACGTGTACACACCTGTGCACATGCGTACACA
EMPV1_36030	AGGATCCAGTGTTCACTGCTTGTAGCTTGGGTTATTGCTGTGTGTGGATTTGACCCCTGG
EMPV1_36031	CCTTTGGAATCTGTACCTGAAGGGATAATCTCGAGGACTCTAAAGTGTCTGTGTAACCCT
EMPV1_36032	GCCTATGTGGAAAAAATGAATCACACGTTCCAGGCCAGCTCAGAGATATTCACCAGGAGCA
EMPV1_36033	CCACATCACAGGGGTCCTCCATGCAATTTCCAGCAGAATAACCTGAATAGTGAGAGTGGG
EMPV1_36034	GCTCTCTGAAATCAGCTTAAATGCATGGCTCGCTACTCAAAGGTTCCAGAGAACTCCGCG
EMPV1_36035	CATCTGTGAGTTCTACCTCAGTTGCCTCCCAAATGAAGACTCGTTCCCCTTTGGAGTCTC
EMPV1_36038	GTACTGAGTCACAAGTCCGCTAGTACTCTCGATGGTTTGAAGACACACAGATTTTTTTTT
EMPV1_36039	CCTTCTGCAGTATGACGTCTGAGCCCTTGTCTTGGTGGAAGTGAGAGACACAGAGAGGTA
EMPV1_36040	GGAGGGCTTTGGAGCAGAAACTCCTACTGGCTGGTGGGAACATAAATTGCTATAGTTTAG
EMPV1_36041	GGCCGCGGATTCCCGCAGGGACCGATTCATTCAGAGGATCTTTAAGCTAAGTACAATTGA
EMPV1_36042	TTCCAGTATGCACAGGAGGTTTGGGCAGAGTGAACCCCTATCCGTCTTTCATGGTCTTTC
EMPV1_36043	GAGACTTACACATTGGGATTCATGGTTACTGCTAATAGTGGCTTTATTTCTGTGGCATCC
EMPV1_36044	CTCATGTGACTCTCCTATTCCATCTGTTTCTTCGGGACCATCTTCACCTCTTTCAGCCAC
EMPV1_36046	TTGGGTCTCAATCAGCATTACCCGAGCTTTAAACCGCCTCCATTTCAGTACCATCACCGC
EMPV1_36047	CCAGGATCTAGTCTGTCTGGAGATGAAAGTGAGGGAAAGATGCTGGTACCTAGAAGCTCC
EMPV1_36049	GGTTAGAACGGGTTACTCAGGGGAGTCAGTTGGAAGGGGCGACAACCTCCCCACCAAAGT
EMPV1_36051	AGAAATATCCTGACTCACAAGGACACGACAGGAAATCTGGAGAACCCAGTGGTAGAGTCC
EMPV1_36055	GCCATTGACCACTTTTACTGTGACACTCGCCCACTTCGGAGAATATCTTGTTCTGATGTC
EMPV1_36056	TCCCTGAGACCCTTTAACCTGTGAGGACATCCAGGGTCACAGGTGAGGTTCTTGGGAGCC
EMPV1_36058	ATGTGCACCACCCTCTTCCTGCTCAGCACCTTGGCCATGCTCTGGCGCCGCCGATTCGCC
EMPV1_36059	GGCAGCAGCTCCGAGACCAACTGGATGAAGTGGAGAAAGAAACTAGATCGAAACTCCAGG
EMPV1_36060	GATGCAGATGTGAAAAGCAGTAGCTCCGTATCGAGCACATTGACAAATGGAGGTGTCCGC
EMPV1_36061	AGTGCTCTACAGCTGGCAAAGCGTTTAGCCCCAGACCAGATACCGTTTATATCAAAAGAA
EMPV1_36062	TCCTTAGTCCTCACCCCTTCCTGCTTCGGTGGCTGCCTTACTGCAGAACCAATGTCCTTT
EMPV1_36063	CAGCGACACTGCTGCTCAATCCTCCAGCATCCTTAGATGAACTTTAACCTGTTTGGGCAG
EMPV1_36064	GTCCCTCAAGGGAAAAGCTTAAACCATAACAGAGGAGCCTTTATCTTCCTGAGGCTCCTT
EMPV1_36069	GGGGTGGAATTTGGGAGTCTTCAGAGTGGGGTTGCTTTTATTTTGCATGTGGGAGATGTG

EMPV1_36070	GCACTCATTGACTCAACCGTAGAGACTGGCCTCATATTGAAACTGTCCTATTGTGAGCCC
EMPV1_36071	TCTGGTGCCTAACGTGGCCTTGGACTTGACAGTCACCAGGGTGAAAGATCCAAGGCTCAA
EMPV1_36072	AAACAGCAAGAGCTACGACTTGAACTAGAGCGGAAAGTAAGGCAAGAGCTGGAGCATCGG
EMPV1_36073	GCACCTCTTCGGTTTCGTGGCCTTCATGCTGTTCATGTTTTGGGTAGGAGAGATTTACCC
EMPV1_36074	CAGCTGTGGATTATATTTGACCAGAACAAAAGACAGATGGGTTCCGGCGATGCCTATCCT
EMPV1_36078	TCCTCAGAGTATAAGTACAATAAGCCAGTCTTCCTCAGGAACGCCAGGCAGTGGACAGAG
EMPV1_36079	TGGCATTGCAAGCGTTGCTTTAACAGCTGGAGCAATAAAAAGGAAGAAGCCAGCCTTCTT
EMPV1_36080	TGTCGGAAATTCACAAGATGGAGGAAGTTTCTGGAACCAAGGGGCGCCAGGAGAGAAATG
EMPV1_36081	TCTTGCCTAATTGCTCTGGCTAGGACTTCCAGTACAGGCATACCTTGGAGATATTACAGG
EMPV1_36082	GTCTAGGGGAACTGGTTGGAGGGAAGGTGAAGTTCAATGATGCTCTTGATTTCAATCCCC
EMPV1_36084	GAGCATGAGTTTTCCTAATAAAGCGGTCTTGCTAACGTCATAAGTTAGCTGGTGGATATC
EMPV1_36085	GGTTATCTGTCCTGGAGAAGGACTTTGAGGAGTTACCAACCTTAACTCTGCTACTCAGAG
EMPV1_36087	CTACATCTGCTCCAACCAGATCATGGGCTGGGGTGAGAAAGCCATTGAGATCCGCTCGGT
EMPV1_36090	AGAGCTGGAGAAAGATGCTGAAGGCTCCTAGGGAGCCGTCGGAAGGCCACATGACCAAGA
EMPV1_36092	GAACCTAACAGGTCTGGTATCTTCAGAAAATTCATCCAAGGCAACAGATGAGACGGGTGT
EMPV1_36094	AAAGCCTCACCTTGCTGGCATCTCGCTGGAGCAGCGACCTCACCAGCTGTCTCACGTCTA
EMPV1_36095	TGAGAGAGAACTCTGCCACACCTAGTGGGACTTGTGCGGAATTTACCAGAAACCCAGACA
EMPV1_36097	GACTGCAGACACAGGTGTAACTTTATCCTTCCTCGTTGTGGGGACCTAGGAGACAAGAGA
EMPV1_36098	CTTTACTGGCCCTCTCTTGCATCCCTGCCCCTGGAATTGAGCTCTCCTGTTCCATATTGA
EMPV1_36099	AGTTGGGTGTTCTGGTTTTACTGTGGGGTGATGGAGAGCACTGAGGCTTTGCCGTTAGCT
EMPV1_36100	GCAGCAGGATGCTTAAAGCCCAAGTTCCCCTTCTTAAGTGTAAAGAGATCTGCGCTACTC
EMPV1_36101	TGGAGACCCCAGTTCCTCTCTCACATCCTGTCCGGAGAGGCGCCTCCACTCCGGGAATAT
EMPV1_36103	GGAAGAAGCCAACTCTAAGCCACTAACATACAGGAAGGTTGTTTCCTTAAACAAGAGTCA
EMPV1_36104	CAGGTAACAGGCCAGGAAGTGATGGAGAGTCACTATTGGTGGAGATTCTGGTTTCCACGT
EMPV1_36107	TTGGCATTCCTTGTAATATTAAAATCACAGACACACCACCACTGCTCTTTGAGGTGTCCT
EMPV1_36108	CGACACGAGCCATGGTCTTTGCCTTGCTTTAAGTTGTGGGGAATTCCTGAAAAGTGGTCG
EMPV1_36109	CACCACCTCCAGTAAGAAAGTGGAGAGGGAAGACTGTGCTATCAGAAATGGCTTGTGCAG
EMPV1_36110	ATAAAGTAAATGTAACCCTTGGGGACTGACCATAAATGAGTATGTGGGACCCTCTGCGGG
EMPV1_36111	TGGTGTTCTCTCCTCTACCACCTACCTCTTCCCTGCCCTGGTGCGGGCAAGGAGAGGGTG
EMPV1_36112	GGGACTGAACCCAAGACCCCATAGATACTAGTCATAGATACTGAGCCAGGAGAGGAACTC
EMPV1_36113	TTCCAGCTGCCATTCTGTGCAACCTACAAGATTGACCATTTCTTCTGTGACCTGGCTCCC
EMPV1_36114	AAGCTGCATCTGTGACCTACACAGCATCTTGCATCTACATCGTATCTTGCAGCAATGCTG
EMPV1_36116	TACTTTGATTGATGTCACATGCCTTGCAGTTCCTCCGTCGTGTTCTCAGGGTTGAGTGGC
EMPV1_36117	TTTCGGGGTCGTGCGCTGCCCACAGCCTGCTCATCACCACGGAAGGGAAGCTGTGGAGCT

EMPV1_36118	GTCTGTGTCCACCAAAGACTCACTGATTGCCGGAGAGGCCTTGTCTCTTCTTGTGACGTG
EMPV1_36124	TTTCTGAAGTGCTCCTTTTATTATTTTCATATTCAAGTTACTATCTCCCCTGGCGTTTCT
EMPV1_36125	CCCCCAGGTTATAATGAAATCCTAAGCGTATCCATATAGCTTCAAAGTGTAGCCTTCACC
EMPV1_36126	GGGATGGATACGCCCCATCATGTATCCCCATGCAGAGTATCAGGCAGAACCTACCATCTA
EMPV1_36127	ATGCCGCCTAAGTTCAACCCCAACGAGATCAAAGCCATGTACCTGAGATGCACCAGTGGA
EMPV1_36128	GAAGCATTTCCAGAGTGCCAATTTAATAACCTGACTAGCTTTTTAGTATAACCATCCGCC
EMPV1_36132	CCCTATGTAAAGCGTTCCGTCAAAGTCAAGAGCCTGAGCGACATGACAGCAAAGGAGAAG
EMPV1_36133	TGCCAATATGCCAGCTTGGACATCAGTGTTTGTTGGATCTGTTTGACCAATTTGCACCGG
EMPV1_36134	CGAAATTTTGAAGCAGAAGCTAAGTTCACTCACATCTGCAAGATGGAGGCAGCTGCAGTG
EMPV1_36135	ACGTGATGTGGGAATATAAATGGGAGAACACGGGGGATGCTGAGCTGTACGGGCCTTTCA
EMPV1_36136	CAGTTCACTGAGAAATTACAAGCCGAAGTGAAAGCTTCCCAGGAGCAACTTGAAGCCCAT
EMPV1_36137	AAGGGATGTTACAGACCAGCCTTCCTGCTTCCCACCATGCCTTCATCTTGACAGAAGGAG
EMPV1_36139	CCTCCAGTCTTTCCTTCCTCAGCTGTAGAATAGATGATGACATATACCTACCACTGATTG
EMPV1_36140	GGAAGGTTTGCCTCTCTCAGGCTTAGATATAGTCTCTTAGTCTGACATCTGATAAAGAGT
EMPV1_36141	AAGAGCGACCCTGCAGCCTCCCTCGGGCTCAGCCGCCTCCAGCCTGGAGCGCCAAGGTCT
EMPV1_36144	TCCTGGAAAGAGAAGTGCAGTTGTAATGGGAAGAGGGAGACGGCGAGATGACATAGCTCG
EMPV1_36149	CTAGAATCGTCGAGTAGGCATGTACTTTCTTCTGAACGCGGTCTCCGCCAAGGTGTTGGA
EMPV1_36151	ATTCCTGGGGACTCACCGATCTTTTCGACCCACTCAAAGCTAACTTGAAGGGAATTTCAG
EMPV1_36154	AACGCAATCCAGGCGCTTTCCTGGAAGGACTATAGGCAGTGCTGGGGAATCAGGAATATT
EMPV1_36155	GTGGTACCGAGCCCCTGAGCTCCTGTATGGTGCCCGCCAGTACAACCAGGGTGTTGACCT
EMPV1_36157	CCTAGAAGTAGAATTGCTAGATCATATGTGGTAAGGGCTCCATTTCTCCACATCTTTGGA
EMPV1_36158	AGCCTCTCTCAGGAGATCCACCGCCTCTCCGAGAGCATAGCCCAGATATACCGCGGTTCT
EMPV1_36160	GGAAACTACACTCTGAGAGTTGATTGTACCCCCCTGATGTACAGCCTGGTGTACAACCTA
EMPV1_36165	CTCCTATGTGTACCAGGCCCTGGCCTTCAACTTGGATCGCGAGGATATGGCCTTGAAGCC
EMPV1_36166	GCCATGGGCCTTTACTGTACGTGCTGGATCTTACCAAGGTGATGTATTCTTCTGCTTTGC
EMPV1_36169	CCAAAGAAACCACAACCGCCAAGAGCAGAGTGCCTTCTGCTGGGGATGTGGAGAGAGCCA
EMPV1_36170	ACAGATTCAACTTCACTCCCTCACACTACCCTCCCCCTCCAGACCGTCACTTTCAAGGAT
EMPV1_36171	CAAGAAAATGAAAATATCTGCAGTAGTTTTTAAATTACAGCTATACCTTGAAGGGGGGGA
EMPV1_36172	GGTGTTGTTCACTAACAGGTTCCACAGGTAGAGCTGTATGATCTTGAGGAGACCCCAGTG
EMPV1_36173	TTTCCCAGGCTCTCCTGACGCAGGCTGCTTTCCTGAGCTGAATGTGTCCCTTTCTGAGGG
EMPV1_36174	TAAAAGAGGAGAAGGTGGAGGAGAGAGACCTGCTCTCTGACCTGCAGGACGTCAGCGACA
EMPV1_36178	AATGAATATTTTGGTAAGGGTTCTTACTCCTGTGCTGGGTTTCTTTCTCCAGATGTTGCC
EMPV1_36179	CTGTGCTATACAGCAGGTCCCCACTGACTGATCTAAATTTCATTTTGACGAAGGTATTAC
EMPV1_36180	TTCAGTTGCTCAGATGTAACGAGAAACTCAGCTATGCTGCCCTAAGCAGCCTGCAGACCT

EMPV1_36182	CCTGCAACTTTACAAAATTCGTTGATGAGATCCAAACAGTTTTCTGGTAGCATCTTAGGA
EMPV1_36183	CAAACTTGATCACCCTAACTTCGTATTTTTTGATACGCACTTTGCAGGATGACCTCAGGG
EMPV1_36184	GGCATGGCAGGAGATTCAGGGTACAGAACCACCCAAGCCAGTCTATACAACCTACAGTTT
EMPV1_36186	CTGCAGTGGCAGGAAACGCACCACCCAACCTCAACATGTCACCTCGGGGAGGAGGAGCCA
EMPV1_36187	CTGTTCTCCAAAGTACACTATGACTTATAATTACGACACTGCCGGTGGCTCTCAGAGGTC
EMPV1_36188	AACGTGTCAGAGGCAGATACAGAAAGGAGAAAGGCTTTCCTGACTAGGACATGGAGCTCC
EMPV1_36190	ATCAATCACTGCCACAGCCACTCCAAGGCCACAGAGAACAGGGCAAAGGAGCAAGTCTAT
EMPV1_36192	TCTGAAAACAGCAGTCTGCTCCCTTGTCCTGCTACCTCCCCAAACTCGCCTGCTGGCGTT
EMPV1_36193	AAAACCTTAAACCCTTTTCCCCTCTGTTGCCACTATTGAAAGCCACTGCTGTGGAGTGGG
EMPV1_36194	CCGCACAGTTCTGCTATCGATCCAGGCTTTGTTAAGCGCTCCCAATCCAGATGATCCATT
EMPV1_36195	AGGTGACAGCAAATATTCTAAACCCATGAGCGACTCAAGGAAATCAGAGCTCTAAGTGGG
EMPV1_36200	GGGAGATGGTGGAGTATGCAAGCTGTTCTCAGTCACACATACAGTAGAGGGGAAATGCAG
EMPV1_36201	CTATGATGATTTCTCTGGTTAGGGACAGCATGCAGAATGGACAGCATCCAGACAGGACCC
EMPV1_36203	ATCGGGTGCTGGAACCCTTGGGAGACTTTACCATCTTTCCACAAAAACCAAAAGTGTGCC
EMPV1_36204	TGGAGGACAAGGACACCAAGGCCCGTCTGGTGCTCACCTCTGGGATCATCTTCGTCCTCT
EMPV1_36205	TGCGGGCATGTCTCCCTCACTAGACTGAGGCTCTGTGAGGTTCACGGGTTCTGCCCTATT
EMPV1_36206	AGTACGTGGAAATGTCTTGGTTGGAAGCTGTAATAGTCAGCCCTCCTTACTGGGCTCCAG
EMPV1_36207	AGCAACATCAGGCTGGCTGTGCTGGGCACCCGTGCTCTTGTGTGCAATCTCAGGATGAAA
EMPV1_36210	CATCTTCCTCCTCTCCGTGGGCACCGCTCTGCTGGGCCTGAAGGAGGCGCTGCTGTTCCA
EMPV1_36211	AGGACTGTTGTGTAGATTAGGAGACAAAGGCTGCAGATCGTGGGATTTCAGGGACTCCAG
EMPV1_36212	TCCTGATCCTGTTGATTACTGACACAGACAAAAGAAAGGATCTTGGTCTTGTTACAGCTG
EMPV1_36213	GTGCATAGGCGAGTTGAATGATGACCCAAGGAGTGACCCCAAATTTACATATACTGGAAC
EMPV1_36214	CCCAGGTGACCACTTAATGCAGTGTGATTGAGCACCAAGGAAATAAGCAAACTTTCCCAG
EMPV1_36216	CTTGGGCAGTGGCCCCAAATTGCTATGACAACCAATGAACCAGCTATGTGTACTGGTATC
EMPV1_36217	CATGGATGCATGGGTGCGACTGCCTCTGTGTACAGTGCAGACATCCTGAAGAACATCACT
EMPV1_36218	TAGTAGCCGGTGACCACAGCCATGCTCCTTCTCTCTTCTGCTCTCCCATCTGCGAAATAA
EMPV1_36219	AGCTAGTGGCTGCTTCCTACGCATTTGGTGGAGCTAACTCTGCTATTCAGACGGGGAATG
EMPV1_36220	TGTCTCAGTTCCGATAGTTTGGAACTGGGTGAAATGTCTGGCCAGACTATGTCTTCATTC
EMPV1_36221	GCTGCACTTTCCAAAAAGCGCTTCGCTCTGCAGAGCTTTGCTGCCCTCAAAGGTCAGAAA
EMPV1_36222	GGATTGACCTTGTATTTGATCCTGTGCTAAGAGTGACTACACAATGCCAGGCCTCAGGAG
EMPV1_36223	TGTGATTGAATCTCCTCGCACGCCGTATCATTGTTTTTATTATGTATTTTATGAAAGAAA
EMPV1_36225	GTGGAAGCTGTCCTCGCAGAAGCTGGGATTTTTCCTAGTGACTTTTGGCTTTATTTGGGG
EMPV1_36226	AGCAGCTGTCAACTCACCTGTTGTGTCTTGGAAGATGAAGGCTCCAGAAAAGAAGCCCCT
EMPV1_36230	CATTCTGCCCACTCCACAGTAGACAGTCCAGTCTCCCTCACTGCAAGTCATTTGGTAAGA

EMPV1_36235	ATTACAGCGCTGGCCAGCACTCAGCTCTGTAAGGAGCTAACCTCATGGGTCAGAAGGCCA
EMPV1_36236	ATCCAGTGAGGATGACAGGTGGGTGGCAGGTGCATGTGCAATAATCGGGGCTCAGGAGAA
EMPV1_36240	AGCTCTGCCACTTGGTACTTGAGATCGGAGAAGAAAATGGCCCCGCATTGATGAAGCCTG
EMPV1_36241	ACTTCGAGGTCAAAGCATTCGCCACAGACAGTGCAGATACCGGAGAGGACAAAATTCCCA
EMPV1_36244	TTTCTGTCGTTGTTGGACACGGACGTGAAGAACCCTCGCTACGACATGCAGGGAGACATT
EMPV1_36245	AGCCTGGTCACTACCATCCTTGAGTAACCTCTGGGCTTAGCTTCTCCACCTGCAAAACGT
EMPV1_36246	GACACAGGGAGTGCTGCCATTGAGAGTGTTCAGGGGGCAAAGAACGTGGACATCATTGTT
EMPV1_36247	AAACTGCTGCTTCATGTTATTGTGCTATGTGCACCAGGGGTTTCTGTTCATGCGGGGGGA
EMPV1_36248	GGTTGTAAATTAACCCACAGTGTTTCTGACAGTCTCTTTGGCTGGGAGCTGAGTGGCAGA
EMPV1_36249	CCTTAGGCATCCAAATATTCTCAGACTGTATGGTTATTTCCATGACGCTACCAGAGTTTA
EMPV1_36250	TGATGTACATGGCCGTGACCCCGATGTTAAATCCCTTTATCTACACTCTGAGAAATGGGG
EMPV1_36251	CAGGCTGCAAACATTCAGGACCTACAGGTTCCCCGTTTTGTCAGTTCTTTTATTGTCCTG
EMPV1_36252	CTGCTACAGGATTTCAGGGATTTCTCTTTTGGGGGTTCTTTTCTGGGTAGCTCAGGCATT
EMPV1_36253	CCCTCCCCCTATTTTATACCCATTCTTTCCTTTGATTGAGATAGTGTAAAGTGCACTGAG
EMPV1_36257	GAAGACTGTTCATTGCGGGCTCTTGAAACAGAAGAAGGAGGCCTGTCCTGAGAGATTCAG
EMPV1_36258	TCGTTCCCAAAGCATTTGAAAAAGTATTCTATCTTCCAGGCTGGCTTTCTGTATTTACTC
EMPV1_36261	CACTGGTTTTGCCAACTCCGTATTGCAGACAGTGCTCATCTTCCTTTTACCACTCTGTGG
EMPV1_36263	TGACAGTGATGGGGAGCTGGAATCCAGATACTCCTCCGGGTATTCCTCTGCAGAGCAGGT
EMPV1_36264	GAAAACCAGGTGGTCATCAGCTTTGGACAGGAGGAGCTGGATTCAGGAAAGCTCTCTTAC
EMPV1_36266	CTGAGCCTTAGATGCCCTTCCCAATGGCCCCAGCCTTTTTGTCTTACTCTGGAACTGGAA
EMPV1_36269	ACGAAGGCCATTCCCCTGAGGAAGCACAGCATCGCGCCGCCGGGAGGGCGCTGCGCTCTG
EMPV1_36270	CTTCAACATCTACAAAAGGATCTTCACTGACATGGTGAGCTCCCCTGGGACCAACAGTGC
EMPV1_36272	ATAACGGGTAGTAGCAGAGTCCTCCTCGGTTGGTATCGTTCACATAGACGATGGCTGCAG
EMPV1_36275	TCAAGCTCTCAAGATCATTTGGTATCCATAAAATTGGCTCCTTCAGTCAACACAAATAGC
EMPV1_36279	CGTTCAGACCCTGGTCAACTCTGTTAATTCCAGTATCCCCAAAGCCTGTTGTGTTCCCAC
EMPV1_36280	TGGTCCCCGGGGGGGGCCACCACTCCGGCCTCTCTTGGGGCCCGCACTCTCGCTCCGGGC
EMPV1_36282	GGCCTGTGTTTTCTCCATCCTCCTCTCTGCCCTAATAACTCTTCCTACAGTCCCTTCAAG
EMPV1_36283	GTTACTCCCAATCGTTTTGCACAGCGAACATCAAAGTAAGCATTCGGATGCCCTGAGGCC
EMPV1_36287	CCTGGGTCTGCCGCTGGCTGGGGTACATGGTGGTCTGATCATGCTTCTTTCAATTATACT
EMPV1_36289	TCCACAGGACTGGTGGAAACACAAACTCCGCCCTTGGAGGGTGCACACAAGATTTCATGT
EMPV1_36290	CAGCACGGCATCTTGTGATTTTGGACAGCGAAAGACATGTACGGTTTCTTATGTATGTGG
EMPV1_36292	ACAGTGGGTTGGGGATTCAGTGTTGCTGCAGCTGTGGCTTAGACTAAGGTCGCAACTACG
EMPV1_36293	AAGCTTCTGTGTTATTGTCCCATGTGTTTTCCTTGCACATCGTCTCCCCTTCTGCGGGAC
EMPV1_36295	CTCTCAAGGCAAAACCTGGACTTGGAGGAGGAACAGGTGGCATCCGAGGAAACCTTGCTT

EMPV1_36298	ACCTGCTTCTCTCTCCTTATCCTGAGTCCCTGTCTCTGCAACTGTTCGGGAATCTGTTTC
EMPV1_36299	CAGCTGTGAGGGGAGTGTGGCAGGGAGGAAGAAGAGAGTGAGAGTGGAACAGTTTTGTAT
EMPV1_36300	CTGTAGCAACAACCAGAGCCACTGCAGTGACATCAGATCCTTAACCTGTTGAGCTGCTAG
EMPV1_36301	TGGGACGCCCAGGACTACCACCCGCTCATCTTCCAGAACGCCAACATCACCTTCGTGAGC
EMPV1_36302	CCGTTTTAACCGTCTAAGCACCCCAACACATACCTGATGTGCCTCAGATTCTAAAGGGGC
EMPV1_36304	ACTTAGTGGACTGCTTGCAGGTCCTTTTGCTCATGAATAATACACTGTATGGGCTGGCCA
EMPV1_36305	AGCACACTAAGAACAGTCACTTCACATCAAGTCTGCCATCCCTCATCCACAGATCTTGGG
EMPV1_36306	TACAGTCGCGATGTGCCTCTGCCCCCTCGGCAAGACCTCAACGCCCCCGACCTCTATATT
EMPV1_36309	TAAACGACTTTCTGAGCGTGAAGCATGAGGGGGTTGGAATGAGGGGGTAGGCTTTCAGAC
EMPV1_36311	TGCTGGCTATGCTCCCTTCAGAAAACAGTTCTAAGAGTACTGATGAGGATGATGCTCGGG
EMPV1_36312	AATCTGTTTCTGTCCAACTCCAGGCTCAGCGAAACTGCTCCGAGACGAATACTGCGCACT
EMPV1_36313	TCTAAGGACCTGGTGAGGATTAGCCGTTTGACTTACTGGGGGCATTAAACCAAGGTTGCG
EMPV1_36314	AGTTGAGAGTTAACTTTTATTGGGGACAAAATGAGGACTTGAGCCTGGGAGGCAGCATCC
EMPV1_36317	CAGACTTGGTCACGGACTTGGACTTCTCTCCGTTTGACGATTTCCTCCTGGCCACAGCCT
EMPV1_36318	TCATGTGTTATGTGGATGGGCTCTTACCTCCACTTGGACTTTCTGAGTTAAGAGCCTCTT
EMPV1_36319	ACAAAGCATTGGATAAGGCCCGGTTAAAAAGCAGAGACATCCAGTTGGCTGAGGCACACC
EMPV1_36323	TCTTTTCTCATACCCCACATCCAAGCCATCCTCAAGCCCTGGCAGCTCAGCCTCAGAATA
EMPV1_36324	TCGCCTGCTCGGCTTTAATTTCTGCATCTGTGAAATGGGGATAAGCAGACTCACCCAGAG
EMPV1_36325	CTCAGCCTAAACTTATGTCCAGGCAAGAGCTCCTTGTGTGTCCAGGCCTTAAGGAAATCC
EMPV1_36328	ATGCCTGTGCCTCACCGACTGTGTCCCCCTGTTCCACAGCCACCTGGCCTTGTCTGTCAT
EMPV1_36329	AGAATTGGTCATGGATGAAGCTCTTTTTCAAGATGAAGCCACTGCTCCGCTCGGCACAGG
EMPV1_36330	GAGGTGAGGAAAAACAGGAGGTAACACAAGGTGATAGCAGGTTAAACCTCGTTCATGACA
EMPV1_36332	ATCAGTTATTGCTTTGTAACATCACTCATGATTTAATTTGATATGAAATCAGAATTAGAA
EMPV1_36333	AATGTTCTTCCGGCAGCAGGATGAGATTCGGAGGTTGAAAGAGGAGCTGGCCCAGAAGGA
EMPV1_36335	CTGATGTTGCTGCAGATGTGGCATGGGTTGCAGCTGTGGTTCAGATTAGATCTTTGGCCA
EMPV1_36338	TTGGAGAAATGTCAGGTCTGTGGTTGTGCCCTGGAGCAAAGGTCATAGGAGAGGACACTG
EMPV1_36339	CCCTTGCAAAGATGACGAGTCTCTCCTTCACCCTGGAAACCTGACCAGTACTTCAGATGA
EMPV1_36343	TGAAGTGCTATGACCAGGAGAAATCTTATCAGGCACGTTGTGTTTGACTTTGGTCCTTGG
EMPV1_36344	TTTCCTTCTGCACCGTAATCTTTGTGTTCAAGACGCCCGGGGAGGAAAACGGGACCTGAT
EMPV1_36348	ATGCTTTTGCAAACTCTCCTCCCAGCGAAGGAGAAAGGCCAGTGTGACTGTGCATGTTGG
EMPV1_36349	TGCAGTTGTAGTCTATGCCACAGCCACAGCAACACTGGATCTATGCCACAACTGTGACCT
EMPV1_36352	CATGTCTTCTGAGAATCATTGTGGTGTTCTTGCTCGTGTGATTTTAGAGTCAGCGGGGAC
EMPV1_36355	GGGCTGAGGACCTGGGCTTTCCCGCCCCATCACGCTGCCCTGGCCCCTGCGGGCGAGGCC
EMPV1_36357	AGTTCTGAGCCGGCCCAGCATCATTCATCAGCAGAACTGGGCAACCTGCTTACTGTCGAA

EMPV1_36358	CATCATTTACAGAAGAAGGTGGAAGAGAAGCGAGATGGGCTTCCAGGAGCTATACCAGGG
EMPV1_36359	TCCTCACCACCACAAGGGGCCTCTCCTGTGCCTGAGAGTTCTCTCACTGCTGACCTCTTA
EMPV1_36360	TCCAGATCATTCTGCCTTTGCTAGGCTTTTGCTTAGAGCCGTCTTTCCATCATTATCACC
EMPV1_36361	TTCTTGGATCTGCAAAGAACGCTTTTCTTATGTTGCTGCCCCAGCCTCTCCCCTACACCT
EMPV1_36363	GACTCAAACGGATAAGAGTCCTCCCACTCCTGGAACATTTGGCGACGTTCGTTTCATGGT
EMPV1_36364	GTGAACCAGGTCCTCGCCAGCCGGTACGGGATCAGAGGATTTCCTACCATCAAGATCTTT
EMPV1_36366	TCTTGTTGTCATCAAACTGGCCAACACTGGCTACAAGTCCGTCTACGGCAGCGACTGGTT
EMPV1_36367	TCAGGAGGATGATGATGCCAAGGGCACAGAGAGTCCCTGCGCAGATGAGCCCGCCAACTC
EMPV1_36368	TCCGGGAGGATCAATTGGAGCCAGTTACCAAAGCGGCACAACAGGAAGAATTGGAAAGGA
EMPV1_36371	GCCATGACCAAATTCACATGATTCATAGTCCTTAAATATGTATTACTTGGTGTACAGACT
EMPV1_36374	CATTCTGAAGACTACGTTCGCTCCTATAACTATGAAGGAAAAGGGTCTGTGGCTGGCTCG
EMPV1_36376	AAACATAGTGTTAGGGTCGTTTGGGACCTGGGATGGGCACAGGTTCATTGACTTGGGGCT
EMPV1_36377	CTTCTTCGCCTGTCCCAGCAAGGCTGAGTTTTCCCGGAACATCATGAACATCATCGACGT
EMPV1_36378	GAAGCTCTTCGTGGGAGGGCTCAACTTCAACTCAGATAAGCAGGCTCTGGAAGACCACAT
EMPV1_36379	ACCAGCTTCCCCTGACACATTAAGAGAAGATCACAGTTGATGGAACGAACAAACTGCCTC
EMPV1_36380	GCTGTTCGAGAGAGCAACCTTCTTGGTCATAGCAGCTGCAGGAAGCCTTCGACCAGGCGT
EMPV1_36381	GGGAATGTTACTCCCGAAACTTATCTTCCTGTGCCAAACTGAAGGGGATAAGACACCTAC
EMPV1_36383	TTGGGTTTGCGCCGACGCTCCACAGCACCAGACATCCCCAGCTTGGTCGGAGAGGCACCT
EMPV1_36384	CCAGAGTCAGTATGTTCCTATTTCCTTGAGGCCAGTTCTTGGAATTGTGTAAGACGGAGC
EMPV1_36385	AGATACGGATTTGGCCCCACACCCCTTTGCTTTTGATGGAGGCCTGGGATGAGCATGTGA
EMPV1_36386	AAGATGATCAACACCCTGCAGCAGCTGCTCCTGCAGACAGGTGCCCAGAGCTACCACACG
EMPV1_36388	GGTCTGCCAGCCATAGCCTGGACCCAGTATCCATTTTCATGCTGCTCTTAATCACAGAAC
EMPV1_36389	CCAAGTAAATGCTTTGCAAGAGAAGGCTGGCATCTGCGATCTGGTCCAGGAGCAAGTTCA
EMPV1_36390	AGCATGCCTGCCCGTCCTGGCTGTGCCTTAGTTGGTGTGTATGTGTTTGTGCTTGTGTTT
EMPV1_36391	CCAGATCTCTGTGCTCAGTGGAGGGAAGGCCAAGTGCTCGCAGTTCTGCACGACGGGCAT
EMPV1_36394	TCCAGACTTTCAACATCTGCTACGCAGAGACTGGTTTGCTTGGCGCTCACTTTGTCTGTG
EMPV1_36395	CCGAGGTGCCTGTTGTGACACCTTCCTTAATGACATAATGGTGTTCATTGCAGCTGGGCT
EMPV1_36396	AGGGGAAGAACAAAAACTATGCCTCCACAGCGCTTCCCAACCAGTTCAGCTATGCGTGGT
EMPV1_36397	ATGTCTTTCAGACAACAATCTCTTAAGAATGAGGGGGACATTGAAAATACCCTTTCTCAG
EMPV1_36398	CTCGAGTACCTAGCCCTAAAGTGGTACTTGGCAGCCAACCCGACCAGGAATGTGGATTTT
EMPV1_36399	ATGGTGCAGGTGCTGACACAAATTACAGCTCCAAGTCGATGAGGCAAGACGGAGGCTATG
EMPV1_36400	CACTGTCTTTACTTCCTGAATGGGTTGTCCATGAACTTGTTCCCCTGGACCGCTGTATCC
EMPV1_36401	TGGTACCCAAGGGGAGAGGGAGTGGGAGGAACAAATTAGGGGTACGGGGTTAACAGGTAC
EMPV1_36402	TGCCCCTTCAGTGGGAGGGTGGTCAAGTAACTCATCGTCATCTGTGACTCCAGGTAGTTA

EMPV1_36403	ATACTAGCCGGGTTCGTATCCACGGAGCCACAATGGAAACTCCCAGAGCTGTGTTTTTTT
EMPV1_36406	GCGCTGCTCTATCTTGATGTCTCTTTGCTGGTTGTTTGGATTCCTTAGTTACTCCGTCTC
EMPV1_36412	CTACCTGGTGGGGCTGTTTGAAGACACGAACCTGTGTGCCATCCACGCCAAGCGCGTGAC
EMPV1_36413	GAGATCCAGGGGGGCTACATGGACATGTACCGACGCAACCAGGCGCTGGAACAGGAACTT
EMPV1_36414	GAAGCTGCACAAGGGCTTTTGTAAGCATAGCTGATGGTTTTCAACACCAGCCTTCAAACG
EMPV1_36415	CAGATGGCCTGATTGATGTTCCATAACCATAAGAAGTAGTTAGAAACTCATGGAAAGTCT
EMPV1_36417	TCAGCAGTGTTGGTCATTTTGGTGCCAGGTTGATGGACAGCTTCATTGGGCAGCACTGTT
EMPV1_36419	CACTGGTTTTGCCAACTCCGTATTGCAGACAGTGCTCATCTTCCTTTTACCACTTTGTGG
EMPV1_36420	TGCTTTCCAAGGAACTAACCCTACCCCTTGTCAGGCAGTAAGAACTTCCTCCACTAGCCT
EMPV1_36421	GAATTCCTGAAAGCACCACCAACAGCCCTCTGGAAGGTTCCCATTAAACTTTCAGCCAGC
EMPV1_36423	CCAGGGACCCGCAGTTCTGGCACAGGTTTGGGCAGAGTGAGACAGTCAAGAAAAGCTGTT
EMPV1_36427	AGGGCACAAGGCGGCTTCTCTGGGAAGTTGGTACCTGGGTGATACTGAGAACTGGAGAGT
EMPV1_36429	GTGAAGACAACGCCAAAAATGTGTTTTTCTCACCCATGAGCCTCTCCTCCACGCTGGCCA
EMPV1_36430	AGACTCTGGGATCTGGAACATGCTCTGGGCTGTAAGGGCAAGATGGCAGGTAGTTAGGAC
EMPV1_36431	GCAGACCCGCTACACAATGACAGAGGGAACTCACAGCCACACTGTAAAAATCACCCGTTT
EMPV1_36432	CATGATGCACATGGCTTGAGATATCAGTGGGGCGGCTCCCATAGCAACAAGAAGCTTTTG
EMPV1_36435	GGCAATGTAGTGTGTAATGCATCTCTGCAGGTGACCAGAGCCATTCTCTGTATGAGGTAA
EMPV1_36437	TGTAAGCTGTGGTGTAGGTCGCAGACATGGCTTTGATTTGGCATTGCTGTGGCTGTGGCT
EMPV1_36438	CTCTTACTCTGATCCTCTTTTCTGCCTCCTCTTCCACATTTAAGGTCTATGTGATTACAC
EMPV1_36441	GCTCCCACAGGGGAATAAACAGAATGAACCCAAACTTTCAAAAATAGCTCCCCGCCTCCC
EMPV1_36442	CAGCTACTGGTCTAATAAACACAGCCAGCCCTTTTCTGTCCTACAACATCTCTCTCAGAG
EMPV1_36444	GGCATTTCATACTTTGAAGATTCTTATGGTCTGTGTGCTTTCACTATCGTTAAGACTCCT
EMPV1_36445	ATAGCCCTATTCATGTATCTGGGAACTGGCTGGTGTAGTGCTGTGTTGTTACTGTCAGGA
EMPV1_36447	AGGGAGTTCTCTCAATCACAGGGTAAGATCACTGTTGGAATCAGGGGAAGTGTGTGCACC
EMPV1_36449	CAGCTATGGAATTTGCCTTTCCTAAGGAATTTGCTAATAGATGCCTAAACCCAGAAAGGG
EMPV1_36451	GGATGGTCCTGGAAGAGCCTGCCTTTGTGTGGTGGATTCGAATAAAGGGCATCATGAGAT
EMPV1_36453	GCATGCACCCATCCAAATGCTCTCTTCCCAGCATCCACTAAATAGGACCTTGACTTGGTG
EMPV1_36454	GTGGTTTAACATGACCCAGGAGGTCAGGAAGGCAATTCTCACATCATTCAGAGGAAGCGG
EMPV1_36458	TGAACACTCAGATGGGTGCATGTCATCTTCATTTGCCCCTCGATGCATGTGACTCTTGCT
EMPV1_36459	TGGAGCTGTTTGAGGAGCAGAATGGCAGCTTGCAGGAACTGCACTACCTCATGACCATCA
EMPV1_36461	AATCAGGGAGACACAAGGAGGCTGCTGTCATAGGCCAGGCAAGAACGATGGCGGCTCAGA
EMPV1_36462	GCTGAGGCCTAGCATTTTAGTGATTACTAAGCACTTCTGTCAGTCTTTGAGAAAAGGACA
EMPV1_36463	CGACCGGACCTCGACCGGCTCGTCTATGTTGCCAATCGACTCGGCGTGGCGTCGGTCGTG
EMPV1_36464	AGTACCTTGTCACCACCCCACAAGGGAGCAAGGCCCTGCACTTGGAGAAAGGATCCTAAG

EMPV1_36466	CAAGGCCCCCATGCCAAGCTCTGCCTCCAAGAATCTCCACGAAGCCCCAGTTTAAAATCT
EMPV1_36469	AACATTGAGAGCCTCATCTGCAAGGTAGGGGCCGGTGGACATGCCCTGTCAGCTAGTGCT
EMPV1_36470	AGCGTGTAATAATTTGGACTGTTGCTCCCCCTTACCCCTGCACCCCCCATTAAGTGAGAG
EMPV1_36471	GAGTCAAGAGGAGAAGCGGACATGAGTGTCAGAGCTGGTGGTTGTGTCCTTCCTACTAGT
EMPV1_36475	ACCTTGCGAGATGCTTGGATATGTGGGCATCGAGGCTGTGCTGGATCAACTGAAGATCAA
EMPV1_36476	AAGAACCATTTCTTCATGTGCTTTTGCCATTTCATTAAAATTTACACTGATGTTCACAGG
EMPV1_36477	GAGAAAATAAGCGAGGGCCTCAGGGCCCCCCTGAATGCCTTCCTGCACCGGACGGGCCAG
EMPV1_36478	CGGCCTCTTTTCCTGAGATGGAGAACTGGGAGGTAATCCACTTGCTCAGTGTGGAATCAA
EMPV1_36479	TGCTGCAGAAGGATGTGGGAAAGCGGCTGCAGGTTGGCCAAGAACTTATAGACTACTTCT
EMPV1_36481	CTCTGGTCCCAGTGTTTGACCTTGAGCCACATCCTGGTGTTCTAGTTGGCAGTCAGGTTA
EMPV1_36484	GGAGTAGATTCAAGATGGCCACATGACGCTGACGGGAGAACTAACGACCTTCTTCACTGA
EMPV1_36485	TGGGGAGAAGGTCTGCCTGGAGGTGAGCAGCTGCCTTTCCCAGTGTGGGTTCAGTCCCCT
EMPV1_36486	GGGATTTAGTTCAAGACAGCGCCCAGATGGGATGTGCGTGTGTTTAAGATGACATCGCTG
EMPV1_36490	AGGAGAAGGTATTTGCTTATAAAAACCCAAGGAAATGGAGGTCCCTCTGTGGCTCAGCAG
EMPV1_36491	CTATTGCTACTACCACCATGACTACTAGTGCTGCCTCTACCACCACCACCATTACTCCTA
EMPV1_36493	TCCGGAAATGGTCATCTTTATCCAAGCTCACTGTCCCAGATAACTGCAGCCAGGGTGGCA
EMPV1_36495	CTGGTGATGCACCTGACCCTCGTCCACATGGAGCGCAACCCGTACGTGCGCATTCTGCAT
EMPV1_36497	TGGCTTTGAGCGATTTCATGCATTAATTCAAAAAAAATCATTCAGAAGTTTATCAGAATT
EMPV1_36498	GTGAACATTTAGCATCTGTCTAGTGATGAGAATACAGTATTTGTCAGCTACAAAAGCTTG
EMPV1_36500	CTTGAGAAGGATACCAGTGCAGCCGTGAAGCAGGCAGCCCAGATTGCCATCAAAGTCATT
EMPV1_36501	CCTTCAGACGTCCTTGTTTTCCAGCTGCTTTCTTCCGGGACGAGCATTTTAAAGCAGGAA
EMPV1_36502	GCAACAGAAACACCCAGAGGATGTGCTCAGCTACCCCCAAATATTTGCAGCTGGGATGTT
EMPV1_36504	TGTGTGTGTGTGTGTGTGTGTGTGTGTGGACTGGAGCGCACACATTTCCACACACTTACAAG
EMPV1_36505	TGCCCGCTCAGGGAAATTCTCATCCCGGTATTGGTCGATGTGACGGGCTTAAACCAACTA
EMPV1_36506	TGTGTGGGGAAGACCCCTGGGGCCAGGGAAGTCCTGGGTTTCAACTCCCAGATTTAGGGT
EMPV1_36507	CAGCTTCCATGCTGTTACCTTCTTTGGATTTTCTCTTTGTCTTGGGAGGCTGCGTAGCGC
EMPV1_36508	TCAGAGACATTAAATGACAGTGTGTCCCAAATTCCTGACAGGGCTTACTACAGTCTGCAG
EMPV1_36510	CTAGAAGACTGCAAATCAACACTGCAACGAGGTACCACTTGAACTCTGGTCAGAATGGCC
EMPV1_36511	GTGGACTGACTGGTCAATGGGGACCTGCTGTGTAGCATAGGGAACTCTACCCAACATTCT
EMPV1_36514	CCGAAAGCGTTACAGCGCAATACCAGGGAAAACAGCCATGAGATCAGCTTCTGCAAACAC
EMPV1_36518	GGCACACTCCAAGGAAATACAAGAGAAGTCCTGCACAAATTCACTGTGGATCTCCCAAAG
EMPV1_36520	CATATGTGACTTTGCTGACTCCTTTCCTCCTCAGGATAGAGATGCTCAGACTCAAAGCTA
EMPV1_36522	TGTAGGATCTTCGGCTTCAGTCCCGGTGTGGGAGGCGAGAGTAGTCATGGCGATGTTTAA
EMPV1_36524	AGCATTCGAAATAAGAAGGGTCAGTCACCACTTGATCTCTGTCCTGATCCAAGTCTCTGC

EMPV1_36525 TTGCGTGCATCGAGCTACTGGCTGTGAGTTTGCGGTGAAGATCATGGAGGTGACAGCCGA
EMPV1_36527 GAACGGCAGGAAAGGATTATGAACGAAGCCAAGAAGCTTGAGAAAGACCTAATAGATTGG
EMPV1_36528 GAAGCAGGCAGCTCTGTGCAATCAGTGGGTCAGTTTATTATAGGGTCACTTCCTCACAGG
EMPV1_36529 ACAGTGGTTTTCAACCCTGGCTGCACCTGAGAACAACCAAAAGAGTCCTTTTCCCAGAGC
EMPV1_36530 TCGAATAGCCCTGTTAGATTTTTGATCTGCCTTTGGTGTATGTGGGGGTGGCAGGAGAGT
EMPV1_36531 CCAAGATGAAATCAATAAAAATGTCATGTCAGCATTTGGCCTAACAGATGATCAGGTTTC
EMPV1_36532 ACTCGGGGCATCGATGGCGCTAAGAAGGAGATTGCAATGATTAAAGTAGCTGCCCCCCGG
EMPV1_36535 AAACTTTAGGCCATTTACCAATGGTCAGAACCAACCAACCAATGTAATTACATTTTCAGA
EMPV1_36536 CAGTGAGGTTTTCAGAATTGGTAGTGAGAGAAGAGCAGACGAAATGTACTATATGAATGA
EMPV1_36537 CTCTCTCTTACAACAGCCAGCGTAATACCAGCCCTTCCTCAGACCCCCGCCAGAAGAATT
EMPV1_36538 TGGGACTGAAGGACGTGTTCATTATCTTGAAGGTTGTAATGGTTTGACGGTATCTACATG
EMPV1_36539 CTGGAGCATATGGCAATGAGATGTTTAATTTTTGAGGACCTACCAGGCAATCTTCCACAG
EMPV1_36540 CTATCCAGGCAGAAGAGGAAGAAGCCCCAGGAGACAAGCAGCTCAAGAAACAGGAGAAAA
EMPV1_36541 AGGAGGACAGCGGTGTGGAAGTGTATTACCGGGAAGGAGAAGAGGAGGTGGAAGAGACAG
EMPV1_36544 AGGAACAACTGAGGCGCGCAGAGCAAGAAGAGGAACAGAGAGGTCAAAGGCAGAGAGACA
EMPV1_36547 TGTACCAAGGGTTGAGTGATTCCCTTTCGGCAGTGAGATTGTGGGTGTTCAGTTGGGATC
EMPV1_36550 CTAAACATCGTATTTACTCTGTGTGCAATGAGGTGCTAGAAAAGCCACATGGCAATGCCG
EMPV1_36551 GAGTTCCTCTCCTGGCTCAGTATCTATGACTAGTATCTATGGGGTCTTGGGTTCAGTCCC
EMPV1_36554 TGGCCCTCTAAGTCCATCTTCACATCACCTGCACTTTCATAAGGGCATTGGTGGACTCAC
EMPV1_36558 CTTCCACCTCTTCCTCTTCAAGCTCATCTAGAACGAGTACTGCGGCCCTGGCGCCAAGCC
EMPV1_36559 CATGGATTTCAGAACCCCTGACCTACTGGATGTGTGGCTGGAGCCCCCAGAAGATGTCTT
EMPV1_36562 TGAAATCCAGGTTCCAGGTGAAGGACAGGTTGTCTCAGCCTAAGTCTATCCCACATAGTG
EMPV1_36565 GATCCAGACGACAGGGAGGAGGGAGCTGCTTCTACGGCTGAGGAGGCAGCTAAGAAAAAA
EMPV1_36567 GACACCAAAATCATCCTGGAGACCAAGAGCAAGACCATTTACAAGCTGAACGGCGTGTCC
EMPV1_36568 TCAATCGGGATCTTCCTTTTCTCGGAGTCGAATCTTCCCCACGTCTCTTCACAGAACCCG
EMPV1_36569 CCCAGAGACCCAGGAACTATCACATTTGTGGCAGGAGAATGAAAGAAAAGTCTAGTGCTC
EMPV1_36571 GCTTCAGTGAACTTGGTTTGGGGTCATACACGCCAGTGGGACTGATCCAGAACTTACTGG
EMPV1_36572 ATGGAGCCCAGCCAAATCTTGGTGGGATTTTATAAACATGCTTACCTCTTGCTTTGAGGG
EMPV1_36573 GCAGTGTTACTGCTCAGTGCCCAAATAAGATGTGCTTATGTGGTTATTGCTATTGTCTTG
EMPV1_36574 GATTTCACCTTAAGTTCATTTCTGGACTCGAAACCCGGAGACGCAGCTGGTGGAATGGTG
EMPV1_36575 GGCCATAGCTAAAGCCCGTCAGACCTTTGACCGAGACGGGTCTGAAGCAGGGCTTATTAA
EMPV1_36576 AAGGAAGCCCTCGCCGCAAGCCGGCGTCCGGGTCGGCACAAAACACGCCGCCACTAAAAA
EMPV1_36577 TCTGCTCCTGTTCTCAGTTCTTTAGTACCCGTCTTTGGAGAGTGTCGTTGTTCTCCCTTC
EMPV1_36579 GCAGCCTGAACATCAAAGCCACATTCTACGGGCTCTACTCCATGAGTTGTGACATCCAAG

EMPV1_36582 GGACATGACCGCATCTACATAGCCACAGTGAGCAGGTTGTTTGGTGTGCTAAAGCTGATG
EMPV1_36584 GACTGAAGCCCCATGTGTTTTTCATGCACACACTAATCCACACTCAGCCTCCAGCAACTC
EMPV1_36586 TACCACTTCGTCCTCACTAGGATGGCTAAAATGAAAGCAGACAAGGCGTTCCTTTGGCGG
EMPV1_36588 GTCTGCTTCGACCAGGTGAGTTTCCTGCTCACCAAAAGTCAGTTGTATTTATTTTAAGCC
EMPV1_36589 CCCCCTACCCTTGTTCTCCTTGCCTTTGTGAATGTTAACCCTGTTTCTTGCTCTGGACTG
EMPV1_36590 CAGCCCTGAAGGTCAGCCCCTTTGGAGGCATTTCACAGGCAAAGAGAATTTTACTAGAAG
EMPV1_36592 TGTGCAGGATGCCACGGGACCAGCGGGGGCCTGGGACCCTGGCAGAGCCAATGCAGGTCA
EMPV1_36593 GACAAGTTTAGGCAGAAATTAGAGGAGACCTTAAGAAAATCCCAGGGCAAACCTGGCTAG
EMPV1_36594 TCACAAGGGGGTGATGGTTACTATGGGCGAGAATGTGATTGGTGGTCTGTGGGTGTTTTC
EMPV1_36595 AGTGAAGGAAACTGGATCTTGGGGGCAGCCTTCTCTGTAGGTTCTGGCTCTATGCATGCT
EMPV1_36597 GAGGACGCACAATCCATTTGTTCCTCCTTGGAAAAGATGCTTTCTAAAAACATGGAACTG
EMPV1_36600 GAGTAGAACACCTTAACACCGGCAATTATCCCCAAAGTAATCCGAAAACAAGCGCAATTC
EMPV1_36604 GTGGTACTTTGGCAAGATCACCAGACGCGAGTCAGAAAGGTTACTGCTCAATGCGGAGAA
EMPV1_36607 GGAGTCTGTCCTGAGCTGAACAGTGGCGTGGACGTGATTAGGAACAGAAACACCTGAGAA
EMPV1_36609 CACCTCCTGTGTTTTGAGATGGCTAGATAAGGTCTTTGTGAGCAGGGCAAGGCTGTGAAG
EMPV1_36610 AGAAGGCCCTACCACCACGAGTCCTTGTTGACTCAGATAGAGCCCCCTTCATCAGTAACT
EMPV1_36613 CTGCCCTTCGTGAATCTTTTTGCGGTCTGGGCTTGCTGTACATAACTCAATAGCCGGAAG
EMPV1_36614 TTAGAGTGGAGGTTGGTCTGACCCCCCAGTGTCAGTCTGTCCTGAGCAGGAGGGACAGAT
EMPV1_36616 CGTCTCTATTACTGGACAATAGTTGCTTGGCGCTTCGTCCTGCTCTGGTAATTCTGTGGC
EMPV1_36617 GGGAGGGAAGACACTGTTTGTTATTTCAAGGCCTTCCGTGAAAACTCCATCTCAGAGGAC
EMPV1_36619 TACCTTCAACCCCCACTTCTCTCTCTTTTCAACGGATATGAGACCCTGAGGACCCCAAAG
EMPV1_36620 GGTCTGTATCTGCCTGATTGCTGGTCCGTATGTCTATGGGTTCCTTAGTGGCCTGATGGA
EMPV1_36621 CACAGCACTTGTGAGATATTTTTCCTTGGTGAAAACTGACCACTTTATGATGATCTACCC
EMPV1_36622 AGTATCTACTTTGAGGCCAGCAGAGAGCAGAGTTTTGTCATTACAGCAGCCCCTACCTGG
EMPV1_36623 AAAGTTGCAGGATAGGATAATAAGGAAAACAATAGGGCCTCAAGGAGGGACATGGGTTCC
EMPV1_36624 GCCTTGTGGATCAGCCCACCAAGCAAATGGCTCTACATCTAATACTAGAAAGGAGACAGC
EMPV1_36625 GTGTGTTAAGTGCAATGACCTATTTAAGGTAAACCCATCCCACCCACGCCAATTCGGGGC
EMPV1_36626 GGCAGGTGCGATGCTTCAGTACATCTGTGGTCAGGCCATTTGCCAAGCTTGTGAGGCCAC
EMPV1_36631 AAGCTCAAGTTATTCAAGATAGCGGCCGAAAAGCACCAGCATCTGTACCGCCTGGCGATG
EMPV1_36633 GGCTCCGAAGAAGGAGCCTGAACTTACAGAGACACATAGATTTGGCGTTGACTTCCAGAT
EMPV1_36634 TGGGCCAGCTAGAATCCCATCTTGGTTCCATCAAGTTCACTTCCTCTGCTTACCTCCTCC
EMPV1_36636 AGAAGAGGCACCACTGTTAGTCCCCATTGGATTATCAAGGAAGGGAATCCATGTTCACAC
EMPV1_36638 GCCTCCTATGGGTCAAGTAGTTGCAGGATGAGAGTGAGGAATAGACGAAGACCGAAACCC
EMPV1_36640 ATGACTCATTGTAGTAGACGTTGATTCTCTCCAGTTGGAGCGCGGAGTCACCCACGTAGC

EMPV1_36642	GGTAAATAGGGGAGTCAGACTTCACGCAGATGACACTGGAGCAAAGACTTGAAGGAAGAG
EMPV1_36644	CTGAAAACTCAGAAAGAACGTGATTTTCATCGAATGCACCACAAGCGAGTAGTCCAGGAG
EMPV1_36646	CGCCTGACGCTTTTCCAAATGTGAATGCCGCCGTCCCTTACAATGAGTGCAGATTCGAGA
EMPV1_36647	AGTGACAGTGGAGGTCTTTTGCTTCTTGGCTTCCAGTGCTGGGTTACTGGGGTGCATCTT
EMPV1_36648	ATTTGTGTTGCTGTGGCTGTGGCATAGGCTGGCATCTACAACTCCCATTCAAACCCCTAG
EMPV1_36651	CTGCTCTGTTTCCGTCGCTGTCTTCTTCCCTCACATGGATCTGGTCGGAGTTGCATCCCC
EMPV1_36653	AGGCCTCTCATAACAAAATGATGAATCCCAGCTCCCACAGCCACCCTGGACACGCTCAGT
EMPV1_36655	GAAACTCCACCAAAGTGCGTAATGCCATGATCCAGAAGAGCATCCTCAGCATTGAGCAGG
EMPV1_36656	GTTTGATGCAGAACAAAGACGGGTCAAGTTCATTAACATGAATGGGCTTATGGAGGATCC
EMPV1_36658	TCCAGATGTGGAACCTGAGGCTTGACAACCAGATCCCAGGGCTGACGATTTCTAAAGCCT
EMPV1_36660	GATGTGGGGGAACATGTACTGATGTGTACTGCTGATGTGACTGGAAAGGGGGCAGCTTTT
EMPV1_36661	GACCCTGGGCCCCATTCAACTGGCATATACTTATTCTCTGGAGACAGCAATTCATTTCAC
EMPV1_36662	ACCTGCAGTTGGAAAATGCTACAGAGTATGCGATCCTTCACTTCCCACAGGCCAAACCCC
EMPV1_36664	TACTATTCACACTCCTCCAGATGCTCCAAATATCATACACAAATGAGCAGGGCCACGGTG
EMPV1_36665	AAATCCTTTGGCCCCAAAGAGCTTCTGAAAACCATCAGTTAAAGTGTCATACCATCCTGG
EMPV1_36666	AGAATCTCCTGACCCATACCCTATCCATACCTCACCACCAGACCTCTTGGTTCCAGACTA
EMPV1_36667	CAACTCGGCTATCCTGGGCTCTGAAAAACTGTCATCACTGGCGTCGCTGTATCGATCACT
EMPV1_36668	GGGATTACTGTGGATGATGTGATAGCTGCCGGGGTTCAACTGTCAGGAGTGTTTGCTAGT
EMPV1_36669	AGACGGTAGTTCCCGGACTGGTACCCCCCGCTGGAAGTGAACTCAATCACTGACAACACC
EMPV1_36671	GTTCAGATACACATTTCCTAGAGCTCATGTCTTTTCTCCTGGCTGTGGTGACACTGATGG
EMPV1_36672	GTCTGATTTCAGCTCCACAAGAGGAAGATGCGCGCTTTGTTTTCCCACACTTATTGTCAC
EMPV1_36675	GCTCTTTGGCCAGACGTTCCCCACACAGCATGATCTGTTCAGTGTGCATTTTGATTGTAA
EMPV1_36676	TCTCATTTAAAAGCGGCCGCGCAGGCGCAGTGAGCCGAAATGCGAACTTAGGCTGTTACA
EMPV1_36677	GTGACCTGCTGAAAGAAGTTTGAGAAGCTACATTTACTGAGCACCGACCATGTGCCGGGC
EMPV1_36678	GTCACAGGAAATCAGGGGTGGTGAGGTGGCATTTGAGGATTGGCGTGTTTGGAATCATTG
EMPV1_36681	CCGGCGGAGATGGTGAAAGAAGTTGAAGAGACACACAGGAACAGCCCTGATTTTGAAAGC
EMPV1_36682	CCTCTGCCCCCACAATATTTTGGCCTGCACAAGATTAGTACATTTCAGGAATATCTGACC
EMPV1_36683	TAGTGACCCTCTCTGGCCTGTCCCATCTGCATAGTTTGGTTAAGCATGGACAGAGGCATA
EMPV1_36685	TGCAGCAGGGTATTATCGACATGGTCTTAGCCACCGAAATGACAAAGCACTTCGAGCATG
EMPV1_36686	TTTACCAGGGGCTGTGGTGGAGAAGGAATCGGGAGCTATTGCTTAACAGGTACAGAGTTC
EMPV1_36687	AGTAGAAGCCGCAGCGAGAACCTCCTGTATCCAAAGTCAGGCACCTAAATGGCTGGTAGA
EMPV1_36688	AACGACATGCTCGATGGCTAGAGACCCTCAACTCTCAACCCGAGATGCTTATTTGGAACG
EMPV1_36689	ACATCAACGAGTGCCTGGTCAACAATGGAGGCTGCGACCACTTCTGCCGCAACACCGTGG
EMPV1_36690	TCAAGGTCAGGGGCCAGTCACAGCCCAGATTTACCAGAGACCGGCCTGAAGGCTTGGTGA

EMPV1_36691	CCATCAGAGCTGACAAACTAGAGCAGAACTCTAAACCCTAGCGATGGTCTGAAACCAGGG
EMPV1_36692	TAGATGGCCCCGGAGTAGTCCCACAATGAGATGATTTCCGTCGTATTCCAGATCCGGGAG
EMPV1_36694	GGCCGGATGTCACCAAGTAAGTGCCACATAATCAAGCCTGACAGGTGAAGAACGTTGAAC
EMPV1_36695	AGCTGGCCCATGGCTGGCGTTGCTTTTCTTACACCCGCTCTCTGAACAAATGTCATCCAT
EMPV1_36700	GTGTGTTTTAGTGTGTTCAGTGATGATGTCCCTAGTCCCCAGCTAGATGCTTCTTGGCCG
EMPV1_36701	GGCTTGGGGACAAAGGGGGTTCTTTTCCAAGGAAGTCTGTGATACTGTTTGGTCTCTAGA
EMPV1_36703	CACTCAACAGGGGCCACATCTCCACCTGCAGCGACTTGACCGCTTCCGTGTCCTTTTCGT
EMPV1_36704	CAGCACTCGGCTGCCGCCTTCCCCCTCCTGCCAGACATGCACGCCGCGGTCACGGAATTA
EMPV1_36705	TGTGTCCCACATAGGTGCCATTTTAGCCTTCTACACACCTGCGGTCATCTCCTCGGTCAT
EMPV1_36706	CCCTGAGTTTGCAACCCGTAGCATGGCAGCAGTACTTGCAGGGACAACAGAAGCAATTTT
EMPV1_36707	CGTGCATGTATTGAGTACCTACTGTATGCCAGGTACAAGAATCTCGGGCCTCATGGAACA
EMPV1_36708	CCCTTTGTCATAAAGACACCTGTCATATTGGATTAGGTCCCACCCTAATGACCGCATATG
EMPV1_36710	GAAGCAGGAAGAACAACGGCGGAAGCTGGAGCAGCAGGTGGCACTAATGGAGACCCGACA
EMPV1_36711	GAATGGAGCTGTGTGACTATACAGAGACCTTATAACAGACACATGCTGTCCCCTGCCAGG
EMPV1_36712	CCCCATCCTTCAACAGCTGAAGCAAAGGCTCCAACACCAAAGTTTGACTTATTAGCCTCA
EMPV1_36713	GAGGGTTGGGAATGGTGAGATGAGGGAGAGGAAAGCCAAGGCAAAGGTACATATTAAGAT
EMPV1_36716	GACTTTAAAGGTGGGGGTAGAGGAGTTAGGCAACTATAGACCGTATATGGCGAGGCACGT
EMPV1_36719	AGAGCATCTCTTCTTGGATGTGAGCAAAGGAGGGCCAGAAAACGGCCAGCATAGATGAGA
EMPV1_36720	CAGCTATGGGACACAGCAGGACAAGAACGATTCAGAAAGAGCATGGTACAGCACTACTAC
EMPV1_36721	CCAGACCATTGTCAGTTCCTATGAAAGCCATGCTGAATATATCTGAAGGCTGTAGAAGCC
EMPV1_36722	CCTCAATTTTGTGTGGGGAAGAGGAGACTCCGAAGGCAAGGTCTCTACCCCCGCCTTTTA
EMPV1_36724	CCAGGCAAGGAGATGAGACACCATCTACAAATAATGGAAGCGATGATGAGAAAACGGGAC
EMPV1_36725	TGCTGTGTATCCTTCCAGGCAGGGACTCTGGCTCGGTCATCTTCCTTCCCACTGGGCTGT
EMPV1_36728	AGGCTTTCTGTCTCCCCAGGGCCTTTGCCCCTGCTGTTCTTCACCTTGTGGCCCTCTCTA
EMPV1_36730	GGGTGAGGATCAGACTTGGCTACCAAAGGACTTTCCATTGTCCCTGAAAACCTGACCACA
EMPV1_36731	AGGTACCATATCTTGTTTCTACTGTTCCCCCAGGATGGAGTTGGCCTGGCACACAGTGGT
EMPV1_36732	TCCAAGATGCACACGGAGACCATCAAGCCCCACAAGTGCCCGCACTGCTCCAAGACCTTC
EMPV1_36733	TTCCCACTGTGATTGGCATGTCTACAAGGGCTGTCCCAAGTCAATGGAAAGGGAAAGGGT
EMPV1_36734	GGCAATAGTTTCATCATCTGTTGGGAACTGGTATATCTGAACACCATTGCTGACCAGTTC
EMPV1_36735	GGTATCTGTTTTATTTCAATCTGCAGCATTATTTCAATCTCTTTAGTATTTCTTGCAGGG
EMPV1_36738	AGATACTTCTCAATCTTCCACCAGAGCGTCTTGAATTCCACCAGCCCCAAGGTGCCTGTC
EMPV1_36741	GCAGTTCCAATGAAGTAGCTTTGTCTCTGCCTTCCAAATGCCCTGTGTTCTCGGGTTTGG
EMPV1_36742	ATGACCATGACCCTACACACCAAAGCGTCCGGCATGGCCCTGCTGCACCAGATCCAAGCC
EMPV1_36743	GCAGCAGATATAAAGCCTGTCGACCCTAACTCAGGCTACCCAACTGACATCCTTGCCATT

EMPV1_36744	GGGACTGGCAGCGGGTCCCAGGGCGGCGCTGTTCACGGTCGGGTCCCCTCCGCACGGCGC
EMPV1_36747	GGCTTTCTACTCTCATTGAGCCACTTACTGCTATGAAGGTCTTTAGTGTCAGAAGGACCC
EMPV1_36750	CCTCTGAGTTTCTGGGTCAATTTGGCAGCAAATCATCTGGCCTTTGGTGCCTTCAGCACT
EMPV1_36754	GCAACCACAGCTTTAGAATTGGTGGCTGGGGACAGACTCTATAATGTTGTAGTCGACACG
EMPV1_36756	TCTTTGTAAAATCAGGAACTGGACAAGACCCCGGACAAAAGGTCGGATCGTAGGGGTCCT
EMPV1_36757	TAGCTGAGTGTCCCGCGGGGCCCGAAGCGTTTACTTTGAAAAAATTAGAGTGTTCAAAGC
EMPV1_36758	GGGGGACAGTCCTTTTTCACACATCTAGATCTTTTCTGAAGTGGTGGAAATTGGCAGCTG
EMPV1_36760	GTGAGGATAAACAGACCTTCTTACAGGACTGTGAGGATGATGGGGAAACAGCAGCAGGTG
EMPV1_36761	TTTTAGGGCCTCACCTGTAGCACATGGAGTTTCCCAGGCGAGAGGTTGAATTGGAGCTAC
EMPV1_36763	ATATCATAGTCTCACTCTCCTCAGATTAAGTTTCACAGCATTTGGTCACCCCAGCACTGC
EMPV1_36764	TCTCCTCTTTTATCGCTCTTCATCAGGGTCCAGGCTATGGAGCAGGGCCATTTAGTTTGC
EMPV1_36766	GAACAGCCTGATGAAGAGTACGCAACAGCCAAACTCTCTGATAAACTGCTGTCTTTCACA
EMPV1_36768	ATTGCCATTGTGGGTCTTTCCCTGTGTCTGATAATATCAAGCCATGGTCAACTGAGAAGG
EMPV1_36770	GTGGCGGCCTCAGCCCAGGGAGAAGGGGAGACACCTGTGACCGCCCAAGGACTTCGGTGA
EMPV1_36771	CACTGCGCTAGAACCATCCATTGTGATAGAACCATCCGTTGTGATAGAACCATCCATTGC
EMPV1_36777	CCCGCAGTTTTACATTTGAATTACTCACCCTGTGTCTGGGCTCACTAAGGTTCAGGTTCC
EMPV1_36780	TGCCAATCTTGGAGAGCATGAATTTGTGGAGGAGAAAACCAAAGTTGCCAAAGTTAGTCA
EMPV1_36782	GCTACACTCAGGCCTGTGATTCACTCAGAACTGAGTTGTGTGCACTACGTTAGGAGGAAG
EMPV1_36783	GCTGTATTAAAGCCATCTCCACTAGCTTTGATGACCGTTTCTTGCTGTCTGCTGGAGCAG
EMPV1_36784	CAGCTCCACCTGTGGATCAGAATCTCCACTTCCCTCAAGATCAGGCTGAGCACCATTGCC
EMPV1_36787	TTTCCCACTTTAGAGAACGGAGCGGTGGAAACGTTACATGATTAGGCCAAGGCCGCACAG
EMPV1_36790	GTCAGATCCTTAGCAAGGCCAGGGATCAAACCGCATCCTCATGAATAGTGGTTGGGTTCT
EMPV1_36791	TTCAGCAACCATCTCCTCTTACCTGCGCTCCAGGCTCTTCCCCAAGAGCCAACTGCCTGA
EMPV1_36792	AAGACGGAAATCAATGGGGCCAGGGGTCAGCAGGAAGTCGCTACAGGACTCTAAATCTAG
EMPV1_36793	GAGAAACAAAATGAAGAAGGCTGGTTTGCTCTTTCTACTCACGTGTCATAAGTTAGGTTG
EMPV1_36795	AACGCCATCACCACTGTCCTGCCGCCCAGGACCAGCCAGCCCAGCGCGCTTCATCCACCA
EMPV1_36796	CCTCCCCCACCTCCACCATCCACATCAATCAGAAATGGCTTCCAAGACTCTTCATGTGAA
EMPV1_36798	AAGACTTCTCCCTCCCACAGCACCTTAGGACATATCACTGCCCTACAACATTCCTAGGGG
EMPV1_36800	TAAAGTAACAACCAGCCTTCTGGTGGCACACCCAGCCACTCAAACTCACCCAGAGGACAT
EMPV1_36801	CCCCAAATGTGCATGCTTTTGGTAGTGGCTGCTTGGATAACTGGAGTGACTCATGCTGTC
EMPV1_36803	TTTCCTCTAGCTGCTCTCAAGTGCCAGTGTCCACTCCTCTACTCCCTTCTCACCCAAGTG
EMPV1_36804	GAGCTGCTAGATGCAGTTCTATCTCCCACCCCTTGTTGGATTTGGAGATTCTAAGTGATG
EMPV1_36806	CAGCACCGTCAAGCACGCCTTGTCCATCTGGCTCAGTGTCATAGTTTTCGGCAACAGAGT
EMPV1_36807	TCTCACCCTGATAAGTAGACACATCACGTGTTGCTCCTGCGAGTCTCCTGGGGACGTGTT

EMPV1_36809	GGTCATGTCCGGGCTCATGCAGATGCTGCTGCTGAAGGTGTCGGCCCACATCACGGAGCA
EMPV1_36810	TCCCCATCCATTCAAGCCACAACTGTCTTCTGGTGGCATCTTCTCTCCAGGACTTGCCTT
EMPV1_36811	GGATTGCAGCTTCTGGGAGCGGCTCTTGGCTCGGCAGTGTCGAATCTATGAGCTTCGAAA
EMPV1_36814	AACAGTTTTTGGAGTGTGCCCAGAACCAGGGTGACCTTAAGCTTTGTGAGGGTTTCAGCG
EMPV1_36820	CGGTTTTCAGCCATCCTCAGACAGTGGTGCTGTGATAAGCCAGCCTCACAGGTCATCCTC
EMPV1_36822	AACTCCAGGGAACAGCTGATCCTAAGGCTTCGGCCGAGAGGGCAGGAGTGAGGATGGGAA
EMPV1_36823	CAGCACCACAAAAGCAATGTCAGCATCACGGTAAAGCAGTGAAGAGCAGTTTTCCTGTGC
EMPV1_36826	TCCACTGCGACTCCGGCTGGGGTGGGAGGAAGGGGAGCACCCGCCGCTCAGAACCTGCTG
EMPV1_36829	GATGAGTGAATTCCAGTCACAGATCAGTTCTAACCCTGAGCTGGCAGCCATCTTTGAAAG
EMPV1_36831	CCAGCGGCAAGGTGCGGCCTGAAATCATCATTGAGCATGGAAACCGCAGCTACAAGTATT
EMPV1_36833	TAACCCCACTAAGCAATGCCAGGGATCAAACCCAAGTCGACGCTAGTCAGGTTCGTTAAC
EMPV1_36834	GGATTTAGACTCTTTCTTGGATGTGCTCTGGTAGGTTAACGATGGCCCTAAATTCCTCGC
EMPV1_36836	ATAATCCTGAGAATCTGGCCACCCTGGAGCGCTATGTGGAGACACAGGCTAAGGAGAATG
EMPV1_36837	AGGTCACAGCCCGGAGGGGAAGGCAGAGAACACAAAGCTCATTCTGGAAACAGCCACACA
EMPV1_36838	TAACATTTGTGCCAGGGCCGGCCTTTTAGTCAGATACATCAGTTTCATCTATGAGAGTCC
EMPV1_36839	GAGCTGTTCTACTCCCGTAGCTGGACGTCCTTTTTGTACTGTTTCGCCAGCTAGCATCTA
EMPV1_36843	GGTCCTAGTTGGGTTATCTACTTCCTATAGAGTCGTGTGTATCTGTTAACCCCAAGCTCC
EMPV1_36844	GATGTAGGTGCCTGAGGATAAAACAGAGTGGCAGGAATTATACACAGACAGAAGAGGGGC
EMPV1_36845	ATCAGACCCGCCTCCAGGAGAACTCTGAATTTGCAGCTAATCTCGGGGAAGAGAAAGAGA
EMPV1_36846	GGACCTACCAGGCCCTGGATGAGCTGATCCAGTCCCGAGACTCTCTCACCTTTTCCAACT
EMPV1_36849	GGCCTGGACCGGGAGGAACAGCGAGGGGCGGCGAGCGAGGCTGGGTCCCGGCACGCCCCC
EMPV1_36850	AGATTGTCAACATTGCCCGGCAGATGCGGCACCGCTCTTTAGCTAGAGAGCTTTCTGGAA
EMPV1_36852	TGGTGATAGCTGTGGTCCTGGGTACCATGGCTATTTGGAGATCAAGTTCAGGGAACAACA
EMPV1_36854	CTGCCCGGAGCTCTGTCCGGATGCAGTATTGAGACTGATGGACCAGTGGTGGGCGTACGA
EMPV1_36856	GACAGTTTTCAGTCGTACAGGAGGGTTTTAGCATAGGAAATGTGGTCCTAGCCCTCGGAG
EMPV1_36858	ATCCAGGCAAGATCAGAGAAGAACAGGGCTGAGGAGGAGAAGAATGAGGAGGGGGACACA
EMPV1_36859	GATTAACTGAGCACCTGCTATGTGCCAGGAGTGGTGAATAGGACAGACTTGATCTCTGTT
EMPV1_36860	GCTCCTACCAATGCCAAGACCCAAGGGTGCTCACTGCCTGTATAGAAAACACTGGAATAC
EMPV1_36861	TTCAGCCTTCCCTTTGGCCTTGGGATTCAGTGAGGAACAATTTGAGAAGTGCCCTGACAG
EMPV1_36862	ATGCCTGTAATGACATTCGAGTGAACATCTGGTATGGACTCTTTGTCCTGATGGCAACAC
EMPV1_36863	GCCATAGCATCCCCATCCTCAGAGTGGCCTCATACCTCCACTGTTTTATATTCGAGTGCA
EMPV1_36865	TTTACTCTTCACAAGAGGACACACACGGGAGAGAGGCCCTTCCAATGCAGTCTTTGTGCC
EMPV1_36867	AGTACATGCAGGCCGTCATTCAGTATGGGATGATAGTGTCCTGGCAAGAGATGGAGTACG
EMPV1_36870	CTGTGGCTGGATGAGATACAGCATGCAGTCAATGAGGCCAACAAGGACAAGGACAAAGCA

EMPV1_36873	CCCCAAAAGGTATGTTCAGAGACTACAGTTTCAGAAACACTGACTTATGAAACTGAAGTC
EMPV1_36874	TGACCTTGGCCCGGGCCTCCCGGGATGCCTCTGCTTCTGCAGCCATGGCTCTCTGGAGCT
EMPV1_36875	AAGGTCCTCCTTATTCCCCTGACTGGGCTGCTTACGGCAAAAAGCCTCAAAGATGTGCAA
EMPV1_36876	GGATAATGGGAGACTGCCTGAGCTGTGCAGATCTCCTGATGACAGGGTGCAGCCCTGTGT
EMPV1_36877	GTGGTGTTGTGGGCTCTGTGTCTGGGCGTGTTCTCATACGTGAAGGTGGCTACCAGCTCC
EMPV1_36879	CTTCTGGGGGCTGTTCCCAGACACAGAATCCCAGGCTGACCAAGCTGGACCAGATTTCAA
EMPV1_36881	CCACCGGCACTTAACTCACTTTTTATTAGCTATCTACAGCGAGAGGCTACAAACCCCCCT
EMPV1_36882	TGAGAAAGGAATACACCTACAGTGGAACAGAGCTCCCAGAACACCTGCTGAACCCCAGCA
EMPV1_36883	TTGGAGAAGGTGGCCGTCCCGAAGACCTATGACTACGTCTTCCACTGGAACATGCTCAAA
EMPV1_36885	ATGGGGACACCTGTCACAAGCTCAAGTACAGCTTCATCCAATTCAGAAAGTCTGTCTGCA
EMPV1_36886	GGAGTTATTTGGGTTTTGACACTGGAGTTGCGCCAAATAAGCATCAGAAATGTAAGATGC
EMPV1_36887	TTCCTTGGGTCTGAGCCAGCTCGGGATAACAACCTCTTTGTGCCCACACCAGTGCGGAGA
EMPV1_36889	TGAAGGAAGTCAACCGCAGCAATCACACCTACACCCTCGTCAGCAAGCTGAGCCTCCCCA
EMPV1_36891	TACCAACAGAGGGAGAGAACTTTGGAAGAAAGGACCATCTTTACAACAGCGAAATTTGAC
EMPV1_36892	CATGGGTGCTCATCTCAATGCCTATACCTCCAGAGAGCAGACTGTATATTATGCCAAAGC
EMPV1_36895	TCTGGCTGACCCCAGAGCCCACACTCTTACCTAGAATGGAAATTTAGCAGAGTTTTCTGC
EMPV1_36897	GGCACAAAACTGTCTGTGGGAGCCATCGGTCTGGCGTTTTATAATGGACTCTGGGCATAT
EMPV1_36901	CAGTCTCCAGTCTGCCAAGGGGTCGCTTGCTAAATCCTCCACTGAGGAAGAGGAAAAGGC
EMPV1_36902	CCTTAAAAGATGGTCTCAGCCACATCAGGCCCCCTAGGTCCAGAAAACTCCCAAGGCCAA
EMPV1_36904	ACGCGGTTGCTGGCGACGATTGTAATGATCTTGTGTTTGGTACTTACCCTGTGTGCTGCT
EMPV1_36905	AGCCATTAGCATGTACCTTCAGCCCCCTTCCTCCATCTCTACAGACCAGCCCAAGTTCAT
EMPV1_36909	CTGCTAATTGCAACTGGGACTGAGAAGAAGTCAGGAAGACACAGAGGTACATTAGATGGG
EMPV1_36912	ACGGCCTTTTCCTTCGACCTGAGGATCGCCCGGCTGGAGGAAAAAGTGAAAACCTTGAAA
EMPV1_36918	CTGTATGCCAACAGTTTCCTCTGATAGTTTGACTGATTTAGGATATCTGCTGTATGATGC
EMPV1_36919	ACCTGTGTCTGCACAGTTCAGCATAGAGCCTGTACCTAATACCCAAGGGAGAAGGAAAGG
EMPV1_36922	CCACAATCAGGAACTGTTTTCATCTTTGGTACTTAGGGAAGGTCTAGGAGGCTGAAGCCC
EMPV1_36923	GAAGACCGTATCCCAGAATTTGAAGGACCTAGGGCCCATCTAAGCCAGCATGGCCTCCCT
EMPV1_36924	ACTCGACCTCTAGGTCTCTCACGCCCTGGAACCTGGACAGAGGGTGCTGGTTGTGGACGA
EMPV1_36925	ATCCAGTCTACACACCAATAGGCTGGCACAAGCTTTGGGACACTCTGGACCCTGAAACCT
EMPV1_36926	CTGTTTCTCAGCGATGCCTTTTCTTAAGAAGATGCAGGCGGGGCCCATGGGAAACTGCAT
EMPV1_36927	TGAAGAAGTGAAAAGCTTGGACAGCAACGGTGAATAAGGAACTGGTTTCCCTGATGCGGG
EMPV1_36929	CCATCGACGGCTTAGAAGATCTGGTACATGGACAACTGTTTGGACCGATGGACTTACCAG
EMPV1_36930	CTTGGGTATGAAGGACCAAACGCAGTGACTAAAGTAGATAAAATCCCCCTACCCTCCTGG
EMPV1_36932	CGTGGAAATGAGGCATCAGACCATCTGACGTTACCAGTTGGTTGAATGTGTCCACTGTGG

EMPV1_36933	GAGCCCCTTGTACTTTCATGGGAACTTCAAGATTGGCTTTCTGTTTCTGCAAAAAGACTA
EMPV1_36934	TTTGATTCGAAAAATGAAGCTCTCAGCCAGCTGTGTGGGGAGGCTGTCTGCTGACTGTTC
EMPV1_36935	GTACACGTGCAAGGAGTGCGGCAAGGCCTTCAGCCAGAACTCGTCCCTTGTGCAGCACGA
EMPV1_36936	AGATAGGTCTCTCTCATTCCCAAACCCCTGCTCTTTTTATACCCCAAGGCTCCATTTGGG
EMPV1_36937	TTCTGTGACCTTTCCTCAGGTTCGACCTCCATCCCAGCACTACCGCCAAGGTGTTTATGT
EMPV1_36939	GCTACATTGCCAAAGCTGTGCTGAGAATGAAGTCAAGGGCAGGCCAGCGGAAAGCAATCA
EMPV1_36940	TTGGTCAGTTGGGTGCATCATGGGAGAAATGATCAAAGGTGGTGTTTTGTTCCCAGGTAC
EMPV1_36941	CACATCGTGGTAGAGGCGGCCGCTCTAGCAGCAGACATCAGCCACGCACCTGACATTGTT
EMPV1_36942	TATGACTGTCCTATTTTAGATGAAGATAGAGTTGTTGATGAGTTAGAAAACCAAATGAGT
EMPV1_36943	TCCAAGATGCACACGGAGACCATCAAGCCCCACAAGTGCCCGCACTGCTCCAAGACCTTC
EMPV1_36945	GATCCCAAAGGCCACAAAGAAATACATCTGGGCAAGGCACTGGGCAAAGGGGATGGTCTT
EMPV1_36946	CATGATGCAGTATGCATAGCACATTCTGTCCTTCCTGAACTGGACCACATAGTCATAGCT
EMPV1_36947	TACGGGGAGGAAGCAGAGGTCTGACTGTGGTGGGTAGTGTGCTTCTGGCACGTTTAAGAA
EMPV1_36948	ACTACTGGATGTGTTTTCAGAGCTAGTGGATATGGTTTGCTTAGAACTGTGATTGCTGTC
EMPV1_36949	GCCTTGGCTGGCCAGGCTTCAGAGGAACAAGGGGAGATTATTAAAGTGAAGGTGAAAGAA
EMPV1_36951	AGGAGTTCCTGCTGTACGGTTAATGATCCGGCTTGTCTCTGTAGAGGCTCCAGTTTGATC
EMPV1_36953	GTCCCGGGGGCCCTCGCAGACCTGCCTTCCCGCGCCGGCCCATCTCCCCCTTGGCGCCCG
EMPV1_36954	CCAAAGCTGAGGCCAACTGACTAACCCAGGAAAAGCCCAGGAAAGTCCCAGATCTTGCTT
EMPV1_36955	GGTTTCCTTTTATTAGCAGGGACTGGAATCATGGTGCTTTGTGGTAGGACTTTTGGCCCT
EMPV1_36956	GCCTCTCTTAAGGATGAGCCCAGTTAGCAAGAATGTGACTGTAAGCAAGCAGAGAGCCCT
EMPV1_36957	ACTCTAAACCTGAACCATGACTAAACCCACCATAAATCAGTGTAGCACATCACTACAGTC
EMPV1_36958	CCCATGCTGCAGACTGTGGCCAAGAACAAGGACCAGGGCACCTACGAGGACTATGTCGAA
EMPV1_36959	GTTTTGTCAGCCAATCTCTCCACGCAATTTATACATTGAAGCCCTTACTCTTAGTACCTC
EMPV1_36964	TATGTCAGACATAGCAATTTAATGTTGGAGGACCTGGATAAGGCCCAGGAGGAAATACTG
EMPV1_36966	CTCCTGCTCTGCGGGCAGCTTTTTGCAGCAGAAACCGGCAATGAGGCCACAGATGCCACA
EMPV1_36967	ACAGCAGGGACCCCAGCAGACTTACACCTCTGTGCTTGTGGAGCTTATGTTCTCATGGAT
EMPV1_36968	CTCTAGTGGAAGAGGAAGACCAACACATGAAATTGTCCCTTGGAAGCAGCGAAATGGGCC
EMPV1_36971	CACATTTATACCTGCTTTGTGGGGTTGGTGTAGGATTGGAGATAATGTAGGCAGAGTGCC
EMPV1_36972	ACCTGAAAGACTTTCTCGCCTCCCACTCGAGTGGACCAAGCACACGGTGCACGTGGAGTG
EMPV1_36975	GACTATATCCAGACAGACGCCATCATCAACTATGGAAATTCCGGAGGCCCATTAGTAAAC
EMPV1_36977	GGCATACTTGTAGGCAGAGCTGGGTTCTGGAGAGGATGATTGCAGGGCTAGGTTCTGACA
EMPV1_36979	GTCATGAACTTGGAGTGAAATCAGAGCCTCGCCATGGACAAAAGGAGAGAATAGGGTTTC
EMPV1_36981	GGAGCCTCTGGGAGTCACATTAAGTCATGAGTCTGCACCATATTGGAAAAACACCTGTTC
EMPV1_36985	CTGTGTGTCATGGTCCAAAAGTGCGAAGGTCATTACTGCTCAAAAAGTAGAAAACCCAGA

EMPV1_36989 AAGAAGCAGAGCAGCGGGATAGTGGCCGACCTCAGCGAGCACAGCCTGAAGGATGGAGAG
EMPV1_36990 TCCTGGAAGTCTCCTCTGACCACCTGTTGCCCACCATATCCTGTTCCCGAATCTTACTAC
EMPV1_36991 AAGCCTTGGAGGGTTTCATTGCCGTAGTGACCCAGGATGGCGACATGATCTTTCTGTCGG
EMPV1_36992 CCAGCCAACTTCTGAGTCCAACATACTTGCCAAACCACCTCTTTCATGGGGTGCAAGCCA
EMPV1_36993 TCATACCTAATCAGAGCTTAAAACCTAATGAATGCCAGGATCTTAAAGGAGTCTCACACC
EMPV1_36995 ACCAACGAGCAGTTACAGATACATTATAAGGTATGCTATGCACGGGTTCTTGATTATAGA
EMPV1_36996 TTCTGAGCTCTGCGTCTTGGTGAGAGCAGGATGGATCAGCTGTGTAGGCTGTAGTTGAGA
EMPV1_36997 GTCTCAGTGCATTAAAGGATCTGTGGTTGCTGGTTGCTGCATCTGTGGCATAGGTTGGGT
EMPV1_36998 CAGGTTTATTAATGCCCGGAGAAGAATAGTGCAGCCCATGATAGACCAGTCCAACCGAGC
EMPV1_37000 AGATCCCAAATGGACCCTCTATAAAACCGGCCTGTGCTCACTCCCTCAGCTTCCCTGGCA
EMPV1_37001 CAGGTAGCATTCATGCCAGCGCAGGAGACAGGATGCCAGACAATTAAACATCATACCAAG
EMPV1_37002 GAGGATGAGATCGAGGCTCTCATGAAAGCAGCAGGTGTAAATGCTGAGCCTTTCTGCCAG
EMPV1_37007 CTCATTTTACACGAGGAAACAGGCACAGAGAGAGGTTAAGGAACTCGCCCAAGCTCACAC
EMPV1_37008 TCCCTCTCTTAAGAAACATAAGTAAGGAGTTACTGTCGTGGCTCAGCAATAATGAGCCCG
EMPV1_37010 TTGTCACTGGCGTCCCAGCCTGCACCAGGCAGCAGGACATCTGGGGACTTGGAGTTTCTA
EMPV1_37012 TCAGCAGCTCCCTCTCCAGGCTGAGTTGATTCTTCTTTCAGCTGGCAAAACCAAATATTG
EMPV1_37013 GTGTTGTGTCCATGTCTGTTTCATATAAAGAAAAGTCTGAGGGGAGAGACACAAAAGTAT
EMPV1_37014 AAGCTGAGGGTCAACCCAAAAGGGTCTGCGGCAAGTGGGATACAGTCCTGCCCACCGATT
EMPV1_37018 GACATCCACTCCGTCCCAACCCCTCCTCTTTCTCAGGGCAGATCTGGTGATTGGAATAAA
EMPV1_37019 ATCTATGCCTTTGTGTCCGCGGTCTGGCTAAAGCTCAAGCTCCTTAGGCTCTCCTTCAGA
EMPV1_37021 GTAGCTGGGGAAGAAGCTGGTTCATACTTAGGGCATATTCAGACAAAAATAGGTCCAGCG
EMPV1_37022 ATCCCCCACGAAGGCTGGGGAGAGAACTGGACTGAAAGAAGCCCTGGAAATTCTGGGAAA
EMPV1_37025 AAGGCTAGCTGGGCCTGGAATCTCCCTCTTTGTTCAGAGCCTTCCAGTGAAGCCAGCTAT
EMPV1_37028 GATGCTCAAGACTTGGCAGACCTGAACTTCAGCGAGCTTGATGCTGTCAGGATACCTGGT
EMPV1_37029 CCCTTCATCAGGGGATGGTGAGATCAGGGAATGGGGTAGACTTTGAGATTCATAGCCAGT
EMPV1_37033 TGTGTAGCCGCATCGCCTGCCACACGGAGCCTGTCATGGGACTGGATGTTGATTCTCAGA
EMPV1_37034 GCTGGAGCACAGTGTAAAAGGAAGAGTTTGAGAGATAGGCAAGGGCCAGACTGTACAAGC
EMPV1_37035 CTGCTCGCAAATGTCTGCATGTTAGCCCCTCCTATGCTAAACCCCATCATTTACGGGATC
EMPV1_37037 ACAGAATGCCTTGCAAATGCAAAACCAAGCAACACAAAGGGATACTGAATATTCATGGGA
EMPV1_37038 CTGGGTCAGACATCTATTATATACTCTCGTAGAGCTCCGTCTTCTTCCTCTGTCTGTTCC
EMPV1_37040 CCCCATACTATCTCTGAATCACGTAGAAGAAGATGGGGAGGTTAAAGATTTTGGGCAAGA
EMPV1_37042 TCCTTGTTCAGTGGGTTAAGGATCCGGCATTGCCGCAAGCAGCGGCGAAGGTCACAGATG
EMPV1_37043 CCATCCAAACCAGGCTGGGCTTCTGTCAAATCTGAAAAAATCGCTCCTAGGATGAGTGCC
EMPV1_37045 TTCAAGAAGCCAACCTGAGGGGTTCCAACGTGAAGGGCGCCATCTTTGAGGAGATGCTGA

EMPV1_37046	AAATGGATCGCGATCGCACAGAATCCCCTCTGCACTTCCCTGGTAAGCTCTTCTCGATCC
EMPV1_37047	AAGGATTACTTCAGGACAAGCATAGCACCAAGGGGCTGGGCAAGATGGCTCCCAGTCCTT
EMPV1_37049	GCTCAGAGGGACAAGGTTAATAAAGGTGTGTGGATGAGCAGATACTGCAACTGTCCGCAC
EMPV1_37051	GAGAGGGGATATCTTTGCTTTGCTCCCAGTCTCAAGGGGAAGCATTTGGTGAGCTGTAGA
EMPV1_37052	TGTGCCTCTGCTCCCAGCCTTACAACTGCTAACAAGAAAAGCTCAGCACCAGGAGAGACT
EMPV1_37053	GGAAGTATAGCCCAGAGGCTGAAATCCAAAAGCTGTAAAAAGGAATTCAGTGGAGGGGGC
EMPV1_37055	ATCCCCATGGAGCATCAGAAGATTCCTAGATTTCAGACTCACCGCTCTGGCTCTTTCTCT
EMPV1_37056	TCGGAGAGTCGTTCTTTCTTGAGTGACGGGGGAGAAGCGCTGAACCCCAATCGAGCCAAA
EMPV1_37062	GACGTGTAAGTATGTGCCGTACAATATTTGGGACATACTTATACTAAAAAGTATTTGTTG
EMPV1_37065	TCTCCTTGGAAGGTCAAGGTCAGGCTTCTGTGCAGGTCTATTTCCTAAGACCTATCAGCC
EMPV1_37066	TCTGGACCCCTCGGCCCCCTCCCACCAAAGGGTTCACCACACACTTGAATGTACAACCCA
EMPV1_37067	GCTCCAATGAGCTTCACTACTTGGCAATATTCTGTGGGTGGTGCGATATATCAATGCCAG
EMPV1_37068	GGCGTCGAGATCGGATAAATAACAGTTTATCTGAGTTGAGACGACTGGTGCCAACCGCTT
EMPV1_37074	GATAGGGAAACTGAGGCACGGAGCACTTACGGTGCTTGCTAGTATGTGATAGGTCCAGAA
EMPV1_37075	GCACAGATGGAGTGATAATTTACGTGGCTGAAAACATCTCATCTCTCCTTGGCCACCTAC
EMPV1_37076	TACAAGATTTCCATCCCAAACCCCTAGCTCATCCCCCTCCAACCTGTCTCCTTTGGTAAC
EMPV1_37077	ACAGGCTTTAAGAAGTACATGCAGTCGCAGTTCCTTGTGGCTCGGCGGTAATGAACCCGA
EMPV1_37080	ATTGGACAGGTCAGCCTATGCCTGAGGGCTTTGACCTGGATATCATGCATGGCCTTCTAA
EMPV1_37081	CTAGCTCAGGTTGGAAAGCATGTGTGCTGGGTTGAAAAGAGTCTTGGTCTAAGAAGAGGG
EMPV1_37082	CGACGACAACAGCGGTCATCACTACTCGGAAAACGACGGTCACCACGACTCCAAAAACAA
EMPV1_37083	CTTCTGTTCTGGGCCCACTGTCACACCGTTGCCAACCAATTCTACAGCTAAAACCACAAC
EMPV1_37085	TCTAGCCTTTCCCTGGCTTCGCTGAACACGGGCGATAACGAGAGCATGCAGTCTGGCAAG
EMPV1_37088	AGCCTGGGGTCAGAATCACTGGCTGAATCTCGCTGCTTGCTTCTTGCTCAGCCTACATGT
EMPV1_37089	CATCAGTTACATTTGATGCAGGATCAGTCAGGTCTGGAGGCGAGTGCCTGCCAGCAGCTT
EMPV1_37091	GCTCTGACTTTGAGAAACTGAAGCCTGGTTATGTGGACGAGATCCCTGAGAAGATGAGGC
EMPV1_37092	TGTGCAGCCAGTCTTGGACATGTGTGCGAGTCAGAACCCTAAAGACCATGAAGTGAGCGG
EMPV1_37093	AGCTCCAGGTTCGAAGGTCCAGCCCAGTGACCCAGGTGAAAAAGATGATCCAGATGAAGA
EMPV1_37095	AGCTGGAGGGAATATCAACGCTCGAGATGCTTTCTGGTGGACCCCACTGATGTGTGCTGC
EMPV1_37096	TTTTGATTTTGCATCCAGTTTCTACTCCTCTCTGCCCTCGTAGCACTGCAACAGCCACAG
EMPV1_37097	AGGACGAGCAGCTGAGGGCCCTGGTGAAGCAATTTGGGCAGCAAGACTGGAAGTTCCTGG
EMPV1_37099	GGCCAGATTAAAGCATAGCCAGTGCCACCAGTTGAGATGCTGAAACACAGATTTTGAGTG
EMPV1_37105	TCTCATCTGGCTGTGGTTTCACTGTTCTATGGCTCTGTACTGGTCATGTATGGGAGCCCA
EMPV1_37106	TTGGCCTTCTCCTGTCTGGAGGTATCCAACCTCAACCTCAATCAGAGAGACCGGATTGCC
EMPV1_37109	ACTGGGCTGGTTTAGATGAAGATGAAGATGCACATGTCTGGGAGGATAATTGGGATGATG

EMPV1_37110	GCATGTGAAAGTGGGCCCGTGGCTGCCTTTTTTCAGAAGATCAAAGGTGAACTCCCTGTT
EMPV1_37112	AGGGAGGAAGCTCGTCGCCGACCTCGCCCTCCTACTCTACGCCCGGCACGTCCCCTGCAA
EMPV1_37115	AGCCGGACGTGATGTACTTTCTGCTCTTCCTCTGGCTCCTGGTTTACTGCCTGCTGCTCT
EMPV1_37118	TAAGGCTGGGAAGGACTCCGGAAAGGCCAAGACAAAGGCGGTTTCCCGCTCGCAGAGAGC
EMPV1_37122	GCTCCAGCCCCACTTCTTAAGCATTCTTACTCCTCTTCTGGGGCTAATGTCAGCATCTCT
EMPV1_37125	TGATCATCCCTGTTCGCTGTTTCACGTGCGGCAAGATCGTCGGCAACAAGTGGGAGGCCT
EMPV1_37129	TTTTACAAAAAACCTGTTCTGGAGGCTGCGTGGGAGCCTGGCTGGAAGAAGGAACCGGCT
EMPV1_37130	ACTTCCCTCCTGCAGCTGAGACTCCATAGCCCAGGAGGAGCACCTCGACCATCAGCTGCA
EMPV1_37132	ACTGAGGGTGCAGATTCCAAGGCTCATTCTAAGGGGAACGGAGAAAATCGGAACGCTTGC
EMPV1_37136	GCAAATGGCTTTTTGTCTTGCTAGTTTGCCTCAGTGAATACCTTCAATTCTGTGTCCTAG
EMPV1_37137	GGGCAGCCTCCAGCCAATAACTAGCAAGAAACCGAAGACCTCAGTTCAATATCCCTTCAA
EMPV1_37138	AGGTTTTTCCAAGGCTGTTTTTAGTCCACTGACGGTTATTGAAGGAGGTGGCCCAGGTGG
EMPV1_37139	AAGCACTGCCGATCTGGAGGCTGCCCTCAGAGCAGCCGGAGCCTAAGCGCTCTACCAGCT
EMPV1_37141	CTCGCGAGAGGGCCCGGCCGCCGGAAGGGGAGGCGACCCGGAAGTGCCCGCCCGGCCGCC
EMPV1_37144	GCTAGACCATCTCTGCAGGTTCCCCAAACCATTGCTGTTTTGGACAGTAAACCAAATACC
EMPV1_37146	CTGTTAATGCTAATTGTGATAGGGGTTTTTACCTCCGTCTGACTCCTACATGTTAGCATT
EMPV1_37149	CTGCAGCACCTCTGCTCTTGCTTGTTTCTTGTCTCCACATTCTTCGAGGTGACAGGGAGC
EMPV1_37150	CTCACTCGGTCGGTGCTGTGAGTGAGCGATTCGTTTTCTTTCTTCCAGAAGATCTGTGGT
EMPV1_37151	CCAGCGACTCCTCATCCTCTGAGCGGCTGGCAGTGCCTGATGCCATCAGGGATGAATCTA
EMPV1_37153	GACTGACGTTCTGCCGAAGTAATGTCCTTCCACATACCTGTTGTGAACACATCGGCTTGG
EMPV1_37154	ACTGAGACCTGTCAGAGGAGGACCTGTAGCCTTCCCGGGGCAAAGACCATCTTTTAGAAA
EMPV1_37155	AAGGTGGACACGAGAATCACCATCCAAGGCGAGGAGATCAGTGCCCCCATTTGCATCGCA
EMPV1_37156	TCCCAACTGCTCGGATACCCATAGCACAAATCTCTGCCGGTGTCTACTTCACCATCAAGA
EMPV1_37158	ATCAGGGAAGCGGAGACTCTTAAGTGTAACGGAGAAATAGCTCAGCCTCCTTCTTCCCGA
EMPV1_37159	CTGGAAACGGAGCTTAGAAAGAAAATTGAAGAACAAAATCAACTCGTTTGCAAAATGAGC
EMPV1_37160	GGTCTGCTGGTGCTACAGTATTTTTCTCCTGGAATACTCTTGATTCATCACAGGTCACCA
EMPV1_37161	GGAAGGCACCCATGAAAAGAGGAACCAGAATTTGTCTGGAAGACTGTGGATCACAAGGGC
EMPV1_37163	CTAACCCATCTGAAACTGAATCTGAGCGTAAAGATGAGCTAAGTGATTGGTCGTTGGCAG
EMPV1_37164	CTTTGATAGCTGCACCATGCCAAATAATCACCAACACTTCTACACTGCTAAGAAACTACC
EMPV1_37165	TCCGATTTATTAACCTTGCAAGAACTCTTCAGGCACATATGGAAGATCTCGAAACTTCAG
EMPV1_37168	AAAATACAGCTTAGTCTAGGAGGTTGCACACTGTCCGAGCCCTTTTCCAGGACAACACGG
EMPV1_37169	ACCTCAGACTCCGGTGGAAGCGACGTGGACCTGGACGCCCCTGACAGCAAGCCCTTCTCC
EMPV1_37170	TGGACTGCCACTCATTCCAGGGCTCCCAGCATCCACACGTACTTCCATCATTTTCCCCCT
EMPV1_37171	CTTTGGGAACAATTTTTACTCACCGTTTGACCCATCGTCGGCTACTCGGCCCGGAGAATT

EMPV1_37173	TCGAGGAAGTACAGGTTGGTGTTGTCTGGAGGCAGGTGGACAGCTTTTGTCTTGGCTTCA
EMPV1_37174	TTTGACAAGGTCAGACTCCATATTGCACAATCCAGTGGTCCCTGCTCTGTCCCCACTCTC
EMPV1_37176	TTGAATATAGGGACATAGTCACCCCAACTAAACCCAGGTCCTATCAGTGAGGAGGGATAC
EMPV1_37177	TCATAATCCCTTGGTGGGACATGGGATGTTGTTAAAGCTTCCAGGTGGTTGCAGTGGTCC
EMPV1_37178	CAACGTCAGGGACGCCGCCTGCTACGTGTGCTGGGCCTTCGCTCGCGCCTACGAGCCTCA
EMPV1_37182	ATGAGGGACTTTCAGGGGCAGCTGTGTTTTCTGACTCAGTCATAATGCCCCTAAAAATCC
EMPV1_37183	CGACAAGGTAAATGATGACTTCTACTCCAAGCGACGGCACCTGGCCGAGCTGGCTGCCAA
EMPV1_37184	CAGGGGTCGGTACCCCCAACCCGCATATTGTTCAAGGGTCAACTGTATATCTTTTGAAGC
EMPV1_37186	AGTAACCTGGGTCCAGGGCCTCGGTGGTGCGATGTCCCTCACCCACCCCTGAAGATCCCA
EMPV1_37189	TTTCCTCGGCACGGCCTGCATGCTCTGGTTGCTCGACTTCCTGTGCATCCGCAAGCATTT
EMPV1_37190	TAGGTTAACATGGTCTGCATTTTTCAAGTTTTTGGTTCGGGCGAGGGGTGGGCAGTGGAC
EMPV1_37191	GCTTGCTTCAATGTCACCTGAAGAGCCTATTAAAACAGATTGTTGGAGTGCCTGTTGTGG
EMPV1_37192	GAACAGGCATTCTCATCGCACATACTGGAACCAATAGAAGAACTTTCAGAGGAAGAAAAA
EMPV1_37199	CTTTGGTCATAGCTAGATGCTCCCGGAGTTGCTCACCTCTTCTTGCCTCGTGCAAAGGAG
EMPV1_37200	CCTTCATAAGACTGGCAGTGACCTTCTGTAAAATGTGTGCTTGGTTGCATTCACCTCCCC
EMPV1_37201	AAGTACCTCGACATCCATTCCATGCAGAGGCTGGAGAAGACAACCAACGTGGAGGAGATG
EMPV1_37202	ACTACATAGGACACAAGGGCAAGTTTGGACACGAGTTTCTGGAGTTCGAGTTTCAGCCGG
EMPV1_37204	TTGGAGAAGATGGGCAACAAATGATTCTGAGGCTTGGGGGCTGCTTGGACTTACTCACAG
EMPV1_37205	CATCCACAAGCTGAAGAAGGCCTGCAGGGTGGAGATGGAGGCTGAGCAGCAAGGCCAGCT
EMPV1_37206	GCAGAGCTAGGAGCTAGGAGAGCTTAAAAACAGGTACAAAGATGCAAGTAAACACTGCTA
EMPV1_37207	TGGCTTCTTGGAAATGTTGATGAGCTCCTTAAGGCGGGGCACACCCAGGGTCACGTTCTT
EMPV1_37208	GCTGTCCTCCTTGTCAGAGGGCTGACCACCATGCCAAATGTGTAGGAATAAAGCATTCTG
EMPV1_37209	GCAGGTGTGTCAGGAGAACCAGCTCATCTGCGAACCTTCCTTCTTCCAGCATCTCAACAA
EMPV1_37212	GTGGGAAAGCCTTCTGTCATAGGTCATCTCACTACTGGTTAAAGCTCCACTACAACGTGC
EMPV1_37215	GTCAGAGCCTTCAGAGGGAAGGAACCTGGATGACACCTTGACTTTAGCCCGGTGGATTTT
EMPV1_37218	ACAGAGATTCAAATTGGAGAAAAGAATAAGGGTATAGTTATAAAAATAGAATGCTCTGTC
EMPV1_37222	TCTGTAGATGGAGGCACGGATTAGAAAATCCCCTGGAAGCCCCTACAGCTCCCAAATCTG
EMPV1_37223	ACCTTTCCAACTCCCTCACCATCATCCCAGTGTCTTATGCCTTCATTATTGCCACCGTCC
EMPV1_37224	TGACACCAAAGCTACAGTGAGACCGGCACAGCAGGGGCCATGAAGAACCGTTTACCTTTA
EMPV1_37226	GATGAAAGCAGTGGACACCGTGGAACAAAATGGTATGCACACAAGGAGGGTCTGGACCAC
EMPV1_37227	GCCAGCAAACCTGGTAGTGGCTCAATTAGGCAAAGTGTGGGAAGCTTACTTCTGTTCTCC
EMPV1_37232	GCTTTCAAGTCCAGCTATGACTGTATCGTACATGAGAAAAATCACATTGGCGAAGGGCCC
EMPV1_37235	ATTACACATCTGCTGGTGATGAAGCTGGACGGCAAACAGAAGGACCTCCACTCAGGAAAG
EMPV1_37236	CACACTGCGTGGGTTAAAGATCCAGTGTTTCCATGAGCTGTGATGTAGGTCGCAGACACA

EMPV1_37237	TTCCCCTTCATCCAACAGGACTCAGAGGAGCCATGATGAGGAGGCTGGGGCGTGGCTTCT
EMPV1_37239	ACGGTGCAGTTCTCCCAGCCTGCCTGTTTCCCGCCCAGGCACAAAGGGCGCAAACCGAAA
EMPV1_37242	CAGAGTTCACCAACCGGAACCCCCGGAATTTGGAGCTCTTAGCAGTAGCCAGAAAAGAGC
EMPV1_37243	TTTTCCCTCACTTCAATCCTGCCTCCTCCTTGCCCTCCTTCCACAGAGCCCATCTTGAGA
EMPV1_37244	CCGTCTTCTTACTGACGAGCAAAATGAAGTTAGACAAGAAATGGCCAAGTTGCAGGACAA
EMPV1_37248	GGGTGTTTCCCATGTACCTCATGGCCTGTTCATCCAAAATGATGGCAGAAGCCACAGAAC
EMPV1_37250	GCCACAACCGAACTCTATCAGTATTTACTACAGCCATTCCGAGACATGCGAGAACTTGCC
EMPV1_37251	TACGGAAATGGCAAAACTCTTGATTTCAGGTGTGCCGAGGGCATGGGTAGATGAACTGTT
EMPV1_37252	AGACTCTGGCTTCCTGGTATATTCATACAACAAGTCAGGGATATTGCATCCAGATTCTTC
EMPV1_37253	TGCTTCAGGAGGTTGGCTACTCTAGAAAGCTCTGCCTTTTCCTTGTACACGTTGCTAAGG
EMPV1_37254	TGTCTCCCTATTTTTTTGTACAAGCATAGGAGTGTACTTCAGCGCTGCTGCTACCCACGG
EMPV1_37255	CTGGAGCCAGGGAGTGATTGAAATCAGGGAGTGTGAGATGTAAGCTAGTAAATACAGGAA
EMPV1_37256	GGGGCTCTACTGAGCTGCAGGAATAGTTGGTGTGTGTCAGGTATTATTGTAAGACTCGGG
EMPV1_37260	AGCGCTTGACATCGCTGAAGGAGCACATCAAGTATTGCCACGAGAAGAATGAGGAGAACT
EMPV1_37262	CAGGCAGAGTTACATAACATCACTATATCTTACTTTCTTTGTAGAATGGGGAGTGTTGTG
EMPV1_37263	CTCACCAGGTCCCGCTCCATTTAAAACCAAAGAGCCATCCTCTCGGATCTGGAAAATACA
EMPV1_37264	CATACCCTCAACAGCACTTTTATCCCTTTGCATCTTGGCAAAGGACTTGCATTTTCACTT
EMPV1_37265	GCAGTCAGCCCTGGACCACTGACTCCCAAACGTATCTTGTTGTAAGCATTATGTGGAGCT
EMPV1_37266	CTCTTCCACACAAAAATAAGGTGCCATGGAACAGGCTGTTCCCAAATGCTGACTCCAAAG
EMPV1_37267	GACCATGGCTTTGCTTCTCAAACAGGCCGAACTACACCTGAGGACTTGCGGGTTAATTTG
EMPV1_37268	GATGAGGGAGAAAGAAAAAGCCCAGAAAATCAGCTACATGATCAGGAGATTCTGAGCCTC
EMPV1_37269	TTAACCACCAAGACCTTCCCAAGTCCTACCTAGGGGGTATAGATCAGCAGCACAGAGTCC
EMPV1_37270	ATGCTGAGCTCCGACTTCAGGCCCAGCCTGCCCCTCCCCCACTTCAACAAGCACCTGCTG
EMPV1_37271	TCAGCACAAGCCACTCATGCGCTGGAACACCACGACCCGTGGAAGAAATACTCAGAGGAT
EMPV1_37272	TGCCACCATGAGGACAATCTCTCCACATTAATTTCCAGGGTTGCCACCCAGCGGAAAACT
EMPV1_37276	GGCAGAATGGCAGTAGGGTTTTCCCCATTTTGGTTTCGGAAGGACAGGAATAGTGTGTTT
EMPV1_37277	GTGTCCTCGCAAGGTCGCTTTGCAGAAGGGGAAGCGCGCGTATGTGACAGGACCCTCGTT
EMPV1_37278	TCCAAGCTGCCCTGATGTCCAGGACTTTAGAAACTGTAAGGTCACCTCGGAGCCTCTGAA
EMPV1_37279	GGCATCTCCCTGATCACAGCTAATTGTCAAAATAGTGTGGGGTCGTTTATAACGAGAAGC
EMPV1_37280	GTGAACCATCCCAGTTACAAGAATATGCTAAGCTCCGACTAGCTCTGCTCAAAGTCTTAC
EMPV1_37282	AAAGCGGAAGAGAGTGGACGTAGAAGAATCCGAGGCCAGCAGCAGTGTCAGTATCTCTCA
EMPV1_37283	GACGCCACTTTCTCCTCCTTTGCCCCTTAGTGGTATCTGAAACATGCTCCCCAAAGCAAT
EMPV1_37284	CTACGGCCGCTGATTTCAAGCAGGAGCTCAGGCGGAAAAGCAAATTGGTTGAAGAACCAA
EMPV1_37285	AAAAAGAAGCTGGATGTGTGTGGACAGAGCGGGCTGGAGGTAGGGAGGTTGCCCACTAAG

EMPV1_37288	GCTGATGGAGAAGCCTGATATAGTGGTGGGGACCCCATCTCGGATATTAAACCACTTGCA
EMPV1_37291	AGAGACCTAGGGATAGAGGGTCCGAATTGCCTGGCGGTCGACCAGCTGTCATGGCCGACT
EMPV1_37293	GAGACCGGTAACGTGGCCAACCTCATTAGCATCTTCGGTTCCAGCTTCTCAGGACTCTTA
EMPV1_37297	ACACTCAGTAAGTATTGGCTGTTGTTTCTGTCACTCATTTCTGCTGAGTCGGCGGGACTC
EMPV1_37301	GGATCCCGGCTCGCCTCAGGGCTTTTGTTAGTGATTCCTTGTGTGTATGAAGGATTGCAA
EMPV1_37302	GGGTAGAAGTGAGCCCTTCCTCTCTGTCATCCCCACCGTATTCTTTATCCCACTTTCACA
EMPV1_37304	ATTACAGCCAGTTCCGGAAGGTCAGATAGACTTAGGGTGTCTCGATTTGGACTCCAGCCA
EMPV1_37305	AGCCAAGGAAGAAGCACCAAAGGCAGAGACGGAGCAGAAGGAGGGATTGGAGGGGAAGCA
EMPV1_37307	GGTTTGTTTGAGCAAGTATCCTGGTGCATACGAAAAAAGATATCAATTCTCCTCAATTAG
EMPV1_37309	GTTCAGTGGACGTGGCGAGACAAAGTCTGCGGAGGGAAAGGTAGCTGTTAAAGAGTCACT
EMPV1_37310	GCACGCTGGTTTTGTAGATCAGCCTCCCGTAGCACCAGCATAGGTTCTGTGTTATCCAGA
EMPV1_37312	TGTCTCCTCACCAAGCTCTCCAAGTTCCTGCGGGACATGATCTGGGCAGTGCGCAGCTTT
EMPV1_37313	TCCAGAACCAGGATGGCCACCTTCTATACGATTATGCTTCTTGAGAACATGATCCTCGTG
EMPV1_37314	AGCTTCAGGATGGTTCGTGCTAAGAGCTGGACCCTGAAGAAGCATTTTGTAGGCTACCCT
EMPV1_37315	GCAAAAAGTCAAATGGAAGGTACACAGCGACTCTGGATGCAACCACTATGCACAGCACCC
EMPV1_37316	GTCAGGTACTAGTGACAATCCCCTACCTATACAGCCTCTTTTTGTCCTTGCTGACCATCA
EMPV1_37319	CCTTCCTGACCGTGACCGGAACGGACTTCAGTGAGAAATGTGGAAAGACACCGACCTCAG
EMPV1_37320	TTTTACTACTCTGGGTCATCACACAAGGCCAAAAATCAAGTCAGCAGGCCTTACCTGCAG
EMPV1_37321	AAGGCCCATCTGCGGCGCCTGGATCGCCGGTGGACCCTGGGCGGGATGGTCAACAGGCAG
EMPV1_37322	GCTGCTGGAATGTGTAGGTGCCCTCAATGGCTCAGTAGCCAGTGTAGATCCTGTCATTTG
EMPV1_37323	GCTACCCATCTAGCCCTGTAGCCCAAGAGATGTCGCAATGTGTGGCAGAGCTGGCAGGGC
EMPV1_37325	CTACTCGCCTCAGCCCCAGGCACCACCATGACCGGCTCCTGCTGCGCCCAGCCCTGCTGC
EMPV1_37328	TGCACCCAGTGCCGCCACCAGTTCTGCAGTGGCTGCTACAATGCCTTTTATGCCAAGAAT
EMPV1_37333	GGTTTCTATGTCAACACTTTCCAGAGCATCGCGGGCTTGGAGGAGAACTTCCACAAGGAA
EMPV1_37334	CATGGCTGTGGATATGGCATCTTCCTCACATTGGGCTATGGCTGTGGCTGCAGAAGCTAC
EMPV1_37335	AGTGGTTCCAGCTATGACAACCCTCAGCATTGAAAGCAGCGCAGGGAGAAGCACTGAGTT
EMPV1_37336	GTATGGGAAACGTGACAGCCCAGACGCTCTTCTCTCCAAACTGCTCTTCCCTGAAGGCGA
EMPV1_37339	GGCGCGAAGGGGCCTCCCTAAGTTATTCATCTCCTCTCGCTGCTGCTGCTCTGGGAGGGA
EMPV1_37340	AGGTGACACTAGAAGGGGACAAGGTGAAAGTGGAGCGGGAGATCGACGGGGGCCTGGAGA
EMPV1_37341	CACTAAGGTTAGGATCTTCTAAGTGTATAACTGTCTCCTAAGCCCATCACTGTGGCACAC
EMPV1_37342	CCCAACCACAGGAGAATATGGGACCGTGTAAAACAATGGAGAACAAGAGGAAATTGTCAC
EMPV1_37345	GTGCCTATCTATAGGTAAACTGATTCATAAGCTCTAGCATGTTTGGCCCTCTTCTCCACT
EMPV1_37347	CAGAGTTGCCGTATGATACAGGAATCCTACTTCTGGGGATATATCCAAAGGAAACTGTAA
EMPV1_37348	ATGTGGTGTCAGTAAATGGCTGCTGTGCTAGTCCCTGAATTAGCCTGTAGACTGTAGGGC

EMPV1_37349   TACTACAGGCCATTCCACCTTCTGACCAGGCTCATTTGTTCCCCCAGGGTCACTTTCAGT
EMPV1_37350   TGTCCGACACCTTGGCCGAGGGCATGACGCTGAAGGAGTTGAGGATGCGGTCCGGGAACT
EMPV1_37352   CTGGCGCAGTGTGTCCACGTGCTCCTGGGTAGAGAGACACAGGCCTCTTGGAAGTGCAGC
EMPV1_37354   TCATTCTGGCCACCCTGGCCCTGGAGCTGCTCTGTTCCCTGACGTGCCTGCTTGTGTACA
EMPV1_37355   AGATACTGAGAGGAACAGAACCTGAAGCTGAAGGTATGTTTGCCATGAATGCGGATGGAG
EMPV1_37356   ACTTGCTCAGCACACAGCCACACGGCACTCCCGCACACACAACTCACGTGCCGTTTCACT
EMPV1_37357   AACATGGGCTACGTACAGCAGCGCCGGGAGTCCAGCCTGAAGTACCAGACTCGCTGCATA
EMPV1_37365   ATCTCATGCGCTGCGTGGATCTCTACAACCAGGCCCAGTCCAAGTGGTTTGAGGAGATGG
EMPV1_37366   GCTGAGAGCACCAGCCACATGGAAGTATCTGAACGGAGACCATCAAGAAGACATTAGGAG
EMPV1_37370   TCGACTGTTGACCGCCGAAGGACCACTGCTATCAGGGAGGACAATAATTCACTTTTGCCC
EMPV1_37371   CAGTTCCTGCAGCCGCTTTTGTGTTTCAGTTAATTCCACTTTCGCCTGTTCTGCTTCATG
EMPV1_37372   GGCATCCCGGCCCACGTCATCCAGCTGGGGCCTGTCCGTTGTGTTCACTGGAACCAGGCC
EMPV1_37374   TCAGACATTGCTGACCCTGGCCTCCACCGAGGAGACGGCAGAGGGGCCCACATCCCTGTG
EMPV1_37376   GGCTACATTCATGCCCTTTCCTCCCAACACCCAGGCCAAATTCTCCCATTGATTTTAGGC
EMPV1_37378   GACGTCAACCTGCAGACAAAGCTGGTGCTTGTGGAGAGACTCGTGCGGCCTTCCTGATGA
EMPV1_37379   TTCATTTTTGGAGATCCTTCTGTGGCATGAGATCAACAGTGTCTCTCTAGCAGCAGGGAC
EMPV1_37382   AGGTCACGTCTCTGCAGTTACACAGCTCACGAGTGCCTGCTGGGGTGGAACCTGGTCTGT
EMPV1_37383   AATGCAGATTCAGAATCAGCTGGGCCTATGATTCTGCATTTCTGACAAGCTCCCAGGGGT
EMPV1_37387   AGGTGCTCATTGTGTTTCTACTGCTCTCCTGGGCAGTTGGGTTCATACATACCCACAAGCC
EMPV1_37388   GGAACAAAAGACATTTCAAAAAACCTTAGTTAAAATCCTCAGAGGTAAGAGCAGCTATTG
EMPV1_37389   TCGGGTCGCTTCACGGTTGTGGATTAAGACCTCGGGGCCGACCAAAGGGGAGCAAAAACA
EMPV1_37392   CTCCATGTGGAGCTTCGCACTGAGACACAGCGAACTCTTAGCAGAAGCAAGGACATAGCT
EMPV1_37393   CCAGCACACACAACCATAGCTTTCAGTGTTTTTGAACTCTTCATTTACCTGGATGGTGCC
EMPV1_37394   CACCTAGGAAGGCCTCTCCCAAGGCTGACAGTTTCCCCATGCTTTAGCGCCTTCAATGAA
EMPV1_37395   GCCAGGACCTTATCCAAATTTGTGGGGCTGCCGACGGGATTCGCCTTTTCAATACTCTTA
EMPV1_37396   TGTAGAGCAGCGCCGCCTGGTGGACGGCAGCTTAAAGGAAGGGGAATTAAGTTACTTTTA
EMPV1_37398   GAAGGTGGGGAGCTCTCTCTCAGATACTGAAAGTCAAAGGATCAAGACAAAAATGGATGG
EMPV1_37400   GATTGTTGCAAACATACTTTCAGGAAGATCCCGATACGCCCATGTCCTTCTTTGACTTTG
EMPV1_37401   CCACTTCAACCAAAGGATTTCTGTCCCCAGGATCACGTAGCCCTAAATTTAAGAGCTCAA
EMPV1_37403   AGCGACCTTCTGTCACACATCTCCTTGACCATCCATTTATTCAGAGAGTCCACGGGAAGG
EMPV1_37405   GCCAAGGCCAGAAAGTGTGGAAGGTGATGGTGCAACCCATCAACGTCATCTTCAGATACT
EMPV1_37406   TTACTGGGGAGCTTCGTGGATCTATTTGGTCTCTCCGCCCCTCCCCCCACTGGTCTTTAC
EMPV1_37409   TATCCATGAGGTTTTATCCCTGGCATCGCTCAGTGGCTTAAGGATCCGGCGTCAGATGCA
EMPV1_37410   CCAAGGCCCCCCAACCCAAGGTGCATCTTCCCTGGAGCCCTGGAGACTTGTTGGAGAAAG

EMPV1_37411	ATCCTGCAGCGAGTAGAAGACAACACCCTCGTCTCCTACGATGTGTCTTCGGGGGCCGCG
EMPV1_37412	CTTTAGATTGGGCCCTTGGAAGTTAAAGTTAGAAAGAGCTTCAAAGATAAGCTGACACAC
EMPV1_37413	GGCACCACAGCCAGAAGTAACTTATTTTGTGCTAGTATCCGCACAAGAAAAAGAATCGGC
EMPV1_37414	TGACACTTTTGAGGAGAGGGTGTGCTCAGAACTTAGGCAGGATACAGTAGGCCAGGTATG
EMPV1_37418	ACTATGGTATGTCTGTGTCCGAAAAATGTTATAAGGTCGAGATAGTAAAGCGAAGCCCGC
EMPV1_37419	TGACAAATGCTCAACCAGCAAAGGCTGTTATGACCCAGCAGAGCCAGGACGTGAACTCAG
EMPV1_37420	GGTGGGTGTTCGCAGAATGTTTACAAATACTCCTTCCTCCTCTCTTTGGGGTTTGCTGCC
EMPV1_37421	CCACCTCCAGGAGCAGCAGTTGTAGAGAATGATAAGAGAGGACAGCCACTAAAGTCTGGA
EMPV1_37422	ATTCTGAGGCATTTCCTGTAGTGGAGTGATGGGTGCGTGGGAAGAAGTCACCCTTTTCCA
EMPV1_37423	CAGCTTGGGACTAACCCCAACAGGCGTCCTTGTTTTTGAAGGAGAGACCAAAATTGGCTT
EMPV1_37424	GCTGGGCTAAATGACTTGTTGAAGCCTCTTTCCGACTCTTTAACCTTGAAAAGCAAACCA
EMPV1_37425	AACAGGAGTGACCGTGCAAGCCCTTTCCACGGTTCCGGTTATGTTTAGCTACTGGGCCAA
EMPV1_37426	AGCCGTAGCTGCTGCTTCCGATGACTGACACCCTCCCTATGACGGCTCTACACATCCGAA
EMPV1_37427	GCCGAAGGGCTTCAGCAAGTGGAGTCTTGTGCCGTTACATGGTATAACCAGGAAAAATTA
EMPV1_37428	TAATTGGGTTATTTGCATTTTCATTAACTGTAGGAGTGCATTTTGTATTTTGGATGTAGG
EMPV1_37431	GGTGCAAAGACGGCATCATCTTCTTCTGTCTTACGTTGACCAAGGCCAAGTTCCTTTTTC
EMPV1_37435	ACTTACTGCCCAAGTGGTGACGGTGGTGCTCCTTTGTGCTTCTGCTTTTTCTGGCACACT
EMPV1_37436	CCCAGGAGTGGGATTGTTGGATCATGTAGCAGTCCTATACTTAGCTTTCTGAAGTACCTC
EMPV1_37437	ATGTTGTGGGGCGGTTGTTAGGTTTTTGCCTCTGTAAAGTGCCTTGCAGGATAACCAGGC
EMPV1_37438	AACCCCCAGACCCTTGCCTCATTTCAAACACCCTGCCCAGCATATCCCTGTCCTCGTTTT
EMPV1_37439	AGGCTTACACAGGGGGCCTAGAGCTGATGGAAGGCAGGAAGCAGTATGACGAGCTCTGCA
EMPV1_37440	ATTTTTAACAGAGACTGTCATGGAAACATTGTTACTCAGGAAATCTTGTGAGGATCCACC
EMPV1_37441	GGTGAAGATGAAGAGGAGTTGTCTGAATTGCAACTTCGTCTTTTGGCTCTTCAGTCAGCC
EMPV1_37443	TCATGCCAACTGATAAATGGGCAGAGGCCAAAAGCGGGTAGTTCAGAGCTGTGACATCCA
EMPV1_37444	ACATTGCGACTCTTCGCAAGTACACTTACGCCAAGCACATTCTGGCCAAGCTGGAGAAGT
EMPV1_37445	TCTCACTTCAGTGGATGACAGCCTTGCACAGGAATATCAGAAGGAACTCTGGGAAGCCAA
EMPV1_37447	CAATTTCCTGACTCGAGTTAGTGGCATTGGTCCATCTGCTGCAAGGAAGTTTGTAGATGA
EMPV1_37450	ACGTGTGCTCAGAAGGACTTTGCGTGCCTGCCTTCAGTTTGTCGGAAAAGCCCATTTCGA
EMPV1_37451	AGGGGTCCCAGGGGTCCACAGAGATGTGTTCTCTCCGCTTCCAGTCAAAAGAAGACTTTA
EMPV1_37452	TTGCGGAAGCCCTTTGTGTCTCCATTAACAGCAGCTTGTCGACACTGGGGCTGGTCATCA
EMPV1_37453	AAGGGATTCATGTTTCCAGCCACGCTTCATCAATGTCAGGCTGTGATGGTCAGCTATTGC
EMPV1_37455	TTTTTAGCCACAAGTACCAGACATGGGCCCACTGGTATCCACCTGAACTGCAGGCTTACA
EMPV1_37456	AGCTTTTGCACAGCAAAGAAAACTATATACAAAATGAAAAGACAACATAAAGACTGAGAG
EMPV1_37457	AACATTAATGAAAGACTTCAGAAGAAATACGTATACCAACTGCAGCCTTATCAAGTATAT

EMPV1_37458	GGTGTCACAGGAGACCACCTGAGGTCAGAAGACAATCAGGGGTGACGTCCGCCGCAGAGA
EMPV1_37459	GACTACAAAGGCACAGCTCAACCTAATAGTTCCCAACTTCCCCACAGTACAAGGAGCAGC
EMPV1_37461	ACTCAGTGCATCATTTACAGACTCTGATGCAATCCGGGGTTGACAGGCAGGAGGCATGGA
EMPV1_37462	CAGGAGGCAGCTGTTCTTGTTCTCCCCATTTCTCAGACGAGCAAGCTGAGGCTTAGAGAA
EMPV1_37463	TCCATATAGAAGGCTCTGTCTGTGCAACTGGCAGCAAGTCACTGTTTTCAGGCATTTTCC
EMPV1_37466	GGGTGCCAGCGTTTGGTTGTCTTTCCCTAGAGTTTTTAACATCCTCTCTCTGCTTTGCCC
EMPV1_37467	TCCCTCTCGCTGGACGTGCTAAGTGTGCTCGGGAAGCTGAGAGAGACACCAACAGACGAA
EMPV1_37468	ATGGGAGTGGATTTGACTTGTCCTTTTGGCGTCTCCTCAGCCTGTGGAGCCCAGGCCTTT
EMPV1_37471	CAGACACAGAAATCAATTCAGTCTTGATGTATCCTGTTCCTTTAGGGCCGTCAAGTTCCA
EMPV1_37472	AAGTTCAGCTTTACTCCGACCTGCTGGAATTTTCTCCTTGGCAGTGCCCAACTGACTGTC
EMPV1_37474	AGGGTACCACGCTGAAGGTGTTCATGATCCGGTCCGGGAACTCCTCGCGGATCTTGCTGA
EMPV1_37476	CTACCTGAAGCTAGATGGTTCATTTCTCTGGAAATTTTTCCTCTCTTTTCCTCTGTTGCC
EMPV1_37478	TTCTATCTGGAGAACCTCTTCGGACAAACCGAGTCCTTCCTGCTATTCCAAGCCAGCGAG
EMPV1_37480	AAGATCCTGGAAGCCCTGCACGACCCGAAGACTAAGGTCACGGAGGCGCTGGTGGCAGAA
EMPV1_37483	GTGCTAGGAGATGTCCTTCAGATTTGAGCTACAACATCAAACCCTTCCCTGGGTCTCCAG
EMPV1_37484	GAATCAATGTCGTAGTCCAGTCTACGAATTGGAAGACGGAGGATCCCACAGAGTTGGGTG
EMPV1_37487	TGGGTTCGAGTTCTGGAAACAACTGTGCGCCGAGCATGGCATCAGCCCCGAGGGCATCGT
EMPV1_37490	GGCAGAGCGTGCTTACATTATGGTCTGGCTCTTGATTTGCTTTCACTCCCTTTGGTTAAC
EMPV1_37493	GGTTTGCATGCAGCTCCCACCTCTACCCCTTGCTCCTCGCTGTACATCGTGCTTTTTGTT
EMPV1_37495	ATTGGCTTTCCTTGCAGCCTTTGAGCGCCCCTGGGTTGGGGACCATGGTTCTCTTTTCCA
EMPV1_37497	ACTTTGTGCTAGCCATCGTCATCCTCGTGTCTCCTCTCTCAGCCACGGTCCTTTCCTATG
EMPV1_37500	TTTTGCTGGAGCCAGCGTCCACTGAGGCTCTCTCCTGGGGACTCATCCACAGCCCTTCCT
EMPV1_37503	AGGCACTGGAGGAGAAAAGACAAGTCCACTTAGCCAGGACCAAGCAGAGACATCGTGACA
EMPV1_37504	TGATGTTCTTCTCCAGCTTTGTCACCAGCTTGGGGGCTTTCGTGGTCATTTCATCCATCC
EMPV1_37506	GACTGGCCTTTCTGTTCTGACGAAGACTGGAACATCAAATGCCCTTCTGGCTGCAGAATG
EMPV1_37507	GTAAATTATCAAATCACAATTCCAGAGCCACACTTGCCTGTCTCAGAAGAACGGTGGGCA
EMPV1_37509	AATATACCTACAGGCAAACTGCACACCACGGTGGGAGGAGCAAGCCGAGACTGGGTTAAA
EMPV1_37511	CTGGTAGCCTGAAACCACCGTTAGTAATGAGCTCAGTGAGGAGAAAAGATTTTTGCATGC
EMPV1_37513	TCTCAAAGCACATCCAGCTTACCCCAAAATCAGAAAGGATATACTTGATAAGGCCCGTGC
EMPV1_37514	GCTTGGGGAGAGTTATTCTCTACGCAGTAAGTGTTCCAAGGGGTTGGACATTGTTTATGA
EMPV1_37515	CCCTGAGACCCTAACTTGTGATGTTTACCGTTTAAATCCACGGGTTAGGCTCTTGGGAGC
EMPV1_37516	AGAAAAATCGAGAGGAAGATCCCTCATTATTGTGGCAGGTGTTTGGCAGTGCCACAGGCC
EMPV1_37518	CCTCCAATACTAGGACACTTCGATTCCCCTTGCTCAGTTCCTTCTGGACTCTGGAGACGA
EMPV1_37519	GAACAGGAGAGAATCATAGAACTGGCAGTGAAATATACAAACCACCCTCCTGCTCTCTGG

EMPV1_37520 CATACCTGGAATTCCACAAGCAAACATCTGATGGAAATTGGTTAATGGTTCATCGAACAG
EMPV1_37523 AAGCAGCGATTGAGAAACAGAAATCAGCCTTCATCCGTTGGGGAATAATGGCAGACTGGG
EMPV1_37524 CTAGGTGGCACAGCAGGCTCCTCGTTCTGTCCTTGTCACTCTTGCTCTGTACCACACAGA
EMPV1_37527 ACCCTGCCCTCCTCACGACCAAACTAGGGTGCTATGGGAGGTTCTCCATCCTGCGGCTAA
EMPV1_37528 AAGTTTTCTACCACTCTCTACTTCTGCTCTGGCTCCTTCCTTTGCAAGGCTAACCCACAC
EMPV1_37537 TGGTGTGTGAGGGGGGGCAGGGATTATCATATGGATCGATTTCTACGTATTGCTAAGTTG
EMPV1_37539 TTAATGTTGACAGCTCAAATAAATGGAGGAAGGATCAGAGTTCCCTTCGTGGCACGGCGG
EMPV1_37544 AGAGGCCCTGATTCTGATGTGACCAGGCCTATATTGTGGAGAATGATCAGTACACATTGT
EMPV1_37547 CCTCATCTGCACATCCACCTTGCTGAGGCCCATGCACAGACTATAGCAACTAATTCCTCT
EMPV1_37548 TAACTGTTCACAGCTGTCTGAACCGCCAGTGGACCAGGAGCAGCTTGGAGCTTTAACTTT
EMPV1_37549 GATTCCTTGTCCATCGTCCTCCTCTACACACTGCAAGCCATCTGCCATAGTCTGTAACTG
EMPV1_37552 GCTCTGCATCCACAAGGATGGTTGTAGAACTGCAGACCCATCTGTATCACATGATGGATC
EMPV1_37553 GCTGTTCAAGCGGCAACTAGACAGACAAACAAATGAAAGTGCCTTGTGGTTTCTCATGCC
EMPV1_37554 CATCACAACCAGCCATTCCTTGGGCACCAAAGTCAGATCCGTTTGATACAGTAACAGGCC
EMPV1_37555 GTCACGGATCAGTTGGAATCAGTGCTCACCCCATCCCCGTTGTTTTGAACATGACTTTCC
EMPV1_37556 GAAGCCCCACATGTATGATTATTCCTCAGCATAGCTTGGAAGGTCAGCGATCAAGAGCTG
EMPV1_37557 AGAAGGAAAATAACCATTCTGTATTAACTGCTTATCTATTGTGGGAATTGTGATAAGTGC
EMPV1_37558 GATTACCCTGTGTTTACAGATTGAGTCCTGTGGTGAGGCCATTTTTACAGAAGAGTTAGT
EMPV1_37562 GTAGTGAGGTTACACAATATCCCACCACTCTTACACTGGGGCCAGAGATGTGCTGTAGAG
EMPV1_37563 TTCATCCCCAGACCTCCGATTTAGTATGGCTCTGTGTGACGAGGAGAAAGCTTTCCGACA
EMPV1_37565 TGGGGTGTTGGTGGCTGTGGCCTGGGTGGGATCCTGTGTGCATTCTTTAACTCAGATTTT
EMPV1_37566 GTTTCGAGTGGGACAAGGATTGATAGAAAGGTTTACAAAAGATACTGCAAGGTTCAAGGA
EMPV1_37567 CAGAAACAAGTCTACAAATAGCAAATGCTGCAACGGGTGTGAAGAAATGGAACCCCCCTT
EMPV1_37568 GCAGCCACAACAAGTACATGGGGAAGTCATTCCCAGGGGTCTATGACGCTCTGTTTGATA
EMPV1_37570 GGTTCTGGCATTTATGACCCTTGTGAAAAAGAAGCCACTGATGCTATTGGGCATCTAGAC
EMPV1_37572 GATTGCAGTTGTGGGGGCCGGTGTGATGGGGCTCTCCACTGCTGTGTGCATTTTCAAATT
EMPV1_37573 GGCACCCGGTAGTGACGTGCAATGGTTATAAGCTCTTGATCTGTGAGTTTCCCGACAGTT
EMPV1_37576 ACAGTGGCAGCCTTTCAACAGGAGTGGGGAGACGGAAGAAGGAACCGATATGAGGTACTT
EMPV1_37577 TCCTTTTCATTTTCCAAGTTCATTGCAGAGAAAAAATGCATGGCTGTCACCACTGACCTCC
EMPV1_37578 CGCCTCCGAGCCGCGCGCGCTCGACTGGGTGGCCAGTGCCAACCTGTTGGATGACATCAT
EMPV1_37580 CTGTTTTGTTCATATGGTTTTCCTGTGGCCAGAATAGTGCCCAACCCCAGGCACACAGTC
EMPV1_37582 GACATTGACAGTGCCATCGAGGTCTTCACACAAGCTATCCAGTGGTATCAAAGCCATCAG
EMPV1_37585 CGTAAAGGACTACGTTATTAGTGAGGCTTTACCACTGCACAGACCTGACCTTTTGAGACC
EMPV1_37586 GAGCTGGCTGTCCAGAATGAAGTGGGCCTGGTCGATAACCCTCTGAAGATTTCCTGGTTG

EMPV1_37588 TCTCGTTTCATGGATGTTTCCTTGCGTAGCTTCTCCCTTTGTTGTGACCTGTTGAGCCGG
EMPV1_37590 CAAGTGAGGAGGAACGTATCAGACTTCAACAGGAATTAAGTCTCGTGAAACAGGAGGTTG
EMPV1_37592 CATCCCTTTCTGAAATTGGCCAAACCATTATCCAGCTTGACACCACTGATCATGGCAGCT
EMPV1_37593 AGCAAGTGCTCAGGCTTCTTAGAGGCCCACAAGGGGAGTGAAATGATTGTCCTCGGCTTT
EMPV1_37595 GAAACTCCAAATTACATGTATTATACCACTTGTGGTCCCATGAGTTCCCTGAGGCTCTGT
EMPV1_37597 AGGGAAGCCAGACACCCAAGTTCAAGTCCTTAGTGATGTGTGCAGGGCTGAAGGGATGAA
EMPV1_37599 TTTTCAACGAGCCAAGAACTCGGCGCCCTGTGTGATATTCTTTGATGAAGTGGATGCCTT
EMPV1_37600 GAGGTCGGAACCTCAGCAAGTACTGAAGTATGTGGGCCTAGAAGACTCTGTCCTTTTGGA
EMPV1_37602 CCAGCCTATGCCACAGCCACAGCAACTTGGATCTGAGCCACATCTGCAAACTATGCCATA
EMPV1_37604 CCTTCCAGTTTGAGAAGCCAAGGAAGGAAACAGGGAAAAATGTCGCCATGAAGGCCGAGA
EMPV1_37608 GTTGGGTGTACATGCTCAAAATTGTCTTACTAATAGGGGAGCATGATCAACACAGTTTGG
EMPV1_37609 AGATGCCACTTGGATCTGGCATGGCTGTGGCTGTGACTTCAGCTCTATTAGAACCCCAAC
EMPV1_37613 AGCTGGCTTAAAGGCTGGGGTCATTGGTTGTGGAGGTTTTGCTGCTTTCTCGGCTGCAAT
EMPV1_37614 TCACCTTCCCCGTACTCAACCAAGCTGTCGGCACTTTCTGTGACCTGCATATCCCTGTTA
EMPV1_37615 GAGAGAACCTCTTTTAAACGGCACCTTGGTCGGGCCTGGGGGAAAGGTTATCCCTAATTT
EMPV1_37617 CAGCTGTGGCATAGATTGCAGTTGCTGCTCAAACTTGATCCCTGACCAAGGAATTTCCAT
EMPV1_37618 CGGTCCCCGGATTGGTCAGGTTTTAACAAGCGCCTCCTTTGCAACCAAATCTCAGAGTAA
EMPV1_37619 CTGAGAGATCATCCAGTCTAGCTGCCTAGTGTGACAGAGCAAGAGACAGAGGCCCAAAGA
EMPV1_37627 AGATAGCTCCTCCAAGAAGACAAAGTCTAGTTCAGAGGAGAGTAGATCCGAAATATATGG
EMPV1_37629 ACACAAGTGAATGGACTATGGGTATGTCTTGTGCTTCAGGTGAGTGGGTCAGATTTGTCC
EMPV1_37630 CTGCCATCACTACATGTAGGGGACTGGCTGAACATGGGCGCCTACACCGTGGGCATGGGT
EMPV1_37631 CATCCTTGGGTCCTATACCCTGGTTCTCAGATCTGTGCTTCAAGTTCCTTCTTCAGCTGG
EMPV1_37635 GACTGACACTCCAGGCGGCTTTGCCTTTCCTCCTGCTTCCTCATCTTCAGACTTCATTAC
EMPV1_37637 CGGAGCCTCACCAACAAGAAGCTCGAAGTCCTGCTTGCACTTTTGCATTTGCCTAACAGA
EMPV1_37642 ATGATCAGCAAGATGCTGAAGATGCAGATGCTGGAGGACGAGGACGACCTGGCCTACGCG
EMPV1_37643 ACTACTCAGATGCTCCAATGACACCAAAGCAGATTCTGCAGGTCATAGAGGCAGAAGGAC
EMPV1_37644 GTCTTCCAAAGCCCCTGCTTCTCCAAAAACTCTTGTGAAAACACATGGTTCAGTGCCGCC
EMPV1_37645 CAAAGGGGGCGCCAAGCGCCACCGCAAGGTGCTGCGGGACAACATCCAGGGCATCACCAA
EMPV1_37647 GTCTCCAAGTATGGTCCCAAACGAAATACTTGGTCCCTGCCCAGTGCTCATGGAACTACA
EMPV1_37648 CCCTGTGGACAAACTTCCAAACAAAACGTTGAAGACTTGCTGTGTTACCTCCAGAGCCCC
EMPV1_37652 TCTCTTTAACGCGAGAGGAAGCGATGCAGAGGGGTGGAAAATGGCAGAGCTGCAGATGTT
EMPV1_37653 CAGAAGAACTCGGCGATCAACCCAAAGCACCACAGATAAAGAACAAAGCAGACTTGCAGG
EMPV1_37656 GACAAATTGTTAAGAGCCTTAAAAGCCCACCTTAAAAATGAATTAAAAGAAGAAAAAGAG
EMPV1_37658 GGTGATATAGCAAGTCCCTGTGCACCATCCCCACAGACTAACCCACTCACCCCTGCAATT

EMPV1_37659	GGCAAATGTGTAATAACCAACAACGTATAGCACAGAAACAAGAAGTGTTTTAAGTCTAGT
EMPV1_37663	AGAGCAACTGACAGTCAAAAAGCACAAGGAAACTAAGAACCATGAGCACTGGTCAGCAGG
EMPV1_37666	GCCACTACTGAGGTAGACGACATGCAACAGGATATTGAGCAAGTTTGGGAGGAGCTATTA
EMPV1_37667	GATGGCCATGTTATAGAGAACGAGTGTCTGGGCATGGCTGTGTTGCCCATGTCCCTCTAT
EMPV1_37669	AGAGGGGCTGGGTTCTGACTTTGAATCTGGCAGCCACCATTTACCAGCCGGGTGATCTTA
EMPV1_37670	TGTGAGATCGGCAGTGGCATTGGCAGTGGCATTGGCGGTGGCCAGGAGGGTAGCAGTGGA
EMPV1_37671	GCTGCTCATTGAGAGGAGTGCCATCTACACCAAGGTCTATTCCCGAGCTGGCCTTGCTCT
EMPV1_37672	ATCTGGCATTGCTGTGAGTTGGGGTGAAGGTTGAAGACACGGCTCAGATCCTGCATTGCT
EMPV1_37675	GCTAAGCTAAGCTATCGGGCCCATACCCCGAAAATGTTGGTTCATACCCTTCCCATACTA
EMPV1_37677	TCATGGGGGCTGGTTGAAAGGCTAATGGGACTAACAGGAAGGGTGTGAGAAGCCTGGATT
EMPV1_37678	CGATTACTGACATACTGAAATTGGCATACATATCTTTTTCATGCATCTAACAGAATGGAC
EMPV1_37680	GACCGAATTGGCAACCACTAAAAGATGTGATTGAACAGTTACGTATGACCAGCCCGGAGC
EMPV1_37683	CCAGGGAACTCCTATTACAGTTTTTATTCAACAGTCTGCTAAAAGTCACACCTGGTGCGG
EMPV1_37684	TGGGCGAAAGAAACAGGCGGTTGCTCTAATTCGGCTGCCGGGCAAGTGTAAAAGCCAACT
EMPV1_37686	CGTTGCCGAGAGATGGATGAGCAGATCAGACTGATGGACCAGAATCTGAAGTGTCTGAGT
EMPV1_37689	TCCAACTTCCCCTTTGACAACCTGGAGACCCTTACCCTTACCAACAACCCCTGGAAGTGT
EMPV1_37691	ATGACCCCGTCCCCTCCCACCCTGGTTCCTTCCACGTTGAGAAAATAAATGAATAGCCCA
EMPV1_37692	GAATCACAGACCTCTCAGATCCATCATACAGTGGCCTTCAAAGATACCCCCAAGGCTCCA
EMPV1_37693	ACAGTTGGAGTAGAATCACACTTGAGTGGTCTATAGCAGTGGTCATCCTTGGGCCTCCTG
EMPV1_37694	GGGCATGGCACTGATGGCTTTCGTCTCTTCTGGATGTTCACTTTCCTGATGGTGTTTCCT
EMPV1_37695	ATTGCATAGTGTCCTTTGAGCTCATGCAGGGCCCACTTGATGTCCTGACTGCTCAGCATT
EMPV1_37698	CACACTGGAGAGAAACCGTTCAGATGTGTGGAGTGTGGCAAAGGCTTCAGCTATAGTTCT
EMPV1_37699	CATAGAGACCAGAGAGATTGCTGCGTTGAAAGAGTCAGCCCATCTTCTCCAGAGGTCTGT
EMPV1_37701	AAGAAGCTGAATTAGGGCTTCCATTTTCCCCGCTTTGTGATCGGAAGTCGACGATGGTGG
EMPV1_37703	TTCCCCAAACTGACAATTATAGGGGAAGAGGATCTGCCTCCTGAAGAAGTGGATCAGGAG
EMPV1_37710	TGAGGGTCCAGACACTACTGCCTTGTCTCCAGCAGAAGGGACCGGTTTTCTGCGCCGTGT
EMPV1_37712	CTGCCCTGGGCCCCATGTTATATGGGATCCCGCATAAAACAAACACACAACATGTGTATT
EMPV1_37714	GATTCAGACATTCATACAGCTCGAGGAAGATTTGAAAGAGGACGATGAATCATTAAGAAA
EMPV1_37715	TCCACAAAGCCAATGCTGCAAAGGATAGTGAAGCCCAGGAAGCTGCTCTGAGTCGTGAAA
EMPV1_37721	CTGAGCACAGCCTCCTTGAACAAGACAGAGAAGGAACATGGACAGTGGTGAGCAAGGTGT
EMPV1_37723	TTGTCTCTCTTTCTGACTTAATATTGTAAAAGTGGCCCTATCACCCAAAGCAATCCACAG
EMPV1_37724	CTGCAGCATGTTCGTTTGCCATTGCTTAGTCCCAAATTCCTGGTGGGCACTGTTGGCTCT
EMPV1_37725	AACATCCCGAAAGCCGATGAGATCCGGACCCTCATCAAGGACGTGTGGGACACTCGCGTA
EMPV1_37728	ATCAAGTCCAACATCGATGCCCTGCTGGGTCGCTTGGAGCAGATTGCTGAGGAGCAGAAA

EMPV1_37729 CCCACCCCTCCCGGTGTTGTTATTAAACGTCTGTGGAGCTTCTGCTGCAAGGAACAAAAA
EMPV1_37731 TACCTGCTGGGATGGAGGAGGCTGGCCAGAGCAGGGAGGAGATTTCTGCTCAGATGCTTT
EMPV1_37732 AGTTATGCTCGAGATGAAGGCTCTGGAATCTTCGGACCTCACCGAAGTCATGGTTTACGG
EMPV1_37733 GCTATGTAACCCAGTTTTGGCTGAAGGGGGCTTATGAAAAGCATATGAAGCACAGTCTTC
EMPV1_37734 TGCTACTGCTGCAACCAATGGATATTTTGGAGCTGCTTCTCCCCTAGCTGCCAGTGCCAT
EMPV1_37735 AACAGGACAGTGCCCGCACCAGACAACCTGCCCAACTACGAGAACACAGTGGTTTTTTCT
EMPV1_37736 ATGAGCTCTATGCCCAGAAACTGAAGTACAAGGCCATTAGCGAGGAGCTGGACCACGCCC
EMPV1_37739 GGGAAAAGGGGAGCAAAGGGAAAACAGGCCGAAGTGACCAAGCAAGAGACAAAAGATCTG
EMPV1_37741 CCCATCAGTAACACCTAGCATTAGTACCATTGTGCTTTAGGGGTGGTGCCCAAGATAGAC
EMPV1_37744 CTAGGTTTGAATTGCCACTGAGCACTCTAGCTCCAAAAAGCCTTCCAGAATTTCCTCAGG
EMPV1_37746 GCCAGGCCCTGCGTGGCTACTAGCTAAGTGCACTGGTTTTGGGGAAATTTGGTAGTTTTA
EMPV1_37747 GGAAACGCTGGAAGCAAGTGTCGGCCCAAGGGAAACGGAAGAGAAAGTGCGCGATCTCCT
EMPV1_37748 AGACGGTTTGGGGTCTTGGGCCTTCTCCCCAGCCTCCCTCGGTTATTTATGTTTCTGTTT
EMPV1_37754 TACTTAAAGGTATGGGGATGGTATTCCAATCAGCTGTCTGAACTCGCTGAAGTACAGGCC
EMPV1_37755 CAAAGCCACAGAAGCTTGTACGGTCTCCTGCAAGAATATCCCCTCACATTAAAGATGGAG
EMPV1_37756 GGAGCTTCTGGGTGCTTTGTCTCAGGCTCAGGCTCTGGTGCAATCTTATCTCTCTCCCTT
EMPV1_37759 AAATTAGGATGTGATATATACTGATGGTTCTGGGTCTGGAAAGGGGTGAGGGACCAAAGG
EMPV1_37760 TTAATCTGCAGGAACTGTACAGGATTTGCCCTCTTTAGGCAAACTGTGGCTGGGGGGAAG
EMPV1_37761 TGCTCTGATAACCACAGGCAGCTATGGGAAAGAGCGGGAAAAACAAGGTGTCCCCAAGAA
EMPV1_37762 TGACTCAGAAAGCAGCCATGATGACCACCAGTCCTACCAAGGAAACCCTGCCTTAAATCC
EMPV1_37763 CAGCGCTTTGAAGGCACAAGAGGCTTAGGAGAGGAAGCATTAGGAGCAGCAGCCATAGTA
EMPV1_37764 GGTAACACAGTGATTCACTTGGATCAAGCACTAGCCAGAATGAGAGAATATGAGCGTATG
EMPV1_37769 TCACAACCACCTGGGAATTAGGTACGAGCCAGAGTCTCATTTTACCCCTGAGAAAACCAC
EMPV1_37771 AGATGGGCAGCTGAAGATCATGTCCTTCGTGATACCGATGAAAATCGGAGATTACAGCGT
EMPV1_37772 TATGGTGCCTGGCAAAGGGCCCAGCTTCCCAGAGCCATTACGAGAAGAGCGAGACTTAGA
EMPV1_37773 TTTCTCTAGAAAGTGCTAAGACCTCACCACACTGGGCCTACAATTGTGCCTGGCTGCTCT
EMPV1_37776 CCAGCTCTACTTCAAAACCCAAGCCCAAAACCCAGAACCCCAACTCCAGGTCCTAGCAAA
EMPV1_37777 GTGGTCTCCAGCATCAGAATTCAACACTGCTTACCTCCTCCCAATAAGCCTGCCATGTGT
EMPV1_37778 CCTCACAGGGTTCTCCACCCAGAGAGACTTGCCCAGCTTCAGGTTGAGGTGTTGAAAATT
EMPV1_37781 GACCATCGGCATCCGCCAAGACGATGTCCACTACATCCAGAAGTACAACTGCTTTAGGAA
EMPV1_37784 ACACCTGTGTGCTGCAAGCCCGTGTGCTGTGAGGCCTCCCCCTGCTCAGACTCTTCCTAC
EMPV1_37785 AACAGGCAAAGGGAAAGCGGCCCCAGTGAACGACCAAATAGAGAAGTGGCTCCTGTAGAA
EMPV1_37788 TGTACGATAACATGCCGTCTCCGAAAATCTTTGCACGCTACCCGCCTGCTGACAGGAAGG
EMPV1_37790 CTCACCCTGGCTGTGGCAAGAAGTTCTATTTATCCAACCACCTGCGGCGGCACATGATCA

EMPV1_37791	GGTGGTGCGTCAGCTCATCCTCTTTGTATTTTCTCTCAGCAGTAGTATTCCAGCCAAGTG
EMPV1_37792	GCAGAAATGATGGCTAAGTAGCCTATATAGGATTGTGAAACTCAGAGCAATATTCTGTTG
EMPV1_37795	CTACCCTAAGGAGATCTCCCTGAACTGGCAGCGCGAGGGCCAGGACCAGAGCCAGGACAT
EMPV1_37797	TGTAATCACAGGTACATTGGAATGTCTTTAGGTGGGAGGTGCCTCTGGGAGGGATTCTGC
EMPV1_37798	CTTACACACCATCCAAACTGAAGACCGAGTCTGGTCCATTGCTATCAGCCCATTGCTCAG
EMPV1_37799	AGTGCTGCTCTGCCTCTAATTTGCTGTGGGATCTTAGGCAAGTCACTGTCCCAGTCTGAG
EMPV1_37800	TGCTGCATCTCTATTTTCTTTAAGGCTTCAGGCGGCCATCCCAGGCAGGATTGAGCACTG
EMPV1_37802	CCTTAATAGAGCTTTGTACTTCGCCTGCGATAATGTCCTGTGGGCCGGAAAGTCTGGACT
EMPV1_37804	CACTTGTGGCCTAGCTGATGTCTGATGAGTATTGTCAGTGTGCAGAAGTCTTAACCCCAG
EMPV1_37805	GTCCAGACGATTAGAAAACAGGAAACTGCAAGAATTATTGGGTACTATTATGAGTAATGG
EMPV1_37806	CCCTCAGCTTTACAATGGGACATGGGGCAGATTTCACACTAAGAGACTGGGCACAAGGAT
EMPV1_37808	GTGAGGATGTCTTGGAGAAGAGCTGTGGTCCTTCTAATGTTATGGTTCATAGGGCAGGCC
EMPV1_37809	TCATGTTACAGAAGCTGAGGTGTGGGTTTTTTGTTGGAGTTCATTTATCGTTAGAATGTC
EMPV1_37810	GAAGCTGTCTAGTGGTATTTACTGGAAGGAGTTGGCCGCTGCGTGGATGACACTCTGAGA
EMPV1_37811	TAGCAGGTTTCCGCTAGAGCCTCCATCCTCTTCATCTCTAAATTTCCTATAGCCCCTGGG
EMPV1_37812	AGAAGGCTGCTCAGAACTCCCTGGGAAGGTGAAATAAACCCAGCCCCTGCTGAACTCTTT
EMPV1_37813	GTCCTTGCTTCCTTCTCTGATTTAGATGCAGTCGTCACTGAAGGTGGGGAGAATTTTAGC
EMPV1_37814	CTCTTGCACCTCTCAGAAGATATCTGCCTCTTGTGCTTGGATGTTTAGTACATCTGATGG
EMPV1_37815	GCCTCCCCAAGTGAAGGATGAGTCTACAGTTCTTTTCAGCTTCTAAAGCATCCACACCCC
EMPV1_37816	GGTCACCACTACATGACTAGCACCTAGCACAGCACGTGACAGGTGGAAAAGACTCTATCA
EMPV1_37817	GCCTGTTCTGTGAATTGGCATTTTGGATGCCTTCATGGACTATGAGGCTTTTGCAAGGCT
EMPV1_37820	GATATGTGGTTAATCTTACTGTATGTATACTGATCAACATGTCAGGTATGATGCCAGAAC
EMPV1_37823	CTCTGCTTTGTCGAATGTGTTCCCTCTTCCTTAAAGCACACCTAGGGCTTGCCAGCTGAC
EMPV1_37825	CTGGTGACCTCTATCTCCAACTGGGGTTCCAACTGCCAAAGAGAAACTCAGCCCTCCTCT
EMPV1_37826	CCTAAGCCACAGCTACAATACCACCAGACACAAGCCACATCTGAGACCTATGCCACAGCT
EMPV1_37828	ACGTCAACAACTGGCTGCAGGTCAGCTTCACTGCCAAGCACGCCAACAAGGCCAAGGTGT
EMPV1_37833	CATATTCAACTCCGTGAAAAGACACTAGCCCAAATGGATCTATGCTAAGGAAAGCGACCC
EMPV1_37834	TGGATTGCGGGAGAAGATCCACCACTTGGATGACATGCTTAAGAGCCAGCAGCGGAAAGT
EMPV1_37835	CCTGAGGATGGCGTGCTGGTTCATGGGATGTTCATGGATGCTTCTCGATGGGACAATAAG
EMPV1_37836	GAATGGATAAGCAACAAGGTTCTACTGTATAGCCGAGGGAACTGTAGCCAGGCTCTTAGG
EMPV1_37841	ACCCTGATTCTTCAGTTTTCTGCCTGGTGACACTGACATTAAGTAGTTGGGGGGACAGTC
EMPV1_37842	GAAGGCTCGGACGGCTCTGAGTGTGAGACCTTTCGCAAATGCTTCAGGCAATTCTGTTAT
EMPV1_37845	GCAATAACTTAACCTCTCTGGTCCTCAGTTGTCCTGTCTGATAATAACAGCTTCTACCTC
EMPV1_37847	TTACCACTGCTGGCTAAAGAAGGAAAACTCTACCTGTTGCTCACCCTCCGGTCTGACAAG

EMPV1_37850 CGGCCGTGTTTACGTATATAATGGCAGACAGATCACCTTGCGAGAGGTGAGAGGCTAATG
EMPV1_37852 TCTCTAACATCCTTCACGTCAGCTCCTTGGAGAGCAGGTCCAAAGCTTTCACCACCTGCA
EMPV1_37854 TTTACTGGTATGCTAAGCAAGCACTTAGCCTCTCTCCCTTGTGGAATGGAGCACTGGTCG
EMPV1_37855 GGAAAAGCTTCTTGTCTCTTTCGAGATACTGAGCACTGTGGAAGCACTGAAAATTGTTGG
EMPV1_37856 TTTGTCAGACTGGGAAGAACAGCACAGGAACCTTGGAGAACTTTGAGGTGGCTCCTGGAA
EMPV1_37857 GATGAACTACTTCTTGGAAGTGGAGATTGGCCGAACCACGTGCACCAAGTCCCAGGCCAA
EMPV1_37858 TGCAGCGTGGGTCTGGATAAGAACACAATGCAGGATCAAGAGCATATCACGGAGCATCCA
EMPV1_37860 AACCCTCAGGTTCATCTGCACACTCAAGTTTGAGAACCACTGTCTTGGCCCGATGAGTAG
EMPV1_37861 AGATCAGCAACGCCGTCCTCATCATCTTTGTCAGCTACTTTGGCAGCCGCGTGCACCGTC
EMPV1_37862 GCCTAATCCGAGGCTGTGGCAATTAGAATAGGAAGGGTCATTAATTCTTTGACTGCTGAC
EMPV1_37863 AAGGTTTGGTTCTTTTTCTTTGCGTTCATGTCCTGGACCTGCTTCGAGACCGAATCGGGC
EMPV1_37865 CAAGGGGGTGTACAAAGGAGACTCCACCCACCAGGAGCCATAATAGCTTAAAAATCGGAC
EMPV1_37867 TGCAACATTGGGATTAATGTCAAGTACCACTTGTAATGACTATCAAGTATTACCCAAAAC
EMPV1_37868 ACTGCAGAGGAATCTGGTGAATGTCAGGGAGTATGTAGGTATTTGGTGAGAATGACCCGG
EMPV1_37869 CCCCAGCATTGAAAGGTCAGCAGAGTATCCTAACACTTGTACCAGAACCGCCTCTTCTGT
EMPV1_37870 GTGTGTGTGTGTGTGTCCTTGCTCGGGCCTGGCACTGCTAGACACCACCCACGAACAACT
EMPV1_37872 CCAGCTACCCCATGTGTTACTGCTACAGCTGGAAACAACTTTCTTCAAATTACCTGGTGG
EMPV1_37873 GAGCCAAACCCCTCGCCTCAGAGTCCCCTTTGACCTCTGCAAAATTCTGGAAAATATCAG
EMPV1_37874 TTGCCTCAAACTCTCTTGTGATGGGCAACGTGGAAGAGGCTCTTATAGCCCTGACCAACT
EMPV1_37876 GAAGGTTGAGCTTTTAACTTGAAGGGCTCTAGGGCAATCCAAACACTGCCTGAAGGTCTG
EMPV1_37877 GATCAACAAAATTGATAAACCCCTAGCCAGACTTCTCAAGCAAAAAAGAGAGAGGACTCA
EMPV1_37878 AAACCATGCAATGCCAGTGACCCTCAGTGTCCCCCTGACGGCCGTTACCAATTCAACACT
EMPV1_37879 ACAGCGTTGCTGAAGTTATTAGAGAAGACCTGCGGCCCAACCCCTTGCAGTACTACCTGT
EMPV1_37882 CTGGGTTGTCCTATCTTGTGGATGATCCCCTGTTATCTGTACGAACCTCCACCATCAAGA
EMPV1_37883 GGACATAGATGACACGCACGTCTTCGTAATAGCAGAGTTGGTTAATGTCCTCCAGGAGCG
EMPV1_37884 CCCCAGTCCTGTGGAACTTCCATTGAACAAACTTAATGAACACAATGTACTAGTACTAAG
EMPV1_37885 TAGAGTCTGTGACTAGCAAAAGGACCCCGACAGGGAAAGTTCTCCAATGCCCTTGCCTTT
EMPV1_37889 CTCCCATGTAATTTCTTTGTCCTTATTCAGTCTCTCTAAATTGCCCTCTCCCAGCCCCCC
EMPV1_37890 GTGTGTTTGGGGAAGGAGAAATAATCAGGCATTTCCAAGTACTATGAGGGTTCCTGGGAG
EMPV1_37891 ACTCGGGTTTAGTGAAAACCCAACACTGAGTGAAAGGAGGGGATTACAGTTTGCTGTGGG
EMPV1_37893 AAGAGGTGTCAGTTGGGGGGCGAATATATGGGGTGCATCGGGTGATCTTGGCTGCTGTTA
EMPV1_37894 AGGCTGAGCAAGCCAGGAAGGGAAGGATTCTGTTTGTGGTGTGTATGTGCATGCACCCAA
EMPV1_37895 GAGTCTGCTTCTGGTTGGTAGCTGGTTCGTATGTCATGGGCTCAATGAATGCTTCTGTGC
EMPV1_37896 CACCCATAGTTGCTCTGTGCTCTCAACTTGGAATACCTCAAAACATGCTAGTTTTATGTC

EMPV1_37897 TTCCAGTAAATGGGCCAAGGGGGAAATACAGGCAATCGGGGCTTTGGGTACTGAGAAGAG
EMPV1_37898 GGCAGGCAGATAATATAAATGAACAAAAGAGGCAGGGTCACTAGGGTGCGGTGGGGTCTG
EMPV1_37902 CCACAATCGGAACTCCGGGTTGGCTGTTTATGTAGAAGCGTAATTGGGAGCCCTCTCTCT
EMPV1_37903 GCACATCAGGCAGAGGGGTGGAGAGCATTTTTGGTTTTGTTTCTGAAGTGGTGCCTGTAC
EMPV1_37904 TGCCATTACAGAGGGCCACCTAGATCGGAGCATTTTCCACGGGGCTATCATGGCTATCTT
EMPV1_37906 GGAGACCAGTTAAGAGTGTTGTTGCAGTAATTTAGATGGGAGATGCTACAGCCTGTGGGC
EMPV1_37907 GACTTGGGCTCATATCCACAATACTTGGTATAAGGTAGTAGGTTGTTGGGGGTGGGGAGG
EMPV1_37908 TGACAGCAGACCCTCCTCCCGGCAGGACTCAGAGAAGAATATCCATCTCCCAGGAACATG
EMPV1_37910 TCAAGAACTCACCTGTCACCAAGCTCTGTGGTTTCTTCCATCAGAGAGAGATCTGGGCAG
EMPV1_37912 CTCAGACAGGAGTTCCTTGTGCAAAACGCTGCACGCACAACTCCATTCAAGACAGCAGCA
EMPV1_37913 TCTCGACCCCAGTGCACAGTGAGGGTCAAGCTGCTGCAGGACAGCATCTCTTCGCTCCTG
EMPV1_37915 AATCTTTGTTATCATAACATTCAGCAAACACATAGTGGAGCAGATGGTGGCTTTCATTGG
EMPV1_37917 TAAATATTCAGCAATGTTCAGGAGCACTAGCCAATATGATTTGAGGAAGAGGAAATGAGG
EMPV1_37920 ATCCCTTTTTCGTCGGGGTCCAGTACCACCCCGAGTTCCTGTCCAGACCCATCAAGCCTT
EMPV1_37921 TGACTTCGGCCTGCTTTTCAGAGACCAGGGGAGACGTGGCGGGAGGGGAATACTTAGTTT
EMPV1_37922 TGACTTGTGGCCAAAGGAAACACGGGAGGAGGGGTGGGAGAGGGCATGGTCAGTCTAAGA
EMPV1_37923 CCCAGAAGGGGCAGCGGGCAGCATGAAGGCACCAGCGGACCCTGTGGAGGCTGCCAGGAT
EMPV1_37925 CTAGCTTGCAGGACATACAAAAACAGCCACAGCAGGATTTGGCCTTGGGCAGTAGCTGCT
EMPV1_37927 ACTCTCGTTTCTAAAACCATTTATCAAAAGCTCAGGGCTTGAGTGGGTTGATGGACACAC
EMPV1_37928 GAGTAAAAGCCTTGGCATTATTCTTGTCATTCCAGGGAGGTCAGTGAAGCCCCGGCAGAT
EMPV1_37929 GGCATCTTCAGCCAAAGTCCAATACATGAAGAGTCCTGCCACTGCTGGGAAGAGACCCAA
EMPV1_37932 GCAGCATTTTGCTTTTGCTCTTTCTTTACGTGCTTCAGGGCATTCCACTGGGCCTGGCAG
EMPV1_37937 CCTGTCTCTCTGACCTCCTCTGGTCTGACAGTTATTCATATTTTCCTAGTTTATGATGAC
EMPV1_37938 GGGCCTGGTGATTGCTTGGTGCGTGCATGGGCTCCTCACATAATCCCCGCTGACAGCAGT
EMPV1_37939 GACTTTGGTTTCCGCAGCCGGATCTCAGAGCACCTGCTGGATGTGGATGTGCTCTCCTCC
EMPV1_37940 AGTACCCCACGCCGCGGTTGTGGTGCCCGTAGGCCAGCAGCACGTTGTACAGGGTTCTCT
EMPV1_37941 CTGCCTGTCGATCTAAAAGGGATGATGCAGTGGTGTAGCCTTTCCTGCTTGGTGTGTAAC
EMPV1_37943 GTGTCATTACAGAGGCAGCGTTTACCAACCTGGAGAGCTCATTCCAACACCCTCAGGCTT
EMPV1_37944 TCCATGGTCATTAACTCCTCTGCCCCCACTCCATTCGCTTGGTTGTTCATACATGACAGC
EMPV1_37947 CTTCAGAGATGCCGCCACACTGTTTAGGTCGGACCTTCCGGCTTTCACCAACTTGTTCTT
EMPV1_37948 CATTCACACCTTCCCACCACCCAAGGATCTAGCGAATCCAGGGCCCTCTCTGCAGCTTTA
EMPV1_37949 GGTGGAACACAGGGCGAATGTGCAGGTCTGCATTTGAGTTTGACAGAGAAGCATTCTGTG
EMPV1_37953 TAGGCTATAGATCAGCAGCCCAACCCCGAGTTACACATTTCTCAGCTTGGGATACACGGG
EMPV1_37955 GCTCCATGATACGAATATGGAACTGACAGATCTTAAACTACAGTTGGAGAAGGCCACCCA

EMPV1_37957	AGTTTTAAACCCGGCTCCAGAAAAAGCACGGTTTCATCTCACATCGCAGCGTTGCGCGTT
EMPV1_37959	GCCTTAGGCAAGTCATACCTCCCTGTGCCTCGGTCATTTCCTGTCCTAATGAGCAGGGCT
EMPV1_37960	GATGATGAGAAGAAGGCAATCAGGATGGTTTTTCAGTCTCCATTGTGCATATGTGACTCC
EMPV1_37961	TGGTTCTCCTATCTTGATGCCAGGATGCTGTGTGTAGGCATGAGAATGTGTACTTGGGGC
EMPV1_37963	TGGCTTGCACCCCATGAAAGAGGTGGTTTGGCAAGTATGTTGGACTCAGAAGTTGGCTGG
EMPV1_37966	ATTGCCAAAGCAATCCTGAGGATAAAGAAGCAGGAGGCATAACTCTCCCAGACTTCAGAC
EMPV1_37967	GGGACTGATGGCATCTTGAGCTCGTTATTTTGACATTGATTTTTCTTTGGAGCGGAGGCA
EMPV1_37968	AGCAATCCTTAACGTGTACCTGCCTAACAGCAACGCTTGAAAACAGGTGAGACAAGAGCC
EMPV1_37969	TCCAGCCTTTCCTCCTCCCCATTACCATCAAGCCTCATCTGAGTATGCTTGTTTTGCTCC
EMPV1_37970	GGGGGCATGTGCTTGCTCATTTTTTCCATACTGATTGTCTCCTACATTGTCATCCTGAGC
EMPV1_37971	GTGATATCGATGGCCAGACTCCGAAGTGAACTTGCCATGCCGGGGTGATGGAGTGAATAA
EMPV1_37973	CCTGCTGCTGGACCTAGTGTGATACATCTGTCCTCTGCTTTACCCGTGAAGTGATTAAAC
EMPV1_37975	CCTCCTGGCCTTCGCCAAACGCCGAAACTGCTGCATTGGCTGCCGCACTGTCCTCAGCCA
EMPV1_37976	GACCAATGCCACCTGCAATTATGAGCCTAAATCAGAGAATGGTGTAGACGTGGCCATGGG
EMPV1_37977	AAGAGAAGCACAAAACCAGCTTTCAGCCCTTAGCTCAGCTTCCCTTTCTTCTGGTGGCAG
EMPV1_37978	AGAAATCCTGCACACACTGGCTTCTCCACATACCTCCTACACCAGCCCTAAGCAACTACT
EMPV1_37980	CAGAAAATTCATAGAAATGGGTTTCATTGATGAAAAAAGAATAGCCATATGGGGCTGGTC
EMPV1_37981	CAGTGTTGTCAAACGTGTTGCTCCAGGAGCTACCAACATGATAGGTTACATGGGGCAGAC
EMPV1_37983	CAGCGTTTGTTCTGTGGATGAAGACGCAGGAGGGTGACAGGGACAGGCTCTCTTGATAAA
EMPV1_37984	TTTCTTGTTGCTGCTGGTTTACCCCGAGAAGCAGAGACTCCCCGAGGCCTTGCACGGATT
EMPV1_37985	CTTGCTCTTCGGACACCAGGATAGGCTAAGGTGTGAGGTATCGTCCTCAGGGCTTGTTTT
EMPV1_37987	GCCTCTGTGATCTTGGTGCAAGATGTGATCCATGCTCTTGCTATTCTAATAACAGGTCTG
EMPV1_37989	TCCGATTAGACACCTGTGAGGACGGTTTGAGCCAGCTCCTCTGGAAAGTGGGTTCAGGAA
EMPV1_37990	ATAGTGGCGTATAAGTGGAGAGGCACAGATGATTTCAGGACCTACCTCTGGCATAGTCTG
EMPV1_37993	TTAGGACTACTCGACCTGTAGTGACGTCCCGACATCCTGGAGGTCTTCCTGGAAGGGGAG
EMPV1_37996	GATCTCCTCCCTCCTGGTCATCCTAGTGTCCTACCTATTGATCCTGATAGCCATATTTCG
EMPV1_37999	TAACAAGAGCCTGAGCCGTGTGATCCCTGTGCTACATCCCTTTCTGCCCAAATGAACAGT
EMPV1_38001	AGGGTCACTAGGCCCGATCTCTTGCCTGACTGCCTCTTGCTGCTGTTTCATACTGTTTCC
EMPV1_38002	CTGAAGATAACTCAGAGGATGAGATTAGCAACCTGGTGAAGCTGGACCTGCTGGAGGAGA
EMPV1_38003	CGGTACAGGCCTGGGACCTTGTGGATGGATTTTGGTGGCTGTATCATTCTTGTTCACTGT
EMPV1_38005	TGGTCTCCTGGTGGAGGTGGTATTACAAAACAGGAGGGGACTAGAAGTACTGACCTCAGA
EMPV1_38006	TTTGCATGGCCTTCAGGGGACAGAGAAACAAGTTTAGAGATACCATGGGCAGCCAGTCAG
EMPV1_38009	GCTTGTGGCTTGCACACGGCTGACAGTTCAGCAAAGACCTGTTTCTTCAGGTTCATTTCC
EMPV1_38010	CTCTATACCACTGGTTTTTAAATGATATCACTGCCTTGTCTGAAAACCTCTGGTGGCCCC

EMPV1_38011	ATGGGACAGGTTATTTGAGCTCCATCTATTGAATGGTGTGTAGGGGTGAGGGGGCCTGGT
EMPV1_38012	TTAGGGCCTCATCACCCAGCGAGCTATTTTGAGCCAGCTTGTTTTCCCCAAAGGGGCCCT
EMPV1_38014	TGGACAGCCAGCCCCATCTGGAAGACTAGTGATTTTGTTGTTGTCTCACTGCGCTCAACA
EMPV1_38016	GACACAGTGATGTATTTTGCAGCAGGGGTGTATGGTGGAGCTTCCCTCACTGGAATTCTT
EMPV1_38017	GTACCTTCACTTAGGTAGAGACCCCAAGACACCAGAAGAAGTTACTCGGACGGTGAACAC
EMPV1_38018	TATTTGAGCCATAGCTGTGACCTACATGGCTGGATCCTTTAACCCACTGCTCTGGGCTGA
EMPV1_38019	ACAGCACGTGTTTTACAAGAGTTCCACCAGGGCCTAGGAATATGTGCCATTCTTCCCAAA
EMPV1_38020	ACTCACAAGGAGGATACATAATGTACAGCCGAATCTGCAATCTCCTATTTTGTCAGCTGC
EMPV1_38022	ATGTCTGAGCGCCATCCACTGCTTTCATGAGGCCCGCCTGGACGACGAGTGTGCCTTCTA
EMPV1_38024	CATCCAGAAGCTGATGGACAGACTGAAGAATCTGGGGGCCGATCATGTTGTCACAGAAGA
EMPV1_38025	GGTGGCCATCACGGTTTCGAGCATTGTCCTGCTGATGACACCTTTCTGCTTGGTTCTCAT
EMPV1_38028	TTGGAAGGGCTCTACTCTGACTCTGCTTAATAGAATGAGTCTGGCTTAGGGCAGTGGGAG
EMPV1_38031	GTGGAGCTAGGTGAGGTTATGAAGACAGGCAAACCTGGAACTTTATCCCTGTGGACCTAG
EMPV1_38032	CAGCAAGCGAGCTGTCGGAATAAGACGCAAAACCCGAGCATAAAGTCTGCCTCAATTCCA
EMPV1_38036	TGGTGTTCCTACTGATGAGGAGCAGGCGACTGGGCTGGAGAGGGAGGTCATGATGGCCGC
EMPV1_38037	CACCATCAGGAGATGAACACCTGTGTGACCCTCTGTCCTGCTGGGTTTTATGCTGATGAA
EMPV1_38038	GCCCATAGTTTGTGCAGAATCTAATCAGTCTCTTTGGGGCTTTCCAAGGGCAAAATTTGG
EMPV1_38039	TTGTGGTTGGTTATACCCCCACTGGAAATCCAATCTTCCGTAACCCCTGCACAGAACAGG
EMPV1_38040	GCTCCAGTGAGCATGCGACCTTTTGGCTTTGGGCGTGTTAGAGATGCTTACATGTGTGCA
EMPV1_38041	AAAAGGACGAGCTTCTCGTGGCTCTGGAGAGAACGATCCGACCTGACGTGGAACAGATGT
EMPV1_38045	CGGATGCTTGTAGGATCGTCTCTTTGTTCCAGGGTCTGAAATGTCACTGTGTTCCAACTT
EMPV1_38047	GCAGTTCCTCAACCGGGAGGACATCGGCATCATCCTCATCAACCAGTACATCGCAGAGAT
EMPV1_38049	CTGAGAAGCCCCCATTCTCCTTGGAGTGTTCCCACTGCTGCAAAGAACAAATAAACCCAG
EMPV1_38051	GGAGCTCCCTTCGCTGTTGTGCAGCAGAAACAGATTCTCAGTTCATTTTTACTCCCACTG
EMPV1_38052	GCTGCTGTATTCTGCATCGAGCCTGGTTTTGCCATCCCCCCATGATTATACATGACTATG
EMPV1_38053	CAGTTCAGTCAGTTCTTCCTGACAGCATAACACGACAAATACACAAGACCTCAAACATGC
EMPV1_38056	CCACTTATTTCAAACGAGGCCATAGGAGACAGTTGTGACTCAAATTGTTCTATCCTGTAT
EMPV1_38057	GCTCCACCCAGAGAAGCTCACTTCGTGAGAACCAACGGAAAGGAGCCTGAACTGTTGGAA
EMPV1_38061	GCAGATTCTGACTCAGTAGTTTTGGAGTAGGCTCTGGGATTCTGCATTTCTAACAAGCTC
EMPV1_38063	GCGATTATGGCTTTGTCTACTCCTCCCCACCTTCACCCCCACCTCCAAGTTTATGTTGCA
EMPV1_38064	GGCTGGGATATCATCATATACTGTAAGTTTGCGATGAGACACTACAGTATAGATGATGTA
EMPV1_38065	CCTGGTACCTGAACTGGCCCTGCTGCGTTTTGTGGTAAAAGATTATGACTGGAAATCCCG
EMPV1_38066	CCTCACACTGGAGAACAAATTCACAGAGCAAAGGGAGCCCCAGTCTACAATACTGACAGG
EMPV1_38069	AATTATGATTTCCCTCTTACCCTGCAAGACTACATCCACAGAGCAGGGAGGGTGGGCCGT

EMPV1_38070 CCACCCAGGTAAGAAAACCCTGGAAATAAATATTAGCGGTCTGATCACCTACAAGAGGGA
EMPV1_38071 CCTCCATCCCCGGCCACCAGATCCTGAACAACACCCTGCCCGCGCACGTCCCCTCCGTGA
EMPV1_38073 GGGGTTTTAGGAGGGGTGCCAGATATGGACGAGTTGCCTCCATCCATTGTGCTTTGTAAA
EMPV1_38075 CCTCTCGCAGGGATCTGGAGAACTTCCTAAAGTGGCTACAAGAAGCAGAGACCACGGTTA
EMPV1_38078 GAGGCTCCATAGGTTCATTTATCCATACTGTCTACCCTATGCACTTTCCCATCTGTGGGT
EMPV1_38080 CTTGTGGTTACCCACTTTTTCACATATGTGAACCCCTTCCTGTCTCTTCTTGCTTTAGAC
EMPV1_38082 CTTGAAGAGTTAGAAAAATGATGAAAAAGCTCCTTGGAGCAAAGCTGCTGACAACTACCC
EMPV1_38084 TTTCCTCCAACATCCAGGGCTTTACTGCCAACACTGATACTGGGAACCAAGGGCCACTCT
EMPV1_38086 TTCCTGGATTTCGGGGCAATGGGGAGGGCGGGCATCTCTCAGGGGGCTTCCCTTCTTGAT
EMPV1_38088 CAGTGTTTTCATAATACAGTAGTCCTGTTTGGTAAAGGCATACCTGAGTTAAGTACACTG
EMPV1_38093 GACTGCGCTTCCCAGTGCGCTGTTGACGTGGAGATGTGAATGCCTACTGCTTAAGATATC
EMPV1_38098 GCTGGTTAGACCATCCTTACTTCCAGTCTCTGAACCAACAGCCCCGTGAGATAACAAACC
EMPV1_38099 AAGCCGGAATTGATTGCATAAGGCACAGTCTCTTTGGGCATGCAGAGTAAACAACCTTGT
EMPV1_38100 AGCGGCCAGCTTCATTGAGGAGCTGACGTCCATTTTTAGAGAAGCCGCAAAGCCAAGAAA
EMPV1_38102 CCTGAGATCATCCTGGGCAAAAAAGGTGGAGGGGTCCAGCCGCACAGACATTGAAAGGCA
EMPV1_38103 GGACTGGCCTCTTGATGCTGGAAGACCAGCTTGACTGAAAAATTAGAACTGGGATGGAGC
EMPV1_38104 GCAAGATCAAATTCTTCGACCTGCCCAGCAAATCTGTCATCTCCCTGGTGTTTGACCTGG
EMPV1_38105 TCGAAACCCAGTAGGAAACTATCAAATTCATGTCTGGACCACAACGCCATGCATCCTTCG
EMPV1_38107 GCTCTTAAGGATGAAAACTGGGACTGCTTTATATTCCATGATGTGGACCTGGTGCCTGGG
EMPV1_38108 GGCTCAGACAGTCAAGTACACAGTAAACTTTATGGATGCAGAAGAGGCAGATCTCCACAG
EMPV1_38109 CCAGAGACATTCCATGGGGACCTGTCAGTAGGTGACATGAACCTTGATCAGCTGGTTAGG
EMPV1_38111 GAGCAATTCCTAACGATTCTGCCCACTGAGATAGAGACCTGGGTGAGGGAACATTGCCCA
EMPV1_38112 CTTTGCTGCATTTGTTTGGGATTCCAGAGGGAAGTGGTGGGAATAAGGTGGCTGATTTCA
EMPV1_38115 GAGTGGTGGCCGCCCTTTCTGGATGTTTCCCTTTCTGCTTCAAAAATTGAGAAACCGGCC
EMPV1_38116 GAGTTTTCTAAGCGAACCAAAGGCATCGCAGTCCAGGCTGAGAATCTTGTGAAGGAAGCT
EMPV1_38117 ATGGCATATGATCGTGCCGTGGCCATCACCCGCCCACTTCATTACACAACAGTCATGAGA
EMPV1_38118 GAGCTACAGAAATCGATGTGGTGATTAACAGGACCTTGGTGCTAACAGGCCAGTGGGAGG
EMPV1_38119 TGAATATGATGACATTGCACAAGTCTGTGTGGTATCTTCAGTCATTAAAGTGGAAAGCTG
EMPV1_38122 CATGACAGCTTCTGCTGTTCTGCCATTCCCGTTGTTTCTGGGGGAAAGGGTACTACTCAG
EMPV1_38124 TAAGAAAGGTCAGTGTTTGAGGGGCAAAGACAAATGCATCATGGAACAAGGTCCTGGATG
EMPV1_38127 CTGGTGAGCCATTGAGGGGGTTTAGATGTTTGTAAACAAGTCACCACAGTACATCTGCTC
EMPV1_38128 GGAGCTAGAGTTCATGGTAACTGTCAACAGTGGGCTCCTTTCTGTGGGCTCCTTTGTCTT
EMPV1_38129 TTCTCGGTACTTCACATGGTACAGGCACTATCCTGGGAAAGGCCCTGAGTTGCTACTGTA
EMPV1_38130 GCACTTCGGAGGATTGGAGAGTTGAGAGAGGAGCTCCAAATGGACCAGCAAGCAAAAAAA

EMPV1_38131	CGTCCGGGTGTGAAAGGCCACCAATAACGCTTGAGGCAGGACAAGGTGTTAACCAACTTC
EMPV1_38132	AGATTCCGACACAAAGAGGTGGTCCATCTGTGCAGGTTGAGGAATGGGCTGAACGTGCTA
EMPV1_38133	ATTGATAGTCACTTTTTAACACAGCAGCCCTTTGCCCACCTAGGAATAATGGAGGGTGGG
EMPV1_38134	GTTTCATCCATCATAAAGGGCCGCATGGACTTGTTCCTATCCACCAGATGAGTTCTGACC
EMPV1_38138	ATCCAACCTCCTCAATTAGGCAGCGTAAATATACATTACTCTGAATTGTCCCTTCCTGCA
EMPV1_38139	GAAGCTGGAGGAAAGGGGCTTTGACAAGGCTTGTATTGTCCTTGGCCAGTTTCTGGTGTT
EMPV1_38140	TGTGTCCACCTTTGGCATGGACCTGTTATGTATCGTCCTCTCCTATGTGCTCATTCTGCG
EMPV1_38141	CTGGGCGGGGTTGTGGGGGTGAGAACCGGATGTGGGGACCCTCTTCCTTCAGTAAGTAAA
EMPV1_38142	CATGGATTTCAGAACCCCTGACCTACTGGATGTGTGGCTGGAGCCCCCAGAAGATGTCTT
EMPV1_38147	ATCTTGAGCCACACCATCAAAGCTTACAATGCCGAAGACAACGAGAAGTTCAAGACGATA
EMPV1_38148	TCTGTGCTTTAGAGGTGGTGTTGCCATCCTATGAGTGCAGAAGTACTGGAGTTGGCATGG
EMPV1_38149	GAGCCACAACAGGAGCTCCAGCCTTGGATTGTGGTCTTGAATTTAGTTGTGTACCCTGAG
EMPV1_38151	AGTGAGCACCCCTGCTGCTGAGTTTTGAGTTTAAGTGACATGGCCATAGTGTTCCATTGC
EMPV1_38153	TACAGACCACCGGCTGGCCCAGCGTCGTCACCTGCCTCAGCAACTGGTTTGGAAAAGGGA
EMPV1_38154	CTCCGGTGTGGGACCCTCTCGATCCTGTCTGTTTTCTTAATGGCCACATGGTAGCTTCTA
EMPV1_38155	CTGGGTCCTAGAGCCCCTTGGCCGGCGCTCGCGTTCGAAACCGTCACATCCCTTGGAAAA
EMPV1_38156	TTCCTCCCCCTGTTTATTTCTCAGGGTTGTTTTGCTAGAACCCCAGCAGGACCTGTGTGT
EMPV1_38158	GAGGAGGACTCCATCCTGGCTGTGAGGAAATACTTCCACAGACTCATCCTCTATCTGCAA
EMPV1_38159	GTCGTCAGATACCTCAGCTGTTGCTGGAAGGCGAGGGAGGAGCAGAATAGAGTGAGCACA
EMPV1_38160	CCAATCCAGAGATGGCGCCACTTTTAAACTGGTGGTGAAATGGAAATTGCAGAAAAACTG
EMPV1_38161	GTCTCGCAAGCCAACAGTACATCTAAAGACTCCACCAAAGGCCACATCTTCACCAAATAT
EMPV1_38164	CTCTGGTTTTCCCAGCCTGAGATGAGCCTCGCCCCCAAAGGAAGCAATAAGGTCGCTGTT
EMPV1_38165	GGAGCCTCTGTGTCATTCACGTGTCTGTCTCCACAGTTGTGCAAAATGGCAGATTCTACC
EMPV1_38166	TGTGCATGGAGAATCTGGTGAATGGGGTTACCAAGGAGAGCAAGGGTTTCCAGGCTCACA
EMPV1_38167	CCATGTCACCGACTTCATCTTCTTGTGTTTGAGCAACATACATATTACTTTCCACACGGG
EMPV1_38168	TGGGTGCCGGCTAGGCCCTTGGGGAGATGCCTTCCTCCTGCCGTCAGGGGATCTAGGGTT
EMPV1_38172	GTGTTTAATTTGAGCCCTGTCAGTTTGATTAATGTGTGTCTTGGTGTGTTCCTCCTTGGG
EMPV1_38173	GCTGGTACCGTGGTTATGAGTACTTAAGGGAGCACTTGTACGAGGGTTAGTTGTGTGGGA
EMPV1_38174	CCCATATTTATTCCAGGTGGCTAAGTGCTGAGCTTCTGTTTCCCTGTTTATCTTCCTCCC
EMPV1_38175	CTGATGTCAGACAAAGTAAATACTAGACCTTTGCCCTTGGTGCCATCCTTTGCTCATTTC
EMPV1_38176	CCAAGGGTATCCCGATAGGGGCACTCGAATGCCTAACTGCGATAAATAAGGCAGTGGGAA
EMPV1_38177	TATGAGGAGTACAGTGGCCTCAGTGATGGTTACGGCTTCACCACCGACCTATTTGGAAGA
EMPV1_38178	CAACAAGGCGGTCAAGTGTGCAGAGGTGATCATTCCCCTGCTGAGCAGCGTCAATGTCTC
EMPV1_38179	GTAACTACCAGAAGTCTGCACACATGCTTCTGCAATCTAACTGTAATAAGACTATAGGGG

EMPV1_38182	AGACGAACAGCCGGTACTATTAGACGTTGTGGCTGTTGTTGTTGTACTCCAGCTTGTGCC
EMPV1_38183	TGGCCTGAAGGAGCTCTACGAGAAGGGGCCCCCTAATGCCTTCTTCCTCGTCAAGTTCTG
EMPV1_38184	AGTGATCAAGGTCAGGGGTCAAACCCAGATTCTCACAGACACCGTGTTGGGGTCTTAACC
EMPV1_38185	CTGGCTCCAGTCTTGACAGTCTCTGCTGTGTTATACCCCGCCACTTCTCACACTTATGAC
EMPV1_38186	CATGTCTGTGGCCCTTATCTGAAGGAGTAATTTGTATGGTGAAGTAGCTTTTCTATGTCC
EMPV1_38188	ACCAGTCCCAGGCGAAGTCCCAATCTCTGCATCTTCTACCAGCAAGGACATAGTCAAGGT
EMPV1_38189	GTCCTATCACTCCACAGGGTACTGGGTTCTTGGCAATACAGTCTGTCAGTGGCATAATGC
EMPV1_38190	GGATCGCTAAATGGCTGATTCCAAGAAGTTAGTTTCAGTGAATTCCTGTGGCTCTCATCT
EMPV1_38192	ACCTAGATGAGTGTGCCTTTCCGGGAGTCTGCCCCTCGGGAGTCTGCGCCAACACTGCCG
EMPV1_38193	ACGCTCGGGGTGGCAGGACAAGCCGATTATTGGAAATGCCCCTTGTTACCAGAGAAACTC
EMPV1_38199	GTTGTGAACTGCCTTCACTCTTTCCTCTGTCCTGCACTGATACCACAGCTAACACGATCC
EMPV1_38200	TCAGAAGCTGTCATCTCACAGTTATTATGTACTAATCTGCTTCTCTTGCAAGGACCCAAT
EMPV1_38201	AATGTAAGGACTTTATCCTTTCTGCTTGATACAGCAGGGGTGCCCTACTAGCTGGTCCAG
EMPV1_38202	GTGTGGCTGAGACGAAAGGTCACAGACTTGGGCATTTGTCATTTTCTCACCATGTGACCC
EMPV1_38204	GCTTACAAGGCAAACCTCAGGCCAGAGCGGCTGCATGAGCGGCTTATCAAGAATGATCAT
EMPV1_38205	GAGCCAGTCATACATATACACATGCACTCACTCCTTTTTTCACATCAGCCTCCATCACGC
EMPV1_38206	TACTGCAGCCTGCATCCCCACGAACGTTTGAGCTATCCATCACACTCCTCAGCAGACATA
EMPV1_38209	GCCTTTGTGTGTCTGTGTCGGTGGCTATGTGTTTGTGCTTTGGAGCCCATGTGTCTGCAT
EMPV1_38211	CACAAGTCCTATAGTCGTTTCTTACTTTTCGGATATGGACGAGCTACGCATGTTCGATGG
EMPV1_38212	GAAAAGCTGAAAGATTTTCTCTAAGATTAGGAACAAGACAAGGATGTCTACTCGTGCCAC
EMPV1_38213	TCAGGTAGAGGCAAAACACAGCAATGCACGATGCATTCGGGGACCCACATACAGTTTGGT
EMPV1_38215	TTTTTTAAAGGTCGCATCCTTGGCATATGGACGTCCCGGGCCAGGGGTTGAATCCGAGCT
EMPV1_38216	CAAGAACTGGCCTCAAAGAGATGGGAACAAACCGACCCTGGAACTGAAGATGAACTGTGC
EMPV1_38217	GTGCTCTTCCTGTTCGTCTGCGGCCTCCTGCCCTGGATTGACAACATCGCCCACATCTTT
EMPV1_38219	TGCTTGATCTGCTTCACCTCTCCACCAAGCCCTGCTCACTCATCTCCCACCCAGAATCTT
EMPV1_38220	ACCAGCGTTCTATTTTGCCACGGAGTCAGGCGGCCCCCCTTCAGTATCAACCTCTTTAAG
EMPV1_38221	CCGCTCCTGAAACAACCATAGCAACACCAGATCCAAGCCATATCTGCGAACCTTGCCACA
EMPV1_38222	TATTGGCTGAGAGACACACGTGCCACCAAAAACGACCCTGAGCCAGACCAATTATGGGCT
EMPV1_38223	TTTAATTGAAGCCTTAACCTCTGCTTTATGCTCTTGTGGGAGGCGACGGGGCAAGGGAGC
EMPV1_38225	GCCTCTGAGGAACTCCTGAAGCAGCACTACATTGACCTGAAAGACCGGCCCTTCTTCCCG
EMPV1_38226	TTAGATTCAAAAGAATGGCCGTATTGTTCCCAGTGTCCAGCAGTATGGAATGGCTCCGGA
EMPV1_38229	CCTTCTTGCCACCTTATGTCCATTCTCTTTGCTTGCTAATCTGCCGAAAGGAGAGAGGCA
EMPV1_38230	TGGTTACTGAACCTGCAGCATTTGACATTGGGAGCGCAGATAGGAGAGGGAGAGTTTGGA
EMPV1_38231	AAGGGAACGGGCTTGGCGGAATCAGCGGGGAAAGAAGACCCTGTTGAGCTTGACTCTAGT

EMPV1_38233   AGCCACAGCAACACTAGATCCGAACCACGTCTGCAACTTATACCCAGCTCACGGCAACTC
EMPV1_38235   CAATCGCCCCCTTCTTGCCCTGGGGGCCCAGGATGTAGATATTGTACAAAGGTTTCTAAA
EMPV1_38236   CACACCAGATCCTAAATGTGCCGCTTTTCTCCTTCCCTGAGGGTATGTGTTCAGTACCTG
EMPV1_38237   CTCTTAGAGAATAACCTTCCTGCTCCAGCCAAGCACTGGCAAAGGCTAAGTGAAGCATGC
EMPV1_38239   GTGTGACCCTGAGTAAGACCAAGAACAGATGGGTGGAGACTTGAGGTAGGACCCTATTAT
EMPV1_38240   GCCTGCACATTTGGGGCCCCTTAGGGTAGATCACTTCCACTTTGCCGTCAATTATTTCCT
EMPV1_38241   ATTTGCTTACAAGGCAATTCCACTTCTCCTCAAGACCGTGCTGGGCCATGTCTTGTCACC
EMPV1_38243   GGTCATGCACCCTTTCCCTGCTTTCACGCAGCACCTCTGAAATGGCAGTATCTCATTGTC
EMPV1_38244   CCAACACCTCTTCCACATTTCTCGTCCTGCAAGAAAGCAGTAAAGGCCCAAAGAAGGTCC
EMPV1_38245   TGACTGGATGAGCCCTGTATGCACAGAAATACAAGCCTGGGAGCCTCTCATCTGACTTCA
EMPV1_38246   AGATATCTATGCTTTATGTGTGGCCTATCCTGAACCCCTTGGAGAAAGACTTTATACAGA
EMPV1_38247   TCCTCACCCCCCACCCTCGGTCGCCGGCTGTCGGCCCTTTTGTCTGTCTGTGTCTGGGGA
EMPV1_38248   TAGGTTGTCTCAGCTTAATGGCAGAGCCTGGCCTAGGTTGGTGGCCTGGCAGATCCTTCA
EMPV1_38249   GGAAACGGAGACGATAACACTGCTGCATATCAGAATGAGAGGGAACACTTGTCCCTGAGC
EMPV1_38250   CAACCTGGATTCTCATGGATCGTAGTTGGATTCCTTAGCACTGAGACACGATGGGAACTC
EMPV1_38251   TGACCAGTCACCTCAACTCCACTTCCGTTGATCCCTACGACAGACACCTGTTGCCTAACT
EMPV1_38253   TGAATATCCAGCAGTGATTGTGGAGCCAGTTCCAAGTGCCAGATTAGAGCAGGGCTATGC
EMPV1_38255   AATTGTGTTGACAGCCTGCTCACAGCTGCCCTCCCCCTTCCATACCTTAATACAGCATCT
EMPV1_38256   GCTGCTTCGCATCAAGTATTACGTAATTTGATCCTCAAGAGACCGTTCAAGGAATGAGGC
EMPV1_38257   AAGTGCCAAGGCCCAGGAGGAGTTTGGTGCTTCTCCCTCCAGTCACCCAGTCTTGAGAAA
EMPV1_38258   AGGAACTTCAGGGAAGGAAGCAGACTCCTGTGTGTTGGGGGCAGTCGAGTCCCAGTCCTA
EMPV1_38259   GGGGTGTTTACTCTTCTCTAAACATTACCATTTGCTGCTACGTGTGCTGCTAACTGAGGG
EMPV1_38260   ATAAAGGTGGATTGAGATTTTCTCTCTTAGCCCCTTGTCTCCCCGTGTGGAATGGTGGGG
EMPV1_38261   GTTTTCATCATTTTTTCATTACAGACAGAGCTACATCTTGCTATGAGATACAGAGATTCC
EMPV1_38262   GGCTTGGAGCTGGCTCACCGTCTCCCGCAGTAGGAAACGTGTCACAAACTGGTCCACTTT
EMPV1_38264   TAGGCACCCCATCAACAGAGGCTGACCTGGGTTCAGTAAAGGAGGAAAATGTGGCAGAGA
EMPV1_38265   CCTTTCGACCAAAATTCCTGGTGCCCAATGGCTGTGCTCACTCGTCAGCTCAGGGCCTGA
EMPV1_38266   TGAAGGCACTGCACGAGGGCACTACAAAGGGCAATGGGTTGGCAAAGTCATCCAGGTTTA
EMPV1_38268   CAGGGTTATGCATTCACGGCTTTGCCAGTCTCTTGCAGGTATGGCTTGGTTGAGCGGAAT
EMPV1_38269   ACAAATCCCCAGAGTCAGACTGGAGCAGTTGGGAAGCTGAGGGCTCATGGGAGCAGGCTT
EMPV1_38270   GTGTCCATGCAGCCCTTTTGCAGGAGGACTACTGGTTTGAAGCGCTGAGTGCCAGGCCTC
EMPV1_38273   TTTGCGGGTCCAGGTGCATGCTGCCTCCCTCCCCAAGGCCAAAACCTCAATAAACTGCCT
EMPV1_38276   TACATGTGCAGCCCTGTCCGTGGAGGTTTGCTCAACCCAAACCCAGAAGCACCTGAGAGC
EMPV1_38277   TTCCCTACCCGGCTGCCCCGACACCCCATGCAGTCCCCAGAGGTCAAACTCACGGACCTT

EMPV1_38279	TTCGAGGATAGTCCTTGGATGCAGTAAGGAGGCAGGTTTGAGACAGCCTTTTCAGTGGTT
EMPV1_38288	CATAGTCAATAGAAGAGGCACCTGAGACCATCTGGGTCAGTTCTTAATGTGATGACAGTC
EMPV1_38289	TACAGTGACCAACCATTCTGGTTTCCCTGGGACTGAGGGGCTTCCTGGATATGGGACTTT
EMPV1_38291	CTATTTTTGTCCTCTCCAGAAAGTAAATGGTTGAAATGGCATTGTCTAATCCAGTGGCAC
EMPV1_38293	CATGCAGAGACTCTTCTTCCACTGCTTTCTCTCATGGGCTACTTCAAAGACAAGGAGCCC
EMPV1_38298	ACATTAACGTCAGGTCCCTGGAAAGAATCGCTCTGCACTTCAACAGGCCCTTTCTGTTCG
EMPV1_38300	CCTTTGATATTTCTCTATACATGGCCATCTCCCTCCATACCCCTGGATAAGTTTCTGGCC
EMPV1_38302	CGCGTGGTCCTCCTGGGGTTATTCATACAGTTGAGATCTTATCATCGTTGCTGCTGTCTG
EMPV1_38304	CCACCCCCGGTAGCAAGAAGCATGCATAGTGCCTAAATCTCTTCTCCAATAAAAGGAACC
EMPV1_38305	ACCACCGTCACCACGCAGACCATTCTCGGACACTGCCTTCTCTTTGTCTATTTTCCCCAA
EMPV1_38307	CCTTCTGACAAGCTGATTTTCTCTCATTCTAGCATCAGAAGGCAGTGGGCCTAATCTCGG
EMPV1_38308	ATGTGCGTTTCTGTGTGCCGAGGATGAGGTAGATGATGAGGGTGAGGCTAGGGTTTGAGA
EMPV1_38309	GGAAGTCTTTCTCCCAGAACTCCTGCCTCATCATCCACAAGAGAACTCACATGGGCAAGA
EMPV1_38310	TCTTGAGAACGACATTAGAACTTTCTTTCCTGAATATACCCATCAACTCTTTGGGGATGA
EMPV1_38315	CCCACCTCTGCAGTATGGTTCATGTCTCAGTGTTACGTGCCCTGGAATGTTTTTCACGTC
EMPV1_38316	TTTCCTTGGTGGGCACCCCAGGACTGTGGCGACACGCACTGTGGGGAATGTTTAATAAAA
EMPV1_38317	TCTAAGAGTCACAGTCCAGGCAGCATCCTTTCAGGTTAACAGTGGGCCAAGCCAGCATTA
EMPV1_38318	TGGAGGCCCATGATATTGTGGCATCAAAGTGTTATGAATCACCTCCATCAAGCCCGGAAA
EMPV1_38319	CTACTAGGTTACACAACAGCAACAGACTGGGGGCTGAGGACTAGAAATTTGGGGTGAACC
EMPV1_38321	TGACTACCTGTTTAAGCTGCTTTTGATTGGTGACTCGGGCGTGGGCAAGTCATGTCTGCT
EMPV1_38323	AAGGGGTGTCTGTTCTCACCACTTCAGTTGCCCCTTTGCTTAATCCGTTCATTTATACAC
EMPV1_38324	GCCCCTGGAGCCTGAGTAATGAAAACATCCTGTTAAGCTTCAAGACCTTCAGGCAAGTGG
EMPV1_38326	TGTGTGGCCATGGACAAATCACTGCCCCTCTCTGAGCCTCGGGAAAAGCAGAGTCTTCTG
EMPV1_38328	CATATGCCCTCCCACACACTGAAGAAACAAGGAAGATGCCTTTCAAGCCTCACTGGGCCT
EMPV1_38329	ACATGTCGTCGCCAGTACCATAGAGAGCTGGCGGCCGAGAGAGCGAGGCCGAGCCAGGGG
EMPV1_38330	AGATGGCTCTCGGTATCTTTCCTCCCCATCTTGTCCTTGAGCTAAATGTCTTGGTTACTC
EMPV1_38331	CCTCGATTGGTCACCTGGGGGTCCAGGGAGAGGGGCTGGGACCATGCCTCTTATCCTCCT
EMPV1_38332	GCTCTTCGGAGTAGACTTGGAAACCGACCTGGCCCCTGACCTCCTGTTAGCGGAAATCGA
EMPV1_38333	GAGCTGTGGGGCCTTTTGCAGATGAAACTCTGGATGTATTTGCAGATGTGTTGTAGATGC
EMPV1_38336	TACATATATACATTCACACACACGCACGTCTCGTTTCTTTTATAAGTTGCTTTCTCCCTC
EMPV1_38341	CCTACCTTGTGAATGGAACTCATACTGGCCCCATGCCATTTCCCTTTTCTGGGCTCAGTT
EMPV1_38343	GCGCTTGGAAGAGCTAGTAAAATTCCTTAGGGACTGCCTGCTGCGACGCCGTACGCTCTT
EMPV1_38345	CATTGTCACCTGAAGTAAATGGCCTACTGGTAGAACTGTGCCTGGACAAGCATGAGAGCC
EMPV1_38346	GAGACTCCTACTGTCACCTGGCTGAGGTCGCTGCTGGTGATGAGAAGAAAGAGATTCTGA

EMPV1_38347	GCAGACTGGTTGCAGAAGAATATTTGAAGTTTTTTGATTTTACAGGAATGACACTGGATC
EMPV1_38348	TGATCCAGCCCTCCAGGGAGCAGGTGCAGAGGCTCATAGCGGAGCATCCCCCACACCTGA
EMPV1_38349	GGACTTGTGTATATACACATGCACAATAACCCCACGAGCATGTTTATAGTCAGTGGATGG
EMPV1_38350	GAATCACTGCATTGCATGCTATTCCAAAGTTCTGTGTAGCTGGATTGTGCTAGCTACTCT
EMPV1_38352	ACAGTGTGCCGATGGGGGCACCCATCTCTGTTTAAATAGCCAGGGAGTCTGGGATGTCAA
EMPV1_38355	GTACGGGATGCTCTTCTCAATCCGCTCCTTTGTCAGCAAGATGTCCCCGCTAGACATGAA
EMPV1_38357	CTCAATTCCAAGATGCCTTCTGTAATTAAAATTCACACCTTAGTAGAGAGTTTGAAACTT
EMPV1_38359	GCTGTATTCCCCGGAGGTTGGTCATCTTACATTCCCACCAGCAGTATGTGAAAATGGCAG
EMPV1_38360	GTCTGGTCTCGCCACACCCTCCCCATCACAGACCTGCACTGCGGCTTTGGGGGGCCCCTT
EMPV1_38361	CTTTTCCATTAAGACAACGCAGTTACTTCATCCAGCTTTGAAAACCAACACAATGGCTTT
EMPV1_38363	GGTAGATGTTTCCACACCCAAATCAACCAGAAGATCTGTAAGAAGTAGCAGCACGCCTCC
EMPV1_38364	GTCTGAGCAATTCACTTCCCCACCCTTCCCTACTCCCCAACTAAACCATTTCTTCAAACT
EMPV1_38365	AGAAGAGCATGGGGTGGCCCTCCTGGCTTACACGGCCAACAGCCCCCTGCACAAAGAATT
EMPV1_38366	CAAACTAAAGCAGAAGTTGGAAGCTCATATGGAAAAACTAACAGAGGTCCAGGAAGAATT
EMPV1_38368	GTGAGCTGCCCTCTCTCTACCCTTTGTCTTGCTCTGATGTATCTGCAAGTTTTACTGCCC
EMPV1_38369	TGTACAACAGTAAACTCCTCCGTTGTAACCAGCCGTCACGTCTCCATCTCCATCTCTTCC
EMPV1_38370	GTGGCCAACTTCTACTGTGACCTTGCCTCTTTGCTCAAGCTGTCCTGTTCTGACATCCAC
EMPV1_38371	CTCACAGAGCAGAAGGACATAAACTTCCCCAAGGGCTTCTGTGCAGGATGGCTAATGACA
EMPV1_38372	GGCCATGACCGCTCTTCTGTGAACACAATGTGAAAGCAAGGTGCTCTGGTTCTGACCGTC
EMPV1_38376	GTTCTTACTCCTGTGCTGGGTTTCTTTCTCCAGATGTTGCGTTCATCACGTCCGTGACCT
EMPV1_38377	AGGTTAGAGAGGAGATGAAGTGGGAATTTTGGAGAGTGTTTGTCAGATGCAGCCCTCCGG
EMPV1_38380	CTTTGAAGAAAACGTCCTGCCTCCGAAGAACTAATTGGTGCAATGTAACGGGAAGGCGGC
EMPV1_38382	CCCCCAGCACTTCCTGGATGAGAACGGGCAGTTTAAGAAGAATGATGCTTTTGTGCCCTT
EMPV1_38384	ACTGGAAAACAACAATGACCATTCAAGACCAGACATTCAAATTCAAGCCATGAGACTGGC
EMPV1_38385	CACAGACGAAGCACTCGAGGACCAAATTCCCATTGGTTCTGTTCGTCTCCCAGCCCCCCA
EMPV1_38386	ACATTGCCTCCAAGCCATCTGGTATCAAAAGCACATAACATTCCCAGTAGCACAGCTGCC
EMPV1_38387	CAAGGAAAGCATGCTCCAGTGGCGCAATCGGTTAGCGCGCGGTACTTATACAGCAGTATA
EMPV1_38388	AGATCGTGTATTTCACCGCTACATTCCCCTACGTGGTCCTCGTTGTACTGCTGGTGCGTG
EMPV1_38389	GGTTTGGTATTTGATCCCAAAGAAGGCCAAGAGATTGCAAGTGTGAGTGATGATCATACC
EMPV1_38390	GCCGGGTGAATGGGAGAGTACTTGTGTGTTTCATAACAGCCATGATCCCCTTGATAGGTG
EMPV1_38393	TCTCTGCGGCGGACGTCACCCCTGATTGTCTTCTGACCTCAGGTGGTCTCCTGTGACACC
EMPV1_38394	GCGCAAATCAGTTCGGGGTTGCATTGTGGATGCCAATCTAAGTGTTCTCAATTTGGTCAT
EMPV1_38395	AACCTAATTTGTGGCCTCCTGACTTTTAAGGAAGCAAGTGTGCAGCCATTACACACAGCC
EMPV1_38398	GCGGTTGCAGTCCGATACCCTGTAGATCATAGGTGGCAAGAATCGTGACGTCTGAAAGGT

EMPV1_38399	GTGGAAACTCCCAGGAATGTACTAGGTCTCAGCCATCTCTGTAAGCCAGAGTCAGCAAAT
EMPV1_38401	GCACATGCATCTTCCATTGCCCCAAGACACATAGCACCTTGTGAGAGCCCATATAATTTA
EMPV1_38402	CCTTGCGGCAGGTGTGGGTTTTAAGACTGGTTGACCGGGATCTTTAATAGCGGTGTGGAA
EMPV1_38406	TTTACCCAAAGACCTCAAATCTAAACTGCCCCAGTCTGCATCTCCTCCTAGCCCTTCTCC
EMPV1_38407	GATCTGCAGAAACGAATTGCTGAAATGGAAACTCAAAAGGAAAAAATTCATGAAGATACC
EMPV1_38410	TAAAGTTCTGAGAGGATGACACATGGATTCAGAGGTGACTGCAGGTCAGAGAGGGCTTCC
EMPV1_38411	CAACCGTCCTGCTAGCTGGTGGTCCTTTTGCTGGCATTCTTTACTCCTACTCTAAAATAG
EMPV1_38412	CCAGGAAGTGCTCATTGAGATGACTGATGGGGGAGTGGACTATTCCTTTGAGTGTATTGG
EMPV1_38414	TCATCCTTGGGTAGGGGTGCGCGTGTGTATGCACAGGTGTTCCACCCAGAGGCTATCTGA
EMPV1_38415	ATTACACTACCCTGCCTCTTCTCCACGAGAGGCAGCGGGGTGTAGTGGATAGAGCACGGG
EMPV1_38416	GCCTGGGAACACTGGACCTGATCCTCCCTCCATTCCCCCATTTTTGTGCTTCTAGTTTGT
EMPV1_38417	GGCCCTATATAACCCTGTTCCATCATCCCTCTGATCTCATCCTGTAACCCTCTATTGTAC
EMPV1_38420	GAAGATGAGTATTGCCCTCCACCCTCCCCTCAAAAAAGTGTTCATTAAACTTAACTACAC
EMPV1_38421	CCACTATCATGGCCGTGCAATTCGATGGGGGCGTGGTTCTAGGAGCCGACTCCAGAACAA
EMPV1_38422	CTCTGGCGCCCTCGTGTGGACATCTGCCAGAACAGCCCCTCTCAGGCTGGCCTCCTCTCT
EMPV1_38425	CTGTTCCATGTGGCTGTAGGACTCAGGTCCAAGTTTCCTTACTGATTGTCAACTAGGAGG
EMPV1_38426	CACTATACAACCATTATGAACTGGCGGCTGTGTGGCCTGCTAGTAGGAGTGTCATGGGTA
EMPV1_38427	CCCTCTAAGGATTTCTTTCCTAGCTGAAGGCCATGCTCAGTGTGTTAAAGCCCCAGAAGC
EMPV1_38428	ATATTTCCAACCAGGATCTTCGCGCATTCACGGCACCAGAGGTTCTTCAAAATCAGTCAC
EMPV1_38430	GGCAGCAAGTGATCCACGTCACTCAAGACCTAGACACGGACCTGGAAGCCCTCTTCAACT
EMPV1_38435	CCTCCAACAGGAGGACGAGGCTCTCATGCAGAAACACAAGCCCAGCAGGATGAAAATCCT
EMPV1_38436	ACGGGACGCGGTGCAGTGTTGTTCTTTCCCCTGCCAATATTGCACTCGTCCCGGCCTCCG
EMPV1_38439	CGAACTCCCTTTTTCCTCTTGGAAATTTGAGTTGCATCAGATTCGCTCTGCCGACCAGCC
EMPV1_38441	TATGATACCCAAAGTAGAGTGGGCGGCGCTTCTGGAGGCGGCCGACACCGTGAGGACCCT
EMPV1_38443	CAAGAACTGTAGATCTCTGTCGGAAGGCTGAAGCAACAGGAGTTTCCTGGATTACAGTCC
EMPV1_38444	GCAGTGCTGCATGGGACAGAAGCAGGCTCAGCAAGTAATGGAAGGGACAGGTGCCAGTTA
EMPV1_38445	TGGCATCACACGATCACCCCAAGAACTCAAGGGAATTAAGCCCATATGTCCTGAGCCTTG
EMPV1_38452	TCCCATCCACACCTTGCTGGCCAACTTCTACCTCCTCATTCCTCCAATCCTCAATCCCAT
EMPV1_38453	AGTGCGCTGTAGCCAGAGGCCCCCTCTTTCCTTGTGGGCTGGATGGGTTCTTGAGTGTAA
EMPV1_38455	GCACTGCCCTAAAATCACCATGGCAACAGATGCATCTGTTCTTTGAACGGGGTGGGGTAT
EMPV1_38456	TAGAGGAAACCTGAGATGATGGGTCAGGGCAGTAGTTATGCACTTAGGTCACTGTAGCCC
EMPV1_38460	CTCACGCTCTGCGAACCAACTCCTCCGTTTCCAGAATTTGAGTTCAACAAAGGCAGCGTC
EMPV1_38461	AGACCATCTCACTGGCACTCATCCTCTTCAGCAACTACTACGCCTTCTTCAAGCTGCTGC
EMPV1_38463	CTCCGGGGGCAGAGGTTTCCCTTTGTGTCTTCATGACTTTTTGTTGCCTGTTGTAACCAG

EMPV1_38465	AAGGTGAAGGAGAAGAACACGATCCTCTGGAGACAGACCAGCCACGCGGATTGCTTAGCT
EMPV1_38468	CATTCCACAGTATTCCATCCCCAGCAGCTATCACAAACTCAAAGCTCTCATGGCACAGCG
EMPV1_38470	CAGAGTATGCACTCCCTAGTTTAGTCCCTGACCTACTCTTTCTACTGTATCATATCCAGG
EMPV1_38471	GGATGGGTCCAAACAGCTTTCTGAGCTTCATCTGCTCTACACCTATCTTCCTGGACGCCA
EMPV1_38472	TGTACATGCCAATCGAAAACCTCAGACTTCGGAAAAGTACCATGCAGCACCCAGACCCAC
EMPV1_38473	TGGGTCGTCGCATCTTCCAGAACACCCATGCCATCGACAAATACTCCCGCTTGATATTCC
EMPV1_38474	CTTTTGTGGCCTGTGGCTCGGCAGCATTTCTTATCTGTCTGTAGTTGGATGTGGCCAGGT
EMPV1_38476	ACGGTGAGGCATTCAGCCAGCACTTGCAGGCAGTGAAGATCCTGGCCGTGAAAGATGTCA
EMPV1_38477	CAAAAGCAGGGGAACCTGACATCATGCCTCTCCCAATGTGATGTAATTATACACTCTCAC
EMPV1_38478	GTTCAGGGCTATTGTTTATAACCCGAATCTGAAGTTCCTGAGCCTGAATTGTGCGCCCCA
EMPV1_38479	TACTTAGAAATAATTTGCTCTCAGATTAAGCCAACCTCCTACTCCCCAAACAGAACTTCC
EMPV1_38480	AGCTGATGACAAGGGCCGCACCGCTCTCCACTTTGCCTCCTGCAACGGCAATGACCAGAT
EMPV1_38481	TGAGAAAGAGAGAGCCCAAGACAAAGCTGCCGCTGGGGATAACACTGCTGCCATGGCCAG
EMPV1_38484	GGTTTATCCATGGCTTAGAGGACTGGGTTATACATCCTTGGGTATATTTTTAATGGGATT
EMPV1_38486	TTTCCAGGTCTGAGGCCCTTGCAAGTCTACAGGAGGTGTAAACCCGAAGGCCGAGATCCA
EMPV1_38487	GCATCCGAATGAAACAGATGGCAAAAGCACTGCTATACATGCAAAACTCTTAGCACCTGA
EMPV1_38488	AGCCTTTACTCCCGTTGAGAGTTTTCAACACCAGTTGATTGAGCTTCAGGCTTTGCAACG
EMPV1_38489	TTCCTGTGTTGCTCTTAAAAAGGGATCCTCCACCTTCCCGGACCACAGGGTCAAGGTAAC
EMPV1_38491	GTGGTTTTGATTTGCTTTTCTCTAATGACAAATGAGCACCTTTTTGTGTGCTTGTTGAAC
EMPV1_38496	CGTCTTGGTGACAGCTCTACTGGGAAAACCTGGGGAGCCAGATCTTTTGAGAGCACCAAA
EMPV1_38497	CGACAGCATACTGGGGTGGAACCGATTTTCTTCAGCAACTTGACTGTCGTATCTTGGTCC
EMPV1_38498	GACTCAATGCCTTTAACAGAAATACCTGAAGCTGTGGCTGTAGATGAGAGCCAGTCGGAG
EMPV1_38500	AACATGCGCTTTGAGACTAGCCACTCTGGAGAATTTCCTGTTACACACGGGACAGGTGGG
EMPV1_38502	TCCAGTCGCCTACTTCCCCTACTTCAACAGGACCTACCTGGCGGTGGCCGCATCACTTAA
EMPV1_38503	AAGGGGTTTCCTGCAGAATTTGCCATGTACTTTTGTGGTGGACTGCGCTTTGAGGAAGCC
EMPV1_38505	TGTGCCCCAGAGTTGAAGTGATGCATGTCCACCACACAGCCATCCTCCTTCTGGTCTAGA
EMPV1_38506	AGCGAATTTGTAGTTGAAGTTAAGTCAGATAAGCTCCCAGAAGAGATGGGCCTCCTTCAG
EMPV1_38507	TGTTTTAGTGTGAGTTACCGTTACTGTATTTGTTTATTGTAAAGGTGGACATTTAGCGTT
EMPV1_38508	CACAGTCACACTATTGGAAAGTGATAGAGCCAGGAGCTGATGCCAGGGATGGCAATGGCT
EMPV1_38509	GGATGCTGTCATGATACAATGGTAGAGTGGAGTAGCTGTAACAGAGAACTTGGGCTCAGA
EMPV1_38510	TGATGGGATCCTTACTTCATGTTTTTGGTGCATTCCTACACTCTGAAATCAGCCCTGCAC
EMPV1_38511	TTTCCAAAACAAACCCTCAATGCTGCTAGGATTTCCCCCTCCCCACTTCTGAATAGGCCA
EMPV1_38513	CTTAAGCCAGGGCCATAATCTGTCTCCCAGCCACTCTGTAGCTTTTCTTTCATACTAATG
EMPV1_38519	CAGGATTTAACTGGTTCCTTTGCCCAGGGTCTCACAGGGCTGCAATCAAGGTGTCATCCA

EMPV1_38520 CTACTGGAACCTGTTCTGCTTTTGGGCAAGGAACGATTTGCTGGTGTGGACATCCGAGTC
EMPV1_38522 ACGATTTGGCAGATCAACTACATGCTGCAGTTGGTGCTTCTCGTGCAGCTGTTGATGCTG
EMPV1_38523 ATGTAACAGACTCAGCAATGTTAAAGGGGATTTCTGAAGCCAACTCTGGAGTCTGCAGGC
EMPV1_38524 CCACTCCTTTTACACTTGTTAGTGGATGCTCTTGTGCTAGAGGGCTTGTCCTTCTTCCTC
EMPV1_38527 CCTTTGAAGAAAGCTGTTAGAATGATGGGAGCACCTAACCTAATAGCAGACAGTATGGAA
EMPV1_38528 AGTTTCCTCCTCTAGCGATTATCCCACCTTAACACATGTTGGCTGTGTCACTTGGGTCTC
EMPV1_38530 CGAGAACGGTGGAGGAAGCCAACATACTCAGATAGAGCACAAAGTAGTCAAGAAGGAAGA
EMPV1_38531 GGATCTGGCTTTGCCGTACCTGTGGCATAGCATTACAGATGCAGCTTGTATTTGATTCCT
EMPV1_38532 TGGCAGGACGGCCACAGAGGTATGTTTTCCTTTTTAGCTCAGGAAGGTATGTCAGGCTCA
EMPV1_38533 AGCGAAGTCCTCCATGCCAGTGGTTGGAATCATGGTCGGCTTCCTACTGCTCTTGATCGT
EMPV1_38534 TGCGTCGGCAAGTAAGGCCCATGTCAGATAAAGTCGCTGGGAAAGTCACTCGGAAGCTGA
EMPV1_38535 GGGGCTGTCTATCCCCATCTGTCGTAAAGGCACCAAACAGTGCTGTCTCTATCAATAAAT
EMPV1_38536 GGAGCTTCTAGGTACCAGCATCTTTCCCTCACTTTCATCTCCAGACAGACTAGATCCTGG
EMPV1_38538 AAAGTGATTCAACAGCCTTCTCTGCCACCACCCCCACCACCTGACAGCCTGGATCAAACA
EMPV1_38539 AGCCTGAGTCCAGTCATTCCTTTGACACCGATAAAGTGGCTTCCATATTTTATACCCTGG
EMPV1_38540 CTGGGATCAGATTACCCTAGACGGAAACTGGAAAAATCCCTGACTTGGTCAAACCATGGG
EMPV1_38541 CTCACCCACGTTCACTCCAGCCTTCCCATTCAGTCCTTCTGGAACTCGTAATCTTTGTTG
EMPV1_38542 GAGGACCTGAAGTCCCCTTTGTTACTTCTGAATCAGTATTGCTCTCCTGGCAAGCAGACA
EMPV1_38543 CTGGGATGGATGTTGAAAGCAGCCTTGATGTGCCCAGAGCAAAGCACGTGTGCCACACAT
EMPV1_38546 CTCTTAAATATAATGCCAAGATTATATAAAGAATATATAAGGAACATAAATTATTCTTAA
EMPV1_38548 GCCTGTAGATTTATTACCATTTTCCAAATTTAGCTCCCGGCACGCACAGATCCCACCCCC
EMPV1_38549 CTCATGGAAGCAGATCAAGAACAAGCTGATAGAGATACAGTGGTGGCCAGGTTGAGCTTA
EMPV1_38551 TCGCAAGCTTCCAGGTGTTGTCCCTTTCTGCTAGAGTCACATAGGTGTGCGTAATTCCTC
EMPV1_38553 AAGACACCTACATGCTACAGCTACTGGTCATTGCTGACAGTGGCCTCCTGTCCCTAGTCT
EMPV1_38557 GGTAACATAAAGGTCTCTGCTTATTTGGTTGAGCAATCTTATTGTTGAAGTCTGCCATTT
EMPV1_38558 TCGAGGTCAGTCATCGTTACTCGCAGCGGCGCCATTTTGCCTAAACCGGTGAAAATGTCC
EMPV1_38559 ATGCCTCGGTACTGCCTCTTTGGGGACACTGTCAACACCGCATCTCGGATGGAGTCTACA
EMPV1_38561 AAGTGGAGATTTCTTGGCTCTGAGAGGAAAAGGCCACGCCGTGTAACTGCACCTATGTCT
EMPV1_38563 GATTCAAATGTTAGTCCAGAGAGGTTATGAATCTGACCCAGTGAAAGCCTGGAAAGAAGC
EMPV1_38565 AGCTCAGAGATTCCTCACTGTCAGCACTGGGAGATGGGCAGTACAGAGGGCACAGAACTT
EMPV1_38568 CGGAAACATATTGAAGTTGAGGAAGAAAGAGCCAGACCGCTAGTCCCCTTGCTATCAGCC
EMPV1_38569 AGCAGATGTTCCCACTTGTGATAGCTTAACCCCAGGTACTCTACAATTGGTTTCAAAGTT
EMPV1_38571 CTTTAACTCTTGAGCCTACCTGGGGAGTGGGGGTGGTGAGGATGGAGGTGCTATAGGCTA
EMPV1_38573 ATGGATCGCAACAGTGATGAGTACCGGCAGGGCAGAGAGAGGAACTACATGGCTGTGAAA

EMPV1_38578	CAGTTACAGTGGGGAGTTCCAGCTGAGTGTGGAATGTGCCAACAAGACCAAGAGTGACCT
EMPV1_38579	TGGGGGTTTGGTGAGGTAGGTTGGAGGGTAGCATTGTACGCCAGTAATCTCTCGGCTTAT
EMPV1_38583	ACCTGCTTTCCTGGCAGAGTGAGAATTTTACTGGGTCCTGCCTCACCGAGGGAACTCATT
EMPV1_38584	GCATTGGATGTTGCCTGTTCTCGCCAGCAGCTTACAGAGCTTCCTGATAATGGAGTGCCT
EMPV1_38585	ATCTTACTCTGGTGGGGAGATCAAGGACTCAACCTGCTCCGCTTCTCCCAGCCCCATCTA
EMPV1_38586	ACAGACACTGGCCCAGGGGAGTTGGAAACCTGTGACAGCATCTTAGACTCTTTGGAGCTT
EMPV1_38588	TAGAGGTGATGAAAGAATACCAGGGGCCGAGGCGCCCAGTGCTGTAGGAAGCTGTCGTCA
EMPV1_38589	TCCCCACGCAGCAGACTTCGTGCGGCCCCGGGCCAGGCTACCTGGTGCCTGCTCGCATGA
EMPV1_38590	CCAGAATTTCCTATAAGAATTAGGGCACTGCATATATTTTAAGAGCAGGTTGGAATGGTG
EMPV1_38593	GAGTCATCCTGTTCTATGGCTCAGCCTTTCTTCTCTATCTCATGCCCATCTCAGGATCAC
EMPV1_38595	GCTAAGAGAAAAGAGGATTCAGGAAAGATAAAACTTTTGCATCATTGGATGCCTGAAGAC
EMPV1_38596	TCTCCCCAGAGCAGGAGAGTGAAGGTTGCAGGAGCTTATCGCTGAAGTCGGCCACGTACA
EMPV1_38598	TAGTCATTGGATATTGATATGCTGTGCTGTCAGAAAACCAGACTAAGATAGAAACCTTTA
EMPV1_38602	TTCTGGTCCAGCAGCAACTGCAACAGGAACTGCCACAGCGTGATTGCACTCTCCATACCT
EMPV1_38603	CAGGCAATTAAAGAAGGTGGAGCAGGTGGTGGTTTTGGCTCCCCCATGGATATCTTTGAT
EMPV1_38604	GCCGATGAACAAGATAGAGAGGAGCATACTACACGATGTAGTAGAAGTGGCTGGTCTGAC
EMPV1_38606	TCCCTGGAGCACTTCTTTGAGCTTCCCACTTGGGGCCTGATATTTAAGGGTCTGTTTCGG
EMPV1_38607	AGCCTTCCTTTCTGTCCCTCGGGCTTTCAGCAAAATTGTGTAATGACTCCAGGCGGACTT
EMPV1_38608	TGGCTGCTCTTGAAGCTGTTGGGAAATCAGAAAATGATCTGGAAGGGCGGATAGTTTGTG
EMPV1_38609	TCGGGCTGCTATCCGCTAGGAGAACAGGTACACTCCCTATTGTGTTCTGCTTTGCATTTC
EMPV1_38610	GGGGCAATTGTACTTCAAAGTAATGTGAACTCTGATGATGACCACCTCATTGGGCTCTAG
EMPV1_38613	GGGCTCTACTCTCAAAGGTTGGAAGTATCAGGAGATACATTTTTGGCAGCCAGGCCATGC
EMPV1_38620	AGCAGGAAACCTTCTGCCTCTTTGACATTGGGAAAAATTTCAGCAACTCCCAGGAACACC
EMPV1_38622	GAAGAACGATACAGATACAGGGAGTATGCAGAAAGAGGCTATGAGCGCCACAGAGCAAGC
EMPV1_38624	TGTGCCCAACTCCAAAAGTTCATGGCTCATGGTCAAGGTGGGCTCTGTCTTTTACACGGT
EMPV1_38625	GGCTGTCAAGCTTACGGTAGTGAATACAAATTTCCAAAAACTGTGATTGTCCCTCGAAAG
EMPV1_38626	TTGCTGAGTCTTGAGGGTGGAGCCTCTATCAACGGGAAGAGCGGCCTCGTAAAAGAGACT
EMPV1_38628	TTTATCGATGGTTGTTCAGCAGCTAGGTGTGATTTGCGAGTGCTCGTGAGAGGACACGAG
EMPV1_38630	ACGTGTACCACCCTAGAAGTTAGGGCTCCTCTCCCAGGGAACTGGTGGCGATAACTGTTA
EMPV1_38631	GTGACCCCACCTCCATCACCTTCTCCCCCTGCTAAGAAGACTCAGAAAAAAGGCACAACT
EMPV1_38632	CTCTTGCCTCTGTTCCTGTCTCCTTTCTAGCCTCTCCTGCGAATCAGCTGTGTCAACGTT
EMPV1_38633	CCGTGGGGCCAATTTTATAACTCAGATTTTGCTGAGACCAGGAGCATCTGATTTAACAGG
EMPV1_38635	GGGAAAAATCAAAGCGACTTGGGCTCTGATGACTGAAATTCTCTAGGGCCACAGGGTTGC
EMPV1_38636	CTGTGGTCGCACGTAGCAGGAAGAATACAACTTAAGCAAAGGGGGGCATCTGGCAAGCTT

EMPV1_38638	CAACATCTTCCTTCTTGTACCACCACGTGCATGACATTAGAGCCCTGATTCCCCAGCGTG
EMPV1_38639	ATCCTGAGTCCCCTGGTGCCCCCTTAAACAGTGCAAAGTCCCAAGCCCTGAACATTCTTC
EMPV1_38641	GAGAGAAGCCTGCTACGAGTATTCTGTCCAGTCAGAGGTGCCTCCTTATTTGCCTCTCCA
EMPV1_38642	GACAGCAGTTTTATCACAGCAGTATCTCAAGTTGTAGTATGGGGAAATCTTGGTCAGTGC
EMPV1_38643	CTGCAAGAAGAAAAGCTCCAAGTCGCAAGCCTCCGCCACACACTTGAAGGCCCCACCGTA
EMPV1_38646	CCATCTTCCCAGATGAACTATTTGCGTTGTGGGACTTATTTTTGAAACCACATTGCAAGG
EMPV1_38647	GGGTCCTCTACAGCAGCAGTGTGGGCTCATCCCCTATTCAAATCTCCCAAACAATTTAAC
EMPV1_38650	GGGAGGGAAACACGGAGAAGTTTTTCATCATTGAGCAATCGCTTTAAGTAACTGGCCTGT
EMPV1_38652	AGAGAGAACCTGGAGGTGAAGATGGTACAGAGACCTGGGCACCTGGAGAAGAAAGTCAGA
EMPV1_38653	GTGGTCATTCATTTTACTGACGGGGCGGACGGAGACCTGGCAGACTTACAAAGAGCGTCT
EMPV1_38654	CCATATGGTCTGACAGTTTCACTTCCAGGTTCCTATGTCCACAGTAGCTGAAAGCAGCTT
EMPV1_38656	CCTTCGATGAGATCAACTCCCTAGAAATCTTTCCCGCTTTGGAAGTGCCTGCTCCAGCTG
EMPV1_38659	GTATCTGGTACATCAACTTCTTTGGGTGTGAGACGCACGCGATGCTGCCAAATGACCAGT
EMPV1_38660	GGACGGGAAGGCCCAGCTGGTGCTGCTGGACCACGGGCTCTACCAGTTTCTGGATGCAAA
EMPV1_38661	TTTCTGAGCCTTGGTTTCCCCATCTGTGAAACGGGGGTGATAGAACTTGGTGCCCAGAGT
EMPV1_38662	GGCAAAATAACATTTAAGACTGAAATCTTTTCATGCAAGTGAGTGAGCTCCCAAACACCC
EMPV1_38663	GTCAAGCCCAAGTTCCCCAACATCCCCAAGCATTTCGCTAATACAAAGCACTTCCCCTGC
EMPV1_38664	TCATGGCGTTCAGCCCCTGGCAGATCCTGTCCCCCGTGCAGTGGGCGAAATGGACATGGT
EMPV1_38665	CTTATGTACCGGGCACCTGATCATCAAGCCAAAGACACGGGTGGCTGGTTTCCTAAACGC
EMPV1_38666	GGCTGGGTATTGCAGCTCTTGGATTTGCAGGTCGCTATGCATTTCAGATCTGGAAACCTC
EMPV1_38668	GGCCAGAATTGGCATCACGGCTGTCACACACCAGAATAAGATTTTGAACAGCATCCAGGC
EMPV1_38673	GTGCCCAGAACGTGGTTTTCCTCCCTGCACTCAATCCTCTCACGTGTATTGCCAGACATT
EMPV1_38676	GGTGTGGACCTCCCCTGAGTCACTGGGTAAAATACAGAAACCACTGCTACCACCAAACTC
EMPV1_38677	ATTCCTCTACCGCCCGCAAATCCAACCTCATGTGGCCTATGACCTGTGCTTCATCCATGA
EMPV1_38678	TGGCCACAGCAGGTGATGACAGGCAAGGCCATGTGGCATATTGATAGCTACTGTAAACAT
EMPV1_38683	CTTTCTTCGCTGTCTGGAAAAAAGGCTCCTCCCTGACTTTGAAAGGCTGAGGCGCAGCGT
EMPV1_38684	AGTTACTAATCTAACTAACTGTAACCGGTTGAACAACTGAACCCAAAGGGTGCAAAGTGG
EMPV1_38687	GAGATGCAGGTGAGCAACAGGTGGTATCACAGGAACAGAGCAACACCCATGATGCTTCTT
EMPV1_38688	CTGAGCTTCTTTTGGCATCTCTAACATGGGGATAATCATACCAGCCTCTCAGGGCCATGG
EMPV1_38694	GGAGGTTATGGCAACATTCTCCTGTGATATTTCAATTACAGTCCGAGACCCTGGTCTGGC
EMPV1_38696	TCCAGAGTCTCACCGTCATGCATCATTCAGACATTTCACATCCCAGAGGAGCACAACACA
EMPV1_38698	GGAAAGTACTCAAAAGTGTCAGAAAGAAAACAAAGCAAACCATCTCATCCAAATTTAAAA
EMPV1_38699	AGAGCACAGTGTAGTCTGGTTAATCTGGTGGCTGTCACTCTGAACCTGTTTCACTGAGAA
EMPV1_38700	ATGCACACTGTATCCAGGTTAGGAGTTAAAGTCAACACCCTCACGCATTGAGCCTCTGCA

EMPV1_38701	GAGACAAAGAAGCCACAATGAGATGGTAGGAGGGGTACTTTCACAATATAGGCAATCCCG
EMPV1_38703	CCCTGCTTCCAACAAAGTCTCAAGGACAGGGTGGCCACAGTGCAGGCACCAAGGGTGCCA
EMPV1_38704	TGTAAACAATCTTCGACTTTCCCTTATGGATAATCAGAATGTCAGTAAAGAATTTCAAGC
EMPV1_38706	GTTTGCTATGTGAAGCTTACCTGAGATGGTGGCAGCTTCAAGATTCGAGCCGGATCACTG
EMPV1_38707	GACGAGGTTGGTGGAAGCCTGGGGTTAGTCCTCAGGAGTTCTGTAAATCATGTTAAGAGC
EMPV1_38709	CCCTCGGGCACTCAAAGAAATCCAGAAATTTGCCATGAAGGAGATGGGAACTCCGGATGT
EMPV1_38711	ACGAAGCCCGGAATCTGGAACACTTCCAGTGCAACTTCCTGCACGTGAACTCTGTCAAAT
EMPV1_38712	CTCGCCATGTAAATACAGATTTAATTAACACTTGCAACCTGTGAAGATGCAAAAACTTAA
EMPV1_38713	ACTATAATGGCTTCAAGCAACGAAACCTTAGTTGTCAATAGGGTGACTGGAAATTTCAAG
EMPV1_38715	CATGTCTACAGTTCTCAGACATGTGGTCAAATACTCTTCAGAGCCATCAAATGTCGATGG
EMPV1_38716	ACATGTCCCCAGCCAGGGATCAAACCCACACAGCTCTCACAGACTTATTAGCAGGTTTTG
EMPV1_38717	ACCTAGACCAGGGATCAAATCTAACCGGCAACTACAACCAACGCCACAGCTACAGCAAGA
EMPV1_38719	AGAGCTGTTCTGTGTGGCTGCATCTCTGTCCTCAATTTTCCATTAGCTGAACCATTCCTG
EMPV1_38720	GGCACAGTTGGGAGTGAAACCTGTGGCTAAGAAAGTTGAGAGCACAAAGAAGTCCTCAGA
EMPV1_38721	GGGCGTTCAACACAGACCACCAGACCCAGCTCCTTTATCAAGCATCAGCCATGATGGTAG
EMPV1_38722	TCCTTTCCCTGGCTCTTTGCCTCCATTTCACGCAGCATACAACAAGGTTGCCAGCTACCA
EMPV1_38723	TGTGAAAGTGGGATAAAGAATACGGTGGGGATGACAGAGAGGAAGGGCTCACTTCTACCC
EMPV1_38725	CTACAAACGATTAACTATGGGAGGGACGACGAGAGGTGAGGGGAGCAGACGACAAGGACA
EMPV1_38726	GACAGGTATGCGCTGTTCTCTTAAATTTCCCCTGGGTTGAGCCTCACATCCTAACACTCC
EMPV1_38729	CCAAGGTTATTTGGCAGCTAACCCGAGGTTTGGATCATTGCCTAAAGTTGCACTTGCTGG
EMPV1_38730	CACCACGTCTGACCCACACAGCGTCACTCCCACACGTACTCCCTGACTCTGATGAGGTTA
EMPV1_38731	GTGGGTTGAGGGAGGATGGAGCTGTTAGCACTGGAGAAATGTAATGGGGTTAAAGTCAGC
EMPV1_38733	AAGACTACGTCCTCGGTGGTTTCTGGTCTCTCACGCAGCTGTCTTCATCCTGGACTTTGT
EMPV1_38735	AGATGAGTGGCTGAAGCCAGAACTCTTTCGAATAGGTGCCAGCACCCTCCTAGCAGACAT
EMPV1_38736	TTCAAAATGCACCAAAGCAGAAGAGAACTTGCATTTGATAACTGCAAACATTCACTCAAA
EMPV1_38737	AGGAGTATTATCTTTCTAGCCACTAAGTATATGTTTGGGATTGATGAAAAAGCTAGGAAC
EMPV1_38738	TCTAGGAATGTGACACTTGAGCCAGGGAGTGGTTGTGTGTGCCAAAGGGACCGTGTGATT
EMPV1_38741	GGCTGCCACTGTCTGGATCATTGGTTTTTTCCATGCCCTGATGCACTCCATAATGACCTC
EMPV1_38744	CCCCCTACAAATTGTAATTCCTGAAATACTGCTGCTTTAAAAAGTCTCACAGATTATATG
EMPV1_38745	ATGAAACGTCAGCAAGACTTTTGTAGATATAGACAAAACTTACATGGAAAAACAAAGCAA
EMPV1_38747	AGAGGTAAATGGTTGCAGTTTTGTGGTGAGAACAGGAAAGCCTACAAATTTATTAAGGGA
EMPV1_38752	GTGTGGCAAGATGAATTTTCCCTAAGTGCAGCAGCGTCGCCAGTGCCAGATAAAGTCTTC
EMPV1_38753	ATTGCAATGGTGGATACCTAACCCACCGGATTGAGGCCAGGGATCAAATCTGCATCCTCA
EMPV1_38755	AGGACCGGCACGTGCGCGTCCTCATCTTCAGAAGTGGAGTGAAGGGTGTATTCTGCGCAG

EMPV1_38756	GGATAAGAAGGAAGATGGAGGTGTTCCCTAGTGGCCTCATGGGTTAAGAATCAGAGGGTC
EMPV1_38757	CTGCAAGGACATAGCAGTAGTATAGAAACTGTGAAAGAAAAATTATTGAACTTTACCCCA
EMPV1_38760	CAGCGATTGTGGCCAGGGTCTTGGAGCTGCACCACGGTCAACAGAGCCGAGAGAAGGATA
EMPV1_38762	CAGGCAGGTGGCCAACATGACCAAGCCTACCACAATCATCGAAGTGAATGGGGACACAAT
EMPV1_38763	GACCCCCTGCTGGAAGATGCCCCTAAGCACCTGCCCAGCTGCCCCAACAAGGGCTTTGTA
EMPV1_38765	ATGCCAGATCTAAGCCACATCTACGACCTACACCACAACCCATGGCAACACCAGATCCTC
EMPV1_38768	CCCAAGTCTTCCACAAACCTAGGAAAGCAGTGGATGGGGATGGCAGACCTAGATGTACTA
EMPV1_38769	TTCGCGCCAAGGAGTACTTTGAGTTCTTCCAGAGCAACCCCATGAACAGCCTGCCTGCTG
EMPV1_38770	GGCAGTGGCGGGGCTGTGGCTCCAGCATTGCTTTCTTCTCGAGGACAATATGAACATTAT
EMPV1_38771	ATTGGGACGTCGTACACTTGTCATTTCAGTGCCTGGACAAAGCCACAGAAGAGGATAGCC
EMPV1_38772	TGCACTGGATTTCTGGCTGACGGGGAAGAAGGCTACGCCGAGACCATGGCCCAGATTCTT
EMPV1_38773	CCTTGGTGAATCTGGCCTGGGTAGGGTCTTGCATACATTCTTTAGCACAGATTCTCTTGA
EMPV1_38775	CCCAACTTGACAGTGAAAAAGCCCCATACGTCGCACCCCGGAAGTGGAAGACAGATCTTT
EMPV1_38777	CTTCTGCTCCACTTCTGGTTGCCAGGAGACAGCTAGACGCGCAGTTGCTATTAAATGAAC
EMPV1_38778	ACAATGTTGTTTCCCTTCTAATCAGATGCACACAGCGACTGAAGAATCCAGGAGCCCCAC
EMPV1_38779	CAAGCAAGAGCTTCAGCAAGTTACGGTTGGGTGGAGGGAGAATGAGGAAGAGCAACTGAA
EMPV1_38781	CACGATGATAGATCCTGGAAGGTTTTCCATGTGGCCAGGTTTAGCTGATGTCACGTCCCT
EMPV1_38783	GTGAGCACCCGTCTGTGTATCTCTTTCACTGGACATTCAGCACTGAGATCTTGGCTGGGT
EMPV1_38784	AGCTGAACAAAGAGTCAGGTGTTTGAAGATGGTGGGAGTAGAGGCTGTGGCTAGGGCACA
EMPV1_38787	AGTTTCCACGAACGGAGCATACCTCTCTGAGCTCTATCTCTAGAGCCGATATTTAGCCAC
EMPV1_38789	CGTACAACGCCCCTCGCCCCCGCAAGTTCTTACTGCAACATGGAAAGCTAGAAAATAACA
EMPV1_38797	GGGTGTTGAGTGTGCTTGTTGCTGTTGGGATGTTATTGCTTCTGGTCCTTCTCAATGGGC
EMPV1_38799	GAAGATGTACCAAGAGAGATGCTGGGATTTCTTCCCGGCTGGCGATTGTTTCCGCAAGCA
EMPV1_38801	TGAAAAAGACCCCATCAGAGCACAGACCTGTTTCAGTGTCCTCTACCTCCCGAAGTACTC
EMPV1_38802	GCCCAGGGCAACGTTGGACCGCCTTTTACTTTGTAAAGTTAGTATTTATTCCCCTTTAGG
EMPV1_38809	ATGGGTGTGTTGCACGTGGGTTAGAAGGATCTGTTAGGTGGAGGTTTGCACTTGCTTGGG
EMPV1_38810	CGTCGTTCCCTGAGCACAAGCTGTACGGAGCCGTGTTTACAGACGTGCCATTTAGAAAAT
EMPV1_38811	TGGGAAAAAGGCATATACTACAAGCCAGGAAATTTATGAAGCAGTTAAATCATTGTGGAC
EMPV1_38813	CAGTCTTCCATGAAGCTATCAAAGCCACATTTTCACCTTAACTCGAGGCATCGTGCATGG
EMPV1_38815	AGGAAGAGTCAGAGTTCAATCAGCAAAGAAGCAGATCCACCAGGAGTTATGGGCTGAGGG
EMPV1_38817	CAGCAGGTTAAATATCCAACTTTGTTGCTGTAGTACTTTGGCTTGCTCCTGTGGTGTTGG
EMPV1_38818	CGAAGGTGGATTGGGATCAGCTTGGAAGAGATTGAGAGCATTGACCAGGAGATAATGATC
EMPV1_38819	CAGTGATCTTGCAGTGATCAATACTGACAGCCACTCCATTACTGGACCCACCGCCGTTGA
EMPV1_38822	CTGCAGTGACTACACCAGATCCTTACCCAACTGTGCCACAAGGAAACCAAAGACATCTTA

EMPV1_38825	CCACACCAGCGTGAACTTTAGATGAAGAAGTCAATCAGACGTTTGTACAGTGGGAGCCAC
EMPV1_38826	AAAGATAGCAGGGGTGATTCAACAGTGAGAGACAATCCAGGTAGCCATCAGGGTCTAATC
EMPV1_38829	AGTTTGTGAAATACAAGGGTGGTACTCCCTGGAAGTGGGATGCCCAAAAGCCACCGACTA
EMPV1_38831	CTCACAGTTCACCACTCTCTTGGTAGAGGCATTATACATCCACACCTCTGACATAACCTC
EMPV1_38832	ATCTTGTATTTTCACTGCTGTGGCTTGGGTCATTGCTGTGGCATGGGTTTGATACCTGGC
EMPV1_38833	CGGAGCTTATTTAATGAAAAGTCCCGTAGATTGAAAAGCCAAACATGAGGGTAGCAGGTG
EMPV1_38834	GAGAGTGTGCTCTGTAGCGGTTTCCTTCCGAGCAGGTGGTGACTGCTCCTTGGAGAGCAA
EMPV1_38840	GTTGACACACGCAGTTTGTAAGCTTCTTGAACGACGAAGGAAGTTGAAGCCTAAGAGGAA
EMPV1_38841	TGAGACCTCGGGCTACAACACGGGACTCGGGCGCTGCTCTGACCCCTCGTGTCTTGTGTT
EMPV1_38844	CACGCTTTTTGTAAATAAACTGAAAGTGAATTCAAAACCAGAAATCATCTCCTCCACTGC
EMPV1_38845	ATGAAACTCAAAGAGAGGTGATGGGAGCCAGACTGAGCTGGACGGCACAGCAATACCCTT
EMPV1_38846	CCCTGCAAGCAACATAGTTATGGAACATAAGCTTTGAGTGGAACGAGGATGGGGGAAGCG
EMPV1_38848	GTTGGGCAAGACCCAGAGGCAGCTACACCCCCATTCAAAGTGCAGTCATCCTTATAGAGT
EMPV1_38849	GCAAGAAGACAACAAAGAGATGCCTTAAAGGACAAAAGATGGAGGAGGAAGGCACAGAAG
EMPV1_38850	TCACAAACCCAAGTTCAAGTGTCCTGAGTTTCCAATAGCAGGAAGGTAATGGTAGCCAGC
EMPV1_38852	CTGCTGCTCATCTGCCTGTCCTACGTTTTCATCCTTGCTGCCATCCTTCAGGTGCCCTCT
EMPV1_38853	TGCTTCATTACTTTGCTCGGAATTTCTTCGCTCCACTGTTGCCTGTGGGTTTTGAGGATG
EMPV1_38854	ACACTAGCACACCACTTTTACTTAGGGAGGAAGGACAGTTACCCAAACGAGAAGGAGGGG
EMPV1_38859	AGGTCCGCGATCCAGAACCTCCACTCTCACCATCCCTTTGCTGGTGTAGGTAGGGATGGT
EMPV1_38860	AGTGAATACGCCCGGCCCCACGTCATGTCTCCTGGCAATCGCAACCATCCATTCTACGTG
EMPV1_38865	GAATTCATGACAGTTTTCACATTGTATTATCAATATGTGGAAATAATTGTCTGCTCTGAG
EMPV1_38866	AGACGTGTACTTCGATGTCTCCATCCACCAGCTGCTACAAGAGAACAAGCCAAACAAGGG
EMPV1_38867	ATAGGCTCAAGATCAAAACAGCATACCCTCTAAGCTCAGATAGACTGTTGACTCCAAGGG
EMPV1_38868	ATGAGTGTACTACCCTTTCTACCTCGTCGTCCATTGTAGGGATTAAGTGTGGGCAGCAGC
EMPV1_38869	GGGACCTGGAAGCTCTCACCATTTGGACGGAGGAGCAAAACTACAGACCCAAAGTCAATT
EMPV1_38870	ACTATTCCCTGAGGTCCCTAAGGTCTCATTGCTGAAACTCCCCAGCTCCCTGGAGCACAC
EMPV1_38872	ACACCATGGGCTGCTGTGGCTGTGGAAGTTGTGGTGGTTGCGGTGGCTGCAGCGGCGGCT
EMPV1_38876	CTAGGTAGTGTTGACTCTCCCAGGTAAAGAACGTGCAGCATGAACGACCATGAAAGTCCA
EMPV1_38877	ACTCCACATTGGTGTTTTCAGAACTAGGGGTATGTTTGGTATTCCCTGGTGTTGCAGCAG
EMPV1_38879	GAACGCTGGGACACCAAACATTATCCTGTAAAGACCACCCCTCATCTCCTCAGCTAACTC
EMPV1_38881	AGCTATGGCAGTGCGGATCCTTAACCTACTGCACCACAGGAGGGAACCCCTAACATCATT
EMPV1_38883	TGGCTGGAGAGACTGAAACAAGATCAGTATGGCGACATCTACAGCTTTCACATCCATGTC
EMPV1_38884	CTCAATCTCTCAACCTGACTGCGAAAAGTGGACTGCTGTTAGCTCTTGTAGCATTCACCA
EMPV1_38885	GTGCCTGAACCAAGATCTGAACAGAAGGTGTCCAACATATTTGATGATCCTTTGAACGCC

EMPV1_38887	GTTTTCAGTTAATGCACGTACAATATTCATTTGCTTGGTTGTGCTACCTCTCAGGAGTAA
EMPV1_38888	AACATTTTACTATTGGCGTACTAACCTAGGGTATCATAAGACTCTCGCCCAATCAGTGAG
EMPV1_38889	TGGACGGTGCCACGCAGACCCGGACCGGATTCACGTGCACCACCTCATTGCGGCCCAGTT
EMPV1_38890	CAACCTGGTGAGGAGCGTTTTCACTTCCAAGTGATAGTTCGCAGACATCAAATGACATTC
EMPV1_38892	TCATGACCAGGGTGACCTTCTGTGGGTCCCGGAATATCCACTACATCTTCTGTGAGATGT
EMPV1_38894	AACAGGACCCCGTATGTCACCACCACCGTGTCTGCCCACAGTGCCAAGGTTAAAATAGCA
EMPV1_38895	TCATTTAAAGGTGGGAGTGGGAACAGGGACAGGAAGGCCTTGGTAGAAGGCAGTATCTAC
EMPV1_38896	TCTGATCATCCAGTTCTTCATCCTCCGGACCTTTGGGCAGCAGCTGCAGAAGAGCATGCT
EMPV1_38897	TTCTGTTAGAGAAGGGTTTCTGTATAACTCATGAATGCTTGGCCATAATGTAACTATTCC
EMPV1_38900	AGCCAAATCAGAACACCTCAGCAACCCTTGTATTTGAGAGTTTCAGCAATTTCAGAAACC
EMPV1_38901	AAGGATAAGGAAAAGCCAGAGAGTGAGGCAAAGCCCAAAGAAGGAAAGCCAGAGAGCCAG
EMPV1_38904	GAGACAGAAGTGGTTGCCCAGCTCGCTGCGTTTAAACCAGTTGGTGGGGAAATTGAAGTA
EMPV1_38905	AGAGATCGCACCCGATAGAGATCATTGGCTCGCATGTTTACGTCATCCTTGTGAGCGTTC
EMPV1_38906	TTCTGACTCTACTAAGTGGTGTCAATACCCATCCTGCGGATGGGACAGTGGCTCCTTAGG
EMPV1_38907	ATACCTTTCTCTCACCAGCCCCCAGCTTATACCCTTCTCTCCAGTATCCTTCCTCTAGCC
EMPV1_38908	GTGTCTCCAGCAGCGGGGTCACCAGCAGTGGCGTCTCCAGCAGTGGCATCTCCAGCAGCA
EMPV1_38909	ATCAGTGAGCAGACATGGGCAAATGCTACAAAGAACAAGACTGGAGCTGCCCCATCACTG
EMPV1_38911	CAGGCCGAGCCTGCGCTGGAATCCAAGCTGGAGCCAGAACCTCCCCAGGACCCCGAGGAA
EMPV1_38913	AAGAAGGTGGCGTCTGGTCCATCTGTATAAAACTGACAGGAGGGTCAGGGCTCGTCCGTA
EMPV1_38914	GCAGTTGCCAGAAGAAGGACCAGGAGTAACGATAAGCTAGAACCTATTAGAGATGTGTTT
EMPV1_38915	AGCACCTGGGGATGTTGTTTAAATGAAGAGTCATTTTGGGTAGGTATGAGAGGACCTAAG
EMPV1_38917	CACTCAGTTAAGAGGTACAGGAGAGAAAAGCCTCCAAGAAAATAGCCCTCGTGAAGAGGC
EMPV1_38918	CCACCTCTCCTCAACCCTAAACCTAACAGAGAACTATAATCCTGTATTCCTCTAACCCTG
EMPV1_38919	AGGAAGGTCAGAGAAAATAAGAACATACAATTTTCCACAGAATCGGGTCACGGATCACCG
EMPV1_38920	TCAAACTGCTGTATATGAGGAGCTCGAAGATGAAACAAGAAGGCTCTGTGATGTCAGGCC
EMPV1_38921	GGAAGATGAATCTAGTTGCTCTAGTGAAGAAGATGAGGAAGATGATTCAGAATCAGAAGC
EMPV1_38923	TTCAGTAGCTTCATCAGCCCTTCCAGCTGGACCATCAGCTCCCGCCTGCTCTCCTGCAGC
EMPV1_38926	CCAAAACAAGGGTGTTGCCCCAGGAGGATATTGCCTGCCACCTGCTCAGCTTCTGCCTAG
EMPV1_38928	AGGACCTCCTTGTCCAAAGCCCTGTGACCATACTGCTATCCAGGGACAATTCTGTCACCA
EMPV1_38930	AAGCCAGCTTTTAGGGGGTTCAGTCAGCTTCCTTCTTGGGCAAAACAGTGGCCTCCAGAT
EMPV1_38931	TGTGGTGATTGCCGTGCTCACCTTCGCCATCAGCGCCAGCACCGTCTTCATTGCCTTGAA
EMPV1_38932	TTCAGTGAAAGCCGCAAAGCAGCCAAAACCAGCCTCTCCCCCCTCCTGGAGTTTGTATAT
EMPV1_38933	GGTCGATTGCAGCAGGAGAGAGTAGCAGCCTTTCTGCCAGCACATTGGTCTCAGATTCAT
EMPV1_38935	CTCCAGTTCCTTTGTCCCTGTCTCGTACCTTTGTTGTCAGCAGAACAGAGTTGTTAGCAG

EMPV1_38936 ATGGAAGACAGGCCTCCGTCACATGGCAGGATCCATCTATATTTTTGCCGTCACACACAG
EMPV1_38938 ACCTCCTCCATGCTCCTTGCTCCCTCTTGGGGCAGAATTCTTAGCCTGTATGTCTTTTGA
EMPV1_38939 CTGGGTGTATAATTTGCTATGCACCTGAAACTAGCACAACATTGTAAGTCAACTATGCTC
EMPV1_38943 AGTGGGATCCCCTGCCACCATCCTCTACACAGGCCTGGATGGTCACCTCAGTAGATACCA
EMPV1_38945 AGCCTTCTTCTGGTTGGTGTCTCTGCTGCTTTCCTCCCTAGTTTGGTTCATGGCAACAAC
EMPV1_38946 AAACACCTGCTACTGTAACTCTGTGCTTCAGGCCTCGTACTTCTGCCGGCCTTTCCGGGA
EMPV1_38947 TAGGTCTTAGTTTTGTCATCTGTTTCCAGGGAGATGACCCGGAGGCAGTTGTGAGGGTGA
EMPV1_38948 GGTAGCACCACCAAATGCTGACATGGAACAAGGATTCCAAGAAGGAGTTTCAAATGCTAG
EMPV1_38949 CGTAGCTTCTGGGACTATATCAGAGTGGCTTGCCGGAAAGTTTCACAGAATTGTATCTGC
EMPV1_38952 GTCTTAAGCTACTGAGATTCGGGGGTTGTTTCTTACCATCATACCCAGCCCTTTCCTGAT
EMPV1_38955 CAGCTGCATAAACGAGCTGAGAAGATAGCCGTGATGCTGATGGAGAGAGGGCATCTGCAG
EMPV1_38957 GGAAGGACTTCAGGATGAATACGTGCACCCCCCACCCCAGAGGAGCCTTCATGTTGGAGC
EMPV1_38958 CTGTCCAGACTACTCTCAAGTTTACATACAGTGAAAAATATCCAGATGAAGCCCCCCTTT
EMPV1_38959 ATAAAAGGAAGTGGGGACAGATTGGCAGCGCAGAGTCCTTGGCCTGTTTCACTCCTGCTT
EMPV1_38960 GATAAATGACAGGCCCGCAGCATCTCGGCGCGGCGGACGGCGCTGCGGGTCTAAGTGAGA
EMPV1_38961 CAGTCCTTCAGCCCACTGGCTGTGTATGTCATCTTCAGAGTAAGGGCATCTTCAGTGGAT
EMPV1_38963 AAGCACAATGGTGGGGTTGAGCTCCGGGCTGAGTCCAGCCCTCATGAGCAACAACCCTTT
EMPV1_38964 AGTGGTTGTGATTATGCTTCCACTCCTGTGCATTCTGGTCTCTTATGGCTACATTGGGGC
EMPV1_38965 AGACCTGCGCCTGGAGAACTATCCTATCCCTGAACCAGGCCCAAATGAGGTGTTGCTGAA
EMPV1_38967 TGCTCCTTGCCAGAGAATCTGTAAACGCTGAGCGGCTGTTAAAAACTGAGACTTGAGCAG
EMPV1_38970 CCCTTTCCTTCATTCTGGCCTCTTATTTCCGCATCTTTGCCACCATATTGAGAATTCGCT
EMPV1_38971 TGCCTCTTAAATCCAAACTCTTCCACTTAGATAACAAGGAGGCTGCACAAAGCAGAATGC
EMPV1_38972 GCCCTGGACCACTTTTCCAGATGCCTCTTCCTGGCTCTGCGGGAGCTGCAGCTAGGCTTT
EMPV1_38974 GGAAGGGGGATGTATCTTCCACTGAGATGGATGGGCCACTTTTCAGGTCAAAGGATTCTG
EMPV1_38975 CTGTACGTTTTATCTGTACGGAATATCTGGTAGTGTGCAGTGGGGAGGAGGGTGTTGATG
EMPV1_38976 CTTCTGGATTCTGAAAAACAAGCTCTCCTCACTCAACAATGATGTGTCCACTCTCCGGAA
EMPV1_38977 GAGTAGCTACTGAAACATGACTGAGTTTTAGCACCCTGAGAACTGTACAGTGGGTGTCTC
EMPV1_38978 CCATAGAGCCCCAGAAGGAGGAAGCCGATGAGAATTATAACTCGGTCAACACAAGAATGA
EMPV1_38979 TCCAGTGGACAGAGAGGAGGCGGGGTTGTGCTACACAGCATCTGGGCCTGACCTCCTTTT
EMPV1_38987 ATGTCTGACCTCCCTGGCCCACCTTCAGCAAGAATCCTGTTAGGTCTGTTTAGCCAGATC
EMPV1_38989 GTTAACTCAGGTTTCAGCGCTTTGGATTTTGAGCGTGGTGTGTGGCCAAGTGGATGGAGA
EMPV1_38990 TGTGCAGCCTGGTCCTTTGAGGTGTGTCCTTGGCATCCCCATATGAAACTTCTGCAGGAT
EMPV1_38991 TTCCTCAGCTGCCAGAAGGAAGATGAACCCCTGGTATATATCCTATCAGACCGTCCCTCC
EMPV1_38992 ACGACCCTCCGCAACCTGAAGCTGGACTACCTGGACCTCTACCTTATCCACTGGCCCACT

EMPV1_38993	CTTCTGGAAGCTGGTTTCACATGGTGGCTTAGATTTTTCCATCTTTGTATCTAGCACCAT
EMPV1_38994	GTCCGACGTGGTCCTCTTACATGAGAAGATGTGCAACATGCAGCAGTGGGCAAAAGAGCA
EMPV1_38995	TCTTCATTTACTCACAGGGGCACTCTGCAACCCAGAGAGGGTGGGGGGGTATATGTGTAA
EMPV1_38996	ACATTTAGCTGGACCTTGTTATCTACGAGTTTTGCATCCTCAGACACAGAGGACTGACCG
EMPV1_38998	CCTCAGGCTCTCGGGTTGGGCTTTCGTCTTCTGGGTCATTGAAAGAGGAGCTTGATTGTT
EMPV1_38999	TCAATGATGCCCAGTCCTAATTCTCACGCTGCAGGCTTCAAAGGGTTTGGACTAGAAGGG
EMPV1_39000	GAGATGGTTTTTTCATTAGAAAAGTTTGTGCGGTCAATTCGAGAACTAGGACTTCCAATC
EMPV1_39001	CACCACCTCTGGCCATCCCACCCCTTTCCCTAAATTACTCTTGACCAAAAGATTCCTCAC
EMPV1_39002	GTCGGTTCTGTGTGGAACTGATTCGCAGTTCCATGGGCTTTGGCCTCACATTAAGTGGAG
EMPV1_39006	TTAAGGATCCAGCTTTACCGTGAGCTGTAGGTCACAAACGTGGCTGGGATCCTACTTTGC
EMPV1_39007	ATGGGCAGCCAGGCATAATAGGCTGGAGTCTGGGCAAGGTGAGAAGGTCAACACACAGGA
EMPV1_39010	AGCCTGGGCTCACCTCCCTCTTTTACCACTTAGTTGCCGCGTGACTTTGGGCGAATTCCT
EMPV1_39011	GACCAAGACTTAGGCGAGTGCTTTCTGAGACTCAAAATACCCCATACCCTTGGCAGACAG
EMPV1_39012	CTCCCAGGATATGCGAGAGGCTTTACTGGGCTGTCTGGTGTTCCTCCTCAGCCAGAGACA
EMPV1_39015	CATGATGGGAACTCCTGGGATTTTCTTTTTCTTTGGGGGGTGAGAAAGGGCAATCGGTTT
EMPV1_39018	ATACACTGGCTGGCTGTGAACGGGCGGACAGAACTACTCCATGATCTTGTACAGCACGTC
EMPV1_39019	CAAAAAGAGGCTTGAGACTCAAAACTGTAGATTCCTGCTTCCAAGACCTCAAGGAGAGCA
EMPV1_39020	GCGTAGGTAGCAGAGGATTAGTACCATAGCCTGAAAACAAACCCAAGCATCATCAGGGCG
EMPV1_39026	TGTTCTTGCATCTTGTGCTTGGCTGTTTTGCGACTAGACCCTCTCTTTGCTGCGAACCTC
EMPV1_39028	CTTGGCAAGTGGAAATTCCCGGGCTAGGGATCAAATCAATGATACAGTAGAGATTTGAGC
EMPV1_39030	TTTTGCCATGTTCAGTTAAAAGTGCACAGTCTGTTACAGGTTGACACATTGATTGACCTG
EMPV1_39033	CAAAGCATCCGTCCTCTCCCAAGCCAGCTCTACCTAGGGAGCACTGCACTTCAGCCCTTA
EMPV1_39037	CAAGCCCTGACTGAGCAGAGAGACATCAGCATTAGCCAATAGGGGCAACTCCTAGATCTA
EMPV1_39038	ATCATGAGTGTATTGCTGTTCATTGAGCACTCAGTGGAAGTGGCCCACGGCAAGGCTTCC
EMPV1_39039	AGTAACCGTTCGCCTTCCGAAGTCGTGCAGTGGTCCAACCACAGGGTGATGGAGTGGTTG
EMPV1_39040	CAAATGCTCCCCAAAGTGGTATAGAGACCCAATTTACTGGCATTTGCTAAATGGCATTAG
EMPV1_39041	TAAAGGTGAGGTCTTGACAAACTGCCAGAGGCTCAGTGTGGACCGTTCTGAGAGTGAGAC
EMPV1_39042	CATTTCAGAGCCCTGCCGCCTGAATGCCTTTTGTCTGCTCTTTGTCTTCAACTCTCCTCT
EMPV1_39045	GACCATGGGCATGTGGCAATCACCTAATACACACGGCGACAAGCGAACACACAACTATCT
EMPV1_39046	CGGGGCTTTGAGGAAAAGGGGATAAAGAATGACAAGCCTGAGGACCAGCTGAGTAAAGAG
EMPV1_39048	TACTTCTCACCAGCACTTAGATTTAGACTGGCCAGCCGACTTGCGAGGAGACGTGGCAGT
EMPV1_39049	CCTGGAACCACAGGCATTTAGTTAAAGGTGAAGCCTTGTCTCCCACCACTTTGTAGAAAA
EMPV1_39057	AGTAAAGCCAAGGTCCTAAGCATGGCCTTCAGGTCCTGTGTGATACGGCTGTTGTCTCCT
EMPV1_39059	CATCTTCCTCATCTTCAACATCCAGAATGCCTGAATCCTGTTCAGACTTTGGGCTAGTGC

EMPV1_39061 CCCAGAAGCCACTGCAAGAAATGTATAGAGATCATGTTTGGAGCAGAGGGTGCATTGCCT
EMPV1_39063 GTCCTTATTTAGGAAATGTAGACTAATGAAAAAGCCAGGATTTACACTTATTGCTGCCCC
EMPV1_39064 GTCAAGACAGGCTGAAAAACCAGGAAGCAGAAGTACGATTCCAAGAAGATCGACTGGTCC
EMPV1_39066 ACTGGATTTACAGTTTTCTGAGGATGAGAAGAGGTTGTTGCTGGAGAAGCAGGCCAGCGG
EMPV1_39067 AGGGCCTCCCTATGGCCGGCTCTGGTTGGAGATGGTACAGTTTTATTGTACAGGTGCTAA
EMPV1_39069 TGGGACCCGTGCCACCCACCTTCGTTTTCCTGCCCAAGCACAAGGCTAATGCCTACAAGT
EMPV1_39071 ACTCGTCATTGCATCCTATGTTGGTGGCCTTTTGAATGCTTCTGTGCACACAGTGGCCAC
EMPV1_39072 TTCTTGACCTCTTTGAAGACAACTACAATGGCTCAGCAAGCAGCGGCCCGGCACCCGAAA
EMPV1_39073 TGGCAGACCCGGGTTCCAGCCCGCCGGCGCGACGCCTCCAACCTTCCAACCCGGAAGAAC
EMPV1_39074 AATGTTGAGGAGAGGCGGAGTGAACAGCATGCAAAAGAGGCATGAGGAAGAATGGCTGAG
EMPV1_39078 TGATTCCCCAGCATTTGGAATAAAAGAGGTGACTGACACAGAGCTGCATGGGAAAGTGCC
EMPV1_39079 GGTATGTGAGCCTTCTTTGGTACTCTTACACTCAGGCCCCTCAAATGTTACAGGTGGGTC
EMPV1_39080 CTAGGTCCACAGGGATAAAGTTCCAGGTTTGCCTGTCTTCATAACCTCACCTAGCTCCAC
EMPV1_39088 GAGGACCGTAGGGCTGAATGGGGACTAGTTGGTAATAGAGAAAGGATCAGTGGCCTAGAA
EMPV1_39089 ATCAAAAGTTTCTCTTCCCACGGTAGGTTCCCCTGGGTCTTTCTCGGTTGGTCTTGGCCG
EMPV1_39091 CAGAAGCTCTGGCAGGGATAAGCAGGACGCCCAACTGGAACGTATACTAAATGTAATCAG
EMPV1_39092 GTCTTATTTGATGTGTGCATACCTCTGTAAATAGGTGACAGTAGACCATAGCTGACATTT
EMPV1_39098 ACCATGCAGAGCACAGAGGACCCCACTGAAGGGTACAAATAACTTTCAACAGGACTCGAG
EMPV1_39099 GCATCCCTGATTTCATGCATTCCCAGCTTGAAAGCCCAGCCATATTACTCTAGTCCCTCC
EMPV1_39102 GGAGCAGGAACTCCTGGTTCTTTCACTACAGTTAAGAACTCCTGGTTCTTTCACTACAGT
EMPV1_39103 GGTGAATACAATGTTTACAGCACATTCCAGAGCCATGAACCCGAGTTCGATTACCTGAAG
EMPV1_39104 GAGACATAATCAGCTGTGTTGCGAGACACCACCTACTGTCACTGTACATGTAAAATCAGG
EMPV1_39105 GGGGCAGTCATCAACCCCATTTTTTTGTCCTTATTCCTATGGAAGCTGTATATGTTCTCT
EMPV1_39106 ATACTCACCTGGCTGGGACGCCAAGTTCTTGTACCTCGCCATGAGGTACTTGGCCACCTT
EMPV1_39107 CAGGGAGTAATCTCCAAACATCTCATGGAGTTTTCTATTGCAGGGAGTAATCTCCAAAAT
EMPV1_39108 ATGCACGAAATCCCTAGAAGTCGCCTGCGACCGCCTCCCCGCTCCCAACCCCAGAGAGGG
EMPV1_39110 ATCACTAACCCTAATCCTATCCCTAATTTTAACAGAGAGGAATGAACTGTTAGCTATTGG
EMPV1_39113 GAAAAACCAGTGAGGAAGCTGTTAGAAACCAGGTACCATCTAATTGGCTCTGGGCGGTGC
EMPV1_39114 CTTCCTTCACGGTAGCCAGAGGTGGCGTGTCTCTTGGATTTGAATGGGGTTGTACTTAAC
EMPV1_39115 GCTGACATGCTGACCGGTGATGAACAAGTATGGAAGGAAGTGCAAGAATCCCTGAAAAAA
EMPV1_39116 AGCATTGTGATGCCCCCATCTGTCTGTATGAACAAGGCAGAGACAGCTTTGAGGACGAAG
EMPV1_39117 GCTTGAGAAACAAATCCTGATGCAGTTTGACTCTAAAGATCCCAGATCATAATGTGGTGC
EMPV1_39118 CCCTGTTGCAGCAAGGAGAAGATCCCTGGAAGGTAGAGAAAGAAAGTCCTGGAAGCTTCT
EMPV1_39121 AGCACCAGCATCACTTGGGAGCCCACCCCAGAGCCATTGAATTCTGCTCTCCTTTTAACA

EMPV1_39122	CTTCGACAAGCACTGCGACCGCAAGCAGCCCAAGTACTGGCCCGTCATCCCGCTCAAGTT
EMPV1_39123	AAGGGAGCTTCGTTGGTGACTTCAACATACAGGGTGTGCTGGGTTTTGGCCTCGAAATCC
EMPV1_39124	TTGGTGTATCTGCACTACTGCCATCGGAAGGGTGCTGTGCTCCTTCAGACTGCAACCCTG
EMPV1_39125	ACCCTCCAAAGGTCCATTCTACACTTTATTTCAGGCTGGACATGAACAGTTCCCTACTAG
EMPV1_39127	GCGTCCAACATGTAAAGAAGTTGTCAGCAGAGGAGATGACAATTATACTTTGTCCTAAGC
EMPV1_39128	CCCGTTTTTAATGTGCTCCCAAGTTAGTGATGGCAGTGGACACTTTGTAGTCAGTGCCAC
EMPV1_39129	CAGAGCAGCAGTATAACAGACTTGTGGACTACATCACAAAGACCTCTTGTCACCTGGCCC
EMPV1_39130	GCCAGGAGAATGCTTTGTCAGTGATGACGGGGATGTGGTGACATTGGTGATTCTGATGAG
EMPV1_39132	AGTGGCAGCAGCTGTGCCGCCAGCGTGGAGCTGGAGGCGAGAGGGTGTGGAGAGAAGGGA
EMPV1_39134	AGCGTTCCCTTCGCGTTCGCTGCTGCTCCTGGGCACCCCAACCCCCGATTCCCATCAGCT
EMPV1_39135	TGCTGAACAGATTAAGGAGTGGATCCTCCGCCTCTGTGAGAAGAACTGCGGGAAGGGCCT
EMPV1_39136	GAAGTGGCTTCTCATTGCCATGGTTGGTAGAGTGAGGTGCCTTCGGTTTCTCATTTGGAG
EMPV1_39137	CCCCATGTGTCTGGATGAACGATATTATTCCAGGGCAAAGCACATGTAAAGCAGTTTGTT
EMPV1_39140	CCCATCCTGTCGCGCCACAGCGGGAACTCCAGCATCAGAGATTTTTCATCAAGACAAAAA
EMPV1_39141	GGCACCAGAGGGTGCCCTTATTTCTCCCCACTCAACTGACTCTAAGACAAAGTTTCGTCT
EMPV1_39142	CGATGTTGCGGAACCCTCAAGAGATGTAGATGTATGTCCTAAGATGGTTAGTCTTACTGT
EMPV1_39144	GCATGGAGGAGTTCATAGGATTGACTAGAAGTTTGGAGAAGTAGACTCAGGAGTCAAGGC
EMPV1_39145	AGCTGCTACTCTGTCCTTCTCCTACTGTGGCTCCCGAGAAATATCCCAGTTCTTCTGTGA
EMPV1_39147	TTCTTGACCCAGCACCGGAACTTGAACCACACCAAACAGCGTCTTCTGGAGGTTGCCAAC
EMPV1_39148	TCAGAGCGTTCCATCTGTGCACCAACCATCCTCTCCACTGTAGCTCCTTCACTTCTCTGT
EMPV1_39149	TAGTGGAGAACATAAGTCTGCATAAAGTCTGCAGGGCGGTTCACAGCGAGGTACCAAGGG
EMPV1_39150	GGCTGGGTCCCAAATACTTTTAAAATGACCATTTTCTGTGGCCTTGCACTTGCATTTCTG
EMPV1_39152	TCTTTCACCACCCTTTCCCTGTATGTGGCTGTAGACAGAAGTAGCCCACACAGTAAGCAC
EMPV1_39153	TGCTCTGGCGCTGCTTCAGCACCGTGGTCATCTTCCTGTTCCTGCTGGACGAGCAGACGA
EMPV1_39155	CGCTCAGGATGAATGGTTTTAGCTGCGTACTTACACATGCCCAGCTCCTGGCAAGCACTT
EMPV1_39156	TGATACTGCTCTTCCGAGTCCTCAGACACCGAATCTACGAGGTCACCCACCAGTTTGTGA
EMPV1_39159	CTGCCGGATCCTTAACCCACTAAGCGAGGGACCGAATTTGCATCCTCACAGACTCTATGT
EMPV1_39160	TGGTTTACGCCTCGCCATGGTTCGTCTGCCTCTGAAGTGTCTCCTCTGGGGCTGCTTTTT
EMPV1_39161	TCAGAGAAACACTTCCAACAACTGGGACGCTGCCTTCGGTCATCCCCAGGCCTACCAAGG
EMPV1_39163	ATAGAATGCGACCCTGCTATGAAGCAGTTTTTCCTGTACTTGGATGAGTCCAGTGCCCTG
EMPV1_39164	GAAAGGCCAGGACCTCCTACAGCATGAAAACTTAGATCATTACAGGGGACAACACACATG
EMPV1_39165	AGCACGATAGCAGTGGATACTGCTCGGGCTGGCTGGTGGACAAGGTGGAGGTCACCAACA
EMPV1_39166	GACCAGGGTCACGGAAATTCTAATAACTGAGGAGGCTTTTATCCTGGCGACTTCTAGACT
EMPV1_39168	TATCCATCTCCCGTTGCTACTCACTGGAATGCTTCAACTATTACATAGTGGCACTATATT

EMPV1_39169	GTTCGACCCTTGAATAGTGAAGGATCCCTCAATATTTTACTTTTGGAGACAATTAGATTA
EMPV1_39170	TATGCCTATGGTGTCATGGATCGGGGTTACTCCTATGACCTGGAGGTGGAACAGGCTTAT
EMPV1_39171	AGAGTCTGTTTGGGGAGCGTGGAAAGCATCTTTTATACCTGGTGGCTGTCCCTGTATGGA
EMPV1_39172	CAACCAGCAGCCCAAGCTCCAGATGAGAGTCCCTGCCGTCAGGTTAAAGACACCCACCAA
EMPV1_39177	CCTGATACGCAGGTGCTTGATGTCCCCGAAAATACCCAAGGAGAAGCAACAAGTTCTGAA
EMPV1_39180	GACTTGCAGATACCGGAAATCTGTGTGGCTCCAAGTTCCCTGCTAGCGAGAGAATGTGTA
EMPV1_39182	AATTCTTCCTGGATGGACTTGGTTCTCTTCAGGTGGGCTCCTGCTCAGATGTCGTTGTGG
EMPV1_39183	AGGCCTCCTGCTCGCGGATTCACACTTGAGACAGCCACTCCAACAACAGTAATAATACAG
EMPV1_39186	TCACCCCGCTTCCATGAGTACTGTCTACTCCCACCCTCCTGCAACTCCCTTTTGGAGTCT
EMPV1_39190	CAGAACAAGAAGTGGTTTTACTTCGGCAGCAGTTACTGGCTCTAAGGCAGGCTCTGGCCA
EMPV1_39191	TTTCTGAAGAAGCTGATTGGGTGACAAGCAGGGAAGAGTCGTGACTGCCCCGGCCTGCCA
EMPV1_39192	GATGAATATACTTCTCCTCTTCCTGGAGGACGAGGCTCTCATGAGGACTCATCAGCCGGG
EMPV1_39193	TCCTTGAAAGCAGAGACCACATCTGTCCATCATCTTCCTTCTTAAGGGTTACAGTTGAGG
EMPV1_39194	GCATCTCCTGCTAACATAAAGAAGCTGGAGAGCCGGTACCACGAACTATCACTAGCCCTG
EMPV1_39195	GCATTAGCCGAAATGCACCCCATATTTTTAGCCCACGGGCCTGCCTTCCGAAAGAACTTC
EMPV1_39198	CAAGGACAAGAGTCTTTTGGGAACACGTGAAGTTTTAAATGAGGAAGATGAAGTGCAGCC
EMPV1_39201	AAGAATACAAAGAAGATGGGAGTGGGGAAGGCGTCTGCAAGTGATGGGTCCTCCTTCGTG
EMPV1_39202	GCTCCAGTGAGCATGCGACCTTTTGGCTTTCGGCGTGATAGAGATCCTTACAAGTGTGCA
EMPV1_39203	TGAGGTGGGACAGCGGTTGGGGTAGAGGGGACACAACAGGGTTATGGGGGTTCTTTAAGT
EMPV1_39206	CCCCAGCCAGTTCTGGGAGCAGGAGCTGGAGAGCTTACACTTTGTCATCGAAATGAAGAA
EMPV1_39211	GTAAGAGAGGCATTAAAGGGACAGATCAAAGGAATGCTCTAGACTCTTGGGATGGGCGGG
EMPV1_39214	AAATGAGTCTAGGTCTGAAGGCCGCGCGGTGACTAAATACAAGGGGAGAATGGAGGGAGA
EMPV1_39215	AAGCTTGGCGCAAGACTCTCGGGACCTGTGGTTCCCATCTACTGGTGGTGTTCCTCTTCT
EMPV1_39216	CTGCAAGCAGCAGAAAATGAGAAAGTGAGGTGGGAGCTAGAAAAGACGCAACTCCAACAG
EMPV1_39217	CCCGCATTCACATCCTCATTGCCAATATGTACCTTCTGGTACCGCCTTTTCTCAACCCGA
EMPV1_39219	AAGCAGCAATGCTGGTTCTTACCATTTTCCTTCTTAGCAAAAAGATAGGGAGCCCAGGGG
EMPV1_39221	CTGGATGAGCCCCAGAGAGTTGGAAGCTCTGAGAACCAAGTGGAAGACCCCGCATTAAGT
EMPV1_39223	AGAATGCAAACACATTCTAGCCTTTAGAGCTCAGTGGCTCGCCAGCCGGCCAAGAAAGAT
EMPV1_39225	CACTGTCTTCATCATTCAAGGCCTACGCTCAGGTGGTCCCTCTAGACACCAAGGGTCCTT
EMPV1_39226	CTGCAAGAGCAGGGTAGAGGAGTAAGCTTCAAAGTACTGATTCCTGAGCAGCCCTAGCCT
EMPV1_39228	CGCAAAGTGGTCTTGTTAGATTAAGATGTAGCGGGAAGGCGGGTCTGAGGAAGCTCACAT
EMPV1_39229	CAATGTGGACGACTCGGCGCTCCCCTCGGTGGCCCAGCTGGCTGGGCGGTTTAGGGAACA
EMPV1_39230	GTCCAGTTCTCCAATGGGAAGCTGCAGAATCCAGGCAACATGCGCTTCACCTTGTACAAG
EMPV1_39231	GCTCTATGTGGCCACAGAGGTCAGTTCTAAGATAAAGGCCTACAAACCATATGGAAGAAG

EMPV1_39232 GTAGCAAGTGGGAGCAAGTAGTTCATACAACCACTCTGGGAAGTAGTTTGGCAGTTTTCT
EMPV1_39233 CCCAACAAGGAGAATGCAGTGTGGAGACATCTCTAGGGCCGACTATTTTAACCAGTGAAG
EMPV1_39234 TTCTCTCATGTAAAATGCCCATTTCAGGGCCTTTGCACCTGCTGCACCACATGCCTGGAA
EMPV1_39235 GATCATCAGTGGGAAGCAGTCACTGTGCCTGAGGAAAAAGTGCAAATATACAGCATAGAA
EMPV1_39238 AAGGCTACTCAGCCTATTACTGTGAAGGGGAGTGCTCCTTCCCGCTGGACTCCTGCATGA
EMPV1_39240 TTCAACAGCCTGAACTATGATGTTGCGGCCAAGAAGAGAAAGAAGGACATGCTGAACAGC
EMPV1_39242 AGTGGTTGCTGCCTCTGAGTTCTCAATCCCAACTTTTCTTGATAATCTACCCAGACACCA
EMPV1_39244 AAACGGCGTCAACTGGAGTTCGGGGAAAGGGTACAACTACAGCTACAAGGTGTCGGAGAT
EMPV1_39245 AGAATCAGACTGGAATCCATGAGGATGCGCATTTGACCCCTGGTCTCGCTCAGGGGGCTA
EMPV1_39246 CTGGATCTTATGTAGTGGAAGTTATATCTCCAGCTTATAGGTTTGATCCCGTCCGAGTGG
EMPV1_39250 TGACCTGAGCAGAGAGAAGTGTTTGAGCTGTGTGGTGGCCTGTAAACTTCCTGGTTCCAG
EMPV1_39251 GAAAAACATGTAGATTATAAGAATGTGCAGCTTTTATCCCAGTTTATTTCTCCTTTTACT
EMPV1_39254 AAACGACAGACTGAGCAAAATTGCTTGCAAACGTGGCACAGAGTTAGCACTCCACACCCC
EMPV1_39257 CCAGATCAGAGTTGACTGCAATAATGAAAGGAGTGTCCACACTAAAACCTTACAGAATAC
EMPV1_39259 TGTGATTGGTGCTCCTCTTCTGACTGGACTCCTTAGTCTCGGAGGGATGAAGCTGGGCAT
EMPV1_39261 CCAAACGTAATCTACCCATGTGATCCAGCAGTCGCATTCCTTGGTATTCATCCAAAGCAG
EMPV1_39262 CTTCCACCGTCACAGTCAGGGCCTCTTTCTGCTGATCATCCCACACTGGCTTGAGAAAAT
EMPV1_39263 AGTCACCATAATCAAGGTGCCAGTAATGTGGCATTACACACATTTCGACATAATACCTGG
EMPV1_39265 TAATGGTAACAGCAGTAGCTGGAGCAGTCTTGGTTTAGAAGGAGATTTGTATGAGGACAG
EMPV1_39267 GACCTCAAACGTATCTATATCTGTACATGTACAAGAACCGTCAAAAATTATTCACAGAAC
EMPV1_39268 GGTTTGGGAAAGAAGCGTCCTATGATACACTTGCTGGTTAGTTGGGGGTGTATAAGGCCC
EMPV1_39269 TGACTCCTAGCCTGGAAACTGCGATATGCCACACATGCAGCCCTAAAAAACAAACAGACA
EMPV1_39272 CGAAGCTGAAACCTTGGGGCCTTTTTGAGGATCTAGTCGAGAAGTTTGAGTGCTCTCAGG
EMPV1_39273 GGTCCTGGACAATGGGTGGGGACATGTTCAACGCCAGGTTCATTCGAAACTTGCAGGAAA
EMPV1_39274 AGTTGAAATGTGCCTCTTTCTGGACAGTGGTAGCCAAAGAATGGATTTTCATTCAAAGGC
EMPV1_39275 AGGATGAGCAAGGCGACAGTGAGGACGATCAGGAGGGTACCAGGGAGGATGACTTCAAAA
EMPV1_39276 GATGGGGGCTCGTATTATCCATGCTGATGACTGACTAACTGAAAGTTGAGTGTTGGGGGC
EMPV1_39277 CCAAGCTGTGATCAGTATAAATTACCAGGATGTCCCAGGGACTTTAGCCCCGTGTGTGGC
EMPV1_39278 CCAGCTTCACGGGAGCAGAGGGCCTCCTCAGAAGGGGAGCCCTTGCTGCTTCCCCGTCTG
EMPV1_39279 AATGCACACTTGCCTTTCTTCATTACCCAGACTTGGATGGCCTTGTGGGGGTTCAAGGGA
EMPV1_39280 TACTTCTGGACCTGCCCCACTCAGATGGGGAGGCCCAGGCGAAGAAGTTAGGGAAGAGCT
EMPV1_39281 AGAGTTTGAGCGTGTCTTGCCTTTGCTCTCTGCGTTCCCGTTTTTGACCTCCCCATCTGT
EMPV1_39282 CAAGCCAGATGATGACACAGACATGTATAATACTCCTTATACATATAATGCAGGCCTGTA
EMPV1_39283 GCCATCTGAACCCATGCTCTTTGATGCTTCATTCGTCGGGCATGTGTAAAGCACACTAAC

EMPV1_39285 AGGGGCTGGCACCTAGGGAGCTGCACAAATGAAGCAGGTGCCAAAGCCGTGGGTGAAAAA
EMPV1_39288 GTAATCCTTACGCAACTCAGGAAACAAGAGATGGAGCAGATGGTGCTTTTTATCCAGATG
EMPV1_39292 TCTACTTGGCCCTCTCCAAGGGTTCTACCCTGTGCCTCAACCAGTTGCCCCAGTTTACAA
EMPV1_39293 CCTGGGTGACGTGGTCCAGGTGGTTGGTGCCTACAATCAGTGTCCAGTCGTCAGGTTCAT
EMPV1_39294 CATCACTCTGTCCTATGTCCAAATATTCATGGCAGTGTTTCGTCTGCCCCAGAAGGAGGC
EMPV1_39295 GGGGTGATGACAGTAGTTGGGATCTCGATGAATGGAGTAAAGACTAAGGGAGCTAATGTT
EMPV1_39297 AAGCCTTTGGTAAAGCCACATGTGAAGGATGACTGAGATAATTCCAAGGAGCTATGGGGA
EMPV1_39300 GTGGCTCAGCAGGTTATGAATCCCACTCTTATCCTTGAGGATGCAGGTTTGATCCCCGGC
EMPV1_39301 TCGCTCAGTGGGTCAAGGATCCAACCATTGCTGTGGCTGTGGTGTAGGCCGGCCGTTGTA
EMPV1_39303 CCCCCAGGAGGGAATTCTGGAAGATATGCCTGTGGATCCTGACAATGAAGCTTATGAAAT
EMPV1_39307 AAATAACTTAGGGAACCCCTTCCACCCTGCCCTCCTCTTCAGGGCAACCTTCCATAGTGA
EMPV1_39308 AGTTCTCCTCATTCTTCTCGTGGCAATACTTGATGTGCTCCTTCAGCGATGTCAAGCGCT
EMPV1_39310 TATGAGTAGATGGTCCCCAAGTTCCCCCAGATCATCATTTACGCAGCTCCACCATTCAGC
EMPV1_39311 GGACGTCCGCCGGCGCTTAAGTGATCACTATAATCGAATACCCGCGGCCCCCATGGCGGC
EMPV1_39312 AGCCTCAGCCCCTGTAATCCCACTAGATTTTTCAAGTCAGCTTTCCTGTTCCCTGCAGTG
EMPV1_39315 GCTGGTCAGCTGGAGCTATGACAAAACATGCACCCACAGCCTTTACACAGCCTTCACCAA
EMPV1_39316 CCTATGATATGAGGTCCTTGAGAATCCTGTCTAATCTCCTGGTCAGAACCAAGCTCCCTT
EMPV1_39317 GCATTGCTGTGGCTGTAGCATAGGCCATCAGCTGTAGCTTTAATTTGACCCCTAGGCTGG
EMPV1_39319 CACAAATGGCCCTTATGCTTCTGGGAGGACGGGGGCAGGATTTGCGCCGCCTCCTGAGCG
EMPV1_39320 ACTTGAGTTTCATGTTTACAGTGGCTCCCCTTGTCCCTCAGGTCCGTGGGCATCAATGCA
EMPV1_39322 GGAAGTCCTCTTATGTCAGACCTTGGGAGAGTCTTGTATGCAGTAGCCTGGACACAGCTT
EMPV1_39323 GAAGCTGTAAAGCCGTGCATTCAAGAATCACAAGGAACCCCCTAAGCAAATAAAACATGC
EMPV1_39324 TGTGACAGTGCCAGATACTGGTTCTATTTGGGAACTCCAGCCAACAGCCTCACAGCCTAC
EMPV1_39328 AGCTGCTACTCTGTCCTTCTCCTACTGTGGCTCCCGAGAAATATCCCAGTTCTTCTGTGA
EMPV1_39330 ACACCCAGAACATCGACGGGCTTGAGAGAGCGTCTGGCATCCCTGCCTCAAAGCTGGTTG
EMPV1_39331 AGGAGCGTCCTGTGATTCCCTTTGTCACTCGTTGTTGCCGAAGACTGAACTGTGTCGGAA
EMPV1_39332 GGTACCAAGATGATACCCTGTGACTTCCTTATCCCGGTCCAGACACAGCACCCGATACGG
EMPV1_39333 TACCACGTCCACGAGTGTGTTTAATCAGCACCAAGTCGATGTGGGAAGACGGAGGCTATG
EMPV1_39335 CCCCTCTCCCTTTCTCCTGGGAGGTCTCCTATTCTTTTCCTGGGTTTGTACTCTCTTTAG
EMPV1_39336 GGGAATCAACTATAAAAATTGGGGACGACTTGTGAAGCCCAAGGGTGGGAATCCAGCTGC
EMPV1_39337 CATTCCCCCCTGCAAGAAATCTCGGAGGGAGAAGCCAAGAGAAACAGGGCCATTAGGAAA
EMPV1_39338 ATCCTGAAACCTGCAAGTGCCCACTCTCCAACTCAAGTGAGAAGCCGGGGAAAATCCCTG
EMPV1_39339 GGAAGGAAACGACCAAGAGTGTTTTATTGCCTTTGCCCGACTATTCAGTGGTGTGGCTCG
EMPV1_39340 TATGAGTTAGCGCTGTCCATCTCTGGAGTGCTGATCTTGCTGCCCCTGTCTCTCATT

EMPV1_39342	TTTCATTCACAGCGCTCACGTCCACGCCCGCCGGAGGGCCGCTTCCATTTTGAAAAATTA
EMPV1_39344	TGACTGTGACCAGAAGAGAACTGCCCTTTAGATGCACCTATCAGAGACTCGGAGGTGCCT
EMPV1_39345	AGCAGCTTTACCAGAACCTTGGTGCTCTTTCACCTCAAAGCCCAGGACGGGAAAATAGAC
EMPV1_39347	CCTGAAGAGGTTGCCAAGAAGCCCCTACGTCCTACCGCCTTAATTCCAGCAGAAGCTCCT
EMPV1_39350	CCCATCCTCATCATCTCTCTTCTTGTCCTGGGGCTCCATCCTCATCATCTCTCTTCTGGT
EMPV1_39351	TGACAGGCTCGTTATTATCAAAGAATCTTGAGCAAATCCCTTTGATTCACACTGTATGTC
EMPV1_39352	TAAGTACCCATCAGTGAGCAGTGAGACGGATCGAAGCCGCTATGTTGCAGTGTTCCAGGA
EMPV1_39354	GACCTTGACATGTGGAGAAACCCAGAAGAGGAAGGTGAGAATGGAGATAAGGTCGTCTGC
EMPV1_39356	TGGCCCACTGGTGAGGAAACGTTAATATCTCAGCCTTGGGTAGAGGTAATCAGAACACAC
EMPV1_39359	GGAAAGCAAAAGGATGGGACGTCTTTCGGAGAGTATGGCGCCTGGTACAATGCCTGCAAA
EMPV1_39360	TAATTGGAAGGAAAGCAGAAGTTGCTCTCGGGAGTGCCTGACATGTATTCTGGATGCTAC
EMPV1_39364	CTGTGCTGCCCACGTCTGCACTATACTTGTCTCCTACACGCCTGCACTGTTTAGCTTTCT
EMPV1_39366	GACGACCCTGCGGGTGAAGCAGGCCTGCCGGTGGCGCTCAGCCCCGAGCCCTCTGCCTTC
EMPV1_39367	CGAGAAGCTACAGGCAATCTCACTATACCTCGTTGGGACAGTGGATAAGAGAATGTAGTC
EMPV1_39368	TACCACGTCCACGAGTGTGTTTAATGTCGTCCAAGTCGATGAGGCAAGACGGAGGCTATG
EMPV1_39369	TTAACCCTTCCTCTGGGAATGTTGTTTCTGATCTGGGCTCTTGGTCTCTGATGCTCGAGG
EMPV1_39371	CATAGGCACTGAAGGTGGAGGGATGGACCAGTCCATATCATTTCTTGCAGAAGAAGGAAC
EMPV1_39372	ACAGGAGTTCCAGTTGTGGCTCAGCAGATTAAGCACCCAGCTAGTATCCATGGTGATGCC
EMPV1_39374	GCTGTGGCATTTCTTCATTAACATAGTTGTAATGTTCCTAGGGCAGACCTTTTCTTCATA
EMPV1_39375	TCTGTGGGAAACGGTATAAGAACCGGCCAGGGCTCAGCTACCACTACACCCACACCCACC
EMPV1_39378	CTGAATCCCTGAGTGCAGCCAGATTCCTTGGTCTTTTCCATATTAGGCAGGACAGAACCC
EMPV1_39380	TCTGTCTTCGAATAACACTGACCTCATGGGTGGTCGTCAGCCTCAACAGCCTCCTCTACA
EMPV1_39381	TCAGCCGGGTTCTGGCTCCGCGAACCGGCGATGAATCAGCCCGATTCCATTCAAAAGAGT
EMPV1_39382	AAGTGGGGACCAGGTCTAATGCCAGTGATGGTGTTGAATGTGTGTACAACAGCTCTGCTG
EMPV1_39384	CGGAGACCACTCTCCTTGATTCCACCTCCTTCAAAAAGCCCCCTTCATCTTCATATTGCC
EMPV1_39389	GCTGCTACAGCTCGTTCAAAAGAAAAACACCCTGTGGCCGAGGGAGGCTGATCTCATGAT
EMPV1_39396	CCAACATCAAAAGTGTAATCAGCCTCATTGGACCACTGGTCAGAAATGTCTGTGTTACGT
EMPV1_39399	CTGTCTCTCAGGAGCGAAGATCTGATTGCTGAATTTGCCCAAGTCACAAACTGGTCCAGC
EMPV1_39401	TCTGTGTGCACACATGTGTACATGATATGTGTATATATATATGTGTATACACACACACAC
EMPV1_39402	ACCCCAGGCTGAAAGGGGAGGGCTTTCTGGTCTACCTAAGTTTTCTATGGCGCTCAAAAG
EMPV1_39405	CACGGTGCCGGTGGAGAAGTTCCTTGCCATTTTGGACTTTATGATTACGCCCCTCCTGAA
EMPV1_39408	CGGAGGGTGAGCGGCTCGACGGGGCGGTTTGGCGGGGCACCGGGAGCTTCGGCCTCTGGG
EMPV1_39409	CAGGAAGGAGCAGATGAGCTGCAGAAGATGCTTCAGGAAGATGAGTTGCAAGATGCAGTG
EMPV1_39410	AGGCACCACGGGGGGCAACCAAGGCCAGGAGTTTGTCATGAAGAAAAGGACAAAGGAATA

EMPV1_39412	TGCCCTTCACCACCCTCTTTCCAATTCTCGATCTTGCTCCCAAGCGTTCTGGGTTCTACC
EMPV1_39413	AGTTCTTGACTTCGAGTTCGCTGACCACACATCGCCAGATATGCCCTGGATTTGACTTGG
EMPV1_39414	TGGATCTCCTGGACCAAGGGCTGCAGGTCCATGTGGTAGTAGATGCCTGCACCTCCCGCA
EMPV1_39415	GATATCCGTGTTGCTCAAATACTCCTGCCGCTCGCCACCATTCTAGGGCTTTTCAGCAAA
EMPV1_39416	CCTGAAGAACCATTACATTGATGACCACAGCTGGACCCTTTTTGAGCATCTGCCTCACAT
EMPV1_39417	CTACAACGCATGTGCCCTGTGGAGCCAATCGTAAGGAGGGATGGAATACGAGGCATATCT
EMPV1_39420	ATGACCCTAAGAAGGACAAACACTTCTAGGGCACTGTTAGGTTTAAGTCCACTCCCCGCC
EMPV1_39421	GAGCCCTGCCTTTTTCTACTGGTTCCCCCTAATGTTTGAAAGAGGCATAAGGCTCCTGGT
EMPV1_39423	AGGGCCGTTGAAGGCATCCAGCTCTTACTGCCTAGTTTGAAGAAGGGTACATGGGCCTTA
EMPV1_39424	GCAGTGCTCTAGTACCCAACAGGTCTGGGAGTGCTAATATCCTGCCTGTGGCTGTAAATC
EMPV1_39425	TCTGGCTCCTTCTTCCAGTCTCTCCCGCAGGCCTTTTGATTCCACAAGGGCAGAGATCAT
EMPV1_39427	GGTACTTTCAGGACACCTTATGCAGACACGAGATCTTGTACAACCAGATAAACCTGCAAG
EMPV1_39430	TTCAAGTCATCCTCATATTTATTGGATACGCCAGTGCCAAACATCACGTGGCCATTTGGG
EMPV1_39431	TAGAAAGAGACCTTATGACCAGTACAGCAGAGACCGCGACCACCGGGGACACAGAGATTA
EMPV1_39432	CTCAACAGCTCTGACTCAACCAGAAACAACAGTGTCTCCCCAAAATCAACTGTCAGAGGG
EMPV1_39435	AAGGAGGAAGGCCCTGAGATGGAGATCAAGGTTGAGGGAGTGAGAGTGTTCGGAGCCAGG
EMPV1_39437	AAATCTTGTGGATGTCAAGGGCTACACGGCCCTCCACTATGCTGCCTTTGGTCCGAATAT
EMPV1_39439	CTGCTTTCCTTCTGTGGCTGGCTTAAGAACATGCTTGTCAAAATACATTCTCACCAGGCG
EMPV1_39445	AAGAAAGCCAGGGAGGCCGCAGCGGCCAGCAAGAGCCTGAACGTGACCGTGGACGTGCAG
EMPV1_39446	TGGGAACCAAGCCCAGCCAACCCTTGCTGAACCCAATAGAGCCATAGTCAATATATAGGC
EMPV1_39447	TCGTCTGCGAGAGGAAGCTGCTATGAAGGCCAAGACAGAGTAGCAGAGATACCCATGTTG
EMPV1_39448	TTGCTGTCTCTGTCGCTCTTTCCACTTTTTCTCCTGCTGCGTCGAGGTGTGTCTGCTTTC
EMPV1_39449	TGGCTGGTGTGTAGCCATTCAGTTTGATTTTGATATTGTTTTCTGAGAACTTCTGTAGAT
EMPV1_39451	CCAGACGGGATCCTGGATGTCATTCTCCTTGTCTTTGTGCTCTCTTCTATTCATCCTGAC
EMPV1_39452	TTTTGTAATGTGGTCAGTGGTCATGAAAGGCCCCAGTGAGAAGGCAACAGCTGAGTAGAG
EMPV1_39453	GGAAGGCAACTGGTCTCTAGTGTTTGAAAAGCTGTGGCCTAAAAGTGGCTGGGTTGGCCA
EMPV1_39454	CCTATCGCCTACGTTCCTCGCCGGTTTCCATGGTTCATCTGGCTGATATGCTGAACTTCT
EMPV1_39456	CACAGACATCATCTCAGCCCTGGTGACCAGCACATTCATCATCGAGAAGCAGCCCCCTCA
EMPV1_39458	GATAAGATCAATGCCCTCATTAAAGCAGCAGGTATAAATGTTGACCCTTTCTGGCAAGGC
EMPV1_39459	GATGTACGGGATGCTCTTCTCAATCCGCTCCTTTGTCAGCAAGATGTCCCCGCTAGACAT
EMPV1_39461	GCAGAGAGGAAGGGCCGCCGCAGTCCCCGGACCGCACCAGCAGCGATGCGCAGCCGGAGT
EMPV1_39462	GAACAGACGAAATACCATAGGATGTATTAAGCAACATAGAATTCCAACGGAAGGGTTTCC
EMPV1_39463	GGGATTGCTTACACCTGACACTCAGAAAGGTGGTGAGACCCCTGGATCAGAACAATGGAA
EMPV1_39468	CCCCCGAGGAAAAGGATGCTGTAACTGGTTTGAATTCAGGTCTAAGCGGAGAAGCGAAAC

EMPV1_39469	GAACCTCAAGGCAGAGTAAAGCTGATGCCCTAAGGAAGCTGCCAAAGTCACCATGGACCA
EMPV1_39471	TAACGGAGTTTCGGACGGTCCTCGTCTAACAGGTCGTGCTGCTCAACAGCCTGCAGCAGT
EMPV1_39472	GAGCACTCCAAACTTTCCTCCTCATCCCCTCTCCTGCTATTGATGGCATAATCTGTACGG
EMPV1_39475	ACAGTCCCACCAGAGAGGGGGCTTAACCCTGGACAAAGATGGGACACGCTTGTATTCTTT
EMPV1_39479	ACTTGTTGGGACACGTGACTAGAAGTCAGGGCCCTAAGCAGCGCTCGACGTAGAGGACGA
EMPV1_39480	TTTTTCCTGATTCACACGGAGATGTTTTGTAGGTTCATGGTCCACCCTGGAAGGGCCTCG
EMPV1_39481	GTTAACTGGTTCTCCCTAAGTCTCAAAGTTTCTATTTTGTTCCACCATGTCATTGTGCAC
EMPV1_39482	GATTTTGAGGATGGTCTCTGCAGTGTGATTCAAGATCAGAGTCTGCGACTTGGCTGGACC
EMPV1_39483	CGGCTGCAAGAGACAGCTGTCCCGGTCATTTAAATGCAAGTCTCCAAAGCGTCAATAACG
EMPV1_39484	AAATGAGTCTCAGAGGGCCCAATGGAGCACTGCCCCAGGCGAGCTCCTCTGGCCTCTCCT
EMPV1_39485	TACTGGATGGGATTCCTGCCAATGTAATCAGAAACAGCCCCCAGTATATTGCTGCCCCAC
EMPV1_39486	CTTGTCTAATTACAACGAAAGCCACCCAAAGGACCAAAGGAGGATGCCAAGGATGGCCGC
EMPV1_39487	CCCACTCAGGGGAAGGAACCTCTTGCAAAGCTTAATGATCCTATTAGTTACTCTTTTGTT
EMPV1_39489	CGGCATTCTTTCTGCTGTTTTGACTGAGTAACTTCCATTACACTATTGCCTAGATTACTT
EMPV1_39492	AGCCCTCACTATGCCAAGAAGATTGGTTTCGTGTTTCTCCTAGCACTCTCATCACACACA
EMPV1_39493	CTTGCCCTCGGGCTTACTCTCAGTGCTTTCTAGGTAAAACTGCCTTGAATTCCCTTTCCT
EMPV1_39494	TTTTTCTCTTTGAATTTCATCCATATGTGGATGTGGCTTCAAGTAGAAATCAACAGCTCG
EMPV1_39495	GAGGAGAGAGATCCCTTCTGACATGGCCTTGTTGGCATCCCCAGCACTCAGTAAGTATTT
EMPV1_39496	GACCTAGGGTTTTTTAGAGATGACAGAGTGGGGCTGAAAGACAGGGGTTCCAAGAGGCCT
EMPV1_39497	TTTCCCTGAAACTCTTTGGTCCATTCCCATTGGCGCCTGGGCTTAACGAAACCCGCTTGT
EMPV1_39498	ATCATTACGGACCTATTCCTGGGATTCCTGTAGGATCAACTTGGAGATTTAGAGTTCAGG
EMPV1_39499	CAGGTTTTTGTATGGCCAAAACATTTTCAGAAGAGTCCCAGTTAACCTTCTAAGTAGCCC
EMPV1_39501	CACGGACAATGGGCAGGTTTGGCCTTCCGAGGACCAAGGGGCCCCCATATCATTCTACAA
EMPV1_39503	CCACCAGGAAACCCCCCTAGAAGAATGGGTCGAATTAATCATCTCCGTGGCCCTAATCCT
EMPV1_39504	CTGCTGAGCCACCAGGAAACTCCTCTAGTCCACGTTTGATGGATGTTTTCAGTTTTTGCT
EMPV1_39505	GACAGTCTGGCGTTTGCATGTGGAAGTTAAGCTTTGGGAGGAAGCAGCATGGGGGTTATT
EMPV1_39509	GGGCAGATAATCACCTGTCTGTCTGCTCTCCTTCTGAGGGCAAATCCACACTTAAATAAA
EMPV1_39511	GTAGCACTTTTGTGGGGACATGCAAAGATGGTCAACGTACGTAAAACCCGAAGGACTTTC
EMPV1_39513	GACTCCCCAGAGTCATGCCCTTCAGCCTCATCACCACATCCCCATGGTGCCAGCTCAACA
EMPV1_39517	GAGCCTAAAATGCAACATAGTTTTAGAGATGAACTCGCCACCGTCACATAAATGCCCACG
EMPV1_39518	CATTTGTAAAGCTGTTAAACTACCATGAAAATATACTCACACACAGCTTTATGTCACCCC
EMPV1_39519	AACACGTGAGAAGAAGGACGATTCCGCTTAGGGTTCCCGCTGAAAGCTTCGCAATAGGAC
EMPV1_39520	CAGTGAGTCAGGCCATCACAACTTCTGACACCCAGACATCTTCATCAGGAGGAAGGCTGT
EMPV1_39522	ACTGTCTAACGGCTGCACCGAGGCCGGAGGCGGGGGCACCACTAGCTGAGCACGTGGTCT

EMPV1_39524	ACACAGTCGTTGCCCTGCCTTCTCAGCAGCAAGGCTAGCTCCAGGAGCCAGGTGAATTAG
EMPV1_39525	TTGCCAAAGGCTACTGTACTCCTCTAGTTAGAAAAATTCGCTCAGATCTTCAGAGGACAC
EMPV1_39526	AAGCTCGGCAGCCTCAAGCAGCGCTAGCTGCAGAAGTAGAAGGAGAAGCAGCAGCAGCAA
EMPV1_39529	GCATCACGCTTGTGGCTGAACTGGATCTGGGATTTGCCAGGGCTTTAGCGTCTGATTGAT
EMPV1_39532	GGAGACATGGGTTCCTGTGTTGCTCATGTTCGGTGCTGTCTCTGTAAAGTAACTCAAGGT
EMPV1_39533	TGGAAGACCTCTGGTGGTGGGCGTCTCGACTCTGTCCCCTGAGTTTTAAGGTTGAGATTT
EMPV1_39534	AACACTTCCTCTTTGCAGATCACAAGCCTAGCCAATGCCACCACCAGACAGGACCGATTT
EMPV1_39536	CGAAGGAGCTTTTTGGAGTGTTGAATGCCTTCTAAAATTTGATTGTGGTTGTGGTTGCTG
EMPV1_39540	CAGCCTGATTCTGAGGACTTGGTGAAATGTATGGCCTATGTGGGGCAAGGCAGCCAAGTT
EMPV1_39541	AGCGTATGCTGTCTGGGTCCGTGATGCTTGTGGTTCCTTTCGATTTTCTCTGCTCGCTGT
EMPV1_39542	TCCAGCTTCCAGAGGTTATCTGCATTTCCTGGCATGTGGCCCCTTCTTCTACCTTCAAAG
EMPV1_39543	CCTGCCCTTAGGCTATTAGAGTGCTTTTCTTTCAAGCATAGAGTGCCAAAAGTGCCTCGC
EMPV1_39544	GCTCTTTTTGTCCACCATGAGCTCCCAGACTTGTCAGAATTTTTCCACTGAGGTGACGGC
EMPV1_39545	CTCCTACTGACATAGCTACCTGTGATGTACTTCCTTAGGGCTTTTAAGGATGAGCCATTC
EMPV1_39546	TGGCAGAGTCCTGTTGCCTAGAGATGGATAAGCAGGTCAGACTGATGGACCAGAACCTGA
EMPV1_39548	CTCTCTCAACTTCATCGACTCCACCAACACTGTCACACCCACCGCCTCCTTCAAACCCCT
EMPV1_39549	AGAGCAAACCACCAACAAGTGACACCAACAAGGCCAAAGCAATGGTGCCAGAGTAGTTGG
EMPV1_39551	CTATTTTGTCTGATATGATGTTAGTGTTGCAACTCCTGCTTTCCTGTCATGTCTATTGGC
EMPV1_39552	CCTCCTCTTCATTCTGCTTATCTCACAGCATTTGGGTGTGGCTTGTTGCGACTGATAAAG
EMPV1_39553	GAAAAGGCTGGCTTGGGAGATGCCACAATAAAGCCCCAGGCCCAATATTCTATCTGCTCT
EMPV1_39554	ATCAAATCCAACTACCAAGGCCTCCCAGTACGTTGTGTGAAGAGCATCATTCGACAGGTG
EMPV1_39556	TTGGGCACCTTTACGTGACTCACTCCTTACAAATGTAAATGCACGAGCTCGCGGTAGGTC
EMPV1_39557	TAATTTTCTGCGGGGTGCTGCCAGGCCGAATCAAGTGAACAGAATTCCCGATCCAGAAAG
EMPV1_39560	CTAACTTCCTCGCCACAAAGGTCTGACCAGTAAGGGCAGATTAAGGACAGACAATCATGG
EMPV1_39562	GCACTCACTTGCAAAAAGGCTCCTGTGATAGAGCTTCTCTTCTCTACTTTAAACCCTGTG
EMPV1_39563	TCTGGACCCACCTCGCACATGTGTCACATCGTTTCCCCTGCCCTTCCTGGCGTGCACAGG
EMPV1_39565	CAGTTCTGTGGAATGTGGGCTGAGTTGTCCCTTTACATGTGGTCCAGGAAATGTGAGGTC
EMPV1_39569	TTCATGAGGAATCAGATGCTCCTTTGCTGCCCCCAAGAGTTTACTCCACGGAGAACCACC
EMPV1_39571	TTGCAGGCTGACAGTGCCTGGAAGTGATCTTGGTGTCCTCGGGTTACCAATAAAGCTGTT
EMPV1_39572	GGAGTTTCCGAATCTTAAGCGTATACGTGTGCATCTCATGCTCATACTAACGTCATGATG
EMPV1_39573	TCCATGGACAAGCACTGGCGGGGACATGCAGTGTCTGAGAAGGATTCTCACCTGAGATGC
EMPV1_39574	ACAGTCTGCACCCCCGGAGGACGTTCCGTGACATGAACACAGGCTTTACTTGAGCTGGAA
EMPV1_39576	CTGGGGATATTTCATTAGGAAGTGTTCATGCACGGAATATAACGTTACAAAGCAAGCTGG
EMPV1_39579	AGGTGAGGCTGCTGATTCTAGAGAACATCCTTCTGAACCCCGCTTACGACGTCTACCTCA

EMPV1_39580	ATTCAGTGATGGAGAGGAGGAAAATGGGCTGGTTGGATTGTGGTCTTGGTGCCTCGCAGT
EMPV1_39581	AAGGTTAGACTCCATATTCTTTGAGCCCTGCCTCCACTGATCCAAGGAGAAAAATTCCCC
EMPV1_39586	CTCTGGGCTGGCACACAGTTCAAAGATGTTTCTCAACAGACTTCAGGAGGATGAAGTTAA
EMPV1_39587	GATCCAAAACATACGGAACAGCTCTGTGAGGATGAAGTTAGAACCGGAGTCTTACTTGGA
EMPV1_39592	TTGCTCCTCAGTCCCTTCCTTTCAGGGGTTGCTGCTCTTTGTAAAATGTGCTGGTGGTGG
EMPV1_39593	GCGGGACAGGAGGGTAAATTCTCGGTAGGGACAATCCAGTCAATGGCCAGTTCAGTAATA
EMPV1_39594	GCAGGCAGAGAGAATGCCTACCTGAGGGGGCTCCTTTTCCTTCATGTACAGTTCAAGTAA
EMPV1_39595	GGCATGGATGACAGCAGGCTTAGCAGTGGAAAGTAGGGTGGAGACTAGAAGACTGTTCGA
EMPV1_39596	AGACGAACAGCCGGTACTATATCTGCAACAGGCTGTTGTAGTTGAACTCCAGCTTGTGCC
EMPV1_39598	CCGTACGTCGGATATGGTCAAGATAACCAGGTCGAGAAGATCACGCAGGCATCAAATAAT
EMPV1_39599	ACCTTGAAAAGAGTGACTTTTGCTCTGCCAGATGATGAGGAACCTGAAGATACAGATGTC
EMPV1_39600	CATACCAGCTTTTCCATGTAAGTATTATCTATTTTGCAGGTGGGGAGCTGGGGCTCAGAG
EMPV1_39601	ACAGGAGATGAAACACTCTGCTCTCTTTCAGATTTGCTCCGCTTCACCCCTCTGATGCGT
EMPV1_39602	GAGATTTTTAGATTTTCCTACAATGTTTGTGTCCTTAGATTAGAATGCACTCTGTGAAGT
EMPV1_39603	AGCAGCCTCTGCGATAACAAGGCCACGTGGTCACTAGCACACAAACAGCGGAGCTCCAAT
EMPV1_39605	ATCATCCCCTCTGGTTGGACACCCCTAGGGGTCAGTGGTCAACGAGGAGATGATGTGACT
EMPV1_39607	AGAATCTCTCTTTCTGTTAGACTGTGCGCCTTTGTTTCCTGTGACGTGGAGAGTGCCTGC
EMPV1_39608	TCTGGATCCTCCTGATCATGCCCTCCACAAATCATCCTGTTTCAGCATCTAGCAGTTCTC
EMPV1_39609	TATACCTGGGGAAACTGGGTATAGACTAGGGAAACAACCTGCTTTGGCATAGGCTCCGTG
EMPV1_39610	AGGCTGTTGAGAGCATCTTTAGCTATAGCGCCCTGCTGTGGGCTTGGTGCACCGTAAACA
EMPV1_39611	GCCTGGATTAGTGGGATACTCCATGCCACTGTGCAGATTCACTTTGTGCTTGACTTGCCC
EMPV1_39613	GTCATAAGTGTGAGAAGTGGCGGGGTATAACACAGCAGAGACTGTCAAGACTGGAGCCAG
EMPV1_39615	AGGGTCCAAGCAAGGTGGAGCGTGAGGACAGAGGCCCTGTGACATCAGGGATCCCAGTGA
EMPV1_39617	TGGAGATCATCCGAGGTTGTCAAGGCCTGAACGTGGTGGGTTGTGATCTTGTGGAAGTTT
EMPV1_39618	TCATCAGAATTGGGGATCGGGGGGCAAGTGATGAGAGGAGCTCATAGTGGATTTCCTGGT
EMPV1_39620	CTCCCCTTCACTGGGAAGAAACACGTTAACAAAAGCACATCCCTCCCGATGTCACTCGTA
EMPV1_39621	CTCTGAGAACACTCTCCCATCCTACTACTCACTTGAAAGCCCTGCTCTACTTTCAGCTGC
EMPV1_39623	CTAGATCTCCCCAACGATGAGAGTCCAGGTGGTAGCTGGGGCTTTCCTAAGAATGGGCTT
EMPV1_39625	GACCTTGTCCAGATCACACAACCGGTAAGTGGCAGAGACTGAAAGCCACAGCATTGCCTA
EMPV1_39626	ACAATGCAAATACATGAAAGCCATTTACTCCAGGGAAATTCTTCAGGGCCCAGGTTTCCC
EMPV1_39627	CCTCCCAGTGTGTACAGTTACTCTGCTAGGAGACTTGCCCCTGTAAATCCCAACATCTGT
EMPV1_39628	TCCTGTGACCGTGACTGTGACCAATAACACGGAGAAGACCGTGAAGAAGATCAAAGCACT
EMPV1_39630	GGCGAGTAGGACCTATTAAGCCTTCAGGACCCATCACAACAACACCCTATGATGATAAAA
EMPV1_39632	ATGTGATTGGAGCTAGAAGAGCTTCCTGGAGAATAATCAGCAGCATTGAACAGAAAGAAG

EMPV1_39633 AACGGCAGTCGCTGTCTCCCTTTCGTATGAAACCTGCCGGCGTATCTACAACATGGAATT
EMPV1_39634 GTGTCCCAACCGTGATGACGGCAAATAAAACGAAAAAGAAATTCCAGTACCTGCTGGAGT
EMPV1_39635 GTGCAGAATGTTTGTAAGTCTTACCGAGACACGTGTCAGCTGCGGCTGGAGGACCTGCTT
EMPV1_39636 CCAAGGTATTGGTAGCCTTATATGAAGAACCTGAGAAACCTAATAGTGCTTTGGATTTTT
EMPV1_39637 AAGGCCCTTGCTGCCAGACCTTCAGCTTTTGCTTCAACACTGTGTCTTCGTTATCCATTG
EMPV1_39638 TAAGGGCTGAAGTGCAGTGCTCCCTAGGTAGAGCTGGCTTGGGAGAGGACGGATGCTTTG
EMPV1_39640 TTTTCAGCGTTCCAAGGAGGAGAAGGAGCAGAAGATGCTGGTCCTGGAGGAGGCCCGGGC
EMPV1_39642 CTAACTCAGGTATACCAGCTGCACAGAGAGCAACGTCAGTTGATTACAGTTCCTTTGCAG
EMPV1_39646 GCCCTCTTTCGGGGTTCTCGAATCCTTGAGGGCGCAGTCTCATTTCTTTGCCTTTCTATT
EMPV1_39647 TATGGGATACAGCCCTGGTGTAAACAGCCCAGGCCACTTGCAGGTTGTGCAAATCAGAGT
EMPV1_39648 GGCCATTCTGACCAGAGTTCAAGTGGTACCTCGTTGCAGTGTTGATTTGCAGTCTTCTAG
EMPV1_39649 TCTCCCCTGTGAAGATCTGCGACTTTGACCTGGGCAGTGGCATCAAACTCAACGGGGACT
EMPV1_39650 CTGGTTCTCACGAATATCAGAGAGGCACCTGGAGCAGTATTCGTCTATTTATCAGAGGCG
EMPV1_39651 CAGGGCAGTCAGATCCCTGTGTTTGCTTTTCACTAAGAACTCAGCAGTGATATCTCTGGG
EMPV1_39652 AGATTGGCCGCAATGGGACTCTAACCTTCGGGGATGTGGATACAAGTGATGAAAAGTCGG
EMPV1_39653 TCATTCCAGATTTCTTCTGTGACCTGGGACCCCTGATGAAGTTGTCTTGCTCTGACACGC
EMPV1_39654 TGAGCGACTTCATCGGGGATGCGGATGCAAACCTTCAAAGGATCATCAGGGAGTGTGGAA
EMPV1_39655 GAAGAGTAGTAGTCATACTGCACCACCTCCTTCTCTAACGTGTTCTCAATACCTCCATTC
EMPV1_39657 CTCTGGCTCAGTGCTTGGACTGGTGACTCTAAAATCTACAATAGCACCAACTATCCAACC
EMPV1_39659 TGGGTGTAAAGGATATGGGGTTTAGATGGTCTATGGTGAAGGTGAAGAAGGGGTCTCAAG
EMPV1_39663 GGGTGGGGGCGTGGAACAAGGTCTTCATGAGTTCACCATCCTTAGACCAAAGTTCAGAAT
EMPV1_39664 GCTTAAATATCCACTCACAGGGCTTAGGAACAACCACACAACCAATGAGCACATCTCCTG
EMPV1_39666 GTGGAAAGTCGTTCTTTGTGAAGTCCAACCTCACTGAACATCAAAGAATTCACACAGGAG
EMPV1_39667 CCTGGCTATGCAGTACTACATCCAGGATAGGGGTGACAGGCGGGAGGTGTACCGGATCCT
EMPV1_39668 AGCCGGTACCACGAACTATCACTAGCCCTGGACTCCAGCAATCTAACAAACAAACAACTC
EMPV1_39670 ATGACTGTAGTTATCCTCTTCTATGGGGCTGCTATCTACACCTACATGCTCCCCAGCTCC
EMPV1_39671 CTGGGGGAGATAAATCCAAGAAAGGGGTAAAGCGGAAGAAGATTTCTGAGGAGAGTGGGG
EMPV1_39676 TCCACAAAGCCCCTAAGAAGAAAGGCATCAAGTCCTCCATCGGCCGCTTGTTTGGCAAGA
EMPV1_39677 GTTCATGAATCGCTTGGCATTAGCCATCGAGCCTCTGCTGGACGCAGCCCCCGTAGACAT
EMPV1_39678 TTCCCTTTGTTCACTCGGCTCCAGCCACACCAGCCTTTGTGCGCCTCTTAAAAAGCACCA
EMPV1_39679 TGAAGATAACAGAAGAAGCTGGGCAAGCCCGGTCTACACGGAAGCAGATGGAACATTTCC
EMPV1_39680 TGATGTGAAGAAGGTGGAGGAGGTGGTCTATGATCTCTCCATCCGAGGCTTCAACAAGGA
EMPV1_39681 TTTTGGATCGGTTGGCTGTGCCTGGGGAATGGAGTTAAATGGGGATACTCTGGGGACTGT
EMPV1_39682 ATAGTGATACTGTATTTAATGTGAATGAACCTGCCGCAACAAAGTTAGAAGAAATGCTTG

EMPV1_39683	TGAAGTTCGGGCCCACCGCTTGAACTTGCGGATTCCTGAGCGACTCCTTCTCCTTCCTCT
EMPV1_39684	TATACAGCTTTCAGTGGTATAAGCAGAGGCCTGGGGGGAGTCCCGTCTTCTTGATGTTGT
EMPV1_39685	AGGTTCAAGCTCCTCCAGGAGTATGTGTATGAGCGGGAAGGGAACACGGAGGAAGATGAA
EMPV1_39686	GGGAGTTTGAGGATTTGCTGAGCAGCATTGATGAGGTGTCAGAGGCATCTCTGGAGACAT
EMPV1_39687	TTTCAGGGGATTGTGTGCGCTGGGAACGGGCTTCCCCAGGGTGATTACTTAAGTACCCTA
EMPV1_39689	CCCACCCAGCCTTTTGACTTTTGCAGACACTTTTTAACTGTGTTCCTTACTGCCACCACA
EMPV1_39690	CCTGGAGCCCAGCATTCGGATGAGAAAGGTGCTAAAGAAATTATCCTGGACGATGACGAG
EMPV1_39691	GTGAAACCTTGGGATCTGTCTTCTTTCTAAACCACCACTTCCCATCAGGAAGGCGGAGAG
EMPV1_39692	CATGACTTCAACCCGGATGTGCAGGCTGCTTTTCAGAAGGTGGTGGCTGGTGTGGCCAAT
EMPV1_39693	GGTCTCAACGTTATCCTTTTGTTCCAAAGATGTCCTGTAGCCAATAGCTGCAATGAACAC
EMPV1_39695	GCCATCGTGCTCTTGTAAGCCTTCTGTTTTCTTCTGATTGGCACATTTGCAGCTCGTTGC
EMPV1_39697	GCTCAGCACGGCTCTGAAGCCCCCAAAATAAGTATGTTCTTCGTTAACTGGTGTGGGTTG
EMPV1_39698	GGTCTCAGGTTTTGCCTGTTTTGGAAGCCTCAAGTCCAGACGTGAAACCTAGCATCAAGA
EMPV1_39699	TTCGCCACGTACCTGAACTTCTGCCGTTCCCTGCGCTTTGATGACAAGCCTGACTACTCG
EMPV1_39701	ATCCTCGGCGATGTCTTCTTGGGTAGCTACCTGGCCGACTTCGACGGTGGCGTCAGAAAA
EMPV1_39702	CTATGGAACGAGTCTAGGGGTCTACTTCACCTCTGCTGTGACCCATTCTCCCCATAGAAT
EMPV1_39703	TCAGAACTATTGATTCGGAGTTAGTGAGTCAGGAAGCCAGGAGCAGTCTAGGAGAAGCCG
EMPV1_39705	AGAGACCGAACCAAAAACAGTGCCACCTAGAAGTCACATCTTGCATTCAGACATCAAGTC
EMPV1_39709	AGATCTGTGAGACCTTCACCCGAGATGCTTGTTCCCTGCTGGGGCTTTCTGTGGAGTCAC
EMPV1_39710	GTATGGCAAGCACCAACCCTGCAAAGTGACACAATACAAGAAGGGCAAGGATTCACTGTG
EMPV1_39711	CCCTGGATAATTGCCATTTTGAACCCCCTCTTACACACCCTTATGATGACCCGCCTACAC
EMPV1_39712	GAATGTTTCTTTATCAGACGAGGACTTTGAAATATAGCCCTGCAGCACCATTATGACAAC
EMPV1_39716	CAGCCGGGGTATGTGGCTGACATATGCATTGGGAGTTGGCTTGCTTCATATCGTTTTACT
EMPV1_39717	ACAAGTGCGAGAACACACCGGGCTCTTACTACTGCAGCTGCACCATAGGCTTCCGGCTCT
EMPV1_39719	CACGTGAAGGAGATTGAGCGCCTGCAGAAAGAAATTGAGCGGCACAAGCAGTCAATCAAG
EMPV1_39720	CTCCAGTTCCTGGGGTTTTTATGCTGGTGTCTGGATCACAAGAAAGGCTGTGGTTTGTGC
EMPV1_39722	AGAAGATGAGATCCTTGCCCTGAAGAACCCTGACTTTGTCTGGAGACCTTGCCACACGTC
EMPV1_39723	TGTGATAGAGGTGCTGATGGTCTCGAACAGTGGTCTGCTCTCTCTCATCTGCTTCTTGGT
EMPV1_39724	CCATGGTAATCAAGAGGGGGTTCTCTGAGACATTTCTCAAGGTACAATTTGAGTGTCACT
EMPV1_39726	AAGCAGGGATCATGGGGTCGTTGGCCAGGAAGTCCAGGTCGAAGAAGTGGCCCTCCTGGT
EMPV1_39727	ATCACTGTGTATGAAATCGCCGTGTACATTGGTGACATCGTCCTGCTCCTTCTCCCCATC
EMPV1_39729	GGGAAAAAGAACAAAGCTGGAAGAATAACCCTCTGAGACTTGAGACAGTGCTACAAAGCT
EMPV1_39732	CTCCTCCTGGTCACCAGCCTGGTGGCTGTGTTGCTGCTAGAGGCAGGCTCAGCCCCAACA
EMPV1_39733	CTGTAACCATTTCTTAAAGTGCATTTGAAATGCTCCTTTGGAAACATAGAACTTCTAAAT

EMPV1_39734	AGGTTGAAGTTCCGACTAATTGGACGCTTCTCAACTAGTCTGGAGGGGGTGTTTGCTGCA
EMPV1_39737	CACGGTCCCTGGGCGGCCTCTTTAAGGGAGGGGGCGGAGCCACGTGGAAGGATCGCGAGG
EMPV1_39738	GCAGTGCCACTTCTGCATCTTTGCCTTTTATGCCGTTTGTGATGGGTGGTGCAGCATCAT
EMPV1_39739	GAATTCCCACTGTGGTGCTGTGGGTTAATGATCTGGCTTGTCTTTGTGGAGGCGCTGATT
EMPV1_39741	GAAGAGCAAATGGGGAGGGACTTTCAACTTCGAGAGGGCAGAGCCCAGTCTGTTTTGCTT
EMPV1_39743	GAAAGAATAAAGGTTTCAAAGGCATCAGAGGACCTCGTGTCCTCCTGCGAGGAACATGCC
EMPV1_39745	GTACAGAATGTGGCAAAGCCTTTAAGTGGCCTGGAGGTCTTACTCAGCATCAGAGAATTC
EMPV1_39746	GCAGAAATGAGCACCATTTTGCCGTAGGACATGTATTTTGTGGTCTGAGTCTCCTAAGGA
EMPV1_39747	CTTCCAATGTTCACGTTCACCGCAGGGAATGCCCTAAAAGCCATTATTGCCATGGATACA
EMPV1_39748	GCCACGAGGCAAATGTAGTCTCTCCCAGCCTGGACCCTCTGTCAACAGTCCACATAGCAG
EMPV1_39751	CTGCTAATAAAACAATAGTTCCTGCAATGCTACCACAGCTCACCCCCACCCTGGTGTCAC
EMPV1_39752	CTGAGGAGCCACTATCACACGGGAAACCTAGCAAGCTCTTCTAAAATGGGCCAGAGTCTT
EMPV1_39753	GCTTAACATCACAGAGCCTCCCTGAAATGAGTTGATAAAATGCCTATACAGCCTTCATTC
EMPV1_39756	GTCTTGTCGTCCTTTAGACGAATAGCAACACACAGCGGCTGCAGAAGCCACACTTTGAAG
EMPV1_39758	AACTACCCTCCGGTGGAAGCAAGAAGACATTTCAGCCACTGTCATCCAAAAGGCCTATCG
EMPV1_39759	TTAAAACGTTCACATCCCCAAAACGGGGCTGCCCGCTTGCTTGCCCTTCCTTGGTGTGCC
EMPV1_39760	TTTTTTTTTTTTTTTTTGCCAGACCTGGGGCACATGGAAGTTCCCAGGGCAGGAACTGAA
EMPV1_39761	CACTCCCTGCCCCTGCAGAGCTTCCTGCTCAAACCCGTTCAGCGCATCCTCAAGTACCAT
EMPV1_39762	CTGCCAGTGTGAAAGAGCTTGTTTTCCTACTGCAGACCTCAAGAAGAATTAGGACAATGT
EMPV1_39763	CGGAAAGCAGGAGATAGACTCCAGAATACAGACCCTTCATGAGTTCAACAAAGTAACTTG
EMPV1_39764	CCACAGAGAGGGGGATGCTCAGATCGGAGGTGCATTTCCACATGCTGGAAAAAGTCATGA
EMPV1_39767	CTGCTGCTCATCCTTGGGACCTCCCTGGAGGTGGAACCTTTTGCCAGCTTGTCTGAGGCT
EMPV1_39768	TCTTTAGCTTTACCAAACTTTCCAGTACTACCTCTCCCCGTGGGCACTTGCAATGGGGTC
EMPV1_39771	GTGACACAGTGGAAATGAATCTGACTAGGAGCCATGAACTTTCGGGCTTGATCCCTGGCT
EMPV1_39772	TGGTTTCAGCAAGTCCTGTGGAGTCACCGTGTTCCTGAAGCTGCTGTGTCCCCTAGTTGA
EMPV1_39773	TGTCCCACTAAAGCAGGAGAAAAGTTGTAATAATAGCGGAGGATTAGAAACTTAAAACAC
EMPV1_39774	CCTCTCTCTGGTGGAGGCCAGTGGAGCCAGGATGAGGCCATGAGTGATGGATGGAACCAT
EMPV1_39775	CCTTCAGTTGGATGTCTTTTAGGCACTAGACTCCACAGAGCAGGACAAGAAAATCAGAGG
EMPV1_39778	GGTCAGAAACAAAAACGCAGATCAATCAACTCCAAAATTCCTGCTCCAAAGGAAGGTCTC
EMPV1_39779	AGAGACAAAGTAGATTAGTGGTTGCCTGCGGCTGGGAGTGGCAATTGGGAGTGACTGCAA
EMPV1_39780	GTCTTGTAATTTTGTAGCAAGAGAAAATAAGCGTCCCTCAGGACGGCGAGCTGCGGAAGC
EMPV1_39781	ACGGATGACCTCTGTCGTCAAACATCGTGTTGCTGGAACCCACATTCGCTTCCTGTGTTG
EMPV1_39782	CTATTGGGGCGACAGGTGGTACCTGCAATCAGACCAGTGGGCAGTGCTCCTGCAAGATAG
EMPV1_39783	GTGTATAACGAGTATTAGCTGAATGAGGAGACTACACTTGCTCTCAAGGAACTTGGTAGG

EMPV1_39784	CTCCTGTCATTGTGCCTGTAGGATCAGGGTTGGAAGAGTCTGGGCTGTTTTTAGACCTTC
EMPV1_39785	TGAAAAAGAAAGCAGATTGGCCAGGCAACAGAGAGAAGTCATGCAGGAAGAAGAGGAGGA
EMPV1_39787	ATAATTACGTGGTTTGCAGGGTCCTGTGTGAGTTCCCCTTACCTCTGGGCCTTACCTGCA
EMPV1_39788	CGGGTTCAATTTGGGGGCAATAATATTTCATGTGTGGCAGTATGAACTGTGTAAACTATC
EMPV1_39791	CAGTTGGTTTTTGCCTGCTGGTGTGGAGGTTTCATCCACTCTATAACACAGGTCACACTG
EMPV1_39792	GTCACGCGGTGCCAAGTACCATTCTTGCAAATGATTCTTTGATACAACTGACCAGTGCTT
EMPV1_39795	CCTATACTCTTCGGTTTACAACGTGTTCTGCTCATTCTCGGCCTGTTCTTCACTGGCCTC
EMPV1_39796	TATCCTGGCACATAGTTTCCCACCAGGGAGATAGACAGTTGAGGAGAAATGCTCTTCCTC
EMPV1_39797	GCTAAGTGTCCTAGCACTTATCAGGTTGTATTATCATTGTCCGTGTCTATGGCTCTCGTC
EMPV1_39799	AGCCACGATGGGAACTCCTCCAGAGCAGAAATAATTTTCAGGGGTGCCTCATTCCCACTT
EMPV1_39801	TCCTTGGGAACAGAACCTCCCTCTTCCTCTCCAGCACCAGCAGTCGAGCCCACCTTTGCA
EMPV1_39802	CCTAGAATGAAGACTGTGATGTTGCAGGGAAGAGCCCAGCCAATCCAGAACCGGCATATA
EMPV1_39804	AGATGAGAAGATCAAGAACCGCCTGGACCCTCTGCGGGAGATGCAGAAGCACTTGGGGAA
EMPV1_39805	GGAAGATGGTACAAGGCCAGGTAATGCTGAATCCTTGGTCCACTTTTGTCCAGAGCTCAA
EMPV1_39806	AGAGAGATTCCAGCCTGATGGTGACCAAGGTAGTGGTTGACAGTGGCTCTCTGCTACACA
EMPV1_39807	CTCCTTGCAGAGTGCAGCCAATCTCTGCTAACCACCTGGAGCCCCCTTTCCAGATTATTA
EMPV1_39809	ATTAAATGGTTTCAAGCAGAGATGGGCAGCAAGGGGTCACCTAATACAGTACCCAGTCCT
EMPV1_39810	GGGAATTTGTGGCAGGATTCGTCCAGTTCAGGGTGTTTAATAACGAGAGAGCAGCCAACG
EMPV1_39811	CATTCACAAAGTAAAGGTGGGCTCTTCGTTCCTAACTTCTGAGCCTCCCCAGTCTGCTGA
EMPV1_39813	GAAGTGGCAAGAAATTACAGACGGAGTGGGAACTGAAGCAGAGGCAGCTGGTATGGATTC
EMPV1_39815	AGTCTAATTGGCTTTCTACTAATCTGAAAGCCGTGCTGCTCAGCAATGACAGTCCTTTGA
EMPV1_39816	AGGTCATGGACATTAATTCAAGCCTGAGTAGCAGTTGCTGTTGGTCACCTGTTCGGGAGG
EMPV1_39817	CCAGTGTTTACAAGTTGACCACTATTTGACTTCGTTTCATTGTCCACGTAAAATAGCTGC
EMPV1_39818	TAAAGTTCCAAGTGCTTTGGCACCTGCCTCCCAGGAGCCCTCCCCTGCTGCTTCTGCTGA
EMPV1_39820	AAGACGTGACACCAAAAGGACCCTAGATGGTGCCCCCAGCTGAAAATTCCTGGTCTAGAT
EMPV1_39821	TCTGAATGGCTGTCCTGACCACTAACTCAAACTCATCACCCAGCGAGGACGTTGCCCTTA
EMPV1_39823	AAATGAAGAAGAGCTCGTCAAGTTCATGTGCGGCGAGAAGCAGTTCTCCGAGGAGCGAAT
EMPV1_39827	AAGATGGAGGTCTGGGCAAGACTTTGGGACCAGACTCCTCCCGCCTGTTTCCCAGGCCCA
EMPV1_39829	ACATCACCTCCTCCCTCTGCTACTTAACCCAGCTGCTTCGAGGACCCAATGCTCAGAGTA
EMPV1_39831	GAGATGAGAAAAATACATCCTGAACAAGGAGGAAAGAAGTCAAAAGAGGGTACATATGGG
EMPV1_39832	CTGCTGCCGCCAGGAGATGTACATTGATCTGCAGGGGATGAAATGGGCTGAGAACTGGGT
EMPV1_39833	AACCTCTTCTGCTGCCTGTACCGCTCCTGGTGCCACAACCCGGTCACTACAGTGTCGCTG
EMPV1_39834	GTCGCTGGGAACAGTGCGGTTGGACACAAGTTTGCCTGGGAGTGTTTTCCTTTTGTCAAT
EMPV1_39835	CATGCAGTGTCTCCCAAACCTGGAAGAGATAAAATTGGCCCTGGACCTGTATAAGCTGTC

EMPV1_39841 ATAACTGGTAGGTGTGCGCAGCCTGACCTGGATTTACTGACAGGCATTGAAAGGATCCAC
EMPV1_39842 TGAGAATCCTGCCTTCTGACCGAAATCCAACCACTCCCTGGTAACTGTCTGAAATAAGTG
EMPV1_39843 CTTCTAACCTGGAAGGCCAGAGTGATGAAAGAGCATTGTTGGACCAGCTGCACACACTCT
EMPV1_39844 CCTGCCAAGCGAATTTCTGGCAAAATGGCACTGAATCATCCATATTTTAATGATTTGGAC
EMPV1_39846 CAAAAACATCTTTCGCGTTCGCTTCCAAGACGCCTCTGCAGGCCAGTCCCACACTCTCCA
EMPV1_39847 GGGGTTTGTCTTATCCCTAGTTATCCTCCATCGGCATTCTCCCACGACCCACACCCTAAA
EMPV1_39849 GGGAGGTACTTATGCTAAAAACATGTAATCATTGTTTATTTGAACTTCCGCTCCAACTGG
EMPV1_39852 TTCTTGAAATTAAGTTCTCCACAGCTCCTTGCATGAGTGCATCCACTGCTTGGTATCAGC
EMPV1_39853 GCCTTCTAGAAACAGAAATACCTTTTGCATATTCTTTTACTGTTCTGACTTTTTAAGACC
EMPV1_39854 CGGAGGACCTGAAGAAGAAGCAGAAAAACCTGTGAAAACTAAGACTGTTTCTTCCAGTAA
EMPV1_39855 CAGTCCCTGACCCCTTGAAGGAGATAACAAGCATTTTACTTCAACTCCATTTCGAGTGGG
EMPV1_39856 ATATCTGCTTCCTGCACCCCATCTACTGCAATGCACACATCTTGTGGCCTCACTGGATAG
EMPV1_39857 TCCCCTCCCGGGGACCAAGGTATGCAACCAGACCTGCATTAGTTTTCTGGTGCTACTGTA
EMPV1_39858 AGCTCCGAGTCCATCATGCTGAGTAGGGGACCGTGTTCTGGGACTCCCCGACGGCTGCCC
EMPV1_39860 GTGGAAAATACTGGAGATTGTGATGGATCGTGTTTCAAAATTCAGGCGGCCAGGCTTTGG
EMPV1_39861 CAAGAAGGGCTCGAAGAAAGCCGTCACCAAAGCCCAGAAGAAGGACGGCAAGAAGCGGAA
EMPV1_39862 GGAGTGGATGGCCGCCCTGCGCCGAGCCAGTTACGAATTCATGCGAAGGAGCCTCGTCTT
EMPV1_39863 GCCACATGACAAGGTTAGTAAACAGGAATACTAGAACTACGTTTTGCATGATGCACAAGC
EMPV1_39864 GGGAGCTATTATAGAAGTTGTCTTTTCTGTTTACATGGAAAAAGCAGATGGGAGTGACCC
EMPV1_39865 AGGAAATAATGAACCCATGGCAGAGCAGCAATCCAAGCCACTGCAGTGACAACACCGGAT
EMPV1_39869 TTATGAAGCTCCTGAGAAAGTCCCCTCCATCTGTTTCCTCACCTCTAAGCACTGGAAGCG
EMPV1_39870 CAGAAACCATCGCGAACCAAGCTTGCCAAGTGGAGAAGTCGGTTTACACACGTCGTTTCA
EMPV1_39871 TCACCCTCTTAGGCCTGCTCGGGTACCTCCATTTTGTGAAGATTGATCAGGAGACTCTGT
EMPV1_39872 GTTCTGGCTGGTACACAAAACTTCCTGTGGAACCTGGGACAAATCTGGTAATAACGGTTG
EMPV1_39876 GGTGAGGATTTTAATTACATTGTTGCATTTTTCCTTGGAACAGCAGCCTGCCTTTATCAG
EMPV1_39877 ACGCTATAGGCAACGAAAAGGAACAAAGAAAAGAAGACAACAGAGGAGAGCTTCCGCTAG
EMPV1_39879 AGGAAGCTTTTGGCAACACAGAACTACATGGCATCAGTATTGTTTTCATGGCCTCTGGGG
EMPV1_39881 CTTGGAAGCTACTTCACTTGCTGAATGGGAAGGAGGTGGGAGAGAATTTACAAGTCGTAT
EMPV1_39882 TGATGGCCCAGCTGAGGTTCTGTGGCCCCAACCACATTGACCACTTTTACTGTGACTTCA
EMPV1_39883 ACCGTGTTGGAGAATAGAAGCAGGAAGGATGTAACTTGGATAGTGGGGAGTGTGTCTGGG
EMPV1_39884 CGCTGATACCTTCCCCAAACCCAGTCAGCAAGAATAACCCTACAGTTGCTCGTCAATGGA
EMPV1_39885 AGCCGAGAAGTGCAATCCAAAATTGAGAAGTGCAGTGTGGCCCTGATGTAGGGCGCCCGG
EMPV1_39886 TCAGAGGTGCCAGAGAGTGCCATTTTTGCATTCCCACTCTGACTTATAGCATGTCGAGGA
EMPV1_39887 GGTGACCGAGTGATCAGGTAGAGATATTACAGATACTGCATTGACCTAAGAAGAAACTAG

EMPV1_39888	CTCCTTGTGCATTGACATGGGATGAGTCCTTCAGCCTGGGCTCGGATGGACCCTGCCTCT
EMPV1_39892	TAGAGAAGCGGGAAAGGGTCGCGAGGCCCTGGGTTGCAGCAGCCCCAGCACCAAGCATGT
EMPV1_39893	ACAGAATTGATGGAACTACAAGAATTTTATGACCCAGATACAGTGGAACTTATGACCTGG
EMPV1_39895	CGTCAACAGAGCCAGCGGGTGTCACCATTACAACCATCACGATACTTACATCCCACTTGA
EMPV1_39897	GGTAGCATGGAAATTATCTGAGGTGTCTTTTGTATGATCGTGCTTTGGCCAGGGTGGACA
EMPV1_39898	CTTGATGCTCAGGAATGCATTCTACCACCAACTCGTCCAACTGACTAGCCGTTGCCCCTG
EMPV1_39900	GGCCAAGGTTGTCAACTCCTCTCAAACACACCTACAAGTTCAGGGCCAAGAGTTATTAAG
EMPV1_39901	CTGGTAGCTTGCAGGTGGAGGATCCAGTCAATGTTTTTATGGCTGTGGCTCCAGTCACTG
EMPV1_39902	AGGACACGATATGAGGTTAGCCGATGTTTGAGTAGAACCTAGACACTTCCAGGGCTTCAG
EMPV1_39904	TCCTCTACTCATCCATCATGTCACCCTCTCTCTGTGGTCGGATGGTGCTGGGGTCCTATA
EMPV1_39906	GGCAATATCGGATCCTGAAACCACTGAGAGAGGCCAAGGGATCAAACTGGCATCCTTAGG
EMPV1_39907	AGCTCCAGAATTCACCTTTGGATAGCCACGCTCCACCGAATTTGCACCTACGCAGCTCAA
EMPV1_39910	TCTTGTTCGTTCGCTGGAAGAGGGTGAGGGCTCATGAGGGTGAATACAGCACGGTTCCAA
EMPV1_39912	TGGTCAACTGGGCTACTCCTCAAAGGCCCACTTGGAATCAGGGAACAAACTGTAGTCTCC
EMPV1_39913	CCTGCAGCCGGCCGCGTATTCACTTGTGATTAAACGAATTCGCGCGGCCGGCATGGCGGC
EMPV1_39915	GTGAACATGGATGACCGGTTTGGGCAGATCATGATTGAGAACCTGCGGCGGCGACAGTGT
EMPV1_39916	GCAAAGCAAAAGGATGTCTCAGGCGGATCCTAGCAGTTAAGGATTCAGCATTGTCACTGC
EMPV1_39917	TAAGTGGCACAAACAGAGCACCAGCACGAGGAATAAACATGAGCTCCTCCTCCTCCTGGT
EMPV1_39920	CCCTAGGAACACGACCATTTACATCGTGAATCAAAACAATACTGGACAGCATGTGGAACC
EMPV1_39921	TTGAACTCTGATAGACTGACGTCAGGTAAGCTTGTCCTGTTGGAATGACGGCAGCAGAGC
EMPV1_39922	CAATCGCTGCAGCTACATCGACTTCCTGAACGTGGTGACCTGTGTCAGCTGCAAGCACAA
EMPV1_39923	AGTCGTCAAGATCCTGGTGGGCATTGTGGGTCGCAGCTTTGCCGTCATATTTCCTGTTGT
EMPV1_39925	ACTGGCAAGAGAGGATGCCGCTGTGGAAAGACCCAGAAATTCACGCTGGTGCCAGGTTTT
EMPV1_39926	TTACAGTTTCCTTTGGATATATCCCCAGAAGTAGGATTCCTGTATCATACGGCAACTCTG
EMPV1_39927	CTTACTCCGGCGGATACCCTCCGGTGTACGCAATGATGCCTGGGCCCCCAATGATGAGAC
EMPV1_39929	CACTGAAGAGCACTGTCTACCTGAACTATATGGCTATTGAGCACAACGTGTCCTTCCCCG
EMPV1_39930	TTTTTGATGGGCTCACAAGGACTCAGTGTGTCTGTTCCCCCAGAGCTCAGTGCAGAGGGA
EMPV1_39931	CCGGGAACCAGTTTTTTCTCCTGGCACCCTGTATTCATGGCCTTGGCGTTCTGCCTCTGC
EMPV1_39932	TAGGACTTTGCTCTGTAACCTGCCCACAGATACCCACTCAGGACAAATCTTCAATTTGCC
EMPV1_39933	AGCTGGAATTGTCAAAACTGGACAAGGCGGGTTCTCACCTCCGGCTGCTGATGGATGCGT
EMPV1_39934	TAGAACCAGGCACTGCCGAGCGTGGAGCCACAGTGGTGGTGTGTCTCTGGTTTCCCAGTT
EMPV1_39936	CCTAAGGAGTACATATCACTAGCTGTTTCACAGCTCACAGAAAGTATGTATTCAGGAGCC
EMPV1_39937	GGGGGGGTCCATTCAGAAATCACCAAACATAAAGGAAAACAAGTCAGAAAAACTACAACC
EMPV1_39939	TGTCTCTCATCAATTCTGAGTCCCCAAAGGTGAAATAAACCACATCTCGCTCAGCTGCAG

EMPV1_39941	TAATCAGAAGGAAGAGGAAAGAAGGACTTGTCGACTTTGGTATTTTGCTGTTAGGTGGCC
EMPV1_39942	TGTGTAGGGGAGAGGTATGTGGTCAGGCCACTCAAGGTGGCTATTCCCTTGCTCCCGGTA
EMPV1_39943	CAAATCACCTAACCTCTCGAGTCCGTTTCCTCCTCCCTAAACGCAAAGGAGAAGCCTTTC
EMPV1_39944	GAGAGGTCCAAGTGCTTGAGGTTCAGCATAGGGTGGCACGAAATCTTCTCCAACTTGTTG
EMPV1_39947	GATCATCTTCAAAGGGCACGAGAATGTGGAGGCCGCGCAGGCAGAGTACATTGAGAAGTT
EMPV1_39950	GGCCTCCTGCTTTGGAAATGCCACAGCGATTGGGAAACCGAGTGCTTCTGGTTAAGAATT
EMPV1_39951	GGCCAAGCGTAGCCCCAAGCATCACCCACAGAGGGCTCGGAAGAAGATAGAAGGGCTCGT
EMPV1_39953	CAGTAGCAGCCAGCATAGAGCAGCTTCTGGAGAGGCAGTGGAGTGAAGGACAACAGTTTT
EMPV1_39954	CCGAGCCCCTGGAGCAGGGACCACGAGAGGTTTTTCTATTACATTTTTCTACTCTAAGCT
EMPV1_39955	ACTTCCCAAATCCTACTGGCGCAGCCCCCCCAAGCGCCAGGTCGATCGTGAGGTAAAAGT
EMPV1_39956	TTCTCCTGCCCCTCCACATCACACACTATGTGGCAAATTCAAGTTTATGAAAAGGACCGG
EMPV1_39957	AATAAATAACAGTGCTTGCTTCCAAGATGGAGAGTTAGGTGGTTGGGGTGGACAGAAAGG
EMPV1_39961	AGAGCAGCTCCCCTCCAAGGCTCCCCACTTTGTGCCTTTAGTAAACACTGTGCTTTGTAT
EMPV1_39962	AAGGAGAAGCGTGAGACGGAATTCAAGGGGAGAAGGTGTCTCTTCCTCACATGGCATCTT
EMPV1_39963	CAGTAATGTAATTTGTAATCTGGCCTTTATCATCTCTTTGCCTTCCCCTGAATTAGCATA
EMPV1_39965	CGTTTCCAGGTGAAGATTGGGTACAATGCGGAGCTCATTGCAGTGTTTAAGAGTCTGCCC
EMPV1_39967	AGACGCAGTGAGTTCACACAATATAGCAAACATGCCCCATCACCAGGGACGACCGCCCTT
EMPV1_39968	GAGTCAGAATGTCCCTGCATAGTTTGTGCTTATTAAGTTTGCTGTTATGTTTCAGGAGCC
EMPV1_39970	ACAGTGATTCCATCTTGACAGCCTCTGGCTGGGAGGTGTGATGGCTGAGAGGGAGGACAG
EMPV1_39972	GCACCTATTAGAATGCTACCCGCTGTGTTGGCACTAGAAGAGGCGGATGATGTATTAGGG
EMPV1_39975	TCCCCGCTTCCTGATCTGCTCTTTGTTCCCACAATGTCTCTTCTTCGTTCTCCCAGGACA
EMPV1_39977	GGGTGTCTTGGTTGGCTGTGTATGTATCTCCCAGGAGGTGGGACACTGGCTAATAAAAAC
EMPV1_39979	CCTGGGGTTGGTTGTTGTGAGAATTAAATGGATACAAATTGCCAGGCACACCAGAGGTGC
EMPV1_39983	TGATAGCCAAGTGGGACTACACGGCCCAGCAGGACCAGGAGCTGGACATCAAGAAGAACG
EMPV1_39987	ATCAAGGACAAAAGGCCAGGTTGCAGCCCCTTTGGAGAGAATGGTACAGGACTGGCTGGA
EMPV1_39989	TTTCCACCTGTGGGTCTCACCTTTCAGTGGTTTCCTTGTTCTATGGCACAGGCCTTGGAG
EMPV1_39990	GACATGCACGAGATCCTGAGAAACCTCCAGTCTGAAGTCACTTTTCCAAGGTAACAGCCC
EMPV1_39992	CTCAGTTCAGAGTTGCCACATTTTGAGTGCTCAGTAGCTGCATCTGTTTAGTGTCCACCG
EMPV1_39993	CAGCGATGATGGAAAAAGACACCCAAACACCTCTTTCTGTCCGAGTGCTTGCTGTGACGT
EMPV1_39996	GGAGCCCCTGTGGATTCTGGGGGATGTCTTCCTCAAGGAGTATTACTCTGTCTACGACAT
EMPV1_39998	TTGGACTCTGAAAACCGGGCCTTGACAGTTGGTGTAGAAATGATGCTCACCTCGTGCTGA
EMPV1_40001	CCTAAGGGGATCTGTGGGACATCAGTGAAAATCGCCATTGATACGGGGTACCGGCACATT
EMPV1_40004	TGACTGTCTTAGCGGATGCCTTCTATGGAGTCATAAACTTCGTGCTTACCCTGGCGTCTT
EMPV1_40005	CTCTCCTGCCCCCTATCGAAGAATTTCAAGCCAGTGAAAGATGACTCAGACCCAGAGCTC

EMPV1_40006	GGATCTCTTGTGGGTGTGTATTTACTTGCTCCATCAACTCACTTTGCAGAGCGGGGAAGC
EMPV1_40008	GTCTCGGGCTGGCTTTCATTATCATCAGAGGGTTCATGCAGGATTGAGGCCATATGAGTG
EMPV1_40009	GAACACGAGCAAGGTCCTGGGAGCCAGGTGAGGTTCAGGGTTACATAAGTGTAGTTAACA
EMPV1_40010	GTGGCTCTCAGTGTTCGGTGGGATAATATTGAAAGAGTTCACAAACTGGCTGCAGCTGGC
EMPV1_40012	AGGGATATCACATACTGACAGTGCCCAGGACCGCAACAGAGACTTGACAGCCTTAAGTGT
EMPV1_40013	TTCTCAGTTGTAAATAGTTGTGTGTTTTTGTTTGACTGGTGGGTGGCGGGAGGCGGGGAG
EMPV1_40017	TAGACACTTTCTCTGAAGATTTGAAAGGGCAGAACAGGAAGATGAGTGTGCTGCAGGAGG
EMPV1_40019	CTTCCCCTCGGATGCAGATGGGAAAGCGATGGCAATCGCCAATGACCAAAAGTGGTAGAC
EMPV1_40020	TTTCTCCTCTGTGTTCCTGACTCCAACAGTCCATGGCTCAAGGTCAACATGGCCTCTGTC
EMPV1_40021	GAGACCAAAGGAAATAGAACTAACAATCACAGAAACCCTGTGGGACCATGTGCTAATGGC
EMPV1_40022	CTACAGTGACGAAGTACGGAATGAGCTCCTAGGGGATGATGGGAATTCCTCAGAGAACCA
EMPV1_40025	GCATGGTCCTTCTCTTCGTACACGATGGTGCTCAGTTTTGCCTCCGTCAGTTCACACTGT
EMPV1_40027	GTTGCTGTGACTGGGAAGTTGACCCCTGTGCTTTTTACAGCTTGTTTCTCCTCCCACTAC
EMPV1_40028	GGCAGGCACCTTGGGGCTGGTGGAATTCAAGACGCTCTGGTGGAAGATTGAGAAGTATCT
EMPV1_40029	TGTCGCTTGGATCCTGCTTTGTCCCCATTCCTTTCCATTCGGCCTGACCTTCTTTTCCTG
EMPV1_40030	TGAGCCATTTGATTCTTTTGGGGAGGAACCACGTAACTTTTTGAGGACAGGTGCTGTGGC
EMPV1_40034	AGAGAGGAGTTCTTCCAGGCAGCTGTCCAGATTTGGAGACTGTACTTAAAGCCAAATACT
EMPV1_40035	CCCAGCCACCAACTCCTGTCTGGTTTCATGTCGTTTTCTATCTGGCAGTAGGGTCTGTAT
EMPV1_40036	CCACAGTAAGTGGTAGAACTGAAATTCAAACCAAGGTCCATCTGAGTCAAAGTGCAATGG
EMPV1_40037	ATAACGTTCTTTCAGTCCTGTTCCTTGGGGTAGGCCAGCCTTGAAGCCACCGTGCAGTCT
EMPV1_40038	CACGAAGCAGAAAACTGAGATATGGGAAATTAGGAACCGTCGACCTAAGTGAGAGGGAGG
EMPV1_40039	CAAGGTGGAACAATCCAGATTTCTAACCCAGGATCTGATGGTGTTCAGGGACTGCAGGCA
EMPV1_40042	TATTGGACAAGACCACAAACAAATGCAAAGGCTATGGCTTTGTGGACTTTGACAGTCCTT
EMPV1_40043	ACCCAGCATTATCTCACAGTAACCAAATTTCCAATTCTTCAGCTGATGGTTGAAAAAGAA
EMPV1_40044	CCTGGTCAAGTCTATCAGCCTAACCTACCTGGCTACACTGATCTCTTCTGAGTCTTAACC
EMPV1_40045	TCGGCCTCTGGTGAGGACAACACCCATCACTAAACATGTCCCCTTTTGTAACTGAGCTGT
EMPV1_40046	AAGTTCTTCTTCCAGATGTCCCAGCCCATCCCCTCCTATCTCATCGCTTTGGCCATCGGA
EMPV1_40048	GTCTCAGACAATGTTTGGGACATGCTTATGATAAATATTTCTTGTCTATCTGAAATTCAA
EMPV1_40049	TAAAATGGTCAGTTAGGTGTGGCCATAGGAGACAGAGGAGAAGTTCAGGAAAAAAATGAAG
EMPV1_40050	AGTTCCCTCCCCAAAATTTCATTGGAATGAGAGTAGTGACCCACCCTCAAAGTTCACTGA
EMPV1_40055	CGTTGATACCCCGTTTATAGATGAAGAAGCTGGGGCTTGCTGGAGTTAGCGTCTTGTCAG
EMPV1_40056	CTTCCGACACACAGGCCAGGGCCATCTTGTCCGTACCTGCATCATGGGTATCCCAGCAGC
EMPV1_40057	GGCTTTCACTGACTTCTGCTTGAGGATTTGTAGTCTGTTGCGGTGCTTCAGTGGTTCAAG
EMPV1_40058	GGAGATATTTCTACCGTTACTCAAAACTATCTTGTAGGCCTTGTGAAGCAGGAATGTCCC

EMPV1_40059	GCCAGGTCTGCGTACTCTTGTGCCTAGCACTTAAGAATAAAAGCTAATACTTACCTAGGG
EMPV1_40060	CTGCAGTGGTGTTCCCTTTGTAGATGCTGCAGGGGCTAGAGACGTGGTTCAGGTGAGGAA
EMPV1_40064	GACTAGCAGGTAAGCCTGGCTCAGTATCCTGTCAAATTACGGCTTTTATCCTGGGTCCTG
EMPV1_40065	AACGCCGGGGTACCAACCAGCAGTTTTGCAGGGGTAGGAGATGAGAATAGCTTCCAATTC
EMPV1_40066	TTTTCTCCCATTCCCTGGGGGGAGAGTGTGCAGTTGGTGCTTTCTTTAATTCCCTCGTTG
EMPV1_40068	CACCTCCGTGCTTACTACCAAGGGTGGAGTTAAGGAGAAAGCTGGCTAGTGTAACAACTC
EMPV1_40070	AGGGCGGTCAGTCCCTTCATTAGGGTATTCAGTAGTTTCCAAAGCCCTGTGTCATGTCTC
EMPV1_40071	TCAGTATTTGAGCTGAGTCTCCCTCCTGCCGACCTCCAGCCAGAGCAGAATCAGTATTGG
EMPV1_40075	CTTAAGAGCACATTTTACAATATGGAATAAGCTATTGGAAAGGTCTCGGAAAATGGTAAC
EMPV1_40076	CAGACTGTGGGAATCTTGGCGTGTAGAGTTAGGGAGCTTGAATCCCTCCTTTAGCGCAAT
EMPV1_40078	TACCAAAGTGGGGTATTTGGAGGTGGCGTGTTCTGCCACTCTTCCCTGTGATCTAAGTAG
EMPV1_40079	CACTTGTTCACACAGAAAGACGTATGCAAGAGTCTATAGGCAGGTGTATGGACAGTCACA
EMPV1_40082	GCCTCTTACCTGGTCTCTCTAGCAAATTCAAACATCCTTTACTAGTCTCTGTCCAGGCTC
EMPV1_40083	ATCTAGCCCAGTAAGATGAAACTTGAGAACATCAGAATGGTGCCCCTGGGCCAAGCCCTT
EMPV1_40084	TGTGCTACCTCCTGAACCAGCCCATGCTACCTCTTCAGAGCCTTGTCCAAAGTCACATCT
EMPV1_40085	TGGTTCCTGGGATCAGTGGATGGCTTTATGCTCACACCAGTTACCATGACCTTCCCTTTC
EMPV1_40088	GGGAACTCCATGTGGGGCATTCCTTAGCCACAAATCTAGCCTCATTAAACATCAGAGAAG
EMPV1_40089	TGCTTCAGCTTTCTTGAATGATTGAGAGCAGTGATTTTCCATCTGTTTGCAGGCACCCCT
EMPV1_40092	ACCAGGCAGGTGACCAACATGACCAAGCCTACCACAATTATCAAAGTGAATGGGGACACA
EMPV1_40093	TGGTTCCTAGGAGTTCTGCTGTCACAAAAGTTCCATCAGTGGGGCCTGGGCAGTTTAATG
EMPV1_40094	CTGACCATGCCCACTCCATCTTCTTCCAAGGGACATAACTCTTATACTTCCCAGACTCCC
EMPV1_40095	GGTCAGCCGAGACAGGAATATCGAAATCAAAACTCCAAGAGGACCTGAACGTTAAAATAA
EMPV1_40097	TTCTCTCTTCCAGTCATGTCTGAACTTGTTCCCTCAGCAGGGCTAAAAAGAGCAGCCATG
EMPV1_40099	CCTCTCTTCCCCCAGGCCCAACTTTCTCACTCAAGTCGCCCATCGAACTAACTGTCAGAT
EMPV1_40101	CAGGTAGCCTACATTTAATCATGCAGCGGATGCACGCCAGCTTGAAAAATTGCTGCAGGC
EMPV1_40103	ATTGTGTGTAAGATGTATGTGTACGAGGGTAGGTGGGGTGTGTTGCTGTGGGAGCAGATC
EMPV1_40105	GCCTGCTTACTCCCAAAGTAGAGAAAGTAAATTGAAACTACCCATGGCCACTAGCTGGTC
EMPV1_40107	TTACCTCAGACAACTGCCCTTTTCTTCCCTAGTACCCAGATGGCCTGGAATCTCCCTTGA
EMPV1_40108	TGCTGCTGGCTTTTTGTGGAGTAGCTAAATGGTAAGATGGCCTCCGAGAAGCACAGTTAG
EMPV1_40109	TAAAGTGTCCAACTGTCCTGGTTTGCCTGGGACTGAGGCAGGGCTCAGTTTTAAAACTGG
EMPV1_40110	CAGGTGCACTGTATGTGGCTGGGGTTGCACTGATGGTAGAACACGCCCCATCTAAATGAG
EMPV1_40111	CCGTACAGATTATGCCATCAATAGCAGGAGAGGGGATGAGGAGGAAAGTTTGGAGTGCTC
EMPV1_40112	GATCCTGCAGACCCACCGAGCCGTGCTGGAAATGAAGGTGAACCACAAGGGCTATAATTA
EMPV1_40113	CCTTCTCTGCCCTTCTCTGACCTGCTCTGTGCTTGGAGACTGACCCTATGGATGACAATT

EMPV1_40114 GAAAACAGAGGCATTGGTGGCTTCAAGGGTTAGGAGATATTCATTCCGTGCCTTAATCGA
EMPV1_40115 GACGTGTGCACAGTGTGTTATGACAGGAACTGTTAAAGAATCCAAGCAGATTGCACATTC
EMPV1_40117 CGTCTGTGACCGTTAAGAGCCTGATTTCCTATAAAGAAGCACAGAGGCTTCCTTGGCTGC
EMPV1_40120 AGAAAACAAGAGAAGGAGATTTTTGAGGGACAAACCCTGTTGGTTTTGACAGGCCGGTGA
EMPV1_40122 TTCCTACTCTCGGTGGCTGCTATTTCTGACCTTGTGGGGCGAGTGGCTTCTGGGTGTCTA
EMPV1_40131 AGAGGCCCCTGAATTCTGTACAAAGAGCACAAATGTTGTTAGAGGGTGGGACTGGGGACT
EMPV1_40137 CCCATGATTACTACATTACCTGGCTTATACCTGCTGTCAGTTGGAGTGGTCAAACCGGCC
EMPV1_40140 GGTTTGCTTTCAACTTTGGGAGGTTCTTCCTCTTCCTCGTACTCCTGCTCTGACTCTGCT
EMPV1_40141 AGTGCAAAGGAACAATGCAGGCAGATGCAGCCACCAATCCCAAAGGCAGAGAACATAGGG
EMPV1_40142 GCAGGCTCTCTCTGCCGACCAGCAAAAGATGAATGTGATTTTCCTGAAACGTGTACTGGC
EMPV1_40143 CAAGAGTTCATTGTTTGAGGTTCGTGCTCTTGGCTCCGGGGCTGGGACCGTTCACTAAAT
EMPV1_40147 CCAGTGCAAGACATTAACACAGCACAATCAGGGAATCGGGCGGAAGCAACCATTTCACAA
EMPV1_40150 TACAGAAGTCTGAGTTTTCTCACGCCTGGGGTTCTGGGATTCCGAGAAAGAGCTCCCGGA
EMPV1_40151 TACATGGCACCTCTGTGTCCTGGTCACTCCTGTCACTCATGTCCTGATTCTGTGCATCAG
EMPV1_40152 GGTTCAGAGTTAGCTTCAAGCAGTAGATACCTGACCAAGACAGTGGAATTGGCATGGGAC
EMPV1_40155 ACCAACAACAGGGAGTTCCCATCAGCAGAAGCAAATCCAGCTAGGAACCATGGGGTTGCA
EMPV1_40156 CGGGAACAGGGACCGGATGAAACTGACTGATTTTAACTTCTTGATGGTGTTGGGGAAAGG
EMPV1_40159 CTGAGATCTTCTGGCTTCTCTCTTTGAAAATTGATCATGCTTATCATTATCAAATCATGC
EMPV1_40160 AAGTGCATGTCTGAGGAAGGAGTGAAAACTCCAGAGGGCCACACAGTGGGAAGAAGGCCT
EMPV1_40161 CAGACCAGGAGACAGAGGCACTGCAAGCAGACCGAGCAAAAGCAACAGAGGAGAAGAGCT
EMPV1_40163 TTGATTCTCCAGCACACCGTGGACATCAGGTCGTCTGTGATTGGTTGAGGTGGTTTTCTG
EMPV1_40164 AGGGCACCACGCTGAAGGTGTTCATGATGCGGTCGGGGTACTCCTCCCGGATCTTGCTGA
EMPV1_40166 TGTGGATGAGAAGCCTGACTCCCCCATGTATGTGTATGAGTCCACAGTCCACTGCACCAA
EMPV1_40168 CTCTGATCTCTAAACACCAGTCCATGAAGGAAGCTCCACAGTTGAAATCCTTTTGCCGAG
EMPV1_40169 TGAAAAGAGCTGGGCACATTGCAGATGGTCTGACCCTAGACTGGAGTTGGTTCCTCTTCA
EMPV1_40170 CCGGGCGTGGTAACTGGAGTGTCGAGTCCTTGTCATATGTTACATTGTTCGAAGTCACGT
EMPV1_40172 GTCCCCTTCCTGCCTCTTTCATAGCATATCTTACCACTCTGCTGCTCTGTATCCCAATCT
EMPV1_40173 AAAAGCAAACTCGAGGAGTTCCCGAGTAGCTCCGCGGGTCACGGGGCCAGCATGATCACA
EMPV1_40175 GAAGGAAACCCTATGGGCTCAGCCTTGCAGGCATATGTTAATTTTAAAGCGATGTCAGTC
EMPV1_40177 TTTTCCTCCACCCCCACCTCCACCTCTAGATGATTCCAGTGCTCTCCCATCTGGATCTGG
EMPV1_40179 CCCCTTCTGGTCCCCACGCTTTGGTATAATATATATACTTCTCCATACACAGACCTCTGC
EMPV1_40181 TATGACAAGCGGATCGTCCTCTGGGACATCGGGGTGCCCAACCACGATTATGAATTCCAG
EMPV1_40183 ATGTTTCCTGAAATGTCCATCCACTTATCCAGGCCCAATGGAACGTCTGCCATGCTTCTG
EMPV1_40185 GCACCAAATATAGATGAAAAAGTAGATCTTCATTTTATTGCATTAGTTCACGTAGATGGG

EMPV1_40186	CAAAGTCGGATGACCCACCTCCTGACAAACACCTACTCTTCACTGCGGCAACAGCTAGAA
EMPV1_40187	AGAAAGGAGAACTGGGCTTTCTCCAAGATGCGGAGGTTGAGGTTTTTCCAGACCCATCCT
EMPV1_40188	TCCCTGTGGAAACATCTCAAGAAGATGAAGTGCAATGGCAAAGGATCCCAAAATCCCAGC
EMPV1_40190	CAGTCACAGACATACATTCCAAACCCACAGAGATCCCCTCATGTAGTCCCACATTCTGAG
EMPV1_40191	GGCAATACAGCTAACTGAAGAACTCAAAGCCAGTGATGTACTTGCCAGGTTTCTCAGCCA
EMPV1_40192	AGCAGAAAGCGGAAGAATTGAAAGCAGCTGACCAAGTCTCGTGACAGCCTGGGCGGGGGC
EMPV1_40193	TGATAAGCCCACCGCTGCAGACATGTTCCCAGGCACCCACCGAAGGCCTTCCTTATGCAG
EMPV1_40194	CATCTCAAATTTCCTAGGATATCCTCTTGCTGGTTCTCACACACACTATCTCATTAATGG
EMPV1_40196	TACCGTTCAGTGGCTTCCTGAAGTGACTAAACCGGAAGGAAAGGATTATGCCCTTCATTG
EMPV1_40198	CTCATCAAATTCCTTCTTTTACAGTTAGGTTTTACTGGCATTTAAATCCTCACCCTCAGC
EMPV1_40200	GCAAGTGGCCATGTAAGCCTGTAACCTTCAGTTGCATCGTATGTAAGACCACCTCATGGG
EMPV1_40201	CCTACTCTAGGCCTGGGAGCCCTCATTCTCTCTCCATATTGGGCCTTGATTTATGCTGAG
EMPV1_40203	CGAGAGAGTCAAGTCCCTAAGGTGGAGGTGATTGCGAAAATCCTAGATTTTATTAGAGAG
EMPV1_40206	CACTGCAGAGACAACCTGGGATCCTTGACGAGCTGTGCCACAATAGGAATGCCCTAAGCT
EMPV1_40207	GGTTGTGTATATGACAGTTCAGTTGAGATGTATAGTTACGATTTGGCAGATTTCCTACGT
EMPV1_40208	GATACCAGGATGAGGCTGCCTCTGAGTATTTGCACAGAAATTGAAGATAGAAAACTCAAA
EMPV1_40209	GGAGAAGGAGGGTGTGATGTGCTACTGCCTCTTTCCTTGCTTCCAGGTTTTTCTGGATGT
EMPV1_40210	GGAGCTGACATAAAGTCTGTGAGGATGTGCATTCAATCCCTGGCTTCACTCAGTGGGTTC
EMPV1_40211	TCGTGGACCTTTTGAGAAACAACTCACCCTTGGGGAGGCCGAGGTTCATCCATTGCGGCT
EMPV1_40212	CCGTCCATCTACAGGGAGTACCCAAGGAGTCAAACTGAGCAAAGAGCCTTAAGAAGAAGG
EMPV1_40215	GTGCCAGCGGCCCCTTTTGAAGGAGTTGTGGACCCAGTATGAGTGCTGCCAGATATTGAT
EMPV1_40218	CTCCCTTTAACTTTAACATGGGAGTGCTTTCTGTGAGTTTTAAAGTAAGTGCTTCCATGT
EMPV1_40220	CTATACTATCTCAACGATGGCTACTACGGCGACTTCCGTGTCTTCCTCCGGGATCCTGAA
EMPV1_40223	AAACACTACCATCCTGGCTGAGTTTGCCACGACCTGGTGGACAAACTTCAAGCGAAGGTC
EMPV1_40224	CCTGTGAAGCTAACTGGACCACAGGATGTAGGAAATGATACGAACATCGAGGGGAAATGG
EMPV1_40226	ATGCAGCGTTGTGTCGGCAGGTGACGTGTGTTCTTCGGGTCCTCGTATGAATAAAAGGCT
EMPV1_40227	CAGTGACGAGGAGAAGTCTTGGTACTTGATTCCCTCTGGCATGCTGGGTATTCCTTGATA
EMPV1_40228	AGTTGACCGGGTCTTGATTTTTCCCAAGGCAGGGGGTACCATAATCATTCAGGGAGAAGG
EMPV1_40233	CCTGAGGACGTCAACCTGCGTGTCATCTCGGAATATCAGAAGCTCTTTCCTGACATTCCC
EMPV1_40236	AGGCAAATATGAGTGCAAAGCTGCCAATGAGGTCTCCTCGGCGGACGTCAAACAAGTCAA
EMPV1_40237	AGCTGGGCTCAACTTCTGTTTCTTCCCGCTTGTAATCATAAAGCCCATTTTGGACCAGGA
EMPV1_40238	CCCGACGCCACCACCTGCGCCCGCTCCGGCGCCGCCTTTGTCCGGAGCTTCGCCGCCAAG
EMPV1_40240	TCTGATGCCCGCTTCGTGGCTGCGGTTCTCACCATCGCTTTCAATCCACTCTTCCGGAAT
EMPV1_40241	CTGTGTTTAGCCAAGTAGCCTGGGGGGAACAACTACCCTAAACTCCGTCAGATGTTCTTC

EMPV1_40242   CCTCACAGCTGGCTGCGGTCGTGTGACTCTGGGATGTGAATGAAAATCATCAGAACAACA
EMPV1_40243   AACAATGTCCCTGACCTGGAGCTCAACCCGATCCGATCCAAAATCGTCCGTGCCTTCTTT
EMPV1_40245   GGGGACAATTATGTATCTCCAACCATCATCTTCTGGGTCTATGGATAAGGGGAAAATCTC
EMPV1_40246   ACCTGCTAAGCTCTGAGGTGACACAGCAAATCACCATCCACTGCCTTAACATGACCGTGT
EMPV1_40247   TCTACTGTTTCAGTATCTCTAGGGGTGGAACCCAGGAATCTGCATTTTCAAGCTTCCCGC
EMPV1_40248   ATACTGGCTGCTCTCAGCCGCGCTTAACGCACTTTAGCTCTTAGCTCTCACCAGGGAATT
EMPV1_40250   GACGATGATCCTTATTGTGTGGCCTGTTTTGGAGAACTCTTTGCACCCAAGTGCAGCAGC
EMPV1_40251   AATTGCAAGATTCCATGTCCATATTCAAGTATCTCCGGTACTTCACGCGCACCATCCTGG
EMPV1_40253   GGATCCTCCAGAAGCTGACTCTGAGACAAGGTTTTAAAGTTCAGGTAGTTTACTGGCGAG
EMPV1_40254   GACCCAAGCCCCACAGTGCCTGGTCCAAGGGGATGAGCCCTCGAAGGGAGCTGGGCCCAA
EMPV1_40255   TCGTCTGGAGTTCTTACGGGGTGTGCCCTTTTGCAGGCATGATTGGTTCACTCGCTGAAT
EMPV1_40258   CACATGAATATGTCTGTGGTGGAAAAGCAGGTGAAGCGACACCTTGAATCTACATTCTCC
EMPV1_40260   AAGCTGGGAAGCAGGGATTGGAGATGAACGAGGGTGTTGCGTCTGGATGACGTGTGATGT
EMPV1_40262   AATGTAGAAGGCTTGGTGGGAGGTCTCAGCTGCCAAGGCTCGGTCATTCCATTTGTTGCT
EMPV1_40263   TTCTGTTCAAGCCACAGCAGAACCAATCCCCACCACTTTCCTTGTCCATGCCAGGACCAC
EMPV1_40264   AATTGCTGCTTCTCCGACACCATCCCCGAGGTGCCCTGGTGCTTCTTCCCCATGTCCGTG
EMPV1_40265   TTGGGGCCTCGGTCCCCGACACTCCCGGTGGCACCCGTGTTTCCCATGAATCCTTGCTCT
EMPV1_40266   CTTTGATGGGGATGCTTGCCTCTTCTCGGACGAGTCTGACCATGTTACCAAGGAGCATGT
EMPV1_40267   CTGGCCTGTGTCCCCAAGTCGGCCATCTGTCTGAGCTTGAGGTGAGCAGAGGGCTTGGGA
EMPV1_40268   ATAGAGTTTGCCACCGAGAGCTCTGCCCAGGCCGCCGTGGAGCTGGACAACAGTGTTTTT
EMPV1_40269   ATTTCGACAGCGAGAGGGGAAACTCTACCTGATCGACTTGGCCGGCTCAGAGGACAACCG
EMPV1_40271   CAGAGTTGTAACTTGCTTTCCTGAGAGTTAAAGGATTACCGCCAACACACAGAGCCCCTC
EMPV1_40272   CAGCTGCTCTCAGACATCTTCACATAGTAAATGCCGGGAGTTCCAGGAAACTTCACAGGC
EMPV1_40274   CCCCACAATTTTTGCAACTATTACTGGTCCATGAAACCCAAAAGTCTGGGAACCACTGCC
EMPV1_40275   CCGTGTCGGACCTGTATGCCATGAACTTCAATCCCGTCATCTCCAGAAAGGACATCACAG
EMPV1_40278   TCCGGGAAAGCCCCAATTCTTCCCCATCGACATCAACGTGAGAGCTGTGTAATCTAAGGC
EMPV1_40279   TAATCTGGATTTGAAAGGTCTGGCCGAAGCCACCATGCTAGGGACCCATCCTGCCCTAAA
EMPV1_40280   CTGCAGGTGCTGAATGGGAGGGAGAACTGGCGGGAGCCTTAGCCGGGGGGACGAATCTGA
EMPV1_40282   TCCCACGGTTCCCAGGGTTTTGCACGAAGCTATCGGACTTGAGGCATGAACAGGAATTCA
EMPV1_40285   CCTTACCCAAGTGTTCAGATTGATCCCAGTCCTTTCAGAAAAATGCAACCCCCTTGCTCC
EMPV1_40287   CCATCGACGGCTTAGAAGATCTGGTACATGGAGAACTGTATGGACGGATGGACTTACCAG
EMPV1_40288   GAGCTCTAGTCTATGCTAGACACTCCATAAGGCATATATATACACTATGTCACTTAATCC
EMPV1_40289   CGGCCGTTACTTCTGACCACATCACAAGCTTTGCCCCTCATAAATGCTAACTGCAGGACT
EMPV1_40292   GATCAGTTTCTGACAACTCCAGATGGTGATGAGAAGGACGTAACACAGGAAAACTCTGAG

EMPV1_40294	GGTAGGTGTCCGTAACAGGGGCCAAGAGGACAAATGCTTAGAAATCACTTTCAGGTCTGG
EMPV1_40295	CAACAAAATATAGTTTGTAACTATGGATGGGAGGCTTTTCACCTAGCCTGAGAAAATCAG
EMPV1_40298	AATAAACGGCCACGACTTTTTGAAATACTTGGTAGTGTCGCCTCTTGGGGCACGAGTCCC
EMPV1_40300	AAAGGGATTCCTCTCAGACTTTTGGTTCCCCTTCAGTCAAAGCCAAAGATGACCGGCTGC
EMPV1_40305	CATCTGGCTGCTCAGTTTGGACATACCTCAATTGTTGCTTATCTGATAGCGAAAGGACAG
EMPV1_40306	GTGATGACAGATATGCTGAGAAAGGCCACCATTTGTGTAAGGAAAGGGTCAAACATGATC
EMPV1_40307	GTGTGTGCCTGTGTGGACCGTGTCATCTTCCAGCATGATAGGATTCGACTGTTTTGGTGT
EMPV1_40309	AATATCACGGTCGGTTGAAATGTTGATCCTGGGCCGATTGGTTATTGGCCTCTTCTGCGG
EMPV1_40310	TCTCAGAGGACCGAACCAGCATCCACCCAGCAGGAGATCAAGACAGAAGCTGGAAAAGAA
EMPV1_40311	CTTGCCCTGACTCCGTGGAGAGTGTATCACCTGATTTCCGTCATAATACTTGGGCGTGTT
EMPV1_40312	TGAAGCTCATTCATCACGAGAGACAGCTTAGAAGAATGTTCTCTTGGGGCTTAAAGATGC
EMPV1_40313	AGCTGCACTGGCAAGAGAACGGACCCACCTTTTCTAACACGCCTTACCGCTACCAGGCCT
EMPV1_40314	TAGCTTAGGGGGAGTGCCTTTTCCTAATCGCTAAGCGGAGGCTAAATGCAGCACAGCTTT
EMPV1_40315	GCCCTCAGAAAAGGACCCTCCTATCCATTCTTCATACCGTACTGGGGTGAGGTTTCTAAC
EMPV1_40316	AGGAAGTGACAGAAGAAAGACTGAAAGCTGAGCAGGAAGCATTTGAGAGGAAGATCCGCC
EMPV1_40317	TATGATGGATCTGTGAAGTGTTCCTTGAATATCTCTGCCCAGAGTGCAACAATGAGGCCG
EMPV1_40319	ACCGGTGTCCCCCATTAACATGGAAGACCAGGAGCGCATCAAAGTAGAGCGCAAGCGGCT
EMPV1_40321	GTGGTACCCTGAAAACTGAAAACTACAGCTCCAAGAATGAAATTAGGAAAAGTAGAAATG
EMPV1_40323	CACTCTCTGCCACTATATCCAGAAGAGGATTCTGGAAATAAAACTGGGGTTGGACCTCTC
EMPV1_40324	TGCATTCCGTATGACAGCTATGGTTATTTCATGAGGAACACTTAACAGGACGCTGACTCC
EMPV1_40326	GAGCTGCTGCCTCCATTGCTGGAGCGTGCCACCGACCTGCTCCTGGACCACATTCCGTGA
EMPV1_40331	CCCTCAGTGACATCTTTCTGCTCTTCAAGAGTTCCGATTTCATCACTCGTGACTTCACCC
EMPV1_40332	GTGGAACAGGGGAAAATGGTTGCTGTCTTTTATACTACAGTAATTCCCATGCTGAACCCC
EMPV1_40333	GCTTAATGGACTCTGGAAGTGGTGATGTTAGATGGAATCTGCGGCACGTGAAGAGTTGCC
EMPV1_40337	CCCTGGGAATGACGCTCGGCATCAAACTGCCATAGTTTTGAGCATTTGTGCTTTTTCTGG
EMPV1_40338	GCTGCATGTGCCTTTGAACTTTCCTACTGCCTGTGTCTCTTTCTGGAAACTAGGTGTCAC
EMPV1_40339	TTGAGCACCCCCTGTGGAACCTGCACGCCTTGCGCAACGACATCGCCCTGCTGAAGCTGG
EMPV1_40340	GGGCCTGCTGGCCAATTGAGCTATAATCTCATGGACACATACAGTCATCAGGCACTGAAG
EMPV1_40341	CTCACCTGCTCCTCTCTGGGCTGCAGCTTTATAATCTCTCAGGGAAGCTCCTGAATGATA
EMPV1_40343	AGTAACTGTGCTGTCTGTTTGGCTTTACGGTCCTCCCTCTACAGGCACCATCACACTCTT
EMPV1_40345	TTCCTGACCTTCCTTCGGACAGAGGGAGCCCGCCTGCGGATGAAGACGCTGTTGCAGATG
EMPV1_40346	GTCCACTTACCAGCTGTGGACAAAGGCCAAGGGACTGCGCTAAACCCATGAAACCGTATC
EMPV1_40347	CATGCTGGTGAATGCAGCTGATGACCCCTTGGTGCATGAATGTCTTCTGAGCATTCCAAA
EMPV1_40348	TTTCTGTGGGCTGAAGGCATCCACATTGCCTCCTTCCAGTGTTTTGCAAGTGTGCGAACC

EMPV1_40349	ATGGGCCTGTGTGTGGAGTGTGAAAACCTGGTGCCCATGAATGCCCCCATCTGTGTGGTG
EMPV1_40351	TAGATGATCATGATGGCGGCTGGAGGGAGACGACGGCGGCGCTAGCTTAGCAGGAACCGG
EMPV1_40352	AGCAGTTCCTGTCACGCCTCTTCCTGTTTGTGGCCCTGGTGATCATGTTCTGGCTATTGA
EMPV1_40353	GCTGGGGGATAGAGTGCATGGAGGGTGGAAAATGATGAAGGAGGTGCTTTATATCAATTC
EMPV1_40356	CCATCATGTTGTGTCAAATGGGTTCAGCCTACAGCCAACATGGGTTCTTTTATGAAGCAG
EMPV1_40357	AATCGTTGACACTGCCAACCAGATTCTTTAAAACCATGGTCCGCCAGCGCTTCCCTGGGA
EMPV1_40358	TTCTGTAGTGAATTCCGTCCAGGTTCTTACCTCAAAGGCTGCCATCTTGGAAGCTGCTCC
EMPV1_40363	ACAAGTCGGGTTCATTTCTGCTGAGCCGCAACAGGAACTCCTGTCTGTTTCTTAATCGCC
EMPV1_40364	GCTTCCCGACTACCTGATCTTCACCGGAGCGGACGTCATCTTGACGGTGGAGTTCGAAAT
EMPV1_40365	AGAGGCCTAAAAGCCACAGGTAGAGAGAACCCAGACCTTGGCACCCTCAGATCATTGGAA
EMPV1_40367	GGAGGGAAACCCACCCACACTACTTCCCTGAGCCGGCGACTGATAAGAAACTACTCCTTT
EMPV1_40368	CGGAAAACGACGGTCACCACGACTCCAAAAACAACAACTACCACCACTCAGAAAACGACG
EMPV1_40371	TGGGAGCAGCTAAGCTAAAGGAGCTGAGTCGGAGAACTACCTTCATCCGTGTCACCCAGC
EMPV1_40372	CAGCTGGTATTATCCAAGTTGTTGAGTGAACGTGCGAGGTTGGACTATCTTCTTGCCTTG
EMPV1_40373	CACCGCCCTTGTGACAGTGTTTGCTCACCTCTTCTGTGTCCCCTTTCCTGTCTCATAGTT
EMPV1_40374	GTATGTGGCCCAGGCTAGCTGTCCAGTGTGATGATAAAAGGCCATGGCATAATAACTCCC
EMPV1_40375	AGTGTAAGCATATCCCATACAAGATTTAGGAGATACTTATGCTAAAAATTATTCAGTCAG
EMPV1_40376	CCCTTCAAGTGTCATAGCCAGGGTCCTGTTAACCTGCTCATTAACCCACGCTGTGTTCCT
EMPV1_40380	CAATTTGGCTGATGTGCCTCTGATAGGGAGTAGGCATGGGGTTGCACCAAGGGGAAAGTA
EMPV1_40381	ACAGCGTGGCACGAAAGAAGTGGGGAGAAGATTGAAGGTTTTTAACGTCTTGATTGCCAG
EMPV1_40385	TGTCCATGTCGCCTCGGTCGCCCGGTGTCTGCGCTTGACCATGTTGCACTGTTTGCATGC
EMPV1_40386	CAGATGAACGATGTGATGGCCGAGTTAGCCAAGGAGCACCCGCAAGTTTCCTTTGTGAAG
EMPV1_40387	ATACTACTGAGATGTATGGCAGAGACACCTTTTTCCGGGCTTTGACGAGCATGACACGGT
EMPV1_40389	TCTGAGCAGCGACAAGACAGCATCTCTGATTGGACAGCTCCAGAAGATCGCCAAGAAGGA
EMPV1_40392	GTATTTTTCCGGAGACCCGGTTGTCGGTTTCAGGACACAGGACAATGCTCCGGCCTAAAA
EMPV1_40393	TGTCCCTTACGAGGGCTGGGGGCTTTGGGCTGTGCTGGAAAGCACTCTGTGTAGACAACA
EMPV1_40394	AGGACCTGCGTTGCTGCTATTCCTCGTCTGCTTCCCGATGGGATGTCAAAACTTGAACTT
EMPV1_40395	GAGAAGATATACAGCGACTGTGCCTGGGAGACTGTCAGAGTGGAAATCACAAGACCCTTG
EMPV1_40396	ATGTTGTGCCAATTTCTGCTGTAGAGCAAGTGTGTGTATACACACACACACACACACACA
EMPV1_40397	TGGGATCAGTATCTACAGGAGAAAAAAAGGGAACTTAAAAATGAAATCTGGGAATATTCT
EMPV1_40398	CTAGACCGATGAGAAAGAATCCCAGACCACAGGTCAGGAAGAAGGCATTGGGCAAGTCAT
EMPV1_40401	TATCCACCAGCGTTTTCCCTTAGCTCTCAAGGCTCCACCTTCCTGGGGTTGAGCTGAAAT
EMPV1_40402	AACGACGTCTTGCCCACAGCCGTGTCCCCCAGCAGCGCGATCCGAAACTGGTAGCGGAAG
EMPV1_40406	TCGGCTGAACAGCCTAGCAGGAGTGTTGACGAAGAGCTGAGTAAAAAATCCCAGAAGTGG

EMPV1_40407	GGATAGAGGACCTGTCCCATTCATCTTTGTATACTTTGCCATTCTTAATATTGTATACTG
EMPV1_40408	TTAACGAGGAGCCCTTCCTTGGGCTTCCTTTGTGCCCATGACATTGACAGGTCAGAGGAC
EMPV1_40410	TGACCAAAGAGGGGCCAAATACTCAAACAGTTGGGGCAGAGTGCTCTGAAAGGCAGTTTG
EMPV1_40411	AGCTCTGTGACTTCTTCAGAGGTCCCTGTTCATGCGGCAGTTCTGTGCGCCACACCCCTT
EMPV1_40414	GAGCTGAAGACATGGACGCAGGCGAGAAGGTGAAGGCGCGTACACACAGAGGCAGGAATT
EMPV1_40415	TCCCTAAGGACCTCCCCCCATTTCTCTCATGCAAGCCATTAATCTGGAGACAGAGATGAG
EMPV1_40416	GGCTGCAGTCTTTGCTGTGGCCATCCTTTGCATTTCCAGCCCATTTTTACTCATTGCTTC
EMPV1_40417	GATGAAGTCACCCACCACATTAAACAAAGTCTGAGATGGGGAGTTTCAGATAACCGAATG
EMPV1_40419	TTAGATGTGAGGTCCTCCACTCCGACCTCTTACCAGGCTCCCAAGCACTTTCACATCCCA
EMPV1_40420	ACGGCCTCAGAAGGCAGAGGCCCGTGTCCCGCTGGGCAGCCCCTCCAGACACGCATACGA
EMPV1_40422	TTGAGGCTTTTCCGGCAGAGATCAGGAAATCAACATCTTGGAGATGCTGCGCTGTGTAGG
EMPV1_40426	AGATATCCACTGCTTATGACCAACGTGGTATGTGGGCAACTTGTGGCCTCTGCTTGGGTT
EMPV1_40429	ATTGCCTGAAAGAAAGATCAATAGAAAAAGCATCAGTCATTCCCAGTCTTACTGAATATG
EMPV1_40430	GTGCTCACAGTGCAGGTAGGTTTTGCATTACTCTACTGTAGTGTGGGCACTTCCAGTACT
EMPV1_40433	AATGTTATCTGCATTGATGATTTCTCCAGCACCAGGTATCAAGGGAATGCCCTGTGCACC
EMPV1_40437	AAATCTTGGACAATGTATACAAGCCATGAAGACATTGGGTATGATTTTGAAGATGACCCC
EMPV1_40443	TGAGCTGTGGTGTAGGTAGCACATTGCTCAGATCCCACATTGCTGTGACTGTGGCATAGG
EMPV1_40444	ACATAGAGCAATTCCCATAGGCATCCAGGGTGTCACTCACAGGGCTCGGGACAGACAACA
EMPV1_40445	CATGAATGTCAACACCTGTATTCACGGAATCCTAGCCATCTGGATCAGTGGGGTCGTCTC
EMPV1_40447	TGGGAAAAGGTGGCGCCAAGCGCCACCGCAAGGTGCTGCGAGACAACATCCAGGGCATCA
EMPV1_40448	GCTGGATCCACACTAGTGGTGTCTCTTACCACGATAGTAGTGTCCTATGTGTCTATCCTG
EMPV1_40450	GGCGGGTACCACGTGTACCGGTTGGAAAGCACCCCCCAGTGTAATCTGAGATATTGCACA
EMPV1_40451	GCAACGTTCACCAGCCACCAGCGGAAGGAAGAGCAGAAATGAAAGTAGCTTCTGCAATTG
EMPV1_40452	CTCGTCCTACACCTCGCCGTGAAGACTCTAGGCGAACTGAAGGAGCTCTTTACCCTGGAT
EMPV1_40453	GTGGGGAGGGTCTTTTCTGCCCAAACTACAAACTGGAAATGCTGCTGCCCCAAAGCACAA
EMPV1_40454	GGTAGTGTGAAAGATTTTGGCATTCAGCCAGTGGAAGATAAAGGAGCATGTCGCCTCCCG
EMPV1_40455	GGAAACCCTTCCGTTGGAATTCTATGTTGCTTAATACATCCTATGGTATTTCGTCTGTTC
EMPV1_40458	CTTTGTCGAAGTCTGTAAGCGGGAAGGAAGTGACCCCGAGAAGGGAGTGGACCGGCTCCT
EMPV1_40461	GAGAGTACAGTTCCATCTACCTACATGTTTCTTCTTGTCATCCCGATAGCTCTCCTTGGC
EMPV1_40462	GGATCCAAATTACATCTGTGACCTACACCACATCTCATGGCAACGCGGGATCCTCAAGTC
EMPV1_40464	AATCCGAGGCCAGGCAATTAGGCAAAGTGTTGTCTCCCCACACGGAGCTAGAAGTCAAGA
EMPV1_40466	GTGGGCTTCCATACGTGGAGGTGCTCTGTAAAAATCGCTACGTGGGAAGAACCTTCATTC
EMPV1_40467	GGGGTTGAGGGTAAAATCAGGTGTAGGGAAAGTTAGTTCACTTGTGAGGACTCAGAGATT
EMPV1_40470	GTATTGCTACTGTTTCAGTGCAAAAATGTACTAGCCAATACGCTTAAGTGTGTGGCCCAC

EMPV1_40471	TTCCTGCTCTCCCACCCACGCCTCAGCAACAGCCACCCTTGAATAATCACATGATCTCAC
EMPV1_40472	GCCATTCCAGTCAGCAAACAGCTGGAAGGACCGCATTCTCCCAACAAGAAAAGACACAAA
EMPV1_40476	GTTGTTGCCACCGTTCGCGTAATACCTCTCCAACCCTTTCGATGCTTCTACTTCTTCACG
EMPV1_40477	CAGCCGTCACCATATTCTACGTAGCTCTTTTCTTCATGTACCTCAGGCCTCCTTCAGAGG
EMPV1_40478	CTCCAGCATTTGGAGAGTATGCCTGTGCATGAAAGGTAGAAATCTTGCCATATCAGGGGC
EMPV1_40479	GGAGTTCAATGCGAGTTCTTGGGGATGTTGTCAGGAGACCTCCCATTCATAGGAGAAGTT
EMPV1_40483	CTTTCTGCACTCTGGTCCCTTCTACTGCTGCTCTGGTTCCTTGTTGGGGGAGCCTTTCCA
EMPV1_40484	CCCCGGTAAAGTGTTACCTTGGGTAATGGTTCGTGTGCGTCCTGCTGAAAAGAAATTGAG
EMPV1_40485	GCAGCTAACCCAGTGTACCTTAGTGTGCATGTTGGTGCTGAAGTCTGTTCTCACTGAACC
EMPV1_40488	AGATGGTGGAGATCTCGGAGGACTGCTCCTCACTCCATGACCGCTTCCACTTCAGTGAGA
EMPV1_40489	TCTGTGACCTCACTGTTCTCTTGAAGTTGAGCTGTTCAGACACCTCCCTCAATGATCTGG
EMPV1_40496	CATTGTGGCTGTGGACTGGAAGCACCCGAGGATCACATCTTTCGTCATACCCGAGCCCTC
EMPV1_40497	AAGATCAGAATGTAGACTGTGCGGTTCCAGGGGAGAATCAATACCTGTCAGAGGGGGGAA
EMPV1_40500	AACTCCGCGGTGGGATTCCAGAACTTCCCGGAGGTCGTGGCTCCTAGAAAGGCCATGGGT
EMPV1_40501	TGCCCTGCACCACCTCCGGTTCCCGGCTACAGGCTTTCCAGGGGGAGGTTAGAAATAGAT
EMPV1_40507	TCTCTAGAATGCTGTCTCCACTCTGGGGGCAGGAGAACGCCTGGAAAAAGACAGAAGGAT
EMPV1_40508	GACAGTTTTGTCACCAGACAGGCAAGTGAAAACCGTTCACGAGAGACTATGGCACACGCT
EMPV1_40509	GGACAGCTTTGAAAATTCAGGGACTAAAGAAAGAGCTTTACTTAGAAGACTGGAACTGGG
EMPV1_40512	CTAGGCTAAGGAACGGCTAGGTATTGGAATTAGTGCCAAGGAAGGGTGTTTCCCAAGAGG
EMPV1_40514	ATTCCAGGTCTGGAGGAACGTGAGAAATGGAACTGTCAGCGCAAAGGTGGGGAGGAGACT
EMPV1_40516	TAATCCAGACCTCGACTATCTGCGCAAGCTCTTTACTGACCTTCTTGGTCACCCTGCGTC
EMPV1_40517	AGCACGGTAGACATGACTGGAGTCCCGACCTTGGCTAACCTTCAGAAAGGAGTCCAGTTT
EMPV1_40518	CTTGACTTCTTCCTGAACCTAGATTGGCATGGGAAATCCCTTCCTGATAACACAAGACCC
EMPV1_40521	CGACATCTACGACAAGGTGTCAGGCGAGATGCAGAAGAAGGGCATCGACTGCGAGTGCCT
EMPV1_40522	GAAGATGCTTTGGCAATGTTTAAGGTTCTTACGCAGAGATGCACACATCATCAACGATCG
EMPV1_40525	CTGGGCCAAGGATCAAACCAGCGCCACTGCAAATGGTGACAATGCCTGGTCCTTAACCCC
EMPV1_40526	CGGGGACAGCAGAGTTTATTACGCAGGCAGTTGTCCCTCTATCCTTACTTCTCTATGGGA
EMPV1_40527	ATCTGATGAATTGCCTTCTTGCACTGGGCTATACTGGAGGCCTGTGGGTGGTAAGAAGAC
EMPV1_40529	GATGGTGGCAGTGACCTCCTGTTGATCGATGATCAGGGTGTACCCTACACAGTCTCTGAA
EMPV1_40530	CGGGTCCACTGGGATCGTGTCTTCTTTTCTCAGGTGTCAGGTATCAGCTCTTATGTGTGA
EMPV1_40531	GCTTCTCTGGGTGGGGGCCAAGCCTGCACAGCTGATGCTGGGCTTTATGGGCGTCACGGC
EMPV1_40533	CGATGGCTGGAATGATAGCAAATGTACGGTAGAAAACGCCTGGATCTGTGAGAAGCCCTC
EMPV1_40536	CCCTGGAGTTTCCGGTTCAGGTTTGCAATGGGGGCCTCAGAATTACACCTGCGTCAGCTT
EMPV1_40537	GGTTCTTGGAGGGTGAGTAGGAGAAAGTTGGAGAATGGGCAGCCTAAAGGATAATTGAAC

EMPV1_40538	ATCACGGGGAGAGGGGCCTTAGTGATCCTAGACTGTTTCTGTTTGGTCCCCACTGATCAA
EMPV1_40540	ATTGCTGTGGTCCTAGGTGTCATTTGGGGAGTGCTACCCTTGCGAGGTTTCTTGGGAATA
EMPV1_40541	TGTGCTCAGCTCTGTTTTCTTCTTTCTCTCATGTCCTCTTTGTCGTTCCTCCCGCTGCCC
EMPV1_40542	CATGAATGTCAACACCTGTATTCACGGAATCCTAGCCATCTGGATCAGTGGGGTCGTCTC
EMPV1_40543	TGTATCTAGGTTTAGCAGTTGTAGGGCTACGGCTTTCCTCGGGTTTTGTACCAGTGTCCC
EMPV1_40548	CAAGCAGGCAACCGGAAAGATGTTCTCTGTAGAAGGGCCATAACCTGAAGCAAAGTCACT
EMPV1_40550	AAGCTCTCACAGCCTTCACTTCCCATAGAGGATTCCCAGGATCTGTATAAGGCCTGCCCA
EMPV1_40552	CGGGAAACAGCAAATCCAACAGATCGGACTTCATCAGGCTGGGGTCTCGCTTCAGGTTCA
EMPV1_40554	ATAGGAACATTATACACAAACCTGCAGAGTGGACCTTAATTGCTTTGGTGTTGCTCTCAT
EMPV1_40555	CACAGAGTGGTGAGAAATTCAGGTACGACAACATGAGGACACAAGTGTTTGTCCGGCCTG
EMPV1_40556	AGGCTGTGCCCCACATCACAGATGAGGAGACTGAGGCTTAGCAAAGGCGAGTAAGTCCCT
EMPV1_40557	AGTCCTGAACATTGCCTGACCACGGATTGGGTACATCTCTGGTACATATGGTTGCTGGTG
EMPV1_40558	AGAGGAGATGAACCTCGTGGAGATCCTGCCCCCTGCAGGCCAGGACAACAGGAAGAGAAA
EMPV1_40561	GGAAACACTATATTTGCCAGCTCACCTAGACCCTGGATAAGCAGGGAGCTTGAGAGCCAA
EMPV1_40562	TCCAACGCATTAACTAAGGAGGCCACCTGGTCCACCTATGGCCCCAGCAAACATCAGAAT
EMPV1_40565	GTGGACCTATCTGGAACCTCTGTACAGCATGATGCTCCAGCAATTGGCCCTGCTTCAGCA
EMPV1_40570	CGCTTCATGCCTTACGTGGGAAGGACAGCAATTCCAGGGAAAAACTGCCATTGTGGAGAA
EMPV1_40571	CTACCACTGGCTGACTGTCATCGCCGTGCCAGTCTTCTACAACTGGTGTCTGCTTGTGTG
EMPV1_40572	CACCCATTGAGAGGACTGCATCTCCTAACCTGTCAAAAGAATCTGTATCATCCTTCCTCC
EMPV1_40573	CCCCACCACTGGCTCTACGTTCTAGCTGATTACAATTATCTGTCCCAAACTTGAGTGGAG
EMPV1_40577	CATGTCTTCCAGCAAATCTGTGTCTTCCAAATAATCCAGGTCTTCCGGACAATTCGGGTC
EMPV1_40578	GCAGGGCTACATAAGCCTGACTCTGACTCCTTCCCAGGTCTACCAATGGTCATCAACAAA
EMPV1_40579	TGCCTTTTCTATCCAATTCTGGCCCTGGGAGAAGGAAGCCATTCGCGATGCTGGTTTGTC
EMPV1_40580	GGCGCTGGAGAAGGAAAAGAAGTCTAAGAAGTCAAACTGCAAGTTTAATTTGGACCTTAC
EMPV1_40581	CACCCTGCCTTTTAATGGGGAGTGAGGACCACACCTGTCTTTTGCAAGTGGATGAGCCCA
EMPV1_40582	CCCATGTCTATCTTGCTTGCCCAGACCTCTCTAGTCCTTCTCATGCACTCTGTTACCACT
EMPV1_40583	AGTACTTCTACACTGCCTTGTCCCGACCTGCAATCAGCGAGCCATGGTATATCACTGTGG
EMPV1_40584	CGGCCTTTGTTCTTGAAATTTTTGAGCCGTTGGTTGTCCAGTTTCTCGCTGTCCGCCATG
EMPV1_40585	ATCCAATCTTCGATCCTCCCCGTCTGGCCCTGGAAGCTGCCCTCACATTCCAACGATGAA
EMPV1_40586	GGGCTCTGCATAAGTAATTCACTCTACTCTTCCATGATTCAGAGGGTACAATTAACTCTG
EMPV1_40587	CCAGGCTGTGGTGGCCAAAAAGAAGAACAAGATCACCCACGTGCAGTTCAACCCCATCCA
EMPV1_40588	ACTCCATGTGGACTGTGCCCCAATTTCCAGTGGAGATGCTGTTACCTTTGACGGCGACCA
EMPV1_40590	GAGATTGTGATGTTCACTTTTATTGGCTGTGTTGTGGGCTGTAGCATCGTCACTGTCCTC
EMPV1_40591	ACTACTGGGAAATTCAAACTTTCCCGAGTGCTGAAGTAGCAAAGCAACAGAAAGAGTGAG

EMPV1_40592	TGATCACCCAGATCCGCCTGGAGCACGACGTCAACATCCAGTTTCCTGATAAGGACGATG
EMPV1_40593	CTTGGGATTTCCATGACCCCCCTCTCTGGACCTGGTATGTAGACCTCATTCCTATAGCTG
EMPV1_40594	GTAGCTTCTGCAGCCACAGCCATAGCCCAATGTGAGGAAGATGCCATATCCACAGCCATG
EMPV1_40596	ATGAGAAGGGAAAATCTGGGAAGTGATGTGGTGGGGTGTGTTGTGCTGGCAGTTGGTGAC
EMPV1_40597	CCTCGTTCTGTCTTGCTTTATGGACCCCACCTGAGGCGAGCGTCGGTCGCCCGCCCTCGA
EMPV1_40598	GTCCTCTGACCTGTCAATGTCATGGGCACAAAGGAAGCCCAAGGAAGGGCTCCTCGTTAA
EMPV1_40599	AGTGTACCTTACTGTTTGGCAACCGCCCCCACCCTGCCCCTTTTCAGAATCTTCAACCAT
EMPV1_40600	CCAGAACGTCTACACGGGCGACATCATCAGTGGCCTCCACGTTACAACCAACTTCACGGT
EMPV1_40602	TAAATTCCAAGAGCCCCAGCCCCAAAGGAACCCCAAGTCCTCAAGAATGAAAGGTGCCAG
EMPV1_40604	GCCAGTTACCATGCATAGATCCGCAAAAGAGTTGGATTTCCACGTCAGCTCCCACTGTAG
EMPV1_40605	GTCTCATCTTCCCCAAAGAAGGATGTGGTAGCCTCAGTAATGTACACTGTGGTCACACCT
EMPV1_40606	GGTTTGCTTCAAGTGTTTAAGTCTAAACATTTCTTTTTTGTTATGTAACAGGTACATCCC
EMPV1_40607	GGACCGGCTCCTGGGCCTGGGCAGCTCCAGTGGCTCTGCAGGGCGAGTGGCGCTGTCACG
EMPV1_40611	AGCGGTTTGAACCTTAATCACACAACTCATAACCCCATCCCTAGTAGTCAGGGCCCCGCT
EMPV1_40615	TCTCATGTCTCCCAGCTTGCCTGAGCCTGCGTTAGTGAGATGCAGGTTTGGTCCAAGTCA
EMPV1_40616	GCTGTCAATCATGCGACATTTGCAGCACAAACAAAGTGTTGGAGTGTGGGGTGGCACGTT
EMPV1_40617	CAATTGGTTATATCACTACTCCTGGGCCTGTTTGCCAGTTAATGTGGTCTGGAGTCTCTC
EMPV1_40619	CGCTGGAGACTCTGATATATGCCAAAACTTGAGAATGCGTGTTCTGCAGGAAGAGCTGGC
EMPV1_40621	TCAGTGAGGACTTGGGGAATGCTACACAACCTAGAACTTGGCTCTCTTGAACTGAGTGGC
EMPV1_40622	GACTCCTCATTATTCTCCTTGGTGGGTGAGTGTGGCCTGAAGGGGCTTGGTCTCAGATTT
EMPV1_40624	TACAAAAGTCACAGGGGTGAAGGGACAGGGCAAAAGTTGAGCCAGATAGCAGCATTCAGC
EMPV1_40629	GCAAGTCAGAGATCGTTGTACAATTCCCCTGCAGTGACCGATTCAAGCCATCTTACGTCC
EMPV1_40632	CCTGTTGCTCAAACACCGTTGGACTAAACCGTTCAGTTAATAGATATTTTTGCATCCACA
EMPV1_40633	CCCTCCAAAGGCAGTATTCACTCATCTCTGGCCCCATCCTCACCCCCACACTCACACTCA
EMPV1_40635	TGACCTTGTACCACTTACAACAGACGTCAGCTCAGGCCACCACGCCAGTATCTGCCTCCT
EMPV1_40636	AAAAGAGAGAAGAGGAAAAGAGGAGATCAGAATACAAGTCATACCGGCTGCGAGCCCAGC
EMPV1_40637	TCAACTTCTGAAGGTTCATCTTCCAACCCTGCGTTCTCTGCTGAGTGTCTGCATTTAGCA
EMPV1_40638	GCTGGGCATCTCGTTGATGACCCTATTCCTTTTTGTCATCATTTTGGCAGTCTCTAGTGC
EMPV1_40639	GAAGGAACAGGGTGGGCCGAGAGGCAGGAAATTGTCCCTTTATCCTCTTCCGGGGCCTCT
EMPV1_40640	TCAAGATGCCAGAGAAAGATGGGGGAGGTGAAGCAGATATGAGCAAAGCAGTCAAGCCAG
EMPV1_40641	GCCCATTGAGAAGGACCAGAAGCAATAACATCCCAACAGCAACAAGCACACTCAACACCC
EMPV1_40642	TCGTTGCTGGATCGCAGTTATCGCTGACCTGGACATGGAGTCAAGGGCCCAGGAGGAAAA
EMPV1_40645	TGGTCATCTTCATCCTCCTGACCTTCCTCTACGCCTACTGGACCACGTGAGCCTGAGATT
EMPV1_40647	TCCTGCAGTAGGTTAGGGCCCATGTTAGGGACTGGCACATCTTGAGACTGGTTTAGGATA

EMPV1_40649 CCCCCGGACTGCCACTATGTCAAGGTTGTCAGGAATGTTTTGCAGTTCTAATGCCAGAAG
EMPV1_40651 TTGGTTCTGAAGCTCTGTGGAGAAGGCAGGTGGGAGCTGACCAGAATCAAAGTGGAATGA
EMPV1_40652 CAGACACGACGACTTCGCTCGGAGGCATATCGGACCCGGGGACAAAGACCAGAGGGAGAT
EMPV1_40653 CATGATCTCCACGCTGCTGCGCGAGACCCCCAGCTTCGGCGTCTACTTCCTCACCTACGA
EMPV1_40654 TTCTGGAGTTTTGCAAGTCCAAGGACATCGTTCTAGTTGCCTATAGTGCACTGGGATCCC
EMPV1_40655 CAAACAGTGTGGTCTTGGTGGTTTGGGGCTAGAGAGCACTGACAAAATACTATTTAAGCT
EMPV1_40656 TGGTTCTGACGTCCAAGCAGCAGAGTCTGGAAACCGCCATGTCCTTCGTGGCCAGAAACA
EMPV1_40657 GGCTTCGGTGCTGGGGAGCGGCCCCCGGGCGGGCCTCTGCTCTGGCCCCTCCTGGGGCCC
EMPV1_40658 CCCCCATTTTATTCCTGACTCCTGGGAAAATGCAGAGAACTGTATTGAGAGCTAGGCGAG
EMPV1_40660 GGACATACTCAGCAGAGAAAGGCTGGGATGACAGTTTCTGGAAGGTGATTGGTTTGGAAG
EMPV1_40661 GATTCTAGTCTCTCCTGTTCACGGTTTCCTGTTACACAGTACCTAGCTCGTAGTAGACAC
EMPV1_40662 GCGTCGAGAGCGAGAACGCATTAGAATAATTCGTGAACGGGAAGAACGGGAACGCTTACA
EMPV1_40663 TGCCAAACTTAGTATTTGGAACTTTAACTCTGAGTTCCTTTCTATAGTTCCCTTTGTAAC
EMPV1_40666 TGGTGTACACAATTTAAATAGTCTCCCGTACATATATGTTCACACTGGGATTTGGTTTAC
EMPV1_40667 TTACTCAATGTCTCAATCCGTAGGACAGAAAAGGGCACCCATTAACCTCTGCTCCTGGCC
EMPV1_40671 CTCACCCACCCCAGCAGAAGTACCTTCACAAGACACCCTGCTGCTTTGTTTGCACACTAA
EMPV1_40672 ATACACACGATTTGAGAAGATTGGACAAGGTGCTTCGGGCACTGTGTACACTGCAATGGA
EMPV1_40675 GGAGAAAGGACTTGTTTGGCTGGGGGTTGTCTCTAGTGAGAGTCACAAGGTTTAGAACCT
EMPV1_40676 GCAAAGTGGACAGCCCCGTTCTTTGGTAAACTGGGAATGTAGAGCTAGCCAACGTATCAC
EMPV1_40680 TGACAAGGTGACAGAAGCATCTTCAAACAATTTTGTTGCTATTAAAATCGATACCAAAAG
EMPV1_40681 TGCAGCTGAAGATCCGTGAAGTGCGCCAAAAGATCATGCAGGCGGCCACTCCCACAGAAC
EMPV1_40683 CTCCAAAGGCAAGTCCAAGCCATCCAAGCAGGTCCAGAAGGAGCAGAAGGAACAGCGGAA
EMPV1_40684 TTGACCACATTGTGGACTGCATCGTGGACTTCCAGCAGAAGCAGGGTCTGAGTGGGCACA
EMPV1_40685 GGCCTGAGGCCAGTTCTAGTGATGAAAAGATGAACTGGGCTTTTCTGACTCCATGTTGTC
EMPV1_40686 TTTAAATAATACAAAAATAATACAAACAGCCATTTGTTTCAACACTTAATTTTACATAAG
EMPV1_40689 ACAGTGCTGGGTAAGTCCTGACTGAAAGTAGCACAGCCAAACCTGTGAGGGAAGAGAAGT
EMPV1_40690 TGACCCGAGGTCACAACCAGCAGAGCTCCGGCTGTACTGGGAGTTTCGATGAGTCCATAA
EMPV1_40691 GGATTAGACCCAGACCTAGACTCCTGGAGCAGGTCCCGAACCAATGATTTGGCAGATTTT
EMPV1_40692 GGGATAGCCTATGGAAAGAAGGGCCTACATGACGTTAAGAATGCTGAGCTTGCTCTGTTC
EMPV1_40693 TGATCAGAAGTTTTGGGTAGTTATGGAGTTGAGATAAGAGATCTCATTGGCTAAGCTGGG
EMPV1_40695 GTTACCTAGCTTTCCTGGGCTGTTGAAGGATTTGAATTTCTGTGTGTCTACTTGCTGCAG
EMPV1_40698 GTTGTCACTGCTACTTGGATGGGGACCTGAAAGGAATTTTTATAGCTCCTCCTGGGACCC
EMPV1_40702 GCCTTTTCCCTGCCCTATAATATTTCTCTCATTCCCCCTCTCCTAAGAGCACTCCCTCAA
EMPV1_40703 AGGTATTTCCTCAGAGCACATCTCATTGTCACCAGGGTCCTTGCTCCCACCCTGTCACCA

EMPV1_40704 GGTATCATAATTCCAGGTTATCCTCCAGTCTCTCTTGTTTACAGCTCCCTTACTGCTATC
EMPV1_40705 AAACATAGTGGCCCTTGTGCATGACAGTGGAGACACTGAGGACGAGGAGAATGACATCCT
EMPV1_40706 CTACCAGAAAGTGAATGAGAAGATTGAAACGTCAATAAACCTCGCATGGACGGCCGGCAG
EMPV1_40707 AATGGATTCGTTCCCGTCCAGGAGCCCCTTCCTGCGCTTTCTCCGTTGGCCACTAATTTA
EMPV1_40708 GGACCACATCTATTTCATTCTCGGGATAAGCTGAGGACGTGTACCATTTTGCAGTAACGA
EMPV1_40712 CAGCTGTGACAAGGGGCTGTCAATATCCATGGGCAGGCAGATGCTCACCTTCATGTAGCT
EMPV1_40714 AAGAGGAGGTGAATTGCACACAATCCCGATTCCAAACATGGGAACGAGCGGAGGGCTTCT
EMPV1_40715 CTTGGGAAACACAGGAAGGAAATTCTTAACTGCCATGAAGTAGATGGATTCCCAGTCCAC
EMPV1_40717 GGACAGCAGAACTACTCGTCAGCTACACTTAGTCACAAAGATGTTCTTCCAGACAGCTTG
EMPV1_40718 GCCACCAACAACCCCAAATTATCTTACATCCACCGCTTGGCTTTTCGAAGTGTTCCTGCC
EMPV1_40719 TGCCCACTGTGACCCTTGCTGGTGTGGGAGTAGCTAAGTCTGTTGTCAGGCTTCCTGTTT
EMPV1_40720 ACAGCTGCCAACAGGTTACGGAAAGAAGGAGGCCAGTATGGTTTAGTTGCTGCCTGTGCA
EMPV1_40722 ATGAAGGAGTATGGGCATGGTTGGGGCATGCTGACCTTAACTGGAATGTTCCCCACTGCT
EMPV1_40723 GGGGCACTGAGAAGGGAGGCCAGCGCCGAGAGTGAAGAAAACCGCTGTTTTAATATATTT
EMPV1_40724 TCTGATCCCTCCTTCAGGACCTCCCTCTCACCTCTCCACTCGTCCCCGCTTCCAGGAGCT
EMPV1_40725 CATCTGGAGTCCCAGAGCCTCAGCATCCAGTGGGTGTATAATATCCTCGACAAGAAGGCT
EMPV1_40727 CCTCGGGATTATTTTCTCTTACACTCAAATTGTCTCCTCCGTTCTGAGCATGCCATCAGC
EMPV1_40730 GAAAGAGTTGAGTGATAAACAGTCTTCCAGCATGTAAATGTCATTGACTTCTGACCTGAC
EMPV1_40732 ATACTGAGATCAGCCTTATCCTTCCTGCGGTGTGTGGACTTCTGAGTAAGAGCCTGGTCT
EMPV1_40734 TTTGCATCGGGGGAAATTATGTACCTCCAACCATCATCTTCTGGGTCTATGGATAAGGGG
EMPV1_40735 AGGGCTTGAACATCCAGAGTGTGATTTCCGTAGAGCAGCCCCCTCTTTTCTAGCACATAG
EMPV1_40736 ACAAGCGGGAGCTCAGGCCCTTGTACATTTTGGCTCTGCCTGCTTAAGCCCTCCTGCCCG
EMPV1_40737 CGAAAGGAACTTCGAAAAAAGTTGCCTGTGTCACTGTCCCAGCAGAAGCTGCTGACATGG
EMPV1_40739 CTGGTTTGTTCCTGAGGCCCAGATGCATGTCTGTTCTTGAATTCTCCTGATACTCCAGTG
EMPV1_40740 TGGGGAAGAACATTGACAAGTGGATCATTGCACTGCTGAAGGGCCTGGCGGCTGTTAAGA
EMPV1_40746 TCTAGCCTTCCTGCTTTTCAGCACAGAAGCCAAACCTTGAATGACCCTGCGGTGTGATGG
EMPV1_40748 CATTTGAGGAGGGAGTGAGTCTAGGGCCGCTGCTTGGGGAATCCTCGGTTTTCAATCTTT
EMPV1_40749 GCCTTGTCCTTCACTCCAAGGGAACAGTGGATTAGATAGCAGGAATCTTCCCAGTCCCTT
EMPV1_40750 ATGACAGCCGCCTGCCGCCTGCGTTCTTCAGCGCTCTGGTGTGTGACATGCTCCACTTCT
EMPV1_40752 GGACTTGAACATATGACAGATTTATTGAAGGAAAGGGATATAACTTTAAGACACCTTCTG
EMPV1_40753 TAATAACCTCTGCTACAGCACCTTTGAGGTGATGGGGGATACTCTGACCGAGAGCCAGAC
EMPV1_40754 GGCCAAGTCGTCTGCTGTCCTCTTCAGATTTAATAGTGTTCAGTCCGTCCTGCACCTGCT
EMPV1_40755 CCCCCCGAGAACTCCTACCAGTTTTTTCTTTCTGGTTTCCGGCATTATGGTCAAATCTAG
EMPV1_40757 CGGAGAAAGGAGACCCAAAGATGACCAGGTGTTTGAAGCCGTGGGGACTACAGATGAATT

EMPV1_40758	ATACTGATGGACGTCTAATCTGGGCAGCCCTGTAGAATCAGTTTCCTCCCAGCTGTGTCA
EMPV1_40761	GACACTGACAGGATAGGGGGCTTGCCTGCAGGGAACTTGGAGTCTGAGCTTCTCTTGGAA
EMPV1_40762	TGTGGAAGGGCTTTCAGCAGGCGTGTGTCCCTTACTATACACATGAGAACTCACACTGGG
EMPV1_40764	ACGAAAGCTGGAAATAGGAATTGAAGGGCTCGTGAGGGGGCCTGGGAGATTCGATGGAGT
EMPV1_40766	GTCGTCAGCCAGAGCACCCCCTGAGCTAGAACTTGCTGGAAGAGTTGAATGTGATTCTAA
EMPV1_40767	CACCGAAATCTAGGGCCTTGGCATCCAGCTCTGCGGGGGCTCTACTGAATAGGGGCAGTA
EMPV1_40770	AGGACTCCATCCTGGCTGTGAGGAAATACTTCCACAGACTCACCCTCTATCTGCAAGAGA
EMPV1_40771	GGTCATCTCTTTATCCCTTTCCTAGTGTCATACCCATTTCCAGAAACAGTGACTTTCTCC
EMPV1_40773	GATGGACAGACTTATCTTAATACAGTGGTGGCCTATGATCACCAGACAAATGAGTGGACC
EMPV1_40774	CTGATTCAGTAAATCTGGATTGGGGCCCAAGATTCTGCACTTCTAACAAGCTCCCAGATG
EMPV1_40777	AGAAAGCTGGATCAGACATTTCCCTAGAGTTGTGGCACCTGGTGAAAATTGGAGTGGGCC
EMPV1_40783	AGAGATGGGGGAAGAAGGGCGGCGGGTCCAGCGGTTGCTGAGGCTGAAGCTCCGGCTCCT
EMPV1_40785	TGGAGCCAGCACACCTGCATCTTACAATTGGCTGTGTGGACTCGGGGCAGCTCTTTTTAT
EMPV1_40786	ACAGATCTGCTGGAGCCGCCCCAAGCAGGCATTTCTCTCCTGCTCCATGAGCCTTCTTAA
EMPV1_40787	GGTTCACCCGTTATCACAGTGTTTGCAACTAGGAGTGATTGTGATCTTCGTCACAGGCGG
EMPV1_40788	CCTGGAGCAGCCAGGGTCCCGTTGGATAAGTAGTTTGGGTTTAAGGTCCAAATGGCTTTC
EMPV1_40789	GGTGCCCAACCCCAGTAAGCAGTTGATACAGAATTGTGACAGAAGGTTGCAAGTTAACAC
EMPV1_40790	GTGGAACACATGTCAAAGCCATGTCCATTGAAGAATCTTCCAACGGGAGTCTCTCTGTAG
EMPV1_40791	CTGTGGTTGGACTTAGCCTCTGCGTCTCAGAGTGTACCCCAGTGCATGGCCCTCTTCTGA
EMPV1_40792	AGTGGTCTGGACTGCTTGCTACAATCTTTGAGAAGTTCCGTCTTGTTGCTGAACTTGCAG
EMPV1_40793	GTCCCCCTCTCTCTACCCCTGTAAGCCATTGCAAACTTGACATTTAGTTGGATGCCTGAC
EMPV1_40795	GTCGAATGACCGGGCCGCCGCAGGCATGGTATGGAAACCAAAATCCTGTGAACCAATTCG
EMPV1_40796	TGGGGACTCTTACACTATCTCAACTTCCTTTTCTAGCTCCTTCAGACAACCCTCGAGTGG
EMPV1_40799	GCTGTTTAGGATATTGATATTCATATTACAAATAGTTTCTCAGTGGCTACATAGCCTTTG
EMPV1_40806	GTATCTATGGAAAAGTGAAAGGCCACTCTACCACTCAGCCATCGACAAACCAGTGGTCTA
EMPV1_40807	TCTCCTGCACAGACACAGCATTCCTAGAGCTCATGGCATTTTTCTTGGCAACATTCACGC
EMPV1_40808	TGATATCCGCTATATGTAGCCCAGCCACTCATCTGGGCTTCCCTTGCACCCTCTCTGCCT
EMPV1_40809	TAAGGATCCAGCATTGCCGTGAGCGGCAGTGTATGTAGGTCACAGAGGTGGTTCAGATCT
EMPV1_40810	ATGATGAAGGGTTTTTTGGACCAGCCCTTCCTCCTGGATTTAAAAAGCAGGATGATTCTC
EMPV1_40811	AAAGCTAGAACTTGCGAGCTTTCCCGCATTGACATCTGGACTGGGTGTGACAGGCTTGCT
EMPV1_40812	GGCCCTTGTGAGAAGCAGCATTTATGTGAATAGACAGGCATGGTTCTGCAGATGCTCAGG
EMPV1_40813	CCTCAGGTCTGTACTCAAATGGCTGTCACTGTCTGGATCACTGGTTTTTTCCATGCCCTG
EMPV1_40815	CTCCTCACACAGCATGAGAACATCAAAAACGAGATCGACAACTACGAGGAGGACTACCAG
EMPV1_40818	GCAAGCTCCAACATTGGGGACTCGTTCAGAAGATGATACATCAGACTAAATCCTGGTGGC

EMPV1_40820	ACTCTGCCCATCACACTATGGGTGGAATTTTCTCCTGAAAAGGTGGGCCCTAGAAGCCTC
EMPV1_40821	AGCAGAGGCGTCGGAGGTGGCGGGTGGAGCCCAGGAGCCTGGGGAGCCGGCAGCAAGTGA
EMPV1_40824	CGAGACCTTTTGATACCTGCTTGACCAGGATTTGGATAGAGGAAGAAGAATTGCTGGAGG
EMPV1_40825	GCCAGGTGGATTTCTGTGCCTCCAATGTTCTGAATCACTATTACTGCGACTATGGTCCCC
EMPV1_40828	TGCAGCGACCCTGGCGCGTCCGGGAGGAGGACGCAGCTCAGAACTGGCGACGGCGTTCTA
EMPV1_40830	CCTGGGAACTGGAGTGCTCCTGGTGTGTCTGGGAGCTTAGTGAAGGAAAGGGAGTGAGAG
EMPV1_40832	CGGGCGGAGATTGCAGAGAGGTCAGTAACTAAATTGGAGAAAAGCATTGAAGACTAAGAA
EMPV1_40834	TGAGCTCTGGGATTGTGGCGGCGACCCAAAGTTCGAGTCTTGCTGGCCAGCTCTGATGAA
EMPV1_40835	TTAAAGGCTTCGGCGCTGTTTCTGAAGGTGCAGACGCTACAATCCCAAAAGCCTTCGTCT
EMPV1_40836	ACGGAGACCACACCCTTTCCAGACTACTCTTCCCCTACCTGACCCCAGCATAGTTTAAGT
EMPV1_40840	GCCGTGGCAGCCAAGAAGCGTTTCAGTGTATACATTACAGAGTCACAGCCTGACTTGTCA
EMPV1_40842	CCCAGCTTTCAGCAGGATGGACCAGTAAGACTAGATAAAGCCTGGTTTCTGTGAGGATGT
EMPV1_40843	GTGTGTCGAGTACACCATTTACCTTTCGAACTGTTGGTTGCAGGGGTCTGCATTCATTCC
EMPV1_40844	GAGCTGTGTTTTAGGAACCAAATCCCACACTTTCAGTCCTAGAGCAATAGAGGCCCTGGA
EMPV1_40845	CCTGGATTGCATCATCAGAGAAAATACACCCAGTCATTTTGCGGTGAAAACATAATGATG
EMPV1_40847	CCTGTATTGTCTCTGGAGTGGGAGGAACAATCATTTACCACTATTTGGGGTTCCAGGTAG
EMPV1_40850	CTTGCCTCTCTCCGCTCAGTTTCTGGAGGATCCGTGGCTAGAAGTCATGCATTGAAGTAC
EMPV1_40851	AAGGACAAACACGTTGGCTGCCTTTGCTTGCATGGATGACCTACCGTACCACGTACCACT
EMPV1_40852	CTTGAACATCAGTTTTCACTCCCTTGAAAGGCTCGGAGGGTGAGGCACAAAATCCAGGAC
EMPV1_40853	TGTCAAATGACCTTCAACACCCAGATTCCTGCCCTCCGTTGCTTCATGAGCCCCTCCATA
EMPV1_40855	AATTCCTGCCTCTGTGTGTACGCGCCTTCACCTTCTCGCCTGCGTCCATGTCTTCAGCTC
EMPV1_40857	GGAGACATTAAAGGCAGACCCTGGAGATTGTGGGTTCGGTTTCAGACCACTGCAATGAAG
EMPV1_40858	TGGCAGAGTACAGAAGTTCTCAGGCCAGGGAGCGAACTGGTTGCCAAAGCTGTAACCAGA
EMPV1_40859	GAAAGGGGTCAGAAGCGAAAACGAGAACCTGAAGACGAGGGAGAAGATGATGACTAAGTG
EMPV1_40861	CATCGCAAGCAGGGTATCTCATTGCAGTTCTGTGTTTTCCTCCTGGTAATGATGCTGAGC
EMPV1_40865	CCATTTGATTTCACACCAGAAAACTACAAGAGGATAGAGGCAATTGTAAAAAACTACCCA
EMPV1_40866	TTTGCACTGTGAAGAGGGTTTAAATTTCAGTGTAGCGAGGTGGTGGCAGAGGAATGAAAG
EMPV1_40867	GTGCAAGTGTTTAGCTTCCGCTGGTTTGTGCACGATTTCAGCACCGAGGACAGCGCCACA
EMPV1_40868	TACAACGAGGTGGCGGATGAGCTGGTGGCAGAATTCAGCGCTGCCGACAACCACATCTTA
EMPV1_40869	GTGATAGGTCACACTGGTGGAGTATTTCATTGTTCCTAGCACTGTTCTAAGTGCCTTACG
EMPV1_40870	GTTTACAGTGACTTTTATCCTGTGCCACCTCCCTACAGTGTTGCTACCTCCCTTCCTACG
EMPV1_40871	ACCAAGGTGCAGCTGAAGGAGCTAGAGAAGGAGTATGCGGCCAGCAAGTTCATCACCAAA
EMPV1_40875	CGTTAACCACTGCGCCACGATGGGAACTCCCCAGTAGTTCATTTCTTGCTCCATGTAACA
EMPV1_40876	TCACTAAGAAAACTGAGTTGACTGAAGAAGAAAAGAATCGCATAAAATTTCTGAACAAAA

EMPV1_40877	TTGGCCCTCGAGCCTCAGTCCCACTACAGCTCCAGAAGCTTCCATCTGATCCTCAGAGAC
EMPV1_40884	GACTTTCACAAAGAAGACTCCTTAGGCATCTCAGATTTCACGGATTGGAGGGCAATCTGG
EMPV1_40885	GGAGAAGGTGAGCACGACATCTTACTCCTCAGGCATCTGATCCACCTCCCAGAACATTTA
EMPV1_40886	CTGGAGCATCATCAGTATGTTAAACCTAAAATGCAGGGCCAAGCTTCCCTTGGAGAGCAG
EMPV1_40887	TGGGCTTGTTTCGGATCAATGAGTCGGCCGAGGGGGAGATCGTCATTGATGACGTCAACA
EMPV1_40888	CTGATTCAAGTCTTCAATCACATTTTACTCAATGCACTTAATTTGGAATTGCCAAGTGAA
EMPV1_40891	TAGCACTTCAGCCCTGAGGAATCCCGGGAAGCAGGACTCTCCAGGACGAGCTTTATCATT
EMPV1_40892	AATTTCTAGAGTTTGAAGCCCCGAAAACCTTGTCCCAGTGGAAGAAGGACCACAATCCAG
EMPV1_40893	AGGCTGGCAGTGGGTCAGTGGAAACCTACTGGGAAGATGTCTCTTAATGTAACAGTTCTC
EMPV1_40894	TAGCTTCTCTGCTCCAACCTCCTTTTGGAGGAACGGGAGGAAACCCTGAAGACATGTTCC
EMPV1_40895	TTTCAGATAAACAATGAATGATTTTTTTGGTACAACTGTGTCCCAAGTATTGCATGGGAC
EMPV1_40903	TGTCACCATCAAAGAAGGCTCACAGCTGAAGCAGCAGATCCAGTCCATCCAGCAGTCCAT
EMPV1_40904	GAGGCTACTCTTCCAGCTCCACCCCTCAGCAGTCTAATTACAGCACCTCCAGTAACAGTA
EMPV1_40905	GTGAAGAATGCCAATGGTATTTTGAATGAGGGTTACATTGAATCTATAGATGACTTCAGG
EMPV1_40907	CCTATGACATGCCCAGCACTGTACCAAATCTTTCTTTAAACTACCAGCTGAAGTTGCATC
EMPV1_40908	TTTATTACATAAGGGATGCAGCCTTGCCCACAGTCCAGTATACAGGGTCCCCACTCACAG
EMPV1_40909	CTCCTGCCCTGAACCCCTAAATGCAAGCAACTGGGCAGAAATTCACATGTGGGTAAGAGT
EMPV1_40910	CCCCAAAGGTTCTACTCTTTAAGCCAGGGGATGGAGTTGGTGAGTCATTAAGAAGAGATG
EMPV1_40911	TCTCTGGGATTTCAAATTGTACCTGTGTGCCCCCTCAGGGAATTGGATGTCCTGATTCAC
EMPV1_40913	AGCGTGGAGGATGCACAGATGCACACGGTCACTATCTGGCTCACAGTGCGTCCTGACATG
EMPV1_40914	AGAAAATAAACCACGGACCCGGAGCATGAAATGTGCCAAGGTGTCTTATACTGAGATAGC
EMPV1_40915	CCGCTCCAGCCCCCTCTTCTATTTAAAGACCACCCGGTCAACCCCGCCCCCGCCCCGCCC
EMPV1_40918	AAAATTGGAGCCTGCCCTTCAGCCCATAAGCCTCTTTTGGGAACTGAGAAGTGCGTCTGG
EMPV1_40919	CTCCAAGAAAGGCCTCAGGTTCAAAGGACTGAAGTGAATGTCAACCACGTCCTGATTTAC
EMPV1_40920	TTGTCATGCGCGTCAGTACTTCATTCCTTTTACCAAGGAGTCCAAGGAGGTCCCACCGTG
EMPV1_40921	TCCCTGGGGAGCTGGTGGTACATGAGCTCTTCTCCAGTGTCCTCCAGGAGATCTGTGATG
EMPV1_40922	TCAGGATCGACATACAGAGGCTGTGAAATGTACTGAGAAGACACAGGATCACGTGCAAAA
EMPV1_40924	ATCTAGACCAGGAATTTTCAGCTGGGGGCACCATCTAGGGTCCTTTTGGTGTCACGTCTT
EMPV1_40926	CACACGATAAGAAAAGCCTTCATGTATCCCCACCTACAGTGACCTTGCTTCCCAGTGACG
EMPV1_40930	TTCCATGAACACCAAACCTTCCCTACTGCCGTGTGCAGAAGCCCTTGAATACAGAGCCCA
EMPV1_40931	CCCTGGGGTCATCATTTGGAAGGCGGTTTGGCCCCACATTTGTAGATAACAGTAACATGG
EMPV1_40934	CTCCCTTGGCAGCCCACCCATGCCAAAGAGGGGAATGCCTTTTCACCAGTTTTTATTCCT
EMPV1_40935	ACATGTCCCTTGGATGTTGGCTGAGGTCTTTCACTGAGGAGCCATCATTTGCTCCAGGGA
EMPV1_40937	ACAATGTTCACCCGCTTTTCGCAGAGGAACCCCCAATCAAACTTCCCAGTGTACCCTCTA

EMPV1_40939	CTAGCAGCTGAGGTGTGAGGGCTCCCTGTGCCTGAGTGATTCTACTTGTTATGAAGTCTC
EMPV1_40946	GCAGGTCGAAAGGAAGCATCACCAGCATCCCGTTGTGGTCCATGAACAAGGCAGCTTCGT
EMPV1_40947	TCCAGGCGTTTCTTTATGCTCTGGGGTCGTAGCAGTTGTCCATGCATCCTTCAAGTGTTT
EMPV1_40949	GTTTTGGCACTTAAGAGGGGTAAGCTGGCTCTGTGTTCACCTTGGCAGGCAGGCGGGTGT
EMPV1_40950	ATAGCCTCGAATCTCCCGCCAGCCCCGATGACTCAGGCCTCACGCTGTACCTGGTGGTGG
EMPV1_40951	CTTGGTAGACAATGCCCTGAAAGACCAAGGAGTGATGGTGCTGTGTGAGGCCCTGAAGAA
EMPV1_40952	CACTGTGGAGCCTGGAATTGTCGGAATGGATGAACTAAGAAGCGTTGAGGCTACCAGGCA
EMPV1_40954	ATGAACTCTGGAGGAAGGAGGCAGGAAGCAGCAGAGCCCAGGGGTAGAAGGGCTCACAGA
EMPV1_40956	TGGCCGACCTGGCCAGCAGCGGGTCCCTGGCTCGCATCCTGCAGCACTTCTGCGCCGAGA
EMPV1_40957	AGATGCTCATTGTGTTTCTACTGCTCTCCTGGGCAGTTGGGTTCATACATACCACTAGCC
EMPV1_40959	TCAGGATCCGGGCTCATCCGTCATTCCACCTCTATCGCTCACTGCATCATCCCAATTCTT
EMPV1_40960	AGAGGTCCAGAAGAGAGAGCAGAGGTAGTGTGGGAAGTTATACCACCTATGTGCAGCATC
EMPV1_40961	TAGTGAGCCCATGTTTACGATCGTCTACCCCAAGCTCTTTCCCCTGAGAGAGACACCAAT
EMPV1_40963	GCACATAGCACCAACGAGAAACCGGGTGGTGAAGGCTTGAGAAATGAGAGCTCCTAACTT
EMPV1_40966	ATCGCTTGACAATATCTTATTGTCGGAGTAGTGGTCCTGGGGGCCAGAACGTTAACAAAG
EMPV1_40967	CAGTTTGAGGCAGCAGAGTGTAAAGGAGTGTGACTCCAGTGATGTTCTGAAACTGGGCTG
EMPV1_40969	TGAACAGTACAGCGAATACACAGGCTATGCTGAAACATATCGTTGGGCCCGGAGCCATGA
EMPV1_40971	ATCTGATGTGAAGTTGGCTACCCAGCAGTCTAGTCAGGCTGTAGAATGTTTAACTCAGGG
EMPV1_40972	AATCCCCTCAAGTGCAGAGCCCAGGCTGAGTTGTTCTCCGAGGGGTTACAAGGCTTGAAA
EMPV1_40973	ATGGGTGGCTCTGGGAAGGAATTAACTGTGGGTCAAGTGCATTTTACGATGATGGCTTCC
EMPV1_40974	TTGCTCTACGACCTACCCATGAGTCCCTCTACACTGAACCCCAGAAGGTTATTAGGAACG
EMPV1_40976	CACTGTGTTCCTGTGTCTGAAGTTCTTCCTGCTGCCACCTAAGCTACAAGTGAGTGACCT
EMPV1_40979	GGGTGTCGTAAACCTTGGTATTCAGGCCTCTGCCCTTTGGTAGCAGTATGACCATTAAGC
EMPV1_40981	AACAAAATGCCCAGAGGAACAAGCAAGGCCCACAGCCCGGGGTCAGAGATGAAACCTGTA
EMPV1_40982	TTCAAATGGTGAGGGGACCATGTGGTTAGTCAGCTCGGTCTGGGTGGCTTAAAAAACAGG
EMPV1_40983	TGTGTCTTCAACCTACACCACCACAGCTCACAGCAATGCCAGATCCTTAACCCACCGAGT
EMPV1_40984	TGGACAAGGACCCAGTCTCCAGATCCCAGCAAGTTACCTTTTCTGAATGGCTCCCGGTTT
EMPV1_40985	CCGTTGTAAGGGGCGTTTTCAACTGGCATCTACAGTTCGAATGGGATCGTGTTTTCTCTT
EMPV1_40986	TGCTACCTTATTCGGGCTGCCTAGGACATGTGGAGAGATGCATTGGTAACTGTTCAGGAG
EMPV1_40987	GCAAGGATCGAATTGCACATGGCAGGTTCTACAGAAGGGTTGGCTGTGATCTCTCTCCAT
EMPV1_40988	GGATGTGTGCCTGGGTCACCTTGCTATACAGTAGAAAACCAATGGAGCCCTATCAACCAG
EMPV1_40990	AGCAGCCCCATTGTACATTCCGGTCAATAAGCCAAACCTCTTGGCACTATACCCCTTCCC
EMPV1_40991	AAACAGCCAAGCTTCCAAAGATCCTGTAGCATCCTGTTCCCTGAGTAGTAAGTCTGGAGC
EMPV1_40992	CACTCAGCCAACTTATCCATACTTTGGGACTAGTAGCTGTTCTTACTTTCCAAATGAGGC

EMPV1_40994	CGTGGCTTCTAGTCAGATTCGTTAACCACTGAGCCACGATGGTAACTCCAACAGGTTCAT
EMPV1_40995	GGCTTTTTGGAGAAGTTTTTACAGATAACATGAGGCGGGACTTTGGGTCTGGAGCCACGG
EMPV1_40996	TGTAACCTATGATTTTGTGGAAGATGAGGAAGGCGAGGGAGATGACTACGGTGGCGTGCT
EMPV1_40997	TGGCCCAAGTAAAGCGGTGGCCAAAATCCATTCTCTCATCAATGCTGCGTACGTTAAGGA
EMPV1_40998	TTCTAAAATCTGCTTCAAGCTGTCTCTGGACTCTAGGTTAGGATGGATCAGCCTCGGCGG
EMPV1_41000	AAGAAAGAGAAAGGCAGTCTGCTTCCCCCAGCCCTGTTTTGTTTTCTCTAATTGGTCCTG
EMPV1_41001	TCAGTGTCACTTGACGATGCAGATTCCTCAGGACCTGTTTTCCGTGGGCTTAGCTTTACC
EMPV1_41002	TTTGATTTCAGTGCCTGGATACCCAATGTTCCCACCACCATGCGGCTCCCCCCGCCTACC
EMPV1_41003	TTCCCTACTCACCCCCTGCTAGTATCAGACCACCTGAGCATGGAAGACAACGCTTTACAA
EMPV1_41004	ACAGAGCTTTTAACCTGGCCTCAGAAGCCCTGCAGTATCACTTCTAGCATATTCTGTTAG
EMPV1_41005	ATGTGGGTTCAATCCCTCCTCGCTCAGTGGGTTAAGGATCTGGCATTGTCCATGAGCTGT
EMPV1_41006	GGGTAGAGTTTACATTTAAGTATGCCAGTGTCTGACCAGTTTCTTAATGTATACAGTTGT
EMPV1_41007	CTGAAGAAATGACGGAGTGGAGAATGTAGAAAAGTGAAGGTGTATGATTCTGGAACAGAG
EMPV1_41008	CTCCCTGGGAGCCTAGGACATTAAGGATCTGGTGATGTCACTGCTATGGCATAGGTTTGA
EMPV1_41009	TGCGGACATCCGGGTCAACAGCTGGTATGCCTTTGCTGTGGTTTTGCTCATCGTTGTGAT
EMPV1_41011	GTGCTGCGCGCCCACGAAGCTCAACGCCATCTCGGTTCTTTACTTTGATGACAACTCCAA
EMPV1_41012	TTGTCATCTCAGATACTCTTCCTACTGGGTTTACATCTCACGGTCCCAGAGCAGCAGCCT
EMPV1_41014	CACCTGCAGTGAGCAAGAAGACATAGGGAGCAACACGTTCCAGTTTGCGGAACTACAATG
EMPV1_41017	AAACTCAAATCCTCTGAGGACTGACTCTGCCAACACACAGGATGGTGACAGACACCAGAG
EMPV1_41020	TACTCGCTGGCCATGGCCAAGCAGAAGCTGCCCAAGAAGAAGAACAAACACATCCCCCGG
EMPV1_41023	TTCCTGTTTACTTATTTTTCTTTGGAAGCCCACACCTGCCAAATATGAAAGCTCCAGGGG
EMPV1_41026	AAGGCTTTGGCTGTTTGTAGGGAACCTGAGTTAGGCTGAGACACTGACCTTGAACTTTGT
EMPV1_41027	GGTTGGCCACCACCTCTTCAACTTGCCATCTGAGAACACACCTGAATGGTAGAGCTGAGT
EMPV1_41031	CATGGCCAGCTCCATTCCCCTGTATTAGCCATGAAGGGCAAAGGTTTATCATCATCTCTT
EMPV1_41032	CCGAAACTTCCTGGTGCGAGCCTCTTGCCGCCTTCACTTAGAACCTGGGAAAGAATATCT
EMPV1_41034	GTGGGCCAGACTGAATCCATTTCTATTGCATCCACAGGGTCCTAGACATCCTGAAGCCTT
EMPV1_41035	TCTTCCAAGGAGCTGAAGAAAGAGTGTGTTACCAGTGTGAGGGTGAAGATGAGTCCCAGC
EMPV1_41036	TGGTACCGGCAACATCCTGGCAAAGGACCTGAATTATTGATCGCAGCACGTTCTAGTTCA
EMPV1_41041	ACACTTGCGCTGCCATACAGGCGACAGACCATTCATCTGTACCTGGTCTTACTGTGGGAA
EMPV1_41042	AGGCCTCCAGCTTACAGGCAACAGCAGCAGAAAAAACACTTAATTCACAGAGGAAAGCAG
EMPV1_41043	ACTTTCATGACACAAATGCAAGTGAAGGTGATGTTGGCCTGGTGGACCCTGCAAAGCTGG
EMPV1_41044	GGAGGCAGTTGACACCCTTCCCCACAGAGGTCATCATGCTGGTTATGTGTGCATTGTTTA
EMPV1_41046	CCTGCACGCGCCGCACGGTGAGCTCCAGCACCTCTGCGTTCTCCAGCTTGGCCTGCACCT
EMPV1_41050	CAACACTGTCGCCCCTACCACACTTTTTGGGATAAGGGTTCACTCTTGAACTTGGCTGTC

EMPV1_41051	GAACGATTTAAGATGAATGGCTGCTACTGGAAATCGGAGGTCTGCAACCTAAGACCGGCC
EMPV1_41054	CAGTGCTGCTCTGTAAATGAGATGATTTTAGCATCAAAGCCCGGAAGCTGGAGGTGACCT
EMPV1_41056	ATCCAGAAGCACATGAAGTTTGCAACTTTCCACCCTGCCCATTTTTGTAAAACTGCAGTC
EMPV1_41058	TGTACGCCTCCATGACAGTATTTCTGAAGAAGGGTTTCACTACCTCGTGTTTGACCTTGT
EMPV1_41059	AGACGAACAGCCGGTACTATATCTGGTTGTGGCTGTTGTAGATGTACTCCAGCTTGTGCC
EMPV1_41061	TAGGTGTGAAGTTGCTGGGTCCTAAGGTAACCGCCTTTCGCCTTTTGAGGAACTGCTGGA
EMPV1_41064	TCTGCTCTCCACGGGGTGACTAGGGACTTAGCAGTAAGCCTGGAAAGAGAGGTGAAGAGT
EMPV1_41068	TGCTGAAGTAACAAACATCCCCCAACCTCAGATGCTGACTTCTTGCTCAAGGTGCATGGC
EMPV1_41070	GGCTGTGCAAGGAAGCCAGCTACCTGGTGCGCAACAGTGAGACCAGCAAGAATGACTTCT
EMPV1_41071	ACGGAGGACAACAAGCGCCGGACTCTCCAGAGGAATGATATCGCCATGGCAATTACAAAA
EMPV1_41074	GGTCCCCTGCAACAAAACATTTATAAAGAATTCTCGGGGGAGAGGACAAGATGGCGGAGG
EMPV1_41075	CCTGTGAGCCCAGATCCTCTCTTCTTTGGCATGGGGCCCTAGCAAGATGCTGTGAATATT
EMPV1_41076	CTGCTGTCTCTTACTGTCGATGTGGATTCTCTTGCTGAGGTGGATGATGGAATGGCTTCC
EMPV1_41079	CCCTCTTCCAGTCGGTTAGCTGTCTAGGCTATCTGTCCAGCTGTTAACTTGAAATGCTCG
EMPV1_41081	CCTCCTCCCGGTTATGTAAGCTTGAAACTCAGCCCCAAGTGAATGGATGGGGAACAAAAG
EMPV1_41082	CCCACCAGTATGAGGGTTCCAACTTCTCAACTAGGTCACCAACACTTGTTTTTAGCAGCT
EMPV1_41083	GGGAGGACCTATACCCATAATAGAATTATGGCACTCATTTCTGACAGTGACCGTGAAATC
EMPV1_41084	TTCGTACTAAAGGGTGAATGGATGGGGGCAAAACATAGAGCGTAACTCTGTGAAGAAGGC
EMPV1_41086	TTGGGGTAGTAGGCTAGATATTTGGGACAGAATTTAGCTGAACCCTTCATACCTTTAGCC
EMPV1_41088	CCGCCTTGTATTCACAAGATAACACAGTACCATCTCAGTGCAAGTTCATCTGACAACTGA
EMPV1_41089	TGCAAAAATCCTAAGAAGCTAAGACCTCTCCATCCCTTGTTCCCAGTCTCCTGAGTCAAG
EMPV1_41090	TTCGCAGGACCACGAGAACACGATCGAAACCTTTGGAATACTGGCGAGGGGAGCGAATAA
EMPV1_41091	AGCTGATTTTCTCACCTCCTCTGCAACCCTAGGTAAATAAGCAGACACCTCTGTACGGTG
EMPV1_41093	GTATGTTTGCACAGGTGAGTGGAGACAGAATACAGGAGGTGAAAATCCTACAGGGGAGCT
EMPV1_41094	CCCAAGTCCTCTTCTTGCACTCACTTGCAAAAAAGCTCCTGTGATAGGGCTTGTCTTCTC
EMPV1_41095	CTGCCCAACTAGGATGGACAGGAGAGAGGGACTTGATTTGCTAAAGCCGGTTTTGGAGAA
EMPV1_41096	AAGGTGGCCTCTTTGGAGGAGGTGCCCTTTGCAGCTGCCTCAATCGGACAGGTGCACCAG
EMPV1_41097	CCTTCTGGAAATCACTGACCCACAGGACACAGCCGCCGTCTGTGCAAGTGGCCAAAACCA
EMPV1_41098	CTTCTGCCCATTTTTTCTCGGATGGGGTTTGCTTTTTTGATATTGAGCTGCAGAAGGTGT
EMPV1_41100	CGCTGCAGACACGGATGGTTCAGAAGAGAACACCGTTTCTGGGCATCCTCAGCCCCACTA
EMPV1_41101	GCTCATCGGCTTCACCCTGATGTTCTGGAGCGAGTTCACGGCCTCCTTCCTCTTATTCCT
EMPV1_41102	CTCTTCTCCCCAACGGACCCTTGTATGAAGCTGAAATGCCAAGTTCTTTCCCCAGAGGAA
EMPV1_41104	GTCCATTTCTTCTAGGTTGTCCATTTTATTGGCGTGTAGTTGTTTATAGCAGTCTCTGAG
EMPV1_41106	TTAACCAGGCAGCCCCCCTTTGCCCCTGGAAAATCCACACTGTTCTCTCTGCACTATGAA

EMPV1_41107	TTCCACAGTAACCGGATCTATGACTACAACAGTGTCATCCGCCTGTACCTGGAGCAGCAG
EMPV1_41108	TGGAGCTGCATATTATTATATCCAGTTTGATTTTGCTCATTGAAAACTCTGCCTTGAGTC
EMPV1_41109	GGTTGGCAGACGTACTGAGACAATTAGAATGCCTTATTTCAGAGCAATGGGAGAAACTAC
EMPV1_41110	GGGACCTCTTGGCAGTTTCTGATTGAGGCAGTGAACATTTTATGGAAATAGAGAGCTGTG
EMPV1_41111	GCTTAGGTACCAGGCTTCAGTGCTCAGGTCCTCCTACGATAATCCCGTGGATATTAAGTA
EMPV1_41115	TTTATGGGGAAAACAGACCCACATGCCCCACGCCATCAACAGCAGTCTGTGCTCTTGGTT
EMPV1_41120	GCTCTGGATTAGGCTTTGGCTTAAGGGAGTGTTGTGGCTGGTTTGATCTTCTATCCAGAC
EMPV1_41121	TTTAACTACTAACAGCAGAGGTATTTGGGAAGAGCGCTACCACTGCCCTGGGCTACCAGT
EMPV1_41126	AAACAGGGAGAGCCAACCCAGAGCTCGTGATCCTGTCTGTTTTTATAGCCCAGAGAAAAA
EMPV1_41128	ACGTAGCCGCGGACGACGTGGCTCTGGATGGAGAGCACCCGGCACTCCTGCTCCATGCCT
EMPV1_41130	GCACACCATTTGACAAGGCTTAAACTGGAGAATGACACCTCACTCAAGTCCTTAAAACAC
EMPV1_41131	GGCCATCTTTGTTCCTCACCCAGATAGCTAGTACAGAAGGTGTACTAAAAAATACGTTTG
EMPV1_41134	CCCCTATACAGGTTGAAATAATGCCCAAAATACTGAGCTCACCAGACTGTGCAGCAGGTA
EMPV1_41135	TGCTAACCAAAGTAAAAATTGCAGATCAGCCATCAGAAATGGGGCCAAGATCAGGTCCCC
EMPV1_41138	GGAGGAGACAAGACAAAGCAAATGAACACATATATCATATAAAATCAGGTTATATTAGGA
EMPV1_41139	GCCTGTTTCCACGAGGTACTGGCCTCTTCGTGTTCACACCATGTGCCACTATATTTTGCA
EMPV1_41140	AACAGCATCGCTGATGAGTGGCCCGGTGGCATCGTGGCCCAGTCCACAGCCAGGGGGCTC
EMPV1_41142	AATCAGCAGTAAAGCAGTGGGTGTTGTTTGGGCTCTGGGAAAGCAGCATGATTGGTCCAC
EMPV1_41145	TCACACACTCCTGAACCCTCCTTGGAGCTGCCTTGTAGAAACTAGAGAGAAGGGAGAGGA
EMPV1_41146	CACTGAGTAGTGGAAGAGGGGGCTACGTGGGGTGAATAGACCACATTGAAAGGGCACAAT
EMPV1_41149	GTTTGCAGCTGAAAGCATGTTTTGTCCATACCGGTGTTTATAACCTTGGAACTCCCAGGG
EMPV1_41150	CATCTAGAAGATTCAGCAGCCATGTGGTCCCTCCCACAGTCAACTAGCTCCTTGGAGAGA
EMPV1_41151	GAGATGAGCTGCCCAGAAACCATCCCTCAGATTCAGAGAGATCTGGCACAGTTCCCTAGT
EMPV1_41154	GTGGCTTACCTGCAACTCGTGTAATTGGAAGTGGTTGTAATCTAGACTCTGCCCGTTTCC
EMPV1_41155	GGACGTTTTCAAGGTCACTTACTGCTCTCAGGGGAACGGATAAAACCAAAGCAAGAGAAG
EMPV1_41157	GAGAAGAAGGAGAAAGTGACCACAGAGGATGGTCCAGACAGTGGCCAGGAGATAAAAGGA
EMPV1_41158	ACCTACACAGTCTCAGCACACTGATCGTCTGAGGTGCTTCCTATTCACCTTGAGAGGGTT
EMPV1_41159	TGGGGGAAGAGGGTCGGGAAGGCTAAGGATCGCTGTGCTGAATGTTTCTAGGGTACCAGT
EMPV1_41162	AATTTGCCCCAAATGAGAAATTCCAGTAGCTGCCACTTCAGACCAGGGGGTCCCAGACTG
EMPV1_41163	ACGCATCATGACAAAACAACTGTGCACTGGCCTCATCGTCTTTGGGTGGTCCTGTGGGTT
EMPV1_41164	CCTGTCAGTGGGACTGAATGGTCTGAGTGTTCAGAAGTGGTTGGGGAAGTGGAACGTTGC
EMPV1_41167	AAAAACTCCTTGCTGCCCCTCTCCCCACCCAAGTCTGAGCCACCTGACATGACCTTGTTT
EMPV1_41168	TCATCTAGAGCATCTGTAACATCCAGAGCACCTATAACATCCAGAGCATCTGTAACTTCC
EMPV1_41169	TGACAAGGTATACTGTGTTGATGCCCCTTTTAGAATTCGTTGCAAACGGGCTCCAGGTAG

EMPV1_41171	CTTTGGACATCCTACACCGTGGACAGAAATGACAGTTTCTCTGCAGAAGACTTTTCCAAC
EMPV1_41172	CTTCTCCACCTCAGACCCTACCACAGAATTTGAAAAGATCCACACCTCCATCAGGATCCC
EMPV1_41173	CATGGAGAGGTGACCCTGACAGTGAAGCTGATAGCATAGACAGTGATCAAGAAGATCCCC
EMPV1_41174	CCATGGCACGAGAAATATAAGAGTAGGAAAGGGAATCTGGTGTAGTCTGGAGCTAGTGAC
EMPV1_41175	GGTGCTGTAGTTCCCAGCTCCTTCCTGGTTTTTCTAATCTCTCCTTCCAGGGAACAGGAA
EMPV1_41177	ACTCTAAAGAGCGGTGGGGGTACACCTCCAAGTGGACAAATGACTACTCCATGGTGTTGA
EMPV1_41178	AGTTTTGACTCCTCCTGGGTATGGAAATGTCCTGCTGTCCATGCAACTGCTGGGCTGAGC
EMPV1_41182	CAGTCTGGGACCCCCTGGTCTGAAGTGGCAGCTACTGGAATTTCTCATTTGGGGCAAATT
EMPV1_41184	CTTCTTCAACTGCATTCACAACAGACAAGGAGTCCCGGGGAGCAGAAGTTGCCGATACCA
EMPV1_41185	ATGTGATCTTGAGAAACCAAGAGGTGATGCTACTTGTCTGGTGCTTCTGGTGCCTCCTGG
EMPV1_41186	GTTGCTCTCAGCATAGGATCTTGTTGAAGTTGAAAGAATGCCTGGTAAGAAATCCCGATA
EMPV1_41190	TGAGGGGTTTCCTTATTTTGGCCTAGGGCTTCCGCTGTCCCCCCATCGTTGCCCCCAAGA
EMPV1_41193	CTCCTGAGCCCCACCTTCATTCTTCTGTCCTGAGTCTCGATATGAACATTCACCCTTGTC
EMPV1_41194	CCTCTGTTGTTGGGTATCTGGCTTATTGATTATCCTTCCACCCCTTGGCCTGAGTCTCCA
EMPV1_41195	CACTCTTATCATGAATCCTCGGGGCTGTACTCAAATGGCTGTCACTGTCTGGATCATCGG
EMPV1_41196	GAAGATCTGACAGATGTACTCCTGGGTGTAGCGGGACCTGCTGAGCAGTGCCATGAGATG
EMPV1_41197	CTTCAAGCCAGAAAGTCAGCATGGCCGGAAAGATCCGAGAAGGGGATTGATTGTAGGAGT
EMPV1_41199	TATAGTCAATCTTGCTTCTTTCACGAGTGTGTTCAAGGACTAGCTCTGGTTAGTCTAGGG
EMPV1_41200	GTGGGGCGTGTATGAAGGAGCCACAGAGGCTGGTGGTTTGCTAGTTCTTTTGTGTTTTGA
EMPV1_41201	TGACTGTGGCCGAGCAGTATGTCAGCGCGTTCTCTAAACTGGCCAAAGACTCCAACACTA
EMPV1_41204	GGATGCCAGCAGTGATGAAGAGCAGGATGATAAATCCTCCACTGAGGAAGAGGAAAAGGC
EMPV1_41205	CAGCTCAAAATGGGGACAGGATGAATTCCAAAAAGCCACAGCCCCAGCTTCAGAGCAGCT
EMPV1_41207	AGACAGTCACGAATTACATTATGCTCAAAGAAGTCTTTCTGGAGATCTGGCCTTAGGTAT
EMPV1_41208	CCTCAGTTTCTAACACTTGTTCCTACTTTCCCTTTACCATCAAGCCTCCGAGGGATGCCT
EMPV1_41210	TGTCAACTGCTCGGTGTATGTGCAGGTGGATGTGTGCAGGGGAAGCCCTGCTAATGTCAC
EMPV1_41211	GCAGGCAAAACAGACTATTGACTCTGTTATGATTTAAGACATGGTGATGAAGTGCTCAAC
EMPV1_41212	TATCATCTCGGGCAGTGAGGACCACACGCTCGTGGTGTTCGACCGCCGAGCCAACAGCGT
EMPV1_41213	TTCCCCCCAACCATACCCACAGGCAACACGGCCATGGGTTTTCAGATTCAGTGTCTAGAA
EMPV1_41218	GTTGGAGAAAATTCAGAAAGAAAAAGCCCTTCTCCAGGAGCTGGAAGATTTGGAACTGGG
EMPV1_41220	GAGAAGGCGTGTGTCTGCCAGCTGGGGAGAGAGGCCTCAGAAGAGACCAACTCTGGAATA
EMPV1_41222	GCAGAACTTGCTACAGCTACAATGCCTGGTAAAGAATATGCGCAGGGGCTTATTCAACTG
EMPV1_41223	AGGTGGCACTGGTCAAAAACGGGGTCAAAATCCTGCACACCAAGGACGGTTACACGAGCT
EMPV1_41224	GGAAAACAGAGTAGGGGCCTGCAGATCAAAGTCCCTTGGAAATCCTTCTGCCCTAGACTT
EMPV1_41226	GCTAAAGGCAGGTCAGGATGATCACAAAGTCAAGGGTGAGGAGATTTTTGCTAAGCTGAC

EMPV1_41228	ATATTGAGACCCAGCTGAAGAAATTCACACCCTCATGCTCAAGGCAGCAGTCCGCCCCAA
EMPV1_41229	GCTCTAACCACTTGCTATCTACTATTAGGCCCTGCCTTTTCCCCAGTGTGCGCTGCTTCT
EMPV1_41233	TAAGAAAAAACTGCAAGAGAGAGCTGAAGAGGAGGAAAGAGAAAGGAGGGCTGCTGCACT
EMPV1_41234	TAGCATGCTTGCTAGAGTCCTCATTGCAGAAGGTCATTGTTTGGTCCCAAGTTGTCACCA
EMPV1_41235	TACCCTGAGCCAGGCCGCACCCTCAGGCCAGCCCTCTCCACCATGCAGACCCCTGATGGA
EMPV1_41237	CTAAAACTCAGTGGAACAAAAATGAATGGGCAGCACCCTCTCTGGAATGGGAGCGGTGTG
EMPV1_41238	AGCAGGATTTCATGCTCCAAAGCTGCTACTGTTTATCAACAAGCAGTCCACTCAGTGCCA
EMPV1_41239	AAGGGCAAAGAAGCCCCGGGAGGGACAGACTATCAGTGTCGAGAATGCAACAAGAGGTTT
EMPV1_41241	GAGGATGTCTAGTGGACGCTGAGGAAGCTTCCCTCAAGTACAGCTTAAAACCTTAGTGCA
EMPV1_41242	CGTCGCGGCCTCGCCCACCCCTTGAGAGTCTGTGTTTTCCTTTGCTGTTTTTAATTCAGA
EMPV1_41245	TTTTGGGAGAAGGATCCCACCAGCCTGGCCTTATGAAAGTTGCCATCCTGGGCAGGCGTT
EMPV1_41247	TTGCTACCAGACCCAGCTCAGTGACTGGCACACCGGTCTCACGGACTGCTGCAATGACAT
EMPV1_41248	AATTGAAGTTGCCAGGGAAGTGCTTTCAGATGGAAGTCCCAAGTCCATATGCCTTTTGGC
EMPV1_41249	GGCCTCTTTGTGGCAGACCTGAAATTTCTGCTTGGTGTGTTAACTTTCTGCCCCTAGAGC
EMPV1_41250	TTTTCTGAGCAACCCCCCTGGCGTGGTGCTTGGTCTTGCCAGCCAGGGACCCCTGCTGAT
EMPV1_41253	TTCTGGACCCTCCACAGGACATTTTTTCCGCGCCCTTGCAGAGGACAGAATCTTAAAGAG
EMPV1_41255	AGGGTTGGCCTAGATGACGACAAAGGCCCTTCCTGTTCCCCAAACACCACAATTCTATGA
EMPV1_41256	TTCTCCAGGAAGAAGGTGCTTTCTGCTTACTGACTTAGGCAATACACCAAGGGCGAGATT
EMPV1_41257	ACCATTTTACCCCGACTCCATATATGTTGCCACAAAACACTTTTACACACTTTTCTCCAG
EMPV1_41258	GGGCCCAGATCAATTATACCTTAATGAAGTAGCCCAGGTTGTTCTATCCTGAGGCCTGGG
EMPV1_41261	TTGAGACTCCTTGGCTTCACCCCTCCTCCTACCTGGCGCTGCAGCTGGTGGTGCTTCTGT
EMPV1_41266	TAGCAGAAGACTTCCTATGCAACAGTGTCCCTGAGAACATGTGACCCGGTGATTCTCCTG
EMPV1_41268	CTCCAGAAACCCCTACCAAAACTTAGTTGTTTTCAGATGCCATTCCTGAATCTATGGTTG
EMPV1_41270	ACCTGCCGGCTTATGGTTTCAATGCTGGATAGAGATATGTCCGGCACAATGGGTTTCAAT
EMPV1_41272	TCGATGCCTACAGACACCTGTGAGAACGATAGCAGACTTATTTTCATTGCCCTTCCTGCC
EMPV1_41273	GGTTATTATGTCTTTGGTTTCCCCCTTGGAGTATCCCTGATGTTTGCTGCTCATCATGGG
EMPV1_41274	AAAGTGCAAAATGCAGGGAGCAGCCCACAGGCCCAGGGTGATGAAATGCACCAGAGATAT
EMPV1_41275	TTATGGGCTTTGCTGCCCCCTTCACCAACAAGCGAAAGGCTTACTCCGAGCGTAGGATCA
EMPV1_41276	GGCCTTCCACTTTCGGACTAGTAGCAGTACAACATATACCCATGGCAAAGATGAACTATG
EMPV1_41277	AGCGCGGAGCCCGAGCCAAGGGCGTTCCAAGAAACTGTGTGTCTGTTTTCTGTACCAAAA
EMPV1_41278	GAATGTTAGCCTATGTAAGGCATCTGGTTTTGGTGGGACCTAGGGTGAAACTGAGGACTC
EMPV1_41279	CGCCACGACGGGAACTCCCATCCTTACTTCTAAGGTTGAGTTTTGTAGGAGTACTAGCTG
EMPV1_41280	CTCCTGCCTGGCCACTTCCCCATGCTCTTGTTTGTACATTCACTTCTCCTTCCTGTAGTT
EMPV1_41281	AACTGGTAATGCTCCGGCCTCTCGCGGCCTCCCTACTACCGCTCAGAGAGTCGGTGTTCC

EMPV1_41284	ACAGCTTGAGCAAACTCCAACCACCCATTCCAGTCAACCTCCAGTCCACCTGTAGCCAAT
EMPV1_41285	AAGGAAGGCACCTCCCACATAGAGACTAGTGATCTGAAACGAACTGAACGAGGGCGGACA
EMPV1_41286	GTATCAAGAGGCTTTTAACCGCAACAGAACCACAGCATTTTGCCCCATGTATATCTATGC
EMPV1_41288	GTGTGTGTGTGTGCATGCATGTGCATGTATATGCATATACTTCCTTTATCACATACTGGC
EMPV1_41289	CACCCAGAGATATCAGCTGTCAGTGGAAGACCAGTGTGACTTCATTGGATACGTTTAGTG
EMPV1_41290	CCCACTGCACCAAGGCAGGAACTCCTTATTTGTATACTTTTTGATGATGGCCATTCTGGC
EMPV1_41294	GAGCTGAGGGTGACCTGGAAGAGAAACGGGGTGCCCGTCTCCAGTGGGCTGCACAGCTTT
EMPV1_41296	CCGCGGCGCAGGACGATGTGAACACAGATTCCAAGACGTGGTCCTTTTTTGTAGTTCTTA
EMPV1_41297	AAACGAGCAGGTTCTCGCCTGTTGGTCTGATTGTCGTTTTTACCCAGCCAAAGTCACTGC
EMPV1_41298	GCCTGAAATTAAGCCAGCAATACCTCTCCCTGGACCTGAACCAAAACCAAAGCCTGAACC
EMPV1_41299	CAGGTATGTAAACCTTTTTTCTAGTGATAAGTACCTTTGAAAGTGGGTGTTCTTGCACAG
EMPV1_41304	CCACGAATGAGTCTTAAGCTGAATGTTGTCTCTTTTTGGTAGATAAGTCATTCATACTAA
EMPV1_41305	ATGGGAAGGCCCTAAACAAACATTCCCAAGGTCGGTCCTCTTGGCCACAGACAGATGTAT
EMPV1_41306	GGTAAATATTGAAAGCTCCAGGCATCGAAATCAAGAGCAGAAGCATCCTCAGCCACAACC
EMPV1_41308	TTCGACTCCAGGACGACCGTATACTGATGAATGGACAGCCTCAGCCTCGTGACCATGGCT
EMPV1_41310	GCCAATAAGAGTTGATTATTCCTCATCCTCATTTTCATTCTCCTGACCAGAGCCTTCCGA
EMPV1_41313	GTAGTTGCAGGCATAGCCAATCATGGGACAATTCTCAAACACGTGACCTTTGCCTCCTGC
EMPV1_41314	GAAATTCCTTCCTGGACCCCACCAAACCCTCTTCAGTAATTGGGAGAAACTGAAAATGTT
EMPV1_41317	GCACACTGACCTGCCAGCCTTGATTATCTCCTTCCTCCCTTTCCCACCAGGTATTAGATT
EMPV1_41319	GCTCCTTTCAATCAAGGTGCAGTGATTCCAGGCTTTGGATGTAGAAGCTCTGAGCAGAGG
EMPV1_41323	ATTTATTCACTGCCCCGAGTCATATGGACTTCCTCAGCCAGGGACCAGATCCAAGCCACA
EMPV1_41324	GTGCCACCCCCTCCGGAAGACCTTTATGCCATCATTAGTGGTTAAGAAGCAACGAGCCAA
EMPV1_41325	ACCGGCAGTGGGACCAGTAGCTCTGTGTTGGGCAGCATAACACCATGTCCCTTCAAATTT
EMPV1_41326	TTGAAGGTACCAGCGTCCTTGATTTTCCAGCTTCAAAGAGAGCTGCCTCAAGTAGGGGGA
EMPV1_41328	GGTGGCAAGACCTGTTCAGAATCGTGTTCAGGATTTTTGACAATATGAAACTCCCTGAGC
EMPV1_41330	GGAGCATTTATAACACAAATCACGCAGAGACAGGAGGTGGTAGAAGGTTCCCAGTGAAAG
EMPV1_41331	AGCCTGTCCACGTGAAGCTGTGAAGCCAGGCACTGACTTCTCCCTAGCTGTGAAAGTTTT
EMPV1_41333	ATGCATTTCAAGGTAAGTCGCAAACTTCAGCTCACTTTCCCCTAAATACTTCAGCAGGGA
EMPV1_41334	ATGGCACACGGACGGCCCGCACCACCACAGTGGAGTCAAGTTTCGTGAGGCGCTCGGAGA
EMPV1_41335	GCTGACCTGGTACGCTGTAAGCTCATGGATGTAACTGGTGGCTTGGGCACTGATGAACTT
EMPV1_41337	CTAGAGCTTCACACCTAAACTCAGCTCGCCTTGAAGGAGATAACACTATGATTTGGGCAG
EMPV1_41338	GGCGCACCCTGCATTGTCTGATGAAAGTAGTTCGAGGGGAGAAAGGGAACCTTGGAATGA
EMPV1_41340	TCATCTTTATGGGGTTTAAGCTTGGGTAGGAGCAGATGGGGTGTGAGCTGGGGATTTGGG
EMPV1_41343	CAAGTTAGACCACGTGGTGACCATCATCAAGGGAAAGGTGGAGGAGGTGGAGCTGCCGGT

EMPV1_41344	TGACCGCCACTTTCCTGGGTAGGGATGGATAAAGGGGCACAGAGAGCAATCACTGGGGAA
EMPV1_41346	TCCCATTAGCGAGGAGCAAGACAAAAAGGCTGAGGAGAAAGTGAGCCTGAGGGGAAAGAG
EMPV1_41347	TTGGGCCTTGCGGGTGCATTCATGTAATCTGACTCTTGAGCTTTATGATGGAGTCTTCAA
EMPV1_41350	GACTTTGATGCAGCGGATAATGGTAGAGAAAGGGGGTGGGGGTACTGACCTTGGTATAAG
EMPV1_41351	CCTCACTTACTGGATTAAAGATTCAGAGCTGTTGCAAGCTGCTGCATAGGTCACAGGTGC
EMPV1_41353	CCTCTGAGGAGCTCGGCACCAACAAGGCCACCCTGGTGTGTCTAATAAGTGACTTCTACC
EMPV1_41354	GTCGGGAGTGCCCTTCAGATGAATGTGGTGCTGGAGTTTTTATGGCCAGCCACTTTGACA
EMPV1_41355	GGGGTGTCTACAAGTTTGGGCCTTCTTTTGGTTCTGCCTGTGTGCCGCAAGTGAATAAGA
EMPV1_41356	TCTCAAATGCTCATGGAACATTCTCAAGAATCGACCACATATTGGGACACAAAGCTAACC
EMPV1_41359	AGGTCTTAGATGAGAAGGAGTCCATTTCTTCAGCCTCTCTTGCTGGATCTAGCTTGCCTG
EMPV1_41360	CCATCAAGTACACCATCTCGGGCGAGGGCGCTGGGACCATCTTCCTGATCGATGAGCTGA
EMPV1_41361	GTTTTTGGTGTCTACGATCTTGACCACTTTAGATACATCATAGGAGTGGAATCACACAAC
EMPV1_41362	GCACACTCTTCCTCAACTTAGCTGTTTTATTCTGAACACGTTTGAGTTTGCCACAGTCTT
EMPV1_41364	CCATCCTTCCTGTGCTGCCGCCTCTCCCCCACAGTTGCTAGTTCTGCTTAAAAATGTAAA
EMPV1_41365	CAGAGTCCCTGCTCTTCCACTCACAGCCTTGGGCAACTTAGCCTCCCAGTATTTCAGATT
EMPV1_41370	ACTATTCATTATGGAACCGTCTCGGGGCAACCCTGGCAAACGGAGACCGCAGTGAGGAAG
EMPV1_41371	GTTAACAAACGTTGACGCTTCACTACGTCCTGGAGTCTGGATCTGAAAACCGTGTTTGTC
EMPV1_41372	GAAAGTCCAAGTCTGGAATCACGAAGCACAGCTGTCACTCGAACATCAAGTATTCACCAG
EMPV1_41374	CTGAACAGCCGGTGGATAGAGTTCTGCCAATTGCTAAGTGAGAGACTTAACTGGCTGGAG
EMPV1_41375	ACTACCCATTCTACCCTGGCTCCAGATCCTGAGGCTATCCATGAGTGGCCCAATGACAGA
EMPV1_41379	TGCCAAATTCTGGGAAATCCAGGGCTCCTCCTTAAAGATTCCCAATGTAGAACGGCGGAT
EMPV1_41381	TTGCGATACCTTCTAGCACTAGCTCAATGGCAGAGTGGACCAATGAAGATCCCCATCTAA
EMPV1_41383	GCAAATTCAGCAAAGTTGCAGGATAAAAAATCAACACAGAAAAATCAGTTCCGTTTCTAG
EMPV1_41384	CTGAGCAGCAGTCTTTAGTGTTAGTGAAGAGGCTCCTAGCAGTTTCAGTATCCTGCATCA
EMPV1_41385	GACACTCTGTTGGGTCCTTACCCTGCAGAGCCACGACAGGTATTCCTGTTATTTATTCTT
EMPV1_41388	CCAGGAGATTTGGGTAGGAATGGGGTTGATGGGATTCATCCTCTAAAAAAACAGGTGTGC
EMPV1_41391	ACCGAACCATAGTCATCACTTCTCATCCAGGTCAGATTGTCAAGCATGGAGATATCAAGT
EMPV1_41392	TGTCAGGGCTTACCTTTTTATTTTTAGGGTGCCGTTGAGTGCTTCCAGGGAGATGGAGGC
EMPV1_41393	GCAGGAGCAGCTGGTTTACAGTGTCCTGTCAATTTTCTACTGTACAGCAAGCACCAGAAA
EMPV1_41395	CCGGGACAGGGATGTCTGCCTGGTAATAGAGGACTTGAAACAGAAAGCAAGGGAATATGA
EMPV1_41399	TCAATACATGAAGCTATGGAGTTCCCACTGTGGCACAACAGGATCAAGTGCTGGGATGCC
EMPV1_41400	TTGACCCTGAAGCATGGCTGGCGAACTGGTGCAATTATCCCAGAATTAAGATCTGAGCTC
EMPV1_41401	CAGTACCTGGAAAGAAATGGGGGAGCAGAGGGAGTTACAGGAAGGCACATACGTGATGGT
EMPV1_41403	GTTGGAGGATCCGTTGTTTCATCGTCACATTAGAATCAGGAGTCTAAACCAGCCACAGCC

EMPV1_41404   ACAAGTACAGACCGGAGACAAAGCAAGAGAAGAAGCAGAGCTTGCAGGCCGGAGCAGAGA
EMPV1_41405   ATGAGCATACGGTCCATCTCCCACAACTTGCTCCTGGTCCTCAGATACGTGATACGTCTG
EMPV1_41406   TGCTTCAAAGGAGATAGCAGAAAATGCTCTGGGGAAACACAAGGAAAGAATAGGGCACAG
EMPV1_41408   CCTTCAGAGTGTTGAGGCATTAGATCCAGAACAACCTATTATCTCCCTGCGTGAAGCTGG
EMPV1_41409   AGGGTACCACGCTGAAGGTGTTCATGATCCGGTCCGGGAACTCCTCGCGGATCTTGCTGA
EMPV1_41411   TTGGTGGAACTGTTGCCCTTTGCATTGGACTTCTCACATCTGTCTACCTGGCCACTTTGC
EMPV1_41412   AAAAGAGTATCAAGAGAGAAAGCAAATTCAAAAAGCCCTCAGATCATGTACCTGGAGCAA
EMPV1_41414   GGATCCACCCAGAAATAGACAACCCTGAGTACTCCCCTGATAGCAACATCTATGCCTATG
EMPV1_41415   CTCAACCTAGGCGCAGCTTCCTCCTCTTGTCTGTCTCATGTGTGCTCTTTTTCTACCAGT
EMPV1_41417   GAAAAGTAAGGTTATAGCCATAGAAATGACAGCAGAGGGTTCCCTGAACTTAAATTGCAC
EMPV1_41418   TCTACTGCACATACACGGCATTCCAGGCACTGCTCCAGAGGCCACAACTTATTGTCAAGA
EMPV1_41421   CCTCTCTCTCAGCTCCATTTGCTTTATAAGGAAGGTCAGCTTTGCCAAGTTCTTGCTGTC
EMPV1_41423   TGTGTGTTAAGCTTCTACCCCATCTGCTCGATAGGCCCCCAGTACAGAGGAAGCAGTGAT
EMPV1_41424   CCCATATTCTGTGCCGAGGCCTCTGTTGATAGCTCTATTCTGGGAACAGACCCTCTATCT
EMPV1_41425   GCCTCTGCCTCGCCGGCGACCACTTCCCTGAAGAAAGGTTACCAGCATGGGGGGTGGCCA
EMPV1_41427   TTCTGTGATGCCCAAACTATACACCACTACACCTGTGAACTGCCTGCTCTTTTCCCCCTG
EMPV1_41432   GATAACAGAGCCAAGAATGCTGGAGTTACGATCCAAGACACTCTCAACACGTTGGACGGC
EMPV1_41433   TCAAGTTGTGTTCACTAGCCTGTTAAGGCATGAGCCAATGGTAAACTGACAGTGTCCCCT
EMPV1_41434   GAGGTAAATCTTACAGTATTTTGCCCCTCTCCGCACCCCAGGACTGGTTCCCTAGGAGTT
EMPV1_41437   CATTGATAGCGTCAATTATGAAAAGTAGCCAGAGGATTCTTGGGCATTCCTATTGCTTTC
EMPV1_41438   CCAAGCGGGACAGTGCTTGATACAGGAACAACATTTACAACTCATTCCAGAACCCAGGCC
EMPV1_41440   GTAGATATTGTGGATGTTCTTGTCAGCTTAGGCCAGGTCAAGGCCAGGGATGTAGGACAG
EMPV1_41443   ATCTCCCCATCCTCCCACATCTCCATCTCCCAAGAAGCCTCGGGGCCCTTCTCTCCTACT
EMPV1_41444   GTCACAAAGAGTCTGCTGGGTGCTGGTAGCAGTCCCCTATCTTTACAGTGTCTTTCCTTC
EMPV1_41445   CTGGGACTGTCGCCAGACTTCGGGAAGCAGTGTCTATGGAAACGAAGATCCCCACTGATC
EMPV1_41449   ACTCTTGGTTAAGCTTCTCCCTGTGTTCATCTGTTTTTCTCCCGAGTTTGTTGAGCATCG
EMPV1_41451   GGAAACTCTCCCTTGTGCTTGGAAACCCCAAATCCAAGACTGAGGTGGTCAGATGGTCAG
EMPV1_41453   CGGCTCCGAGCCGCCTCCGAGGGACGCAGGCCGAGGGCGGAAGGCAAACACACCAGCGGC
EMPV1_41454   TCAGGTGCATTACCACTGAGCTATGACAGGAACTCCCAAGTAAAGTTCTGATCCCACTGG
EMPV1_41456   CCACATTTCAAGGGCTCCATAGCTACATTTGGTTAGTGCCTGTCATATTGGAGAATGCAG
EMPV1_41457   GTTTTTCTCAAAGAAGGAACCCTGATGAAGCTATCTCGGAAAGTCATGCAGCCCCGAATG
EMPV1_41458   CCAACTCGCAGCAGATAAGAGATGCTCACAGCAAAGTGATGGAAAAGGTGCAGAACAGAG
EMPV1_41459   TTTTAAAGCACTGAGCCACAACACATGTTCGGGTACCTAAGCACCATGGGATCCAAGTAC
EMPV1_41460   TGGTGGGGTGTATGGGTAGGAGGCAGGTCCTAGGGTGGGGATCCTTTTCACAATGAATCA

EMPV1_41461 TGGCCACATTTGCTATCACCACTCTGTGGCTTGGGCCTTGTGATATTGTCTACCTGTGGT
EMPV1_41462 CATGACAATGCCCAAGGCCATACATGCTTAGTGGTTGAGCTAGAACTAAAAGCCACGGCT
EMPV1_41464 CCTGTGGTGAGAACTGTGCATCCAGCACCTGGCACAGACACTCTGGAGGAGGATGCACGA
EMPV1_41465 GACGTGGCTGCCCTTCTCATGGAAAACTGTAATGAGTTCGTGGGCCTGTGGCTGGGCATG
EMPV1_41466 CACCATGGCAATGTGGTGTACCTATTTGAAAGAGACTGTAGTGTGCAGAGGAGATATCAG
EMPV1_41469 AGTTTACTGTCAATGCTGTCAACTAAGTGGCCTGCAAGTATCTCTGGTTTAAGGAGAAGT
EMPV1_41470 CTCCTTCTCCTACTGTGGCTCCCGAGAAATACCTCAATTCTTCTGTGATGTCCCAGCACT
EMPV1_41471 CCATACGGTGTAGTGTGCAAGTATTTTCAGCGAGGGTACTGTATTTACGGAGACCGCTGC
EMPV1_41472 AGAAGCGTGGATTTTGTTGTAAAGAACTCAGCCTTGGAGTTCCTGTTTTGGCTCAGCGGA
EMPV1_41474 GCTCCGAGCTCGGAACTTTGACCTGCAGAAATCAGAGGCCATGCTCCGCAAGTACATGGA
EMPV1_41475 TTGATAGTCAGGACGGAGGCTTGGTTTGCAGAAGCTGTCATTTGCCCCCGAAACACAGTG
EMPV1_41476 TACAGGGGAAGCAGTTTTACTGTAGGGAGTGGGTTTTCCACAAGCTTCAGCATTGCCTCC
EMPV1_41477 CGTCTGTTCGTGTCATCTTTGGTCTCTTTCATCAGCCCCTTAGAGTTTTCAGAGGACAGG
EMPV1_41478 ATCCCACGCATCAGAGCATCCTCAAGCCCTGCCCCACTCCCTGCCCTGCAAAAGCCAAGT
EMPV1_41480 CCGGGAAGAGCAGAATTTGGTGGCTTATCCCCATGATGGAAAAATCTATTTCTGCACCTC
EMPV1_41481 TGGGTGAGCTGCCCATTAAGGACTGTGCTCAGGGTGGGAAAAAGAGGAAATCTGAAGGCA
EMPV1_41485 CAACTCCAAACACAGCGAGAACAAGTGGTGGGCAGGAGATTGACAGCTAAGTAGCAGGGT
EMPV1_41487 GGATGGTACTGGCATAAAAACAGACCAGGGGACCAGAATGGAGAGCCCAGAAATAAACCC
EMPV1_41488 TGCGTGGGAACAGTGAACACTCATACCTCCAACATTCAAGACAAACCCAGAGAAGAGGAG
EMPV1_41489 AGCCGGGGCTGATGCCCTTGCAACCCAACCTGGACTTCATGGACACTTTTGAGCCTTTCC
EMPV1_41491 TAAATAATGTCAGAAAGGATGATCCAATGCTTTTGTCTTCAGGGAGAGTGCAGCTGGTTG
EMPV1_41494 ACACTTGCGTTCATTGCGGATGTCAAAACCAACAAGCACCGGATTAAGCAGGCTGTGACG
EMPV1_41495 GGAAGGAAACGACCAAGAGTCTTTTATTGCCTTTGCCCGAGTATTCAGTGGTGTGGCTCG
EMPV1_41496 TACACTGGTGTGAATGACAAGGTCAAATGCTTCTGTTGTGGCCTGATGCTGGATAACTGG
EMPV1_41497 AAAAGCAGAGGCAGCGATAGAAAAGCAGAAGATTTCCAGGGTGGGCAGATGGATTGAGGG
EMPV1_41499 ATGGAGAGAGATCACAGCCAACCCTTCTGTAGAACCTGCCATGTGCAATTCGATCCTTGC
EMPV1_41500 CTGAAGAGGAGACTGAACAGGTGTTGGGCAATTAGCAGACTCCCCCAACTGAGACCCTGT
EMPV1_41502 GGATTCAGGGTGGGAAAATCAGGATACTGGCCCTAAATAGTAAAGCATGTGTCAAGAGGG
EMPV1_41503 GCATCATTACCAGGGTAAACCTGCCTGGTCCAGCATGCATTCATCATCTATTGTGACTGC
EMPV1_41505 TCTCATAGACTTGTGAGCATTATACACCACCATGTAGTCCCTCTTCTACTAGGGGCCACC
EMPV1_41510 CTCCGGTGCCTACTGAGCTGATATCAGTTCTCATTTTACACACTGGCTCAGTTCAGCAGG
EMPV1_41511 GGACAAATAGTTGGTGACAAGGTGCTTTAGTGCCCCAGTGTAAGAAGGTACAGGGAGTCC
EMPV1_41512 AGCTGTCAGAAATCTTTGATCCCACGCGAGAGTGCATGAACTCAGAGCTCTTGGAGGAGC
EMPV1_41513 GGCAATCGATATATCTGCCACAGGACTCACGGTCAGAATACATAAAGAAGCTTACATCCC

EMPV1_41514	CAGCGCTCCCAAGATGCCACCAATCCCATTGCACCACCGTGGGAACTCCAAACACTTTTT
EMPV1_41515	CTTCAGTGGTTGAAACCTATCTTCATGTCCTGAATCAATGGCTATTTCTGTCCCTTTTGG
EMPV1_41516	CCAGCTGAGATGGAAGCATTCAAACAAAGGCATTCTTACCCTGAGAGATTAGTTCGCAGC
EMPV1_41519	CCAGTATCCTGAAGAAGGTCCACAGCTTCTGCTGAGAGCCTCGAAGGTCAATGATAAGAG
EMPV1_41520	CTTAATGTTCATGCTTTGGAGAGTGTCGTAGCCCCAGACGTGTAGTTGCCCTTAGGTCCT
EMPV1_41522	TTAGTGTAAGGAGTTTAAAGGAAATACTCAAACTGGGTCTCATCGTCTGCCCGTTTGGGC
EMPV1_41524	CGGTGGTGACGAGGTCGTTTAAATAACGACTGTTAACAATGGCCAGAAGCCTGAAGAATT
EMPV1_41528	CTGGCCCCATTTCGGCATTTCCCATTACCCTCCACACTCCAAAAGGAAACCTCACTTCTA
EMPV1_41529	GAACGAGATAAAGTGATCTCTGTGTTCTACTCAGCAGTGACACCCATGCTGAACCCCATC
EMPV1_41530	AAAAGGAGATGCTGCTTCATTCGGGCCTCCAGCTTGGTACATCCAAAAGCCACACAGGCT
EMPV1_41531	ACTGCTGCCAGGGGTGCCTTTCCTGTGATGAGACAGCATGGTTAGCTGTCAGTGTAGACA
EMPV1_41536	CAAACGTGCTGACATCTATGCAATGGGCTTAGTATTCTGGGAAATAGCTCGACGATGTTC
EMPV1_41537	TGCAAGTGAGGAAAGCAAGTCACAAGAAAGATTGATAGAGTTCCCACTGTGGCTCGGTGG
EMPV1_41538	CAAAAATAGCCATTATGAAGAAATTCAACAAGCTATAAGAAAACTCAGAAAGGCATCCAC
EMPV1_41540	CCAGAGTCAGGGCTTCACTGCTGAAGTAAGAAAATAACATGTGCTTATCTCAAAAGAAGC
EMPV1_41541	CCTGGAAAAGGCTGAGATCTTGTTAAGCTTTGAAGCTGGGTGGAAAGCAGTTAATGGACT
EMPV1_41542	GCATTCCAGGGTTTTTTCATCTTATGTTTTGGAATGCTCTTGGATAATAAGCTTCGACAC
EMPV1_41543	ATCTGGATCATGGCAGCTGGGTATTAGGTTGGGAATGCTAGGCTGGGAGGAGGAAGAACA
EMPV1_41544	CTGGCTAGTGTGGCTTTGCATTGCCTGCTTCTTCTTTGACTTTTCCTCAACAGGAATCCC
EMPV1_41546	ACACGTTGTTCATGCACCTCTTTGTGGCCGTGGATGAGTATTCTGTTGGCTGCTGCAAAG
EMPV1_41547	GTCACCCAAGATATCTGCTGTATAAGCAATGAGGACCTGGTGCATAAACATGGGGCAACA
EMPV1_41548	CCCCTTTCCTACATCCAAGGACTGTCTCATAGAAATCTGCTGGGAGATGACACCACAGAC
EMPV1_41551	TCTCTGTTCTTTGGATCGGGGGCAATTATGTACCTCCGACCATCATCTTCTGGGTCTATG
EMPV1_41554	CCCGAGTCAGAAAGGAGGCCTGGTAGTGCTGAAGGCCTGTGACTACGGTGACCCTGATCA
EMPV1_41557	TGACCCCACAATTAGTTTGCAAACAGTGAAGCTCATGAAAGAAGGCTTGCAGGCCGCTGG
EMPV1_41558	GTGGCAAGTACCACCTTACGGAGTATACGGGCAGCCATGGAATCAACAAGGATTTGGAGT
EMPV1_41560	GTTTGCATCTACTAACATCAAACTCCCAGTCCATCCCACTGCTTGGCGACAAGTCTCTTC
EMPV1_41566	CACCGCACTCATCCTTCAGTCTCTGATTTGGCCCCTCTTCTCATCAGGTGCCTCAGCTTA
EMPV1_41567	TGCAATCCAGGCACAGAGGAGCATTTGAACTGGCTTACACTGGCTTTGTGAAACTCACTG
EMPV1_41568	AGTTGCATGGACACAAACCAAGGAATGCCTGGAGCCAGCAGAACCTGGAAGAAGCAAGGA
EMPV1_41570	TAAGGAAAATGCCAGAATAGAGTCAGAGGGACGTCGTATGGTGGTCGGAGGCATGTGTTG
EMPV1_41571	TGCCATCAAGCCATTCCAAGGCAATAGTTAGAATACAGAAATCTCTCCATGAGGGTTTGG
EMPV1_41572	GATATCCACTGCTTATGACCAACGTGGTATGTGGGCAACTTGTGGTCTCTGCTTGGGTTG
EMPV1_41573	GCGAGGAATTTAGGGCCATCGTTAACCTACCAGAGCACATCCAAGAAAGAGTCTAAATCC

EMPV1_41575 AGCTTTGAGTCTCGTCTTAGCTGGGTGGTGTGGCCAGAACAGCTCCCTCTTCTCGGTACT
EMPV1_41576 CAATGGAGAAAAAGACTGTCTCTTCAGTAAGTAGTGCTGGGAAAACTGGACAGCTACATA
EMPV1_41580 CTGGGGTCTTGTAGGCTGAGTTTCAGGGGTGGTTTTGGACTTTAGGACCATTAGCCTCCT
EMPV1_41581 AGCAGGAGAGCCCAGGGCGGCAAAGACATCCTTTGCAAGGTCAATATCTGGGGTCTTGAA
EMPV1_41583 ATGGCTCCACTGAACCGCCCAGCCCCTGTTGAAGTCACATACAGGAACATGAGATTTCTT
EMPV1_41584 ATCTTCGGCCAGACCACAGCCAACGCCATCTTTGTGGACCTCTTGTCTTAACCAGTACCT
EMPV1_41586 ATCTTTGGGAAGTGAGACGGGGTTATATTTATGATTCAACCTCTTGGGGTGAAGGTGGAC
EMPV1_41587 CATCACTGTGGTTGTAGTCTTCAAGGTCACTGCTCCAGACGAAAGGATGCGGCAGTGGTT
EMPV1_41589 GTGCTGAACATTTTACCTTTGCACTGTACCCCCAACCACTTTGGATGGCTATTGCAAATT
EMPV1_41590 TGCCGCCCCGTCTCAGCTTTGCCCTGTGTCCACTGACACTGGTGCCGTCAGGGTTGGCTT
EMPV1_41591 CCCATGCAATAGTTGGGACATACTTATACTAAAACACTCTTCGTTCTTTATCTGAAATTC
EMPV1_41592 AAAACAAGTTCGGGTTCCGACGCGGGGAGTTTTGCCGGAGCAGTACTTCAGGCTTTGTCA
EMPV1_41593 ACATTTCTCCCTACAAGGCCTCAGTCGCTCATCCTGGCAAGTCAGAGACAAGCAGGCTAA
EMPV1_41594 GGACAGGTGGGTCTTTAATGGTGGCATATGGGTTTTCAGAGCTGCTTAGGGAGCAGTTAC
EMPV1_41596 TCCTCATCCGGGCAGCAGCTACAAAGGAGAGATTTGAAGGGCAAGGTGAAAGCCTAGGAA
EMPV1_41597 AAGCAATCAAGGTCATATGGACACGGATGTTAGGGTGACCGACTCTCCCTCATCTCCTGG
EMPV1_41598 AAGAAGGAGAAGAGCTGCCCAGTGGGTGACCCAAATCAGATGAAGTGGACAAATGGCTAC
EMPV1_41599 CAGGAGCTGCTTTATTTAACAATCCAACATCTTCTTCAAACTCTGATGCAAACACTGACG
EMPV1_41601 AAGAGAAGCTTAGTGAGGTCCTGTTCAGTGGGAGGCAGCAAGGGTGGGAACATGTCTTCA
EMPV1_41603 ACATAGAAACTGGTAGACAGAGGCTGCAGGAGAGTTCACATCAGCGGTGGGGACAGGCCA
EMPV1_41605 AGGACCTCATTGCTTATCCACTCCAGGTGCAATAGTTGGCATCAACTAACCCCAAACTCC
EMPV1_41609 GGTGGTGGAACTTTCATAGTCAATGTTCACAGTATTGTGCAGGGGCATCAAGAAAACTGC
EMPV1_41610 CCAGCCCCAATCCCAGAATTTTTCCAAGCTCCCTGACCTTATGAAGTTTTCACCCCCCAA
EMPV1_41612 GTTTGCAGACCCCACTGAGGGGTTAGGGGTCTGCTCACTCAGCAATAAACAACTGTTTCA
EMPV1_41613 CCGTGAAATTGTGACTTAACAGAAATAGTTATGGCCAAAGAGGCTCCAATCAAGGCTCTC
EMPV1_41614 TCCGGAGTCGCTGCAGCAGCCGGCCAAGTTCGACGAAGCAGAGCAGGTGTGGGTGTGGGA
EMPV1_41615 CCTGCTTTTAGGGATGAGGAGGACTTCTTTACGGAGGGATGGTGGTCCAGGACATGAAGG
EMPV1_41617 CCTGGTACCCGCACCCTCATCCCCACTCACATATTCCTGGAGCAGCTGCTCAGCATATTT
EMPV1_41622 ATGACAGTGTGGTGGGCAAGCTCCTGTACGCCATAGAACATGAACAGCATCCTGACCTCT
EMPV1_41625 ACGGCACCGGTAGTTGCTGGACAGCGTGGTAGTCGACCTCCTGAACATTGTCAACCAGAA
EMPV1_41627 GCAATGGAGCCAAGCAGAGCTAGGACATGGAGAGAAAGAAACAAAGTCCTGGCCACATGA
EMPV1_41629 CCATGCAATCAGTTTGGAGAATCGGAGCCCCGCCCAAGCAAGGAAGTATTATCTTTTGCA
EMPV1_41632 CTGAGTGAGTTTCTCAAGCCAAACATTTTGAAATAAAGGAATGCTGGTGGGCTGAATAGG
EMPV1_41636 CCACCCTCAAAATCGATGGCGATCCCTCCAAGACGAGCTGAAGAGTAGATTAAGGGTGTT

EMPV1_41637	GGTTGGGGGCTTCGTCCTGGGTCTGCTCTTTGTTGCTGTGGGGCTGTTCATCTACTTCAA
EMPV1_41638	GGAGGGCACCCGGACTGGAGCTGAGTGTAAACAAACCATGAAGACCCAGAGATGTAAGAT
EMPV1_41639	GCACTACCCCGTCATCATGAACCCACGCTTTTGTTGCCTCTTAGTATTGGTGTCTTTTTT
EMPV1_41640	CTCTTGAGCCTCATCCCAGTCTCTTGGTACAACCACTTCTTGGCAGATCGCGCCGTCCTG
EMPV1_41642	GTACTCCAACGAGGACACACTCTCTGTGGCGCTGCCGTACTTCTGGGAGCACTTTGATAA
EMPV1_41643	GGCAGCCAGCGAGATGACTGACTTGCAAAGAAGCAGGAGTGTGGGCGGCCTGCACCAGAA
EMPV1_41644	AGCCATAAAGGTCAGTCTCACACCTACGTCCCGGCCAGCATGGCACCAGATTCGTAATAA
EMPV1_41645	ACGATTTCAGAACACCTGCTCTCCTTGCTGTCACTTCTCCACGTGACTCCCAGCCCACGA
EMPV1_41646	AATCATTCCATATACCTCAGTGTGTGTGTGGCCATCCCAAACCCCCAGTCCATCCCTTGC
EMPV1_41647	CCCCGCTTCCCTTCTCAATTCCTAGACGACGGCAGTATCAGCGTGCTCTCGTAAATAGGA
EMPV1_41648	GTTGGAATGAAATACAGAAACCTAATCCTGAAACCTGGGGGATCGCTGGACGGCATGGAC
EMPV1_41649	ACGTCGTGTCCACTGGTGACTGGTTACAACCGTGTGATTCTTGCGGAGTTTGACTACAAC
EMPV1_41652	TGCGGCACCAGCGGCAACAATCAGTTGGCAGAGAAGGTCAGATTGCGCCTTCGATACGAA
EMPV1_41653	GAGGTAGATGTGCTCCTGATGGAATTGTACTTTACGTGTTTGAGTTACTGTGTCGCTGGG
EMPV1_41654	TCATTTGCAGAGACACAAGCTGAATTAATGGCCCTGAAGAGCAGAATGAATAACGCAGAG
EMPV1_41656	GCTCTAATGCCTTCTTGGGGCATTAAGTTAATTGAGGTAAAATAATACCAATAACTTGAT
EMPV1_41657	ACTATGTAACGTGGACCCATAACCTCGGTGGACTCCTGATAACGCTGTTGAAACGGTTCC
EMPV1_41659	ATGTTGCAGCTCATTCCTCCCTTCCAGTGCCGAAGACACTGCCAATCTGTGGCGATGCCA
EMPV1_41662	GCCATCTGGTCACACAAATAAGGAGCTTGTCCAAACGCTTTTGATGTCCTTTCTGCTCAC
EMPV1_41664	GGCTCCTAGAAGCCTCTGGATGTTTTCTCTTGTTTTTGGCAAAAATTGCTGAAATCTGCT
EMPV1_41666	ACTTAGCCATGATCGCGAGCCAGACAGAAGTACAACACCTCCATTGCCACCTCTGCCATT
EMPV1_41667	AGAAGATGAAGTCTCCCAGAGTAAGATCACCATTGTTGGAACCGGTGCTGTAGGCATGGC
EMPV1_41670	ATAGCCAAGAGAGAGAAACTGATTTTGGAAATAGAAAAGGAGTCACGCCGCATGGAAGAG
EMPV1_41672	TCTCTACACTTCGGCCATGTTCTCTTACATGAACCCCCGTAGCACCCATGGTCCTGACAA
EMPV1_41673	CCCAGCGACACAGTAACTCAAACACGTAAAGTACAATTCCATCAGGAGCACATCTACCTC
EMPV1_41674	CCCAGCCATGTGGTATCTGTTAAAATGTCTCAGGTCTGCTATTAAATGAAGGCAGTCTGG
EMPV1_41677	GTGCACCCCCAGAAGCTGTTGATGCCAAGATGAAAGTGAAGCAGTGTGCAAATGGCATCT
EMPV1_41678	ACCAGCTCCTCTGAAGTTTGGCGAAATATGAGTTATTAAACCTACGCTCAGATCAAGTTA
EMPV1_41680	CAAGCCTGGACCTTTGCTGTCTTCCTTATGGACTGAACCATCACCATCTTTGTCATCTTC
EMPV1_41681	CAGTGTTTGGGGACATTGAAGTCACTTGTCTCGCAGTGGGGAGGCTACTGAGGGGTAGCA
EMPV1_41682	CTTGTCTTCTCTGTATCTTCTCTGTCACTGGAGTCAGGAAAGCCTGGGTTCCATTCAGGC
EMPV1_41684	CTGCAAGGTGACTAGCATGTTCTGTGAATCTGCCATTCCTAAAAATTTGATAAACACTCA
EMPV1_41685	ACCGAGACAAAAGCGACTCAGAAACCGAGGGCCTAGTGTTCTCCCGGGATCTCAACAAAG
EMPV1_41686	GATAGATGTTAAGCTCTTGCTTTTTAGTGGGAGGGATTGGGAGCTTGAGGTTAATGGATG

EMPV1_41687	GCTCTGGAATAGTCCGTTGCATAGCTGCTCTATAATTTAGCCATTTTCCTGTTGTTGGAC
EMPV1_41688	AAGTCGCTTAAGCACTGCGGCCCCACCTCTGCCCTCAAAGGAGGTAACTCAGTCCAGAAG
EMPV1_41693	CCTTACTGTGCGAACTCTGTGCTTTAAGAGTGTGAGAAATGGCTTGGGGAGGGTAGCCCT
EMPV1_41694	TTACTAACTTGACAATCATGTCTGGCCCCCCTCACTCCCCAGCCACTGCCAAACCTGATA
EMPV1_41695	ATGAGCCGACATAGCCGGCTCCAGAGGCAAGTTCTGAGCCTGTACCGCGAACTGCTGCGC
EMPV1_41696	TTGCGATCCGTGGTGATGAAGAGTTGGATTCGCTTATCAAGGCCACCATAGCTGGGGGTG
EMPV1_41698	ATTTAAAGAATTTCCTCCCACAGTGATACAGCCCTTGACACAAACCCCTCTCCTGGGGGC
EMPV1_41699	GTTCGGCGCCTTCTGCTTTGGCCTACGTTGTCACATACTCGCTCTGAGAAACTTGCATGT
EMPV1_41701	TGATCTTGGCCTCTCGGATGGCAGCACCAGTTACAAGAACCTCACGCTCAAATTCCACAA
EMPV1_41708	CCTGTGCACCGGCCTCTCCTGCTACTCCCGGTTCCCGGAGCTAGAAAGCCCTGGGCTCAG
EMPV1_41709	GCCTTGTGTGCCAAAGATCCTGTGAAGGAGAGAAGAGCACATGCACGACAGTGTTTACTA
EMPV1_41710	TCATACATGAACAGCTTTGCTAACCCTTGCTACTCGGCCTGGAGACTGGACATAGGACCA
EMPV1_41711	CATATACTGGTTGGGCTCCAGCCATGGAGAAAATCCATGGACATGTGTCTGCCCACCTGA
EMPV1_41712	GCTCGAAGCATTGAATGTGAAGTTGTTAAAAAAGATAAACAAATGAAGATATTAGAAAAC
EMPV1_41713	GCAAATTGGAGACAATCTGATTGTCCCTGGAGGAGTGAAGACCATCGAAGCCAATGGGAA
EMPV1_41714	CAACCTACAGCATCGTTTGCAGATGCGGCTGGTATCCTAGAAATGGCGCCAACTATTACC
EMPV1_41715	AGGCAGTGCAAGAAACGGCCCCGAGCAGTTGGATCAGGAAACTCTCATATCATCTCACTA
EMPV1_41718	AACGTCGCCGTATTGCTCTGAAGAAACAACCTACCAAGAAAAATATGGAAGAGGCTGCAG
EMPV1_41719	ATGGACATGGAAGGAGATTGCAACATCTGGATCGTGAAGCCAGGAGCCAAGTCCCGTGGA
EMPV1_41723	AGTTTGCGAGAAACTGTCTCAACTTGAAAGATGCTGATTCTACAGAATTTGAGCCAAATG
EMPV1_41724	GGCAAGTTCCCTTCCTTGCTGACCCACAATGAGAATATTGTGGCTAGAGTTGATGAAGTC
EMPV1_41725	GCCCGCCCTTCTCTATTTGGTGCCATTTACCTTATTGCCACTCCTCACAATGGCCTATTT
EMPV1_41726	ATTCAGGGAAAGCAGGTTGTCAGACCCTTGTGGAGCCAGCAGACAATAGGGGCAGCAATT
EMPV1_41727	TACCTGCCGGGCAGCTGGCAGGGTGTGTTGCACACAGCGTTGGTGCATGTTAAATAAAAT
EMPV1_41729	GATCTTTTTCGCACCAAATCCAAAAATGAAGAAATCCAGCTTGAACTGGGAAAACTGGAG
EMPV1_41730	TTTTATTACGGCCACACCTGCCGCATACGGAAGTTCCGGGCTAGGGATCGAATTAGAGCT
EMPV1_41732	GGAGCAGCCCCAGCTATCAGTCAGAAAGGAAACCAGAGACCACAGTCCTAAACCTGTAAA
EMPV1_41736	AGGAGCTGGCTCAGATGCGGAAACAGTGCCTGGACTATCACTACAGAGAGATGGAGGCGC
EMPV1_41737	CTGGAGCCGCTACTACCAAGGATCTCAAGGGGTAATATTTGTATTAGACAGTGCCTCTTC
EMPV1_41742	GTGGTGGAGACCGGTAGGGTCCCCTTTCCCTAATTTACTCTTGACCTAAAGATTCCTCAC
EMPV1_41744	TTCTGACCCCAGTGCTGAGTCTGTCCCTTTCCCTGACCATATAGACAGTTCTAGACTCCT
EMPV1_41745	CAAGAACCTAGTGACAGGAAACCAGAGTCAGAGCAGGAGACACCCATGTAGGGTGAGCCT
EMPV1_41747	CCAGTGTAATCACAAGGTCCATATAAGAGGAGGCAAGAGGAGTGAGAGTCAGAGGAAAGG
EMPV1_41748	TGCTGGCTCCACACAGAAGAAGGATTTATATGGACCTTTCTTGGTCCTGTCTGCACCATC

EMPV1_41749	AGGATGCCCCGTACCCCCTACTTCATGCCCTTGCTGCTACTGCTGCTGCTCCTCTCACTT
EMPV1_41751	CCTAAAGACAGTGTTGCAGGAGGTCAGAAAAAATCCTCCACCTAACATCCCCCAAGGGTC
EMPV1_41752	GAAAGTAGCCTCTCAGACTCAAAGGCCTTATCTCACCGCTGGTACCTCAATTTATCACCC
EMPV1_41753	CTGGCACTGGTTCGGCTATCCTGCCTCATTTCCACCATGCGTTCAGATGACTGGTTTTTA
EMPV1_41754	AATACTTCCACAGACTCACCCTCTATCTGCAAGAGAAGAACTACAGCCTCTGTGCCTGGG
EMPV1_41755	GCACCGACTAACTATGGATTCTGCTTTAAGCCTAACAAAGCGGGAGGGCCCCGAGACCTG
EMPV1_41756	CCGGGCAGCTCCCAGTTTAAATTTTCTTGCTCTGCGTCCAAGCTAATATCCCTTCACACC
EMPV1_41757	AGCAGATGAGGATCTAGTAAAGCAGTGTCGGACTCCACCCCAGCAGAAATCACCCACCAT
EMPV1_41759	CCTGGCCGACTCCCACCCAGCAACATTCGACATTCCCATTCTATCAGCAGAAAATGCTGC
EMPV1_41761	CCAGTAACTTCCTGCATTATGTGGTCATTTAGCTGAGCTTCCCACTGATAGCTGGCATCA
EMPV1_41762	GCTCCTTTTTGCTGTCTTGTGAGTCTTAGCCAGTGATCCAGGTGCCCAAGGAGAGAGTCT
EMPV1_41763	TGGAGAACCAGAGCAGCAGCTTCCTCAAGAGTATGGAACTCAACGTGCTGGACTCACTCA
EMPV1_41764	GAATATAGAGAGCATCTACGCGCTAAACAGCCAGCTACTCCGAGACCTGGACAGCTGCAA
EMPV1_41765	TCAGCCTTTCCACGTTGCAGAGGTGTTTACTGGTCAGTTGGGCAAGTTCGTACCGCTGAA
EMPV1_41766	GCTAGTAGCATCACCAAAGAGCTGCAGGGAAAAGAGCTAATGCTTAATAACCTTCAGGAA
EMPV1_41767	CTCCCAGCAGATCCGGTCACACCTCTCTGCTTTAAGCCTTAGCCAACTAGTGACAAGTAA
EMPV1_41768	AGGAGGCAGCAGTGTAGACCCTGTTTTTCTCACAGAAGAGGCTGGAAAGGACAAGCCCA
EMPV1_41769	GGAACTTCCATGTGCCACAGGTATGGGCCCACAAAGCAGAGGAAATAAAAGCACATACTA
EMPV1_41772	ATCTGCTCGACAAACTCGTCCTTGCTAATGTACGCACAGCGATGACCAACCGAGCAGGCT
EMPV1_41773	CAAGTGCTTAGAGCAAGTGAGGAAAATCCAGGCTGATGGCGCCGAGCTGCAGGAGAGGCT
EMPV1_41774	CGCATTGAACCTGAAGAAGGCAGCCTCGCAGGGGGAACATGGATCACAGTCTTTTTTGAT
EMPV1_41776	AGGTTGGAGAGACTACACCTGACAAACTTTTCACTCTTATAGAAGTGGAATGTTTAGGGG
EMPV1_41777	GGTCAAAATTCAGTCCCCACTTTAGCTCAGGTTTCTGTAGCTGGATCAGGCACCGGAAGA
EMPV1_41779	GACACTGTACAGCATGGTGTGGAAGGATTAATATACCTCCTCACTGAAAGCTCAAAGCTC
EMPV1_41780	CTTCTGCGCTTCCTGGTGTCTGAGCAGGCCTCACTGCTACTCTTCAGTGATGGGACGGTA
EMPV1_41781	GTTCATCTCTTTCTTTCCCAAGCTGTGGTAGCCAACAAAAAGCAGAGTAAAATTCTAGTG
EMPV1_41782	GATGGCTCTCACGGCCGCCAGCCCCCCGGAGGTGGTCCGTGCGGCGCAGAAGGACGACTA
EMPV1_41783	GCGACAACCGTATCTCGGTCAGTTAGAGATATGTTGTTTTGCAGATGACGAGGAGATCCC
EMPV1_41787	TCCAAGGTTCTGTGACTTTGCTGGGCTTCCCCTTTCTGAGCTGGTTTACAGGTGCCTCAG
EMPV1_41789	CTTCAGTATCAGAGCCTGAGTTTTATCTCGAACCTTGTCTTTGGCATCTCCCAATCTGTC
EMPV1_41791	ACGGGCTCTCCACCTTGCTTACGTTTTCAAAGTCCTCTTCCTCGTCGCTGTCTATCCGAT
EMPV1_41793	CCTAGCCTGGAGTCTGGGACATAAGTCCACCCCAGCCTGGAACCTGGAATCAGGATCCCA
EMPV1_41794	GTGCATCATGACAATTTCCAGAACGGGCAAGCTGGATCTGGTTGGACTGATGAGACCGTT
EMPV1_41796	TCCATGTGGTTCACCTCAGCACTGCATTTGCCAAAATTGACGAGGCCTTGGGGCGCCCGA

EMPV1_41797	TGGCACATTGGATGTCTTGGTTGGTTTGTCGGATGAACTGGCTAAACTGGATGCATTTGT
EMPV1_41799	CCAGTGGCTTCACGGGTGAATTCTACCAAACATACAAAAGGAACTTATACCCATCCTCCT
EMPV1_41802	CCCAGGAACTGAGTCTTAATTAGGCAAAGTCAATCAGGATATGCCCTCACCTTGCCAGGG
EMPV1_41803	GGTACCAACAGCACCCGGGCTTGGCCCCCAAATTCCTGATTTATTATGTCAACACACGGG
EMPV1_41805	AGGATAAGGCACCGTCCTGCTTCGTCCCCTCCTGTTTCACATTTTCAGGGTGTTTCATCG
EMPV1_41807	AAGGATTTACTCCTCAATGAAATTCCCAGCAGCACCGTGTCGCTCTGCTCCAGGAGGACG
EMPV1_41808	GAAACTCAGCTCCCACCATGCCTCCACCTTGCACGGATTTTTGGACTCAGATGAATCTAG
EMPV1_41809	AAGGAAGATGGAAGATTGAGAGTTTCCCCCAGTCAAAGTGCCAAACATGTGTCCTTGCAC
EMPV1_41812	CCCCAGTGGTGCACATCTTAAAAGTTCTGTCTGACCACAGGAATTCAATGGTGGGTATAA
EMPV1_41813	TGACTGTCACTTAACTTGTGGAACCCAGCTCCCTACCTGGTCCTTGTCTCCAAGCACCTT
EMPV1_41814	GGAGGCCAGAGGTAGTGGGATGAAATATTCAAAGTGATGAAAGAAAAAGTCTGTCAACCA
EMPV1_41820	TTGTTAATTGCTTAGAGCAGGCTTACCACAAGCCCCAGGAGGACTGCAGAGCTGCCCACA
EMPV1_41825	TTAAAGGTGGCATGAGACCAGGCAAGAAGGTGTTAGTGATGGGCATCGTAGACCTCAACC
EMPV1_41826	GGCAAAAATGGCAGATACGTGGGGATGTGTTACGTGATGTGGCTGAGATTGTAAAGGGGC
EMPV1_41827	TACCACGTCCACGAGTGTGTAATTACAGCACCTAGTCGATGAGGGTAGACGGAGGCTATG
EMPV1_41829	ACAAGCAGGTAAACAGACACCTGGAGCACCAACAGGAAAGACTAGGGGTCGGAAGGACAA
EMPV1_41832	GAATCAGCTCACCTTCTGCGACTCAAATATCATCCATCACTTTCTCTGTGACCTCAGTCC
EMPV1_41834	GATTGCACACCAAGCAGTGAAAATAGTGTTTGACTCTATTGACAACCTAGAGGCGGCGCC
EMPV1_41835	CCCTACTTAGGGGCGGGATGTCGCTCCGAGGTCAAAACCTGCTCCGAGGTGGACGAGCCG
EMPV1_41836	AGAAGACTCCTCTCAGATCTCTGACTTCAGGAAGCATAATTAACCCTTGGCCCAGCTGCC
EMPV1_41838	GCGGTTGCCTGGATCATTGGTCTAACTCACTCATTGGCCCAATTGGCTTTCGTAGTCAAC
EMPV1_41839	TGCCTTTTCCTGTGCGCTTTGCTACCATGTTCTCTGATTGCCTTGGTGTCTCTGTCAGTC
EMPV1_41841	CGCCTGGGAGCACTGGACATAGACCCAAAAAAAGGAAAGGACAGCCTTCCACAGACAAAT
EMPV1_41843	GATCATGGTCAGAGTGATGCTCGTGGTCGGAGTGATGCTCGTGGTCATGGTGTATATGGA
EMPV1_41846	TTGGGTTATGAACCTGACAAGTATCCATTAGGATGCAGGTTCCATCCCTGACCTCGCTCA
EMPV1_41847	GTGTGGTGGTGCAGCTTGGGAAGACAGGGGAGTGCTGTAGTTCTCTCTTTAGGACTCAGT
EMPV1_41848	CCCACCAGCATTTAATATGATGCCATGTAAATTACTGAAGGATAAGAAGTGGGTACCAAG
EMPV1_41849	GGAGATTGTCCGGCTCAAGCCTCTGCTCCAGCTCTTGGGTCTCTGCTGACGGCTTCCTGA
EMPV1_41851	GTCTAGGTCAGGACAAAACATTGGAATGGATCAGCCTTCTCTGGGTTCTGCTTTCCTCCC
EMPV1_41852	CCACACGGTCGTGGGCATGTCGCAAATGGACTCGCACAAGCGCAAGCACGAGAAGCAGGA
EMPV1_41855	GAGGGACCACCACCATTCTCATTCTTATCCCACTCAATCAAAGGAACTCTCGGAACGAGG
EMPV1_41856	TCCCTACAGAATGCTTCTTTCTCACCCTGCACGCCCACCACCTCTCTATTCTGCCGAGCT
EMPV1_41857	AGTGTTTGATAAAGTCTCGCAGACACGGTGAGGGCTCGTTGCAACTGCAACTGACAGGGA
EMPV1_41858	GGGGACTGAATCCACACTACAGCAATGACAACACTCGATCCTTAACCTGCTGCACCACAA

EMPV1_41859 AGTGTTGGGTCTGGGTTTTGCTTTACTCTGAAGTCCTTGTGTCCCTGCTGCAGAGCTACA
EMPV1_41861 GCCCTGCTGTAAAAGGAGAATAAAGAATTGGCAGAAGTAGCAGAACATGTCCAGTTTATG
EMPV1_41862 AATATTACTTGTCATCGTCATTGTCCTCATCGTTGTCCTCGTCGGCGTGAAGTCTCACTG
EMPV1_41864 TGGTGGTCATACAGTCATCCACCGTTCTCCACATCCTCCTCTGCCTGGTTTCATTCCTGT
EMPV1_41865 TAGACTCCTTGCTCCGTGGGTTACTGGTGTTGCGGCTGTCCTTTTGTACTGACTTGGAGA
EMPV1_41866 CCAGCGGTGTCCTCCTGATTCTGTTCACACTTTACCCTGAAAGCAGGGATTCTTAACCTG
EMPV1_41868 CTTGGGAAGAAAGCCTCCGGACTTGTTTGGACTCCCAGGATTTTGTTCACATCAAGCCAG
EMPV1_41871 AGGGAAAGAAGACAGATTCAGTGGTGTCCTGGCAGGGATAGAGGAAAGGCTCAGAGACCT
EMPV1_41872 TTGGCTAAACGCCTCAAGAAGGAGAAAGTAAATGTTGACATTATCAATTTTGGGGAAGAG
EMPV1_41873 GGGAGCAGACAGTAATATTACCAACAAAGCAGGGGAGAAACCCAGTGATGTGGCTAAGAG
EMPV1_41878 GTCTTGTGAGTATGGTGTCAAACCATTTTGGCAGTGGCCGCCAAGAGAGGGCATTGGGTA
EMPV1_41879 GTATCTTTAAAATCACCTGTTGGCATGTGTGTGTCTCCCCAGCTAGACGGAGGGCTTCTG
EMPV1_41883 GGGCCGTAGTCCTAAACAGACGTTTCTCCAAAGAAGACATGGATGGCCAACAGACACAAG
EMPV1_41885 TATCAGGAGTCTTACTGGTTCCTATGCCAAATTACCTAGCCCAGAGCCAAGCCTGAGTCC
EMPV1_41886 GGAACTTTCATCCTGCTGTCCTGGGAAAACACTCTGCTTGGTATTCACCTTCCTCTTACT
EMPV1_41888 TCAAAGTCAGCCTGACCTTATTGGAGACAAGTTAGTAGGAGGGCTGCTTTCCCTCAGCCT
EMPV1_41889 AAGAGGAACTCAGGTGGAAATAGAGGGAACTATGTTCAGGCTGCAAGCTGACTTGTGTGG
EMPV1_41890 GTATCCATCTCCCGTAAACTCGCTGCACACCCCACCTCTCCACCGGCACAATGTCTTCAA
EMPV1_41893 AAGCTGCTTGTACTGTCAGTGAGCTTCCAAACACATCATTAACCCAGTGGTGACTCGCGA
EMPV1_41897 GCTAGCAAAACTTAGTCTTTTGGATAAAGACAGCAAAAGGCAGAGTAGGAAGCTTCCCAC
EMPV1_41899 AACCCTTACAGTTCTGCTAAACTCAATAAATCGCTAGATGTACACTGAGCAGTGAGATGC
EMPV1_41901 GCTCTAAAAGCCATTGACTGGGTAGCCTTAGGAGAGATCATACCCCAAAACCAGAAGGCC
EMPV1_41902 TCCGTATGAAGAATGTAAGAGGAGGAGGAGTACAAGGGTCTATGACCCTCCTGATGCCCC
EMPV1_41904 TATACAACCATCATGAATTGGAGAGTGTGTGGGCTACTACTGGGAGTGTCATGGGTGGGA
EMPV1_41907 CCTGCTGCTTTGCTGAGTTCGTTGATCAGTTTGAGTAGTTTTTATGCAAGGTCCTTTGGG
EMPV1_41908 AATGAGCTAAAAACATTAAGAAAGTAACATAAGCTAGGAATGCCTAAGCAAAATATATGC
EMPV1_41910 TGGAAGGGCCCCCTGTTCTTCCGGTTTGTCAGCACACTGGTAGACCCAGACTTGGCTATT
EMPV1_41912 AAAGCTGCATTTCAGGGCGCAGCGCTCTGCGAGACGAGAAGGGGTCAGAGCTACTGGAGT
EMPV1_41913 AGCTATGTCCACGCCAATGACATCTACCAGGTCACTCCTGCCTACCGCCTTGGCTCCAAT
EMPV1_41914 GTGAACCTCATCACAGCAGATCGTGTGTCCCCACTCCATGAAGCTTGTCTCGGAGGTCAT
EMPV1_41917 ATCCTATGATGCTACTGGACGAGGTCGTGGTACACGTGAGATGCCCTTTTCGAGAGAGAG
EMPV1_41921 CGGTTAAAGGGTTTGGGCTCCCGTAGCAAACTAATACTAGATAATAAGGAAATGGGGGTG
EMPV1_41922 AGAGAAGATTGCACAGCTTTCTTCCAGGGCTCAGGAAATAATTAAAAGCTGGGCTGTCAC
EMPV1_41923 GTAGGTCTCTTTCACTTGGCTTCAGGTGGTACTTCTGGGAGCGGTGGTAACTTCTCTATG

EMPV1_41929	TATTAGCAAGAAGAGCTGCCGCCGTGTAGACAAGAGGCCACTGACAAGTTGGGATGTGCC
EMPV1_41931	TTTCTGGGTGGCCTGGTGGTCACCATCTTCCTGGACATCGTCCACATCAGCATCTTCTAC
EMPV1_41933	CGAATTCCCCAACGCGCTCTACACCCACTGCTACAACTGGCCTGAGATGAAGCTCTTCGT
EMPV1_41935	GCCATGGCTGAGACCTTTTACCTTTCTAATATTGTGCCTCAGAATTTTGATAATAATGCT
EMPV1_41936	TCATTCTGTAGCAGAAGAAGAACTGCTGAACATGTTGCCAGCGATGCAGAAAGATGATCC
EMPV1_41939	GGACTGGCCTTATTCCGGGACAAAAACAATGTATTCTTTTGAAAGGGGTGTGTAAGGACC
EMPV1_41940	ACATCCAGGCAGTGTATGTGCAGAATGTGGTCAAGTTGTATGCGGCCATCCTGCAGCAGA
EMPV1_41943	CTCCTGGCTCATTCCCCAGACCTCTTTCCTCAGCAACGTCTGTGGACTTGGAATTGGGCT
EMPV1_41944	GCATATATTTTGCTTAGGCATTCCTAGCTTATGTTACTTTCTTAATGTTTTTAGCTCATT
EMPV1_41947	ACACCATCTGCTCCCCCTGGCTATAACATTGCCGTCAAACCAGATCAAATCCAGTACACC
EMPV1_41948	CCTGAAGGAGCTGAGGGTGGGGAATAGATTTTTGGAGTCATTTAAGCGAGATGTCCTGAG
EMPV1_41951	GCAGTTTCCAGTGAGGATGGAGGGAGGAGGTACATGGAATGGTGGTGGAGTCCTTATAAA
EMPV1_41955	AACAGCTACAGGAGCTGCTTGTGTACTAGAAGTATTGGCATTAGTTACAGGAGCCATGGG
EMPV1_41957	GGTTGGGAAAGGTCGACATCCTCGTAAACTGTGCAGCTGGGAACTTCCTGTGCCCCGCCA
EMPV1_41959	ATGGCAAAAAGACTCCGCGGGGAGAAGCCCAATGCGAGGTGCTGCAGACTGGTTTGTCCA
EMPV1_41961	ATGAGGTGTCAGGCCTCTGCCTCTCCAAGGACACAGAAGAGAGAAGCCCAGTCAGTGCAA
EMPV1_41964	ATCAAGGAACTCAGGACCCTCGGACGGCCAACTAGGGTCAGGGTCTGTGTGTGGCTTCTC
EMPV1_41965	AGTCCTTGTGCATGACCCCCAGTGCTTGCCTATATTCTCTCTTGATGGGCTGGATCCTTC
EMPV1_41966	ACAGGATGAGGAGGAAGCACGTTCATCCCAAATTCAAAGACCACTTTTACAAGAACCTGC
EMPV1_41968	TTGTACCCTCATTCAGTGACATGAGCACTTCTCCAAACAAGCTAATACAAGGGCAAACAC
EMPV1_41969	GAGAATAAAAACATTGTCACTCCACAGTTCCTATAATCTATTCTGGACATTGGAGACACC
EMPV1_41970	CATCCAAGGGGAAGCAGCCGTTGTCATCCGCCTCAATGGACAGCGGCTTCCCACAGTCCT
EMPV1_41972	TCAAGGAAGGCCTGTGTAACTCTGACACCTCTCCAACGTACTGGCATTCACAGCGTGATG
EMPV1_41973	ATAATTCATGCAATGGGTGCTAATCGCCCGCTCGCCAGCCGCCGTTCAGTTGCAGTACTC
EMPV1_41974	CCTAAAAGTCAGTTGAGTAAATGCAATAAATATTGGTTGCTCATATATGCCAGAAGTAAC
EMPV1_41975	CAACCAATGATCCAGCAAATGAAGGCACGTCCACACTGGAGCCCTCATATCCATAGTTGG
EMPV1_41977	ACATCTTTCTAGACACAAGCCCACCACTCACGTCCAGCACAGGGGAAAGGAGGTGTCTTA
EMPV1_41978	CTCTCTTCCGAAACACCAATGTGGAAGACGTTCTCAATGCCCGAAAACTGGAGCGGGACT
EMPV1_41979	GGGCAAGGAGTGGGATGGACAGGGAGTTTGGAGTTAGTGGATGCAAACTGTTGCATTTGG
EMPV1_41980	GCTGCTGTTGGGAGACCCTGGTCTGCACTCTATCTGTATGCTTACTGAAGGGAGCGCAGG
EMPV1_41981	GCCCCTGTGGGCCAGTGCTTCAGGTTGTTTTCCAGAGGTGGTGGTTTATTCATATTGTTC
EMPV1_41982	TTACTTAGACTAACAAGCCCCAGCTGTTCACAGCCCAGAGGCCTACCTCCTACCTCATTT
EMPV1_41985	TGACGCCCGCATCACTGAGATTTACGAAGGCACCAGTGAGATCCAGAGGCTGGTGATCGC
EMPV1_41986	GGGTCATTCAGAACATTTAGAAGCTAGTTAGTGGGGGGAGTTCGCTTCATGGCTCAGTGG

EMPV1_41988	TGGTAATGCTCTCCTTTTATTGGTTCTGGTACTATTTATGACTCTTCATTCTGTGAGAGC
EMPV1_41989	GGCAGCTTTCTCGAAGTCTCCCTTCCACCATTAGAGCCACTGGGCACAACTTCAGTAACT
EMPV1_41990	ATTGGCAGGTACTGGAGTTCCCTGGTGGCTCAGTTGGTTAAGAATATGGCATTGTCACTG
EMPV1_41991	GGAGGCAATGACATCCTGATGTGTTCCTTGATCTTTGTGCTTTGGAGCTGGCGAAAGGTC
EMPV1_41993	AACATTCCCATCGGCAGAAGCGTAGGGAAAGGACTTCTCTGGGGACCCAACATGAGACTT
EMPV1_41995	AGCATGCTGAAGAGGAGGCAAATGAAGAAGATGTACTGGGAGATGAAACAGAGGAAGAAG
EMPV1_41998	CACGTGTGCTCACAGCATCTCTGCCGTAGTTGGAATTGCCCATGTTTATGTCGGTCACTT
EMPV1_42000	CAGGGTAAAGTGTTGCAATGCAGACAGCTCATTCTCAGCTGATTCAGAAAACAATCATAC
EMPV1_42002	GACGGCCTCATCCTTATGACAGTAACTCCAGTGATCCAGAGAATTGGGATCGGAAATTGC
EMPV1_42004	TGTACCACCCCCCATGCTGCCCCTGTCCCGCTGGCTGAGATCTGTGGGGCTCTTCTTGCT
EMPV1_42005	GAGGGAAGACGGAGAAGCCATAATTGCTGGTATTTTGGTGGGCCTGAATGTCACTGATGC
EMPV1_42006	GGCTCCTGAATACATACTTTCTGTGAGCTGTGAAACAGCTAGTGATATGTACTCCTTAGG
EMPV1_42007	CCACTGCCAGGGATTTCCAACTAGATAGCCATGTGACTTCCTTAATGACTTGGGGGACAA
EMPV1_42008	CTTCTTCTAGGACCAGCGCAGGACAAATGTCAAGAACTCATGTGCAAGTGTGACCAGGCG
EMPV1_42009	TGACAGGTCTTACTGGCACAGGCCACTTCCGGGCATGTTCTCACAATCTGGTTGAAAACC
EMPV1_42010	AAGGGAGGTAGGGCTTCTACCGTTCTGTCTAGATTTCCCAGGTCTCTCCCATTCTACTTG
EMPV1_42014	GTAGCCCCTTGGCACTGCTGTTGTCATTAGGAAAAGCTATTGTGGCTTCAGTCAAGGATG
EMPV1_42017	GTGTGATTCTGATTGTTGTCATGTGAAAAGTACAAATATCCCCACTCGGTTATGCTCCAG
EMPV1_42018	AAGGAAGATGGAAGATTGAGAGTTTCCCCCAGTCAAAGTGCCAAACATGTGTCCTTGCAC
EMPV1_42019	CCAGGGCTCATGGAGGGACATACAGATGTTTTGGCTCCTACAACAAGCACGCATGGTCTT
EMPV1_42021	TTACTTTGACCTGCGAATTGGAGATGAAGAAATAGGCGGGGTGGTGATCGGTGTCTTTGG
EMPV1_42026	GGGAAGACTAAGTTAATCCAGTTCCCAAGAACACCAGCTGAATGAGGCTTCAAGAGTCAG
EMPV1_42028	CCTAGAGCAGCATCTAAAATGACACCAGAGGTCTCAACAAACATGACTTTCACAACCTTG
EMPV1_42030	CTCATTCTCCTCTAAGTGGTGTCTAGGCTTTTCCATGTGACAGTATTTGCTTTGGGCCTG
EMPV1_42031	CCCATTTTGGGTTGTGGTGCCTGTGTATCTTTGCCCGTCTGGTCTCCAGTTGGTGGAATT
EMPV1_42032	GCAGTATTAGGAAAGGGGCTGACGCGGTGAAGAAACAGATGCTCCAAAGCAAGATGGTCT
EMPV1_42033	GCCCTCTGGTGGCAGAGCTCGGGAGGCTGCTTGTAATCGGGCAGAAGGAGAGCGAGCTGT
EMPV1_42035	TGGATCGACCATACGCATTGCCCAGATATTGAGGCCCATGAATCCTCGGGAATTATCTTT
EMPV1_42036	GACTGATTTTAGAAGAGTAGGTGTTCTTGCCCATTATTACACATGTGGGACTGGCCTTCC
EMPV1_42037	ATCCCACCCTATTTAGTCCTCTCCTGTAGTCCCAGCCCTGTGTGATGACCTCTTTGGCAA
EMPV1_42039	GCCAACCCAGTGTTCAGACTACCTGTTCAGGGGGCTCTGAATGTGTACAGTAGTCTGCAC
EMPV1_42040	TAAATGCAAAAGTGGTCGGGGGTCCACACGGAGACAAAGACAAAATGGTTGCGGTCGCCT
EMPV1_42042	ACCAGCGAGCCAAAGTTTTCATTCACGATTGTCTGCCCCCTGTGTAGACTGACAGGGAGA
EMPV1_42043	CCAAGTGCTCTTTTTCTATGTGAAGTACTTGGTGCTCTTCGGCGTCCCCGCTCTGCTCAT

EMPV1_42044 AGACCTGCAGTTATATACTACAGCTGTTGGTTTCAAGCAGAGGCCCAGAGGACTGTCTTC
EMPV1_42045 GGGCTTTTCCGCAGGCCAAGTCCAGCTTATAGACCCAATCAAAAAAGAAACTAGCAAACT
EMPV1_42046 GAGAAGAACCCCCAGCAATCAGACCACTGTAATACCCTCTGCTTCTCCTGTACCACATAG
EMPV1_42047 GTCTGGGATCCTGAAAGCAGTGCTGTCCATGTATGGCTTTCGAGAACTTGAAATATGACT
EMPV1_42048 GTACTTTGCCAAAAAAAATGAGGAAAGAAAGAAACATAAAATAGAAGCTAAATTACGAGC
EMPV1_42050 CCTAAGCCTGGAGCTTTATTTTTCAGCAGTGTCACTTGTATCTGTGGAATACTGGTGATG
EMPV1_42054 TTAATCTCCCACAACTTCTTTTTCCGGAAAATGTAATGCAGAATAAAGATAATACCCTCG
EMPV1_42055 TGTACAACCACGAGACCCACCTCCAGAGCGATCCTGAGTATATCGGGGAAGAATTTCAGA
EMPV1_42056 CACTGATACTCCCAACCATGATTTCTCTGCCTACTTCTGACAATTTGGACCTCTGGGCCG
EMPV1_42058 TCTTCATGCAAGAAGTAAGGCTTTCTGTTCCACTGCATTTGTAAATAAACTTGCTGATGC
EMPV1_42060 TTGCTCGCGTTATGTTTCTCATCCGAATATAAGACGAACAAAAGGTTTGTCTGAGGGCAG
EMPV1_42061 AGAGATCACGTTTCCCGACCAGACTGTGAAGACCTTATTCGCTGACGGGCAAGAAGAAAG
EMPV1_42062 GAGTTCATTAGCTTATTGGAGGACGCTCCTACACGTGGTTAGAAGAATACATACGTGTGG
EMPV1_42065 TATCCTGGGAGATGCCAGCAGACAACATTACAAGGGGGAGAGTCGTAGGGTATAACGTCA
EMPV1_42067 GATGGATCCCAGGGAGGGTTTCTCCTCAGAAAGGCGGGGGCCCAAACCTGGAGAGGAAAA
EMPV1_42068 TGTAGTCCTGCTTGCCCGCGTGTTTCCAGGGGATCCGGAACATGCTCTTCTGGTCGTTCT
EMPV1_42069 GGTACAGAGAAGATAGCCTAGGGAGTTCCCTGGTGGTCTAAGTAATGAGGACTCAGCACT
EMPV1_42070 ATCTTGCAGGAGACCTGTTCTTGCTTCACTGGGGGGCCTGGAAAAAGATGTCAGAAGCCA
EMPV1_42071 TTCGAGATGAAGTGGAGAAGTACAACCGAAATGGAGTCAATGCCCTACAGCTGGACCCAG
EMPV1_42072 TACATTCCACCTGTCCTTCGTCCCTCCTGGGAACCGTGCTGTCAGTATGTGGCTCTGAGT
EMPV1_42074 ACCTGGTGGCATTAACTGTGCATGTGCCTGTGGAGTGAATTGGGGCGACTGCCCTGTGAC
EMPV1_42075 CATCATGCCTATTTCAGTTCTCAATTGGAGACAAACTATGCCAAAAAAGTCACCAGTTAG
EMPV1_42078 TGTCCTGGAGGAGCTGGATCTGTCCCATAACATCCTAGCCCACCTCTCGGGAGCCGCTTT
EMPV1_42079 TGTTGGCGGGGTTAAAGGGGAGGGTAGGAGAGAAGGGATTTATCTGTTTGGTTGTGACGG
EMPV1_42084 CACCTGTTCACTCTGTTAGGAAGTATTTTGAATGTATACACTCATCCAGCTGAGGCTTCC
EMPV1_42085 TTCATGATCCTACTGCAGGCAGACCTCAGGCCGTTGAATAAGACCAGCCCTTTCGCAGGA
EMPV1_42087 CTGTGGAGTTCAGCTAGGCTCCTGACACAGGGAAGAGTGCGCATCAAAAATATTTCAGTG
EMPV1_42089 TGAGTTGTCTGCGGTTCGCGGCCCGCGCCCAAGCCGCCGCGGACCACGAGGTGGGCTGAA
EMPV1_42090 TAGAGGGTTCCAAACCTTAACTAACCAATAGTTGCTTCCTGTGCTAAGGTTAATGACTTC
EMPV1_42094 AACACCCCATCTGTAACTCCATACCAACCTGGCTGCCGATATTATGCACCAAGCACCCAG
EMPV1_42095 CTTGCCCCACCTCCAGTCCATTCTCCACAGGCAGGAGAGAACAATCTTTTTCAAGACATT
EMPV1_42097 GCCATAGGAAAAGGATACAGAGTAGAGTTTCATGAACCTGAAACTTGGTGGAAATTTGAT
EMPV1_42098 TGCTTAAGGACGTGCGGCTCATCAGCGCCAAGACTGGCTATGGAGTCAAAAAGTTGATCT
EMPV1_42100 GGCCATGAGAACAGTACGGATTCTCTTAGTCAAGTCTTTGACTTCTGTGGGAAAAGCAGT

EMPV1_42103	ACGTGACCAAGGACTTCTACCGGGCCTACGACTTCATGTGCATGGGGTTCGTGCTGCTCT
EMPV1_42104	GAGATGCTGGAGCGATGTGGCTACGGCGAGTCGCTGGGAACGCTGTCTCCCTACTCTCAG
EMPV1_42108	CAAACCCAGGTCTGCTGACTCCTAGTCCAGTGCTTGTGATGGCTGGTGGGCCCTGAACTA
EMPV1_42111	AAGCTTCCGACTGATCCTCATGTGCGTGGTGACGCTGCTCCTGTTCGCCATGGAGCTGGT
EMPV1_42112	CGGCTTCGCTCCCCAAGTCCTCGACACACACTATAACACTGAGAACGACGTGGAGATCAT
EMPV1_42113	GCTCTCAAGACACTCACTGTTGAACCCCTTGCACCACAGCAGGAACTCCCCAAATAATCA
EMPV1_42115	GTAGAGACACCAAGACACCCACAACTCTCCCCAAGAGCATCCCCGTTTCTGTTCCTGGAA
EMPV1_42116	TCATTTCCTGGTTACCCCAAGGAAGGGAGGTTCCTAAACCCTAGCCAAGCCTGAGTGGCA
EMPV1_42117	GGCCCTGAAAGCCCACTGAACACCTTCGTGTAAACTCAGAAATACATCACAGGGGGGACT
EMPV1_42119	CAGACCTCTTCAGTCTGATAGACACTGACTTCAAAAGGGAGACAGTGAAAATACTTAAGG
EMPV1_42120	TCCTTGTGCTATGGGACGAAGATTTCCATTTCTTTGCTGGCTGTTAGCTGGAGGCCATCC
EMPV1_42123	CCGTTGTTTAAAAGTTTAACAATCGAGGGTGTTCCCGTTGTGACTCAGTGGTAACGAGCC
EMPV1_42125	ACAGGAGAGAGTGAGCCAGAGCCACGATTAGGTTACTCCCGGTAGAGTCAGGAACTTTAT
EMPV1_42126	GGACCGCGGGCCGCGCCTTCGCTCACGCAGGTCGCGCCGCGGTGCAGACGCTGCGCCGGG
EMPV1_42127	GATTGGCTGAGTTGTGAGCACCTCTCCTTCCGATGATTCGGCTCTTCTCGCCTCAGTCTT
EMPV1_42128	CACTGAACTCTAAAGCTGATTCTTTGAGAGACTAATAAAATTTATAAACCATTAGCCAGC
EMPV1_42130	TTTGACAGGGAGTAAAGTTGTGTCCAGTGTCGTGGGTGTTAGTGTGCCCTCCCCCCTTCA
EMPV1_42131	GTGACTCACATGGGGCAGACCCCTTGCTGCACGGGAAGACGGACATTGAAAAAGATAATT
EMPV1_42134	GCCTCTTTGCGCCGATACTTCTACAACTCTACGTCCGCTGAGTGTGAGCTCTTTATGTAC
EMPV1_42135	CACTTGCTACACCTTGAACTGTACTAAGAGAAACAGACAGCCAGGAGTCCAGGTTCATGC
EMPV1_42136	AATGGCCCCAACCTCAGCTTCCTCACAGCCCTGGTGCTACTCAGCTGCAATGCCATCTAC
EMPV1_42138	ACTAGTCGGATTCGTTTCCTCTGCACTACAATGGGAACTCCCTATGCTGTATTTTTACAG
EMPV1_42139	CATTTTCTCCATGCCAAACCTAACAAATTCAAATGAACTAGGGAGAGCCAGCTACTCGCC
EMPV1_42143	ATGTGGGGAGAGATACCGCCAACTCCGTTTCTTTGGAGACCACACATCAAACCTCAGCAT
EMPV1_42147	GTGGGCGAGATCATCAAGCGTTTTGAGCAGAAGGGATTCCGCCTCGTTGCCATGAAATTC
EMPV1_42150	CTTGAAACTTTTCAATGTGAGCGTAAGAGGTTGGTGCCAGCTATGCGCTGGCCAAAAGAT
EMPV1_42151	GAACCCTCGTGTAGAGCACTTGCTTTGTGTTAAACCTCCTGTCTCCATGTGAAACTGACC
EMPV1_42152	CACGTGGACAGGGCTAATCACATCATGCAAAAATTGGCCTTGAACATGAAGAATATCCGC
EMPV1_42153	AGCAATAAATGCCAGCTCCAGAAAGGTCCTGGGTTTGGGCGACCCAGCTCCATCCCTTCA
EMPV1_42154	AAATGAATGCCTCGTGTCAGGTCCTGCAGTGAGCAGTTTTTGCGATGTAACAGCTAGAGT
EMPV1_42157	CCTCCTCTTTCAGGTACACTGACTGCTGTGGAAAGTTTTCTGACTCTACATTCAGGACCT
EMPV1_42158	CCCAGGCTACCAGATTGTGGTGAATCCACCTAAACTCCAAAACTGGCAGGACTGACATCA
EMPV1_42160	CATGTCTAGTGTACACAGCAGAGGTTCCGTGGATGTTCTGATGAAGCGTGGAAAATGATG
EMPV1_42161	TGGACCAGTCTGTAAAACAACATCGGGAAATCTTTCCAGGCAGCAACGTCACGCACTTTG

EMPV1_42163 ACGAAAGCTTCCAGTGGCTGCGGCACACAACGCCTACACCTTCTTCTAAGCACCCCTTTA
EMPV1_42164 AAAGGCCATGAAGTTCTCCTGTGGCCCAGTGGGCTGAGGATCCAGCATTGTCACTGTTGT
EMPV1_42166 GCAGTCATTCTTACAATAAATACAGAAGTGCTAAACGGACCATCAGCACAGTGGCCGAAG
EMPV1_42168 TGTTAGGGGTGGCCGCGACAGATCCCGGACGAGCCCCCAAATGTAAGACCAGCAAAAAGA
EMPV1_42170 ATCCAGAGAAAGGCGTGTTTCAGAGTGTTCATATTCGGGATGCAGAAAACATCGCAAAAG
EMPV1_42171 TTCAAGAAAGACAGCACGCAAACCTACCTGCCTTGACTGCTGGCCATCCCAAGGGCAATT
EMPV1_42172 GTGGGAAAGCCTTCACGTGGCAGAAAACCTTTCAGAAACACATGATAACTCACAATGGAG
EMPV1_42174 TACGTGGTGATCCCCACACGACGCTCCACTGCTGAAGCCTTCCAGATCGTGCTGTCCCAC
EMPV1_42175 CCATATGTGGCTCTTGAGCACCTGAAGTGTGGCGAGTGTGACAAAGGAACAGGATTTTAC
EMPV1_42178 GCCCACAGCTTTAGCCAGCGACCAAATTAGAGGCAACTTGTTAGCACAGAATCTCTCATC
EMPV1_42180 AGGGCCAGGCTTTCCTGCTCCGTGAGCTGCCATTTCCGAATTTCCCGTCTCTAGTTAAAA
EMPV1_42181 ATGCATTTTTTGGATATTATGACACACTCTTTTGTGAAGTGTTTTGTCAGGCATCTTGCC
EMPV1_42182 AAGGTCTGTCATCTGCTGGTTGTGATTGTATATGTGGGAGCATTTATTGGTGCCTGGGCC
EMPV1_42183 TGCTGTTGCCCTCTCTCCACCTCGAGGCCTTGGACCTGCTCTCTGCGCTCATCCTCGCGT
EMPV1_42186 GGCATGATACTTACATTTGTTGTTCTGGTGGTGATGATAAAGGGGGAACCTGGTCTCCTC
EMPV1_42187 TGCCTCAGCCAACCAGGGAGCAGACTCCGGGCACAGGAAAATCACATCTGCAGCGAAGGA
EMPV1_42188 GGCTCTGAGCTCTTTCACTTCAGAGTGTCTGTCGTTCCAGGGGTCACGTTTTGTAACTTA
EMPV1_42189 CATCACTCTAGCCAAACTCTCCGTCATCCCTGTGTGGCGAGGCTCCTGGTGGAGCAAGAT
EMPV1_42192 ATTCTTTAGCAGCAAGTCTAATCTCATTCGACACCAGGAAGTTCACACAGGAGCAAGGCC
EMPV1_42193 TCCTGTGGGAAGAGCTAGAGCTGGTGCGGGAGGAAGTGACCTTCATTTATCAAAAGCTCC
EMPV1_42194 CTGCGTGCTCTTCACTGTCATGGACCACGACTGGCTGTCCACCAACGACTTCGCAGGGGA
EMPV1_42196 ACAGACTTGGGTTCGAGAGCATCATCCAGAGAATGGAGAGGAGGCAGTGACCGTTCTAGA
EMPV1_42197 GGCTTTGAGAACAAAGACCCTCAATTCTGGGAAGAACCACACCTATGACCCTCCGCAGTC
EMPV1_42198 CTAAGGTTAGGGGTACAAAAGGGAGTGCTGCATGATCGCAGGGCAGATAATTGCTCCCCA
EMPV1_42200 AGAAGTAAATGAGTGTCCCCGTGAGTGCAACCCAGACAGGGTGGGGGGCTATATGTGTAA
EMPV1_42202 TCCACTGGCTCTGCCCCTGGAACCTTTGAGGCCTCCCACCCTACTCCTGCCAAGCCCAGC
EMPV1_42204 GAAGCCCCAGCAAGGGTTCCTCTCCAGCTTCTTTACCGACAACCAGAAGTGCCAGCTGAT
EMPV1_42207 GTGTGCGAGGTTGGTGTGCAGCTCTTGGGCTGTGGCCTTTCTGGATGCACTCATCAACAT
EMPV1_42209 CAGGAAAAAATATATTAAGACAAATCTAAGACAAATGACCATGGAAACAATTGACTCTCA
EMPV1_42211 GATGAATTTCCTGGGGCTAAGTTCTTGCAGGCATTTTCAGTGCTGAGTTCTGGCTCCGTC
EMPV1_42213 GTGTGAGAGGCTTTCTAAAGTACGAGATCAACTTGGACAGGAATTGGAGGAACTCACAGC
EMPV1_42214 GGAGTCAGAGAAAAACACCGTGGTGGAAGGAGGAGAGGTTCGTTTCAACGGGAAAGGCAA
EMPV1_42215 AGAAGGAACCTTTTGCGGCAGGAGAATGGGGGTGTTGATAAAGCTGATGGCACGTCGCTT
EMPV1_42216 CAAGCACCAGCGCTCCGGCTGCAGGGGCAAGCACCAGCGCTCCATCCGCTGCAAGCACCA

EMPV1_42217	AGTAAGAGAGGGAGAGACAAGGGTGGGTATTTTAGTGAAGCCCCCCAGACGCCTGACTTA
EMPV1_42218	GGAACACATAGGCCCACCACTCTCTGACATCAAAGACAGCAATATCTTCTCATATCCACC
EMPV1_42219	TTATGTGAAATGGTATTATCAAATATCATTTATGCTATGAGAACTGAATGTATTGGTGAT
EMPV1_42220	GAGGAACTGGCCTGAAGCATCCCCACACCTTGAGGTTTATTTGAATATGTCAGCTCTCGG
EMPV1_42226	CAAACCCGCTGAACATACGCCACCCACAGGGACTTCTAAATGACAGACAACCATGGAGTC
EMPV1_42227	TTTTGGTGCTTGTCCTCACTACCACTTTGACCTTCCCTCCCTATCCTGCCCTGTCTCCTT
EMPV1_42228	CTCGACAAGCCACCGGAGCTGTGCAAATGAGAATCAAAAATGCCAACAGCCACCACGACA
EMPV1_42231	ATGACATAATGGTGTTCATTGCAGCTGGGCTGCTGAGTGGTGGTCCATTCATTGGAATAC
EMPV1_42232	CGGTCAGTCCTCTGTGTCTGAGGATGCAAAACTCGTAGATAACAAGGTCCAGCTAAATGT
EMPV1_42234	GCATCAGTGGGTTCGTGACCTTTGGAGCTGCCGTGGACCCTGATGAACTCGTGTCGTACC
EMPV1_42236	CAAGCTGATCGAGCAGCCGGACATCGTGGCGCTGTTGGAGGTCAAGATGAACGAGATGAA
EMPV1_42239	CTCCCGAGTGACGGTGTCCGGGAGGTAAAGAAGCTGCATGATAAGTGCCATTACTACTGT
EMPV1_42241	ATCTGCCGGGGCTTCACTGACCTCCCTGGAATGACAAGAATAATGCCAAGGCTTTTACTC
EMPV1_42242	CTACAGCCAGGACGACACAGACAACCTGCTCCGTCTCACGCAGTACAAGATCTGCAACAA
EMPV1_42245	TATTATTTGGAAGCCATGGAGACAACAGTGTACAAGGGGATTTCGAGGAAAGCACGCTGC
EMPV1_42248	CAAGTTCTTGGACCGTAGCCCCTCTATGCTTAACTGGAAAGTGACCCTCCACTGCCACCT
EMPV1_42250	CCCTGGGACTGAGGGCCTTTTGGTGCTAAAATTAGGAGAGTTCTGGGCAAACCAGCTTGA
EMPV1_42251	TTGCGGTACCCATCAGGGGCATCTTTCAGAGTCCTGGGTCCTTGAGTAGCCTTGTCTCAT
EMPV1_42252	CGTATGTCGAACACGGTCACATGAAGGGGAAGTCATTGTTTTTGGTGGGTGCGCCAATAA
EMPV1_42253	GCAGACACCAAGGTTGCCAGGCGCTATGGGGACTTCTTCATCCGACAGATTCACAAATTT
EMPV1_42254	CCTACCAAGTTCCTTGAGAGCAAGTGTAGTCTCCTCATTCAGCTAATACTCGTTATACAC
EMPV1_42255	GTTCCTACTTCTTCAATTTTAGGGCTGATCTGGATTTCTGTCATGTGGGAAGCAGTTGCC
EMPV1_42256	AACAAATACCATGAGCTCAGGACGTTATTCCCTCACCCACTTCAGTGACCTCAGGGCGCT
EMPV1_42257	CTGAGTTCCCACCCAGGAACATTGTGAACAATAAGATGCTTGACTTAGAGGTTTTCCTGC
EMPV1_42260	CCTTGGTTTTCCCGAGTGAGAGTGAGATCGAGGACAAGAGAATCCATGTGGAGCTGGACT
EMPV1_42261	TGCAGGATATCAAAGCTCGCGAGAAAGATGGCAAGGGGCGGACGGCGTCTCCCGTCAACT
EMPV1_42263	AATGGGAAGTGCCTGGGCAGGAAGCAGAGACAGTGACCCTGGACTGTACATATACTACTA
EMPV1_42269	AATGCCATTCTTACTTTTGGGGCTATGTAAACACAGGCAGGGGGCCAAATCAGCCCATGG
EMPV1_42270	CCAGGTCTACGTCCCATCCTGATTTGCTGAGTGAGGTTGTGCGAATGGTCAAATTGGTAC
EMPV1_42272	GAGAGCAGAGTGGACAGAACATATTCCTGGGATTGGGCTGGAAAGAAAGCCCACCATCAA
EMPV1_42275	TGACTTTGCAGGTTGCTCACTCCTTTCCCAGCACTTTCCCACTGAAGTTTCCCTCACCTG
EMPV1_42277	TGAGCAGTTGTTCACACCAATTTTGGGTTCGCCAGTGACGCCCCCTGGAATAACAGAGGA
EMPV1_42278	GAGACACAAGGTGAGCAAAGGCTGTTGGAAAAATGGTGTTGATAGACTTGCTAGACGCAG
EMPV1_42279	TTGGGTCCTGAGGGGCGGGCTGTGGCTGTGGTGCAGGCCAGTGGCTACATCTCTGATTCA

EMPV1_42281	AAGCAGTCAAATTTGTCTGATTCAACTGAAAAGGACAGGCTTCAGATGGCTGAAGATGAA
EMPV1_42282	AAACACTGGTCACAAGAACTCAAGGAACCAAAATTGCATCTGATGGCCTCAAGGGTCGTG
EMPV1_42283	TTCAGTTGCTCTTCCTCATTCTCCCTCCACCCAACCGTAACTTGCTGAAGCTCTTGCTTG
EMPV1_42284	CTGATATTAAGAAACAGGCTGAAATTGCTCACTTATATATTGCATCTCTTGCTGACCCCC
EMPV1_42285	TGCCACTGCAGCTAGAATCCAAGTCGGCCCTGAGTCCAAAAAATGGCGTCTACATCAGGA
EMPV1_42287	TATGCCGACACGAGGTTCGCTATGGCTGTCTGCGGGAAGACGAGTGCTTTTATGCACATA
EMPV1_42288	AGAGTGTGGAGCCTCCCGCCTCGGCACCATGGCTGGGCTCATCAAGAAACAGATCCTGAA
EMPV1_42290	TGGGTAATTCCAGACTTTTACTGTGAACTTGCTCAGGCCCTCACACTTGCCTGCTCAGAC
EMPV1_42291	CCGTCCAGCTGATCCAACAGTCAGAAATGCGACGGAAAAGTGATCTGCTCCGAACTCTGA
EMPV1_42294	CTGATGACTTCCGGAAGCTGATAGATGGGTATAAACTGATGACTTCTGGAAGCTTCTATG
EMPV1_42296	GCATGCAAAAATGAATTCGCCCCTCCCACCTCTTTCTTCAGCTAATGGAAAACTTAGGGG
EMPV1_42297	AGGACTCCATCCTGGCTGTGAGGAAATACTTCCACAGACTCACCCTCTATCTGCAAGAGA
EMPV1_42299	CTCCGACGCAGCTCGTTTGCAATTGTGTGTGCCATCTGCTGTGTTAGAACTAGGAAAGGA
EMPV1_42300	TGTCCCTCTGCCTTCCCTCCCGGGGCCCTACTACTTGAATCACTGCATCTAATTTGTCTA
EMPV1_42302	GGAGAAATCATCCAGGACTATGACAGTGATAAACTCTTCCCAGCCTACGGCTTTGGGGCC
EMPV1_42303	CCTCTCCTGAAACCCCTCCTTTTGCTTCCTTATGCCGCCAACACGGTCTGATTAAACTCA
EMPV1_42305	CATTGTCACCTGGAGATCTCCCACCTCTGTTTCAATACTGACGTCTGAGGTCACTGAGGG
EMPV1_42306	TTTAAAGCCACGGACAATGGCACAGCTTACGTCAGTGGGAGATGCAAGATGAGTACCAGG
EMPV1_42307	CCTGCCCAAGTTTCATGGCCCTAGTATCTCCTGGTTTGCACCAATAACCAACACCAAACC
EMPV1_42310	GACATTCTAGCTGTAGGAACAGAGAGCAGATGACCGTACAAGGGGCACAAGAGAACTCTC
EMPV1_42312	AATAGCTTCTCAGGTACTTTATGACCGACGGCCTGACTGATGGGAAGCAGAGAGAGGCTC
EMPV1_42314	CACTTTCTTGATACGCACATGTTTCTACTCTACTCTGACCTTCCTTAGTTCTTGGCTACC
EMPV1_42315	TTGTACTCTAAGGAATATCCAGTCAAAGATGCAGAGCGGGAGCTGTCAGACAATACCAGC
EMPV1_42319	AGCGAGGGGTTGGGCTGAGCCGCACTTGGGATACCGCGCGGACCATCTGCTAGGACTCGG
EMPV1_42320	GAACTTCGACGAGCTGGACAGGTCTAGGGAGGTTGTGCAGGAGCTCATTGACGAGTACCA
EMPV1_42321	CAGGTTATTGCAGGTTTGGCTATGGGCTTCTTAATGCAGTTCTTATGAGTTGTTATGGTG
EMPV1_42322	AGATCTAGCATGGCCATGAGCTGTGGTGTAGGTTACAGATCCTGCATGGCTATGGCGTAG
EMPV1_42323	TCTCCTTGAGGGGCCTTAGCCTCTGCCCAGGACTCTCTCGTGACCAGGAGGCTGAGGCCC
EMPV1_42325	TGCTTTTACTGTGCTCGTGGACAACTATGTGAAGGAGGAAGAAGGCACTGGGGTTGTGCA
EMPV1_42326	GGTGGATCCTCACAAGATTTCCTGAGTAACAATGTTTCCATGACAGTCTCTGTTAAAAAT
EMPV1_42327	CTCTCAACATCAACATTGAGGACATGGAACTTTGGTGGTACAGGGACCAGCTTTCCCCAG
EMPV1_42328	GGATGTGGGTCACTTATTCATTGTCAGGAGGCAGTTTGGGGACTGTCATGTGTCCAGCTG
EMPV1_42330	CTTGAGATTCAGACTCTTCGCAGCGACCCTGTCTGGGACCGATCAGTCGGTCCTGAGGAT
EMPV1_42331	GATGCTATCTGCAACGAGGTGGGAACCACAAGAGGATGTAGGGTTAAAGACTTATTAGAC

EMPV1_42333	AGAACTGAAGAGCAGCCCTGAGGCGTGAACCCAGAAATTCCACAGACACTCCAGGTTGAT
EMPV1_42334	AAGAGAAGCGACTGCTGCCCCCTGAGGACAGCCCCCTGTGGCAGTATCTGCTGAGCCGCT
EMPV1_42336	GGTTCCGTTGGTTTAACTGAAGTTCATAAATATTCAGACATTTGCTGGGGGAAAGAAACG
EMPV1_42338	CTTCTGACGTGCCTTTTGCACCCCTCCCATTAGGACAATCAGTCCGCTCCCATTTGGGAG
EMPV1_42339	ATCCCAAAGTGGGCCCGGGAGAAGATCGTCAATGGCATTCAGCTGGGGATCCATTTGTAT
EMPV1_42340	CTCCATTTTGACCCCTAACCTGGGAAACTTCCATATCTGCGGGTGCGGCCCTAAAAGGAA
EMPV1_42341	ACTCCGACCTCGAGCAGAAGTATTGGGAAGCCCTAAACTCGGAGCAGTGGGACCCTGAAG
EMPV1_42342	CTGTTTGACAACCTCCCACTCTTCTATCGGCTCGGACTGTGCTGGGGCAGGGGTCCGAGC
EMPV1_42344	ACAGCCCAAAACACTGCAACTCAGAAGGCAGCCCAAATATCCTCGGAAAAGCGCCCCTAG
EMPV1_42346	CCTCTCCTGCTCATTGCCTCACCTCCACTCACTGCTACACACACTTCTGCTGAATCAACT
EMPV1_42349	GGAGAATTTACACCATGAGAGAGGTTGGTCAACACTTACAGCCCGATTTCAAGCGCCACC
EMPV1_42350	CGAGGAGACTGGAACTTTGCGCACTGGCCCCTTTCCCCCATTTTTACGCTTAAATACATC
EMPV1_42351	GCCCTGCTGTCGCTGCGTGCTGTCCGCGAGATGCGCCTCAGCAACGGCAAGCTCTACTCT
EMPV1_42353	TGTCCCTCTTTTATGGGACCCTTTTTGTCATGTATGCCCAGCCGGGAGCGGTGGAGTCCA
EMPV1_42356	CTGCTCCTGAGGCCTGATTTGTCTCCTTACATTGGCATCCCTTATAGAAACAGGAAGAGC
EMPV1_42357	CAGTCCAATAGCAGGGATCACCTCAATGACAAGGAAAGTAAGGAGAGCAGTGTGGAAGGC
EMPV1_42359	GCTTTGTGGGACTTGTGGAAAGTGTGCTTAATTCCATTGTCGTTGTCACTGTGTGGCGGC
EMPV1_42360	TGCTCCTACAGGATCCAGAGCCGGGACACCAAGTGTGGGGACAACCAGCTCCCATGCAAG
EMPV1_42361	GGGATGGGGAAAAATACTGGTTCTGTGAAAATACCCCCTTTCTCCATTAGTGGCATGCTC
EMPV1_42362	CCACCAAGAAAGTGAGTCCCAGCAGAGATCAGCTGAATGTGTCTATAGAATGGAGGCAGA
EMPV1_42364	AAGATGTTTTGATCCCTAAAATTATGAGAGAAAAGGCCCGAGAGAGGTGCTCTGAACAAG
EMPV1_42365	GACCTTGAGCCACTGCAGATGGATTCTTAACCCACTGTGCCACAGCAGGAACTCTCACAA
EMPV1_42366	ATCCATCTTGCTCCTTCTGGGTGATGCCACGTGAAGGAAGGTGACGGTACTGGACACTAT
EMPV1_42367	GCTCCCCACTATGCCCTCGAATCTAGTGGGTCTTGATGTATTTGTCTGGTAGGCGTAAAT
EMPV1_42369	TTAAGTTGTTCTTTCCCTGTGCTTGATGGAAAACAGTGGCACGTGGGCTTGTCTGCTAGC
EMPV1_42370	ACCTCGTTTTTCACTTCTTAATAAACAGCTGAGGATGAGCTGAGGCCGCTGGTGCCCTGA
EMPV1_42371	GTCTCGTCACTTCCCTTAGCACCTGCTTCATGTGTATTCTTAAACCTCCACCTTGTGGCC
EMPV1_42372	GACAAGAAGACAGAAGAGTTCTTCTCTGTGGTGACTACAGACTAGAGGAATGCTCTACAG
EMPV1_42373	AGGAGCTGGACAATGACAGCTTCTCCTATGATGAGGAGTCTGAAAACCTGGACCGAGAGA
EMPV1_42376	AATCTCATTAAAAAATGTAGAGGTTAACAGATATTTTCTAACTACTTTTAAACATGGGGA
EMPV1_42377	GGCCCTGTAGGCCCTTTGAGGACTGGTTTTTACTCTGTAATGATTTTAGGGAGCATAGGG
EMPV1_42378	TGGATTGAGGTGCAGTTCCTGAAGAAAGCAGTTGATGTCCTCTGCCAGTGTCGTGCCACA
EMPV1_42379	ATCCACTTGGAGATCAAGCCGGCCATCCGGAACCAGATCATCCGCGAGCTGCAGGTACTG
EMPV1_42380	AGGCACACCAGAAACTTGCTGCCATCTCAGGGTAGGAGACTCTTTGCATTGCTTGCCAAT

EMPV1_42383	AAAGCAGCTGTCTTTAGCTGGAAGAGAGAAGCTGTGTTGTGAGCTGCGCAACAGCAGTAC
EMPV1_42384	ACAGAAAGGTGGGACAGATGATGGCTGTTACGTCTTGGTTGGGAGCATCAGTAAACTCCC
EMPV1_42386	GCCTGGCATGTTTGAAGATCAGCAGGGAGCCGGTGTGGCTTAAGAAGAATGAGCACTTAC
EMPV1_42387	TGGGCATGACCCTGAGCTACTTGTTCCGGGAGCCGGCCACCATCAACTACCCATTTGAGA
EMPV1_42389	TGCGGCTGCAGGACAAGAAGCTGCCGCCTTAGCTGTCCGAGATCTCGGACGTCCACGAAT
EMPV1_42390	CAACAGTCTCAAAGCAAGACCAAGATCCAGATCTTACTCTAGCACCTCCATAGAAGAAGC
EMPV1_42391	CTGAATTCCATGATAAGTCTGAAAACTGACTTGAGGTGTTACTGATCTGGGTTCTTGGCC
EMPV1_42392	CTCAGAGAGCCATTGCTGTTTTTATTGCCCGTCTAAGCTCCTTGTCTTCAAGTGGGTCAG
EMPV1_42394	CCACCCAGGACCAGCTCAGCCAGTCAGAGAGGTACCTGTACGGCTCACTGGCCACACTGC
EMPV1_42399	AGCATTCCCACGGCTCAGCCATGCTCCTGCTGCTCATCCCAGTGCTTGAGATGGCTTTTG
EMPV1_42400	TGTGAAGCTATTCCAATTTGTTAAGACATCTTTAATCTCTCAGGCAGTTGAACTTCATAC
EMPV1_42401	ACGTATGTGTATACATATTACTATGTATGTGCATATATACCTACATATAAACACCCTTAT
EMPV1_42402	GATAATTCCATCCCACTGTTAACATAACTTGTATGTGGAAGGGAGAATGGGACTTGTTAG
EMPV1_42409	ATCCAAAATGCAAGCTCAGGCTCCAGTGGTCATTGTGACTCAGCCCGGAGGTGGCTCCAT
EMPV1_42410	AGAGACATCTGCAGCAGAAGAAATGGAGACGGAGACAGAGTCCACGGCTCTCCAGGAAAA
EMPV1_42411	CCTGGGAGGATTGTCACCAAGTGCCTTTGAGTTTTGGAAGGCCAGAGATGCAAAGTTCAC
EMPV1_42412	GAATCAGGTATGGGCCCTTTTGCTGTGAGGTCATAAAGAGTAGCCGCATAATGAATGAGG
EMPV1_42415	GCAAGACCATCGACAACAAGGCTCTGTACAACATCTTCTCGGCGTTCGGCAACATCCTCT
EMPV1_42417	TGACGTCTGGAGCATTGGCTGCATTCTGTTTGAGTACTACCGGGGCTTCACACTCTTCCA
EMPV1_42418	GCCTTCATCTGCCCGAGCCAGAGACCATGCTGTTATTATGTATGTTTGCTTCCCAAAGCC
EMPV1_42421	TCCTTCGCGGAGAACTTCTCCACCACCAGCAGCAGCTTCGCGTACGACCGCGAGTTCCTC
EMPV1_42422	CCCTGGTGCCCATGGACTCTTCCTTCTCCCTCATCTCGGTCTCGCACGCGCTGTATGACA
EMPV1_42424	TGGCTTTGCCACTGATAATAATGAAGGCCCGTCTTAGGGAACACCCCTCTGTTGGTACCT
EMPV1_42425	GCTTTCGATCACGATTCCTCACGGCTGCTTTATGGAAACCGAGTGAAGGATTTGGTCCAC
EMPV1_42426	ATCAGGCAGGGTACCGTTGGCCTTGCTCTTGGGTTCCTGGCTTGGTCAGCACTGATTCCT
EMPV1_42427	GGCCTGGGTAAACTGGTTAATTGCGGGAGGCACCTTCAGCCGCTTATAGAGTATGGCCCT
EMPV1_42431	ACTCAAGGATGTCGCCTTATCCTCAGGGAGTGCCTGCACCTCTGCATCCTTGGAACCTTC
EMPV1_42432	TAAGGTGATCTTTCCTGATGTAGAGCTGGAGGATGTCACCAACTCTGGGCTTAAGGTCCG
EMPV1_42436	CTGACTCGTTGAAGAATGGAACAGAAGATGCTGGGGACGCAGATGAGTTTTGGAAGGACC
EMPV1_42437	ACATCAAGGAGAAACTCTGCTGTGTCACCCTGGACTTCAAGCAGGAGATAGCCACCACCA
EMPV1_42439	GCCACCAAGTATCTGAAAGACGTCACTCTACAGAAGCAATGTGTGCCATTCCGTCGTTAC
EMPV1_42441	AGGCTGAGCGGAAGCTGAAGCCCAATCCCAGTGTGGGTGGGGAAGCAATGTCTCTGGGAC
EMPV1_42443	ATTAGGGGAACTTTTATTCCCTGATACTTGTGTTGAAGGACGCAGTCATCAGTGCTGGCT
EMPV1_42444	TTGGTTAAAGATCCCACATTGCTGTGAGTTGGTGGGTTGGTCGCAGACGAAGCTTGGATC

EMPV1_42445	GGATACTGCCCTTGTCAATGTTGGTTTTATAAGCACATTCTCTCTGTTAGACTTGTCAAG
EMPV1_42448	GCTCTGGAAGCACAAGTGAAGATGAAAACAGGAAGGCTGGTGTCTCTGAGTGCACAGAAC
EMPV1_42453	TCCTCTCCGCCTGCTGTGATTTGAGGCCACCTCTGGGACCAGACTCCTAAGAGATGAACT
EMPV1_42455	AGAACCCAATTTACACCTGTGTTCCAAGGACTGATTCAGGAACTGGGCAGAGGCAGCACT
EMPV1_42458	AATTTCAGACATTCTTTGCGAGCCAAGAGGGCCGTACCAAACCCAGAAGAAGTTACTCCT
EMPV1_42459	CCTCAACTGCTCATGGATGCTAAACACATCTTTCCCGTCACTTTTCCTTTCAACCCATCC
EMPV1_42460	TTCTGCCTGTCTTGGACTGGCTCCCCAAATACCGGATCAAGGAATGGCTGCTTAGTGATA
EMPV1_42463	ATTACATTGACCTGAAGGACCGTCCCTTCTTTGCTGGCCTGGTGACGTACATGCATTCAG
EMPV1_42464	GAAGTGTTAAGTAGATCACTGAGAGAGGTGGATAATGACTGCCATGAAGAGTTGGCCAGG
EMPV1_42465	TTTCTGTCATGGTTGGGCTTCTACCCCACGGACACCAACGGCTCTTACCCTGGCAATGAC
EMPV1_42466	GAGTCCAAATGGATACTCTTGGGTCTTTAGAAAATAGTCCCATGCTCGGATGGAGTAGAG
EMPV1_42467	ATATACTTTGTCATTCTCCGTCTTTTAATTGGTATGCTTAGACCATATACATTTGATATA
EMPV1_42468	GATATTTCAGTATAGATATTATCAAAGAAAAGCTCTGAACTAAACTGAGCATGAGCTTTG
EMPV1_42469	TCTCCCCATCGGGTTGGCAAGAGCAGGGAAGGCATTTGAGCTTAGTTCTCATTGGCTCTT
EMPV1_42470	GGGGCTGTGCCGGGTAGAGAGGGCCACGGGAGATAAGAGCTTTTCACCCCTCACCACCTT
EMPV1_42471	ATGTGGGAAGGAGGCTTTTGCTTCTACTTGCTGGTGGAAACTGCCTGGTTCACTCACCCT
EMPV1_42472	GACTTCAACCTGATCGGATGGGGCTCCTGGATCATCTACCCCAAGCAGTACAATGCCTAT
EMPV1_42474	GGTCTGGGAACAAAGGGCACGGAGACGGTGTACAGTGAAATCCGGAAAGCTGACCCTGAT
EMPV1_42477	TTGGGGCCTCACTTTTTGACTGTAGCTTGTTGGAAGGCACATTTTTAACATTTCGAGCAG
EMPV1_42478	CCTAGCTGCTATGGCAGTTGCTAATCAAGATATGACTACTGCAGAAATAGCCTATGCAGC
EMPV1_42480	CATAATGGATGCCGGGTGGCCCCAGCCCTATCTTTTGACAGGAAAGTAACATCCTCCCAT
EMPV1_42481	AGAACCCTCAGGGCTCCCACAAAGCCAGAGTGCCCTGGCCCTTGAGCCGCTCCCGATGCC
EMPV1_42484	CCTACACACTGCAGGAAGCACAGCTCGGTATCATGGACAGCCAGATCTGTACGATGTTTT
EMPV1_42493	CAGAGGTCTGCGCCAGGGAAGATGAAATCGATCAGCCTACCCATCCTTGAACCTGACTTT
EMPV1_42496	GGTTCTCTTATTTTGAAAGAGTATCGGTGGTAAGTTAGTTCCATACACAGATAGCATTTA
EMPV1_42497	GTGATGGTCAAGTGGACTTCGAGGAGTTTGTGACGCTCCTGGGACCCAAGCTCTCTACCT
EMPV1_42498	TGCATCAGCCTGACCAACCTGGATGGCTCGCCGGCTTCACACGAGGTGCTGCAGTGTGTT
EMPV1_42499	CAGGGCTCTAAAACAAGCCTAAGAAGACTGGGCAGTAATGAAGATACTCAAAGTCTGCCC
EMPV1_42501	GTTATATCCACGCAGCCTTCGGACTGTAATACAACTGTAAGACCTGCCAAGCCACCTGTG
EMPV1_42503	TGAGACGACATTGGACACTGCCCCCAGATATAAGAAGCATCACTTCCTTCAGATGGCGGT
EMPV1_42504	CCAGGCCACTCAGGACATTTATAGCTTCTGTTTCCCCATGACCCCTTTCTGTCTGAACTC
EMPV1_42506	TGGGCATCTTACAGCTGTTCAGCTAGCTTCTTCTTTTATCAGTCTTCTCATCCTGGATCT
EMPV1_42510	AAGGCAAGAAAGGTTAAATAAGACACTGAAGGCCCCATTAGCCAATCTGGATTCAGGATC
EMPV1_42511	GAGGCAGCAAGGTCCACCAGGGTGAAAAGGTAATCAGTACAAGAAAGATGAGGCAGGTTG

EMPV1_42512	GACCGTATTGCAATCAGATCAACAGCTCCTGGATAATTGCATGATTCTCGCCAGAGACTT
EMPV1_42513	AAATAGTTGGTAAGGCCATCCTGGTGCAAACTAGCTGAACTTCAACCCTGTCATCCACGA
EMPV1_42516	CATTTTAAGTGGGTTCAGCCATATGCCACCCTCATACCAGCTCCGCTTCATTCCTGTATA
EMPV1_42517	TAAGCCCTGCAAAGCCAAAAACTGTGGAAATCAGCCTAGAAAGTCCTCACTTACGGCCCC
EMPV1_42519	ACGCGTGTTTGTTTCATAAGCGTCTTGCCCATTGGTAGGTGGGGAGGGTGCCTTGTTTGG
EMPV1_42520	CAGTCCCCAGCCAGCTTCCTCAGCTCCATATTACCATCACAGCCTGATTACAGTAGCAGT
EMPV1_42521	CCATTAAAGCCACCTAAATAGCGATATCCTGGCACTGATATGTAACACTTTCTTAAAAGG
EMPV1_42523	CAGGGGTAGATGGGTCTTTGAATTTGATGACCCGCCAAGATCCCTTTCAGTACCAAGATC
EMPV1_42527	TTTCTAGCCCAGTGGTTCTCCAACCAGCCAAGGGGCTTGTCTCCTCCACATAGGATCCTA
EMPV1_42528	CTGAGCCCAGGAGCATGATCACAATCATCACGGGCATCTGTTTGGACAACTCTAGGAATT
EMPV1_42529	TCCAACTTAGAGAAGGGGCTCCAAGGGGATGTCTAGGAACCAGAGCAATCCAGAGACTGT
EMPV1_42530	AAGGTGGCACGAGGCTGGGCATGTGCCTGGTCCCAGACAGTGTCTAAAGAGTGTCAGACA
EMPV1_42531	GTGTTCCCACTATGGATTTCAAGTCTGTCTCAGAGCAACAGTCCAAGACCAGGACCTCTT
EMPV1_42533	CCACCACTTAAACGTGGATGTACTTGCTTTCAAACTCTAAAAGTAAGTGCTTCCATGTTT
EMPV1_42534	GATGAAGAGTTGCGAGGTGGGCAGACCCCAGGGCTAGTTATCATTTCTCAGGGGAAAAGG
EMPV1_42536	TTGACACATGCAGCACTGCCGCTGCAGCACGCATTGAAGAGGGGCGTGGCTCCGTGAACT
EMPV1_42537	GTGGTGAGCAAGTGTGTGATGGATGGTAAATGTATTTTCCTGAACTGTCATTGCCCCCAG
EMPV1_42539	CTCAAGTACACATGGAACATTCTCCCAGATCACACAATGGGCGCAAATCAAGCCTTGGTA
EMPV1_42542	CTCTCTTCCAAGCACATAAACGTGAAGAAGCCCTCAAGGAGAAAGTCAAGGCAAACCGGC
EMPV1_42545	TACCTTCTGAGTCAGCTTATGTGGTTCTGCAACTTTGCCGCTGTCACCTGCAAGACTTCG
EMPV1_42548	CTTGAGCCAGAGCCACGCAGCTAAACTGCTTCTAGACTCACAACCCTCAGAAACTATGAG
EMPV1_42549	ATAAATACTCATTGCTCATCTGTATCCCCAATGTCTGGAGGTTGTGGTCTAGCCAGTGGC
EMPV1_42551	GGGTGGAGCCGCCGCAGGTGCAGATCTTGGTGGTAGTAGCAAATATTCAAACGAGAACTT
EMPV1_42552	GCCTAACCAGGGCAGCCTGAACTCCCAGGAATGGTTGTATGATGACGGATGAAGCATAAA
EMPV1_42553	GTGCTGCTGACCACAGGCATGTTTGTTGGTGGATTTCTGGGTTTTCTACTGGACAACACC
EMPV1_42554	CCCTCCAGCTCATGACCCTGTTTGGCCTTTCTTGGGGAGCAGATGGGATATTCCCACTCA
EMPV1_42555	GCCTCATCCTAGTCCCAGCCCTGTGTTCTATTTCTAAGGTTTGATGGTGGGGACATGAGT
EMPV1_42556	GGGCCACCTGAGAGTCACCCCAAGGGCATGGACAAGCTTATTGCTTTGGTGTATACTTTT
EMPV1_42558	TTCGGCATAAAGATTACTTGGAGCGGAAGGCGACTGAGAAGCAGCAGATGCAAGAAGGAC
EMPV1_42559	CAGAACATCCTTACAATCCAGCAATCTCACTCCTGGGGATATATGCGGAGAAAACTATGA
EMPV1_42560	GTTGGTCAGTAGAACTTTTAACACTGAGAAGCCTCCAACATGGTAACAACTCAGTGCTGC
EMPV1_42569	ACCTTAGCTTCCAGCAGGACCAAATGACACAGACTTTTGGCTTCAGGGATCCGGAGATCA
EMPV1_42570	GGCCAAAGGCCAGGACGGGCATAGTGGAGTTTCGTTTTTGAGCTGAATTCTCGTCGTAAA
EMPV1_42571	TCCTGGCTGTGCTGGGAGGCCTCTATGCCTTCTTCCTGTTCGAGAGCCTTTTCAACCTCT

EMPV1_42572	TTGTCCAGACTGCTGCTCAGGTTGCCAGCTTTGAGACTGATGAACGCTACCATCACCGAA
EMPV1_42574	CAGATCTCCTGTTAAGATGTGAAAGCTTCAGAATGCTGCTGTGAACACCATTTCAGAGCC
EMPV1_42575	ATCACTGTGTATGAAATCGCCGTGTACATTGGTGACATCGTCCTGCTCCTTCTCCCCATC
EMPV1_42576	AGAATTCAAGGCGGATTTCTTCGTGTGTCCCGAGGAAGGCGGCAACATCGCATTCCATTT
EMPV1_42579	GCCTGGACATGCATAGACTGTGGTGGGAGGATCCGAGCTTTGACGTCTATGAGTTACAGT
EMPV1_42581	CCCACATCAATACTGAATTCTTATGTTAAGTGGCTCTCACCTGATTTCAACACAAAACCC
EMPV1_42583	CCCTGGGGAACTTTCCTGATGGGTTTTTGCTGTTCTGAGGGTTTCAGTGAGGGCCTTGCT
EMPV1_42586	CACCAAATGGAATGTCTCCCTGATGATTTGCTGTTGTGTATCTTCACTGTAGGGCTGGTA
EMPV1_42589	GAGCAAAGGCCCCTGCTCTCTTCGCTCTTCTCCTCATCATTTGCATTGGGCATTACTGCC
EMPV1_42590	ACCTGACTCGTTAGTCTCAGTGTGGCGATAATAATGTGGAAAATGCTCTGCATTTTTATT
EMPV1_42591	CATCAAAGCTGGACGACTCCTGTCCTACATTCCCTATCAGCTATACAGCAAGCAGTCCAC
EMPV1_42594	AGATCTCTCTGCTGAAGGGGGCCACCTTTGAGCTGTGCCAGCTGAGATTCAACACGGTGT
EMPV1_42595	ATGTCAGAGACCCGCTCTGGCTCTTGCAGTCACAGTGACAGCCTCAGCTGTGCACCCAGA
EMPV1_42596	CTGACATGTACCTCCCTGTTCTCAGTTCCTAAGCGCTCCTCTCCCTACTTCAAAGGGTAT
EMPV1_42597	TGGTCACCTGAAAAGGTCTTGTTGCACACGCCCACGACCATCTACACGCTGCAGGACTAT
EMPV1_42598	AGTCCACTCCACTTCTACCGGGACTGGGTCTGCCCCAACAGGCCCTGCATCATCCGAAAT
EMPV1_42599	TGCTGGATTTCTGCAAGTCAAATGACATTGTTTTGGTTGCCAATGCTGCCCTGGGATCCC
EMPV1_42601	GCCTGGGGCAGGACCCACCACAAAAGCCAAGTTTTAGAAATCCGAGCATTAAAGGAAGAC
EMPV1_42603	CACAAGTCTTTTAAGTTGGCCTCGAGCATAAAGCAAGCTACCAGAATGCCCAGTCCTGGC
EMPV1_42604	TGGCTCGCCGCATCCGCGGGGAAAGAGCTTAAATCTCCTGGCATCTTCCTTCATCTAATA
EMPV1_42605	ATTCAGAGCTCGGCAGGAAGTGGGACCGGTGCTTGGCGGATGCGGTCGTGAAGATAGGTA
EMPV1_42608	GGGCAAATACTTCTGGTTTACTCAGAGCCAGGATTTTTATATGGTTCCACAGGGACTCCC
EMPV1_42609	TTTTCTTTGGCGCTCCACAATCTTTCTCCCTCCTTGAGTTTCTTGACGCCCCCATCCCCT
EMPV1_42610	TTCTGGATTGGTTCCATCAATGCTTTGTCCCCCGTAATGAAGTACCGTGCAGGAAACAAG
EMPV1_42611	TGAGCAGGCTTGTTTGGTCAGACATTATACCCAAACTTTGGTAGACCACAGAATGCTTTG
EMPV1_42614	CTGAGAGAGACAGGTATGGAGTTAGTTGGAGTTAGCTGGGTGATTCGGTTGTTCATGGGC
EMPV1_42615	AGCCTTCCACCTCGGGAAATGCCCGAATCCTCCAGTGCAGGAGAATTTTGACGTCAATAA
EMPV1_42620	GGTTGCTCAGGGATACACACAGGAAGTAAGAAACTCCTTCCCAGAGATATGGTTTAGGCT
EMPV1_42622	GACGTTTTCCCAGGCAGAGATGCTCAGGCTCCTTGTTTACCCACGGAACTCAGCAGACCT
EMPV1_42623	AGGCAACCTGTATTTCGATTGCACTCTGCATGACTCCACATACTACTGGTGTTCCGTAAC
EMPV1_42624	GTGGAGTGGACGAGGCCGGCTGTGCCACCTTGTGCGCCCCGCACGAGTTCCAGTGCAGCA
EMPV1_42626	AATACAGACCTGCTCCTTTCCAAGGGGGTAAAGCTCGCGCCAAGGCCAAAACCCGGTCCT
EMPV1_42628	ATGGACCAGAAGAATACCTGGAGGATGACAGAACCAAGTCACTCCTCAGGACCACGAGAA
EMPV1_42629	ACTATTTCCGAAGCCATAGTTGCTGAAGAGGATGAGGAGGGACGGTTGGACCGGCAGATT

EMPV1_42631	CAGCTCACACTCAGCTCATAAATGTCTCACAGCCCCACATCTCGCACTTGTCACACAATC
EMPV1_42632	GTCCCTTCTCGCCCATCTCGGCTCTCGATTTGACACAGAACACGCTGACTCCACCAGCTT
EMPV1_42633	CCACTCTTACTAACACCTGACGGTAGCTACCCCTGAGCGGGTTACTCTGGATGAATCACT
EMPV1_42636	AACTCCTCCCTGCTGACCCCAGACTGCAGCAAGCGCTATTCCTGTTCCTCAAGCACCGGC
EMPV1_42637	TTTGCACTACAGCTTCCACGGACGCCACTCCAAAAAGGGGCAGGTTGCCAAGTCGGACCT
EMPV1_42639	CCTGCTCAGCCCAGGGAAGAACTAACATGACTAACCAGGCCCATCCCAATGCTTGTTAAC
EMPV1_42642	AGCCCACTGAAATTTACCCTGTGTCCATTTGTGACCCTTCCAGTCAGAAATTAGGACCCC
EMPV1_42644	CAGAAAAGTACAGTCTTCCAAGTGCAAACCCCAAGATGAATAGAAAAGATGAATGGACCA
EMPV1_42645	GTACCTAGCGCCGAACGACCCGTGCTTTAGCACAAGCGAACCGTGTACTACCTCATCCAA
EMPV1_42646	CAAGCAGTACGCGGGCAAGCATGTCCTTTTTGTCAATGTGGCTACCTATTGTGGTCTGAC
EMPV1_42647	CAAGCCTTCAGCCAAAGAAGAGGTGGACATTAACAAAGGAGTATCTGTGCTCACTACATC
EMPV1_42650	TCGGTCAATATTTTACCCAGCTCTGCCAGGTACTAAGAAATGCAAGAAGAATTTACGACG
EMPV1_42652	TCCACTTCGACGGGAAGAGTTTATACCAGTGACTTTGAGCCCGTCACCAATCCCAAATTT
EMPV1_42653	CCTGATCCTGTCATGAGTTACCTTTGTCGCCAGTATCACATTCATGAAGTTCCTGTAGGA
EMPV1_42654	GCGCCGGGTGGAGCTGGCCGTCATGCTGAACTTGACAGAGAGACACATCAAAATCTGGTT
EMPV1_42655	TGACTGTGGATCAGCGAGCTGCCATCATTCAGAGGTTTCGGGATGGAAAGGAGAAAGTTC
EMPV1_42659	TGGGATCTACCTCAGGAGGCCAAACAGGCATTGGAACTTCTGGGAAAATGGAAGCCAATG
EMPV1_42660	CAACCAGCCTGAGCAGTTTGACTGGGCCATCAACGACCGCATCGACGAGATCGTGAGCTT
EMPV1_42661	TGTGATTAGGAAACTCAGGTGACTAGCATGTCAAGGGGTCCTCTGCTGCCACTTCCTCCT
EMPV1_42663	AGCGGGAACCGGGTGAGAAGGTCCAGACCCGCAGGAGAGGCCGGGCTCCATCAGGAGGAG
EMPV1_42664	TCATGAATGCTGTCACAGGTCAGGCACTGTGCAAGCTCTGCTAATAAGGAGGCGTTAGAC
EMPV1_42667	CATGTGACCTACATCATCAGCTTCCACATTTTTGTAAGGGCAAATCTCTGTAGTAAGCCC
EMPV1_42668	GATCTGGAGGTTGAACTTTCCTTCCGAAAGGGGGAGCGCATCTGCCTGATCCGCAAGGTG
EMPV1_42669	GGCTTTCTCTGTGTGAATTCTCACCTTAGACTTTCTCTGCGCGCGTGAACTGAATGCTGA
EMPV1_42671	ATACAGAGTTCTACATGCGGACCTTCGGTCATGTGCGCCCAGAGCCCCCTGGCCAGGCCA
EMPV1_42674	ATGAAGCGCTCGTGGCAGATTTTTGTCCTGTGGGTCTTCTGGGTGTTCATCTTGTGGCTG
EMPV1_42676	TTGTGGAAACATTAGACAAGTGTTTTGAAAATGTCTGTGAACTGGATTTAGTTTTCCATG
EMPV1_42678	TCCTGGCACAAATGAGAATCTCCCCTTTCTCCTGCCTGGACCACAGAAGGGACTTTGGAT
EMPV1_42681	GTGTGTGGGTGTTGAAGTGGTGCAAAGAGGAAGACCAGAGAGGGGAGTAAGGTGTCCTTT
EMPV1_42684	AAATACAGGAAGCCCAAAATCTATGAACTGAGTGTTCAGTTAGCTTTGCCCACTGAGTTC
EMPV1_42687	AAAAGGCGGCGCCAAGCGCCACCGCAAGGTGCTGCGAGACAACATCCAGGGCATCACCAA
EMPV1_42689	GATCTTGCCTCTGTCTATTGCTTTCTGACCGTGTTTAGTCCTTATATGATGGGAGCCCTG
EMPV1_42690	CTTAGTGGGCAACCTCCCTACCTCCAGCCCGCTCTGTCCACACACATCCAGCTTCTTTTT
EMPV1_42691	GGCGCGCTGAGGCGAGAGTGCGCGCCCTGGAGAGTGCCGCCTTCGCTTCAGGCCAACTTT

EMPV1_42692	CACTTGGGGATGTTTGGGTCTGATATCGGTCAAGTGAAAGAGGGCACAATATGCGGTCCA
EMPV1_42693	GACAGATAATAACAAGTGTCAGCGAGGATGTGGAGAAACTGGAACCCTCAGACACTGCTG
EMPV1_42695	AAGAAAACGTTCCTGACTTTCGGTAAGGAAGTAAGGATGATGCCAAGCTGGACCTGGGCG
EMPV1_42696	GTCTGAGTCCACAGACCAGGAGAAACACGTGTAGACTGTCAGCACTTTGGTTAATCTCTG
EMPV1_42697	AACCTGTGGACAAAGTGGCTGCCCTGAGGGAGTTCCGGGTGCTGCACACTGCCCTGCACA
EMPV1_42698	ACTTTACGGTCATCCTTCAGAGGCCATTCAAACTGGTGGAAGAGAGATTTCTGCGGTGGC
EMPV1_42700	CCCCCTAACAGGTGTAGCCCCACTCAATTGCTAGGCTGCTCATTTTCACTATGATGAGAG
EMPV1_42704	CGTCCTCGCCTGCTCCTGTGGACTTCATTTCAGTAACCCGTGGACAAGACCAGTGATGTT
EMPV1_42706	TGGACCCTATCCCCTCCAAGGGCAAGAAGGGCTCCTCAAAGGAGGAGAGTACCTTACAGG
EMPV1_42707	GTCCCGCTCGTTTTATGCTCAGTCTGTGTCCTCTGCACCAGGTGAGCCATTTAGAACTGG
EMPV1_42709	TCATGATGCCACAGGGACAGGGACAGGGATGGGTGATGAAGCTTGTGAGATGTGTGGATT
EMPV1_42711	AAGAGCTGTTTCCATCTCCCATACCTGATTCTGTCTCCCAGTGTTGGATTCTGCCAGTGG
EMPV1_42712	GATGCGTACGATTCTTAAGGACCACGAAGTCTAGAGATCAAAGTACACTCTCTGCTGGAC
EMPV1_42713	GTTGAGTGACGCGAAGCTCCACTGCTCAGATCCTAAGCCCGCCCAGGCGTCTCCCGCCGA
EMPV1_42714	CTTGGTGGTCTCAGGCTTGGCGGCCTCAGTCTTGGTGACCTCGGGCTTGGTGGCCTCTGT
EMPV1_42716	TAAGGTGCTTGCTTCCCCTGTTTTGTGGGGAGAATAGAGGGAATGAGAACTAGAGGTGGG
EMPV1_42717	GTGGGTCCTTGAGGCCTACCAGTGTGTAAAGGTGTTCAAATAAAGGGCTGTTCCTACAGG
EMPV1_42723	TTTCTGCCCCCAAGCCAACTCTTCCCAGACTTTTAGCCACCCACAAACCCTTCCAGAAGA
EMPV1_42724	CGTGTTGACGTTCTCGCCTTACTAGCTCAGCAACTGCTGTTACACTTCAAGCACATGTTT
EMPV1_42725	CAAGCAGGCAGAGTGCGAGCAGCGCGGGATCCTTTCCTTCCTTTGGGCCAAATTCTCCAG
EMPV1_42726	AAGGTGATGAAGGCCAATGGCAGAGGTGATCCTGTGCTGTCCTTCTTTATTCAGGGCAAC
EMPV1_42727	TTGGCAGGCAGAGCTCCGCCCCCGCCTCGCCTGCCGCGAGCGCCGCCGGCCTGGCCGGGC
EMPV1_42728	GAGCTGAATGTGCTTCTTCTGTTCACGTTTCTTCTTCATACATGGAGTGGGTGTGCCAAG
EMPV1_42729	TCACTGTGCCTCCGTGTTGCTCAGTTGAAACAATAAGGCAGCTTCTGGGACAGAGAGGAT
EMPV1_42730	CGGATGGATCTCGCTTGGAACAGACTTGTGTTATCAGCCCTTCTCGTGACCTTCTATCCT
EMPV1_42732	TTACCCCCAACCTTGGCCATTACCCCAGTGACTTCATGACTCCCCACCACTTTGGACTTT
EMPV1_42733	TTTTGCTTCTGTGTGGCACTTGGGCTATTGGCCTCCTTCACGCAGTGGTCCAGTTAGCTT
EMPV1_42734	GAAAATGAAGCCACTTACAGGTCAGTCCTAAGAGGGAGAGAGACCAGCCTTCAAGAACGT
EMPV1_42735	CCATGCCGGGATTGAAATTTAGTGACAAGAAAGGGGCAGTTCACCCGGCGTCAGAGAACT
EMPV1_42736	TTAAAGATGATCTGAAAACCAAGTATCCAACTCAGTCTGGCCAACCGCCGGTGCTCGGGT
EMPV1_42740	AATGGCTTCCACAGAGAAGAGTGGTGAGAAGGGTCACTCTGCATTCAGTGAGGTGGTGAC
EMPV1_42746	TACCTCAGCTGTGTCAGGCAGGACTATCACAAGGCAGTGAGGCAAAGAAAGGAGTTTAGG
EMPV1_42747	CCAGATGTATCAACACTATAGCCTTTGGATGTCCCTCCCTGTTTCAAAGCACTTAGCGGC
EMPV1_42748	ATAACTCTCTGAAAGCTTGGCGTCCAAGATCAGTATATGCATTAAGCCGGGCAGGGACCG

EMPV1_42750	AGATTCCTCGGAACCAGATGGGCAAGGCGAACAAGCGGGAGCTCGTCAGGCAGTTCTACC
EMPV1_42751	TTCCTGAAGAACCTAGACACAGGGCATCCTGTTTTCTTTTCCAACCATTGAGCCTGTCAG
EMPV1_42753	ATTGCCCCAGTGTCACCTGAGGAGCATCACGTCATTCCCACACTTTAGAAAGATCTCAGG
EMPV1_42754	CACCAGGTAAATACCTATCATCACTTTGCATGGAATCTCTTTAATTGTATGACACCGCCC
EMPV1_42755	TGTGTAGGAGGCGTCTGAGCCCTTGGCTGTAGGTTCCTTGAACTGTGTGTAGCATTTCCA
EMPV1_42757	CGGTGACCCGACGGCAGTGTGGCGCCATGGCGTCGCCCTTCAGCGGGGCCGTGCAGCTGA
EMPV1_42759	CCAGCTAGTTGTACAGACCACAACTGAAGAAAGACGAAAACTGGGTAATATCTTGGAGCG
EMPV1_42760	CTGGGCCGTGAACTACCTGCCCTTCTTCATGGTGGAGAAGACGCTCTTCCTCTACCACTA
EMPV1_42762	AAGAGTCTCCCAGGATCGACTACCCCAAACTCCCTTCTCACGTAACACAGTGCCCACTTT
EMPV1_42763	ATGCCAAAGATGTTCCCTCATTGAAGGATGTTGAAGAGACCATGATAGAGACAATTGCCC
EMPV1_42766	ATATGTGTTCAAAAAGATGAACTTGACCGTCTCTGCAAACAACAGCTAACTTCACAGGCT
EMPV1_42767	AAGCTTTCGCGCCCATTCAGGAGATGATGACTTACGTGCAGTTTGCTAACGATGAGTGCG
EMPV1_42768	CCAGCACTGCTTAGACTGTCATGCATTGATACTCATGCTAATAAGCTGACCCTCATGATC
EMPV1_42769	TCAGCGACACAGAGCTAGTGAGGGGCATTGCTAGGAGAGGTCTGGGGATAGTCTAAATCA
EMPV1_42772	TTCCCCAGCACCACCACTGCCATCTGTTATGTCTCCTAGCAGGGTGGCAGCTAGTCGACT
EMPV1_42773	AATATCTGGAGGTGGCCAAGCACTTCAAACCTCATGGGTTCTCCGGCGACAAAGCTAAGG
EMPV1_42775	AAGCCCAACATTTTTGTTGCTTCCACACCCCAAGGGCCTTTCTCTCTGTTCTGGGAGGAT
EMPV1_42776	GTTTCATGCAGCATTTGAGACCTACTTATACTAAAAATGTTGGTTGTTTATCTGGAACTC
EMPV1_42777	TGAATAAATGCTGCCCTAGGGCCAGAGAAGCTGAGCCTCTGCTCCTGAAAGCTCGCTATC
EMPV1_42778	AAAAGAGAAAACTCGGCTGCGGGCATCTCCCAACCCTACTTTGGAGCACTTCTACCTGAC
EMPV1_42779	CGTGTATTGAGAACACCCAATCGAGCGGAAGAGTTTTTGGTTTCTGCGTTTAGAGCCGCC
EMPV1_42780	AGGCGGCGCCGCACAGGACGGAGCGGAAGTGCTCGGCTGCTCGTTCTAGGACCGAGAGAA
EMPV1_42782	CGAGTCACACGCAGAAATGTGGAACCCCCTTCACGAAACCGACTCGACCTCTGTCGCCTG
EMPV1_42783	AAGAAGTTTGCTGGTGGAGTTCTGGATGTGGAGCAATGGGATTGGCAGCATCTTAGGAGC
EMPV1_42784	GGTGAGAACCCTGCAAGTCACTCTTATGTCCTCAACAAAACCAGAACAGCTGCCGATGTG
EMPV1_42785	CCCAGAGCAGGCAGTGGTATAGTTCAGGATTAATAACTCATTGGGAGTCTTTCAAGATCA
EMPV1_42786	ACTCGAGGAGAAAGCTGACAGGCGTGTGTCTTTGAGAAACATGACTCTCTTGGCAACAAT
EMPV1_42791	CTGGTCTTACCCTCTGCGCTGATGTGATCCACAAAGTGCTCCGAATGGAAACTGCTTATG
EMPV1_42792	TGGTCTTATTTCCCATCACAGAGTTCACACTGGCGAGAAGCCCTACACTTGTATCGAGTG
EMPV1_42794	GGCTCTTCACATGGTATATTCTCCCTCTTTTGTGCATTATCTTGCTCCTCAAAATGGCCC
EMPV1_42796	TCAAGAGTGGGTGCAAAAGATTTTAGCCCAAATGCCTTCATCCTTTGAGGTCCAAAGTGT
EMPV1_42797	CCCCGCTGAAGGAGCGCATCTCGCTGCCCTCGCGGAGGAGCTTCTACGTGTACGCGGGCA
EMPV1_42798	GAGTAAGGATTGTGTGCGTTTAACGGCCTACCTAGCTTTCTTCCTCTGTGATGACATTTC
EMPV1_42799	AGGAGCTGCTGAGGGCGATGATGAGGAAGGCTGAGCTGGAAATCAGCAGCAAAGTGATCG

EMPV1_42801	TCTAAATCCAAGACTCCACGCTCCCCAATTTTGTGGGTTCAGTAACCTCCATCCTGGGAG
EMPV1_42802	AACTTCATCAGATCCTGTCTCACCCTTCTCTGTTCCTCCCAAGCGGTGGCCTCATTCTCC
EMPV1_42806	AACCACAACCGGCGAACCATGGCCGACCACAAAAGAAGCAAAGGCATGATTCCGTCCTGA
EMPV1_42807	GGCCACTGGTCATGCCCGAACTAGATCTTTGCCCATTTGGGTCTGTTTGTGAGTTCTGTT
EMPV1_42810	AGCCTGGTGGCATCCACTTCCCTTTTCCCTTCACACCCTATCCCATCCAGAAGGACTTCA
EMPV1_42811	CTGGCAGTTGAGCTTATACCTCCATTCTGAGGCAACCAAAGAGCCCCAGGCAATGCTTTG
EMPV1_42812	CCACTCAGAGGAATTGTTGGACCAGATTATGTGACTGGATCAAATCTTCCCAGTCATTCC
EMPV1_42814	GCCTTGAGCCTGCAGAAGTGCTTATTATGGTCAGCCTCTGCTACCGGAACATTCATTACA
EMPV1_42815	GGTGATGGCCAGGGTTGAGGACCCAAGGTTTCACCAAGGACCTGCATTTCAAAGTTGTAA
EMPV1_42817	GGAGCGATCCACCCGGGAGCTGTGTCTCAACTTCACCGTTGTCCTAATTACAGTTATTCT
EMPV1_42819	CCCCAGGGTCCACGAGCTCAAGATTGAAGAATATGTGCAGCGGGGGCTACAGCGGGTGGG
EMPV1_42824	TTTCAAAAACATTCACACCGTCGCCAAAGCCTTCAAGCTCCTCGACGTCAACAAGACGGG
EMPV1_42825	TGCTTCAATCATAAGTCCCCAGGTGTGTGTTGCTCTTGTGATGGTTTCCTGGGTTGTGGG
EMPV1_42826	AGCTGCACCCACGGCATATGAAGTTCCCAACAAAGGATCAAATCCGAGCCACAGCTGTGA
EMPV1_42827	AACACACCTAGGTACTGGAAGCTGATTTCTCAACAGAAGTATTTAGGAAGACATACCTAC
EMPV1_42828	TTTGTGTGACAAACAGCCAATTGGGAGGCTGCTTTTCCGGCAGTTCTGCGAAACCAGACC
EMPV1_42830	AATCAGCCTTTGCTACGGACCCGGGGGGGCGTGCCAAGTAGTCTAGGACCAATCCGTGAC
EMPV1_42833	TCTTTGCGAGGAAGTGGCGTCGGACAAAGACATGCCATTCAGCAAACAAACCATTGCGGC
EMPV1_42834	ACCCTCTCAGGTCCTCACACAGGTCTGGTGTCTGTGCTAAGTCCTGCCGGAGCTACTCCT
EMPV1_42835	CTGTCTTCTGTATATACCCTGTAGATCCGAATTTGTGTAAGGAATTTTGTGGTCACAAAT
EMPV1_42838	ATGGCCTGCTGTTCAACCAGCTTCTGTGGATTTCCCATCTGCTCCACCGGTGGGACCTGT
EMPV1_42840	ACCGTGTCTAACCGGGAACAGGAGGATCTCTGCCGAGGAATTGTCCAGCTCTCTTTCAAT
EMPV1_42842	GCCTCCTAGCATGTATTAATTAAGAGAATTGCCTTCCGGAGGCTGTGGAAAAGCAGTGGG
EMPV1_42843	ACTGGGGCTTCCTGCTGACATTTAGTGCCCACTTTTGTCCTTCTTGTCCTTCAGAGGTCT
EMPV1_42845	ATGATTTTATCGAGAGCGTGGTGACAGCAGCCTGCCAGCTTGCTCGGCATCGCAAGTCCA
EMPV1_42847	TCTTGCCCGGCTTTTACAGCAGCTTGTGTTCCTCACCGACAGCACTCGTTTTCACACCTA
EMPV1_42849	ATGAGACATCGGCTCGTCTGCTCTTCATGGCTGTCAAGTGGGCCAAGAACCTGCCTGTGT
EMPV1_42850	GCTGCACCAGCAGCATATGGAGGATGCCACAGCCACAGCATCATGAGATCCAGGCTGCAT
EMPV1_42851	GGATGTTTCCCGATATCTGCGCTTCATACCAGAGACCCCTCTTCCGAGAAAACCAGCAGA
EMPV1_42853	ATGCTTTCCCGTTCAGCATTGAACTTGCCTCGAGGCAGAGGGAGGCCTGAGGACGGTGCT
EMPV1_42854	CTCTTCTCCAGTCAGTGCAGGAGCAGACAGAGAAGACCCTCACCTCGGAGGAGCTGAGGC
EMPV1_42855	ATCTGCATGGCCTACTATGAGAGCCACTACAACACCACAGCTCAGACCAACCTGGAGGAT
EMPV1_42856	TGCCCTCCCTGCGGGGAGACCTCATTGTCGAGTTCAAAGTTCGCTTCCCACATAGTTTAA
EMPV1_42858	GAGTCTCAAATTGTATAATGTTTGGGAAGAAAAAGACACATACCTGAGAGTAAACTTTAT

EMPV1_42859	TCATACAGCGCAAGAGGGAGCTGTGGCCCCCCGAGAAGGAGGACCAGCGCCAAGAGCTAG
EMPV1_42860	GCTGGTTTTGTGGAGCAGCTTTGTACATATCAAGGAGGCAAAGAAAGCAGTAAGTGTGAC
EMPV1_42862	AAGAAAGCTCATTTCCAACCAAGTCATGGGGGCTATGCCAAGGCCAAGGTTCTAGCTGGA
EMPV1_42863	GCCACTTTTGAAACCATACCCACATCAGTAGAATTTTTGGAATGCTTTCATTTGCTTCAG
EMPV1_42864	GCTTCCAACCGTTTCGGCTTGAGTTTTTGATGTTGCTGTGGGTCTATGGCGGTCCTTTGA
EMPV1_42865	CTGGTGCTTAGAAAGCCTGGCCTTATTTTGCGTTGAAATGTCTTCCTCTACAGTTTCTGC
EMPV1_42866	TGTCTCTGCTCTTACAAACGCCTCCACAGTTACTGCTACCTGCTCCCTGGAAACAACACT
EMPV1_42867	TCCCAGATGCCTTGCAATCACACAAGGCTGCAAACACTCAGAAAGCGCACATGAATTTTG
EMPV1_42868	GGGAGGGAGACTGAGCCTCTGCAGCAGGAAAGGCCTGGGGCCGAAACAACATAAAGATAA
EMPV1_42869	TCCTCACAGACGCTATGTCGGGTTCTTACCCTGATGAGCTGCAATAGGAACTCCTGAACT
EMPV1_42870	GCAGATTCGTATTTGAAGAACTTTGCTTCTGACCGATCTCACATGAAGGATTCCAGCGGG
EMPV1_42872	CTTCTCATTAAGGAAGACCAGGAGTTCCCTCTCAGTGCAGTGGTTTCAGGATCTGGCATT
EMPV1_42874	CAGCTCAGTGGAGTTTGGGGCTGATCCACTCCTACTCTTTGAGAACTGCCACACCTTCAA
EMPV1_42875	AGTTGGCAGTTCACTTAACCTTTTCATATTTGCATAATGGCCAAAAGGCTTGCAGATCTG
EMPV1_42876	TGCTGGCATTGTGGCCGAGCTGCAGGCCACTGACTTGAGGAACTTCCGGGCAGTGTTCAT
EMPV1_42879	GAAATGAGTGGTCGTAAATCAAAGAGTAGCAACTTAATACAAGCAGAATACCTTTCACAG
EMPV1_42882	TCAGCTCGCCTTGAAGGAGATAACACTATGATTTGGGCAGAGGAAGTGACAAAATCTGAA
EMPV1_42885	ATCTCAGAGGAGTGGTGGAGCTGGTGGCCTGGAGATCCTGGAGATTGAAGCTAGTTAGTT
EMPV1_42887	ACGTATGTGTTGGTCCTGTTTCTGGGATGTCCCCCTCAGTCCTGGGCTTTTGAATCATCC
EMPV1_42888	GACCAGTAGGACCGATTTCCAGCAGAGGAGATGGAGACGGATATTCCTGTAGATGTCATG
EMPV1_42889	TACGTATGTTCCATGTAATGTTTGAGATATACTTATGCTAAAAGTTACTTGTAGGTCTGG
EMPV1_42890	TCTGCAGGAGTATGGGACCAGAATAGGGAAGACGCCCGTGTACGACCTTCTCAAAGCCGA
EMPV1_42892	ACACAGTGGCAGGCAAGAGACAAGCTGTGTTGAAGGCACTCGGTGACAGGTACAATGGAC
EMPV1_42894	TCTGCAACTGGAGCCAAGAAAGTGAAGATGGCCACCAAATCATGCCCGGAATGCGACCAA
EMPV1_42895	GCAGACCAGGATCCGACTGTCCGGCAGAGCGTGGAACACCTGCATCCAGGGCACGCGGGA
EMPV1_42897	CAGCAAGGATAAACAGCAACCTGTCCTTAGGATGTAGGCAGGCCAGAAACAGCCCATAAG
EMPV1_42898	CAGTAACTATGTGGACCAGGAAACTCATGACCAACCCACTGCTTCAGCAGAAACAAACGG
EMPV1_42902	CTCCCAGGAAGAAAACTATTGGAGAATGCTCGCTATCACTCAGAACTCTTAGCACACAGG
EMPV1_42906	ATGATGACACTGCCACCTCTGACTTCTGCCTCTGGCCTTCCACTCTCAGTAAGAAAAGCC
EMPV1_42908	GACACAGTTAAAGTAATGGTTGTCATGCTTGCAATTTGTTTTCTTGCAAGATCAGATGGG
EMPV1_42909	CTCAGCTTCCATTACCTGTCCTTCCTCATAGATGGTAGGTCCCAGTGTAGTTAAGCATTC
EMPV1_42910	AAATACTGCTGTAATACTGTCATACAAAGTGAGTAAGAGTAACAGAGAGGAGAAGACATA
EMPV1_42912	GATCGTGTCTACTGGATAGATGGGGAAAATGAAGCAGTCTATGGTGCCAATAAATTCACT
EMPV1_42913	GGATTCCCAGATGCCACTTTTCCATGTGTAAACTCCGTGAAAGCTCATGCCACTTGAGTT

EMPV1_42914	CTATCTGGACCCCGAAGTTCATGACCAACCGACTACTTTCACGGAAACAAATGATCCTCC
EMPV1_42915	GACCGAGTCTTCTCCACAAAAGAAGCAGTGCCCTGAAGCAGTTGCTAGAAGTAGTTTTGG
EMPV1_42916	GTATTCGAAAAGCCACCAAGCATCTGAAGGATGTCCCTCTACGGAAGCAAAGTGTGCCAG
EMPV1_42917	TGAGAGGGAGGAGCAAGCCCCCAGCCGAGCTGGCTGGTTCCAGGAGAAAAAGCCAGGACT
EMPV1_42918	CTCTCCGGCTCGCTCCTCCTCTTCTCCAGTTCGTCATAAATGGTGAGGATACTGAACGAG
EMPV1_42920	GAAAACAAGTCCCCCAAACCTGTTTCCAAACTTGGCCCACCCCGGAGAAGCAAGTCAGAT
EMPV1_42921	CAGGTCGTGTGGCACTCCATGCAGGACGAATTTATCCGCCAGTACAAGCATTTTGAAGGT
EMPV1_42923	CTGGAAATCCACTCTCCACATCTGTCTGCCAGTGCCTGTCTCAGAAAATCAGCGAACTGA
EMPV1_42925	TGCTAATGGCCTCCAGCGCCTTCTTGCCATCGAAGACCGGAGAGGACAGGATGAGGGTGA
EMPV1_42926	TTGTGTCACAGGCCAGCAGCTGAAGCTCAGATAAACCCCTGGCTCAGGAACAATCATGTG
EMPV1_42927	CGGGGCCAGAGAACCCTTTCCCGGGGGTGTTAGAAATGTGTCTGATGAGAGGATGAATAA
EMPV1_42928	CGGAGCATCTTGGGAAAAGGCTTCCCCTGTGATGATTGTACTGCTGTTTGTAAGTTTGAC
EMPV1_42929	CCTTTTCTGGCTACCCTATGGCACATGGAAGTTCTCTGGGATCAGACCCAAGTTGCAGTT
EMPV1_42931	TCCATTATGCGATGACTGCAGAGCTTCTGTGCTTCCTTGTCCCCATACCCACATGCTGAG
EMPV1_42932	AACTTCCAGCTGGTACTCCACCCAGTGAGCAGCTGCCACCTTTTCCAGGACCTTCATTAA
EMPV1_42934	AGAGCCCCACGGTGAAGGCGCTTTTGGGCAAGACCGGCGTCTCACCCATTCTCCCTTTCT
EMPV1_42935	TGCATAGCACCTTGCCCACGCGCACACCCAGCCGCACGTCCTCAAAGCGCAGCCCTAGGT
EMPV1_42936	CTCGATGAAGGAATAGGGTGCTCTGGACTCGATGAAGGAACAGTATGCTCTGTACCCAGC
EMPV1_42937	ACATGTTCGTGAAACTCAGGAGCCAGGAGTGCGACTGGGAGGAAATCTCCGTCAAGGGTC
EMPV1_42938	TCGGCTGGGACCGAAGGGCTCGGACGAGACCGCACAAGTTGTGCCGCACCTGCTAAGGAA
EMPV1_42940	TCAGAGTTTCCACTCACCGGTTGTTGACAGATGGTACAATTCCAAGGCCCTGCCCACTGA
EMPV1_42943	ATCTTCATGGGCCTCATGCCCCACGTCCAGGGCATCTTCCTCTATGGCATCGGGCGTGTT
EMPV1_42946	GAGGGAGTCTACTTACGACCCATCACTGCCCACATCCCACCAGAGCAGCTCATCCTGGAA
EMPV1_42947	ATGGGGGTGGTGCCACCACCAACACCAATGGGGATGCTCAATCAGCAGTTGACACCTGTT
EMPV1_42948	GATCGCTGATCAAGTCTCCACCCCACATCCCCACTCTGAAATGTGACTCGGCATTGTATT
EMPV1_42949	GCCTACCATAACCCAGATGCAAGGACATATGAAGATAAAAGGGCTGTTGTATCTCTAGAC
EMPV1_42952	AGAACTGGATGCTGTTAAGCATTTTCTTAGGAGTTTATGGCCTCTGCATGGGTGGGGGCG
EMPV1_42953	CTCCAGGACGAAAATTTTGTCAGTAAAGAAGAGTTCCAGGCAGTGGAAAAAAAGCTGGTG
EMPV1_42954	TCCTACACTGGTCAGGACTACAGTGCACAGGGAAATGCTGCGAAGATTTCATTGGATCAG
EMPV1_42955	GTAACAAGACACAGCATACCAGGCCTGCCACTCAAGTTATTTATGGTAAGTCTCAGCTTC
EMPV1_42957	GCTTTGTTTTAAAAAGATGTTGGTAACCAGTCAGTGTCTAATCCTTGGGATGTTGGATGG
EMPV1_42958	TTCGTAGAGCTGGACACGAACTTGCCCGCCGACCGCTTGGCCGCAGGACTGGAGAAGCGG
EMPV1_42960	CTTTAGTGGTCCTACCAATGCCAGTTTTGGTGATGGACTCAGCACAGGTGCTGGTTTCAG
EMPV1_42961	GGTGCATTTCGCGCTCTTCGTGTCGGTGCTCTTCTGGCTGCTGACCCTGGGCCTCTACTT

EMPV1_42962	TGGCCCGGGTTATACCTGATGCTCACGTATAAGACGAGCAAAAAGCTTGTTGGTCAGAGG
EMPV1_42965	CACCCAGGTTCAGAGACCCGTCCAGTAAACCCCCGTTAGGAGAGAACTAAACTCACTGAT
EMPV1_42966	CCCACATGACTTGGCAGGAATCAGGAGAGGTAAGATTTGATCCCAGGTCACGTTTAGCTG
EMPV1_42969	TCAAATACTACATCCCAGAAGTTGTTTACACTGGCGTCTACCCGCCTTATGCAGGGGGCG
EMPV1_42971	CTCCCCATCAGAGGCATCAGGATGAAATTTGCCGTGTTGACGGGGTTGGTTGAAGTTGGA
EMPV1_42974	GTGATGCTTGTCGACTCCTCATAAGTAAGAACTAAAGAACAGGGCAGTGGTCGCAGTTCC
EMPV1_42976	CCAACAGATTTCCTGGGGTTGGATTCGATGTGTGAGTGTCCTCAAGAATGACCTTGGAGT
EMPV1_42977	CCTCAGAGGGTTCAAAAATGCATGGCACATGAGACAGAGCAGTCAATACATCCATGTTGG
EMPV1_42978	AAGGAAAGACAGGAGTCCAAGATTCTGATGGAAAAGATGGAACTTGAAGTGGCAGAGAGA
EMPV1_42979	GTTACAGGTTGAAACTGCAGCACAGGTTTGATACCCAGCCTAGGAACTTTCCACATGCTA
EMPV1_42983	CCCAGGTTTTGCAAGATGCCTCTCTTCCTTGTCATTTTGTCCGTTTTCATCACAGCTGAC
EMPV1_42986	CCTTGACTGAGAACAACTGATACTGATGGTGCATTATCACCCATCTAAGGTGGCAGCAGC
EMPV1_42987	GGCAGATTCCTGTGCCACATCTACTATCTACTAAACATGAATCTTTGACATGGGGTCCAG
EMPV1_42988	GGGGAGTGGGGGAGAAGAGAACTGTCCTTAGCCTTGACCCTGGGAAGCTCTTTAAATTAT
EMPV1_42990	AGTGGGCCCCGGCCCTAAGTCTGTCCCGCCGGACCCGCGCTTCGGAGCTCCCCATCTTCT
EMPV1_42991	GAAGGCATTTCTTCTCGGGGGGCACTGCCAGGGGGTGTCATAGACTGAAAGGCAGCTCAA
EMPV1_42992	AGTCAAGGCCAGGGTTGGAGATCCCCATCAGCCCGGAAATAAGCACAGGTTCTCTGAAAT
EMPV1_42993	CAAGGTAGCCTAGGTACGTACTTGCCTCCAAGGGCCTCATTCCTCTGCTGTTACTTATGA
EMPV1_42996	TGTCAGGAGAAAACCCCAGTCAGTTCAGACCTCTTGGAAAGAGTCCCATCTCTGGTCAAG
EMPV1_42997	TGACGATACTAAGACTATGGAGAAAGAGAAGACCCATCGTGAAAGACAGCGTGGAGGTGC
EMPV1_42998	CTTAGATATTATAGTACTAAGTGAAGGATATACAAAGAATGATTTTGCCATCCATACACT
EMPV1_43000	CGGCCATGATCTTCATGTACGTTCGGCCCAGAGCCATTGCTTCGTTTAACTCCAACAAAC
EMPV1_43001	TGCTCCAGCAGACCATCTGCCAACGCTTTGCCAACAAGACAGTGCTGACCATTGCCCACA
EMPV1_43002	ACTCATGGCCTTTAAGGGAAGAGCAAGGTGGGAAGATTCACTGAGGACTTAGGTATTCAC
EMPV1_43003	TCTGCCCAGCAGGTGCCCCAGGTACAAGGTCAGATCTGCCAGAAGTTTGCCTTTGTTTTT
EMPV1_43004	GGATTATAGTGCTGCATTGTCTGGAGTTAGCACCTCTTGGACTGAATCGTTTGTCTAGAC
EMPV1_43006	GGTTTGGCCAAGGTCACACCTCATCAGAAATAGCAGAAGCAGGCTTGGACTTTTGCTCAG
EMPV1_43009	AGACTGTAGGATAACTCTAACCAAGTATTTTGAGGCTGAACCCACAAGTTTGGTGGCAGG
EMPV1_43011	AGAAGTCAGAGCACACTATTTTCATGGAATAAGGTAGTCAGAGGGGGCAACTTCACTCCC
EMPV1_43014	TCCCTCGCCATCTCTTCTTCCTCACGCTGTCGGACACAAACTATTAAGTCAGGCGTCAAT
EMPV1_43016	GTCAGAAATCAGGTGATACTTGCTGTTCGTCAGGAATATGTCGAGCTTGGAGATCAGCTC
EMPV1_43019	ACCTCAATTCCCGACTCGGAAAGCAATCTGGATCAGAACTGTTTGTCTCGCTGCAGCACA
EMPV1_43020	TACAGGCCATGGTGCATGGGGTGACCACAGAGGAGTGCCAGGCGGCCTTGCAGAGCCACA
EMPV1_43021	GACAGCTGGACCCTCCTTGCAGAGCTAAGACTCTCATTGAATCGAATGTGGTGGAGATCT

EMPV1_43022	CATAGCACTTTTGTCAGGGCAAATTACGAAGACTGCAAGATTTAGCAGGTGTGGCCGTCT
EMPV1_43023	GATACCCAAAAGCAGACACAGTGCCAGGCTCATAACACTTAATAAAAAATGAAGAGGCTC
EMPV1_43027	GGCAGTGCACAGAAACCACCCGCTGGGAAAGTCCAGATAGTCTCCAAAAAAGTGAGCTAC
EMPV1_43029	CTGGTCTTACTTGTCCCGATAATGAATCCTTCTGTGGCCAAGCAAGCAGACAAGTTCCCC
EMPV1_43030	AAGGAGGACCTGTGCTCCAAGTTCGGGATGGACCTGAAGCGGGGGATGCTGCTGCGGCTT
EMPV1_43032	TGGCAAGGGAGGGGAATGGGATGGAATGTGGGTGGTTGTCAAGATGTTCTACTGGGAGTT
EMPV1_43033	TGAAGAACTGTCTTGTCAATCAGCTCAGGATAGCAAATTGTATATGCTCTGTATTGCAGC
EMPV1_43035	ACCAGGGATCTTTGAGCTCCTACTCCTGGGGCCCTTCCTCACCTCAGCCTGCTCCTCTGA
EMPV1_43039	ACTACAGTTGTGCCCTCTCCAACAACTTTGCCCGCTCCTTCCACCCCATGCTCAGATATA
EMPV1_43043	GCAGAAGAGGATCTCCGAACGCTGTTTGCTAACACTGGGGGCACTGTGAAAGCATTTAAG
EMPV1_43044	GTCTTTGGTGCTGCGATCCTGGCGGTAGCAATTTGGCTGAGAGAAAACAAAGATAGTCAT
EMPV1_43045	GGATGTCAAAAAGCGAGAATATCCAGGTTTCGGTTGGATCTATTTTAAGGGGAACCTGCC
EMPV1_43046	ATGCTCATGGAACATTCTCAAGAATCGGCCACATATTGGGACACAAAGTGAATCTCAGTA
EMPV1_43049	GCTGTATTTACCCACAATTCTGACCTTGGTGCATTTGGCCATTTCATTGCAAATCCAATC
EMPV1_43050	CAACTTAAGAGGAATGAGGAAGACGCTTTGTAAACTTAGAACCAGGGTACAAGGCGAGGG
EMPV1_43051	CCTTCATGTATTTATAGTCTAAGCCAGCAGGAAGGGTCAGAATTCGTCACAGCCAGTGAG
EMPV1_43052	ATAGGGCTAGCATCCTTATAAGAGTAAGAGACACCAGCACTCATGCTCTCGCTCCAGCAC
EMPV1_43056	GGGCCCCACAGTATTTTGATAGAAGTGGAGAAGTTGGAGGTGCAGTGTATGTCTACATGA
EMPV1_43058	CCACACCGGTCAGTGATAATTGGGAAGTAATGACAAAGAACTGACTCTGGAACTGTTGCC
EMPV1_43061	AAGTACACAGCCAGAGTGACGGTGGGCAGCAAGGAGGTGACCCTGAACCTCTACGACACT
EMPV1_43064	GTTGGGCTCGATTGTTAACTGCATAATCCTCTGGTCTGTATAGAAAGGGCTGCAGTGTGC
EMPV1_43065	CCCTATGATGCAAATGGTGGCATGTTTAATGTTGTCCCAGAGGTCCCTTATACTGTCCTC
EMPV1_43066	AGATGGACAAGCCACAGGGTGGAAGGAGCTGCATCTGAGTCACCACTGGAGAATAATTAC
EMPV1_43067	CCGAGAAGCTGTACTACGACAAAGAGCAGAAAGTGTCAATGACAGACAACATCAGGCCCC
EMPV1_43069	CAGAGACATTTTTGATGAATGGGAGCTCCTGACGTAGTTCAGTGGTTGATGAACCCGACT
EMPV1_43072	GCAAGAAGCACGACCTCACAGAGTCCCTTGATTGGCAGGGAAAGTACCACCTTGCTCTTA
EMPV1_43073	GAATCCTCAGGGAACGAACCCAGGCCACATAATTTAACAATGCTCTAGGCAGACTCCACC
EMPV1_43080	CGGAAAAGCTCGCGCTAAAGCTAAGACTCGCTCTTCTAGGGCTGGTCTCCAGTTTCCAGT
EMPV1_43081	AGACTGGGACAGAGGAGGGCAAGGGCTGCTTACAGATGCTCATGCAAACTGATTCATTCC
EMPV1_43083	TTTCTGAGATGCAAGCTGATTAAAAGGCACTGAAGAGCAGAAGGATTAATGCAGAGGAAC
EMPV1_43087	CGGGAATTGCAAGCAATATATCTATCTTAGTATTGTGTGTGCTGACGAGAGCCTCCGTGG
EMPV1_43088	CTAGCACAGAGGCTATGGAACCTACCTAAATTTCATTCAGGAACAGGAGAAAAACAAGCC
EMPV1_43089	GTGCTGCTCCCCGAGGTGGTCTGTTTCACACTTAAAGTCCATCCCCCAAGTTTCCATCTT
EMPV1_43090	AGAACTAGTTTGCTTATCAATCGTATGAACGAATGGCTGGTTGAAACAATCTTTCCTTAC

EMPV1_43093 ATGCCATTCGTCTCCCTCCAGAATTGGTCAACATAGCACTCTTTGGCTTTGGCAGTGAAG
EMPV1_43094 TTACCAAAAGCAAGTCTGAAGTGCAGGGACTAAAGTCCCGCCCTGCACCCCAAATAGGGA
EMPV1_43095 GGCTGCAGTCTTTGTTGCGGCCACCCTTTGCATTTCCAGCCCATTTTTACTCATCATTGC
EMPV1_43096 CCTCCACTTGTTCTGGCTGCTGCAAACGTGGTGAGGAACATCAGCTATAAATATCGGGAG
EMPV1_43098 CAGCTGGTTCCAGCAGACACCAGGCCAGCCTCCCCGACAGCTGATCTACCAAACAAACAG
EMPV1_43099 CTGAGAATGAAGACCCTCAGCCGGAGTTGTACCACCCAGTGAAAAAAGGATCTGCCCTGG
EMPV1_43100 GGCCTTCCCAAGCCCATATTGTGCACCCAAAAGCTGTCAAGTGGCTGAGATGGCTGTTTT
EMPV1_43102 TTCATGAGGAATCAGATGCTCCTTTGCTGCCCCCAAGAGTTTACTCCACAGAGAACCACC
EMPV1_43103 TGGCCCCAGGAATGAGTCACCCAGGAACTGAGCTCTAGCAAAGCAGGTGATTGGTTCAGG
EMPV1_43104 TGGCCAGGGGCATCCTCTCAACTACCGGGTTCCAGTCTCTAAAGTGACAAATAACAAGTC
EMPV1_43105 TTGGTGGAGAGTCCTTAGCAGTTGCCCAGAATACATATGCTGTGAGTTGGTTTAAAGGCA
EMPV1_43106 CACCCCAAGGTAAGAACCCAGGATAGTGGAAGCCTGAAGAGGCTCTGAGGGACACCAATA
EMPV1_43107 CCCTGGACCCTGAAAAGCTCATACAATGCCCCTATGATAAAAATCACCAGATCAGGGCCT
EMPV1_43108 ACACTGGGCAAGGGTGGTCATCCTGAAATACATGTCCAGGCTCTTGTTTGTCTACGACGT
EMPV1_43112 TTGATTGAAGACCTCCTCAACTGGAATAACTTATACATTGCTGGAAGACTCCAGAAACCG
EMPV1_43113 TACCGTGAGACTGTCAGTGAGGAGTCAAATGTGCTCTGCTTGTCTAAGTCCCCAACCAAG
EMPV1_43114 AACTACTACCTTGGGTCGTTTCTTTGGCATTCCCACTACTGGGTTTAGTGCCTTTGGGCC
EMPV1_43115 GGAGCTCGCTATGATCAAGAAGAAAATCACATAGTGACGGGACAGTGCAAACTCAGCCGC
EMPV1_43119 GGGAATCAAATAGGGATGATAAAGCATCCTGGGCTCTCAAAAGCAGCATCAGGTACTACC
EMPV1_43122 GCCTCTGTGGATCTCACAAACAAGGTCACGCGCTGCAGGGACTGCAACCTCGTGAAGAGA
EMPV1_43123 TCTTCCGGGTAAACGTCCCATCCCTAGGCCAAGAGGTGAGGAGGATCGAAGACAGTATTA
EMPV1_43125 TCACATGAAGAGTCTTGGAAGCCATTTCTGATTGATGTGGATGGTGGAGGTGGGGGAGGT
EMPV1_43126 CTGTCAGCCAAATTGGGGAAGCAAGTGTTTGCTGTATCTATTTGACCCTGAAAAGGCAAG
EMPV1_43128 CTATACAGACTCCTAGGAGGAGAAAGGAGTGGAGAAGGTATTACTATCCACATAGTTTAC
EMPV1_43129 TGCAGCTTCTGGCCTTCACAACAGTCACATCAATGCCAGATCCAAGCTGAGTTTGCAACC
EMPV1_43130 ATTCAGGGAATAGCTGGAGAAGAGTGGGACAGGAAAATGACGTCGGAGAAGGTGACAGGG
EMPV1_43131 TCCTACAATGTGGCTCTCATCACCGGGGTCAAGTCCAGGCAGTGGTTTTGGACAGGAATT
EMPV1_43132 CTTAAGGATAACAAAGCATCATCATTCAGTTCCCGCTGTGGCGGCGTGGGTTCAAAATCC
EMPV1_43135 GACTTGTGATGAGAGCACTGGAGGATGGTGGAAGGTATTGAGGTGGCGAGATCATCAGGA
EMPV1_43136 ATTGCTGAAGCCTTTAAGAAGGCCCATGAGACCGGGGACAAAGTGGTCAAGGAGGTCACT
EMPV1_43137 TAATTTTTCAAAATGGAAGCGGCCCTCCGGCGGGCGTGGACGTGAGCGCTGTGAATGAAA
EMPV1_43138 TTGAGGATGCGGTAGTTCTTCTTCCATGAGCTGGAACGTGTGCCCAAATCCAACCGTTGA
EMPV1_43140 ACCTAACACACTGGTGAGAGCTAGCATTAGCATGAGCAAGCACACTTTTCTAGGCCCATC
EMPV1_43143 TGAGGCCCTGGAAGAAGCACTCCACCTTTGGAGATGTGAGAAAGCTTATCACTGAGGAGT

EMPV1_43144	GGAACTATTGCTAAAGTCTGTCCCTGCAGTATGCTTCCTTCCAACCGGAGTTTCCCTAGC
EMPV1_43145	GTTCGAGATCAACTTCTTGTCGGAGGCGGGGGACATTGTCTTCCACATCAAGCCCCGCTT
EMPV1_43146	CTGGGGAGAAAACCCATAGATGTAAAGATTGGGGAAAAGCCTTTGACAGGACATCAGAAC
EMPV1_43147	TTTGAAGTCGCTTCTCCGGCCCCCAAGCTTGGCAAGTGGGCGGAGTCTTCGCACTTAAGG
EMPV1_43148	AGCAGTGTGCGACAGCCTGCCTGGCCCATGGGGCCTGCAGGATGAAGAGAGAGGGAAGGG
EMPV1_43149	ACATGCAACAGGACTTCAAATCTTCCCTTTGAGAATTCCGCTCTGTGAATGAGTCCCCTG
EMPV1_43150	GCACCTCGCCCATGTCTATCCTGTAGTAAAGAATCAAATGTTGATTGAGCAGAATTAGGC
EMPV1_43151	GTGGCATGAAGATTGTCCTTATCAGCAGATCCCAGGACAAACTGGACCAGGTTTCCAGTG
EMPV1_43152	GGGGTCCAATCGAAGCTGTAGCTGCCAACCTATGACAGTGCCACAACAACACAGGATCTT
EMPV1_43153	GATCTGGCCCTGCAGCATCCAAAGATCCCTGTGTGGCTAGTGTATCTTGCTGCCACTTTT
EMPV1_43155	GTGCACTGATTGTTTCCAAGCTTTTTTGCCTGTGCACTATCGCTATCATCGGCCACATTG
EMPV1_43156	GGGAAAGGAATCATGCTTGGTAGAGCAACTTCATCAACCACCAGGGGATCATTGCCATGA
EMPV1_43157	GTGCTTTTGTACTGGATCTGCCGAAGCATCTGTCCAGCTTGGTATCCTGTGAAAAGTTTG
EMPV1_43158	CAAGACCAGTCCTGGAGCTACCTCGTGCTGGAAATCTTCATAACAGTTCAGTGTTTCGTG
EMPV1_43159	AACTATTAGGTGGTGGGGGGCTAGGGGTAGGGCCCAGACCACAGGGATGATGACGATTCT
EMPV1_43160	CGAGAGTGGTCGTGCCATGTCTGAGTTGAAGGAGCAGCCACATCTCCCTTACAGCTTGTA
EMPV1_43162	GGGACAAATCCCGTTAATAAGTAAGAGGATTTGTGCTTGGCTCTGTCACATGCCACTTTG
EMPV1_43163	ACCTCGGAGTTGACCAGAAGGATCTCCGTTGTTTTCACGAGGCTGTAAGTAGCGATGGTG
EMPV1_43165	TGACCAACTGGACTGGGATCCGGTGCCCAAACTCATAGAAGGGATCACCGTTTACCACCA
EMPV1_43166	AAAACGACAGTTTTGCTGGCACGTGAAGCTGCAGTTTCCTCAGAGTCAAGCCGTGTACGT
EMPV1_43167	GGAAGCTTGAGTCTTTTTTGCTGGGAGGTTTGCTCTCCTGTCACCTAGCCACTACATGTC
EMPV1_43168	GGTCACCTTATTTAAAGACATCTTCCCACCTGTACTGGAGAAGCCAACCATGGCAGTCAT
EMPV1_43172	AGCTTGGTTTGGACTCAGAAGTGTTACCTGTCCGGGGAGACCATCCAGCTTCCAAGAACA
EMPV1_43173	CAAATGCTCCATGAACAATCAGTACCCATGGATGTACAAAACTGAGCTTATCAACATGGG
EMPV1_43174	TCCTCAGGGGAATCCACGGCCCTTTCCTCATCATCGCCCCGCTCTCCACCATCACCAACT
EMPV1_43177	GTGAAGCACGTCTCATTTATTTCTACAAGGCCTGGGACACAGGAAGCTGCCCGAACTTCA
EMPV1_43178	AGCACTGAAAGAGAAGATTGAATCTGAAAAGGGGAAAGATGCCTTTCCTGTAGCAGGTCA
EMPV1_43179	TTTCAACAGAAGGGGGATATGTGGTTCTAGATGAGTCTTTCAATATTGGATTGAAATTTG
EMPV1_43180	GAAACTCCCAAAGGATGTTTCAGCAGGTTTTAAGATCTCTTTCTAGAAGCACCAGTTAGG
EMPV1_43181	CTCCAGCTGCCATGTCCAGCCCACTGTTCCCAGTTCCTGTGTACGCCCCGCAGGCCCCAG
EMPV1_43182	ATGTTGCCTTAATCTCCCAAAGGGCTGATGGTGTATGTGTAATCTGCTTCTGTGTGGTGC
EMPV1_43186	CCCTGGTCTTCCAACCCTCCCATCTGTCTCCAGAATCTTAATCTGTTCCTTTCTTTCCCT
EMPV1_43187	TCTCAGCACTAGGTTACCTGCTCACAGCTCAAACTGGCCCACATTTGGAGGCTCAGCCAT
EMPV1_43188	TTTTCTTCCGGCCTCTCACGTTCCTATTTGTATGTGTCCCTAGAAGTGAAGTGGGTCACC

EMPV1_43190	TGTACAAGTCTGCAGCTCAGAGGAGATAGCTGACGTATAGACGAAAAATTGTGGAGACTG
EMPV1_43191	TGTATAATGGACACTCCTCTGTGCTATGTGGACAGTGCCATCCCATGCAACCACAAGGAC
EMPV1_43196	GCTCCGCAGAGGTGCGTTCCACTCTGGGTAAGCCCAATATGTGAAAATCCAAATAGTCAA
EMPV1_43197	TGTGTCCTCGCTGGATGAGCTAGTGGAACTGAACGAAAAGCTCCTGAAGTGCCAGGAATT
EMPV1_43198	AGGACGTGGTTCAGAGAGCCTTAGCACTTGCACATGGTATTTGATGGAACATTTGAGAGC
EMPV1_43199	TGCAACATTAGCCAAGAGGTGGGTTCTGTTTTCCAGGTGAGACGTTTTGCTCCACGACAG
EMPV1_43201	TGGCAGGTCTCTGTATTGCTATTTCGAAAGGTCATTTTTATGTTGAATCAAAGTTAGTCC
EMPV1_43202	CACTTCTACCCTATACTCCTGGTTTAGATGTGGCTGGAATAGTAGAAGCTGTCGGAGAGC
EMPV1_43203	AACAGCAGCATTTTTTACCAACCCATTGCTCGAAGCACATTTGGACCTTGGCACCCCTTG
EMPV1_43204	GTGCTGGAATTGACTTAACGAACTTTAACGTGGAAGGACAGGGCTGGGGTAGGTTCCAAC
EMPV1_43205	AGAACCCACCAGCCAGAAGCCCAGATAACTGTCTGCCCAAGGCTGTGTGATGGAAATACA
EMPV1_43206	AAATGTGGGCTGTCACATGGGAACACACAAAGTAGCCAGAGAGACCGGGTGCAACCAGTA
EMPV1_43207	GTGGAAACGGAAACCAGGTCACAGATGGACATAAAGATGTGCAAAGGCCCAACAGTTCCC
EMPV1_43208	GTCTTCTTCAGAGTCAATCCTTACAGCTCCAGGAACAGGAGAAACGCTTGACAAAGAAAG
EMPV1_43209	TCTCAACTTTGGTGGGATCAGGGTGGGCAACGGACCGACAGAGGAGGATGCTGAGATTAT
EMPV1_43211	GAAAGAAGTAGGAGCAAATTCTGCCTTAAAGCCCTAAAGGTGATCATCCCAAAGTACAGC
EMPV1_43213	TCTGGAAAAGCTGAAGGTGATGCAGAGGAAAGAGCACCGCAGCCAGGGAACGTGGGTCCT
EMPV1_43215	CCTGTCAACATTCTCTTCCCTAAACCATGGCCAGCCATTATGCAGTAGATCCAAACTCTG
EMPV1_43216	GCTGAGCGGAATGCAGTGATCGTGAGCCTTTGCTTCCAGCAGGATCTTTAACGCAAATCT
EMPV1_43219	GCCAGGAACAGTCTTCACGGGGACAAAGAATTTTATCTATTCCAATTTGGCCCCCAAACC
EMPV1_43220	ATGAGCCTGAAATAGCAGAGCCCCACACAGCAACAAGCGATCCATTTCTGGATTCTCTCC
EMPV1_43221	TTGTCACCACACTCTGCAACCAAGGGGGAGAGAGGTGTTAACTTCCAAAACACATGGGGG
EMPV1_43222	CTTCTATCTCGGAAAAATCCGAAGCTTGTTGATGCAGAGTACACCAAAAATCAGGCCTGG
EMPV1_43223	TTTAATGAAACCGGCTTGGCCATAACCTTCACCTTGACACCCTGCATCTGCACGTGATGG
EMPV1_43225	CTCCTGCCTGGGCTGAGCAAACCGCTTCTCCGGGTAGATCTCCTTCAACCAGCCCTCAAA
EMPV1_43227	AAGAAATGTAGACTATCATGATGTGAAAGTTATATGAAATTAAAATTTCAGTGTGTATTA
EMPV1_43229	TCTTGACCTTACGTCCTCTTAACAACGTTTGGGTCTAGGAGCCAGATTGCTTTTTCATCC
EMPV1_43233	AATGCACACATCAAAACCACAACAAGCGGCCACTTCACACTCATTAGGATGGCTATTAAG
EMPV1_43236	GGTCTCCACTTTCTAGGGCTCAGTGGTTTGAATGGTCGGAGTTCTGTCTGTATCAAGTGC
EMPV1_43237	GCCAAGCTGGCACCAGAATTTGCCAAGAGGAATGTTAAGATGATTGCCCTTTCAATAGAC
EMPV1_43238	CTGAGGATATCATGAAAATGGCATGTGGAATGCAGGCTTACTATTCCTGACTTTGTCAGG
EMPV1_43240	TCTGTGTCCTGGTGGGGGACTTTGCACAAGCCGCAGACTCTGGGTAATTTCTGTGACTCT
EMPV1_43241	CCCCCTCCCACCTCTAAAAACAAAATGCCTGAATATTCGGAGTAGATATTCCTGATTTGA
EMPV1_43243	CTTTGACCGCCTACACAATGCCCACAAAGTGTTGCTCAGTGTGGCGTGCATCCTAGCCCA

EMPV1_43251	GGGCTGAGCTGTGCTACAGCCATTGCCAAGGCCTATCCTCCCACGTCCTTGTCCAGGAGC
EMPV1_43253	GCCATTGTCCGTGTAGTTATGTCTGTCAGATTTGAATGGTCACGTCCCGACCTCAGGCGT
EMPV1_43255	GGACACATATGACCATGACCTACCTCTTTTTCTCATCTCCTCCCACCATCCTCCTCTGTC
EMPV1_43256	AGGTGTGAGCTCCATTCGTTCACATCTCCATGTCTTTGTACTTGCTGGTCCCTCCACCTG
EMPV1_43258	AGACTGATTGTCATATGTCGTTTGTGGGATCCGTCTCAGTTACTTTATAGCCATACCTGG
EMPV1_43259	CAGACTTCAGACACGAAAATTATTAGACGTGACATGCCGGATCGCACCCTTGCAGCTCAC
EMPV1_43260	GACTGAGGACTCAACCCTGCTGAAGAGAAAGCTGGAGGAACATCTGCAGAAGCTGCATGA
EMPV1_43261	GACCCAATGAGATACACCATTTTGCTTGTCATGTGCCTCCACTCTTGAAGTTGGCCTGTG
EMPV1_43262	TTGAAATTGAATGGTTCCATGTGGCTAGAGGCTGCTGAATTTGTTGGACAGTGCAGCTCT
EMPV1_43263	CATCTACTGCAATCTTCCGTTTTACAAGGTCATTTACTTCCTGTTTTAGTCCCGATTCTC
EMPV1_43265	TTTTACACAAAGTGTCCTGGAGCAACACCCTCATCATGCCGGACAGACAGTAGACAGGAC
EMPV1_43267	CAAGCCTGGTGCTTCTGAGCCTTGCATTTCCTCTTCATGTAAGAACACTGGCAGCATGGT
EMPV1_43270	CTGATAGATTTTCAGTGGAAGCTGGGAATGGCCGTGAGCTCGGACAGCTGCAGATCACTT
EMPV1_43272	ACAGAGCAACAACAAAAACAGTCTCGTAAGCCCAGCCCAAGAGCCTGCGCCCTTGCAGAC
EMPV1_43273	TGCCTGGAGGTTTGCACTCTTCAAATCTGGAGACGCGGCTCCTGTGATTTACCGGGACGT
EMPV1_43274	AAACCCAGGGAAAAAAGCATGGCGTGAGTGCTCTAAGGATAGACCTACGGTATTCTAGAG
EMPV1_43275	ACAGACATGTACAACGCCCACCTCACCAAGGATTACACCGTGCGTGGCCTCCTGGGCAAG
EMPV1_43277	GTGGTTATGTCAATCTGCAGAGAGTACAGATGATCATGTTAGCAGTTGGTGAAGTTGAGG
EMPV1_43278	CAAATCCGTGTGCACTTGTGCCGAAAGTGCCAGTGGAACCAAATATAACTCCTCCACCAG
EMPV1_43280	GGTTTCGGGGTGGATCTGACATCCCTGAGTGTATGTGGTGAACCTGAATTTGCCTTGGGT
EMPV1_43285	TCATAATTAGTCATAATACATCTTTGATAGAACTCTTGACACATTTACTATCTGTCAACC
EMPV1_43286	ATTGTGCTGGGTTTTACAATGGGTTTAATATAGCGGGGTGCCAAAGGGCAGGAGGGGAAG
EMPV1_43291	CCACCAGTTGTGTAGGTTGAAGACATGGCTCGGATCTGGCATTGCTGTGGCTGTGGTGTA
EMPV1_43295	AGCTGAGGGCTGGGTCTTTGCGGGCGAGATGAGGGTGTTGGTTCAACTGGCCTACAAAGT
EMPV1_43297	TGTCTACGACAATGCAGACAATAAGCTGGCCCTGGTGGAAGAGAATGGCACCTTCGAGCT
EMPV1_43298	TCTTTATGGGGTAGTTGGAGCTGAAAGCAGGTCTGTACCTGTTCAGCATTTGGCAGGATG
EMPV1_43300	GATTGGGGGGATCCTGCACTCCACTGTCCAGATTTTTTTCACACATGAGTTGACCTTCTG
EMPV1_43302	AGGCCGTTGGTATCTTACAAGGCAGCAGCATCAGTAGAGAGTGAAGAAGTGATTGCGTGG
EMPV1_43303	CTTCTATGTCTATTTACAGCCTGTGTCCTCCTACACCGTCAGGGACCACATGGCAACAAT
EMPV1_43305	ATAGGTCTCAGAAGCAGCTCAGATCCGGTGTTGCTGTGGTTGTGGCATAGGTAGCAGCTG
EMPV1_43307	CTGTCTAATAGATCTGCAGGCCAGAAGTCTGAAAGCAACATGTCAGCAGGGCTATGCTCC
EMPV1_43308	CACAGCTCTCTATTTCCATAAAATGTTCACTGCCTCAATCAGAAACTGCCAAGAGGTCCC
EMPV1_43309	CTCCTGACTTTCAGAGCAGCCTGGAGAGACCAGAGCTGGAGCTCAGCAGACATTCACCCA
EMPV1_43313	GTGGCACTTAGGACATGAATCCCAGGCGATCCGACTCTTTGAGTCTGTGCTTTCAAACAC

EMPV1_43314	AGTCAGGTACGCGGAGGTGCCGGCTCAGGACGGTCGCATCCCGCTTCCGGGCCAGTGAGG
EMPV1_43315	CAGATACAGATGGTTATTGATTCAGGGGTTGTCCCATTTCTTGTGCCTCTTCTGAGCCAC
EMPV1_43316	TATGCCAAGACACAGCCACGCAAGATCCAAGCCGCACATGCAACCTACGCTGCAGCTTGC
EMPV1_43317	TATATGCAGCTCAGACGTATGCCGTGCCCAATGTGGAGAAAACCGTGAGGGACTATCTTC
EMPV1_43318	TTTCCTTCTCAGACTGACTCTTGGTTCCTCTTTTCCTCGACAGCATGAAGCCGCTGGGAG
EMPV1_43322	CCAGTGGAAAGTGTGATCTGAAGAAACAACACAGTGTCACTCAGGCCACTCAGACAAGCC
EMPV1_43323	TTTTCCTCATTATCTGCCCCCAAGAGCTGGATTTTTCCCTCCACCCCCACATCCTGAAAG
EMPV1_43324	TTACTTTTTAGGAGCTGGGTTCCCATCAGACACACTCTTCATGGAAAGCAGCCTTGCGCC
EMPV1_43325	GTTACCTGACTTTGGAGAGCGGGACAGTGCCATTGTTGGAAGATACAGCATCCAGAATTG
EMPV1_43327	TTTATGGTGGGCACTGTGGTAAGGGGACAGGATACAGCGGTGGAAAAGACAGACCAAGGA
EMPV1_43328	CATGCCTTTAGCTCAGTTTGTGTCTCTGCAAAATATTTTCTATTTTTTCTTGGTTCCTCC
EMPV1_43331	AAGCCGAGTGGATTAATTTTTCAACACTGTGCCTGGCAACATGCCTACAGAGTCATCTGT
EMPV1_43332	GGGTTTCTATGTCAACACTTTCCAGAGCATCGCGGGCTTGGAGGAGAACTTCCACAAGGA
EMPV1_43333	GGTAGAGGGCACCGAATTCCAGAGCATCTACAGCCTGGACAAGCTCTACCCCGAGTCGCG
EMPV1_43337	GTATCTAATTCATGAATGTAGTTTTACTATAGGATGTCATTGTAAATGGAGCAAAGTACA
EMPV1_43338	CTCCATGGTAACATGCACATCCTGTTTACACCTCCATCCGGGCAAGTTGGTCTAAGCTAG
EMPV1_43339	CCAGCTCAGACCTGCTCCTCCCGCCAAGCCTCTCCCGGTGCTGAAGCCCAAGCAGGTTGT
EMPV1_43340	GGCGACAGCACGGCGACTCCAGCGCAAACTAGCAACTCTCAACTCTGGAAGCTCAGAATA
EMPV1_43341	TTTTCTCGTATCTGTTCAGAGGGAGGAACAGGAAGCATCAGGCAGCTCCAACAAGGATGC
EMPV1_43342	GGCCACTTCTGTTGTAGGCAGAATAATGCTCCCCAGTGATGTCTATGACCTAATCCCTGG
EMPV1_43344	TCAATTCCGATGGTATGATTGCTTCAAACACGAGTCATTGCCGCAGACTTGCTGCTAAGG
EMPV1_43347	TTTCAACTCCATCGCCCACCCTGATGCTGTAGTTTGCTTTTACATCACTTAAGAGCCAGC
EMPV1_43349	GATAAGGTCTTACAGGATTCCTTGTCTATGCATTCTTCTGATTCTCCTTCAAGTTCTCTA
EMPV1_43353	AGGTCAGTGAATTTTCAGCAGGACTGATATGGTTAGGGTGGTGACACAGGGCTGCCTTCT
EMPV1_43354	TGGTGGTGTGTTTCACTGTTTTCGGAGGCCTGATGGCATTTAACTACAACCGTGTGCTCC
EMPV1_43355	AACCACAGTTACATCTACATGCCTGCCTTTTCTATGAAGACGGGGACAGAGCCATCCCTC
EMPV1_43357	AACAGGAACGGCTGCCCTAATCACAGGACCGCAACAGCTGGAGAAAGGACTTAGTAACCT
EMPV1_43358	GTGTCTCTCAGCTCAAAACTCTGGGCAAATAGCGAATCTGCTGGCTTTCTAGCCACATCC
EMPV1_43360	GACAGTCAAACACTGCGCCTTGTCCCTTGTGGGAGAACCCATCATGTACCCAGAAATCAA
EMPV1_43363	TTGTGAACATCTCGTACAACCATACAAGAACCTGAAGTCCTGGGCCAGTGTCAATCGGGG
EMPV1_43365	GTGTGTGTGTGTACGCACATGCGCAGGATTTATGCAGTATAGGCACAAAGCCCACAGGCA
EMPV1_43366	GAGCATTGAGGACTGATACTACGTGACTACAAGACTGGCTATAAAGCTCCAATAATCAAG
EMPV1_43367	CTAGACAGAGGCTTGGTGAAGGATAAGATGACGATGGTGCCACTCAAGGAAAACAGTGCC
EMPV1_43368	CCCACACCTTTTCCTCTTCTTCTTGGCCGGGTTGGCTCCGCCAGCTCCCCCTGAGGAGGA

EMPV1_43369	GTCAAGTGGTCATGGAAAGCCACTCTAATGAGGTGGTACAAGAGTAGAGACCTGAGTGAG
EMPV1_43370	GGCTCTGGAGAAAGATTACGAAGAGGTGGGCGTGGACTCGGTAGAAGCAGAGGCTGAGGA
EMPV1_43371	AAACCCACCTATCAAACCTGACCCAACTTAGAGCTGCTGGTGACATGCTGTCCACGTGTG
EMPV1_43372	TGACCACTTCTGTGACGTGGAGGCTGTCAGGGAGTATCTGCTACGGAAGCAAGCGTTGAA
EMPV1_43373	AGGCATTGGGGAAGCCTCAGTACCAGCAAGACATCGCCTCCGTTTCTGAAGAATGGAAGA
EMPV1_43375	CTGCCTAGGATGGGCGGTTGGTTCCAGATTAAGTTGAAACACTGAGGTGTTGGTACTGCT
EMPV1_43376	TACTACCGATTTTCCCGGTAACTATTCGGGCTATGATGATGCCTGGGACCAGGACCGCTT
EMPV1_43378	CAGAGCATTCCAGTTGTTAATGATGAACAGAGAGGTGATTTTTCCTATATTGGCAATTTG
EMPV1_43379	GAATTTGGTCTGTATGCCAGTTCCTCTTCTACAGGTTCCAGAGCCTCCCTTTGTGAGCAG
EMPV1_43380	GGAATGTGTGAGCCTGAAAGGAGCTGCAGCATTAATGAAGACATTGGCCTGGGTTCAGCT
EMPV1_43381	AACCCCATGTCTACACAGAATAAGTTGGAGGCAGTGCCTTCCTTCTTCTCACCCAAGAAG
EMPV1_43383	GCCTTCAGATTTCTTCTTTCCTGATAGCGTGGTCCTGGCAATACACACATTTTGCTCTTC
EMPV1_43384	TGGTGCTGCCTGACTGCTCCTTCTGGATTCCCTCTTCCATCTTCCCCACCAAAAGAAACA
EMPV1_43385	CTGCAGGAGGACGACGCTAGTTCAGCACTTTCTTGCATTGCTGAGTCCCTGAAATTCTAC
EMPV1_43387	CAATACACAGAATGGGAGGGAATATGTGCAAATGAAGTGGCCAACAAGGGACTCAGCTCC
EMPV1_43389	ACTCTGCAAAACAATGGTTGTCTGCTGTTGGCTGGCAACAATTATGATTATCCTCCCTCC
EMPV1_43391	TAAAAGTGGCAGAAAGTCAATGTGTGGGGCCCCGTGGTCCCTTGAAGCTTCCCGACTACA
EMPV1_43392	AGTGGGAAGGATCAAAGTAGGAGGGAGTTAACATTATTATGCTTTTCTTGGGTGCCAGGC
EMPV1_43395	GCCGTAATAGAGCCATGTAGGGAATGCACTGATTGCATGTAAATTCTGGCTGGAAAACCC
EMPV1_43396	GGGGGTGGGGATGGTCATTGGATAAGTTCCACCTTCTTTCTGTGTACTGGTCACTCACCT
EMPV1_43397	GATCCAGCTTCATGTTGCCTTGTACTTGGTGCTATGTGGGTTCAAGCCATGGTCCTTCTC
EMPV1_43398	CTGTGTCGTAGCTGTGATGGACAGAACAAAGGTGGGAACTGTGGAGCAGCTCAGATTGAG
EMPV1_43401	GTGAACACACTGCCAATCTCAGCATGAATCTGGTCTGACAACTACCTGGACAAGAAGGGG
EMPV1_43402	CCCCCACTGCCTGGAAGCCCTGCAAGAGCTGTACAAGCACATCCACAGGTATTACGACCA
EMPV1_43403	CTGGGTCTGGCCGTCCGAAGTCATCTCCAACTGGTCAGGTATCTACTATAGAAAGGTTCA
EMPV1_43405	CTACCAGCTCCTGCTGACATATGGACATCCCTCTTGCTTGTTTGGTGCCAGGAAGAATTC
EMPV1_43407	AGAAACACAGGGAAGCACTGTTGTGTGGAACTTGTGGCAGGCACTGACTTTCAGCCCAGA
EMPV1_43408	ATTAGAGAATTTGTGAAATAAGAGTTGTGGTGCCCGGGTGGGATGGGGCTGGGGGGACAA
EMPV1_43409	GGCTACAAGATGGACGACCTGCTGACCTCATATGTGCAGACATTGATGAGTGCTGTGAAC
EMPV1_43410	GGAGGAGATGGAGCTGAGAATTGAAACGCAGAAACAGACCCTTAATGCCCGAGATGAGTC
EMPV1_43411	ACTTGACAACTTTATTACAGTGTGAATTAATTGCACTGCTAAGTAGGAAGAGGTGTAACT
EMPV1_43413	GGCCCAGGGAGAGGTGCTCCCGTGTACATACGTGTAACTGATGCTCTGCCAGTGGTCACA
EMPV1_43414	TATCTTCCAATTGCAAATGTTGCAAGGATAATGAAAAATGCCATACCTCAAACGGGAAAG
EMPV1_43415	GCACCTGCAAGAGATGACCAAGATGGCCAGCCGCCCTTTCGCCAAGGTCACGGTCTGCTT

EMPV1_43416 CTGCCAAGCTGAGTGGGAGGGCAAGGTCGAGGCTGCCTGGGGTGTTTGTCCCTCCTTGGG
EMPV1_43418 TTCGGCTGCTGAAGAAAGCCCTGGAAAAGCATGCAGACGAGAACCTGGTCATTCTCTTCA
EMPV1_43419 GGTGAAGGTTCATGTTGGTGTCAAACCAGTTCCTGCCATCTTGTCACTTGTGCACACTGA
EMPV1_43422 TGCCCAGTGTACATTCATTGATGCTTATACCAAATAGGGCATGTGTGGCTGGCAGCTGGC
EMPV1_43423 CTGGGGCAAACCGTCTCCCTGTGGGTGACCCCCAAGGGGCTTTGCACACAGCGGTCATCA
EMPV1_43425 ACATGGGGCCCCTGCAGGCTGGTCTCTCTGCTTCCCACAGCTGAAAGGAGATTGAGACTA
EMPV1_43426 AGGCCCTTCTAGAGGCTCAGGGACTCAGCAGAAGCTCAAGGCACTTTGGAGCCAACTTGT
EMPV1_43427 AAACAAGAGAACGAACGACGGGGAGACCAGCGAAAAGAGCGGGGGGAGGAGGCGAGGGAA
EMPV1_43428 AGGTTGAACTCTCACAGTTGTAGAGACAAGGCCTGGTCCTTAACCTGCTGTGTCACAGCA
EMPV1_43429 TAAGTCCATAAATAGTAACTTTAGAGTAATGCAACTCCTAACAATAAGAAAATATTAGGC
EMPV1_43432 TAGGTCTCAGCTGCGTTGTTTCAGAGTGGACGTCTGGTTGGCATCTTCGAGAAGTAGTTC
EMPV1_43433 AACAAAGTACAGAGGAGGGTCTGGGTGTTTGACCAGTGCCCTCAGCCCCACAATGCTTTA
EMPV1_43434 CCAGAGGCCTGAATCTGGTGATGTTGTTGAGGGCAACCTTGAGGTGACTTTCATTACTCC
EMPV1_43436 TGGAAGTCCCCTAGGTGTACGAGAGCATCTAGCTTTTCTTGATCGCGTCAAGATACCATC
EMPV1_43439 GGCGTGGCCTGGCTCCAGCCACTCAAGGTGTCGTAACCAAGGAACGACACCGAGAGATCA
EMPV1_43440 CAGTTTGGTTTCTAGCCATCCAGTTGATTTAGCAGGAGAATGCAGGGCTTTTTAGCAGCT
EMPV1_43443 CAGCAAGACTACGCACCTCTCTCCAGCCCACTGAACACTCATTACTGAACAAGGAAGAGG
EMPV1_43444 TTCTCTCCACTCCTATGCATGATTTTCAGTACAGTCACAAAGAGAGGGGGGAAGGAGGGC
EMPV1_43446 AAGCAGAAAACCAAGGCGTGGGTTGGCTCCCTGGTGAAGCAGCACAAGGAGGCGCTGAAA
EMPV1_43447 CATCAGAGCCACTGTCTTCCAGTACCCCTTGGGCTGGCCACCTGTCCAGGAGCTGCCTCT
EMPV1_43448 AGGCTGGTGCTTTCATACTCTTCTTTACCGTTTCCAACAACCATGATTCCCCTGTCCCTG
EMPV1_43450 CCCAGCTGTCCTTTTGTGATGACAATACCATTGACCACTTCTTCTGTGACCTTAGTGCCC
EMPV1_43451 GGAGGAAGGTATGCCTATCAAAGCCAAATAAGAACAGAGAAAAATAACCAAGGACTTAGC
EMPV1_43453 TCGGAGAAATGGAATTTCGGTAATGCTGGCTCATTGAGAGAACTGACTTGGGATGTATGG
EMPV1_43454 CGGTTACCCCCACATCACCTTCGAGAACATGATCGTAGACAACACCACCATGCAGCTGGT
EMPV1_43455 GGTCTCCCTGTGTTTGTGTCCTTGAAGCCGAGCCCCGCCCAGGACGGGGAACTGGATTCC
EMPV1_43456 TCAAACCACCTCCTCTTCCTCTCCGTGCAGCTGGGACTTTAGCTCGCGTTGCTATGGTTA
EMPV1_43457 GACCAGCAACAGTGGCAAAGACGAGAGCAGCGAGATAGTCAGGAAGAAGAGCAGTTGCAA
EMPV1_43460 GCTGTCCTGTGTCAAGCAGACATCATACCAGCTCTCCCTGTCCTCATTCTGTCATTTGCG
EMPV1_43464 GGAAGAACTCAGATGTCAGGGCACAGCTATGATTACGACACTCTCTTGCAATCCAGAGGC
EMPV1_43466 CACCACTAAGGTCCGAAACATCTTGTCTTCTGCAAGGGGGAGTCGGGGGTGATACGTTTT
EMPV1_43470 TTCAGGAACAGCTGAATGGTTTGAACCTACAACTTGGGTTCTGGAAAAAGGGTCCCCCTT
EMPV1_43471 TCAGAAGCACAGCCCCTTACCTGAGAAAGCCGTTCCACTCATTGCTCCAGGCTCTGCCAG
EMPV1_43474 CAGCATTTGAGGGTGACGATGGATTCTGTGTGTTCGAGAGCAATGCCATTGCCTACTACG

EMPV1_43475	ATGCAGCGTGCCATCCTACATCTGGGTCAGTAGTGCCTTGTGGGGTGTCGAGGAGAACCC
EMPV1_43476	AGCGCTCTCCGGAGCAGGGTCAGACCACAAGGAGGAGCCGGACAGTTTGCCTCCAAGAAA
EMPV1_43478	ATCTGGTTGTGAGTTAGAAGAGGTGCCATTAAATTGAGGGCCCAGAACACCCCTGAAGAG
EMPV1_43479	TACCATGTAAGGATGCACACTGGAGAGAAGCCATGTAAATGCAAGCAATGTGGGAAAGCC
EMPV1_43481	ACCCTGTCTGAGGGCGTGGCCCCTGTGTGTTTGTATTTTAACCACTGCTTCACGTCTCAA
EMPV1_43484	CAGGGCCACTAGAACCTTATCATGCAAGTGGAGTGAAATAAGCTATGCGTGAAGCTCTCA
EMPV1_43485	ATTGAGACTTCACTCCTCCTTTCCTGACGACTCCTAAGTCTGACACTGGAAGTGAGATGG
EMPV1_43486	TCGCTACTCTGGACGCAAAGCAGTCATCGTAAAGAACACTGATGATGGCACCTCAGACCA
EMPV1_43487	GTGAACAAGGAGGAACAGGCGGATGAGAACCAAACCGAGTGGCTGTTGGAGGAGATCCAG
EMPV1_43488	CCGCTACCGGGAGGAAGAACTGACTGTGGTGGAAGAAGTGGATGACGACAAGAGAAGGCT
EMPV1_43489	AGTCTGCCTGACTGAAGTTTCTGAGGTGTTAGTGAGTGTCCATGAGGCTACCAACCTCAC
EMPV1_43491	TCCTACATTGCTCATCGGACCACCCCTAAGCCTTCAAGATGGTCAGCAAGGCCAGCAGAC
EMPV1_43492	CACCATTACAGCGTGCACCACCCCCAATGAGAAGCAAAAATCCAGGACCTTCCCAAGGGA
EMPV1_43493	ACCAAAAGCCCATCCAAACAGAAATGTAGAAGCAGTGATTGTGGCTTGGGAGCCTGGGAG
EMPV1_43494	GAAGGGCCACCTGTAGTATTTGCCTTGGTTTGGGCGGGGTACAGTGGATGTGAATACTGT
EMPV1_43495	TTCACAGAAGAACAGGCTGAGCTGACACCTCACTCCTATCTGGACAGGGAAACCTCCCTT
EMPV1_43496	TGAAACCAGTTCTGTCCAAGCAGACCACGGGCAAGGCCACCAACCTTTGTCCTCAGTTAT
EMPV1_43497	GGAAATGACAGTGAAGGCCCACTATGTGAATTGCTTTTTTTCTTCCTGACTGCCATCTTC
EMPV1_43500	GTGTGGCTCGTTACTCACAGTATAAAACCAATCCAGAATGAGGAGAGTGTGGTGCTCTTT
EMPV1_43501	AGCAACCCCAGCTGCTGCAGTTGTATCCTTAATCTACTGTGCCATAGTAGGAACTCCCAG
EMPV1_43502	GTAGAGGGATAGATAAATAGACTCGTGGAAGAGACAGGGTGCAGATCATGCCTTCATCCC
EMPV1_43503	TGCTCTAATGCAATGGAACCAGAGACTGCCCAGCCAAGGCAAAAAGAGGGGTCCCAGTCT
EMPV1_43507	CTCTCAGATCCACTTCTGCCATGGTCCTTTTGAAGTTACCACTGAATCCTCTCGCTCCTT
EMPV1_43513	CCCTTTCAGTACACAATACATGCCAACATCTGTCCCGCTTCCCCTGGTCACTTGAATGCA
EMPV1_43515	CTCCCTTTTCTATGTATCTTCAAGCCTCACCTACTTCCGGCCCAAGTCCACTAATTCCCC
EMPV1_43517	CTTGGTCCTTGGCTAAGCAGGGCTTTTCTCCCAGAAATCTGTCTGCCTCAGCATTGCAGT
EMPV1_43518	CTAATCCACAGCTACAGCCACACCAGATCCAAGCCGCATCTGCAACCCACAATGCAACTT
EMPV1_43519	AGGGCTGGCTTTCTGACACCTCAGAAGTCCCACTTTGTGTCTGTCTCAGCGATTCTTGAT
EMPV1_43520	GCTCGGAGAGCCGGGCGCCTGCCACCGCAGAGACTCCAAGTGAGGAAATTGATAATAGAA
EMPV1_43521	GGGGAGACGTTAGTTTTGGTGATGTAAGCTTATGCATTTGTATCCCAGTGATTGCACATA
EMPV1_43522	ACCACTTAGCGTGGCGTAGAGCGGCCAGAGCGTCAAGGCATTTTTGCCTGTCCAAGACGT
EMPV1_43523	CTAGTGTGAATAACCCTCGAAAAATGATTGATGCTGTTGTGACATCTCGGAGTGAGGATG
EMPV1_43525	GTAACTAGGGTGGGTGTGGGTTTCCAGACAGTCAAGAAATATCTGTGTTACTTAGGAGCC
EMPV1_43526	GCATCCAGTGTTCCTTCAGGTTCGTGTAGATCATAGCTGGGTTTCGATCCTTGAGTGCTC

EMPV1_43527	GAAGTGGGACCTGGGGCTACAGCACAGAGGAATTGGTATGCCTTGCTGACTTTGTCCATT
EMPV1_43528	GGGATGTACCTGTTACATAACAAAAAAGAAATGTTTAGACTTAAACACTTGAAGCAAACC
EMPV1_43529	TCCTGGCACTACATAATCACAGCCACAGAGACGTCCATTCATAGTAAGCCCAAGCTCACC
EMPV1_43531	CCTATGTGCTTCTTCTGTCTCCCTAAACTGAGGATATCTGTGTCAGAGTTCTCACCACTC
EMPV1_43532	AGAGCTTTGCTGCCCTCAAAGGTCAGAAAGCTTCTCCTCCCAGCCCTGACAAAAAAGCTA
EMPV1_43533	GTCGGCTGTAGCCCCAGGGTGTGGTTGAAATGGATTACTGCCTAGCTGAGCCAAAACGTT
EMPV1_43534	ATGCCGAACCCTATGGAGGTACCTTAGAAATCGCTACGTAGAACTACCCTATGACCCAGC
EMPV1_43536	GGCTTCCTACCTTGCAACAAAATAATTGCACAAACTCCTTAGTGCCGATTCCGCCCACAG
EMPV1_43537	GAGCTCTGTGAGTTTAGGCCTTATTCCTGTCCGTGCCCTGGTGCTTCCTGTAAATGGCAA
EMPV1_43538	ATTAGCACCATTTTATCACAGAATGACAAGGGTTTAGAGACACAGTGTTGCAATGTCAGC
EMPV1_43539	CGTGGAGACGAAATGCGTGACACGTGCTGCACTGGATCTCAGAGAATAAGATTAACACCC
EMPV1_43541	ACATTCCCCAGAAAGGGTCATCATGGCCTGCAAGAAGGGGTGCTCCAAGTGGTCCCTTGT
EMPV1_43542	GAGGATGACAAGGACTGCAAAGAAGAGGAAAACAAAAGCAGCTCTGAGGGTGGAGATGCC
EMPV1_43544	GCTGTGTCATCCAGGGAAGAGATTCAGGTGGTCTGAACCAGCAGGCAAAGACAGCAAAAT
EMPV1_43545	GGTGATCAGTTATGAAGAAGGGAAAGCATTAGCAGAATCTTGGAATGCAGCTTTTTTGGA
EMPV1_43546	CCAGACTCTCCTTTGGCCAGCCGTTCCCTCTCCTTTTCTTCCAAACTTCCATAATAGATG
EMPV1_43549	CTATATGTCCAAGATGGGCAACCCCTTCCTGACCCAGTGGCAGCGGCGGTACTTCTACCT
EMPV1_43550	CGCCGCCGCCGCCGCCCGCTCCCGAGAACGCGCCCGGGGTCGCGCATGCGCAGGCGCGCG
EMPV1_43552	TGTTGGGCTTCTGGTTCAGGGAATTGCCCACCACATGAAATTCAATCACACTCCTGCGCT
EMPV1_43553	TCCCATACCGTGTCCACTTAGAATGTTTGAAATGCTGGTAGTTCCTGAACAAGAGTACCC
EMPV1_43554	GCAGGAAGCCCAGGATGCTGTGGACTCAGAGGGCCTTGATGATGGTCAGGTTGTTGTAGT
EMPV1_43555	CTCACACACGCTTTTCCTCAAGCCAACCGTCAGACTTTCATAGGCAATACAAGTGCTTTC
EMPV1_43557	TTGCTGTGATGCACCCTCTGACCTATGCAGAAACCTTGATCGATTACCACCTGTGCTCTC
EMPV1_43558	ATAAAAGGCTATTTGCTTCTGTGTTTTTGGCAGGCTTCCAGCCTCTCGGCTAGCACAGGG
EMPV1_43560	ATCCAATATGATGGGTGTCCATACAAGAAGCTGAGACACACCCACACCCACGGAAGACCA
EMPV1_43561	CATGGGATTGTTTTCATAATTTCTCTTTCTGATAGTTGTTAGTAAACAGAAATGCAACAG
EMPV1_43564	CCAAGTGCTGAGACTCGCTTGCACTGACACTTCTGTCCTAGAGTTCCTCATGATCTGCAA
EMPV1_43565	GTGAAGATGACCCTGTCCAGACACTGTTACACATCTATTTTCATCACCAGACGCTGGGCC
EMPV1_43566	GGGCCTCAGCGGAGTTTATAGACAAGCTAGAATTCATCCAGCCGAATGTCATCTCTGGGA
EMPV1_43568	GCTGAATAAATCAACAGTATGGACCCGTGGCTCCCTGAAAATTGCCTTCCACAGGTGCTG
EMPV1_43569	GACAACCCTTGTCAATATCGAATGTGGCAGAGGCAGCACCAAGACATGCCTGTACCAAGA
EMPV1_43570	GGTAGGTGATGCATAGAAGAAAGGGAGAGATCAAATACATGGAAATGCACAGTGAGCTGG
EMPV1_43571	GTTCCCTCCTAGTCTCCAGGAGCCCACCCCTCACAGGAAAGCCTCAAATCTAAAACATAT
EMPV1_43572	GCCAAAACTTGGCAAGGGTGATGTAAAGGATAAGTTTGAAGCCATGCAGAGAGCCAGGGA

EMPV1_43574	GTCAGAATGATGTTTTCCATACTATCCTCAAGAACCTGAAGCTCAAGGACCGAGGGACCC
EMPV1_43575	CAGTCTCAAGCTAGTACAGGTAGCCTGAGAGTCTAGAGGCCTTTAAGGTTAAAGGAATGT
EMPV1_43576	CGAATTGAAGACCAGTAGGCTGAAGACGAGAGGATTTTGAGGAGGTGTTGGCAGTAGTCC
EMPV1_43577	CCAGGGCTGGGAGTGAGCCAAGAACAGAATAATCAACACATCACACATCACACAGGCCCA
EMPV1_43578	CCACTAAAACTCTGTGAGAGAGTCACAAAGGTTCTGAATTGGATTAGGGAACAACCTGAA
EMPV1_43581	CAGAGTGGACACTTAGAACTCTGGGTCTCTTATCCGTGATCTGGAACATGGTGGTCTTGC
EMPV1_43582	GACATTGATGAGGTCAATGCCCTGAAGCTACAAGTGGACCAGTGGAAAGTGCCCACGGGC
EMPV1_43586	TACCGGGGGCTATGCCGCCCTTTCAAGGTTTTTCAATAGTAGGTGTTTGCGTTTCCAACC
EMPV1_43587	GCCTGCCTATGACTTGGAAATGACCTGTCTGTGATGAGAGGGGAAAGGAGTAGGTGAGAA
EMPV1_43589	GGAGTGCTGGCAAAATTCTAGCTATTGTAAATGGAGTGCTGGCACAATTCTTGGTGTGGG
EMPV1_43590	GACGTAGAGTTTCACTCCAGGTCAACCAGGCTCATACATGACCGTGCTAGAAAATGCTCC
EMPV1_43591	CCCTATCGATATTGTCTCTGCTGTGTAAATAGCTCTGAGTAGTGCAATATTGCTTATAGG
EMPV1_43593	GAGGCTGCAAGCAGTGGAAAAGTCACCAGATGTCCATCTCCAATTGCTCAAGAAGACCAG
EMPV1_43596	CATGGAATAGCTACCTTCTCTTCCAGGCCACAGCTCATCAGAGGGGGAAAGGCAATGATG
EMPV1_43597	TTGGGATTTGAACGAGTATTGACCAGTGGATGTGACAGTTCGGCACTAGAAGGGCTACCC
EMPV1_43598	CACAGCCTGTCTGTACTGCACATCCCAAGTCAGAACCAAGTCGGCCAGACCTACTCGCAA
EMPV1_43599	AACAAATGCACATACACTGAAAAGTAGCATCTGGTAAGCTTTCAACATGAGTGTCTTTTC
EMPV1_43601	GCCGCCTCGGCGCTGGACGCCGACTGCCGCCTGAAGCAGAACCTGTGCCTAGCGGGCCCC
EMPV1_43602	GCTGGTCATCTTCACTGTCTACTCCTCCACCATGTCCTCGTCCATCGTCTACCGTGTGGC
EMPV1_43606	CCTGGAAAACCACATCAAAGCCAATCACCACCTCCCAAAACAAGGTCTATGAGGAATAGC
EMPV1_43607	ATTACAATGCGTCCAGTGGCAAGTTCGTCTGCGGCGTGCCAGGGATCTACTACTTCACCT
EMPV1_43608	AGTATCTGGACCCCGTTTCATTTTTACACGAACCTGGGGAAAACCCTGTCTCCTCTGGAG
EMPV1_43610	TGTTCATCTGTACTATGTCGGTGGAGAGGTGTATGCCGAGTGCCTCAGTGACAGCAGCAT
EMPV1_43611	AAATACCCGCCTCGGAAAGACTTGGTCCGAGCTGTGTCCATCCAGACGGGCTACCTCATC
EMPV1_43612	CGCGACAGAAAGTTCCGCGAAGAGGAACAGCTCCGTCAGGAAAGGGAAGAACAGCGACTT
EMPV1_43614	AGGGATGTTATAAAGATTGAGTTTATCATGTTTGTGAAAATGCTTACCCAGGAGGTGGGC
EMPV1_43616	CCCGCACGGTCCGGCCGGCCATGCTGGCGGGGCTGGGGCCGAGGCCGAGCCCGCGGCTGG
EMPV1_43617	AGCTCCTCAGCCCAGGATGTCTTCCAGGTCACCATTTCCGAGAGCCAGGATAAGTGCTTT
EMPV1_43619	AAGCACAAGGAGTTGAGTGGTCTTGAGAAGACTCCATCACAACAAAGGAAAAAAAATCAGT
EMPV1_43620	GTGGTTATATCAAAGCACAGAAGTATAACAAACTTCCCCTTGTGACTGCTGCCTCTGAAC
EMPV1_43621	AGCACGAATGCGGCGACGCGGACTGTCAGGAACCCCCCGAAAACCCCTGCGACTGTCATA
EMPV1_43624	GGTGCTATTGCCTAGAGGGATCAATCCTGTCGAAAGACTGGTGAGTTGGTAGCTGTAGAG
EMPV1_43625	GGCATAGCTTTGGTGATGCGAGTGGATGGTGCTGTGAGCTCTTGGTTCAGGCAGTTAGTT
EMPV1_43627	GTGTATGGAGCTTTCCAAGAAAATTGCCGAGCGACTGGGGGTGGAGATGGGCAAGGTGCA

EMPV1_43628	TTTCTTCCTGTCCCAAGGGTCTGCGGGCCAAGAGGAAGGATTGCTCTTTTTCAGCTGTCA
EMPV1_43629	GTTTACAGCATCTTCTCCCACCCTCCTCACCTCCCTCAGCCTCATACACAACTTTTTGGT
EMPV1_43630	TCAGGTTGGTGTTACCTATATGGGGCCGTGGTCCATGTTCAGTCCTGGGGCCTTGAAAAT
EMPV1_43631	AGACCCCTTGGAGGAACCCCAGCCTTTTCTTGAGTCAACATGGGCACCTTGGTTGGAACA
EMPV1_43632	TTTGTCCTCTCCTGTTGGGCTGGAGGTTTGTTCATCATAATCCCTCCACTTAGTCTAGGC
EMPV1_43638	CAAAGCTCATGCTCAGTTTAGTTCAGAGCTTTTCTTTGATAATATCTATACTGAAATATC
EMPV1_43639	GGTCTAATAGCCATGAGCTCCTTTGTGCTCTTGCTGGTCTCCTACATGGTCATCCTGGTT
EMPV1_43640	AGAAAGAGCTCAACCATATGCTGACGGACACGGGGAACCGAAAGGCTGCTGACAAGCTTA
EMPV1_43645	AGGAAACACCTGAACCAATATTTCCCAGCCCTTTGTCAGAACAAACCTCAGTGCCTCTCC
EMPV1_43647	GCTTGATGGCTGGGAATGAGTGGAGGAAGAATGGAAACTTAGACCTAGTTCACCCCTGAG
EMPV1_43649	ACCAGGTCTGCCCGGAAGAGGATTCCCAGGGTTTCCAGGGACCAAAGGAGAGAAAGGTTC
EMPV1_43650	TTCGATGAGGTGGACAAAGCCCATCCAGATGTGCTGACCATCATGCTGCAGCTGTTTGAC
EMPV1_43655	CTGAGGTTAGTATGGGTTCTGTGGCTCAGATTCCCTCTGGTCCTTGCTGTATGCAGAGAT
EMPV1_43656	AATGAGAAAGAAAAACCACAGGCTAATGTCGCTTCAGGTCTGCCAACACTTGGTGCCGGC
EMPV1_43657	TGTGAGAAATGCGCCAAGGATTGCGTGTGTAAAGGTGGAGAGGGGGCCGAAACTGAGGAA
EMPV1_43659	GTAATGGACAGTGATTAACAGCCAACACCAGCTCTCCCAGGCACCAAGCCACAAGCAAGA
EMPV1_43662	TCATGAACCCCCATCTCTGTGGCTTGTCGGTCCTGGTATCCTTGTCCATCAGCCTTTTGA
EMPV1_43663	GAGGGTACACTGTCAAAATCTCTGGGGACTTGAGAACTTCCCGAGCATCCCTCTGCCTTT
EMPV1_43665	CCGCAGGGAAAATGAGGGACTTTTGGGGGCAGATGTGTTTCCATTCCACTATCATAATGC
EMPV1_43668	CACCTGGAGAACATTCACAACTATTTTCAGTGTTTATCTGCTGAGAATCACGGTGTAGTT
EMPV1_43669	GTGCAGAGATGCTGCTTCCCGCCAAAGACTGTGAATTTTAAGCCTGACTCAGTGGACTCT
EMPV1_43671	GTGAAATACGAGAGGTGTACACATGACGGCCACAGTAGCTGGCTTCAGAATGCCTGTTCT
EMPV1_43672	AAGGCAGATATTTTGTCAAGGGGCTTTGCATTCAAACTGCTTTTCCAGAGCTATGCTAGG
EMPV1_43674	CGGATGAGAACAGGATGTGGTGAAACCGTAAACAGTCACGGATGCCAAATGATAGGCAGT
EMPV1_43675	CCCCATGACTCCTGTCCGGTATCAAAGACAGGCCCCAGGTTTCATTTTATCCCTGTGTAT
EMPV1_43679	TCTGTCTTTCAAGGAACCAGCACGGCAACACTACTTCTCCAAGCATGACAACCGCACTTC
EMPV1_43680	CTGTTAATGTGTCCCCTTGTTCAGACTTGCGTGTACCTAGCTCTTCTGTCCCCAGTGTGG
EMPV1_43683	GAACCCTCTGCCTCTCACTCCTATTTATTAAGCGTCACACTACCCAGGAGATACACTAGC
EMPV1_43684	ACCTACAAGGAATCCGAGGGTGCCCTCAATGAACACATGACCAGCTTAGCCTCTGTCCTA
EMPV1_43685	CCTAAGGGCTGCTGTTTCTCGAGCTATTGCGATATGGTTCTTTCCTGGGCCTGTCTTAGA
EMPV1_43687	GAAGATTTATTGCTGGGCTCTCTGTTCTATTCCACTGGCCTATCTGTCGGTCCTTATGGC
EMPV1_43689	CTTCAGCCTGGAGGTGCTGAAGGTGGTTGGCATTCATAAGAAAGTGGCCCAGACCATTCA
EMPV1_43690	GGATATTGTTTCTTTGCCTACTAGAAGTATGAGGGTGCTGTGTCAGGAGACTTACATTGA
EMPV1_43692	CATGGGCCTGCATGTTCATAGGAGCACTGTTGACAAAGACATGGATACAGCCTAAACGTC

EMPV1_43693	GAAAGAACAGGAAGCAGAGGCCTACAGACAGAAGTTAGAAGCCATGACTCGTCTTCAGAC
EMPV1_43695	AGGTTGGAAGTGACAAATTGGGTCTCCTTGGGTCTTAGGCCTGCCAGATTTTGGACTAGA
EMPV1_43696	GGGATTCTAATGTCTGGCCCCAGATGAGGAAGTCATGTCAGGGCATAGATCAACAGCCAA
EMPV1_43697	ATGCATGACTGCCATATCAAGGAGGATGAGTTCCTGCACCTGCCGGCACATCTGCGTGTC
EMPV1_43700	CTCATAGCTGGAGGGGGTTCTTCATGCAGCCCTGTCCTTTCAAGAAAACAAAAAGGGAAG
EMPV1_43701	GATTTTGGGAAGTCCCTTCACCTCCCTATAGCCATCTCCAGGGTGACAAACCCTAGTGTA
EMPV1_43702	CTCGGCTGTGGTTCTTTCACCGGCTCTTTTCTGGAATATTATGCAGCTGACATAAGCTAC
EMPV1_43705	GAAAAGGAGCAGTCGGAACAGAATGCCATCGACATATGTGAGCTCTGTACCTGCAGAAAC
EMPV1_43706	CGGGAGGAACAATTTGATCCAGCAAAAAACTGCAGCAGCAATGCTTGCCCAGCAAATGCA
EMPV1_43708	TGAGAAGTACTGCCAGCTAGAGAACGCCCACCTCGTGGTCATCAACTCCAGAGAGGAGCA
EMPV1_43710	GCAGTTGTGCGGAAAGTTTGAGTGTTTGGAAACTCATGAGGAGTCTGGCAGAGTGGTCCT
EMPV1_43714	CTTCTCCTTCAGCCAGTGAAATGGACAGTTGTTCACAAAACTCATCTGCCAAGCCCGTAA
EMPV1_43715	TCCGGAGTCATCGCCAGGAGTTGGAAGGCGAAGAACAAAGACTCCACATACATTTCCACA
EMPV1_43716	TCTTGACCGCTGGTGTGATGTGATGGAGCGTGTTTTTCTACTGAAGTGGGCACGGAGTTT
EMPV1_43717	ATGTCCGAGGCCATGCCGCTGTACACGCTGTGTAAGGAGGACTTAGAGAGTATGGACAAA
EMPV1_43721	TGACCCAGCAGTGTGTTATGGTTGTGAGCCGAGAGCAGAGGAGTGAGGACAGAGGTAGTG
EMPV1_43725	CCTCAACAGCAACAGAGAGACAATGCGGTCCTCTCATTATGCACATGCTCTAGGATCGTT
EMPV1_43726	TGTCTCTATGGCTGCTACGTTCACTCTTCCATCGTCCCCACATTCCATCGGCGCTGCTAC
EMPV1_43727	GAAGGTGCCGTATGTAGATTAGTGGGCAGGAGCAGGCATAGGCAATGATTCTCTTAGGGA
EMPV1_43729	CCTATAGTCGCTCTTCCTCCATGTCTTCCATTGATCTAGTCAGTGCCTCTGACGATGTTC
EMPV1_43732	CAAGATTGTCGCAAATTTTGCACTACTGGCATTGACGGAGCCATGACAATTTGGGATTTC
EMPV1_43733	CCATCGTGCATGTTGCTGGTTTTGATTGTCACTCTTTTAATGACGGTGAGAAGCTTGGAC
EMPV1_43735	GGGGAATGGGATTCTGGCAGTCGAGGTATTTCCCAAGTTAGAGGGCTTGAAAGTGTCTCA
EMPV1_43737	TATAATATGGCGGCAGCGACGGCGGCCTGAACTCTAGGGAGCCTGGGTGATTTTGATGCT
EMPV1_43738	TTCAGCTTGCTGAAGGAGCTCACCAAACTCATCAGTATCCATTGGAGAGGCAGATCCTGG
EMPV1_43740	CCCCTGCTACTGTGGAGCCATCAGCCATAATATTTGTCAAGCTGAAGATGCTAGTCCTCC
EMPV1_43741	GTTTCTGGTCATCCCTGAGTGCCCAGCACCCCTCTTAGGTAGAGACTTATTGACCAAGAT
EMPV1_43742	CACAGTTCCTGGTCTTCTTCTTCTCTCGCCTAAGACCCAACCGCAGCGGCCGCTACGAGA
EMPV1_43746	ATGGATCTTGGTCGCCATGCCAGCCGCCGCTTCTTCAACTGGTTTTACTGGAGCATCAAT
EMPV1_43747	GAGTGACAGTTGATGGCGAATTTCTGCATTACATTTTCCCCTTCCAGTTTCTGGATTCTC
EMPV1_43748	TATCAAGTATGATTTCTATGTCTCCTGCCGAGGCCACCTCTTTTCTCGACAGCACCTGGC
EMPV1_43749	CCTAGCTTGTGAAGAGTGTACGTTTCCAAAGAGCGAAGGGAGAGGGAAATCTGGAGTGTG
EMPV1_43750	TTGAAACCACGGTACCAGCTGAGGAATAGCATAACCAGGCTTGGAGTTAGGAGCACCGAC
EMPV1_43752	GAGCTGAACTTGGAGGATGTATAGGCTTTCAAGGGTAGAGTGAATGCTCTAGGGTCTTGC

EMPV1_43753	AGACCCCTTCCTTCAGGGCAGTTGACTGTAAGTAATCTCTGGCCTTGATTGTGACCATTC
EMPV1_43756	GTCACTGGACGCATTCATTATGAGGTCGATGACCAGATTGGAGGCCCCCTCCTTGTCAAG
EMPV1_43758	GTATCCCGGACTCCTGATCCTATTAATGACCCAACTGCATGAAACACGTAGGGCGGACTC
EMPV1_43759	TGTGTGTGTGTGTGTGTGTGTGTGCGCGCGCACGCGCTCAAGCACAGGCAAGCATGTGCACA
EMPV1_43760	GCAGGACGGTGTACGCGCAGATGCACAACGAGAAGGAGCAGGAGATGACGAGCCCCGTGA
EMPV1_43769	ATGCTGGCAAGATTCGCTTCCACGACCTCCAGGGTGAGAAGCACTACAACCGGGGTTTTG
EMPV1_43772	ATAAGCGACGTGGTTGACACCCCGATGCGTGCCCAAGGATAAGGCCATTAAGAAGTTCGT
EMPV1_43776	CCCCGAGTGCGAGATCGAGCGTCCTGAGCGTCTGACCACAGCTCTGGAGCGCCTGCGGCA
EMPV1_43780	TGGCAGTAACTAGACCAGCCAAGTGCGGAAGGTCAAAGATTTAGGAGGGATCTTGGATTA
EMPV1_43782	TAGTGACGAAGTTACCAGAAGCTGATGAGCTGGGAGCGGGGAAAGGTCGGCATTTCAGTA
EMPV1_43783	GAACATCAAGGTCAAGCTGGAGGCTGAGATCAACACCTACCGCCGCCTCCTGGAAGATGG
EMPV1_43784	ATCATCCCTTCCTCTGTCGATGGACGCGGCGCTTGTTTCCACGTCTCGGCTTGCTGTGTT
EMPV1_43791	AGCTGGACCTCGTCAGCCGCCTCGCCAAGGAACATGGGGCTTTTGATGCTGTGAAGTGCA
