# Supplementary figures and images for: Development of a porcine (Sus scofa) embryo-specific microarray: array annotation and validation
Source: BMC Genomics. 2012 Aug 3;13:370. doi: 10.1186/1471-2164-13-370 (PMC3468353; doi:10.1186/1471-2164-13-370)

## Array-Array Intensity Correlation

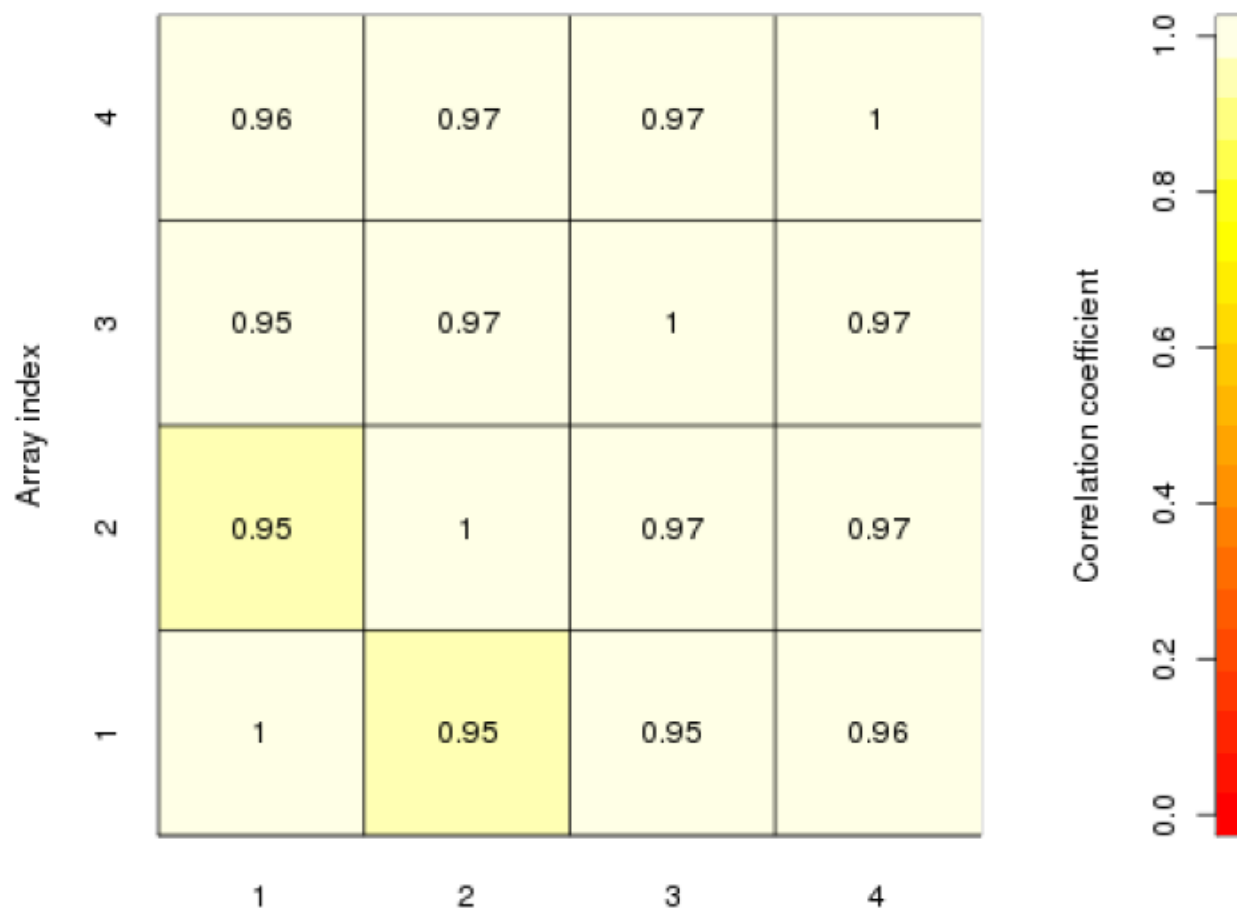

### Array Index

1. Spike-in A
2. Spike-in B
3. Spike-in C
4. Spike-in D

Supplement: Additional file 11 — Array-array intensity correlation associated with labelled spike-in RNA mixed with labelled COC aRNA. PDF file containing the correlation index from two biological COC samples. A: COC1 labelled with Cy3 and COC1 labelled with Cy5; B: COC2 labelled with Cy3 and COC2 labelled with Cy5; C: COC1 labelled with Cy3 and COC2 labelled with Cy5; D: COC2 labelled with Cy3 and COC1 labelled with Cy5. [file 1471-2164-13-370-S11.pdf]
