# Supplementary material for: Development of a porcine (Sus scofa) embryo-specific microarray: array annotation and validation
Source: BMC Genomics. 2012 Aug 3;13:370. doi: 10.1186/1471-2164-13-370 (PMC3468353; doi:10.1186/1471-2164-13-370)
Supplement: Additional file 16 — OR genes expression in COC and embryos. PDF file containing the spot intensity values extracted from microarray data in COC and embryos related to olfactory receptor gene symbols. [file 1471-2164-13-370-S16.pdf]

Probe ID	Gene_Symbol	Mean intensity in COC	Mean intensity Embryos	Expression condition
EMPV1_05268	OR9Q2	6.839148	6.961849	in both
EMPV1_40418	OR51S1	6.870413	6.976416	in both
EMPV1_37494	OR52R1	6.819017	7.011617	in both
EMPV1_20907	OR5D13	6.763874	7.065205	in both
EMPV1_09103	OR5AR1	6.753858	7.073603	in both
EMPV1_35772	OR6X1	6.776291	7.074715	in both
EMPV1_30175	OR5B12	6.845796	7.106134	in both
EMPV1_31271	OR6C6	7.171631	7.10807	in both
EMPV1_43600	OR4C45	6.82034	7.121959	in both
EMPV1_25805	OR4K5	6.788527	7.125371	in both
EMPV1_30390	OR52E5	6.778026	7.132514	in both
EMPV1_17487	OR1J2	6.759699	7.139805	in both
EMPV1_12623	OR8B12	6.807487	7.145586	in both
EMPV1_08011	OR10AG1	6.787332	7.147023	in both
EMPV1_04006	OR6N2	6.703125	7.154738	in both
EMPV1_35138	OR10G6	6.956086	7.169368	in both
EMPV1_07022	OR51C1P	6.943733	7.179326	in both
EMPV1_04877	OR51V1	7.100996	7.187248	in both
EMPV1_01066	OR52N5	6.704545	7.188436	in both
EMPV1_06833	OR5D16	6.834589	7.18908	in both
EMPV1_19320	OR13A1	6.880293	7.192325	in both
EMPV1_35418	OR2Y1	6.739343	7.220759	in both
EMPV1_03531	OR2H1	6.854165	7.227471	in both
EMPV1_10635	OR13H1	6.702459	7.262485	in both
EMPV1_28338	OR2K2	6.733938	7.273568	in both
EMPV1_02456	OR13C8	7.073036	7.281062	in both
EMPV1_05773	OR5J2	6.82848	7.294357	in both
EMPV1_12407	OR1N1	6.927236	7.296257	in both
EMPV1_00441	OR10R2	6.915026	7.325862	in both
EMPV1_34694	OR9G4	7.032089	7.341058	in both
EMPV1_10415	OR4C5	6.80731	7.344398	in both
EMPV1_32879	OR6K6	6.818036	7.364491	in both
EMPV1_21356	OR10J1	7.034591	7.390859	in both

EMPV1_09419	OR8D1	6.788324	7.394308	in both
EMPV1_32363	OR10Z1	6.995251	7.403728	in both
EMPV1_16786	OR4E2	6.842628	7.433012	in both
EMPV1_04586	OR8B8	7.028431	7.464629	in both
EMPV1_10384	OR4S2	6.921332	7.502022	in both
EMPV1_43245	OR14K1	7.112813	7.516088	in both
EMPV1_40463	OR10A4	6.957746	7.518001	in both
EMPV1_06735	OR8G5	7.281311	7.53682	in both
EMPV1_08355	OR1J1	7.259571	7.549838	in both
EMPV1_18448	OR10V1	7.498271	7.552791	in both
EMPV1_27294	OR3A1	7.09436	7.587522	in both
EMPV1_33044	OR14A16	7.072624	7.599299	in both
EMPV1_01145	OR5B2	7.393008	7.641653	in both
EMPV1_24505	OR2Z1	7.680132	7.701306	in both
EMPV1_24598	OR2B11	7.319828	7.718648	in both
EMPV1_02221	OR10P1	7.026767	7.719477	in both
EMPV1_16634	OR52H1	7.69202	7.764929	in both
EMPV1_41690	OR6K2	7.007557	7.783503	in both
EMPV1_28598	OR7G3	7.493132	7.832918	in both
EMPV1_13751	OR51A7	7.353949	7.9057	in both
EMPV1_11772	OR56A1	7.326478	8.048461	in both
EMPV1_07007	OR51D1	8.315179	8.460223	in both
EMPV1_24052	OR51G1	8.237912	8.827682	in both
EMPV1_24702	OR2G2	9.36049	9.045728	in both
EMPV1_18813	OR5D18	8.661377	9.135075	in both
EMPV1_17759	OR7A10	6.80791	9.978891	in both
EMPV1_23744	OR6Q1	6.737675	siganl < background (6.96)	Only in COC
EMPV1_12309	OR13G1	6.738702	siganl < background (6.96)	Only in COC
EMPV1_41683	OR52E4	6.782903	siganl < background (6.96)	Only in COC
EMPV1_23463	OR2G3	7.89026	siganl < background (6.96)	Only in COC
EMPV1_42353	OR9I1	siganl < background (6.69)	6.962221	Only in embryos
EMPV1_33931	OR10Q1	siganl < background (6.69)	6.964814	Only in embryos
EMPV1_23493	OR7D4	siganl < background (6.69)	6.969238	Only in embryos
EMPV1_33294	OR1D5	siganl < background (6.69)	6.971211	Only in embryos

EMPV1_15392	OR8B3	siganl < background (6.69)	6.971532	Only in embryos
EMPV1_20675	OR12D2	siganl < background (6.69)	6.972082	Only in embryos
EMPV1_25738	OR4X1	siganl < background (6.69)	6.973453	Only in embryos
EMPV1_24867	OR2B6	siganl < background (6.69)	6.974047	Only in embryos
EMPV1_14148	OR7E24	siganl < background (6.69)	6.974275	Only in embryos
EMPV1_16896	OR1M1	siganl < background (6.69)	6.975642	Only in embryos
EMPV1_28248	OR7E24	siganl < background (6.69)	6.97837	Only in embryos
EMPV1_03824	OR7D4	siganl < background (6.69)	6.978506	Only in embryos
EMPV1_16939	OR52B6	siganl < background (6.69)	6.978914	Only in embryos
EMPV1_36043	OR4F6	siganl < background (6.69)	6.979095	Only in embryos
EMPV1_22303	OR8H1	siganl < background (6.69)	6.981087	Only in embryos
EMPV1_11579	OR6M1	siganl < background (6.69)	6.982171	Only in embryos
EMPV1_35105	OR4C11	siganl < background (6.69)	6.984603	Only in embryos
EMPV1_43303	OR1L8	siganl < background (6.69)	6.985143	Only in embryos
EMPV1_09044	OR9G1	siganl < background (6.69)	6.985547	Only in embryos
EMPV1_19676	OR10A7	siganl < background (6.69)	6.985951	Only in embryos
EMPV1_08813	OR4K15	siganl < background (6.69)	6.985951	Only in embryos
EMPV1_09274	OR52H1	siganl < background (6.69)	6.985951	Only in embryos
EMPV1_32659	OR6C2	siganl < background (6.69)	6.987028	Only in embryos
EMPV1_42290	OR7A10	siganl < background (6.69)	6.987387	Only in embryos
EMPV1_36419	OR2Y1	siganl < background (6.69)	6.987387	Only in embryos
EMPV1_27057	OR8B12	siganl < background (6.69)	6.987476	Only in embryos
EMPV1_11353	OR52N4	siganl < background (6.69)	6.99007	Only in embryos
EMPV1_06890	OR2T6	siganl < background (6.69)	6.990516	Only in embryos
EMPV1_11766	OR8B3	siganl < background (6.69)	6.990561	Only in embryos
EMPV1_34904	OR4C46	siganl < background (6.69)	6.991497	Only in embryos
EMPV1_09341	OR51T1	siganl < background (6.69)	6.992654	Only in embryos
EMPV1_22751	OR6B1	siganl < background (6.69)	6.993454	Only in embryos
EMPV1_42207	OR4M1	siganl < background (6.69)	6.995584	Only in embryos
EMPV1_09543	OR12D2	siganl < background (6.69)	6.99859	Only in embryos
EMPV1_03145	OR7D4	siganl < background (6.69)	6.999427	Only in embryos
EMPV1_15661	OR2G2	siganl < background (6.69)	6.999692	Only in embryos
EMPV1_17850	OR1S1	siganl < background (6.69)	6.999824	Only in embryos
EMPV1_08808	OR6C3	siganl < background (6.69)	7.000264	Only in embryos

EMPV1_14397	OR2L8	siganl < background (6.69)	7.001408	Only in embryos
EMPV1_11850	OR12D2	siganl < background (6.69)	7.001671	Only in embryos
EMPV1_33159	OR7A17	siganl < background (6.69)	7.00347	Only in embryos
EMPV1_00446	OR11A1	siganl < background (6.69)	7.005089	Only in embryos
EMPV1_39215	OR2G2	siganl < background (6.69)	7.0064	Only in embryos
EMPV1_38892	OR1D2	siganl < background (6.69)	7.007708	Only in embryos
EMPV1_27438	OR10H4	siganl < background (6.69)	7.008796	Only in embryos
EMPV1_04342	OR2B6	siganl < background (6.69)	7.011747	Only in embryos
EMPV1_29091	OR2AG1	siganl < background (6.69)	7.012786	Only in embryos
EMPV1_26510	OR4F21	siganl < background (6.69)	7.014644	Only in embryos
EMPV1_03284	OR2A7	siganl < background (6.69)	7.015118	Only in embryos
EMPV1_32197	OR11H12	siganl < background (6.69)	7.015506	Only in embryos
EMPV1_30618	OR13C2	siganl < background (6.69)	7.016625	Only in embryos
EMPV1_07276	OR2D3	siganl < background (6.69)	7.017657	Only in embryos
EMPV1_02653	OR13F1	siganl < background (6.69)	7.023732	Only in embryos
EMPV1_03959	OR4K1	siganl < background (6.69)	7.024073	Only in embryos
EMPV1_34897	OR4A47	siganl < background (6.69)	7.024669	Only in embryos
EMPV1_21374	OR10D4P	siganl < background (6.69)	7.024839	Only in embryos
EMPV1_35331	OR8K3	siganl < background (6.69)	7.025902	Only in embryos
EMPV1_28128	OR2S2	siganl < background (6.69)	7.028065	Only in embryos
EMPV1_19151	OR6C76	siganl < background (6.69)	7.029418	Only in embryos
EMPV1_33109	OR10S1	siganl < background (6.69)	7.029418	Only in embryos
EMPV1_00119	OR51I1	siganl < background (6.69)	7.030601	Only in embryos
EMPV1_23891	OR2T34	siganl < background (6.69)	7.030854	Only in embryos
EMPV1_01513	OR5R1	siganl < background (6.69)	7.030854	Only in embryos
EMPV1_18781	OR2T3	siganl < background (6.69)	7.032372	Only in embryos
EMPV1_37035	OR52B4	siganl < background (6.69)	7.032372	Only in embryos
EMPV1_23024	OR52N4	siganl < background (6.69)	7.038784	Only in embryos
EMPV1_08300	OR5V1	siganl < background (6.69)	7.040284	Only in embryos
EMPV1_01247	OR51I1	siganl < background (6.69)	7.040284	Only in embryos
EMPV1_16997	OR8J3	siganl < background (6.69)	7.040534	Only in embryos
EMPV1_36615	OR4K17	siganl < background (6.69)	7.040534	Only in embryos
EMPV1_17588	OR13C2	siganl < background (6.69)	7.041158	Only in embryos
EMPV1_01889	OR12D3	siganl < background (6.69)	7.042072	Only in embryos

EMPV1_12794	OR51L1	siganl < background (6.69)	7.042736	Only in embryos
EMPV1_09211	OR5AN1	siganl < background (6.69)	7.045056	Only in embryos
EMPV1_15744	OR1J4	siganl < background (6.69)	7.045139	Only in embryos
EMPV1_19603	OR8U9	siganl < background (6.69)	7.045139	Only in embryos
EMPV1_18294	OR52A5	siganl < background (6.69)	7.045139	Only in embryos
EMPV1_15066	OR5B21	siganl < background (6.69)	7.046379	Only in embryos
EMPV1_38199	OR8S1	siganl < background (6.69)	7.047122	Only in embryos
EMPV1_25266	OR4C46	siganl < background (6.69)	7.047534	Only in embryos
EMPV1_38624	OR5L1	siganl < background (6.69)	7.047945	Only in embryos
EMPV1_40825	OR6C4	siganl < background (6.69)	7.047945	Only in embryos
EMPV1_41672	OR10AD1	siganl < background (6.69)	7.048481	Only in embryos
EMPV1_36890	OR51E2	siganl < background (6.69)	7.04951	Only in embryos
EMPV1_29390	OR51F2	siganl < background (6.69)	7.049592	Only in embryos
EMPV1_19259	OR5B2	siganl < background (6.69)	7.050578	Only in embryos
EMPV1_27930	OR1L8	siganl < background (6.69)	7.050988	Only in embryos
EMPV1_00364	OR4K15	siganl < background (6.69)	7.05148	Only in embryos
EMPV1_34126	OR51F2	siganl < background (6.69)	7.051644	Only in embryos
EMPV1_37895	OR8B8	siganl < background (6.69)	7.052791	Only in embryos
EMPV1_23090	OR6C6	siganl < background (6.69)	7.055568	Only in embryos
EMPV1_11601	OR12D3	siganl < background (6.69)	7.055731	Only in embryos
EMPV1_11153	OR51V1	siganl < background (6.69)	7.056383	Only in embryos
EMPV1_43639	OR4K14	siganl < background (6.69)	7.056586	Only in embryos
EMPV1_10142	OR2S2	siganl < background (6.69)	7.057684	Only in embryos
EMPV1_19129	OR9Q1	siganl < background (6.69)	7.058172	Only in embryos
EMPV1_01322	OR4F16	siganl < background (6.69)	7.058172	Only in embryos
EMPV1_17547	OR8S1	siganl < background (6.69)	7.059146	Only in embryos
EMPV1_08818	OR51B2	siganl < background (6.69)	7.060565	Only in embryos
EMPV1_08463	OR14I1	siganl < background (6.69)	7.06198	Only in embryos
EMPV1_10571	OR56A3	siganl < background (6.69)	7.062222	Only in embryos
EMPV1_21178	OR4K14	siganl < background (6.69)	7.064601	Only in embryos
EMPV1_36250	OR1A1	siganl < background (6.69)	7.067855	Only in embryos
EMPV1_36395	OR7A17	siganl < background (6.69)	7.068336	Only in embryos
EMPV1_21130	OR5A2	siganl < background (6.69)	7.069456	Only in embryos
EMPV1_42346	OR1L8	siganl < background (6.69)	7.071213	Only in embryos

EMPV1_16229	OR4C6	siganl < background (6.69)	7.071692	Only in embryos
EMPV1_35544	OR13C9	siganl < background (6.69)	7.072966	Only in embryos
EMPV1_30589	OR1M1	siganl < background (6.69)	7.072966	Only in embryos
EMPV1_16682	OR1J4	siganl < background (6.69)	7.073404	Only in embryos
EMPV1_10041	OR2G6	siganl < background (6.69)	7.073841	Only in embryos
EMPV1_23660	OR6C3	siganl < background (6.69)	7.074437	Only in embryos
EMPV1_27349	OR4F6	siganl < background (6.69)	7.074636	Only in embryos
EMPV1_40426	OR10X1	siganl < background (6.69)	7.075984	Only in embryos
EMPV1_07771	OR2T8	siganl < background (6.69)	7.077883	Only in embryos
EMPV1_14605	OR12D2	siganl < background (6.69)	7.080369	Only in embryos
EMPV1_14154	OR8A1	siganl < background (6.69)	7.080369	Only in embryos
EMPV1_26661	OR2C3	siganl < background (6.69)	7.083709	Only in embryos
EMPV1_35824	OR4F4	siganl < background (6.69)	7.084962	Only in embryos
EMPV1_00482	OR52M1	siganl < background (6.69)	7.085432	Only in embryos
EMPV1_02144	OR3A3	siganl < background (6.69)	7.086839	Only in embryos
EMPV1_18534	OR10G3	siganl < background (6.69)	7.087073	Only in embryos
EMPV1_01009	OR52A1	siganl < background (6.69)	7.089954	Only in embryos
EMPV1_22542	OR8J3	siganl < background (6.69)	7.093019	Only in embryos
EMPV1_05101	OR5AZ1P	siganl < background (6.69)	7.09553	Only in embryos
EMPV1_38016	OR7A17	siganl < background (6.69)	7.095954	Only in embryos
EMPV1_14930	OR1D2	siganl < background (6.69)	7.097648	Only in embryos
EMPV1_35289	OR7G1	siganl < background (6.69)	7.098916	Only in embryos
EMPV1_38553	OR4K13	siganl < background (6.69)	7.099606	Only in embryos
EMPV1_17417	OR11H4	siganl < background (6.69)	7.099913	Only in embryos
EMPV1_22092	OR6C1	siganl < background (6.69)	7.106134	Only in embryos
EMPV1_07401	OR7A10	siganl < background (6.69)	7.107311	Only in embryos
EMPV1_15578	OR8B3	siganl < background (6.69)	7.108297	Only in embryos
EMPV1_01991	OR51V1	siganl < background (6.69)	7.112152	Only in embryos
EMPV1_23584	OR2C1	siganl < background (6.69)	7.112641	Only in embryos
EMPV1_25305	OR52K2	siganl < background (6.69)	7.115685	Only in embryos
EMPV1_12509	OR1J4	siganl < background (6.69)	7.120992	Only in embryos
EMPV1_19750	OR10X1	siganl < background (6.69)	7.120992	Only in embryos
EMPV1_04764	OR5R1	siganl < background (6.69)	7.121884	Only in embryos
EMPV1_40727	OR7G2	siganl < background (6.69)	7.123036	Only in embryos

EMPV1_27288	OR5D18	siganl < background (6.69)	7.124631	Only in embryos
EMPV1_27560	OR5P3	siganl < background (6.69)	7.125149	Only in embryos
EMPV1_33387	OR4C46	siganl < background (6.69)	7.126	Only in embryos
EMPV1_43632	OR6C2	siganl < background (6.69)	7.127035	Only in embryos
EMPV1_38268	OR2G3	siganl < background (6.69)	7.136606	Only in embryos
EMPV1_04711	OR52A5	siganl < background (6.69)	7.136716	Only in embryos
EMPV1_01146	OR1A1	siganl < background (6.69)	7.136716	Only in embryos
EMPV1_17101	OR2T12	siganl < background (6.69)	7.13748	Only in embryos
EMPV1_08683	OR6C2	siganl < background (6.69)	7.141254	Only in embryos
EMPV1_07966	OR7D4	siganl < background (6.69)	7.141544	Only in embryos
EMPV1_01378	OR8D1	siganl < background (6.69)	7.141942	Only in embryos
EMPV1_00983	OR4K14	siganl < background (6.69)	7.148315	Only in embryos
EMPV1_17775	OR10R2	siganl < background (6.69)	7.151034	Only in embryos
EMPV1_43662	OR7E24	siganl < background (6.69)	7.151998	Only in embryos
EMPV1_21360	OR2F1	siganl < background (6.69)	7.153672	Only in embryos
EMPV1_29445	OR2AE1	siganl < background (6.69)	7.154276	Only in embryos
EMPV1_32175	OR6C1	siganl < background (6.69)	7.157575	Only in embryos
EMPV1_01381	OR52H1	siganl < background (6.69)	7.158777	Only in embryos
EMPV1_14533	OR5AN1	siganl < background (6.69)	7.158989	Only in embryos
EMPV1_08562	OR51I2	siganl < background (6.69)	7.160012	Only in embryos
EMPV1_22291	OR5D14	siganl < background (6.69)	7.160788	Only in embryos
EMPV1_21633	OR2T2	siganl < background (6.69)	7.161527	Only in embryos
EMPV1_08413	OR5M3	siganl < background (6.69)	7.162372	Only in embryos
EMPV1_26724	OR10J5	siganl < background (6.69)	7.162793	Only in embryos
EMPV1_38140	OR51I2	siganl < background (6.69)	7.162793	Only in embryos
EMPV1_05746	OR52K1	siganl < background (6.69)	7.165458	Only in embryos
EMPV1_28403	OR6C1	siganl < background (6.69)	7.166787	Only in embryos
EMPV1_14604	OR6C74	siganl < background (6.69)	7.166927	Only in embryos
EMPV1_38581	OR2B2	siganl < background (6.69)	7.166927	Only in embryos
EMPV1_39989	OR7C2	siganl < background (6.69)	7.170412	Only in embryos
EMPV1_34902	OR8U8	siganl < background (6.69)	7.173672	Only in embryos
EMPV1_39904	OR5AN1	siganl < background (6.69)	7.175918	Only in embryos
EMPV1_04049	OR2S2	siganl < background (6.69)	7.180595	Only in embryos
EMPV1_17923	OR9K2	siganl < background (6.69)	7.181794	Only in embryos

EMPV1_01834	OR2T2	siganl < background (6.69)	7.182136	Only in embryos
EMPV1_40590	OR5R1	siganl < background (6.69)	7.182888	Only in embryos
EMPV1_37105	OR11G2	siganl < background (6.69)	7.183229	Only in embryos
EMPV1_33272	OR1N2	siganl < background (6.69)	7.184799	Only in embryos
EMPV1_23444	OR6K3	siganl < background (6.69)	7.185037	Only in embryos
EMPV1_38128	OR4F15	siganl < background (6.69)	7.185105	Only in embryos
EMPV1_11283	OR11H1	siganl < background (6.69)	7.187757	Only in embryos
EMPV1_17537	OR4F15	siganl < background (6.69)	7.188605	Only in embryos
EMPV1_20134	OR7E24	siganl < background (6.69)	7.188775	Only in embryos
EMPV1_41163	OR11G2	siganl < background (6.69)	7.191211	Only in embryos
EMPV1_41195	OR12D2	siganl < background (6.69)	7.192122	Only in embryos
EMPV1_00545	OR4F16	siganl < background (6.69)	7.192729	Only in embryos
EMPV1_28045	OR7A10	siganl < background (6.69)	7.194345	Only in embryos
EMPV1_15819	OR6C75	siganl < background (6.69)	7.194412	Only in embryos
EMPV1_35382	OR5AN1	siganl < background (6.69)	7.195219	Only in embryos
EMPV1_39294	OR52A1	siganl < background (6.69)	7.20134	Only in embryos
EMPV1_18407	OR2AE1	siganl < background (6.69)	7.203103	Only in embryos
EMPV1_27708	OR2B6	siganl < background (6.69)	7.203866	Only in embryos
EMPV1_18581	OR2L8	siganl < background (6.69)	7.205358	Only in embryos
EMPV1_32201	OR10G3	siganl < background (6.69)	7.210112	Only in embryos
EMPV1_09222	OR52R1	siganl < background (6.69)	7.21146	Only in embryos
EMPV1_05911	OR51G2	siganl < background (6.69)	7.211657	Only in embryos
EMPV1_32651	OR10X1	siganl < background (6.69)	7.217281	Only in embryos
EMPV1_32340	OR6C68	siganl < background (6.69)	7.220759	Only in embryos
EMPV1_42733	OR4F6	siganl < background (6.69)	7.22853	Only in embryos
EMPV1_21993	OR5AC2	siganl < background (6.69)	7.229011	Only in embryos
EMPV1_04765	OR8J3	siganl < background (6.69)	7.232209	Only in embryos
EMPV1_04236	OR4M1	siganl < background (6.69)	7.233038	Only in embryos
EMPV1_30615	OR4C46	siganl < background (6.69)	7.23393	Only in embryos
EMPV1_29836	OR5V1	siganl < background (6.69)	7.234567	Only in embryos
EMPV1_06613	OR10G7	siganl < background (6.69)	7.234948	Only in embryos
EMPV1_09698	OR51Q1	siganl < background (6.69)	7.234948	Only in embryos
EMPV1_30578	OR2A5	siganl < background (6.69)	7.235202	Only in embryos
EMPV1_15920	OR5B12	siganl < background (6.69)	7.239417	Only in embryos

EMPV1_17631	OR4C46	siganl < background (6.69)	7.241404	Only in embryos
EMPV1_33939	OR56A3	siganl < background (6.69)	7.244987	Only in embryos
EMPV1_25558	OR4P4	siganl < background (6.69)	7.245926	Only in embryos
EMPV1_27922	OR4K1	siganl < background (6.69)	7.247334	Only in embryos
EMPV1_11535	OR52B6	siganl < background (6.69)	7.248271	Only in embryos
EMPV1_24241	OR11H6	siganl < background (6.69)	7.250515	Only in embryos
EMPV1_01003	OR5AN1	siganl < background (6.69)	7.251851	Only in embryos
EMPV1_32264	OR11G2	siganl < background (6.69)	7.251913	Only in embryos
EMPV1_24531	OR6C68	siganl < background (6.69)	7.252192	Only in embryos
EMPV1_19884	OR1J4	siganl < background (6.69)	7.256124	Only in embryos
EMPV1_34875	OR8B2	siganl < background (6.69)	7.25785	Only in embryos
EMPV1_07026	OR5AC2	siganl < background (6.69)	7.258619	Only in embryos
EMPV1_38426	OR4C13	siganl < background (6.69)	7.260033	Only in embryos
EMPV1_10274	OR5AR1	siganl < background (6.69)	7.260033	Only in embryos
EMPV1_30131	OR52B2	siganl < background (6.69)	7.265203	Only in embryos
EMPV1_08784	OR13C8	siganl < background (6.69)	7.265325	Only in embryos
EMPV1_32966	OR1L4	siganl < background (6.69)	7.271304	Only in embryos
EMPV1_13877	OR7A17	siganl < background (6.69)	7.272724	Only in embryos
EMPV1_35600	OR9K2	siganl < background (6.69)	7.273568	Only in embryos
EMPV1_34920	OR4K14	siganl < background (6.69)	7.273568	Only in embryos
EMPV1_09840	OR5AN1	siganl < background (6.69)	7.276515	Only in embryos
EMPV1_24766	OR56A4	siganl < background (6.69)	7.281121	Only in embryos
EMPV1_04615	OR52L1	siganl < background (6.69)	7.282908	Only in embryos
EMPV1_17449	OR6B3	siganl < background (6.69)	7.284957	Only in embryos
EMPV1_03097	OR4S1	siganl < background (6.69)	7.287179	Only in embryos
EMPV1_33766	OR9K2	siganl < background (6.69)	7.289569	Only in embryos
EMPV1_29228	OR2S2	siganl < background (6.69)	7.289658	Only in embryos
EMPV1_34764	OR7D4	siganl < background (6.69)	7.293918	Only in embryos
EMPV1_36671	OR6C75	siganl < background (6.69)	7.29617	Only in embryos
EMPV1_24454	OR56A4	siganl < background (6.69)	7.296345	Only in embryos
EMPV1_04940	OR13D1	siganl < background (6.69)	7.301378	Only in embryos
EMPV1_16974	OR1J1	siganl < background (6.69)	7.301842	Only in embryos
EMPV1_27135	OR8K5	siganl < background (6.69)	7.301842	Only in embryos
EMPV1_08334	OR52D1	siganl < background (6.69)	7.301871	Only in embryos

EMPV1_14677	OR2Y1	siganl < background (6.69)	7.303087	Only in embryos
EMPV1_28939	OR2T12	siganl < background (6.69)	7.305916	Only in embryos
EMPV1_32670	OR5K2	siganl < background (6.69)	7.307413	Only in embryos
EMPV1_38964	OR1J4	siganl < background (6.69)	7.308159	Only in embryos
EMPV1_43261	OR10H1	siganl < background (6.69)	7.309422	Only in embryos
EMPV1_00709	OR4F6	siganl < background (6.69)	7.313311	Only in embryos
EMPV1_05363	OR1J1	siganl < background (6.69)	7.314764	Only in embryos
EMPV1_01243	OR7A10	siganl < background (6.69)	7.322154	Only in embryos
EMPV1_35045	OR2G6	siganl < background (6.69)	7.324628	Only in embryos
EMPV1_12337	OR56A3	siganl < background (6.69)	7.327122	Only in embryos
EMPV1_15391	OR6C65	siganl < background (6.69)	7.328659	Only in embryos
EMPV1_23649	OR5L1	siganl < background (6.69)	7.33114	Only in embryos
EMPV1_39923	OR52H1	siganl < background (6.69)	7.331501	Only in embryos
EMPV1_07439	OR9I1	siganl < background (6.69)	7.334221	Only in embryos
EMPV1_24245	OR4C3	siganl < background (6.69)	7.340592	Only in embryos
EMPV1_30480	OR10A3	siganl < background (6.69)	7.344125	Only in embryos
EMPV1_32885	OR56B4	siganl < background (6.69)	7.347859	Only in embryos
EMPV1_21698	OR52D1	siganl < background (6.69)	7.351303	Only in embryos
EMPV1_09561	OR5L1	siganl < background (6.69)	7.351357	Only in embryos
EMPV1_04781	OR51G1	siganl < background (6.69)	7.35368	Only in embryos
EMPV1_01927	OR4X2	siganl < background (6.69)	7.35465	Only in embryos
EMPV1_07250	OR2Y1	siganl < background (6.69)	7.35723	Only in embryos
EMPV1_24292	OR4C6	siganl < background (6.69)	7.35841	Only in embryos
EMPV1_00251	OR7A10	siganl < background (6.69)	7.360309	Only in embryos
EMPV1_16604	OR10H2	siganl < background (6.69)	7.361697	Only in embryos
EMPV1_30590	OR7A10	siganl < background (6.69)	7.36215	Only in embryos
EMPV1_24548	OR1J4	siganl < background (6.69)	7.366931	Only in embryos
EMPV1_29603	OR4L1	siganl < background (6.69)	7.367434	Only in embryos
EMPV1_41572	OR10X1	siganl < background (6.69)	7.378174	Only in embryos
EMPV1_20076	OR5R1	siganl < background (6.69)	7.384531	Only in embryos
EMPV1_29362	OR8B3	siganl < background (6.69)	7.384996	Only in embryos
EMPV1_06963	OR14I1	siganl < background (6.69)	7.387445	Only in embryos
EMPV1_13552	OR4P4	siganl < background (6.69)	7.387856	Only in embryos
EMPV1_02061	OR51F1	siganl < background (6.69)	7.389706	Only in embryos

EMPV1_35922	OR5A2	siganl < background (6.69)	7.392931	Only in embryos
EMPV1_15905	OR6C70	siganl < background (6.69)	7.395531	Only in embryos
EMPV1_34151	OR4L1	siganl < background (6.69)	7.397309	Only in embryos
EMPV1_09069	OR5R1	siganl < background (6.69)	7.399565	Only in embryos
EMPV1_28186	OR6C68	siganl < background (6.69)	7.40098	Only in embryos
EMPV1_10680	OR9G1	siganl < background (6.69)	7.404432	Only in embryos
EMPV1_41904	OR4C46	siganl < background (6.69)	7.404633	Only in embryos
EMPV1_33344	OR9I1	siganl < background (6.69)	7.404633	Only in embryos
EMPV1_06256	OR2H2	siganl < background (6.69)	7.405135	Only in embryos
EMPV1_40734	OR8B4	siganl < background (6.69)	7.407191	Only in embryos
EMPV1_10903	OR4L1	siganl < background (6.69)	7.408417	Only in embryos
EMPV1_26961	OR4F4	siganl < background (6.69)	7.412976	Only in embryos
EMPV1_04162	OR7D4	siganl < background (6.69)	7.43972	Only in embryos
EMPV1_33737	OR2M3	siganl < background (6.69)	7.440055	Only in embryos
EMPV1_39328	OR2M3	siganl < background (6.69)	7.441131	Only in embryos
EMPV1_35499	OR52D1	siganl < background (6.69)	7.442252	Only in embryos
EMPV1_42182	OR8B8	siganl < background (6.69)	7.443634	Only in embryos
EMPV1_21420	OR1N2	siganl < background (6.69)	7.444728	Only in embryos
EMPV1_33085	OR1J4	siganl < background (6.69)	7.464907	Only in embryos
EMPV1_26450	OR14C36	siganl < background (6.69)	7.468825	Only in embryos
EMPV1_10700	OR4C46	siganl < background (6.69)	7.472722	Only in embryos
EMPV1_19537	OR2M3	siganl < background (6.69)	7.47728	Only in embryos
EMPV1_23013	OR2A2	siganl < background (6.69)	7.484199	Only in embryos
EMPV1_34130	OR4E2	siganl < background (6.69)	7.487476	Only in embryos
EMPV1_02494	OR4K2	siganl < background (6.69)	7.488819	Only in embryos
EMPV1_35303	OR10G2	siganl < background (6.69)	7.488863	Only in embryos
EMPV1_23851	OR2M3	siganl < background (6.69)	7.49036	Only in embryos
EMPV1_35721	OR6C2	siganl < background (6.69)	7.495451	Only in embryos
EMPV1_12873	OR52K2	siganl < background (6.69)	7.505089	Only in embryos
EMPV1_34471	OR6Q1	siganl < background (6.69)	7.509904	Only in embryos
EMPV1_11805	OR1J4	siganl < background (6.69)	7.513262	Only in embryos
EMPV1_36620	OR5M8	siganl < background (6.69)	7.514859	Only in embryos
EMPV1_39145	OR2M3	siganl < background (6.69)	7.518087	Only in embryos
EMPV1_19746	OR4F6	siganl < background (6.69)	7.519438	Only in embryos

EMPV1_10796	OR2A20P	siganl < background (6.69)	7.528213	Only in embryos
EMPV1_00735	OR2M3	siganl < background (6.69)	7.528319	Only in embryos
EMPV1_20972	OR4C46	siganl < background (6.69)	7.532813	Only in embryos
EMPV1_35662	OR8B4	siganl < background (6.69)	7.533381	Only in embryos
EMPV1_15361	OR1J4	siganl < background (6.69)	7.535899	Only in embryos
EMPV1_00929	OR1J2	siganl < background (6.69)	7.538825	Only in embryos
EMPV1_03397	OR4P4	siganl < background (6.69)	7.541158	Only in embryos
EMPV1_23821	OR4A47	siganl < background (6.69)	7.543711	Only in embryos
EMPV1_11366	OR10G4	siganl < background (6.69)	7.552668	Only in embryos
EMPV1_30739	OR4B1	siganl < background (6.69)	7.555812	Only in embryos
EMPV1_38852	OR8S1	siganl < background (6.69)	7.557258	Only in embryos
EMPV1_20809	OR8S1	siganl < background (6.69)	7.559146	Only in embryos
EMPV1_04842	OR4F21	siganl < background (6.69)	7.560605	Only in embryos
EMPV1_04090	OR4F21	siganl < background (6.69)	7.560807	Only in embryos
EMPV1_05901	OR7A10	siganl < background (6.69)	7.568235	Only in embryos
EMPV1_28328	OR11H6	siganl < background (6.69)	7.569715	Only in embryos
EMPV1_32296	OR5C1	siganl < background (6.69)	7.572011	Only in embryos
EMPV1_08693	OR52E6	siganl < background (6.69)	7.574715	Only in embryos
EMPV1_01163	OR6P1	siganl < background (6.69)	7.591507	Only in embryos
EMPV1_14079	OR1L8	siganl < background (6.69)	7.596147	Only in embryos
EMPV1_35935	OR52K2	siganl < background (6.69)	7.605906	Only in embryos
EMPV1_24236	OR5P3	siganl < background (6.69)	7.612679	Only in embryos
EMPV1_27316	OR51G2	siganl < background (6.69)	7.623036	Only in embryos
EMPV1_14896	OR4C6	siganl < background (6.69)	7.626702	Only in embryos
EMPV1_06993	OR2T29	siganl < background (6.69)	7.628989	Only in embryos
EMPV1_04022	OR1J2	siganl < background (6.69)	7.630643	Only in embryos
EMPV1_13764	OR2A2	siganl < background (6.69)	7.631011	Only in embryos
EMPV1_13111	OR1J1	siganl < background (6.69)	7.635075	Only in embryos
EMPV1_20538	OR8B4	siganl < background (6.69)	7.636606	Only in embryos
EMPV1_02049	OR11G2	siganl < background (6.69)	7.647149	Only in embryos
EMPV1_29447	OR2M2	siganl < background (6.69)	7.652461	Only in embryos
EMPV1_22433	OR7D4	siganl < background (6.69)	7.652603	Only in embryos
EMPV1_13417	OR10J3	siganl < background (6.69)	7.652853	Only in embryos
EMPV1_25494	OR8B2	siganl < background (6.69)	7.65296	Only in embryos

EMPV1_42825	OR4K2	siganl < background (6.69)	7.660259	Only in embryos
EMPV1_34949	OR10J5	siganl < background (6.69)	7.669925	Only in embryos
EMPV1_29226	OR52E8	siganl < background (6.69)	7.683161	Only in embryos
EMPV1_05451	OR1E1	siganl < background (6.69)	7.695454	Only in embryos
EMPV1_06701	OR1J1	siganl < background (6.69)	7.702438	Only in embryos
EMPV1_03887	OR9I1	siganl < background (6.69)	7.709881	Only in embryos
EMPV1_12669	OR6M1	siganl < background (6.69)	7.714098	Only in embryos
EMPV1_01029	OR4L1	siganl < background (6.69)	7.716303	Only in embryos
EMPV1_02658	OR1J2	siganl < background (6.69)	7.725718	Only in embryos
EMPV1_01841	OR4B1	siganl < background (6.69)	7.72853	Only in embryos
EMPV1_39727	OR2AE1	siganl < background (6.69)	7.736789	Only in embryos
EMPV1_16988	OR4F16	siganl < background (6.69)	7.742254	Only in embryos
EMPV1_28777	OR4P4	siganl < background (6.69)	7.742317	Only in embryos
EMPV1_02145	OR4D10	siganl < background (6.69)	7.748723	Only in embryos
EMPV1_32405	OR14J1	siganl < background (6.69)	7.754331	Only in embryos
EMPV1_29726	OR4L1	siganl < background (6.69)	7.76782	Only in embryos
EMPV1_39723	OR4Q3	siganl < background (6.69)	7.778612	Only in embryos
EMPV1_11764	OR4S1	siganl < background (6.69)	7.781121	Only in embryos
EMPV1_01430	OR5P3	siganl < background (6.69)	7.792305	Only in embryos
EMPV1_02570	OR5P3	siganl < background (6.69)	7.796783	Only in embryos
EMPV1_24902	OR6M1	siganl < background (6.69)	7.834138	Only in embryos
EMPV1_14571	OR11H4	siganl < background (6.69)	7.839396	Only in embryos
EMPV1_21898	OR2T1	siganl < background (6.69)	7.84136	Only in embryos
EMPV1_22796	OR5R1	siganl < background (6.69)	7.842607	Only in embryos
EMPV1_38368	OR2M5	siganl < background (6.69)	7.844985	Only in embryos
EMPV1_25298	OR4K1	siganl < background (6.69)	7.845436	Only in embryos
EMPV1_16833	OR10G9	siganl < background (6.69)	7.848972	Only in embryos
EMPV1_16802	OR10G9	siganl < background (6.69)	7.853154	Only in embryos
EMPV1_40489	OR1J4	siganl < background (6.69)	7.85841	Only in embryos
EMPV1_04481	OR1J4	siganl < background (6.69)	7.878799	Only in embryos
EMPV1_07357	OR9I1	siganl < background (6.69)	7.893951	Only in embryos
EMPV1_04703	OR7A10	siganl < background (6.69)	7.894754	Only in embryos
EMPV1_12053	OR2M2	siganl < background (6.69)	7.896103	Only in embryos
EMPV1_36293	OR52H1	siganl < background (6.69)	7.899603	Only in embryos

EMPV1_03151	OR6Y1	siganl < background (6.69)	7.907892	Only in embryos
EMPV1_05222	OR2H2	siganl < background (6.69)	7.922684	Only in embryos
EMPV1_25088	OR8K5	siganl < background (6.69)	7.930253	Only in embryos
EMPV1_00892	OR2M3	siganl < background (6.69)	7.935122	Only in embryos
EMPV1_22987	OR6Y1	siganl < background (6.69)	7.963055	Only in embryos
EMPV1_06996	OR7A10	siganl < background (6.69)	7.986826	Only in embryos
EMPV1_41194	OR6C74	siganl < background (6.69)	7.990605	Only in embryos
EMPV1_43602	OR52B6	siganl < background (6.69)	8.008056	Only in embryos
EMPV1_16280	OR11H4	siganl < background (6.69)	8.036181	Only in embryos
EMPV1_18445	OR1S2	siganl < background (6.69)	8.055975	Only in embryos
EMPV1_12262	OR9I1	siganl < background (6.69)	8.077765	Only in embryos
EMPV1_22598	OR10Z1	siganl < background (6.69)	8.092235	Only in embryos
EMPV1_38117	OR1A1	siganl < background (6.69)	8.156069	Only in embryos
EMPV1_29471	OR3A4	siganl < background (6.69)	8.175073	Only in embryos
EMPV1_34215	OR8S1	siganl < background (6.69)	8.193049	Only in embryos
EMPV1_39340	OR2Z1	siganl < background (6.69)	8.216939	Only in embryos
EMPV1_20349	OR1J4	siganl < background (6.69)	8.263853	Only in embryos
EMPV1_22160	OR2H1	siganl < background (6.69)	8.285892	Only in embryos
EMPV1_03469	OR4S1	siganl < background (6.69)	8.34707	Only in embryos
EMPV1_38300	OR4F21	siganl < background (6.69)	8.390257	Only in embryos
EMPV1_06131	OR1E1	siganl < background (6.69)	8.390532	Only in embryos
EMPV1_19261	OR4F21	siganl < background (6.69)	8.412411	Only in embryos
EMPV1_37024	OR52J3	siganl < background (6.69)	8.429719	Only in embryos
EMPV1_37497	OR6B3	siganl < background (6.69)	8.435983	Only in embryos
EMPV1_43300	OR4C3	siganl < background (6.69)	8.440832	Only in embryos
EMPV1_19944	OR5P3	siganl < background (6.69)	8.471738	Only in embryos
EMPV1_27580	OR51F2	siganl < background (6.69)	8.659642	Only in embryos
EMPV1_31962	OR7A10	siganl < background (6.69)	8.716475	Only in embryos
EMPV1_09882	OR7A10	siganl < background (6.69)	8.730296	Only in embryos
EMPV1_24577	OR2H1	siganl < background (6.69)	8.807517	Only in embryos
EMPV1_19327	OR52E2	siganl < background (6.69)	8.849997	Only in embryos
EMPV1_13297	OR4C16	siganl < background (6.69)	8.972602	Only in embryos
EMPV1_15725	OR4B1	siganl < background (6.69)	8.98446	Only in embryos
EMPV1_16317	OR4D2	siganl < background (6.69)	8.988673	Only in embryos

EMPV1_16660	OR13H1	siganl < background (6.69)	9.041298	Only in embryos
EMPV1_16651	OR2AT4	siganl < background (6.69)	9.20863	Only in embryos
EMPV1_30410	OR4B1	siganl < background (6.69)	9.224223	Only in embryos
EMPV1_32442	OR56A1	siganl < background (6.69)	9.268016	Only in embryos
EMPV1_37996	OR8K5	siganl < background (6.69)	9.305166	Only in embryos
EMPV1_36905	OR2B11	siganl < background (6.69)	9.313439	Only in embryos
EMPV1_07576	OR1A1	siganl < background (6.69)	9.386384	Only in embryos
EMPV1_10946	OR4B1	siganl < background (6.69)	9.606487	Only in embryos
EMPV1_37970	OR4C3	siganl < background (6.69)	9.661186	Only in embryos
EMPV1_17815	OR4B1	siganl < background (6.69)	9.694068	Only in embryos
EMPV1_36098	OR1A1	siganl < background (6.69)	9.694929	Only in embryos
EMPV1_34164	OR8B4	siganl < background (6.69)	9.701685	Only in embryos
EMPV1_08382	OR4N2	siganl < background (6.69)	9.830342	Only in embryos
EMPV1_41865	OR7E37P	siganl < background (6.69)	9.898475	Only in embryos
EMPV1_37565	OR4C16	siganl < background (6.69)	10.91687	Only in embryos
EMPV1_06511	OR4C16	siganl < background (6.69)	11.14043	Only in embryos
